# Abstracts from the World Congress of Cardiology/Brazilian Congress of Cardiology 2022

**DOI:** 10.5334/gh.1165

**Published:** 2023-03-07

**Authors:** The Editorial Team (on behalf of the World Heart Federation)

**Affiliations:** 1 On behalf of the World Heart Federation, Switzerland

**Keywords:** Cardiology, Global Health, Heart Health, Cardiovascular Disease, Conference

## Abstract

These are the abstracts from the combined 77^th^ Brazilian Congress of Cardiology, together with the World Congress of Cardiology, held in October 2022. From 1950 to today, the World Heart Federation’s World Congress of Cardiology (WCC) has been a key event on the cardiovascular calendar, offering a global perspective on cardiovascular health and bringing together thousands of cardiology professionals from all over the world with one common goal: to reduce the global burden of cardiovascular disease and help people live longer, healthier lives.

107880

Modality: E-Poster Researcher – Non-case Report

Category: EPIDEMIOLOGY AND HEALTH POLICIES/GLOBAL HEALTH

## Mortality in a 3-Year Follow-Up of Congestive Heart Failure Due to Chagas Disease in Women and Men

ANTONIO DE PADUA MANSUR^1^, Carlos Henrique Del Carlo^1^, José Antonio Ramos Neto^1^, André Barbosa de Abreu^1^, Airton Roberto Scipioni^1^, Antonio Carlos Pereira Barreto^1^

(1) Insituto do Coração – HC FMUSP

**Background:** Chronic Chagas cardiomyopathy (CCC) is one of the leading causes of congestive heart failure (CHF) in Latin America and carries a high morbidity and mortality burden. Previously, it was believed that there was no epidemiological and clinical evidence of a gender-associated risk of death in patients with CCC.

**Purpose:** To analyze the mortality of congestive heart failure due to CCC in women and men.

**Methods:** From February 2017 to September 2020, we followed a cohort of patients with CHF (Framingham criteria) due to CCC in a single-center outpatient clinic. Appropriate serologic tests defined Chagas disease. Baseline data included clinical characteristics and echocardiographic findings. Statistical analyses were performed with the Kaplan-Meier (K-M) method to analyze time-to-event data and the Cox proportional hazards methods to search for predictors of death.

**Results:** We studied 733 patients, mean of 61.4 ± 12.3 years, 381 (52%) males. Females were older (63.0 ± 11.9 vs. 60 ± 12.4 years; p = 0.01), had a higher baseline mean left ventricular ejection fraction (LVEF) (44.5 ± 14.6% vs. 37.3 ± 14.8%; p < 0.001), and a lower left ventricular diastolic diameter (LVDD) (56.7 ± 8.9 vs. 62.4 ± 9.4 mm; p < 0.001). Over a 3-years follow-up period, 168 (44%) men and 126 (36%) women died (K-M: log-rank p = 0.002; Figure). Women had more implantable pacemakers (PM) (26.1% vs. 16.5%; p = 0.002) and men more implantable cardioverter-defibrillators (ICDs)(20.7% vs. 12.5%; p = 0.003). Heart transplant occurred in 10.8% of men and 7.4% in women (p = NS). Cox regression for death adjusted for age, previous myocardial infarction, diabetes, previous stroke, chronic kidney disease (CKD), atrial fibrillation, PM, ICD, heart transplant and LVEF, showed, in descending order, previous stroke (HR = 2.4; 95%CL:1.5–3.6), diabetes (HR = 2.0; 95%CL: 1.3–3.1), and CKD (HR = 1.8; 95%CL:1.3–2.6) as the main predictors of death in men, and in women diabetes (HR = 2.2; 95%CL:1.4–3.4), previous stroke (HR = 1.8; 95%CL:1.1–2.9), and CKD (HR = 1.7; 95%CL:1.1–2.7).

**Conclusions:** Women had a better prognosis than men but similar predictors of death. Control of diabetes and prevention of stroke and CKD could significantly reduce the death rate in CHF due to CCC.

108498

Modality: WHF Abstracts – Young Researcher

Category: EPIDEMIOLOGY AND HEALTH POLICIES/GLOBAL HEALTH

D: 14/10/2022    H: 16:50/17:50

L: Auditório 14

## Relationship between Sociodemographic Index and Ischemic Heart Disease in Brazil in the Period of 2000–2019

JOSÉ LUCAS PERES BICHARA^1^, José Lucas Peres Bichara^1^, Luiz Antônio Viegas de Miranda Bastos^1^, Paolo Blanco Villela^1^, Glaucia Moraes de Oliveira^1^

(1) Universidade Federal do Rio de Janeiro – UFRJ

**Introduction:** Cardiovascular diseases (CVD) are the main causes of death in the world and in Brazil, and ischemic heart diseases (IHD) are one of the main responsible for these statistics. Previous studies have already suggested a relationship between the evolution of IHD mortality rates and socioeconomic indicators.

**Objective:** To relate the evolution of IHD mortality rates and the sociodemographic index (SDI) from 2000 to 2019 in Brazil and in its federative units (FUs).

**Methods:** Ecological time series study of deaths from IHD in Brazil. Crude and standardized mortality rate for IHD were analyzed by sex, age group and FU between 2000 and 2019. Data were correlated with the SDI. Deaths and population were taken from DATASUS to estimate crude and standardized mortality rates per 100,000 inhabitants (direct method with Brazilian population in 2000). The SDI for each UF was extracted from the Global Health Data Exchange website.

**Results:** In the period, there were 1,968,160 deaths from IHD in Brazil, 58.19% of which were male. The national SDI ranged from 0.538 in 2000 to 0.64 in 2019, with constant growth in the period. Concomitantly, the age-standardized mortality rate for IHD decreased from 46.12/100,000 inhabitants to 36.42/100,000 inhabitants. Thus, IHD has become the leading cause of mortality in the country. In the FUs, all states in the North and Northeast regions showed improvements in the SDI, however, the best indicators continued to be concentrated in the other regions. In 2000, the highest mortality rates from IHD were found in the South, Southeast and Midwest regions of the country, but they showed a significant reduction. When evaluating the variation of the standardized mortality rate in the period, it was noted that the FUs with the best SDI were responsible for the greatest drops (graph 1).

**Conclusion:** During the period, the country showed a significant improvement in socioeconomic indicators accompanied by a reduction in IHD mortality rates. When evaluating the FUs, it was noted that those with better socioeconomic indicators were able to obtain a greater reduction in these mortality rates.


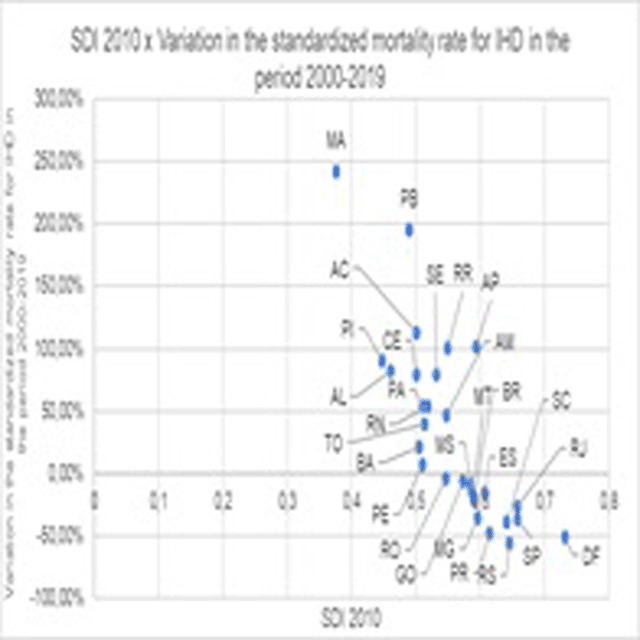


108502

Modality: WHF Abstracts – Researcher

Category: NEGLECTED CARDIOVASCULAR DISEASES

D: 14/10/2022    H: 13:50/14:50

L: Auditório 14

## Association between Autonomic Denervation, Myocardial Hypoperfusion, Fibrosis and Ventricular Arrhythmias in Chagas Cardiomyopathy

ADRIANA SOARES XAVIER DE BRITO^1^, Renata Junqueira Moll-Bernardes^1^, Martha Valeria Tavares Pinheiro^1^, Paulo Henrique Rosado de Castro^1^, Gabriel Cordeiro Camargo^1^, Adriana Pereira Glavam^1^, Sergio Altino de Almeida^1^, Fabio Paiva Siqueira^1^, Marcelo Teixeira de Holanda^2^, Luiz Henrique Conde Sangenis^2^, Fernanda de Souza Nogueira Sardinha Mendes^2^, Andrea Silvestre de Sousa^2^

(1) D’Or Institute for Research and Education (IDOR), Rio de Janeiro; (2) Evandro Chagas National Institute of Infectious Diseases, Oswaldo Cruz Foundation

**Introduction:** Chagas disease continues to be an important cause of morbidity and mortality in Latin America. Sudden cardiac death (SCD) represents the most dramatic course of Chagas cardiomyopathy (CC), and it is closely related to the presence of ventricular arrhythmias and myocardial dysfunction. However, there is also a high incidence of SCD when left ventricular ejection fraction (LVEF) is normal or mildly depressed. Unfortunately, despite its significant mortality, there is no clear recommendation for early cardio-defibrillator implantation in patients with CC. Ideally, the risk of SCD should be evaluated in earlier stages of the disease, but important questions remain unresolved regarding its pathophysiological mechanism and the diagnostic tools. New imaging parameters to identify the genesis of arrhythmia such as fibrosis, inflammation and dysautonomia can be a promising strategy.

**Purpose:** We tested the correlation between the extent of myocardial sympathetic denervation, myocardial perfusion, fibrosis and the severity of the ventricular arrhythmia in patients in the early phase of CC.

**Methods:** Twenty-nine patients with CC and LVEF >45% prospectively underwent magnetic resonance (MRI), SPECT imaging of myocardial sympathetic innervation using 123Iodine-MIBG (MIBG) and rest myocardial perfusion with 99mTc-sestamibi (MIBI), and were divided into two groups: arrhythmic group (n = 15): >120 ventricular ectopic beats and/or Non-Sustained Ventricular Tachycardia (NSVT) and non-arrhythmic group (n = 14): <120 ventricular ectopic beats without NSVT on 24-h Holter monitoring.

**Results:** Compared to non-arrhythmic, the arrhythmic group had significantly higher denervation score (mean ± SD 23.2 ± 18.7 versus 5.6 ± 4.9; p < 0.01), higher summed rest perfusion score (mean ± SD 4.7 ± 6.8 versus 0.3 ± 0.6; p < 0.05) as well as the higher mismatch in the innervation/perfusion score between MIBG and MIBI images, which evaluates the extent of denervated but viable myocardium (mean ± SD 18.4 ± 17.5 versus 5.3 ± 4.7; p < 0.01). There was a correlation between myocardium denervation (r 0.555; p < 0.01), hypoperfusion (r 0.562; p < 0.01), and interstitial fibrosis evaluated by extracellular volume at MRI.

**Conclusion:** The combination of different imaging parameters to assess autonomic innervation, myocardial perfusion and fibrosis may allow better understanding of the pathophysiology and risk stratification for SCD in the early stages of CC.

108593

Modality: WHF Abstracts – Researcher

Category: NEGLECTED CARDIOVASCULAR DISEASES

D: 14/10/2022    H: 13:50/14:50

L: Auditório 14

## Progression of Electrocardiographic Abnormalities in Elderly Chagas Disease Patients During 14 Years of Follow-Up: The Bambui Cohort Study of Aging

BRUNO OLIVEIRA DE FIGUEIREDO BRITO^1^, Bruno Oliveira de Figueiredo Brito^1^, Emilly Malveira de Lima^2^, Elsayed Z. Soliman^3^, Maria Fernanda Lima-Costa^4^, Antonio Luiz Pinho Ribeiro^1^

(1) Hospital das Clínicas and Faculdade de Medicina, Universidade Federal de Minas Gerais, Belo Horizonte, Brazil; (2) Department of Statistics, Instituto de Ciências Exatas, Universidade Federal de Minas Gerais, Belo Horizonte, Brazil; (3) Department of Epidemiology and Prevention, Wake Forest School of Medicine, Winston-Salem, NC, USA; (4) Instituto René Rachou, Fundação Oswaldo Cruz, Belo Horizonte, Brazil

The electrocardiogram (ECG) plays a key role in the evaluation of Chagas disease (ChD). The ECG shows progressive abnormalities that indicate worsening myocardial damage. There is no information about the evolution of the ECG of elderly individuals with ChD.

**Objective:** To compare the evolution of electrocardiographic abnormalities in Trypanosoma cruzi chronically infected individuals to that of the non-infected (NChD) elderly in a follow-up of 14 years of the Bambui Cohort Study of Aging in Brazil.

**Methods:** A digitally recorded 12-lead ECG of each individual was obtained at the baseline examination in 1997, in 2002 and in 2008, and was classified by the Minnesota Code criteria. The influence of ChD on the ECG evolution was assessed by the semi-competing risks methods considering a new ECG abnormality as the primary event and death the terminal event. The group with ChD was compared with the NChD group. A Cox regression model was conducted separately in ChD and NChD groups, in a landmark point at 5.5 years until the end of follow-up. The individuals of both groups were compared according to the following categories: No major: individuals without major abnormalities in the first and second visits; Maintained major: individuals who had the number of major abnormalities in second visit equal to the first visit, More major: individuals who had major abnormalities in the first visit and gained more abnormalities in the second visit and New major: individuals who had no abnormalities in the first visit and gained any major abnormality in the second visit.

**Results:** Among the 1,462 participants, 557 had ChD. The median age was 68 years for patients with ChD and 67 for patients without ChD. Chagas disease was independently associated to the occurrence of new major ECG abnormalities in the multivariate analysis HR: 2.89 (95% CI 2.28–3.67). Compared with the group of ChD No major, the risk of death was HR: 2.48 (95% CI 1.43–4.28) for the Maintained, HR: 1.95 (95% CI 1.04–3.69) for the New major, and HR: 2.77 (95% CI 1.57–4.88) for the More Major. Compared with the group of ChD Maintained major, the risk of death was HR: 0.79 (95% CI 0.44–1.42) for the group New Major and HR: 1.11 (95% CI 0.66–1.89) for the group More Major.

**Conclusion:** Even in advanced ages, the patients with ChD have higher risk than NChD of developing new abnormalities in the ECG. It is enough for individuals with ChD to have ECG abnormalities for their risk of death to increase.

108671

Modality: WHF Abstracts – Young Researcher

Category: EPIDEMIOLOGY AND HEALTH POLICIES/GLOBAL HEALTH

D: 14/10/2022    H: 16:50/17:50

L: Auditório 14

## Comparison of Adoption of Guideline-Recommended Medical Therapy for Heart Failure Across Global, National, and State Essential Medicine Lists

GAUTAM SATHEESH^1^, Mohammad Abdul Salam^2^

(1) The George Institute for Global Health, Hyderabad, India; (2) The George Institute for Global Health, University of New South Wales, Sydney, New South Wales, Australia

**Introduction and/or rationale:** Heart failure (HF) is a leading global health burden that disproportionately affects patients in low- and middle-income countries with weaker health systems, such as India. Underutilization of guideline-recommended medical therapy (GRMT)—causing large gaps in evidence and clinical practice—can be mitigated through the adoption of GRMT in the EMLs.

**Objectives:** To compare the adoption of GRMT across the global (WHO) EML and the national and state EMLs of India.

**Methods:** We collated a list of medicines recommended by American (American College of Cardiology Foundation, the American Heart Association, and the Heart Failure Society of America; 2017), European (European Society of Cardiology; 2021), and relevant Indian guidelines (Cardiological Society of India; 2018). We assessed the adoption of these medicines in WHO EML (2021), India’s latest national EML (2015), Ministry of Health and Family Welfare’s free medicines list (2020), and 23 Indian state EMLs. We only included ‘evidence-based medicines’, i.e., those supported by relevant randomized controlled trial data for HF. Therefore, we did not include medicines listed as ‘commonly used’ without relevant evidence. We also excluded medicines listed for use in selected patients only (e.g., milrinone, levosimendan, nesiritide etc.).

**Results:** The adoption of GRMT by WHO EML 2021 was 48% (n = 17), ranging from 75% (beta-blockers) to 0% (SGLT-2 inhibitors). GRMT adoption by Indian EML was 35% (n = 11), ranging from 50% (mineralocorticoid-receptor antagonists) to 0% (SGLT-2 inhibitors). On average, Indian state EMLs contained 36% [18% (Punjab)–53% (Rajasthan)] of GRMT. The lowest adoption (16%; n = 6) was observed in the Ministry of Health’s free medicines list, which omitted several major first-line medicine classes. Newly included GRMT, including angiotensin-receptor neprilysin inhibitors and SGLT-2 inhibitors, are yet to be listed in global and national EMLs. Further, at least one emergency medicine was included in all EMLs.

**Conclusion:** Inclusion of GRMT for HF remains suboptimal in the global as well as the national and state EMLs of India. EMLs guide medicine selection and procurement in limited-resource settings. Considering India’s increasing cardiovascular disease burden and the potential for EMLs to improve availability and affordability of GRMT, optimizing India’s national and state EMLs and particularly its public sector (free medicines) list, is vital.

109112

Modality: WHF Abstracts – Young Researcher

Category: NEGLECTED CARDIOVASCULAR DISEASES

D: 14/10/2022    H: 16:50/17:50

L: Auditório 14

## Myocardial Perfusion Disturbance in Experimental Chronic Chagas Disease: The Role of Endothelial Inflammatory Activation

DENISE MAYUMI TANAKA^1^, Camila Godoy Fabricio^1^, José A. Marin-Neto^1^, Antônio Carlos Leite de Barros Filho^1^, Luciano Fonseca Lemos de Oliveira^3^, Jorge Mejia^4^, Rafael Ribeiro Almeida^2^, Maria de Lourdes Higuchi^2^, Edecio Cunha Neto^2^, Minna Moreira Dias Romano^1^, Marcus Vinícius Simões^1^

(1) Medical School of Ribeirao Preto – University of São Paulo, Sao Paulo, Brazil; (2) Heart Institute (InCor), Faculty of Medicine – University of Sao Paulo, Sao Paulo, Brazil.; (3) Physiotherapy Department – Federal University of Minas Gerais, Brazil; (4) Hospital Israelita Albert Einstein, Sao Paulo, Brazil.

**Background:** Microvascular myocardial perfusion defect (MPD) is frequent in chronic Chagas cardiomyopathy (CCC) and may be involved with the progression of left ventricular systolic dysfunction (LVSD). However, the histopathological meaning of MPD in CCC and its correlation with endothelial activation is scarce.

**Purpose:** To investigate the correlations between MPD detected in vivo with functional and histopathological changes in the model of CCC in hamsters.

**Methods:** 24 female hamsters were studied 8-months after intraperitoneal infection with 35,000 trypomastigote forms of T. cruzi. All animals were submitted to rest high-resolution 99mTc-Sestamibi-SPECT myocardial perfusion scintigraphy and echocardiography in vivo. The area of MPD was assessed by calculating polar maps using dedicated software (MunichHeart®). After euthanasia, we performed a histopathological study of cardiac inflammation and fibrosis and mRNA expression for TNF-alfa and ICAM to assess inflammation and endothelial activation, respectively.

**Results:** 17 animals presented MPD (71%) – extension ranging from 1.4 to 30.3% of LV surface. Animals with MPD present lower values of LVEF (38.5 ± 11.2%) when compared with animals without MPD 48.4 ± 9.1%, p = 0.04), and a trend to higher intensity of myocardial inflammation in animals with MPD (540.4 ± 153.6 cell/mm^2^) vs. without MPD (409.6 ± 130.3 cell/mm^2^), p = 0.09. In addition, animals with MPD presented a higher ICAM (0.02 ± 0.01) expression when compared with animals without MPD (0.01 ± 0.01, p = 0.02). There was no difference between groups regarding the extent of fibrosis. There was a negative correlation between individual values of MPD with LVEF (R = –0.6, p = 0.001), wall motion score index (WMSi, R = 0.5, p = 0.007), and the number of mononuclear cells (R = 0.5, p = 0.01). Moreover, an analysis based on myocardial segments (n = 312) showed that segments with MPD (n = 54) in comparison to those without MPD (n = 258) presented a higher number of mononuclear cells (608 ± 299.9 cell/mm^2^ and 478.3 ± 201.1 cell/mm, respectively, p < 0.0001) and higher WMSi (1.8 ± 0.9 and 1.2 ± 0.4, respectively, p < 0.0001.

**Conclusions:** MPD is a common finding in the experimental model of CCC in hamsters and is correlated with inflammation, endothelial inflammatory activation, and systolic ventricular dysfunction. These results suggest that MPD may be an in vivo surrogate marker for inflammation with potential translational implications for monitoring disease activity.

109166

Modality: WHF Abstracts – Researcher

Category: EPIDEMIOLOGY AND HEALTH POLICIES/GLOBAL HEALTH

D: 14/10/2022    H: 13:50/14:50

L: Auditório 14

## Regional Variation in Women’s Awareness of Cardiovascular Disease and its Risk Factors

MELANIE B. TURNER, MPH, FAHA^1^, Laura L. Hayman, PhD, MSN, FAHA^2^, Dhruv Kazi, MD, MSc, MS, FAHA^3^, Laxmi S. Mehta, MD, FAHA^4^

(1) American Heart Association; (2) University of Massachusetts Boston; (3) Beth Israel Deaconess Medical Center; (4) The Ohio State University Medical Center

**Introduction:** Cardiovascular disease (CVD) is the leading cause of death (LCOD) in women globally, causing ≈9 M deaths annually. Global community awareness data of CVD and its risk factors are limited.

**Objective:** Determine global awareness of CVD incidence and risk factors among women and examine regional variation in awareness.

**Methods:** An online native language survey was administered among nationally representative samples of women ≥18 years of age in 50 countries across 6 global regions. The survey was administered June-July 2021 using the YouGov real time omnibus service. The survey included an open-ended question regarding LCOD for women in the country (coded as CVD, cancer, or other/don’t know), and a multiple choice question on CVD risk factors. Results were weighted by country, then combined by region.

**Results:** Of 24,100 women, 15.5% reported CVD as LCOD (regional range 6.9–35.0%), 49.4% cancer (23.9–62.9%) and 18.0% other/don’t know (9.1–34.4%). Cancer was perceived as LCOD in all regions except South Asia and Middle East and North Africa where other/don’t know was the most common response. Regarding CVD risk, 69.5% identified family history as a risk (regional range 51.0–77.1%). Most recognized hypertension, overweight, high cholesterol, and stress. Awareness of diabetes as a CVD risk was low (32.0%, 26.5–44.1%).

**Conclusions:** Awareness of CVD as LCOD among women is low in all global regions. Most women recognize hypertension, high cholesterol, and overweight as risk factors, but awareness of diabetes as a risk factor is low. Efforts are needed in all regions to create awareness of lifetime CVD risk in women, focusing on modifiable risk factors. As an increasingly prevalent global chronic condition, diabetes merits raised attention in community heart health education.


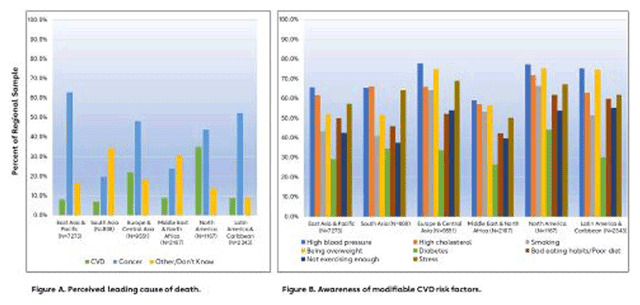


109180

Modality: WHF Abstracts – Researcher

Category: EPIDEMIOLOGY AND HEALTH POLICIES/GLOBAL HEALTH

D: 14/10/2022    H: 13:50/14:50

L: Auditório 14

## The Impact of Country- and Individual-Level Socioeconomic Factors on Maternal and Neonatal Outcomes in Women with Peripartum Cardiomyopathy: An Esc Eorp Registry Study

CHARLE ANDRE VILJOEN^1^, Karen Sliwa^1^, Peter van der Meer^2^, Alice M Jackson^3^, Mark C Petrie^3^, Cecile Laroche^4^, Jolien W Roos-Hesselink^5^, Petar Seferovic^6^, Alexandra Frogoudaki^7^, Bassem Ibrahim^8^, Hasan Al-Farhan^9^, Johann Bauersachs^10^

(1) Cape Heart Institute, Department of Medicine and Cardiology, Faculty of Health Sciences, University of Cape Town, South Africa; (2) Department of Cardiology, University Medical Center Groningen, University of Groningen, The Netherlands; (3) Institute of Cardiovascular and Medical Sciences, Glasgow University, Glasgow, United Kingdom; (4) EurObservational Research Programme, European Society of Cardiology, Sophie Antipolis, France; (5) Department Cardiology, Thoraxcenter, Erasmus Medical Center, Rotterdam, the Netherlands; (6) University of Belgrade Faculty of Medicine, Belgrade, Serbia; (7) Attikon University Hospital, Athens, Greece; (8) North Cumbria University Hospitals, Carlisle, UK; (9) Iraqi Board of Medical Specilazations, Baghdad Heart Center, Iraq; (10) Department of Cardiology and Angiology, Medical School Hannover, Hannover, Germany

**Background:** Peripartum cardiomyopathy (PPCM) is a global disease associated with substantial morbidity and mortality. The aim of this study was to analyze to what extent country- and individual-level socioeconomic factors were associated with maternal and neonatal outcomes.

**Methods:** In 2011, >100 national and affiliated member cardiac societies of the European Society of Cardiology (ESC) were contacted to contribute to a global registry on PPCM, under the auspices of the ESC EORP Programme. We investigated the characteristics and outcomes of women with PPCM and their babies according to individual (income and educational attainment) and country-specific (Gini coefficient [GINI], health expenditure [HE] and human developmental index [HDI]) socioeconomic status.

**Results:** 739 women from 49 countries were enrolled (Europe [33%], Africa [29%], Asia-Pacific [15%], Middle East [22%]). Women from countries with low HDI had lower income and educational attainment (p < 0.001). Low HDI was associated with greater LV dilatation at time of diagnosis (p < 0.001), but LV ejection fraction (LVEF) did not differ according to HDI, HE or GINI. Countries with low HE prescribed guideline-directed heart failure therapy less frequently (p < 0.001). Low HE was associated with more frequent mortality (p < 0.002), whereas HDI and GINI were not. Women from countries with low HDI and low HE had significantly less recovery of LV function (p < 0.001). Analysis of maternal outcome as per highest level of educational attainment showed significant differences in LVEF at 6 months (43.7 ± 12.9% [primary], 46.5 ± 13.0% [secondary], 48.9 ± 11.7 [tertiary education] respectively, p = 0.022). Low maternal income, irrespective of region of origin, was independently associated with poor outcome (OR 1.99 [95% CI 1.1–3.6] for composite of maternal death, re-hospitalization, or LV non-recovery). Neonatal death was more prevalent in countries with low HE (p = 0.009) and low HDI (p = 0.023) but was not influenced by maternal sociodemographic parameters.

**Conclusion:** Maternal and neonatal outcomes depended on country-specific socioeconomic characteristics, with a greater prevalence of maternal and neonatal deaths in women from countries with low HE. Globally, women with low income and lower levels of educational attainment had poorer outcomes, irrespective of region. Attempts should be made to improve patient education, and allocation of adequate health resources to improve maternal and neonatal outcomes in PPCM.

111430

Modality: WHF Abstracts – Young Researcher

Category: NEGLECTED CARDIOVASCULAR DISEASES

D: 14/10/2022    H: 16:50/17:50

L: Auditório 14

## Factors Associated with Peak End-Tidal Carbon Dioxide Pressure During Exercise in Patients with Chagas Cardiomyopathy and Systolic Dysfunction

WHESLEY TANOR SILVA^1^, Matheus Ribeiro Ávila^1^, Pedro Henrique Scheidt Figueiredo^1^, Lucas Frois Fernandes de Oliveira^1^, Vanessa Amaral Mendonça^1^, Ana Cristina Rodrigues Lacerda^1^, Vanessa Pereira Lima^1^, Henrique Silveira Costa^1^

(1) Universidade Federal dos Vales do Jequitinhonha e Mucuri UFVJM

**Introduction:** In the functional assessment of patients with Chagas cardiomyopathy, peak oxygen consumption (VO2peak) and ventilatory equivalent of carbon dioxide (VE/VCO2 slope) have already been shown to be parameters of clinical and prognostic relevance. However, other variables assessed by the Cardiopulmonary Stress Test need to be investigated. The peak end-tidal carbon dioxide pressure (PETCO2 peak) has been prominent in the therapy of patients with heart failure. However, its behavior is still unknown in patients with Chagas cardiomyopathy and systolic dysfunction.

**Objective:** To verify, in patients with Chagas cardiomyopathy and systolic dysfunction, the association between peak PETCO2 and functional and echocardiographic parameters.

**Methods:** Seventy-six patients with Chagas cardiomyopathy and systolic dysfunction (49.9 ± 10.8 years, 60% male, NYHA I to III) were recruited and underwent clinical evaluation, echocardiography and Cardiopulmonary Stress Test. Systolic dysfunction was defined as a left ventricular ejection fraction (LVEF) of less than 52 or 54% for men and women, respectively. The variables of interest were PETCO2 peak, VO2peak and VE/VCO2 slope (on Cardiopulmonary Stress Test) and LVEF and left ventricular diastolic diameter (LVd) (on echocardiogram).

**Results:** In the sample, the mean peak PETCO2 was 33.6 ± 4.9 mmHg. In the correlation analysis, PETCO2 peak was associated with VO2peak (r = 0.355; p = 0.008), VE/VCO2 slope (r = –0.626; p < 0.001) and with LVEF (r = 0.299; p = 0.029). There was no correlation between peak PETCO2 and VEd.

**Conclusion:** These results suggest that peak PETCO2 is associated with important clinical and functional parameters of patients with Chagas cardiomyopathy and should be used in the management of patients with Chagas cardiomyopathy.

112213

Modality: WHF Abstracts – Young Researcher

Category: NEGLECTED CARDIOVASCULAR DISEASES

D: 14/10/2022    H: 16:50/17:50

L: Auditório 14

## Association of Clinical and Genetic Profile in Adults with Aorthopathy – Retrospective Cohort Study with Prospective Follow-Up

JULIANA DA ROCHA FERREIRA^1^, Juliana da Rocha Ferreira^1^, Julia Passarelli Perreira^1^, Marcelo Machado Melo^1^, Helena Cramer Veiga Rey^1^, Glauber Monteiro Dias^2^

(1) Instituto Nacional de Cardiologia – INC; (2) Universidade Estadual do Norte Fluminense Darcy Ribeiro – UENF

Aortopathies are a silent disease with a high natural fatality rate. Multiple genes have been linked to hereditary aortic thoracic disease (HATD), and it is believed that 30% of people have a deleterious mutation. This study aimed to uncover genetic variations related to aortopathies using genomic and molecular analysis. Seventy-nine people with aortopathies were studied using clinical data, target-NGS (tNGS), and the Sanger sequencing. The ACMG classification was used to find causal variations. This study defined a severe phenotypic population. Aortic dissection occurred in 49.4% of patients diagnosed at 44.59 years old, mainly after 40, and required surgery at 72.2%. Seven pathogenic variants (PV), 4 likely pathogenic variants (LPV), and 22 variants of uncertain significance (VUS) were discovered. tNGS found 10.37 percent of ATDs in this cohort. Direct sequencing of the fibrillin-1 gene (FBN1) yielded 37.5% diagnostic yield for Marfan syndrome suspect individuals, with two PV and one LPV. PV/LPV variants were identified in 6 genes (ACTA2, FBN1, MYLK, SMAD3, TGFB2, TGFBR2), being FBN1 the most prevalent (6 var). Seven PV/LPV are novel variants. Patients with VP/PPV had a younger mean age (39.3 years vs. 44.4 years) and a larger mean aorta diameter (39.3 years vs. 44.4 years) (6.56 vs 5.66 cm), however no statistic significance was achieved. Both groups required surgery (81.8%), with the VP/PVP group having a worse prognosis and severity. An emphasis is placed on clinical suspicion in selecting the genetic test to use and increasing the yield.

111396

Modality: Best Abstracts Oral – Researcher

Category: ATHEROSCLEROSIS/CARDIOVASCULAR RISK FACTORS/CARDIOVASCULAR PREVENTION

D: 14/10/2022    H: 10:40/11:40

L: Auditório 14

## Inclusion of Cognitive Measures Improves the Predictive Performance of Score2 for Future Cardiovascular Events

MARTIN BOBAK^1^, Steven Hageman^2^, Hynek Pikhart^1^, Abdonas Tamosiunas^3^, Andrzej Pajak^4^, Ruzena Kubinova^5^, Wentian Lu^1^

(1) University College London (UCL), United Kingdom; (2) Utrecht University, Utrecht, The Netherlands; (3) Lithuanian University of Health Sciences, Kaunas, Lithuania; (4) Jagiellonian University Medical College, Krakow, Poland; (5) National Institute of Public Health, Prague, Czech Republic

**Introduction:** The cardiovascular disease (CVD) risk prediction model SCORE2 uses several biomedical and behavioural factors. It is likely that other factors may also contribute to CVD risk prediction. Cognitive impairment and CVD are associated and share some risk factors. While the causality of this relationship remains unclear, adding cognitive measures to risk prediction may be useful for predicting future CVD events.

**Objective:** To test the hypothesis that including simple measures of cognitive functions improve the prediction of SCORE2 for future CVD events.

**Methods:** We used data on 13,391 Polish, Lithuanian and Czech adults without CVD at baseline (45–69 years) from the Health, Alcohol and Psychosocial factors In Eastern Europe cohort. Incident cases of myocardial infarction (MI), stroke, CVD mortality and composite (fatal and non-fatal) CVD events were identified over a 10-year follow-up. Baseline cognitive measures included immediate and delayed word recall (verbal memory and learning), animal naming (verbal fluency) and letter cancellation (attention, mental speed and concentration). Using competing-risks regression, relationships between cognitive functions and CVD outcomes were examined. Improvement in Receiver Operating Characteristic (ROC) was used to compare predictive performance for CVD events of models including SCORE2 variables combined with cognitive tests vs SCORE2 alone.

**Results:** Incidence rates of MI, stroke, CVD mortality and composite CVD events were 5%, 4%, 4% and 11%, respectively. All four cognitive measures were inversely associated with CVD outcomes; e.g. one standard deviation increase in immediate word recall score was associated with a 28% and 17% reduction in risk of CVD mortality and composite CVD events, respectively. Adding cognitive measures to SCORE2 variables improved the ROCs for prediction of MI, CVD mortality and composite CVD events. E.g. for the composite CVD measure, inclusion of all four cognitive measures increased the ROC area from 0.6948 to 0.7030 (p < 0.001) and similar improvement was seen for word recall.

**Conclusion:** Including even a single (and simple) cognitive test in assessment of cardiovascular health can improve prediction of future CVD risk and it may be potentially feasible to do so in general practice.

110777

Modality: Best Abstracts Oral – Researcher

Category: HEART FAILURE/CARDIOMYOPATHY/TRANSPLANT

D: 14/10/2022    H: 10:40/11:40

L: Auditório 14

## Acute Heart Failure in Patients with Chagas’ Cardiomyopathy in Comparison to Other Etiologies: Results of the I Brazilian Heart Failure Registry (Breathe)

MARCUS VINICIUS SIMÕES^1^, Pedro Gabriel Barros e Silva^2^, Denilson Campos Albuquerque^3^, Renato Delascio Lopes^4^, Luis Eduardo Paim Rohde^5^, Lidia Zytinsky Moura^6^, Fabiana Goulart Marcondes-Braga^3^, Evandro Tinoco Mesquita^3^, José Albuquerque de Figueiredo Neto^7^, Ricardo Mourille Rocha^8^, João David de Souza Neto^9^, Mucio Tavares Oliveira Junior^10^

(1) Faculdade de Medicina de Ribeirão Preto – USP, Ribeirão Preto, SP, Brasil; (2) Brazilian Clinical Research Institute (BCRI), Sao Paulo, Brazil; (3) Sociedade Brasileira de Cardiologia, Departamento de Insuficiência Cardíaca – DEIC, Rio De Janeiro, Brazil; (4) Duke Clinical ResearchInstitute, Durham, United States of America; (5) Hospital de Clínicas de Porto Alegre, Porto Alegre, Brazil; (6) Santa casa, Curitiba, Brazil; (7) Centro de Pesquisa Clínica doHospital Universitário da Universidade Federal do Maranhão (CEPEC-HUUF, Sao Luis, Brazil; (8) PedroErnesto University Hospital, Rio De Janeiro, Brazil; (9) Messejana Hospital, Fortaleza, Brazil; (10) Heart Institute (InCor), University of São Paulo Medical School, Brazil

**Background:** Chagas cardiomyopathy (CC) is a prevalent cause of heart failure in Latin America countries. Studies describing clinical manifestations and outcomes of heart failure associated to CC are scarce.

**Purpose:** Report the results of the I Brazilian Heart Failure Registry (BREATHE) addressing the clinical and laboratorial characteristics, and outcomes of patients with acute heart failures (AHF) due to CC in comparison to other etiologies.

**Methods:** BREATHE was a multicenter nationwide prospective registry, enrolling 3,013 adult patients hospitalized with AHF, median follow-up of 346 days. We proceeded the comparative analysis between 261 (8.7%) patients with CC and 2,752 (91.3%) patients with other etiologies, concerning clinical, demographic, cardiac structure/function on Echocardiogram, death rate or heart transplantation during hospital stay and death rate at 3, 6 and 12 months after discharge. The categorical variables were compared by using Fisher Exact test and the continuous variables were compared by using Mann-Whitney test. A multivariate logistic model was used to estimate the odds ratio of CC in 12-month mortality adjusted for clinically relevant variables.

**Results:** CC patients, in comparison to other etiologies, were younger (60.6 ± 13.9 vs 65.7 ± 15.7 y.o., p < 0.001), presented lower systolic blood pressure (108.3 ± 26.1 vs 128.3 ± 30.3 mmHg, p < 0.001), lower heart rate (77.3 ± 22.1 vs 88.5 ± 23.2 bpm, p < 0.001), higher rate of jugular vein distension (54.8% vs 38.9%, p < 0.001) and hepatomegaly (47.9% vs 25.6%, p < 0.001), higher rate of “cold and wet” clinical hemodynamic profile (27.2 vs 10.6%, p < 0.001); larger diastolic left ventricular (LV) diameters (65 [57–72.8] vs 59 [51–66] mm, p < 0.001), and lower LV ejection fraction (25.4 [19–36]% vs 37 [27–54]%, p < 0.001), with higher rates of dobutamine use (23.8% vs 6.8%, p < 0.001); presented higher rate of death or heart transplantation during hospital stay (11.1% vs 17.4%, p = 0.004), and higher cumulative death rate after discharge at 3-months (16.5% vs 10.8%, p = 0.017, at 6-months (25.7% vs 17.5%, p = 0.006, and at 12-months (40.8% vs 27.8%, p < 0.001). In a multivariate analysis, CC was independently associated with 12-month mortality risk with odds ratio = 2.02 [95% IC: 1.47;2.77].

**Conclusions:** Patients hospitalized with AHF with CC etiology, in comparison to other etiologies, presented higher-risk profile that was associated with a poorer outcome during hospital stay and after discharge.

111321

Modality: Best Abstracts Oral – Researcher

Category: ACUTE AND CHRONIC CORONARY DISEASE/THROMBOLYSIS

D: 14/10/2022    H: 10:40/11:40

L: Auditório 14

## In-Hospital Program to Systematize Chest Pain Protocol (In-Hope)

PEDRO GABRIEL MELO DE BARROS E SILVA^1^, Ana Amaral^3^, Antonielle Figueiredo Macedo^4^, Celso Musa Correa^5^, Eduardo Zincone^6^, Marcelo Paiva Cury^7^, Gustavo Augusto Lopes Rosa^8^, Alexandre de Matos Soeiro^9^, Carlos Alexandre Lemes de Oliveira^10^, Augusto Celso De Araujo Lopes Junior^11^, Adriana Bertolami^7^, Renato Delascio Lopes^12^

(1) Hospital Samaritano Paulista; (2) Cardiologia Americas; (3) Hospital Pro-cardíaco; (4) Hospital da Luz; (5) Americas Medical City; (6) Hospital Santa Helena; (7) Metropolitano Lapa; (8) Ipiranga Mogi; (9) Incor HC FMUSP; (10) Hospital Paulistano; (11) Hospital Monte Klinikum; (12) Brazilian Clinical Research Institute

**Background:** Chest pain is a major cause of medical evaluation at emergency department (ED) and demands observation in order to exclude the diagnosis of acute myocardial infarction (AMI). Recent algorithms using high-sensitivity cardiac troponin assays at 0 h and 1 h are accepted as a rule-out/rule-in strategy but there is a lack of validation in specific populations.

**Methods:** IN-HOPE was a multicentre prospective study that included patients admitted to the ED due to suspected symptoms of AMI at 16 sites in Brazil. All patients followed the standard approach of 0–3h but, in addition, blood samples were also collected at 0 and 1 hour and sent to a core laboratory to measure high sensitivity troponin T (hs-cTn T) Elecsys (Roche). Troponin <12 ng/L with a delta <3 was considered rule out while a value ≥52 and/or a delta ≥5 was considered rule in for AMI. The main objective of the study was to assess the accuracy of 0–1 h rule-out/rule-in algorithm in comparison to the standard of care (0–3h). All patients were followed for 30 days. In addition to the prospective cohort, a retrospective analysis was performed assessing all patients with hs-cTn T measured during 2021 but not included in the prospective cohort.

**Results:** A total of 5.497 patients were included (583 in the prospective and 4.914 in the retrospective analysis). The prospective cohort had a mean age of 57.3 (±14.8) and 45.6% of females with a mean HEART score of 4.0 ± 2.2. By the core lab analysis, 71.6% would be eligible for a rule-out approach while 7.3% would fit the rule-in criteria. At 30 days, no death or AMI occurred in the rule-out group while 64.9% of the patients in the rule-in group were considered as AMI. In the retrospective analysis, 1.091 patients had a troponin value <5 ng/L without cardiovascular deaths in this group. Among all 4.914 patients, the 30-day risk of AMI or cardiovascular death increased according to the level of troponin: 0% in the group <5 ng/L, 0.6% between 5 and 14 ng/L, 2.2% between 14 and 42 ng/L, 6.3% between 42 and 90 ng/L and 7.7% in the level ≥90 ng/L.

**Conclusions:** In this large multicentre Brazilian study, a 0–1h algorithm was effective for classifying as rule in or out almost 80% of the patients. The rule-out protocol had high negative predictive value regardless clinical risk scores. Categories of levels of hs-cTn T also showed good accuracy in discriminating risk of the patients with a very favourable prognosis for the group with values <5 ng/L.

112023

Modality: Best Abstracts Oral – Researcher

Category: CARDIOVASCULAR SURGERY

D: 14/10/2022    H: 10:40/11:40

L: Auditório 14

## Perioperative Cardiovascular Events and Mortality After Cardiac Surgery Across the Spectrum of Chronic Kidney Disease

LUIS EDUARDO PAIM ROHDE^1^, Marcio R. Martins^1^, Luis E. Rohde^1^, Flávia K. Borges^2^, Andre Lamy^2^, Richard Whitlock^2^, P. J. Devereaux^2^, Carisi A. Polanczyk^1^

(1) Hospital de Clinicas de Porto Alegre, Post Graduate Program in Cardiovascular Sciences and Cardiology, UFRGS; (2) McMaster University, Hamilton Health Sciences

**Background:** Previous studies addressing the association of chronic kidney disease (CKD) and prognosis after open-heart surgery had limited sample sizes and retrospective designs.

**Methods:** We investigated the association of preoperative renal function and in-hospital mortality, and major cardiac and cerebrovascular events (MACCE) in patients enrolled in the prospective multicentric VISION Cardiac Surgery Study. Patients were divided in 5 CKD stages according to preoperative estimated glomerular filtration rate (eGFR in mL/min/1.73 m^2^): Stage I(>90; n = 1914), Stage II(60 to 89; n = 8122), Stage III(30 to 59; n = 3406), Stage IV(<30; n = 352) and Stage V(dialysis; n = 227).

**Results:** 15,837 were included in the current analysis (71% male, 66% hypertensive and 20% >75 y.o.). Mortality and MACCE during the first 30 days occurred in 480(3%) and 1727(11%) patients, respectively. Logistic regression models adjusted for EuroSCORE demonstrated increased 30-day mortality in CKD Stage III (odd ratio[OR], 1.82; 95% confidence internal[CI], 1.36–2.41), CKD Stage IV (OR, 2.62; 95% CI, 1.66–4.15) and in patients in dialysis (OR, 3.56; 95% CI, 2.17–5.85) In analysis across the whole spectrum of renal function (Figure), mortality was increased particularly when eGFR was <45 mL/min/1.73 m^2^, while MACCE risk was observed in less severe stages of CKD.

**Conclusion:** In this contemporary cohort, CKD was significantly associated with morbidity and mortality after open-heart surgery.


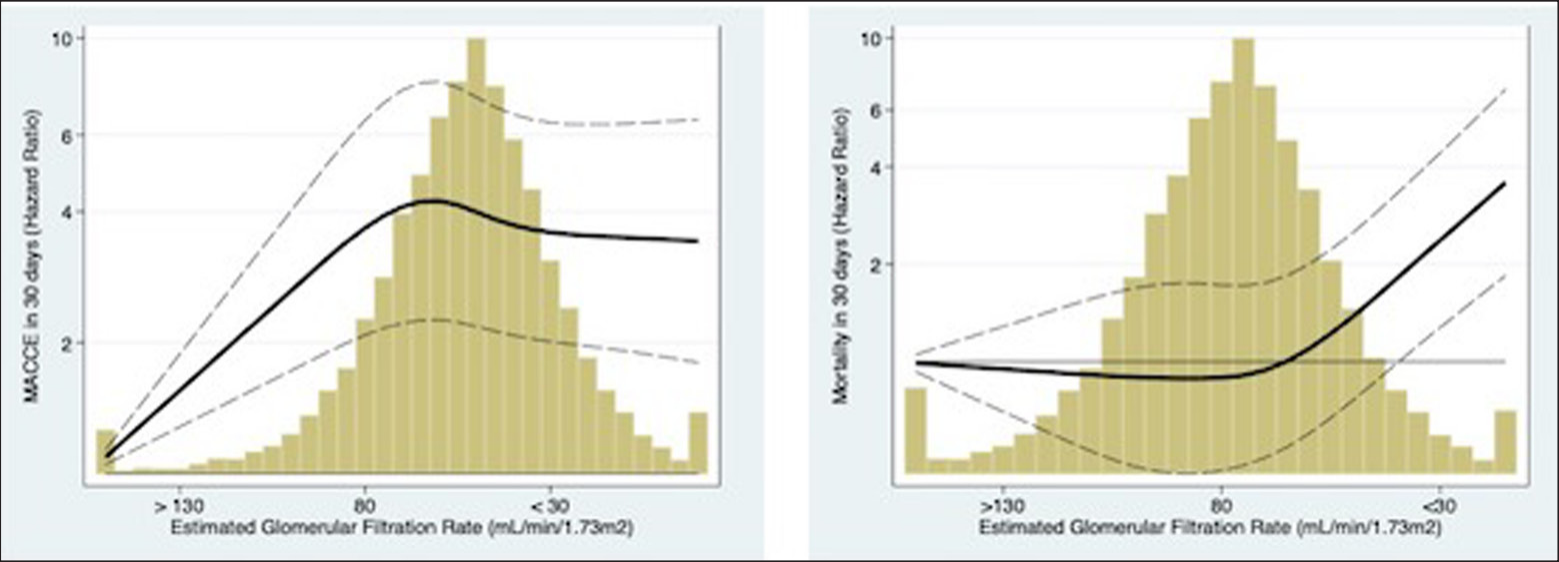


112135

Modality: Best Abstracts Oral – Researcher

Category: DIGITAL HEALTH/INNOVATION

D: 14/10/2022    H: 10:40/11:40

L: Auditório 14

## Combining Short- and Long-Term Predictive Windows Improve Risk Estimation of Adverse Cardiovascular Outcomes Among Individuals with Premature Acute Coronary Syndromes

LUIZ SÉRGIO FERNANDES DE CARVALHO^1^, Gustavo Alexim^5^, Ana Claudia Cavalcante Nogueira^3^, Marta Duran Fernandez^1^, Tito Barbosa Rezende^4^, Sandra Avila^4^, Ricardo Torres Bispo Reis^6^, Alexandre Anderson Munhoz Soares^3^, Andrei Carvalho Sposito^4^

(1) Clarity: Inteligência em Saúde; (2) Universidade Católica de Brasília; (3) Instituto Aramari Apo; (4) UNICAMP; (5) Hospital de Base do Distrito Federal; (6) Universidade de Brasília

**Introduction:** Among individuals with premature acute coronary syndromes (prACS, <55 years-old), prevalent risk factors and the magnitude of their impact on recurrent ischemic events critically differ from older subjects. In that sense, it is plausible to develop risk prediction rules specific for young individuals. Our aim was to evaluate potential improvements in risk prediction quality among prACS by (i) developing models specifically in prACS subjects versus in the global cohort; (ii) splitting predictive rules into two models (short- and long-term prediction windows [STWm and LTWm]) versus a global follow-up model (GFm).

**Methods:** Consecutive individuals with ACS who undergone coronarography up to 48h after hospital admission from January/2011 to February/2020. 6341 subjects (2242 with prACS) admitted into public hospitals in Brasília (Brazil). The observation window in STWm and GFm included the first 48h upon hospital admission, and LTWm included all in-hospital information. prACS cohort was divided into train/validation-set (70%, n = 1569) and test-set (30%, n = 673); global cohort was divided in training/validation-set (70%, n = 4439) and test-set including the 673 prACS subjects. Models were repeated over five cross-validation folds and then assessed in the test-set. C-statistics was the evaluation metric for STWm and time-dependent concordance (Ctd-index) for LTWm. STWm evaluated the occurrence of in-hospital cardiovascular deaths and recurrent ACS (MACE) and LTWm estimated events occurring post-discharge from index ACS hospitalization considering time-to-event with competing risks (MACE versus non-cardiovascular deaths).

**Results:** Median follow-up of 6.7 years (95%CI 5.6–7.2). Among prACS and older subjects, respectively, in-hospital MACE occurred in 180 and 493 individuals, post-discharge MACE in 454 and 881; and post-discharge non-cardiovascular death in 47 and 285. The best strategy was to design models specifically in prACS individuals combining STWm and LTWm. Among prACS subjects STWm and LTWm, respectively, TabNet and DeepHit yielded the best C-statistics [0.921 (95%CI 0.889–0.953)] and Ctd-index [0.722 (95%CI 0.678–0.760)], while the best Ctd-index in GFm was 0.681 (95%CI 0.654–0.703). There was very low concordance among top predictors of MACE for prACS versus global cohort, as well as for STWm versus LTWm.

**Conclusions:** Risk prediction in ACS is optimized by using specific rules for prACS and combining short-term and long-term prediction windows.

109042

Modality: Best Abstracts Oral – Young Researcher

Category: CARDIORESPIRATORY PHYSIOLOGY/BASIC SCIENCE

D: 14/10/2022    H: 09:00/10:00

L: Auditório 14

## Landscape of Heart Proteome Changes in Exercised Aortic Stenosis Rats

GUSTAVO AUGUSTO FERREIRA MOTA ^1^, Sérgio Luiz Borges de Souza^1^, Danielle Fernandes Vileigas^2^, Vitor Loureiro da Silva^1^, Paula Grippa Sant’Ana^1^, Licia Carla Silva Costa^3^, Silméia Garcia Zanatti Bazan^1^, Marília Afonso Rabelo Buzalaf^4^, Lucilene Delazari dos Santos^5^, Marina Politi Okoshi^1^, Mariana Gatto^1^, Antonio Carlos Cicogna^1^

(1) São Paulo State University, UNESP, Botucatu Medical School, Brazil; (2) University of São Paulo, USP, Institute of Chemistry, Brazil; (3) University of Campinas, UNICAMP, Institute of Biology, Brazil; (4) University of São Paulo, USP, Bauru Dental School, Brazil; (5) São Paulo State University, UNESP, Biotechnology Institute, Brazil

**Introduction:** The beneficial effect of aerobic exercise training (ET) on cardiac remodeling induced by aortic stenosis (AS) has been observed in experimental studies. However, the mechanisms involved in cardiac function improvement are not fully understood. In this study we investigated the myocardial proteoma in rats with AS subjected to ET.

**Methods:** Wistar rats (n = 60, 21 days) were divided into 2 groups: operated control (C) and supravalvar aortic stenosis (AS). AS was induced by the insertion of a stainless-steel clip, 0.60 mm, around the ascending aorta. Two weeks after surgery, rats were assigned into 4 groups: C, exercised C (C-Ex), AS and exercised AS (AS-Ex). Exercised rats underwent treadmill exercise 5 days a week for 4 months, at 60% of the maximal functional capacity. Two and 18 weeks after surgery, rats were subjected to echocardiogram. Functional capacity was analyzed by treadmill maximum exercise testing and blood lactate concentration. Myocardial proteome was assessed by label-free shotgun approach using mass spectrometry. Protein expression was quantified by Western blotting. Statistical analysis was performed by ANOVA or Kruskal-Wallis; significance level of 5%.

**Results:** Two weeks after AS induction, AS rats had diastolic and systolic dysfunction and concentric hypertrophy. At the end of the protocol, AS maintained the same remodeling pattern; the AS-Ex group presented lower left atrium diameter-to-aortic diameter and lactate concentration; and higher E/E’ ratio, percentage of midwall fractional shortening, and functional capacity than AS. After enrichment analysis by Gene Ontology, myocardial proteome analysis showed higher expression of proteins related to glycolytic pathway, oxidative stress, and inflammation, and lower expression of proteins associated with beta-oxidation in AS than C. The AS-Ex had higher expression of proteins related to mitochondrial biogenesis and lower expression of proteins related to oxidative stress and inflammation than AS. Expression of proteins of physiological and pathological hypertrophic pathways did not differ between groups, except for p-p38, which was higher in the AS than C group.

**Conclusion:** Aerobic exercise training improves cardiac remodeling and mitochondrial biogenesis, and attenuates oxidative stress and inflammation in rats with ascending aortic stenosis. Financial support: CNPq, FAPESP, and UNESP.

108618

Modality: Best Abstracts Oral – Young Researcher

Category: PHYSICAL EDUCATION

D: 14/10/2022    H: 09:00/10:00

L: Auditório 14

## Coronary Inflammation by Computed Tomography Pericoronary Fat Attenuation in Young Male Anabolic Androgenic Steroids Users

FRANCIS RIBEIRO DE SOUZA^1^, Carlos Eduardo Rochitte^1^, Douglas Carli Silva^1^, André Matheus Rodrigues Gomes^1^, Marcelo Rodrigues dos Santos^1^, Guilherme Wesley Peixoto da Fonseca^1^, Antonio Carlos Battaglia Filho^1^, Kelly Thayane Souza Correa^1^, Maurício Yonamine^2^, Rosa Maria Rodrigues Pereira^3^, Carlos Eduardo Negrão^1^, Maria Janieire de Nazaré Nunes Alves^1^

(1) Instituto do Coração do Hospital das Clinicas da Faculdade de Medicina da Universidade de São Paulo (InCor, HCFMUSP); (2) Faculdade de Ciências Farmacêuticas da Universidade de São Paulo (FCF/USP); (3) Laborátorio de Reumatologia e Metabolismo Ósseo da Faculdade de Medicina da Universidade de São Paulo (FMUSP)

**Background:** The illicit use of anabolic androgenic steroids (AAS) has been associated with diminished cholesterol efflux mediated by HDL, remarkable decrease in high-density lipoprotein (HDL) plasma concentration and subclinical coronary artery disease (CAD). Inflammation is the key to the atherogenic process associated with atherosclerotic plaque vulnerability. The pericoronary mean fat attenuation (pFAM) has emerged as a marker of coronary inflammation and can predict future cardiovascular events, which is measurable from standard coronary computed tomography angiography (CCTA). However, whether AAS abuse has a role in pFAM in young male AAS users is unknown.

**Purpose:** The aim of this study was to evaluate whether AAS abuse could leads to higher pFAM and premature coronary inflammation in young male AAS users.

**Methods:** Twenty strength-trained AAS users (AASU) age 29 ± 5 yr, 20 age-matched strength-trained AAS nonusers (AASNU), and 10 sedentary controls (SC) were enrolled in this study. Coronary inflammation was assessed by pFAM-CCTA in the right coronary artery (RCA), left anterior descending artery (LDA) and left circumflex coronary artery (Cx).

**Results:** pFAM in RCA was significantly higher in AASU compared with AASNU and SC (–64.59 ± 9.45 vs. –79.21 ± 6.67 vs. –80.97 ± 7,91 Hounsfield Units (HU), respectively, p < 0.001]. Also, the pFAM in the LAD was higher in AASU compared with AASNU and SC (–72.83 ± 7.21 vs. –79.41 ± 6.72 vs. –80.97 ± 7.72 HU, p = 0.006). However, no difference to pFAM in the Cx between AASU, AASNU and SC (–74.30 ± 5.85 vs. –79.77 ± 7.13 vs. –78.19 ± 5.82 HU, respectively, p = 0.069) was found.

**Conclusion:** This study indicates that AAS abuse may be associated with higher pFAM and premature coronary inflammation in the RCA and LAD. In addition, the higher pFAM may be linked to early development of CAD in young AAS users.

109119

Modality: Best Abstracts Oral – Young Researcher

Category: DIGITAL HEALTH/INNOVATION

D: 14/10/2022    H: 09:00/10:00

L: Auditório 14

## Utilizing Automated Machine Learning for Rheumatic Heart Disease Detection in Doppler Echocardiography

KELSEY BROWN^1^, Pooneh Roshanitabrizi, PhD^3^, Alison Reese, MS^1^, Andrea Beaton, MD^2^, Emmy Okello, PhD^4^, Joselyn Rwebembera, MMed^4^, Peter Lwabi, MMed^4^, Emma Ndagire, MMed^4^, Craig Sable, MD^1^, Marius George Linguraru, DPhil^3^

(1) Children’s National Hospital; (2) Cincinnati Children’s Hospital Medical Center; (3) Sheikh Zayed Institute for Pediatric Surgical Innovation; (4) Uganda Heart Institute

**Introduction:** Rheumatic heart disease (RHD) is the number one cause globally of morbidity and mortality from heart disease in children and young adults. The mitral regurgitation (MR) jet length on color Doppler echocardiography is an important index for diagnosis, but its measurement and interpretation are variable.

**Objective:** Develop an automatic machine learning approach to identify and measure the MR jet length on color Doppler for RHD detection. Methods/Design: We used 316 echocardiograms in video format from 95 children (mean age 12 ± 2 years; range 5 to 17 years) with DICOM color Doppler images of the mitral valve taken from parasternal long axis (PLAX) and apical 4 chamber (AP4) views. All echocardiograms were independently reviewed by an adjudication panel which consisted of four expert pediatric cardiologists to determine maximum MR jet length and diagnosis (RHD positive or not). Among 95 cases, 29 were normal and 66 had RHD. Our automated method included. (1) Selection of frames during ventricular systole using a convolutional neural network architecture based on the ResNet-50. (2) Localization of left atrium using convolutional neural networks with LinkNet structure. (3) Measurement of MR jet length using image color analysis. (4) Detection of RHD by applying a generalized regression model based on the maximum MR jet length measured on each view and maximizing the balanced accuracy using cross validation.

**Results:** Machine learning selected the correct systolic frame with an average accuracy of 0.95 (sensitivity 97%/specificity 93%) and 0.94 (sensitivity 94%/specificity 94%) for the AP4 and PLAX view, respectively. It localized the atrium with an average Dice coefficient of 0.89 and 0.9 for the AP4 and PLAX view, respectively. We estimated the length of the MR jet with an average absolute error of 0.33 ± 0.4 cm (p-value = 0.15 compared to manual measurements). Our deep learning approach performed similar to or better than previously published manual methods for categorization of RHD positive vs negative. The accuracy of RHD detection was 0.84 (sensitivity 86%/specificity 79%).

**Conclusion:** Our automatic method has the potential to reliably detect RHD as accurately as expert cardiologists. This innovative approach holds promise to scale echocardiography screening for RHD and greatly expand prophylaxis to prevent progression of RHD globally.

109213

Modality: Best Abstracts Oral – Young Researcher

Category: CARDIO-ONCOLOGY

D: 14/10/2022    H: 09:00/10:00

L: Auditório 14

## Impact of High-Intensity and Moderate-Intensity Training on Cardiometabolic Variables in Oncological Patients with Newly Diagnosed Cardiovascular Disease. Randomized Clinical Trial

JAVIER ELIECER PEREIRA RODRIGUEZ^1^, Fernando Rivera-Theurel^2^, Jorge Antonio Lara-Vargas^3^, Devi Geesel Peñaranda-Florez^1^, Pedro Pereira-Rodriguez^4^, Karla Dominguez-Gomez^1^, Kelly Perez-Diaz^1^, Dafne Palacios-Toledo^1^, Hiady Rivera-Lopez^1^, Alondra Mijangos-Dolores^1^, Isaias Sánchez-García^1^

(1) Centro de Estudios e Investigación FISICOL; Colombia; (2) University of Toronto; Canada; (3) Cardiofit; México; (4) Clínica Medical Duarte; Colombia

**Introduction:** Cardio-Oncology aims to prevent and treat cardiac dysfunction induced by anti-oncological therapies. One emergent strategy is the physical activity, which has been considered an important strategy to improve quality of life and outcomes in the susceptible population.

**Objective:** Determine the cardiovascular effects of Moderate Intensity Continuous Training (MICT) and High Intensity Interval Training (HIIT) on oncological patients newly diagnosed with cardiovascular disease (CVD).

**Methodology:** Randomized controlled trial with sample of 690 with oncological patients with a new diagnose of CVD (heart failure and ischemic heart disease) distributed in 3 groups (MICT, HIIT and control group). We identified the echocardiogram, cardiac biomarkers, stress test, clinical and hemodynamic parameters, hematological samples were identified. In addition, 6-minute walk, anthropometry, quality of life, fatigue, sarcopenia. The tests were performed pre and post 36 sessions of 70-minute training. Then, descriptive statistics were carried out to estimate, the normality of the data was assessed by the Kolmogorov-Smirnov test and the indication of specificity was evident for all analyzes. Also, the ANOVA analysis of variance (one-way analysis of variance) was used, and subsequently, post hoc tests with Tukey test. In all cases, a significance level was 5% (p = <0.05).

**Results:** After an structured exercise program, there was an increase in left ventricular ejection fraction (LVEF) (EG1: 40 ± 5 vs 44 ± 4%; EG2: 41 ± 4 vs 47 ± 3%; CG: 40 ± 3 vs 40 ± 1%; p < 0.05). In fact, higher values of functional capacity (VO2peak) were noted in the experimental group 2-HIIT (7.0 ± 5.2 vs 9.7 ± 3.7) compared to experimental group 1-MICT (8.0 ± 4 vs 8.5 ± 3) and control group (9.0 ± 5.0 vs 9.2 ± 4.0). Other parameters results incuded systolic blood pressure (135 ± 10 vs 127 ± 2 mmHg), diastolic blood pressure (85 ± 5 vs 80 ± 2 mmHg), maximum heart rate (166 ± 10 vs 179 ± 5bpm) and quality of life (106 ± 8 vs 73.2 ± 11). It was possible to show significant changes in all variables comparing HIIT vs MICT groups (p = <0.05), and also compared to the control group.

**Conclusions:** HIIT and MICT in oncological patients with newly diagnosed CVD improved the LVEF, functional capacity (VO2peak), hemodynamic parameters (systolic and diastolic blood pressure), exercise tolerance, depression, anxiety, strength, Cancer related fatigue improved as well as other specific cancer variables such as sarcopenia, and quality of life.

109929

Modality: Best Abstracts Oral – Young Researcher

Category: ATHEROSCLEROSIS/CARDIOVASCULAR RISK FACTORS/CARDIOVASCULAR PREVENTION

D: 14/10/2022    H: 09:00/10:00

L: Auditório 14

## Effectiveness of Influenza Vaccination as a Secondary Prevention Therapy for Coronary Artery Disease: A Systematic Review and Meta-Analysis of Randomized Clinical Trials

LETICIA MARA DOS SANTOS BARBETTA^1^, Letícia Mara dos Santos Barbetta^1^, Eduardo Thadeu de Oliveira Correia^1^, Ronaldo Altenburg Odebrecht Curi Gismondi^1^, Antônio José Lagoeiro Jorge^1^, Evandro Tinoco Mesquita^1^

(1) Universidade Federal Fluminense; (2) Instituto Cardiovascular do Complexo Hospitalar de Niterói; (3) Centro de Educação e Treinamento Edson Bueno – UnitedHealth Group; (4) Hospital Niterói D’Or

**Introduction:** Previous randomized trials showed conflicting results regarding the effectiveness of influenza vaccination on cardiovascular outcomes in patients with coronary artery disease (CAD). These studies were not adequately powered to show the effectiveness of influenza vaccine on major adverse cardiovascular events (MACE); all-cause mortality or cardiovascular mortality. Therefore, this up-to-date meta-analysis combines data from randomized trials to assess the effectiveness of influenza vaccination on endpoints in patients with CAD.

**Methods:** We performed a search of the Cochrane Controlled Register of Trials, Embase, MEDLINE, www.ClinicalTrials.gov and the World Health Organization International Clinical Trials Registry Platform from inception to September 2021. Two authors performed the screening, quality analysis and data extraction. A p-value <0.05 was defined as statistically significant. This study protocol is available in PROSPERO under the following registration number CRD42021282917.

**Results:** We identified 355 records through search of databases. After duplicate analysis, 258 articles remained, of which 239 were excluded based on title and/or abstract analysis. Nineteen full-text articles were assessed for eligibility, and 5 articles were selected for inclusion. Five trials were included in this meta-analysis, comprehending 4238 patients, from which 2116 were controls and 2122 received influenza vaccination. Influenza vaccination reduced all-cause mortality compared with placebo (RR 0.50, CI 0.29–0.88, p = 0.02). However, in a prespecified subgroup analysis, influenza vaccination only reduced all-cause mortality in patients with acute coronary syndrome (ACS) (RR 0.44, CI 0.23–0.82, p = 0.01), but not in stable CAD (RR 1.02, CI 0.32–3.31, p = 0.97). Moreover, influenza vaccination reduced cardiovascular mortality compared with placebo (RR 0.54, CI 0.37–0.80, p = 0.002). Regarding MACE, influenza vaccination was effective compared with placebo (RR 0.65, CI 0.48–0.88, p = 0.005), especially in ACS (RR 0.58, CI 0.39–0.87, p = 0.007), although it was not effective in stable CAD (RR 0.91, CI 0.54–1.54, p = 0.72).

**Conclusions:** The results of this meta-analysis shows that influenza vaccination is effective to reduce all-cause mortality, cardiovascular mortality and MACE among patients with CAD, especially those with ACS. Healthcare strategies must be outlined to deliver this cheap and effective intervention in order to reduce hard outcomes for CAD patients.

109253

Modality: Best Abstracts Oral – Scientific Initiation

Category: COVID-19 AND CARDIOVASCULAR SYSTEM

D: 13/10/2022    H: 15:40/16:40

L: Auditório 05

## Endothelial Dysfunction, Hypercoagulable State, and Thrombosis in Patients who Died from Covid-19

NICHOLAS VINCENT LEE^1^, Nicolas Henrique Borges^1^, Thiago Mateus Godoy^1^, Sabriany Nunes Mendes^1^, Anna Flavia R S Miggiolaro^1^, Rafaela C Zeni^1^, Lucas Baena Carstens^1^, Marcos Roberto Curcio Pereira^1^, David Batista Wiedmer^1^, Marina Luise Viola de Azevedo^1^, Lúcia de Noronha^1^

(1) PONTIFICIA UNIVERSIDADE CATÓLICA DO PARANÁ – PUC/PR

**Introduction:** Vascular endothelial cells are involved in the immune response mediated by SARS-CoV-2, which, when activated, has a significant influence on cell stability, culminating in edema, thrombosis, and endothelial dysfunction. The pathways responsible for this endotheliopathy process are still being discussed in the literature. Therefore, the analysis of the vascular events involved in the disease is necessary to concatenate findings that justify the cardiovascular repercussions.

**Objective:** Evaluate the tissue expression of endothelium dysfunction markers, Intercellular Adhesion Molecule 1 (ICAM-1), angiotensin 2 (ANGIO-2), interleukin-1-β (IL1β), and Von Willebrand Factor (VWF) and correlate with endothelial activation/dysfunction in the vascular endothelium of lung samples from COVID-19 patients and compare with H1N1 and control cases.

**Method:** The study analyzed post mortem lung samples (COVID-19 group = 20 cases; Group H1N1 = 10 cases and control group = 11 cases) through immunohistochemistry, using the primary monoclonal antibody of anti-ICAM-1, anti-ANGIO 2, anti-IL1β, and anti-VWF. The immunostained slides were scanted and selected by blinding. Then, software was responsible for quantifying the tissue expression of ICAM-1, ANGIO-2, IL-1β, and VWF obtained in each case. The findings were compared using the Kruskal-Wallis nonparametric test.

**Results:** The histopathological characteristics of pulmonary vascular damage caused by H1N1 differ from those observed in the COVID-19 group. For ICAM-1, an increase in expression was observed with statistically significant (p < 0,0001) when comparing the COVID group with both the control and H1N1 groups. The same pattern was repeated in the expression of IL-1β. Although, for ANGIO-2 and VWF, there was no statistical significance when comparing the COVID group with H1N1 (p > 0,05), when comparing the COVID group with the control group, the expression of these markers was higher (p < 0,05). In addition, there are no significant fibrinous thrombi or neutrophilic endotheliitis present in patients infected with H1N1.

**Conclusions:** Our results demonstrated endothelium activation and dysfunction secondary to cytokine storm. The endothelial injury and the state of hypercoagulability caused by COVID-19, when added to the blood stasis present in bedridden patients, culminating in the formation of the Virchow triad, capable of elevating the chances of systemic thrombotic events and cardiovascular repercussions.

111707

Modality: Best Abstracts Oral – Scientific Initiation

Category: HEART FAILURE/CARDIOMYOPATHY/TRANSPLANT

D: 13/10/2022    H: 15:40/16:40

L: Auditório 05

## A New Score Based on Machine Learning to Predict In-Hospital Death in Patients with Acute Heart Failure: The ML-HF Score

JORGE TADASHI DAIKUBARA NETO^1^, Jorge Tadashi Daikubara Neto^1^, Matheus Bissa Duarte^1^, Gustavo Sarot Pereira da Cunha^1^, Leonardo Henrique dos Santos de Melo^1^, Michelle Bozko Collini^1^, Carolina Ruschel Senger^1^, Jessica Tamires Reichert^1^, Raphael Henrique Déa Cirino^1^, Sabrina Bernardez^2^, Fábio Papa Taniguchi^2^, Miguel Morita Fernandes-Silva^1^

(1) Hospital de Clínicas – UFPR; (2) Hospital do Coração – HCor

**Introduction:** Acute Heart Failure (AHF) has high mortality and identifying those patients with worse prognosis helps guiding their management. However, current available prognostic assessment scores have sub-optimal performance, so they are rarely used in clinical practice.

**Objective:** Developing and validating a machine learning-based prognostic score to predict in-hospital death in patients with AHF and compare its performance with the Acute Decompensated Heart Failure National Registry (ADHERE) and the Get With the Guidelines–Heart Failure (GWTG-HF) scores.

**Methodology:** We included patients admitted with AHF in 17 brazilian hospitals participants of a multicenter study from 2016 to 2019. Clinical, laboratory and echocardiographic data and the WHOQOL-BREF quality of life questionnaire at hospital admission were used as covariates. The outcome was in-hospital death from any cause. Machine learning prediction models – using Random Forest, Gradient Boosting Machines, and Deep Neural Networks – were applied in 70% of the sample (training set) to develop the score (ML-HF score), which was validated on the 30% remaining of the sample (test set).

**Results:** From 2657 patients hospitalized for AHF, we included 887 [59% men, 61.6 ± 14.5 years, ejection fraction 41.8 ± 17.2%, 84 (9%) died] who had complete data. The five most important variables of the ML-HF score were: Physical Health Domain quality (WHOQOL-BREF), serum sodium, serum urea, serum creatinine and systolic blood pressure at hospital admission. In the test set, the ML-HF score showed good model calibration (Hosmer-Lemeshow test p value = 0.124) and good model discrimination area under the ROC curves [(AUC) = 0.739 (95%CI,0.652–0.825)], which was better than the GWTG-HF [AUC = 0.601 (IC95%, 0.459–0.743)], p = 0.05 and the ADHERE [AUC = 0.594(IC95%, 0.445–0.742)], p = 0.05 scores (figure).

**Conclusion:** We developed and validated a score using machine learning to predict in-hospital death in patients with AHF, which outperformed the ADHERE and GWTG-HF scores.


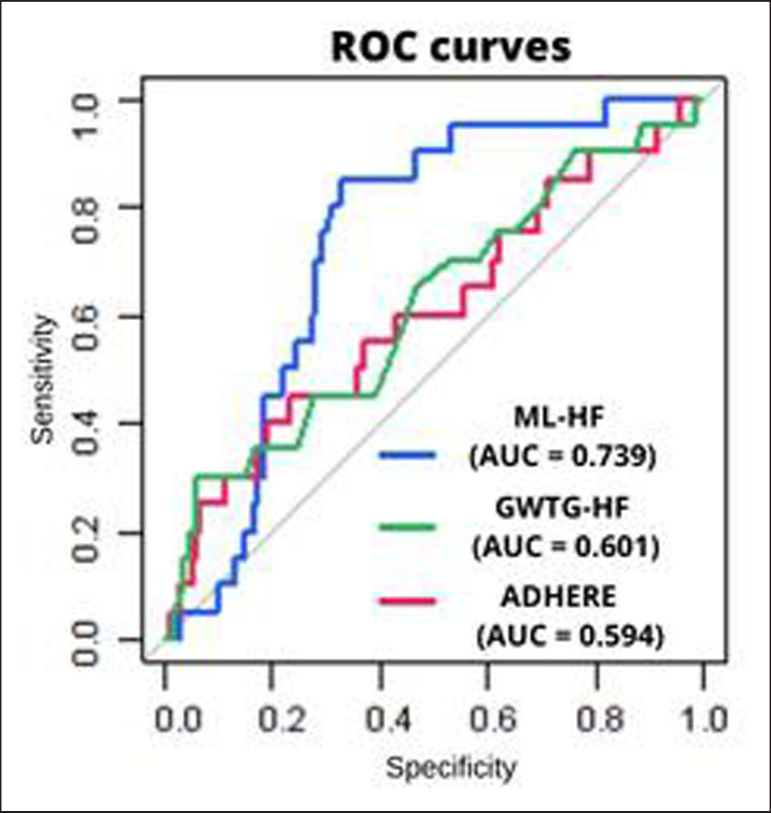


111880

Modality: Best Abstracts Oral – Scientific Initiation

Category: EPIDEMIOLOGY AND HEALTH POLICIES/GLOBAL HEALTH

D: 13/10/2022    H: 15:40/16:40

L: Auditório 05

## Heart Transplant: Trends in Brazilian Epidemiology between 2008 and 2021

BEATRIZ SALES DE FREITAS^1^, Ana Carolina Sampaio Freire^1^, Caio Resende da Costa Paiva^1^, Isabel Gomes da Silva^1^, Gabriel Haiek Fernandes^1^, Gabriela Gonçalves Almeida^1^, Maria Luíza Marinho de Sá de Paula Lima^1^

(1) Universidade de Brasília (UnB)

**Introduction:** Heart transplant (HT) is one of the main surgeries by which patients with advanced and refractory heart failure can prolong their lives. Nonetheless, its complexity requires a well prepared staff and abundant financial resources, which can be a challenge in developing countries such as Brazil. In this scenario, searching for trends in epidemiology and regional disparities is key to scale the problem and propose interventions.

**Objectives:** To analyze data of HT in Brazil’s different regions between January 2008 and December 2021.

**Methodology:** Epidemiological inquiry based on data related to mortality rates and annual HT absolute numbers from 2008 to 2021 in Brazil. The data was obtained through the public domain national platform TABNET from the Informatic Department of the Brazilian Universal Healthcare System. A comparative analysis of data was made, in addition to complementary research in recent literature.

**Results:** From January 2008 to December 2021, 3.368 HTs were performed in Brazil. The main causes for HT were Ischemic Cardiomyopathy, followed by Congestive Heart Failure and Chagasic Cardiomyopathy. The majority of HTs were in the Southeast (n = 1792). In contrast, in the North, which doesn’t have specialized centers, no surgeries were performed (n = 0). It is possible to recognize an increase in the absolute number of procedures throughout the period analyzed followed by a decrease in the last two years. This decrease may be a consequence of the COVID-19 pandemic. Despite the upward trend described, the number of capacitated teams or centers remained fairly the same from 2014 to 2021. Regarding the mortality rates, 13,62% of the patients who underwent HT died in the procedure or in post-op care at the health service. In general, these mortality rates have been declining, going from 20,99% in 2018 to 9,27% in 2021, a possible effect of the evolution in transplant techniques. HTs represent a challenge for Brazil’s health system. In December 2021, 321 patients were waiting for the surgery. Compared to kidney, cornea and liver transplants, HT boasts one of the higher discrepancies between performed versus demanded transplants.

**Conclusion:** HT is a highly complex procedure that requires proper logistics, training, resources and specific post-op care, representing a challenge for Brazil’s public health system. Considering the demand of HTs in Brazil, it is necessary to create new centers and to train new teams, especially in the North.

112045

Modality: Best Abstracts Oral – Scientific Initiation

Category: EPIDEMIOLOGY AND HEALTH POLICIES/GLOBAL HEALTH

D: 13/10/2022    H: 15:40/16:40

L: Auditório 05

## Hypercholesterolemia and Mortality from Ischemic Heart Diseases in American Men

LUIZ FELIPE FAÇANHA RAMOS^1^, Karen Tássia Façanha Ramos^1^, Hildeman Dias da Costa^2^, Ayrison Melo Sousa^3^, Reny Wane Vieira dos Santos^1^

(1) Universidade Federal do Amapá; (2) Universidade Federal de Rondônia; (3) Centro Universitário UNINORTE

**Introduction:** The increase in the amount of low-density lipoproteins (LDL cholesterol) or hypercholesterolemia is considered one of the factors in the occurrence and mortality of cardiovascular and cerebrovascular diseases, especially ischemic heart disease (IHD).

**Objective:** To relate hypercholesterolemia and IHD mortality in men in the countries of America in the year 2018.

**Methodology:** This is an analytical ecological study of geographic distribution with secondary data from the Health Information Platform for the Americas (PLISA), of the Pan American Health Organization (PAHO), of the year 2018, on the averages of total cholesterol and fractions and the mortality rates due to IHD in men in the countries of the American continent. The rates were standardized per 100,000 inhabitants with a confidence interval (CI) of 95%.

**Results:** Guyana had the highest IHD male mortality rate (228.9; 95% CI 175.8–312.7) in 2018 in America, followed by Haiti with the 2nd highest rate (190.3; 95% CI 124.6–278.8). In contrast, Peru has the lowest rate (46.6; 30.4–66.6) in the same year. In addition, it was shown that Guyana has men with the highest mean LDL cholesterol (x̄ = 3.81; 95% CI 3.56–4.05). It is noteworthy that Venezuela was the country with the lowest mean LDL cholesterol (x̄ = 3.23; 95% CI 3.03–3.41), despite having the third highest mortality rate in South America (130.9; 95% CI 98.9–169.1).

**Conclusions:** There is a positive linear correlation between high LDL cholesterol and mortality in men from IHD in America, as Guyana has the highest mortality rate and the highest mean LDL cholesterol in men in 2018. This suggests that public policies should be directed to combat hypercholesterolemia that can significantly increase mortality from ischemic heart disease.


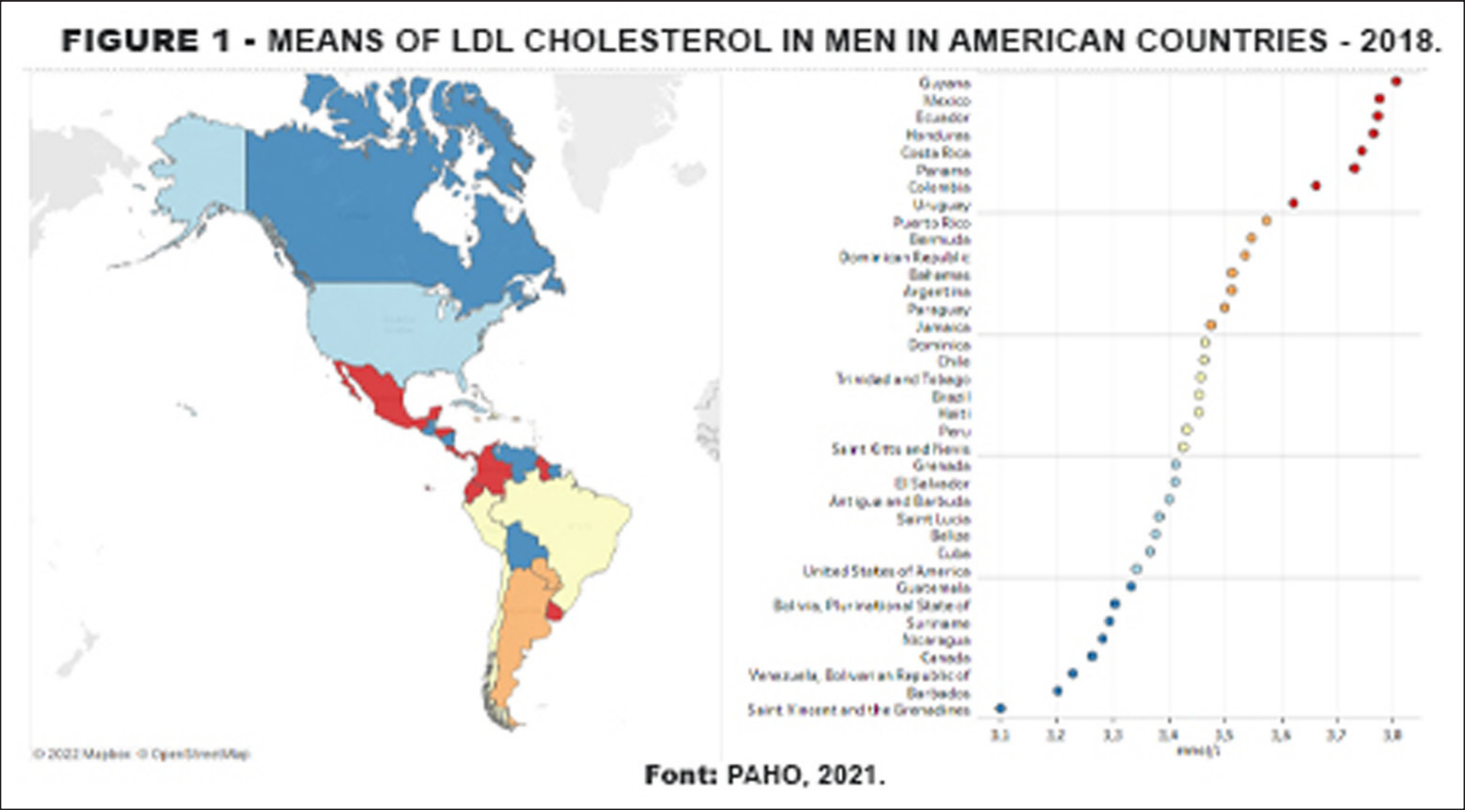


112186

Modality: Best Abstracts Oral – Scientific Initiation

Category: HEART FAILURE/CARDIOMYOPATHY/TRANSPLANT

D: 13/10/2022    H: 15:40/16:40

L: Auditório 05

## Regional Characteristics of HFrEF in Brazil: Rosa Dos Ventos Study

DHAYN CASSI DE ALMEIDA FREITAS^1^, Jefferson Luiz Vieira^2^, Sabrina Bernardez Pereira^3^, Fabiana Goulart Marcondes-Braga^4^, Wilson Nadruz Junior^5^, Silvia Marinho Martins Alves^6^, Gabriela Arcoverde Wanderley^7^, Jessica Tamires Reichert^8^, José Albuquerque de figueiredo neto^9^, Alana de Oliveira Castro^9^, Miguel Fernandes da Silva Morita^8^, Odilson Marcos Silvestre^1^

(1) Universidade Federal do Acre; (2) Hospital de Messejana – Dr. Carlos Alberto Studart Gomes; (3) Hospital do Coração; (4) Instituto do Coração – Hospital das Clínicas da Faculdade de Medicina da Universidade de São Paulo; (5) Universidade Estadual de Campinas,; (6) Universidade de Pernambuco; (7) Pronto-Socorro Cardiológico Universitário de Pernambuco; (8) Universidade Federal do Paraná; (9) Universidade Federal do Maranhão

**Background:** There are still no studies that characterize regional differences in HFrEF in Brazil.

**Objective:** To compare regional characteristics in the socioeconomic, clinical, and treatment aspects of HFrEF in Brazil.

**Methods:** Rosa dos Ventos is a cohort study in all Brazilian states which will include 3,000 patients with outpatient HFrEF (EF < 50%). We carried out a cross-sectional study to compare the characteristics of patients (n = 853), according to the Brazilian region of origin: North (n = 115), Northeast (n = 351), Center-west (n = 140), Southeast (n = 77) and South (n = 170). We investigated socioeconomic characteristics, clinical presentation of HF, and use of at least four medications of guideline-directed medical therapy (GDMT) in HF (beta-blockers, mineralocorticoid antagonists, SGLT2 inhibitors, and ACE inhibitors or ARBs or ARNIs). We used Student’s T and Chi-square statistical tests.

**Results:** Comparing the patients, the youngest belonged to the southeast region (55 ± 14, p < 0.001), and were less frequently white (19%, p < 0.001) and with lower monthly familiar income (R$ 2176 ± 3015, p < 0.001) in the Northeast region. There was a higher percentage of chagasic etiology in the Central-West region (42%, p < 0.001) and of ischemic etiology in the North region (39%, p = 0.021), the latter region had the highest mean ejection fraction (37 ± 8, p < 0.001). The use of at least four GDMT drugs was more frequent in the Northeast (23%, p = 0.033) and South regions (22%, p = 0.033), with a higher frequency of ARNI use in the South (45%, p < 0.001).

**Conclusions:** The Rosa dos Ventos Study aims to determine regional differences in HFrEF, and the results will help in planning its prevention and treatment. These preliminary data suggest regional differences in the etiology and use of therapy.


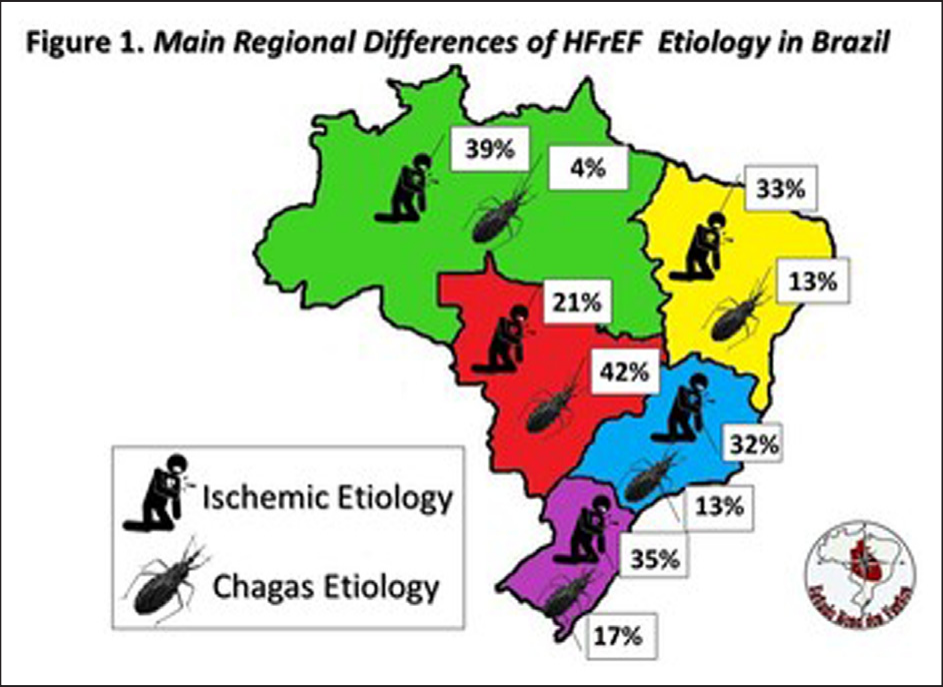


108365

Modality: Best Poster – Researcher

Category: ATHEROSCLEROSIS/CARDIOVASCULAR RISK FACTORS/CARDIOVASCULAR PREVENTION

D: 14/10/2022    H: 10:00/10:40

L: Área de exposição de pôsteres

## Comparative Effectiveness and Cost-Effectiveness of Cardioprotective Glucose-Lowering Therapies for Type 2 Diabetes in a Middle-Income Country: A Modeling Analysis

ANA CLÁUDIA CAVALCANTE NOGUEIRA^1^, Joaquim Barreto^2^, Beatriz Luchiari^2^, Isabella Bonilha^2^, Luiz Sérgio Fernandes de Carvalho^3^, Andrei Carvalho Sposito^2^

(1) Escola Superior de Ciências da Saúde – ESCS; (2) Atherosclerosis and Vascular Biology Laboratory (Aterolab), Cardiology Division, Universidade de Campinas – UniCamp; (3) Clarity Healthcare Intelligence

**Background:** We sought to compare the effectiveness and cost-effectiveness of cardioprotective glucose-lowering therapies in individuals with T2D in a middle-income country.

**Methods:** A systematic search was performed for randomized clinical trials published until April 2021 reporting the incidence of MACE for pioglitazone, GLP1A, or SGLT2i. Using date from two national cohorts of T2D, we developed a Markov model to estimate the outcomes for each treatment based on incremental cost-effectiveness ratio (ICER) and the disease-adjusted life years [DALYs] gain per dollar spent projected over a lifetime horizon using a 5% annual discount rate.

**Results:** 157 RCT including 267,508 patients and 176 active arms were considered. Compared with sulfonylureas, SGLT2i, GLP1A and pioglitazone reduced the relative risk of non-fatal MACE with HR of 0.81 (95% CI 0.69 to 0,96, p = 0.011), 0.79 (95% CI 0.67 to 0,94, p = 0.0039) and 0.73 (95% CI 0.59 to 0.91, p = 0.0057), respectively. Pioglitazone resulted in incremental effectiveness of 0.2339 DALYs per patient, at a mean incremental cost of US$1660 and a US$ 7,082 (95% CI: 4,521; 10,770) incremental cost per DALY gained, when compared to standard care. The addition of SGLT2i or GLP1A led to more evident (0.261 and 0.259, respectively) but with higher ICERs [US$ 12,061 (95% CI: 7,227; 18,121) and US$ 29,119 (95% CI: 23,811; 35,367) per DALY gained, respectively]. Compared to SGLT2i and GLP1A, pioglitazone had the highest probability of being cost-effective based on the estimated maximum willingness-to-pay threshold.

**Conclusions:** The three therapies bear similar effectiveness in reducing cardiovascular events. In a middle-income country, pioglitazone presents a higher probability of being cost-effective followed by SGLT2i and then GLP1A.


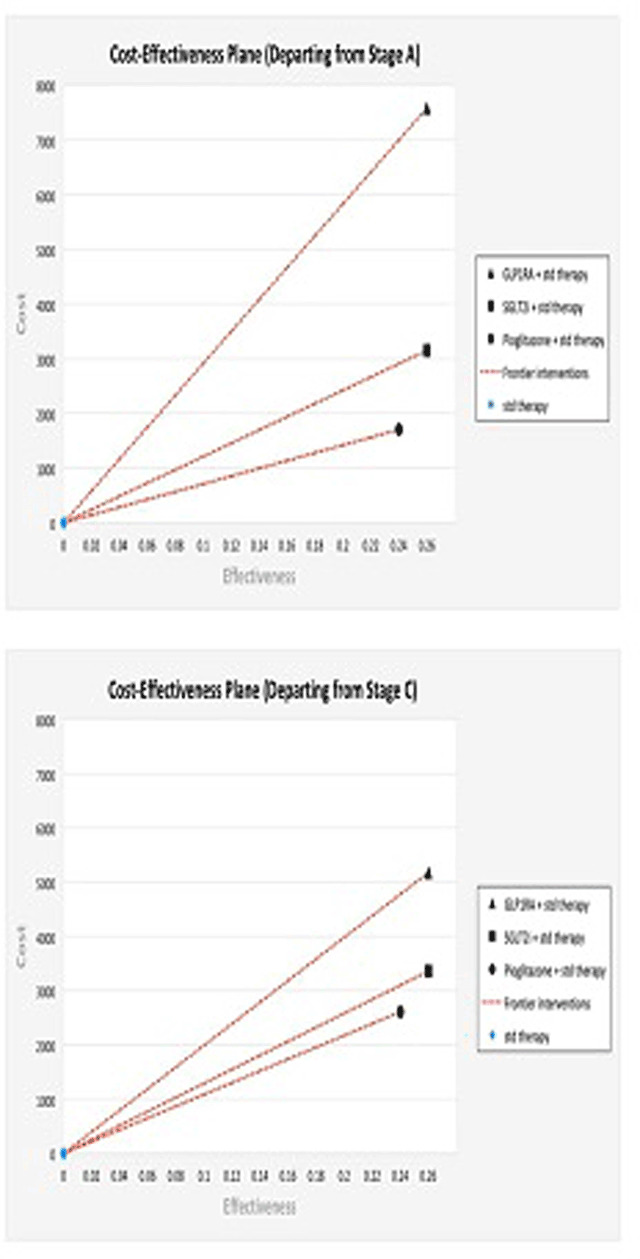


109342

Modality: Best Poster – Researcher

Category: HEMODYNAMICS AND INTERVENTIONAL CARDIOLOGY

D: 14/10/2022    H: 10:00/10:40

L: Área de exposição de pôsteres

## Distal Transradial Access for Coronary Procedures: A Prospective Cohort of 3,991 All-Comers Patients from the Distraction Registry

MARCOS DANILLO PEIXOTO OLIVEIRA^1^, Lélio Lemos Pinto Neto^1^, Ednelson Navarro^2^, Adriano Caixeta^1^

(1) Universidade Federal de São Paulo, UNIFESP; (2) Hospital Regional do Vale do Praíba

**Background:** Distal transradial access (dTRA) as a refinement of the conventional transradial approach has several potential advantages in terms of patient and operator comfort, faster hemostasis, and lower risk of proximal radial artery occlusion. We aim to describe our prospective real-world experience with dTRA as default for routine coronary angiography and percutaneous coronary interventions (PCI) in a broad sample of all-comers patients.

**Material and methods:** From February 2019 to April 2022, 3,991 consecutive all-comers patients submitted to coronary angiography and/or PCI via dTRA have been enrolled into the DISTRACTION registry.

**Results:** Mean patient age was 63.36 ± 13.3-year-old, most male (65.4%) and with acute coronary syndromes (48,8%) at admission. Overall, 843 (20.1%) patients had non-ST-elevation myocardial infarction, 799 (20,0%) had ST-elevation myocardial infarction, and 114 (2.6%) presented in cardiogenic shock. There were only 94 (2.3%) access site crossovers, in only 79 (1.9%) patients dTRA sheath insertion could not be obtained. Right dTRA was the most frequent access (80.1%), followed by redo right dTRA (10.8%), left dTRA (8,1%) and simultaneous bilateral dTRA (0.7%). In 2,210 (60.0%) of all patients, PCI was performed and left anterior descending was the most prevalent target coronary territory (29.1%). No major adverse cardiac and cerebrovascular events and no major complications directly related to dTRA were recorded.

**Conclusions:** The adoption of dTRA as default for routine coronary angiography and PCI in a real-world fashion of all-comers patients by experienced transradial operators appears to be feasible and safe.


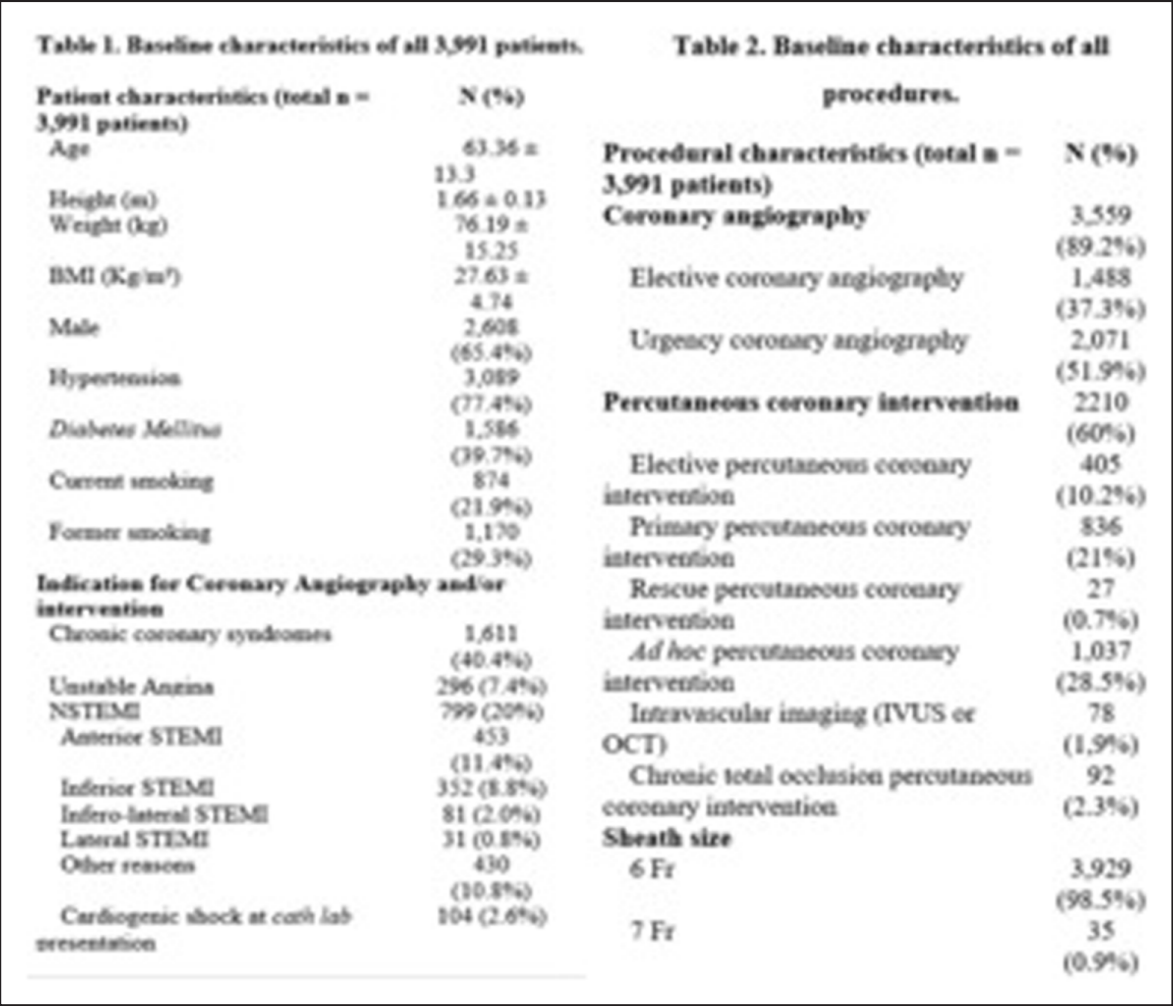


111318

Modality: Best Poster – Researcher

Category: DIGITAL HEALTH/INNOVATION

D: 14/10/2022    H: 10:00/10:40

L: Área de exposição de pôsteres

## Digital Personal Walking Plans Increase Physical Activity Levels of Australian Adults

ELIZABETH ANNE CALLEJA^1^, Elizabeth A Calleja^1^, Dr Amanda K Buttery^1^, Teresa Gadaleta^1^, Sheree Hughes^1^, Jarrod Leggett^1^, Associate Professor Trevor Shilton^1^

(1) National Heart Foundation of Australia

Physical activity is an important modifiable risk factor for the prevention and management of cardiovascular disease and can reduce the risk of morbidity by 35% [1]. However, only 15% of Australian adults meet the physical activity guidelines [2]. A national walking program was rolled out across Australia led by a national voluntary group-based community walking program in 2007. In response to the COVID-19 pandemic, we aimed to develop a digital program option for people to walk independently. Personal Walking Plans (PWP) were launched in Australia as a free six-week web-based digital program for adults to address this need.

**Methods:** We designed personal walking plans on behaviour change principles and theory. This included four graded walking programs with strengthening and flexibility exercises, supported with online instructional videos and motivational messaging via email or text (SMS) messages. A digital self-report survey was conducted on enrollment to the program, and after completion at six weeks. These were completed between 11th of May and 30th June 2021. Survey questions included established items on participant goals, physical activity levels, influence of motivational messaging (text and email) and intention to continue walking following program completion.

**Results:** Of 2338 (mean 65 years; 87% female) participants completing the survey, 74% completed the program. Of these, 86% achieved their goal and 69% met the Australian physical activity guidelines by the end of the six-weeks. On average, participants increased the number of days they engaged in physical activity from 2.7 days to 4.4 days per week by the end of the six weeks. Strength exercise sessions increased from 0.9 days to 2.4 days per week. Participants reporting they paid attention to motivational messages increased their physical activity (30min physical activity, 4.4 days, strength 2.4 days) more than those who ignored/did not receive texts (30min physical activity, 3.9 days, strength 2.0 days). Nearly all (99%) reported that they would continue walking following the intervention.

**Conclusion:** A six-week digital personal walking plan was effective in improving participants’ attainment of the Australian adult physical activity guidelines. Motivational text messaging and emails appeared to enhance engagement with the program and physical activity levels. Long-term follow-up surveys at 6 and 12 months will evaluate if benefits are sustained over time.

111421

Modality: Best Poster – Researcher

Category: CARDIOVASCULAR IMAGING

D: 14/10/2022    H: 10:00/10:40

L: Área de exposição de pôsteres

## Myocardial Blood Flow Reserve by CZT Gamma Camera Increases Accuracy of Obstructive Coronary Artery Disease Detection

RONALDO DE SOUZA LEAO LIMA^1^, Andre Luiz Bezerra^2^, Claudio Domenico^2^, Andrea Rocha de Lorenzo^2^

(1) Fonte Imagem; (2) Universidade Federal do Rio de Janeiro

**Introduction:** CZT cameras have higher sensitivity for photon detection, as well as higher temporal and spatial resolution. These have enabled que noninvasive quantification of myocardial flow reserve (MFR), which may increase the accuracy of myocardial perfusion SPECT (MPS) for the detection of obstructive coronary artery disease (CAD). This study aimed to compare the accuracy of CZT MPS and of MFR for the detection of obstructive CAD.

**Methods:** 66 patients with CAD (>50% obstruction) detected at invasive coronary angiography or CT angiography underwent dipyridamole MPS and MFR evaluation within 30 days. A 1-day protocol (rest-stress) was used to quantify MFR. The acquisition of dynamic rest and stress images was initiated simultaneously to 99mTc sestamibi injection (10mCi e 30mCi, respectively), both lasting for 11 minutes, followed by 5-minute imaging. Pharmacologic stress with dipyridamole (0,56 mg/kg for 4 minutes) was performed with the patient positioned in the CZT camera. The images were processed and time-activity curves were generated, calculating global and regional MFR in a semiautomatic software. A global or regional MFR <2.0 was considered abnormal. The MPS perfusion images were classified as normal or abnormal and perfusion scores were calculated. The images were interpreted by experienced physicians blinded to the results of MFR and coronary angiography/CT.

**Results:** Mean age of the population was 63 ± 3 years, 51.5% male. Hypertension, hypercholesterolemia and diabetes were the most frequente risk factors (80.3%, 45.4%, and 42.4%, respectively). Thirty patients (45.5%) had single-vessel CAD, 28 (42.4%) 2-vessel CAD and 8 (12.1%), triple-vessel CAD. Among the 110 vessels with obstruction, 67 had perfusion abnormalities in MPS and 81 had reduced MFR, while among the normal vessels, 76 had normal MPS and 74 had preserved MFR. The sensitivity of MFR (73,6%) was higher than that of MPS (60.2%), without significant changes in specificity (86,4 vs 84.1%).

**Conclusions:** MFR in the CZT camera is an absolute, physiologic, quantifiable measure which is more sensitive for the detection of obstructive CAD than perfusion abnormalities in MPS, especially in patients with multivessel CAD.

111997

Modality: Best Poster – Researcher

Category: COVID-19 AND CARDIOVASCULAR SYSTEM

D: 14/10/2022    H: 10:00/10:40

L: Área de exposição de pôsteres

## Predictors of Venous Thromboembolism in COVID-19 Patients: Results of the COVID-19 Brazilian Registry

WARLEY CEZAR DA SILVEIRA^1^, Manuela Furtado Sacioto^2^, Luiza Margoto Marques^2^, Mateus Chaves Ferreira^1^, Beatriz Figueiredo Lima^1^, Maria Izabel Alcântara Cunha^4^, Bruno Barbosa Miranda de Paiva^1^, Marcos André Gonçalves^1^, Polianna Delfino Pereira^1^, Ana Beatriz de Castro Feres^2^, Magda Carvalho Pires^1^, Milena Soriano Marcolino^1^

(1) Universidade Federal de Minas Gerais (UFMG); (2) Faculdade Ciências Médicas de Minas Gerais (FCMMG); (3) Institute for Health Technology Assessment (IATS/CNPq); (4) Centro universitário de Belo Horizonte (UNIBH)

**Introduction:** COVID-19 patients present a high incidence of venous thromboembolism (VTE), which requires early recognition and treatment. In those patients, the diagnosis of pulmonary embolism is a challenge, as its symptoms and signs overlap with the ones of the severe acute respiratory syndrome (SARS). Several studies have tried to identify risk factors of VTE in hospitalized COVID-19 patients, as a path to promote prevention, early diagnosis and treatment. However, such studies have shown inconsistent results.

**Objective:** To identify VTE predictors by both logistic regression (LR) and machine learning (ML) approaches and report the incidence of thromboembolic complications in COVID-19 and their prognostic impact.

**Methods:** This substudy of a large Brazilian COVID-19 Registry included consecutive COVID-19 adult patients from 16 hospitals, admitted between March and September, 2020. Symptomatic VTE was confirmed by objective imaging. LR analysis, tree-based boosting and bagging were used to investigate the association of variables upon hospital presentation with VTE.

**Results:** Among 4,120 patients (median age was 61 years [IQR, 48–72] years-old, 55.5% men, 39.3% critical patients), VTE was confirmed in 6.7%. In multivariate LR analysis, obesity (OR 1.50, 95%CI 1.11–2.02); being an ex-smoker (OR 1.44, 95%CI 1.03–2.01); surgery ≤90 days (OR 2.20, 95%CI 1.14–4.23); axillary temperature (OR 1.41, 95%CI 1.22–1.63); D-dimer ≥4 times above the upper limit of reference value (OR 2.16, 95%CI 1.26–3.67), lactate (OR 1.10, 95%CI 1.02–1.19), C-reactive protein levels (CRP, OR 1.09, 95% CI 1.01–1.18); and neutrophil count (OR 1.04, 95%CI 1.005–1.075) were independent predictors of VTE. Temperature at admission, SF ratio, neutrophil count, D-dimer, CRP and lactate levels were also identified as predictors by ML methods. Patients with confirmed VTE had higher mortality (28.4% vs 18.5%, p < 0.001) and had a higher frequency of mechanical ventilation support (58.4% vs 26.4%, p < 0.001) and renal replacement therapy (21.5% vs 9.7%, p < 0.001), when compared to the group without confirmed VTE.

**Conclusion:** By using ML and LR analysis, we showed that D-dimer, axillary temperature, neutrophil count, CPR and lactate levels are risk factors for VTE in COVID-19 patients. Therefore, we suggest that patients presenting these risk factors at admission should be more closely monitored for VTE development, considering the importance of prevention, early diagnosis and treatment of VTE.

108390

Modality: Best Poster – Young Researcher

Category: CARDIORESPIRATORY PHYSIOLOGY/BASIC SCIENCE

D: 14/10/2022    H: 16:10/16:50

L: Área de exposição de pôsteres

## Doxorubicin-Induced Cardiotoxicity Attenuation by Omega-3 Fatty Acid Supplementation in Rats is not Mediated by Sphingomyelin-Ceramide Pathway

MARINA GAIATO MONTE ^1^, Carolina Rodrigues Tonon^1^, Anderson Seiji Soares Fujimori^1^, Ana Paula Dantas Ribeiro^1^, Katashi Okoshi^1^, Paula Schmidt Azevedo^1^, Marcos Ferreira Minicucci^1^, Leonardo Antonio Mamede Zornoff^1^, Sergio Alberto Rupp de Paiva^1^, Bertha Furlan Polegato^1^

(1) Faculdade de Medicina de Botucatu

**Introduction:** Doxorubicin (DOX) is widely used effective chemotherapy drug; however, it can cause cardiotoxicity which is a very serious side effect. There is no effective therapy for cardiotoxicity. Omega-3 fatty acid (O3) supplementation may act in the sphingomyelin-ceramide pathway. We aimed to evaluate the influence of O3 in attenuating DOX-induced cardiotoxicity.

**Methods:** Male Wistar rats (n = 60) were divided into 4 groups: control (C), administration of O3 only (O3), DOX only (D), and DOX and O3 (DO3). O3 (400 mg/kg/day, gavage) was administered for 6 weeks. DOX (3.5 mg/kg, IP, once a week) was administered for the last 4 weeks of the experiment. At the end of 6 weeks, rats were submitted to echocardiogram and euthanized (thiopental 120 mg/kg, ip). Statistical analysis: 2-way ANOVA (pi: p value for the interaction between DOX and O3; pD: p value for the effect of DOX; pO3: p value for the effect of O3).

**Results:** Group D exhibited increased left atrium diameter/aorta diameter ratio (C 1.31 ± 0.11; D 1.45 ± 0.11; O3 1.36 ± 0.11; DO3 1.27 ± 0.11; pD = 0.467, pO3 = 0.028, pi < 0.001) and decreased left ventricular fractional shortening (C 0.57 ± 0.07; D 0.46 ± 0.07; O3 0.56 ± 0.08; DO3 0.53 ± 0.08; pD = 0.002; pO3 = 0.164; pi = 0.046) compared to Group C, characterizing diastolic and systolic dysfunction, respectively. DOX increased neutral sphingomyelinase activity (nSMase, C 2283 ± 412; D 2879 ± 680; O3 2461 ± 639; DO3 3319 ± 284 UI fluorescence; pD < 0.001, pO3 = 0.087, pi = 0.461) and decreased myocardial nSMase protein quantification (C 0.05 ± 0.03; D 0.04 ± 0.02; O3 0.05 ± 0.02; DO3 0.03 ± 0.02 arbitrary units; pD = 0.009, pO3 = 0.455, pi = 0.275). There were no differences between groups in myocardial ceramide deposition evaluated by immunohistochemistry.

**Conclusion:** O3 supplementation attenuates DOX-induced diastolic and systolic dysfunction with no changes in neutral sphingomyelinase activity or expression in the myocardium. Financial support: FAPESP 2018/25677-7 and CNPq 407201/2021-1.

108392

Modality: Best Poster – Young Researcher

Category: CARDIORESPIRATORY PHYSIOLOGY/BASIC SCIENCE

D: 14/10/2022    H: 16:10/16:50

L: Área de exposição de pôsteres

## Netosis is Involved in the Pathophysiology of Acute Doxorubicin-Induced Cardiotoxicity

MARINA GAIATO MONTE ^1^, Carolina Rodrigues Tonon^1^, Tatiana Fernanda Bachiega Pinelli^1^, Anderson Seiji Soares Fujimori^1^, Ana Paula Dantas Ribeiro^1^, Nayane Maria Vieira^1^, Natalia Fernanda Ferreira^1^, Danilo Malmonge Barbosa Luciano^1^, Ronny Peterson Cabral^1^, Katashi Okoshi^1^, Bertha Furlan Polegato^1^, Leonardo Antonio Mamede Zornoff^1^

(1) Faculdade de Medicina de Botucatu

**Background:** Doxorubicin (dox) is used in the treatment of several types of cancer. However, cardiotoxicity is a common side effect of the drug. The pathophysiology of cardiotoxicity is not clearly understood. Neutrophils produce attractant substances such as NETs (neutrophil extracellular traps), that are involved in immune response and inflammation which could mediate myocardial extracellular matrix remodeling.

**Purposes:** To analyze the role of NETs in the pathophysiology of acute dox-induced cardiotoxicity.

**Methods:** 60 male Wistar rats were allocated into 3 groups: Control (C), Dox (D), and Dox + DNAse (DD). D and DD groups received an intraperitoneal injection of dox 10 mg/kg, and 2h later, DD received a subcutaneous injection of DNAse 20 mg/kg (NETs inhibitor). Rats were submitted to cardiac function evaluation and euthanasia 48h after dox injection. Statistical analysis: one-way ANOVA.

**Results:** D showed increased NETs production compared with C and DD (C = 2997 ± 810; D = 5955 ± 1906; DD = 4108 ± 1674 pg/mL; p < 0.001). Transthoracic echocardiogram showed no differences in systolic parameters, but isovolumetric relaxation time corrected by heart rate was higher in D and DD than C (C = 46 ± 4.6; D = 51 ± 7.9; DD = 51 ± 7.7, p = 0.039). Additionally, in isolated heart study, area under curve for diastolic pressure-volume ratio was reduced in D, indicating lower ventricular compliance, compared with C and DD (C = 827 ± 74; D = 670 ± 109; DD = 966 ± 218; p = 0.007). Dox induced increased malondialdehyde myocardial concentration in D and DD compared to C (C = 48 ± 28; D = 73 ± 32; DD = 82 ± 25 nmol/mg of protein; p < 0.05). Regarding extracellular matrix, dox increased and DNAse attenuated collagen in cardiac tissue (C = 2.88 ± 0.97, D = 3.51 ± 0.7, DD = 2.99 ± 0.66%; p < 0.05). Additionally, dox increased matrix metalloproteinase activity (MMP)-2 (C = 1.01 ± 0.28, D = 2.04 ± 0.47, DD = 2.36 ± 0.6; p < 0.001), but DNAse did not interfere with this parameter. Evaluation of protein expression of |Type 2 and 4 MMP tissue inhibitors (TIMP) showed no differences between groups.

**Conclusions:** Dox-induced cardiotoxicity is associated with diastolic dysfunction, cardiac fibrosis, increased MMP-2 activity, and oxidative stress. NETs are involved in the pathophysiology of dox-induced cardiotoxicity. Netosis inhibition improved diastolic function, associated with decreased myocardial collagen content. However, this effect was not mediated by oxidative stress, MMP-2 activation, or TIMP-2 and –4 protein expression.

109335

Modality: Best Poster – Young Researcher

Category: CARDIO-ONCOLOGY

D: 14/10/2022    H: 16:10/16:50

L: Área de exposição de pôsteres

## Resistance Training Preserves Left Ventricle Ultrastructure and Function in Doxorubicin-Induced Cardiotoxicity

RENATA ALVES^1^, Cláudia de Morais Sequeira^1^, Jefferson Fernandes Evangelista^1^, Ana Lucia Rosa Nascimento^1^, Cristiane Matsuura^1^

(1) Universidade do Estado do Rio de Janeiro

**Introduction/aim:** It is well known that doxorubicin (DOX) elicits toxic effects on the heart limiting its use in cancer treatment. Here, we investigated the effects of resistance training on left ventricle (LV) ultrastructure and function in DOX-induced cardiotoxicity in rodents.

**Methods:** Male adults Sprague Dawley rats were divided into three groups (n = 10): control, DOX that remained sedentary (DoxSed), or DOX submitted to resistance training (DoxTr). Resistance training (5 d/wk for 8 wks) consisted of climbing a ladder with weights placed on the tail, with progressive increase in the training load (number of repetitions and weight). DOX was administered for 10 consecutive days (1 mg/kg/d, i.p.) and it initiated concomitantly with training. At the end, cardiac function was measured by echocardiography, and LV fragments were processed for transmission electron microscopy.

**Results:** There was a reduction in mortality in DoxTr compared to DoxSed (20 vs. 38%, P < 0.001, log rank test). The decrease in LV ejection fraction observed in DoxSed was attenuated in DoxTr (control: 76 ± 1; DoxSed: 64 ± 1; DoxTr: 71 ± 1%; P < 0.05, one-way ANOVA). The most striking effects were seen in LV ultrastructure (Fig 1). The control group showed a normal structural arrangement, with myofibrils arranged in parallel, preserved sarcomeres with uniform distance between Z lines. Intact mitochondria were seen in parallel arrangement to the myofibrils. The cardiomyocytes of the DoxSed group showed severe cellular disruption, with fragmentation of the myofibrils, disappearance of some sarcomeres, increased electron-lucid cytoplasmic content and the presence of autophagosomes, and degenerated mitochondria. Resistance training resulted in positive effects on the ultrastructural morphology of cardiomyocytes, with intact mitochondria and large areas of preservation of the structural organization of the sarcomere, although it was still possible to observe a non-linear arrangement and reduction in the density of myofibrils.

**Conclusion:** Resistance training can be a non-pharmacological strategy to prevent the deleterious effects of DOX on the heart.


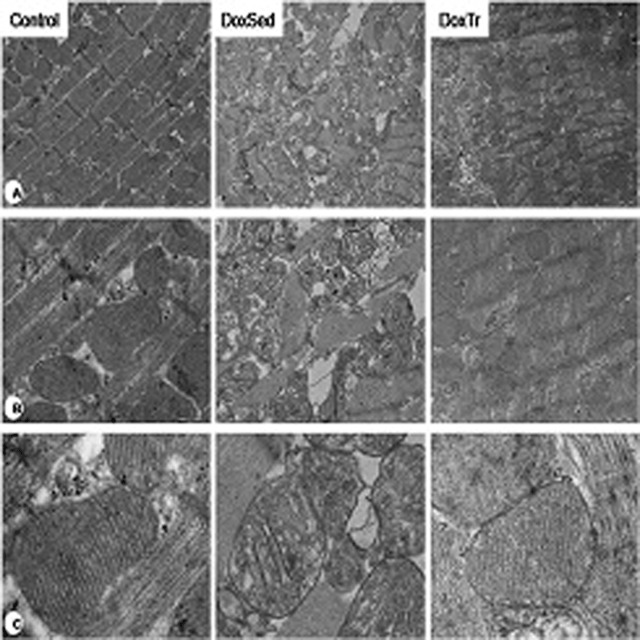


110132

Modality: Best Poster – Young Researcher

Category: CARDIOLOGY OF SPORTS, EXERCISE, ERGOMETRY AND CARDIOVASCULAR REHABILITATION

D: 14/10/2022    H: 16:10/16:50

L: Área de exposição de pôsteres

## Physiological Electrocardiographic Findings in a Brazilian Cohort of Young Football Players: B-Pro Foot Ecg Pilot Study

FILIPE FERRARI^1^, Henrique C. da Silva^2^, Luiz G. M. Emed^3^, Guilherme D. Dilda^4^, Haroldo C. Aleixo^5^, Márcio Dornelles^6^, Fernando Bassan^7^, Felipe E. F. Guerra^8^, Frederico P. L. Coimbra^9^, Mateus F. Teixeira^10^, Anderson D. da Silveira^11^, Ricardo Stein^1^

(1) Hospital de Clínicas de Porto Alegre (HCPA), Universidade Federal do Rio Grande do Sul (UFRGS), Porto Alegre, RS – Brazil; (2) Universidade do Estado do Pará (UEPA), Belém, PA – Brazil; (3) Hospital Cardiológico Costantini, Curitiba, PR – Brazil; (4) Hospital das Clínicas da Universidade de São Paulo (USP), São Paulo, SP – Brazil; (5) Universidade Federal de Minas Gerais (UFMG), Belo Horizonte, MG – Brazil; (6) Grêmio Foot-Ball Porto-Alegrense (GFPA), Porto Alegre, RS – Brazil; (7) Universidade do Estado do Rio de Janeiro (UERJ), RJ – Brazil; (8) Clínica Biocardio, Natal, RN – Brazil; (9) Hospital de Urgências de Goiânia, Goiânia, GO – Brazil; (10) Clube de Regatas Vasco da Gama, RJ – Brazil; (11) Hospital de Clínicas de Porto Alegre (HCPA), Porto Alegre, RS – Brazil

**Introduction:** The 12-lead ECG is a useful tool for screening cardiac abnormalities in athletes. We aimed to describe physiological ECG findings in young Brazilian football players (YBFP) based on the “2017 International Criteria for Electrocardiographic Interpretation in Athletes”.

**Methods:** Cross-sectional/descriptive study. Intra-group differences were estimated by linear models or binomial and multinomial logistic regressions.

**Results:** 3.490 YBFP from 41 clubs, aged 15–35 years (median: 19 y) were evaluated. 1.668 were Caucasians, 1.154 Mixed-race (MR) and 668 Afro-Brazilians (AB). Prevalence: sinus bradycardia (50%), incomplete RBBB (12%), first-degree AV block (3%), Mobitz type I AV block (0.1%), and increase QRS voltage for left or right ventricular hypertrophy (34% and 15%, respectively). ST-elevation followed by T-wave inversion confined to V1–V4 leads were identified in 2% of AB. Early repolarization (ER) was present in 35% of athletes (AB versus Caucasians and MR: P = 0.002 and P = 0.004, respectively), which was similar to the PR interval (P < 0,001 for both comparisons). There was no difference between Caucasians and MR for ER or PR intervals. For all remaining variables, there was no difference among races.

**Conclusions:** This is the first large study to describe the prevalence of physiological electrocardiographic findings in YBFP. Further studies comparing the frequency of these findings with the prevalence observed in other cohorts are welcome.

112183

Modality: Best Poster – Young Researcher

Category: HEART FAILURE/CARDIOMYOPATHY/TRANSPLANT

D: 14/10/2022    H: 16:10/16:50

L: Área de exposição de pôsteres

## Improving Prognostic Assessment in Heart Failure: The Interplay between Nyha Classification and Cardiopulmonary Exercise Testing

PEDRO HENRIQUE DE BORBA ENGSTER^1^, Pedro Henrique de Borba Engster^1^, André Zimerman^1^, Anderson Donelli da Silveira^1^, Marina S. Borges^1^, Thomas U. Schaan^1^, Gabriel C. de Souza^1^, Giovanni Donelli Costa^1^, Luís Eduardo Rohde^1^

(1) Hospital de Clínicas de Porto Alegre; (2) Universidade Federal do Rio Grande do Sul

**Background:** For patients with heart failure (HF), the validity of the New York Heart Association (NYHA) functional class to assess prognosis may be limited when compared with the objective cardiopulmonary exercise test (CPET).

**Purpose:** To investigate the prognostic value of NYHA classification and CPET parameters.

**Methods:** We included the first CPET of every adult patient with HF who in a tertiary care center in Brazil. NYHA class was determined on the day of CPET or during the prior ambulatory visit. NYHA and Weber classes were stratified into “favorable” (NYHA I or II; Weber A or B) or “poor” (NYHA III or IV; Weber C or D), and subjects with discordant classes were compared in a survival analysis. Primary endpoint was all-cause mortality at 5 years. We used a Cox proportional hazards model to estimate the probability of death in 5 years according to relative peak VO2 and NYHA class, adjusted for age and sex.

**Results:** We included 855 patients, of which 30% (255) were classified as NYHA I, 43% (368) as NYHA II, 24% (202) as NYHA III, and 4% (30) as NYHA IV. Mean age was 56 years (±13), 42% (359) were female, and mean LVEF was 36% (±15). Mean relative peak VO2 ranged from 19.6 (NYHA I) to 14.0 (NYHA IV) ml/kg/min. Patients with poor NYHA and favorable Weber classes displayed similar rates of all-cause mortality as patients with favorable NYHA and poor Weber classes (hazard ratio 1.54 [95% CI 0.88–2.70]. In the multivariable model, both NYHA and relative peak VO2 significantly predicted mortality in 5 years after mutual adjustments (Figure 1). The distinction between NYHA I and II did not, however, improve prognostic assessment.

**Conclusions:** Physician-assigned NYHA class and objective CPET measures provide complementary prognostic information, and NYHA classification may be particularly limited for mild cases of HF. These findings question the use of NYHA as the main determinant to guide HF therapy.


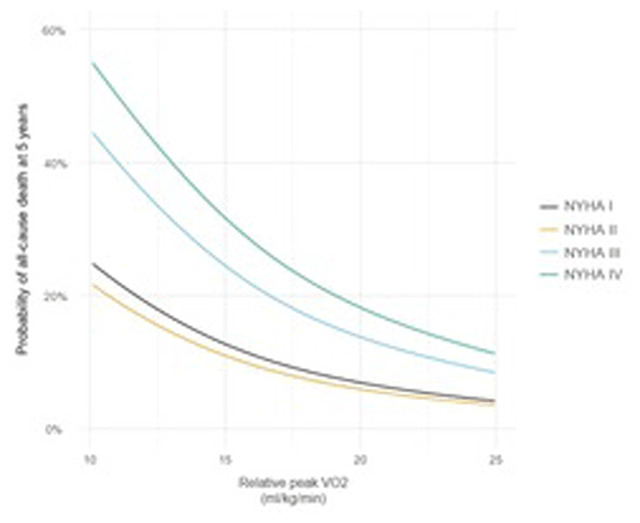


108597

Modality: Best Poster – Scientific Initiation

Category: DIGITAL HEALTH/INNOVATION

D: 13/10/2022    H: 10:00/10:40

L: Área de exposição de pôsteres

## Prediction Factors for Events in the Remote Monitoring of Implantable Electronic Cardiac Devices. Not for all, but Essential to Many!

MARIA EDUARDA QUIDUTE ARRAIS ROCHA^1^, Bruna Sobreira Kubrusly^2^, Fernanda Pimentel Arraes Maia^2^, Rodrigo Carvalho Paiva^3^, Alessia de Alencar Araripe Gurgel^3^, Davi Sales Pereira Gondim^1^, Juvêncio Santos Nobre^2^, Luis Gustavo Bastos Pinho^2^, Ana Gabriela Ponte Farias^2^, Maria Camila Timbó Rocha^3^, Eduardo Augusto Quidute Arrais Rocha^3^, Francisca Tatiana Moreira Pereira^2^

(1) Universidade de Fortaleza (Unifor); (2) Universidade Federal do Ceará (UFC); (3) Centro Universitário Christus (UNICHRISTUS)

**Introduction:** The remote monitoring (RM) of implantable electronic cardiac devices (IECD) has shown advantages in the reduction of morbidity and mortality. The early detection of alterations and the possibility of treatment before the occurrence of symptoms has been a great attractive of this follow-up method. Meanwhile, not all the groups benefit, there are costs associated to these devices and the large number of events may determine an overload to the professionals. This work aimed to identify the predictive factors of larger benefit during the IECD follow-up through RM.

**Methods:** This is a cohort prospective study. The statistical analysis used logistic regression models, with p < 5% for statistical significance. The explanatory variables were selected by a stepwise selection method based on the Akaike Information criterion as a measure to choose the best explaining model. The analyzed variables were age, sex, functional class (FC) ≥ II, ejection fraction (EF), type of IECD, sustained ventricular tachycardia and red alert transmission, indicating important events detections. Three different models were created, with the response variable given by, respectively, elective therapy change (model 1), urgent therapy change (model 2) and whether event detections prevented hospitalizations (model 3).

**Results:** There were 119 patients, with mean age of 72 ± 14 years, 69.8% males. The most common pathologies were ischemic cardiomyopathy 28.6%, dilated cardiomyopathy 22.7% and Chagas disease 6.7%. The groups using a biventricular pacemaker(PM) (75.0%, p = 0.02), reduced EF (76.5%, p = 0.01) FC ≥ Il (75.0%, p < 0.01) had the larger rates of events. The red alert transmission variable was statistically significant in the three models (p-values of, respectively, 0.048;0.007 and 0.048), while the presence of ventricular tachycardia was statistically significant in two models, with p-vales of p = 0.039 (changes in elective therapy) and p = 0.039 (prevented hospitalizations). The FC ≥ II was associated to the urgent therapy change (p = 0.047).

**Conclusion:** Patients with biventricular PM, reduced EF, and more advanced FC had higher event rates in the RM of IECD. The detection of alerts considered by the system as important (red alerts) was associated with changes in immediate, elective conduct and was able to reduce hospitalizations.

109148

Modality: Best Poster – Scientific Initiation

Category: HYPERTENSION/RENAL DENERVATION

D: 13/10/2022    H: 10:00/10:40

L: Área de exposição de pôsteres

## Inflammatory Markers and Chronic Kidney Disease

JOÃO GABRIELL BEZERRA DA SILVA^1^, João Gabriell Bezerra da Silva^1^, Gabriela da Silva Nascimento^1^, Hugo Farah Affonso Alves^1^, Lucca Hiroshi de Sá Kimura^1^, João Gabriel Rega do Nascimento Vallaperde^1^, Vitor de Melo Nolasco^1^, Bianca Botelho Viegas^1^, Carlos Filipe dos Santos Pimenta^1^, Bernardo Chedier^1^, Arthur Fernandes Cortez^1^, Elizabeth Silaid Muxfeldt^1^

(1) Universidade Federal do Rio de Janeiro – Hospital Universitário Clementino Fraga Filho – ProHArt

**Background:** Resistant hypertension (RHT) defined as an uncontrolled blood pressure (BP) despite the use of 3 or more antihypertensives presents a high cardiovascular (CV) morbidity and mortality and high prevalence of chronic kidney disease (CKD). High BP levels and kidney injury appear to be strongly associated with inflammatory biomarkers.

**Objective:** To evaluate the relationship between inflammatory markers and subclinical and established CKD in a large cohort of patients with RHT.

**Methods:** Cross-sectional study that evaluated 423 resistant hypertensives (30.5% male, mean age 64.0 ± 10.8 years) submitted to renal function assessment (albuminuria dosage and evaluation of glomerular filtration rate calculated from the CKD-EPI formula) and dosage of inflammatory markers: TNF-alpha, MCP-1, E-selectin and PAI-1. Socio-demographic characteristics, anthropometric measurements and CV risk factors were recorded. We considered subclinical CKD those patients with moderately high albuminuria (30–300 mg/g) and/or GFR between 30 and 60 ml/min/1.73 m^2^ and established CKD those who presented albuminuria >300 mg/g and/or TFG < 30 ml/min/1.73 m^2^. Variance analysis compared serum levels of the 4 inflammatory markers and bivariate analysis compared patients with and without subclinical and established chronic kidney disease.

**Results:** The prevalence of established CKD was 7.3% (31 patients) and subclinical CKD was 47% (187 patients). Patients with subclinical CKD were older and with higher arterial stiffness (higher pulse wave velocity). TNF-alpha (7.1 [4.4–8.6] vs 51, [3.2–7.5]) and MCP-1(284 [220–379] vs 260 [185–359] were significant higher in this group of patients. When we analyzed patients with established CKD, we observed that they have higher BP levels and that TNF-alpha values (7.8 [5.6–14.0] vs 5.6 [3.5–8.3]) and E-selectin (54.4[41.2–61.3] vs. 47.8 [32.0–65.3]) were significantly higher in this group.

**Conclusion:** Among the inflammatory markers evaluated, which was most strongly correlated with subclinical CKD were TNF-alpha and MCP-1, while those with established CKD have higher TNF-alpha and E-selectin levels, possibly pointing out that the MCP-1 is an earlier marker of kidney injury.

111855

Modality: Best Poster – Scientific Initiation

Category: EPIDEMIOLOGY AND HEALTH POLICIES/GLOBAL HEALTH

D: 13/10/2022    H: 10:00/10:40

L: Área de exposição de pôsteres

## Mortality from Cardiovascular Diseases and Policies to Reduce Salt Consumption in South American Countries: An Ecological Study

LUIZ FELIPE FAÇANHA RAMOS^1^, Hildeman Dias da Costa^2^, Reny Wane Vieira dos Santos^1^

(1) Universidade Federal do Amapá; (2) Universidade Federal de Rondônia

**Introduction:** Mortality from cardiovascular diseases (CVD) is aggravated by arterial hypertension, as a consequence of high consumption of table salt. Worldwide salt intake is above the 5 grams (2 grams of sodium) per day recommended by the World Health Organization (WHO), and its consumption varies from 8.5 to 15 grams per person.

**Objective:** To relate CVD mortality with policies to reduce salt/sodium consumption in South American countries in the year 2019.

**Methodology:** This is an analytical ecological study of geographic distribution with secondary data from the Information Platform in Health for the Americas (PLISA), from the Pan American Health Organization, of the year 2019, with CVD mortality indicators and policies to reduce salt/sodium consumption in South American countries. In addition, mortality rates (MT) were standardized per 100,000 inhabitants, with a confidence interval of 95%, classified by quintile. These data were tabulated in the Microsoft Excel software and the variables were analyzed in the GNU SPPP software version 1.5.3, using the Pearson Correlation Test, with a significance level of 5%.

**Results:** In 2019, in South America, the highest CVD mortality rates occurred in Guyana (TM = 448.3; 95% CI 363.3–548.5; Q5) and Suriname (TM = 270.8; 95% CI 212.1–337.6; Q5), on the other hand, the lowest mortality occurred in Peru (TM = 77.8; 95% CI 56.9–103.9; Q1) and in Chile (TM = 95. 5; 95% CI 74.4–118.9; Q1). It is noteworthy that the countries that had the highest mortality are the nations that did not have any (n = 0) initiative to reduce salt/sodium consumption in 2019, however, the countries with the lowest mortality were those that developed 3 policies to reduce this excessive consumption. It is evident that Brazil developed 2 reduction policies, but had a high number of deaths from CVD (TM = 217.1; 95% CI 189.9–239.9; Q3). Analyzing the bivariate correlation of the two variables, a negative Pearson coefficient was obtained (r = –0.59; p = 0.043).

**Conclusion:** Countries that developed more initiatives to reduce excessive salt consumption had significantly lower mortality rates from CVD, showing the importance of reducing this consumption to avoid hypertensive complications that lead to death. Thus, the inversely proportional relationship between the number of these policies and the number of deaths from CVD is confirmed.

111704

Modality: Best Poster – Scientific Initiation

Category: CARDIOVASCULAR PHARMACOLOGY

D: 13/10/2022    H: 10:00/10:40

L: Área de exposição de pôsteres

## Cardiovascular Outcomes in Long-Term with Prasugrel Versus Clopidogrel in Acute Coronary Syndromes: Real-World and Paired Analysis for Propensity Score

PIETRA ARISSA COELHO MATSUNAGA^1^, Evellyn Cristiny Pereira Marinho Bezerra^1^, Júlia Fonseca da Silva Saad^1^, Lara da Silva Soledade^1^, Ana Cláudia Cavalcante Nogueira^3^, Alexandre Anderson de Souza Munhoz Soares^3^, Gustavo Alexim^2^, Luiz Sérgio Fernandes de Carvalho^5^, Renato Barros^4^

(1) Universidade Católica de Brasília UCB; (2) Hospital de Base do Distrito Federal HB-DF; (3) Instituto Aramari Apo; (4) Hospital Regional de Sobradinho do Distrito Federal HRS-DF; (5) Clarity Healthcare Intelligence

**Introduction:** Prasugrel is an thienopyridine antiplatelet agent, which can be an important alternative to clopidogrel in patients with Acute Coronary Syndrome (ACS) treated through percutaneous coronary intervention. In spite of the role of clopidogrel in these conditions, there are no long-term real-world studies comparing clopidogrel versus prasugrel.

**Objective:** To evaluate the long-term risk of major adverse cardiovascular events (MACE) in patients with ACS who are making use of prasugrel or clopidogrel.

**Methods:** This study is a retrospective analysis of all the individuals that were admitted with the principal diagnosis of acute coronary syndrome within the period of 2013 to 2015 and were submitted to cardiac catheterization in public hospitals of Brasilia, FD, Brazil. The total group was divided into two: the first group being of patients who took clopidogrel and the second group formed by patients on prasugrel. The primary clinical outcome was comprised by cardiovascular (CV) deaths and recurrent ACS (MACE). Multivariate analyses on 16 covariates were carried and followed by a Propensity Score Matching analysis (PSM).

**Results:** A total of 2,491 patients with a principal diagnosis of ACS were included (1,616 with clopidogrel and 875 with prasugrel) and monitored for a median of 6.5 (IQR 1.9) years with 742 primary events. Clopidogrel was associated with high rates of MACE in 12 months (20.1% vs 11.8%, p < 0.001, 428 MACE) and 24 months (23.8% vs 17.7%, p < 0.001, 540 MACE) compared to prasugrel, but the curves equalized at 36 months (25.9% vs 26.6%, p = 0.740) and remained equalized until median follow-up time. In multivariate analysis, prasugrel showed a hazard ratio (HR) 0.61 (95% CI 0.47–0.78 at 12 months), 0.78 (95% CI 0.63–0.96, p < 0.001 at 24 months) and 0.94 (95% CI 0.78–1.1 at 36 months). The results were maintained in the PSM analysis. The mean time on prasugrel in this cohort was 22.1 (IQR 5.9) months.

**Conclusion:** Stemming from these findings, despite the already known role that prasugrel has in reducing the risk of MACE in ACS, these results do not seem to be sustained in the long term. The data reveal that after the interruption of drug use there was an increase in the amount of major adverse cardiovascular events in the second and third year of monitored patients in the prasugrel group. Therefore, the benefit of using prasugrel is neutralized over time.

111863

Modality: Best Poster – Scientific Initiation

Category: CARDIORESPIRATORY PHYSIOLOGY/BASIC SCIENCE

D: 13/10/2022    H: 10:00/10:40

L: Área de exposição de pôsteres

## Is a Non-Invasive Method for ECG And HRV Assessment Capable to Detect Ventricular Dysfunction and Sympathetic Denervation?

RAFAEL DIAS DE BRITO OLIVEIRA^1^, Denise Mayumi Tanaka^2^, Thayrine Rosa Damasceno^1^, Camila Godoy Fabrício^2^, Alessandra Arantes Resende^2^, Eduardo Elias Vieira de Carvalho^2^, Dawit Albieiro Pinheiro Gonçalves^1^, Enrico de Francisco Magnani^1^, Mariana Duarte de Souza^1^, Marcus Vinícius Simões^2^, Luciano Fonseca Lemos de Oliveira^1^

(1) Universidade Federal de Minas Gerais – UFMG; (2) Faculdade de Medicina de Ribeirão Preto – FMRP/USP

**Introduction:** Denervation of atrial parasympathetic and ventricular sympathetic fibers is common in chronic Chagas cardiomyopathy (CC) and may be associated with increased ventricular arrhythmias and sudden death. However, it is unclear if the heart rate variability (HRV) present potential to detect these alterations.

**Objective:** We aimed to evaluate the applicability of a low-cost, non-invasive, and unrestricted method to identify animals with ventricular sympathetic denervation and severe cardiac dysfunction in an experimental model of CC in hamsters.

**Methods:** In the study’s first phase, 10 control hamsters were used to validate the non-invasive electrocardiogram (ECG) acquisition system using a vest. Signal quality and heart rate variation were evaluated in anesthetized and dobutamine-infused animals. The vest was also compared to the acquisition of invasive electrodes in conscious animals. In the second phase, HRV was analyzed in an experimental model of CC (CC; n = 6) and its respective controls (CT; n = 6). Myocardial function and morphology were evaluated by two-dimensional echocardiography in dedicated equipment (VEVO2100). The intensity of ventricular fibrosis and/or inflammation was assessed by the extent of perfusion defects at rest using high-resolution myocardial perfusion scintigraphy, acquired by locally constructed equipment and validated system of small animal images.

**Results:** The ECG acquired by the vest showed excellent signal quality, detecting both the increase in HR during dobutamine infusion and HRV in awake animals. In CC animals, left ventricular ejection fraction (LVEF) correlated with indicators of parasympathetic activity (LF; r = 0.72, p = 0.01) and sympathetic activity (HF; r = –0.72, p = 0.01), as well as with the LF/HF ratio (r = 0.70, p = 0.01). In addition, a significant correlation was observed between left ventricular perfusion defect (LVPD) with LF (r = 0.72, p < 0.00), HF (r = –0.72, p < 0.00), and LF/HF (r = 0.71, p = 0.01). A significant correlation was also observed between left ventricular diastolic diameter (LVDD) and LF (r = 0.59, p = 0.04) and HF (r = –0.59, p = 0.04).

**Conclusion:** The low-cost, non-invasive and unrestricted vest method effectively detected ECG signals from animals at rest, during dobutamine infusion and in motion. The vest was also able to detect autonomic changes resulting from ventricular dysfunction, and was sensitive in identifying sympathetic denervation resulting from myocardial fibrosis and/or inflammation.

107748

Modality: E-Poster Researcher – Non-case Report

Category: CARDIOVASCULAR INTENSIVE CARE/CARDIOVASCULAR EMERGENCIES

## Focus Echocardiography for Prediction of Adverse Intrahospital Outcomes of Acute Pulmonary Thromboembolism in COVID-19 Pandemic – Single Center Experience

IRENA MITEVSKA^1^, Elena Grueva^1^, Irina Kotlar^1^, Emilija Lazarova^1^, Marijan Bosevski^1^

(1) University Cardiology Clinic

**Introduction:** In patients with pulmonary embolism (PE), assessment of right ventricular (RV) function on transthoracic echocardiography has been shown to be an independent predictor of 30-day mortality.

**Objective:** We wanted to assess how echocardiographic assessment for RV function compares with other PE prognostic parameters used in ICU during the COVID-19 pandemic.

**Methods:** This is retrospective cohort study of patients with confirmed PE hospitalized in Intensive Care Unit between January and December 2020. All unstable patients underwent 2-dimensional FOCUS echocardiography before CT pulmonary angiography (CTPA). FOCUS included assessment for right ventricular dilation, McConnell’s sign, septal flattening, tricuspid regurgitation, and tricuspid annular plane systolic excursion (TAPSE). Adverse outcomes were defined as shock, respiratory failure requiring intubation, death, or major bleeding. We have evaluated FOCUS markers of PE diagnosis and prognosis, as well as independent ICU mortality predictors.

**Results:** We studied 47 patients, with mean age 58.6 ± 19.4 years. Eight patients (17%) had massive PE (3 patients positive for COVID-19) and 39 (83%) had sub massive PE confirmed by CTPA. Twelve patients (25,5%) were tested for COVID 19 with PCR assay, and 3 come positive (12.5%). Eight patients were high risk with shock (17%), 29 were intermediate high risk (61.7%) and 10 patients were intermediate low risk (21.3%). Abnormal RV function with TAPSE <17 was found in 32 patients (68%). Five high risk patients died within 72 hours, resulting in an overall ICU mortality rate of 10,6% and 62.5% for patients with cardiogenic shock. We found FOCUS echocardiography 93% (95% confidence interval [CI] = 72% to 98%) sensitive and 69% specific (95% CI = 55% to 81%) for PE. Mean TAPSE values in patients who died were 13+–/2, comparing to 18+/–2, p < 0.001 in survivals. Multivariate logistic regression analysis showed thrombolytic therapy OR 2.145 (95% CI: 1.105–4,512), TAPSE <14 OR 2.893 (95% CI: 0.932–3.241), and acute renal failure OR 1.821 (95% CI: 1.105–4.762) as an independent mortality predictors.

**Conclusions:** RV dysfunction is significant predictor of intrahospital adverse PE outcomes. A negative FOCUS examination significantly lowers the likelihood of PE in most unstable patients. Focus echocardiography is very useful tool for fast assessment of RV function, risk stratification and PE management decision in unstable patients during the COVID-19 pandemic.

107749

Modality: E-Poster Researcher – Non-case Report

Category: CARDIOLOGY OF SPORTS, EXERCISE, ERGOMETRY AND CARDIOVASCULAR REHABILITATION

## Effects of a Mountain Ultramarathon on Biochemical Markers of Myocardial Damage and Ventricular Dysfunction in Young and Inexperienced Runners, Unaccustomed to Altitude

ROQUE DANIEL GONZÁLEZ^1^, Cayetano Bellomio^2^, Mercedes Ríos^2^, Carlos Ramiro Castellanos^1^, Manuel Parajón Viscido^2^, Roque Daniel González^1^

(1) Instituto de Cardiología Tucumán; (2) Sociedad Tucumana de Medicina del Deporte

It has been reported that ultramarathons can elevate plasmatic levels of troponins and natriuretic peptides. The mechanisms and significance of these findings is debated. The objective of this study was to investigate the behavior of these markers before and after a mountain ultramarathon in young and inexperienced runners, unaccustomed to altitude. 20 runners (36.7 ± 6.3 years) were evaluated (6 females). They reported 4.0 ± 3.4 years competing in ultramarathon with a training load of 43.7 ± 16.8 km/week. The plasmatic values of troponin T (Trop-T HS) and N-terminal pro-brain natriuretic peptide (NT Pro-BNP) were determined before, immediately after the race and 24 hours later. The race was completed in 637.9 ± 197.5 minutes, and 55.1 ± 7.3 kilometers were covered. Both markers raised significantly after the competition. There was a statistically significant difference between the values of both determinations before the competition and at the end of the race (p < 0.0001) and between these and those after 24 hours (p < 0, 0001). At the end of the run, 95% of the sample presented NT Pro-BNP values above the diagnostic cut-off point of heart failure. All athletes presented values above the upper reference limit for Trop-T HS and 80% exceeded the diagnostic cut-off value for acute myocardial infarction. Troponin levels showed an inverse relation with weekly training (p = 0.04). There was a significant association between relative intensity of exercise and NT Pro-BNP levels after the competition (p = 0.001), where the group that completed the race with a heart rate higher than 80% of its maximum, significantly raised NT Pro-BNP.

**Conclusions:** Trop-T HS and NT Pro-BNP significantly raised after the race. The extreme conditions and some characteristics of the athletes (poor habituation to altitude, short experience and relatively low volume of training) could justify these results. The clinical significance and mechanisms of these findings are still unknown, but the uniformity and brevity of the responses, as well the absence of clinical manifestations, suggest that they could represent an adaptive phenomenon.


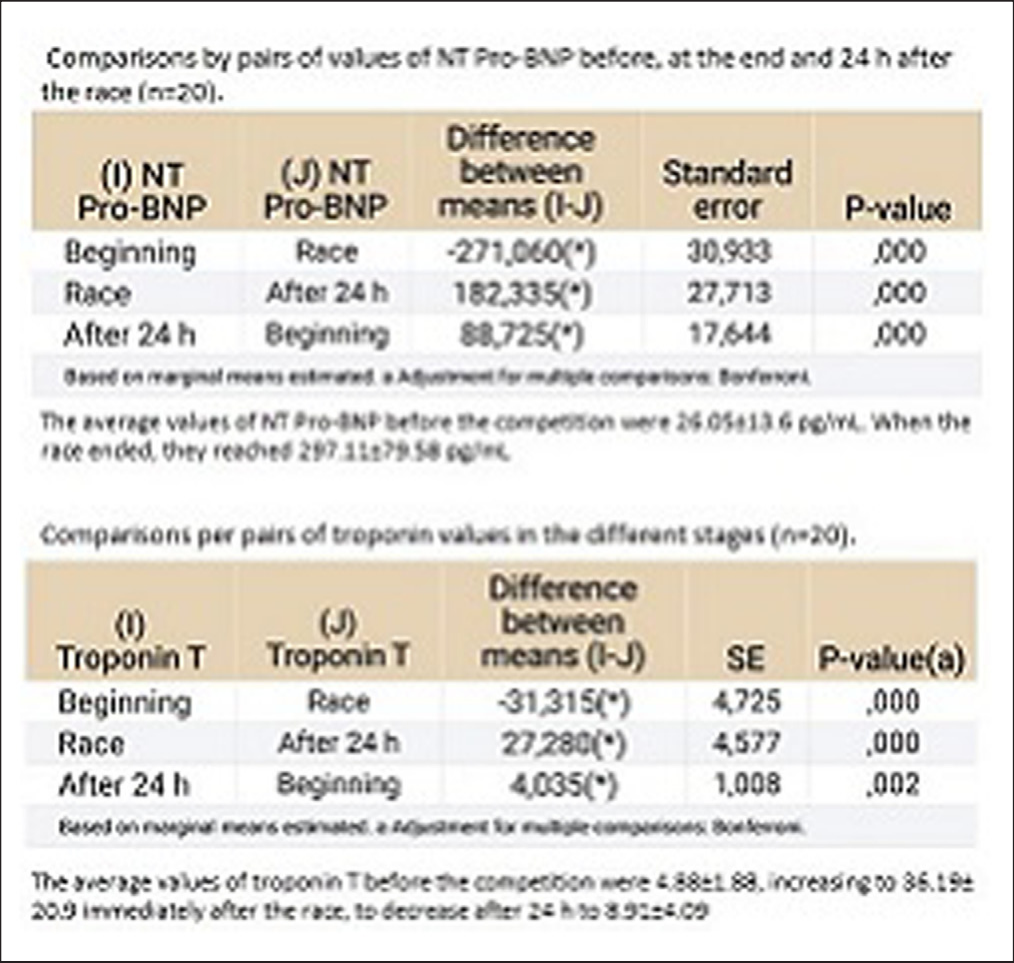


107765

Modality: E-Poster Researcher – Non-case Report

Category: ANTICOAGULATION

## Anticoagulation Control, Outcomes and Associated Factors in Patients Receiving Warfarin in Long-Term Care in Africa: A Systematic Review

TAMRAT ASSEFA^1^, Gobezie Temesgen^1^, Dejuma Yadeta^2^, Legese Chelkaba^1^, Teferi Gedif^1^

(1) School of Pharmacy, College of Health Sciences, Addis Ababa University, Addis Ababa, Ethiopia; (2) School of Medicine, College of Health Sciences, Addis Ababa University, Addis Ababa, Ethiopia

**Background:** Oral anticoagulation therapy with warfarin requires frequent monitoring level of anticoagulation by the international normalized ratio (INR).

**Aims:** This systematic review aimed to systematically summarize anticoagulation control, treatment outcomes, and associated factors in long-term patients receiving warfarin in Africa.

**Methods:** The literature search was conducted in PubMed, Cochrane Library, African Journal of Online databases, Google Scholar, and Google. An advanced search strategy was computed to retrieve relevant studies related to anticoagulation control and outcomes. Duplication, title and abstract screening, and full-text assessment were conducted in Covidence software. Study quality was assessed using the Joanna Briggs Institute Critical appraisal quality assessment tool. The systematic review is registered in PROSPERO (CRD42021260772) and performed based on the PRISMA guideline.

**Results:** Out of 298 identified articles, 18 articles were eligible for the final review and analysis. The mean of 39.4 ± 8.4% time in therapeutic range (TTR) (29.4% to 57.3%), 36.7 ± 11.5% TTR (range 25.2–49.7%) and 46% TTR (43.5–48.5%) was computed from studies that determined TTR by Rosendaal, direct and cross-section-of-the-files methods, respectively. The lowest percentage of TTR was 13.7%, while the highest was 57.3% was observed in this review. The highest percentage of patients (32.25%) who had TTR ≥ 65% was reported in Tunisia, but the lowest percentages were in Namibia (10%, TTR ≥ 65%) and Kenya (10.4%, TTR ≥ 70%). Generally, 10.4–32.3% of study participants achieved desired optimal anticoagulation level. Regarding secondary outcomes, 1.6–7.5% and 0.006–59% of patients experienced thromboembolic complications and bleeding events, respectively. The presence of chronic comorbidities, taking more than two drugs, and the presence of medications that potentially interact with warfarin were the frequently reported predictors of poor anticoagulation therapy.

**Conclusion:** Oral anticoagulation control was suboptimal in patients taking warfarin as evidenced by low TTR in Africa. Therefore, there is an urgent need for further improving oral anticoagulation management service.

107767

Modality: E-Poster Researcher – Non-case Report

Category: COVID-19 AND CARDIOVASCULAR SYSTEM

## Thromboembolism and its Management in Hospitalized COVID-19 Patients using Modified Caprini Risk Score: A Single Center Experience in Ethiopia

TAMRAT ASSEFA^1^, Ashenafi Teklu^1^, Alfoalem Araba^1^

(1) School of Pharmacy, College of Health Sciences, Addis Ababa University, Addis Ababa, Ethiopia

**Background:** Reports indicated association of COVID-19 with coagulation dysfunction and venous thromboembolism (VTE) prevalence was in the range of 20–40%. All patients with COVID-19 who are hospitalized should receive pharmacologic prophylaxis unless they have contraindications.

**Aims:** To assess VTE risk, incidence, and its management in patients with COVID-19 admitted to Tikur Anbessa Specialized Hospital (TASH).

**Methods:** A retrospective study was conducted among 146 COVID-19 patients admitted to TASH. A pre-tested data abstraction format was used to collect patients’ clinical information and VTE risk by using the modified Caprini Risk Score in COVID-19. We used Statistical Package for the Social Sciences (SPSS) version 26 for data analysis. Descriptive statistics were used to summarize the findings and binary logistic regression analysis to assess the association between the variables of interest.

**Results:** Out of 146 patients, 57.53% were males and the mean age was 45.56 ± 18.17 years. All patients were at risk of developing VTE. The most often observed VTE risk factors were being COVID-19 symptomatic (88.40%), serious lung diseases (56.2%), and age >40 years (52.10%). The incidence of VTE was 23 (15.75%) and majorly (91.3%) occurred in highest VTE risk (≥5 scores) patients, >40 years patients, and in patients with severe COVID-19 symptoms (100%). However, parenteral thromboprophylaxis was prescribed only for 98 (67.12%) patients. Out of 23 patients who developed VTE, 15 didn’t receive prophylaxis, and the remaining 8 received thromboprophylaxis. Unfractionated heparin (UFH) was the most widely used prophylaxis. For patients who developed VTE, the majority of them (86.96%) were given a therapeutic dose of UFH.

**Conclusion(s):** All patients with COVID-19 were at risk of developing VTE. Only one-third received thromboprophylaxis. The incidence of VTE was high and majorly occurred in patients that didn’t receive prophylaxis.

107796

Modality: E-Poster Researcher – Non-case Report

Category: CARDIAC ARRHYTHMIAS/ELECTROPHYSIOLOGY/ELECTROCARDIOGRAPHY

## Cardioneuroablation Controlled by Extracardiac Vagal Stimulation for Treatment of Carotid Sinus Syndrome

JUAN CARLOS ZERPA ACOSTA^1^, Jose Carlos Pachon Mateos^2^, Enrique Indalecio Pachon Mateos^1^, Carlos Thiene Cunha Pachon^2^, Juan Carlos Pachon Mateos^1^, Tasso Julio Lobo^1^, Tomar Guillermo Santillana Pena^1^, Felipe Augusto Ortencio^1^, Ricardo Cardneiro Amarante^1^, Maria Zelia Cunha Pachon^2^

(1) Hospital do Coração – São Paulo; (2) SEMAP – Serviço de Eletrofisiologia, Marca-passo, Desfibriladores, Ressincronizadores e Arritmias Prof. Dr. José Carlos Pachón M.

**Background:** Carotid sinus syndrome (CSS) is a rare condition caused by sudden autonomic nervous system imbalance and Vagal overactivity that may lead to sinus arrest, AV block and syncope with severe symptoms commonly treated with pacemaker (PM). Since 2005 the Cardioneuroablation (CNA) procedure has been applied to aim vagal denervation through endocardial radiofrequency removing the cardio inhibition by ablation.

**Objective:** To study the outcome of CNA controlled by extracardiac vagal stimulation (ECVS) in symptomatic functional CSS.

**Methods:** Prospective, controlled study of 8 patients with symptomatic CSS, positive carotid sinus massage (CSM) and normal atropine response. Biatrial RF-ablation of the neuro endo-myocardial interface guided by filtered endocardial electrograms, fractionation, and 3D anatomical mapping, identifying type-1 AF-Nests (AFN) related to the 4 main ganglionated plexuses with FlexAbility Abbott irrigated catheter(30W/43°C/30s). ECVS(5s) at the right and left jugular foramen and CSM was performed before, during, and at the end of the ablation, to guide the level of denervation defining the higher CNA effect and end of the procedure, analyzing induced pauses and AVB. The endpoint was to eliminate complete pauses and AVB caused by ECVS and CSM.

**Results:** Patients mean age was 58,6 (±11,8). Pre-CNA ECVS and CSM response were sinus pauses (5,4 ± 1,3secs) and transient AVB 3/8. Post-CNA, ECVS, CSM and Atropine responses were completely abolished in 8/100%. There was observed immediate sustained HR increase (59.6 ± 12,7/81,7 ± 5,3bpm) as acute result. In a follow up of 28,1 (±11,8) months there were no syncope recurrence. 1 patient had positive CSM at 6 months FU, without spontaneous syncope and had PM implanted.

**Conclusion:** Bi atrial CNA vagal denervation based on AFN/GP ablation guided by abolishment of the vagal effect by ECVS and CSM response was effective for eliminating syncope recurrence in all the patients with CSS.


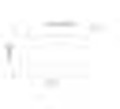


107798

Modality: E-Poster Researcher – Non-case Report

Category: ATHEROSCLEROSIS/CARDIOVASCULAR RISK FACTORS/CARDIOVASCULAR PREVENTION

## Influence of Severe Chronic Periodontitis on Left Ventricular Function, Inflammatory Markers and Microvascular Reactivity in Hypertensive and Normotensive Patients

RITA DE CÁSSIA CASTELLI DA ROCHA^1^, Sérgio Emanuel Kaiser^1^, Márcia Regina Simas Torres Klein^1^, Cyro José Morais Martins^3^, Ricardo Guimarães Fisher^2^, Maria de Lourdes Guimarães Rodrigues^1^, Débora Valença^1^, Carolina Vicente Teófilo^2^, Carolina de Castelli da Rocha Carneiro^3^

(1) CLINEX – UERJ; (2) FACULDADE ODONTOLOGIA- UERJ; (3) HUPE

Epidemiological studies suggest that chronic periodontitis (PD) associates with an increased risk of developing cardiovascular disease, whereas both share a link through inflammatory mediators. Arterial hypertension is a major risk factor for cardiovascular diseases, and it is believed that periodontal disease may favour its development. The relationship between PD and inflammation has been studied through biomarkers and assessment of endothelial function. Several conditions associated with subclinical inflammation seem to promote subtle changes in left ventricular systolic function, that can be detected by speckle tracking technology coupled with echocardiography. This study sought to explore, among hypertensive (HT) and normotensive (NT) individuals, the possible associations between severe chronic PD, inflammatory biomarkers, carotid intimal-medial thickness, and left ventricular systolic and diastolic function. The study consisted of a cross-sectional analysis with control for potential confounding factors, of 88 non-smoking, non-diabetic individuals of both sexes, among which, there were 50 AH under pharmacological treatment, and 38 NT. All underwent dental evaluation with a probe bag protocol aimed at identifying severe periodontitis, as well as laboratory tests (lipid profile, glucose, high-sensitivity C-reactive protei (HsCRP), interleukin-6 and tumor necrosis. They were broken down into 4 groups:AH/no PD; G3(N = 23):NT+PD; G4(N = 15):NT/no PD. Results: Mean age was 49.94 ± 7.32 (Range 35–60) years, 43% women; 39% non-white. Blood pressure (BP) mmHg: G1:136/82; G2:136/79; G3:125/79; G4:131/76 (p < 0.004 for systolic BP G3 vs G1). Mean BP was significantly higher in hypertensives (p < 0.01) regardless of severe PD. HsCRP (mg/dL) was significantly lower in G4 (p < 0.003); Intimal-medial thickness (IMT) was not significantly different among groups. Parameters of diastolic function did not differ significantly among groups regardless of the presence or absence of hypertension or PD. Global longitudinal strain (GLS) was also similar among groups; G1:–21.11 ± 2.24 G2:–21.33 ± 2.67 G3:–20.80 ± 3.17 G4:–21 ± 2.82 (p = 0.94). There were no significant differences in proportion of GLS ≥ –18 among the 4 groups. Since all HT patients were treated, no correlation systolic or diastolic BP and GLS was found. In conclusion, severe chronic PD was not independently associated with IMT, diastolic function nor with subclinical left ventricular dysfunction in the sample studied.

107816

Modality: E-Poster Researcher – Non-case Report

Category: CARDIORESPIRATORY PHYSIOLOGY/BASIC SCIENCE

## Methotrexate Associated with Lipid Core Nanoparticles Modulates Microrna-1 Expression that Prevents Left Ventricular Cardiomyopathy in Mice with Marfan Syndrome

MARIA CAROLINA GUIDO^1^, Priscila Oliveira de Carvalho^1^, Natalia de Menezes Lopes^1^, Aline de Oliveira Silva^1^, Leonardo Jensen^1^, Roberto Kalil-Filho^1^, Lygia da Veiga Pereira^2^, Francisco Rafael Martins Laurindo^1^, Raul Cavalcante Maranhão^1^

(1) Instituto do Coração – HCFMUSP; (2) Instituto de Biociências – USP

**Introduction:** Patients with Marfan syndrome (MFS), a disease caused by mutation of the fibrillin-1 gene, are vulnerable to left ventricular (LV) cardiomyopathy. MicroRNA-1 (miR-1) has involved in cardiomyopathy, but the role of miR-1 in MFS is unknown. Methotrexate (MTX), a chemotherapeutic agent, when associated with lipid nanoparticles (LDE) that mimic LDL composition, markedly increased cell uptake, which enhances the pharmacologic properties and diminishes the toxicity of the drug. LDEMTX showed powerful anti-inflammatory and anti-proliferative effects on rheumatoid arthritis and atherosclerosis rabbit models. In rats submitted to acute myocardial infarction, LDEMTX contributed to the reduction of LV remodeling.

**Objective:** To investigate the effect of LDEMTX on miR-1 expression in the development of LV cardiomyopathy in MFS mice.

**Methods:** MFS and wild-type (WT) mice were allocated to the following groups: WT and MFS, both untreated; MFS-MTX, MFS treated with commercial MTX; MFS-LDEMTX, MFS treated with LDE-MTX. The treatment occurred weekly at a dose of 1 mg/kg ip, between the 3rd and 6th month of life. After 12 weeks, the animals were submitted to echocardiography, morphometry and, miR-1 and proteins expressions of LV.

**Results:** LDEMTX did not improve the LV diastolic dysfunction in MFS mice. LDEMTX reduced LV hypertrophy by decreasing the interventricular septum and posterior wall thickness and the myocytes diameter. Collagen volume fraction in subendocardial, interstitial and papillary muscle areas was also diminished. The protein expression of caspase 3 and hypoxia-inducible factor 2α were lower, whereas VEGF and angiopoietin 1/2 were higher in LDEMTX group. Compared to MFS and MFS-MTX groups, MFS-LDEMTX increased miR-1 expression. The expression of miR-1 had a negative correlation with pro-apoptotic (r2 = 0.48; p < 0.01), cellular hypoxia (r2 = 0.45; p < 0.01), fibrosis (r2 = 0.40; p < 0.01) and myocyte hypertrophy (r2 = 0.24; p < 0.05) markers; while had positive correlation with pro-angiogenic marker (r2 = 0.50; p < 0.01).

**Conclusion:** Although LDEMTX had no effect on diastolic dysfunction, LDEMTX treatment improved cellular changes in LV cardiomyopathy in MFS mice. LDEMTX had beneficial effects on miR-1 modulation by increasing angiogenesis and decreasing apoptosis, cell hypoxia, fibrosis and hypertrophy of LV. This is the first study that shows therapeutic efficacy in the cardiomyopathy of MFS mice, under the modulation of miR-1.

107826

Modality: E-Poster Researcher – Non-case Report

Category: DYSLIPIDEMIA

## Paraoxonase 1 (PON1) Activity in Patients with Familial Hypercholesterolemia and Subclinical Coronary Artery Disease

FABIANA CORDEIRO JULIANI^1^, Fátima Rodrigues Freitas^1^, Viviane Zorzanelli Rocha^1^, Márcio Hiroshi Miname^1^, Ana Paula Chacra Marte^1^, Wilson Salgado^1^, Raul Dias Santos^1^, Raul Cavalcante Maranhão^1^

(1) Heart Institute of the Medical School, University of Sao Paulo – Brazil

**Introduction:** Familial hypercholesterolemia (FH) is a monogenic disease characterized by elevated LDL-C from birth and increased risk of premature coronary artery disease (CAD). Accumulation of LDL-C in plasma may impair anti-atherosclerotic actions attributed to HDL. In addition to HDL particle size, metabolic aspects related to the antiatherosclerotic protection of HDL should be considered beyond simple determination of HDL-C, such as antioxidant action of HDL-associated paraoxonase 1 (PON1) that protects LDL and other lipoproteins from oxidative stress.

**Aim:** To assess whether PON1 activity and HDL particle diameter are altered in individuals with FH with or without subclinical CAD.

**Methods:** Twenty patients with genetic diagnosis of heterozygous FH and subclinical CAD documented by coronary CT angiography and 20 patients with heterozygous FH without subclinical CAD, matched by age, sex and BMI were enrolled. After 30-day interruption of lipid-lowering drugs, plasma lipids and apolipoproteins (apo) B and A-I were determined by commercial kits. LDL-C was calculated using the Friedewald formula. PON1 activity was determined by spectrophotometry. HDL diameter was determined by laser light scattering method.

**Results:** LDL-C, non-HDL-C and apo B were equally high. There were no differences in triglycerides, HDL-C or apo A-I. PON1 activity and HDL diameter also did not differ between the two groups.

**Conclusion:** Among patients with FH, the presence of subclinical CAD was not associated with significant differences in plasma lipids and apo’s and in either HDL size or activity of PON1. In the quest for factors not related to high LDL-C that can facilitate or protect against the development of atherosclerosis in FH patients, other defects of HDL functions should be investigated.


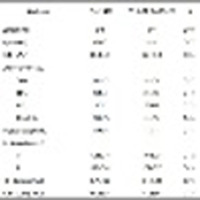


107879

Modality: E-Poster Researcher – Non-case Report

Category: CARDIOVASCULAR IMAGING

## Analysis of Regional Speckle Tracking and Tissue Doppler Strain in Identification of Coronary Arteries Responsible for Induction of Ischemia During 4D Stress-Echo with Adenosine Triphosphate in Patients with Stable Coronary Artery Disease

NIKOLAI NELASOV^1^, Dmitry Safonov^1^, Nikolai Nelasov^1^, Alexander Zubov^2^, Maria Ovrulova^1^, Maxim Morgunov^1^, Roman Sidorov^1^, Olga Eroshenko^1^, Anna Nechaeva^1^, Georgiy Chudinov^1^, Anna Bazilevich^1^, Elizaveta Palenaja^2^

(1) Rostov State Medical University, Russian Federation; (2) Donetsk National Medical University, Ukraine

**Introduction:** Analysis of left ventricular myocardial strain parameters during stress-Echo showed promising results in detection transient myocardial ischemia. Various echocardiographic modalities of strain analysis can be used: speckle tracking longitudinal (Ls), circumferential (Cs), radial (Rs), area (As) strain and tissue Doppler longitudinal strain (TDI Ls). However, it is still debatable which of these modalities better detects stress-induced ischemia in regions of vascularization of obstructed coronary arteries. Objectives. The objectives of this study were: 1) to explore which of the methods (Ls, Cs, Rs, As or TDI Ls) can more frequently reveal regional transitory deformation disorders and identify coronary arteries responsible for induction of ischemia in patients with stable obstructive coronary artery disease in dynamics of left ventricular 4D stress-Echo with adenosine triphosphate; 2) to compare the efficacy of conventional method of visual evaluation of local contractility and above mentioned strain modalities in detection of ischemia-associated obstructed coronary arteries.

**Methods:** 32 patients (male 29, mean age 58,2 years (95% CI: 58,2–60,2 years) with stable angiographically documented coronary artery disease (single vessel obstructive disease was detected in 6, multi vessel – in 26 cases) underwent left ventricular 4D stress-Echo with adenosine triphosphate. Criterion of strain impairment during stress-test was an increase in values by 5% in at least 2 adjacent myocardial segments using Ls, Cs and TDI Ls methods, by 7% using As method, and decrease in strain value by 10% using Ra modality.

**Results:** During stress-test Ls revealed appearance/increase of strain disorders in the regions of vascularization of obstructed coronary arteries in 71.9%, Cs – in 65.6%, Rs – in 59.4%, As – in 75.0% and TDI Ls – in 62.5% of patients, while visual assessment of LV myocardial contractility revealed impairment of contractility in the territories of vascularization of obstructed coronary arteries only in 31.2% of patients (differences with strain methods in all cases were statistically significant).

**Conclusion:** All methods of regional strain analysis (Ls, Cs, Rs, As, TDI Ls) can identify coronary arteries responsible for induction of ischemia during 4D stress-Echo with adenosine triphosphate in patients with stable coronary artery disease more effectively then visual evaluation of myocardial contractility; the best results can be obtained by applying As.

107881

Modality: E-Poster Researcher – Non-case Report

Category: HEART FAILURE/CARDIOMYOPATHY/TRANSPLANT

## Mortality and Predictors of Death in Women and Men with Congestive Heart Failure with Preserved, Mildly Reduced, and Reduced Ejection Fraction

ANTONIO DE PADUA MANSUR^1^, Carlos Henrique Del Carlo^1^, José Antonio Ramos Neto^1^, André Barbosa de Abreu^1^, Airton Roberto Scipioni^1^, Antonio Carlos Pereira Barretto^1^

(1) Insituto do Coração – HC FMUSP

**Background:** Congestive heart failure (CHF) is one of the leading causes of death from cardiovascular disease and years lived with disability. Studies showed that women had better survival than men despite higher hospitalizations in women. However, little is known about differences in mortality and predictors of death in women and men with heart failure with preserved (HFpEF), mildly reduced (HFmrEF), and reduced ejection fraction (HFrEF).

**Methods:** From February 2017 to September 2020, we analyzed the mortality and the predictors of death in women and men with CHF (Framingham criteria). Baseline data included clinical characteristics and echocardiographic findings. Statistical analyses were performed with the Kaplan-Meier (K-M) method and the Cox proportional hazards methods to analyze death rates and search for predictors of death for women and men.

**Results:** We studied 12,015 patients, mean of 63.8 ± 14.3 years, 6637 (55%) males. Females were older (64.9 ± 14.8 vs. 62.8 ± 13.8 years; p < 0.0001), had a higher baseline mean left ventricular ejection fraction (LVEF) (49.8 ± 18.9% vs. 42.6 ± 15.4%; p < 0.001), and a lower left ventricular diastolic diameter (LVDD) (54.1 ± 9.0 vs. 60.1 ± 9.6 mm; p < 0.001). Over a 3-years follow-up period, 1543 (23.2%) men and 1051 (19.5%) women of the cohort died (K-M: log-rank p < 0.0001). Cumulative incidence of death was higher in men (K-M: log-rank p = 0.0002) with HFrEF but similar for HFmrEF and HFpEF (Figure). Cox regression for death adjusted for age, ischemic, idiopathic, hypertension, Chagas, valve, previous myocardial infarction, diabetes, previous stroke, chronic kidney disease (CKD), atrial fibrillation, and LVEF showed that CKD, previous stroke, and diabetes were the main predictors of death for all phenotypes of LVEF in women and men.

**Conclusion:** Women had a better prognosis than men in HFrEF but similar mortality for HFmrEF and HFpEF. Control of diabetes and preventing stroke and CKD could significantly reduce the death rate in women and men with all CHF phenotypes.

107882

Modality: E-Poster Researcher – Non-case Report

Category: HEART FAILURE/CARDIOMYOPATHY/TRANSPLANT

## Mortality and Predictors of Death in Women and Men with Congestive Heart Failure of Different Types of Cardiomyopathies

ANTONIO DE PADUA MANSUR^1^, Carlos Henrique Del Carlo^1^, José Antonio Ramos Neto^1^, André Barbosa de Abreu^1^, Airton Roberto Scipioni^1^, Antonio Carlos Pereira Barretto^1^

(1) Insituto do Coração – HC FMUSP

**Background:** Congestive heart failure (CHF) is one of the leading causes of death from cardiovascular disease and years lived with disability. Studies showed that women had better survival than men despite higher hospitalizations in women. However, there is evidence of a gender-associated risk of dying of heart failure of different types of cardiomyopathies (CMP).

**Purpose:** To analyze the mortality of CHF due to different types of CMP in women and men.

**Methods:** From February 2017 to September 2020, we analyzed the mortality and the predictors of death in women and men with CHF (Framingham criteria) in five types of CMP (ischemic, idiopathic, hypertensive, Chagas, and valve disease). Baseline data included clinical characteristics and echocardiographic findings. Statistical analyses used the Kaplan-Meier (K-M) method and the Cox proportional hazards methods to analyze death rates and search for predictors of death for women and men for each CMP.

**Results:** We studied 12,015 patients, mean of 63.8 ± 14.3 years, 6637 (55%) males. For all patients, death occurred in 27.5%, 15.3%, 17.5%, 40.1%, and 24.4%, respectively, for ischemic, idiopathic, hypertensive, Chagas, and valve CMPs (p < 0.0001). HFrEF was more prevalent in idiopathic (51.2%) and Chagas (49.9%), and for HFpEF valve disease (80.4%), hypertensive (51.9%), and ischemic (44.4%). Compared to men, women were older, had a higher baseline mean left ventricular ejection fraction (LVEF), and a lower left ventricular diastolic diameter (LVDD) for all five types of CMP (p < 0,001). Over a 3-years follow-up period, the cumulative incidence of death was higher in men with ischemic CMP (38% vs. 31%; p = 0.037) and Chagas CMP (48% vs. 38%; p < 0,001) but similar for idiopathic, hypertensive, and valve disease CMP (Figure). Cox regression analysis for death, for each CMP, adjusted for confounders such as age, sex, previous myocardial infarction, diabetes, previous stroke, chronic kidney disease (CKD), atrial fibrillation (AF), any cardiac surgery, cardiac pacemakers, and LVEF showed that men were an independent predictor of death only for Chagas CMP (HR = 1,28; 95%CL: 1.08–1.43; p = 0.009).

**Conclusion:** Men were at a higher risk of death in ischemic and Chagas CMP but not for idiopathic, hypertensive, and valve CMP. The other main predictors of death were similar for all types of CMP, namely CKD, stroke, diabetes, and AF.

110849

Modality: E-Poster Researcher – Non-case Report

Category: DIGITAL HEALTH/INNOVATION

## The Fuzzy Logic Approach in the Prevention of Sudden Cardiac Arrest

MICHAŁ LEWANDOWSKI^1^

(1) National Institute of Cardiology, Warsaw, Poland

**Introduction:** Sudden cardiac death (SCD) constitutes a major clinical and public health problem, whose death burden is comparable to the current worldwide pandemic. The presented abstract and project encompasses the following topics: available rescue systems, wearable electrocardiograms (ECG), detection and transmission technology, and a newly developed fuzzy logic algorithm (FA) for heart rhythm classification which is state-of-the art in the field of SCD prevention. Project “PROTECTOR”, the Polish Rapid Transtelephonic ECG System to Obtain Resuscitation for development of a rapid rescue system is presented.

**Methods:** From a database containing RR interval series of the recorded arrhythmia events and controls, stored in the defibrillator memory and archived on PC during systematic control visits, RR data was chosen consecutively on the basis of full data availability, i.e., RR interval series with simultaneous intracardiac electrogram (IEGM) prints. Then, the heart rhythm classification of the recorded event was assigned into one of the six diagnostic categories of the developed algorithm. The total number of RR recordings evaluated for statistical analysis came to 298 (obtained from 183 pts). Sensitivity and specificity of the proposed algorithm were calculated on the basis of the widely accepted criteria. If a lethal arrhythmia is detected on the basis of FA, the system produces an alarm signal audible for bystanders and transmits the alarm message along with location to the emergency medical center. Phone guided resuscitation can be started immediately because an automated external defibrillator (AED) localization map is available. An automatic, very fast diagnosis is a unique feature of the PROTECTOR prototype.

**Results:** The sensitivity and specificity of the tested algorithm were 100% and 97.8%, respectively. Internet data transfer opens potential application of the presented heart rhythm classification in telemedicine. The presented methodology is completely new ECG-based technique in the diagnosis and treatment of cardiac patients.

**Conclusions:** The rapid detection of SCA is based on a processor characterized by 100% sensitivity (as measured in the pilot studies). An integrated circuit which implements FA has already been designed and a diagnosis is made within few seconds, which is extremely important in ischemic brain damage prophylaxis. This algorithm could be implemented in many mobile devices: smartphones, tablets etc.

107971

Modality: E-Poster Researcher – Non-case Report

Category: CONGENITAL AND PEDIATRIC CARDIOLOGY

## Reference Ranges of Filling Time and Systolic-to-Diastolic Time Index of the Left Ventricle, Right Ventricle and Interventricular Septum using Both Spectral and Tissue Doppler of Fetal Heart between 20 and 36+6 Weeks of Gestation

NATHALIE JEANNE MAGIOLI BRAVO-VALENZUELA^1^, Alberto Borges Peixoto^3^, Mattar, Rosiane^2^, Antonio Fernandes Moron^2^, David Baptista da Silva Pares^2^, Gabriele Tonni^4^, Edward Araujo Júnior^2^

(1) FEDERAL UNIVERSITY OF RIO DE JANEIRO-RJ, Brazil; (2) FEDERAL UNIVERSITY OF SAO PAULO-SP, Brazil; (3) Federal University of Triangulo Mineiro-MG, Brazil; (4) Prenatal Diagnostic Service, Department of Obstetrics and Gynecology, Istituto di Ricerca a Carattere Clinico Scientifico (IRCCS), AUSL Reggio Emilia, Italy

**ABSTRACT Purpose:** To establish reference values for the systolic-to-diastolic duration ratio (SDR) of the left ventricle (LV) using spectral Doppler, as well as for the SDR’ of the interventricular septum (SEP), LV, and right ventricles (RV) using tissue Doppler of the fetal heart.

**Methods:** This prospective and cross-sectional study evaluated 374 low-risk singleton pregnancies from 20 to 36+6 weeks of gestation. The ventricular filling time (FT) was obtained from LV inflow using spectral Doppler. Tissue Doppler was used to assess the FT of each ventricle by placing the cursor at the atrioventricular junction marked by the mitral and tricuspid valves, respectively. SDR was calculated as the sum of the isovolumic contraction time (ICT) and the ejection time (ET) divided by the sum of the isovolumic relaxation time (IRT) and the ventricular FT. We used regression analysis to obtain the best-fit model polynomial equation for the parameters. The concordance correlation coefficient (CCC) was used to assess intra- and inter-observer reproducibility.

**Results:** SDR and SDR’ LV showed a progressive decrease with gestational age (GA); the SDR’ RV and SDR’ SEP did not show a significant decrease with advancing GA. The SDR LV (r = 0.29, p < 0.0001), SDR’ RV (r = 0.21, p < 0.0001), SDR’ LV (r = 0.20, p = 0.0001), and SDR’ SEP (r = 0.25, p < 0.0001) showed a significant weak positive correlation with fetal heart rate. The inter-observer SDR’ SEP measurements demonstrated poor reproducibility (CCC: 0.50), whereas intra-observer SRD LV measurements demonstrated moderate reproducibility (CCC: 0.78).

**Conclusions:** Reference values for SDR SEP, LV, and RV using spectral and tissue Doppler of fetal heart were established between 20 and 36+6 weeks of gestation.


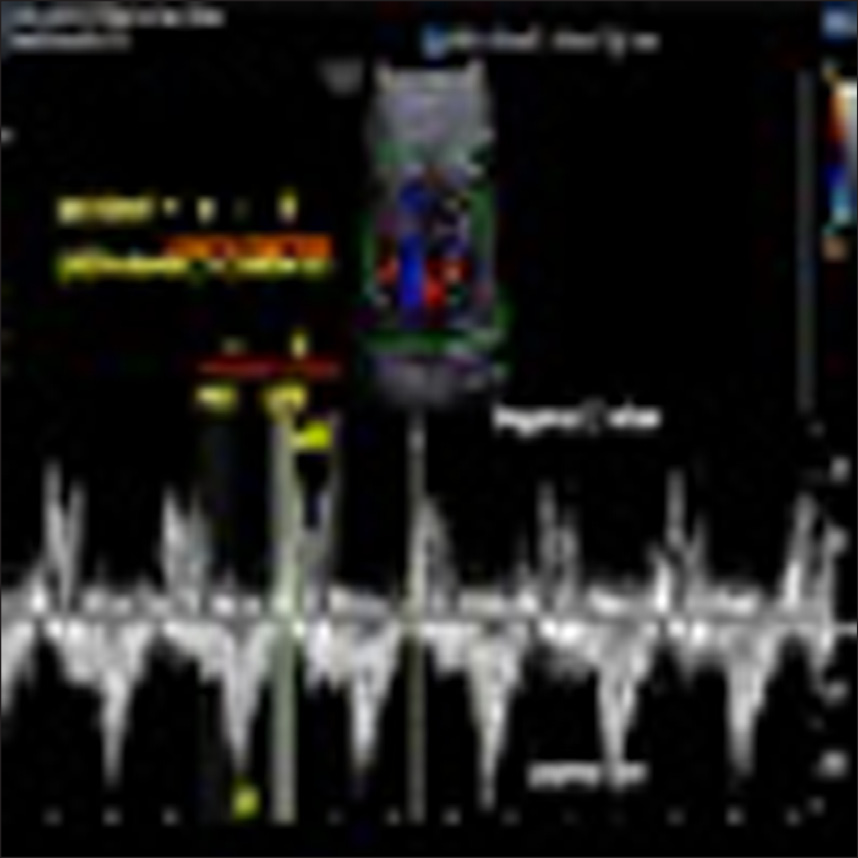


108035

Modality: E-Poster Researcher – Non-case Report

Category: EPIDEMIOLOGY AND HEALTH POLICIES/GLOBAL HEALTH

## Impact of the COVID-19 Pandemic on Hospitalizations, Mortality Rate and Length of Stay in Patients with Pulmonary Embolism (PE) in Brazil

AUREO DO CARMO FILHO^1^, Rogerio Gomes Fleury^1^

(1) Hospital Universitário Gaffrée e Guinle – UNIRIO/EBSERH

**Objectives:** The novel coronavirus (SARS-CoV-2) may predispose patients to arterial or venous thrombosis due to excessive inflammation, platelet activation, endothelial dysfunction, and blood stasis. Pulmonary embolism as one of the most serious manifestations of this systemic framework worries those who are dealing with COVID-19 daily. The need for isolation and the home office can favor the development of risk factors for PE such as a sedentary lifestyle, immobility, obesity and smoking. The present study aimed to assess the influence of COVID-19 on hospitalizations, mortality rate and length of stay for pulmonary embolism in Brazil.

**Method:** We used the database from the Department of Informatics of the Brazilian Health System (DATASUS), through the website https://datasus.saude.gov.br/. We evaluated the number of hospitalizations, deaths and length of stay of patients for PE in Brazil and their relationship with the beginning of the COVID-19 pandemic in Brazil (March 2020) based on data from January 2018 to December 2021.

**Results:** From January 2018 to December 2021, 38902 patients were hospitalized for PE in Brazil, 23706 (60.9%) were female and 15196 (39.1%) were male. There was a slight increase in the average number of hospitalizations, but there was no statistical difference in the average total number of hospitalizations comparing 2018–19 with 2020–21: 9655 ± 624 and 9797 ± 201 (p = 0.804). The number of deaths and mortality rate also did not show important differences for the same period, with 1684 ± 66 deaths and a mortality rate of 17.5 ± 0.43 for 2018–19 and 1805 ± 142 and a mortality rate of 18.4 ± 1.1 for 2020–21 (p = 0.428 and p = 0.415 respectively). The mean hospital stay was similar, with 9.1 ± 0.3 days in 2018–19 and 8.5 ± 0 days in 2020–21 (p = 0.205). Regarding the age group, the number of hospitalizations is higher between 60–69 years old with 7495 cases and an annual average of 1874 ± 59 and the group over 80 years old with the highest mortality rate 34.7 ± 1.5.

**Conclusion:** In the context of the COVID-19 pandemic, the number of hospitalizations due to PE remained stable in the period studied from 2018 to 2021, with a predominance of female patients (60.9%). The number of deaths and mortality rates also remained stable, as well as the average length of hospital stay. Advanced age was an important factor for both hospitalization and mortality.

108036

Modality: E-Poster Researcher – Non-case Report

Category: EPIDEMIOLOGY AND HEALTH POLICIES/GLOBAL HEALTH

## Influence of the COVID-19 Pandemic and Vaccination on Deaths from Stroke in the Elderly Population in Brazil

AUREO DO CARMO FILHO^1^, Rogério Gomes Fleury^1^

(1) Hospital Universitário Gaffrée e Guinle – UNIRIO/EBSERH

**Objective:** Cardiovascular diseases are increasingly becoming a health problem in Brazil and worldwide. They are related to important acute complications such as stroke, which have high morbidity and mortality, especially when identified late. Taking into account the difficulty of the population’s access to health services in brazilian pandemic scenario, the present study aimed to assess the impact of COVID-19 pandemic and vaccination on stroke deaths in the elderly population in Brazil.

**Method:** Using data from the Civil Registry Information Center in Brazil, through the website https://transparencia.registrocivil.org.br/especial-covid, we evaluated the number of deaths from stroke in Brazil in the elderly (age above 60 years) and its relationship to the COVID-19 pandemic from January 2019 to December 2021.

**Results:** From January 2019 to December 2021, 239771 patients above 60 years old died of stroke in Brazil, 120107 (50.1%) were male and 119664 (49.9%) female. There was a slight decrease in stroke deaths in the comparison between 2019 and 2020, from 79806 to 79435 with a further increase in 2020 to 80530. The age group with the highest number of deaths was 80–89 with an average of 25807 ± 519, followed by 70–79 with an average of 25180 ± 1012. When we compare the monthly average of deaths and the beginning of the pandemic in Brazil (March 2020), the average was: before 656 ± 44 and after 673 ± 23 (p = 0.27). We also performed an analysis with the start of vaccination in Brazil in January 2021 and found a mean number of monthly deaths of 673 ± 23 before and 671 ± 46 after (p = 0.89).

**Conclusion:** In the context of the Brazilian COVID-19 pandemic scenario, the number of deaths from stroke in the elderly population fell slightly from 2019 to 2020, with a new increase in 2021. The number of deaths remained similar for the years studied, with the age group of 80–89 years old as the highest number of cases followed by the 70–79 group. Neither the COVID-19 pandemic nor vaccination changed the monthly average deaths from stroke in the Brazilian elderly population.

108037

Modality: E-Poster Researcher – Non-case Report

Category: EPIDEMIOLOGY AND HEALTH POLICIES/GLOBAL HEALTH

## Impact of the COVID-19 Pandemic on Deaths from Stroke in Young People in Brazil

AUREO DO CARMO FILHO^1^, Rogerio Gomes Fleury^1^

(1) Hospital Universitário Gaffrée e Guinle – UNIRIO/EBSERH

**Objectives:** The cardiovascular diseases are an important healthcare problem in Brazil and worldwide. They are related to acute complications such as stroke, which have high morbidity and mortality, especially when identified late. The difficulty of the population’s access to health services in brazilian pandemic scenario associated to isolation and home office can favor the development of traditional risk factors for stroke, most often neglected by young people, as sedentary lifestyle, obesity, smoking and poor nutrition. The present study aimed to assess the impact of COVID-19 on stroke deaths in young people in Brazil.

**Method:** We used data from the “Portal da Transparência”, a platform managed by the National Association of Registrars of Natural Persons (Arpen-Brazil), through the website https://transparencia.registrocivil.org.br/especial-covid. We evaluated the number of deaths from stroke in Brazil in young people (aged 20 to 59 years) and its relationship with the beginning of the COVID-19 pandemic in Brazil (March 2020) analyzing data from January 2019 to December 2021.

**Results:** From January 2019 to December 2021, 57,965 patients between 20 and 59 years old died from stroke, 33,764 (58.2%) were male. There was an increase in the number of total deaths over the years studied from 17506 to 19042 and 21417. The average monthly deaths in the period before the pandemic in Brazil was 1451 ± 80, in contrast to 1711 ± 117 after the beginning of the pandemic (p < 0.001). The age group with the highest number of deaths was 50–59 with a mean of 10246 ± 788, followed by 40–49 with a mean of 5215 ± 488.

**Conclusion:** According to official data on deaths from strokeI in young adults between 20 and 59 years old, there was a progressive increase from 2019 to 2021, with a predominance of male patients. The COVID-19 pandemic contributed to this increase significantly. Age remains an important risk factor and older age groups continue to be the most affected.

108038

Modality: E-Poster Researcher – Non-case Report

Category: EPIDEMIOLOGY AND HEALTH POLICIES/GLOBAL HEALTH

## Impact of the COVID-19 Pandemic on Deaths from Acute Myocardial Infarction (AMI) in Young People in Brazil

AUREO DO CARMO FILHO^1^, Rogerio Gomes Fleury^1^

(1) Hospital Universitário Gaffrée e Guinle – UNIRIO/EBSERH

**Objectives:** Cardiovascular diseases are one of the main causes of death in Brazil and worldwide, and AMI is its main exponent. During the pandemic of the new coronavirus, health systems had to adapt to adequate care of these patients. The need for isolation and home office can favor the development of traditional risk factors for AMI, most often neglected by young people, such as a sedentary lifestyle, obesity, smoking, excessive consumption of alcohol, stress and poor nutrition. In addition, and taking into account the difficulty of the population’s access to health services in the Brazilian pandemic scenario, the present study aimed to evaluate the impact of COVID-19 on deaths from AMI in young people in Brazil.

**Method:** We used data from the “Portal da Transparência”, a platform managed by the National Association of Registrars of Natural Persons (Arpen-Brazil), through the website https://transparencia.registrocivil.org.br/especial-covid. We evaluated the number of deaths from AMI in Brazil in young people (aged 20 to 59 years) and its relationship with the beginning of the COVID-19 pandemic in Brazil (March 2020) with data from January 2019 to December 2021.

**Results:** From January 2019 to December 2021, 64,512 patients between 20 and 59 years old died from AMI, 44,940 (69.7%) were male. There was an increase in the number of total deaths over the years studied from 20075 to 20706 and 23731. The average monthly deaths in the period before the pandemic in Brazil was 1680 ± 123, in contrast to 1862 ± 181 after the beginning of the pandemic (p = 0.001). The age group with the highest number of deaths was 50–59 with a mean of 13568 ± 766, followed by 40–49 with a mean of 16710 ± 533.

**Conclusion:** According to official data on deaths from AMI in young adults between 20 and 59 years old, there was a progressive increase from 2019 to 2021, with a predominance of male patients. The COVID-19 pandemic contributed to this increase significantly. Age remains an important risk factor and older age groups continue to be the most affected.

108078

Modality: E-Poster Researcher – Non-case Report

Category: HEART FAILURE/CARDIOMYOPATHY/TRANSPLANT

## Outcomes of Pregnant Women with Non-Compaction Cardiomyopathy: Cases Series from Incor- Registry

WALKIRIA SAMUEL AVILA^1^, Daniel VInicius Rodrigues Pinto^1^, Vera Maria Cury Salemi^1^, Walkiria Samuel Avila^1^

(1) Instituto do Coração do Hospital das Clinicas da Faculdade de Medicina da Universidade de São Paulo; (2) Instituto do Coração do Hospital das Clinicas da Faculdade de Medicina da Universidade de São Paulo; (3) Instituto do Coração do Hospital das Clinicas da Faculdade de Medicina da Universidade de São Paulo; (4) Instituto do Coração do Hospital das Clinicas da Faculdade de Medicina da Universidade de São Paulo

**Background:** Non-compaction cardiomyopathy (NCC) is a genetic congenital disease, with adverse clinical manifestations. The circulatory overload and hypercoagulability status of pregnancy, both can lead to serious complications.

**Purpose:** To study the maternal and fetal outcomes of pregnant women with NCC.

**Methods:** Out of 495 pregnant women with heart disease studied, four of them had a diagnosis of NCC confirmed by echocardiography and/or cardiac magnetic resonance exams. Before pregnancy, the symptoms were angina pectoris, syncope, and three cases with symptomatic arrhythmias, two of them underwent radiofrequency ablation. In the multidisciplinary follow-up, the therapy management was adjusted for pregnancy.

**Results:** The Table below presents the characteristics of patients and the maternal-fetal evolution. There were no deaths, but three patients required hospitalization for treatment of heart failure and cardiac arrhythmias, right away emergency cesarean section resulting premature babies. In the follow-up-12months, no changes were observed in the clinical or myocardium remodeling of NCC patients. The investigation of newborns did not identify any case of NCC.

**Conclusions:** The heterogeneity of NCC expression and the proportion of maternal and fetal complications, observed in this series, discourage pregnancy in patients with NCC. However, if pregnancy occurs, it should be followed into a tertiary hospital with a multidisciplinary team on standby.


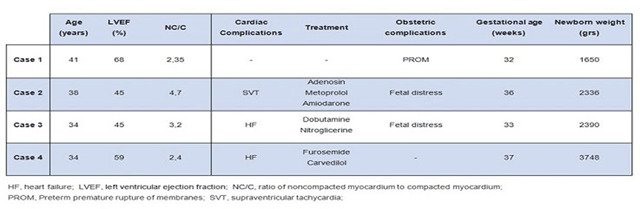


108095

Modality: E-Poster Researcher – Non-case Report

Category: CONGENITAL AND PEDIATRIC CARDIOLOGY

## Global Prevalence of Depression and Anxiety in Adolescents and Adults with Congenital Heart Disease: A Systematic Review and Meta-Analysis

PHILIP MOONS^1^, Liesbet Van Bulck^1^, Koen Luyckx^1^

(1) KU Leuven – University of Leuven, Belgium

**Background:** Living with congenital heart disease (CHD) and the required invasive treatments are believed to have an impact on the psychological health of afflicted individuals. Therefore, mental health in persons with CHD is an area of concern. The epidemiology of depression and anxiety in CHD, however, is not fully understood and literature seems to report contradictory findings.

**Objective:** We aimed (i) to estimate the proportion of depression and anxiety in adolescents and adults with CHD around the globe; and (ii) to explore differences in depression and anxiety rates between people with CHD and the general population.

**Methods:** We conducted a systematic literature review and meta-analysis, registered in PROSPERO (CRD42021228395). Searches were performed in PubMed, Embase, Cinahl and Web of Science from their inception to March 6, 2021. We identified 91 studies, which enrolled 180,176 patients in total. A random effects meta-analysis of single proportions was conducted according to the DerSimonian-Laird method. Hedges’ gu (g) was calculated to compare the level of depression and anxiety with the general population.

**Results:** The pooled estimated proportion showed that 21.1% of the people with CHD had ≥mild depressive symptoms and 10.3% had ≥moderate depressive symptoms. When looking at the clinical diagnosis of depression, 10.3% had actual depression, and 16.1% had a lifetime episode of depression. For anxiety, 42.7% had ≥mild anxiety symptoms and 21.9% had ≥moderate anxiety symptoms. A clinical anxiety disorder was found in 12.7%, and 23.3% had a lifetime episode of anxiety disorder. The mean scores on questionnaires assessing symptoms of depression (g = 0.215; 95%CI = 0.062–0.368) or anxiety (g = 0.292; 95%CI = 0.165–0.419) were significantly higher in CHD than in the general population. However, this higher symptom score was not reflected in a significantly higher proportion of CHD patients having ≥mild depressive symptoms (p = 0.654), ≥moderate depressive symptoms (p = 0.869), ≥mild anxiety symptoms (p = 0.055) or ≥moderate anxiety symptoms (p = 0.287), when using the predefined cut-off scores.

**Conclusions:** This meta-analysis showed that there is a high proportion of depression and anxiety in persons with CHD. Although patients with CHD had higher scores of depressive and anxiety symptoms than people from the general population, the prevalence of depressive or anxiety symptomatology was not higher in CHD.

111457

Modality: E-Poster Researcher – Non-case Report

Category: SPIRITUALITY AND CARDIOVASCULAR MEDICINE

## High Spirituality Score Protects Against Burnout and its Consequences Among Brazilian Cardiologists

MAURO RICARDO NUNES PONTES^1^, Mario Borba^2^, Lucélia B. N. C. Magalhães^3^, Alvaro Avezum^4^, Emilio Moriguchi^5^, Roberto Esporcate^6^, Sérgio Lívio Menezes Couceiro^7^, Hermilo Borba Griz^8^, Fernando A. Lucchese^9^

(1) Nucleo de Pesquisa Clinica do RS; (2) Cardioclinica do Vale, Lajeado; (3) UniFTC-Faculdade de Ciência e Tecnologia; (4) Hospital Alemão Osvaldo Cruz; (5) Hospital de Clínicas de Porto Alegre; (6) Faculdade de Ciências Médicas, UERJ; (7) Universidade Federal Fluminense; (8) Hospital Santa Joana, Recife; (9) Hospital São Francisco, Santa Casa de Porto Alegre

**Purpose:** Burnout among physicians is frequent and has several consequences for physician and patient. We hypothesized that high spirituality protect against burnout.

**Methods:** A survey with cardiologists, Aug 2018, asked about Burnout, demographics, spirituality, religiosity, job characteristics. We measured Burnout by 3 validated questions (three MBI -Maslach Burnout Inventory- domains). Subjects were Burnout + (B+) if they were in the top 3 scores (“>once a week”) on emotional exhaustion and/or depersonalization. Demographics, Spirituality score, DUREL religion index, job aspects, were correlated with B+ or B–. We evaluated consequences of burnout (suicidal ideation, stress, anxiety, depression measured by the DASS-21 form). Multivariate models evaluated the adjusted impact of spirituality over burnout and its consequences. Statistical analysis with SPSS version 23.

**Results:** 1000 survey forms, 40.5% response rate; 39 excluded (4 incomplete, 35 graduated £2y); final sample, 375 cardiologists; age 48.8 ± 12.6y, male 57.3%. Burnout rate was 34.6%. Variables associated with Burnout in bivariate analysis: civil state (divorced/single 28.9% B+, married 6.8%, P = 0.020), years from graduation (B+, 20.3 ± 13.1y; B–, 25.3 ± 12.5y; P < 0.001), age (B+, 45.4 ± 13.1y; B–, 50.0 ± 12.4y; P = 0.001), duty hours/week (Q1 to Q4; 24.0%, 27.8%, 46.5%, and 60.9%; P < 0.001), and night shifts/week (0 to 3; 30.7%, 38.2%, 41.7%, and 71.4%; P = 0.001). Gender (P = 0.295), Spirituality score (P = 0.099), DUREL (P > 0.05 for all 3 domains), postgrad (P = 0.701) and subspecialty (P = 0.668) not associated with B+. Consequences of Burnout: suicidal ideation (B+, Yes 10.9%; B– Yes 1.3%; P < 0.001), DASS Global score (B+, 18.1 ± 11.7; B–, 8.1 ± 6.8, P < 0.001), and DASS subscales of Anxiety, Stress, and Depression (P < 0.001 for all) were all associated with B+. In a multivariate regression model (Table 1), Number of Duty Hours per Week was associated with Burnout (OR = 1.79, 95% CI 1.34–2.41, P < 0.001), while Spirituality Score (but not Religiosity by DUREL index) protected against Burnout (OR = 0.87, 95% CI 0.77–0.98, P = 0.043).

**Conclusions:** High number of duty hours per week increases Burnout among Brazillian cardiologists, while high Spirituality protects them against Burnout.

108248

Modality: E-Poster Researcher – Non-case Report

Category: CARDIOLOGY OF SPORTS, EXERCISE, ERGOMETRY AND CARDIOVASCULAR REHABILITATION

## Impact of Existing Cardiovascular Risk Factors on the Improvement of Exercise Tolerance in Patients with Coronary Artery Disease After PCI and Cabg Undergoing Early Post-Hospital Cardiac Rehabilitation

EDYTA SMOLIS-BAK^1^, Ilona Kowalik^1^, Jurand Sloniewicz^1^, Ewa Rydzewska^1^, Iwona Korzeniowska-Kubacka^1^, Jadwiga Wolszakiewicz^1^, Rafal Dabrowski^1^, Edyta Smolis-Bak^1^

(1) National Institute of Cardiology

**Background:** Regular physical activity is crucial for treatment and prevention of cardiovascular diseases. Adaptive changes in response to regular physical exertion show individual variability, which depends on a number of internal and external factors. The aim of the study was to assess the effectiveness of early post-hospital rehabilitation and to analyze the factors which may influence the exercise tolerance in patients with coronary artery disease (CAD) treated with PCI and CABG.

**Material and methods:** The study included 334 patients (pts), men 75.1%, with CAD, treated with PCI (87.4%) or CABG (12.6%). Mean age of the study group was 61.1 ± 10.9 years, mean BMI: 27.6 ± 4.1 kg/m^2^, mean LVEF: 56.1 ± 7.2%. The main risk factors for CAD were: dyslipidemias: 76%, hypertension: 64.7%, smoking: 28.7%, diabetes: 22.5%. The rehabilitation procedures lasted for 6–8 weeks. Pts participated in endurance training and general conditioning exercises with elements of resistance trainings. Before and after rehabilitation, all patients underwent a symptom-limited exercise test (ETT). In order to identify factors influencing the increase in tolerance of training loads, patients were divided into 2 groups: group 1 (198 pts, 59.3%) – pts with the training load which increased ≥50%, group 2 (136 pts, 40.7%) – pts with training loads which increased less, by <50%.

**Results:** After rehabilitation, a significant improvement in the ETT parameters was observed: 5.7 ± 1.3 vs. 6.8 ± 1.7 METs, p < 0.001; the resulting load predicted for age: 81.5 ± 22.5 vs. 93.7 ± 22.89%, p < 0.001; tests duration: 352 ± 106 vs. 436 ± 136 sec., p < 0.001 and in the training loads obtained: 46.7 ± 10.9 vs. 71.4 ± 22.4 Watts, p < 0.001. On the other hand, after rehabilitation, a significant increase in the number of pts with supraventricular (24.2% vs 34.7%, p < 0.001) and ventricular (34.2% vs 40.5%, p < 0.02) arrhythmias was observed. There was no correlation between the increase in training load tolerance (Watt) and the METs increase in the final ETT. The increase in the tolerance of training loads of ≥50% during rehabilitation was significantly influenced by: younger age, p < 0.001, gender (men), p < 0.001, EF (>50%), p < 0.003, absence of diabetes, p < 0.003, lower initial values of training loads, p < 0.003. The increase in workload (MET > 50%) in the final ETT was significantly influenced by age (younger patients), BMI (thinner subjects) and lower workload (MET) in the baseline test. Other factors: smoking, hypertension.

108254

Modality: E-Poster Researcher – Non-case Report

Category: HEART FAILURE/CARDIOMYOPATHY/TRANSPLANT

## Sexual Dysfunction in Patients After Implantation of Left Ventricular Assist Device, LVAD

EDYTA SMOLIS-BAK^1^, Edyta Smolis-Bak^1^, Zbigniew Lew- Starowicz^2^, Monika Szymanska^2^, Ilona Kowalik^1^, Ryszard Piotrowicz^1^, Eliza Noszczak^1^, Rafal Dabrowski^1^

(1) National Institute of Cardiology, Warsaw, Poland; (2) Postgraduate Medical Education Center, Warsaw, Poland

**Background:** Data show that cardiological patients (pts), after achieving an improvement in the functioning of the cardiovascular system, begin to expect improvement also in the field of sexual health, which is important in partner relationships, mental health and quality of life. The aim of the study was to analyze the occurrence of sexual disorders and to assess the impact of sexual rehabilitation and physiotherapeutic education on sexuality in pts after left ventricular assist device (LVAD) implantation.

**Material and methods:** The study included 50 men after LVAD implantation, mean age 51.6 ± 12.8 years. 18 pts did not complete the study (heart transplant –5 pts, resignation –10 pts, death –3 pts). The pts were assessed by a sexologist in terms of the quality of sexual life and erectile dysfunction. All pts received therapeutic interventions: sexological counseling, education (positions during intercourse, safety recommendations) and physical activity. Additionally, pharmacological treatment of erectile dysfunction was started in 15 pts. Upon entry into the study, the quality of sexual life, sexual needs (Mell-Krat scale) and sexual function (International Index of Erectile Function, IIEF-5) were assessed retrospectively (before the onset of the disease) and actually. Additionally, exercise tolerance was assessed using the 6-minute walk test (6-MWT). The tests were repeated after 6 months.

**Results:** After the implementation of sexual and physical activity education, significant improvement in exercise tolerance assessed by 6-MWT [m] was observed: 350 (310–445) vs. 450 (400–500), p < 0.001. The quality of sex life in the whole group significantly worsened after LVAD implantation compared to the period before the onset of the disease (40 [40–41] vs 33.5 [12–40] vs 34 [26–40]). Mild erectile dysfunction was observed in all the pts, both before onset of the disease, after LVAD implantation and after 6 months. The number of pts with sexual dysfunction increased significantly after LVAD implantation (9.4% vs. 53.1%) and remained at a similar level for 6 months. There was a significant reduction in the frequency of sexual intercourse after LVAD implantation and a significant increase in it after pharmacological treatment, but its level did not reach the level before the disease (3 [2–4] vs 1.5 [0–2.5] vs 2 [1.0–2.5]). The improvement in the quality of sexual life and the reduction of erectile dysfunction were significantly dependent on age and LVEF – bett.

108346

Modality: E-Poster Researcher – Non-case Report

Category: COVID-19 AND CARDIOVASCULAR SYSTEM

## Cardiovascular Mortality in Brazil During the COVID-19 Pandemic: Comparison between Underlying and Multiple Causes of Death Analysis

LUISA CAMPOS CALDEIRA BRANT^1^, Pedro Cisalpino Pinheiro^1^, Luiz Guilherme Passaglia^1^, Eduardo David Gomes de Sousa^2^, Carla Valença Daher^2^, Roberta Maria Leite Costa^2^, Antonio Luiz Pinho Ribeiro^1^, Bruno Ramos Nascimento^1^

(1) Faculdade de Medicina e Hospital das Clínicas, Universidade Federal de Minas Gerais, UFMG, Brazil; (2) Department of Specialized and Thematic Healthcare, Ministry of Health, Brasilia, Brazil

**Introduction:** COVID-19 has impacted cardiovascular (CV) mortality worldwide, possibly due to avoidance of healthcare, health system collapse, and the direct CV effects. Data from Brazil are scarce and restricted to some locations or early phases of the pandemic.

**Objective:** To evaluate the impact of COVID-19 pandemic on mortality for CV diseases (CVD), stratified by age, sex and region of Brazil, comparing underlying (UC) and multiple causes (MC) of death.

**Methods:** Ecological, time series study analysing the age-standardized death rates for CVD, from epidemiological week (EW) 10/2020 to EW 11/2021, using data from the Mortality Information System (SIM-SUS). CVD was defined as in Chapter IX, ICD-10, and considered if reported as UC or MC of death (UC + other causes reported in any line of death certificate), in separate analyses. Observed was compared to expected data (mean for the same EW of 2017–2019). Risk ratios (RiR) were analysed and 95% confidence intervals were calculated.

**Results:** Age-standardized mortality rate for CVD as UC of death in the pandemic was 136.8 (95%CI 136.3–137.3)/100,000 inhabitants, 7% (RiR = 0.93; 95%CI 0.93–0.93) lower than expected (147.1; 95%CI 146.6–147.6)/100,000. There was an increase in out-of-hospital mortality (6%) (RiR = 1.06; 95%CI 1.05–1.07) and in deaths with ill-defined CVD causes (49%) (RiR = 1.49; 95%CI 1.47–1.50), particularly in the out-of-hospital setting (58%). The increase in out-of-hospital deaths was more pronounced in the North (RiR = 1.27; 95%CI 1.25–1.28) and Northeast (RiR 1.18; 95% CI 1.17–1.18) regions, both with less structured health systems. Conversely, in MC of death analysis, there was a 4% increase in CV mortality (observed: 225.1 (CI95% 224.5–225.8), expected: 217.2 (95%CI 216.5–217.8)/100,000, RiR: 1.04 (95%CI 1.03–1.04), noticeably in the out-of-hospital setting (11%). In MC of death analysis, the steeper increasing trends also ocurred in the North region (RiR = 1.13; 95%CI 1.12–1.15), and in individuals >60 years (RiR = 1.05; 95%CI 1.05–1.06), and among men (RiR: 1.06; 95%CI 1.05–1.06) vs. women (RiR: 1.02; 95% CI 1.01–1.02).

**Conclusions:** During the pandemic, mortality rates for CVD as UC of death reduced in Brazil, contrasting with an increase when MC were considered. Higher out-of-hospital mortality – resulting from social distancing and overwhelmed health systems – exaberbation of existing CVD by COVID-19, and competing causes of death may have accounted for this pattern.

108258

Modality: E-Poster Researcher – Non-case Report

Category: CARDIOLOGY OF SPORTS, EXERCISE, ERGOMETRY AND CARDIOVASCULAR REHABILITATION

## Does Early Hospital Rehabilitation Contribute to the Reduction of Falls in Patients After Cardiac Surgery?

EDYTA SMOLIS-BAK^1^, Kamil Szczesniak^1^, Ilona Kowalik^1^, Grzegorz Skorupski^1^, Lukasz Lasecki^1^, Jerzy Osak^1^, Magdalena Furmanek^1^, Hanna Szwed^1^, Rafal Dabrowski^1^

(1) National Institute of Cardiology, Warsaw, Poland

Balance and coordination disorders are factors provoking falls in the elderly. Their fast and precise identification is therefore of vital importance. The aim of this work was to assess the risk of falls and the influence of early hospital rehabilitation on the balance and coordination parameters in patients after cardiac surgery.

**Material and methodology:** The study was conducted on 207 patients: 44 women and 163 men aged 18–87 (mean 60.2 ± 14.0), who participated in comprehensive cardiac rehabilitation following cardiac surgery. The risk of falls and patients’ functional status were tested twice with the use of the FallSkip device: after the initial mobilization and before the discharge from the ward.

**Results:** After rehabilitation, improvement was noted in the whole group in all analysed functional parameters and in the reduction of the risk of falls (p < 0.0001). A significantly higher risk of falls was observed in women in comparison with men (p < 0.001), and in individuals aged >70 in comparison with patients aged <70 (p < 0.001). There were no differences observed in the risk of falls of patients in the groups with the BMI <30 and >30, both before and after rehabilitation. After rehabilitation the risk of falls decreased significantly in men (p < 0.001) and in individuals aged <70, (p < 0.001), as well as in patients with the BMI <30, p < 0.001, and >30, p < 0.012. There was a tendency for better results in men in all tested parameters.

**Conclusions:** The proposed comprehensive rehabilitation model after cardiac surgery significantly improved the effectiveness of gait, time of reaction in response to audio stimuli, increased muscular strength of lower limbs and significantly reduced the risk of falls. The FallSkip equipment is useful in the assessment of the risk of fall and of the level of functional parameters in patients after cardiac surgery.

108351

Modality: E-Poster Researcher – Non-case Report

Category: COVID-19 AND CARDIOVASCULAR SYSTEM

## The Indirect Effect of COVID-19 Pandemic in Hospitalizations for Cardiovascular Diseases in Brazil: Reduced Numbers with Higher In-Hospital Deaths

LUISA CAMPOS CALDEIRA BRANT^1^, Luiz Guilherme Passaglia^1^, Pedro Cisalpino Pinheiro^1^, Eduardo David Gomes de Sousa^2^, Carla Valença Daher^2^, Roberta Maria Leite Costa^2^, Antonio Luiz Pinho Ribeiro^1^, Bruno Ramos Nascimento^1^

(1) Faculdade de Medicina e Hospital das Clínicas, Universidade Federal de Minas Gerais, UFMG, Brazil; (2) Department of Specialized and Thematic Healthcare, Ministry of Health, Brasilia, Brazil

**Introduction:** The COVID-19 pandemic impacted hospitalizations for other causes due to change in populations’ behaviour, as a result of fear or adherence to social distancing, or overwhelmed hospitals. Low and middle-income countries may have higher negative impact because of less resilient health systems. Previous studies in Brazil showed a reduction in hospitalizations for cardiovascular diseases (CVD). However, they were restricted to some cities or the first three months of the pandemic.

**Purpose:** Evaluate the impact of COVID-19 pandemic in the number and severity of hospitalizations for CVD in the Brazilian public and universal health system during 2020 and 2021, considering differentials by age, sex, region of the country.

**Methods:** Ecological, time series study analysing the number of hospitalizations, number and proportion of intensive care unit (ICU) admissions and in-hospital deaths for CVD, from epidemiological week (EW) 10/2020 (first confirmed COVID-19 case) to EW 21/2021, using data from the Hospitalization Information System (SIH-SUS). CVD was defined as in Chapter IX from ICD-10. Observed data was compared to the mean for the same EW of 2017–2019. Risk ratios (RiR) were analysed and 95% confidence intervals were calculated.

**Results:** A 16% (RiR 0.84; 95%CI 0.83–0.84) reduction in hospitalizations for CVD was observed in the pandemic period (n = 953,025). However, higher proportion of ICU admissions (RiR 1.09; 95%CI 1.08–1.09), and in-hospital deaths (RiR 1.14; 95%CI 1.14–1.15) revealed greater severity of hospitalized individuals, which may have ocurred due to delayed admissions, or disrupted pathways of care. Two peaks of reductions in hospitalizations were observed, possibly resulting from different drivers: the first, at the beginning of the pandemic, may have resulted from deferred hospitalized treatment, while the second may represent competing demands with COVID-19, resulting in even higher proportion of deaths (Figures 1 and 2). Women, older individuals and those living in the least developed North and Northeast regions were mostly negatively impacted, exacerbating vulnerabilities in CVD care.

**Conclusions:** The COVID-19 pandemic disrupted CVD care in Brazil. Health policies must address hospitalizations by CVD during future pandemics, including reorganizing pathways of care, public campaigns about symptoms that require hospitalization, and greater availability of ICU beds, besides a plan to face the rebound effect for deferred procedures.

108357

Modality: E-Poster Researcher – Non-case Report

Category: ACUTE AND CHRONIC CORONARY DISEASE/THROMBOLYSIS

## Evaluation of Angiographic Patence of the Guilt Artery, in Patients with Acute St-Elevation Myocardial Infarction, using Double Antiplatelet Aggregation

MARCOS VINICIUS COELHO DUTRA^1^, CARLOS ALBERTO KENJI NAKASHIMA^1^, LILIAN BELINASO^1^, VIVIANE DE SA PEREIRA^1^, RICARDO PHILIPE ZAGO^1^, ANTONIO DEJAIR ACOSTA PAZZINI^1^, VANESSA DELMIRO DOS SANTOS^1^, CASSIO PERFETE^1^, ERASMO SIQUEIRA^1^, DALTON BERTOLIM PRECOMA^1^

(1) Hospital Angelina Caron

**Background:** Early reperfusion of the ischemic myocardium is the most important advance in the last 30 years when it comes to acute myocardial infarction (AMI) with ST-segment elevation (STEMI), which can be in the mechanical form by angioplasty, or pharmacologically with use of thrombolytic, respecting the indication of each one. Antiplatelet therapy, from the studies with acetylsalicylic acid as in the Isis-2 study, demonstrating a 23% reduction in mortality when used alone in acute coronary syndrome (ACS), the addition of low molecular weight heparin, to more recent publications with the new adenosine diphosphate blockers and thrombin receptor blockers, corroborating the reduction of outcomes in the acute scenario of post-angioplasty AMI (thrombosis reduction), and making dual antiplatelet aggregation (DAPT) an essential part of the treatment of ACS. Few trials provide us with information comparing whether such medications, used as indicated in STEMI, were able to maintain the patency of the affected vessel pre-coronary angiography.

**Objective:** To compare, between the two groups analyzed (pre-treatment with clopidogrel vs. ticagrelor) the patency of the culprit coronary artery assessed by TIMI angiographic flow analysis by coronary angiography.

**Methods:** A total of 3287 medical records of patients admitted to a chest pain unit of a tertiary hospital in the metropolitan region of Curitiba – Paraná were analyzed from March 1, 2019 to March 1, 2021. Of the 3287 patients initially evaluated, 384 patients were selected with a diagnosis of STEMI, 16 patients were excluded due to lack of data in their medical records and 334 patients were analyzed until hospital discharge.

**Results:** Of all the cases that used ticagrelor, 65.1% had occluded vessels and of the cases of clopidogrel, 65.8% of the affected vessels remained occluded. Therefore, no significant difference was found between clopidogrel and ticagrelor regarding the probability of being occluded (p = 0.585).

**Conclusion:** We can conclude that in the analyzed sample, there was no statistical difference between the use of clopidogrel and ticagrelor in the patency of the vessel affected by the occlusion.

108384

Modality: E-Poster Researcher – Non-case Report

Category: HEMODYNAMICS AND INTERVENTIONAL CARDIOLOGY

## Independent Clinical and Echocardiographic Predictors of Restenosis After Percutaneous Mitral Balloon Commissurotomy Followed for 24 Years

RAFAEL ALEXANDRE MENEGUZ MORENO^1^, Nisia Lira Gomes^1^, Alfredo Nunes Ferreira-Neto^1^, Auristela IO Ramos^1^, Zilda Meneghelo^1^, J. Italo Franca^1^, Amanda GMR Sousa^1^, Sérgio Luiz Navarro Braga^1^, Dimytri Siqueira^1^, J. Ribamar Costa Jr.^1^

(1) Instituto Dante Pazzanese de Cardiologia; (2) Universidade Federal de Sergipe; (3) Hospital Primavera

**Background:** Mitral valve stenosis (MVS) is one of the most common structural heart diseases in developing countries, primarily due to rheumatic disease. Percutaneous mitral balloon valvuloplasty (PMBV) has been, since its introduction in 1984, the preferred option of treatment for such disease. However, restenosis is presented with an approximate incidence of 20%. Echocardiographic scoring of the mitral apparatus has been the main tool used to indicate and foresee the possible result of the procedure. The objective of this study was to enlight risk factors of mitral valvular restenosis in a significant number of patients submitted to percutaneous mitral balloon commissurotomy for the treatment of mitral stenosis (MS), particularly when secondary to rheumatic heart disease.

**Methods:** This study reports the vast experience of a single center high volume tertiary institution where 1.794 consecutive patients were treated with PMBC between 1987 and 2011. The primary endpoint was to determine the independent predictors of this untoward event, defined as loss of over 50% of the original increase in maximum valve area (MVA) or MVA < 1.5 cm^2^.

**Results:** Mitral valve restenosis was observed in 26% of the cases (n = 483). Mean population age was 36 years old, with most patients being female (87%). Mean follow up duration was 4.8 years. At multivariate analysis independent pre-procedural predictors of restenosis were: left atrial diameter (HR: 1.03, 95% ci: 1.01–1.04, p < 0.01), pre procedure maximum gradient (HR: 1.01, 95% ci: 1.00–1.03, p = 0.02) and higher wilkins scores (HR: 1.37, 95% ci: 1.13–1.66, p < 0.01).

**Conclusion:** In the very long term follow-up, mitral valve restenosis was observed in a quarter of the population undergoing PMBC. Preprocedure echocardiographic findings, including left atrial diameter, maximum valve gradient and high Wilkins scores were found to be the only independent predictors of this deleterious event.

108388

Modality: E-Poster Researcher – Non-case Report

Category: ACUTE AND CHRONIC CORONARY DISEASE/THROMBOLYSIS

## Evaluation of Perioperatory Complications by Bleeding in Kidney Transplantation Patients with Dual Antiplatelet Therapy

LILIAN BELINASO^1^, Camila Richter^1^, Carlos Gustavo Marmanillo^1^, Carlos Alberto Kenji Nakashima^1^, Erasmo Junior Toledo Siqueira^1^, Dalton Bertolim Précoma^1^, Marcos Vinicius Coelho Dutra^1^, Viviane de Sá Pereira^1^, Luan Gabriel Paese^1^, Sérgio Antonio López^1^, Rômulo de Lima Moreno^1^, Rodney de Oliveira^1^

(1) Hospital Angelina Caron – HAC

**Background:** As the prevalent kidney transplant population grows, there is an increasing need to quantify the risk of medical conditions to minimize complications. Bleeding events are a cause of hospitalization and contribute to the morbimortality of these patients.

**Objective:** To evaluate perioperative bleeding complications due to the use of dual antiplatelet therapy (DAPT) in kidney transplantation patients.

**Methodology:** Patients who underwent kidney transplantation between January 2019 and May 2021 were included (n = 372) and divided into 3 groups: control group without antiplatelet therapy (n = 230); patients with perioperative antiplatelet therapy only with acetylsalicylic acid – ASA (n = 123); and patients with perioperative DAPT – ASA and clopidogrel (n = 19). The primary outcome was the rate of bleeding in patients on antiplatelet therapy compared with patients without antiplatelet therapy. Secondary outcomes included location and timing of bleeding, post-bleeding acute myocardial infarction, perioperative transfusion requirement, surgical reintervention, renal explantation, and mortality.

**Results:** Patients with DAPT were a mean age of 59.63 ± 6.94, were more insulin-dependent diabetes and they were all hypertensive (P > 0,002), significantly higher data compared to the other groups. These patients bled significantly more (P > 0.005), mainly at the surgical site (64,7%); 35.3% of the patients required surgical reintervention (p = 0.0013). In this study, most bleeding events (73.6%) occurred within one week after transplantation and 80% required blood transfusion (p = 0,0001).

**Conclusion:** Kidney transplantation can be safely performed without interrupting perioperative ASA therapy and prophylactic anticoagulation. There is an increased risk of bleeding requiring blood transfusion and surgical reintervention when the patient is on DAPT, but without an increase in acute myocardial infarction, renal explantation and short-term mortality.

108408

Modality: E-Poster Researcher – Non-case Report

Category: CARDIOVASCULAR PHARMACOLOGY

## Effect of Long-Term Sodium-Glucose Cotransporter 2 Inhibition on Cardiac Remodeling in Rats with Type 1 Diabetes Mellitus

EDER ANDERSON RODRIGUES^1^, Camila Moreno Rosa^1^, Dijon Henrique Salome De Campos^1^, Felipe César Damatto^1^, Gilson Masahiro Murata^2^, Lidiane Moreira de Souza^1^, Amanda Bergamo Gonçalves de Castro Rêgo^1^, Leiliane Rodrigues dos Santos Oliveira^1^, Patrícia Aparecida Borin^1^, Katashi Okoshi^1^, Marina Politi Okoshi^1^

(1) Botucatu Medical School, Sao Paulo State University, UNESP, Botucatu, Brazil; (2) 2 LIM29, Division of Nephrology, University of Sao Paulo Medical School, Sao Paulo Brazil

**Background:** Sodium-glucose cotransporter 2 (SGLT2) inhibitors have beneficial effects on the cardiovascular system in diabetes mellitus (DM) patients. However, as most clinical trials were performed in type 2 DM, the effects of SGLT2 inhibitors in patients with type 1 DM still need further clarification. In this study, we evaluated the effects of long-term treatment with the SGLT2 inhibitor dapagliflozin on cardiac remodeling, myocardial function, and energy metabolism in rats with type 1 DM.

**Methods:** Male Wistar rats were divided into three groups: control (C, n = 15); DM (n = 15); and DM treated with dapagliflozin (DM+DAPA, n = 15) for 30 weeks. DM was induced by streptozotocin; DAPA was added to the rat chow (5 mg/kg/day). Cardiac performance was evaluated by echocardiogram and myocardial function in isolated left ventricular (LV) papillary muscle preparations. Myocardial energy metabolism enzyme activities were evaluated by spectrophotometry. Statistical analyzes: ANOVA and Tukey or Kruskal-Wallis and Dunn.

**Results:** DM+DAPA had lower glycemia than DM [C 112 (108–116); DM 531 (522–535)*; DM+DAPA 267 (179–339) mg/dL; p < 0.05 vs C and DM+DAPA]. Echocardiogram showed that DM and DM+DAPA had left atrium and left ventricle dilatation with systolic and diastolic dysfunction; in DM+DAPA, the changes were attenuated in relation to DM. Developed tension and +dT/dt were higher in DM+DAPA than DM in basal condition. After inotropic stimulation with post-pause contraction, extracellular calcium concentration elevation, and isoproterenol addition to the nutrient solution, +dT/dt and –dT/dt were higher in DM+DAPA than DM. Hexokinase, phosphofructokinase, and pyruvate kinase activity was lower in DM than the C. Phosphofructokinase and pyruvate kinase activity was higher in DM+DAPA than DM.

**Conclusion:** Long-term dapagliflozin treatment attenuates cardiac remodeling and myocardial dysfunction and preserves hexokinase, phosphofructokinase and pyruvate kinase activity in rats with type 1 diabetes mellitus.

108797

Modality: E-Poster Researcher – Non-case Report

Category: ATHEROSCLEROSIS/CARDIOVASCULAR RISK FACTORS/CARDIOVASCULAR PREVENTION

## Cardiovascular Risk Assessment in Patients with Psoriasis: Comparison of Different Clinical Scores and Their Associations with Subclinical Biomarkers Such as Pulse Wave Velocity and Carotid Intima-Media Thickness – a Pilot Study

MARCELA MATTOS SIMÕES^1^, Angélica Navarro de Oliveira^3^, Ricardo Simões^1^, Karina Braga Gomes^2^, Bruno de Almeida Rezende^4^, Marcus Vinícius Bolivar Malachias^3^

(1) Post-Graduate Program in Health Sciences – Faculty of Medical Sciences of Minas Gerais-MG, Belo Horizonte/MG, Brazil; (2) Department of Clinical and Toxicological Analysis, Faculty of Pharmacy, Federal University of Minas Gerais, Belo Horizonte/MG, Brazil; (3) Institute of Hypertension of Minas Gerais, Belo Horizonte/MG, Brazil,; (4) Department of Physiology and Biophysics, Institute of Biological Sciences, Federal University of Minas Gerais, Belo Horizonte/MG, Brazil

**Background:** Psoriasis is an inflammatory skin disease that can have systemic consequences such as cardiovascular disease (CVD).

**Objective:** To evaluate commonly recommended clinical risk scores and their associations with pulse wave velocity (PWV) and intima-media thickness (IMT), recognized subclinical cardiovascular (CV) biomarkers, in individuals with psoriasis without apparent CVD.

**Methods:** This is a cross-sectional observational pilot study involving 44 male subjects, 11 of whom had moderate to severe psoriasis, according to the Psoriasis Area Severity Index (PASI) > 10 and the Dermatological Quality of Life Index (DLQI) > 10, and 33 healthy subjects (control group). Groups were assessed using the Global Risk Score (GRS), Atherosclerosis Cardiovascular Disease (ASCVD) by Pooled Cohort Equations and the SCORE system, in addition to measurements of PWV and IMT. Comparisons between groups and associations between clinical risk scores and biomarkers measurements were performed considering a significance level of 5%.

**Results:** The psoriasis group (PG) had a higher CV risk estimated by GRS (p = 0.001) and ASCVD (p = 0.025), but not by SCORE (p = 0.289), as well as higher PWV (p = 0.033) compared to the group control (GC). ASCVD (p = 0.014) and SCORE (p = 0.009) were associated with PWV in the psoriasis group. GRS, ASCVD and SCORE showed a positive association with PWV in relation to the total sample. Clinical risk scores showed no significant association with carotid IMT in the psoriasis group.

**Conclusion:** Patients with psoriasis without apparent CVD have a higher cardiovascular risk when evaluated by clinical scores GRS and ASCVD. ASCD and SCORE were associated with subclinical findings such as higher PWV.

108731

Modality: E-Poster Researcher – Non-case Report

Category: CARDIOGERIATRICS

## Association between Maximal Aerobic Power, Isometric Muscle Strength and Functional Capacity in Elderly Women

LEANDRO BRASIL REGO^1^, Leandro Brasil Rego^1^, Rodrigo Villar^2^, Danilo Salles Bocalini^3^, Gustavo Allegretti João^4^, Ruth Caldeira Melo^1^, Bruna Trindade Souza^1^, Francisco Luciano Pontes Júnior^1^

(1) Laboratório de Fisiologia do Exercício e Envelhecimento, Escola de Artes, Ciências e Humanidades da Universidade de São Paulo, São Paulo, Brazil; (2) Cardiorespiratory & Physiology of Exercise Research Laboratory, Faculty of Kinesiology and Recreation and Management, University of Manitoba, Winnipeg, Manitoba, Canada; (3) Laboratório de Fisiologia experimental e Bioquimica, Centro de Educação física e Desporto, Universidade Federal do Espirito Santo, Vitoria, Brazil; (4) Laboratório de Fisiologia do Exercício, Faculdades Metropolitanas Unidas, São Paulo, Brazil. São Paulo, Bra

**Introduction:** Aging is associated with a decline in cardiorespiratory and musculoskeletal systems that may compromise maximum aerobic power and muscle strength, fundamental aspects for the maintenance of functional capacity in the elderly.

**Objective:** To verify the correlation between maximal aerobic power, isometric muscle strength, and functional capacity in elderly women.

**Methods:** The sample consisted of 30 elderly women aged 60 years or more (X = 65.4 SD = 2.8). To determine the maximal aerobic power, we performed ergospirometric test (gas analyzer Metalyzer II, Cortex®, Germany) on a treadmill (centurion® 300) with constant 1% inclination, initial speed of 4 km/h and 1 km/h increases every minute. The maximum strength was evaluated in lower and upper limbs by isometric contraction in a portable dynamometer (Lafayette Instrument Company, USA). The functional capacity was evaluated by the tests: TUG- (Timed Up and Go), TC 10 m (10 m walk) and SLC (Sit and Stand Up Chair). In all tests 3 measurements were taken and the mean was computed. In the statistical analysis, significance was set at 5% and the assumptions of normality, homogeneity, and sphericity were confirmed with the Shapiro-Wilk, Levene, and Mauchly tests, respectively, and parametric techniques were used.

**Results:** There was a positive and strong correlation between peak VO2 (mL/kg/min) vs. lower limb strength (r = 0.62; p = < 0.001); negative and strong correlation between peak VO2 (mL/kg/min) vs. TUG (r = –0.63; p = < 0.001); moderate and negative correlations between peak VO2 (mL/kg/min) vs. TC10 m (r = –0.49 p = 0.005); peak VO2 (mL/kg/min) vs. SLC (r = –0.52 p = 0.003) and between lower limb strength vs. TUG (r = –0.50; p = 0.005); TC10 m (r = –0.50; p = 0.005); SLC (r = –0.48; p = 0.006). The multiple linear regression showed an R2 49.8% of the result of the dependent variable aerobic power and the following values of the independent variables lower limb strength (B = 0.25; p = 0.012) and “TUG” test (B = –2.161; p = 0.008).

**Conclusions:** It was demonstrated a positive correlation of aerobic power with isometric muscle strength (lower limb) and a negative correlation of these variables with performance in functional tests. Furthermore, isometric muscle strength (lower limb) and the TUG test were important predictors of maximal aerobic power.

108420

Modality: E-Poster Researcher – Non-case Report

Category: EPIDEMIOLOGY AND HEALTH POLICIES/GLOBAL HEALTH

## Cardiovascular Health Control in the Family Health Strategy

GILBERTO ANDRADE TAVARES^1^, Gilberto Andrade Tavares^1^, Joathan Borges Ribeiro^5^, Marcos Antonio Almeida-Santos^4^, Antônio Carlos Sobral Sousa^1^, José Augusto Soares Barreto-Filho^1^

(1) Postgraduate Program in Health Sciences, Federal University of Sergipe; (2) Federal University of Sergipe; (3) Rede D’Or São Luiz, Hospital São Lucas, Division of Cardiology; (4) Tiradentes University; (5) Postgraduate Program in Adult Health Nursing, University of São Paulo; (6) Hospital Sírio-Libanês

**Introduction:** In Brazil, the Unified Health System (UHS) regulates public health care and has in the Family Health Strategy (FHS) the main strategy of primary care. In 2010, the American Heart Association (AHA) proposed to check seven cardiovascular health (CVH) metrics, with goal of reducing CVD deaths in the U.S. by 20% until 2020. The results of the FHS regarding the CVH of the Brazilian population are not known.

**Objective:** Evaluate the control of CVH among adult patients assisted by the FHS in the municipality of Aracaju, Sergipe, Brazil. METHODS A cross-sectional study was conducted using the 7 metrics in CVH among patients treated by the FHS. Those metrics being graded at the “ideal”, “intermediate” and “poor” level. The variable “CVH control” was dichotomized into “Controlled” (>five metrics at the ideal level) and “Uncontrolled” (<five metrics at the ideal level).

**Results:** In our study, 32.5% were at ideal level. The majority of the sample (62.75%) were at intermediate level. At the poor level, 4.5% was found in the sample. In the multivariate analysis, after adjustments, <45 years of age (RRa 1.61 (IC 95% 1.15–2.28)), females (RRa 2.07 (IC 95% 1.20–3.60)) and following health guidance from family members and neighbors (RRa 1.28 (IC 95% 1.15–2.28)) were associated with “Controlled” CVH. Having a higher number of children reduces the chance of having “Controlled” CVH by up to 9% (RRa 0.91 (IC 95% 0.84–0.95)).

**Conclusions:** We demonstrated that, only 32.5% of the patients had controlled CVH; the adjusted multivariate analysis showed that being under 45 years of age, female and sharing decisions about their health with neighbors and family members are associated with “Controlled” CVH. Having more children reduces this association.

108421

Modality: E-Poster Researcher – Non-case Report

Category: NURSING

## Spatial Distribution of Chronic Chagas Disease in Terms of Clinical Stages in a Reference Service in Northeast Brazil

CAROLINA DE ARAÚJO MEDEIROS^1^, Maria Beatriz Araújo Silva^3^, André Luiz Sá de Oliveira^4^, Maria das Neves Dantas da Silveira Barros^1^, Maria Elisa Lucena Sales de Melo Assunção^1^, Maria da Glória Aureliano de Melo Cavalcanti^1^, Tayne Fernanda Lemos da Silva^1^, Gênova Maria de Azevedo Oliveira^5^, Cristina de Fátima Velloso Carrazzone^1^, Zulma Maria de Medeiros^2^, Sílvia Marinho Martins^1^, Wilson de Oliveira Júnior^1^

(1) Ambulatório de Doença de Chagas e Insuficiência Cardíaca-PROCAPE-UPE; (2) Programa de Pós-graduação em Ciências da Saúde – FCM-UPE; (3) Faculdade de Enfermagem Nossa Senhora das Graças – FENSG-UPE; (4) Instituto Aggeu Magalhães, Fundação Oswaldo Cruz – Recife –PE; (5) Secretaria Estadual de Saúde de Pernambuco – PROGRAMA SANAR-SES-PE

**Introduction:** Chronic Chagas disease (CCD), caused by Trypanosoma cruzi. In Brazil, the Northeast Region is one of the endemic areas for CCD. The geoprocessing allows the identification of patterns and trends in spatio-temporal distributions.

**Objective:** To analyze the spatial distribution of CCD in terms of clinical stages in the reference service in the Northeast region of Brazil.

**Methods:** Ecological population-based approach, the unit of analysis being the municipalities where the population with CCD from 2016 to 2018. The State of Pernambuco is composed of 184 municipalities and 05 mesoregions: Metropolitan Region, Zona da Mata, Agreste, Sertão and Sertão do São Francisco. The classification used the Clinical stages listed in the “I Latin American Directive for the Diagnosis and Treatment of Chagas Heart Disease” (A, B1,B2,C and D). The analyzed indicator was the average annual rate of occurrence.

**Results:** The 801 chronic cases, the mean age was 62 ± 12.0 years, with a predominance of females (60%). As for the clinical stages classified as A: 22.5%; B1: 43.5%; B2: 8.5% and C: 25.5%. The mean CCD occurrence rates in terms of clinical stages, the respective standard deviation (SD): A: mean = 0.76; SD = 2.1 in Zona da Mata and Sertão; B1: mean = 1.6; SD = 4.4 in Zona da Mata and Sertão; B2: mean = 0.23; SD = 0.82 in Zona da Mata, Sertão and Sertão do São Francisco;C: mean = 0.67; SD = 1.27 in Zona da Mata, Agreste Sertão and Sertão do São Francisco as shown in the figure.

**Conclusions:** The spatial distribution of CCD showed spatial heterogeneit, there were clusters in two mesoregions in stages A and B1, while in stages B2 and C in 4 mesoregions indicating the persistence of CCD in these areas, requiring priority health surveillance actions and strengthening of the assistance decentralization.


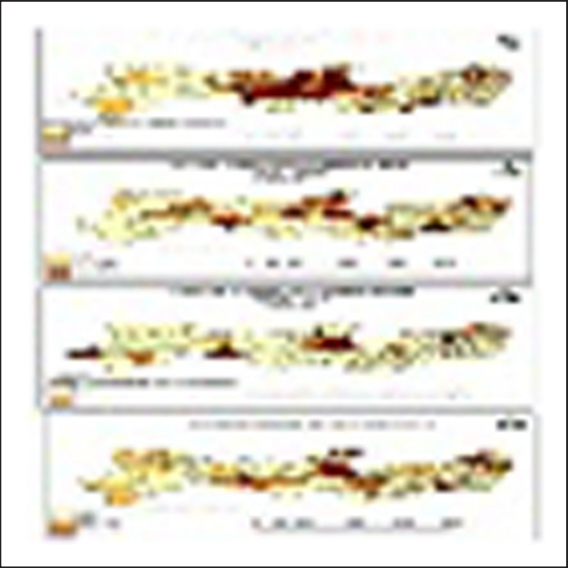


108422

Modality: E-Poster Researcher – Non-case Report

Category: DIGITAL HEALTH/INNOVATION

## Cardiovascular Health Monitoring Mobile Phone Application Development

GILBERTO ANDRADE TAVARES^1^, Matheus Henrique Costa Xavier^2^, Filipe Euclides Gobatto^2^, Iara Victoria dos Santos Moura^2^, Virna Anfrizio Souza^2^, Wictor Hugo De Souza Silva^2^, Eleonora Ferraris de Gaspare^5^, Gledson de Carvalho Santos^4^, Fabio Batista Santos^4^, Virgílio Antônio Cardoso Faro^4^, José Augusto Soares Barreto-Filho^1^

(1) Postgraduate Program in Health Sciences, Federal University of Sergipe; (2) Federal University of Sergipe; (3) Rede D’Or São Luiz, Hospital São Lucas, Division of Cardiology; (4) Tiradentes University; (5) Parma University

**Introduction:** The Cardiovascular Diseases have been the main cause of death in the world with a slow decrease in mortality rates in most countries. In 2010, The American Heart Association (AHA) defined 7 metrics for a Cardiovascular Health in order to reduce Cardiovascular mortality by 20%. The Mobile Health Tools are able to support shared clinical decision making, telemonitoring feedback and improve patient’s adherence to medication regimen.

**Objective:** Demonstrate the development and applicability of the application “Cardiovascular Health” for mobile phones according to the parameters defined by The AHA.

**Method:** The method chosen is the User Centered Design, Dart programming language, Flutter framework and Firebase database. The application was developed using Scrum, with 2-week sprints in which tasks were divided into small deliverables in order to avoid rework and make the process more agile. The research team monitored the project closely and decided about what was done by the group.

**Results:** Each parameter considered ideal, evaluated as “Good”, following those requirements, will provide the patient 1 mark. If the participant scores between 5 and 7 marks, will be classified as “Good” Cardiovascular Health; if the score is between 3 and 4 marks, the classification will be “Can be improved”; and if the score is null or under 2 marks, it will be defined as “Needs to be improved”. Hence, he will have his “Cardiovascular Health” classified, generating PDF reports.

**Conclusion:** The “Cardiovascular Health” application comprises all the components to measure Cardiovascular Health and will be able to provide physicians and other healthcare workers with better decision making about Cardiovascular Health of the treated population.

109090

Modality: E-Poster Researcher – Non-case Report

Category: EPIDEMIOLOGY AND HEALTH POLICIES/GLOBAL HEALTH

## Relationship between Heart Attack and Revasculation Procedure Events on Drug Adhesion in Individuals with Heart Diseases: Population Survey in Brazil

ROMERO HENRIQUE DE ALMEIDA BARBOSA^1^, Emerson Silva de Jesus^1^, Eldys Myler Santos Marinho^1^, Johnnatas Mikael Lopes^1^

(1) Universidade Federal do Vale do São Francisco

**Background:** Medication adherence is imperative for adequate care of chronic cardiac health conditions due to their disabling and death potential. However, this self-care characteristic does not develop without professional support and may not be homogeneous in the entire population.

**Objective:** To identify the existence of differences in medication adherence in people with a history of acute cardiac events.

**Methods:** Cross-sectional population-based study of the National Health Survey (NHS) in Brazil in 2019. The sample with 90846 interviews in people over 15 years old. Diagnosis of heart disease was given by the presence of heart failure, arrhythmia, angina, history of myocardial infarction. The independent variables were coronary artery bypass graft surgery (yes/no) and myocardial infarction (yes/no). Medication adherence due to cardiac condition was treated as an outcome. Data analyzed by stratifying the prevalence of the outcome and estimating the prevalence ratio, taking into account the complex sampling of participants and a confidence interval of 95%.

**Results:** We identified 4732 (5.1%;4.8–5.3) individuals with some type of heart disease in the NHS, of which 1383 (29.2%;27.0–31.4) reported at least one episode of myocardial infarction. Revascularization was performed in 1483 (28.9%;26.8–31.0) of individuals with heart disease. Approximately one-third of participants with heart disease do not adhere to medication regularly [3284 (31.4%:29.1–33.7)]. However, it is possible to state that individuals who underwent revascularization procedure [1297 (87.9%;85.4–90.0)] have more medication adherence than those who did not undergo. The occurrence of acute myocardial infarction increases the probability of greater adherence to medication by 13.3% in those who underwent revascularization and 27.5% in those who had an infarction and did not undergo revascularization. In relation to individuals with heart disease and without infarction and revascularization events, the probability of adherence is 59.10%.

**Conclusion:** Acute and harmful cardiac events such as myocardial infarction and revascularization seem to be associated with greater medication adherence in this population. This refers to a scenario of low self-care supported by monitoring of cardiac conditions, requiring greater attention and the proposition of mitigating strategies on the part of health professionals.

109088

Modality: E-Poster Researcher – Non-case Report

Category: EPIDEMIOLOGY AND HEALTH POLICIES/GLOBAL HEALTH

## Prevalence, Outcomes and Costs of Patients with Renal Dysfunction in the Emergency Department of a Specialized Cardiology Hospital

FARID SAMAAN^1^, Louis Nakayama Ohe^1^, Lívia Gâmbaro^1^, Renata Viana^1^, Emmanuel Almeida Burdmann^2^

(1) Instituto Dante Pazzanese de Cardiologia; (2) Universidade de São Paulo

**Introduction:** Chronic kidney disease and acute kidney injury (AKI) are important complications of heart diseases. In developing countries, epidemiological and cost information on the interaction of these conditions are scarce.

**Objectives:** To determine the prevalence, costs and outcomes of patients admitted for acute coronary syndrome (ACS) with renal dysfunction and AKI.

**Methods:** The study was based on a prospective database analysis of patients admitted for ACS to a Brazilian public hospital specialized in cardiology between 7/16/2018 and 12/31/2019. Renal dysfunction was defined as an estimated glomerular filtration rate (eGFR) <60 ml/min/1.73 m² at hospital admission. Community-acquired and hospital-acquired AKI were defined as a fall and an increase of ≥0.3 mg/dl in serum creatinine from baseline, respectively.

**Results:** 1295 of the 1620 patients had a confirmed diagnosis of ACS (median age 64.2 [56.5–70.6] years, 65.4% male, 82.7% had hypertension, 45.5% diabetes and 22.6% renal dysfunction). The imaging diagnosis of ACS was coronary angiography in 84.3% and the treatment was performed by angioplasty, only clinically and by myocardial revascularization in 47.3%, 40.0% and 12.7%, respectively. Hospital- and community-acquired AKI occurred in 43.9% and 2.3% of patients, respectively. Compared with patients admitted with eGFR ≥60 ml/min/1.73 m², those with eGFR <60 were older (70.6 vs. 62.5 years, p < 0.001), had a higher prevalence of hypertension (92.1% vs. 80.0%, p < 0.001) and diabetes mellitus (60.6% vs.54.5%, p = 0.010), had a higher incidence of AKI (65.0% vs. 51.4%, p < 0.001) and higher: median amount reimbursed for hospitalization (1,344 [366–2,103] vs. 1,334 [290–2,018] dollars, p = 0.034), median length of stay (5 [3–10] vs. 4 [2–7] days, p < 0.001), death within 30 days (4.1% vs. 1.4%, p = 0.004) and death within 12 months (9.2% vs. 2.9%, p < 0.001). Patients with AKI, compared to those without this condition, were older (65.6 vs.63.3 years, p = 0.008), had lower eGFR on admission (78.1 ml/min/1.73 m² vs.86.1 ml/min/1.73 m², p < 0.001) and greater: median amount reimbursed for hospitalization (1,334 [301–1,865] vs. 1,724 [973–2,549] dollars, p < 0.001), median length of stay (6 [4–13] vs. 3 [2–5] days, p < 0.001) and death within 12 months (4.8% vs. 2.3%, p = 0.032).

**Conclusions:** In patients with ACS at a cardiology referral hospital, renal dysfunction on admission and AKI during hospitalization were frequent and associated with worse clinical and economic outcomes.

108437

Modality: E-Poster Researcher – Non-case Report

Category: HEART FAILURE/CARDIOMYOPATHY/TRANSPLANT

## Growth Differentiation Factor-15 is Associated with Greater Severity in Patients with Chronic Heart Failure

DIANE XAVIER DE ÁVILA^1^, Gustavo Rodolfo Moreira^1^, Angelo Michele Di Candia^1^, Victoria Depes Scaramussa^1^, Najla Cassibi Cavaliere^1^, Fernanda Turque Martins^1^, Maisa Passos Vieira^1^, Humberto Villacorta^1^

(1) Universidade Federal Fluminense

**Background:** Natriuretic peptides (NP) are the gold standard biomarkers in HF. However, new biomarkers have emerged, with additional effects to NP in relation to prognosis. Growth differentiation factor-15 (GDF-15) is a marker of oxidative stress and inflammation that may contribute to the prognosis of patients with HF.

**Objectives:** We sought to evaluate the distribution of GDF-15 values, the characteristics of patients with high values and the relationship between their values and NT-proBNP in patients with chronic HF.

**Methods:** This was a cross-sectional study of patients with chronic HF who were on guideline-recommended medical therapies. Patients with signs and symptoms of HF and LVEF <50% were included. The dosage of NT-proBNP was performed using the Elecsys® system (Roche, Basel, Switzerland) and GDF-15 by the sandwich immunoassay method with monoclonal antibodies (Elecsys®, Roche, Basel, Switzerland). Patients were grouped according to GDF-15 values above and below the median. Correlation analysis was performed between GDF-15 and other non-normal continuous variables, using the Spearman method.

**Results:** A total of 67 patients were included. Etiology was predominantly non-ischemic, 61.2% were male, with mean age of 61 ± 13 years. The median GDF-15 values were 1413 pg/mL (interquartile range 1044–2554) and NT-proBNP was 759 pg/mL (168–2354). Patients with GDF-15 values above the median had higher creatinine values (1.32 mg/dL [0.85–1.71] vs 0.91 [0.78–1.08], p < 0.001), higher NT-proBNP values (2071 pg/mL [508–5350] vs 463 [87–962], p = 0.001) and lower LV ejection fraction (30% [27–43] vs 45% [30–54], p = 0.08. There was a positive correlation between GDF-15 and NT-proBNP (r = 0.46, p < 0.001), creatinine (r = 0.56, p < 0.001) and left atrial volume (r = 0.32, p = 0.037).

**Conclusion:** Even after adequate treatment, some patients with chronic HF had high levels of GDF-15. GDF-15 identified patients with greater severity. GDF-15 may be a useful tool in the management of HF patients.

108438

Modality: E-Poster Researcher – Non-case Report

Category: CARDIAC ARRHYTHMIAS/ELECTROPHYSIOLOGY/ELECTROCARDIOGRAPHY

## Growth Differentiation Factor-15 is Associated with Severity Parameters in Patients Admitted to the Hospital with Atrial Fibrillation

ANGELO MICHELE DI CANDIA^1^, Diane Xavier de Ávila^1^, Gustavo Rodolfo Moreira^1^, Humberto Villacorta^1^

(1) Universidade Federal Fluminense

**Background:** Growth differentiation factor-15 (GDF-15) is a marker of oxidative stress and inflammation and is increased in a number of cardiovascular disorders.

**Objectives:** We sought to assess the relationship of GDF-15 with severity parameters in patients hospitalized with atrial fibrillation (AF).

**Methods:** Fifty hospitalized patients with a primary or secondary diagnosis of AF from two hospitals were included. GDF-15 was measured at baseline, as well as cardiac and inflammatory biomarkers. A comparison of baseline characteristics was performed in groups with high (above median) and low (below median) GDF-15 values. Correlations were made between the GDF-15 and other variables, using the Spearman method.

**Results:** Twenty-nine (58%) patients were male and the mean age was 68.5 ± 17.3 years. Twenty-eight (56%) had permanent AF and 18 (36%) had HF with reduced ejection fraction (LVEF <40%, HFrEF). The median GDF-15 was 2724 pg/mL (interquartile range 1116–5139). Patients with high GDF-15 values were older (77.5 ± 9.7 vs 59 ± 18.5 years, p = 0.0001) and more likely to have hypertension (92.3% vs 70%, p = 0.018) and permanent AF (69.2% vs 37.5%, p = 0.016). They also had higher NT-proBNP values (4006 pg/mL [2156–7023] vs 816 [174–3814], p = 0.0015), creatinine (1.2 [0.95–1.7] vs 1, 0 [0.75–1.15], p = 0.017) and C-reactive protein (3.1 [1.55–7.32] vs 1.25 [0.55–2.45], p = 0.008). GDF-15 was higher in patients with HF (n = 34 [68%]) vs non-HF (3126 [1737–6457] vs 774 [565–4074], p = 0.01). Patients with HFrEF had higher levels of GDF-15 as compared with those with LVEF >40% (3019 [2012–7299] vs 1533 [784–4674], p = 0.05). All risk scores were higher in patients with high GDF-15, namely CHA2DS2-VASC (4.5 ± 1.6 vs 2.9 ± 2.3, p = 0.008), HAS-BLED (2.4 ± 1.3 vs 1.3 ± 1.1, p = 0.0029), ORBIT (3.5 ± 1.5 vs 1.7 ± 1.5, p = 0.0003) and MAGGIC (20.8 ± 12.3 vs 11 ± 11.2, p = 0.006). There was a direct correlation between GDF-15 and NT-proBNP (r = 0.5, p = 0.002), D-dimer (r = 0.52, p = 0.002) and age (r = 0.43, p = 0.002).

**Conclusion:** GDF-15 values were associated with parameters of greater severity in AF. GDF-15 may be useful as a prognostic factor and may possibly aid in the indication of anticoagulation in AF.

108439

Modality: E-Poster Researcher – Non-case Report

Category: HEART FAILURE/CARDIOMYOPATHY/TRANSPLANT

## Relationship between Gdf-15, Urinary Sodium and Markers of Renal Function in Patients with Chronic Heart Failure

GUSTAVO RODOLFO MOREIRA^1^, Diane Xavier de Ávila^1^, Angelo Michele Di Candia^1^, Victoria Depes Scaramussa^1^, Najla Cassibi Cavaliere^1^, Maisa Passos Vieira^1^, Fernanda Turque Martins^1^, Humberto Villacorta^1^

(1) Universidade Federal Fluminense

**Background:** Patients with heart failure (HF) often have altered renal function. Urinary sodium is a marker of diuretic resistance and is associated with a worse prognosis in HF. Growth differentiation factor-15 (GDF-15) is a marker of oxidative stress and inflammation and is a prognostic predictor in HF.

**Objectives:** We sought to assess the relationship of GDF-15 with renal function parameters and with urinary sodium in patients with chronic HF.

**Methods:** We undertook a cross-sectional study of patients with HF from specialized outpatient clinic. Patients with signs and symptoms of HF and LVEF <50% were included. An echocardiogram was performed and blood samples were collected, which were frozen for the final study, where the performance of GDF-15 in the prediction of renal outcomes in HF will be evaluated. The dosage of NT-proBNP was performed using the Elecsys® system (Roche, Basel, Switzerland) and GDF-15 by the sandwich immunoassay method with monoclonal antibodies (Elecsys®, Roche, Basel, Switzerland). Analysis of the relationship between GDF-15 and baseline renal parameters was performed. Correlation analysis was performed between GDF-15 and continuous variables, using the Spearman method.

**Results:** Sixty-seven patients were included. The etiologies of HF were hypertension, diabetes mellitus, alcoholic cardiomyopathy and idiopathic cardiomyopathy. Forty-one (61.2%) individuals were male, with a mean age of 61 ± 13 years. Median GDF-15 values were 1413 pg/mL (interquartile range 1044–2554). Patients with GDF-15 values above the median had lower urinary sodium values (88 mEq/L [53–121] vs 112 [76–171], p = 0.06), lower glomerular filtration rate (GFR) (63.9 ± 29.1 vs 91.2 ± 25 mL/min/1.73 m^2^, p = 0.001) and higher creatinine levels (1.32 mg/dL [0.85–1.71] vs 0 .91 [0.78–1.08], p < 0.001). There was no significant difference in relation to urinary albumin (21 mg/L [7–126] vs 15.1 [5.8–41], p = 0.30). The urinary albumin/creatinine ratio was higher in the group above the median, but did not reach statistical significance (38 mg/g [11–110] vs 12.5 [4.9–29], p = 0.13). There was a direct correlation between GDF-15 and creatinine (r = 0.56, p < 0.001) and an inverse correlation with urinary sodium (r = –0.39, p = 0.005) and with GFR (r = –0.55, p < 0.001).

**Conclusion:** GDF-15 correlated with lower urinary sodium levels and worse kidney function in patients with chronic HF. Future studies should address whether GDF-15 is a predictor of worsening renal function overtime.

108440

Modality: E-Poster Researcher – Non-case Report

Category: COVID-19 AND CARDIOVASCULAR SYSTEM

## The Impact of Cardiovascular Risk Factors and Renal Disease on Outcomes in Patients Hospitalized with COVID-19: An Observational Study from Two Public Hospitals in Brazil

DIANE XAVIER DE ÁVILA^1^, Ana Luiza Carraro de Souza^2^, Gabriel Alverca Meyas^2^, Mayara Cristina Villela Santos^2^, Jonatas da Costa Mendonça^2^, Luciene Maria Mendes da Costa^2^, Beatriz de Paula Sousa^2^, Maria Victoria Borges de Oliveira^2^, Julia Correia Cardoso Guimarães^2^, Julie Xavier de Ávila Guedes^3^, Ulisses Oliveira de Melo^1^, Humberto Villacorta^2^

(1) Hospital Municipal Che Guevara; (2) Universidade Federal Fluminense; (3) Universidade Estácio de Sá

**Background:** Cardiovascular risk factors are prognostic factors in COVID-19 and have been scarcely studied in Brazil.

**Objectives:** To assess the impact of cardiovascular risk factors on the outcomes in patients admitted for COVID-19.

**Methods:** From July 2020 to February 2021, 200 patients from two public hospitals were enrolled. Patients were included if they had: typical symptoms or signs of COVID-19, a positive real-time polymerase chain reaction test (RT-PCR) for COVID-19, and age above 18 years old. This was a prospective, observational, longitudinal study. Data was collected within 24 hours from admission. The primary endpoint was a combination of hospital mortality, mechanical ventilation, hemodialysis or hospital length of stay >28 days.

**Results:** There were 98 (49%) events during the hospital course and 72 (36%) died in the hospital. Patients with a primary endpoint were older and more likely to have a history of hypertension, diabetes, chronic obstructive pulmonary disease (COPD) and chronic kidney disease (CKD). Vital signs at admission associated with events were diastolic blood pressure, respiratory rate and oxygen saturation in ambient air (O2Sat). Serum creatinine >1.37 mg/dL at admission had sensitivity of 51.6 and specificity of 82% to predict the primary endpoint, with an area under the curve (AUC) of 0.68. In multivariate analysis, age, diabetes, CKD, and COPD were independent predictors of the primary endpoint. Age and CKD were independent predictors of in-hospital mortality.

**Conclusion:** Cardiovascular risk factors, such as diabetes and CKD are related to a worse prognosis in patients hospitalized with COVID-19 in Brazil.

108452

Modality: E-Poster Researcher – Non-case Report

Category: CARDIAC ARRHYTHMIAS/ELECTROPHYSIOLOGY/ELECTROCARDIOGRAPHY

## Remote Monitoring of Pacemakers and Defibrillators – Effective and Safe in Brazil?

EDUARDO ARRAIS ROCHA^1^, Luís Gustavo Bastos Pinho^1^, Juvêncio Santos Nobre^1^, Maria Eduarda Quidute Arrais Rocha^3^, Marcela Sobreira Kubrusly^2^, FernandaPimentel Arrais Maia^4^, Eduardo Augusto Quidute Arrais Rocha^2^, Maria Camila Timbó Rocha^3^, Pedro Barbosa Duarte Vidal^1^, Vitor Olímpio Coimbra^2^, Bruna Sobreira Kubrusly^1^, Ana Rosa Pinto Quidute^1^

(1) Universidade Federal do Ceará; (2) Universidade Unichristus; (3) Universidade de Fortaleza – UNIFOR; (4) Universidade Federal do Ceará – Campos Sobral

**Introduction:** The remote monitoring (RM) of Implantable electronic cardiac devices (IECD) has become a common follow-up modality in many countries given its effectiveness, safety, facilities for the patient and possibilities of early interventions. In Brazil, however, this form of follow-up is an exception. This work aims to demonstrate the findings, evolution and peculiarities of IECD follow-up in a tertiary center in Brazil.

**Methods:** This is a cohort, prospective study, involving 119 patients, followed in room office visit at 6 months interval with daily RM, with mean age 72 ± 14.2, ejection fraction 55% (34.5/57%), 57.1% in functional class ≥II, 30.2% with pacemakers (PM), 42.8% with defibrillators (D), 3.3% with biventricular pacemaker (CRT) and 22.7% with CRT-D. The logistic regression was used to obtain evidences that the RM is associated with the following outcomes: immediate changes in therapy, elective changes in therapy and avoidance of hospitalizations.

**Results:** Events were detected in 63.9% of the cases in 29.5 ± 23 months of follow-up; 16% presented more than 6 events; 36.1% no events and 27.7% had more than one event detected. The most common events were: 18.5% with alterations in the electrode/battery or in the thoracic impedance parameters; 33.6% with ventricular arrhythmia and 44.5% with supra-ventricular arrhythmias. It was observed that 86.5% of patients and 91.6% of physicians reported feeling secure with this method of follow-up. The outcomes found were: 23.5% needed immediate changes in therapy, while 44.5% needed elective changes. The RM was important to the beginning or maintaining of the anticoagulation in 16.9% and avoided hospitalization in 19.3%. Through the use of logistic regression, there are evidences that the RM had an statistically significant impact in the elective changes of conduct (p = 0.03), immediate changes of conduct (p = 0.007) and reduced hospitalization (p = 0.04).

**Conclusion:** The RM was considered effective and safe in the following-up of patients with IECD in some regions of Brazil. This method of follow-up allowed for many early interventions or electives ones which made therapeutic handling and increased safety of the patients.

108459

Modality: E-Poster Researcher – Non-case Report

Category: CARDIOVASCULAR IMAGING

## Potential Clinical Relevance of Left Atrial Strain in the Identification of Undetermined Diastolic Dysfunction in Patients with Chronic Kidney Disease Under Conservative Treatment

JAIME AFONSO SOUSA NETTO^1^, Arise Garcia de Siqueira Galil^1^, Andre Pereira Duque Estrada^2^, Marcus Gomes Bastos^1^

(1) Universidade Federal de Juiz de Fora-UFJF; (2) Universidade Federal Fluminense-UFF

**Background:** Half of the patients with heart failure (HF) have diastolic dysfunction (DD) and a preserved left ventricular ejection fraction (LVEF). The indexed left atrial volume (ILAV) is one of the main components in the assessment of DD. However, there are controversies regarding the use of the left atrial strain (LAE) to assess DD.

**Objectives:** To evaluate the use of LAE in the identification of indeterminate diastolic dysfunction (IDD) not detected by ILAV, in patients with chronic kidney disease (CKD) stages 3B to 5 in conservative treatment and LVEF.

**Methods:** Prospective cross-sectional study that evaluated 114 patients with CKD stages 3B to 5 under conservative treatment and who presented LVEF through transthoracic echocardiography (TTE). The patients were divided into two groups, with and without left ventricular hypertrophy (LVH). The analysis of the strain of the left atrium was evaluated by the speckle tracking method from the apical section of 2 and 4 chambers, evaluating the deformation curves of the left atrium (LA).

**Results:** LAE increased the detection of DD in the group with LVH (p < 0.01). The mean glomerular filtration rate (GFR) (in mL/min/1.73 m^2^) in patients with and without LVH was 32.6 ± 11.9 and 40.4 ± 13.9, respectively (p < 0.05). The LAE found was lower in the LVH group 20.3 ± 5.11 vs 26.1 ± 9.1, (p < 0.05).

**Conclusion:** In patients with non-dialysis CKD stages 3B to 5 with LVH and LVEF, in the studied sample, the use of LAE potentially increases the identification of indeterminate cases of DD.

108717

Modality: E-Poster Researcher – Non-case Report

Category: HEART FAILURE/CARDIOMYOPATHY/TRANSPLANT

## Influence of Type 2 Diabetes Mellitus on Mortality from Congestive Heart Failure Due to Chagas Disease in Women and Men

ANTONIO DE PADUA MANSUR^1^, Carlos Henrique Del Carlo^1^, José Antonio Ramos Neto^1^, André Barbosa de Abreu^1^, Airton Roberto Scipioni^1^, Antonio Carlos Pereira Barretto^1^

(1) Insituto do Coração – HC FMUSP

**Background:** Chronic Chagas cardiomyopathy (CCC) is one of the leading causes of congestive heart failure (CHF) in Brazil and carries high morbidity and mortality. Type 2 diabetes mellitus (T2DM) is associated with a higher cardiovascular risk in women than men. This study aimed to analyze the influence of T2DM on CHF mortality from CCC in women and men.

**Methods:** From February 2017 to September 2020, we followed CCC’s cohort of outpatients with CHF (Framingham criteria). Specific serological tests diagnosed Chagas disease. Baseline data included clinical features and echocardiographic findings. Statistical analyzes used the Kaplan-Meier method for time-to-event data and Cox proportional hazards methods to look for predictors of death.

**Results:** We studied 733 patients mean age of 61.4 ± 12.3 years, 381 (52%) were male. T2DM was present in 46 (15%) women and 40 (12%) men. Women with T2DM, compared to men with T2DM, had a higher mean age (62.7 ± 12 vs. 59.7 ± 12.5 years; p < 0.001), had a higher mean left ventricular ejection fraction (LVEF) (42.8 ± 14.3% vs. 37.3 ± 14.3%; p < 0.001) and a smaller left ventricular diastolic diameter (58.2 ± 8.5 vs. 61.9 ± 8 .6 mm; p < 0.001). Over a 3-year follow-up period, 26 (65%) men and 30 (65.2%) women with T2DM died, and mortality were higher in T2DM patients compared to nondiabetics (Women: log-rank p < 0.001; Men: log-rank p < 0.001; Figure). Cox regression adjusted for age, previous myocardial infarction, T2DM, previous stroke, chronic kidney disease (CKD), atrial fibrillation (AF), and LVEF showed, in descending order, T2DM (HR = 2). .19), stroke (HR = 1.81), AF (HR = 1.80), CKD (HR = 1.72), and reduced LVEF (HR = 1.29) as the most important predictors of death in women and, in men, stroke (HR =), DM2 (HR = 2.01), CKD (HR = 1.83), AF (HR = 1.58) and reduced LVEF (HR = 1.24).

**Conclusions:** CHF mortality from CCC was similar in women and men with T2DM. T2DM neutralized the protection of women compared to men concerning mortality from CHF.

108719

Modality: E-Poster Researcher – Non-case Report

Category: NURSING

## Quality of Life and Depression in Patients with Heart Failure

LEANDRO LOUREIRO BUZATTO^1^, Tarsila Perez Mota^1^, Leticia de Lana Pereira^1^, Mayumi Alves Hayafugi^1^, Daisa de Mesquita Escobosa^1^, Marina Barros de Melo^1^, Carolina Ivo de Araujo^1^, Carla Manuela Pereira de Araujo^1^, Marcelo Franken^1^

(1) Sociedade Beneficente Israelita Brasileira Hospital Albert Einstein

**Introduction:** The adaptations and changes in the lifestyle of patients with heart failure (HF) is the main reason why the quality of life of these individuals becomes fragile. Studies show the high prevalence of depression and associate this condition directly with HF. The relationship between changes in quality of life, the occurrence of depression and the presence of HF result in unfavorable outcomes for the patient, justifying the performance of the present study.

**Objective:** Identify factors associated with quality of life and depression in patients with heart failure.

**Method:** This is a descriptive exploratory cross-sectional study, in patients aged over 18 years, hospitalized with a diagnosis of systolic HF and who had a recent hospitalization between the period March 2017 to December 2020. Patient Health Questionnaire (PHQ2) and Kansas City Cardiomyopathy Questionnaire (KCCQ-12) were administered 30 days after discharge by telephone contact. The analysis considered the relationship between the scores of both questionnaires and the following variables: gender, diagnosis, functional class, previous atrial fibrillation, previous diabetes, previous acute myocardial infarction and previous arterial hypertension.

**Conclusions:** Female patients with heart failure showed a greater association with the depression outcome (p = 0.001) and the reduction in quality of life showed a decline once associated with diagnosis, functional class and age.


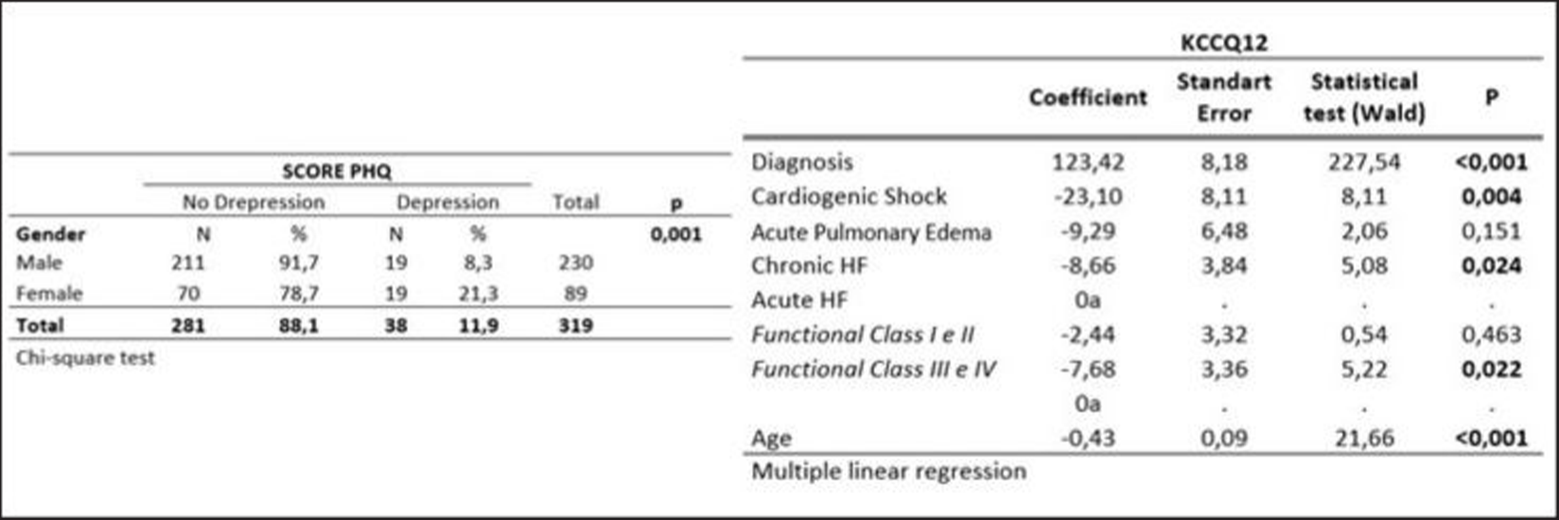


108484

Modality: E-Poster Researcher – Non-case Report

Category: NURSING

## A Transition Program is Effective in Improving Adolescents’ Knowledge about their Congenital Heart Disease: The Stepstones-CHD Trial

EWA-LENA BRATT^1^, Mariela Acuna Mora^1^, Carina Sparud-Lundin^1^, Sandra Skogby^1^, Åsa Burström^2^, Markus Saarijärvi^1^, Katrina Hanseus^3^, Annika Rydberg^4^, Shalan Fadl^5^, Eva Fernlund^6^, Kalliopi Kazamia^7^, Philip Moons^8^

(1) Institute of health and care science, University of Gothenburg, Sweden; (2) Karolinska Institute, Department of Neurobiology, Care Sciences and Society, Stockholm, Sweden; (3) Department of Pediatric Cardiology, Children’s Heart Center, Skane University Hospital, Lund, Sweden; (4) Department of Clinical Sciences, Umeå University, Umeå, Sweden; (5) Department of Paediatrics, Örebro University Hospital, Örebro, Sweden; (6) Department of Clinical and Experimental Medicine, Linköping University, Division of Pediatrics, Crown Princess Victoria Children’s Hospital, Linköping University Hospital, Linköping, Sweden; (7) Children’s Heart Center Stockholm-Uppsala, Karolinska University Hospital and Akademiska University Hospital, Sweden; (8) KU Leuven, Department of Public Health and Primary Care, Leuven, Belgium

**Introduction:** Adolescents with congenital heart disease (CHD) need to acquire knowledge about their heart disease and treatment. This is fundamental for them to implement health promoting behaviors and know how to manage their CHD. Amongst other things, transition programs aim to equip adolescents with this knowledge. However, evidence on the effectiveness of such interventions is limited.

**Objective:** To investigate if a transition program is effective in improving disease-related knowledge of adolescents with CHD.

**Methods:** The STEPSTONES (Swedish Transition Effects Project Supporting Teenagers with chrONic mEdical conditionS) transition program is an 8 components intervention. The central component was a transition coordinator (specialized nurse) who had three consultations with the participants during a 2y period. A randomized controlled trial (RCT) was conducted in two CHD centers in Sweden. Participants were randomly assigned to the intervention (IG; n = 70) or control group (CG; n = 69). Knowledge was measured at the age of 16 y (T0; baseline), 17y (T1) and 18.5y (T2) using the Knowledge Scale for Adults with Congenitally Malformed Hearts questionnaire, psychometric evidence supported its validity and reliability. The total score ranges from 0–7, with higher scores denoting a higher CHD-related knowledge. Change in score between T0 and T2 was analyzed using Fisher’s non-parametric permutation test unadjusted between the two groups. Analyses were performed on the Full Analysis Set (FAS).

**Results:** In total, 114 participants were included in the FAS, of which 54 were in the IG and 60 in the CG. At baseline, the mean knowledge score in the IG was significantly lower (3.7 ± 1.6) than in the CG (4.4 ± 1.5) (p = 0.03). At T2, the knowledge score was significantly higher in the IG (5.0 ± 1.3) than in the CG (4.5 ± 1.5) (p = 0.045). In line with this, the change over time (T0–T2; 2.5y) was significantly different between the two groups (Mean difference = 1.22; 95%CI = 0.60–1.82; p = 0.0002). The effect size was 0.74, indicating a moderately large effect.

**Conclusion:** This RCT showed that the STEPSTONES transition program was effective in increasing disease-related knowledge in adolescents with CHD, which is an important aspect in preparing them for the transition to adulthood and the transfer to adult care.

108485

Modality: E-Poster Researcher – Non-case Report

Category: CARDIO-ONCOLOGY

## 18F-FDG PET/CT in the Early Diagnosis of Cardiotoxicity: Evaluation of the Best Site to Obtain the Cardiac SUV Index

DIEGO RAFAEL FREITAS BERENGUER^1^, Gustavo Freitas Alves de Arruda^1^, Mayara Laís Coêlho Dourado^1^, Felipe Alves Mourato^2^, Monica de Moraes Chaves Becker^1^, Roberto de Oliveira Buril^1^, Brivaldo Markman Filho^1^, Simone Cristina Soares Brandão^1^

(1) Universidade Federal de Pernambuco (UFPE); (2) Real Hospital Português de Beneficiência em Pernambuco

**Objective:** To evaluate the behavior of the standardized uptake value (SUV) of the radiopharmaceutical 18F-FDG before, during and after chemotherapy (QT) at different cardiac sites, as well as to measure the degree of reproducibility for the method in the context of cancer treatment follow-up.

**Materials and Methods:** Retrospective cohort including lymphoma patients who underwent 18F-FDG PET/CT before, during and/or after chemotherapy. The uptake behavior through the mean and maximum SUVs was evaluated in four cardiac sites and in control sites in the aorta and liver. Twenty exams were randomized for reproducibility analysis by two examiners who were blinded to each other’s results. Each one of them did the evaluation in a second moment to elucidate the intra-observer reproducibility.

**Results:** A significant increase in SUVs was observed in all cardiac sites in the interim and final moments (post-terminus of QT) when compared with pre-QT SUVs. The left ventricular (LV) free wall was the cardiac region with the greatest increase in 18F-FDG uptake (Figure). As for the reproducibility, it was possible to verify substantial results for the reliability of the measurement of SUVs, both intra and inter-observer (Figure).

**Conclusions:** 18F-FDG Cardiac uptake increased along QT, with the LV free wall being the site with the greatest increase. The reproducibility analysis showed high intra- and inter-observer correlation values.


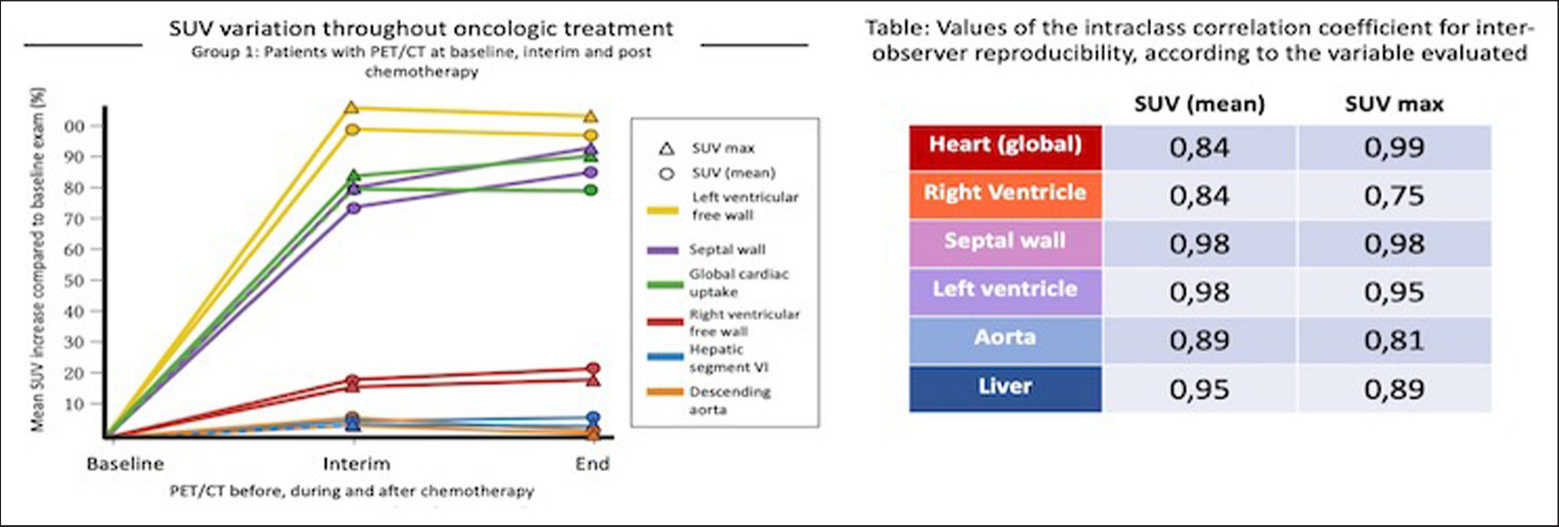


108487

Modality: E-Poster Researcher – Non-case Report

Category: NUTRITION

## Investigating Perception on Obesity for Prevention of Cardiovascular Diseases

RAMPHUL YOGESHWAREE^1^, Chan Sun Marie France^1^, Cheeneebash Jayrani^1^

(1) University of Mauritius, Faculty of Medicine and Health Sciences

**Introduction:** In Mauritius, a rapidly developing island in the Indian Ocean, 89% of the deaths are due to non-communicable diseases, out of which 33% are caused by Cardiovascular Diseases. With the prevalence of obesity which has increased from 10.3% in 2004 to 19.1% in 2015, cardiovascular complications linked to obesity are expected to increase, based on the well-established relationship between obesity and cardiovascular diseases.

**Objective:** This study investigated the perception on obesity of Mauritian police officers for tailor-made health interventions. No such study had previously been conducted in Mauritius.

**Methods:** This cross-sectional study was carried out using a pre-tested survey instrument, based on the Health Belief Model with obesity-related questions pertaining to participants’ perceived susceptibility, severity, barriers, benefits, cues to action and self-efficacy. The study population consisted of random sample police officers, with the calculated sample size being 384. Ethical clearance was obtained from the relevant research ethics committee.

**Results:** There were 384 respondents, consisting of 240 men (62.5%), and 144 women (37.5%) with age span from 18 to 59, 77.3% of the study population being in the age group 26 to 50. The participants’ mean score on the perceived severity scale was 3.8 out of five points (±1.2), while their mean score on the perceived susceptibility scale was 3.0 out of five points (±1.2). A stepwise multiple regression analysis showed that the susceptibility score (dependent variable) and sex, age, ethnicity, socioeconomic position and alcohol (independent variables) were significant at 95% confidence interval.

**Discussion:** The lower mean susceptibility score among women as compared to men and among those who are overweight/obese as compared to those with normal Body Mass Index is of great concern. The findings of this study need to be the basis for the development of customized health interventions for the primary prevention of cardiovascular diseases. The vulnerability of women to cardiovascular diseases needs to be emphasized to make women more cautious on their health.

**Conclusions:** In light of the findings of this study, we recommend primary prevention with integrated approach for promotion of healthy eating and physical activities, with a particular focus on women who need to be sensitized on their vulnerability to cardiovascular diseases.

108490

Modality: E-Poster Researcher – Non-case Report

Category: HEART FAILURE/CARDIOMYOPATHY/TRANSPLANT

## Prognostic Impact of Right Ventricular Dysfunction After Acute Decompensated Heart Failure. the Forgotten Ventricle

BRUNO REZNIK WAJSBROT^1^, Ana Luiza Ferreira Sales^1^, Andre Luis Sales Feitosa^1^, Carolina Pereira de Barros^1^, Felipe Neves de Albuquerque^1^, Marcelo Imbroinise Bittencourt^1^, Pedro Pimenta de Mello Spineti^1^, Roberto Esporcatte^1^, Denilson Campos de Albuquerque^1^, Ricardo Mourilhe-Rocha^1^

(1) State University of Rio de Janeiro – Pedro Ernesto University Hospital

**Introduction:** Right Ventricle (RV) failure is found in as many as fifty percent of heart failure (HF) patients. Echocardiographic parameters are studied with the aim of refining prognosis of HF, but isolated prognostic impact of the “forgotten ventricle” is not often studied.

**Purpose:** The objective of this study is to define echocardiographic parameters associated with post-discharge mortality after admission for ADHF.

**Methods:** This is a study in a single center, transversal, cohort of hospitalized patients with ADHF, aged >18 years, conducted at a University Hospital in Brazil. Data was collected from September 2019 to September 2021. Patients in renal replacement therapy before admission or with cancer in palliative care were excluded. Continuous variables were analyzed with Mann-Whitney U test, categorical variables with log-Rank test. Analysis was made using SPSS software.

**Results:** A total of 200 patients were enrolled. The mean age was 62 years (SD ± 14.5), 65.9% male, 63% with hypertension, 29.1% with diabetes and 32.8% with ischemic heart disease (IHD). Three month and a year mortality was respectively 8.4% and 39.5%. HFrEF was observed in 67.2%, 14.4% HFmrEF and 18.4% HFpEF. Severe mitral regurgitation (MR) was observed in 22.5% RV dysfunction in 47%. Median ejection fraction, end-systolic (ESD), end-diastolic diameter (EDD) and systolic pulmonary arterial pressure (sPAP) was respectively 33% (24–45.5), 49 mm (38.25–56), 59 mm (52–65) and 44.5 mmHg (32.75–57.25). Only RV dysfunction was associated with reduced long-term mortality (p = 0.038). Average follow-up time was 459 days.

**Conclusion:** RV disfunction has an isolated prognostic impact on long term survival after admission for ADHF. Trials that focus on therapies for RV function might reduce mortality for HF patients.


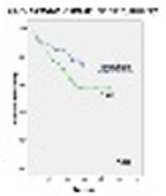


108496

Modality: E-Poster Researcher – Non-case Report

Category: CARDIOVASCULAR SURGERY

## Efficacy of Patient Blood Conservation Strategies and Reduction of Plasma Transfusions and Their Complications in Coronary Artery Bypass Graft Surgery

ANTONIO ALCEU DOS SANTOS^1^, Gilmara Silveira da Silva^2^, Flávia Cortez Colósimo Bastos^2^, José Francisco Baumgratz^2^, José Pedro da Silva^2^, Luciana da Fonseca da Silva^2^, Rodrigo Moreira Castro^2^, Rodrigo Freire Bezerra^2^, Carlos Eduardo Panfilio^1^, Isabel Cristina Céspedes^1^

(1) Escola Paulista de Medicina – Universidade Federal de São Paulo (UNIFESP), São Paulo, SP, Brazil; (2) A Beneficência Portuguesa de São Paulo Hospital, São Paulo, Brazil (BP-SP)

**Introduction:** Allogeneic blood is an increasingly scarce resource in blood banks around the world due to its increasing demand [1]. Cardiac surgeries represent an excessive consumption of hemocomponents [2]. Plasma transfusions are associated with more risks than benefits [3,4]. The rational use of allogeneic blood has become a worldwide necessity, aiming to reduce morbidity and mortality and hospital costs [5–7]. Hence the importance of scientific educational measures, encouraging procedures, techniques, and drugs to reduce allogeneic blood transfusions in coronary artery bypass graft surgery (CABG).

**Objective:** To verify whether clinical and surgical strategies for conserving the patient’s own blood are effective in reducing plasma transfusions and their complications in CABG.

**Methods:** Retrospective cohort study of patients undergoing CABG. A total of 4923 patients were studied from a database, 3010 patients from the year 2010 (group 1) and another 1913 patients from the year 2012 (group 2). For one year and six months, biweekly multidisciplinary meetings were held for scientific updates on transfusion practices, focusing on learning and implementing patient blood conservation strategies, based on two main pillars: 1. Reduction of blood loss (meticulous hemostasis and optimal use of antifibrinolytics); 2. Optimization of physiological tolerance to anemia (supplementary oxygen therapy, more restrictive transfusion management, treating anemia). After these educational measures to conserve the patient’s blood, a new data collection was carried out for a comparative analysis of the variables of group 1 and 2.

**Results:** A total of 377 patients received plasma transfusions, with a significant reduction from 13.1% (282 patients) in group 1 to 6.7% (95 patients) in group 2, p < 0.001. There was also a significant reduction in infection (mediastinitis) from 3.0% (group 1) to 1.4% (group 2), p < 0.05. There was no difference in the total length of hospital stay and intensive care unit stay in the two groups. The mortality expected by the Euroscore (2.35 vs 2.44, p = 0.215, groups 1 and 2, respectively) in the preoperative period of the patients showed no significant difference in both groups.

**Conclusion:** Patient blood conservation strategies focused on reducing blood loss and optimizing physiological tolerance to anemia resulted in reduced plasma transfusions and postoperative mediastinitis-like complications in patients undergoing coronary artery bypass graft surgery.

108503

Modality: E-Poster Researcher – Non-case Report

Category: EPIDEMIOLOGY AND HEALTH POLICIES/GLOBAL HEALTH

## Impact of the COVID-19 Pandemic on Coronary Angioplasties in Brazil

AUREO DO CARMO FILHO^1^, Rogerio Gomes Fleury^1^

(1) Hospital Universitário Gaffrée e Guinle – UNIRIO/EBSERH

**Introduction:** Coronary artery disease represents one of the main causes of death in Brazil and worldwide. Rapid diagnosis is important for effective treatment. The difficulty of accessing referral medical centers during the COVID 19 pandemic influenced the management of these diseases. Coronary angioplasty (CA) as an option for the management of patients with coronary disease was also impacted during this period. The present study aimed to evaluate the influence of COVID-19 on coronary angioplasties in Brazil.

**Method:** We used the database from the Department of Informatics of the Brazilian Health System (DATASUS) through the website https://datasus.saude.gov.br/. We evaluated the number of hospitalizations, deaths, costs and length of stay of patients undergoing CA in Brazil and their relationship with the beginning of the COVID-19 pandemic in Brazil (in 2020) based on data from 2018 to 2021.

**Results:** From January 2018 to December 2021, 302572 patients were hospitalized for CA with stent in Brazil with an annual mean of 75643 ± 4742. Meanwhile, 43870 were hospitalized for primary angioplasty with an annual mean of 10968 ± 278. There was an 8.5% decrease in CA averages for the years before the beginning of the pandemic compared to after the beginning of the pandemic, from 79022 ± 4214 to 72264 ± 2008 (p = 0.177). The mean number of primary angioplasty also decreased (1.8%) from 11067 ± 287 to 10868 ± 332 (p = 0.587). Despite the decrease in the number of angioplasties, there was a 13% increase in the mortality rate in this period in CA from 4.3 ± 0 to 4.9 ± 0 (p = 0.013). In cases of primary angioplasty, there was a 4.5% decrease from 6.8 ± 0.1 to 6.5 ± 0.2 (p = 0.200). The mean hospital stay was 8.5% shorter in CA (p = 0.069) and 8% (p = 0.057) in primary angioplasty. Regarding the costs (in brazilian reais) per hospitalization, there was a slight increase both in CA with 1.6% (p = 0.137) and primary angioplasty with 1.3% (p = 0.133).

**Conclusion:** In the context of the COVID-19 pandemic, hospitalizations for CA and primary angioplasty decreased by 8.5% and 1.8%, respectively, comparing the period before the beginning of the COVID-19 pandemic and after. However, we observed a 13% increase in CA mortality rates over the same period while a 4.5% decrease in primary angioplasty rates. The average hospital stay decreased by about 8% for both PTCA and primary angioplasty while costs increased slightly in both cases.

108501

Modality: E-Poster Researcher – Non-case Report

Category: EPIDEMIOLOGY AND HEALTH POLICIES/GLOBAL HEALTH

## Impact of the COVID19 Pandemic on Coronary Artery Bypass Graft (CABG) Surgery in Brazil

AUREO DO CARMO FILHO^1^, Rogério Gomes Fleury^1^

(1) Hospital Universitário Gaffrée e Guinle – UNIRIO/EBSERH

**Introduction:** Coronary artery disease represents one of the main causes of death in Brazil and in the world, whose diagnosis and treatment often require speed and appropriate technologies. The difficulty of accessing health systems during the COVID19 pandemic influenced this entire process. Myocardial revascularization surgery (CABG), one of the options for the management of patients with coronary artery disease, was also impacted during this period. The present study aimed to assess the influence of COVID-19 on (CABG) in Brazil.

**Method:** We used the database from the Department of Informatics of the Brazilian Health System (DATASUS) through the website https://datasus.saude.gov.br/. We evaluated the number of hospitalizations, deaths, costs and length of stay of patients undergoing CABG using cardiopulmonary bypass (CPB) in Brazil and their relationship with the beginning of the COVID-19 pandemic in Brazil (2020) based on data from 2018 to 2021.

**Results:** From January 2018 to December 2021, 65827 patients were hospitalized for CABG with CPB in Brazil. There was a decrease in these surgeries from 18987 in 2018 and 19272 in 2019 to 14641 and 12927 in 2020 and 2021 respectively. Surgery averages for the years before the beginning of the pandemic dropped 27.9% compared to after the beginning of the pandemic, from 19130 ± 201 to 13784 ± 1211 (p = 0.025). Despite the drop in the number of surgeries, there was an 8.8% increase in the mortality rate in this period, from 11.9 ± 1.3 to 12.9 ± 0.6 (p = 0.405). The mean hospital stay was slightly shorter (6.5%), with 25.9 ± 0.6 days in 2018–19 and 24.2 ± 0 days in 2020–21 (p = 0.064). Regarding costs (in brazilian reais) per hospitalization, there was a slight increase (1.2%) from 26332 ± 353 to 26655 ± 131. (p = 0.350).

**Conclusion:** In the context of the COVID-19 pandemic, hospitalizations for CABG with CPB decreased by almost 30% when compared to the period before the beginning of the COVID19 pandemic and after. However, we observed an 8% increase in mortality rates in the same period associated with a slight increase in hospitalization costs. The length of hospital stay remained similar.

108521

Modality: E-Poster Researcher – Non-case Report

Category: HEMODYNAMICS AND INTERVENTIONAL CARDIOLOGY

## Distal Transradial Access as the Route of Choice for Primary Percutaneous Coronary Intervention in a Large Cohort of Patients in a Real-World Registry

MARCOS DANILLO PEIXOTO OLIVEIRA^1^, Lélio Lemos Pinto Neto^1^, Ednelson Navarro^2^, Adriano Caixeta^1^

(1) Universidade Federal de São Paulo, UNIFESP; (2) Hospital Regional do Vale do Praíba

The ANGIE (Anatomical sNuffbox for Coronary anGiography and IntervEntions) randomized study compared efficacy and safety between distal transradial (dTRA) and right conventional transradial (cTRA) approaches, observing an association of dTRA with a 2-fold lower risk of occlusion of the proximal radial artery on Doppler ultrasound at 60 days. The dTRA, however, resulted in a 4-fold higher access crossover rate (21.8% vs 5.5%), mainly due to failure to insert the guidewire, as well as longer times to obtain access and perform the procedure. (dTRA vs cTRA: 120 vs 75s and 14 vs 11min, respectively), in addition to a higher radiation area-dose product (~10%). It is therefore questioned whether such limitations associated with the dTRA could impact its incorporation into the primary PCI, which must be performed in a timely manner and by experienced operators. default approach for Coronary Angiography and IntervenTIONs, eventosclinicos.gov.br Identifier: RBR-7nzxkm), among 3,991 patients consecutively submitted to coronary angiography (89%) and/or PCI (60.3%) via dTRA (80.1% by dTRA right), 917 (23%) were due to STEMI. In this subgroup, there were only 17 (2%) access crossovers (failure to insert the sheath wire), 4 of them made possible by contralateral dTRA. Thus, despite the retrospective nature and potential selection biases not documented in this series, it seems to us to be feasible and safe to incorporate dTRA as the route of choice for primary PCI in patients with STEMI. Large randomized trials are still needed and expected to assess the limitations and advantages of this potentially disruptive technique in such a challenging scenario.


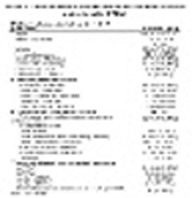


108540

Modality: E-Poster Researcher – Non-case Report

Category: PHYSIOTHERAPY

## Cardiovascular Risk and Mental Status of Students and Staff at a Public University: Cross-Sectional Study

ISABEL CRISTINA SILVA SOUSA^1^, Brunnella Alcantara Chagas de Freitas^1^, Kelvin Oliveira Rocha^1^, Luciana Moreira Lima^1^

(1) Universidade Federal de Viçosa – UFV

**Introduction:** Mental stress, represented by disorders such as anxiety and depression, has figured in the current scenario, along with modifiable and non-modifiable risk factors, as a risk factor for cardiovascular diseases (CVD).

**Objective:** To describe the global cardiovascular risk and mental status of students and employees at a public university.

**Methods:** This is an observational, descriptive study, whose sample consisted of students, teachers, and staff, aged 18 or over, of both sexes. Data collection was conducted during the months of August and September 2021, using an online questionnaire contemplating the data necessary to calculate the global cardiovascular risk by the Framingham Score and by three instruments of screening of the metal status: Beck Depression Inventory (BDI), Beck Anxiety Inventory (BAI) and Self-Reporting Questionnaire (SQR-20).

**Results:** A total of 247 individuals participated in the study, 171 (69.2%) were female and 76 (30.8%) were male. Regarding age, 120 (48.6%) were under 30 years old, 118 (47.8%) were between 30 and 60 and 9 (3.6%) were over 60 years old. There were 157 (63.3%) students, 49 (19.8) professors and 41 (16.6%) staffs. Female participants had a lower 10-year risk of developing CVD (p < 0.001), higher scores for anxiety (p < 0.001), depression (p < 0.001), and mental distress (p < 0.001) when compared to male participants. In the stratification by age group, dichotomizing by gender, individuals younger than 30 also had a lower risk of developing CVD in 10 years (p < 0.001), higher anxiety scores (p = 0.003), depression (p = 0.007), and mental distress (p = 0.004) when compared with participants aged 30 years or older, regardless of gender. The parameters age, body mass index, total cholesterol, LDL, and triglycerides were significantly higher in professors and staff when compared to students (p < 0.01). However, significantly higher scores of depression, anxiety, and mental distress were observed in students when compared to staff and professors (p < 0.001). In addition, the group of professors had a higher risk of CVD in 10 years than staff and students (p < 0.001).

**Conclusions:** Women have higher scores for mental health disorders compared to men. The younger the age, the higher the scores for mood disorders, anxiety, and mental distress, regardless of sex. The 10-year CVD risk was not correlated with mental health markers in the sample studied.

108584

Modality: E-Poster Researcher – Non-case Report

Category: EPIDEMIOLOGY AND HEALTH POLICIES/GLOBAL HEALTH

## A Lifetime Hazard Analysis on the Interaction between Comisa and Cardiometabolic Disorders

MIGUEL MEIRA E CRUZ^1^, Cristina Salles^2^, David Gozal^4^, José Fausto Pinto^5^, Isabel Rocha^3^

(1) Centro Europeu do Sono; Sleep Unit, Centro Cardiovascular da Universidade de Lisboa, Lisbon, Portugal; (2) International Center on Clinical Sleep Medicine and Research, Bahiana School of Medicine and Public Health, Salvador, Brazil; (3) Cardiovascular Autonomic Function Lab, Centro Cardiovascular da Universidade de Lisboa, Lisboa, Portugal; (4) University of Missouri, Columbia, United States; (5) Dpt Coração e Vasos, Centro Hospitalar Universitário de Lisboa Norte, CAML, CCUL, Faculdade de Medicina, Universidade de Lisboa, Lisboa, Portugal

**Introduction:** While the associations between Sleep Apnea and cardiovascular and metabolic dysfunction are now well established, the interactions between insomnia and cardiometabolic disturbances are less well understood. Notwithstanding, when both sleep disorders occur as in COMISA patients, it is possible that the morbid consequences will be exacerbated, albeit while displaying age and sex dependencies. We therefore examined 3 groups of COMISA patients of markedly different ages to assess whether the presence of COMISA interacts differentially with cardiovascular risk during the lifespan.

**Methodology:** A total of 850 patients were enrolled in this study: Pediatric (n = 50; ages ranging from 9 to 19 years), Adult (n = 685; 31–60 years) and Elderly (n = 115; ≥65 years). Obstructive Sleep Apnea and Insomnia were assessed with validated age-adjusted questionnaires for each group and high-risk COMISA was established when high risk for OSA as well as high risk for Insomnia were present.

**Results:** Among the Pediatric cohort (58% males), 9 (18%) subjects were high-risk for COMISA (age was 13.6 ± 3.3 years; BMI of 23.0 ± 6.9 Kg/m^2^). Neither hypertension nor CVD were present in this age group; 28% were obese (BMI >95% for age), 11.1% consumed alcohol regularly and 54.4% were sedentary (no physical activity). In the Adult cohort, 25.3% were high-risk for COMISA (49.8 ± 13.8 years; BMI of 31 ± 6.3 Kg/m^2^). 76.5% were hypertensive or reporting cardiovascular disease, 13.3% were diabetic and 60.3% were sedentary, 8.4% were cigarette smokers, and 46.9% consumed alcohol regularly. Of the Elderly cohort, 22.6% of patients (11 males, 42.3%) were with high risk for COMISA. Their mean age was 70.6 ± 6.6 years and BMI of 28.7 ± 5.1 Kg/m^2^, with 61.5% being hypertensive or reporting the presence of cardiovascular disease, 21% were diabetic, 65,4% were sedentary and 7.7% were cigarrete smokers.

**Conclusions:** The presence of high risk COMISA is frequent across all ages and may confer increased risk for cardiovascular and metabolic disorders, particularly after reaching adulthood and beyond. It is likely that both the sedentary lifestyle, and alcohol consumption may contribute to this risk, but the presence of COMISA may add an additional risk factor for cardiometabolic complications. Further research is needed to explore how the concurrent presence of Sleep Apnea and Insomnia exacerbates cardiovascular and metabolic risk in larger clinical populations subjected to more precise diagnostic methods.

108589

Modality: E-Poster Researcher – Non-case Report

Category: ATHEROSCLEROSIS/CARDIOVASCULAR RISK FACTORS/CARDIOVASCULAR PREVENTION

## Diagnostic Properties of Diagonal Ear Lobe Crease in Coronary Artery Disease

SÉRGIO LUIZ ZIMMERMANN^1^, Sérgio Luiz Zimmermann^1^, Leonardo Campanelli Steinhausen^2^, Bruno Luiz Mueller^2^

(1) Hospital Santa Isabel, Blumenau; (2) Universidade Regional de Blumenau

**Background:** Coronary angiography is the most accurate method for diagnosing coronary heart disease, but it envolves exposure to radiocontrast agents and radiation, which can increase morbidity and mortality. Thus, it is necessary to study additional markers to identify of coronary artery disease (CAD), such as the diagonal ear lobe crease (DELC).

**Objectives:** To determine DELC’s diagnostic properties in the identification of coronary disease and its association with comorbidities.

**Methods:** A cross-sectional study with 278 patients over 18 years old, who underwent coronary angiography performed by at least 1 of 2 interventional cardiologists. The presence and absence of PLL and associated comorbidities was determined by two trained examiners.

**Results:** PLL was only associated with older age (p < 0.05). PLL had a sensitivity of 76.2%, a specificity of 17.6% in the identification of CAD and an overall accuracy of 58.3%.

**Conclusion:** We concluded that the diagnostic accuracy of PLL was lower when compared to the scientific literature, acting as a low quality marker for the identification of patients with CAD. In our study, there was no association between PLL and CAD.

108592

Modality: E-Poster Researcher – Non-case Report

Category: EPIDEMIOLOGY AND HEALTH POLICIES/GLOBAL HEALTH

## Hypertension and its Risk Factors in Male Prisoners: A Cause for Concern?

DR OSUNKWO DAMARIS AMARACHUKWU^1^, Dan-Nwafor Chioma^3^, Chukwuma David Umeokonkwo^3^, Patrick Nguku^2^, Emmanuel Nna^4^

(1) Department of Internal Medicine, National Hospital Abuja; (2) African Field Epidemiology Network Nigeria Country Office; (3) Nigeria Field Epidemiology and Laboratory Training Program, Abuja, Nigeria; (4) Safety Molecular Pathology Lab, The Molecular Pathology Institute, Enugu

**Background:** Hypertension is on the increase in sub-Saharan Africa and it is one of the risk factors for cardiovascular morbidity and mortality. Prisoners are at a greater risk for hypertension because of prevalent tobacco use, unhealthy diet, and incarceration-induced stress. We investigated the prevalence and the risk factors associated with hypertension among Kuje Prison inmates in the Federal Capital Territory (FCT), Nigeria.

**Materials & Methods:** A cross-sectional study was conducted among 180 male prisoners. WHO’s Stepwise approach to surveillance of chronic disease risk factors was used to conduct the study. Data collected at the various steps included: demographic and behavioral risk factors (Step 1); Blood pressure and anthropometric measurements (step 2) and Random blood sugar (Step 3). Body mass index (BMI) was determined. Data were analyzed using Epi-Info version 7. Odds of hypertension among participants with risk factors were calculated, p-value less than 0.05 was considered significant.

**Results:** The mean systolic blood pressure was 131 ± 15 mmHg, diastolic 81 ± 12 mmHg, BMI 24.8 ± 3.6 and RBS 97.1 ± 21.8 mg/dl. Overall, 60 (33.3%) were overweight, 13 (7.2%) obese, 65 (36.1%) were prehypertensive, 57 (31.6%) were hypertensive and 1(0.6%) diabetic. The risk for elevated systolic BP was significantly higher among respondents that has first degree relative with hypertension (OR = 5.6; CI = 1.6–19.9), ≥45years (OR = 7.5: CI = 1.6–35.9), insufficient physical activity (OR = 4.3; CI = 1.5–12.5; P = 0.007) and increased BMI (OR = 9.1; CI = 2.7–31.1).

**Conclusion:** There is a high prevalence of hypertension among prison inmates in FCT, age >45 years, insufficient physical activity, family history of hypertension, and obesity were found to be independent risk factors for hypertension.

108634

Modality: E-Poster Researcher – Non-case Report

Category: HEART FAILURE/CARDIOMYOPATHY/TRANSPLANT

## Effect of Antihypertensive Treatment on Left Ventricular Diastolic Function in Subjects with Obstructive Sleep Apnea and Hypertension: A Randomized Controlled Trial

JULIANO AFONSINO JORGE^1^, Murilo Foppa^1^, Fábio Tremea Cichelero^1^, Deniz Martinez^1^, Marcelo Balbinot Lucca^1^, Geórgia Pante^3^, Flávio Danni Fuchs^1^, Sandra Costa Fuchs^1^

(1) Graduate Studies Program in Cardiology, School of Medicine, Universidade Federal do Rio Grande do Sul (UFRGS), Porto Alegre, RS, Brazil; (2) Division of Cardiology, Hospital de Clínicas de Porto Alegre (HCPA), Porto Alegre, RS, Brazil; (3) School of Medicine, UFRGS, Porto Alegre, RS, Brazil

Obstructive sleep apnea (OSA) has been associated with left ventricular (LV) diastolic dysfunction. Treating HTN with diuretics could reduce fluid retention and rostral fluid shift in patients with OSA, leading to changes in LV diastolic parameters. We compared the effects of diuretics or amlodipine on echocardiographic LV diastolic parameters.

**Methods:** Patients with HTN and an apnea-hypopnea index between 10 and 40 events/hour were randomized to receive chlorthalidone plus amiloride (25 mg/5 mg) daily or amlodipine (10 mg/daily) for eight weeks. The changes on echocardiographic LV diastolic function parameters were the primary outcome.

**Results:** 62 participants completed the study. Systolic and diastolic BP (24h) decreased without between-treatment differences at the end of study. Nighttime SBP dipping was higher in the diuretic group than the amlodipine group (P = 0.01). The following deltas were found between diuretic and amlodipine groups, respectively: septal E/e‘ ratio, –0.20 ± 0.36 vs. 0.08 ± 0.36 (P = 0.6); lateral E/e‘ ratio –0.14 ± 0.22 vs. 0.66 ± 0.38 (P = 0.07); and average E/e‘ ratio –0.19 ± 0.21 vs. 0.43 ± 0.32 (P = 0.1).

**Conclusion:** Patients with OSA and HTN treated with diuretics or amlodipine showed similar reductions in BP and LV structural measurements. The trend for the association of diuretics to have a greater effect on diastolic parameters and nocturnal SBP dipping suggest it can have beneficial cardiac effects in the long-term management of BP.


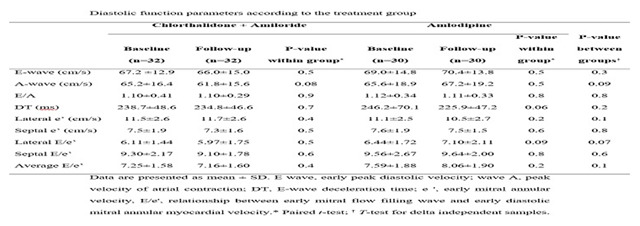


108659

Modality: E-Poster Researcher – Non-case Report

Category: PERICARDIUM/ENDOCARDIUM/VALVOPATHIES

## The Frequency, Associated Factors and Progression of Heart Valve Disease in Chronic Kidney Disease Patients

SANDRO GONÇALVES DE LIMA^1^, José Arthur Viana de Oliveira Pimentel^2^, Manuella de Amorim Silva^2^, Maria Eduarda Cavalcanti Tompson^2^, Andréa Bezerra de Melo da Silveira Lordsleem^1^

(1) Hospital das Clínicas – Universidade Federal de Pernambuco – UFPE; (2) Centro Universitário Maurício de Nassau – UNINASSAU

**Introduction:** Cardiovascular diseases are a major cause of death in patients with advanced chronic kidney disease (CKD). Valve calcification (VC), mitral and aortic, associated with significant valve disease is a predictor of cardiovascular mortality, coronary artery disease and arrhythmias in these patients.

**Objectives:** To assess the frequency of heart valve disease, its progression, and associated factors in patients with CKD.

**Methods:** A total of 568 medical records of patients treated between 2007 and 2021 at the cardiology-renal outpatient clinic at a university hospital were analyzed, of which 347 were included due to the presence of CKD and heart valve disease. The explanatory variables analyzed were: age, gender, VC, glomerular filtration rate (GFR), dyslipidemia, coronary artery disease, systemic arterial hypertension, diabetes mellitus, C-reactive protein, secondary hyperparathyroidism and laboratory data on mineral and bone metabolism. To assess the association between categorical variables, the Pearson’s chi-square test or Fisher’s exact test was used.

**Results:** 50.7% were female. The most frequent risk factor was systemic arterial hypertension (83.9%) and 25.1% of patients were diabetic. Mitral valve disease was observed in 81.6%, followed by aortic valve disease (66%). Regurgitant lesions were the most frequent: mitral (75%) and aortic (40.2%). Around half (48.1%) of those with heart valve disease were in the 30–39 age group, confirming the precocity of heart valve disease in CKD. A progression of heart valve disease was observed in 122 patients (35.2%). Valve calcification was significantly associated only with the progression of aortic valve disease (p = 0.01). The variables significantly associated with mitral valve disease were secondary hyperparathyroidism (p = 0.03), GFR (p = 0.02) and total cholesterol (p = 0.02). None of the variables analyzed were significantly associated with aortic and pulmonary valve disease. Secondary hyperparathyroidism (p = 0.019) was also significantly associated with tricuspid valve disease, as well as altered levels of triglycerides (p = 0.017).

**Conclusion:** The frequency of heart valve disease, particularly mitral and aortic, is high in patients with CKD. The factors significantly associated with heart valve disease were: secondary hyperparathyroidism, GFR, total cholesterol and altered triglyceride levels. The progression of valve disease was observed in 35% of patients and was significantly influenced by VC.

108733

Modality: E-Poster Researcher – Non-case Report

Category: CARDIORESPIRATORY PHYSIOLOGY/BASIC SCIENCE

## Impact of Air Quality During Sleep on Cardiorespiratory Dynamics: An Exploratory Study

MIGUEL MEIRA E CRUZ^1^, Joana Belo^2^, Mariana Picado^3^, Nuno Canha^5^, Carla Viegas^2^, Susana Marta Almeida^4^

(1) Centro Europeu do Sono; Sleep Unit, Centro Cardiovascular da Universidade de Lisboa, Lisbon, Portugal; (2) H&TRC- Health & Technology Research Center, ESTeSL- Escola Superior de Tecnologia da Saúde, Instituto Politecnico de, Lisboa, Portugal; (3) Instituto Português de Oncologia de Lisboa, Lisbon, Portugal; (4) Centro de Ciências e Tecnologias Nucleares, Instituto Superior Técnico, Universidade de Lisboa, Lisbon, Portugal; (5) Centre for Environmental and Marine Studies, Department of Environment and Planning, University of Aveiro, Aveiro, Portugal; (6) NOVA National School of Public Health, Public Health Research Centre, Universidade NOVA de, Lisboa, Portugal; (7) Comprehensive Health Research Center (CHRC), Portugal

**Introduction:** Inadequate sleep and poor air quality are both associated to an increased cardiovascular risk. Sleep quality and structure are also vulnerable to environmental influences. Typically, sleeping environments have low ventilation rates, leading to pollutants accumulation. Though during nighttime and early morning there is a circadian propensity to cardiorespiratory events, environmental factors which may aggravate this risk should be taken into account. The aim of this study is to test the associations between ais pollutants, specifically CO and CO2 and heart rate dynamics as an indicator of cardiovascular autonomic function.

**Methodology:** Ten men followed specific inclusion criteria: age between 25 and 40 years old, healthy individuals, non-smoking, without children below five years and without sleeping, cardiac and respiratory problems and whose households are within Lisbon area, were recruited. An unattended polysomnography (PSG) was performed during 2 weeknights in a row. The second night PSG’s results were used in order to minimize the first night effect. For the propose of this study the data related to heart rate (HR) were collected, namely HR acceleration index (HR Acc index), mean of HR (HRmean) and minimum of HR (HRmin). IAQ monitoring was based on a comprehensive multi-pollutant assessment where chemical, in particular carbon dioxide (CO) and carbon monoxide (CO), were assessed through real time instruments. Non-parametric statistics were applied, namely Spearman correlations, to analyze potential associations between sleep and environmental parameters. The level of significance considered was α = 0.05.

**Results:** The mean age was 33.9 ± 5.20 years. HR and IAQ parameters showed a moderately positive correlation between CO exposure and HR Acc index (rs = .635) and HRmin (rs = .0,667) and a highly positive correlated between CO exposure and HRmean (rs = .0,71); also a moderately and positive correlation between CO2 exposure and HR Acc index (rs = .668) awas observed as well as a highly positive correlation between CO2 exposure and HRmin (rs = 713) HRmean (rs = .0,794).

**Conclusions:** Results from this preliminar study suggest that high levels of CO and CO2 may increase the HR Acc index, HRmin and HRmean, raising awareness on the possible impact of indoor air quality on cardiovascular autonomic modulation. Further studies are needed to confirm such findings and to establish their clinical relevance within cardiorespiratory health.

108720

Modality: E-Poster Researcher – Non-case Report

Category: ANTICOAGULATION

## The Relationship between Baseline Characteristics and Ischemic Stroke in Patients with Atrial Fibrillation After Transcatheter Aortic Valve Replacement Treated with Edoxaban or Vitamin K Antagonists

CHRISTIAN HENGSTENBERG^1^, Martin Unverdorben^2^, Helge Möllmann^3^, Nicolas M Van Mieghem^4^, Holger Thiele^5^, Peter Nordbeck^6^, Tienush Rassaf^7^, Raul Moreno^8^, Roxana Mehran^9^, Irene Lang^1^, Roland Veltkamp^10^, George D Dangas^9^

(1) Department of Internal Medicine II, Division of Cardiology, Vienna General Hospital, Medical University, Vienna, Austria; (2) Daiichi Sankyo, Inc., Basking Ridge, NJ, USA; (3) Department of Internal Medicine, St. Johannes Hospital, Dortmund, Germany; (4) Department of Cardiology, Erasmus University Medical Center, Thoraxcenter, Rotterdam, the Netherlands; (5) Department of Internal Medicine–Cardiology, Heart Center Leipzig at University of Leipzig, Leipzig; (6) Department of Internal Medicine I, University Hospital Würzburg, Würzburg & Klinikum Links der Weser gGmbH, Bremen, Germany; (7) Clinic for Cardiology and Vascular Medicine, Essen University Hospital, Essen, Germany; (8) Department of Cardiology, Tübingen University Hospital, Tübingen, Germany; (9) Zena and Michael A. Wiener Cardiovascular Institute, Mount Sinai Hospital, New York, NY, USA; (10) Division of Brain Sciences, Imperial College London, London, UK; Department of Neurology, Alfried Krupp Krankenhaus, Essen, Germany

**Introduction:** In ENVISAGE-TAVI AF (NCT02943785) edoxaban was noninferior to vitamin K antagonists (VKAs) for the composite endpoint of net adverse clinical events in patients with atrial fibrillation after transcatheter aortic valve replacement (TAVR).

**Objective:** To evaluate the association between baseline patient characteristics and ischemic stroke (IS) incidence.

**Methods:** This on-treatment analysis of ENVISAGE-TAVI AF included patients that received ≥1 dose of the study drug over the period treatment and ≤3 days after interruption or discontinuation. Baseline demographic and clinical characteristics were stratified by IS incidence. Numerical variables were compared using one-way analysis of variance; categorical variables were compared using Fisher’s exact test. Stepwise logistic regression with 30 independent predictors determined patient characteristics associated with first IS event.

**Results:** Of 1377 patients included in the on-treatment analysis, 41 (3.0%) experienced IS during the on-treatment period (edoxaban, n = 19; VKA, n = 22). Most IS events occurred within 6 months of TAVR for both the edoxaban (57.9%) and VKA (68.2%) arms. Differences in baseline demographic and clinical characteristics are shown for patients who did or did not experience IS while receiving treatment (Table). Significantly more patients who experienced IS had a history of systemic embolic events (SEE; P = 0.015). Only a history of SEE was independently associated with IS (P = 0.006). Body mass index (P = 0.052) and history of myocardial infarction (P = 0.074) were numerically, but not statistically, associated with IS.

**Conclusions:** In this on-treatment analysis of the ENVISAGE-TAVI AF trial, few patients experienced IS. Patients with history of SEE may be at increased risk of IS following TAVR.


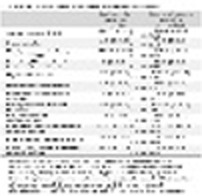


109072

Modality: E-Poster Researcher – Non-case Report

Category: COVID-19 AND CARDIOVASCULAR SYSTEM

## The Impact of Hospital Acquisition of COVID-19 on Cardiac Patients

CRISTIANE DA CRUZ LAMAS^1^, Mariah Rodrigues Paulino^1^, Léo Rodrigo Abrahão dos Santos^2^, Ingrid Paiva Duarte^2^, Marcelo Goulart Correia^1^, José Alfredo de Sousa Moreira^1^, Letícia Roberto Sabioni^1^, Fabiana Bergamin Mucillo^1^, Rafael Quaresma Garrido^1^, Bruno Zappa^1^, Stephan Lachtermacher Pacheco^1^, Andrea Rocha de Lorenzo^1^

(1) Instituto Nacional de Cardiologia; (2) Universidade do Grande Rio

**Introduction:** Reported nosocomial acquisition of COVID-19 has varied between 5 to 41%. Our aim was to describe the demographic, clinical and laboratory features and outcomes of patients with cardiac disease hospitalized with COVID-19 in a reference cardiology institution in Brazil, comparing the patients with hospital-acquired COVID-19 (HACOVID19) with those who had non-hospital acquired (NHA) COVID-19.

**Methods:** This is an observational retrospective study of consecutive adult patients admitted, between March and September of 2020 with a diagnosis of SARS-CoV-2 infection confirmed by RT-PCR. Data was collected as per the International Severe Acute Respiratory and Emerging Infection Consortium and complemented with a cardiovascular questionnaire. HA COVID-19 was considered when the patient had a negative nasopharyngeal swab on admission and a positive one 14 or more days later. Statistical analysis was performed using the Jamovi 1.6 and R 4.0.1.

**Results:** One hundred twenty-one patients with a confirmed diagnosis of COVID-19 were included. There were 20 patients (16.5%) with HA-COVID19 and 101 (83.5%) with NHA-COVID-19. Patients who developed COVID-19 in hospital had as reasons for admission: decompensated heart failure (35%), acute coronary syndrome (20%), cardiac surgery (CABG, valve replacement or aortic surgery) in 25%, complete AV block, pacemaker dysfunction, stroke and hematologic disease (5% each). The median(IQR) length of stay of HACOVID19 was 29 days (18.5–60.2) vs 16 (7–31)days. Median age was 64 years (61.8–69.3) for HA-COVID-19, and 63 (52–72) for NHACOVID19. There was no difference between patients with HACOVID-19 and those with NHACOVID-19 regarding the presence of heart disease, COPD, obesity, complicated diabetes mellitus, heart valve disease, coronary artery disease, and systemic arterial hypertension. However the 2 groups were significantly different regarding laboratory features (serum creatinine, D-dimer, ferritin, AST). HACOVID patients had more dyslipidemia (15/20 (75%) vs 49/101 (48.5%), P = 0.030] and chronic renal failure [7/20 (35%) vs 15/101 (15%), p = 0.033]. Most importantly, mortality was significantly higher in HACOVID-19[10/20 (50%) vs 19/101 (18.8%), p = 0.003].

**Conclusions:** Cardiac patients who acquired COVID-19 in hospital had longer hospital stay and higher mortality, which highlights the impact of the pandemic on the outcomes of cardiac patients admitted to hospital for cardiac, non-COVID related reasons.

108800

Modality: E-Poster Researcher – Non-case Report

Category: NEGLECTED CARDIOVASCULAR DISEASES

## Evaluating the Value of Point-of-Care NT-proBNP for the Screening of Pregnancy-Related Heart Failure – the Preg-HF Study

CHARLE ANDRE VILJOEN^1^, Mahmoud Al-Naili^2^, Jean Jacques Noubiap^3^, Alice Jackson^4^, Karice Hyun^5^, Andrea Neves^6^, Clovis Nkoke^7^, Charles Mondo^8^, Juliet Nabbaale^9^, Julian Hoevelmann^10^

(1) Cape Heart Institute, Faculty of Health Sciences, University of Cape Town, Cape Town, South Africa; (2) Division of Cardiology, Groote Schuur Hospital, Faculty of Health Sciences, University of Cape Town, Cape Town, South Africa; (3) Centre for Heart Rhythm Disorders, The University of Adelaide, Adelaide, Australia; (4) British Heart Foundation Glasgow Cardiovascular Research Centre, University of Glasgow, Glasgow, G12 8TA, Scotland; (5) School of Health Sciences, Faculty of Medicine and Health, University of Sydney, Westmead 2145, Australia; (6) Gynecology and Obstetrics, Hospital Geral José Macamo, Av. OUA, 1100, Maputo, Mozambique; (7) Department of Internal Medicine, Buea Regional Hospital, Buea, Cameroon; (8) St. Francis Hospital Nsambya, P. O. Box 1799, Nsambya Road, Kampala, Uganda; (9) Uganda Heart Institute, Plot No. 1, Upper Mulago Hill, P. O. Box 37392, Kampala, Uganda; (10) Klinik für Innere Medizin III, Kardiologie, Angiologie und Internistische Intensivmedizin, Universitätsklinikum des Saarlandes, Saarland University Hospital, Homburg (Saar), Deutschland

**Introduction:** Cardiac disease is an important cause of maternal mortality worldwide. However, diagnosing heart failure (HF) during pregnancy remains challenging. Patients with HF present with symptoms that are often attributed to the physiological changes of pregnancy. Although the measurement of natriuretic peptides has been recommended as a cost-effective screening test for HF, its value in predicting underlying structural heart disease on echocardiography during pregnancy is unclear. We aimed to evaluate the accuracy of point-of-care (POC) NT-proBNP to predict echocardiographic evidence of structural heart disease in pregnant women. Methods All consecutive consenting pregnant women with symptoms of HF, who underwent echocardiography at a tertiary hospital in Cape Town, South Africa, between March 2021 and March 2022 were compared with asymptomatic pregnant women. Demographic and obstetric data were collected, as well as clinical and echocardiographic parameters. POC NT-proBNP was measured; a receiver operating characteristic (ROC) curve was used to determine the level of NT-proBNP that would have the best predictive value for detecting structural heart disease on echocardiography.

**Results:** We included 121 women with a median age of 31.3 years (IQR 24.9–36.4), gravidity of 3 (2–4), mostly in their third trimester of pregnancy (76.9%). Symptomatic women (66.9%) presented mainly with dyspnoea (90.8%) and peripheral oedema (50%). Overall, the median POC NT-proBNP was 108 pg/ml (60–470) but was not statistically different between symptomatic and asymptomatic participants. However, NT-proBNP levels were significantly elevated in those with left ventricular (LV) dilatation (395 [86–1668] vs 80.5 [60–303], p = 0.001), left atrial enlargement (417 [98–1211] vs 78 [59–167], p < 0.001), LV systolic dysfunction (539 [94–2582] vs 80 [60–323], p < 0.001), diastolic dysfunction (300 [77–1450] vs 80 [60–322], p = 0.038), mitral regurgitation (247 [60–794] vs 84 [60–323], p = 0.027) and pericardial effusion (448 [84–1487] vs 80 [60–303], p < 0.001). An NT-proBNP of <250 pg/ml had the highest negative predictive value (79.3%) to rule out structural heart disease (ROC area 0.66).

**Conclusion:** In this cohort of pregnant women with symptoms HF, POC NT-proBNP identified those with structural heart disease with acceptable discrimination. POC NT-proBNP testing might be particularly useful as a screening test in settings where pregnant women do not readily have access to echocardiography.

108804

Modality: E-Poster Researcher – Non-case Report

Category: CARDIOLOGY OF SPORTS, EXERCISE, ERGOMETRY AND CARDIOVASCULAR REHABILITATION

## Health Coaching for Patients in Cardiac Rehabilitation: Results of a Program to Promote Healthy Lifestyle Habits

DANIELE BARRIONUEVO KALLAS^1^, Lia Kanae Okita Buschinelli^1^, Eneas Antonio Rocco^1^, Amanda Barbuio Teixeira^1^, Luana Talita Diniz Ferreira^1^, Raquel Yuri Mori^1^, Bianca Sprovieri Moraes^1^, Gabriela Macoppi Carreiro^1^, Gabriela Zanussi Barreto^1^, Camila dos Santos Arcas^1^, Felipe Lopes Malafaia^1^, Patricia Canteruccio Pontes Vianna^1^

(1) Hospital Samaritano Paulista

**Introduction:** Health Coaching (HC) is an approach for changes in health behaviors and self-care. The British Association for Cardiovascular Prevention lists health behavior change, education, and management of risk factors related to physical activity, diet, weight control, alcohol and tobacco among the 6 central components of cardiac rehabilitation (CR) programs. Thus, interventions based on changes in health behaviors are justified as part of medical care.

**Objective:** To evaluate the impact of a HC Program in lifestyle of patients under CR immediately after and 6 months after the intervention.

**Methods:** Patients in CR of a specialized institution were invited to voluntarily enroll in a HC Program in the dimensions of diet, physical activity, stress management and sleep. The intervention was composed of 6 weekly coaching sessions, with trained health professionals, lasting 1h30, made 100% remote by the telemedicine of the institution, where patients were gathered in groups of up to 4 people, plus the Health Coach. Fantastic Lifestyle Questionnaire (Fantastic) was applied before, immediately after and 6 months after the intervention. Sessions addressed wellness, ambivalence, behavioral change process, thoughts and beliefs, relapse prevention and habit support plan, and patients were invited to create health goals with the Smart framework to a day-to-day practice. Between sessions they received educational materials about the four dimensions.

**Results:** The intervention took place between August 2020 and March 2022. 31 patients completed the program. Results of the Fantastic are at Table 1.

**Conclusions:** Interventions for health behavior changes seem to be promising for patients in CR and seem to last after 6 months.


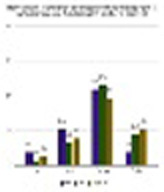


108810

Modality: E-Poster Researcher – Non-case Report

Category: COVID-19 AND CARDIOVASCULAR SYSTEM

## NKG2A Expression Among CD8 Cells is Associated with COVID-19 Progression in Hypertensive Patients: Insights from the Brace Corona Randomized Trial

RENATA JUNQUEIRA MOLL BERNARDES^1^, Sérgio Costa Fortier^3^, Andréa Silvestre de Sousa^4^, Renato D. Lopes^6^, André Feldman^8^, Guilherme D.A.S. Arruda^9^, Denílson C. de Albuquerque^1^, Ariane V. S. Macedo^7^, Olga Ferreira de Souza^1^, Fernando A. Bozza^1^, Ronir Raggio Luiz^5^, Emiliano Medei^2^

(1) D’Or Institute for Research and Education, Rio de Janeiro, Brazil; (2) National Center for Structural Biology and Bioimaging, Federal University of Rio de Janeiro, Brazil; (3) Patological Anatomy Laboratory, Rede D’Or São Luiz, São Paulo, Brazil; (4) Evandro Chagas National Institute of Infectious Diseases, Oswaldo Cruz Foundation, Rio de Janeiro, Brazil; (5) Public Health Studies Institute – IESC, Federal University of Rio de Janeiro, Brazil; (6) Brazilian Clinical Research Institute, São Paulo, Brazil; (7) Hospital São Luiz Jabaquara, São Paulo, Brazil; (8) Hospital São Luiz Anália Franco, São Paulo, Brazil; (9) Hospital São Luiz São Caetano, São Caetano do Sul, Brazil; (10) Hospital Sino Brasileiro, Osasco, Brazil; (11) Hospital Villa Lobos, São Paulo, Brazil; (12) Hospital São Luiz Morumbi, São Paulo, Brazil

**Background:** Cardiovascular comorbidities and immune response dysregulation are associated with 2019 coronavirus disease (COVID-19) severity. Reduced populations of lymphocytes, including T, B, and natural killer cells, and increased neutrophil counts have been detected in patients with COVID-19. We aimed to explore the key immune cell profile and understand its association with disease progression in patients with hypertension hospitalized due to COVID-19.

**Methods:** Immune cell populations in cryopreserved peripheral blood mononuclear cell samples were obtained from 156 hypertensive patients who were included in the BRACE CORONA randomized trial. The primary outcome was progression to severe disease, according to a modified World Health Organization Ordinal Scale for Clinical Improvement, during hospitalization. The probability of progression to severe disease was estimated using a logistic regression model that included clinical variables and immune cell subsets associated with the primary outcome.

**Results:** During hospitalization, 11 (7.1%) patients showed progression to more severe COVID-19; 3 of these patients died. Obesity, diabetes, oxygen saturation, lung involvement on computed tomography (CT) examination, the C-reactive protein concentration, total lymphocyte count, proportions of CD4+ and CD8+ T cells, CD4/CD8 ratio, CD8+ human leukocyte antigen DR isotope (HLA-DR) median fluorescent intensity (MFI), and CD8+ natural killer group 2 member A (NKG2A) MFI on admission were associated with progression to severe COVID-19. According to our predictive model, the risk of progression to severe disease reached 86.2% in the presence of three clinical variables (obesity, diabetes, reduced oxygen saturation, or >50% CT lung involvement) combined with increased CD8+ NKG2A MFI.

**Conclusions:** This study demonstrated that increased CD8+ NKG2A MFI at hospital admission, in combination with some clinical variables, is associated with a high risk of COVID-19 progression in hypertensive patients. These findings reinforce the hypothesis of functional exhaustion of T cells with the increased expression of NKG2A in patients with severe COVID-19, elucidating how severe acute respiratory syndrome coronavirus 2 infection may break down the innate antiviral immune response at an early stage of the disease, with future potential therapeutic implications.

109063

Modality: E-Poster Researcher – Non-case Report

Category: ATHEROSCLEROSIS/CARDIOVASCULAR RISK FACTORS/CARDIOVASCULAR PREVENTION

## Improvement of Heart Rate Variability as a Surrogate Measure of Cardiovascular Autonomic Function in Comisa Patients Treated with a Mandibular Advancement Device

MIGUEL MEIRA E CRUZ^1^, Luis Vicente Franco de Oliveira^3^, Monica Gomes^2^, Lilian Giannasi^2^

(1) Centro Europeu do Sono; Sleep Unit, Centro Cardiovascular da Universidade de Lisboa, Lisboa, Portugal; (2) Universidade Estadual de São Paulo – UNESP, São José dos Campos, São Paulo, Brazil; (3) Centro Universitário de Anapolis-UniEvangélica, São Paulo, Brazil

**Introduction:** COMISA refers to the frequent co-occurrence of Insomnia and Sleep Apnea and has been linked to increased cardiovascular risk where autonomic dysfunction play an important role. Therefore, improving cardiovascular autonomic function (CAF) is a clinically relevant target to be achieved in these patients. While mandibular advancement devices (MAD) are recommended as an effective alternative for obstructive sleep apnea control, the benefits of such therapeutic tool in COMISA remains elusive. In this pilot study we aimed to test the null hypothesis that MAD will have no effect on heart rate variability (HRV) as a surrogate of CAF in COMISA adult patients.

**Methodology:** Patients with COMISA, as defined by OSA + Sleep Latency >30min, were treated with PMPositioner oral appliance (OAm) for their sleep disordered breathing and were evaluated after 3 months of OAm therapy, been retrospectively compared regarding major clinical and polysomnographic outcomes. Sleep variables were evaluated by PSG and Heart rate variability (HRV) was accessed and analized by rythmography.

**Results:** 12 COMISA patients (6 males), mean age = 49.7, mean BMI = 25.9, mean neck circumference = 38.9 were enrolled. Insomnia and OSA were confirmed by both clinical criteria (validated questionnaires) and PSG parameters. All patients were treated with a PMPositioner oral appliance for sleep disorder breathing. The AHI was reduced from 22.7 ± 12.7 to 4.0 ± 3.5(p < 0.0002), IDO was reduced from 18.8 ± 14.0 to 3,5 ± 2.0 (p < 0.02), sleep latency reduced from 63.1 ± 49.4 to 21.8 ± 21.4 (p < 0.2), reaching a normal level. The time-domain and frequency–domain parameters were significant for RR interval and both Fast Fourrier Transform and Wavelet spectral method, respectively.

**Conclusions:** The rejection of the null hypothesis allow us to confirm the impact of MAD in HRV of these patients. This is the first study showing a positive and clinically relevant effect of MAD therapy on both respiratory and insomnia related therapeutic outcomes in COMISA together with an improvement of CAF and therefore on cardiovascular risk as a major concern among these patients.

109361

Modality: E-Poster Researcher – Non-case Report

Category: ATHEROSCLEROSIS/CARDIOVASCULAR RISK FACTORS/CARDIOVASCULAR PREVENTION

## Pulse-Wave Velocity in Down Syndrome Individuals

ANNE GERYMAIA OLIVEIRA DE MELO SILVA^1^, ANNE GERYMAIA OLIVEIRA DE MELO SILVA^1^, NEILA ANDERS AIDAR^2^

(1) SECRETARIA DE SAÚDE DO DISTRITO FEDERAL; (2) MEDCOR

**Introduction:** The interest in understanding what accelerates the degradation of cardiovascular function in some individuals, is matched by the need to understand what protects other subjects. Pulse-wave velocity (PWV) is a measurement of arterial stiffness that is an independent predictor of cardiovascular risk. It can be measured simply and noninvasively by modern oscillometric methods.

**Objectives:** To evaluate vascular aging variables and arterial stiffness in adult patients with trisomy 21, treated at the Distrito Federal Down Syndrome Reference Center – Crisdown.

**Methods:** Transversal study of PWV in 28 adults with Down syndrome. The central hemodynamic parameters of blood pressure, pulse wave velocity (PWV), and heart rate-adjusted increase index (Alx@75) were estimated using the oscillometric device Arteris-AOP (Cardio Sistemas Comercial e Industrial Ltda, São Paulo, Brazil), ANVISA 10361059013. The device performs the analysis through a cuff installed on the patients‘ left arm. The software was used for data analysis comparing with a database with the Brazilian population matched by sex, age, weight, and height.

**Results:** Twenty-eight patients with Down Syndrome ranging from 18 to 64 years old, being 18 female and 10 male, were evaluated. For data analysis, they were divided into groups of 18 to 24 years (N = 13), 25 to 34 years (N = 6), and over 35 years (N = 9). There was no statistical difference between the groups regarding mean BMI (30.7 + 4.3 Kg/m^2^), mean central systolic pressure – CSP (85.6 + 10.5 mmHg), mean central diastolic pressure – CDP (62.3 + 10.1 mmHg), mean cardiac output (3.4 + 0.5 l/min) and mean total cholesterol serum level (171.7 + 35.3 mg/dl). The mean PWV was 5.2 m/s (SD 1.4) and the mean augmentation index was 17.5% (SD 8.5). There was a statistical difference between the groups in relation to pulse wave velocity and augmentation index, which were significantly higher in the group aged over 35 years. Compared with a database of the typical Brazilian population, the level of PWV in adults with Down Syndrome was within the average, even in the group aged over 35 years.

**Conclusion:** The findings suggest that adults with Down syndrome have a pattern of central arterial hypotension. The lack of relationship of PWV, an independent predictor of adult cardiovascular events, with its traditional determinants, including arterial pressure suggests Down syndrome–specific phenomena may alter such relationships in this population.

109071

Modality: E-Poster Researcher – Non-case Report

Category: NURSING

## Instrument to Guide Nurses in the Physical Examination of Patients with Suspected Low Cardiac Output

MIRIAN FIORESI^1^, Juliana Mitre da Silva^1^, Cândida Caniçali Primo^1^, Maria Edla de Oliveira Bringuente^1^, Bruno Henrique Fiorin^1^, Karolini Zuqui Nunes^1^, Lorena Barros Furieri^1^, Walckiria Garcia Romero Sipolatti^1^

(1) Programa de Pós-Graduação em Enfermagem, Universidade Federal do Espírito Santo

**Introduction:** The concept of decreased cardiac output is found mainly in the nursing and medical fields. As a nursing phenomenon, it is present in the NANDA-I and ICNP classification systems. Nurses are the professionals who stay the longest in direct care of people, so they commonly identify clinical changes and signs of deterioration of patients‘ health at all levels of health care. Therefore, a data collection instrument can optimize risk prediction in adults and facilitate clinical thinking and nursing care for patients with this condition.

**Objective:** To develop an instrument to assist nursing in the physical examinations of a suspected low cardiac output patients.

**Methods:** This is a methodological study developed in three stages: literature scope review, pilot instrument construction and validation by judges. The instrument was constructed following the principles of Pasquali’s elaboration of instrument. Content and face validation was based on content validity index (CVI) and was considered as inclusion criteria CVI greater than or equal to 0.8. The judging population consisted of nurses with a minimum degree of specialist and minimum experience of two years in the field of nursing cardiology. The items that made up the instrument were evaluated as: 1- Adequate, 2- Needs adequacy and 3- Inadequate, within the criteria of clarity, relevance or representativeness and comprehensiveness.

**Results:** After the validation process, the instrument was composed of the following nine items, called clinical indicators: consciousness status, respiratory status, activity tolerance, fluid volume, gastric status, sensory alteration of cardiac origin, heart rate and rhythm, blood pressure and tissue perfusion. Each item can be scored from 1 to 3 in ascending order of severity and by summing the total points the patient risk can be stratified into: minimum risk (9 to 12 points), intermediate risk (13 to 18 points) and high risk (19 to 27 points). If the patient is tired with minor exertion (eating) and/or presents with chest pain that does not cease at rest and therapy, he/she should be classified as high risk even if the patient has a score below 19 points.

**Conclusion:** An instrument to assist nursing in the assessment was developed and validated to classify the risk of decreased cardiac output in adults that can support nursing care and favor the clinical reasoning of nurses in the nursing process to patients who present signs and symptoms of this phenomenon.

108869

Modality: E-Poster Researcher – Non-case Report

Category: CARDIOVASCULAR IMAGING

## Correlation between Myocardial Strain, Mechanical Dyssynchrony, and the Presence of Myocardial Fibrosis in the Mild Cardiac Form of Chagas Disease

POLYANA EVANGELISTA LIMA^1^, André Maurício Souza Fernandes^2^, Marta Silva Menezes^1^, Edmundo José NassriCamara^2^, Rafael de Castro da Silva^2^, Heverton Garcia de Oliveira^3^, Sarah Rodrigues de Assunção Vaz^3^, Alane Mota dos Santos^3^, Matheus Pereira Barreira^3^

(1) Escola Bahiana de Medicina e Saúde Pública; (2) Universidade Federal da Bahia; (3) Universidade Federal do Vale do São Francisco

**Introduction:** Early identification of myocardial damage appears to be important in the approach to patients with Chagas disease (CD). Echocardiography with strain obtained by speckle tracking (STE) and the evaluation of myocardial fibrosis (MF) through cardiac magnetic resonance imaging (CMRI) may be promising diagnostic methods in this regard.

**Objective:** Evaluate myocardial involvement, specifically in the chronic mild cardiac form of CD using strain by STE and MF by CMRI, as well as their correlations.

**Methods:** A cross-sectional study analyzed patients with the chronic mild cardiac form of CD (preserved ejection fraction) who underwent STE strain echocardiography and CMRI.

**Results:** Twenty-one participants were included (female: 62%, age: 54 +/– 5 years). The prevalence of MF by late myocardial enhancement (LME) was 50%. Global longitudinal strain (GLS) was decreased in 17 patients (81%) with a median of 14.1% (interquartile range 12.1–16.3). The average T1 mapping values were high in patients with CD (993 +/– 163 ms). T1 mapping was significantly correlated with GLS (r = 0.634; p = 0.015). Furthermore, the mechanical dispersion index obtained by strain was increased (>55 ms) by 84%, with the largest area under the receiver operating characteristic curve (AUC 0.696; 95% confidence interval 0.412–0.981) for fibrosis discrimination by LME.

**Conclusion:** The present study suggests that myocardial strain and T1 mapping behave as early markers of myocardial damage in mild chronic CD. The mechanical dispersion index was elevated, and it was the parameter that most correlated with myocardial fibrosis by LME.


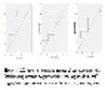


108876

Modality: E-Poster Researcher – Non-case Report

Category: ATHEROSCLEROSIS/CARDIOVASCULAR RISK FACTORS/CARDIOVASCULAR PREVENTION

## Screening of Cardiovascular Problems in 129 Adults with Down Syndrome

ANNE GERYMAIA OLIVEIRA DE MELO SILVA^1^, NEILA ANDERS AIDAR^2^, MARIA CAROLINA VIANA VALE^1^

(1) SECRETARIA DE SAÚDE DO DISTRITO FEDERAL; (2) MEDCOR

**Introduction:** Down syndrome continues to be the most common chromosomal condition, with rising prevalence and increased survival. With increased longevity, such individuals are susceptible to differing cardiovascular disorders.

**Objectives:** Describe cardiovascular disorders in adults with Down Syndrome.

**Methods:** Descritive and observational study of 129 adults with cytogenetically and/or clinically proven Down syndrome. For each individual, carers were interviewed to elicit a past history of any medical condition and to elicit any symptoms suggestive of an ongoing medical illness. A standard physical examination was undertaken.

**Results:** 129 adults with Down syndrome participated in this study, sex distribution being 65 (50.3%) males and 64 females. The mean age of the sample population was 29 years; range 18–64 years. The individuals were living in their family homes. The commonest cardiac disorders were cardiac congenital malformation in 43 (33.3%), dyslipidemia in 37 (28.7%) subjects, cardiac arrhythmia in 12 (9.3%) and dysautonomic disorders in 10 (7.7%). The commonest cardiac malformations were valvular diseases in 21 (16.3%) atrioventricular septal defect in 13 (10.0%), interatrial septal defect in 11 (8.5%), interventricular septal defect in 9 (7.0%), patent ductus arteriosus in 9 (7.0%). The mean rate was 74 beats per minute (SD 14), the mean systolic arterial pressure was 118.7 mmHg (SD 21.6), the mean diastolic arterial pressure was 77.2 mmHg (SD 16.2) and the mean oxygen saturation level was 95% (SD 4). The mean body mass index was 30.8 kg/m^2^ (SD 15.4), overweight and obesity was observed in 67.6% of individuals. The mean cholesterol was 175.1 mg/dl (SD 46.6). Other important clinical disorders was hypothyroidism in 52 (40.3%) individuals, epilepsy in 17 (13.1%) and diabetes mellitus in 6 (4.6%). Screening of obstructive sleep apnea (OSA) was positive in 20 cases (15.5%), being 13 (10.0%) considered severe OSA and 7 (5.4%) moderate OSA. Carotid ultrasound was performed on 26 individuals and measurement of intima-media thickness was normal in all cases.

**Conclusion:** Overweight/obesity, dyslipidemia and cardiac defects were frequently. Considering that Down syndrome presents with chronic hypotension, it is reasonable to propose that the prolonged reduction of arterial distending pressure may contribute to functional preservation of the arteries in patients with Down syndrome.

108881

Modality: E-Poster Researcher – Non-case Report

Category: CARDIOVASCULAR IMAGING

## Native T1 Mapping and Myocardial Fibrosis in the Mild Cardiac Form of Chagas Disease

POLYANA EVANGELISTA LIMA^1^, Rafael de Castro da Silva -Silva^1^, André Maurício Souza Fernandes^2^, Marta Silva Menezes^1^, Edmundo José NassriCamara^2^, Matheus Pereira Barreira^3^, Alane Mota dos Santos^3^, Sarah Rodrigues de Assunção Vaz^3^, Heverton Garcia de Oliveira^3^

(1) Escola Bahiana de Medicina e Saúde Pública; (2) Universidade Federal da Bahia; (3) Universidade Federal do Vale do São Francisco

**Introduction:** Chronic Chagas cardiomyopathy presents as chronic myocardial inflammation that causes progressive tissue destruction and extensive fibrosis. Cardiac magnetic resonance (CMR) is the gold standard noninvasive test to assess myocardial fibrosis (MF) using the technique of late myocardial enhancement (LME). CMR has recently been used to perform new evaluations with parametric mapping (T1 and T2). It is possible that the use of the T1 mapping technique generates complementary and even prognostic information in the evolution of patients with CD.

**Objective:** Describe the T1 mapping values obtained with CMR in the evaluation of patients with CD and their correlation with the LME technique.

**Methods:** This cross-sectional study analyzed patients with the chronic mild cardiac form of CD who underwent CMR. Eligible patients underwent CMR to analyze MF by 2 techniques: the LME technique, which was categorized as present/absent for MF, and T1 mapping time, expressed as average +/– standard deviation.

**Discussion:** Sixteen patients composed the study population; 81% female, and the mean age was 54.3 +/– 5.3 years. Mean left ventricular ejection fraction was 65.3% +/– 5.4%. The prevalence of MF in the sample using the LME technique by CMR was 50%. The T1 mapping values were high in patients with CD without ventricular dysfunction, with mean of 993 +/– 163 ms. The medians and interquartile range (IQR) of T1 mapping were: 1033 (IQR 998–1081) in group I and 1010 (IQR 1002–1047) in group II. In the MF group, the times were even higher, as found in other dilated cardiomyopathies.

**Conclusion:** These data reinforce the ability of T1 mapping to identify MF more completely and earlier when compared with LME and suggest that this technique is a marker of early cardiac involvement by CD.


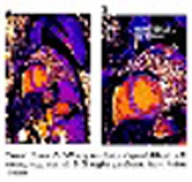


108908

Modality: E-Poster Researcher – Non-case Report

Category: EPIDEMIOLOGY AND HEALTH POLICIES/GLOBAL HEALTH

## Epidemiological Crossover in the Elderly in Brazil: Time Trend Analysis of Heart Failure Mortality from 1996 to 2019

EDUARDO PITTHAN^1^, Vânia Hirakata^2^, Natanael Alves de Lima^1^, Thiago Emanuel Rodrigues Novaes^1^, Pamela Sandri^1^

(1) Universidade Federal da Fronteira Sul Passo Fundo; (2) Instituto de Cardiologia do Rio Grande do Sul

**Introduction:** In Brazil, the clinical-epidemiological scenario of Heart Failure (HF) has shown significant changes in recent decades. The main factors that have affected the change in profile were the exponential increase in the elderly population and the impact of new therapies.

**Objective:** To evaluate the trend of variation in gross mortality rates in HF in Brazil from 1996 to 2019, comparing with the variation in the growth of the elderly population stratified by age groups.

**Methods:** Refers to an ecological, descriptive and quantitative study that used data available on DATASUS on March 8th 2022 regarding to mortality in the elderly due to HF, in Brazil, from 1996 to 2019, divided into three age groups: 60 to 69 years, 70 to 79 years and 80 years or more. The gross mortality rates in these variables were calculated per 100,000 inhabitants, based on the population variation in Brazil in the years chosen for the study, made available by IBGE in a consultation that happened on March 8th 2022.

**Results:** The analysis of mortality from HF over 80 years, in 1996, shows a rate of 770.2/10^5^ inhabitants and showed a significant drop in 2019, being 324.4/10^5^ inhabitants. Making a drop of 57.9% per 10^5^ inhabitants. On the other hand, in the same period, the population of this age group has increased 172.5%. The analysis of mortality from HF from 60 to 69 years, in 1996, showed a rate of 87.6/10^5^ inhabitants and showed a significant drop in 2019, being 26.6/10^5^ inhabitants. Making a drop of 69.6% per 10^5^ inhabitants. On the other hand, in the same period, the population of this age group has increased 120.7%. In the 70–79 age group, in 1996, it had a mortality rate of 248.1/10^5^ inhabitants and showed a significant drop in 2019, being 79.6/10^5^ inhabitants. Making a drop of 67.9% per 10^5^ inhabitants. On the other hand, in the same period, the population of this age group has increased 121.3%.

**Conclusion:** The results of this study demonstrate downward trends in the mortality rate from HF in the elderly in Brazil in recent decades. In contrast, there has been an exponential increase in the elderly population in all ages studied.


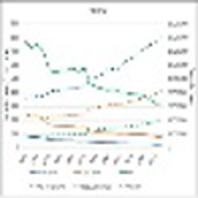


108942

Modality: E-Poster Researcher – Non-case Report

Category: CARDIOVASCULAR SURGERY

## Does Long-Term Storage in 4% Formaldehyde Changes the Expression of Immunophenotypic Markers of Glutaraldehyde-Treated Bovine Pericardium?

LUIZ FERNANDO KUBRUSLY^1^, Douglas Mesadri Gewehr^2^, Alexandre Gelás Haddad^1^, Victor Daniel Falkenbach Tenius^1^, Fernanda Prehs Izar^1^, Allan Fernando Giovanini^1^, Fernando Bermudez Kubrusly^3^

(1) Mackenzie Evangelical School of Paraná, Curitiba, Paraná, Brazil; (2) Denton Cooley Institute of Research, Science and Technology, Curitiba, Paraná, Brazil; (3) Curitiba Heart Institute, Curitiba, Paraná, Brazil

**Introduction:** The bovine pericardium is a biological tissue widely used as a biomaterial for tissue engineering applications. Glutaraldehyde and formaldehyde are frequently used in these reticulation processes to improve the material’s resistance and preservation.

**Objective:** The objective was to evaluate the impact of long-term storage in 4% formaldehyde on the quantitative expression of immunophenotypic markers of glutaraldehyde-treated bovine pericardium.

**Methods:** Bovine pericardium (BP) patches used were produced and supplied by Braile Biomédica®. We performed a histological and immunohistochemical analysis comparing two patches of BP, one manufactured in 2009, thickening 0.35 mm/measuring 35 cm^2^, and another manufactured in 2020, thickening 0.36 mm/measuring 99 cm^2^. BP were fixed in a 10% formalin solution for 24h, embedded in paraffin blocks, trimmed and mounted on histological glasses. Sections were stained with H&E, Weigert and picrosirius red and immunolabeled with vimentin, laminin 5, collagen I and collagen IV using a standardized protocol. Images were captured using light and polarized microscopy and the area of antibody signal was quantified using Image J Software.

**Results:** Histological analysis of the patches showed no autolysis or significant changes. In immunohistochemical analysis, collagen I and IV was diffused throughout the connective tissue of the patches. In 2020 specimen, collagen I occupied an area of 21.36% and collagen IV an area of 24.67%, while in the 2009 specimen, only 15.87% (collagen I) and 12.02% (collagen IV). Laminin was not reacted between the specimens. Immunopositivity for vimentin differed markedly between patches occupying a 54% area in the 2020 patch and a 13% area in the 2009 patch.

**Conclusion:** We observed no expressive differences in immunophenotypic expression between 2009 and 2020 bovine pericardia, except for the higher expression of the vimentin in the 2020 bovine pericardium patch.

108955

Modality: E-Poster Researcher – Non-case Report

Category: CARDIOGERIATRICS

## Epidemiological Transition in the Elderly in Brazil: Time Trend Analysis of Heart Failure Mortality by Gender from 1996 to 2019

EDUARDO PITTHAN^1^, Vânia Hirakata^2^, Pamela Sandri^1^, Guilherme Sommavilla^1^

(1) Universidade Federal da Fronteira Sul Passo Fundo; (2) Instituto de Cardiologia do Rio Grande do Sul

**Introduction:** Heart failure (HF) presents epidemic characteristics with considerable impact on morbidity and mortality, especially among the elderly. In Brazil, HF is responsible for high mortality rates.

**Objectives:** To evaluate the variation trend of HF gross mortality rates by gender in the elderly in Brazil, comparing with the variability of population growth by age group and gross mortality rate from all causes in recent decades.

**Methods:** An ecological study that used data available on DATASUS on March 2022, regarding mortality by gender in elderly people with HF in Brazil, from 1996 to 2019, stratified into three age groups: 60 to 69 years, 70 to 79 years and >80 years. The gross mortality rates were calculated in these variables per 10^5^ inhabitants, comparing with the variation of the population in Brazil in the years that were studied, made available by the IBGE in a consultation on March 2022.

**Results:** In the analysis of HF mortality in Brazil in men aged 60 to 69 years, there has been a decrease of 67%, falling from 98/10^5^ inhabitants to 32/10^5^ inhabitants. In the same age group, among women, the gross death rate has decreased 72%, falling from 77/10^5^ to 21/10^5^ inhabitants. In contrast, the male and female population aged 60 to 69 has grown 119% and 121%, respectively. In men aged 70 to 79 years, the mortality rate has dropped from 269/10^5^ to 94/10^5^ inhabitants, with a drop of 65%. Moreover, among women in this age group, there has been a drop of 70%, from 229/10^5^ to 68/10^5^ inhabitants. In contrast, the male population grew 112% and the female population 129%. In the age group of more than 80 years, in the same period, male mortality has decreased 54%, falling from 741/10^5^ inhabitants to 339/10^5^ inhabitants. In women, there was a drop of 60%, falling from 785/10^5^ inhabitants to 315/10^5^ inhabitants. In contrast, the male population has grown 151% and the female population, 187%.

**Conclusion:** This study demonstrates trends of a significant decrease in the mortality rate from HF in elderly people of both genders in Brazil in the recent decades. This trend is more pronounced in women aged 60 to 79 years.


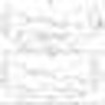


108973

Modality: E-Poster Researcher – Non-case Report

Category: EPIDEMIOLOGY AND HEALTH POLICIES/GLOBAL HEALTH

## Evaluation of Health Literacy in Patients with Systemic Arterial Hypertension in Northeastern Brazil

ROMERO HENRIQUE DE ALMEIDA BARBOSA^1^, Luana Resende Cangussú^1^, Eduardo Antonio Sartori Alho^1^, Matheus Rodrigues Lopes^1^

(1) Universidade Federal do Vale do São Francisco

**Introduction:** Health literacy comprises the cognitive and social skills that determine an individual’s ability to obtain, process, understand and use health and medical information to make decisions that are relevant to their own health. Cardiovascular diseases are the leading causes of death in Brazil and Systemic Arterial Hypertension (SAH) contributes directly or indirectly to 50% of these deaths. Poor health literacy can impact in the management and control of these comorbidities leading to significant losses in quality of life.

**Objectives:** To evaluate the level of health literacy and the quality of life of patients with systemic arterial hypertension users of the Public Health System in a municipality in the Northeast region of Brazil.

**Methods:** Cross-sectional analytical observational study carried out with 105 patients with SAH through the application of the Short Assessment of Health Literacy for Portuguese-speaking Adults (SAHLPA-18), Short Test of Functional Health Literacy in Adults (S-TOFHLA) and the Mini- Quality of Life Questionnaire in Arterial Hypertension (MINICHAL).

**Results:** It was found that about 60% of the interviewed patients did not have adequate health literacy in both tests to measure the level of health literacy. It was found that some factors such as age, economic class and education were associated with inadequate health literacy (p < 0.01). In the assessment of quality of life, using the MINICHAL, 46.7% of patients reported that hypertension interferes with quality of life. It was also possible to show that the time of diagnosis and economic class influenced the quality of life of patients (p < 0.05).

**Conclusion:** A relevant portion of patients with SAH did not have an adequate level of health literacy, which reflects the difficulty in understanding and processing health information and can impact the therapeutic management of the disease.

108986

Modality: E-Poster Researcher – Non-case Report

Category: HEMODYNAMICS AND INTERVENTIONAL CARDIOLOGY

## Amplatzer Cardiac Plug – (Abbott Vascular) Sizing for Occlusion of the Left Atrial Appendage Based on the Evaluation of the Landing Zone Measurements, Assessed by Three-Dimensional Reconstruction of the Computed Tomographic Angiography

EDGARD FREITAS QUINTELLA^1^, Leonardo Hadid^1^, Márcio José da Costa Montenegro^1^, Luiz Kohn^2^, Dinaldo Cavalcanti Oliveira^5^, Gustavo Lycurgo^4^, Paulo Antonio Marra da Motta^4^, Maximiliano Otero Lacoste^3^

(1) Instituto Estadual de Cardiologia Aloysio de Castro (IECAC); (2) Universidade Estadual do Rio de Janeiro, Hospital Pedro Ernesto (HUPE); (3) Hospital Copa Star (Copa Star); (4) Hospital HOME; (5) Universidade Federal de Pernambuco (UFPE)

**Introduction:** The left atrial appendage(LAA)percutaneous closure presents itself as a promising non-pharmacological alternative to anticoagulation. However, due to the anatomical singularity of the LAA, the accurate sizing and selection of the ideal prosthesis becomes a complex task.

**Objectives:** In an attempt to mitigate the risks of inadequate sizing and to enable better standardized choice, this study proposes a new technique for sizing the Amplatzer Cardiac Plug prosthesis(ACP-AMULET)(Abbott Vascular).

**Methods:** The current proposal was based on the consensus between experienced operators, who recommend that sizing should be performed by measuring the area and perimeter of the prosthesis landing zone(LZ), according to the measurements acquired by three-dimensional reconstruction of the LAA neck planes by Computed Tomography Angiography(CTA). In addition, a consolidated table was created from the manufacturer’s recommended data, where the range of the different sizes and oversizing was accounted for to enable anchorage and safe implantation. Hence, after the proper sizing of the LZ measurements, the results are crossed with the table and choice is made with greater precision.

**Discussion:** The rationale for the proposal derives from the irregularity and heterogeneity of the LAA shapes, making the use of bidimensional sizing methods based on the average of the diameters a less accurate decision-making criteria of prosthesis selection. Although TEE is still the most widely used method, it is also subject to biases such as echocardiographer experience and volume status, which may restrict the widespread treatment success when used as a stand-alone decision-making strategy. Moreover, by performing the choice through CTA, it allows a lower dependence of choice during the procedure, avoiding its possible biases.


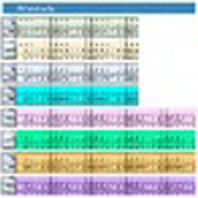


108992

Modality: E-Poster Researcher – Non-case Report

Category: CARDIOGERIATRICS

## Analysis of Gait Speed and Risk of Falls in the Elderly

ANGELA SICHINEL^1^, JOLIANE ALVES DE MORAES ROTILLI,^1^, LUCI MATSUMURA^1^, MARILENA INFIESTA ZULIM^1^, LUCIANE PEREZ DA COSTA^1^, CLAUDIA GONÇALVES GOUVEIA^1^, CAMILA SICHINEL SILVA DA CUNHA SOUZA^1^, MARIA LÚCIA SALAMENE DE OLIVEIRA KROLL^1^, GABRIELLA P PELLIZZER^1^, MARCIA MARIA DA COSTA^1^, MATHEUS PORTOCARRERO PETERLINKAR^1^, ERIVALDO ELIAS JR^1^

(1) SAO JULIAO HOSPITAL-HSJ; (2) SAO JULIAO HOSPITAL-HSJ; (3) SAO JULIAO HOSPITAL-HSJ; (4) SAO JULIAO HOSPITAL-HSJ; (5) SAO JULIAO HOSPITAL-HSJ; (6) SAO JULIAO HOSPITAL-HSJ; (7) SAO JULIAO HOSPITAL-HSJ; (8) SAO JULIAO HOSPITAL-HSJ; (10) SAO JULIAO HOSPITAL-HSJ; (11) SAO JULIAO HOSPITAL-HSJ; (12) SAO JULIAO HOSPITAL-HSJ

**Introduction:** The risk factors of falls in the elderly are consequences that occur due to internal or external changes; The internal modifications are those physiologically associated to the aging process, emphasising difficulties walking, lack of muscular strength, movement range restrictions, balance issues and lack of physical exercise; The external ones are those that depend on social or environmental circumstances that represent difficulties to the elder.

**Objective:** To analyze the relationship between gait speed and the occurrence of falls amongst elderly that participate in the MAE Project (Multidisciplinary Assessment in the Elderly).

**Method:** A cross-sectional study was carried out with 109 elderly individuals associated to the MAE Project, 72 of them (66,05%) being female and 37 (33,94%) being male. The data collection took place at the physiotherapy outpatient clinic of São Julião Hospital, from April of 2017 to November of 2019, through application of the gait speed test, in which was measured the time, in seconds, the patient coursed the distance of 20 meters, not taking into account the 5 first meters because it is the acceleration period, nor the 5 last meters which is the deceleration period, and a questionary which contained name, sex and the question: Have you had falls in the last 12 months?

**Results:** The sample was composed by 109 elders. 41 of those (37,6%) reported having falls in the last 12 months. Amongst those, 32 (78,04%) were female and 09 (21,95%) were male. Of the elderly that presented falls, 31 (75,60%) showed reduced gait speed, taking into consideration that the normal gait speed score, which ranges between 1,2 meters per second (m/sec) and 1,4 m/sec. Amongst the elderly with reduced gait speed, the female sex predominated with the number of 25 (80,64%) and average age of 70 years.

**Conclusion:** Gait speed showed significative relationship with the occurrence of falls, being spotted reduced gait speed on more than half of the elders that had falls. However, when analyzing the study sample, in which less than half of the elderly has had falls, it was concluded that active elderly people have better results, that is, with the practice of physical activities, the functional capability increases and so the risk of falls is reduced.

108994

Modality: E-Poster Researcher – Non-case Report

Category: CARDIOGERIATRICS

## Bnp Plasmatic as Predictor of Heart Failure Mortality in Study Cohort in South Brazil

EDUARDO PITTHAN^1^, Vânia Hirakata^2^, Juarez Barbisan^2^

(1) Universidade Federal da Fronteira Sul Passo Fundo; (2) Instituto de Cardiologia do Rio Grande do Sul

**Introduction:** The diagnosis of HF in elderly patients in clinical basis is difficult due to comorbidities. The BNP is used as a diagnostic and prognostic tool in HF but is not sufficiently studied in the elderly.

**Hypothesis:** To evaluate the association of plasma BNP levels with diagnostic accuracy of HF and longterm prognostic validation.

**Methods:** Six hundred and thirtyfour consecutive patients presenting with suspected HF in the Emergency Room of a tertiary hospital in southern Brazil participated in this cohort study. All patients underwent BNP measurement (Biosite POCT) as the institution protocol were included between March 2008 and September 2014. Following the Gold Standard for diagnosis of HF consisting of history and physical examination ECG chest Xray echocardiogram Uni and Two Dimensional Color Doppler. The sample was divided into 3 groups: SHF (Systolic Heart Failure) HFPEF (Preserved Ejection Fraction Heart Failure) and NHF (No Heart Failure) and patients were followed for 78 months. The study endpoint was mortality identified by the certificate of death of the Mortality Information Service (SIM).

**Results:** Most patients (59.6%) were female the mean age was 77 ± 8 years 40.5% over 80 years. The majority (46.8%) had a diagnosis of HFPEF and BNP median 335 pg/ml 25% presented SHF and BNP median of 573 pg/ml and 28% did not meet the criteria for NHF and median BNP 45 pg/ml (KruskalWallis’s test p < 0.005). Half of the deaths were caused by HF. In the group with SHF was 79 deaths (49%) with a BNP median of 800 pg/ml and the 80 survivors median 383 pg/ml (p < 0.005). In HFPEF group occurred most deaths that computed 157 deaths (52% of the group) with a median of BNP 380 pg/ml and 140 survived with a median 245 pg/ml (p < 0,005). The NHF group had 40 deaths (22% of the group) with BNP median of 59 pg/ml and 138 survivors median of 36 pg/ml (p < 0,005). Survival analysis in 78 months (6.5 years) was performed by KaplanMeier curve. Two hundred seventh six deaths were recorded in the total group (42%). The group SHF with a 27month mean survival time (MST) the group HFPEF with a 52month MST the NHF group more than 50% survived.

**Conclusions:** The BNP level showed association with the mortality index. BNP is an independent prognostic biomarker for longterm mortality in patients with HF in all ages.

109019

Modality: E-Poster Researcher – Non-case Report

Category: CARDIOGERIATRICS

## Applicability of BNP as a Biomarker of Prognosis for Longterm Mortality in a Comparison between Non-Elderly, Elderly and Major-Elderly Patients in South Brazil

EDUARDO PITTHAN^1^, Vânia Hirakata^2^, Juarez Barbisan^2^

(1) Universidade Federal da Fronteira Sul Passo Fundo; (2) Instituto de Cardiologia do Rio Grande do Sul

**Introduction:** Btype natriuretic peptide (BNP) is used as a shortterm biomarker for prognosis in patients with heart failure (HF). The prognostic role for longterm mortality is insufficiently studied.

**Hypothesis:** To validate the BNP test as a biomarker of longterm mortality prognosis in patients with suspected HF, comparing the non-elderly 80 years old.

**Methods:** The sample consisted of 634 patients with suspected HF, attended at the emergency room between March 2008 and September 2014. The efficacy of BNP to identify patients with heart failure and the association between the level of BNP (POCT Biosite) and longterm prognosis were evaluated. The study was divided into three age groups, Elderly (E) 60–79 years (46%), major-elderly ≥80 years (38%) and Non-Elderly <60 years old. Cause of death was identified through a search of death certificates in registry offices, informed by the Brazilian Mortality Information System.

**Results:** Most patients were white (93%), female (64%), with a mean age of 77 (±8.6) years. HF was diagnosed based on a new gold standard that considered the Framingham and Boston criteria, plus echocardiography and ECG. HF was present in 340 patients (53%). Most of these patients (63%, n = 216) had HF with PreservedEjection (HFPEF). In bivariate analysis a BNP > 180 pg/ml was associated with a higher risk of mortality. In multivariate analysis BNP > 180 pg/ml remained associated with increased risk of mortality, with an HR of 3.4 (CI95%: 1.2 to 9.6;p80 years group with HF was 595 pg/ml, with a 27month mean survival time (MST) and 47% mortality rate. The median BNP for the Elderly between 60–79 years group was 369 pg/ml, with a 52month MST and 38% mortality rate. The Non-Elderly group BNP was 222 pg/ml, with an MST >50% and 26% mortality rate.

**Conclusions:** The BNP level showed association with the mortality index. BNP is an independent prognostic biomarker for longterm mortality in patients with HF in all ages.


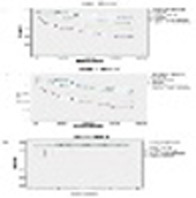


109029

Modality: E-Poster Researcher – Non-case Report

Category: ATHEROSCLEROSIS/CARDIOVASCULAR RISK FACTORS/CARDIOVASCULAR PREVENTION

## Triglycerides/HDL-Cholesterol Ratio in Patients Admitted to the Department of Cardiology of Hospital San Martín

GRACIA LUZ DON^1^, Eugenia Machbeth^1^

(1) Hospital San Martín, Paraná, Entre Ríos, Argentina

Dyslipidemia has been identified as the risk factor that has the greatest impact of suffering ischemic heart disease. Triglyceride/HDL-cholesterol ratio (TG/HDL-C) was proposed as the best marker of insulin resistance and is considered an atherogenesis marker. It is obtained from triglycerides (mg/dl)/HDL-cholesterol (mg/dl) calculation. The cutoff point of 3.0 correlates with insulin resistance and cardiovascular risk, >3.5 with small and dense LDL-C particles, coronary atherosclerosis and risk of myocardial infarction. The present study was conducted to evaluate the relationship between TG/HDL-C index and cardiovascular diseases that led to hospitalization in the Department of Cardiology of San Martín Hospital. Method Ninety-one patients admitted to the hospital over a four-month period were evaluated according age, gender, TG/HDL-C ratio (triglycerides in mg/dl and HDL-C in mg/dl), BMI, admission blood glucose, coronary lesions and discharge diagnosis Results 30 women, 59 men and two without declaring gender were analyzed. The highest concentration of patients corresponds to ages between 60 and 69. Risk factors of the studied population were: • Hypertension 66% • Diabetes Mellitus 25% • Smoking 20% • Dyslipidemia 19% • Overweight/obesity 67% Variable evaluated -Percentage TGL/HDL Index >3.5 IMC 25–29,9 51% IMC > 30 60% Admission Blood glucose 111–199 mg/dl 61% Admission Blood glucose >200 mg/dl 54% Severe coronary lesions >70% DA: 56% CX 72% CD 72% discharge diagnosis: ischemic heart disease 74% heart failure 48% Previous myocardial infarction 81% history of coronary artery bypass grafting or angioplasty 50% Dyslipidemic patients 52% Total evaluated patients 74% Conclusions TGL/HDL-C index >3/>3.5 is observed in hospitalized patients with high prevalence. This demonstrates the impact of insulin resistance and phenotype B on severe cardiovascular complications such as ischemic heart disease and heart failure.

109036

Modality: E-Poster Researcher – Non-case Report

Category: HEMODYNAMICS AND INTERVENTIONAL CARDIOLOGY

## Mitral Balloon Valvuloplasty: Risk Factors for Lack of Success, Severe Mitral Regurgitation and Major Complications

IVANA PICONE BORGES DE ARAGÃO^1^, Ricardo Trajano Sandoval Peixoto^1^, Rodrigo Trajano Sandoval Peixoto^1^, Caio Teixeira dos Santos^1^, Raul Ferreira de Souza Machado^1^, Thaís Lemos de Souza Macedo^1^, Ivan Lucas Picone Borges dos Anjos^1^, Sara Cristine Marques dos Santos^1^, Edison Carvalho Sandoval Peixoto^1^

(1) Universidade de Vassouras

**Background:** Mitral balloon valvuloplasty is not Always successful and free from complications.

**Objectives:** To determine the independent risk factors for an unsuccessful procedure, severe mitral regurgitation and major complications in mitral balloon valvuloplasty.

**Methods:** Longitudinal prospective study of 518 mitral balloon valvuloplasties performed between July 6, 1987 and December 31, 2004, on 429 (82.8%) female patients and 89 (17.2%) male patients with a mean age of 37.5 ± 12.8 years. Major complications were considered to be: perforation with cardiac tamponade, stroke and severe mitral regurgitation per procedure. The continuous variables were transformed in categorical variables and the chi-square or Fisher exact tests to compare the categorical variables, and logistic regression and multiple logistic regression were used to identify independente factors for predicting success, incomplete procedure, severe mitral regurgitation and major complications.

**Results:** Success was noted in 452 (94.2%) procedures, with major complications occurring in 22 (4.2%) patients, of which ten were severe mitral regurgitation; there were no per-procedure deaths, with four (0.8%) in-hospital deaths. In the multiple logistic regression, lower age predicted success in the procedure; the only variable that predicted an incomplete procedure was the initial period of the procedure, and a score >11 points predicted severe per-procedure mitral regurgitation. There was no independent predictor of major complications in this study.

**Conclusions:** Success was related to younger patients, an incomplete procedure to the initial period of the procedure and severe per-procedure mitral regurgitation to an echocardiography score >11 points.

109044

Modality: E-Poster Researcher – Non-case Report

Category: ATHEROSCLEROSIS/CARDIOVASCULAR RISK FACTORS/CARDIOVASCULAR PREVENTION

## Women Self-Knowledge Screening for Cardiovascular Risk Factors in Different Age and Labor Actives Populations

IVANA PICONE BORGES DE ARAGÃO^1^, Lívia Liberata Barbosa Bandeira^1^, Simone Aparecida Simões^1^, Tatiana Soares Spritzer^1^, Caio Teixeira dos Santos^1^, Raul Ferreira de Souza Machado^1^, Thaís Lemos de Souza Macedo^1^, Ivan Lucas Picone Borges dos Anjos^1^, Sara Cristine Marques dos Santos^1^, Vanessa de Freitas Marçolla^1^

(1) Universidade de Vassouras

Cardiovascular disease (CVD) may be clinically different in women compared to men being underdiagnosed and treated. Worldwide, CVD and stroke are the leading causes of death in females reporting 8.6 million deaths/year in the literature. The objective of this study was to identify the self-knowledge (SK) and prevalence (P) of risk factors (RF) for CVD and stroke in female populations of different age groups and work activities: students of basic cycle medical students (group MS), Police Pacifying Units Police (PPU) (group PPU) and government employees (group GE).

**Methods:** Cross-sectional, observational study of P of RF for CVD and stroke in female populations of different ages and labor activities between: group GE-27/09/13 and 10/24/2013; group PPU-10/05/2013 and 10/10/2013; groups MS-06/2016 and 12/2016; through the filling of a similar and anonymous questionnaire with 30 objective questions of quick answers about SK of RF: age, stress level, smoking, hypertension (H), dyslipidemia, sedentary lifestyle, obesity, diabetes. weight, height. pregnancy, menopause, gynecological (G/Y) and cardiological/year (C/Y) consultations. A positive response or ignorance equaled one point. Considered a risk group: women with ~2 points for positive or unknown response.

**Results:** A total of 961 women interviewed were divided into groups MS (total 159), PPU (602) and GE (200), respectively: mean age 20.62, 28.1 and 44.3; high stress 44%, 31%, without report; smoking 3.8%, 7.0%, 16%; H 2.5% (1.3% unaware), 7% (3%), 13% (3%); 76.7% had they cholesterol levels measured (10.0% total cholesterol >200 mg/dl and 33.3% did not know, 62.9% did not know HDL <40 mg/dl), 76.0% (7% and 59%, 87%), 95% (22% E 25%, 62%); 89.9% had measured blood glucose, 76%, 88%; S 45.3%, 53%, 36%; BMI calculated 88.7% (weight and height reported) 12.57% ;?25 and 0.0% ;?30, BMI 51% being 23% ;?25 and 0.0% ;?30 and 49% being 17% ;?25 and 8% ;?30; they did consultations G/Y: 79.9%, 90.0% 98% and C/Y: 7.54% 12% and 33%; score i?2: 98.75%, 97.0%, 74.0%.

**Conclusion:** Most women, in different age groups and work activities, were at risk of developing CVD and stroke due to the high prevalence of RF or their lack of knowledge, after applying a similar questionnaire. It was highlighted the importance of primary prevention and awareness programs.

109046

Modality: E-Poster Researcher – Non-case Report

Category: HEMODYNAMICS AND INTERVENTIONAL CARDIOLOGY

## Percutaneous Mitral Valvuloplasty with Inoue Versus Balt Single Balloon. Results, In-Hospital Evolution and Cost Effectiveness

IVANA PICONE BORGES DE ARAGÃO^1^, Ricardo Trajano Sandoval Peixoto^1^, Rodrigo Trajano Sandoval Peixoto^1^, Caio Teixeira dos Santos^1^, Raul Ferreira de Souza Machado^1^, Thaís Lemos de Souza Macedo^1^, Ivan Lucas Picone Borges dos Anjos^1^, Sara Cristine Marques dos Santos^1^, Edison Carvalho Sandoval Peixoto^1^

(1) Universidade de Vassouras

**Objective:** To compare the results, in-hospital evolution and cost of 468 percutaneous mitral balloon valvuloplasties (PMBV) with Inoue balloon (IB) and single Balt balloon (SBB).

**Methods:** IB group (IG): 73 procedures and SBB group (BG): 395 performed between 06/1987 and 12/1999. Mean age: IG 37.1 ± 10.1 years and BG 37.3 ± 12.8 (p = 0.71745); 59 women in IG and 327 in BG (0.685255); NYHA functional class in IG and BG, respectively: I in 4 and 4 patients, II in 23 and 87, III in 40 and 265 and IV in 6 and 39 procedures (p = 0.010929). Atrial fibrilation: 7 in IG and 55 BG (p = 0.315511). Echocardiographic score 7.2 ± 1.2 IG and 7.3 ± 1.5 BG (p = 0.958911). Mitral valve área (MVA) Echo pre-PMBV: 0.98 ± 0.19 cm^2^ IG and 0.94 ± 0.21 BG (p = 0.143954).

**Results:** Within-group comparison IG and BG, respectively: Pre-PMBV: mean pulmonar pressure (MPP) 33.9 ± 13.5 and 38.6 ± 14.3 mmHg (p = 0,007662); mitral gradient (MG) 17.3 ± 6.4 and 19.8 ± 7.0 mmHg (p = 0.013180); MVA Gorlin pre-PMBV 0.90 ± 0.20 and 0.91 ± 0.21 cm^2^ in BG (p = 0.8228449). Post-PMBV: MPP 25.3 ± 8.6 and 27.2 ± 10.6 mmHg (p = 0.261415); MG 5.9 ± 3.1 and 5.5 ± 3.7 mmHg (p = 0.083664); MVA Gorlin 1.98 ± 0.46 and 2.04 ± 0.40 cm^2^ (p = 0.419208). Complications: 5 cardiac tamponade in BG: 3 treated by surgery with 2 deaths, 2 with pericardial drenage without death. 1 stroke in BG. Severe mitral regurgitation (MR) 1 patient of each group, treated by surgery. Calculated cost of both technique 2 consecutive years with reuse and price of acquision at current prices demonstrated: IB technique U$1,286,32 and SBB U$309.94 for procedures.

**Conclusions:** Both techniques were efficients. IG less symptomatic; MPP and MG were higher in BG; results post-PMBV were similar. MR were similar. Other complicaion only in BG. Lower cost for material acquisition in BG.

109047

Modality: E-Poster Researcher – Non-case Report

Category: HEMODYNAMICS AND INTERVENTIONAL CARDIOLOGY

## Prior Surgical Mitral Commissurotomy and Echocardiographic Score Influence in Mitral Balloon Valvuloplasty. Immediate Post Procedure Results

IVANA PICONE BORGES DE ARAGÃO^1^, Ricardo Trajano Sandoval Peixoto^1^, Rodrigo Trajano Sandoval Peixoto^1^, Caio Teixeira dos Santos^1^, Raul Ferreira de Souza Machado^1^, Thaís Lemos de Souza Macedo^1^, Ivan Lucas Picone Borges dos Anjos^1^, Sara Cristine Marques dos Santos^1^, Edison Carvalho Sandoval Peixoto^1^

(1) Universidade de Vassouras

**Introduction:** Percutaneous mitral balloon valvuloplasty is effective in mitral stenosis.

**Objectives:** To evaluate prior mitral surgical commissurotomy (PMC) and echocardiographic score (ES) in the results and complications of mitral balloon valvuloplasty (MBV).

**Methods:** From 1987 to 2013, 526 procedures with Inoue balloon, double or single Balt balloon technique; 480 without PMC named primary MBV group (PMBVG) and 46 that have been submitted to PMC, the PMC group. The PMCG was older than PMBVG (42.7 ± 12.4 vs 36.9 ± 12.5 years, p = 0.0030). Gender, atrial fibrilation and NYHA functional class were similar. In PMBVG and PMCG, respectively, ES were 7.2 ± 1,4 and 7.7 ± 1.5 points (p = 0.0158) and mitral valve area (MVA) 0.94 ± 0.21 and 1.00 ± 0.22 cm^2^ (p = 0.0699).

**Results:** Pre-MBV: mean pulmonary artery pressures (MPAP) were 37.8 ± 14.2 and 37.6 ± 14.4 mmHg, p = 0.9515; mean gradient (MG) 19.6 ± 6.9 and 18.3 ± 6.9 mmHg, p = 0.2342; MVA 0.90 ± 0.21 and 0.93 ± 0.19 cm^2^, p = 0.4092, respectively, whem compare PMBVG and PMCG. Post-MBV: MPAP were 26.8 ± 10.2 and 26.6 ± 10.9 mmHg, p = 0.9062; MG 5.4 ± 3.5 and 6.3 ± 4.2 mmHg, p = 0.1492; MVA 2.04 ± 0.42 and 1.92 ± 0.41 cm^2^, p = 0.0801, respectively. Mitral regurgitation (MR) were similar pre and post-MBV. Severe MR post-MBV in 10 patients: 8 in PMBVG and 2 in PMCG, p = 0.2048. As there were not found significant diferences, the total group were divided in ES ≤ 8 and >8 groups: Pre-MBV: MPAP 37.5 ± 13.9 and 39.3 ± 16.6 mmHg, p = 0.4041; MG 19.7 ± 6.8 and 18.3 ± 7.3 mmHg, p = 0.1753; MVA 0.90 ± 0.21 and 0.94 ± 0.20 cm^2^, p = 0.0090 respectively. Post-MBV: MPAP 26.7 ± 10.1 and 28.0 ± 10.6 mmHg, p = 0.3730, MG 5.5 ± 3.6 and 5.5 ± 3.3 mmHg, MVA 2.06 ± 0.42 and 1.90 ± 0.40 cm^2^, p = 0.0090.

**Conclusion:** The groups with and without prior mitral commissurotomy in MBV were compare and no differences were found in pre- and post-procedure, as mean pulmonary artery pressure, mean mitral gradient, mitral valve area, and mitral regurgitation. Although PMCG was older, with higher ES, its hemodynamics datas were similar. Whem the entire group was divided based on echo scores, those with echo scores >8 had highse MV (p = 0.0090). and smaler mitral valve areas post-valvuloplasty. The valve anatomy were more important than prior commissurotomy.

109049

Modality: E-Poster Researcher – Non-case Report

Category: HEMODYNAMICS AND INTERVENTIONAL CARDIOLOGY

## Percutaneous Mitral Balloon Valvuloplasty: Single Versus Inoue Balloon. Long Term Follow-Up

IVANA PICONE BORGES DE ARAGÃO^1^, Ricardo Trajano Sandoval Peixoto^1^, Rodrigo Trajano Sandoval Peixoto^1^, Caio Teixeira dos Santos^1^, Raul Ferreira de Souza Machado^1^, Thaís Lemos de Souza Macedo^1^, Ivan Lucas Picone Borges dos Anjos^1^, Sara Cristine Marques dos Santos^1^, Edison Carvalho Sandoval Peixoto^1^

(1) Universidade de Vassouras

**Introduction:** The single balloon (SB) is the less expensive technique to perform mitral balloon valvuloplasty (MBV).

**Objectives:** This study aimed to demonstrate that MBV with the Balt single (BSB) and Inoue ballon, the wordwire accepted technique, had similar outcome and long-term follow-up (FU).

**Methods:** From 1987 to 2013, 526 procedures were performed, being 312 with a FU, 56 (17,9%) with Inoue balloon (IB) and 256 (82,1%) with BSB. The mean FU 156 ± 144 months, p < 0.0001. Univariate analysis (UA) and multivariate Cox analysis (MCA) to determine independent predict variables of survival and event free survival (EFS) of death, cardiac surgery and new MBV, in both techniques groups.

**Results:** In IB and BSB groups there were, respectively: female 42 (75.0%) and 222 (86.7%); mean age 37.3 ± 10.0 (19 to 63) and 38.0 ± 12.6 (13 to 83) years, p = 0.7138; sinus rhythm 51 (91.1%) and 215 (84.0%), p = 0.1754; echo score (ES) 7.6 ± 1.3 (5 to 10) and 7.2 ± 1.5 (4 to 14) points, p = 0.0528; echo mitral valve area (MVA) pre-MBV 0.96 ± 0.18 and 0.93 ± 0.21 cm², p = 0.2265; post-MBV mean MVA (Gorlin) were 2.00 ± 0.52 and 2.02 ± 0.37 cm², p = 0.9554; MBV dilatation área 6,09 ± 0,27 and 7,02 ± 0,30, p < 0,0001. At the end of the FU, there were in IB and BSB groups, respectively: echo MVA 1.71 ± 0.41 and 1.54 ± 0.51 cm², p = 0.0552; new severe mitral regurgitation in 5 (8.9%) and 17 (6.6%) patients, p = 0.5633; new MBV in 1 (1.8%) and 13 (5.1%), p = 0.4779; mitral valve surgery in 3 (5.4%) and 27 (10.4%), p = 0.3456; deaths 2 (3.6%) and 11 (4.3%), p = 1.000; cardiac deaths 1 (1.8%) and 9 (3.5%), p = 1.000; ME 5 (8.9%) and 46 (18.0%), p = 0.1449. In UA and MCA the BSB or IB technique do not predict survival or EFS. The independent risk factors to survival were: age <50 years (p = 0.016, HR = 0.233, 95% IC 0.071–0.764), ES ≤ 8 (p < 0.001, HR = 0.105, 95% IC 0.34–0.327), MBV dilatation area (p < 0.001, HR 16.838, 95% IC 3.353–84.580) and no mitral valve surgery in the FU (p = 0.001, HR0.152, 95% IC 0.050–0.459). Independent risk factors to EFS: no prior commissurotomy (p = 0.012, HR = 0.390, 95% IC 0.187–0.813) and post-MBV MVA ≥ 1.50 cm² (p = 0.001, HR = 7.969, 95% IC 3.413–18.608).

**Conclusion:** In 25 years, survival and EFS were similar in BSB and IB technique. Independent predictors of surviva: age < 50 years, ES ≤ 8 points, MBV dilatation area > 7 mm^2^ and no mitral valve surgery in the FU. Independent predictors of EFS: no prior commissurotomy and post-MBV MVA ≥ 1.50 cm².

110916

Modality: E-Poster Researcher – Non-case Report

Category: ATHEROSCLEROSIS/CARDIOVASCULAR RISK FACTORS/CARDIOVASCULAR PREVENTION

## Assessment of Lipoprotein(A), Apolipoprotein B, Apolipoprotein A-1, and Apo B/Apo A1 Ratio in Predicting the Severity of Premature Coronary Artery Disease (CAD) in North India

SATYENDRA TEWARI^1^, SOHAM CHAUDHARI^2^, ANKIT SAHU^3^, ROOPALI KHANNA^4^, SUDEEP KUMAR^5^, NAVEEN GARG^6^, ADITYA KAPOOR^7^

(1) Sanjay Gandhi Post Graduate Institute of Medical Sciences, Lucknow (India); (2) Sanjay Gandhi Post Graduate Institute of Medical Sciences, Lucknow (India); (3) Sanjay Gandhi Post Graduate Institute of Medical Sciences, Lucknow (India); (4) Sanjay Gandhi Post Graduate Institute of Medical Sciences, Lucknow (India); (5) Sanjay Gandhi Post Graduate Institute of Medical Sciences, Lucknow (India); (6) Sanjay Gandhi Post Graduate Institute of Medical Sciences, Lucknow (India); (7) Sanjay Gandhi Post Graduate Institute of Medical Sciences, Lucknow (India)

**Introduction:** CAD is one of the principle cause of mortality and morbidity. Considerable data suggests involvement of Lipoprotein (a), apolipoprotein B, apolipoprotein A-I, its ratio and abnormal lipids.

**Objectives:** To compare the risk factor profile, clinical presentation, and angiographic severity of CAD in young patients (Age ≤ 45 years) and elderly patients (Age > 45 years). To correlate the level of Lipoprotein (a), Apolipoprotein (a), Apolipoprotein (b) and Apo B/Apo-A1 with the angiographic severity of CAD.

**Methods:** This a retrospective, observational, single-centre study performed at tertiary care hospital. Angiographically proven CAD patients of all age groups were enrolled.

**Results:** In our study, 714 patients (12.49%) belonged to group 1 (age ≤ 45 years) and 5003 patients (87.51%) belonged to group 2 (age > 45 years). Mean value of age(years) of study subjects was 56.92 ± 9.8. Distribution of gender was comparable between age group <=45 and >45 years. (Female:– 17.23% vs 17.49% respectively, Male:– 82.77% vs 82.51% respectively) (p value = 0.863). Proportion of patients with number of vessels involved:– 1 (single vessel) was significantly higher in age group <=45 years as compared to >45 years (1:– 79.97% vs 76.31% respectively). Proportion of patients with number of vessels involved:– 2 (double), 3 (triple) was significantly lower in age group <=45 years as compared to >45 years (2:– 19.33% vs 21.75% respectively, 3:– 0.70% vs 1.94% respectively). (p value = 0.017) There is statistically significant rise in the level of Lp (a) (mg/dL) and Apolipoprotein B/Apolipoprotein A-1 ratio with number of vessels involved in age group <=45 years and >45 years (p value < .05).

**Conclusion:** Elevated Lp(a) is strongly associated with the development of high-risk vulnerable plaques that are prone to rupture. The ratio of Apo B to Apo A-I represents the ratio of total atherogenic to antiatherogenic lipoproteins. We observed a gradual increase in Lp(a) and Apo-B/A1 levels with the increasing number of stenotic vessels and an independent association between Lp(a) and Apo B/Apo A-1 with the severity of CAD after adjusting for other possible confounding factors. Effort should also be made to assess the atherothrombotic risk due to the Lp (a) particles on one hand and to apolipoproteins on the other hand. It is also essential that Lp (a), apo B, and ratio of abo B/apo A as an independent risk factor should be integrated into the treatment guideline.

109096

Modality: E-Poster Researcher – Non-case Report

Category: EPIDEMIOLOGY AND HEALTH POLICIES/GLOBAL HEALTH

## Prevalence and Risk Stratification of Chronic Kidney Disease in a Tertiary Cardiology Hospital: A Cross-Sectional Study

FARID SAMAAN^1^, Gianna Mastroianni Kirsztajn^2^, Ricardo Sesso^2^

(1) Instituto Dante Pazzanese de Cardiologia; (2) Universidade Federal de São Paulo

**Introduction:** Chronic kidney disease (CKD) is common, preventable and silent in its early stages. Therefore, early detection of this condition in the population at risk, through laboratory tests, is essential.

**Objectives:** To estimate the CKD prevalence and perform its risk stratification in a tertiary health service specialized in cardiology.

**Methods:** The study was cross-sectional and based on laboratory records of patients from a public hospital specialized in cardiology. The evaluated tests were serum creatinine and urinary albumin/creatinine ratio (ACR, random sample) performed on an outpatient basis between 01/01/2021 and 12/31/2021. Duplicate exams and patients under 18 years of age were excluded. The estimated glomerular filtration rate (eGFR) was calculated using the CKD-EPI creatinine equation. CKD was defined by eGFR <60 ml/min/1.73 m² and classified into the following stages: 3a (45–59 ml/min/1.73 m²), 3b (30–44 ml/min/1.73 m²), 4 (15–29 ml/min/1.73 m²) and 5 (<15 ml/min/1.73 m²). Albuminuria was classified into three levels: A1 (<30 mg/g), A2 (30–300 mg/g) and A3 (>300 mg/g). According to the CKD risk map, individuals with simultaneous creatinine and ACR measurements were stratified into low, moderate, high or very high risk.

**Results:** The sample consisted of 36,651 patients in whom the same number of serum creatinine results and 19,031 ACR results were evaluated (median patients‘ age 72.5 [51.0–73.6] years, 51.3% male). The prevalence of CKD was 30.9% and patients with stages 3a, 3b, 4 and 5 corresponded to 15.3%, 10.2%, 3.6% and 1.7%, respectively. CKD was more frequent in older age groups: 18–29 years (2.5%), 30–44 years (8.4%), 45–59 years (25.5%), 60–74 years (30 .7%) and ≥75 years (56.8%) (p < 0.001). Patients with albuminuria categories A1, A2 and A3 were 71.5%, 22.6% and 5.9%, respectively. ACR ≥ 30 mg/g was not associated with age: 18–29 years (23.3%), 30–44 years (23.4%), 45–69 years (26.0%), 60–74 years (28 .5%) and ≥75 years (36.9%) (p = 0.671). Patients with simultaneous measurements of serum creatinine and ACR were 19,031 and their distribution in the CKD risk categories was: low (52.0%), moderate (23.8%), high (13.1%) and very high risk (11.2%).

**Conclusions:** The results showed that CKD is present in about 30% of the patients assisted in the cardiology institute evaluated. In up to half of the patients, the risk of major outcomes such as hospitalization, need for renal replacement therapy, and death was moderate, high, or very high.

109100

Modality: E-Poster Researcher – Non-case Report

Category: CONGENITAL AND PEDIATRIC CARDIOLOGY

## Pulmonary Vascular Remodeling in Monocrotaline-Induced Pulmonary Arterial Hypertension: Morphometric Analysis

LUIZ FERNANDO KUBRUSLY^1^, Douglas Mesadri Gewehr^2^, Jéssica Ferreira Eduardo^1^, Izabele Maria Geri^1^, Allan Fernando Giovanini^1^, Fernando Bermudez Kubrusly^3^

(1) Mackenzie Evangelical School of Paraná, Curitiba, Paraná, Brazil; (2) Denton Cooley Institute of Research, Science and Technology, Curitiba, Paraná, Brazil; (3) Curitiba Heart Institute, Curitiba, Paraná, Brazil

**Introduction:** Pulmonary arterial hypertension (PAH) is a high severity vascular disorder with fast progression, which clinical spectrum is related to the reduction of the pulmonary arteries lumen with an increase in pressure and vascular resistance. These changes result from many mechanisms such as medial hypertrophy, concentric intimal fibrosis and necrotizing vasculitis, which are responsible for the remodeling of pulmonary artery wall, the reduction of pulmonary compliance and leads to cardiac muscle overload.

**Objective:** To evaluate the severity of pulmonary arteriopathy in an animal model of Monocrotaline-induced Pulmonary Arterial Hypertension, through histological morphometric analysis of pulmonary vessels.

**Methodology:** Morphometric analysis was performed in Zen 3.2 Software and 50 PAH animal model slides, with random selection of 10 arterioles and 10 pulmonary non-arteriolar vessels for each animal. The gravity was delimited through three pulmonary systems elements (1) medial layer thickness, (2) microvascular muscularization of non-muscle vessels, and (3) obliteration of muscle arterioles and non-muscle vessels. We also assess cardiac overload thought right ventricular freewall thickness and the right ventricular chamber area.

**Results:** There was a gradual onset of pulmonary disease in MCT animals, whose evolution was followed by histological findings of pulmonary arteriopathy, including arterioles’ muscularization, medial layer hypertrophy, concentric and “plexiform-like” laminar and non-laminar neointimal lesions. There was an important increase in the thickness of the tunica media and the degree in microvascular muscularization through the experimental groups compared with the control. There was a significant increase in luminal obliteration of muscle arterioles and non-muscular vessels, measured by the luminal obliteration ratio. The right ventricular hypertrophy (HVD), assessed by measuring the thickness of the right ventricle, had increased significantly on 30-to-37-day groups, represented almost twice the value of the control group. Besides that, there was a significant dilatation of the Right Ventricular Chamber on 30-to-37-day groups.

**Conclusion:** The MCT application proved the effective inducing PAH, being able to generate changes in vessels through the lower exposure group. Furthermore, lungs lesions are intensified over time, and the observation of plexiform lesions were not registered in a similar model.

109135

Modality: E-Poster Researcher – Non-case Report

Category: DIGITAL HEALTH/INNOVATION

## Algorithm for Identification of Limitations of Life Activities in Individuals with Heart Disease: Validation for the Brazilian Population

ROMERO HENRIQUE DE ALMEIDA BARBOSA^1^, Emerson Silva de Jesus^1^, Eldys Myler Santos Marinho^1^, Johnnatas Mikael Lopes^1^

(1) Universidade Federal do Vale do São Francisco

**Background:** Heart disease burdens individual health, limiting life activities such as people’s productive capacity and quality of life. In addition to understanding the clinical characteristics of heart diseases, identifying subjects at risk prior to the installation of limitations helps in directing preventive therapeutic and multiprofessional actions.

**Objective:** To develop an algorithm to screen people with heart disease in an outpatient setting for their risk of future functional life activity limitations (LAL).

**Methods:** Cross-sectional study with data from the 2019 National Health Survey in Brazil. The general sample consisted of 90846 interviews, being selected individuals aged 18 years and some type of heart disease diagnosed for more than a year by physicians. The outcome was current usual LAL due to the disease. Independent variables were grouped into cardiac, self-care and social morbidities. The data were analyzed using the CHAID-type decision tree algorithm, and the sample was divided into training (70%) and testing (30%). Sensitivity (S), specificity (SP), positive (PV+) and negative (PV-) predictive values for the outcome were estimated.

**Results:** 4711 (5.3%;95%CI: 5.0–5.6) individuals have a diagnosis of heart disease, of which 4409 (94.4%; 95%CI:93.4–95.3) have been diagnosed for more than one year and mean age of 59.31 (95%CI: 58.31–60.31) years. 2374 (49.2%; 95%CI 46.5–51.8) of those with heart disease reveal LAL. 2830 observations were used to train the algorithm and 1171 to test it. Five attributes remained in the final model, two of morbidity, two of self-care and one of social, generating 13 nodes and a thickness of 3 in the decision tree. An algorithm with S = 81.9%, SP = 43.2%, PV+ = 58.94%, PV– = 70.54% was obtained. In the test, S = 83.8%, SP = 43.6%, PV+ = 60.67% and PV– = 72.04%.

**Conclusion:** The algorithm has a relevant rate of true positives, however it has a low rate of true negatives for LAD. As the damage caused by LAL and its high prevalence are much greater than the damage caused by the provision of care and follow-up, its application in clinical and community contexts in Brazil is justified.

109138

Modality: E-Poster Researcher – Non-case Report

Category: ATHEROSCLEROSIS/CARDIOVASCULAR RISK FACTORS/CARDIOVASCULAR PREVENTION

## Morphological and Morphometrical Aspects of the Aortic Wall in Equine (Equus Caballus, L. – 1758)

LUCIANO DE MORAIS PINTO^1^, Marcelo José Böck^1^, Vitor Pires Pereira^1^, Marcelo Leite da Veiga^2^, Claudia de Melo Bertoncheli dos Santos^3^, Luciano de Morais-Pinto^1^

(1) Laboratório de Design Anatômico – Universidade Federal de Santa Maria; (2) Laboratório de Morfofisiologia Experimental – Universidade Federal de Santa Maria; (3) Laboratório de Anatomia Patológica – Hospital Universitário – Universidade Federal de Santa Maria

Aortic rupture is relatively common in horses. However, architectural differences have not yet been considered in the pathogenesis of aortic catastrophe in this species. Thus, our objective was to describe the morphological and morphometric aspects of the tunica intima (TI) and media (MT) in the aortic bulb (AB) and ascending aorta (AA). 52 healthy aortas from adult horses were processed according to standard optical and scanning electron microscopy protocol. TI and TM thickness as well as aortic lamellar units were digitally measured. The aorta was pale yellow and had a rigid texture and distinct thicknesses. The AA wall was thicker than the BA, in totality and in the thickness of the layers (Tab.1). The lamellar units were better defined in AA and arranged in interleaved layers of elastic lamellae, smooth muscle cells and collagen fibers, while in BA the trilaminar aspect was not maintained and the muscle tissue bands were arranged in mosaic. The increase in the thickness of the aortic wall in the BA-AA direction suggests that blood flow is clockwise helical. It is assumed that blood pressure was higher in the septal AB for the left AA; from left BA to right AA and from BA to septal AA. As dissection/rupture involves failure of tissue structure, it is expected that a better understanding of the normal tissue architecture of the aorta in horses will contribute to future studies of the pathogenesis involving aortic catastrophes in animals and humans.


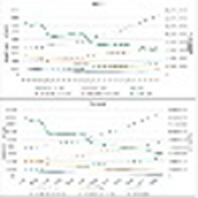


109147

Modality: E-Poster Researcher – Non-case Report

Category: CARDIORESPIRATORY PHYSIOLOGY/BASIC SCIENCE

## The HDAC Inhibitor Valproic Acid (VPA) Reduces Heart Damage of the Shr (Spontaneously Hypertensive Rat) Through Epigenetic Cardiac-TRH Modulation

SILVIA INES GARCÍA^1^, Maria Silvina Landa^1^, Maia Aisicovich^1^, Mariano Luis Schuman^1^, Ludmila Soledad Péres Díaz^1^, Graciela Giardino^2^, Carlos Jose Pirola^1^, Silvia Inés García^1^

(1) Molecular Cardiology Laboratory, Institute of Medical Research-IDIM University of Buenos Aires-CONICET; (2) Experimental Medicine Laboratory, Hospital Aleman, Buenos Aires

Cardiac TRH (cTRH) induces cardiac damage and its inhibition attenuates heart injury in different models: SHR and cTRH overexpression (Schuman 2011,2014); Angiotensin II infusion and doxorrubicin cardiotoxicity (Peres Diaz 2018,2020) and LVH induced by obesity or leptin infusion (Aisicovich 2019,2021). We described that cTRH inhibition increase LVEF% and preserve cardiac function after acute MI in rats (Schuman 2021). Histone acetylation (HDACs) modulates gene expression by epigenetic alterations as DNA methylation. VPA, an FDA approved drug for bipolar disease and epilepsy, protects heart against MI injury (Tian 2019). As VPA is an inhibitor of HDACs, we hypothesized that inhibition of HDACs with VPA might attenuate LVH and fibrosis in SHR by the modulation of cTRH. 7 w-old male SHR and WKY received VPA and hearts were used after 10w of treatment. BP, LVH index and cTRH expression significantly increased in SHR. VPA slightly attenuated (p < 0.05) the higher SHR BP, without effect in WKY. LVH index decreased (p < 0.05) only in SHR. LVPWT significantly decreased (p < 0.05) only in SHR. As hypothesized, VPA normalized cTRH mRNA expression in SHR (WKY = C:0.61 ± 0.7vs VPA:0.41 ± 0.97; SHR = C:5.72 + 0.9 vs VPA:0.61 + 0.9, p < 0.05) and TRH IHQ confirmed these results (p < 0.05) which brought a strong reduction in fibrosis in LV, by decrease in BNP and 3-collagen expression in SHR+VPA. This was confirmed by Masson’s an d Sirius Red stainings (p < 0.01). SHR offspring born from VPA-treated parents with a 2-week washout period before mating, wich never received VPA, surprisingly had a significant decrease in hypertrophy (LVW/BW) vs offspring of both SHR untreated parents, showing a transgenerational inheritance. Indeed, SHR showed a decrease in % TRH promoter methylation (Increase in gene expresion) that is significantly reverted in offspring of VPA treated parents (methylation-specific PCR). Consistently, we observed a decrease in cTRH expression (p < 0.05), and therefore, a significant reduction (p < 0.05) in BNP and type 3 Collagen expression, despite the high BP in VPA parents offsprin. To sum up, we described for the first time that VPA treatment modulates cTRH gene and consequently attenuates heart fibrosis and hypertrophy in the SHR, without affecting BP. We show an epigenetic modulation of VPA over cTRH promoter that could be responsible not only of cardiac alterations during VPA treatment, but beyond affecting next generation with results still unknown.

109150

Modality: E-Poster Researcher – Non-case Report

Category: NUTRITION

## Relationship between Obesity and Cardiovascular Diseases in Adults with Obstructive Sleep Apnea Syndrome (OSAS)

RENATA BORBA DE AMORIM OLIVEIRA^1^, Renata Borba de Amorim Oliveira^1^, Ingrid Beranger da Costa Pereira^1^, Ana Paula Menna Barreto^1^, Monica Feroni de Carvalho^1^

(1) Centro Multidisciplinar UFRJ-Macaé

Obstructive Sleep Apnea Syndrome (OSAS) is a very common disorder of breathing during sleep, characterized by recurrent episodes of total interruption (apnea) and/or partial interruption (hypopnea) of ventilation resulting from collapse of the upper airways. Obesity, systemic arterial hypertension and cardiovascular diseases are risk factors of the disease, being related to a major risk of morbidity and worsening of the already installed disorder. The objective of study was to assess the frequency of obesity by different anthropometric measures of total and central body adiposity in adult individuals with OSAS, and its association with excessive daytime sleepiness and severity of the syndrome. The present study was a prospective observational study, in which adults who underwent polysomnography for the diagnosis of OSAS were evaluated. Were evaluated variables: socio-demographic, sleep-related complaints, degree of daytime sleepiness, which was evaluated by the Epworth Sleepiness Scale developed by Johns (1991) and validated in Brazil by BERTOLAZZI et al. (2009), and also to risk factors for OSAS and anthropometric: weight, height, Body Mass Index (BMI) and Waist Circumference (WC). In the 64 patients evaluated, only 43 subjects (67.2%) were diagnosed with OSAS and entered the study. Regarding the classification of OSAS, 44.2% presented mild degree, 23.2% moderate and 32.6% severe degree. Excessive Daytime Sleepiness (EDS) was present in 72.1% of the patients. The classification by BMI is 11.6% of eutrophy, 18.6% of overweight, 25.6% of obesity grade I, 27.9% of obesity grade II and 16.3% of obesity grade III. Regarding the classification of WC, 62.8% presented a much increased risk. Analyzing the morbidity profile of the studied population, 39.50% had systemic arterial hypertension and 11.60% cardiovascular disease. There was a statistically significant association between obesity and WC (p = 0.001), otherwise was not found between obesity and severity disease (OSAS) (p = 0.76) or with SDE (p = 0.127). To conclude, the studied group presented high prevalence of obesity, arterial hypertension and cardiovascular diseases and changes in weight and in the health condition are important to manage symptoms and aggravation of the OSAS and cardiovascular disease.

109167

Modality: E-Poster Researcher – Non-case Report

Category: HEART FAILURE/CARDIOMYOPATHY/TRANSPLANT

## Cardioprotective Effect of Cannabidiol (CBD) in Cardiomyopathy and Heart Failure. From their Molecular Pathways to Clinical Translation

GERARDO GARCIA-RIVAS^1^, Omar Lozano^1^, Christian Silva-Platas^1^, Judith Bernal-Ramírez^1^, Hugo Alves^1^, Carlos Jerjes-Sánchez^1^, Guillermo Torre-Amione^1^

(1) TECNOLOGICO DE MONTERREY

Increasing evidence points to CBD as a promissory therapeutic molecule for diverse inflammatory pathologies, including cardiovascular diseases such as coronary disease and myocarditis. Our study evaluates the application of CBD as a potential therapy for heart failure (HF). Here we assessed the cardioprotective effects of CBD administration in a non-ischemic HF model in mice. Every 3rd day a dose of 1 mg/kg of synthetic CBD was administered via a subcutaneous injection. After four weeks of treatment, the animals were euthanized, and their heart was collected for further analysis. Fibrosis development and myocyte hypertrophy was determined by histopathologic imaging quantification. Additionally, to explore the CBD-antihypertrophic mechanisms, we used cultured cardiomyoblasts to explore the role of cytosolic and mitochondrial oxidative stress and NF-kB pathway intermediates. We developed a multi-center, double-blind, placebo-controlled, randomized trial for the clinical translation, considering the preclinical data. The primary objective of this study is to evaluate the effect of CBD on the prevention of cardiovascular and COVID-19 complications in patients hospitalized for COVID-19. We observed a significant reduction of fibrosis and cell hypertrophy in CBD-treated animals. The expression by qPCR of BNP and collagen1 as a pathological remodeling marker was significantly reduced when CBD was administered in contrast with the HF mice. Pro-inflammatory cytokines expression was observed in IL1b and IL6 expression. At the cellular level, a significant reduction in cell area was observed in CBD treated groups even when exposed to Ang II, achieving sizes comparable to those of the controls. CBD significantly reduced the generation of mitochondrial ROS entities in our hypertrophy model and showed an increase in NF-kB activation that can be reversed by CBD treatment. Also, CBD normalizes the expression of mechanical stress and remodeling markers such as BNP, TGFa, SOD2, IL6, and IL10 involved in inflammation. 23 Non-critical patients hospitalized within the previous 48 hours who tested positive for COVID-19 within seven days and have a prior history of cardiovascular disease (CVD) and significant risk factors for CVD were enrolled. Preliminary results indicate the safety of CBD in patients with COVID-19 infection and cardiovascular disease.

109186

Modality: E-Poster Researcher – Non-case Report

Category: CARDIOVASCULAR IMAGING

## Usefulness of Strain in Chagas Disease in Patients with Ventricular Arrhythmia and Normal Ejection Fraction

NATALIA CARRO^1^, Graciela María Rousse^1^, Cabrejos Gustavo^1^, Matilde Del Campo Contreras^1^, Leandro Parrilla^1^, Nudelman Ezequiel Adolfo^1^, Rubio Edgardo^1^

(1) Division of Cardiology. Echocardiography Laboratory, Hospital of the Government of the city of Buenos Aires

**Introduction:** Global Longitudinal Strain (GLS) is a sensitive method for the early detection of myocardial damage. The latest AHA classification of Chagas disease (ChD) includes, in stage BI, a population with preserved Ejection Fraction (EF) heterogeneous due to ECG changes and/or segmental wall motion abnormalities. The evaluation of abnormal Segmental Longitudinal Strain (SLS) and its comparison with MRI has not been sufficiently evaluated in this population.

**Objective:** In patients (p) with ChD in Stage BI the aim was1)To evaluate the usefulness of GLS in comparison with p with the indetermined form of ChD or Stage A 2) To compare abnormal SLS with cardiac MRI in detecting the anatomical substrate of the arrhytmia.

**Methods:** Transversal, monocentric study. 55p were included January 2018-March 2020: 17p with ChD in Stage B1 with frequent ventricular arrhythmia, cardiac Echo-Doppler with Longitudinal Strain assessment, and gadolinium-enhanced cardiac MRI (Group B); they were compared with 38 p Stage A of ChD (Group A). The association between Strain and abnormal cardiac MRI in group B patients was analyzed.

**Results:** 2 p were excluded due to inadequate ultrasonic image quality. The p from Group B compared to those from Group A were older, had higher Left Atrial volume, lower MAPSE, lower TDI, lower absolute value of GLS (18.3 +– 2.3% stage B vs 21.81 +– 2.5% stage A, p < 0.001) and similar EF. Through ROC curve between group A and B patients, the best cut-off point GLS was determined, a value of –20.5% with sensitivity of 93% and specificity of 70%, and area under the curve of 0.83 CI 95% (0.71–0.95). A cardiac MRI was performed in 11 out of 16 p; in 6 of them it was abnormal: due to late enhancement (4 p), edema plus enhancement (1 p), and thinning and akinesia (1 p). 5 of the 6 p with pathological cardiac MRI presented abnormal SLS and none of the p with normal cardiac MRI. The association between cardiac MRI alterations and SLS abnormal evaluated by means of the Kappa index was 0.82.

**Conclusions:** In our limited population: 1.GLS was significantly lower in in group B1.2. The accurate association between cardiac MRI and abnormal SLS shows the latter as a useful alternative for the diagnosis of the anatomical substrate of arrhythmia.

109181

Modality: E-Poster Researcher – Non-case Report

Category: CARDIAC ARRHYTHMIAS/ELECTROPHYSIOLOGY/ELECTROCARDIOGRAPHY

## Randomized Comparison between Ultrasound Guided Axillary Vein and Cephalic Cutdown for Cardiac Stimulation Devices Implantation

RITA ELIZABETH IBARRA CASTILLO^1^, JORGE LUIS ARBAIZA SIMON^2^, JOSE LUIS LASO BAYAS^1^

(1) Hospital de Especialidades Carlos Andrade Marín; (2) Hospital Vozandes Quito HVQ

**Introduction:** Cephalic vein cutdown (CVC) and subclavian vein puncture are the most widely used techniques for leads insertion during the implantation of cardiac stimulation devices (CSD). Lately, axillary vein access (AVA) has emerged as an alternative. Many approaches have been described for AVA, including the ultrasound (US) guided technique which seems to be fast and safe.

**objective:** Our purpose was to assess whether the US guided puncture of the axillary vein was as efficient as the CVC for CSD implantation.

**Methods:** This was a multicenter prospective trial. All patients undergoing first pacemaker or implantable cardiac defibrillator implantation were submitted to an US scanning of the site of implantation to determine if the axillary vein was visible. Only those with a clear axillary vein were included. None of the operators had previous experience in US AVA. All procedures were planned as ambulatory. We randomized 76 patients to either US AVA (n = 38) or CVC (n = 38). We included only patients with CSD with ≤2 leads. Primary endpoint was success in introducing all the intended leads through the selected access. Secondary endpoints were: complications during the first week after the implantation, time of fluoroscopy, duration of the procedure, use of local and general anesthesia and need of hospitalization. We used X squared and T-tests to compare results from categorical and continuous variables. Analyses were performed according to the intention-to-treat principle.

**Results:** There were no differences in the demographic characteristics. The mean age of all patients (n = 76) was 70.8 years, with 56.6% (n = 43) being male. In the AVA group, access success was achieved in all the subjects (n = 38), and in 78.9% of patients (n = 30) the vein was gained with US. The mean of attempts to gain the first access was 1.8. In the CVC group, the access success was lower, 76.3% (n = 38, p = 0.002). Regarding the rate of complications, there were no differences (AVA 5.2%, CVC 7.8%; n = 76, p = 0.64). Furthermore, we found no differences in time of X-ray exposure, procedure duration, quantity of local anesthetic used, need of general anesthesia or hospitalization (n = 76, p > 0.05).

**Conclusion:** We found that, in comparison to CVC, US AVA was a more efficient technique, allowing successfully insertion of all leads from a conventional bicameral CSD, without increasing complications or issues of the implantation procedure. Our results are in concordance with other randomized clinical trials.

109233

Modality: E-Poster Researcher – Non-case Report

Category: ATHEROSCLEROSIS/CARDIOVASCULAR RISK FACTORS/CARDIOVASCULAR PREVENTION

## Melatonin and NLRP3 Deficient Mice Protect Heart Mitochondria from Aging. Role of NRF2

MIGUEL MEIRA E CRUZ^1^, Germaines Escames^2^, Jose Fernandez Martinez^2^, Dario Acuña Castroviejo^2^

(1) Centro Europeu do Sono; Sleep Unit, Centro Cardiovascular da Universidade de Lisboa, Lisbon, Portugal; (2) Centro de Investigación Biomedica, Departamento de Fisiologia, Facultad de Medicina; Instituto de Biotecnologia, Universidad de Granada, Spain

**Introduction:** Cardiovascular diseases (CVD) constitute the leading cause of death in the world, and aging is by far the major risk factor for cardiac dysfunction. Aging involves subclinical inflammatory activation, with NF-kb and NLRP3 inflammasome as the main components of such response. But inflammation also induces a pro-oxidative response, responsible for the oxidative damage to the cell and mitochondria, leading to a bioenergetic failure. In turn, melatonin exerts profound anti-inflammatory and antioxidant properties, with the mitochondria as the main intracellular target. Here, we analyzed the impact of the NRLP3 inflammasome in the myocardial aging and the role of melatonin in preventing it.

**Methodology:** For this study, we used wild-type and NLRP3-deficient mice of 3, 12, and 124 months of age, with and without melatonin treatment.

**Results:** The absence of NLRP3 prevented the age-dependent myocardial failure and mitochondrial impairment, affecting the Bax/Bcl2 ratio. The Nef2-dependent antioxidant response was unaffected by the lack of NLRP3. In wild-type mice, melatonin treatment produced counteract the age-dependent damage, providing similar protective features than the absence of NLRP3. Melatonin also improved mitochondria structure and enhances the Nrf2 antioxidant response.

**Conclusions:** NLRP3 inflammasome-dependent chronic inflammation during ageing is main responsible for myocardial loss of function, causing proapoptotic phenomena, free radicals’ formation and oxidative damage, and mitochondrial bioenergetic deficit, suggesting a molecular target for cardiac protective therapies. Melatonin, which reaches all subcellular compartment of the myocardial, blocks the NLRP3 inflammasome response and maintains the integrity of the heart, doing it a drug of choice for this age-dependent preventive therapy.

109239

Modality: E-Poster Researcher – Non-case Report

Category: CARDIOVASCULAR IMAGING

## Correlation of Left Atrial Volume Increase with Ischemic Stroke and Transient Ischemic Attack

KLEIBER MARCIANO LIMA BOMFIM^1^, Alan Alves de Lima Cidrão^1^, Pedro Braga Linhares Garcia^1^, Leonardo Miranda Macêdo^1^, José Doriberto Freitas^1^, Giovanna Rolim Pinheiro Lima^1^

(1) HOSPITAL REGIONAL DO SERTÃO CENTRAL

**Introduction:** Stroke is one of the main causes of morbidity and mortality, currently representing the second leading cause of death worldwide. Regarding the etiologies, the cardioembolic type is responsible for 14 to 30% of the ischemic cases and presents higher mortality and worse functional complications. In terms of structural heart diseases, the increase in left atrial volume is an independent factor for stroke, in which the chance of thromboembolism is 20% per year.

**Objective:** To evaluate left atrial volume increase, determined by transthoracic echocardiographic study, as an independent variable for risk of ischemic stroke and transient ischemic attack.

**Methods:** This is a quantitative descriptive, cross-sectional, prospective cohort study conducted between April 2020 and April 2021 in the stroke unit of a tertiary public hospital. Data analysis was performed using the statistical package SPSS, version 22 (IBM – 2017), in which it was possible to perform descriptive analysis to characterize the sample, student’s t test for independent samples, correlation analysis and simple linear regression. The calculation of the left atrial volume was performed by two-dimensional echocardiography in the apical projections of two and four chambers, indexed by the body surface.

**Results:** The study included 308 patients from the State of Ceará who had ischemic stroke and transient ischemic attack. Age ranged from 50 to 98 years (M = 72; SD = 10.8), being 53.7% male and 46.3% female. Most had a diagnosis of ischemic stroke 93.9% and 6.1% transient ischemic attack. Results revealed that the variable “left atrial volume” did not have a normal distribution (K-S (308) = 0.46, P < 0.05; S-W(308) = 0.55, P < 0.05). The Pearson correlation analysis between left atrial volume and diagnosis of ischemic stroke/transient ischemic attack evidenced a positive linear correlation (0.021).

**Conclusion:** The present data demonstrate a positive correlation between increased left atrial volume and the occurrence of ischemic stroke/transient ischemic attack.

110338

Modality: E-Poster Researcher – Non-case Report

Category: CARDIAC ARRHYTHMIAS/ELECTROPHYSIOLOGY/ELECTROCARDIOGRAPHY

## Atropine Test in Predicting Cardioneuroablation Results

JC PACHON-M^1^, Enrique I. Pachon-M^1^, Juan C Zerpa-Acosta^2^, Carlos TC Pachon^2^, Tomas G Santillana-P^1^, Tasso J Lobo^2^, Felipe A Ortencio^2^, Ricardo C Amarante^1^, Juan C Pachon-M^1^

(1) São Paulo University; (2) São Paulo Heart Hospital; (3) Semap Arrhythmia’s Service

**Background:** Vagal tone predominates most of the time. Therefore, isolated sinus heart rate(HR) is higher than the in-situ sinus rate. Atropine stops vagal action by blocking muscarinic receptors. Cardioneuroablation (CNA) also abolishes vagal action by eliminating the postganglionic vagal neuron, causing HR increase. Knowing previously whether HR will increase with CNA is very important since CNA is not indicated if there is no atropine response.

**Objective:** To assess the correlation between HR and Wenckebach point(PW) determined by the atropine test (AT) and that one resulting from CNA.

**Methods:** 76p, 41 females (54%), 14–67 (37 ± 13.2) years, with reflex functional bradyarrhythmias (neurocardiogenic syncope) or non-reflex (sinus dysfunction, vagal AF or bradytachycardia) submitted to CNA controlled by extracardiac vagal stimulation. AT was performed with IV infusion of 0.04 mg/kg. CNA was performed by ablation of the P-point, the presumed 4 main GPs areas and spare AF-Nests, at least 02 days post-AT. The maximum HR and PW obtained under the atropine and at the end of the CNA were compared.

**Results:** There were significant HR and PW increases with atropine and with CNA. The increases were smaller with CNA probably because CNA also eliminates sympathetic fibers and AT does not.

**Conclusion:** There is a strong correlation between HR and PW determined by atropine and those resulting of CNA. Thus, atropine test can be used as a good predictor of the CNA result.


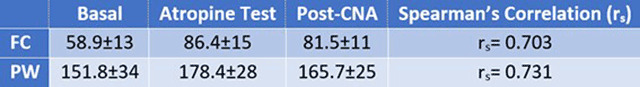


110339

Modality: E-Poster Researcher – Non-case Report

Category: CARDIAC ARRHYTHMIAS/ELECTROPHYSIOLOGY/ELECTROCARDIOGRAPHY

## Cardioneuroablation Based on Atrial Fibrillation Nests Ablation

JC PACHON-M^1^, Enrique I Pachon-M^1^, Carlos TC Pachon^2^, Tasso J Lobo^2^, Tomas G Santillana-P^1^, Juan C Zerpa-A^2^, Felipe A Ortencio^2^, Maria Zelia C Pachon^3^, Ricardo C Amarante^1^, Juan C Pahon-M^1^

(1) São Paulo University; (2) São Paulo Heart Hospital; (3) Semap Arrhythmia’s Service

**Background:** Symptomatic reflex and non-reflex functional bradyarrhythmias are related to vagal hyperactivity. Cardioneuroablation[CNA] provides vagal denervation through endocardial RF ablation of the post-ganglionic vagal neurons. Neuro-myocardium interface identification, stepwise denervation control and denervation validation are critical. Several findings have shown that the Arial Fibrillation-Nest[AFN] are intrinsically related to the vagal innervation.

**Objective:** To verify whether CNA based on AFN ablation promotes enough vagal denervation compared to a control group with non-AFN ablation.

**Method:** Prospective, controlled study with 54 p gathered in two groups: AFN group (AFNG-24 p) with functional or reflex bradyarrhythmias treated with AFN ablation, and a control group (CG-30 p) with anomalous bundles, ventricular premature beats, atrial flutter, nodal reentry and atrial tachycardia, treated with conventional ablation (non-AFN ablation). Ablation of AFNG was targeted to AFN detected by fragmentation of the endocardial electrograms and/or by Fractionation Software. Vagal response was evaluated before, during, and post-ablation by 5s non-contact vagal stimulation at the jugular foramen, through the internal jugular veins[ECVS], analyzing longest RR, pauses, and AV block induced by ECVS. All patients had current guidelines arrhythmia ablation indications.

**Results:** There was found a high density of AFN over the P-Point (right half of the left interatrial septum), over the presumed areas of the main 4 ganglionated plexi, over the coronary sinus roof, and over the Waterston’s groove. Pre-ablation ECVS induced sinus pauses, asystole and transient AV block in both groups, showing normal vagal response (p = 0.94). Post-ablation ECVS in the AFNG showed complete elimination of the cardiac vagal response in all cases (Pre/Post-ablation ECVS = p < 0.01), demonstrating acute and complete vagal denervation. In the CG, vagal response remained practically the same as pre-ablation (p = 0.31) showing that non-AFN ablation caused no significant denervation.

**Conclusion:** CNA based on AFN ablation attained an excellent degree of acute vagal denervation while non-AFN ablation did not cause denervation. This fact suggests that AFNs are directly related to vagal innervation and may be a good alternative for CNA. Additionally, ECVS was remarkably valuable for CNA control.

109246

Modality: E-Poster Researcher – Non-case Report

Category: COVID-19 AND CARDIOVASCULAR SYSTEM

## Variables Associated with Bleeding and Thrombotic Outcomes Among Patients Admitted to the Hospital with COVID-19 and Elevated D-Dimer: Insights from the Action Randomised Clinical Trial

PEDRO GABRIEL MELO DE BARROS E SILVA^1^, RD. Lopes^4^, RHM. Furtado^5^, AVS. Macedo^1^, B. Bronhara^1^, E. Ramacciotti^6^, PA. Martins^7^, AL. De Oliveira^7^, VS. Nunes^7^, LEF. Ritt^8^, AT. Rocha^8^, L. Tramujas^2^

(1) Brazilian Clinical Research Institute; (2) IP-HCor; (3) Hospital Samaritano Paulista; (4) Duke Clinical Research Institute, Durham, United States of America; (5) Hospital Israelita Albert Einstein, Sao Paulo, Brazil; (6) Loyola University, Chicago, United States of America; (7) Hospital Estadual Dr Jayme Santos Neves, Serra, Brazil; (8) Cardio Pulmonary Hospital, Salvador, Brazil

In the ACTION trial, therapeutic anticoagulation did not show benefit on mortality, days of hospitalization and oxygens therapy at 30 days among patients with COVID19. However, this strategy was associated with higher rate of bleeding and a potential reduction in the rate of clinical thrombotic events. The current analysis evaluated which variables were independently associated with both outcomes in order to help the identification of the risk for thrombotic and hemorrhagic events among patients with COVID19.

**Methods:** A total of 615 patients hospitalized with COVID-19 and elevated D-dimer were randomly assigned to prophylactic anticoagulation (mainly in-hospital heparin) or a therapeutic-dose strategy that used in-hospital rivaroxaban 20 mg/day for stable patients, or full-dose enoxaparin for unstable patients, followed by rivaroxaban through 30 days. One patient withdrew consent and was not included in the analysis. The current analysis tested baseline clinical characteristics and laboratorial exams one by one with independent logistic regressions for the composite of bleeding (major bleeding and clinically relevant nonmajor bleeding) and thrombotic events (venous thromboembolism, myocardial infarction, stroke, systemic embolism, and major adverse limb events). Significant variables (p < 0.05) were selected to adjust several multiple logistic models. Final models were chosen based on Akaike information criterion and therapeutic anticoagulation was included in the final model based on the primary results of the trial.

**Results:** The model for bleeding events showed an accuracy of area under the curve (AUC) of 0.635 while the model for thrombotic events had an AUC of 0.725. Level of respiratory support (especially invasive ventilation) was associated with both outcomes in the multivariable analysis. Beyond respiratory support, level of creatinine and history of coronary disease were also independently associated to the risk of thrombotic events. When the utilization of therapeutic anticoagulation (mainly with rivaroxaban) was included in the multivariable analysis, this variable was strongly associated with higher risk of bleeding (model AUC of 0.718) but was not associated with lower rate of thrombotic events.

**Conclusion:** Variables associated with higher risk of thrombotic events commonly are associated also to bleeding complications. The models developed in ACTION may help the selection of patients with better balance of risk vs. benefit to anticoagulation.

109274

Modality: E-Poster Researcher – Non-case Report

Category: EPIDEMIOLOGY AND HEALTH POLICIES/GLOBAL HEALTH

## Trends in Deaths from Ischemic and Hemorrhagic Cerebrovascular Disease in Brazil from 2000 to 2019

ALESSANDRO ROCHA MILAN DE SOUZA^1^, Bernardo Queiroz de Carvalho Souza^1^, Davi da SIlveira Barroso Alves^1^, Glenda Borges de Lacerda^1^, Paulo Henrique Godoy^1^

(1) Universidade Federal do Estado do Rio de Janeiro – Federal University of the Rio de Janeiro State

**Introduction:** Cerebrovascular diseases (CBVD) and cardiovascular diseases represent the leading cause of death in the world. In Brazil, these diseases correspond to 32% of deaths per year.

**Objective:** To analyze the trend of causes of death due to ischemic CBVD (ICBVD) and hemorrhagic CBVD (HCBVD) in Brazil, from 2000 to 2019.

**Method:** Ecological study, in which the data of deaths due to CBVD, according to ICD 10, were obtained from the Mortality Information System. The codes for CBVD were divided into: ICBVD, HCBVD and CBVD not specified as ischemic nor hemorrhagic (NSCBVD). The age groups in years were divided as 20–39, 40–59, 60–79, and 80 or above. Crude and standardized rates were estimated according to sex and age group. The variation in mortality rates was also estimated, comparing 2000 and 2019.

**Results:** There were 1,766,788 deaths from CBVD in the period. The following distribution by causes was observed: ICBVD – 362,666; HCBVD – 409,079; NSCBVD – 1,009,927. The rates in all causes were higher in males. In the HCBVD higher death rates were observed in younger age and in the ICBVD in the older age. Stability trend was observed in the ICBVD, with a small decrease in females and a small increase in males, and a decrease trend in HCBVD and NSCBVD for both sexes.

**Conclusion:** In general, the trend of deaths from ICBVD and HCBVD seems to be decreasing in Brazil, for the period studied, although this trend is lower in ICBVD.


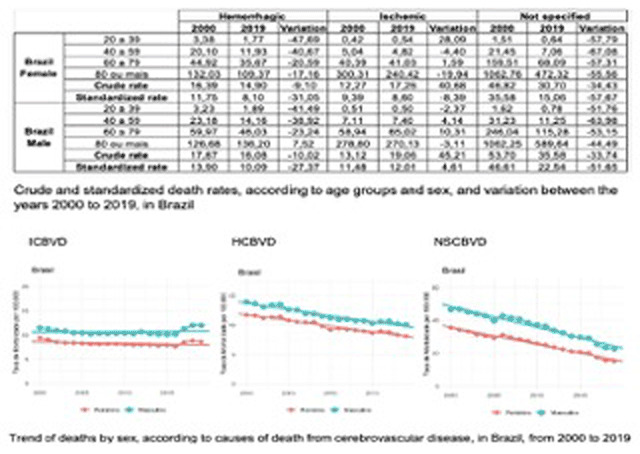


109281

Modality: E-Poster Researcher – Non-case Report

Category: PERICARDIUM/ENDOCARDIUM/VALVOPATHIES

## Infective Endocarditis in Hemodialysis Patients

CLAUDIO QUERIDO FORTES^1^, Natália Rodrigues Querido Fortes^3^, Plínio Resende Jr^4^, Juliano Carvalho G. de Almeida^4^, Roberto Muniz Ferreira^4^, Ana Claudia Pinto de Figueiredo Fontes^4^, Luiz Felipe de Abreu Guimarães^4^, João Roquette Fleury da Rocha^4^, Vlander Gomes da Costa Jr.^4^, Marina da Costa Carvalheira^4^, Ronir Raggio Luiz^2^, Mauro Paes Leme de Sá^5^

(1) Hospital Universitário Clementino Fraga Filho HUCFF/Universidade Estácio de Sá UNESA; (2) Instituto de Estudos em Saúde Coletiva IESC; (3) Hospital Universitário Antonio Pedro HUAP/Hospital Universitário Clementino Fraga Filho HUCFF; (4) Hospital Universitário Clementino Fraga Filho HUCFF; (5) Hospital Universitário Clementino Fraga Filho HUCFF/Instituto do Coração Edson Saad ICES

**Background:** Infective endocarditis (IE) is one of the most dreaded infectious complications in hemodialysis (HD) patients.

**Methods:** Descriptive analysis of HD and non-HD patients with IE.

**Results:** Of the 505 patients (540 episodes) admitted to university hospital between 1978–2021 with definite IE according to the modified Duke criteria, 54 patients (57 episodes) had undergone HD and 451 (483 episodes) had not. Vascular access for HD was central catheter in 75.4% and 49,1% had arteriovenous fistula but some of them with fistula failure. The mean age of HD patients was not statistically different from that of non-HD patients (47.5 vs 43.3, p 0.117). More female gender (57.9% vs. 34.6%, p = 0.001) was observed in HD patients. Diabetes mellitus was significantly more frequent in the HD-patients (36.8% vs. 6.6%, p < 0.001), while intravenous drug use (0% vs 13.9%, p 0.029) and prosthetic valve (7.0% vs 20.7%, p 0.013) were more commonly in non–HD-patients. The mitral valve was the most affected (50.9% vs 51.1%, p 0.773), followed by aortic valve (38.6% vs 43.1%, p 0.416) and tricuspid valve (19.3% vs 13.3, p 0.212). The proportion of Enterococcus spp. was significantly higher in HD group than in non-HD group (33.3% vs. 5.4%, p < 0.001). Staphylococcus aureus was the second most frequent one (29.8% vs 22.0%, p 0.183). Valve replacement for active IE was less frequently performed among HD patients but without statistical significance (35.1% vs 42.2%, p 0.300). In-hospital mortality was significantly higher in hemodialysis than in non-hemodialysis patients (52.6% vs. 37.7%, p 0.030).

**Conclusions:** IE is a serious complication in HD patients. Enterococcus spp. is the most common causative organism in this group. Mortality is very high and significantly higher than in non-HD patients.


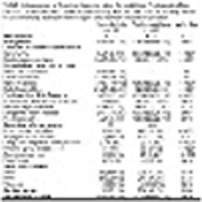


109299

Modality: E-Poster Researcher – Non-case Report

Category: ATHEROSCLEROSIS/CARDIOVASCULAR RISK FACTORS/CARDIOVASCULAR PREVENTION

## Network to Control Atherothrombosis: Main Results of the Neat Registry

PEDRO GABRIEL MELO DE BARROS E SILVA^1^, Renato Delascio Lopes^4^, Charlene Troiani^5^, Rodrigo Pinto Pedrosa^6^, Marcelo Nakazone^7^, Sérgio Luiz Zimmermann^8^, Rodrigo Morel^9^, Ricardo Reinaldo Bergo^10^, Dalton Bertolim Précoma^11^, Lucas Tramujas^1^, Ricardo Pavanello^1^, Eduardo Ramacciotti^12^

(1) IP-Hcor; (2) Brazilian Clinical Research Institute; (3) Hospital Samaritano Paulista; (4) Duke Clinical Research Institute, Durham, United States of America; (5) Hospital Regional de Presidente Prudente; (6) PROCAPE; (7) Fundação Faculdade Regional de Medicina São José do Rio Preto; (8) Clínica Procárdio; (9) Hospital Ana Nery; (10) Hospital Santa Lucia – Hospital do Coração de Poços De Caldas; (11) Sociedade Hospitalar Angelina Caron; (12) Loyola University, Chicago, United States of America

**Background:** There is limited contemporary prospective real-world evidence of patients with chronic arterial disease in Latin America.

**Methods:** The Network to control atherothrombosis (NEAT) registry is a national prospective study of patients with known coronary (CAD) and peripheral arterial disease (PAD) in Brazil. A total of 2,015 patients were included among 21 sites from September 2020 to March 2022. The follow-up of all patients was one year by the protocol. Patient characteristics, medications under use and laboratorial data were collected. The primary objective is to assess the utilization of evidence-based therapies (EBT) at baseline.

**Results:** From the total of patients enrolled, 56.6% had isolated CAD, 29.6% had PAD and 13.8% had both diagnoses. The overall mean age was 66.3 (±10.5) years and 65.7% were male patients. The median glomerular filtration rate was 76.4 [57.2–96.1] and 72.3% of the patients had an evaluation of microalbuminuria which was detected in 6.2% of the cases. Regarding EBT, 4.0% were not using any antiplatelet and/or anticoagulant therapy but only 0.9% were using low dose of rivaroxaban (2.5 mg BID); 5.0% were not using statins and 55.6% of the patients were not using high intensity statin therapy; ACE inhibitors or ARBs were used in 76.2% of the overall population while, among patients with isolated CAD, 10.3% were not using betablockers. Among diabetic patients, 67.8% were using metformin and only 12.5% were using SGLT2 inhibitors and/or GLP1 agonists. Regarding the targets for secondary prevention, 33.0% had a body-mass index between 18.5 and 24.9; 44.4% were doing at least 150 minutes of exercise per week; 15.7% continued to smoke; 41.0% had a blood pressure <130 × 80 mmHg; 38.7% and 14.7% had LDL-cholesterol below 70 and 50 mg/dl, respectively. Among diabetic patients, 41.2% had a glycated haemoglobin <7%. Patients with PAD had lower use of EBT and lower percentage of patients on target of risk factors control. Among all cases without use of EBT, the main barrier identified was related to the physician perception that did not consider a formal medical indication of these therapies.

**Conclusion:** Our findings highlight that the contemporary practice still has important gaps in the treatment of patients in secondary prevention, especially among patients with PAD. Populational interventions addressing these gaps have the potential to produce a major impact, reducing the burden of atherothrombotic complications in Brazil.

109312

Modality: E-Poster Researcher – Non-case Report

Category: CARDIO-ONCOLOGY

## Cardioprotection Strategy Based on Positive Troponin Detection During Treatment of HER-2+ Breast Cancer

FERNANDO PIVATTO JÚNIOR^1^, Marco Aurélio Lumertz Saffi^2^, Guilherme Oliveira Magalhães Costa^1^, Vinícius Henrique Fritsch^1^, Eduarda Foresti Englert^1^, Ângela Barreto Santiago Santos^2^, Géris Mazzutti^2^, Pedro Emanuel Rubini Liedke^2^, Andreia Biolo^1^

(1) Universidade Federal do Rio Grande do Sul (UFRGS), Porto Alegre-RS, Brazil; (2) Hospital de Clínicas de Porto Alegre (HCPA), Porto Alegre-RS, Brazil

**Introduction:** Patients with positive troponin (Tn+) during breast cancer treatment are considered to be at high risk for cardiotoxicity, and cardioprotection with ACE±BB is indicated. Doubts persist about the ideal time to collect Tn.

**Objective:** To compare the incidence of cancer therapy-related cardiac dysfunction (CTRD) in the Tn+/onset of cardioprotection and Tn-/no additional intervention groups. Patients and methods. Prospective cohort including consecutive female patients with HER-2+ early breast cancer who consulted at the institution’s breast cancer outpatient clinic between march/19-march/22. CTRD: drop in LVEF > 10 p.p. to <53% (ASE/EACI). Tn collection was performed together with the lab tests requested by Oncology before the 1st and 2nd cycles of trastuzumab (TTZ), in addition to 3 months after its initiation. Tn+: TnTus ≥ 14 ng/L or TnIus > 15.6 pg/mL. It was not considered as Tn+ if baseline Tn+ without previous cancer treatment or if absence of increase >20% after its beginning. Patients with Tn+ were referred to the institution’s Cardio-Oncology outpatient clinic to begin cardioprotection, as were those with CTRD. Comparison between groups: Fisher’s exact test. P < 0.05 was considered statistically significant.

**Results:** We studied 46 patients, mean age 53.1 ± 13.1 years, 21 (45.7%) in a therapeutic protocol including doxorubicin (ACdd-TH). Regarding risk factors, 21 (45.7%) had a history of smoking, 18 (39.1%) were obese and 15 (32.6%) had hypertension. Of the 138 troponins analyzed, there were 18 (13.0%) Tn+, the majority being detected before the 2nd cycle of TTZ (12/18, 66.7%). Of the total number of patients, 12 (26.1%) had ≥1 Tn+, with the majority (7/12, 58.3%) having only one of the three collected. In the Tn- group (n = 34), only 6 (17.6%) had hypertension and used ACEi/ARB as treatment. The incidence of CTRD was 10.9%, 8.3% in the Tn+ group and 11.8% in the Tn- group (P = 1.0).

**Conclusions:** Although patients with Tn+ had a higher risk of CTRD, there was no difference in the incidence in those with Tn+/onset of cardioprotection in relation to those with Tn-/without additional intervention. This finding suggests that the cardioprotection strategy based on the detection of Tn+ collected together with the lab tests requested by Oncology may have been effective, equating the occurrence of this adverse event between the groups.

109383

Modality: E-Poster Researcher – Non-case Report

Category: ATHEROSCLEROSIS/CARDIOVASCULAR RISK FACTORS/CARDIOVASCULAR PREVENTION

## Association between the Number of Cardiovascular Risk Factors and Coronary Calcification

PAULO EDUARDO BALLVÉ BEHR^1^, Rafael Vianna Behr^1^, Leonardo Henrique Bertolucci^1^, Gabrielly Burkhard Vilasfam^1^, Lara Helena Zortéa^1^, Luiza Zwan Dutra^1^, Maiara Both^1^, Mariana Saadi de Azevedo^1^, Paulo Ernesto Leães^1^, Fernando Antônio Lucchese^1^

(1) Hospital São Francisco – Santa Casa de Misericórdia de Porto Alegre

**Introduction:** Traditional risk factors (RF) are used to predict the probability of cardiovascular events. However, it is still uncertain whether they are able to predict the probability of subclinical atherosclerosis. Therefore, our objective is to evaluate the association between the number of RF and coronary calcification measured by the calcium score (CAC).

**Methods:** Cross-sectional study, including patients seen as outpatients between 2012 and 2020, aged between 45 and 75 years, in primary prevention. To assess coronary calcification, the CAC percentile (PCAC) was used, considering PCAC > 75 as an important calcification. The RF evaluated were: hypertension, diabetes, current smoking, dyslipidemia and family history (FH) for coronary artery disease.

**Results:** 444 patients were included, mean age 59 ± 7 years, 54% female, all Caucasian, 54% hypertensive, 41% dyslipidemic, 9% diabetic, 11% smokers; 59% with FH. Table 1 shows the association between the number of RF and coronary calcification. The higher the number of RF, the higher the prevalence of PCAC > 75 (p < 0.01) and the lower the prevalence of PCAC = 0 (p < 0.01). Compared to patients without RF, the prevalence of PCAC > 75 was 1.86 times higher in patients with 1 risk factor (CI 0.60–5.81), 3.59 times higher with 2 RF (CI 1.20–10.76), 3.89 times higher with 3 or more RF (CI 1.27–11.90). However, even in patients with zero, 1, or 2 RF, significant calcification was observed in 9.1%, 16.9%, and 32.6% of patients, respectively. In multivariate analysis, smoking [PR 1.68 (CI 1.16–2.43)] and FH [PR 1.96 (CI 1.37–2.79)] were independent RF.

**Conclusion:** There was an association between the number of RF and coronary calcification. However, a considerable percentage of patients with none or fewer RF had significant coronary calcification.


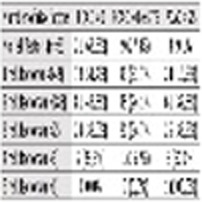


109364

Modality: E-Poster Researcher – Non-case Report

Category: HEMODYNAMICS AND INTERVENTIONAL CARDIOLOGY

## Distal Transradial Access for Post-Cabg Coronary and Surgical Grafts Angiography and Interventions

MARCOS DANILLO PEIXOTO OLIVEIRA^1^, Lélio Lemos Pinto Neto^1^, Ednelson Navarro^2^, Adriano Caixeta^1^

(1) Universidade Federal de São Paulo, UNIFESP; (2) Hospital Regional do Vale do Praíba

**Background:** Post-CABG coronary and grafts angiography (CGAG) and interventions (PCI) have historically been performed via classic transfemoral approach. Particularly for those with left internal mammary artery (LIMA) grafts, left standard transradial access (lsTRA) represents a feasible alternative, with significant fewer vascular complications, but it has ergonomic disadvantage for the operator because of the need to bend over the patients, especially in obese ones. Distal transradial access (dTRA) may provide important advantages, including shorter hemostasis and greater patient and operator comfort, mainly for left dTRA (ldTRA). We aim to describe the feasibility and safety of right and left dTRA for post-CABG CGAG and PCI.

**Material and methods:** From February 2019 to February 2022, 151 consecutive post-CABG patients submitted to CGAG and/or PCI via dTRA have been enrolled.

**Results:** Mean patient age was 67.81 years old. Most were male (82.1%) and had chronic coronary syndromes (59.6%). Overall, 40.3% had acute coronary syndromes. Distal RA was successfully punctured in all patients, always without ultrasound guidance. All procedures involving LIMA grafts were done via ipsilateral ldTRA. We had only 9 (6.0%) access site crossovers. Successful dTRA sheath insertion was then achieved in 94% of all patients, mostly (66.9%) via ldTRA and with standard 6Fr sheath (98%). Distal and proximal RA pulses were palpable in all patients at hospital discharge. No major adverse cardiac and cerebrovascular events and no major complications related to dTRA were recorded.

**Conclusions:** dTRA for routine post-CABG CGAG and PCI by experienced transradial operators appears to be feasible. Further randomized and larger trials are needed to assure clinical benefits and safety of this new technique.


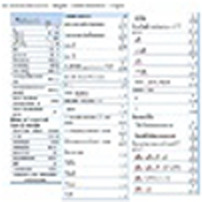


109367

Modality: E-Poster Researcher – Non-case Report

Category: ATHEROSCLEROSIS/CARDIOVASCULAR RISK FACTORS/CARDIOVASCULAR PREVENTION

## Correlation between Nonalcoholic Fatty Liver Disease and the Severity of Acute Coronary Syndrome: A Retrospective Study

MÁRIO CLAUDIO SOARES STURZENEKERM^1^, Mauricio Montemezzo^2^, Dalton Bertolim Précoma^2^, Paola Gonçalves Moreira de Oliveira^1^, Bruna Karas^1^, Ana Carla Dlugosz^1^, Alice Magro Koscianski^1^, Larissa Almeida Busnello^1^, Francielle Nocera Viechineski^1^, Camilla Mattia Calixto^1^, Júlia Henneberg Hessman^1^

(1) Universidade Estadual de Ponta Grossa; (2) Pontifícia Universidade Católica do Paraná

**Introduction:** Cross-sectional studies have widely described the relationship between non-alcoholic fatty liver disease (NAFLD) and atherosclerotic coronary artery disease. A clinical study reported an increased risk for cardiovascular events in NAFLD patients, and this relation was suggested in a retrospective study. However, the role of NAFLD in atherosclerotic disease remains inadequately elucidated. Furthermore, the potential correlation between NAFLD severity and the clinical expression of acute coronary syndrome (ACS) remains undetermined.

**Purpose:** To assess whether there is a correlation between NAFLD severity and ACS severity.

**Methods:** Were selected data of 99 adult patients without previously known coronary artery disease or liver disease, without a history of significant alcohol consumption and other common causes of secondary steatosis, who presented to the emergency room with chest pain between March 2015 and March 2016. The diagnostic criteria for acute myocardial infarction with ST-segment elevation (STEMI) were based on ST-elevation ≥1 mm in ≥2 contiguous leads (2 mm for leads V1 to V3). The acute myocardial infarction without ST-segment elevation (NSTEMI) diagnostic was established in patients who did not meet the criteria for STEMI and who had elevated necrosis markers (creatine kinase-MB isoform and troponin I). Unstable angina (UA) diagnostic was established in patients who did not meet the criteria for STEMI and NSTEMI but had more than three cardiovascular risk factors and typical thoracic pain. The presence of steatosis and its degrees were assessed using ultrasound, and the diagnosis of NAFLD was based on the presence of steatosis and clinical history.

**Results:** UA, NSTEMI and STEMI diagnosis were established in 40, 33 and 26 patients, respectively, and NAFLD was observed in 30%, 66.6% and 76.9% of these patients. The diagnostic of STEMI and NSTEMI were more frequently than UA in NAFLD patients (P < 0.01, p < 0.001). NAFLD diagnosis and its degrees were significantly correlated with the three presentations of ACS (P < 0,001) for both. The STEMI and NSTEMI diagnosis frequency was similar in NAFLD patients. Diabetes, obesity and hypertension were not correlated with NAFLD diagnosis and degree.

**Conclusion(s):** In this study, NAFLD diagnosis and its degrees were significantly correlated with ACS and its severity. Therefore, NAFLD could be considered a potential risk marker for coronary atherosclerotic disease progression and instability.

109382

Modality: E-Poster Researcher – Non-case Report

Category: NURSING

## Value-Based Chronic Care Model Approach for Patients with Arterial Hypertension and Diabetes Mellitus in Brazil: A Cross-Sectional Study

DALMA ALVES PEREIRA^1^, Priscila Valverde de Oliveira Vitorino^2^, Katarinne Lima Moraes^3^, Vanessa da Silva Carvalho Vila^2^, Marina Aleixo Diniz Rezende^2^, Maria Alves Barbosa^1^, Claudia Regina de Oliveira Zanini^1^, Virginia Visconde Brasil^1^

(1) Federal University of Goiás – UFG; (2) Pontifical Catholic University of Goiás – PUC Goiás; (3) Brasília University – UnB

**Background:** Support for self-management of chronic conditions is essential in the development of patient-centered care. The use of integrated care models that focus on the person and not only on the disease represents a viable solution for effective care. The Chronic Care Model is based on the relationship between motivated and informed users, and a proactive and prepared health team. According to this model, support for self-management is associated with significant results, especially in hypertension and diabetes. It includes the Patient Assessment of Care for Chronic Conditions (PACIC) instrument to assess and monitor the integrated care and support for self-management, as perceived by the patient.

**Aim:** To assess the quality of care for people with arterial hypertension and diabetes mellitus treated at a specialized service, according to the Chronic Care Model.

**Methods:** Cross-sectional study, carried out in a reference outpatient clinic of hypertension care in Brazil. Eighty-two people with diabetes and hypertension were evaluated, with a follow-up of at least 5 years. Participants completed the 20-item PACIC, as well as measures of demographic and clinical aspects during the nursing appointment. PACIC has 5 scales and 3 of them assess support for self-management. Higher scores (>3.0) indicate greater involvement in self-management and team support. For analysis, the Mann-Whitney Test, Spearman Test, and a significance level of 0.05 were used.

**Results:** The mean age was 68.98 ± 8.79 years, female (82.93%), and median 4 years of study (IQR 3–8). Most parents (70.73%) did not study. The median time from diagnosis of diabetes was 8 years (IQR 6–12). Less than half of the patients had controlled glycated hemoglobin – HbA1c (34.15%) and blood pressure (36.59%). There was a positive correlation between the time of diabetes diagnosis and the (HbA1c) value. The Overall PACIC mean was 3.4 (IQR 2.8–3.8). The scales scores that map onto self-management support were Patient Activation – 3.0; Problem-Solving/Contextual – 3.0 and Goal Setting/Tailoring – 3.8. Patients with controlled (HbA1c) were significantly different on the Goal Setting/Tailoring scale. Having parents with some schooling was significantly different on both the Overall PACIC and Patient Activation scale.

**Conclusion:** Quality of care was high considering the overall PACIC, but was moderate on scales that assess greater patient participation and involvement in decision-making and treatment plans.

111107

Modality: E-Poster Researcher – Non-case Report

Category: DIGITAL HEALTH/INNOVATION

## Cost-Effectiveness Analysis of Pare de Fumar Conosco: An Electronic Decision-Making Tool for Quitting Smoke

ROBERTA DA SILVA TEIXEIRA^1^, Arise Garcia de Siqueira Galil^2^, Ana Paula Cupertino^3^, Francisco Cartujano-Barrera^3^, Fernando Antonio Basile Colugnati^2^

(1) Instituto Nacional de Cardiologia – INC; (2) Universidade Federal de Juiz de Fora – UFJF; (3) University of Rochester Medical Center – URMC

**Introduction:** E-health technologies such as the Pare de Fumar Conosco software can be effective for smoking treatment. This intervention is a web-based tool for decision-making about quitting smoking, drawn on principles of social cognitive theory, comprising motivational messages, behavioral change support, and pharmacotherapy use. Efficacy and effectiveness evaluations are not enough. Before being adopted in the health system, technologies must be adequately evaluated in the economic sphere.

**Objective:** To evaluate the cost-effectiveness of the Pare de Fumar Conosco software versus the standard of care according to Brazilian Ministry of Health guidelines.

**Methods:** We developed an analytical decision model for engagement in the smoking cessation counseling group and smoking cessation under service providers‘ perspectives and the public health system. We measured costs by primary and secondary sources and a randomized clinical trial for effectiveness. The temporal horizon adopted was one year. The TreeAge Pro Suite 2018 software guided the development of the analytical decision model. The analysis comprised the incremental cost-effectiveness ratio (ICER), deterministic sensitivity analysis, and Monte Carlo probabilistic analysis (1,000 simulations). As the outcomes are intermediary, no willingness to pay threshold was adopted.

**Results:** The software had a lower cost and greater effectiveness than its comparator. ICER for the engagement and smoking cessation were dominant in both perspectives adopted ($ – 464, 125.36 to $ – 58,348.50). Quadrant II (lower cost and higher effectiveness) presented higher percentages (53,6% to 82,5%) on Monte Carlo simulations.

**Conclusions:** Pare de Fumar Conosco software is a cost-saving technology for smoking treatment. This cost-effectiveness analysis provides objective and explicit data that support the decision of health managers in evaluating the adoption of a promising web-based decision tool for smoking cessation.

109401

Modality: E-Poster Researcher – Non-case Report

Category: HEART FAILURE/CARDIOMYOPATHY/TRANSPLANT

## Results of Comprehensive Rehabilitation Program of Patients After Left Ventricular Assist Device Implantation

TOMASZ CHWYCZKO^1^, Edyta Smolis-Bak^1^, Laura Zalucka^1^, Agnieszka Segiet-Swiecicka^1^, Ewa Piotrowicz^1^, Joanna Was^1^, Magdalena Niedolistek^1^, Malgorzata Sobieszczanska-Malek^1^, Tomasz Zielinski^1^, Mariusz Kusmierczyk^1^, Ryszard Piotrowicz^1^, Rafal Dabrowski^1^

(1) National Institute of Cardiology, Warsaw, Poland

**Background:** The novel method of comprehensive rehabilitation after LVAD implantation was developed in our institution in order to rehabilitate the growing LVAD patients population. Study group: 47 recent LVAD (32 HeartMate III, 15 HeartWare) recipients (19–68 years, mean 58,7 years, 43 men) participated in the rehabilitation program. 4–5 week program included supervised endurance training on a cycle ergometer (5 times per week). The training was programmed on the basis of the cardiopulmonary exercise test results. Other exercises included: resistance training, general fitness exercises with elements of equivalent and coordination exercises (every day). The hospitalization was followed by 6–8 weeks of individual exercises performed at home. 6-minute walking test (6MWT), cardiopulmonary exercise test (CPET) and prognostic biomarkers of heart failure: NT-proBNP, Galectin-3 and ST2 were investigated at the beginning and at the end of the rehabilitation program.

**Results:** Table 1. An increase of 6MWT distance, higher maximal workload, peak VO2 and upward shift of anaerobic threshold in CPET were observed in all patients. Significant reductions of NTproBNP, ST2 and galectin-3 levels were observed. There were no major adverse events during rehabilitation.

**Conclusions:** Comprehensive novel rehabilitation in LVAD recipients is safe and results in significant improvement of functional tests and biomarkers of heart failure.


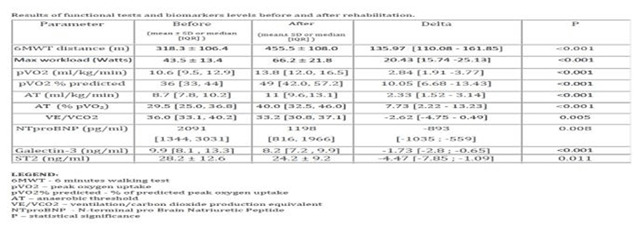


109413

Modality: E-Poster Researcher – Non-case Report

Category: NUTRITION

## Evaluation of Red Wine Influences on the Gut Microbiota and Trimethylamine N-Oxide

ELISA ALBERTON HAAS^1^, Mario Jose Abdalla Saad^2^, Andrey Santos^2^, Nicola Vitulo^3^, Wilson José Fernandes Lemos Junior^4^, Aline Maria Araújo Martins^5^, Carolina Raíssa Costa Picossi^6^, Desiderio Favarato^1^, Renato Simões Gaspar^1^, Peter Libby^7^, Francisco Rafael Martins Laurindo^1^, Protasio Lemos da Luz^1^

(1) Instituto do Coracao (InCor), Hospital das Clinicas HCFMUSP, Faculdade de Medicina, Universidade de Sao Paulo, Sao Paulo, SP, Brazil; (2) Department of Internal Medicine, State University of Campinas (UNICAMP), Campinas, SP, Brazil; (3) Department of Biotechnology, Verona University, Verona, Italy; (4) Faculty of Science and Technology, Libera Università di Bolzano, Bolzano, Italy; (5) Department of Medical Science, University of Brasilia (UnB), Brasilia, Brazil; (6) Institute of Chemistry, University of Sao Paulo, Sao Paulo, SP, Brazil; (7) Division of Cardiovascular Medicine, Department of Medicine, Brigham and Women’s Hospital, Harvard Medical School, Boston, MA, USA

Gut microbiota profile closely relates to cardiovascular diseases through mechanisms including the reported deleterious effects of metabolites, such as trimethylamine-N-oxide (TMAO), highly studied as a diagnostic and therapeutic target. Moderate red wine (RW) consumption is reportedly cardioprotective, but RW effects on gut microbiota and plasma TMAO are not fully understood. We conducted a randomized, crossover, controlled trial involving 42 males, average 60 yr with documented coronary artery disease, comparing 3-week consumption of 250 mL of RW/day, 5 days/week, with an equal period of alcohol abstinence, given adequate washouts. RW consumption significantly remodeled gut microbiota, with a difference in beta diversity. Plasma metabolomic analysis (n = 20) revealed changes after RW consistent with improved redox homeostasis modulation, such as an increase in precursors of riboflavin and ascorbate metabolism. In contrast, plasma TMAO did not differ with the RW intervention and TMAO levels showed a low intra-individual concordance over time. Thus, modulation of the gut microbiota may contribute to the putative cardiovascular benefits of moderate RW consumption.


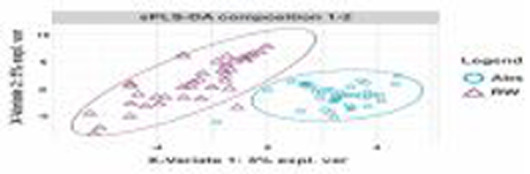


109418

Modality: E-Poster Researcher – Non-case Report

Category: DIGITAL HEALTH/INNOVATION

## Deep Learning and High-Performance Computing for Screening Patients with Depression in Primary Care

ERITO MARQUES DE SOUZA FILHO^1^, Erito Marques de Souza Filho^1^, Helena Cramer Veiga Rey^2^, Rose Frajtag^2^, Daniela Matos Arrowsmith Cook^3^, Lucas Nunes Dalbonio de Carvalho^1^, Antonio Luiz Pinho Ribeiro^4^, Samuel Santana de Oliveira^5^, Filipe Silva Neves Nogueira dos Santos^5^, Jorge Amaral^6^

(1) Universidade Federal Rural do Rio de Janeiro; (2) Instituto Nacional de Cardiologia; (3) Pró-Cardiaco; (4) Universidade Federal de Minas Gerais; (5) Senai Cimatec; (6) Universidade do Estado do Rio de Janeiro

Cardiovascular disease and depression are prevalent. Patients with cardiovascular disease are more depressed than the normal population. Depression is associated with an increased risk of cardiovascular disease (CVD) and death. People with CVD who are also depressed fare worse than patients who are not depressed. Depression’s intensity influences one’s risk of death and other cardiovascular problems. Depression is a multifactorial disease that has a high socio-economic impact. Currently, many patients do not receive adequate treatment or diagnosis. In this context, the present work proposes a Deep Learning model for diagnostic screening of patients in primary care using clinical, laboratory, and sociodemographic data. These data were obtained from a telecardiology project of the Cardiovascular Disease Research Network from 2016 to 2018. Due to the complexity and processing demand, we used a high-performance supercomputer (OGBON – Senai/Cimatec) in the hyperparameter search process, training, and testing the models. We utilized a cross-validation strategy to aim at a better evaluation of the external validation of the model. We designed SMOTE and TOMEK links for data augmentation. We implemented the models in Python and used the Optuna package in searching best parameters. We used the area under the receiver operating characteristic curve, recall, precision, F1-score, and accuracy in models evaluation. Our best model reached, respectively, 0.81, 0.76, 0.88, 0.81, and 0.76. These results indicate that Deep Learning has great potential in terms of screening patients with depression based on data from routine use in primary care.


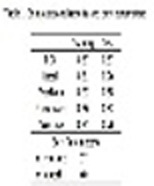


109428

Modality: E-Poster Researcher – Non-case Report

Category: ATHEROSCLEROSIS/CARDIOVASCULAR RISK FACTORS/CARDIOVASCULAR PREVENTION

## Smoking Cessation is Associated with Short-Term Improvement in Vascular Health in a Cohort of People Living with HIV/AIDS in Rio de Janeiro

RODRIGO DE CARVALHO MOREIRA^1^, Anny Rodrigues2^2^, Beatriz Leonardo^1^, Daniel Arabe^1^, Sandra Wagner^1^, Beatriz Grinsztejn^1^, Valdilea Veloso^1^, Antonio Pacheco^3^

(1) Evandro Chagas National Institute of Infectious Diseases; (2) Universidade Federal do Rio de Janeiro; (3) Programa de Computação Científica, Oswaldo Cruz Foundation

**Background:** Smoking is highly prevalent in people living with HIV/AIDS (PLHA) producing detrimental effects in different organs and leading to illness. There is limited evidence about pharmacological interventions for treating nicotine dependence in PLHA. We examined if Nicotine replacement therapy (NRT) is an option for smoking cessation and ameliorates vascular health in this specific population.

**Methods:** From December 2019 to October 2021, we prospectively enrolled PLHA who were actively smoking in our center. The primary outcome of interest was to assess the effect of NRT plus counseling on smoking cessation and endothelial function measured by brachial artery flow-mediated dilatation (FMD). Statistical analysis evaluated the change in %FMD (Δ%FMD = %FMD at week12-%FMD at baseline) to test the hypothesis that Δ%FMD would improve among participants who quit smoking compared to those who relapse. To confirm the results, we have run multiple linear regression to account for classical cardiovascular (CV) confounders. Results are presented in medians (interquartile ranges) and percentages.

**Results:** We included 115 participants with median age of 45.5 years (IQR = 36.4–54.8); 22 (20.4%) had hypertension, 9 (8.3%) had diabetes and 30 (27.8%) had dyslipidemia, almost half were smoking 20+ cigarettes/day (41.7%). Individuals were living with HIV for a median of 10.9 years (5.7–17.4) and were on antiretroviral therapy for 8.6 years (3.7–13.6) with median Nadir of CD4 of 307 (153–490.5). Baseline of median brachial artery diameter was 3.6 mm (IQR = 3.2–4.1). Unadjusted analysis showed that years of smoking, younger age and white race were associated with poor %FMD (75th per centile). After 12 weeks 29.6% participants quit smoking. Comparison of Δ%FMF showed that among participants adherent to therapy, there has been an increase in Δ%FMD when compared to those who relapsed (1.11% [0.29–2.93] vs –0.15% [–1.8–0.91], p < 0.001). After adjustment for CV factors, multiple linear regression showed that participants who quit smoking present a mean 2.53 (p = 0.007) points increase in Δ%FMD in comparison to those who continued to smoke.

**Conclusion:** This study provides evidence that a strategy of NRT and counseling is effective for smoking cessation in PLHA and improves their vascular health in a short period of time. This reinforces the importance of the widespread anti-tobacco programs in HIV clinics and the expected impact lowering incidence of future cardiovascular events.

109452

Modality: E-Poster Researcher – Non-case Report

Category: HEART FAILURE/CARDIOMYOPATHY/TRANSPLANT

## Iron Supplementation in LVAD Patients with Iron Deficiency and Anemia – Comparison of Treatment Strategies

TOMASZ CHWYCZKO^1^, Laura Zalucka^1^, Agnieszka Segiet-Swiecicka^1^, Edyta Smolis-Bak^1^, Ilona Kowalik^1^, Malgorzata Sobieszczanska-Malek^1^, Tomasz Zielinski^1^, Anna Borowiec^1^, Mariusz Kusmierczyk^1^, Rafal Dabrowski^1^, Ryszard Piotrowicz^1^

(1) National Institute of Cardiology, Warsaw, Poland; (2) National Institute of Oncology, Warsaw, Poland

**Background:** Iron Deficiency (ID, ferritin <100 ng/mL or 100–300 ng/mL with transferrin saturation <20%) is present in up to 70% of LVAD (left ventricular assist device) recipients. It causes severe anemia, impairs exercise tolerance, and may worsen the prognosis. Aim of the Study: To determine, which method of iron supplementation: oral or intravenous, is more efficient in LVAD patients.

**Methods:** In 47 recent LVAD recipients (19–68 years, median age 58.7 yrs, 43 men) iron parameters were investigated. 35 patients (74.5%) were diagnosed with ID and 44 patients (93.6%) had anemia. 27 patients were treated with intravenous iron (ferric carboxymaltose, average dose 1248 mg), 9 pts were treated with oral iron, 11 pts did not receive iron (high ferritin level). Blood morphology and iron management markers: serum iron level, transferrin, ferritin, transferrin saturation – TSAT, were measured before and 3 months after the therapy.

**Results:** Resolution of ID was observed in 19 pts from IV Iron Group (73.1%), and in one patient from Oral Iron Group (12.5%), p = 0.002. 8 pts from no treatment group developed ID. Intravenous repletion was more efficient than oral supplementation in ID resolution: OR = 16.29, 95% CI = 2.25–338.1, p = 0.017. IV treatment was more effective than no treatment strategy in anemia resolution: OR = 18, 95% CI = 3.03–159.4, p = 0.003.

**Conclusions:** The study proves the advantage of intravenous over oral iron treatment in LVAD patients.


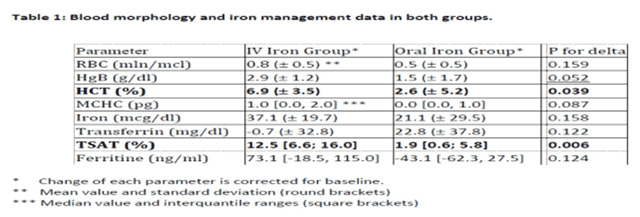


109460

Modality: E-Poster Researcher – Non-case Report

Category: ATHEROSCLEROSIS/CARDIOVASCULAR RISK FACTORS/CARDIOVASCULAR PREVENTION

## Association between Family History for Coronary Disease and Coronary Calcium Score

PAULO EDUARDO BALLVÉ BEHR^1^, Rafael Vianna Behr^1^, Leonardo Henrique Bertolucci^1^, Gabrielly Burkhard Vilasfam^1^, Lara Helena Zortéa^1^, Luiza Zwan Dutra^1^, Maiara Both^1^, Mariana Saadi de Azevedo^1^, Paulo Ernesto Leães^1^, Fernando Antônio Lucchese^1^

(1) Hospital São Francisco – Santa Casa de Misericórdia de Porto Alegre

**Introduction:** It is still uncertain how the family history (FH) for coronary heart disease (CAD) should be assessed: at any age or through the inclusion of an age cutoff. In this work, we evaluated the association between FH at any age and coronary calcium score (CAC). Additionally, we analyzed the influence of the number of family members on the probability of having significant subclinical atherosclerosis.

**Methods:** Cross-sectional study, including patients seen as outpatients between 2012 and 2020, aged between 45 and 84 years, in primary prevention. For FH, parents or siblings were considered, with sudden death, AMI, coronary angioplasty or CABG, at any age. To assess coronary calcification, the CAC percentile (PCAC) was used, with PCAC > 75 being considered important calcification.

**Results:** 509 patients were included, mean age 60 ± 8 years, 54% female, all Caucasian, 55% hypertensive, 36% dyslipidemic, 11% diabetic, 11% smokers; 58% with HF for CAD. The table shows the distribution of PCAC according to FH. Compared to patients without FH, the prevalence of PCAC > 75 was 1.65 times higher in patients with FH (CI 1.20–2.26); 1.59 times higher with a family member with CAD (CI 1.14–2.22); 1.58 times higher with two family members (CI 1.00–2.48); and 2.52 times higher with three or more family members (CI 1.51–4.23). The prevalence of PCAC > 75 with 1 or 2 family members was 31%, while with 3 family members it was 50% (p = 0.053). In addition, the greater the number of family members, the greater the prevalence of PCAC > 0 (p = 0.002).

**Conclusion:** In this study, the presence of FH for CAD was associated with a higher prevalence of significative coronary calcification. In addition, the greater the number of family members, the greater the chance of coronary calcification.


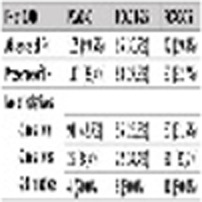


109873

Modality: E-Poster Researcher – Non-case Report

Category: HEMODYNAMICS AND INTERVENTIONAL CARDIOLOGY

## Initial Experience with Left Atrial Appendage Occlusion using the Amplatzer™ and the Watchman™ Dedicated Devices, in a Single Center from Brazil

SERGIO GUSTAVO TARBINE^1^, Costantino R Costantini^1^, Costantino Ortiz Costantini^1^, Shibata Vinicius^1^, Marcos Denk^1^, Rafael Macedo^1^, Marcio M. Luize^1^, Everton Cardoso Dombek^1^

(1) Hospital CArdiologico Costantini

**Background:** The Amplatzer™ and the Watchman™ are dedicated devices for percutaneous left atrial appendage (LAA) occlusion. This is an elective procedure planned to avoid thrombus-embolizaton in patients with atrial fibrillation, unable to use anticoagulation.

**Objectives:** The aim of the study was to describe the initial experience with both devices for percutaneous LAA occlusion.

**Methods:** This is a single-center study of patients undergoing percutaneous LAA occlusion. Inclusion criteria considered a formal contraindication for oral anticoagulation, previous history of stroke due to INR lability, left atrial thrombus in use of NOACs, and patient preference. All procedures were done under general anesthesia and transesophageal echocardiography (TEE) guidance. Transthoracic echocardiography was performed during the first 24hs after the procedure in order to rule out complications. Further follow-up was done with clinical visits and TEE.

**Results:** Between 09–2010 and 10–2021, patients with a mean CHA2DS2-VASC of 4.6 ± 0.8 and Has-bled of 4,5 underwent LAA occlusion with the Amplatzer™ device (24) and the Watchman™ device (8). Both were successfully implanted in 32 patients (100%), 75,1 ± 2,7 yrs old, 75% male, without any procedural stroke or device embolization. TEE showed complete LAA sealing in all patients with no residual leaks. Pericardial effusion needing successful pericardiocentesis in 3 patients. During follow-up, 1 patient had minor retinal embolization and 3 patients died (1: cancer; 2: not related osteomyelitis; 3: chronic renal failure.).

**Conclusion:** In this initial series of patients, both devices showed a good acute and short-term performance considering feasibility and safety regarding the successful implantation rate and the low incidence of complications.

109495

Modality: E-Poster Researcher – Non-case Report

Category: ATHEROSCLEROSIS/CARDIOVASCULAR RISK FACTORS/CARDIOVASCULAR PREVENTION

## Effects of Weight Loss on Lipid Transfer to HDL in Grade III Obese Individuals After 1 Year of Bariatric Surgery

WILSON PASCOALINO CAMARGO DE OLIVEIRA^1^, Fatima Rodrigues Freitas^2^, Maurício Tavares Costa^2^, Aline de Oliveira Silva^2^, Roberto Kalil Filho^2^, Marco Aurélio Santo^3^, Raul Cavalcante Maranhão^2^

(1) Department of Clinical Analysis – FCF-USP; (2) Laboratory of Metabolism and Lipids – InCor; (3) Department of Clinical Analysis – FCF-USP/Discipline of Surgery of the Digestive System at HCFMUSP

**Introduction:** Obesity leads to decrease in HDL-C, which is an independent risk factor for cardiovascular events. Bariatric surgery is an effective treatment for grade III obesity, which leads to decreased LDL-C, triglycerides and increases HDL-C. The impact of bariatric surgery on anti-atherosclerotic functions of HDL, such as transfer of cholesterol from other lipoproteins to HDL was not yet explored.

**Aim:** To evaluate the effects of weight loss by bariatric surgery on the transfer of lipids to HDL and plasma lipids and apolipoproteins (apo) 1 year after bariatric surgery.

**Methods:** Fifteen individuals with grade III obesity (43 ± 6 years, BMI 49 ± 3 kg/m^2^, 14 women) were evaluated before and 1 year after bariatric surgery. Blood samples were obtained after a 12-hour fast. Lipid transfer to HDL was measured by in vitro assay, using an artificial emulsion labeled with 3H-cholesteryl ester and 14C-cholesterol as lipid donor. Lipids, apo A-I, apo B, glucose, insulin, and C-reactive protein (CRP) were determined by commercial kits. Insulin resistance was estimated by HOMA-IR index. HDL diameter was measured by laser light scattering method.

**Results:** As expected, BMI decreased after surgery (49 ± 6 vs 35 ± 5 kg/m^2^; p < 0.0001). Total cholesterol (174 ± 43 vs 154 ± 25 mg/dL; p = 0.022), LDL-C (105 ± 34 vs 78 ± 19 mg/dL; p = 0.007), non-HDL-C (128 ± 39 vs 91 ± 19 mg/dL; p < 0.0001), triglycerides (113 ± 39 vs 72 ± 19 mg/dL; p = 0.0012) and apo B (105 ± 27 vs 78 ± 13 mg/dL; p < 0.001) decreased after surgery. On the other hand, HDL-C (46 ± 9 vs 62 ± 10 mg/dL; p < 0.0001), apo A-I (136 ± 24 vs 157 ± 25 mg/dL; p < 0.0029) and diameter of the HDL (8.88 ± 0.39 vs 9.12 ± 0.34 nm; p < 0.05) were increased. CRP (9.77 ± 6.23 vs 1.75 ± 1.90 mg/dL; p < 0.0001) and HOMA-IR index (10.4 ± 12.2 vs 2.07 ± 0.97; p < 0.05) decreased compared to pre-surgical values. Transfer of unesterified (3.64 ± 0.86 vs 3.79 ± 1.05%) and esterified cholesterol (3.42 ± 0.48 vs 3.20 ± 0.52%) to HDL were not changed after surgery.

**Conclusions:** The weight loss 1 year of after bariatric surgery improved plasma lipids, including HDL-C and promoted reduction of insulin resistance and systemic inflammation associated to grade III obesity, and all those changes conceivably contribute to the diminished cardiovascular risk reported in the literature. However, the surgery did not impact the transfer to HDL of both cholesterol forms, which is also involved in atherogenesis.

109526

Modality: E-Poster Researcher – Non-case Report

Category: CARDIOVASCULAR SURGERY

## Intermittent Aortic Cross-Clamping with Hypothermia in Coronary Artery Bypass Grafting Surgery: Troponin Evaluation and Outcomes

ASSAD MIGUEL SASSINE^1^, José Carone Filho^1^, Elisa ito mendes de Andrade^1^, Ramon Ott Vargas^1^, José Silva Henrique^1^, Márcio Luiz Roldi^1^, Lúcio Pereira Guarçoni^1^, Dalton Vinicius Menin^1^, Carlos Alberto Sancio Junior^1^, Ana Carolina Simões Ramos^1^, José Carone Junior^1^, Schariff Moyses^2^

(1) Hospital Evangelico de Vila Vleha; (2) Instituto de Cardiologia Do Espirito Santo

**Introduction:** Coronary artery bypass grafting (CABG) surgery is an important weapon in the therapeutic arsenal of coronary artery disease. The aim is to perform a safe procedure, with myocardial preservation and a low rate of perioperative complications. The purpose of this study is to evaluate myocardial injury and clinical evolution in patients undergoing CABG in a referral hospital in Espírito Santo state, where intermittent aortic clamping in hypothermia is used as a myocardial protection strategy.

**Methods:** Observational, cross-sectional and prospective study were developed in a referral Hospital in Espirito Santo state.

**Results:** 108 patients were included in the study between April and December 2019. The mean age of patients was 65 years. The mean EuroSCORE II was 2.54 and the mean Society of Thoracic Surgeons (STS) score was 1.54. Most patients were male (62%) and 75.9% had hypertension. Approximately half of patients had diabetes mellitus and dyslipidemia. Less than a fifth of the sample reported prior acute myocardial infarction. Mean cardiopulmonary bypass (CPB) time was 56 minutes and the average period of cross-clamping time was 43 minutes. Cardiac troponin I alterations were associated with death when considered from 6.1 ng/mL on the 1st postoperative day; values >6.1 ng/mL were related to longer CPB and cross- clamping times.

**Conclusion:** The technique of intermittent aortic cross-clamping in CABG surgery proved to be safe, with a low rate of postoperative morbidity and mortality. New studies, including analyzes with a larger sample, should be carried out to better understand this relationship.

109552

Modality: E-Poster Researcher – Non-case Report

Category: ATHEROSCLEROSIS/CARDIOVASCULAR RISK FACTORS/CARDIOVASCULAR PREVENTION

## Is the Acc/Aha Risk Score Able to Predict Coronary Calcification?

PAULO EDUARDO BALLVÉ BEHR^1^, Rafael Vianna Behr^1^, Leonardo Henrique Bertolucci^1^, Gabrielly Burkhard Vilasfam^1^, Lara Helena Zortéa^1^, Luiza Zwan Dutra^1^, Maiara Both^1^, Mariana Saadi de Azevedo^1^, Paulo Ernesto Leães^1^, Fernando Antônio Lucchese^1^

(1) Hospital São Francisco – Santa Casa de Misericórdia de Porto Alegre

**Introduction:** The ACC/AHA 10-year Atherosclerotic Cardiovascular Disease Risk Score (ACC/AHA Score) is one of the most used to assess the risk of MI or stroke. However, it was not evaluated whether it is able to identify individuals with greater or lesser coronary calcification. Therefore, our objective is to assess whether the risk categories of the ACC/AHA Score are able to predict coronary calcification.

**Methods:** Cross-sectional study, including patients seen as outpatients between 2012 and 2020, aged between 41 and 69 years, in primary prevention. Patients were divided into the four risk categories of the ACC/AHA Score: low (<5%), borderline (≥5% and <7.5%), intermediate (≥7.5% and <20%) and high (≥20%). Coronary calcification was measured by the Calcium Score (CAC), using absolute values (in Agatston), and CAC ≥ 100 was considered a significative calcification.

**Results:** 518 patients were included, mean age 59 ± 8 years, 52% female, all Caucasian, 54% hypertensive, 59% dyslipidemic, 11% diabetic, 11% smokers. The Table shows the distribution of CAC according to the ACC/AHA risk category. The increase in risk category was associated with a progressively higher prevalence of CAC ≥ 100 (p < 0.01), just as the reduction of category was associated with a higher prevalence of CAC = 0 (p < 0.01). Compared to low-risk patients, the prevalence ratio for CAC ≥ 100 was 2.35 in borderline-risk patients [(CI 1.44–3.82) (p = 0.01)]; 2.88 in intermediate risk [(CI 1.89–4.39) (p < 0.01)] and 4.32 in high risk [(CI 2.73–6.82) (p < 0. 01)].

**Conclusion:** In a population sample from southern Brazil, we observed that the ACC/AHA Score categories are able to predict the probability of significant subclinical atherosclerosis, assessed by coronary calcification.


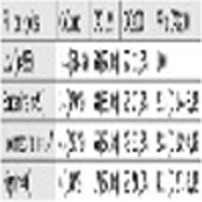


109571

Modality: E-Poster Researcher – Non-case Report

Category: NURSING

## Development of Nursing Technology for the Management of Tachycardias in Intensive Care: Experience Report

ÉRICA SOBRAL GONDIM^1^, Emiliana Bezerra Gomes^1^, José Hiago Feitosa de Matos^1^, Ana Camila Gonçalves Leonel^1^, Sarah de Lima Pinto^1^, Amanda da Costa Sousa^1^, Antônia Elizângela Alves Moreira^1^, Raynara Augustin Queiroz^1^, Ana Luiza Rodrigues Santos^1^, Mariane Ribeiro Lopes^1^

(1) Universidade Regional do Cariri – URCA

**Introduction:** Critical care units are characterized by the continuous monitoring of physiological parameters essential to the maintenance of life and the immediate conduct of health interventions. Subtle changes in organic functioning variables are indicative of potential risks to homeostasis, which can cause events that culminate in interruption of blood flow to vital organs and cardiorespiratory arrest. Heart rate, the number of beats per minute that determines the effectiveness of blood pumping from the heart to the other organs, is one of the vital parameters in controlling the proper functioning of the cardiovascular system. Although it may indicate a cardiac disorder itself, tachycardia is also present in non-cardiac conditions common in the Intensive Care Unit (ICU), such as delirium, shock, sepsis, anxiety, pain, inadequate management of sedation and respiratory distress. In this context, a nursing guide was developed to approach tachycardias in the ICU, seeking to optimize the time to restore hemodynamic balance and prevent cardiorespiratory arrest.

**Objective:** The present study, therefore, aims to report the experience of an intensive care nurse in the development of an instrument that classifies tachycardias according to intervention priorities and action packages.

**Method:** This is an experience report on the development of nursing technology by the professional, whose 12-year experience in intensive care and immersion in studies on the subject provided subsidies to identify priorities and conduct them properly according to their own professional skills.

**Results:** The elaborate script identified cardiac and non-cardiac causes, directing a direct nursing intervention to each cause and its time of completion according to the required urgency, citing in each intervention the subsequent steps and the professionals who must be activated according to the limits of professional exercise.

**Conclusions:** The development of this technology, based on the technical competence and experience of the professional nurse, followed an approach, although simple, practical and directed to the most prevalent causes in the ICU. The instrument resulting from this perception demonstrates practicality and applicability in routine care and nursing in the prevention of adverse events in intensive care units, optimizing the quality of care provided.

109608

Modality: E-Poster Researcher – Non-case Report

Category: NURSING

## Hasca – Hypertension Registry in Children and Adolescents: Pilot Study of National Database Viability

JACQUELINE VAZ ALENCAR^1^, Caroline Naidon Coelho^1^, Clarissa G. Rodrigues^10^, Liliana Fortini Cavalheiro Boll^1^, Luiza Junqueira Trarbach Lovato^2^, Renata Póvoas^3^, Nicole Saldanha de Souza^5^, Emily Justiniano^6^, Fernanda Consolim-Colombo^7^, Katia de Angelis^8^, Maria Claudia Irigoyen^3^, Danielle Irigoyen da Costa^9^

(1) Instituto de Cardiologia da Fundação Universitária de Cardiologia (IC/FUC) – Porto Alegre, RS – Brasil; (2) University of Technology Sydney (UTS) – Austrália, Sidney; (3) Instituto do Coração da FMUSP (InCor) – São Paulo, SP – Brasil; (4) Universidade Nove de Julho (Uninove) – São Paulo, SP – Brasil; (5) Universidade Federal de Ciências da Saúde de Porto Alegre (UFCSPA) – Porto Alegre, Rio Grande do Sul – Brasil; (6) Hospital de Clínicas de Porto Alegre (HCPA) – Porto Alegre, RS – Brasil; (7) Universidade Nove de Julho (Uninove) – São Paulo, SP – Brasil; (8) Universidade Federal de São Paulo (Unifesp) – São Paulo, SP – Brasil; (9) Pontifícia Universidade Católica do Rio Grande do Sul (PUCRS) – Porto Alegre, Rio Grande do Sul – Brasil; (10) Global Research and Innovation Network (GRINN) – Curitiba, Paraná – Brasil

**Introduction:** Multicenter registries that represent the real-world provide important information, but there are few studies describing how to implement it.

**Objective:** To describe the feasibility pilot project of a database on systemic arterial hypertension in children and adolescents in a reference hospital.

**Methods:** Prospective, observational study to document and assess the feasibility of a multicenter registry of systemic arterial hypertension in children and adolescents. 3 steps were performed. Step 1: ethical aspects, contact with the school management, project pitch meeting and team training. Step 2: participants and workflow, variables included, action at school (screening and confirmatory phase). Step 3: evaluation of protocols, variables analyzed.

**Results:** Sample composed of 80 students, average age of 15.73 ± 0.77 years, 77.5% female and 22.5% male. The mean BMI was 23.58 ± 4.11 kg. Students who presented at least two altered measured pressures in the screening phase were: altered BP 42 (52.5%) and normal BP 38 (47.5%). In the Confirmatory Phase: 22 (32.8%) maintained altered BP. These will be monitored at a specialized center. The data was analyzed with the “REDCap” software. After the viability of the Registry, other centers participated through training. Data quality reports were generated for quality control.

**Conclusions:** The description of the methodology makes HASCA possible, enabling other centers to standardize data collection and promote the development of new health technologies, assisting in public policies on systemic arterial hypertension in children and adolescents.

109786

Modality: E-Poster Researcher – Non-case Report

Category: DIGITAL HEALTH/INNOVATION

## Accuracy Index of Artificial Intelligence Platform in the Preparation of Electrocardiography Reports in Adult Intensive Care Unit

FIRMINO HAAG FERREIRA JUNIOR^1^, Rosa Maria da Costa Simões^1^, Diany Priscilla Oliveira^1^, Carolina Vieira^1^

(1) Hospital Geral de São Mateus

**Objective:** To analyze the accuracy index of electrocardiogram reports performed in Intensive Care, reported by an artificial intelligence platform, compared with reports performed by cardiologists in person. The electrocardiograms were randomly assigned and reported “blind” to the results provided by the artificial intelligence, and then both results were compared.

**Report:** Thirty (30) electrocardiograms of different patients were analyzed, randomly chosen, admitted to an intensive care unit, with a baseline diagnosis of cardiovascular diseases. Among the electrocardiograms analyzed, artificial intelligence was able to accurately diagnose 16 (sixteen) cases of inactive zone/myocardial infarction, 2 (two) cases of ST segment elevation, 2 (two) cases of atrial flutter, 5 (five) cases of atrial fibrillation, 5 (five) cases of diffuse changes in ventricular repolarization, 1 (one) case of left ventricular overload, 1 (one) case of anterior superior divisional hemiblock. Artificial Intelligence was able to identify electrode failures, as it did not consider the report. There was 100% compatibility of the artificial intelligence reports with the reports prepared by the cardiologists in person.

**Conclusion:** The introduction of new technologies such as artificial intelligence producing reports in electrocardiography is of significant importance, due to the reliability of the reports presented, not different when compared with reports prepared by experienced cardiologists in person, being therefore an important tool in helping to physicians in identifying and confirming the diagnosis, especially for non-specialists, often present in emergency services and intensive care units.

109612

Modality: E-Poster Researcher – Non-case Report

Category: DIGITAL HEALTH/INNOVATION

## Use of Capnography in Differentiating Non-Heart and Non-Pulmonary Disease as Cause of Dyspnea in Hospitalized Individuals

ALEXANDRA CORRÊA GERVAZONI BALBUENA DE LIMA^1^, Caroline Barreto Cavalcanti^1^, Paula Fernandes Freitas Lima^1^, Barbara Cunha Barreto^2^, Bruno Ramos Carneiro^2^, Gabriela de Oliveira Silva^2^, Maria Alice Ramalho Bragatto^2^, Luís Moreira da Silva de Azevedo Meireles^3^, Sergio Henrique Rodolpho Ramalho^4^

(1) North Wing Regional Hospital – Brasilia – Brazil; (2) School of Health Sciences – Brasília – Brazil; (3) MDI Industrial, Salvador – Brazil; (4) Clinical Research Center of the Brasilia of the Dasa Hospitals Network – Brasilia – Brazil

**Background:** Rapid differentiation the cause of dyspnea from other heart or respiratory causes, is very important for choosing an appropriate therapy. Capnography is a non-invasive and accurate method to measure end-tidal carbon dioxide (PETCO2) and can help physicians in some critical situations. Although this is not used in many emergency situations and it is not used routinely in the emergency department, its application is increasing in many emergency situations, such as patients undergoing mechanical ventilation, procedural sedation and analgesia, pulmonary disease, heat failure, shock, metabolic disorder and trauma.

**Aim:** To evaluate the PETCO2 in differentiating heart and/or pulmonary disease from non-heart and non-pulmonary related dyspnea in hospital setting.

**Methods:** This was observational prospective study performed in the North Wing Regional Hospital, Brasilia, Brazil, August 2021–Febuary 2022. 254 Adults, conscious and spontaneous breathing hospitalized individuals were evaluated and divided in four groups of patients: heart disease (heart failure, ischemic heart disease) group 1 (n = 54), pulmonary disease (chronic obstructive pulmonary disease, asthma) group 2 (n = 68), heart and pulmonary disease group 3 (n = 74) and non-heart and non-pulmonary disease group 4 (n = 56). PETCO2 was measured by a portable capnograph, using a nasal catheter, without supplemental oxygen.

**Results:** The groups were similar in age (group 1 62.2 ± 15,7 vs group 2 64.5 ± 18.0 vs group 3 62.0 ± 15.6 vs group 4 66.9 ± 17.4, p = 0.33) and gender (male, group 1 23% vs group 2 26% vs group 3 28% vs group 4 22%, p < 0.82). The PETCO2 was lower and similar in the heart and/or pulmonary groups and higher in non-heart and non-pulmonary group (group 1 29.2 ± 6.9 vs group 28.6 ± 5.7 vs group 3 28.7 ± 5.8 vs group 4 31.2 ± 5.0, p = 0.05). The PETCO2 in non-heart and non-pulmonary disease (area under the curve, 0.63; 95% confidence interval, 0.57–0.71, p = 0.03).

**Conclusion:** The PETCO2 was able to screen individuals without heart and pulmonary disease in treatment of dyspnea in a public hospital. Capnography can be an easy, cost-effective and non-invasive tool to evaluate dyspnea and does not require cooperation of the patient. Other studies are necessary to different heart from pulmonary disease as cause of dyspnea.

109619

Modality: E-Poster Researcher – Non-case Report

Category: COVID-19 AND CARDIOVASCULAR SYSTEM

## Myopericarditis Detected by Magnetic Resonance in Long COVID-19

RENATA JUNQUEIRA MOLL BERNARDES^1^, Julia Barroso^1^, Andréa Silvestre de Sousa^1^, Eduardo Schaustz^1^, João Dario Mattos^1^, Emiliano Medei^1^, Olga Ferreira de Souza^1^, Denilson de Campos Albuquerque^1^, Ana Cristina Baptista da Silva Figueiredo^1^, Mariana Tortelly^1^, Juliana Ferreira^1^, Gabriel Cordeiro Camargo^1^

(1) D’Or Institute for Research and Education, Rio de Janeiro, Brazil

**Background:** The pathophysiological mechanisms associated with cardiac symptoms in post-acute COVID-19 are still poorly understood. A high prevalence of myocardial injury, associated with higher in-hospital mortality has been reported and one of the postulated mechanisms is an autoimmune inflammatory response. Cardiovascular magnetic resonance (CMR) is the best non-invasive method for the assessment of myocardial and pericardial inflammation, allowing the detection of edema and fibrosis. AIM To characterize the presence and prevalence of myopericardial inflammation in a population with a high prevalence of cardiac injury, recently discharged from COVID-19 hospitalization.

**Methods:** In this prospective study, 190 hospitalized COVID-19 patients with clinical or laboratory cardiovascular abnormalities were included in a multicentric registry between November 2020 and December 2021. After discharge, all patients were contacted and 48 consented to return for CMR exam.

**Results:** Of 48 included patients, 33 (68.8%) were male, the mean (SD) age was 57.0 (18.9) years, 24 (50%) were hypertensive and 12 (25%) had diabetes. Chronic cardiac disease was reported in 6 (12.5%), asthma in 4 (8.3%), and chronic obstructive pulmonary disease in 3 (6.3%). Thirty-one patients (64.6%) were overweighed and 37 (77.1%) had myocardial injury detected by increased troponin levels. The median (IQR) time interval between hospital admission and CMR was 74 (26–157) days. Myocardial late gadolinium enhancement (LGE) was observed in 20 (41.7%) patients, including 5 with subendocardial, 11 mesocardiac, and 4 with transmural LGE. Patients with transmural and subendocardial LGE were diagnosed with ischemic heart disease (5), hypertrophic myocardiopathy (2), and right ventricular muscular band (1). Fourteen patients (32.6%) had pericardial LGE, and the prevalence of this finding reduced from 41.7% to 30% and 11%, according to the time interval from hospital discharge to the CMR exam (0–3, 3–6, and 6–12 months, respectively). Small pericardial effusion was detected in 11 (25.6%) patients, and the frequency decreased from 37.5% to 10% and 11%.

**Conclusion:** There is a high incidence of post-COVID myopericardial inflammation in patients who presented with cardiac abnormalities during acute illness, particularly myocardial injury. These findings indicate that myocardial injury may be related to late myocardial inflammation and might explain some of the long-term cardiovascular symptoms of COVID-19.

109651

Modality: E-Poster Researcher – Non-case Report

Category: DIGITAL HEALTH/INNOVATION

## Remote Monitoring Application for Patients with Heart Failure: Pilot Randomized Clinical Trial

ANA CARLA DANTAS CAVALCANTI^1^, Ana Carla Dantas Cavalcanti^1^, Lyvia da Silva Figueiredo^1^, Jessica Santos de Souza Leal^1^, Mellissa Barreto Oliveira Da Silva^1^, Flavio Luiz Seixas^1^, Caroline Barboza Braga^1^, Kelly Maria Augusta Tavares Bentes^1^, José Paulo de Mello Gomes^1^, Paula Vanessa Peclat Flores^1^, Evandro Tinoco Mesquita^1^

(1) Universidade Federal Fluminense

**Introduction:** Heart failure is a clinical condition associated with morbidity and mortality, and it is necessary to manage the disease, relieve symptoms, prevent hospitalization and reduce mortality. Therefore, the use of mobile applications represents a strategy to optimize care.

**Objective:** To compare self-care, quality of life, and depressive symptoms in patients with chronic heart failure using a mobile app with conventional follow-up.

**Method:** The study was carried out in two phases, the first, a technological development study, and the second, a (pilot) randomized clinical trial. For the application development, the following software was used: Android Studio, Flutter and the Android operating system. Prospective validation was carried out with experts in heart failure through the System Usability Scale (SUS). In the pilot study, patients were divided into two groups. Patients in the intervention group used the application for 30 days, and patients in the control group used the conventional telephone follow-up. Outcomes were assessed using the European Heart Failure Selfcare Behavior Scale, the Minnesota Living with Heart Failure questionnaire, and the Beck Inventory. Data were analyzed using SPSS v.24 and a repeated-measures ANOVA. The study was registered in ReBEC (RBR-2w7wkb) and approved by an Ethics Committee.

**Results:** The “Card.io” application features, as a resource, the sending of notifications and alarms reminding the patient of a certain action defined by the professional. The prospective validation was performed by 39 experts, mostly nurses (89.7%), with a mean age of 33 years, mostly female (82.1%), and 35 (89.7%) residing in Brazil. The application was considered excellent based on the SUS score. The pilot clinical trial was carried out with 42 patients. There were significant differences in the interaction regarding depressive symptoms (p = 0.016), self-care (p = 0.019), and quality of life (p < 0.001).

**Conclusion:** The Card.io application for remote monitoring effectively improves the quality of life of patients and offers an alternative for healthcare professionals, with an innovative and low-cost proposal that can be implemented in healthcare services.

111068

Modality: E-Poster Researcher – Non-case Report

Category: ACUTE AND CHRONIC CORONARY DISEASE/THROMBOLYSIS

## Prognostic Relevance of Quality of Care Indicators in the Treatment of Acute Myocardial Infarction

RICARDO MOURILHE-ROCHA^1^, Bruno Reznik Wajsbrot^1^, Marcelo Luiz da Silva Bandeira^1^, Julia Paulo Mourilhe Rocha^1^, Thiago Matos Barcellos^1^, Flávia Prado Fialho Santos^1^, Eric Costa de Almeida^1^, Roberta Siuffo Schneider^1^, Ricardo Mendes Carneiro^1^, Claudia Lanzillotti Weksler^1^, Fernando Oswaldo Dias Rangel^1^, Daniel Xavier de Britto Setta^1^

(1) HOSPITAL PRÓ-CARDÍACO

**Background:** Measuring the quality of care indicators enable the recognition of weaknesses in health care. Based on this assessment, it will be possible to improve outcomes.

**Objectives:** To evaluate the quality of care based on ventricular dysfunction, length of stay, coronary angioplasty and hospital mortality rates.

**Materials and Methods:** Observational, retrospective, cohort study of 398 patients admitted with a diagnosis of AMI, with and without ST-segment elevation between January 2018 and January 2022; 69.3% male with a mean age of 65 years. Data were analyzed by the SPSS software.

**Results:** They had the following comorbidities: 47.5% dyslipidemia, 77.9% hypertension, 50.5% previous CAD, 41.2% diabetes, 8.5% heart failure, 37.2% stroke, 15.1% coronary artery bypass surgery, 29.6% previous AMI, 10.1% chronic kidney disease, 43.0% smoking, and 10.3% atrial fibrillation. There were 31.2% with STEMI and 68.8% with NSTEMI, with 83.4% in Killip I, 8.4% in II, 3.1% in III, and 5.0% in IV. The median length of stay was 4 days (IQR 3–8) in the overall population, 4 days in NSTEMI and 5 days in STEMI; p = 0.02. The rates of events at discharge in the overall population and subgroups were: hospital mortality = 7.5% (4.7%, NSTEMI and 13.7%, STEMI; p = 0.002), ventricular dysfunction = 34.4% (28.1%, NSTEMI and 49.1%, STEMI; p < 0.001), being mild dysfunction in 15.5%, moderate 12.6% and severe 5.3%. The angioplasty rate was 79.1% (74.4% NSTEMI and 89.5% STEMI; p < 0.001).

**Conclusions:** The mortality rate was higher than predicted by the Killip score. The incidence of ventricular dysfunction was high, but it was not possible to determine how many of these patients presented ventricular dysfunction prior to the event. The length of hospital stay was within the recommended range and the coronary angioplasty rate was also high.

111069

Modality: E-Poster Researcher – Non-case Report

Category: PHYSIOTHERAPY

## Functional Capacity of Individuals Post Infection by COVID-19 Treated for Cardiopulmonary Rehabilitation in Home Based Format

ANA INÊS GONZÁLES^1^, Ana Inês Gonzáles^1^, Guilherme Michels^1^, Jackson da Silva Gullo^1^

(1) Universidade Estácio de Santa Catarina – São José

**Introduction:** Studies on “Long Covid” suggest that long-term persistent clinical manifestations can last even months after infection. In this sense, post-covid rehabilitation programs are essential. Due to the restrictions imposed by the pandemic, carrying out rehabilitation programs in closed environments has become a concern, leaving home rehabilitation as the only option, also called Home Based (HB).

**Objectives:** To verify the benefits of a post-covid-19 cardiopulmonary rehabilitation program in Home Based format.

**Method:** Longitudinal, interventional study, with an accessibility sample, with individuals of both sexes, ≥30 years old, with a clinical diagnosis of COVID-19 infection by the reverse-transcriptase polymerase chain reaction (RT-PCR) method, already completed, and who have remained with cardiopulmonary complications. Patient recruitment occurred publicity on social media and radio. The recruited individuals were submitted to the 30-second sit-and-stand test (30CST) and the 2-minute stationary gait test (2MWT) remotely during the pre- and post-rehabilitation program period. The intervention protocol took place synchronously, in a home-based format, using video calls via Whatsapp app, based on: 1) respiratory kinesiotherapy exercises, 2) aerobic training; 3) resistance training with localized muscle strength exercise, 4) stretching, two days a week for approximately 60 minutes, and individuals performed rehab with the help of tutors. The Borg Subjective Scale was used to monitor the intensity of the exercises, and should remain between 3 and 4 points.

**Results:** Twenty-eight individuals were treated, 12 men (43%) and 16 women (57%), mean age of 50 ± 5 years, who performed 16 ± 1.2 sessions. There was a mean improvement of 8 ± 1.2 elevations (p = 0.002) to 30CST and 10 ± 2 elevations (p = 0.01) of the dominant limb to TME2 in the post-intervention values, with Borg subjective scale to 2MWT falling from 5 to 3 during testing after intervention.

**Conclusions:** A home-based post-covid 19 rehabilitation program promotes improvement in functional capacity, proving to be an applicable and beneficial intervention method for these patients.

110374

Modality: E-Poster Researcher – Non-case Report

Category: COVID-19 AND CARDIOVASCULAR SYSTEM

## Exercise Limitation Mechanisms in COVID-19 Survivors

FERNANDA SAHAR LUCAS VIDAL DOMECG^2^, Fabricio Braga da Silva^1^, Marcelo Kalichsztein^3^, Gustavo Nobre^3^, José Kezen^3^, Gabriel Espinosa^1^, Christiane Prado^1^, Marcelo Faccio^1^, Illan Gotlieb^3^, Ronaldo Leão Lima^2^

(1) Laboratório de Performance Humana; (2) Universidade Federal do Rio de Janeiro; (3) Casa de Saúde São José

**Background:** Limited exercise capacity (LEC) is a prevalent complaint in COVID-19 survivors. The cardiopulmonary exercise test (CPET) is the gold standard in determining LEC mechanisms.

**Objectives:** To analyze LEC mechanisms in COVID-19 survivors of different clinical severities and compare them with a non-COVID-19 control population.

**Materials and methods:** Cross-sectional analysis of CPET was performed between July/19 and March/21. Patients were divided into 3 groups: mild COVID-19(L-Cov; outpatient treatment); Severe COVID-19(G-Cov; hospital admission) and non-COVID-19(N-Cov; CPET performed before the start of the pandemic). LEC was defined as VO2 <85% of predicted at peak exercise and classified as: cardiocirculatory(CC), ventilatory(VEN), mixed(MIX; a combination of CC and VEN), or aerobic deconditioning(AD). Furthermore, the prevalence of findings suggestive of vascular-pulmonary involvement(Vas-Pul) was compared between the groups. Based on the values of end-tidal CO2 pressure and the ratio between ventilation and CO2 production, patients were classified at the first ventilatory threshold according to the probability of Vas-Pul involvement as non-suspected, suspected, likely, and very likely.

**Results:** 702 patients were included(61.1% men; 52.1 ± 14.3 years), 310(44.2%); 305(43.4%) and 87(12.4%) N-Cov, L-Cov and G-Cov, respectively. Table 1 shows the LEC mechanisms between the groups(χ2 = 3.76; df = 6;p = 0.709). Table 2 shows the probability of Vas-Pul involvement(χ2 = 34.26; df = 6; p < 0.001). In the posthoc analysis, a probable and very likely pattern was higher in the G-Cov group (p < 0.05).

**Conclusion:** AD was the main mechanism of exercise limitation, followed by cardiocirculatory, ventilatory and mixed limitation (CC+Vent.).


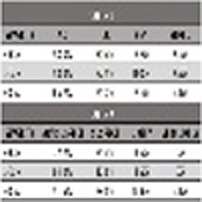


110375

Modality: E-Poster Researcher – Non-case Report

Category: COVID-19 AND CARDIOVASCULAR SYSTEM

## Early Triage Echocardiography to Predict Outcomes in Patients Admitted with COVID-19 – a Multicenter Study

BRUNO RAMOS NASCIMENTO^1^, BRUNO RAMOS NASCIMENTO, Maria Carmo Pereira Nunes^1^, Nicholas Ollberding^2^, Allison Hays^3^, Pranoti Hiremath^3^, Federico Asch^4^, Chad League^4^, Chris Fung^5^, Laurie LeBouef^5^, Craig Sable^6^, Andrea Zawacki Beaton^2^

(1) Serviço de Cardiologia e Cirurgia Cardiovascular e Centro de Telessaúde do Hospital das Clínicas da UFMG, Belo Horizonte – MG, Brazil; (2) The Heart Institute, Cincinnati Children’s Hospital Medical Center, University of Cincinnati School of Medicine, Cincinnati – OH, United States; (3) Cardiology, The Johns Hopkins Hospital, Baltimore – MD, United States; (4) The Heart and Vascular, MedStar Washington Hospital Center, Washington – DC, Unites States; (5) Cardiovascular Center, University of Michigan Hospital, Ann Harbor – MI, United States; (6) Cardiology, Children’s National Health System, Washington – DC, United States

**Introduction:** Cardiac involvement impacts prognosis of COVID-19, especially in critically ill patients. We aimed to assess the prognostic value of limited cardiac assessment by bedside triage echocardiography (echo) in patients admitted to emergency departments (ED) in the United States with COVID-19.

**Methods:** Patients admitted in 4 reference US EDs with confirmed COVID-19 underwent triage echo within 72h of symptom onset, with remote interpretation. Clinical and laboratory data, as well as COVID-19 symptoms, were collected. A comprehensive echo protocol, with quantitative assessment, was applied in high-resource units, while low-resource EDs utilized a focused qualitative protocol. Association between echo variables, demographics and clinical data with all-cause mortality and intensive care unit (ICU) admission was assessed; factors significant at p < 0.10 were put into multivariable models.

**Results:** A total of 399 patients were enrolled (137 from low-resource EDs), 41% women, with a mean age of 62 ± 16 years. Mean oxygen saturation on presentation was 92.3 ± 9.2%, and the average number of comorbidities was 2.1 ± 1.7. Compared to survivors, non-survivors were older (68 ± 12 vs. 60 ± 17 years, p < 0.01), had lower oxygen saturation (88.8 ± 12.0% vs. 93.1 ± 8.2%, p < 0.01), were more likely to have a chronic condition (2.6 ± 1.6 vs. 2.0 ± 1.7 comorbidities, p = 0.01) and had lower LV ejection fraction (50.3 ± 19.7 vs. 58.0 ± 13.6, p < 0.01). 101 (25%) patients had moderate/severe LV dysfunction and 131 (33%) had moderate/severe RV dysfunction. Older and lower oxygen saturation were independently associated with death (OR = 2.14 (95%CI 1.06–4.32) and OR = 0.67 (95%CI 0.53–0.85), respectively) and ICU admission (OR = 0.65 (95%CI 0.46–0.94) and OR = 0.48 (95%CI 0.37–0.64)). No echo variables, including LV and RV function, were independent predictors of outcomes. The mortality model comprised of age, sex, count of co-morbid conditions, and oxygen saturation, had a C-statistic = 0.68 and Brier score = 0.14. The inclusion of LV/RV dysfunction, and pericardial effusion did not improve performance (C-statistic = 0.67, Brier score = 0.15). A similar pattern was observed for the ICU admission model.

**Conclusion:** In patients admitted with COVID-19 and undergoing early echo triage, independent predictors of death and ICU admission were age and oxygen saturation. Despite differences in baseline LVEF, no echo variables were independently associated with unfavorable outcomes.

109741

Modality: E-Poster Researcher – Non-case Report

Category: HEMODYNAMICS AND INTERVENTIONAL CARDIOLOGY

## Patent Foramen Ovale: Percutaneous Closure of the Defect as a Therapeutic Alternative for Patients Over Sixty Years of Age

MARCELO SABEDOTTI^1^, Bibiana Guimarães Maggi^2^, Gabriel Almeida Krul^2^, Elisa Rocha Nonemacher^2^, Rafaela Oliveira Leite^2^

(1) Unimed Nordeste; (2) Fundação Universidade de Caxias do Sul

**Introduction:** Percutaneous patent foramen ovale (PFO) closure effectively prevents embolic ischemic stroke. However, most studies do not include patients over sixty years of age. This group of patients usually has other causes of ischemic stroke, the main ones including cardiac arrhythmias, small cerebral vessel disease, and atherosclerosis. The present study aims to support the hypothesis that PFO closure is safe in patients over 60 years of age.

**Objective:** To assess the safety of percutaneous PFO closure in patients over sixty years.

**Methods:** A retrospective, single-center cohort study. Patients aged over sixty years who underwent percutaneous PFO closure to prevent recurrence of cerebrovascular events at Hospital Unimed Nordeste do the Rio Grande do Sul, between September 2019 and January 2022 were included.

**Results:** From June 2019 to March 2022, 15 patients were submitted to percutaneous PFO closure (Table 1), with a mean age of 70 ± 7 years, 60% were female. All patients had PFO with ISA, transcranial ultrasound (TU) with more than 100 high-intensity signals (HITS), Holter without atrial fibrillation, and hematological investigation without coagulopathies. As for medications, 53.3% of patients were being treated with Acetylsalicylic Acid (ASA) associated with clopidogrel and 46.7% using anticoagulants being 26% Dabigatran, 13% Apixaban, and 6% Rivaroxaban. Two days before the procedure and three months after, all remained with ASA and clopidogrel. There weren‘t any complications during the procedure. In 30 days, echocardiographic control showed a foramen completely occluded, without a residual shunt.

**Conclusion:** The procedure for closing PFO in patients over 60 years of age is effective and safe. Careful evaluation is important, including shunt quantification by TU, exclusion of coagulopathies, arrhythmias, and atherosclerotic causes of ischemic stroke. A high-risk PFO morphology can also influence the decision to carry out closure. Data from studies in patients over 60 years of age are limited and additional studies are needed to assess the benefits of this treatment as the population grows old.


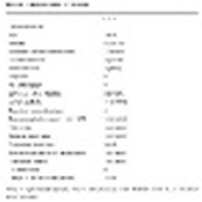


109758

Modality: E-Poster Researcher – Non-case Report

Category: ATHEROSCLEROSIS/CARDIOVASCULAR RISK FACTORS/CARDIOVASCULAR PREVENTION

## Association between Sex and Therapeutic Targets of Lipid Profile in Individuals with Previous Acute Myocardial Infarction: Subanalysis of Dica-Nuts Trial

ERLON OLIVEIRA DE ABREU SILVA^1^, Erlon Oliveira de Abreu-Silva^1^, Angela C. Bersch-Ferreira^1^, Rachel H. Vieira Machado^1^, Lucas R. Silva^1^, Erica R. Ribeiro Sady^1^, Debora H. Kodama Miyada^1^, Bernardete Weber^1^, Aline Marcadenti^1^

(1) Hcor Research Institute (IP-Hcor), Hcor, São Paulo, São Paulo, Brazil.

**Background:** Differences between sexes regarding therapeutic targets of lipid profile in populations at very high cardiovascular risk are not fully elucidated.

**Objective:** To evaluate the association between sex and therapeutic targets for LDL-cholesterol (LDL-c) and non-HDL-cholesterol (NHDLc) in individuals diagnosed with acute myocardial infarction (AMI).

**Methods:** Cross-sectional analysis with baseline data from a multicenter randomized clinical trial (DICA-NUTS Trial, NCT03728127) conducted among subjects ≥40 years with previous AMI (60 to 180 days). Sociodemographic, clinical and lifestyle data were collected using specific questionnaires. Therapeutic goals of LDL-c (<50 mg/dL) and NHDLc (<80 mg/dL) were defined according to American Heart Association guidelines. Binary logistic regression was used to evaluate potential associations adjusted for confounders.

**Results:** In total, 471 individuals with a mean age of 59.6 ± 9.3 years were evaluated; 72.2% were men, the estimated monthly household income was US$ 180.00, 10.2% were smokers and 61.8% had AMI with ST-segment elevation (STEMI). The prevalence of married individuals was higher among men compared to women (61.5% and 45.8% respectively, P = 0.001), and among women there were higher proportions of high levels of physical activity (35.9% vs. 22.9%, P = 0.003) and from lower social classes (D/E classification) (68.7% vs. 53.2%, P = 0.002). The use of simvastatin was more frequent (52.4%), followed by rosuvastatin (21.4%) and atorvastatin (21%), with no difference between men and women. Among men, 14.4% and 18.2% had LDL-c and NHDL-c concentrations in line with the recommended targets; these prevalences were higher than those observed in women (LDL-c: 6.9% [P = 0.028]; CNHDL: 8.4% [P = 0.007]). After adjusting for age, marital status, social class and physical activity levels, there was an association between male sex and achieving therapeutic targets for NHDL-c (OR 2.04; 95%CI 1.003–4.15), but not for LDL-c (OR 1.84; 95%CI 0.84–4.03). However, being in lower social classes conferred a 74% lower chance of reaching LDL-c targets (OR 0.26; 95%CI 0.07–0.92) and 81% lower chance of reaching NHDL-c targets (OR 0.19; CI95% 0.06–0.59) regardless of age, sex, marital status, and levels of physical activity.

**Conclusions:** There are differences between sexes regarding therapeutic targets for LDL-c and NHDL-c. However, social class appears to be a major determinant.

109784

Modality: E-Poster Researcher – Non-case Report

Category: HEART FAILURE/CARDIOMYOPATHY/TRANSPLANT

## Trabecular Bone Score and Body Composition in Elderly Patients with Heart Failure

RAFAEL BUARQUE DE MACÊDO GADÊLHA^1^, Mirela Ávila Litvin^1^, Lucian Batista de Oliveira^1^, Cybelle Luza Costa^1^, Jessica Myrian de Amorim Garcia^1^, Francisco Bandeira^1^

(1) Hospital Agamenon Magalhães

**Introduction:** Heart failure (HF) is associated with a higher risk of osteoporotic fractures, with few studies evaluating the factors related to this increase. The trabecular bone score (TBS) is a tool to assess bone microarchitecture, appearing as an independent predictor of fracture risk and gaining greater importance in populations where there is an increased risk of these events even with preserved bone mineral density (BMD).

**Objectives:** To evaluate TBS in elderly patients with HF, comparing it with BMD results and with echocardiographic and body composition parameters.

**Methodology:** Observational, cross-sectional and analytical study involving elderly individuals (≥65 years) diagnosed with HF, followed up in a cardiology service from August 2020 to August 2021. Patients with contact isolation, lipodystrophies or body mass index >37 kg/m² were excluded. All were submitted to dual energy X-ray absorptiometry (DXA) for analysis of body composition, BMD and TBS.

**Results:** Sixty patients were evaluated, with a mean age of 73.4 ± 5.3 years, 51.7% of which were female. The mean left ventricular ejection fraction (LVEF) was 46.5 ± 16.1%, with 40% having reduced LVEF (<40%). BMD showed osteoporosis (T-Score ≤ –2,5) in 38.3% and osteopenia (T-Score between –1.0 and –2.5) in 46.7%, while TBS showed bone degradation (TBS < 1,230) by 35% and partial degradation (TBS between 1.230 and 1.310) by 30%. There was a statistically significant association between some degree of degradation indicated by TBS with lower lumbar spine BMD (0.99 ± 0.22 g/cm² × 1.21 ± 0.24 g/cm²; p < 0.001), femoral neck (0.78 ± 0.15 g/cm² × 0.88 ± 0.08 g/cm²; p < 0.001) and total femur (0.86 ± 0.16 g/cm² × 0.98 ± 0.13 g/cm²; p = 0.006). There was no association between degraded/partially degraded TBS and lower LVEF means (47.49 ± 16.42% × 44.50 ± 15.66%; p = 0.523). Regarding body composition, an association was observed between degraded/partially degraded TBS with lower means of lean mass in the upper limbs (4.47 ± 1.04 kg × 5.16 ± 1.50 kg; p = 0.041).

**Conclusion:** Low BMD and changes in bone microarchitecture are frequent in elderly patients with HF. Degradation of bone microarchitecture was associated with a reduction in appendicular lean mass.

109799

Modality: E-Poster Researcher – Non-case Report

Category: DIGITAL HEALTH/INNOVATION

## Accuracy Index of Artificial Inteligence Platform in the Preparation of Electrocardiography Reports in Adult Intensive Care Unit

FIRMINO HAAG FERREIRA JUNIOR^1^, Rosa Maria da Costa Simões^1^, Diany Priscilla Oliveira^1^, Carolina Vieira^1^

(1) Hospital Geral de São Mateus

**Objective:** To analyze the accuracy index of electrocardiogram reports performed in Intensive Care, reported by an artificial intelligence platform, compared with reports performed by cardiologists in person. The electrocardiograms were randomly assigned and reported “blind” to the results provided by the artificial intelligence, and then both results were compared.

**Results:** Thirty (30) electrocardiograms of different patients were analyzed, randomly chosen, admitted to an intensive care unit, with a baseline diagnosis of cardiovascular diseases. Among the electrocardiograms analyzed, artificial intelligence was able to accurately diagnose 16 (sixteen) cases of inactive zone/myocardial infarction, 2 (two) cases of ST segment elevation, 2 (two) cases of atrial flutter, 5 (five) cases of atrial fibrillation, 5 (five) cases of diffuse changes in ventricular repolarization, 1 (one) case of left ventricular overload, 1 (one) case of anterior superior divisional hemiblock. Artificial Intelligence was able to identify electrode failures, as it did not consider the report. There was 100% compatibility of the artificial intelligence reports with the reports prepared by the cardiologists in person.

**Conclusion:** The introduction of new technologies such as artificial intelligence producing reports in electrocardiography is of significant importance, due to the reliability of the reports presented, not different when compared with reports prepared by experienced cardiologists in person, being therefore an important tool in helping to physicians in identifying and confirming the diagnosis, especially for non-specialists, often present in emergency services and intensive care units.

109805

Modality: E-Poster Researcher – Non-case Report

Category: DIGITAL HEALTH/INNOVATION

## Cardiobreath Application Slow Deep Breath for Health and Performance

CLAUDIA FETTER^1^, Maria Cláudia Irigoyen^3^, Liliane Appratto de Souza^1^

(1) Instituto de Cardiologia do Rio Grande do Sul; (2) TECNOPUC/RS; (3) Incor/USP

CardioBreath is an application for mobile and tablets available at Google Play and Apple Store (only in Portuguese). This application prescibes, teaches and evaluates slow deep breath for health and performance. The main objective of Cardiobreath is to offer to users an option for practicing respiratory exercises at rates slower than individual spontaneous rate in order to increase vagal modulation, decrease arterial pressure, increase strenght and resistance of respiratory muscles and cardiorespiratory fitness. Together these benefits may represent great achievements in health and performance. Handling CardioBreath App allows the user to be in touch with their own biological signals like respiratory rate and heart rate. For slow breath exercises prescription, some biological information like age, gender, weight, height (and body mass index is calculated inside the app), and including other conditions like smoking, sedentary life style, hypertension, diabetes, obstructive sleep apnea and cardiopaty make a score that drive users to their more reccomendable band of respiratory exercise. Users may chose on position: sit, standing or lying. Audio lessons are available about posture and respiratory technique (victoriuous brath/ujjayi pranayama from yoga). The prescription includes basic, intermedium and advanced exercises in order to identify progression stages. There is the option for the user to chose the respiratory exercise rate (cycles per minutes). The respiratory frequencies of exercise may vary from 15 to 2 cycles per minute using a metronome, either visual as bell sign, or audio lesson recorded. Other option is four frequencies of exercise with respiratory hold (or pause), either in inspiratory or expiratory hold, with metronome, bell or audio lesson. Users may follow their progress by a graphic of exercise respiratory rate X time of exercise, and other functionality available is heart rate before and after the exercises, which are also represented in graphic. And the most easy lesson of the application is available at the first screen, is the 17 minutes guided relaxation based on a specific miofascial and visceral relaxation. CardioBrreath was created in 2018 and a second version is being developed through a grant (Programa doutor Empreendedor/FAPERGS/CNPQ SEBRAE) and will be availabe in June. Any other information may be found at www.cardiobreath.com and includes more functionalities and gamification in order to increase adherence of users.

109813

Modality: E-Poster Researcher – Non-case Report

Category: DIGITAL HEALTH/INNOVATION

## Main Diagnoses Found and Potential for Acertivity in the use of Artificial Intelligence Platform in the Preparation of Electrocardiogram Reports in Adult Intensive Care Unit

FIRMINO HAAG FERREIRA JUNIOR^1^, Rosa Maria da Costa Simões^1^, Diany Priscilla de Oliveira^1^, Carolina Vieira^1^

(1) Hospital Geral de São Mateus

**Objective:** To analyze the main diagnoses found through the use of an artificial intelligence platform for electrocardiogram reports performed in adult intensive care. Patients were randomly selected from November 2021 to March 2022.

**Results:** Seventy-one (71) electrocardiograms of randomly chosen patients admitted to an intensive care unit with a baseline diagnosis of cardiovascular disease were analyzed. Among the analyzed electrocardiograms, artificial intelligence was able to accurately elaborate the main diagnoses: 13 (thirteen) patients had electrocardiographic alterations compatible with diffuse alteration of ventricular repolarization; 09 (nine) patients had a diagnosis of acute myocardial infarction with ST segment elevation; 12 (twelve) patients had atrial fibrillation; 7 (seven) patients had a diagnosis of subepicardial ischemia and 01 (one) patient had a diagnosis of atrioventricular block. Of the cases analyzed, 28 patients had an electrocardiogram within the normal range. Artificial intelligence was able to identify electrode failures, as it did not consider the report. There was 100% compatibility of the Artificial Intelligence reports with the clinical diagnoses of the patients.

**Conclusion:** In addition to the precise diagnosis that allowed guiding directed procedures in each case, artificial intelligence was significantly important due to the reliability of the reports presented, helping physicians in the identification and confirmation of the diagnosis, becoming an important tool in the approach of critical patients for physicians not specialists working in intensive care units

110123

Modality: E-Poster Researcher – Non-case Report

Category: NURSING

## Transitional Care Program for Patients with Heart Failure and Recent Hospitalization Improves Self-Care, Quality of Life and Knowledge of the Disease: Experience of a Public Center in Brazil

JULIANA DE MELO VELLOZO PEREIRA TINOCO^1^, Ana Carla Dantas Cavalcanti^1^, Bruna Lins Rocha de Padua^1^, Beatriz Paiva e Silva de Souza^1^, Tereza Cristina Felippe Guimarães^2^, Evandro Tinoco Mesquita^1^

(1) Universidade Federal Fluminense; (2) Instituto Nacional de Cardiologia

**Background:** Heart failure (HF), a syndrome that requires complex therapeutic regimens, is associated with high rates of hospitalization. Transitional care programs are crucial for encouraging patients to practice self-care and for minimizing preventable readmissions. These interventions are essential in promoting self-care for HF hospitalized patients. However, brazilian clinical evidence on patients from The “Sistema Único de Saúde” is still scarce.

**Objective:** To compare the effect of intervention with transitional care versus conventional hospital follow-up on self-care skills, knowledge of the disease, quality of life and depressive symptoms of patients hospitalized with HF.

**Methods:** A blind, randomized clinical trial of 74 patients with HF admitted to two quaternary-care hospitals in Rio de Janeiro was conducted. Criteria for inclusion: age ≥ 18 years; clinical diagnosis of HF, irrespective of etiology; hospitalization for decompensated HF. Criteria for exclusion: hemodynamic instability; neurological/cognitive impairment reported in medical records; participation in previous studies involving educational interventions; perioperative hospitalization; transfer to another hospital; undergoing preparation for heart transplantation. The intervention group (IG) received transitional-care follow-up in the form of five encounters during hospitalization and weekly post-discharge telephone calls for four weeks. The control group (CG) received conventional hospital follow-up. Outcomes scored: self-care, quality of life, knowledge of the disease and depressive symptoms within 30 days post-discharge. Blinding was achieved by designating separate teams for evaluation, randomization, and intervention. Sample size calculation was based on a pilot study of 70 patients. Data were treated with repeated-measures ANOVA and Fisher’s exact test.

**Results:** Thirty days post-discharge, the scores for IG patients, compared with CG counterparts, were higher for self-care maintenance (74.3 vs. 44.2; p < 0.001), self-care confidence (79.3 vs. 56.4; p < 0.001), knowledge of the disease (41.3 vs. 27.5; p < 0.001), and lower for quality of life (42.1 vs. 64.5; p < 0.001). There was no effect on self-care management skills and depressive symptoms.

**Conclusion:** The transitional-care program improved the outcomes self-care maintenance, self-care confidence, knowledge of the disease and quality of life. Study is registred in Brazilian Registry of Clinical Trials (RBR-2dpc6b).

109845

Modality: E-Poster Researcher – Non-case Report

Category: NEGLECTED CARDIOVASCULAR DISEASES

## Use of Pet-Ct to Investigate the Genesis of Ventricular Arrhythmias in Chagas Disease

RENÉE SARMENTO DE OLIVEIRA^1^, Renée Sarmento de Oliveira^1^, Renata Junqueira Moll Bernardes^1^, Adriana Soares Xavier de Brito^1^, Paulo Henrique Rosado de Castro^1^, Martha Valéria Tavares Pinheiro^1^, Sergio Salles Xavier^2^, Otacilio da Cruz Moreira^2^, Flavia Vernin de Oliveira Terzi^1^, Andréa Silvestre de Sousa^1^

(1) Instituto D’or de Pesquisa e Ensino; (2) Instituto Oswaldo Cruz/Fiocruz

**Background:** Chagas cardiomyopathy is the most frequent and potentially severe manifestation of Chagas disease, with the highest morbidity among neglected tropical diseases. Although endemic in Latin America, it has become a global problem due to the migration of individuals to non-endemic areas. Sudden death is the main mechanism of death in chronic Chagas cardiomyopathy, being associated with ventricular tachycardia and may even occur in patients in the early stages of heart disease. In addition to fibrosis, persistent inflammation may be implicated in the genesis of arrhythmias. Strategies that identify patients at greater risk of developing complex ventricular arrhythmias in their different mechanisms of presentation would be able to guide the prophylaxis of sudden death more appropriately in chronic Chagas cardiomyopathy. Recent studies showed promising results using radionuclide imaging for the identification of areas of inflammation in the myocardium of patients with non-ischemic cardiomyopathies, such as sarcoidosis.

**Objective:** To correlate the presence of persistent myocardial inflammation with FDG-18F and DOTATOC-68Ga PET-CT with the severity of ventricular arrhythmias and the presence of parasite in patients with chronic Chagas‘ heart disease.

**Methods:** Two groups were included, totaling 24 patients. Group 1 consisted of patients with sustained ventricular tachycardia who required implantable cardioverter-defibrillator (ICD). Group 2 was the comparison group composed of patients at the same stage of Chagas heart disease (B1, B2 and C) but without complex ventricular arrhythmia. All patients underwent FDG-18F and DOTATOC-68Ga PET-CT, Holter, and polymerase chain reaction for trypanosoma cruzi.

**Results:** The presence of the parasite in chronic Chagas cardiomyopathy was higher in patients from the ICD group (66.7%) compared to group 2 (33.3%). There was no statistical difference between the uptake of FDG-18F and DOTATOC-68Ga by PET-CT in both groups. Physiological increased uptake of FDG-18F may have jeopardized the analysis in some patients.

**Conclusion:** The persistence of chronic parasitemia in Chagas‘ heart disease may be implicated in the genesis of arrythmia, increasing the risk of sustained ventricular arrhythmia; however, larger studies are necessary to investigate this association. PET-CT with FDG-18F and DOTATOC-68Ga are not currently recommended as markers or inflammation in Chagas cardiomyopathy.

111116

Modality: E-Poster Researcher – Non-case Report

Category: SPIRITUALITY AND CARDIOVASCULAR MEDICINE

## Willingness to Forgive in Patients with Chronic Coronary Syndrome

ADELLE CRISTINE LIMA CARDOZO^1^

(1) Federal University of Sergipe; (2) Rede D’Or São Luiz – São Lucas Hospital; (3) Primavera Hospital

**Introduction:** Despite advances in treatment, chronic coronary syndrome (CCS) persists as an important cause of morbidity and mortality worldwide. There is evidence of the influence of the disposition to forgive in the processes of illness and healing of patients with cardiovascular disease, including myocardial ischemia.

**Objectives:** To evaluate the willingness to forgive in patients with CCS and its association with Spirituality/Religiosity.

**Methodology:** This is an observational, cross-sectional, analytical study, whose sample included patients with CCS assisted at the cardiology outpatient clinics of three hospitals in Sergipe. Two scales were applied: DUREL (Duke Religious Index) and the BMMRS (Brief Multidimensional Measure of Religiosity and Spirituality), whose domain number 3 assesses forgiveness. This domain is composed of three items: I) disposition to self-forgiveness; II) disposition to forgive those who offend us; III) belief in God’s forgiveness. Each item has an ordinal scale from 1 to 4, corresponding to responses: Never, Rarely, Often, and Always, respectively. The level of forgiveness is determined from the sum of the three items and ranges from 3 to 12. Student’s t test was used for comparison between the groups, with the significance level set at 0.05.

**Results:** Fifty-three patients with CCS were included, of which 50.9% were female. The mean age of patients was 62.2 ± 10.5 years. Regarding the clinical profile, 81.1% were hypertensive, 80.0% dyslipidemic and 46.2% had diabetes mellitus. Regarding the religious profile, 65.4% were Catholic, 23.1% Evangelical, 5.8% Spiritualist, 1.9% Umbandaist, 1.9% Atheist, and 1.9% believed in God but did not belong to any religion. The patients‘ religiosity was evaluated using the DUREL, and was divided into organizational, non-organizational, and intrinsic religiosity. Patients with high levels of organizational religiosity, non-organizational religiosity, and intrinsic religiosity showed higher levels of forgiveness compared to patients with low levels of religiosity in these categories (11.1 vs. 9.8; p < 0.05) (10.8 vs. 7.0; p < 0.001) (11.0 vs. 8.7; p = 0.001).

**Conclusions:** The results show that CCS patients who have higher levels of religiosity are more likely to forgive themselves, forgive others, and believe in divine forgiveness when compared to patients with lower levels of religiosity.

109864

Modality: E-Poster Researcher – Non-case Report

Category: CARDIOVASCULAR SURGERY

## Risk Score Elaboration for Stroke in Cardiac Surgery

ELLEN HETTWER MAGEDANZ^1^, João Carlos Vieira da Costa Guaragna^2^, Luciano Cabral Albuquerque^4^, Fernanda Lourega Chieza^3^, Brenda Gonçalves Donay^1^, Luize Mancuso Silva^1^, Jasseane De Borba Sparremberger Vitt^1^, Luiz Carlos Bodanese^1^

(1) PUCRS; (2) Hospital Divina Providência; (3) Santa Casa de Misericórdia de Porto Alegre; (4) Hospital São Lucas PUCRS

**Introduction:** Stroke is a complication that causes considerable morbidity and mortality during the heart surgery postoperative period (incidence: 1.3 to 5%; mortality: 13 to 41%). Models for assessing the risk of stroke after heart surgery have been proposed, but most of them do not evaluate postoperative morbidity.

**Objective:** The aim of this study was to develop a risk score for postoperative stroke in patients who undergo heart surgery with cardiopulmonary bypass.

**Methods:** A cohort study was conducted with data from 4,862 patients who underwent surgery from 1996 to 2016. Logistic regression was used to assess relationships between risk factors and stroke. Data from 3,258 patients were used to construct the model. The model’s performance was then validated using data from the remainder of the patients (n = 1,604). The model’s accuracy was tested using the area under the receiver operating characteristic (ROC) curve.

**Results:** The prevalence of stroke during the postoperative period was 3% (n = 149); 59% of the patients who exhibited this outcome were male, 51% were aged ≥ 66 years, and 31.5% of the patients died. The variables that remained as independent predictors of the outcome after multivariate analysis were advanced age, urgent/emergency surgery, peripheral arterial occlusive disease, history of cerebrovascular disease, and cardiopulmonary bypass time ≥110 minutes. The area under the ROC curve was 0.71 (95% confidence interval 0.66–0.75).

**Conclusion:** We were able to develop a risk score for stroke after heart surgery. This score classifies patients as low, medium, high, or very high risk of a surgery-related stroke.

110935

Modality: E-Poster Researcher – Non-case Report

Category: CARDIOLOGY OF SPORTS, EXERCISE, ERGOMETRY AND CARDIOVASCULAR REHABILITATION

## Physical Active Patients with Stable Coronary Heart Disease Show Distinct Gut Microbiota Profiles

ELISA ALBERTON HAAS^1^, Elisa Alberton Haas^1^, Wilson José Fernandes Lemos Junior^2^, Laura Treu^3^, Andrey Santos^4^, Mário José Abdalla Saad^4^, Francisco Rafael Martins Laurindo^1^, Protásio Lemos da Luz^1^

(1) Instituto do Coracao (InCor), Hospital das Clinicas HCFMUSP, Faculdade de Medicina, Universidade de Sao Paulo, Sao Paulo, SP, Brazil; (2) Faculty of Science and Technology, Libera Università di Bolzano, Bolzano, Italy; (3) Biology Department, University of Padova, Italy; (4) Department of Internal Medicine, State University of Campinas (UNICAMP), Campinas, SP, Brazil

**Background:** Regular physical activity supports cardiovascular disease (CVD) prevention through mechanisms that include: influences on inflammatory profile, on the autonomic nervous system and endothelial modifications. One potentially beneficial and overlooked mechanism of physical activity is its effects on the gut microbiota.

**Methods:** This was a cross-sectional cohort analysis of 39 participants of a larger cohort (registered at ClinicalTrial.gov, NCT03232099), evaluating their baseline characteristics. Participants had established coronary artery disease (CAD), were male, aged 46–69 years, had BMI < 30, were stable and non-symptomatic. The cohort was divided into two groups: 21 physically active (PA) participants and 18 physically inactive (PI). The gut microbiota was evaluated with 16S amplicons, and the level of self-reported physical activity was calculated in metabolic equivalents (MET)-hour/week score (METs.h/wk). Patients were considered physically active when levels of Mets.h/wk were >5. A 3-day food frequency questionnaire obtained nutritional information regarding total calories, macronutrients and micronutrients.

**Results:** The gut microbiota profile of PA and PI individuals were clearly separated by sparse partial least squares discriminant analysis. The main taxa that differed the PA group were Ruminococcaceae_UCG-014 and Coprococcus_2 eutactus, which are associated with reduced visceral adiposity and with butyrate production, respectively. Meanwhile, nutrient consumption was similar in the two groups.

**Conclusions:** In patients with CAD, physical activity was associated with different and putative beneficial gut microbiota profile. These findings indicate that gut microbiota modifications further support the healthy mechanisms promoted by physical activity to reduce CVD.


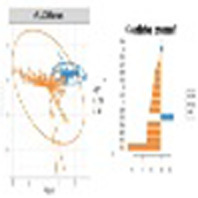


109902

Modality: E-Poster Researcher – Non-case Report

Category: NURSING

## Validation of an Educational Strategy for Capacitation of the Nursing Team on Management of Oral Anticoagulants

ADRIANA DA COSTA COELHO^1^, Dasymar Martins da Silva Lucas^1^, Milena Preissler das Neves^1^, Geovane de Kassio Nunes^2^, Renata Flavia Abreu da Silva^3^

(1) Federal Hospital for State Servants – HFSE; (2) Hospital Pro Cardiac; (3) Federal University of the State of Rio de Janeiro – UNIRIO

**Introduction:** The oral anticoagulants are widely prescribed drugs for treatment and prophylaxis of thrombotic diseases, and the antagonists of vitamin K, such as Warfarin, the main protagonists of this pharmaceutical class. Anticoagulation therapy requires specific knowledge from the nursing team for its safe management and training in service is necessary for the improvement of a qualified care, and constitutes a foundation between practice performed by the professionals and their instrumentalization.

**Objective:** Describing the validation of an educational strategy for capacitation of the nursing team on the management of oral anticoagulants.

**Method:** Methodological study with elaboration of tools such as word search puzzles and a checklist to be performed by the nursing team in the administration of oral anticoagulants, that was based on an integrative review. The theoretical validation of the products by experts was made by online questionnaire. The content validity index acceptable must be higher than 0.70.

**Results:** The 16 experts agreed to take part in the research and evaluate the tools. The subject’s profiles were: nurses with professional degrees between 14 and 20 years (43,8%) and working with anticoagulants between 8 and 11 years (37,8%); 8 (50%) of them participated in scientific events about oral anticoagulants in the last 5 years. The Content Validity Index by the experts was 0.83. The suggestions were accepted and modified for the final version of the tools. The statements that showed greater agreement (93,4%) were the ones stating the Content, that included nursing care management. The one that had the minor agreement was the item about the Title clarity (73,4%). Despite the high level of agreement, some experts suggested a few improvements to the word search puzzle, such as: replace the “Stop” sign carried by the character for an “Attention” one, and re-elaborate some sentences.

**Conclusion:** The word search puzzle has been theoretically validated, therefore it should be used as an educational strategy for the patient’s care in use of the oral anticoagulation by the nursing team.

109914

Modality: E-Poster Researcher – Non-case Report

Category: PHYSICAL EDUCATION

## Viability of Hemodynamic Measures and Psychossocial Factors Screening in Gym Centers: Pilot Study

CLAUDIA FETTER^1^, José Antonio Caçapietra Reteguy^1^, Juliana Bertoletti^1^

(1) Instituto de Cardiologia do Rio Grande do Sul

**Introduction:** Sedentary lifestyle presents deleterious effects over human health and this condition may be reverted through the regular practice of physical exercise. Regular exercise practice offers benefits beyond physical health, with evidence to the improvement of psychosocial factors such as anxiety, depression and stress. Gyms are places destined to the practice of physical exercises, although most of it does not perform the follow-up of benefits to physical and mental health. Besides that, the strong appeal to the search of the perfect body very prevalent in this environment may exert a reverse effect of dissatisfaction with body image.

**Method:** This pilot study about the viability of follow up of hemodynamic measures (blood pressure and heart rate) psychosocial factors through the DASS-21 (anxiety, depression, stress) and (brazilian scale of silhouette (body image) in gyms recruited 24 sedentary individuals for 4 weeks (men and women) with two weekly sessions of resistance exercises, and evaluated these psychosocial factors in order to verify the efficacy of the exercises and the viability of the follow up. 24 individuals, 12 women with mean age of 51 years and 12 men with mean age of 45 years took part in this research, assessed in one moment (T1) pre wash out of 4 weeks maintaining the sedentary condition, moment T2 immediately before initiating exercise and moment T3 at the end of the exercise intervention. The instruments used for the assessment were: Depression, Anxiety and Stress Scale (DASS-21) and the Brazilian Scale of Silhouettes for Adults. Data analysis was performed by GEE and significance level of p 0,05.

**Results:** The results of the assessments of T3 were significant in relation to T2 for the variables Body Mass Index (BMI), Systolic Arterial Pressure (SAP) and Diastolic (DAS) and the three variables of DASS 21(anxiety, depression, stress) and relation of actual silhouette to body mass index, both for women and men.

**Conclusions:** These results suggest that the practice of two weekly sessions of resisted exercise for 4 weeks may have a beneficial effect over psychosocial factors and that it is viable and recommended to implement the follow up of these variables in the context of gyms. Besides, the difference between actual silhouette and BMI very much closer at moment T3 suggests that the positive effect of exercise plays an important role over body image. Data found about blood pressure are promising but better understanding is needed.

109930

Modality: E-Poster Researcher – Non-case Report

Category: CARDIOLOGY OF SPORTS, EXERCISE, ERGOMETRY AND CARDIOVASCULAR REHABILITATION

## Resistance Exercise and Ischemic Preconditioning on Endothelial Dysfunction Caused by Ischemic/Reperfusion Injury in Young Adults and Elderly

FÁBIO TANIL MONTREZOL^1^, Daniel Bannel^2^, Helen Jones^2^, Alessandra Medeiros^1^, David Low^2^

(1) Federal University of Sao Paulo; (2) Liverpool John Moores University

Ischemic events can cause damage. However, reperfusion can cause more damage. This cluster of injuries are named ischemia/reperfusion injury (IRI). Studies show that short intermittent periods of ischemia can produce beneficial effects, named ischemic preconditioning (IPC), this strategy can reduce the area and injury severity. In humans, IPC have shown reductions on the deleterious effects of an IRI, including in the myocardium. In animal models, previous IPC can preserve systolic function after an IRI. IPC can be applied only remotely (R-IPC) in humans to preserve the myocardium. Then, resistance exercise can be a potential toll to produce such effect. The present study aimed to compare the effects of R-IPC and resistance exercise in lower limbs on the vascular function after a bout of IRI. 13 participants aged 23.61 ± 2.95 (Young group – YG) and 7 participants aged 62.83 ± 2.63 (Elderly group – EG) were evaluated. The protocol was approved by the Liverpool John Moores University (19/SPS/025). Participants showed at the lab in 3 non-consecutive days with interval of at least 72 hours during the morning. The sessions were randomized. Session structure was: participants rested for 20 minutes, then underwent to a brachial artery flow-mediated dilation analysis (FMD), then received the experimental treatment: Control visit rested for 40 minutes, R-IPC visit, cuffs were placed at the medial part of the thighs then inflated for 5 minutes at a pressure of 220 mmHg and deflated for 5 minutes for 4 times, at the Squatting visit performed 5 minutes of squatting without load with cadence of 20 squats per minute and rested for 5 minutes for 4 times. After, for the IRI, a cuff was placed in the humeral midpoint and inflated at 220 mmHg for 15 minutes, after deflation, the reperfusion occurred for 15 minutes. Then, FMD was repeated. 2-factor analysis of variance was performed, p ≤ 0.05. Results are shown in figure 1. We concluded that IRI can produce temporary vascular dysfunction, such dysfunction can be abolished by R-IPC in young adults and in elderly resistance exercise can abolish deleterious effects of IRI.


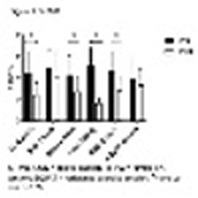


109955

Modality: E-Poster Researcher – Non-case Report

Category: NURSING

## Multicenter Registry of Hypertension in Children and Adolescents (Hasca): A Redcap Approach

JACQUELINE VAZ ALENCAR^1^, Emily Justiniano^6^, Nicole Saldanha de Souza^5^, Liliana Fortini Cavalheiro Boll^1^, Luiza Junqueira Trarbach Lovato^2^, Renata Póvoas^3^, Danielle Dias^8^, Fernanda Consolim-Colombo^4^, Katia de Angelis^8^, Maria Cláudia Irigoyen^3^

(1) Instituto de Cardiologia da Fundação Universitária de Cardiologia (IC/FUC) – Porto Alegre, RS – Brasil; (2) University of Technology Sydney (UTS) – Austrália, Sidney; (3) Instituto do Coração da FMUSP (InCor) – São Paulo, SP – Brasil; (4) Universidade Nove de Julho (Uninove) – São Paulo, SP – Brasil; (5) Universidade Federal de Ciências da Saúde de Porto Alegre (UFCSPA) – Porto Alegre, Rio Grande do Sul – Brasil; (6) Hospital de Clínicas de Porto Alegre (HCPA) – Porto Alegre, RS – Brasil; (8) Universidade Federal de São Paulo (Unifesp) – São Paulo, SP – Brasil

**Background/Introduction:** Hypertension is asymptomatic in childhood and adolescence. We used REDCap to organize the Multicenter Registry of Hypertension in Children and Adolescents (HASCA) survey. Nine Teaching/Health/Research, distributed in different regions, participated in data collection at the national level.

**Purpose:** This study analyzes the national data reported from the REDCap platform and optimizes the obtained results.

**Methods:** This is a Longitudinal observational multicenter study of the registry type using the REDCap platform to implement data from 9 national centers, with children and adolescents aged 7 to 18 years, both genders, from public or private schools, with altered blood pressure indicative of hypertension in any level. On the REDCap platform, the input of the data occurred in different stages: – Screening: form for collecting identification data, anthropometric data, and blood pressure values. – Confirmatory: confirmation of blood pressure values. – Registry: one-year follow-up and systematic data collection. The data were collected electronically and inserted in the REDCap software, a safe methodology. The study follows ethical principles and guidelines for good clinical practice.

**Results:** The HASCA Registry CRF consists of a total of 180 questions. Screening data were collected from July 2018 to December 2019, with 4398 Brazilian children and adolescents, of which 915 had high BP at least twice.

**Conclusions:** It is possible to make a report with HASCA information generated throughout Brazil by Regions, Coordinating Centers, or by Schools to identify where and which children have high blood pressure levels and need to be monitored.

109983

Modality: E-Poster Researcher – Non-case Report

Category: SPIRITUALITY AND CARDIOVASCULAR MEDICINE

## Application of the Story Drawing in the Research on Spirituality in the Field of Health: Adhesion to the Treatment of Arterial Hypertension and Diabetes Mellitus

GILMARA RIBEIRO SANTOS RODRIGUES^1^, Fernanda Sampaio Novaes^2^, Gabriella da Cruz Silva Dias^2^, Cláudio Luiz Anunciação Santos Junior^2^, Paula Silva Peixoto^2^, Luciana Feitosa^1^

(1) Universidade Federal da Bahia; (2) Escola Bahiana de Medicina e Saúde Pública

**Introduction:** Spirituality as an object of study of science in the health area is a recent approach. It consists of an individual’s strategy to transcend the process of illness, losses and existential emptiness. It is anchored in Brazilian culture and is part of common sense, influencing beliefs, behaviors and worldview. It was considered the assumption that there is a possibility of spirituality influencing the adherence to treatment of people with arterial hypertension and diabetes mellitus.

**Objectives:** To identify whether the representations of people with arterial hypertension and diabetes mellitus about religiosity/spirituality can influence non-adherence to treatment and describe the religious/spiritual practices of these people.

**Method:** Qualitative and quantitative exploratory research, carried out at a philanthropic medical center in the city of Salvador, Bahia, Brazil, in 2019. Participants were individuals over 18 years of age with arterial hypertension and diabetes. Data were collected through semi-structured interviews and the Drawing-Story with Theme procedure. A form consisting of three sections was used, the first with an approach to sociodemographic and clinical characterization data. The second with the guiding questions: (1) How does religiosity/spirituality influence adherence to the treatment of arterial hypertension and diabetes mellitus? (2) What religious/spiritual practices do you use related to DM and AH treatment? (3) What kinds of religious/spiritual practices do you think other people use related to DM and AH? (4) What types of religious/spiritual beliefs can interfere with adherence to treatment for AH and DM? (5) Do you consider that there is any religious/spiritual practice that can contribute to non-adherence to the treatment of AH and DM? And the third contains the guidelines for the design of a themed story. Thematic content analysis was used.

**Results:** The data from the interviews were aggregated into two categories: (1) Spiritual/Religious Practices that Assist in Adherence to Treatment and (2) Treatment Dissociated from Faith.

**Conclusion:** There are several representations about spirituality/religiosity and adherence to the treatment of arterial hypertension and diabetes, however, it is clear that people believe in curing the disease and have hope regardless of the conduct they choose for their treatment.

110030

Modality: E-Poster Researcher – Non-case Report

Category: DYSLIPIDEMIA

## Reference Intervals of Total Cholesterol and Fractions for Brazilian Adults According to the National Health Survey

ANA CAROLINA MICHELETTI GOMIDE NOGUEIRA DE SÁ^1^, Elton Junio Sady Prates^2^, Pedro Cisalpino Pinheiro^3^, Deborah Carvalho Malta^1^

(1) Graduate Program in Nursing, Federal University of Minas Gerais (UFMG). Belo Horizonte, MG, Brazil; (2) School of Nursing, Federal University of Minas Gerais (UFMG). Belo Horizonte, MG, Brazil; (3) Faculty of Medicine, Federal University of Minas Gerais (UFMG). Belo Horizonte, MG, Brazil

**Introduction:** The reference values of laboratory tests are defined by two threshold classes, the reference intervals (RI) derived from a healthy population and the clinical decision limits, in which a medical decision is recommended. Lipid reference limits were established by clinical studies of cardiovascular outcomes in which clinical decision limits were defined. It should be noted that lipid reference values were collected from studies in developed countries and lipid levels are influenced by demographic, environmental, genetic, ethnic, lifestyle factors and chronic diseases. Therefore, even having defined decision limits, it has been encouraged to build lipid RIs specific to the population that will be applied. However, determining RI is a challenge because it requires methodological rigor, such as a representative sample of the population and care in collection and analysis. In Brazil, international IR are used. The National Health Survey (PNS) carried out laboratory tests, thus, it was possible to establish, in an unprecedented way by non-parametric methodology, RI of total cholesterol (TC), low-density lipoprotein (LDL) and high-density lipoprotein (HDL) for Brazilians.

**Objective:** To estimate RI of TC and fractions for Brazilian adults.

**Methods:** Cross-sectional study, with data from the PNS, between 2014–2015, in 2,976 adults. To establish RI, exclusion criteria were applied (pregnancy; smoking; chronic diseases), outliers were removed (Tukey’s method) and partitioned (gender; age; race/color). RI were estimated considering 95% of healthy individuals, the lower limit (LL) corresponded to the 2.5th percentile and the upper limit (UL) to 97.5. Differences were evaluated using the Mann Withney and Kruskal Wallis tests (p ≤ 0.05).

**Results:** HDL RI (mg/dL) were higher in women (29–74) than in men (25–67). Men had lower TC and LDL RI (mg/dL) between 20 and 39 years (115–239; 51–151) compared to 40 and 59 years (131–250; 57–156) and 60 years or older (125–252; 64–156). Women had higher RI for TC and LDL (mg/dL) at age 60 years or older (128.5–264; 59–172) and for HDL (mg/dL), UL were higher between 40 and 59 years (29–75) than at 20 to 39 years old (29–74) and in white race/color (29–75) compared to brown (29–73).

**Conclusion:** Own lipid RI evidence the health conditions of Brazilian adults and can support the adequate identification of dyslipidemias and prevention of cardiovascular disease.

110043

Modality: E-Poster Researcher – Non-case Report

Category: NEGLECTED CARDIOVASCULAR DISEASES

## T1 Mapping CMR Imaging Techniques and Their Association with Ventricular Arrhythmias and Sudden Death in Patients in the Early Stage of Chagas Heart Disease

MARTHA V T PINHEIRO^1^, Renata Junqueira Moll-Bernardes^1^, Gabriel Cordeiro Camargo^1^, Marcelo Teixeira de Holanda^2^, Luiz Henrique Conde Sangenis^2^, Andrea Silvestre de Souza^2^

(1) ID’Or; (2) FIOCRUZ; (3) Instituto Nacional de Cardiologia

**Background:** Chagas disease involves progressive myocardial inflammation leading to myocardial fibrosis, which may predispose to sudden cardiac death. Although focal fibrosis can be detected by late gadolinium enhancement (LGE) in cardiac magnetic resonance (CMR), this technique is not sensible to detect diffuse interstitial fibrosis.

**Objectives:** The goal of this study was to assess the prognostic value of extracellular volume (ECV) by CMR in predicting ventricular tachycardia (VT) and sudden death in a 4-year follow-up period in Chagas disease.

**Methods:** This prospective cohort study included patients in the early stages of Chagas disease. Myocardial fibrosis assessment by CMR with measurement of ECV was performed. The patients were followed on an outpatient basis and submitted periodically to electrocardiogram and Holter for a period of 4 years. The combined primary outcome was cardiac sudden death, sustained ventricular tachycardia, or cardioverter-defibrillator (ICD) implantation.

**Results:** A total of 47 patients were included; the mean age was 58,6 + 10,4 and 44,7% were male. The ECV maintained an association with the presence of non-sustained VT, a surrogate outcome, even after adjustments for fibrosis mass and left ventricular ejection fraction assessed by CMR. Over the follow-up, 3 patients died suddenly (6,4%) and 2 (4,3%) had sustained VT. These patients had a mean of 45,2 + 21,1 non-sustained VT events (group 1) versus 2,29 + 1,0 (p = 0,001) in the group which not reached the combined endpoint (group 2). In group 1, the mean ECV value was 29,5 + 3,8 and 26,7 + 3,4 (p = 0,04).

**Conclusion:** ECV could be an early marker of increased risk of ventricular arrhythmia in the early stage of Chagas disease, presenting an independent association with NSVT, as a predictor of adverse outcomes.

110045

Modality: E-Poster Researcher – Non-case Report

Category: HEMODYNAMICS AND INTERVENTIONAL CARDIOLOGY

## Long Term Clinical Outcomes After Implantation of Absorb BVS in a Real World Setting, with Pre Dilatation and Guided by Intravascular Imaging

SERGIO GUSTAVO TARBINE^1^, Costantino R Costantini^1^, Costantino O Costantini^1^, Vinicius Shibata^1^, Marcos Denk^1^, Rafael Macedo^1^, Marcio M. Luize^1^, Everton CArdoso Dombek^1^

(1) Hospital Cardiologico Costantini

**Background:** The safety and performance of the Absorb Bioresorbable Vascular Scaffold (BVS) has been previously demonstrated with clinical data. However, these trials included patients with simple lesions. The Absorb III trial demonstrated an excess of adverse events following BVS implantation. Aiming to evaluate clinical outcomes, we analyzed the treatment of real world patients using optimal technique and intravascular image guidance in all cases, at long term follow up from a single center.

**Methods:** Observational retrospective study, in a single Brazilian center, from 12/2014 to 12/2017, including 128 patients treated with BVS implantation. Safety and efficacy outcomes were analyzed in the in-hospital and late follow-up (5 years).

**Results:** All 128 patients completed 4 years 3 months follow-up. Mean age was 58,2 years, 85,9% of the patients were men, and 28,1% were diabetic. Regarding clinical presentation, 54,6% had stable angina or silent ischemia. Intravascular imaging (IVUS-OCT) was used in all cases. Lesion preparation included balloon PTCA, and when necessary Cutting balloon and PTCRA. Device success was achieved in 100% of cases with 99,2% overall procedure success rate (1 case of sub acute thrombosis). Long term major adverse cardiovascular events rate were (including hospital stage): Cardiac death 1,5%, acute stent thrombosis 0,78%, MI 2,34%,TVR 16,4%.

**Conclusions:** The analysis of this cohort of pts, in a real world setting with more complex scenarios, showed so far to be safe and effective at late follow-up using an enhanced technique, including intravascular imaging in all cases. Wether these results are durable beyond 5 years will be reported. Keywords: Percutaneous Coronary Intervention, Absorbable Implants/utilization, Everolimus, Coronary Artery Disease, Clinical Evolution.

110059

Modality: E-Poster Researcher – Non-case Report

Category: EPIDEMIOLOGY AND HEALTH POLICIES/GLOBAL HEALTH

## Acute Myocardial Infarction Care Program and Its Impact on Mortality in the Public Health Network

ANA KAROLINA QUEIROZ DE SOUZA RICARDO^1^, Natanael Barbosa dos Santos^3^, Sara Carolline Gomes de Araújo Lima^2^, Patrícia Caldas de Oliveira^1^, Eliel Bezerra da Silva Júnior^1^, Antonio Fernando Barros Pereira Junior^4^, Isabela de Angelles Floro Alonso^1^, André Arthur de Souza Lima^3^, Marcelo Menezes Malta^1^, Bibiana Toshie Oniki de Mendonça^2^, Monteiro Pires Bastos Junior^3^

(1) Hospital do Coração de Alagoas; (2) Centro Universitário Tiradentes de Alagoas; (3) Centro Universitário Cesmac; (4) Universidade Estadual de Ciências da Saúde de Alagoas

**Introduction:** Cardiovascular diseases are important causes of morbidity and mortality worldwide. In 2019, in Brazil, 95,557 deaths were due to acute myocardial infarction, the main etiology. Treatment systems in STEMI aim to improve care and survival; there is a lack of data on their performance.

**Objective:** To evaluate the impact of the institution of a STEMI care program on mortality in the public health network of Alagoas.

**Methods:** Observational, analytical and cross-sectional study, authorized by the CEP (opinion No. 3,283,780), data tabulated between January 2017 and June 2019, analyzed using the Mann-Kendall test, comparison of factors by regression and multiple logistic regression. Census sample of 712 participants.

**Results:** The mean age was 64.3 (±13.1) years and 58.3% were men and 31.3% of the sample was treated; the main cause of non-treatment was symptomatology >12h in 43.5% of the untreated. The prevalence of risk factors (RF) was: 68.3% hypertensive, 36.5% diabetic and 29.4% smokers. The in-hospital death rate was 5.6%. Temporal trends were observed for treated and untreated groups: at time 1, increasing trend for both treated (p < 0.001) and untreated (p = 0.04); while at time 2, there was no increasing or decreasing trend (p > 0.05); at time 3, there is an increasing trend (p < 0.001) for both groups. At time 4, for treated patients, there is a significant decreasing propensity (p = 0.02). Time 5 reinforces a decreasing trend (p = 0.008). Times 6 and 7, no trend was observed. However, at time 8, there is a decreasing trend in the treated group (p = 0.009). At time 9, there is a significant drop in time (p = 0.003).

**Conclusion:** It is evident that the care network is viable and allows better access to STEMI treatment. Hospital mortality was similar to systems in developed countries. There is a need for population education revealed by the significant increasing trend of pre-hospital time. There was an improvement in care for the treatment of STEMI, demonstrated by the significant downward trend at times 4, 5 and 8. In addition, the data contribute to helping the construction of public health policies, as well as guiding prevention strategies, especially for those with RF.

110062

Modality: E-Poster Researcher – Non-case Report

Category: HYPERTENSION/RENAL DENERVATION

## Pulse Wave Velocity Response from Renal Denervation – A Systematic Review

LÍLIAN SOARES DA COSTA^1^, Valerio Fuks^4^, Maria gabriela Pimenta dos Santos^2^, Julia Resende de Oliveira^2^, David Ferreira de Lima Duarte^2^, Antonio Carlos Eberienos Assad Filho^2^, Gabriela Gama Zagni Jardim^3^, Paola Pugian Jardim^4^, Andréa Vaospasse Cocco Faria^4^

(1) Universidade Estacio de Sá/IDOMED, Campus Città e Presidente Vargas e Instituto Estadual de Cardiologia Aloysio de Castro/IECAC; (2) Universidade Estacio de Sá/IDOMED, Campus Città; (3) Universidade Estacio de Sá/IDOMED, Campus Vista Carioca; (4) Instituto Estadual de Cardiologia Aloysio de Castro/IECAC

**Introduction:** Renal denervation (RDN) has been shown to be effective in reducing BP in treatment-resistant hypertension. Ambulatory blood pressure (ABPM) and central blood pressure (BP) are better predictors for overall cardiovascular risk and mortality than brachial BP. Renal denervation (RDN) has been shown to reduce these pressure parameters, but data on central ambulatory BP and arterial stiffness (AS) analysis after RDN are limited and have to be highlighted.

**Objective:** A systematic review on the role of arterial stiffness (AS) in renal denervation (RDN) treated-resistant hypertensive.

**Methods:** PubMed and Scielo databases, using the descriptors “denervation” AND “hypertension” AND “pulse wave velocity”. Twenty-two citations were identified and being considered.

**Results:** Some studies sought to investigate the effect of RDN in patients with treatment-resistant hypertension according to the established definition and confirmed by 24-h ABPM. These data indicate that RDN may reduce office and 24-h ABPM substantially in patients with moderate treatment-resistant hypertension. Central ambulatory BP is reduced and ambulatory assessed averaged daytime pulse wave velocity (PWV) improved after RDN and total vascular resistance decreased. Multivariate analysis showed that short-term effects on PWV were BP-related, whereas during 6 months follow-up, improvement of PWV becomes BP-unrelated. RDN improves peripheral and central blood pressure as well as AS and, thus, may improve cardiovascular outcome. The BP changes were associated with reductions in peripheral resistance, whereas cardiac output, plasma renin, aldosterone levels and renal function remained unchanged. The observed effects were not explained by an increased intake of antihypertensive medications. RDN did not result in a statistical significant effect on end organ damage 12 months after treatment. The statment that the arterial stiffness beneficial was observed during follw-up, assumed the supposition that an improvement of arterial mechanical properties coul be related to a reduced sympathetic arterial drive.

**Conclusion:** Extended assessment of AS can help improve patient preselection for renal sympathetic denervation and identify which subgroup of hypertension patients will benefit by sympathetic modulation. RDN improves brachial and central ambulatory BP, arterial stiffness, and total vascular resistance, indicating an improvement of cardiovascular outcome.

111669

Modality: E-Poster Researcher – Non-case Report

Category: ACUTE AND CHRONIC CORONARY DISEASE/THROMBOLYSIS

## Potential Risk of Early Hospital Discharge After Acute Coronary Syndromes: Observations from Secure-Pci Trial and Accept Registry

PEDRO GABRIEL MELO DE BARROS E SILVA^1^, Otavio Berwanger^2^, Renato Nakagawa^1^, Thiago Macedo^3^, Lucas Tramujas^1^, Dalton Precoma^4^, Oscar Dutra^5^, John H Alexander^6^, Christopher B Granger^6^, Renato D. Lopes^3^

(1) HCor Research Institute, São Paulo, Brazil; (2) Hospital Israelita Albert Einstein, São Paulo, Brazil; (3) Brazilian Clinical Research Institute, São Paulo, Brazil; (4) Pontificia Universidade Católica do Parana, Curitiba, Brazil; (5) Instituto de Cardiologia Fundação Universitária de Cardiologia do Rio Grande do Sul, Porto Alegre, Brazil; (6) Duke Clinical Research Institute, Duke University Medical Center, Durham, NC, USA

**Background:** In clinical practice, there is large variability in hospital length of stay among cases of acute coronary syndrome (ACS). Even in ACS patients without major complications, the ideal timing for hospital discharge is unknown.

**Objective:** To assess the risk of clinical outcomes within 30 days among patients who did not have a major cardiovascular complication in the first 48 hours after the onset ACS.

**Methods:** SECURE PCI trial and ACCEPT registry enrolled patients admitted with ACS (with or without ST elevation). Patients who had a major cardiovascular event (MACE) within 48 hours were excluded from this analysis since these patients would not have been considered eligible for an early discharge. The rate of MACE between 48 hours and 30 days was described in the overall population and according to the type of ACS and timing of PCI. MACE was defined as myocardial infarction, stroke or death. Multivariable analysis was used to identify factors associated with clinical outcomes. The cases of MACE between 48h and 30 days in the SECURE-PCI trial were further evaluated by source documents and classified according to clinical stability before the occurrence of the events in order to identify patients that indeed would be potentially eligible for early discharge in clinical practice.

**Results:** From the 9,244 patients enrolled in the both studies, we did analyze 8,684 (93.9%) patients who had a confirmed ACS and did not have a MACE within 48 hours. At 30 days, the rate of MACE was 4.1% varying from 2.4 to 4.8% according to type of ACS. In the multivariate analysis, the following variables were associated with the risk of MACE: Age (HR 1.25 per 10 years increment; P < 0.01); Diabetes (HR 1.44; P = 0.02); Type of ACS (STEMI HR 3.56; P < 0.01; NSTEMI HR 1.68; P = 0.049) and PCI within 24 hours (HR 0.36; P < 0.01). The overall rate of death at 30 days was 2.4% but was 0.9% among patients who underwent PCI within 24 hours and were stable in the first 48 hours.

**Conclusion:** Patients with ACS undergoing PCI in the first 24 hours who are stable in the first 48 hours after hospital admission presented low risk of MACE in 30 days (mortality <1%). Advanced age, Diabetes, STEMI and PCI after 24 hours were associated with worse 30-day outcomes among patients without MACE after 48 hours of hospitalization due to ACS. Our findings suggest that early discharge of patients around 48 hours seems safe and reasonable and might save costs and resources utilization.

110086

Modality: E-Poster Researcher – Non-case Report

Category: NURSING

## Effect of Motivational Interview on the Self-Care of Patients with Heart Failure: A Randomized Clinical Trial

PAULA VANESSA PECLAT FLORES^1^, Paula Vanessa Peclat Flores^1^, Ana Carla Dantas Cavalcanti^1^, Lyvia da Silva Figueiredo^1^

(1) Universidade Federal Fluminense – UFF

**Introduction:** Heart Failure is a complex syndrome that demands strict commitment to the therapeutic regimen. In addition to the monitoring strategies that will be used, to achieve better results it is vital to motivate the patient to incorporate new habits into the routine of daily life.

**Objective:** To analyze the effectiveness of the motivational interview in the self-care of patients with chronic heart failure.

**Method:** A randomized, multicenter clinical trial where the intervention group and the control group were followed for 60 days in each of the centers. A total of 130 patients were included, where the intervention group received 3 appointments per motivational interview, with a 30-day interval, while the control group maintained the conventional follow-up in the clinics they attend. The data were evaluated through the Self Care Heart Failure Index 6.2, before and after the intervention, in each of the centers. Among the several statistical calculations performed, the use of mean, median, simple frequency, chi-square, t-test, mann whitney and wilcoxon is highlighted and the effect of the intervention was calculated by Cohen’s d.

**Results:** The global sample (Brazil + Uruguay) has 118 patients, 59 in the control group and 59 in the intervention group. Self-care, evaluated through the sub-scales of maintenance, management and confidence, presented Cohen’s d 0.6723 and p-value (<0.001), indicating medium and significant effect; d of Cohen 0.5086 and p-value (0.187), which means a medium effect but not significant; d of Cohen 0.9877 and p-value (<0.001), showing a high intervention effect with significance.

**Conclusion:** Data from these studies showed that motivational interviewing is effective in self-care of patients with chronic heart failure. Motivational interviewing is a low-cost, effective approach that can be applied by trained professionals who work clinically with patients with heart failure.

110122

Modality: E-Poster Researcher – Non-case Report

Category: ATHEROSCLEROSIS/CARDIOVASCULAR RISK FACTORS/CARDIOVASCULAR PREVENTION

## A Prospective Study on Adherence to Secondary Prophylaxis for Rheumatic Fever using Benzathine Penicillin G in Mozambique

ZAKIR OSSMAN^1^, Edna Lichucha^1^, Alcides Munguambe^1^, Lucio Ribeiro^1^, Karen Sliwa^3^, Albertino Damasceno^2^, Ana Olga Mocumbi^1^

(1) Instituto Nacional de Saude – INS; (2) Núcleo de Investigação, Departamento de Medicina, Hospital Central de Maputo, Mozambique; (3) Cape Heart Institute, University of Cape Town

**Introduction:** Delivery of regular long-acting intramuscular benzathine penicillin G (BPG) injections is the most effective method for secondary prophylaxis to prevent recurrence of acute rheumatic fever (ARF).

**Objective:** We aimed to assess the adherence rates to BPG regimens for prevention of ARF in a low-income setting.

**Methods:** Between November/2017 and October/2018 we profiled a cohort of patients with RHD on secondary prophylaxis at two hospitals in Mozambique; then we prospectively assessed adherence to secondary prophylaxis using monthly BPG injections. Cultures obtained from throat swabs collected at the 12th month of prophylaxis on 78 patients selected randomly, and were examined by gram stain, catalase test and CAMP test to detect Group A Streptococcus.

**Results and Discussion:** We enrolled 121 patients, mostly adolescents (mean age 20.8 years; SD 5.9) and females (77; 63.6%). Isolated or combined mitral regurgitation was the commonest lesion (107 patients; 88.4%), followed by aortic regurgitation (54; 44.6%) and mitral stenosis (21; 17.4%). The mean follow up was 19 months. The adherence rate was 86.8%, corresponding to 2228 injections applied out of the 2555 expected; only 1708 (66.8%) were administered at the expected date and 59 patients (49%) were fully compliant. Testing for GAS throat colonization in patients randomly selected on month 12 was negative. One fatal event occurred in relation to BPG injection, not fulfilling criteria for anaphylaxis.

**Conclusions:** RHD patients on secondary prophylaxis in urban hospitals in Mozambique had good adherence and acceptable compliance to monthly BPG injections. The incidence of ARF during follow-up was low and no classic anaphylaxis occurred. Further research is warranted to address gaps in delivery of secondary prophylaxis.

110435

Modality: E-Poster Researcher – Non-case Report

Category: COVID-19 AND CARDIOVASCULAR SYSTEM

## Relationships between Brachial-Ankle Pulse Wave Velocity and Left Ventricular Longitudinal Strain Three Months After COVID-19 Pneumonia

YAROSLAVSKAYA E.I.^1^, Migacheva A.V.^1^, Krinochkin D.V.^1^, Shirokov N.E.^1^, Gorbatenko E.A.^1^, Korovina I.O.^1^, Osokina N.A.^1^

(1) Tyumen Cardiology Research Center, Tomsk National Research Medical Center, Russian Academy of Sciences

**Introduction:** Data regarding the influence of arterial stiffness on longitudinal myocardial strain has been scarce. This is especially important for patients who have undergone a complicated course of COVID-19.

**Objective:** To investigate the associations between brachial-ankle pulse wave velocity (baPWV) and left ventricular (LV) longitudinal strain 3 months after COVID-19 pneumonia.

**Methods:** 369 patients (52 ± 11 (from 19 to 84) years; 50.9% women) 3 months ± 3 weeks after discharge after COVID-19 pneumonia were prospectively enrolled into the study. All participants underwent conventional echocardiography, including 2D speckle-tracking echocardiography. Measurements of baPWV were made at the same day as echocardiography in 322 patients. Parameters of LV global longitudinal strain (LV GLS) and segmental longitudinal strain were studied in 296 patients with optimal visualization quality in echocardiography. Both baPWV and LV longitudinal strain were measured in 243 patients.

**Results:** Three months after discharge, obesity was noted in 46.5% of patients, cardiovascular diseases were diagnosed in 73.4%. Arterial hypertension occurred in 71.5% of patients, coronary artery disease – in 22.5%. The median baPWV were 13.3 [11.8; 15.1] cm/s and 13.4 [11.9; 15.1] cm/s for the left and right sides, respectively. Mean LV ejection fraction was 67.8 ± 5.0%, mean LV GLS was –19.6 ± 2.5%. The baPWV showed a weak correlation with longitudinal strain of LV basal level (r = 0.289 for the right side and r = 0.272 for the left side, both p < 0.001) and of LV mid level (r = 0.229, p < 0.001 for the right side and r = 0.218 for the left side, p = 0.001). The baPWV showed a correlation of medium strength only with the longitudinal strain of LV basal anterior segment (r = 0.303 for the right side and r = 0.309 for the left side, both p < 0.001). There were no correlations between baPWV and strain of LV apical level segments.

**Conclusion:** In patients after COVID-19 pneumonia 3 months after discharge baPWV increase associated with deterioration of LV longitudinal strain at basal and mid levels, more pronounced in LV basal anterior segment.

110457

Modality: E-Poster Researcher – Non-case Report

Category: ACUTE AND CHRONIC CORONARY DISEASE/THROMBOLYSIS

## Adherence to the Acute Coronary Syndrome Care Guidelines: A Program of the Best Practice in Cardiology

LUIZ GUILHERME PASSAGLIA^1^, Luiz Guilherme Passaglia^1^, Carolina Teixeira Cunha Érika^1^, Erika Nunes de Oliveira Rodrigues^1^, Flavia Mariana Mendes Diniz^1^, Darkiane Fernandes Ferreira^1^, Tiago Almeida de Oliveira^2^, Maria Augusta Duarte Abreu^2^, Regina Bicalho Gomes de Faria^2^, Pedro Henrique Coelho Pinto^2^, Antonio Luiz Pinho Ribeiro^2^

(1) Hospital das Clínicas da Universidade Federal de Minas Gerais, Belo Horizonte, MG – Brasil; (2) Faculdade De Medicina da Universidade Federal de Minas Gerais (UFMG)

**Introduction:** In Brazil, low adherence to care guidelines is one of the reasons for the high mortality from acute coronary syndrome (ACS). In view of this, the Brazilian Society of Cardiology (SBC) in partnership with the American Heart Association and with the support of the Ministry of Health developed the Best Practices in Cardiology Program (BPC Program), whose objective is to evaluate the rates of adherence to the SBC guidelines for in institutions of the Unified Health System before and after the implementation of the project.

**Method:** Prospective observational study with data collection from May 2016 to December 2021. The performance measures analyzed were drugs prescribed at hospital discharge. The primary outcome of the study consisted of the evaluation of performance measures with a minimum stipulated target of 85% of global adherence.

**Results:** In this sample, 932 patients were included, with a mean age of 61.6 ± 12.1 years, 69.2% of whom were male, with a low level of education (77.1% with incomplete secondary education or less) and low family income (91.5% with ≤5 minimum wages a month). The main comorbidities were current or previous smoking (67.1%), arterial hypertension (65.7%), diabetes (29.7%), and dyslipidemia (23.0%). In the characterization of ACS, ST-segment elevation infarction corresponded to 68.3%, with Killip classification ≤2 in 75.5% of the sample. Most of the patients (92.2%) underwent coronary angiography, which showed the following pattern of coronary disease: 24.1% trivascular, 16.9% bivascular, 17.5% univascular, with the anterior descending artery being affected in 21.8% of the cases. Coronary angioplasty of the culprit’s vessel was performed in 64.9% of the cases, and only 5.4% of the sample was referred to coronary artery bypass graft surgery. Analysis of performance measures showed aspirin (98.5%), beta-blockers (94.5%), angiotensin-converting enzyme inhibitors or angiotensin II receptor blockers (96.3%) and statins (96.6%) at hospital discharge. There were 27 deaths during hospitalization (2.9%).

**Discussion:** The predominance of males, age ≥ 50 years, and high prevalence of comorbidities show characteristics of ACS patients already known by epidemiological data extensively published in the medical literature. Performance measures indicate good adherence to ACS care guidelines in the hospital.

**Conclusion:** Adherence to quality programs such as the BPC Program is an essential step in improving care for patients with ACS.

110441

Modality: E-Poster Researcher – Non-case Report

Category: CARDIOLOGY OF SPORTS, EXERCISE, ERGOMETRY AND CARDIOVASCULAR REHABILITATION

## The Extreme Prognosis Value of Cardiopulmonary Exercise Testing in Heart Failure

DIANE XAVIER DE ÁVILA^1^, Ricardo Vivacqua Cardoso Costa^2^, Salvador Manoel Serra^3^, Marcelo Westerlund Montera^2^, Evandro Tinoco Mesquita^1^, Alexandre Siciliano Colafranceschi^2^

(1) Complexo Hospitalar de Niterói; (2) Hospital Pró-Cardíaco; (3) Instituto Estadual de Cardiologia Aloysio de Castro

**Introduction:** Cardiopulmonary exercise testing (CPET) is a well-established clinical tool to predict outcome, stratifying cardiovascular risk and help to select candidates for heart transplantation (HTx) or left ventricular assist devices (LVAD) in patients with chronic heart failure (CHF).

**Purpose:** To evaluate CPET measurements in advanced CHF patients that are being considered for HTx or LVAD and its association to early mortality regardless of the performed procedure.

**Methods:** Maximum intensity CPET was performed on a treadmill and ramp protocol in 73 patients with CHF and reduced ejection fraction, NYHA functional classes III and IV between 2012 and 2021. Measurements derived from CPET were the following: peak V‘O2, V’O2 at the anaerobic threshold (AT), percentage of the V’O2 of the anaerobic threshold in relation to the peak, the slope of oxygen consumption efficiency (OUES), Oxygen uptake efficiency plateau (OUEP), VE/VCO2 slope, maximum heart rate (HR), circulatory power (CP), and the relation (VE/VCO2 slope)/(VO2peak).

**Results:** Seventy-three patients, 75% males. Mean age of 70 ± 9 years. Almost half (47%) had ischemic etiology. There were no complications related to CPET. Ten patients were transplanted, six had an intracorporeal LVAD implanted and the reminder (57 patients) were kept in supervised physical rehabilitation program. There were 12 deaths, 2 in HTx, 2 in LVAD, 8 in the rehabilitation group. Mean follow-up among the survivors was 43 months ± 40,6 and it was 12,1 ± 10,3 months in those who died. CPET derived measurements between survivors and non-survivors, respectively, were as follows: V‘O2 peak (mL.kg–1.min–1): 12,6 ± 4,6 and 8,5 ± 2,8 (p = 0,001); the V’O2 AT (mL.kg–1.min–1): 9,8 ± 3,3 and 6,0 ± 3,0 (p = 0,0004); VE/VCO2 slope: 34,2 ± 12,1 and 67,3 ± 65,6 (p = 0,0002); R peak: 1,1 ± 0,2 and 1,0 ± 0,1 (p = 0,009); T½, in seconds: 136,1 ± 47,8 and 167,9 ± 78,5 (p = 0,03); HR reduction at the first minute 16,7 ± 13 and 7,2 ± 5 (p = 0,009); OUES (L.min–1): 1,2 ± 0,4 and 1,0 ± 0,3 (p = 0,104) and CP [(ml O2.kg–1.min–1).mmHg] 1.530 ± 671,6 and 960,6 ± 363,6 (p = 0.005), OUEP (ML/L): 34,2 ± 8,3 and 25,1 ± 8,3 (p = 0.001) and the relation VE/VCO2 slope and V‘O2 peak were 3,1 ± 2,0 e 11,4 ± 19,5 (p = 0.001).

**Conclusion:** The predisposition to early death could be stratified by V‘O2 peak, V’O2 AT, VE/VCO2 slope, T½, recovery HR, CP, the relation VE/VCO2 slope)/(V‘O2 peak) and notably, OUEP can be more one powerful predictor for early intervention due to its association with severity.

110448

Modality: E-Poster Researcher – Non-case Report

Category: COVID-19 AND CARDIOVASCULAR SYSTEM

## The use of Laboratory and Instrumental Parameters for Prediction of Long-Term Events in Patients with Cardiovascular Pathology and Type 2 Diabetes Mellitus who Underwent COVID-19 Associated Pneumonia

PETELINA T.I.^1^, Musikhina N.A.^1^, Garanina V.D.^1^, Avdeeva K.S.^1^, Sharoyan Y.A.^1^, Gapon L.I.^1^, Yaroslavskaya E.I.^1^

(1) Tyumen Cardiology Research Center, Tomsk National Research Medical Center, Russian Academy of Sciences

**Introduction:** By given pandemic status, the viral infection has shown how SARS-CoV-2 can affect patients involved in other noninfectious epidemics that have been gaining momentum for years.

**Objectives:** To perform a prospective analysis of blood biomarkers in patients with cardiovascular (CV) diseases (CVD) who underwent COVID-19 associated pneumonia with and without type 2 diabetes mellitus (DM2); to assess the nature of their relationship with instrumental parameters and to identify indicators of long-term adverse CV events.

**Methods:** Out of 380 patients with SARS-CoV-2 associated pneumonia participating in the study, we used data of 65 patients. They were divided into 2 groups: group 1 included patients with CVD: arterial hypertension (AH) in combination with coronary artery disease (CAD) without DM2 (n = 45), group 2 – patients with CVD and DM2 (n = 20). Patients were examined at baseline and 3 months after discharge. We evaluated parameters of general blood test; biochemical parameters; instrumental parameters – ABPM, PWV in right and left elastic arteries, echocardiography with the study of left ventricular (LV) apical myocardial strain, computer tomography of lungs.

**Results:** The analyzed leukocyte parameters and their index coefficients – increase in neutrophils/lymphocytes ratio and decrease in lymphocytes/CRP ratio were more significantly changed in DM2 group. Patients in both groups had a significant excess of baseline max CRP concentrations with decrease in parameters after 3 months, but with persistent excess values in group 2 (p < 0.0011). 3 months after discharge patients with DM2 had levels of hs-CRP, IL-1b and TNFa (p < 0.05) that exceeded both the reference values and those in group 1, which reflected the presence of more pronounced vascular inflammatory potential for possible adverse events in this group of patients in post-COVID period. In addition, the exceeding values of NT-proBNP, SBP and DBP 24, PWV-L and apical LV myocardial strain in group 2 compared to group 1 may be indicators of occurrence and progression of adverse CV events in patients with DM2.

**Conclusions:** In groups of patients with AH and CAD, which differ only by the presence of DM2, it is clearly seen that comorbid condition can significantly affect the development of adverse CV complications due to increased inflammatory potential of blood parameters, increased stiffness of the vascular wall, and the presence of myocardial longitudinal strain of LV apical segments.

110901

Modality: E-Poster Researcher – Non-case Report

Category: DIGITAL HEALTH/INNOVATION

## Virtual Cardiac Rehabilitation Post Coronary Artery Bypass Graft (CABG) – Real World Experience from India

ALBEN SIGAMANI^1^, PALLAV SINGH^1^, NUMEN CARE TEAM^1^

(1) NUMEN HEALTH

Coronary Artery Disease (CAD) is the number one killer of cardiovascular diseases; yearly, nine-million deaths. CABG was first done in India in 1975; the surgery has grown exponentially, to nearly 100,000 every year. Ischemic cardiomyopathy (IC) and delays in undergoing an evidence-based recommended CABG contribute to significant mortality and morbidity over five years of survival (>35% composite event rate). Cardiac rehabilitation, an evidence-based, effective secondary prevention, is scarce in India; there is just one seat for every 30,000 new IC patients. Numen health is an online digital health platform that organizes virtual cardiac rehabilitation. The program is delivered at the patient’s home via a mobile application. Referrals are received from cardiologists and cardiac surgeons at the time of discharge following a coronary artery event or procedure. On establishing contact and intent to perform cardiac rehabilitation, the patient is subjected to several online tele assessment sessions to help the patient and the care team arrive at a baseline of their health status. The discharge summary of their most recent admission is used for setting Specific Measurable, Achievable, Relevant, and Timely (SMART) Goals. All patients are categorized as per age, gender, known history of pre-existing chronic diseases, medication consumption pattern, physical activity input, potential energy output, detailed analysis of the daily diet, habits, and identification of possible nutritional deficiencies. The program is subscription-based, and the fee is collected either monthly. Between February 2021 and April 2022, 83 patients post CABG have been referred to the program. This represents 10% of the overall number of patients who participated in the program and were referred from 15 cities and 33 doctors across India. They spent a median of 90 days (ranging between 30 to 210 days) on the program and had 600 interactive sessions with the assigned health care manager and other health experts. The age was between 38 and 72 years; 53 (64%) were males. 35(42%) had baseline type 2 diabetes, 8(10%) had significant comorbidity. There were zero deaths, and 35(42%) had achieved their assigned goals. Digital health can bridge the widening gap of providing cardiac rehabilitation to the growing prevalence of IC in India. Virtual cardiac rehabilitation effectively delivers comprehensive secondary prevention to patients post CABG and any coronary event or intervention.

110458

Modality: E-Poster Researcher – Non-case Report

Category: HEART FAILURE/CARDIOMYOPATHY/TRANSPLANT

## Adherence to the Assistance Guidelines for Heart Failure: A Program of the Best Practice in Cardiology

LUIZ GUILHERME PASSAGLIA^1^, Luiz Guilherme Passaaglia^1^, Carolina Teixeira Cunha Érika^1^, Erika Nunes de Oliveira Rodrigues^1^, Flavia Mariana Mendes Diniz^1^, Darkiane Fernandes Ferreira^1^, Tiago Almeida de Oliveira^2^, Maria Augusta Duarte Abreu^2^, Regina Bicalho Gomes de Faria^2^, Pedro Henrique Coelho Pinto^2^, Antonio Luiz Pinho Ribeiro^2^

(1) Hospital das Clínicas da Universidade Federal de Minas Gerais, Belo Horizonte, MG – Brasil; (2) Faculdade De Medicina da Universidade Federal de Minas Gerais (UFMG)

**Introduction:** In Brazil, low adherence to guidelines is one of the reasons for the high mortality from heart failure (HF). In view of this, the Brazilian Society of Cardiology (SBC) in partnership with the American Heart Association, and with the support of the Ministry of Health, implemented the Best Practices in Cardiology Program (BPC Program), whose objective is to evaluate the rates of adherence to the SBC guidelines in institutions of the Unified Health System before and after the implementation of the project.

**Method:** Prospective observational study with data collection from May 2016 to December 2021. The performance measures analyzed were the medications prescribed at hospital discharge and the scheduling of a return appointment. The primary outcome of the study consisted of the evaluation of performance measures with a minimum stipulated target of 85% of global adherence.

**Results:** In this sample, 431 patients were included. The mean age was 59.0 ± 14.6 years, 55.0% were men and 85.4% had a previous diagnosis of HF. The main comorbidities were arterial hypertension (43.4%), atrial fibrillation/flutter (41.1%), diabetes (26.0%), acute myocardial infarction (16.2%), and hypothyroidism (16.0%), with a high rate of current and/or previous smoking (51.0%). The main etiology of HF was Chagas (25.7%), followed by idiopathic (23.0%), ischemic (22.3%), and valvular disease (15.3%). The predominant hemodynamic profile was wet and warm (67.4%), followed by wet and cold (25.0%). At admission, 61 (14.2%) patients underwent heart transplantation. The mean left ventricle ejection fraction was 35.9% (±17.0), and 83 (19.3%) patients died during hospitalization. The analysis of performance measures showed a return appointment schedule (94.6%), beta-blocker (94.8%), angiotensin-converting enzyme inhibitors or angiotensin II receptor blockers (97.3%), and spironolactone (80.9%) at hospital discharge.

**Discussion:** The clinical characteristics of patients with a high prevalence of comorbidities reflect the profile and complexity of patients hospitalized at the hospital and highlight the need for multidisciplinary care. The values of the performance measures indicate good adherence to HF care guidelines, except for the prescription of spironolactone at hospital discharge.

**Conclusion:** Adherence to quality programs such as the BPC Program is an essential step in improving care for patients with HF.

110472

Modality: E-Poster Researcher – Non-case Report

Category: NEGLECTED CARDIOVASCULAR DISEASES

## Relationship between Urinary Norepinephrine, Fibrosis and Arrhythmias in Chronic Chagas Heart Disease with Preserved or Mildly Reduced Ejection Fraction

EDUARDO MARINHO TASSI^1^, Emilia Matos do Nascimento^1^, Marcelo Abramoff Continentino^1^, Basilio de Bragança Pereira^1^, Roberto Coury Pedrosa^1^

(1) Universidade Federal do Rio de Janeiro (UFRJ)

In Chronic Chagas Cardiomyopathy (CCC), studies are needed to identify arrhythmogenic risk factors in patients where moderate to severe ventricular dysfunction is not present. The direct relationship between sympathetic neural activity and norepinephrine is known. In patients with heart failure, sympathetic hyperactivity is persistent. However, in CCC, there are indicators that the sympathetic system is heading towards exhaustion as cardiac involvement progresses.

**Objectives:** To verify the dependence between frequent ventricular extrasystoles (FVE), left ventricular ejection fraction (LVEF), extension of fibrosis by cardiac magnetic resonance (CMR) and urinary norepinephrine (NOREPI) measurement in CCC with preserved or minimally compromised LVEF.

**Methods:** The presence of ventricular extrasystoles >720 in 24h was analyzed on Holter. In CMR, the biventricular ejection fraction, presence of segmental alteration and detection and quantification of fibrosis mass using the delayed enhancement technique were evaluated. NOREPI was measured using the Muskiet method. The correlation coefficient matrix was calculated to measure the predictive ability of the variables to predict another, with a significant p-value of p < 0.05. A total of 59 patients were included. Mean age was 57.9 + 10.94 years. FVE was detected in 28 patients. The variable fibrosis showed a negative correlation with the LVEF found (R of –0.61) and with NOREPI (R of –0.68), as well as the variable FVE showed a weak negative correlation with the LVEF (R of –0.33) and to NOREPI (R of –0.27). LVEF, on the other hand, showed a positive correlation with NOREPI (R of 0.83). The variables fibrosis, LVEF and NOREPI showed a high power of association with each other according to Cramér’s V statistics (fibrosis and NOREPI 0.64, LVEF and NOREPI 0.63 and fibrosis and LVEF 0.53).

**Conclusion:** In patients with CCC with preserved or slightly reduced ejection fraction, it is confirmed that the arrhythmogenic substrate is already present in this population, with dependence between NOREPI levels, LVEF and myocardial fibrosis, but not with the presence of FVE (>720 in 24h).


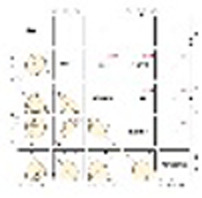


112420

Modality: E-Poster Researcher – Non-case Report

Category: CONGENITAL AND PEDIATRIC CARDIOLOGY

## Customized Expandable Polyurethane Stent Valve, Implanted by Catheter. Strategies for Pulmonary Artery Approach

MIGUEL MALUF^1^, Miguel Angel Maluf^1^, Alfredo Eyer Rodrigues^1^, Maria Eduarda Siqueira^1^, Ana Fatima Salles^1^

(1) Universidade Federal de São Paulo; (2) Hospital Alemão Oswaldo Cruz – SP

**Background:** Patients with tetralogy of Fallot, undergoing Right Ventricular Remodeling, in childhood, with or without pulmonary valve reconstruction, evolve, in the late follow-up, with pulmonary insufficiency and Right Ventricular dysfunction, requiring the implantation of a pulmonary prosthesis. The anatomical variations of the pulmonary artery, associated with the presence of calcifications, dilations, or stenosis as a result of surgeries performed, require adequate planning in the surgical approach for Transcatheter Pulmonary Valve Replacement – TPVR.

**Material:** A new Expandable Polyurethane Stent Valve, implanted by catheter, in pulmonary position has been developed and approved in Biocompatibility, Physical, Hydrodynamic, Fatigue, Experimental, and Ultrastructure Study of explanted sheep prostheses after 24 months of follow-up, analysis, following ISO 5840-3, 2015.

**Method:** In a group of 45 adult patients, in the late follow-up of surgical correction of Tetralogy of Fallot, with late follow-up, at São Paulo Federal University, with an indication for TPVR, they were classified into 6 groups according to the anatomical aspects of the pulmonary artery, analyzed by CT Angiography and scheduled minimally invasive procedure intervention, TPVR: Tipe.1: Pulmonary Valve Insufficiency (PVI) Tipe.2: PVI + Pulmonary Trunk (PT) stenosis Tipe.3: PVI + PT aneurysmal dilation Tipe.4: PVI + PT + Right Pulmonary Artery (RPA) + Left PA (LPA) stenosis Tipe.5: Pulmonary prosthesis dysfunction Tipe.6: Valved Conduit (VC) disfunction Through post-processing images by CT Angiography, it was possible to enlarge them to their natural size, followed by 3D printing, inelastic and transparent plastic mass, keeping the interior of the hollow cardiac cavities. Custom prosthesis manufacturing: 3 transverse diameters (TD) are measured: TD1: At the level of the Pulmonary Ring; TD2: In the middle third of the TP and TD3: At the level of the origin of the RPA and LPA. Also, a longitudinal measure (LM), allows for knowing the length of the prosthesis.

**Conclusion:** The strategy for the treatment of residual defects, after correction of the Tetralogy of Fallot, is based on the need to have customized devices to obtain successful and lasting results.

110529

Modality: E-Poster Researcher – Non-case Report

Category: CARDIOVASCULAR IMAGING

## Evaluation of Cardiac Fibrosis using T1 Mapping and Cardiovascular Risk in Patients with Non-Alcoholic Fatty Liver Disease

FLAVIA VERNIN DE OLIVEIRA^1^, Gabriel Cordeiro Camargo^1^, Martha Valério Tavares Pinheiro^1^, Adriana Soares Xavier de Brito^1^, Renee Sarmento de Oliveira^1^, Daniella Braz Parente^1^, Renata de Mello Perez^1^, Rosana Souza Rodrigues^1^, Ana Maria Pitella de Souza Leite^1^, Gilberto Portela^1^, Andréa Silvestre de Sousa^1^, Renata Junqueira Moll Bernardes^1^

(1) INSTITUTO D’OR DE PESQUISA

**Introduction:** Non-Alcoholic Fatty Liver Disease (NAFLD) is the principal cause of chronic hepatic disease in developed countries, reaching almost 30% of the adult population. It has a large spectrum of histological presentation, from steatosis to cirrhosis. It is a multisystemic disease, affecting a variety of organs, including heart and vascular system, and these are the principal cause of death for these patients, beyond the hepatic disease. Interstitial fibrosis is a factor of worse prognosis for heart and liver, individually, but the correlation between cardiac fibrosis and liver fibrosis has not been studied yet.

**Purpose:** To define the frequency and grade of early myocardial fibrosis in patients with NAFLD and correlate with the intensity of hepatic fibrosis.

**Methods:** Forty-four patients with NAFLD were included; prospectively, the patients performed echocardiogram, myo-intimal carotid measurement by ultrasound, coronary calcium score by computed tomography, cardiac and hepatic magnetic resonance imaging using elastography and T1 mapping sequences; twenty-eight were allocated in the group without fibrosis or with discrete hepatic fibrosis (Group 1) and sixteen patients with significant hepatic fibrosis in Group 2, defined by the magnetic hepatic elastography.

**Results:** The mean age was 57.9 years and 59% were women. The group with significant hepatic fibrosis had also more cardiac interstitial fibrosis, compared with Group 1. The median of myocardial ECV for Group 1 was 22.7% and for Group 2 was 26%, with p = 0.002. The correlation between hepatic and cardiac fibrosis was 0.45, with p = 0.02, even when adjusted for age and body mass index. We also found a negative correlation between hepatic fibrosis and the Global Longitudinal Strain of the left ventricle (r = –0.36 and p 0.016) and with the volume of the left atrium (r = 0.30 and p = 0.04).

**Conclusion:** The study suggests the presence of a correlation between hepatic fibrosis in patients with NAFLD and the presence of diffuse interstitial myocardial fibrosis detected by the cardiac T1 mapping magnetic resonance.

110556

Modality: E-Poster Researcher – Non-case Report

Category: CARDIAC ARRHYTHMIAS/ELECTROPHYSIOLOGY/ELECTROCARDIOGRAPHY

## Factors Associated with Improvement of Left Ventricular Function After Catheter Ablation for Atrial Fibrillation in Patients with Heart Failure: A Multicenter Experience

MARCOS ROBERTO QUEIROZ FRANÇA^1^, André Assis Lopes do Carmo^1^, Gustavo de Araújo Silva^1^, André Dias Nassar Naback^1^, Reynaldo de Castro Miranda^2^, Anna Terra França^3^, Henrique Barroso Moreira^2^, Vitor Freitas Fontes^1^, Leandro Garambone de Cerqueira^1^, Lucas Ruback^1^, Antonio Luiz Pinho Ribeiro^1^, Bruno Ramos Nascimento^1^

(1) Serviço de Cardiologia e Cirurgia Cardiovascular e Centro de Telessaúde do Hospital das Clínicas da UFMG, Belo Horizonte – MG, Brazil; (2) Hospital Felício Rocho, Belo Horizonte – MG, Brazil; (3) Hospital Mater Dei, Belo Horizonte – MG, Brazil

**Introduction:** Catheter ablation is a well-established therapy for atrial fibrillation (AF), with a promising impact on heart failure (HF) outcomes. We aimed to evaluate the impact of AF ablation on echocardiographic and clinical parameters in patients with HF in Brazil, and to assess factors associated with improvement of the left ventricular ejection fraction (LVEF).

**Methods:** Patients diagnosed with HF and LVEF <50%, who underwent radiofrequency AF ablation in 5 high-volume centers, were prospectively enrolled. All patients underwent standard transthoracic echocardiography before the procedure and during clinical follow-up, and the analysis by the examiner was considered. The primary outcome was LVEF normalization (LVEF ≥ 50%) at follow-up. Clinical, echocardiographic, and procedure-related variables associated with the primary outcome were assessed by univariate logistic regression. P-value <0.05 was considered significant.

**Results:** From 2018 to 2022, 77 patients were included, being 52 (68%) males, mean age 66 ± 12 years. Of these, 27 were in NYHA functional class 3/4, 64 (83%) had persistent AF, and only 3 had previous ablations. Pre-procedural LVEF was 38 ± 7%, with 24 (31%) having LVEF < 35%. Complications occurred in only 3 patients (2 in vascular access and 1 endocarditis). In the 13 ± 11 month follow-up, there was a substantial improvement of the LVEF to 54 ± 14% (p < 0.001), and 54 (70%) achieved the primary outcome of LVEF normalization. Fifty-three patients (69%) had LVEF improvement of ≥10%, only 7 remained in NYHA class 3/4, and AF recurred in 13 (17%). Three patients died during follow-up, and none had LVEF improvement. Predictors of LVEF normalization were: pre-ablation LVEF (OR = 1.23, 95%CI 1.11–1.36), baseline left atrial diameter (OR = 0.86, 95%CI 0.79–0.94), ischemic (OR = 0.29, 95%CI 0.08–0.98) and Chagasic (OR = 0.14, 95% CI 0.03–0.78) etiologies and prescription of amiodarone (OR = 0.26, 95%CI 0.09–0.78) and ACEi/ARB (OR = 0.24, 95%CI 0.07–0.81, p = 0.02). In the multivariable model, independent predictors were: baseline LVEF (OR = 1.20, 95% CI 1.06–1.27, p = 0.005), left atrial diameter (OR = 0.88, 95%CI 0.77–0.99, p = 0.03), ischemic (OR = 0.13, 95%CI 0.02–0.85, p = 0.03) and Chagasic (OR = 0.08, 95%CI 0.01–0.76, p = 0.03) etiologies.

**Conclusion:** AF ablation in patients with HF resulted in remarkable echocardiographic and functional improvement, with low complication rates. Less clinical severity and less morpho-functional impairment were associated with LVEF normalization.

110563

Modality: E-Poster Researcher – Non-case Report

Category: PHYSIOTHERAPY

## Effects of Mindfulness Meditation Associated with Transcranial Direct Current Stimulation on Heart Rate Variability in the Elderly

EVELYN COSTA CRUVINEL^1^, Aline Teodoro Mendes^1^, Denise Mayumi Tanaka^2^, Fernanda Regina Moraes^3^, Gustavo José Luvizutto^1^, Marilita Falangola Accioly^1^, Luciana Duarte Novais Silva^1^, Douglas Reis Abdalla^4^, Eduardo Elias Vieira de Carvalho^1^

(1) Programa de Pós-graduação em Fisioterapia da Universidade Federal do Triângulo Mineiro.; (2) Departamento de Clínica Médica da Faculdade de Medicina de Ribeirão Preto da Universidade de São Paulo.; (3) Curso de Fisioterapia da Universidade de Uberaba.; (4) Departamento dos Cursos de Saúde do Centro Universitário Talentos Humanos.

**Introduction:** Aging is a physiological process that leads to decreased heart rate variability. Physical training is an established therapy that induces increased heart rate variability, and transcranial Direct Current Stimulation (tDCS) potentiates this beneficial effect. However, a large part of the elderly population does not have access to adequate physical training programs. Instead, meditation is an easily accessible and useful tool to induce small increases in heart rate variability. However, no studies have evaluated the effects of the association of meditation with tDCS on heart rate variability.

**Purpose:** To evaluate the influence of meditation associated with tDCS on heart rate variability in the elderly.

**Methods:** Eleven older adults of both sexes, aged 69.1 ± 7.2 years, were studied. Heart rate variability was collected in the supine position, in a quiet environment, free from the circulation of people and external influences, lasting 80 minutes, with 5 minutes of initial rest to control heart rate, followed by recording the RR intervals at 20 minutes baseline, 15 minutes meditation, 20 minutes tDCS and 20 minutes final/post- tDCS. The meditation technique was mindfulness, applied by a qualified and experienced therapist; tDCS was applied by an anodic current of 2 mA, following the international standards for the system (EEG10–20). Outcomes were analyzed using the ANOVA model with fixed effects.

**Results:** It was observed a significant increase of the heart rate variability indexes with the techniques association: RMSSD from baseline (27.2 ± 35.9 ms) to post-meditation (33.6 ± 43 ms) and post-tDCS (36.7 ± 50.7 ms), p = 0.04; SDNN from baseline (23.8 ± 26.3 ms) to post-medidation (27 ± 28.3 ms) and post-tDCS (31.8 ± 34.5 ms), p = 0.0001; Low-frequency component from baseline (369.6 ± 602.4 ms^2^) to post-meditation (352.8 ± 599.1 ms^2^) and post-tDCS (672.2 ± 1034.1 ms^2^), p = 0.0001; High-frequency component from baseline (207.4 ± 375.5 ms^2^) to post-meditation (599.7 ± 1068.5 ms^2^) and post-tDCS (896.8 ± 1958.7 ms^2^), p = 0.0009; SD1 from baseline (19.2 ± 25.4 ms) to post-meditation (23.8 ± 30.4 ms) and post-tDCS (26 ± 35.9 ms), p = 0.02; e SD2 from baseline (27.4 ± 27.6 ms) to post-meditation (28.9 ± 27.2 ms) and post-tDCS (36 ± 34 ms), p = 0.0001.

**Conclusion:** The results of the present study documented for the first time that the application of anodal tDCS can significantly enhance the beneficial effects of meditation on heart rate variability indexes in the elderly.

110632

Modality: E-Poster Researcher – Non-case Report

Category: CARDIOVASCULAR IMAGING

## Left Ventricular Global Longitudinal Strain Three Months After COVID-19 Pneumonia

YAROSLAVSKAYA E.I.^1^, Krinochkin D.V.^1^, Shirokov N.E.^1^, Gorbatenko E.A.^1^, Osokina N.A.^1^, Migacheva A.V.^1^

(1) Tyumen Cardiology Research Center, Tomsk National Research Medical Center, Russian Academy of Sciences

**Background:** COVID-19 can result in myocardial injury in the acute phase. The late cardiac consequences of complicated course of COVID-19 is limited.

**Purpose:** To study the prevalence of disorders and the relationship of left ventricular global longitudinal strain (LV GLS) in patients with proven COVID-19 pneumonia 3 months after discharge from the hospital.

**Methods:** 369 patients underwent a comprehensive clinical examination 3 months ± 3 weeks after discharge after COVID-19 pneumonia. The mean age was 52 ± 11 (from 19 to 84 years), 50.9% women. Standard echocardiography and myocardial strain assessment were performed. LV GLS was studied in 296 (80%) examined patients with optimal visualization quality. LV GLS was considered reduced when its value was above –18%.

**Results:** 3 months after discharge, obesity was noted in 46.5% of patients, cardiovascular diseases were diagnosed in 73.4%. Arterial hypertension (AH) occurred in 71.5% of patients, coronary artery disease – in 22.5%. LV GLS was reduced in 25.1% of the patients. LV GLS showed no relationship with the patient age, NYHA functional class and LV ejection fraction (EF). Reduced LV GLS was independently associated with AH (OR 1.174; 95% CI 1.056–1.305; p = 0.003), obesity (OR 1.295; p < 0.0001), male sex (OR 0.734; 95% CI 0.657 –0.821; p < 0.001). LV GLS showed positive correlations of medium strength with echocardiographic parameters: length of the right (r = 0.308) and left ventricles (r = 0.306), LV mass (r = 0.326), anterior-posterior left atrial (LA) dimension (r = 0.303, all p < 0.0001). LV GLS showed a weak positive correlation with the severity of lung lesions during hospitalization (r = 0.134; p = 0.032), diastolic blood pressure (r = 0.230; p < 0.001), LV posterior wall thickness (r = 0.255, p < 0.001) and interventricular septum (r = 0.188, p = 0.001).

**Conclusions:** Reduced LV GLS 3 months after COVID-19 pneumonia was observed in 25.1% of survivors and is associated with AH, obesity, and male sex. LV GLS was not associated with the patient age, NYHA functional class and LV EF. The correlation between impaired LV GLS and the severity of lung lesions in hospitalization was weak, the correlations with the ventricles length, LV mass, and LA anterior-posterior dimension were of medium strength.

110778

Modality: E-Poster Researcher – Non-case Report

Category: CARDIOGERIATRICS

## Functional Sarcopenia Phenotype Predicts Mortality in Older Adults with Cardiovascular Disease at 1 Year. Data from the Sarcos Study

ALBERTO FRISOLI JUNIOR^1^, Elaine Azevedo^1^, Amanda kimura^1^, Angela Paes^1^, Valdir Ambrosio^1^

(1) Universidade Federal Sao Paulo

The diversity of concepts of sarcopenia makes it difficult to use in clinical practice. Low performance on functional tests (functional sarcopenia) has been shown to be associated with severe outcomes, while low muscle mass (anatomic sarcopenia) has controversial results. Functional sarcopenia has phenotypes determined by the accumulation of physical dysfunctions, whose predictive value for mortality is not yet established.

**Objective:** To evaluate if Functional Sarcopenia phenotypes are associated with mortality in elderly people with cardiovascular disease (CVD).

**Methods:** SARCOS is a prospective cohort study on Sarcopenia and Osteoporosis with mortality in elderly with cardiovascular disease.

**Sample:** Elderly outpatients of the Federal University of São Paulo – Brazil. Physical functions were evaluated by: walking speed (WS) in 4.5 mts, the time to sit and stand up from chair test (CST) and hand grip strength (HGS), by dynamometer. Appendicular muscle mass (AMM) was measured by DXA. AMM/height2, HGS, WS and CST were evaluated in quartiles. Functional sarcopenia (FS) phenotype was characterized according to the presence of weakness (HGS < 26 Kgf M, <16 Kgf W), low WS (LWS, if WS < 0.5 m/s) and high time to CST (HCST > 25s): Robust = 0, Pre- Functional sarcopenia = 1, Functional Sarcopenia ≥2. Mortality was assessed by telephone contact at 6 and 12 months. Significant mortality variables and low AMM according to EWGSOP II* and FNIH# were used in the regression.

**Results:** Among the 555 subjects, 57.5% (n = 319) were women, mean age 77.91(7.27). Weakness occurred n = 151 (27.2%), LWS n = 156(28.1%) and HCST n = 129(23.2%). The prevalence of phenotypes: Robust n = 281 (50.6%), Pre FS n = 155 (27.9%), and FS n = 119 (21.4%). Mortality was 4.7% (n = 26), of which, n = 5 (19.2%) were robust, n = 7 (26.9%) Pre FS, and n = 14 (53.8%) had FS, (p < 0.001). The FS phenotype was characterized by being female, higher age, lower HGS and WS, higher Time to CST, higher functional loss (IADL and ADL), lower depression score, lower AMM/h2 and higher percentage of total body fat, compared to other phenotypes. In regression analyses for mortality, Pre FS OR: 1.84 (0.45–7.49, p = 0.390)* and OR: 2.30 (0.56–9.48, p = 0.247)#; and FS OR: 4.98(1.35–18.40; 0.016)* and OR: 7.06(1.71–29.09; p < 0.007)#.

**Conclusion:** In the older adults with cardiovascular disease, Functional Sarcopenia phenotype predicts higher mortality compared to the other phenotypes, regardless of low muscle mass and CVDs.

110761

Modality: E-Poster Researcher – Non-case Report

Category: HEART FAILURE/CARDIOMYOPATHY/TRANSPLANT

## Frailty and 5-Year Mortality in Patients with Congestive Heart Failure and Implanted Devices for Cardiac Resynchronization Therapy

SOLDATOVA A.M.^1^, Kuznetsov V.A.^1^, Enina T.N.^1^, Bogdanova D.S.^2^, Benzineb F.T.^2^

(1) Tyumen Cardiology Research Center, Tomsk National Research Medical Center, Russian Academy of Sciences; (2) Tyumen State Medical University

**Purpose:** To access the prevalence of frailty and its impact on long-term prognosis in patients with implanted devices for cardiac resynchronization therapy (CRT).

**Methods:** 77 patients with congestive heart failure (74% men, 26% women; mean age 58.7 ± 10.7 years) with NYHA class II–IV were enrolled. Frailty index (FI) was calculated using 31 parameters (the ability to perform daily activities, clinical status, laboratory markers, comorbidities). Based on the median of FI value patients were identified as not frail (<0.375; n = 41; 53%), and frail (≥0.375; n = 36; 47%).

**Results:** The mean follow-up period was 38.0 [21.0; 66.0] months. The 5-year survival was 87.8% for not frail patients compared to 52.8% for frail patients (Log rank p < 0.001). In univariate analysis the presence of frailty was significantly associated with long-term mortality (HR 6.108; 95 CI 2.207–16.907; p < 0.001). After adjustment for age, gender, NYHA class, left ventricular ejection fraction, presence of left bundle branch block and QRS width frailty remained a significant factor associated with 5-year mortality (HR 5.763; CI 95% 1.837–18.083; p = 0.003).

**Conclusions:** Frailty is widespread in patients with heart failure and implanted devices for CRT and it has an independent association with the risk of 5-year mortality.

110798

Modality: E-Poster Researcher – Non-case Report

Category: PHYSIOTHERAPY

## Level of Knowledge and Safety Measures Adopted by Physiotherapy Professionals Working in Pilates Studios in Care Provided to Patients with Cardiovascular Diseases

PRISCILLA MORETTO^1^, Priscilla Moretto^1^, Tatiane Boff Centenaro^3^, Gabriela dos Santos^3^, Fernando Rosa Inácio^1^, Ana Inês Gonzáles^1^

(1) Centro Universitário Estácio de Sá de Santa Catarina; (2) Centro Universitário para o Desenvolvimento do Alto Vale do Itajaí – UNIDAVI; (3) Universidade do Estado de Santa Catarina – UDESC

**Introduction:** In physical exercise programs aimed at Cardiovascular Rehabilitation, it is important to emphasize the adequate monitoring of these patients for care in the event of a cardiovascular event. Given the above, questions arise whether these care and monitoring are performed by physical therapists who work with the Pilates Method in patients with Cardiovascular Diseases (CVD’s) and if it occurs in an adequate and safe way.

**Objectives:** To assess the level of knowledge and safety measures adopted by physiotherapy professionals working in pilates studios regarding the care provided to patients with cardiovascular diseases.

**Methodology:** Cross-sectional research, with a non-probabilistic and quantitative sample, where professionals with active CREFITO, certified in Pilates and active in the method in the states of Brazil were invited. Those who did not provide all the data were excluded from the research. There was the development of a self-response questionnaire containing information related to the professional and the assessment of the level of knowledge regarding the approach and safety measures adopted with the Pilates Method in exercise programs for patients with CVD‘s. Professionals were invited to respond digitally and by publicizing CREFITOS and universities in Brazil. The TCLE was considered electronically signed when the volunteer accepted to participate in the research through an electronic form (Google Forms). The analysis of the study data was carried out using descriptive statistics, based on measures of central tendency (mean and standard deviation), performed in the SPSS statistical program for Windows® version 2.0.

**Results:** 134 physical therapists answered the questionnaire, 98 (73.1%) do not have a training course in the cardiovascular area, 81 (60.4%) have practical knowledge in cardiopulmonary resuscitation, 68 (50.7%) are not up to date regarding rehabilitation guidelines and approximately more than 90% of professionals consider it important to check safety measures regarding vital signs before, during and after each session. In the event of any cardiovascular intercurrence, only 69 (51.5%) of the professionals stated that their team is prepared to assist.

**Conclusion:** Most professionals do not have training to care for this type of patients, and have little knowledge of updating in the area, at the same time they consider it necessary to measure vital signs and perception of effort in the sessions.

110832

Modality: E-Poster Researcher – Non-case Report

Category: COVID-19 AND CARDIOVASCULAR SYSTEM

## Neutrophil/Lymphocyte Ratio is Correlated with Levels of Inflammatory Markers and is Significantly Reduced by Smoking Cessation

MAKI KOMIYAMA^1^, Yuka Ozaki^1^, Yoichi Sunagawa^2^, Yasufumi Katanasaka^2^, Masafumi Funamoto^2^, Kana Shimizu^2^, Hajime Yamakage^1^, Noriko Sato-Asahara^1^, Hiromichi Wada^1^, Tatsuya Morimoto^2^, Koji Hasegawa^1^

(1) Clinical Research Institute, National Hospital Organization Kyoto Medical Center, Kyoto, Japan; (2) Division of Molecular Medicine, School of Pharmaceutical Sciences, University of Shizuoka, Shizuoka, Japan

**Background:** Inflammation of blood vessels is key in the process of coronavirus disease 2019 (COVID-19) aggravation. Besides, risk factors for COVID-19 such as cardiovascular disease is strongly associated with smoking habits. Previous studies have reported that neutrophil to the lymphocyte ratio (NLR), the inflammation marker, is associated with the onset and prognosis of cardiovascular (CV) disease. Smoking is a potent CV risk factor, and smoking cessation significantly reduces CV risk. However, the effects of smoking cessation on the NLR remain unknown.

**Purpose:** The purpose of this study is to demonstrate the effect of smoking cessation on the NLR.

**Methods:** Among smokers visiting our smoking cessation clinics, we examined changes in the NLR and CVD biomarkers before and after smoking cessation. A total of 389 individuals (301 men and 88 women) were enrolled in the study.

**Results:** The median NLR was significantly reduced after successful smoking cessation (before: 1.8 [1.5, 2.5], after: 1.7 [1.3, 2.4]; p < 0.001). In a linear regression model adjusted for sex, percent change in NLR comparing before and after smoking cessation was significantly and positively correlated with percent changes in C-reactive protein (β = 0.260, p = 0.001), α1-antitrypsin-low density lipoprotein (β = 0.151, p < 0.05), and serum amyloid A-low density lipoprotein (β = 0.325, p < 0.001).

**Conclusion:** Our study demonstrated for the first time that smoking cessation significantly reduces the NLR in tandem with markers of inflammation and oxidative stress. These results may suggest that smoking cessation improves the risk of developing cardiovascular diseases and more severe symptoms of COVID-19. Moreover, smoking cessation recovers airway ciliary clearance and immune function as early as one month. Thus, smoking cessation awareness is strongly encouraged as part of the public health measures aiming to limit the global impact of COVID-19.

110803

Modality: E-Poster Researcher – Non-case Report

Category: HYPERTENSION/RENAL DENERVATION

## Prevalence of Arterial Hypertension in a Sample of Adults in Luanda: A Comparative Analysis Based on the Most Recent American Guidelines

HENRIQUE COTCHI SIMBO MUELA^1^, Guilherme Passassi^1^, Angelina Clara Cassoma Francisco^1^, António Gerson Bastos Francisco^1^, Isaura da Conceição Almeida Lopes^1^, Gade Satuala Vasco Miguel^2^

(1) Department of Physiology, Faculty of Medicine, Agostinho Neto University, Luanda, Angola; (2) Cardiothoracic Surgery Department, Girassol Clinic, Luanda, Angola

**Introduction:** In 2017, the American College of Cardiology/American Heart Association (ACC/AHA) published a new guideline (JNC 8) on the treatment of hypertension (HTN) that changed the diagnostic standard of high blood pressure (BP) from a BP threshold of 140/90 mmHg, as set by the seventh report of the Joint National Committee (JNC 7) to a BP of 130/80 mmHg.

**Objective:** To carry out a comparative study on the frequency of HTN and the profile of BP levels in a sample of adult inhabitants in Luanda, Angola, based on the most recent American HTN guidelines.

**Methods:** We carried out a cross-sectional study was, which included 1480 individuals who participated in the study on high blood pressure between May 2018 and June 2019, and who were recruited at Shopping Xyami, Angolan Public Television (TPA) and Congolenses’ market. Sociodemographic, anthropometric and clinical data were collected. Each participant had his/her BP taken with three measurements on the arm, after a 5-minute rest, using a semi-automatic Omron® device (Kyoto, Japan), with a 1-minute interval between. HTN was defined as the mean of systolic blood pressure (SBP) ≥140 mmHg and/or diastolic blood pressure (DBP) ≥90 mmHg according to JNC 7 and the mean of SBP ≥130 mmHg and/or DBP ≥80 mmHg according to JNC 8 or use of antihypertensive drugs for both guidelines.

**Results:** The sample was composed mostly by men (69.30%), young people (mean 39.74 ± 11.55 years old, range 18 to 94 years old). Alcohol consumption (74.6%) and overweight/obesity (44.9%) were the most prevalent risk factors. The prevalence of HTN was twice as high using the criteria proposed by the JNC 8 guideline compared to the cutoff points proposed by the JNC 7 (60.20 vs. 34.70; X2 = 519,03; p < 0.001). Both the knowledge and the treatment as well as the control of HTN were worse when using the JNC8 guideline definitions compared to JNC 7 guideline (X2 = 519,03; p < 0,001). Age, weight and alcohol consumption were the main predictors of blood pressure changes.

**Conclusion:** The use of JNC 8 definition resulted in a significant increase in the prevalence of high blood pressure, placing the BP of a significant proportion of adults in the hypertension range.

110804

Modality: E-Poster Researcher – Non-case Report

Category: HYPERTENSION/RENAL DENERVATION

## Prevalence, Awareness, Treatment and Control of Arterial Hypertension in a Sample of Residents of Luanda, Angola

HENRIQUE COTCHI SIMBO MUELA^1^, Angelina Clara Cassoma Francisco^1^, Guilherme Passassi^1^, António Gerson Bastos Francisco^1^, Isaura da Conceição Almeida Lopes^1^, Gade Satuala Vasco Miguel^2^

(1) Department of Physiology, Faculty of Medicine, Agostinho Neto University, Luanda, Angola; (2) Cardiothoracic Surgery Department, Girassol Clinic, Luanda, Angola

**Background:** Arterial hypertension (HTN) is an important public health challenge worldwide. Data regarding the burden of hypertension is essential for the development of effective strategies for both treatment and control. Studies on the prevalence and awareness of hypertension in Sub-Saharan Africa (SSA) and especially in Angola are still scarce.

**Objective:** To assess the prevalence, awareness, treatment and control of HTN in a sample of dwellers of Luanda.

**Methodology:** We carried out cross-sectional study in a sample of dwellers of Luanda, Angola. Sociodemographic data and cardiovascular (CV) through questionnaires. Blood pressure was measured with semi-automatic sphygmomanometer (Omron®, Model HEM-7131-E), after 5 minutes resting and seated. Three blood pressure (BP) measurements with a 1-minute interval between were used to determine SBP and DBP in each patient. The average of the two last blood pressure readings was used for the analysis. Hypertension was defined as SBP and/or DBP ≥ 140/90 mmHg or current use of antihypertensive drugs. Controlled hypertension was defined as blood pressure <140/90 mmHg under antihypertensive drugs.

**Results:** Between May 2018 and June 2019, we carried out a cross-sectional study including 1480 individuals was carried out. The majority of the sample was young (mean age 39.74 ± 11.55 years old) and men (69.30%). Alcohol consumption (74.6%) and overweight/obesity was the most prevalent risk factors. The HTN prevalence was 34.9% in the sample. Among the hypertensive patients, 245 (47.4%) were aware of their disease and only 190 (36.8%) were under hypertension treatment; among those were taking drugs, only 68 (35.8%) were under control, defined as BP <140/90 mmHg.

**Conclusion:** HTN prevalence was high; the awareness, treatment and control levels were low.

110806

Modality: E-Poster Researcher – Non-case Report

Category: ATHEROSCLEROSIS/CARDIOVASCULAR RISK FACTORS/CARDIOVASCULAR PREVENTION

## Neutrophil-Lymphocyte Ratio and Coronary Artery Disease Risk in Patients Undergoing Coronary Angiography

HENRIQUE COTCHI SIMBO MUELA^1^, Fernando Muhongo Sandala^1^, José António Tito^2^, Guilherme Mendes Lima Franco^1^, António Gerson Bastos Francisco^1^, Isaura da Conceição Almeida Lopes^1^

(1) Department of Physiology, Faculty of Medicine, Agostinho Neto University, Luanda, Angola; (2) Cardiology Department, Sagrada Esperança Clinic, Luanda, Angola

**Background:** Inflammatory response is one of the main mechanisms in the pathogenesis of atherosclerosis and its progression. The neutrophil-lymphocyte ratio (NLR) has been proposed as an inflammatory biomarker and potential risk and prognosis predictor in cardiovascular disease (CVD).

**Objective:** To assess the association between the neutrophil-lymphocyte ratio and the severity of coronary artery disease.

**Methods:** An observational and retrospective study was carried out, including 56 patients aged ≥18 years (41 men and 15 women) who underwent diagnostic and/or therapeutic coronary angiography from 2014 to 2019 at Sagrada Esperança Clinic, Luanda, Angola. Demographic data, risk factors and comorbidities, biochemical tests and full blood count were collected from the patients’ medical reports. The NLR was calculated as the ratio between the total neutrophil and lymphocyte counts from the patients‘ full blood count. For the statistical analysis purposes, the sample was divided into two groups according to the median of NLR (median: 2.02): patients with NLR ≤ median and patients with NLR> median. The statistical significance was set at 5%.

**Results:** The group of patients with CAD was relatively older than the group without CAD, although without any significant difference (58.49 ± 8.76 vs. 54.74 ± 8.7, p = 0.137). Likewise, the age was similar between groups considering the level of NLR (57.89 ± 8.60 vs. 56.54 ± 9.12, p = 0.571). The risk of having CAD and it being obstructive was twice as high in the group with NLR above the median (NLR > 2.02) compared to the group with a lower NLR (OR: 2.25, CI: 0.722–1.012 and 2.17, CI: 0.717–6.550, respectively). However, in general, our data suggested that there was no association between the neutrophil-lymphocyte ratio and the presence of CAD, nor with its severity (χ2 = 1.991, p = 0.259 and χ2 = 0.760, p = 0.562, respectively).

**Conclusion:** The risk of CAD occurrence, as well as the occurrence of obstructive CAD was twice higher in the group with higher neutrophil-lymphocyte ratio.

110817

Modality: E-Poster Researcher – Non-case Report

Category: CARDIOVASCULAR IMAGING

## The Role of NT-Probnp and Echocardiographic Parameters in Patients with Acute Exacerbation of COPD

SASHA KJAEVA ANASTASOVA^1^, Prof, d-r Srbinovska -Kostovska^1^, D-r. Paljoskovska-Jordanova^1^, D-r. Shehu^1^, D-r.Risteski^1^, D-r.Debreshlioska^2^, D-r. Angelovska^2^, D-r.Nivicka-Kjaeva^3^, D-r.Kjaev^4^

(1) University Clinic of Cardiology, Skopje, North Macedonia; (2) University Clinic of Pulmoallergology, Skopje, North Macedonia; (3) University Clinic of Ophthalmology, Skopje, North Macedonia; (4) University clinic of Gynecology, Skopje, North Macedonia

**Aim:** The aim of the study was to evaluate which echocardiographic parameters in assessment of right ventricular function may contribute to risk stratification in patients with severe/very severe chronic obstructive pulmonary diseases (COPD) in correlation with biomarker Nt-proBNP.

**Material and methods:** We have analyzed 38 patients with severe/very severe COPD, stages 3 and 4 according to Gold classification. All patients with heart diseases were excluded from the study. Additionally, all patients were divided into two subgroups: patients with acute exacerbation and chronically stable patients. Echocardiography, analysis included several parameters with emphasis to right chamber echo parameters in correlation to natriuretic peptide. Special emphasis was given to 3 predominant echocardiographic parameters: TAPSE, S’ wave assessed by tissue doppler of the free wall of the right ventricle and FAC%.

**Results:** Of a total number of 38 patients, 81,6% had elevated values of NT-proBNP and 18,4% had values of NT-proBNP in normal ranges. Analysis of the average values of NT-proBNP showed much higher values in the group of patients with acute exacerbation where the average value of NT-proBNP was 786,73 pg/ml in correlation with chronically stable patients, where NT-proBNP was 141,17 pg/ml. All 20 patients with acute exacerbation had elevated NT-proBNP values (above 125 pg/ml). In the group of patients with acute exacerbation, 13 patients (65%) out of 20 had S’ wave from the tissue doppler below 0,095 m/sec and 8 patients (22,6%) had TAPSE below 16 mm. The group of chronically stable patients (who were 18 in number), showed not convincing results. In this group 61% (11 patients) of the patients had elevated NT-proBNP, 4 of them had S’ wave from the tissue doppler below 0,095 m/sec and TAPSE was in normal range(>16 mm). FAC was below 35% in one patient, where NT-proBNP was 345,4 pg/ml.

**Conclusion:** In patients with chronic obstructive pulmonary diseases (COPD), echocardiographic parameters TAPSE and S’ wave assessed from the Tissue Doppler of the free wall of the right ventricle can be significant prognostic markers for disease monitoring. These parameters, especially S’ wave, can also be a marker for acute deterioration of the condition of the patients with COPD.

110833

Modality: E-Poster Researcher – Non-case Report

Category: PERICARDIUM/ENDOCARDIUM/VALVOPATHIES

## Long-Term Follow-Up in Patients with Rheumatic Mitral Stenosis and Severe Pulmonary Artery Hypertension Submitted to Percutaneous Mitral Balloon Commissurotomy

RAFAEL ALEXANDRE MENEGUZ MORENO^1^, J. Ribamar Costa Jr.^1^, Nisia Lira Gomes^1^, Auristela IO Ramos^1^, Alfredo N. Ferreira-Neto^1^, Dimytri Siqueira^1^, Mercedes Maldonado^1^, Zilda Meneghelo^1^, Cesar A. Esteves^2^, Sérgio Braga^1^, Amanda Sousa^1^

(1) Instituto Dante Pazzanese de Cardiologia; (2) Universidade Federal de São Paulo (UNIFESP)

**Background:** Percutaneous balloon mitral commissurotomy (PMBC) is an attractive therapeutic approach in patients with mitral stenosis. The aim of this study was to assess the immediate and long-term clinical, echocardiographic and haemodynamic outcomes of PMBC in patients with severe pulmonary hypertension (PH).

**Methods:** Among all procedures (in more than two decades of experience), PMBC was performed from 1987 until 2011 at a single-center in 147 patients who had significant PH defined as baseline pulmonary artery mean pressure (PAMP) (systolic pulmonary pressure >75 mmHg). All-cause mortality, need for mitral valve replacement (MVR) or new PMBC, and valve restenosis were evaluated during follow-up yearly.

**Results:** Mean age was 33.8 ± 12.8 years and 83.6% (123 patients) were women. Primary success was achieved in 89.8% of the patients (132 patients). Mitral valve area (MVA) increased from 0.83 ± 0.17 cm^2^ to 2.03 ± 0.35 cm^2^ (p < 0.001), and at 20-years, mitral valve area was 1.46 ± 0.34 cm^2^ (p = 0.235). Systolic pulmonary artery pressure decreased from 87.0 ± 6.0 mmHg to 60.0 ± 0.9 mmHg (p < 0.0001). The rates of all-cause mortality, need for MVR, new PMV, and valve restenosis were 0.67%, 20.0%, 8.78% and 30.4%, respectively, in long-term follow- up (mean 15.6 ± 4.9 years).

**Conclusions:** PMBC is a safe and effective technique for the treatment of patients with mitral stenosis and PH. A significant decrease in pulmonary pressure was observed after commissurotomy. Although there was a gradual decrease of MVA at long-term follow-up, most patients remained asymptomatic and without major adverse events.

112450

Modality: E-Poster Researcher – Non-case Report

Category: COVID-19 AND CARDIOVASCULAR SYSTEM

## Trends in Cardiovascular Services and Procedure Volumes Across Different Phases of the COVID-19 Pandemic: An Analysis of 2019–2022

FATHIMA AAYSHA AAYSHA CADER^1^, Ishmum Zia Chowdhury^2^, Saidur Rahman Khan^1^, M. Maksumul Haq^1^, Mashhud Zia Chowdhury^1^

(1) Ibrahim Cardiac Hospital & Research Institute; (2) BIRDEM General Hospital

**Introduction:** The impact of the COVID-19 pandemic on cardiology services globally has been variable, with a paucity of contemporary data comparing pre- and post-pandemic trends over a long period in a South Asian setting.

**Objectives:** We aimed to report annual trends of cardiovascular services delivery and procedure volumes from 2019 to 2022, to assess the impact of the COVID-19 pandemic, and recovery of services at a tertiary cardiac centre in Bangladesh.

**Methods:** Data on patient visits (outpatient and emergency), admissions, procedures and catheterization volumes were collected from January 2019 to February 2022 via hospital electronic records. Differences for each month of the preceding year were expressed as a percentage (%Δ). Trends (2019 to 2022) were graphically depicted via line diagrams.

**Results:** Following significant reductions of all cardiology services in 2020 (1), particularly ER visits (Δ–59.5%; p = 0.0), cardiology services delivery and procedure volumes had recovered, reaching almost pre-pandemic levels by Q1 of 2021 (Figure 1). A steep and significant decline of admissions and procedures was seen in March–April 2021 as compared with 2020, coinciding with the Delta variant surge (2). By Q4 of 2021, patient visits and procedures, both outpatient and catheterization volumes had once again reached near pre-pandemic levels (3). During the Omicron surge in early 2022, a small decline in outpatient visits (Δ–10.9%) and outpatient procedures (Δ–6.83%) was observed in January–February. However, in-patient admissions (Δ4.39%) and catheterization laboratory procedures (Δ5.7%) showed a rise in February 2022 as compared with the preceding month, with ER visits showing the steepest rise (Δ 41.9%). Notably, although ER visits remained relatively blunted post-pandemic (2020–2021), this trend was not reflected in outpatient visits/procedures, in-patient admissions and catheterization procedures, all of which increased to pre-pandemic levels by the end of 2021 (1–3). This may be explained either by patients presenting at index to the outpatient department, instead of the ER; or, a higher proportion of patients being directly referred to our centre for admission from the peripheries.

**Conclusion:** Two years on from the pandemic, cardiology services and cath lab volumes have reached almost pre-pandemic levels in 2022, except for ER visits which remain low, albeit gradually rising.

110840

Modality: E-Poster Researcher – Non-case Report

Category: ACUTE AND CHRONIC CORONARY DISEASE/THROMBOLYSIS

## Pretreatment with P2Y12 Inhibitors in ST-Elevation Acute Coronary Syndrome (STEACS). Should We Keep Doing?: Systematic Review and Meta-Analysis

LUIZ FERNANDO KUBRUSLY^1^, Douglas Mesadri Gewehr^2^, Taiane Belinati Loureiro Kubrusly^3^, Fernando Bermudez Kubrusly^3^, Luiz Fernando Kubrulsy^1^

(1) Mackenzie Evangelical School of Paraná, Curitiba, Paraná, Brazil; (2) Denton Cooley Institute of Research, Science and Technology, Curitiba, Paraná, Brazil; (3) Curitiba Heart Institute, Curitiba, Paraná, Brazil

**Background:** The practice of pretreatment with oral P2Y12 inhibitors in ST-elevation acute coronary syndrome (STEACS) remains common; however, its association with improved cardiovascular outcomes is unclear, since no large RCT has addressed this issue.

**Objective:** We aimed to evaluate the association of oral P2Y12 inhibitor pretreatment in STEACS patients with cardiovascular and bleeding outcomes.

**Methods:** PubMed, MEDLINE, Embase, Cochrane, Scopus, Web of Science were systematically searched for studies that compared pretreatment with P2Y12 versus no pretreatment in STEACS, and reported efficacy and safety outcomes. A meta-analysis using a fixed and random effects model was used to calculated outcomes of interest. Heterogenicity was assessed with I2 statistics.

**Results:** A total of 3 RCTs and 14 observational studies assigning 91,771 patients to either pretreatment (65,598 patients) or no pretreatment (26,171 patients) were included. Follow-up ranged 7 days to 19 months. Medications included clopidogrel, prasugrel and ticagrelor. At 30 days, P2Y12 pretreatment was associate with lower 30-day mortality (risk ratio [RR], 0.71; 95% CI, 0.56–0.91; p = 0.006; I2 = 75%), stent thrombosis (RR, 0.33; 95% CI, 0.12–0.95; p = 0.04; I2 = 83%), 30-day major bleeding (RR, 0.81; 95% CI, 0.74–0.90; p < 0.0001; I2 = 0%). No difference in the incidence of 30-day myocardial infarction (MI), target vessel revascularization (TVR), MACE (death/MI/TVR), stroke, pre-PCI TIMI 0 and post-PCI TIMI 0–2 was observed. Subgroup analysis for 30-day mortality, including only randomized studies, indicated a trend in favor of no pretreatment, but without statistical significance (RR, 1.49; 95% CI, 0.89–2.52; p = 0.13; I2 = 38%).

**Conclusion:** In this study, pretreatment with oral P2Y12 inhibitors among patients with STEACS before cath lab, compared with treatment once coronary anatomy is known, was associated with decreased all-cause mortality and bleeding risk.

110852

Modality: E-Poster Researcher – Non-case Report

Category: DIGITAL HEALTH/INNOVATION

## Nanumsmart: An Innovative Nanofunctionalized Polymerless Endovascular Platform

CHRISTIANE DIAS MAUÉS^1^

(1) Smart Vascular Technologies EI; (2) Federal Government of Brazil ME/MCTI

In recent years, nanotechnology has been a strong ally to medical technology advances, and in this sense, one of the greatest applications concerns drug carrying and releasing systems. The use of nanostructured systems to carry out drug delivery allows the development of new systems for an efficient transport of these drugs, where a controlled release is achieved to the diseased tissues of living systems. These drugs are linked to nanostructures in a controlled way, commonly called functionalization, along with other attached molecules – biomarkers. Smart nanoparticles as liposomes could still be functionalized for a better targeting to the target tissue, preventing the side effects of the drug they carry. As a pioneer project, this privilege holds a non polymeric multidrug eluting coronary stent, which spatial structure is represented by six configurations, associated to a biological matrix (coating) in which nanoparticles, more precisely functionalized liposomes, are stored and grouped into sequential layers, each one differing the type of pharmacological agent to be applied, as well as increased coefficients of biodegrability applied to this support matrix, conferring continuously and selectively control of drug releasing pulse. In this sense, nanofunctionalized liposomes, in addition to being cell membranes models, act as excellent biocompatible systems for formulation, carrying and release of drugs to organic tissues.


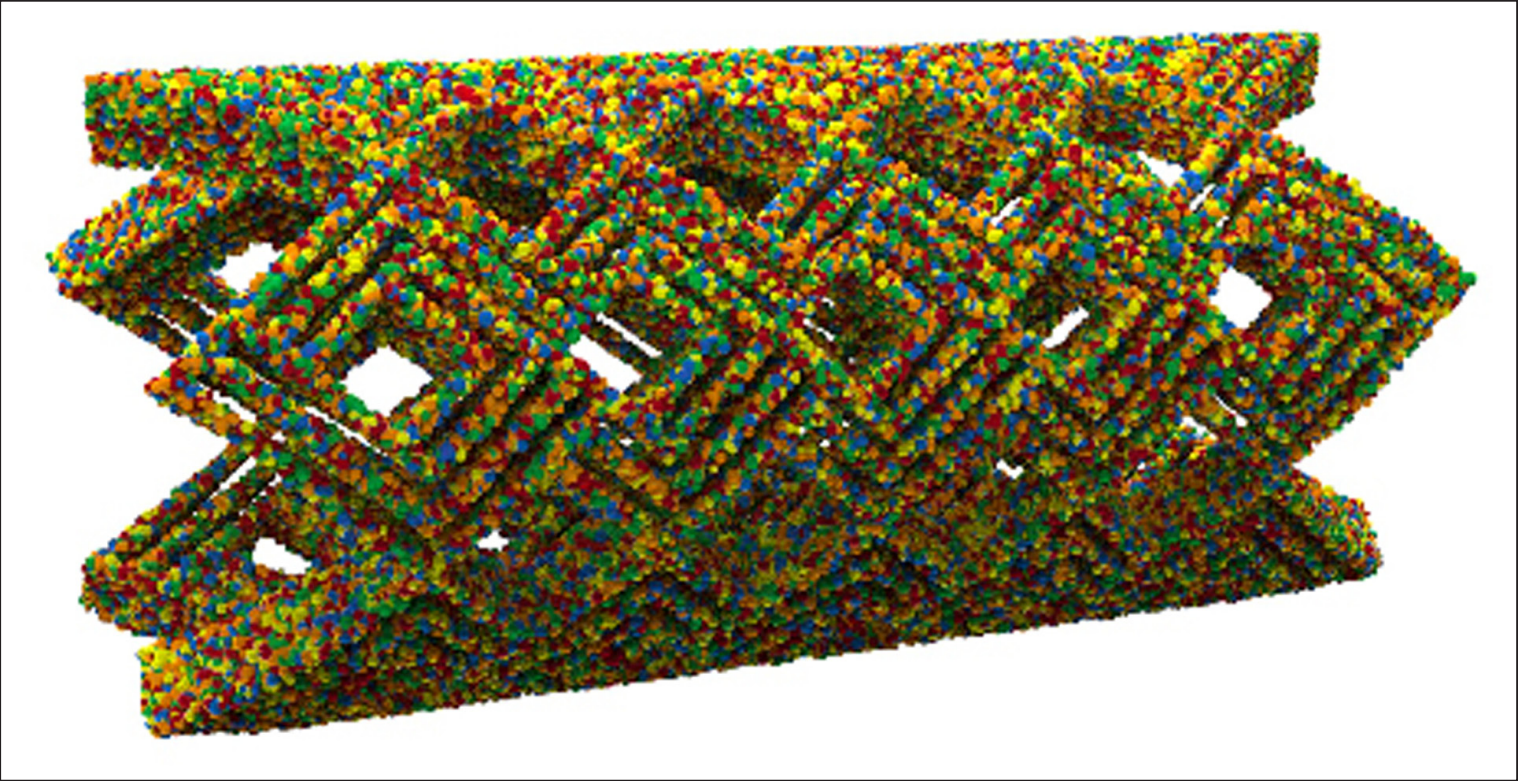


110846

Modality: E-Poster Researcher – Non-case Report

Category: DIGITAL HEALTH/INNOVATION

## RR Interval Analysis Program as a Predictor of Interventions in Implantable Cardioverter-Defibrillator Patients

MICHAŁ LEWANDOWSKI^1^, Ilona Kowalik^1^

(1) National Institute of Cardiology, Warsaw, Poland

**Background:** Prediction of sudden cardiac death remains a significant challenge. There is some evidence that ventricular ectopic activity could be regarded as a predictive marker.

**Objectives:** We carried out an analysis to explore whether premature ventricular complexes (PVCs) are a risk factor in implantable cardioverter-defibrillator (ICD) interventions.

**Materials and methods:** The study method was a RR interval series analysis (n = 184) of arrhythmic events and controls from the ICD. Study group consisted of patients with a mean age of 55 ± 27 years; 74% of them were male, 85% were secondary prevention patients, 62% had coronary artery disease (CAD), 15% hypertropic cardiomyopathy (HCM), 15% dilated cardiomyopathy (DCM), and 8% diseases of other etiol¬ogy. The mean follow-up time was 64 months (range: 3–126 months). The study population was divide into patients with at least 1 appropriate intervention ventricular tachycardia/ventricular fibrillation (VT/VF) (group A, n = 101) and controls without interventions (group B, n = 83). The number of PVC/4000 RR cycles, the shortest coupling intervals between a PVC and preceding R as well as the number of PVCs of very short (180–220 ms), short (220–280 ms) and different cycle lengths (CL) as well as the incidence of short-long-short (SLS) sequences were compared.

**Results:** The number of PVCs/4000 RR cycles was significantly higher in group A (263 ± 32 compared to 43 ± 17, p < 0.0001). The mean shortest PVC CL was significantly shorter in group A (320 ± 13 compared to 400 ± 38, p = 0.029). The number of PVCs with a very short CL was 1 ± 0.4 compared to 0.1 ± 0.1 (p = 0.028). The number of PVCs with a short CL was 5 ± 1.2 compared to 0.6 ± 0.4 (p = 0.0007) in groups A and B, respectively. The incidence of SLS sequences was significantly higher in group A than in group B (67 (94% of patients) and 4 (33% of patients) respectively (p < 0.0001)).

**Conclusions:** Significant differences were found in the characteristics of PVCs and SLS sequences between patients with appropriate ICD interventions and controls. A newly developed basic computer program called PCRR was applied for RR interval analysis. This simple method could be a predictor of PVC burden, heart rhythm variability and life-threatening arrhythmias in different populations.

110878

Modality: E-Poster Researcher – Non-case Report

Category: HEART FAILURE/CARDIOMYOPATHY/TRANSPLANT

## Genotyping of Patients with Hypertrophic Phenotype in a Latin American Cardiovascular Reference Center

MARIA JULIANA RODRIGUEZ GONZÁLEZ^1^, Andres Felipe Barragán Amado^1^, Maria Fernanda Tamayo Tamayo^1^, John Alexander Ramirez^1^, Julián Gelves^1^, Esther Campos^1^, claudia Patricia Jaimes Castellano^1^

(1) Lacardio

**Introduction:** In the setting of hypertrophic phenotype, the hypertrophic cardiomyopathy (HCM) is the most prevalent genetic disorder and responds to the 60% of the cases; with variable penetrance and expression. Genotyping is a step in its approach to defining prognosis and establishing phenocopies. Mutations in some genes encoding sarcomere proteins like cardiac myosin-binding protein C and β-myosin heavy chain are the most prevalent affected, representing 30–40% of all pathogenic or likely pathogenic variants.

**Aim:** Describe the genotyping of our first cohort of hypertrophic phenotypes in a reference heart failure clinic.

**Method:** Data was taken for in-Hospital patients irrespective of the cause of hospitalization or out of hospital who required a cardiovascular imagen test; the only requirement was to have a cardiac magnetic resonance or transthoracic echocardiography with a hypertrophic phenotype defined by any wall over 15 mm or 13 in patients with a family history of HCM between 01/01/2019–31/12/2021. Descriptive statistics are presented as median (range) or % of affected patients.

**Results:** Patients with hypertrophic phenotype were 45, the average age was 54 years (ds: 19,73), 52% were women, 37% had a previous diagnosis of hypertension, the MYH7 was detected in 36% of the overall patients, and the total gene mutations were 55 and the unknown significance variants represented the 69%. Nine of these patients had multiple mutations and only three patients had non-sarcomere genes mutation (they had late-onset Fabry disease, amyloidosis, and Noonan). On the imaging aspects, the average diameter was 17,86 mm (ds: 4,16), the most affected segment was anteroseptal basal with 45% and the average left ventricular ejected fraction was 59,3 (ds: 15,01).

**Conclusion:** These results provide important knowledge about genotyping patients with hypertrophic phenotype, to determine the possible diagnosis, the gene mutation, the type of variant, and the significance. This study shows that in our population, the pathogenic variants represent 27% of the total, similar data in comparison with the available publications. This is the first study in Colombia that reveals genotyping in hypertrophic phenotype could let us establish some mutations that carry a worse prognosis and establish family approaches.

111055

Modality: E-Poster Researcher – Non-case Report

Category: PSYCHOLOGY

## Gender Differences in Nicotine Dependency and Depressive Tendency Among Smokers

MAKI KOMIYAMA^1^, Hajime Yamakage^1^, Noriko Satoh-Asahara^1^, Yuka Ozaki^1^, Tatsuya Morimoto^2^, Yuko Takahashi^1^, Koji Hasegawa^1^

(1) Clinical Research Institute, National Hospital Organization Kyoto Medical Center, Kyoto, Japan; (2) Division of Molecular Medicine, School of Pharmaceutical Sciences, University of Shizuoka, Shizuoka, Japan

Depressive tendency and nicotine dependency are factors related to the failure of smoking cessation. Women generally have a higher depressive tendency and difficulty in smoking cessation than men. However, the impact of sex differences on the relationship between nicotine dependency and depressive tendency remains unclear. We evaluated 727 patients (496 men and 231 women) who visited our outpatient clinic for smoking cessation therapy and compared various parameters measured between sexes during consultation. Age, duration of smoking, and daily cigarette consumption were significantly higher in men during the first visit. Women had significantly higher self-rating depression scale (SDS) scores and took significantly more antidepressant drugs than men. The SDS score significantly correlated with the Fagerström test for the nicotine dependence score and with daily cigarette consumption in women, but not in men. Thus, the present study demonstrates the differential relationship of depressive tendency with tobacco use or nicotine dependency in men and women, which might reflect sex differences in response to psychological stress. We previously reported that depressive tendencies of patients with neurosis improve even in the initial stages of the smoking cessation treatment (i.e., within two weeks after starting the treatment), but further studies are required for the sex difference for the improvement.

110894

Modality: E-Poster Researcher – Non-case Report

Category: PERICARDIUM/ENDOCARDIUM/VALVOPATHIES

## A Multicenter Case Series of Late Prosthetic Valve Endocarditis and Analysis of Risk Factors for Mortality

CRISTIANE DA CRUZ LAMAS^1^, Giovanna Ianini Ferraiuoli Barbosa^1^, Rafael Quaresma Garrido^1^, Wilma Félix Golebiovski^1^, Clara Weksler^1^, Diego Santos^2^, Caio Sambo^2^, Vitor Milczwski^2^, Milena Paixão^3^, Flavio Tarasoutchi^3^, Tânia Mara Varejão Strabelli^3^, Rinaldo Focaccia Siciliano^3^

(1) Instituto Nacional de Cardiologia, Rio de Janeiro, Brasil; (2) Department of Infectious Diseases, Hospital das Clínicas, University of São Paulo Medical School, São Paulo, Brazil; (3) Heart Institute (InCor), Hospital das Clínicas, University of São Paulo Medical School, São Paulo, Brazil

**Introduction:** Prosthetic valve endocarditis (PVE) is found in 10 to 30% of contemporary series of infective endocarditis (IE) and has a high mortality rate. Our aim was to describe cases of late PVE and analyze mortality-related risk factors.

**Methods:** Two quaternary cardiac surgery institutes were the study scenarios. Data were collected from 2011–2021 in site 1, and 2006–2021 in site 2. Patients were prospectively identified using site-specific procedures to ensure consecutive enrollment. Only adult patients with definite IE according to the modified Duke criteria were included. Studied variables were those present in the International Collaboration in Endocarditis case report forms. Statistical analysis was performed with the SPSS v26 software. Variables with p values less than 0.1 were included in the model.

**Results:** There were 250/559 (44.7%) episodes of LPVE in site 1 and 77/438(17.6%) in site 2 during the study periods. Males accounted for 207/327 (63.3%) of patients, mean age ± SD was 55.5 ± 17.3 years. Rheumatic valve disease was present in 161/326 (49.4%) and congenital heart disease in 29/327(8.9%). Healthcare-associated IE was present in 83 (25.4%) and 132/326(49.4%) patients were in heart failure (NYHA class III/IV). Etiologic agents were defined in 275(84.1%) of cases, and oral streptococci were responsible for 107/327(32.7%). Death was the outcome for 101/327(30.9%). Risk factors for death were assessed by multivariate analysis and those found to be statistically associated were: age over 60 years (OR = 2.4; 95% confidence interval [95%CI]: 1.2–5; p = 0.014), heart failure (NYHA class III or IV) (OR = 4.1; 95%CI = 1.8–8.3; p < 0.0001), the presence of perivalvular abscess/fistula/perforation on admission echocardiography (OR = 2.8; 95%CI = 1.3–6.1; p = 0.009) and being referred from another hospital (OR = 2.6; 95%CI = 1.25–5.4;p = 0.011).

**Conclusion:** Previous series have described that late PVE is found in older patients, but our cohort shows younger patients and a high frequency of rheumatic valve disease. A quarter of patients acquired IE in a healthcare setting, highlighting the relevance of this mode of acquisition and the extra care needed in infection control in patients with prosthetic valves. Similarly to other studies, destructive disease and heart failure were associated with death, and mortality was higher than in native valve IE.

110902

Modality: E-Poster Researcher – Non-case Report

Category: DYSLIPIDEMIA

## Plasma Lipoprotein(A) Concentration Among Acute Myocardial Infarction Patients

KU MING YING^1^, Shirley Tan Siang Ning^1^, Crystal Tan Sing Yee^1^, Tiong Lee Len^1^, Saiful Shakirin bin Rosli^1^, Hwang Siaw San^2^, Ong Tiong Kiam^3^, Alan Fong Yean Yip^3^

(1) Sarawak General Hospital; (2) Swinburne University of Technology Sarawak Campus; (3) Sarawak Heart Centre

**Introduction:** Lipoprotein(a) [Lp(a)] is known to have prothrombotic, proinflammatory and proatherogenic properties contributing to Atherosclerotic Cardiovascular Disease (ASCVD) development and progression. Previous studies have shown ethnic differences in plasma Lp(a) levels.

**Objective:** We aim to determine the plasma Lp(a) concentrations among Malaysians with Acute Myocardial Infarction (AMI).

**Methods:** This prospective study involved AMI patients admitted to a tertiary hospital in Sarawak between August 2018 and March 2019. Blood samples were collected during hospital admission and stored at –80°C. Plasma Lp(a) concentrations were determined by quantitative sandwich enzyme immunoassay technique (Elabscience, USA).

**Results:** A total of 240 AMI patients were recruited, with mean age of 56.4(±10.90) years and 88.8% males. Ethnic distributions were 42.9% Malay, 30.4% Chinese and 26.7% Iban/Bidayuh. The mean Lp(a) concentration and baseline LDL-C level were 5.3(±7.49) mg/dL and 3.2(±1.15) mmol/L respectively. Mean Lp(a) concentrations among Malay, Chinese and Iban/Bidayuh patients were similar [5.9(±6.69) vs 5.3(±10.46) vs 4.5(±3.80) mg/dL; p = 0.340]. Patients with or without prior statin therapy demonstrated no significant difference in Lp(a) concentration [5.7(±9.71) vs 5.1(±5.53) mg/dL; p = 0.521]. Only 1.7% of patients were found to have Lp(a) >30 mg/dL. Patients presenting with 1-year MACE (AMI, stroke or cardiovascular death) were found to have significantly higher Lp(a) compared to those who did not [7.9(±7.67) vs 4.96(±7.41) mg/dL; p = 0.042].

**Conclusions:** Our study showed that the mean Lp(a) concentration among our AMI patients were lower compared to other published studies. However, those who experienced 1-year MACE had significantly higher Lp(a) concentration compared to the rest of the study population.

110904

Modality: E-Poster Researcher – Non-case Report

Category: CARDIORESPIRATORY PHYSIOLOGY/BASIC SCIENCE

## The Role of NT-Probnp in Patients with Acute Exacerbation of COPD

SASHA KJAEVA ANASTASOVA^1^, Prof, d-r. Srbinovska-Kostovska^1^, d-r Pajloskovska-Jordanova^1^, d-r Shehu^1^, d-r Risteski^1^, d-r.Debreshlioska^2^, d-r Angelevska^2^, d-r.Nivicka-Kjaeva^3^

(1) University clinic of cardiology, Skopje, North Macedonia; (2) University clinic of pulmo-allerology, Skopje, North Macedonia; (3) University clinic of ophthalmology, Skopje, North Macedonia

**Aim:** The aim of the study was to evaluate chronic obstructive pulmonary diseases (COPD) in correlation with natriuretic peptide biomarker Nt-proBNP with special emphasys to the subgroup of acute exacerbation patients with COPD. All patients analysed were hospitalized.

**Material and methods:** We have analysed 94 patients with COPD devided in 4 groups according to Gold classification system. Gold class I (18 pat.), Group II (18 pat.), Gold class III and IV (28/30 patients). Additionally, all patients were divided into two subgroups: patients with acute exacerbation and chronically stable patients. From the whole group of 94 patients, 63 were patients with acute exacerbation while 31 was were classified as chronically stable patients.

**Results:** Natriuretic peptide was measured in all 94 patients with the average value above reverence ranges in all pts and it was 236.27 pg/ml. Gold class I/II included patients only with acute exacerbation. In both groups (Gold I/II), values od Nt-proBNP were at a higher level (159, 62 pg/ml). The average value of Nt-proBNP in Gold class III, in the group of acute exacerbation was 492,60 pg/ml versus 147,48 pg/ml in the group of chronic stable patients while in Gold class IV these values were 307,44 versus 151,74 pg/ml. The average values in acute patients are far higher then in the chronic group of stable COPD patients with in appropriate Gold class. Such a difference is almost 3,5 times higher in Gold class 3 and 2 times in Gold class 4.

**Conclusion:** According to the results here is a clear and statistically highly significant difference in the values of Nt-proBNP in relation to acute and chronic COPD patients, especially in Gold class III and IV. In the acute group, this is all due to rich inflammatory process and deterioration of the lung function which is replicated on the right heart cavities resulting in high release of Nt-proBNP. Deterioration of lung function leads to right heart strain and weakness and increased excretion of Nt-proBNP.

110924

Modality: E-Poster Researcher – Non-case Report

Category: PERICARDIUM/ENDOCARDIUM/VALVOPATHIES

## A Contemporary Prospective Cohort of Adult Patients with Infective Endocarditis in Brazil: Distinct Features

CRISTIANE DA CRUZ LAMAS^1^, Nícolas de Albuquerque Pereira Feijóo^2^, Thatyane Veloso de Paula Amaral de Almeida^2^, Mariana Giorgi Barroso de Carvalho^2^, Gabriel Santiago Moreira^1^, Léo Rodrigo Abrahão dos Santos^2^, Rafael Quaresma Garrido^1^, Bruno Zappa^1^, Giovanna Ianini Ferraiuoli Barbosa^1^, Wilma Félix Golebiovski^1^, Marcelo Goulart Correia^1^, Clara Weksler^1^

(1) Instituto Nacional de Cardiologia, Rio de Janeiro, Brasil; (2) Unigranrio-Afya

**Introduction:** Infective endocarditis is a severe disease, with high mortality. Its features in a developing country such as Brazil differ substantially. Our aim is to describe a series of adult patients with IE cared for in a cardiac surgery referral center, highlighting its specificities.

**Methods:** Adult patients with definite IE according to the modified Duke criteria were included from 2006–2021 using the International Collaboration in Endocarditis case report form. Patients were identified prospectively using site-specific procedures to ensure consecutive enrollment. Statistical analysis was performed using the Jamovi 1.6 and R 4.0.1 softwares.

**Results:** There were 435 episodes of IE in the study period, and mean age ± SD was 47.8 ± 17.3 years; 283(65.1%) were males. Community-acquired IE occurred in 284 (65.4%), hospital-acquired in 112 (25.8%). Early prosthetic valve IE (PVE) accounted for 47/435(10,8%) cases and late PVE for 77(17,7%). Previous 170 (39.3%) had previous cardiac surgery, congestive heart failure in 173 (40.0%), chronic renal failure in 91 (21.0%), coronary artery disease in 60 (14.0%), diabetes mellitus in 55 (12.6%). Main predispositions to IE were rheumatic valve disease (RVD) in 133 (31.5%), valvular prosthesis in 28.5%, congenital heart disease in 61 (14.0%), previous IE in 51 (11.8%) and intravenous drug use in 5 (1.2%). Vegetations were seen in the mitral valve in 214 (49.4%) and aortic valve in 180 (41.6%), in intracardiac devices, in 33 (7.6%). Fever was seen in 91.7%, new regurgitant murmurs in 54.4%, embolism in 48.3%, splenomegaly in 20.4%; Osler’s nodes, Janeway lesions, subconjunctival hemorrages and splinters were seen in less than 5% each. CRP levels were high in 82.3% and ESR in 58.9%. Blood cultures were taken in 98.6% of episodes, but were positive in only 67.1%. The most frequently isolated pathogens were viridans group streptococci, VGS(21%), S.aureus (11%), and enterococci(11%). Main complications were acute heart failure, in 263 (60.7%), acute renal failure (33.9%), myocardial abscess (22.6%). Surgery was indicated for 373 (86.1%) and effectively done for 316 (79.8%). Inhospital mortality was 109/425 (25.6%).

**Conclusions:** In our quaternary center in Rio, left-sided IE predominated, with negative blood cultures or VGS as main findings. RVD was the main predisposition. Surgical indication was frequent, due to referral bias, and mortality was overall similar to the literature.

110942

Modality: E-Poster Researcher – Non-case Report

Category: CARDIOLOGY OF SPORTS, EXERCISE, ERGOMETRY AND CARDIOVASCULAR REHABILITATION

## Impact of Early Exercise-Based Cardiac Rehabilitation on Hostility, its Behavioral Components, and Disease Perception in Patients After Myocardial Infarction

IWONA KORZENIOWSKA-KUBACKA^1^, Anna Mierzyn’ska PhD^2^, Ewa Rydzewska PhD^1^, Edyta Smolis-Bąk PhD^1^, Rafał Dąbrowski MD, PhD^1^

(1) National Institute of Cardiology, Warsaw, Poland Department of Coronary Artery Disease and Cardiac Rehabilitation; (2) Department of Rehabilitation. Medical Centre of Postgraduate Education Gruca Orthopaedic and Trauma Teaching Hospital, Otwock, Poland

**Introduction:** Hostility and its behavioral components, anger and aggression are psychosocial risk factors for coronary heart disease. The impact of exercise training on these negative emotions in patients after myocardial infarction (MI) without psychological support, has been poorly studied.

**Objective:** The purpose of the study was to evaluate the effectiveness of physical training on the level of negative emotions, the cognitive aspect of adaptation to disease, and physical capacity in patients after MI who participated in cardiac rehabilitation.

**Methods:** We enrolled 60 post-MI men and women in the study. All of them underwent an 8-week training program(TP) consisting of 24 interval trainings 3 times a week, started on average 40 days after MI. Before and after completion of TP, patients underwent a symptom-limited exercise test with analysis of maximal workload, duration, HR and BP at rest and during effort. The Buss-Perry Aggression Questionnaire assessing the level of negative emotions and the Brief Illness Perception Questionnaire were performed with results analysis in the entire group and subgroups of men, women, patients under 60 years of age (younger) and over 60 years of age (older).

**Results:** After TP a significant reduction in the general level of negative emotions was found only in the group of younger: 67.8 ± 4.6 vs 63.9 ± 3.7 points, P < 0.01. Furthermore, a significant reduction in the sense of the impact of the disease on life was found in younger 6.96 ± 0.5 vs 5.48 ± 0.5points, P < 0.01. Furthermore, there was a significant improvement in overall adaptation to the disease in the subgroup of women from 40.6 ± 2.2 to 35.7 ± 1.9 points, P < 0.05. Physical capacity increased significantly in all studied groups.

**Conclusions:** Participation in rehabilitation based on exercise trainings without psychological intervention not only improved physical capacity, but also beneficially contributed to a decrease in negative emotions and had a positive effect on disease adaptation especially in younger patients under 60 years of age after MI.

110949

Modality: E-Poster Researcher – Non-case Report

Category: CARDIOVASCULAR IMAGING

## Evaluation of Echocardiographic Parameters for Right Heart Function and Pulmonary Hypertension in the Progression of Chronic Obstructive Pulmonary Disease

SASHA KJAEVA ANASTASOVA^1^, Prof.D-r.Elizabeta Srbinovska-Kostovska^1^, D-r Elena Grueva-Nastevska^1^, D-r Savetka Paljovskovska-Jordanova^1^, D-r Dean Risteski^1^, D-r Danica Petkoska-Spirova^1^, D-r Enes Shehu^1^, D-r Irina Angelovska^2^, D-r Angela Debreshliovska^2^

(1) University clinic of cardiology, Skopje, North Macedonia; (2) University clinic of pulmo-allerology, Skopje, North Macedonia

**Aim:** The aim of the study was to evaluate the echocardiographic parameters used to assess right ventricular function and pulmonary hypertension in patients with chronic obstructive pulmonary disease (COPD) according to their specificity and sensitivity and disease progression.

**Material and methods:** We have analysed 94 patients with COPD (Gold class I–IV). The 13 echo-cardiography parameters important for assessment of right ventricular function and pulmonary hypertension due to their sensitivity and specificity and progression of the disease were evaluated: basal dimension of the right ventricle(DV bazal), right atrium(DA), right atrial area(DA area), S‘wave of the right ventricle of TDI, TAPSE, functional area change (FAC%), (SPAP), Vmax of tricuspid regurgitation, acceleration time of pulmonary artery (AT), pulmonary vascular resistance (PVR), myocardial performance index of the right venricle (MPI), global strain of the right ventricle(GL strain), collapsibility of vena.cava inferior >/<50%.

**Results:** Predictors of disease progression with high specifity and sensitivity are the parameters: MPI DV TDI, Global strain of DV and collapsibility of v.cava inferior less then 50%. Predictors of disease progression with high specifity and low sensitivity are: DV bazal, DA, DA area, S TDI, TAPSE, FAC, SPAP, V max TR. Predictors of disease progression with low specifity and high sensitivity are parameters: shortened acceleration time of the pulse Dopler of the pulmonary valve and the development of pulmonary vascular resistance.

**Conclusion:** Echocardiography is a non invasive and useful method for evaluation and follow up the patients with COPD. All this indicates that the values of certain echo parameters can help us detect disease progression with high sensitivity, specifity or both.


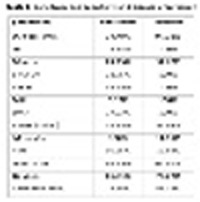


110955

Modality: E-Poster Researcher – Non-case Report

Category: CARDIAC ARRHYTHMIAS/ELECTROPHYSIOLOGY/ELECTROCARDIOGRAPHY

## Extended Electrocardiographic Screening for the Subcutaneous Implantable Cardioverter-Defibrillator (SICD)

MICHAŁ LEWANDOWSKI^1^, Rafał Waligóra^1^, Paweł Syska^1^, Ilona kowalik^1^

(1) National Institute of Cardiology, Warsaw, Poland

**Background:** The condition for SICD therapy is passing an electrocardiographic screening test (ECG-s). A patient is considered suitable for implant if at least one surface ECG lead (sense vector) is acceptable for all tested postures.

**Objective:** Identifying the rate of failure, the predicting factors and clinical importance of extended ECG-s with right sided lead position and lying on the left and right side of the body.

**Methods:** ECG-s is based on a manufacturer-specific measurement tool. We performed this test in 50 consecutive pts with indication for SICD using standard protocol(lead at the left sternum border: sitting, standing and lying) and extended for right parasternal lead location and 2 additional position: lying on the left and right side. We analyzed age, gender, BMI, heart rate, rhythm(sinus/atrial fibrillation), QRS duration, PR interval, corrected QT interval(QTc), echocardiography parameters, etiology.

**Results:** ECG-s failure occurred in 4/50(8%) of patients. Screening results: 1 vector failure rate with the standard device and electrode positions: primary(P) 12%,seconadary(S) 17%, alternative(A) 40%, P vs. A and S vs. A p < 0,001. Testing with an electrode positioned on the right sternum border- 1 vector failure: (P) 10%,seconadary(S) 17%, alternative(A) 48%, P vs. A and S vs. A p < 0,001. In case of failure on left side 50,14 and 6% of pts passed the test with the right sided lead position for P, S and A vector respectively, p < 0.001. Identified factors predicting ESG-s passing vs. failure included: QTc interval 445,32 ± 35,4 vs. 480,6 ± 37,6 ms for any vector(p = 0,034), BMI:26,7 ± 4,7 vs. 29,9 ± 4,7 for vector A(p = 0,008), EF: 43,3 ± 20,1 vs. 34,0 ± 16,2 for vector A(p = 0,027). Sense vector failure rate with the left parasternal lead location lying on the left and right side of the body was concordant with lying on the back, p = 0,65. For the right parasternal lead location the rate failure was lower lying on the right side for all sense vectors: P, S and A(p = 0,045; 0,083 and 0,020) respectively.

**Conclusions:** ECG screening failure rate for SICD is comparable with previous reports. The right parasternal lead location screening enables important clinical findings before implantation and in case of lead reposition during procedure, thus we recommend to screen routinely on both sternum borders. Some additional body position testing: lying on the left and right side of the body, mimicking sleeping/rest could be an important element to determine eligibility for SICD.

110961

Modality: E-Poster Researcher – Non-case Report

Category: CONGENITAL AND PEDIATRIC CARDIOLOGY

## Interventions, Complications and Survival Among Transvenous and Subcutaneous Implantable Cardioverter-Defibrillator Patients Under Thirty Years of Age

MICHAŁ LEWANDOWSKI^1^, Paweł Syska^1^

(1) National Institute of Cardiology, Warsaw, Poland

**Background:** Young implantable cardioverter-defibrillator (ICD) recipients present high rate of inappropriate shocks(IS) and complications. Some of them seem to be underestimated. The subcutaneous ICD system was developed to eliminate lead-related complications and was proved to be effective. S-ICD appears to be a good therapy option and an alternative to transvenous implantable cardioverter-defibrillator (TV-ICD) in pediatric and young patients population. Aims To report on our clinical experience with TV-ICD and S-ICD therapy in patients under thirty years of age.

**Methods:** We reviewed database of ICD recipients in our institution between 1996 and 2021(25 years) and have chosen 115 pts consecutively implanted up to and including the age of 30 years. We retrospectively analyzed the rate of appropriate and inappropriate interventions, lead complications rate, infection rate, mortality and treatment options.

**Results:** The study group: 84 TV-ICD(age: 6–30, BMI: 16,3–22,2) and 31 S-ICD (age: 15–30, BMI; 15,6–31,1) patients. The mean follow-up in analyzed groups was 159 ± 48 and 36 ± 2 months respectively. Abnormal ventricular function: EF < 30 occurred in compared groups: 12/84(14%) and 5/31(16%) respectively, p = 0,3. 24/84pts(28%) received ≥1 appropriate therapy(AT) for VT/VF(ATP or shock) in TV-ICD and 3/31pts(10%) in S-ICD groups. 25/84 pts(30%) had one or multiple (IS) and 0% in compared groups respectively, p = 0,02. There were 18/84 (21.5%) ventricular lead dysfunctions(reimplantation of a new system) in TV-ICD and 0% in S-ICD groups, p = 0,025. An infection rate (endocarditis or device pocket) was 6/84 (7%) in TV-ICD group with complete system removal and 1 pts (3%) wound infection in S-ICD group successfully treated with antibiotics. Mortality rate was 6/84 pts (7%), caused by ventricular lead dysfunction, end stage heart failure or heat transplantation fatal result in TV-ICD group. There was no death in S-ICD group.

**Conclusions:** Endocardial ICD implantation in children and young adults is a feasible and effective procedure in a 25-year follow-up but the rate of complications is high in this population. S-ICD recipients did not experienced lead failures, systematic infections or inappropriate shocks. S-ICD appears to be a good therapy option in life-threatening arrhythmias, preventing from some serious T-ICD complications in young patients. The method and follow-up period effect is present between the TV-ICD and S-ICD in analyzed groups.

110968

Modality: E-Poster Researcher – Non-case Report

Category: EPIDEMIOLOGY AND HEALTH POLICIES/GLOBAL HEALTH

## Less Physical Activity and Increased Screen Time are Associated with Higher Stress Levels at School in Lithuania Adolescents

PRANAS SERPYTIS^1^, Egle Majauskiene^1^, Pranas Serpytis^1^

(1) Vilnius University, Faculty of Medicine, Lithuania; (2) Vilnius University, Clinic of Cardiac and Vascular Diseases, Centre of Cardiology and Angiology, Lithuania

**Introduction:** Physical activity is associated with improved health outcomes for adolescents. Many of the benefits are observed with an average of at least 60 min of moderate-to-vigorous intensity physical activity daily (1). Unfortunately, more than 80% of school-going adolescents did not meet current recommendations for daily physical activity (2).

**Objective:** We aim to analyze physical activity trends and their association with stress, weight, screen time, and other factors in Lithuania adolescents.

**Methods:** A prospective observational cross-sectional study enrolled students from High Schools in Lithuania’s capital city. A survey was administered to a cohort of students aged 13–18 years. Data from a questionnaire about behavioral risk factors, academic performance, stress level at school, and physical activity were collected. Stress levels were measured by 10 points system. Data were analyzed using SPSS v28 statistical package.

**Results:** The study population consisted of 589 adolescents (61.4% female and 38.5% male participants). The mean age was 15.79 ± 2.81 years. Adolescents engage in physical activity for 3.30 ± 1.91 days a week. Only 18.34% of adolescents’ (12.98% female vs 26.87% male; p < 0.001) physical activity lasts more than 420 min a week (60 min daily). Adolescents who exercise more, experience less stress at school (p < 0.001). Whereas screen time lasts 6.80 ± 3.23 hours daily and is associated with higher stress levels at school (p < 0.001). Smoking and alcohol consumption were significantly more common among girls (smoking: 22,37% vs 22,70%; p = 0,632; alcohol: 27.73% vs 21,15%; p < 0.001).

**Conclusions:** More than 80% of adolescents in Lithuania did not meet recommendations for daily physical activity. Girls are less active than boys and tend to have more addictions. To avoid health consequences, it is necessary to strengthen education about cardiovascular risk factors and a healthy lifestyle benefits.

110978

Modality: E-Poster Researcher – Non-case Report

Category: CARDIO-ONCOLOGY

## Effect of Exercise on Cardiorespiratory Capacity After Bone Marrow Transplantation in Oncohematologic Patients: A Pilot Study

LOURENÇO SAMPAIO DE MARA^1^, Mariana Kleis Pinto da Luz Lodi^3^, Suellen Cristina Roussenq^3^, Magnus Benetti^2^

(1) Curso de Pós Graduação em Cardio-Oncologia SBC/INC/INCA, Rio de Janeiro, RJ, Brasil; (2) Universidade do Estado de Santa Catarina-UDESC Centro de Ciências da Saúde e do Esporte – CEFID, Núcleo de Cardioncologia e Medicina do Exercício Santa Catarina, SC, Brasil,; (3) Centro de Pesquisas Oncológicas – CEPON, Santa Catarina, SC, Brasil

**Introduction:** Bone marrow transplantation (BMT) remains the best clinical alternative for the treatment of leukemias and chemotherapy-refractory lymphoid cancers. There is an increase in the survival of these patients, but with cardiovascular sequelae, which can be minimized by the effects of exercise.

**Objective:** To analyze the effect of exercise on the gain of cardiovascular capacity in patients undergoing allogeneic BMT.

**Methods:** Pilot study of a randomized controlled clinical trial with the Brazilian Registry of Clinical Trials, registry RBR-887gqvs. Fifteen patients with leukemia and lymphoma who were eligible for BMT participated, nine were randomized to the exercise intervention group (EIG), and six to the control group (CG). The EIG performed aerobic and resistance exercise during hospitalization, followed by a cycle ergometer protocol at Day Hospital, three times a week until the 100th day after BMT, with assessments of cardiovascular capacity by the six-minute walk test, which occurred before BMT or time 1 (T1), at hospital discharge or time 2 (T2) and 100 days post BMT or time 3 (T3). Data were evaluated using the SSPS software version 20.0.

**Results:** The mean age of patients in the EIG was 38.7 (±8.7) years with 78% men and for the CG, 41.7 (±15.4) years with 50% men, with no difference between the groups (p = 0.860). In the comparison between EIG and CG regarding cardiorespiratory functional capacity, there was no difference in T1 (727.5 ± 160 × 640 ± 209; p 0.232) and in T3 (885 ± 379.1 × 790 ± 162.8; p 0.794) and an increase in functional capacity in favor of the EIG in T2(813.7 ± 192 × 530 ± 209.3; p 0.009). Comparing the distances covered with the predicted value within the groups, it was observed that the EIG covered distances significantly greater than the predicted in the three different times: T1 (598.9 ± 39.7 × 727.5 ± 164.9; p = 0.038); T2 (598.9 ± 39.7 × 813.7 ± 192.7; p = 0.011); T3(598.9 ± 39.7 × 885 ± 379.1; p = 0.039). The CG covered a greater distance than predicted only in T3 (578.5 ± 21.9 × 790 ± 162.8; p = 0.046). There was no significant difference between the average distance covered predicted for the EIG and the CG (598.9 ± 39.7 × 578.5 ± 21.9; p = 0.131).

**Conclusion:** Patients who perform an exercise program during and after hospitalization for allogeneic BMT benefit from a gain in cardiorespiratory capacity.

110982

Modality: E-Poster Researcher – Non-case Report

Category: HEART FAILURE/CARDIOMYOPATHY/TRANSPLANT

## Impact of a Specialized Heart Failure Therapeutic Protocol In-Hospital Outcome and 30-Day Readmission in Patients with Acute Heart Failure

MARCELO WESTERLUND MONTERA^1^, Louise Freire^1^, Fabiola Traverso^1^, Beatriz Robert^1^, Isaura Rocha^1^, Daniele Cordeiro^1^, Ana Maria Medeiros^1^, Ana Amaral Dutra^1^

(1) Hospital Procardiaco

**Introduction and/or Fundamentals:** The treatment of acute decompensated heart failure (ADHF) in clinical practice is usually carried out by a cardiologist not specialized in heart failure (HF). The implementation of specialized HF protocols (HFP) in the treatment of ADHF has shown benefits in improving the outcomes and quality of care.

**Objectives:** To assess the benefits of HFP versus usual care(UC) in patients with ADHF in terms of clinical outcomes in in-hospital mortality(IHM), length of stay(LS), and readmission at 30 days(R30d), and the quality of care through the analysis of the medication prescription rate at hospital admission and discharge.

**Materials and methods:** Retrospective non-randomized study of a cohort of 834 pts with ADHF and HFrEF(LVEF < 50%) admitted to a private hospital in Rio de Janeiro, between 01/2015 and 12/2021 381 pts were submitted to HFP and 453 pts to the UC defined by the attending physician. All pts were evaluated for their clinical characteristics, LVEF by echocardiogram, natriuretic peptide levels. The outcomes evaluated were:LS, IHM and R30d. The rate of prescription of beta-blockers (BB) and converting enzyme inhibitors or angiotensin II receptor blockers (ACEI/ARB) and spironolactone(spiron.) were also evaluated at hospital admission and discharge.

**Results:** There was no significant difference between the groups in terms of age, sex, ADHF etiology, systolic blood pressure at admission, BNP and NT-proBNP levels and LVEF. HFP pts had a lower rate of IHM(11% vs 20%;p < 0.0001), and LS(7.4 ± 6.9 vs 10.5 ± 12.8 days;p < 0.001), and with no significant difference regarding R30d (4.3% vs 7.2%; p = 0.2). At admission the HFP pts showed a higher rate of ACEI/ARB prescription (45% vs 36%; p < 0.006) and higher dosage of intravenous furosemide (120 ± 60 mg vs 60 ± 30 mg, p < 0.01) and no difference in BB prescription rate(75% vs 70%; p < 0, 15). At discharge, the HFP pts had a higher rate of prescription of ACEI/ARB (89% vs 43%; p < 0.0001) and spiron. (50% vs 27%; p < 0.0001) and furosemide(72% vs 62% p = 0.05) and no difference in BB prescription rate(87% vs 84%; p = 0.2).

**Conclusions:** The treatment of pts with ADHF with HFP compared to UC, had an impact on improving in-hospital clinical outcomes with lower IHM and LS and they also had a better quality of care with a higher rate of prescription of ACEI/ARB and higher dosage of intravenous furosemide at admission, and a higher rate of prescription of ACEI/ARB, spiron. and furosemide at hospital discharge.

110986

Modality: E-Poster Researcher – Non-case Report

Category: HEART FAILURE/CARDIOMYOPATHY/TRANSPLANT

## Differential Diagnosis between Takotsubo Cardiomyopathy and Acute Myocardial Infarction at Hospital Admission

MARCELO WESTERLUND MONTERA^1^, Louise Freire^1^, Arnaldo Rabischoffsky^1^, Amarino de Oliveira^1^, Luiz Antonio Carvalho^1^, Ana Amaral Dutra^1^

(1) Hospital Procardiaco

**Objectives:** Identify the clinical characteristics and complementary tests that allow the differentiation of Takotsubo from Acute Myocardial Infarction (AMI) with ventricular dysfunction, at hospital admission.

**Materials and methods:** Retrospective study of a cohort of patients admitted to a private hospital in Rio de Janeiro, from 2010 to 2020, with a diagnosis of Takotsubo and AMI with ventricular dysfunction (LVEF < 40%). The variables evaluated were: 1) clinical characteristics: age, gender, clinical presentation, presence of decompensating factor (DF), systolic blood pressure (SBP) and heart rate (HR). 2) ECG changes: T wave inversion, ST segment changes, pathological Q wave; 3) changes in the echocardiogram (ECO) regarding the location and type of segmental contraction 4) Levels of TpI, BNP, NT-proBNP, leukocytes and CRP. All patients initially underwent coronary angiography. The t test for independent samples, chi-square and logistic regression were used.

**Results:** 65 pts were evaluated with Takotsubo and 75 pts with AMI. There were no differences between the groups regarding age, clinical presentation, SBP and HR. There were also no differences regarding the levels of BNP and NT-proBNP, TpI, leukocytes and CRP at admission. Pts with Takotsubo had a higher prevalence of females (84.6% vs 13.8%, P < 0.0001) and identification of a DF in 78% of Takotsubo pts. The ECG in the pts with Takotsubo showed a higher prevalence of T wave inversion (38% vs 12.5%; p = 0.0005) with no differences in the other ECG alterations compared to the pts with AMI; The ECHO of the pts with Takotsubo showed a higher prevalence of akinesia in Apical (69% vs 32%; P < 0.001), anterior (23% vs 5.5%; P < 0.003) and Lateral (32% vs 2.7%, P < 0.0001)walls. In the logistic regression, the variables that showed high significance and predict the diagnosis of Takotsubo were:female (OR:9.8;CI:3.1–30.8); T wave inversion on ECG(OR: 5.8; CI: 1.45 to 23.71); Apical (OR:36.12;CI:4.8–271.7) and Anterior akinesia (OR:17.3;CI:2.8–109.8) on ECHO.

**Conclusões:** In the differential diagnosis of Takotsubo from AMI on hospital admission, the presence of female Pts, with DF, T-wave inversion on ECG, anterior or apical akinesia on ECHO, strongly suggest the diagnosis of Takotsubo in the face of AMI with ventricular dysfunction.

110998

Modality: E-Poster Researcher – Non-case Report

Category: DIGITAL HEALTH/INNOVATION

## Microrna in the Cardiac Variant of Fabry‘s Disease: New Biomarker?

ANDREA VIRGINIA FERREIRA CHAVES^1^, Débora Nascimento da Nóbrega^3^, Marcelo Santos Veloso^4^, Eveline Barros Calado^5^, Manuel Markman^2^, Anabel Lima Vieira^2^, Milca Dantas da Silva^1^, Brivaldo Markman Filho^5^, Luydson Richardson Silva Vasconcelos^3^, Dinaldo Cavalcanti de Oliveira^5^

(1) Rarus – Serviço de Doenças Raras de Recife; (2) Hospital Agamenon Magalhães, Recife, PE; (3) Instituto Aggeu Magalhães; (4) Hospital Alfa, Recife; (5) Universidade Federal de Pernambuco – UFPE

**Introduction:** Molecular investigation of hypertrophic cardiomyopathy (HCM) through genetic panels makes it possible to identify phenocopies with left ventricular hypertrophy (LVH), as Fabry disease (FD). New biomarkers are being studied, including microRNAs (miRNAs).

**Objective:** To investigate miRNAs as pathogenicity markers in the cardiac variant of FD.

**Methods:** A total of 131 patients (pts) with HCM who underwent genetic investigation for FD were included, of which 76 were through GLA sequencing gene and 55 by the genetic panel for HCM.

**Results:** In 7 pts (5.34%) mutations were found in the GLA gene and the following variants were identified: 1 c.967C>A(p.Pro323Thr), 4 c.352C>T(p.Arg118Cys) and 2 c.937G>T(p.Asp313Tyr). Pts with mutations in the GLA gene were submitted to the panel for HCM: 3 pts (p.Arg118Cys) had mutations in other genes and in 4, there were no other detecting variants. The family screening revealed 17 individuals with mutations in the GLA gene, and 2 siblings were identified with LVH with the c.937G>T(p.Asp313Tyr) variant. The pts and the carriers of variants in the GLA gene were submitted to the determination of lyso-Gb3 and the analysis of the following miRNAs: miR-1291, miR-214, and miR-505. In the comparison between individuals with and without LVH, there was no significant difference for miRNAs, however, there was a tendency for overexpression in individuals with LVH, especially for miR-1291. The expression of miR-214 in subjects with LVH was significantly lower than in subjects without LVH (p = 0.0416). There was a significantly higher expression for miR-505, both in carriers of variants in the GLA gene without LVH and in those with LVH (p = 0.0195). The miR-505 and miR-214 showed a significant and directly proportional correlation with lyso-Gb3 in pooled pts (with and without LVH). All subjects had normal lyso-Gb3 levels.

**Conclusion:** In this study, provides evidence that increased miR-505 and miR-1291 expression and miR-214 under-expression in those with LVH may be pathogenic markers in FD. Further investigations of these microRNAs with a larger number of individuals are necessary.


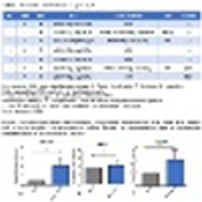


111032

Modality: E-Poster Researcher – Non-case Report

Category: HEMODYNAMICS AND INTERVENTIONAL CARDIOLOGY

## Femoral Arterial Puncture Guided by Fluoroscopy Versus Puncture Guided by Anatomical Parameters Alone: A Prospective Randomized Clinical Trial

INGRID LOUREIRO DE QUEIROZ LIMA^1^, RICARDO ZAUTIH SILVA^1^, FABIO AUGUSTO PINTON^2^, JOSE MARIANI JUNIOR^3^

(1) HOSPITAL IRMANDADE SANTA CASA DE SAO PAULO; (2) HOSPITAL SIRIO LIBANES; (3) HOSPITAL ISRAELITA ALBERT EINSTEIN

**Objective:** To evaluate whether puncture guided by fluoroscopy was more effective for accessing the common femoral artery (CFA) than puncture guided by anatomical parameters alone.

**Background:** Although radial vascular access is becoming the preferred route of access for most procedures in interventional cardiology, CFA remains the major vascular access used in practice. Properly accessing the femoral artery reduces the incidence of vascular complications; however, in some cases, the anatomical parameters may not adequately guide the appropriate route of access to the CFA.

**Methods:** Single-center, prospective, controlled, randomized study, performed between June and December of 2015, evaluated 158 patients subjected to elective coronary angiography guided by fluoroscopy or angiography guided by classical anatomical parameters alone. In all patients, angiography was performed at the site of vascular access and was considered appropriate if the access route to the CFA was above the bifurcation of the CFA and below the inferior epigastric artery.

**Results:** Seventy-nine patients underwent puncture guided by anatomical parameters alone, and 79 underwent puncture guided by fluoroscopy. Puncture guided by fluoroscopy achieved greater success rates than did the use of anatomical parameters alone (92.4% vs. 64.6%; p < 0.001). In all groups analyzed, the one guided by fluoroscopy had more appropriate punctures than did the group using anatomical parameters alone. The independent predictors of inadequate puncture were age > 65 years and puncture guided by anatomical parameters alone.

**Conclusions:** Puncture guided by fluoroscopy increased the likelihood of adequate access to the CFA compared with puncture guided by anatomical parameters alone.

111048

Modality: E-Poster Researcher – Non-case Report

Category: ATHEROSCLEROSIS/CARDIOVASCULAR RISK FACTORS/CARDIOVASCULAR PREVENTION

## Correlation between Carotid Artery Disease (CAD) and Peripheral Arterial Disease (PAD)

PALJOSKOVSKA JORDANOVA SAVETKA^1^, D-r Danica Petkoska Spirova^1^, D-r. Sasha Kjaeva Anastasova^1^, D-r Dejan Risteski^1^, D-r Emilija Lazarova Trajkovska^1^, D-r Enes Shehu^1^, D-r Aleksandra Eftimova^1^, Prof. D-r Marijan Bosevski^1^

(1) University Clinic of Cardiology

**Introduction:** Peripheral arterial disease (PAD) is a condition in which the arteries are narrowed and they can’t carry as much blood to the outer parts of the body. Carotid artery disease (CAD) causes a narrowing of the major blood vessels that supply the brain. Both are known to be specific manifestations of atherosclerosis. We aimed to evaluate patients with documented CAD who also have PAD and investigate the risk factors that should be considered when talking about these vascular diseases.

**Material and methods:** This study included a population of 1031 patients with documented carotid artery disease is defined as a presence of plaque or stenosis confirmed with B-mode ultrasonography. 66,6% were men and 33,5% were women. The average age was 64.44 years. We investigate how many patents with CAD have PAD and evaluate the potential risk factors.

**Results:** This analysis reveals that 1030 out of 1031 patient (99,9%) have CAD and 1023 out of 1031 patient (99.2%) also has PAD. The most common risk factor is HTA in 1026 patients or 99.5% followed by diabetes mellitus in 1025 patients or 99.4%, tobacco smoking in 958 patients or 92.9%, HLP in 958 patients or 86.8% and also obesity found in 351 patients or 34%.

**Conclusion:** PAD is strongly associated with carotid artery disease. According to our study, these diseases seem to occur more commonly in men that women. We can conclude that the most prevalent factors that can influence the risk of developing an outcome are hypertension and diabetes mellitus. In accordance with this, these patients need more intensive medical management to prevent complications.

111244

Modality: E-Poster Researcher – Non-case Report

Category: ACUTE AND CHRONIC CORONARY DISEASE/THROMBOLYSIS

## Acute Coronary Syndrome During COVID-19 Pandemic: What Changed? a Single Center Retrospective Database

CONRADO ROBERTO HOFFMANN FILHO^1^, Conrado Roberto Hoffmann Filho^1^, Benjamin Massao Harada^1^, João Paulo Souza Brighenti^1^, Michele Mendonça^1^, Beatriz Roca^1^, Gilmar Sidnei Erzinger^2^, Esther Botelho^1^, Gabriel Erzinger^2^, Jaqueline Barp^1^, João P.P. Ribeiro^2^

(1) Hospital Regional Hans Dieter Schmidt; (2) Univille Universidade da Região de Joinville

**Introduction:** Acute myocardial infarction remains the leading cause of death in Brazil and worldwide with high mortality rates, especially when adequate treatment is not performed. During the period of the COVID-19 pandemic, the demand for care services caused by acute coronary syndromes (ACS) was reduced by several factors.

**Objetives:** The present study was carried out to evaluate the management of our institution in the treatment of ACS during the COVID-19 pandemic.

**Methods:** Retrospective cohort study of patients treated at a tertiary center from 01/01/21 to 01/31/21, through the analysis of hospital’s electronic medical records. 582 patients were selected. SPPS software was used. Student’s T test to compare numerical variables. Categorical variables were expressed as frequencies and percentages and compared using the chi-square test. Pearson’s correlation used as needed. Quantitative variables were expressed as mean and standard deviation.

**Results:** Mean age did not differ between men and women 62.1% and 62.8% respectively. The ACS were divided in unstable angina (UA) in 30.6% and 32.1%, Non ST Elevation Myocardial Infarction (NSTEMI) 36.5% and 31.5% and ST elevation myocardial infarction (STEMI) 32.3% and 36.2%. Among coronary lesions >70%, Left anterior descending 61.4% and 64.1%, right coronary 45.6% and 49.3%, circumflex 28.9% and 35.8%, left main >50% 1.1% and 6.3%. The presence of left main lesion was higher in men. Significant positive correlation was observed between age and left descending lesion p = 0.04. In-hospital death was greater among older (67.7 and 61.9 years respectively) p = 0.003. Despite the number of deaths being significantly higher (p = 0.001) in STEMI (4.6%) compared to NSTEMI (2.4%) and UA (0.5%), the mean age was significantly lower in the first p = 0.03. Angioplasty was the most used treatment with 68.2% of cases and myocardial revascularization in 5%, p = 0.0005. Regarding treatment, about 87% used ASA, 80% antiplatelet drugs, statins 88%, ACEI/ARB 88.9% beta-blockers 85.6%, spironolactone 16.5% and insulin 8.6%. Comparing our data with literature, we found that the in-hospital mortality rate is similar (7.6%), the mean age is lower (62% and 68% respectively). The number of coronary angioplasty is higher (68.2% and 38.5%). And the number of revascularizations is lower (5% and 7.8% respectively).

**Conclusions:** The pandemic reduced the number of patients who went to the hospital, however the standard treatment of ACS was maintained.

111065

Modality: E-Poster Researcher – Non-case Report

Category: ACUTE AND CHRONIC CORONARY DISEASE/THROMBOLYSIS

## In-Hospital Mortality Risk Predictors of Acute ST-Elevation and Non-ST-Segment Elevation Myocardial Infarction

RICARDO MOURILHE-ROCHA^1^, Daniel Xavier de Britto Setta^1^, Marcelo Luiz da Silva Bandeira^1^, Thiago Matos Barcellos^1^, Flávia Prado Fialho Santos^1^, Eric Costa de Almeida^1^, Julia Paulo Mourilhe Rocha^1^, Bruno Reznik Wajsbrot^1^, Roberta Siuffo Schneider^1^, Ricardo Mendes Carneiro^1^, Vitor Hugo Mussi Campos^1^, Fernando Oswaldo Dias Rangel^1^

(1) HOSPITAL PRÓ-CARDÍACO

**Background:** Acute myocardial infarction is a prevalent disease with high morbidity and mortality. The recognition of mortality predictors allows the identification of patients at higher risk of death.

**Objective:** To identify the risk predictors of in-hospital mortality.

**Materials and Methods:** Observational, retrospective, cohort study of 398 patients admitted with a confirmed diagnosis of AMI, with and without ST-segment elevation (STEMI and NSTEMI) between January 2018 and January 2022. Data were subsequently analyzed by SPSS software.

**Results:** Among the comorbidities analyzed, mortality in the presence of previous diseases was heart failure (HF) occurred in 20.6% and 6.3% in those without HF (p = 0.03); previous stroke (stroke) in 21.4% and without stroke of 6.5% (p = 0.04); with atrial fibrillation (AF) 22% and without AF in 5.9% (p < 0.01). The mortality found in patients with different characteristics was: Killip I was 2.5% and in Killip >= 2 was 31.7% (p < 0.001); EF > 50% was 1.2% and EF <= 50% was 14% (p < 0.001); STEMI was 13.7% and NSTEMI was 4.7% (p = 0.02); without angioplasty was 13.3% and with angioplasty was 6.1% (p = 0.027). Binary logistic regressions were performed to check if such factors are independent predictors of risk, being statistically significant: ejection fraction <50%, OR 6.2 95% CI (1.6–23.6), p = 0.007; and Killip classification >= 2, 95% CI (3.0–25.7), p < 0.001.

**Conclusions:** The predictors of mortality were the presence of EF <= 50% and Killip >= 2. Such factors are related to the extent of myocardial injury caused by AMI and the presence of previous heart disease. Early recognition of these circumstances may allow the development of care aimed at reducing this outcome.

111071

Modality: E-Poster Researcher – Non-case Report

Category: PHYSIOTHERAPY

## Whole-Body Vibration Exercise in the Management of Cardiovascular Diseases: A Systematic Review

ANA INÊS GONZÁLES^1^, Ana Inês Gonzáles^1^, Amanda da Silva^1^, Gabriella Lavarda do Nascimento^1^, Mario Bernardo-Filho^2^, Danúbia da Cunha de Sá-Caputo^2^, Anelise Sonza^1^

(1) Universidade do Estado de Santa Catarina (UDESC) – Centro de Ciências da Saúde e do Esporte (CEFID); (2) Universidade do Estado do Rio de Janeiro

**Introduction:** In the management of cardiovascular diseases (CVD), the adequate exercise prescription is an essential condition. In this scenario, the whole body vibration exercises (WBV) appear as a promising therapeutic resource.

**Objective:** To investigate the effects of WBV alone or associated with other types of exercises in the management of CVD.

**Methods:** This study is a systematic review following the PRISMA guidelines and registered PROSPERO under number CRD42021230663. The databases used were PubMed, Cochrane, PEDro, Lilacs, and Science Direct, from the beginning of the databases until January 2021. The descriptors related to WBV and CVD used were: [(Hypertension OR Heart Diseases OR Heart Failure OR Coronary Artery Disease OR Myocardial Infarction) AND (Whole Body Vibration OR Whole-Body Vibration)]. The following inclusion criteria were determined:studies such as controlled and randomized clinical trials, quasi-randomized controlled trials, comparative studies with or without simultaneous controls, case studies, case series with ten or more consecutive cases, cross-sectional studies, and pilot studies. The population was composed by adults (≥18 years), of both sexes, with a clinical diagnosis of CVD, submitted to intervention with the exercise of WBV. Studies in Portuguese, English, and Spanish were included. The selected studies were assessed for quality, risk of bias, and level of evidence.

**Results:** Three studies were included, with CVD submitted to WBV as an isolated intervention (35 females/12 males). There was variation in the parameters applied in the WBV. There were no abnormal cardiovascular responses in the three studies, and no adverse events were reported. The reactive hyperemia index (RHI) showed a significant increase after WBV in several subgroups and the muscle strength measure showed a significant increase in muscle strength when compared to the control group in the two studies. There were improvements in the parameters of systolic and diastolic blood pressure, pulse pressure, augmented pressure, augmentation index, AIx adjusted to 75 beats per minute, first systolic and second systolic peak and mean arterial pressure.

**Conclusion:** The use of different IVC protocols, alone, to improve hemodynamic, cardiovascular, vascular/arterial and muscular parameters in individuals with CVD is plausible and can be considered a safe and effective training resource.

111077

Modality: E-Poster Researcher – Non-case Report

Category: CARDIOLOGY OF SPORTS, EXERCISE, ERGOMETRY AND CARDIOVASCULAR REHABILITATION

## Implementation of a Home-Based Cardiac Rehabilitation Program in Brazil: An Effectiveness Analysis

ANA INÊS GONZÁLES^1^, Ana Inês Gonzáles^1^, Yolanda Gonçalves da Silva Fontes^1^, Daiane Pereira de Lima^1^, Tatiane Boff Centenaro^1^, Tales de Carvalho^1^

(1) Universidade do Estado de Santa Catarina (UDESC) – Centro de Ciências da Saúde e do Esporte (CEFID)

**Introduction:** Cardiac Rehabilitation is considered an effective strategy in the treatment of individuals with Cardiovascular Diseases and has substantive evidence base on its benefits. However, in Brazil there is a predominance of programs based on rehabilitation centers that provide access to a minority of patients, providing low adherence to rehabilitation. Given this situation, alternative models are plausible to be tested, with home-based CR being a possibility to expand access and adherence of individuals.

**Objective:** To compare adherence, quality of life (QoL), functional capacity and perception of barriers in patients undergoing center-based (CB) and home-based (HB) CR.

**Methods:** Individuals with Coronary Artery Disease, randomized into two groups (CB and HB), were submmited to a 12 week of aerobic aerobic training with walking and patient education. Exercise prescription was individualized, based on heart rate (HR) determined by the ventilatory thresholds of the cardiopulmonary exercise test. In all, 36 physical exercise sessions were proposed. The sessions in the CB group were all supervised and monitored in person and in the HB only 3 supervised (one per month) and 33 at home (streets, squares, parks), with remote monitoring and monitoring (asynchronous). Adherence to sessions was evaluated by the average percentage of sessions performed. QOL and functional capacity were measured before and after 3 months using Short Form 36 and peak oxygen consumption, respectively. Barriers were verified after 3 months by the Scale of Barriers for CR (EBRC).

**Results:** 21 (32.3%) individuals participated in the study, distributed in CB (N = 11) and HB (N = 10). HB showed higher adherence in the number of prescribed sessions (p < 0.001) and extra sessions (p = 0.001). Regarding QoL, only a significant intergroup difference was observed. In the HB group, in the domains of functional capacity (p = 0.04), limitations due to physical aspects (p = 0.04) and emotional aspects (p = 0.04). In the CB group, regarding the functional capacity domains (p = 0.01) and limitations due to physical aspects (p = 0.01). There was no difference between groups and between groups for peak VO2 values. Regarding the perception of barriers, the CB group had the highest total score (p < 0.01).

**Conclusions:** HB adherence was higher, with better results in the emotional aspect of QoL and lower perception of barriers.

111076

Modality: E-Poster Researcher – Non-case Report

Category: ACUTE AND CHRONIC CORONARY DISEASE/THROMBOLYSIS

## Quality of Care: Getting with the Guidelines in Patients Hospitalized with Acute Myocardial Infarction

DANIEL XAVIER DE BRITTO SETTA^1^, Marcelo Luiz da Silva Bandeira^1^, Bruno Reznik Wajsbrot^1^, Julia Paulo Mourilhe Rocha^1^, Thiago Matos Barcellos^1^, Flávia Prado Fialho Santos^1^, Claudia Lanzillotti Weksler^1^, Roberta Siuffo Schneider^1^, Ricardo Mendes Carneiro^1^, Vitor Hugo Mussi Campos^1^, Fernando Oswaldo Dias Rangel^1^, Ricardo Mourilhe-Rocha^1^

(1) HOSPITAL PRÓ-CARDÍACO

**Background:** Acute myocardial infarction (AMI) is a prevalent disease with high morbidity and mortality. The measurement of quality indicators in health care enables the recognition of weaknesses and the construction of action plans to improve care. One of the main indicators is the utilization rate of survival modifying medications at the time of hospital discharge, whose internationally recommended goal is at least a 90% utilization rate.

**Objectives:** To evaluate the rate of use of survival modifying drugs in AMI care [ASA, P2Y12 inhibitors, beta-blockers, statins and iECA or ARB (the latter in the presence of ventricular dysfunction) at hospital discharge].

**Methods:** This is an observational, retrospective, cohort study of 398 patients admitted with a confirmed diagnosis of AMI with and without ST-segment elevation between January/2018 and January/2022. Data were collected by a trained healthcare professional team and subsequently analyzed by SPSS software.

**Results:** The diagnosis of STEMI (68.8%) was predominant. Drug utilization rates at discharge in the general population and subgroups were: AAS = 96.9% (97.6% in NSTEMI and 95.2% in STEMI; p = 0.23), P2Y12 inhibitors = 93.5% (93.9% in NSTEMI and 92.5% in STEMI; p = 0.59), beta-blockers = 86.9% (81.3% in NSTEMI and 83.8% in STEMI; p = 0.4), iECA or ARB = 82.9% (71.0% in NSTEMI and 60.0% in STEMI; p = 0.23), statins = 93.8% (93.5% in NSTEMI and 94.3% in STEMI; p = 0.79).

**Conclusions:** The ASA, P2Y12 inhibitor, and statin utilization rates were within the recommended target. However, the usage rates of beta-blockers and ACEI or ARB were lower. The recognition of these indicators allows the initiation of strategies to expand the use of these drugs and improve outcomes in the medium and long term.

111102

Modality: E-Poster Researcher – Non-case Report

Category: SPIRITUALITY AND CARDIOVASCULAR MEDICINE

## Spirituality/Religiosity in the Cardiology Outpatient Clinic: How are we 02 Years After the Publication of the Updated Cardiovascular Prevention Guideline of the Brazilian Society of Cardiology (Spirituality and Health)?

ADELLE CRISTINE LIMA CARDOZO^1^, José Icaro Nunes Cruz^1^, Giulia Vieira Santos^1^, Jade Soares Dória^1^, Camille Marques Aquino^1^, Juliana Maria Chianca Lira^1^, Philipi Santos Soares^1^, Diego Maldini Borba de Lima^1^, Mariano César de Souza Reis^1^, Antônio Carlos Sobral Sousa^2^, Enaldo Vieira de Melo^1^, Joselina Luzia Menezes Oliveira^2^

(1) Federal University of Sergipe; (2) Rede D’Or São Luiz – São Lucas Hospital; (3) Primavera Hospital

**Introduction:** Several studies analyze the influence of Spirituality/Religiosity (S/R) on cardiovascular diseases, considered the leading causes of global morbidity and mortality. The brazilian Updated Cardiovascular Prevention Guideline (2019) shows the importance of S/R in healthcare and recommends its approach in clinical practice.

**Objectives:** To describe the clinical profile and S/R in cardiology patients and to evaluate the acceptance of the S/R approach during the medical consultation.

**Methods:** Cross-sectional, descriptive study. The sample included patients from cardiology outpatient clinics of three hospitals in Sergipe (Brazil). A questionnaire of self elaboration and two scales were applied: Duke Religiosity Index (DUREL) and Brief Multidimensional Measure of Religiosity/Spirituality (BMMRS). The values obtained in the constructs and domains of the scales were entered into the database so that they scored directly proportional to the level of S/R.

**Results:** 130 patients were included in the study (mean age 60.6 ± 11.2 years). 62.3% were female. The prevalences of hypertension, dyslipidemia, coronary artery disease, and diabetes mellitus were 73.8%, 59.2%, 40.8%, and 35.4%, respectively. 97.6% of the patients believe in God and 79.8% believe that S/R helps to cope with the diseases. 96.9% would like to have S/R addressed in a doctor’s appointment, however 78.1% stated that this topic was never addressed by their doctors. 96.9% said they were Christian (76.2% Catholic, 19.8% Evangelical, and 2.4% Spiritualist), 3.8% said they had no religion, and 0.8% were Umbanda believers. The domains with the highest mean scores on the BMMRS were “Values and Beliefs” (7.1 ± 1.2; scale maximum: 8.0) and “Religious and Spiritual Overcoming” (25.0 ± 2.9; scale maximum: 28.0). The means of the constructs organizational religiosity, non-organizational religiosity, and intrinsic religiosity (DUREL) were 4.1 ± 1.5, 4.6 ± 0.8, 13.8 ± 2.1, respectively, all of them considered high.

**Conclusions:** Cardiology patients had a high level of religiousness in this study. Almost the totality of the sample believed that S/R should be addressed during medical consultations. Despite this, most stated that their physicians never addressed these topics during consultations, denoting a mismatch between clinical practice and the recommendation of the Guideline.

111090

Modality: E-Poster Researcher – Non-case Report

Category: CARDIAC ARRHYTHMIAS/ELECTROPHYSIOLOGY/ELECTROCARDIOGRAPHY

## Predictors of Mortality in Patients Hospitalized for Acute Atrial Fibrillation

RICARDO MOURILHE-ROCHA^1^, Bruno Reznik Wajsbrot^1^, Ricardo Mendes Carneiro^1^, Eric Costa de Almeida^1^, Julia Paulo Mourilhe Rocha^1^, Thiago Matos Barcellos^1^, Flávia Prado Fialho Santos^1^, Claudia Lanzillotti Weksler^1^, Ana Amaral Ferreira Dutra^1^, Tiago Azevedo Costa Mattos^1^, Fernando Oswaldo Dias Range^1^, Daniel Xavier de Britto Setta^1^

(1) HOSPITAL PRÓ-CARDÍACO

**Introduction:** Atrial fibrillation (AF) is the most common sustained cardiac arrhythmia in clinical practice. Epidemiological data have shown that AF is a huge public health problem due to its association with a high risk of death and functional loss. Characterizing mortality predictors allows identifying patients at higher risk for evolution to death.

**Objective:** To identify risk predictors for in-hospital mortality in patients with atrial fibrillation.

**Methods:** Retrospective and consecutive cohort of patients hospitalized for acute atrial fibrillation between January 2018 and December 2021 at a tertiary hospital in Rio de Janeiro. Demographic, clinical, laboratory and mortality data were analyzed, obtained from a database collected by reviewing electronic medical records and subsequently performing univariate and multivariate statistical analysis using SPSS 20.0.

**Results:** There were 819 patients, with a male predominance (57.6%), mean age of 71 +– 14.4, 72.6% aged >65 years, 66.4% hypertensive, 21.7% diabetic, 12% with HF, 8.2% with stroke or previous TIA, 21.6% with previous coronary artery disease and 8.6% with CABG surgery. The median of CHA2DS2-VASc was 3 and the median of HASBLED was 2. There was reversion to sinus rhythm in 69.9% of the patients. The general mortality of the population was 0.9%. Mortality in patients with CHA2DS2-VASc >= 3 was 1.2% vs 0 in those with CHA2DS2-VASc <3 (p = 0.02). In patients with CHA2DS2-VASc >= 2 or +, 0.9% died vs no death in patients with CHA2DS2-VASc <2 (p = 0.092). Analyzing the VASc variables (from CHA2DS2-VASc), if variables are present it increases mortality by more than 3 times (0.5% vs 1.8%; p = 0.071). Another important parameter was age >= 75 years, which almost triples mortality (0.5% aged < 75 years vs 1.3% aged >= 75 years (p = 0.193). All other variables showed no relationship to the mortality.

**Conclusion:** The only predictor of mortality in this population was CHA2DS2-VASc >= 3. Early recognition of higher-risk patients can provide a more targeted care plan aimed at early discharge without increasing negative outcomes.

111113

Modality: E-Poster Researcher – Non-case Report

Category: SPIRITUALITY AND CARDIOVASCULAR MEDICINE

## Spirituality/Religiosity and Resilience in Patients with Chronic Coronary Syndrome

ADELLE CRISTINE LIMA CARDOZO^1^, José Icaro Nunes Cruz^1^, Jade Soares Dória^1^, Camille Marques Aquino^1^, Gabriela de Oliveira Salazar^1^, Bruna Souza Matos de Oliveira^1^, Diego Maldini Borba de Lima^1^, Giulia Vieira Santos^1^, Juliana Maria Chianca Lira^1^, Antônio Carlos Sobral Sousa^1^, Enaldo Vieira de Melo^1^, Joselina Luzia Menezes Oliveira^2^

(1) Federal University of Sergipe; (2) Rede D’Or São Luiz – São Lucas Hospital; (3) Primavera Hospital

**Introduction:** Despite advances in treatment, chronic coronary syndrome (CCS) persists as an important cause of global morbidity and mortality worldwide. Spirituality/Religiosity (S/R) has been pointed out as an important dimension in health care, including cardiovascular diseases.

**Objectives:** To evaluate S/R in patients with CCS and its association with Resilience.

**Methods:** This is an observational, cross-sectional, analytical study. The sample included patients from cardiology outpatient clinics of three hospitals in Sergipe. The patients included were divided into two groups: I) CCS; II) Control. Three questionnaires were applied: Duke Religiosity Index (DUREL); Brief Multidimensional Measure of Religiosity/Spirituality (BMMRS), and Connor-Davidson Resilience Scale for Brazil-10. The scales were quantified ordinally according to the progression of the degree of S/R, so that the higher the average on the scale, the higher the level of S/R. Mean scores on the scales were compared using Student’s t-test. Demographic characteristics and comorbidity profile were described in terms of means and frequencies and compared between groups using the Chi-square or Fisher’s exact test.

**Results:** Were included 130 patients, of whom 53 (40.8%) belonged to the CCS group and 77 (59.2%) to the Control group. Ages were not different between the CCS and Control groups (62.2 vs. 59.4 years; p > 0.05). There was also no difference between the groups for the following variables: marital status, work status, diabetes mellitus, hypertension, sedentary lifestyle, and obesity (p > 0.05), however, dyslipidemia was more prevalent in the CCS group (80.0% vs. 50.7%; p < 0.001). According to the BMMRS, patients in the CCS group believe that “God punishes them” more than control patients (3.1 vs. 3.5; p < 0.05). In addition, the CCS group believed that “people in their religious communities would offer them less comfort in difficult times” than patients in the Control group (2.8 vs. 3.2; p < 0.05). There were no differences between the groups for the DUREL or the Resilience Scale.

**Conclusions:** The results suggest that patients with CCS are more likely to believe that “God punishes them” and that “people from their religious communities would offer them less comfort in difficult times”. There was no correlation between S/R and Resilience. Studies with larger sample sizes are needed to better understand this last association.

111123

Modality: E-Poster Researcher – Non-case Report

Category: HEART FAILURE/CARDIOMYOPATHY/TRANSPLANT

## The Importance of Chloride in Chronic Heart Failure

MARTA AMORIM^1^, Diana Ferrão^1^, Marta Carreira^1^, Fernando Nogueira^1^, Pedro Ribeirinho Soares^1^, Sergio Madureira^1^, Rita Gouveia^1^, Catarina Elias^1^, Ana Neves^1^, Joana Pereira^2^, Patrícia Lourenço^3^

(1) Heart failure clinic of the Internal Medicine Department of Centro Hospiralar e Universitário São João; Faculdade de Medicina da Universidade do Porto; (2) Heart failure clinic of the Internal Medicine Department of Centro Hospiralar e Universitário São João; (3) Heart failure clinic of the Internal Medicine Department of Centro Hospiralar e Universitário São João; Cardiovascular Research and Development Unit of Faculty of Medicine of Porto University

**Introduction:** Low Chloride (Cl) has been associated with worse prognosis in heart failure (HF). Still, results are controverse and mainly based in old Randomized controlled trials.

**Objectives:** To assess determinants of Cl levels and to study the prognostic role of Cl in a contemporary real-world chronic HF population.

**Methods:** Retrospective analysis of chronic ambulatory HF patients followed in a HF clinic. Adult patients with history of HF with left ventricular systolic dysfunction (LVSD) attending the clinic between January 2012 and May 2018 were included. Patients with no Cl measurement were excluded. Follow-up: until February 2022. Primary endpoint: all-cause mortality. Multivariate linear regression analysis to assess independent determinants of Cl level. The prognostic impact of Cl was evaluated by Cox-regression analysis. Cl was assessed both as a categorical (cut-off 101 mmol/L – percentile 33.3) and as a continuous variable. Multivariate adjustment was performed accounting for: age, gender, LVSD, NYHA class, arterial hypertension, diabetes, atrial fibrillation, ischaemic aetiology, BNP, renal function, serum sodium, loop and thiazide diuretics and evidence-based therapy.

**Results:** We studied 859 patients. Mean age 70 years, 66.3% male, 44.9% had ischaemic HF and 46.7% had severe LVSD; 84.3% were on renin-angiotensin system inhibitors (RASi), 92.8% on beta blockers and 29.1% on mineralocorticoid receptor antagonists. Cl ranged from 85 to 116 mmol/L. Independent determinants of decreased Cl levels were Diabetes, severe LVSD, higher NYHA class and lower natremia. RASi medication associated with higher Cl and loop and thiazide diuretics predicted lower serum Cl. During a median follow-up of 56 (29–90) months 464 (53.4%) patients died: 45.9% in those with Cl > 101 mmol/L and 66.6% in the remaining, p < 0.001. Patients with Cl ≤ 101 mmol/L) had a multivariate adjusted HR of death of 1.27 (95% CI 1.02–1.57), p = 0.03 compared to those with higher Cl. Per each 2 mmol/L increase in Cl levels the all-cause death HR was 0.94 (95% CI: 0.89–1.00), p = 0.048.

**Conclusions:** Low Cl is an independent predictor of all-cause mortality in HF with LVSD. Patients with Cl ≤ 101 mmol/L have a 27% increased risk of death and per each 2 mmol/L Cl increase there is a 6% decrease in the death risk. Main determinants of lower Cl are diuretic use, concomitant diabetes, worse LVSD and higher NYHA class; use of RASi and higher sodium levels are independently associated with higher Cl levels.

111134

Modality: E-Poster Researcher – Non-case Report

Category: HEART FAILURE/CARDIOMYOPATHY/TRANSPLANT

## Effect of Prolonged Oral Pentoxifylline in Patients with Chronic Chagas’ Cardiomyopathy: Results of a Prospective, Randomized, Double-Blind Pilot Study

MARCUS VINICIUS SIMÕES^1^, Káryta Suely Macedo Martins^1^, Antonio Carlos Barros-Filho^1^, Minna Moreira Dias Romano^1^, Denise Mayumi Tanaka^1^, Paulo Louzada-Junior^1^, Edecio Cunha-Neto^2^, José Antonio Marin-Neto^1^

(1) Faculdade de Medicina de Ribeirão Preto – USP, Ribeirão Preto, SP, Brasil; (2) Incor – Instituto do Coração – FMUSP

**Background:** The essential histopathologic lesion in chronic Chagas’ cardiomyopathy (CCC) is a relentless low grade myocarditis with increased production of cytokines. Recent experimental studies have suggested that pentoxifylline, an immunomodulator that reduces the production of TNF-alfa, may be associated to a better evolution of CCC.

**Purpose:** We tested the effect of pentoxifylline in cytokines profile and cardiac function in patients with CCC.

**Methods:** This is a prospective double-blind clinical study, enrolling 38 CCC patients (age: 60 +/– 13 y.o., 66% male, LVEF = 48 +/– 7%), randomized to pentoxifylline (PTX, n = 19), 400 mg 3x/day for 6 months or placebo (PLC, n = 19). At baseline and post-treatment, patients underwent assessment of serum cytokines (IL6, IL10, TNF-alfa) and 2D-Echo with measurement of LVEF, indexed LV end diastolic volume (LVEDVi), LV wall motion score index (WMSi), tricuspid annulus systolic excursion (TAPSE). Mixed effects ANOVA for repeated measures was used to test the treatment effect between groups.

**Results:** The table summarizes the results. We observed a strong trend toward reduction of TNF-alfa and increase of IL10 values in the PTX group, but no significant effect in cardiac function variables.

**Conclusion:** Despite its immunomodulator effect, pentoxifylline was not associated to significant improvement in cardiac function in patients with CCC.


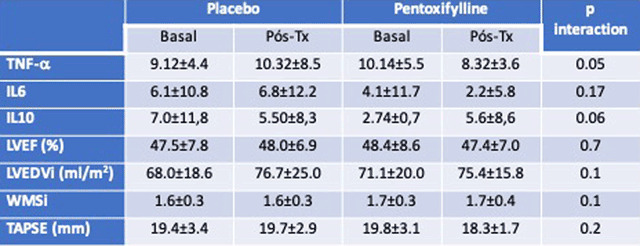


111145

Modality: E-Poster Researcher – Non-case Report

Category: PHYSIOTHERAPY

## Indirect VO2 Analysis in Patients with Metabolic Syndrome

JULIANA RIBEIRO GOUVEIA REIS^1^, Laissa de Cássia Alves^2^, Karine Siqueira Cabral Rocha^2^, Alessandro Reis^3^, Juliana Ribeiro Gouveia Reis^1^

(1) Instituto Pró-Vida; (2) Centro Universitário de Patos de Minas; (3) Hospital Santa Casa de Misericórdia de Patos de Minas

Metabolic syndrome is characterized by a set of cardiovascular risk factors such as arterial hypertension, insulin resistance, hyperinsulinemia, glucose intolerance/type 2 diabetes, central obesity and dyslipidemia. People with this condition have low functional capacity. The cardiopulmonary stress test is considered the gold standard in the assessment of physical capacity, however, it requires specific equipment and a specialized team. Therefore, the Shuttle walking test becomes a more practical option to estimate VO2 max in this population. The purpose of this study was evaluate the measurement of indirect VO2 in people with metabolic syndrome and compare it with predicted values for normality. This is a cross-sectional study with a quantitative approach. The inclusion criteria for the study were patients with metabolic syndrome who were being treated at the State Center for Specialized Care in Minas Gerais/Brazil, aged between 18 and 59 years. Patients with SatO2 < 88%, severe arterial hypertension, aortic coarctation, and decompensated heart failure were excluded. Patients underwent anthropometric assessment and then the Body Mass Index was calculated. The test was started after analyzing the contraindications and the VO2 obtained and the maximum expected VO2 were recorded. The results were analyzed using descriptive statistics, mean and standard deviation using the Wilcoxon test. The significance level was set at 0.05. The project received approval from the Research Ethics Committee under opinion number 3,153,984. Thirty patients participated in this research, 15 men and 15 women. The mean age of the male group was 55 years (±3.6) and the mean age of the female group was 49 years (±3.6). The mean BMI of the male group was 32.45 (±3.44). The female group had a mean BMI of 33.77 (±4.31). Regarding the VO2 of male patients, an average VO2 value of 12.84 (±2.20) was observed, while the average VO2 maximum was 72.90 (±2.12), with p value <0.05. In female patients, there was a lower value compared to males, with an average VO2 obtained of 12.52 (±2.20), and VO2 max with an average of 36.31 (±4.49), with a value of p < 0.05, also with mean values below the male participants. It is concluded that individuals with metabolic syndrome have low VO2 max values when compared to the expected values for this population and women have lower rates.

111149

Modality: E-Poster Researcher – Non-case Report

Category: CONGENITAL AND PEDIATRIC CARDIOLOGY

## Nutritional Status and Prevalence of Breastfeeding in Preterm Infants with Congenital Heart Disease Pre-Surgery in Southern Brazil

VIVIANE PAIVA DE CAMPOS^1^, Ana Carolina Krauspenhar Gluszczuk^2^, Nathalia Jacques Pereira^2^, Francisca Moura Strebel^3^, Fernanda Lucchese-Lobato^4^

(1) Instituto de Cardiologia/Fundação Universitária de Cardiologia Programa de Pós-graduação em Ciências da Saúde: Cardiologia; (2) Universidade Federal do Rio Grande do Sul; (3) Universidade Federal de Ciências da Saúde de Porto Alegre; (4) Irmandade Santa Casa de Misericórdia de Porto Alegre

**Background:** About 320,000 preterm (PT) infants are born in Brazil annually. Of these, it is estimated that 10–15% have congenital heart disease (CHD). Infants with CHD are born with normal body weight and lose it substantially after birth. The stunted growth and low weight characteristic of CHD infants are associated with poor developmental and surgical outcomes. Due to the challenges that infants with CHD face they are less likely to breastfeed, which leaves them more vulnerable to a diminished immune system, incidence and severity of diseases, mortality, prolonged hospital stay, and developmental delays.

**Objective:** To verify the prevalence of breastfeeding in PT infants with CHD admitted to a reference children’s hospital in Southern Brazil between 2019–2022.

**Methods:** This is a descriptive cross-sectional retrospective study. Data from medical records of PT infants (<37 weeks gestational age) with CHD, ages 0–12 months submitted to surgical procedures between 01/19 to 03/22, were analyzed. Infants with genetic syndromes were excluded from the analyses. Demographic, anthropometric, and clinical data were collected from electronic charts and entered into the REDCap data platform. Statistical analyses were performed using the SPSS v. 25 software.

**Results:** The 62 infants included in the study were mainly late PT (34–36 weeks and 6 days; 68%), female (53%), white (73%), acyanotic CHD (58%), and SUS users (77%). Seventy percent of all infants had appropriate weight for gestational age at birth. Most infants were formula fed (73%), while only 27% received breast milk, either exclusively (3%), complemented with solids (5%), or mixed breast milk and formula (19%). Fifty-two percent used tube feeding exclusively. Only 13% of tube-fed infants received breast milk. At the time of surgery, 65% were severely stunted, 56% severely underweight and 50% extremely thin/thin in BMI.

**Conclusions:** The findings suggest that premature infants with CHD in Southern Brazil are more likely to have an adequate nutritional profile at birth with increasing deficits and significant weight loss in the pre-surgical period. Due to the low prevalence of breastfeeding and high use of exclusive tube feeding, we conclude that it is of utmost importance to encourage breastfeeding and nutritional programs to promote weight gain and better immunity before surgery. Future multidisciplinary programs should focus on milk bank collection and inclusive breastfeeding pre-surgical protocols.

111197

Modality: E-Poster Researcher – Non-case Report

Category: ACUTE AND CHRONIC CORONARY DISEASE/THROMBOLYSIS

## Feasibility and Safety of Prehospital Thrombolysis in Stemi by Paramedics using Motorbike Ambulance in India – Mission Delhi (Delhi Emergency Life Heart Attack Initiative): A Pilot Study

RAMAKRISHNAN SIVASUBRAMANIAN^1^, Chandini Suvarna^1^, Praveen Aggarwal^1^, Sandeep Seth^1^, Neeraj Parekh^1^, Meenakshi Sharma^2^, Sheikh Vamik^1^, Ambuj Roy^1^, Ganesan Karthikeyan^1^, Sandeep Singh^1^, Rajiv Narang^1^, Balram Bhargava^2^

(1) All India Institute of Medical Sciences (AIIMS), New Delhi, India; (2) Indian Council of Medical Research (ICMR), New Delhi, India

**Background:** Outcomes of ST elevation myocardial infarction (STEMI) are suboptimal in India. Pre-hospital thrombolysis is an established practice in developed countries for timely reperfusion in STEMI. Mission DELHI (Delhi Emergency Life Heart Attack Initiative) is a pilot project for pre-hospital thrombolysis using motorbike ambulance service by trained paramedics at the patient’s doorstep in a selected geographical area of New Delhi. To the best of our knowledge, this is the first study of prehospital thrombolysis in India.

**Objective:** In this pilot phase, we report the safety and feasibility of prehospital delivery of care in STEMI. Methods We covered a geographical area of 5-km around our institution. A command center was set up and the patients were required to call a dedicated helpline number. Upon initial screening, a motorcycle ambulance was dispatched to the caller’s location. A brief history, physical examination and an ECG were obtained. ECG was electronically transmitted to the command center and thrombolysis with Tenecteplase was given at patient’s doorstep after evaluation of the ECG by a cardiologist.

**Results:** A total of 26 STEMI patients (mean age 56.2 years; 88.5% male) were treated either at home (46.2%), public places (23.1%), place of work (15.4%) or at small clinics (15.4%). Time taken to reach the patient location was 15.1 ± 6.1 min, and call to ECG time was 25.0 ± 6.8 min. Out of the 26 patients with STEMI, 15 patients were thrombolysed at a pre-hospital location with a door to needle time of 31 ± 11.1 min. Out of these, 13 patients had successful thrombolysis and had undergone further MI management. There was one death in a patient with cardiogenic shock. We also managed a total of 60 patients diagnosed with NSTEMI.

**Conclusions:** In this pilot study, we have demonstrated the feasibility and safety of a telemedicine supported pre-hospital thrombolysis by paramedics at the patient’s location. Such an approach is likely to significantly improve thrombolytic therapy usage and reduce ischemic times, and thereby could result in significant improvements in STEMI outcomes in a country like India.

111208

Modality: E-Poster Researcher – Non-case Report

Category: CARDIOVASCULAR IMAGING

## Role of New Echocardiographic Modalities for Assessing Myocardial Evaluation of Fabry Disease Patients

JOSÉ LUIZ BARROS PENA^1^, Isabel Cristina Gomes Moura^1^, Marcos Victor Prosdocimi Diniz^1^, Fabiano Maia Linhares^1^, Pedro Manuel Marques Cristóvão^1^, Eduardo Back Sternick^1^, Amanda Marcos de Oliveira^2^, Gabriela de Campos Viveiros^2^, Rafaela Santos Garcia^2^, Eduardo Felipe Souza de Deus^2^, Tatiane Pires da Silva^2^, Tarcila Paoli Marques Moreira^2^

(1) Faculdade Ciências Médicas de Minas Gerais; (2) Hospital Felício Rocho

**Introduction:** Due to its various clinical manifestations, Fabry disease (FD) is a commonly misdiagnosed rare condition. Treatment depends ideally on early disease detection and timely enzyme replacement therapy to improve prognosis. Echocardiography may be an efficient noninvasive technique to detect structural and functional heart consequences resulting from the disease. This study aimed to determine whether new echocardiographic modalities may contribute to more improved diagnoses.

**Material and methods:** Prospective and transversal study of 15 patients (Pts) with FD. During one examination, conventional transthoracic, bi-, and tridimensional echocardiography as well as strain/strain rate speckle tracking were measured. Specific echocardiographic data were obtained and exported to a workstation for subsequent evaluation. Statistical analyses were performed with 5% significance using the R program, version 3.1.3.

**Results:** Nine Pts (60%) were male, and the mean age was 39 ± 11.9 years. Pts presented increased left atrium volume index, mean left ventricle (LV) septum, and posterior wall thickness. LV hypertrophy was found in varying degrees in 8 (53%) FD Pts. Mean myocardial mass value measured by the 4D technique was 110.9 ± 8.1 grams. A binary appearance of LV endocardial border (“binary sign”) and hypertrophy of the anterolateral papillary muscle were detected in 9 (60%) of Pts. Mitral regurgitation was present in 4 (26.7%) Pts, mitral valve prolapse in 2 (13.3%), and aortic regurgitation in 1 (6.7%). Four (26.7%) Pts presented RV hypertrophy. Global mean longitudinal 2D strain measured –19.5 ± 3.4%, and 4D circumferential strain measured –17.7 ± 3.5%. We identified peak systolic strain reduction in basal and mid inferolateral segments in 10 (66.6%) Pts. By analyzing the bull‘s-eye plot.

**Conclusion:** Echocardiography is an accurate, robust tool in Fabry disease evaluation. Compared to conventional measurement techniques, advances in 3D and speckle tracking echocardiography have augmented and improved anatomical and functional data.

111251

Modality: E-Poster Researcher – Non-case Report

Category: ATHEROSCLEROSIS/CARDIOVASCULAR RISK FACTORS/CARDIOVASCULAR PREVENTION

## The Role of Check Up in the Prevention of Cardiovascular Diseases

FERNANDA SANCHES AGUERA GROCHOCKI^1^, LAÍS SANCHES AGUERA^1^, CRISTINA PELLEGRINO BAENA^1^, JOSE ROCHA FARIA NETO^1^

(1) PONTIFÍCIA UNIVERSIDADE CATÓLICA DO PARANÁ – PUCPR

**Introduction:** Cardiovascular diseases are the leading cause of mortality in the world. The identification and correction of modifiable risk factors are the cornerstone of cardiovascular prevention. However, this purpose in performing periodic health assessment (check-up) in asymptomatic individuals still has its effectiveness questioned.

**Objective:** To analyze whether the periodic health assessment can be a tool for the control and treatment of cardiometabolic risk factors and whether the impact of this assessment is different according to the presence of these factors.

**Methods:** A retrospective cohort was carried out, including data from patients seen consecutively in 2015 and 2016, at a check-up service in Curitiba, Paraná. The cardiometabolic profile of the patients was evaluated and a comparison was made between variables of interest obtained in 2015 and 2016. It was also evaluated whether the variation from one year to the other was different between patients who presented cardiometabolic alterations in the first year compared to those who presented variables without alterations. Microsoft Excel and SPSS Statistics programs were used for calculations, using Student’s T test for paired samples or Wilcoxon test, according to normality tests.

**Results:** A total of 478 patients were included, 80.3% of whom were male and with a mean age of 46.29 years (SD ± 8.06). The following altered variables showed improvement: triglycerides (p < 0.05), HDL (p < 0.001), LDL (p < 0.001), non-HDL (p < 0.001), fasting glucose (p < 0.001) and glycated hemoglobin (p < 0.001). Evaluating altered anthropometric data, there was an improvement in Body Mass Index(BMI) (p < 0.01), Systolic Blood Pressure (p < 0.001), Diastolic Blood Pressure (p < 0.001) and male waist circumference (p < 0.05). Analyzing the altered variables, there was a change to values within the normal range in 21% of cases of total cholesterol, 29% triglycerides, 26% HDL, 27% LDL, 32% non-HDL, 7% BMI, 87% systolic blood pressure, 84% Diastolic blood pressure, 10% waist circumference, 54% blood glucose, 43% glycated hemoglobin. (p < 0.001).

**Conclusion:** Periodic health assessment seems to have a positive impact on the control and treatment of cardiovascular risk factors. This effect was mainly observed in patients who already had altered laboratory and anthropometric variables in the first evaluation.

111254

Modality: E-Poster Researcher – Non-case Report

Category: CARDIO-ONCOLOGY

## Myocardial Strain by Speckle-Tracking and Evaluation of 3D Ejection Fraction in Drug-Induced Cardiotoxicity‘s Approach in Breast Cancer

DANIANE RAFAEL^1^, Juliana Bueno Refundini^1^, Adriana A. da Silva^1^, Ana Cristina Camarozano^1^, Claudio Leinig Pereira da Cunha^1^, Sérgio Lunardon Padilha^1^, Admar Moraes de Souza^1^, Ana Karyn Ehrenfried de Freitas^1^

(1) Universidade Federal do Paraná – UFPR

Chemotherapeutics brought greater survival to breast cancer patients, however, they can generate cardiotoxicity with important dysfunction. The echocardiogram (Echo), has enabled early detection of myocardial dysfunction. Our purposes was to obtain, with Echo, the incidence of left ventricle dysfunction after chemotherapy, analyzing the Ejection Fraction (EF) in 2 dimensions-Simpson Method (2D) and in 3 dimensions (3D), and to identify early contractile changes using the Longitudinal Strain – Speckle-Tracking (ST). A prospective study studied 37 women, mean age 48 ± 11 years with breast cancer in the beginning of chemotherapy between 2013 and 2015. Echo was performed at baseline, after 3 and 6 months, evaluating 2D EF and 3D EF in addition to the contractile evaluation using the ST and peak systolic velocity at the base of the lateral wall (lateral S’) on tissue Doppler. The patients were also evaluated for signs, symptoms, possible risk and protective factors contributing to myocardial dysfunction overall. Dyspnea was the only symptom detected (23%). We observed that the altered ST in the 1st exam proved to be a predictor of 3D EF reduction in the 3rd exam (p = 0.049). The lateral S‘ reduced at 6 months, corroborating the change in systolic function. Both risk and protective factors did not affect the parameters evaluated. There was a progressive decrease in EF after exposure to cardiotoxic chemotherapeutic agents, which became more evident by 3D analysis. The evaluation of the ST showed significant changes associated with the period of administration of anthracycline, and an ST altered in the beginning of the treatment was a predictor of EF reduction at 6 months of treatment. These findings support the importance of these new Echo techniques in the follow up of these patients.


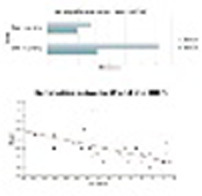


111263

Modality: E-Poster Researcher – Non-case Report

Category: PERICARDIUM/ENDOCARDIUM/VALVOPATHIES

## Prevalence of Silent Brain Infarct in Mitral Stenosis and its Predictors

AMBUJ ROY^1^, Ambuj Roy^1^, Nitish Naik^1^, Sharma Sanjiv^1^, Sandeep Singh^1^, Gautam Sharma^1^, Ramakrishnan Lakshmy^1^, Rohit Bhatia^1^

(1) All India Institute of Medical Sciences (AIIMS)

**Background:** Rheumatic mitral stenosis (MS) is associated with significant morbidity, especially ischemic stroke(IS) and continues to be very prevalent in developing countries. Clinical predictors of IS are poor and is common even among those not in atrial fibrillation (AF). Silent brain infarct (SBI) is an important predictor and surrogate of future ischemic stroke. In this study, we assessed the prevalence of SBI in patients with MS in normal sinus rhythm (NSR) and AF.

**Methods:** Consecutive patients with more than moderate mitral stenosis underwent non-contrast MRI. Standard MRI sequences and definitions for infarct were used. Patients with prior intervention and surgery for MS were excluded as were those with prior clinical IS or transient ischemic attack (TIA), left atrial clot, and severe concomitant other valval lesions.

**Results:** Of 211 patients who underwent MRI, 140 were in NSR and 71 in AF. 59% were females, 88% had severe MS, 23%, 24% and 53% were in NYHA Class I, II and III respectively. A total of 44 (21%) patients had SBI. The baseline variables in those with normal MRI scans and with SBI were as in Table 1. Only NYHA functional class and not AF was significantly different in patients with SBI.

**Conclusions:** SBI is common among patients with MS including those in NSR. Only NYHA class was different in the two groups. Better predictors of IS beyond rhythm are needed in patients with MS to decide on IS preventive strategies.


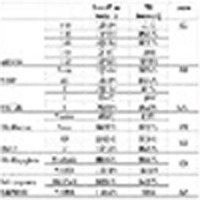


111280

Modality: E-Poster Researcher – Non-case Report

Category: CARDIOVASCULAR SURGERY

## Preoperative Physical Status and In-Hospital Outcomes After Cardiac Surgery

ABISAI DOS SANTOS SANTANA^1^, Giovana Côrte Real Ruffier^2^, Mauro Felippe Felix Mediano^1^, Daniel Arthur Barata Kasal^1^

(1) Instituto Nacional de Cardiologia; (2) Universidade do Estado do Rio de Janeiro

**Introduction:** The assessment of physical performance in the preoperative evaluation of open-chest heart surgery (OHS) can help to establish the most appropriate moment for the intervention. There are noninvasive and simple methods that can be used at the bedside, in order to evaluate physical performance in these patients.

**Objective:** To evaluate physical performance in the preoperatory period of elective OHS and establish associations with surgical recovery outcomes.

**Methods:** Adult patients subjected to elective OHS, either coronary artery bypass grafting (CABG) or valvular replacement (VR) surgery, at a quaternary hospital were recruited for the study. Physical performance was measured with 30-s chair-stand-test (30sCST), timed up and go test (TUGT) and hand grip, performed during surgical admission, before the intervention. Surgical risk was estimated with the Euroscore II risk score. Clinical data were obtained using medical records. The outcomes evaluated were mechanical ventilation time (MVT), admission time (AT), and in-hospital death (IHD), and their associations with the physical tests were evaluated with either logistic (IHD) or linear (MVT, AT) regressions (p < 0.05).

**Results:** One hundred sixty-six individuals were evaluated, most patients were male (65%), aged 58.3 ± 11.3 years, and with body mass index of 27 ± 4.4 kg/m^2^. Left ventricular ejection fraction (Teicholz) was 59.4 ± 15.5%, and New York Heart Association functional classes I, II, III and IV were 8, 70, 53, and 3%, respectively. Euroscore II was 3.1 ± 2.1, 98% of surgeries employed extracorporeal circulation, and IHD was 7.2%. In the unadjusted analysis, the hand grip was associated with IHD (OR 0.90; p = 0.007), but the association was not significant after adjustments for age and sex, or Euroscore II (OR 1.00 p = 0.93 and OR 0.92 p = 0.06, respectively). None of the physical tests were associated with either AT or VMT.

**Conclusions:** The present results suggest a lack of association between the 30sCST and TUGT physical tests and the recovery variables after OHS studied. Preoperatory hand grip offered an association with mortality after surgery, but significance was borderline after adjustment for Euroscore II. The contribution of physical fitness before OHS to surgery outcomes may be evaluated with additional methods and in particular groups such as the elderly in future studies, in order to explore this important association.

111769

Modality: E-Poster Researcher – Non-case Report

Category: CARDIAC ARRHYTHMIAS/ELECTROPHYSIOLOGY/ELECTROCARDIOGRAPHY

## Analysis of the Impact of Implantable Cardioverter-Defibrillator Therapy in Patients with Hypertrophic Cardiomyopathy

LUCAS CARVALHO DIAS^1^, Luiz Eduardo Montengro Camanho^2^, Eduardo Benchimol Saad^2^, Charles Slater^1^, Luiz Antonio Oliveira Inacio Junior^2^, Gustavo Vignoli Santos^2^, Ricardo Mourilhe Rocha^1^

(1) Hospital Universitario Pedro Ernesto; (2) Hospital Pró-Cardíaco

**Introduction:** The hypertrophic cardiomyopathy (HCM) is an autosomal dominant genetic disease, strongly related to the occurrence of malignant ventricular arrhythmias and sudden death.

**Objective:** Describe the clinical data and the occurrence of appropriate and inappropriate therapy in a subgroup of HCM patients with implantable cardioverter-defibrillators.

**Methods and results:** In a cohort of 720 consecutive and retrospective patients, monitored from March 2006 to December 2021, with 57 of this total being diagnosed with HCM. The male sex was found in 42 (74%), and the average age was 54.6 years. ICD implantation by primary prophylaxis occurred in 49 (86%) patients and in 8 (14%) by secondary prophylaxis. The report of unexplained syncope was observed in 50 (88%) patients. At the time of ICD implantation, the records showed that 51 (89%) patients were in NYHA functional class 1 and the average LVEF was 59,4%. The electrocardiographic analysis showed that the mean QRS was 118 ms and that 25 (44%) had an LBBB pattern. During the ICD monitoring period, 11 patients (19%) were observed with a record of VF/VT and appropriate therapy. Inappropriate therapy was observed in 7 patients (12%), all in the AF group, which occurred in 25 (44%) patients throughout the record.

**Conclusion:** Despite the not insignificant number of inappropriate therapies, the ICD has been shown to be an effective therapy in preventing sudden cardiac death and in the clinical management.


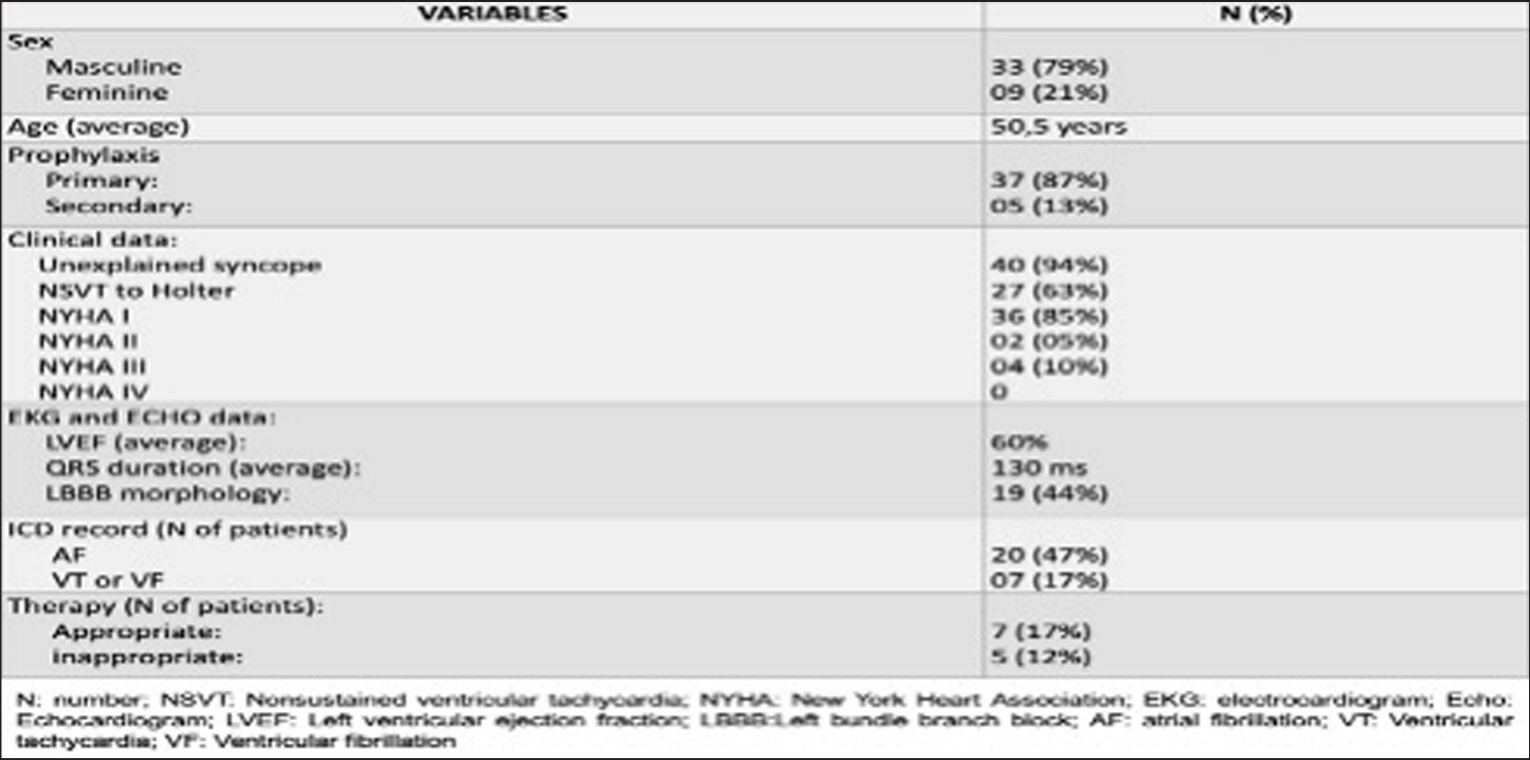


111563

Modality: E-Poster Researcher – Non-case Report

Category: ATHEROSCLEROSIS/CARDIOVASCULAR RISK FACTORS/CARDIOVASCULAR PREVENTION

## Digital Therapeutic as a Tool to Manage Cardiovascular Risk Factors: Preliminary Results of a Brazilian Cohort

CAMILA MACIEL DE OLIVEIRA^1^, Roberto Luis Zagury^3^, Luiza Borcony Bolognese^4^, Davi Casale Aragon^5^, Livia Mandina da Graça Couto^2^, Marcela Carvalho^2^, Thaíza dos Anjos^2^, Mercedes Balcells^6^, Clemente Nobrega^2^, Chunyu Liu^7^

(1) Universidade Federal do Parana (UFPR); (2) Klivo LLC; (3) Instituto Estadual de Diabetes e Endocrinologia Luiz Capriglione (IEDE); (4) Pontificia Universidade Catolica de Minas Gerais (PUC-Poços); (5) Universidade de São Paulo (USP-RP); (6) Massachusetts Institute of Technology; (7) Boston University

Digital therapeutics, an emerging type of medical approach, is defined as evidence-based interventions through qualified software programs that help prevent, manage, or treat chronic diseases. We aim to describe the clinical impact of a digital therapeutic product of a Brazilian startup company that focuses on individual needs as an essential shift for the ongoing management of patients with chronic conditions such as diabetes, hypertension, and dyslipidemia. This program is a model of behavior change based on an intensive lifestyle intervention method and seeks to manage cardiovascular risk factors in adults aged ≥ 18 years. It was sponsored by health plans and healthcare provider organizations and was free for patients. During the management process, patients were remotely supervised by nurses on a weekly basis via phone calls intended at health education and assessment of laboratorial outcomes. An app was developed to improve the communication between patients and nurses, but e-mails and text messages were also used. To improve control of glycemic events in patients with diabetes, a glucometer was sent to the patient’s home and was connected to the app. When abnormal glucose parameters were detected, the patient was contacted according to an established protocol. Outcomes of interest were evaluated at baseline and after 3 months. For a total of 2544 patients (53.35 ± 13.45 years; 50% male) distributed in 21 Brazilian states (40% in São Paulo), it was possible to verify significantly improved glycemic and lipid profile (total cholesterol and LDL-cholesterol) after follow-up for 3 months. A1c decreased (9.13 ± 2.10 versus 7.14 ± 1.69; p < 0.01), and so did fasting glucose (127.19 ± 51.96 versus 122.51 ± 42.40; p = 0.03), total cholesterol (176.66 ± 50.11 versus 161.36 ± 45.72; p = 0.01), and LDL-cholesterol (100.42 ± 40.11 versus 89.87 ± 33.82; p = 0.03). No difference was observed for HDL-cholesterol or triglycerides in this sample. This program has shown promising findings related to glycemic and lipid profiles, providing insights into the management of cardiovascular risk factors, which will allow us to reshape this unique therapeutic approach for the Brazilian population continuously.

111319

Modality: E-Poster Researcher – Non-case Report

Category: HEART FAILURE/CARDIOMYOPATHY/TRANSPLANT

## Characteristics and Clinical Outcomes of Patients with Heart Failure with Preserved Ejection Fraction: Insights from the First Brazilian Registry of Heart Failure (Breathe)

PEDRO GABRIEL MELO DE BARROS E SILVA^1^, Denilson Campos Albuquerque^3^, Marcus Vinícius Simões^5^, Renato Delascio Lopes^6^, Conrado Hoffmann^7^, Paulo Roberto Nogueira^8^, Helder Reis^9^, Fabio Akio Nishijuka^10^, Lidia Zyntynski Moura^11^, Fernando Bacal^3^, Evandro Tinoco Mesquita^3^, Múcio Tavares de Oliveira Junior^3^

(1) IP-Hcor; (2) Hospital Samaritano Paulista; (3) Sociedade Brasileira de Cardiologia; (4) Hospital Copa D’Or; (5) Hospital das Clínicas da Faculdade de Medicina de Ribeirão Preto da Universidade de São Paulo; (6) Brazilian Clinical Research Institute; (7) Hospital Regional Hans Dieter Schmidt; (8) Fundação Faculdade Regional de Medicina de São José do Rio Preto; (9) Hospital de Clínicas Gaspar Viana; (10) Hospital Naval Marcílio Dias; (11) Irmandade Santa Casa de Misericórdia de Curitiba; (12) Instituto do Coração (INCOR HC FMUSP)

**Background:** Heart failure with preserved ejection fraction (HFpEF) accounts for almost half of the HF cases. However, little is known about the characteristics, medical therapies, and long-term prognosis of patients hospitalized with HFpEF in Latin America.

**Methods:** BREATHE was a nation-wide prospective registry that included patients hospitalized due to acute heart failure (AHF) in Brazil. In-hospital management as well as 12-month clinical outcomes were assessed. In the current analysis, patients were classified according to left ventricular ejection fraction (LVEF) in 3 groups: <40% (HFrEF), 40 to 49% (HFmrEF) and ≥50% (HFpEF).

**Results:** A total of 3,013 patients were included with a median follow-up of 346 days. In 1,204 patients, the LVEF was assessed during the first 24 hours of hospitalization with 28.1% of patients classified as HFpEF. The patients with HFpEF were older (70.5 ± 16.4 vs 63 ± 15.9; P < 0.01) had more female patients than HFrEF ones (57.5% vs 30.4%; P < 0.01). Among comorbidities, hypertension (81.4% vs 70.5%) and atrial fibrillation (40.5% vs 27.2%) were more common in patients with HFpEF (both P < 0.01), while history of myocardial infarction was more common in HFrEF (18.9% vs 29.4%; P < 0.01). The creatinine was 1.5 ± 1.0 mg/dL and similar among the 3 groups but the level of BNP/NT-ProBNP was >2 times lower in the HFpEF population. The wet-cold profile occurred in 5.9% of the patients with HFpEF and in 12.6% of patients admitted with HFrEF (P = 0.04). The main cause of decompensation was poor adherence in the HFrEF patients, and infection in HFpEF. At hospital admission, almost 54% of HFpEF patients were using renin-angiotensin-aldosterone inhibitors or beta-blockers, and 19.5% were using spironolactone. There was no enhancement in the use of these medications during the 12 months follow-up. Intra-hospital mortality was similar among the 3 groups of LVEF (average 10.5%). After discharge, the mortality and hospital readmission rates at 365 days were not statistically different in all groups (23.3 deaths for 100 patient years with 51% readmission rate at 12 months in the HFpEF group).

**Conclusions:** In this large multicentre nationwide Brazilian prospective registry of AHF, patients with HFpEF presented different clinical characteristics but similar prognosis compared to patients with lower ejection fraction during one year of follow-up. Improvement in the medical care is necessary to minimize complications in this high-risk population.

111345

Modality: E-Poster Researcher – Non-case Report

Category: COVID-19 AND CARDIOVASCULAR SYSTEM

## Spatial-Temporal Deep Learning for Automatic Detection of Echocardiographic Predictors of Mortality in Patients Hospitalized with COVID-19

BRUNO RAMOS NASCIMENTO^1^, Washington Luis S. Ramos^2^, Gisele Lobo Pappa^2^, Edson Roteia Araújo Júnior^2^, João Francisco Barreto da Silva Martins^2^, Wagner Meira Júnior^2^, Sander Luis Gomes Pimentel^1^, Juliane Franco^1^, Kaciane Krauss Bruno Oliveira^1^, Maria Carmo Pereira Nunes^1^, Antonio Luiz Pinho Ribeiro^1^, Erickson Rangel do Nascimento^2^

(1) Serviço de Cardiologia e Cirurgia Cardiovascular e Centro de Telessaúde do Hospital das Clínicas da UFMG, Belo Horizonte – MG, Brazil; (2) Departamento de Ciência da Computação da Universidade Federal de Minas Gerais, Belo Horizonte – MG, Brazil

**Introduction:** Cardiac involvement seems to impact prognosis of COVID-19. Bedside echocardiography (echo) holds promise for early prediction of unfavorable outcomes, and artificial intelligence may be an additional tool to overcome personnel limitations. We propose a spatial-temporal deep learning-based approach for automatic prediction of mortality of inpatients with COVID-19 with echo images.

**Methods:** Patients admitted in 2 reference hospitals in Brazil in 90 days with confirmed moderate and severe COVID-19, based on the Berlin criteria, underwent clinical and laboratory evaluation, and focused bedside echo (GE Vivid IQ), following admission, with remote interpretation by telemedicine in Brazil and the US. Independent echo predictors of all-cause mortality were assessed, after adjustment for clinical variables. Our image dataset consists of 737 videos (Mpeg) collected from included patients in 3 different probe positions: apical 4-chamber and parasternal long and short axis. It was used to predict the patients’ outcome (discharge or death) in a 10-fold cross-validation procedure manner. We used a 2-stream deep neural network composed of two identical ResNet-18 Convolutional Neural Networks (CNN) – originally developed for Rheumatic Heart Disease diagnosis – followed by attention units to extract relevant spatial and temporal features from RGB and Optical Flow frames. A final softmax layer is used for classification, and accuracy measures are presented with 95% confidence intervals (CI).

**Results:** Total 163 patients were enrolled, mean age was 64 ± 16 years, 107 (66%) were admitted to intensive care and in-hospital mortality was 34% (N = 56). Independent predictors of mortality, after adjustment for clinical and demographic variables, were age ≥ 63 years (OR = 5.53, 95%CI 1.52–20.17), LVEF < 64% (OR = 7.37, 95%CI 2.10–25.94) and TAPSE < 18.5 mm (OR = 9.43, 95% CI 2.57–35.03), C-statistic = 0.83. During training, we rescaled videos to 224 × 224 pixels and used temporal jittering to select a final clip with 32 frames per video in random batches of size 6. Our proposed method achieved an exam-wise accuracy of 61.21% (95%CI 50.87–71,55), sensitivity of 59.31% (95%CI 36.12–82.5) and specificity of 65.18% (95%CI 45.08–85.28) for mortality.

**Conclusion:** Automatic detection of high-risk echo findings in COVID-19 inpatients at bedside seems feasible and, with more research, can improve mortality prediction at the point-of-care, especially in low-income settings.

111379

Modality: E-Poster Researcher – Non-case Report

Category: NEGLECTED CARDIOVASCULAR DISEASES

## Urban Chagas Disease: Identification of High Risk Patients

EZEQUIEL J ZAIDEL^1^, Lara Gheco^1^, Luis Guillermo García Chamorro^1^, Erna Florencia Segovia^1^, Diego Carvallo Claros^1^, Elena Vargas Parra^1^, Agustín Monzón^1^, Brian Perotti^1^, María Inés Sosa Liprandi^1^, Álvaro Sosa Liprandi^1^

(1) Sanatorio Güemes, Buenos Aires, Argentina

**Introduction:** Migratory movements have led to Chagas disease (CD) being diagnosed in non-endemic areas. There are few epidemiological studies on the prevalence and behavior of the disease in urban areas.

**Aim:** To describe the clinical profile of patients with CD evaluated in an urban area and to identify the frequency of high risk cases.

**Methods:** Retrospective cohort study of patients who were hospitalized or evaluated in outpatient clinics, in a third level center from Buenos Aires. Patients over 18 years of age with a diagnosis confirmed by serology were selected. The patients were evaluated by clinical examination and complementary studies in accordance with current standards. A descriptive analysis was performed and the risk scores of Viotti, Rassi, Pinho Ribeiro, and Sousa were analyzed.

**Results:** Data from 46 patients were analyzed, with a mean age of 61 ± 11 years, and 41% female. Main origin of patiets was nothern provincies from Argentina, as well as Bolivia and Paraguay. The diagnosis was made mainly by medical check-up due to risk factors for ECH (30%) or by finding in blood banks (10.8%). The median time of evolution from the date of diagnosis of the disease to the cutoff made for this analysis was 6 years (IQR 1–12). Only one case in this cohort had received trypanocidal treatment. The prevalence of cardiomegaly was 51% by radiography, but the diastolic diameter by echocardiogram was 51 mm ± 7 mm with a mean ejection fraction of 58%. 21% presented right bundle branch block, 4% left anterior hemiblock, 18% atrial fibrillation, and 6% low voltage and ventricular extrasystoles. Fifty percent had symptomatic heart failure, 23% had implanted cardiac devices, and 17% had cerebrovascular accident (CVA). No cases of digestive or neurological pathology were found. By Viotti score, 41% had a high risk of progression. Using Rassi score, 10% had a high risk of mortality, but by the Pinho Ribeiro score, only 1 patient had a high risk. When analyzing the risk of stroke, 16% of the cohort presented high risk by Sousa score, however, anticoagulation in this cohort was prescribed only to subjects with atrial fibrillation or those who had already had a stroke.

**Conclusions:** The evaluation of patients with urban CD is a growing challenge for cardiologists from urban settings: Using current scores, the proportion of high risk cases was variabe, highlighting the need of better stratification tools.

111386

Modality: E-Poster Researcher – Non-case Report

Category: CARDIAC ARRHYTHMIAS/ELECTROPHYSIOLOGY/ELECTROCARDIOGRAPHY

## Syncope and Pre-Syncope: Predictor of Unfavorable Outcomes in the Electrophysiological Study in Chronic Chagas Heart Disease

IEDA PRATA COSTA^1^, Eduardo Arrais Rocha^1^, Almino Cavalcante Rocha Neto^1^, Roberto Lima Farias^1^, Daniele Melo Leopoldino^1^, Cristiane Liberato^1^, Ronaldo Vacsoncelos Távora^2^

(1) Hospital Universitário Walter Cantidio – universidade Federal Ceara; (2) Hospital de Messejana _ SESA/CE

**Introduction:** Cardiovascular death is the main cause of death in chronic Chagas‘ heart disease (CCC). Syncope in CCC can be caused by bradyarrthymias (sick sinus syndrome-SSS and conduction disorders) or ventricular arrhythmias.

**Objectives:** To evaluate the association of syncope and pre-syncope with important unfavorable outcomes (SSS, severe conduction system disorders – HV >70 ms and VT/VF) in the electrophysiological study (EPS) in patients with CCC.

**Methods:** This is a prospective cohort study, including 52 patients with CCC from the postgraduate’s research project, 48 of whom underwent an EPS with or without previous use of antiarrhythmic drugs; with a mean age of 57 + 10.2 years; 62.5% male. PTs were classified into two groups: I- With syncope/pre-syncope and II- Without syncope. The chi-square test and Fisher’s exact test were used for statistical analysis.

**Results:** The clinical characteristics were the mean: Rassi score was 8.43 + 4.8 points (39.5% low risk); 43.7% used antiarrhythmics; 8.3% had Functional Class III/IV and 79.1% (38pt) had syncope/pre-syncope. In group I, we observed that 31.2% (10pt) were at low risk of Rassi and 71.8% (23pt) had altered EPS (1 by SSS, 1 by prolonged HV and 21 by VT). In group II, we found 56.2% (9pt) low risk and 25% (4pt) had altered EPS. Unfavorable outcomes in the EEF were greater in group I than in group II (p = 0.002). Comparing the risk of Rassi and the presence of altered EPS, in the groups we have: Group I- Low risk- 60%, Intermediate- 71.4% and high- 80% altered EPS (p = 0.83). Group II – Low risk – 11%, Intermediate 42.8% altered EPS(p = 0.57).

**Conclusions:** Syncope/pre-syncope was a predictor of unfavorable outcomes in the EPS. The presence of altered EPS was similar in the different Rassi risk scores in both groups.

111401

Modality: E-Poster Researcher – Non-case Report

Category: NURSING

## Teaching Strategy on Electrocardiogram in Undergraduate Nursing

ÉRICA SOBRAL GONDIM^1^, Antonia Elizangela Alves Moreira^1^, Ana Camila Gonçalves Leonel^1^, Amanda da Costa Sousa^1^, Emiliana Bezerra Gomes^1^

(1) Universidade Regional do Cariri – URCA

**Introduction:** The electrocardiogram (ECG) subsidizes an assertive nursing care plan aimed at cardiac conduction abnormalities. But there are difficulties in performing and interpreting the test, which demonstrates the need for teaching strategies for its understanding and skills development.

**Objective:** To report a teaching strategy on ECG shared between a professor, a student in teaching internship and an undergraduate teaching assistant.

**Method:** Experience report of theoretical and practical teaching about ECG, carried out in October 2021 with 36 undergraduate nursing students, in a public university in Northeast, completed in two phases: theoretical, (skills training) and practical (simulation and case studies).

**Results:** The implementation of the strategy used theoretical-practical association in a transversal way, which stimulated the development of knowledge, skills and attitudes, emphasizing the creativity and improvisation demonstrated due to the scarcity of materials at the public university. Finally, the promoters of the teaching strategy shared the potentialities and difficulties perceived in the process. The main potentiality was the motivation demonstrated by the students as a result of the interactivity and dynamics of the class, which stimulated their interest and active participation in both moments of the strategy. The biggest obstacle was the scarcity of resources, making it necessary to use low-fidelity simulation to replace the equipment and electrodes themselves. The heterogeneity of the learning process was also mentioned as a difficulty, since there are different ways to build knowledge aimed at different audiences, but it was evident from the students‘ reports that the strategy guided the necessary aspects of the teaching theme.

**Conclusion:** Reporting the experience of teaching with simulation allowed the promoters to improve their strategies based on the students‘ feedback and the development of the activity. We suggest studies that enable the development of skills with the use of dynamic and participatory strategies.

111415

Modality: E-Poster Researcher – Non-case Report

Category: HEMODYNAMICS AND INTERVENTIONAL CARDIOLOGY

## Ultra-Minimalistic TAVR During COVID-19 Pandemic

SEBASTIÁN DARÍO PERALTA^1^, Marcelo Omar Bettinotti^1^, Carlos Maximiliano Giuliani^1^, Guillermo Jubany^1^, Luis Murillo^1^, Juan Grieve Bruno^1^, Ezequiel José Zaidel^1^, Luis Carlos Sztejfman^2^, Matías Sztejfman^2^

(1) Sanatorio Güemes, Buenos Aires, Argentina; (2) Sanatorio Finochietto, Buenos Aires, Argentina

**Introduction:** Current transcatheter aortic valve replacement (TAVR) procedures may be performed with an ultra-minimalistic approach which improves hospital resources. Aims The aim was to describe in hospital length of stay (LOS) and vascular events of TAVR procedures using ultra minimalisitc approach prior and during the COVID-19 pandemic.

**Methods:** Consecutive TAVR cases from two centres between Jan 2019 and June 2021 were analyzed. Ultra-minimalistic approach included: all in one (AIO) femoral single puncture, conscious sedation, self-expanding valves, and femoral closure devices. Intra-pandemic cases were considered since March 2020 according to country’s COVID 19 uptake. Conventional descriptive and comparative statisitics were performed with Epi Info V7.

**Results:** 37 cases were included, 21 (56%) before the pandemic and 16 intra-pandemic. Mean age was 82 (6) years and 59% were female. 73% had no prior conduction abnormalities, 35% had concomitant peripheral artery disease, 8% porcelain aorta. Median EuroScore was 5.8 (IQR 4.2–8), STS 5.4 (IQR 3.6–7.2), mean ejection fraction 56% (SD 11%) and mean gradient at baseline 45 mmHg (SD 12). A self-expandable valve was used in all cases. A predilatation was used for all cases. There were no deaths, no bleeding events, no strokes nor infections, 1 case of vascular complication (iliac dissection, resolved during the same procedure) and 1 case of permanent pacemaker implantation (2.7%). The rate of mild paravalvular leak was 43% and moderate leak 5.4%. Median length of stay was 2 days (IQR 2–3). 16 cases (43%) were performed intra-pandemic, without differences in procedural duration (58 minutes vs 56 pre-pandemic, p = 0.56), nor in LOS (median = 2 days in both groups, p = 0.24). 16% of the cases were discharged at day 1.

**Conclusion:** Ultra-minimalisitc TAVR was safe and associated with short LOS prior and during COVID-19 pandemic. Physicians and patients must consider these findings in the context of the pandemic with risk of in-hospital infections and low healthcare capacity. These results must be considered also for TAVR cost-effectiveness analysis.

112184

Modality: E-Poster Researcher – Non-case Report

Category: CARDIOVASCULAR IMAGING

## Myocardial Perfusion and Fractional Flow Reserve by Computed Tomography and Scintigraphy in Patients with Ischemia by Exercise Stress Test

SERGIO RODRIGO BERALDO^1^, Sergio Rodrigo Beraldo^1^, Tiago Augusto Magalhaes^1^, Augusto Hiroshi Uchida^2^, Rafael Willain Lopes^3^, Douglas Carli Silva^4^, Erico Luiz Camacho^5^, Gabriel Nunes Rodrigues^5^, Paulo Roberto Maia^6^, Ana Clara Beraldo Muniz^6^, Carlos Eduardo Rochitte^1^

(1) Computed Tomography and Cardiovascular Magnetic Resonance Sector from Heart Institute (InCor), University of Sao Paulo Medical School.; (2) Hospital Israelita Albert Einstein; (3) Nuclear Medicine Sector of Hospital do Coração -HCOR; (4) Siemens Healthineers; (5) Corpus Computed Tomography and Magsul MND Nuclear Medicine; (6) Sapucai Valley University School of Medicine

**Background:** Patients with low to intermediate probability of CAD presenting exercise treadmill test (ETT) consistent with myocardial ischemia is a common scenario and may lead to other complementary tests, frequently myocardial scintigraphy (SPECT).

**Purpose:** We sought to evaluate the diagnostic performance of myocardial perfusion (CTP), and Fractional Flow Reserve Derived from Computed Tomography – (cFFR) compared to SPECT in a population of patients with inducible ischemia on exercise treadmill tests (ET), using coronary computed tomography angiography (CTA) as the reference method.

**Methods:** Sixty patients (58.4 ± 9.2, 37 men (61.7%), underwent clinical evaluation and two non-invasive imaging exams: SPECT and CTA with CTP during stress with dipyridamole.

**Results:** SPECT showed significantly lower diagnostic accuracy than CTP (AUC 0.62 vs. 0.72, p < 0.001). The cFFR and minimal luminal area showed higher diagnostic accuracy than perfusional methods (AUC = 0.86 and 0.90). Coronary calcium score (CAC) showed AUC of 0.72 for the detection of obstructive CAD by CTA. In a per-patient analysis, CAC score and CTA were the strongest predictor of revascularization (AUC 0.87 and 0.94 p < 0,001).

**Conclusions:** CTP alone presented higher accuracy than SPECT in patients with positive ETT with low or intermediate probability of CAD. Adding the cFFR and CAC data increases the diagnostic accuracy by computed tomography. These results suggest the use of CTP, cFFR or CAC in clinical practice might be adequate alternatives to SPECT in this scenario.

111437

Modality: E-Poster Researcher – Non-case Report

Category: CARDIOVASCULAR SURGERY

## Determinants of Vasoplegic Syndrome After Cardiac Surgery in Patients not using Angiotensin Converting Enzyme Inhibitor or Angiotensin Receptor Blocker

GABRIEL ASSIS LOPES DO CARMO^1^, Bárbara Carolina Silva Almeida^1^, Gabriela Zamunaro Lopes Ruiz^1^, Renato Braulio^1^, Ana Cristina Carioca^1^, Fábio Morato Castilho^1^, Cláudio Leo Gelape^1^, Bruno Rodrigues Pereira^1^, Luiza Moreira Gomes^1^, Ana Carolina Sudário Leite^1^

(1) Universidade Federal de Minas Gerais (UFMG)

**Introduction:** Vasoplegia after cardiac surgery is a well described complication, closely related to increased mortality. It is associated with use of angiotensin converting enzyme inhibitor or angiotensin receptor blocker. Therefore, we routinely discontinue these medications before elective surgeries. However, we still face several cases of vasoplegia.

**Objective:** Describe clinical characteristics associated with vasoplegic syndrome after elective cardiac surgery.

**Methodology:** Prospective cohort analysis of cardiac surgery patients in a public hospital in Brazil between 2016 and 2021, excluding heart transplant.

**Results:** We enrolled 406 patients, median age 57 (46;66) and 214 (52,7%) females. 287 (70,7%) had valve surgery, 99 (24,4%) coronary artery bypass graft, 29 (7,1%) congenital heart correction, 17 (4,2) aorta procedures and 33 (8,1%) other cardiac surgeries. Univariate analysis showed that age, 64 (57;73) vs 55 (44;65), p < 0,001, chronic obstructive pulmonary disease, 47,1% vs 16,5%, OR = 4,51 (1,68–12,15), p0,004, renal replacement therapy (RRT), 57,1% vs 17%, OR = 6,49 (1,42–29,66), p = 0,021, and aortic surgery, 41,2% vs 16,7%, OR = 3,49 (1,28–9,50), p = 0,018, were associated with vasoplegia. After multivariate analysis, age, OR = 1,068 (1,041–1,095), p = 0,005, RRT, OR = 5,77 (1,12–29,69), p < 0,001, and aorta procedures, OR = 3,45 (1,17–10,17), p = 0,025, remained statistically significant.

**Conclusion:** Our study shows that older age, RRT and aortic procedures are associated with higher incidence of vasoplegia in elective cardiac surgeries. All these factors are non-modifiable, but could add some information in the decision to perform a surgery in selected patients, as well as planning best intra and post operative monitorization strategies. Therefore, the diagnosis of vasoplegia could be made as soon as possible and appropriate therapy could be started earlier.

111439

Modality: E-Poster Researcher – Non-case Report

Category: CARDIAC ARRHYTHMIAS/ELECTROPHYSIOLOGY/ELECTROCARDIOGRAPHY

## Contribution of the Vectorcardiogram in the Differential Diagnosis of Brugada Electrocardiographic Pattern

BRUNA AFFONSO MADALOSO^1^, Nelson Samesima^1^, Nancy Maria Martins de Oliveira Tobias^1^, Caio de Assis Moura Tavares^1^, Horacio Gomes Pereira Filho^1^, Mirella Espanhoto Facin^1^, Carlos Alberto Pastore^1^

(1) Clinical Unit of Electrocardiography – Instituto do Coração (INCOR), Hospital das Clínicas FMUSP

**Background:** The electrocardiogram (ECG) is a powerful tool for differential diagnosis among a group of pathologies called J-wave syndrome. The vectorcardiogram (VCG) can be used as a complementary method to the ECG in several dubious alterations.

**Purpose:** We carried out a VCG analysis; after conceiving a novel parameter (JT-distance), that quantified the visual VCG change observed, in most Brugada type-1 patients and that allows diagnosis of the Brugada ECG pattern.

**Methods:** We selected ninety-six ECGs (test cohort) with J-point elevation in V1/V2, ECG superior leads and VCGs, all performed on the same day. The VCG measurement by Frank method (JT-distance) was designed in transverse and right sagittal planes by three lines drawn 1) at the final third of the QRS loop, comprehending J-point; 2) at the initial portion of the T loop; 3) a parallel of the J-point line at the beginning of T loop. JT measure was determined by the distance between parallels. A validation cohort of thirty-five patients was also established.

**Results:** JT-distance ≥1.5 mm (transverse plane) and JT-distance >1.25 mm (right sagittal plane), differentiated Brugada type-1 from type-2, early repolarization and others, with 95% sensitivity,68% specificity (p < 0.05). JT-distance <1.5 mm (transverse plane) and JT >1.25 mm (right sagittal plane) had 100% sensitivity, 85% specificity (p < 0.05) for Brugada type-1 diagnosis (Figure 1). Validation cohort showed JT distance ≥1.5 mm (transverse plane) could differentiate both Brugada types from others with sensitivity of 61%, specificity of 94%, (p = 0.0009). When JT distance (transverse plane) was <1.5 mm and in right sagittal plane was >1.25 mm, we found sensitivity of 83%,specificity of 94%, for Brugada type-1 diagnosis. (p = 0.001). Both cohorts showed very similar Cohen’s kappa levels (0.65 vs 0.77, test and validation cohorts, respectively).

**Conclusions:** The novel vectorcardiogram measurement (JT-distance) presented a new diagnostic criterion to identify Brugada pattern with a practical and possible reproducible method. Nevertheless, prospective studies should also be performed to confirm these findings.


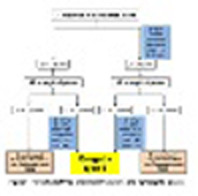


111440

Modality: E-Poster Researcher – Non-case Report

Category: ATHEROSCLEROSIS/CARDIOVASCULAR RISK FACTORS/CARDIOVASCULAR PREVENTION

## Low Adiponectin as a Risk Factor in Cardiovascular Diseases (CVD) in Obesity

ARCHNA SINGH^1^, Sakshi Shukla^1^, Sandeep Aggarwal^1^

(1) All India Institute of Medical Sciences, New Delhi

**Aim:** Adiponectin is a cytokine produced by adipocytes that act on specific receptors of several tissues through autocrine, paracrine, and endocrine signaling mechanisms. Adiponectin circulates in different oligomeric isoforms, with different biological effects. Circulating levels of adiponectin decline under conditions of metabolic stress, including obesity and metabolic syndrome, and are associated with decreased adiponectin signaling. Adiponectin has insulin-sensitizing effects and antiatherogenic properties. Higher adiponectin has been associated with decreased CVD risk.

**Methods:** We divided female participants into 3 categories based on their BMI: normal weight control (C) = 24.28, overweight (OW) = 26.28, and non-diabetic obese (NDO) = 39.40. We estimated adiponectin mRNA expression (n = C: 31, OW: 21, and NDO: 67), serum levels (n = C: 44, OW: 29, and NDO:75) and fasting lipid profile. The Kruskal-Wallis and one-way ANOVA tests were applied for between-group analysis.

**Results:** Overall group analysis for adiponectin mRNA levels and serum levels showed no significant differences; however, a significant difference was noted between C and NDO groups at both mRNA (0.0000) and serum (p-value: 0.0032) levels. A significant difference was seen between groups for all lipid profile parameters i.e., HDL (mean: C: 54.67, OW: 47.79, NDO: 42.28), LDL (mean: C: 96.54, OW: 97.73, NDO: 113.17), TC (mean: C: 165.82, OW: 154.92, NDO: 184.84), TG (mean: C: 114.56, OW: 107.40, NDO: 151.78) and VLDL (mean: C: 22.92, OW: 21.47, NDO: 32.73). Adiponectin gene expression correlated negatively with serum adiponectin levels and with HDL and ApoA1 levels.

**Conclusion:** Lower adiponectin levels could contribute to an increased CVD risk in obesity, an effect that could be mediated via changes in HDL.

111474

Modality: E-Poster Researcher – Non-case Report

Category: HEART FAILURE/CARDIOMYOPATHY/TRANSPLANT

## Characteristics and Prognosis of Patients with Heart Failure with Recovered Ejection Fraction

CARLOS HENRIQUE DEL CARLO^1^, Antonio Carlos Pereira Barretto^1^, Mucio Tavares de Oliveira Junior^1^, Alfredo José Mansur^1^, Antonio de Padua Mansur^1^, Sergio Jallad^1^, Juliano Novaes Cardoso^1^, André Barbosa de Abreu^1^, José Antonio Ramos Neto^1^, Roberto Kalil Filho^1^

(1) Heart Institute (InCor) University of Sao Paulo Medical School

**Background:** Heart failure (HF) is a clinical syndrome with high morbidity and mortality. Optimal treatment can lead to improvement in ventricular remodeling in HF with reduced ejection fraction (HFrEF).

**Objective:** To analyze the characteristics and prognosis of patients (pts) with HFrEF who evolved with HF with recovery ejection fraction (HFrecEF): left ventricular ejection fraction (LVEF) >40%. We also analyzed the outcomes of pts with any degree of LVEF improvement.

**Methods:** We analyzed the electronic medical records of outpatients treated at a tertiary medical center in São Paulo-SP (Brazil) diagnosed with heart failure (ICD-10: I50) in 2017 and followed up until December 31st, 2020. There were included 4068 pts with an initial echocardiogram (ECHO1) presenting LVEF ≤40% (HFrEF). Data from a second evolutionary echocardiogram (ECHO2) were analyzed and pts were reclassified into HFrEF, HFrecEF, or any LVEF improvement (LVEF in ECHO2 > ECHO1). The clinical characteristics and prognosis of pts with respect to HFrecEF and those with any degree of LVEF improvement were analyzed.

**Statistical methods:** Mann-Whitney U test, chi-square test or Fisher’s exact test, univariate and multivariate logistic regression analysis, and survival using the Kaplan-Meier method (p = Log-Rank).

**Results:** Of the 4068 pts studied, age 61.3 ± 13.4 years, 65% male, with regard to etiology: idiopathic cardiomyopathy (46.6%), ischemic (27.5%), hypertensive (14.2%), Chagas disease (7.6%), valvular (4.2%); the mean initial LVEF: 30.0 ± 6.7%. Comparing LVEF in ECHO1 and ECHO2, 2594 pts (63.8%) showed any degree of LVEF improvement and 1250 (30.7%) evolved with total or partial LVEF recovery. Pts with HFrecEF had a higher initial mean LVEF: 32.2 ± 6.6% vs 29.0 ± 7.5% (p < 0.001). The ECHO2 showed an important improvement in the LVEF among pts with HFrecEF (50.9 ± 7.5% vs 29.4 ± 6.4%, p < 0.001). Female gender, hypertensive and valvular etiologies were associated with HFrecEF, while male gender, chagasic etiology, and history of stroke were independently associated with non-recovery of LVEF. Pts with HFrecEF had lower mortality (16.5% vs 30.1%, p < 0.001). The reduction in mortality was also observed in pts with any LVEF improvement (21.9% vs 33.0%, p < 0.001).

**Conclusion:** HFrecEF was observed in 30.7% of pts with HFrEF and was accompanied by an improvement in prognosis. A reduction in mortality was also observed in pts with any degree of LVEF improvement.

111477

Modality: E-Poster Researcher – Non-case Report

Category: COVID-19 AND CARDIOVASCULAR SYSTEM

## Impact of the COVID-19 Pandemic on the Incidence of Extra-Hospital Cardiovascular Deaths in Brazilian Capitals

ANA CAROLINE DARIVA CHULA^1^, Ana Caroline Dariva Chula^1^, Márcia Olandoski^1^, Elias Teixeira Krainski^2^, Lucas Henrique Olandoski Erbano^1^, Bruna Olandoski Erbano^1^, Raisa Natalia Dotto^1^, Lucas Baena Carstens^1^, Nicolle Amboni Schio^1^, Amanda Zanlorenzi^1^, Rafaela Lima Camargo^1^, José Rocha Faria-Neto^1^

(1) Pontificia Universidade Catolica do Parana – PUC PR; (2) Universidade Federal do Parana – UFPR

**Background:** Many countries have reported an increase in the number of out-of-hospital deaths from cardiovascular diseases during the COVID-19 pandemic, which may be due to factors intrinsic to the disease or social conditions arising from the pandemic.

**Objective:** To analyze possible differences in the temporal trend of out-of-hospital cardiovascular deaths during the COVID-19 pandemic in the year 2020 in Brazilian capitals.

**Method:** This is a time series, whose data were collected on the websites https://transparencia.registrocivil.org.br/especial-covid, based on the Civil Registry Information Center, and https://opendatasus.saude.gov.br/dataset/bd-srag-2020, from the Influenza Epidemiological Surveillance Information System. On the first website, the numbers of deaths from COVID-19 and deaths from out-of-hospital cardiovascular causes were collected, week by week, from the 2nd to the 35th epidemiological week of 2019 and 2020, in Brazilian capitals. On the second website, data on hospitalizations for COVID-19 in the capitals were collected daily between March and October 2020, by date of first symptoms and by date of hospitalization itself, and tabulated weekly for combination by epidemiological week. The 2020 time series of total out-of-hospital cardiovascular deaths (TMCV-EH) was considered as the outcome. The time series effect of COVID-19 data was tested to explain the variation across the studied weeks of 2020. COVID-19 hospitalization data were tested as potential predictors of MCV-EH, as were death data from COVID-19. The TMCV-EH in the corresponding period of 2019 was used for comparison.

**Results:** There was an increase in the TMCV-EH in 2020 compared to the non-pandemic year of 2019 and a correlation in the variation of these deaths with the COVID-19 data, with a peak in late April and early May when the relative risk, in the sum of the data, reached twice. There was also heterogeneity in the distribution of MCV-EH when analyzing each capital, with a predominance in the north and northeast regions. The highest relative risks stand out, compared to 2019, in peak periods in some capitals such as Salvador with 3.8, Recife with 3.6, São Luis with 5.6, Belém with 7.5 and Manaus with 9. 5 times. Rio de Janeiro presented a relative risk in the peak period of 2.2 times and São Paulo of 1.6 times.

**Conclusion:** There was an increase in the number of out-of-hospital cardiovascular deaths during the COVID-19 pandemic, in Brazilian capitals.

111486

Modality: E-Poster Researcher – Non-case Report

Category: COVID-19 AND CARDIOVASCULAR SYSTEM

## Impact of the COVID-19 Pandemic on Hospital Admissions for Acute Coronary Syndromes and Mortality from Cardiovascular Diseases in the State of São Paulo

MARIANE VENTUROLI FERREIRA BENAVENTE^1^, Márcia Olandoski^1^, José Rocha Faria Neto^1^

(1) Pontifícia Universidade Católica do Paraná – Escola de Medicina

**Introduction:** During COVID-19 pandemic, several countries reported reduction in hospital admissions for cardiovascular diseases (CVD), including acute coronary syndromes (ACS), in addition to excess cardiovascular mortality due to direct and indirect effects of the pandemic. In Brazil, there are studies demonstrating reduction in hospitalizations, increase in in-hospital lethality and excess deaths from CVD. There are no studies evaluating the impact of the pandemic on hospitalizations and mortality from CVD in the state of São Paulo, the country’s most populous state, which concentrates the largest number of COVID-19 cases and deaths. Understanding this impact is essential for the implementation of public health strategies to reduce morbidity and mortality from these conditions.

**Purpose:** The aim of this study was to assess the impact of the pandemic on ACS admissions and mortality from CVD in the state of São Paulo.

**Methods:** We performed a retrospective observational study by analyzing the number of ACS hospital admissions – angina pectoris (AP) and acute myocardial infarction (MI) and the number of deaths from CVD – MI and unspecific cardiovascular causes (UCVC), in-hospital and at home, between January 1st, 2020, and December 31st, 2021. Data from 2018 and 2019 were used for comparison.

**Results:** In 2020, there was a 10% decrease in hospitalizations for ACS (18% for AP and 4% for MI) compared to 2019. In 2021 reduction was of 12% (27% for AP and 2% for MI). In 2020, there was a 10% decrease in deaths from MI (11% in-hospitals and 7% at home) compared to 2019, while in 2021 there was a 12% increase in the number of MI deaths compared to 2020, 11% in-hospitals and 15% at home, reaching pre-pandemic levels. In 2020, there was a 50% increase in deaths from UCVC (33% in-hospitals and 127% at home) and in 2021 there was an increase of 11% in the number of these deaths, 11% in-hospitals and 10% at home. The decrease in ACS hospitalizations was followed by increase in deaths from MI and UCVC causes in both 2020 and 2021.

**Conclusion:** In the state of São Paulo the pandemic had impact reducing ACS hospitalizations and increasing deaths from CVD, specially UCVC and at home, which remained above baseline levels throughout the analyzed period.

111495

Modality: E-Poster Researcher – Non-case Report

Category: PSYCHOLOGY

## The Role of a Psychoeducation Intervention on 1-Yr Follow-Up of Rehospitalization, Quality of Life, and Post Traumatic Growth, in Heart Failure Patients: A Randomized Clinical Trial

MARCIA MOURA SCHMIDT^1^, Filipa Waihrich de Oliveira^1^, Camila de Matos Ávila^1^, Brenda Pereira Nunes^2^, Fernanda Lucchese-Lobato^3^

(1) Instituto de Cardiologia/Fundação Universitária de Cardiologia; (2) Hospital Geral de Caxias do Sul; (3) Hospital da Criança Santo Antônio, Irmandade Santa Casa de Misericórdia

**Background:** Heart failure (HF) is a systemic disease characterized by deterioration of the heart. There are high rates of morbidity, mortality, and re-hospitalization. Cardiology Societies recommend the inclusion of self-care in the treatment.

**Objective:** This study aimed to verify whether a psychoeducation intervention can reduce hospital readmissions, improve quality of life, and promote post-traumatic growth in patients with HF at 1-yr follow-up.

**Methods:** Parallel randomized clinical trial study with HF patients from a regional hospital in Southern Brazil. Patients were invited to join the study at their first outpatient appointment after hospital discharge. All participants completed the WHO quality of life (WHOQOL-BREF) and the post-traumatic growth inventory (PGI) questionnaires at both pre- (T1) and 6 months post-(T2) intervention assessments. Randomization envelopes were opened after the first interview. The patients in the control group (CG) continued to carry out their regular outpatient consultations per medical instructions. Patients in the intervention group (IG), in addition to the regular visits, had two additional individual follow-ups, of 1 hour each, with an interval of 7 days in between, to promote health and psychological education. About one year later, hospital readmission was assessed through medical records and phone call patient report. Statistical analyses were performed using SPSS software v. 24.0.

**Results:** A sample of 142 patients was recruited at T1 (72 in the CG and 70 in the IG). While at T2, 19 dropped out, and 123 patients (63 in the CG and 60 in the IG) were reassessed after 315 ± 198 days. The participants were 65% male, with 64 ± 11 years old, 58% had low income and 67% had less than high school. The risk of readmission was reduced by 54% (p = 0.050). There was an improvement in the total quality of life (p = <0.05) and positive psychological growth (p < 0.001) in the IG at T2.

**Conclusions:** The intervention proved to be protective for patients with HF in a regional hospital in Southern Brazil. A 2-session psychoeducational intervention was effective in reducing readmission rates by half compared to the CG, as well as improving quality of life and promoting positive psychological growth. Future brief culturally sensitive psychoeducation programs should be implemented in Brazilian hospitals to improve knowledge about HF and patient self-care, reducing the burden that HF creates to our health system. NCT 04870918.

111496

Modality: E-Poster Researcher – Non-case Report

Category: ACUTE AND CHRONIC CORONARY DISEASE/THROMBOLYSIS

## Initial Learnings of Prehospital Management of NSTEMI: Report from Mission Delhi

DR. CHANDINI SUVARNA^1^, Praveen Aggarwal^1^, Ramakrishnan Sivasubramanian^1^, Sandeep Seth^1^, Neeraj Parekh^1^, Sheikh Vamik^1^, Ambuj Roy^1^, Ganesan Karthikeyan^1^, Sandeep Singh^1^, Rajiv Narang^1^, Balram Bhargava^2^, Meenakshi Sharma^2^

(1) Chandini Suvarna; (2) Praveen Aggarwal; (3) Ramakrishnan Sivasubramanian; (4) Sandeep Seth; (5) Neeraj Parekh; (6) Sheikh Vamik; (7) Ambuj Roy; (8) Ganesan Karthikeyan; (9) Sandeep Singh; (10) Rajiv Narang; (11) Balram Bhargava; (12) Meenakshi Sharma

**Background:** Non–ST-segment elevation myocardial infarction (NSTEMI) are common manifestations of coronary artery disease (CAD) which is the leading cause of death in India. Despite advances in the treatment of ACS, pharmacologic therapy remains underused and is often delayed. Mission DELHI (Delhi Emergency Life Heart Attack Initiative) is a pilot project for pre-hospital thrombolysis using motorbike ambulance service by trained paramedics at the patient’s doorstep in a selected geographical area of New Delhi.

**Objective:** We sought to determine the feasibility and safety of prehospital care and risk stratification of NSTEMI.

**Method:** We covered a geographical area of 10-km around our institution. A command center was set up and the patients were required to call a dedicated helpline number. Upon initial screening, a motorcycle ambulance was dispatched to the caller’s location. A 12 lead ECG was obtained as soon as possible after first medical contact by paramedic and electronically transmitted to a dedicated command center. Evaluation of the ECG was done by cardiologist. Patients with suspected NSTEACS, administration of platelet aggregation inhibitors, anti-cholesterol drugs and transferred to the emergency department for further diagnostic assessment and therapeutic decision. On site troponin testing was done when needed. Patients with persistent symptoms despite initial therapy may be transferred directly to a catheterization laboratory.

**Result:** A total of 60 NSTACS patients (mean age 57.7 years; 68.3% male) were treated either at home (66.7%), public places (15%), place of work (11.7%) or at small clinics (6.7%). Time taken to reach the patient location and take an ECG was 27.9 ± 8.2 min. Nearly 56.7% of patients received a loading dose of aspirin, clopidogrel and atorvastatin pre-hospital. Nearly two third of patients (46.7%) received medication for HTN, followed by 66.7% of patients given cardiac medications and 60% patients given other medications including antacid/antiemetic.

**Conclusions:** In this study, we have demonstrated the prehospital acquisition and transmission of ECG by paramedics was feasible. An early diagnosis of the majority of cases with NSTEMI and initiation of basic pharmacotherapy was possible prehospitally. Such an early diagnosis and risk stratification could improve outcome, which needs to be assessed in larger studies.

111914

Modality: E-Poster Researcher – Non-case Report

Category: COVID-19 AND CARDIOVASCULAR SYSTEM

## SARS-COV-2 Variants and Clinical Outcomes

ILIANA REGINA RIBEIRO MENEZES^1^, Giovanni Possamai Dutra^1^, Letícia de Sousa Peres^1^, Nathalia Duarte Camisão^1^, Mariana Moreno Canário da Silva^1^, Renata Mexias Abdala Felix^1^, Thiago Moreira Bastos da silva^2^, Anna Butter^1^, Henrique Custódio Goudar^1^, Bruno Ferraz de Oliveira Gomes^1^, João Luiz Fernandes Petriz^1^, Gláucia Maria Moraes de Oliveira^2^

(1) HOSPITAL BARRA D’OR; (2) UNIVERSIDADE FEDERAL DO RIO DE JANEIRO

**Introduction:** There is evidence that the clinical outcomes of COVID-19 vary according to its variants. Given the high risk of infection-related mortality, recognizing the outcomes in each variant may contribute to understanding the severity of the disease.

**Objective:** To evaluate the association of mortality, myocardial injury, lung parenchyma involvement and use of mechanical ventilation, and length of hospital stay with the most prevalent variants of COVID 19 in Brazil.

**Methods:** Patients admitted to an intensive care unit during the COVID-19 pandemic, with an RT-PCR confirmed diagnosis of COVID-19 were included. The following outcomes and clinical features were analyzed in each period: mortality, length of hospital stay, myocardial injury, lung parenchyma involvement, and use of mechanical ventilation (MV). We divided our sample according to periods where some variants were more prevalent: March to October/2020 (beta); November/2020 to July/2021 (P.1); August to December/2021 (delta) and January to April/2022 (omicron). The variables were analyzed using the chi-square test (categorical) and ANOVA (continuous).

**Results:** 1454 patients were included, mean age = 59.8 ± 17.0 years, 62.6% men. Occurred 269 deaths (18.5%) during the study period (mean follow-up = 338 ± 209 days). We observed a greater association with mortality in the beta variant: beta (23%), P.1 (16.6%), delta (16.6%), and omicron (12.1%) with p = 0.015. Myocardial injury was more prevalent with the omicron variant: beta (32.7%), P.1 (49.2%), delta (53.5%), and omicron (60.6%) with p < 0.001. Lung involvement >50% was more common in omicron: beta (13.0%), P.1 (14.3%), delta (13.6%) and omicron (27.6%) with p < 0.001. No difference was observed in MV need. The average length of hospital stay was higher in beta: beta (20.6 days), P.1 (14.3 days), delta (13.5 days), and omicron (9.6 days), p < 0.001. Finally, the Average time of MV use was higher in beta and P.1: beta (21.4 days), P.1 (21.1 days), delta (14 days), and omicron (9 days).

**Conclusion:** The beta variant was associated with greater mortality and length of hospital stay, while the omicron variant was associated with myocardial injury and shorter mechanical ventilation time in patients hospitalized in intensive care for COVID 19.

111523

Modality: E-Poster Researcher – Non-case Report

Category: CARDIOVASCULAR IMAGING

## New Non-Invasive Assessment Model of Coronary Lesions by Computer Tomography Fractional Flow Reserve Compared to the Invasive Technique Under Hyperaemic and Basal Conditions

DIEGO LOPEZ OTERO^1^, Alberto Otero Cacho^2^, Brais Diaz Fernandez^1^, Maria Bastos Fernandez^1^, Xoan Sanmartin Pena^1^, Vicente Perez Munuzuri^2^, Alberto Perez Munuzuri^2^, Jose Ramon Gonzalez Juantey^1^

(1) Universitary Clinical Hospital of Santiago de Compostela, Cardiology. CIBERCV, Santiago De Compostela, Spain; (2) FlowReserve Labs S.L. Santiago de Compostela

**Purpouse:** To validate a new model of Fractional Flow Reserve obtained from a coronary computed tomography angiography (CCTA) (FFRct) (figure 1A), as well as obtaining new emerging parameters, less studied, such as ΔFFRct (Figure 1B), wall share stress (WWS) (figure 1C) and stenosis resistance (SR).

**Methods:** We included patients referred by the chest pain unit for ischemic heart disease screening, selecting for invasive study those with at least one coronary lesion >50% in the CCTA. Patients with unadequate CCTA interpretation were excluded. To eliminate possible biases, the results of the invasive FFR were unknown at the time of applying this new model to obtain the FFRct value.

**Results:** 26 patients (32 lesions) were included. 86.6% were males and a mean age 57.4 ± SD 11.7 years. In most cases, as these were low-risk patients, and critical disease was excluded, a FFR value >0.80 was obtained. To study the correlation between invasive FFR values and those obtained by the computational model, Spearman’s ρ coefficient and R2 were calculated. There were no false negative or positive cases. The correlation was high in all the parameters studied (>0.6), being positive for the FFRct and negative for ΔFFRct, WWS and SR. The plot in Figure 1D compares the FFRct under steady hyperaemic conditions as a function of the invasive FFR, note the good agreement between the numerical and invasive methods.

**Conclusions:** Our new model was shown to have a good correlation with the invasively measured FFR in this serie of low-medium risk patients. The clinical usefulness of the new parameters in isolation is yet to be determined, but they could be helpful in cases where the FFRct value is in the gray area (0.75 ≤ FFRct ≤ 0.8), specially the ΔFFRct.


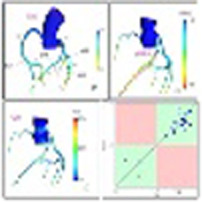


111547

Modality: E-Poster Researcher – Non-case Report

Category: NEGLECTED CARDIOVASCULAR DISEASES

## Short Term Outcomes of Chagas Disease Patients Hospitalized Due to COVID-19

EZEQUIEL J ZAIDEL^1^, Álvaro Sosa Liprandi^1^, Joaquin Perea^1^, Matías Ariel Oliva^1^, Pablo Perel^2^, Kavita Singh^2^, Dorairaj Prabhakaran^2^, Lana Raspail^2^, Karen Sliwa Hahnle^2^

(1) Sanatorio Güemes, Buenos Aires, Argentina; (2) World Heart Federation

**Introduction and aims:** Vulnerable patients with chronic Chagas Disease (CD) may have worse outcomes if hospitalized due to COVID-19. As CD is a neglected tropical disease, it is under-represented in international COVID-19 studies. Current data is scarse and with diverse results. The aim of the present analysis was to describe the clinical profile of patients with known chronic CD during COVID-19 hoapitalization and to analyze if CD patients had different clinical outcomes.

**Methods:** WHF Covid-19 and cardiovascular (CV) Disease registry is a unique COVID-19 prospective registry, as it included cases from different continents and economic strata. CD was a specific subgroup of analysis, and different centres mainly from latin america included CD cases in the registry. A conventional descriptive and comparative analysis was performed.

**Results:** A total of 5313 Covid-19 patients were enrolled in 40 hospitals from 23 countries. Among them, 36 were reported as chronic CD cases. The mean age of CD was similar to non-CD patients (56.4 ± 15 vs 57 ± 16 years, p = NS) but the proportion of women was higher (61.1% vs 40.4%, p = 0.011), and had higher rate of baseline heart failure (16.6% vs 5.4%, p < 0.01), prior pacemaker (3.5% vs 1.1%, p < 0.01) and use of CV drugs (75% vs 46.1%, p < 0.01). During hospitalization, the rate of invasive ventilation was higher in CD (8.3% vs 7.5%, p < 0.01), as well as inotropes requirement (8.3% vs 6.8%, p < 0.01), and ventricular arrhythmias (11.4% vs 1.09%, p < 0.01) or heart blocks (11.4% vs 1.4%, p < 0.01). Regarding outcomes, in-hospital death was 5.7% in CD patients and 13.2% in non CD patients (P = 0.38), and no additional deaths occurred at 30 days (0% vs 2.6% in non CD cases, p = 0.054).

**Conclusions:** Altough a limited number of Chagas disease patients were included, they seem to have more CV disease at baseline than non-CD patients, more CV events during COVID-19 hospitalization but similar short term outcomes.

111530

Modality: E-Poster Researcher – Non-case Report

Category: CARDIOVASCULAR IMAGING

## Impact of the COVID-19 Pandemic on the use of Myocardial Perfusion Imaging for the Assessment of Coronary Artery Disease: A Multicenter Study

ANDREA ROCHA DE LORENZO^1^, Ivanete Costa^2^, Augusto Santos Tavares^3^, Gabriel Grossman^2^, Rafael Lopes^3^, Ronaldo SL Lima^1^

(1) Clinica de Diagnóstico por Imagem; (2) Hospital Moinhos de Vento; (3) Hospital do Coração-HCOR

**Background:** The COVID-19 pandemic- and more specifically, the lockdown period- caused unquestionable effects on non-COVID healthcare, with reductions in the access to exams and treatments. As myocardial perfusion imaging (MPI) has a well-established role in the evaluation of coronary artery disease (CAD), the diagnosis of CAD might have been affected, with possible long-term adverse consequences. Understanding of the temporal trend of MPI utilization is central in this context.

**Objective:** This study aimed to evaluate the impact of the COVID-19 pandemic on MPI performance and results in 3 Brazilian, Nuclear Cardiology laboratories.

**Methods:** Consecutive patients, with or without known CAD (prior myocardial infarction or revascularization), who underwent stress/rest MPI at 3 Brazilian private Nuclear Cardiology laboratories were studied. The number of tests, indication (diagnostic vs known CAD), frequency of abnormal tests, and frequency of ischemic MPI (those with reversible perfusion defects) were registered in three 30-day time intervals: immediately pre-pandemic, lockdown, and post-lockdown. Variables were compared using Fisher’s exact test; p < 0.05 was considered statistically significant.

**Results:** From a total of 1027 MPI studies, 443 were pre-pandemic, 127 were during lockdown (a 71% reduction), and 457 were post-lockdown. Diagnostic tests were 70% of the total before, 61% during lockdown, and 67% after (p = 0.05). The frequency of abnormal MPI tests increased in patients without known CAD in the lockdown period, returning to prior levels after lockdown (11%, 19%, and 11%, p = 0.05), although ischemic tests remained stable (7%, 9%, and 8%).

**Conclusions:** A large reduction of MPI performance occurred during lockdown, with less diagnostic tests. There was no significant difference in the frequency of myocardial ischemia among the 3 intervals, although abnormal MPI tests increased during lockdown in patients undergoing MPI for diagnostic reasons. The altered pattern of MPI utilization caused by the pandemic might have future effects on CAD diagnoses, and therefore should be further and continuously assessed.

111536

Modality: E-Poster Researcher – Non-case Report

Category: EPIDEMIOLOGY AND HEALTH POLICIES/GLOBAL HEALTH

## The Perception of a Group of Parliamentarians on Cardiovascular Diseases and Their Impact on Public Health in Brazil

SORAYA ARAÚJO^1^, Patrícia Vieira^2^, Andrea Bento^3^, Carolina Cohen^3^, Regina Próspero^4^, Amira Awada^4^, Sônia de Castilho^5^, Vanessa Pirolo Vivancos^6^

(1) Cardiovascular Advocacy Group; (2) Brazilian Association of Family Hypercholesterolemia; (3) Collaborate with the Future; (4) Rare Lives Institute; (5) ADJ Diabetes Brazil; (6) Botucatuense Association of Diabetic Care

The Cardiovascular Disease Advocacy Group (GAC) promoted a campaign of Cardiovascular risk awareness named “Faça de Coração – Teste seu Risco Cardiovascular”, as a part of the campaign for World Heart Day. Our group applied a poll to stakeholders at the Legislative Assembly of the State of São Paulo- Brazil to mobilize one of the critical stakeholders to this day. This type of approach provides quick evidence of knowledge from the people who are responding and, at the same time, provokes reflections on the subject. The aim of this study was to verify the perception of parliamentarians about cardiovascular diseases (CVD) and their impact on public health in Brazil. Method: A poll conducted by the GAC in the Legislative Assembly of São Paulo, from 06/09 to 30/08 of 2021 was applied. The poll consisted of eleven multiple-choice questions (five questions about their health status; two questions about public policies; four questions about specific knowledge of CVD) available on the Googles Forms platform. The questions were presented to the 94 parliamentarians who compose the Legislative Assembly of São Paulo (LASP) in virtual visits, witnessed and sent by WhatsApp and e-mail. A descriptive analysis was performed. Results and conclusions: Of the 94 members within a current mandate in the Legislative Assembly, 70 responded to the poll. 84,3% were male; 62,9% aged 36 to 59y; 18,6%, 18 to 35y; and 18,6% aged >60y. In the self-report health status, all are non-smokers, 74% practice physical activity; 52,9% made health check-up; 80% know about their cardiovascular risk; 14,3% has hypertension; 4,3% High Cholesterol, 2,9% Diabetes and 1,4% Obesity. 42% be a part of any collegiate of parliamentarians at LASP to find the best strategies to promote health. Most respondents demonstrate a good knowledge of cardiovascular disease. It is very worrying to note that 91,4% do not believe that the current public policies are effective in the management and control of CVD. However, this is the first step to changing this scenario.

111548

Modality: E-Poster Researcher – Non-case Report

Category: HYPERTENSION/RENAL DENERVATION

## Arterial Stiffness Caused by Hypertension Leads to Baroreflex Dysfunction

MARIANO DUARTE^1^, Mariano Duarte^2^, Analia Aquieri^1^, Javier Coyle^1^, Claudio Yaryour^3^, Carlos Reyes Toso^2^

(1) Laboratorio Hipertensión Arterial, Hospital de Clínicas Facultad de Medicina UBA; (2) Laboratorio de Fisiopatología Cardiovascular UA 2 Facultad de Medicina UBA; (3) División Urgencias Hospital de Clínicas Facultad de Medicina UBA

**Introduction:** The rapid regulation of blood pressure allows the continuous adaptation of the cardiovascular system. This function is carried out by baroreflex (BR). Despite the hypertensive (HT) damage to the arterial wall and BR structures are located within it, the impact of hypertension (HYP) on the BR function has not been fully studied. We hypothesized that HYP has decreased BR function caused by increased arterial stiffness (AS).

**Materials and methods:** In prospective study, 101 patients over 65 years of age were included; G1: HT G2: Normotensive (NT). BR function: A continuous rhythm strip in lead DII of electrocardiogram and the Valsalva maneuver was performed. Chronotropic response at the end of the test was established by the lowest R-R recorded up to 5 beats post-Valsalva. According with AHA, BR function was established by the greatest variation in heart rate (smallest R-R) within 5 immediate beats over Valsalva. Results 2 populations: A-BR Nomofunction: increase 10 and 30 beats. B-BR hypofunction, increase <10 beats. Assessment of AS by means of pulse wave velocity (PWV): It is performed by Complior System device, the “gold standard” for the non-invasive measurement.

**Results:** 101 patients (43 men) were studied, 76 of them were HT (147.2 ± 15.7 mmHg) and the rest NT (130.2 ± 10.8 mmHg) p < 0.05. BR hypofunction was observed in 60 of the 76 HT and only in 3 of the 25 NT. PWV was higher in the HT vs. NT (10.54 vs 8.58 m/s) p < 0.001.

**Conclusions:** As hypothesized, 8/10 in G1 had decreased BR function and only 1/10 in G2. Best predictors of BR hypofunction were sought by multivariate analysis were HYP and AS. So, AS could be one of the causal links between HYP and BR hypofunction and would explain the HT baroreceptor desensitization.


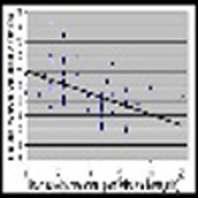


111558

Modality: E-Poster Researcher – Non-case Report

Category: ANTICOAGULATION

## Treatment Satisfaction and Convenience with Edoxaban and Vitamin K Antagonists in Patients with Atrial Fibrillation After Transcatheter Aortic Valve Replacement: An Analysis of the ENVISAGE-TAVI AF Trial

CHRISTIAN HENGSTENBERG^1^, Nicolas M. Van Mieghem^2^, Rosa Wang^3^, Xiaomei Ye^4^, Ling Shi^4^, Shien Guo^4^, Cathy Chen^3^, James Jin^3^, Xin Ye^3^, Martin Unverdorben^3^, George Dangas^5^

(1) Department of Internal Medicine II, Division of Cardiology, Vienna General Hospital, Medical University, Vienna, Austria; (2) Department of Cardiology, Erasmus University Medical Centre, Thoraxcenter, Rotterdam, the Netherlands; (3) Daiichi Sankyo, Inc., Basking Ridge, NJ, USA; (4) Evidera PPD, LLC, Bethesda, MD, USA; (5) Zena and Michael A. Wiener Cardiovascular Institute, Mount Sinai Hospital, New York, NY, USA

**Introduction:** ENVISAGE-TAVI AF (NCT02943785; N = 1426) was a randomized, open-label trial that compared edoxaban vs VKAs in patients with atrial fibrillation (AF) after transcatheter aortic valve replacement (TAVR). Noninferiority was shown with edoxaban vs VKAs for the primary endpoint, net adverse clinical events. The effect of edoxaban or VKA-based therapy on patient-reported treatment satisfaction remains unknown.

**Objective:** To assess patient-reported treatment satisfaction and convenience with edoxaban vs VKAs in patients with AF after TAVR.

**Methods:** The Perception of Anticoagulation Treatment Questionnaire 2 (PACT-Q2) is a validated patient-reported outcome instrument that was used to assess patients’ satisfaction and convenience with their anticoagulant treatment. A mixed-effect model for repeated measures assessed the least squares mean difference (LSMD) in PACT-Q2 scores between edoxaban- and VKA-treated patients. Cohen’s effect size (ES) evaluated clinical meaningfulness of the differences, defined as ES ≥ 0.2.

**Results:** A total of 1107/1426 (77.6%) patients (edoxaban, n = 585; VKA, n = 522) had evaluable PACT-Q2 data and were included in this analysis. Edoxaban- vs VKA-treated patients had similar baseline characteristics. Edoxaban-treated patients reported significantly higher overall PACT-Q2 scores for treatment satisfaction than VKA-treated patients (LSMD [95% confidence interval], 6.1 [4.5–7.7], P < 0.05), with a clinically meaningful difference (ES, 0.4 [0.3–0.5]). Results were similar in the convenience dimension (Figure).

**Conclusions:** ENVISAGE-TAVI AF patients found edoxaban to be more convenient and had greater treatment satisfaction than those who received VKAs. For patients with AF after TAVR, edoxaban may elicit an improved patient experience compared with VKAs.


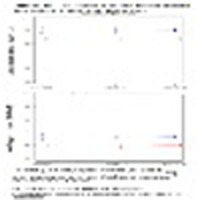


111569

Modality: E-Poster Researcher – Non-case Report

Category: COVID-19 AND CARDIOVASCULAR SYSTEM

## Impact of the COVID-19 Pandemic on Acute Myocardial Infarctions Admissions and Outcomes (2020–2021): Report from Large Tertiary Cardiac Centre

FATHIMA AAYSHA AAYSHA CADER^1^, Ishmum Zia Chowdhury^2^, Syeda Rifat Mahmud^1^, Isha Abdullah Ali^1^, Saidur Rahman Khan^1^

(1) Ibrahim Cardiac Hospital & Research Institute; (2) BIRDEM General Hospital

**Introduction:** There is little data from South Asia on the impact of the COVID-19 pandemic on acute myocardial infarctions (AMI) admissions and outcomes, particularly in the two years following the onset of the pandemic.

**Objective:** We aimed to compare AMI admissions and outcomes over two time periods since the onset of the pandemic at a tertiary cardiac care centre with catheterization laboratory facilities and referrals from across the country.

**Methods:** In this cohort study conducted at our tertiary cardiac centre, we collected and compared data of all patients diagnosed and admitted with AMI over two time periods: between March–August 2020 and March–August 2021. Patient demographics, diagnostic and therapeutic strategies and in-hospital outcomes were statistically analysed and compared by the student’s t test and chi-square tests, as appropriate.

**Results:** A total of 380 AMI admissions were included, 82 (21.6%) in 2020 and 298 (78.4%) in 2021 over the same time periods. Significantly more men presented in both years (69.5% and 83.2% in 2020 and 2021 respectively; p = 0.005). No significant differences in cardiovascular risk factors or type of AMI were seen across both time periods (49.7% vs 52.4% STEMI in 2020 vs 2021). Late presentation of STEMI was 30.5% vs 23.2% in 2020 and 2021 respectively (p = 0.13). 35.9% were vaccinated against COVID19 in 2021 (p < 0.001). Significantly more patients developed cardiogenic shock (40.2% vs 23.8% (Odds Ratio [OR] & 95% confidence interval [CI] 2.15 (1.29–3.61); p = 0.003); heart failure (76.8% vs 57.4%; OR 2.46 (1.40–4.32); p = 0.001), cardiac arrest (19.5% vs 9.7%; OR 2.25 (1.15–4.38); p = 0.015) and required ventilation (19.5% vs 9.1%; OR 2.43 (1.24–4.78); p = 0.008) in 2020, as compared with 2021. In-hospital mortality was numerically higher in 2020 (17.1% vs 9.7%; OR 1.91 (0.96–3.81); p = 0.063). AMI patients were significantly less likely to undergo coronary angiography (54.9% vs 71.1% for 2020 vs 2021; p = 0.005) and percutaneous coronary intervention (35.4% vs 44%; p = 0.001) during index admission in 2020. 45% of STEMI patients were given fibrinolysis overall, with fewer in 2020 as compared with 2021 (20.9% vs 47.2%).

**Conclusion:** In the immediate aftermath of the pandemic, fewer patients presented with AMI in 2020, as compared with similar time periods the next year, in 2021. Significantly fewer patients underwent angiography and PCI in 2020. AMI patients had improved outcomes in 2021, as compared to 2020.

111588

Modality: E-Poster Researcher – Non-case Report

Category: HYPERTENSION/RENAL DENERVATION

## Linear and Nonlinear Analyzes of Autonomic Modulation in Controlled and Uncontrolled Resistant Hypertensives

TATIANE DE AZEVEDO RUBIO^1^, Bruno Rodrigues^1^, Silvia Elaine Ferreira-Melo^1^, Lucia Helena Bonalume Tacito^2^, José Fernando Vilela-Martin^2^, Moacir Fernandes de Godoy^2^, Heitor Moreno-Junior^1^, Juan Carlos Yugar-Toledo^2^

(1) University of Campinas – UNICAMP; (2) Faculty of Medicine of Sao Jose do Rio Preto -FAMERP

Among the blood pressure (BP) regulation mechanisms in RHTN, the expression of autonomic modulation plays an important role. Linear and non-linear analyzes of heart rate variability (HRV) assess the autonomic modulation of the cardiovascular system. Resistant hypertensive patients may present with autonomic dysfunction with different degrees of impairment. The objective of the present study was to evaluate the impairment of the autonomic function of controlled and uncontrolled resistant hypertensive patients, as well as those using beta-blockers. Assessments of autonomic function were performed in 49 resistant hypertensive patients aged between 53 and 82 years.

**Results:** Uncontrolled RHTN patients (U-RHT) present a reduction in HRV and greater expression of sympathetic activity in relation to parasympathetic activity, demonstrated through a statistically significant reduction of the variables SDNN, RMSSD, SD1 and SD2 when compared to the group (C-RHT). In addition, despite the use of beta-blockers, both in RHTN + beta-blockers (RHT+BB) and U-RHT patients, the autonomic balance shows negative changes, that is, lower HRV, compared to the controlled RH group.

**Conclusion:** RHTN patients present a reduction in HRV due to the occurrence of a reduction in parasympathetic activity and a relative increase in the sympathetic component. These results corroborate the importance of interventions on the autonomic nervous system.


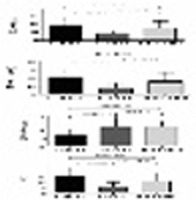


112313

Modality: E-Poster Researcher – Non-case Report

Category: COVID-19 AND CARDIOVASCULAR SYSTEM

## Association of Biomarkers with Mortality in Patients Hospitalized for COVID-19

NATHALIA DUARTE CAMISÃO^1^, BRUNO FERRAZ DE OLIVEIRA GOMES^1^, JOÃO LUIZ FERNANDES PETRIZ^1^, GLAUCIA MARIA MORAES DE OLIVEIRA^2^, ANNA BUTTER^1^, HENRIQUE CUSTÓDIO GOUDAR^1^, RENATA MEXIAS ABDALA FELIX^1^, THIAGO MOREIRA BASTOS DA SILVA^2^, MARIANA MORENO CANÁRIO DA SILVA^1^, LETÍCIA DE SOUSA PERES^1^, ILIANA REGINA RIBEIRO MENEZES^1^, GIOVANNI POSSAMAI DUTRA^1^

(1) Hospital Barra D’or; (2) UNIVERSIDADE FEDERAL DO RIO DE JANEIRO (UFRJ)

**Introduction:** The pandemic caused by the SARS-Cov-2 virus brought the need for and importance of identifying biomarkers that may indicate a worse prognosis and an increase in the long-term mortality of these patients.

**Objective:** Identify biomarkers associated with long-term death in patients hospitalized by COVID-19.

**Methods:** Retrospective study with hospitalized patients with a confirmed diagnosis of COVID-19. The following biomarkers, collected during hospitalization, were analyzed: d-dimer (admission, peak, and discharge), ultrasensitive troponin (admission and peak), C-reactive protein (admission and peak), and platelet count (admission and peak). We used the ROC curve and the area under the curve (AUC) analysis to determine the biomarkers with the best association with all-cause mortality.

**Results:** A total of 1454 patients were included, mean age of 59.8 ± 17.0, 62.6% were men. There were 269 deaths (18.5%) during the study period (mean follow-up = 338 ± 209 days), and 44.7% of patients had myocardial injury. We observed that the highest AUC was obtained with the discharge d-dimer (AUC = 0.900) and the peak d-dimer (AUC = 0.870). The cutoff point proposed by the Youden index was, respectively: 1945 ng/dL (sensitivity 89%/specificity 73%) and 1987 ng/dL (sensitivity 81%/specificity 88%).

**Conclusions:** We observed a high association of mortality (in and out of hospital) with the peak d-dimer obtained before hospital discharge. This data highlights the need for attention to patients who present high levels of this biomarker at the hospital discharge and may indicate the need for preventive therapies for thromboembolic events.


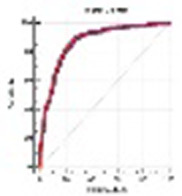


111595

Modality: E-Poster Researcher – Non-case Report

Category: HEART FAILURE/CARDIOMYOPATHY/TRANSPLANT

## Exercise Oscillatory Ventilation Prevalence and 2-Year Mortality Prediction in Chronic Heart Failure Patients: Are There Differences between Ben-Dov, Corrà, and Leite Definitions?

MARLUS KARSTEN^1^, Gustavo dos Santos Ribeiro^2^, Luís Fernando Deresz^3^, Elisabetta Salvioni^4^, Dominique Hansen^5^, Piergiuseppe Agostoni^6^

(1) Universidade do Estado de Santa Catarina (UDESC); (2) Universidade Federal de Ciências da Saúde de Porto Alegre (UFCSPA); (3) Universidade Federal de Juiz de Fora (UFJF); (4) Centro Cardiologico Monzino (CCM); (5) Hasselt University (UHASSELT); (6) University of Milan (UNIMI)

**Background:** Exercise oscillatory ventilation (EOV) is an abnormal phenomenon observed in chronic heart failure (HF) patients and is associated with a greater risk of adverse cardiovascular events.

**Purpose:** To compare the EOV prevalence of 3 EOV definitions and to predict 2-year all-cause mortality in HF patients.

**Methods:** Cardiopulmonary exercise test data from 233 HF patients were analyzed. Two blinded reviewers identified EOV according to the definitions of Ben-Dov, Corrà, and Leite. Prevalence data were compared by the Cochran’s Q test and McNemar post hoc test. The relative risk of death and Kaplan-Meier survival analysis were applied in a 2-year follow-up. Sensitivity and specificity to predict 2-year mortality were determined by the receiver-operating characteristic curve.

**Results:** EOV prevalence was 16.7, 17.5, and 8.4% by Ben-Dov, Corrà, and Leite’s definitions. There was no difference in EOV prevalence between Ben-Dov and Corrà. Both were different from Leite (p < 0.01). The 2-year death risk was 1.9 (1.0–3.7), 2.8 (1.6–5.2), and 2.0 (0.9–4.3) applying Ben-Dov, Corrà, and Leite’s definitions. Table 1 shows the sensitivity and specificity to predict 2-year mortality.

**Conclusion:** EOV definition’s choice directly affects the number of EOV cases. Ben-Dov and Corrà definitions seem to have similar predictive power. Corrà definition shows better sensitivity to predict 2-year mortality.


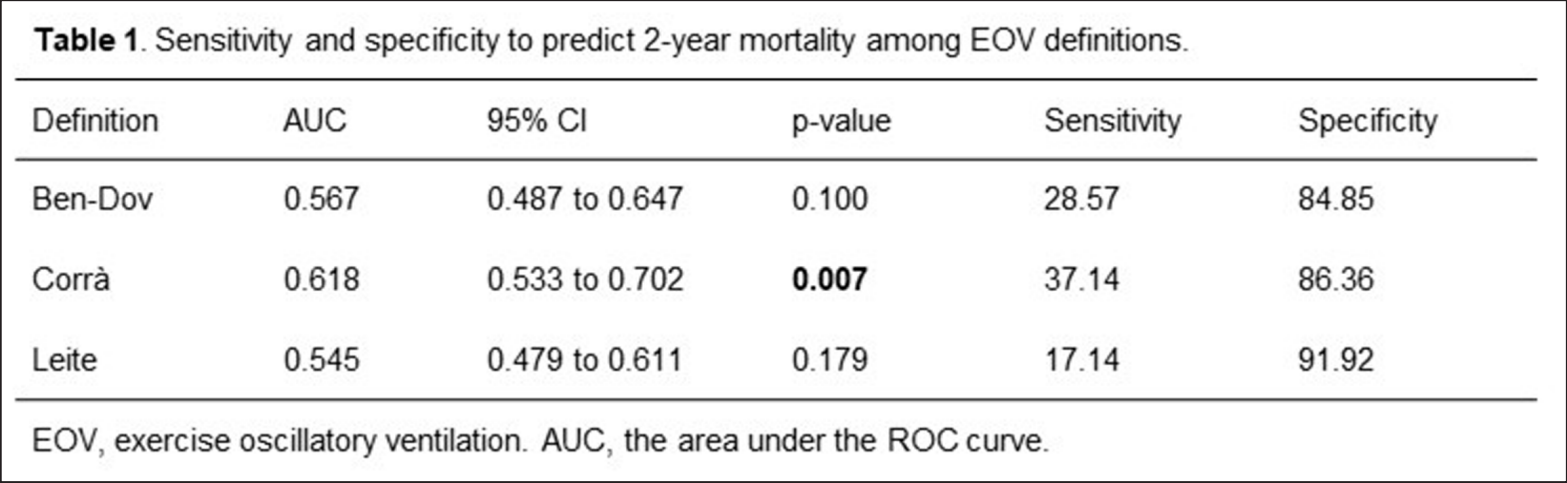


111589

Modality: E-Poster Researcher – Non-case Report

Category: PSYCHOLOGY

## Line of Care for Value-Based Surgical Valvular Disease: The Influence of Surgery in the Physical, Psychosocial and Mental Health Improvement Aspects from the Patient‘s Perspective

SIRLEI PEREIRA NUNES^1^, Sirlei Pereira Nunes^1^, Bellkiss Wilma Romano^1^, Flávio Tarasoutchi^1^, Danielle Misumi Watanabe^1^

(1) InCor – Instituto do Coração do Hospital das Clínicas da FMUSP

**Introduction:** The high mortality from cardiovascular diseases compromises significantly the Quality of Life (QoL) of patients (pacs) in all aspects, especially physical, psychosocial and mental health, making it necessary to rethink care in this population, implementing care, carried out and shared by a team of multidisciplinary.

**Objective:** Verify the surgical influence the valve on the improvement of QoL at perspective of pacs.

**Method:** Retrospective randomized observational longitudinal study of pacs surgical valve disease from a public health institution in the State of São Paulo, by a multidisciplinary team: clinical and surgical cardiologists, nurses, psychologists and others. Psychology assessment occurred in three moments: preoperative(preop), postoperative (posop) in the nursery ward and postoperatively (posop B), six months after the procedure from hospital discharge. 995 pacs were evaluated in preop, 517 posop in nurse and 144 pacs in posop B, March/2018 to February/2020, using semistructured interview technique and the SF 36 instrument that measures QoL from the individual’s perception of their general health status- score from 0 to 100.

**Results:** The results showed an improvement in QoL the SF 36, emphasizing physical, social, emotional and mental health aspects, see table 1(one).

**Conclusion:** Value-based care through the surgical valve disease line of care was effective in providing pacs with na improvement in their QoL.


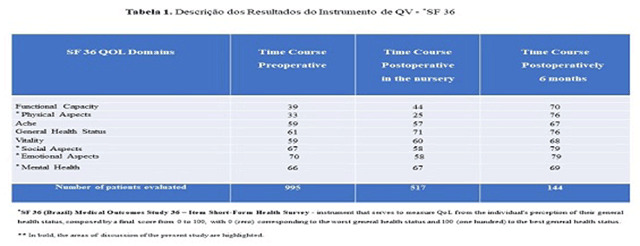


111644

Modality: E-Poster Researcher – Non-case Report

Category: NURSING

## Factors Associated with High Time Spent in Sedentary Behavior in Nursing Students

FERNANDA CARNEIRO MUSSI^1^, Flávia Silva Ferreira^1^, Maria Cecilia Gallani^4^, Jules Ramon Brito Teixeira^2^, Thiago Ferreira Sousa^3^, Francisco José Gondim Pitanga^5^, Fernanda Michelle Santos Silva^1^, Brenda Silva Cunha^1^

(1) Escola de enfermagem, Universidade Federal da Bahia, Salvador, Bahia, Brazil.; (2) Escola de Enfermagem, Universidade Estadual de Feira de Santana, Feira de Santana, Bahia, Brazil.; (3) Centro de Formação de Professores, Universidade Federal do Recôncavo Baiano, Amargosa, Bahia, Brazil; (4) Faculté des sciences infirmières, Université Laval, Québec, Québec, Canada.; (5) Escola de Educação Física, Universidade Federal da Bahia, Salvador, Bahia, Brazil.

**Introduction:** Sedentary behavior (SB) has been associated with chronic disease and all-cause mortality even in sufficiently active people. College students are referred to as a group exposed to SB, but little is known about this behavior in nursing students.

**Objective:** To estimate the amount of time nursing students, spend in sedentary behavior in their daily lives and to examine the association between time in sedentary behavior and sociodemographic, academic, and behavioral variables.

**Method:** Cross-sectional study, with 286 university students. Pearson’s Chi-square or Fisher’s Exact test and Multiple Logistic Regression were used. Sedentary behavior ≥8 hours per day (h/day) was common and risks were corrected by the Delta estimation method, obtaining the prevalence ratio and 95% confidence interval. A statistical significance of 5% was adopted.

**Results:** Sedentary behavior ≥8h/day was identified for 53.5%. Older college students were 33.0% less in sedentary behavior ≥8h/day compared to younger students; with ≥3 hours of the out-of-class study showed 1.23 times more sedentary behavior ≥8h/day compared to those with <3 hours; with ≥4 subjects showed 1.58 times more sedentary behavior ≥8h/day compared to those with ≤3; insufficiently active showed 1.25 times more sedentary behavior ≥8hr/day compared to those who met the recommendation and who used sleep medications showed 1.46 times more sedentary behavior ≥8h/day compared to those who did not.

**Conclusion:** It is necessary to combat sedentary behavior especially in younger college students, with accumulation of subjects, sleeping drugs, and insufficiently active.

111660

Modality: E-Poster Researcher – Non-case Report

Category: CARDIAC ARRHYTHMIAS/ELECTROPHYSIOLOGY/ELECTROCARDIOGRAPHY

## An Electrocardiographic Index in Aortic Root Enlargement and Ventricular Hypertrophy with Phi-Guided Chest-Wall Thickness Derived Measure

JOSÉ RAMÓN LANZ-LUCES^1^, Fernando Augusto Alves da Costa1^1^, Luís Fernando Escobar Guzman^2^

(1) Instituto Paulista de Doenças Cardiovasculares (IDPC); (2) Hospital Beneficência Portuguesa de São Paulo (BP)

**Introduction:** Dilated aortic root (DAo) is a risk for cardiovascular events besides left ventricular hypertrophy (LVH). There is a lack of a useful index pondering both scenarios.

**Objective:** To evaluate an electrocardiographic index in DAo and LVH comparing echocardiographic consensus values and those derived using the wall chest thickness (CT).

**Methodology:** 631 patients, 236 hypertensive (HT) and 395 non-hypertensives (nHT), the diameter of the aortic root was based on the golden number (Phi) and the derived formula CT × 1.33 = Ao, using a cut-off >15% for enlargement. The index stemmed from the R + S amplitude sum in leads D1 + D2 + D3 and for values <23 mm. The index was also compared with Cornell and Sokolow-Lyon-Rappaport indexes and stablished criteria for Dao and LVH using T-test and CH2 test as appropriate.

**Results:** The index prevalence was similar between groups (HT:42.7 vs. nHT: 37%, p = 0.15). There were differences in echocardiographic measurements (aorta, septum, posterior wall, and ventricular mass, p < 0.05). The index failed to be related to LVH, on the contrary it was associated to DAo derived from CT in the general population (OR: 2.189 CI95%: 1.546–3.100, p < 0.001) and in NTH (OR:3.010 CI95%: 1,869–4.847). Similar results for actual Dao criteria in the NTH (OR:3.078 CI95%:1.224–7.738, p = 0.012). Cornell index showed similar odds however, the test-sensibility was far less than the novel index, 8.4% vs 71.2%.

**Conclusion:** This novel index was advantageous separating those with a DAo based CT measure along with literature consensus criteria for non-hypertensive patients.

111670

Modality: E-Poster Researcher – Non-case Report

Category: CARDIOVASCULAR SURGERY

## Coronary Artery Bypass Graft or Percutaneous Coronary Intervention in Young Patients with Acute Coronary Syndrome and High Atherosclerotic Load: A Paired Analysis by Propensity Score

LUIZ SÉRGIO FERNANDES DE CARVALHO^1^, Alice Pacheco Santos^4^, Ana Carolina Machado Rodrigues da Cunha^4^, Guilherme Albuquerque Gruber^4^, Ana Claudia Cavalcante Nogueira^2^, Alexandre Anderson de Souza Munhoz Soares^2^, Gustavo Alexim^3^

(1) Clarity Healthcare Intelligence; (2) Instituto Aramari Apo; (3) HRS-DF – Hospital Regional de Sobradinho do Distrito Federal; (4) UCB – Universidade Católica de Brasília

**Introduction:** The epidemiology of acute coronary syndromes (ACS) has gone through dramatic changes in recent decades, with an increase in its incidence in young individuals (under 55 years, ACSy) and a relative decrease in older individuals (ACSo). The management of ACS in young patients with moderate to high atherosclerotic load after the primary event still needs elucidation, since these individuals retain a long life expectancy.

**Objective:** To compare clinical outcomes and cost of care in individuals with premature CAD and moderate to high atherosclerotic load undergoing Coronary Artery Bypass Graft (CABG) or percutaneous coronary intervention (PCI).

**Method:** The study population consisted of the retrospective B-CaRe:QCO assortment, which includes all patients admitted with ACS in public hospitals in Federal District between 2013 and 2015 and who underwent cardiac catheterization with SYNTAX score >28. Outcomes were adjudicated with death certificates and medical record data. The primary clinical outcome was composite by CV death and recurrent ACS (MACE), followed by secondary endpoints: all-cause death and AMI. To assess indirect and direct costs, we evaluated, in international dollars (Int$) per year, the cost of lost productivity due to illness and death, ambulatory costs, and costs of new hospitalizations. Multivariate and propensity score paired (PEP) analyses were performed.

**Results:** Among 1088 subjects (111 treated by CABG and 977 with PCI) aged <55 years accompanied for 6.2 (IQR 1.1) years, 304 primary events were observed. MACE was observed in 20.7% of the CABG group and 28.8% of the PCI group (p = 0.037). All-cause deaths were seen in 8.1% of the CABG group and 6.6% (p = 0.450) of the PCI group. AMI was seen in 6.3% of the CABG group and 15% of the PCI group (p = 0.014). On multivariate analysis, PCI was associated with higher hazard ratio (HR) for MACE, 1.227(95% CI 1.004–1.499; p = 0.04577), and in PEP HR = 1.268 (95% CI 1.048–1.548; p = 0.0271) compared to the CABG group. Although we observed no statistical differences in direct costs, the cost of lost productivity was higher in the PCI group (Int$4,511 (IQR 18062)/year vs Int$3,578 (IQR 13198)/year; p = 0.049] compared to CABG.

**Conclusion:** In younger, high-atherostatic load ACS patients, the surgical approach is associated to lower occurrence of major long-term adverse cardiovascular effects, besides being associated to lower indirect costs.

111703

Modality: E-Poster Researcher – Non-case Report

Category: PERIOPERATIVE EVALUATION

## Myocardial Injury After Non-Cardiac Surgery Prediction using Machine Learning

BRUNO FERRAZ DE OLIVEIRA GOMES^1^, Thiago Moreira Bastos da Silva^2^, Leticia de Sousa Peres^1^, Iliana Regina Ribeiro Menezes^1^, Nathalia Duarte Camisão^1^, Mariana Moreno Canário da Silva^1^, Renata Mexias Abdala Felix^1^, Giovanni Possamai Dutra^1^, Anna Butter^1^, Henrique Custódio Goudar^1^, João Luiz Fernandes Petriz^1^, Gláucia Maria Moraes de Oliveira^2^

(1) Hospital Barra D’Or; (2) Universidade Federal do Rio de Janeiro

**Background:** Myocardial injury in non-cardiac surgery (MINS) occurs in 10–25% and increases mortality in 30 days. Prediction tools can help in the creation of preventive strategies.

**Methods:** Retrospective cohort study including all non-cardiac surgery patients admitted to a postoperative care unit who stayed at least one night in this unit and had at least one measurement of high-sensitive cardiac troponin. Clinical characteristics and the occurrence of MINS were assessed. All variables were included in the classification tree statistical model, a machine learning method, where variables with p < 0.05 were included in the analysis.

**Results:** 2230 patients were included, mean age = 63.7 ± 16.2 years, and 55.6% women. The prevalence of MINS was 9.4%. The classification tree is available in Figure 1. The variables selected by the model were: age, RCRI score, high-risk surgery, previous myocardial infarction, and creatinine. This model has an accuracy of 90.6%.

**Conclusion:** Age, RCRI score, high-risk surgery, previous myocardial infarction, and creatinine were predictors of MINS and were included in the classification tree. This model exhibited high accuracy and may be useful in the early identification of these patients.


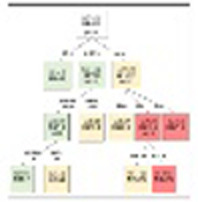


111681

Modality: E-Poster Researcher – Non-case Report

Category: HEART FAILURE/CARDIOMYOPATHY/TRANSPLANT

## Epidemiological Profile of Patients with Heart Diseases with Indications for Palliative Care

MARIA TERESA CABRERA CASTILLO^1^, MILENA DAVI NARCHI^1^, FLAVIA CUNACIA D‘EVA^1^, ROBERT TANAKA^1^, ADRIANA FUCCI^1^, DEBORA CLOTILDE CAROLLA^1^, LUISA MURAKAMI^1^, FARID SAMAAN^1^, RUI FERNADO RAMOS^1^, GUSTAVO BERNARDES DE FIGUEIRDO OLIVEIRA^1^, ARI TIMERMAN^1^

(1) INSTITUTO DANTE PAZZANESE DE CARDIOLOGIA

**Introduction:** Cardiovascular disease (CVD) is the leading cause of death in Western countries, with heart failure being the final pathway of heart disease. Despite this, few patients are referred for Palliative Approach (PA). There is no consensus about the indication or the right time to refer patients to PA, whose main objective is to improve the patient’s quality of life.

**Objective:** Characterize the epidemiological profile of patients with heart diseases with indication for PA in a hospital specialized in cardiology.

**Methods:** This is a cross-sectional study with analysis of 554 patients included for PA from Feb 2018 to Feb 2022. Frequency, median, percentage, mean, maximum and minimum value were used for statistical analysis.

**Results:** The sample comprised of 554 patients (P), for PA 270(57%) were male, mean age 71 ± 14 years, 179(37%) Catholic, 91(20%) Evangelical, With most frequent medical history of arrhythmia 242(51%), ventricular dysfunction 297(62%), Coronary artery bypass graft surgery 109 (23%) and stroke 175(15%). The most frequent clinical complains were: Dyspnea 329(59.30%), Lower limb edema 96(17.39%), diagnosis at the time of PA was renal failure 351 P (74.4%) and of these 148 (31%) needed hemodialysis, Heart failure 177(31.9%) (HF), Sepsis/Septic shock 146(26,3), stroke 113(20%). The mean ejection fraction was 40%. As for performance in activities of daily living we used the Palliative performance Scale (PPS) where 282 (71,1%) needed life support. We evaluated the prognosis with the PPI scale, 345(56.8%) with high short-term mortality. Regarding treatment, 277(58,7%) of patients with PA had CVC, 277(58.7%) received broad spectrum antibiotics, 196(41.5%) dobutamine, 138(30%) noradrenaline, 164 (34.7%) on mecanichl ventilation 130(27.5%) on hemodialysis 116(24.6%) sedated, 81 (8%) with IAP. Among these patients 295(52.9%) died during hospitalization and 126 (22%) were able to be discharged. Only 26(4.7%) did not accept the PA protocol, the most frequent site of the PA was 245(51.1%) in the ICU, followed by the ward with 227 (48%) P.

**Conclusion:** In our sample we identified that patients with heart disease in PA still receive futile interventions and therapies, and the most frequent site of palliation is the ICU. This shows a later indication for PC, in patients with advanced HF and at the end of life. Strategies and institutional protocols are needed for an earlier indication, in order to promote integral care and better qualy of life.

111684

Modality: E-Poster Researcher – Non-case Report

Category: CONGENITAL AND PEDIATRIC CARDIOLOGY

## Maternal Supplementation of Omega-3 is Safe for the Fetal Heart? a Randomized Clinical Trial

PAULO ZIELINSKY^1^, Paulo Zielinsky^1^, Júlia Foresti^1^, Débora Raupp^1^, Daniela Babinski Guimarães^1^, Kelly Zucatti^1^, Vitória Gomez^1^, Vitória Aragon^1^, Izabele Vian^1^, Eduarda Bonamigo^1^

(1) Institute of Cardiology of Rio Grande do Sul (IC/FUC)

Omega-3 (DHA) has been reccomended to adequate fetal development during gestation. Since it is a substance with intense anti-inflammatory action, such as dietary polyphenols, its effects upon the fetal heart and circulation are unknown and safety of its administration during gestation is not yet established. This study was designed with the purpose to investigate if maternal supplementation of omega-3 is followed by alterations of flow dynamics on fetal ductus arteriosus, at the final trimester of pregnancy, through a double blind, placebo-controlled, randomized clinical trial by groups. Pregnant women between gestational 27 and 28 weeks without cardiac alterations at fetal echocardiography were included, with exclusion of those taking nonsteroidal anti-inflammatory drugs or other substances with this effect, such as polyphenol-rich foods. The intervention group received supplementation of 450 mg of DHA/day in gastroresistent capsules and was compared to the placebo group, after 8 weeks. The participants in both groups were submitted to fetal Doppler echocardiography, assessment of dietary polyphenol and omega-3 consumption, as well as serum prostaglandin levels. Intergroup and intragroup data were evaluated. The study ended up with 24 participants in each group. After 8 weeks, Doppler echocardiographic parameters of ductal flow and serum prostaglandin levels in both groups did not show significant intergroup differences (systolic velocity: p = 0.59; diastolic velocity: p = 0.53; pulsatility index: p = 0.29; serum prostaglandin levels: p = 0.40) The expected intragroup differences as a result in increase in gestational ages were present. The results of this study suggest that omega-3 supplementation is safe at the final third of pregnancy, not causing alterations in the dynamics of the fetal ductus arteriosus flow, at the doses used, despite its potential anti-inflammatory action.

111685

Modality: E-Poster Researcher – Non-case Report

Category: CARDIORESPIRATORY PHYSIOLOGY/BASIC SCIENCE

## Gene Therapy with Mirna-205 Selected from Aerobic Exercise Training Promotes Cardiovascular Protection in Arterial Hypertension

TIAGO FERNANDES^1^, Fernanda Roberta Roque^1^, Vander José Neves^2^, João Lucas Penteado Gomes^1^, Andre Casanova Silveira^1^, Graziela Amaro Vicente Ferreira Saraiva^4^, Suliana Mesquita Paula^3^, Camila Paixão Jordão^5^, Rodrigo Souza^4^, Luciana Venturini Rossoni^3^, Maria Urbana Pinto Brandão Rondon^4^, Edilamar Menezes Oliveira^1^

(1) Laboratory of Biochemistry and Molecular Biology of Exercise, School of Physical Education and Sport, University of Sao Paulo; (2) President Tancredo de Almeida Neves University Center; (3) Department of Physiology and Biophysics, Institute of Biomedical Science, University of Sao Paulo; (4) School of Physical Education and Sport, University of Sao Paulo, Sao Paulo; (5) Heart Institute

Arterial hypertension (AH) is a multifactorial syndrome characterized by high levels of blood pressure. Aerobic exercise training (ET) has been used as an important non-pharmacological treatment of AH, since it mitigates cardiovascular remodelling and blood pressure; however, the mechanisms involved are poorly understood. Our analysis of the miRNA’s expression profile by microarray, we selected the miRNA-205 for being one of the most differentially expressed miRNAs in AH, being neutralized by ET, and for targeting many genes involved in the vascular process. Therefore, we aim to evaluate the therapeutic potential of ET and gene therapy with AAV9-miRNA-205 treatment in spontaneously hypertensive rats (SHR), and the circulating miRNA-205 expression in AH patients. SHR aged 6 months and the respective controls Wistar Kyoto (WKY) were divided into four experimental groups (n = 7/group): SHR, trained SHR (SHR -T), WKY and trained WKY (WKY-T). The swimming ET consisted of 10-week, 1 × day/5 × a week/10 weeks. ET promoted reduction in blood pressure in SHR and resting bradycardia in trained animals. We observed a reduction in VO2max accompanied by cardiac hypertrophy and skeletal muscle atrophy. Moreover, these animals showed an increase in wall:lumen ratio of the femoral artery and muscle arteriole. ET corrected these changes in the peripheral vessels. AH increased 300% miRNA-205 levels by real-time PCR paralleled with a decrease of target genes VEGF, Akt, Bcl-2, eNOS, Itga5, TGFα and p70S6K levels by western blot compared to WKY group in skeletal muscle. In contrast, ET counteracts miRNA-205 and target genes levels toward control. Also, we used an adeno-associated virus 9 (AAV9) delivery methods to inhibit miRNA-205 in cardiovascular and muscle systems. Interestingly, AAV9-mediated miRNA inhibition (single systemic injection of AAV: 2 × 1012 viral genome particles) reduced AH-induced high levels of blood pressure compared to AAV-9 control around 25 mmHg in SHR. Indeed, miRNA-205 inhibition attenuated cardiac remodelling in SHR. In AH patients, the circulating levels of miRNA-205 were higher when compared to the normotensive group. Together, these results support the hypothesis that the morphofunctional changes arising of AH may be regulated by miRNAs and target genes; and ET participates in restoring the cardiovascular system. Thus, AAV9-mediated miRNA-205 inhibition appears as perspectives for the potential therapeutic use of miRNAs in the treatment of AH.

111930

Modality: E-Poster Researcher – Non-case Report

Category: ACUTE AND CHRONIC CORONARY DISEASE/THROMBOLYSIS

## 2016 Left Ventricular Diastolic Function Guideline Have Better Prognostic Value Than 2009 Guideline in Patients with Acute Coronary Syndrome

RAFAEL MODESTO FERNANDES^3^, Carolina Aslan Ribeiro Brito^5^, Carolina Costa da Silva Souza^5^, Vitor Queiroz de Castro Souza^5^, David Le Bihan^4^, Andrea A. Vilela^1^, Rodrigo B. M. Barretto^2^, Elizabete S. Santos^1^, Amanda G. M. R. Sousa^1^, Ari Timerman^1^

(1) Dante Pazzanese Institute of Cardiology, São Paulo, Brazil; (2) Instituto do Coração (INCOR), University of São Paulo, Brazil; (3) D’Or Institute for Research and Education (IDOR), Hospital Aliança, Salvador, Brazil; (4) Grupo Fleury- São Paulo; (5) Bahiana School of Medicine and Public Health, Salvador, Brazil.

**Background:** Cardiovascular disease is the leading cause of death in Brazil and worldwide. In the context of diastolic dysfunction, echocardiography is the best non-invasive diagnostic method. However, the comparison of the two main guidelines for evaluate left ventricular diastolic function in acute coronary syndrome (ACS) is scarce.

**Objective:** To compare the prognostic value of both guidelines of American Society of Echocardiography, from 2009 and 2016, in patients with ACS.

**Methods:** This is a sub-analysis of a prospective cohort observational study with 109 patients admitted to the emergency with ACS. The follow up was performed within 1 year and combined outcome was cardiovascular death or new heart failure. We used the non-parametric Spearman method to assess the correlation between the categories of diastolic function according to the guideline used. The Cox model and the Log rank test with Kaplan-Meier curves were used to compare the prognostic value of categorizing patients according to the guideline used. The results were expressed as hazard ratio with a confidence interval of 95%.

**Results:** The mean age was 63 years ±11, most male patients (73.4%) and the predominant color of patients was white (60.4%). Among the main risk factors, the most frequent for coronary artery disease was dyslipidemia (78%), followed by systemic arterial hypertension (77.1%) and sedentary lifestyle (65.9%). The study identified a mean borderline ejection fraction, a high E/E’ ratio, and LV diastolic and systolic volumes presented means above normal. The most common electrocardiographic change on admission was T-wave inversion (45%), and NSTEMI was the main clinical diagnosis (74%). The categories in which there was a greater disagreement between the guidelines were grade II diastolic dysfunction and normal function. The correlation between the diagnosis of diastolic dysfunction when compared to the use of the 2009 and 2016 guidelines was weak (R = 0.56 by Spearman’s method; p < 0.001). By the Kaplan-Meier curves, dividing the groups into with or without LA pressure elevation, the distinction between the evolution of the two groups is significant when performed by the 2016 guideline (Log Rank = 8.17; p = 0.04).

**Conclusion:** The current guideline (2016) for the assessment of left ventricular diastolic dysfunction showed a higher prognostic value of combined outcome of cardiovascular death or new heart failure within one year, when compared to the guideline of 2009 in patients.

111722

Modality: E-Poster Researcher – Non-case Report

Category: DIGITAL HEALTH/INNOVATION

## Artificial Intelligence Technology-Based Capnography Waveform Processing and the Presence of Heart or Pulmonary Disease in Hospitalized Individuals

ALEXANDRA CORRÊA GERVAZONI BALBUENA DE LIMA^1^, Caroline Barreto Cavalcanti^1^, Paula Fernandes Freitas Lima^1^, Barbara Cunha Barreto^2^, Bruno Ramos Carneiro^2^, Gabriela de Oliveira Silva^2^, Maria Alice Ramalho Bragatto^2^, Ilan Sousa Figueirêdo^3^, Wenisten Jose Dantas da Silva^3^, Erick Giovani Sperandio Nascimento^3^, Luís Moreira da Silva de Azevedo Meireles^4^, Sergio Henrique Rodolpho Ramalho^5^

(1) North Wing Regional Hospital – Brasilia – Brazil; (2) School of Health Sciences – Brasília – Brazil; (3) Manufacturing and Technology Integrated – Campus SENAI CIMATEC, Salvador, Bahia, Brazil; (4) MDI Industrial, Salvador, Bahia – Brazil; (5) Coordinator of the Clinical Research Center of the Brasilia of the Dasa Hospitals Network – Brasilia – Brazil

**Background:** Dyspnea is challenge in hospitalized individuals. The reasons for dyspnea are often multifactorial and the evaluation may use different and expensive methods (ex.: arterial blood gas, natriuretic peptide measurement, echocardiogram, computed tomography). This objective of this study was to assess the accuracy of an artificial intelligence (AI) capnography waverform and the presence of heart or pulmonary disease using the AI technology.

**Methods:** In this prospective observational study, of patients admitted to a public hospital in Brasilia, Brazil, from September 2021 to February 2022, deep learning algorithms were developed and validated using capnography waveforms acquired from patient monitoring. The classification model was a binary classifier of individuals with healthy patients and sick patients (heart disease or pulmonary disease or heart and pulmonary disease) by analyzing biosignal waveforms. We assessed the model performance using area under the receiver operating characteristic curve (AUC-ROC), Accuracy, Sensitivity, Recall, F1-score, and Specificity.

**Results:** Among them, 286 cases were taken as the experimental group, 61 (21%) heart disease, 69 (24%) pulmonary disease, 85 (30%) heart and pulmonary disease and 71 (25%) without heart or pulmonary disease (healthy patients). The AI model revealed a true positive (sick patients) of 88% and a true negative (healthy patients) of 82%. Therefore, the AI capnography waveform algorithm showed an average performance of 86% of Accuracy, 85% of Specificity, 85% of Recall and 83% of Precision, 84% of F1-score, and 85% of AUC-ROC.

**Conclusions:** Capnography is a non-invasive, low-cost, and easy-to-use equipment. The capnography waveform could predict the presence or absence of heart or pulmonary disease based on biosignals acquired using noninvasive patient monitoring. The research showed that the design of AI information processing can assistant the clinical diagnostic and evaluation of patients.

111727

Modality: E-Poster Researcher – Non-case Report

Category: PERICARDIUM/ENDOCARDIUM/VALVOPATHIES

## Immediate and Long-Term Predictor Score of Success After Percutaneous Mitral Balloon Commissurotomy

RAFAEL ALEXANDRE MENEGUZ MORENO^1^, Luiz Eugenio B. Prota-Filho^1^, Caio C.V. Queiroz^1^, Fabricio C. Wohnrath^1^, Felipe A.C. Carboni^1^, Gisele R.C. Silva^1^, Joselyn I.P. Castro^1^, Wandemberg S. Silva^1^, Auristela I.O. Ramos^1^, Nisia L. Gomes^1^, Sergio L.N. Braga^1^, J. Ribamar Costa Jr^1^

(1) Instituto Dante Pazzanese de Cardiologia

**Background:** Percutaneous mitral balloon commissurotomy (PMBC) remains the preferred treatment for patients with severe symptomatic rheumatic mitral stenosis (MS) and suitable anatomy. The objective of this study was to propose a new score for the prediction of immediate and late success.

**Methods:** This is a retrospective, single-center, single-arm registry encompassing all 1915 consecutive patients with rheumatic mitral stenosis recruited and referred to PMBC between August 3rd 1987 and July 19th 2010. All data were previously collected and recorded in a dataset. Clinical status was determined according to the New York Heart Association (NYHA) classification. Long-term outcome was a composite of incidence of major adverse cardiac events (cardiovascular death, new PMBC or mitral valve repair surgery) up to 24 years of clinical follow-up (from 1988 until December 3rd, 2011), including cardiovascular death, need for new PMBC, or mitral valve replacement surgery.

**Results:** Mean patient age was 36.8 ± 12.9 years, most (86.4%) were female, and Wilkins score was between 9–11 in 49.1% of patients. In the multivariate analysis, the predictors of immediate success were age (odds ratio [OR], 0.98; 95% confidence interval [CI], 0.96–0.99; p = 0.01), left atrium size (OR, 0.96; 95% CI, 0.93–0.99; p = 0.01), mean pre-procedure mitral gradient (OR, 0.93; 95% CI, 0.89–0.96; p < 0.001), intermediate Wilkins score 9–11 (OR, 0.62; 95% CI, 0.40–0.94; p = 0.02), and high Wilkins score ≥12 (OR, 0.35; 95% CI, 0.16–0.76; p < 0.01). For prediction of late events, age (hazard ratio [HR], 0.98; 95% CI, 0.97–0.98; p < 0.001), New York Heart Association class III–IV (HR, 1.50; 95% CI, 1.18–1.92; p < 0.001), left atrium size (HR, 1.02; 95% CI, 1.02–0.04; p < 0.01), and high Wilkins score ≥12 (HR, 2.02; 95% CI, 1.30–3.15; p < 0.01) were significant. Two nomograms were developed using significant predictors from the model (one for immediate results and another for long-term results).

**Conclusions:** In this large population, not only the Wilkins score, but also clinical and hemodynamic features, seem to be relevant in predicting immediate and late success for patients with rheumatic MS who underwent PMBC.

111724

Modality: E-Poster Researcher – Non-case Report

Category: HEART FAILURE/CARDIOMYOPATHY/TRANSPLANT

## The Impact of Rapid Titration of Beta Blockers on Functional Capacity in Patients with Advanced Heart Failure and Analyzed Through the 6-Minute Walk Test

DOMINGOS SAVIO BARBOSA DE MELO^2^, Domingos Savio Barbosa de Melo^2^, Antonio Carlos Pereira Barreto^1^, Aristea Izabel de Oliveira^2^, Rodrigo Savio de Oliveira Melo^2^, Breno Domingos de Gusmao Melo^2^

(1) Heart Institute- InCor-FMUSP; (2) Ergocardio Medicina Diagnosis and Research

**Background:** Rapid up-titration of beta-blockers (BB) in the treatment of heart failure (HF) during hospitalization has yet to be tested.

**Objectives:** The purpose of this research was to evaluate the correlation between the rapid up-titration of beta-blockers and functional capacity in patients hospitalized for HF using the 6-minute walk test (6MWT) as a parameter.

**Methods and Results:** 92 patients with advanced HF (New York Heart Association [NYHA] IV) and left ventricle ejection fraction (LVEF) <45% were hospitalized for clinical compensation and followed-up for one year after discharge. The patients were distributed into two groups: 46 in the treatment group (TG) and 46 in the control group (CG). During hospitalization, they were divided randomly for BB rapid up-titration with an increase of dosage every two days (TG), or by the usual treatment regime (CG). Statistical analysis used: Student’s t-test, Mann-Whitney, Chi-square and Fisher’s exact test, Kaplan-Meier for survival, using the Log-rank test for comparison and the hazard ratio (HR) calculated with the Cox model. P < 0.05 was considered significant. The exercise tolerance analysis was performed at the entrance, three months and one-year intervals and the walking distance was stratified into four levels (NV): NV1 <300 meters (m), NV2 = 300–375 m, NV3 = 375–450 m and NV4 > 450 m. At the entrance moments, three months and one year the distance traveled for the TG/CG were, respectively, 206.62 ± 33.54/185.09 ± 32.32 meters, 384.55 ± 77.54/336.55 ± 65.23 meters, and 422.17 ± 98.57/387.41 ± 76.66 meters. It was verified that there was an increase in walking distance for the two groups studied (p = 0.002). However, the distance walked in the TG was significantly higher than in the CG (p < 0.001).

**Conclusions:** The rapid up-titration of beta blockers dosage during hospitalization of end-stage HF patients compared to the usual treatment regimen in advanced HF is safe, effective, and promotes increased functional capacity through 6MWT analysis.

111743

Modality: E-Poster Researcher – Non-case Report

Category: HEART FAILURE/CARDIOMYOPATHY/TRANSPLANT

## Evaluating the Trial Strategy for Dosage Application of Beta-Blockers and Their Impact on the Quality of Life using the Minnesota Living with Heart Failure Questionnaire (MLHFQ)

DOMINGOS SAVIO BARBOSA DE MELO^2^, Antonio Carlos Pereira Barretto^1^, Aristea Izabel de Oliveira^2^, Rodrigo Savio de Oliveira Melo^2^, Breno Domingos de Gusmao Melo^2^, Domingos Savio Barbosa de Melo^2^

(1) Heart Institute- InCor-FMUSP; (2) Ergocardio Medicina Diagnosis and Research

**Background:** Rapid up-titration of beta-blockers (BB) in the treatment of heart failure (HF) during hospitalization has yet to be tested.

**Objectives:** Our purpose for this research is a new model for administering beta-blockers in patients with end-stage HF in order to, after clinical compensation of the patient, promote more rapid dosage increments than the conventional method or usual care procedures. We also sought to ascertain whether or not this model for administering beta-blockers could modify the impact of HF in relation to the enhancement of the quality of life (QOL) as measured by the Minnesota Living with Heart Failure questionnaire.

**Methods and Results:** 92 patients with advanced HF (New York Heart Association [NYHA] IV) and left ventricle ejection fraction (LVEF) <45% were hospitalized for clinical compensation and followed-up for one year after discharge. The patients were distributed into two groups: 46 in the treatment group (TG) and 46 in the control group (CG). During hospitalization, they were divided randomly for BB rapid up-titration with an increase of dosage every two days (TG), or by the usual treatment regime (CG). Statistical analysis used: Student’s t-test, Mann-Whitney, Chi-square and Fisher’s exact test, Kaplan-Meier for survival, using the Log-rank test for comparison and the hazard ratio (HR) calculated with the Cox model. P < 0.05 was considered significant. The pcts responded to the questions of the MLHFQ. This battery of 21 items uses a scale for answers from (0–5). Total compilation of scores varied from 0 to 105. A lower score reflects a better QOL. Among the total group of 92 patients studied, initially, LVEF average was 27.02%, end-diastolic diameter average was 66.15 mm, and end-systolic diameter average was 57.04 mm. For the TG, the average dosage of Carvedilol reached 34.37 mg/per day; and with the CG, 13.99 mg/per day. In the initial evaluation, the pcts showed an average score of 82 points in the two separate groups evaluated. With treatment, the TG moved to a score of 63, while the CG scored 73. There was a reduction of 23% in the MLHFQ score for the TG in relation to the CG group (p < 0,001).

**Conclusions:** Beta blockers rapid optimization dosages during hospitalization of end-stage HF patients is safe and promotes an enhanced patterns of QOL as measured by MLHFQ, indicating that more elevated dosages of BB is probably responsible for this positive outcome than that observed with a usual treatment schedule.

111747

Modality: E-Poster Researcher – Non-case Report

Category: NEGLECTED CARDIOVASCULAR DISEASES

## Value of Ventricular Premature Beats and Non-Sustained Ventricular Tachycardia in Predicting the Presence of Myocardial Fibrosis in Chronic Chagas Cardiomyopathy

ANDRE SCHMIDT^1^, Maria Fernanda Braggion-Santos^1^, Marcel Koenigham Santos^1^, Gustavo Jardim Volpe^1^, Henrique Turin Moreira^1^, José Antonio Marin-Neto^1^

(1) Faculdade de Medicina de Ribeirão Preto – USP

**Introduction:** Myocardial fibrosis (MF) identified in Cardiac Magnetic Resonance (CMR) is a recognized prognostic marker in Chronic Chagas Cardiomyopathy (CCC). Since CMR is not widely available, it seems relevant to identify markers of MF in other methods that may indicate the need for a CMR in this neglected disease.

**Objective:** We sought to investigate the relationships between the presence of ventricular premature beats (VPB) in an Electrocardiogram (ECG), Non-sustained ventricular tachycardia (NSVT) on a Holter exam, and the presence of MF at CMR.

**Methods:** Consecutive CCC patients with an ECG, Holter, and CMR within one year were selected from our institution database, and the presence of VPB on ECG, NSVT on Holter, and MF on CMR were collected, as well as demographic and clinical data. Descriptive statistics, Fisher exact tests, and logistic regression analysis were performed. A 5% level of significance was established.

**Results:** Inclusion criteria were fulfilled by 121 patients, 53% females, 56 ± 14 years, and most (93%) were in NYHA class I or II. The mean left ventricular ejection fraction was 48 ± 14%. Amiodarone was used by 22 (18%) patients at the physician’s discretion. Twenty-three (19%) patients resting ECG presented VPBs, and 38 (31%) patients had NSVT on Holter. MF was identified in 94 (78%) CCC patients. Concomitant occurrence of VPBs on ECG and NSVT on Holter in the same patient was low (12%) but MF was present in all of those with both VPB and NSVT. VPBs in ECG indicated an odds ratio (OR) of 7.9 (CI: 1.02–61.9; P = 0.04) of having MF, as 22 (96%) out of 23 with VPB had it. NSVT on Holter presented an OR of 16.9 (CI: 2.2–129.8; p = 0.007) as 37 (97%) out of 38 patients with NSVT had MF on CMR. Amiodarone use did not significantly (p > 0.05) influence the results of each exam to predict MF.

**Conclusions:** Electrocardiogram and Holter may present findings indicating MF occurrence in CCC. Although rare in resting ECGs, VPB significantly increases the odds of finding MF. The presence of NSVT has a much higher chance of finding MF in a CMR exam. These findings may indicate a subgroup of CCC patients that should be followed closely due to the possible presence of myocardial fibrosis.

111933

Modality: E-Poster Researcher – Non-case Report

Category: PHYSIOTHERAPY

## Evaluation of Overtraining Syndrome in Patients from a Cardiac Rehabilitation Program

ALTAIR ARGENTINO PEREIRA JUNIOR^1^, Ana Inês Gonzales^2^, Darlene Aparecida Pena^1^, Tales de Carvalho^3^, Alexandro Andrade^3^, Sabrina Weiss Sties^1^

(1) Centro Universitário Avantis – UNIAVAN; (2) Centro Universitário para o desenvolvimento do Alto Vale do Itajaí – UNIDAVI; (3) Universidade do estado de Santa Catarina – UDESC

**Introduction:** In the context of cardiac rehabilitaion, the practice of regular physical exercises is considered a mandatory therapeutic strategy, since it significantly reduces the morbidity and mortality of patients with heart disease. However, especially in individuals with low exercise tolerance, the overtraining syndrome could occur.

**Objective:** Evaluate the presence of signs of overtraining syndrome in patients with coronary artery disease undergoing cardiac rehabilitation.

**Methods:** Seventy-seven adults participated in the cardiac rehabilitation program were analized. For early detection of overtraining syndrome was used the Brunel Mood Scale. Data were evaluated by descriptive statistics.

**Results:** In this study, the prevalence of overtraining syndrome among patients with coronary artery disease was 11.68%. The tension domain score was 4.44 (±3.25), depression 4.22 (±3.07), anger 0.78 (±1.09), fatigue 4.89 (±3.52) and mental confusion 3.33 (±2.87) were high, while the vigor domain (9.67 ± 3.24) was low, indicating signs of overtraining syndrome.

**Conclusion:** Some patients with coronary artery disease showed signs of overtraining syndrome. Taken together, these results indicate this target population requires attention to the need for adopt strategies aiming at preventive measures.

111935

Modality: E-Poster Researcher – Non-case Report

Category: CARDIOVASCULAR IMAGING

## Left Atrial Minimum Volume is Better Correlate of Left Atrial Dysfunction by Two-Dimensional Strain

RAFAEL MODESTO FERNANDES^3^, Rafael Modesto Fernandes^3^, Andre Moises de Oliveira Nunes^5^, Hayala Machado Cavalcante Conceição^5^, Loren Lacerda Rodrigues^5^, David Le Bihan^4^, Andrea A. Vilela^1^, Rodrigo B. M. Barretto^2^, Elizabete S. Santos^1^, Amanda G. M. R. Sousa^1^, Ari Timerman^1^

(1) Dante Pazzanese Institute of Cardiology, São Paulo, Brazil; (2) Instituto do Coração (INCOR), University of São Paulo, Brazil; (3) D’Or Institute for Research and Education (IDOR), Hospital Aliança, Salvador, Brazil; (4) Grupo Fleury – São Paulo; (5) Bahiana School of Medicine and Public Health, Salvador, Brazil.

**Background:** The analysis of left atrial (LA) function can add important information to the understanding of cardiovascular diseases. Therefore, to assess its functioning the use of volumetric measurements is adopted. The maximum LA volume is the most echocardiography parameter used, however, some studies demonstrated that minimum LA volume is better to correlate with atrial dysfunction.

**Purpose:** The purpose of this study was compare the accuracy of the phasic volumes of the left atrium in determining LA dysfunction identified by the two-dimensional strain.

**Methods:** This observational, cross-sectional study admitted 109 participants with diagnosis of acute coronary syndrome non-ST-segment elevation. The exams were performed within 72 hours of admission. LA volume was defined by the mean of measurements performed in the four and two-chamber apical windows using Simpson’s method. The phasic volumes of the left atrium were measured using an electrocardiogram synchronized with the device. Reservoir LA strain less than 21% was the cut off to LA dysfunction.

**Results:** The phasic LA volume variables analyzed were maximal (LAVMAX), minimal (LAVMIN) and before atrial contraction (LAVBAC) volume, and each variable indexed for body surface (LAVIMAX, LAVIMIN and LAVIBAC). Therefore, a Roc curve was generated for each volume variable to assess which would be more accurate in predicting left atrial dysfunction. The Youden index was used to determine the cutoff point for each one of them. The areas under the Roc curves were: 0.83 (LAVIMIN), 0.81 (LAVMIN), 0.78 (LAVBAC), 0.76 (LAVIMAX), 0.74 (LAVIBAC) and 0.71 (LAVMAX).

**Conclusion:** This study concluded that phasic volumes were good determinants of left atrial dysfunction identified by 2D strain. The minimum left atrial volume was the better correlated with left atrial dysfunction.

111782

Modality: E-Poster Researcher – Non-case Report

Category: HEMODYNAMICS AND INTERVENTIONAL CARDIOLOGY

## Clinical and Angiographic Predictors of Troponin Elevation After Non-Complicated Angioplasty: A Cross-Sectional Study

INGRID LOUREIRO DE QUEIROZ LIMA^1^, THIAGO CAVALCANTE^1^, LUIZ CARLOS DE LIMA^1^, FERNANDO LUIZ WESTPHAL^1^

(1) UNIVERSIDADE FEDERAL DO AMAZONAS; (2) HOSPITAL DO CORAÇÃO FRANCISCA MENDES

**Background:** Troponin T (TnT) and troponin I (TnI) are myocardium necrosis biomarkers very sensible and specific used to evaluate a myocardial injury, and their rise after coronary angioplasty correlate with worst prognoses. Complications associated with this procedure are a well-recognized cause of elevated myocardial necrosis biomarkers. In its absence, the exact causes of elevated troponin are merely speculative.

**Objective:** To evaluate the relationship between troponin elevation and clinical and anatomical variables after coronary angioplasty in the absence of complications.

**Method:** It is an epidemiological study evaluating 124 patients submitted to coronary angioplasty from December 2018 to December 2019 in the catheter laboratory of University Hospital Francisca Mendes, using clinical variables and troponin doses before, 6 hours and 12 hours after the procedure.

**Results:** Of all 124 analyzed patients, 63,3% were male and mean age of 63 years old. The mean implanted stents per patients was 1,66. Hypertension was the most prevalent comorbidity. Eighty patients (64,5%) presented high troponin dosage (compared to 99th percentile) and 32 (25,8%) showed troponin dosage above five times the 99th percentile. When analyzing all patients presenting troponin dosage above 99th percentile, none of the clinical characteristics were significantly related to troponin alteration. While analyzing procedure-related characteristics, we observed that troponin elevation above 99th percentile is significantly correlate with the number of implanted stents, the number of treated arteries (p < 0,001 and p = 0,046 respectively), procedure duration (p = 0,032), and contrast media volume (p = 0,008). The number of implanted stents and number of treated arteries were still associated with troponin elevation above five times the 99th percentile.

**Conclusion:** Clinical characteristics were not associated with troponin elevation above the 99th percentile. However, the procedure duration, contrast media volume, number of treated arteries, and number of stents implanted are significantly associated with troponin elevation when studying the procedure characteristics. Amongst the subgroup of patients with troponin levels above five times the percentile, clinical history of smoking, the number of implanted stents and stent length were significantly associated with the elevation of this biomarker above five times the 99th percentile.

111805

Modality: E-Poster Researcher – Non-case Report

Category: CARDIOVASCULAR SURGERY

## Predictors of Mortality in Cardiac Valve Surgery – a Prospective Cohort

GABRIEL ASSIS LOPES DO CARMO^1^, Bárbara Carolina Silva Almeida^1^, Gabriela Zamunaro Lopes Ruiz^1^, Renato Braulio^1^, Ana Cristina Carioca^1^, Fábio Morato Castilho^1^, Cláudio Leo Gelape^1^, Bruno Rodrigues Pereira^1^, Luiza Moreira Gomes^1^, Ana Carolina Sudário Leite^1^

(1) Universidade Federal de Minas Gerais (UFMG)

**Introduction:** Rheumatic valve disease (RVD) has a high prevalence in Brazil, but remain poorly studied. Different from developed countries, valvar cardiac surgeries are the most performed procedure in several public hospitals in Brazil. However, impact of RVD on cardiac surgery is not well established.

**Objective:** Describe baseline characteristics and prognosis of cardiac valve surgery patients in a public hospital in Brazil.

**Methodology:** Prospective cohort analysis of cardiac surgery patients between 2016 and 2021, excluding heart transplant.

**Results:** We enrolled 416 patients in the study, median age 57 (47;67) and 377 (49,8%) females. Overall, 287 (69,2%) procedures were elective, while 115 (27,7%) were urgent and 13 (3,1%) emergencies. Most procedures, 330 (79,3%), were isolated valve surgery, followed by combined valve and coronary artery bypass surgery (CABG), 36 (8,6%), valve and aorta, 19 (4,6%), valve and congenital, 8 (1,9%), valve, CABG and aorta, 2 (0,5%), and valve plus other cardiac surgery in 21 (5,1%). 147 (23,3%) had previous cardiac surgery. Diabetes was present in 69 (16,6%), 47 (11,3%) non-insulin dependent and 22 (5,3) insulin dependent. 25 (6%) had a history of previous myocardial infarction, 40 (9,6%) had stroke, 21 (5%) chronic pulmonary disease, 141 (43,9%) atrial fibrillation, 143 (44%) had rheumatic valve disease and 8 (1,9%) were on renal replacement therapy. Heart failure was diagnosed in 303 (73,5%) and 50 (12,1%) had a NYHA IV classification. Median ejection fraction was 63% (55;68), PSAP was 42 (31;55) and creatinine 0,98 (0,8;1,15). Observed in-hospital mortality was 13,0%. Univariate analysis showed that age, 67 (56;74) vs 56 (46;66), p = 0,002, endocarditis, 14 (30,4%) vs 40 (10,8%), OR = 3,60 (1,77–7,31), p < 0,001, mitral replacement plus tricuspid repair, 18 (23,4%) vs 26 (11,6%), OR = 2,34 (1,18–4,63), p = 0,013, and non-elective procedures, 27 (21,1%) vs 27 (9,4%), OR = 2,57 (1,44–4,60), p = 0,001 were related to increased mortality. Rheumatic etiology was a protective factor, 17 (9,3%) vs 37 (15,9%), OR = 0,54 (0,29–0,99), p = 0,045. In multivariate analysis, only age, OR = 1,058 (1,025–1,091), p = 0,001, endocarditis, OR = 4,51 (1,717–11,855), p = 0,002, and mitral replacement plus tricuspid repair, OR = 2,903 (1,325–6,362), p = 0,008, remained statistically relevant.

**Conclusion:** Our study shows that older age, endocarditis and mitral replacement combined with tricuspid repair are associated with increased mortality after cardiac valve surgery.

111816

Modality: E-Poster Researcher – Non-case Report

Category: CARDIOVASCULAR INTENSIVE CARE/CARDIOVASCULAR EMERGENCIES

## Implementation of Telecardiology Service and ST-Elevation Myocardial Infarction in the State of Sergipe, Brazil: A Pilot Study

JOSÉ EDIVALDO DOS SANTOS^1^, José Victor Furtado Jacó de Oliveira^2^, Maria Letícia de França Oliveira^2^, Rafael Alexandre Meneguz-Moreno^2^

(1) Hospital de Urgências de Sergipe; (2) Universidade Federal de Sergipe – Campus Lagarto

**Introduction:** The implementation of Telecardiology (TCL) in the state of Sergipe, Brazil, is a viable, promising and innovative strategy. Regarding specifically STEMI (ST-elevation myocardial infarction), it is known that mortality is directly proportional to the first medical contact to balloon time. The objective of the study is to evaluate data regarding patients with STEMI regulated by TCL service in Sergipe and referred to a tertiary hospital.

**Methods:** This is a cross-sectional, retrospective, descriptive study, reporting data from June 2021 to April 2022 involving TCL service. Data were collected from electronic medical records, through the e-SUS program.

**Results:** The resulting analysis population included 444 patients presented with STEMI during 11 months, being 288 (65%) male, mean age 61 years old, ranging from 27 to 102 years. Patient had previous hypertension (55%), diabetes mellitus type 2 (32%), dyslipidemia (12,8%), smoking (12,8%), previous coronary artery disease (5,6%), obesity (4,0%) and 68 (15,3%) reported no comorbidities. From all patients presented with STEMI during the analyzed period, 294 (66,2%) patients were referred to immediate PCI (percutaneous coronary intervention), 51 (11,4%) had been submitted to thrombolytic (TB) therapy, and 99 (22,2%) were not treated with reperfusion therapy within 12 hours of symptoms onset, yet 47 (47,4%) from these 99 patients were referred to urgent coronary angiography after TCL cardiologists evaluation. Among TB group, 32 (62,7%) patients were referred to a CICU (Coronary Intensive Care Unit) immediately after reperfusion, 2 (4%) were referred to rescue PCI and 17 (33,3%) have not been transported. The reasons for choosing TB therapy were, as follows, long distance (21), no transport available (6), no CICU vacancy (6), choice of attending physician (5) and not informed (13). There were 22 (5%) deaths among all patients, 4 from PCI group, 4 from TB group and 14 from the other group.

**Conclusion:** At the beginning of TCL service in Sergipe, there were some patients that still have no access to PCI. However, with the consolidation of TCL, this flowchart was reorganized and since October 2021, there was no lack of vacancy. Therefore, TCL has improved the quality of STEMI care in the state of Sergipe (northeast Brazil). Strategies must still be implemented to increase PCI rates, such as improvement on medical education, in order to recognize symptom and trigger the TCL service more quickly.

111830

Modality: E-Poster Researcher – Non-case Report

Category: CARDIOVASCULAR SURGERY

## Clinical Characteristics and Prognosis of Cardiac Surgery Patients in a Public Hospital in Brazil

GABRIEL ASSIS LOPES DO CARMO^1^, Bárbara Carolina Silva Almeida^1^, Gabriela Zamunaro Lopes Ruiz^1^, Renato Braulio^1^, Ana Cristina Carioca^1^, Fábio Morato Castilho^1^, Cláudio Leo Gelape^1^, Bruno Rodrigues Pereira^1^, Luiza Moreira Gomes^1^, Ana Carolina Sudário Leite^1^

(1) Universidade Federal de Minas Gerais (UFMG)

**Introduction:** Knowing baseline clinical information is important to compare results between different institutions. Brazil has a particular socioeconomic scenario and disease profile that make users of public health system (SUS) unique.

**Objective:** Describe baseline characteristics and prognosis of cardiac surgery patients in a public hospital in Brazil.

**Methodology:** Prospective cohort analysis of cardiac surgery patients between 2016 and 2021, excluding heart transplant.

**Results:** We enrolled 633 patients, median age 58 (48;67) and 377 (49,8%) females. Most procedures, 406 (64,1%), were elective, followed by urgent, 109 (31,4%), and emergent, 28 (4,4%). Most surgeries were valvar, 416 (65,5%) followed by coronary artery bypass surgery (CABG), 188 (29,6%), aorta, 43 (6,8%), congenital heart disease, 33 (5,2%), and other surgeries, 50 (7,9%). 147 (23,3%) had previous cardiac surgery. Diabetes was present in 133 (20,9%), 88 (13,9%) non-insulin and 45 (7,1) insulin dependent, myocardial infarction in 76 (12%), stroke in 59 (9,3), chronic pulmonary disease in 29 (4,6%), atrial fibrillation in 150 (23,6%), rheumatic valve disease in 134 (21,1%) and 13 (2%) were on renal replacement therapy. Heart failure was diagnosed in 370 (58,9%) and 56 (8,8%) had a NYHA IV classification. Median ejection fraction was 62% (54;68), PSAP was 38 (29;51) and creatinine, 0,97 (0,8;1,18). Mean and median EUROSCORE II were 3,978 (±6,591) and 1,8 (0,990;4,020), respectively. Observed in-hospital mortality was 13,3%.

**Conclusion:** Our results show a much higher than predicted mortality according to EUROSCORE II. However, our population has some important differences from that evaluated in EUROSCORE II study, especially a higher prevalence of rheumatic valve disease. Also, more commonly patients were operated on an urgent basis. Our population had more similarities with the InsCor Risk Assessment study performed in Brazil, which found a general mortality of 8,9%, but included only CABG and valve surgeries. Due to these specific baseline characteristics, local developed score would be more appropriate while evaluating cardiac surgery candidates in Brazil.

111860

Modality: E-Poster Researcher – Non-case Report

Category: CARDIOLOGY OF SPORTS, EXERCISE, ERGOMETRY AND CARDIOVASCULAR REHABILITATION

## Cardiopulmonary Exercise Test Variables and its Association with Quality of Life in Patients with Chronic Chagas Cardiomyopathy: Results from the Peach Study

MARCELO CARVALHO VIEIRA^1^, Fernanda de Souza Nogueira Sardinha Mendes^1^, Paula Simplício da Silva^1^, Gilberto Marcelo Sperandio da Silva^1^, Flavia Mazzoli-Rocha^1^, Andrea Silvestre de Sousa^1^, Roberto Magalhães Saraiva^1^, Vitor Barreto Paravidino^2^, Luiz Fernando Rodrigues Junior^3^, Alejandro Marcel Hasslocher-Moreno^1^, Pedro Emmanuel Alvarenga Americano do Brasil^1^, Mauro Felippe Felix Mediano^1^

(1) Evandro Chagas National Institute of Infectious Disease, Oswaldo Cruz Foundation, Rio de Janeiro, RJ, Brazil.; (2) Institute of Social Medicine, State University of Rio de Janeiro, Rio de Janeiro, RJ, Brazil; (3) Department of Research and Education, National Institute of Cardiology, Rio de Janeiro, RJ, Brazil

**Introduction:** The association between functional capacity and quality of life (QoL) in individuals with chronic Chagas cardiomyopathy (CCC) is still poorly investigated, with the few studies including indirect measures of functional capacity, limiting the validity and the interpretation of the results.

**Objective:** The present study aimed to evaluate the association between functional capacity (quantified by cardiopulmonary exercise test [CPET]) and QoL in individuals with CCC.

**Methods:** A cross-sectional analysis using baseline data from PEACH study, a randomized clinical trial that evaluated the effects of exercise training in patients with CCC, was performed. QoL was assessed using the SF-36 questionnaire. Sociodemographic, anthropometric, clinical, cardiac function and maximal progressive CPET variables were retrieved from PEACH study dataset. Generalized linear models adjusted for age, sex, and left ventricular ejection fraction were performed to evaluate the association between CPET variables and QoL.

**Results:** After adjustments for potential confounders, VO2peak and VO2AT were both positively associated with physical functioning and physical component summary (PCS). Double product was positively associated with physical functioning, general health perceptions, and PCS, whilst heart rate recovery <12bpm at the first minute (HRR) was negatively associated with physical functioning, role limitations due to physical problems, bodily pain, and PCS. VE/VCO2 slope presented a negative association with all mental scales of SF-36: vitality, social functioning, role limitations due to emotional problems, mental health, and mental component summary. HRR <12bpm was negatively associated with vitality and mental health. Double product was positively associated with vitality. The CPET variables that most explained the QoL variation in the adjusted models were VO2AT (50% for physical functioning and 36% for PCS), VO2peak (31% for physical functioning and 21% for PCS), VE/VCO2 slope (45% for mental health and 31% for mental component summary), and HRR < 12 bpm (20% for vitality).

**Conclusions:** The association between CPET variables and QoL reinforces the importance of CPET inclusion for a more comprehensive evaluation of individuals with CCC. Intervention strategies aiming to improve functional capacity may also promote additional benefits on QoL and should be incorporated as a treatment strategy for patients with CCC.

111901

Modality: E-Poster Researcher – Non-case Report

Category: CARDIOLOGY OF SPORTS, EXERCISE, ERGOMETRY AND CARDIOVASCULAR REHABILITATION

## Exercise Training on Quality of Life in Patients with Chronic Chagas Cardiomyopathy: From the Peach Study

MARCELO CARVALHO VIEIRA^1^, Fernanda de Souza Nogueira Sardinha Mendes^1^, Paula Simplício da Silva^1^, Gilberto Marcelo Sperandio da Silva^1^, Flavia Mazzoli-Rocha^1^, Andrea Silvestre de Sousa^1^, Roberto Magalhães Saraiva^1^, Fernanda Martins Carneiro^1^, Luiz Fernando Rodrigues Junior^2^, Alejandro Marcel Hasslocher-Moreno^1^, Pedro Emmanuel Alvarenga Americano do Brasil^1^, Mauro Felippe Felix Mediano^1^

(1) Evandro Chagas National Institute of Infectious Disease, Oswaldo Cruz Foundation, Rio de Janeiro, RJ, Brazil.; (2) Department of Research and Education, National Institute of Cardiology, Rio de Janeiro, RJ, Brazil

**Introduction:** Physical exercise has been described as an efficient and safe strategy to improve quality of life (QoL) in heart failure (HF). However, there is a lack of information about its influence on the QoL of patients with chronic Chagas cardiomyopathy (CCC).

**Objective:** The present study aimed to investigate the effect of physical exercise training on the QoL of patients with CCC.

**Methods:** PEACH study was a single center, superiority randomized parallel-group clinical trial of exercise training versus a control group with no exercise training. The sample comprised Chagas disease patients with CCC, left ventricular ejection fraction <45% or HF symptoms (CCC stages B2 or C). QoL was assessed at baseline, after three months, and at the end of follow-up (six months) using SF-36 questionnaire. Patients randomized for the intervention group performed physical exercise (aerobic exercise, strength training and stretching exercises) for 60 minutes, three times a week, for a period of six months. Patients in the control group were not provided with a formal exercise prescription. During the study, patients from both groups underwent monthly appointments with the same cardiologist, and with other specialists, if necessary. In addition, patients in both groups received identical nutritional and pharmaceutical counseling during the study. Longitudinal analysis of the effects of physical training on QoL, considering the interaction term (group × time) to estimate the rate of changes between groups in the outcomes (represented as beta coefficient), was performed using mixed linear models. Analyses were adjusted to the baseline QoL values.

**Results:** There were significant improvements in physical functioning (β = +10.7; p = 0.02), role limitations due to physical problems (β = +25.0; p = 0.01) and social functioning (β = +19.2; p < 0.01) scales during the first three months in the exercise group compared to control group. No significant differences were observed between groups after six months of follow-up.

**Conclusion:** Exercise training was associated with short-term improvements in physical and social aspects of QoL and should be incorporated as a treatment strategy for patients with CCC.

111898

Modality: E-Poster Researcher – Non-case Report

Category: HEART FAILURE/CARDIOMYOPATHY/TRANSPLANT

## Hypertrophic Cardiomyopathy Carriers Characteristics in a Cardiogenetics Investigation Service

BRÁULIO CRUZ MELO^1^, Larissa Rebeca da Silva Tavares^1^, Irlaneide da Silva Tavares^3^, Antônio Guilherme Cunha de Almeida^1^, Antônio Carlos Sobral Sousa^2^, João Vitor Andrade Pimentel^2^, Emerson de Santana Santos^2^, Enaldo Vieira de Melo^2^, Giovanna Medeiros Resende^1^, Evelen Rouse de Souza Santos^1^, Diego Henrique da Silva Tavares^1^, Joselina Luzia Menezes Oliveira^2^

(1) Universidade Tiradentes; (2) Universidade Federal de Sergipe; (3) Hospital Primavera

**Introduction:** Hypertrophic Cardiomyopathy (CMH) is the most common genetic disease in the world, and can lead their carriers to death, therefore making it’s correct diagnosis fundamental, along with the follow-up and genetic counseling of patients and their family.

**Methods:** This is a cross-sectional, prospective and analytical study, with patients diagnosed with CMH in Sergipe between January 2021 and April 2022, being also subjected to genetic study.

**Objective:** To describe the patient population under CMH investigation.

**Results:** 80 patients were studied, 49 (61%) being of male gender, with the average age of 49. MRI was performed in 50 patients and positive for CMH in 45 (90%); 71 patients have resting echocardiogram, 60 of which cover CMH criteria. The most relevant echocardiographic variant for CMH is septal thickness, with an average of 15,5 mm on echocardiogram and 16,5 on MRI. It’s obstructive form was present at the MRI in 20% of the cases (10 patients), ischemia in 18 (39% in the septal wall), and fibrosis in 40 patients (80%). The doppler echocardiogram under physiological stress was performed in 21 patients, 11 on the treadmill, 2 on the bicycle and 6 on both methods which made possible the identification of 7 individuals with labile obstructive form. Finally, in the genetic study, the most found genes were MYBPC3 and MYH7 (28 and 12%, respectively), in agreement with whats already described in literature, in addition to 30% of cases being identified as phenocopies.

**Conclusion:** The CMH can appear more frequently than expected and present variable expressiveness and penetration. Ocasionally, phenocopies and dislead it’s diagnosis. The echocardiographic evaluation under physiological stress can identify higher risk individuals (labile obstructive CMH).

111910

Modality: E-Poster Researcher – Non-case Report

Category: NEGLECTED CARDIOVASCULAR DISEASES

## Electrocardiographic Abnormalities in Chronic Chagas Cardiomyopathy and its Correlation with Presence and Burden of Myocardial Fibrosis and Left Ventricular Ejection Fraction Measured by Cardiac Magnetic Resonance

ANDRE SCHMIDT^1^, Maria Fernanda Braggion-Santos^1^, Marcel Koenigkam Santos^1^, Gustavo Jardim Volpe^1^, Henrique Turin Moreira^1^, José Antonio Marin-Neto^1^

(1) Faculdade de Medicina de Ribeirão Preto – USP

**Background:** Chronic Chagas’ cardiomyopathy (CCC) is a consequence of several insults resulting in myocardial fibrosis (MF). Detection of MF is possible noninvasively through cardiac magnetic resonance (CMR). However, it is an expensive and not widely available method.

**Objective:** This study aims to correlate ECG Selvester score (or QRS score) with quantification of MF and left ventricular ejection fraction (LVEF) through CMR analysis, in patients with CCC.

**Methods:** Retrospective analysis of ECG and CMR exams, performed within one year of 194 patients with CCC. Rassi score was evaluated in 171 patients. Continuous variables were presented as median and interquartile range (IQR)and categorical data were summarized as percentages. Spearman’s rank correlation coefficient was used to evaluate the correlation between estimated MF by QRS score and quantified MF by CMR, and LVEF. Bland-Altman plot was applied to compare percentage of MF estimated by QRS score and measured by CMR. Patients were divided into tertiles based on QRS score values and Kruskal-Wallis test was applied to compare MF and LVEF among the groups. Receiver Operating Characteristic curves (ROCcurve) were generated to define cut-off values with the best accuracy to identify MF >= 10% of left ventricular mass, MF >= 12.3 g, LVEF < 50%, or LVEF < 35%. According to Rassi score classification, patients were separated in 3 risk groups, and QRS scores were evaluated by the Kruskal-Wallis test. P < 0.05 was considered statistically significant.

**Results:** A total of 98 participants were women. Median age was 56 years(IQR: 44–67). Correlation between QRS score and MF by CMR was moderate (r = 0.45,p < 0.001), as well as the negative correlation between QRS score and LVEF (r = –0.42, p < 0.001). Bland-Altman plot showed worse agreement between methods for higher values of QRS score, and a trend to overestimate percentage of MF. QRS score >= 5 was correlated to MF >= 12.3 g, MF >= 10% of left ventricular mass, and LVEF < 50%. QRS score >= 6 was associated to LVEF < 35% with good specificity. Patients classified as high risk by Rassi score had a median QRS score of 5 points (IQR: 3–9).

**Conclusion:** Higher QRS score in CCC had a moderate correlation with higher burden of MF and worse LVEF. QRS score values tend to overestimate percentage of MF, whereas lower values of QRS seem to have a better correlation with CMR. Selvester scores above 5 or 6 points are associated with more LV impairment, and potentially related to worse outcomes.

111923

Modality: E-Poster Researcher – Non-case Report

Category: HEART FAILURE/CARDIOMYOPATHY/TRANSPLANT

## Predictors of Status Epilepticus After Heart Transplantation

GABRIEL ASSIS LOPES DO CARMO^1^, Bárbara Carolina Silva Almeida^1^, Gabriela Zamunaro Lopes Ruiz^1^, Renato Braulio^1^, Ana Cristina Carioca^1^, Fábio Morato Castilho^1^, Cláudio Leo Gelape^1^, Bruno Rodrigues Pereira^1^, Luiza Moreira Gomes^1^, Ana Carolina Sudário Leite^1^

(1) Universidade Federal de Minas Gerais (UFMG)

**Introduction:** Neurological complications after heart surgeries are relatively frequent. Status epilepticus (SE) is particularly relevant due to its association with increased mortality. However, little is known about its pathogenesis and risk factors.

**Objectives:** To evaluate the incidence of SE and identify its predictors after heart transplant.

**Methods:** Prospect cohort of patients referred to heart transplantation in a university health facility in Brazil.

**Results:** Between April of 2014 and November of 2021, 211 patients were referred to heart transplantation, median age 51 (40;58) and 73 (34,4%) females. In the early postoperative period, 28 (13.27%) patients had SE. Univariate analysis showed that INTERMACS 1 or 2 profile, OR = 4,88 (2,03–11,71), p < 0.001, and cardiorenal syndrome on renal replacement therapy (RRT), OR = 3,87 (1,59–9,39), p = 0.009, were associated with SE. Multivaltivariate analysis showed that only the INTERMACS 1 or 2 classification remained associated with SE, OR 3.88 (CI = 1.43–10.51), p = 0.008. The Hosmer-Lemeshow test had a value of p = 1.000. The occurrence of SE was not associated with increased mortality, OR 1.65 (CI = 0.61–4.45), p = 0.393.

**Conclusion:** The occurrence of SE was only correlated with the INTERMACS 1 and 2 profile, a well know critical hemodynamic state with low cardiac output (CO). SE may be related to sudden increase in CO after cardiac transplantation, leading to an imbalance in cerebral blood flow. In the present analysis, SE was not correlated with higher mortality, possibly due to the small sample size.

111961

Modality: E-Poster Researcher – Non-case Report

Category: CARDIOVASCULAR IMAGING

## Correlation between Modified Selvester Score and Fibrosis Mass After Acute Phase of Myocardial Infarction

MARIA CATARINA DE MELO DIAS GUERRA^1^, Augusto Hiroshi Uchida^1^, José Antônio Franchini Ramires^1^, Eduardo Cavalcanti Lapa dos Santos^1^, Renata Ávila Cintra^1^, Jürgen Beuther^1^, Nevelton Heringer Filho^1^, Tiago Augusto Magalhães^1^, William Azem Chalela^1^, Guilherme Garcia^1^, Leonardo Filipe Benedeti Marinucci^1^, Carlos Eduardo Rochitte^1^

(1) Instituto do Coração (InCor – FMUSP)

**Introduction:** Myocardial infarction (MI) size is a key predictor of prognosis in post-MI patients. Cardiovascular magnetic resonance (CMR) is the gold standard test for MI quantification but is still limited due to restricted availability in daily routine. Previous studies have documented the capability of different electrocardiogram (ECG) markers for prognosis assessment soon after ST elevation myocardial infarction. Selvester QRS scoring detects scar, although the reported performance varies. A simplified version of this score showed comparable predictive value for infarct size in a prospective study including 201 patients at a median of 2 days after a revascularized MI (area under the curve = 0.64 vs. 0.67).

**Methods:** Retrospective, observational, single-center study. The sample was obtained through analysis of medical records with a record of clinical diagnosis of MI who underwent CMR to assess myocardial viability. Those who did not present delayed enhancement with an ischemic pattern on CMR and those whose CMR revealed a diagnosis other than MI or did not have satisfactory image quality were excluded. Patients reporting a new acute coronary syndrome in the interval between the available ECG and the CMR were also excluded. The ECG performed 7 days after the infarction and with a maximum interval of 1 year from the CMR were analysed by an evaluator with extensive experience in electrocardiography.

**Results:** The mean age of patients was 61.3 years and 75% were men. Conventional infarction identification through the presence of a pathological Q wave occurred in 172 of the 245 patients included (70.2%). In the subgroup of 192 patients in which CMR was already analyzed, the median modified Selvester score was 5 and the fibrosis mass was 23.2% ± 11.3 of the left ventricular mass. There was no statistically significant correlation between the modified Selvester score and fibrosis mass on CMR.

**Conclusions:** After seven days of acute coronary syndrome, the modified Selvester score was not a predictor of fibrosis mass. This correlation had been previously described in smaller studies that evaluated this score at admission of acute MI. Intraventricular conduction disorders and ventricular hypertrophy are factors that may impact the correlation between the Selvester score and fibrosis mass.

111962

Modality: E-Poster Researcher – Non-case Report

Category: HEART FAILURE/CARDIOMYOPATHY/TRANSPLANT

## Validation of SCAI Shock Classification in a Cohort of Heart Transplant Patients

GABRIEL ASSIS LOPES DO CARMO^1^, Bárbara Carolina Silva Almeida^1^, Gabriela Zamunaro Lopes Ruiz^1^, Renato Braulio^1^, Ana Cristina Carioca^1^, Fábio Morato Castilho^1^, Cláudio Leo Gelape^1^, Bruno Rodrigues Pereira^1^, Luiza Moreira Gomes^1^, Ana Carolina Sudário Leite^1^

(1) Universidade Federal de Minas Gerais (UFMG)

**Introduction:** The Society for Cardiac Angiography and Interventions (SCAI) shock classification was published in 2019, but not yet validated in patients referred to heart transplant.

**Objectives:** To evaluate the association between different groups of the SCAI shock classification and the occurrence of death among patients undergoing heart transplant.

**Methods:** Prospect cohort of patients referred to heart transplant in a university health facility in Brazil.

**Results:** Between April 2014 and November 2021, 211 patients underwent heart transplantation, median age 51 (40;58) and 73 (34.4%) females. Baseline population characteristics were similar among the 5 different groups of the SCAI classification, with the exception of insulin-requiring diabetes mellitus (15.1%, 21.4%, 1.5%, 8.3% and 5.6%, p = 0.038), use of intra-aortic balloon prior to surgery (0.0, 4.6%, 20.0%, 55.6%, p < 0.001), days of hospitalization before surgery [0 (0;0); 1.5 (0;22); 30(15;59); 30(15;60); 39 (27;44)], platelet count [191000 (159250;246500); 214500 (172500;287500);221000 (176000;285500); 191000 (146000;250000); 184500 (139500;252500)] and RNI [1.51 (1.22; 2.39); 3.07 (1.65; 4.51); 1.46 (1.23; 2.37); 1.30 (1.12; 1.55) and 1.34 (1.18; 1.65)] respectively for groups A, B, C, D and E. Death occurred in 4 (7,4%) patients in group A, 2 (14,3) in group B, 4 (6,2) in group C, 15 (25,0%) in group D and 7 (38,9%). Compared to group A, patients in classification B had OR 2.083 (0.341–12.733), p = 0.595, those in group C OR 0.820 (0.195–3.444), p = 1.000, group D OR 4.167 (1.288–13.481), p = 0.012 and group E 7.955 (1.979–31.972), p = 0.004, for death. The ROC curve for the SCAI rating was 0.695 (0.590–0.799). Patients classified as D or E had an OR 4.832 (2.146–10.879), p < 0.001. Multivariate logistic regression analysis showed that group D or E classification was the only variable associated with mortality, OR 4.028 (1.397–11.610), p = 0.010.

**Conclusion:** SCAI shock classification D or E was associated with increased risk of death after heart transplantation. The model had a limited capacity of discrimination, probably due to similar risk of death in groups A, B and C.

111972

Modality: E-Poster Researcher – Non-case Report

Category: ATHEROSCLEROSIS/CARDIOVASCULAR RISK FACTORS/CARDIOVASCULAR PREVENTION

## Leisure-Time Physical Activity and Cardiovascular Risk in Brazilian Adults: National Health Survey

ANA CAROLINA MICHELETTI GOMIDE NOGUEIRA DE SÁ^1^, Elton Junio Sady Prates^2^, Deborah Carvalho Malta^1^

(1) Nursing Post Graduate Program. Nursing school, Federal University of Minas Gerais (UFMG). Belo Horizonte, MG, Brazil.; (2) School of Nursing at the Federal University of Minas Gerais (UFMG). Belo Horizonte, MG, Brazil.

**Introduction:** Cardiovascular diseases (CVD) are the main cause of morbidity and mortality in the world and in Brazil and are caused by unhealthy lifestyles, genetic factors, social and health inequalities. These diseases cause disability and loss of quality of life. Consequently, this situation exacerbates the social and economic burden for society, governments and health services. Evidence has identified that physical inactivity is associated with increased coronary risk. However, there are still scientific gaps about the association of leisure-time physical activity (LPA) and cardiovascular risk in Brazil, even in view of the magnitude of CVD. The National Health Survey (PNS) performed laboratory tests and blood pressure (BP) measurements, thus, it was possible to explore, in an unprecedented way, the effect of AFL practice on cardiovascular risk in a representative sample of Brazilian adults.

**Objective:** To analyze the association between AFL practice and cardiovascular risk in Brazilian adults.

**Methods:** Cross-sectional study, with data from the PNS, between 2014–2015, in 8,952 adults. Prevalence and 95% confidence intervals were estimated for the practice of LPA stratified by sex, according to the Framingham risk score and its components. It was defined as sufficient AFL, the practice ≥150 minutes per week of light or moderate intensity or 75 minutes per week of vigorous intensity. The risk score was classified as low, medium and high. Differences were evaluated using the chi-square test or analysis of variance (p ≤ 0.05).

**Results:** The highest prevalence of sufficient LPA practice was associated with low cardiovascular risk in men (17.9%; 16.0–20.0) and women (12.0%; 10.9–13.4) (p ≤ 0.05). For risk score components, sufficient AFL prevalence was higher: in men aged 18 to 29 years (10.43%; 8.9–12.1), with high-density lipoprotein (HDL) between 35–44 mg/dL (9.8%; 8.5–11.3), total cholesterol (TC) between 160–190 mg/dL (11.2%; 9.8–12.8), untreated BP < 120 mmHg (11.4%; 9.9–13.1), who do not have diabetes (25.5%; 23.6–27.6) and are non-smokers (23.3; 21.4–25.3); and in women with HDL between 50–59 mg/dL (4.43%; 3.8–5.2), untreated BP < 120 mmHg (11.50%; 10.24–12.89) and not are smokers (16.2%; 14.9–17.5) (p ≤ 0.05).

**Conclusion:** The practice of AFL is associated with a decrease in cardiovascular risk in Brazilian adults. Thus, it is important to encourage the practice of AFL in the country. These results can support CVD prevention actions and policies.

111973

Modality: E-Poster Researcher – Non-case Report

Category: HEMODYNAMICS AND INTERVENTIONAL CARDIOLOGY

## Impact of Antegrade Dissection and Re-Entry Technique on Coronary Chronic Total Occlusion Percutaneous Interventions

SEBASTIÁN DARÍO PERALTA^1^, Marcelo Omar Bettinotti^1^, Guillermo Jubany^1^, Ezequiel José Zaidel^1^, Luis Murillo^1^, Juan Grieve Bruno^1^, Luis Carlos Sztejfman^1^, Matías Sztejfman^1^, Carlos Maximiliano Giuliani^1^, Ramón Gomes Marquez^1^, Pedro Piccaro de Olivera^1^, Alexandre Schaan de Quadros^1^

(1) LATAM CTO registry

**Background:** The newest techniques for chronic total occlusions (CTO) percutaneous coronary interventions (PCI) may improve technical success.

**Purpose:** To describe safety and efficacy of ADR (antegrade dissection and reentry) technique as initial revascularization strategy.

**Methods:** Multicentric registry from Latin American countries. We analyzed baseline characteristics and outcomes of cases using ADR as primary strategy or bailout of antegrade wire escalation (AWE). Retrograde approach cases were excluded.

**Results:** From 1875 patients analyzed, 50 were planned primary ADR (pADR) and 1825 planned primary AWE (pAWE). pADR was preferred in older patients, with a history of revascularization (CABG: pADR 33.3% and pAWE 13.4%, p < 0,001; PCI: 66.6% and 48.8% respectively, p = 0.012). Longer CTOs (30 mm [22–41] and 21 mm [15–30], p < 0.001), and moderate or severe calcification (62% and 42.6%, p = 0.008) were associated with the selection of pADR instead of pAWE. There was a significant correlation between increasing J-CTO score (Chi2 = 37, df = 5, p < 0.001), and use of pADR. pAWE had a success rate of 88.45%, and pADR of 76.67%. For pAWE and bailout ADR, the use of a dedicated device was related to the highest rates of success (92.31% and 82.69%, respectively). Short term outcomes were similar between groups.

**Conclusions:** In Latin America, ADR was safe and effective, both as primary or bailout strategy, even when used for higher complexity lesions. The use of dedicated devices was related to a higher success rate.

111999

Modality: E-Poster Researcher – Non-case Report

Category: ACUTE AND CHRONIC CORONARY DISEASE/THROMBOLYSIS

## Health Care Assistance to Patients with Acute Myocardial Infarction with St-Segment Elevation in Public Service in Salvador: Has There Been a Change During the COVID-19 Pandemic?

POLLIANNA DE SOUZA RORIZ^1^, José Victor de Sá Santos^3^, Murilo Jorge da Silva^3^, Victor Luis Peixoto Pereira Botelho^3^, Marcelo Vincenzo Sarno Filho^3^, Bianca Aparecida Colognese^3^, Márcio Andrade Barreto Filho^4^, Rhanniel Theodorus Helhyas Oliveira Shilva Gomes Villar^1^

(1) Protocolo IAM – SAMU Salvador; (2) Hospital Ana Nery; (3) Universidade Federal da Bahia; (4) Escola Bahiana de Medicina e Saúde Pública

**Introduction:** An integrated healthcare system plays an important role in ST Elevation Myocardial Infarction (STEMI) assistance. Published data emphasize that the number of hospitalizations, diagnostic and therapeutic procedures performed in STEMI assistance in the pandemic context have decreased.

**Objective:** Evaluate if there was a change in STEMI care assistance by the Acute Myocardial Infarction Protocol (AMIP) team, as reperfusion strategies and mortality in Salvador’s health public system during the pandemic of Covid 19.

**Methods:** Ambispective cohort of STEMI patients attended by the AMIP in Salvador. Epidemiologic data, assistance times, reperfusion strategies and intra-hospitalar mortality were compared between pre-pandemic period (jan/2019–feb/2020) and pandemic (march/2020–dec/2021) – groups I and II, respectively. Statistical significance was considered for p < 0.05.

**Results:** Group I was composed of 542 patients and group II of 932. The mean age and male sex frequency were similar in both groups. There was a higher frequency of hypertension (73.2% × 67.8%, respectively; p = 0.03) and typical A/B pain (91.2% × 85%, respectively; p = 0.01) in group I. Other comorbidities, clinical presentation, Killip and GRACE showed no differences between groups. Controversially, there was no difference in the median symptom-first medical contact time [I–120 (50–270)min × II–123 (60–268.5)min, p = 0.32]. Door-to-ECG time was even shorter in group II [29 (14–70)min] compared to I [35 (16–92.7)min], p = 0.01. There was a shorter door-to-needle time in group II compared to I [111.5 (76–174.8)min × 140 (91.8–202.3)min, respectively; p = 0.002] as well as door-to-balloon time [219 (169.3–317.8)min × 253 (175.8–356.5)min; p = 0.03]. Regarding reperfusion strategies, more patients underwent Percutaneous Coronary Intervention (PCI) in II × I (78.3% vs. 73.5%, p = 0.04), due to a higher frequency of pharmaco-invasive strategy in the second group (II–17.7% × I–3.2%, p < 0.001). There was no difference for primary PCI between groups (57.3% I × 58.8% II; p = 0.64). In-hospital mortality did not differ between the groups (I–17.3%) and (II–16.1%), p = 0.56.

**Conclusion:** During the pandemic, there was a switch in the reperfusion strategy supported by AMIP in order to offer more room to fibrinolysis. The troubled scenario caused by the COVID-19 pandemic did not have a major impact on mortality of patients with STEMI in Salvador.

112000

Modality: E-Poster Researcher – Non-case Report

Category: EPIDEMIOLOGY AND HEALTH POLICIES/GLOBAL HEALTH

## Newborn Pulse Oximetry Screening Prevalence in a Nationwide Complex Survey Sample: An Assessment of Congenital Heart Diseases Early Detection Program at the Regional Level in Brazil

ARN MIGOWSKI ROCHA DOS SANTOS^1^, Arn Migowski^1^, Gustavo Tavares Lameiro da Costa^1^, Helena Cramer Veiga Rey^1^

(1) Instituto Nacional de Cardiologia (INC)

**Introduction:** In Brazil, early neonatal mortality is responsible for more than 50% of deaths in the 1st year of life and congenital heart diseases accounts for 40% of malformations. Newborn pulse oximetry screening(POS) test for critical congenital heart defects between 24–48 hours of life is non-invasive, easy-to-perform and highly specific.

**Objectives:** To estimate the prevalence of POS in Brazil, as well as identify the factors associated with it and the prevalence of positive screening results.

**Methods:** Prevalence was estimated based on results of the most recent National Health Survey, a nationwide population-based cross-sectional Brazilian study. The adjusted marginal prevalence ratios were estimated from a logistic regression model based on Wilcosky & Chambless approach(R prLogistic package).

**Results:** The prevalence of POS was 66.3% (95% CI: 65.5–67.1; N = 3,140,023). The screening prevalence in children born in private funded hospitals (PFHs) was higher than in the National Unified Health System (SUS): 78.1% (76.7–79.5) vs 61.1% (60.2–62.1), respectively. Major regions comparison shows important differences. In the North the prevalence in PFHs (64.9%; 59.7–70.1) was lower than in the South (82.5%; 79.4–85.6) and the Southeast (81.5%; 79.3–83.6) and it is even lower when compared with SUS in the North (44.0%;42.4–45.6). An ordinance was published in Aug 2018, allowing SUS to finance after-screening diagnosis to investigate congenital heart diseases, increased the prevalence of screening in SUS: 57.6% (56.2–59.1) before vs 64.6% (63.3–65.9) after the ordinance. Prevalence of positive screening tests was 9.2%(8.9–9.5) in SUS and 7.8% (7.3–8.3) in the PFHs. The proportion of these newborns that underwent complementary exams after screening was lower in SUS than in the PFHs (40.8%; 40.5–41.1 vs 57.2%; 56.7–57.7). In the multivariate model, the main independent predictors of POS were the prevalence of other newborn screening tests, specially the red eye reflex (PR = 2.03; 1.90–2.16) and newborn hearing test (PR = 1.75; 1.64–1.87).

**Conclusions:** Inequalities were found in the prevalence of screening between major regions, as well as in SUS and PFHs. Government financial incentive has reduced this inequality between public and private, although the percentage of complementary exams after positive screening was also unequal. The main independent predictors of screening prevalence were those related to the organization of health services.

112004

Modality: E-Poster Researcher – Non-case Report

Category: ATHEROSCLEROSIS/CARDIOVASCULAR RISK FACTORS/CARDIOVASCULAR PREVENTION

## Out-Of-Office Measurement of Blood Pressure: Practice, Patient Adherence and Impact on Blood Pressure Control in a High-Complexity Public Outpatient Facility

ANTONIO GABRIELE LAURINAVICIUS^1^, Antonio Gabriele Laurinavicius^1^, Alessandra Cristina Vieira^1^, Julia Luzo Elias Thame^1^, Rafael Gonçalves Zimmer^1^, Henrique Marino de Medeiros^1^, Jorge Tadeu Campos Paixão^1^, Fernanda Consolim Colombo^1^, Marcio Gonçalves Sousa^1^

(1) Instituto Dante Pazzanese de Cardiologia

**Introduction:** Out-of-Office Measurement of Blood Pressure (BP) is recommended in addition to office BP for the diagnosis and follow-up of hypertensive patients. Ambulatory Blood Pressure Monitoring (ABPM); Home Blood Pressure Monitoring (HBPM); and Self-Monitoring of BP (SMBP) are the currently available options and their indication may vary according to the context. The aim of the present study was to assess how Out-of-Office Measurement of BP takes place in clinical practice and its impact on BP control in a public tertiary outpatient clinic.

**Methods:** We evaluated 225 consecutive patients seen at a high-complexity public outpatient facility (mean age: 66.7 ± 11.9 years; female: 62.7%). All patients were routinely requested to perform SMBP according to a prespecified institutional protocol. ABPM and HBPM were indicated for selected cases at the discretion of the attending physician. Patient Adherence to Out-of-Office Measurement of BP was labeled into 5 possible categories: a) No Measurement; b) ABPM; c) HBPM; d) Adequate SMBP e) Inadequate SMBP. Patient Adherence was also stratified according to sex, age, number of antihypertensive drugs, schooling, length of follow-up at the facility, comorbidities and availability of BP monitor at home. Rates of BP control were related with Patient Adherence, as well as with the aforementioned variables.

**Results:** 87.5% of the study population reported having a BP monitor at home. However, adding up the 5 possible categories, adequate Out-of-Office Measurement of BP was available in only 46.7% of the sample (40.9% of the patients did not bring any measurement; 13.8% underwent ABPM; 32.9% adequate SMBP; 12.4% inadequate SMBP; 0% HBPM). Availability of a BP monitor at home (p < 0.001) and the number of antihypertensive drugs in use (p = 0.019) were strongly associated with adherence to SMBP. Prevalence of smoking was 2 folds higher (7.5% vs 3.4%) in those who returned without SMBP. Rate of BP control based on office BP was 42.6% (79.5% of the sample was under ≥3 classes of antihypertensive drugs). Out-of-office BP measurements were not associated with higher rates of BP control (p = 0.377), but allowed to identify a White Coat Effect (WCE) in 1 out of 3 patients with uncontrolled BP according to office BP (WCE prevalence: 29% among uncontrolled patients vs 3.9% among controlled ones.

**Conclusions:** Out-of-Office Measurement of BP is still an unmet need in the treatment of hypertension.

112035

Modality: E-Poster Researcher – Non-case Report

Category: CARDIO-ONCOLOGY

## Avaliation of Glycolytic Metabolism and Myocardial Deformation of the Left Ventricle in Patients with Lymphoma, Pre and Post Chemotherapy with Anthracyclines

MONICA DE MORAES CHAVES BECKER^1^, Gustavo Freitas Alves de Arruda^1^, Diego Rafael Freitas Berenguer^1^, Roberto de Oliveira Buril^1^, Felipe Alves Mourato^1^, Paulo José de Almeida Filho^2^, Brivaldo Markman Filho^1^, Simone Cristina Soares Brandão^1^

(1) Universidade Federal de Pernambuco; (2) Real Hospital da Beneficência Portuguesa

**Introduction:** Studies have suggested that the assessment of cardiac 18F-FDG uptake may be an early metabolic marker of cardiotoxicity. Echocardiography is the method of choice for the evaluation and follow-up of patients undergoing chemotherapy (CT) with anthracyclines, especially through the Global Longitudinal Strain (GLS) technique, which would change earlier than the drop of the left ventricular ejection fraction (LVEF) in patients with reduced myocardial performance.

**Objective:** The objective of this study was to show the cardiac metabolic and functional behavior pre and post-CT with anthracyclines, in patients with lymphoma and to evaluate the relationship between metabolic alteration and cardiac performance.

**Methods:** 18F-FDG PET-CT and strain echocardiography were prospectively performed before and after CT. On PET-CT, the maximum standardized uptake (SUV) value of 18F-FDG was measured in the interventricular septum (IVS) and aorta – blood pool. An increase above 30% in the uptake of 18F-FDG in the IVS was considered significant. On echocardiography, LVEF and GLS were measured. After CT, patients were divided into two groups according to the drop in GLS ≥15% from baseline. Data were compared pre- and post-CT and between groups.

**Results:** Twenty-four consecutive patients were selected (mean age 44,5 ± 18 years, 58,3% female and 66,7% with non-Hodgkin’s lymphoma). The mean LVEF did not show significant difference before (63,5% ± 4,6%) and post CT (64,9% ± 4.4%). Subclinical left ventricular dysfunction (GLS drop ≥15%) occurred in seven (29,2%) patients, during or after CT. Significant increase in IVS FDG uptake occurred in 13 (54,2%) patients. The pre-CT median SUV maximun SIV/SUV maximun aorta ratio increased from 0.84 (IQ 0.73–1.01) to 1.06 (IQ 0.84–1.93) after CT, p = 0.02. However, when we compared this relationship between the groups, there was no significant difference.

**Conclusions:** In this casuistic, we did not observe a significant reduction in LVEF after chemotherapy. However, about 29% of patients presented a decrease in cardiac performance assessed by the strain echocardiography and more than half had a metabolic change in IVS. The relationship between metabolic alteration and subclinical cardiac performance needs to be better clarified.

112042

Modality: E-Poster Researcher – Non-case Report

Category: CARDIOVASCULAR SURGERY

## Superior Periareolar Access for Minimally Invasive Cardiac Surgery

ADRIANO MÁRCIO DE MELO MILANEZ^1^, Marina Coelho Feitosa^2^, Yury Pifano Varela^2^, Josué Viana de Castro Neto^3^, Flávio Duarte Camurça^1^

(1) Cardiovascular surgeon, Cardiovascular Surgery Department, Hospital Antônio Prudente, Fortaleza, Ceará, Brazil.; (2) Medical school student, The University of Fortaleza – UNIFOR, Fortaleza, Ceará, Brazil; (3) Cardiovascular surgeon, Professor of Surgery, The University of Fortaleza – UNIFOR, Fortaleza, Ceará, Brazil.

**Introduction:** The Right Inferior Periareolar Access (RIPA), also known as the Brazilian Technique, has been progressively applied in minimally invasive cardiac surgery (MICS) for atrioventricular valve and septal defect procedures. It provides good functional and aesthetic results, while using a similar fourth intercostal access when compared to the usual inframammary minithoracotomy. We aim to describe a Right Superior Periareolar Access (RSPA), performed one intercostal space above the RIPA, with advantages.

**Methods:** The RSPA consists of a videoassisted minithoracotomy in the 3rd intercostal space through a superior periareolar incision of 3 cm enlarged by a wound protector (Image 1). The cardiopulmonary bypass was established through a femoral platform and cannulation of the right internal jugular vein was added when necessary. A 5 mm port was inserted through the 2nd intercostal space for non dominant hand instrumentation initially and for clamp placement afterwards. The camera port was inserted through the 5th intercostal space. This technique was performed in 39 patients: 30 with atrial septal defects, 5 submitted to mitral valve reconstruction and 4 undergoing mitral valve replacements. Twenty four were females. The mean age was 40.

**Results:** The RSPA provided adequate surgical fields for all procedures. The cardioplegia infusion in the ascending aorta was easily done. Conversion to full sternotomy was necessary in one patient due to bleeding. The median lenght of intensive care unit stay was 1 day and the median postoperative hospital stay was 3 days. There were no postoperative wound infections and no postoperative mortality. The RSPA provided easy access to cardiovascular structures, contributing to effective, functional and safe procedures. The resulting surgical scars were very subtle, and did not compromise the areola.

**Conclusion:** The MICS procedures applying the RSPA technique were effective and safe. Other remarkable advantages were adequate access to the aorta and atriums, low lenght of stay in the intensive care unit and in the hospital and good aesthetic results.


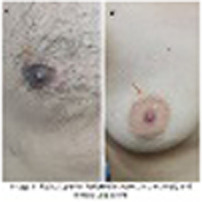


112055

Modality: E-Poster Researcher – Non-case Report

Category: CARDIOVASCULAR IMAGING

## Prevalence of Fragmented QRS and its Correlation with Fibrosis Mass After Acute Myocardial Infarction

MARIA CATARINA DE MELO DIAS GUERRA^1^, Augusto Hiroshi Uchida^1^, José Antônio Franchini Ramires^1^, Eduardo Cavalcanti Lapa dos Santos^1^, Renata Ávila Cintra^1^, Jürgen Beuther^1^, Nevelton Heringer Filho^1^, Tiago Augusto Magalhães^1^, William Azem Chalela^1^, Guilherme Garcia^1^, Leonardo Filipe Benedeti Marinucci^1^, Carlos Eduardo Rochitte^1^

(1) Instituto do Coração (InCor – FMUSP)

**Introduction:** Fragmented QRS (fQRS) is an easily evaluated electrocardiographic parameter and has been associated with alternation of myocardial activation due to myocardial scar.

**Methods:** Retrospective, observational, single-center study. The sample was obtained through analysis of medical records with a record of clinical diagnosis of myocardial infarction (MI) who underwent cardiac magnetic resonance (CMR) to assess myocardial viability. Those who did not present delayed enhancement with an ischemic pattern on CMR and those whose CMR revealed a diagnosis other than MI or did not have satisfactory image quality were excluded. Patients reporting a new acute coronary syndrome in the interval between the available electrocardiogram (ECG) and the CMR were also excluded. The ECG performed 7 days after the infarction and with a maximum interval of 1 year from the CMR were analysed by an evaluator with extensive experience in electrocardiography.

**Results:** The mean age of patients was 61.3 years and 75% were men. Conventional infarction identification through the presence of a pathological Q wave occurred in 172 of the 245 patients included (70.2%). Fragmentation of the QRS complex was identified in 102 of the 245 patients included (41.6%). The prevalence of fQRS was significant even in smaller MI and no correlation was found between the presence of fQRS and the mass of fibrosis on CMR. A higher prevalence of fQRS was observed in infarcts predominantly in the lateral and anterior walls (figure 1).

**Conclusions:** This study revealed a significant prevalence of fQRS in patients after the acute phase of infarction, even in those with a lower mass of fibrosis. Although the analyzed sample of lateral infarctions is still limited, the high prevalence of fQRS may be a relevant finding, considering the low sensitivity of the classic criteria for identifying lateral infarction on the ECG. Figure 1 – Prevalence of QRS fragmentation according to the wall most affected by the infarction.

112063

Modality: E-Poster Researcher – Non-case Report

Category: CARDIOGERIATRICS

## The Clinical Features of Acute Heart Failure in Octogenarians

ALEXANDRA CORRÊA GERVAZONI BALBUENA DE LIMA^1^, Luana de Oliveira Alves^1^, Laila Morais Nahass Franco^1^, Pedro Pinto Machado^2^, Pedro Henrique Parcianello Teixeira^2^, Luciana Bartolomei Orru D’Avila^1^

(1) North Wing Regional Hospital – Brasília – Brazil; (2) School of Health Sciences– Brasília – Brazil.

**Background:** Heart failure (HF) increases significantly in relation to advancing age. With aging, not only do age-related, morphological, and physiological cardiovascular changes predispose to HF, there is also increased prevalence of comorbid conditions that compound cardiac limitations (e.g., renal insufficiency) and others that tend to overwhelm limited cardiovascular reserves (e.g., infections and ischemia). The HF among very old adults, the prevalence, the complications in acute HF demand more studies.

**Objective:** Evaluate the clinical features and prognostic evolution of individuals 80 years and older with acute HF.

**Methods:** Retrospective and observational study of patients 80 years or older, hospitalized with acute HF at North Wing Regional Hospital, Brasília, Brazil, January 2017 to May 2020. Patients who did not meet the clinical criteria of HF, with missing or incomplete information in the medical record or were not accessible by telephone were excluded. The minimal follow-up were 12 months and final date were 05.21.2021. Clinical profiles, clinical history, blood test, and mortality were analyzed. The patients were divided into groups: 80 to 84 years and ≥85 years.

**Results:** A total of 119 individuals (66 and 53) were included, 45% and 47% male, 92% and 83% hypertensive. The mean age of the groups were 81.7 ± 1.3 years (80–84) and 88.6 ± 3.5 years (85–97), hemoglobin 11.9 ± 2.4 mg/dL and 10.8 ± 2.0 mg/dL, creatinine 1.44 ± 1.9 mg/dL and 1.78 ± 1.4 mg/dL, left ventricular ejection fraction 51.8 ± 15.7% and 58.6 ± 11.8%, and follow-up 29.1 ± 25.9 days and 28.4 ± 24.6 days. The presence of pulmonary congestion 40% and 45%, peripheral edema 45% and 43%, and atrial fibrillation (AF) 34% and 35%. During the hospitalization, hemodialysis 15% of both groups, vasoactive drugs 28% and 18%, intensive care unit 45% and 39%, and mortality rate 21% and 18%. In multivariate analysis deaths were related to hypertension (HR = 0.54, 95%CI = 0.29–0.98, p = 0.046), pulmonary congestion (HR = 3.38, 95%CI = 1.97–5.79, p = 0.000), peripheral edema (HR = 4.12, 95%CI = 2.30–7.38, p = 0.000), AF (HR = 0.58, 95%CI = 0.34–0.98, p = 0.043), and hemoglobin (HR = 0.84, 95%CI = 0.75–0.95, p = 0.007).

**Conclusion:** In our study simple clinical features as pulmonary congestion, peripheral edema and hemoglobin showed association with mortality. Consideration of disease prognosis according to factors that predict mortality can help to better define the care plan and promote palliative and supportive care.

112078

Modality: E-Poster Researcher – Non-case Report

Category: EPIDEMIOLOGY AND HEALTH POLICIES/GLOBAL HEALTH

## Sedentary Behavior in Adolescents from Public Schools of Goiás During the COVID-19 Pandemic

PRISCILA VALVERDE DE OLIVEIRA VITORINO^1^, Alice PInheiro Ribeiro^1^, Edison Nunes Pereira^2^, Gustavo Carvalho Marcelino^1^, Ana Carolina Arantes^2^, Ademir Schmidt^1^, Weimar Kunz Sebba Barroso^2^

(1) Pontifícia Universidade Católica de Goiás; (2) Universidade Federal de Goiás

**Introduction:** Sedentary behavior (SC) is a habit present in worldwide, with increasing percentages in the last decade, especially among adolescents. With the COVID-19 pandemic, CS increased, especially due to social isolation and school closures.

**Objetives:** Carachterize adolescens regarding sociodemographic and economic conditions, aspects related to school and work and general health; classify adolescents in terms of SC and compare CS according to gender.

**Method:** Analytical cross-sectional study, conducted with adolescents (10 to 19 years), enrolled in 19 schools of Goiás. Electronic data collection took place from November 2020 to August 2021. The form contained questions about personal, sociodemographic, lifestyle and CS. Two cutoff points were considered for SC: ≥6 and ≥ to 3 hours/day. The project was approved by Ethics Committee. Descriptive analysis and comparisons were performed using pearson’s Student and Chi-square T tests. Significance level p < 0.05 was considered.

**Results:** We evaluated 167 students with a mean age of 15.3 DP 2.1 (Table 1). The frequencies of SC 89.1% and 52.7% at the cutoff points of 6 and 3 hours/day, respectively.

**Conclusions:** The sample was characterized by adolescents who did not work, were mainly from social classes B and C and had a low frequency of bad health habits. There was no difference in the frequency of SC in relation to the variables evaluated.


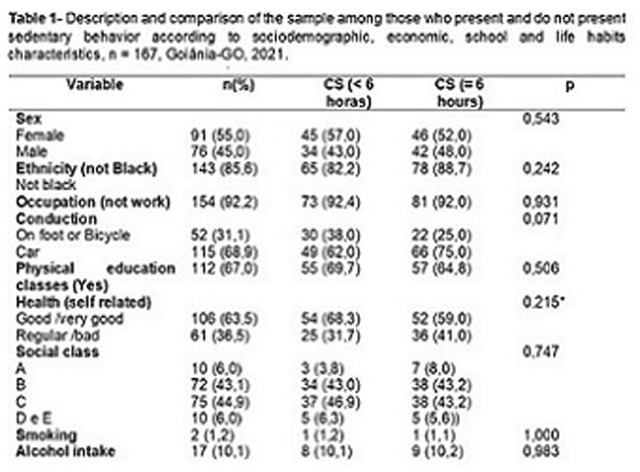


112101

Modality: E-Poster Researcher – Non-case Report

Category: SPIRITUALITY AND CARDIOVASCULAR MEDICINE

## Religiosity in Adhesion to Treatment of Pilgrims with Hypertension

CÉLIDA JULIANA DE OLIVEIRA^1^, Héryka Laura Calú Alves^1^, Gabriela de Sousa Lima^1^, Érica Sobral Gondim^1^, Emiliana Bezerra Gomes^1^

(1) Universidade Regional do Cariri – URCA

**Introduction:** Hypertension evolves slowly and is asymptomatic, so it does not usually receive the proper care, resulting in poor adherence to treatment and an increase in cardiovascular events. It is a disease that requires changes in lifestyle for adherence to treatment, which is influenced by several factors, such as religiosity, which includes people’s beliefs, attitudes and values. Therefore, the pilgrimage of Padre Cícero stands out, a religious manifestation that attracts thousands of faithful to the municipality of Juazeiro do Norte-CE, in which the attendance to pilgrims because of the elevation of blood pressure is a constant, making it necessary to study the relationship between religiosity and adherence.

**Objective:** To investigate the influence of religiosity in the treatment adherence of hypertension by pilgrims.

**Method:** Cross-sectional, qualitative, carried out in Juazeiro do Norte, between September 9 and 15, 2018. The question was: “Does religion influence you to comply with treatment for high blood pressure?”. The data collected were organized into thematic categories. The study was approved by the Ethics Committee under opinion number 2,753,051.

**Results:** 338 pilgrims self-reported with hypertension were investigated, male (62.43%), retired (71.06%) and mean age of 64.5 years. When questioned about the influence of religion on treatment, three categories emerged: Category 1 “Faith”: Faith was answered as the influence of religion on compliance with treatment, either alone or in a complementary way, also as a cure for hypertension. Category 2 “I pray for improvement”: Prayers are the most cited as influencing treatment, for comforting, improving thoughts and guiding behaviors, such as waking up to pray and taking medication. Category 3 “Religion is above all”: Church and masses were cited as contributors. Pilgrims report feeling good and associating religious practices with treatment, combining medication intake times with church participation. The people who are in this space represent a support network, especially the priest, by encouraging self-care related to the treatment.

**Final Considerations:** It was noticeable that the pilgrimage and its elements influence the perception of health, illness and treatment. Thus, they can be used as beneficial means of promoting health and adherence to therapy through strategies that enhance the spiritual dimension.

112117

Modality: E-Poster Researcher – Non-case Report

Category: PHYSICAL EDUCATION

## Exercise Training in Patients with Chagas Cardiomyopathy: A Systematic Review of Randomized Controlled Trials

FÁBIO SILVA MARTINS DA COSTA^1^, Henrique Silveira Costa^2^, Lucas Frois Fernandes Oliveira^2^, Matheus Ribeiro Ávila^2^, Pedro Henrique Scheidt Figueiredo^2^, Mauro Felippe Felix Mediano^3^, Luciano Fonseca Lemos de Oliveira^1^, Manoel Otávio Costa Rocha^1^

(1) Universidade Federal de Minas Gerais – UFMG; (2) Universidade Federal dos Vales do Jequitinhonha e Mucuri – UFVJM; (3) Fundação Oswaldo Cruz – FIOCRUZ

**Introduction:** Patients with Chagas cardiomyopathy (ChC), in addition to having a worse prognosis compared to other cardiomyopathies, can have important consequences, including reduced functional capacity and quality of life. The exercise training (ET) has emerged as an adjuvant option for treatment of ChC, relieving dyspnea and fatigue caused by heart failure. This study aimed to discuss the main findings of effect of ET in patients with ChC, focusing on the functional capacity, hemodynamic and autonomic function, musculoskeletal system, myocardial function and morphology, and health-related quality of life (HQoL).

**Methods:** This is a systematic review using the descriptors: (“Chagas disease” OR “chronic Chagas cardiomyopathy”) AND (“exercise training” OR “cardiac rehabilitation”). A literature search of the MEDLINE, LILACS, Web of Science, CINAHL, Scopus, PEDro and EMBASE was performed with no data or language restrictions. The study was registered in PROSPERO database (CRD CRD42017064912) and edited following in guidelines of the Preferred Reporting Items for Systematic Reviews and Meta-Analyses (PRISMA) statement.

**Results:** A total of 5 papers (screened from 1.169 studies) reached the inclusion criteria and were included in the present review. The patients with ChC submitted to ET presented improvement of functional capacity (increase in the VO2peak and the walked distance in the 6-minute walk test, but did not reduce the ventilatory efficiency given by VE/VCO2slope), hemodynamic and autonomic function (reduce the HR at rest but do not change the blood pressure; slight increase in the HRV in patients with reduced LVEF and better improvements in the autonomic balance given by increase in the parasympathetic activity and reduction in the sympathetic activity over the heart in patients with preserved LVEF and autonomic dysfunction), musculoskeletal system (increase in the cross-sectional and number of type I fiber, reduce the genes expression involved in the muscle atrophy process, increase skeletal muscle perfusion) and the HQoL (improve domains of vitality and emotional aspects). However, no changes in the myocardial function and morphology were observed.

**Conclusion:** In conclusion, the ET as a low-cost non-drug therapy option seems to provide important benefits in association with the classic treatment of patients with ChC, however, more studies are warranted to confirm these results.

112137

Modality: E-Poster Researcher – Non-case Report

Category: CARDIAC ARRHYTHMIAS/ELECTROPHYSIOLOGY/ELECTROCARDIOGRAPHY

## Safety and Effectiveness of Conduction System Pacing (His-Bundle Pacing and Left Bundle Branch Pacing) as a First Option for Cardiac Pacing

CHARLES SLATER^1^, Luiz Eduardo Camanho^1^, Eduardo Benchimol Saad^2^, Luiz Antonio Oliveira Inacio Jr^2^, Lucas Carvalho Dias^1^, Gustavo Vignoli Santos^2^, Ricardo Mourilhe-Rocha^1^

(1) State University of Rio de Janeiro (UERJ), Rio De Janeiro, Brazil; (2) Hospital Pro Cardíaco, Rio De Janeiro, Brazil

**Background:** Conduction system pacing (CSP) (His bundle pacing and left bundle pacing) is a group of techniques intended to achieve cardiac pacing with a narrow QRS complex. The safety and effectiveness of this technique are not yet entirely understood.

**Purpose:** To describe the implant findings and safety profile of CSP as a first option after 4 years in a single center.

**Methods:** In a period of 42 months, 214 patients were submitted to CSP as a first strategy to restore AV synchrony (pacemakers for AV block or sinus node dysfunction) or as a resynchronization (CRT) strategy (for patients with heart failure and bundle branch block). CSP was implanted in lieu of a conventional right ventricular lead in pacemaker cases, and in addition or in lieu of a coronary sinus lead, in CRT cases, depending on the technical and anatomical findings.

**Results:** The mean age was 76.7 ± 16.4 years, 65% males. 162 patients implanted a CSP lead for a dual-chamber pacemaker, 3 patients for a single chamber pacemaker, 32 patients for CRT-D (CSP lead replacing the coronary sinus lead with a defibrillator), and 13 patients for an optimized CRT (CSP lead plus coronary sinus lead). In 16 patients (7.4%) the technique of choice was His bundle pacing. One patient (0.4%) had subacute lead dislodgement, being submitted to repositioning. 4 patients – intended for CRT (1.8%) didn‘t meet the criteria for His Bundle pacing or left bundle pacing, being submitted to conventional coronary sinus lead placement. There were 10 cases (4.6%) of confirmed lead perforation during the lead septum insertion, with prompt repositioning, all uneventful. No pericardium effusion related to lead perforation was observed. One patient (0.4%) had a pneumothorax, requiring chest tube drainage.

**Conclusion:** Conduction system pacing as a first strategy is a feasible, effective and safe technique, both for pacing and for resynchronization purposes, with complication rates comparable to conventional implantation.

112166

Modality: E-Poster Researcher – Non-case Report

Category: PHYSIOTHERAPY

## Does Respiratory Muscle Strength and Diaphragmatic Mobility Influence Glittre ADL-Test Performance in Patients with Heart Failure?

DANIELLA CUNHA BRANDAO^1^, Bruna T.S. Araújo^1^, Kamyla Maria Alcantara Silva Alves^1^, Armèle Dornelas de Andrade^1^, Daniella Cunha Brandão^1^

(1) Universidade Federal de Pernambuco

**Introduction:** Studies relating respiratory muscle strength and diaphragmatic mobility in heart transplant patients are scarce, especially when correlated with submaximal exercise tests, such as the Glittre ADL-Test, which is still poorly investigated in this population.

**Purpose:** To investigate whether the time required to perform the Glittre ADL-Test correlates with respiratory muscle strength and diaphragmatic mobility.

**Methods:** This was a cross-sectional study with 38 adult individuals aged 21 to 65 years, diagnosed with HF of all etiologies and with reduced ejection fraction. The performance of the Glittre ADL-Test followed the standardization of the literature, using the Total Time to complete the five laps of the test. The assessment of respiratory muscle strength was made by a digital manovacuometer and diaphragmatic mobility was measured by ultrasound in the M. mode.

**Results:** The mean Glittre ADL-Test time was 286.5 seconds and showed significant correlations with maximal inspiratory pressure (MIP) (r = –0.445 – p < 0.01) and maximal expiratory pressure (MEP) (r = –0.531 – p < 0.01) and with diaphragmatic mobility (r = –0.361 – p < 0.05).

**Conclusions:** Our findings suggest an inverse relationship between respiratory muscle strength and diaphragmatic mobility with Glittre ADL-Test performance. If respiratory muscle strength and diaphragm muscle movement are lower, the time taken to complete the test will be longer, suggesting that increased respiratory work may potentiate peripheral muscle fatigue. The impairment of respiratory muscles seems to have important implications on the performance of HF individuals in their daily activities, confirming the need for early identification of patients with respiratory muscle weakness, enabling a targeted intervention in this population. Support: Edital PROPG-UFPE 230760186722020-32; CNPq 421656/2021-7 and FACEPE 0801-4.08/21.

112169

Modality: E-Poster Researcher – Non-case Report

Category: HEART FAILURE/CARDIOMYOPATHY/TRANSPLANT

## Conduction System Pacing as a First Strategy for Cardiac Resynchronization Therapy in Heart Failure

CHARLES SLATER^1^, Luiz Eduardo Camanho^1^, Eduardo Benchimol Saad^2^, Luiz Antonio Oliveira Inacio Jr^2^, Lucas Carvalho Dias^1^, Gustavo Vignoli Santos^2^, Ricardo Mourilhe-Rocha^1^

(1) State University of Rio de Janeiro (UERJ), Rio de Janeiro, Brazil; (2) Hospital Pro Cardíaco, Rio de Janeiro, Brazil

**Background:** Conduction system pacing through direct His bundle of left bundle stimulation is a new alternative to achieve adequate CRT in patients with heart failure with reduced ejection fraction (HFrEF).

**Purpose:** To describe the short and medium term outcomes of CRT through direct conduction system pacing in a single center.

**Methods:** 53 consecutive patients who underwent CRT through direct stimulation of the His bundle or left bundle branch were retrospectively evaluated. All presented in functional class III/IV (NYHA). The CRT response criteria were: improvement of functional class and reverse remodeling criteria (increase in ejection fraction (EF) >10% and/or decrease in left ventricular end-systolic diameter (LVESD) >15%). The patients were evaluated at 1 and 3 months and every six months after the procedure.

**Results:** The mean age was 76,4 ± 14,8 years, 65% males, 42% Ischemic cardiomyopathy. Left bundle branch block (LBBB) was observed in 98% and mean QRS duration was 169 ms; average EF: 28%; mean LVESD and left ventricular end-diastolic diameter (LVEDD) was 53 and 68 mm, respectively. Direct His bundle pacing was the technique of choice in 5 patients, while left bundle branch pacing was used in 48 patients. There were five patients where His bundle pacing of left bundle branch pacing criteria was not met, being submitted to conventional CRT through coronary sinus branches. The mean follow up time was 21 months. Mean QRS duration was significantly shorter after the procedure (128 ms). Improvement of functional class was found in 85,7% of patients. Reverse remodeling criteria was found in 70,5% of patients. 4 patients progressed to end-stage HF and subsequent death.

**Conclusion:** CRT through conduction system pacing is a valid alternative to conventional CRT, with promising results, allowing cardiac resynchronization in patients with HFrEF.

112201

Modality: E-Poster Researcher – Non-case Report

Category: COVID-19 AND CARDIOVASCULAR SYSTEM

## Long-Term Mortality Predictors in Hospitalized COVID-19 Patients using Machine Learning Model

BRUNO FERRAZ DE OLIVEIRA GOMES^1^, Thiago Moreira Bastos da Silva^2^, Leticia de Sousa Peres^1^, Iliana Regina Ribeiro Menezes^1^, Nathalia Duarte Camisão^1^, Mariana Moreno Canário da Silva^1^, Renata Mexias Abdala Felix^1^, Giovanni Possamai Dutra^1^, Anna Butter^1^, Henrique Custódio Goudar^1^, João Luiz Fernandes Petriz^1^, Gláucia Maria Moraes de Oliveira^2^

(1) Hospital Barra D’Or; (2) Universidade Federal do Rio de Janeiro

**Introduction:** Several studies have already scored the predictors of in-hospital mortality in patients hospitalized for COVID-19. 2 years after the beginning of the pandemic, few studies have evaluated predictors of long-term mortality.

**Objectives:** To evaluate clinical and laboratory characteristics predictors of mortality using machine learning techniques.

**Methods:** Retrospective cohort study with patients who were hospitalized with a confirmed diagnosis of COVID-19. Comorbidities, laboratory tests, vaccination status, and clinical characteristics were evaluated. The primary outcome of this study is all cause death occurring in the hospital or after hospital discharge. We used the classification tree model for the survival outcome, available in RStudio 2021.09.0.

**Results:** 1454 patients were included, mean age of 59.8 ± 17.0, 62.6% men. There were 269 deaths (18.5%) during the study period (mean follow-up = 338 ± 209 days). 44.7% of patients had myocardial injury. The following predictive variables were selected by the method: use of mechanical ventilation, age, platelet count at discharge, CRP at discharge, myocardial injury, SAPS3 score, d-dimer peak of hospitalization, dementia, chronic renal failure, COVID-19 vaccination, and use of anticoagulation therapy. The model is available in the figure.

**Conclusion:** In patients hospitalized for COVID-19, patients who required mechanical ventilation, with high CRP at discharge and higher age had the worst long-term prognosis. Other markers have shown promise in predicting more severe patients, such as platelet count at discharge and the occurrence of myocardial injury.


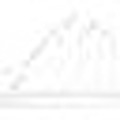


112202

Modality: E-Poster Researcher – Non-case Report

Category: CARDIAC ARRHYTHMIAS/ELECTROPHYSIOLOGY/ELECTROCARDIOGRAPHY

## Ultrasound Guided Vascular Access for Cardiac Devices Implantation – Safety and Efficacy Results

CHARLES SLATER^1^, Luiz Eduardo Camanho^1^, Eduardo Benchimol Saad^2^, Luiz Antonio Oliveira Inacio Jr^2^, Lucas Carvalho Dias^1^, Gustavo Vignoli Santos^2^, Ricardo Mourilhe-Rocha^1^

(1) State University of Rio de Janeiro (UERJ), Rio de Janeiro, Brazil; (2) Hospital Pro Cardíaco, Rio de Janeiro, Brazil

**Background:** Vascular access is the first step to perform cardiac device (CD) implantation. However, vascular access-related complications still remain a major cause of complications in CD implantation, increasing risk, costs, and hospitalization time. Ultrasound has become an important tool in helping vascular access, and its use has increased recently for CD implantation.

**Purpose:** To describe the results after 4 years of US-guided vascular access for implantation of CD.

**Methods:** Over a period of 50 months, 627 patients (average age 77,1 ± 14,6 years – interquartile range: 13 years) were submitted to CD implantation (271 dual-chamber pacemakers, 9 single-chamber pacemakers, 24 CRT-P’s, 167 conduction system pacemakers (His bundle pacing or left bundle pacing), 63 CRT-D’s, 46 dual-chamber ICD’s, 2 single-chamber ICD’s, 4 lead implantations and 41 upgrades for CRT), in a total of 1282 US-guided punctures (longitudinal “out of plane” axillary vein approach) using a “Point of Care” US machine with a 12MHz probe protected with a sterile cover. All punctures were performed on intact skin, followed by an incision. Chest radiography and pulmonary ultrasound were performed in the search for complications immediately after the procedure and the next morning, before discharge.

**Results:** Vascular access was successful in all cases. In 11 cases (1,75%) there was limiting vascular obstruction, requiring contralateral vascular access to complete the procedure. US image also allowed alternative approaches, such as access to a supraclavicular vein (3 cases/0,47%) in patients with complete obstruction of the axillary vein; and cephalic vein puncture (4 cases/0,63%) when the axillary vein was too deep. Pneumothorax was found in one patient (0,16%) through the pulmonary US the next morning and submitted to chest drainage with no further complications. No haemothorax was found in this sample.

**Conclusion:** US-guided vascular access technique allows safe implantation, with complication rates considerably lower than that related in literature.

112215

Modality: E-Poster Researcher – Non-case Report

Category: PHYSIOTHERAPY

## Performance in Glittre ADL-Test Associates with Maximum Functional Capacity in Heart Failure Patients: A Cross-Sectional Study

DANIELLA CUNHA BRANDAO^1^, Jéssica Costa Leite^2^, Bruna T.S. Araújo^1^, Armèle Dornelas de Andrade^1^, Daniella Cunha Brandão^1^

(1) Universidade Federal de Pernambuco; (2) UNIFACISA

**Introduction:** The Glittre ADL-Test appears as one more test option to assess submaximal exercise tolerance in heart transplant patients.

**Purpose:** To verify the association between the time spent in the Glittre ADL-Test with the variables of the Cardiopulmonary Exercise Test (CPET).

**Methods:** A cross-sectional study evaluating 53 male and female subjects with Heart Failure (HF), NYHA (New York Heart Association) II–III and left ventricular ejection fraction <45%. The individuals performed the CPET on a treadmill with ramp protocol to evaluate the maximum functional capacity and on another day the Glittre ADL-Test, considering the total time to complete the five rounds of the test. A minimum interval of 24 hours and a maximum of 7 days between the tests was respected.

**Results:** Glittre ADL-Test time was negatively and moderately associated with peak oxygen uptake (VO2peak) (r = –0.48; p = <0.001) and oxygen uptake at the first ventilatory threshold (r = –0.475; p = <0.001), besides a low negative association with the time to reach VO2peak (r = –0.345; p = 0.014).

**Conclusions:** We can suggest from the results obtained that the Glittre ADL-Test is an accessible method for assessing individuals with HF and correlates with the oxygen uptake measured by CPET. As clinical implications we have: The Glittre ADL-Test can be a fast and economical alternative for estimating the functional capacity of individuals with HF in the absence of a maximal exercise test. Support: PROPG-UFPE n.23076018672/2020-32; CNPq 421756/2021-7; FACEPE 0801-4.08/21

112236

Modality: E-Poster Researcher – Non-case Report

Category: EPIDEMIOLOGY AND HEALTH POLICIES/GLOBAL HEALTH

## Obesitiy and Hypertension in Primary Basic Setting

MONICA AMORIM DE OLIVEIRA^1^, Helena Cramer Veiga Rey^1^

(1) Instituto Nacional de Cardiologia

HTN is the most common, identifiable and modifiable risk factor for cardiovascular disease. The prevalence of SAH is about three times higher in obese patients. Weight loss has an impact on the treatment of HTN and this should be encouraged. The aim of the study was to evaluate the prevalence of obesity in a group of 1000 patients over 18 years of age treated in primary care in the city of Rio de Janeiro and its relationship with HTN. Of the 1014 patients included, 737 (72.68%) were hypertensive. A sedentary lifestyle was present in 65.5% of the population studied and there was no difference between the group of patients with and without SAH. The average BMI of the general population was 29.6 ± 6.2 kg/m^2^. Being 30.1 ± 6 Kg/m^2^ and 27.8 ± 6.5 Kg/m^2^ for hypertensive and non-hypertensive patients, respectively, with statistical significance. Obesity was found in 41.2% of the population studied. The distribution according to the WHO BMI classification can be verified in the table and showing the association between HTN and obesity. The treatment of hypertension in the obese requires a targeted approach to obesity. Adopting a healthy lifestyle facilitates weight loss, increases responsiveness to antihypertensive drug therapy, and produces independent beneficial effects on factors. did not present SAH, as a measure to prevent its development.


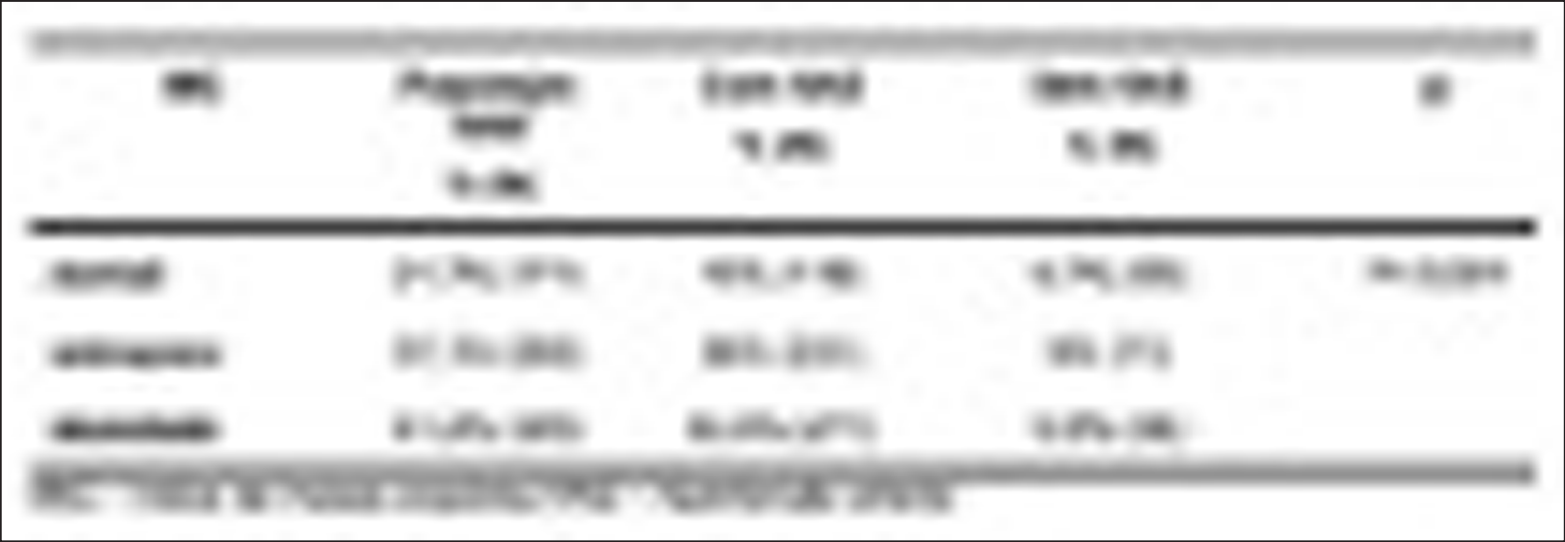


112240

Modality: E-Poster Researcher – Non-case Report

Category: CARDIAC ARRHYTHMIAS/ELECTROPHYSIOLOGY/ELECTROCARDIOGRAPHY

## Stereotactic Radiotherapy for the Treatment of Recurrent Refractory VT in Chagas Disease Patients

CRISTIANO FARIA PISANI^1^, Rodrigo M. Kulchetscki^1^, Bernardo Salvajoli^2^, Marina P. Mayrink^1^, Ligia Arteaga^2^, Jason Cook^3^, William Stevenson^3^, João Salvajoli^2^, Mauricio Scanavacca^1^

(1) InCor – Heart Institute – University of São Paulo Medical School; (2) ICESP – Instituto do Cancer do Estado de São Paulo; (3) Vanderbilt Heart

**Background:** Stereotactic radiotherapy (SBRT) is a new therapeutic option in cases of recurrent ventricular tachycardia (VT) in patients with structural heart disease where there is a contraindication or a therapeutic failure of the catheter ablation (CA).

**Objectives:** To describe the initial experience of SBRT for recurrent VT in patients with Chagas Cardiomyopathy (CCM) in whom catheter ablation is not an option.

**Methods:** The target sites and SBRT doses were planned based on imaging exams and on bipolar voltage maps from previous CA procedures and new electrophysiologic study was also performed aiming to evaluate the exit site of induced VT.

**Results:** Five patients with CCM and recurrent VT underwent SBRT from July 2021 to April 2022. Most patients were male (60%), mean age 61,4 ± 5, years and EF 22% (Q1: 20 Q3:45). One patient (20%) had two prior catheter ablation and the others only one. The median number of VT episodes in the six and two months prior to SBRT was 13 (Q15.5–Q348.5) and 11 (Q1:2.5;Q3:26.5), respectively. The mean PTV (planning target volume) was 82 ± 17 mL and the ITV (internal target volume) was 29 ± 5 ml, with safe constraints regarding the esophagus and stomach. In a median FU of 194 days, 2 (40%) patients presented VT recurrence. No patients died. The median number of VT episodes reduced from 24 (4.75;52.25) to 2 (0;8.5) (P = 0.068).

**Conclusion:** SBRT is safe and seems to be effective in Chagas Disease patients


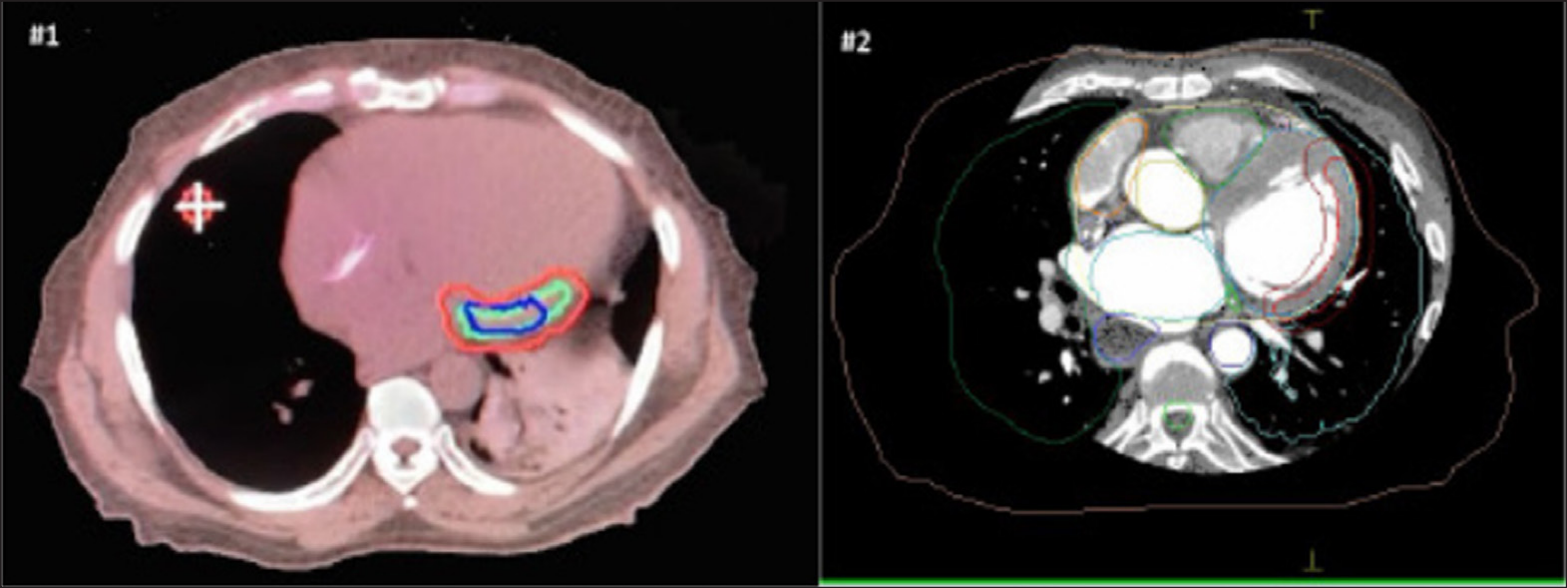


112483

Modality: E-Poster Researcher – Non-case Report

Category: DIGITAL HEALTH/INNOVATION

## Remote Blood Pressure Monitoring in Patients with Chronic Heart Failure

ANDREY GARANIN^1^, Andrey Garanin^1^, Dmitriy Duplyakov^1^, Irina Mullova^1^, Oksana Shkaeva^1^, Polina Duplyakova^1^

(1) Samara State Medical University

**Introduction:** The pandemic of a new coronavirus infection has shown the need for the development of telemedicine technologies, especially remote medical monitoring using telemonitoring of vital body functions. In some regions, this approach is also justified by the factor of distance and the shortage of certain categories of medical workers.

**Objective:** Decrease of repeated hospitalizations and mortality in patients with chronic heart failure by means of remote monitoring system including arterial pressure monitoring.

**Methods:** 401 patients, who were randomized into two equal groups comparable by sex, age, left ventricular ejection fraction, functional class of heart failure, six-minute walking test results, body mass index, baseline systolic and diastolic blood pressure, and heart rate of the underlying disease and hospitalized for myocardial infarction or decompensation of chronic heart failure, were examined at two research centers. In group 1, remote blood pressure monitoring using oscillometric method was performed using certified tonometers with the ability to transmit the measurement results via cellular communication channel to the research center. Based on the results of the data obtained, the physician was able to contact the patient and adjust the previously prescribed treatment. Group 2 patients performed self-monitoring of blood pressure. All the patients were observed for 3 months. Treatment in both groups was prescribed according to the latest recommendations of the European Society of Cardiology.

**Results:** Group 1 showed a tendency to blood pressure lowering from 130 mmHg to 125 mmHg. In contrast, Group 2 showed an increase in blood pressure from 125 mmHg to 130 mmHg. Group 1 showed 4 hospitalizations related to acute cardiac pathology with a total duration of 30 days; group 2 showed 13 hospitalizations with a total duration of 133 days (p = 0.027; OR; 95% CI 3.4; 1.1–10.8). Total mortality was 6 in group 1 and 11 in group 2 (p = 0.226; OR; 95% CI 1.9; 0.7–5.3). Cardiovascular mortality was 3 in group 1 and 10 in group 2 (p = 0.052; OR; 95% CI 3.5; 0.9–12.9).

**Conclusions:** Remote management of patients with chronic heart failure, including blood pressure monitoring, over 3 months demonstrates a significant reduction in hospitalizations and a trend toward lower total and cardiovascular mortality.

112516

Modality: E-Poster Researcher – Non-case Report

Category: CARDIOVASCULAR PHARMACOLOGY

## Beneficial Effects of Aliskiren-Loaded Polymeric Nanoparticles in the Cardiovascular System of Hypertensive Rats

OLGA PECHANOVA^1^, Andrej Barta^1^, Martina Cebova^1^

(1) Centre of Experimental Medicine, Slovak Academy of Sciences

**Background:** Aliskiren, a renin inhibitor, has been shown to exert cardio-protective, reno-protective, and anti-atherosclerotic effects independent of its blood pressure lowering activity. However, relatively high dose and frequency of the treatment, which is needed for beneficial effects of the drug, may incur several side effects such as high blood potassium levels, particularly when used with ACE inhibitors in diabetic patients. We hypothesized that gradually released aliskiren from polymer-based nanoparticles could at least partially solve the problem of bioavailability. Therefore, we aimed to determine the effects of aliskiren-loaded polymeric nanoparticles on blood pressure, nitric oxide synthase (NOS) activity and structural alterations of the heart and aorta developed due to spontaneous hypertension in rats.

**Methods:** Twelve-week-old male spontaneously hypertensive rats were divided into the untreated group, group treated with powdered aliskiren or aliskiren-loaded nanoparticles (25 mg/kg/day) and group treated with nanoparticles only for 3 weeks by gavage. Blood pressure was measured by tail-cuff plethysmography. NOS activity, eNOS and nNOS protein expressions, and collagen content were determined in both the heart and aorta. Vasoactivity of the mesenteric artery and wall thickness, inner diameter, and cross-sectional area (CSA) of the aorta were analyzed.

**Results:** After 3 weeks, blood pressure was lower in both powdered aliskiren and aliskiren-loaded nanoparticle groups with a more pronounced effect in the latter case. Only aliskiren-loaded nanoparticles increased the expression of nNOS along with increased NOS activity in the heart (by 30%). Moreover, aliskiren-loaded nanoparticles decreased vasoconstriction of the mesenteric artery and collagen content (by 11%), and CSA (by 25%) in the aorta compared to the powdered aliskiren group. In conclusion, aliskiren-loaded nanoparticles represent a promising drug with antihypertensive and cardioprotective effects.

112557

Modality: E-Poster Researcher – Non-case Report

Category: CARDIOVASCULAR PHARMACOLOGY

## Randomized Control Trial Comparing Efficiency between Losartan and Amlodipine to Black Hypertensive Cameroonian in Yaounde

NGO YOUMBA EP NTEP GWETH^1^, Marie Ndjie Ndzana^1^, Liliane Mfeukeu Kuate^1^, Chris Nadège Nganou Gnindjio^1^, Valerie Ndobo^1^, Sylvestre Efonle Ngoh^1^, Samuel Kingue^2^

(1) HOPITAL CENTRAL DE YAOUNDE (HCY); (2) HOPITAL GENERAL DE YAOUNDE

Randomized control trial comparing efficiency between losartan and amlodipine to hypertensive Cameroonian in Yaounde SUMMARY.

**Introduction:** High blood pressure is the first chronic disease in the world and which also increases the cardiovascular risk. The reduction of this particularly high risk in patients from sub-Saharan Africa should be the main goal of care in our setting. Numerous studies on the choice of a better antihypertensive treatment of arterial hypertension have been conducted but the inherent specificities of our populations, in particular a lower renin activity, a tendency towards sodium-water retention and vascular hyperreactivity, impose a customization of the pharmacological treatment in Africans. Although there is a difference in response to antihypertensive classes in African subjects who respond better to amlodipine than to losartan, few study in our context has yet demonstrated this to the best our knowledge.

**Objective:** The main objective of this study was to compare the efficacy of a amlodipine blocker and losartan receptor blocker in Cameroonian hypertensives at the Yaounde Central Hospital.

**Methods:** This was a randomized, controlled clinical trial phase IV. It involved patients with grade 1 or 2 clinical hypertension, naive to antihypertensive therapy. The intervention consisted in starting the recommended average doses in monotherapy for 12 weeks (84 days) of losartan 50 mg in single dose per day in group 1 and amlodipine 5 mg in single dose per day. Group 2. The follow-up were scheduled 2 weeks. The increase in monotherapy doses occurred from the fourth week (S4) when the blood pressure goal was not reached with losartan 100 mg once a day and amlodipine 10 mg once daily for group 1 and 2, respectively. The patients for whom BP was still not controled despite the increase in monotherapy doses adding a low dose of hydrochlorothiazide at 12.5 mg at eight week (S8) to achieve the blood pressure goal. Compliance assessment was done through Morisky’s questionnaire and telephone calls.

**Results:** In all 46 patients initially included, 40 were selected including 21 women and 19 men. The mean age was 54.0 ± 5.5 years in the losartan group 1 and 58.05 ± 7.1 years in the amlodipine group2 (P = 0,350). The two groups were substantially comparable at inclusion for the clinical measurement of blood pressure, the biological parameters tested namely lipid profile, blood ionogram, blood glucose and serum uricemia. After intervention, the mean SBP.

112572

Modality: E-Poster Researcher – Non-case Report

Category: CARDIAC ARRHYTHMIAS/ELECTROPHYSIOLOGY/ELECTROCARDIOGRAPHY

## The First Brazilian Cardiovascular Registry of Atrial Fibrillation: The Primary Results of the Recall Study

PEDRO GABRIEL MELO DE BARROS E SILVA^1^, Renato Delascio Lopes^1^, CR. Hoffmann Filho^4^, MA. Cavalvante^5^, C. Miranda^6^, RB. Esper^7^, G. Lima^8^, L. Zimerman^9^, O. De Souza^11^, A. Fagundes^12^, E. Saad^10^, R. Teixeira^12^

(1) Brazilian Clinical Research Institute; (2) IP-HCor; (3) Duke Clinical Research Institute, Durham, United States of America; (4) Hospital Regional Hans Dieter Schmidt, Joinville, Brazil; (5) University of the West of Sao Paulo, Presidente Prudente, Brazil; (6) Hospital Madre Teresa, Belo Horizonte, Brazil; (7) Prevent Senior, Sao Paulo, Brazil; (8) Institute of Cardiology of Rio Grande do Sul, Porto Alegre, Brazil; (9) Hospital de Clínicas de Porto Alegre, Porto Alegre, Brazil; (10) Cardiologia Americas; (11) D’Or Institute for Research and Education, Rio De Janeiro, Brazil; (12) Sociedade Brasileira de Arritmias Cardiacas, Sao Paulo, Brazil

There is limited prospective real-world evidence of patients with atrial fibrillation (AF) in Latin America.

**Methods:** RECALL was the first nationwide prospective study of patients in Brazil with known atrial fibrillation. A total of 4,585 patients were included among 89 sites from April 2012 to August 2019. The follow-up was one year by the protocol. Patient characteristics, medications under use and clinical outcomes during the follow-up were collected.

**Results:** From the total of patients enrolled, 41 were excluded from the analysis since they did not have a confirmed diagnosis of AF. The median age was 70 (61–78) years, 46% were women and the majority of the cases were permanent AF (53,8%). The mean CHA2DS2VASc was 3.2 ± 1.6 and the median HASBLED was 2 [2–3]. The most common risk factor was arterial hypertension (77.9%). The median heart rate was 74 [65–85] and the mean ejection fraction was 52.2 ± 2.6 (%). Only 4.4% of patients had history of previous ablation and 30.4% were using anti-arrhythmic. At baseline, 22.0% did not use anticoagulants and 9.1% did not use any antithrombotic therapy. The most common anticoagulant was vitamin K antagonist (VKA) (62.6%) while the remaining 37.4% were direct oral anticoagulants. The main reasons for not using an oral anticoagulant were physician judgment’s (low risk of stroke – 24.6%) and difficult to control (14.7%) or perform INR (9.9%) while patient preference and adverse event represented respectively 5.3% and 4.1%, respectively. Only 42.5% of the INRs at baseline were between 2 and 3. During follow-up, the use of anticoagulants and INR in the therapeutic range increased to 87.1% and 59.1%, respectively. The use of antithrombotic therapy changed not only during follow-up but it was different also according to the CHADSVASC of the patients. Regarding clinical outcomes in one year, 17.8% of patients were hospitalized due to AF and the rates of death, stroke, systemic embolism and major bleeding were 5.76 [5.12–6.47], 2.77 [2.32–3.32] 1.01 [0.75–1.36], 2.21 [1.81–2.70], respectively. Among VKA users, the rate of mortality and bleeding was higher in the group with time in therapeutic range below 60%.

**Conclusion:** RECALL represents the largest prospective registry of patients with atrial fibrillation in Latin America. Our findings highlight important gaps in the treatment of patients which can inform clinical practice and help to guide future interventions to improve the care of these patients.

112591

Modality: E-Poster Researcher – Non-case Report

Category: CARDIOVASCULAR SURGERY

## Coronary Artery Bypass Grafting Surgery Improves Frailty at 6-Months Follow-Up: An Insight of the Fragile Clinical Trial

OMAR ASRUBAL VILCA MEJIA^1^, Bianca Meneghini^1^, Ligia Cristina Fonseca Hoeflinger^1^, Fabiane Leticia de Freitas^1^, Bruno Mahler Mioto^1^, Luis Augusto Ferreira Lisboa^1^, Luis Alberto de Oliveira Dallan^1^, Alexandre Chiapina Hueb^2^, Mauricio Landulfo Jorge Guerrieri^2^, Rodrigo Coelho Segalote^3^, Felipe Cosentino^3^, Fabio Biscegli Jatene^1^

(1) Instituto do Coração do Hospital das Clínicas da Faculdade de Medicina da Universidade de São Paulo; (2) Hospital das Clinicas Samuel Libanio; (3) Instituto Nacional de Cardiologia do Rio de Janeiro

**Background:** The world is getting older. This change in demographics increases the overall prevalence of coronary artery disease. The growing number of elderlies is not only related to an increase in the number of coronary heart diseases but also frailty, a syndrome that affects at least 10% of the elderly population and is an exceedingly effective parameter for correlating with the adverse effects of aging. Regarding CABG surgery, randomized controlled clinical trials have mainly focused on low-risk, elevated-risk, or high-risk patients, but not on frail patients.

**Purpose:** The aim of this study was to evaluate the impact of coronary artery bypass graft surgery on the frailty index of patients in the 6-month follow-up.

**Methods:** This was a subanalysis of the national, multicenter, randomized, controlled trial called the FRAGILE clinical trial. We analyzed 62 patients aged 60 years or older undergoing coronary artery bypass graft surgery with and without cardiopulmonary bypass in 8 main cardiac surgery centers in Brazil. Fried’s Frailty Criteria were used to classify patients into frail, pre-frail, and non-frail. The parameters evaluated were unintentional weight loss, self-reported fatigue, physical activity level, grip strength, and gait speed. We divided the patients into two groups: the off-pump CABG group (n = 30) and the on-pump CABG group (n = 32). We compared each group for frailty variables before and after surgery using the Mann-Whitney test and Stuart-Maxwell marginal homogeneity using the R software. The study was approved by the local Ethics Committee and all patients signed the informed consent.

**Results:** We observed an improvement in the frailty of patients undergoing CABG. Overall, the number of pre-frail patients doubled in 6 months after surgery (from 11 to 22 patients), and the number of frail patients decreased from 19 to1 patient. Nine patients included in the study no longer had any degree of frailty after 6 months of surgery. No patient was classified as non-frail preoperatively in both groups because it was one of the exclusion criteria of the study. There is no difference between the groups. Regarding the frailty criteria, all tests showed differences in the pre and post-surgical comparison, except the unintentional weight loss variable.

**Conclusion:** CABG played a role in improving frailty at the 6-months follow-up. Both techniques, on-pump and off-pump CABG, showed similar results when comparing frailty features before and after CABG surge.

113890

Modality: E-Poster Researcher – Non-case Report

Category: COVID-19 AND CARDIOVASCULAR SYSTEM

## Apixaban for Prophylaxis of Thromboembolic Outcomes in COVID-19 – the Primary Results of the Apollo Randomized Trial

RENATO DELASCIO LOPES^1^, Pedro Gabriel Melo de Barros e Silva^2^, Ariane Vieira Scarlatelli Macedo^2^, Alexandre B. Cavalcanti^4^, Regis G. Rosa^6^, Otávio Berwanger^7^, Viviane C. Veiga^8^, Luciano C.P. Azevedo^9^, Murillo O. Antunes^10^, Otávio Celso Eluf Gebara^11^, Eduardo Ramacciotti^1^, Alvaro Avezum^12^

(1) Brazilian Clinical Research Institute, Sao Paulo, Brazil; (2) Duke University Medical Center, Durham, NC, USA; (3) Hospital Samaritano Paulista, São Paulo, Brazil; (4) HCOR Research Institute, São Paulo, Brazil; (5) Brazilian Research in Intensive Care Network; (6) Hospital Moinhos de Vento, Porto Alegre; (7) Academic Resaearch Organization (ARO) – Hospital Israelita Albert Einstein; (8) BP–A Beneficência Portuguesa de São Paulo; (9) Hospital Sírio Libanês Research and Education Institute; (10) Hospital Universitário São Francisco de Assis na Providência de Deus and Irmandade do Senhor Bom Jesus dos Passos da Santa Casa de Misericórida de Bragança Paulista, Bragança Paulista; (11) Dasa-Hospital Santa Paula, São Paulo, Brazil; (12) International Research Center, Hospital Alemão Oswaldo Cruz

The efficacy and safety of anticoagulation in the early phase of COVID-19 is unknown.

**Methods:** APOLLO is an academic-led, multicenter, randomized, double-blinded clinical trial that planned to enroll 1000 patients from 30 sites participating in the Coalition COVID-19 Brazil initiative. Eligible patients with a confirmed diagnosis of COVID-19 with onset symptoms up to 10 days at increased risk for thrombotic complications were randomized to apixaban (2.5 mg twice daily) or placebo for 30 days. The primary outcome was the number of days alive and out of the hospital at 30 days. Secondary outcomes included hospitalization for bleeding (key safety outcome), hospitalization for cardiovascular/pulmonary causes (key secondary efficacy outcome).

**Results:** The trial was stopped prematurely due to slower than expected recruitment and low overall event rates. Among 411 (41% of the initial sample size) patients randomized (207 in apixaban and 204 in the placebo group), the mean (+/– SD) age was 44.0 years (±14.0), 58.4% were women, three patients from the apixaban group withdrew the informed consent, and all the remaining 408 patients completed the 30-day follow-up. The mean time (+/– SD) from symptom onset to randomization was 6.2 (2.4) days. There was no significant difference in the number of days alive and out of the hospital in patients assigned to apixaban and placebo (29.6 ± 1.6 and 29.5 ± 2.4 days, respectively; mean difference 0.18, 95% confidence interval [CI] –0.22 to 0.58; P = 0.38). Among patients assigned to apixaban versus placebo, there was also no significant difference in secondary endpoints including all-cause hospitalization (4.9% vs. 6.9%; odds ratio [OR] 0.70, 95% CI 0.30–1.61), and hospitalization for cardiovascular/pulmonary causes (4.4% vs. 6.9%; odds ratio [OR] 0.63, 95% CI 0.26–1.48). There was no hospitalization due to bleeding and no arterial and venous thrombosis in either study groups. One patient died (in the placebo group) due to a non-cardiovascular cause.

**Conclusions:** In symptomatic, but clinically stable outpatients with COVID-19 and additional risk factors for thrombotic complication, treatment with apixaban 2.5 mg twice daily compared with placebo did not improve the number of days alive and out of the hospital. No major bleeding was observed in the trial population. While the power is limited due to not reaching the intended sample size, our results do not support the routine use of low dose apixaban for outpatients COVID-19.

113909

Modality: E-Poster Researcher – Non-case Report

Category: CARDIOVASCULAR PHARMACOLOGY

## Risk of New-Onset Stroke in Patients with Type 2 Diabetes on Sodium-Glucose Co-Transporter-2 Inhibitor User: A Population-Based Cohort Study

GWO-PING JONG^1^

(1) Chung Shan Medical University Hospital; (2) Chung Shan Medical University; (3) Taoyuan Armed Forces General Hospital; (4) National Defense Medical Center; (5) Central Taiwan University of Science and Technology; (6) Taichung Armed Forces General Hospital

**Background:** Epidemiological evidence suggests the association of diabetes with an increased risk of stroke. Clinical studies have investigated the effects of sodium-glucose co-transporter-2 (SGLT2) inhibitors on new-onset stroke (NOS), but the results are inconsistent.

**Objectives:** To determine the association between the use of SGLT2 inhibitors and NOS in patients with type 2 diabetes mellitus (DM).

**Methods:** We conducted a nationwide retrospective cohort study based on the Taiwan Health Insurance Review and Assessment Service database (2016–2019). The primary outcome of the assessment was the risk of incident stroke by estimating hazard ratios (HRs) and 95% confidence intervals (CIs). Multiple Cox regression was applied to estimate the adjusted HR of NOS. Subgroup analysis was also conducted.

**Results:** Among the 232,101 eligible patients with type 2 DM aged ≥20 years, SGLT2-inhibitor users were compared with non-SGLT2-inhibitor users based on age, sex, and the duration of type 2 DM matching at a ratio of 1:2. The event rate per 10 000 person-months was 9.20 (95% CI 8.95 to 9.45) for SGLT2-inhibitor users and 10.5(10.3–10.6) for non-SGLT2-inhibitor users. There was a decreased risk of NOS for SGLT2-inhibitor users (adjusted HR 0.85, 95% CI 0.82–0.88) compared with non-SGLT2-inhibitor users. Results for the propensity score-matched analyses showed similar results (adjusted HR 0.87, 95% CI 0.84–0.91 for both SGLT2-inhibitor users and non-SGLT2-inhibitor users).

**Conclusion:** The risk of developing NOS was lower in patients with SGLT2-inhibitor users than in non-SGLT2-inhibitor users. The decreased risk of NOS in patients with type 2 DM was greater among patients with concurrent use of statins, biguanides, thiazolidinediones, and glucagon-like peptide-1 receptor agonists. We, therefore, suggest that the long-term use of SGLT2 inhibitors may help reduce the incidence of NOS in patients with type 2 DM.

107880

Modality: E-Poster Researcher – Non-case Report

Category: EPIDEMIOLOGY AND HEALTH POLICIES/GLOBAL HEALTH

## Mortality in a 3-Year Follow-Up of Congestive Heart Failure Due to Chagas Disease in Women and Men

ANTONIO DE PADUA MANSUR^1^, Carlos Henrique Del Carlo^1^, José Antonio Ramos Neto^1^, André Barbosa de Abreu^1^, Airton Roberto Scipioni^1^, Antonio Carlos Pereira Barreto^1^

(1) Insituto do Coração – HC FMUSP

**Background:** Chronic Chagas cardiomyopathy (CCC) is one of the leading causes of congestive heart failure (CHF) in Latin America and carries a high morbidity and mortality burden. Previously, it was believed that there was no epidemiological and clinical evidence of a gender-associated risk of death in patients with CCC.

**Purpose:** To analyze the mortality of congestive heart failure due to CCC in women and men.

**Methods:** From February 2017 to September 2020, we followed a cohort of patients with CHF (Framingham criteria) due to CCC in a single-center outpatient clinic. Appropriate serologic tests defined Chagas disease. Baseline data included clinical characteristics and echocardiographic findings. Statistical analyses were performed with the Kaplan-Meier (K-M) method to analyze time-to-event data and the Cox proportional hazards methods to search for predictors of death.

**Results:** We studied 733 patients, mean of 61.4 ± 12.3 years, 381 (52%) males. Females were older (63.0 ± 11.9 vs. 60 ± 12.4 years; p = 0.01), had a higher baseline mean left ventricular ejection fraction (LVEF) (44.5 ± 14.6% vs. 37.3 ± 14.8%; p < 0.001), and a lower left ventricular diastolic diameter (LVDD) (56.7 ± 8.9 vs. 62.4 ± 9.4 mm; p < 0.001). Over a 3-years follow-up period, 168 (44%) men and 126 (36%) women died (K-M: log-rank p = 0.002; Figure). Women had more implantable pacemakers (PM) (26.1% vs. 16.5%; p = 0.002) and men more implantable cardioverter-defibrillators (ICDs)(20.7% vs. 12.5%; p = 0.003). Heart transplant occurred in 10.8% of men and 7.4% in women (p = NS). Cox regression for death adjusted for age, previous myocardial infarction, diabetes, previous stroke, chronic kidney disease (CKD), atrial fibrillation, PM, ICD, heart transplant and LVEF, showed, in descending order, previous stroke (HR = 2.4; 95%CL:1.5–3.6), diabetes (HR = 2.0; 95%CL: 1.3–3.1), and CKD (HR = 1.8; 95%CL:1.3–2.6) as the main predictors of death in men, and in women diabetes (HR = 2.2; 95%CL:1.4–3.4), previous stroke (HR = 1.8; 95%CL:1.1–2.9), and CKD (HR = 1.7; 95%CL:1.1–2.7).

**Conclusions:** Women had a better prognosis than men but similar predictors of death. Control of diabetes and prevention of stroke and CKD could significantly reduce the death rate in CHF due to CCC.

107731

Modality: E-Poster Researcher – Case Report

Category: HEMODYNAMICS AND INTERVENTIONAL CARDIOLOGY

## Two Unusual Treatment of Type III Coronary Perforation with Manufacture Coated Stents According to the Reality of a Public Hospital in Brazil. Report of Two Cases

FREDERICO LOPES DE OLIVEIRA^1^, Giulliano Gardenghi^2^, Adriano Gonçalves de Araújo^1^, Maurício Lopes Prudente^2^, Flávio Passos Barbosa^1^

(1) Hospital de Urgências Governador Otávio Lage; (2) Hospital Encore; (3) Hospital Samaritano de Goiânia

**Introduction:** Coronary perforation is a potentially fatal complication during percutaneous coronary intervention. Although rare (0.2 to 0.6% of procedures), it is life-threatening, which justifies its immediate diagnosis and correction.

**Objective:** We report two cases of coronary perforation and alternative management of current practice. Case reports: CTO 78 years old, admitted on 04/16/2020 and WlSO, 61 years old on 01/12/22, men with previous AMI of more than 12 hours of evolution (CTO with 36 hours and WISO with 18 hours), still in typica pain refractory to intravenous nitrate, dual product control and potent analgesia, thus opting for invasive stratification in both cases. After anterior descending recanalization, a type III rupture with the presence of pericardial contrast was observed. Heparin action was immediately reversed, the balloon was inflated with low pressure and the echocardiography and cardiac surgery teams were contacted. Manufactured coated stents were made (CTO with balloon catheter segment and WISO with a sterile adhesive skin dressing) successfully correcting its perforations.

**Discussion:** In coronary perforation, reversal of anticoagulation and occlusion of the bleeding orifice are essential to prevent tamponade until definitive treatment (lately only direct suture of the vessel by open thoracotomy). However, with the evolution of materials and techniques, it has been proven that ballooning at low pressures and protamine infusion have high success rates in punctual occlusions. Circulatory assist devices and decompressive pericardiocentesis (Marfan puncture) are still needed, increasing costs, morbidity and mortality. In case of failure, polymerized mesh stents (Graft stent) are able to “seal” the leak to pericardial sack, however, their value is high and their numbering is restricted. Thus, alternatives manufactured stents with venous grafts, parts of coronary balloon catheters, sterile skin patches (such as Tegaderm®) or even intentional infusion/embolization of autologous fat are good examples of low cost. Other techniques that are also costly, such as the use of microcatheters for infusion of “coils” and the implantation of successive smaller-caliber stents (decreasing the lateral holes), are also used.

**Conclusion:** In the cases reported here, the procedures presented were able to contain the bleeding. We understand that disseminating this knowledge, we can contribute with other colleagues in solving this complication.

107875

Modality: E-Poster Researcher – Case Report

Category: CARDIO-ONCOLOGY

## Pediatric Cardiac Myxoma in Carney Syndrome- Case Report

NATHALIE JEANNE MAGIOLI BRAVO-VALENZUELA^1^, Eliane Lucas^4^, Juliane R Sousa^2^, Fernanda C Lemos^3^, Patrícia S Correia^2^

(1) Curso de Pós-Graduação em Cardio- Oncologia da Sociedade Brasileira de Cardiologia, Instituto Nacional de Cardiologia e Instituto Nacional do Câncer, Rio de Janeiro, RJ, Brasil; (2) Hospital Federal de Bonsucesso (HFB), Rio de Janeiro-RJ, Brazil; (3) Federal University of Rio de Janeiro, Rio de Janeiro-RJ, Brazil; (4) Faculdade de Medicina do Centro Educacional Serra dos Órgãos (UNIFESO), Teresópolis-RJ, Brazil

**Introduction:** Cardiac myxomas are the most common primary cardiac tumors in adults but rare in children. However, in Carney syndrome, myxomas develop at an earlier age and tend to recur more frequently. SC is considered a familial multiple endocrine neoplasia (such as primary adrenal nodular disease, cardiac and cutaneous myxoma, testicular neoplasia) that results from PRKAR1A gene mutations.

**Case report:** We present the case of a 14-year-old male patient who was admitted to our hospital for exercise-induced fatigue, acute chest pain, and mental confusion. His mother reported a history of high blood pressure since the age of 5 that worsened in the last 2 months. His maternal family history was positive for atrial myxoma. A physical examination revealed skin hyperpigmentation, obesity, and hirsutism. Chest radiographic and electrocardiographic findings were normal. Fundoscopy revealed grade II hypertensive retinopathy. Elevated serum ACTH levels were consistent with primary hypercortisolism. The transthoracic echocardiography showed the image of a single, hyperechoic, and irregular mass located in the left atrium (Figure 1). The mass was voluminous, pedunculated, and adhered to the anterior leaflet of the mitral valve, causing obstruction of the left ventricular inlet blood flow. Surgical resection of the tumor was performed, and the histopathological features confirmed the diagnosis of myxoma. Screening for Carney complex was performed. Testicular ultrasonography and abdominal tomography revealed testicular microcalcifications and adrenal tumors, respectively. The patient underwent an uneventful adrenalectomy and was discharged from the hospital on corticosteroid therapy. PRKAR1A gene sequencing showed heterozygosity for the nonsense variant R228X in the patient and his mother.

**Conclusion:** Atrial myxoma justifies surgical resection due to the risk of cardiovascular embolization, complications, and sudden death. Pediatric cardiac myxomas should raise the suspicion of a Carney complex. The early diagnosis of this syndrome contributes to the optimization of the treatment of these patients and can have a positive impact on their management and prognosis.

107905

Modality: E-Poster Researcher – Case Report

Category: HEART FAILURE/CARDIOMYOPATHY/TRANSPLANT

## Focal Myocarditis Simulating ST-Segment Elevation Myocardial Infarction in Patients with Berger’s Disease

ANTONIO TANAJURA GOMES NETO^1^, Bruna Aparecida Souza Machado^1^

(1) Clínica Médica Santa Helena – CMSH

**Introduction:** The clinical presentation of myocarditis is variable and often mimics myocardial infarction. The diagnosis of acute myocarditis is frequently empirical and is made based on clinical presentation, electrocardiographic alterations, elevated cardiac enzymes, and theabsence of epicardial coronary artery disease. We report the case of a 46-year-old patient with a history of IgA nephropathy (NIgA) admitted for atypical acute precordial pain associated with ST-segment elevation in the inferiorwall lead.

**Description:** A 46-year-old man with a history of NIgA, SAH, PLD, obesity (BMI 34) and sedentary, using anlodipino/losartan (2.5 mg/50 mg daily), succinato de metoprolol (50 mg daily) and simvastatin (20 mg daily). He was a non smoking and had no family history of coronary heart disease. The patient complained of chest pain into his right hemithorax over the 2 days, stabbing, intermittent, of moderate intensity that traveled into his sternum and right shoulder. The patient looked anxious and high blood pressure (200 × 100 mmHg). His vital signs were stable. Performed ECG and measurement of myocardial necrosis markers (MNM). He was classified as MNM and after improvement he was discharged in a reasonably good condition. ECG: showed ST-segment elevation in the inferior wall. Lab: Hb/Ht: 16/54 U: 35 Cr: 1.39 CKMB: 12 U/L (VR <5 U/L) Troponin: 13 ng/ml (VR: Between 0 and 0.5 ng/ml) Proteinuria 4,488.96 mg/24h Urine volume 1,120 ml Negative thrombophilia investigations. Echo TT: Preserved global and segmental LV systolic function, LV concentric remodeling, and type I diastolic dysfunction. Cate: Myocardial bridge with mild systolic constriction. Cardiovascular magnetic resonance (CMR): Presence of late mesocardial enhancement (non-ischemic pattern) in the inferior basal and inferolateral segments of the left ventricle. ID: Myocarditis.

**Conclusion:** When we came across such a case, some questions were raised, suchas the attempt to correlate myocarditis with the nephrotic picture. The reported case and publications raised bring to light the discussionon the importance of CMR in the diagnosis of acute myocarditis.


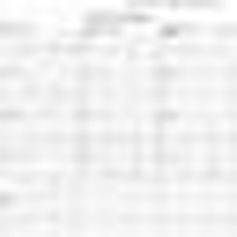


108022

Modality: E-Poster Researcher – Case Report

Category: COVID-19 AND CARDIOVASCULAR SYSTEM

## COVID-19 Vaccine-Induced Myocarditis: Case Report

VICTOR RODRIGUES RIBEIRO FERREIRA^1^, Amanda Pupim Assunção Toledo^1^, Maria Fernanda Aguilar de Azevedo^2^, Maria Christiane Valéria Braga Braile Sternieri^1^

(1) Braile Cardio; (2) Beneficência Portuguesa de São José do Rio Preto/SP

**Introduction:** The global race for a safe and effective vaccine against SARS-CoV-2 (COVID-19) has challenged scientists around the world. The mRNA-based vaccine was quickly linked to cases of myocarditis. Thus, doubts arose about the evolution of the cases and the therapeutic approach. A descriptive study recently published highlighted 1,626 reports myocarditis among the 192,405,448 individuals vaccinated with mRNA based vaccine and registered in the Vaccine Adverse Event Reporting System.

**Case Description:** SKP, 35 years old, healthy, immunized with a booster dose of the BNT162b2 SARS-CoV2 mRNA vaccine and, after 5 days of administration, developed dyspnea on exertion. Laboratory tests rule out acute COVID-19 infection and elevated NTproBNP (2161 pg/ml) indicated acute heart failure. Echocardiography revealed left ventricular (LV) dilatation, reduction in LE ejection fraction (LVEF = 32%), moderate mitral regurgitation and pulmonary hypertension. The Speckle Tracking study revealed reduced strain rate (–9.6%). Cardiac magnetic resonance imaging confirmed myocarditis, revealing mesocardial late enhancement in several segments, with reduced systolic function and mild pericardial effusion. The standardized treatment was optimized doses of Bisoprolol (10 mg/day), Sacubitril/Valsartan (400 mg/day) and Spironolactone (25 mg/day). There was complete remission of symptoms after 5 weeks of treatment. After 14 weeks, a new echocardiographic study revealed normalization of all previously detected alterations.

**Conclusions:** Writing the history of a disease as it unfolds before our eyes is a challenge for contemporary medicine. Findings are built on everyday experiences and case reports have proved to be valuable in the last two years. Vaccine-induced myocarditis still lacks studies regarding its epidemiology and pathophysiology. But we must stick to reality: vaccination remains the most effective means of combating SARS-CoV2. The data are categorical in affirming the benefits of vaccination for public health and the damage related to this pharmaceutical agent is much lower than the damage caused by viral infection in non-immunized peop.


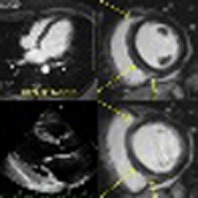


108531

Modality: E-Poster Researcher – Case Report

Category: CARDIO-ONCOLOGY

## Rare Etiology of Heart Failure: Primary Cardiac Angiosarcoma

ELIAS ANTONIO YUNES^2^, Andre Luiz Marins Vera Cruz Porto^1^, Erika Ferreira de Moura Porto^1^, Carlos Eduardo Cordeiro Soares^1^, Wolney de Andrade Martins^2^

(1) Dr. Beda General Hospital, IMNE group, Campos dos Goytacazes, Rio de Janeiro State, Brazil; (2) Postgraduate Course in Cardiology SBC/INC/INCA, Rio de Janeiro, Rio de Janeiro State, Brazil

**Introduction:** Among neoplastic tumors, secondary implants are 132 times more common than primary cardiac tumors (PCT), which have an incidence of 1.38 cases/100,000. More than 90% of TCPs are benign and only 5% to 6% are malignant.

**Case Description:** Female patient, 24 years old, previously healthy, started progressive dyspnea on exertion 30 days ago. She presented dyspnea with mild exertion, paroxysmal nocturnal dyspnea and lower extremity edema, in addition to intermittent fever spikes. Echocardiogram showed left ventricle with normal dimensions and function, ejection fraction = 69%, large tumor mass occupying almost the entire right atrium (RA). Following the investigation, MRI was requested, which showed an extensive mass with invasion of the RA roof, fixed, heterogeneous, irregular, filling almost the entire cavity, extending to the hepatic and superior vena cava, measuring 106 × 100 × 76 mm. A CT-guided biopsy was performed and the histopathological report indicated partially necrotic tissue fragments with proliferation of pleomorphic epithelioid cells forming slits and vascular channels, which associated with positive immunohistochemistry for CD31 and CD34, led to the diagnosis of angiosarcoma. The tumor was considered unresectable and the proposed conduct was chemotherapy with paclitaxel alone for 21/21 days. Staging with chest, abdomen and pelvis CT also showed bone metastasis in the thoracolumbar spine and hip, in addition to mediastinal invasion. After 2 months, she presented a spontaneous femur fracture. She underwent 7 cycles of chemotherapy and died 10 months after the onset of symptoms due to pulmonary sepsis.

**Conclusion:** This report presents a rare case of malignant PCT in a patient with unusual sex and age group, initial clinical presentation with heart failure, extremely aggressive and with a poor prognosis. The increase in PCT diagnoses is attributed to the advances in imaging technology and the use of multimodality. In malignant PCT, sarcomas represent 64.8%, of which 2/3 are angiosarcomas. It most often affects male patients in the fourth decade of life, and in 75% the RA. They can have important hemodynamic and arrhythmic consequences, depending on their size and location. Despite advances in diagnostic methods and cancer treatment, in case of these tumors there are serious limitations. The median survival at 12 months is 45%, but in cases without resection, it drops to 3.8 months.

108362

Modality: E-Poster Researcher – Case Report

Category: HEMODYNAMICS AND INTERVENTIONAL CARDIOLOGY

## Approach to Kounis Syndrome in Coronary Cineangiography

FERNANDA CRISTINA CASTANHO^1^, Claudia Catelan Carlomagno^2^, Glaucia de Farias Correia^2^

(1) Hospital Regional de Sorocaba “Dr. Adib Domingos Jatene”; (2) Curso Intensivo de Cardiologia Clínica

**Introduction:** Kounis syndrome is characterized by the coexistence of cardiac signs and symptoms with allergic clinical manifestations. The pathophysiological mechanism involved is believed to include mast cell activation with release of inflammatory mediators that induce coronary vasospasm and/or rupture of the atheromatous plaque. Among the molecules involved, several cytokines and chemokines, histamine, arachidonic acid products, platelet activating factor, neutral proteases, tryptase and cathepsin-D can be identified. Cardiac clinical manifestations differ depending on the syndrome subtype: type 1 results from coronary spasm in normal coronary arteries; type 2, by spasm or rupture of plaque, in coronary arteries with previous atherosclerosis; type 3, the hypersensitivity reaction leads to previously implanted drug-eluting stent thrombosis.

**Case report:** LFS, 67 years old, male, hypertensive, after contrast injection during coronary angiography, evolved with dyspnea, chest pain, hypertension, skin rash and diffuse coronary spasm. Intracoronary injection of nitroglycerin and intravenous injection of hydrocortisone and diphenhydramine were performed with clinical improvement and resolution of pain. Control coronary angiography demonstrating resolution of the spasm without fixed strictures. After clinical stabilization and initiation of drug therapy with diltiazem, he was discharged asymptomatic and without skin changes.

**Discussion/Conclusion:** Among several proposed mechanisms of immediate hypersensitivity to iodinated contrast, the following stand out: direct effect on the membrane related to osmolarity or chemical structure of the iodinated contrast medium molecule; complement system activation; direct bradykinin formation and IgE-mediated mechanism. In the face of sudden chest pain associated with symptoms of allergy or anaphylaxis, the possibility of Kounis syndrome should always be considered. The approach to the patient must be directed to the coronary event and the allergic reaction that induces it. Vasospasm is the primary mechanism, nitrates and calcium channel blockers should be considered first-line therapy. Corticosteroids play an important role in the treatment of allergic reactions and adrenaline is the mainstay of anaphylaxis treatment, however, it can worsen ischemia. Kounis Syndrome is not uncommon, but it is underdiagnosed and there are no specific clinical guidelines for treatment.

108516

Modality: E-Poster Researcher – Case Report

Category: CARDIAC ARRHYTHMIAS/ELECTROPHYSIOLOGY/ELECTROCARDIOGRAPHY

## R Over T Phenomenon During Vagal Maneuver

THIAGO ALEXANDRE FERREIRA PIRES^1^, LUDMILLA DA ROCHA FREITAS VIEITAS^1^, HUGO ANDRADE SANTOS^1^, BRUNO SANTANA BANDEIRA^1^

(1) HOSPITAL CAXIAS D’OR

**Introduction:** Supraventricular tachyarrhythmias are frequent pathologies in the emergency department and their approach must be systematized and prepared for possible complications. Degeneration of ventricular rhythms is uncommon but potentially serious.

**Case description:** Young patient, without comorbidities, comes to the emergency room with tachycardic palpitations for 30 minutes. Physical examination showed no changes, BP 120 × 80 mmHg. ECG showing regular, narrow QRS tachycardia with a heart rate of approximately 250 bpm. Corrected normal QT interval. A modified Valsalva maneuver was performed, evolving with a brief period of wide QRS polymorphic tachycardia, with subsequent reversion to sinus rhythm with normal QT interval. Normal laboratory tests.

**Conclusions:** Polymorphic ventricular tachycardia (PVT) is rare, with various causes classified according to the QT interval. In the case of prolonged QT, we have the Torsade of bridges. In cases where the QT is normal, a ventricular extrasystole (VES) with a short coupling interval, which is not pause-dependent, causes PVT. In the case in question, the patient had a narrow QRS tachyarrhythmia, suggestive of tachycardia due to nodal reentry, with hemodynamic stability, and the vagal maneuver was chosen as the initial approach. Evolved with PVT with spontaneous reversion to sinus rhythm. PVT started from a ventricular extrasystole that appeared at the end of the T wave, giving rise to the R over T phenomenon. This is an event that can occur in the VES, when it appears in the T wave of the anterior QRS, generating a conduction with a high probability of developing ventricular tachycardia/fibrillation belonging to grade 5 of the Lown classification. This is an unusual event that can occur during an attempt to reverse arrhythmias.


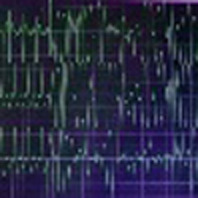


108522

Modality: E-Poster Researcher – Case Report

Category: CARDIOVASCULAR SURGERY

## Bacterial Endocarditis Due to Coronary Fistula: Case Report

LARISSA NETO ESPINDOLA^1^, Christian Martins Macedo^2^, Beatriz Sant‘ana Isaías Rodrigues^1^, Elise Sant‘ana Isaías^1^, Paula Oliveira de Andrade Lopes^1^

(1) Curso Intensivo de Revisão em Cardiologia Clínica; (2) Hospital Santa Izabel

**Introduction:** Coronary fistulas are rare conditions (0.002% of the population), comprising 48.7% of all coronary anomalies. The pathophysiology and clinical presentation depend on the magnitude of the flow through the fistula and its location. They can cause angina (3–7%), dyspnea (60%), endocarditis (20%, usually associated with Streptococcus viridans), syncope, palpitations, congestive heart failure (HF –19%) and cardiac arrhythmias. They can lead to aneurysmal dilatation of the involved coronary artery.

**Case report:** A 46-year-old male patient suffered trauma to the elbow in May/2018, with local phlogosis and recurrent episodes of fever. In September/2018, he had atypical chest pain and increased fever frequency. He sought medical attention and, after auscultation of a heart murmur, a transthoracic echocardiogram (ECHO) was performed, which showed left ventricular dysfunction and severe aortic insufficiency. Therapeutic treatment for HF was initiated, it evolved with significant decompensation, requiring hospitalization. He was discharged on a surgical schedule for valve replacement. He was referred for preoperative cardiac catheterization, which revealed a circumflex artery (CX) aneurysm with a fistula to the right atrium (RA). Transesophageal ECHO showed significant aortic insufficiency secondary to endocarditis and CX fistula to the superior vena cava (SVC). Coronary computed tomography angiography confirmed atrial branch of the CX with fistula to RAand SVC. Negative blood cultures. Patient used Gentamicin + Ceftriaxone. He underwent aortic valve replacement (biological) and ligation of the fistula drainage ostium in the SVC and the proximal ostium next to the CX. Blood transfusion and vasoactive amines was performed in the immediate postoperative period.

**Conclusion:** Coronary angiography is the main diagnostic method. In some cases the origin and relationship of the fistula to adjacent structures can be difficult to determine and non-invasive techniques can be used. There is general agreement that symptomatic patients should be treated. Closure of fistulas in the proximal segment of the coronary vessel is highly recommended due to the greater propensity for aneurysm and rupture. The prognosis of treated patients is excellent, although it depends on the severity of the shunt and on complications such as HF, pulmonary hypertension and bacterial endocarditis. Recurrence rates after surgical treatment are around 25%. Long-term follow-up is essential due to the possibilit.

108530

Modality: E-Poster Researcher – Case Report

Category: CARDIOVASCULAR INTENSIVE CARE/CARDIOVASCULAR EMERGENCIES

## Native Aortic Valve Infective Endocarditis with Interventricular Septum Dissection: A Case Report

LARISSA NETO ESPINDOLA^1^, Christian Martins Macedo^2^, Elise Sant‘ana Isaías^1^, Beatriz Sant‘ana Isaías Rodrigues^1^

(1) Curso Intensivo de Revisão em Cardiologia Clínica; (2) Hospital Santa Izabel

**Introduction:** The global impact of the disease due to infective endocarditis (IE) is practically unknown. IE is a heterogeneous syndrome whose incidence is influenced by multiple factors of the patient himself that determine the risk of infection, including anatomical conditions, injection drug use, in addition to social factors.

**Case Description:** Male patient, 17 years old, using an orthodontic appliance for 1 year, sought the Emergency Unit due to asthenia with 9 months of evolution. Bradycardial, electrocardiogram showing total atrioventricular block (TAVB) and transthoracic echocardiogram (TTE) with signs of right coronary sinus rupture and interventricular septum dissection (IVS). Transesophageal Echo also showed bidirectional flow in the neocavity that formed within the IVS with the presence of perfusion (use of echocardiographic contrast). Vegetation was not seen. Physical examination without peripheral IE stigmata. Antibiotic therapy was started for IE, although blood cultures were negative (patient with dental abscess and visit to a “practical” dentist for braces implantation). Surgical treatment was chosen to close the IVS and excision of possible infected material. During the procedure, an aspect suggestive of a chronic process was observed, with a cavity within the IVS already endothelialized. Opted to occlude entry orifice and reapproximate IVS walls. Definitive pacemaker was implanted (patient on TAVB and with extensive septal lesion). Patient kept under antibiotic therapy for 28 days, when he was discharged in good condition and asymptomatic, after a new TTE confirmed the absence of flow in the topography of the neocavity observed in the preoperative examination.

**Conclusion:** The clinical presentation of IE encompasses a wide spectrum of symptoms, being influenced by multiple factors (pathogen virulence, persistence of bacteremia, extent of tissue destruction (valvular and perivalvular), hemodynamic sequelae, septic embolization, consequences of circulating immune complexes. Due to the close proximity between the atrioventricular node and the proximal intraventricular conduction system and the aortic valve and root, perivalvular extension of infection in this valve is the most common cause of new atrioventricular block of any degree. driving is also a multivariate risk predictor of death associated with IE.

109857

Modality: E-Poster Researcher – Case Report

Category: CARDIOVASCULAR IMAGING

## Huge Atrial Myxoma in an Asymptomatic Patient

JULYANA GALVAO TABOSA DO EGITO^1^, 2. Rafael de Almeida Torres^1^, 3. Manoela Falsoni^1^, 4. Remulo José Rauen Jr^1^, 5. Guilherme Barreto Gameiro Silva^1^

(1) Hospital Santa Cruz – Curitiba –PR- Brazil

Primary cardiac tumors are rare, and about 75% are benign. Myxoma is the most frequent primary cardiac tumor and its most common location is in the left atrium (LA) (75%). Approximately 70% of patients with LA myxoma have cardiac symptoms. The most frequent symptom is dyspnea (70%). The size, location and mobility of the myxoma will determine the symptomatology and severity of valve obstruction, 12% of myxomas are completely asymptomatic. Although cardiac myxomas are histologically benign, they may be lethal because of their strategic position. Therefore surgical removal should be performed as soon as possible. Case report: Female patient, 53 years old, previously dyslipidemic and with a positive family history of coronary artery disease. She was asymptomatic when she underwent a chest tomography due to the diagnosis of covid-19. The exam resulted in increase in the dimensions of the LA and a voluminous hypodense formation. Physical examination showed telediastolic murmur +/4+. A transesophageal echocardiogram described: Echogenic mass occupying the entire atrial cavity measuring 62 × 46.2, suggestive of myxoma. Preoperative coronary artery CT, showed the size and irrigation of the Myxoma (figure 1). The patient underwent removal of the myxoma without intercurrences. The presence of a murmur in a young and previously healthy patient should not be neglected and it is mandatory to complement it with imaging tests for diagnostic elucidation.


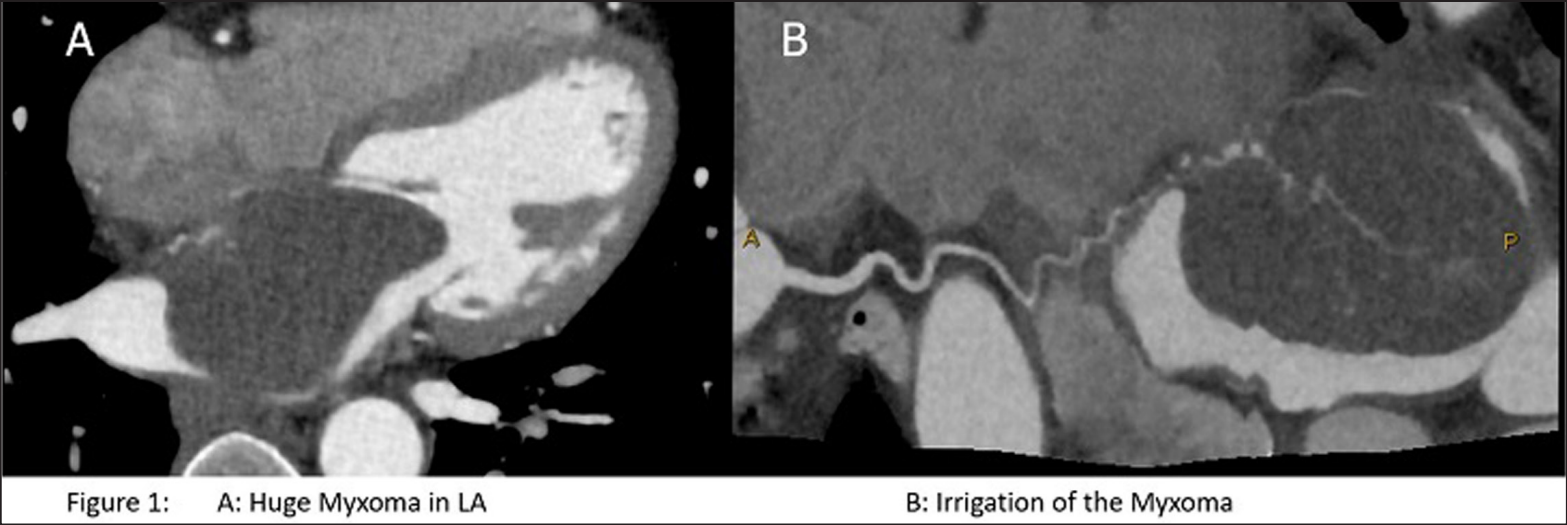


108647

Modality: E-Poster Researcher – Case Report

Category: HEMODYNAMICS AND INTERVENTIONAL CARDIOLOGY

## Subacute Valve Thrombosis After Transcatheter Aortic Valve Replacement in a 86-Year-Old Man with SARS-CoV-2 Infection Successfully Treated with Rivaroxaban

JOAO LUIZ DE ALENCAR ARARIPE FALCAO^1^, Lara Lins Afio Ponte^2^, Breno de Alencar Araripe Falcao^3^, Sandra Nivea dos Reis Saraiva Falcao^1^, Ana Carolina Pereira Nogueira de Alencar Araripe Falcao^4^

(1) Universidade Federal do Ceará; (2) Universidade de Fortaleza; (3) Hospital de Messejana Dr. Carlos Alberto Studart Gomes; (4) Faculdade Christus

**Background:** Transcatheter aortic valve replacement (TAVR) is indicated for treating symptomatic severe aortic valve stenosis (AS) with intermediate-to-high surgical risks. Few reports are available on managing leaflet thrombosis after TAVR with worsening heart failure.

**Case Summary:** A previously fully vaccinated for SARS-CoV-2 infection 86-year-old man with severe AS received a successful TAVR with Edwards Sapien 3 valve. It was prescribed aspirin 100 mg at hospital discharge. Three weeks later, patient developed flu-like symptoms and fatigue. An RT-PCR test diagnosed SARS-CoV-2 infection. D-dimer test was elevated. Serum creatinine increased to 1.9 mg/dl. A new transthoracic echocardiogram showed a subacute aortic prothesis disfunction with mean transaortic pressure gradient of 50 mmHg. Rivaroxaban 20 mg substituted single antiplatelet therapy to treat presumed subacute leaflet thrombosis. After 45 days of anticoagulation a follow-up echocardiogram showed a decrease in mean transaortic valve pressure gradient to 8 mmHg. Symptoms of heart failure has improved gradually. It was planned to mantain anticoagulation for 3 months.

**Conclusion:** A worsening heart failure or an increase in transaortic valve pressure gradient, especially in the early months after TAVR, suggest subacute leaflet thrombosis. SARS-CoV-2 infection may have triggered this thrombotic event. Subacute thrombosis after TAVR can be resolved after using NOAC as in our present case.

108847

Modality: E-Poster Researcher – Case Report

Category: CARDIOVASCULAR IMAGING

## Dysfunction of Metallic Prosthetic Valve in Mitral Position by Native Posterior Leaflet

POLYANA EVANGELISTA LIMA^1^, José Verissimo dos Santos Neto^1^, Fabio Antonio Amando Granja^1^, Matheus Pereira Barreira^1^, Alane Mota dos Santos – Santos, AM^1^

(1) Universidade Federal do Vale do São Francisco

**Introduction:** Infective endocarditis (IE) is a serious pathology with a high mortality rate. Early diagnosis is essential, since delayed or inadequate treatment can lead to serious complications.

**Case Report:** A 47-year-old patient with mitral valve prolapse evolving to severe mitral valve insufficiency underwent valve replacement surgery with a metallic prosthesis. Five months later, he presented with progressive dyspnea NYHA III/IV for 1 month associated with fever peak. Transesophageal echocardiogram was performed, showing mobile mass in the ventricular surface, measuring 15 × 18 mm, and dysfunction of the metallic prosthesis with stenosis. He underwent cardiac surgery that revealed maintenance of the posterior cuspid of the mitral valve, inadvertently preserved along with the subvalvar apparatus that obstructed the prosthesis, locking the posterior hemi-disc. Usually, the vegetation of IE is positioned upstream of the valve; in this case, it would be positioned on the atrial side of the mitral prosthesis. Following the new guidelines, other imaging modalities, such as positron emission tomography with 18F-fluorodeoxyglucose and single photon emission computed tomography have gained importance in defining IE.

**Conclusion:** We report one of the presentation forms of early dysfunction of a metallic prosthesis in mitral position. This is a case that would possibly have benefitted from the use of new imaging modalities for diagnostic elucidation.


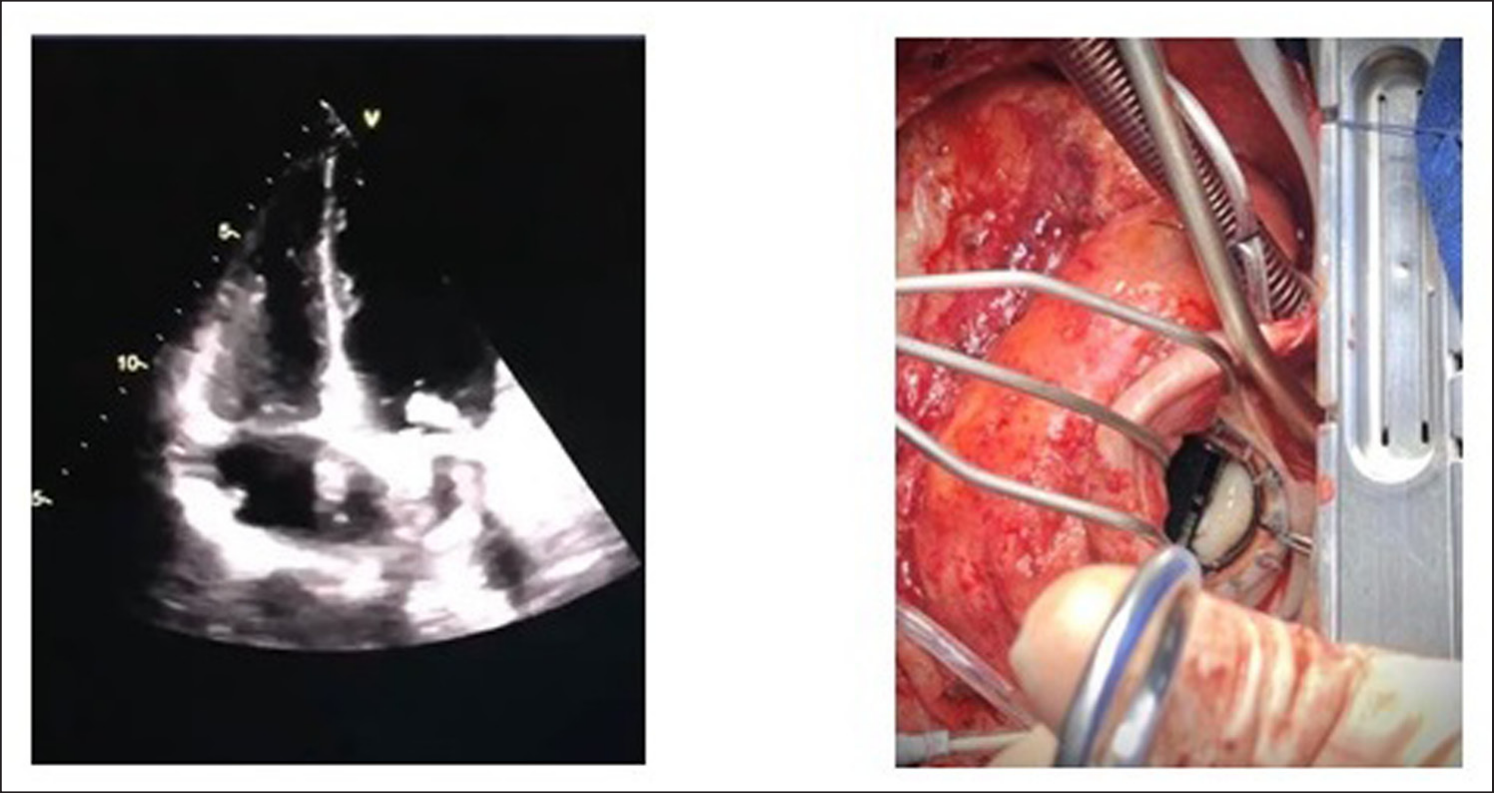


108883

Modality: E-Poster Researcher – Case Report

Category: CONGENITAL AND PEDIATRIC CARDIOLOGY

## Unilateral Lung Whiteout in a Infant: A Rare Case of Isolated Unilateral Absence of Pulmonary Artery

MARÍLIA PEREIRA^2^, Lívia Camarinha Franco de Menezes^2^, Flávia Gurgel^3^, Anna Esther Araujo e Silva^2^, Ana Flávia Malheiros Torbey^1^

(1) Universidade Federal Fluminense; (2) Hospital Universitário Antônio Pedro; (3) Hospital Getúlio Vargas Filho

**Introduction:** Isolated unilateral absence of the pulmonary artery (UAPA) is a rare congenital malformation, prevalence range from 1 in 200,000 to 1 in 300,000. Clinical presentation is variable, making its diagnosis a challenge. Some patients have no symptoms and others may have heart failure and pulmonary hypertension (PHT).

**Case description:** 1-year-old boy with a history of pneumonia at 2 months of life, presented to the pediatric emergency room with fever, cough and dyspnea. The chest radiograph showed unilateral whiteout of the left lung with hyperinflation of the right lung, and deviation of the mediastinum to the left. The diagnosis of comunitary pneumonia was made and penicillin initiated. Despite the important clinical improvement, the radiographic image remained and the patient was transferred for investigation of sustained pulmonary hypotransparency. At admission the vital signs were HR of 84 bpm, RR of 24 rmp, SatO2: 97%; breath sounds were decreased on the left side. The bronchoscopy was performed and showed reduction in the caliber of the division of the left main bronchus, making it difficult to visualize the subsegments, and a pulsation in the inferior right third of the trachea without any obstruction. The Doppler echocardiogram revealed a structurally normal heart with a right aortic arch and no visualization of the left pulmonary artery. CT angiography confirmed the absence of the left pulmonary artery.

**Conclusion:** Isolated UAPA is a very rare congenital malformation and the absence of the left pulmonary artery is twice less frequent than that of the right pulmonary artery. Later age at presentation may result in higher risk of morbidity and mortality. The early diagnosis can prevent the development of PHT, recurrent pulmonary infections and hemoptysis.


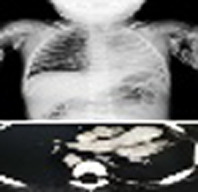


108902

Modality: E-Poster Researcher – Case Report

Category: PHYSIOTHERAPY

## Inspiratory Muscle Training in Heart Failure with Preserved Ejection Fraction Patients in a Home-Based Cardiac Rehabilitation Programme- A Case Report

RENATA CRUZEIRO RIBAS^1^, Sabrina Costa Lima de Andrade^1^, Jessica Blanco Loures^1^, Estêvão Lanna Figueiredo^2^

(1) RC Fisioterapia – Reabilitação Cardiovascular; (2) Instituto Orizonti

**Introduction:** Dyspnea and poor exercise tolerance are frequent in Heart Failure with preserved ejection fraction (HFpEF). Respiratory muscle (RM) weakness is an independent predictor of low ventilator efficiency during exercise and contributes to poor prognosis in this population. Inspiratory Muscle Training (IMT) enhances the benefits to aerobic and peripheral muscle training by improving RM fatigue, exercise tolerance and functional status, increasing quality of life (QoL) and reducing mortality. IMT is recommended as part of a cardiac rehabilitation (CR) programme in HFpEF patients.

**Case report:** An 86 year old hypertensive man with reduced functional status on the setting of Covid-19 pandemic lockdown developed HFpEF, with increasing dyspnea (NYHA’s functional class III), peripheral and pulmonary congestion. In sinus rhythm. Ejection fraction 65% and signs of diastolic dysfunction on echocardiogram, elevated NTproBNP. Patient was enrolled in a home-based CR programme for 12 weeks, with 4 sessions/week – 2 supervised and monitored sessions and 2 non-supervised. Medical therapy was optimized (diuretics, espironolactone, empagliflozin, anlodipine, telmisartan). Supervised and monitored sessions included IMT with POWERbreathe (PB)®, set on 30%–40% of S-index, weekly adjusted, and aerobic associated with muscular resistance exercises. The non-supervised sessions included IMT and muscular resistance exercises. Patient was submitted to functional assessment before starting CR, and after 12 weeks, that included dynamic inspiratory strength (S-index), peak inspiratory flow (PIF) and inspiratory volume measured with K5 PB®, Incremental Shuttle Walk Test (ISWT) and 30-second sit-to-stand test (STS). Dyspnea and self-perceived exertion were assessed with the modified Borg Scale and QoL by Minnesota Living with HF Questionnaire (MLwHFQ).

**Results:** Compared to baseline, there was an increase in the % of the predicted S-index (Δ55%), in PIF (Δ2.39%), in inspiratory volume (Δ0.28%), in ISWT (Δ200 m) and in STS (Δ7 repetitions), with reduction in self perceived exertion (very hard to quite hard). There was an important decrease in MLwHFQ (Δ36 points) related to a better perception of QoL. NYHA’s functional class improved and NTproBNP decreased significantly.

**Conclusion:** IMT in CR context, associated with optimized medical therapy, contributed to functional improvements, reducing dyspnea, and amelliorating QoL. This case illustrates how CR can change patient’s prognosis.

108931

Modality: E-Poster Researcher – Case Report

Category: COVID-19 AND CARDIOVASCULAR SYSTEM

## Multisystem Inflammatory Syndrome (MIS-C) by SARS VOC-2 and Chikungunya Associated with Left Ventricular Dysfunction – Case Report

FIRMINO HAAG FERREIRA JUNIOR^1^, Camila Louro Mota Branco^1^

(1) Hospital Geral de São Mateus

**Objective:** To describe a case of Multisystem Inflammatory Syndrome (MIS-C) associated with SARS COV-2 and Chicungunya associated with left ventricular dysfunction in a child admitted to a children’s intensive care unit in a public hospital in the city of São Paulo.

**Case report:** A.S.P.S., female, mixed race, 9 years old, was admitted to the pediatric intensive care unit with fever, dysuria, recent-onset arthralgia, with confluent and hyperchromic micropapular lesions, accompanied by choluria and abdominal pain. On physical examination, she was hypoactive, tachypneic, acyanotic, contacting and oriented. Complete blood count and urine analysis without significant changes. Antibiotic therapy was introduced with Ceftriaxone 2 g/day, Hydrocortisone and clinical support. Serology was requested for SARS COV-2, in addition to serology for Dengue, Chicungunha, Zika and leptospirosis, which showed detectable results for SARS COV-2 and Chicungunha. Abdominal US showing free fluid in the cavity without involvement of target organs. A transthoracic echocardiogram was performed, which showed moderate myocardial dysfunction with associated pericardial effusion without signs of restriction. Opted for the association of pulse therapy with methylprednisolone 10 mg/kg/weight for 3 days, associated with immunoglobulin 1.0 g/kg/weight for 10 days. The control echocardiogram showed preserved ejection fraction, normal cardiac dimensions and mild laminar pericardial effusion. He evolved with progressive improvement of the condition, being discharged asymptomatic and in satisfactory clinical conditions.

**Conclusion:** Multisystem inflammatory syndrome is a rare but serious disease in which children with COVID-19 develop a systemic inflammatory process. Cross-association with other types of viruses is rare, but evident in the literature. Recovery of left ventricular function is very common, and occurs within a few days of starting treatment. The clinical evolution of patients treated early has been favorable, as in the case described above.

108979

Modality: E-Poster Researcher – Case Report

Category: HEMODYNAMICS AND INTERVENTIONAL CARDIOLOGY

## Percutaneous Treatment of Rupture of the Interventricular Septum After Acute Myocardial Infarction: A Possibility

MARCELO SABEDOTTI^1^, Bibiana Guimarães Maggi^1^, Leandro Gazziero Rech^1^, Rafael Massuti^1^, Rafaela Oliveira Leite^2^

(1) Hospital Geral de Caxias do Sul; (2) Fundação Universidade de Caxias do Sul

**Introduction:** Rupture of the interventricular septum (RIVS) is a rare complication of acute myocardial infarction (AMI), which occurrence has decreased due to early intervention of AMI. Despite surgical advances, mortality of RIVS remains high, and such complications may be indicated by a new heart murmur and sudden hemodynamic impairment.

**Case report:** A 70-years-old male with diabetes, dyslipidemia, systemic arterial hypertension, and previous coronary artery bypass surgery, was hospitalized with an acute coronary syndrome without ST-segment elevation. Physical examination demonstrated a holosystolic murmur and diffuse precordial fremitus, with no signs of left heart failure. The patient was stable and promptly referred for early intervention. The coronary angiography demonstrated a subtotal left main coronary artery (LMCA) injury, patent left internal thoracic artery with total occlusion of the anterior descending artery and first diagonal branch. Circumflex artery was dominant, and the first marginal branch had a subtotal injury at the origin and an occlusion of the saphenous graft. Ventriculography demonstrated RIVS on the inferior sept and preserved left ventricular systolic function, with drug-eluting stent implanted on the LMCA towards the circumflex-anterior descending artery, protected by the left internal thoracic artery. The RIVS percutaneous closure was scheduled for the following day. Transthoracic echocardiogram (TTE) identified the RIVS on the lower portion of the muscular sept, with an 11 mm diameter, mild hemodynamic consequences, a 55% Simpson ejection fraction and inferior akinesia. The patient evolved with cardiogenic shock on the day of the procedure, demanding volemic reposition and analgesia. Control TTE demonstrated an increase of the RIVS to 22 mm. We performed the RIVS occlusion as scheduled, through an arteriovenous strap from the right femoral vein to the left femoral artery. The introducer was placed anterogradely through the defect to the ascending aorta. The measurement of the VSD could be performed using a sizing balloon, but due to severe periprocedural instability, we decided on the use of a VSD prosthesis sized according to echocardiographic and angiographic controls. The procedure was successful, with a dramatic hemodynamic improvement after prosthetic placement. The patient was discharged after five days.

**Conclusion:** RIVS percutaneous treatment after AMI is a therapeutic alternative for high-risk surgical patients.

108982

Modality: E-Poster Researcher – Case Report

Category: COVID-19 AND CARDIOVASCULAR SYSTEM

## Myocarditis and Pulmonary Thromboembolism in a Patient Diagnosed with COVID-19: Case Report

CRISTIANE KOPLIN^1^, João Artur Amaral Guarienti^2^, Felipe Lisboa Frota^3^, Fabiana Amaral Guarienti^4^

(1) MD from Federal University of Rio Grande do Sul, Emergency Departament of Public Health Foundation of Novo Hamburgo, Brazil; (2) MD from University of Passo Fundo, Rio Grande do Sul, Brazil; (3) Professional in Physical Education, Physical Activities and Exercises for the Prevention, Treatment of Diseases and Health Promotion Postgraduate Program of Albert Einstein Hospital, São Paulo, Brazil; (4) Geriatrics and Gerontology Postgraduate Program of Pontifical Catholic University of Rio Grande do Sul, Brain Institute, Porto Alegre, Brazil

**Introduction:** This report describes the case of a patient diagnosed with COVID-19 who presented with symptoms of myocarditis and pulmonary thromboembolism (PTE).

**Case description:** Female patient, 35 years old, arrived at the emergency in June 2020 with flu-like symptoms. History of allergic rhinitis, asthma, grade II obesity (BMI: 37.2 Kg/m²) and miscarriage in 2017. Requested RT-PCR SARS-CoV-2 with positive result. The patient had a good evolution with symptomatic treatment. She returned after two months with burning chest pain and vomiting. On arrival she was hypertensive (BP: 190/120 mmHg) and the electrocardiogram showed no acute ischemic changes. However, myocardial necrosis markers, troponin T and CK-MB, were positive in three measurements. The case was initially managed as an acute myocardial infarction without ST-segment elevation. Coronary Angiography showed no coronary lesions. The Transthoracic Echocardiogram showed an LV with increased wall thickness (Fig 1). The patient also had increased inflammatory markers (C-Reactive Protein and ESV). Treated with analgesics and antihypertensives, she had an improvement of the symptoms. She was discharged with outpatient follow-up for suspected myocarditis. After three months the patient returned with dyspnea for 3 days. She had 82% O2 saturation on room air, BP 150/110 mmHg and no other findings. Pulmonary artery CT Angiography showed signs of PTE (Fig. 2). As she had a Pulmonary Embolism Severity Index (PESI) of 55, indicating a low risk of severe morbidity, she was released and maintained on oral anticoagulation.

**Conclusions:** The pathophysiology of cardiovascular complications associated with COVID-19 is not completely understood. Myocardial viral infection and cytokine storm are etiological theories. This patient had typical signs of myocarditis with a history of recent viral illness, indicating an important relation between COVID-19 and myocardial damage. The thrombus tendency in COVID-19 may involve two mechanisms: a hypercoagulable state responsible for large vessel thromboembolism and direct endothelial injury responsible for microvascular thrombosis. In this report, PTE may have an etiologic link with previous SARS-CoV-2 disease. Interestingly, the relationship between COVID-19 and cardiovascular complications seems evident. Therefore, there is a need of additional studies to elucidate the pathophysiology of cardiovascular impairment related to COVID-19.

109004

Modality: E-Poster Researcher – Case Report

Category: ATHEROSCLEROSIS/CARDIOVASCULAR RISK FACTORS/CARDIOVASCULAR PREVENTION

## Subclavian Vertebro Steal Phenomenon in the Right Vertebral Artery: A Case Report

ROMERO HENRIQUE DE ALMEIDA BARBOSA^1^, Gabriela Santos Andrade^1^, Nayara Ribeiro Máximo de Almeida^1^, Poliana kalinne Simões de Melo Barbosa^2^, Adirlene Pontes de Oliveira Tenório^1^

(1) Universidade Federal do Vale do São Francisco; (2) Instituto Médico São Francisco

**Introduction:** The subclavian vertebro steal phenomenon(SVSP) occurs when a stenosis or occlusion in the brachioencephalic trunk or subclavian artery proximal to the origin of the vertebral artery causes partial or complete reversal of blood flow from the ipsolateral vertebral artery. It usually behaves asymptomatically, in up to 80% of patients, but may evolve with symptoms of vertebrobasilar insufficiency. Atherosclerotic disease is the most common cause and the left side is more commonly affected in a 4:1 ratio with respect to the right side.

**Case description:** I.M.S., female, 66 years old, Body Mass Index: 26, from Delmiro Gouveia-AL, diabetic, hypertensive, chronic renal failure on dialysis. Due to frequent dizziness and syncope, she had her first medical visit on 07/10/19, denying previous neurological and cardiologic symptoms. After suspension of beta-blockers and hydroelectrolytic and metabolic correction, Holter was requested, which showed advanced atrial ventricular block, being referred for definitive pacemaker implantation. After implantation, she returned for consultation on 24/01/20, hemodynamically stable, but with persistent vertigo. Doppler echocardiography of the carotid and vertebral arteries was requested, which revealed a totally reverse flow in the right vertebral artery, compatible with SVSP with permanente retrograde pattern. A computed tomography angiotomography of the cervical artery and venous was ordered on 01/21/2021, showing a mixed plaque located in the proximal right subclavian artery at the origin of the right vertebral artery, with an irregular surface, determining stenosis between 50 and 70%, with an extension of 2 cm. After evaluation by the vascular surgeon to analyze the need for the procedure, supra-aortic trunk angioplasty was indicated as treatment for SVPS.

**Conclusions:** The SVSP in the right vertebral artery is of infrequent occurrence, about 10% of cases, and that in about 5% of patients present symptoms, the report of this case can contribute to the identification of such condition and differential diagnosis of vertigo and syncope.


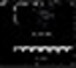


109073

Modality: E-Poster Researcher – Case Report

Category: CARDIOVASCULAR IMAGING

## Left Atrial Mixoma: An Unusual Presentation

PEDRO ANTONIO GALDEANO^1^, PATRICIA REGINA ALVES GALDEANO^1^, GABRIEL ANTONIO STANISCI MIGUEL^1^, FERNANDA NOGUEIRA BUENO RODRIGUES ALVES^1^

(1) CLINICORE; (2) CARDIOLIFE

**Identification:** Patient 52 years old, female, white, natural from anápolis-goiás. Case report: patient presented in 2014 a complaint of palpitations with effort associated with episode of transient loss of movement in lower left left seeking medical assistance. Requested echocardiogram that demonstrates a large oval image (figure), regular outlines, measuring 03 × 03 cm, inside the left atrium with bulleting to the inside of the left ventricle adhered to the interatrial septum, and the hypothesis of left atrial mixoma being proposed. Patient after clinical stabilization was referred to heart surgery with complete removal and diagnostic confirmation by anatomological and pathological left atrial mixoma. In the year 2021 (07 years after surgery) in control examination with echocardiogram, a regular contour mass was again identified, measuring 07 × 07 mm adhered to the interatrial septum (figure) with probable diagnostic hypothesis of recurrence of left atrial mixoma.

**Discussion:** Cardiac mixoma is the most common primary tumor of the heart and is mainly located in the left atrium. It predominates in the female sex and the age group around 50 years old, being rare in children. Symptomatology depends on the size, shape, mobility and location of the tumor. The treatment is surgical removal, which most of the times is curative.

**Conclusion:** Recurrence of cardiac myxoma after surgical removal is uncommon. This is due to the excision of its implantation base in the heart walls. If this resection is not broad, there is a possibility of the tumor recurrence. In the present case, the patient was followed annually without presenting residual image to echocardiography. Mixoma recurrence in followed cases is uncommon, which aroused the interest of the authors.


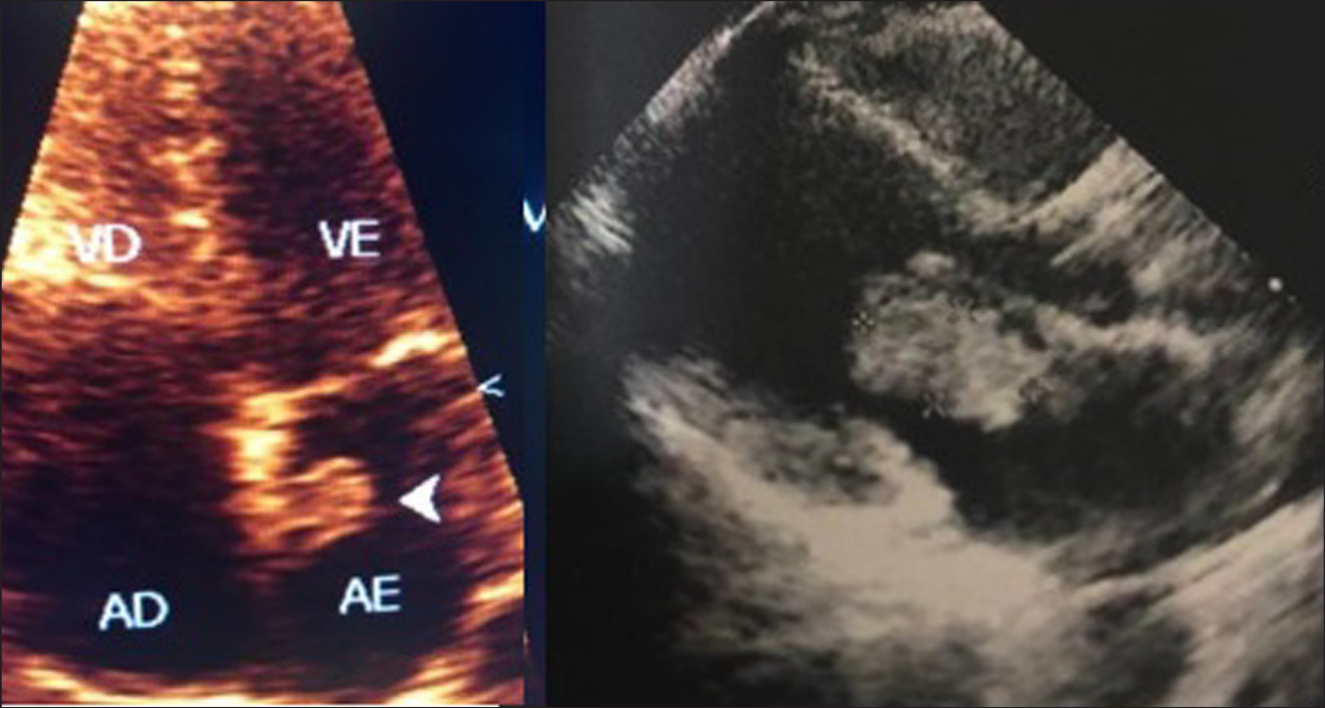


109330

Modality: E-Poster Researcher – Case Report

Category: CARDIOVASCULAR SURGERY

## A Rare Mitral Valve Tumor

DEINER PAULO MARTINS RESENDE ^1^, Alberto Rodolpho Hüning^1^, Giuliano Minor Zortéa^1^, Márcia Castilhos Puchalski^1^, Marcela da Cunha Sales^1^

(1) Santa Casa de Misericórdia de Porto Alegre (ISCMPA)

**Introduction:** Benign intracardiac tumors are uncommon entities, among which blood cysts are even rarer. They are usually located in the heart valves or adjacent structures and have a congenital in origin, disappearing spontaneously with time and rarely found after the first year of life. These tumors can cause left ventricular outflow tract (LVOT) obstruction, valve dysfunction, pulmonary embolism, and coronary obstruction. From 1960 to 2021, only 54 cases of blood cysts located in the mitral valve have been reported.

**Case report:** We present a case of a 36-year-old man, asymptomatic, who sought a cardiologist for routine evaluation. The electrocardiogram was normal. The transthoracic echocardiogram showed a rounded structure, with hyperechogenic edges, hypoechogenic center, measuring 2.1 × 1.9 cm (3,4 cm²), located in the chordae of the anterolateral papillary muscle of the mitral valve, without obstruction of the LVOT. Cardiac magnetic resonance imaging showed a sessile mass attached to the head of the anterolateral papillary muscle, 24 × 20 × 19 mm, hypointense and absence of contrast uptake. The patient was referred for cardiac surgery, where 2 cysts were identified, completely attached to the mitral valve and subvalvular apparatus. Resection of the anterior leaflet, chordae, top of the anterolateral papillary muscle and cysts were performed, followed by implantation of a bioprosthesis.

**Conclusion:** Intracardiac blood cyst are more commom in newborns and extremely rare in adults. Surgery is usually indicated due to the risk of embolization and other potential complications, as well as to define the exact diagnosis.


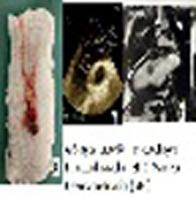


109566

Modality: E-Poster Researcher – Case Report

Category: CARDIO-ONCOLOGY

## Cardiac Involvement as an Early Sign of Hereditary Transthyretin Amyloidosis Manifestation in Carrier of the 107Val Mutation in the TTR Gene: A Case Report

ANTONIO AUGUSTO XIMENES^2^, Carolina Thé Macêdo^1^, Jemima Araújo Silva Batista^4^, Marcela Machado Costa^6^, Tonnison de Oliveira Silva^2^

(1) Curso de Pós-Graduação em Cardiologia da SBC/INC/INCA; (2) Hospital Cardio Pulmonar, Rede D’Or; (3) Hospital São Rafael, Rede D’Or; (4) Universidade Federal do Vale do São Francisco, UNIVASF; (5) Faculdade Estácio de Juazeiro; (6) Escola Bahiana de Medicina e Saúde Pública, EBMSP

**Introduction:** Hereditary transthyretin amyloidosis (ATTRv) is a severe disease transmitted by an autosomal dominant inheritance associated to more than 100 mutations in the transthyretin gene. The ATTRv phenotypic expression depends mainly, but not exclusively, on the type of mutation, with neurological and/or cardiological involvement most frequently. The improvement of the disease prognosis by the new drugs available relies on early diagnosis and treatment. The best strategy for the earliest diagnosis is based on knowledge about how the disease manifests itself in each mutation. However, due to the incomplete penetrance of mutations, detection of an initial stage of the disease is challenging. Val107 (Ile107Val) mutation is a rare form of ATTRv, characterized by neurological and cardiological involvement, with neurological impairment manifesting earlier and being more severe. We describe a case with cardiological alterations as the initial manifestation of the disease.

**Case:** 20/12 Asymptomatic 58-year-old male with Val107 mutation. He is is part of a group of 27 carriers of the Val107 mutation, followed up by our group. The institution protocol for the asymptomatic carriers routinely includes cardiological tests. Normal physical examination. Bilateral carpal tunnel syndrome confirmed by electroneuromyography Protein electrophoresis – no monoclonal band. Echocardiogram (Echo) – Left Ventricular ejection fraction (LVEF) 65%/Global Longitudinal Strain (GLS) = –17%. Holter – rare supra and ventricular ectopy. Cardiac Ressonance – mild pericardial effusion, increased left atrium. 99 mTc-PYP bone scintigraphy – Perugini grade 3 cardiac uptake and H/CL ratio = 1.56. Tafamidis was introduced. 22/03 Echo: LVEF: 70%, GLS:–20%, apical sparing pattern.

**Conclusion:** In the scarce literature available on Val107 ATTRv, the neurological impairment is the most common form of initial manifestation, being cardiac involvement observed later. However, this case points out that cardiological tests should also be included early in the monitoring strategy of asymptomatic Val107 carriers for diagnosis of ATTRv.


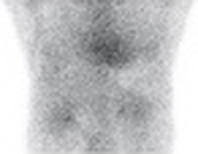


111119

Modality: E-Poster Researcher – Case Report

Category: HYPERTENSION/RENAL DENERVATION

## Serious Systemic Arterial Hypertension Secondary to Paraganglioma – Case Report

ELIZABETH CARDOSO DOS SANTOS^1^, Vera Maria Lúcia Cury Salemi^2^, Fernanda Willemann Ungaretti^1^, Juliana Gil de Moraes^1^, Roberto Kalil Filho^1^

(1) Sociedade Beneficente de Senhoras Hospital Sirio Libanes; (2) Instituto do Coração

Paragangliomas are rare tumors originating from cells from the neuroendocrine system, and like pheochromocytoma, they are catecholamine producers. However, they usually differ in their location, which is extra-adrenal. Regarding the clinical presentation, the classic triad consists of episodic headache, palpitations and sweating. Therefore, the objective is to describe an unusual clinical case and the general aspects of this entity.

**Case report:** A 29-year-old man with previous retroviral disease and arrhythmia is admitted to the emergency room with headache and an episode of emesis, with blood pressure levels of 247 × 152 mmHg. He reports episodes of morning headache associated with nausea and palpitations for 3 weeks. He was evaluated by the neurologist, with normal eye fundus and cranial computed tomography, and was hospitalized for blood pressure control and investigation of secondary systemic arterial hypertension. Laboratory tests were performed, the results of which showed significantly increased levels of vanillylmandelic acid, catecholamines and urinary metanephrines, and complementary tests such as: abdominal computed tomography that showed a voluminous solid lesion with necrotic areas and heterogeneous post-contrast enhancement centered on the right adrenal store, measuring approximately 6.5 × 6.0 × 5.0 cm. The mass displaces the inferior vena cava, trunk and posterior right portal branch, without signs of vascular invasion, presents extensive contact with the upper pole of the right kidney, displacing it inferiorly, but maintains a cleavage plane, suggesting the possibility of pheochromocytoma. Abdominal magnetic resonance imaging showed a solid mass in the right adrenal, indeterminate, suggesting that, in addition to pheochromocytoma, we consider carcinoma as a possibility. Whole-body PET-CT showed marked glycolytic hypermetabolism in the right adrenal mass, suspicious for a neoplastic process. He underwent resection of a peritoneal mass, right hepatectomy and partial resection of the inferior vena cava with primary reconstruction. The immunohistochemical profile was compatible with succinate dehydrogenase deficient paraganglioma. After tumor excision, the patient evolved with good blood pressure control and is currently asymptomatic.

**Discussion:** Once there is a clinical suspicion of pheochromocytoma/paraganglioma, laboratory tests and imaging tests should be performed, and surgical removal of the tumor remains the only curative treatment.

109606

Modality: E-Poster Researcher – Case Report

Category: CARDIOVASCULAR SURGERY

## Major Recurrent Ventricular Pseudoaneurysm in a Patient with Behçet

MARINA PINHEIRO ROCHA FANTINI^1^, Juan Mario Vaca Pereira Vrandecic^2^, Felipe Magalhães Becker^1^, Larissa Maria Ferrarez Faria^4^, Carlos Eduardo de Menezes e Souza Filho^5^

(1) Hospital Mater Dei; (2) Santa Casa de Belo Horizonte; (3) ECMO MINAS; (4) Faculdade Ciências Médicas de MG; (5) Faculdade Federal de Minas Gerais

**Introduction:** Behçet Syndrome (BS) is an autoimmune systemic vasculitis of variable caliber and with different phenotypes. It classically courses with ulcerative, papulopustular and nodular erythema-like lesions on the skin, mucous membranes and genital region. Among its presentations, angio-behçet has venous and, rarely, arterial vascular involvement. These arterial presentations, despite affecting approximately 4% of patients, represent a unique characteristic of BS, whose aneurysms potentially affect peripheral, visceral and pulmonary arteries. This case report presents a 16-year-old boy with recurrent left ventricular pseudoaneurysms since 2020.

**Case Report:** ELP, male, 16 years old. He was diagnosed with Behçet’s Syndrome in 2020 when he started presenting oral and genital ulcers, constitutional symptoms, hepatosplenomegaly, fever and night sweats. He underwent surgery to correct a left ventricular (LV) pseudoaneurysm on 5/29/20, did not improve and presented an aortic pseudoaneurysm and a new left ventricular apex pseudoaneurysm on examination on 8/28/20. As a result, he started pulse therapy with methylprednisolone and cyclophosphamide on 9/2/20 and surgical correction of ventricular and aortic pseudoaneurysms on 9/8/20. Treatment with pulse therapy was maintained, with 9 sessions with cyclophosphamide, until the finding of a new ventricular pseudoaneurysm on 10/01/21, when the corticosteroid was changed to infliximab. This new pseudoaneurysm had important dimensions (12.1 cm × 7.6 cm in its largest diameters), presence of LV shunt to the pseudoaneurysm, with a peak gradient of 64 mmHg and maximum velocity of 4.01 m/s, ejection fraction of (EF) 62.17% and preserved biventricular systolic function. He was admitted on 01/18/22 with decompensated heart failure with a warm and humid profile, associated with anasarca, EF of 37.5% and LV and right ventricle dysfunction. The conduct was clinical treatment and enrollment for urgent heart transplantation.

**Conclusion:** This report discusses a rare case of recurrent left ventricular aneurysm in a 16-year-old boy, with recurrent cardiac manifestations despite immunosuppressive therapy and corrective surgery. The patient’s condition shows that Behcet’s Syndrome may present as an aggressive cardiac form with more severe manifestations.

109690

Modality: E-Poster Researcher – Case Report

Category: CONGENITAL AND PEDIATRIC CARDIOLOGY

## Myocardial Revascularization in a Patient with an Anomalous Left Coronary Trunk with a Trajectory Posterior to the Pulmonary Artery and Anterior to the Aorta

MARINA PINHEIRO ROCHA FANTINI^1^, Henrique Loiola Duarte^5^, Pedro Henrique Cavalcanti de Albuquerque Sá^5^, Leonardo Nunes Coelho Fantini^1^, Vinícius Loureiro^2^

(1) Hospital Mater Dei; (2) Santa Casa de Belo Horizonte; (3) ECMO MINAS; (4) Faculdade Ciências Médicas de MG; (5) Faculdade Federal de Minas Gerais

**Introduction:** Anomalous coronary artery emerging from the contralateral sinus is a rare congenital anomaly. Some variations incur increased risk of adverse cardiovascular events, with sudden death often being the initial manifestation of the disease. This report presents a case of a patient with a variant of anomalous coronary artery with a poor prognosis, requiring surgical myocardial revascularization.

**Case Report:** SASL, female, 59 years old, diagnosed with anomalous coronary artery in 2014. On that occasion, coronary angiography was performed after the atypical chest pain, which showed an anomalous origin of the left coronary trunk, without atheromatous problems. She also has arterial hypertension, dyslipidemia and hypothyroidism, and uses Olmesartan, Hydrochlorothiazide, Duloxetine, Levothyroxine, Rosuvastatin and Alprazolam. On 01/2022, she had angina pectoris and dyspnea on exertion. She underwent an exercise stress test, which was positive for ischemia. Coronary computed tomography angiography was performed, which showed the left coronary trunk with anomalous origin in the right coronary sinus, path posterior to the pulmonary artery and anterior to the aorta, calcified plaque in the ostium of the anterior descending artery, determining luminal reduction of 25 to 49%. After evaluation, The Heart Team of Hospital Felicio Rocho opted for surgical intervention. She was admitted electively on 03/03/2022 for myocardial revascularization by the construction of a bridge from the mammary artery to the anterior descending artery using cardiopulmonary bypass and under balanced general anesthesia and erector spinae plane block. Extubated while still in the operating room, she was referred to the intensive care unit (ICU) in good clinical condition. She was discharged from the ICU on the 2nd postoperative day (POD) and discharged from the hospital on the 7th POD. The postoperative echocardiogram showed preserved biventricular systolic function, mild aortic insufficiency, laminar pericardial effusion and left ventricular ejection fraction = 71%. She is currently asymptomatic and in outpatient follow-up.

**Conclusion:** This report highlights the situation of a patient with an anomalous coronary trunk who had her cardiovascular prognosis positively modified by means of surgical myocardial revascularization. This demonstrates the importance of diagnostic investigation and surgical intervention in these patients, in order to avoid negative outcomes such as sudden death.

109701

Modality: E-Poster Researcher – Case Report

Category: CONGENITAL AND PEDIATRIC CARDIOLOGY

## Myocardial Revascularization in a Patient with an Anomalous Left Coronary Trunk with a Trajectory Posterior to the Pulmonary Artery and Anterior to the Aorta

MARINA PINHEIRO ROCHA FANTINI^1^, Felipe Becker Magalhães^1^, Carlos Eduardo de Menezes e Souza Filho^5^, Larissa Maria Ferrarez Faria^4^, Murilo Malta Batista^2^

(1) Hospital Mater Dei; (2) Santa Casa de Belo Horizonte; (3) ECMO MINAS; (4) Faculdade Ciências Médicas de MG; (5) Faculdade Federal de Minas Gerais

**Introduction:** Anomalous coronary artery emerging from the contralateral sinus is a rare congenital anomaly. Some variations incur increased risk of adverse cardiovascular events, with sudden death often being the initial manifestation of the disease. This report presents a case of a patient with a variant of anomalous coronary artery with a poor prognosis, requiring surgical myocardial revascularization.

**Case Report:** SASL, female, 59 years old, diagnosed with anomalous coronary artery in 2014. On that occasion, coronary angiography was performed after the atypical chest pain, which showed an anomalous origin of the left coronary trunk, without atheromatous problems. She also has arterial hypertension, dyslipidemia and hypothyroidism, and uses Olmesartan, Hydrochlorothiazide, Duloxetine, Levothyroxine, Rosuvastatin and Alprazolam. On 01/2022, she had angina pectoris and dyspnea on exertion. She underwent an exercise stress test, which was positive for ischemia. Coronary computed tomography angiography was performed, which showed the left coronary trunk with anomalous origin in the right coronary sinus, path posterior to the pulmonary artery and anterior to the aorta, calcified plaque in the ostium of the anterior descending artery, determining luminal reduction of 25 to 49%. After evaluation, The Heart Team of Hospital Felicio Rocho opted for surgical intervention. She was admitted electively on 03/03/2022 for myocardial revascularization by the construction of a bridge from the mammary artery to the anterior descending artery using cardiopulmonary bypass and under balanced general anesthesia and erector spinae plane block. Extubated while still in the operating room, she was referred to the intensive care unit (ICU) in good clinical condition. She was discharged from the ICU on the 2nd postoperative day (POD) and discharged from the hospital on the 7th POD. The postoperative echocardiogram showed preserved biventricular systolic function, mild aortic insufficiency, laminar pericardial effusion and left ventricular ejection fraction = 71%. She is currently asymptomatic and in outpatient follow-up.

**Conclusion:** This report highlights the situation of a patient with an anomalous coronary trunk who had her cardiovascular prognosis positively modified by means of surgical myocardial revascularization. This demonstrates the importance of diagnostic investigation and surgical intervention in these patients, in order to avoid negative outcomes such as sudden death.

109781

Modality: E-Poster Researcher – Case Report

Category: ACUTE AND CHRONIC CORONARY DISEASE/THROMBOLYSIS

## Myocardial Infarction with Non-Obstructive Coronary Arteries (Minoca): A Case Report of a Young and Athletic Woman

FRANCISCO ITALO DUARTE KUMAMOTO ^1^, Maria Alice Oliveira Magalhães Teixeira^1^, Maria Eliza Soares Gadelha de Oliveira^1^, Eduardo Franco Correia Cruz Filho^1^, Ana Carolina Navarro Henriques Cabral^1^

(1) Hospital Memorial São Francisco

**Introduction:** Myocardial Infarction With Non-Obstructive Coronary Artery (MINOCA) is a special type of Acute Myocardial Infarction (AMI). It is considered a working diagnosis, because the disease behaves similarly to a conventional AMI until the Coronary CT Angiography is done, which does not show lesions greater than 50% of the lumen in a main epicardial vessel, and this possibility should be considered until no other clinically overt specific cause is proven. Compared to coronary obstruction, It most often affects young women without comorbidities, such as the case reported.

**Case Report:** A 35 year old female, without comorbidities, entered in the ER in 2019 complaining of anginal chest pain, palpitations, intense sweating, drowsiness and high SBP of 180 mmHg. Based on the past medical history, the physical examination was normal and biomarkers were abnormal. A coronary CT Angiography showed an eccentric thrombus in the distal midsegment of the descendant anterior artery, which determined luminal stenosis below 50%. Changes in the left marginal branch, including left ventricle with subendocardic hypoattenuation of the anterior and septal walls of the apical region were also detected. According to the patient, the ECG was abnormal. The transthoracic echocardiogram showed an anterior and apical hypokinesis of the left ventricle. The cardiac catheterization presented an isolated lesion of the descendant anterior artery, which determined luminal stenosis below 50%. She was overtreated with aspirin, captopril, furosemide and low molecular weight heparine and was followed up by a cardiologist over 2 years. She remained asymptomatic until February 2022, when was admitted in the ER, reporting typical anginal pain. The cardiac troponin-1 was abnormal (2.8 ng/ml). She was admitted to the CCU. The electrocardiogram was normal. The coronary CT Angiography did not show any obstructive lesion and the cardiac catheterization was normal. The Magnetic Resonance Imaging (MRI) showed the presence of Late Gadolinium Enhancement (LGE) in subendocardial area, suggesting previous AMI. Today, she was discharged, with aspirine, statin and beta blocker.

**Conclusion:** Young patient without classic cardiovascular risk factors with apical wall AMI confirmed by magnetic resonance imaging without obstructive lesion, which in our view is compatible with MINOCA.

109836

Modality: E-Poster Researcher – Case Report

Category: CARDIAC ARRHYTHMIAS/ELECTROPHYSIOLOGY/ELECTROCARDIOGRAPHY

## Defibrillator in a Pregnant Woman with Arrhythmogenic Right Ventricular Cardiomyopathy: Concerns About Procedure, Pregnancy and Delivery

ERIKA OLIVIER VILELA BRAGANCA^1^, Érika Olivier Vilela Bragança^1^, Graziela Oliveira de Avelar^1^

(1) RitmoCheck

**Introduction:** 35-year-old woman with Arrhythmogenic Right Ventricular Cardiomyopathy (ARVC) with non-sustained ventricular tachycardia, right ventricular enlargement, left ventricular dysfunction (LVEF 46%) and important cardiac fibrosis (45% of late enhancement in cardiac MRI) and an ICD was indicated to primary prevention. Preoperative tests showed a positive betahCG and obstetric consultation confirm the pregnancy. After sharing making decision against radiation exposure and sudden cardiac death, we decided to move on to procedure of implantation of an ICD. The ionizing radiation is absorbed by any part of the body and the pregnant woman receive protective apron against lead in her belly, neck, head and eyes. The procedure was performed using fluoroscopic view and a dual chamber ICD was implanted. The total time was sixty minutes, the radiation time was ninety seconds and the burden of radiation was 1.6rads and the acute electronic measurements were very good. The post operative period was suitable and no cardiovascular or obstetric complication was noted during the pregnancy. Betablocker has not been discontinued. At 38 weeks the patient went into labor and the route of delivery chosen was the vaginal one. The patient underwent epidural anesthesia and she gave birth a female newborn of 3,315g weight and an Apgar score of 9/10. Only ventricular ectopics beats was seem during this period of time and no device abnormality. The puerperium phase was uneventful.

**Discussion:** There are a few studies of pregnancy with ICD managed and there are actually no guidelines for this issue. As regarding ARVC only sixty pregnancies were identified between 1968 and 2016 and only 17% having an ICD. ARVC is associated with a low rate of major cardiac events and vaginal delivery appears safe. X-ray exposure of less than 5 rads has not been associated with an increased chance for birth defects.

**Conclusion:** The ICD is a relative contraindication during pregnancy, nonetheless the procedure and delivery should be carefully planned and protected.


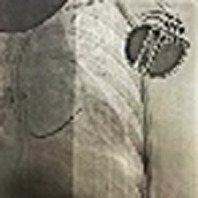


109823

Modality: E-Poster Researcher – Case Report

Category: CARDIO-ONCOLOGY

## Carcinoid Heart Disease and the Best Time for Valve Surgical Approach – Case Report

LARISSA ARLETE MOSKO^1^, Bruna Bozelli Leopoldino Barion^1^, Michael Malca Sepúlveda^2^, Sanderson Antonio Cauduro^1^, Celso Soares Nascimento^2^

(1) Hospital Erasto Gaertner HEG; (2) Hospital Angelina Caron HAC

**Introduction:** Neuroendocrine tumors (NET) are neoplasms derived from enterochromaphin cells that produce neurotransmitters and neuromodulators. Its incidence is 2–5 new cases per 100,000 inhabitants/year. Skin flushing, diarrhea, telangiectasia and cardiac lesions are symptoms that characterize Carcoininoid Syndrom (CS). The Biopsy defines the diagnosis. Cardiac involvement is due to an increase in the right heart caused by tricuspid and pulmonary insufficiency. Valve replacement can improve functional capacity.

**Case Description:** A 50-year-old male patient was admitted in 2017 to oncology hospital Erasto Gaertner with diarrhea, urticariform lesions and 15-kilo weight loss. He denied comorbidities and use of medications Upon examination, palpable mass in the abdômen was noticed. Hepatic nodulus were found on CT scan. A biopsy of these lesions revealed neoplasm of metastatic neuroendocrine pattern. Search for the primary site was carried out unsuccessfully. The initial echocardiogram showed an increase in the right chambers, with preserved right ventricular (RV) systolic function and severe tricuspid insufficiency. Despite the use of somatostatin analogs (SA), in 2018, the patient presented signs of right heart failure, hence a proposal for surgical approach, patient turned it down. Chemoembolization the liver lesions was performed to decrease serotonin hypersecretory. In 2021, he presented cardiac decompensation with RV dysfunction on echocardiogram. Back then, the patient agreed to tricuspid valve replacement, but in post-op even with the use of SA, the decompensation evolved to refractory cardiogenic shock due to RV failure, causing death.

**Conclusion:** The carcinoid heart (CH) is a therapeutic challenge not only regarding risks of valvar surgical intervention, but also because of the systemic effects of CS itself triggered by surgical trauma. NGUYEN et al. evaluated 240 patients who underwent valve surgery. In this study, as well as in other analysis of smaller groups, most of the patients who were assessed received SA in pre and post op, modifying surgical evolution. Furthermore, early surgery improved short and long-term outcomes. In this case report, the patient refused to undergo early valve replacement surgery, and approach with RV dysfunction was performed, which probably contributed to the unfavorable outcome. Patient selection, the care of a multidisciplinary team and SA infusion protocols, seem to be the main points for success in the CH approach.

109832

Modality: E-Poster Researcher – Case Report

Category: CARDIOVASCULAR SURGERY

## Ressection of Infected Thoracic Endograft using Extra-Anatomical By-Pass and Revascularization of Left Carotid

EDMILSON CARDOSO DOS SANTOS FILHO^1^, Diogo Alves Cardoso^3^, Simone Cristina Soares Brandão^1^, Manuela de Sousa Moura Fe^1^, Fábio Antônio Amando Granja^1^

(1) Hospital das Clinicas da Universidade Federal de Pernambuco; (2) Instituto do Coração de Pernambuco; (3) Faculdade Pernambucana de Saúde

**Introduction:** Thoracic stent graft infections are devastating complications related to the treatment of aortic aneurysms. Definitive treatment requires a strategy of revascularization, explantation of the infected prosthesis, and debridement of all infected tissue. It represents a flaw in the minimally invasive approach to endovascular treatment and puts the patient at an increased risk of morbidity and mortality.

**Case description:** A 42-year-old female patient, HIV + with an undetectable viral load, was admitted complaining of asthenia, cough, and intermittent fever (39–40°C) for 9 days. Hospitalized 6 months earlier due to COVID-19 infection, she evolved with pericardial effusion with purulent cardiac tamponade, and a pericardial window was performed. Had endovascular treatment for a traumatic descending aortic aneurysm 13 years ago. Despite an inconclusive investigation for an infectious focus, the patient initially presented a clinical and laboratory response to empirical ABX (cefepime + teicoplanin). After 10 days of treatment, she returned to present febrile peaks associated with an increase in WBC and CRP. PET-CT showed increased uptake and periprosthetic gas, suggesting infection with the presence of biofilm. The surgical approach was performed, through a sternotomy, an extra-anatomical by-pass with a Dacron prosthesis soaked in Rifampicin, between the ascending and descending aorta through of diaphragm, with re-implantation of the left common carotid, without the use of cardiopulmonary bypass (CPB), closed the sternotomy, left thoracotomy was performed with removal of the prosthesis and all the infected tissue.

**Conclusion:** Infections in thoracic endoprostheses can be approached safely and with good results in a single procedure, without the need of CBP.


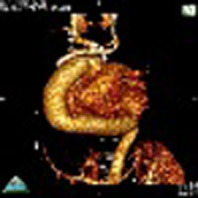


109991

Modality: E-Poster Researcher – Case Report

Category: CONGENITAL AND PEDIATRIC CARDIOLOGY

## Everolimus Reduces Multiple Cardiac Rhabdomyomas in Newborn: Case Report

SERGIO ALEXANDRE PEREIRA GONÇALVES^1^, Talita Nolasco Loureiro^1^, Jessica de Cassia dos Santos Peloso^1^, Alice Pereira Duque^1^, Raquel Tavares Boy^1^

(1) ¹Hospital Universitário Pedro Ernesto (HUPE/UERJ), Rio de Janeiro, RJ, Brazil.

**Introduction:** Primary cardiac tumors are rare, with a prevalence range of 0.001%–0.03%. Rhabdomyomas are the most common type of cardiac tumors in children. They are often associated with tuberous sclerosis complex and can cause right or left ventricular outflow tract obstruction, severe heart failure and arrhythmias. Everolimus, an inhibitor of mammalian target of rapamycin (mTOR), is suggested to be a promising therapeutic strategy for the involution of rhabdomyomas.

**Case report:** Male newborn (NB), diagnosed with multiple rhabdomyomas by fetal echocardiography, born by cesarean section, gestational age 39 weeks and 3 days, weight 3,405g, head circumference 36 cm, length 50 cm, APGAR 5/9. NB required positive pressure ventilation, orotracheal intubation in the delivery room and received care from neonatal intensive care unit. Thereafter, NB was extubated on the same day and maintained on non-invasive ventilation. On echocardiography: multiple rhabdomyomas, left ventricular (LV) enlargement with severe dysfunction and heart failure. During hospitalization, dobutamine, captopril and digoxin were administered. Everolimus 4.5 mg/m²/day beginning on third day (D3) after birth with clinical, laboratory and echocardiographic tumor involution follow-up and monitoring side effects. Hypochromic macules, presence of astrocytoma in fundoscopic examination and central nervous system tumors in magnetic resonance imaging determined the Tuberous Sclerosis Complex diagnosis. NB presented a 71.2% reduction of cardiac tumor in the LV (7.64 cm² vs. 2.20 cm²) and a 248% increase in ejection fraction (EF) determined by Simpson’s method, from D3 and after 12 weeks of Everolimus use, respectively. After hospital discharge, Everolimus was discontinued due to the restriction of purchasing high-cost drugs. After 13 weeks of Everolimus interruption use, the cardiac tumor showed an increase of 98% (4.37 cm²) but without reduction in EF (53.8%). Currently, the patient remains using captopril (0.08 mg/kg/day) and digoxin (0.01 mg/kg/day) daily and followed-up by pediatric cardiology.

**Conclusion:** Despite being in a phase II clinical trial, Everolimus still improved the involution of cardiac tumors, providing clinical improvement of the heart failure condition and reducing the risks of an extended hospitalization.

110333

Modality: E-Poster Researcher – Case Report

Category: CARDIOVASCULAR INTENSIVE CARE/CARDIOVASCULAR EMERGENCIES

## Occlusive Myocardial Infarction (OMI) Without the Classic Electrocardiographic Criteria for ST-Segment Elevation: Importance of Recognizing the Aslanger Pattern in the Emergency Department

JEAN MAX FIGUEIREDO^1^, Tulio Rangel Cardozo^2^, Caroline Roberta Torquato^2^, Daniel Xisto Ferreira Lordani^2^, Otávio Henrique Correa Siqueira Chagas^2^

(1) Universidade Iguaçu; (2) Hospital Municipal de São João de Meriti; (3) Hospital Geral de Nova Iguaçu

Male, 72 years old, with oppressive chest pain 2 hours before arrival at the emergency department. History of myocardial infarction 6 years ago, hypertension, smoking, and diabetes. ECG showing ST elevation on D3 without ST elevation on D2 or aVF, ST segment depression with terminal positive T wave in V4 and V5. V1 with ST segment higher than in V2. This electrocardiographic pattern was first described by Aslanger in 2020 and indicates right coronary occlusion in the presence of multivascular atherosclerotic coronary artery disease. The coronary angiography performed after one week showed occlusion of the right coronary artery and severe multivessel lesions including the left main coronary artery. We present here a case report in which the patient presented with occlusive myocardial infarction without the classic electrocardiographic expression of ST-segment elevation in two or more contiguous leads. Two questions need to be answered in the future: 1- In the emergency department, should patients with a clinical picture of acute coronary syndrome and electrocardiographic patterns highly suggestive of coronary occlusion, such as Aslanger or De Winter, be promptly thrombolysed when emergency coronary angiography is not available? And 2- Is it time to change the nomenclature from acute myocardial infarction with ST-segment elevation and acute myocardial infarction without ST-segment elevation to, respectively, occlusive myocardial infarction (OMI) and non-occlusive myocardial infarction (NOMI)? References 1-Aslanger E et al. J Electrocardiol. 2020. 2-Meyers HP et al. Int J Cardiol Heart Vasc. 2021.


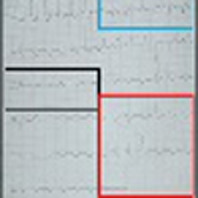


110150

Modality: E-Poster Researcher – Case Report

Category: COVID-19 AND CARDIOVASCULAR SYSTEM

## Recurrent Supine Cardioinhibitory Cough Syncope in a Critically Ill COVID-19 Patient

NÁGELA SIMÃO VINHOSA NUNES^1^, Joelma Dominato^1^, Paulo Roberto Benchimol Barbosa^1^, Alfredo de Souza Bomfim^1^

(1) Complexo Hospitalar de Niterói; (2) Hospital Universitário Antônio Pedro

**Background:** Reflex syncope is the most common cause of syncope in all age groups and the classic triggers are orthostatic stress, pain, fear and phobia. Cough is an uncommon trigger and when it triggers vagal reflex with syncope it is classified as situational syncope. We report a case in which asystole followed by syncope was always triggered by cough or tracheal aspiration in a patient with critical COVID-19.

**Case:** Female, 40 years old, hospitalized for 40 days with COVID pneumonia, tracheostomized and attached to respirator. She was on daily hemodialysis due to acute renal failure. The cardiology service was contacted to assess recurrent asystole followed by syncope that occurred during airway aspiration or coughing, without concomitant drop in oxygen saturation. Baseline electrocardiogram was normal. 24-hour Holter and continuous telemetry showed numerous sinus pauses of up to 25 seconds (Figure). Beta-blocker had recently been introduced and she was on continuous dexmedetomidine infusion. Staff was oriented about the need to avoid hypovolemia, suspend beta-blocker and dexmedetomidine and aspirate the airway with a transcutaneous pacemaker turned on demand, until the events were controlled. In the following days, after these measures, no new episodes of asystole were recorded.

**Discussion:** The causal mechanism of the pauses in this case is compatible with the cardioinhibitory vagal reflex triggered by the Valsalva maneuver due to persistent cough or tracheal aspiration. In the initial phase of the Valsalva maneuver, there is an increase in intrathoracic pressure, that is reflected to vessels in the neck, in addition to a drop in venous return to the heart, which can cause both stimulation of vessel baroreceptors and cardiac mechanoreceptors (Bezold-Jarish reflex). The occurrence of overshoot pressure in the final phase of Valsalva maneuver also can trigger vagal reflex, leading to a reflex cardioinhibitory situational syncope. Recognizing the triggers (hypovolemia and Valsalva maneuver) followed by withdrawal of drugs that can potentiate both triggers and the vagal reflex (dexmedetomidine and beta-blockers) may be essential for the resolution of similar cases in critically ill patients infected with SARS-CoV2, during the COVID-19 pandemic.

110845

Modality: E-Poster Researcher – Case Report

Category: CARDIOVASCULAR INTENSIVE CARE/CARDIOVASCULAR EMERGENCIES

## Triple Vessel Spontaneous Coronary Dissection – Not Just Another Heart Attack

ASHOK TAHILYANI^1^, Dr Hannah Gower^2^, Dr. A. Kotecha^2^

(1) Royal Devon Universoty healthcare NHS foundation Trust.; (2) Royal Devon and Exeter Hospital

**Background:** Spontaneous coronary artery dissection (SCAD) is an uncommon but important cause of non-atherosclerotic acute coronary syndromes (ACS) and sudden cardiac death[1]. There is very little data that exists in regards to patient clinical characteristics, risk factors, treatment and outcomes. SCAD can be easily missed, as patient characteristics and management differ substantially from typical ACS cases[1]. Results of revascularisation with percutaneous coronary intervention (PCI) and coronary artery bypass grafting (CABG) are suboptimal1. Conservative management is the preferred option with spontaneous healing of the dissection in the majority of uncomplicated cases[1].

**Case Presentations:** We present an unusual case of an active 61-year-old male who presented to a local district general hospital with worsening continuous chest pain for 24 hours and arm stiffness, following a week of indigestion-like chest pain. He has noticed increasing fatigue and shortness of breath with exertion in the preceding 7 days. Coronary angiogram revealed triple vessel SCAD of the left anterior descending (LAD), left coronary circumflex (LCX) and right coronary artery (RCA) with long segments of severe, smooth, tubular narrowing, with distal vessels appearing to be normal calibre and disease free. No PCI was attempted. The diagnosis was confirmed with CT coronary angiogram (CTCA).

**Conclusion:** This case highlights an unusual case of triple vessel SCAD, which emphasises the need to recognise SCAD as a cause for ACS presentations as the management differs from classical atherosclerotic ACS cases. It also highlights that whilst management is preferably conservative, revascularisation options may be indicated and PCI is the preferred revascularisation option. However, it is associated with significant challenges and has low reported success rates of <50%.

111456

Modality: E-Poster Researcher – Case Report

Category: CARDIO-ONCOLOGY

## Case Report: Multiple Masses in Right Cardiac Chambers Manifesting as Pulmonary Thromboembolism

LUIZ MARCELO CRUZ^1^, Davi Dorval Pereira Cordova^1^, Mariana Ribeiro dos Reis Arruda^1^, Márcio Costa Silveira de Ávila^2^, Alexandre David Ribeiro^3^

(1) Hospital Tereza Ramos; (2) Clínica Pulsar; (3) Clínica Neurocor

Cardiac tumors are rare and 80–90% are benign in adults. About half are myxomas, especially in the fourth and sixth decades of life with a slight predominance in women (1.5:1). May be asymptomatic or it can present with respiratory and embolic symptoms such as pulmonary thromboembolism (PTE) if they are in right chambers and also sudden death. Woman, 43 years old, obese, smoker, pulmonary emphysema, deep vein thrombosis in 2016, COVID negative, hospitalized due to pleuritic pain, hemoptysis and hypoxemia one month ago. CT angiography showed involvement of the left pulmonary artery and its branches to the left lower lobe. Echocardiogram showed pulmonary hypertension, enlarged right chambers and supposed thrombi in the right ventricle (RV). Despite anticoagulation, there was no reduction in lesions in RV, neither with fibrinolysis with alteplase. Cardiac resonance showed RV insufficiency and isointense lesions, without enhancement, irregular contour, the largest with 24 × 12 mm in RV output, 28 × 8,5 mm in papilar musculature region of the tricuspid valve and 17 × 4,7 mm in septum, raising hypothesis of myxomas. PET-CT showed no contrast uptake by the lesion. Due to probable benignity of the lesion, the patient opted for conservative treatment and evolutionary control, discharged with home oxigen and warfarin. One year later, exams showed no lesions growth. Although rare and generally benign, cardiac tumors should be part of the differential diagnosis of PTE.


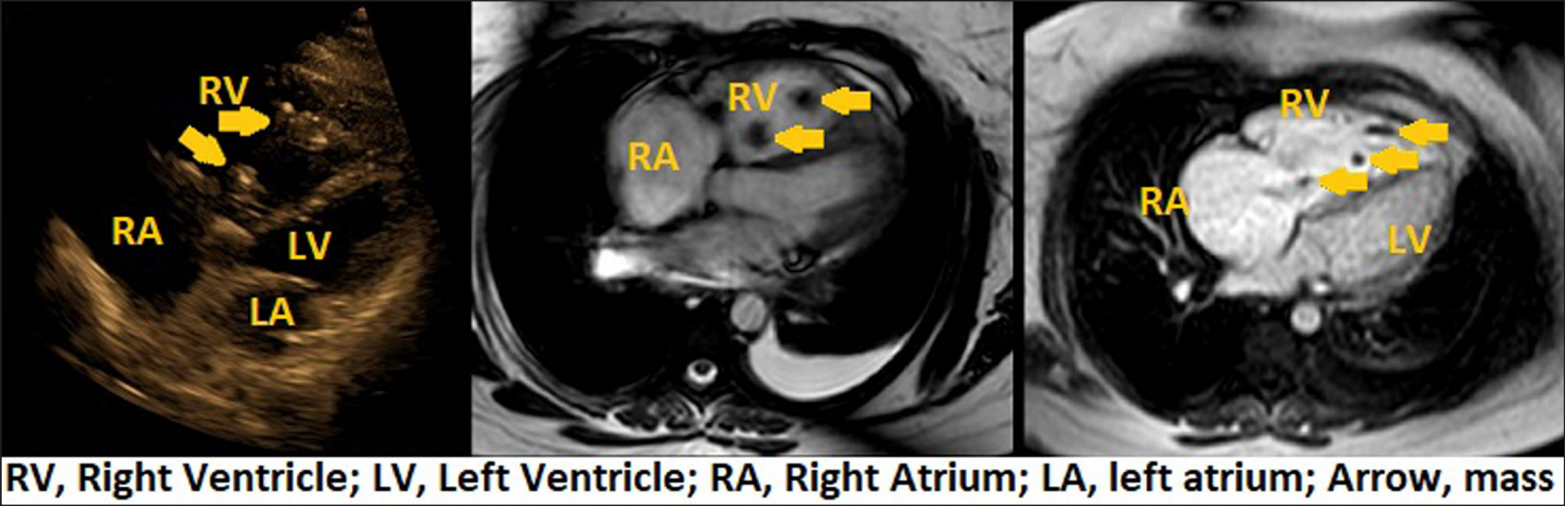


111538

Modality: E-Poster Researcher – Case Report

Category: CARDIO-ONCOLOGY

## Transcathehter Valve Implantation in a Patient with Severe Aortic Stenosis Enabling Bone Marrow Transplant for Poems Syndrome Due to Plasmacytoma, a Case Report

DIEGO RAUL ROMERO CAWEN^1^, diego romero cawen^1^, eduardo schlabendorff^1^, vanessa santos dos santos^1^, euler roberto fernandes manenti^1^

(1) hospital mãe de deus; (2) instituto de medicina vascular mãe de deus

One of the purposes of the cardio oncology team is to provide medical solutions for the oncological patient that enables him to receive the most indicated treatment for his pathology. Patients with heart disease also need optimal cancer treatment and cancer diagnoses. When cardiac surgery is indicated, hard decisions must be made and a multidisciplinary point of view promotes a qualified opinion. We present a case of a 50 year old male with a history of pain and paresthesia of upper and lower limb extremities, defined as sensory-motor non demyelinating polyneuropathy. He had a medical history of type 2 diabetes mellitus and hypertension. In April of 2018 he was hospitalized due to a severe aortic stenosis with surgical indication. Cardiac catheterization showed normal coronary arteries. In the preoperative evaluation a serum monoclonal peak was seen in proteinogram. Medullogram revealed 1.5% of non-clonal plasma cells. His whole body MRI showed an expansive lesion located in the proximal diaphysis of the right tibia, measuring 7.0 × 2.0 cm. Biopsy findings indicated a plasmacytoma. VEGF levels were 1294 ng/l indicating POEMS. He underwent radiotherapy and received dexamethasone, with a non satisfactory response. Sternum and fifth dorsal vertebra infiltration were detected in PET-SCAN. Systemic treatment was indicated and at this time patient was symptomatic from his AS. He suffered an acute pulmonary edema during mediastinal biopsy. His echocardiogram revealed a bicuspid aortic valve with severe aortic stenosis. After consultation with the heart team, the patient was judged to be an EuroScore II intermediate. We therefore decided to perform TAVI to reduce the interval between AS treatment and cancer treatment, preventing malignant tumor progression. In addition, benefits of TAVI have been reported in cancer patients because it does not require cardiopulmonary bypass. These include reducing the risk associated with tumor bleeding secondary to anticoagulant disorders and administration of anticoagulants, and reducing tumor dissemination with immunosuppressive and inflammatory effects of the cardiopulmonary bypass. After the procedure, the patient had a favorable evolution and a marrow transplant was performed. This point-of-care treatment was conducted due to the expertise of the multidisciplinary heart team.

111005

Modality: E-Poster Researcher – Case Report

Category: HEART FAILURE/CARDIOMYOPATHY/TRANSPLANT

## Danon Disease: A Case Report

PAULO LEÃO ANDRADE^1^, Larissa Neto Espíndola^2^, Maria Cristina Costa de Almeida^3^, Maria Gabriela Costa de Almeida^3^, Fernando Carvalho Neuenschwander^4^

(1) Hospital São Vicente de Paulo, Itabirito-MG; (2) Hospital Santa Izabel, Salvador-BA; (3) Centro Universitário de Belo Horizonte; (4) Instituto Orizonti, Belo Horizonte – MG

**Case Presentation:** A 17-year-old male, asymptomatic, amateur soccer player, sought a cardiologist after the sudden death of his maternal uncle at age 43 due to acute myocardial infarction, confirmed at necropsy. Apparently, he did not show any muscular, cognitive, cardiological or other system involvement. The electrocardiogram performed in the office met criteria for left ventricular (LV) hypertrophy and suggested the presence of ventricular preexcitation, such as the presence of delta wave and a short PR interval. Holter monitor showed preserved sinus automatism, ventricular preexcitation, infrequent ventricular extrasystole. Transthoracic echocardiogram showed hypertrophy of the middle and apical segments, suggesting the possibility of apical hypertrophic cardiomyopathy (HCM). He had normal LV ejection f n but incipient signs of systolic dysfunction (reduced longitudinal global strain(–13%). He underwent cardiac magnetic resonance which showed asymmetric HCM with areas of late enhancement in approximately 25% of the myocardial mass, in addition to increased thickness of the right ventricular free wall with small areas of late enhancement in its apical portion and signs of increased trabeculation. In view of the findings of HCM + ventricular preexcitation, we chose to perform a genetic test, which confirmed a mutation in the LAMP2 gene. Opted to implant an implantable cardioverter defibrillator for primary prophylaxis of sudden cardiac death. The patient is doing well, with no signs or symptoms of heart failure and no shock therapies to date.

**Discussion:** Mutations in X-linked lysosome-associated membrane protein gene (LAMP2; Danon disease) produce a cardiomyopathy in young patients that clinically mimics severe HCM due to sarcomere protein mutations. However, the natural history and phenotypic expression of this rare disease is incompletely resolved and its identification may have important clinical implications. Early identification of Danon disease in male is extremely important for patient care because rapid clinical deterioration leading to cardiac death occurs in young men before 25 years of age, in general. Heart transplantation is the reliable treatment once the occurrence of heart failure and should be considered as early as possible due to its rapid progression. New hope is emerging with the possibility that gene therapy could change the course of this dreaded disease.

111024

Modality: E-Poster Researcher – Case Report

Category: HEMODYNAMICS AND INTERVENTIONAL CARDIOLOGY

## Percutaneous Treatment of Pulmonary Vein Stenosis After Atrial Fibrillation Catheter Ablation: The Key to Succeed!

FABIO RODRIGO FURINI^1^, Carlos Antonio Abunader Kalil^1^, Anibal Pires Borges^1^, Oscar Ivan Lopez Leon^1^, Valter Correia de Lima^1^

(1) Santa Casa de Misericórdia de Porto Alegre

**Introduction:** Pulmonary vein (PV) severe stenosis post atrial fibrillation (AF) catheter ablation is a rare event (<0.5% of treated veins) that can cause dyspnea, pneumonia, pleural effusion, hemoptysis, pulmonary hypertension and even death. Percutaneous stenting PV, instead of balloon angioplasty alone, is effective in order to reduce the chances of restenosis. Stents implanted with 8 mm or larger are related to better outcomes.

**Case Description:** 60 y/o patient with AF since 2019 and two ablations of the arrhythmia. Three months after the ablation he presented with tiredness and CT scan which showed a pulmonary infiltration into the bronchus from the basilar left pulmonary trunk. The investigation ruled out neoplasia. A 3D reconstruction of the image showed the complete occlusion LIPV and a critical stenosis of the LSPV, with reference diameters of 15 mm on the last one. Therefore, we only address the LSPV. The procedure was performed in the cath lab by the interventional cardiology and electrophysiology teams. Invasive measurements of pulmonary pressures showed postcapillary pulmonary hypertension (mean arterial pulmonary pressure of 38 mm Hg and wedge pressure of 37 mm Hg) and the translesional pressure gradient measured by TTE Doppler US was 27 mm Hg on LSPV. Sequential dilatations were performed, and finally a 12 mm diameter balloon with a Palmaz Genesis XD 1019 was fully expanded at the lesion. Angiography showed an excellent angiographic result and the translesional pressure gradient by Doppler fell from 27 mm Hg to 5 mm Hg (See Figure). The patient was discharged from the hospital on the second day after the procedure.

**Conclusion:** PV stenosis after AF catheter ablation is a rare and insidious event. Treatment can be challenging and require multidisciplinary expertise.


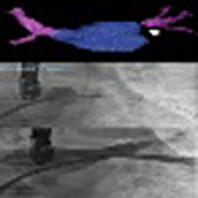


111030

Modality: E-Poster Researcher – Case Report

Category: COVID-19 AND CARDIOVASCULAR SYSTEM

## St-Segment Elevation Myocardial Infarction in the Absence of Obstructive Coronary Artery Disease (Minoca) in a Patient with SARS-COV2 Infections: A Case Report

INGRID LOUREIRO DE QUEIROZ LIMA^1^, RODRIGO FERNANDES CASTRO^2^, TALITA CARVALHO^2^, ANGELO BRUNO PAGOTO^2^, CARLOS ROBERTO ROHENKOHL EVANGELISTA SANTOS^2^

(1) UNIVERSIDADE FEDERAL DO AMAZONAS; (2) HOSPITAL DO CORAÇÃO FRANCISCA MENDES

**Background:** Since the pneumonia outbreak in December 2019 in the province of Wuhan, China, the SARS-COV-2 infection has spread to hundreds of countries and emerged as a pandemic crisis. In addition to the fact that underlying cardiovascular conditions have a negative impact on the prognosis of these patients, the virus can also affect directly the cardiovascular system through myocardial injury, cardiac failure, arrythmia, myocarditis and shock. Hypercoagulability state is also described due to the SARS-COV2 infection. Series of patients with myocardial infarction without coronary artery disease were described and intracoronary thrombus was described as a possible cause.

**Case Report:** A 58 y-o male was admitted in a public hospital in Amazonas/Brazil due to acute respiratory syndrome on May 11th, with low oximetry. He received oxygen support with nasal catheter O2 and remained stable for 8 days. On May 18th he presented a syncope without prodrome at the clinical ward. EKG showed a ventricular tachycardia, promptly reverted by electric cardioversion. The new EKG showed sinus rhythm and ST-elevation in inferior and lateral leads, immediately treated with thrombolytics. The coronary angiogram performed three days after the event showed coronary arteries without obstruction. Cardiac MRI evidenced transmural infarction in inferior and lateral walls.

**Discution:** Myocardial injury is observed in some patients due to SARS-COV-2 infections and it can be caused not only by plaque rupture but also by inflammatory storm and massive citokyne release, coronary spasm, microthrombus or vascular an and endothelial direct lesion. The absence of coronary artery disease in the angiogram and the previous history of thrombolytic treatment of this patient points toward a possible intracoronary thrombus causing the myocardium infarction. The evidence of a transmural contrast enhancement in the cardiac MRI reinforces the diagnostic of myocardial infarction and helps differentiating it from a myocarditis.

112421

Modality: E-Poster Researcher – Case Report

Category: ACUTE AND CHRONIC CORONARY DISEASE/THROMBOLYSIS

## Pinch Off Syndrome: A Rare Case Reported

GISELLE LAURITZEN DUARTE^1^, Andre Sansonio de Morais^1^, Sergio Medeiros da Silva Junior^2^, Daniel Cavalcanti de Carvalho^3^, Flavio Roberto Azevedo de Oliveira^4^

(1) Hospital Dom Helder Camara (HDH); (2) Hospital Dom Helder Camara (HDH); (3) Hospital Dom Helder Camara (HDH); (4) Hospital Dom Helder Camara (HDH)

**Introduction:** The central venous catheter is a device used in critically ill patients in emergencies and ICUs. Among the vascular complications, Pinch-off syndrome is related to puncture of the subclavian vein and occurs when the catheter is compressed between the clavicle and the first rib, causing laceration, fracture and embolization to the right chambers or pulmonary circulation, with high rates of serious complications, including death. We report the case of a patient with a finding of a foreign body in the coronary sinus during an echocardiogram performed in the context of heart failure decompensation.

**Case Description:** SJF, male, 62 years old, with heart failure (HF) with reduced ejection fraction (EF = 29%), admitted to the hospital with decompensated HF profile B, underwent transthoracic echocardiography (TTE) with image being visualized irregular, mobile, hyperechoic in the right atrial cavity, measuring about 5 cm, which seemed to be attached to the interatrial septum, or coming from the coronary sinus, with mobile elements attached to the extremity – neoplastic mass? thrombus? catheter tip covered by thrombus? A chest tomography was performed and a linear image with high density located in the topography of the coronary sinus and right atrium was visualized, a nonspecific finding, which may be related to a foreign body (catheter fracture?). The case was discussed at a clinical meeting, and the diagnostic hypothesis of Pinch-off Syndrome was raised, with a probable etiology due to a fractured catheter in an internment performed 2 years ago in another Service. Anticoagulation was started and a hemodynamic approach was chosen. Before the procedure, a new TTE did not identify images suggestive of thrombi adhered to the catheter. The interventional procedure was uneventful, with rapid removal of the foreign body and confirmed to be a catheter tip. The patient evolved hemodynamically stable and was discharged asymptomatic with optimized treatment for HF.

**Conclusion:** Pinch-off syndrome is associated with high rates of serious complications. The finding of an embolized catheter fragment on imaging is typical. The removal of the fragment is mandatory and the hemodynamic procedure is the first option. The thrombotic burden associated with the catheter initially made it difficult to define the nature of the fragment and data from the retrospective anamnesis were essential for the conclusion of the diagnosis.

111143

Modality: E-Poster Researcher – Case Report

Category: COVID-19 AND CARDIOVASCULAR SYSTEM

## Post-Covid Syndrome and Systemic Arterial Hypertension (SAH) in a Young Adult: A Case Report of the Functional Status After a Cardiopulmonary Rehabilitation Program

ANA INÊS GONZÁLES^1^, Ana Inês Gonzáles^1^, Lucas Lemonie Zunino^1^, Josie Budag Matsuda^1^, Luis Otávio Matsuda^1^

(1) Centro Universitário para o Desenvolvimento do Alto Vale do Itajaí (UNIDAVI)

**Introduction:** Previous Systemic Arterial Hypertension in individuals affected by COVID-19 can lead to a worse prognosis, increase complication rates and reduce functional capacity. In view of this, exercise-based cardiopulmonary rehabilitation may be able to promote functional improvement. This study reports the case of a male patient, post infection by COVID-19, with a previous diagnosis of Systemic Arterial Hypertension, submitted to a cardiopulmonary rehabilitation program.

**Case Description:** V.N.F, 24 years old, male, diagnosed with Systemic Arterial Hypertension prior to SARS-Cov-2 infection, referred to a cardiopulmonary rehabilitation program, due to fatigue and lack of physical conditioning in usual life activities 4 months post infection. Pre- and post-intervention assessment: Post-COVID-19 Functional Status Scale (PCFS), 1-minute Sit and Stand Test (SST1), 2-minute Stationary Gait Test (2MWT) and 6-minute walk test (6MWT). The rehabilitation program was carried out for 8 weeks, twice a week, based on: 1) aerobic exercises on a treadmill, 2) strength and muscular endurance exercises, developeded in a progressive and individualized way. Patient started the first session with continuous aerobic exercise for 10 minutes, at 3.5 km/h, without incline, reaching Borg 4, heart rate of 119 bpm (61%) and muscle strengthening exercise in upper limbs with an elastic band, reached Borg 3 for dyspnea. Patient completed 100% of the sessions performing, in the last session, aerobic exercise in running for 20 minutes, at 6.5 km/h, without incline, final Borg of 0.5 with heart rate of 148 bpm (75%) and 8 minutes in circuit associating exercise bike (load 4) interspersed with squat on a trampoline, with final Borg of 0. After 8 weeks, the following data were evidenced: PCFS (Grade 1 vs Grade 0), SST1 (18 repetitions/final Borg 5 vs 23 repetitions/Borg end 0), 2MWT (66 elevations/Borg end 5 vs 94 elevations/Borg end 0), 6MWT (510 m vs 552 m), respectively. The patient completed 100% of the intervention sessions.

**Conclusions:** The results show that performing cardiopulmonary rehabilitation for 8 weeks was able to improve the functional status of a young adult individual with arterial hypertension and symptoms of post-covid syndrome.

111266

Modality: E-Poster Researcher – Case Report

Category: CARDIOVASCULAR IMAGING

## Positioning of the Left Ventricular Lead Target by Gated Spect Determining Reverse Ventricular Remodeling

ERIVELTON ALESSANDRO DO NASCIMENTO^1^, Fernando de Amorim Fernandes^1^, Zhuo He^3^, Weihua Zhou^3^, Claudio Tinoco Mesquita^1^

(1) Universidade Federal Fluminense – UFF; (2) Instituto Estadual de Cardiologia Aloysio de Castro – IECAC; (3) Michigan Technological University – MTU

A 66-year-old female with ischemic heart failure had an ejection fraction (EF) value of 14%, with left bundle branch block, and was considered functional class IV despite optimized therapy. Subjected cardiac resynchronization therapy (CRT) with left ventricular (LV) lead positioning targeted by the last viable segment was determined by GATED SPECT. After CRT, the EF value was 57%. LV contraction was successfully analysed by radionuclide ventriculography at first, however, the addition of GATED SPECT phase analysis and its subsequent validation, has shown excellent potential in determining left ventricular mechanical dyssynchrony. Studies that report on the placement of the LV lead are consistent with the findings of the last contracting segment identified by GATED SPECT phase analysis and show significant clinical improvement. Recent VISION CRT data demonstrated that dyssynchrony improvement, but not target lead placement, predicts clinical outcomes in CRT. Parameters of the LV geometry obtained by GATED SPECT phase analysis were able to predict a super-response to CRT. This report demonstrates a super-response with a significant change in the LV eccentricity index and LV shape in both LV end-systole and end-diastole, denoting reverse remodelling after CRT target by GATED SPECT.


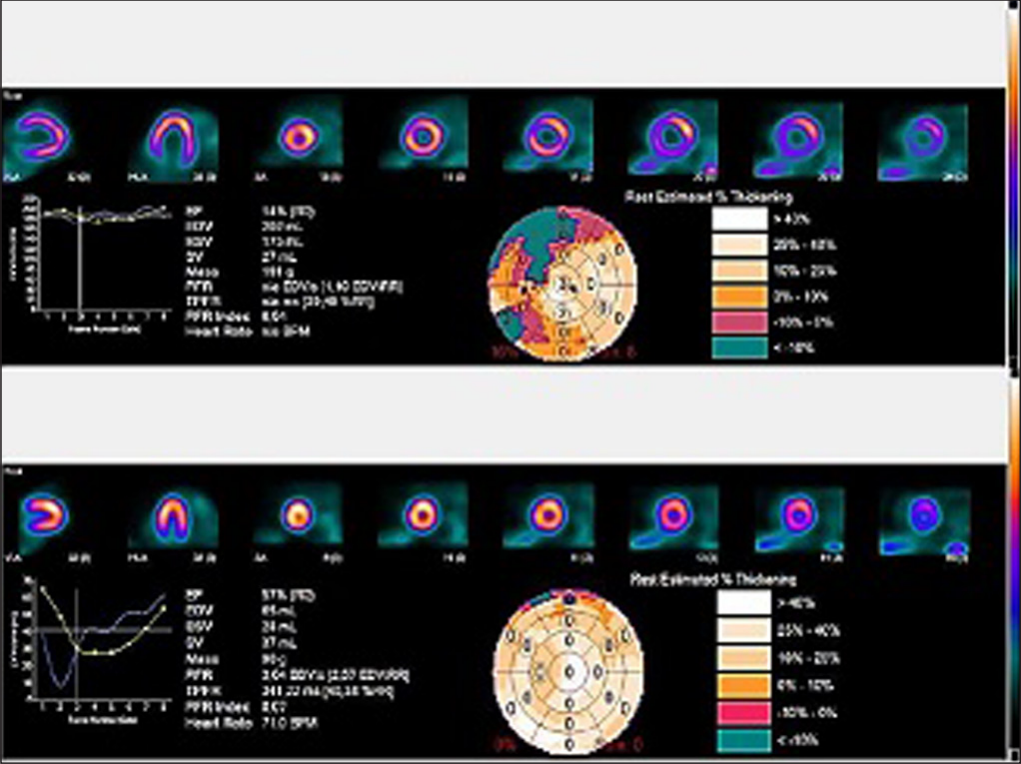


111276

Modality: E-Poster Researcher – Case Report

Category: CARDIO-ONCOLOGY

## Cardiac Amyloidosis and Multiple Possible Cardiotoxicities in the Same Patient Making it a Complex Diagnosis

VANESSA SANTOS DOS SANTOS^1^, Eduardo Schlabendorff^1^, Diego Romero Cawen^1^, Euler Fernandes Manenti^1^

(1) Hospital Mãe de Deus (HMD)

Cardiac amyloidosis has been historically difficult to diagnose due to lack of specific symptoms and necessity of biopsy. In the oncologic population this becomes even harder, since there may be multiple confounding factors related to the essential neoplasm and its treatment cardiotoxicities. We present a case of a 72 year old male that developed symptomatic supraventricular arrythmias and congestive heart failure (CHF). He followed with repeated episodes with hemodynamic instability. Attempts to ablate the arrhythmia were unsuccessful and because he had a left atrial tachycardia, we chose to ablate the atrioventricular node and implant a dual chamber pacemaker in the deep interventricular septum. To rule out ischemic heart disease, he underwent a cardiac resonance which showed signs of global left ventricular systolic dysfunction due to diffuse hypokinesia with an ejection fraction of 35% and extensive areas of discontinuous delayed enhancement, predominantly mesocardial and subepicardial (non-ischemic pattern), diffusely compromising the left ventricle. These findings suggested an inflammatory process of the myocardium (post-infectious or drug related) or an infiltrative cardiomyopathy – presumably amyloidosis, although not a typical imaging presentation which usually appears with predominance in the subendocardial areas. Amyloidosis was pursued and after ruling out light chain amyloidosis by laboratory tests, he performed a pyrophosphate scintigraphy. The result was a heart to contralateral (H/CL) ratio of 1,7 (a ratio of >1,5 is considered to strongly suggest transthyretin amyloid deposition). He also performed genetic testing and we are waiting the identification of the mutation. His treatment for CHF was optimized and he was discharged with improved symptoms. His past medical history was notable for a non-Hodgkin’s lymphoma diagnosed in 1983 and treated with chemotherapy, which included an anthracyclic, and radiation therapy of the mediastinum. In 2013 he was diagnosed with a prostate cancer and underwent prostatectomy, followed by androgen deprivation therapy (ADT) with an GNrH antagonist, also with known cardiotoxicity. All of these aggressors may have contributed to the ventricular dysfunction and appearance of arrythmia, presumably being of multifactorial etiology. But we highlight the need to pursue the diagnosis of amyloidosis as the correct identification of this disease is crucial for the initiation of potentially life-saving treatments.

111305

Modality: E-Poster Researcher – Case Report

Category: HEART FAILURE/CARDIOMYOPATHY/TRANSPLANT

## Restrictive Desmin Deposition Cardiomyopathy

CARLOS MANOEL DE CASTRO MONTEIRO^1^, Laís Olivo Rossi^1^, Luísa Carvalho Benedito^1^, Raul Cordeiro Pessanha^1^, Mireya Mendonza Rodrigues^1^

(1) Hospital Samaritano Paulista

**Introduction:** Heart failure (HF) is the end of many diseases that affect the heart, which explains its increasing prevalence. Restrictive cardiomyopathy is the least prevalent form of cardiomyopathies. This case report aims to discuss restrictive cardiomyopathy, addressing a rare etiology that is desmin deposit.

**Case description:** Male patient, 26 years old, with cardiomyopathy diagnosed 3 years ago, evolving for 2 months, with swelling in the lower limbs and progressive dyspnea. He was admitted to our institution with dyspnea on minimal exertion and signs of low cardiac output, being diagnosed with HF profile C. Family history: father, aunt and paternal cousin have cardiomyopathy. The ECG showed atrial tachycardia with RBBB and the echo showed significant increase in both atria, slight increase in both ventricles and significant diffuse involvement of the RV and LV with EF = 45%. Magnetic resonance imaging (MRI) of the heart showed late non-ischemic enhancement. Given the clinical picture, family history, echocardiogram and “MRI”, genetic testing was indicated. The complete exome sequencing identified a heterozygous pathogenic variant in the “DES” gene related to a restrictive cardiomyopathy secondary to the accumulation of desmin, which is the most important intermediate filament of skeletal and cardiac musculature, functioning as a protein cytoskeleton. Cardiac manifestations can occur in the form of restrictive cardiomyopathy, conduction system disease, arrhythmias, and sudden death. The patient presented worsening heart failure, requiring vasoactive amines, “IAB” and “ECMO”, being listed on the heart transplant waiting list. Transferred to a transplant center, undergoing heart transplantation, however, in the postoperative period, he presented sepsis, progressing to death. In this clinical case, a pathogenic variant associated with autosomal dominant/recessive cardiomyopathy was identified. The genetic test elucidated the etiology of cardiomyopathy, the refractoriness of heart failure to clinical treatment and optimized hemodynamic support, included the indication for heart transplantation (HT).

**Conclusion:** This clinical case reports the importance of genetic tests in the etiological diagnosis of cardiomyopathies and “HT” is considered a therapeutic option in patients with advanced and refractory HF.

111327

Modality: E-Poster Researcher – Case Report

Category: ATHEROSCLEROSIS/CARDIOVASCULAR RISK FACTORS/CARDIOVASCULAR PREVENTION

## Impact of Daylight Saving Time Transitions in Cardiovascular and Metabolic Acute Events: A Preliminary Exploratory Study

MIGUEL MEIRA E CRUZ^1^, Mauricio Machado da Rosa^1^, Michele Gomes da Rosa^1^, Dario Acuña Castroviejo^2^, Amélia Feliciano^3^

(1) Centro Cardiovascular da Universidade de Lisboa, Lisbon School of Medicine, Lisbon, Portugal; (2) Centro de Investigación Biomedica, Universidad de Granada, Granada, Spain; (3) Faculdade de Medicina, Universidade Católica, Lisbon, Portugal

**Introduction:** Daylight saving time (DST) imposes a twice-yearly hour shift. This circadian challenge has been associated to detrimental health effects including an increased incidence of acute myocardial infarction following the springtime transition and increased ischemic stroke following both DST transitions. While cardiovascular consequences of DST are still controversial, the possibility that DST may impact metabolic disorders was never discussed before. We aimed to investigate a possible relationship between cardiovascular and metabolic acute events and DST transitions.

**Sample and Methods:** All acute cardiovascular and metabolic events occurring during the 3 days before (pre transitional period) and after (post transitional period) the spring and the autumn shift were extracted from the records of two main hospitals in Lisbon, Portugal. The variation between the number of cardiovascular and metabolic events observed in the post transitional and pre transitional periods from both ocasions were statistically compared.

**Results:** From the total of 492 patients (47.2% males) recurring to the hospital either during 2018 spring time transition or same year autumn time transition, with a mean age of 70.36 ± 16.1 years old, 89% presented with cardiovascular events whereas 11% presented with metabolic events. The variation amplitude from the 3 preceding days to the 3 subsequent days was +8.33% for cardiovascular and +50% for metabolic events in the spring transitional period. The same variation in the autumn transitional period was +16.13% and –65%, respectively.

**Conclusion:** Results from this preliminary study corroborate the belief that a conflict between DST and cardiovascular health could be expected in both DST transitional periods although with important seasonal differences. Further, a relevant impact on metabolic status was also showed for the first time. While these results may reflect an impact of the circadian misalignment on cardiometabolic functions, the clinical meaning of such interaction may rely on further complex interaction with circannual oscillations from seasonal timing systems for which future exploration is warranted.

111419

Modality: E-Poster Researcher – Case Report

Category: ACUTE AND CHRONIC CORONARY DISEASE/THROMBOLYSIS

## Acute Myocardial Infarction by Spontaneous Coronary Dissection

ANA AMARAL FERREIRA DUTRA^1^, Isabelle Mendes Rodrigues Salomão^1^, Luanna Damasceno Amaral de Sousa^1^, Louise Freire Luiz^1^, Bruna Pereira de Mendonça^1^

(1) Hospital Pró Cardíaco

**Introduction:** Spontaneous coronary artery dissection (SCAD) is a rare cause of acute coronary syndrome (ACS), which can lead to sudden death. SCAD is an important differential diagnosis of chest pain and should be considered especially in young women with few risk factors for coronary artery disease (CAD).

**Case report:** Patient, 63 years old, female, grade IV obesity and arterial hypertension, and positive family history of CAD. Admitted to the emergency department of a quaternary hospital with hypertensive emergency (BP 230 × 92 mmHg) associated with typical chest pain, at rest, radiating to the back and upper limbs, with no improvement and/or worsening factor. She reported an intense episode of anxiety the night before the pain. Physical examination showed no changes in cardiopulmonary auscultation, blood pressure was similar in the upper limbs and no lower limb edema. A loading dose of acetyl salicylic acid (ASA) was performed and nitroglycerin infusion was started. The admission electrocardiogram showed: sinus rhythm, ST infra of DIII and aVF. First troponin of 312 ng/mL (ultrasensitive) and other tests without changes. Bedside echocardiogram with preserved biventricular systolic function, with no change seen in the ascending aorta. The patient evolved with chest pain, refractory to clinical treatment, and therefore, an emergency hemodynamic study was chosen. Coronary angiography (CAT) showed spontaneous coronary dissection (diagonal artery). We opted for conservative treatment with dual antiplatelet therapy (ASA and Clopidogrel), high-potency statin and strict control of the dual product (heart rate and systemic blood pressure).

**Discussion/Conclusion:** The incidence of SCAD is higher in young women and presents as ACS, it is extremely important to make the differential diagnosis of chest pain. It may be related to fibromuscular dysplasia, pregnancy, puerperium and hormone replacement. The approach to patients should follow the normal ACS protocol, but it should be taken into account that CAT can aggravate the dissection. Recent studies have shown benefits with conservative treatment in low-risk DEAC, as most cases evolved with spontaneous healing of the intramural hematoma after 30 days.

111501

Modality: E-Poster Researcher – Case Report

Category: CARDIO-ONCOLOGY

## Tumor Thrombus Ascending Through the Inferior Vena Cava and Extending Into the Right Atrium Successfully Removed by a Multidisciplinary Surgical Team in a Young Patient with Advanced Testicular Cancer

EDUARDO SCHLABENDORFF^1^, Vanessa Santos dos Santos^1^, Diego Raul Romero Cawen^1^, Marcelo Haertel Miglioranza^1^, Euler Roberto Fernandes Manenti^1^

(1) Hospital Mãe de Deus de Porto Alegre

**Introduction:** Tumor thrombus is a rare complication of testis cancer and needs appropriate treatment including chemotherapy and surgery.

**Case report:** A 23-year-old man was hospitalized in October 2019 with epigastric pain, dyspnea, tachycardia and tachypnea without hypoxemia. The EKG showed a S1Q3T3 pattern. Laboratory revealed mild anemia, leukocytosis, normal troponin and BNP levels. D-dimer levels were extremely high. Point-of-care echocardiogram followed by a 3d echocardiogram showed a large mobile mass compatible with a thrombus inside the right atrium that incursed towards the right ventricle in diastole. CT angiography revealed a probable tumor thrombus originated in the left renal vein, entering the inferior vena cava and ascending into the right atrium. There is also an increase in volume of the left testis and a voluminous expansive mass next to the left renal hilum most likely associated with lymph node conglomerate. Anticoagulation was started even though CT scan was inconclusive for pulmonary embolism. The next day, the patient presented clinical worsening with signs of low output. Due to the high chance of response with cytotoxic chemotherapy in testicular tumors, chemotherapy with Bleomycin, Etoposide and Cisplatin (BEP) was guided by the oncologist. The patient underwent a left radical orchiectomy. After 4 courses of BEP chemotherapy the tumor markers normalized. A surgical team composed of cardiovascular, urological and oncological surgeons performed a triple surgery at the same surgical moment to remove the tumor thrombus using extracorporeal circulation (figure above). It was also performed left nephrectomy and retroperitoneal and pelvic lymphadenectomy. Advanced testicular cancer was diagnosed with a clinical stage of pT2-Nx-MO-S3, which has a poor prognosis. The pathological examination revealed a mature teratoma. The patient has been disease-free since surgery.

**Conclusion:** A multidisciplinary approach is necessary to treat patients with tumor thrombus secondary to advanced testicular cancer.


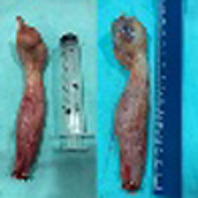


111659

Modality: E-Poster Researcher – Case Report

Category: NURSING

## Nurse‘s Role as a Scientific Educator in a Project to Care for AMI with ST Elevation in the Public Health Network

JOSELITO ADRIANO DA SILVA^1^, Nathalia Pereira^1^, Paola Begliomini^2^

(1) Allm Inc; (2) Boehringer Ingelheim

**Introduction:** Acute Myocardial Infarction (AMI) has a high rate of morbidity and mortality in Brazil and worldwide. In 2017, DATASUS recorded that AMI was responsible for 10% of hospitalizations in the Unified Health System (SUS) and 7% of all deaths in the country. In view of the magnitude of the disease and the benefit of the treatment being time-dependent, in 2019 the Sprint Project was started: a public-private collaboration initiative aimed at optimizing care for patients who enter the service with AMI with supra of ST through the development of care networks in SUS units, promoting rapid diagnosis and the appropriate choice of therapeutic strategy in a timely manner.

**Methods:** This is an experience report on the role of the nurse as a Scientific Educator in the Sprint Project, which acts as an interlocutor and facilitator of information within the public service of care and manager of the results that are obtained through the use of a mobile application. that allows communication between the services involved, for example UPA and hemodynamics.

**Results:** Currently the project is in 4 states and 06 cities promoting the integration between emergency services and the reference hemodynamics center, as agreed with the municipal/state health departments. With a strategic role, the nurse who works as a Scientific Educator must have experience in the care of patients with AMI and will actively work with the leader (“Champion)” of the network and the services involved, supporting the structuring of the care network in all areas. the phases. Project actions include: project presentation, diagnostic evaluation of the service from the physical structure to the service flow, analysis of improvement opportunities, construction of a multidisciplinary plan, training and supply of tools according to needs, periodic monitoring of networks and management of quality of care indicators based on the European ST-supra AMI Guideline.

**Conclusions:** It is concluded that the role of the Scientific Educator nurse is strategic and visionary, being considered by the care network as a partner to assist in the organization of the AMI care flow, strengthening the bond with the services involved, thus minimizing delays in treatment. and reducing mortality from AMI with ST elevation. The nurse’s role is, therefore, fundamental in the periodic maintenance of the project, reducing errors, engaging the professionals at the end and contributing to improve the journey of the patient with AMI.

111696

Modality: E-Poster Researcher – Case Report

Category: PERICARDIUM/ENDOCARDIUM/VALVOPATHIES

## Mitral Valve Infective Endocarditis with Septic Embolization in a Young Patient: A Case Report and Review of Literature

CAMILA RICHTER^1^, Priscilla Vicente Lista^1^, Carlos Alberto Kenji Naashima^1^, Jessica Woehl^1^, Thammy Lethicia de Sousa Silveira^1^

(1) Hospital Angelina Caron

**Introdution:** Infective endocarditis (IE) is a serious disease that requires treatment by a multidisciplinary team, involving cardiologist, infectious disease specialist and cardiovascular surgeon, as well as neurologists in cases of involvement of neurological events.

**Case Report:** DB, 39 years old, male, no previous diseases. No vices. The patient was admitted in-hospital with fever, headache, evolving into mental confusion and decreased level of consciousness and strength in legs. Moreover, the cardiac auscultation presented a more audible systolic murmur in the mitral focus. The cranial tomography revealed right frontal and left temporal ischemia (figure 3). The sorologies were required and were negative for the hepatitis B, C, Human Immunodeficiency vírus (HIV) e syphilis. Meningitis was ruled out after liquor evaluation. Janeway lesions were observed in the toes (figure 4). A bedside investigation was performed with Phelcomâ portable retinograh and Roth spots were identified (figure 1). Blood culture was positive in less than 24 hours for Staphylococcus aureus. Transesophageal echocardiogram confirmed suggestive image of mitral vegetation on the atrial face (figure 2). The mitral valve was replaced by a biological prosthesis.

**Conclusion:** This case report showed the critical course of the IE, presenting with the worst possible manifestations for the disease. The surgical treatment is the principal recommendation for this case.


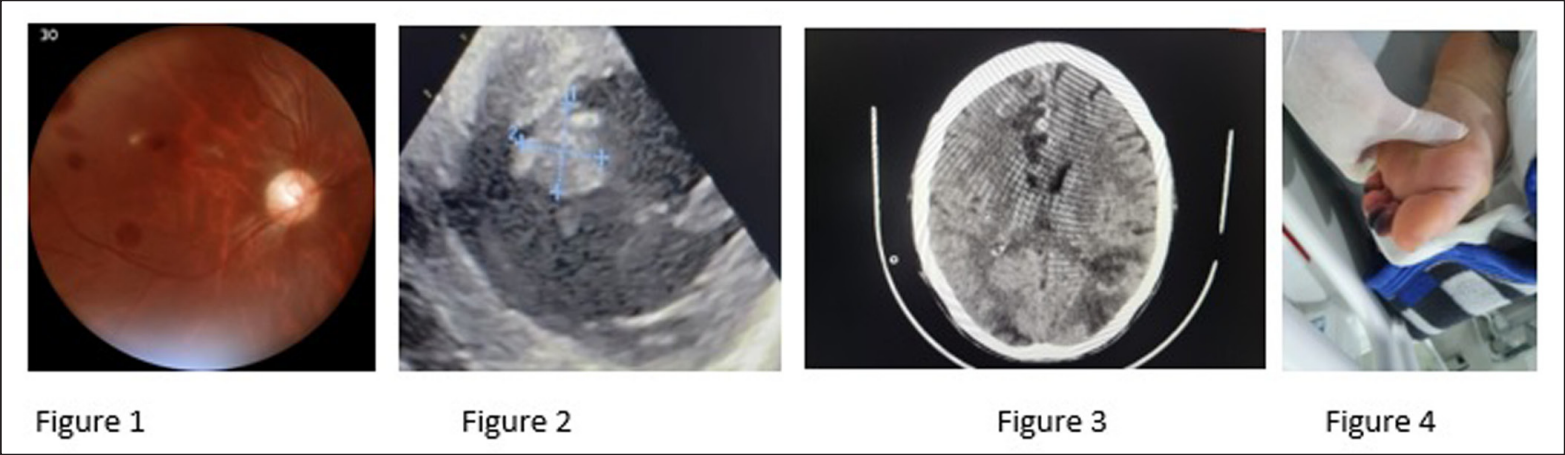


111728

Modality: E-Poster Researcher – Case Report

Category: PERICARDIUM/ENDOCARDIUM/VALVOPATHIES

## Pseudoaneurysm Due to Coxiella Burnetti Endocarditis Successful Treated with a Vascular Plug

CLAUDIO QUERIDO FORTES^2^, Paula de Medeiros Pache de Faria^1^, Ilan Gottlieb^1^, Luiz Antônio Carvalho^1^, Valdo José Carrera^1^

(1) Casa de Saúde São José; (2) Hospital Universitário Clementino Fraga Filho – Universidade Federal do Rio de Janeiro

**Introduction:** Infective endocarditis (1E) with negative blood cultures represents a small percentage of the cases, however, in many of these episodes it is possible to identify the microorganism causative through serological and/or molecular methods.

**Case Report:** A 65-year-old male with a bicuspid aortic valve, diagnosed with 1E with persistently negative hemocultures, complicated by aortic paravalvular abscess evidenced by transesophageal echocardiography (TEE) and by aortic angiotomography. Empiric treatment with ampicillin plus sulbactam associated with ceftriaxone was started and valve replacement by biological prosthesis was performed. After 6 weeks on antibiotics therapy, he was discharged. Three months later fever returns and after an extensive investigation PCR for Coxiella burnetii was positive in blood and in the specimen from hepatic biopsy and IgG phase I, II and IgM phase I was higher than 1;1024. Treatment with doxycycline and hydroxychloroquine was started. At that time TEE and angiotomography evidence an abscess which, in few days, resulted in pseudoaneurysm. As Eurocore was too high and there were technical difficulties for a reoperation it was chosen to use a vascular plug with which a successful closure of the communicating orifice was achieved. Patient was discharged with oral treatment and after 4 months of clinical and echocardiographic follow up he is still doing well without any sigh of recurrence.

**Conclusion:** When the Euroscore is too high and the surgical procedure almost impossible to be performed, the use of devices such as the vascular plug might be a plausible alternative, as it was for the patient here reported.

111773

Modality: E-Poster Researcher – Case Report

Category: HEMODYNAMICS AND INTERVENTIONAL CARDIOLOGY

## Analysis of Intracranial Compliance During Transcatheter Aortic Valve Implantation: Case Report and New Perspectives

ROBERTO ABDALLA FILHO^1^, Hugo Bertipaglia^1^, Cledicyon Eloy da Costa^1^, Maurício Marson Lopes^1^, Gustavo Frigieri^2^

(1) Hospital Samaritano de Campinas, Campinas, SP, Brasil; (2) Brain4care, Brasil

The connection between cardiac alterations and brain function is an area still under inquiry. The use of tools for neurological monitoring of patients with heart dysfunction may represent a possibility to achieve better outcomes. This report will discuss the use of a new non-invasive intracranial compliance monitoring tool for patients with heart dysfunction prior to and following transcatheter aortic valve implantation (TAVI).

**Case description:** A 80-year-old patient with progressive fatigue with marked aggravation during the past year. The image tests showed aortic valve fibrocalcification with severe stenosis and severe atheromatosis in the aorta, that contraindicated de transfemoral access for TAVI and made the transapical access the eligible option. Intracranial compliance (ICC) monitoring was done prior to TAVI and post-procedure.

**Results:** The patient showed improvement in the peak gradient, which went from 125 mmHg before the procedure to 14 mmHg after, and at the peak speed initially of 5.58 m/s and 1.88 m/s after TAVI. The ICC of the patient presented values altered before TAVI, with a Ratio of P2/P1 of 1.38 and time to peak of 0.27, values suggestive of intracranial hypertension. After the procedure, ICC was normalized, with a P2/P1 ratio of 0.71 and a time to peak of 0.14 (p < 0,001).

**Conclusion:** The noninvasive system was able to show alterations in intracranial compliance with cardiac alterations, as well as improvement after TAVI.


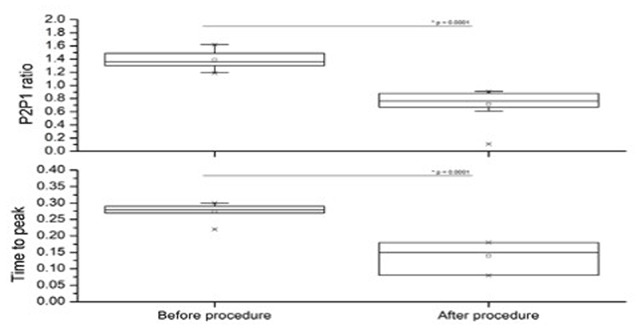


111787

Modality: E-Poster Researcher – Case Report

Category: CARDIOVASCULAR INTENSIVE CARE/CARDIOVASCULAR EMERGENCIES

## Aortoesophageal Fistula Caused by Thoracoabdominal Aortic Aneurysm Rupture: A Case Report

NINA NOVAES AZEVEDO^1^, Bruno Daniel Ferrari^1^, Fernanda Corrêa de Oliveira Lima^2^, Flávia Corrêa de Oliveira Lima^2^, Victor Sandi Mori Salvador^2^

(1) Curso Intensivo de Revisão em Cardiologia Clínica – Rio de Janeiro; (2) Hospital Regional de Presidente Prudente – HRPP

**Introduction:** Aortoesophageal fistulas (AEF) are rare and require rapid diagnosis. They represent a major therapeutic challenge because of their high morbimortality; 2/3 of these are a consequence of thoracic aortic rupture, resulting from aneurysms or traumas.

**Case description:** M.E.M.R., 87 years old, female, from São Paulo countryside, was admitted with hematemesis to the Emergency Room in a private hospital in the area. Personal history included arterial hypertension and diabetes mellitus. On physical examination, cardiac and pulmonary auscultation showed no findings, no carotid bruits, Glasgow 15, no focal neurological deficit. After clinical stabilization, the conduction of Upper Digestive Endoscopy showed esophageal aortic fistula, which was non-communicating, thrombosed and without active bleeding. The computed tomography of the chest showed a thoracoabdominal aortic aneurysm of approximately 70 mm in diameter, with the presence of extensive intramural thrombus from the abdominal aorta to the ascending aorta trunk, without signs of acute dissection, with esophageal compression. Assessment through vascular surgery indicated no intervention with endoprosthesis, due to her advanced age. Because of her hemodynamic stability, a second analysis showed that intervention was recommended, and she was inserted in the Vacancy Regulation Center (CROSS). The day before the procedure, a new bleeding led to her death.

**Conclusion:** AEFs are rare and fatal presentations of thoracoabdominal aortic ruptures. The patient’s advanced age was a decisive factor in indicating the intervention, together with her hemodynamic stability. Despite the high morbimortality of AEFs, the effectiveness of the entire system of early diagnosis, stabilization, and treatment is essential for a complete patient care, often being successful, and sometimes unsuccessful.

111820

Modality: E-Poster Researcher – Case Report

Category: CARDIO-ONCOLOGY

## Pericardial Effusion and Mediastinal Mass

FLÁVIA CRISTINA CARVALHO DE DEUS ^1^, Aurora Felice Castro Issa^1^, Claudio Domênico Sahione Schettino^2^, Carlos José Coelho de Andrade^3^

(1) “Curso de Pós-Graduação em Cardiologia da SBC/INC/INCA, Rio de Janeiro, RJ, Brasil”; (2) Hospital Pró Cardíaco; (3) Instituto Nacional do Câncer – INCA

**Introduction:** Mediastinal masses with pericardial effusions constitute a great challenge that shows the importance of the Cardio Oncology specialty.

**Case Description:** S.R.P., 73 years old, female. Admitted to the emergency room with progressive dyspnea. Stable vital signs, but dyspnea with saturation 88%. Performance Status of 2. Laboratory with anemia, hypoalbuminemia and elevated C-reactive protein. Echocardiogram showed preserved biventricular function, strain – 16, presence of a mass anterior to the mediastinum (15 × 13 cm), pericardial effusion without restriction and bilateral pleural effusion. Angioresonance showed right paracardiac mass that insinuates into the right atrium and extends to the aorta. It was requested an Oncologist Opinion. FDG PET scan showed hyper uptake in the right mediastinal mass with SUV 24 (Standardized Uptake Value). Values greater than 2 are suggestive of malignancy. CT-guided liver biopsy was suggestive of Sarcoma. Doxorubicin 75 mg/m^2^ was started. The electrocardiogram was monitored for the risk of QT interval prolongation and serial measurements of troponin and BNP were performed. Immunohistochemical panel result changed the diagnosis to GIST (Gastrointestinal stromal tumors) and Imatinib 400 mg orally once a day was started. Genetic biopsy test did not show mutations. Follow-up CT scans and echocardiogram showed no response to Imatinib. She presented with severe headache after the tomography and sudden coma with ventricular bleeding and unfortunately evolved to death. GISTs are sarcomas of cells of the gastrointestinal tract (cells of Cajal). They have CD 117 surface markers (C-kit) involved in tumor growth. They are rare tumors, diagnosed incidentally, occur between 50 years old and are most frequent in the stomach and small intestine. Only 5% in other locations. About 30% progress to malignancy. The final diagnosis is based on immunohistochemistry and genetic tests. The treatment is surgical resection and therapy with tyrosine kinase receptor inhibitors. The first choice is Imatinib at a dose of 400 mg per day. Early tumors have a 90% survival at 5 years, but tumors larger than 11 cm and metastatic have a worse prognosis.

**Conclusion:** Mediastinal masses with cardiac involvement are of high risk for patients. Although rare, GISTs should always be considered. The union between Cardiology and Oncology is essential for the best treatment in these cases.

111924

Modality: E-Poster Researcher – Case Report

Category: ATHEROSCLEROSIS/CARDIOVASCULAR RISK FACTORS/CARDIOVASCULAR PREVENTION

## Comisa and Associated Cardiovascular Risk: A Case Report of a Successful Combined Intervention with Pap and CBTI

MIGUEL MEIRA E CRUZ^1^, Ana Brito^2^, Verona Mihai^3^

(1) Sleep Unit, Centro Cardiovascular da Universidade de Lisboa, Lisbon School of Medicine, Lisbon, Portugal; (2) Guy’s & St Thomas NHS Foundation Trust, London, UK; (3) Manchester University Foundation NHS Trust, London, UK

**Introduction:** Insomnia and obstructive sleep apnoea (OSA) are the two most common sleep disorders globally, and co-exist in up to 50% of patients in clinic. The risk of cardiovascular and metabolic disorders (CMD) is often independently associated with insomnia and OSA, and such risk seems to be aggravated when these conditions are comorbid (COMISA). Recent data shows that collaboration of psychology and physiology-based Sleep Medicine strategies may help improve patient-centric care.

**Case description:** 54 years old male patient with diagnosis of resistant hypertension, complaints of snoring and difficulties in initiating and maintaining sleep underwent sleep investigations in our department. The sleep study performed was consistent with the diagnosis of severe OSAS. The positive airway pressure (PAP) therapy was initiated and the patient was 100% compliant with the therapy. The response was excellent, with a residual Apnoea and Hypopnea Index (AHI) of 1.3/h and improved Epworth Sleepiness Scale from 11 to 5/24. Despite these good results, the patient still had problems initiating and maintaining sleep, presenting an Insomnia Severity Index (ISI) score of 13. He was at that point included in a Cognitive Behavioural Therapy program for insomnia (CBTI). After CBTI intervention, the patient presented a residual ESS of 4/21 and an ISI of 3, indicating no signs of clinical insomnia. The compliance with PAP remained excellent and the patient reported improvements on sleep quality, therapy management, as well as overnight awakenings. The blood pressure became normalised at 120/80 mmH2O.

**Conclusion:** Despite the high co-morbidity and impact of COMISA, there are many unknowns concerning the pathophysiology and optimal therapeutic management of this condition. The optimal use of PAP and tailored CBTI showed significant results not only on resolving the OSAS and Insomnia but also by reduction of cardiovascular risk, with blood pressure being normalised. Therefore, the combination of sleep apnoea therapy and of behavioural sleep therapies, such as CBTI, can be the solution to address this complex condition.

111950

Modality: E-Poster Researcher – Case Report

Category: HEART FAILURE/CARDIOMYOPATHY/TRANSPLANT

## Incidental Finding of Arrhythmogenic Right Ventricular Cardiomyopathy in 72-Year-Old Man – Should we Consider Cardio Defibrillator Implantation for Primary Prophylaxis?

RAFAEL MODESTO FERNANDES^1^, Luciana Cunha Weber^1^, Vitor Queiroz de Castro Souza^2^, Loren Lacerda Rodrigues^2^, Mariana Baptista Guedes^1^

(1) D’Or Institute for Research and Education (IDOR), Hospital Aliança, Salvador, Brazil; (2) Bahiana School of Medicine and Public Health, Salvador, Brazil.

**Background:** Described by Fountaine and Marcus in 1982, arrhythmogenic right ventricular cardiomyopathy (ARVC) is a genetic disease characterized by progressive replacement of myocardial cells by fibrofatty tissue. This is a leading cause of sudden arrhythmic death in young people and athletes.

**Case:** A 72-year-old man presented with heartburn, without relief after use of antacids. He denied chest pain, dyspnea, history of drinking or smoking. Past medical history was positive for stable coronary artery disease, non-specified arrhythmia, hypertension and hyperlipidemia. Physical examination was normal. The diagnosis of non-ST-elevation myocardial infarction (NSTEMI) was established based on T wave inversions in inferior and anterior leads at electrocardiogram and an abnormal ultrasensitive troponin level. A pharmacological stent was placed in the mid-segment of the right coronary artery to treat a sub occlusive during the invasive coronary angiography. Transthoracic echocardiogram (TTE) revealed preserved left ejection fraction but severely compromised right ventricle (RV), not corresponding to angiography. Cardiac magnetic resonance imaging corroborated the findings of the TTE, segmental changes and RV aneurysms, in addition to the reduced RV ejection fraction. These findings were supportive of ARVC. Holter identified frequent ventricular premature beats, however without complex arrhythmias. After discussion with the Heart Team, the patient was classified as low risk, despite the important involvement of the RV. He was discharged and kept under outpatient follow-up without the need for implantable cardio defibrillators (ICD). Further screening was advised for family members.

**Discussion:** Patients with ARVC usually present symptoms during the second to fifth decade of life. Advanced age poses a challenge for the diagnosis of the disease, as symptoms could be disguised as those of NSTEMI. In order to prevent misdiagnosis and improve risk stratification, more information is needed on the natural history of these patients. In this case, the decision to implant the ICD is challenging, considering the patient’s age, degree of RV involvement and absence of complex arrhythmias.

112096

Modality: E-Poster Researcher – Case Report

Category: COVID-19 AND CARDIOVASCULAR SYSTEM

## What are the Effects of Cardiovascular Rehabilitation on Exercise Tolerance, Fatigue and Quality of Life in Patients with Limiting Symptoms in the Post-COVID-19 Period in a Reference Service in Northeastern Brazil?

DANIELLA CUNHA BRANDAO^1^, BRUNA THAYS SANTANA DE ARAÚJO^1^, JULIANA ANDRADE FERREIRA DE SOUSA^1^, Maria Inês Remígio^1^, Armèle Dornelas de Andrade^1^

(1) Universidade Federal de Pernambuco

**Introduction:** The main limiting symptoms in the post-COVID-19 period are dyspnea, fatigue and reduced exercise tolerance. In these patients, cardiovascular rehabilitation can improve functional capacity, reduce deconditioning after a prolonged stay in the intensive care unit, and facilitate return to work.

**Objective:** To verify the effects of cardiopulmonary rehabilitation consisting of aerobic and continuous resistance training of moderate intensity on pulmonary function, respiratory muscle strength, maximal and submaximal exercise tolerance, fatigue and quality of life in post-COVID-19.

**Methods:** This is a quasi-experimental study with a protocol of 12 outpatient intervention sessions in a reference service for COVID-19 in Pernambuco. Adults over 18 years of age (N = 26) diagnosed with COVID-19 and discharged from hospital at least 15 days before the first assessment were included. Participants performed continuous moderate-intensity aerobic and resistance training twice a week. Maximum and submaximal exercise tolerance, pulmonary function, respiratory muscle strength, fatigue and quality of life were assessed before and after the intervention protocol.

**Results:** Cardiopulmonary rehabilitation improved maximal exercise tolerance, with an increase of 18.62% in peak oxygen consumption (VO2peak) and 29.05% in time to reach VO2peak. The VE/VCO2 slope reduced by 5.21% after the intervention. We also observed an increase in submaximal exercise tolerance (increase of 70.57 meters in the 6-minute walk test, p = 0.001), improved quality of life and reduced perceived fatigue after the intervention.

**Conclusions:** In this work, cardiopulmonary rehabilitation improved pulmonary function, respiratory muscle strength, maximal and submaximal exercise tolerance, fatigue and quality of life in patients with limiting symptoms in the post-COVID-19 period. Aerobic and continuous resistance training of moderate intensity proved to be effective in the recovery of these patients.

112015

Modality: E-Poster Researcher – Case Report

Category: HEMODYNAMICS AND INTERVENTIONAL CARDIOLOGY

## Percutaneous Treatment for Calcified Coronary Lesions using Intravascular Lithotripsy Assessed by FFR and OCT: Putting all Together

LUIS AUGUSTO PALMA DALLAN^1^, Marcelo Harada Ribeiro^3^, Cristina Silveira^3^, Gabriel Tensol Rodrigues Pereira^1^, Guilherme Ferragut Attizzani^1^

(1) University Hospitals Cleveland Medical Center; (2) InCor – Instituto do Coração – HCFMUSP; (3) SOS Cardio, Florianopolis, SC, Brazil

**Introduction:** Patient 70 years old male caucasian, with multiple comorbidities, including hypertension, diabetes, obesity, dyslipidemia, CKD 3B (GFR 37, Creat 1.8), S/P prostate cancer, and severe AS (LVEF 55%, mean grad 50, Vmax 4.6, DI 0.19) with NYHA Class II symptoms including dyspnea with minimal exertion, worsening fatigue over the past 3 months, and intermittent chest pressure. In summary, the patient met indications for AVR. Coronary angiography pre-procedure showing a severely calcified lesion in proximal/mid/mid-distal LAD with angiographic moderate-to-severe lesions (60%). Per Heart Team discussion, elective FFR with possible PCI to LAD and elective TAVR.

**Procedure:** In this case, assessment through FFR, OCT and Intravascular Lithotripsy contributed to simplify a potential high-risk procedure. FFR confirmed the severity distally to sequential lesions and indication for PCI. The images evidenced by OCT completely changed the patient’s treatment. OCT was especially important to determine the anatomy of the lesion and adequate planning. Evidence of heavy proximal circumferential calcification and severe lesion in mid-distal LAD that would not be assessed through angiography. If the proximal lesion was not pre-treated, probably would have resulted in underexpansion of the stent with higher rates of stent failure. As per evidence of severe lesion in mid-distal LAD, it mostly would not be assessed only based on angiography. In addition, OCT also allowed for adequate treatment planning, with pre-dilatation and ideal stent sizing, with a satisfactory final result evidenced by excellent stent expansion and without signs of dissection of the stent edges. Intravascular Lithotripsy contributed to simplify a potential high-risk procedure such as a rotational atherectomy in proximal LAD in a patient with severe AS and no possibility of protected PCI with Impella due to severe aortic stenosis. Three days after discharge, the patient was submitted to elective TAVR successfully.

**Conclusion:** We report the importance of physiology and intravascular imaging on a successfully OCT-guided PCI using intravascular lithotripsy: FFR confirmed the severity of the lesions and indication for PCI; OCT provided important information for adequate treatment planning and execution of the procedure, minimizing the risk of stent underexpansion; and Intravascular Lithotripsy contributed to simplify a potential high-risk procedure.

112041

Modality: E-Poster Researcher – Case Report

Category: CONGENITAL AND PEDIATRIC CARDIOLOGY

## Holt Oram Syndrome Associated with Biventricular Non-Compaction Cardiomyopathy

ERIVELTON ALESSANDRO DO NASCIMENTO^1^, Romero Ribeiro Duque^2^, Vinícius de Queiroz Aguiar^2^, Fabrício Vilhena de Castro Souza^2^

(1) Instituto Estadual de Cardiologia Aloysio de Castro – IECAC; (2) Centro Universitário de Volta Redonda – UniFOA

Holt Oram Syndrome (HOS) is characterized by the presence of upper limb abnormalities associated with cardiac malformations and cardiac conduction disorders, being an autosomal dominant hereditary disease, present in 1 in 100.000 individuals. Cardiac malformations, in general, are related to defects in the septation of the heart, with interatrial communication ostium secundum and interventricular communication being the most common. Non-Compaction Cardiomyopathy (NCC) is a rare condition characterized by increased trabecculation in one or more segments of the ventricles, especially the left ventricle. NCC can be found isolated or related to multiple phenotypes of cardiac structural alterations and arrhythmogenic alterations, however, the association with HOS presents a low number of case reports in the literature. We report a case of HOS associated with biventricular NCC. A 17-year-old male student with HOS and deformities in both upper limbs and previous atrioseptoplasty, was referred to the arrhythmology ambulatory complaining of “weak beats” and suspected NCC after performing a transthoracic echocardiogram. In the evaluation by the specialist, the patient had a heart rate between 42 and 45 beats per minute, and a 24-hour Holter and cardiac MRI were requested to assess bradycardia and suspicion of NCC. The 24-hour Holter presented periods of atrioventricular dissociation with bradycardic junctional escape. MRI showed increased endocardial trabeculate in the right ventricle and increased subendocardial trabeculation in the left ventricle (LV), with diagnostic impression of LV dilation associated with criteria of non-biventricular compaction, with preserved systolic function and absence of local fibrosis. Given the diagnosis of bi-ventricular NCC, the patient is followed up by the arrhythmology ambulatory to evaluate bradycardia with serial examinations. HOS associated with NCC presents a reduced number of reports in the literature, with presentations commonly affecting only the left ventricle. Thus, the case reported describes a patient diagnosed HOS related with bi-ventricular NCC, manifesting with atrioventricular blockade.

112175

Modality: E-Poster Researcher – Case Report

Category: HEART FAILURE/CARDIOMYOPATHY/TRANSPLANT

## Super Response in Resyncronization with Direct Left Bundle Branch Stimulation and Right Ventricle Depolarization Delay Correction. Case Report

RAONI DE CASTRO GALVÃO^1^, João Paulo Velasco Pucci^1^, Ofir Gomes Vieira^1^, Edvagner Sérgio Leite de Carvalho^1^, Carlos Eduardo Duarte^2^

(1) Centro de Ritmologia de Brasília; (2) CARE: Centro Avançado de Ritmologia e Eletrofisiologia; (3) Hospital Santa Lúcia

Cardiac resynchronization (CRT) has been an established procedure for over 20 years. Recently, direct stimulation of the cardiac conduction system has been gaining strength. Recent studies already show them with results similar to traditional CRT. We report a case submitted to CRT with direct stimulation of the left bundle branch followed by correction of the right ventricular depolarization delay with stimulation of its region with the greatest delay, in inferior wall activation.

**Case Report:** Female 77y, idiopathic cmp, LVEF 23%, increased LV diameters, diffuse hypokinesia and absence of fibrosis in the LV walls. ECG with LBBB and QRS 170 ms. Evolved with decompensated HF, recent hospitalizations. After clinical compensation, a CRT was implanted on 04/2021. First ventricular lead was implanted in the deep interventricular septum, after mapping the HIS bundle. During threshold test, there was selective stimulation of the LBB, with a RBBB morphological QRS, axis at 80° and width of 130 ms. By direct pacing of the left bundle branch, we took advantage of the other ventricular lead to correct the RV conduction delay generated by LBB pacing. After finding a delay of 90 ms, we chose to fix the second ventricular lead in the inferior wall of RV. Joint ventricular pacing resulted in a QRS of 108 ms, QRS axis –20°. Transthoracic echocardiogram at 45 days post-op showed an increase in LVEF to 48%, LV dimensions at 55/40 mm. At the moment the patient remains in NYHA-I. A 6 month ECHO-TT showed a LVEF 54% and preserved LV dimensions, patient remaining in NYHA-I.

**Discussion:** This report proposes a RV-CRT to correct a RBBB QRS morphology generated by direct stimulation of the LBB. About 30% with CRT are non-responders. Whether due to a non-ideal LV lead position, unfavorable coronary sinus anatomy, phrenic stimulation, etc. In this sense, CRT by direct stimulation of the HIS-purkinje system corrects many adversities in a simpler procedure to be performed. In this case, complete correction of previous LBBB encouraged us to use the other electrode to correct the RV activation delay generated by the LBB stimulation, resulting in a narrower QRS, allowing clinical response and an important and early LVEF increase. This report corroborates the literature by showing that stimulation of the conduction system is a real alternative to patients with indication for CRT.

112185

Modality: E-Poster Researcher – Case Report

Category: HEMODYNAMICS AND INTERVENTIONAL CARDIOLOGY

## Percutaneous Coronary Intervention for Ostial Right Coronary Artery Chronic Total Occlusion using Rotational Atherectomy Through the Subintimal Space

LUIS AUGUSTO PALMA DALLAN^1^, Luis Augusto Palma Dallan^2^, Cristina Silveira^1^, Carlos Augusto Campos^2^, Michael Megaly^3^

(1) SOS Cardio, Florianopolis, SC, Brazil; (2) InCor – Instituto do Coração – HCFMUSP, São Paulo, SP, Brazil; (3) Department of Cardiology, Willis Knighton Heart Institute, Shreveport, Louisiana, USA

A 78-year-old man with previous coronary artery bypass surgery (CABG) 10 years ago presented for right coronary artery (RCA) chronic total occlusion (CTO) percutaneous coronary intervention (PCI) for medical refractory angina after prior failed attempt. Coronary angiogram patent vein grafts to left anterior descending (LAD) and ramus arteries with early septal collaterals to occluded RCA (Figure 1, A). RCA occlusion was ostial and severely calcified (Figure 1, B). After crossing the Fielder XTa wire (Asahi Intecc, Japan), the lesion could not be crossed with different balloons or microcatheters. Therefore, we knuckled a RotaWire floppy (Boston Scientific, USA) subintimally beyond the proximal cap and performed rotational atherectomy (RA) with 1.25 mm followed by 1.5 mm burrs at 180,000 rpm (Figure 1, C). We then could advance a Finecross microcatheter (Terumo, Japan) along with a Pilot 200 wire (Abbott vascular, USA). We then crossed retrograde through septal collaterals and performed guide-extension assisted reverse controlled antegrade-retrograde dissection tracking and reentry (reverse CART) (Figure 1, D and E) followed by completion of PCI and good final angiographic result (Figure 1, F). Hospital stay was uneventful and the patient was free from angina at 6-month follow-up. Our case describes the modification of ostial CTO lesions with RA after DR, which has not been described before. Calcification remains one of the major challenges during CTO PCI.1 Different modalities can be used to modify calcium including RA. RA use after dissection-reentry (DR) techniques within the main vessel have been previously reported, but still carries a very high-risk of perforation and is advised against. The implementation of DR techniques has played a key role to make possible wire crossing even in severe calcified occlusions and increase the overall success rate. Although with DR, the pathway is easier as you go around calcium, the vessels might remain non-compliant requiring calcium modification. For ostial modification of CTO lesions after DR techniques, the authors recommend using high-speed (180,000 rpm) with very short runs and mainly in straight segments. Our technique represents a bailout strategy after failure of other techniques. It still carries a high risk of perforation or aortic dissection and benefits should outweigh the risks when making the decision to proceed.

112316

Modality: E-Poster Researcher – Case Report

Category: ATHEROSCLEROSIS/CARDIOVASCULAR RISK FACTORS/CARDIOVASCULAR PREVENTION

## Vasospasm and Acute Coronary Syndrome

NATHALIA DUARTE CAMISÃO^1^, MIGUEL ÂNGELO RIBEIRO^1^, ANDRÉ PAZOS TEIXEIRA^1^, NATÁLIA DOMINGUEZ PAES LEME DE SOUZA^1^, JOSÉ HERBERT DA SILVA PALHANO^1^

(1) Hospital Norte D’or

**Introduction:** Vasospastic angina is a variant form of angina pectoris in which angina occurs at rest with transient changes in the electrocardiogram and exercise capacity preserved. It is involved in many clinical scenarios such as stable angina, sudden death, acute coronary syndrome, arrhythmia or syncope. Smoking is a well-known precipitating factor in this clinical setting.

**Case Report:** Male patient, 58 years old, systemic arterial hypertension, smoker (smoking history 25 years – pack), in outpatient follow-up due to long-standing chest pain but with significant worsening in the last two months. Transthoracic echocardiogram (TTE) with preserved left ventricular systolic function, no segmental changes, no intracavitary overloads. Electrocardiogram with alteration of repolarization in the antero septal wall. Myocardial scintigraphy without perfusion changes, without ventricular dysfunction. Guided lifestyle changes and smoking cessation. Admitted to the ER 6 months later with oppressive chest pain radiating to the left upper limb, profuse sweating, associated with dynamic ST-segment change in the high lateral wall and increase in myocardial necrosis markers (ultrasensitive troponin peak of 38520 ng/L). He continued smoking. TTE similar to that performed previously, without significant changes. Coronary angiography was performed: significant vasospasm in the circumflex artery reversed after nitroglycerin, no obstructive lesions, ventriculography with infero apical hypokinesia. A calcium channel blocker was introduced with good clinical evolution without a new episode of chest pain during hospitalization.

**Conclusions:** Although the pathogenesis of coronary artery spasm has not been fully elucidated, different pathogenic mechanisms have been proposed, such as vascular smooth muscle cell hyperreactivity, endothelial dysfunction, vascular inflammation, altered autonomic nervous system response, and oxidative stress. These variables can be modified if it’s possible to detect early precipitating factors such as smoking in this case.

107810

Modality: E-Poster Young Researcher – Non-case Report

Category: ACUTE AND CHRONIC CORONARY DISEASE/THROMBOLYSIS

## Late Follow-Up of Patients with ST-Elevation Myocardial Infarction Treated by Pharmaco-Invasive Strategy

MARINA PASSOS PIZZITOLA^1^, Vinícius Nasser de Carvalho^1^, Pedro Luiz Homem de Mello^1^, Paula Santiago Teixeira^1^, José Roberto Pineda Pietrobon Redini Martins^1^, Filippo Mancini Diotto^1^, Bruna Regina Cogo^1^, Adriano Henrique Pereira Barbosa^1^, Iran Gonçalves^1^, Pedro Ivo de Marqui Moraes^1^

(1) Universidade Federal de São Paulo – Unifesp

**Introduction:** The risk of new cardiovascular events after an episode of ST elevation myocardial infarction (STEMI) conditions patients to a high-risk profile and reinforces the need to expand epidemiological knowledge about the long-term follow-up in this group.

**Objectives:** To evaluate the rates of all-cause and cardiovascular mortality, reinfarction and ischemic stroke in the late follow-up of STEMI patients treated by the pharmaco-invasive strategy (PhIS).

**Methods:** Data from a county network for the treatment of STEMI from January-2016 to December-2019 were analyzed, containing 1,170 consecutive patients undergoing fibrinolysis (97% tenecteplase) in county hospitals and systematically transferred to the tertiary center for cardiac catheterization and continuity of care, according to the PhIS. Post-discharge follow-up was performed on an outpatient basis or via telephone call to those who were absent, with approval by the institutional Research Ethics Committee. Numerical variables were described as median and interquartile range.

**Results:** The baseline demographic profile of the 1,170 patients evaluated was 348 (29.7%) women, age 59 [51–66] years, 654 (55.9%) hypertensive, 324 (27.7%) diabetics, left ventricular anterior wall infarction in 559 (47.8%), door-to-needle time of 68 [41–110] minutes and fibrinolytic-catheterization time of 16.5 [7.3–23.1] hours. In-hospital mortality was 60 (5.1%) cases. Loss of post-discharge follow-up occurred in 251 (21.4%) patients who were not located, so 919 were analyzed with a median follow-up of 2.6 [1.7–3.2] years. The overall mortality rate was 50 (5.4%), with 34 of the deaths (68.0%) attributable to cardiovascular causes, and the rates of reinfarction and ischemic stroke were respectively 56 (6.1%) and 15 (1.6%).

**Conclusion:** Although the elevated loss of follow-up constitutes a limitation for the analysis, patients from a county network for the treatment of STEMI based on PhIS had a high risk of recurrence of cardiovascular outcomes.


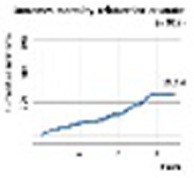


110502

Modality: E-Poster Young Researcher – Non-case Report

Category: EPIDEMIOLOGY AND HEALTH POLICIES/GLOBAL HEALTH

## Spatiotemporal Distribution of the Mortality of Coronary Arterial Disease Amongst Women in the State of Paraná/Brazil between 2008 and 2017

GIOVANNA MENIN DA SILVA ^2^, Marcelo Aguilar Puzzi^1^, Luciano de Andrade^1^, Amanda de Carvalho Dutra^1^

(1) Universidade Estadual de Maringá; (2) Universidade Federal de São Paulo

Coronary artery disease (CAD) remains the leading cause of death in women. The negative impact of CADs can be reduced through preventive care with interventions that address from the population to actions that have an individual character. Thus, combining information regarding the occurrence of CAD in women with the spatiotemporal variation constitutes an instrument that enables the development of strategies to identify areas of greater risk for developing the disease, the increase in risk over time and proposition of policies that favor interventions to reduce morbidity and mortality.

**Objective:** To analyze the spatiotemporal distribution of mortality rates of adult women with CAD in the State of Paraná – Brazil from 2008 to 2017.

**Methodology:** This is a descriptive, observational, retrospective study with the purpose of analyzing the spatiotemporal distribution of inequalities, with the use of geospatial health statistics, based on secondary mortality data in this period.

**Results:** There is a correlation between the annual and spatiotemporal rates, demonstrating that the analyzed covariates lead to municipal inequalities, mainly, income, education and access to a medical specialist, showing that comprehensive health care can disadvantage the survival of these patients. SMR greater than 1, correlates with higher income (p = 0.381), higher education index (p = 0.056), higher health index (p = 0.115), higher number of cardiologists (p = 0.232), higher rate of exercise test (p = 0.056) is considered high risk, corresponding to 75%, 50%, 25%. The correlation between greater accessibility, greater number of cardiologists, greater exercise testing and greater health index in Paraná with 75% of a SMR with greater risk.

**Conclusion:** Public health policies that serve everyone with equity, observing their loco-regional particularities should be encouraged and taken as a goal in the management of conflicts that affect women unequally.


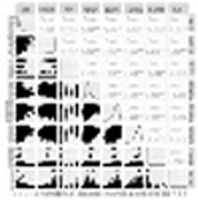


107823

Modality: E-Poster Young Researcher – Non-case Report

Category: CARDIOVASCULAR IMAGING

## DNA Damage Induced by Ionizing Radiation from Myocardial Perfusion Scintigraphy: A Real Threat?

ANNA PAULA ARPINI BOTELHO^1^, Júlia Passarelli Pereira^1^, Raiana Andrade Quintanilha Barbosa^1^, Aniele Moritz^1^, Marcelo Goulart^2^, Glauber Monteiro Dias^1^, Andrea De Lorenzo^1^

(1) Instituto Nacional de Cardiologia

**Background:** Patient exposure to ionizing radiation has grown due to increases in medical imaging, raising concern about potential risks. Among cardiac imaging tests, myocardial perfusion scintigraphy (MPS) is vastly employed. Overall, the link between radiation from MPS and cancer has not been considered consistent; however, further evidence obtained from studies at the molecular level is desirable.

**Objective:** To study the effect of ionizing radiation on the DNA of patients undergoing MPS using the comet assay, a method for detection of DNA damage, and the activation of repair genes in response to potential DNA damage.

**Methods:** Twenty-nine patients ≥18 years without acute diseases, recent surgery, cancer or autoimmune diseases were studied. MPS was performed with Tc-99 sestamibi (15–20 mCi) in a 2-day protocol. Peripheral blood was collected immediately before radiotracer injection at rest and 60–90 minutes after injection for the analyses. Single-cell gel electrophoresis (comet assay) was performed with blood lymphocytes to detect strand breaks, which determine a “comet tail” of variable size, visually scored by 3 observers in a fluorescence microscope after staining (0: no damage, no tail; 1: small damage; 2: large damage; 3: full damage). A damage index was calculated as a weighted average of the individual scores. The expression of repair genes CDKN1A, BBC3, XPC, GADD45a was evaluated with RT-PCR (QuantStudio 5, Thermo Fisher Scientific).

**Results:** After radiotracer injection, there was a significant, although small, increase of the damage index and of classes 1–3 of damage, even though most patients remained in class 0 (Figure A: top: percentages of damage classes before and after radiotracer; bottom: plot of DNA damage index from individual patients). There was no significant difference in the expression of CDKN1A, BBC3, XPC, GADD45a after radiotracer injection (Figure B).

**Conclusion:** DNA damage was detected in lymphocytes after a single radiotracer injection for MPS, without activation of repair genes. This suggests that MPS with the current protocols may not result in relevant DNA damage.


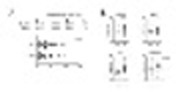


107829

Modality: E-Poster Young Researcher – Non-case Report

Category: CARDIAC ARRHYTHMIAS/ELECTROPHYSIOLOGY/ELECTROCARDIOGRAPHY

## Evaluation Electrocardiographic in Rats Infected with Plasmodium Falciparum and Submitted to Phytotherapy with Ammbaka

CUSTÓDIO JOSÉ GASPAR^1^, Miracelma de Assunção Pedro Alexandre^1^, Amélia Carlos Cazalma^1^, Jordão Augusto Trajanno^2^, Luciano Júlio Chingui^1^

(1) Universidade Metodista de Angola (UMA); (2) Casa de Caminho André Luís

**Introduction:** Malaria is a multisystem, non-contagious infectious disease caused by protozoa of the species Plasmodium falciparum, vivax, P. maleriae, P. ovale Stephens. Worldwide, malaria is characterized as the main cause of morbidity and mortality, in Angola malaria is caused mainly by Plasmodium falciparum, that is, 87% of cases.

**Objectives:** The objective of the present work is to evaluate and describe the effect of AMBAKA on the biological system of albino rats infected with Plasmodium falciparum.

**Material and methods:** This is an experimental/animal study, developed at the Methodist University of Angola. Eighteen male albino rats, aged 3 to 4 months, were used. The animals were divided into 3 experimental groups of n = 6: Control group, malaria and malaria treated with Ammbaka. For the induction of malaria in animals, human blood infected with the protozoan Plasmodium Falciparum was used. Each animal received an intraperitoneal injection of 0.05 ml of infected blood. 15 days after infection, microscopy was performed to evaluate parasitemia, for treatment, Ammbaka was used for 3 consecutive days, each animal received a dose of 0.7 ml intraperitoneally. On the fourth day, microscopy was performed to assess the absence of the parasite. The results were submitted to the Kolmogorov-Smirnov normality test, followed by ANOVA and post Tukey test with a significance level of 5% (p < 0.05).

**Results:** The results reveal that the animals developed malaria and Ambaka was able to eliminate plasmodium falciparum, the animals with malaria developed incomplete left bundle branch block, acute myocardial infarction and right atrial overload, both the malaria and the malaria group treated with Ammbaka caused insulin resistance, Ammbaka caused a reduction in adipose tissue.

**Conclusion:** The proposed protocol was efficient for inducing malaria, Ammbaka can be used as a therapeutic model for malaria, Ammbaka did not change cardiac contractility, Ammbaka generated metabolic disturbance and depreciated the weight of periepididymal fat. The study revealed that malaria significantly compromises the functionality of the heart muscle, as these changes found can lead to death.

111453

Modality: E-Poster Young Researcher – Non-case Report

Category: PHYSICAL EDUCATION

## Longitudinal Associations of Physical Inactivity, Overweight and Obesity with the Development of Cardiometabolic Multimorbidity: A Nationally Representative Cohort Study

WILLIAM YANG ZHAO^1^, Li He^2^

(1) The George Institute for Global Health, China; (2) College of Physical Education and Sport, Beijing Normal University, China

**Background:** Cardiometabolic multimorbidity is increasingly common and is associated with adverse health outcomes. Physical inactivity and obesity are associated with poor health and psychological well-being. This study aims to identify the longitudinal associations of physical inactivity, overweight and obesity on the development and worsening of cardiometabolic multimorbidity over time.

**Methods:** Using a four-wave nationally-representative sample from the China Health and Retirement Longitudinal Study, we analyzed 17,708 participants ≥45 years with and without cardiometabolic multimorbidity. The development of Cardiometabolic multimorbidity was measured as the accrual of additional conditions over an 8-year period. Poisson-distributed Generalized Linear Models (GLM) were used to estimate the association of cardiometabolic multimorbidity with physical inactivity, overweight and obesity.

**Results:** 22.48% of included participants had cardiometabolic multimorbidity at baseline and 35.28% at follow-up. 4,102 of 9,927 (41.32%) participants without multimorbidity and 819 of 2,879 (28.45%) with existing multimorbidity developed new condition/s. Physical inactivity was significantly associated with cardiometabolic multimorbidity (relative risk [RR] = 1.254, confidence interval [CI]: 1.136–1.385), complex multimorbidity (RR = 1.614, CI: 1.348 1.933), and worsened multimorbidity (RR = 1.214, CI: 1.032–1.427). Overweight & obesity was also significantly associated with cardiometabolic multimorbidity (RR = 1.705, CI: 1.574–1.846), and complex multimorbidity (RR = 2.156, CI: 1.852–2.510), and worsened multimorbidity (RR = 1.385, CI: 1.217–1.576).

**Conclusion:** Physical inactivity, overweight and obesity are associated with the development and worsening of cardiometabolic multimorbidity over time. They support the recent National Institute for Health & Care Excellence (NICE) Guidance on multimorbidity that suggests that patients with multimorbidity should be identified and targeted for interventions to improve health outcomes.

107888

Modality: E-Poster Young Researcher – Non-case Report

Category: CARDIOVASCULAR PHARMACOLOGY

## Effect of Colchicine use on Inflammatory Markers, Myocardial Injury and Pain Control in the Postoperative of Heart Surgery Patients: A Study Conducted in a High-Complexity Cardiovascular Hospital in Southern Brazil

ALLANA MAYCHAT PEREIRA OLIVEIRA^1^, Bárbara Swarowsky Tabach^2^, Leonardo Silveira Nascimento^1^, Dannuey Machado Cardoso^3^, Silvio Augusto Ortolan^1^, Lara de Matos^1^, Júlia de Moraes Costa^2^, Francisco Coelho Lamachia^1^, Caroline Brand^2^, Lester Krann Motta^1^, Leonardo Dorneles de Souza^1^, Dulciane Nunes Paiva^2^

(1) Hospital Santa Cruz. Santa Cruz do Sul, RS, Brazil; (2) Universidade de Santa Cruz do Sul. Santa Cruz do Sul, RS, Brazil; (3) Centro de Ensino Superior Dom Alberto. Santa Cruz do Sul, RS, Brazil

**Introduction:** Coronary artery bypass grafting is the most frequently used cardiac surgery (CS) for the treatment of symptomatic coronary artery disease. Because this is a major surgery, it is related to an increase in the inflammatory response in the postoperative (PO) period. Pain is one of the main complications of sternotomy.

**Objective:** To verify the effect of colchicine on inflammatory markers, myocardial injury and pain control in the PO of CS.

**Methods:** Randomized, double-blind, placebo-controlled clinical trial, carried out in a high-complexity cardiovascular hospital in southern Brazil. Patients undergoing CS and admitted to the intensive care unit (ICU) were evaluated. Patients of both genders, aged between 18 and 45 years and hemodynamically and neurologically stable, were included. The exclusion criteria were: emergency CS, patient with pacemaker, need for antibiotic therapy, presence of atrial fibrillation in the preoperative period and renal failure. Pain perception was assessed using the Visual Analogue Scale and troponin and C-reactive protein (CRP) levels. The patients were randomized into a control group, which received placebo, and a colchicine group, which received 0.5 mg 1x/day if body weight was up to 70kg or 0.5 mg 2x/day if body weight was over 70kg. Colchicine and placebo were administered preoperatively, restarted in the PO, and continued until outpatient reassessment (30 days after the CS). Patients were evaluated preoperatively, in the immediate PO, in the late PO (before discharge from the ICU) and 30 days after the CS.

**Results:** The sample consisted of 43 patients (24 males and 19 females), of which 21 were allocated to the colchicine group and 22 to the control group. The mean age of the colchicine group was 63.9 years (±2.9) and 63.4 years (±3.0) in the control group. There was no statistical difference between the groups regarding variance in troponin, CRP levels and pain perception in the four assessments. However, in the analysis of individual prevalence, the colchicine group had 51.6% of respondents in relation to the decrease in CRP levels, which was observed in 48.4% of the control group.

**Conclusion:** There was a higher individual prevalence of responders to colchicine on CRP levels compared to individuals who received placebo, with no difference in the prevalence of responders between the groups regarding myocardial injury markers and pain control in the PO of CS.

107849

Modality: E-Poster Young Researcher – Non-case Report

Category: EPIDEMIOLOGY AND HEALTH POLICIES/GLOBAL HEALTH

## Mortality from Heart Failure in the Elderly in Brazil: Temporal Trend Analysis from 2013 to 2019

NATANAEL ALVES DE LIMA^1^, Natanael Alves de Lima^1^, Thiago Emanuel Rodrigues Novaes^2^, Eduardo Pitthan^2^

(1) Universidade Federal da Fronteira Sul; (2) Universidade Federal da Fronteira Sul

**Introduction:** Heart Failure (HF) is a complex clinical syndrome and represents the final stage of the natural history of heart diseases of multiple etiologies. The increase in survival has impacted the exponential increase in the elderly population. The elderly in Brazil have a high rate of incidence and prevalence of HF, morbidity and mortality and hospitalization, demanding pressure and high costs in the Brazilian Health System.

**Objective:** To evaluate the trend of variation in crude mortality rates from HF in the elderly in Brazil, comparing age groups.

**Methods:** Refers to an ecological, descriptive and quantitative study that used data available on DATASUS (https://datasus.saude.gov.br/informacoes-de-saude-tabnet/) on March 8, 2022 referring to to mortality in the elderly due to HF, in Brazil, from 2013 to 2019, divided into two age groups: 60 to 79 years and 80 years or older. Crude mortality rates were calculated in these variables per 100,000 inhabitants, based on the population variation in Brazil in the years chosen for the study, made available by the IBGE (https://www.ibge.gov.br/) in a consultation carried out in 08 March 2022.

**Results:** The analysis of mortality from HF over 60 years, in 2013, showed a rate of 55.7/10^5^ inhabitants, and showed a small drop in 2019, being 51/10^5^ inhabitants. Making a drop of 8.4% per 10^5^ inhabitants. On the other hand, in the same period the population of this age group increased by 94.4%. The analysis of mortality due to HF over 80 years, in 2013, showed a rate of 60.9/10^5^ inhabitants and showed a small drop in 2019, making a drop of 1.5%. On the other hand, in the same period, the age population increased by 95.3%.

**Conclusion:** The results of this study demonstrate that there has not been a significant change in the mortality rate from HF in the elderly in Brazil in recent decades. The trend is more pronounced in the range above 60 to 79 years.


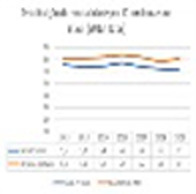


108558

Modality: E-Poster Young Researcher – Non-case Report

Category: CARDIOVASCULAR PHARMACOLOGY

## Effects of Euterpe Oleracea Mart. (Açaí) on Platelet Aggregation in Healthy Individuals

WANDA VIANNA MURY^1^, Wanda Vianna Mury^1^, Mariana Siqueira de Medeiros^1^, Marcela Anjos Martins^1^, Cláudia de Morais Sequeira^1^, Angela de Castro Resende^1^, Cristiane Matsuura^1^

(1) Universidade do Estado do Rio de Janeiro

**Introduction:** Euterpe oleracea Mart. (açaizeiro) is a plant typical from Brazil, rich in polyphenols, which has vasodilator properties, prevents endothelial dysfunction and improves metabolic profile. However, its role on platelet function is not known. Platelets are essential for the maintenance of vascular hemostasis, but they may also participate in thrombus formation when hyperactivated, contributing to the pathogenesis of ischemic diseases. Thus, the objective of this study was to investigate the effects of açaí stone hydroalcoholic extract (ASE) on platelet aggregation.

**Methods:** The blood from 15 healthy young men was collected, centrifuged and the isolated platelets were incubated with 10, 50 or 100 μg/mL ASE for 15 min (according to each experiment). Platelet aggregation was measured in platelet-rich plasma (PRP) and whole blood. In order to assess the effects of ASE on the platelet inhibitor nitric oxide (NO), we measured cGMP levels. Additionally, cAMP levels were quantified, the second messenger of platelet inhibitor prostacyclin. To compare the different ASE concentrations, the one-way ANOVA test with repeated measures was used. In case of significant F, a Holm Sidak post-test was performed. When only one concentration of ASE was tested, the paired t-test was applied. Statistical significance was considered when p ≤ 0.05.

**Results:** ASE inhibited collagen-induced PRP aggregation induced by collagen (baseline, 82.7 ± 5.6, ASE50, 54.0 ± 7.2, ASE100, 31.4 ± 4.1%, p < 0.0001), ADP (baseline, 46.1 ± 7.5, ASE50, 32.1 ± 4.1, ASE100, 17.6 ± 2.6%, p < 0.0001) or thrombin (baseline, 103.4 ± 4.5, ASE 50, 77.2 ± 8.9, ASE100, 53.4 ± 7.1%, p = 0.0002), as well as in whole blood under stimulation with collagen (baseline, 28.0 ± 4.0, ASE50, 19.6 ± 4.4, ASE100, 14.8 ± 2.5 Ω, p = 0.008). ASE increase cGMP levels (baseline, 0.67 ± 0.19, ASE50, 1.50 ± 0.39, ASE100, 1.64 ± 0.49 pmol/108 cells, p = 0.020). Similarly, there was an increase in cAMP levels (baseline, 9.8 ± 1.8, ASE50, 13.9 ± 2.3, ASE100, 15.3 ± 2.6 pmol/108 cells.

**Concluison:** These results indicate that ASE has a potent inhibitory effect on platelet aggregation. In this way it is possible that ASE has the potential to be used in the prophylaxis and treatment of diseases associated with platelet hyperaggregability, although the mechanisms underlying this process need to be fully clarified.

107969

Modality: E-Poster Young Researcher – Non-case Report

Category: COVID-19 AND CARDIOVASCULAR SYSTEM

## Long Term Prognostic Value of Troponin Elevation on Admission After Hospitalization for COVID-19

GABRIEL SALIM SAUD DE OLIVEIRA ^1^, João Roquette Fleury da Rocha^1^, Bárbara Mariz Ferreira Passos^1^, Ismael Magalhães Matos de Faria^1^, Ana Carolina Rodrigues Lado^1^, Ricardo Antônio Correia Lima^2^, Pedro Paulo Noguères Sampaio^2^, Juliano Carvalho Gomes de Almeida^1^, João Mansur Filho^2^, Roberto Muniz Ferreira^1^, Lúcia Helena Alvares Salis^1^, Nelson Albuquerque de Souza e Silva^1^

(1) Federal University of Rio de Janeiro, Edson Saad Heart Institute, Brazil; (2) Samaritano Hospital, Botafogo, Rio de Janeiro, Brazil

**Introduction:** Several biomarkers have demonstrated prognostic value in patients with Coronavirus Disease-2019 (COVID-19), primarily during hospitalization. Troponin I (TnI) has been widely studied in this context, though its association with outcomes after the initial hospitalization period remains unknown.

**Objectives:** To determine the association between high sensitivity TnI elevation above the 99th percentile upper reference limit on admission in hospitalized patients with COVID-19 and long term survival among those who were successfully discharged.

**Methods:** Medical records from consecutive patients with confirmed COVID-19 admitted to a single institution between March and July 2020 were retrospectively analysed. Clinical data and TnI values were collected and correlated with long term mortality after the index hospitalization.

**Results:** Among 230 patients, 194 survived until hospital discharge, of which 149 had TnI values on admission and were included in the analysis. Median age was 65 years (52–78) and 56.4% were male. Cardiovascular disease (CVD) was present in 12%, and 36.2% were treated in the intensive care unit (ICU). Troponin elevation occurred in 21 patients (14.1%), and 9 (6%) died after a median follow up of 631 days (612–643). Troponin was associated with death in the univariate (OR 16.7; 95% CI 3.8–73.6, p < 0.001) and multivariate analyses (OR 8.6; 95% CI 1.5–49.6, p = 0.02), after adjusting for age, previous CVD, ICU admission, C-reactive protein and creatinine values. Figure 1 represents the Kaplan-Meier survival estimates after hospital discharge according to TnI elevation.

**Conclusions:** Although most patients have a favourable outcome after hospitalization for COVID-19, cardiac injury on admission appears to remain predictive of long term survival after discharge.


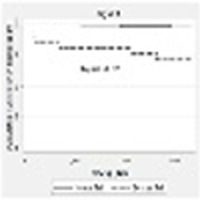


107978

Modality: E-Poster Young Researcher – Non-case Report

Category: ACUTE AND CHRONIC CORONARY DISEASE/THROMBOLYSIS

## Validation of the Grace Risk Score to Predict In-Hospital and 6-Month Post-Discharge Mortality in Patients with Acute Coronary Syndrome

VÍTOR BONIATTI NEVES^1^, Vítor Boniatti Neves^1^, Raquel Melchior Roman^2^, Tiago Vendruscolo^2^, Gilberto Heineck^2^, Carlos Alberto Santos de Mattos^2^, Eduardo Ilha de Mattos^2^, Luiz Carlos Pereira Bin^1^, Karine de Lima Sírio Boclin^3^, Marcelo Fialho Roman^1^

(1) Faculdade Meridional (IMED), Passo Fundo, RS – Brazil; (2) Hospital de Clínicas de Passo Fundo (HCPF), Passo Fundo, RS – Brazil; (3) Universidade Estácio de Sá (UNESA), Rio de Janeiro, RJ – Brazil

**Background:** The wide range of clinical presentations of acute coronary syndrome (ACS) makes it indispensible to use tools for risk stratification and for appropriate risks management; thus, the use of prognosis scores is recommended in the immediat clinical decision-making.

**Objective:** To validate the Global Registry of Acute Coronary Events (GRACE) score as a predictor of in-hospital and 6-month post-discharge mortality in a population diagnosed with ACS.

**Methods:** This is a prospective cohort study of consecutive patients diagnosed with ACS between May and December 2018. GRACE scores were calculated, as well as their predictive value for in-hospital and 6-month post-discharge mortality. The validity of the model was assessed by two techniques: discriminative power using the area under the receiver operating characteristic curve (AUC) and goodness-of-fit, using the Hosmer-Lemeshow (HL) test, at the 5% level of significance.

**Results:** A total of 160 patients were included, mean age 64 (±10.9) years; of which 60% were men. The risk model showed to have satisfactory ability to predict both in-hospital mortality, with an area under the curve (AUC) of 0.76 (95% confidence interval [CI], 0.57–0.95; p = 0.014), and 6-month post-discharge mortality, with AUC of 0.78 (95%CI, 0.62–0.94), p = 0.002. The HL test indicated good-fit for both models of the GRACE score.

**Conclusion:** In this study, the GRACE risk score for predicting mortality was appropriately validated in patients with ACS, with good discriminative power and goodness-of-fit. The results suggest that the GRACE score is appropriate for clinical use in our setting.


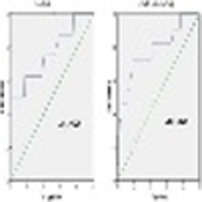


108053

Modality: E-Poster Young Researcher – Non-case Report

Category: EPIDEMIOLOGY AND HEALTH POLICIES/GLOBAL HEALTH

## Mortality from Diseases of the Circulatory System in Brazil and its Relationship with Social Determinants Focusing on Vulnerability

LUIZ ANTONIO VIEGAS DE MIRANDA BASTOS^1^, Luiz Antonio Viegas de Miranda Bastos^1^, José Lucas Peres Bichara^1^, Paolo Blanco Villela^1^, Gabriela da Silva Nascimento^1^, Gláucia Maria Moraes de Oliveira^1^

(1) Universidade Federal do Rio de Janeiro UFRJ

**Introduction:** Deaths from diseases of the circulatory system (DCS) and ischemic heart diseases (IHD) are declining, but slowly in developing countries, emphasizing its probable relationship with determinants of social vulnerability.

**Objectives:** To analyze the temporal progression of mortality rates of DCS and from 1980 to 2019 and the association of the rates with the Municipal Human Development Index (MHDI) and Social Vulnerability Index (SVI) in Brazil.

**Methods:** We estimated the crude and standardized mortality rates of DCS and IHD and analyzed the relationship between the obtained data and the MHDI and SVI. Data on deaths and population were obtained from the. The MHDI and the SVI of each federative unit were extracted from the websites Atlas Brasil and Atlas da Vulnerabilidade Social, respectively.

**Results:** The age-standardized mortality rates of DCS and IHD showed a downward trend nationwide, which was unequal across the federative units. There was an inversely proportional relationship between the standardized mortality rates of DCS and IHD and the MHDI. The downward mortality trend was observed when the indices were greater than 0.70 and 0.75, respectively. The SVI was directly proportional to the standardized mortality rates of DCS and IHD. An upward mortality trend was observed with an SVI greater than 0.35.

**Conclusion:** Social determinants represented by the MHDI and the SVI were related to mortality from DCS and IHD across the Brazilian federative units. The units with most development and least social inequalities had the lowest mortality from these causes. The most vulnerable die the most.


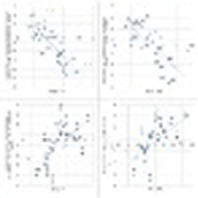


108060

Modality: E-Poster Young Researcher – Non-case Report

Category: HYPERTENSION/RENAL DENERVATION

## Methotrexate Associated with Lipid Nanoparticles Prevents Interstitial Fibrosis of the Left Ventricle in Spontaneously Hypertensive Rats

ALINE DE OLIVEIRA SILVA^1^, Maria Carolina Guido^1^, Mauricio Tavares Costa^1^, Natalia de Menezes Lopes^1^, Amanda de Almeida Silva^1^, Priscila Oliveira Carvalho^1^, Raul Cavalcante Maranhão^1^

(1) Instituto do Coracao, Hospital das Clinicas HCFMUSP, Faculdade de Medicina, Universidade de Sao Paulo, SP, BR.

**Background:** Systemic arterial hypertension is a major risk factors for cardiovascular diseases. Interstitial fibrosis is directly associated with ventricular dysfunction in spontaneously hypertensive rats (SHR). Incorporation of methotrexate (MTX), a folic acid inhibitor, into lipid nanoparticles (LDE) increases cellular uptake and decreases MTX toxicity. LDE-MTX showed potent anti-inflammatory and anti-proliferative effects in rabbits with atherosclerosis and rheumatoid arthritis. In rats submitted to acute myocardial infarction, LDE-MTX reduced infarct size and left ventricular (LV) dysfunction.

**Aim:** To investigate the effect of LDE-MTX treatment on the prevention of cardiac remodeling that occurs in SHR rats.

**Methods:** Three groups of 6-week-old male rats were studied: 1) Control (CT, n = 8), Wistar-Kyoto rats weekly treated with I.P injection of saline solution; 2) SHR-LDE (n = 8): SHR rats treated with injection of LDE only and 3) SHR-LDE-MTX (n = 8): SHR rats treated with LDE-MTX (1 mg/kg intraperitoneally, weekly, for 20 weeks). After the treatment period, echocardiography was performed and the animals were euthanized for LV morphometry and protein expression by Western blot.

**Results:** Compared to CT, SHR group presented LV dilation, represented by increase in systolic and diastolic diameters; cardiac hypertrophy due to increased LV posterior wall thickness and relative heart weight. SHR also had reduced ejection fraction and LV shortening, indicating LV systolic dysfunction. In SHR-LDE, there was increase in collagen content in the interstitial region of the LV, possibly resulting from an increase in type 1 collagen, which indicates increase in fibrosis of LV. LDE-MTX had no effect on LV dilation, cardiac hypertrophy or LV systolic dysfunction in SHR rats. However, LDE-MTX decreased LV interstitial fibrosis. In order to identify possible mechanisms associated with LDE-MTX and the reduction of interstitial fibrosis, we quantified the expression of angiotensin II receptors type 1 (AT1) and 2 (AT2) in the LV of the animals. However, there was no difference in the expression of AT1 and AT2 between the 3 groups.

**Conclusion:** LDE-MTX treatment reduced LV interstitial fibrosis in SHR rats, possibly by reducing type 1 collagen expression, despite having no effect on LV systolic dysfunction. It is possible that the reduction in LV fibrosis achieved by treatment with LDE-MTX reduces the damage of hypertension on cardiac function when the animal reaches more advanced.

108092

Modality: E-Poster Young Researcher – Non-case Report

Category: CARDIOGERIATRICS

## Atrial Fibrillation and Hospital Mortality in Octogenary Patients Included in the Sepsis Protocol from January 2018 to December 2020

MICHELE OURIQUES HONORATO^1^, Juscelio Trajano de Souza Filho^1^, Luiz Frederico Bezerra Honorato Junior^1^, Nathalia Watanabe^1^, Gabriela Machado Goulart^2^

(1) Hospital Sírio Libanês; (2) Universidade do Extremo Sul Catarinense

**Introduction:** Atrial Fibrillation (AF) is the most common sustained cardiac arrhythmia, affecting about 2 to 4% of world population, and in patients hospitalized in intensive care units, this incidence can go up to 23%. Advanced age is a risk factor for the development of AF, and those above 80 years of age become more susceptible to its deleterious effects. The impact of AF in septic patients is reflected in worse clinical outcomes and the identification of the triggering factors can be a target of future prevention and treatment strategies.

**Objectives:** To verify the relationship between the development of AF and the mortality in patients over 80 years of age included in the sepsis protocol and to identify the risk factors that contribute to the development of AF in this population.

**Methods:** Retrospective observational study, with a review of medical records and inclusion of 895 patients aged 80 years or older, included in the sepsis protocol of a high-complexity hospital in São Paulo/SP, from January 2018 to December 2020.

**Results:** The incidence of AF in the sample was 13%. After multivariate analysis, using multiple logistic regression, it was possible to demonstrate an association of mortality, in the studied population, with the SOFA score OR 1.21(1.09–1.35), higher values of C reactive protein – OR 1.04 (1.01–1.06), need for vasoactive drugs – OR 2.4 (1.38–4.18), use of mechanical ventilation – OR 3.49 (1.82–6.71) and mainly AF – OR 3.7 (2.16–6.31).

**Conclusion:** In the great elderly patient (80 years of age and older), septic, the development of AF was shown to be an independent risk factor for in-hospital mortality.


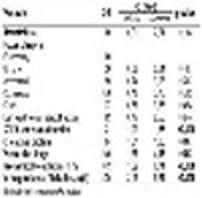


108625

Modality: E-Poster Young Researcher – Non-case Report

Category: SPIRITUALITY AND CARDIOVASCULAR MEDICINE

## The Clinical-Hemodynamic Profile and the Impact of Spirituality on the Quality of Life of Patients with Heart Failure Admitted to a Hospital in the Countryside of Sao Paulo State

IEDA BARRETO MOURÃO BERTINI^1^, Sofia Gonçalves Tonoli^1^, Reinaldo José Gianini^1^, Emerson de Albuquerque Seixas^1^

(1) Pontifícia Universidade Católica de São Paulo (PUCSP)

**Introduction:** Heart Failure (HF) is a disease where the heart is unable to properly pump blood around the body to support the metabolic needs. HF is associated with frequent underlying symptoms and complex treatments, which compromises a quality of life. The suffering can be lessened with a holistic approach focused on spirituality, which has been shown to affect the health and wellbeing of people with chronic diseases.

**Objective:** To assess the clinical-hemodynamical profile of patients with HF to test if spirituality correlates with quality of life.

**Methodology:** A cross-sectional study was undertaken, where two questionnaires were applied: i) Minnesorta Living with HF Questionaire (MLHFQ), to address the quality of life of patients with HF and ii) Functional Assessment of Chronic Illness Therapy-Spiritual Well-Being (FACIT-Sp-12), regarding the spiritual wellbeing of chronic patients. The patient’s classification according to the New York Heart Association (NYHA) and the Stevenson hemodynamic profile were also used. Statistical tests were performed using STATA®, through Pearson’s correlation and linear regression. A total of 41 individuals ≥18 years old with HF were interviewed.

**Results:** Most respondents were male (53%) and the mean age of respondents was 66 years old. The mean HF etiologies identified were hypertense (37%), ischemic (34%) and valvular (12%), with the majority of patients falling into NYHA class III (41%) and Stevenson category B (90%). According to the FACIT-Sp-12 classification suggested by the authors of this study, as there are no other classifications in the literature, 2.4% showed low levels of spirituality and 65.8% showed high levels. According to the MLHFQ, 24.4% of respondents have great quality of life and 22% have a bad one. When both questionaires were correlated, there was a significant r coefficient of –0.45, showing a significant inversely proportional relationship (p-value 0.003), meaning higher the levels of spirituality are associated with lessend symptoms in patients with HF. However, when FACIT-Sp-1 was correlated with both the NYHA and the Stevenson classification, no stastistically significant relationships were found.

**Conclusion:** A holistic approach focused on spirituality in patients with HF could have a positive effects on their quality of life through lessened symptoms. However, there is no evidence to suggest that spirituality has an effect on either the functional status or hemodynamic profile of patients.

108163

Modality: E-Poster Young Researcher – Non-case Report

Category: CARDIORESPIRATORY PHYSIOLOGY/BASIC SCIENCE

## The Role of Liraglutide on Cardiac Function in an Experimental Model of Acute Doxorubicin-Induced Cardiotoxicity

CAROLINA RODRIGUES TONON^1^, Paola da Silva Balin^1^, Marina Gaiato Monte^1^, Danilo Malmonge Barbosa Luciano^1^, Natalia Fernanda Ferreira^1^, Marcos Ferreira Minicucci^1^, Paula Schimdt Azevedo^1^, Sergio Alberto Rupp de Paiva^1^, Leonardo Antonio Mamede Zornoff^1^, Marina Politi Okoshi^1^, Katashi Okoshi^1^, Bertha Furlan Polegato^1^

(1) Faculdade de Medicina de Botucatu

**Background:** Cancer is one of the main causes of morbidity and mortality worldwide. Despite doxorubicin (DOX) being an effective chemotherapy drug, DOX-induced cardiotoxicity is the most severe side effect, limiting treatment and worsening quality of life. There is no effective treatment for preventing DOX-induced cardiotoxicity. Glucagon like peptide-1 (GLP-1) analogs such as liraglutide reduce oxidative stress and inflammation.

**Purpose:** To evaluate the role of liraglutide in acute DOX-induced cardiotoxicity in rats.

**Methods:** 62 male Wistar rats were allocated into 4 groups: Control (C), DOX (D), Liraglutide (L), and DOX + Liraglutide (DL). The animals in L and DL groups received a subcutaneous injection of 0.6 mg/kg liraglutide daily while C and D groups received a subcutaneous injection of an equivalent volume of saline daily, for 2 weeks. After 12 days, D and DL groups received an intraperitoneal injection of DOX (20 mg/kg). Rats were submitted to echocardiogram and isolated heart study 48 hours after DOX injection. Statistical analysis: one-way ANOVA.

**Results:** In the first 12 days, L and DL had lower food ingestion and body weight than C and D. After DOX treatment, body weight was lower in D and DL than C and L and lower in DL than D. In isolated heart study, DOX decreased +dP/dt (C 4518 ± 1469; D 2083 ± 534; L 4125 ± 647; DL 2150 ± 399 mmHg/s; p < 0.001) and -dP/dt (C 2679 ± 886; D 1292 ± 504; L 2896 ± 496; DL 1413 ± 285 mmHg/s; p < 0.001), with no changes between DL and D. In echocardiogram, D and DL exhibited lower peak systolic annular velocity at tissue Doppler [C 3.7 (3.2–4.2); D 3.1 (3.0–3.5); L 3.8 (3.4–4.0); DL 2.9 (2.7–3.0) cm/s, p < 0.001], and posterior wall shortening velocity (C 39 ± 4.5; D 32 ± 2.5; L 41 ± 5.0; DL 30 ± 2.9 mm/s; p < 0.001) than C and L. Liraglutide did not change these variables. D and DL had decreased E waves (C 77.2 ± 10.5; D 56.6 ± 9.0; L 74.3 ± 7.4; DL 52.5 ± 9.6 cm/s; p < 0.001) and A (C 56.9 ± 13.8; D 46.7 ± 10.4; L 56.3 ± 16.6; DL 39.8 ± 10.3 cm/s; p = 0.002) than C and L. DL showed increased isovolumetric relaxation time normalized to heart rate (C 54 ± 4.9; D 58 ± 8.0; L 51 ± 6.1; DL 65 ± 8.8; p < 0.001) than C, L, and D.

**Conclusion:** Liraglutide administration does not attenuate doxorubicin-induced acute cardiac dysfunction.

108578

Modality: E-Poster Young Researcher – Non-case Report

Category: PERICARDIUM/ENDOCARDIUM/VALVOPATHIES

## Percutaneous Mitral Commissurotomy for Mitral Stenosis Patients: A 20 Years Follow-Up

RICARDO JORGE DOS REIS ALVES PINTO^1^, Miguel Martins Carvalho^1^, Tânia Proença^1^, Catarina Costa^1^, Ana Filipa Amador^1^, João Calvão^1^, Catarina Marques^1^, André Cabrita^1^, Luís Santos^1^, Mariana Paiva^1^, Filipe Macedo^1^

(1) Centro Hospitalar Universitário São João, Porto, Portugal

Percutaneous mitral commissurotomy (PMC) is a viable alternative to mitral valve (MV) surgery in the treatment of clinically significant mitral stenosis (MS). Our aim was to evaluate the long-term results of PMC in patients (pts) with rheumatic MS and to compare events concerning pulmonary hypertension (PH) presence. We analysed all pts between 1991 and 2008 with rheumatic MS undergoing PMC. MACE was a composite of all-cause mortality, MV re-intervention or cardiovascular hospitalization. A total of 124 pts were enrolled: 87% were female, mean age of 46 ± 11 years (yrs) and mean follow-up (FUP) of 20 ± 6 yrs. Before PMC, 34% were in NYHA class ≥ III, 81% had Wilkins score ≤8, all had preserved biventricular function, 83% presented PH; mean transvalvular gradient (TVG) and mitral valve area (MVA) were 12.8 mmHg and 1.0 cm^2^. Most of the procedures were successful (91%) and without major complications (94%), with mean MVA improvement of 0.9 cm^2^ and reduction of 8.5 mmHg in TVG and 9.7 mmHg in PASP after PMC. During long-term FUP, 42% of pts were re-interventioned and 24% died. In pts non-submitted to re-intervention, TVG and PASP remained similar throughout FUP, while MVA reduced over time, yet still statistically superior to baseline MVA (1.6 vs 1.0 cm^2^, p < 0.001). In time-to-event analysis, approximately 80% of pts kept uneventful after 10 yrs; after 30 yrs, >20% continued MACE-free and 50% alive. Regarding PH before PMC, there was no significant difference in MACE or mortality (p = 0,846 and p = 0.661, respectively). After long-term FUP, pts maintained reduction in TVG and PASP and a smaller but significative improvement in MVA. Most pts were MACE-free after 10 yrs and half were alive after 30 yrs. There was no difference in all-cause mortality or MACE concerning PH presence.


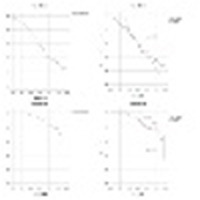


108271

Modality: E-Poster Young Researcher – Non-case Report

Category: DIGITAL HEALTH/INNOVATION

## Validation of a New Method for Monitoring the Electrocardiographic Signal and Diagnosis of Heart Arrhythmia

ERIC SANTOS RODRIGUES DE OLIVEIRA^1^, Larissa Araújo de Lucena^1^, Vitor Martinez Silva^1^, Antonio Lucas Arruda de Oliveira^1^, William Santos Rodrigues de Oliveira^1^, Nestor Rodrigues de Oliveira Neto^1^

(1) Hospital Universitário Onofre Lopes (HUOL) – Universidade Federal do Rio Grande do Norte (UFRN)

**Introduction:** Rhythm disorders often present intermittently, making it difficult to record them. However, the electrocardiogram (ECG) performed during the event allows diagnosing the type of arrhythmia. Several studies have shown that portable devices can diagnose rhythm disorders, making these devices a practical alternative to the expensive and poorly available equipment. Thus, we hypothesize that our low complexity and cost device can monitor cardiac rhythm and diagnose cardiac arrhythmias with a single-lead ECG.

**Objectives:** To evaluate a low-cost system’s accuracy and technical quality for recording ECG and diagnosing cardiac arrhythmias.

**Methods:** This is a validation study where outpatient or hospitalized patients are selected based on continuous cardiac monitoring and standard 12-lead ECG. Patients who had cardiac arrhythmia underwent a 12-lead ECG, and then the heart rhythm was recorded with a lead similar to CM5 through the new device. The device comprises a biological signal amplifier associated with filters connected to the smartphone. We will categorize the records into six rhythm groups, which will give us its diagnosis accuracy by comparing the device and the 12 lead ECG registers.

**Results:** Presently, there are 30 records made with the device. Of these, 29 were of satisfactory quality. 1 was excluded for artifact influence. The general profile of the patients from the study was the median age of 55; 15 were men, 13 women, 1 undetermined; 12 had no comorbidities, 1 case of hypertrophic cardiomyopathy, 4 congestive heart failure, 4 hypertension, 3 diabetes mellitus. The rhythm profile of the registers is 15 with sinus rhythm, 7 bradycardia, 5 atrial fibrillation, 1 supraventricular or ventricular ectopy, 1 narrow qrs tachycardia, no wide qrs tachycardia so far. The device diagnosis accuracy is undetermined as we have not compared the device registers and the 12-lead ECG yet.

**Conclusions:** The partial results show a low-cost mobile device that records in satisfactory quality and can be a tool for diagnosing rhythm disorders. According to the initial analysis of 30 tracings, the quality was satisfactory in 96.67% of the cases. Our solution is an efficient lower price alternative to other existing devices. The main limitation of this study is the result’s initial state, which makes it challenging to verify the performance of the tested device. Thus we only accessed its ability to perform tracings of good quality.

108355

Modality: E-Poster Young Researcher – Non-case Report

Category: HEMODYNAMICS AND INTERVENTIONAL CARDIOLOGY

## Percutaneous Coronary Intervention in Late Presentation Acute Myocardial Infarction Without Viability. Effects on Left Ventricular Remodelling and Contractility – Rationale and Design

BARBARA PORTO VALENTE^1^, José de Ribamar Costa Junior^1^, Ibraim Masciarelli F Pinto^1^, Bruno Pereira Albuquerque Coelho^1^, Gabriela Carolina Santamaría Naranjo^1^, Luciano de Figueiredo Aguiar Filho^1^, Paul Salvador^1^, Tiago Senra^1^, Vivian Lerner Amato^1^, José Roberto Tuma da Ponte Junior^1^, Ricardo Pavanello^1^, Pedro Silvio Farsky^1^

(1) Instituto Dante Pazzanese de Cardiologia

**Introduction:** The potential benefits attributable to late reperfusion fall under the “open artery hypothesis”, in which the stunned peri-infarction zone after revascularization restores blood supply, improving its contractility. A study showed that late coronary recanalization after myocardial infarction (MI), irrespective of the viability status, improved left ventricular ejection fraction (LVEF) and myocardial contractility mainly in the segments adjacent and distant to the infarction.

**Purpose:** To evaluate whether late recanalization in patients with STEMI without viability by Cardiovascular Magnetic Resonance (CMR) can reduce the reverse remodeling. Viable cases will be kept in the registry.

**Methods:** This is a prospective randomized controlled trial, with at least 6 months follow-up. Patients with STEMI not reperfused between 24 hours and 28 days with IRA with lesion greater than 50% with segmental dysfunction and absence of viability on CMR are eligible for inclusion. Patients with previous MI, cardiomyopathy or clinical instability are excluded. Participants randomly assigned (1:1) to PCI and Optimal Medical Treatment (OMT) or only OMT. Expected outcome is the change on reverse remodeling of the end systolic volume at 6 months likewise the improvement in segmental contractility at 6 months. The sample size of 35 patients in each group provides 80% power with a two-sided significance level of 0.05. Descriptive statistics and statistical inferential methods are employed to measure changes between the groups. The trial has local ethical review board approval.

**Results:** 28 patients have been already enrolled.

**Conclusion:** This trial will provide insights into the potential benefits of opening the IRA in segments without viability, improving contractility of surrounding myocardium.


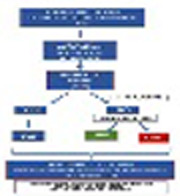


108396

Modality: E-Poster Young Researcher – Non-case Report

Category: CARDIOVASCULAR PHARMACOLOGY

## Effects of Sodium Glucose Co-Transporter 2 Inhibition on Muscle Energy Metabolism in Infarcted Rats

LIDIANE MOREIRA SOUZA^1^, Bruna Brasil Brandao^2^, Luana Urbano Pagan^1^, Mariana Gatto^1^, Felipe Cesar Damatto^1^, Eder Anderson Rodrigues^1^, Patricia Aparecida Borim^1^, Gilson Masahiro Murata^3^, Leonardo Antonio Mamede Zornoff^1^, Katashi Okoshi^1^, Marina Politi Okoshi^1^

(1) Botucatu Medical School, Sao Paulo State University, UNESP; (2) Joslin Diabetes Center, Harvard Medical School, Boston, MA, USA; (3) Institute of Biomedical Science, Sao Paulo University, USP

**Introduction:** Skeletal muscle energy metabolism is commonly altered in heart failure patients, with a metabolic shift from oxidative to glycolytic muscle fiber. These changes contribute to reduced functional capacity. Sodium glucose co-transporter type 2 (SGLT2) inhibitors improve cardiovascular outcomes in both diabetic and non-diabetic patients, as well as those with and without heart failure. However, the effects of SGLT2 inhibitors on skeletal muscle during heart failure have not been established. The aim of this study was to assess the metabolic effect of empagliflozin (EMPA) on skeletal muscle of rats with myocardial infarction (MI)-induced heart failure.

**Methods:** One week after MI induction or simulated surgery, male Wistar rats were divided into four groups: Sham (n = 10), Sham+Empa (n = 12), MI (n = 10), and MI+Empa (n = 09). EMPA was added to rat chow (5 mg/kg/day). Rats were supplied with ad libitum water and chow for 12 weeks. Infarct size was measured by histological analysis. Metabolic enzyme activity in the soleus muscle was assessed by spectrophotometry. Statistical analysis: ANOVA and Tukey, and Student’s t tests.

**Results:** Only rats with infarction size greater than 35% of total left ventricle area were included in this study. Infarction size did not differ between infarcted groups (MI 41.8 ± 4.2; MI+Empa 40.7 ± 5.7 of total left ventricle area). In the MI soleus muscle, metabolic enzyme activity of glucose-6-phosphate-dehydrogenase, citrate synthase and beta-hydroxy-acyl-dehydrogenase was higher than the Sham group. These changes were not observed in the MI+Empa group. MI+Empa had lower hexokinase, phosfructokinase, and pyruvate kinase activity (glycolytic metabolism enzymes), and lower citrate synthase and glucose-6-phosphate-dehydrogenase activity than MI.

**Conclusion:** Chronic treatment with SGLT2 inhibitor empagliflozin prevents metabolic abnormalities in skeletal muscle in infarcted rats.

108368

Modality: E-Poster Young Researcher – Non-case Report

Category: ACUTE AND CHRONIC CORONARY DISEASE/THROMBOLYSIS

## Very Long-Term Mortality After ST Elevation Myocardial Infarction (Stemi) Complicated by Cardiogenic Shock

GABRIEL KANHOUCHE^1^, Jose Nicolau^1^, Talia Falcão Dalçoquio^1^, Mauricio Felippi de Sa Marchi^1^, Roberto R Giraldez^1^, Remo Furtado^1^, Luciano Baracioli^1^, Roberto Kalil Filho^1^, Fabio Sandoli de Brito Junior^1^, Alexandre Abizaid^1^, Henrique Barbosa Ribeiro^1^

(1) Heart Institute of Sao Paulo – InCor/HCFMUSP

**Introduction:** About 5–10% of myocardial infarctions develop severe form of presentation, with cardiogenic shock (CS). Despite evidence showing higher in-hospital and short-term mortality in patients with STEMI complicated by CS, long-term follow-up data is lacking.

**Hypothesis:** The aim of this study was to assess very long-term prognosis of survived patients with STEMI complicated by CS.

**Methods:** STEMI patients (n = 1,444) were included (1998–2017) and followed for up to 17.6 years (median 5.4 [2.5–9.9] years). Long-term survival was assessed by Log-Rank test.

**Results:** Compared to STEMI without CS, patients who presented CS (n = 118) had no significant differences regarding age (62.0 ± 11.8 vs. 60.7 ± 12.7; p = 0.31) and female sex (28.0% vs. 25.1%, p = 0.51), but had lower left ventricle ejection fraction (38.8 ± 13.0% vs. 49.7 ± 13.1%, p < 0.001) and higher mean baseline creatinine levels (1.4 ± 0.6 mg/dL vs. 1.2 ± 0.7 mg/dL, p = 0.002). Of note, CS patients presented higher in-hospital mortality (42.2%) compared to those without CS (OR = 28.5; 18.1–44.7, p < 0.001). Long-term survival was lower in patients with CS (10.7 ± 0.6 vs. 13.3 ± 0.2 years, log rank = 0.016). By multivariate analysis adjusted for baseline characteristics the main factors predicting mortality were age (HR = 1.06; CI95% 1.05–1.07, p < 0.001), previous MI (HR = 1.34;CI% 1.06–1.69, p = 0.01), history of heart failure (HR = 1.65; CI95% 1.15–2.37, p = 0.007), stroke (HR = 1.67; CI95% 1.12–2.47, p = 0.01), diabetes (HR = 1.41; CI95% 1.14–1.74, p = 0.001) and the presence of CS (HR = 3.65; CI95% 2.92–4.56, p < 0.001).

**Conclusion:** In a very long-term follow-up after a STEMI, CS increased ~4-fold the mortality rates. After hospital discharge, CS remains an important independent risk factor for death in the follow-up. Our findings reinforce the importance of intensive clinical monitoring with optimal medical and non-pharmacological therapy for the remarkable risk factors in the very long-term post-STEMI complicated by CS.

108369

Modality: E-Poster Young Researcher – Non-case Report

Category: ACUTE AND CHRONIC CORONARY DISEASE/THROMBOLYSIS

## Viability Assessment as a Strategy to Reduce Long Term Events in Left Anterior Descendent Artery with Chronic Total Occlusion

BARBARA PORTO VALENTE^1^, Maria Júlia Silveira Souto^1^, Manuela Gomes de Aguiar^1^, Gabriela Carolina Santamaría Naranjo^1^, Ismar Junior Peinado Lijeron^1^, Rodrigo Urdan^1^, Lucas Ferreira Marcondes Lemos^1^, César Henrique Morais Alves^1^, Kelvin Henrique Vilalva^1^, Maria Isabel Del Monaco^1^, Luciana Uint^1^, Ricardo Pavanello^1^

(1) Instituto Dante Pazzanese de Cardiologia

**Introduction:** Late intervention of a chronic total occlusion (CTO) in stable patients is not routinely recommended by randomized trials. Previous studies have confirmed the negative effect of CTOs on prognosis.

**Purpose:** To evaluate if a strategy of viability assessment (VA) to guide revascularization in the left anterior descendent artery (LAD) CTO can reduce 5-year clinical outcomes.

**Methods:** Retrospective cohort with at 5-year follow-up of 223 patients with LAD with CTO without any other significant lesion. Patients with previous myocardial infarction, cardiomyopathy or clinical instability were excluded. The primary outcome was the composite end point of myocardial infarction (MI), death, new LAD revascularization, heart failure hospitalization (HFH) and severe arrhythmias.

**Results:** We identified 223 patients with LAD CTO as a single lesion. Only 53 (23.7%) had VA to guide therapy compared to 170 (76.2%) with no VA (NVA). The mean ejection fraction was higher in the group with NVA (54.5 +/– 13.1 vs 41.5 +/– 11.8 with p < 0.001). The occurrence of angina was more common in the NVA group (64.7% vs 30.2% with p < 0.001), mainly due to Canadian Cardiology Society II (71.8%). The VA group was more frequently maintained in optimal medical therapy (OMT) (54.7%) compared to the NVA group (23.5%). NVA group underwent revascularization more often than the VA group, PCI was performed in 59.4% vs 41.5% and CABG in 17.1% vs 3.8% respectively. The primary outcome occurred in 5.7% in the VA group compared to 22.4% in the NVA (p = 0.056).

**Conclusion:** VA is a feasible strategy to reduce the need of unnecessary interventions. There was a marginal reduction of the total number of events in the VA group in the 5-year follow-up, mainly with respect to reducing the need for new LAD revascularization.


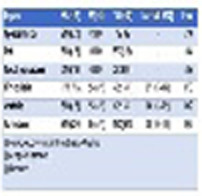


108397

Modality: E-Poster Young Researcher – Non-case Report

Category: HEMODYNAMICS AND INTERVENTIONAL CARDIOLOGY

## Prognostic Value of Autonomic Nervous System Imbalance in Heart Failure Patients with Low Left Ventricular Ejection Fraction Receiving Cardiac Contractility Modulation

ALLA PAVLOVSKAYA^2^, Elena Lyasnikova^1^, Ekaterina Zhabina^1^, Maria Trukshina^1^, Victoria Galenko^1^, Larisa Prokopova^1^, Tatiana Lelyavina^1^, Marianna Wander^1^, Evgeniy Mikhaylov^1^, Maria Sitnikova^1^

(1) Almazov National Medical Research Centre; (2) Semashko Republican Clinical Hospital

**Introduction:** Experimental data have showed the effect of cardiac contractility modulation (CCM) on the autonomic nervous system (ANS) activity. However, there are limited data regarding the prognostic value of ANS and its imbalance assessed by heart rate variability (HRV) in patients receiving CCM. Objective. To evaluate association of HRV with prognosis in heart failure patients with HFrEF and sinus rhythm undergoing CCM therapy.

**Methods:** This retrospective study included 52 patients with HFrEF, II/III fc, being on optimal drug therapy more than 3 months (age 53 ± 10.4 years, 86% men, CAD 73%, CABG/PCI 54%, ICD 21%, sinus rhythm 100%, LVEF 25 ± 6%, peakVO2 16.8 ± 4.8 ml/min/kg, NtproBNP 1087.5 [706.6;1752.7] pg/mL), who underwent CCM device implantation with further followed by a team of heart failure specialists. HRV parameters were calculated using 24-hour ECG monitoring before device implantation. The relationship between pre-implant time-domain (SDNN) and frequency-domain (LF, HF, LF/HF ratio from 24-hour; VLF from a 5-min segment of the resting period) and the endpoint of 3-year all-cause death was analyzed.

**Results:** During a mean 36 ± 2 months of follow-up, 11 patients reached the endpoint. All patients were divided into 2 groups depending of the SDNN duration. Initially, SDNN <100 ms was observed in 18 patients (34.6%). The Kaplan-Meier analysis using the log-rank test showed worse prognosis among patients with low SDNN (p = 0.01). LF, HF, VLF during 5-min were significantly higher in the subgroup of surviving patients (p = 0.02, p = 0.04 and p = 0.001, respectively) and low VLF (≤179 ms^2^) was associated with an increased risk of HF-death (p = 0,005).

**Conclusions:** Our preliminary findings indicate that baseline ANS dysfunction (quantified by low HRV indices) in patients with HFrEF receiving CCM-therapy was associated with the all-cause mortality and cardiac events within 3 years. The future of risk stratification of events in HF patients undergoing CCM lies in the integration of HRV data with other prognostic factors, imaging methods and laboratory biomarkers. It might help in optimizing qualifications for this treatment.


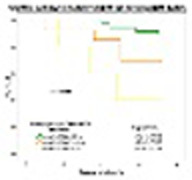


108387

Modality: E-Poster Young Researcher – Non-case Report

Category: EPIDEMIOLOGY AND HEALTH POLICIES/GLOBAL HEALTH

## Epidemiological Profile of Hospitalizations for Acute Myocardial Infarction in Brazil from 2010 to 2019

HILDEMAN DIAS DA COSTA^1^, Hildeman Dias da Costa^1^, Luiz Felipe Façanha Ramos^2^, Leo Christyan Alves de Lima^3^, Matheus Akira Suzuki de Oliveira^1^, Mateus Viana Osório de Barros^1^, Matheus Rodrigues Gomes^3^, Mathews Barbosa Santiago^4^, Milena Stephanie Alves Matos^3^, Júlia de Ávila Gutierrez^5^, Laura Jane França Lacerda^3^, Maria Beatriz Mourão^8^

(1) Universidade Federal de Rondônia; (2) Universidade Federal do Amapá; (3) Centro Universitário São Lucas; (4) Centro Universitário Uninorte; (5) Faculdades Integradas Aparício Carvalho

**Introduction:** Myocardial infarction, or heart attack, is the death of cells in a region of the heart muscle due to the formation of a clot that interrupts blood flow in a sudden and intense way. Fast and efficient care proves to be of fundamental importance to reduce patient morbidity and mortality.

**Objective:** To analyze the epidemiological profile of hospitalizations for acute myocardial infarction on an emergency basis in Brazil between 2010 and 2019.

**Methods:** This is a cross-sectional epidemiological study, in which data were obtained from the Department of SUS Informatics – DATASUS. The variables researched were: total hospitalizations, sex, color/race, age group, deaths and mortality rate. The research period was delimited between the years 2010 and 2019.

**Results:** 911,607 hospitalizations were recorded. Males reported 578,246 hospitalizations, and females 333,361. The white color/race recorded 372,948 hospitalizations. The most affected age group was 60 to 69 years old, with 266,378 hospitalizations. The total number of deaths was 109,295. The mean mortality rate was 11.99. Mortality rates in 2010 and 2019 were 13.46 and 10.37, respectively.

**Conclusions:** The epidemiological profile of hospitalizations was characterized by male, white individuals aged between 60 and 69 years. Although the number of hospitalizations and deaths have increased, mortality rates from AMI have been decreasing over the years.


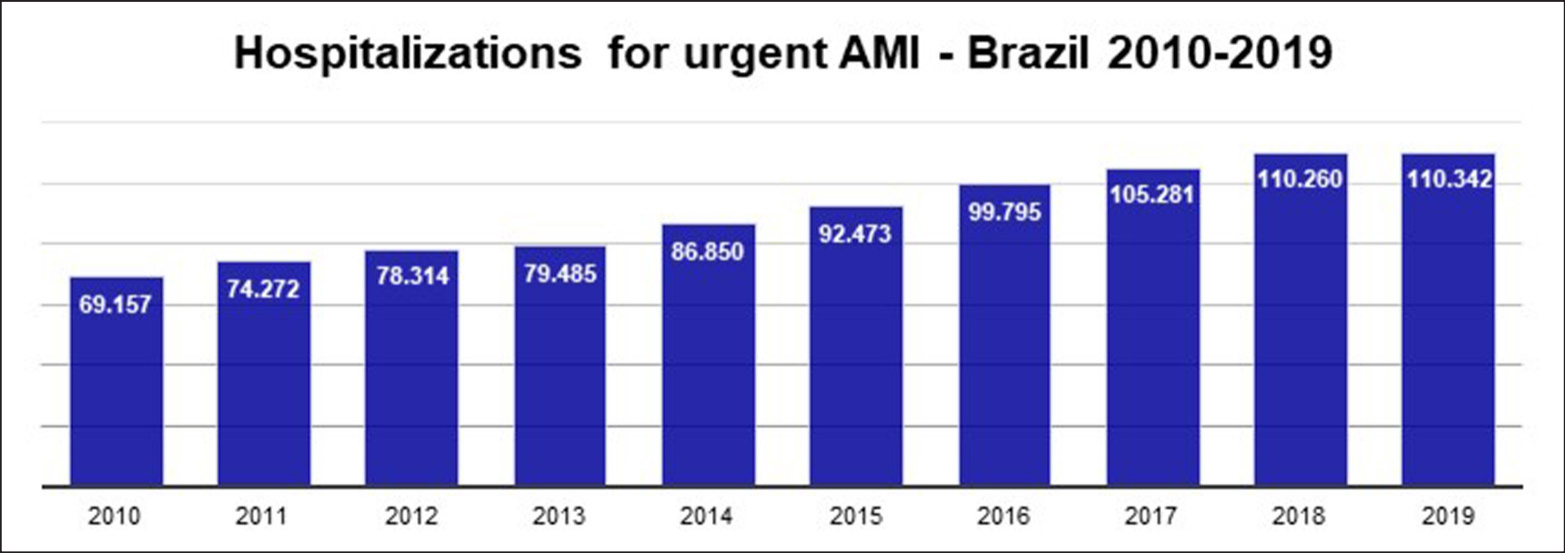


108400

Modality: E-Poster Young Researcher – Non-case Report

Category: PSYCHOLOGY

## Behavioral Cardiology: Smoking in Hospitalized Brazilian Heart Disease Patients

LEONARDO SANTOS DE SOUZA^1^, Leonardo Santos de Souza^1^, Silvia Maria Cury Ismael^1^

(1) Hospital do Coração de São Paulo

**Background:** Considering the complex public health challenge of preventing tobacco-associated diseases as the main cause of morbidity and mortality, understanding the risky health behavior variables thus enabling their proper management. Few Brazilian studies have investigated this issue in the in-hospital setting and in supplementary health frameworks.

**Objectives:** To characterize and assess the variables involved in the smoking behavior of in-patients with heart disease.

**Methods:** Exploratory cross-sectional study approved by the Research Ethics Committee (CAAE nº44662921.0.0000.0060) that assessed 211 tobacco-addict patients with heart disease of which 77.7% were men, with a mean age of 60.5 years (±11.4), evaluated in the last five years by the psychology service of a private hospital in the city of São Paulo. Data collected retrospectively through the Smoker Profile Questionnaire, the Fagerstrom test, the Stages of Behavior Change and Reasons for Smoking Scale were submitted to descriptive analyses as well as non-parametric tests, Chi-Square and Fisher’s Exact tests, using the R Core Team software (2021) and setting a p-value <0.05 as statistically significant.

**Results:** There was a prevalence (p = <0.001) of married men (72%) professionally active (82.3%) with higher education (75%). Consumption had been 20 cigarettes a day for 39.4 years (±13.4) with ineffective cessation attempts (M = 1.9 ± 1.8). Smoking was adhered to for pleasure (85.8%), stress reduction (81.5%) and physical dependence (62.1%), despite motivation to quit (82.9%). In addition, the respondents reported difficulty not smoking when they were sad (46.2%), angry (49.8%) or anxious (60.8%). There was an association (p < 0.001) between time of active smoking, age, hypertension and coronary artery disease, as well as cronic obstructive pulmonary disease (p < 0.001), overweight (p = 0.027), acute myocardial infarction (p = 0.022). Nicotine dependence was associated with chronic alcoholism (p = 0.012) and longer hospital stay (p = 0.032). Weight control (p = 0.003) and stress reduction (p = 0.018) were the main reasons for women with heart disease to keep smoking.

**Conclusion:** Smoking in patients with heart disease has multiple functions and respondent-operant interactions, which makes smoking more resistant to change and demands a thorough functional analysis and comprehensive interventions still during hospitalization, for an efficient secondary prevention.

112369

Modality: E-Poster Young Researcher – Non-case Report

Category: EPIDEMIOLOGY AND HEALTH POLICIES/GLOBAL HEALTH

## Prevalence of Cardiovascular Diseases at the Center of Medical Specialties of Cesupa (Cemec), from January to December 2013

GIOVANNA BOLINI BRAZÃO^1^, ISABELLA ROCHA GONÇALVES^1^, RAISSA MARIA CHAVES LOBATO^1^

(1) Centro Universitário do Estado do Pará (CESUPA)

**Introduction:** Cardiovascular diseases (CVDs) affects the heart and blood vessels, with the ability to compromise other vital systems, representing important causes of deaths worldwide. In Brazil, they are a major public health problem, being associated with several risk factors. To change this situation, it is necessary to know about the epidemiological profile of CVDs at regional level, in order to assist prevention and intervention in modifiable risk factors, through public policies and health actions.

**Objectives:** To verify the prevalence of cardiovascular diseases in the Center for Medical Specialties of Cesupa – CEMEC outpatient clinics; to identify risk factors in the clinical history and their correlation with the development of CVDs; to identify the epidemiological profile of patients affected by CVDs.

**Methods:** The prevalence of CVDs at CEMEC, in the metropolitan region of Belém-PA, during 2013, was evaluated through an observational and cross-sectional study, carried out from the analysis of medical records of the cardiology outpatient clinic in the referred period.

**Results:** The study analyzed the medical records of 152 patients; 40.8% of these were male and the other 59.2%, female. In terms of the neighborhood of origin of these patients, Marambaia (16.4%) and Mangueirão (6.5%) stood out with the largest number of residents who are users of CEMEC’s cardiology outpatient clinic. The history of previous illness, medications in use, life habits, signs and symptoms at the time of consultation and other variables of each patient were analyzed, in order to record, create statistics and understand the needs of the outpatient clinic.

**Conclusion:** Certainly, the poor filling of the medical records collected and the great demand for cardiac care in CEMEC draws attention. In addition, the close correlation between CVDs recorded with the risk factors prevalent in the study population suggests an important criterion in which future local policies can be developed.


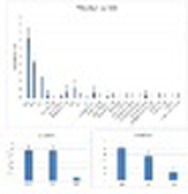


108424

Modality: E-Poster Young Researcher – Non-case Report

Category: DYSLIPIDEMIA

## Algorithm for Familial Hypercholesterolemia Detection and Screening in Lithuanian Population

URTE ALIOSAITIENE^1^, Zaneta Petrulioniene^1^, Egle Skiauteryte^2^, Dovile Gabartaite^1^, Emilija Meskene^1^, Juste Staigyte^1^, Viktoras Sutkus^2^, Rimante Cerkauskiene^1^

(1) Vilnius university faculty of medicine; (2) Vilnius university hospital Santaros Klinikos

**Background and aims:** Familial hypercholesterolemia (FH) is highly underdiagnosed and undertreated congenital disorder worldwide. In this study we aim to establish an algorithm for most efficient FH detection and screening in Lithuanian population.

**Methods:** Since 2016 more than 220 FH patients have been included in Lithuanian FH registry, which is based on Lithuanian High Cardiovascular Risk (LitHiR) primary prevention programme and established as a part of The European Atherosclerosis Society (EAS) Familial Hypercholesterolaemia Studies Collaboration (FHSC) global FH registry. Adults with clinically suspected FH (LDL-C ≥5 mmol/L) were referred to specialist lipid centre, where detailed personal and familial anamnesis, physical examination, evaluation of laboratory and instrumental tests have been performed and secondary causes of dyslipidemia have been excluded. The clinical diagnosis of FH according to Dutch Lipid Clinic Network (DLCN) criteria were determined. Patients with DLCN score ≥6 and/or LDL-C ≥6,5 mmol/L were referred to genetic testing. Cascade first-degree relatives screening were initiated if an index-case meets DLCN criteria for definite or probable FH.

**Results:** During the period of 2016–2021 data of 220 patients (120 females, 100 males) were included in the analysis. Mean age at FH diagnosis were 45,3 (±12,6) years with significant difference between genders: 48,17 (±12,68) years in women and 41,73 (±11,5) in men (p < 0,001). According to DLCN criteria definite FH has been diagnosed for 56 subjects (25,5%), probable FH – 65 patients (29,5%), possible FH – 68 subjects (30,9%) and 31 patients (14,1%) has been included in unlikely FH group. Results of genetic testing have been received for 126 patients: no mutation or variant of unknown significance (VUS) had been detected in 83 patients (65,87%), LDL receptor mutation has been found in 24 patients (19,05%) and ApoB receptor mutation – in 19 subjects (15,08%).

**Conclusions:** The algorithm we use for FH detection seems to be valuable tool for recognizing patients who are in the highest probability of having FH gene mutation. High percent of undetected mutations may be associated with a number of genetically tested patients who were included in possible and unlikely FH groups. Therefore, it could be assumed that in the first instance patients who meet criteria for probable and definite FH should be referred for genetic testing and initiation of cascade screening should be started at this point.

108432

Modality: E-Poster Young Researcher – Non-case Report

Category: ATHEROSCLEROSIS/CARDIOVASCULAR RISK FACTORS/CARDIOVASCULAR PREVENTION

## Atherosclerosis, Deep Vein Thrombosis and Chronic Venous Insufficiency: Is There a Link? a Case – Control Study

JOSE PESENTI^1^, Leticia Gonzalez^2^, Francisco Vargas^4^, Gonzalo Martinez^5^, Prakash Saha^6^, Marcelo E. Andia^2^

(1) School of Medicine, Pontificia Universidad Católica de Chile, Santiago, Chile; (2) ANID-Millennium Institute for Intelligent Healthcare Engineering, Santiago, Chile; (3) Biomedical Imaging Center, School of Medicine, Pontificia Universidad Católica, Santiago, Chile; (4) Vascular Surgery Department, School of Medicine, Pontificia Universidad Católica, Santiago, Chile; (5) Cardiovascular Division, School of Medicine, Pontificia Universidad Católica, Santiago, Chile; (6) School of Cardiovascular Medicine & Sciences, King’s College London, London, UK

**Introduction:** Atherosclerosis, deep vein thrombosis (DVT) and chronic venous insufficiency (CVI) have been seen as different entities, however thrombo-inflammation could be the link between them. In this study we explore this relationship in patients with DVT, CVI and controls.

**Methods:** We recruited 7 patients with DVT and 9 with CVI from the Vascular Surgery Department of our University Hospital and 9 healthy controls matched for gender and age. We registered their cardiovascular risk factors and obtained blood tests for hs-CRP, Circulating Endothelial Cells (CEC), ICAM-1 and CXCL7 as biomarkers of inflammation, endothelial and platelets activation. Carotids plaques were classified according to the AHA MRI classification. We used Principal Component Analysis (PCA) and Hierarchical Clustering Analysis (HCA) to study the combined effect of all variables.

**Results:** There were no significant differences between the groups for inflammatory markers and cardiovascular risk factors (Fig.A). However, using PCA and HCA we identified 2 clusters: cluster 1 with all the controls, one DVT and three CVI patients; and cluster 2 with only DVT and CVI subjects (Fig.B). There was a significant difference between carotid plaque complexity between both clusters (Fig.C).

**Conclusions:** Our study supports the link between inflammation, DVT and atherosclerosis, and contributes to consider CVI as another risk factor for atherosclerosis. Acknowledgment: FONDECYT 1180525.


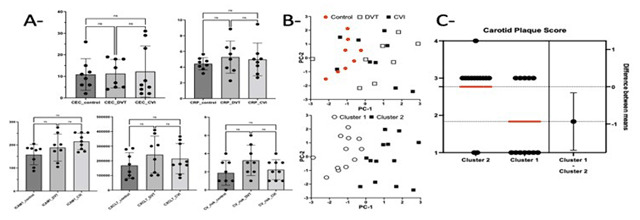


108463

Modality: E-Poster Young Researcher – Non-case Report

Category: HYPERTENSION/RENAL DENERVATION

## Educational and Drug Interventions for Blood Pressure Control in Kidney Transplant Recipients Based on Ambulatory Blood Pressure Monitoring

RAFAEL NAUFEL DE SÁ REBELO^1^, Rafael Naufel de Sá Rebelo^1^, Cibele Isaac Saad Rodrigues^1^

(1) Pontifícia Universidade Católica de São Paulo

The goal of this study was to compare blood pressure (BP) control, pre-and post-educational and therapeutical interventions in kidney transplant recipients (KTR) obtained by office (OBP) and ambulatory blood pressure monitoring (ABPM) at baseline and 6 months later. It is an observational, and interventional cohort study with 33 adult hypertensive and non-diabetic KTR. Data collection included three medical appointments bimonthly. Statistical analysis included a paired t-test, Student’s t-test, and Fisher’s test. We observed that OBP presents mean values higher than those obtained by the ABPM (p < 0.05) and high prevalence of atypical BP curve, especially white coat hypertension (46.1%), and 90% showed abnormal nocturnal dipping. Association of drug adjustments plus lifestyle changes was the most needed intervention (88%). The diastolic OBP significantly decreased after 6 months (p = 0.040). Patients were more likely to follow the diet changing (69,3%) than the physical activity program (34.6%). Dietary modification was associated with a significant reduction of ABPM mean systolic BP. This study showed high prevalence of hypertension, detected different phenotypes using ABPM, and implemented personalized actions to achieved BP control. These changes proved to be effective after a 6-moths showing that ABPM is an important strategy for BP diagnosis and follow-up in KTR.

112340

Modality: E-Poster Young Researcher – Non-case Report

Category: CARDIOGERIATRICS

## Heart Disease in People Older than 95 Years – the Cardiogeriatric Clinic Experience in Mexico

MARIANA GAMBOA ESPARZA^1^, Ana Mercedes Andrade Aguilar^2^, Arturo Moreno Pérez^1^, Natalia Capistrán Páramo^1^, Henry De Las Salas Pérez^1^, Carlos Alberto Guillén Rosaldo^1^, Luis Alberto Lasses y Ojeda^1^

(1) Instituto Nacional de Cardiología Ignacio Chávez INCICH; (2) Hospital General Regional No. 46 IMSS

**Introduction:** Very old patients form an increasing population in cardiology consultation, then specialized geriatric care is needed.

**Objective:** To describe cardiovascular and geriatric profile in patients older than 95 years with heart disease.

**Methods:** We performed a cross-sectional study collecting data through clinical record and a telephone survey to complete a geriatric comprehensive assessment (GCA) and cardiovascular variables of patients older than 95 years attending a tertiary referral hospital during 2021.

**Results:** Of a total 74 patients older than 95 years, 36 participated in this study. 19 were deceased and 19 were unreachable. Mean age is 97 ± 1.79 years, and 75% were female. Mean years attending Cardiogeriatric Clinic is 6.2 ± 3.46. The most common diagnosis was hypertension (75%) followed by arrythmia (61%). Atrial fibrillation was the most common arrythmia (40%). 36% of patients have pacemaker. Ten patients are anticoagulated and in reduced dose. Only 3 participants visited emergency room in the last year. None of them was for bleeding. Prefrailty is present in 42% and Frailty in 53% of patients. Sarcopenia screening was positive in 69%. 13.8% had 2 or more falls. All patients have caregivers, and the majority self-reported a good health status. Even thought, 33% have depression and 28% have dementia.

**Conclusions:** Geriatricians trained in heart diseases may improve outcomes in this population. Implementation of a Cardiogeriatric Clinic in a tertiary referral hospital is preparing new physicians’ generations to design interventions for patients older than 95 years, especially in frailty, sarcopenia and falls.


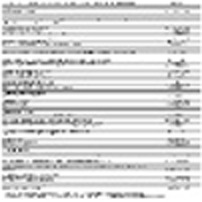


108471

Modality: E-Poster Young Researcher – Non-case Report

Category: EPIDEMIOLOGY AND HEALTH POLICIES/GLOBAL HEALTH

## Heart Transplantation in Brazil: An Epidemiological Study of Surgeries in the Last Ten Years (2012–2021)

HILDEMAN DIAS DA COSTA^1^, Hildeman Dias da Costa^1^, Luiz Felipe Façanha Ramos^2^, Leo Christyan Alves de Lima^3^, Matheus Akira Suzuki de Oliveira^1^, Mateus Viana Osório de Barros^1^, Mathews Barbosa Santiago^4^, Júlia de Ávila Gutierrez^5^, Laura Jane França Lacerda^3^, Ayrison de Melo Sousa^4^, Erica Mayara Gama Pinheiro^1^

(1) Universidade Federal de Rondônia; (2) Universidade Federal do Amapá; (3) Centro Universitário São Lucas; (4) Centro Universitário Uninorte; (5) Faculdades Integradas Aparício Carvalho

**Introduction:** According to the Brazilian Association of Organ Transplantation, the heart is the third most transplanted organ in Brazil, behind only the kidney and liver. Structural problems in the Brazilian health sector and the resistance of families to authorize donations are some of the main obstacles to performing surgeries.

**Objective:** To analyze the epidemiological profile of heart transplant surgeries in Brazil in the last 10 years.

**Methods:** This is a cross-sectional epidemiological study. Data were obtained from the Informatics Department of the Unified Health System – DATASUS. The variables studied were: total number of transplants, states that performed the most surgeries, average length of stay, deaths and mortality rate. The research period was delimited between 2012 and 2021.

**Results:** 2,732 heart transplant surgeries were performed. In 2012 and 2021, 193 and 226 transplants were performed, respectively. São Paulo and Minas Gerais were the states that performed the most surgeries: 852 and 400, respectively. The average length of stay was 17.8 days. The total number of deaths was 311. The average mortality rate was 11.38.

**Conclusions:** The epidemiological profile of transplants was characterized by surgeries that took place mainly in the states of São Paulo and Minas Gerais, with an average length of stay of hospitalizations of 18 days. The reduction in the number of surgeries in 2020 and 2021 may reflect the pandemic caused by COVID-19.


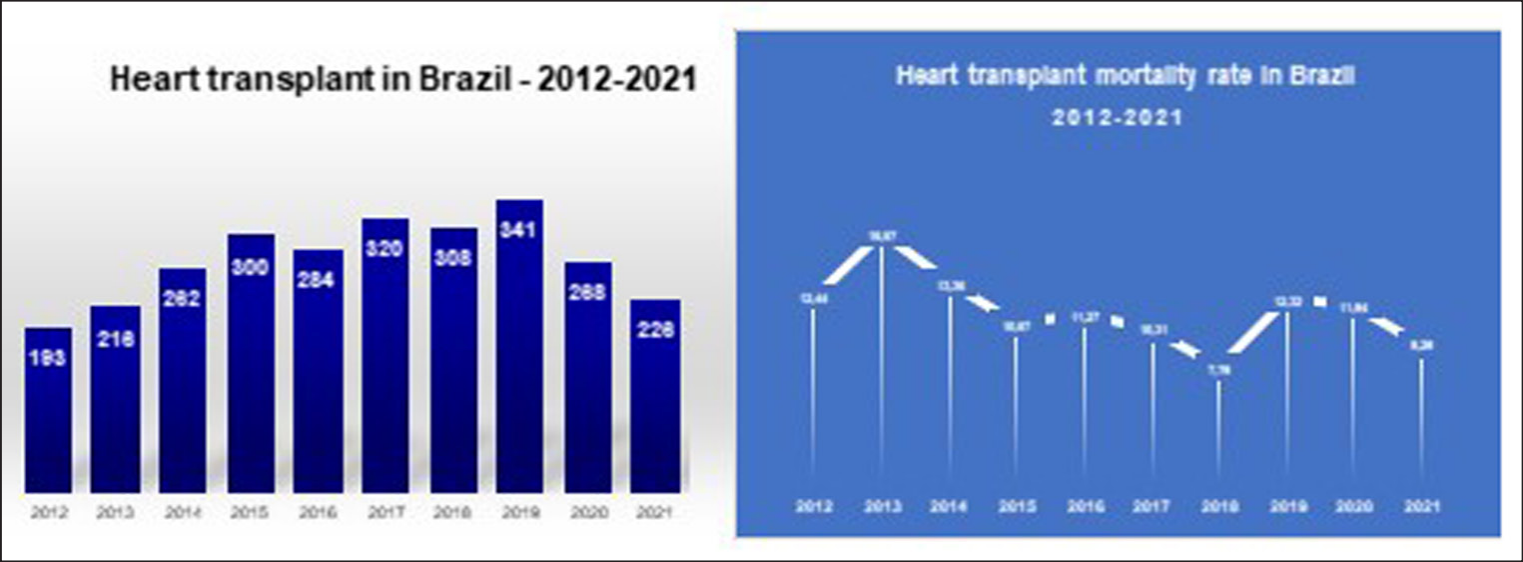


108481

Modality: E-Poster Young Researcher – Non-case Report

Category: EPIDEMIOLOGY AND HEALTH POLICIES/GLOBAL HEALTH

## Trend of Mortality from Heart Failure in Brazil: 1998–2019

VILMEYZE LARISSA DE ARRUDA^1^, Vilmeyze Larissa de Arruda^1^, Lúbia Maieles Gomes Machado^1^, Jaqueline Costa Lima^1^, Pãmela Rodrigues de Souza Silva^1^

(1) Universidade Federal de Mato Grosso (UFMT)

**Introduction:** Heart failure (HF) is characterized by decreased cardiac output and/or high filling pressures at rest or on exertion. HF is considered the most prevalent cardiovascular disease and the main cause of hospitalization in the Unified Health System.

**Objective:** To analyze the trend of mortality from HF in Brazilians aged 50 years and over, within 21 years.

**Method:** Ecological study with time series analysis of mortality from HF in Brazil, according to regions and federation units, in individuals aged 50 years and older in the period from 1998 to 2019. Deaths that had HF as the underlying cause (International classification of diseases –10: I50), that occurred during the study period were included in the study. Statistical analyzes were performed using the Stata 11.1 program. The HF mortality rate per 100,000 inhabitants was calculated. In the trend analysis, the Prais-Winsten regression was used.

**Results:** Between 1998 and 2019, 567,789 deaths from HF were recorded in adults over 50 years of age, which corresponds to an average rate of 75.5 per 100,000 inhabitants. The trend was decreasing by sex, regions and in 23 FUs. The highest observed mortality rates occurred at older ages in all regions of the country.

**Conclusion:** The trend of mortality rates from HF in adults aged 50 years and over among the federation units and Brazilian regions was decreasing over 21 years. There was an increasing trend in mortality from HF in the northern region and in the category other health facilities.

108598

Modality: E-Poster Young Researcher – Non-case Report

Category: ATHEROSCLEROSIS/CARDIOVASCULAR RISK FACTORS/CARDIOVASCULAR PREVENTION

## Association of Eight Genetic Polymorphisms to High Blood Pressure and Obesity in a Sample of Brazilian Adolescents. Rio De Janeiro Study II

YASMIN LEMOS ROLLEMBERG CRUZ MACHADO^2^, Flávia Lopes Fonseca^2^, Rossana Ghessa Andrade de Freitas^2^, Pedro Pimenta de Mello Spineti^2^, Erika Maria Gonçalves Campana^2^, Roberto Pozzan^2^, Maria Eliane Campos Magalhães^2^, Dayse Aparecida da Silva^1^, Andréa Araujo Brandão^2^

(1) Roberto Alcântara Gomes Biology Institute, State University of Rio de Janeiro (UERJ); (2) Faculty of Medical Sciences, State University of Rio de Janeiro (UERJ)

**Introduction:** Several genetic polymorphisms have been investigated as potential risk predictors of cardiovascular (CV) diseases and their comorbidities mainly in adults from genetically homogenous populations; although their CV complications correlated with the presence of obesity and high blood pressure (BP) since childhood.

**Objective:** To evaluate the association of eight SNPs in genes related to the renin-angiotensin-aldosterone system, endothelial function and leptin-melanocortin pathway with high blood pressure (BP) and obesity in a sample of admixed Brazilian adolescents from a longitudinal study.

**Methods:** 1,054 Brazilian adolescents aged 10–15 years, both genders, were genotyped for rs699 (AGT), rs1799998 (CYP11B2), rs1799983 (NOS3), rs9939609 (FTO), rs1137101 (LEPR), rs3746619 and rs3827103 (MC3R), and rs17782313 (MC4R) through customized Multiplex Minisequencing System.

**Results:** Prevalence of high BP and obesity were 13.5% and 41.4%, respectively. Minor A allele of rs3746619 (MC3R) was more prevalent in black (P = .001), in hypertensive (P = .017) and correlated with increased systolic BP (P = .023). G wild-type allele of rs1799983 (NOS3) was more prevalent in obese (BMI, P = .002; WC, P = .027), correlated with increased BMI (P = .020) and was associated with obesity in non-adjusted (OR = 0.713, P < .001) and adjusted regression models (OR = 0.730, P = .001). Variants rs3827103 (MC3R), rs699 (AGT) and rs1799998 (CYP11B2) presented different genotypic distributions according to color, gender, WC and BP status (P < .05). No other SNP was associated with elevated BP, obesity or their quantitative traits.

**Conclusion:** The G wild-type allele of rs1799983 (NOS3) was associated with increased BMI and risk for obesity in admixed Brazilian adolescents with high BP.

108492

Modality: E-Poster Young Researcher – Non-case Report

Category: CARDIOVASCULAR SURGERY

## A Comparison of the 2021 Ckd-Epi Creatinine Equation and Cockcroft-Gault Formula in Patients Undergoing Cardiovascular Surgeries

STELLA DE SOUZA VIEIRA^1^, Bianca Kajimoto Magalhães^1^, Andressa Muzzo de Souza^1^, Marcelo Arruda Nakazone^1^, Lilia Nigro Maia^1^, Maurício Nassau Machado^3^

(1) Faculdade de Medicina de São José do Rio Preto – FAMERP; (2) Escola Paulista de Medicina – Universidade Federal de São Paulo – UNIFESP-EPM; (3) fundação Faculdade Regional de Medicina de São José do Rio Preto – FUNFARME

**Introduction:** Although the use of the Cockcroft-Gault (CG) formula is no longer recommended, the ease of application still makes it widely used. Recently, the Chronic Kidney Disease Epidemiology Collaboration (CKD-EPI) equation was updated to disregard the race statement, a non-biological construct.

**Objective:** We evaluated the strength of agreement between the CG formula and the 2021 CKD-EPI equation and assessed the impact of disagreement between estimated glomerular filtration rates (eGFR) methods on outcomes.

**Methods:** A total of 2,855 adult patients who underwent cardiac surgeries (isolated coronary artery bypass grafting or heart valve surgery) were analyzed. Linear regression, Bland-Altman plots and Cohen’s Kappa statistics were used to determine the strength of agreement between the eGFR determinations. Cumulative survival curves were constructed to demonstrate the impact of renal function categories on mortality.

**Results:** Sixty-one percent (1,748) and 23% (658) of the patients were classified by both equations as eGFR ≥60 and <60 mL/min/1.73 m², respectively. Sixteen percent of the patients were classified as discordant eGFR. There was a significant positive linear correlation (Spearman’s rho, 0.817; P < 0.001) between the eGFR based on the CG formula compared with eGFR according to the 2021 CKD-EPI equation. The Bland-Altman plots showed that the mean difference between the methods was 2.5 with the 95% limits of agreement ranging from –30.8 to 35.8, but the one-sample T test of the difference showed disagreement between the eGFR methods (P < 0.001). Patients with prevalent eGFR 60 to <90 and 45 to <60 had modest strength of agreement (K = 0,389 and K = 0,365; respectively) and the percentage of agreement was 69.7% and 80.6%, respectively. There was only one agreement classified as good in the stage 5 (K = 0.729), but this group represented only 2.4% of our cohort. The Kaplan-Meier curves showed cumulative survival rates at 30-day of 95.8%, 91.5% and 83.9% for patients with eGFR ≥60, discordant eGFRs, and eGFR <60 mL/min/1.73 m² (P < 0.001).

**Conclusions:** Despite of a positive linear correlation, the means of the differences between the two equations is statistically significant and the CG formula shows only modest strength of agreement with the 2021 CKD-EPI equation, limited to the prevalent strata of renal function. This finding suggests that the CG formula should be no longer used in cardiac surgery settings to estimate glomerular filtration rate.

108495

Modality: E-Poster Young Researcher – Non-case Report

Category: EPIDEMIOLOGY AND HEALTH POLICIES/GLOBAL HEALTH

## Relationship between Sociodemographic Index and Cerebrovascular Disease in Brazil in the Period of 2000–2019

JOSÉ LUCAS PERES BICHARA^1^, José Lucas Peres Bichara^1^, Luiz Antônio Viegas de Miranda Bastos^1^, Paolo Blanco Villela^1^, Glaucia Moraes de Oliveira^1^

(1) Universidade Federal do Rio de Janeiro – UFRJ

**Introduction:** Cardiovascular diseases (CVD) are the main causes of death in the world and in Brazil, and cerebrovascular diseases (CVD) are one of the main responsible for these statistics, a fact aggravated by the aging of the population. Previous studies have already suggested a relationship between the evolution of CVD mortality rates and socioeconomic indicators.

**Objective:** To relate the evolution of CVD mortality rates and the sociodemographic index (SDI) from 2000 to 2019 in Brazil and in its federative units (FUs).

**Methods:** Ecological time series study of deaths from CVD in Brazil. Crude and standardized mortality rate for CVD were analyzed by sex, age group and FU between 2000 and 2019. Data were correlated with the SDI. Deaths and population were taken from DATASUS to estimate crude and standardized mortality rates per 100,000 inhabitants (direct method with Brazilian population in 2000). The SDI for each UF was extracted from the Global Health Data Exchange website.

**Results:** In the period, there were 1,925,765 deaths from CVD in Brazil, 50.54% of which were male. The national SDI ranged from 0.538 in 2000 to 0.64 in 2019, with constant growth in the period. At the same time, the age-standardized mortality rate for CVD decreased from 49.81/100,000 inhabitants to 30.98/100,000 inhabitants. In this way, CVD has become the second leading cause of mortality in the country. In the FUs, all states in the North and Northeast regions showed improveme.


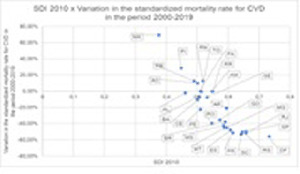


108510

Modality: E-Poster Young Researcher – Non-case Report

Category: COVID-19 AND CARDIOVASCULAR SYSTEM

## Impact of COVID-19 on Cardiac Surgeries in a Tertiary Care Center

FERNANDA AREJANO VAUCHER^1^, Maria Antonia C. da Silva^1^, Maria Eduarda M. B. Busko^1^, Vanessa C. de Lima^1^, Thaísa R. F. Basagla^1^, Ana Karyn E. de Freitas^1^

(1) Irmandade Santa Casa de Misericórdia de Curitiba (ISCMC)

**Background:** The infection caused by COVID-19 in patients undergoing cardiac surgery leads to higher mortality and clinical complications in the postoperative period. In addition, most patients are older and have several comorbidities, which are factors of worse prognosis for disease progression.

**Objectives:** This study aims to identify the impact of COVID-19 infection on the mortality of patients undergoing cardiac surgery within a 30-day period, as well as the incidence of clinical complications.

**Methods:** We retrospectively evaluated 213 patients undergoing elective cardiac surgery or in the context of urgency and emergency, from January to December 2020, in a tertiary referral center. Of these, those who tested positive for COVID-19 within 30 days before or after the procedure were compared with those who did not have the infection in the period, considered the control group.

**Results:** Of the 213 patients included in the study, 27 (12.6%) were diagnosed with COVID and 186 (87.3%) did not have the disease. Of the total, 95 were women (45%) and 118 were men (55%). The mean age was 62 years. There was no significant difference between the groups regarding epidemiological data. Mortality was significantly higher in the COVID-19 group (33% vs 15% patients in the control group). Complications such as arrhythmias (37% × 19%), prolonged ICU time (8 ± 8 days × 7 ± 7 days), total hospital stay (25 ± 16 days × 16 ± 19 days), respiratory failure requiring orotracheal intubation (30% × 1.1%), mechanical ventilation time (3.23 ± 7 days × 1.83 ± 5.48 days) and venous thrombosis (11% × 1.6%) were also more prevalent in the COVID group –19. The others showed no statistically significant difference, although they were more frequent in the group of patients with COVID-19.

**Conclusion:** COVID-19 infection in the perioperative period of cardiac surgeries is associated with higher rates of morbidity and mortality. Delaying elective surgeries in coronavirus-positive patients can help reduce the risk of complications, along with effective strategies to decrease in-hospital infection.

108514

Modality: E-Poster Young Researcher – Non-case Report

Category: PERICARDIUM/ENDOCARDIUM/VALVOPATHIES

## Long-Term Success in Percutaneous Valve Commissurotomy – is Wilkins Score Over 9 a Definitive Limit?

JOÃO MIGUEL MARTINS DE CARVALHO^1^, Ricardo Alves Pinto^1^, Tânia Proença^1^, João Calvão^1^, Ana Filipa Amador^1^, Catarina Marques da Costa^1^, André Cabrita^1^, Catarina Marques^1^, Carla de Sousa^1^, Mariana Paiva^1^, João Carlos Silva^1^, Filipe Macedo^1^

(1) Centro Hospitalar Universitário São João

Percutaneous valve commissurotomy (PMC) is an established treatment in patients with significative mitral stenosis (MS). Although rheumatic MS incidence has decreased in the last century, it remains a prevalent pathology worldwide. The Wilkins score (WS) is a reference in echocardiographic assessment of MS; a score ≤8 is considered a predictor of treatment success and score between 9 and 11 is a “grey zone” (WGZ) in which doubts persists regarding PMC success. The purpose of our study was to evaluate the early and long-term results of PMC in patients with rheumatic MS and to compare long-term events between patients with WS ≤ 8 and patients in WGZ. We retrospectively analysed all patients between 1991 and 2008 with significative rheumatic MS undergoing PMC. Data were collected at baseline and during long-term follow-up. MACE was defined as a composite of all-cause mortality, mitral valve re-intervention or cardiovascular hospitalization. In our cohort, 124 patients were included. Most were female (87%), mean age at the time of repair was 46 ± 11 year-old and mean follow-up was 20 ± 6 years. Before the procedure, 81% had WS ≤ 8 and 19% were in WGZ. Both groups had similar baseline characteristics, namely age at first intervention, NYHA class and follow-up time. All patients had preserved biventricular systolic function, 83% presented PH, mean transvalvular gradient (TVG) and mitral valve area (MVA) were 12.8 mmHg and 1.0 cm^2^, respectively. Most of the procedures were successful (91%) and without complications (94%). Mean MVA improvement was similar in both groups [0.9 cm^2^ in WS ≤ 8 and 0.8 cm^2^ in WGZ, t(102) = 0.173, p = 0.863]; there was also no significative difference in TVG and PASP reduction after PMC. During long-term follow-up, re-intervention and mortality occurred in 40% and 23% in WS ≤ 8 and in 50% and 29% in WGZ, respectively, and none of these differences was statistically significant (p = 0.389 and p = 0.544, respectively). Concerning time-to-event analysis, approximately 80% of patients kept uneventful and >90% alive after 10 years in both groups and no significant difference in M ACE events and all-cause mortality between WS ≤ 8 and WGZ was observed (Log Rank, p = 0,419 and p = 0.950, respectively). PMC was safe and effective in clinically significant rheumatic MS in both WS ≤ 8 and WS 9–11, with similar MVA improvement. After 10 years, approximately 80% of patients were MACE-free and >90% alive in both groups. There was no difference concerning WS groups.

108515

Modality: E-Poster Young Researcher – Non-case Report

Category: CONGENITAL AND PEDIATRIC CARDIOLOGY

## Destination Therapy or a Bridge to Transplant? – Long-Term Outcome in Fontan Procedure Patients

JOÃO MIGUEL MARTINS DE CARVALHO^1^, Ricardo Alves Pinto^1^, Tânia Proença^1^, Catarina Martins da Costa^1^, Ana Filipa Amador^1^, João Calvão^1^, Catarina Amaral Marques^1^, André Cabrita^1^, Sofia Cardoso Torres^1^, Cristina Cruz^1^, Filipe Macedo^1^

(1) Centro Hospitalar Universitário São João (CHUSJ), Porto, Portugal

The Fontan procedure (FP) is considered a palliative surgical technique used for complex congenital heart disease (CHD) patients not suitable for biventricular repair. Currently, these patients’ life expectation is extended but they experience high morbidity and mortality risks and their long-term management is challenging. Our aim was to evaluate the morbidity of these patients after a long-term follow-up. We collected a retrospective cohort of patients palliated with FP, that were followed in an adult CHD outpatient clinic born between 1980 and 2001. Clinical and echocardiographic data were collected. A time to adverse event analyses was performed. A total of 26 patients were enrolled, with a median follow-up of 17 years. The median age was 24 (IQR 22–30) years-old, 35% were female. As for the cardiovascular risk factors, none had hypertension or dyslipidaemia, 3,9% had diabetes and 11,5% were smokers or previous smokers. The main anatomic abnormalities that lead to the FP were single ventricle (53,8%), followed by pulmonary atresia (23,1%), double outlet right ventricle (11,5%), ventricular septal defect (3,9%) and Ebstein anomaly (7,7%). The majority of patients were previously submitted to a shunt (42,3% with a Blalock-Taussig and 19,3% with an atrial septostomy); the most prevalent surgical technique was the cavopulmonary connection (69,2% extracardiac, 15,4% intracardiac), followed by the atriopulmonary anastomosis (11,5%). The systemic ventricle was morphologically left in 84,6%. A fenestration or a residual shunt persisted in 30,8%. The mean basal oxygen saturation was of 95%, with 3,9% of patients being cyanotic. The majority of patients were asymptomatic (69,2%), with a normal ventricular function in those with a systemic left-ventricle (91,7% of patients), and a moderately impaired in those with a systemic right-ventricle (50% of patients); more than moderate AV valve regurgitation was present in 7,7%. Pulmonary hypertension was observed in 7,7%. Atrial arrythmias were present in 11,5%. Liver disease affected 50% of patients (1 patient with an hepatocarcinoma) and protein-losing enteropathy was present in 7,7%. One patient was submitted to a re-FP, due to a Fontan circulation obstruction; another patient was submitted to a heart transplant. Regarding the time-to-adverse-events analyses, more than 65% of patients were event-free during the first 15 years. However, after 20 years of FP more than 60% of patients presented with an adverse event.

108517

Modality: E-Poster Young Researcher – Non-case Report

Category: HEART FAILURE/CARDIOMYOPATHY/TRANSPLANT

## Cardiac Remodeling in Rats with Myocardial Infarction Submitted to Sleep Restriction

BRUNNO LEMES DE MELO^1^, Brunno Lemes de Melo^1^, STELLA DE SOUZA VIEIRA^1^, Ednei Luiz Antonio^1^, Helenita A. Oliveira^1^, Ighor L. Teixeira^1^, Andrey Jorge Serra^1^, Paulo José Ferreira Tucci^1^

(1) Universidade Federal de São Paulo; (2) Faculdade de Medicina de São José do Rio Preto

**Introduction:** Sleep is an active and heterogeneous physiological process, evolutionarily conserved, which plays a critical role in survival. It is presumed that sleep has the main function of restoring the physiological and biochemical balances that change during the waking period. It is also crucial for the tissue regeneration process and metabolism. It is known that sleep deprivation or restriction (SR) generates negative physiological effects on the body. Sleep deficit promotes patent neuroendocrine and metabolic changes in humans and laboratory animals, such as reductions in the levels of thyroid hormones, thyroid-stimulating hormones, growth hormones, and testosterone, as well as increases in adrenocorticotropin, corticosterone, adrenaline, noradrenaline, and dopamine. Added to this are the hyperactivation of the sympathetic nervous system and the stimulation of a pro-inflammatory state. These implications lead to obvious clinical repercussions, by which the cardiovascular system is also affected. Evidence indicates that the decrease in sleep hours is associated with an increased risk in the development and worsening of cardiovascular diseases (CD).

**Objective:** To determine if SR enhances cardiac remodeling after Myocardial Infarction (MI) in male rats; to verify if the treatment with β-blocker can attenuate the damages resulting from the SR after MI.

**Method:** Male Wistar rats were used, the animals were divided into 5 groups (SHAM:submitted to sham surgery; MI:submitted to MI; SR: submitted to SR; MISR: submitted to MI and SR; MISR+βB:IMRS treated with β-blocker), in the groups submitted to the IM technique, only animals that had infarction affected >37% of the LV.

**Results:** The MISR group had pulmonary congestion; the MI resulted in an increase in the diastolic area (LVDA) and systolic area (LVSA) of the LV, in the SHAM and SR groups there were no significant changes. The MI group demonstrated a decrease in the LV cross-sectional area fraction (LVAF) and an increase in nuclear volume; increased LV end-diastolic pressure (EDP) values were identified in the MI and MISR groups vs. Sham and MI vs. SR.

**Conclusion:** The use of β-blocker was effective in slowing down post-infarction myocardial remodeling under SR conditions. These effects were observed in the LVAF, PDF, and pulmonary congestion preservation data. Thus, it is concluded that the β-blocker administered under conditions of adrenergic hyperactivity caused by SR was effective in attenuating cardiac remodeling.

108525

Modality: E-Poster Young Researcher – Non-case Report

Category: NUTRITION

## Effects of Cocoa Consumption on DNA Damage, Blood Pressure, Body Composition and Biochemical Parameters of Resistant Hypertensive Individuals: A Pilot Study

THAIRINI DE SOUZA MIGUEL^1^, Letícia Machado Santos^1^, Jorge da Silva Pinho Junior^1^, Anna Paula Arpini Botelho^2^, Julia Passarelli Pereira^2^, Sergio Girão Barroso^1^, Glauber Monteiro Dias^2^, Andrea Cardoso de Matos^1^, Grazielle Vilas Boas Huguenin^1^

(1) UNIVERSIDADE FEDERAL FLUMINENSE; (2) INSTITUTO NACIONAL DE CARDIOLOGIA

**Introduction:** Cardiovascular diseases (CVD) are the main causes of death in the world and hypertension is the main triggering factor of CVD. Some individuals are resistant to standard treatment for hypertension, and resistant arterial hypertension (RAH) is characterized. Cocoa has been shown to be a food with interesting repercussions on cardiovascular health, attenuating blood pressure, improving glycemic and lipid profile, body composition, in addition to possibly protecting against DNA damage. However, it is not yet known whether these effects are reproduced in the RAH.

**Objective:** To investigate the effect of cocoa powder consumption on DNA damage, blood pressure, glycemic and lipid profiles and body composition in patients with RAH.

**Methods:** Randomized, double-blind, placebo-controlled clinical trial. Twelve participants were randomized into a control group (CG = 6) or intervention group (IG = 6). The GI received 30g of cocoa powder for 60 days. The GC received 30g of maltodextrin, colored and flavored, for 60 days. Blood glucose, HbA1c, triglycerides, total, HDL and LDL cholesterol, weight, BMI, waist and hip circumference were analyzed at times T0, T30 and T60. DNA damage analysis was performed using the comet assay, at T0 and T60. Descriptive statistical analysis was performed using the GraphPad Prism software. When applicable, ANOVA test was performed. Results were presented as mean and standard error and the difference was considered significant when p ≤ 0.05.

**Results:** Participants were 59 ± 8.27 years old and 58.33% were women. At T30, there was a significant decrease in HbA1c (p = 0.0531) in the IG (5.325 ± 0.3065%) compared to the CG (6.520 ± 0.3865%). We observed a trend towards attenuation of DNA damage at level 1 (T0 21.75 ± 14.73–T60 10.50 ± 5.18). This result is inverse in the placebo-supplemented group, where we observed a trend of increased damage (T0 7.17 ± 1.41–T60 14.33 ± 4.47). The other parameters studied did not change.

**Conclusion:** Supplementation with cocoa powder, by individuals with RAH, was beneficial in reducing HbA1c levels, without altering fasting glucose. As for DNA damage analysis, cocoa supplementation seems to add some protection to DNA, at damage level 1. More studies need to be carried out to better elucidate the issues addressed in the present study.

108527

Modality: E-Poster Young Researcher – Non-case Report

Category: ATHEROSCLEROSIS/CARDIOVASCULAR RISK FACTORS/CARDIOVASCULAR PREVENTION

## The Role of Primary Care in Controlling an Important Cardiovascular Risk Factor

BRUNO GIUDICE D’AVILA^1^, Bruna de Matos Bauer^1^, Rosane Hermann^2^

(1) Irmandade Santa Casa de Misericórdia de Porto Alegre – ISCMPA; (2) Atenção Primária, Prefeitura Municipal de Brusque-SC

**Introduction:** Smoking is an important isolated risk factor for cardiovascular disease and the leading cause of preventable morbidity and mortality worldwide. Such relevance has demanded from the health systems the elaboration of strategies to face this problem. This article aims to describe the successful experience of a multidisciplinary team of a Brazilian Public Health Unit in the fight against smoking during a follow-up period of 21 months.

**Methods:** The strategy used for smoking cessation treatment involved group activities and psychosocial interventions with an emphasis on the cognitive-behavioral approach. Drug treatment with bupropion and nicotine patches was used according to medical evaluation and in accordance with the guidelines of the Brazilian Ministry of Health. A survey was carried out to evaluate users motivated to quit smoking and interested in participating in the proposed activities, in addition to evaluating the existence of cardiovascular comorbidities. The limit of up to 15 participants per group was defined. The activities were carried out in 10 sessions, the first four being weekly and the follow-up sessions after 15, 30, 60, 90 and 180 days, from February 2017 to November 2018. The actions were carried out by a team composed of a doctor, a nurse, community health agents and with the support of a nutritionist, a psychologist and a physical educator. All the professionals involved had previously completed a training course for the development of actions according to the clinical protocols and therapeutic guidelines of the Brazilian Public Health System.

**Results:** During the study period, 54 patients were selected to participate in the activities. In this group, there was a predominance of females, a prevalence of arterial hypertension of 59% and diabetes of 18%. 74% of individuals reported at least one previous unsuccessful attempt to quit tobacco use. At the end of the observation period, 70% of the participants remained abstinent. There were 4 evasions due to change of residence territory and 12 due to resumption of tobacco use.

**Conclusion:** This study highlights the remarkable potential of primary care in controlling one of the main risk factors for cardiovascular disease. It is possible to observe that even low-cost interventions for the Health System, as long as they are planned considering local sociocultural factors, can provide satisfactory results.

108534

Modality: E-Poster Young Researcher – Non-case Report

Category: EPIDEMIOLOGY AND HEALTH POLICIES/GLOBAL HEALTH

## COVID-19 on Brazil: Correlation between Socioeconomic, Demographic and Health Services Structure and Mortality and the Proposal of a Vulnerability Score

LUKA DAVID LECHINEWSKI^1^, Lucas Henrique Olandoski Erbano^1^, Bruna Olandoski Erbano^1^, Nicolle Amboni Schio^1^, Raisa Natalia Dotto^1^, Amanda Zanlorenzi^1^, Lucas Baena Carstens^1^, Rafaela Lima Camargo^1^, Fernanda Leticia Perez Moraes^1^, Márcia Olandoski^1^, José Rocha Faria-Neto^1^

(1) Pontifícia Universidade Católica de Curitiba

**Introduction:** COVID-19 pandemic rapid spread caused pressure over health systems and impaired assistance to other diseases, as observed by the reduction of acute coronary syndromes hospital admissions. In Brazil the number of cases and deaths related to COVID-19 happened quite differently through the Brazilian states (BS).

**Objectives:** Identify socioeconomic, demographic and health structure services indices that had association with a higher death rate on the initial months of the pandemic and propose a score to identify the most vulnerable states based on these indicators.

**Methods:** This population based ecological study analyses data from the 100th days after the first registered death for COVID-19 in the 27 BS and correlate with socioeconomic, demographic and health structure indices. The vulnerability score (VS) of COVID-19 death was composed by the indices that presented a Pearson’s linear correlation >0,4. We separated the BS on 3 groups, according to the cumulative death rate/100.000 inhabitants at the 100th day after the first death in each state.

**Results:** The indicators with higher correlation coefficient were: the number of hospital bed/100.000 inhabitants (0,61), the Social Vulnerability Index (0,52), percentage of population <40 years old (0,51), population with tap water (0,49), number of doctor/100.000 inhabitants (0,44) and life expectancy at birth (0,43); these indices built the VS. The overall correlation coefficient between the final score and the number of deaths/100,000 inhabitants on the 100th day after the first death in each state was 0.63. The correlation was 0.44 for states with higher deaths (group 1), 0.71 for intermediate profile (group 2) and 0.85 for states with lower death rates (group 3).

**Conclusions:** Socioeconomic, demographic and mainly health structure services indices before the pandemic correlate with COVID-19 mortality. The VS proposed has good correlation with the cumulated death rate/100.000 inhabitants at the 100th day after the first death. As a underdeveloped country, identify the most vulnerable states is strategic to better allocate supplies and health investments.


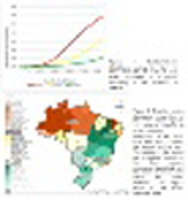


108567

Modality: E-Poster Young Researcher – Non-case Report

Category: CARDIAC ARRHYTHMIAS/ELECTROPHYSIOLOGY/ELECTROCARDIOGRAPHY

## Close Protocol with Center-Specific Ablation Index Target in AF Ablation

TÂNIA FILIPA GALVÃO PROENÇA^1^, Ricardo Alves Pinto^1^, Miguel Martins Carvalho^1^, Catarina Costa^1^, Carlos Xavier Resende^1^, Pedro Diogo Grilo^1^, João Calvão^1^, Ana Filipa Amador^1^, Gonçalo Pestana^1^, Ana Lebreiro^1^, Luis Adão^1^, Filipe Macedo^1^

(1) Centro Hospitalar Universitário São João

Pulmonary vein (PV) isolation is an established treatment for atrial fibrillation (AF). A contact force (CF)-guided ablation protocol respecting region-specific criteria of lesion contiguity and lesion depth (‘CLOSE‘ protocol) has been associated with high incidence of acute durable PV isolation and a high single-procedure arrhythmia-free survival at 1 year. Differences in ablation index (AI) targets exist between centers, and its optimal value remains unknown. In the present study, we sought to evaluate the safety and outcomes of our local ablation protocol. We retrospectively analyzed 37 patients with paroxysmal AF who underwent antral PV encircling using a CF–sensing catheter in a tertiary center from January 2018 to November 2019. Radiofrequency (RF) was delivered targeting interlesion distance ≤6 mm and ablation index (AI) ≥380 at posterior wall and ≥500 at anterior wall. Cavotricuspid isthmus (CTI) ablation was performed if previous typical atrial flutter (AFL) was documented. PV isolation was documented with entrance- and exit block and the use of adenosine. Recurrence was defined as any AF, atrial tachycardia (AT), or AFL >30 s on 24-hour Holter monitoring or 12-lead ECG; or symptoms recurrence. 37 consecutive patients (70% male, median age 53 years) underwent antral PV encircling. 30 were taking antiarrhythmic drug (AAD) and 12 were submitted to previous cardioversion (11 with successful CV to sinus rhythm). Procedure and fluoroscopy time were 100 +/– 19.6 min and 5 +/– 2.0 min, respectively. Mean AI values, CF and RF time per lesion, were as follows: anterior left PVs – 490 +/– 21, 9.5 +/– 1.9 g, 32.0 +/– 5.1 sec; posterior left- 383 +/– 11, 10.53.5 g, 28.8 +/– 5 sec; anterior right – 498 +/– 15, 12 +/– 2,9 g, 32.3 +/– 4.8; posterior right – 384 +/– 12, 10.3 +/– 2.2 g, 27.9 +/– 3.1 sec. Incidence of first-pass and adenosine-proof isolation were 97% and 95%, respectively. Touch up lesions were applied to ensure isolation, with 100% success at the end of the procedure. CTI ablation was performed sucefully in 22%. 76% were discharged on AAD and 24% maintained AAD at one-year follow-up. At 12 months, single-procedure freedom from recurrence was 89%. Only one patient had an acute complication, a femoral haematoma that solved with local compression. Our experience with “CLOSE” protocol with specific AI target supports that an ablation respecting the referred predefined criteria for lesion depth and contiguity results in safe and efficient outcomes.

108570

Modality: E-Poster Young Researcher – Non-case Report

Category: COVID-19 AND CARDIOVASCULAR SYSTEM

## Myocarditis, Pericarditis and COVID-19 Vaccination – a True Concern?

TÂNIA FILIPA GALVÃO PROENÇA^1^, Ricardo Alves Pinto^1^, Miguel Martins Carvalho^1^, Filipa Amador^1^, Catarina Costa^1^, João Calvão^1^, André Cabrita^1^, Catarina Marques^1^, Ana Pinho^1^, Luís Santos^1^, Paula Dias^1^, Filipe Macedo^1^

(1) Centro Hospitalar Universitário São João

Myocarditis and pericarditis are inflammatory diseases affecting 1 to 10 persons in 100 000 persons/year. Anti-SARS-Cov-2 vaccines are being widely used worldwide, and concerns about its association with a higher risk of cardiac inflammatory syndromes are being raised. We compare the prevalence and severity of myocarditis and/or pericarditis admissions before and after Covid-19 vaccination. We retrospectively analysed all patients admitted to a Cardiology Department due to myocarditis and/or pericarditis during COVID-19 pandemic and compared the clinical data between two 10-months periods, before and after Covid-19 vaccination. Pre-vaccination period (PVp) was defined from March to December 2020 and vaccination period (VCp) from January to October 2021. A total of 23 patients were enrolled, 12 in PVp and 11 in VCp, with a median age of 28 (IQR 22–43) year-old. Of total, 44% had perimyocarditis, 30% myocarditis and 26% pericarditis. In VCp there was significatively more man admitted (50% vs 90%, p = 0.019). Concerning clinical data, the two periods were similar. Median CRP was 68 mg/L and, in perimyocarditis or myocarditis, median high-sensitivity troponin I and BNP were 10 250 ng/L and 38 pg/mL, respectively. The majority of patients had ECG abnormalities, mostly ST-elevations (39%) and repolarization abnormalities (20%). At presentation, left systolic function was preserved in all patients in PVp group, while 27% had mildly to moderate reduced ejection fraction in VCp (p = 0.052); all of them improved to normal function. Of the 17 patients with myo or perimyocarditis, 14 were submitted to MRI (median 6 days after the event): 60% had high signal intensity in T2 weighted sequences suggestive of oedema and 73% had LGE enhancement compatible with myocarditis. Median left and right ventricular ejection fraction by MRI were both 61%. In VCp group, 55% of patients were previous vaccinated (3 patients with mRNA-1273, 1 with BNT162b2 and 2 with JNJ-78436735 vaccines). The median time between last vaccine administration and event was 73 days (2–112). In VCp group performing cardiac MRI, 40% of vaccinated patients had LGE enhancement (p = 0.038). There was no difference in the incidence of patients admitted. During two equal periods before and after Covid-19 vaccination, there was no difference in the incidence of patients admitted with myocarditis and/or pericarditis. Curiously patient with previous Covid-19 vaccination exhibited less LGE-enhancement on cardiac MRI.

108605

Modality: E-Poster Young Researcher – Non-case Report

Category: NURSING

## Innovations in Telecardiology: Activities Developed by Nursing Residents in a Reference Hospital in the State of Pernambuco

TAYNE FERNANDA LEMOS DA SILVA^1^, Larissa dos Santos Brandão^1^, Raquel Maria Alexandre da Silva^1^, Maria Tayná Silva Feitosa^1^, Monaliza Evelyn Pereira de Sousa^2^, Gislainy Thais de Lima Lemos^1^, Rodrigo Manoel do Nascimento^2^, Dara Stephany Alves Teodorio^2^, Hortênsia Paula Bernardino Ribeiro^1^, Renata Santos de Oliveira^3^, Dulcineide Gonçalo de Oliveira^3^, Simone Maria Muniz da Silva Bezerra^4^

(1) Enfermeira Especialista em Cardiologia pelo Pronto Socorro Cardiológico de Pernambuco – PROCAPE/UPE; (2) Residente de Enfermagem em Cardiologia Uniprofissional e Multiprofissional do PROCAPE/UPE; (3) Núcleo de Saúde da Secretaria Estadual de Saúde de Pernambuco – NET-SES/PE; (4) Coordenadora do Programa de Residência Uniprofissional e Multiprofissional em Cardiologia do PROCAPE/UPE

**Introduction:** In Brazil, cardiovascular diseases account for 30% of all deaths, with about 1,000 deaths per day. In 2017, the Ministry of Health (MS) launched a projector called National Telediagnostic Offer, expanding the remote diagnosis service to the poorest areas of Brazil.² The Telecardiology Network of Pernambuco aims to meet the demands on the Clinical Management of Cardiovascular Diseases in its municipalities, with the issuance of online electrocardiogram reports (Tele-ECG) and teleconsultations. This network works in partnership with a multiprofessional residency program of a hospital recognized by the MS as one of the four cardiology reference institutes in Brazil, located in the state of Pernambuco.

**Objective:** To describe the results of the activities carried out by nursing residents in cardiology in a referral hospital in the State of Pernambuco.

**Methods:** This is a descriptive, observational study based on the activities carried out by nursing residents of a multiprofessional program in cardiology, from March 2020 to February 2022.

**Results:** During the cardiology residency program, nurses had the opportunity to be pioneers in Teleassistance in cardiology services, in partnership with the Pernambuco State Health Department (SES-PE). The following were implemented: Telemedicine-based service for the follow-up and monitoring of patients treated with anticoagulant therapy and the Tele-ECG service in primary health care in the municipalities of Pernambuco. The residents established the outpatient department flowchart and carried out training for the multidisciplinary team. Up to now, 8,712 teleconsultations were performed from June 2020 to February 2022, and of these, 3,202 were performed by nursing residents. In the Tele-ECG service, from 185 municipalities, 56 (30.3%) are already issuing electrocardiogram reports and 52 (28.1%) are still in the implementation period. In total, 14,349 reports were issued (from October 2020 to March 2022), of which 14,121 had been classified as an elective electrocardiogram and 122 as an emergency report. Therefore, the joint work of the multiprofessional residency program and the SES-PE constitutes a success when considering the high productivity attained by the project so far and assuring the access of patients from the countryside of Pernambuco to specialized cardiology services.

108577

Modality: E-Poster Young Researcher – Non-case Report

Category: HEMODYNAMICS AND INTERVENTIONAL CARDIOLOGY

## Twenty-Years After Percutaneous Mitral Commissurotomy in Women with Mitral Valve Stenosis

TÂNIA FILIPA GALVÃO PROENÇA^1^, Ricardo Alves Pinto^1^, Miguel Martins Carvalho^1^, João Calvão^1^, Ana Filipa Amador^1^, Catarina Costa^1^, Catarina Marques^1^, André Cabrita^1^, Cátia Oliveira^1^, Mariana Paiva^1^, João Carlos Silva^1^, Filipe Macedo^1^

(1) Centro Hospitalar Universitário São João

Rheumatic fever in childhood used to be the major cause of valve disease in women. Despite the reduction in its incidence, nowadays rheumatic mitral stenosis (MS) remains an important healthcare problem. We retrospectively analysed all women with clinically significant rheumatic MS submitted to percutaneous mitral commissurotomy (PMC) between 1991 and 2008 and compare long-term events between patients with and without pulmonary hypertension (PH). A total of 108 women were enrolled, with a mean age at the time of PMC of 46 ± 11-year-old and a median follow-up of 21 (IQR 17–26) years. Before the procedure, all patients had preserved biventricular systolic function; 86% presented PH, median transvalvular gradient (TVG) and mitral valve area (MVA) were 12.0 mmHg and 1.0 cm^2^, respectively, Most of PMC were successful (91%) and without major complications (94%), with a median MVA improvement of 0.9 cm^2^ and reduction of 9.0 mmHg in TVG and 8.0 mmHg in pulmonary artery systolic pressure (PASP). During long-term follow-up, 43% of patients were submitted to re-intervention (most of them surgically) and 25% died. In patients non-submitted to re-intervention, TVG and PASP remained similar with early post-procedure evaluation (p = 0.241 and p = 0.964, respectively), while MVA reduced over time, yet still statistically superior to baseline MVA (1.5 cm^2^ vs 1.0 cm^2^, p < 0.001). Concerning time-to-event analysis, approximately 80% of patients kept uneventful after 10 years; after 30 years, more than 20% continued MACE-free and almost 50% were alive. Regarding PH presence at time of PMC, there was no significant difference in MACE events and all-cause mortality (Log Rank, p = 0,711 and p = 0.689, respectively). PMC was safe and effective in clinically significant rheumatic MS in women.


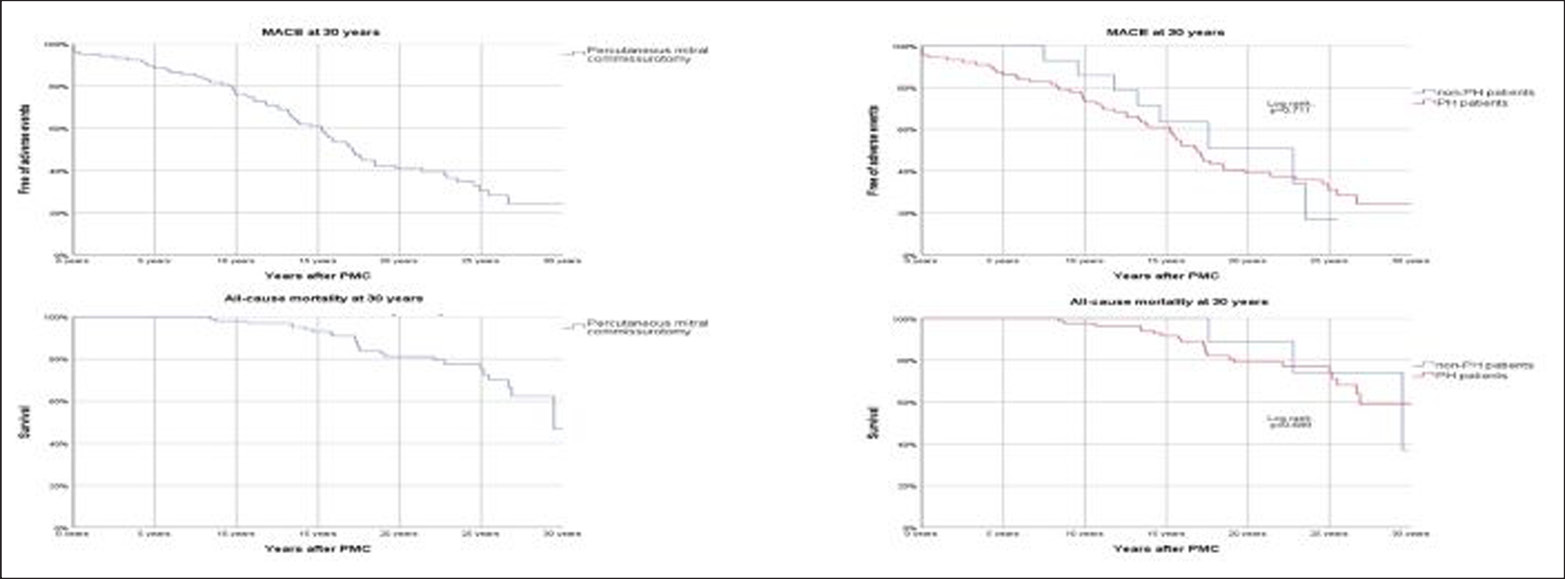


108585

Modality: E-Poster Young Researcher – Non-case Report

Category: CONGENITAL AND PEDIATRIC CARDIOLOGY

## Noacs Safety and Efficacy in Adult Congenital Heart Disease

RICARDO JORGE DOS REIS ALVES PINTO^1^, Miguel Martins Carvalho^1^, Tânia Proença^1^, Catarina Costa^1^, Ana Filipa Amador^1^, João Calvão^1^, Catarina Marques^1^, André Cabrita^1^, Cátia Oliveira^1^, Cristina Cruz^1^, Filipe Macedo^1^

(1) Centro Hospitalar Universitário São João, Porto, Portugal

**Introduction:** Adult Congenital Heart Disease (ACHD) patients are an increasing population with known high risk for thromboembolic events. Validated scores as CHA2DS2-VASc and HAS-BLED are uncertain in this population. Although apparently safe, data is scarce about the use of non-vitamin K oral anticoagulants (NOAC) in this population. Its use remains off-label and more studies are warranted.

**Purpose:** To evaluate all patients on-NOAC followed in an ACHD outpatients clinic and observe its safety and efficacy in thromboembolic prevention during a median follow-up of 34 months (IQR 7–60 months). Major bleeding was defined according to types 3 to 5 in Bleeding Academic Research Consortium (BARC) scale. Adverse event was defined as at least one of the follows: death, stroke, myocardial infarction, systemic embolism or major bleeding.

**Results:** A total of 65 patients on NOAC were included, with a mean age of 52 ± 14 year-old, 66% were female. Most frequent ACHD were atrial septal defect (22%) and tetralogy of Fallot (TOF, 22%), followed by atrioventricular septal defect (17%) and transposition of great arteries (9%). Regarding cardiovascular risk factors, 37% had hypertension, 23% had dyslipidaemia, 9% had diabetes, 8% were smokers or previous smokers and 23% had obesity. Most patients had preserved biventricular function, 20% presented systemic ventricle systolic dysfunction and 12% subpulmonic ventricle systolic dysfunction. Atrial fibrillation or atrial flutter (AF/AFL) were the major reasons for anticoagulation (94% of patients); the remaining were on NOAC due to previous ischaemic stroke, intra-cardiac thrombus or deep venous thrombosis. At the time of NOAC initiation, 49% of patients had a CHA2DS2-VASc score ≥2 (median 1, IQR 1–3) and median HAS-BLED score was 0 (IQR 0–2); 43% were medicated with apixaban, 29% with rivaroxaban, 22% with edoxaban and 6% with dabigatran. During a median follow-up of 34 months, none of the patients had ischaemic complications or major bleeding and one patient died after pulmonic prothesis dysfunction surgery. Concerning time-to- adverse-event analysis, all patients kept uneventful after 2 years and more than 95% continued event-free after 8 years on-NOAC.

**Conclusion:** In an ACHD population on-NOAC, the major reason for anticoagulation was AF/AFL. After a long-term follow-up, most patients maintained event-free after 8 years. This report highlights the safety and effectiveness of NOAC in ACHD patients.

108588

Modality: E-Poster Young Researcher – Non-case Report

Category: CARDIORESPIRATORY PHYSIOLOGY/BASIC SCIENCE

## Development of Low-Cost Multiplex SNP Genotyping Methodologies for Association Studies Related to Cardiovascular Risk

YASMIN LEMOS ROLLEMBERG CRUZ MACHADO^2^, Gizella da Cunha Rodrigues^1^, Rossana Ghessa Andrade de Freitas^2^, Maria Eliane Campos Magalhães^2^, Ricardo Mourilhe-Rocha^2^, Andréa Araujo Brandão^2^, Denilson Campos de Albuquerque^3^, Dayse Aparecida da Silva^1^

(1) Roberto Alcântara Gomes Biology Institute, Rio de Janeiro State University (UERJ); (2) Faculty of Medical Sciences, Rio de Janeiro State University (UERJ); (3) Department of Cardiology, Pedro Ernesto University Hospital/Rio de Janeiro State University (UERJ)

**Introduction:** The role of genetic variants in multifactorial diseases has been widely investigated through genome-wide association studies, mainly replicated in genetically homogeneous populations. However, the replication of such associations in smaller assays requires an alternative molecular methodology more accessible to the scientific community as a whole. Customized Multiplex assays including informative genetic markers offer a single, low-cost and practical option.

**Aim:** To develop a cheaper Multiplex methodology based on SNaPshot® technology for genotyping 21 SNPs clinically relevant in genome-wide studies and their application in clinical samples from an admixed population.

**Methodology:** Four custom Multiplex Panels involving 21 SNPs in genes related to cardiovascular risk factors were constructed for genotyping 1,064 buccal swabs and 528 blood samples, differently preserved. The resulting genotypes were validated by Sanger sequencing.

**Results:** The full genetic profile was achieved in 72–92% among the panels considering both swabs and blood samples. Marker’s performance ranged from 81–100%. Panels reached above 90% of confident genotyping calls in samples stored for 2 years or more, regardless of their type. The agreement rate between genotyping methods ranged from 97.5–100%.

**Conclusion:** The customized Multiplex Panels represented an efficient molecular methodology to genotype SNPs with high sensitivity, efficiency, and low cost from different biological samples stored for years.

108608

Modality: E-Poster Young Researcher – Non-case Report

Category: EPIDEMIOLOGY AND HEALTH POLICIES/GLOBAL HEALTH

## Epidemiological Profile of Hospitalizations for Acute Myocardial Infarction in Children and Adolescents in the Last 10 Years in Brazil

MATHEUS AKIRA SUZUKI DE OLIVEIRA^1^, Matheus Akira Suzuki de Oliveira^1^, Júlia de Avila Gutierrez^2^, Hildeman Dias da Costa^1^, Luiz Felipe Façanha Ramos^3^, Karen Tássia Façanha Ramos^3^, Mathews Barbosa Santiago^5^, Leo Christyan Alves de Lima^4^

(1) Universidade Federal de Rondonia; (2) Centro Universitário Aparício Carvalho; (3) Universidade Federal do Amapá; (4) Centro Universitário São Lucas; (5) Centro Universitário Uninorte

**Introduction:** Acute myocardial infarction is a pathology that affects all age groups and should be prevented even in less common age groups, such as children and adolescents, in order to reduce morbidity and mortality rates. The points to be observed are care with the prevention of cardiac risk, and the adoption of healthy lifestyle habits throughout childhood.

**Objective:** To describe the epidemiological profile of hospitalizations for acute myocardial infarction among children and adolescents in Brazil between 2012 and 2021.

**Methods:** Cross-sectional epidemiological study. Data were obtained from the informatics department of the unified health system – DATASUS. The variables studied were: total number of hospitalizations, sex, color/race, deaths and mortality rate. The age group surveyed was individuals from zero to 19 years old. The period was delimited between 2012 and 2021.

**Results:** 2,542 hospitalizations were recorded. Males reported 1,472 hospitalizations. The brown color/race registered 867 hospitalizations. The most affected age group was adolescents between 15 and 19 years old, with 1,182 hospitalizations. The total number of deaths was 172. The average mortality rate in the period was 6.77.

**Conclusions:** The epidemiological profile of hospitalizations was characterized by brown adolescents aged between 15 and 19 years. The reduction in the number of hospitalizations in 2020 and 2021 may have been influenced by the COVID-19 pandemic.


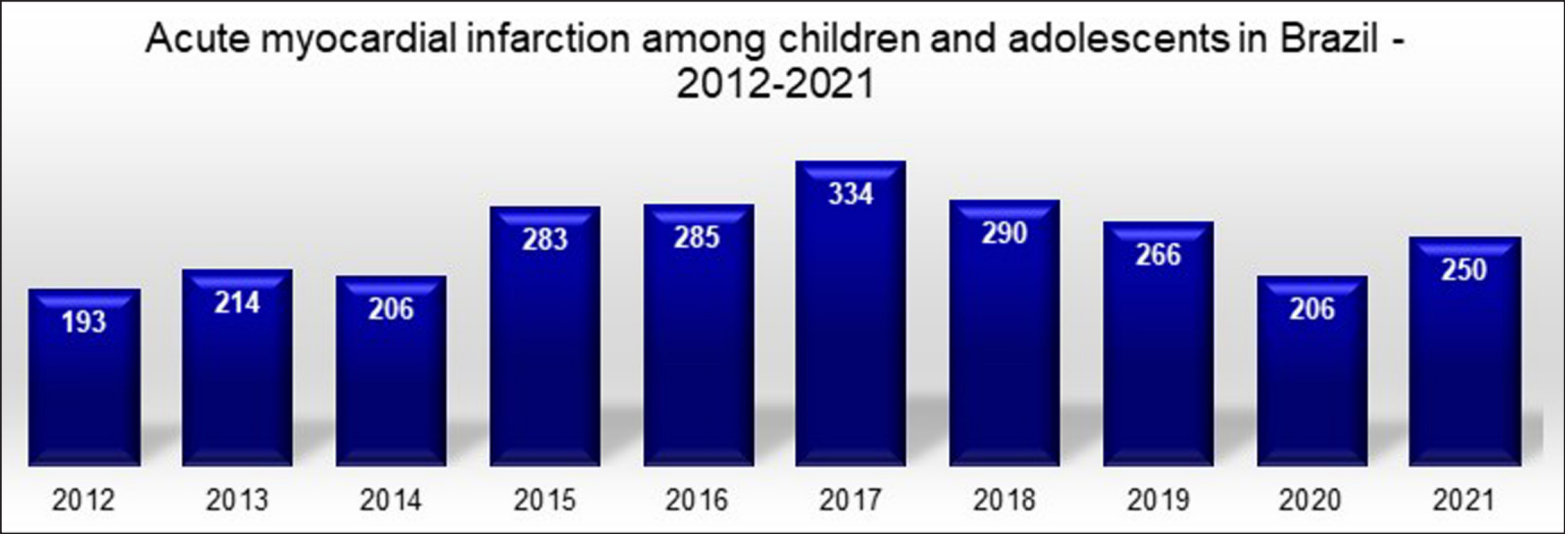


108617

Modality: E-Poster Young Researcher – Non-case Report

Category: CARDIOVASCULAR INTENSIVE CARE/CARDIOVASCULAR EMERGENCIES

## Association between Lung Ultrasound Findings, SCAI Shock Classification and Clinical Outcomes in Patients with ST Segment Elevation Myocardial Infarction

GUILHERME PINHEIRO MACHADO^1^, Fernando Luis Scolari^1^, Gustavo Neves de Araujo^3^, Alan Pagnocelli^1^, Angelo Chies^2^, Marco Vugman Wainstein^2^, Rodrigo Vugman Wainstein^1^

(1) Hospital de Clinicas de Porto Alegre (HCPA); (2) Universidade Federal do Rio Grande do Sul (UFRGS); (3) Imperial Hospital de Caridade

**Background:** Incidence and mortality in cardiogenic shock (CS) remains high despite current management. Improvements in early diagnosis and risk stratification are warranted in order to prevent CS in ST segment elevation myocardial infarction (STEMI).

**Objectives:** Our aim is to evaluate the association between pulmonary congestion evaluated by lung ultrasound (LUS), Society for Cardiovascular Angiography and Interventions (SCAI) shock classification and clinical outcomes in patients admitted with STEMI.

**Methods:** Prospective cohort study of STEMI patients treated in a tertiary care hospital in Brazil. LUS was performed immediately before coronary angiography. Development of cardiogenic shock in the first 24 hours and in-hospital mortality were retrospectively evaluated.

**Results:** A total of 582 included patients. Mean age was 61 ± 12 years and 373 (64.1%) were male. After 24 hours of admission, SCAI shock stage A was present in 361 (62%) patients, while 115 (19.8%) were class B, 44 (7.6%) class C, 58 (10%) class D, and 4 (0.7%) class E. There was an association between increasing number of positive LUS zones and the SCAI shock classification (P < 0.001). We also found strong association between number of positive zones in lung ultrasound and CS (OR = 1.4 (95% CI 1.3–1.5, P < 0.001), SCAI shock stages (OR 1.3 (95% CI 1.2–1.4, P < 0.001) and in-hospital mortality (OR 1.3 (95% CI 1.2–1.4, P < 0.001). Additionally, presence three or more positive LUS zones was associated with increased mortality, P (log-rank) < 0.001.

**Conclusions:** Lung congestion evaluated by admission LUS was significantly associated with increment in SCAI shock stage, development of CS and in-hospital mortality in STEMI patients.


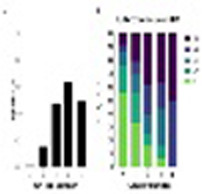


108636

Modality: E-Poster Young Researcher – Non-case Report

Category: CONGENITAL AND PEDIATRIC CARDIOLOGY

## Cardiac Arrest on Persons Under the Age of 20, from 1996 to 2019

THAYANNE MENDES DE ANDRADE^1^, Mariara Lopes da Costa Marques^1^, Sofia Almeida Guerra^1^, Thaís Rocha Salim^1^, Glaucia Maria Moraes de Oliveira^1^

(1) Universidade Federal do Rio de Janeiro (UFRJ)

**Introduction:** Cardiac arrest (CA), in pediatrics, has high mortality and severe neurological sequelae. Information on the causes and mechanisms of death in children under 20 can provide theoretical support for improving health in childhood and adolescence.

**Objectives:** To carry out a population analysis of mortality rates (MR) from basic and multiple causes of death, in children under 20 years of age, of both sexes, from 1996 to 2019, and to identify the frequency of description of CA in death certificates (DC) of these individuals and their places of occurrence, to promote strategies to improve the prevention of deaths.

**Method:** An ecological time-series study, from 1996 to 2019, of children deaths under 20 years of age, in which MR and we evaluated proportional mortality (PM) due to underlying cause of death. We analyzed the percentage of CA description in the DC (any lines) and the place of occurrence. Also, we described MR per 100,000 inhabitants and PM due to underlying cause of death under 20 years of age, by sex, and age group. The percentages of death from underlying causes were calculated when CA was described in any line of parts I and II of the DC, by age groups, and the percentages of death from underlying causes, according to their place of occurrence. Data were taken from DATASUS, IBGE and SINASC.

**Results:** In Brazil, from 1996 to 2019, there were 2,151,716 deaths in children under 20 years of age, with a MR of 134.38 per 100,000 inhabitants, with higher death rates among male neonates. Two hundred forty-nine thousand three hundred thirty-four had CA described in any DC line, corresponding to 11.6% of these deaths. We defined four patterns for the underlying cause of death when CA in the death sequence. In the neonatal period, perinatal causes; in children under five years, respiratory system diseases; 5 to 14 years, neoplastic and hematological; and in adolescents aged 15 to 19, external causes. The central place of occurrence of these deaths was in the hospital.

**Conclusion:** The highest MR of underlying causes of death, in those under 20 years of age, in Brazil, from 1996 to 2019, was due to perinatal and external causes. When we evaluated multiple causes of death, the leading underlying causes of CA were respiratory, hematological, and neoplastic diseases, with higher in-hospital mortality. It is crucial to understand deaths sequence events and implement teaching strategies to pediatric cardiopulmonary resuscitation.

108644

Modality: E-Poster Young Researcher – Non-case Report

Category: NURSING

## Patients‘ Perception of Educational and Musical Nursing Intervention Before Emergency Cardiac Catheterization

LETÍCIA DE CARVALHO BATISTA^1^, Maria Do Perpétuo Socorro de Sousa Nóbrega^1^, Marina de Góes Savetti^1^, Rita de Cássia Gengo e Silva Butcher^1^

(1) School of Nursing University of São Paulo

**Objective:** To evaluate the experience and acceptability of an educational and musical nursing intervention to reduce anxiety in patients undergoing unscheduled catheterization.

**Method:** Qualitative study was carried out in a highly specialized cardiology hospital in the city of São Paulo. In the study, 15 patients diagnosed with the acute coronary syndrome were treated and submitted to unscheduled catheterization at the emergency unit. All patients received the nursing intervention, consisting of a musical and educational component called Education and Music Intervention to Reduce Anxiety (EMIRA), prepared according to the methodological framework of complex intervention by Sidani and Braden. The interviews were recorded, transcribed, and analyzed according to Bardin’s content analysis. Data collection took place from October to September 2021.

**Results:** The analysis of the interviews allowed the grouping into three categories, 1) EMIRA Intervention: a new experience that helps reduce anxiety; 2) EMIRA intervention: an experience that generates satisfaction; and 3) EMIRA Intervention: anxiety alleviating experience. Participants have suggested using EMIRA to promote a feeling of relaxation and satisfaction.

**Conclusion:** EMIRA seems to be an acceptable and potentially relaxing intervention for patients awaiting unscheduled catheterization in the emergency department.

108674

Modality: E-Poster Young Researcher – Non-case Report

Category: HEART FAILURE/CARDIOMYOPATHY/TRANSPLANT

## Prevalence of Reduced Left Ventricular Ejection Fraction in the Brazilian Amazon Basin and Diagnostic Usefulness of B-Lines by Lung Ultrasound

LUAN OLIVEIRA MATOS^1^, Anna Engell Holm^2^, Laura C Gomes^3^, Alma Wegener^2^, Karine O Lima^1^, Molly Dam Kaagaard^2^, Isabelle V. M. Vieira^1^, Claudio Romero Farias Marinho^3^, Rodrigo Medeiros de Souza^1^, Tor Biering-Sørensen^2^, Odilson M Silvestre^1^, Philip Brainin^1^

(1) Universidade Federal do Acre; (2) Herlev-Gentofte Hospital; (3) University of São Paulo

**Background:** B-lines by lung ultrasound (LUS) indicate presence of extravascular lung water and has been associated with reduced left ventricular ejection fraction (LVEF) in patients with heart failure. We aimed to describe the prevalence of reduced LVEF in a community sample without a history of heart failure and to evaluate the usefulness of B-lines by LUS in this setting.

**Methods:** In a cross-sectional study we examined a random sample of adults (≥18 years) from a community in the Northwestern part of the Brazilian Amazon (June-December 2020). All participants underwent state-of-the art echocardiographic image acquisition and 8-zone LUS by a medical doctor. No patients had known heart failure, recent chest trauma or clinical signs of infectious disease. Reduced LVEF was determined by Simpson’s biplane method and defined as <45%. We assessed the mean of B-lines across all zones. Logistic regression models were applied to investigate reduced LVEF and B-lines.

**Results:** A total of 551 participants were included (39% men, mean 41 ± 15 years) who had a mean LVEF of 57 ± 5%. From this group 16 (3%) had LVEF <45%, corresponding to a prevalence of 29/1000 adults with reduced LVEF. Participants with reduced LVEF were older, had higher blood pressure and more frequently smoked. Number of B-lines by LUS was significantly higher among participants with reduced LVEF compared to those with normal LVEF (mean B-lines 4 vs 1, P = 0.002. In logistic regression models, adjusted for clinical and cardiovascular risk factors, presence of a single B-line was associated with 1.18 higher odds of having reduced LVEF (95%CI 1.06–1.31, P = 0.002).

**Conclusion:** The prevalence of reduced LVEF was 29/1000 adults in a community from the Amazon Basin without a known history of heart failure. B-lines by LUS were significantly more present in participants with reduced LVEF. As LUS is feasible in resource limited settings, may be conducted by non-medical personnel and by the use of handheld devices, this may be useful to identify patients with reduced LVEF in rural communities where echocardiography is not available.

108747

Modality: E-Poster Young Researcher – Non-case Report

Category: CARDIORESPIRATORY PHYSIOLOGY/BASIC SCIENCE

## Neutrophil Peptidylarginine Deiminase 4 is Essential for Detrimental Age-Related Cardiac Remodeling and Dysfunction in Mice

STIJN VAN BRUGGEN^1^, Sirima Kraisin^1^, Jore Van Wauwe^1^, Paolo Carai^1^, Thilo Witsch^2^, Kimberly Martinod^1^

(1) Centre for Vascular and Molecular Biology, Department of Cardiovascular Sciences, KU Leuven, Leuven, Belgium; (2) Department of Cardiology and Angiology I, Heart Center, Faculty of Medicine, University of Freiburg, Freiburg, Germany; (3) Department of Cardiology, University Hospital Basel, University of Basel, Basel, Switzerland

Increasing age is the highest risk factor for cardiovascular disease and heart failure (HF) development, and HF is a leading cause of death in the elderly. In our study, we aimed to investigate the specific role of neutrophils and neutrophil extracellular traps (NETs) on cardiac remodeling and decreasing function with increasing age. For this, neutrophil-specific PAD4 deletion (Ne-PAD4-/-) mice were created and aged for 2 years together with their littermate controls (WT). After 2 years we could clearly identify a decrease in both systolic (LVEF) and diastolic (E/A ratio) cardiac function in WT mice, while this age-dependent cardiac dysfunction was absent in Ne-PAD4-/- mice. In addition, cardiac function of old Ne-PAD4-/- mice was comparable to young controls. In order to explain this decreased function, hearts were evaluated histologically which showed an increase in cardiac collagen deposition in old WT as compared to old Ne-PAD4-/- mice. Additionally, we found increased levels of circulating CXCL-1, a chemokine responsible for neutrophil recruitment to sites of inflammation in old WT mice, as well as increased levels of plasma IL-6. From this we can conclude that neutrophil PAD4, and in extension likely NETs, are essential for age induced cardiac remodeling, possibly through the increased recruitment of additional neutrophils to the heart, in response to an elevated state of chronic inflammation, which is the case during natural aging.


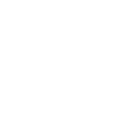


108681

Modality: E-Poster Young Researcher – Non-case Report

Category: ATHEROSCLEROSIS/CARDIOVASCULAR RISK FACTORS/CARDIOVASCULAR PREVENTION

## Clinico-Epidemiological Profile of Cardiac Admissions at a District Level Hospital in South Africa: A Cohort Study

LILLIAN LIZE ENGELBRECHT (MARRIED SURNAME: GILES)^1^, Nasief van der Schyff^3^, Mark Engel^1^

(1) University of Cape Town (UCT); (2) Groote Schuur Hospital (GSH); (3) Victoria Hospital Wynberg (VHW)

**Introduction:** Sub-Saharan Africa (SSA) is experiencing a change in its pattern of disease prevalence, and it has been projected that non-communicable diseases (NCD) will overtake infectious diseases by the year 2030. Despite the fact that more than 80% of deaths from NCD’s worldwide are estimated to occur in low-and middle-income countries, the majority of available research is from first world countries. Due to SSA’s own rich genetic, geographic, social and cultural diversity, this available first world research is not applicable to our context, highlighting the need for more relevant local studies.

**Objectives:** To better understand the clinical and epidemiological characteristics of patients admitted with cardiac disease in our district, in order to inform and guide relevant clinicians and policymakers.

**Methods:** We conducted a retrospective records review of all patients admitted with a primary cardiac diagnosis to a district level hospital in South Africa between 1 September 2020 to 30 November 2020.

**Results:** Our final analysis is still in progress, but we can report some preliminary findings at this time. During the three month period, we collected data from 236 cardiac admissions at our district-level, 52 medical bed, hospital. The age of patients ranged from 22 to 95, with a mean age (standard deviation) of 60 (±14.79) years. The length of stay ranged from under 24 hours, up to 26 days, with a median length of 3 days. Hypertension (76%), Smoking (55%), and Diabetes (42.7%) were the top 3 cardiovascular risk factors noted. More than a third of diabetic patients (36.2%) had a glycated haemoglobin (HbA1c) of greater than 10.1%. Acute decompensated heart failure (45.8%) and acute coronary syndromes (40.1%) were responsible for the vast majority of admissions. Admissions were most often precipitated by prior inadequate therapy (29%), defaulting medication (17.8%) or uncontrolled hypertension (14.8%). There was a 6.7% readmission rate noted during the three month period. The inpatient mortality rate was 7.8%.

**Conclusion:** This study showed that cardiovascular disease (CVD) can affect a wide range of ages, and reiterated the well known link between CVD and hypertension, smoking and diabetes. Poorly controlled hypertension was the third most common precipitant for admission, and a third of diabetic patients had poor glycaemic control, highlighting important areas for intervention in order to reduce the readmission and mortality rate.

108688

Modality: E-Poster Young Researcher – Non-case Report

Category: PHYSIOTHERAPY

## Effectiveness of Structured Exercise Program on Insulin Resistance and Quality of Life in Type 2 Diabetes Mellitus – a Randomized Controlled Trail

SAMPATH KUMAR AMARAVADI^1^, G Arun Maiya^1^

(1) Department of Physiotherapy, Manipal College of Health Professions, Manipal Academy of Higher Education, Manipal, Karnataka, India; (2) Department of Physiotherapy, College of Health Sciences, Gulf Medical University, Ajman, United Arab Emirates.

**Background:** Impaired glucose control & Insulin resistance are reported to be risk factors for the development of cardiovascular diseases. In the Indian population, Insulin resistance is one of the strong reasons for altered glycemic control, even though exercise training is found to be effective in glycemic control along with standard medical care. There is a dearth of literature on the effectiveness of exercise training on insulin resistance and quality of life in patients with Type 2 diabetes mellitus.

**Objective:** To determine the effects of a structured exercise program on insulin resistance, glycaemic control, functional capacity, and quality of life in patients with Type 2 diabetes mellitus. Design: Randomized, controlled trial. Setting: Diabetic Foot Clinic, Department of Physiotherapy & Department of General Medicine, Kasturba Hospital in Manipal, Karnataka, India. Participants: 160 participants aged between 30–65 years with Type 2 diabetes mellitus. Intervention: A set of structured exercise programs (aerobic, resistance, and combined) along with the standard hospital care was performed 3–5 times weekly for 12 weeks. Measurements: Primary outcome measures: Fasting Insulin Level, Homa-IR, Six-minute walk test (6MWT), and WHOQOL-BREF questionnaire at baseline and 12th week. Secondary outcome measures: Body composition analysis, Fasting Blood Sugar, Postprandial Blood Sugar, Glycated Hemoglobin (HbA1c), and GPAQ questionnaire at baseline and 12th week.

**Results:** The mean age of the participants in the study group was 56.05 ± 8.77 and the control group was 53.90 ± 10.20, the mean duration of diabetes in the study group was 12.74 ± 8.67 years and the control group was 11.46 ± 5.11 years, mean body mass index 25.32 ± 3.16 in the study group and 26.27 ± 3.63 in the control group. Significant differences have been observed in Homa-IR (p = <0.001), Fasting insulin (p = <0.001), Fasting blood sugar (p = <0.001), Postprandial blood sugar (p = <0.001), Glycated hemoglobin (p = <0.001), WHOQOL-BREF (p = <0.001), Six-minute walk test (p = <0.001) in the study group when compared with the control group.

**Conclusion:** In the present study, we found that a 12-week structured exercise training program is effective in decreasing insulin resistance and improving the quality of life in type 2 diabetes mellitus. We also found that a 12-week structured exercise training program is effective on functional capacity and glycemic control in type 2 diabetes mellitus.

108714

Modality: E-Poster Young Researcher – Non-case Report

Category: CARDIOVASCULAR PHARMACOLOGY

## New Rock Inhibitor Improves Cardiovascular Function and Pulmonary Artery Remodelling in Pulmonary Arterial Hypertension

TADEU LIMA MONTAGNOLI^1^, Jaqueline Soares da Silva^1^, Bruna de Souza Rocha^1^, Bianca dos Santos Carlos do Nascimento^1^, Bruno Eduardo Dematte^1^, Gabriel Fonseca Gomide^1^, Carlos Alberto Manssour Fraga^1^, Gisele Zapata Sudo^1^

(1) Universidade Federal do Rio de Janeiro

**Background:** Pulmonary arterial hypertension (PAH) is characterised by increased pulmonary arterial pressure and vascular thickening, which ultimately leads to right ventricle (RV) failure. Current therapies lack efficacy in preventing disease progression and lead to poor survival of patients at moderate-to-high risk. Rho-activated protein kinases (ROCK) play a key role in smooth muscle contraction and proliferation and cardiomyocyte hypertrophy and survival, and ROCK inhibitors are potential candidates for treatment of PAH. This study assesses the efficacy of a new ROCK inhibitor, LASSBio-2065, on cardiac and vascular function of monocrotaline (MCT)-induced PAH in Wistar rats.

**Methods:** PAH was induced after a single intraperitoneal injection of 60 mg/kg monocrotaline (MCT). After 2 weeks, PAH was confirmed by Doppler echocardiography and rats were randomly divided in groups (n = 6) and treated with vehicle or LASSBio-2065 (60 μmol/kg/day, i.p.) for 14 days. Wistar rats which received a single saline injection and treated with vehicle were used as controls. At the end of treatment, hemodynamics and cardiac function were evaluated by echocardiography and RV catheterization and tissues were collected for histologic and morphometric analysis. Data was analysed by One-way ANOVA followed by Dunnett post-test and presented as mean ± standard error.

**Results:** LASSBio-2065 reduced the pulmonary acceleration time-to-total ejection time ratio (26.7 ± 2.0 vs. 21.7 ± 1.6; p < 0.05) and increased RV output (64.2 ± 6.1 vs. 39.1 ± 8.0; p < 0.05), thus alleviating pulmonary vascular resistance and RV afterload. Arterial and cardiac remodelling were prevented by LASSBio-2065, as indicated by reduced medial wall thickness of distal pulmonary arterioles (60.7 ± 1.1 vs. 68.5 ± 3.9; p < 0.05), short axis RV-to-left ventricle (LV) internal area (85.8 ± 11.4 vs. 115.2 ± 17.8; p < 0.05) and RV Fulton index of hypertrophy (44.8 ± 2.3 vs. 55.6 ± 2.3; p < 0.05). The new ROCK inhibitor also improved the RV diastolic function, restoring both RV end-diastolic pressures (5.3 ± 0.8 vs. 11.1 ± 1.9 mmHg; p < 0.05) and Tau (16.9 ± 2.2 vs. 27.1 ± 2.8; p < 0.05) to control levels. Echocardiographic analysis revealed no significant changes in heart rate and LV diastolic and systolic functions between control and PAH groups (p > 0.05).

**Conclusions:** The data demonstrate that LASSBio-2065 significantly improves RV function and cardiovascular histology in PAH.

108722

Modality: E-Poster Young Researcher – Non-case Report

Category: CARDIAC ARRHYTHMIAS/ELECTROPHYSIOLOGY/ELECTROCARDIOGRAPHY

## Syncope by Intermitent Av-Block with Normal Electrophysiologycal Study Treated with Cardioneuroablation

JOSE MIGUEL CAYO MONTES^1^, JOSE MIGUEL CAYO MONTES^1^, JOSE CARLOS PACHÓN MATEOS^2^, JUAN PACHÓN MATEOS^1^, JUAN CARLOS ZERPA ACOSTA^1^, ENRIQUE I. PACHÓN MATEO^1^

(1) HOSPITAL DO CORAÇÃO; (2) SEMAP

**Introduction:** Functional atrioventricular block (BAV) can generate pre-syncope and syncope to the point of compromising patients‘ quality of life, and pacemaker implantation does not resolve the condition in some cases, it is important to clarify the diagnosis, especially in young and without structural heart disease.

**Objective:** To perform cardioneuroablation (CNA) of the parasympathetic cardiac ganglia to modulate the vagal influence on the heart and thus eliminate the cardioinhibitory response on the AV Node, treating syncope due to functional BAV, as an alternative therapy to pacemaker implantation.

**Methods:** CNA was performed in 8 patients under 50 years of age, who had syncope due to BAV, normal electrophysiological study and positive atropine test. The mapping of the parasympathetic ganglia, the neuromyocardial interface and the mapping of AF nests was performed with the Navix-St-Jude system. These regions and those anatomically related to the 4 ganglia were treated with endocardial radiofrequency (RF) ablation. Extracardiac vagal stimulation (ECVS) is used for the end point of the procedure, demonstrating the absence of parasympathetic activity on the sinus node and AV node, proving vagal denervation in these structures.

**Results:** Imediate and persistent increase in heart rate was observed after CNA (HR = 61, final HR = 78 bpm), Wenckebach point increase (PW:100->148 ppm) p: < 0.05. In the short and long term follow-up (after 05 years) the patients had no more syncope episodes. Holter monitoring performed at 03 months of follow-up in these patients did not record pauses or BAV, and the R-R variability was predominantly sympathetic (SDNN = 30 ms).

**Conclusion:** Syncope and normal EPS may be caused by functional origin; Functional AV block may be treated by Cardioneuroablation without pacemaker implantation; Extracardiac vagal stimulation was fundamental to evaluate the CNA step by step.


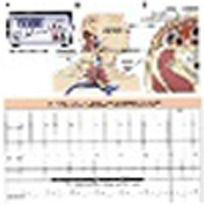


108734

Modality: E-Poster Young Researcher – Non-case Report

Category: HEMODYNAMICS AND INTERVENTIONAL CARDIOLOGY

## Prevalence of Contrast-Induced Nephropathy in Cardiopathy Patients from a Hemodynamics Sector

GABRIELA STOCHERO^1^, Gabriela Stochero^1^

(1) Unimed Noroeste/RS

**Introduction:** Contrast-induced nephropathy (CIN) represents a form of acute kidney injury that occurs in patients undergoing diagnostic and therapeutic medical procedures using iodinated contrast. In the last decade, CIN has been identified as the third leading cause of acquired acute renal failure (AKI) in hospitalized patients, reaching 12% of cases. CIN is defined as an absolute increase in serum creatinine ≥0.5 mg/dL or a relative increase of 25% from baseline creatinine within 24 to 72 hours after exposure to the contrast agent and in the absence of an alternative cause.

**Objective:** To determine the prevalence of CIN in patients with heart disease undergoing diagnostic and/or treatment angiographic procedures.

**Method:** Prospective, quantitative study carried out in the hemodynamics department of a large hospital located in the northern region of Rio Grande do Sul, Brazil. To determine the sampling of this research, a sample size calculation was performed, with an error of 5%; confidence level 90%; population 253 patients, maximum percentage of CIN 12%, totaling 79 individuals.

**Results:** The sample consisted of 52 (65.8%) men and 27 (34.2%) women. The mean age was 65.9 ± 9.52 years. No patient in the study underwent dialysis therapy, at least 72 hours after contrast use. The prevalence of contrast-induced nephropathy was 30.38%, totaling 24 patients. It was identified that 51 (64.6%) patients who underwent contrast-enhanced procedures did not receive prophylactic measures related to the prevention of CIN, while 28 (35.4%) received hydration with saline solution (SF) 0.9% intravenously. The volume of contrast used ranged from 50 to 500 mL, with a mean volume of 171.97 ± 91.27 mL. Comparing patients with and without CIN, patients who developed nephropathy were more hypertensive, had a higher percentage of reduced left ventricular ejection fraction and a higher incidence of heart failure. Patients who developed CIN had more complications after the procedures, corresponding respectively to 25% (n = 6) versus 3.6% (n = 2); (p = ≤ 0.001).

**Conclusion:** A high prevalence of contrast nephropathy was evidenced, despite the patients having few risk factors, which highlights the need for preventive measures and reduction of the volume of contrast.

108749

Modality: E-Poster Young Researcher – Non-case Report

Category: CARDIOLOGY OF SPORTS, EXERCISE, ERGOMETRY AND CARDIOVASCULAR REHABILITATION

## Barriers to Maintaining an Aerobic Exercise Routine (AER) for People Living with Prediabetes and Diabetes: Six-Month Follow-Up

ANA PAULA DELGADO BOMTEMPO BATALHA^1^, Josiane Aparecida de Almeida^3^, Isabela Coelho Ponciano^3^, Dara Marília Damasceno^2^, Ana Carolina Oliveira Pierangeli Vilela^2^, Tamara Rafino de Castro^2^, Ana Carolina Silva d’Ornelas^2^, Angélica Jesus de Assis^2^, Camila Alves Quintino de Souza^2^, Samantha de Jesus Carolina^2^, Mariana Balbi Seixas^1^, Lilian Pinto da Silva^1^

(1) Graduate Program in Physical Education, Faculty of Physical Education and Sports, Federal University of Juiz de Fora, Juiz de Fora, Brazil; (2) Faculty of Physical Therapy, the Federal University of Juiz de Fora, Juiz de Fora, Brazil; (3) Graduate Program in Rehabilitation Sciences and Physical-Functional Performance Faculty of Physical Therapy, Federal University of Juiz de Fora, Juiz de Fora, Brazil

**Background:** Although it is widely known that physical exercise contributes to blood glucose control and reduces cardiovascular risk factors, adopting and maintaining an aerobic exercise routine (AER) is still a challenge for people living with diabetes as it requires overcoming several barriers. Therefore, it becomes relevant to identify these barriers and correlate them with adherence to AER.

**Methods:** Participants of a randomized pilot study involving a 12-week exercise intervention aiming to maintain an AER with at least 150 min/week in moderate-intensity were follow-up for six-month post-intervention. The follow-up objective was to support the participants in maintaining the AER through monthly structured phone calls; in the five calls, they answered a Likert scale about the impact of barriers on AER (No impact – 0 and extreme impact- 10) and reported minutes AER/week. The ten pre-established barriers are presented in Table 1. The participants were divided into two groups: G1) participants with AER ≥ 150min/week and G2) AER < 150min/week on average. The median for each barrier was calculated, and the Mann-Whitney test was used to compare the barriers between G1 and G2. The Spearman test analyzed the association between the interest variables.

**Results:** During the six-month follow-up, 29 participants (55% male, 58.5 ± 9.0 yr, 10.3% prediabetes, 20.7% Type 1 diabetes, and 69% Type 2 diabetes) answered the phone calls. The average time of AER/week was 178 minutes, and 58% of participants reported AER ≥ 150 min/week during the follow-up. Table 1 shows the barriers comparing G1 and G2. Fatigue and lack of: company, space, and equipment had less impact on AER in the G1 than G2. A negative and moderate correlation was identified between fatigue, lack of time, lack of company, lack of space, and lack of equipment with the average time of AER/week (r = –0.50; –0.41; –048; –0.54; –0.55; P ≥ 0.05, respectively).

**Conclusion:** Although some barriers were related to a short average time of AER/week, most participants could maintain more than 150 min/week of AER in moderate-intensity six months after a 12-week exercise intervention.


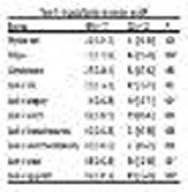


108759

Modality: E-Poster Young Researcher – Non-case Report

Category: CARDIOLOGY OF SPORTS, EXERCISE, ERGOMETRY AND CARDIOVASCULAR REHABILITATION

## Diabetes Knowledge and Glycemic Control in Patients with Type 1 and 2 Diabetes

ANA PAULA DELGADO BOMTEMPO BATALHA^1^, Josiane Aparecida de Almeida^3^, Juliana Gomes Soares^2^, Bárbara Faria Filgueiras^2^, Isabela Coelho Ponciano^3^, Ana Carolina Silva d‘Ornelas^2^, Angélica Jesus de Assis^2^, Camila Alves Quintino de Souza^3^, Samantha de Jesus Carolino^2^, Tamara Rafino de Castro^2^, Mariana Balbi Seixas^1^, Lilian Pinto da Silva^1^

(1) Graduate Program in Physical Education, Faculty of Physical Education and Sports, Federal University of Juiz de Fora, Juiz de Fora, Brazil; (2) Faculty of Physical Therapy, the Federal University of Juiz de Fora, Juiz de Fora, Brazil; (3) Graduate Program in Rehabilitation Sciences and Physical-Functional Performance Faculty of Physical Therapy, Federal University of Juiz de Fora, Juiz de Fora, Brazil

**Background:** Some studies have shown that diabetes patients with higher disease knowledge are more likely to adhere to the treatment and achieve behavior change, which is critical for effective glycemic control. Therefore, this study aimed to compare diabetes knowledge between patients with type 1 (T1D) and type 2 diabetes (T2D) and investigate the correlation between this outcome and glycated hemoglobin (A1c) levels.

**Methods:** Participants from different phases of the Diabetes College Brazil Study (NCT03914924) – (1) validation of questionnaires, n = 106; (2) pilot randomized trial, n = 18; (3) feasibility study, n = 7) – whose glycemic control has been assessed by A1c levels measured up to three months before been enrolled in one of the phases of Diabetes College Brazil Study were included in the present study. The diabetes knowledge was assessed by DiAbeTes Education Questionnaire (DATE-Q) total and each item scores. Mann-Whitney test was used to compare DATE-Q total scores between patients with T1D and T2D, and the Spearman test analyzed the association between DATE-Q total score and A1c level.

**Results:** One-hundred and thirty-one diabetes patients (55 ± 14 yr; 59% female; 74% T2D; 48% with effective glycemic control (A1C < 7%); 23.6% elementary school non-concluded, 23.6% high school concluded and 17% postgraduate; average house income of 4 Brazilian minimum wages) participated in the study. The median and interquartile range of A1C level and DATE-Q total score were 7.2% [6.2–8.3] and 14 [12–16], respectively. Patients with T1D presented higher DATE-Q scores compared to those with T2D (total score: 16 [14–18] vs. 14 [11–15], P < 0.01; item scores: item 2, P = 0.01; item 3, P < 0.01; item 4, P = 0.03; and item 12, P = 0.03). There was no significant correlation between A1C levels and DATE-Q total scores as well as between A1C levels and DATE-Q each item scores.

**Conclusion:** The result of higher diabetes knowledge in patients with T1D is predictable since it is recommended to provide at least some education as part of this disease’s primary care. Although the non-association between DATE-Q scores and A1C levels found refutes our study hypothesis, it reinforces findings from other studies considering that diabetes control is related to disease type and treatment and psychosocial conditions rather than diabetes knowledge.

108807

Modality: E-Poster Young Researcher – Non-case Report

Category: CARDIOVASCULAR PHARMACOLOGY

## Antihypertensive Effect of New N-Acylhydrazones

BRUNA DE SOUZA ROCHA^1^, Jaqueline Soares da Silva^1^, Júlia Galvez Bulhões Pedreira^1^, Eliezer Jesus Barreiro^1^, Gisele Zapata-Sudo^1^

(1) Universidade Federal do Rio de Janeiro (UFRJ)

**Introduction:** Systemic arterial hypertension is a multifactorial condition with structural and/or functional changes in target organs, heart, brain, kidneys and vessels. The search of new strategies for the prevention and treatment of cardiovascular diseases, led to the design and synthesis of new N-acylhydrazones derivates, to identify agents with vasodilator activity and antihypertensive effect. New compounds containing selenium, which has antioxidant properties, which could provide reduction of the oxidative stress associated with hypertension.

**Objectives:** To investigate the vascular and antihypertensive effects of new N-acylhydrazones.

**Methods:** Protocols were approved by Animal Care and Use Committee at Universidade Federal do Rio de Janeiro n. 017/19. Vascular reactivity was evaluated using isometric tension recording of pre-contracted thoracic aorta from male Wistar rats (220–250 g) after exposure to increasing concentrations of each derivate (0.1–100 µM) and was calculated the half maximal effective concentration (EC50). Two compounds (LASSBio-2062 and LASSBio-2063) were selected to investigate the antihypertensive effect in spontaneously hypertensive rats (SHR, 12–14 weeks old), through the measurement of mean blood pressure (MBP) and heart rate (HR) after intravenous administration of 10 µmol/kg.

**Results:** The Table 1 shows EC50 of the compounds for the vasodilator effect in aorta in the presence and absence of endothelium. All derivates tested demonstrated vasodilator activity. LASSBio-2062 and its N-methylated analogue, named LASSBio-2063, were more potent than the prototype and LASSBio-2063-induced vasodilation occurs regardless of endothelium integrity. LASSBio-2062 and LASSBio-2063 (10 μmol/kg; n = 5, each) reduced MBP in SHR from 132.0 ± 9.2 to 82.1 ± 12.6 mmHg and from 129.7 ± 4.5 to 96.0 ± 14.6 mmHg, respectively (p < 0.05). LASSBio-2062 but not LASSBio-2063 reduced HR from 248.1 ± 12.1 to 146.5 ± 21.0 bpm.

**Conclusion:** The vasodilator activity induced by the new N-acylhydrazones could explain their antihypertensive effect.


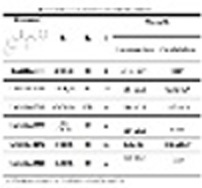


108809

Modality: E-Poster Young Researcher – Non-case Report

Category: HEART FAILURE/CARDIOMYOPATHY/TRANSPLANT

## Short-Term Outcomes of Patients with Combined Systolic and Diastolic Heart Failure

AHMAD GILL^1^, Omar Al-Taweel^1^, Dalia Hawwass^1^, Chowdhury Ahsan^1^

(1) University of Nevada, Las Vegas (UNLV)

**Introduction:** There is limited research on patients with combined systolic and diastolic heart failure.

**Objective:** In this study, we compare the short-term outcomes of patients with combined systolic and diastolic heart failure to patients with diastolic heart failure.

**Methods:** This was a retrospective cohort study. We queried the National Inpatient Sample database from 2016 to 2018 and identified adult patients admitted with a principal diagnosis of combined systolic and diastolic heart failure or diastolic heart failure. We analyzed the categorical and continuous variables by Pearson’s chi-squared and Student t-test respectively. Multivariable logistic regression, adjusted for age, gender and comorbidities, was used to compare mortality. The comorbidities adjusted for included coronary artery disease, chronic kidney disease, chronic obstructive pulmonary disease, hypertension, obesity and type 2 diabetes mellitus.

**Results:** 598,250 patients met our inclusion criteria. Patients with combined systolic and diastolic heart failure had 1.54 times higher odds of suffering in-hospital mortality compared to patients with only diastolic heart failure (aOR 1.54, 95% CI: 1.43–1.66; p < 0.001). When separated by race, combined heart failure patients had higher mortality rates amongst White (3.9% vs 2.7%, p < 0.001), Black (1.9% vs 1.6%, p < 0.001) and Hispanic (3.0% vs 1.8%, p < 0.001) patients. Patients with combined heart failure were predominantly male (57.6% vs 38.2%, p < 0.001), had longer hospital stays (5.8 days vs 5.1 days, p < 0.001) and increased hospital costs ($56,544.60 vs $41,666.02, p < 0.001). These patients also had higher rates of comorbidities such as coronary artery disease (46.2% vs 35.1%, p < 0.001) and prior myocardial infarction (12.9% vs 7.0%, p < 0.001), but decreased rates of hypertension (28.4% vs 33.2%, p < 0.001), obesity (16.5% vs 22.6%, p < 0.001) and type 2 diabetes mellitus (24.0% vs 26.2%, p < 0.001).

**Conclusion:** Patients with combined systolic and diastolic heart failure had higher odds of suffering in-hospital mortality compared to patients with only diastolic heart failure. Our findings illustrate that combined heart failure patients require close monitoring in the inpatient setting. Additional studies should explore if specific races are at increased risk for prolonged hospitalization and mortality.

108813

Modality: E-Poster Young Researcher – Non-case Report

Category: HYPERTENSION/RENAL DENERVATION

## Is Home Blood Pressure Monitoring Useful in Diagnosing Hypertension in Young Adults in Primary Care?

TAISSA LORENA DOS SANTOS^1^, Taissa Lorena dos Santos^1^, Tomás de Souza Mello^1^, Ana Rachel Buchar Cervasio^1^, Bruna Gopp Botelho^1^, Carlos Augusto Parente Macedo Moura^1^, Karine da Silva Guimarães^1^, Ana Cristina Tenório da Costa Fernandes^1^, Daniela Fiuza Gomes Monteiro^1^, Elizabeth Silaid Muxfeldt^1^

(1) Universidade Estácio de Sá, School of Medicine, Campus Vista Carioca. IDOMED

**Background:** Recent guidelines have recommended out-of-office blood pressure (BP) measurement to improve the accuracy of the diagnosis of hypertension (HTN) but there are few studies evaluating its use in the younger population and in the scope of primary care.

**Objective:** To assess the degree of agreement between office BP and HBPM, evaluating their use in a young population assisted in primary care.

**Methods:** This is a cross-sectional population study with adults between 20–50 years old registered in the Family Health Strategy. Sociodemographic, anthropometric data and CV risk factors were recorded. Office BP was the mean of 2 measurements and HBPM followed the 7-day protocol. HBPM < 135 × 85 mmHg and office BP < 140 × 90 mmHg were considered normal, identifying the 4 phenotypes: Normotension (controlled office BP and HBPM); white-coat HTN (uncontrolled office BP and controlled HBPM); masked HTN (controlled office BP and uncontrolled HBPM) and sustained HTN (uncontrolled office BP and HBPM).

**Results:** 475 individuals were included (male sex: 38%; mean age: 37.6 years ±8.8 years, of which 93 (20%) had their diagnosis modified after HBPM (43 with white-coat and 47 with masked HTN). In multiple logistics regression, male gender (OR 3.87: 95%CI 1.70–8.82) was independently correlated with white-coat HTN, while obesity and prehypertension increased the risk of masked HTN by 2 and 5.5 times, respectively. Uncontrolled office BP has high specificity (89%) and low sensitivity (49%) for detecting sustained HTN, with a low concordance in the diagnosis of HTN (kappa = 0.388) Among men with uncontrolled office BP, 55.6% had a diagnosis of white-coat HTN and among women, 37.1%.

**Conclusion:** HBPM was a useful procedure in the diagnosis of hypertension in a young and apparently healthy population in primary care, especially in those with uncontrolled office BP to identify white-coat HTN and in those with high normal office BP, especially if they are male or obese for early diagnosis of masked HTN.

108820

Modality: E-Poster Young Researcher – Non-case Report

Category: HYPERTENSION/RENAL DENERVATION

## Changes in Clinical Profile of Refractory Hypertensives After the Introduction of Spironolactone

TAISSA LORENA DOS SANTOS^1^, Taissa Lorena dos Santos^1^, Carlos Filipe dos Santos Pimenta^1^, Victor Margallo^1^, Hugo Farah Affonso Alves^1^, Lucca Hiroshi de Sá Kimura^1^, João Gabriel Rega do Nascimento Vallaperde^1^, Vitor de Melo Nolasco^1^, João Gabriell Bezerra da Silva^1^, Bernardo Chedier^1^, Elizabeth Silaid Muxfeldt^1^

(1) Universidade Federal do Rio de Janeiro – Hospital Universitário Clementino Fraga Filho – ProHArt. UFRJ – HCUFF

**Introduction:** Refractory hypertension (RfHTN) is defined as uncontrolled blood pressure despite the use of 5 or more anti-hypertensive drugs. It is considered the extreme phenotype of resistant hypertension (RHTN).

**Objectives:** To characterize prevalence and clinical profile of patients with RfHTN among a historical cohort of patients with RHTN in 2 moments: before and after the introduction of spironolactone.

**Design and Methods:** A cross-sectional analysis was performed during the pre-spironolactone period (before 2005) with 1,048 participants with RHTN (72.3% women, average age: 61.2 ± 11.3 years). All of them were submitted to a standard protocol including clinical and laboratory tests, 24-hour ABPM, echocardiogram, and pulse wave velocity. In a second analysis, refractory patients were evaluated after the introduction of spironolactone (post-spironolactone period – after 2005). The statistical analysis included bivariate comparisons between patients with RHTN and those with RfHTN, as well as logistic regressions to assess independent correlations of RfHTN pre- and post-spironolactone.

**Results:** In the initial cross-sectional analysis (pre-spironolactone), RfHTN prevalence was 14%. Age < 60 years, smoking, obesity and left ventricular hypertrophy (LVH) were independently correlated with RfHTN. After the introduction of spironolactone, prevalence increased to 17.6%. It was observed that refractory patients using spironolactone presented less aortic stiffness, lower prevalence of LVH, cerebrovascular disease and peripheral arterial disease (PAD), what might indicate a reduced cardiovascular risk despite the lack of blood pressure control. Refractory patients have also presented higher magnitude of white-coat effect, reflecting an exacerbated sympathetic activity. Age < 60 years and lower prevalence of PAD were correlated with RfHTN in the post-spironolactone period.

**Conclusion:** The use of spironolactone seems to reduce cardiovascular risk despite the lack of blood pressure control.

108831

Modality: E-Poster Young Researcher – Non-case Report

Category: ATHEROSCLEROSIS/CARDIOVASCULAR RISK FACTORS/CARDIOVASCULAR PREVENTION

## Consumption of Ultra-Processed Foods and Risk of Death from Noncommunicable and Cardiovascular Diseases: Elsa-Brasil Cohort

FERNANDA MARCELINA SILVA^1^, Luana Giatti Gonçalves^1^, Luisa Campos Caldeira Brant^1^, Maria de Fátima Haueisen Sander Diniz^1^, Alvaro Vigo^2^, Maria de Jesus Mendes da Fonseca^3^, Sandhi Maria Barreto^1^

(1) Universidade Federal de Minas Gerais, UFMG; (2) Universidade Federal do Rio Grande do Sul, UFRGS; (3) Escola Nacional de Saúde Pública, ENSP/Fundação Oswaldo Cruz, Fiocruz

**Introduction:** Changes in dietary patterns related to food processing have been associated with an increase in the incidence of several noncommunicable diseases (NCDs). Greater consumption of ultra-processed foods (UPF) was associated with a higher risk of death from all causes and cardiovascular diseases (CVDs), but results are controversial, especially for CVDs mortality.

**Objectives:** To investigate whether the consumption of UPF is associated with a higher risk of death from NCDs and CVDs in Brazilian adults after eight years of follow-up.

**Methods:** A total of 14,011 adults from the baseline (2008–2010) of the ELSA-Brasil cohort study, aged between 35 to 74 years and free of CVDs were included. Food consumption at cohort insertion was obtained using a food frequency questionnaire and the NOVA classification was used to estimate the contribution of UPF, in grams/day, on the total dietary. The following baseline variables were used for adjustments: sex, age, investigation center, schooling, physical activity, smoking, excessive alcohol use, total energy intake, body mass index (BMI), and reported diagnosis of diabetes, hypertension, and use of lipid-lowering drugs. The association between UPF consumption and time to death from NCDs and CVDs was investigated using Cox regression models, after adjustments. Restricted cubic spline regressions were used to test nonlinearity. The participants were followed from the first year of the cohort until the date of death, lost to follow-up, or December 31, 2018, whichever came first.

**Results:** After a mean follow-up of eight years, 331 deaths from NCDs and 116 from CVDs occurred. The proportion of UPF in the diet was 16.1% (10.5–23.4). After adjustments, the results suggested that a 10% increase in UPF consumption increased the risk of death from NCDs by 15% (95% CI: 1.03–1.29). These results were maintained even after adjustments for total energy intake and BMI and for comorbidities. However, no association was observed between UPF consumption and CVDs mortality.

**Conclusions:** An increase in UPF consumption was associated with a higher risk of death from NCDs. These results support public policies aimed at reducing UPF consumption and encouraging the consumption of unprocessed foods for NCDs prevention. However, a longer follow-up period may be necessary to assess the association between UPF consumption and cardiovascular mortality.

109110

Modality: E-Poster Young Researcher – Non-case Report

Category: COVID-19 AND CARDIOVASCULAR SYSTEM

## Cardiovascular Changes in Pediatric Patients with COVID-19: A Systematic Review

VICTOR LUIZ ROCHA PIRES^1^, Murilo Sousa Ramos^1^, Beatriz Rihs Matos Tavares^1^, Quelvin Claiton Souza Costa^1^, Grasiely Faccin Borges^1^

(1) Universidade Federal do Sul da Bahia (UFSB)

**Introduction:** Children affected with COVID-19 are mostly asymptomatic, in this way, they do not belong a group of risk for this disease, due to mild symptoms, a better prognosis and low lethality. Recent studies describe that some children were affected with Multisystem Inflammatory Syndrome owing to COVID-19 and facilitated complications, such as mitral regurgitation, vasculitis, myocarditis, valve regurgitation, pericardial effusion, and electrocardiographic changes, thus increasing hospitalization and care intensive.

**Objective:** To analyze the incidence of cardiovascular changes in pediatric patients associated with COVID-19.

**Methodology:** This is a systematic literature review study, conducted according to the Preferred Reporting Items for Systematic Reviews and Meta-Analyses (PRISMA) methodology. Researches and studies were performed in SciELO, LILACS, PUBMED, MEDLINE and BVSalud. Original articles on cardiovascular changes in pediatric patients, whose ages ranged from 0–18 years old, with complications repercussions for COVID-19, in the period from 2020 to 2022, were included.

**Results:** Of the 40 studies found, three were included, one research has pointed out that a third of pediatric patients who had COVID-19 had coronary changes; Kawasaki disease and the multisystem inflammatory response stand out, which occurred in most patients, followed by cardiogenic shock and other shocks. In the imaging tests, it was observed 53% had left ventricular dysfunction, 27% had right ventricular dysfunction, and the others evolved with low normal systolic function and pericardial effusion. Laboratory standards showed an increase in the mean level of partial thromboplastin time and C-reactive protein, as well as hydroelectrolytic repercussions, which were related to prognosis and mortality.

**Conclusion:** There are a few studies that analyzed children affected by COVID-19, and these indicate the possibility of pediatric patients to develop serious cardiovascular complications after infection by SARS-CoV-2.

110853

Modality: E-Poster Young Researcher – Non-case Report

Category: ATHEROSCLEROSIS/CARDIOVASCULAR RISK FACTORS/CARDIOVASCULAR PREVENTION

## Vascular Responses to Mental Stress in Postmenopausal Women with Treatment-Resistant Hypertension: The Role of Nitric Oxide/Cgmp

AMANDA SAMPAIO STORCH^1^, Helena Naly Miguens Rocha^1^, João Dario Mattos^1^, Larissa Lírio Velasco^1^, Juliana Mentzinger^1^, Gabriel Fernandes Teixeira^1^, Rebeca Lial Rosado^1^, Antonio Claudio Lucas da Nóbrega^1^, Ronaldo Altenburg Odebrecht Curi Gismondi^1^, Natália Galito Rocha Ayres^1^

(1) Fluminense Federal University

**Introduction:** Patients with treatment-resistant hypertension (TRH) usually present mental stress (MS)-induced impairments in the endothelial dysfunction. We suggest that the decrease in nitric oxide (NO) bioavailability evoked by MS may aggravate vascular function in those patients.

**Objective:** To determine the effects of increasing NO bioavailability during MS through inhibition of phosphodiesterase-5 on vascular response in those patients.

**Methods:** It is a cross-sectional, randomized, crossover, double-blind, and placebo-controlled protocol (CAAE: 79958017.3.0000.5243). In two experimental sessions, postmenopausal women with TRH (61 ± 5 years) underwent a randomized oral administration of PDE-5 inhibitor (iPDE5; sildenafil 50 mg) or placebo (PL). After 30 minutes, the patients were submitted to MS (modified Stroop Color-Word Test) for five minutes. Flow-mediated dilatation (FMD; vascular ultrasound) and pulse wave analyses (applanation tonometry) were measured at baseline, immediately after (MS), and 30 minutes after MS (MS30). Blood samples were collected at baseline, during MS, and MS30 to measure coagulation (coagulometry), oxidative stress (colorimetry), and nitrite/nitrate concentration (chemiluminescence). Heart rate (HR; electrocardiogram) and BP (photoplethysmography) were continuously monitored throughout the protocol. Data were presented as mean ± standard deviation. Two-way repeated-measures ANOVA was used to compare baseline, MS, and MS30 measurements between sessions. Paired Student’s T-test was performed to compare the delta values, followed by effect size Cohen’s D test.

**Results:** Preliminary results showed that MS increased the HR and systolic BP (p < 0.05) in both sessions. Baseline FMD was higher in the PL session (p = 0.02). However, there was a reduction in FMD at MS and MS30 in the PL session (p < 0.01 vs. baseline), while FMD was higher at MS30 in the iPDE5 session (p = 0.05 vs. baseline; p < 0.01 vs. PL). Additionally, the FMD response at MS30 was higher in iPDE5 compared to PL session with a medium effect size (p = 0.02; Cohen’s D:0.64). Nitrate concentration was higher at baseline and during MS in the iPDE5 session (p = 0.05 vs. placebo). No differences were observed throughout the protocol or in the response at MS30 in pulse wave analysis, coagulation, oxidative stress, and nitrite measurements.

**Conclusions:** Present data suggest that the PDE-5 inhibition attenuates the deleterious effects of MS on endothelial function in patients with TRH.

108864

Modality: E-Poster Young Researcher – Non-case Report

Category: COVID-19 AND CARDIOVASCULAR SYSTEM

## Myocardial Injury and Prognosis in Hospitalized Brazilian COVID-19 Patients: Results from the Brazilian COVID-19 Registry

HANNAH CARDOSO BARBOSA^1^, Maria Auxiliadora Parreiras Martins^1^, Manuela Furtado Sacioto^2^, Jordana Cristina de Jesus^3^, Karina Cardoso Meira^3^, Luiz Guilherme Passaglia^1^, Milena Soriano Marcolino^1^, Carísi Anne Polanczyk^4^

(1) Universidade Federal de Minas Gerais UFMG; (2) Faculdade Ciências Médicas de Minas Gerais FCMMG; (3) Universidade Federal do Rio Grande do Norte UFRN; (4) Institute for Health Technology Assessment (IATS/CNPq)

**Background:** Cardiovascular complications of COVID-19 represent an important aspect of the disease’s pathogenesis and prognosis. Troponin levels are commonly abnormal in hospitalized patients with COVID-19 and they are associated with poorer prognosis. Evidence on the prognostic role of troponin and myocardial injury in COVID-19 in-hospital patients from Latin America are still scarce. This study aimed to fill this gap by evaluating myocardial injury as an independent predictor of in-hospital mortality and mechanical ventilation requirement in COVID-19 hospitalized Brazilian patients.

**Methods:** This cohort study is a substudy of the Brazilian COVID-19 Registry, conducted in 31 Brazilian hospitals in 17 cities. Consecutive patients admitted with confirmed diagnosis of COVID-19 between March and September 2020 were included. Medical records were reviewed to collect data regarding patients’ demographic and clinical characteristics; COVID-19 symptoms; clinical assessment upon hospital presentation, third and fifth days of hospitalization; laboratory, imaging, and electrocardiographic data; in-hospital medications, treatment, and patient outcomes. Models for the primary outcomes were estimated by Poisson regression with robust variance. For the multivariate analyses, two predictive models were estimated to evaluate the role of elevated troponin on the primary outcomes: in-hospital mortality and mechanical ventilation requirement.

**Results:** Overall, 2,925 patients were included in this analysis. The median age was 60 (IQR 48–71) years old. The proportion of patients with comorbidities was higher in the group of people with cardiac injury. Laboratory tests brain natriuretic peptide, creatine phosphokinase, lactate dehydrogenase, N-Terminal pro-brain natriuretic peptide and C-reactive protein presented higher median values in patients with myocardial injury and this condition was a significant independent predictor for risk death and mechanical ventilation requirement. Patients with myocardial injury represented by elevated troponin levels, presented a higher risk of death relative risk (2.03 [1.60–2.58]) and mechanical ventilation requirement relative risk (1.87 [1.57–2.23]) when compared to controls.

**Conclusion:** Cardiac injury showed to be an independent predictor of in-hospital mortality and need of mechanical ventilation in hospitalized COVID-19 patients. The design of strategies involving the continuous monitoring of troponin levels as risk biomarkers in thes.

109466

Modality: E-Poster Young Researcher – Non-case Report

Category: HEMODYNAMICS AND INTERVENTIONAL CARDIOLOGY

## Real-World Comparison between Inspiron Sirolimus-Eluting Stent and Other Third-Generation Drug-Eluting Stents in Patients with ST-Elevation Myocardial Infarction Submitted to Primary Percutaneous Coronary Intervention (Instemi Registry)

GUSTAVO NEVES DE ARAUJO^3^, Guilherme Pinheiro Machado^1^, Marcia Moura^4^, Anderson Donelli da Silveira^1^, Pedro A. Lemos^5^, Alexandre Schaan de Quadros^4^, Marco Vugman Wainstein^2^

(1) Hospital de Clinicas de Porto Alegre (HCPA); (2) Universidade Federal do Rio Grande do Sul (UFRGS); (3) Imperial Hospital de Caridade; (4) Instituto de Cardiologia do Rio Grande do Sul (IC-FUC); (5) Hospital Israelita Albert Einstein

**Introduction:** Coronary drug-eluting stents are continuously developing, and the current gold standard consists of new metal alloys with thinner struts and bioresorbable polymers. Our aim was to compare a new ultrathin strut, sirolimus-eluting stent (Inspiron®) with other third-generation drug-eluting stent platforms in patients with ST-elevation Myocardial Infarction (STEMI) submitted to primary percutaneous coronary intervention (PCI).

**Methods:** The present work assessed clinical outcomes from a STEMI multicenter registry, from two centers in southern Brazil. Patients were considered for inclusion if they were admitted with STEMI and were submitted to primary PCI. All patients included received third generation DES, and Inspiron® was compared with five other stent platforms. A propensity score matching (PSM) adjusted for age, diabetes, admission Killip classification, cardiac arrest and creatinine was computed to generate similar groups in relation to clinical and procedural characteristics.

**Results:** From January 2017 to January 2021, 1711 patients have undergone primary PCI and 1417 patients matched our entry criteria (709 in the Inspiron and 708 in the other third generation DES). Rates of hypertension (60 vs. 65%, p = 0.042), chronic kidney disease (2.7 vs 6.6%, p < 0.001), admission cardiogenic shock (5.2 vs. 9.5, p = 0.002) and cardiac arrest (1.0 vs. 7.2, p < 0.001) were lower in Inspiron group. After PSM, the study sample was comprised of 706 patients (353 in the Inspiron and 353 in the other third generation). Differences in baseline characteristics described above have lost significance. The rates of new revascularization (OR 0.52, CI 0.21–1.34, p = 0.173), stent thrombosis (OR 1.00, CI 0.29–3.48, p = 1.000), new revascularization (2.0 vs. 3.7%, OR 0.52, CI 0.21–1.34, p = 0.173), mortality (HR 0.724, CI 0.41–1.27, p = 0.257) and MACE (OR 1.170, CI 0.77–1.77, p = 0.526) were similar between groups after a median follow up of 17 months.

**Conclusion:** Our findings support that Inspiron is safe in patients with STEMI, with similar outcomes after propensity score adjustment compared to well stablished third generation drug-eluting stents in the treatment with primary PCI at a long-term follow-up.

108891

Modality: E-Poster Young Researcher – Non-case Report

Category: ATHEROSCLEROSIS/CARDIOVASCULAR RISK FACTORS/CARDIOVASCULAR PREVENTION

## Altered Neck Circumference as an Indicator of Cardiovascular Risk Among Hypertensive Patients Treated in Primary Health Care

LUCAS CAUNETO SILVEIRA^1^, Patrícia Pereira de Almeida^2^, Marselha Marques Barral^1^

(1) Hospital e Maternidade Therezinha de Jesus – HMTJ; (2) Universidade Federal de Juiz de Fora – UFJF

**Introduction:** Excess body fat is a modifiable risk factor associated with chronic non-communicable diseases, including systemic arterial hypertension (SAH). Regarding the assessment of nutritional status, anthropometric measurements are inexpensive; among which the neck circumference (NC) has been used as an indicator of cardiometabolic risk, as it estimates the accumulation of fat in the upper segment of the body. In addition, NC is considered an accessible measurement, it does not change with food intake and can be measured without the need to remove clothes.

**Objectives:** To identify the presence of altered neck circumference among hypertensive patients treated in primary health care.

**Methods:** Cross-sectional study conducted from September 2019 to March 2020 with adults and elderly treated in primary health care in the municipálity of Guidoval-MG. We collected socioeconomic, lifestyle, health history data through a questionnaire and took anthropometric measurements. We measured neck circumference with an inelastic tape; with the participant standing and the head in a straight position, the tape was positioned at the midpoint of neck height. For the classification of NC, we used the cutoff point proposed by Zanúncio et al (2017), which considers NC>39.5 cm for men and NC>33.3 for women associated with cardiometabolic risk. The diagnosis of SAH was self-reported and confirmed in the electronic medical record. The study was approved by the Research Ethics Committee of the Federal University of Viçosa, under protocol number 3,189,427. We analyzed data using descriptive statistics, with calculation of mean, standard deviation and prevalence.

**Results:** The sample consisted of 361 individuals with a prevalence of systemic arterial hypertension of 40.2% (n = 145). Among hypertensive patients (n = 145), the mean age was 58 ± 12.6 years and 91 individuals (62.8%) were identified with altered neck circumference, the majority being female (n = 74). Thus, there was a high prevalence of hypertensive patients with altered neck circumference measurements.

**Conclusion:** Our findings are in agreement with the literature, which states that NC constitutes an indicator of cardiometabolic risk and its alteration is associated with other chronic diseases such as SAH and diabetes. Therefore, due to its facility and low cost, NC measurement can be adopted in health services, especially in places where there is a scarcity of human and financial resources.

109084

Modality: E-Poster Young Researcher – Non-case Report

Category: COVID-19 AND CARDIOVASCULAR SYSTEM

## Complications Associated with Myocardial Injury in Patients with COVID-19

JOÃO DARIO MARTINS DE MATTOS^1^, João Dario Mattos^1^, Eduardo Schaustz^1^, Andréa Silvestre de Sousa^2^, Emiliano Horacio Medei^3^, Denílson C. de Albuquerque^1^, Olga Ferreira de Souza^1^, Fernando A. Bozza^1^, Gabriel C Camargo^1^, Juliana Ferreira^4^, Ronir Raggio Luiz^3^, Renata J Moll-Bernardes^1^

(1) D’OR Institute Research & Education (IDOR); (2) National Institute Of Infectious Diseases (Oswaldo Cruz Foundation); (3) Federal University of Rio de Janeiro; (4) Hospital Copa D’OR

**Background:** Myocardial injury (MI), detected by elevations in cardiac troponin is common in hospitalized patients with COVID-19, and has been associated with adverse outcomes and mortality; however, there is still some controversy regarding the role of this biomarker, and routine measurement of troponin levels to increase risk stratification is not recommended. Aim: To explore the association between MI with mortality and complications in hospitalized patients with COVID-19.

**Methods:** Consecutive adult hospitalized patients with COVID-19 were included prospectively in a multicenter registry (n = 3246). Troponin levels were normalized to the 99th percentile upper reference limit (URL) and presented as ratios. Myocardial injury was defined by troponin >1 × URL. Troponin ratios were categorized as normal (≤1x URL), mildly elevated (>1 to ≤3x URL), moderately (> 3 to ≤10x URL) and severely elevated (>10x URL). Clinical data was collected from electronic medical records. Patients were prospectively followed until hospital discharge or in-hospital death.

**Results:** Myocardial injury was associated with increased in-hospital mortality (22.7% vs 5.5%) and most cardiovascular adverse outcomes, such as thromboembolic phenomena (4.0% vs 2.6%; p = 0.047), myocarditis (4.8% vs 1.2%; p < 0.001), heart failure (6.5% vs 1.3%; p < 0.001), myocardial ischemia (9.1% vs 1.4%; p < 0.001), sepsis or septic shock (20.1 vs 9.2%; p < 0.001), acute renal failure (21.8% vs 7.4%; p < 0.001) and respiratory failure requiring mechanical ventilation (36.5% vs 10.8%; p < 0.001). From a total of 3246 patients in our cohort, 1770 (54.5%) did not have troponin measurement during the first week of hospitalization. In-hospital mortality was higher in patients with no troponin measurement compared to patients with no myocardial injury (11.9% [95%CI, 10.5–13.5] vs 5.5% [4.3–7.0]), highlighting the negative predictive value of this biomarker. Some complications were also more prevalent in patients with no troponin measurement, compared to patients with normal troponin levels.

**Conclusion:** Our findings suggest that troponin is an important biomarker that may improve risk prediction and resource utilization in hospitalized patients with COVID-19.

108920

Modality: E-Poster Young Researcher – Non-case Report

Category: EPIDEMIOLOGY AND HEALTH POLICIES/GLOBAL HEALTH

## Social Determinants of Outpatient Healthcare Utilization and Related Costs Among Patients with Rheumatic Heart Disease: A Longitudinal Study in Uganda

XINPENG XU^1^, Jenifer Atala^2^, Andrea Z. Beaton^3^, Rosemary Kansiime^2^, Miriam Nakitto^2^, Emma Ndagire^5^, Haddy Nalubwama^2^, Emmy Okello^5^, Emily Chu^6^, Hui Miao^7^, David A. Watkins^8^, Yanfang Su^8^

(1) School of Public Health, Nanjing Medical University, Nanjing, China; (2) Department of RHD Research, Uganda Heart Institute, Kampala, Uganda; (3) Department of Cardiology, The Heart Institute, Cincinnati Children’s Hospital Medical Center, Cincinnati, OH, United States; (4) Department of Pediatrics, School of Medicine, University of Cincinnati, Cincinnati, OH, United States; (5) Division of Cardiology, Uganda Heart Institute, Kampala, Uganda; (6) Interlake High School, Bellevue, WA, United States; (7) Vanke School of Public Health, Tsinghua University, Beijing, China; (8) Department of Global Health, University of Washington, Seattle, WA, United States; (9) Department of Medicine, University of Washington, Seattle, WA, United States

**Introduction:** Rheumatic heart disease (RHD) is prevalent in socially disadvantaged settings, placing a severe burden on patients and their households. Many studies have highlighted the relationship between social determinants and RHD disease development, but there is limited understanding about social determinants of healthcare utilization and related financial and time costs among RHD patients.

**Objective:** To investigate social determinants of healthcare utilization and related costs among RHD patients in Uganda.

**Methods:** This was a retrospective longitudinal study involving patients with RHD recruited from the Uganda National RHD Registry. Our survey included 87 patients from the registry stratified by 3 districts in Uganda. Between December 2018 and February 2021, data were collected. A random-effects model was used to examine the associations between social determinants and healthcare utilization, as well as financial and time costs.

**Results:** Among the social determinants, increase of an additional person per bedroom is associated with the reduction of outpatient spending by 1.73 USD per visit (P < 0.05), owing primarily to a reduction in direct medical spending (1.25 USD; P < 0.01). RHD patients under the age of 18 had 3.2 more outpatient visits annually (P < 0.01), and their per-visit time costs and those of caregivers were 2.5 hours (P < 0.05) and 4.9 hours (P < 0.05) more than those of adult RHD patients, respectively. More educated individuals spend 2.5 USD less on nonmedical cost per visit (P < 0.05). In Western Region, which is underdeveloped in Uganda, the number of annual outpatient visits by patients with RHD were 4.8 higher than those in the Central Region(P < 0.01); RHD patients living in western region faced 3.8 USD higher direct medical costs per outpatient visit (P < 0.1) and more missed work hours (1.9 hours, P < 0.1).

**Conclusions:** RHD patients underutilized RHD secondary prevention and experienced high healthcare expenditures. Crowding, age, and geographical characteristics all have significant correlation with RHD patients’ healthcare utilization and costs. Targeted measures, such as expanding housing construction and improving housing maintenance, reducing barriers in RHD secondary prevention among adult patients, ensuring the supply of medicines in economically worse-off areas, should be taken to ensure that equitable and affordable medical services are available to those RHD patients who are most in need of secondary prevention services.

108924

Modality: E-Poster Young Researcher – Non-case Report

Category: HEART FAILURE/CARDIOMYOPATHY/TRANSPLANT

## Impact of Systemic Lupus Erythematosus in Patients with Hypertrophic Obstructive Cardiomyopathy

AHMAD GILL^1^, Emily He^2^, Omar Al-Taweel^1^, Dalia Hawwass^1^, Chowdhury Ahsan^1^

(1) University of Nevada, Las Vegas (UNLV); (2) Loma Linda University (LLU)

**Introduction:** Although there is extensive literature present on hypertrophic obstructive cardiomyopathy (HCOM), there is limited data on HCOM patients with systemic lupus erythematosus.

**Objective:** In this study, we investigate the short-term, in-hospital outcomes of HCOM patients with and without systemic lupus erythematosus.

**Methods:** We queried the National Inpatient Sample database from 2016 to 2018 to identify adult patients admitted with a principal diagnosis of HCOM and a secondary diagnosis of systemic lupus erythematosus. We analyzed the categorical and continuous variables by Pearson’s chi-squared and Student t-test respectively. Multivariable logistic regression, adjusted for age, gender, comorbidities, hospital bed size, hospital region and hospital teaching status was used to compare mortality. The comorbidities adjusted for included atrial fibrillation, coronary artery disease, chronic kidney disease, heart failure, hypertension, obesity and type 2 diabetes mellitus.

**Results:** 9395 patients met our inclusion criteria, of which 35 patients had both HCOM and systemic lupus erythematosus. Patients with HCOM and systemic lupus erythematosus had 7.08 times higher odds of suffering in-hospital mortality compared to patients with solely HCOM (aOR 7.08, 95% CI: 1.21–41.32; p = 0.03). When separated by race, HCOM and systemic lupus erythematosus patients had a higher mortality rate in White patients (21.7% vs 1.6%, p < 0.001), but decreased mortality rates in Black (0% vs 2.9%, p = 0.67) and Hispanic (0% vs 2.0%, p = 0.73) patients. Patients with both HCOM and SLE were predominantly female (85.7% vs 58.6%, p = 0.15) and had lower median household income (71.4% vs 23.6%, p = 0.02). These patients also had higher rates of comorbidities such as coronary artery disease (42.9% vs 28.0%, p = 0.05), chronic kidney disease (14.3% vs 0.8%, p < 0.001), chronic obstructive pulmonary disease (42.9% vs 9.8%, p < 0.001) and prior myocardial infarction (14.3% vs 5.0%, p = 0.01), but decreased rates of atrial fibrillation (0% vs 28.1%, p < 0.001), heart failure (14.3% vs 28.4%, p = 0.06) and hypertension (28.6% vs 42.8%, p = 0.09).

**Conclusion:** Patients with HCOM and systemic lupus erythematosus had higher odds of suffering in-hospital mortality compared to patients with solely HCOM. Our findings illustrate that patients with HCOM and systemic lupus erythematosus are a vulnerable population that require additional medical support in the acute care setting.

108930

Modality: E-Poster Young Researcher – Non-case Report

Category: CARDIORESPIRATORY PHYSIOLOGY/BASIC SCIENCE

## Increased Myocardial Retention of Mesenchymal Stem Cells and Improved Cardiac Function Post-MI by Pre-Conditioning Exercise Training

STELLA DE SOUZA VIEIRA^1^, Brunno Lemes de Melo^1^, Ednei Luiz Antonio^1^, Fabio Navarro Marques^2^, Daniele de Paula Faria^2^, Paulo José Ferreira Tucci^1^, Andrey Jorge Serra^1^

(1) Laboratório de fisiologia e fisiopatologia cardíacas. Universidade Federal de São Paulo (UNIFESP); (2) Instituto de radiologia, Hospital das clínicas da FMUSP, Universidade de São Paulo

**Introduction:** Stem cell (SC) therapy is a promising approach to improve post-myocardial infarction (MI) cardiac remodeling, but the proinflammatory microenvironment may lead to SC loss and, therefore, may have a negative impact on therapy. It appears that exercise training (ET) improves myocardial microenvironment for SC transplantation.

**Objective:** We tested the effect of ET on post-MI retention of adipose-derived SC and its combined effects on the inflammatory microenvironment.

**Methods:** Female Fisher-344 rats (12 weeks) were randomized: SHAM; MI (MI); MI with SC (sMI); MI with ET and SC (esMI). Rats were trained 9 weeks prior to MI, followed by SC transplantation. Results MI led to left ventricular (LV) dilation and dysfunction; myocardial hypertrophy and fibrosis and increased expression of pro-inflammatory genes compared to SHAM rats. On the other hand, SC rats (sIM and esIM) exhibited better LV morphology and function; inhibition of hypertrophy and fibrosis; and attenuation of pro-inflammatory cytokines in the myocardium compared to MI rats. A positive correlation is observed between the expression of cytokines (interleukins 1β and 10, tumor necrosis factor α, and transforming growth factor β) and retention of SC. There was a correlation between cytokine expression and myocardial ADSCs retention. The. ET enhanced the beneficial effects of ADSCs in infarcted myocardium, which was associated with higher ADSCs retention.

**Conclusion:** ET enhanced the beneficial effects of SC post-MI, which was associated with higher SC retention. Cytokine analysis suggests improvement in ET-linked myocardial microenvironment based on its anti-inflammatory action.


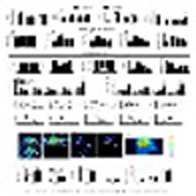


108953

Modality: E-Poster Young Researcher – Non-case Report

Category: CARDIOVASCULAR SURGERY

## Personalized External Aortic Root Support (PEARS): First Cases in America

IURI SCHWAAB^1^, Felipe Borsu de Salles^2^, Lucas Krieger Martins^1^, Rafael de Oliveira Ceron^1^, Marisa Fatima dos Santos^1^, Diogo Ferrari Centenaro^1^, Ari Tadeu Lirio dos Santos^1^, João Carlos Vieira da Costa Guaragna^2^, Renato Abdala Karam Kalil^3^

(1) Instituto de Cardiologia de Porto Alegre; (2) Hospital Divina Providência; (3) Hospital Hospital Moinhos de Vento

Aortic Root replacement is the standard treatment for Aneurysm in Marfan syndrome, despite the morbidity of this kind of surgery. Personalized External Aortic Root Support (PEARS) is a novel approach for this condition, which consists in applying an pre-tailored external prosthesis. Initial reports demonstrate low in-hospital morbidity and good mid-term aortic-specific complication prevention.¹ We present the first three cases of PEARS in America. Patients were 2 male and 1 female, aged 30–41 years, with previous Marfan syndrome diagnosis and Aortic Root Aneurysm (ranging 47–52 mm). All patients had normal ventricular and kidney function, with no other systemic disease. All patients received standard aenesthesic monitorization and median sternotomy. Case #1 was submitted to PEARS and mitral valve repair (quadrangular resection and complete ring annuloplasty). He is a 33 years old male patient, with 52 mm aortic root aneurysm, mild aortic valve regurgitation and severe mitral regurgitation. Postoperatively Aortic Root size reduced to 42 mm, valvar function improved to only trace aortic and mitral valve regurgitation. Other 2 cases were submitted to PEARS off pump and were extubated in the operating room after the procedure. Case #2 is 41 years old, male, with 48 mm aortic root diameter and mild aortic valve regurgitation. After surgery aortic size was reduced to 43 mm and had complete correction of aortic regurgitation. Case #3 is 30 years old, female, with 47 mm aortic root diameter and trace of aortic valve function. Postoperative echo-cardiogram demonstrated reduction in aortic root aneurysm to 43 mm and stable valvar function. There was no surgical adverse event nor in-hospital clinical complication. Patients didn’t require vasoactive drugs nor had significant postoperative bleeding or laboratory abnormalities. Intensive care unit stay was 2–3 days and in-hospital stay was 4–8 days. Postoperative echo-cardiogram demonstrated normal ventricular function, general reduction in aortic diameter and improvement of valvar function. PEARS demonstrate feasibility and safety in this initial surgical series. Long-term results may support the inclusion of PEARS as a therapeutic alternative for patients with Marfan syndrome and aortic aneurysm. REF: Van Hoof L, Rega F, Golesworthy T, et al. Personalised external aortic root support for elective treatment of aortic root dilation in 200 patients. Heart 2021; 107: 1790–1795.

108962

Modality: E-Poster Young Researcher – Non-case Report

Category: CARDIOVASCULAR PHARMACOLOGY

## Activation of Adenosine Receptor Reduces Inflammatory Process and Cardiac Remodeling Induced by Myocardial Infarction

BIANCA DOS SANTOS CARLOS NASCIMENTO^1^, Jaqueline Soares da Silva^1^, Bruna Rocha^1^, Tadeu Lima Montagnoli^1^, Eliezer de Jesus Lacerda Barreiro^1^, Rodolfo do Couto Maia^1^, Gizele Zapata-Sudo^1^

(1) Universidade Federal do Rio de Janeiro – UFRJ

**Introduction:** Treatment of acute myocardial infarction (AMI) is based on strategies to relief pain and increase survival. This work investigates the effects of a new agonist of adenosine receptor (AR) named LASSBio-1860, in experimental AMI, in order to improve cardiac function.

**Methods:** Protocols were approved by the Ethics Committee for the Use of Animals at Universidade Federal do Rio de Janeiro (n°103/17). Experimental AMI was induced by ligation of the anterior descending coronary artery in male Wistar rats (180–200 g) which were randomly treated orally with either vehicle (DMSO) or 70 μmol/kg of LASSBio-1860. After 7 days of treatment, hemodynamic parameters were measured by catheterization and hearts were collected for evaluation of cardiac inflammation (TNF-alpha, p38 MAPK and iNOS) and remodeling (ERK-1/2 phosphorylated) markers using Western blot and immunohistochemistry analysis.

**Results:** Left ventricle thickness was increased after AMI from 0.11 ± 0.04 to 0.29 ± 0.02 cm (p < 0.05) and reduced to 0.21 ± 0.01 cm after treatment with LASSBio-1860. AMI impaired ejection fraction to 45.2 ± 1.3% (Sham: 91.5 ± 0.5%, p < 0.05) which was partially reversed to 60.1 ± 10.3% after treatment. The agonist of AR normalized the increased filling pressure of AMI animals from 31.3 ± 6.3 (Sham: 18.6 ± 3.7) to 15.9 ± 3.9. Increased LV end systolic pressure was detected after AMI, changing from 83.8 ± 12.5 (Sham) to 104.7 ± 1.1 mmHg, and was recovered to 95.3 ± 21.4 mmHg by LASBio-1860. Elevated collagen deposition from 3.6 ± 0.9 to 31.5 ± 4.29% was observed in AMI hearts, which the treatment attenuated to 26.8 ± 1.6%. AMI increased the ratio of phosphorylated-to-total ERK-1/2 from 0.78 ± 0.01 to 0.89 ± 0.01 while LASSBio-1860 reduced to 0.79 ± 0.01 (p < 0.05). Similarly, cardiac content of p38 increased to 1.58 ± 0.36 after AMI but reduced to 1.17 ± 0.43 after LASSBio-1860. AMI also promoted increased p38 nuclear labeling from 9.9 ± 3.9 to 40.8 ± 3.3%, which was decreased to 29.9 ± 1.7 after treatment. Expression of iNOS increased in AMI from 9.0 ± 4.1 to 25.3 ± 3.9 and LASSBio-1860 improved to 14.2 ± 0.6%.

**Conclusion:** Activation of AR by LASSBio-1860 led to improvement in AMI-induced cardiac remodeling and cardiac function.

109017

Modality: E-Poster Young Researcher – Non-case Report

Category: EPIDEMIOLOGY AND HEALTH POLICIES/GLOBAL HEALTH

## Analysis of Epidemiological Indicators of Syphilis in Brazil between 2010 and 2021 and its Cardiovascular Implications

PEDRO TOSCANO PAFFER^1^, Vitória Maria Terra Lopes^1^, Camila Santiago de Castro^1^, Kamille Didier Melo Almeida^1^, Silvio Hock de Paffer Filho^1^

(1) Faculdade de Medicina de Olinda (FMO)

**Introduction:** Despite the number of syphilis cases has decreased in recent years due to the advent of antibiotics and public policies for the prevention of Sexually Transmitted Diseases, cases of cardiac syphilis are still reported worldwide, especially in developing countries. The cardiac sequelae of syphilis appears approximately 10 to 20 years after the initial infection. Understanding the relation between syphilis and its cardiovascular repercussions, its important to analyze the epidemiological indicators of syphilis in Brazil and its potential risk, with a view to preventing these complications.

**Objective:** Analyze the epidemiological indicators of syphilis in Brazil and its potential risk of cardiovascular complications Methods Epidemiological, descriptive study with data obtained by SINAN (Information System on Notifiable Diseases). The platform was used to search for cases of acquired syphilis between 2010–2021, specifying by region, age group and gender.

**Results:** Between 2010 and 2021, 911179 cases were reported in Brazil. The majority of the cases were reported in the South-east region, with 471767 cases (51%); followed by the South region, 203969 (22%); North-east region 121524 cases (13%); Mid-west region 63057 (6%) and northern region 50862 (5%). 549201 patients were male (60%). The age group with the highest incidence was between 20–39 years old, with 518970 cases reported (56.96%). Among people aged 60 years or older, 75368 cases (8.27%) were reported. In the year 2021 alone, 64279 cases were reported Discussion A total of 911179 cases of acquired syphilis were reported between 2010 and 2021, with its peak in 2018. Males had the highest incidence, with 549201. Syphilis infection has been linked to certain behavioral and social factors, including incarceration, multiple or anonymous sexual partners, sexual activity related to illicit drug use, and other high-risk sexual network dynamics. Given this large increase in the number of cases, there is a risk that in the future cases of cardiovascular involvement will increase. The most significant cardiovascular complication of cardiovascular syphilis is syphilitic aortitis, which leads to aortic aneurysm formation in most cases, and aortic valve insufficiency and coronary ostial stenosis in a minority of patients.

**Conclusion:** The high incidency and cardiovascular implications the diseases carries, requires prevention, monitoring and adequate treatment, with awareness policies for the risk groups

109033

Modality: E-Poster Young Researcher – Non-case Report

Category: CONGENITAL AND PEDIATRIC CARDIOLOGY

## Descriptive Analysis of the Correlation between the Prenatal and the Occurrence of Anomalies in the Cardiovascular System in the Period of 20 Years

RENATA PINHEIRO MARTINS DE MELO^1^, Isabela Aragão Colares^1^, Mariana Salles Ballalai^1^, José Levi Tavares Cavalcante^1^, Gabriel Sousa Santos^1^, Helena Raquel Nogueira de Oliveira^1^, Marcelo Brito Cavalcante^1^, Gabriel Coelho Brito Dias^1^, Weiber Silva Xavier^1^, Sandra Nívea dos Reis Saraiva Falcão^1^, João Luiz de Alencar Araripe Falcão^1^

(1) Universidade Federal do Ceará (UFC)

**Introduction:** Congenital anomalies are the fourth leading cause of neonatal death and, according to the World Health Organization (WHO), the most common and serious congenital disorders are heart problems, neural tube defects, and chromosomal abnormalities. In this sense, despite all advances in obstetrics and improvements in prenatal care for pregnant women, the prevention of mortality in neonates due to defects in the circulatory system remains a challenge to be overcome.

**Objective:** Analyze, during the period from 1999 to 2019, the proper performance of prenatal care, correlated to the occurrence of deaths from cardiovascular defects in neonates.

**Methodology:** Descriptive analysis, obtained from the DATASUS platform, of the frequency of prenatal care, the frequency of births with congenital anomalies and how many of these disorders are related to defects in the cardiovascular system and mortality rate in children under 1 year old due to diseases of the circulatory system.

**Results:** During the period from 1999 to 2019, in Brazil, 8702 neonates died due to defects in the circulatory system and 3,443,087 women did not perform prenatal care or did it inappropriately. In addition, 30,734 children, whose mother didn’t perform prenatal care or it was performed inappropriately, were born with a certain congenital anomaly, which represents 21% more than those who had congenital disorders, but prenatal care was being performed. Furthermore, of a total of 170,392 cases of births with anomalies, 17.3% were characterized by defects in the cardiovascular system.

**Conclusion:** From the information above-mentioned, it is possible to correlate the inadequate performance of medical attendance during pregnancy and the occurrence of congenital defects, such as disorders in the circulatory system, because, with the performance of prenatal, the pregnant woman can be treated for a certain pathologies, such as hypertension and diabetes, to avoid harmful consequences to the fetus and to prevent the occurrence of premature birth, since premature neonates are twice as likely to have heart abnormalities. In addition, in case the fetus is already with the disturb, the prenatal allows to early diagnosis and treat the condition, increasing the survival percentage. In resume, performing prenatal care correctly, is essential for the prevention or the successful treatment of defects in the circulatory system in neonates and for the reduction of mortality in children under 1 year old.

109057

Modality: E-Poster Young Researcher – Non-case Report

Category: CARDIOVASCULAR IMAGING

## Diagnostic Value of Cardiovascular Magnetic Resonance in Acute Peripartum Cardiomyopathy (PPCM)

JULIAN HOEVELMANN^1^, Charle André Viljoen^3^, Stephen Jermy^4^, Jacqui Cirota^5^, Sarah Kraus^5^, Karen Sliwa^2^, Ntobeko AB Ntusi^4^

(1) Department of Internal Medicine III, Cardiology, Angiology and Intensive Care Medicine, Saarland University Hospital, Homburg (Saar), Germany; (2) Cape Heart Institute, Faculty of Health Sciences, University of Cape Town, Cape Town, South Africa.; (3) Division of Cardiology, Groote Schuur Hospital, Faculty of Health Sciences, University of Cape Town, Cape Town, South Africa.; (4) Cape Universities Body Imaging Centre, Faculty of Health Sciences, University of Cape Town, Cape Town, South Africa; (5) Department of Medicine, Groote Schuur Hospital, Faculty of Health Sciences, University of Cape Town, Cape Town, South Africa.

**Introduction:** Peripartum cardiomyopathy (PPCM) is characterised by left ventricular (LV) dilatation and systolic dysfunction developing towards the end of pregnancy or in the first five months postpartum. Cardiovascular magnetic resonance (CMR) allows for comprehensive evaluation of myocardial structure, function, and tissue characteristics. There is a dearth of studies investigating utility of CMR in PPCM.

**Purpose:** To evaluate diagnostic benefit of multiparametric assessment of myocardial oedema, fibrotic burden, and strain impairment in PPCM using CMR.

**Methods:** Eighteen consenting women with newly diagnosed PPCM and 20 female, age-matched healthy controls (HCs) underwent CMR imaging on a 3T MR scanner. A comprehensive, contrast-enhanced CMR protocol was used. Images were evaluated qualitatively and semi-quantitively for the presence of late gadolinium enhancement (LGE).

**Results:** Patients with PPCM (median age of 34.5 years [IQR 25–38]) presented with severely impaired LV ejection fraction (LVEF) of 31.4% (IQR 19.6–37.9) and reduced right ventricular (RV) ejection fraction (RVEF) of 37.2% (IQR 21.6–51.7). LGE was present in 13 (81.2%) PPCM patients and included linear or circumferential mid-wall, patchy and diffuse patterns (LGE mass 19.1g [IQR 15.0–26.5] vs. 11.4g [8.8–13.2] in HCs, p < 0.001). Patients with PPCM had significantly higher T1 times (1369.3 ms [IQR 1343.7–1409.7 vs. 1207.8 ms [IQR 1194.8–1241.3], p < 0.001) and ECV (36.5% [32.7–37.0] vs. 27.5 [26.3–28.5], p < 0.001) compared to HCs. RV dysfunction (present in 61.1% of PPCM cohort) was associated with significantly higher ECV (37.0% [IQR 36.5–38.4] vs 33.4% [IQR 28.5–37.0], p = 0.05 and higher T1 (1409.0 ms [IQR 1349.0–1443.0] vs. 1311.3 ms [IQR 1299.3–1369.3], p = 0.015) compared to those with preserved RV function. LV fibrosis was not significantly different between PPCM patients with and without RV dysfunction. LGE mass correlated negatively with LVEF and RVEF (r = –0.540, p = 0.001; r = –0.568, p < 0.001), respectively. There was a strong positive correlation between LGE mass and native T1 (r = 0.619, p < 0.001), LGE mass and GLS (r = 0.638, p < 0.001) and moderate correlation with ECV (r = 0.528, p = 0.001).

**Conclusion:** For the first time, we report a high prevalence of myocardial fibrosis in well-phenotyped patients with newly diagnosed PPCM. Increased LGE mass was associated with severe impairments in LV strain, LVEF and RVEF. RV dysfunction was associated with significantly higher ECV and native T1 times.

109059

Modality: E-Poster Young Researcher – Non-case Report

Category: HEART FAILURE/CARDIOMYOPATHY/TRANSPLANT

## Global Prevalence of Mortality and LV Recovery in Women with Peripartum Cardiomyopathy – A Systematic Review and Meta-Analysis

JULIAN HOEVELMANN^1^, Mark E Engel^3^, Elani Muller^2^, Ameer Hohlfeld^4^, Michael Böhm^1^, Karen, Sliwa^2^, Charle André Viljoen^5^

(1) Department of Internal Medicine III, Cardiology, Angiology and Intensive Care Medicine, Saarland University Hospital, Homburg (Saar), Germany; (2) Cape Heart Institute, Faculty of Health Sciences, University of Cape Town, Cape Town, South Africa; (3) Department of Medicine, Groote Schuur Hospital, Faculty of Health Sciences, University of Cape Town, Cape Town, South Africa; (4) South African Medical Research Council, Cape Town, South Africa.; (5) Division of Cardiology, Groote Schuur Hospital, Faculty of Health Sciences, University of Cape Town, Cape Town, South Africa

**Introduction:** Peripartum cardiomyopathy (PPCM) remains a major contributor to maternal morbidity and mortality worldwide. The disease is associated with various complications mainly occurring early during its course. Reported adverse outcomes include decompensated heart failure (HF), thromboembolic complications, arrhythmias and death.

**Purpose:** We aimed to systematically summarize the outcomes of women with PPCM across different geographical regions.

**Methods:** We performed a comprehensive search of all articles published between January 2000 and June 2021 on a number of electronic databases. All cohort, case-control and cross-sectional studies, as well as control arms of RCTs reporting on the in-hospital complications and 6- and/or 12-month outcomes of PPCM were considered eligible.

**Results:** Forty-seven studies (4875 participants across 60 countries) met the eligibility criteria. Hemodynamic and echocardiographic parameters were similar across all continents. In-hospital mortality was reported as 1.9% [95% CI 0.5–4.0] across all regions. About 10% of patients received invasive ventilation, 21.5% inotropic and 3.1% received mechanical support, respectively. Left ventricular (LV) thrombus complicated 9.0% [95% CI 6.5–11.9] of patients and all-cause embolic events occurred in 6.1% [95% CI 3.8–8.9]. All-cause mortality was 8.0% [95% CI 5.5–10.8, I2 = 79,1%) at 6 months and 9.8% [95% CI 6.2–14.0], I2 = 80.48%) at 12 months respectively. Overall, 44.4% ([95% CI 36.2–52.8], I2 = 91.7%) of patients recovered their LV function within 6 months and 58.7% ([95% CI 48.1–68.9], I2 = 75.8%) within 12 months, respectively. The lowest rate of LV recovery was reported by studies conducted in the Middle East (13.6% [95% CI 9.5–18.1], 3 studies), whereas the highest rate of LV recovery was reported for patients from Europe (56.8% [95% CI 38.1–74.7], 6 studies, I2 = 93.3%). All-cause mortality was highest in Africa and Asia/Pacific. Europe and North America reported the highest prevalence of LV recovery. Frequent prescription of beta-blockers, ACE-I/ARB and bromocriptine treatment was associated with significantly lower all-cause mortality and better LV recovery.

**Conclusion:** We identified significant global differences in prescribed treatment, prevalence of in-hospital complications and 6- and 12-month outcomes. Frequent prescription of guideline-directed HF therapy was associated with better outcomes. Timely initiation and up-titration of HF therapy should be strongly encouraged.

109066

Modality: E-Poster Young Researcher – Non-case Report

Category: ACUTE AND CHRONIC CORONARY DISEASE/THROMBOLYSIS

## Cost of Managing Stroke in a Prospective Observational Cohort of 50 Acutely Hospitalized Cases in Maputo Mozambique

IGOR SAMUEL DOBE^1^, Neide Canana^1^, Simon Stewart^4^, Ana Olga Mocumbi^2^

(1) Instituto Nacional de Saúde; (2) Hospital Geral de Mavalane; (3) Universidade Edurdo Mondlane; (4) Torrens University Australia, Australia

**Introduction:** Stroke is an often-deadly condition responsible for substantive great physical, social and economic limitations for survivors and their family globally. In LMIC, Unfortunately, there is a paucity of data describing its economic impact in sub-Saharan Africa. Our study aimed to evaluate the cost of stroke-related management and care during and post-hospitalization in Mozambique.

**Methods:** We performed a prospective, cost-of-illness study (from the provider and patient perspective) of acute presentations of stroke to a first referral urban public hospital in Maputo, Mozambique. From June to December of 2019, 50 consecutively admitted patients were enrolled, data were collected from medical files and through interviews to patients or caregiver using semiquantitative questioner during hospitalization and by home visits in 28-days after discharge. Applying a bottom-up approach, direct and indirect costs were calculated. Data were presented as median, interquartile intervals, except financial data presented also as medians and ranges. All available costs were computed for the year 2019, the year of analysis.

**Results and discussion:** In total 80 patients with acute stroke were admitted to ward during the study, of those, we consecutively recruited 50 patients comprised by 28 women (56%), a median age of 60.5 (38.3; 68.4) years, of whom 22 (44%) were discharged with diagnostic of hemorrhagic stroke. Median hospital stay was 7.0 (4.0; 8.0) days. 10 (20%) patients died within 28-days after discharge. Direct cost (Hospital stay, Medication, laboratory tests), for all cases was US$ 35 302,52 and the average direct cost per patient was US$ 706.05. Indirect cost (travel to hospital, family visits) during hospitalization and within 28-days post-discharge was US$ 22.00 and US$35,58, respectively, loss of productivity was very high in the informal sector, quality of life in all five dimensions were seriously compromised. The cost of stroke in Mozambique is particularly high considering, the relatively young age of affected cases.

**Conclusion:** This study highlights the importance of understanding the costs of stroke for the system and for the patient. Routine register-based data can be used for accurate productivity cost estimates of health shocks.

109098

Modality: E-Poster Young Researcher – Non-case Report

Category: PHYSIOTHERAPY

## Predictors for Indication of Non-Invasive Ventilation in the Postoperative Heart Surgery in Adults

JÉSSICA GONÇALVES DE LIMA^1^, Victoria Maria Garcia de Medeiros^1^, Fernando Gomes de Jesus^1^, Ana Gabriella Arena de Sá^1^, Thaísa Sarmento dos Santos^1^, Mariana de Oliveira Carvalho^1^, Lucas Araujo de Carvalho^1^, Marcus Vinicius de Souza Amaral^1^, Juliana Rega^1^, Claudia Rosa^1^, Mauro Felippe Felix Mediano^1^, Luiz Fernando Rodrigues Junior^1^

(1) National Institute of Cardiology

**Introduction:** Non-invasive ventilation (NIV) has been used for prophylaxis/treatment of pulmonary/respiratory complications during postoperative period (PO) of cardiovascular surgery. However, the indiscriminate use of prophylactic NIV may burden health systems due to the exacerbated use of human and material resources, resulting in a worsening of the standard of care provided.

**Objective:** To identify the predictors for NIV indication in patients in the postoperative period of cardiovascular surgery.

**Methods:** This was a retrospective cross-sectional study. Data from 614 patients submitted to cardiovascular surgery at the Instituto Nacional de Cardiologia (Rio de Janeiro-RJ), from October/2018 to March/2020, was obtained from the Physiotherapy Service database, stored on the Research Electronic Data Capture platform (REDCap) and complemented based on the review of physical records. Preoperative variables such as demographic data, comorbidities, functional class, cardiac function and risk assessment for cardiac surgery were collected. Intraoperative variables were: type/complexity of surgery, fluid balance, time on cardiopulmonary bypass (tCPB), time of aortic clamping and surgical complications. Variables evaluated in the PO were: level of consciousness, pain, water balance, imaging, laboratory and blood gas tests, tracheobronchial secretion, mechanics and pulmonary/respiratory function before and after extubation, extubation attempts and failures, and use of NIV after extubation. The univariate and multivariate logistic regression model (P < 0.05 considered significant) was used to verify the association of possible predictors with the NIV indication in the postoperative period of cardiovascular surgery.

**Results:** The preoperative: age (OR:1.05; P = 0.005); the intraoperative: tCBP (OR:1.01; P = 0.046); and the postoperative variable: partial pressure of carbon dioxide (PCO2) before extubation (OR:1.08; P = 0.045) were independently associated with the need for NIV in the postoperative period of cardiovascular surgery.

**Conclusion:** Age, tCPB and pre-extubation PCO2 are independent predictors of NIV need in patients in the postoperative period of cardiovascular surgery, so that the higher age, tCBP or PCO2, the greater the chances for the patient needing NIV, a finding that may encourage the choice of patients eligible for early intervention with NIV, and the creation of a score/calculator for supporting bedside decision about the use of prophylactic NIV.

109101

Modality: E-Poster Young Researcher – Non-case Report

Category: CARDIOVASCULAR IMAGING

## Protocol for the Safety of Dobutamine Stress Echocardiography in a Large Unselected Population

ISADORA SUCUPIRA MACHADO^1^, Tereza Cristina Pinheiro Diógenes^1^, Marilia Esther Benevides de Abreu^2^, Ana Gardenia Liberato Ponte Farias^2^, Marcia Maria Carneiro^2^, José Sebastião de Abreu^1^

(1) Clinicárdio Métodos Diagnósticos; (2) Cardioexata de Fortaleza

**Background:** Adverse effects and serious complications can occur during dobutamine stress echocardiography (DSE) in a conventional protocol (CP).

**Objective:** To assess the safety of DSE using a modified protocol (MP).

**Methods:** Prospective collection of data from patients undergoing DSE, administering dobutamine in up to four stage. Atropine could be administered from the fourth stage in the CP and, as a routine, concomitant with the beginning of the third stage in the MP. At the end of the exam or to control arrhythmia, metoprolol was administered to the CP and esmolol to the MP. In the event of typical angina, the examiner defined the therapy in the CP, while in the MP, if necessary, a nitroglycerin solution could be infused over a period of three to twelve minutes. A p-value <0.05 was considered statistically significant.

**Results:** Among 17,811 DSEs, 9,121 were included in the MP. Hypertensive peak (1% vs 0.4%; p = 0.0001) and non-sustained ventricular tachycardia (0.6% vs 0.1%; p = 0.0001) were more frequent in CP, while supraventricular tachyarrhythmia (1.9% vs 3%; p = 0.0001) and atrial fibrillation (0.8% vs 1.3%; p = 0.003) in the MP. These arrhythmias reversed spontaneously or with medication. Nitroglycerin was administered in 76 MP cases. Ventricular fibrillation and acute coronary syndrome occurred in CP. There was no acute myocardial infarction, sustained ventricular tachycardia, ventricular fibrillation, cardiac rupture, asystole, or death with MP.

**Conclusion:** The MP for the DSE is a safe option in the application of this methodology.

109102

Modality: E-Poster Young Researcher – Non-case Report

Category: EPIDEMIOLOGY AND HEALTH POLICIES/GLOBAL HEALTH

## Chronic Rheumatic Heart Disease Among Children and Adolescents in Brazil: Study of the Epidemiological Profile of Hospitalizations between 2010 and 2020

MATHEUS AKIRA SUZUKI DE OLIVEIRA^1^, Matheus Akira Suzuki de Oliveira^1^, Júlia de Ávila Gutierrez^3^, Karen Tássia Façanha Ramos^2^, Luiz Felipe Façanha Ramos^2^, Leo Christyan Alves de Lima^4^, Laura Jane França Lacerda^4^, Hildeman Dias da Costa^1^, Mathews Barbosa Santiago^5^, Ayrison de Melo Sousa^5^

(1) Universidade Federal de Rondônia; (2) Universidade Federal do Amapá; (3) Faculdades Integradas Aparício Carvalho; (4) Centro Universitário São Lucas; (5) Centro Universitário Uninorte

**Introduction:** Rheumatic heart disease is caused by damage to the heart valves and muscles that arise from inflammation and scarring caused by rheumatic fever. Rheumatic fever mainly affects children in developing countries, especially where poverty is widespread. About 2% of deaths from cardiovascular diseases in the world are related to rheumatic heart disease.

**Objective:** To describe the epidemiological profile of hospitalizations for chronic rheumatic heart disease among children and adolescents in Brazil between 2010 and 2020.

**Methods:** Cross-sectional epidemiological study. Data were obtained from the information technology department of the Unified Health System – DATASUS. The variables researched were: total hospitalizations, sex, color/race, age group, deaths and mortality rate. The age group surveyed was individuals from zero to 19 years old. The research period was delimited between the months of January 2010 and December 2020.

**Results:** There were 5,909 hospitalizations in the studied period. In 2010 and 2020, 627 and 290 hospitalizations were recorded, respectively. Males reported 3,127 hospitalizations, and females 2,782. The brown color/race registered 2,115 hospitalizations. The most affected age group was 15 to 19 years old, with 2,136 cases. The total number of deaths was 194. The average mortality rate was 3.28.

**Conclusions:** Hospitalizations for chronic rheumatic heart disease show a decreasing curve in recent years in Brazil. The epidemiological profile of hospitalizations between 2010 and 2020 was characterized by male adolescents, brown, aged between 15 and 19 years. The number of hospitalizations in 2020 may have been influenced by the pandemic caused by COVID-19.


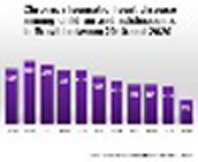


109103

Modality: E-Poster Young Researcher – Non-case Report

Category: CARDIOGERIATRICS

## The Kansas City Cardiomyopathy Questionnaire Evaluation in Elderly Patients with Diabetes and Pre-Diabetes

CAROLINA JERONIMO MAGALHAES^1^, Matheus Dantas Soeiro^3^, Sabrina Barreto Braga Pires^3^, Maria Eduarda Borges Matias^3^, Ellen Beatriz Sobral^3^, Carolina De Carvalho Moury Fernandes^3^, Jessica Myrian de Amorim Garcia^2^, Francisco Bandeira^1^

(1) UNIVERSIDADE DE PERNAMBUCO; (2) HOSPITAL AGAMENON MAGALHAES; (3) FACULDADE PERNAMBUCANA DE SAÚDE

**Introduction:** Diabetes mellitus (DM) and heart failure (HF) often co-exist, and the number of people with both diseases is increasing due to ageing and better treatment. DM contributes to the progression of HF, commonly associated with worsening of symptoms. Quantifying the quality of life (QOL) in HF is extremely important especially in the elderly, in order to maintain good health and personal satisfaction through life.

**Objectives:** Evaluate the QOL of elderly based on the Kansas City cardiomyopathy questionnaire (KCCQ) associated with DM, pre-DM and normoglycemia in a large tertiary center.

**Methods:** A single-center analysis of in-patients aged ≥65 years presenting with HF from August 2020 to January 2022 in a Brazilian cardiology teaching hospital. KCCQ was performed considering QOL 15 days prior to admission. Scores scaled 0–49 represented poor health status, and 50–100 good health status. Based on glycated haemoglobin (HbA1c) the elderly were diagnosed with DM, pre-DM or normoglycemia.

**Results:** A total of 182 patients were studied. The mean age was 73 ± 6 years, 54.3% were men. Study population had 88.8% prevalence of hypertension and 51.5% of chronic kidney disease. The mean level of HbA1c was 6.6 ± 2.0%, minimum of 4.1% and maximum of 14.7%. Based on HbA1c, 45.8% had DM, 23.6% pre-DM and 30.6% normoglycemia. Mean score for KCCQ was 54.6 ± 19.65. QOL was considered good in 52% patients and bad in 48%. There was no association between HbA1c levels and poorer QOL in the elder patients.

**Conclusion:** We found no differences in QOL specific evaluation for HF in elderly patients with or without DM or pre-DM.

109114

Modality: E-Poster Young Researcher – Non-case Report

Category: CARDIO-ONCOLOGY

## Clinical and Epidemiological Profile of Patients Followed in a Specialized Outpatient Clinic of Cardioncology

VINÍCIUS PEREIRA DANTAS^1^, Rafael Alves da Silva^1^, Ademar Alexandre de Morais^1^, Rosiane Viana Zuza Diniz^1^, Kelton Dantas Pereira^1^, Kelson Kemuel Confessor de Sousa^1^

(1) Hospital Universitário Onofre Lopes – HUOL UFRN

**Introduction:** Considering the aging of the population and a common occurrence of risk factors, it is increasingly probable that a patient may have both cancer and cardiovascular disease. The cardiotoxicty is one of the main and most feared complications of cancer treatment. It can manifest itself in several ways including, with heart failure (HF) being the most frequent and most severe. So, oncologist and cardiologists should undertake the therapeutic option for a cancer patient to minimise cardiotoxicity without compromising anticancer efficacy.

**Aim:** Identify the cardiotoxicity risk in cancer outpatients referred to the cardio-oncology clinic in a tertiary hospital and determine the treatment plan at the referral time.

**Methodology:** Cross-sectional, observational and descriptive study, conducted between September 2018 and September 2021, involving 60 adults, at the first visit in a cardio-oncology clinic of a tertiary hospital.

**Results:** The majority of patients were women (68.3%, n = 41), white (35%, n = 21). The mean age was 56 ± 15.5 and the most frequent aetiology was breast neoplasia (40%, n = 24). The levels of high to very high for cardiotoxicity risk were prevalent (71.6%, n = 43). At the first visit, 45 patients (75%) had already started chemotherapy and/or radiotherapy treatment against cancer and 38 (63.3%) of the patients were diagnosed with heart failure.

**Conclusion:** The risk for cardiotoxicity in this population was high, as well as the presence of heart failure and use of anti-neoplastic treatment prior to consultation in a specialized outpatient clinic. The optimal timing and mechanisms for referral need to be studied and implemented. Referral to the specialist in cardio-oncology allows an early recognition of subclinical signs and symptoms, detecting and preventing CV side effects in cancer patients.

109139

Modality: E-Poster Young Researcher – Non-case Report

Category: DYSLIPIDEMIA

## High-Dose of Potent Statins May not be Sufficient to Achieve Therapeutic Goals of Ldlc and Non-Hdl Cholesterol in Patients at Very High Cardiovascular Risk

GISELI CASARINI^1^, Natan Alevato Donadon^1^, Gabriel Tamanaha Pacheco^1^, Rafael Ferreira e Bringel^1^, Sara Regina Alcalde Domingos^1^, André Árpád Faludi^1^, Daniel Branco de Araujo^1^, Natasha Soares Simões dos Santos^1^, Adriana Bertolami^1^, Luiz Paulo Bastos Schmidt^1^

(1) Instituto Dante Pazzanese de Cardiologia

**Introduction:** High-dose of potente statins are first-line and mandatory therapy to reduce LDL-c among very high cardiovascular risk patients. The Brazilian Guideline on Dyslipidemia and Prevention of Atherosclerosis of the Brazilian Society of Cardiology (SBC) recommends a target of LDL-c below 50 mg/dL or non-HDL cholesterol (NHDLc) <80 mg/dL.

**Objective:** Evaluate the amount of very high-risk patients using the initial treatment recommended by the brazilian guideline that achieved the LDL-c and NHDLc therapeutic goals.

**Methods:** This is a cross-sectional observational study that included 1122 very high cardiovascular risk patients (significant atherosclerotic disease with or without clinical events or obstruction ≥50% in any arterial territory), treated in outpatients clinics between January and March of 2022, in a tertiary care hospital in Brazil, using atorvastatin 40 mg or 80 mg daily. Exclusion criteria were: use of atorvastatin in a dose lower than 40 mg/daily or use of other statins. Data from the electronic medical record were collected regarding lipid profile, such as LDL-c and NHDLc, age and sex. RESULTS A total of 1122 patients were evaluated and 1012 were included. Mean age was 68.8 years (SD 9.2), 634 (62.4%) were men. Regarding statin use, 613 (60.6%) patients used atorvastatin 80 mg/daily and 399 (39.4%) used atorvastatin 40 mg/daily. Average LDL-c was 83.1 mg/dL (SD 29.5) and NHDLc was 113.5 mg/dL (SD 35). The mean TC was 152 mg/dL (SD 29.5), HDL-c was 39.5 (SD 11.8) and of TG was 154.9 (SD 85.6). Ninety two (9%) patients had LDL-c <50 mg/dL and 133 (13.14%) patients had NHDLc <80 mg/dL.

**Conclusion:** The low amount of patients in this population that achieved LDL-c and NHDLc target shows that with high-intensity statins monotherapy may not be sufficient. These data suggest that in very high-risk patients, combined lipid-lowering therapy, in the initial phase, should be considered.

109153

Modality: E-Poster Young Researcher – Non-case Report

Category: ANTICOAGULATION

## A Nationwide Service of Tele-Electrocardiography: The Telehealth Network of Minas Gerais

MILENA SORIANO MARCOLINO^1^, Maria Cristina da Paixão^1^, Leonardo Bonisson Ribeiro^1^, Paulo Rodrigues Gomes^1^, Breno Max Horta Melo^1^, Gabriela Miana de Mattos Paixão^1^, Antonio Luiz Pinho Ribeiro^1^

(1) Centro de Telessaúde do Hospital as Clínicas da Unniversidade Federal de Minas Gerais

**Introduction:** Cardiovascular diseases are the main cause of death in Brazil and in many developing low- and middle-income countries (LMIC). Access to specialized cardiac care remains precarious in most of these places.

**Objective:** To describe the experience of the Telehealth Network of Minas Gerais of developing and implementing the Brazilian Nationwide Service of Tele-Electrocardiography.

**Methods:** Remote points, located primarily on primary healthcare units, received a standard computing system including a computer, connected to the internet, and a digital electrocardiogram (ECG). ECG signal is captured by the computer, using in-house developed software, and uploaded to a central server, where they are pre-processed, including automatic ECG measurements. A web-based interface is available for the cardiologists to analyze and interpret ECG exams, including tools for magnifying and re-measuring the ECG waves and intervals and a support system for improving the quality of the report. Quality is regularly audited. This system, initially implemented in the state of Minas Gerais in 2006, has been implemented in other Brazilian states since 2017.

**Results:** The service is available 24 × 7. Emergency exams or those with extreme abnormal automatic findings are prioritized and reported in less than 10 minutes; the others are analyzed in less than 2 hours. Since 2006, over 6 million ECGs have been analyzed and the system is now available in 1199 cities in 12 Brazilian states. The majority of the remote points are in primary care centers, but some are in small hospitals and emergency care units. The system is cost-effective, having generated economy of over R$ 345,000,000 with the reduction of referrals to other cities, and allows access to a basic cardiological exam in places where it was not available. More recently, the dataset of ECGs was organized and linked to mortality data (the CODE study) and is being used in epidemiologic and artificial intelligence studies.

**Conclusions:** Telehealth tools can be used in LMIC countries to provide cost-effective access to high quality ECG diagnosis in large scale. This kind of service can also help to build datasets for relevant research for local communities and the development of advanced artificial intelligence. The availability of these tele-ECG systems may have impact in cardiovascular care in LMIC.

109170

Modality: E-Poster Young Researcher – Non-case Report

Category: CARDIOVASCULAR IMAGING

## Prognostic Value of Stress Cardiac Magnetic Resonance at 3 Tesla in Patients with Known or Suspected Coronary Artery Disease

SOMLUCK PUNAWAKUL^1^, Pairoj Chattranukulchai^2^, Monravee Tumkosit^3^, Yongkasem Vorasettakarnkij^2^

(1) Department of Medicine, Faculty of Medicine, Chulalongkorn University, Bangkok, Thailand; (2) Cardiac center, King Chulalongkorn Memorial Hospital, Thai Red Cross Society, Bangkok, Thailand; (3) Department of Radiology, Faculty of Medicine, Chulalongkorn University, Bangkok, Thailand

**Background:** Adenosine stress CMR is playing an important role in diagnosis of coronary artery disease (CAD). Objectives To assess the prognostic value of adenosine stress CMR at 3 Tesla to predict clinical outcomes during two years of follow-up in patients with known or suspected CAD.

**Methods:** A retrospective cohort study in patients who underwent adenosine stress CMR at 3 Tesla between 1 Sep 2017–31 Aug 2019 at a tertiary hospital in Bangkok, Thailand. The primary outcome includes composite outcomes of CAD-related death, non-fatal MI, and revascularization.

**Results:** Total of 633 patients were analyzed (age 66.21 ± 12.60 years, 53.7% male, 31.9% known CAD). The result of CMR was positive in 215 cases (33.97%), and 118 patients (2.7%) had myocardial scar. We observed 5 CAD-related deaths, 10 non-fatal MI, and 94 revascularizations within 2 years of follow-up. Positive adenosine stress CMR demonstrated a significant association with both primary outcome (43.4% vs. 2.9%, p = <0.001), and secondary outcome (61.4% vs. 25.4%, p = <0.001). The severity of perfusion defect was significantly associated with the primary outcome in both patients with and without myocardial scar groups. Among the patients without scar, primary outcome in the no ischemia, mild/moderate, and severe ischemia group are 2.38%, 16.28%, and 53.03% (p = <0.001), respectively. Among the patients with scar, the primary outcome were 14.29%, 22.73%, and 70.21% (p = <0.001), respectively (Figure 1).

**Conclusions:** In this cohort study, positive adenosine stress CMR at 3 Tesla is a powerful tool to predict clinical outcomes in patients with known or suspected CAD. The severity of myocardial ischemia and the presence of myocardial scar could help identifying high-risk patients. More study Further studies are warranted.


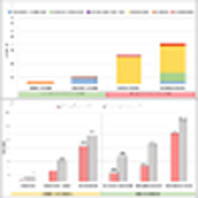


109179

Modality: E-Poster Young Researcher – Non-case Report

Category: CARDIOLOGY OF SPORTS, EXERCISE, ERGOMETRY AND CARDIOVASCULAR REHABILITATION

## Effects on Depression and Anxiety Comparing Resistance Training According to Trained Muscle Group in Low Functional Capacity Heart Failure Patients

JAVIER ELIECER PEREIRA RODRIGUEZ^1^, Jorge Lara-Vargas^2^, Fernando Rivera-Theurel^3^, Magalli Diaz-Bravo^1^, Eduardo Leyva-Valadez^2^, Luis Fernando Ceballos-Portilla^1^, Arizbeth De Jesus-Guerra^1^, Salvador Aguilar-Nava^1^, Sebastian De Marcos-Sanchez^1^, Guadalupe Montserrat-Pineda^1^

(1) Centro de Estudios e Investigación FISICOL. Colombia; (2) Cardio Fit. México; (3) University of Toronto. Canada

**Introduction:** Heart Failure patients (HF) are characterized by decreased functional capacity and quality of life. The prevalence of depression of 25% in newly diagnosed HF and up 50% in advanced stages of HF. Anxiety prevalence in HF patients can up to 40%.

**Objetive:** Determine the difference between strength training of upper versus lower limbs in the depression and anxiety scale of patients with heart failure with low functional capacity within a cardiac rehabilitation program.

**Methods and materials:** Prospective randomized study with 412 cardiac rehabilitation participants comparing 2 groups (Aerobic exercise (60–80%) + Group 1: MMSS Strength (40–60%) and Group 2: MMII Strength (40–60%). An exercise stress test, a 6-minute walk, a 1-repetition maximum test, depression and anxiety with the HAD test were performed. Measurements were done after the 24 sessions of 60 minutes and in the absence of psychological intervention. In the statistical analysis, normality was evaluated by means of Kolmogorov-Smirnov and, subsequently, the analysis of variance ANOVA.

**Results:** 411 finished the intervention and it was possible to determine that both groups improved the depression levels GE1: 21%vs11%; GE2: 27%vs7%; p = 0.001 and anxiety GE1:13%vs7%; GE2:15%vs4%; p = 0.001.

**Conclusion:** Strength training combined with aerobic exercise reduces depression and anxiety in HF.


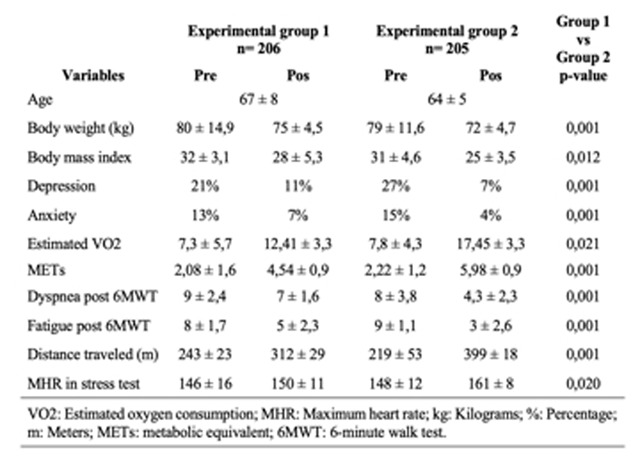


109183

Modality: E-Poster Young Researcher – Non-case Report

Category: CARDIO-ONCOLOGY

## Carvedilol or Exercise Training: What is the Best Approach to Prevent Baroreflex During Doxorubicin Treatment?

FILIPA DANIELA COSTEIRA MACHADO^1^, Ana Afonso^1^, Filipa Machado^1^, Ângela Amaro-Leal^1^, Miguel Meira-Cruz^1^, Isabel Rocha^1^, Vera Geraldes^1^

(1) Faculdade de Medicina e Centro Cardiovascular da Universidade de Lisboa

Doxorubicin (DOX) is a highly effective anticancer drug, but its effectiveness is offset by dose-related side effects, including autonomic dysfunction and cardiotoxic effects. Exercise training (ET) and β-blockers have been suggested to prevent cardiotoxicity. ET reduce sympathetic activity and increase parasympathetic activity. Beta-blockers reduce sympathetic activation and improve cardiac function. Despite extensive research it is not yet clear which therapeutic regimen is best suited to prevent or reduce DOX side effects. In our work, we compare the efficacy of two approaches: carvedilol (CVD) and a non-pharmacological intervention, an exercise protocol (ET) with treadmill training in an animal model of DOX. Female Wistar rats were divided into 4 groups: Doxorubicin (DOX; ip cumulative dose 16 mg/kg, 1 time/week, for 4 weeks), DOX with ET (DOX+EX;treadmill, 25 cm/sec for 30 min, 5 times/week), DOX with CVD (DOX+CVD:10 mg/kg, orally, 5 times/week, for 4 weeks) and controls (CTL ip with saline). At the end of the protocol, animals were anaesthetized, and blood pressure (BP), electrocardiogram, heart rate (HR) and respiratory rate (RF) were recorded. Baroreceptor and chemoreceptor reflexes were tested, and baroreflex gain and sensitivity, and chemoreflex sensitivity were calculated. HRV was determined to estimate sympathetic and parasympathetic activity. Our results show that DOX treatment induced a significant decrease in systolic BP (CTL:150.1 ± 5.338; DOX:103.1 ± 10.19 mmHg, p = 0.0127) and mean BP (CTL:129.0 ± 3.520; DOX 90.38 ± 10.08 mmHg, p = 0.0072), and in HR (CTL:379.5 ± 54.34; DOX:288.3 ± 53.11 bpm, p = 0.0284), compared with the CTL group. The ET protocol normalises systolic BP (122.4 ± 29.85 mmHg), mean BP (94.51 ± 20.08 mmHg) and HR (350.4 ± 22.18 bpm). Administration of CVD during DOX treatment significantly restored HR to near-physiological levels (368.6 ± 16.23 bpm p = 0.0359). DOX treatment resulted in an increase in baroreflex gain compared to the CTL group. CVD treatment, similar to the ET effect, decreased baroreflex gain compared with the DOX group (DOX:4.738 ± 2.771; DOX+EX:0.5046 ± 0.06773; p = 0.0473 and DOX+CVD:0.5516 ± 0.05672 bpm/mmHg; p = 0.0376) and also restored BEI, BRS and SDNN to normal values. No significant changes in chemoreflex sensitivity, RF and frequency domain indices were observed. Overall, these results suggest that CVD and ET improve baroreflex through HR modulation. However, ET can be considered the best strategy as it acts on both autonomic branches.

109234

Modality: E-Poster Young Researcher – Non-case Report

Category: NUTRITION

## Effect of Ketogenic Diet Vs. Recommended Diet on Body Composition and Muscular Functional Capacity in Obese Individuals

JOÃO MOTARELLI^1^, Juliana Tieko Kato^1^, Marilia Andrade Papa^1^, Maria Cristina de Oliveira Izar^1^

(1) Universidade Federal de São Paulo

**Introduction:** The reduction of body mass (BM) in obese individuals has been considered a modifiable risk factor for cardiovascular diseases (CVD) morbidity and mortality. However, the potential benefits of ketogenic diet in BM reduction may be compromised due to the reduction off strongh predictors of CVD such as lean mass (LM) and muscle strength, which may inadvertently affect the individual’s muscular functional capacity (MFC).

**Objective:** To evaluate changes in body composition and MFC in physically inactive obese patients undergoing one of two different low-calorie diets over a 6-month period.

**Methods:** We included 25 physically inactive patients with grade I or II obesity, of both genders, with a mean age of 40 years, followed for 6 months through periodic medical and nutritional care, allocated to the Dukan’s Diet (DD) group (n = 11) who received the nutritional recommendations specified by the Dukan Diet (ketogenic diet) or the Recommended Diet (RD) (n = 14), who received nutritional guidelines based on the 2014 Food Guide for the Brazilian Population. Double absorbance x-ray (DEXA) was used for analysis of LM, and Body Fat (BF); Biodex® Multi-joint System 3 isokinetic dynamometer was used for MFC analysis of the knee extensor muscles (Peak torque at 60°/s – PT and its variable corrected by BM – PT/BM). Food intake was quantified via 3-day dietary recall and ketosis analyzed through urine stripes. Analyzes were performed at baseline, 05, 30, and 180 days.

**Results:** Significantly higher consumption of protein and lower consumption of carbohydrates and fiber were observed in the DD group compared to RD as the presence of ketosis in urine (in 05 and 30 days). At the end of the study, it was observed that both interventions promoted significant reductions in BM (DD P < 0.001; DR P < 0.001) and BF (DD P < 0.001; RD P < 0.001), however, only DD presented significant reductions of LM (P < 0.031), PT (P < 0.001), PT/BM (P < 0.001). Comparison between groups at 180 days showed significantly lower measures for BF (P < 0.031) in the DD group.

**Conclusion:** DD promoted more expressive reductions in BF and LM at the expense of a loss of MFC even with a high protein intake. Longer-term studies are needed to understand whether the reduction in MFC could lead to functional impairment and/or increased risk for CVD in this population as a result of the practice of this diet.

109235

Modality: E-Poster Young Researcher – Non-case Report

Category: CARDIOLOGY OF SPORTS, EXERCISE, ERGOMETRY AND CARDIOVASCULAR REHABILITATION

## Preoperative Lung Cancer Patient Risk Assessment with Cardiopulmonary Exercise Testing

BIANCA FELDMAN^1^, Rodrigo Mazza^1^, Bruno Dias^1^, Gabriel Moraes^1^, Joao Magalhães^1^, Carlos Gil Ferreira^3^, Tatiane Montella^3^, Anderson Nassar^2^, Gustavo Nobre^2^, Marcelo Kalichsztein^2^, Leandro Toledo^1^, Fabricio Braga^1^

(1) Laboratório de Performance Humana(LPH); (2) Casa de Saúde São José (CSSJ); (3) Oncoclínica

**Background:** The role of aerobic capacity as a predictor of surgical risk (SR) has been highlighted, especially in cancer patients. Cardiopulmonary exercise testing (CPET) is the gold standard in this assessment.

**Objective:** To characterize and identify risk predictors among lung cancer (LC) patients evaluated with the CPET.

**Materials and methods:** Cross-sectional analysis of LC patients who underwent CPET as part of the SR assessment for lung resection. Before CPET, all patients performed rest spirometry to determine forced vital capacity (FVC) and end-expiratory volume in the first second (FEV1). SR was classified by an algorithm based on peak exercise VO2 (pVO2) and the slope of the ratio between minute ventilation (VE) to carbon dioxide (VCO2) output (VE/VCO2 slope) in low, medium, high and very high SR. A multivariate logistic regression model (MLRL) was created to identify predictors of low SR.

**Results:** Ninety-eight patients were analyzed (44.9% men, 71 ± 9.1 years). The mean pVO2 was 16.7 ± 4.2 ml/kg/min (77.9 ± 17% of the predicted values), and the mean VE/VCO2 was 36.6 ± 6.4. The FVC and FEV1 were 2.66 ± 0.8 L and 2.08 ± 0.65 L, respectively. According to the SR, 38 (38.6%), 31 (31.6%), 26 (26.5%) and 3 (3.1%) were classified as low, moderate, high and very high, respectively. Low SR patients were younger (67.8 +/– 9.3 vs. 73.1 ± 8.6 years [p = 0,005]), showed a higher FVC (2.87 ± 0.88 vs. 2.52 ± 0.73 [0.043]) and FEV1 (2.34 ± 0.70 vs. 1.89 ± 0.55 L [p = 0.001]), and trended to a lower COPD prevalence (25 vs. 43.5% [0 = 0.075]) than other risk patients. In the MLRL, age (OR = 0.93 [95% CI 0.88–0.98]) and FEV1 (OR = 24.4 [95% CI 2.2–266.3]) were independently associated with a low SR.

**Conclusion:** (1) More than 60% of lung cancer patients are not at low risk when assessed by CPET; (2) advanced age and reduced FEV1 increased the odds of a higher SR. These data can guide prehabilitation strategies to reduce perioperative morbidities for LC patients.

110340

Modality: E-Poster Young Researcher – Non-case Report

Category: CARDIAC ARRHYTHMIAS/ELECTROPHYSIOLOGY/ELECTROCARDIOGRAPHY

## Pulmonary Vein Reconnection Patterns in Atrial Fibrillation Catheter Ablation Redo Procedures: Single-Center, Cross-Sectional Study

GABRIEL ODOZYNSKI^1^, Isabella Bianco^3^, Helcio G. Nascimento^1^, Andrei Lewandowski^1^, Maurício L. Spessatto^1^, Clóvis Froemming^1^, Grazyelle Damasceno^1^, Alexander Dal Forno^1^, André Pacheco Silva^1^, André d‘Ávila^1^

(1) Hospital de Cardiologia SOS Cardio; (2) Instituto de Cardiologia de Santa Catarina; (3) Universidade do Sul de Santa Catarina

**Introduction:** Different electro-anatomic recurrence patterns were previous described. Yet, pulmonary vein (PV) electric reconnection to left atrium is the typical finding after atrial fibrillation (AF) catheter ablation recurrence.

**Aims and Method:** We aim to evaluate AF ablation recurrence patterns in a cross-sectional, single-center study of recurrent AF patients who had underwent to a 2nd ablation procedure (REDO).

**Results:** Of 47 enrolled patients to REDO, 31 patients had paroxysmal AF (≤7 days-PAF) and 16 patients persistent AF (>7 day ≤1 year –AF-Per). PV reconnection were confirmed in 21 (68%) and 16 (62%) patients of PAF and AF-Per. From 113 targeted PVs in PAF and 58 in AF-Per groups– 35 (30%) and 18 (31%) of PVs were reconnected, respectively. 10 (32%) PAF and 6 (37%) in AF-Per patients have maintained PV isolation during REDO procedure. 6 (19%) and 3 (18%) patients had 1 reconnected PV, 11 (35%) and 6 (37%) had 2 reconnected PVs in PAF and AF-Per groups. Only 2 patients in PAF and 1 in AF-Per have presented all PVs reconnected during REDO procedure (2 left common trunk). Right PVs were reconnected in 11(61%) of PAF patients. Cavo-tricuspid isthmus flutter ablation were performed in 6 patients in PAF group. Left atrial appendage was electrical isolated in 1 AF-Per patient during REDO procedure. At the first procedure, left atrium posterior wall ablation was performed in 6 (19%) and 8 (50%) of PAF and AF-Per. The posterior wall reconnection was confirmed in 2 AF-Per procedure. Left common trunk were reconnected in 2 (6%) and 2 (12%) in PAF and AF-Per.

**Conclusion:** PV reconnection is the typical finding in patients with recurrent AF after catheter ablation. Right PVs reconnection was the common described pattern.


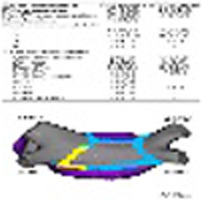


109269

Modality: E-Poster Young Researcher – Non-case Report

Category: ATHEROSCLEROSIS/CARDIOVASCULAR RISK FACTORS/CARDIOVASCULAR PREVENTION

## Differences in the Compositions of the Gut Microbiota in Patients with Acute Myocardial Infarction in the Presence or Absence of Type 2 Diabetes Mellitus

JULIANA TIEKO KATO^1^, João Henrique Fabiano Motarelli^1^, Andrey Santos^2^, Mario José Abdala Saad^2^, Maria Cristina de Oliveira Izar^1^, Carolina Nunes França^1^, Henrique Andrade Fonseca^1^, Francisco Antonio Helfenstein Fonseca^1^

(1) Universidade Federal de São Paulo; (2) Universidade Estadual de Campinas

**Introduction:** There is clear evidence that the composition of the gut microbiota (GM) plays an important role, not only in extracting energy from food, but also as a key endocrine organ, producing metabolites that impact the pathogenesis of type 2 diabetes mellitus (DM). Despite advances in metagenomics techniques, it is still difficult to establish a microbial signature in DM due to numerous confounding factors, such as the association with other pathologies, diet, drug and geographic location.

**Objective:** To identify changes in the GM composition in individuals with DM or without DM [prediabetic (PDM) and non-diabetic (NDM)] in the first 24 hours of acute ST elevation Myocardial Infarction.

**Methods:** Eighty-five patients were included, metagenomic analysis was performed through stool using a technique that is based on DNA extraction and amplification of the 16S rRNA gene. Data were processed by the Miseq Illumina platform and analyzed using the Illumina 16S Metagenomics software. Other analyzes were performed using the SPSS version 21. The significance level was p < 0.05, and when necessary, the p-values of the fecal samples were adjusted using the False Discovery Rate (FDR) multiple comparison test. Subjects were divided into groups according to glycated hemoglobin values, <5.7% (NDM), 5.7 to 6.5% (PDM) and >6.5% (DM).

**Results:** Relative abundance analysis showed significant differences between the phyla Firmicutes (P = 0.05) and Verrumicrobia (P = 0.02) and the genus Akkermansia (P = 0.03) between the NDM vs DM groups. When the values were adjusted by FDR, we found a significant increase in the phylum Firmicutes (P-FDR = 0.035) and class Clostridia (P-FDR = 0.041), as well as a decrease in the phylum Verrucomicrobia (P-FDR = 0.035) and its class Verrucomicrobiae (P-FDR = 0.045) in NDM when compared to DM.

**Conclusion:** The present study showed that the composition of the intestinal microbiota differed between the NDM vs DM groups after acute myocardial infarction, with a duality in the results, where an increase (Firmicutes and Clostridia) and a decrease of bacteria considered beneficial (Verrucomicrobia) were observed.

109255

Modality: E-Poster Young Researcher – Non-case Report

Category: HEART FAILURE/CARDIOMYOPATHY/TRANSPLANT

## Application of the Prisma Methodology in a Systematic Study on the use of Radiofrequency Ablation as an Alternative for the Treatment of Obstructive Hypertrophic Cardiomyopathy

ANA LÍGIA VALERIANO DE OLIVEIRA^1^, Ana Lígia Valeriano de Oliveira^1^, Andressa Pimentel Afiune^1^, Jhenefr Ribeiro Brito^1^, Vitória Lorrane dos Santos^1^, Thais Aratk Marques Taia^1^, Danilo Borges de Sousa^1^, Anna de Paula Freitas Borges^1^, Andressa Morgado Parreira^1^, Lucas Eduardo Almeida França^1^

(1) Pontifícia Universidade Católica de Goiás

Obstructive hypertrophic cardiomyopathy is a genetic disease capable of causing changes in the structure and function of the myocardium muscle. Hypertrophy results in left ventricular obstruction and symptoms of heart failure, exertional syncope, or sudden cardiac death. Radiofrequency myocardial septal ablation is a treatment option for symptomatic and drug-refractory cases. It is a less invasive method and can be used for septum reduction in patients at high risk for surgeries such as myectomy and alcohol ablation. The study seeks to evaluate the feasibility, efficiency and outcome of treatment with radiofrequency ablation in obstructive hypertrophic cardiomyopathy. This is a systematic review based on the methodological recommendations Preferred Reporting Items for Systematic Reviews and Meta-Analyses – PRISMA. The identification and selection of articles was conducted by two independent researchers between October and December 2021 on the platforms PubMed, Scientific Electronic Library Online and Latin American and Caribbean Literature. The filters used were “human” and “published in the last 10 years”. The included studies were in English and Portuguese. The descriptors used were obstructive hypertrophic cardiomyopathy, treatment and radiofrequency ablation that were associated with the Boolean operator AND. We found 66 articles, after applying the inclusion and exclusion criteria, we selected 10 articles that were used in this review. The studies analyzed showed a reduction in the left ventricular outflow tract gradient, septum thickness, mitral regurgitation volume and pro-BNP. Furthermore, ablation showed the ability to suppress ventricular tachycardia in all patients. There was improvement according to the NYHA functional class in four articles, with statistical significance. It was analyzed in a study that the procedure, combined with imaging techniques with a mixture of intracardiac echocardiography and CARTOSound, enabled a more effective mapping of the affected area and avoided damage to the conduction tissue. Finally, the radiofrequency septal ablation technique proved to be a safe and less invasive option, with a shorter hospital stay. Radiofrequency septal ablation allows a significant reduction in the left ventricular outflow tract gradient, in addition to improving the NYHA functional class, thus being an alternative to alcohol septal ablation and myectomy, but later studies comparing it with others invasive methods become necessary.

109270

Modality: E-Poster Young Researcher – Non-case Report

Category: NURSING

## Driveline Dressings Used in Patients with Heartmate and Their Relationship with Local Complications up to 30 Days Post-Operative

RAFAELA BATISTA DOS SANTOS PEDROSA^1^, Natalia Balestra^2^, Roberta Cunha Matheus Rodrigues^1^, Adryel Vieira Caetano da Silva^2^

(1) Faculdade de Enfermagem da Universidade Estadual de Campinas; (2) Hospital Sírio-Libanês

**Introduction:** Driveline infections are common in patients with left ventricular assist devices and an important cause of morbidity and mortality. Nurses play a fundamental role in the care of the driveline, however, scientific evidence regarding dressing standardization is lacking.

**Objective:** To describe the categories of dressings used in the driveline of patients using the HeartMate (HM) device and the incidence density of local complications (infection, bleeding and pressure injuries) within 30 days of the postoperative period.

**Methods:** This was a retrospective cohort of patients hospitalized in the Intensive Care Unit (ICU) after HM II and III implant surgery in a private hospital in the state of São Paulo, Brazil, between 2015 and 2021. The data source was the hybrid medical records of the participants. We performed a descriptive analysis and Fisher’s exact test and statistically significance level of 5% was considered. Incidence density was calculated for infection, bleeding and pressure injury. The study project was approved by the local Ethics Committee (No. 4,593,315).

**Results:** The results showed a high variability of dressings with 22 different categories being recognized and for each participant, an average of 2.2 ± 1.0 dressings. The dressing categorized as C6 (Chlorhexidine, Excilon, Gauze and IV3000) was the most used (45.4%), followed by C13 – ChloraPrep, Biatain Ag, Gauze and IV3000 (31.8%) and C9 – Protosan, Excilon, Gauze and IV3000 (22.7%). Bleeding was more frequent in categories C6 (9.0%), C11 – ChloraPrep, Gaze, Excilon and IV3000 (9.0%) and C13 (9.0%), while pressure injury was identified in only two categories. Subjects using FlexiTrak stabilizer had the highest rate of bleeding (50.0%) and those using Hollister had the highest infection rate (61.1%) and pressure injuries (11.1%). The participants who presented bleeding had higher median values of cardiopulmonary bypass time (112.5 minutes) when compared to those without bleeding (100 minutes); p = 0.029. Infection incidence density was the highest, 43.6/1,000 person-days, mainly in men (55.5/1,000 person-days), using the Heartmate III (48.3/1,000 people-day) and who used two or more categories of dressings (47.2/1,000 person-days).

**Conclusion:** The infection was the complication with the highest incidence density; the findings point to a standardization with the use of chlorhexidine, absorbent foam impregnated with silver and transparent film, as well as the use of a stabilizer.

109282

Modality: E-Poster Young Researcher – Non-case Report

Category: ATHEROSCLEROSIS/CARDIOVASCULAR RISK FACTORS/CARDIOVASCULAR PREVENTION

## Differences in the Composition of the Intestinal Microbiota in Patients with St-Segment Acute Myocardial Infarction

JULIANA TIEKO KATO^1^, João Henrique Fabiano Motarelli^1^, Andrey Santos^2^, Mario José Abdala Saad^2^, Maria Cristina de Oliveira Izar^1^, Carolina Nunes França^1^, Henrique Andrade Fonseca^1^, Francisco Antonio Helfenstein Fonseca^1^

(1) Universidade Federal de São Paulo; (2) Universidade Estadual de Campinas

**Introduction:** The study of the gut microbiota (GM) in coronary artery disease is an emerging area, because metabolites generated by GM seem to be involved in the progression of the disease, with the proatherosclerotic metabolite N-oxido-trimethylamine (TMAO) being the best described. Currently GM is considered a potential therapeutic target, but few studies have evaluated the changes and influence of GM composition in post-acute myocardial infarction.

**Objective:** To identify changes in the GM composition of individuals in the first 24 hours (baseline), 30 and 180 days after ST-segment elevation acute myocardial infarction.

**Methods:** 173 patients were included and 355 stool samples were sequenced, the metagenomic analysis was performed using the technique that is based on DNA extraction and amplification of the 16S rRNA gene, the data were processed by the Miseq Illumina platform and analyzed using the Illumina software 16S Metagenomics. Other analyzes of non-parametric variables were performed using Friedman test for paired samples, using SPSS 21 version. TMAO levels were measured in urine by liquid chromatography with a mass spectrometer from 23 subjects. The significance level was p < 0.05, and when necessary the p-values of the fecal samples were adjusted by the False Discovery Rate (FDR) multiple comparison test.

**Results:** The analysis between times showed a more inflammatory profile at baseline when compared to 30 days, with a decrease in beneficial bacteria belonging to the phylum Bacteroidetes (P-FDR = 0.04) and genus Faecalibacterium (P = 0.04), in addition to the increase in Proteobacteria considered to be pro-inflammatory bacteria (P-FDR = 0.04). However, over time, GM showed an improvement in 180 days when compared to baseline, with a significant increase in bacteria producing butyrate, a short-chain fatty acid with a crucial role in human health, phylum Bacteroidetes (P = 0.01), family Lachnospiraceae (P-FDR = 0.04) and genus Faecalibacterium (P < 0.01), in addition to the increase in Prevotella (P = 0.02). Interestingly, other important analyzes of the composition of GM, showed a worsening of richness and diversity decreased at 30 and 180 days when compared to baseline, in addition to the increase of TMAO over time (P = 0.03).

**Conclusion:** A more inflammatory profile was observed at baseline with improvement at 180 days after infarction, however TMAO increased over time, accompanied by a decrease in richness and diversity.

109304

Modality: E-Poster Young Researcher – Non-case Report

Category: HEART FAILURE/CARDIOMYOPATHY/TRANSPLANT

## The Time between the Emergency Room Admission, Diuretic Infusion, and the Readmission Risks Among Patiens with Decompensated Heart Failure

NICHOLLAS COSTA ROSA^1^, Nichollas Costa Rosa^1^, Maria Antonieta Moraes^1^

(1) Programa de Pós Graduação em Ciências da Saúde: Cardiologia da Fundação Universitária de Cardiologia

**Introduction:** The treatment of Heart Failure (HF) with diuretics substantially impacts this syndrome’s prognostic. Data suggest that patients at a higher risk of furosemide intake in the early stages of the disease can decrease the mortality rates.

**Objetivos:** Monitor the time between the patient’s admission into the emergency room and were administered the first dose of diuretics among patients with HF and their risk of readmission within 30 days of hospital discharge using the LACE.

**Methods:** Prospective and retrospective cohort study conducted with decompensated HF patients, supported by echocardiographic data, of both sexes with ≥18 years old, treated in a cardiology emergency (ES) from August 2018 to December 2018. 2019. The parameters used were diuretic infusion time, length of stay in the ES, and readmissions. The risk of readmission was classified using the LACE score as low (0 to 4), moderate (5 to 9), and high (>9) risk. The Chi-square test check for categorical variables. The Mann-Whitney test for the time spent in the SE and time of diuretic infusion.

**Results:** We analyzed 361 medical records of mostly male patients (56%), aged 68 ± 12 years. There was a predominance of ischemic HF (49%), reduced ejection fraction (57%), functional class III (73%), and hemodynamic profile B (81%). After hemodynamic stabilization (60%) were discharged, and (39%) required 6 (4–12) days of hospitalization. The time between admission and infusion of the diuretic was 87 (60–128) minutes. Of these, 26.3% infused up to ≤60 and 73.7% in >60 minutes. The 40 mg dose of diuretic was used in 72% of them. There was a significant relationship between receiving a diuretic for ≤60 minutes and staying in the ES < 6 hours (early discharge), p < 0.05. The LACE score showed that 62% of patients had a moderate risk of unplanned readmission within 30 days. Patients at high risk for readmission (37%) were associated with altered pulmonary auscultation and the presence of jugular swelling (p < 0.05). After 6 months, 15% of the patients died, 9% lost contact and 75% were followed up. Of these, 37% were readmitted, 21% within 30 days and 46% within 180 days.

**Conclusion:** The time between admission and intravenous infusion of diuretics in this population with acute HF was sub-optimized; however, patients who received the drug earlier were in the emergency department for a shorter time. The risk of readmission through LACE was effective in 34% of patients.

109307

Modality: E-Poster Young Researcher – Non-case Report

Category: HEART FAILURE/CARDIOMYOPATHY/TRANSPLANT

## Prevalence of Genetic Alterations in Individuals with Cardiomyopathies Without Defined Aetiology in a Tertiary Hospital

DIOGO MACIEL SILVA AZEVEDO^1^, Diogo Maciel Silva Azevedo^1^, Kelson Kemuel Confessor de Sousa^1^, Fabio Mastrocola^1^, Rosiane Viana Zuza Diniz^1^

(1) Hospital Universitário Onofre Lopes – HUOL

**Introduction:** Cardiomyopathies are a heterogeneous group of structural and functional disorders of the heart muscle, with different etiologies and phenotypic expressions, in the absence of congenital, valvular, coronary diseases or arterial hypertension compatible with the myocardial alterations found. The diversity of clinical presentation makes the etiological definition difficult, and pathogenic genetic alterations are important in this definition.

**Objective:** To determine the prevalence of genetic alterations in individuals with cardiomyopathies without defined aetiology in a tertiary hospital.

**Method:** This is a retrospective, observational and longitudinal study involving 39 individuals with cardiomyopathy without an initially clarified diagnosis, inhospital and outhospital patients, follow-up in a tertiary hospital during the years 2020 to 2022, with identification of the genetic profile and phenotype of presentation assessed by echocardiographic parameters. Data were presented as means and proportions.

**Results:** The mean age was 45.7 years+–14.5 years, with an even distribution regarding gender (51% men). The prevalence of positivity in the genetic test was 33.3% and the most frequent presentation phenotype was hypertrophic cardiomyopathy (53%), followed by dilated cardiomyopathy (33.3%). Among the 21 individuals with hypertrophic cardiomyopathy, 47% had a positive genetic test, with the MYBPC3 and MYH7 genes being the most frequently identified in pathogenic or probably pathogenic mutations, representing 70% of positive cases.

**Conclusion:** The prevalence of genetic alterations in our population was high, especially in those with a hypertrophic phenotype, reinforcing the importance of determining the genetic profile and tracking family members in these patients.

109309

Modality: E-Poster Young Researcher – Non-case Report

Category: CARDIOVASCULAR IMAGING

## Histopathological Changes in Endocardial Layer is Related to Myocardial Perfusion Defects in Chronic Chagas’ Cardiomyopathy

CAMILA GODOY FABRICIO^1^, Denise Mayumi Tanaka^1^, José Antonio Marin-Neto^1^, Luciano Fonseca Lemos de Oliveira^2^, Alessandra Arantes de Resende^1^, Fernando Fonseca França Ribeiro^1^, Alexandre Todorovic Fabro^1^, Sabrina Setembre Batah^1^, Jorge Mejia Cabeza^3^, Minna Moreira Dias Romano^1^, André Schmidt^1^, Marcus Vinícius Simões^1^

(1) Medical School of Ribeirao Preto – University of Sao Paulo, Sao Paulo, Brazil; (2) Physiotherapy Department – Federal University of Minas Gerais, Brazil; (3) Hospital Israelita Albert Einstein, Sao Paulo, Brazil

**Background:** Chronic Chagas’ cardiomyopathy (CCC) have showed, in previous studies, a high prevalence of microvascular myocardial perfusion defects (MPD) that may participate in the myocardial damage process. However, there is a scarcity of studies correlating the histopathological (HISTO) changes with MPD in CCC.

**Purpose:** We aimed at testing the hypothesis that MPD is more closely related to HISTO changes occurring in the endocardial layer in an experimental model of CCC in hamster.

**Methods:** We investigated 23 female hamsters infected with 3.5 × 104 trypomastigote forms of T. cruzi, and 6 control non-infected. Animals underwent rest high-resolution 99mTc-Sestamibi SPECT myocardial perfusion scintigraphy, and rest transthoracic echocardiogram. The area of MPD was assessed through the calculation of polar maps by using Munich Heart® software. MPD was defined by areas with 99mTc-Sestamibi uptake <50% of the maximum pixel uptake value. After euthanasia, counting mononuclear cells for inflammation was performed by Weibel reticule in Hematoxylin-Eosin staining. Fibrosis was quantified by fraction area method using Red-Picrosirius staining. The analysis was topographically performed at three left ventricular myocardial wall layers: endocardial, mesocardial and epicardial. A total area of HISTO parameters was also computed, by summing up the respective areas of all myocardial layers.

**Results:** Fourteen infected animals exhibited rest MPD (61%), and presented, in comparison to animals without MPD and to controls higher inflammation in total (1.09 ± 0.55, 0.93 ± 0.43, 0.41 ± 0.32, respectively; p = 0.03), endocardial (1.05 ± 0.50, 0.75 ± 0.44, 0.38 ± 0.22, respectively; p = 0.02), mesocardial (0.64 ± 0.44, 0.55 ± 0.33, 0.11 ± 0.08, respectively – p = 0.02) and epicardial layers (1.57 ± 0.97, 1.50 ± 0.73, 0.73 ± 0.89, respectively; p = 0.03). However, only the inflammation in endocardial layer presented positive correlation with individual values of MPD area (R = 0.55; p < 0.01). In addition, the total fibrosis area presented no difference between animal groups, but the endocardial layer presented a larger fibrosis area in the group with MPD (0.67 ± 0.18) compared to controls (0.45 ± 0.15; p = 0.03).

**Conclusion:** Our results indicate a close relationship between MPD and HISTO changes in the endocardial layer of left ventricular myocardium, consistent with the notion that endocardial is the myocardial region more sensitive to the effects of myocardial ischemia in CCC.

109311

Modality: E-Poster Young Researcher – Non-case Report

Category: CARDIOVASCULAR SURGERY

## Degree of Functional Independence in Patients Submitted to Cardiovascular Surgery After Discharge from Intensive Care

PAULINE ELOISE MARIANI^1^, Marco Antonio Vinciprova Dall Agnese^2^, Grasiele do Amaral Martins^2^, Maria Antonieta Pereira de Moraes^1^

(1) Instituto de Cardiologia/Fundação Universitária de Cardiologia – IC/FUC; (2) Universidade Federal de Ciências da Saúde de Porto Alegre – UFCSPA

**Introduction:** The functional capacity and degree of independence of patients undergoing cardiac surgery can be compromised by the length of stay in the Intensive Care Unit (ICU).

**Objective:** To analyze the behavior of functional independence domains in patients undergoing cardiovascular surgery and its correlation with the length of stay in the ICU.

**Methods:** Prospective cohort study, conducted with patients undergoing coronary artery bypass graft surgery and valvular surgery alone or in combination aged ≥50 years. Outcomes: self-care, work ability, mobility and eliminations were evaluated using the Barthel Index and the Karnofsky Scale at hospital discharge and at 90 days by telephone.

**Results:** Preliminary analysis of 172 surgical patients showed a predominance of men (70%), hypertensive (58%), dyslipidemic (43%), smokers (37%), aged 63 ± 11 years. Coronary artery bypass graft surgery was the most prevalent (57.6%), with cardiopulmonary bypass time of 82 ± 31 minutes. The length of stay in the ICU among all patients was 6 ± 2 days. The Barthel index showed that the functional impairment was lower at discharge (86%) and 90 days (64%), that is, they showed greater independence right after the intervention and after 3 months they were a little more dependent. The Karnofsky scale showed that the degree of dependence was similar at discharge and at 90 days after the intervention, 76% and 78% respectively. The most prevalent domains of functional status decline were self-care 11.4 ± 1.6 and mobility 15.8 ± 2.6. However, a very expressive inverse correlation was observed (r –0.152 and p < 0.05) with statistical significance, regarding the functional impairment after discharge from the ICU and at the 90 days of follow-up.

**Conclusion:** Partial results indicate that there is a reduction in behavior in all domains of functional independence and an inverse correlation between the length of stay in the ICU and the functional impairment of these patients. Thus, long periods of stay in the intensive care unit seem to be inversely associated with the development of functional impairment in relation to an individual’s basic daily activities.

109313

Modality: E-Poster Young Researcher – Non-case Report

Category: NUTRITION

## Acute Ingestion of Nitrate-Rich Beetroot Juice Reduced Blood Pressure and Inhibited Platelet Aggregation in Healthy Male Individuals

JEFFERSON FERNANDES EVANGELISTA^1^, Renata Alves^1^, Thaiane Lopes da Silva^1^, Juliana Silva Figueredo^1^, Cristiane Matsuura^1^

(1) Universidade do Estado do Rio de Janeiro

**Introduction:** Beetroot is a functional food that has been gaining prominence due to its cardioprotective effects, in part attributed to its high nitrate content. Its effects are associated to the reduction of nitrate to nitric oxide, an important molecule involved in vascular homeostasis.

**Aims:** To examine the effects of acute beetroot juice (BJ) ingestion on platelet aggregation, blood pressure (BP), and the expression of proteins involved in platelet activation in healthy adult men.

**Methods:** Nine men (28 ± 6 yr old, 78 ± 8 kg, 1.77 ± 0.05 m), eutrophic and normotensive participated in the study. The experimental procedure consisted of only one visit in the morning. After 15 min of rest, BP was measured and blood was collected to measure platelet aggregation and protein expression by Western Blot. After that, the subjects ingested 500 mL of BJ (~400 mg of nitrate). After 2.5 h, a new measurement of BP was performed, as well as blood collection. Platelet aggregation was measured in washed platelets, platelet-rich plasma and whole blood using collagen (5 µg/mL) and ADP (10 µM) as agonists. Paired Student’s t test was used to compare pre- and post-BJ conditions.

**Results:** Acute BJ ingestion reduced ADP-induced platelet aggregation in platelet-rich plasma (–29%), but was not altered when induced by ADP and collagen in whole blood and washed platelets, as well as collagen in PRP. Regarding BP, we observed decreases in systolic and mean BP (–10% and –6%, respectively) after 2.5 h. Protein expression of phosphorylated protein kinase B (Tyr 437/434) was lower after BJ ingestion (–19%), but the expression of phosphorylated-endothelial nitric oxide synthase (Ser1177) and vasodilator-stimulated phosphoprotein were not altered. All results are shown in Table 1.

**Conclusion:** Our findings indicate that the acute ingestion of nitrate-rich BJ can be a strategy for controlling BP and inhibiting platelet aggregation in healthy individuals, although the topic still lacks further investigation, including an understanding of the its mechanisms and its effects in populations with increased cardiovascular risk.


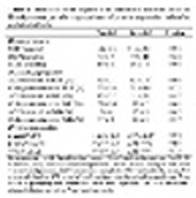


109320

Modality: E-Poster Young Researcher – Non-case Report

Category: HYPERTENSION/RENAL DENERVATION

## Arterial Stiffness: The Damage and Control

MATHEUS TOSCANO PAFFER^1^, Pedro Toscano Paffer^1^, Rafaella Kelly Bello Wanderley^1^, Silvio Hock Paffer FIlho^1^

(1) Faculdade de Medicina de Olinda

**Introduction:** Arterial stiffness is a new risk factor for cardiovascular diseases. It’s relation to aging is well known, being a condition to the development of hypertension (HTN).

**Objective:** Evaluate the arterial stiffness of elderly patients and its correlation to HTN.

**Methods:** 31 very elderly patients were submitted for evaluation of the central blood pressure via a Mobil O’Graph of IEM device.

**Results:** 31 very elderly patients were analyzed (older than 80 years old), from which 78.1% were females. At the average age of 85.8 (+–5.20), and with average BMI of 25.96 (+–4.32). With an average arterial systolic blood pressure of 143.06 mmHg (+–22.71) and the diastolic of 74.83 mmHg (+–9.30). The average central systolic blood pressure of 155.19 mmHg (+–32.88) and the diastolic of 80.96 mmHg (+–20.79). The average PWV (Pulse Wave Velocity) was 13.34 m/s (+–1.44). From the analyzed patients, 67.74% had altered their PWV values. Discussion The prevalence of HTN increases the aging and the occurrence of arterial stiffness plays an important role in this process. Even though this finding is not common, it shouldn’t be considered a benign occurrence, because it’s the main cause of HTN development. It’s a condition prevalent in the elderly population, as observed in this cohort, but it can be reversed with the correct treatment for HTN.

**Conclusion:** Arterial stiffness is a strong risk factor and important for the development of great cardiovascular diseases and its correlation with HTN is well known. Although the elderly population presents higher PWV rates, with an increase of arterial stiffness, it can be controlled and even reversed with optimized treatment of HTN.

109328

Modality: E-Poster Young Researcher – Non-case Report

Category: ATHEROSCLEROSIS/CARDIOVASCULAR RISK FACTORS/CARDIOVASCULAR PREVENTION

## Carotid Intima Thickness and Atherosclerotic Cardiovascular Disease Among Type 2 Diabetes Individuals: Results from the Brazilian Diabetes Study

JOAQUIM DE PAULA BARRETO FONSECA ANTUNES DE OLIVEIRA ^1^, Nestor Martins^2^, Sheila Tatsumi Kimura-Medorima^1^, Sofia Vitte^1^, Thiago Quinaglia^2^, Barbara Assato^1^, Otavio Rizzi Coelho Filho^2^, Jose Roberto Matos-Souza^2^, Wilson Nadruz^2^, Andrei Carvalho Sposito^1^

(1) Laboratory of Atherosclerosis and Vascular Biology, Cardiology Division, Unicamp; (2) Cardiology Division, Unicamp

**Introduction:** The accumulation of Apo-B containing lipoproteins in the subendothelial layer is an initiating step of atherosclerotic cardiovascular disease. In the carotid artery, this event is assessed noninvasively by arterial layers width measurement using B-mode ultrasound. Due to technical limitations, the intima-media thickness (C-IMT), and not the carotid intima (C-IT) width, where atherosclerosis manifests, has been pursued as a marker of disease with a negligible incremental value when compared with traditional risk factors prediction models.

**Objective:** We sought to determine the prognostic value of C-IT for cardiovascular outcomes.

**Methods:** This was a predefined analysis of the Brazilian Diabetes Study, an ongoing, single-center, prospective cohort of T2D individuals aged 30 years or more. Carotid ultrasound was performed by two trained physicians in each common carotid artery to obtain three manual measurements of far-wall C-IT, media (C-MT), and C-IMT. C-IMT above the 75th percentile for age, gender, and race group, and C-IT or C-MT above the sample’s median were deemed as high. Cardiac CT was used to calculate the coronary artery calcium score, and a value above 0 Agatston was considered diagnostic of coronary artery disease (CAD). The primary outcome was major adverse cardiovascular events (MACEs) as a composite of death, myocardial infarction, stroke, myocardial revascularization, and limb amputation.

**Results:** Among 402 individuals (Age: 58 + 7.6 years; 55% male; median T2D duration: 7.7 years) followed for 1676 days, 5 (3.1%) MACEs were reported in the low-IT, and 15 (10.1%) events were disclosed in the high-IT groups, with an HR of 3.11 (95%CI: 1.13, 8.58; p = 0.029). The prevalence of CAD was higher among high-IT than in low-IT group (58.4% vs 78.4%, p < 0.001), with an age-, and sex-adjusted OR of 2.29 (95%CI: 1.09, 4.85; p = 0.029). C-IMT and C-MT were not related to neither MACE nor CAD.

**Conclusions:** C-IT, but not C-IMT nor C-MT, was related to incident MACEs and to coronary artery disease in T2D subjects. C-IT should thus be considered when assessing cardiovascular risk in T2D.

109345

Modality: E-Poster Young Researcher – Non-case Report

Category: NEGLECTED CARDIOVASCULAR DISEASES

## Indicators for Situational Screening of Rheumatic Fever in Brazil

CAMILA POLIS BELLOTT^1^, Gustavo Tavares Lameiro da Costa^1^, Matheus dos Santos Silva de Oliveira^1^, Cristiane da Cruz Lamas^1^, Renato Cerceau^1^

(1) Instituto Nacional de Cardiologia – INC; (2) Fundação Oswaldo Cruz – FIOCRUZ; (3) Universidade Federal do Rio de Janeiro – UFRJ

Rheumatic Fever (RF) and Rheumatic Heart Disease (RHD) once again stood out internationally in 2018 in the panel proposed by the World Health Organization (WHO) held in Assembly, which set a deadline for its control and eradication. A document that guides public policies for RF was organized. Contains indicators for situational tracking of the national and subnational context. The worldwide incidence of RF is 19/100.000. It is estimated 30.000 new cases/year. There are about 33 million people with RC in the world, resulting in 300.000 deaths annually. In order to assess Brazil’s adherence to RF care, we applied the WHO tool. A search was carried out in public data and websites of medical societies. Data not found was requested in digital public services. It was possible to identify some national data, such as the number of cardiac surgeons (1.9/100.000 habitants), interventionists (0.46/100,000 habitants), echocardiography equipment (10572), cardiology services (40207). However, there is no mention of the value considered adequate. Overall mortality from RHD is low (0.16/100.000 habitants in 2014) but 3.9% of hospitalizations resulted in death, and 17% of those hospitalized for more than 90 days. As a comparison, in New Zealand, a reference country in the screening, control and treatment of RF, the annual mortality is 4.9/100,000 habitants. We were unable to quantify the acute cases and patients undergoing outpatient follow-up, there is only a record of hospitalization by international disease code involving RHD. It was impossible to count adherence to secondary prophylaxis, since there is no specific code for the application of Penicillin for RF. We saw that primary care is the most lacking in information. We highlight the absence of a code of procedure in the application of Penicillin for RF, non-accounting of acute cases and patients on secondary prophylaxis, recurrence of RF and mandatory notification of new cases. Is the incidence and prevalence of RF therefore falling? Or does the scarcity of data affect the measurement of the real epidemiological situation in Brazil? The Brazilian Society of Cardiology already highlights the importance of RF/RHD in our country. The socioeconomic impact of RHD must be reported to the government to include it in important public policies with the creation of a National Plan to combat and manage, training of health teams and guarantee of medication and adequate clinical follow-up to the patient.

109350

Modality: E-Poster Young Researcher – Non-case Report

Category: DIGITAL HEALTH/INNOVATION

## Machine Learning for Predicting Readmission in the Coronary Care Unit

DAYANNA DE OLIVEIRA QUINTANILHA PALMER^1^, Pedro Gemal Lanzieri^1^, Ronaldo Altenburg Odebrecht Curi Gismondi^1^, Flavio Luiz Seixas^1^, Guilherme Schittine Bezerra Lomba^1^

(1) Universidade Federal Fluminense (UFF)

Electronic health records (EHRs) are a great opportunity for big data research and professionals from the Coronary Care Unit (CCU) can benefit to support clinical practice. This study aims to analyze epidemiological information from patients admitted to the CCU, the readmission impact on mortality, and the factors associated with readmission. We retrospectively analyzed patients 18 years or older, admitted to the Beth Israel Deaconess Medical Center, Boston, between 2008 and 2019. Data were accessed through the Medical Information Mart for Intensive Care, a public data bank. Patients without discharge data were excluded. Demographic data, vital signs, and lab were evaluated. Posteriorly, patients were divided into two groups: one of the patients who were readmitted and one of the patients who weren’t. Factors associated with readmission were evaluated by statistical methods and machine learning. From an initial group of 53,150 patients, 8,015 were selected. The CCU mean period of stay was 2.8 days and the readmission rate was 7.42%. The mortality rate was 28.73% among those readmitted, while among those not readmitted, it was 18.61% (P-value < 0,001). The readmission rate was mainly influenced by the CCU length of stay, hospital length of stay, respiratory rate, creatinine, hematocrit, chloride, sodium, anion gap, temperature, and calcium. EHRs research can help define best practices and resource management. Machine learning was able to identify more relevant variables. Limitations include the need for longer analyzed periods and a broader data collection.

109362

Modality: E-Poster Young Researcher – Non-case Report

Category: CARDIAC ARRHYTHMIAS/ELECTROPHYSIOLOGY/ELECTROCARDIOGRAPHY

## Cryoablation of Pulmonary Veins for the Treatment of Patients with Atrial Fibrillation After Coronavirus Infection (COVID-19)

ALFREDO AURÉLIO MARINHO ROSA FILHO^3^, Lucas Brandão Cavalcante^2^, Alice Wanderley Rosa^2^, Briane Alcântara Vieira Pasini^3^, Alfredo Aurélio Marinho Rosa^1^, Edvaldo Xavier Ferreira Júnior^1^

(1) Santa Casa de Misericórdia de Maceió – SCMM/AL; (2) Centro Universitário Tiradentes – UNIT/AL; (3) Hospital Universitário Professor Alberto Antunes – HUPAA/UFAL

**Background:** Coronavirus infection preferentially affects the upper airways and mainly the lung parenchyma, however, due to systemic infection, some patients have atrial fibrillation even in the absence of myocarditis or myocardial damage. Atrial fibrillation in most patients does not respond to drug treatment.

**Objective:** To present the results of cryoablation for electrical isolation of pulmonary veins in patients with persistent atrial fibrillation after coronavirus infection.

**Material and methods:** Between March 2020 and March 2022, 53 cases of cryoablation were performed for the treatment of atrial fibrillation, with 15% (8) of patients having atrial fibrillation after coronavirus infection. In all patients, atrial fibrillation did not respond to pharmacological therapy. In these patients, the echocardiogram showed a normal left atrium size, age ranged from 46 to 67 years with a mean of 59 years. The patients did not have structural heart disease or pulmonary sequelae of COVID-19. Of the 8 patients, 62.5% (5) were male and 37.5% (3) were female. All patients had a serological marker of previous infection assessed by IgG serology. Patients were referred to the electrophysiology laboratory, under venous sedation, submitted to transseptal puncture and then an angiography was performed to locate the pulmonary veins and introduction of a balloon catheter where an application of 04 minutes in each pulmonary vein was performed, temperature ranged –51 to –62 with an average of –55 degrees Celsius. In the trans and postoperative period, no complications were recorded.

**Results:** In 100% (8) of the patients, all four pulmonary veins were isolated. There were no complications such as esophageal and/or phrenic nerve injuries and all patients returned to sinus rhythm.

**Conclusion:** The disease caused by the coronavirus can lead to serious cardiac complications, such as uncontrolled atrial fibrillation, refractory to optimized drug treatment. In this sample, the experience of Cryoablation of pulmonary veins proved to be a safe alternative for patients with atrial fibrillation after coronavirus infection.

109380

Modality: E-Poster Young Researcher – Non-case Report

Category: NURSING

## Cardiometabolic Risk Factors Among Nursing Students: Just Knowledge Does not Chance Behavior!

YASMINN BENEVIDES ADBA^1^, Anne Caroline Lisboa Marinho^2^, Andressa Teoli Nunciaroni^1^, Bruna Ribeiro Almeida Magalhães^1^, Caio Victor Chagas das Virgens^1^, Clauanny Caroliny Bicego Reis Nogueira^1^, Débora Novais Lopes^1^, Judson Vieira de Carvalho Pereira^1^, Juliana Resende Correa Lima^1^, Luan Wesley Marques Máximo^3^, Renata Flávia Abreu da Silva^1^, Yasmin Rocha de Almeida^1^

(1) Federal University of the State of Rio de Janeiro – UNIRIO; (2) Federal University of Rio Grande do Norte – UFRN; (3) State University of Piauí – UESPI

**Introduction:** In the context of high prevalence of Cardiovascular diseases (CVD), conditions faced by nursing students such as high curricular workload, stress, lack of time, long commute to university, access to food with low nutritional value, deserve attention in view of primary prevention.

**Objectives:** To identify the cardiometabolic risk factors and its knowledge among nursing students.

**Methods:** Cross-sectional, descriptive and quantitative study. An online self-instructional questionnaire was used for sociodemographic characterization and identification of cardiometabolic risk factors. The knowledge of cardiovascular risk was assessed from five statements about: modifiable risk factors, prevention of CVD, protective behaviors implementation and the contact way with the topic. Nursing Students from a Federal University were enrolled. Data collection occurred from September, 2021 to March, 2022. Data was analyzed through RStudio Software. The study was approved by the local Ethical Committee.

**Results:** The study included 117 nursing students with 24.5(±5.7) years on average. Most(75.4%) were female, single(92.9%), with family income between two and four minimum wages*(59.6%), living with relatives(87.5%). Most of the students were attending 5th and 6th semester(22% and 20%, respectively). Regarding the time spent commuting to the university, 63.4% reported between 30 to 120 minutes. The risk factors identified in the sample are reported on Table 1. 74.6% declared CVD family history. Results relating to knowledge are presented on Figure1. *1 minimum wage = US$253.35.

**Conclusions:** The study shows cardiometabolic risk factors among undergraduate nursing students, even when knowledge about the topic is reported. Knowledge does not mean implementing protective behaviors.


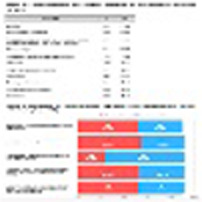


109410

Modality: E-Poster Young Researcher – Non-case Report

Category: PHYSIOTHERAPY

## Predictors of Functional Status in the Immediate Postoperative Period of Cardiovascular Surgery

VICTORIA MARIA GARCIA DE MEDEIROS^1^, Jéssica Gonçalves de Lima^1^, Fernando Gomes de Jesus^1^, Ana Gabriella Arena de Sá,^1^, Thaísa Sarmento dos Santos^1^, Cristianne Rafael Campos,^1^, Lucas Araujo de Carvalho^1^, Marcus Vinicius de Souza Amaral^1^, Juliana Rega^1^, Claudia Rosa^1^, Mauro Felippe Felix Mediano^1^, Luiz Fernando Rodrigues Junior.^1^

(1) National Institute of Cardiology

**Introduction:** Patients in the postoperative period (PO) of cardiovascular surgery (CS) may have reduced functional status during hospitalization. However, little has been studied regarding preoperative, intraoperative and PO factors that may be related to better functionality at the time of discharge from the intensive care unit (ICU).

**Objective:** To identify pre, intra and PO predictors of functional status of patients under PO of CS.

**Methods:** Retrospective cross-sectional study. Data from 614 patients was obtained from the Physiotherapy Service database (stored in REDCap) and reviewed in medical records of the Instituto Nacional de Cardiologia (Rio de Janeiro-RJ), from October/2018 to March/2020. Preoperative variables such as age, sex, body mass index (BMI) and comorbidities were collected. Intraoperative variables were: type/complexity of surgery, fluid balance, time on cardiopulmonary bypass, time of aortic clamping, and surgical complications. The variables evaluated in the PO were: level of consciousness, pain on admission, fluid balance, imaging tests, blood gases, laboratory tests, mechanical ventilation and pulmonary function before and after extubation, peripheral muscle strength, extubation attempts and failures. Functional status was quantified at ICU discharge using Functional Status Score for the ICU (FSSICU). The univariate and multivariate linear regression model (P < 0.05 considered significant) was used to verify the association of possible predictors of functional status.

**Results:** The PO variable: obesity (BMI ≥ 30 kg.m^–2^; β = –2.8; CI: –5.4 –0.3; P = 0.026) was negatively associated with functional status in the ICU; while the postoperative variables: pain on admission (β = 11.2; CI: 1.8–20.5 P = 0.02) and peripheral muscle strength at discharge (β = 0.3; CI: 0.2–0.4; P = 0.001) were directly and independently associated with functional status at discharge; Furthermore, muscle strength at the time of extubation were directly and independently associated with strength at discharge (β = 11.1; CI: 0.1–0.2; P = 0.007).

**Conclusion:** Pain intensity on admission and peripheral muscle strength at ICU discharge predict functional status at discharge, while muscle strength at the time of extubation was evidenced as an independent predictor of muscle strength at discharge, and a suggestive intervention target for preventing functional status loss in adults submitted to cardiac surgery.

109426

Modality: E-Poster Young Researcher – Non-case Report

Category: HEMODYNAMICS AND INTERVENTIONAL CARDIOLOGY

## Impact of Small Stent Diameter in Patients with St Elevation Myocardial Infarction

GUILHERME PINHEIRO MACHADO^1^, Gustavo Neves de Araujo^3^, Arthur Cabareira Baptista^4^, Mariana Rabolini^4^, Luiza Atanazio^4^, Alan Pagnoncelli^1^, Angelo Chies^2^, Wagner Azevedo^2^, Rodrigo Wainstein^1^, Marco Vugman Wainstein^1^

(1) Hospital de Clinicas de Porto Alegre (HCPA); (2) Universidade Federal do Rio Grande do Sul (UFRGS); (3) Imperial Hospital de Caridade; (4) Pontifícia Universidade Católica do Rio Grande do Sul (PUCRS)

**Introduction:** In patients submitted to elective percutaneous coronary intervention (PCI), treatment with small stents diameters (SD) has been associated with worse outcomes. However, the impact of small SD on outcomes in patients with acute myocardial infarction in scarce.

**Methods:** This was a prospective cohort study that included patients with STEMI submitted to pPCI admitted to a tertiary university hospital between April 2011 and December 2021. Patients were categorized into groups based on SD. Small SD was considered <2.50 mm. Patients who underwent implantation of multiple stents were assigned to the study group according to the smallest stent size used. The primary clinical outcome was major adverse cardiovascular events (MACE) defined by death, in-hospital MI, stroke, and stent thrombosis and target vessel revascularization. Secondary outcomes included MACE and each individual outcome at in-hospital, 30 days and long-term period.

**Results:** From 1458 Patients admitted with STEMI in the study period, 1238 were included and 468 (34.4%) were women. Mean age was 63.4 ± 12.8 years in small stent diameter vs 60.4 ± 11.7 years (p = 0.009). Patients with small SD had a higher prevalence of diabetes (36.9 × 25.3%, p = 0.003) and previous acute myocardial infarction 11 × 17%, p = 0.04). In multivariate analysis, stent diameter <2.5 mm remained independent predictor of MACE (odds ratio [OR] 1.6 95% confidence interval [95% CI] 1.08–2.42; p = 0.018); mortality (OR = 1.78, 95%CI = 1.092–2.878; p = 0.018, and target vessel revascularization (OR = 1.963; 95%CI = 1.019–3.612; p = 0.036).

**Conclusion:** In this prospective cohort study of patients with STEMI treated with PCI, a small stent diameter <2.5 mm was associated with increased rates of MACE, mortality and target vessel revascularization.


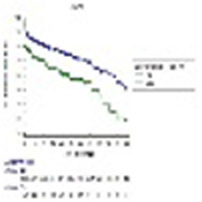


109427

Modality: E-Poster Young Researcher – Non-case Report

Category: CARDIORESPIRATORY PHYSIOLOGY/BASIC SCIENCE

## Resistance to Obesity Promotes Metabolic Alterations and Cardiac Hypertrophy Without Changes in the Reactive Oxigen Species

JANETE CORRÊA CARDOSO^1^, Amanda Rangel Madureira^1^, Vinicius Valois Pereira Martins^1^, Ramon dos Santos^1^, Suellem Torezani Sales^1^, Ana Paula Lima-Leopoldo^1^, André Soares Leopoldo^1^

(1) Universidade Federal do Espírito Santo (Ufes)

Resistance to Obesity is associated with the complex interaction of stringent and environmental factors, conferring the ability to gain mass gain and body fat deposition, even when eating high-calorie diets. Considering that there are numerous gaps in the literature on the metabolic processes that explain Resistance to Obesity, specifically in relation to oxidative stress, the purpose of the study was to investigate whether obesity-resistant rats develop elevated reactive oxygen species in cardiac tissue. Wistar rats (n = 71), aged 30 days, were initially randomized into two groups: a)Standard diet (n = 35) and b)High-fat diet (n = 36). The experimental protocol consisted of two moments: obesity induction (4 weeks) and characterization of resistance to obesity (10 weeks). After obesity detection, the animals were redistributed into three groups: Control (C), Obese (Ob) and Obesity-Resistant (ROb). Was analyzed: Nutritional profile, metabolic changes, cardiac mass and Oxidative stress. The comparison of the experimental groups was performed using ANOVA, complemented with Tukey’s multiple comparisons test. The level of significance considered was 5%. Body mass showed a significant difference between the standard diet and high-fat diet groups in the 4th week of the experimental protocol, characterizing obesity. In the 4th week, after the characterization of Resistance to Obesity, there was a significant difference in body mass between groups C, Ob and ROb. Ob and ROb groups showed a significant increase in caloric intake (p < .001; p = .0005) in relation to the C. Ob group showed a significant increase in final body mass (p < .0001; p = .03), in retroperitoneal fat pad (p.0001; p = .006), sum of corporal fat deposits (p < .0001; p = .02) and in reactive oxygen species (p = .001; p = .0006), in relation to groups C and Rob. The area under the glycemic curve (p = .02), insulin resistance index (HOMA-IR) (p = .02) and basal glucose (p = .01) were elevated in the Ob group in relation to the C. Resistance to Obesity also promoted an increase in HOMA-IR when compared to C. Total cardiac mass (p = .0004 and;p = .01), right (p < .0001; p = .002) and left ventricles (p = 0.002; p = 0.03) the cross-sectional area (p < .0001; p < .0001) and cholesterol levels (p = .0001; p = .001) in were significantly elevated in the Ob and Rob groups compared to the C group. In conclusion, Resistance to Obesity promotes metabolic alterations and cardiac hypertrophy without changes in reactive oxygen species.

109458

Modality: E-Poster Young Researcher – Non-case Report

Category: ATHEROSCLEROSIS/CARDIOVASCULAR RISK FACTORS/CARDIOVASCULAR PREVENTION

## Copper Overload Causes a Reduction of Vascular Reactivity in Diabetic Rats

KAROLINI ZUQUI NUNES^1^, Dalton Valentim Vassallo^1^, Julia Antonietta Dantas da Silva^1^

(1) Universidade Federal do Espírito Santo – UFES

**Introduction:** It is a known fact that diabetes mellitus is associated with several cardiovascular abnormalities such as increased arterial stiffness and endothelial dysfunction. In addition to these changes, enhanced plasma copper concentration is also characteristic in individuals with diabetes. Although there are theories that the increase in plasma copper concentration and the development of cardiovascular problems are related, to date the mechanisms that explain this correlation are still unclear.

**Objective:** The present study seeks to understand the effects of chronic copper overload on vascular reactivity in isolated segments of the thoracic aorta of diabetic rats.

**Methodology:** This is an experimental study, in which about 44 12-week-old Wistar rats were used, weighing approximately 200g, obtained from the central vivarium of Federal University of Espírito Santo (UFES). The experimental protocols were approved by the university’s animal use ethics committee No 22/2019. The animals were divided into four experimental groups: Control (CT); Copper (Cu); Diabetes Mellitus (DM); Diabetes Mellitus + Copper (DM+Cu). Type 1 diabetes was induced via a single injection of streptozotocin 50 mg/kg/i.v., and the animals were treated with twice the recommended daily dose of copper (1.8 mg/kg). They were then killed after 30 days of treatment, and the thoracic aorta was removed for vascular reactivity experiments. Statistical tests, one-way and two-way ANOVA were used to examine the outcomes.

**Results:** The main findings show a reduction in vascular reactivity in the DM+Cu group: Using an inhibitor of nitric oxide synthase (L-NAME), an increased bioavailability of nitric oxide was suggested, explaining the vasodilation; employing an inhibitor of NADPH oxidase activity (Apocynin) and an enzyme that degrades hydrogen peroxide (Catalase) we showed the participation of hydrogen peroxide as a vasodilator factor; the potassium channels blockade (tetraethylammonium) suggests the participation of the channels for potassium as vasodilators; and the use of selective angiotensin II AT1 receptor antagonists (Losartan) suggests that angiotensin II could be acting on AT2 receptors as vasodilators.

**Conclusion:** Copper overload in diabetic rats causes a reduction of the aorta vascular reactivity of, which can be explained by the action of nitric oxide, hydrogen peroxide, and potassium channels, which are potential vasodilators, thus explaining the mechanism of our main discovery.

109470

Modality: E-Poster Young Researcher – Non-case Report

Category: PSYCHOLOGY

## Quality of Life and Mental Disorders Profile Among a General Population of Pre-Hypertensive and Hypertensive Patients

JOÃO GABRIEL REGA DO NASCIMENTO VALLAPERDE^1^, Bianca Botelho Viegas^1^, Fernanda Oliveira de Carvalho Carlos^1^, Victor Margallo^1^, Vitor de Melo Nolasco^1^, João Gabriel Bezerra da Silva^1^, Bianca Zattar de Mello Barreto^1^, Marcus Vinicius Serejo Borges Vale da Silva^1^, Daniela Fiuza^2^, Karine Guimarães^2^

(1) Universidade Federal do Rio de Janeiro – Hospital Universitário Clementino Fraga Filho – ProHArt; (2) Universidade Estácio de Sá – UNESA

**Introduction:** Symptoms of depression and anxiety may interfere in the quality of life of individuals diagnosed with hypertension.

**Objectives:** To investigate the association between anxiety, depression and quality of life among pre-hypertensive and hypertensive patients.

**Materials and methods:** A cross-sectional study including 89 pre-hypertensive (n = 15) and hypertensive (n = 74) participants. All of them were submitted to a standard protocol with collection of sociodemographic data, cardiovascular risk factors, BP measurement and mental health evaluation. For depression symptoms, it was used the Patient Health Questionnaire-9 (moderate/severe depression ≥10 points); for anxiety, the Generalized Anxiety Disorder Assessment-7 (moderate/severe anxiety ≥10 points); for quality of life, the World Health Organization Quality of Life instrument. Bivariate analyses were performed to compare participants’ characteristics according to the presence of anxiety, depression, and quality of life. Logistic regression assessed the main variables independently associated with moderate/severe anxiety and depression and reduced quality of life.

**Results:** The average age was 50.2 ± 11.2 years old and 52.8% of the participants were female. Blacks and browns were the majority (53.9%). 21% of the participants have low education (Elementary School) and 64% have family income ≤3 minimum wages. Sedentary lifestyle (43.8%) and dyslipidemia (47.2%) were the most common risk factors among this population. Moderate/severe depression was found in 46.6% of the participants and moderate/severe anxiety in 43.8%, with no differences between men and women. The mean score of quality of life was 87 [63–111]. The presence of moderate/severe depression, moderate/severe anxiety and worse quality of life was not associated with sociodemographic conditions nor traditional CV risk factors, but they were associated with each other. Moderate/severe depression increased elevenfold the risk of worse quality of life (OR = 10.9; IC95% 2.81–41.65; p < 0.001). Moderate/severe anxiety (OR = 6.41; IC 95%; p < 0.004) and worse quality of life (OR = 11.19; IC 95%; p < 0.001) were independently associated with moderate/severe depression. The independent variable associated with moderate/severe anxiety was the presence of moderate/severe depression (OR = 6.37; IC 95%; p < 0.004).

**Conclusion:** The presence of depression and anxiety symptoms were associated with each other and with worse quality of life of individuals evaluated.

109473

Modality: E-Poster Young Researcher – Non-case Report

Category: DYSLIPIDEMIA

## Functional Aspects of the HDL Particle: A Comparative Study between Patients with Refractory Angina, Non-Limiting Angina, and Healthy Individuals

SARAH FAGUNDES GROBE^1^, Maurício Tavares^1^, Thauany Tavoni^1^, Pedro Gabriel Senger Braga^1^, Raul Cavalcante Maranhão^1^, Luis Henrique Wolff Gowdak^1^

(1) Instituto do Coração da Faculdade de Medicina da USP – InCor HCFMUSP

**Introduction:** HDL particle plays an important role in atherosclerosis for its complex metabolism and relationship with other pro-atherogenic lipoproteins. Patients with refractory angina (RA) usually presente advanced atherosclerosis of the coronary arteries. So far, no biochemical markers have been found to explain the greater atherosclerotic involvement in patients with RA compared with patients with non-limiting angina (NLA).

**Objective:** To compare the activity of paraoxonase 1 (PON1) and the HDL particle diameter in patients with RA or NLA with healthy individuals (HI).

**Methods:** 35 patients (15 RA and 20 NLA) and 20 HI were included. Plasmatic concentrations of lipids were determined using commercial kits and ApoA-1 and ApoB by the turbidimetric method. The HDL particle diameter was measured by light scattering method and PON1 activity by the spectrophotometric method. One-way ANOVA was used for statistical analysis, and a P-value < 0.05 was considered statistically significant.

**Results:** There were no differences in age, sex, and body-mass index between groups. The lipid profile was similar between groups, except for higher levels of HDL-cholesterol and ApoA-1 in HI (see Table). There were no differences in ApoB concentrations, HDL particle size, and PON1 activity.

**Conclusion:** As expected, patients with coronary atherosclerosis had lower ApoA-1 and HDL-cholesterol levels than HI. However, the HDL particle size and the PON-1 activity were similar among patients with RA or NLA. The search for markers of plasma lipid metabolism able to discriminate between patients with RA and NLA is of interest. They may mirror metabolic alterations related to the genesis of RA and become therapeutic targets.


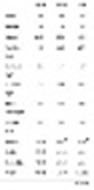


109481

Modality: E-Poster Young Researcher – Non-case Report

Category: CARDIAC ARRHYTHMIAS/ELECTROPHYSIOLOGY/ELECTROCARDIOGRAPHY

## Ablation of Anomalous Pathways Manifested in the Mitral Annulus Through Transradial Access with Early Hospital Discharge Due to the COVID-19 Pandemic

ALFREDO AURÉLIO MARINHO ROSA FILHO^3^, Lucas Brandão Cavalcante^2^, Alice Wanderley Rosa^2^, Briane Alcântara Vieira Pasini^3^, Dandara Dias Gomes Cunha^3^, Alfredo Aurélio Marinho Rosa^1^, Edvaldo Ferreira Xavier Júnior^1^

(1) Santa Casa de Misericórdia de Maceió – SCMM/AL; (2) Centro Universitário Tiradentes – UNIT/AL; (3) Hospital Universitário Professor Alberto Antunes – HUPAA/UFAL

Ablation of manifest anomalous pathways in the mitral annulus through transradial access with early hospital discharge due to the COVID-19 pandemic.

**Introduction:** Ablation of anomalous pathways on the left or in the mitral annulus can be approached by transseptal puncture, by retrograde aorta approach or through the transradial approach. Due to the shortage of infirmary beds during the Covid-19 pandemic, we chose to perform transradially due to early hospital discharge.

**Material and methods:** Between January 1998 and December 2021, 3,535 catheter ablations were performed. In 29,9% (1059) patients were treated for manifest anomalous pathways, of these 54.3% (575) were male, with ages ranging from 03 to 75 years and a mean of 31.24 years, success was obtained in 94, 9% (1005) of cases. Our service had previously performed 16 cases of transradial ablation. During the COVID-19 pandemic and the difficulty for the hospitalization of patients, between May 2020 and July 2021, 8 cases of ablation of manifest anomalous pathways manifested in the mitral annulus were performed, 62,5% (5) were anterolateral and 37,5%(3) Left postero-septal. 62,5% (5) patients were male and the age ranged from 24 years to 56 years with a mean of 40 years. The approach was made through the right radial artery, under local anesthesia, the patient was submitted to venous sedation, introduction of a 7Fr introducer and placement of only the exploratory catheter. Mapping performed always during sinus rhythm and after ablation performed tests with IV adenosine. The catheter was removed and a local pneumatic dressing was applied for 2 hours and replaced with a conventional dressing for 24 hours. All patients were selected after performing Doppler imaging of the radial and ulnar arteries. COVID-19 test were negative for all patients.

**Results:** In the 8 patients ablation was successful (100%), the procedure time ranged from 40 minutes to 80 minutes with an average of 55 minutes. No complications were recorded during or after the procedures, all patients were discharged from hospital 3 hours after the intervention. At outpatient follow-up, all patients are asymptomatic and have an ECG without ventricular pre-excitation.

**Conclusion:** Faced with the COVID-19 pandemic and lack of hospital beds for elective admissions of patients with left manifest anomalous pathways, the transradial approach was a safe option providing early hospital discharge.

109502

Modality: E-Poster Young Researcher – Non-case Report

Category: EPIDEMIOLOGY AND HEALTH POLICIES/GLOBAL HEALTH

## The COVID-19 Pandemic Impact on Stroke Notification in Brazil: An Epidemiological Study

DÉBORAH ESTEVES CARVALHO^1^, Déborah Esteves Carvalho^1^, Anna Laura Lima Larcipretti^2^, Aline Dias Bedetti^2^, Julia Regina Assis da Rosa^2^, Bárbara Alves de Abreu Rocha^2^, Leonardo Brandão Barreto^3^

(1) Universidade Tiradentes, Aracaju, Brasil (UNIT); (2) Escola de Medicina, Universidade Federal de Ouro Preto, Ouro Preto, Brasil (UFOP); (3) Departamento de Clínica Pediátrica e Adulto, Universidade Federal de Ouro Preto, Ouro Preto, Brasil (UFOP)

**Introduction:** The Coronavirus Disease 2019 (COVID-19) pandemic has placed a drastic strain on healthcare services, requiring the reallocation of resources, and possibly affecting the quality of stroke monitoring and care.

**Objective:** This is an epidemiological, descriptive, and retrospective study to analyze the profile of stroke in Brazil from January 2018 to December 2021. In addition, we aim to investigate the impact of the pandemic on stroke monitoring, including the possible reasons for the underreporting of cases.

**Methods:** The results were obtained by collecting data from the SUS Hospital Information System (SIH/SUS). Also, a review of the literature on this topic was performed, using the Pubmed database.

**Results:** Stroke is one of the main causes of mortality and morbidity worldwide. Although an increased number of stroke cases was expected due to cardiovascular events caused by the SARS-CoV-2 infection, the data show otherwise. Between 2018 and 2019, there was an increase of 6,116 in the number of cases. In contrast, a drop of 12,889 and 5,490 in the number of cases between the years 2019 and 2020, and 2020 and 2021, respectively, was observed. A significant decrease in the number of cases was observed in the Northeast region and in the elderly population. The literature describes many possible reasons for the underreporting. The most present hypothesis was fear of infection by the virus, which may have prevented many from seeking emergency medical care, impacting directly on the notification of stroke cases. Moreover, studies indicate that social distancing makes the recognition of stroke signs more difficult since the latter is facilitated by person-to-person interaction. Also, the pandemic requires the reallocation of healthcare resources to be used for COVID-19 care, it can create gaps in stroke care, affecting its quality.

**Conclusions:** Thus, the decreased number of stroke cases observed between 2019 and 2021 may be a result of underreporting, which could be a direct impact of the COVID-19 pandemic on stroke care and monitoring.

109510

Modality: E-Poster Young Researcher – Non-case Report

Category: CARDIAC ARRHYTHMIAS/ELECTROPHYSIOLOGY/ELECTROCARDIOGRAPHY

## Contact Force Sensing Technology in Atrial Fibrillation Catheter Ablation: A Prospective, Single-Center, Cohort Study

GABRIEL ODOZYNSKI^1^, Isabella Bianco^3^, Helcio G. Nascimento^1^, Andrei Lewandowski^1^, Maurício L. Spessatto^1^, Clóvis Froemming^1^, Grazyelle Damasceno^1^, Alexander Dal Forno^1^, André d‘Ávila^1^

(1) Hospital de Cardiologia SOS Cardio; (2) Instituto de Cardiologia de Santa Catarina; (3) Universidade do Sul de Santa Catarina

**Introduction:** The use of contact force (CF) sensing catheters aims to provide safety and efficacy to atrial fibrillation (AF) ablation by permanent pulmonary veins electrical isolation.

**Objective and Method:** This study sought to evaluate the AF catheter ablation outcomes (recurrence rate and adverse events) in a single-center, prospective cohort of 380 patients underwent to first-ever procedure using CF sensing technology. Data were analyzed based on digital AF ablation database/SysCardio®. Recurrence was defined as any atrial arrhythmia documented – episodes of 30s or longer after blanking period (90 days).

**Results:** Of 380 enrolled patients, 290 patients had paroxysmal AF (≤7 days-PAF) and 90 patients persistent AF (>7 day ≤1 year–AF-Per). The mean age was 63 ± 11 and 67 ± 10 years, respectively. 68% and 80% of patients were men in the PAF and AF-Per groups. There was no difference among PAF and AF-Per groups regarding, hypertension and diabetes mellitus, coronary artery disease and previous stroke (16 patients). The CHA2DS2-VASc was 1.8 ± 1.4 vs 2 ± 1.5 in PAF and AF-Per, respectively. After the first procedure, 44 patients (15%) and 22 (24%) had recurrence in PAF and AF-Per groups (Fig.1). Considering a sequential redo procedure, these rates decrease to 5 and 8% in PAF and AF-Per, respectively. There were 15 minor primary adverse events from 427 procedures (7 inguinal hematomas, 6 pseudoaneurysms [1 surgical] and 2 pericardial effusions, no surgical drainage need). There were no major complications. There were no deaths related to the procedure.

**Conclusion:** Initial outcomes using CF sensing technology catheter to AF ablation demonstrate a cumulative recurrence rate of 5% and 8% in patients with PAF and FAP-Per. The procedure is safe, no major adverse events were recorded in our study.


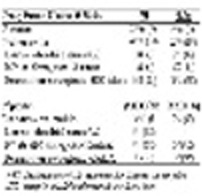


109527

Modality: E-Poster Young Researcher – Non-case Report

Category: CONGENITAL AND PEDIATRIC CARDIOLOGY

## The Cone Procedure to Ebstein‘s Anomaly Repair: Clinical and Echocardiographic Outcomes

NATÁLIA ALBERTIN DOS SANTOS^1^, José Cícero Stocco Guilhen^1^, Omar Alonzo Pozo Ibanez^1^, Vivian De Biase^1^, Renata Burini Chaccur^1^, Jaime da Conceição Padeiro Junior^1^, Luciana Fonseca Martins^1^, Wanda Teixeira Moreira do Nascimento^1^, Wagner Knoblauch Santos^1^, Maria de los Angeles García Andrade^1^, Daniela Lago Kreuzig^1^, Simone Rolim Fernandes Fontes Pedra^1^

(1) Instituto Dante Pazzanese de Cardiologia

**Introduction:** Ebstein’s anomaly occurs due to failure in the delamination of the septal and posterior leaflets with apical displacement of the functional annulus, dilatation of the atrialized portion of the right ventricle, thinning of the free wall, dilatation of the true annulus and redundant anterior leaflet with multiple fenestrations. The “Cone” reconstruction of the tricuspid valve, described by Silva et al, results in complete coaptation of the valves with excellent results in the medium and long term.

**Objectives:** In this study, clinical and echocardiographic data of patients undergoing surgical repair using this technique was reviewed.

**Methods:** Pre and post-operative (PO) records and echocardiograms of 8 patients operated on from 2018 to 2022 were reviewed.

**Results:** The median age of the group was 22.5 (2 to 42 years). In the pre-operative 3 patients had Wolf-Parkinson-White ventricular pre-excitation and 6 had atrial septal defect associated, 6 had moderate to severe dyspenia, with only one being cyanotic. All had severe tricuspid regurgitation (TI) and 6 had moderate to severe right ventricular dilatation. In the immediate PO, moderate TI was detected in 2 and mild in 6. In 1 patient, Glenn anastomosis was added to the Cone procedure due to hemodynamic instability and hypoxemia immediately after bypass. In the post-operative follow-up time, median of 11.5 months, all patients were asymptomatic and TI increased from mild to moderate in only one case. One patient required pacemaker implantation due to complete atrioventricular block.

**Conclusion:** In this small cohort, the Cone procedure resulted in significant improvement of the tricuspid regurgitation, no mortality and low morbidity with significant improvement of the clinical status.

109533

Modality: E-Poster Young Researcher – Non-case Report

Category: HEART FAILURE/CARDIOMYOPATHY/TRANSPLANT

## Epidemiological Profile of Chronic Rheumatic Heart Disease Hospitalizations in the North Region in Brazil between 2011 and 2021

LEONARDO LEÃO BARROS^1^, Ludmila Oliveira dos Reis^2^, Mariana Lassance Maya Palheta^1^, Maria Luiza do Socorro Alves Lucas^3^, Juliana de Sousa Tavares^1^

(1) Universidade do Estado do Pará – UEPA; (2) Universidade Federal do Pará – UFPA; (3) Centro Universitário do Estado do Pará – CESUPA

**Introduction:** The chronic rheumatic heart disease describes a group of heart diseases that can occur as a result of rheumatic fever. It is the most common cause of heart failure in children and young adults. In Brazil, these disorders generate high costs for the health system, with large number of hospitalizations resulting from this pathology. Besides, in the North region there is a high prevalence of this comorbidity.

**Objective:** To evaluate and analyze the epidemiological profile of patients undergoing hospitalization for chronic rheumatic heart disease, in addition to determining their incidence and prevalence, in the period from 2011 to 2021 in Northern Brazil.

**Method:** This research is a cross-sectional, analytical, and retrospective study, with observation of the period from 01/2011 to 12/2021. The materials used as a source were collected by the informatics department of the Brazilian Unified Health System on hospitalizations for rheumatic heart disease in the North region of Brazil and three variables were analyzed: age group, sex and race.

**Results:** There were a total of 3.891 hospitalizations. The male population corresponded to 47,87% (1.863) of the hospitalizations, the female population corresponded to 52,12% (2.028). The races: brown, “no information”, white, black, yellow and indigenous, corresponded respectively to: 60,62% (2.359), 30,40% (1.183), 5,98% (233), 2,18% (85), 0,56% (22) and 0,23% (9). For age groups, the following ranges in years: “< 19”, “20 to 59” and “60 and +”, corresponded respectively to: 12,02% (468), 64,53% (2511) and 23,43% (912) of the hospitalized population.

**Conclusion:** Rheumatic heart disease (RHD) is a condition with a significantly larger prevalence in women. Different studies show the ratio to be 2:1. In this study, women indeed corresponded to a larger number of hospitalizations (52,12%). But, the ratio found was only 1,08:1. No relation between race and RHD was found in studies. The higher prevalence in the brown race (60,62%) may be a reflection of the ethnic distribution of the region. RHD has a higher prevalence in the 25 to 45 years range. This is consonant with the findings of this study, which showed that the adult population with age between 20 and 59 corresponded to 64,53%.

109554

Modality: E-Poster Young Researcher – Non-case Report

Category: COVID-19 AND CARDIOVASCULAR SYSTEM

## Autonomic Dysfunction Post-COVID-19 in Obese: Cross-Sectional Study

ALICE PEREIRA DUQUE^1^, Luiz Fernando Rodrigues Junior^1^, Humberto Batista de Macedo Junior^1^, Daniel Arthur Barata Kasal^1^

(1) Education and Research Department. National Institute of Cardiology. Rio de Janeiro, RJ, Brazil

**Introduction/Objective:** Autonomic dysfunction, a commonly underdiagnosed clinical entity with important cardiovascular implications, can occur in post-COVID-19 Syndrome. However, the entire impact of obesity on this clinical entity is still unknown. Thus, we aimed to assess if obesity can be a predictor of autonomic dysfunction in post-COVID-19 patients.

**Methods:** Cross-sectional study recruited employees from a public hospital in Rio de Janeiro. Inclusion criteria: age ≥ 18 years and previous diagnosis of COVID-19. Exclusion criteria: physical inability to perform the autonomic test. Clinical, anthropometric and sociodemographic variables were collected. Volunteers were asked about the presence of cardiovascular symptoms (lipothymia, syncope, palpitations, dyspnea or chest pain) after COVID-19, and classified according to body mass index (BMI) in non-obese and obese (BMI < 30 and ≥30 Kg/m², respectively). Autonomic dysfunction was defined as the presence of orthostatic hypotension (OHYPO) and/or orthostatic hypertension (OHYPER). Systolic (SBP) and diastolic (DBP) blood pressure variation was evaluated after 15min of rest followed by 3min of orthostasis. Variations of SBP ≥ 10 mmHg and DBP ≥ 5 mmHg were considered as alterations. The association between variables was assessed using Fisher’s exact test and logistic regression adjusted by sex, age and medications that may influence autonomic function. P < 0.05 was considered significant.

**Results:** In 55 volunteers evaluated, none required hospitalization and only 10.9% (N = 6) had post-COVID-19 symptoms. The time between COVID-19 diagnosis and the evaluation was 405 [380–421]days. In total, 87.3% (N = 48) had autonomic dysfunction. Of these, 76.4% (N = 42) had OHYPER and 20% (N = 11) had OHYPO. In common to both groups, 9.1% (N = 5) had both alterations. Non-obese represent 82.5% (N = 33) of the participants who had OHYPER, against 17.5% (N = 7) obese volunteers (P = 0.001). Obese individuals represent 32% (N = 17) of our sample. Of the volunteers who had OHYPO, 72.7% (N = 8) were obese, against only 27.3% (N = 3) non-obese (P = 0.002). Obesity exhibited a positive association with the occurrence of post-COVID-19 OHYPO (OR = 12.4; P = 0.009).

**Conclusion:** Obesity increases in 12 time-fold the risk of developing post-COVID-19 OHYPO. The high prevalence of post-COVID-19 OHYPO in obese individuals occurs regardless of the presence of symptoms and the infection severity, suggesting that obesity could be a predictor of post-COVID-19 autonomic dysfunction.

109640

Modality: E-Poster Young Researcher – Non-case Report

Category: CARDIOVASCULAR INTENSIVE CARE/CARDIOVASCULAR EMERGENCIES

## Cardiopulmonary Resuscitation Procedures Addressed Through Telesimulation to Health Professionals

GISELLE OLIVEIRA AZEREDO^1^, Renata Flávia Abreu da Silva^1^, Andressa Teoli Nunciaroni^1^, Vanessa de Almeida Ferreira Corrêa^1^

(1) UNIVERSIDADE FEDERAL DO ESTADO DO RIO DE JANEIRO (UNIRIO)

**Introduction:** Training in Cardiopulmonary Resuscitation through Telesimulation is challenging, due to its theoretical-practical peculiarity. However, gaps in the knowledge of health professionals can be filled through this remote education strategy. It is worth mentioning the actions described in the Cardiopulmonary Resuscitation protocol, which, if they have any problems in their compliance, can negatively influence the patient’s clinical outcome.

**Objective:** To analyze Cardiopulmonary Resuscitation procedures addressed through Telesimulation to health professionals.

**Methods:** Descriptive, cross-sectional, retrospective and quantitative study. We included secondary data from a remote course database with the telesimulation strategy and application of pre and post-tests instruments. The questionnaires items were grouped into four thematic areas: Identification and management; analysis of cardiac rhythms; medication administration; leadership. Participants were invited to provide their data. Descriptive and inferential statistics were used for data analysis. The study was approved by the Research Ethics Committee, with number: 4,945,123/2021.

**Results:** 35 groups were formed with the participation of 227 health professionals. The pre-test, which corresponded to a situational diagnosis, showed that the leadership action had the highest number of hits, with 93 (96.8%), 47 (97.0%) and 70 (90.9%) and the action referring to the administration of epinephrine had the highest number of errors, with 11 (11.4%), 5 (9.20%) and 13 (16.8%) correct answers. The answers corresponded to the questionnaires applied in the three telesimulated scenarios, namely, hospital scenario (n = 96), psychiatric scenario (n = 54) and emergency scenario (n = 77). However, there was a progressive increase in correct answers of 47% and 69% between pre-test and post-test 1, respectively. In addition to the significant increase in their overall hit mean from 4.66 (1.67) to 6.85 (1.69), respectively (p < 0.0001). As for post-tests 1 and 2, there was an increase from 69% to 80%, and an overall average of correct answers from 6.85 (1.69) to 8.04 (1.55), respectively (p < 0.0003).

**Conclusion:** The Telesimulation method, via remote course, seems to contribute to Cardiopulmonary Resuscitation procedures learning, considering the number of correct answers in the sample and the contexts studied.

109654

Modality: E-Poster Young Researcher – Non-case Report

Category: CARDIAC ARRHYTHMIAS/ELECTROPHYSIOLOGY/ELECTROCARDIOGRAPHY

## Increased Qt Dispersion in Depressed Patients Without Cardiovascular Disease: Systematic Review and Meta-Analysis

VITHORIA VIDOTTI NEVES^1^, Julio Cesar Tolentino Junior^1^, Sérgio Luis Schmidt^1^, Alan Marques Joaquim^1^, Vithória Vidotti Neves^1^

(1) Hospital Universitário Gaffrée e Guinle

**Background:** Depression is linked to increased cardiac mortality risk. Dispersion of QT interval is an indirect electrocardiography (ECG) expression of ventricular repolarization heterogeneity. Increased QT dispersion is related to a higher risk of arrhythmias and sudden death. Therefore, depression may be associated with increased QT dispersion even in cardiac-healthy patients.

**Objective:** We conducted a meta-analysis to investigate whether QT dispersion could be increased in depressed patients without cardiovascular disease.

**Methods:** A systematic review following the Preferred Reporting Items for Systematic Reviews and Meta-Analysis (PRISMA) guidelines was performed by two independent reviewers. A systematic search of MEDLINE and EMBASE from 1990 to 2022 was performed. An all-fields search for index terms (“depression” OR “depressive” OR “depressed”) AND (“QT dispersion” OR “autonomic nervous system”) was done. We included all articles that evaluated the relationship between QT dispersion on electrocardiogram and depression in patients without cardiovascular disease. Articles of revision, meta-analysis, editorials, and case reports were excluded. Outcome measures were either crude QTD or heart rate-corrected QT (QTcD) dispersions. The effect model was analyzed by MedCalc statistical software. The percentage of variability in the set of effect sizes due to true heterogeneity was tested with the I2 statistic.

**Results:** The reviewers identified 677 publications. Four articles were included in the final analysis, which encompassed 267 cardiac health subjects (131 depressed and 136 non-depressed). There were 198 (74.1%) women. Age means did not differ between depressed and control groups. All studies reported significantly higher values of QTD and QTcD in depressed patients. As I2 values for crude QTD and QTcD were 79.49% to 83.48%, the random-effects model was used in this meta-analysis. The standardized mean difference was 1.493 (95% Confidence interval: 0.786–2.201; standard error: 0.36; Z-value = 4.135, p < 0.001) for crude QTD (Figure1). For QTcD, the standardized mean difference was 1.441 (95% confidence interval: 0.739–2.143; standard error: 0.356; Z-value = 4.042, p < 0.001). respectively.

**Conclusion:** Depression is associated with increased QT dispersion in healthy cardiac subjects. Our data suggest that depression could be associated with changes in cardiac electrophysiological properties, which may predispose these individuals to cardiac arrhythmias.

109666

Modality: E-Poster Young Researcher – Non-case Report

Category: CARDIAC ARRHYTHMIAS/ELECTROPHYSIOLOGY/ELECTROCARDIOGRAPHY

## Proposal of a New Algorithm for Tachyarrhythmias’s Management During Pregnancy Including Drug Safety

BRUNA SANTI DOS SANTOS^1^, Eduardo Bartholomay^1^, Adriano Kochi^1^, Karina Andrade^1^, Bruna May^1^, Thaís Nicola^1^, Cristiano Jaeger^1^, Daniel Souto Silveira^1^, Eduardo Schlabendorff^1^, Cícero de Campos Baldin^1^, Euler Manenti^1^

(1) Hospital Mãe de Deus

**Background:** Due to advanced maternal age, cardiovascular risk factors, and the successful management of congenital heart disease conditions, the prevalence of cardiovascular disease complicating pregnancy is rising. When arrhythmias are present, they may increase fetal and pregnant morbidity and mortality. The use of medications during pregnancy requires a thoughtful approach, balancing fetal and maternal risk. Therefore the management of tachyarrhythmias during pregnancy including drug safety should be a core knowledge area for all physicians who work with emergency, obstetrics or cardiology.

**Objective:** Our purpose is to facilitate the approach of tachycardias in pregnant women by presenting a new algorithm based on the current evidence regarding the management of this pathology including drug safety in this group.

**Methods:** Formulation of a new algorithm for tachyarrhythmias’s management during pregnancy including drug safety.

**Results:** The new algorithm proposed is presented in the figure.

**Conclusion:** Besides rising prevalence of cardiovascular disease complicating pregnancy, any arrhythmia’s treatment must be carefully evaluated, aiming at the safety of the fetus and the mother, preventing both from taking unnecessary risks. We expect that the new algorithm presented considering drug safety in pregnant women facilitates the approach of tachyarrhythmias in these patients.


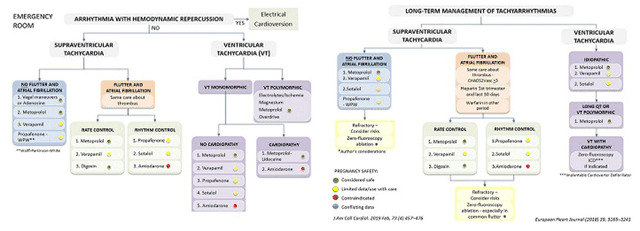


109674

Modality: E-Poster Young Researcher – Non-case Report

Category: NURSING

## Application for Decision-Making About Nursing Diagnoses of Patients with Heart Failure: Methodological Study

ALYNE SANTOS BORGES^1^, Alyne Santos Borges^1^, Ana Carla Dantas Cavalcanti^1^, Flávio Luiz Seixas^1^, Paola Pugian Jardim^1^

(1) Universidade Federal Fluminense

This study developed and evaluated an application for clinical decision-making in the care of patients with heart failure, registered at the National Institute of Industrial Property. As a general objective to develop and evaluate a mobile application based on the classification of nursing diagnoses from NANDA International Inc. for clinical decision-making in the care of patients with heart failure. As specific objectives: to map the defining characteristics of priority nursing diagnoses; develop an application for mobile devices for clinical decision making, and; to evaluate the usability of the application for clinical decision-making in the care of patients with heart failure.

**Method:** This is a methodological study, carried out in three phases. In the first phase, the conceptual definition was carried out from the survey of the nursing diagnoses of the previously validated Nursing Consultation Instrument of the Heart Failure Clinic Coração Valente. A scope review was carried out with the objective of mapping the nursing diagnoses of patients with heart failure. In the second phase, the Nursing DiagnosIC mobile application was developed through meetings of a scientific committee composed of researchers from Nursing and Computer Systems. In the third phase, an evaluation of the usability of the application was carried out by expert nurses and Information Systems undergraduates, using a predictive and prospective technique.

**Results:** From the scope review, 41 articles, 14 nursing diagnoses and 147 defining characteristics of patients with heart failure were mapped. The application has 19 screens, 205 defining characteristics, 27 nursing diagnoses, subdivided into 11 domains, using the version (2018–2020). The evaluation of the application was carried out by two groups containing a total number of 16 participants. The Nursing DiagnosIC was considered to have good/excellent usability (75.3%). The evaluated heuristics received 55 points considered high usability.

**Conclusion:** With the developed and validated application, it is expected to impact the care of patients with heart failure through a technology for clinical decision-making, with easy access to the accurate judgment of nursing diagnoses of patients with heart failure.

111070

Modality: E-Poster Young Researcher – Non-case Report

Category: CARDIO-ONCOLOGY

## The Association between Arterial Hypertension and Cancer Worsens Physical Capacity, Tumor Progression, Cachexia, and Heart Damage

LUIS FELIPE RODRIGUES^1^, Bruno Rocha de Avila Pelozin^1^, Edilamar Menezes Oliveira^1^, Tiago Fernandes^1^

(1) School of Physical Education and Sport (EEFE), University of São Paulo (USP).

**Introduction:** Cardiovascular diseases followed by cancers are the leading causes of death in the world. The bidirectional relationship between the diseases may potentiate cardiac damage and tumor progression, however little is known about their association. Our work aimed to investigate the impact of the association between arterial hypertension (AH) and cancer (CA) on physical capacity, tumor progression, cachexia percentage, and cardiac morphology in rats.

**Method:** Wistar Kyoto (n = 18) and SHR (n = 18) male rats at 12 weeks old were assigned into 4 groups: Wistar Kyoto control (WKY), control tumor (WKY-T), SHR control (SHR), and SHR tumor (SHR-T). Walker-256 tumor cells (2.5 × 106 cells in 0.5 mL of phosphate-buffered saline) were subcutaneously injected into CA groups while control rats were injected with a vehicle. We assessed: tumor weight and growth, cachexia percentage (CP), grip strength, exercise tolerance, cardiomyocytes cross-sectional area (CSA), and left ventricular (LV) mass by LV: tibia length ratio.

**Results:** SHR-T animals showed greater tumor growth (160%; p < 0.0001) and weight (96%; p < 0.05) compared to WKY-T group. In addition, SHR-T showed a higher CP (10 ± 1.96%; p < 0.001) compared to WKY-T group (2.84 ± 0.42%). The SHR showed a higher maximal muscle strength of hind limbs (2345 ± 81 g; p < 0.05) compared to WKY group (2033 ± 70 g). Otherwise, the association between AH and CA reduced the muscle strength in the SHR-T (1662 ± 94 g; p < 0.05) compared to the WKY-T group (1990.00 ± 35.29 g). Regarding the physical capacity, both groups with CA WKY-T (457 ± 44 m; p < 0.001) and SHR-T (297 ± 50 m; p < 0.001) developed exercise intolerance compared to WKY (695 ± 40 m); whereas the association between the diseases had even greater impairment in performance compared to CA alone. As expected, AH promoted cardiac remodeling by an increase in LV weight (23.9 ± 0.44 g/mm; p < 0.05) compared to WKY (21.96 ± 0.51 g/mm). However, both CA groups WKY-T (20.17 ± 0.44 g/mm; p < 0.0001) and SHR-T (20.54 ± 0.5 g/mm; p < 0.0001) showed a reduction in LV weight compared to WKY. This reduction in LV weight was accompanied by a reduction in cardiomyocytes CSA in the SHR-T (399 ± 11 µm^2^; p < 0.05) compared to SHR group (458 ± 12 µm^2^), highlighting that the combination of both diseases contributes to cardiomyocyte atrophy.

**Conclusion:** Our data highlight the harmful effects of the bidirectional relationship between AH and CA on physical capacity, tumor progression, cachexia, and cardiac damage.

109709

Modality: E-Poster Young Researcher – Non-case Report

Category: HEART FAILURE/CARDIOMYOPATHY/TRANSPLANT

## Adaptation and Validation of the Edmonton Symptom Assessment System for Patients with Heart Failure in Palliative Care

PAOLA PUGIAN JARDIM^1^, Ana Carla Dantas Cavalcanti^1^, Bruno Bompet dos Santos^1^, Alyne Santos Borges^1^, Camila Achao Rosa^1^, Paula Vanessa Peclat Flores^1^

(1) Universidade Federal Fluminense (UFF)

**Introduction:** Heart failure (HF) is considered a complex syndrome, characterized by a wide range of symptoms. Because it is a progressive chronic disease that limits life, there is a growing recognition of the important role of palliative care in HF. Symptom control is one of the goals of palliative care, but its assessment in HF remains a challenge. The Edmonton Symptom Assessment System (ESAS) scale has been used for this purpose in cancer patients, however, to our knowledge, there are no validated scales for use in monitoring the severity of HF symptoms.

**Objective:** To adapt and validate the content of the ESAS for use in patients with HF in palliative care.

**Method:** Methodological study divided into three phases: 1– Definition of content through a scoping review of the main signs and symptoms of HF in palliative care; 2– Adaptation of the ESAS content, based on the symptoms found in the scoping review and on the symptoms of the original version of the ESAS; and 3- Validation of the content through a committee of 30 expert judges. For analysis, the content validity index ≥0.80 was adopted.

**Results:** The first version of the ESAS for use in IC (ESAS-IC) consisted of 33 symptoms. The ESAS-IC was evaluated by 16 nurses, 6 cardiologists, 4 physiotherapists, 2 psychologists, 1 nutritionist and 1 educator, 2 of them were palliative care professionals. Most specialists were female (83.3%), with a median age of 35 years old and an interquartile range of (32–41.5) years. The group consisted of 14 masters, 8 specialists, 7 doctors and 1 bachelor, with a median of experience working with HF of 7.5 years and an interquartile range of (3.75–10.25) years. After analysis, 23 symptoms were validated and after suggestions from experts, the second version of the ESAS-IC was composed of 20 symptoms (pain, shortness of breath, tiredness/weakness, sadness, sleep, anxiety, swelling/edema, cough, loss of weight, feeling of well-being, dizziness, urinary incontinence, numbness/tingling, weight gain, problems with sexual interest/activity, self-esteem concerns, mental confusion, Improved ability to perform activities of daily living, spiritual well-being and memory).

**Conclusion:** This study adapted and validated the ESAS content for use in patients with HF. However, there is still a need for further validation. The use of the ESAS-IC can contribute to improving the health outcomes of patients with HF, in terms of quality of life, symptom control and readmissions, among others.

109726

Modality: E-Poster Young Researcher – Non-case Report

Category: HYPERTENSION/RENAL DENERVATION

## Assessment of Religiosity and Spirituality in the Care of Patients with Systemic Arterial Hypertension: Is There an Influence?

STEPHANIE RODRIGUES SCHAFF^1^, Elise Souza dos Santos Reis^1^, Mario Augusto Cray da Costa^1^, Cassiano Ianke^1^

(1) Universidade Estadual de Ponta Grossa – Paraná

Systemic arterial hypertension (SAH) is a public health problem and an important risk factor for cardiovascular diseases. Religiosity/spirituality (R/S) are practices that can interfere both in adherence and in the therapeutic goals of certain diseases.

**Objective:** To evaluate the degree of influence of R/S in the drug treatment of SAH.

**Method:** Study carried out at the cardiology outpatient clinic of a high complexity hospital (HAC) and a basic health unit (UBS) from September to December 2020 and August to December 2021, respectively. A total of 240 patients were evaluated using accredited scales to assess R/S and treatment adherence. Patients were divided into two groups: group with R/E and group without R/E. The p value was considered significant with p < 0.05.

**Results:** In the general group there was a predominance of females (68.4%) in the group with R/S and males (54.1%) in the group without R/S (p: 0.006). White skin color prevailed in both groups with statistical difference (79.3% × 67%, p: 0.069). The other sociodemographic characteristics had no statistical difference between the groups. Regarding therapeutic adherence, the prevalence of high adherence was obtained both in the group with and in the group without R/S (82% × 69%, p: 0.026). The group with R/S from HAC achieved greater achievement of therapeutic goals (52.8% vs. 35.5%, p: 0.033). At the UBS, there was a high degree of adherence in both groups, with a statistical difference (83.3% × 63%, p: 0.011).

**Conclusion:** Hypertensive patients with R/S had a greater adherence to treatment.

109730

Modality: E-Poster Young Researcher – Non-case Report

Category: HYPERTENSION/RENAL DENERVATION

## Treatment with Aliskiren Reverses Ultrastructural Changes Caused by Hypertension in Model 2K1C

LUCIANA LONTRO ALVES^1^, Carolina da Silva Valentim^1^, Priscila Gomes Pereira^1^, Raíssa Leal de Carvalho dos Santos Cunha^1^, Kíssila Rabelo^1^, Bianca Torres Ciambarella^1^, Ana Lucia Rosa Nascimento^1^, Jorge José de Carvalho^1^, Jemima Fuentes Ribeiro da Silva^1^

(1) Universidade do Estado do Rio de Janeiro (UERJ)

Renovascular hypertension (RH) is characterized by an increase in systemic blood pressure due to partial or total obstruction of the renal artery leading to overstimulation of the renin-angiotensin-aldosterone system. RH is potentially reversible and the system blockers are the ones chosen to control blood pressure. We aimed evaluate the antioxidant enzymes profile and tissue remodeling in the carotid artery of Wistar rats induced to HR by the two kidneys one clip (2K1C) model proposed by Goldblatt. Animals were divided in groups: SHAM; 2K1C; 2K1C+A. Treatment was administered via orogastric gavage with Aliskiren (10 mg/kg/day) for 30 days. Was performed electron microscopy and immunohistochemistry for collagens, metalloproteinases (MMPs), superoxide dismutase 1 (SOD1), catalase and glutathione reductase (GSH). Collagen I had greater staining in the hypertensive groups and aliskiren decreased your distribution. Collagen III was more expressed in the SHAM and the 2K1C+A present reduced expression. MMP2 show in the 2K1C an irregularity in the distribution and a more intense expression and the treatment decreased the marking. MMP9 2K1C showed more intense marking in concentrated areas, the treatment had no changes. Both catalase and GSH showed greater immunostaining in the SHAM group compared to the hypertensive groups. SOD expression was predominant in groups with hypertension. Oxidative stress enzymes showed no differences with treatment. Electron microscopy 2K1C generated alterations in endothelial cells, observed: pyknotic nucleus, poverty of organelles and decrease in their adhesion. We visualized fragmented and disorganized elastic lamellae. The treatment, these characteristics were not observed, indicating that Aliskiren contributed to the preservation of the carotid ultrastructural tissue. The SHAM group, on the other hand, presented ultrastructural characteristics expected for a control group. In conclusion, hypertension generated structural and ultrastructural changes in the carotid artery, that were reversed or preserved with the administration of Aliskiren. Oxidative stress had no effect with treatment.


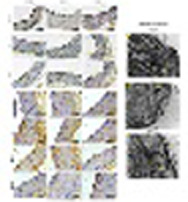


109737

Modality: E-Poster Young Researcher – Non-case Report

Category: NURSING

## Usability of Cardiac Defibrillators by Healthcare Professionals

PAULA RODRIGUES DOS SANTOS PIRES^1^, Monaliza Gomes Pereira^1^, Renato Dias Barreiro Filho^1^, Renata Flavia Abreu da Silva^2^

(1) National Institute of Cardiology – INC; (2) Federal University of the State of Rio de Janeiro – UNIRIO

**Introduction:** Technological advances and the increase in healthcare complexity have made it important to emphasize the concept of usability, which can be understood as the ability of a product to be used in the face of a specificity to users, objectives and context. The usability of high-risk medical devices, such as defibrillators, is highlighted due to their clinical importance in their proper management and their potential to cause harm to patients and professionals who operate them.

**Objective:** To identify the usability of defibrillators focusing on the recognition of their model type, wave type and the ideal selection of the load to be used.

**Method:** Descriptive study with a quantitative approach carried out with healthcare professionals in a public system hospital, located in the city of Rio de Janeiro (RJ), Brazil (BR), whose data collection took place between March and May 2021. The population of the study were healthcare professionals, namely, nurses, physicians, nursing residents and medical residents, allocated in care for adult patients and operating the cardiac defibrillator in clinical practice. There were no exclusion criteria. Sampling was done for convenience, with the sending of a semi-structured online questionnaire, which was divided between questions about the professional profile and about the operational usability and technical domain of the defibrillator in use in the subject’s sector. The study was approved by the Research Ethics Committee and received number 4,489,535 and 4,499,886.

**Results:** 58 healthcare professionals took part, being 40 (69%) nurses, 14 (24%) doctors and 4 (7%) not identifying their professional category. When questioned, 34 (59%) healthcare professionals answered correctly about the defibrillator in their unit. The majority, 45 (78%), could not report the defibrillator wave in their sector. As for the energy load, 25 (43%) participants were unable to relate the manufacturer’s recommendation for the defibrillator in their unit and 28 (48%) subjects were not able to inform the American Heart Association’s recommendation, both in situations of shockable rhythms.

**Conclusion:** Gaps were identified in the knowledge of healthcare professionals attending in the study regarding the use of the defibrillator, necessary for its handling, which may negatively influence its usability.

109904

Modality: E-Poster Young Researcher – Non-case Report

Category: CARDIAC ARRHYTHMIAS/ELECTROPHYSIOLOGY/ELECTROCARDIOGRAPHY

## Case Series of Patients Undergoing HV Measurement Simultaneously with Transcatheter Aortic Valve Replacement

PATRICIA FERREIRA DEMUNER^1^, Daniel de Magalhães Freitas^1^, Giulliano Gardenghi^1^, Silvio Roberto Borges Alessi^1^, Mauricio Lopes Prudente^1^, Fernando Henrique Fernandes^1^, Ellen Gonçalves Guimarães^1^, Débora Rodrigues^1^, Max Weyler Nery^1^, Ricardo Curado de Oliveira e Silva^1^, Larissa Xavier Alves de Oliveira^1^, Fernando Araújo Cintra Canedo^1^

(1) Hospital Encore

**Introduction:** Transcatheter aortic valve implantation (TAVI) is one of the treatments of choice for patients with severe aortic stenosis. In the last decade, there have been technical and technological advances, but there has been no decrease in the number of cases requiring permanent pacemaker implantation (MPD): those with total atrioventricular block (TAVB) or second-degree Mobitz type II. One of the predictors of the need for MPD is the development of a left bundle branch conduction disorder, which can be diagnosed even in the subclinical form in an invasive way by measuring the his-ventricular interval (HV). Data on the impact of left bundle branch block after TAVI are scarce and treatment is individually tailored leading to marked differences in clinical management.

**Objective:** To describe a series of 7 cases of patients undergoing TAVI simultaneously with the electrophysiological study (EPS), in order to immediately assess the need for MPD after implantation.

**Material and Methods:** 7 patients were evaluated (4 males; mean age 80 years; mean BMI 23.3) whose data were collected in the Tasy® electronic medical record system and tabulated and analyzed by excel®. The EPS was performed with a quadripolar diagnostic catheter and positioned in the bundle of His to measure the HV interval, when the HV measurement exceeded 70 ms, MPD implantation was indicated. For TAVI, the technique used was the same in all cases, with transfemoral access, under sedation and local analgesia, Sapien®, Corevalve revolute R® and Evolut® prostheses, sizes ranging from 20 to 34 mm.

**Results:** Among the patients analyzed, 4 evolved with left bundle branch block after TAVI. MPD implantation was indicated for 1 patient, the other patients underwent removal of the provisional MP while still in the hemodynamics room. The patient with an indication for MPD underwent implantation of the device during the same hospitalization, without intercurrences. The HV interval ranged from 46 to 58 ms (average 53,28) before the intervention, to 52 to 84 ms (average 62,8) after, approximately 18% positive variation. Only one patient died of an etiology unrelated to the procedure. No FES-related complications have been described.

**Conclusion:** The FES strategy during TAVI seems to be a viable strategy to stratify patients for the risk of TAVB and 2nd degree Mobitz type II AVB in an early way, already referring them to appropriate treatment without demonstrating an increase in the risks related to the procedure.

109753

Modality: E-Poster Young Researcher – Non-case Report

Category: HYPERTENSION/RENAL DENERVATION

## Residential Blood Pressure Monitoring: Analysis of a Database of 1474 Patients

MATHEUS TOSCANO PAFFER^1^, Matheus Vieira Cabral Figueiredo^1^, Carmen Beatriz Oliveira Silva Veras^1^, Francyelle Maria Barbosa Fonsêca^1^, Pedro Toscano Paffer^1^, Ramon Barbosa Barros^1^, Tatiana Maria Toscano Paffer^1^

(1) Faculdade de Medicina de Olinda

**Introduction:** Hypertension is a highly prevalent disease, being one of the main risk factors for cardiovascular events. With just the measurement of blood pressure in the medical office, white coat hypertension can occur, clinical condition characterized by abnormal blood pressure values in the office, but with values considered normal by home blood pressure monitoring (HRPA). The aim of this study is to analyze a HRBP database from a private cardiology clinic, showing cases of white coat hypertension.

**Methods:** Evaluate the results of HMBP exam of 1474 patients from a private cardiology clinic in Recife/PE, Brazil. The device used in all patients was the Omron 705 CP Blood Pressure Monitor.

**Results:** A total of 1474 patients, of which 57.8% were women, with a mean age of 53.4 years (+–16.17). The mean office measurement of systolic blood pressure was 130.68 mmHg (+–18.54) and diastolic 81.83 mmHg (+–11.55), the overall mean systolic pressure of the HRBP exam was 123.99 mmHg (+–14.3) and diastolic was 76.5 mmHg (+–8.99). Among this total, 196 patients (13.3%) who were diagnosed with white coat hypertension were analyzed separately. Of these 196, 50.5% were men and the mean age was 56 years (+–15.15). Mean office systolic blood pressure averaged 148.5 mmHg (+–11.79) and mean diastolic blood pressure of 90 mmHg (+–8.78). The overall mean of systolic blood pressure was 125 mmHg (+–6.78) and diastolic pressure was 76.4 mmHg (+–6.44).

**Conclusion:** White coat hypertension has a significant prevalence in the total number of patients evaluated, evidencing the importance of using HMBP for the diagnosis of arterial hypertension. Therefore, it is important to follow the guidance of the main hypertension guidelines around the world, such as the European one, which guide the use of this test in the diagnosis of arterial hypertension.

109764

Modality: E-Poster Young Researcher – Non-case Report

Category: ANTICOAGULATION

## Anticoagulation and Clinical Outcomes in Atrial Fibrillation: Retrospective Analysis of a Regional Hospital

ESTHER BOTELHO SOARES DA SILVA^1^, Conrado Roberto Hoffmann Filho^1^, Michele Tavares Mendonça^1^, Juliana Alzira Gonzales Oliveira^1^, Beatriz Vieira Roca^1^, Rafaela Louise Sales^1^, João Paulo Souza Brighenti^1^, Joao Paulo Panato Ribeiro^1^, Gabriel Erzinger^1^, Gilmar Sidnei Erzinger^1^, Jaqueline Barp^1^

(1) Hospital Regional Hans Dieter Schimidt

Atrial fibrillation (AF) is the most frequent cardiac arrhythmia affecting 37.5 million worldwide. It’s a major risk factor for ischemic stroke and has strong associations with other comorbidities. Age is one of the greatest risk factors to develop AF. Life expectancy is increasing as does AF incidence and prevalence, affecting approximately 10% of subjects over 80 years. Effective anticoagulation reduces the burden of disabilities and death caused by stroke. Assess real-world data from our institution and provide tools to improve our effectiveness in diagnosing and treating this disabling disease. Retrospective study based on medical records. The inclusion criteria were AF and atrial flutter. From May 2021 to January 2022, we analyzed one thousand medical records of patients treated at our institution. Most of the population was male (58.4%), white patients (96.4%). The presence of comorbidities was frequent, especially arterial hypertension and heart failure (80.8% and 52.3%, respectively). Breathing disorders were present in 36.3% while diabetes corresponded to 32.8%. Coronary artery disease 28,1%, kidney disease 12,8%, cerebrovascular disease 8.7% and tobacco use 8.6%. 33.3% of patients had paroxysmal AF, 50.6% permanent, 8% persistent, 5.3% flutter and 2.8% indeterminate. The mean age for men was 66.9 years and for women 69.9 years. Male mean CHADSVASC was 3.2 HASBLED 1. Average female CHADSVASC was 4.4 HASBLED 2. The BMI for men was 28.4 kg/m² and 29.3 kg/m² for women, mean CKD EPI was 65. Comparing the number of strokes a greater rate was observed with warfarin versus DOAC (20.27% and 17.83% respectively). Regarding bleeding, the rate was 5.3% with DOACS and 6.37% with warfarin. The total death rate with DOACS was 10.36% and with warfarin 15.83% with an absolute risk reduction of 5.47% (p = 0,003). In the stroke analysis, patients treated with warfarin had a higher rate when compared with DOAC (p = 0,021). In this study of a public health care service, it was observed that a large percentage of patients were using DOAC therapies. It was also observed that the rate of bleeding and stroke was lower with the use of DOACs, however without statistical significance by chi squared test. The overall death rate was shown to be reduced in favor of DOAC therapy over warfarin. In the final analysis, comparing stroke patients, those treated with warfarin had a higher rate when compared with DOAC. One explanation could be the lack of adequate adjustment of the INR.

109789

Modality: E-Poster Young Researcher – Non-case Report

Category: CARDIOVASCULAR PHARMACOLOGY

## Nanomedicine Associated with Coronary Artery Disease: A Theragnostic Method?

GUSTAVO SANTOS PORFIRO^1^, Gustavo Santos Porfiro^1^, Leonardo Favaro Pereira^1^, Lucas Dalvi Armond Rezende^1^, Helena Alves de Andrade Ribeiro^1^, Fernando Luiz Torres Gomes^1^

(1) Universidade Federal do Espírito Santo

**Introduction:** Cardiovascular diseases are the most common cause of death around the globe, with approximately 17.9 million people dying from heart attacks in 2021, representing 31% of global deaths. The rupture of atheromatous plaques is a major health problem and the main cause of heart attacks. Despite new technological advances for the diagnosis and treatment of coronary artery disease, early detection remains static, so that, commonly, myocardial infarction is the first clinical manifestation of this involvement. Thus, nanotechnology has been opening new horizons in the theragnostic perspective for the improvement and prevention of cardiac conditions such as coronary artery disease.

**Objective:** To perform an integrative literature review to establish the main findings and relationships between coronary artery disease and nanoparticles.

**Methods:** The method of integrative literature review was adopted, covering the period from January 2022, in the PubMed and Cochrane Library databases, using the descriptors: nanoparticles, coronary artery disease, and nanomedicine.

**Results:** Sustained evidence was found about the miRNA of atherosclerotic plaque and the synthesis of nanoparticles, being characterized with low toxicity and anti-inflammatory properties, attenuating atherosclerosis, which would ease the cases of myocardial infarction due to atherosclerotic obstruction.

**Conclusion:** The use of antibodies and peptides that recognize markers associated with cardiovascular disease has allowed the development of a more current generation of selective nano-systems. However, further studies on how nanoparticles can act in the intra-atheroma environment are needed to improve the understanding of nanomedications, generating important therapeutic contributions.

109790

Modality: E-Poster Young Researcher – Non-case Report

Category: SPIRITUALITY AND CARDIOVASCULAR MEDICINE

## Comparison of Religiosity/Spirituality of Hypertensive Patients between Primary Health Care and High Complexity Hospital

STEPHANIE RODRIGUES SCHAFF^1^, Elise Souza dos Santos Reis^1^, Mario Augusto Cray da Costa^1^, Cassiano Ianke^1^

(1) Universidade Estadual de Ponta Grossa – Paraná

**Introduction:** Systemic arterial hypertension (SAH) is a public health problem and an important risk factor for cardiovascular diseases. Religiosity/spirituality (R/S) are practices that can interfere both in adherence and in the therapeutic goals of certain diseases.

**Objective:** To compare the degree of influence of R/S in the drug treatment of SAH in hypertensive patients who adhere to religious practices in a high-complexity hospital (HAC) and a basic health unit (UBS).

**Method:** Study carried out at the cardiology outpatient clinic of a HAC and UBS from September to December 2020 and August to December 2021, respectively. A total of 155 patients were evaluated according to accredited scales to assess R/S and treatment adherence. Patients were divided into two groups: group with R/S from HAC and group with R/S from UBS. The p value was considered significant with p < 0.05.

**Results:** There was a predominance of females in both the HAC and UBS R/S groups (60.7% × 78.8%, p: 0.016). Regarding marital status, there was a predominance of married people in both groups (53.9% × 68.2%, p: 0.003). The other sociodemographic characteristics had no statistical difference between the groups. Regarding therapeutic adherence, the prevalence of high adherence was obtained both in the group with R/S from the HAC and from the UBS (80.9% × 83.3%) with no statistical difference. With regard to therapeutic goals, both groups achieved goals in more than half of the interviewees (52.8% × 56%).

**Conclusion:** Hypertensive patients with R/S from both HAC and UBS had greater adherence to treatment and achievement of therapeutic goals by more than 50%.

109796

Modality: E-Poster Young Researcher – Non-case Report

Category: CARDIOLOGY OF SPORTS, EXERCISE, ERGOMETRY AND CARDIOVASCULAR REHABILITATION

## A Walking Diary in Phase-1 Cardiac Rehabilitation: A Randomized Clinical Trial

GABRIELA LAGO ROSIER^5^, Gleide Glícia Gama Lordello^1^, Patrícia Alcântara Doval de Carvalho Viana^2^, Luiz Eduardo Fonteles Ritt^3^, Gilson Soares Feitosa Filho^4^

(1) Bahiana School of Medicine and Public Health; (2) Hospital Santa Izabel, Santa Casa da Bahia; (3) Hospital Cárdio Pulmonar; (4) Hospital Aliança; (5) UniFTC

**Introduction:** Inpatient rehabilitation is extremely important for patients recovering from cardiac surgery. Although a walking diary is routinely used in clinical practice, it has yet to be adequately tested and reported in the literature.

**Objectives:** To establish whether use of a walking diary affects the number of steps taken following cardiac surgery and whether this is related to the patient’s level of cardiac anxiety.

**Methods:** An open, controlled, randomized clinical trial was conducted at a referral hospital in cardiology with adult patients submitted to elective valve and/or coronary surgery, who had no motor impairment. Following discharge from the intensive care unit, all the participants used a pedometer to register the number of steps taken over five consecutive days. Twenty-nine individuals were randomized to an intervention group to use the walking diary as treatment strategy, while twenty-three were allocated to a control group. Statistical significance was defined as p < 0.05. A statistician blinded to patient allocation conducted the analysis on an intention-to-treat basis.

**Results:** The groups were similar regarding their demographic, clinical and surgical characteristics. Mean age was 59.3 ± 13 years, most participants (76.9%) were male, and the most common type of surgery was myocardial revascularization (57%). There was no difference between the groups regarding the total number of steps taken: control group = 1,496 (477.5–2992.5) vs. intervention group = 1,468.5 (494.2–2,678) (p = 0.902).

**Conclusion:** Use of the walking diary had no effect on the number of steps taken and was unassociated with the level of cardiac anxiety in inpatients following cardiac surgery.

109797

Modality: E-Poster Young Researcher – Non-case Report

Category: DIGITAL HEALTH/INNOVATION

## New Method to Fix Temporary Pacemakers in Order to Reduce Cable Displacements and Loss of Pacing

HEIGLON ESTEVÃO BONELLA DENTI^1^, Vítor Boniatti Neves^2^, Edimar Lima^3^

(1) Universidade Federal da Fronteira Sul – Campus Passo Fundo; (2) Faculdade IMED; (3) Hospital de Clínicas de Passo Fundo

Transvenous temporary pacemaker (PM) is very useful for patients with severe bradycardia. Studies that analyzed its complications estimated that the rate of loss of capture is up to 43%. They have also found that the displacement of the PM cable requiring repositioning is one of the most common complications, occurring in 32% to 38% of the procedures performed. Cable displacement occurs because kits manufactured to install the devices do not have effective methods to lock in the pacemaker cable. This study proposes a simple, inexpensive and effective change in the temporary transvenous pacemaker kit (please see image attached). The original kit has a thread (blue color in the image attached) that locks the cable at the distal end of the contamination protection cap, but between this thread and the introducer the cable is relatively loose (it does not have a good fixation). Currently, the thread (in blue as seen in the image attached) is kept at the distal end of the protective cap as it is produced. The change this study proposes is to remove the blue thread from the distal end and to adapt this thread inside the plastic to the proximal end of the protective cap, securing it to the end of the introducer. Thus, it is possible to handle and to reposition the cable, if necessary, without losing the sterile environment. On the other end, instead of the thread, one could fix the plastic with micropore so there is no displacement of the protective cover over the cable which could accidentally reduce the sterile area. In this way, the cable is locked at the end of the introducer and does not allow displacements. With the proposed change, the sterile area of the cable is kept inside the protective cover, and it is still possible to loosen the cable if necessary and retighten after handling it because the blue thread will remain inside the protective cover (wrapped by sterile plastic). We concluded that this is an effective way to assemble the transvenous PM kit (from the attached photo) to avoid the loss of pacing due to cable displacement. This modification can be carried out when manufacturing the kit.


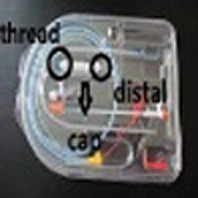


109798

Modality: E-Poster Young Researcher – Non-case Report

Category: CARDIOVASCULAR SURGERY

## Systematic Review of Transcatheter Versus Surgical Aortic Valve Replacement in High-Risk Patients with Severe Aortic Stenosis: The Choice of Treatment Influences Mortality Rates Over the Years?

LEONARDO TORREÃO BEZERRA CAVALCANTI^1^, Glaudir Donato Pinto Junior^1^, Ana Karolina Bento da Silva^1^, Lívia Farias de Holanda Furtado^1^, Manassés Almeida do Nascimento^1^, Gustavo Gomes Santiago^1^, Lucas Caetano da Silva^1^, Andressa Alves de Carvalho^1^, Wanessa Alves de Carvalho^1^, David Cesarino de Sousa^1^, Marcela Lukerli Araujo Paulina da Silva^1^, Evellyn Pereira de Melo^1^

(1) Federal University of Paraíba, UFPB

**Introduction:** Transcatheter aortic valve replacement (TAVR) has emerged as an alternative treatment for patients with aortic stenosis, especially at high surgical risk (HSR). However, the procedure is not always available at lower complexity health centers. Further, controversies remain regarding the impact of TAVR on mid- and long-term mortality compared to surgical aortic valve replacement (SAVR).

**Aims:** To compare TAVR versus SAVR mortality over the years after the procedure from randomized clinical trials (RCT) with patients at HSR.

**Methods:** We searched Medline, Embase, LILACS and SciELO on March 27th, 2022, for TAVR versus SAVR RCTs. Were included studies that provided mortality data in follow-up for both interventions performed in HSR patients. Were excluded duplicates and studies that did not meet the inclusion criteria. An independent review was performed by two authors following the PRISMA protocol.

**Results:** After applying the eligibility criteria, 20 studies remained to be reviewed. These studies included follow-ups from days up to 5 years. Considering mortality within 30 days, there was no significant difference between patients undergoing TAVR or SAVR. However, some studies marked a greater association of perioperative complications, such as bleeding and atrial fibrillation, linked to SAVR invasiveness. Still, major vascular events and even intraoperative deaths were seen in TAVR intervention. Regarding mortality from 1 to 3 years after the procedure, the studies diverged: while some showed no difference, others reported a reduction in mortality in the TAVR group. This result was also found in studies evaluating patients with diabetes and patients with chronic lung disease. Moreover, prosthesis-patient mismatch (PPM) was significantly lower in TAVR compared to SAVR, which in severe PPM was related to increased risk of death at 2 years. Nevertheless, paravalvular regurgitation was found to be more frequent in TAVR, which was associated with lower survival rates. Overall, at follow-ups of more than 3 years, there was no significant mortality difference between the groups.

**Conclusions:** For 1 to 3 years follow-ups, it was outlined an advantage for TAVR, especially in the groups with diabetes and chronic lung disease. However, the complications arising from both interventions do not seem to be associated with statistically different mortality rates, especially at longer periods of follow up.

109984

Modality: E-Poster Young Researcher – Non-case Report

Category: ATHEROSCLEROSIS/CARDIOVASCULAR RISK FACTORS/CARDIOVASCULAR PREVENTION

## Cardiovascular Risk Associated with Anabolic Steroid Use: A Systematic Review

EDUARDO FRANCO CORREIA CRUZ FILHO^1^, Eduardo Franco Correia Cruz Filho^1^, Ana Carolina Souza Diniz^1^, Fernando de Paiva Melo Neto^1^, Emanuel Francisco de Carvalho Pinto^3^, Lucas Vinicius Rafael Figueiredo^1^, Lorena Souza dos Santos Lima^1^, Laís Nóbrega Diniz^3^, Gustavo Nóbrega de Melo^2^, Gustavo Soares Fernandes^1^

(1) Centro Universitário de João Pessoa – UNIPÊ; (2) Faculdade de Ciências Médicas da Paraíba – FCM; (3) Faculdade de Medicina Nova Esperança – FAMENE

**Introduction:** Anabolic androgenic steroids (AAS) are widely used for cases of osteoporosis, hypogonadism, Turner syndrome, hormone therapy in climacteric women, and are even used without indication as a form of treatment to increase physical performance. However, its use has several important adverse reactions in the cardiovascular system, often fatal to the individual, which make its use controversial and in most cases contraindicated.

**Objective:** The present study aims to evaluate the cardiovascular risk of AAS use.

**Methods:** A systematic literature review study, based on the PRISMA methodology, conducted with published articles on the Virtual Health Library and the United States National Library of Medicine (PubMed), between 2017 and 2022. The descriptors utilized were “Cardiovascular Disease” and “Anabolic Agents”, and their variations, associated with the Boolean operators “AND” and “OR”.

**Results:** Initially, 57 articles were selected, which were chosen for manual selection based on their abstracts. In this way, duplicated articles and whose themes did not fit the objectives of the work were excluded, thus, 07 articles were chosen for the present work. Of the 07 articles involved, 05 are literature reviews, 01 meta-analysis and 01 case report. Among the studies evaluated, 100% highlighted the negative cardiovascular effects of steroid use, of which 85% evaluated these risks in the general population, while 15% focused on the risks for postmenopausal women using hormone therapy. As for the substance’s manifestations, the main alterations presented were: dyslipidemia (86%), coronary heart disease (86%), arterial hypertension (57%), cardiomyopathy (42%) and arrhythmia (42%). The meta-analysis that specifically evaluated the woman, compared tibolone with placebo and no treatment, especially evaluating its impact on lipoprotein (a) and the overall lipid profile.

**Conclusion:** It is important to have well elucidated the cardiovascular risks due to the indiscriminate use and in supraphysiological doses of AAS, which in the long term can lead to negative and more severe outcomes. Furthermore, in order to evolve in this topic, more evidence-based therapeutic approaches are important, with clear objectives on the use of AAS and the risks of atherosclerosis, infarction and myocardial spasms and arrhythmias.

110051

Modality: E-Poster Young Researcher – Non-case Report

Category: CARDIOVASCULAR PHARMACOLOGY

## Cardiovascular Impact Glp-1 Receptor Agonist in Patients with Diabetes Mellitus Type II using Glp-1 Receptor Agonist

EDUARDO FRANCO CORREIA CRUZ FILHO^1^, Eduardo Franco Correia Cruz Filho^1^, Camila Araújo Novais Lima^1^, Anna Julie Medeiros Cabral^1^, Gabriel Lucena de Carvalho Soares^1^, Waneska Lucena Nóbrega de Carvalho^2^

(1) Centro Universitário de João Pessoa – UNIPÊ; (2) Universidade Federal da Paraíba

**Introduction:** Diabetes Mellitus (DM) type II is a metabolic disease with a high incidence, affecting 370 million people worldwide. DM II generates systemic changes, allowing a series of secondary disorders, among them, the increase in cardiovascular risk. Adequate therapeutic management is relevant, mainly related to cardiovascular protection. In this context, the use of agonists of the peptide similar to Glucagon I (GLP-1) has been increasingly highlighted, both in diabetics and in patients with heart disease.

**Objectives:** To analyze major cardiovascular events in patients with type II DM using GLP-1 agonists.

**Methodology:** This is a systematic review. The searches were performed using the PubMed database with works published in the last five years. The Health Sciences Descriptors used in the searches were: “Glucagon-Like Peptide-1 Receptor”, “Diabetes Mellitus, Type 2” and “Cardiovascular Diseases”, linked to the Boolean operator “AND”. To ensure a better structuring and organization of the results, the PRISMA recommendation was used. Initially, 116 articles were selected, which were manually selected based on their abstracts, excluding duplicate articles and whose themes did not fit the objectives of the work. This final selection resulted in 02 articles.

**Results:** A meta-analysis using 360 articles and 56004 patients with type II DM evaluated the occurrence of non-fatal myocardial infarction, non-fatal stroke and death from cardiovascular or undetermined causes (Major Cardiovascular Adverse Effects – MACE). In that study, a significant 12% reduction in MACE was found for patients using GLP-1 receptor agonists. In addition, another cross-sectional study with 4076 patients with type II diabetes, which assigned patients to the use of efpeglanatide (2717) or placebo (1359), showed that major adverse cardiovascular events occurred in 189 patients (7.0%) using of efpeglanatide and in 125 patients (9.2%) on placebo.

**Conclusions:** In summary, GLP-1 agonists are currently widely studied, with effects in more than one area of interest and no longer a glucocentric perspective. Therefore, the most recent studies have shown an important application of this drug in patients not only with diabetes, but also with heart disease. The main point of attention is the reduction of MACE, as it represents a reduction in cardiovascular risk in patients with type II DM.

109884

Modality: E-Poster Young Researcher – Non-case Report

Category: CARDIAC ARRHYTHMIAS/ELECTROPHYSIOLOGY/ELECTROCARDIOGRAPHY

## Qt Interval and Associated Measures Predict Prognosis of Patients with Acute Stroke?

CATARINE BENTA LOPES DOS SANTOS^1^, Catarine Benta Lopes dos Santos^1^, Marcelo Bender Angst^1^, Sergio Ferreira de Ferreira^1^, Sheila Ouriques Martins^1^, Maurício Pimentel^1^

(1) Hospital de Clínicas de Porto Alegre

**Background:** The stroke is a relevant cause of mortality and disability around the world. Electrocardiogram is part of the clinical examination of patients with stroke and its alterations are used for the etiological and prognostic evaluation of the patients. Changes in ventricular repolarization assessed by measuring the QT interval and its associated variables have been recently studied in stroke patients.

**Objectives:** To evaluate the association of measures of the corrected QT interval (QTc), QTc dispersion, Tpeak-T-end dispersion (Tpe-d) and ratio Tpe/QT with mortality and neurologic disability (Rankin Scale) in ischemic stroke patients in the hospital discharge and within 3 months.

**Methods:** Retrospective cohort study including patients admitted with acute ischemic stroke in a tertiary university hospital. The measurements of the QTc, QTc-d, Tpe-d and Tp-e/QT intervals were performed by experts physicians. The outcomes evaluated were: total mortality and the Rankin Scale at hospital discharge and within 3 months. The comparison between groups was performed using the Kruskal-Wallis test.

**Results:** A total of 170 patients were included, predominantly female (53%), with a mean age of 64.4 ± 12.4 years. The length of hospital stay was 14 ± 21 days. Mortality during hospitalization was 11.17% and the total at 3 months was 14.1%. The results are shown in the table in milliseconds.

**Conclusion:** This cohort showed that QTc was higher in stroke patients who died within 3 months and those with worse Rankin at hospital discharge. The ratio Tpe/QT was lower in those patients who died during hospitalization. The assessment of ventricular repolarization expressed by the QTc interval and associated measures can identify more severe patients who need implementation of optimized treatment.


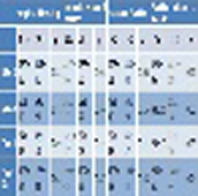


109886

Modality: E-Poster Young Researcher – Non-case Report

Category: CARDIOVASCULAR IMAGING

## Assessment of Miocardial Strain in Aortic Stenosis

RAÍSSA GABRIELA VIEIRA DA CÂMARA BARROS^1^, RAISSA GABRIELA VIEIRA DA CÂMARA BARROS^1^, EVELINE BARROS CALADO^1^, ELLEN MAGALHÃES LISBOA ALVES^1^, MONICA DE MORAES CHAVES BECKER^1^, LUCIA MARIA VIEIRA DE OLIVEIRA SALERNO^1^, ROBERTO DE OLIVEIRA BURIL^1^, JOSÉ RENATO E MELO FIGUEIROA^1^, Eduardo Cavalcanti Lapa Santos^1^, TACITO CUNHA LIMA^2^, ROBERTO VIEIRA DA CAMARA^2^

(1) UNIVERSIDADE FEDERAL DE PERNAMBUCO- UPE; (2) UNIVERSIDADE FEDERAL DO RIO GRANDE DO NORTE

Aortic stenosis (AS) is the most common primary valve disease in the US and Europe. The gold standard diagnosis of AS is to perform a echocardiogram (ECO) and the measurements to classified the severity are: maximum jet velocity (Vmax), maximum and mean transvalvular pressure gradient (GM) and aortic valve area by the continuity equation. The miocardial strain assessment through the global longitudinal strain (GLS) is one of the most promising techniques for the early detection of reduced left ventricular ejection fraction (LVEF) and has become useful in AS scenario by correlating with the severity of AS. The GLS is a technique to assess the myocardial deformation suffered by myocardial fibers through Speckle tracking (normal value is below –18%). Several studies have already shown that the reduction in GLS precedes the decrease in LVEF, with relative reductions of 15% in GLS predicting a subsequent decrease in LVEF. This study aims to detect early changes in ventricular systolic function not only based on LVEF by Simpson’s method, but also through the assessment of the GLS in the AS and to correlate the pattern of changes in the GLS with the degree of stenotic impairment observed. General objective: Assess global and segmental LV systolic function through myocardial strain in aortic stenosis. Secondary objectives: Characterize the distribution pattern of the GLS in the AS; correlate the degree of AS with the myocardial GLS; correlate LVEF by Simpson’s method and GLS.

**Methods:** This is an observational, descriptive, cross-sectional study.

**Results:** A total of tirty patients were obtained, with a prevalence of 33% of major AS, 36.7% of moderate AS, 16.7% of mild AS and 13.3% of paradoxical AS was evidenced. An inverse relation between the severity of the AS and the GLS could be seen, that is, the more severe the stenosis, the lower the GLS value. We observed an average GLS in important AS of –17.6%, in moderate AS of –18.86%, in mild AS of –19% and in paradoxical AS of 16.85%. Regarding the pattern of segmental distribution of GLS values, there was a greater involvement of the mid-basal segments than the apical segments of the LV.

**Conclusion:** The present study suggest a progressive impairment of LV systolic function assessed by the GLS as the severity of AS progresses, even though there is no direct impact on the LVEF measurement what can be primordial on the decision of early valve replacement despites the severity of AS founded on ECO.

109890

Modality: E-Poster Young Researcher – Non-case Report

Category: HEART FAILURE/CARDIOMYOPATHY/TRANSPLANT

## Alcohol Ablation Versus Surgical Myectomy in the Treatment of Obstructive Hypertrophic Cardiomyopathy: A Systematic Review using the Prisma Method

ANA LÍGIA VALERIANO DE OLIVEIRA^1^, Matheus Araújo Borges^1^, Gabrielly de Souza Correia^1^, Eduarda Tatico Lagares^1^, Lucas Eduardo Almeida França^1^, Isabela Castro Pereira^1^

(1) Pontifícia Universidade Católica de Goiás

**Introduction:** Alcoholic septal ablation (ASA) consists of a selective infusion of high-grade alcohol in a septal branch that supplies the basal interventricular septum. It is a technique used as an alternative to surgical myectomy in obstructive hypertrophic cardiomyopathy (HOCM). The procedure seeks to create an iatrogenic infarction and, as a consequence, reduce left ventricular outflow tract (LVOT) obstruction. AAS has been improved over time, with benefits similar to myectomy.

**Objective:** To analyze the benefit of treating hypertrophic obstructive cardiomyopathy with alcohol septal ablation as an alternative to myectomy.

**Methodology:** This is a systematic review following the Preferred Reporting Items for Systematic Reviews and Meta-Analyses – PRISMA methodology. The literature search was carried out between January and February 2022, using the Scientific Electronic Library Online (SciELO), PubMed and Latin American and Caribbean Literature (Lilacs) databases. Thirty-three articles were selected, in English and Portuguese, excluding those that did not fit the objectives of this review and the methodological recommendations. The filters used were “human” and “published in the last 10 years”. The descriptors used were “obstructive hypertrophy cardiomyopathy”, “myectomy” and “alcohol ablation”.

**Results:** Rigopoulos et al., found that threatening arrhythmic events seem be rare after ASS and occur more in patients with an estimated very high risk of sudden death. One study confirmed these findings, finding ventricular tachyarrhythmia or 30-day sustained ventricular fibrillation in only 7% of patients. ASS is a therapeutic alternative in patients with advanced age or comorbidities and showed shorter hospital stay and reduced post-procedure pain. Naidu et al., concluded that ASS results were similar to those of myectomy up to 8–10 years, in 90% of patients. showed to be able to reduce LVOT and relieve symptoms. Another relevant finding was the need for experienced services to perform SSA so that complications are less and the greatest therapeutic effects.

**Conclusion:** AAS has become an alternative to myectomy that can be considered in carefully selected patients. Data show similar functional and hemodynamic benefit to myectomy, in addition to shorter hospital stay, reduced pain and associated complications.

109942

Modality: E-Poster Young Researcher – Non-case Report

Category: COVID-19 AND CARDIOVASCULAR SYSTEM

## Decreased T3 Levels as a Marker of Clinical Worsening in Patients with Sars-Cov-2 and its Correlation with Serum Troponin at Diagnosis – a Retrospective Cohort Study

VITOR FEUSER DA ROSA^1^, Guilherme Pinheiro Machado^1^, Victor Henrique Ignácio de Souza^1^, Georgia Martina Chichelero^1^, Simone Magagnin Wajner^1^

(1) Hospital de Clínicas de Porto Alegre

**Introduction:** The SARS-COV-2 pandemic had a great impact on the morbidity and mortality of affected patients. T3 has a direct impact on cardiomyocytes, causing myocardial injury in patients with SARS-COV-2. Clinical observations have revealed a relatively high (up to 64%) prevalence of sick euthyroid syndrome among COVID-19 patients, with some exhibiting a profound decrease in thyroid hormone levels. We know that low levels of T3 in sick patients occur early, correlate with disease severity, and that normalization of hormone serum concentrations is related to clinical recovery.

**Objective:** The objective of the study is to describe the changes in thyroid hormones present in patients diagnosed with COVID-19. The primary outcome of the study is to assess the relationship between T3 levels and the degree of cardiac involvement.

**Methods:** Samples from PCR positive patients for COVID-19 who were referred for admission to the ward or clinical ICU of a tertiary hospital were included. Serum samples stored in the COVID-19 biobank at Hospital de Clínicas de Porto Alegre were used. Half of the samples collected in the year 2020 and half collected in the year 2021 were requested. In the requested serum, T3 levels were measured on arrival at the hospital and around day 7 of hospitalization.

**Results:** The number of patients enrolled in the study was 119. The mean age of patients was 61 ± 29 years. About 31.09% of the patients in the study died and 68.90% were discharged from the hospital. Among patients who died, T3 on the 1st day of hospitalization had a mean of 52.01 ± 16.48 and among patients who were discharged, the mean T3 on the first day of hospitalization was 62.25 ± 22, 14. On arrival at the hospital, among those who died, the mean troponin was 623 ± 152 and among those who were discharged was 176 ± 34.5. The T3 levels on the 7th day of the patients who died had an average of 42.66 ± 7.22 and of those who were discharged it was 60.98 ± 21.53.

**Conclusion:** The study seeks to evaluate two tests that are relatively possible to obtain in patients admitted to hospitals of great complexity in the context of the COVID-19 pandemic, with the objective of helping medical teams in taking conducts and evaluating the prognosis of these patients. It is possible to infer that patients who have T3 levels below 50 have a higher risk of death associated with elevated troponin values, regardless of other clinical factors.

112331

Modality: E-Poster Young Researcher – Non-case Report

Category: CARDIAC ARRHYTHMIAS/ELECTROPHYSIOLOGY/ELECTROCARDIOGRAPHY

## A New Machine Learning-Based Model for Risk Stratification of Patients Undergoing Ventricular Tachycardia Ablation

PETER VAMOSI^1^, Marton Tokodi^1^, Patrik Toth^1^, Ferenc Komlosi^1^, Istvan Osztheimer^1^, Peter Perge^1^, Katalin Piros^1^, Zoltan Sallo^1^, Nandor Szegedi^1^, Bela Merkely^1^, Laszlo Geller^1^, Klaudia Vivien Nagy^1^

(1) Semmelweis University Heart and Vascular Center, Department of Cardiology

**Background:** Monomorphic ventricular tachycardia (VT) is a life-threatening condition. Catheter ablation is an effective treatment method for many patients, but significant variability is observed in postprocedural mortality, due to the high burden of comorbidities and other factors. Therefore, there is a high need for an accurate risk stratification system.

**Aim:** We sought to implement a machine learning pipeline to predict 1-year all-cause mortality in patients undergoing VT ablation.

**Methods:** For 265 consecutive VT ablation patients at our center, we processed procedural, demographic and medical history data, their laboratory and echocardiographic findings (63 parameters). To predict 1-year all-cause mortality, several supervised machine learning models were trained and evaluated using 5-fold cross-validation with applying recursive elimination to identify the optimal subset of input features. We quantified their performance with the area under the receiver operating characteristic curve (AUC); we identified the most important predictors by Shapley values. Finally, we used topological data analysis to visualize patient subgroups with different mortality risks.

**Results:** 57 (22%) patients died during the 1-year follow-up. The best predictive performance was achieved by a random forest model with 18 input features [AUC: 0.73 (95% CI: 0.68–0.78)], which significantly outperformed previously published risk scores (I-VT [AUC: 0.63 (95% CI: 0.55–0.70), p < 0.001], PAINESD [AUC: 0.63 (95% CI: 0.55–0.71), p = 0.009]). The most important predictors were mitral E-wave deceleration time, cardiac resynchronization therapy, age, electrical storm, and hemoglobin concentration. In the topological network based on the input features of the above model, we identified five groups with differing clinical characteristics and mortality rates (Figure).

**Conclusions:** Our machine learning model could effectively predict 1-year all-cause mortality in VT ablation undergoing. Thus, it facilitates the identification of high-risk patients and the personalization of treatment and follow-up strategies, ultimately leading to improved outcomes.


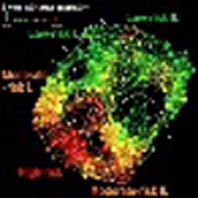


109975

Modality: E-Poster Young Researcher – Non-case Report

Category: HEART FAILURE/CARDIOMYOPATHY/TRANSPLANT

## Literature Review: Left Ventricular Noncompaction of the Myocardium

CAROLINE TEIXEIRA BERNARDI^3^, Beatriz Sadigursky Nunes Cunha^1^, Rafael Fortes Locateli^1^, Giana Bevilacqua Schmitz^1^, Bárbara Müller Rettore^1^, Gustavo de Lemos Souza^1^, Sergio Rosas de Barros Neto^1^, Ângela Quatrin Campagnolo^1^, Lucca Corcini Biscaino^2^, Pedro Miguel Mariussi^3^, Sérgio Luís Pereira Furtado^1^

(1) Universidade Federal de Santa Maria – UFSM; (2) Universidade Franciscana – UFN; (3) Hospital de Clínicas da Faculdade de Medicina da Universidade de São Paulo – FMUSP

**Introduction:** Left ventricular noncompaction (LVNC) is defined as a pattern of prominent trabeculae, intra-trabecular recesses, and a thin compacted layer on the left ventricular (LV) wall. It is an entity with yet unclear physiopathology, and may be congenital or acquired. The prevalence ranges from 0,014 to 1,3%.

**Objective:** Summarizing LVNC’s main findings of clinical manifestations, etiology, diagnosis, prognosis and treatment.

**Methodology:** Our research was conducted on PubMed with using “Isolated noncompaction of the ventricular myocardium”, and papers from the last 5 years were selected. 188 results were found and a selection was made from their abstracts and afterwards from reading the full article. The final number of selected articles for this review was 58.

**Results:** LVNC’s clinical presentation may range from an asymptomatic entity to more serious manifestations, such as arrhythmias, heart failure and embolic events. Genetics play an important role in this condition, although a definitive correlation between genotype and phenotype has not yet been established. LVNC is similar to other cardiomyopathies, genetically heterogeneous and more frequently inherited as a dominant autosomal disorder or related to the X chromosome. There are no gold standard criteria for its diagnosis, which leads to many false positives. Transthoracic echocardiography, cardiovascular magnetic resonance and computerized tomography are the most frequently used imaging methods, allowing for morphologic and functional evaluation. Patients with LVNC present a significantly higher risk of fatal arrhythmias – requiring implantable cardioverter-defibrillator (ICD) – death, heart failure and cardiac transplantation. Patients with these presentations have an increased risk of death in 6 years of 47–75%. Furthermore, a higher prevalence of this pathology was observed in other genetic conditions, such as Ebstein’s anomaly (15%) and aortic coarctation (3%), among others. A definitive treatment is not presented in any guidelines, and the orientation is to avoid and treat heart failure, thromboembolism and arrhythmias caused by the condition.

**Conclusion:** The lack of sufficient knowledge of its physiopathology, the still restricted understanding of the genetics and the diagnostic challenges are obstacles in fully understanding LVNC, and further studies are required to better characterize this pathology.

109976

Modality: E-Poster Young Researcher – Non-case Report

Category: CARDIAC ARRHYTHMIAS/ELECTROPHYSIOLOGY/ELECTROCARDIOGRAPHY

## Catheter Ablation in Early Rhythm Control in Patients with Atrial Fibrillation and Heart Failure with Reduced Ejection Fraction: A Systematic Review

ANA KAROLINA BENTO DA SILVA^1^, Lucas Caetano da Silva^1^, Gustavo Gomes Santiago^1^, Leonardo Torreão Bezerra Cavalcanti^1^, Glaudir Donato Pinto Júnior^1^, Lívia Farias de Holanda Furtado^1^, Manassés Almeida de França^1^, Andressa Alves de Carvalho^1^, Wanessa Alves de Carvalho^1^, David Cesarino de Sousa^1^, Evellyn Pereira de Melo^1^, Marcela Lukerli Araújo Paulina da Silva^1^

(1) Universidade Federal da Paraíba

**Introduction:** The association between atrial fibrillation (AF) and heart failure with reduced ejection fraction (HFrEF) increases the risk of cardiovascular complications. The Early Treatment of Atrial Fibrillation for Stroke Prevention Trial (EAST-AFNET 4) demonstrated that early rhythm-control therapy generated favorable outcomes in patients with AF.

**Objective:** This study sought to investigate whether there is benefit in catheter ablation (CA) based rhythm-control in patients with AF and HFrEF compared to other strategies.

**Methods:** Clinical trials and randomized clinical trials found in the Medline, Embase, Cochrane, and Scopus databases that analyzed the use of CA in early rhythm-control in patients with HFrEF and AF — paroxysmal, persistent or diagnosed in less than 12 months — between 2012 and 2022 were included. Articles that contemplated long-standing persistent AF and did not indicate left ventricular ejection fraction or New York Heart Association (NYHA) functional class were excluded. An independent review was performed by three authors following the PRISMA protocol.

**Results:** Of the 41 articles found, 4 were selected for this review. Of these, 3 articles chose a similar primary outcome — composition of cardiovascular events, including death and hospitalization due to worsening of HF. Nevertheless, the results were discordant. In the CASTLE-AF trial, CA altered primary outcome and death and hospitalization for HF. In the CABANA trial, however, there was a significant variation only in isolated criteria. In a subanalysis of EAST-AFNET 4, Rillig et al., 2021 indicates that the early rhythm-control therapy positively impacted primary outcomes, but that CA alone showed no significant difference compared with the antiarrhythmic control strategy. Jones et al., 2013 chose to approach AF from a functional perspective — VO2max and impacts on quality of life after CA, with favorable results after 3 months of the procedure.

**Conclusion:** Although CA is a promising alternative for early rhythm-control therapy in patients with AF, there is no precision on cardiovascular morbidity and mortality outcomes when HFrEF is associated. The variability of results found in this review indicates the need for even more robust future clinical trials in order to outline the possible benefit of the optimized indication of CA in this group.

109981

Modality: E-Poster Young Researcher – Non-case Report

Category: CARDIOLOGY OF SPORTS, EXERCISE, ERGOMETRY AND CARDIOVASCULAR REHABILITATION

## The Role of Cardiopulmonary Exercise Testong (Cpet) in Predicting Morbidity Outcomes in Grown-Up Congenital Heart (Guch) Disease – a Preliminary Study

LAIZ TEIXEIRA PONTES^1^, Alice Cunha Darzé^1^, Pamela Castro Pereira^1^, ALmir Ferraz^1^, Ana Luíza Guimarães Ferreira^1^, Carlos ALberto Cordeiro Hossri^1^, Carolina Christianini Mizzaci^1^, Flavia Bernardes Morais^1^, Guacira Grecca^1^, Susimeire Buglia^1^, Ediele Carneiro Brandão^1^, Rica Dodo Delmar Buchler^1^

(1) Instituto Dante Pazzanese de Cardiologia

**Introduction:** Given the advances in diagnosis and clinical-surgical therapies, congenital heart defects are increasing their prevalence in the adult population. Yet, there is still no consensus on the best way to follow up with these patients. Among the validated tools for monitoring patients with heart disease, the Cardiopulmonary Exercise Test (CPET) plays an important role. However, the use of this test for monitoring the population of Grown Up Congenital Heart (GUCH) still lacks studies. This study aimed to assess the correlation between ergospirometry variables and the severity of the GUCH population measured by echocardiographic aspects.

**Methods:** A retrospective cohort with 248 GUCH (Table 1) over 18 years old, was referred to CPET, from 2015 to 2021, in a tertiary hospital in the state of São Paulo. Patients were sequentially included, and those in functional class IV or with contraindications to CPET were excluded. Ergospirometry variables were analyzed in association with ventricular function – estimated by echocardiogram in the same period.

**Results:** Most patients were in functional class I and II (86.3%). In the echocardiographic findings, 40% had pulmonary hypertension and almost all had preserved left ventricle function. The CPET showed a median VO2 peak around 69% of predict. Other parameters are summarized in Table 2. CPET variables were able to stratify the severity of GUCH, mainly by pulmonary hypertension. Comparing CPET data and imaging diagnosis, VE/VCO2 slope >32 and OUES <60% were related to the presence of pulmonary hypertension.

**Conclusion:** The CPET is an important resource for prognostic and diagnostic definition in the evolution of GUCH patients.


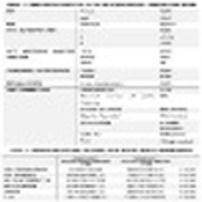


110000

Modality: E-Poster Young Researcher – Non-case Report

Category: EPIDEMIOLOGY AND HEALTH POLICIES/GLOBAL HEALTH

## Evaluation of the Rate of Arterial Hypertension Control in the Basic Health Strategy: A Systematic Review

GUSTAVO GOMES SANTIAGO^1^, Ana Karolina Bento da Silva^1^, Leonardo Torreão Bezerra Cavalcanti^1^, Evellyn Pereira de Melo^1^, Marcela Lukerli Araujo Paulina da Silva^1^, Andressa Alves de Carvalho^1^, Wanessa Alves de Carvalho^1^, David Cesarino de Sousa^1^, Glaudir Donato Pinto Júnior^1^, Lívia Farias de Holanda Furtado^1^, Manassés Almeida do Nascimento^1^, Lucas Caetano da Silva^1^

(1) Universidade Federal da Paraíba

**Introduction:** Systemic Arterial Hypertension (SAH) is a multifactorial condition defined by persistent elevation of blood pressure (BP). It is considered an important public health problem due to its high prevalence and an independent cardiovascular risk factor. Studies show higher morbidity and mortality from cardiovascular disease (CVD) among individuals with high BP. Between 2008 and 2017, 667,184 deaths attributable to SAH were estimated in Brazil and it is estimated that more than 30% of Brazilians have SAH. However, despite the high prevalence, it is considered that the control of SAH in Brazil is still poor.

**Objective:** To assess the rate of control of SAH at the level of the Basic Health Strategy and the socioeconomic indicators of cardiovascular risk and mortality.

**Methodology:** For the production of this integrative literature review, articles were searched in PubMed and SciELO databases, using the following descriptors: “Primary Health Unit” and “Hypertension”. The following inclusion criteria were used: articles from 1996 to 2020; studies in English and Portuguese. The following exclusion criteria were used: studies that did not correlate the two descriptors; published outside the period from 1996 to 2022; studies not available in English, Spanish and Portuguese. 732 articles were found, 5 of which included randomized double-blind studies, literature review and clinical trials.

**Results:** BP values were considered controlled in most studies (systolic blood pressure lower than 140 mmHg and diastolic blood pressure lower than 90 mmHg). However, some studies have adopted different values for BP control and different BP targets depending on the target group that hypertensive patients are in (elderly, diabetic, post-infarction, post-stroke, etc). The rate of SAH control at the primary health care level in Brazil, according to the studies evaluated, ranged from 20 to 53.9%. There was a great diversity of techniques adopted to measure the control rate, such as repeated BP measurements by different devices (oscillometric, auscultatory, etc.), active search in medical records and in databases. There was variation in the BP control rate according to the Brazilian states.

**Conclusion:** There is a wide variation in the frequencies of SAH control rates. Consequently, further studies are needed for a better care strategy and reduction of cardiovascular morbidity and mortality.

110036

Modality: E-Poster Young Researcher – Non-case Report

Category: DIGITAL HEALTH/INNOVATION

## Initial Experience of the Telehealth Center at the University Hospital of the Federal University of Piauí: Results from the Tele-Icu

PATRYCK ARAUJO DANTAS DA SILVA^1^, Carlos Eduardo Batista de Lima^1^, Igor Denizarde Bacelar Marques^1^, Victor Eulálio Campelo^1^, Maria Susane Filgueira Barreto Ferreira^1^, Lucas Cortez Macedo^1^, Lina Madeira Campos Melo^1^, Alessandro Aita^1^, Ginivaldo Victor Ribeiro do Nascimento^1^, Paulo Márcio Sousa Nunes^1^

(1) Hospital Universitário da Universidade Federal do Piauí

**Introduction:** Teleconsulting activities in remote intensive care units can improve the quality of care and health indicators in regions with less access to specialists. In times of the Covid-19 pandemic, this technology has been increasingly used in assistance actions in health services.

**Objective:** To evaluate the clinical-demographic profile and the remote coverage of the telehealth service in the studied population.

**Methods:** The ICU telehealth system consists of a base at the HU-UFPI and a analysis system called Gestor Saúde that keeps the peripheral units connected in a teleconsultation platform, allowing simultaneous consultation with the on-duty physician in the remote unit. From August 2020 to February 2022, all data from teleconsultations in intensive care performed in the telehealth service of the Hospital Universitário of the Federal University of Piauí provided at the Floriano, Bom Jesus, São Raimundo Nonato and Parnaíba units were evaluated. Data on age group, sex, unit of origin of care and clinical diagnosis of patients were analyzed. Other variables included were the use of vasoactive drugs and the need for oxygen therapy. The results were tabulated in an Excel spreadsheet and presented in a descriptive way.

**Results:** 2114 teleconsultations were carried out in the ICU, most of them in male patients over 80 years old, followed by the age group from 60 to 69 years old. Most diagnoses were sepsis, with a lower proportion of patients with Covid-19 infection and traumatic brain injury. Among the centers assisted, the one with the highest number of consultancies was the city of Floriano, which already had a link with the UFPI due to a similar service previously implemented. Patients from 68 cities in Piauí were assisted, demonstrating the coverage of the project.

**Conclusion:** In this initial experience of the telehealth center in the ICU of the HU UFPI, we observed the shortcomings that places far from large health centers present, such as, for example, a shortage of specialists in intensive care, internal operational difficulties and complementary exams. The frequent contact with HU-UFPI specialists helped to improve processes and transmit the quality culture that has been proven to improve results in the ICU. We encountered technical difficulties during implementation, but they did not prevent the process from progressing in most centers.

110040

Modality: E-Poster Young Researcher – Non-case Report

Category: PHYSICAL EDUCATION

## Comparison of Cardiac Chronotropic Response of Cardiopathes Participating in Cardiac Rehabilitation During Endurance Training and Resistance Training

RENATA MARIA BEGNI AFONSO^1^, Dáira Karoline Silva de Sousa^1^, Thiago Nascimento dos Santos^1^, Juliana Magalhães Santos^2^, Rafaela Rodrigues de Sousa^2^, Thaiane Campos Martins^2^, Luana Cristina Pereira Pacheco^11^, Lucas Heitor Moura^2^, Renato Luiz de Alvarenga^3^

(1) Instituto Carlos Chagas; (2) Cardioclin; (3) UFRJ

**Introduction:** Resistance training in Cardiac Rehabilitation is still underused, even though the low strength and muscle mass levels of this population. This is due to the fear of a greater cardiovascular risk in relation to this model, although it is already understood in the literature that heart rate (HR) is more responsive to the time of muscle stimulus than the applied load, presenting higher mean values in resistance training than in resistance training.

**Objective:** To compare the HR behavior of cardiopaths trained during resistance and endurance exercise.

**Methods:** Eighteen cardiac patients (65 ± 12,9), practitioners of Cardiac Rehabilitation, clinically stable and medicated with beta adrenergic blockers, performed their usual training on a horizontal exercise bike Movement RT230 (Brazil), between 10 and 15 minutes and 40% and 80% of reserve FC. Soon after, they performed resistance training in the Buick leg extension (Brazil): 10 submaximal repetitions and after 2 min of interval, 5 more submaximal repetitions with the addition of 20% of the previous load. HR was measured using the Polar Verity Sense transmitter tape (Finland), before and shortly after each exercise model, and a ∆HR (final – initial) was calculated. For statistical analysis, the Friedman test and the Bonferroni post-test were used, with p < 0.05.

**Results:** Table 1 shows the highest HR value for the group of exercise bike compared to the others, which was confirmed with p = 0.018 and p = 0.000 when compared to 10 repetitions and 5 repetitions, respectively.

**Conclusion:** A greater spread of resistance training is encouraged in Cardiac Rehabilitation programs, in order to provide eligible cardiac patients with all the cardiovascular and peripheral benefits that resistance training makes possible.

110050

Modality: E-Poster Young Researcher – Non-case Report

Category: NUTRITION

## Arterial Stiffness Associated with Sympathetic Hyperactivity in Obese Individuals with Moderate to Severe Obstructive Sleep Apnea

SAMANTA MATTOS CARDOSO^1^, Samanta Mattos Cardoso^1^, Michelle Rabello Cunha^1^, Marcia Regina Simas Torres Klein^1^, Mario Fritsch Toros Neves^1^

(1) Universidade do Estado do Rio de Janeiro

**Introduction:** Obstructive sleep apnea (OSA) is known to be an independent cardiovascular risk factor. The presence of OSA and obesity may have synergistic effects on the progression of cardiovascular disease.

**Objective:** To evaluate sympathetic tone and vascular disease in obese patients with moderate and severe OSA.

**Methods:** Individuals of both sexes, aged 40–70 years and body mass index (BMI) ≥30 and <40 kg/m^2^, submitted to assessment of heart rate variability (HRV), central parameters by Mobil-O-Graph and carotid ultrasound. The sleep study was performed through a portable home sleep test device (WatchPAT).

**Results:** Patients (n = 76) were divided into two groups based on the apnea-hypopnea index (AHI): mild-absent (MA) group (AHI <15 events/h, n = 30) and moderate-severe group (MS) (AHI ≥15 events/h, n = 46). The mean age was higher in the MS group (50 ± 6 vs 54 ± 8 years, p = 0.022) and BMI was similar (35 ± 3 vs 34 ± 3 kg/m^2^, p = 0.239). As expected, the MS group presented higher oxygen desaturation index (3.4 ± 2.2 vs 17.6 ± 12.7 events/h, p < 0.001) and respiratory disturbance index (12 ± 4 vs 33 ± 14 events/h, p = <0.001). Systolic blood pressure (119 ± 13 vs 127 ± 15 mmHg, p = 0.004), pulse pressure (41 ± 8 vs 46 ± 10 mmHg, p = 0.026), cardiovascular risk (6.4 ± 3.8 vs 11.9 ± 9.5%, p = 0.012) and cardiometabolic age (48 ± 6 vs 52 ± 9 years, p = 0.033) were significantly higher in the MS group. The SD2/SD1 ratio (1.4 ± 0.4 vs 1.7 ± 0.6, p = 0.064) and the low frequency/high frequency (LF/HF) ratio (0.85 ± 0.51 vs 1.53 ± 1.76, p = 0.051) were higher in the MS group, although not reaching statistical significance. The pulse wave velocity (PWV) (6.9 ± 0.7 vs 7.7 ± 1.2 m/s, p = 0.003), normalized PWV (7.1 ± 0.8 vs 7.7 ± 1.6 m/s, p = 0.046), PWV adequacy (–0.14 ± 0.42 vs 0.07 ± 0.37, p = 0.030), vascular age (47 ± 6 vs 53 ± 9 years, p = 0.005), mean carotid intima-media thickness (cIMT) (0.59 ± 0.08 vs 0.66 ± 0.13 mm, p = 0.008) and maximum cIMT (0.64 ± 0.09 vs 0.71 ± 0.15 mm, p = 0.015) were significantly higher in the MS group. PWV was significantly correlated with LF/HF ratio (r = 0.611, p = <0.001) in the MS group.

**Conclusion:** In this sample of obese individuals, moderate to severe OSA was associated with sympathetic hyperactivity and evidence of accelerated vascular aging with arterial stiffness and subclinical atherosclerosis.

110944

Modality: E-Poster Young Researcher – Non-case Report

Category: HEART FAILURE/CARDIOMYOPATHY/TRANSPLANT

## Multidisciplinary Heart Transplant Outpatient Clinic: A Pilot Study of Pharmaceutical Care

LIDIA EINSFELD^1^, Leticia Orlandin^1^, Jacqueline Kohut Martinbiancho^1^, Thalita da Silva Jacoby^1^, Simone Dalla Pozza Mahmud^1^, Nadine Clausell^1^, Livia Adams Goldraich^1^

(1) Hospital de Clinicas de Porto Alegre, Brasil.

**Background:** The role of clinical pharmacists as part of multidisciplinary teams for solid organ transplant care has been discussed worldwide. Particularly, heart transplant recipients have a complexity of medication regimens, which greatly impact on both patient and transplant program outcomes.

**Aim of the study:** To evaluate the participation of clinical pharmacists in the multidisciplinary heart transplant outpatient clinic through the identification of drug therapy problems (DTPs) and interventions in the pharmacotherapy based on pharmacist recommendations.

**Methods:** In this descriptive pilot study, pharmacists provided direct patient care in the weekly heart transplant clinic from January to December 2021. Each patient seen at a pharmacist consult visit received a comprehensive assessment using a systematic drug therapy review process (Figure 1). Pharmacist’s activities were documented in electronic charts, and data, collected prospectively. DTPs and pharmacist interventions were classified according to the PCNE.

**Results:** A total of 207 clinic visits to 70 patients were performed and 223 DTPs identified, leading to a median of 2 [0, 9] interventions per consult visit. In more than a third of the opportunities, patients received a dose adjustment recommended by the pharmacist (39.2%). Frequent interventions included laboratory monitoring (15.9%) and medication deprescribing (9.3%). Immunosuppressants were the drugs most commonly involved (35.6%), followed by electrolyte supplements and antihypertensive agents (15.1% and 13.7%, respectively).

**Conclusion:** Our study was the first to describe pharmaceutical care of ambulatory heart transplant recipients in Brazil. Pharmacist-led interventions can potentially contribute to the management of challenging post-transplant pharmacotherapy.


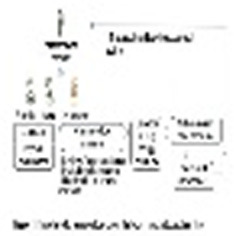


110056

Modality: E-Poster Young Researcher – Non-case Report

Category: HEART FAILURE/CARDIOMYOPATHY/TRANSPLANT

## Microcosting of Intravenous Diuretic use Strategy in Day Hospital for Patients with Heart Failure

DAYANNA MACHADO PIRES LEMOS^1^, Ana Paula Beck Da Silva Etges^3^, Nadine Oliveira Clausell^2^, Lívia Adams Goldraich^1^

(1) HOSPITAL DE CLÍNICAS DE PORTO ALEGRE; (2) UNIVERSIDADE FEDERAL DO RIO GRANDE DO SUL; (3) Instituto de Avaliação de Tecnologias em Saúde

**Background:** The use of intravenous (IV) diuretics to treat congestion in outpatients with heart failure (HF) seems to be as effective in maintaining clinical stability as usual hospital therapy, and can potentially reduce costs.

**Objective:** To assess the cost of a day hospital strategy for IV diuretic in patients with HF.

**Method:** Cost study was carried out in a public and university hospital in Brazil. Time-driven Activity-based Costing was applied to calculate the total cost of services and their composition per patient, in addition to an exploratory analysis of a day hospital schedule for HF.

**Results:** Data of 68 sessions of IV diuretic from 20 patients during 2020 were analyzed. On average, 80 mg of furosemide was administered in sessions lasting 39 minutes; 11 patients had a single session of IV diuretic, while 2 patients underwent 19 and 21 sessions each. The annual median cost per patient was US$ 34 (min. US$ 30; max. US$ 515) (Figure). Costs was attributed to professionals (58%), exams (23%), physical structure of the unit (10%), consumables (5%) and medicines (3%). A hypothetical schedule with 48 monthly sessions would cost US$ 1,464.

**Conclusions:** This study contributes with novel information involving microcosting of a day hospital strategy for IV diuretic treatment in patients with HF. This data can generate subsidies for the incorporation of such potentially cost-saving strategies in a sustainable manner.


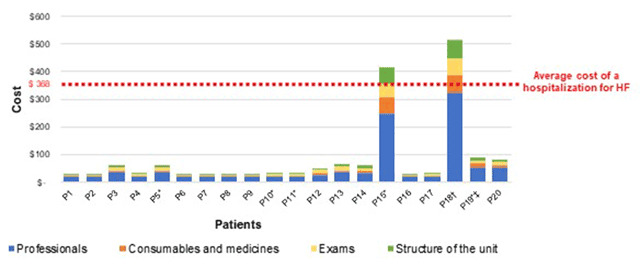


110183

Modality: E-Poster Young Researcher – Non-case Report

Category: HEART FAILURE/CARDIOMYOPATHY/TRANSPLANT

## Association of Cyp11B2 Rs3097 C> T and Rs3802228 G> a Polymorphisms with Reduced Ejection Fraction Heart Failure in a Population of Mixed Ancestry

GIZELLA DA CUNHA RODRIGUES^1^, Felipe Neves de Albuquerque^2^, Gustavo Salgado Duque^2^, Ariene Brito do Amaral^2^, Yasmin Lemos Rollemberg Cruz Machado^1^, Marcelo Imbroinise Bittencourt^2^, Denilson Campos de Albuquerque^2^, Dayse Aparecida da Silva^1^, Ricardo Mourilhe-Rocha^2^

(1) Laboratório de Diagnóstico por DNA, Instituto Roberto Alcântara Gomes, Universidade do Estado do Rio de Janeiro, Rio de Janeiro, Brasil; (2) Hospital Universitário Pedro Ernesto, Universidade do Estado do Rio de Janeiro, Rio de Janeiro, Brasil

**Introduction:** Heart failure (HF) is a clinical and multifactorial syndrome that affects approximately 26 million people around the world. HF is generally subclassified according to the left ventricular ejection fraction (LVEF) into 3 categories: HF with preserved ejection fraction (LVEF ≥ 50%), HF with mid range ejection fraction (LVEF 41%–49%), and HF with reduced ejection fraction (HFrEF, in which the LVEF is ≤40%). The pathophysiology of HFrEF is complex, and it is usually preceded by direct injury to the myocardium or a previous disease that leads to reduced ventricular contraction. The renin-angiotensin-aldosterone system (RAAS) is an important target to study the pathophysiological mechanisms involved in HF development.

**Objective:** The main objective of this study was to determine the association of rs3097 (C>T) and rs3802228 (A>G) single nucleotide polymorphisms, present in CYP11B2 gene, with HFrEF. Ancestry informative markers of polymorphisms insertion/deletion were also determined to identify individuals’ ancestry and exclude spurious associations regarding the selected genetic targets.

**Methods:** A total of 185 unrelated patients with HFrEF were selected, in addition to 124 unrelated volunteers without cardiovascular diseases. Data provided by HGDP-CEPH reference, containing profiles from African, European, and Native American populations were used to perform ancestry analysis estimations.

**Results:** The control and patient groups were aligned according to gender (HFmen = 66% versus Cmen = 55%, p = 0.06) and age (HF = 50.6 ± 11.6 versus C = 48.5 ± 8.9, p = 0.09). A statistically significant association can be observed in relation to the genotypic frequencies of rs3097 C>T between HF group versus controls when using the codominance (p = 0.0031), dominance (p = 0.0007) and overdominance (p = 0.0035) models. There were not observed any association regarding the rs3802228 A>G polymorphism. The analyses involving the ancestral relationships between the groups are still being carried out.

**Conclusion:** The preliminary results suggest a possible link between the T allele of CYP11B2 rs3097 C>T polymorphism and HFrEF.

110087

Modality: E-Poster Young Researcher – Non-case Report

Category: PHYSICAL EDUCATION

## Health Education for Rheumatic Heart Disease Prevention and Early Detection at a Highly Prevalent Peri-Urban Community in Mozambique

EDNA SUSANA LOPES LICHUCHA^1^, Adjine Mastala Fumo^1^, Andrea Neves^3^, Euridsse Amade^1^, Keila Jamal^4^, Karen Sliwa^5^, Ana Mocumbi^1^

(1) Instituto Nacional de Saúde (INS), Maputo – Mozambique; (2) Universidade Eduardo Mondlane (UEM), Maputo – Mozambique; (3) Hospital Geral José Macamo, Maputo – Mozambique; (4) Mozambique Institute for Health Education and Research, Maputo – Mozambique; (5) Cape Heart Institute, University of Cape Town – South Africa

Abstract Rheumatic Heart Disease (RHD) usually follows repeated episodes of acute rheumatic fever (ARF), which complicates untreated group A streptococcus (GAS) infections in susceptible individuals. RHD is a neglected disease that affects socially and economically disadvantaged young people in endemic areas, with a more malignant course in Africa, where it is an important cause of premature mortality, including indirect maternal mortality. We report an approach to increase awareness and promote health education of high-risk populations in a low-income setting.

**Methods:** The activities were implemented in peri-urban Maputo, Mozambique from February/2019 to February/2020. We trained 21 maternal health professionals in RHD screening and implemented a bi-monthly joint cardio-obstetric clinic involving health education sessions run by six patients living with RHD (PLRHD) trained as peer-educators. At Matchikitchiki Primary School we painted outdoor RHD educational walls (September–October/2019) and trained 40 teachers to support RHD book coloring and video workshops.

**Results:** The Cardiac-Obstetric Clinic assisted 127 women, of which 21 (17.3%) with RHD. Six trained PLRHD delivered 17 health education sessions to 127 patients. We trained 864 children (375 colored the book), 357 watched and discussed the educational video, and 232 were involved in outdoor activities using the RHD wall).

**Conclusions:** Training of teachers, maternal health professionals and PLRHD allows creation of awareness and early detection of RF/RHD in high-risk populations. These strategies may foster decentralization of early diagnosis and prevention of RF/RHD in highly prevalent communities in Africa.

110129

Modality: E-Poster Young Researcher – Non-case Report

Category: CARDIOVASCULAR INTENSIVE CARE/CARDIOVASCULAR EMERGENCIES

## Ecg Protocol: Evolution of Care Times for Patients with High Risk Acute Coronary Syndrome in Emergency Rooms in Salvador-Ba

RAYLANE MARQUES DE BARROS CRUZ^1^, Pollianna de Souza Roriz^1^, Daiane Dias de Jesus^1^, Marta Gabriela Moura Lopes^1^, Tatiana de Sena Leitão^1^, Rilary Silva Sales^1^, Shirley Casais Reis^1^

(1) Serviço de Atendimento Móvel de Urgência – SAMU

The electrocardiogram (ECG) is essential for the stratification of Acute Coronary Syndromes (ACS), in which the finding of ST-segment elevation reflects coronary occlusion and indicates the need for reperfusion therapies. In order to optimize the door-to-ECG time, the team responsible for the evaluation of cases with high risk ACS in the city of Salvador-BA, entitled “Protocol-IAM” (P-IAM), developed the “ECG Protocol”, easy to apply in the risk classification – implemented in the Pre-hospitalar Emergency Care Units (UPA) of the municipality since July/2020. The time elapsed between the ECG and the P-IAM trigger (ECG-trigger) is also relevant as it starts P-IAM attending.

**Objective:** To compare the door-to-ECG and ECG-trigger with high-risk ACS in patients at the UPA in the city of Salvador-Ba before and after promoting ECG Protocol in the city.

**Method:** This is a cross-sectional and descriptive study; The P-IAM database was used, referring to users diagnosed with high-risk ACS treated at the UPA in Salvador, between 03/04/2017 to 12/31/2021. The analyzed data were the mean and median (minutes) of the Symptom-Admission, and ECG-Trigger times; total number of patients on reperfusion therapy.

**Results:** A total of 1,679 patients were attended by the P-IAM accessing the public health system through the UPA during this period. Of these, 1,416 (84%) were diagnosed with ST-segment Elevation Myocardial Infarction (STEMI), with an increase of 58% comparing 2017 to 2021, followed by reperfusion therapies (171 in 2017 to 310 in 2021). Median times are seen in figure 1.

**Conclusion:** After the disclosure of the ECG Protocol, there was an increase of P-IAM team activations; greater sensitivity of the emergency network in identifying patients with STEMI, as beneficiaries of the program.


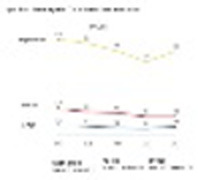


110139

Modality: E-Poster Young Researcher – Non-case Report

Category: ANTICOAGULATION

## The use of Oral Anticoagulants and Strategies to Improve its Adherence Among Outpatients with Heart Disease

ANDRESSA TEOLI NUNCIARONI^1^, Gabriella Sena do Nascimento Santos^2^, Renata Flávia Abreu da Silva^1^

(1) Alfredo Pinto Nursing School, Federal University of the State of Rio de Janeiro – UNIRIO; (2) State University of Rio de Janeiro- UERJ

**Introduction:** Among the drugs that make up the treatment of cardiovascular diseases, the class of oral anticoagulants (OAC) stands out. In Brazil, the main representative of the class is sodium warfarin, due to its low cost and distribution by the Brazilian Unified Health System. The treatment with OAC is complex and requires specific care related to prescription, guidelines for use, taking the drug, and monitoring of efficacy. Thus, adherence to treatment is necessary to ensure its full effectiveness and achieve positive prognosis.

**Objectives:** To identify OAC adherence among outpatients with heart disease and to recognize strategies for increasing adherence among non-adherents.

**Methods:** Cross-sectional quantitative study, conducted in the Anticoagulation Outpatient Clinic of a large Cardiology Federal Hospital, located in Rio de Janeiro, Brazil. Data collection occurred from March to July 2021. We included 76 patients in outpatient follow-up, older than 18 years of age, using OAC. Adherence to OAC was measured through the Instrument for Global Assessment of Medication Adherence (IAGAM), already validated for Brazilian culture and for the assessment of adherence among patients using OAC. Data was analyzed through RStudio Software. The research was approved by the local ethics committee under evaluation number 4.531.072 of 09/02/2021.

**Results:** Considering only medication adherence rate, the participants had averages above 99% when asked about taking the medication on the day, week, and month prior to data collection. However, when associating the number of pills taken with the care required, only 86.84% implement adequate care. These numbers represent the overall adherence measured by IAGAM. Strategies reported by participants to increase adherence were use of an alarm clock; placing the prescription in a visible place, such as the refrigerator and bedside table; keeping the medicine box next to bed, with the schedule written on it; taking the medication associated with routine activities, such as before going to work; family members being responsible for administering the medication, thus remembering the schedule and the correct dose.

**Conclusions:** The assessment of adherence to OAC should consider, in addition to taking the pills, the care needed to improve the drug efficacy. Strategies to enhance OAC adherence might target the underlying care.

110182

Modality: E-Poster Young Researcher – Non-case Report

Category: CARDIOLOGY OF SPORTS, EXERCISE, ERGOMETRY AND CARDIOVASCULAR REHABILITATION

## St-Segment Elevation and T-Wave Inversion Confined to V1–V4 in Young Soccer Players: Prevalence and Differences from Afro-Brazilians to Ghanaians

FILIPE FERRARI^1^, Henrique C. da Silva^2^, Artur H. Herdy^3^, Luiz G. M. Emed^4^, Felipe E. F. Guerra^5^, Haroldo C. Aleixo^6^, Guilherme D. Dilda^7^, Fernando Bassan^8^, Anderson D. da Silveira^9^, Ricardo Stein^1^

(1) Hospital de Clínicas de Porto Alegre (HCPA), Universidade Federal do Rio Grande do Sul (UFRGS), Porto Alegre, RS – Brazil; (2) Universidade do Estado do Pará (UEPA), Belém, PA – Brazil; (3) Instituto de Cardiologia de Santa Catarina, Santa Catarina, SC – Brazil; (4) Hospital Cardiológico Costantini, Curitiba, PR – Brazil; (5) Clínica Biocardio, Natal, RN – Brazil; (6) Universidade Federal de Minas Gerais (UFMG), Belo Horizonte, MG – Brazil; (7) Hospital das Clínicas da Faculdade de Medicina da Universidade de São Paulo (USP), São Paulo, SP – Brazil; (8) Universidade Estadual do Rio de Janeiro (UERJ), Rio de Janeiro, RJ – Brazil; (9) Hospital de Clínicas de Porto Alegre (HCPA), Porto Alegre, RS – Brazil

**Introduction:** Afro-Caribbean athletes may present some peculiarities in resting 12-lead electrocardiogram, such as T-wave inversion (TWI) confined to V1–V4 preceded by ST-segment and J-point elevation. However, the prevalence of this finding in young Afro-Brazilian football players (YABFP) is unknown.

**Purpose:** To compare the prevalence of ‘domed’ ST-elevation and TWI in V1–V4 among YABFP with young Ghanaian black football players (YGBFP).

**Methods:** A visual analysis was performed, as the data from the YABFP were raw data and those from Ghana were aggregated data. A forest plot was constructed with the point estimate and 95% confidence intervals.

**Results:** 668 YABFP (mean age: 21 years) and 159 YGBFP (mean age: 19 years) were evaluated and compared. The average height and weight were similar (178 cm and 73 kg for YABFP, and 175 cm and 68 kg for Ghanaian players). Ghanaians had a significantly higher prevalence of ‘domed’ ST-elevation and TWI in V1–V4 than YABFP (16.7% versus 2.4%, respectively).

**Conclusion:** YABFP presents a low prevalence of ‘domed’ ST-elevation combined with TWI in V1–V4, and African players had an almost 8-fold prevalence of this finding compared to Afro-Brazilian players.


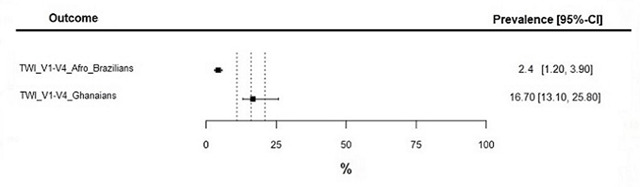


110181

Modality: E-Poster Young Researcher – Non-case Report

Category: CARDIOLOGY OF SPORTS, EXERCISE, ERGOMETRY AND CARDIOVASCULAR REHABILITATION

## Prevalence of Abnormal Electrocardiographic Findings in a Brazilian Cohort of Young Football Players: B-Pro Foot ECG Pilot Study

FILIPE FERRARI^1^, Henrique C. da Silva^2^, Luiz G. M. Emed^3^, Artur H. Herdy^4^, Guilherme D. Dilda^5^, Felipe E. F. Guerra^6^, Haroldo C. Aleixo^7^, Fernando Bassan^8^, Frederico P. L. Coimbra^9^, Mateus F. Teixeira^10^, Anderson D. da Silveira^11^, Ricardo Stein^1^

(1) Hospital de Clínicas de Porto Alegre (HCPA), Universidade Federal do Rio Grande do Sul (UFRGS), Porto Alegre, RS – Brazil; (2) Universidade do Estado do Pará (UEPA), Belém, PA – Brazil; (3) Hospital Cardiológico Costantini, Curitiba, PR – Brazil; (4) Instituto de Cardiologia de Santa Catarina, Santa Catarina, SC – Brazil; (5) Hospital das Clínicas da Faculdade de Medicina da Universidade de São Paulo (USP), São Paulo, SP – Brazil; (6) Clínica Biocardio, Natal, RN – Brazil; (7) Universidade Federal de Minas Gerais (UFMG), Belo Horizonte, MG – Brazil; (8) Universidade Estadual do Rio de Janeiro (UERJ), Rio de Janeiro, RJ – Brazil; (9) Hospital de Urgências de Goiânia, Goiânia, GO – Brazil; (10) Clube de Regatas Vasco da Gama, Rio de Janeiro, RJ – Brazil; (11) Hospital de Clínicas de Porto Alegre (HCPA), Porto Alegre, RS – Brazil

**Introduction:** A 12-lead resting electrocardiogram is a useful tool for diagnosing pathological conditions in athletes. The prevalence of electrocardiographic abnormalities in young Brazilian football players (YBFP) is unknown.

**Purpose:** To describe the prevalence of abnormal electrocardiographic findings in YBFP based on the “2017 International Criteria for Electrocardiographic Interpretation in Athletes”.

**Methods:** Cross-sectional/descriptive study. Intra-group differences were estimated by linear models or binomial and multinomial logistic regressions.

**Results:** 3.490 athletes from 41 Brazilian clubs, aged 15–35 years (median: 19 years) were evaluated. 1.668 were Caucasians, 1.154 were Mixed-race (MR), and 668 Afro-Brazilians (AB). T-wave inversion in the inferior leads (4%), high lateral leads (0.5%), V5 (2.4%), V6 (2%), and V5–V6 (2%) were identified. Prolonged corrected QT interval (0.1%), QRS ≥140 ms (0.1%), premature ventricular contractions (0.2%), PR interval ≥400 ms (0.03%), Wolff-Parkinson-White pattern (0.06%), and a suggestive case of a type 2 Brugada pattern were also observed. Other abnormalities were not observed. Overall, 216/3.490 (6%) YBFP had electrocardiographic changes considered to be abnormal.

**Conclusion:** This is the first large electrocardiographic cohort of YBFP described. In it, a prevalence of approximately 6% of abnormal findings was identified. Further evaluation in all these cases is indicated.


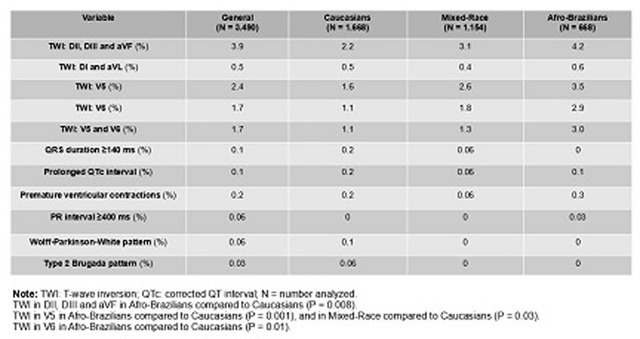


110364

Modality: E-Poster Young Researcher – Non-case Report

Category: HEART FAILURE/CARDIOMYOPATHY/TRANSPLANT

## Clinical and Epidemiological Behavior of Cardiopatic Pregnant Women in a Cardiological Reference Hospital in the Brazilian Amazon

LUCIANNA SERFATY DE HOLANDA^1^, Maria Elizabeth Navegantes Caetano Costa^1^, Maria Talita Rodrigues Pinto Campos^1^, Laíse Braga Vieira^1^, Vitor Bruno Teixeira de Holanda^1^, Ivy de Almeida Cavalcante e Silva^1^

(1) FUNDAÇÃO HOSPITAL DE CLÍNICAS GASPAR VIANNA DO PARÁ

**Background:** Studies on heart disease in pregnant patients show that it is the main indirect cause of deaths of pregnant women who are in the pregnancy-puerperal cycle. In addition, heart disease can lead to other problems that depend on the clinical and epidemiological state presented during pregnancy.

**Objectives:** To describe the clinical and epidemiological behavior of pregnant with heart disease registered at Hospital das Clínicas Gaspar Viana, from January 2017 to December 2019.

**Methods:** An epidemiological, retrospective and cross-sectional study, by analyzing the medical records of 133 pregnant women with heart disease admitted to HCGV in the aforementioned period. Excel 2007 and Bioestat 5.3 software were used for descriptive statistics; the results were evaluated using the categorical variables of the sample acquired by the G and Chi-Square Adherence Tests and G and Chi-Square Independence Tests.

**Results:** There was no difference between the age groups of the patients, where 81.2% did not have a personal morbid history; the most frequent diagnosis among the others was atrial septal defect with 14.3%. 80.5% are aware of their heart disease and are followed up. In turn, the functional class with the highest proportion was NYHA I with 72.2%. Finally, 60.2% had no problems with pregnancy, childbirth and postpartum. At the time of delivery, a statistically significant majority (*p = 0.0003) of patients were between 35 and 39 weeks of gestation (66.9%). Cesarean delivery was the one with the highest proportion (70.7%) and statistically significant (*p = 0.0008), in relation to vaginal delivery (27.1%). The majority of live births were statistically significant (*p < 0.0001).

**Conclusions:** This study is relevant because due to the scarcity of information about heart disease in pregnant woman in the State of Pará, considering that each one pregnant presents peculiarities, mainly on maternal and perinatal results or clinical and epidemiological profile of then.

110370

Modality: E-Poster Young Researcher – Non-case Report

Category: HEMODYNAMICS AND INTERVENTIONAL CARDIOLOGY

## The Effect of Statin Therapy on the Prognosis of Patients who Underwent Transcatheter Aortic Valve Implantation (Tavi)

BRENDA GABRIELE SMANIOTTO RAULIK^1^, Mateus de Miranda Gauza^2^, Rodrigo Ribeiro e Silva^2^, Júlia Opolski Nunes da Silva^2^, João Pedro Ribeiro Baptista^2^, Caroline Figueiredo da Silva^2^, Marcelo Pitombeira de Lacerda^2^, Gibran da Costa Reis^3^

(1) Universidade Positivo; (2) Universidade da Região de Joinville; (3) Hospital Regional Hans Dieter Schmidt

**Introduction:** In many cardiovascular diseases and procedures, statin therapy has been associated with a reduction in cardiovascular risk for future events. However, the effect of statin therapy for patients undergoing TAVI is not well documented and there are no randomized clinical trials evaluating this association. Considering the long-term mortality after TAVI not to be very satisfactory, it Is of great interest to find alternatives to reduce mortality after this procedure.

**Objetive:** Evaluate the prognostic factor associated with statin therapy in patients who underwent TAVI.

**Methods:** A systematic review was caried out using three data bases: Medline, Embase and the Cochrane library. We included observational studies that evaluated major adverse cardiovascular events, death and major bleeding in patients using statin therapy prior to TAVI. The terms used in the search strategy included “tavi”, “tavr” and “statin”. We found an initial number of 140 articles published until April 2022. After exclusion of duplicates and other studies that did not fit our objectives, 15 articles were included in our analysis.

**Results:** In our review, 15 observational studies were included in the analysis. From them, 7 studies reported a reduction in all-cause mortality and cardiovascular mortality in patients using statin therapy. 3 studies reported a dose-dependent reduction in long-term mortality, with 1 paper indicating no reduction in the short term with any intensity. Only 1 case-control study found no reduction in mortality in the long-term nor the short term. The effect on mortality may even extend to octogenarian population.

**Conclusion:** Statin therapy appears to be related with a reduction in long-term mortality rates after TAVI procedure, in dose dependent manner.


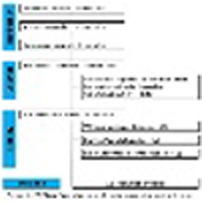


110416

Modality: E-Poster Young Researcher – Non-case Report

Category: NEGLECTED CARDIOVASCULAR DISEASES

## Hospital Admission for Acute Rheumatic Fever and Investment in the Treatment of Pharyngotonsillitis in Brazil: Is There a Relationship?

NATHALIA LIMA SCHRAMM DOS SANTOS^1^, Nathalia Lima Schramm dos Santos^1^, Lucas Santos Silva^1^, Isabele Carolina Tokumoto^1^, Rebeca Magalhães Araújo^1^, Lucas de Almeida Santos Rocha Pereira^1^, Ana Layse de Sá Gonçalves Torres^1^, Pedro Emanuel de Jesus Ferreira^1^, Fernando Gassmann Figueiredo^1^, Vanessa Arata Figueiredo^1^

(1) Universidade Estadual de Feira de Santana (UEFS)

**Introduction:** Rheumatic Fever (RF) is a multisystemic disease that, although preventable, still represents a common sequel of pharyngotonsillitis (PT) caused by group A beta-hemolytic streptococcus. In Brazil, there are an estimated ten million cases of PT per year, of which thirty thousand culminate in cases of RF. Its clinical importance is due to the sequelae of this condition, with biological impacts, as it is the main cause of mitral stenosis in the country and also socio-economic impacts, as it is related to a third of the heart surgeries in Brazil.

**Objective:** To analyze the relationship between hospital admission rates for acute rheumatic fever per 100,000 inhabitants and the ratio between hospitalization costs of patients with pharyngitis and acute tonsillitis over the total number of cases of hospitalization for rheumatic fever in Brazilian states in the periodo of January 2008 to May 2021.

**Method:** This is an ecological epidemiological study, whose data were obtained from the DATASUS database and from estimates from the Brazilian Institute of Geography and Statistics for the year 2020. The data were tabulated in the Microsoft Excel program, in which the rates and measurements of the linear regression test were calculated (Pearson’s correlation coefficient and p-value).

**Results:** During the study period, there were 49,056 hospital admissions for acute rheumatic fever in Brazil. The age group from 40 to 69 years old stands out, which has the highest number of hospitalizations (n = 20,733). Among those under 20 years old, there is a range of 10 to 14 years old with 4,673 hospitalizations. After performing the correlation between hospital admission rates for acute rheumatic fever and the ratio of hospitalization costs of patients with pharyngotonsillitis by the total number of hospitalizations for RF, a significant negative correlation was observed (r = –0.4 and p = 0.03888). The analysis of the distribution of hospital admission rates by RF by state highlights Pernambuco with 68 admissions per 100,000 inhabitants; Goiás with 50 and Ceará with 41. The correlation between the mortality rate and costs per hospitalization was also negative, although negligible (r = –0.2 and p = 0.03893).

**Conclusions:** The results show a relationship between the low investment in the treatment of acute pharyngotonsillitis and the higher rates of hospitalization for rheumatic fever.

110420

Modality: E-Poster Young Researcher – Non-case Report

Category: NURSING

## Diagnoses, Interventions and Nursing Outcomes in Immediate Postoperative Period of Cardiac Surgery in Adults: A Systematic Scoping Review

MARYANNA CRUZ DA COSTA E SILVA ANDRADE^1^, Ana Carla Dantas Cavalcanti^1^, Juliana de Melo Vellozo Pereira Tinoco^1^, Isabelle Andrade Silveira^1^

(1) Universidade Federal Fluminense – UFF

**Introduction:** Nurses working in cardiology intensive care units need to improve their knowledge about diagnoses, interventions and nursing outcomes of cardiac surgery patients. Despite, there are no studies that have mapped these concepts in immediate postoperative period context.

**Objective:** To map diagnoses, interventions and nursing outcomes for adult patients in immediate postoperative period of cardiac surgery.

**Method:** Scope review based on Joanna Briggs Institute manual and guided by Preferred Reporting Items for Systematic reviews and Meta-Analyses extension for Scoping Reviews (PRISMA-SrC) carried out on bases: Biblioteca Virtual em Saúde, Cochrane Library, EBSCO, Epistemonikos, Embase, National Institute Heath and Care Excellence, PMC, Pubmed, Scielo, Science.gov, Scopus e Web of Science. Data were mapped considering Population of adult cardiac surgery patients with sternotomy, in the context of immediate postoperative period and with concept of interest in diagnoses, interventions and nursing outcomes.

**Results:** 23 studies were identified for extraction in the end of selection process. 70 diagnoses, 57 interventions and 47 nursing outcomes using standardized language were mapped, summarizing an unprecedented way the three essential components for the nursing process of this clientele. Five scales for pain assessment, three for neurological assessment, one for predicting patient outcome and one for measuring severity were also mapped.

**Conclusion:** The steps of nursing process were presented in systematic way for the patient in the immediate postoperative period of cardiac surgery through diagnoses, interventions and nursing results representing the affected needs of this clientele. Thus, main implication of this review for nursing care is to guide nurses‘ critical thinking.

110482

Modality: E-Poster Young Researcher – Non-case Report

Category: ACUTE AND CHRONIC CORONARY DISEASE/THROMBOLYSIS

## The Impact of the Heart Team on Chronic Coronary Artery Disease: Long-Term Follow-Up

LUHANDA LEONORA CARDOSO MONTI SOUSA^1^, Bruno Mahler Mioto^1^, Nilson Tavares Poppi^1^, Julio Yoshio Takada^1^, Luís Henrique Wolff Gowdak^1^, Carlos Augusto Homem de Magalhães Campos^1^, Luis Roberto Dallan^1^, Amanda Mazetto^1^, Miguel Antonio Moretti^1^, Luiz Augusto Ferreira Lisboa^1^, LUIZ ANTONIO MACHADO CESAR^1^, Luciana Oliveira Cascaes Dourado^1^

(1) Instituto do Coração (InCor) do Hospital da Clínicas da Faculdade de Medicina da Universidade de São Paulo (HCFMUSP)

**Introduction:** The Coronary Heart Team (CHT) is based on shared decision-making, between of the non-interventional, interventional cardiologist and cardiac surgical. The focus of defining the best treatment in a individualized way, based in scientific evidence. It has class I recommendation, evidence level C guidelines. However, the impact of this intervention is still poorly understood.

**Objective:** To assess the impact of the CHT decision: clinical treatment (CT) percutaneous coronary intervention (PCI) and coronary artery bypass graft surgery (CABG) in reducing primary composite outcomes: Acute Coronary Syndrome (ACS) and Cardiovascular Death (CV) and symptom improvement: angina class (CCS) and heart failure (HF) class in patients with chronic CAD according to the degree of agreement with the CHT.

**Methods:** Prospective, single-center cohort study, mean follow-up of 41.1 ± 20.7 months. From a cohort of 500 patients discussed in CHT from 2015 to 2020, we evaluated 113 patients, whose final treatment (FT) was defined by the CHT of a tertiary hospital between august 2015 and november 2016. Fisher’s exact test or the chi-square test was used. Student’s t test for paired samples. The agreement was when estimating the Kappa coefficient, p < 0.05 indicated statistical significance. Stata/SE Program v.14.1. StataCorpLP, USA and SPSS 20.0.

**Results:** The mean age was 62.6 ± 9.8 years, 62.8% were male, diabetic (63%) and a history of AMI in 53.1%. The mean EF was 49.7%. Angina CCS II–IV 57.2%, HF class II–IV 49.1%. They were multivessel in 65.6% and main left coronary >50% associated in 19.47%. There was agreement between the proposed treatment (PT) by the referral cardiologista and the CHT Ƙ = 0.19 and good agreement between the CHT and the FT Ƙ = 0.62. CABG was the predominant FT and with the highest agreement with the CHT 88.1%, followed by PCI (79.2%) p = 0.005. Total mortality was 10.2%, CV death 5.6% and ACS 27.7%. There was a higher incidence of primary composite outcome when PCI was performed in disagreement with CHT p = 0.001, no difference for the other strategies. In 72% of the cases, angina improvement occurred (p = 0.001). For HF class, only the cases in agreement with the CHT had an improvement of 74.4% p = 0.008.

**Conclusion:** Final treatment in agreement with the CHT was associated with a lower incidence of primary composite outcome and significant improvement in long-term symptoms.

110487

Modality: E-Poster Young Researcher – Non-case Report

Category: CARDIOVASCULAR IMAGING

## Acceleration/Ejection Time Ratio as a Predictor Factor of Aortic Valve Stenosis

SARA ABOURADI^1^, Abouradi Sara^1^, Bendahou Hajar^1^, Mackonia Noel^1^, Tamir Mehdi^1^, Bennouna Ghali^1^, Habbal Rachida^1^

(1) CHU IBN ROCHD CASABLANCA

**Background:** Several factors may contribute to higher risk in patients with presumably moderate AS. Acceleration time (AT) and ejection time (ET) increase in parallel with AS severity, however AT/ET ratio in identification of high-risk subjects among asymptomatic mild-moderate AS is unknown The aim of the present analysis was to investigate the association between higher AT/ET ratio and outcomes in mild-moderate AS.

**Methods:** Were included all patients with asymptomatic patients with presumably mild-moderate AS followed at the hospital of Ibn Rochd cardiology departement service in casablanca from October 2017 to June 2021, Patients were grouped according to the optimal AT/ET ratio threshold to predict cardiovascular death and heart failure hospitalization. Outcome was assessed in Cox regression analyses, and results are reported as hazard ratio and 95% CI.

**Results:** The mean age was 65 ± 10. Higher AT/ET ratio was significantly associated with lower systolic blood pressure(p = 0,02), lower left ventricular ejection fraction(p = 0,01), low flow (p = 0,02), and with higher left ventricular mass (p = 0,03) and higher peak aortic jet velocity (p = 0,01). AT/ET ratio ≥0.34 had a higher risk of cardiovascular death and heart failure hospitalization (hazard ratio: 1.98 [95% CI, 1.50–3,8]). In patients with moderate AS, AT/ET ratio >0.36 was associated with higher rate for cardiovascular death (p = 0,01) and HF hospitalization (P = 0.035).

**Conclusions:** In asymptomatic moderate AS higher AT/ET ratio was associated with increased cardiovascular morbidity and mortality.

110488

Modality: E-Poster Young Researcher – Non-case Report

Category: PERICARDIUM/ENDOCARDIUM/VALVOPATHIES

## Assessment of Myocardial Energetic Efficiency in Asymptomatic Aortic Stenosis

SARA ABOURADI^1^, SARA ABOURADI^1^, NOEL MACKONIA^1^, HAJAR BENDAHOU^1^, SOUKAINA ZAHRI^1^, ABDNASSER DIGHIL^1^, RACHIDA HABBAL^1^

(1) CHU IBN ROCHD CASABLANCA

**Introduction:** Low myocardial energy efficiency (MEE) is undoubtedly associated with left ventricular dysfunction (LV) of the myocardium. the objective of this study was to investigate the effect of MEE in patients with asymptomatic aortic stenosis (AS).

**Methods:** Were included all patients with asymptomatic aortic stenosis followed at the hospital of Ibn Rochd cardiology departement service in casablanca from October 2017 to June 2021. MEE was calculated from Doppler stroke volume/([heart rate/60]) and indexed to LV mass (MEEi). Outcome was assessed in Cox regression analysis and reported as HR and 95% CI.

**Results:** The mean age was 60 ± 14 years mean ejection fraction: 50 ± 9%; mean aortic valve area: 1.04 ± 0.45 cm^2^; MEEi <0.35 mL/s per gram was associated with increased cardiovascular mortality [HR 1,98 (95% CI 1.60–3.28)] (both p < 0.02). This relationship persisted after multivariate adjustment and in the subgroups including more severe AS, higher body mass index, lower ejection fraction and presence of hypertension. (p = 0.01).

**Conclusions:** In patients with asymptomatic AS, Low myocardial energetic efficiency is associated with increased mortality in aortic stenosis regardless of other associated factors.

110489

Modality: E-Poster Young Researcher – Non-case Report

Category: PERICARDIUM/ENDOCARDIUM/VALVOPATHIES

## Asymptomatic Aortic Stenosis: What to Choose between a Conservative Attitude or An Early Surgery

SARA ABOURADI^1^, SARA ABOURADI^1^, HAJAR BENDAHOU^1^, NJIE MALICK^1^, AMRI MERYEM^1^, AZZOUZI LAILA^1^, HABBAL RACHIDA^1^

(1) CHU IBN ROCHD CASABLANCA

**Background:** Aortic stenosis is a very common pathology, several studies lean towards rapid surgical management, The aim of the study the objective of the study is to determine the appropriate attitude between rapid or conservative surgery.

**Methods:** Were enrolled all patients with asymptomatic patients with severe aortic stenosis (defined as an aortic-valve area of ≤1 cm^2^ with either an aortic jet velocity of ≥4 m per second or a mean transaortic gradient of ≥40 mm Hg) followed at the hospital of Ibn Rochd cardiology departement service in casablanca from October 2017 to June 2021 Patients were classified according to early surgery or to conservative care according. The primary end point was death during or within 30 days after surgery.

**Results:** The mean age was 65 ± 10 In the early-surgery group there was no operative mortality. The primary end-point event occurred in 3 patient in the early-surgery group (2%) and in 12 patients in the conservative-care group (14, 3%) (hazard ratio, 0.07; 95% confidence interval [CI], 0.03 to 0.24; p = 0.002). Death from another cause occurred in 7 patients in the early-surgery group (10,5%) and in 16 patients in the conservative-care group (19,06%) (hazard ratio, 0.22; 95% CI, 0.11 to 1,90, p = 0,02).

**Conclusions:** Early surgical aortic-valve replacement has a significantly lower risk of operative mortality or death from cardiovascular causes during the follow-up period than conservative care among asymptomatic patients with severe aortic stenosis.

110490

Modality: E-Poster Young Researcher – Non-case Report

Category: CARDIO-ONCOLOGY

## Cardiotoxicity: Therapy for HER2+ in Patients Diagnosed with Breast Cancer in a Private Institution in the Amazon

MARCELLO FACUNDO DO VALLE FILHO^1^, Marcello Facundo do Valle Filho^1^, Odilon Pereira Velho Filho^1^, Vanessa Mendes Moreira^1^, Thalita Giovana Diniz Silva^1^, Giovanna Piva^1^, Lia Mizobe Ono^2^, William Hiromi Fuzita^2^, Monica Regina Hosannah da Silva e Silva^1^

(1) Faculdade Metropolitana (FAMETRO); (2) Oncologia Sensumed e Instituto Sensumed de Ensino e Pesquisa Ruy França (ISENP), Manaus-AM.

**Introduction:** Heart disease and cancer are the main causes of morbidity and mortality in the industrialized world. Breast Cancer is a heterogeneous disease with different prognoses, therapeutic protocols and treatment outcomes. Identification of HER2+ on immunohistochemistry and/or FISH indicates a need for targeted therapy with HER2+ receptor blockers. However, its use may be related to cardiotoxicity, which may be manifested by an asymptomatic decline in left ventricular ejection fraction (LVEF) and/or by the occurrence of symptomatic heart failure, diagnosed through echocardiography. One of the therapies involves pertuzumab (a monoclonal antibody) associated with trastuzumab (which can also be applied alone) and docetaxel, with improved overall and progression-free survival. Considering normal LVEF above 55%, a fall below 10% may be completely reversible, while in those above 10%, reversal may occur in 91% of cases. Even in the presence of cardiotoxicity, 70% to 80% of patients continue to receive targeted therapy.

**Method:** Cross-sectional, observational, descriptive and retrospective study of 697 patients diagnosed with breast cancer at Oncologia Sensumed in the city of Manaus-AM where 69 patients with HER2+ were selected.

**Results:** Of the 69 patients, 45 (65%) received transtuzumab monotherapy, with a mean of eight doses (minimum of 3 and maximum of 17) at 21-day intervals, 16 (23%) received combined triple therapy. All ejection fractions measured quarterly by echocardiogram were within the normal range, ranging from 61% to 75%. Eight patients (12%) did not use any of the three drugs due to previously diagnosed medium and high-grade heart failure.

**Conclusion:** The assessment of LVEF and the presence of heart failure (HF) before starting treatment for breast cancer with HER2+ influences the therapeutic protocol and treatment outcome. In addition, maintaining a quarterly evaluation elucidates data that indicate maintaining, discontinuing or establishing new options for treatment of both cancer and reduced LVEF and HF. Therefore, even with cardiotoxic effects, therapy directed at HER2+ in breast cancers can bring benefits to the prognosis, provided that they are monitored and evaluated quarterly through echocardiography and anamnesis to identify symptoms.

110496

Modality: E-Poster Young Researcher – Non-case Report

Category: CONGENITAL AND PEDIATRIC CARDIOLOGY

## Epidemiological Profile of Pediatric Heart Failure (HF) Hospitalizations in Pará from 2017 to 2021

PEDRO ARTHUR RODRIGUES DE OLIVEIRA^1^, Pedro Arthur Rodrigues de Oliveira^1^, Marcello Vieira dos Santos^1^, Daniely Maués Beliqui^1^, Laura Coutinho Viana^1^, José Wilker Gomes de Castro Júnior^1^, Jéssica Lorena Alves^2^, Juliana Miranda Benício^3^, Juliane Costa Santos^3^, Lucian Herlan da Costa Luz Fernandes^3^, Lígia Maria dos Santos de Oliveira Vieira^1^, Amanda Carício Gomes^1^

(1) Centro Universitário do Estado do Pará (CESUPA); (2) Universidade Federal do Pará; (3) Centro Universitário Metropolitano da Amazônia (UNIFAMAZ)

**Introduction:** Heart failure (HF) is a syndrome characterized by the inability of the heart to maintain sufficient cardiac output for the body’s metabolic needs, leading to ventricular dysfunction. In the pediatric population the most common cause of HF is congenital anomalies, such as cardiac malformation. Other less frequent causes are myocardiopathies, myocarditis, and arrhythmias.

**Objectives:** For this reason, this paper aims to conduct an evaluation of the epidemiological profile of hospitalizations for heart failure iciency in Pará (Brazil) in the period from 2017 to 2021 in pediatric patients.

**Methodology:** A descriptive, retrospective, quantitative study was conducted based on secondary data provided by the Hospital Information System (SIH) of the SUS Computer Department (DATASUS). The collected information was stored and tabulated in the Microsoft Office Excel™ program.

**Results:** Among the 1,577 cases found after analyzing the evaluated period, there are the years 2017 with 327 cases (20.73%), 2018 with 379 cases (24.03%), and 2019 with 311 notified cases (19.72%), being the 3 most incident years of the investigated period. The municipalities with the highest number of hospitalizations for heart failure were: Belém (79.64%) followed by Breves (1.83%) and Santarém and Altamira (1.5% each). Moreover, it was identified that mixed race, with 1,130 cases (71.65%), female gender, with 798 cases (50.60%) and the age group of less than 1 year, with 578 cases (36.65%) are the most affected epidemiological variables. After evaluating the cases reported, it was noted that 104 cases (6.59%) died.

**Conclusion:** It was observed, therefore, that patients under the age of 1 year had a higher prevalence of hospitalizations for heart failure, which may be associated with hypertrophic congenital heart diseases. Moreover, the considerable amount of deaths is evident, reflecting the need for attention to pediatric HF.

110500

Modality: E-Poster Young Researcher – Non-case Report

Category: HYPERTENSION/RENAL DENERVATION

## Inflammatory Markers and Refractory Hypertension

CARLOS FILIPE DOS SANTOS PIMENTA^1^, Taissa Lorena dos Santos^1^, Camila Bello Nemer^1^, Gabriela da Silva Nascimento^1^, Hugo Farah Affonso Alves^1^, Lucca Hiroshi de Sá Kimura^1^, Bernardo Chedier^1^, Arthur Fernandes Cortez^1^, Elizabeth Silaid Muxfeldt^1^

(1) Universidade Federal do Rio de Janeiro – Hospital Universitário Clementino Fraga Filho

**Introduction:** Refractory hypertension (RfHT) is defined as an uncontrolled blood pressure (BP) despite the use of 5 or more antihypertensives, including spironolactone and it is considered an extreme phenotype of resistant hypertension (RHT). High BP levels lead to RAAS stimulation, sympathetic hyperactivity and endothelial dysfunction with consequent production of pro-inflammatory cytokines.

**Objective:** To evaluate the relationship between inflammatory markers and refractory hypertension in a large cohort of patients with RHT.

**Methods:** A cross-sectional study that evaluated 423 resistant hypertensives (30.5% male, mean age 63.9 ± 10.8 years), of which 62 (14.6%) diagnosed as RfHT. All of them were submitted to the dosage of inflammatory markers: TNF-alpha, MCP-1, E-selectin and PAI-1. Socio-demographic and anthropometric characteristics and cardiovascular risk factors (CV) were recorded. Variance analysis compared serum levels of the 4 inflammatory markers and bivariate analysis compared patients with RHT versus RfHT.

**Results:** Patients with RfHT are younger, with higher prevalence of smoking, higher levels of albuminuria and higher prevalence of chronic cerebrovascular and renal disease stages 4 and 5. PAI-1 values (126 [108–162] vs 118 [94–153] were higher in those with RfHT, although they did not reach statistical significance. The other biomarkers evaluated showed no association with the diagnosis of RfHT.

**Conclusion:** Among inflammatory markers, PAI-1 was the one that correlated most strongly with refractory hypertension. Our study is the first one to demonstrate PAI-1 association with treatment refractoriness.

110881

Modality: E-Poster Young Researcher – Non-case Report

Category: HYPERTENSION/RENAL DENERVATION

## Cardiovascular Events Associated with White Coat Hypertension

ALAYN KLEBER FREIRE DA SILVA JUNIOR^1^, Adelyanne Santos Barbosa^1^, Alayn Kleber Freire da Silva Junior^1^, Bárbara Maria Oliveira da Silva^1^, Guilherme de Azevedo Guedes^1^, Marco Antônio Mota Gomes^2^

(1) Centro Universitário CESMAC, Maceió, AL, Brazil; (2) Centro de Pesquisas Clínicas Dr. Marco Mota, Maceió, AL, Brazil

**Introduction:** Systemic Arterial Hypertension is a chronic disease with a multifactorial condition characterized by blood pressure (BP) levels above or equal to 130 and/or 80 mmHg. The combination of BP measurements in the office and Home Blood Pressure Monitoring (HPBM) and Ambulatory Blood Pressure Monitoring (ABPM) made it possible to define BP standards, including White Coat Hypertension (WCH). HAB is characterized by the elevation of BP in the office during repeated visits, concomitantly with normal values of BP out of the office, verified through ABPM and HMBP.

**Objective:** This study aims to identify and analyze evidence about cardiovascular events associated with White Coat Syndrome.

**Methodology:** A literature search was performed using the descriptors: White Coat Hypertension, Cardiovascular and Events together with the Boolean operator “AND” in MedLine/PubMed, Lilacs and Scielo databases. The use of these descriptors allowed the retrieval and restriction of articles. The filter of the last 5 years was used. The inclusion criterion used was the presence of keywords in the title or abstract of the articles. The following were excluded: letter to the editor, dissertations and theses. Reading steps: titles, abstracts, case reports and full articles.

**Results:** Six articles were selected, and it was possible to observe that in a significant part of them, patients with WCH, in general, presented a higher level of cardiovascular alterations when compared to the group of normotensive individuals. Although these alterations were smaller when compared to individuals with sustained hypertension, individuals who suffer from WCH may manifest several anomalies, such as increased LV mass, thickness of the interventricular septum, structural alterations in the carotid artery and greater LA diameter. Because of these changes, WCH has been associated with negative physiological changes, such as sympathetic hyperactivity, which is related to increased cardiovascular risks. Considering this, some studies proposed to analyze the effectiveness of treatments with antihypertensive drugs, but in most of these, there was no reduction in cardiovascular events.

**Conclusion:** In view of the aforementioned arguments, it can be inferred that the cardiac and vascular damages of patients diagnosed with WCH are greater when compared to normotensives, and further studies are needed to develop effective ways to reduce the problems brought about by WCH.

110591

Modality: E-Poster Young Researcher – Non-case Report

Category: CARDIOVASCULAR SURGERY

## Impact of Preoperative Pulmonary Hypertension on Early Surgical Death in Patients Undergoing Mitral Valve Surgery Due to Rheumatic Disease

GUSTAVO PINHEIRO SANTANA^1^, Gustavo Pinheiro Santana^1^, Rodrigo Morel Vieira de Melo^2^, Tainá Teixeira Viana^1^, Ana Luísa de Aguiar Almeida Silva^2^, João Pedro Martins Moreira Granja^2^, Osvaldemar Regis Do Nascimento Junior^1^, Danilo Sousa Sampaio^1^, Thais Harada Campos^1^, Edmundo José Nassri Câmara^1^, Luiz Carlos Santana Passos^1^

(1) Hospital Ana Nery, Salvador – Ba (HAN); (2) Universidade Federal da Bahia (UFBA)

**Introduction:** Rheumatic valve disease is still a health problem worldwide. Pulmonary hypertension (PH) may occur with the gradual progression of mitral stenosis and regurgitation in this condition. Whether or not PH is associated with early surgical mortality in this specific population remais controversial.

**Objective:** In a population of patients undergoing cardiac surgery for rheumatic mitral valve disease, evaluate the impact of preoperative PH on early surgical mortality.

**Methods:** This is a retrospective cohort carried out from January 1, 2017 to December 30, 2020. All patients over 18 years of age who underwent cardiac surgery to correct rheumatic mitral valve disease with functional tricuspid regurgitation in an echocardiogram performed up to 6 months before surgery were included. Systolic pulmonary artery pressure (sPAP) value was also defined by preoperative echocardiogram evaluation. The primary outcome was surgical mortality.

**Results:** 144 patients were included. The mean age was 46.2 (±12.3) years with 107 (74.3%) female individuals, the median left ventricular ejection fraction was 61.0% (55–67) and sPAP was 55.0 mmHg (46–74), with 45 (31.3%) individuals with right ventricular dysfunction. The predominant valve disease was mitral stenosis (74.3%). The prevalence of severe tricuspid regurgitation was 47.2%. The total in-hospital mortality was 15 (10.4%) individuals. sPAP was independently associated with early surgical death RR 1.04 (1.01–1.07), p = 0.003. To determine a sPAP cut-off that indicates higher mortality and help decision making in clinical practice, we performed an analysis through the ROC curve (area 0.70, p = 0.012). The estimated value of 73.5 mmHg has the highest accuracy in our model for predicting early mortality.

**Conclusion:** In patients with rheumatic heart disease who will undergo mitral valve surgery, pulmonary hypertension is associated with higher early mortality. Values above 73.5 mmHg predict higher risk and, in this part of the population, additional measures to control intraoperative and immediate postoperative pulmonary hypertension should be considered.


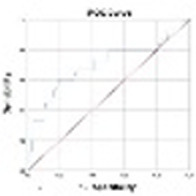


110617

Modality: E-Poster Young Researcher – Non-case Report

Category: CARDIOVASCULAR IMAGING

## Association between Phase Angle and Coronary Artery Calcium Score in Patients on Peritoneal Dialysis

FABRICIO MOREIRA REIS^1^, Maryanne Zilli Canedo Silva^1^, Nayrana Soares Carmo Reis^1^, Fabiana Lourenço Costa^1^, Caroline Ferreira da Silva Mazeto Pupo da Silveira^1^, Alejandra Del Carmen Villanueva Mauricio^1^, João Carlos Hueb^1^, Rodrigo Bazan^1^, Pasqual Barretti^1^, Luis Cuadrado Martin^1^, Silméia Garcia Zanati Bazan^1^

(1) Faculdade de Medicina de Botucatu – Universidade Estadual Paulista

**Introduction:** The phase angle has been used as a nutritional marker and predictor of mortality in patients on peritoneal dialysis. The coronary artery calcium score has showed to predict the incidence of acute myocardial infarction and death from cardiovascular disease in these patients. However, the association between phase angle and coronary artery calcium score in patients on peritoneal dialysis is not well established.

**Objective:** To evaluate the association between phase angle and coronary calcium score in patients on peritoneal dialysis.

**Methods:** This was a cross-sectional study with patients on peritoneal dialysis, followed up at a University Hospital, between March 2018 and August 2019. Phase angle was evaluated by unifrequency bioimpedance. The coronary artery calcium score was calculated based on cardiovascular computed tomography, considering positive when greater than or equal to 100 Agatston and negative when less than 100 Agatston.

**Results:** We evaluated 44 patients on dialysis, with mean age of 56 years and 11.7 months. In a univariate analysis, an association was observed between the coronary artery calcium score, the presence of atherosclerotic plaque in the carotid artery, and the difference in systolic blood pressure in the upper extremities with the phase angle. In the hierarchical multivariate logistic regression, only the coronary artery calcium score maintained an association with the phase angle in the final model.

**Conclusion:** The phase angle is associated with a positive coronary artery calcium score in patients on peritoneal dialysis, and despite other factors, may be useful as a risk marker for coronary artery disease in this population.


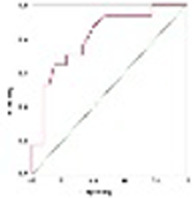


110620

Modality: E-Poster Young Researcher – Non-case Report

Category: CARDIOVASCULAR IMAGING

## The Left Atrium as a Predictor of Coronary Calcification in Patients on Peritoneal Dialysis

FABRICIO MOREIRA REIS ^1^, Maryanne Zilli Canedo Silva^1^, Nayrana Soares Carmo Reis^1^, Fabiana Lourenço Costa^1^, Caroline Ferreira da Silva Mazeto Pupo da Silveira^1^, Alejandra Del Carmen Villanueva Mauricio^1^, João Carlos Hueb^1^, Rodrigo Bazan^1^, Pasqual Barretti^1^, Luis Cuadrado Martin^1^, Silméia Garcia Zanati Bazan^1^

(1) Faculdade de Medicina de Botucatu – Universidade Estadual Paulista

**Introduction:** Cardiovascular disease (CVD) is the leading cause of death in patients with chronic kidney disease (CKD) undergoing peritoneal dialysis (PD). The assessment of coronary calcification by coronary artery calcium (CAC) score has shown to predict the incidence of acute myocardial infarction and death by CVD in those patients. Left atrial volume has also been associated with acute myocardial infarction and cardiovascular events.

**Objective:** To assess the association between left atrial volume and CAC score in PD patients.

**Methods:** A cross-sectional study with PD patients, followed up between March 2018 and August 2019, including demographic, clinical and laboratory data collection; assessment of nutritional status by anthropometry and bioimpedance; protocol of residual renal function and adequacy of dialysis (Kt/V); doppler echocardiogram, carotid ultrasound, pulse wave velocity, ankle-brachial index and CAC. Patients were divided into two groups: positive or negative CAC score. Comparisons between groups were performed using the Chi-square, Student t or Mann-Whitney test (p < 0.05).

**Results:** 44 patients on PDP, mean age of 56 years. Main cause of CKD was hypertension. Median time on dialysis therapy was 11.7 months. In the univariate analysis, a significant association of CAC score was observed with variables such as age, diabetes mellitus, over hydration, femoral pulse wave velocity, left atrial volume index, E/A ratio and left ventricular mass index, but in the multivariate analysis, only the left atrial volume index was related to a positive CAC score, odds ratio of 1.71.

**Conclusion:** The left atrial volume index is associated with positive CAC score in patients with CKD undergoing PD, and it can be used as a risk marker for coronary artery disease in this population.


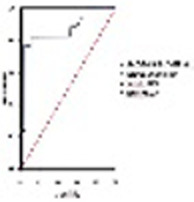


110669

Modality: E-Poster Young Researcher – Non-case Report

Category: NUTRITION

## Dietary Indicators of Carbohydrate Quality and Blood Pressure in Brazilian Adolescents

VANESSA PROÊZA MACIEL GAMA^1^, Camilla Medeiros Macedo da Rocha^4^, Edna Massae Yokoo^6^, Amanda de Moura Souza^7^, Katia Vergetti Bloch^7^, Rosely Sichieri^5^

(1) Instituto Federal de Educação, Ciência e Tecnologia Fluminense – IFFlumnense, Av. Souza Mota, 350, Parque Fundão, CEP 28060–010, Campos dos Goytacazes, RJ, Brazil;; (2) Instituto de Saúde Coletiva, Universidade Federal Fluminense – UFF, Rua Marques de Paraná, 303, 3° andar, Centro, CEP 24030–210 Niterói, RJ, Brazil;; (3) Curso de Especialização em Nutrição Clínica, Instituto de Nutrição Josué de Castro, Universidade Federal do Rio de Janeiro – UFRJ, Av. Carlos Chagas Filho, 373 – bloco J 2° andar, Ilha do Fundão, CEP 21941–902, Rio de Janeiro – RJ, Brasil.; (4) Instituto de Alimentação e Nutrição, Centro Mutidisciplinar – UFRJ – Macaé, Universidade Federal do Rio de Janeiro, Av. Aluizio da Silva Gomes, 50, Novo Cavaleiros, CEP 27930–560 Macaé, RJ, Brazil;; (5) Instituto de Medicina Social, Universidade do Estado do Rio de Janeiro – UERJ, Rua São Francisco Xavier, 524, Pavilhão João Lyra Filho, 7° andar, CEP 20550–900 Rio de Janeiro, RJ, Brazil;; (6) Departamento de Epidemiologia e Bioestatística, Universidade Federal Fluminense – UFF, Rua Marques de Paraná, 303, 3° andar, Centro, CEP 24030–210 Niterói, RJ, Brazil;; (7) Instituto de Estudos em Saúde Coletiva, Universidade Federal do Rio de Janeiro -UFRJ, Avenida Horácio Macedo, s/n, Ilha do Fundão, CEP 21941–598, Rio de Janeiro, RJ, Brazil;

**Introduction:** The consumption of foods and meals with high glycemic index (GI) and high glycemic load (GL) have been suggested as risk factors for chronic diseases, including hypertension. Population-based studies that have investigated this association in adolescence are still scarce.

**Objective:** To evaluate the association between dietary indicators of glycemic response and changes in blood pressure among adolescents in the Study of Cardiovascular Risk in Adolescents (ERICA).

**Methods:** A sample (n = 69,057) of students (12 to 17 years) from public and private schools, representative of Brazilian adolescents living in cities with 100,000 inhabitants or more was evaluated. Prevalence of hypertension was estimated, as well as mean systolic and diastolic blood pressures (mmHg) stratified by sex and age group and mean dietary indicators of glycemic response: daily GI, average GI, GI of the first meal of the day, daily GL, average GL and GL of the first meal of the day, according to sex and age group. Linear and logistic regression models stratified by sex and adjusted for age and body mass index were estimated to assess association between dietary indicators of glycemic response and systolic, diastolic blood pressures and hypertension.

**Results:** The mean age was14.7 years and 56% female. The prevalence of hypertension was 9.1%, almost twice more frequent among boys (12.3%), compared to girls (6.5%). The mean systolic blood pressure was 110.6 mmHg and the mean diastolic blood pressure was 65.8 mmHg. Boys had higher mean dietary indicators of glycemic response (GI of first meal, daily GL, average GL, and GL of first meal) compared to girls (p-value < 0.05), except for mean GI and mean GL. There was a positive association between GI of the first meal with systolic blood pressure in boys.

**Conclusions:** Factors related to carbohydrate metabolism and the nutritional value of the diet should include GI and GL of individual meals, beyond the global indicators of glycemic response such as daily GI and GL. Further studies are needed to deepen the relationship between the glycemic response from food intake.

110759

Modality: E-Poster Young Researcher – Non-case Report

Category: COVID-19 AND CARDIOVASCULAR SYSTEM

## Assessment of Cardiac Adverse Events Following COVID-19 Vaccination by Tissue Speckle Tracking

SRISAKUL CHAICHUUM^1^, Shuo-Ju Chiang^2^, Masao Daimon^3^, Su-Chen Chang^2^, Chih-Lin Chan^2^, Chu-Ying Hsu^2^, Ching-Li Tseng^1^

(1) Graduate Institute of Biomedical Materials and Tissue Engineering, Taipei Medical University, Taipei, Taiwan; (2) Division of Cardiology, Department of Internal Medicine, Taipei City Hospital Yangming Branch, Taipei, Taiwan; (3) Department of Cardiovascular Medicine, The University of Tokyo Hospital, Tokyo, Japan

**Background:** Cardiac discomfort has been reported periodically in individuals who received COVID-19 vaccines. Transient cardiac symptoms are imperatively monitored to evaluate changes in heart function.

**Objective:** This study aimed to evaluate the role of myocardial strains in the early assessment of the clinical presentations after COVID-19 vaccination.

**Methods:** In total, 121 subjects who received at least one dose of a COVID-19 vaccine within 6 weeks were recruited. Serological markers and echocardiography were assessed. Two-dimensional speckle tracking echocardiography (2D-STE) was implemented to analyze changes in the left ventricular myocardium.

**Results:** After vaccination, 66 individuals (55.4 ± 17.4 years) developed cardiac discomfort, such as chest tightness (n = 59, 89%), palpitations (n = 23, 35%), dyspnea (n = 21, 32%), and chest pain (n = 6, 9%). All had normal serum levels of creatine phosphokinase (93.0 U/L; IQR, 66.5–137.5), creatine kinase myocardial band (10.4 U/L; IQR, 2.3–14.7), Troponin T (6.0 ng/L; IQR, 4.0–8.5), N-terminal pro b-type natriuretic peptide (44.5 pg/mL; IQR, 20.2–79.5), platelets (222.0 × 10^3^/𝜇L; IQR, 199.2–275.5), and D-dimer (0.3 mg/L; IQR, 0.1–0.4). Echocardiogram presented left ventricular ejection fraction in the symptomatic group (71.41% ± 7.12%) and the control group (72.18% ± 5.11%) (p = 0.492) were normal. However, the symptomatic group had a greater ratio of mitral peak velocity of early filling (E) to early diastolic mitral annular velocity (e’) than the normal group (9.32 ± 3.56 vs 7.74 ± 2.34, respectively; p = 0.005), correlated with e’ (8.64 ± 3.96 vs 10.25 ± 3.58, respectively; p = 0.023). Use of 2D-STE showed that global longitudinal strain (GLS) and global circumferential strain (GCS) were reduced in the symptomatic group (17.86% ± 3.22% and 18.37% ± 5.22%) compared to the control group (19.54% ± 2.18% and 20.73% ± 4.09%) (p = 0.001 and p = 0.028).

**Conclusions:** COVID-19 vaccine-related cardiac adverse effects can be assessed early by 2D-STE. The prognostic implications of GLS and GCS enable evaluation of the subtle changes in myocardial function after vaccination.


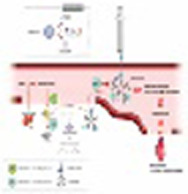


110780

Modality: E-Poster Young Researcher – Non-case Report

Category: ANTICOAGULATION

## Cost-Effectiveness of Rivaroxaban Versus Warfarin in New Onset Atrial Fibrilattion After Coronary Artery Bypass Graft Surgery (Task POAF): A Prospective, Single Center and Randomized Trial

MARCEL DE PAULA PEREIRA^1^, Eduardo Gomes Lima^1^, Fabio Grunspun Pitta^1^, Luis Henrique Wolff Gowdak^1^, Bruno Mahler Mioto^1^, Luiz Antonio Machado Cesar^1^, Henrique Trombini Pinesi^1^, Omar Asdrubal Vilca Mejia^1^, Fabio Biscegli Jatene^1^, Roberto Kalil Filho^1^, Francisco Carlos da Costa Darrieux^1^, Carlos Vicente Serrano Junior^1^

(1) Instituto do coração – Universidade de São Paulo

**Introduction:** New post-operative atrial fibrillation (POAF) in the setting of coronary artery bypass graft (CABG) is an entity observed in up to 40% of procedures. Despite this, there is little evidence about the use of direct oral anticoagulants in this scenario.

**Purpose:** Our aim was to compare cost-effectiveness of a strategy using rivaroxaban versus the standard therapy after POAF.

**Methods:** This is a single center, randomized, prospective and open-label study comparing two anticoagulation strategies: Warfarin associated with bridging with enoxaparin versus rivaroxaban after POAF. Medications started during hospitalization and continued up to 30 days. Primary endpoint was the assessment of cost-effectiveness. Costs were analyzed from the perspective of supplementary health in Brazil and converted to US dollars. Quality-adjusted life year (QALY) were assessed using the SF-6D questionnaire. Safety endpoint was any bleeding using the international society of thrombosis and haemostasis.

**Results:** In a tertiary hospital, 51 patients who underwent CABG and presented with POAF were randomized. Twenty-six patients were randomized for the warfarin group and 25 for the rivaroxaban group. Both groups had similar baseline. The median of the primary cost-effectiveness outcome was $ 1347,7 in the warfarin group and $433,0 in the rivaroxaban group (p < 0.001), as shown in Figure 1. The result of the SF-6D questionnaire showed no statistical difference between groups. Bleeding rates were 26% in warfarin and 12% in rivaroxaban group (p = 0.18).

**Conclusion:** In this study with a 30-day follow-up, anticoagulation strategy with rivaroxaban in patients with POAF was more cost-effective the standard therapy using warfarin.


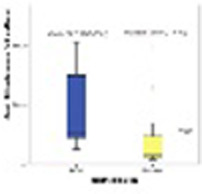


110933

Modality: E-Poster Young Researcher – Non-case Report

Category: ANTICOAGULATION

## The use of Anticoagulant for Preventing the Formation of Thrombosis in Patients who Have Undergone Plastic Surgery: A Systematic Review

MARCELLO VIEIRA DOS SANTOS^1^, Cassiane da Silva Portela Pinto^2^, Jessica Lorena Alves^3^, Pedro Arthur Rodrigues de Oliveira^1^, Izadora Avelar Neto^2^, Daniely Maues Beliqui^1^, Roberta Louise dias Rodrigues^1^, Adilson talis Ferreira dos Santos^1^

(1) Centro Universitário do Estado do Pará; (2) Universidade do Estado do Pará; (3) Universidade Federal do Pará

**Introduction:** Thrombosis is formed from clots in the circulatory system, and is related to venous stasis, trauma, surgery, and others, and can evolve to more fatal conditions, such as pulmonary thromboembolism. To avoid this condition, it is necessary to start anticoagulant therapy early. This condition is recurrent in the postoperative period of plastic surgery, being aggravated by the non-use of elastic stockings, the stimulation of early ambulation, the use of anticoagulants, and the failure of risk analysis. Therefore, this paper discusses anticoagulants in the postoperative period of plastic surgery as a way to prevent thrombosis.

**Objectives:** To analyze the use of anticoagulants as a prophylactic form for thrombosis formation in the postoperative period of patients undergoing plastic surgery.

**Methods:** This is a systematic review of literature, based on the recommendations of the Preferred Reporting Items for Systematic Reviews and Meta-Analyse – PRISMA. We used the term “(anticoagulants) AND (thrombosis) AND (disease prevention)” in the LILACS, PubMed and Scielo databases, obtaining initially 65 publications and after the PRIMAS guidelines, 7 remained for analysis.

**Results And Discussions:** Throughout the studies, it was seen the presence of Virchow’s triad in the postoperative period of plastic surgery, characterized by venous stasis, endothelial injury and hypercoagulation. For this, it was observed, only the Caprini score as recommended for risk assessment in the postoperative period. Regarding anticoagulants, it was shown vitamin K antagonists, Factor Xa inhibitors (heparins), direct oral Factor Xa inhibitors (rivoraxaban) being the first line of choice, however there are still many discussions about the ideal propedeutics, leading to its irrational use, since its excess can bring bleeding and smaller doses do little effect.

**Conclusion:** Thus, the diversification of methods for the evaluation of venous thromboembolism (VTE) were shown to be necessary in plastic surgery, since only one method of choice was analyzed, which ends up hindering the patient’s risk assessment. Similarly, the creation of a protocol by the Brazilian Society of Plastic Surgery, addressing VTE in the postoperative period could standardize chemoprophylaxis and make it more effective. Finally, greater follow-up of patients at higher risk by a hematologist also shows promise for improving the efficacy of therapy.

110782

Modality: E-Poster Young Researcher – Non-case Report

Category: HEMODYNAMICS AND INTERVENTIONAL CARDIOLOGY

## The Overestimation of the Prevalence of Mismatched Aortic Prosthesis After Transcatheter Implantation by Echocardiography

FERNANDO COUTO PORTELA^1^, Sayuri de Souza Yamamura^1^, Francisco Expedito de Albuquerque Filho^1^, Marcus Vinícius Alves Gomes^1^, Luis Felipe Mousinho Lima de Carvalho^1^, Marco Túlio Hercos Juliano^1^, Márcio Mesquita Barbosa^1^, Ana Cláudia Conrado de Oliveira^1^, Gustavo Travassos Gama^1^, Márcio Mendes Pereira^1^

(1) UDI Hospital

**Introduction:** The valve prosthesis-patient mismatch (PPM) is characterized by a small effective orifice area (AOE) in relation to the body surface, resulting in high transvalvular gradients. The presence of MPP in the scenario of transcatheter aortic valve implantation (TAVI) has different characteristics according to the type of prosthesis (balloon-expandable or self-expanding). Knowing the real prevalence of PPM in a real-world sample is very important since such presence impacts survival, functional recovery, hospitalization for heart failure and valve durability.

**Objectives:** To determine the prevalence of PPM in a sample of patients undergoing TAVI, using predicted AOE and AOE measured by the continuity equation. Associate the presence of PPM with several clinical, echocardiographic, tomographic variables and with the type of prosthesis used.

**Method:** An observational study was performed with 26 consecutive patients undergoing TAVI in a private hospital. PPM was defined as present if AOE <0.85 cm^2^/m^2^ (<0.70 cm^2^/m^2^ if obese). The predicted PPM was obtained from data provided in the medical literature according to the size and prosthesis used.

**Results:** All 26 patients who underwent TAVI within the 9-year period were included in the study. The mean age was 78.9 ± 8.3 years and 57.7% were male. The measured prevalence of PPM was considerably higher (41.7%) than predicted (15.4%). The mean residual gradient was 18.7 ± 4.4 mmHg in patients with PPM and 10.5 ± 4.5 mmHg in patients without PPM (p = 0.029). The rate of PPM in balloon-expandable prostheses was significantly higher than in self-expanding prostheses (42.9% vs. 5.3%, p = 0.018). Patients with PPM had a smaller area of the aortic annulus measured by tomography than those without PPM (3.34 ± 0.16 cm^2^ vs. 5.06 ± 1.95 cm^2^, p = 0.014).

**Conclusion:** The use of the AOE calculated by the continuity equation overestimated the prevalence of PPM in patients undergoing TAVI. PPM was more prevalent in balloon-expandable prostheses than in self-expanding ones. Reduced aortic annulus was another factor associated with PPM.

111196

Modality: E-Poster Young Researcher – Non-case Report

Category: COVID-19 AND CARDIOVASCULAR SYSTEM

## Modified CHA2DS2VASC Score as Prognostic Risk of Developing Severe COVID-19

YURI CAVALCANTI ALBUQUERQUE TENORIO^1^, Priscila Alves da Silva^2^, Ana Lívia de Oliveira Barros^2^, Gabriel Noé Albuquerque Paffer Cruz^2^, Nicole de Lima Larré Barbosa^4^, Carolline Cavalcante de Melo^3^, Ana Clara Valente de Lima Melo^3^, Arthur Henrique Fernandes Rodrigues^2^, Edecio Galindo de Albuquerque^1^, Antônio Everaldo Vitoriano de Araújo Filho^2^, Francisco de Assis Costa^2^

(1) Hospital Veredas; (2) Centro Univesitário Tiradentes; (3) Universidade Federal de Alagoas; (4) Universidade Estadual de Ciências da Saúde de Alagoas

**Introduction:** Covid-19 was declared a pandemic in early 2020 by the World Health Organizations (WHO) and it is a respiratory disease caused by SARS-CoV-2 virus. One of its main complications is the development of coagulopathies, given that the infection promotes increased thrombin formation and hypercoagulability state. The modified CHA2DS2-VASc score is used to estimate the risk of thromboembolic stroke in atrial fibrillation, as well as the thrombotic risk in other heart diseases. The abbreviation stands for congestive heart failure, hypertension, age, diabetes, previous stroke/transient ischemic attack. The risk factors that make up the m-CHA2DS2VASc score could also be recognized as risk factors for mortality in patients with COVID-19. The aim of this study is to identify the association between m-CHA2DS2VASc score in mortality and hospital staying in Covid-19 patients to use it as prognostic score of severe disease.

**Methods:** Case-control study in a philanthropic hospital in the Brazilian state of Alagoas, selecting 103 hospitalized patients from 2020 to 2021 aged more than 18 years old and diagnosed with SARS-CoV- 2 infection. The statistical analysis was calculated using Fisher’s Exact test.

**Results:** The mean m-CHA2DS2VASc score. was 1,74 (SD 1,53) and mean age was 53.81 years (SD 17.37). There was a significant relationship between CHA2DS2VASc score and mortality rate (p = .003) and hospital staying (p = .009).

**Discussion:** The results lead us to associate m-CHA2DS2VASC score to risk of death (any cause). Another similar study with 864 patients also found this relation with m-CHA2DS2VASc score and death (p < .001). Other authors suggest that m-CHA2DS2-VASc score can be used to discern patients with higher thromboembolic risk. Also, higher m-CHA2DS2-VASc score is associated with a higher need for mechanical ventilation and a greater risk of death.

**Conclusion:** There was a relationship between m-CHA2DS2VASc score and development of severe covid-19 and mortality according to the population analysed and other similar studies.


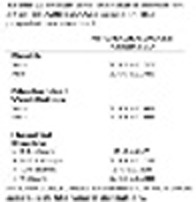


110794

Modality: E-Poster Young Researcher – Non-case Report

Category: NUTRITION

## Epigallocatechin-3-Gallate (EGCG) Supplementation in Heart Failure: Systematic Review of Animal Experiments

YURI CAVALCANTI ALBUQUERQUE TENORIO^1^, Thayrone Romário da Silva Santos^1^, Francisco de Assis Costa^2^

(1) Hospital Veredas; (2) Universidade Federal de Alagoas

**Introduction:** Epigallocatechin-3-gallate (EGCG) is a natural flavonoid present in green tea leaves with important therapeutical effects such as antioxidant properties, reduction of free radicals involved in numerous chronic diseases. The literature identifies a strong correlation between EGCG supplementation and the treatment and prevention of cardiovascular diseases; consequently, this flavonoid is a potential adjuvant for patients with several clinical conditions, such as arterial hypertension, cardiac heart failure (CHF), arrhythmias, chronic coronary disease, or acute myocardial infarction. Therefore, this paper aims to identify correlation between EGCG extracted from green tea leaves and treatment of CHF.

**Methods:** A systematic literature review was developed selecting seven original articles, focusing on laboratory experiments in animal models.

**Results and discussion:** The results found that EGCG supplementation promoted treatment of CHF and cardiac remodelling prevention from reducing heart weight (lower p was < .0001), increasing left ventricle ejection fraction (lower p was < .01), decreasing protein expression levels of hypertrophy markers, such as brain natriuretic peptide (BNP) in animal models (lower p was < .01), and reducing mortality in one study (p < .05).

**Conclusion:** Therefore, it was possible to conclude that there is a positive relationship between EGCG supplementation with the treatment and prevention of CHF. However, although laboratory tests in animal models have positive results, multicentered randomized clinical trials in the population are necessary to define the actual association of this substance in humans.


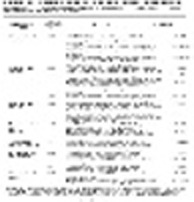


110809

Modality: E-Poster Young Researcher – Non-case Report

Category: CARDIO-ONCOLOGY

## Cardio-Oncology on Demand: Applying a Protocol to Classify and Identify Patients to be Referred to a Cardio-Oncologist

GISELE PLAÇA RODRIGUES OLSON^1^, Eduardo Schlabendorff^1^, Euler Manenti^1^, Jairo Lewgoy^1^

(1) Hospital Mãe de Deus, Porto Alegre/RS – Brasil

**Summary Base:** Cardiovascular diseases (CVD) and cancer (CA) are the main causes of death in Brazil. Its incidences are associated with rising in life expectancy and various common risk factors. With the improvement of CA treatment and the increasing number of survivors, the event of CVD in these individuals has also been increasing, due to the impacts of the anticancer drugs in the cardiovascular (CV) system, which is called cardiotoxicity. Cardio-oncology becomes essential for early identification, treatment, and prevention of such complications. Our practice suggested a protocol capable of stratifying cancer patients according to the risk of cardiotoxicity.

**Purpose:** The purpose of this study was to estimate the number of patients that were at elevated risk for cardiotoxicity, at their first oncology visit. And the amount of them were referred for an evaluation in cardio-oncology.

**Methods:** We reviewed 104 medical records of patients who had their first appointment at the oncology department of Hospital Mãe de Deus, in Porto Alegre/RS, from June 1 to August 31, 2019. To stratify them, we filled out an appropriated protocol based on guidelines.

**Results:** We have identified that 87 (83%) patients at elevated risk for cardiotoxicity, which should be evaluated by a cardio-oncologist. Only 15 (17.2%) were referred. The risk determinants are in the figure.

**Conclusion:** We have shown that most patients seen at the first visit to an oncologist had a substantial risk of cardiotoxicity, but only few were actually referred to the cardio-oncologist department. This initiative encourages initial cardiologic evaluation routine in patients who will undergo therapies with potential cardiotoxicity.


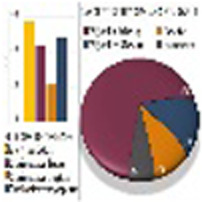


110812

Modality: E-Poster Young Researcher – Non-case Report

Category: ACUTE AND CHRONIC CORONARY DISEASE/THROMBOLYSIS

## Long-Term Outcomes of PCI vs CABG in Diabetic Patients with Left Main Coronary Disease: A Meta-Analysis of Randomized Controlled Trial

PEDRO EMANUEL DE PAULA CARVALHO^1^, Thiago Mamede Alencastro Veiga^1^, Gabriel Venturim Porto^1^, Felipe Sarsur de Lanna Machado^1^

(1) School of Medicine, Federal University of Minas Gerais

**Introduction:** It has been recognized that diabetic patients with complex coronary artery disease have poor outcomes when undergoing Percutaneous Coronary Intervention (PCI) compared to Coronary Artery Bypass Grafting (CABG). However, among diabetic patients with unprotected Left Main Coronary Artery (LMCA) disease, the optimal method is not established.

**Objectives:** Evaluate long-term outcomes after PCI with drug-eluting stents, as compared with CABG in diabetic patients with unprotected Left Main Coronary Artery (LMCA) disease.

**Methods:** MEDLINE, ClinicalTrials, and Cochrane databases were searched for randomized clinical trials (RCTs) that reported outcomes after PCI with drug-eluting stents vs CABG in unprotected LMCA disease among the diabetic population. We chose a minimum follow-up period of 3 years. Trial level Risk Ratios (RRs) and 95% confidence intervals (CIs) were pooled by random-effects modeling. The risk of bias was estimated by assessing funnel plots. PRISMA-IPD guideline was followed to present the data.

**Results:** Four RCTs including 1080 patients were identified, of whom 553 (51.2%) underwent PCI. The risk of composite endpoints of death, myocardial infaction, stroke or revascularization was higher after PCI compared with CABG (RR 1.30, 95% CI [1.09–1.56], P = 0.0037, I2 = 0%). There was no difference for individual endpoints of deaths, cardiovascular death and myocardial infarction (RR 1.32, 95% CI [0.94–1.85], P = 0.1131, I2 = 0%; RR 1.29, 95% CI [0.76–2.18], P = 0.3405, I2 = 0%; RR 0.94, 95% CI [0.61–1.45], P = 0.7881, I2 = 0%, respectively), however the risk of stroke was higher in CABG group (RR 0.41, 95% CI [0.18–0.94], P = 0.0351, I2 = 0%) meanwhile the risk of any revascularization was higher in PCI group (RR 1.99, 95% CI [1.44–2.75], P < 0.0001, I2 = 0%). There was no difference between groups with respect to graft occlusion or stent thrombosis (RR 0.66, 95% CI [0.19–2.31], p = 0.5162, I2 = 64,6%).

**Conclusion:** A higher risk of revascularization was associated with PCI compared to CABG in diabetic patients with left main coronary disease. This finding affects the composite endpoint that favors CABG. However, the risk of stroke was greater in patients submitted to CABG and no significant difference was found between the other outcomes evaluated (MI, cardiovascular death, and total deaths) when analyzed individually. These findings could help with the decision-making between PCI and CABG in diabetic patients with unprotected LMCA disease.

110830

Modality: E-Poster Young Researcher – Non-case Report

Category: HEMODYNAMICS AND INTERVENTIONAL CARDIOLOGY

## Clinical-Angiographic Profile and Procedural Outcomes in Patients Undergoing Percutaneous Coronary Intervention of Unprotected Left Main Coronary Artery Disease: An Observational Study

GUSTAVO PAES SILVANO^1^, Rodrigo Pinheiro Amantéa^1^, Guilherme Pinheiro Machado^1^, André Luiz Theobald^1^, Alan Pagnoncelli^1^, Luiz Carlos Corsetti Bergoli^1^

(1) Hospital de Clínicas de Porto Alegre (HCPA)

**Introduction:** Left main coronary intervention is increasingly used as a treatment option for unprotected left main coronary artery (ULMCA) lesions due to technical improvements in percutaneous coronary intervention (PCI), stent technology and new guideline recommendations.

**Objectives:** To report the clinical profile, angiographic status, and procedural outcomes in patients undergoing ULMCA PCI.

**Methods:** A prospective observational study including all consecutive patients with ULMCA who underwent PCI at a tertiary hospital in Southern Brazil between January 2017 and February 2022. Data including clinical-demographic profile, angiographic details and procedural complications were analyzed.

**Results:** A total of 206 patients were investigated, of which 63.1% were male, with a mean age of 67.7 ± 11.4 years. Hypertension and diabetes were the most common risk factors, present in 81.1% and 44.2% of patients, respectively. Smoking, previous acute myocardial infarction, chronic kidney disease, heart failure and peripheral vascular disease were present in 43.9%, 22.3%, 22.3%, 15.5% and 11.7% patients respectively. Chronic stable angina was the most common mode of presentation (32.5%), followed by non-ST-segment elevation myocardial infarction (23.8%), ST-segment elevation myocardial infarction (16.5%) and unstable angina (15%). The percentage of patients with single-, double-, and triple-vessel coronary disease (in addition to left main stenosis) was 28.6%, 20.9%, and 39.3%, respectively. Isolated ULMCA lesion was present in 11.2% patients and left main bifurcation lesions in 70.9%. Majority of the procedures were performed via femoral approach (63.6%). The median stent number placed was 2 (1–3) and the median contrast volume used was 240 mL (175–300). Predilatation with balloon was performed in 90.8% of patients and postdilatation in 96.1%. Pre-PCI ultrasound was used in 31.2% of patients and 42.9% following stent implantation. Procedure related complications occurred in 19 (14.6%) cases. Most common among these were distal embolization (3.4%), side branch occlusion (3.4%), and no reflow phenomenon (2.4%). Procedure related mortality was 1.0% and occurred exclusively in acute coronary syndrome patients. Among the 18 patiens who died during hospitalization, 12 (66.7%) had cardiogenic shock at presentation.

**Conclusion:** ULMCA PCI was safely performed and presented excellent results in this single-center prospective registry.

110843

Modality: E-Poster Young Researcher – Non-case Report

Category: ATHEROSCLEROSIS/CARDIOVASCULAR RISK FACTORS/CARDIOVASCULAR PREVENTION

## Three-Year Follow-Up of Chronic Coronary Syndrome Patients in a Specialized Center in Brazil

HENRIQUE TROMBINI PINESI^1^, Eduardo Martelli Moreira^1^, Cibele Larrosa Garzillo^1^, Eduardo Bello Martins^1^, Fabio Grunspun Pitta^1^, Paula Martins Paulino Bolta^1^, Carlos Alexandre Wainrober Segre^1^, Desiderio Favarato^1^, Roberto Kalil Filho^1^, Fabiana Hanna Rached^1^, Eduardo Gomes Lima^1^, Carlos Vicente Serrano Junior^1^

(1) Instituto do Coracao, Faculdade de Medicina, Universidade de Sao Paulo

**Introduction:** Incidence of cardiovascular events in patients with Chronic Coronary Syndrome (CCS) may vary significantly between geographical regions. Although populous, Brazil is often underrepresented in international registries.

**Objective:** This study aimed to describe the quality of care and 3-years incidence of cardiovascular events and associated prognostic factors in CCS patients in a tertiary public health care center in Brazil.

**Methods:** Patients with CCS (either previous revascularization, myocardial infarction, or stenosis >50% in at least one epicardial coronary artery) presenting for clinical evaluation were enrolled and followed for at least 3 years. The main endpoint was the composite of MI, stroke, or death. We also evaluated prescription, symptoms, and laboratory records.

**Results:** 844 patients with mean age of 65 (±9.4) years, 31.3% women, were included. Previous MI was present in 60.9%, previous CABG in 28.8%, and previous PCI in 44.5%. Diabetes was prevalent in 49.6% and high blood pressure in 86.7%. The use of aspirin was prevalent in 90.9% of the patients, 21.2% with dual antiplatelet therapy, and only 7.1% with no antiplatelet agent. Statins were prescribed to 94.4% of the patients, with high-intensity statin therapy in 68.2%. At a median follow-up (FU) of 881 days, we recorded 70 events of primary composite endpoint, with a 3-year estimate event incidence of 8.3%. Age (1.54, 95%CI 1.32–1.90), stroke (2.62, 95%CI 1.18–5.84), LDL (1.10, 95%CI 1.03–1.18) and left ventricular function (0.84, 95%CI 0.76–0.93) were the main prognostic factors in multivariate analysis. At admission, angina was present in 29.1% of the patients. This percentage dropped to 16.3 after follow-up. On the other hand, there was no improvement in the LDL levels.

**Conclusion:** CCS patients at our institution had a 3-year incidence of the primary composite endpoint of 8.3%, the prescription of antiplatelet therapy and statins were high, and LDL-cholesterol was the main modifiable risk factor of worse prognosis.

110847

Modality: E-Poster Young Researcher – Non-case Report

Category: HEART FAILURE/CARDIOMYOPATHY/TRANSPLANT

## Prognostic Value of Pulse Pressure in Heart Failure with Reduced Ejection Fraction

DIANA SEIXAS FERRÃO^1^, Joana Pereira^1^, Pedro Ribeirinho Soares^1^, Marta Soares Carreira^1^, Marta Amorim^1^, Catarina Elias^1^, Rita Gouveia^1^, Sérgio Madureira^1^, Ana Neves^1^, Jorge Almeida^1^, Patrícia Lourenço^1^

(1) Internal Medicine Department, Centro Hospitalar e Universitário de São João

**Introduction:** Pulse pressure (PP) is the difference between systolic and diastolic blood pressure. Increased PP is a well-recognized cardiovascular risk factor but is less well established in HF, particularly with reduced ejection fraction (HFrEF).

**Objective:** To study the prognostic role of increased PP in HfrEF.

**Methods:** Retrospective cohort study of adult (>18 years) ambulatory patients with chronic HFrEF that were followed in a specialized HF clinic between January 2012 and May 2018. Patients with preserved ejection fraction and those with recovered ejection fraction were excluded. Patients with PP ≤50 mmHg and PP >50 mmHg were compared. The 50 mmHg cut-off corresponded to the median value of PP distribution and to the midst of the normal range. Patients were followed-up to 5 years and all-cause mortality was the endpoint. A Cox-regression analysis was performed to study the association of PP with 5-year mortality. Interaction between PP and age (cut-off 80 years) was tested. The analysis was further stratified according to age strata: <80 years and ≥80 years. Adjustments were made considering sex, hypertension, diabetes, atrial fibrillation, ischaemic aetiology, systolic dysfunction severity, heart rate, systolic blood pressure (SBP), diastolic blood pressure (DBP), renal function, B-type natriuretic peptide (BNP) and evidence-based therapy.

**Results:** We studied 854 chronic HF patients. Mean age was 71 years and 65% were male. HF was ischaemic in 46.0% of the patients and 51.3% had severe systolic dysfunction. Median (Q1–Q4) PP was 51(40–64)mmHg. Patients with PP >50 mmHg were older, had higher SBP, less systolic disfunction and more often medicated with renin-angiotensin-aldosterone system inhibitors. During the 5-year follow-up, 391 (45.8%) patients died. Patients with PP >50 mmHg had no difference in death risk compared to those with lower PP: HR = 0.94 (95% CI: 0.77–1.15), p = 0.55. When the analysis was stratified according to age, in the subgroup of patients aged ≥80 years, elevated PP was associated with survival benefit, HR of 0.58 (95% CI: 0.37–0.91), p = 0.02. No association with mortality existed in the group of patients <80 years.

**Conclusions:** Elevated PP was not predictive of mortality in patients with chronic HF and in the subgroup of patients aged ≥80 years, a PP >50 mmHg was associated with lower mortality, so it appears that age influences the impact of PP in mortality in HF.

110858

Modality: E-Poster Young Researcher – Non-case Report

Category: COVID-19 AND CARDIOVASCULAR SYSTEM

## Comparison between Cardiac and Non-Cardiac Patients Admitted to an Intensive Care Unit with COVID-19: Analysis of a Tertiary Center in Brazil

HENRIQUE TROMBINI PINESI^1^, Eduardo Martelli Moreira^1^, Melina de Oliveira Valdo Giugni^1^, Pedro Henrique de Almeida Marins^1^, Eduardo Gomes Lima^1^, Fabio Grunspun Pitta^1^, Ludhmila Abrahão Hajjar^1^, Juliana Carvalho Ferreira^2^, Carlos Vicente Serrano Junior^1^, Cibele Larrosa Garzillo^1^

(1) Instituto do Coracao, Faculdade de Medicina, Universidade de Sao Paulo, Brazil; (2) EPICOV Study group, Faculdade de Medicina, Universidade de São Paulo, Brazil

**Background:** Studies have shown an association between cardiovascular diseases and higher rates of mortality and complications in patients with COVID-19. The aim of this study was to compare mortality and other events in critically ill cardiac and non-cardiac patients with COVID-19 in a referral center intensive care unit (ICU) in Brazil.

**Methods:** We analyzed data from a prospective registry of patients admitted to a tertiary hospital ICU for COVID-19 between March and June 2020. 1501 patients were admitted and 221 had heart disease. Continuous variables were described as mean and standard deviation or median and interquartile range. Categorical variables were described as absolute frequency and percentage. To compare clinical and demographic characteristics, as well as laboratory tests between patients with and without heart disease, the Student’s T test and the Mann-Whitney U method were used. For the occurrence of events, Fisher’s exact test was used.

**Results:** Patients with heart disease were older, and had a higher prevalence of diabetes and chronic kidney disease, compared to non-cardiac patients. On the other hand, the prevalence of cancer and obesity was higher among non-cardiac patients. Blood pressure, heart rate and oxygen saturation were similar between the groups, as well as the need of oxygen and ventilatory support. Patients with heart disease were more frequently using vasoactive drugs on admission. Patients with heart disease had worse renal function and higher cardiovascular biomarkers (troponin, NT-proBNP and D-dimer). Non-cardiac patients had higher values of CRP, leukocytes, lymphocytes and platelets. Surprisingly, in-hospital and ICU mortality were similar in both populations studied. Non-cardiac patients required more frequent orotracheal intubation and had more thrombotic events. In contrast, patients with heart disease had a higher occurrence of atrial fibrillation.

**Conclusion:** In our center, the presence of cardiovascular disease in critically ill patients with COVID-19 was not associated with mortality compared with non-cardiac patients.


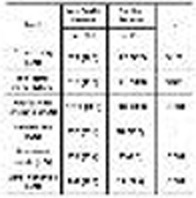


110863

Modality: E-Poster Young Researcher – Non-case Report

Category: CARDIAC ARRHYTHMIAS/ELECTROPHYSIOLOGY/ELECTROCARDIOGRAPHY

## Implantation of a Cardioverter-Defibrillator Associated with Multisite Pacing in Patients with Persistent Left Superior Vena Cava

ALFREDO AURÉLIO MARINHO ROSA FILHO^3^, Lucas Brandão Cavalcante^2^, José Carlos de Souza Neto^2^, Sara Carolline Gomes de Araújo Lima^2^, Alice Wanderley Rosa^2^, Dario de Moura^1^, Virginia Barreto^1^, Marcelo Russo^1^, Fabian Fernandes^1^, Flavio Loureiro^1^, Alfredo Aurélio Marinho Rosa^1^, Edvaldo Ferreira Xavier Júnior^1^

(1) Santa Casa de Misericórdia de Maceió – SCMM, AL; (2) Centro Universitário Tiradentes – UNIT, AL; (3) Hospital Universitário Professor Alberto Antunes – HUPAA/UFAL

**Background:** Persistent left superior vena cava (PLSVC) is a rare venous anomaly, however, in the thorax it is the most frequent venous alteration with a prevalence of 0.3 to 0.5% in the general population. The PLSVC is asymptomatic and in most cases is identified during implantation of the cardiac device.

**Objective:** To present the technique used to implant a cardioverter-defibrillator (ICD) with biventricular pacing (BIV) in patients with PLSVC.

**Material and Methods:** Between March 2007 and April 2022, 776 multi-site cardioverter-defibrillators (ICD-CRT) were implanted, of which 4 patients (0.5%) had SEPVC. All patients had symptomatic HF and refractory to optimized drug treatment with recurrent hospitalizations for HF. The 4 patients were male. They presented LBBB on ECG and ventricular tachycardia on 24h Holter. In all, the anomaly was identified during the procedure. They were then submitted to implantation of ICD associated with stimulation (BIV), during the puncture of the left subclavian vein, the presence of PLSVC was found. It was decided to implant the ICD through the right subclavian vein and introduce the left ventricular lead into the left posterolateral vein through the deflectable sheath. Access to the coronary sinus was obtained by femoral access.

**Results:** In all, the procedure was performed successfully. The procedure time was around 90 minutes. All patients (100%) did not show displacement of the electrodes, being discharged 24 hours after the procedure and in the clinical follow-up, the patients showed clinical and ejection fraction improvement, with an average EF from 24% to 42%.

**Conclusion:** Although PLSVC is a rare and complex anomaly, the implantation of the ICD was possible through the right subclavian artery, not compromising the final result of cardiac resynchronization.

110865

Modality: E-Poster Young Researcher – Non-case Report

Category: CARDIAC ARRHYTHMIAS/ELECTROPHYSIOLOGY/ELECTROCARDIOGRAPHY

## Treatment of Advanced Heart Failure in Octogenarians Through Implantation of a Multi-Site Cardioverter-Defibrillator

ALFREDO AURÉLIO MARINHO ROSA FILHO^3^, Sara Carolline Gomes de Araújo Lima^2^, Lucas Brandão Cavalcante^2^, José Carlos de Souza Neto^2^, Alice Wanderley Rosa^2^, Marcelo Russo^1^, Gustavo Santiago^1^, Fabian Fernandes^1^, Lenine Angelo^1^, Flavio Loureiro^1^, Alfredo Aurélio Marinho Rosa^1^, Edvaldo Ferreira Xavier Júnior^1^

(1) Santa Casa de Misericórdia de Maceió – SCMM, AL; (2) Centro Universitário Tiradentes – UNIT/AL; (3) Hospital Universitário Professor Alberto Antunes – HUPAA/UFAL

**Background:** Heart failure (HF) is considered the final stage of all heart diseases and with the increase in life expectancy of the population, octogenarian patients with HF can benefit from the implantation of a multisite cardioverter-defibrillator (ICD+CR- T).

**Objective:** To present the results of the implantation of multi-site cardioverter-defibrillators in octogenarian patients with heart failure unresponsive to optimized clinical treatment.

**Material and Method:** Between March 2010 and April 2022, 763 multisite cardioverter-defibrillators (ICD+CR-T) were implanted, of which 56 patients (7.34%) were octogenarians with 40 male patients (71.4%). Patients had HF with optimized drug treatment without adequate response, ejection fraction less than or equal to 32%, ventricular tachycardia and syncope. In this sample, they underwent implantation of the ICD+CR-T with access to the coronary sinus via the femoral route. Immediate success criterion coursing with QRS complex narrowing.

**Results:** Of the 56 patients, forty-five (80.3%) responded satisfactorily to the therapy used, with a decrease in the number of hospitalizations and an improvement in the quality of life. Regarding the etiology of heart diseases: 38 (67.8%) were ischemic, 12 (21.4%) were chagasic and 6 (10.8%) had hypertensive heart disease. There were no intraoperative and postoperative complications and the main vein addressed for system implantation was the left posterolateral vein in 32 patients (57.1%). No displacement of electrodes was recorded. In the clinical follow-up, 22 pt (39.3%) received appropriate therapy for ventricular tachycardia. Survival was 100% at the end of 12 months in all patients undergoing ICD+CR-T implantation.

**Conclusion:** It is therefore evident in the analyzed sample that the treatment of heart failure in octogenarian patients undergoing ICD+CR-T implantation showed a satisfactory response in terms of morbidity and mortality.

110870

Modality: E-Poster Young Researcher – Non-case Report

Category: CARDIAC ARRHYTHMIAS/ELECTROPHYSIOLOGY/ELECTROCARDIOGRAPHY

## Comparative Study of Electrophysiological Parameters of Right Ventricular Stimulation Versus Stimulation of the Left Bundle Branch Area

JOSE MIGUEL CAYO MONTES^1^, JOSE MIGUEL CAYO MONTES^1^, JUAN CARLOS ZERPA ACOSTA^1^, JOSE CARLOS PACHON MATEOS^2^, JUAN CARLOS PACHON MATEOS^1^, ENRIQUE INDALECIO PACHON MATEOS^2^, YVAN FLORES TARDIO^1^, CARLOS THIENE CUNHA PACHON^2^, CIBELE MATSUURA DE OLIVEIRA^1^

(1) HOSPITAL DO CORAÇÃO; (2) SEMAP

**Introduction:** Pacemaker (PM) implantation is the treatment of irreversible bradyarrhythmias, normalizing the heart rate, right ventricular apical pacing (RVAP) has been traditionally chosen, but it can induce ventricular dyssynchrony, resulting in a widening of the QRS complex duration. (QRSd) and left ventricular activation time (LVTA) increase, leading to heart failure and/or arrhythmias. In order to prevent these adverse effects, right ventricular septal pacing (RVSP) and more recently left bundle branch area pacing (LBBAP) of the bundle of His were developed, which reproduce more physiological activation of the cardiac conduction system.

**Objectives:** The aim of this study is to compare the electrophysiological parameters of ventricular synchrony (QRSd, LVAT) and other parameters such as QRS axis, R-wave amplitude, threshold and impedance of RVAP, RVSP and LBBAP, in a group of patients with pacemaker implantation indication.

**Methods:** Prospective and controlled study, which included 25 patients with indication for bicameral pacemaker implantation. Using radioscopy, first, the electrode is positioned in the position of the RAVP, using the electrogram recording system and the PM device programmer, unipolar stimulation is performed at this point and the electrophysiological parameters are recorded; QRSd, LVAT in V5 or V6, QRS axis, R wave, ventricular lead threshold and impedance. Afterwards, the ventricular electrode was positioned in the region of RVSP and LBBAP and the same parameters were measured.

**Results:** Electrophysiological parameters. QRSd: RVAP: 162 ± 25 ms, RVSP: 145 ± 20 ms, LBBAP: 109 ± 17 ms (p < 0,05) LVAT: RVAP: 80 ± 11 ms, RVSP: 70 ± 16 ms, LBBAP: 60 ± 11 ms. (p < 0,04). QRS axis: RVAP: –56° ± 10°, RVSP: 45 ± 15°, LBAP: 60 ± 11 ms (p < 0,04). Ventricular lead impedance: RVAP: 603 ± 20Ω, RVSP: 560 ± 25Ω, LBBAP: 805 ± 25Ω (p < 0,03). Threshold: RVAP: 0,75 ± 0.50V. RVSP: 0,75 ± 0.25V. LBBAP: 0.5 ± 0.25V (p < 0,05). R wave: RVAP: 10,1 ± 2 mV RVSP: 9,9 ± 3 mV LBBAP: 10,9 ± 6 mV(p: 0,06) There were no adverse events during the 6-month follow-up.

**Conclusion:** The unipolar LBBAP achieved lower QRSd and LAVT, normal QRS axis, lower pacing thresholds, slight increase in ventricular lead impedance, there were no significant difference in the R wave sense compared to RVAP and RVSP. LBBAP is a safe technique that preserves ventricular synchrony, with better electrophysiological parameters compared to RVAP and RVSP, and could be considered as the first choice of ventricular pacing for patients.

111120

Modality: E-Poster Young Researcher – Non-case Report

Category: NURSING

## Evaluation of Case Study Discussion in Cardiology Nursing: Instrument Validation

JOAO PEDRO DA HORA SILVA BARROS^1^, Júlya de Araujo Silva Monteiro^1^, Andressa Teoli Nunciaroni^2^, Renata Flávia Abreu da Silva^2^

(1) Universidade do Estado do Rio de Janeiro; (2) Universidade Federal do Estado do Rio de Janeiro

**Introduction:** Active methodologies, such as case studies, are teaching strategies in nursing education, making the student an active agent in learning and the teacher a facilitator.

**Objective:** To validate two evaluation instruments for case study discussion in cardiology nursing.

**Methods:** Methodological, prospective, quantitative study, conducted remotely for content validation of measurement instrument. Two instruments were evaluated: Quantitative, with twenty-seven items and three aspects (Psychosocial, Intellectual and Human/Sensitive) and; Qualitative, with six questions addressing the learner’s interest about case studies. Nurses with experience in cardiology for over two years were recruited and the instruments were structured in Google Forms®, being available by link, with questionnaires in Likert Scale with an area for suggestions from experts. Data analysis was performed using Microsoft Excel® and validation was based on the calculation of the Content Validity Index (CVI). Items with at least 80% approval if more than six experts and 100% approval if less than six were valid. The study was approved by the institution’s Research Ethics Committee under Opinion #4,126,925 on July 1, 2020.

**Results:** The data collection took place in October 2020, obtaining 7 responses from jurors with experience ranging from 4 to 30 years; 3 specialists, 2 masters, and 2 PhDs. In instrument 1, 7 items were not validated, namely: 2 on the Intellectual Aspect and 5 on the Human/Sensitive Aspect; in instrument 2, only 1 item on Pertinence. After changes, a new consultation was made, and 7 responses were obtained, validating all items of the instruments. When calculating the CVI of the instrument (CVI-I), the comprehensiveness, clarity and pertinence were: instrument 1 = 0.99, 1 and 0.97 and; instrument 2 = 1, 0.97 and 1. The validation occurred in Portuguese, not being translated so that there would be no reduction in its characteristics. The simultaneous use of the instruments is mandatory. Some limitations: reduced access to hospitals/universities by COVID-19 and no testing with the public.

**Conclusion:** The validated instruments can be used to evaluate learning through case studies in cardiology. With the interpretation of the results of the instruments, the teachers will be able to recognize the weaknesses and form strategies to overcome them, besides being used in actions of permanent education.

110895

Modality: E-Poster Young Researcher – Non-case Report

Category: ACUTE AND CHRONIC CORONARY DISEASE/THROMBOLYSIS

## The Neutrophil-to-Lymphocyte Ratio on Admission as Important Predictor of In-Hospital Mortality in Acute Myocardial Infarct Patients

VISAKHA REVANA IRAWAN^1^, Frans Wantania^2^, Janry Pangemanan^2^

(1) Department of Internal Medicine, Faculty of Medicine, Sam Ratulangi University/Prof. dr. R. D. Kandou General Hospital, Manado, Indonesia; (2) Division of Cardiology, Department of Internal Medicine, Faculty of Medicine, Sam Ratulangi University/Prof. dr. R. D. Kandou General Hospital, Manado, Indonesia

**Background:** The neutrophil-to-lymphocyte ratio (NLR) in peripheral blood has recently been able to act as a biomarker that can predict worse clinical conditions ranging from infectious diseases to cardiovascular diseases.

**Objective:** The study aimed to determine the optimal cutoff level of NLR level to predict in-hospital mortality in acute myocardial infarction (AMI) patients.

**Methods:** We analyzed the data characteristics of 332 consecutive cases of AMI patients admitted in Prof. dr. R. D. Kandou General Hospital, retrospectively, from September 2020 to December 2021. Patients with acute infections, HIV/AIDS, who used corticosteroids in the last three months, and antibiotics in the last 24 hours were excluded. NLR was computed from the absolute values of neutrophils and lymphocytes from the complete blood count (CBC) on hospital admission. Furthermore, the optimal cutoff level of NLR was analyzed using ROC curves to discriminate survivors versus non-survivors during hospitalization.

**Results:** The value of NLR in predicting in-hospital death (AUC = 0.88, p = 0.0001) was higher than the platelet-lymphocyte ratio (PLR) (AUC = 0.79, p = 0.0001), the neutrophil-monocyte ratio (NMR) (AUC = 0.61, p = 0.075), and the lymphocyte-monocyte ratio (LMR) (AUC = 0.17, p = 0.0001). The optimum cutoff NLR level to predict in-hospital mortality was 7.36, with a sensitivity 72% and a specificity of 90.55%. Patients with NLR levels ≥7.36 had a higher probability of mortality when comparing with those who with NLR levels <7.36 (OR = 24.65, 95% CI (9.5–63.94), p = 0.0001).

**Conclusions:** NLR could be an early and important predictor of in-hospital mortality in AMI patients.

111347

Modality: E-Poster Young Researcher – Non-case Report

Category: EPIDEMIOLOGY AND HEALTH POLICIES/GLOBAL HEALTH

## Standard Treatment Protocols for the Pharmacological Management of Hypertension: Comparison with the Major Clinical Practice Guidelines Recommendations

GAUTAM SATHEESH^1^, Rupasvi Dhurjati^1^, Abdul Salam^2^

(1) The George Institute for Global Health, Hyderabad, India; (2) The George Institute for Global Health, University of New South Wales, Sydney, New South Wales, Australia

**Introduction:** Globally, there are large gaps in the diagnosis, awareness, treatment, and control of hypertension. Using evidence-based standard treatment protocols (STPs) may improve hypertension treatment and control.

**Objective:** 1. To summarize major guidelines recommendations for the pharmacological management of hypertension. 2. To identify, characterize and compare available hypertension STPs with the major guidelines.

**Methods:** We summarized recommendations on the pharmacological management of hypertension from four major hypertension guidelines: American Heart Association, European Society of Cardiology, World Health Organization (WHO), and International Society for Hypertension. STPs were identified from websites of WHO, Resolve to Save Lives, Pan American Health Organization and through expert consultations. We defined hypertension STP as series of steps recommended for treating hypertension with information on target patient group (age, comorbidities, race etc.), blood pressure (BP) thresholds for treatment initiation and intensification, BP targets, drugs/classes for each step, follow-up frequency, target time for BP control, and combination therapy/single-pill combinations.

**Results:** All guidelines, except American, presented a stepwise treatment approach and recommended initiation with single-pill combinations in most patients. All guidelines recommended renin-angiotensin-system inhibitors (RASi), thiazide-type diuretics (TZD), or calcium channel blockers as first-line therapy. Except WHO, no guidelines specified individual drugs or doses. Further, WHO and American guidelines did not mention a target time for BP control. All identified STPs (n = 36) presented a stepwise approach, and 86% (n = 31) consisted of 5 or more steps. Majority (86%) recommended calcium channel blockers as initial therapy, and RASi were the second choice in 69%. TZD was introduced as a third line agent in 72% of STPs, second line in 14%, and first line in 11%. Only eight STPs recommended initiation with combination therapy, and only two recommended single-pill combinations. Contradictory to guidelines, 39% of STPs recommended dose-increase if desired BP control is not achieved. No STPs mentioned a target time for BP control.

**Conclusion:** Several deviations from the major guideline recommendations were observed in the identified STPs. Developing optimal, evidence-based, and context-specific STPs is vital to control hypertension, especially in countries with limited resources.

111165

Modality: E-Poster Young Researcher – Non-case Report

Category: HEART FAILURE/CARDIOMYOPATHY/TRANSPLANT

## Development of Cardiomyopathy in Patients After Liver Transplantation for Hereditary Transthyretin Amyloidosis

FERNANDO SARAIVA CONEGLIAN^1^, Fernando Saraiva Coneglian^1^, Flávio Henrique Valicelli^1^, Fernanda Fernandes Souza^1^, Ajith Kumar Sankarankutty^1^, Wilson Marques Júnior^1^, Caroline Lavigne Moreira^1^, Minna Moreira Dias Romano^1^, Pedro Manoel Marques Garibaldi^1^, Rodrigo Tocantins Calado de Saloma Rodrigues^1^, Marcus Vinicius Simões^1^

(1) Hospital das Clínicas da Faculdade de Medicina de Ribeirão Preto

**Introduction:** Liver transplantation (LT) is an alternative treatment for patients with familial amyloidotic polyneuropathy (FAP), correctly renamed hereditary transthyretin (hATTR) amyloidosis, and is associated with a significant improvement in survival. However, some patients may exhibit progression of the disease latelly, with eventual cardiac involvement. There are few reports on the most common cardiac manifestation in these cases.

**Objective:** To describe the clinical evolution and cardiac involvement in patients who underwent liver transplantation for hATTR in a single interdisciplinary Amyloidosis center.

**Methods:** Observational study of cohort with 8 patients undergoing LT after the diagnosis of hATTR, considering cardiovascular symptoms, ECG, Echocardiogram and therapeutic management.

**Results:** All 8 patients had a diagnosis of hATTR with early-onset V30M mutation, 6 were male (75%). The mean follow-up after LT was 13.5 + 3.9 years and the age at the moment of evaluation was 49.3 + 6.2 years. Cardiovascular manifestations occurred in 4 patients (50%), corresponding in all cases to symptomatic bradycardia with Sinus Node Disease (SND) and need for permanent pacemaker (PP) implantation, with the mean period of cardiovascular manifestation being 14 years. All patients with DNS exhibited symptoms of severe autonomic neuropathy with symptomatic postural hypotension. Only 2 patients(25%) developed echocardiographic alterations compatible with myocardial infiltration by CA and significant increase in the thickness of the interventricular septum (13–15 mm), 1 patient have developed manifestations of heart failure (HF) that started 16 years after LT. This same patient had DNS and PP implant 4 years before the onset of HF.

**Conclusion:** Our results indicate a high prevalence of late development of cardiac organic involvement after liver transplantation in patients with hATTR due to V30M mutation. The preferred form of manifestation of heart disease is symptomatic bradycardia by SND requiring PP implantation. There seems to be an association between the intensity of autonomic denervation and SND, suggesting a pathophysiological link between these abnormalities.

110900

Modality: E-Poster Young Researcher – Non-case Report

Category: HEART FAILURE/CARDIOMYOPATHY/TRANSPLANT

## Opposite Effects of Dapagliflozin and Glibenclamide on Diastolic Function in Patients with Diabetes: Randomized Controlled Trial (Addenda-BHS2)

SHEILA TATSUMI KIMURA MEDORIMA^1^, Daniela Camargo Oliveira^1^, Íkaro Soares Santos Breder^1^, Vaneza Lira Waldow Wolf^1^, Luiz Sérgio Fernandes de Carvalho^1^, Alexandre Anderson de Sousa Munhoz Soares^1^, Joaquim de Paula Barreto Fonseca Antunes de Oliveira^1^, Daniel Batista Munhoz^1^, Jessica da Silva Cunha Breder^1^, Wilson Nadruz Junior^1^, Thiago Quinaglia Araújo Costa Silva^1^, Andrei Carvalho Sposito^1^

(1) Atherosclerosis and Vascular Biology Laboratory (Atherolab), Cardiology Department, State University of Campinas (Unicamp), Campinas, SP, Brazil.

Dapagliflozin significantly reduced cardiovascular death and heart failure endpoints, however sulfonylureas and metformin are the only oral antidiabetics available in Brazilian Primary Care Public Health System. This trial was planned to evaluate their effect on diastolic function and to investigate possible mechanisms underlying this change.

**Methods:** The Assessment of Dapagliflozin effect on Diabetic Endothelial Dysfunction of brachial Artery – Brazilian Heart Study 2 trial is an investigator-initiated prospective clinical trial, single-center, active-controlled, open-label, phase-4 randomized trial. High cardiovascular risk patients with type 2 diabetes were randomized to 12-week treatment with Dapagliflozin (DAPA) or Glibenclamide (GLIB) on top of Metformin, in glucose-lowering equivalent regimens. Echocardiogram was performed at randomization and at 12 weeks. Serum nitrate was analyzed by NO chemiluminescence analyzer.

**Results:** 97 patients completed the study, including 61% males, median age 59(10) years, mean LVEF 62.9% ± 8. After 12weeks, E/e’ratio difference significantly reduced in DAPA group, while increased in GLIB arm [-0.17(1.9) vs 0.87(2.3), p = 0.001]. Tissue Doppler e’ velocities did not change significantly, but E velocity increased with GLIB [0(19.7) vs 4.0(16.0), p = 0.016]. LV mass and LV septum reduced in DAPA group, although without statistical significance. Exploratory secondary analysis was performed with clinical variables tested in multivariable binary logistic regression analysis, with E/e’ ratio improvement as dependent variable. Nitrate raising was an independent predictor of diastolic function improvement (B = 1.098; p = 0.024; Exp(B) = 2.999), adjusted for age, sex, body surface area and randomization group. This multivariable model classifies correctly 70,1% of the patients (model R-squared = 0,27; p-value < 0,001).

**Conclusion:** Dapagliflozin significantly improves diastolic function by reducing E/e’ ratio, while Glibenclamide worsens. In our study, E/e’ improvement was significantly predicted by serum nitrate raising after 12-weeks, offering important clues on the pathophysiology evolved in SGLT2 inhibitors myocardium protective effect.

110947

Modality: E-Poster Young Researcher – Non-case Report

Category: DIGITAL HEALTH/INNOVATION

## Hypertrophic Cardiomyopathy: Identification of the Molecular Profile and Treatment of Phenocopies

ANABEL LIMA VIEIRA^1^, Renan Figueiredo de Freitas^1^, Maria Beatriz Siqueira de Araújo^4^, Sérgio José Siqueira de Araújo^3^, Ândrea Virginia Ferreira Chaves^2^

(1) Hospital Agamenon Magalhães, Recife, PE; (2) Serviço de Referência em Doenças Raras – RARUS, Recife, PE; (3) UniNassau, Recife, PE; (4) Faculdade Pernambucana de Saude, Recife, PE

**Introduction:** Hypertrophic cardiomyopathy (HCM) is the most common genetic heart disease (1:200). The development of genetic panels for cardiomyopathies has allowed for the diagnosis of phenocopies, the receipt of adequate genetic counseling and, most importantly, the identification of diseases with specific treatment.

**Objective:** To present the genetic characterization of a group of patients (pts) with an echocardiographic diagnosis of HCM.

**Methods:** This was a cross-sectional observational study that looked at pts who had been diagnosed with HCM and had a molecular panel for HCM. The genes analyzed were cardiac α-actin, desmin, filamin C, alpha galactosidase A, lysosomal-associated membrane protein 2, myosin-binding protein C, cardiac β-myosin heavy chain, myosin light chain 2, myosin 3, myopaladin, phospholamban, Protein kinase A γ subunit, non-receptor tyrosine-protein phosphatase type 11, troponin C1, troponin I3, troponin T2, α-tropomyosin 1 and transthyretin.

**Results:** The panel was performed on 25 pts with HCM. The age ranged from 13 to 91 years, mean 39.3 years. Regarding gender, 52% were women. Variants were found in cardiac β-myosin heavy chain genes (sarcomeric, chromosome 14, autosomal dominant and recessive) in ten pts (40%), filamin C in four pts (16%), myosin-binding protein C in three pts (12%), desmin in two pts (8%), myosin light chain 2 in one pts (4%), α-tropomyosin 1 (TPM1) in one pts (4%), troponin T2 in one pts (4%), non-receptor tyrosine-protein phosphatase type 11 (Noonan syndrome – phenocopy, chromosome 12, autosomal dominant) in a pts (4%), lysosome-associated membrane protein 2 (Danon’s disease – phenocopy, X chromosome) in a pts (4%), alpha galactosidase A (Fabry’s disease – phenocopy, X chromosome) in one pts (4%). In one pts two mutations were found and in another three concomitant mutations. In one pts, no mutation was identified.

**Conclusion:** The molecular panel was used to provide adequate genetic counseling to the pts. Three pts with HCM related phenocopies (Noonan syndrome, Danon’s disease, and Fabry’s disease) were identified. The diagnosis of Fabry disease allowed for the recommendation of specific treatment, which had a direct impact on the natural history of this pts and their families. Because the panel used was limited to 18 genes, no mutations were found in three pts. Expanded panels improve the probability of genotype identification and the possibility of effective therapy.

110957

Modality: E-Poster Young Researcher – Non-case Report

Category: CARDIOGERIATRICS

## Thyroid Dysfunction and Presence of Atrial Fibrillation in Elderly Patients with Heart Failure

ANABEL LIMA VIEIRA^1^, Marina Souto da Cunha Brendel Braga^1^, Amanda Valério Galindo^1^, Caio Correia da Silva^1^, Rafael Buarque de Macedo Gadelha^1^, Matheus Dantas Soeiro^2^, Sabrina Barreto Braga Pires^2^, Jessica Myrian de Amorim Garcia^1^, Francisco Alfredo Bandeira e Farias^1^

(1) Hospital Agamenon Magalhães, Recife, PE; (2) Faculdade Pernambucana de Saúde, Recife, PE; (3) Universidade de Pernambuco, Recife, PE

**Introduction:** Thyroid hormones have both direct and indirect effects on cardiac cells, so both excess and deficiency will influence the cardiovascular system. Atrial fibrillation (AF) is one of the cardiac manifestations of thyroid dysfunction (TD), particularly hyperthyroidism, and arrhythmia with clinical significance is more common in the context of thyroid diseases.

**Objectives:** To assess the relationship between thyroid dysfunction and the presence of atrial fibrillation in elderly patients with heart failure.

**Methodology:** An observational cross-sectional study. The medical records of 118 patients 65 years old admitted to the cardiology ward with HF from August 2020 to October 2021 were analyzed. For qualitative variable comparisons, SPSS v.25.0 was used, and the assumed significance level was 5%; for quantitative variables AF × TD, the chi-square test was used.

**Results:** There is a mean age of 72.8 years (ranging from 65 to 96 years), with the majority of patients being male (54.2%). There are 36 patients (30.5%) with TD present. 47.2% of these patients are male, 97% have Systemic Arterial Hypertension, 46.9% have Diabetes Mellitus (DM), and 25% are pre-DM. Nonetheless, 38.9% have HFrEF, 19.4% have HFrEFi, and 41.7% have HFrEFp, with an average LVEF of 48.16% (standard deviation: 16.8); 5.7% have coronary artery disease, and 26.3% have a history of stroke. In the TD group, 32 patients (88.88%) had subclinical hypothyroidism, while 4 (11.11%) had clinical hypothyroidism. In this group, the mean thyroid-stimulating hormone (TSH) level was 11.26, with (standard deviation: 13.69). 12 patients (33.3%) in the TD group had AF. Only 11 (13.4%) of the non-DT group had AF. The mean TSH in AF patients was 8.50, with a standard deviation of +–14.7, compared to 4.03, with a standard deviation of +–6.17 in non-AF patients (t(116) = 2.267; p = 0.013). The comparison of TD and AF revealed a close relationship between the two variables: 10.2% of the elderly with TD had AF, while only 9.3% of the sample (69.5%) did not have TD (Chi-Square = 6.325a; p = 0.012).

**Conclusions:** According to the study sample, there is a statistically significant relationship between thyroid dysfunction and atrial fibrillation in elderly HF patients, corroborating what has previously been established in the literature. Among the possible thyroid dysfunctions, this study found a stronger link between AF and hypothyroidism, both clinical and subclinical.

110972

Modality: E-Poster Young Researcher – Non-case Report

Category: CONGENITAL AND PEDIATRIC CARDIOLOGY

## The Neurodevelopment of Infants with Congenital Heart Disease in Southern Brazil: A Sample Characterization of a Pilot Study

RITA CASSIANA MICHELON^2^, Caroline Engster da Silva^2^, Ludimila Silveira Parker Lopes^1^, Claudia Costamilan Paes^2^, Fernanda Lucchese^1^

(1) Santa Casa de Misericórdia de Porto Alegre (ISCMPA)/Hospital da Criança Santo Antônio (HCSA); (2) Instituto de Cardiologia/Fundação Universitária de Cardiologia (IC/FUC)

**Introduction:** Congenital Heart Disease (CHD) has a prevalence of about 1 case per 100 live births nationwide in Brazil. Infants with CHD are at greater risk of having developmental delays. In 2012, the American Heart Association (AHA) proposed guidelines for the evaluation and intervention of neurodevelopmental delays in infants with CHD. Multidisciplinary early evaluation and interventions can significantly improve the course of the development of a child with CHD, preventing neurodevelopmental sequelae and increasing quality of life, adaptive skills, and academic and professional achievements later in life.

**Objective:** To present data on the neurodevelopment of infants with CHD participating in a multidisciplinary research clinic seeking to develop low-cost early intervention protocols in Brazil.

**Method:** Fifty-nine infants with CHD under the age of 42 months were recruited from a convenience sample at the Pediatric Cardiology Outpatient Clinic of a reference hospital in Southern Brazil. The information was gathered through parental questionnaires. Infant development was measured by the Bayley-III Infant and Toddler Development Scale.

**Results:** Of the 59 infants recruited, there were 17 drop-outs due to non adherence, schedule incompatibility, fatigue during the assessment or death. Forty-two infants (28 female, at the average age of 10 months). Forty-three infants who underwent surgery had on average 2 procedures, ranging between 1–5 surgeries. Sixty-seven percent of the infants were cyanotic. The average length of hospital stay was 31 days (X–XX range), and eight were preemies. The developmental findings showed that 41% of infants were borderline or delayed in the Cognitive, 31% in Receptive Language, 29% in Expressive Language 29%, 36% in Fine Motor, and 67% in the Gross Motor domains.

**Conclusion:** This study showed high rates of developmental delay in infants with CHD from a public (SUS) outpatient clinic in Southern Brazil. In particular, almost 70% of the sample was at risk or had a delay in gross motor development. These data are congruent with previous research that have shown developmental delays in infants with CHD, in particular gross motor deficits. This evidence corroborates with AHA’s guidelines, and supports the need for multidisciplinary early evaluation and intervention to prevent neurodevelopmental sequelae in low-income infants with CHD in Brazil.

111366

Modality: E-Poster Young Researcher – Non-case Report

Category: CARDIOVASCULAR IMAGING

## Pericardial Involvement is More Transient Than Myocardial in Patients After COVID-19 Infection Detected by Cardiovascular Magnetic Resonance

MARÍLIA MIGUEL DA SILVEIRA^1^, Sérgio Augusto Yukio Hissayassu^1^, Isabela Ramos Ali Ganem^1^, Lívia Hirayama^1^, Raíza Silveira da Costa^1^, Carlos Alexandre Vieira Gomes^1^, Tiago Augusto Magalhães^1^, Adriano Camargo de Castro Carneiro^1^, Carlos Eduardo Elias dos Prazeres^1^, Juliana Matsumoto Bello^1^, Valéria de Melo Moreira^2^, Carlos Eduardo Rochitte^1^

(1) Hospital do Coração (Hcor); (2) Instituto do Coração (InCor), Hospital das Clínicas da Faculdade de Medicina da Universidade de São Paulo

**Background:** Cardiovascular complications in Covid-19 is prognostic. Myocarditis and pericarditis can be precisely detected by cardiovascular magnetic resonance (CMR) and present early and/or later (Covid-19 long). Myocardial and pericardial evaluation by CMR in routine clinical practice is scarce. Therefore, we sought to investigate cardiac abnormalities by advanced CMR including subtle tissue chances in myocardium and pericardium.

**Methods:** We included 303 consecutive patients with confirmed prior Covid-19 diagnosis referred to CMR, performed in a 1.5T scanner (Sola CV edition, Siemens Healthineers) with cine-MR, late gadolinium enhancement (LGE), T1(MOLLI), T2 maps and extracellular volume measurements (ECV) using standard parameters. Early-CMR or late-CMR if CMR was defined as before or after 60 days of the infection, respectively.

**Results:** From the 303 patients, 120 (39.9%) were female, mean age was 47.6 ± 13.8 years and mean body mass index of 27.7 ± 4.9. Symptoms were present in 87 patients (28.7%). Mean interval between Covid-19 infection and CMR study was 155.0 ± 138.6 days for the entire study group, with 78 patients (30.3%) with early-CMR. Myocardial LGE (mLGE) was present in 60 patients (19.9%), with 14.1% in early-CMR and 21.8% in late-CMR (p > 0.05). However, pericardial LGE (pLGE) was more frequent in early-CMR than late-CMR (9.0 vs. 2.8%, p = 0.049). The presence of mLGE was similar between symptomatic vs asymptomatic patients (17.7 vs. 20.8%, p > 0.05). Patients with risk factors for coronary artery disease (including obesity) had significantly more mLGE than those without (26.1 vs. 15.9%, p = 0.039). Native T1 and T2 values were higher in patients with CAD (1041 ± 31 vs. 1005 ± 3.8 and 46.7 ± 2.2 vs 44.3 ± 0.3, p = 0.024 and 0.039, respectively), but ECV was similar. No differences were seen in maps and ECV values between early and late-CMR. Thus, mLGE has similar distribution for symptomatic/asymptomatic and early/late phases after infection. However, pLGE was significantly more frequent in the first 60 days after infection. CAD patients had higher T1/T2 myocardial maps.

**Conclusions:** CMR was able to detect subtle myocardial and pericardial changes in patients after Covid-19. Myocardial involvement was similar regardless of symptoms and time after infection, while pericardial involvement was detected more frequently before 60 days of the infection. This might indicate that pericardial involvement is more transient than myocardial involvement.

110983

Modality: E-Poster Young Researcher – Non-case Report

Category: CARDIAC ARRHYTHMIAS/ELECTROPHYSIOLOGY/ELECTROCARDIOGRAPHY

## An Analysis of the Epidemiology of Conduction Disorders and Cardiac Arrhythmias in Brazil in the Last 5 Years

PEDRO HENRIQUE ALMEIDA WAROL ^1^, PEDRO HENRIQUE ALMEIDA WAROL^1^, TALLES AYRES LIMA^2^, ROMULO RODRIGUES BADINI^3^, NAJLLA DE SALIM RIBEIRO^4^, MARIANNA RAMALHO DE SOUSA^1^, EMILIO PANDELÓ LIMA^1^, EMILIO CONCEIÇÃO DE SIQUEIRA^1^

(1) UNIVERSIDADE DE VASSOURAS; (2) UNIVERSIDADE ESTACIO DE SÁ; (3) UNIVERSIDADE UNIGRANRIO; (4) FACULDADE DE MEDICINA DE PETRÓPOLIS

Cardiovascular diseases (CVD) are the leading cause of death in Brazil. Among such diseases, there are Conduction Disorders and Cardiac Arrhythmias (CBD), electrical changes in the heart that cause changes in its normal rhythm. Even though it is configured as a public health problem, there is a scarcity in the literature regarding the epidemiological aspects of hospitalizations and deaths of this disease, and its analysis is essential in order to prepare health teams to deal with BED. A systematic review of the literature was carried out together with the descriptive, cross-sectional and observational collection of data available in DATASUS – Hospital Information System of the SUS (SIH/SUS) – from 2017 to 2021, evaluating hospitalizations, deaths and pattern of patients: age group, sex and self-reported color/race. In the analyzed period, there were 316,939 hospitalizations for BED with a total expense of 1,391,918,003.70 reais, with 47.5% of these hospitalizations in the Southeast region. The most affected sex was male, with 52.5% of hospitalized cases. The age group with the most hospitalizations was over 50 years old, with emphasis on the 70 to 79 years old group, which corresponded to 25.8% of the total. When analyzing color, white was the most affected with 45.7% of the total, however 20% of hospitalizations did not have this information. As for deaths, in the analyzed period, there were 40,193, with a predominance of males, which corresponded to 54.3% of the total deaths from BED. The age group with the highest mortality was over 60 years, with emphasis on individuals aged 80 years and over, who accounted for 24.8% of deaths. There was an increase in mortality, which in 2017 was 6,854 and in 2021 it reached 8,664. TCACs represent a serious public health problem with high morbidity and mortality. Because of this, and the increase in mortality expressed in the years analyzed by the research, a new dynamics in health is essential. This dynamic must be guided by prevention and health promotion, with effective monitoring carried out by Primary Health Care in association with the care provided by the cardiologist, in order to avoid the development and worsening of these diseases.

111004

Modality: E-Poster Young Researcher – Non-case Report

Category: CARDIOVASCULAR SURGERY

## Analysis of the Results of Minimally Invasive Mitral Valve Surgery in a Brazilian Health Service in the Year of 2021

DANIEL DE MAGALHÃES FREITAS^1^, Artur Henrique de Souza^1^, João Alberto Pansani^1^, Max Weyler Nery^1^, Patrícia Ferreira Demuner^1^, Stanlley de Oliveira Loyola^1^, Maurício Lopes Prudente^1^, Débora Rodrigues^1^, Larissa Xavier Alves de Oliveira^1^, Fernando Araújo Cintra Canedo^1^, Giulliano Gardenghi^1^

(1) Hospital Encore

**Introduction:** The feasibility of minimally invasive mitral valve surgery (MIMVS) has been proven extensively and many centers around the world have been using this technique as a standard approach. Some advantages of the minimally invasive technique, compared to the traditional median sternotomy, are the blood loss reduction, lower morbidity, and shorter intensive care unit and in-hospital stay.

**Objective:** In this study we analyzed the results of MIMVS performed at our institution in the year of 2021 and compare with data from the international literature.

**Methods:** 13 patients were submitted to MIMVS. At our institution, a femoral cannulation is utilized to establish cardiopulmonary bypass; and the incision was made at third or fourth intercostal space starting at the mid-subclavicular line with a length of 5–7 cm. The myocardial protection is done with cold-blood cardioplegia. Data were collected from the patients records and tabulated and analyzed in the Excel software®. We used Mitral Valve Academic Consortium safety definitions for comparison purposes.

**Results:** From the 13 cases, 11 (84,6%) were valve replacement and 2 were of valve repair. Age mean was 59,6 years (±14); 9 (69,2%) were at low risk by STS score, 3 were at medium risk (23,2%) and 1 (7,6%) at high risk. The 30-day complications analysis are shown in table 1.

**Conclusion:** Our results are consistent with the world literature corroborating the feasibility and safety of the technique.


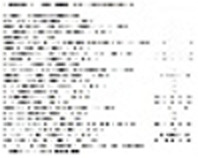


111020

Modality: E-Poster Young Researcher – Non-case Report

Category: DIGITAL HEALTH/INNOVATION

## A Force to be Reckoned With: The Top 75 Twitter Influencers in Cardiac Imaging

NAJAH KHAN^1^, Bindu Chebrolu^1^, Nadeen N. Faza^1^

(1) Houston Methodist Hospital

**Introduction:** Twitter influencers exchange information and stimulate discussions from a breadth of expertise. Cardiovascular imaging with echocardiography (ECHO), cardiac computed tomography (CCTA), single-photon and positron emission tomography (SPECT and PET), and cardiac magnetic resonance imaging (CMR) has gained momentum through social media.

**Objectives:** To analyze demographics of top Twitter influencers and imaging modalities they discuss on Twitter.

**Methods:** Twitter topic scores for “cardiac imaging” (CCTA, CMR, SPECT, and PET) and “echocardiography” were collated for individual accounts on Feb 6, 2022 using the Cronycle monitoring platform (integrated with the Right Relevance application programming interface), generating top 75 influencers. Cardiovascular board certifications were confirmed via country-specific board-certifying organizations. Occupation, gender, location, and imaging modalities discussed by influencers were confirmed via Twitter biographies. Influencers’ academic influence (h-indices) from Scopus were correlated to their topic scores.

**Results:** Majority influencers were male (73%) and board-certified cardiologists (87%), followed by non-cardiovascular physicians (9%), and non-physicians (4%). The most discussed imaging modality was echocardiography by 40% of top influencers, followed by CMR (28%), CCTA (23%), and Nuclear/PET imaging (9%). Most influencers reside in the United States (61%) followed by the United Kingdom (17%), Canada (4%), Italy and Portugal (3%), and other countries (Spain, Romania, Serbia, Peru, Germany, Chile, Columbia, Brazil, and Australia, 1% each). There was a positive correlation between the h-index and Twitter topic score (r = 0.3562, p = 0.0009).

**Conclusions:** While most top influencers in cardiac imaging are board-certified male cardiologists in the United States, other influencers consist of international cardiologists, non-cardiovascular physicians, and non-physicians. There is a vast global representation of cardiac imaging influencers, showcasing the reach and expansive growth of the field. These findings emphasize the diverse representation of cardiac imaging influencers on Twitter, hopefully changing the landscape of this field.

111022

Modality: E-Poster Young Researcher – Non-case Report

Category: ATHEROSCLEROSIS/CARDIOVASCULAR RISK FACTORS/CARDIOVASCULAR PREVENTION

## Peripheral Arterial Disease as a Predictor of Coronary Arterial Disease and Prognosis in Patients with Stage 5 CKD Undergoing Hemodialysis

DANIEL BATISTA CONCIEÇÃO DOS SANTOS^2^, Luís Henrique Wolff Gowdak^2^, José Jayme Galvão de Lima^2^, Luiz Aparecido Bortolotto^2^

(1) Universidade de São Paulo; (2) Instituto do Coração

**Introduction:** There is a need of a simple, inexpensive and reliable noninvasive testing to predict coronary artery disease (CAD) in CKD patients, a population with the higher prevalence of CV events and death.

**Object:** This study analyzed the association between peripheral arterial disease (PAD) and CAD in patients with stage 5 CKD on dialysis.

**Methods:** This was a prospective and observational cohort study in 201 CKD patients on hemodialysis. PAD was defined by either the absence of peripheral arterial pulse by palpation or by ≥50% narrowing by ultra sound Doppler (USD) and CAD by angiography (≥70% narrowing).

**Results:** Palpation and USD predicted the occurrence of PAD (P = 0.0001, OR = 27.37). CAD was observed in 45% of patients with PAD (by palpation) and in 60% by USD. Absence of pulse by palpation predicted CAD with a sensitivity of 55% and a specificity of 76%; USD showed a sensitivity of 62% and specificity of 60%. The risk of combined serious CV events and death was significantly higher in individuals with PAD diagnosed by palpation but not by USD. PAD assessed by palpation also correlated with the occurrence of multivessel CAD and with the probability of coronary intervention.

**Conclusion:** There is a high prevalence of CAD in patients with PAD. Palpation may be used instead of USD for the diagnosis of PAD because is inexpensive and easier to use. Both methods presented a moderate utility to predict CAD but the diagnosis of PAD by palpation was a predictor of combined cardiovascular events and death and was also associated with the severity of CAD and the probability of indication of coronary intervention.

111043

Modality: E-Poster Young Researcher – Non-case Report

Category: EPIDEMIOLOGY AND HEALTH POLICIES/GLOBAL HEALTH

## Resources Management in Brazilian Public Health System: How to Define Priority for Cardiologist Consultation Based on Telecardiology, Risk Classification and Diagnostic Groups?

SHEILA TATSUMI KIMURA MEDORIMA^1^, Eduardo Monteiro Diniz Junqueira^1^, Ana Cristina Seródio^1^, Maria Fernanda Murijo Righi Turatti^1^, Fernanda Melo Gomes^1^, Carolina Rosa Queiroz Fulas^1^, Valeria Cristina Jodjahn Figueiredo^1^

(1) Municipal Health Department (SMS-Campinas), Prefeitura Municipal de Campinas

**Introduction:** The COVID-19 pandemic required special management of health services, once outpatients visits were suspended in most units during the lockdown. In Campinas-SP, since 2006, cardiology matrix support is acknowledged by primary health care as a service to case discussion, EKG evaluation and shared care. Since 2019, our team offers telecardiology through institutional e-mail, which acquired great importance during Pandemics.

**Objective:** Evaluate the content of these e-mails, quantifying diagnostic groups with priority risk classification.

**Methods:** This is a cross-sectional observational study, using data from service registry produced by cardiologists while evaluating cases received from e-mail. This research has been approved by ethics committee.

**Results:** In a total of 2224 cases discussed along 2021, 22.8% had missing data, 10.8% were high risk CAD or unstable CAD, 9.4% had acute HF symptoms, 6.4% intermediate risk chest pain, 5.5% structural chronic heart disease, 4.7% paroxysmal supra ventricular tachycardia and 4.1% had abnormal heart murmur. There were 1089 patients (49% of the cases) classified as priority. More priority cases were in the following diagnostic groups: acute atrial fibrillation (AF) (90.2% priorities), high risk CAD or unstable CAD (90.0% priorities), thromboembolic event (87.5% priorities), syncope (78.0% priorities), post-COVID cardiovascular complications (72.0% priorities), structural chronic heart disease (74.6% priorities), acute HF symptoms (72.7% priorities) and chronic AF (69.2% priorities).

**Conclusion:** Telecardiology in Public Health System of Campinas-SP was a successful experience during the COVID-19 pandemics, allowing to evaluate clinical criteria to guide priority risk classification for cardiology consultations, ensuring equity.


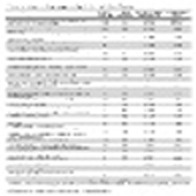


111049

Modality: E-Poster Young Researcher – Non-case Report

Category: EPIDEMIOLOGY AND HEALTH POLICIES/GLOBAL HEALTH

## Telecardiology is a Management Tool for Specialized Care in the Brazilian Public Health System, Campinas-SP

SHEILA TATSUMI KIMURA MEDORIMA^1^, Eduardo Monteiro Diniz Junqueira^1^, Ana Cristina Seródio^1^, Maria Fernanda Murijo Righi Turatti^1^, Fernanda Melo Gomes^1^, Carolina Rosa Queiroz Fulas^1^, Valeria Cristina Jodjahn Figueiredo^1^

(1) Municipal Health Department (SMS-Campinas), Prefeitura Municipal de Campinas

Cardiology matrix support (CMS) is offered to Primary Care services since 2006 in Campinas-SP, allowing closer relationship with the specialists. In 2019, telecardiology was the method used to expand this proposal, through the institutional e-mail for case discussion, clinical orientation, receiving ECG requiring reports and risk classification for cardiology consultation. This method allowed Specialized Care to meet the needs of Primary Care during the COVID-19 Pandemic, allowed priority access to urgent cases and contributed to continued education of Primary Care in Cardiology.

**Objective:** Characterize the CMS performed in Brazilian Public Health System (SUS), Campinas-SP.

**Methods:** This is a cross-sectional observational study with data registered by cardiologists while evaluating clinical cases discussed via e-mail. This research has been approved by ethics committee.

**Results:** In a total of 2224 cases discussed along 2021, the median age was 61 years, most female (N = 1136,51%). A total of 1089(49%) was classified as priority, 538(24%) as intermediate and 46(2%) as low risk. There were 546(24%) referrals with incomplete data, 381(17%) had insufficient data, 809(36%) proposed shared patient care instead of referring the patient to the specialist, 797(36%) received orientation regarding therapeutic adjustments or complementary investigation. The CSM offered cardiology consultation for 477(21%), echocardiography for 303(14%) and exercise testing for 110(5%).

**Conclusion:** Telecardiology was consolidated in Specialized Care at the SUS in Campinas-SP during the COVID-19 Pandemic, allowing quick access from Primary Care to cardiology support. This data will contribute with the analys of the impact of this format of Specialized Care and for future improvements.


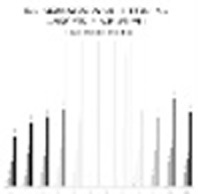


111054

Modality: E-Poster Young Researcher – Non-case Report

Category: DIGITAL HEALTH/INNOVATION

## State Supported Remote Consultations of Cardiologists in Latvia During the Covid Pandemic – Experience and Opportunities

EMMA SOKOLOVA^1^, Aleksandra Jeņičeka^2^, Ieva Bikava^3^

(1) Riga Stradins University, University of Latvia, RISEBA, Riga East University Hospital, Pauls Stradins Clinical University Hospital; (2) Riga Stradins University, RISEBA; (3) Riga Stradins University

**Introduction:** Available data on the doctor-patient remote consultations all over the world shows that those are mostly provided by radiologists, psychiatrists and cardiologists. The regulatory framework of each country, the procedure for organizing and payment procedure for the specialists, the legal aspects and the complexity of the medical sector create specific conditions and the need to develop a model and procedure for providing remote consultation appropriate to each country and institution.

**Objectives:** The aim of the study was to find out, summarize and analyze the experience of state paid remote consultation of cardiologists in Latvia during the year 2021, identify problems and develop recommendations for safe and effective remote specialist-patient and specialist-specialist consultation in cardiology.

**Methods:** The study was conducted through semi-structured interviews with cardiologists of PSCUH, data provided by NHS of Latvia and Specialized Medicine Center (unit of State EMS).

**Results:** The total number of state provided teleconsultations was 2 884 632, from them only 2194 (0,8%) patient-cardiologist consultations were conducted, which for 1,9 billion of Latvian population is a very low indicator. Remote patient-cardiologist consultations remained under 0,1% – compared to consultations of doctors of other specialities. The majority of cardiologists indicated that the first consultations could not be performed remotely over the phone. At the specialist-specialist level, the demand is stable, with around 1,100 consultations each year and accounting for about 20–25% of the total number of remote consultations.

**Conclusions:** The main obstacles to remote visits, especially for first-time visits, are related to two problems: 1) the specialist does not have access to all examinations and results of the patient, and 2) it is not possible to make an objective assessment of the patient. A video call is recommended for remote consultations, as it is able to provide more additional information and patient evaluation options. Patient monitoring through remote consultations would be possible if visits are sufficiently regular (every 3 or 6 months), the patient is compliant, the patient’s condition has not significantly deteriorated, and the necessary examinations and analyzes have been performed and are available to the doctor. At present, procedures have not been developed and defined at the national level for the implementation of telephone consultations.

111073

Modality: E-Poster Young Researcher – Non-case Report

Category: EPIDEMIOLOGY AND HEALTH POLICIES/GLOBAL HEALTH

## Economic Evaluations of Prevention, Screening, Diagnosis, and Medical and Surgical Management of Rheumatic Heart Disease: A Systematic Review

DOMINIQUE VERVOORT^1^, Rachel Livergant^2^, Maryam Shams^3^, Niraj S Kumar^4^, Sameer Hirji^5^, Bobby Yanagawa^1^

(1) Division of Cardiac Surgery, University of Toronto; (2) Office of Global Surgery, Department of Surgery, University of Alberta; (3) Department of Medicine, Stanford University; (4) Department of Medicine, University College London Medical School; (5) Department of Surgery, Brigham and Women’s Hospital, Harvard Medical School

**Introduction:** Rheumatic heart disease (RHD) affects over 30 million people globally and remains one of the most underfunded conditions in global health relative to its disease burden. The lack of access to primary, secondary, and tertiary prevention and care for the majority of the world’s population contributes to these disparities and is a result of a poor understanding of the socioeconomic value of interventions to prevent and manage RHD. Here, we evaluate the current literature to assess economic evaluations of the prevention, screening, diagnosis, and medical and surgical management of acute rheumatic fever (ARF) and RHD.

**Objective:** The objective of this study is to identify the current knowledge and gaps surrounding the cost-of-illness of ARF/RHD and the cost-effectiveness and cost-utility of prevention or care for ARF/RHD.

**Methods:** A systematic review was performed according to the Preferred Reporting Items for Systematic Reviews and Meta-Analyses guidelines using the PubMed/MEDLINE, EMBASE, Web of Science, World Health Organization Global Index Medicus, and EconLit databases to identify original research on economic evaluations of ARF and RHD interventions between January 2002 and December 2021. Variables extracted included bibliometric data, study country income group (high- versus low- and middle-income countries, LMICs), type of economic evaluation, measures of effectiveness, type of interventions, economic outcomes, and the use of decision analysis.

**Results:** Of 3,980 screened articles, 23 were included in the final analysis. Most studies (56.5%, N = 13) took place in LMICs. Primary prevention and care were most commonly studied (52.2%, N = 12), followed by tertiary care (39.1%, N = 9). Cost-effectiveness and cost-utility analyses (65.2%, N = 15) were the most common type of economic evaluation, followed by cost-of-illness studies (34.8%, N = 8). Effectiveness was most commonly measured as quality-adjusted life-years (30.4%, N = 7) and disability-adjusted life-years (17.4%, N = 4). Most studies (78.3%, N = 18) used decision analysis models.

**Conclusion:** The economic burden due to ARF/RHD is large in LMICs, but economic evaluations in LMICs remain limited. ARF/RHD interventions appear consistently but variably effective and cost-effective to prevent and treat ARF/RHD. Increased research, political prioritization, and public health and healthcare funding are needed to address the global burden of ARF/RHD.

111082

Modality: E-Poster Young Researcher – Non-case Report

Category: HEART FAILURE/CARDIOMYOPATHY/TRANSPLANT

## Post-Heart Transplant Dynamic of Cognitive Functioning, the Structure Indicators of Anxiety and Quality of Life in End-Stage Heart Failure Patients

LISAKOVA ANASTASIIA^1^, Maria Simonenko^1^, Petr Fedotov^1^, Larisa Vasilieva^1^, Yulia Sidorovskaya^2^, Olga Tarasova^1^, Svetlana Bagramian^1^, Aelita Berezina^1^, Maria Bortsova^1^, Oleg Mamontov^1^, Yulia Levashkevich^1^, Elena Demchenko^1^

(1) Almazov National Medical Research Centre; (2) Saint-Petersburg state university (SPbU)

**Introduction:** Cognitive impairment and difficulties in coping with negative conditions slows down the rehabilitation. The post-transplant quality of life affects rehabilitation and social adaptation.

**Objective:** To evaluate the dynamics of cognitive and psycho-emotional characteristics of patients with end-stage heart failure before and after heart transplantation (HTx) as a prerequisite for optimization of psychological correction during cardiac rehabilitation.

**Methods:** Study included 60 end-stage heart failure patients (from 07.2019 to 06.2021), 30 of them (mean age–46.9 ± 13.3 yo; 57.1% – male) were examined on pre-HTx and during 6 mo after HTx (1st period – 1–3 mo and 2nd – 4–6 mo), others were excluded due to the various reasons (i.e. still awaiting HTx, death during early-term posttransplant follow-up, etc.). For patients’ examination we used Short Form-36 (SF-36), Luria Memory Words Test, Simple analogies method (for the cognitive sphere) and Integrative Anxiety Test (A. Bizyuk et al.) – the tests validated in Russia. Statistical analysis was performed using Student’s t-tests and repeated measures ANOVA.

**Results:** At the pre-HTx examination the level of long-term memory was lower than at the 3rd mo after HTx, but decreased by the 6th mo (5.5 ± 1.8; 7.8 ± 1.7; 7.3 ± 1.6, p = 0.013 for trends) Social Functioning was decreasing during 6 mo (50.2 –± 7.6; 41.7 ± 6.1 (3 mo); 38.1 ± 10.9 (6 mo), p = 0.011 for trend). Role-Physical Functioning increased to the 3rd mo and dropped to the 6th mo (18.9 ± 17,4; 47.3 ± 40.5 (3 mo); 39.3 ± 34.9 (6 mo), p = 0.01). Mental Health improved to the 2nd period (73.1 ± 15.8 vs 57.1 ± 14.9 pre-HTx, p = 0.011). Physical Functioning increased to the both periods: 30.0 ± 19.1; 68.6 ± 20.4 (3mo); 69.4 ± 22.3 (6 mo), p = 0.004 for trend. General situational anxiety leveled up to the 3rd mo but decreased to the 6th mo (4.5 ± 1.9; 5.9 ± 2.1; 2.6 ± 2.1, p = 0.013 for trend), whereas asthenic component dropped in both periods (6.1 ± 1.9; 4.1 ± 2.5; 3.4 ± 2.5, p = 0.014), alarming assessment of future decreased to the 3rd mo (6.2 ± 2.4; 4.7 ± 2.8, p = 0.046). There were no statistically significant differences in the Simple analogies method.

**Conclusion:** The results can be used for psychological support within rehabilitation of HTx patients. Situational anxiety increased to the 3rd mo, thus, psychocorrection of psycho-emotional states is crucial in 1–3 mo after HTx. Social functioning was dropping in 6 mo after HTx, so psychological rehabilitation is necessary in the 4–6 months after HTx.

111092

Modality: E-Poster Young Researcher – Non-case Report

Category: EPIDEMIOLOGY AND HEALTH POLICIES/GLOBAL HEALTH

## Clinical and Epidemiological Profile of Patients Hospitalized for Acute Atrial Fibrillation

RICARDO MENDES CARNEIRO^1^, Eric Costa de Almeida^1^, Bruno Reznik Wajsbrot^1^, Daniel Xavier de Britto Setta^1^, Marcelo Luiz da Silva Bandeira^1^, Thiago Matos Barcellos^1^, Flávia Prado Fialho Santos^1^, Roberta Siuffo Schneider^1^, Claudia Lanzillotti Weksler^1^, Andre Volschan^1^, Fernando Oswaldo Dias Rangel^1^, Ricardo Mourilhe-Rocha^1^

(1) HOSPITAL PRÓ-CARDÍACO

**Introduction:** Atrial fibrillation (AF) is an increasingly prevalent condition, with an estimate of almost 60 million people worldwide and 2.5% of the Brazilian population, according to data from the ELSA-BRASIL study, which was not exclusively composed by patients hospitalized for AF. Knowing the clinical profile, cardioembolic and hemorrhagic risk allows personalizing the therapeutic approach in different scenarios.

**Objective:** To identify the clinical and epidemiological profile of patients hospitalized with AF in a quaternary hospital.

**Methods:** Retrospective cohort study of 819 hospitalized patients with a confirmed diagnosis of AF, from January 2018 to December 2021. Data were analyzed using the SPSS20 software.

**Results:** 57.6% of the patients were male, with a mean age of 71 ± 14.4 years. They had the following risk profiles: CHADS2 ≥2 in 79%; CHA2DS2VASc ≥3 in 63.8%; age >65 years in 72.6%, systemic arterial hypertension in 66.4%, heart failure in 12%, diabetes mellitus in 21.7%, stroke or transient ischemic attack in 8.2%, coronary artery bypass graft surgery previous in 8.6%and previous coronary artery disease in 21.6%. The median of CHA2DS2VASc was 3, and the median of HASBLED was 2. There was reversion to sinus rhythm in 68.3% of the cases. Of these, 52.5% underwent electrical cardioversion (EVC), 22.5% chemical cardioversion and 25% had spontaneous reversion. Regarding oral anticoagulants administered during hospitalization, most used apixaban (25.6%) and rivaroxaban (12%) and only 1.1% had contraindications for anticoagulation. Regarding antiarrhythmics used during hospitalization, amiodarone (29.1%) was the most prescribed one and, of the heart rate control medications, beta-blockers (50.2%) were the most important ones. Complication rates were low, with only 3 embolic (0.4%), 10 (1.2%) hemorrhagic, and 7 (0.9%) deaths.

**Conclusion:** Despite being a population of high-risk AF patients with a high mean age and CHA2DS2VASc ≥3, only a few complications occurred. CVE was the method of choice for reversion to sinus rhythm and the most used oral anticoagulant was apixaban. The knowledge of this profile can allow the development of specific care plans for this population.

111097

Modality: E-Poster Young Researcher – Non-case Report

Category: CARDIAC ARRHYTHMIAS/ELECTROPHYSIOLOGY/ELECTROCARDIOGRAPHY

## Predictors of Failure of Reversion to Sinus Rhythm in Patients Admitted with Acute Atrial Fibrillation

ERIC COSTA DE ALMEIDA^1^, Ricardo Mendes Carneiro^1^, Daniel Xavier de Britto Setta^1^, Julia Paulo Mourilhe Rocha^1^, Thiago Matos Barcellos^1^, Flávia Prado Fialho Santos^1^, Bruno Reznik Wajsbrot^1^, Roberta Siuffo Schneider^1^, Vitor Hugo Mussi Campos^1^, Andre Volschan^1^, Fernando Oswaldo Dias Rangel^1^, Ricardo Mourilhe-Rocha^1^

(1) HOSPITAL PRÓ-CARDÍACO

**Introduction:** Strategies aimed at reversing atrial fibrillation (AF) to sinus rhythm aim at better symptomatic control and quality of life. Knowing the clinical factors associated with greater success in rhythm reversal remains a challenge.

**Objective:** To assess failure related factors to revert to sinus rhythm in patients with AF.

**Methods:** Retrospective cohort study of 819 patients hospitalized with diagnosis and AF from January 2018 to December 2021. The parameters analyzed were age, BNP, left atrial size, creatinine and left ventricular ejection fraction (LVEF). Data were analyzed by SPSS 20 software.

**Results:** 57.6% of the patients included were male, with a mean age of 71 ± 14.4 years. They had the following risk profiles: CHADS2 ≥2 in 79%; CHA2DS2VASc ≥3 in 63.8%; age >65 years in 72.6%, systemic arterial hypertension in 66.4%, heart failure in 12%, diabetes mellitus in 21.7%, stroke or transient ischemic attack in 8.2%, coronary artery bypass graft surgery previous in 8.6%, previous coronary artery disease in 21.6%. The CHA2DS2VASc median was 3, and the HASBLED median was 2. There was reversion to sinus rhythm in 68.3% of the cases. From the comparison between patients discharged in sinus rhythm and those who remained in AF, the profile of patients who were unsuccessful in reversal were older (mean 70.23 ± 14.6 vs 73.95 ± 13.83), had higher BNP (median 216 pg/ml vs 515 pg/ml; p < 0.001), higher creatinine (median 1.0 mg/dl vs 1.1 mg/dl; p < 0.023), median LVEF of 64.0% vs 62.0%, p < 0.332; and higher left atrial volumes (median 41 ml/m^2^ vs 43 ml/m^2^; p < 0.013)

**Conclusion:** The parameters associated with failure to revert to sinus rhythm were age, BNP, creatinine and left atrial volume. The understanding of this association may allow the elaboration of failure prediction scores in the reversal of patients with AF.

111099

Modality: E-Poster Young Researcher – Non-case Report

Category: HEMODYNAMICS AND INTERVENTIONAL CARDIOLOGY

## Percutaneous Tricuspid Valve-in-Valve Implantation in Failed Surgical Bioprosthetic Valves – Pilot Experience

FILIPPE BARCELLOS FILIPPINI^1^, Cauyna Gurgel Moreira^1^, Tiago Costa Bignoto^1^, Maurício Felippi de Sá Marchi^1^, Gabriel Kanhouche^1^, Antônio Fernando Diniz Freire^1^, Pedro Felipe Gomes Nicz^1^, Renata de Sá Cassar^1^, Henrique Barbosa Ribeiro^1^, Alexandre Abizaid^1^, Santiago Raul Arrieta^1^, Fábio Sandoli de Brito Jr.^1^

(1) InCor – Instituto do Coração do Hospital das Clínicas da FMUSP

**Aims:** Tricuspid bioprosthesis deterioration is a frequent condition and its treatment (Redo tricuspid valve replacement) is complex and associated with high mortality. Percutaneous tricuspid valve-in-valve implantation (TViV) presents as a safe alternative with favorable outcomes. We aim to describe the pilot experience of TViV procedures.

**Methods and Results:** From June 2015 to August 2022, 12 patients were submitted to TViV procedure in one tertiary-care Brazilian hospital. The median age was 30.0 ± 13.1 years old, 50% of patients with previous infective endocarditis and the number of previous cardiac surgery was 3.2 ± 1.5 per patient. The majority of patients had severe heart failure symptoms (NYHA III or IV in 50%) and the congenital disease involved were tetralogy of fallot or Ebstein anomaly in most cases (66.7%). The mean time since last tricuspid valve procedure was 9.5 years, most of the prosthesis were Braile (33%). The principal mechanism of bioprosthetic valve failure (BVF) was regurgitation in 66% and combined prosthesis dysfunction in 75%. Preprocedural echocardiography identified moderate or severe right ventricular systolic dysfunction in 7 (58%), mean tricuspid gradient of 10.4 mmHg and pulmonary arterial systolic pressure of 34 mmHg. The transcatheter heart valve (THV) prosthesis used was either Inovare (Braile) in 10 (83%) or Sapien 3 (Edwards) in 2 (17%), with sizes 24, 26 or 28 mm equally distributed in 9 (75%) of the cases. Balloon pre-dilatation was used in 7 (58%) and post-dilatation of the THV in 2 (17%). Intraprocedural echocardiographic evaluation of mean tricuspid gradient was 3.5 mmHg with no moderate or severe paravalvular leak. Overall technical success was obtained in 11/12 (92%) of the procedures, due to change in access site from jugular vein to transapical approach in one single case. Mean length of stay was 12 ± 5.4 days, with 67% of patients in NYHA I at discharge. No patient died during the median long term follow-up of 29 months, the antithrombotic regimen was vitamin K antagonist in 10 (83%) and late bioprosthesis thrombosis was observed in 1 (8.3%) case during the follow up, in the context of concomitant covid infection. One year echocardiographic evaluation of mean tricuspid gradient was 7.7 ± 2.4 mmHg and 1 case with moderate paravalvular tricuspid leak.

**Conclusion:** The pilot experience of tricuspid valve-in-valve implantation demonstrated safety, effectiveness and marked improvement of symptoms at mid-term follow-up.

111112

Modality: E-Poster Young Researcher – Non-case Report

Category: COVID-19 AND CARDIOVASCULAR SYSTEM

## What’s on Your Plate? COVID-19 and Diet

MASHAAL IKRAM^1^, Mashaal Ikram^1^, Mina Kerolos^2^, Mingxi Yu^3^, Kim Allan Williams^2^, Max Liebo^3^

(1) University of Chicago (Northshore University Health System); (2) Rush University Medical Center; (3) Loyola University Medical Center

**Background:** Food choices have been identified as the leading cause of mortality in the U.S., underpinning many chronic diseases, including cardiovascular disease, diabetes mellitus and cancers. During the SARS-CoV-2 pandemic, nutrition quality, specifically plant-based and Mediterranean diets, have been found to be highly protective from moderate to severe COVID-19 illness. In addition, these diets have been promoted by the 2019 ACC/AHA Primary Prevention Guidelines to reduce cardiovascular disease, which remains the leading cause of death of physicians.

**Objective:** We sought to identify the nutritional habits of medical students and practitioners in two Chicago area academic medical centers. The primary aim of the study was to correlate dietary habits that could correlate with the symptomatic response and infection rates of both SARS-CoV-2 virus and its vaccine.

**Methods:** Medical students, residents, fellows and faculty from Loyola and Rush University Medical Centers with exposure to SARS-CoV-2 patients, and who had received two doses of vaccinations were asked to complete a web-based survey between September 2021 to February 2022. Participants provided their dietary habits, any history of SARS-CoV-2 infection, and occurrence of symptoms after either of their two COVID-19 vaccinations.

**Results:** Of the 274 respondents, 14% indicated that they had contracted COVID-19 prior to the survey. Only 18.% had moderate or severe symptoms with the vaccine. The dietary habits, however, indicated that 72% were eating an unrestricted diet (omnivore), 15% were categorized as semi-vegetarian (intentionally reducing, but not eliminating, animal products), 5% consumed seafood, eggs and dairy, but no red meat, 4% ate eggs and dairy without seafood, 4% ate seafood without eggs or dairy, and 0.3% ate no animal products (figure). Given the relative paucity of categorical dietary diversity, there was no analyzable correlation between viral or vaccine symptoms and nutritional habits.

**Conclusions:** This data indicates that very few (11%) of the surveyed physicians follow ACC/AHA Prevention nutritional recommendations, which would lower both cardiovascular and COVID-19 morbidity and mortality.

111121

Modality: E-Poster Young Researcher – Non-case Report

Category: CARDIOLOGY OF SPORTS, EXERCISE, ERGOMETRY AND CARDIOVASCULAR REHABILITATION

## Cardiac Vagal and Sympathetic Activity in Frail Elderly

LEILA DAL POGGETTO MOREIRA^1^, Tuany Mageste Limongi Zamperlim^2^, Giulia Micali^1^, Camila Martins^1^, Higor Quintas Gonçalves^1^, Thamyres Oliva Bueno^1^, Pricila Helena de Souza^1^, Rodrigo Moreno de Oliveira^1^, Giovanna Dolder^1^, Luciana Gonzalez Viscardi Auad^1^, Adriana Sarmento de Oliveira^1^

(1) Universidade Anhembi Morumbi UAM; (2) Universidade Federal de Juiz de Fora

**Background:** Impairment of cardiac autonomic modulation during the aging process contributes to the development of cardiovascular events, the leading cause of death in the elderly population, and may indicate that the elderly with frailty syndrome have a poor outcome when compared to other elderly.

**Objectives:** To evaluate the cardiac autonomic response to isometric exercise in comparison to baseline and recovery.

**Methods:** After screening, 54 frail elderly (F) and 12 non-frail elderly (NF). Briefly, using Fried’s criteria subject frailty was defined using: handgrip strength, walking speed, unintentional weight loss, report of exhaustion and level of physical activity. The Polar V800 was used to record the RR interval, which was later evaluated by the Kubois HRV Standard. The isometric exercise protocol was performed at 30% of the maximum handgrip strength for 3 minutes. Where appropriate, the Student’s t-test or the Mann Whitney U test was used to compare differences between two independent groups. Statistical significance was set at p < 0.05.

**Results:** The F group has a greater value in the LFun index that is associated with cardiac sympathetic and parasympathetic activity at baseline than the NF group [F:55.3 ± 9.7 vs NF:48.8 ± 7.4; p < 0.02], greater LF/HF value [F:1.2 (0.9–1.5) vs NF:0,9 (0,7–1,2); p < 0.02] and lower HFun index value that is related to cardiac parasympathetic activity [F:44.6 ± 9.7 vs NF:50.9 ± 7.2; p < 0.03]. In the recovery measure, the NF group presented higher values in HFun (REC:40.4 ± 6.0 vs HAND:50.6 ± 12.98; p < 0.05). Additionally, it was observed that at the handgrip moment, group F presented a higher LFun value (HAND:59.7 ± 10.8 vs BAS:55.3 ± 9.7); p < 0.02] and a lower HFun value (HAND:40.1 ± 10.8 vs BAS: 44.60 ± 9.77; p < 0.02). On the other hand, still in the F group, a low HFms index was noted during handgrip [REC:153.5 (61–339) vs HAND:93.9 (57–197); p = 0.02]. For the NF group, the handgrip measurement had higher LFun (HAND:59.4 ± 6.1 vs BAS:48.8 ± 7.4; p < 0.00) and LF/HF [(HAND: 1.73 (1.37–1.98) vs. BAS: 0.99 (0.77–1.20); p < 0.01] and lower for the HFnu index (HAND:40.4 ± 6.0 vs BAS: 50.9 ± 7.2; p < 0.00). There were differences between groups for the HFun index [F:44.6 ± 9.7 vs NF:50.9 ± 7.2); p < 0.03], and the LF index (F:55.3 ± 9.7 vs NF:48.8 ± 7.4) p < 0.03] that were low and high in the F group, respectively.

**Conclusion:** Sympathetic activity is increased and cardiac parasympathetic activity is reduced in the frail elderly at baseline, implying greater cardiovascular risk.

111429

Modality: E-Poster Young Researcher – Non-case Report

Category: EPIDEMIOLOGY AND HEALTH POLICIES/GLOBAL HEALTH

## Impact of the COVID-19 Pandemic on Hospital Admissions and Out-of-Hospital Mortality by Cardiovascular Diseases in the City of Curitiba

DANIELI CRISTINA PIGOZZO^1^, Edson Lessa^2^, José Rocha Faria Neto^2^, Ana Caroline Dariva Chula^2^, Marcia Olandoski^2^

(1) Hospital Santa Casa de Curitiba; (2) Pontifícia Universidade Católica do Paraná

**Background:** Many countries have shown a decrease in the demand for medical care and an increase in out-of-hospital deaths (EH) caused by cardiovascular diseases (CVD) during the COVID-19 pandemic period.

**Objectives:** To analyze possible differences in the temporal trend of EH deaths and hospital admissions for CVD in the city of Curitiba during the period of the COVID-19 pandemic.

**Methods:** Retrospective, observational, time series study. Individuals admitted in Brazil’s Unified Health System network in Curitiba with a diagnosis coded by ICDs I20–I24 and I60–I64 were included. The number of admissions was correlated with the number of EH deaths from CVD and with the number of new COVID-19 cases. The period analyzed was from January 2019 to August 2021. The descriptive analysis was made through graphics.

**Results:** There were 289941 new cases of COVID-19, 13446 hospital admissions and 2336 EH deaths due to CVD. There was a decrease in admissions in the early period of the pandemic (4352 versus 3736 March-December 2019 and 2020) at the expense of fewer admissions for AMI (3307 versus 2435), while those for stroke increased (1044 versus 1301). EH deaths increased mainly from unspecific cardiovascular causes (UCVC). From March to December 2020 this increase was 71% (185 versus 318) compared to the same period in 2019.

**Conclusion:** This study suggests that the COVID-19 pandemic had a negative impact on the evolution of cardiovascular diseases, through a transient reduction in the number of hospital admissions and an increase in the number of EH deaths from CVD. These at the expense of higher mortality from UCVC.

111129

Modality: E-Poster Young Researcher – Non-case Report

Category: ACUTE AND CHRONIC CORONARY DISEASE/THROMBOLYSIS

## Effect of Methotrexate Carried in a Lipid Nanoparticles on Left Ventricular Remodeling and Infarct Size in Patients with ST-Elevation Myocardial Infarction: A Randomized Clinical Trial

ALINE GEHLEN FERRARI^1^, Raul Cavalcante Maranhão^1^, Rocío Salsoso^1^, Vanessa Maria Gomes Taques Fonseca Baldo^1^, Gabriela Liberato^1^, Remo Holanda de Mendonça Furtado^1^, Talia Falcão Dalçóquio^1^, Luciano Moreira Baracioli^1^, Aleksandra Tiemi Morikawa^1^, Cesar Higa Nomura^1^, Thauany Martins Tavoni^1^, José Carlos Nicolau^1^

(1) Instituto do Coração – Hospital das Clínicas da Faculdade de Medicina da Universidade de São Paulo (InCor-HCFMUSP)

**Background:** Inflammation is very important in the pathophysiology of ST-elevation acute myocardial infarction (STEMI), with special role in left ventricular (LV) remodeling. Methotrexate (MTX) is a potent anti-inflammatory drug with a potential benefit in the treatment of STEMI. A formulation of MTX incorporated into lipid nanoparticles (LDE), LDE-MTX, tested in rats with induced myocardial infarction, reduced by 50% the infarct size and improved LV function, without observable toxicity.

**Purpose:** To evaluate effects and safety of LDE-MTX treatment on LV remodeling and infarct size in patients with STEMI.

**Methods:** Randomized, double-blinded, placebo-controlled, proof of concept study. Patients were randomized within 4 ± 2 days after STEMI to receive LDE-MTX (40 mg/m^2^, intravenous) or LDE-placebo weekly for 6 weeks. The efficacy endpoints were change between 90 ± 7 days and baseline, in LV end-diastolic and end-systolic volumes, LV ejection fraction, LV mass and infarct size, measured by cardiac magnetic resonance. The main safety endpoints were serious adverse events and incidence of hematological, renal, and liver dysfunction.

**Results:** Thirty-five patients were randomized (18 to LDE-MTX, 17 to LDE-placebo). Two patients in the LDE-placebo group and one in the LDE-MTX group refused to continue the protocol and were excluded from the final analysis. The main efficacy results are shown in Table 1. There were no significant differences between the groups regarding safety parameters.

**Conclusions:** In patients with STEMI, LDE-MTX resulted in higher reduction in infarct size and lower LV mass loss, despite no differences in LV volumes. No safety issues were observed. These results encourage further clinical studies on this novel Nanomedicine approach for the treatment of patients with STEMI.


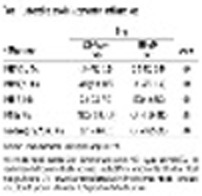


111130

Modality: E-Poster Young Researcher – Non-case Report

Category: CARDIAC ARRHYTHMIAS/ELECTROPHYSIOLOGY/ELECTROCARDIOGRAPHY

## Aethacizinum: Effective and Safe Antiarrhythmic Drug in Patients with Persistent Atrial Fibrillation for Sinus Rhythm Restoration

EMMA SOKOLOVA^1^, Ainārs Rudzītis^3^, Oskars Kalējs^2^

(1) Riga Stradins University, University of Latvia, Riga East University Hospital, Pauls Stradins Clinical University Hospital; (2) Riga Stradins University, Pauls Stradins Clinical University Hospital; (3) University of Latvia, Pauls Stradins Clinical University Hospital

**Introduction:** The improvement in cardiac inotropic function after restoration of sinus rhythm in patients with persistent atrial fibrillation (AF) is very important. Previous studies shows that antiarrhythmic class Ic medications are decreasing inotropic function of the heart.

**Objectives:** To investigate safety and efficacy parameters of widely used antiarrhythmic Ic class medication in patients with atrial fibrillation.

**Methods:** 70 patients with persistent AF underwent bicycle ergometer stress test, rest ECG, 24-hours Holter monitoring, echocardiography and blood tests within 1 day and 4 weeks (±7 days) following electrical cardioversion. We gathered also information considering the quality of life. The investigation protocol included testing with the baseline 100 mg/day aethacizinum treatment.

**Results:** We observed increase in bicycle stress test time from a mean 5,42 minutes to 6,05 minutes (p = 0,005). During Holter monitoring in n = 4 patients (5,7%) atrial fibrillation was observed. Echocardiography showed improve of systolic function from a mean 50% to 55% (p = 0,005), no worsening of diastolic function was observed. There was no significant changes in ECG parameters.

**Conclusions:** No proarrhythmic events were observed during the study. Medication was well tolerated, proved to have high antiarrhythmic efficacy and safety in patients with persistent atrial fibrillation, having no negative inotropic effect on myocardium.

111163

Modality: E-Poster Young Researcher – Non-case Report

Category: CARDIAC ARRHYTHMIAS/ELECTROPHYSIOLOGY/ELECTROCARDIOGRAPHY

## Fragmentation of the QRS Complex and Early Repolarization Pattern and an Mildly Reduced Ejection Fraction

MARIA GORDEEVA^1^, Irina Serdiukova^2^, Alexander Krasichkov^2^, Elena Parmon^1^

(1) Federal State Budgetary Institution “Almazov National Medical Research Centre” of the Ministry of Health of the Russian Federation; (2) Saint Petersburg Electrotechnical University

The key investigation for the assessment of the left ventricular ejection fraction (EF) is echocardiography, however, this method is not a screening method, especially in individuals with mildly reduced ejection fraction (mrEF). At the same time, an ECG is performed in almost all patients with suspected cardiovascular disease and as part of a preventive examination. It is known that traditional ECG-pattern associated with a decrease in EF have a low diagnostic value. Recently, new ECG-patterns associated with depolarization abnormality (fragmentation of the QRS complex (fQRS) and early repolarization pattern (ERP)) have been actively studied. The aim of study is investigated ECG-pattern of depolarization abnormalities (fQRS and ERP) in patients with a mrEF.

**Materials and methods:** The study included 148 patients with ischemic and non-ischemic cardiomyopathy. According to the level of EF, patients were divided into three groups: patients with low EF (lEF) (less than 40%): 31 (25 men, mean age 52.0 +/–15.6); patients with mrEF (49%–40%): 29 (23 men, mean age 54.7 +/– 12.4); patients with preserved EF (pEF) (more than 50%): 88 (57 men, mean age 58.2 +/–12.0) – control group. We used the criteria by Das M. et al, 2006 to identify fQRS and the criteria by Macfarlane P.W. et al., 2015 to identify ERP.

**Results:** In the 1st group (lEF), fQRS was registered in 16 (51.6%) patients, in the 2nd group (mrEF) – in 13 (44.8%), in the 3d (pEF) in 2 (13.6%), p < 0.001. ERP in the 1st group (lEF) was registered in 2 (6.5%), in the 2nd group (mrEF) – in 2 (6.9%), in the 3d group (pEF) ERP – in 11 (12.5%), p = 0,5. As a result of the ROC analysis, a relationship was found between fQRS and an intermediate decrease EF (40–49%).

**Conclusions:** The greatest difficulty for the early detection of heart failure is the group of patients with a mrEF. FQRS has shown its predictive value in identifying these patients. This ECG-pattern must be analyzed to assess the risk of heart failure.

111179

Modality: E-Poster Young Researcher – Non-case Report

Category: CARDIAC ARRHYTHMIAS/ELECTROPHYSIOLOGY/ELECTROCARDIOGRAPHY

## Relationship between CHA2DS2-Vasc Score and P-Wave Indexes and Echocardiographic Parameters in Patients Without Atrial Fibrillation

AMANDA VANESSA DEMARCHI^1^, Luciana Vidal Armaganijan^1^, Dalmo Antonio Ribeiro Moreira^1^, Mariane Higa Shinzato^1^, Kelvin Henrique Vilalva^1^, Pablo Santos Graffiti^1^, Rodrigo Augusto de Miranda Bertin^1^, Murilo Amato David^1^, Mathias Antonio Haruno de Vilhena^1^, Guilherme Dagostin de Carvalho^1^

(1) Instituto Dante Pazzanese de Cardiologia (IDPC)

**Introduction:** The CHA2DS2-VASc score has been used as a predictor of cardiovascular outcomes even in the absence of atrial fibrillation (AF). Several P wave indexes and echocardiographic parameters are associated with a higher risk of developing AF and thromboembolism; however, there are few studies in patients without AF.

**Objectives:** Primary: Evaluate the association between P wave indexes [P wave duration, dispersion and variability, maximum and minimum P wave duration, P wave voltage in lead I, Morris index, PR interval (PRI), P/PRI ratio and P wave peak time] and CHA2DS2-VASc SCORE in patients without AF and valve disease. Secondary: To assess the relation between echocardiographic parameters [left atrium (LA) and left ventricle (LV) size, LV ejection fraction (LVEF), LV mass and LV indexed mass] and CHA2DS2-VASC score in the same population.

**Methods:** A cross-sectional, descriptive and analytical study in which clinical, electrocardiographic and echocardiographic data from 272 patients without AF and valve disease were collected and analyzed. For statistical analysis, the Chi-Square Test, Mann-Whitney U-Test and Spearman Correlation were used with the significance level of 5%.

**Results:** PRI, LA and LV diameter, LV mass and LV indexed mass were positively associated with CHA2DS2-VASc SCORE, while P wave amplitude, P wave voltage in lead I and LVEF were negatively associated to the same score (Table). The presence of the Morris index was related with high CHA2DS2-VASc.

**Conclusion:** The study of the association between P wave indexes, echocardiographic parameters and CHA2DS2-VASc score may be useful in clinical practice to evaluate patients who trend to have a higher cardiovascular risk using clinical parameters and non-invasive methods in patients without AF.


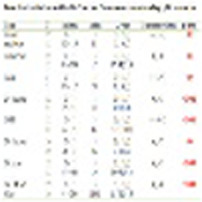


111181

Modality: E-Poster Young Researcher – Non-case Report

Category: COVID-19 AND CARDIOVASCULAR SYSTEM

## Impact of Cardiac, Renal and Hepatic Markers in COVID-19 In-Hospital Patients

YURI CAVALCANTI ALBUQUERQUE TENORIO^1^, Claudia Patricia da Silva Gois^2^, Lamark Melo Silva Moreira^2^, Stephanny Isabelly Pessôa Neri de Araujo^2^, Igor Vieira Lima Alexandre^2^, Priscila Alves da Silva^2^, Larine Ferreira Lira^3^, Edecio Galindo de Albuquerque^1^, Antônio Everaldo Vitoriano de Araújo Filho^2^, Francisco de Assis Costa^2^

(1) Hospital Veredas; (2) Centro Universitário Tiradentes; (3) Centro Universitário Cesmac

**Introduction:** Coronavirus disease (Covid-19) is a pandemic disease caused by SARS-CoV-2 virus first described in Wuhan (China) in 2019. This condition causes general inflammation leading to acute respiratory distress syndrome (ARDS) in severe cases, sometimes requiring mechanical ventilation. The aim of this study is to identify the impact of cardiac, renal and hepatic markers in mortality, hospital staying, development of ARDS, and necessity of mechanical ventilation (MV) in Covid-19 patients.

**Methods:** Case-control study in a philanthropic hospital in the Brazilian state of Alagoas, selecting 103 hospitalized patients from 2020 to 2021 aged more than 18 years old and diagnosed with SARS-CoV- 2 infection.

**Results:** Descriptive of the population analysed was 60.2% of male, 47.6% had hypertension, 26.2% had diabetes, 4.9% had cardiac heart failure, 34.9% had history of smoke, 19% had history of alcohol consumption and 2.5% had history of cancer. There was a positive risk between acute renal failure and death (OR = 2.97), similarly there was a positive risk of requiring MV (OR = 1.74), but there was no relation to hospital staying (p = .456). There was no relationship between hepatic injury and death (OR = .862) and hospital staying (p = .429). On the other hand, there was association to the necessity of MV (OR = 2.86). Finally, there was a relation between cardiac injury and death (OR 19.3) and MV (OR 1.38), but no relation in hospital staying (p = .801). Discussion and

**Conclusion:** The study concluded that cardiac, renal and hepatic markers are related to severe Covid-19 requiring MV in the population analysed. There was association between renal and cardiac markers to death, but not hepatic markers to death.


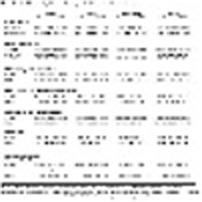


111193

Modality: E-Poster Young Researcher – Non-case Report

Category: PERICARDIUM/ENDOCARDIUM/VALVOPATHIES

## Trends in Early Mortality Rate in Infective Endocarditis

ISABELA GALIZZI FAÉ^1^, Pedro Henrique Oliveira Murta Pinto MD^1^, Fernando Crespo Torres^2^, Sofia Caporalli Barbosa^2^, Gustavo Brandão de Oliveira MD^1^, Rhuan Braga Oliveira^2^, Lucas Bretas de Pádua^2^, Teresa Cristina Abreu Ferrari, MD, PhD^1^, Maria do Carmo Pereira Nunes, MD, PhD^1^

(1) Hospital das Clínicas da Universidade Federal de Minas Gerais; (2) Universidade Federal de Minas Gerais

**Introduction:** Infective endocarditis (IE) is a rare disease associated with substantial mortality. In recent decades, the epidemiology of IE has been changing with an increased age of patients and a crescent number of health-care-acquired IE. However, few studies have examined the contemporary outcomes of IE in this growing population at risk of complications.

**Objective:** The present study aimed to describe the incidence of in-hospital mortality over the past 2 decades and to identify the risk factors for early mortality in a cohort of patients with IE admitted to a Brazilian quaternary hospital.

**Methods:** A total of 334 consecutive patients diagnosed with IE based on modified Duke criteria were prospectively included from 2001 to 2021. Data regarding predisposing baseline condition, laboratory findings, etiologic agents, treatment and in-hospital outcomes were analyzed. The primary endpoint was in-hospital death due to any complication related to IE.

**Results:** The median age was 54 years, 60% men. Cardiac device and rheumatic heart disease were the most frequent predisposing conditions. During the treatment, 78% patients presented adverse events, including worsening of heart failure (HF) (34%), embolic events (13%) and the need for cardiac surgery (43%). The overall in-hospital mortality rate was 34.9% with no changing over this time. In multivariable analysis, the characteristics independently associated with death were embolic events, HF development, increasing age and high CRP levels (C statistic of the model 0.84).

**Conclusion:** Despite recent advances, IE continues to be associated with high in-hospital mortality, without changes in the last 2 decades. Early identification of patients who are at high risk of death may offer an opportunity to improve outcomes in IE.


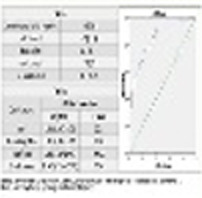


111215

Modality: E-Poster Young Researcher – Non-case Report

Category: ACUTE AND CHRONIC CORONARY DISEASE/THROMBOLYSIS

## Epidemiological Profile of Patients Admitted with Acute Myocardial Infarction Secondary to Spontaneous Coronary Artery Dissection

ISABELLE MENDES RODRIGUES SALOMÃO^1^, Marye dos Santos Xavier Dias^1^, Luanna Damasceno Amaral de Sousa^1^, Alessandra Arnez Pacheco^1^, Larissa Guerra Cunha de Sousa^1^, Daniel Xavier de Brito Setta^1^, Julia Paulo Mourilhe Rocha^1^, Fernando Oswaldo Dias Rangel^1^, Ana Amaral Ferreira Dutra^1^, Louise Freire Luiz^1^, Claudia Lanzillotti Weksler^1^, Ricardo Mourilhe-Rocha^1^

(1) Hospital Pró-Cardíaco

**Introduction:** Spontaneous coronary artery dissection (SCAD) is considered an uncommon cause of acute coronary syndrome (ACS), in which typical chest pain is the main manifestation, being more common in women ≤50 years old. It can be triggered by physical or emotional stress, female sex hormones, inflammatory disorders, fibromuscular dysplasia, and connective tissue disease.

**Objective:** To assess the epidemiological profile of patients with acute myocardial infarction (AMI) secondary to SCAD.

**Methods:** This is a retrospective case series study developed at a Quaternary Hospital in the city of Rio de Janeiro. The database and electronic medical records of 1.200 patients with ACS, admitted between July 2013 and February 2022, were analyzed, in which were selected 0,7% diagnosed with SCAD.

**Results:** Of the 9 patients evaluated, all were female, with a median age of 58 years, 67% had arterial hypertension, 78% obesity, 78% anxiety, 44% smokers, 22% diabetes mellitus, 22% previous AMI and 22% with a positive family history for coronary artery disease. Most (78%) had typical chest pain on admission, with 89% Killip I, 78% non-ST segment elevation AMI, 67% with preserved global left ventricle (LV) systolic function. 11% with severe LV dysfunction and 56% with segmental alteration. Median ejection fraction of LV was 59%. All patients underwent coronary angiography (CAT) within 24 hours of admission, with 67% single-vessel involvement and 44% TIMI III. Conservative treatment, with ASA, clopidogrel, high-potency statin, ACEI and bisoprolol was chosen in 78% of the patients. 22% were treated with angioplasty with drug-eluting stent implantation due to unfavorable coronary anatomy. One patient with extensive and severe lesion of the anterior descending artery and another patient with severe lesion of the marginal artery. 33% of patients had pain recurrence and 22% of whom had recent readmissions due to angina. The length of hospital stay was 5 days. There were no in-hospital deaths.

**Conclusion:** SCAD is more prevalent in females and may be associated with stress factors, smoking and obesity. The approach to patients follows the usual ACS protocol, but it must be considered that the dissection can be aggravated by CAT. Conservative treatment is recommended in most low to moderate risk cases. It is important to perform the differential diagnosis of chest pain in order to avoid underdiagnosis of SCAD.

111760

Modality: E-Poster Young Researcher – Non-case Report

Category: CARDIOVASCULAR INTENSIVE CARE/CARDIOVASCULAR EMERGENCIES

## Admission Bedside Lung Ultrasound Reclassifies SCAI Shock Classification Mortality Risk in Patients with ST-Segment Elevation Myocardial Infarction

GUSTAVO NEVES DE ARAUJO^2^, Gustavo Neves de Araujo^1^, Fernando Luis Scolari^3^, Guilherme Pinheiro Machado^3^, Anderson Donelli Silveira^3^, Felipe Pereira Lima Marques^3^, Rafael Beltrame^3^, Andre Theobald^3^, Alan Pagnoncelli^3^, Rodrigo Vugman Wainstein^3^, Marco Vugman Wainstein^3^

(1) Imperial Hospital de Caridade; (2) Instituto de Cardiologia de Santa Catarina; (3) Hospital de Clinicas de Porto Alegre

**Background:** The new SCAI Shock Classification encompasses patients with progressive severity from stage A (at risk) to stage E (Extremis), and early identification of stepping stages is essential to scaling therapy. Lung ultrasound (LUS) evaluates pulmonary congestion, which may be present even in the absence of other signs of overt shock. Our aim was to evaluate prognosis in patients with and without lung congestion evaluated by LUS among SCAI Shock stages.

**Methods:** Cohort of STEMI patients treated with primary PCI in a tertiary center. LUS protocol consisted of 8 scanning zones performed at admission. SCAI shock classification was evaluated within 24h of admission. Primary outcome was in-hospital mortality.

**Results:** We included 582 patients with mean age of 61 ± 12 years and 373 (64.1%) male. SCAI shock stage A was present in 361 (62%) patients, while 115 (19.8%) were class B, 44 (7.6%) class C, 58 (10%) class D, and 4 (0.7%) class E. In-hospital mortality in patients with SCAI Shock classification A-E was 1.4%, 14.4%, 48.9%, 63.8% and 50%, respectively. Among SCAI B patients, mortality in mild (0–3 positive sites) and severe congestion pattern (4–8 positive sites) were 8.8% and 22.2%, respectively. Mortality in SCAI C patients without of LUS congestion (14.3%) was similar to patients in SCAI B stage (p = 0.583). Mortality in SCAI D patients without of LUS congestion (50%) was similar to patients in SCAI C stage (p = 0.631).

**Conclusion:** Among STEMI patients at risk for cardiogenishock, LUS reclassifies SCAI Shock Classification regarding mortality prediction. Absence of lung congestion was associated with one-step decrease of mortality rates in SCAI C and D patients. While this must be tested in larger cohorts, LUS should potentially be included in SCAI Classification.


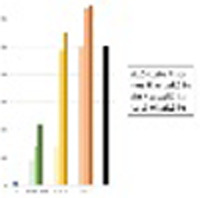


111288

Modality: E-Poster Young Researcher – Non-case Report

Category: CARDIAC ARRHYTHMIAS/ELECTROPHYSIOLOGY/ELECTROCARDIOGRAPHY

## Myocardial Ischemia Predictors in Patients with Left Bundle Branch Block

ANDRÉ PINHEIRO ZYLBERMAN^1^, Lara Teles Alencar Duarte^1^, Allexa Gabriele Teixeira Feitosa^1^, Cláudia Bispo Martins-Santos^1^, Yasmin Juliany de Souza Figueiredo^1^, Cleovaldo Ribeiro Ferreira Júnior^1^, Edvaldo Victor Gois Oliveira^1^, Arthur Leite Lessa^1^, Octavio Morais Veloso^1^, Antônio Carlos Sobral Sousa^1^, Enaldo Vieira de Melo^1^, Joselina Luzia Menezes Oliveira^1^

(1) Universidade Federal de Sergipe

**Introduction:** The left bundle branch block (LBBB) is a degenerative condition of the cardiac conduction system that is easily diagnosed by an electrocardiogram. Its prevalence ranges from 0.2 to 1.1%, increasing with age. As it is a chronic illness, patients may also develop several associated pathologies that require careful investigation, including coronary disease. As a result, the determination of heart ischemia predictors takes its place as an important evaluation, especially considering the exercise test’s diagnostic difficulties in LBBB subjects.

**Objective:** To determine the predictors of heart ischemia in LBBB patients undergoing physical stress.

**Methods:** This is an observational, cross-sectional and analytical study. It was used a dataset of LBBB patients from a private institution, all of which underwent an exercise stress echocardiography. The selection process excludedindividuals with previous coronary events. A total of 15 variables were studied in relation to the test’s result, including: sex, age, body mass index, family background, diabetes mellitus, dyslipidemia, hypertension, obesity, smoking, aortic diameter (AO), left atrium diameter (LA), left atrium volume, left ventricular mass index, ejection fraction and diastolic function. Statistical analysis was performed by the chi-square test (X²) and by the student’s t-test, both considering p < 0,05 as significant. The software SPSS Statistics version 22.0 was used.

**Results:** From the 252 patients included in the study, 115 (45.63%) were men and 137 (54.37%) were women. Ages ranged from 30 to 92 years old, with a mean of 64.07 (±10.93). From the results of the echocardiography, two groups can be clearly identified: positive (n = 64; 25.40%) and negative for ischemia (n = 188; 74.60%). Among the qualitative variables, analysis showed that the male sex was the only associated with heart ischemia (p = 0.01) in LBBB patients. However, among the quantitative variables, AO (p = 0.00), LA (p = 0.02) e left ventricular index (p = 0.02) had significant higher means in the ‘positive for ischemia’ group.

**Conclusion:** Only four of the 15 possible predictors were significant in the present study. Sex was deemed the only important personal factor for the echocardiography’s positive result. However, considering heart measurements as possible predictors, LBBB patients with either a larger atrium or aort, or with a greater ventricular mass were more susceptible to ischemia while facing stress.

111299

Modality: E-Poster Young Researcher – Non-case Report

Category: HEART FAILURE/CARDIOMYOPATHY/TRANSPLANT

## Predictors of Cardiorenal Syndrome Type 1 and Mortality in Patients with Acute Heart Failure: A Case-Control Study

TARCIO SADRAQUE GOMES AMORAS^1^, Giovana Salomão Melo^2^, João Simão de Melo Neto^2^, Christelaine Venzel Zaninotto^1^, Vitor Bruno Teixeira de Holanda^1^, Sheila Santos de Oliveira^1^, Luana da Silva Freitas^1^, Luana Santos Nunes^1^

(1) Division of Cardiology, Gaspar Vianna Clinical Hospital Foundation; (2) UPCEURG, Institute of Health Sciences, Federal University of Pará

**Introduction:** Currently, one of the most common causes of aggravation in hospitalized patients with heart failure (HF) is the worsening of renal function. Approximately one-third of patients with acute HF may develop acute renal injury (AKI); this condition has recently been termed cardiorenal syndrome type 1 (CRS-1).

**Objectives:** To identify predictors of CRS-1 and mortality in patients with acute HF in the Eastern Amazon.

**Methodology:** This was an observational, case-control study in which descriptive and inferential analyses were performed. We included 183 patients with acute HF aged older than 18 years admitted from January 2017 to May 2021 at a public referral hospital in the department of nephrology and cardiology. Patients were categorized into two groups: G1, patients with SCR-1 (n = 72), and G2, patients without SCR-1 (n = 111). The prevalence of sociodemographic and clinical factors was determined for both the groups, and independent predictors of SCR-1 and mortality were identified using the logistic regression model. All statistical analyses were performed using SPSS Statistics for Windows, version 21.0.

**Results:** Among 183 patients, 72 (39.3%) developed SCR-1 and 33 (16.6%) evolved with dialysis AKI. Prevalence of chronic kidney failure (CRF) (p = 0.003), uncontrolled hypertension (p = 0.005), non-adherence to food/water (p = 0.043), advanced age (p = 0.004) and elevation of systolic BP (p = 0.021) was observed in the group with SCR-1. Among the factors that contributed to the worsening of acute HF, only non-adherence to dietary and water intake guidelines was considered a predictor for the incidence of SCR-1 (p = 0.003; OR = 81.45; 95% CI = 4.38–1513.13). For in-hospital mortality, only AKI dependent on renal replacement therapy was considered a predictor for SCR-1 incidence (p = 0.010; OR = 15.5; 95% CI = 1.93–124.35).

**Conclusion:** Non-adherence to dietary and water intake guidelines was considered a risk factor for SCR-1. As it is a modifiable factor, preventive actions in public health become relevant for minimizing the occurrence of SCR-1.

111335

Modality: E-Poster Young Researcher – Non-case Report

Category: HEART FAILURE/CARDIOMYOPATHY/TRANSPLANT

## Effect of Sacubitril/Valsartan on Central Hemodynamic Parameters in Patients with Chronic Kidney Disease in CHF with Type 2 Diabetes

KHOLIKOVA AZIZA OYBEK^1^, Abdullaev Timur Atanazarovich^1^, Kholikova Aziza Oybek^1^, Tsoy Igor Arsenevich^1^

(1) Republican Specialized Scientific and Practical Medical Center for Cardiology

**Purpose of the study:** Evaluation of the effect of sacubitril/valsartan on central hemodynamic parameters in patients with chronic kidney disease (CKD), chronic heart failure (CHF) and type 2 diabetes.

**Material and methods:** Randomization included 42 patients with CHF FC class II–III according to NYHA, EF less than 40% with concomitant type 2 diabetes and CKD stages 3a–3b, mean age 50.4 ± 16.6 years. Every 3 months, all patients were determined the content of urea, serum creatinine, the content of potassium, sodium, magnesium and calcium in the blood, GFR, protein in the urine and blood pressure. CKD in history occurred in 100% of patients, arterial hypertension 90%, diabetes mellitus 100%. Previously, patients received standard baseline cholesterol therapy. After 3–4 days of observation, patients were switched to sacubitril/valsartan by standard dose titration. The follow-up period was 18 months.

**Results:** Patients included in the observation who used sacubitril/valsartan were divided into 2 groups according to CKD stages: CKD group 1 GFR up to 45 ml/min/1.73 m^2^, GFR group 2 above 46 ml/min/1.73 m^2^ Analysis of integral hemodynamic parameters showed the following. The severity of the decrease in SBP, DBP and HR was greater and had statistical power in patients with eGFR ≤45 compared with the group of patients with eGFR >46 ml/min/1.73 m² (∆ SBP 16.9 ± 6.24 VS 10 ± 3.18 mm Hg, ∆ DBP 10.76 ± 3.48 VS 1.72 ± 2.1 mm Hg, ∆ HR 10.38 ± 4.02.24 VS 1.151 ± 3.99 bpm), respectively with an average intergroup t = 2.25; P = 0.03. Those. hemodynamic response was the best in the general group with eGFR ≤45 and was almost completely determined by patients with type 2 diabetes. DBP – 14 ± 4 (t = 2.298; P = 0.03); HR 8.4 ± 4.48 was significantly higher in the group with eGFR ≤45 than in the group with eGFR >46 SBP – 3.34 ± 4.32 (t = 0.701; P = 0.03); DBP – 2.5 ± 2.17 (t = 0.045; P = 0.8), heart rate 6.25 ± 4.47 (t = 1.423; P = 0.105). The content of urea, creatinine in blood serum did not change significantly in both groups (t1 = 0.439408; t2 = 0.220387).

**Conclusion:** In patients with CHF FC II–III according to NYHA, with an EF less than 40% with concomitant type 2 diabetes, therapy with a neprilysin inhibitor/RAAS blockade was not accompanied by a noticeable decrease in hemodynamic parameters in patients with CKD C3a and C3b.

111350

Modality: E-Poster Young Researcher – Non-case Report

Category: ACUTE AND CHRONIC CORONARY DISEASE/THROMBOLYSIS

## Hospital Evolution Comparative Analysis of Percutaneous Coronary Interventions in Acute Coronary Syndrome in Patients with and Without COVID-19 Infection

THALES CARDOSO WHATELY^1^, Joao Gabriel Monteiro Junqueira^1^, Ana Salomé Eurico^1^, Ana Luiza Iannarella Lacerda^1^, Barbara Bezerra de Almeida^1^, Karen Sanae Takehara Vieira^1^, Mayara Bastos Souza^1^, Constantino Gonzales Salgado^1^, João Addison Pessoa^1^, Roberto Pozan^1^, Esmeralci Ferreira^1^

(1) Hospital Universitário Pedro Ernesto; (2) Universidade do Estado do Rio de Janeiro

**Introduction:** COVID-19 is a predictor of mortality in acute coronary syndrome (ACS). In a university hospital, in patients with ACS treated by percutaneous coronary intervention (PCI), an increase in mortality and in-hospital complications was observed. In view of these observations and because there are no data related to PCIs in COVID-19, we developed this work.

**Objectives:** The main objective was to assess in-hospital mortality in patients with and without COVID-19 infection and ACS undergoing urgent PCI. The secondary objective was to evaluate cause of mortality and the association with clinical presentations, risk factors and angiographic findings.

**Methods:** It’s a comparative, retrospective and consecutive study. A total of 598 patients undergoing PCI in ACS were evaluated. Patients were divided into groups: GI with COVID-19 (N = 76) and GII without COVID-19 (N = 522).

**Results:** The mean age was 62 years. The rate of diabetes, obesity, dyslipidemia, smoking, previous coronary artery disease and systemic arterial hypertension (SAH) were similar. SAH was the most frequent factor (GI = 68.8%; GII = 69.2%, p = 0.99). Renal failure and previous coronary artery bypass graft surgery were more prevalent in GI (11.7%, p = 0.005). ST-segment elevation myocardial infartcion (STEMI) was more frequent (GI = 57.1%; GII = 61.5%, p = 0.53), followed by non-STEMI (GI = 35.1%; GII = 25 .1%, p = 0.07) and unstable angina (GI = 6.5%; GII = 13.4%, p = 0.09). We observed a higher frequency of interventions in anterior descending artery (ADA) (GI = 42.9%; GII = 58.8%, p = 0.01), followed by right coronary artery (GI = 13.3%; GII = 34 .9%, p < 0.001) and circumflex artery (GI = 6.5%; GII = 16.9%, p = 0.02). Mortality was higher in GI (GI = 22.3%; GII = 3.6%, p < 0.001) related to severe acute respiratory syndrome (SARS) (GI = 47.0%; GII = 5.3%, p < 0.001). Cardiogenic shock was greater in GII (GI = 41.2%; GII = 78.9%, p < 0.001). Septic shock was similar in the groups (GI = 11.8%; GII = 10.6%, p = 0.9). In multivariate analysis, the presence of COVID, age, renal failure and the involvement of the ADA showed a positive and significant association with in-hospital death.

**Conclusion:** Emergency care and hemodynamic assessment of ACS took on great importance in pandemic. This study proved that presence of COVID-19 was the main predictor of ACS mortality due to SARS and shock. In addition to COVID-19, mortality is related to age, ADA involvement, and renal dysfunction.

111351

Modality: E-Poster Young Researcher – Non-case Report

Category: EPIDEMIOLOGY AND HEALTH POLICIES/GLOBAL HEALTH

## Analysis of Hospital Cardiovascular Mortality in the State of Rio de Janeiro Pre and Per-Pandemic by SARS-COV-2

THALES CARDOSO WHATELY^1^, João Gabriel Monteiro Junqueira^1^, Luciana Ludwig Nigri^1^, Cláudia da Silva Lunardi^1^, Ivana Picone Borges Aragão^1^, Sara Cristina Marques dos Santos^1^, Ana Salomé Eurico^1^, Esmeralci Ferreira^1^

(1) Hospital Universitário Pedro Ernesto; (2) Universidade do Estado do Rio de Janeiro

**Introduction:** The hospital scenario in the state of Rio de Janeiro (ERJ) was overloaded before SARS-Cov-2. Associated with the collapse of the pandemic, there was a reduction in demand for medical services, especially in patients with cardiovascular disease (CVD). COVID-19 represents an additional risk to these individuals. Previous studies have shown lower rates of hospitalization and death from CVD, and an increase in the fatality rate in the pandemic. The lack of epidemiological data related to COVID-19 and mortality in the ERJ led to this study.

**Objective:** Analyze hospital CVD mortality in the health regions of the ERJ in the pre- and per-pandemic period of COVID-19 in patients of the brazilian unified health system (UHS).

**Methods:** Descriptive, population-based, retrospective epidemiological study, using the hospital information system database of the UHS informatics department. The periods evaluated were divided in pre-pandemic (2018 and 2019) and per-pandemic (2020 and 1st quarter of 2021). Mortality data were obtained from the International Code of Diseases, which took place in the ERJ, divided into: ischemic diseases (G1), heart failure and cardiogenic shock (G2), cardiac arrhythmia (G3) and myocarditis (G4). Hospitalizations decreased in the regions of Baixada Litorânea (21.21%), Center-South (14.50%), Middle Paraíba (20.86%), Metropolitan I (18.37%), Metropolitan II (5.32%), North (19.44%) and Serrana (16.38%), and increased in Ilha Grande Bay (7.02%) and Northwest (1.72%). There was a drop in mortality in the Center-South (5.07%), Middle Paraíba (25.38%), Metropolitan I (18.09%), Metropolitan II (2.40%), North (11.02%) and Serrana (11.75%) and increase in Ilha Grande Bay (11.32%) and Northwest (9.28%).

**Conclusion:** Hospitalizations in the CVD groups were reduced, especially in G2 patients. There was a significant increase in mortality caused by myocarditis, probably related to the virus. The health regions with an increase in hospitalizations and mortality were Ilha Grande Bay and Northwest Rio de Janeiro.

111352

Modality: E-Poster Young Researcher – Non-case Report

Category: CARDIOVASCULAR SURGERY

## New Risk Score for Predicting Postoperative Atrial Fibrillation After Cardiac Surgery

JOÃO LINS DE ARAÚJO NETO^1^, João Lins de Araújo Neto^1^, Eduardo Arrais Rocha^1^

(1) Universidade Federal do Ceará

**Background:** Postoperative atrial fibrillation is the most common sustained arrhythmia after cardiac surgery and occurs in approximately 30% of postoperative patients. Because of the large amount of evidence recommending prophylactic treatment and lack of data indicating who should receive such treatment, this study aimed to develop a new predictive score for atrial fibrillation after cardiac surgery.

**Methods:** This retrospective cohort study included 989 adult patients who underwent cardiac surgery, except for heart transplantation and implantation of a ventricular assist device. Patients with previous atrial fibrillation or those using amiodarone were excluded. The analyzed variables were subjected to a univariate analysis of the occurrence of postoperative atrial fibrillation and a multivariate analysis using logistic regression.

**Results:** Statistically significant variables in the multivariate analysis were age ≥60 years (P < .001), left atrial enlargement according to echocardiography (P = .025), inotrope use within 24 hours after surgery (P = .002), and the need for reoperation within 24 hours after surgery (P = .016). The score comprises these four variables and has an accuracy of 77% for predicting outcomes. Scores ≥3 were related to a 34% risk of postoperative atrial fibrillation.

**Conclusions:** The proposed score represents the disease pathophysiology well and will be useful in clinical practice.


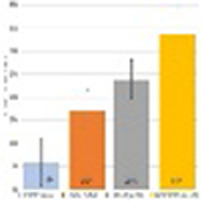


111354

Modality: E-Poster Young Researcher – Non-case Report

Category: CARDIORESPIRATORY PHYSIOLOGY/BASIC SCIENCE

## Cardioprotection by Human Plasma Transfer After Remote Ischemic Preconditioning: Identification of Cardioprotective Humoral Factors Through Omics Techniques

ALBERTO BARRETO GRIMALDI^1^, Gustavo Monnerat^1^, Dahienne Ferreira de Oliveira^1^, Fabio C S Nogueira^3^, Gilberto B Domont^3^, Helena Cramer Veiga Rey^2^, Jose Hamilton Matheus Nascimento^1^, Antonio Carlos Campos de Carvalho^1^, Leonardo Maciel^1^

(1) Instituto de Biofísica Carlos Chagas Filho (Federal University of Rio de Janeiro);; (2) Instituto Nacional de Cardiologia (INC/RJ); (3) Instituto de Química (Federal University of Rio de Janeiro)

Remote ischemic preconditioning (RIPC) provides myocardial resistance to ischemia/reperfusion (I/R) injuries. The protection mechanism by RIPC is suggested to be mediated by humoral factors. However, the identity of these humoral factors remains enigmatic. We aimed to characterize and identify the humoral factors responsible for the cardioprotection induced by RIPC. Human volunteers signed a consent form and answered a health survey. The volunteers were submitted to RIPC protocol (3 cycles of 5 minutes of ischemia alternated with 5 minutes of reperfusion in the arms). The venous blood was collected before (Placebo plasma) and after RIPC (RIPC plasma). The human plasmas were fractionated in different molecular weight ranges and the cardioprotection was evaluated in isolated hearts of rats submitted to 30 minutes of ischemia and 120 minutes of reperfusion in an isolated heart apparatus. Mass spectrometry (MS) was performed in placebo and RIPC plasma. The fraction less than 10kDa from RIPC plasma reduced infarct size in 50% and induced hemodynamic recovery of hearts submitted to I/R compared to control. The fraction less than 10kDa from placebo plasma did not induce protection. Hearts perfused with the fraction greater than 10kDa or total RIPC plasma also had no cardioprotection. MS showed differences in the protein content, including higher content of adenosine and kininogen in quantitative analysis and the presence of 15 putative cardioprotective proteins in qualitative analysis in the RIPC plasma compared with placebo. The cardioprotective humoral factors are in the less than 10kDa fraction of RIPC plasma. Moreover, the cardioprotection by RIPC can be transferred between species. Adenosine, kininogen, and other 15 proteins could be responsible for the RIPC cardioprotection.


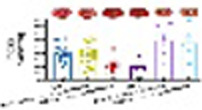


111362

Modality: E-Poster Young Researcher – Non-case Report

Category: HEART FAILURE/CARDIOMYOPATHY/TRANSPLANT

## Analysis of ECG Parameters in Patients with Peripartum Cardiomyopathy

KHOLIKOVA AZIZA OYBEK^1^, Xudoyberganov Otabek^1^, Abdullaev Timur Atanazarovich^1^, Tsoy Igor Arsenevich^1^

(1) Republican Specialized Scientific and Practical Medical Center for Cardiology

**Objective:** To study the features of electrocardiographic changes in patients with peripartum cardiomyopathy in comparison with female patients with dilated cardiomyopathy.

**Material and methods:** ECG and HMECG data of 68 women with peripartum cardiomyopathy and 43 patients with dilated cardiomyopathy were examined. The frequency of occurrence of complete bundle branch block (PBBB) was analyzed. QS phenomenon, atrial fibrillation (AF), ventricular extrasystoles according to Lown’s gradation (PV), AV bdokady.

**Results:** ECG analysis showed that complete blockade of the left bundle branch block (LBBB) occurred in patients with DCMP in 12 (27.9%) (χ2 = 7.061; p = 0.008) cases, and in group 1 in 6 (8.8%) cases, respectively. Blockade of the right bundle branch of His (RBBB) was detected in 1 (1.4%) (χ2 = 0.638; p = 0.008) patients from group 1 and was not detected in patients of group 2. A permanent form of atrial fibrillation was in 4 (9.3%) patients with DCMP, and in 1 (1.4%) (χ2 = 3.756; p = 0.053) cases in patients with PCMP (χ2 = 3.756; p = 0.053). Absence of R wave growth or QS complex in chest leads in 8 (12.7%) and 5 (11.6%) (χ2 = 0.000; p = 0.983) patients in groups 1 and 2. AV blockade of the 1st degree was recorded in 4 (5.9%) cases in group 1, in patients from group 2 AV blockade of the 1st degree in 14 (32.5%) cases (χ2 = 13.797; p = 0.001). Negative T wave in 24 (35.3%), group 2 DCMP in 8 (18.6%) (χ2 = 3.576; p = 0.059) cases. HMECG found that ventricular arrhythmias of grades 1 and 2 were 60 (88%) in PPCM, and high grades: paired VA – in 25 (36.8%) and 23 (53.4%) cases (χ2 = 3.002; p = 0.084), respectively, in groups 1 and 2; group VA – 11 (16.1%) and 14 (32.5%) (χ2 = 4.051; p = 0.045); unstable forms of VT – 6 (8.8%) and 9 (20.9%) (χ2 = 3.304; p = 0.070). A stable form of VT was recorded in one case in both groups.

**Conclusion:** In DCM, PBLBBB and 1st degree AV block are significantly more common. At the same time, high grade ventricular extrasystoles and a negative T wave in the chest and standard leads are significantly more common in patients with PPCM.

111367

Modality: E-Poster Young Researcher – Non-case Report

Category: HEART FAILURE/CARDIOMYOPATHY/TRANSPLANT

## Clinical and Functional Characteristics of Heart Failure in Females with the Familial Form of Dilated Cardiomyopathy

KHOLIKOVA AZIZA OYBEK^1^, Abdullaev Timur Atanazarovich^1^, Tsoy Igor Arsenevich^1^, Mirzarakhimova Saodat Temurovna^1^, Bekbulatova Regina Shavkatovna^1^

(1) Republican Specialized Scientific and Practical Medical Center for Cardiology

**The aim:** To study the clinical and functional features at patients with familial form of dilated cardiomyopathy.

**Material and Methods:** We examined 47 females with the familial form of dilated cardiomyopathy compared with the same of idiopathic dilated cardiomyopathy (n = 50). Examination included: ECG Holter monitoring, echocardiography, test 6-minute walk distance (6MWT).

**Results:** Disease duration was on average 9,4 ± 1,4 and 12,4 ± 1,4 months. (p > 0.05). The length of distance traveled during 6MWT was 264,5 ± 17,3 and 198,4 ± 11,4 m (p > 0.05). Patients with familial form of DCM disease has developed in a relatively early age 36,9 ± 1,6 years, which was significantly lower than in the comparison gr (42,4 ± 1,1 years, p < 0.01). It was shown that AV-blockade I degree was observed with the same frequency in the two grps (12.7% and 14.2%, p > 0.05), however, in the gr with the familial form of DCM in 5 (10.6%) were cases of complete atrioventricular block. Among patients with the familial form of DCM, the LBBB met less frequently (14.8% vs. 21.2%), and AF – more often (19.1% vs. 17.5%, both p > 0.05). The analysis of echocardiographic parameters showed that patients with familial DCM had lower values of ESD (5,6 ± 0, 1 and 6,2 ± 0,1 cm, p < 0.01) and EDD (6,9 ± 0,1 cm and 7,4 ± 0,1 cm, p < 0.01) and EF (37,8 ± 1,8 vs 31,1 ± 1,1%; p = 0.03) compared with patients with idiopathic dilated cardiomyopathy.

**Conclusions:** This form of the disease is associated with younger age, the development of complete atrioventricular block and high rates of left ventricular ejection fraction.

111368

Modality: E-Poster Young Researcher – Non-case Report

Category: CARDIOVASCULAR INTENSIVE CARE/CARDIOVASCULAR EMERGENCIES

## Short Term Outcomes of Intermediate-High Risk Pulmonary Embolism Stratified by Type of Treatment

JULIO LEANDRO BOBADILLA^1^, Cristhian Emmanuel Scatularo^1^, Melisa Antoniolli^1^, Ezequiel Lerech^1^, Ignacio Manuel Cigalini^1^, Ezequiel José Zaidel^1^

(1) CONAREC, Consejo Argentino de Residentes de Cardiología

**Background:** Pulmonary embolism (PE) current stratification includes intermediate-risk patients, in which the specific treatment is controversial yet. The aim of this study was to evaluate short term outcomes of intermediate-high risk patients that received different treatment strategies.

**Methods:** We performed an analysis of a multicenter PE registry. Patients with intermediate-high risk (classification of the European Society of Cardiology) were selected, and clinical variables were stratified by type of treatment received (only antocoagulation or reperfusion with systemic trhombolysis, local thrombolysis, thrombus fragmentation-aspiration or surgical thrombectomy).

**Results:** From 684 consecutive acute PE patients from 75 centres, 178 (26%) were classified as intermediate-high risk. Sixteen percent (n = 28) of this cohort received reperfusion treatment, either systemic thrombolysis (89%) or endovascular treatments, while the rest received only anticoagulation. On multivariable analysis, we found that patients receiving reperfusion strategies were younger (57 ± 17 years vs 68 ± 14; p < 0.001) and had more frequent bilateral PE (78% vs 43.7%, respectively; OR 4.72; 95% CI 1.8–12.3; p < 0.001). No significant differences were observed in total bleeding and major bleeding. 30 days absolute mortality was 3.6% in the reperfusion group and 14% in the non-reperfusion group (OR 0.22, 95% CI 0.02–1.76; p = 0.1).

**Conclusions:** Comparing with anticoagulation only, the use of different reperfusion strategies for intermediate-high risk was safe and showed a trend to improved short-term outcomes, highlighting the need to develop a randomized clinical trial for this specific group of PE patients.

111370

Modality: E-Poster Young Researcher – Non-case Report

Category: CARDIOLOGY OF SPORTS, EXERCISE, ERGOMETRY AND CARDIOVASCULAR REHABILITATION

## Physical Activity Behavior of Prediabetes and Diabetes Patients Three Months Later of the Participation in an Exercise Intervention Concluded During the COVID-19 Pandemic

JOSIANE APARECIDA DE ALMEIDA^1^, Ana Paula Delgado Bomtempo Batalha^2^, Isabela Coelho Ponciano^1^, Mariana Balbi Seixas^2^, Tiago Peçanha^1^, Patrícia Fernandes Trevizan^3^, Raquel Rodrigues Britto^3^, Lilian Pinto da Silva^2^

(1) Graduate Program in in Rehabilitation Sciences and Physical-Functional Performance Faculty of Physical Therapy, Federal University of Juiz de Fora, Juiz de Fora, Brazil; (2) Graduate Program in Physical Education, Faculty of Physical Education and Sports, Federal University of Juiz de Fora, Juiz de Fora, Brazil; (3) Graduate Program in Rehabilitation Sciences, Federal University of Minas Gerais, Department of Physical Therapy, Belo Horizonte, Brazil

**Background:** The COVID-19 pandemic imposed critical social restrictions that affected people’s behaviors and lifestyles as decreased physical activity. At the same time, healthcare systems recommend home-based exercises as a safe and effective alternative to maintain the population’s physical activity levels. Therefore, this study aimed to evaluate motives to maintain physically active and adherence to exercise, as recommended, by patients with prediabetes and type 1 (T1D) and type 2 (T2D) diabetes that participated in a structured exercise intervention delivered partially on-site and partially remote.

**Methods:** This is a cross-sectional study including participants of a pilot randomized trial (NCT03914924) who had completed a 12-week exercise intervention. The exercise intervention was switched from an on-site to a remote delivery in March 2020 due to the social restrictions imposed by the COVID-19 pandemic. All pilot randomized trial participants were instructed to maintain at least 150 minutes of moderate- or vigorous-intensity aerobic physical activity, as measured by the Borg scale, and 2 to 3 sessions/week of resistance exercise. The motives for maintaining physically active were evaluated by the Motives for Physical Activity Measure-Revised scale (MPAM-R), a twenty-six self-administered questionnaire encompassing five motives (enjoyment, competence, appearance, fitness, and social). Exercise adherence, as recommended, was evaluated by the Brazilian Portuguese version of the Exercise Adherence Rating scale (EARS-Br), a six-items self-administered questionnaire containing six items scored from 0 to 24. A score of seventeen is a cutoff point that demarks adequate adherence to the recommended exercise.

**Results:** Fifteen patients (58 ± 11 yrs; 53% female; 13% prediabetes, 27% T1D, 60% T2D) participated in the study. Thirteen participants (87%) reported maintaining physical activity three months after the exercise intervention conclusion. The descending order of median scores obtained in each motive for physical activity was fitness, enjoyment, competence, social, and appearance. The median (interquartile range) of the EARS-Br total score was 17 (13–23), revealing over half, 53% (n = 8), of the participants adhered to physical exercise as recommended at the end of the exercise intervention.

**Conclusion:** Health concerns are the main motive for patients with prediabetes and diabetes to maintain physical activity during the pandemic COVID 19.

111371

Modality: E-Poster Young Researcher – Non-case Report

Category: NEGLECTED CARDIOVASCULAR DISEASES

## Differences in Echocardiographic and Functional Parameters and Quality of Life between the Levels of Physical Activity in Patients with Chagas Cardiomyopathy: A Cross-Sectional Study

WHESLEY TANOR SILVA^1^, Matheus Ribeiro Ávila^1^, Pedro Henrique Scheidt Figueiredo^1^, Lucas Frois Fernandes de Oliveira^1^, Vanessa Amaral Mendonça^1^, Ana Cristina Rodrigues Lacerda^1^, Vanessa Pereira Lima^1^, Henrique Silveira Costa^1^

(1) Universidade Federal dos Vales do Jequitinhonha e Mucuri UFVJM

**Introduction:** Physical training seems to promote important clinical, functional, echocardiographic and quality of life changes in patients with Chagas cardiomyopathy. Thus, it is important to verify the differences in the parameters mentioned in the different levels of physical activity presented by the patients.

**Objective:** To verify the functional, echocardiographic and quality of life differences in different levels of physical activity in patients with Chagas‘ heart disease.

**Methods:** Seventy-eight patients with Chagas cardiomyopathy (53 ± 10 years, 55% male, NYHA I–III, LVEF 53 ± 16%) were selected and evaluated by echocardiography, Cardiopulmonary Stress Test and by Minnesota Living with Heart Failure Questionnaire. Patients‘ physical activity level was assessed by the International Physical Activity Questionnaire (IPAQ), classifying patients as sedentary, insufficiently active, active and very active.

**Resulted:** Insufficiently active Chagas patients (n = 13, 17%) presented younger age (p = 0.019), greater curve of ventilatory carbon dioxide equivalent (VE/VCO2 slope) (p = 0.003) and greater left ventricular diastolic diameter (p = 0.020) in relation to active (n = 38.49%) and younger age (p = 0.023), lower left ventricular ejection fraction (p = 0.017) and greater left ventricular diastolic diameter (p = 0.012) in relation to the very active (n = 27, 34%). There was no difference in quality of life, resting heart rate, blood pressure and VO2peak between the groups. When comparing the active with the very active, there was no difference between any of the variables analyzed. In the total sample, the level of physical activity correlated with the VE/VCO2 slope (r = –0.447; p < 0.001), with age (r = 0.249; p = 0.028), with left ventricular ejection fraction (r = 0.276; p = 0.016) and left ventricular diastolic diameter (r = –0.300; p = 0.009).

**Conclusion:** Insufficiently active chagasic patients had worse values of the main functional and echocardiographic parameters. In addition, the level of physical activity correlated with well-established prognostic markers for this population, however, without influencing the quality of life of patients with Chagas cardiomyopathy.

111372

Modality: E-Poster Young Researcher – Non-case Report

Category: HEART FAILURE/CARDIOMYOPATHY/TRANSPLANT

## The Relationship between Cardial Dysfunction and the Level of ST2 Biomarker in Patients with Dilated Cardiomyopathy

KHOLIKOVA AZIZA OYBEK^1^, Abdullaev Timur Atanazarovich^1^, Gulomov Khumoyun^1^, Tsoi Igor Arsenevich^1^, Akhmatov Yashin Ravshanovich^1^

(1) Republican Specialized Scientific and Practical Medical Center for Cardiology

**The aim of the study:** To assess the relationship between the parameters of cardiac dysfunction according to echocardiography and the level of the ST2 biomarker in patients with dilated cardiomyopathy.

**Materials and Methods of the trial:** The study included 38 patients (28 men and 10 women) with dilated cardiomyopathy, with an average age of 49 years. Depending on the ST2 level at admission, patients were divided into 2 groups: a group with an ST2 level ≥35 ng/ml and a group with an ST2 level <35 ng/ml. In addition to the general clinical examination of patients, an echocardiographic assessment of the state of intracardiac hemodynamics was carried out. The ST2 content in blood plasma was determined by the enzyme immunoassay.

**Results of the study:** The 1st group included 20 people with an increased level of expression ST2 ≥35 ng/ml, the 2nd one – 18 patients with ST2 expression level <35 ng/ml. The concentration of soluble ST2 isoform in blood serum in the 1st group was 77.3 ± 24.7 ng/ml, and in the 2nd – 20.6 ± 7 ng/ml (p = 0.000). According to the results of a comparative analysis of structural of the LV functional state in participants at the time of inclusion in the study, it was found that in patients from the 1 group LVEF was 29.9 ± 4.3% and was less by 2.2% (p = 0.035) in comparison with patients in the 2group (LVEF = 32.1 ± 10.9%). EDD – 72.8 ± 3 mm in the 1group 67.6 ± 9.5 mm, in contrast to the 2 group ESD- 62.1 ± 4 mm, 57.7 ± 10.8 mm, LA- 45.6 ± 3.2 mm, 41.5 ± 4.3 mm, RV- 41.2 ± 6.1 mm, 40 ± 9.2 mm, LVMM – 355.4 ± 118.4 g, 293.3 ± 74 g, respectively, for the 1st and 2nd groups. Analysis of the relationship between the parameters of the structural and functional state of the LV according to echocardiography revealed a weak positive correlation between LVEF (r = 0.193, p < 0.05) with the concentration of sST2, as well as a positive correlation between the concentration of soluble sST2 in serum and the size of the LV cavities (end- diastolic dimensions (EDD), r = 0.467, p < 0.05; end systolic (ESD), r = 0.376, p < 0.05; LA size r = 0.273, p < 0.05), and LV myocardial mass (r = 0.375, p < 0.05).

**Conclusion:** In patients with dilated cardiomyopathy, an increased level of soluble ST2 isoform correlates the left ventricular function; the presence of an increased ST2 level in patients with dilated cardiomyopathy is a significant marker of a worse course of CHF.

111376

Modality: E-Poster Young Researcher – Non-case Report

Category: CARDIOVASCULAR INTENSIVE CARE/CARDIOVASCULAR EMERGENCIES

## Safety of Long-Term use of Intra-Aortic Balloon Pump Therapy in Patients with Advanced Heart Failure Undergoing Heart Transplantation

VANESSA SANCHES CORCIOLI BELLINI^1^, Gustavo André Boeing Boros^1^, Claudia Bernoche^1^, Milena Frota Macatrão Costa^1^, Silvia Helena Gelas Lage Pasqualucci^1^

(1) Instituto do Coração do Hospital das Clínicas da Faculdade de Medicina da USP -INCOR-FMUSP

**Background:** Intra-aortic balloon pump (IABP) is a short-term device that is widely used in patients with advanced heart failure (HF) awaiting heart transplantation (HTx). Complications can be more frequent with prolonged use of the support until HTx.

**Purpose:** Evaluate IABP complications in patients with advanced HF awaiting HTx.

**Methods:** We retrospectively analyzed from 2009 to 2020 in a single center intensive care unit, patients with advanced HF who underwent HTx and received IABP therapy pre-transplant. Patients whose IABP was removed before the transplant were excluded. Prolonged use of IABP was considered over 20 days. We considered IABP relevant complications bleeding, induced thrombocytopenia, infection, and leg ischemia. They were classified as mild or severe. Transfusion or removal for blood complications, replacement and removal for infection and leg ischemia were considered severe.

**Results:** We included 195 advanced HF patients with IABP therapy before HTx. Mean time of IABP onset to HTx was 26 ± 23 days and 107 days was the longest used time. The main cardiomyopathy etiology was Chagas Disease, followed by idiopathic. Left ventricle ejection fraction (LVEF) was 24 ± 6% (TABLE 1). Fifty-two patients had IABP complications. The most frequent complications were induced mild thrombocytopenia and severe infection (TABLE 2). Bleeding occurred in 12 patients and leg ischemia in 5. The IABP replacement occurred in 43 patients and the main reason for replacement was infection. Infection was the only complication that correlated with prolonged use (p = 0.0004). No deaths were attributable to IABP complications.

**Conclusion:** IABP is a safe therapy with low complication rates, most of them minor. Except for infection, complications analyzed were independent of IABP duration.


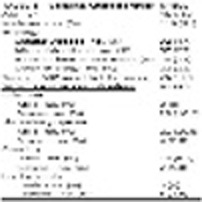


111384

Modality: E-Poster Young Researcher – Non-case Report

Category: DIGITAL HEALTH/INNOVATION

## Virtual Simulation Training as a Tool to Measure the Quality of Medical Knowledge in Hospital Specialized in Cardiology During the COVID-19 Pandemic

MIGUEL BARELLA NETO^1^, Daniel Fontes^1^, Sávio Batista^3^, Jadhe Maillard^4^, Heitor Olegário^6^, Ana Amaral Ferreira Dutra^2^, Louise Freire Luiz^2^, Gabriel Paes^5^

(1) Daktus; (2) Hospital Pró Cardíaco; (3) Universidade Federal do Rio de Janeiro; (4) Universidade Estácio de Sá; (5) Universidade Federal de São Paulo; (6) Universidade Federal de Juiz de Fora

**Introduction:** Virtual training is a tool already used for medical education, adaptation of good practices and training of health professionals to new care flows.

**Goals:** In this work, we report a retrospective observational study demonstrating the results of a Virtual Simulation Training (VST) used as a tool to measure medical knowledge, which allowed a focused face-to-face training intervention during the COVID-19 pandemic in a hospital specializing in cardiology.

**Materials and methods:** During the 3-month period, a VST was performed by the COVID-19 team of the hospital specialized in cardiology, with delivery of a pre and post-test form to evaluate the results. The questions on the form were divided into 5 main criteria: A) Appropriate indication of diagnostic tests for COVID-19 and rational use of resources; B) Recognition of signs of severity and adequate follow-up of patients with suspected COVID-19; C) Use of antibiotic therapy and opening of a sepsis protocol in patients with suspected COVID-19; D) Management of oxygen therapy; E) Airway Management; and F) Cardiovascular emergencies concomitant with COVID-19. The answers to these forms were evaluated according to the percentage of correct answers in good (>90%), medium (between 70–90%) and low (<70%) at the end of the training and, after the results, a face-to-face intervention focused on low performance criteria.

**Results:** The results of the post-test criteria showed Criterion C presenting a good result, Criteria A, B, D and F an average result and Criterion E a low result after the test. VST Criterion E (airway management), due to its low result, was selected for a new face-to-face intervention for airway management. The training consisted of training the entire medical team of the hospital in orotracheal intubation (OTI) through dummies, in airway management. After training, when analyzing the number of complications associated with OTI in the period prior to VST, there was an end to complications of airway management in COVID-19 patients.

**Conclusions:** In addition to being an ally in online medical training, the VST can be a possible tool to show medical training deficits in teams, which may result in choices efficient face-to-face training in specialized teams.

111390

Modality: E-Poster Young Researcher – Non-case Report

Category: EPIDEMIOLOGY AND HEALTH POLICIES/GLOBAL HEALTH

## The Prevalence of Cardiometabolic Multimorbidity and Its Associations with All-Cause Mortality and Premature Mortality in China: A Nationally Representative Population-Based Cohort Study

WILLIAM YANG ZHAO^1^, Lili Song^2^

(1) The George Institute for Global Health, China; (2) The George Institute for Global Health, Faculty of Medicine, University of New South Wales, Sydney, Australia

**Background:** Cardiometabolic disease has been a clinical challenge worldwide and a major public health issue. We aim to examine the epidemiology of cardiometabolic multimorbidity and the association of cardiometabolic multimorbidity with all-cause mortality and premature mortality.

**Methods:** This cohort study utilizes data from the China Health and Retirement Longitudinal Study between 2011 and 2018, which includes 9,712 adults aged 45 years and older. Poisson-distributed Generalized Linear Models were conducted to determine the association of cardiometabolic multimorbidity with all-cause mortality and premature mortality.

**Results:** Overall, the prevalence of cardiometabolic multimorbidity was 32.3% among 9,712 Chinese adults, and increased with age. Compared with the group of none and single disease, cardiometabolic multimorbidity was associated with a higher risk of all-cause mortality (Relative risk ratio [RR] = 1.355, 95% CI = 1.140, 1.611) and all-cause death (RR = 1.601, 95% CI = 1.166, 2.199), after adjusting socio-demographic and lifestyle behavioral covariates. Stratified analyses revealed stronger relationships between cardiometabolic multimorbidity and all-cause mortality (RR = 1.486, 95% CI = 1.126, 1.960) as well as premature mortality (RR = 1.994, 95% CI = 1.137, 3.497) among the females compared to the males. The associations of cardiometabolic multimorbidity with all-cause death (RR = 1.784, 95% CI = 1.317, 2.417) and premature death (RR = 1.781, 95% CI = 1.098, 2.890) were only statistically significant in urban residents.

**Conclusions:** Healthcare systems need to shift from single-disease models to new clinical and public health service delivery models to effectively manage cardiovascular and metabolic diseases as well as multimorbidity.

111413

Modality: E-Poster Young Researcher – Non-case Report

Category: COVID-19 AND CARDIOVASCULAR SYSTEM

## The COVID-19 Pandemic and its Effects on Systemic Arterial Hypertension Phenotypes in the City of São Paulo

MONIZZE VICTÓRIA ROCHA SENTALIN^1^, Wilson Nadruz Júnior^1^, Andrei C. Sposito^1^, Fabiana G. A. M. Feitosa^2^, Audes D. M. Feitosa^2^, Marco A. Mota-Gomes^3^, Weimar S. Barroso^4^, Roberto D. Miranda^5^, Andréa A. Brandão^6^, Eduardo C. D. Barbosa^7^

(1) Universidade Estadual de Campinas – UNICAMP; (2) Universidade Federal de Pernambuco – UFPE; (3) Centro Universitário CESMAC; (4) Universidade Federal de Goiás – UFG; (5) Universidade Federal de São Paulo – UNIFESP; (6) Universidade Estadual do Rio de Janeiro – UERJ; (7) Hospítal São Francisco – Santa Casa de Porto Alegre

**Introduction:** Has been hypothesized pandemic of COVID-19 may have led to inadequate control of cardiovascular risk, like arterial hypertension (HT), due to social isolation and changes in lifestyle habits. However, the impact of pandemic on blood pressure (BP) phenotypes derived from office and out-of-office measures (true normotension, AH sustained, AH white apron, AH masked) is still poorly known.

**Objective:** To compare BP phenotypes before and during the pandemic, and their relation to pandemic’s severity in patients São Paulo city.

**Methodology:** Retrospective study was conducted analyzing clinical characteristics and measures of BP in office and residential measure of BP (RMBP) in 1529 patients who weren’t using anti-hypertensive drugs, from TELEMRPA platform between 2017 and September 2021. Obtained information regarding number of cases and deaths from COVID-19 (SIVEP and SIM/SMS-SP) and medium temperature of São Paulo’s city in same period. Through analysis of multivariate logistic regression – such as adjustment of BMI, age, sex and ambiental temperature – was avaliated relations between SAH phenotypes, pandemic and its number of cases and deaths from COVID-19.

**Results:** There was an increase in sustained HT (28,2% to 42,2%) and decrease in true normotension (44,5% to 31,8%) in individuals evaluated at the pandemic compared to those evaluated before the pandemic. In addition, BMI and age were greater among patients during pandemic (Table). The relation between pandemic and sustained HT persisted (p < 0,001) and the relation between sustained HT and COVID-19 cases (p < 0,001) and deaths (p = 0,015) every two weeks of COVID-19, even after adjustment for confounding factors.

**Conclusion:** There’s a relation between BP phenotypes and COVID-19 pandemic and its severity in São Paulo.


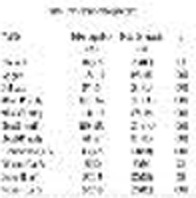


111422

Modality: E-Poster Young Researcher – Non-case Report

Category: ACUTE AND CHRONIC CORONARY DISEASE/THROMBOLYSIS

## Reperfusion Therapies in Patients with Acute Myocardial Infarction with ST-Segment Elevation: A Comparative Study between the Unified Health System and the Private Network

JEFERSON DOS SANTOS^1^, Suelen Maiara dos Santos^1^, Luiz Fernando Souza Santos^1^, Anisia Vieira Souza Fontes^3^, Aline Barreto Hora^2^, Adrielly Meneses dos Santos^2^, Beatriz Silva Ferreira Dantas^2^, Caio Nemuel Nascimento Santos^2^, Luiz Ricardo Gois Fontes^2^, Maria Luíza Leal Paranhos de Oliveira^2^, Andreza Oliveira Almeida^2^

(1) Universidade Tiradentes; (2) Universidade Federal de Sergipe; (3) Centro Universitário Uninassau

**Introduction:** During to the acute ST-segment elevation myocardial infarction (STEMI), the occurrence of clinically relevant events is directly proportional to the time of initiation of reperfusion therapy.

**Objective:** To perform a comparative analysis regarding to the reperfusion therapies used in patients treated in the Unified Health System (SUS) and in the private network.

**Method:** Through the ACCEPT registry database, a project to document the care of acute coronary syndrome in Brazil, patients treated at participating hospitals were selected, whose data were collected from admission to discharge. This study was approved by the Research Ethics Committee of the Federal University of Sergipe, under registration number 302.544.

**Results:** The procedures performed in 1550 patients (Public = 66.4%; Private = 33.6%) with STEMI were evaluated. Coronary angiography was predominant (Public = 89.3%; Private = 94.8%; p < 0.001) and angioplasty (Public = 73.6%; Private = 81.2%; p = 0.001) in both services. Furthermore, the administration of thrombolytics was performed in 21.8% of patients from the SUS and 10% from the private network (p < 0.001). Only 1.8% of the total number of patients underwent coronary artery bypass graft surgery and 16.9% did not undergo any reperfusion therapy.

**Conclusion:** There was a statistically significant difference in the performance of coronary angiography, angioplasty and use of thrombolytics.


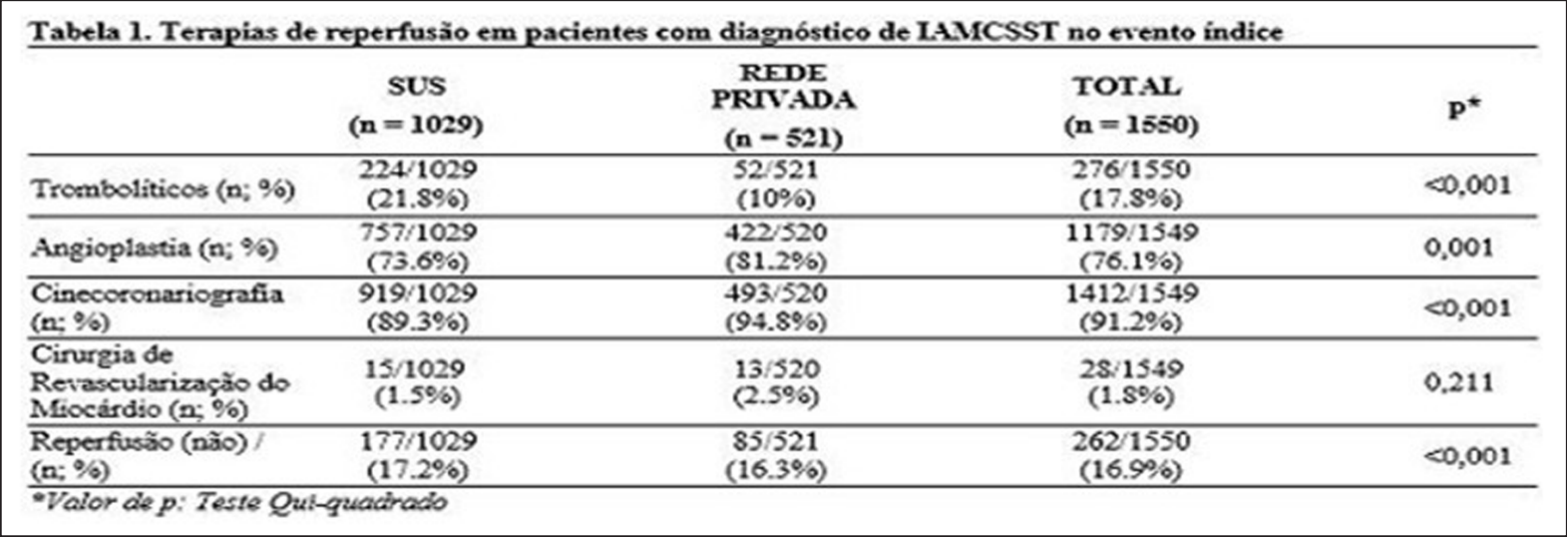


111432

Modality: E-Poster Young Researcher – Non-case Report

Category: EPIDEMIOLOGY AND HEALTH POLICIES/GLOBAL HEALTH

## The Prevalence of Cardiometabolic Disease Multimorbidity and its Associations with Functional Disability in China: A Nationally Representative Population-Based Cohort Study

WILLIAM YANG ZHAO^1^, Siqi Zhao^3^

(1) The George Institute for Global Health, China; (2) The George Institute for Global Health, Faculty of Medicine, University of New South Wales, Sydney, Australia; (3) Yantaishan Hospital of Yantai, Yantai, Shandong, China; (4) Yantai Sino-French Friendship Hospital, Yantai, Shandong, China

**Background:** Cardiometabolic disease has been a clinical and major public health challenge worldwide. We aim to examine the epidemiology of cardiometabolic multimorbidity and the association of cardiometabolic multimorbidity with the onset of functional disability.

**Methods:** This cohort study utilizes data from the China Health and Retirement Longitudinal Study between 2011 and 2018, which includes 9,016 adults aged 45 years and older. Poisson-distributed Generalized Linear Models were conducted to determine the association of cardiometabolic multimorbidity with the activities of daily living (ADL) limitation and the instrumental activities of daily living (IADL) limitation.

**Results:** The prevalence of cardiometabolic multimorbidity was 31.9% among 9,016 Chinese adults, and increased with age. Compared with the group of none and single disease, cardiometabolic multimorbidity was associated with a higher risk of ADL limitation (Relative risk ratio [RR] = 1.297, 95% CI = 1.160, 1.450) and of ADL limitation (RR = 1.165, 95% CI = 1.054, 1.288), after adjusting socio-demographic and lifestyle behavioral covariates. Stratified analyses revealed stronger relationships between cardiometabolic multimorbidity and the onset of ADL limitation (RR = 1.309, 95% CI = 1.138, 1.505) among the females compared to the males. The association of cardiometabolic multimorbidity with IADL limitation was only statistically significant (RR = 1.473, 95% CI = 1.158, 1.676) in urban residents.

**Conclusions:** Cardiometabolic multimorbidity is associated with the greater onset of functional impairment. The Health system needs to shift from single-disease models to new clinical and healthcare delivery models to effectively manage cardiometabolic multimorbidity.

111460

Modality: E-Poster Young Researcher – Non-case Report

Category: CARDIOVASCULAR PHARMACOLOGY

## Assessment of Cholinergic Enzyme Activity as a Possible Therapy Against Damage from Ischemic Heart Disease

JULIA LEAO BATISTA SIMOES^1^, Júlia Leão Batista Simões^1^, Margarete Dulce Bagatini^1^

(1) Universidade Federal da Fronteira Sul

Heart failure (I/R) injury is an important cause of myocardial infarction and heart failure after cardiovascular surgery. Among the therapeutic possibilities, the modulation of the cholinergic system has been highlighted as a prevention of cardiac dysfunction after a cardiac event. The central mechanism of the cholinergic system is related to the stimulation of inflammatory agents in the afferent branch of the vagus nerve. From this perspective, the literature suggests that the greater availability of ACh during and after ischemic deficiencies is associated with a preservation of cardiac function. Thus, the present study was designed to investigate the enzymatic activity of cholinesterases in patients with RHI, given the relationship between Ach and protection against heart problems. Seventy patients with IHD from the Hospital of the Federal University of Santa Maria were selected for the study. Ten milliliters of blood were obtained from each patient and used for biochemical determinations. The protocol was approved by the Human Ethics Committee of the Health Sciences Center of the Federal University of Santa Maria. Thus, the present study demonstrated that the enzymatic activity of AchE and BuChE is increased in patients with IHD, directly responsible for the reduction of ACh in the middle and triggering a worsening in the outcome of the disease.


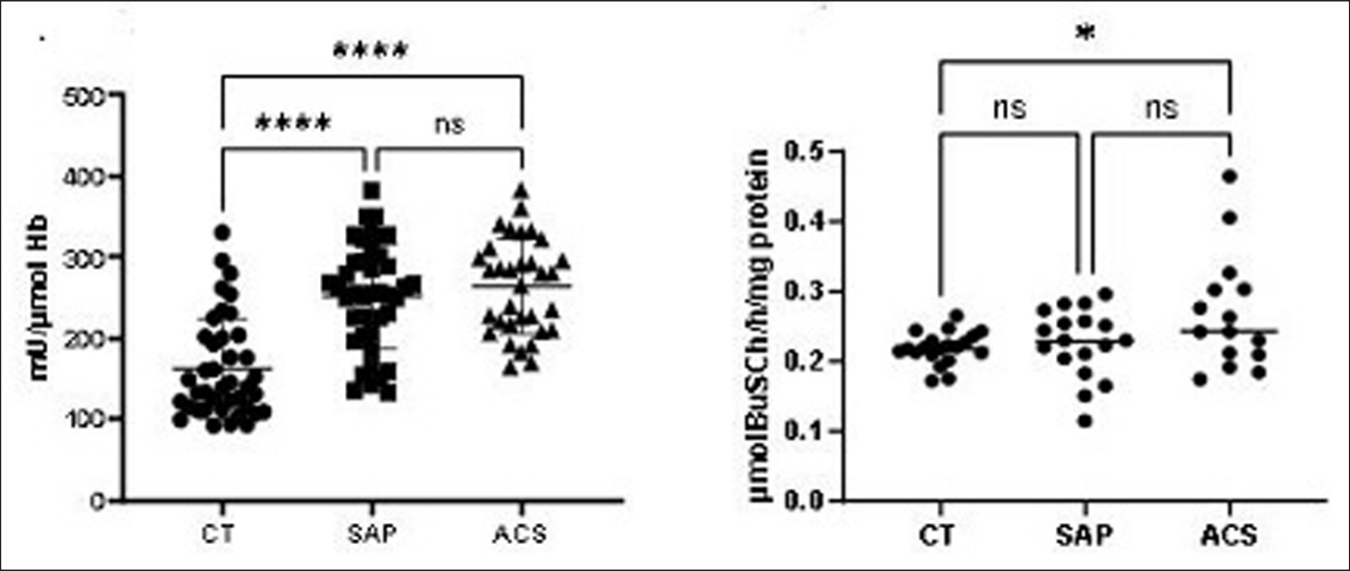


111444

Modality: E-Poster Young Researcher – Non-case Report

Category: HEART FAILURE/CARDIOMYOPATHY/TRANSPLANT

## Association of Potassium Disorders with the Mode of Death in Patients with Chronic Heart Failure

IVNA GIRARD CUNHA VIEIRA LIMA^1^, Ivna Girard Cunha Vieira Lima^1^, Jairo Tavares Nunes^2^, Igor Henrique de Oliveira^2^, Fernanda Ronco^4^, Edimar Alcides Bocchi^2^

(1) Instituto do Coração do Hospital das Clínicas da Faculdade de Medicina da Universidade de São Paulo; (2) Instituto do Coração do Hospital das Clínicas da Faculdade de Medicina da Universidade de São Paulo; (3) Instituto do Coração do Hospital das Clínicas da Faculdade de Medicina da Universidade de São Paulo; (4) Therapeutic Area Leader – CVRM, AstraZeneca, Brazil

**Background:** Previous observational trials suggested a U-shaped association between potassium disorders and clinical outcomes in HF. However, the mechanism and modes of death, as well as the factors influencing this association are not yet fully understood.

**Aim:** Evaluate the association of potassium disorders with all-cause mortality, as well as to describe the association of potassium disorders with the mode of death in patients with coexisting HF.

**Methods:** A retrospective cohort study conducted in a tertiary hospital from January 2013 to December 2020 including 10390 patients. In-hospital deaths were reported by cause and outside of the hospital deaths were considered sudden deaths.

**Results:** Among the causes of in-hospital death HF was reported in 41.7%, cardiogenic shock in 31.2%, and other causes of death in 25.3%. Death outside of the hospital was reported in 12% of patients. The association between serum potassium disorders and mortality was stronger for severe hyperkalemia, adjusted HR (aHR) of 2.06 (1.36–3.12, p < 0.01), and hypokalemia, aHR of 1.93 (1.42–2.63, p < 0.01). Mortality outside of the hospital was more frequent in patients with hyperkalemia (8.7%) in comparison with hypokalemia (4%).

**Conclusion:** Concurrent potassium disorders were associated with mortality in HF. The potassium disorders may have potential to be a new frontier in the management of HF. Out-of-hospital deaths, were more frequently reported in patients with hyperkalemia.


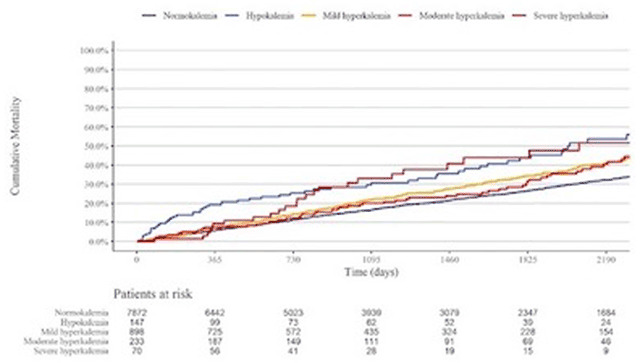


111451

Modality: E-Poster Young Researcher – Non-case Report

Category: NURSING

## Multiprofessional Care Protocol for Patients with Heart Failure During the COVID-19 Pandemic

EMILY JUSTINIANO^1^, Leticia Orlandin^1^, Luisa Mendes Silveira^1^, Simoni Chiarelli Da Silva Pokorski^1^

(1) Hospital de Clínicas de Porto Alegre

**Introduction:** During the SARS-CoV-2 pandemic, the population also suffers from the damage associated with the reduction in the number of outpatient visits and elective exams. In this sense, telemedicine was promoted and expanded, and it served as a strategy in the follow-up and control of patients with chronic diseases, such as heart failure (HF).

**Objectives:** To report the experience of a care protocol, including nursing teleconsultation, during the covid-19 pandemic in patients with heart failure.

**Method:** Experience report of descriptive character constructed from the experience of teleconsultations with patients from a university hospital in southern Brazil during the Covid-19 pandemic. Experience report: In HF, multiprofessional programs focused on care transition, patient education, medication regimen simplification and outpatient follow-up are effective in reducing costs and readmissions. However, during the pandemic period, there was need to reduce outpatient care, which caused a greater team distancing of the patient to the care team which may have contributed to HF decompensation. Faced with this challenge, the nursing team implemented teleconsultation for patients with HF. The team composed of nurses and academic students, physiotherapists and dietitians was trained to use a telephone care protocol based on the application of the European scale, validated for use in Brazil, which evaluates self-care – European Heart Failure Self Care Behavior Scale – EHFScBS. The process begins during hospitalization for decompensated HF, where the patient is evaluated for his/her self-care, receives health education by a multidisciplinary cardiology team, establishes bonding; and has post-discharge follow-up by the team through teleconsultations. In the immediate post-discharge period (5th to 7th day) a telephone call is made with the same questions applied during the hospital evaluation and educational guidelines for self-care are resumed. For the patients who were not hospitalized during the pandemic, teleconsultations were also carried out.

**Conclusions:** The team observed that teleconsultation proved to be efficient for the control of adherence to therapies and it also served as an instrument to reconnect the patient to the team. However, it is worth mentioning that the inability of some patients to use electronic devices, as well as the absence of physical examination during the consultation are some of the difficulties encountered in the use of such strategy.

111487

Modality: E-Poster Young Researcher – Non-case Report

Category: NUTRITION

## Abdominal Obesity Among Smokers with Multimorbidities Undergoing Treatment for Smoking Cessation

FERNANDA SILVA MOTTA^1^, Gabriela Godinho Rezende^1^, Rodrigo Máximo Silveira^1^, Samuel Barud Massensine^1^, Rafael Matoso de Oliveira Figueiredo^1^, Amanda Gonçalves Vieira Martins^1^, Bianca de Fátima Pereira^1^, Moisés de Toledo Vilela^1^, Paula Gouvea Abrantes^1^, Marcela Rabelo^1^, Eliane Ferreira de Carvalho Banhato^1^, Arise Garcia de Siqueira Galil^1^

(1) Internship Department, Medical School of the Federal University of Juiz de Fora (UFJF), Juiz de Fora, Minas Gerais, Brazil

**Introduction:** Abdominal obesity represents an important additional risk factor for both the development of chronic non-communicable diseases, cardiovascular diseases, cancer and diabetes. There is evidence in the literature of the association between smoking and abdominal obesity, in which nicotine acts by increasing insulin resistance and, consequently, is related to the deposition of fat in this region.

**Objectives:** To analyze characteristics associated with abdominal obesity in smokers with multimorbidities.

**Methods:** Longitudinal study, from the “Free Tobacco” group, attended between August/2021 to April/2022, referring to treatment groups consecutive sessions for smoking cessation (with mixed intervention, face-to-face and by telemedicine), composed of a multidisciplinary team, including sessions of cognitive behavioral approach, drug treatment and follow-up. Abdominal obesity was defined as waist circumference (WC) >80 cm for women and >94 cm for men. Light smokers, for use ≤10 cigarettes/day. Abusive use of alcohol, for Audit-C ≥5 points. High risk for Obstructive Sleep Apnea Syndrome (OSAS), Stop-Bang questionnaire ≥5 points. Abnormal systolic blood pressure (SBP), >130 mmHg.

**Results:** 36 users were evaluated, referring to 6 intervention groups. When comparing those with abdominal obesity (60% of the sample) with those who did not, it was observed that these users were predominantly male (p > 0.031), light smokers (p > 0.057) and with alcohol abuse (p > 0.002). As for chronic conditions, they had a higher prevalence of obesity (p > 0.001), OSAS (p > 0.049), diabetes mellitus (p > 0.001), previous acute myocardial infarction (p > 0.001) and peripheral vascular disease (p > 0.001). Allied, higher abnormal SBP (p > 0.021) and increased levels of total cholesterol and LDL-cholesterol (p > 0.021 and p > 0.021, respectively).

**Conclusions:** Among users undergoing treatment for smoking cessation, abdominal obesity was a frequent condition, with the majority of men in abusive alcohol use, but light smokers. Comorbidities associated with vascular damage, as well as lack of lipid and blood pressure control, in addition to OSAS, made up the profile of this population.

111488

Modality: E-Poster Young Researcher – Non-case Report

Category: PERICARDIUM/ENDOCARDIUM/VALVOPATHIES

## Antithrombotic Treatment Before Transcatheter Aortic Valve Implantation: Features and Clinical Impact

CARLOS ROCA GUERRERO^1^, Carlos Roca-Guerrero^1^, Joana Brito^2^, Beatriz Silva^2^, Pedro Carrilho-Ferreira^2^, Claudia Jorge^2^, Pedro Cardoso^2^, Hugo Corte-Real^2^, Marcelo Rizzo^1^, Pedro Canas-Silva^2^, Fausto Pinto^2^, Luís Brás-Rosário^2^

(1) Hospital Universitario Parc Taulí de Sabadell (Barcelona); (2) Hospital Santa Maria de Lisboa – Centro Hospitalar Universitário Lisboa Norte

**Introduction:** Severe aortic stenosis patients frequently suffer other conditions that require antithrombotic treatment. Features regarding different antithrombotic therapy (AT) and their clinical impact on patients admitted for elective transcatheter aortic valve implantation (TAVI) are not well known. Our aim was to describe AT’s diversity and explore its clinical impact in patients admitted for TAVI.

**Methods:** We analyzed retrospectively clinical, laboratory, echocardiographic, angiographic, drug therapy and procedure-related variables from 202 consecutive patients with TAVI between January 2019 and October 2021.

**Results:** This cohort had a mean age of 82,3 ± 5,9 years, 56% male, 83% hypertensive, 62% dyslipidemia, 35% diabetic, 20% chronic kidney disease, 32% had coronary artery disease, 20% previous percutaneous coronary intervention (PICP), 38% Atrial Fibrillation, 18% peripheral arterial disease, 12% cerebrovascular disease. 50% of TAVIs were balloon-expandable and 50% self-expandable. The mean follow up was 11 ± 8 months. 128 (63,4%) patients were under any antithrombotic treatment before admision. 44 (21,5%) under single antiplatelet treatment (SAPT), 11 (5%) under double antiplatelet treatment (DAPT), 9 (9%) under SAPT plus oral anticoagulation (OAC), 2 (1%) under DAPT plus OAC; 62 (30,5%) were under isolated OAC (2 of them with warfarin and the rest with direct OAC). 73 patients (36%) were receiving OAC and 84 (41,6%) were under an antithrombotic strategy different than just SAPT. At the univariate analysis receiving OAC, higher mean hemoglobin during hospitalization and previous percutaneous coronary intervention were all statistically associated with survival at the end of follow up; P < 0,05. At the multivariate analysis adjusted by the previous variables, sex, history of coronary artery disease and age, only PICP, and hemoglobin levels were statistically associated with survival at the end of follow up; P < 0,05.

**Conclusions:** In our patient’s population, AT before TAVI-related admission was frequent and diverse. Previous PCI and bleeding were the only variables independently-asociated with mortality. These findings emphasize the importance of balancing bleeding and thrombotic risk when treating these patients, even before the TAVI procedure.

111494

Modality: E-Poster Young Researcher – Non-case Report

Category: CARDIAC ARRHYTHMIAS/ELECTROPHYSIOLOGY/ELECTROCARDIOGRAPHY

## Novel Predictors of Af Recurrence After Catheter Ablation in Patients with Paroxysmal AF

MARIANA RIBEIRO SILVA^1^, Gualter Santos Silva^1^, Pedro Ribeiro Queirós^1^, Rafael Teixeira^1^, Sara Fernandes^1^, João Almeida^1^, Paulo Fonseca^1^, Marco Oliveira^1^, Helena Gonçalves^1^, Alberto Rodrigues^1^, João Primo^1^, Ricardo Fontes-Carvalho^1^

(1) Centro Hospitalar de Vila Nova de Gaia/Espinho

**Introduction:** Recurrence of atrial fibrillation (AF) after catheter ablation (CA) is estimated to be between 20% and 45% and the prediction of recurrence AF in patients (pts) with paroxysmal AF undergoing CA remains challenging.

**Objectives:** To determine the clinical and procedural predictors of recurrence AF after CA in pts with paroxysmal AF.

**Methods:** Single-centre retrospective study that included all pts with paroxysmal AF who underwent AF CA between 2017 and 2019. Ablation procedures included radiofrequency and second-generation cryoballoon CA. All pts underwent cardiac computed tomography prior the procedure. AF recurrence was defined as any recurrence of AF, atrial flutter or atrial tachycardia >30 seconds (recorded in 12-lead electrocardiogram or Holter) after 90 days of CA. The independent association between clinical and procedural variables and AF recurrence was evaluated with Cox regression analysis.

**Results:** We included 351 pts, 63,5% male and with a mean age of 57,1 ± 11,4 years. CHADSVASC score ≥2 points were present in 43,7% of the pts, median duration of AF prior the intervention was 3 years (IQR 1–6) and most pts were treated with some antiarrhythmic drug at the time of CA (56,9%). Overall, median follow-up was 27 months (IQR 19–39). AF recurrence was present in 96 pts (27,4%), on average, 15,2 ± 10,4 months after CA. Univariate logistic regression identified female gender, thyroid disease, left atrium (LA) enlargement (defined as LA indexed volume >34 mL/m^2^ or LA diameter >38 mm for female or >40 mm for male), coronary calcium score >100, epicardial adipose tissue volume, number of previous electric cardioversions, treatment with antiarrhythmic drugs prior CA and the extent of CA (only pulmonary vein isolation (PVI) or PVI and ablation of other lesions) as predictors of recurrence AF after CA in pts with paroxysmal AF (p < 0,05 for all). Cox regression analysis identified female gender (OR 1,615, 95% CI 1,005–2,597; p = 0,008), LA enlargement (OR 2,084, 95% CI 1,207–3,596; p = 0,008) and coronary calcium score >100 (OR 1,958, 95% CI 1,133–3,385; p = 0,016) as independent predictors of AF recurrence.

**Conclusions:** In our cohort, AF recurrence was significantly higher in pts with LA enlargement, with coronary calcium score >100 and female gender pts.

111499

Modality: E-Poster Young Researcher – Non-case Report

Category: HEMODYNAMICS AND INTERVENTIONAL CARDIOLOGY

## The Impact of Pre-Existing Right Bundle Brunch Block on Short and Mid-Term Outcomes After Transcatheter Aortic Valve Implantation

MARIANA RIBEIRO SILVA^1^, Gualter Santos Silva^1^, Mariana Brandão^1^, Cláudio Espada Guerreiro^1^, Marco Oliveira^1^, Helena Gonçalves^1^, Gustavo Pires Morais^1^, Bruno Melica^1^, Lino Santos^1^, Alberto Rodrigues^1^, Pedro Braga^1^, Ricardo Fontes-Carvalho^1^

(1) Centro Hospitalar de Vila Nova de Gaia/Espinho

**Introduction:** Conduction disturbances after TAVI remains one of the most frequent complications of the procedure. Right bundle brunch block (RBBB) is the strongest risk factor, leading to an increased risk of permanent pacemaker (PM) implantation and is associated with an increased risk of early and late mortality following TAVI.

**Objectives:** To evaluate whether pre-existing RBBB is associated to higher risk of permanent PM implantation and short and mid-term all-cause mortality in patients (pts) undergoing TAVI.

**Methods:** Retrospective study of all pts submitted to TAVI between 2016 and 2018. ECG data before, immediately after the procedure, at day 3 post-TAVI and at discharge were collected, and continuous telemetry monitoring was recorded. We evaluated the rates of temporary and permanent PM implantation during hospital stay and at 1-year follow-up (FUP), ventricular pacing rates in the first visit after permanent PM implantation and all-cause mortality at 30-days, 3 and 6 months and 1-year after TAVI.

**Results:** 220 pts were included, 57,3% female, mean age of 80,7 ± 7,0 years; RBBB prior TAVI was present in 18 pts (8,2%). No significant differences were found regarding baseline characteristics between pts with or without RBBB prior TAVI procedure. Pts with RBBB presented higher baseline QRS duration compared with pts without RBBB (140,0 ± 16,9 ms vs 107,9 ± 26,6 ms; p = 0,002), without differences in QRS duration immediately or at day-3 after TAVI (p > 0,05). High-degree atrioventricular block and complete atrioventricular block immediately after the procedure were more frequent in pts with RBBB (44,4% vs 14,5%, p = 0,004). We found no significant differences in the rates of temporary PM in pts with or without RBBB. Pts with baseline RBBB presented significantly higher rates of permanent PM implantation during hospital stay (55,6% vs 20,0%; p = 0,002) – without differences regarding the timing of PM implantation – and higher rates of PM implantation at 1-year FUP (58,8% vs 21,4%; p = 0,002). The rates of ventricular pacing at the first visit after PM implantation in pts with RBBB was 75,0% (vs 47,2% in pts without RBBB; p = 0,139). No differences were found regarding 30-days, 3 and 6 months and 1-year FUP regarding all-cause mortality, between patients with and without RBBB prior TAVI.

**Conclusion:** Pre-existing RBBB significantly increased the risk of permanent PM implantation but was not associated with a higher risk of short- and mid-term all-cause mortality.

111509

Modality: E-Poster Young Researcher – Non-case Report

Category: ATHEROSCLEROSIS/CARDIOVASCULAR RISK FACTORS/CARDIOVASCULAR PREVENTION

## Biopsychosocial Profile of Female Smokers in the Context of Smoking Cessation Intervention

BEATRIZ STEPHAN FARHAT JORGE^1^, Pedro Drumond Maia^1^, Rodrigo Máximo Silveira^1^, Samuel Barud Massensine^1^, Rafael Matoso de Oliveira Figueiredo^1^, Amanda Gonçalves Vieira Martins^1^, Bianca de Fátima Pereira^1^, Fernanda Silva Mota^1^, Gabriela Godinho Rezende^1^, Moisés de Toledo Vilela^1^, Eliane Ferreira Carvalho Banhato^1^, Arise Garcia de Siqueira Galil^1^

(1) Cardiology Department, Medical School, Federal University of Juiz de Fora – UFJF

**Introduction:** Women exhibit peculiar characteristics that influence the smoking habit. The identification of the female profile is an important tool to be used in the approach to smoking, identifying triggers and factors that prevent cessation or favor relapse.

**Objectives:** To analyze biopsychosocial characteristics among women smokers treated in groups for smoking cessation.

**Methods:** Study with a mixed strategy, cross-sectional and longitudinal follow-up regarding smoking cessation, evaluating smokers of the “Livres do Tabaco” Project, intended for smoking cessation, between 09/2021 and 03/2022. Abdominal obesity was defined as abdominal circumference >80 cm in women and >94 cm in men; neck circumference was abnormal if >40 cm. Normal systolic blood pressure <130 mmHg. Depression, score Patient Health Questionnaire (PHQ-9) ≥9 points. Cognitive deficit, Montreal Cognitive Assessment <26 points.

**Results:** Thirty-six patients were evaluated, 66.7% of whom were women, 56.63 ± 10.80 years old. Comparing women to men, it was noted that women had statistically significant associations regarding sedentary lifestyle (p < 0.030), abnormal neck circumference (p < 0.002), abdominal obesity (p < 0.031), arterial hypertension (p < 0.007), arrhythmias (p < 0.022), cancer (p < 0.057), depression (p < 0.052) and greater cognitive deficit (p < 0.001). Regarding the use of other drugs, marijuana (p < 0.004) and crack (p < 0.002) had negative associations for females. Regarding smoking history, women had a higher frequency of triggers in terms of habits (p < 0.027), while dependence (p < 0.001) and stress (p < 0.001) were higher among men. In addition, they also presented higher nicotine dependence (p < 0.007). Regarding adherence to meetings, it was observed that the first (face-to-face) and the second (remote) were the most frequent among women (p < 0.052 and p < 0.012, respectively). It was women who exhibited the lowest prevalence of cessation, both in the 4th week (p < 0.052) and in the 12th week of treatment (p < 0.012).

**Conclusion:** The present study revealed that female smokers showed cardiovascular risk factors and tobacco-dependent comorbidities more frequently than men. In addition, high nicotine dependence and greater trigger related to habits were evidenced. Greater adherence to meetings was not enough to give women the highest rate of smoking cessation.

111519

Modality: E-Poster Young Researcher – Non-case Report

Category: CARDIOLOGY OF SPORTS, EXERCISE, ERGOMETRY AND CARDIOVASCULAR REHABILITATION

## Exercise Capacity Following Cardiac Rehabilitation Program After Tavi: What is the Role of Mitral Regurgitation?

MARIANA RIBEIRO SILVA^1^, Eduardo Vilela^1^, Ana Mosalina Manuel^1^, Ana Raquel Barbosa^1^, João Almeida^1^, Ana Tavares^1^, Daniel Caeiro^1^, Olga Sousa^1^, Alberto Rodrigues^1^, Pedro Braga^1^, Madalena Teixeira^1^, Ricardo Fontes-Carvalho^1^

(1) Centro Hospitalar de Vila Nova de Gaia/Espinho

**Introduction:** Severe aortic stenosis (AS) and mitral regurgitation (MR) often coexist. However, conflicting data reside concerning the impact of MR on outcomes after TAVI. Also, very few data exist regarding the benefits of a cardiac rehabilitation program (CRP) following TAVI in pts with MR.

**Objectives:** To evaluate the effect of a CRP in functional parameters after TAVI, particularly in pts with MR grade ≥ II.

**Methods:** Retrospective study which included all pts submitted to TAVI between 2014 and 2020 that completed a CRP following the procedure. Cardiopulmonary exercise tests (CPET) were performed after TAVI at baseline (pre-CRP) and post-CRP. We evaluated pre- and post-CRP peak oxygen consumption (pVO2), pVO2 at the anaerobic threshold (AT), respiratory exchange ratio (RER), VE/VCO2 and CPET duration. MR grading severity was assessed with transthoracic echocardiography performed after TAVI and was divided into 2 groups (grade < II vs grade ≥ II).

**Results:** 52 pts were included, 59,6% were male, mean age of 78,6 ± 8,6 years-old. Mean STS risk score was 4,9%. Twenty-seven (51,9%) pts had MR grade ≥ II. Baseline characteristics were similar between pts with MR grade < II vs MR grade ≥ II, with the exception of the prevalence of coronary artery disease which was higher in MR grade ≥ II (p = 0,036). Pts with MR grade < II had higher maximum and median aortic gradients before TAVI (p < 0,05 for all). The mean number of cardiac rehabilitation sessions was 21 ± 7, without differences between both groups. In pts with MR grade ≥ II, there was an improvement in CPET duration after CRP (HF protocol from 03:57 min to 05:02 min; p = 0,017 and modified Bruce protocol from 06:03 min to 06:41 min; p = 0,049) but without significant changes in pVO2 (14,7 mL/kg/min to 14,9 mL/kg/min; p = 0,990), RER or VEVCO2/VO2 ratio. Patients with MR grade < II significantly improved pVO2 (13,8 mL/kg/min to 14,7 mL/kg/min; p = 0,015), and CPET duration with HF protocol from 05:04 min to 06:23 min; p = 0,006 after CRP. There was also an improvement in VEVCO2/VO2 ratio, although not statically significant.

**Conclusions:** Patients with MR grade < II after TAVI who underwent a CRP significantly improved pVO2 and CPET duration. Although pts with MR grade ≥ II did not improved pVO2 after a CRP, an improvement in CPET duration may translate into a clinical benefit in these pts. These results highlight the importance of further research and personalization among this potentially higher risk subset of pts.

111521

Modality: E-Poster Young Researcher – Non-case Report

Category: DYSLIPIDEMIA

## Effects of Vitamin D Supplementation in Children with Childhood Obesity: A Systematic Review of Randomized Controlled Trials

DANNYELLY HYLNARA DE SOUSA CAVALCANTE MAIA^1^, Antônia Gabriela de Araújo^1^, Mariana Roberta Santos de Melo^1^, Maria Eduarda Oliveira Amorim^1^, Josianne Alves de Freitas Maia^1^

(1) Faculdade Nova Esperança de Mossoró (FACENE/RN)

**Introduction:** The worldwide prevalence of obesity is increasing, both in adults and children. When it occurs during childhood, there is an increased risk of developing cardiovascular disease (CVD) in adulthood. Thus, early interventions become necessary to reduce the risk of morbidity and mortality during adult life.

**Objectives:** This study aims to assess whether vitamin D supplementation is efficient to treat childhood obesity.

**Methods:** The present study is a qualitative systematic review, in which we used the prism method for making, we also emphasize that the guiding question was based on the pico strategy. Two databases were used: PubMed and VHL and the descriptors were: “vitamin D” and “Pediatric Obesity”, combined with the Boolean operator “AND”. We obtained as an initial result a total of 354 articles, however, after adding filters, we obtained a total of 49 articles, which went on to remove duplicates and read the title together with abstract in pairs, according to the inclusion and exclusion factors.; secondary and primary studies, with the exception of clinical trials, a study sample not compatible with the pediatric age group and studies in which the relationship between vitamin D dose and obesity was not detailed, resulted in a total of 09 articles. The selected articles went on to read the full texts which resulted in a total of 04 articles which had their data extracted.

**Results:** Most studies did not statistically perceive the reduction in BMI or change in body mass in the supplemented group. In all studies, patients in the supplemented group had an increase in the serum concentration of 25OHD. According to Mazzochi and colleagues, the association of DHA with vitamin D supplements could help improve vitamin D status, body composition and metabolic markers. Most articles also highlight that the dose used in studies may have been insufficient to reach concentrations in obese patients, which would act actively to reduce their lipid profile. However, Alves et al. highlight that supplementation with 1,000 IU/day for 90 days reduced serum TC, LDL-c and LDL-c/HDL-c ratio without altering body composition.


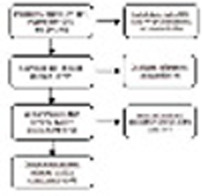


111531

Modality: E-Poster Young Researcher – Non-case Report

Category: HEMODYNAMICS AND INTERVENTIONAL CARDIOLOGY

## Inspiron Real Life Registry

RICARDO CZARNOBAI SOCCOL^1^, Denise Pellegrini^1^, Ricardo Lasevitch^1^, Natalia Lamas Bueno^1^, Carlos Alberto S Mattos^2^, Augusto Lima Filho^3^, Noriaki Takeshita^4^, Silvio Sergio Pontes^5^, Leandro Zacarias^6^, Marcelo Sabedotti^7^, Pablo Teixeirense Tomé^8^, Paulo Caramori^1^

(1) Hospital São Lucas da PUCRS; (2) Hospital da Cidade de Passo Fundo; (3) Santa Casa de Belo Horizonte; (4) Sociedade Hospitalar Angelina Caron; (5) Hospital São Marcos; (6) Santa Casa de Goiânia; (7) Hospital Geral de Caxias do Sul; (8) Instituto dos Fornecedores de Cana

**Aims:** The goal of this study was to evaluate the performance of a Sirolimus-eluting coronary stent, composed of a metallic chromium-cobalt platform with thin struts (75 µm), which releases sirolimus towards a bioresorbable polymer applied only to the abluminal surfaces, in a real-world scenario as a post-marketing clinical follow-up evaluation.

**Methods:** This is a prospective, multicenter, single arm registry of patients treated with the stent in 18 participant sites. All patients with lesions in native coronary arteries treated with the coronary stent were included. Exclusion criteria were target lesion located at a saphenous vein or arterial graft, and use of another stent than study stent during the index procedure. The primary endpoint was the rate of MACE (cardiac death, myocardial infarction and target lesion revascularization) at 12 months of clinical follow-up. Secondary endpoints were target lesion revascularization and stent thrombosis rates at 12 and 24 months.

**Results:** Between June 2017 and January 2022, 2506 patients were included (2991 lesions). The mean age was 63 ± 11 years and 64.2% were male. Clinical presentation was acute coronary syndrome in 53.2%, diabetes mellitus was present in 35.5%, previous myocardial infarction in 27.4% and previous percutaneous coronary intervention in 18.2% of patients. Most of procedures were successful (99.9%) with only one failure related to a vessel perforation followed by cardiac tamponade. To date, 1688 patients have completed the one-year follow-up. The incidence of MACE at 1 year was 3.5%. Cardiac death was 2.2% and non-fatal myocardial infarction was 1.3%. Target lesion revascularization was only 1.1%. Definitive stent thrombosis was 0.3%.

**Conclusion:** In this interim report, the 1-year MACE rate, as well as the individual components of this endpoint were excellent and consistent with previous results available for this stent and others 3rd generation drug eluting stent. The result of this study demonstrates the safety and efficacy of this stent in daily clinical practice.

111542

Modality: E-Poster Young Researcher – Non-case Report

Category: CARDIORESPIRATORY PHYSIOLOGY/BASIC SCIENCE

## Cardiovascular Repercussions Associated with Changes in the Respiratory Pattern: A Systematic Review of the Literature and Meta-Analysis

OLÍVIA FAGUNDES BRUNO^1^, RODOLFO PASSOS ALMEIDA^1^, JULIA DA COSTA ANCIÃES^2^, VICTOR EMANUEL FAGUNDES BRUNO^3^, JOSÉ JARJURA JORGE JUNIOR^1^

(1) PONTIFÍCIA UNIVERSIDADE CATÓLICA DE SÃO PAULO; (2) UNIVERSIDADE FEDERAL DE ALFENAS; (3) PONTIFÍCIA UNIVERSIDADE CATÓLICA DE MINAS

**Objectives:** To evaluate the repercussions of the respiratory pattern of Obstructive Sleep Apnea Syndrome (OSAS) in relation to the occurrence and prevalence of cardiovascular pathologies.

**Methodology:** Systematic review of articles in the databases: Scielo, PubMed, Lilac‘s, NHR and Cochrane. The following search terms are used: Cardiovascular Disease, Apnea and OSAS, selected without restrictions for the language of the articles and for the gender of the observed population, excluding works from the population under the age of 18 years. The specified outcomes were: hypertension, resistant hypertension, Incident coronary heart disease, heart failure in men, cardiovascular death in mild or moderate apnea, cardiovascular death in severe apnea.

**Results:** A joint analysis of stratified disease data revealed 95% confidence, a positive and significant relationship between apnea and cardiovascular diseases, with the treatment of these diseases being a variation that reduces this correlation.

111549

Modality: E-Poster Young Researcher – Non-case Report

Category: HYPERTENSION/RENAL DENERVATION

## Is Office Blood Pressure Underestimating Reality in Pediatric Obese Population?

NATÁLIA ALBERTIN DOS SANTOS^1^, Luiza do Nascimento Ghizoni Pereira^1^, Natália Albertin dos Santos^1^, Simone Rolim Fernandes Fontes Pedra^1^, Carlos Augusto Cardoso Pedra^1^, Oswaldo Passarelli Junior^1^, Márcio Gonçalves de Sousa^1^, Fernanda Marciano^1^, Antonio Gabriele Laurinavicius^1^

(1) Instituto Dante Pazzanese de Cardiologia

**Introduction:** Hypertension (HTN) diagnosis depends on the accuracy and representativeness of blood pressure (BP) obtained by different methods. In pediatric obese patients, office BP can present great variability and does not detect nighttime changes. Ambulatory Blood Pressure Monitoring (ABPM) allows recognition of HTN phenotypes and predicts HTN severity in an earlier and assertive way.

**Objectives:** To compare HTN stages defined by office BP with ABPM, and describe prevalence of masked HTM in pediatric sample from a reference service.

**Methods:** Retrospective cohort of pediatric patients with primary HTN. Patients underwent a detailed clinical history and examination. BP was measured by auscultatory method with technique adequacy. BP was checked at 2 other visits (2 week interval) and the mean BP of these 3 visits was used to classify BP according recommendations. ABPM was performed with pediatric validated device, with a revised report for this analysis, according to guidelines. Mean 24-hours awake and sleep BP and load were considered to identify HTN phenotypes according to 95th percentile for sex, age and height. Diagnosis and stages of HTN based on office BP were compared with ABPM. Patients with sustained HTN had secondary causes discharged after investigation and target organ damage (TOD) was also evaluated.

**Results:** Were included 16 patients with primary HTN, mean age 13 ± 3.3 years, 62% male, 87% obese or overweight (mean weight 89 ± 28.9 kg) and 75% with first degree family history of HTN. Of these, as in Figure 1, masked hypertension was detected in 37.5% (6/16), white coat HTN in 12.5% (2/16), and in 68% of the sample (11/16) ABPM classified HTN at higher stage compared to office BP. Nocturnal HTN was present in 81% (13/16). None patient had TOD and at follow-up, 12 required antihypertensive drugs, with 68% of BP control.

**Conclusion:** For this obese primary hypertensive pediatric sample, ABPM seems to be essential for HTN diagnosis and stratification, evidencing frequent nocturnal changes in BP. Complementary tests to investigate obstructive sleep apnea weren’t done but this could be an associated factor.


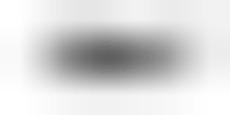


111552

Modality: E-Poster Young Researcher – Non-case Report

Category: PSYCHOLOGY

## Repercussion of Depression on Smoking Cessation Among Smokers with Multimorbidities

PEDRO DRUMOND MAIA^1^, Beatriz Stephan Farhat Jorge^1^, Bianca de Fátima Pereira^1^, Ramon José Moreira Silva^1^, Fernanda Silva Mota^1^, Gabriela Godinho Rezende^1^, Moisés de Toledo Vilela^1^, Paula Gouveia Abrantes^1^, Rafael Matoso de Oliveira Figueiredo^1^, Maria Fernanda Monteiro Lamas^1^, Eliane Ferreira Carvalho Banhato^1^, Arise Garcia de Siqueira Galil^1^

(1) Cardiology Department, Medical School, Federal University of Juiz de Fora, Juiz de Fora – Minas Gerais.

**Introduction:** Smoking is a serious public health problem. Among the several factors that hinder smoking cessation and the maintenance of abstinence, there is the high rate of psychiatric comorbidities, such as depression. Mood disorders are important factors to be considered in the evaluation of smokers, aiming at a better approach to the habit and a greater reduction in it.

**Objectives:** To evaluate the impact of depression on biopsychosocial characteristics among smokers with multimorbidities in the smoking cessation process.

**Methods:** Observational cross-sectional study evaluating medical charts of a public service for smoking cessation (“Livres do Tabaco” Project), between 09/2021 and 03/2022. Depression defined by the score Patient Health Questionnaire (PHQ-9) ≥9 points. Cognitive deficit evaluated by the Montreal Cognitive Assessment <26 points. Abdominal obesity was defined as abdominal circumference >80 cm in women and >94 cm in men. Normal systolic blood pressure (SBP) <130 mmHg.

**Results:** Thirty-six patients were evaluated, aged 56.63 ± 10.80 years, and depression was present in 55.9%. Comparing smokers with and without depressive symptoms, it was observed that those with depression had statistically significant associations regarding obesity (p = 0.007); arterial hypertension (p = 0.035) and uncontrolled blood pressure (p < 0.035); greater cognitive deficit (p = 0.022); COVID-19 infection (p < 0.003). In addition, the occurrence of cardiovascular events also had a positive association in these patients: acute myocardial infarction (p = 0.002); heart failure (p < 0.001); peripheral vascular accident (p < 001); greater hospitalization secondary to COVID-19 (p < 0.001). Regarding smoking history, those with depression had a higher frequency of triggers for stress (p < 0001). Regarding motivation, depressed patients had lower rates (p < 0.056), as well as adherence to meetings and smoking cessation in the 12th week of treatment (p < 0.022 and p < 0.001, respectively).

**Conclusion:** Smokers with depression showed significantly more frequent cardiovascular outcomes. Low motivation reflected in lower adherence to cognitive-behavioral approach meetings and smoking cessation. The association between smoking and depression reveals the need for a multidisciplinary approach to the habit to develop more effective interventions, and to remember that depression is also a chronic disease that should be tracked and treated simultaneously to the process for the success of smoking cessation.

111562

Modality: E-Poster Young Researcher – Non-case Report

Category: PERIOPERATIVE EVALUATION

## Perioperative Profile of Cardiac Biomarkers in Renal Transplant Recipients

RODRIGO PINHEIRO AMANTÉA^1^, Virgílio Olsen^2^, Laura Hastenteufel^1^, Julia Bueno^1^, Santiago Tobar^1^, Lívia Goldraich^1^, Roberto C. Manfro^1^, Flavia K. Borges^3^, Nadine Clausell^1^

(1) Hospital de Clínicas de Porto Alegre (HCPA); (2) Santa Casa de Misericórdia de Porto Alegre; (3) Faculty of Health Sciences, McMaster University

**Introduction:** Cardiovascular risk assessment is important among renal transplant recipients. Biomarkers, such as cardiac troponin and BNP, have been used as adjunctive tools in cardiovascular risk stratification in non cardiac surgeries, but their use in the context of kidney transplantation is not well studied.

**Objectives:** To describe the perioperative profile of cardiac biomarkers in kidney transplant recipients and its association with delayed graft function (DGF).

**Methods:** Prospective cohort study including deceased donor kidney transplant recipients between September 2018 and March 2020 at Hospital de Clínicas de Porto Alegre. We evaluated BNP at hospital admission and 24 hours after kidney transplantation. High-sensitivity cardiac troponin (hs-cTn) was evaluated at admission, 24 and 48 hours after kidney transplantation. Only patients who presented with no major adverse cardiovascular events (MACE) during the first month of follow-up were included in the analysis.

**Results:** We prospectively included 102 patients, of which 52.9% were male, 82.4% were hypertensive and 19.6% had diabetes mellitus. Admission’s BNP median value was 225 pg/ml (IQR 98.6–626 pg/ml), and 24 hours after transplantation was 288 pg/ml (IQR 180–597 pg/ml). Admission’s Hs-cTn above the 99th percentile was observed in 35.3% of patients, and was associated with age at transplantation (OR 1.099/CI 1.043–1.173) and male sex (OR 4.667/CI 1.644–14.838). Hs-cTn’s median change from admission to 24 hours after transplantation was –13.5% (IQR –32–9%) and from 24 hours to 48 hours after transplantation was –1.2% (IQR –17–5%). After transplantation, 52% of the patients presented with DGF, which was associated with concomitant preoperative cardiac troponin above the 99th percentile and BNP above 100 pg/ml (OR 3.989/CI 1.217–15.765).

**Conclusions:** Baseline cardiac troponin and BNP are elevated in a large proportion of kidney transplant recipients. While BNP has an ascending trend after renal transplantation, troponin tends to decrease following surgery. Both biomarkers might be predictive of non-cardiac endpoints such as DGF.


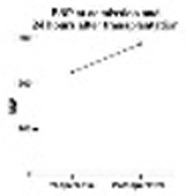


111640

Modality: E-Poster Young Researcher – Non-case Report

Category: CONGENITAL AND PEDIATRIC CARDIOLOGY

## Saving Hearts Saving Brains, a Protocol for Parental Guidance and Early Intervention in Infants with Congenital Heart Disease: A Randomized Clinical Trial

MARIANE LOPES DA SILVA^1^, Débora Gomes da Rocha^2^, Nathalia Bottega Banaletti^2^, Fernanda Lucchese-Lobato^2^

(1) institute of Cardiology, University Foundation of Porto Alegre; (2) Hospital da Criança Santo Antônio, Irmandade Santa Casa de Misericórdia

Children with congenital heart disease (CHD) are exposed to multiple sources of risk for delayed neurodevelopment. Early intervention (EI) studies show positive results in preventing delays. To evaluate the effectiveness of a home-based parent-mediated EI protocol, on the neurodevelopment of children with CHD, in a 6–9-month cohort.

**Methods:** A single-blind randomized controlled trial of a parent-mediated early intervention for infants with CHD. The 6-week cost-effective protocol of activities for early home stimulation was performed by the parents with the support of a specialist. The protocol was remotely guided by a combination of booklet/toy kit/videos, and WhatsApp check-ins. The Bayley-III was used to assess developmental outcomes.

**Results:** Twenty dyads participated in the final evaluation, 10 in the intervention group (IG), and 10 in the control group (CG), with a mean age in months of 6 ± 2, in both groups. Both had greater female participation (58%). The IG performed better than the CG, at T2, in four of the five developmental domains: cognitive (p < 0.05), receptive language (p < 0.001), expressive language (p < 0.05), and fine motor (p < 0.05). The gross motor subscale was not significantly different between IG and CG at T2 (p = 0.58). The CG group did not differ in scores at T1 and T2 in all domains: Cog, Rec lang, exp lang, fine motor, and gross motor (p = 0.9, p = 0.7, p = 0.3, p = 0.3, p = 0.9, p = 0.6, respectively). The IG significantly differed between T1 and T2 scores for four domains: Cognitive, Rec. Lang., Exp. lang. (p < 0.001), and fine motor (p < 0.05). Gross motor performance in the IG did not differ significantly between T1 and T2 scores (p = 0.1).

**Conclusion:** The intervention was effective in improving the developmental scores of the IG in four out of five areas of development at T2. This study showed that a low-cost remote parent-mediated program can be successful in reducing the risks of significant delays in child development associated with CHD, which can also promote long-term academic and professional success in this population. Trial registry: Protocol registered at register.clinicaltrials.gov: NCT04152330.


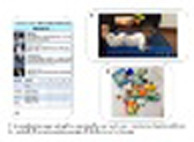


111602

Modality: E-Poster Young Researcher – Non-case Report

Category: ATHEROSCLEROSIS/CARDIOVASCULAR RISK FACTORS/CARDIOVASCULAR PREVENTION

## Effect of Repeatedly Applied Cold-Water Immersion on Subclinical Atherosclerosis, Inflammation, Fat Accumulation, and Lipid Profile Parameters of Volunteers

STEFAN TOTH^1^

(1) SLOVACRIN, Slovak Clinical Research Infrastructure Network, Pavol Jozef Šafárik University, Trieda SNP 1, 040 11 Kosice, Slovakia

**Introduction:** Short-time cold-water immersion (CWI) is associated with significant acute cardiovascular, metabolic, and endocrinological responses. There is, however, no available study following the long-term effect of repeated CWI on atherogenesis, lipid parameters, and fat distribution. This study aimed to explore the suggested protective effect.

**Investigations and methods:** 35 healthy volunteers were followed during the 5-month CWI exposition under standard conditions (three times per week 7–10 min). Neoprene equipment was not allowed; volunteers with followed weight or muscle mass changes over 5% were excluded. Equivalent sham control (N = 30) was included. In the beginning and at the end of the study, blood collection and clinical examinations were made. Lipid and non-lipid parameters, including PCSK9 and hsCRP levels, were quantified. Vascular changes were detected by carotid ultrasound (cIMT) and by echo-tracking for detection of arterial stiffness parameters (PWV; AI; Beta). Liver steatosis quantification was based on the calculation of hepatorenal index (HRI), fat distribution was measured by the quantification of subcutaneous and visceral fat thickness changes.

**Results:** 28 volunteers have completed the given protocol. Significant decrease of cIMT (p = 0.0001); AI (p = 0.0002); Beta (p = 0.0001) and PWV (p = 0.0001) was detected after the long-term repeated CWI. In comparison with the entry values, a significant decrease of hsCRP (p = 0.01) and PCSK9 (p = 0.01) was observed. Liver fat accumulation decreased by an average of 11% in comparison with the entry values (HRI p < 0.001). A significant decrease in LDL; TC; TG and VLDL was followed as well.

**Conclusion:** We suggest a possible beneficial effect of repeated-CWI on atherogenesis, liver fat accumulation, lipid, and non-lipid parameters.

111636

Modality: E-Poster Young Researcher – Non-case Report

Category: CARDIO-ONCOLOGY

## Treatment of Malignant Neoplasms of the Heart, Mediastinum and Pleura in Brazil between 2013–2020

ANA FLÁVIA OLIVEIRA DE SOUZA^1^, Afonso Moraes Melo Júnior^2^, Davi Gabriel Barbosa^3^, Daniel Oliveira da Costa^4^, João Lucas Watrin Braga^5^, Juliana Ayumi Azevedo Kurosawa^6^, Lucas Guimarães Junqueira^7^, Paola Bitar de Mesquita Abinader^8^, Rafaela Oliveira Cardoso^9^, Rafael Silva Lemos^10^, Wanda Maria de França Pires^11^, Luis Eduardo Werneck de Carvalho^12^

(1) Centro Universitário do Estado do Pará (CESUPA); (2) Centro Universitário do Estado do Pará (CESUPA); (3) Universidade Estadual do Pará (UEPA); (4) Universidade Estadual do Pará (UEPA); (5) Centro Universitário do Estado do Pará (CESUPA); (6) Centro Universitário do Estado do Pará (CESUPA); (7) Universidade Estadual do Pará (UEPA); (8) Centro Universitário do Estado do Pará (CESUPA); (9) Centro Universitário do Estado do Pará (CESUPA); (10) Universidade Estadual do Pará (UEPA); (11) Universidade Federal do Pará (UFPA); (12) Oncológica do Brasil

**Introduction:** Intracardiac tumours can be divided into intramural thrombus, primary cardiac neoplasms and metastatic tumours. The management of these pathologies is generally determined by obtaining the tissue pathology, the type of tumour and its degree of involvement. Among the types of treatment, chemotherapy is the main strategy, as well as radiotherapy, immunotherapy and surgery. These neoplasms, when untreated, present a poor prognosis, which highlights the need for epidemiological studies that highlight the therapeutic modalities available today.

**Objectives:** To establish an overview of the treatment of malignant neoplasms of the heart, mediastinum and pleura in Brazil, between 2013 and 2020.

**Methodology:** This is a descriptive, quantitative and retrospective study, whose data came from the DATASUS database. The data were analysed according to the division by federative units and the variables used were the therapeutic modality, cancer staging, year of treatment, age group, gender and total number of cases per year in the selected period.

**Results:** When evaluating the treatment of these neoplasms in Brazil in the period 2013 to 2020, it was observed the prevalence of treatment lasting up to 30 days (82.2%), with the main therapeutic modality being surgery (66.4%), followed by chemotherapy (20.2%), and radiotherapy (13.2%). Among the cases analysed for treatment, staging 3 prevailed (32.7%), followed by 4 (31.4%), and the year 2019 (29.5%) was the one in which the beginning of treatment was recorded the most. Regarding the age range, the ages 55 to 59 years represented 11.3% of cases, followed by 60 to 64 years with 10.9% and the predominance in the male gender (50.9%).

**Conclusion:** It is observed that primary malignant neoplasms of the heart have a poor prognosis and are submitted to palliative treatment, while in metastatic heart tumours the therapy depends on the tumour of origin. Moreover, there is a slight predominance among males and between the ages of 55 and 64 years. Moreover, the TNM stages 3 and 4 were the most prevalent, as well as surgical and chemotherapeutic therapy. Therefore, it is evident the need for continuous scientific and technological advances employed both in early diagnosis and in therapeutic modalities in order to provide a positive prognosis for these patients.

111635

Modality: E-Poster Young Researcher – Non-case Report

Category: ACUTE AND CHRONIC CORONARY DISEASE/THROMBOLYSIS

## Diastolic Dysfunction and Myocardial Ischemia in Chronic Coronary Syndrome

EDVALDO VICTOR GOIS OLIVEIRA^1^, Enaldo Vieira de Melo^1^, Cláudia Bispo Martins Santos^1^, Lara Teles Alencar Duarte^1^, Antônio Carlos Sobral Sousa^1^, Eduardo José Pereira Ferreira^1^, Allexa Gabriele Teixeira Feitosa^1^, Cleovaldo Ribeiro Ferreira Júnior^1^, Irlaneide da Silva Tavares^1^, José Icaro Nunes Cruz^1^, Gabriela de Oliveira Salazar^1^, Joselina Luzia Menezes Oliveira^1^

(1) Universidade Federal de Sergipe

**Introduction:** Left ventricular (LV) diastolic dysfunction is one of the important factors for survival and long-term prognosis in patients with Chronic Coronary Syndrome (CCS). However, the connection between diastolic dysfunction during exertion, Chronic Coronary Syndrome, Myocardial Ischemia (MI) and the profile of increased cardiovascular risk has not been demonstrated over the years. Therefore, there is a need to predict the factors that can aggravate this connection, in order to enable the creation of secondary prevention strategies and decrease the occurrence of unfavorable outcomes.

**Objectives:** To evaluate LV diastolic function and factors associated with the presence of Myocardial Ischemia (MI) in patients with Chronic Coronary Syndrome (CCS).

**Methodology:** A cross-sectional study between January 2000 and January 2022 with individuals with CCS who underwent Physical Stress Echocardiography (FES) in a cardiology referral service. A total of 2000 patients (62 ± 19 years) were categorized according to the presence or absence of MI. Statistical analysis included the chi-square test and t ’s Student using SPSS Statistics version 22.0.

**Results:** We found 937 (46.9%) patients without MI and 1059 (53.0%) with MI – of the latter, 22.3%, 60.7% and 17.1% had induced ischemia, fixed ischemia and ischemia fixed and induced, respectively. There was no association between MI and diabetes mellitus, obesity, physical inactivity (p > 0.05). Myocardial ischemia was associated with: hypertension (77.9% vs. 69.8%), dyslipidemia (77.7% vs. 67.9%), positive family history of coronary heart disease (74.1% vs. 63.8%) and previous myocardial infarction (51.8% vs. 22.3%) – p < 0.001. MI was associated with diastolic dysfunction (p < 0.001): most patients with normal diastolic function belonged to the group without MI (17.6% vs. 6.5%, p < 0.00001), the impaired relaxation was not significant between groups (p = 0.4839), pseudonormal function was more prevalent among patients with MI (28.9% vs. 20.9%; p = 0.0002), as was the pattern restrictive (1.8% vs. 0.1%; p = 0.0007). Left ventricular ejection fraction did not differ between the groups with and without MI (p > 0.05).

**Conclusion:** Arterial hypertension, dyslipidemia, family history and previous myocardial infarction have been shown to be predictors of myocardial ischemia. On the other hand, LV diastolic dysfunction was more prevalent in patients with myocardial ischemia.

112114

Modality: E-Poster Young Researcher – Non-case Report

Category: ATHEROSCLEROSIS/CARDIOVASCULAR RISK FACTORS/CARDIOVASCULAR PREVENTION

## Retrospective Cohort Study Comparing the Incidence of Thromboembolic Events in the Pre-Pandemic and Pandemic Period in a Quaternary Hospital in Rio De Janeiro

ANA CAROLINA PEDROSO ^1^, Andreá Ferreira Haddad^1^, Lara Camporez Menezes Trindade^1^, Antonio Vitor Martins Amaral^1^, Priscila Oliveira Diaz^1^, Bernardo Cleto Teles e Silva^1^, Joao Felipe Tamiozzo Reis^1^, Thaisa Rodrigues Garcia^1^, Alessandra godomiczer^1^, Flávia de Moraes Pedro Moisés^1^, Monica Amorim de Oliveira^1^, Luiz Fernando Simvoulidis^1^

(1) Unimed Rio Hospital

Venous thromboembolism (VTE) is the most common preventable cause of death in hospitalized patients. Hospitalization increases the risk of VTE by 8x, generating important clinical consequences such as deep vein thrombosis (DVT) and pulmonary embolism (PE) that can leave severe sequelae and even being the main cause of death that can be prevented in hospitalized patients. Covid19 is associated with a concomitant prothrombotic state, increasing risk of VTE This is a retrospective cohort that evaluated clinical and surgical patients admitted for at least 48 hours at a private quaternary hospital, where we compared data on thromboembolic events in the pre-pandemic period, from January 1, 2018 to November 30, 2019 with patients hospitalized in the period pandemic from January 1, 2020 to March 14, 2021. All patients were evaluated according to risk stratification for VTE by the physician and prophylaxis was instituted according to the risk found, classified as low, moderate and high risk. Patients with COVID19 were classified as high risk. Patients admitted with DVT and/or PE, arterial thrombosis events and those in palliative care were excluded from the analysis. The events of DVT (distal and proximal), device thrombosis (PICC and AVP) and PE were recorded and all results were confirmed with imaging exams through the EcoDoppler study.

**Results:** In the historical cohort Jan 18 to Nov 19, 114 VTE events were reported in a universe of 81,445 patients, corresponding to 0.14% of hospitalizations in this period. Of these, DVT corresponded to 81.5%, PE 15% and 3.5% central venous catheter thrombosis. There were 23 deaths during hospitalization, corresponding to 23.96% of patients who presented VTE during this period. These events account for 0.03% of in-hospital mortality. In the period from January 1, 2020 to March 14, 2021, 126 events occurred among hospitalized patient ina a universe de 15,000 patients. Of these, 120 (95.2%) were due to DVT and 06 (4.8%) to PE, causing 48 deaths, which corresponds to 38.1% of patients who presented VTE within the in-hospital mortality. VTE is a frequent condition in hospitalized patients and is related to longer hospital stay and in-hospital mortality. It is the most common preventable cause of death in hospitalized patients. We have to consider that SARS-COV2, COVID-19 disease, has been associated with a concomitant prothrombotic state and an increased risk for VTE frequency, therefore, an increase in mortality.

111653

Modality: E-Poster Young Researcher – Non-case Report

Category: CARDIAC ARRHYTHMIAS/ELECTROPHYSIOLOGY/ELECTROCARDIOGRAPHY

## Robotically Navigated Transseptal Puncture System for Left Atrial Catheter Ablation: Pilot Real-Time Virtual Simulation Data

AYA MUTAZ ZEIDAN^1^, Aya Mutaz Zeidan^1^, Yangyang Dai^1^, Zhouyang Xu^1^, Jonathan Behar^1^, Steven Williams^1^, Richard Housden^1^, Aruna Arujuna^1^, Kawal Rhode^1^

(1) King’s College London KCL

Robotically assisted catheter ablation procedures have been shown to be safe, effective and confer both better catheter stability and tissue contact. Despite promising results from robotic cardiac CA systems, all systems require manual navigation for the transseptal puncture access, thus motivating the need to develop a fully robotic TP system. This would potentially improve patient safety, further reduce radiation dose to cardiologists, and pave the way to more complete robotic solutions for CA. The research aim is to present a preliminary design and prototype for a robotic TP system, and demonstrate real-time operation via simulation. By employing computer-aided design (Fusion360, Autodesk, USA) and additive manufacturing methods (Ender 3, Creality, China), a first prototype for a robotic TP system has been designed to achieve the cooperation principle of the transseptal needle and dilator sheath. The prototype implements four degrees-of-freedom to accomplish translation and rotation of the needle and sheath, individually. Safety features include speed and position constraints. The open-source Robot Operating System (ROS 2, Open Source Robotics Foundation, USA) was used for the control architecture. Additionally, ros2_control was used to employ Forward Command Controller for efficient and reliable actuator control. We demonstrated constant positional relationship between the virtual dilator-sheath and the BRK-1 needle, achieved within the ROS 2 graphical interface. In coupling the needle and sheath, the realised system was able to successfully drive to a maximum length of 144.5 mm, with an positioning accuracy of 0.10 mm. The deterministic performance of the system and its real-time functionality exemplified the benefits of leveraging ROS 2. Movement commands were executed within the order of a few microseconds, providing real-time, low-latency operation. This research confirms proof-of-concept for a first prototype real-time control of a Robotic TP system implemented based on the ROS 2 framework. Our next step is to develop a joystick interface and evaluate the system by using an anthropomorphic cardiac phantom.


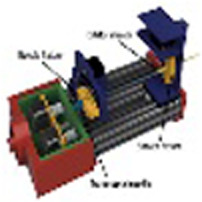


111655

Modality: E-Poster Young Researcher – Non-case Report

Category: CARDIOLOGY OF SPORTS, EXERCISE, ERGOMETRY AND CARDIOVASCULAR REHABILITATION

## Functional Cardiorenal Benefits Induced by Concurrent Exercise Training and Hydrochlorothiazide Treatment in an Experimental Model of Hypertension and Menopause

PIETRA PETRICA NEVES^1^, Maycon Junior Ferreira^2^, Tânia Plens Shecaira^2^, Michel Pablo dos Santos Ferreira Silva^1^, Débora Conte Kimura^2^, Maria Claudia Irigoyen^3^, Guiomar Nascimento Gomes^2^, Kátia De Angelis^1^

(1) Nove de Julho University (UNINOVE), São Paulo, Brazil; (2) Federal University of São Paulo (UNIFESP), São Paulo, Brazil; (3) Heart Institute (InCor), University of São Paulo (USP), São Paulo, Brazil

Cardiovascular diseases are currently the main causes of mortality and morbidity worldwide. Systemic arterial hypertension(SAH)is one of the main risk factors for cardiovascular outcome. Pharmacotherapy associated with exercise training is clinically recommended for the control of blood pressure(BP)to manage SAH. To investigate the effects of concurrent exercise training(CET)and pharmacological treatment with hydrochlorothiazide on hemodynamic and renal parameters in an experimental model of SAH. Female spontaneously hypertensive rats(SHR) were allocated into 4 groups:sedentary ovariectomized(OS, n = 8), trained ovariectomized (OT, n = 8), sedentary ovariectomized treated with hydrochlorothiazide(OSH, n = 8) and trained ovariectomized treated with hydrochlorothiazide(OTH, n = 8). Ovariectomy was performed on the first day of the study. Hydrochlorothiazide(30 mg/kg) was dissolved in the drinking water. The OT and OTH groups were submitted to CET with intensity of 40–60% of maximum capacity,3 days per week, during 8 weeks. In the seventh week, all rats were kept in metabolic cages during 24 hours for urine collection. In the sequence, the rats underwent cannulation of the carotid artery for direct BP recording. Both trained groups showed an increase in their performances when compared to their initial tests, and to the sedentary groups at the end of the protocol(p < 0.0001). Furthermore, OTH group showed an additional increasing when compared to the OT group at the end of the protocol in the maximal load test (p < 0.0001). The OSH, OT and OTH groups presented lower systolic(OSH:189.0 ± 13; OT:179.3 ± 5; OTH:174.1 ± 15), diastolic(OSH:138.9 ± 7; OT:131.3 ± 11; OTH:127.6.1 ± 14) and mean BP(mmHg) (OSH:163.0 ± 9; OT:154.3 ± 13; OTH:149.8 ± 14) when compared to the OS group (SBP:207.6 ± 15; DBP:155.4 ± 18; MBP:180.2 ± 8). The OT group(332 ± 20) presented resting bradycardia (bpm) compared to the sedentary groups(OS:380 ± 25; OSH:374 ± 28). The OTH group showed reduced plasma creatinine levels in relation to the OS group(p < 0.0001). In addition, hydrochlorothiazide combined or not with CET induced higher clearance of creatinine when compared to the OS group(p = 0.0029). Plasma urea levels were similar between groups. The results suggest that the association of CET and pharmacological treatment can promote additional cardiorenal benefits in post menopaused rats, suggesting an important role of the combination of these therapies, probably impacting on quality of life and SAH-related risk factors and morbimortality.

111664

Modality: E-Poster Young Researcher – Non-case Report

Category: EPIDEMIOLOGY AND HEALTH POLICIES/GLOBAL HEALTH

## Distribution of Death from Acute Myocardial Infarction in Brazil

MATHEUS SOUZA DA SILVA^1^, Jéssica Haline de Souza dos Reis^1^, Thalles Franklin Souza Santos^1^, Daniel Ferreira Cunha^1^, Lucca Dal Moro^1^, Carlos Lutian da Silva Andrade^1^, Bruno Artur de Almeida Santos^1^, Lucas Gama Pacheco^1^, Kamylla Batista Brito^1^, Raul Antônio Lopes Silva Campos^1^, João Marcos Rodrigues Silva^1^, Luciano Moura de Assunção^2^

(1) Universidade Federal do Pará; (2) Fundação Santa Casa de Misericórdia do Pará

**Introduction:** Pre-hospital care aims to reduce the time between the onset of an ischemic event in the myocardium and effective treatment. It is at this point that mortality in the first few hours can be reduced (1). Therefore, a good quality of care and adequate management of cases of Acute Myocardial Infarction (AMI) must have a good infrastructure, which is closely related to the number of deaths. Knowing the complexity and differences between the different care contexts by regions, the present study compares the number of deaths from AMI and the number of hospitalizations by regions in Brazil.

**Objectives:** To establish a quantitative analysis on the ratio of the number of deaths to the number of hospitalizations due to AMI and to compare the results by regions of Brazil.

**Methods:** The study is a cross-sectional, retrospective, descriptive and quantitative analysis carried out based on data obtained from the Department of Informatics of the Unified Health System (DATASUS) for Brazil, with a temporal analysis between the period 2015 and 2019.

**Results:** Between 2015 and 2019, there were a total number of 571,473 hospitalizations for AMI in Brazil; being 24,158 in the North Region; 111,422 in the Northeast Region; 283,438 in the Southeast Region; 113,733 in the South Region and 38,722 in the Midwest Region. Of which, 61,499 died in Brazil; 2,892 in the North Region; 13,476 in the Northeast Region; 29,699 in the Southeast Region; 11,392 in the South Region and 4,040 in the Midwest Region. Of the total number of hospitalized patients by region, 10.76% died in Brazil, 11.9% in the North, 12% in the Northeast, 10.47% in the Southeast, 8.79% in the South and 10.43% in the Midwest Region. Thus, there were 9.29 hospitalized for each death in Brazil; 8.3 hospitalized for each death in the North Region; 8.2 hospitalized for each death in the Northeast Region; 9.5 hospitalized for each death in the Southeast Region; 9.98 hospitalized for each death in the South Region and 9.58 hospitalized for each death in the Midwest Region.

**Conclusions:** In view of the results, it can be concluded that the North and Northeast regions have a higher ratio of deaths due to hospitalization due to AMI and do not follow the national average for the same statistics. As a limitation of the study, data referring to out-of-hospital deaths from AMI are highlighted. 1.Myerburg RJ, Castellanos A. Cardiac arrest and sudden death. In: Braunwald E. Textbook of cardiovascular medicine. Philadelphia.

111679

Modality: E-Poster Young Researcher – Non-case Report

Category: ATHEROSCLEROSIS/CARDIOVASCULAR RISK FACTORS/CARDIOVASCULAR PREVENTION

## Cognitive Profile Among Smokers with Chronic Conditions in the Smoking Cessation Process

MARIA FERNANDA MONTEIRO LAMAS^1^, Beatriz Stephan Faraht Jorge^1^, Paula Gouvea Abrantes^1^, Pedro Drumond Maia^1^, Ramon José Moreira Silva^1^, Rodrigo Máximo Silveira^1^, Samuel Barud Massensine^1^, Rafael Matoso de Oliveira Figueiredo^1^, Amanda Gonçalves Vieira Martins^1^, Bianca de Fátima Pereira^1^, Arise Garcia de Siqueira Galil^1^, Eliane Ferreira Carvalho Banhato^2^

(1) Universidade Federal de Juiz de Fora – UFJF; (2) Centro Universitário UniAcademia

**Introduction:** Tobacco is responsible for 4 milion adults death per year. The tobacco problem seems to be concerned with changing the cognitive system as well as the use of cognitive function Impairments skills are designed by psychometrically valid brief screening instruments. Montreal Cognitive Assessment (MoCA), Clock Drawing Test (CDT) and Verbal Fluency (VF) are validated tests that provide information particularly on cognition such as proven activities, planning, decision making and impulse control.

**Goal:** Identify the cognitive profile of smokers participating in a smoking cessation program, using neuropsychological screening tests.

**Methods:** Longitudinal study, “Livres do Tabaco” group, Cardiology Service, Federal University of Juiz de Fora, Juiz de Fora/Minas Gerais. Users with multimorbidities were treated between September/2021 and March/2022, in consecutive treatment groups, mixed intervention (in person and telemedicine), multidisciplinary team, cognitive behavioral approach sessions, drug treatment and follow-up. Definitions: Cognitive deficit, Montreal Cognitive Assessment <26 points (CDT and VF, inserted in MoCA) was used. Low level of education <8 years.

**Results:** Sample of 36 smokers, equivalent to 6 consecutive treatment groups. Women, 66.7%; 8 years or more of schooling, 57.1%; 65.7% were not in a stable relationship. Age, 56.64 ± 10.61 years. Number of cigarettes smoked daily, 22.77 ± 14.64 cigarettes; high nicotine dependence, 68.6%. As for the cognitive profile, there was low performance on the MoCA (79.4%). In the VF, 55.9%; in the CDT, 19.4%. Age was correlated negatively with MoCa (r = –0.508, p = 0.002) and positively with years of addiction (r = 0.791, p = 0.000) and pack years (r = 0.426, p = 0.011). The CDT was negatively correlated with age (r = –0.479, p = 0.008) and years of addiction (r = –0.602, p = 0.000). Verbal Fluency was correlated negatively with years of addiction (r = –0.353, p = 0.040) and pack years (r = –0.409, p = 0.016).

**Conclusion:** The negative and significant association among variables related to smoking history and cognitive tests showed the presence of cognitive impairment in the studied sample. It is possible that the impairment in planning and decision-making capacity is an important barrier to the difficulty of smoking cessation among smokers with the characteristics described.

111678

Modality: E-Poster Young Researcher – Non-case Report

Category: ACUTE AND CHRONIC CORONARY DISEASE/THROMBOLYSIS

## Inequities and Inequalities in the Treatment of Acute Coronary Artery Disease: Comparison between the Private Network and the Public Health System

JEFERSON DOS SANTOS^1^, Suelen Maiara dos Santos^1^, Luiz Fernando Souza Santos^1^, Anisia Vieira Souza Fontes^3^, Aline Barreto Hora^2^, Thiago Vaz de Andrade^2^, Matheus Jhonnata Santos Mota^2^, Victória Rafaela Nunes dos Santos^2^, Carlos Felipe Amado Abud^2^, Jullia beatriz araujo souza^2^, Karenn santos souza cruz^2^, Andreza Oliveira Almeida^2^

(1) Universidade Tiradentes; (2) Universidade Federal de Sergipe; (3) Centro Universitário Uninassau

**Introduction:** Due to the efforts and efforts of the Brazilian Society of Cardiology (BSC), with national records, it is possible to ensure detailed data that allow us to analyze the differences between public and private hospitals in the care provided and outcomes of patients with Acute Coronary Syndrome (ACS).

**Objectives:** To determine differences in patient characteristics, access to well-equipped services, treatment offered, and intra-hospital and 12-month outcomes between public and private hospitals in Brazil for patients with acute coronary syndrome.

**Method:** This is a prospective observational study that included inpatients diagnosed with ACS in 47 Brazilian hospitals. Patients were followed from admission to discharge and then follow-up was performed by telephone contact at 30 days after discharge, 6 months after discharge and 12 months after discharge. Clinical, personal (previous history) and index event data were collected, such as medical prescription and occurrence of major cardiovascular events (cardiovascular mortality, reinfarction and stroke). The current series compared the results obtained by the population using the Public Health System and the population using the private network. Values of p < 0.001 were considered statistically significant. The ACCEPT registry included, from August 2010 to April 2014, a total of 5,047 patients, making it the largest prospective registry ever published with ACS in Brazil. For the present analysis, all patients who completed the 12-month follow-up (n = 4375) were considered. This study was approved by the Research Ethics Committee of the Federal University of Sergipe, under registration number 302.544.

**Results:** Patients treated in the Public hospitals had less direct access to a specialized service, with a greater number having to be transferred (38.9%; p < 0.001), in addition to spending more days in the hospital (after 7 days, 40.7% were still hospitalized p < 0.001) and death in this subgroup also showed a statistical difference, both in the period of hospitalization and at 12 months after discharge.

**Conclusion:** In Brazil the management of patients with Acute Coronary Syndrome is influenced by the type of health service, public or private. Patients were more likely to have worse outcomes in public hospitals.

111682

Modality: E-Poster Young Researcher – Non-case Report

Category: EPIDEMIOLOGY AND HEALTH POLICIES/GLOBAL HEALTH

## Regional Spending on Rheumatic Fever in Adults in Brazil and Mortality Rate: Is There a Correlation?

SILVIO CÉSAR ALVES DO NASCIMENTO JUNIOR^1^, Karimi Mohamed El Bacha^1^, Karine Corcione Turke^2^

(1) Universidade Nove de Julho – São Bernardo do Campo; (2) Faculdade de Medicina do ABC

**Introduction:** Rheumatic fever is a prevalent disease in our country, with an incidence of 30,000 people/year in Brazil. This disease is directly related to environmental and economic factors, and it is known that it is a disease with a higher prevalence in regions with less financial conditions. The disease can be responsible for several long-term consequences. There are few studies that link expenditures of the Sistema Único de Saúde (SUS) with deaths from rheumatic fever in adults.

**Objectives:** Correlate spending on rheumatic fever in SUS and late mortality associated with the disease in different administrative regiões od Brazil.

**Methods:** Ecological study. The data collected through the DATASUS platform. SUS spending on rheumatic fever and deaths from this disease were evaluated through the Sistema de Informações Hospitalares for each administrative region of the country in adults over 20 year old. Obits were adjusted by age group and were stratified by sex, age group and region. For correlations, the Pearson or Spearman tests were used depending on the normality of the data, assessed by Shapiro-Wilk.

**Results:** Throughout the period of 2008 to 2016, were reported 6254 deaths from rheumatic fever in Brazil in adults over 20 years and the central-west region had the highest mortality rate (0,63:100.000). No correlation was found between the overall mortality rate and SUS spending with rheumatic fever (cor: 0.374, p = 0.32). A strong and positive correlation was observed between mortality and outgoing in the northeast region (cor: 0.853, p = 0.003) and central-west (cor: 0.757, p = 0.018). In addition, no correlation was observed between mortality and HDI (cor: 0.552, p = 0.333). No correlation was found by age group or gender.

**Conclusion:** In this study, it was possible to observe that there was no correlation between regional costs and mortality from rheumatic fever in the country, however, central-west and northeast a correlation was found. In view of the above, this correlations show a significant cost associated with higher mortality. In this way, we can assume that the investment these regions receive, there is a right mortality rate from rheumatic fever in adults, what can reflect an ineffective distribution of resources in these places.

111692

Modality: E-Poster Young Researcher – Non-case Report

Category: ACUTE AND CHRONIC CORONARY DISEASE/THROMBOLYSIS

## Inequities and Outcomes in Patients with Myocardial Infarction: Public Network Versus Private Network

SUELEN MAIARA DOS SANTOS^1^, Jeferson dos Santos^1^, Luiz Fernando Souza Santos^1^, Aline Barreto Hora^2^, Andreza Oliveira Almeida^2^

(1) Universidade Tiradentes; (2) Universidade Federal de Sergipe

**Introduction:** In the last decade many scholars have been concerned about the differences in treatment (acute and intermediate) offered by the public and private health systems, particularly for patients with Acute Myocardial Infarction (AMI), who belong to the group cardiovascular disease – the leading cause of death in the world.

**Objectives:** The present study aimed to compare population subgroups in order to evaluate the access to health care based on clinical recommendations and the difference in clinical outcomes of patients affected by Acute Coronary Syndrome (ACS), who participated in the ACCEPT Registry, comparing them with regard to the type of service (Public Network versus Private Network).

**Method:** The present study compared the results obtained by the public system user population and the private network user population. Values of p < 0.001 were considered statistically significant. The ACCEPT Registry included, from August 2010 to April 2014, a total of 5,047 patients, making it the largest prospective registry ever published with ACS in Brazil. This study was approved by the Research Ethics Committee of the Federal University of Sergipe, under registration number 302.544.

**Results:** In both subgroups, there was a predominance of males (Private Network: 65.8%; Public Network: 69.9%). Regarding risk factors, smoking (current or previous) showed a significant difference in patients from the Public Network (63.5%; p < 0.001), as well as dyslipidemia (59.2%; p < 0.001) and sedentary lifestyle. (62.6%; p < 0.001) in patients from the Private Network. Patients treated in the Public Hospitals had less direct access to a specialized service, with a greater number having to be transferred (38.9%; p < 0.001), in addition to spending more days in the hospital (after 7 days, 40.7% were still hospitalized; p < 0.001) and death in this subgroup also showed a statistical difference, both in the period of hospitalization and at 12 months. Patients affected by the group of diseases that constitute the Acute Coronary Syndrome who need to use the Public Health System are more likely to spend more time in hospital and have worse in-hospital outcomes and after 12 months.

**Conclusion:** The need to be transferred to a specialized service seems to contribute to this difference. Thus, in the face of this unequal access to health care, the need for robust, effective and effectively health policies for all is evident.

111694

Modality: E-Poster Young Researcher – Non-case Report

Category: COVID-19 AND CARDIOVASCULAR SYSTEM

## Deaths from Heart Failure and Arrhythmias Recorded in São Paulo from 2010 to 2019. is There an Increase in Incidence After the Beginning of the COVID-19 Pandemic?

SILVIO CÉSAR ALVES DO NASCIMENTO JUNIOR^1^, Natalia Gil Prado^1^, Uelra Rita Lourenço^1^

(1) Universidade Nove de Julho – São Bernardo do Campo

**Introduction:** On March 11, 2020, the World Health Organization declared a COVID-19 (Coronavirus Disease – 2019) pandemic. Data from April 21, 2021 record 14.12.,795 confirmed cases and 381.475 deaths across the country. It is known that there is a correlation between cases of patients infected with COVID-19 and heart disease. However, there are few studies that show an increase in numbers of cases of heart failure (HF) and arrhythmias in adults in the state of São Paulo (SP) after the beginning of the pandemic in Brazil.

**Objectives:** To analyze the incidence of deaths from HF, conduction disorders and arrhythmias registered in the Health Unic System (SUS) from Jan/2010 to Dec/2019, and 2020 when the COVID-19 pandemic was declared.

**Methods:** Ecological study. Data were collected through the DATASUS platform. Deaths from HF, conduction disorders and arrhythmias were evaluated in the SUS through the Hospital Information System (SIH/SUS) for the state of São Paulo in adults over 20 years of age. Deaths were adjusted by year of care and were stratified by sex, age group and administrative region. For the analysis, hypotheses were used, associated with a t-Student test, when deriving the normality of the data.

**Results:** From 2010 to 2019, 283.834 deaths from HF, conduction disorder and arrhythmias were reported in Brazil in adults over 20 years of age, with 76.190 cases registered in the state of SP, mortality rate (16:100.000). There was no correlation between deaths in 2010 to 2019, and 2020, when the pandemic was declared, recorded in the SUS. However, a probability associated with a t-Student test was observed, with a two-tailed distribution (p: 0.00793), with no significant incidence. When stratified by sex, the mean was 7567.63 (M: 49.90%; F: 50.1%), 80% among the population over 50 years old.

**Conclusion:** It was observed that there was no higher incidence of deaths from HF, conduction disorder and arrhythmias in the pre-pandemic period and the total number of deaths in 2020. However, a slight increase was found when compared to the average mortality in the years 2010 to 2020. 2020 between men and women, and also in older populations. Therefore, it is known that more women die from these diseases in SP. In view of this, cross-sectional studies can be carried out to prove whether there is a relationship between the number of deaths from these pathologies in the pre- and post-pandemic.

111695

Modality: E-Poster Young Researcher – Non-case Report

Category: ATHEROSCLEROSIS/CARDIOVASCULAR RISK FACTORS/CARDIOVASCULAR PREVENTION

## Relationship between Neck Circumference, Neck-to-Height Ratio and Arterial Stiffness in Hyperlipidemic Patients

RAPHAELA PAULA PINHEIRO^1^, Carlos Renato de Oliveira^2^, Allice de Souza Rodrigues^1^, Maria Julia Montebeller Meneses^1^, Renato Jorge Alves^3^

(1) Santa Casa de Sao Paulo School of Medical Sciences; (2) Santa Casa de Misericordia de Sao Paulo Hospital, Cardiology; (3) Santa Casa de Sao Paulo School of Medical Sciences; Santa Casa de Misericordia de Sao Paulo Hospital, Cardiology

**Background:** Hyperlipidemia is an important cardiovascular risk factor. Pulse wave velocity (PWV), the gold standard to assess arterial stiffness (AS), can detect subclinical atherosclerosis with accuracy, however, it’s a high-cost tool. Neck circumference (NC) and neck-to-height ratio (NHR), inexpensive and easily obtainable anthropometric measurements, may be associated with AS.

**Aims:** To evaluate the relationship between NC, NHR, and AS assessed by PWV in treated hyperlipidemic patients.

**Methods:** We performed a cross-sectional study with 47 hyperlipidemic patients, over 18 years. Excluded those with cervical anatomical abnormalities. Brachial PWV (bPWV) was obtained by a non-invasive oscillometric method. NC and NHR were assessed. Data were present as frequency (%) or mean ± S.D., used Pearson correlation for analyses, P-value:5%.

**Results:** Sample: 53,2% woman, mean age of 63,6 ± 13,2 years, 63,8% Fredrickson’s phenotype IIa, 63,8% used high-potency statin, 87,2% hipertensives, 14,9% current smokers. On average, patients were overweight (BMI 28 ± 3,5). Mean NHR of 23,5 ± 1,8 cm/m, NC of 37,6 ± 3,8 cm, bPWV of 9,3 ± 2,2 m/s; • bPWV was positively associated with NHR (figure 1), but not with NC (r = 0.083; p = 0.580); • Age (years), a well-established risk factor for increased AS, was strongly correlated with PWV (r = 0.962, p = 0.000) and was also associated with NHR (r = 0.386, p = 0.007).

**Conclusion:** Our findings suggest that NHR, but not NC, was positively associated with bPWV in hyperlipidemic patients and might be an inexpensive potential predictor of arterial stiffness, contributing to the clinical follow-up, and preventing cardiovascular events in this population.


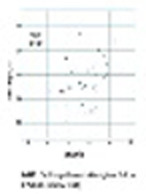


111700

Modality: E-Poster Young Researcher – Non-case Report

Category: ACUTE AND CHRONIC CORONARY DISEASE/THROMBOLYSIS

## Outcomes in the Care of Patients with Acute ST-Elevation Myocardial Infarction in the Public Health System

LUIZ FERNANDO SOUZA SANTOS^1^, Jeferson dos Santos^1^, Suelen Maiara dos Santos^1^, Aline Barreto Hora^2^, Andreza Oliveira Almeida^2^

(1) Universidade Tiradentes; (2) Universidade Federal de Sergipe

**Introduction:** Although we have records that observe patients with Acute Coronary Syndrome (ACS), there is still a lack of studies that show the differences between the public and private service in the care of patients with ACS, in particular the presentation of STEMI (ST-Elevation Myocardial Infarction). The present study aimed to investigate the differences in acute treatment and the presence of outcomes after 12 months of patients affected by STEMI, who participated in the ACCEPT Registry, comparing them with regard to access: Public versus Private Network.

**Method:** The ACCEPT Registry is a prospective observational study that included inpatients diagnosed with ACS in 47 Brazilian hospitals. Patients were observed from admission to discharge and then followed up for 12 months. Clinical data, previous history and index event, such as medical prescription and occurrence of major cardiovascular events (cardiovascular mortality, reinfarction and stroke) were collected. The current study compared the results obtained by the public system user population and the private network user population, specifically affected by the STEMI. Values of p ≤ 0.01 were considered statistically significant. The ACCEPT Registry included a total of 5,047 patients from August 2010 to April 2014, making it the largest prospective registry ever published with ACS in Brazil. For the present article, only patients with STEMI were considered (n = 1550), which corresponds to the most common diagnosis of ACS (35.8%). This study was approved by the Research Ethics Committee of the Federal University of Sergipe, under registration number 302.544.

**Results:** Patients treated in the public health service spent more days hospitalized (after 7 days, 40.7% were still hospitalized) and the death rate in this group was 12.3% in 1 year. Age and diabetes were independently associated with the occurrence of major cardiovascular events. Patients with STEMI who need to use the Public hospitals are more likely to spend more time in hospital and have worse outcomes 12 months after the event.

**Conclusion:** The need to be transferred to a better equipped service seems to contribute to this difference. Thus, there is a need to understand in a better way these variables that impact the dichotomy between services, as well as the implementation of strategies that ensure adequate patient management.

111702

Modality: E-Poster Young Researcher – Non-case Report

Category: HEART FAILURE/CARDIOMYOPATHY/TRANSPLANT

## Independent Assessment of the Atrial Fibrillation – Hypertrophic Cardiomyopathy Score in a Brazilian Cohort

HENRIQUE IAHNKE GARBIN^1^, Fernando Luis Scolari^2^, Felipe Costa Fuchs^2^, Edileide de Barros Correia^3^, Beatriz Piva e Mattos^2^

(1) Post-Graduation Program in Cardiology and Cardiovascular Sciences, Faculty of Medicine, Universidade Federal do Rio Grande do Sul, Porto Alegre, Brazil; (2) Division of Cardiology, Hospital de Clínicas de Porto Alegre, Porto Alegre, Brazil; (3) Division of Cardiomyopathy, Instituto Dante Pazzanese de Cardiologia, São Paulo, Brazil

**Background:** Atrial fibrillation (AF) is a common arrhythmia in hypertrophic cardiomyopathy (HCM), frequently related to adverse outcomes. The AF-HCM point-score is a recently validated predictive model for the assessment of AF risk.

**Objectives:** We sought to independently evaluate the novel AF-HCM score in a tertiary HCM center cohort in Brazil.

**Methods:** A longitudinal HCM cohort followed between 2007–2022 was retrospectively stratified for new onset AF, according to the presence of left atrial dimension (+2 points per 6 mm increase), age at clinical evaluation (+3 points per 10-year increase), age at HCM diagnosis (–2 points per 10-year increase) and heart failure symptoms (+3 points if symptomatic). The AF-HCM score was classified as low (<1,0%/year; score ≤17), intermediate (1,0–2,0%/year; score 18 to 21), and high risk (>2,0%/year; score ≥22) for AF development. Cox regression model and Kaplan-Meier survival free from AF were analyzed, P < 0,05.

**Results:** A total of 116 patients, aged 56 ± 13 years, 64 (55%) females, were followed for 7.0 ± 5.5 years. The left ventricular maximal wall thickness was 18 ± 3 mm, the left atrial diameter 46 ± 6 mm, 46 (40%) subjects were in NYHA class I, 49 (42%) in class II and 21 (18%) in class III. The AF-HCM score stratified risk as low in 13 (11%) individuals, as intermediate in 49 (42%) and as high in 60 (52%). Over the follow-up, 37 (32%) were diagnosed with AF. Among those with newly diagnosed AF, none was classified as low risk, 14 (38%) as intermediate and 23 (62%) as high, P = 0.027. The area under the ROC curve to detect AF was 0.671 (95% CI 0.565–0.777, P = 0.003). The Kaplan-Meier curve showed that patients in intermediate and high-risk were more likely to develop AF [P (Log-Rank) <0.001]. The intermediate and high-risk groups were associated with developing arrhythmia with a hazard ratio of 56.8 (95% CI 3.4–944.6), P = 0.005. The specificity and the negative predictive value were 100%. On the contrary of the previous North-America study population, most patients of the Brazilian cohort were in the high-risk category (37% vs. 52%).

**Conclusion:** The AF-HCM score is a reasonable tool for recognizing patients not prone to develop AF among those stratified as low risk in a Brazilian cohort. However, the model was limited to identify intermediate and high risk individuals in contrast to the previously stratified North-American population.

111708

Modality: E-Poster Young Researcher – Non-case Report

Category: ATHEROSCLEROSIS/CARDIOVASCULAR RISK FACTORS/CARDIOVASCULAR PREVENTION

## Risk Factors Related to the Development of Preeclampsia and the Importance of Early Identification in Prenatal Care

BÁRBARA BRANDÃO LOPES^1^, Bárbara Brandão Lopes^1^, João Joadson Duarte Teixeira^1^, Maria das Graças da Silva Guerreiro^2^, Nádya dos Santos Moura^5^, Ivana Rios Rodrigues^1^, Rebeca Silveira Rocha^3^, Maria Luziene de Sousa Gomes^1^, Weslley Tiago Sousa Alves^4^, Bárbara Gomes Santos Silva^1^, Mônica Oliveira Batista Oriá^1^

(1) Universidade Federal do Ceará – UFC; (2) Universidade Estadual do Ceará – UECE; (3) Maternidade Escola Assis Chateaubriand – MEAC/UFC; (4) Prefeitura Municipal de Fortaleza – PMF; (5) Universidade Federal do Piauí – UFPI

**Introduction:** Hypertensive disorders occupy the first place in the ranking of causes of maternal deaths in developing countries, as well as being responsible for about 14.0% of all maternal deaths in the world, especially preeclampsia (PE). Despite the lack of knowledge about the etiology of PE and the complexity of its pathophysiology, some factors are related to the development of the disease. Therefore, the early identification of risk factors and the correct management of pregnant women at risk of preeclampsia is a decisive factor in the maternal-fetal clinical outcome.

**Objective:** To investigate the risk factors and maternal characteristics for the development of preeclampsia in pregnant women.

**Method:** Prospective cohort study carried out in 18 Primary Health Care Units in the city of Fortaleza, Ceará, Brazil between March 2018 and February 2020. The study population consisted of pregnant women captured in the 1st gestational trimester, being followed up until postpartum. Sociodemographic, clinical and obstetric data were collected using a form and processed using the Statistical Package for Social Sciences (SPSS) version 24.0 and R software version 3.5.5. Study approved by the Research Ethics Committee of the Federal University of Ceará, according to opinion number 2.448.308.

**Results:** Final sample consisted of 146 patients, of which 39 developed preeclampsia (26.71%). No sociodemographic and clinical aspects were associated with preeclampsia. However, obstetric data such as previous hypertensive disorder (p = 0.03), family history of preeclampsia (p = 0.007) and body mass index (p = 0.01), with a mean of 28.52 (SD ± 7,32) had a strong relation with preeclampsia.

**Conclusion:** In prenatal care, the identification of risk factors related to the development of PE is essential, in order to early identify pregnant women at greater risk for the disease and offer them specialized follow-up, with actions aimed at reducing maternal and perinatal morbidity and mortality.

111847

Modality: E-Poster Young Researcher – Non-case Report

Category: ATHEROSCLEROSIS/CARDIOVASCULAR RISK FACTORS/CARDIOVASCULAR PREVENTION

## Office Long Term Systolic Blood Pressure Variability Before and After a Cerebrovascular Event

LARA CABRITA^1^, Ana João Taveira^2^, André Gomes Roque^3^, Estela Cabral^4^, Pedro Damião^5^

(1) USF Fénix de Aveiro; (2) UCSP Mira; (3) USF Santa Joana; (4) USF Atlântico Norte; (5) USF Fénix de Aveiro

**Introduction:** Cerebrovascular disease (CVD) is a major morbidity and mortality cause. Systolic blood pressure (BP) is classically referred to as a predictor of cerebrovascular events (CVE), and clinical attention is generally given to the mean values of SBP. However, several studies have shown that blood pressure variability (BPV) is a predictor of hypertension mediated organ damage, CVE, and all-cause mortality.

**Objective:** Our study aims at describing long term systolic BPV distribution based on different combinations of modifiable cardiovascular risk factors, before and after a CVE.

**Methods:** We performed an exploratory data analysis on a convenience sample of patients with previous CVE (stroke and transient ischemic accident (TIA)), from a single primary care center. Data was obtained from clinical registries in the software in use on our center. The variables included were age, sex, tobacco use, hypertension, dyslipidemia, obesity, overweight, type 2 diabetes and systolic blood pressure. BPV was calculated as the variability coefficient from the closer 3 systolic BP measures pre and post event, to a maximum of 5 years pre and post event. Statistical analysis was performed with R® software and presented in cardinality boxplots. We compared before and after BPV with the Wilcoxon test.

**Results:** Our initial sample included 255 patients with cerebrovascular events, from which only 80 had a minimum of 3 office BP measurements pre and post CVE, and as such were eligible for systolic BPV analysis. Blood pressure target met (defined as systolic BP below 140 mmHg) proportion of patients, pre and post CVE, were 0.475 and 0.508, respectively. No significant differences were found between pre and post event BPV (p = 0.78 global events; p = 0.99 for stroke; p = 0.38 for TIA). No trend was identified for post event reduced BPV for a single or combinations of risk factors.

**Conclusion:** Our study shows that office long term BPV does not vary before and after a CVE. However, in our sample, systolic BP mean proportion of patients that meet the recommended target also remains the same after a CVE, probably meaning a lack of control of this important predictor. This study has major limitations, being observational with a small sample of patients, which renders definite conclusions for the relevance and eventual change of BPV before and after cerebrovascular events. However, it clearly shows that there is a need to improve risk factor goal attainment after a CVE.

111716

Modality: E-Poster Young Researcher – Non-case Report

Category: CARDIOVASCULAR SURGERY

## Rheumatic Mitral Valve Disease: Repair or Replacement?

LUIS HENRIQUE OLIVEIRA PEREIRA^1^, Kelvin Câmara^1^, Tamires Santos Pinheiro^1^, Matheus Mônaco Lemos^1^, Ana Laísa Andrada Oliveira^2^, Maria Eduarda Pereira de Oliveira^3^, Gabrielly Machado Trindade^4^, Marjorie Francisca Raksa^1^, Manoel Flávio Silva Kanisky^1^, Gerlânio César da Silva^1^, Valdano Manuel^5^

(1) Universidade Centro de Ensino de Maringá (UNICESUMAR); (2) Faculdade Pernambucana de Saúde (FPS); (3) Universidade Vila Velha (UVV); (4) Centro Universitário Max Planck (UniMax); (5) Clínica Girassol, Complexo hospitalar de doenças cardio-pulmonares Cardeal Dom Alexandre do Nascimento, Instituto do coração (InCor) do Hospital das Clínicas da Universidade de São Paulo

**Introduction:** Rheumatic heart disease (RHD) remains a public health problem, especially in developing countries. The mitral valve is the main affected cardiac structure, requiring intervention in many cases. The discussion of the best intervention is still a controversy; repair or replacement.

**Objectives:** This systematic review aims to evaluate the survival of patients with RHD submitted to valve replacement or repair.

**Method:** We systematically reviewed the English literature through PubMed, LILACS, SciELO and Google Scholar between January 1, 2021, and February 28, 2022, based on PRISMA methodology. Articles with a sample of at least 30 patients under 66 years-old who underwent mitral valve (MV) replacement or repair were included. The articles were classified by Newcastle-Ottawa Scale.

**Results:** Six studies including 2874 patients were analyzed. Most of the patients were female (2001; 69.6%) with a ratio of 2.30:1 for female:male. The age ranged from 11 to 66 years old, being 10 to 65 years old in the repair group and 12 to 66 years old in the replacement group. The mean follow-up time varied from six to 106 months. In MV repair group, the mortality was 3.2% and reoperation was 6.2%, while in the MV replacement the mortality was 7.5% and 3.2% required reoperation. Patient’s survival was found to be similar (85% for repair and 87% for replacement). About the main complications post-MV repair and MV replacement, respectively, were: stroke (1.8%, 2.5%) and endocarditis (0.5%, 1.3%). One article, composed by 148 patients, compared datas between mechanical and bioprosthetic surgical replacement: mortality (2%, 17%), reoperation (5.6%, 5.2%) and survival in four years (98% ± 2%, 70% ± 10%), in that order. The article also presented that the main general complications were stroke (0%, 5,2%) and endocarditis (1%, 4%).

**Conclusion:** MV repair demonstrated lower mortality in patients compared to MV replacement, even though it had a higher rate of reoperation. These facts support the idea of shared decision with the heart team and the patient, considering their clinical status, life expectancy, measuring risks and benefits of the surgical decision.

111731

Modality: E-Poster Young Researcher – Non-case Report

Category: HEMODYNAMICS AND INTERVENTIONAL CARDIOLOGY

## Benchmarking Analysis of a Single Centre PFO Closure Program: Comparative Study using the Reduce Trial Data

HENRIETTE MÉSZÁROS^1^, Pál Ábrahám^1^, Andrea Ágnes Molnár^1^, Dorottya Pásztor^1^, Sándor Nardai^1^, Béla Merkely^1^, Béla Merkely^1^

(1) Heart and Vascular Centre of Semmelweis University

**Introduction:** Half of the cryptogenic strokes are attributed to persistent foramen ovale (PFO), their transcatheter closure significantly reduces the risk of recurrent stroke.

**Aims:** We aimed to do a comparative analysis of the baseline clinical and follow up data from our PFO closure patients with the data from the largest randomized trial on PFO(441 patients).

**Methods:** We prospectively enrolled 105 patients in our single-centre registry who had PFO-closure between January 2018 and March 2021. We analysed retrospectively their baseline clinical data, PFO anatomy, procedural outcome and prospectively arrhythmias, device-related complications, and recurrent stroke events during a median follow-up time of 585 days. Data was compared to the published data from the PFO-closure arm of the REDUCE.

**Results:** The mean age of the patients(45 ± 10.6 vs.44.4 ± 9.3(p = 0.56); and the distribution of genders was similar(males 64% 67/105 vs.59.2% 261/441; p = 0.44). More of our patients had hypertension(42.9% 45/105vs.25.4% 112/441p = 0.0007), and were smokers(36.2% 38/105vs.14.3% 63/441 p = 0.001), the prevalence of diabetes (4.7% 5/105 vs.4.1% 18/441(p = 0.80) and the proportion of complex PFO-s was similar(57.2% 57/105 vs.42.8% 268/441 p = 0.23). The success rate of closures was similarly high:91.4% 96/105 vs.93.7% 413/441 p = 0.40. Device dislocation occured in 1 patient vs.in 3 patients(0.9% 1/105 vs.0.6% in 3/441 patients p = 0.58). No recurrent stroke occurred in our cohort, while 5 patients (1.2%) had stroke in REDUCE in the first 2 year follow-up period, this difference is non-significant. 1 of our patients(0.9%) had postoperative paroxysmal atrial fibrillation, while 29 patients(6.6%) of the REDUCE cohort, however their follow-up time for arrythmias was longer, therefore this data is not comparable.

**Conclusion:** The short and midterm outcome of our PFO program yielded similar results to REDUCE, despite treating patients of higher cardiovascular risk. Further follow-up is needed to determine the long-term prevalence of atrial fibrillation, therefore we need imaging markers in the future for to predict the developement of this arrhytmia.

111759

Modality: E-Poster Young Researcher – Non-case Report

Category: EPIDEMIOLOGY AND HEALTH POLICIES/GLOBAL HEALTH

## Rheumatic Heart Disease in Brazil: Effects of the Antibiotic Prophylaxis in Preventing Heart Damage. A Systematic Review

GHABRIELA SILVA RABELO^1^, Ghabriela Silva Rabelo^1^, Júlia Souza Siqueira^1^, Lorena Oliveira Carneiro^2^

(1) Universidade Católica de Brasília; (2) Universidade Federal do Goiás

**Introduction:** One of the most prevalent acquired heart diseases in children and young adults in Brazil is the Rheumatic Heart Disease. When the patient has already been exposed to the patogen and wasn‘t treated, the use of antibiotic prophylaxis (AP) prevents further complications.

**Goal:** Given the social/economical difficulties and the high incidence of rheumatic disease in Brazil associated with the prolonged period of antibiotic administration, this study aims to understand the impact of the AP in Brazil in preventing heart damage.

**Methods:** A systematic review carried out in accordance with the recommendations of the PRISMA. The databases used in the study were: SciELO, Lilacs and PubMed. Those search descriptors were used: Rheumatic Heart Disease, Brazil and antibiotic prophylaxis. The inclusion criteria consisted of original articles, of free access, published in English and/or Portuguese and that met the objective of the study. Exclusion criteria included reviews and monographs.

**Results:** The total of 5 articles were screened, 1 of which were duplicate; however, only 3 were selected and included in the review, since the study not selected is a narrative review. A cross-sectional study shows a growth of 215% from 1998 to 2016 of the mortality rate from acute rheumatic fever. A randomized clinical trial with children and adolescents with latent rheumatic heart disease were given intramuscular benzathine penicillin G (BPG) every 4 weeks for 2 years or no prophylaxis (control). Among the participants who completed the study, only 0.8% had echocardiographic progression, compared with 8.2% in the control group. A prospective cohort study followed 593 patients with rheumatic fever for 32 years, starting AP with BPG every 3 weeks, in which 10.4% of patients changed treatment to every 4 weeks after turning 21 years of age. This study was divided into two groups, both with AP, the first with patients without cardiac lesions (41%) and the second with cardiac lesions (59%), among these, 17 patients, in irregular AP, were affected by the worsening cardiovascular; however, no patient on regular AP experienced progression of cardiac damage.

**Conclusions:** This study demonstrates the efficiency of regular use of AP to reduce the recurrence of heart damage and the high rates of rheumatic heart disease in Brazil. However, there are few studies comparing the accessibility and effectiveness of the Brazilian prophylactic scheme in relation to international protocols.

111762

Modality: E-Poster Young Researcher – Non-case Report

Category: ACUTE AND CHRONIC CORONARY DISEASE/THROMBOLYSIS

## Outcomes in Patients with an Episode of Acute ST Elevation Myocardial Infarction: A Comparative Analysis between the Public Health System and Private Networks

LUIZ FERNANDO SOUZA SANTOS^1^, Jeferson dos Santos^1^, Suelen Maiara dos Santos^1^, Aline Barreto Hora^2^, Andreza Oliveira Almeida^2^

(1) Universidade Tiradentes; (2) Universidade Federal de Sergipe

**Introduction:** Cardiovascular diseases represent the main cause of death in Brazil and in the world. Acute Coronary Syndrome (ACS) is one of the presentations of these diseases, with ST elevation acute myocardial infarction (STEMI) being the type with the highest morbidity and mortality.

**Objective:** To assess the correlation between patient outcomes in STEMI episodes between public and private health care networks.

**Method:** The present study used the ACCEPT registry database. Patients with ACS treated in selected Brazilian hospitals were compiled. Only patients with STEMI were considered (Public Network = 1029, Private Network = 521). P-values (Fisher’s exact) were reported to three decimal places. This study was approved by the Research Ethics Committee of the Federal University of Sergipe, under registration number 302.544.

**Results:** Regarding to the clinical outcomes in the first 7 days, there was no significant difference between episodes of reinfarction, stroke and severe bleeding (Table 1). However, there was a difference in death in the first 7 days (Public Network – 3.8%; Private Network – 1%; p = 0.001).

**Conclusion:** It was concluded that there are statistically significant differences between patients who use the Public Network suffering worse outcomes, death showed differences in the two studied moments, while at hospital discharge there was a worse prognosis for SUS users, the other variables were not significant.


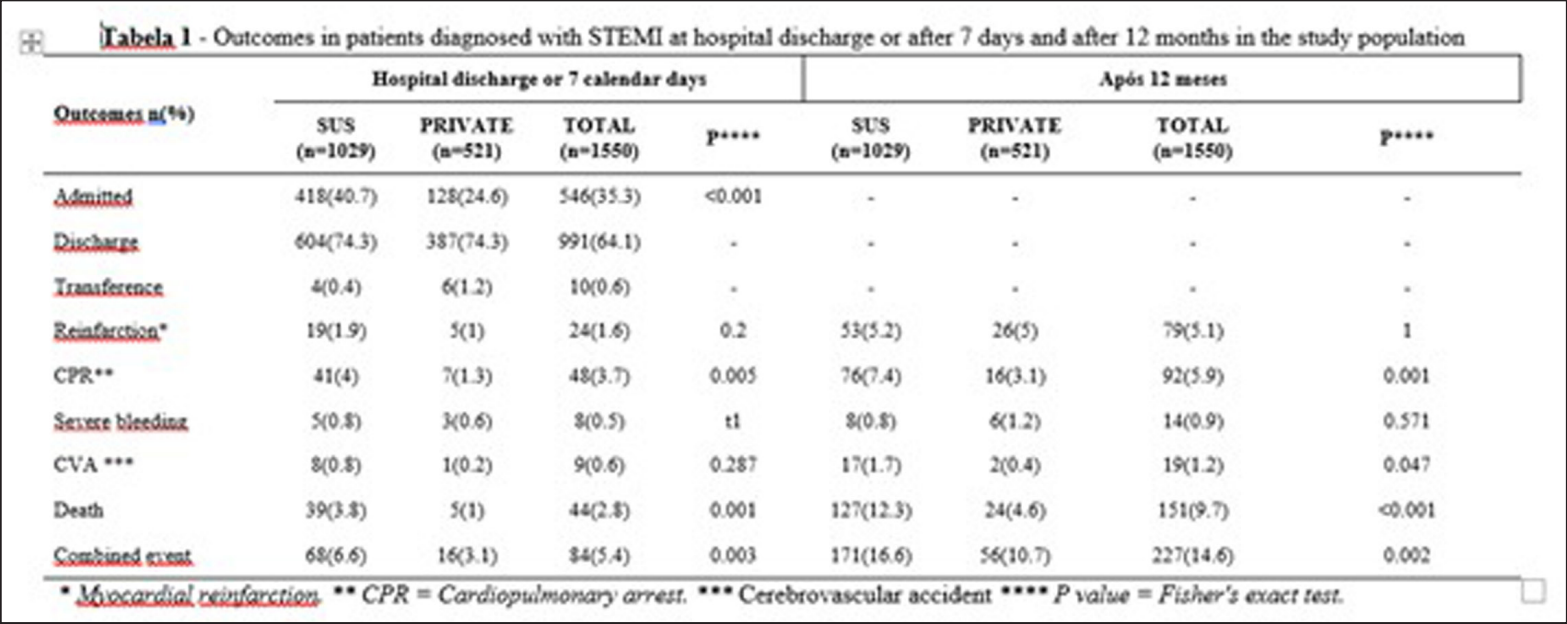


111766

Modality: E-Poster Young Researcher – Non-case Report

Category: HEART FAILURE/CARDIOMYOPATHY/TRANSPLANT

## Chagas Disease-Related Mortality in Brazil from 2010 to 2020: Temporal and Spatial Trends

MARIANA DA SILVEIRA CASTRO^1^, Leonardo Sandrini Costa^1^, Rafaella Quirino Alcântara^1^, Geovana Oliveira de Paula^1^, Felilpe Fonseca dos Reis^1^

(1) Pontifícia Universidade Católica de Goiás (PUC-Goiás)

**Introduction:** Chagas disease is an infectious tropical disease, caused by the flagellate protozoan Trypanosoma cruzi, transmitted by contact with the feces of vector insects, called “barbeiros” in Brazil, and it reamins still quite neglected. It has a greater distribution in Brazil, reaching about 40% of the territory, and it is estimated that 30% evolve to cardiac and digestive form, the main responsible for the high disease morbidity and mortality.

**Objective:** To analyze the number of deaths from Chagas disease reported in the Brazilian population, between 2010 and 2020.

**Methodology:** Epidemiological, retrospective, descriptive and quantitative study, carried out by collecting data from the Mortality Information System (SIM) on the platform of the Department of Informatics of the Unified Health System (DATASUS). Mortality data from Chagas disease in the Brazilian population were analyzed from January 2010 to December 2020. The sociodemographic and clinical variables selected were: region and Federative Unit of residence, year of death, sex and age group.

**Results:** In the analyzed period, the total of 49.574 deaths from Chagas disease in the entire national territory was recorded, with 26.886 being men and 22.684 women. Most deaths occurred in age group 70–79 years, corresponding to 28,2% of the total. The variation in mortality in this 10-year interval followed a specific pattern, with a decreasing rate of 14,6%, with the year 2020 having the lowest rate with 4.165 deaths, while the year 2010 represented the highest number of deaths, with 4.876. According to the Federative Unit residing, it was observed that Southern remained as the largest holder of deaths from Chagas disease in the country, with emphasis on the state of São Paulo. On the other hand, region North presented the lowest number with 2% in relation to the total.

**Conclusions:** It was possible to identify a high concentration of deaths from Chagas cardiomyopathy in the the most populated region of the country, even with a drop in the number of cases in a ten year period. In view of the national perspective, primary care should be further promoted in terms of the access of the Brazilian population to effective diagnosis and treatment of the disease, since they are crucial for improving the quality of life of patients with this disease, as well as for the prevention of its chronic, and even fatal, condition.

111781

Modality: E-Poster Young Researcher – Non-case Report

Category: ACUTE AND CHRONIC CORONARY DISEASE/THROMBOLYSIS

## Prognosis of Acute Myocardial Infarction with ST Elevation and Its Associated Factors: Analyzes between the Unified Health System and Private Networks

SUELEN MAIARA DOS SANTOS^1^, Jeferson dos Santos^1^, Luiz Fernando Souza Santos^1^, Aline Barreto Hora^2^, Andreza Oliveira Almeida^2^

(1) Universidade Tiradentes; (2) Universidade Federal de Sergipe

**Introduction:** Acute ST elevation myocardial infarction (STEMI) is one of the main causes of death caused by cardiovascular diseases. Combined events such as reinfarction, respiratory arrest, severe bleeding and stroke are factors that affect the prognosis of STEMI.

**Objective:** To evaluate events that may contribute negatively to the prognosis of STEMI in the population studied.

**Method:** The present study used the ACCEPT registry database, which selected patients with Acute Coronary Syndrome treated in Brazilian hospitals in all regions. Only patients with STEMI were considered (Public Network = 1029; Private Network = 521). P-values (Fisher’s exact) were reported to three decimal places with p-values less than 0.001 reported as p < 0.001. This study was approved by the Research Ethics Committee of the Federal University of Sergipe, under registration number 302.544.

**Results:** In this present study, the variables: attendance at the Unified Health System (SUS), region, age, systemic arterial hypertension (SAH), diabetes, Congestive heart failure (CHF), current smoking and dyslipidemia were the factors that most contributed to the negative prognosis. (Fig. 1). The variables of female sex, reperfusion with primary and non-primary angioplasty only, and thrombolytic-only reperfusion reduce the likelihood of a negative prognosis.

**Conclusion:** Care in the SUS, SAH, CHF, diabetes and current smoking were factors that most contributed to the negative prognosis.


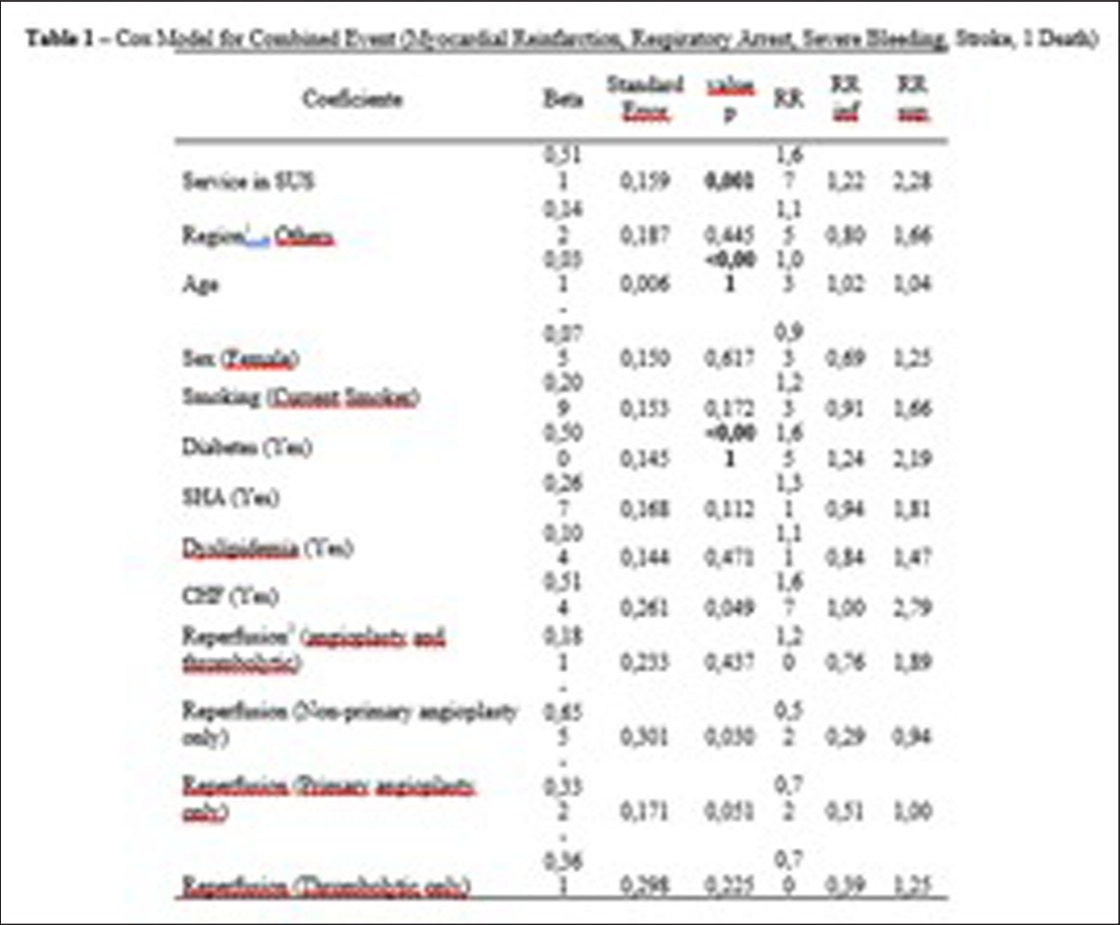


111856

Modality: E-Poster Young Researcher – Non-case Report

Category: EPIDEMIOLOGY AND HEALTH POLICIES/GLOBAL HEALTH

## Cardiac Implantable Electronic Devices in the Brazilian Public Health System: A Real-World Data Study

MIRIAM ALLEIN ZAGO MARCOLINO^1^, Miriam Allein Zago Marcolino^1^, Rodrigo Antonini Ribeiro^2^, Luis E. Rohde^3^, Carisi Anne Polanczyk^4^

(1) Graduate Program in Epidemiology, Universidade Federal do Rio Grande do Sul, Porto Alegre, Brazil; (2) National Institute of Science and Technology for Health Technology Assessment – INCT/IATS (CNPq 465518/2014-1), Porto Alegre, Brazil; (3) Cardiology Center, Hospital Moinhos de Vento, Porto Alegre, Brazil; (4) Graduate Program in Cardiology and Cardiovascular Sciences, Universidade Federal do Rio Grande do Sul, Porto Alegre, Brazil

**Introduction:** Cardiac implantable electronic devices (CIED), including pacemakers (PM), implantable cardioverter-defibrillator (ICD), and cardiac resynchronization therapy (CRT), are interventions long established in the cardiology practice, but restricted for selected patients. They include simple devices to highly complex and costly procedures.

**Objective(s):** To describe the profile of patients that received a CIED covered by the Brazilian public health system (SUS) over a 12-year period.

**Methods:** Retrospective epidemiological study including open data of all hospital admissions with a CIED implantation as primary procedure reimbursed by the SUS between 2008 and 2019, obtained from the Hospital Information System of SUS Informatics Department (DATASUS). Mean annual rate per million population was age and sex standardized by the OMS standard population. All analysis were performed using R.

**Results:** SUS reimbursed 216,440 CIED implant procedures from 2008 to 2019 (mean annual rate of 87.1 per million population), mainly PM (89.3%). Patients mean age was 68.8y (15.3), and were mostly male (54%), residents in the Southwest (45%), South (22%), or Northeast (21%) regions. Arrhythmias diagnoses were present in 87% of the patients, mostly in PM (91%) and ICD (83%). Heart failure was present in 61% of CRT and 38% of CRT-D patients. The mean length of stay was of 4.1 (6.3) days, 1.5% died during hospitalization, and mean hospitalization reimbursement values varied by CIED type (R$8,309 to R$ 56,791). (Table 1)

**Conclusions:** Twelve-year cumulative data indicates that CIED implantation in Brazil was performed mostly on aged patients, males, residents in the Southwest, South or Northeast, PM predominantly. These data might help guide health policy actions on access to CIED therapies.


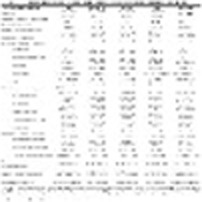


111871

Modality: E-Poster Young Researcher – Non-case Report

Category: ATHEROSCLEROSIS/CARDIOVASCULAR RISK FACTORS/CARDIOVASCULAR PREVENTION

## Overweight Prevalence and Correlation with Arterial Stiffness and Cardiometabolic Risk Factors in Treated Hyperlipidemic Patients

RAPHAELA PAULA PINHEIRO^1^, Carlos Renato de Oliveira^2^, Allice de Souza Rodrigues^1^, Maria Julia Montebeller Meneses^1^, Renato Jorge Alves^3^

(1) Santa Casa de Sao Paulo School of Medical Sciences; (2) Santa Casa de Misericordia de Sao Paulo Hospital, Cardiology; (3) Santa Casa de Sao Paulo School of Medical Sciences; Santa Casa de Misericordia de Sao Paulo Hospital, Cardiology

**Background:** Hyperlipidemia is a well-established cardiovascular risk factor. The overweight is associated with low-grade chronic inflammation and is correlated to cardiometabolic risk factors. Pulse wave velocity (PWV), the gold standard for arterial stiffness assessment, can detect subclinical atherosclerosis with accuracy. Studies suggest an association between PWV and nutritional status. AIM To describe the overweight prevalence, and correlate with arterial stiffness assessed by PWV and cardiometabolic risk factors in treated hyperlipidemic patients.

**Methods:** We performed a cross-sectional study. There were included hyperlipidemic patients, over 18 years. Sociodemographic data and clinical background were collected by a standardized questionnaire and medical records. Nutritional status was determined by body mass index (BMI), considering “overweight” BMI ≥25 kg/m² to adults <60 years and BMI ≥28 kg/m² to elderly ≥60 years. The brachial PWV was assessed by the non-invasive oscillometric method and the patient’s laboratory tests were recently collected by medical records. Data were presented as frequency (%), prevalence(%), or mean ± S.D.

**Results:** Sample: 62 patients. Mean age of 63,9 ± 12,5 years, 53,23% female, 43,5% diabetics, 88,7% hypertensives, 64,5% Fredrickson’s phenotype IIa, 67,7% used high potency statin and 64,5% overweight prevalence, mean BMI of 28,8 ± 3,7 kg/m². Overweight patients presented a lower mean of PWV (8,95 m/s), although higher use of high potency statin (55,65%) when compared to those without overweight (10,06 m/s; 38,46%). The patients with overweight presented higher means of LDL-c (113,7 ± 61,3 mg/dl), total cholesterol (190,3 ± 74,6 mg/dL), triglycerides (202,3 ± 157,7 mg/dL), fasting blood glucose (113,2 ± 40,4 mg/dL), glycated hemoglobin (6,5 ± 1,4%), uric acid (6,2 ± 2,4 mg/dL) and lower means of HDL-c (42,9 ± 11,6 mg/dL), when compared with individuals without overweight (99 ± 30,5; 169,1 ± 47,1; 165,2 ± 100; 108,4 ± 30,3 mg/dL; 6,2 ± 1,0%; 5,7 ± 1,1; 43,1 ± 17,3 mg/dL respectively).

**Conclusion:** The sample had a 64,5% prevalence of overweight, and these individuals presented a worse cardiometabolic profile when compared with individuals without overweight. Contrary to most of the publications, overweight patients presented lower PWV, although they used more high potency statin when compared to those without overweight. Our results may be related to reverse endothelial remodeling, but longitudinal studies can be used to elucidate these correlations.

111884

Modality: E-Poster Young Researcher – Non-case Report

Category: HEART FAILURE/CARDIOMYOPATHY/TRANSPLANT

## The Association of Myocardial Function and Pulmonary Hypertension with Maximal Exercise Capacity in Patients with Chagas and Non-Chagas Heart Failure

JHESSICA MACIEIRA PEREIRA^1^, Thayrine Rosa Damasceno^1^, Rafael Dias de Brito Oliveira^1^, Enrico De Francisco Magnani^1^, Danielle Aparecida Gomes Pereira^1^, Denise Mayumi Tanaka^2^, Eduardo Elias Vieira De Carvalho^2^, Júlio César Crescêncio^2^, Eduardo Rubio Azevedo^2^, Marcus Vinicius Simões^2^, Luciano Fonseca Lemos De Oliveira^1^

(1) Universidade Federal de Minas Gerais – UFMG; (2) Faculdade de Medicina de Ribeirão Preto – FMRP/USP

**Introduction:** Increased pulmonary artery systolic pressure (PASP) and reduced left ventricle ejection fraction (LVEF) in individuals with heart failure (HF) is related to disease progression, morbidity and mortality. On the other hand, booth PASP and LVEF are not related to functional capacity (FC). Patients with Chagas HF are known to present worst prognosis and may develop myocardial dysfunction and pulmonary hypertension (PH). However, the association of FC with cardiac function and PH in Chagas HF is still unclear.

**Objective:** To evaluate the association of FC with cardiac function and PH in individuals with Chagas and non-Chagas HF.

**Methods:** Cross-sectional observational study with 178 patients with HF who underwent clinical examination, cardiopulmonary exercise test (CPET) and 2-dimensional echocardiography. The sample was divided between Chagas (CH, n = 101) and non-Chagas (NCH, n = 77) HF. Data normality was analyzed using the Kolmogorov-Smirnov test. Data comparison was verified with Student’s t test or Mann-Whitney test. The association was performed by using the Pearson or Spearman correlation coefficients.

**Results:** Compared to NCH group, CH group presented higher VO2peak (16.8 ± 6.1vs 13.8 ± 4.3 ml.kg.min–1, p < 0.001) and LVEF (42.6 ± 18 vs 28.9 ± 9.7%, p < 0.001), lower left atrial diameter (LAD; 43.1 ± 8.4 vs 47.7 ± 7.2 mm, p < 0.001), left ventricle diastolic diameter (LVDD; 59.5 ± 9.1 vs 66.7 ± 9.9 mm, p < 0.001) and PASP (37.3 ± 13.4 vs 51 ± 15 mmHg, p < 0.001), and similar ventilatory efficiency given by the VE/VCO2slope (35.5 ± 10.6 vs 35.8 ± 8.2, p = 0.83). In the CH group, VO2peak correlated significantly with PASP (r = –0.60, p < 0.001), LVEF (r = 0.51; p < 0.001), LVDD (r = –0.36, p < 0.001), LV mass index (r = –0.36, p < 0.001), LAD (r = –0.34; p < 0.001), E wave (r = –0.32; p = 0.01) and TAPSE (r = 0.27, p = 0.04). The VE/VCO2slope was correlated with LVEF (r = 0.53; p < 0.001), PASP (r = 0.46; p = 0.001), LVDD (r = 0.42; p < 0.001), LAD (r = 0.33; p = 0.001), TAPSE (r = 0.3, p = 0.03) and LV mass index (r = 0.21, p = 0.047). In the NCH group, VO2peak was only correlated with TAPSE (r = 0.60, p = 0.013) and LAD (r = –0.25, p = 0.04). Moreover, VE/VCO2slope was not correlated with echocardiography variables.

**Conclusion:** In patients with Chagas HF, PASP as well as myocardial function and morphology impacts the functional capacity of the subjects when compared to patients with different etiologies. In addition, the findings suggest that PH may be related to the disease severity and progression.

111891

Modality: E-Poster Young Researcher – Non-case Report

Category: NEGLECTED CARDIOVASCULAR DISEASES

## Profile of Smokers in Abusive use of Alcohol in the Process of Quitting Smoking

SAMUEL BARUD MASSENSINE^1^, Amanda Gonçalves Vieira Martins^1^, Beatriz Stephan Farhat Jorge^1^, Bianca de Fatima Pereira^1^, Fernanda Silva Mota^1^, Gabriela Godinho Rezende^1^, Moisés de Toledo Vilela^1^, Paula Gouvea Abrantes^1^, Pedro Drumond Maia^1^, Ramon Jose Moreira Silva^1^, Eliane Ferreira Carvalho Banhato^1^, Arise Garcia de Siqueira Galil^1^

(1) Cardiology Department, Medical School, Federal University of Juiz de Fora – UFJF

**Introduction:** Smoking and alcohol abuse usually coexist and have synergistic effects, leading those with this duo addiction to worse outcomes than those who only smoke. This culminates in personal and family suffering and high social cost. Then it’s important to evaluate the effects of alcohol consumption in smokers.

**Objectives:** To evaluate alcohol abuse (UAA) with clinical characteristics and smoking history, among smokers during the smoking cessation process.

**Methodology:** Longitudinal study, group “Free Tobacco”, Juiz de Fora/Minas Gerais. Users with multi-morbidity were treated between September/2021 and March/2022, in consecutive treatment groups, mixed intervention (face-to-face and telemedicine), multidisciplinary team, cognitive behavioral approach (ACC) sessions, drug treatment and follow-up.

**Definitions:** UAA, Audit-C ≥5 points. Low level of education, <8 years. Normal systolic blood pressure (SBP) <130 mmHg. Depression, Patient Health Questionnaire (PHQ-9) score ≥9 points. Cognitive deficit, Montreal Cognitive Assessment <26 points. High nicotine dependence, Fagerstrom test ≥5 points.

**Results:** The sample consisted of 36 smokers with multi-morbidity who participated in a group with a multidisciplinary team, intended for treatment for smoking cessation. UAA was found in 20% of the sample. Comparing these patients with those without this use, they were younger individuals (p < 0.049), with a higher prevalence of abdominal obesity (p < 0.002), arterial hypertension (p < 0.002), of cognitive deficit (p < 0.001) and with a tendency to greater use of crack together (p < 0.063). Allied, they had a lower prevalence of depression (p < 0.031), anxiety (p < 0.021), and curiously, sedentary lifestyle (p < 0.049). As for smoking history, they had greater nicotine dependence (0.001), greater triggers for alcohol itself (p < 0.008), for habits (p < 0.003), but not for the use of coffee (p < 0.019). Allied, greater adherence to meetings for ACC, (p < 0.001).

**Conclusion:** The association between smoking and alcohol consumption is associated with the perpetuation of this addiction and increased consumption of illicit drugs. Screening for alcohol use should be encouraged for all smokers, and interventions aimed at encouraging the cessation of both harmful habits should be the target audience. As it is also associated with other unfavorable outcomes, it is necessary to track and discourage alcohol consumption by smokers.

111922

Modality: E-Poster Young Researcher – Non-case Report

Category: PHYSIOTHERAPY

## Association of Myocardial Changes with Functional Capacity and Effects of Aerobic Physical Training in Syrian Hamsters with Chronic Chagas Cardiomyopathy

THAYRINE ROSA DAMASCENO^1^, Enrico de Francisco Magnani^1^, Rafael Dias de Brito Oliveira^1^, Jhessica Macieira Pereira^1^, Denise Mayumi Tanaka^2^, Mariana Duarte de Souza^1^, Jorge Mejia Cabeza^3^, Camila Godoy Fabricio^2^, Alessandra Arantes Resende^2^, Dawit Albieiro Pinheiro Gonçalves^1^, Marcus Vinicius Simões^2^, Luciano Fonseca Lemos de Oliveira^1^

(1) Universidade Federal de Minas Gerais (UFMG); (2) Faculdade de Medicina de Ribeirão Preto (FMRP/USP); (3) Hospital Israelita Albert Einstein

**Introduction:** Chronic Chagas cardiomyopathy (CCC) may present with morphological, functional and myocardial perfusion alterations. However, the influence of these changes in the reduction of functional capacity and the effects of aerobic physical training (APT) on them are still unknown.

**Objective:** To evaluate the association of aerobic capacity with morphology, function and myocardial perfusion as well as to evaluate the effects of APT on such variables in a model of CCC in Syrian hamsters.

**Methods:** Female Syrian hamsters infected with 3.5 × 10000 trypomastigote forms of the Y strain of T. Cruzi (n = 37) and their respective controls (n = 14) were used. Seven months after infection, the surviving animals underwent two-dimensional echocardiography, myocardial perfusion scintigraphy to assess myocardial perfusion defects (PD) and cardiopulmonary testing (CPT). The animals were then divided into 4 groups: Sedentary Chagas (CH-SED, n = 14), APT Chagas (CH-APT, n = 9), Sedentary Control (CT-SED, n = 6) and APT Control (CT-APT, n = 8). The APT was performed for 8 weeks, 5 times a week, for 50 minutes at an intensity equivalent to 50% of the peak velocity evaluated in the TCP. All animals repeated the examinations at the end of the experiment.

**Results:** At baseline, 9 (24%) of the 37 infected animals showed myocardial involvement with reduced left ventricular ejection fraction (LVEF, pANNOVA <0.01), VO2peak (pANNOVA = 0.01) and higher PD (pANNOVA = 0.01) when compared to control and infected animals without cardiac involvement. VO2peak correlated with PD (r = –0.66, p < 0.01), LVEF (r = 0.37, p = 0.02), left ventricular diastolic diameter (LVDD, r = –0.53, p < 0.01) and LV systolic diameter (LVSD, r = –0.49, p < 0.01). In the multiple regression analysis, only PD remained independently associated with VO2peak (r² = 0.42). After the follow-up period, the mixed analysis of variance showed that the groups submitted to APT and the CH-SED group showed eccentric LV remodeling with an increase in LVDD. However, an increase in LVSD and PD, as well as a decrease in LVEF were only observed in the CH-SED group. In addition, the CH-APT group showed an increase in VO2peak and an increase in the maximum distance covered after the intervention.

**Conclusion:** APT was able to prevent the progression of perfusion defects and LV dysfunction, in addition to improving functional capacity, which is determined by morphology, function and myocardial perfusion in an experimental model of CCC.

111952

Modality: E-Poster Young Researcher – Non-case Report

Category: ATHEROSCLEROSIS/CARDIOVASCULAR RISK FACTORS/CARDIOVASCULAR PREVENTION

## Correlation between Postoperative Cardiac Risks Factors, Functionality and Length of Stay

MICAELE FARIAS NASCIMENTO^1^, Beatriz Souza de Albuquerque Cacique New York^1^, Milton Antonio Gonçalves de Oliveira^1^, Kátia Elizabete Galdino^1^, Ana Tereza do Nascimento Sales Figueiredo Fernandes^1^

(1) State University of Paraíba – UEPB

**Introduction:** Cardiac surgery (CS) may be associated with several organic repercussions responsible for the appearance of cardiac risk factors during the postoperative period. These, associated with prolonged hospital length of stay (LoS), may trigger critical manifestations in individuals undergoing this surgical procedure.

**Objective:** To investigate the relationships between postoperative cardiac risk factors, LoS, and changes in functioning state.

**Methods:** Patients undergoing reconstructive, substitutive, or corrective cardiac surgeries were evaluated. The presence of postoperative cardiovascular risks was assessed using the InsCor score, while LoS and functionality were collected from medical records.

**Results:** One-hundred patients with a mean age of 59.2 ± 12.3 years were included. Significant correlations between functionality and both the hospital and Intensive Care Unit (ICU) LoS (p < 0.0001, ρ = 0.56; p = 0.002, ρ = 0.29, respectively), as well as between hospital LoS and the number of comorbidities (p = 0.003, ρ = 0.28) were found. No significant relationships were observed between the number of postoperative risk factors and LoS.

**Conclusion:** Functionality and comorbidities are associated with increased hospital and ICU LoS in patients undergoing cardiac surgery.


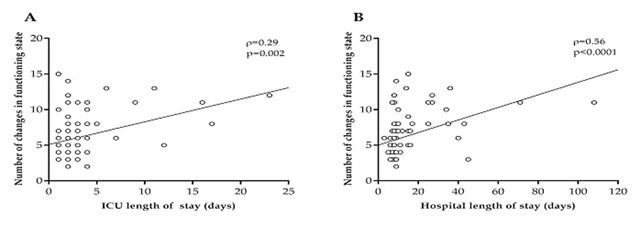


111976

Modality: E-Poster Young Researcher – Non-case Report

Category: HEART FAILURE/CARDIOMYOPATHY/TRANSPLANT

## ICD Indication in Hypertrophic Cardiomyopathy: Which Algorithm to Use?

MANUELLA DE AMORIM SILVA^4^, Marcelo Antônio Oliveira Santos Veloso^3^, Gabriela Cisneiros Arcoverde^4^, Andrea Virgínia Chaves Ferreira^3^, Eveline Barros Calado^1^, Manuel Markman^5^, Lucas Soares Bezerra^3^, Brivaldo Markman Júnior^1^, Dinaldo Cavalcanti de Oliveira^3^

(1) Hospital das Clínicas da Universidade Federal de Pernambuco HC-UFPE; (2) Serviço de doenças raras RARUS; (3) Programa de Inovação Terapêutica da Universidade Federal de Pernambuco – UFPE; (4) Hospital Alfa de Referência ao COVID-19 – HRE; (5) Hospital Agamenon Magalhães – HAM

**Background:** Hypertrophic cardiomyopathy (HCM) is the leading cause of sudden cardiac death (SCD) in youth. The recommendations for prophylactic implantable cardioverter-defibrillators (ICDs) are divergent.

**Objective:** To evaluate the agreement in the indication of ICD in patients with HCM, as per recommendations of the European Society of Cardiology 2014 and American Heart Association 2020, and evaluate fragmented QRS (fQRS) as a predictor of cardiovascular outcome.

**Methodology:** Retrospective cohort with 81 patients evaluated between 2019 and 2021. Patients with HCM ≥16 years old were included. Exclusion criteria: secondary myocardiopathy, follow-up <1 year. Kappa coefficient was used to determine the agreement. Survival and incidence curves were determined by Kaplan-Meier method. A p-value <0.05 was considered significant.

**Results:** The fQRS was identified in 44.4% of patients. There were no differences between patients with and without fQRS regarding clinical parameters, echocardiography, fibrosis and SCD risk. During follow-up of 4.8 ± 3.4 years, there was no SCD, but 20.6% patients with ICD had at least one appropriate shock. Three of the seven appropriate shocks occurred in European Society of Cardiology low-to-moderate risk patients. Three shocks occurred in moderate-risk patients and four in American Heart Association high-risk patients. Overall recommendations agreement was 64% with a Kappa of 0.270 (p = 0.007). C-statistic showed no differences regarding the incidence of appropriate shock (p = 0.644).

**Conclusion:** SCD risk stratification algorithms present discrepancies in ICD indication, both with low accuracy.

111977

Modality: E-Poster Young Researcher – Non-case Report

Category: HYPERTENSION/RENAL DENERVATION

## Descriptive Analysis of the Brazilian Black Population from the First Brazilian Hypertension Registry

MAICON BORGES EUZEBIO^2^, Maicon Borges Euzébio^2^, Priscila Valverde de Oliveira Vitorino^3^, Sayuri Inuzuka^1^, Weimar Kunz Sebba Barroso^4^

(1) UNIVERSIDADE FEDERAL DE GOIÁS UFG; (2) CENTRO UNIVERSITÁRIO DE MINEIROS UNIFIMES; (3) PONTIFÍCIA UNIVERSIDADE CATÓLICA DE GOIÁS PUC GOIÁS; (4) LIGA DE HIPERTENSÃO ARTERIAL LHA HC UFG

**Introduction:** Several studies have demonstrated a higher prevalence and severity of arterial hypertension (AH) in blacks than in whites. However, this is an aspect related to the epidemiology of AH that has been little studied in Brazil.

**Objectives:** To characterize the Brazilian black population of the first Brazilian Hypertension Registry in relation to anthropometric, clinical and pharmacotherapeutic characteristics.

**Methods:** This is a cross-sectional, multicenter descriptive study of the population subgroup of self-declared black from the First Brazilian Registry of Arterial Hypertension, whose collection was carried out from June 2013 to October 2015. A descriptive analysis was performed and the Shapiro- Wilk test was used to verify the data distribution of the variables and adopted as significant p < 0.05. Anthropometric variables were considered: Age, in years; Body mass index (BMI) in kg/m^2^; Waist circumference (WC) in cm. In the clinical variables, the presence of: Diabetes, Dyslipidemia, Cerebrovascular disease, Cardiac disease, Renal disease, Peripheral artery disease, Smoking, Physical Activity, Alcohol dependence, Systolic Blood Pressure (SBP) and Diastolic Blood Pressure (DBP) in mmHg, Blood Pressure (BP) on target (cut-point for BP control <140 × 90 mmHg), Glucose, mg/dL, Total cholesterol, mg/dL, HDL-Cholesterol, mg/dL, LDL-Cholesterol, mg/dL and Triglycerides, mg/dL. As a pharmacotherapeutic conduct, the use of: Acetylsalicylic acid, Clopidogrel, Metformin, Insulin and Statins.

**Results:** Of a total of 2,646 participants (Whites, Blacks, Browns and Yellows) from 45 locations in Brazilian regions, 452 (17.8%) declared themselves to be black. The median age was 62.5 years (54.2–69.7), 57.5% were female; BMI 29.8 ± 5.36 and WC 98 (91–106). Dyslipidemia was the main disease in 182 (40.3%) individuals, followed by Diabetes Mellitus 128 (28.3%) and other heart diseases with 93 (20.3%). Smoking was found in 31 (6.9%) participants and 273 (60.4%) reported performing some type of physical activity. The median of SBP was 139 mmHg (128–151), of BPD was 85 mmHg (80–92.6) and the frequency of controlled casual blood pressure in 233 (51.54%) participants. Statins 167 (36.9%), acetylsalicylic acid 148 (32.7%) and metformin hydrochloride 82 (18.1%).

**Conclusions:** The black population evaluated was predominantly female. On average, the body mass index was above the recommended, most had controlled blood pressure and performed some type of physical activity.

111988

Modality: E-Poster Young Researcher – Non-case Report

Category: COVID-19 AND CARDIOVASCULAR SYSTEM

## Association of Cardiovascular Comorbidities with Outcomes in Patients Hospitalized for COVID-19

LETICIA DE SOUSA PERES^1^, Thiago Moreira Bastos da Silva^2^, Iliana Regina Ribeiro Menezes^1^, Nathalia Duarte Camisão^1^, Mariana Moreno Canário da Silva^1^, Renata Mexias Abdala Felix^1^, Giovanni Possamai Dutra^1^, Anna Butter^1^, Henrique Custódio Goudar^1^, Gláucia Maria Moraes de Oliveira^2^, Bruno Ferraz de Oliveira Gomes^1^, João Luiz Fernandes Petriz^1^

(1) Hospital Barra Dor; (2) UFRJ

**Background:** During the COVID-19 pandemic, cardiovascular comorbidities were associated with greater severity during hospital stay. However, little is known about its association with other long-term outcomes and death.

**Goals:** To assess the association of cardiovascular comorbidities with myocardial injury, lung parenchyma involvement, need for mechanical ventilation, and long-term death in patients hospitalized for COVID-19.

**Methods:** Retrospective cohort study with patients who were hospitalized with a confirmed diagnosis of COVID-19. We evaluated the association of comorbidities diabetes mellitus (DM), arterial hypertension (SAH), chronic kidney disease (CKD) and obesity with the outcomes long-term mortality, in-hospital mortality, myocardial injury, lung parenchymal involvement and use of mechanical ventilation. The variables were evaluated using the chi-square method (categorical variables) and Student’s t test (continuous).

**Results:** 1454 patients were included, mean age 59.8 ± 17.0, 62.6% men. There were 269 deaths (18.5%) during the study period (mean follow-up = 338 ± 209 days). 44.7% of patients had myocardial injury. Obese subjects had greater involvement of the lung parenchyma (>50%) than non-obese subjects (18.5 × 12.1%, p = 0.013). However, they had a lower risk of long-term mortality (OR 0.70; 95%CI 0.51–0.96) as well as a lower prevalence of myocardial injury (OR 0.74; 95%CI 0.59–0.94). We observed an association of DM with long-term mortality (OR 2.30; 95%CI 1.75–3.03), in-hospital mortality (OR 2.21; 95%CI 1.65–2.96), myocardial injury (OR 1.49; 95%CI 1.18–1.87) and need of mechanical ventilation (OR 1.92; 95%CI 1.47–2.50). SAH was also associated with long-term mortality (OR 2.87; 95%CI 2.15–3.84), in-hospital mortality (OR 3.25; 95%CI 2.37–4.44), myocardial injury (OR 2.66; 95%CI 2.15–3.30), need of mechanical ventilation (OR 2.42; 95%CI 1.85–3.17) and pulmonary involvement >50% (17.1 × 11.0%, p = 0.005). Finally, CKD was associated with long-term mortality (OR 3.93; 95%CI 2.39–6.44), in-hospital mortality (OR 3.73; 95%CI 2.24–6.20), myocardial injury (OR 5.73; 95%CI 3.10–10.58) and need of mechanical ventilation (OR 2.61; 95%CI 1.58–4.31).

**Conclusion:** In patients hospitalized for COVID-19, SAH, DM and CKD were associated with long-term mortality, as well as myocardial injury and need of mechanical ventilation. On the other hand, obese individuals had lower mortality and less myocardial injury, despite the greater involvement of the lung parench.

112019

Modality: E-Poster Young Researcher – Non-case Report

Category: COVID-19 AND CARDIOVASCULAR SYSTEM

## The Role of Extracorporeal Membrane Oxygenation in Covid-Related Myocarditis: A Systematic Review

MANOEL FLÁVIO SILVA KANISKY^1^, Gerlânio César da Silva^1^, Kelvin Câmara^1^, Luís Henrique Oliveira Pereira^1^, Marjorie Francisca Raksa^1^, Matheus Mônaco Lemos^1^, Tamires Santos Pinheiro^1^, Ana Laísa Andrada Oliveira^2^, Gabrielly Machado Trindade^3^, Maria Eduarda Pereira de Oliveira^4^, Valdano Manuel^5^

(1) Universidade Cesumar (Unicesumar); (2) Faculdade Pernambucana de Saúde (FPS); (3) Centro Universitário Max Planck (UniMax); (4) Universidade Vila Velha (UVV); (5) Clínica Girassol; Complexo Hospitalar de Doenças Cardiopulmonares Cardeal Dom Alexandre do Nascimento; Instituto do Coração (InCor) do Hospital das Clínicas da Universidade de São Paulo

**Introduction:** COVID-19 can affect the myocardium causing myocarditis. Some patients need ventricular support with Extracorporeal Membrane Oxygenation (ECMO), however, its therapeutic efficacy is not clear yet.

**Objectives:** To know the role of ECMO in patients with myocarditis due to COVID-19.

**Methods:** We systematically reviewed the English literature, searching for patients over 18 years-old on Google Scholar, LILACS and PubMed, using the Preferred Reporting Items for Systematic Reviews and Meta-Analyses (PRISMA), between March 2020 to February 2022. The keywords used were “myocarditis”, “COVID-19” and “extracorporeal membrane oxygenation”. The quality of the articles was assessed by Newcastle-Ottawa Scale.

**Results:** Ten studies including 609 patients were analysed. Most of them were male (70,6%) and 29,4% female. Age range was 18 to 80 years. Mean follow-up was 103 days (10–180), the length of stay at the intensive care unit was 26 days (0,9–51,9) and at the hospital was 33 days (21–56,6). The average ECMO duration was 15 days (6,5–38,5). Its most used type was venovenous, which was applied to 469 patients; only 19 received venoarterial, in accordance with eight studies. Although three studies did not report weaning rate, 214 patients (35,1%) weaned off successfully, whereas it is estimated that approximately 165 (27%) did not wean. The most relevant ECMO-related complications, evidenced in eight articles, were: bacterial infection (207), acute kidney (156), liver injury (69) and multiorgan failure (11). The global mortality was 216 deaths (35,4%) which varied from three to 63 deaths.

**Conclusion:** Patients with severe COVID-related ventricular dysfunction submitted to ECMO achieved considerable results of successful weaning off, even though the death rate had a significant result. In addition, venovenous intervention demonstrated an effective strategy in selected patients. However, ECMO application still needs further studies to validate the benefits above its negative outcomes in these patients.

112022

Modality: E-Poster Young Researcher – Non-case Report

Category: EPIDEMIOLOGY AND HEALTH POLICIES/GLOBAL HEALTH

## Prevalence of Food Insecurity and Its Association with Health Outcomes in Patients with Chronic Chagas Disease

CELSON JÚNIO DO NASCIMENTO COSTA^1^, Paula Simplicio da Silva^2^, Roberto Magalhães Saraiva^2^, Andrea Rodrigues da Costa^2^, Alejandro Marcel Hasslocher-Moreno^2^, Luiz Henrique Conde Sangenis^2^, Marcelo Teixeira de Holanda^2^, Henrique Horta Veloso^2^, Gilberto Marcelo Sperandio da Silva^2^, Fernanda Martins Carneiro^2^, Daniel Arthur Barata Kasal^1^, Mauro Felippe Felix Mediano^1^

(1) Departamento de Pesquisa e Ensino, Instituto Nacional de Cardiologia; (2) Instituto Nacional de infectologia Evandro Chagas

**Introduction:** Chagas disease (CD) is a neglected disease that infected 6 to 7 million people worldwide. Individuals with CD are usually from low socioeconomic status and, therefore, more prone to food insecurity (FI).

**Objective:** To assess the prevalence of FI in CD and its association with clinical forms, nutritional status (NS), comorbidities, and biomarkers.

**Methods:** This is a cross-sectional study including patients diagnosed with CD (confirmed by two serological tests), from both sexes. Patients diagnosed with diseases that affect the immune system, other infectious diseases during the data collection period, those using corticosteroids or anti-inflammatory drugs, cancer patients, pregnant women, and cognitive alterations were excluded. The FI was evaluated according to the Brazilian scale of FI (EBIA 2003). The classification of the clinical form of Chagas disease was obtained following the determinations of the Brazilian Consensus on CD (2016). Parameters established by the Brazilian Institute of Geography and Statistics were used to collect the socioeconomic variables. Anthropometric measurements (weight, height, waist circumference) were collected to assess NS. Biomarkers included lipid profile and plasma glucose. Descriptive statistics included mean (standard deviation for numerical) and percentage (frequency) for categorical variables. Comparisons between participants without and with FI were performed using t-test for numerical and chi-squared test for categorical variables. Linear and logistic regression models adjusted by age, sex, education level, and race were fitted to evaluate the association between FI status and clinical forms, NS comorbidities, and biomarkers.

**Results:** Three hundred sixty individuals (56.1% of women) were included in the study, with a mean age of 60.7 (10.8) years. Of those, 30.8% had FI. Participants with FI were more likely to be women, had a lower per capita income, a lower height, a greater percentage of illiterate/incomplete elementary education, and greater frequency of obesity (Table). No significant association was observed between FI and clinical forms of CD, NS, comorbidities, and biomarkers after adjustments for age, sex, education level, and race.

**Conclusion:** Despite the elevated prevalence of FI among patients with CD, no association was observed for health outcomes. Longitudinal studies examining the impact of FI on health parameters and mortality of individuals with CD are warranted.

112038

Modality: E-Poster Young Researcher – Non-case Report

Category: EPIDEMIOLOGY AND HEALTH POLICIES/GLOBAL HEALTH

## Cardiovascular Premature Mortality between Brazilian Cities with Similar Populations but with Different Human Development Index

CAMILA DOS SANTOS MOREIRA DE SOUZA^1^, Dalmo Valério Machado de Lima^1^, Christianne Bretas Vieira Scaramello^1^

(1) UNIVERSIDADE FEDERAL FLUMINENSE UFF

**Introduction:** Cardiovascular diseases (CVD) are the main cause of death in Brazil and worldwide. Socioeconomic determinants of health are considered independent risk factors for its occurrence. The Human Development Index (HDI) summarizes local human development encompassing health, knowledge and standard of living, being important to investigate if it can be reflected in CVD premature mortality.

**Objective:** Considering the relevance of socioeconomic determinants on CVD deaths, the aim of this work was to evaluate premature cardiovascular mortality (30–69 years of age) in two Brazilian cities with similar populations and different HDI.

**Methods:** The ecological observational study evaluated proportionate CVD premature mortality in two cities of Rio de Janeiro State – Niterói and Belford Roxo – from 2008 to 2019. Populations of these cities encompass about 500,000 habitants and while Niterói presents a very high HDI (0.837), Belford Roxo presents a medium one (0.684), occupying the 1st and the 71st position in the state HDI ranking, respectively. An Excel spreadsheet was used for data analysis whose significance was accepted if p < 0.05. The local Human Ethics Committee approved the work.

**Results:** Proportionate CVD premature mortality was lower in Niterói compared to Belford-Roxo throughout the period studied (27.6 ± 1,3% × 33.5 ± 2.1%; p < 0.01). Pearson coefficient indicates a strong correlation between proportionate CVD premature mortality in these two cities despite the absence of statistical significance (r = 0.4072; p = 0.1888). According to HDI, the mean porportionate CVD premature mortality seems to vary between genders, with Belford Roxo presenting a higher proportion in females (34.2%) while Niterói shows a higher proportion in males (28.3%).

**Conclusions:** Data suggest that socioeconomic determinants may impact CVD premature mortality. It is important to investigate deeply considering ethnicity and gender aspects to allow better discussions of health policies.

112050

Modality: E-Poster Young Researcher – Non-case Report

Category: ACUTE AND CHRONIC CORONARY DISEASE/THROMBOLYSIS

## Access to the Healthcare System of Patients with St-Segment Acute Myocardial Infarction: Has There Been a Change During the Covid 19 Pandemic?

RHANNIEL THEODORUS HELHYAS OLIVEIRA SHILVA GOMES VILLAR^1^, José Victor de Sá Santos^3^, Murilo Jorge da Silva^3^, Victor Luis Peixoto Pereira Botelho^3^, Bianca Aparecida Colognese^3^, Márcio Andrade Barreto Filho^4^, Marcelo Vincenzo Sarno Filho^3^, Pollianna de Souza Roriz^1^

(1) Protocolo IAM – SAMU Salvador; (2) Hospital Ana Nery; (3) Universidade Federal da Bahia; (4) Escola Bahiana de Medicina e Saúde Pública

**Introduction:** The pandemic caused by the coronavirus that started in 2020 changed the pre-established dynamics in health systems and also in the user’s itinerary in the search for medical care. Before, larger general hospitals shared the entrance of patients in the emergency network with pre-hospital units in a similar way.

**Objective:** Identify possible changes in the itinerary of patients with Acute Myocardial Infarction with ST segment elevation (STEMI) in the urgency and emergency care network in the city of Salvador-BA.

**Methods:** An ambispective study with patients with STEMI treated by the Acute Myocardial Infarction Protocol (AMIP) in Salvador during the pre-pandemic (Jan/2019 to Feb/2020) and pandemic (March/2020 to Dec/2021) period. The AMIP is a group designed to assist the entire process of care for patients with suspected STEMI, from diagnosis to reperfusion, in addition to training health professionals for management. Data were collected regarding the unit of origin: fixed pre-hospital (FPH) and general hospital (GH); assistance times; and mortality. Statistical significance was considered for p < 0.05.

**Results:** In the pre-pandemic period, AMIP performed 542 attendings to STEMI, with 25.3% of access through GH. During the pandemic period, 935 visits to STEMI were performed, with the GH being the gateway in 12.0% of the calls (p < 0.001). During the pandemic, there was a higher frequency of activations through the FPH compared to the pre-pandemic period (76.3% × 61.4%; respectively; p < 0.001). Comparing the pandemic to pre-pandemic period, there was a shorter door-to-ECG time [33 (15–77) min × 38 (19–95) min, respectively; p = 0,04] and a shorter door-to-needle time [115 (80–180,5) min × 149 (101,8–206) min, respectively; p < 0,001] in the FPH access. Regarding GH entrance, comparing pandemic to pre-pandemic period, there was a lesser door-to-ECG time [28 (13,5–67) min × 44 (17–149) min, respectively; p = 0,01] and a lesser door-to-ballon time [221 (158,3–294,5) min × 297,5 (203,5–415,8) min, respectively; p = 0,049]. There was no difference in mortality between the groups (15.8% GH × 16.5% FPH; p = 0.92).

**Conclusion:** After closure of general hospitals for non-COVID emergencies during the pandemic, there was a shift in the demand for medical care of patients with STEMI to the pre-hospital units, with shorter care times and without a major impact on mortality in our sample.

112061

Modality: E-Poster Young Researcher – Non-case Report

Category: EPIDEMIOLOGY AND HEALTH POLICIES/GLOBAL HEALTH

## Access to High-Complexity Cardiac Technologies in the Brazilian Public Health System: A 12-Year Real-World Data Study on Cardiac Implantable Electronic Devices

MIRIAM ALLEIN ZAGO MARCOLINO^1^, Miriam Allein Zago Marcolino^1^, Rodrigo Antonini Ribeiro^2^, Luis E. Rohde^3^, Carisi Anne Polanczyk^4^

(1) Graduate Program in Epidemiology, Universidade Federal do Rio Grande do Sul, Porto Alegre, Brazil; (2) National Institute of Science and Technology for Health Technology Assessment – INCT/IATS (CNPq 465518/2014-1), Porto Alegre, Brazil; (3) Cardiology Center, Hospital Moinhos de Vento, Porto Alegre, Brazil; (4) Graduate Program in Cardiology and Cardiovascular Sciences, Universidade Federal do Rio Grande do Sul, Porto Alegre, Brazil

**Introduction:** Cardiac implantable electronic devices (CIED) implantation and other high-complexity procedures in the Brazilian Public Health System (SUS) are restricted to tertiary care centers located in strategic geographic regions that should provide integral access to care with equity to all population.

**Objective(s):** To describe geographic variations in the access to CIED implantation in the SUS over 12 years.

**Methods:** A retrospective epidemiological study including open data of all hospital admissions with CIED implantation as primary procedure reimbursed by SUS from 2008 to 2019, obtained from the Hospital Information System of SUS Informatics Department (DATASUS). The mean annual rate per million (MM) population per region and state of residency was age and sex standardized by the OMS standard population. All analyses were performed using R.

**Results:** SUS reimbursed 216,440 CIED implantations in the period (87.1/MM). Implantation rates varied according to residency region (South 124.9, Central-West 124.6, Southeast 86.7, Northeast 75.4, North 64.6/MM). The national distribution of different types of CIED implants showed distinct patterns of access by the residents of each state. (Figure 1)

**Conclusions:** Although the regionalization principle of SUS should ascertain equity in the access to health care, 12-year data reveal disparities in the access to different types of CIED in Brazil, showing the need of management actions on this field.


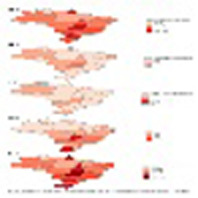


112098

Modality: E-Poster Young Researcher – Non-case Report

Category: EPIDEMIOLOGY AND HEALTH POLICIES/GLOBAL HEALTH

## Epidemiological Profile of Heart Failure in North Region Brazil between 2017 and 2021

JOSÉ PEDRO DA SILVA SOUSA^1^, José Pedro da Silva Sousa^1^, Evaldo da Costa Sá Borges de Rezende^2^, Maria Eliza Alves Teixeira^3^, Juliana Leite de Oliveira^2^, Daniel Sorna Labeca Guerra^2^, Anna Luiza Alves de Oliveira Miranda^1^, José Wilker Gomes de Castro Júnior^1^, Beatriz Siems Tholius^1^, Maria Eduarda dos Santos Lopes Vasconcelos^1^, Matheus Rocha Maia^1^

(1) Centro Universitário do Estado do Pará; (2) Universidade Federal do Pará; (3) Universidade de Gurupi

**Introduction:** Heart failure (HF) is a syndrome that the main characteristic is the inability of heart to pump enough blood that the body’s needs, due to damages in contraction and/or relaxation.

**Objective:** Trace the epidemiological profile of HF patients in the Northern Region of Brazil between 2017 and 2021.

**Methods:** Epidemiological, quantitative and observational study, with a retrospective design based on Health Unic System Informatical Department (DATASUS) data, through access to information on Epidemiology and Morbidity (SUS Hospital Morbidity) on HF from January 2017 to December 2021.

**Results:** The age group with the highest number of hospitalizations, corresponding to 248,343 patients and 27.1% of hospitalizations in Brazil for HF, involved patients between 70 and 79 years of age. In relation to the region, there were 46,403 hospitalizations in the North of the country, with of these, 12,272 (26.4%) were patients between 70 and 79 years. In the analysis between sexes in the same region, the number of hospitalizations among men is higher (58.9% of hospitalized patients). When it comes to the mortality rate, the prevalence is among patients over 80 years old (17.64%) and the North region takes the second position with 12.30% of the total. In the analysis related to sex, in the North, the mortality rate is higher in females (12.97%).

**Conclusion:** Data analysis corroborates the existence of an intrinsic relationship between advanced age and the hospitalization rate of patients with HF in the North region. This scenario is the result of the natural aging of individuals and the bad prognosis of the disease, which the survival rate is 35% after 5 years of diagnosis. In addition, it was observed that the North region ranks second among the Brazilian regions that have the highest mortality rate in patients over 80 years of age, which denotes inefficiency in the hospital management of this group. Furthermore, the study indicated a higher rate of hospitalization associated with men, due to their more negligent lifestyle in relation to health, but it identified higher mortality rates in females, which indicates less efficient therapeutic approaches for women in the management of HF.

112105

Modality: E-Poster Young Researcher – Non-case Report

Category: NURSING

## Digital Interventions for Patients with Heart Failure and the Inequalities: Preliminary Analysis of the Egresos-IC Study

EDMAR GERALDO RIBEIRO^1^, Lilian Cristina Rezende^1^, Júlia Bicas Buback^1^, Tulio Batista Franco^2^, Deborah Carvalho Malta^1^, Luisa Campos Caldeira Brant^1^

(1) Universidade Federal de Minas Gerais UFMG; (2) Universidade Federal Fluminense UFF

**Introduction:** Heart failure (HF) is the main cause of clinical hospitalizations for cardiovascular diseases in Brazil. Hospitalization for HF is related to adverse outcomes, including loss of quality of life, rehospitalizations and death. Digital health interventions (DHI) to improve cardiovascular care is an evolving field, with some studies showing a reduction in adverse outcomes in high-income countries. However, social disparities may be a barrier to the implementation of DHI in low resource settings, particularly due to lower access and education.

**Purpose:** To compare the sociodemographic characteristics of patients discharged from hospital due to HF who refused or accepted to participate in a clinical trial that aims to evaluate the effectiveness of a DHI to improve HF care.

**Methods:** In a randomized clinical trial with patients discharged from hospitalization for HF in a Brazilian capital city, 527 patients were screened in 6 months in 5 public hospitals, which accounted for 73% of hospitalizations for HF in the city. From these, 448 were not eligible, 54 refused to participate, and 79 participants were randomized in the study (43 in the intervention group and 36 in the control group). In a preliminary analysis, sex, age, race, marital status, education, and living in or outside the city were compared between those who refused or accepted to participate in the trial using Fischer’s exact test. A p-value <0.05 was considered statistically significant.

**Results:** Individuals who refused to participate in the study were older (69 vs. 62y, p < 0,001), less educated (53 vs. 30% with less than 4y of education, p = 0.007), with a greater proportion of individuals who self-reported their race as black/pardo compared to white (88 vs. 73%, p < 0.05), and lived more frequently outside the capital city (36 vs. 20%, p < 0.05) than those who accepted. There was no difference between the proportion of females or marital status between the groups.

**Conclusions:** DHI have the potential to overcome major barriers in HF care, such as: access to GDMT care, treatment adherence, and health education. However, our results show that among the eligible population, the most socially vulnerable individuals refused to participate in the trial. As such, DHI studies must evaluate and address the barriers for the acceptance of the intervention to guarantee that DHI in HF care do not increase inequalities.

112108

Modality: E-Poster Young Researcher – Non-case Report

Category: EPIDEMIOLOGY AND HEALTH POLICIES/GLOBAL HEALTH

## Temporal Trends in Cardiac Electronic Implantable Devices Implantation in the Brazilian Public Health System: A 12-Year Real-World Data Study

MIRIAM ALLEIN ZAGO MARCOLINO^1^, Rodrigo Antonini Ribeiro^2^, Luis E. Rohde^3^, Carisi Anne Polanczyk^4^

(1) Graduate Program in Epidemiology, Universidade Federal do Rio Grande do Sul, Porto Alegre, Brazil; (2) National Institute of Science and Technology for Health Technology Assessment – INCT/IATS (CNPq 465518/2014-1), Porto Alegre, Brazil; (3) Cardiology Center, Hospital Moinhos de Vento, Porto Alegre, Brazil; (4) Graduate Program in Cardiology and Cardiovascular Sciences, Universidade Federal do Rio Grande do Sul, Porto Alegre, Brazil

**Introduction:** Cardiac implantable electronic devices (CIED) are technologies long available in the Brazilian Public Health System (SUS). The aging population and increased incidence and prevalence of cardiovascular diseases can impact the rates of CIED implantation, which are essential information for health policy strategies.

**Objective(s):** To describe the temporal trend of CIED implantation in the SUS before the pandemic, according to devices and patient characteristics.

**Methods:** A retrospective epidemiological study including open data of all hospital admissions with CIED implantation as a primary procedure reimbursed by the SUS between 2008 and 2019, obtained from the Hospital Information System of the SUS Informatics Department (DATASUS). The annual rate per million (MM) population per CIED type was age and sex standardized by the OMS standard population. All analyses were performed using R.

**Results:** In the period, 216,440 CIED implants were reimbursed by SUS, with a mean annual rate of 87.1/MM, mostly pacemakers (78.1/MM). Despite the growing number of implants per year, from 14.403 to 20.635 (+43.2%), CIED implantation rate reduced from 92.8 in 2008, to 83.7/MM in 2019 (–9.7%), mainly due to the reduction of pacemaker implants (–12.3%). Cardiac resynchronization therapy (CRT) decreased 57% from 2008 to 2019 (3.55 to 1.53/MM), while CRT with defibrillator implantation increased 3.5 times (0.82 to 2.87/million) and implantable cardioverter-defibrillator (ICD) increased 30%. (Figure 1).

**Conclusions:** There was a decrease in the implantation rates of CIED in SUS, mainly derived from a decrease in the implantation of pacemakers and CRT. The decrease in the implantation rates may be due to an unbalance between the populational growth and the increase of these technologies‘ access in the period. Moreover, implantation of CRT with a defibrillator and ICD increased in the observed period, in accordance with contemporary guidelines.


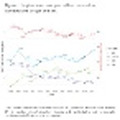


112119

Modality: E-Poster Young Researcher – Non-case Report

Category: CARDIOVASCULAR SURGERY

## Study of the Prevalence of Functional Alterations in the Postoperative Period of Cardiac Surgeries According to the International Classification of Functioning, Disability and Health (ICF)

MICAELE FARIAS NASCIMENTO^1^, Beatriz Souza de Albuquerque Cacique New York^1^, Milton Antônio Gonçalves de Oliveira^1^, Kátia Elizabete Galdino^1^, Ana Tereza do Nascimento Sales Figueiredo Fernandes^1^

(1) State University of Paraíba – UEPB

**Introduction:** Functional changes can now be assessed in a more complete and reliable way through the International Classification of Functioning, Disability and Health (ICF), created by the World Health Organization (WHO), improving communication between interested people and health professionals and bringing an important facility for international comparisons.

**Objective:** To investigate the prevalence of functional changes in the postoperative period of cardiac surgeries based on the ICF codes.

**Methods:** Cross-sectional study, where 100 patients undergoing reconstructive cardiac surgery (coronary artery bypass grafting and valve repair), replacement (valve replacement) and correction (correction of congenital heart diseases) in a public referral hospital in Cardiology were evaluated, with prior permission from the patient regarding the use of their postoperative data. The functional alterations present were evaluated through the ICF, according to the 8 chapters of the Body Functions Component. The data computed through the form used were collected, quantified and presented through means, standard deviation and percentages.

**Results:** The mean age was 59.2 ± 12.3 years. Changes in sensory and pain functions (96%) were the most present postoperatively, followed by functional changes in the cardiovascular system, hematological and immune systems, and respiratory system (31%), mental functions (17%), functions of the digestive system and metabolic and endocrine systems (5%), neuromusculoskeletal and movement-related functions (4%), and changes in genitourinary and reproductive functions (3%). No alteration of voice and speech functions (Chapter 3) was presented in the study patients. Chest pain after the surgical procedure was reported by 98% of the patients, being justified mainly by the sternotomy process performed in cardiac surgeries.

**Conclusion:** Cardiac surgeries have several organic repercussions that can alter physiological mechanisms and may lead to a critical postoperative state, implying the need to reinforce evaluation methods and intensive care in order to establish a good recovery for patients.


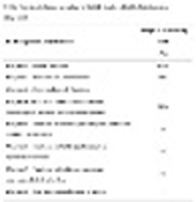


112148

Modality: E-Poster Young Researcher – Non-case Report

Category: ACUTE AND CHRONIC CORONARY DISEASE/THROMBOLYSIS

## Spontaneous Coronary Artery Dissection. Initial Experience of the Brazilian Scalibur Registry

PAULA SANTIAGO TEIXEIRA^2^, Julio Paiva^2^, Alessandra Oliveira^2^, Carlos Campos^3^, J. Ribamar Costa^3^, Henrique B Ribeiro^2^, Milena Fonseca^1^, Bárbara Freitas^1^, Jamil Cade^1^, Breno O. Almeida^1^, Adriano Caixeta^1^

(1) HOSPITAL ISRAELITA ALBERT EINSTEIN; (2) UNIVERSIDADE FEDERAL DE SAO PAULO; (3) INSTITUTO DO CORAÇÃO DO HCFMUSP

**Background:** Spontaneous coronary artery dissection (SCAD) is a non-atherosclerotic, traumatic or iatrogenic epicardial dissection. The pathophysiology is not yet fully elucidated, but is related to triggering factors such as emotional stress, physical stress, or pregnancy. It occurs by intramural hematoma formation or intimal rupture that obstructs the coronary artery, causing acute myocardial infarction, especially in young women (<50 years). Demographic, clinical and angiographic description of SCAD in a Brazilian cohort is poorly explored.

**Objective:** To evaluate the demographic, clinical, angiographic profile and triggers of SCAD in a Brazilian population.

**Methods:** The SCALIBUR registry is a retrospective and prospective study of patients with SCAD involving 22 Hospitals in Brazil. REDcap database review in the period 2010–2022.

**Results:** There were 183 patients with SCAD, with a mean age of 50.15 ± 10.56 years (29 to 84 years) with prevalent incidence in female gender (85%). Patients had none or few risk factors for coronary artery disease, including hypertension (6%), family history of early coronary artery disease (22.5%), mixed dyslipidemia (5%), and active smoking (18%). Ten percent of SCAD cases occurred in the gravid-puerperal cycle. Most cases of SCAD manifested as acute myocardial infarction without supra-ST, NSTEMI (45%), acute myocardial infarction with supra-ST, STEMI (34%), and unstable angina (9%). Among the triggering factors, present in 57.8% of the cases, emotional stress (21%) and menopause (16%) stand out. Fibromuscular dysplasia, not systematically investigated, was observed in a few cases (7%), pregnancy (0.5%), puerperium (9%), physical stressor (5.4%), use of hormone therapy (3%) and psychiatric diseases (2.7%). The anterior descending artery was the most affected vessel, followed by the right coronary and circumflex arteries. SCAD type II occurred in (48.82%). Most frequent serious complications were cardiogenic shock (3.27%) and cardiac arrest (1%).

**Conclusion:** In this large Brazilian cohort, SCAD affected mostly young women with no or few classical risk factors for coronary heart disease and the predominant trigger was emotional stress. It is an underdiagnosed condition, but should be considered in the differential diagnosis of acute myocardial infarction in young women. Demographic and angiographic findings seems to be similar to other ethnicities including in the United States, Canada and Europe.

112149

Modality: E-Poster Young Researcher – Non-case Report

Category: CONGENITAL AND PEDIATRIC CARDIOLOGY

## Identification of a Novel Pathogenic Variant in FBN1 Associated with Marfan Syndrome

JULIANA DA ROCHA FERREIRA^1^, Juliana da Rocha Ferreira^1^, Julia Passarelli Pereira^1^, Anna Paula Arpini Botelho^1^, Marcelo Machado Melo^1^, Glauber Monteiro Dias^2^

(1) Instituto Nacional de Cardiologia – INC; (2) Universidade Estadual do Norte Fluminense Darcy Ribeiro – UENF

Aortic diseases arising in Marfan Syndrome (MFS), such as in aneurysms and dissections of the thoracic aorta, are related to genetic alterations in the FBN1 gene. Databases, such as Universal Mutations-FBN1, ClinVar and The Human Gene Mutation, contain more than a thousand FBN1 mutations associated with MFS. The FBN1 gene, which encodes fibrillin-1, is responsible for the integral production of different protein domains. Possible genetic changes may lead to a weakening of blood vessels, leading to the development of aortopathies. In this study, we present the association of a novel FBN1 variant with MFS. The proband is a man who presented ascending aortic aneurysm and dissection (TAAD) at 42-yr-old, which was surgically treated. Clinical investigations were performed in all family members enrolled in the study. Marfan signs were observed in the proband, daughters and granddaughter. Direct sequencing of the FBN1 gene in the proband identified a novel truncation variant p.(Glu2019Ter) and a cascade screening were done. The variant was classified as pathogenic and causal for MFS according to the American College of Medical Genetics and Genomics (ACMG) criteria and revised Ghent nosology for MFS diagnosis, respectively. Proband’s daughter and granddaughter harbor the variant, however without aortic alteration. This work reports for the first time a patient with the FBN1-p.(Glu2019Ter) variant and its association with MFS/TAAD.

112121

Modality: E-Poster Young Researcher – Non-case Report

Category: CONGENITAL AND PEDIATRIC CARDIOLOGY

## Angiographic Findings in Heart Transplant Patients and Their Correlation with Survival

JOÃO GABRIEL GUEDES DA CUNHA MELLO^1^, João Gabriel Guedes da Cunha Mello^1^, Santiago Raúl Arrieta^1^, Pedro Alves Lemos Neto^1^, Marcelo Biscegli Jatene^1^, Cauyna Gurgel Moreira^1^, Estela Azeka^1^

(1) Instituto do Coração/InCor

**Introduction:** Heart transplantation is an effective option for the treatment of end-stage heart failure. Cardiac allograft vasculopathy (CAV) is considered one of the leading causes of long-term mortality in these patients. The objective of this study is to evaluate the type of angiographic lesions and the impact of CAV on the survival of heart transplant recipients at a referral center.

**Methods:** Retrospective cohort of cineangiography between 2013 and 2022 in patients that underwent heart transplantation. Demographic, clinical and examination data were obtained from medical records as well as the institutional database.

**Results:** The number of transplanted patients during the period of the study was 196, and coronary angiography was performed in 130 (66.3%) of them. Of these (n = 130), 65 (50%) were males, the median age at transplantation was 9.6 ± 7.8 years, and the median age at diagnosis of the first lesion was 6.2 years. CAV was observed in 29 of them (22.3%). The affected arteries followed the given profile: left anterior descending (LAD) 30.9%, right coronary 16.9%, marginal 14.1%, circumflex 12.7%, posterior descending and posterior ventricular 12.7%, diagonal 8.5% and left main coronary artery 4.2%. The CAV classified as moderate and severe were the most common (37.9% each). The event-free survival curve in transplant patients with CAV shows significant sequential drops in 10 years of transplantation, when compared to the curve for patients without CAV (log-rank p = 0.0001). Other coronary angiographic findings correspond to 11.6% of the total of exams, which were, coronary-cavitary fistulas, pulmonary artery fistulas, mediastinal venous plexus fistulas, myocardial bridges, single ostium coronary artery, anomalous origin of coronary artery and thrombi.

**Conclusions:** A prevalence of 22.3% of CAV was observed in transplanted patients that underwent coronary angiography. Moderate and severe lesions were the most common, and the LAD artery was the most affected. CAV has a major negative impact on event-free survival in transplant recipients.


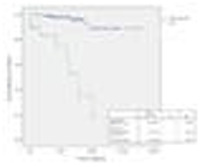


112131

Modality: E-Poster Young Researcher – Non-case Report

Category: HEART FAILURE/CARDIOMYOPATHY/TRANSPLANT

## Contribution of Cardiovascular Risk Factors to Non-Ischemic Left Ventricular Systolic Dysfunction in Patients Referred to Myocardial Perfusion Imaging

MARIA EDUARDA KOSTECKI^1^, Wilson Nadruz Junior^2^, Odilson Marcos Silvestre^3^, Ramon Conde^4^, Daniel Macedo^4^, Ana Carolina de Franca^5^, Giulia Caldeira Gaelzer^5^, Mariana De Nadai Andreoli^4^, Carlos Cunha^6^, Rodrigo Julio Cerci^6^, João Vicente Vitola^6^, Miguel Morita Fernandes-Silva^6^

(1) Hospital Santa Casa de Misericórdia de Curitiba (HSC); (2) Universidade Estadual de Campinas (Unicamp); (3) Universidade Federal do Acre (UFAC); (4) Universidade Federal do Paraná (UFPR); (5) Pontifícia Universidade Católica do Paraná (PUCPR); (6) Quanta Diagnóstico por Imagem (QDI)

**Background:** Cardiovascular risk factors (RF) can lead to left ventricular (LV) remodeling, regardless of the presence of coronary artery disease (CAD). The contribution of each RF to non-ischemic LV dysfunction in the population is uncertain.

**Aim:** To estimate the proportion of non-ischemic LV systolic dysfunction attributable to each CV risk factor in patients undergoing myocardial perfusion imaging (MPI).

**Methods:** This is a cross-sectional study of patients undergoing MPI in an imaging center in Curitiba, Brazil, from 2010 to 2021. We excluded those with known previous coronary artery disease or any perfusion myocardial defects at MPI. Data on traditional CV risk factor, including hypertension, diabetes mellitus, and smoking status, were obtained during medical history before MPI exam. Obesity was defined as body mass index ≥30 Kg/m^2^. LV dysfunction was defined as LV ejection fraction below 50% from rest gated-SPECT. The population attributable fraction (PAF) for LV dysfunction was estimated for each CV risk factor.

**Results:** We evaluated 28,156 patients (60 ± 12 years old, 51% women), 632 (2%) having VL dysfunction. After adjusting for age, sex and body mass index, hypertension (adjusted Odds Ratio [95% Confidence Interval]: 1.34 [1.12, 1.60]), diabetes mellitus (adj OR: 1.43; 95%CI 1.18, 1.74) and smoking (adj OR: 1.69; 95%CI 1.35, 2.13) were independently associated with LV dysfunction, but obesity was not (adj OR: 0.89; 95%CI 0.68, 1.17). Hypertension had the highest PAF for LV dysfunction, followed by diabetes mellitus and smoking.

**Conclusion:** In this large study of patients referred to MPI, hypertension and diabetes mellitus were the CV risk factors that mostly contributed to non-ischemic LV dysfunction in the population, followed by smoking status.

112158

Modality: E-Poster Young Researcher – Non-case Report

Category: COVID-19 AND CARDIOVASCULAR SYSTEM

## Coronary Artery Calcification as Risk Factor for Intensive Care Unit Admission Among Adults with COVID-19

GABRIELE CARRA FORTE^1^, Cristina Carra Forte^1^, Rubens Gabriel Feijó Andrade^1^, Luis Miguel Millan Diaz^1^, Bruno Hochhegger^1^

(1) Pontifícia Universidade Católica do Rio Grande do Sul – PUCRS

**Background:** Severe acute respiratory syndrome coronavirus 2 is recognized as a multisystemic disease. Clinical studies have reported a complex interplay between cardiovascular disease and worse outcomes in COVID-19 patients.

**Purpose:** To evaluate the association between coronary artery calcification (CAC) and intensive care unit admission among COVID-19 patients.

**Methods:** This is a retrospective cohort study design. All eligible patients underwent a chest CT scan, who were admitted in tertiary university hospital, at the first wave from April 01 until December 31, 2020, were included. A positive COVID-19 case was defined as one polymerase chain reaction test positive for COVID-19. Clinical outcomes were collected from electronic medical records. CAC was graded with a semiquantitative grading system and classified as 0 = absent, 1 = mild, 2 = moderate, and 3 = severe. All data were rated in consensus by 2 readers. The readers were blinded for patient outcomes.

**Results:** 441 consecutively admitted COVID-19 patients were included. Among them, 159 (36.4%) had coronary calcification: 80 (18.3%) patients classified as mild degree, 73 (16.7%), as moderate and six (1.4%) as severe. Sixty-four (14.5%) required admission to an intensive care unit; and 54.7% of these required invasive mechanical ventilation. The presence of CAC was associated with intensive care unit admission (OR 4.37, 95%CI 2.46–7.74, p < 0.001), adjusted for multiple confounders.

**Conclusion:** Estimated risks for intensive care unit admission were consistently higher for patients with COVID-19 and coronary artery calcification. In addition, including CAC standard chest CT for these patients may help guide clinical decision and prognosis.

112161

Modality: E-Poster Young Researcher – Non-case Report

Category: NURSING

## Impact of Telemonitoring as a Care Tool in Patients with Heart Failure: Integrative Review

NICHOLLAS COSTA ROSA^1^, Brenda Gonçalves Donay Alves^1^, Melissa Schiwe^1^

(1) Centro Univeristário Ritter dos Reis

**Introduction:** Heart failure (HF) is the final pathway of most heart diseases, it has a great impact on the quality of life of patients with this condition, due to progressive symptoms. Telemonitoring of patients with HF aims to reduce hospitalizations, mortality and improve quality of life.

**Objective:** To identify the impact of telemonitoring as a care tool in patients with HF.

**Method:** This study is an integrative review. The bibliographic search was carried out from August to October 2021. The combination of the following descriptors in health sciences (DeCS) was used as a search strategy: “Telemonitoring” and “Heart failure” crossed using the Boolean connector AND or OR. Randomized clinical trials articles were included, indexed in the aforementioned databases in the last 5 years (2016 to 2021).

**Results:** The search resulted in 285 articles, from the reading of the title and abstract, 60 publications were selected, after reading in full, the final sample consisted of 12 articles. The study by Feijó et al. 2021, had 206 randomized patients and demonstrated that the use of a diuretic adjustment algorithm improves the combined outcome in these outpatients, demonstrating a reduction in HF admissions and clinical instability. The studies by Koehler et al. 2021 and Kalter Leibovici et al. (2017), randomized 710 and 1360 patients respectively, demonstrated that care improved depressive symptoms and had a positive influence on quality of life in patients with HF and moderate depression. The study by Winkler et al. 2021, obtained 1119 randomized patients, as a result obtained a reduction in unplanned hospitalizations and emergency calls could be considered a marker of higher morbidity and mortality. Galinier et al. (2020) and Frederix et al. (2018), randomized 937 and 160 patients respectively, telemonitoring did not result in a significantly lower rate of all-cause deaths or unplanned hospitalizations in patients with HF, but in the study of there was a significant reduction in the number of days of hospitalization for HF.

**Conclusion:** Telemonitoring is a tool that has great potential to improve self-care regardless of the patient’s clinic, however, impacts on readmissions and mortality were not a consensus in all studies. This can be attributed to the different ways of applying telemonitoring and different audiences addressed in each study.

112173

Modality: E-Poster Young Researcher – Non-case Report

Category: ANTICOAGULATION

## Correlation of In-Hospital Mortality in Elderly Patients and Those with Prolonged Hospital Stays between Clinical and Surgical Inpatients Recently Diagnosed with Venous Thromboembolic Disease

BERNARDO CLETO TELES E SILVA^1^, Lara Camporez Menezes Trindade^1^, Priscila Diaz^1^, Antonio Amaral^1^, Ana Pedroso^1^, Joao Reis^1^, Flavia Moises^1^, Alessandra Godomiczer^1^, Monica Amorim^1^, Claudio Carvalho^1^, Thaisa Garcia^1^, Andrea Haddad^1^

(1) Unimed Rio

**Goals:** To evaluate data from patients who had thromboembolic events during hospital stay from Jan 2020 to Mar 2021, correlating length of stay and age with in-hospital mortality in a private quaternary hospital.

**Methods:** Retrospective cohort where consecutive patients admitted to a private quaternary hospital from January 2020 to March 2021 were evaluated, for clinical and surgical hospitalization for at least 48 h. All were evaluated according to risk stratification for VTE by the physician and prophylaxis instituted according to the risk found. Patients admitted with DVT and/or PE, arterial thrombosis events and those in palliative care were excluded from the analysis. DVT (distal and proximal), device thrombosis (PICC and AVP) and PE events were recorded. These outcomes were confirmed with EchoDoppler imaging.

**Results:** We correlated data on thromboembolic events with length of stay and age with an increased risk of in-hospital mortality. In the described period, there were 126 events, of which 120 (92.5%) were DVT and 6 (4.8%). The mean age was 70.8 ± 12.3 years. The age group distribution is shown in table 1. 74 of the patients (58.7%) were male and the mean BMI was 29.6 ± 6.4 kg/m^2^. The most common risk factors were age >65 years (89.7%), immobility or bedridden (49.2%), neoplasia (13.6%) and surgery or trauma (11.1%). Among patients who had VTE during hospitalization, 63.5% were hospitalized due to COVID-19 infection (figure 1), 35% remained hospitalized for 7–29 days (Graph 1) and 49 patients (38.9%) died. A total of 78.5% were hospitalized for >7 days. 48 patients died, of which 60.3% had more than 7 days of hospitalization among patients who had VTE. There is a correlation between length of hospital stay and higher risk of VTE and mortality, especially in patients with COVID19.

**Conclusão:** VTE is a frequent condition in hospitalized patients and is related to longer hospital stay, as well as advanced age with in-hospital mortality. COVID-19 caused patients to stay in hospital longer, which increased the frequency of VTE.

112193

Modality: E-Poster Young Researcher – Non-case Report

Category: EPIDEMIOLOGY AND HEALTH POLICIES/GLOBAL HEALTH

## Hospitalization and Death Secondary to Different Circulatory System Diseases between 2015–2021: A Brazilian National Database Analysis

GIOVANNA LÍDIA GONDIM OLIVEIRA DIAS^1^, Paulo Carvalho Ximenes de Aragão Filho^1^, Joaquim Francisco Cavalcante Neto^1^, Mateus de Sousa Cavalcante^1^, Leandro Cordeiro Portela^1^, Lucas Almeida Magalhães^1^

(1) Federal University of Ceará

**Introduction:** Cardiovascular diseases are the most prevalent comorbidities worldwide and one of the main causes of hospital admissions and mortality, including in Brazil. It is essential to identify the most common conditions in this morbidity group and to guide populations on how to prevent the development of these diseases and their respective negative outcomes.

**Methods:** This is a descriptive, cross-sectional, prevalence study retrospective in nature. The objective is to compare the number of hospitalizations and deaths in Brazil among the main diseases of the cardiovascular system, between 2015–2021. The data used for this work came from DATASUS, in a public electronic data platform of the Brazilian government named “TabNet”. The following informations were consecutively selected: “Epidemiology and Morbidity”, “Hospital Morbidity on SUS (SIS/SUS)”, “General, by hospitalization site – from 2008”, and “Brazil by regions”. The line for table it is classified by “ICD-10 Morb List”, in the column has been selected “Year of attendance” and in the content has been placed “Admissions”, “Deaths” and “Mortality Rate”. The period settled was between “January 2015” and “December 2021”.

**Results:** In Brazil, between 2015–2021, the number of hospitalizations due to circulatory system diseases was 7,714,326, of which 667,815 patients died. Among these hospitalizations, 1,374,104 were due to heart failure – with 156,484 deaths; 1,082,705 due to stroke (ischemic or hemorrhagic) – with 169,224 deaths; 841,559 due to acute myocardial infarction – with 87,432 deaths; 1,017,709 from other ischemic heart diseases – with 27,162 deaths; 457,861 due to varicose veins of the lower limbs – with 1,707 deaths; 440,936 due to conduction disorders and cardiac arrhythmias – with 52,139 deaths; and other morbidities with fewer hospitalizations.

**Discussion:** Analyzing the results presented, the impact of cardiovascular diseases on the public health system is undeniable. Annually, it corresponds to a large number of hospital admissions in Brazil. The comorbidity that caused the highest number of hospitalizations in the last six years was heart failure, followed by stroke. However, stroke has the highest gross number of deaths and the highest mortality rate. In addition, not all diseases with high hospitalization evolve with a significant number of deaths, such as varicose veins of the lower extremities and other ischemic heart diseases (except acute myocardial infarct).

112220

Modality: E-Poster Young Researcher – Non-case Report

Category: PHYSIOTHERAPY

## Comparison between Face-to-Face Rehabilitation and Cardiopulmonary Telerehabilitation in Post Covid

KAMYLLA MARIA ALCANTARA SILVA ALVES^1^, Kamylla Maria Alcantara Silva Alves^1^, Ana Eugênia Vasconcelos do Rêgo Barros^1^, Bruna Thays Santana de Araújo^1^, Talyta Oliveira de Almeida^1^, Daniella Cunha Brandão^1^

(1) UFPE

The pandemic caused by the SARS-Cov-2 virus brought several new challenges in the health area, among them, in the lives of patients, generating a decrease in functional capacity and an increase in the fatigue of patients who contracted the disease, having a direct impact However, as in the context of the current pandemic, the recommendation is to maintain as much social isolation as possible, telerehabilitation has proved to be an alternative resource to help improve the functional capacity of these patients and improve their quality of life.

**Objective:** To compare the effectiveness of face-to-face rehabilitation (PR) and telerehabilitation in relation to quality of life, intensity and impact of fatigue in post-COVID-19 patients.

**Methods:** Patients with a history of hospitalization for COVID-19 were allocated to the face-to-face rehabilitation group, whereas those who were not hospitalized were allocated to the telerehabilitation group. the activities of daily life and, consequently, in the quality of life. Thus, cardiopulmonary rehabilitation has been shown to be of great relevance and efficiency in the recovery of such patients. All participants answered the Medical Outcomes Study Short – Form 36 questionnaire and the Fatigue Pictogram. PR was performed and telerehabilitation was performed via the online platform google meet, during 12 sessions, twice a week. The intervention protocol consisted of flexibility, aerobic, strengthening and breathing exercises. This work is an experimental study with a non-probabilistic sample.

**Results:** 12 patients in each group completed rehabilitation, totaling 24 patients. The mean age was 42.83 ± 14.45 years for the telerehabilitation group and 47.67 ± 10.83 years for the PR group. There was an improvement in quality of life in all domains of the SF-36, both in the telerehabilitation group, with a total score of 267.08 ± 125.50 vs 453 ± 186.03 (p < 0.01), and in the RP group. 298.38 ± 205.61 vs 470.31 ± 142.09 (p < 0.01) with no difference between the groups. Regarding fatigue, a reduction of 66.66% and 100% in the “very tired” category was observed for the telerehabilitation and RP groups, respectively. And the reduction in the impact of fatigue was observed by the greater number of individuals who reported being able to “do almost everything”.

**Conclusion:** It was observed that both face-to-face rehabilitation and telerehabilitation are capable of promoting an improvement in quality of of life, with no differences between them.

112221

Modality: E-Poster Young Researcher – Non-case Report

Category: CARDIOVASCULAR IMAGING

## Thoracic Aorta Calcification and Mortality in COVID-19 Patients

GABRIELE CARRA FORTE^1^, Cristina Carra Forte^1^, Rubens Gabriel Feijó Andrade^1^, Luis Miguel Millan Diaz^1^, Bruno Hochhegger^1^

(1) Pontifícia Universidade Católica do Rio Grande do Sul – PUCRS

**Introduction:** Coronavirus disease 2019 had a great impact worldwide, being an important cause of morbidity and mortality in several countries. Although the typical clinical presentation is acute respiratory syndrome, cardiovascular disease is frequent and reported as an important determinant of poor clinical outcome.

**Purpose:** To evaluate the association of thoracic aorta calcification on in-hospital mortality in COVID-19 patients.

**Methods:** All patients with SARS-CoV-2 infection, who underwent a chest computed tomography (CT) in a tertiary university hospital in southern Brazil, between April and December 2020 were prospectively included. Clinical outcomes were collected from electronic medical records. Thoracic aorta calcification, body composition and bone mineral density was evaluated by low-dose chest computed tomography imaging (chest CT scan), widely performed in moderate to severe SARS-CoV-2 patients. Patients were classified as without calcification (0), having mild calcification (1), moderate (2) or having severe (3) calcification. The readers were blinded for patient outcomes.

**Results:** In all, 441 patients were enrolled in the study. There was a predominance of females (54%), and the mean age was 55 ± 19.2 years. Visceral and subcutaneous fat area was 116.9 m² (55.4–174.0) and 115.3 m² (67.0–181.1), respectively, and muscular area was 102.5 m² (84.2–126.8). The bone mineral density was 171.5 (120.0–219.0)HU. After adjustment for covariates, thoracic aorta calcification [OR 11.45 (95%CI 1.745–75.194); p < 0.001], mechanical ventilation [OR 25.45 (95%CI 3.542–182.972); p < 0.001), visceral fat area [OR 1.01 (95%CI 1.003–1.023); p = 0.014] resulted to be independent predictors of in-hospital mortality.

**Conclusion:** Mechanical ventilation, thoracic aortic calcification and visceral fat area permits to stratify the COVID-19 patients in-hospital mortality risk. In addition, to include the thoracic aorta calcification evaluation on standard chest CT for these patients may help guide prognosis and clinical decision.

112228

Modality: E-Poster Young Researcher – Non-case Report

Category: ANTICOAGULATION

## Use of Direct Oral Anticoagulants (DOACs) in the Postoperative Period of Femur Fracture After Hospital Discharge as Prophylaxis for Venous Thromboembolism

ANA CAROLINA PEDROSO^1^, Ana Carolina Pedroso^1^, Monica Amorim^1^

(1) Unimed rio hospital

Patients with femoral neck fractures have an increased mortality rate. Femoral neck fracture is frequently found in women over 60 years of age and its incidence has been increasing in many parts of the world due to the increase in life expectancy in this age group. Total hip arthroplasty (THA) is one of the most successful surgical procedures in the field of orthopedic surgery. Oral anticoagulants are indicated in the postoperative period (PO) of hip arthroplasty to prevent thromboembolic events. Direct oral anticoagulants (DOACs) were considered by studies to be effective in preventing thromboembolic events, as was enoxaparin. goal Ensure the use of anticoagulant in patients discharged from hospital in the PO of femur fracture, as prophylaxis for venous thromboembolism.

**Methods:** Retrospective cohort from Jan 2019 to Dec 2021 in hospitalized patients with a diagnosis of femur fracture undergoing hip arthroplasty surgery. In a total of 34,167 patients, 373 patients were admitted with a femoral fracture, 28 patients died during hospitalization, and were considered to be patients aged >60 years. All patients were evaluated according to risk stratification for VTE by the physician and prophylaxis was instituted according to the risk found. All patients in the PO period of hip arthroplasty are considered high-risk patients.

**Results:** Of the 34,167 hospitalized patients, 1.1% were due to femoral fractures, of which 7% died during the hospitalization period. Follow-up was performed for 30 days. Of the 345 patients who were discharged from hospital, 100% received anticoagulation with enoxaparin or rivaroxaban or apixaban.

**Conclusion:** Anticoagulation is safe and effective in prophylaxis of thromboembolic events in the postoperative period of femur fracture, showing no risk of increased bleeding with the use of DOACs. Despite the reduced follow-up time of these patients, there was no readmission for VTE.

108565

Modality: E-Poster Young Researcher – Case Report

Category: CARDIOVASCULAR INTENSIVE CARE/CARDIOVASCULAR EMERGENCIES

## Emergent Aortic Balloon Valvuloplasty on a Cardiogenic Shock Patient: A Case Report

JOÃO MIGUEL MARTINS DE CARVALHO^1^, Tânia Proença^1^, Ricardo Alves Pinto^1^, João Carlos Silva^1^, Filipe Macedo^1^

(1) Centro Hospitalar Universitário São João (CHUSJ), Porto, Portugal

Severe aortic stenosis patients who are critically ill are in high risk for mortality. Aortic balloon valvuloplasty (ABV) can be done on emergency, improving hemodynamic status on short-term and can be life-saving. A 59 years old male, with known medical history of hypertension, diabetes, renal cell carcinoma treated with parcial right nephrectomy, had severe aortic stenosis, severe left ventricle dysfunction (left ventricle ejection fraction [LVEF] 15%) and 3 vessel coronary artery disease (50% stenosis on the medium segment of the left anterior descending [LAD], 90% stenosis on the proximal segment of the circumflex, 90% stenosis in the proximal right coronary artery, followed by a occlusion on the middle segment; the distal vessels were in good condition) was awaiting surgery when he was admitted to the Internal Medicine ward on January 2019 with a respiratory infection with hypoxemia and severe systemic inflammatory response syndrome. On the same day, he presented with an inferior ST elevation myocardial infarct and cardiogenic shock. Venoarterial extracorporeal membrane oxygenation (VA-ECMO) and aminergic support were initiated and he was submitted to emergent coronary angiography that revealed progression of the LAD disease, with 90% stenosis on the medium segment. It was decided in Heart Team to perform an emergent ABV; the mean left ventricle-aortic gradient reduced to 10 mmHg. It was decided not to perform LAD angioplasty due to the high procedure risk and the probable need for surgical treatment soon. A week later, the patient was submited to surgery with VA-ECMO support, having performed a coronary artery bypass graft with the left internal mammary artery to the LAD; he didn’t perform aortic valve replacement surgery due to highly calcified aortic root. On the 8th day pos-op, the patient presented with a hemopericardium and tamponade, needing to be submitted to a new surgery. He was off VA-ECMO and aminergic support by day 12 pos-op. On the 21st day pos-op, the patient was submitted to transcatheter aortic valve implantation (TAVI). The pos-procedure echocardiogram showed a normofunctional aortic prothesis and an improvement in LVEF (36%). The patient was discharged, remaining during follow-up in NYHA functional class I. ABV can be a bridge to TAVI or surgery in highly unstable patients, contributing to patient stabilization. It can be done on an emergency basis, can alter the patient prognosis and should be considered in these patients.

108006

Modality: E-Poster Young Researcher – Case Report

Category: ACUTE AND CHRONIC CORONARY DISEASE/THROMBOLYSIS

## Disulfiram-Ethanol Reaction Causing the Deadly Shark Fin Electrocardiogram Pattern

ANDRÉ FILIPE MACEDO ALEXANDRE^1^, Dias de Frias^1^, Andreia Campinas^1^, João Silveira^1^, Severo Torres^1^

(1) Centro Hospitalar Universitário do Porto, Porto, Portugal

Disulfiram is used for the treatment of chronic alcoholism. Yet, it is not a safe drug in patients who still drink alcohol due to disulfiram-ethanol reaction (DER). Acute myocardial infarction due to DER has been rarely reported, and most reports suggest coronary vasospasm as the causative mechanism. We present a case of myocardial infarction probably due to DER, in which “white thrombus” formation was found instead of vasospasm. This 65-year-old man with history of schizophrenia and chronic alcoholism was admitted for out-of-hospital cardiac arrest. After 10 minutes of advanced cardiac life support with four defibrillation shocks, the patient returned to spontaneous circulation and the ECG revealed an anterior “shark fin” STEMI. The patient was referred for emergent coronary angiography, which revealed acute thrombotic occlusion of the left anterior descending artery. Aspiration thrombectomy was performed with recovery of a large “white thrombus”, and a drug-eluting stent was implanted. This patient has been prescribed disulfiram the week before STEMI presentation, yet he was still drinking alcohol while taking the drug. We suspect that DER may have been responsible for plaque instability due to close temporal relationship. This clinical case highlights that DER can not only cause myocardial ischemia by coronary vasospasm, but can also predispose to thrombus formation, leading to extensive myocardial infarction.


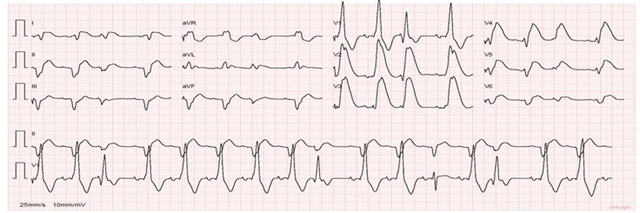


108133

Modality: E-Poster Young Researcher – Case Report

Category: ACUTE AND CHRONIC CORONARY DISEASE/THROMBOLYSIS

## Myocardial Infarction with Aortic Dissection in the Puerperium: Case Report

FRANCIELLY DOS SANTOS VIEIRA^1^, Marina Vitória Silva Costa^1^, Nathália Abdo Zuliani^1^, Jéssica Evangelista de Queiroz^1^, Leonardo Teixeira de Melo^1^

(1) Universidade Federal de Uberlândia – UFU

**Preface:** Cardiovascular diseases are an important cause of gestational and puerperal morbidity and mortality. Because it is rare, aortic dissection is difficult to diagnose during pregnancy and the puerperal period, contributing significantly to maternal mortality due to its high fatality rate, comprising 4% of the causes of maternal death.

**Case Report:** A 38-year-old primiparous patient, on the fifth day of the puerperium (cesarean section), with left renal agenesis, without other gestational comorbidities or complications, presented with severe chest pain with irradiation to the back, upper limbs, neck and jaw. She immediately sought tertiary care, where she was admitted for dyspnea, sweating, bradycardia, and hypotensiveness. Electrocardiogram suggestive of acute myocardial infarction with inferior wall ST elevation. She was referred for coronary angiography, which showed type A aorta dissection associated with acute occlusion of the right coronary artery and right renal artery, without occlusion of supraaortic or mesenteric vessels. She underwent stent implantation in the right coronary artery, followed by emergency surgery with replacement of the ascending aorta using a number 28 Dacron tube and replacement of the aortic valve with a Carpentier Edwards biological prosthesis number 23, replantation of the left coronary artery (Bentall de Bono surgery), associated with implantation of a saphenous vein bypass for the right coronary artery. She remained in the intensive care unit for six days for hemodynamic reestablishment, being discharged on the 11th postoperative day without major complications.

**Conclusion:** Aortic dissection is a rare, life-threatening condition for two main reasons: increased risk of aortic rupture and compromised blood flow from the systemic circulation to major organs by occlusion of the arterial lumen. Pregnancy is one of the main risk factors for arterial dissection among young women. Approximately 60% of them occur related to pregnancy and puerperium, being the third cause of maternal death related to cardiovascular diseases. The therapeutic approach does not differ much from that proposed for other patients, and emergency surgery to replace the ascending aorta with a Dacron tube is the best option in cases of type A acute dissection.

110102

Modality: E-Poster Young Researcher – Case Report

Category: CARDIOVASCULAR IMAGING

## Subclavian and Right Common Carotid Stenosis by Vascular Kinking in a Patient with Symptoms of Transient Ischemic Attack in the Emergency Room – a Case Report

LAÍS ANTONUCCI FERREIRA^1^, Katia Gleicielly Frigotto^1^, Washington Luiz Batista da Costa^1^, Daniela Roberta Alves Silva^1^, Luciano de Figueiredo Aguiar^1^

(1) Hospital São Lucas Copacabana

**Introduction:** Kinking artery is an angular alteration that forms a sharp angle in an artery. Embryogenic and acquired causes can lead to this alteration. Congenital factors are clinically significant in older age, aggravated by arterial hypertension (AH), diabetes mellitus (DM), dyslipidemia, smoking, rheumatological and heart diseases. This alteration is more common in the internal carotid artery, but is rare in subclavian and common carotid arteries. That condition is often asymptomatic, but can present neurologic symptoms, such as mimicking a transient ischemic attack (TIA) due artery stenosis. Diagnostic investigations can be made by anamnestic and clinical data, doppler ultrasonography, computed tomography angiography (CTA), and magnetic resonance angiography (MRA). The therapeutic includes anti-aggregation and anticoagulation therapy, antihypertensives, antilipidemic, cerebral vasodilatation therapy, and surgical treatment if indicated.

**Case description:** The 77-year-old female patient was admitted to the Emergency Room, due dysarthria and loss of upper body strength, with spontaneous improvement in approximately 15 minutes. She has been reported to have diagnosis of HAS, DM, dyslipidemia, parkinson disease, and previous TIA. In the neurological examination she was conscious and disoriented, presenting miosis and ptosis on the right side, without strength deficit. CTA indicated kinking in the right subclavian and common carotid arteries. The patient was treated with acetylsalic acid, clopidogrel, atorvastatin, antihypertensive and antidiabetic therapy.

**Conclusion:** A severe degree of kinking can cause neurological symptomatology and even simulate TIA. The kinking artery should be considered as a differential diagnosis, especially in older patients and/or with risk factors.


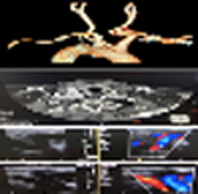


108467

Modality: E-Poster Young Researcher – Case Report

Category: PERICARDIUM/ENDOCARDIUM/VALVOPATHIES

## Irreversible Bilateral Amaurosis Due to Infectious Endocarditis

CYBELLE LUZA COSTA^1^, Cybelle Luza Costa^1^, Paulo Lucas Moraes Pimenta^2^, Fábio Queiroga^1^

(1) Hospital Agamenon Magalhães; (2) Faculdade Pernambucana de Saúde

**Introduction:** Infectious endocarditis (IE) is a serious pathology caused by colonization of the cardiac endocardium, presenting itself in an insidious way, with few specific symptoms, but with catastrophic consequences if not properly treated. Due to its high morbidity and mortality, early recognition and targeted treatment can prevent irreversible sequelae.

**Objective:** Case report of a difficult-to-diagnose infectious endocarditis in a patient assisted at Agamenon Magalhães Hospital in Recife, Brazil.

**Methodology:** Retrospective, qualitative, observational case report through medical chart and literature reviews.

**Case Report:** MSJ, 79 years-old, female, arrives at the Internal Medicine service referred from a reference hospital in ophthalmology for venous antibiotic therapy after a surgical procedure in the left eye, performed for the treatment of Endogenous Endophthalmitis. She went to the hospital due to bilateral eye pain and a drop in visual acuity, evolving with only light perception in the day she sought medical care. She denied previous eye surgery or trauma. She reported being admitted to the ICU for 5 months due to sepsis and bloodstream infection, evolving after discharge with bilateral eye pain. On admission, she was clinically and hemodynamically stable, with bilateral amaurosis, with hospitalization scheduled for completion of broad-spectrum antibiotic therapy for the endophthalmitis. There was no report of fever. During hospitalization, a new systolic murmur in the mitral focus was detected in the physical examination, and a transesophageal echocardiogram (TE-ECHO) and blood cultures were performed to rule out the possibility of infectious endocarditis (IE) as the cause of endophthalmitis, since the diagnosis of this pathology would imply in extension of antibiotic therapy time. Cultures were negative, but this was not valued because of the use of intravenous Vancomycin in progress. The result of the TE-ECHO found vegetation adhered to the mitral valve, confirming the main suspicion of IE, and empirical treatment with Vancomycin and Gentamicin was performed for 6 weeks.

**Conclusion:** IE is a disease of difficult differential diagnosis due to its symptomathological non-specificity. Therefore, it is important to know the guidelines of ESC and AHA, since the non-confirmation of the disease through one does not exclude confirmation by the other, reducing the number of underdiagnosed patients and, consequently, avoiding severe consequences.

108477

Modality: E-Poster Young Researcher – Case Report

Category: CARDIO-ONCOLOGY

## Right Atrial Sarcoma Causing Superior Vena Cava Syndrome and Cardiac Tamponade

VICTOR BAROUKI KORMANN^1^, Carolina Limongi de Oliveira^1^, Marcela Kondo Sato^1^, Claudinei Collatusso^1^, Taiana Emílio Checchia de Lima^1^

(1) Hospital Santa Casa de Curitiba

**Introduction:** Based upon the data of 22 large autopsy series, the frequency of primary cardiac tumors is approximately 0.02%. Primary heart tumors are stated to occur at all ages and to have an equal sex incidence. Three quarters of the primary cardiac tumors are benign and one quarter are malignant. Myxomas and sarcomas were the most common types.

**Description:** Patient, 34 years old, reports that in May 2021 she started with pain in the left scapular region, associated with progressive dyspnea, which became limiting for light physical activities after 3 months. Evolved with edema of the face and upper limbs, severe dyspnea, cyanosis, poor peripheral perfusion, requiring emergency pericardiocentesis. Magnetic resonance showed a tumor measuring 8.3 × 5 × 4.8 cm in the right atrium, compressing the superior vena cava and signs of cardiac tamponade. The patient underwent surgical resection of the tumor and cavity reconstruction with bovine pericardium. Biopsy confirmed the diagnosis of Angiosarcoma, with surgical margins free of neoplastic involvement.

**Conclusion:** Cardiac tumors are rare entities, but they must be diagnosed early due to the high potential for serious complications. In this report, we present a case of right atrial sarcoma, coursing with superior vena cava syndrome and cardiac tamponade, successfully treated surgically. The patient remained asymptomatic, and no metastasis was evidenced on the tomographic study.


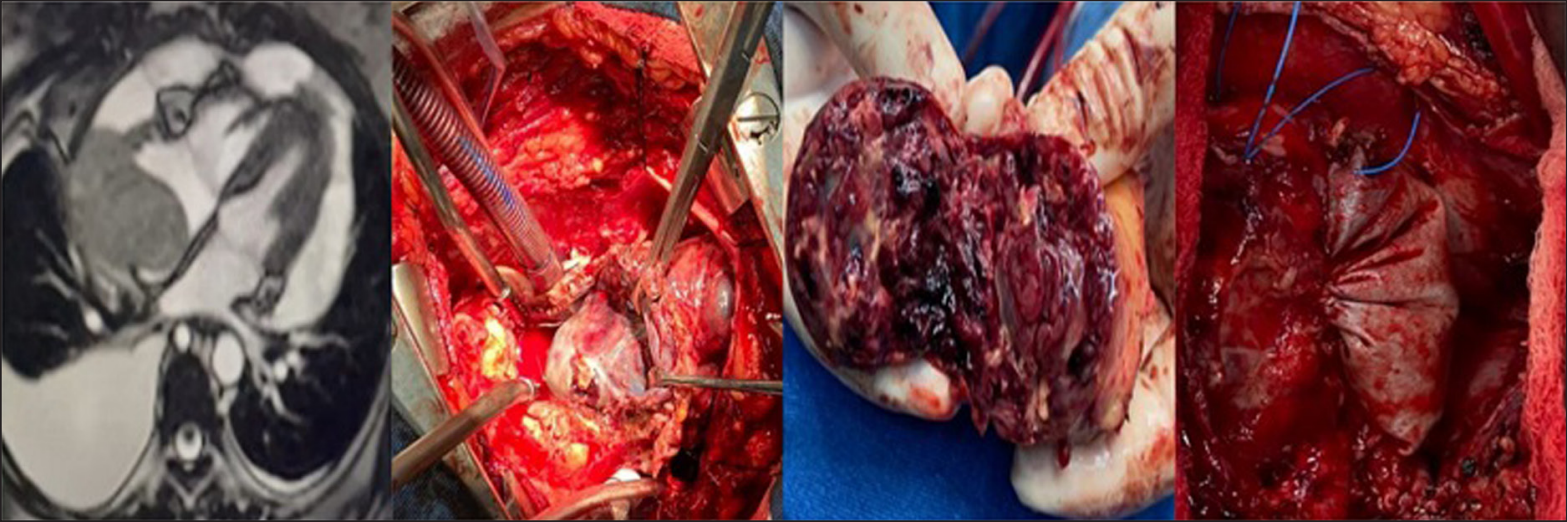


108544

Modality: E-Poster Young Researcher – Case Report

Category: CARDIOVASCULAR IMAGING

## Spontaneous Aortic Intramural Haematoma – Case Report of the Ct Angiography Follow up Before and After Surgery

DANIEL GAMA DAS NEVES^1^, Mario Ricardo Amar^1^, Rafael Vilanova Lima^1^, Alair Augusto Sarmet Moreira Damas dos Santos^1^

(1) DASA – Complexo Hospitalar de Niterói

Aortic intramural haematoma (IMH) in a type of acute aortic syndrome, in which a haematoma is formed inside the aortic wall. In this presentation we aim to report a case of acute aortic IMH diagnosed and treated in our institution, that had findings corroborating one of the etiological theories A 58-year-old female patient is admitted at the hospital after being initially evaluated in another institution of lower complexity. The patient arrived at our institution still complaining of crushing chest pain nonrelated to physical exertion, associated with dull pain in the right upper quadrant of the abdomen. Troponin and CKMB where normal but D-dimer was elevated. The medical team decides to investigate the pain with a computed tomography angiography (CTA) of the thoracic and abdominal aorta. That shows an eccentric dense thickening of the aortic wall dislocating the parietal calcifications inwards in the nonenhanced images. Contrast images showed a small irregularity in the luminal surface about 6 cm from the aortic valve plane, suggestive of a small intimal flap. The medical team then decided to admit the patient to the ICU for close surveillance and strict control the double product. After seven days of hospital care, the chest pain returned. New aortic CTA showed increase of wall thickness, formation of ulcer like projections in the ascending aortic wall covering the same location as the previously described luminal irregularity. The images also demonstrated the formation of pericardial effusion. The attending team then decides that surgery was the best course of action. The surgeons description of the procedure confirmed the location of the intimal flap described in the first CTA. Control CTA performed 10 days after the surgery showed no complications. A new CTA performed a year after the procedure demonstrated complete regression of the haematoma. This case aggregates to the body of evidence that intimal flap is a cause of not only classical aortic dissection, but also of aortic IMH. As better CT scanners becomes more available, and cardiologists, cardiovascular surgeons as well as radiologists become more aware of aortic IMHs, the understanding of this pathology will likely improve, and the diagnosis and treatment criteria may become more defined.

108541

Modality: E-Poster Young Researcher – Case Report

Category: PERICARDIUM/ENDOCARDIUM/VALVOPATHIES

## Case Report of Bacterial Endocarditis Caused by Staphylococcus Aureus with Involvement of Aortic, Mitral and Pulmonary Valves

MARCELO VIAL FELIX DE SOUSA^1^, Maicon Felipe Ribeiro da Cruz^1^, Renata Muller Couto^1^, Silvério Albano Fernandes Júnior^1^, Walter Emanoel Magalhães Rocha^1^

(1) Universidade Estadual de Campinas – UNICAMP

We’ve reported the case of a patient who has presented with symptoms of neurological alterations and, under investigation, was diagnosed with endocarditis by Staphylococcus aureus (SA) involving the mitral, aortic and pulmonary valves. A 53-year-old male sought medical attention due presenting with right arm paresis, right eyelid ptosis and diplopia. During the investigation, he developed fever, lack of appetite, weight loss and diastolic murmur in the pulmonary focus. His exams had shown positive blood culture for SA. In a transthoracic echocardiogram (TTE), vegetations were identified in the pulmonary and mitral valves. After the changes were observed, it was requested a transfer to a tertiary level hospital. In a new TTE, performed in the tertiary service, vegetations were visualized in the pulmonary valve, in the anterior leaflet of a mitral valve and in the right coronary leaflet of aortic valve. After 2 months of vancomycin, the vegetations in the aortic and mitral valves disappeared. Afterwards was performed valvuloplasty in the pulmonary valve with success. Patient maintained in outpatient follow-up without complaints so far. A review of the literature was carried out and it is not uncommon to have 2 valves involved, but the involvement of 3 valves is rare. Multivalvular involvement is related to more severe infections and has higher mortality. Despite the poor prognosis in these cases, the patient in question developed uneventfully.


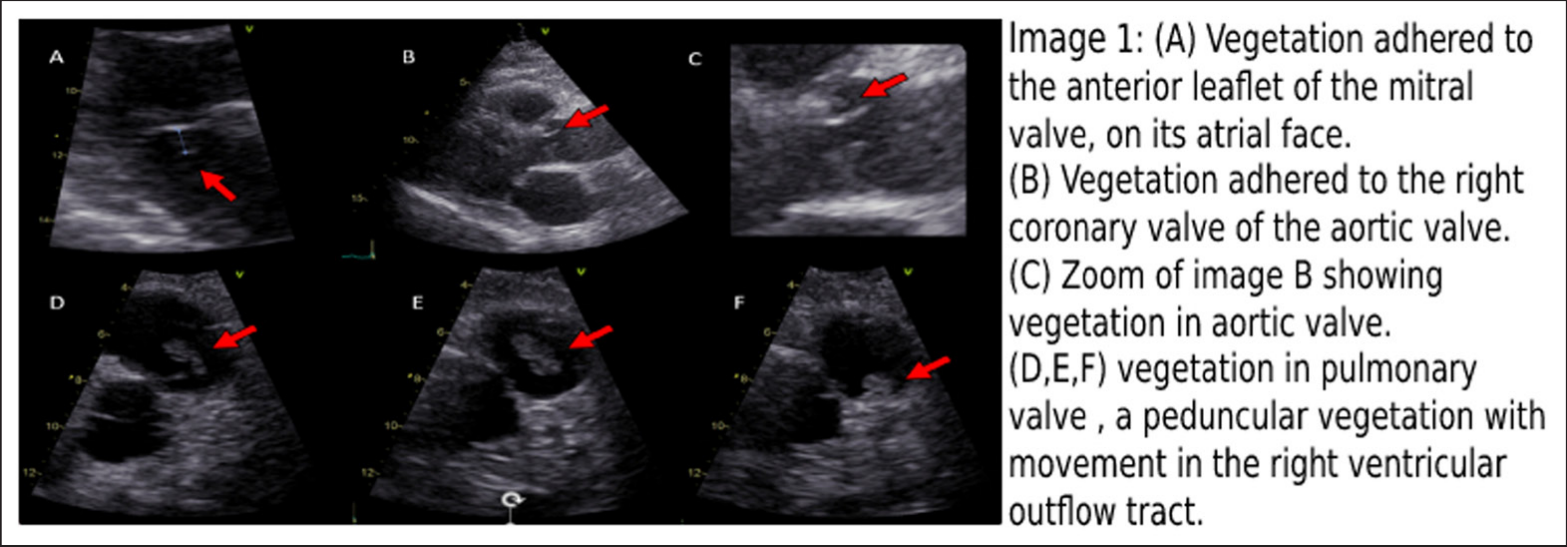


109943

Modality: E-Poster Young Researcher – Case Report

Category: CARDIOVASCULAR INTENSIVE CARE/CARDIOVASCULAR EMERGENCIES

## Stenotic Lesion of the Left Main Coronary Artery as the Initial Manifestation of Large Vessel Vasculitis in a Sexagenarian Patient

PEDRO GUIMARÃES SILVA^1^, BRUNA MITIE IKARIMOTO^1^, GUILHERME RAPOSO DE MEDEIROS^1^, MARILIA TAILY SOLIANI^1^, PAULO ROGERIO SOARES^1^

(1) INSTITUTO DO CORAÇÃO DO HCFMUSP

**Introduction:** Vasculitis is a rare cause for coronary stenosis. However, it can still be responsible for life-threatening conditions. This group of diseases usually present with nonspecific symptoms and involvement of various systems. Here, we expose a case in which its initial manifestation was the development of ischemic cardiomyopathy due to a severe stenotic lesion of the left main coronary artery (LMA).

**Case Report:** A 63-year-old woman without any previously known comorbidities began an outpatient investigation due to a 1-year history of dyspnea and angina under minimal efforts. Dilated cardiomyopathy with reduced ejection fraction (37%) was diagnosed on transthoracic echocardiography and an elective coronary angiography was requested for assessment of coronary disease A 70% focal stenosis in the distal segment of the left main coronary artery was detected, without any other significant narrowing in the other vascular beds. The patient was the admitted to the hospital for an emergent surgical revascularization. A CT angiography of the aorta and its branches was ordered. It showed a circumferential parietal thickening in the transition between the subclavian and axillary arteries to the right, determining significant segmental luminal reduction (>50%); circumferential parietal thickening of the aorta along its entire length, reaching a thickness of approximately 0.3 cm, suggestive of vasculitis. A PET-CT was ordered, which corroborated the diagnosis with the detection of diffuse and heterogeneous increase in glycolytic metabolism, of mild/moderate intensity, suggestive of an inflammatory process compatible with large-vessel vasculitis. Blood tests rendered negative for Chagas, syphilis, ANCA and antinuclear antibody serology. Pulse therapy with methylprednisolone 1 mg/kg/day was initiated and angioplasty of the LMA was performed with the use of 2 drug-eluting stents in the anterior descending and circumflex arteries, following the mini-crush and post-dilation with kissing-balloon techniques. An intra-aortic balloon was used for coronary protection during the procedure. Aspirin and Ticagrelor (60 mg in loading dose followed by maintenance 10 mg per day) were administered as antiplatelet therapy prior to the intervention. A good angiographic result and resolution of the anginal was achieved and the patient was discharged from the ward after 7 days for out-patient follow-up with a rheumatologist.

108609

Modality: E-Poster Young Researcher – Case Report

Category: COVID-19 AND CARDIOVASCULAR SYSTEM

## Acute Coronary Syndrome Followed by Pulmonary Thromboembolism and Identification of a Large Fixed Thrombus between Atria in a Patient with Severe COVID-19 Condition: A Case Report

ALICE MIRANE MALTA CARRIJO^1^, Veronica Perius de Brito^1^, Gabriel Alvarenga Santos^1^, Samuel Gomes Tomaz da Silva^1^, João Lucas O’Connell^1^

(1) Hospital de Clínicas da Universidade Federal de Uberlândia – HC/UFU

**Introduction:** Patent foramen ovale associated with a thrombus in transit represents a rare clinical entity difficult to manage. Its association with other thrombotic events and the hyperinflammatory state of novel coronavirus disease (COVID-19) is the subject of discussion in this study.

**Case description:** A 69-year-old woman with grade III obesity, a history of systemic arterial hypertension, hypothyroidism and chronic venous insufficiency, was hospitalized for severe respiratory condition of COVID-19. On the 16th day of symptoms of COVID-19, the patient was diagnosed with Acute Myocardial Infarction with ST-segment elevation of the inferior wall after presenting with typical anginal pain, radiating to the left upper limb, associated with worsening dyspnea. Treatment was carried out with primary coronary angioplasty with drug-eluting stent implantation in the right coronary artery. One day after angioplasty, a routine transthoracic echocardiogram was performed, and identified preserved global systolic function of the left ventricle and the presence of a thrombus straddling the patent foramen ovale, with mobile segments in the right and left atria (Figure 1A). Full anticoagulation with enoxaparin was initiated, acetylsalicylic acid (ASA) was maintained and clopidogrel was changed to tirofiban to schedule an embolectomy. The patient evolved with clinical stability, improvement in the respiratory condition and resolution of the atrial thrombus, documented on preoperative echocardiography (Figure 1B), without the need for surgical embolectomy. Pulmonary artery CT angiography was performed, and confirmed a pulmonary thromboembolism (PTE). The patient remained without hemodynamic instability and ASA, clopidogrel and oral anticoagulation were maintained after hospital discharge.

**Conclusion:** The identification of thrombus between the atria associated with the presence of patent foramen ovale is a rare event with high mortality rates, especially in the context of severe COVID-19, associated with PTE and Acute Coronary Syndrome. Management in this context is complex and there is still no consensus on the best therapy.


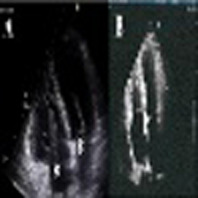


108633

Modality: E-Poster Young Researcher – Case Report

Category: CARDIOVASCULAR SURGERY

## Typical Chest Pain in a Patient with Surgically Corrected Alcapa Syndrome: A Case Report

ANA PAULA DALPICOLLI CORSO^1^, Alessandra Bossardi^1^, Bruna Valduga Dutra^2^, Bibiana Maggi^3^, Leandro Gazziero Rech^4^

(1) HGCS – Hospital Geral Caxias do Sul; (2) Federal University of Rio Grande do Sul UFRGS

**Introduction:** Alcapa Syndrome (anomalous left coronary artery from the pulmonary artery) is a rare congenital disease that presents as a coronary anomaly, consisting of the origin of the left coronary artery (LCA) in the pulmonary trunk. The objective of the report is to emphasize the importance of early diagnosis of cardiac alterations to obtain better prognosis results in surgery.

**Case Description:** A 28 years-old female sought care at the hospital due to tight retrosternal pain radiating to the left upper limb associated with dyspnea worsen on exertion in 2021. Patient reports that she had a typical chest pain episode 3 months ago, but with ECG and normal troponins. Previous history of Alcapa Syndrome, with correction of the anomalous origin of the left coronary artery in 2002. Initial laboratory assessment showed no alteration, with a troponin value of 0.005 (reference value >0.0012), and the patient was in use of furosemide. Transesophageal Echocardiography demonstrated a preserved ejection fraction with mitral valve thickening with a non-dysplastic appearance with mild regurgitation and minimal aortic valve regurgitation. At this point coronary angiography was performed, demonstrating severe stenosis in the anastomosis of the aortic tunneling. After this procedure, they underwent coronary bypass graft without complications with release to the ICU, then to the ward. After 4 days of the intervention, the patient was discharged with instruction.

**Conclusions:** Alcapa Syndrome is a rare disease with an anomalous origin of the LCA from the pulmonary artery. There are two forms of the syndrome, infantile and adult. The adult form is characterized by the formation of a collateral system between the Right Coronary Arteries (RCA) and LCA, with a compensation usually keeping the patient asymptomatic. The patient in the case presented a complication of the tunneling previously performed. Corrected with coronary bypass graft surgery in order to restore coronary flow.


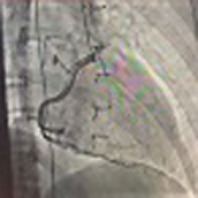


108635

Modality: E-Poster Young Researcher – Case Report

Category: HEART FAILURE/CARDIOMYOPATHY/TRANSPLANT

## Cardiac Amyloidosis: Rare Association of Val142Ile and Val50Met Pathogenic Variants

GIOVANNA MARIN LESSE^1^, Larissa Ventura Bruscky^1^, Edileide Correa de Barros^1^, Jéssika Mayhara Souza Tolentino^1^, Fabiano Castro Albrecht^1^

(1) Instituto Dante Pazzanese de Cardiologia

**Introduction:** Cardiac amyloidosis is a underdiagnosed cause of heart failure. Hereditary transthyretin amyloidosis can occur in more than 130pathogenic variants with variable clinical presentation depending on the type of mutation. They two main phenotypes are peripheral polyneuropathy and amyloidotic heart disease. The variant Val50t is the most prevalent in the world and most important representative of the polyneur phenotype. Val142Ile is the second most prevalent and is mostly expressed with heart disease. We describe a patient who presents a rare association of these two pathogenic variants in composed heterozygosity.

**Case report:** A 66-year-old black male patient from Juazeiro-BA, previously healthy, with a family history of neuropathy and heart disease in two brothers who died with 64 and 73 years old. She had altered sensitivity in the tips of her fingers, hands and feet, burning pain and reduced strength in her left leg and both hands for tree years. She also reported weight loss of 9 kg in two years, dizziness when assuming an orthostatic position and early satiety. Cardiological complaints were just heart palpitations during exercise and lower limb edema. In physical examination she presented with postural hypotension, rediced grip strength and pinching movement in the fingers and atrophy of the intrinsic muscles of the hands. Electrocardiogram: left anterior fascicular block and inactive zone of inferior and anterior walls. Echocardiogram: preserved left ventricle ejection fraction (global longitudinal strain of –8.7%), grade III diastolic dysfunction and enlargement of the left ventricle wall (20 mm) and interatrial septum (9 mm). Pyrophosphate scintigraphy showed pergini grade 3 uptake with a 2.22 rate. Genetic sequencing demonstrated the presence of two pathogenic variants on chromosome 18, both in heterozygosity: Val50Met and VAL142Ile.

**Conclusion:** An association of two pathogenic variants is rare and is associated with a more severe expression of the disease and worse prognosis. In this case the apresentation was mixed: heart disease, polyneuropathy and dysautonomia, with a predominance of the last one. Heart disease symptoms were not exuberant, perhaps because of the physical limitation of the patient. However, the uptake of pyrophosphate on scintigraphy was much more intense than usual, suggesting high degree of deposit and worse prognosis.

110326

Modality: E-Poster Young Researcher – Case Report

Category: CARDIOVASCULAR IMAGING

## Nuclear Medicine in the Diagnosis of Infectious Endocarditis in a Valve-in-Valve Aortic Bioprosthesis – a Case Report

RAFAEL RODRIGUES COUTINHO^1^, Paulo Roberto Dutra da Silva^1^, Arnaldo Rabischoffsky^1^, Nilton Lavatori Correa^1^, Claudio Tinoco Mesquita^1^

(1) Hospital Pró-Cardíaco/RJ

**Introduction:** Prosthetic valve endocarditis (PVE) has an increasing prevalence. In this case report, we sought to highlight the importance of early diagnosis in cases of previous valve-in-valve intervention. We highlight the need for integrated multidisciplinary action in an endocarditis team for the proper diagnosis.

**Case:** Patient 86 years old, male. Hospitalized due to cough and dyspnea associated with low-grade fever and reports that he had a dental procedure two weeks ago. Previous history of heart failure functional class II, TAVI (2004 and 2021) valve-in-valve, Hypertension, dyslipidemia, smoking and alcohol consumption. He performed 2 blood cultures in the current hospitalization, positive for Streptococcus mitis. TTE and TEE without visualization of vegetations or thrombi and preserved ventricular function. CT angiography with absence of aortic vascular occlusion and shows enlarged heart and small bilateral pleural effusion. Normal lower limb venous Doppler. Scintigraphy with labelled leukocytes showing an infectious/inflammatory process in aortic valve prosthesis topography. Initiated antibiogram-guided ceftriaxone with frame resolution.

**Discussion:** The valve-in-valve transcatheter aortic valve implantation (ViV-TAVI) is already a well-established procedure for degenerated prosthetic valves, being also used for patients who would not tolerate open valve replacement surgery. ViV-TAVI is an alternative for high-risk patients, but there is no evidence of lower mortality of patients using this therapeutic approach compared to valve replacement surgery. Nuclear medicine emerges as a useful tool in the diagnosis of infective endocarditis, with two main evaluation modalities in this scenario, PET-CT using the 18-F-FDG agent and SPECT-CT with labelled leukocytes. Leukocytes are deposited in sites where there an active infection, being an interesting tool for suspected PVE. For the diagnosis of PVE, sensitivity and specificity of labelled leukocytes are above 90%, being then included as one of the modified DUKE criteria for infective endocarditis. Therefore, the use of SPECT-CT with labeled leukocytes has proved to be useful in the identification of PVE and should be included in the diagnostic flow of patients with suspected infective endocarditis.

108686

Modality: E-Poster Young Researcher – Case Report

Category: CARDIOVASCULAR IMAGING

## A Challenge Diagnosis in a Rare Presentation of Left Ventricular Pseudoaneurysm Obstructing Left Ventricular Inflow in the Context of Acute Myocardial Infarction

JANINE DAIANA STÜRMER^1^, Raphael dos Santos Silva^1^, Tiago Hansel Basile Vigil^1^, Willer Cesar Bica^1^, Gabriel Soder^1^

(1) Instituto de Cardiologia do Rio Grande do Sul/Fundação Universitária de Cardiologia – ICFUC

**Introduction:** Left ventricular (LV) pseudoaneurysm is a rare complication of myocardial infarction (MI) with high mortality rates. It consists of an intramyocardial dissecting hematoma communicating with the ventricular cavity when an injured myocardial wall rupture. The definitive diagnosis can be made with transthoracic echocardiogram (TTE) in only 26 percent of patients.

**Case report:** A 60-year-old male with a history of smoking habit was admitted with typical chest pain lasting 20 hours. The electrocardiogram showed ST-elevation in I, aVL, V5 and V6 leads (Panel A), suggestive of lateral MI. He received dual antiplatelet therapy and was immediately transferred for coronary angiography, which displayed occluded mid-circumflex coronary artery. During the exam, he presented acute deterioration of hemodynamic state and a TTE was performed. Pericardial effusion was ruled out in subcostal-view (Panel B). At the apical 4 and 5-chamber-view the mitral valve was not visualized and in its topography a muscular band-like image that could suggest rupture of left ventricular free wall (Panel C). Finally, a long-axis-parasternal-view demonstrating a pericardial hematoma compressing the left atrium referring to a LV pseudoaneurysm (Panel D,E) which was confirmed on left ventriculography (Panel F). We theorize that as the pseudoaneurysm progressed he obstructed the LV inflow, as seen by the limited mitral valve diastolic flow at color Doppler (Panel E). The patient collapsed and died few minutes later.

**Conclusion:** LV pseudoaneurysms can present as congestive heart failure, chest pain, arrhythmia or sudden death. Our case is a highly unusual instance of pseudoaneurysm producing severe extrinsic compression obliterating the LV inflow diagnosed with TTE in a sudden cardiac death presentation.


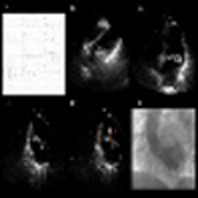


108710

Modality: E-Poster Young Researcher – Case Report

Category: HEMODYNAMICS AND INTERVENTIONAL CARDIOLOGY

## A Rare Embolization of a Chemotherapy Catheter to Coronary Sinus

MATHEUS HENRIQUE MENEZES^1^, Diogo França Souza Camargo^1^, José Alaor da Veiga Junior^1^, Deborah Christina Nercolini^1^, Wilton Francisco Gomes^1^

(1) Instituto de Neurologia e Cardiologia de Curitiba

**Introduction:** Central venous catheters are important devices for medical care. Although they improve life-quality for patients with chronic conditions, complications are not negligible. We report a rare case of embolization of a catheter fragment into the coronary sinus.

**Case:** A 43-years-old woman with a history of breast cancer undergoing chemotherapy through a totally implantable catheter was referred to CT scan due to a chest x-ray suggestive of a catheter fracture and embolization. CT scan images showed an embolized catheter fragment, with one extremity in the coronary sinus and the other in the right ventricle. The patient was referred for percutaneous removal of the fragment. The right femoral vein was punctured and a 10F sheath was installed. We approach the case using the “pigtail through snare” technique, using a 5F pigtail catheter placed into a 20mm loop snare before its insertion. With a 0.035 hydrophilic guidewire, we then positioned the pigtail catheter into the pulmonary artery and, with a pull-back maneuver, we successfully entangled the extremity located into the right ventricle. With coordinated movement, the snare loop was positioned right above the pigtail catheter and the guidewire inside the catheter was pushed, as an extension, allowing for the grasping of the fragment and the pigtail catheter, simultaneously. The fragment was then extracted, pulling out the assembly and sheath as a unit.

**Discussion:** Embolization of central venous catheter fragments is uncommon, occurring in 1.1%–2.1%. The pulmonary artery and right chambers are the most frequent sites of embolization. The coronary sinus is 15–65 mm long structure and the Thebesian valve protects the ostium in almost 88% of people. This demonstrates the complexity and rarity of the case reported, with fragments in the coronary sinus and right ventricle. To our knowledge, only 6 similar cases have been reported before. The “pigtail through snare” technique seems to be a useful tool in these challenging situations.


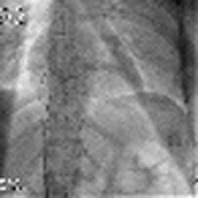


108738

Modality: E-Poster Young Researcher – Case Report

Category: HEART FAILURE/CARDIOMYOPATHY/TRANSPLANT

## Phaeohyphomycosis in a Heart Transplant Patient

SILVÉRIO ALBANO FERNANDES JÚNIOR^1^, Maicon Felipe Ribeiro da Cruz^1^, Marcelo Vial Felix de Sousa^1^, Otávio Rizzi Coelho Filho^1^, Wilson Nadruz Junior^1^

(1) Hospital de Clínicas da Universidade Estadual de Campinas – UNICAMP

**Introduction:** Phaeohyphomycosis is a chronic fungal disease located mainly in the skin, with Fonsecaea pedrosoi being the most frequent fungus. The infection develops after a traumatic inoculation into the skin, and later, as a small papule. They may ulcerate, evolving with lymphatic or hematogenous dissemination. Infections are the main cause of death in heart transplant patients, with fungi being the third predominant etiologic agent.

**Case report:** Male, 44 years old, vigilant, heart transplanted in 2017, using Azathioprine and Tacrolimus. He started with ulcerated lesions began, with exposure of the deep plane in the temporal region and right upper limb. About 2 months later, he developed aphasia and drowsiness. Seen on cranial tomography, frontal expansive lesion on the left, submitted to surgical resection. Diagnosed with Cerebral Chromomycosis, through the result of culture demonstrating the fungus of the genus Fonsecaea sp. Complementary treatment was performed with prolonged use of Amphotericin B during hospitalization and after discharge, replaced by Voriconazole. He was discharged from the hospital with an improvement in his neurological and skin condition.

**Discussion:** Cerebral phaeohyphomycosis corresponds to brain infections caused by dematiaceous fungi. The predominant symptoms are produced by mass effect on the brain parenchyma. The risk of heart transplant patients presenting infection is directly related to the use of immunosuppressive drugs, such as mycophenolate and tacrolimus, which severely damage all components of immunity. Management usually combines surgical and drug treatment, the main antifungals being itraconazole, voriconazole and posaconazole.

**Conclusion:** Phaeohyphomycosis of the central nervous system is a rare pathology in heart transplant patients. The mortality rate among patients with these infections is at least 50%, the high mortality rate highlights the need for aggressive therapy. The case described stands out for the severity of the condition, but with good resolution with combined surgical and antifungal treatment, even in the case of an immunocompromised patient.


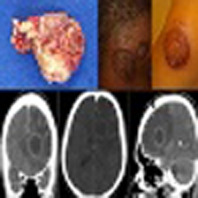


108768

Modality: E-Poster Young Researcher – Case Report

Category: CARDIOVASCULAR INTENSIVE CARE/CARDIOVASCULAR EMERGENCIES

## Intimal Pulmonary Artery Sarcoma: A Differential Diagnosis of Chronic Pulmonary Thromboembolism

FERNANDA MARIA FRANCO CASTRO^1^, FÁBIO MORATO DE CASTILHO^1^, EDUARDO BELISÁRIO FALCHETTO^1^, THIAGO PINHEIRO JUNQUEIRA^1^, NATHÁLIA SIMÕES FERNANDES CAMPOS^1^

(1) Hospital Felicio Rocho – HFR

Intimal sarcoma of the pulmonar artery is a rare and potentially lethal tumor. Its presentation is similar to pulmonary thromboembolism, what makes the diagnosis between these two diseases a clinical challenge. A 50-year-old woman was diagnosed with pulmonary embolism by Computed tomography angiography (CTA) and anticoagulated with warfarin. Despite 8 months of warfarin therapy she remained symptomatic. She complained of dyspnea and fatigue upon mild exertion. An echocardiogram demonstrated pulmonary hypertension and right ventricular dysfunction and the second CTA exam had demonstrated no change in the pulmonary artery filling defects. In view of the clinical worsening a diagnostic hypothesis of chronic pulmonary thromboembolism associated with acute thromboembolism was raised. She was referred for arteriography which showed the appearance of bilateral acute thrombi, unsuccessful mechanical thrombectomy attempt and subsequent local infusion of thrombolytic. With the findings leading towards a diagnosis of acute pulmonary embolism in a patient who was already on anticoagulant therapy, the implantation of vena cava filter was placed. Subsequently, a pulmonary artery endarterectomy was performed, which did not identify a thrombus. The material sent to anatomopathological and immunohistochemical study, has confirmed the diagnosis of intimal pulmonary artery sarcoma. The lesion was partially removed and the treatment with docetaxel and gemcitabine started with a good initial tumor response. After the surgery to remove the tumor, there was an improvement in the right ventricular function and patient’s symptoms. Pulmonary artery sarcomas have no gender preference and generally occur in the fifth decade of life. Histologically, they are mostly undifferentiated or poorly differentiated tumors. The case presented here catch the attention to the need to raise the differential diagnosis in patients who do not respond to the current treatment of pulmonary thromboembolism because the treatments differ markedly and prognosis depends on the early diagnosis and prompt surgical resection of the tumor.


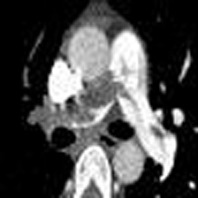


108837

Modality: E-Poster Young Researcher – Case Report

Category: CARDIOVASCULAR IMAGING

## Unicuspid Aortic Valve: A Case Report of a Rare Condition

ELISAMA PIMENTEL ZAMIAN COTIAS^1^, Fabrício Thebit Bortolon^1^, Patrick Ventorim Costa^1^, Herbert Felipe Heimbeck^1^, Berilurdes Wallacy Garcia^1^

(1) Hospital Universitário Cassiano Antonio Moraes (HUCAM)

Unicuspid aortic valve is a rare congenital malformation associated with aortic stenosis and/or regurgitation. This condition can be categorized into two types: unicommissural and acommissural type. This case report presents a 25-year-old man, with a history of heart murmurs, dyspnea only with unusual exertion. His physical examination demonstrated an ejection systolic murmur heard loudest over the aortic valve. Transthoracic echocardiography showed concentric left ventricular hypertrophy with preserved systolic function and dome-shaped aortic valve during systole on a paraesternal long-axis view (Figure 1). Unicuspid aortic valve had nodule calcifications with a lateral zone of attachment to the aorta and an eccentric orifice consistent with unicommissural type on transthoracic and tridimensional transesophageal echocardiography on a short-axis view (Figure 3 and 4). Severe regurgitation at color doppler (Figure 2) and severe aortic stenosis with a maximum velocity of 3.95 m/s, a mean gradient of 38 mmHg and calculated aortic valve of 0.58 cm²/m². In conclusion, echocardiography is a solid imaging method that enables diagnosing morphological anomalies of heart valves, as well as evaluating their functioning.


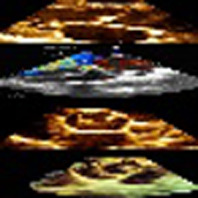


108874

Modality: E-Poster Young Researcher – Case Report

Category: CARDIAC ARRHYTHMIAS/ELECTROPHYSIOLOGY/ELECTROCARDIOGRAPHY

## Severe Gastroparesis After Atrial Fibrillation Ablation by Cryoballoon

PATRICIA MATTOS VIERA DO PAÇO^1^, Olga Ferreira de Souza^1^, Nilson Araujo de Oliveira Junior^1^, João Pantoja^1^, Antônio José Carneiro^1^

(1) Hospital Copastar(HCS)

**Introduction:** Ablation(ABL) is a safe treatment for atrial fibrillation(AF). However, injury of the anterior vagal plexus can occur when energy is applied to the posterior wall of the left atrium, resulting in gastric hypomotility, mostly oligosymptomatic and reversible. The reported case presents a patient with severe gastroparesis after cryoablation (CB).

**Case description:** A 58 years old male patient with recent paroxysmal AF, was submitted to CB (MEDTRONIC ARCTIC FRONT II). The day after, he complained of indigestion and nausea. The symptoms were ameliorated with oral prokinetic drugs. After 10 days, he was readmitted with diffuse abdominal pain and not tolerating food and liquid ingestion. The patient was afebrile, had stable vital signs, marked abdominal distention and tenderness. CT scan showed significant gastric distention and mild diverticulitis(DI). Ciprofloxacin and metronidazole were initiated. Despite the clinical and radiologic improvement of the DI, the gastroparesis(GP) persisted. So, we ruled out any role of the DI in the GP genesis and diagnosed vagal damage due to CB. We started clarithromycin 250 mg BID to enhance gastric motility with excellent response. A new CT showed the resolution of gastric distention and a liquid diet was initiated. After a couple of days, he could eat regularly and get discharged. The figure shows the evolution of the CTs, from right to left side.

**Conclusions:** This case helps us understand the GP related to AF ABL. Besides, he presented a second abdominal pathology, making the diagnosis difficult. The resolution was obtained from an unusual drug for this condition. Despite the protective measures there was an injury to the vagus fibers. After this case, we observed the need for further studies to seek effective protective measures.


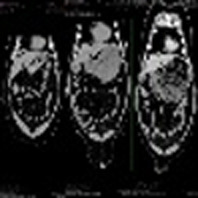


108899

Modality: E-Poster Young Researcher – Case Report

Category: ACUTE AND CHRONIC CORONARY DISEASE/THROMBOLYSIS

## Hypereosinophilia Associated with Spontaneous Coronary Dissection and Refractory Vasospasm: Challenges in Diagnosis and Management

FELIPE REZENDE GIACOMELLI^1^, Marcelo Bettega^1^, Lara do Norte Garcia^1^, Júlia Guidi^1^

(1) Hospital Israelita Albert Einstein

**Introduction:** Acute coronary syndrome (ACS) associated with hypersensitivity represent predominantly eosinophilic and mast cell endothelial and microvascular dysfunction. This spectrum encompasses spontaneous coronary artery dissection (SCAD), non-atherosclerotic cause of ACS, and Kounis Syndrome (KS), defined by the concomitance between allergic reactions, cardiac symptoms and electrocardiographic changes after the triggering event.

**Case report:** Male, 41 years old, with refractory asthma, chronic sinus disease, nasal polyposis, urticaria, IgG immunodeficiency and hypereosinophilic syndrome. It started in August/2021 typical anginal chest pain in the absence of ischemia on electrocardiogram, elevation of markers of myocardial necrosis (MMN) or atherosclerotic lesions on coronary angiotomography. After 2 weeks, he presented with pain, ST and MMN elevation, DACS and vasospasm in coronary angiography (CA). Angioplasty was performed, but it evolved with 2 recurrences of vasospasm refractory to intracoronary nitrate and the need for new angioplasties, totaling 6 stents. Simultaneously, there were allergic skin and respiratory manifestations, intense headache and abdominal pain. Transferred to this service after angina associated with purulent rhinosinusitis. Documented on CA nitrate-responsive vasospasm and patent stents, absence of vasculitis, myocarditis or arteritis on myocardial magnetic resonance imaging and PET-CT. Satisfactory clinical response was observed after corticosteroid therapy and optimization of antianginal agents.

**Conclusion:** ACS and hypersensitivity are correlated with eosinophilic endothelial infiltrates, mast cell activation, and release of cytotoxic agents such as histamine and thromboxane. DACS results in disruption of the intimal layer and expansive intramural hemorrhage. In KS, cerebral and mesenteric territories are also affected and 3 coronary variables are observed:type I includes vasospasm without previous coronary artery disease; type II vasospasm with quiescent atheromatosis and type III in-stent thrombosis. Drugs, food, hives, bronchial asthma and the presence of an intracoronary stent are triggers and treatment consists of corticosteroids, antihistamines and vasodilators. Therefore, allergic angina is an underdiagnosed condition, with high morbidity, mortality and iatrogenic risk, whose treatment remains unspecific. Eosinophilstabilizing therapies and monoclonal antibodies are promising in attenuating inflammatorydependent myocardial injury.

108929

Modality: E-Poster Young Researcher – Case Report

Category: NEGLECTED CARDIOVASCULAR DISEASES

## Acute Myocardial Infarction in a Chagasic Patient: A Rare Cause of Thromboembolism

LEONCIO BEM SIDRIM^1^, Maria das Neves Dantas da Silveira Barros^2^, Rodrigo Brito de Barros Correia e Silva^1^, Fiamma Ferreira Nogueira^1^, Taciana Queiroz Medeiros Gomes^1^

(1) Pronto Socorro Cardiológico de Pernambuco (PROCAPE); (2) Universidade de Pernambuco

**Introduction:** Thromboembolic phenomena are frequent in patients with Chagas heart disease.1,2 Its incidence is 36% in patients with heart failure with this etiology.3 Mortality due to these complications is related, in most cases, to pulmonary and brain embolisms. 4–6 Coronary artery embolisms have been rarely described and their prevalence has not been estimated by clinical studies.2,7 Several anatomopathological and clinical studies have shown a subgroup with higher risk: presence of myocardial dysfunction, presence of intracavitary thrombi, and previous thromboembolic events. 8–10.

**Case description:** Female, 54 years old, history of megaesophagus at 11 years of age and serological diagnosis of Chagas disease 3 years ago. No history of heart disease and no regular use of medication. Transthoracic echocardiogram (TTE) showed preserved left ventricular (LV) diastolic and systolic functions and an ejection fraction (EF) of 71%. For 30 days, she presented chest pain and tightness, in the left hemithorax and radiating to the left upper limb and mandible. Electrocardiogram (ECG) showed ST-segment elevation, measuring 2.0 mm from V2 to V4. Despite looking for the local health service, she was not submitted to emergency coronary angiography or chemical thrombolysis, with spontaneous improvement of chest pain in about 48 hours. After two weeks, the patient sought an outpatient clinic specialized in Chagas disease, reporting what had happened. A new TTE was performed, which showed: dilated LV, with hypokinesia in the anterior (middle), inferolateral (middle) and apical (septal and inferior) septum region, LV EF: 65%. The clinical history and compatible echocardiographic findings allowed the retrospective diagnosis of Acute Myocardial Infarction (AMI). Coronary angiography showed coronary arteries free from atheroma. Ventriculography showed aneurysm at the LV apex. Such findings corroborate the hypothesis of coronary thromboembolism as the etiology for AMI.

**Conclusion:** Chagas disease is always a hypothesis to be raised in thromboembolic phenomena, given its high prevalence. Ventricular aneurysms may represent an important cardioembolic source and may be present even in patients with normal EF. The etiological definition of AMI in this case has an important practical implication for its management, given the need to use anticoagulants as opposed to antiaggregants and statins.

108961

Modality: E-Poster Young Researcher – Case Report

Category: CARDIOVASCULAR IMAGING

## Reversed Cardiorespiratory Arrest in an Adult Woman with Alcapa Syndrome

UANDY DIAS TERRAS^1^, Arthur Gabriel Soares Costa Monteiro^1^, Larissa Amaral de Abreu^1^, Larissa Rosa de Rezende^1^, Solange de Sousa Andrade^2^

(1) UNIVERSIDADE DE RIBEIRÃO PRETO – UNAERP; (2) INSTITUTO DO CORAÇÃO DA FACULDADE DE MEDICINA DA UNIVERSIDADE DE SÃO PAULO

**Introduction:** ALCAPA syndrome (known as Bland-Altman-Garland syndrome) is a rare congenital anomaly in which it occurs in the coronary arteries. We present a case report of ALCAPA syndrome in a 37-year-old female patient, who presented with syncope followed by seizures and cardiorespiratory arrest, remaining in the intensive care unit for 7 days.

**Case report:** Brazilian citizen, female, 37 years old, residing in Santos – São Paulo, presented syncope followed by convulsive crisis and cardiorespiratory arrest during intense physical activity at the gym. Cardiopulmonary resuscitation maneuvers were performed for 15 minutes by the personal trainer until the Mobile and Emergency Care Service arrived, which in the initial evaluation verified a Ventricular Fibrillation (VF) rhythm and performed defibrillation with a return to sinus rhythm, palpable pulse and hemodynamically stable. She was intubated and taken to the Emergency Care Unit. Upon physical examination, it was observed that the patient was in poor general condition, Glasgow 4 and mitotic pupils. After 24 hours, the patient presented with spontaneous breathing, Glasgow 15. The Echocardiogram that showed diffuse hypokinesia of the left ventricle, more accentuated in the apical region, Ejection fraction of 52%. Coronary catheterization was requested, which showed filling of the distal third of the left coronary artery and it was not possible to catheterize the origin of this vessel. Angiotomography of the coronary arteries was then indicated, which showed the origin of the left coronary artery from the trunk of the pulmonary artery. The case was referred to the cardiac surgery team with a diagnosis of Alcapa syndrome and surgical treatment was indicated. Patient underwent cardiac surgery for reimplantation of the left coronary artery in the left coronary sinus with stable postoperative evolution and clinical improvement. One year later, the patient is asymptomatic and performs regular moderate-intensity physical activity.

**Conclusion:** Alcapa syndrome is a rare disease among the group of anomalous coronary arteries, with a high risk of sudden death, as was presented in the case that presented a satisfactory result with the performance of cardiopulmonary resuscitation maneuvers and the correction of this anomaly was fundamental for the evolution and patient prognosis.

108985

Modality: E-Poster Young Researcher – Case Report

Category: ACUTE AND CHRONIC CORONARY DISEASE/THROMBOLYSIS

## Rare Anatomical Anomaly of the Left Anterior Descending Artery Course

VALÉRIA BRAGA SANTIAGO DE SÁ^1^, VALÉRIA BRAGA SANTIAGO DE SÁ^1^, GLAUCIENE FERREIRA DE SOUZA XAVIER^2^, DANIEL MEDEIROS MOREIRA^1^, GILVAN MAGALHÃES PINTO^1^

(1) INSTITUTO DE CARDIOLOGIA DE SANTA CATARINA; (2) UNACON RR

**Introduction:** Coronary artery anomalies are a diverse group of cardiac anatomical congenital disorders presenting a wide variety of clinical manifestations. Patients may be asymptomatic or have varying degrees of symptoms correlating with the anatomical presentation of the anomalous coronary path. In some cases, they may lead to sudden death.

**Case report:** A 43-year-old Venezuelan male patient sought care presenting short-term chest pain episodes for a week. Previous medical history reported systemic hypertension treated with losartan and suffering a transient ischemic stroke 5 years ago. Electrocardiogram showed no ischemic alteration, negative myocardial necrosis markers. Coronary angiotomography presented anomalous origin of the middle and distal segments of the anterior descending artery in a way that left coronary trunk originates the circumflex artery and the proximal segment of the left anterior descending artery (LAD), which originates the 1st septal and 1st diagonal, as usual. However, the medial and distal segments of the LAD originates from the right coronary ostium with its path anterior to the pulmonary trunk until reaching the anterior interventricular groove in the middle segment, providing 2 more diagonal branches.

**Conclusion:** This finding encourages further study and discussion regarding its potential role in the symptomatic report of this case and other similar clinical situations, helping to evaluate the need for some intervention.


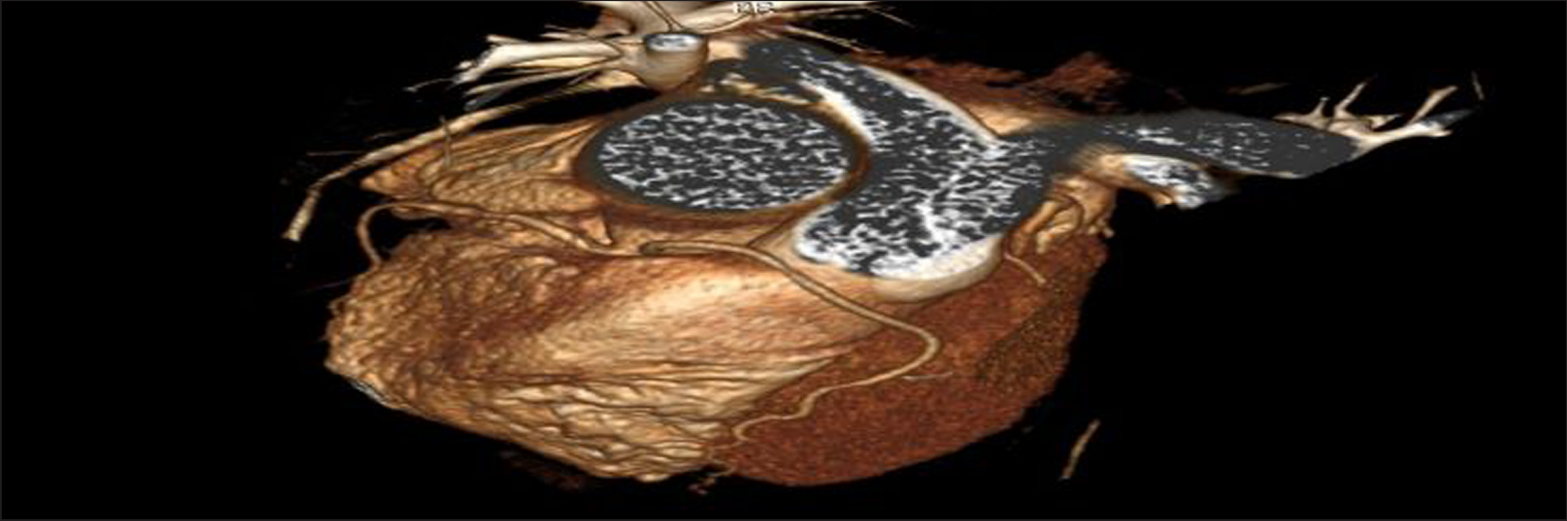


108990

Modality: E-Poster Young Researcher – Case Report

Category: HEMODYNAMICS AND INTERVENTIONAL CARDIOLOGY

## Pulmonary Arteriovenous Malformation as an Etiological Factor of Stroke in Young Patients: What to Think About Beyond the Patent Foramen Ovale

RAFAEL MASSUTI^1^, Bibiana Guimarães Maggi^1^, Marcelo Sabedotti^1^, Rafaela Oliveira Leite^2^, Leandro Gazziero Rech^1^

(1) Hospital Geral de Caxias do Sul; (2) Fundação Universidade de Caxias do Sul

**Introduction:** Pulmonary arteriovenous malformations (PAVMs) are abnormal pulmonary vessels in which an artery is directly connected to a vein, forming a right-to-left shunt and resulting in clinical manifestations such as hypoxemia, predisposition to infections, and ischaemic cerebrovascular accident (CVA).

**Case report:** Patient with a history of right hemiplegia at fifteen years old, normal hematological investigation and magnetic resonance of the encephalon at that time, and transesophageal echocardiogram demonstrating a patent foramen ovale (PFO) with a small passage of air bubbles under Valsalva maneuver, without septum aneurysm. The assistant physician discussed PFO closure, but decided for clinical management with acetylsalicylic acid, 100 mg per day. At 21-year-old, this patient seeks medical care presenting aphasia and right hemiplegia. Brain computed tomography revealed hypodense areas on the right parietal cortex region, and in left white matter, as well as encephalomalacia areas on the left brain hemisphere, suggesting vascular injury as aetiology of the stroke. Transcranial Doppler (TCD) ultrasound revealed more than 100 high-intensity transient signals (HITS), considering the Spencer Logarithmic Scale, a large shunt. Despite the lesion’s anatomy, the patient improved symptoms to the absence of neurologic deficits. PFO closure was discussed once more by the assistant physician team, which opted to investigate differential embolic sources, since a PFO with such anatomical characteristics in a young patient was not considered a high risk for CVA. A pulmonary angiography revealed a massive aneurysmatic PAVM (with 3,3 cm of diameter) on the right lower lobe, nurtured by an interlobar artery branch and drained to the pulmonary vein. We proceeded with the percutaneous embolization of the PAVM through puncture of the right femoral vein and the selective catheterization of the lesion with a Progrear® 2.7 microcatheter. The PAVM was embolized with 8 Microplex® 18 coils, achieving successful occlusion of the defect. A control chest computed tomography angiography 30 days after the procedure revealed the absence of flow through the PAVM, and new TCD ultrasound demonstrated the absence of HITS.

**Conclusion:** Even though PFO is associated with ischaemic events, ischaemic stroke in young patients must be thoroughly investigated, especially when the clinical picture is not compatible with the anatomical alterations. PAVM, although rare, must be remembered.

109221

Modality: E-Poster Young Researcher – Case Report

Category: HEMODYNAMICS AND INTERVENTIONAL CARDIOLOGY

## Transcatheter Aortic Valve Implantation in Patients with Pure Aortic Regurgitation

JORDANA PIRES MENDONÇA^1^, Flavio Rosa Vieira^1^, Rogério Las Casas^1^, Tannas Jatene^1^, Vinicius Daher Vaz^1^

(1) Hospital do coração Anis Rassi

**Introduction:** Transcatheter aortic valve implantation (TAVI) for aortic stenosis has been well documented over the last years. Nevertheless, TAVI for pure aortic regurgitation (AR) it’s stilll challenge due to the absence of calcification of the aortic valve and the high flow state. This features make difficult to anchor the prosthesis in the right spot, that can evolve to acute or late migration with tragic consequences.

**Case Description:** Female 79 y/o fragile lady, at NYHA III on optimal medical therapy. At Physical examination she was tachydyspneic, with severe AR on Doppler, within 100% of the LVOT, the final velocity in the descending aorta was 2.1 m/s, mean gradient (MG) was of 21 mmHg, secondary to hyper flow, PASP of 66 mmHg. Euroscore II 8.39% and STS of 8,5%. showed aortic annulus perimeter of 76.3 mm and mean diameter of 24.3, mean sinus of Valsalva of 34 mm, left main coronary artery height of 12.8 mm and right coronary artery of 11.5 mm with zero aortic valve calcium score. After discussion with the heart team the decision to perform TAVI was made. The procedure was exceptionally performed under general anesthesia and guided by a transesophageal ECO. To secure the pacing during all the deployment a transvenous pacing was chosen. An EVOLUT Pro (Medtronic) #29 was chosen to achieve 19% of oversize. Taking into account the difficulties already anticipates, we did a full paced release. The deployment was done with pacing of 140–160 bpm in order to achieve less than 50 mm/hg of mean arterial pressure until point of no return. After careful checking with angiogram and ECO we did slow full release with 180 bpm and after we kept pacing 120 bpm for 10 minutes. The procedure duration was 69 minutes, with 60cc of contrast and without any major complications. The final MG was of only 4 mmHg and there were no paravalar leak whatsoever. The paciente was discharge from the hospital within 48 hours. At the 30-day follow-up patient has clinically improved markedly, without any further complication.

**Conclusion:** TAVI using self-expanding prosthesis and specific deployments tips may be an alternative for patients with pure aortic regurgitation and high surgical risk or fragile profile.

108998

Modality: E-Poster Young Researcher – Case Report

Category: CONGENITAL AND PEDIATRIC CARDIOLOGY

## Coronary-Cameral Arterial Fistula in a Child: A Case Report

DOUGLAS VINÍCIUS DE OLIVEIRA SANTOS^1^, Iara dos Santos Ferreira^2^, Ana Caroline Mendonça Cardoso^3^, Ana Flávia Reis Costa^4^, Daniela Passos Garcia Campos^5^

(1) Hospital Santa Casa de Misericórdia de Belo Horizonte; (2) Hospital Santa Casa de Misericórdia de Belo Horizonte; (3) Hospital Santa Casa de Misericórdia de Belo Horizonte; (4) Hospital Santa Casa de Misericórdia de Belo Horizonte; (5) Hospital Santa Casa de Misericórdia de Belo Horizonte

**Introduction:** Arterial Coronary-cameral fistulas, congenital or acquired, are rare coronary anomalies, capable of generating serious repercussions in their carriers. We report a case of a 10-year-old child with a coronary-cameral fistula, between the right coronary artery (RCA) and right ventricle (RV).

**Case report:** A 10-year-old female patient with asthma sought medical care denouncing recurrent fevers, associated with prostration and hyporexia, with a month of evolution. On physical examination, a heart murmur was detected, and an echocardiogram was performed suggesting Interventricular Communication (IVC). The patient was fowarded to a high complexity hospital to complete clinical, laboratory and imaging exams, in which evidenced the following: presence of lower dental arch cavities, echocardiographic findings compatible with mobile vegetations, significant regurgitation in the tricuspid valve and coronary-cameral fistula between RCA and RV. The diagnosis of infective endocarditis (IE), ACD-VD coronary-cameral arterial fistula were established, and VSD was ruled out. Antibiotic therapy was instituted (Oxacillin 200 mg/kg for 35 days, Amikacin 15 mg/kg for 22 days) and tooth extraction was performed. The patient evolved with clinical, laboratory and imaging improvement, being discharged with Amoxicillin/Clavulanate for another 7 days, along with Furosemide 40 mg/day, Carvedilol 6,25 mg/day and Beclomethasone 4 g/day. Three months later IE treatment, corrective surgery for a coronary-cameral fistula was successfully performed and all medications were discontinued.

**Conclusion:** Coronary artery fistula with cardiac chambers are rare, being present in 0.002% of the population, representing 0.4% of all cardiac malformations. They can be congenital or acquired, the first being apparently associated with persistence of embryonic sinusoids in the myocardium. Manifestation is very variable, being capable of generating serious repercussions in approximately 19% of patients under 20 years of age, such as infarction, heart failure, cardiomyopathy, arrhythmias and IE. Heart murmur is the main semiological finding, associated with the specific symptomatology of complications. The case exemplifies the challenges of early diagnosis and treatment of coronary-camera fistulas, essential for good outcome, as well as the importance of accurate clinical and echo-Doppler evaluations, given the spectrum of manifestations and differential diagnoses, such as VSD and IE.

109000

Modality: E-Poster Young Researcher – Case Report

Category: HEMODYNAMICS AND INTERVENTIONAL CARDIOLOGY

## Atrial Fibrillation: Alternative for the Management of Anticoagulants in the Preoperative Period

LEANDRO GAZZIERO RECH^1^, Rafaela Oliveira Leite^2^, Marcelo Sabedotti^1^, Rafael Massuti^1^, Bibiana Guimarães Maggi^1^

(1) Hospital Geral de Caxias do Sul; (2) Fundação Universidade de Caxias do Sul

**Introduction:** Treating patients diagnosed with atrial fibrillation (AF) during the use of direct oral anticoagulants (DOACs) and submitted to elective surgeries is a common daily situation, but its management remains a challenge. The correct suspension period of DOACs before interventions varies according to several factors, and recent studies are committed to adopting the best way of managing these patients to minimize risks.

**Clinical picture:** A 72-years-old female was referred to cervical arthrodesis. She had a history of surgical correction of the atrial septal defect (ASD) 20 years before, chronic AF, diabetes, arterial hypertension, and permanent pacemaker due to sinus node dysfunction. The patient was on rivaroxaban 20 mg per day, suspended 72 hours before the procedure. In the transoperative, the patient evolved to cardiac arrest in pulseless electrical activity, which returned to spontaneous circulation after two minutes of resuscitation by following the advanced cardiac life support (ACLS) protocol. In cardiogenic shock, she was referred to urgent coronary angiography, performed 20 minutes after the cardiac arrest, and the exam demonstrated coronary embolization, with a large quantity of thrombus burden, which were aspirated with a dedicated instrument. The coronary was infused with tirofiban. Hemodynamic instability was corrected with norepinephrine and crystalloids replacement, resulting in a favorable and satisfactory evolution of the instability, allowing the extubation on the same day. A transesophageal echocardiogram was performed and demonstrated spontaneous echo contrast in the left atrium and thrombus in the left atrial appendage. Despite the clinical picture, the patient did not present any degree of ventricular dysfunction. She was on enoxaparin (full dosage) during the hospitalization, and after 48 hours, we introduced Dabigatran 150 mg twice a day. The patient was discharged seven days after the occurrence, asymptomatic.

**Conclusion:** Perioperative management of AF patients during the use of DOACs remains a challenge. Recent studies suggest individualizing treatments, which can be discontinued one to five days before the procedure, according to the surgical port, risk of bleeding, drug half-life, and renal function. Individualization remains the best strategy in addition to the possibility of non-invasive procedures for thrombosis evaluation that can corroborate clinical decisions in problematic situations.

109041

Modality: E-Poster Young Researcher – Case Report

Category: CONGENITAL AND PEDIATRIC CARDIOLOGY

## Fistula from the Anterior Descending Artery to the Pulmonary Artery

DAVID EMMANUEL BEDOYA GOYES^1^, Rodolfo Vaz^1^, Luis Antônio Machado^1^, Bruno Mahler Mioto^1^

(1) Instituto do coração – INCOR

**Introduction:** Coronary artery anomalies are congenital alterations that include an extensive group of malformations with a wide variety in origin, course, and distribution. Coronary artery fistula is defined as a very rare anomalous connection between a coronary artery and a cardiac chamber or a main vessel, being present in 0.002% of the population and representing 0.4% of all cardiac malformations, being asymptomatic and benign or could present with symptoms such as chest pain and cardiac dysfunction in young adults, depending on the location. Fistulas are usually diagnosed by computed tomography angiography, coronary angiography, transesophageal echocardiography, or cardiac magnetic resonance imaging. Case report A previously hypertensive, dyslipidemic 45-year-old male patient with symptoms of severe grip-related chest pain with irradiation to the mandible and left arm without other associated symptoms. Attended in the emergency room, electrocardiogram was performed without dynamic changes and markers of myocardial necrosis with slight increase; acute coronary syndrome was considered, so coronary angiography was requested, which showed the presence of an important anterior descending artery fistula (AD) for pulmonary artery trunk (AP) with QP/QS of being programmed into a second moment intervention. The patient was successfully submitted to percutaneous closure of the DA-AP fistula by embolization with interlock coil, successfully, control echocardiogram without segmental alterations, and with preserved ejection fraction.

**Discussion:** Coronary fistulas were first described in 1865, being the first successful surgical correction in 1947; coronary artery fistulas (FAC) are congenital anomalies with an estimated incidence of 0.2 to 0.4% and are the result of persistence of primitive myocardial sinusoidal circulation and primordial epicardial vessels and may be associated with other congenital heart diseases such as arterial canal persistence, tetralogy of Fallot and interventricular communication; however, CAS can also be acquired, secondary to complications of procedures such as coronary angioplasty, myocardial revascularization surgery or after heart transplantation and myocardial biopsy.

**Conclusion:** Coronary fistulas, despite having a low incidence among congenital heart diseases, have become increasingly frequent due to technological advances in diagnostic methods, especially in hemodynamic studies offer the possibility of treatment.

109082

Modality: E-Poster Young Researcher – Case Report

Category: HEMODYNAMICS AND INTERVENTIONAL CARDIOLOGY

## Combined Transcatheter Aortic Valve Implantation and Left Atrial Appendage Occlusion with Cerebral Protection Device

GABRIEL KANHOUCHE^1^, Filippe Barcellos Filippini^1^, Pedro Felipe Gomes Nicz,^1^, Fabio Sandoli de Brito Jr.^1^, Alexandre Abizaid^1^

(1) Heart Institute of Sao Paulo – HCFMUSP

**Background:** About 2–5% of patients undergoing transcatheter aortic valve implantation (TAVI) experience stroke, which substantially increases the in-hospital mortality. Atrial fibrillation (AF) prevalence in patients undergoing TAVI ranges from 16–51% and balancing the risk of cardioembolic and bleeding events represents a major challenge. We report a combined procedure strategy for treating a high bleeding risk patient with AF and severe aortic stenosis (AS).

**Case report:** A 81-year-old woman, with past history of permanent AF, diabetes and previous pacemaker implant, evolved with heart failure class NYHA III due to severe AS. The risk-scores were CHA2DS2VASc = 6 and HAS-BLED = 3 and she was on irregular rivaroxaban use, with one prior gastrointestinal bleeding. Transesophageal echocardiogram (TEE) indicated a severe AS with aortic valve area = 0.6 cm^2^, mean aortic gradient = 56 mmHg, normal left ventricle function and chicken wing morphology in the left atrial appendage (LAA). After careful cardiac computed tomography evaluation, anatomy was deemed suitable for transfemoral TAVI, LAA occlusion and cerebral protection device. During Heart Team discussion, according to the intermediate risk score (STS = 6.8%), frailty, high bleeding risk and suitable anatomy, she was considered to the minimalist combined approach. First, the Sentinel cerebral protection device (Boston Scientific, CA, USA) was positioned in the brachiocephalic trunk and left carotid artery, followed by left transfemoral TAVI with Acurate Neo 2 size 25 mm (Boston Scientific, CA, USA) and right venous transfemoral LAA occlusion with Watchman FLX size 27 mm (Boston Scientific, CA, USA). Intraoperative TEE revealed absence of leak in both devices and no procedural complications. After removal of the Sentinel equipment, many embolic debris were found in the filters. She was discharged 10 days after the procedure, on aspirin use and functional class NYHA I. Clinical follow-up after 30 days was uneventful.

**Discussion/Conclusion:** The combined procedure of TAVI and LAA occlusion with the use of a cerebral protection device followed by single antiplatelet therapy was feasible and safe. Since both TAVR and LAAO are associated with a non-negligible risk of stroke, the use of cerebral protection devices might become the standard of care for this combined approach.

109097

Modality: E-Poster Young Researcher – Case Report

Category: HEMODYNAMICS AND INTERVENTIONAL CARDIOLOGY

## Excimer Laser Coronary Atherectomy as Adjunctive Treatment of Severe Stent Underexpansion with Bulcky Calcification: A Case Report

GABRIEL KANHOUCHE^1^, Mauricio Felippi de Sá Marchi^1^, Marcelo Harada Ribeiro^1^, Carlos Augusto Homem Magalhaes de Campos^1^, Henrique Barbosa Ribeiro^1^

(1) Heart Institute of Sao Paulo – HCFMUSP

**Introduction:** Excimer laser catheter atherectomy (ELCA) has improved significantly in recent years, emitting high-energy ultraviolet (UV) and short wavelength with less penetration and heat emission, ultimately leading to less tissue damage and fewer complications.

**Case report:** We described a real-life case of a 66-year-old male patient with hypertension, diabetes mellitus, dyslipidemia and former smoker. He was submitted to percutaneous coronary intervention (PCI) with bare metal stent in left descending anterior (LAD), coronary bypass in 2001 including right mammary internal artery to right coronary artery. In the past 20 years, was performed innumerous PCIs including a LAD drug eluting stent (DES) overlapping. The last LAD stent presents an acute thrombosis and was managed only with thrombolysis. A new cardiac catheterization was performed because the patients developed chest pain in the last couple months. A severe stent under-expansion in the proximal LAD was observed and ELCA was the device of choice due a overlapping underexpanded stent with bulcky calcification that make crossover the lesion with balloons or others devices impossible. A progressive load of 40/40, 60/60 and 80 mJ/mm^2^/80Hz and saline injection method to prepare the in-stent lesion was performed and subsequent implantation of a DES Firehawk Liberty (MicroPort, Shangai, China) 3.50 × 38 mm from the left main coronary to the LAD.

**Discussion:** ELCA was a precise option in this case because its mechanisms of action allow reaching atherosclerosis beyond the stent without disrupting the stent architecture. The photochemical role makes dissociation of the cell’s molecular bonds, the photothermal effect generated by vibration leads to the softening of collagen and protein fibers of the atheroma and the photomechanical effect generated by the explosion and implosion of the vapor bubbles leads to plaque rupture. In addition to stent under-expansion, ELCA is indicated for in-stent restenosis, calcification, balloon uncrossable lesions, and chronic total occlusion. Similar cases have been shown sporadic in the literature, but it is necessary more publication of successful results with ELCA to remove the past stigma of laser. Thereby more operators could be encouraged to use this useful device.

109141

Modality: E-Poster Young Researcher – Case Report

Category: HEMODYNAMICS AND INTERVENTIONAL CARDIOLOGY

## Alcohol Septal Ablation Before Transcatheter Valve Replacement

MAURICIO FELIPPI DE SÁ MARCHI^1^, Filippe Barcellos Filippini^1^, Antonio Fernando Diniz Freire^1^, Alexandre Abizaid^1^, Fábio Sândoli de Brito Júnior^1^

(1) Instituto do Coração do Hospital das Clínicas da FMUSP (InCor)

**Introduction:** Transcatheter Aortic Valve Replacement (TAVR) has emerged as a less invasive procedure for severe aortic stenosis. It’s not rare to observe concomitant Aortic Stenosis (AS) with left ventricular outflow tract (LVOT) obstruction. This condition is mainly due to ventricular septal hypertrophy, which can potentially impact TAVR result, increasing cardiovascular mortality. We report two cases of patients with AS and high values of intraventricular gradient prior to TAVR. In both cases alcohol septal ablation (ASA) was successfully performed before TAVR.

**Case:** 1 A 75-years-old woman with history of AS, coronary artery disease, paroxysmal nocturnal hemoglobinuria and anemia was admitted in the emergency room (ER) for sudden loss of consciousness. Her initial exams demonstrated aortic stenosis, and a 59-mmHg intraventricular gradient measured at cath lab. Considering this, ablation of the first septal artery branch was executed, with a OTW balloon and 2 mL alcohol. Residual post intervention value of the intraventricular gradient was 7 mmHg. TAVR was indicated after discussion with the Heart Team. After the procedures the patient remained assymptomatic and was referred for out-of-hospital follow-up. Case 2 An 82-years-old woman was referred for TAVR for aortic stenosis. Initial 3D echo showed significant aortic stenosis and a hypertrophic septum of 16 mm. Alcohol septal ablation was performed with an OTW balloon and 1.5 mL alcohol. Initial intraventricular gradient was 100 mmHg intervention and became 24 mmHg afterwards. A transfemoral 23 mm Sapien 3® was implanted. Postprocedural echo reported a 65% EF, absence of dynamic intraventricular gradient, a 23-mmHg maximum and 14 mmHg mean gradient. The patient was discharged after three days and remained in out-of-hospital follow-up.

**Discussion:** Outflow-tract gradients of 30 mmHg or more under resting conditions are independent determinants of symptoms of progressive heart failure and death, especially threating in the context of coexistent AS. Percutaneous alcohol septal ablation is a minimally invasive procedure, which can reduce the left-ventricular outflow gradient, therefore reducing symptoms and mortality in many patients.

109151

Modality: E-Poster Young Researcher – Case Report

Category: HEMODYNAMICS AND INTERVENTIONAL CARDIOLOGY

## Treatment of Post-Endomyocardial Biopsy Fistula between Left Anterior Descending Artery and Right Ventricle with Micro-Coils

MAURICIO FELIPPI DE SÁ MARCHI^1^, Gabriel Kanhouche^1^, Filippe Barcellos Filippini^2^, Luiz Junya Kajita^1^, Alexandre Abizaid^1^

(1) Instituto do Coração do Hospital das Clínicas da FMUSP (InCor)

**Introduction:** Endomyocardial biopsy is the gold standard for the evaluation of graft rejection following orthotropic heart transplantation. It is a relatively safe procedure, with <1% chance of serious complications.

**Case report:** A 69-year-old patient with history of heart transplant in 2018 due to Chaga’s disease underwent a 2-years control myocardial perfusion imaging scintigraphy which demonstrated minor stress-induced apical ischemia. He was asymptomatic and his last endomyocardial biopsy was 2 years ago, with no history of graft rejection. After the scintigraphy finding of ischemia a coronary angiography was performed, which displayed a fistula between the left anterior descending artery and right ventricle with subsequent coronary occlusion. This is a rare complication of endomyocardial biopsy. Fistula occlusion was planned after the scintigraphy finding. The procedure was performed with three micro-coils. First, a 5.5 × 5 mm VortX® (Boston Scientific) was deployed, followed by a 6.5 × 6 mm VortX® (Boston Scientific) and finally a 5.5 × 5 mm VortX Diamond® (Boston Scientific), delivered through a Renegade® (Boston Scientific) microcatheter. Final angiography demonstrated closure of the coronary artery fistula. After the procedure patient remained asymptomatic and was later discharged.

**Conclusion:** We report a seldom case of fistula between left anterior descending artery and right ventricle. This patient was later successfully treated with micro-coils and was discharged for out-of-hospital follow-up.


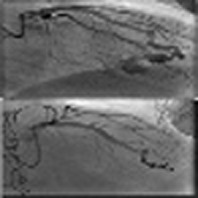


109164

Modality: E-Poster Young Researcher – Case Report

Category: HEART FAILURE/CARDIOMYOPATHY/TRANSPLANT

## Severe Ventricular Dysfunction and Complex Arrythmias as Manifestation of Isolated Cardiac Sarcoidosis: Case Report

OLIVIA SHELLARD JUNQUEIRA FRANCO^1^, Rodrigo Batista Rocha^1^, Bruno Normande Colombo^1^, Rafael Augusto Mendes Domiciano^1^, Guilherme D‘Andrea Saba Arruda^1^

(1) Rede D’Or – Hospital São Luiz Anália Franco

**Introduction:** Cardiac sarcoidosis (CS) is considered the second most common cause of death in sarcoidosis patients globally. Isolated cardiac sarcoidosis (ICS) is defined by the absence of systemic involvement, responsible for 9% of total CS and more common in male patients. Clinical manifestations depend on the location, extent, and activity of the disease and may be evidenced as ventricular arrhythmia, high-grade block, sudden death and heart failure (HF). This is a case report of a young female with ICS initially manifested as syncope.

**Case description:** W.C.M, 30 years, female, was admitted at the emergency room reporting palpitations and an episode of syncope during effort. Eletrocardiogram showed right bundle brunch and first-degree atrioventricular block. Due to an ejection fraction (EF) of 28% evidenced in the transthoracic echocardiogram, a cardiac magnetic resonance was indicated and showed a reduction in thickness and transmural thinning, extensive areas of fibrosis (35,9%) and myocardial edema. The endomyocardial biopsy was compatible with granulomatous miocarditis. Screnning for systemic sarcoidosis was negative. She had frequent sustained ventricular tachycardia episodes and antiarrhytmic therapy was prescribed. Treatment for HF followed current guidelines up to the maximum tolerated doses, in addition to corticosteroids and methotrexate. Hence, an implantable cardioverter-defibrillator (ICD) was implanted to prevent sudden death. After 3 months, patient remained in functional class I, there was improvement in the EF by the transthoracic echocardiogram (44% rate) and lower density of arrhythmias were observed.

**Conclusion:** ICS has poorer prognosis when compared to CS with systemic involvement, presenting itself with lower left ventricular EF, more frequent ventricular tachycardia and ICD indication. The diagnosis may be challenging, due to endomyocardial biopsy low sensitivity. Advanced non-invasive cardiac imaging techniques may allow earlier detection of cardiac involvement. The mainstay of medical therapy for ICS is immunosuppression in addition to therapies for HF manifestations. We have reported a case of ICS with an unusual presentation in a woman with syncope. Early diagnosis, initiation of therapy and recognition of patients in risk of sudden cardiac death and demand ICD prove to be crucial.

109171

Modality: E-Poster Young Researcher – Case Report

Category: CARDIO-ONCOLOGY

## Treatment with Inhibitor of Immunological Checkpoint and Coronary Artery Disease: An Emerging Clinical Problem

NATÁLIA AARÃO BERNARDI^1^, Marina Sahade Gonçalves^1^, Fábio Sândoli de Brito Júnior^1^, Antonio Fernando Diniz Freire^1^, Luana Alencar Fernandes Sampaio^1^

(1) Hospital Sírio Libanês

**Introduction:** The use of immune checkpoint inhibitors (ICI), including programmed death protein inhibitors-1 (anti-PD1), brought large increases in survival, representing a paradigm shift. However, the management of immune-mediated side effects is still a challenge.

**Case report:** A 76-year-old male patient with a history of dyslipidemia and coronary artery disease, was diagnosed with acral melanoma in the calcaneus. He started Nivolumab as the first line of systemic treatment, but before initiation, he underwent coronary angioplasty (AD – 2 stents and Mg-1 stent). After the first infusion of Nivolumab, he developed palpebral ptosis and a condition suggestive of immune-mediated myasthenic syndrome. Because symptoms worsened, it was decided to initiate immunoglobulin. During the first infusion, he presented cardiorespiratory arrest with ventricular tachycardia rhythm. Coronary angiography showed previous stents without complications and important progression of coronary artery disease compared to the examination 6 weeks before, being submitted to new angioplasty (AD – 2 stents, CD – 1stent, and balloon in Dg and VP). Cardiac magnetic resonance showed preserved biventricular function and late basal anterolateral epicardial enhancement, suggestive of a previous inflammatory process, and may represent previous inflammatory cardiomyopathy (myocarditis) or even cardiotoxicity. Data from the literature show that ICIs induce an increase in CD8+ T cells in the atherosclerotic plaque, leading to a change in the predominance of macrophages to lymphocytes in its microenvironment. Therefore, activated T cells favor the production of pro-atherogenic cytokines, contributing to the growth and destabilization of the atherosclerotic plaque.

**Conclusion:** The profile of immune-mediated side effects can directly affect the cardiovascular system, culminating in increased morbidity and mortality in previously frail patients due to oncological diagnosis, thus demonstrating the importance of active surveillance of these patients.


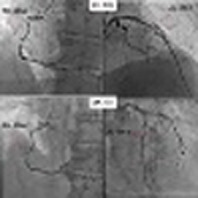


109177

Modality: E-Poster Young Researcher – Case Report

Category: ACUTE AND CHRONIC CORONARY DISEASE/THROMBOLYSIS

## Left Main Coronary Artery Aneurysm: A Therapeutic Challenge

PATRICIA COSTA DE ALMEIDA^1^, Vitor Motta Inacio^1^, Bruno Azevedo da Cruz^1^, Felipe José Monassa Pitella^1^, Gabriel Camargo^1^

(1) Instituto Nacional de Cardiologia

**Introduction:** The objective of this study is to report a rare case of a 15 mm coronary artery aneurysm (CAA) in the left main coronary artery (LMCA) in a patient with trivascular coronary artery disease (CAD), who underwent a surgical approach to CAD and conservative treatment of the coronary artery aneurysm. 68-year-old female patient with arterial hypertension, dyslipidemic and current smoker, with angina and transthoracic echocardiogram showing inferior and apical hypokinesia, with preserved left ventricular systolic function.

**Case report:** The patient underwent stratification for CAD with myocardial perfusion scintigraphy, showing low uptake in the apical and anterior wall and a drop in ejection fraction >10% during exercise, being subsequently referred for coronary angiography with diagnosis of LMCA lesion 95% in the middle and distal third, with the presence of a 15 mm aneurysm, anterior descending artery (ADA) with 70% severe lesion in the middle third, circumflex artery (ACX) of small anatomical importance with 90% ostial lesion, diagonal branch (Dg) with severe lesion at the origin and posterior descending (PD) of the right coronary artery (RCA) with 50% in the middle third. After coronary angiography showed multivascular coronary artery disease, Coronary artery bypass graft surgery (CABG) was indicated and the treatment of the aneurysm was conservative due to technical difficulties.

**Discussion:** Coronary aneurysms are defined as a focal dilatation of a coronary segment at least 1.5 times the size of the adjacent normal segment. The right coronary artery is generally the most affected artery (40%), with the LMCA being the least affected artery (3.5%). In contemporary studies, the incidence of true coronary aneurysms is less than 1%, with a greater predilection for males and for proximal segments of the coronary arteries. The reported clinical case contradicts the epidemiology, showing a rare case of AAC of LMCA in a female patient under investigation for CAD. Most current recommendations on coronary aneurysms are based on small case series or anecdotal reports; AAC involving LMCA, multiple or giant aneurysm.


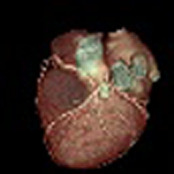


109190

Modality: E-Poster Young Researcher – Case Report

Category: CARDIOVASCULAR SURGERY

## Giant Cardiac Lipoma in the Right Atrioventricular Groove: Case Report of a Rare Lipomatose Site

CATARINA MAARCHON DA SILVA^2^, Rodrigo A. S. Sardenberg^2^, Catarina Marchon da Silva^1^, Andrea Galvão^2^, Pedro Ismael Amaral Silva^2^

(1) Universidade Municipal de São Caetano do Sul; (2) Hospital Alemão Oswaldo Cruz; (3) União das Faculdades dos Grandes Lagos

Cardiac lipomas are rare and benign tumors. We describe a case of a giant lipoma located in the right atrioventricular groove in a 62 year-old female patient who had no related symptoms. The diagnosis was done with an echocardiogram, and confirmed through intra and postoperative histopathology. Chest angio-CT scan was compatible with giant lipoma in the RAV groove or located at the RAV groove, measuring about 9.0 × 4.5 × 5.0 cm, circling the right coronary artery (RCA), ascending aorta and compressing the right atrium, causing dilation. The patient underwent complete tumor resection and was discharged without complications. The combination of an uncommon primary tumor of the heart in a rare location (AV groove) has, according to our researches, has only been reported once in the literature, so far. Cardiac lipomas are so infrequent that no therapeutic guidelines have been established for surgical indications in such cases. They are usually benign, however, tumor embolism, growth capacity, or intracardiac obstruction can cause a dangerous situation for the patient. Consequently, complete surgical resection might be indicated even in asymptomatic patients, varying according to the patient’s or the lipoma’s characteristics.


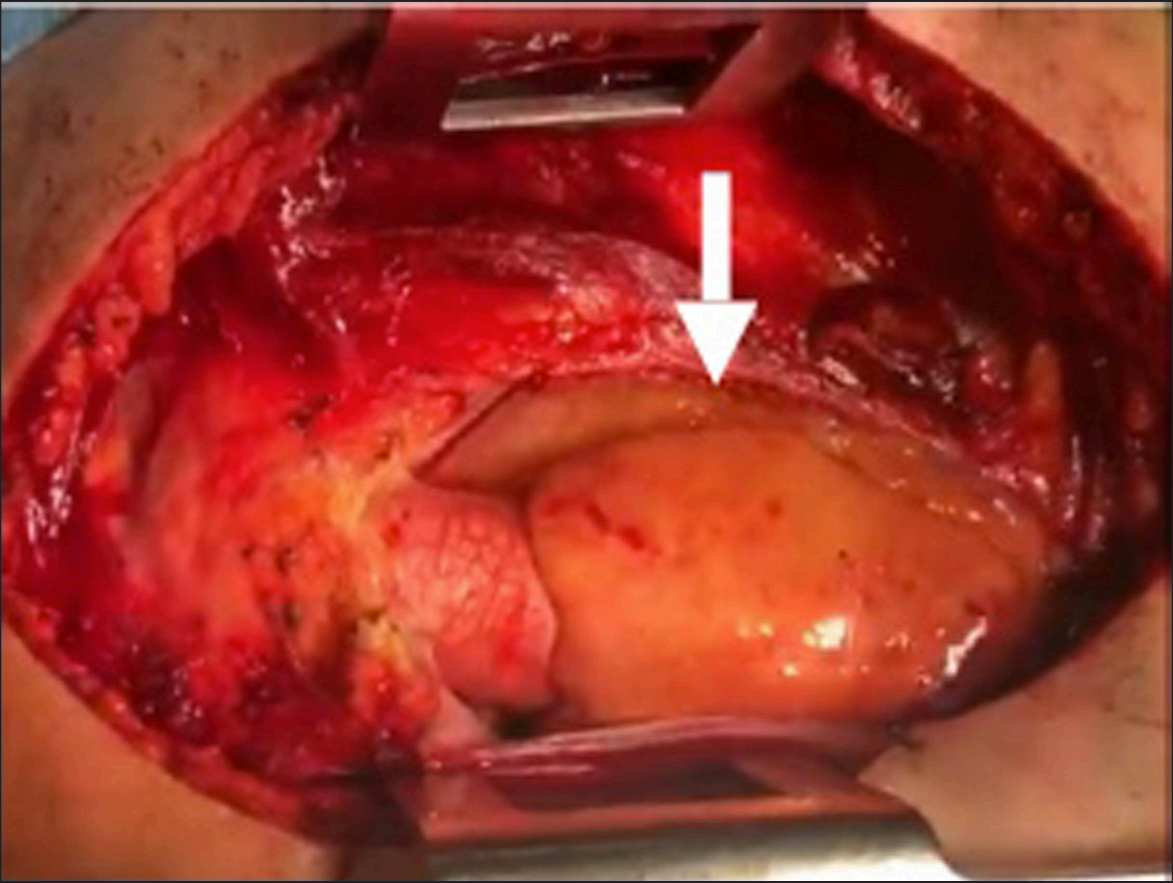


109199

Modality: E-Poster Young Researcher – Case Report

Category: HEMODYNAMICS AND INTERVENTIONAL CARDIOLOGY

## Concomitant Mitraclip Implantation for Severe Mitral Regurgitation and Transcatheter Aortic Valve Implantation with Valve-in-Valve Technique in a Patient with Prior Bioprosthetic Aortic Valve Replacement and Refractory Heart Failure

BRUNA SANTI DOS SANTOS^1^, Eduardo Schlabendorff^1^, Eduardo Keller Saadi^1^, Ana Paula Tagliari^1^, Marcelo Haertel Miglioranza^1^

(1) Hospital Mãe de Deus

**Background:** Transcatheter heart valve therapies such as mitral valve edge-to-edge repair with the MitraClip device and transcatheter aortic valve implantation (TAVI) have been demonstrated as an effective and valid therapeutic option in high-risk patients with heart failure. One-time double-valve treatment appears to be safe, feasible, and an innovative alternative. We present an exceptional case of concomitant MitraClip implantation for severe mitral regurgitation (MR) and TAVI with valve-in-valve technique in a patient with prior bioprosthetic aortic valve replacement and refractory heart failure.

**Case:** An 80-year-old man with hypertension, diabetes, and prior bioprosthetic aortic valve replacement 6 years ago was referred to our hospital with a history of several hospitalizations for decompensated heart failure in the last 6 weeks. Besides clinical treatment optimization, he persisted with recurrent dyspnea in rest. Transthoracic echocardiography revealed severe MR with effective regurgitant orifice 80 mm^2^ and biological aortic valve prosthesis dysfunction with severe aortic regurgitation (AR). The left ventricle was dilated (5.8 cm/3.5 cm) and ejection fraction was 69%. This high-surgical-risk patient was accepted for concomitant transcatheter treatment with Mitraclip and TAVI valve-in-valve by the heart team, and it was well succeeded. After the procedure, the patient’s symptoms improved and on discharge, he was ambulating, without dyspnea in ordinary physical activity.

**Conclusion:** Our case illustrates a success case of concomitant MitraClip implantation for severe MR and valve-in-valve TAVI for treatment of refractory heart failure, with significant improvement of symptoms. We conclude that the combination of the two transcatheter therapies would appear to be a viable and safe approach for managing high-risk patients with concomitant severe AR and MR and refractory heart failure, and it is likely to show better results in clinical improvement if performed before severe left ventricle remodeling with severe dilation occurs due to non treated valve disease.


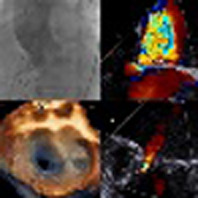


109620

Modality: E-Poster Young Researcher – Case Report

Category: CONGENITAL AND PEDIATRIC CARDIOLOGY

## Sinus Venosus Atrial Septal Defect in Adult Patient – A Case Report

SARAH PINI DE SOUZA^1^, Cíntia Chaves Mattoso^1^, Maria Carolina Terra Cola^1^, Fábio Akio Nishijuka^1^, Thaíssa Santos Monteiro^1^

(1) Instituto Nacional de Cardiologia

**Introduction:** Atrial Septal Defect (ASD) can remain undiagnosed until adulthood. Sinus venosus atrial septal defects (SVASD) account for approximately 5–10% of ASDs. Associated lesions can be found, including anomalous pulmonary venous connection (APVC), persistent left superior vena cava (SVC), pulmonary valve stenosis and mitral valve prolapse.

**Case report:** A 34 year-old male patient with a history of obesity and systemic arterial hypertension, had a known cardiac condition since he was 13 year-old, but lost follow-up and didn’t know any details about the condition. He reported the dyspnea had worsened gradually in the past 20 years, especially after he was diagnosed with atrial fibrillation, when he sought again for medical follow-up. Echocardiogram (Echo) demonstrated SVASD with bidirectional shunt and pulmonary hypertension. The patient had clinical signs of systemic congestion, suggesting right ventricle dysfunction. Right heart catheterization (RHC) was performed to evaluate if the patient was still eligible for ASD closure, and it confirmed SVASD with severe pulmonary hyperflow, increased pulmonary pressures and resistance, but with satisfactory response to vasodilator. The RHC wasn’t able to exclude the presence of partial APVC. The patient was then submitted to heart surgery for the ASD closure and at the procedure, anomalous drainage of two right pulmonary veins was identified, as they were connected to the SVC. For this reason, Warden repair was performed, with redirection of the APVC in the SVC through the interatrial communication into the left atrium. In the postoperative period, the patient complicated with severe pericardial effusion, requiring drainage, and was discharged 21 days after surgery. Postoperative echo showed improvement of pulmonary pressure and right ventricular function. During follow-up consultations, patient presented as NYHA I.

**Conclusion:** Patients with ASD may remain asymptomatic until adulthood, and diagnosis delay can impact significantly in prognosis, as some complications can become irreversible. ECHO is the first line diagnostic technique and RHC is mandatory in cases of signs of pulmonary hypertension. Patients with pulmonary vascular resistance ≥5 wu should be evaluated with extreme caution, considering not to proceed the ASD closure, or leastwise to maintain an interatrial fenestration.

109211

Modality: E-Poster Young Researcher – Case Report

Category: HEMODYNAMICS AND INTERVENTIONAL CARDIOLOGY

## Percutaneous Coronary Intervention of Kawasaki’s Stenosis in an Infant

MAURICIO FELIPPI DE SÁ MARCHI^1^, Cauyna Gurgel Moreira^1^, Gabriel Kanhouche^1^, Santiago Raul Arrieta^1^, Pedro Alves Lemos Neto^1^

(1) Instituto do Coração do Hospital das Clínicas da FMUSP (InCor)

**Introduction:** Kawasaki’s disease is an acute, self-limited febrile vasculitis of unknown cause that predominantly affects children and may lead to coronary dilatation and obstruction. We report the case of a child with extreme coronary aneurysm and stenosis, treated with percutaneous coronary intervention.

**Case report:** A 9-year-old male child was referred to our hospital with chest pain for major efforts. At age of 2, he was diagnosed with Kawasaki’s and received treatment with acetylsalycilic acid (ASA) and Intravenous Immunoglobulin (IVIG). At the time, a computerized tomography angiogram large aneurysmatic dilatations in both the right coronary and the left descending arteries, measuring 11.2 mm and 10.2 mm in diameter, respectively. The patient remained asymptomatic in out-of-hospital care and received ASA and clopidogrel. He remained asymptomatic in clinical follow-up for 6 years. Due to the patient’s complaints, we performed a coronary angiography which revealed a calcified giant coronary aneurysm in the LAD with a 90% focal stenosis before the aneurysm and a giant aneurysm in the RCA with a 60% focal stenosis. After the diagnostic angiography showed signs of coronary stenosis and aneurysm in the left descending artery our Heart Team opted for a new angiographic study with Intravascular Ultrassond. Due to the findings, balloon angioplasty was successfully performed, initially with semicomplacent balloon and finally with a non-complacent balloon. Angiography after the procedure demonstrated reduction of the previously documented stenosis and the patient reported relieved of the symptoms.

**Discussion:** This case demonstrates a rare, albeit reported complication of Kawasaki’s and the treatment option performed. Predisposing factors are not consensual, and an important genetic contribution is observed. Although Kawasaki’s leads to coronary aneurysms in approximately 25% of untreated patients, the timely treatment IVIG has decreased this risk to 3–5% of patients. In our case, despite timely IVIG, the patient still developed two giant aneurysms.


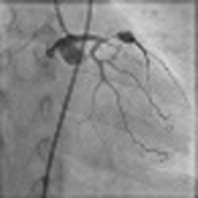


109227

Modality: E-Poster Young Researcher – Case Report

Category: ANTICOAGULATION

## Chronic Management of Pulmonary Embolism in Thromboangiitis Obliterans

MARIANA INOCÊNCIO MARTINHO^1^, Rita Calé^1^, Ana Glória Fonseca^2^, Melanie Ferreira^2^, Hélder Pereira^1^

(1) Cadiology Department, Hospital Garcia de Orta (HGO); (2) Internal Medicine Department, Hospital Garcia de Orta (HGO)

Thromboangiitis obliterans (TAO) is a rare vasculitis of young smokers affecting the small and medium sized vessels of the extremities. Although pulmonary embolism (PE) has been described, risk factors for large vessel occlusion and venous thromboembolism (VTE) recurrence are not known and there is no evidence favoring indefinite oral anticoagulation (OAC) for VTE prevention. Our first patient (pt) is an ex-smoker male diagnosed with PE and TAO at 44yo. He was under indefinite warfarin until 64yo, when OAC was switched to clopidogrel due to absence of VTE recurrence. Within 1 month, he had a massive unprovoked PE. Screening for autoimmunity (AI), antiphospholipid syndrome (APLS) and thrombophilia were negative and he was discharged under indefinite warfarin. At 7y follow-up (FUP) he had arterial ischemia but no VTE recurrence. The second pt is a male smoker diagnosed with TAO at 30yo and previous PE treated with warfarin for 6 months. AI and APLS screening were negative and he had hyperhomocysteinemia associated with MTHFR A1298C heterozigoty. At 36yo he had a submassive central PE treated with indefinite rivaroxaban. At 3y FUP despite arterial ischemia there were no VTE events. Thrombophilia and TAO have been associated and this may correlate with vessel inflammation and peripheral disease severity, but no link has been made to atypical presentation such as large vessel involvement. Hyperhomocysteinemia in MTHFR polymorphisms do not seem to increase VTE recurrence risk in the general population and do not determine prolonged OAC after a first VTE event. To our knowledge, this is the first time that this MTHFR mutation is associated with TAO. Although controversial, chronic OAC has been proposed to alleviate peripheral symptoms with some evidence suggesting that warfarin and rivaroxaban are able to reduce inflammatory markers in TAO. In both cases (the first not having any risk factor other that TAO and the second having hyperhomocysteinemia that alone should not determine secondary prevention), OAC did not prevent arterial events but seemed effective for VTE recurrence, leading us to believe that the proinflammatory state in TAO may warrant long-term OAC after a first event – as already happens with other AI diseases. Regardless, the authors acknowledge that we need more evidence to determine the most suitable approach.

109707

Modality: E-Poster Young Researcher – Case Report

Category: CARDIOVASCULAR SURGERY

## Recurrent Ascending Aortic Pseudoaneurysm – Rare Complication of Rare Disease

BRUNO GONÇALVES GARCIA^1^, Igor Daumas de Souza^1^, Amanda Dias Bomfim^1^, Stephan Lachtermacher Pacheco^1^, Marcelo Machado Melo^1^

(1) National Institute of Cardiology (INC) – Rio de Janeiro, Brazil

**Introduction:** Ascending Aortic Pseudoaneurysm (AAP) is a rare complication in cardiac surgery. It is a discontinuation of layers of vessel, with extravasated blood being contained by neighboring structures. Understanding of this rare event deserves to be discuss, in view of the potential severity of complications.

**Case report:** Male, 46-year-old, with history of bicuspid aortic valve with aortic insufficiency, who underwent metallic aortic valve replacement in 2005. 15 years later, an increase in diameters of ascending aorta (54 mm) was observed. Submitted to implantation of a non-valved tubular graft, and reimplantation of coronary. In immediate postoperative period, patient develops infective endocarditis of aortic prosthesis, treated only with antibiotic therapy. CT angiography of the aorta 1 year after surgery reveals pseudoaneurysm of ascending aorta measuring 8.4 × 5.9 cm extending up to 9 cm to the arch, with partially thrombosed lumen, and extravasation through anastomosis of the left main coronary artery. Although asymptomatic, by the risk of progression and rupture, was submitted to surgical correction, with closure of the orifice using bovine pericardium patch. On control CT angiography 1 and 2 weeks after surgery, new contrast uptake measuring 9 mm was identified in region adjacent on origin of the coronary arteries, without progression. Re-discussion between Heart Team was made, and conservative approach chosen, with periodic short-term follow-up.

**Conclusion:** The case shows a patient with AAP discovered accidentally, after considerable postoperative period of aortic and aortic valve manipulation. Although asymptomatic, there is a risk of generating compressive symptoms of thoracic organs. It can complicate with rupture and bleeding, in addition to being a site of infection, or thrombosis. Predisposing factors are previous manipulation of the aorta, aortotomy site, cannulation points, coronary graft anastomosis, connective tissue diseases, degenerative changes in the aorta, dissection, local infection. Regardless of size, treatment is indicated. Endovascular therapy is possible, but limited in cases of complex anatomy or active infection. Surgical approach can be performed with patch correction, or Dacron conduit. Cardiopulmonary bypass via femoral vessels is suggested for safe sternotomy. There is a risk of recurrence. Although rare, due to relevant complications, PAA should not be forgotten in the postoperative follow-up of cardiac surgeries.

109300

Modality: E-Poster Young Researcher – Case Report

Category: ATHEROSCLEROSIS/CARDIOVASCULAR RISK FACTORS/CARDIOVASCULAR PREVENTION

## Carotid Web: Report of Two Cases

SUELEN DAGOSTIM GISLON^1^, Roberto Gabriel Salvaro^1^

(1) Universidade do Extremo Sul Catarinense (UNESC)

The Carotid Web is a fibrous tissue projection to the carotid artery into the lumen with a pathological characteristic of intimal fibromuscular dysplasia. It is proposed as a mechanism of ischemic cerebrovascular accident (CVA) in which the hemodynamic alteration provoked can cause blood flow stagnation and clot embolisation. The main objective of this search is showed, clinically, the characteristic findings of carotid web in two different patients as well as their imaging tests. APC, female, 78 years old (patient 1), presenting complaints of headache in the occipital region radiating to the frontal region and dizziness for two months. History of Systemic Arterial Hypertension (SAH), dyslipidemia and ischemic heart disease with coronary artery bypass graft surgery in 2017. Due to the history of atherosclerosis and dizziness, an ultrasound of carotid was requested, which showed significant atheromatosis. The evaluation was complemented with carotid angiotomography (left figure), which demonstrated the presence of a linear image with a vertical orientation based on the posterior wall of the right carotid bulb, extending to the site of the internal carotid, determining stenosis in about 70%, probably related to Carotid Web, in addition to partially caicified plaques in the ostium of origin of the left vertebral column, determining stenosis in about 50%. MMF, male, 68 years old, farmer (patient 2), comes for consultation after having seizures with loss of consciousness for a few seconds. History of four CVA, SAH, venous insufficiency in the left lower limb and use of alcohol and tobacco. In investigation of stroke and embolic sources was performed Cranial Magnetic Resonance and Arterial Angio-AM of the neck (right figure), which showed Web Carotid on the right in addition to significant right vertebral lesion. Web Carotid is related to a higher risk of ischemic stroke, being important their identification to carry out the treatment and secondary prevention since the mechanism is also associated with recurrences of stroke, therefore, in some cases, dual antiplatelet aggregation or even anticoagulation is indicated.


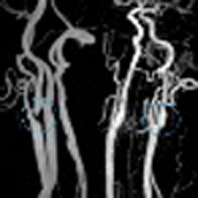


109263

Modality: E-Poster Young Researcher – Case Report

Category: CARDIOVASCULAR IMAGING

## Diagnosis of Infectious Endocarditis in Native Valve by Scintigraphy with Labeled Leukocytes

JONAS PACHECO DO DESTERRO^1^, Rafael Rodrigues Coutinho^1^, Nilton Lavatori Correa^1^, Izabella Caterine Palazzo^1^, Cláusio Tinoco Mesquita^1^

(1) Hospital Pró-Cardíaco

**Introduction:** Infective endocarditis is an infection of the endocardium and is characterized by vegetation, an infected clot of platelets and/or fibrin, containing leukocytes and red blood cells, mainly in valve topography. The diagnosis involves clinical, laboratory and imaging criteria and the prognosis is directly related to the early initiation of treatment. Scintigraphy with MDP-99mTc-labeled leukocytes plays a key role in this pathology.

**Case report:** We report the case of an 85-year-old female patient, hypertensive, dyslipidemic, without history of heart surgery with skin excoriations, fever, poor general condition and tachycardia. COVID19 infection was ruled out and the sepsis protocol started. The patient had leukocytosis with a left shift, elevation of CRP, lactate, massive pyuria and positive blood cultures for Staphylococcus aureus. Cranial, chest and abdomen CT scans showed no signs of infection. The transesophageal echocardiogram showed caseous degeneration in the mitral valve, and it was not possible to rule out the possibility of infective endocarditis (IE). A scintigraphy with labeled leukocytes was requested, which showed anomalous uptake in the mitral-aortic topography, confirming IE in the native valve.

**Conclusions:** In this case report above the scintigraphic method had a crucial role confirming, with high specificity, IE of the native valve and supported the change in the therapeutic management and patient’s prognosis.

109285

Modality: E-Poster Young Researcher – Case Report

Category: ACUTE AND CHRONIC CORONARY DISEASE/THROMBOLYSIS

## Compression of the Left Main Coronary Artery by a Surgical Implanted Aortic Valve Prosthesis – a Case Report

JULIANA JANGELAVICIN BARBOSA^1^, Fabiana Braga Sanches^1^, Rafael de Castro Hendges^1^, Silvio Marques Povoa Junior^1^, Pedro Silvio Farsky^1^

(1) Instituto Dante Pazzanese de Cardiologia

**Introduction:** Cardiovascular disease has been the leading cause of mortality worldwide since the 1960s, including coronary syndrome and valve disease. Surgical implantation of an aortic valve prosthesis is usually a low-risk procedure, but it can be challenging in selected cases. We describe here a compression of the left main coronary artery (LMCA) by a surgical implanted Aortic Valve Prosthesis.

**Case report:** Female patient,53 years old, dyslipidemic, HIV positive, previous coronary artery disease(CAD)-elective percutaneous coronary intervention of the left anterior descending artery in 2020,and surgical aortic valve replacement on May, 2021. On December 24 this patient presented in the Emergency room with chest pain with 2 hours duration. Initial electrocardiogram demonstrated ST-segment elevation in AvR and persistent ST-segment depression in anterolateral wall of up to 2.0 mm. Cardiac catheterization was indicated, demonstrating severe LMCA ostial lesion (90%), suggestive of extrinsic compression by the aortic bioprosthesis. Twenty four hours after the onset of the chest pain, a significant elevation of troponin was detected (12,810 ng/L – Reference value <11 ng/L). After a Heart Team Decision, emergency coronary artery bypass surgery was indicated. The patient was discharged from hospital in 4 days after a successful procedure.

**Conclusion:** Extrinsic compression of the LMCA as a cause of acute coronary syndrome is uncommon. The literature describes cases of coronary obstruction as a complication in patients undergoing transcatheter aortic valve implantation, in cases of pulmonary hypertension and due to aneurysmal structures. However, extrinsic compression of LMCA by a surgical prosthetic valve is a very rare condition, as in this report, that may guide future therapeutic decisions in atypical cases.


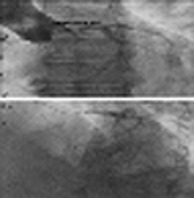


109337

Modality: E-Poster Young Researcher – Case Report

Category: HEMODYNAMICS AND INTERVENTIONAL CARDIOLOGY

## Left Internal Thoracic Artery Fistula to the Bronchial Artery: A Rare Cause of Coronary Flow Theft

MARCELA GOMES DE SOUZA^1^, Alice Mirane Malta Carrijo^1^, Vinicius Ferreira Aratani^1^, Flávia Bittar Britto Arantes^1^

(1) Universidade Federal de Uberlândia (UFU)

**Introduction:** The Left Internal Thoracic Artery (LITA) fistula to the pulmonary artery and its branches is a rare clinical condition, but should be suspected in cases of persistent angina after myocardial revascularization surgery (MRS). From a pathophysiological perspective, there is a pressure difference between the vessels, resulting in coronary flow diversion and myocardial ischemia. Diagnosis requires coronary angiography and treatment may include drug therapy, surgical or endovascular intervention.

**Case Description:** A 75-year-old man, with chronic obstructive pulmonary disease, hypertension, former smoker with a 60 pack-year smoking history. In 2014, the patient underwent MRS with LITA grafts to the anterior interventricular artery (AIA) and bypass graft to the right coronary artery, without complications. Five years later, he developed dyspnea on mild exertion, whose investigation revealed lung adenocarcinoma in the left apex, and a lobectomy was performed. After eight months, he presented with dry cough and dyspnea. The propaedeutics indicated a substernal goiter and heterogeneous thyroid, thus requiring a new surgical intervention in the mediastinum. After two months, he presented with complaints of chest pain on moderate and mild exertion associated with dyspnea. Pharmacological stress echocardiogram showed transient hypokinesia of the left ventricular anterior wall. Coronary computed tomography angiography detected an arterial fistula originating from the LITA following the lateral wall of the heart, confirmed by an angiographic study which indicated its destination for the left bronchial artery, besides blood flow diversion from the AIA. The patient underwent percutaneous embolization and trapping, with Onyx polymer and the release of six Axium micro-coils (Medtronic) into the fistular tract, via left radial access with a modified Seldinger technique. The procedure was successfully performed, and flow was immediately restored to the AIA. The patient was discharged remaining asymptomatic in the early follow-up.

**Conclusions:** Due to the number of MRS procedures performed in the modern era and the preference for LITA as a graft, although rare, the occurrence of fistulas should be considered in cases of persistent angina after MRS. Notably, with the advancement of techniques, endovascular therapy, in addition to sparing patients from the risks of a thoracotomy, has been well documented in the treatment of symptomatic patients with a favorable anatom.

109339

Modality: E-Poster Young Researcher – Case Report

Category: ACUTE AND CHRONIC CORONARY DISEASE/THROMBOLYSIS

## Double Jeopardy: Acute Left Anterior Descending and Right Coronary Arteries Thrombotic Subocclusions in a Young Healthy Testosterone User

MARINA PETSERSEN SAADI^1^, Anderson Donelli da Silveira^1^, Guilherme Heiden Teló^1^, Alan Pagnoncelli^1^, Felipe Homem Valle^1^

(1) Hospital de Clínicas de Porto Alegre

**Background:** Utilization of steroids to improve physical performance increase the risk of adverse cardiovascular events by acceleration of atherosclerosis and thrombogenesis. We report a case of acute two-vessel coronary thrombotic subocclusions in an otherwise young healthy testosterone user.

**Case report:** 37 year-old male, bodybuilder, presented with anterolateral myocardial infarction, six hours progression. He reported steroid use since 2013, using 250 mg of intramuscular testosterone decanoate daily at this time. He was taken to primary percutaneous coronary intervention (pPCI), which depicted highly thrombotic proximal left anterior descending coronary artery (LAD) and proximal right coronary artery (RCA) subocclusions, both with TIMI III antegrade flow. Given the high thrombotic burden and the presence of normal flow, pPCI was deferred and intravenous IIb/IIIa inhibitor and unfractionated heparin were administered for the following 24 hours. Repeated angiography with coronary intravascular ultrasound assessment demonstrated an atherosclerotic plaque rupture at proximal LAD, so PCI with a drug-eluting stent strongly discouraged and aggressive secondary prevention of ischemic heart disease was initiated.

**Conclusion:** The potential increased risk of acute coronary syndromes with exogenous testosterone administration needs to be put in perspective and the testosterone to improve physical performance should be avoided.


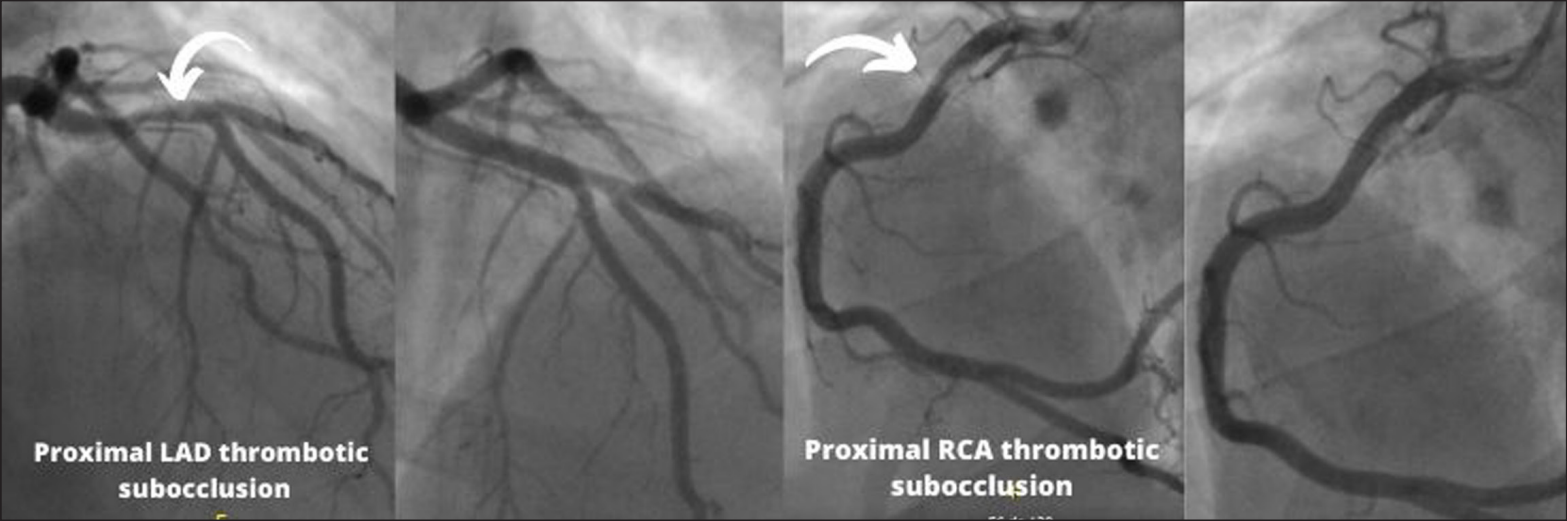


109365

Modality: E-Poster Young Researcher – Case Report

Category: PERICARDIUM/ENDOCARDIUM/VALVOPATHIES

## Ebstein Anomaly in Elderly: Case Report

IZABELLA SILVA FIGUEIREDO^1^, PATRICIA VIEIRA DE SA^1^, CLEICIANE RAMOS CAPEL^1^, ALINE SOUZA DE OLIVEIRA^1^, RAFAEL LUÍS FERREIRA SILVA^1^

(1) HOSPITAL SANTA CASA DE MISERICÓRDIA DE BELO HORIZONTE – HSCM BH

**Introduction:** Ebstein’s abnormality, a rare congenital heart defect caused by malformation in the posterior and septal leaflets of the tricuspid valve, has an incidence of around 1:20,000 births and a prevalence of about 0.5% among patients with congenital heart disease. It causes clear consequences on the right heart, but because the severity of anatomical alteration is variable, the clinical course of the disease is also variable, ranging from intrauterine heart failure to mild manifestations beginning in adulthood.

**Case Report:** Female, 66 years old, history of congenital heart disease, with interatrial communication closure in 2002, atrial block – total ventricular with pacemaker placement in 2012, evolved in early 2022 with dyspnea and edema of the lower limbs of progression of 2 months. He underwent outpatient follow-up with cardiology, diagnosed with Ebstein’s anomaly on the november 2021 echocardiogram, he showed important tricuspid regurgitation and valve alterations suggestive of Ebstein’s anomaly, with indication of surgical correction. Admitted electively on 02/06/2022 at Hospital Santa Casa, she underwent surgery with placement of bioprosthesis in a tricuspid valve on 02/08/2022. In the immediate postoperative period, she developed cardiogenic shock and was extubated after clinical improvement on 02/15/2022. Soon after, he developed fever, leukocytosis, pulmonary congestion. Collected cultures in the catheter tip culture was isolated Staphylococcus capitis, started antibiotic vancomycin. New echocardiogram 25/02/22 that suspected endocarditis or thrombosis in the bioprosthesis. After improvement of the congestive and laboratory condition, transesophageal echocardiogram was performed, seen in the lateral leaflet of the tricuspid thrombus bioprosthesis, discarded endocarditis. Started anticoagulation with xarelto and was discharged from the hospital on 03/10/2022 for outpatient control with cardiology and cardiac surgery.

**Conclusion:** Despite being considered the longest natural evolution of all, often exceeding until the third or fourth decade of life, acquired phenomena such as volume overload and ventricular dysfunction right affect this trajectory to the point of increasing the operative risk and morbidity in the postoperative period with the advent of arrhythmias of difficult control, in addition to the greater deterioration of ventricular function.

109370

Modality: E-Poster Young Researcher – Case Report

Category: PERICARDIUM/ENDOCARDIUM/VALVOPATHIES

## Nonbacterial Thrombotic Endocarditis (NBTE) in a Patient with Clear Cell Papillary Carcinoma with Implant in the Omentum, Peritoneum and Pelvis: Case Report

LUCAS CAUNETO SILVEIRA^1^, Rômulo Teixeira Vidal^1^, Flávio Visentin Pecci Maddalena^2^, Mariana Santos Gomes de Souza^3^, Marselha Marques Barral^1^

(1) Hospital e Maternidade Therezinha de Jesus – HMTJ; (2) Universidade Federal do Rio Janeiro – UFRJ/campus Macaé; (3) Faculdade de Ciências Médicas e da Saúde de Juiz de Fora – SUPREMA

**Introduction:** Nonbacterial thrombotic or marantic endocarditis (NBTE) is a rare condition of non-infectious lesions of the heart valves (mainly aortic and mitral) due to platelet deposition and hypercoagulable state, associated with neoplasms. Epidemiological data show higher prevalence in people with cancer compared to the general population (1.25% versus 0.2%, respectively), most cases are diagnosed in autopsies, with rates ranging from 0.9% to 1.6% and when compared to malignancy, it occurs more in patients with adenocarcinoma. In addition, it affects all age groups, especially in the fourth to eighth decade of life, with no predilection for sex. Overall, treatment is based on systemic anticoagulation and the surgical indications are the same as for infective endocarditis.

**Case Description:** 49 years old female patient was hospitalized due to ischemic stroke. Transthoracic echocardiogram (TTE) showed a mobile filamentary structure adhered to the ventricular face of the aortic valve, along with a moderate aortic regurgitation. The intracranial arterial computed tomography (CT) angiography demonstrated left frontoinsular hypodensity related to ischemic vascular event without further alterations; CT scan of the abdomen and pelvis detected voluminous ascites, multiple soft tissue peritoneal formations, enhanced by contrast, sparse throughout the abdominal cavity and suggesting peritoneal implant. Furthermore, liver hypocaptant images of irregular contours was showed, mainly in segment VII, measuring 2.2 × 1.5 cm; enlarged uterus with heterogeneous density and oval formations suggesting fibroids, large solid-cystic expansive formation in the pelvis, of well-defined edges, with the solid portions enhanced by contrast measuring about 11.0 × 11.8 × 9.2 cm diagnosed ovarian neoplasm. Carotid doppler USG and thoracic aorta CT angiography was normal. Peritoneal biopsy has shown clear cell carcinoma with omentum, peritoneum and pelvis implant; patient status worsened with septic shock and respiratory failure, requiring orotracheal intubation, vasoactive drugs and intensive care unit support. After ten days, brain death was diagnosed.

**Conclusion:** NBTE is a rare disease with high mortality, finding in advanced stages of cancer. Treatment consists in anticoagulation.

109545

Modality: E-Poster Young Researcher – Case Report

Category: PERICARDIUM/ENDOCARDIUM/VALVOPATHIES

## Liver Cirrhosis Secondary to Constrictive Pericarditis: Case Report

FERNANDA MARIA FRANCO CASTRO^1^, Ludmila Stephanie Julio Machado^1^, Mariana Fonseca Bittencourt^1^, Thiago Pinheiro Junqueira^1^, Eduardo Belisario Falchetto^1^

(1) HOSPITAL FELÍCIO ROCHO – HFR

Constrictive pericarditis is a condition associated with the development of constrictive heart failure. It results from a thickening of the pericardium, with restriction of diastolic filling of the heart and consequent decrease in cardiac output. The cause is often unknown, although it may be subsequent to any illness that causes acute pericarditis. Symptoms are related to heart failure, and may also present changes such as severe cachexia, peripheral edema, ascites, pulsatile hepatomegaly associated with congestive liver disease and pleural effusion. Physical examination findings such as pulsus paradoxus, Kussmaul’s sign and pericardial knock may also be presente, but are not pathognomonic of this condition. We present the case of a 56-year-old male patient, melanoderm, hospitalized with a previous diagnosis of liver cirrhosis of undefined etiology, undergoing propaedeutic for liver transplantation. He was under irregular follow-up with a cardiologist, had a previous diagnosis of atrial fibrillation and heart failure, with a report of dyspnea that started 6 months ago, getting worse in the last 4 months associated with increase in ascites. He reported a history of tuberculosis associated with acute pericarditis for about 20 years ago. He performed a transthoracic echocardiogram whose image revealed a pericardium with increased echogenicity, mainly visceral, compatible with significant thickening, suggesting calcified constrictive pericarditis. Patient underwent surgical pericardiectomy with progressive improvement of heart and liver condition. Currently, there is no more indication for liver transplantation. Constrictive pericarditis is associated with elevated hepatic vein pressures, making patients with constrictive pericarditis prone to developing hepatic necrosis and cirrhosis. The clinical features are relatively nonspecific, making it easy to be confused with primary liver cirrhosis. Thus, a high index of suspicion for the diagnosis must be maintained. Some findings on physical examination may generate important clues (elevated jugular venous pressure, Kussmaul’s sign, pericardial knock, and pericardial calcification on chest X-ray). A correct diagnosis is essential, since pericardiectomy is curative if performed early.

109417

Modality: E-Poster Young Researcher – Case Report

Category: CONGENITAL AND PEDIATRIC CARDIOLOGY

## Anomalous Origin of the Left Coronary Artery from the Pulmonary Artery: Case Report

ANDREY ALVES DE FARIA SILVA^1^, Henrique Nunes Pereira Oliva^2^, Mariana Brandão Soares Sousa^1^, Isabela Oliveira Oliva^3^, Rita de Cássia Oliveira Araújo^3^

(1) Universidade Federal dos Vales do Jequitinhonha e Mucurí; (2) Yale University School of Medicine and Unimontes PPGCS; (3) UNIFIPMoc

**Introduction:** ALCAPA Syndrome, also called Bland-Altman-Garland syndrome, is a rare congenital disease characterized by the anomalous origin of the left coronary artery from the Pulmonary Artery (AP), whose probable cause of occurrence is an embryonic alteration that affects cells of the cardiac neural crest during the early stage of embryogenesis. The present article aims to describe the rare case of anomalous origin of the left coronary artery from the pulmonary artery.

**Case Report:** Patient D. P. A, 35 years old, male, electrical engineer, comes to a regularly scheduled review. Electrocardiogram, showed Left bundle branch block. Echocardiogram were performed, and showed akinesia and hypokinesia in lower wall. It was decided to perform a computed angiotomography of the coronary arteries, which confirmed the diagnosis of ALCAPA Syndrome. (Figure 2. computerized tomography angiogram). The patient was submitted myocardial revascularization surgery, using a branch of the mammary artery to connect to the anterior descending coronary artery. In the postoperative period, the patient presented chest pain and dry cough, after 2–3 days he developed bilateral massive pulmonary thromboembolism and was hospitalized for 7 days. Pradaxa was prescribed twice a day.

**Conclusion:** It is concluded that the ALCAPA syndrome in male adults is rare and is not well described in the literature. With the surgical correction employed, the patient evolved to improve his clinical condition. Therefore, knowledge of this condition is extremely important, since the early diagnosis and conduct provide a reduction in the patient’s morbidity and mortality.

**REFERENCES**

1. Angeline P. Revisando a Síndrome de ALCAPA dos Tipos Infantil e Adulto: as Diferenças Estão nos Detalhes. Rev Bras Cardiol Invas. 2007; 15(4).


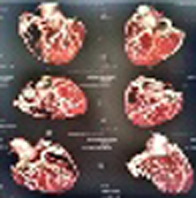


109416

Modality: E-Poster Young Researcher – Case Report

Category: ACUTE AND CHRONIC CORONARY DISEASE/THROMBOLYSIS

## Infarto Agudo Do Miocárdio Por Doença Aterosclerótica Coronariana Em Gestante Jovem

LUAN GABRIEL PAESE^1^, Antônio Dejair Acosta Pazzini^1^, Sergio Antônio López^1^, Rômulo de Lima Moreno^1^, Rodney de Oliveira^1^

(1) Sociedade Hospitalar Angelina Caron HAC

**Introduction:** The incidence of acute myocardial infarction (AMI) in pregnant women is low. Spontaneous coronary artery dissection is responsible for approximately 40% of AMI. Maternal mortality can reach 7%. It usually occurs in the third trimester or the immediate postpartum period. There is usually no benefit of planned cesarean delivery. The treatment is controversial and studies are still insufficient to draw definitive conclusions.

**Case report:** Female patient, 34 years old, gestational age (GA) of 20 weeks, housewife, without comorbidities. Family history with smoker father with ischemic stroke at age 50. She was admitted to the emergency room due to chest pain, sweating and dyspnea. The electrocardiogram demonstrates sinus rhythm, subepicardial ischemia in the anteroseptal wall. Ultrasensitive troponin positive, in ascending curve. The transthoracic echocardiogram presented hypocontractility of the anterior wall, left ventricular (LV) ejection fraction of 46% and reduced global longitudinal strain (–13%). Cardiac catheterization revealed a 95% ostial lesion of the anterior descending coronary artery (AD), with thrombi throughout its extension, LV with anteroapical hypokinesia. She underwent primary angioplasty of AD with Orsiro drug-eluting stent 3.5 × 22 mm and was discharged with therapy for atherosclerotic disease (CAD). The classic presentation of AMI due to CAD is more severe in pregnant women. Unlike the high-risk population, the prevalence of atherosclerotic factors in this type of population is low. The ESC 2018 update guides the use of dual antiplatelet therapy with AAS and Clopidogrel, beta-blockers and nitrates, contraindicating the use of statins, angiotensin-converting enzyme inhibitors and angiotensin receptor blockers. It is recommended that delivery occur at least 2 weeks after the AMI, preferably vaginally. In case of a new pregnancy, it is recommended to wait a period of 12 months. Confirmed AMI in the pregnant woman, there is an indication for coronary angiography, using isosmolar contrast and protective measures. The definition of the use of balloon or stent-eluting is important. The risk of angiography is relatively small compared to its benefit for birth planning.

**Conclusion:** The decision about the treatment to be performed is multifactorial taking into account the GA and the assessment of risks and benefits against the aforementioned drugs. The case had an good maternal evolution with hospital discharge without complications.

109422

Modality: E-Poster Young Researcher – Case Report

Category: HEART FAILURE/CARDIOMYOPATHY/TRANSPLANT

## Hypocalcemia and Heart Failure: Case Series

FRANCIELE LEIMANN^1^, Luiz Cláudio Danzmann^2^, Marciane Maria Rover^1^, Simone Louise Savaris^1^

(1) Instituto de Cardiologia de Porto Alegre; (2) Universidade Luterana do Brasil

**Background:** Heart failure (HF) is a serious health problem with high morbidity and mortality. Among the several etiologies of HF, hypocalcemia is rare, however, it is potentially fatal.

**Case Reports:** We described three cases of female patients aged 51, 58 and 38 years, respectively, with HF secondary to hypocalcemia. Calcium deficiency occurred due to idiopathic hypoparathyroidism in one of them and hypoparathyroidism secondary to total thyroidectomy in the other two. The patients presented with signs and symptoms of HF, besides suggestive clinic of hypocalcemia, with hypoesthesia and paresthesia of extremities. In etiological research findings, serum calcium levels below 5 mg/dL were found. In two cases, there was a prolongation of the QT interval to the electrocardiogram. Furthermore, it was identified a reduction in the ejection fraction of the left ventricle (LVEF) on the echocardiogram in the three cases – at the first case with 30%, second with 19% and third with 28%. After treatment for HF associated with calcium replacement, all the patients presented an improvement of the LVEF (50%, 46%, and 62%, respectively), as well as clinical and functional status.

**Conclusion:** The cases here presented to illustrate this situation, highlighting the risk of hypocalcemia after total thyroidectomy procedure, and also demonstrate improved function systolic and clinical symptoms with correction of hypocalcemia associated with standard therapy for heart failure.

110136

Modality: E-Poster Young Researcher – Case Report

Category: HEART FAILURE/CARDIOMYOPATHY/TRANSPLANT

## Long Covid as a New Etiology for Heart Transplantation: A Case Series

PLÍNIO JOSÉ WHITAKER WOLF^1^, João Manoel Rossi Neto^1^, Marco Aurelio Finger^1^, Carolina Casadei dos Santos^1^, Victor Bemfica de Mello Mattos^1^

(1) Instituto Dante Pazzanese de Cardiologia

**Background:** Long COVID is a condition characterized by long-term consequences persisting or appearing after the typical convalescence period of COVID-19. It may last several months but the duration is still matter of observation. The symptoms and the clinical manifestations are clinically heterogeneous including heart failure (HF) episodes. We describe a case series of 4 patients who had long COVID and evolved to heart transplantation (Tx) indication from January/2020 to February/2022.

**Description:** Mean age was 38 ± 9.5 years, 75% were male and 50% were Caucasian. The whole sample was admitted in NYHA IV functional class. None of the patients had more than one previous comorbidity. All had positive troponin, NT-ProBNP (20234 ± 13373) and c-reactive protein (14,7 ± 13,1). Mean time from COVID-19 infection to admission date was 150 ± 113 days and mean time from admission date to outcome was 112 ± 62,1 days. Mean Ejection Fraction (EF) was 17 ± 3,7% and 75% used Intra-aortic balloon (IAB). Two patients underwent Tx (all still alive), one died prior to Tx and one is still in the waiting list. 75% of patients had thrombotic complications awaiting Tx and all transplant patients had treated cellular rejection and Cytomegalovirus infection (table).

**Conclusion:** HF secondary to COVID-19 due to Long COVID can progress to refractoriness to clinical treatment and Tx is the therapy of choice, showing success, despite high mortality during waiting list.


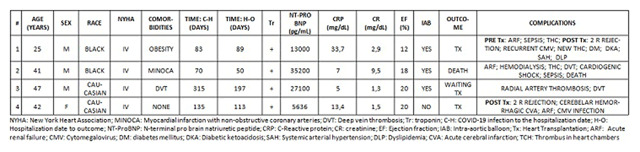


109433

Modality: E-Poster Young Researcher – Case Report

Category: PERICARDIUM/ENDOCARDIUM/VALVOPATHIES

## Post-COVID-19 Pericarditis: Beyond Idiopathic or Viral Etiologies

NATÁLIA GOMES CANDIAGO^1^, Luiza Maria Costi Menta^1^, Rafael Massuti^1^, Tais Regina Bisol^1^, Bibiana Guimarães Maggi^1^

(1) Hospital Geral de Caxias do Sul (HGCS)

**Introduction:** Pericarditis is an usually mild disease with multiple possible etiological factors, including inflammatory diseases, immune responses after heart damages or viral infections, or several other chronic health conditions. Most of these cases, especially viral cases, remain undefined, as idiopathic.

**Case description:** A 62-year-old male presented intermittent symptoms of asthenia, weight loss, thoracic pain, dyspnea, lower limb edema, vespertine fever, night sweats, and syncope on efforts. All symptoms started markedly after an infection of COVID-19, one month earlier. Patient had a familial history of leukemia, multiple sclerosis, and heart failure. The assistant physician team began a thorough outpatient investigation involving exams in search for rheumatic, collagen, neoplastic and infectious diseases. Chest tomography scans demonstrated parenchymal condensation, atelectatic striae, pleural effusion and pericardial effusion. An echocardiography demonstrated valve thickening and elicited suspicion of constrictive pericarditis. The patient evolved within months with recurrent symptoms and worst dyspnea, and so was admitted to the local hospital. A chest angiography demonstrated pulmonary thromboembolism and persistent pericardial effusion, and the patient was transferred to our hospital to assess the need for a pericardiocentesis. At our service, the diagnostic hypotheses of pericardial tuberculosis, other neoplasms and amyloidosis were raised, since the patient persisted with vespertine fever, tachycardia and night sweats during hospitalization. A new echocardiogram did not found constrictive pericarditis, with discrete improvement of findings. Patient evolved with empyema, and a cardiac magnetic resonance imaging found thickened pericardium, pericardial effusion exudate, and protein accumulation. A diagnostic videothoracoscopy was performed, with biopsy of the pericardial tissue and chest drainage. Pericardial biopsy was positive for tuberculosis, and suggestive of both chronic and acute pleuritis. Treatment with RHZE was instituted, and symptoms improved. After removal of chest drains, the patient was discharged, asymptomatic.

**Conclusion:** Not all pericarditis are idiopathic, not even after viral infections. Tuberculosis reactivation after coronavirus infection have been frequent, and physicians must always elicit such diagnosis in light of compatible clinical histories, especially in countries of endemic tuberculosis such as Brazil.

109449

Modality: E-Poster Young Researcher – Case Report

Category: CONGENITAL AND PEDIATRIC CARDIOLOGY

## Coronary Thrombosis and Ectasia in a Young Woman with Turner Syndrome

MARINA SOUTO DA CUNHA BRENDEL BRAGA^1^, Anabel Lima Vieira^1^, Amanda Valério Galindo^1^, Ândrea Virgínia Ferreira Chaves^1^, Dayse de Sena Moreira Alves^1^

(1) Hospital Agamenon Magalhães

**Introduction:** Turner syndrome (TS) is one of the most common chromosomal abnormalities, caused by the partial or total loss of the X chromosome. Among the alterations, cardiovascular disease is more prevalent, and may be congenital or acquired. Mortality rate in young women increase due to associated malformations. In the literature, coronary ectasia associated with thrombosis is not well established.

**Case report:** F.S.G., 44 years old, female, diagnosed with Turner Syndrome and hypothyroidism, without other comorbidities, sought a cardiologic emergency due to typical chest pain, without other symptoms, 12 hours from symptom onset. She denied previous episodes. Electrocardiogram (EKG) on admission showed no acute ischemic changes. Chest radiography without abnormalities. Serum troponin with positive curve (479 –> 1746; RV: 14). Cardiac catheterization was performed with evidence of diffuse coronary ectasia and thrombus in the middle to distal third of the Circumflex Artery. Transthoracic echocardiography (TTE) showed an ejection fraction of 47% with concentric left ventricular remodeling and segmental contractile deficit (basal and medial infero-lateral hypokinesia). It was decided to start full anticoagulation with a schedule of repeat the cardiac catheterization in 07 days, which kept the same pattern. After the result, acetylsalicylic acid and rivaroxaban were started for further reassessment in 01 year. Additional evaluation of atherosclerotic disease showed no changes – lipidogram within the normal range, carotid Doppler ultrasound without plaques, bone densitometry with android/gynoid ratio indicating low cardiovascular risk. Still, chest tomography showed normal aorta.

**Conclusion:** The association of TS with cardiovascular changes, from bicuspid aortic valve and aortic aneurysm to stroke and acute myocardial infarction, is well established in the literature. Coronary ectasia associated with thrombosis was not described as a cause of acute coronary syndrome, as occurred in the case described. This complication corroborates the need for improved cardiological knowlegde of the syndrome, aiming at proper condution and treatment.

109488

Modality: E-Poster Young Researcher – Case Report

Category: NEGLECTED CARDIOVASCULAR DISEASES

## Chronic Constrictive Tuberculous Pericarditis in a Patient with Alcoholic Liver Cirrhosis: Case Report

MATHEUS LIMA LULA GUIMARÃES^1^, Ananda Ribeiro Fretes^1^, Paula Maria Pinheiro Miranda^1^, Letícia Aparecida Braga da Silva^1^, José Sudário Cardoso Neto^1^

(1) Universidade Federal do Mato Grosso

**Introduction:** Tuberculous pericarditis is an important complication of tuberculosis (TB) in developing countries. It affects up to 4% of cases of pulmonary TB and may evolve with fibrosis and calcifications causing chronic constrictive pericarditis.

**Case report:** 58-year-old patient, male, alcoholic, smoker, under follow-up with hepatology due to alcoholic liver cirrhosis. Pathological history: untreated Atrial Fibrillation and treated pulmonary TB. He frequently complained of dyspnea on exertion, orthopnea, lower-extremity edema, ascites, occasional stabbing pain in the left hemithorax, fever, mental confusion, abdominal pain and nausea. Management of decompensated liver cirrhosis was performed, but with progressive worsening of ascites and orthopnea. Therapeutic paracentesis was performed on an outpatient basis, followed by lipothymia after the procedure, therefore, he was hospitalized for clinical compensation. On admission, he was hypotensive, tachycardic, irregular heart rhythm with hypophonetic heart sounds with splitting of the second heart sound, distended jugular veins, right-sided pleural effusion, ascites and lower-extremity edema. Echocardiogram showed preserved systolic function, enlarged hypocontractile right ventricle, significant biatrial enlargement, moderate mitral and tricuspid regurgitation, significant inferior vena cava ectasia, without respiratory variation, and hyperrefringent posterior pericardium. Chest tomography with cardiomegaly with diffuse calcifications in the pericardium, appearing to compress the ventricles and nonspecific mediastinal lymph nodes. Therefore, a diagnosis of constrictive pericarditis was made with echocardiographic criteria and clinical manifestations and of probable tuberculous etiology due to diagnosed pulmonary TB. Pericardiectomy was performed due to refractoriness to the proposed treatments (antituberculous and diuretic therapy). The patient evolved with significant improvement, with dyspnea only on great exertion, resolution of ascites and lower limb edema in subsequent consultations.

**Conclusion:** There is a certain diagnostic difficulty in patients with liver disease due to the clinical similarity, but due to the high prevalence of tuberculous pericarditis and mortality if left untreated, we should consider such a diagnosis in patients with suggestive epidemiology. However, in a world scenario, tuberculous pericarditis is still among the often under-recognized and neglected cardiovascular diseases.

109486

Modality: E-Poster Young Researcher – Case Report

Category: CARDIOLOGY OF SPORTS, EXERCISE, ERGOMETRY AND CARDIOVASCULAR REHABILITATION

## Sudden Death in High Performance Athlete with Bradycardia and SCN5A Gene Mutation

JOÃO ANTÔNIO DA SILVA NETO^1^, João Antônio da Silva Neto^1^, Patrícia Alves de Oliveira^1^, Luciana Sacilotto^1^, Walace Magalhães Barbosa^2^

(1) Instituto do Coração INCOR; (2) Instituto Dante Pazzanese de Cardiologia IDPC

**Introduction:** Sinus bradycardia (SB) at rest is considered normal in athletes and is determined by intrinsic heart rate (HR) reduction and modulations of the autonomic system. However, the possibility of progressive degeneration in association with genetic mutations that determine rhythm alterations should make the marked and persistent BS to be better investigated.

**Case report:** Marathon runner, 28 years, SB with HR <30 bpm associated with first degree atrioventricular block (AVB) and left ventricular hypertrophy (LVH). Diagnosed with atrial flutter (AF) during a period of high-intensity training, which was treated with ablation. After 16 years of follow-up, he had an ischemic stroke (IS), treated with trombolisys, without neurologic sequelae. After developed episodes of sudden loss of consciousness, which was attributed to seizures resulting from the IS. However, the SB with pauses persisted, despite the improvement in the (LVH) pattern. HOLTER showed HR 36-48-80 bpm, BS rhythm, alternating with atrial ectopic rhythm, episodes of type I 2nd degree AVB and pauses of up to 2.7s. TILT-TEST with marked SB, 1st degree AVB and junctional escape, with accentuation of bradycardia and prolonged pauses to orthostasis, without HR response to sensitization with vasodilator, as well as with atropine. Suspecting genetic mutations that cause hereditary primary electrical diseases, a genetic test was collected. However, the patient had sudden death during sleep, before the result, which revealed a mutation of the SCN5A gene.

**Discussion:** SB, prevalent in athletes, may be accompanied by AVB of varying degrees. In this case, in addition to marked bradycardia, the patient had AF. Despite the causal relationship between mutations in the SCN5A gene and the Brugada Syndrome phenotype, there are atypical clinical manifestations that include, in addition to the set of signs and symptoms of Brugada syndrome, AF, sinus node dysfunction, long QT syndrome, dilated cardiomyopathy, and others. Carriers of SCN5A mutations linked to Brugada syndrome or progressive cardiac conduction system degeneration are at increased risk of sudden death.

**Conclusion:** Marked bradycardia in athletes should be investigated and the association with channelopathies leads to phenotypes with aberrant rhythms and progressive degeneration of the cardiac conduction system, which can determine fatal arrhythmias.

109497

Modality: E-Poster Young Researcher – Case Report

Category: COVID-19 AND CARDIOVASCULAR SYSTEM

## Post-Vaccination Myocarditis Against COVID-19: A Case Report

BRUNO MIRANDA MINSKI^1^, RODRIGO BODANESE^1^, RENAN LEOTTE DE SOUZA^1^, NADIA MAYER^2^

(1) Hospital São Lucas – PUCRS; (2) Prefeitura Municipal de Porto Alegre

**Introduction:** Myocarditis is inflammation of the heart muscle resulting from exposure to external antigens or internal triggers, such as autoimmune activation. Due to the coronavirus pandemic and vaccination across the world, cases of vaccine reactions have been reported. Of these, myopericarditis corresponds to approximately 0.1% of complications. As a rule, these cases are related to messenger RNA (mRNA) vaccines.

**Case Description:** A 17-year-old man comes to the hospital with chest pain that started 2 hours ago, with oppression, without irradiation or associated symptoms. Triggered at rest, with inspiration worsening. Previously healthy, without continuous use of medication. Two days before the onset of symptoms, he received the second dose of the COVID-19 mRNA vaccine (Pfizer). The diagnosis of COVID-19 was excluded by RT-PCR. On admission he presented with BP 109/50; HR: 101; SatO2: 98%; RR: 16; BT: 36°C. Cardiac and pulmonary auscultation without significant changes. Absence of signs of congestion. Electrocardiogram (ECG) with sinus rhythm (SR), HR 74, anterior, upper lateral and inferior ST elevation and T wave inversion in the lateral wall. High Sensitivity Cardiac (HSc) Troponin I: 16,752 (RV: 34.2). Given the hypothesis of myopericarditis, he was submitted to Cardiac Magnetic Resonance Imaging (MRI), which showed: extensive late enhancement of a non-ischemic aspect and diffuse mesocardial/subepicardial edema, mainly affecting the apical, medial lateral and basal walls. Pericardial edema, more evident in the middle lateral walls of the LV. EF 44%. Findings consistent with myopericarditis. During hospitalization, he remained asymptomatic, with good clinical evolution, receiving Angiotensin Converting Enzyme Inhibitor, Beta-Blockers and Colchicine. Discharged 5 days after admission, without complaints, with a sharp drop in HSc Troponin (202). Ten days after discharge, new ECG showing RS; HR 62; anterior, superior and inferior lateral T wave inversion. After 42 days ECG of normal pattern. After 90 days of the initial event, a new MRI, which showed: reduction in the extension and thickness of the late enhancement with a non-ischemic aspect in the mesocardium/subepicardium. Virtually complete resolution of myocardial edema and pericardial changes. EF: 50%.

**Conclusions:** The description of adverse effects of the new vaccines is essential to quicly diagnose and manage them in the most appropriate way possible, preventing unfavorable outcomes.

109503

Modality: E-Poster Young Researcher – Case Report

Category: ATHEROSCLEROSIS/CARDIOVASCULAR RISK FACTORS/CARDIOVASCULAR PREVENTION

## Single Coronary Lesion on Young Patient Diagnosed with Axenfeld-Rieger Syndrome

RAMON OTT VARGAS^1^, José Carone Filho^1^, Schariff Moysés^1^, Roberto Ramos Barbosa^1^, Assad Miguel Sassine^1^

(1) Instituto de Cardiologia do Espírito Santo

**Introduction:** Axenfeld-Rieger syndrome is an autosomal dominant genetic disease. Generally, described phenotypes consist in craniofacial and dental abnormalities, ocular anterior segment dysgenesis as well as congenital heart defects, mostly outflow tract diseases. Atrial and ventricular septal defects, valve stenosis and persistent truncus arteriosus are the most frequent cardiac presentations. The occurrence of premature coronary artery disease on these patients is not frequently documented.

**Case Description:** Thirty year old male, diagnosed with Axenfeld-Rieger syndrome for the past twelve years, refers chest pain and oppression caused by mild efforts and daily activity, progressively worsening to limitations of any physical activities and, lately, angina at rest. Patient is eutrophic, practices physical exercise regularly, non-smoker, no diabetes or dyslipidemia and no family history for premature coronary heart disease. After being examined in clinic, laboratory exams were requested, as well as transthoracic echocardiography (TTE) and treadmill stress test. Lab results show normal cholesterol levels, glucose and triglycerides. Liver and renal functions were also normal. TTE showed no abnormalities, but the stress test was interrupted for excess tiredness and probably anginous chest pain on peak effort and recovery. Patient then presented a strong episode of angina at rest, and headed to the Emergency Room. On that occasion, a coronary angiography was requested, and showed a 90% obstruction on proximal left anterior descending artery (LAD). Percutaneous coronary intervention was performed, and a zotarolimus-eluting stent was uneventfully implanted. Patient performed well after the procedure, was discharged to home and returned to normal physical activities, maintaining follow-up in clinic.

**Conclusion:** The occurrence of obstructive coronary lesions on young, low cardiovascular risk patients is always reason for questioning. In the described case, patient bears a rare syndrome, on which ophthalmologic and cardiac outflow tract malformations are frequently described. We hypothesize that the coronary lesion relates to this same syndrome. Further studies are necessary to evaluate this correlation.

109525

Modality: E-Poster Young Researcher – Case Report

Category: HEMODYNAMICS AND INTERVENTIONAL CARDIOLOGY

## Transcatheter Aortic Valve Implantation (TAVI) with Basilica Technique for Surgical Bioprosthesis Failure in Patient with High Risk of Coronary Occlusion

FLÁVIO ROSA VIEIRA^1^, Jordana Pires Mendonça^1^, Tannas Jatene^1^, Rogério Lobo de Andrade Las Casas^1^, Vinicius Daher Vaz^1^

(1) Hospital do Coração Anis Rassi

Bioprosthetic disfunction is a common late complication in patients submitted to surgical aortic valve replacement. Transcatheter Aortic Valve Implantation (TAVI) valve-in-valve is an option to treat this problem according to most recent guidelines. Although rare, coronary occlusion is a potentially fatal complication after TAVI. We present a case in which we used a technique denominated BASILICA (bioprosthetic or native aortic scallop intentional laceration to prevent iatrogenic coronary artery obstruction) to prevent this adverse event. A male patient, 75 years old, hipertensive, former smoker, with ongoing dyspnea NYHA III. Physical exam revealed sistolic and diastolic murmur (3/6+) in the aortic area. Past medical history of coronary artery bypass graft in 2007, surgical bioprosthetic aortic valve replacement in 2009, percutaneous coronary intervention with stent at first diagonal branch in 2012 and 2021 (in-stent restenosis). ECG shows sinus rhythm, first degree atrioventricular block, right bundle branch block and left anterior fascicular block. Transthoracic echocardiogram identified a severe regurgitation and stenosis on the bioprosthetic aortic valve (mean gradient 41 mmHg and peak velocity 4,5 m/s). Although STS Score was 6,14% and euroSCORE II 3,45%, the patient refused his third surgery. Angio CT showed high risk for coronary occlusion (Valsalva sinus mean diameter 24 mm, left coronary height 5,5 mm and VTC 3,5 mm). Heart Team opted for TAVI with the described BASILICA technique. The intraoperatory transesofagical echocardiogram (TEE) showed mean gradient 37 mmHg and severe aortic regurgitation due to rupture in the right coronary sinus leaflet. We used a 0.014 guide wire connected to an electrocautery to perforate and tear the left leaflet. Later after pre dilatation, we finally implanted a Sapien 3 (Edwards Lifescience) # 20 bioprothesis followed by post dilation at higher pressure with a #22 non compliant balloon. The TEE showed mean gradient of 12 mmHg and no paravalvular or central leak at the final procedure. Despite the risk, the left coronary occlusion did not occur. Patient remained for 2 days at the ICU due to an increase in the PR interval, which was sustained, and was discharged 3 days after the procedure. We concluded that BASILICA technique is a feasible alternative to the patients candidates to TAVI in Valve-in-Valve with high risk for coronary occlusion.

109538

Modality: E-Poster Young Researcher – Case Report

Category: CARDIOVASCULAR SURGERY

## Treatment of Severe Mitral Regurgitation Due to Previous Endocarditis in a Patient with Marfan Syndrome and Severe Symmetrical Acute Pectus Excavatum

PEDRO GUIMARÃES SILVA^1^, GUILHERME RAPOSO DE MEDEIROS^1^, MARILIA TAILY SOLIANI^1^, RONEY ORISMAR SAMPAIO^1^

(1) INSTITUTO DO CORAÇÃO DO HCFMUSP

**Introduction:** Pectus exacavatum is one of the most iconic features of Marfan syndrome. In patients who have not undergone thoracic correction during childhood, surgical treatment of valvular heart disease faces an even greater challenge. The following case report presents a patient with significant mitral regurgitation due to previous infectious endocarditis who is submitted to valve replacement along with the correction of his severe acute pectus excavatum.

**Case report:** An 18 year-old male diagnosed with Marfan syndrome at 10 years old presented to the emergency room (ER) in February 2022 with worsening dyspnea and fatigue. He had a severe acute pectus excavatum (Haller index: 8.8), arachnodactyly, and and a positive family history of Marfan syndrome (both his father and brother). Concerning the past medical history, in 2018 he suffered from an infectious endocarditis (IE), treated only with antibiotics at the time, which caused a rupture of the tendinous cords of the mitral valve and a prolapse of both its cusps, along with the formation of a significant vegetation on the atrial aspect of the mitral anterior leaflet. He continued his outpatient treatment for 4 more years, which consisted mainly on the management of the symptoms caused by the the worsening mitral regurgitation After the admission to the ER, a transesophageal echocardiography was performed, showing a significant mitral vegetation, similar to the one described in 2018, of 21 × 6 mm along with severe mitral regurgitation; a left atrium of 230 ml/m^2^; and an ejection fraction was 54%. There was no involvement of the aortic valve or aortic sinus. After thorough assessment, the hypothesis of a recurrent IE was abandoned (low C- reactive protein levels, no fever or other stigma of IE, and negative blood cultures) and the present condition was attributed solely to the worsening of mitral regurgitation. Chest computerized tomography presented a severe pectus excavatum with a pronounced mediastinal deviation to the left, and a total atelectasis of the left lower lung lobe Mitral valve replacement was then performed using a biological prosthesis. After closure of the left atrium, the pectus excavatum was then corrected using the Ravitch technique and Marlex mesh, without the Nuss bar (due to prolonged surgical time and detected intraoperative ventricular dysfunction). The patient evolved well during the postoperative period, with full recovery and relief of symptoms.

109570

Modality: E-Poster Young Researcher – Case Report

Category: NURSING

## The Importance of Telemonitoring in Graft Rejection After Transplantation

EVELYN GOMES OSÓRIO^1^, Kalliza Kary Rodrigues da Costa^2^

(1) Hospital Copa Star; (2) Hospital Copa Star

**Introduction:** Heart transplantation (HT) is considered a therapeutic option for end-stage heart failure (HF) patients¹. The occurrence of rejection is one of the post-transplant complications. Although new immunosuppressive regimens have decreased the incidence of rejection, it still represents one of the key clinical concerns. It is estimated that 25% to 80% of recipients will experience at least one episode of rejection, requiring treatment during the first year after implantation. Rejection is the cause of approximately 10% of deaths in the first year after surgery². The nurse’s role in the post-transplant follow-up and the control of adverse events is of paramount importance to detect new complications early on. The early identification of rejection will impact the prognosis and the maintenance therapy decision for the transplanted patient^3^.

**Description of the Case:** Male, 39 years old, diagnosed with dilated cardiomyopathy without defined etiology about 10 years. Last echocardiogram with ejection fraction (17%), listed for HT, he searched emergency room due to progression of symptoms. He was admitted with acute cardiac insufficiency, requiring inotropic and transfer to HT reference unit where was tested for SARSCOV-2 with a positive diagnosis resulting in removal from the transplant line for 30 days. He returned in line priority subsequently, was transplanted on the 34th day. On the 10th postoperative day, the patient showed rejection grade 3R (severe rejection). The patient evolved with significant clinical improvement but presented infection by cytomegalovirus (CMV), one of the main causes of morbidity and mortality in HT in cases of recurrent or difficult to control infection^4^. After hospital discharge, the patient continued to be followed up by the multi-professional team, made possible through telemonitoring, the nurse’s follow-up to detect early signs of aggravation, monitoring of the serum level in laboratory results to evaluate treatment accession. The treatment was based on oral corticosteroid therapy associated with immunosuppressive therapy. After 3 months of follow-up, the results of the biopsy showed an evolutionary improvement with results of 3R, 2R, and 1R.

**Conclusions:** The outcome of the case was favorable. The advances in telemonitoring and the nurse’s role have shown to be important management and follow-up tools for transplant patients at home, seeking early detection of signs of aggravation and monitoring of vascular graft disease.

109597

Modality: E-Poster Young Researcher – Case Report

Category: CONGENITAL AND PEDIATRIC CARDIOLOGY

## Myocardial Bridge: A Clinical or Invasive Approach?

SÂMIA BADWAN MUSTAFÁ^1^, Sâmia Badwan Mustafá^1^, Bácila Lunks Badwan Musa^3^, Yasmine Badwan Mustafá^2^

(1) Hospital Universitário de Santa Maria; (2) Grupo Hospitalar Conceição; (3) Clínica Médica Bácila Badwan

**Introduction:** Myocardial bridge is a congenital anomaly, usually in the anterior descending artery (AD). It is one of the main differential diagnoses of coronary artery disease, especially when there is a low probability of atherosclerotic disease. It may manifest as angina and, rarely, acute myocardial infarction or sudden death.

**Case description:** B.E, 39, male, military, typical anginal pain for 1 year. No comorbidities, performs physical activities. Physical examination normal. ECG: sinus rhythm, 70 bpm, left atrial and left ventricular overload. Chest X-ray normal. Echocardiogram normal. Prescribed Rosuvastatin 10 mg/day and Ramipril 5 mg. Ergometric test: ST depression. Coronary angiography: large intramyocardial bridge in AD. Clinical therapy was optimized: Nebivolol 10 mg/day, Amlodipine 10 mg, ASA 100 mg/day, suspension of Ramipril. After a year it returns with pain. Myocardial scintigraphy: no ischemic areas. Surgical evaluation requested.

**Conclusions:** A diagnosis of a congenital condition was made based on a typical angiographic finding – systolic reduction in the diameter of the epicardial coronary artery. Although congenital, symptoms are observed later, since, due to the growth of the heart, there is an increase in the systolic tension of the myocardial wall and a reduction in coronary flow due to atherosclerotic processes. Optimized clinical treatment with beta-blockers was started, in order to reduce heart rate, increase diastolic time and reduce systolic compression of the vessel and, thus, improve anginal symptoms; antiplatelet drug to reduce the risk of cardiac events. Nitrates were avoided, as they worsen coronary artery systolic narrowing, which can worsen symptoms. Despite optimized clinical treatment, symptoms persisted, and the possibility of invasive treatment with stent implantation was discussed. However, studies have shown stent stenosis in almost half of patients undergoing angioplasty and need for reintervention in more than one third. Therefore, clinical treatment is the first line, and invasive treatment should only be considered if it is refractory to optimized clinical treatment.


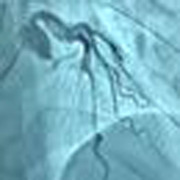


109614

Modality: E-Poster Young Researcher – Case Report

Category: CONGENITAL AND PEDIATRIC CARDIOLOGY

## Tricuspid Valve Dysplasia Mimicking Ebstein‘s Anomaly: A Case Report

AMARILDO BORGES DA SILVA OLIVEIRA^1^, Allan Oliveira Macedo^1^, Ana Caroline Prado Pereira^2^, Jéssica Danicki Prado Fernandes^1^

(1) Hospital Regional de Ceilândia – Secretaria Estadual de Saúde do Distrito Federal (HRC – SES/DF)); (2) Instituto Master de Ensino Presidente Antônio Carlos – IMEPAC Itumbiara/GO

**Introduction:** Ebstein’s Anomaly (EA) is a rare congenital heart disease, characterized by abnormalities of the tricuspid valve (TV) and the right ventricle (RV), with clinical presentation varying from neonatal period to adulthood. Some echocardiographic and anatomical features are essential for diagnosis: apical displacement of the insertion of the TV septal leaflet (≥8 mm/m^2^), redundant elongated anterior leaflet of the TV, and RV thinning, enlargement, and dysfunction. Other comorbidities associated with TV regurgitation and right heart chambers enlargement may be misdiagnosed as EA, such as tricuspid valve dysplasia (TVD), presented in this paper, which aims to correctly identify EA and report a TVD case.

**Case description:** MSL, female, 53 years old, dyslipidemic and diabetic, was admitted to the Emergency Unit with headache, for 4 days, associated with hypotension, sweating, general malaise and diarrhea. Later, she was transferred to a secondary hospital with signs and symptoms suggestive of B-profile decompensated heart failure. During hospitalization, an echocardiography (ECHO) was perfomed, which showed TV with thickened leaflets, apical displacement of the septal leaflet, compatible with EA. Subsequently, a new ECHO was performed with a team specialized in congenital heart diseases, which revealed a TV with redundant and dysplastic leaflets, septal leaflet with normal implantation, however, with a septum attachment with an extension of 7.5 mm/m^2^, not meeting the criteria for EA but for TVD. Surgical correction with valve replacement was indicated.

**Conclusions:** EA is a complex congenital cardiac malformation with a great difference in anatomical and clinical presentations, and may be suspected in cyanotic patients, with tricuspid regurgitation, heart failure and arrhythmias, which are possible manifestations in TVD. Thus, TVD may mimic EA and its management needs to be individualized. Knowing the anatomical and hemodynamic variations, as well as the associated comorbidities, is essential for the correct recognition of cardiac involvement and management of the case, thus optimizing the patient’s longevity.


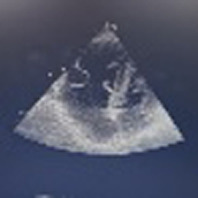


109625

Modality: E-Poster Young Researcher – Case Report

Category: ACUTE AND CHRONIC CORONARY DISEASE/THROMBOLYSIS

## An Acute Myocardial Thrombosis Post – Covid 19 Infection and Vaccination

MARCIO RAFAEL MONTICIELO^1^, Stefano Ramos Farias Leite^1^, Paula Thais Birk^1^, Andrieli Cristina de Oliveira^1^, Leonardo Gheller Zanatta^1^

(1) Hospital de Caridade de Ijuí – HCI

Acute Myocardial Infarction (AMI) is among the leading causes of death in the world. It occurs due to obstruction of coronary vessels, leading to perfusion dysfunction of cardiac areas and consequent myocardial injury, with probable failure if not treated in time.

**Case:** Male, white, 35 year, reporting sudden-onset chest pain, approximately 30 minutes before admission, at rest, radiating to the left upper limb, malaise and sweating. He had previous systemic arterial hypertension and reported COVID-19 disease six months ago. He had been vaccinated for COVID-19 eight days before admission (OXFORD). No alcoholism or smoking. On physical examination, he had stable vital signs, cardiac and respiratory auscultation without alterations and well-perfused extremities. The electrocardiogram (ECG) showed ST elevation in leads D2, D3 and AVF, showing inferior wall AMI. Coronary angiography showed a distal thrombotic lesion in the right coronary artery (TIMI 3). At catheterization time, he was without chest pain and with preserved coronary flow (TiMI 3). We defined for pharmacological treatment with Tirofiban, Acetylsalicylic Acid, Clopidogrel and low molecular weight heparin in therapeutic dose. Some hours after, he returned with chest pain and ECG shows inferior wall AMI. A new catheterization was done and observed a significant reduction of previous thrombotic lesion but now distal occlusion of the posterior ventricular branch was verified. Once again it was decided to maintain conservative treatment due to the risk/benefit relationship. He was discharged from the hospital after complete relief of pain and decrease in the enzyme curve. He was oriented to maintaining double platelet aggregation treatment for a period of 1 year and we requested coronary CT angiography for the return.

**Conclusion:** Studies have shown that patients with covid-19 as well as with vaccine for covid 19 are predisposed to thromboembolic events, such as peripheral and pulmonary thromboembolism, stroke, AMI and acute lower limb ischemia. The fact that these patients had no risk factors for CAD and did not have coronary atherosclerotic plaques raises the possibility that the thrombotic event is associated with the hypercoagulable state of the covid-19/vaccine infection. More aggressive pharmacological therapy, such as fibrinolytics, glycoprotein IIb/IIIa inhibitor and prolonged use of anticoagulants, in these cases, in view of the high thrombotic load, replacing angioplasty, should be considered.

109659

Modality: E-Poster Young Researcher – Case Report

Category: CARDIOVASCULAR SURGERY

## Coronary Artery Compression by Post-Traumatic Left Ventricular Pseudoaneurysm: A Case Report

BRENO DE ALENCAR ANTÃO^1^, Raysa Ramos Santos Negromonte^1^, Anabel Lima Vieira^1^, Maria de Fátima Nunes de Oliveira^1^, Maria Dolores da Trindade Henriques Assunção^1^

(1) Hospital Agamenon Magalhães

**Introduction:** A left ventricular pseudoaneurysm (LVP) it is the result of cardiac rupture contained by adherent pericardium or scar tissue. Unlike true aneurysm, it does not contain endocardium or myocardium. Most of the LVP occurs after transmural myocardial infarction but also occurs after cardiac surgery, trauma, or infection. We describe a case of LVP after blunt chest trauma incidentally occurring with valvular heart failure.

**Case report:** J.S.S., a 60-year-old male was admitted with dyspnea on mild exertion, orthopnea, paroxysmal nocturnal dyspnea, and lower limb edema. He had a mitral bioprosthesis (MB) since 2013 due to a blunt chest trauma caused by motor vehicle accident that led to acute mitral regurgitation (MR) as result of ruptured chordae tendineae. Initial echocardiogram (ECHO) revealed preserved systolic function with moderate tricuspid regurgitation, moderate pulmonary arterial hypertension and dysfunctional mitral bioprosthesis. Pre-surgical catheterization revealed anterior descending artery, 2nd diagonal branch and circumflex artery with significant luminal reduction during systole, without signs of coronary atheromatosis. Coronary angiotomography was requested evidenced a pseudoaneurysm in the basal segment of the anterior wall of the left ventricle with slight extrinsic circumflex artery compression. Patient underwent MB replacement and the LVP, which was below the aortic valve plane, measuring 1.3 cm in diameter, was repaired with implantation of a bovine pericardium patch. There was a postoperative bleeding successfully managed. New ECHO revealed normal implantation and fixation of BM, normal LV systolic function with moderate right chamber insufficiency and without evidence of a pseudoaneurysm. Patient had clinical improvement after procedures and remains in outpatient follow-up.

**Discussion:** According to the literature, up to 80% of blunt cardiac injuries are caused by motor vehicle collisions (MVC) widely ranging from LVP to the ventricular free wall rupture. Untreated pseudoaneurysms have rupture risk (30% to 45%) and high mortality rate (almost 50%) with clinical treatment. Patients with severe MR have a higher risk which requires mitral valve replacement, as in the case described. Correct diagnosis and appropriate surgical management lead to reduced mortality and better prognosis.

109722

Modality: E-Poster Young Researcher – Case Report

Category: CARDIOVASCULAR SURGERY

## Stanford a Chronic Aortic Dissection in a High-Risk Surgical Patient and Decision for Clinical Treatment: Case Report

KELSON KEMUEL CONFESSOR DE SOUSA^1^, Kelton Dantas Pereira^1^, Vinicius Pereira Dantas^1^, Ana Olivia Dantas^2^, Fabio Mastrocola^1^

(1) Onofre Lopes University Hospital; (2) Federal University of Rio Grande do Norte

**Introduction:** Aortic dissection is marked by the delamination of the middle layer of this vessel due to the inflow of blood. According to the Stanford classification, the ascending aorta (type A) or the descending aorta (type B) may be affected. Some studies show that the mortality of patients with type A acute dissection treated surgically is 26%, while that of those not undergoing surgery, due to advanced age or comorbidities, was about 58%. There are no data on mortality in chronic dissections.

**Case report:** A 78-year-old female patient, with obesity, chronic hypertension and frailty syndrome, started with a sudden loss of muscle strength and dysarthria, being diagnosed with ischemic stroke. During investigation of the present clinical scenario, the Transthoracic Echocardiogram showed a Stanford type A dissecting aortic aneurysm measuring 68 mm. An angiotomography of the thoracoabdominal aorta showed a fusiform aneurysm of the ascending aorta, measuring 7.3 cm with signs of chronic aortic dissection, extending from the aortic valve annulus to the infra-renal abdominal segment. The patient was then also submitted to coronary evaluation, which showed multivessel coronary artery disease. In view of the chronic dissection of the ascending aorta, which treatment currently established is to replace the affected aortic segment, and considering the multiple comorbidities peculiar to this patient at high surgical risk, the clinical and surgical team considered that the surgery would bring more risks than the potential benefit. Thus, the patient was discharged with adequate blood pressure and heart rate control.

**Conclusion:** Acute type A aortic dissection is the cardiovascular lesion with the highest mortality if not treated immediately, reaching a rate of 80% in about 14 days, with surgical treatment being one of the only possible approaches for both acute and chronic cases. There are complex and challenging cases such as the one described above in which the risk versus benefit must be weighed, as the patient has already passed the high mortality phase. Furthermore, the current medical literature is not clear about the mortality estimate in the scenario of chronic aortic dissection, which led us to choose a conservative approach in a patient at high surgical risk. This patient has been under follow-up for more than 6 months, an outcome that could be different if the surgical procedure had been chosen at the initial moment.

109749

Modality: E-Poster Young Researcher – Case Report

Category: CARDIOVASCULAR IMAGING

## Loeffler’s Endomyocardial Disease: A Rare Cause of Heart Failure

GABRIELA ASSIS RANGEL DE ABREU^1^, Lucas Feldman Paz de Lima^1^, Angelo Antunes Salgado^1^, Marcos Paulo Lacerda Bernardo^1^, Joaquim Henrique de Souza Aguiar Coutinho^1^

(1) Hospital Universitário Pedro Ernesto

**Introduction:** Loeffler’s endomyocardial disease is a rare disease caused by eosinophil infiltration into the endomyocardial. Heart failure characterizes the clinical picture, secondary to the restrictive behavior of the ventricle, triggered by fibrosis and obliteration of the ventricular cavities. The treatment targets the etiology of eosinophilia, which often remains unknown.

**Case:** Male, 62, started outpatient follow-up in hematology for eosinophilia and splenomegaly. The laboratory showed leukocytosis of 36,090/mm^3^ with eosinophils 21,645/mm^3^ (60%). An investigation was carried out but without etiological elucidation. A transthoracic echocardiogram (TTE) showed biatrial enlargement, preserved biventricular function, type 2 diastolic dysfunction (DD), and increased thickness of the apical segments of both ventricles, suggestive of infiltrative disease. After one month, he presented ascites and dyspnea requiring hospitalization. A new TTE revealed type 3 DD, right ventricular systolic dysfunction with severe tricuspid regurgitation due to coaptation failure, and worsening of ventricular infiltration, obliterating more than 50% of the cavities. During hospitalization, the patient evolved with low cardiac output refractory to pharmacological therapy without improving eosinophilia despite corticosteroid. He then underwent endocardiectomy and biological tricuspid valve replacement; nevertheless, he came to death one day after surgery.

**Conclusion:** Loeffler’s endomyocardial disease is a subcategory of the hypereosinophilic syndrome, with 2D TTE being the primary imaging method for diagnosis and follow-up. In this case, the lack of etiology, corticosteroid refractoriness, and fast progression of biventricular apical obliteration made the case difficult to handle and a worse prognosis.


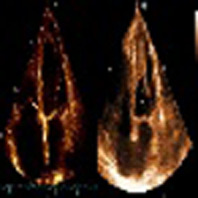


109814

Modality: E-Poster Young Researcher – Case Report

Category: HEMODYNAMICS AND INTERVENTIONAL CARDIOLOGY

## Transcatheter Edge-to-Edge Mitral Valve Repair to Improve Heart Transplant Candidacy in Worsening Pulmonary Hypertension: A Case Report

PEDRO CASTILHOS DE FREITAS CRIVELARO^1^, Nadine Oliveira Clausell^1^, Felipe Homem Valle^1^, Livia Adams Goldraich^1^, Leonardo Hennig Bridi^1^

(1) Hospital de Clínicas de Porto Alegre HCPA

**Background:** Transcatheter edge-to-edge mitral valve repair (TEER) has been utilized as a potential therapeutic strategy in selected patients with advanced heart failure. According to a multicenter retrospective registry, TEER may promote clinical improvement and pulmonary hypertension relief as bridge to transplantation or to candidancy.

**Report:** A 57-year-old male with ischemic cardiomyopathy and recently worsened severe functional mitral regurgitation (MR) was admitted with advanced heart failure and listed for transplant. Clinical status worsening, with intravenous inotropic support increases and temporary mechanical circulatory support followed. Hemodynamic assessments showed heart failure progression with fixed severe pulmonary hypertension and remarkably poor cardiac performance. Transplant was then deemed to be of high risk, and the patient was inactivated on the waiting list. TEER was considered as a “bridge to candidancy” and it was carried with the implantation of 2 XTw MitraClip® (Abbott Vascular) devices. Remarkable reduction on both MR severity and left atrial V-wave pressure were achieved. Functional and hemodynamic status dramatically improved and the patient was reactivated on the heart transplant waiting list at 30-day follow-up.

**Conclusion:** TEER may be a feasible option in selected heart transplant candidates to improve severe pulmonary hypertension.


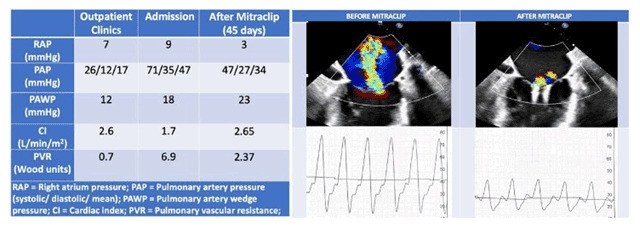


109858

Modality: E-Poster Young Researcher – Case Report

Category: HEMODYNAMICS AND INTERVENTIONAL CARDIOLOGY

## Valve-in-Valve Mitral in Patient with Thrombus in Left Atrial Appendage: The Importance of Brain Protection with Sentinel™ Device and Appendage Occlusion with Watchman™ Prosthesis in the Same Procedure

JOAO CARLOS MATOS PINTO JUNIOR^1^, Luiz Felipe M F Alves^1^, João Luiz Frighetto^1^, Lisley Riano da Silva Pestana^1^, Estevão Carvalho de Campos Martins^1^

(1) Hospital de Força Aérea do Galeão

**Introduction:** Rheumatic valve disease still is a prevalent condition in our country. Even when young, affected patients evolve the need of mitral valve replacement. Subsequent valve dysfunctions, predominantly in mitral valves, generate structural and pathological consequences, primary or secundary. Thromboembolic phenomenons and the increased operative risk in the classical surgical approaches confers a higher risk of morbidity and mortality to those patients. Modern strategies and devices for percutaneous treatment emerge as an option for a better approach.

**Case description:** 78 years old, female, with hypertension and permanent atrial fibrillation. Evolves with worsening of functional class, signs of congestion, requiring hospital admission and intravenous diuretic therapy. This patient have a history of biological mitral valve replacement due to rheumatic valve disease. The first one was in 1968 and the second one was in 2000. She had a subarachnoid hemorrhage event in 2020. Evolves with worsening functional class, with signs of system congestion, requiring the use of intravenous diuretic therapy. After clinical stabilization, a transesophageal echocardiogram was performed, revealing an LA enlargement and a severe degeneration of the biological mitral prothesis with calcified and poorly mobile bases including a eversion of one of these leaflets into the left atrium, causing a severe mitral regurgitation, with a transprosthetic gradient. maximum 21 mmHg and average 9 mmHg, Peak Velocity 2.3 m/s, PHT 179 ms, AEO 1 cm², and presence of thrombus in the left atrium Due high surgical risk, percutaneous treatment was chosen with a VIV – “Valve in Valve” strategy preceded by central nervous system protection with the Sentinel™ device on the brachiouscefalic trunk and the left internal carotid beside Left Atrial Appendage with the watchman™ flex.

**Conclusion:** The treatment of valve degeneration in elderly patients with previous biological valve replacement is still challenging, with the advent of percutaneous treatment (Valve-in-valve), which has brought new perspectives to those patients. The use of devices such as sentinel™ and watchman™ help to prevent one of the main complications, which is embolism to the central nervous system. References: Little SH, Bapat V, Blanke P, Guerrero M, Rajagopal V, Siegel R. Imaging Guidance for Transcatheter Mitral Valve Intervention on Prosthetic Valves, Rings, and Annular Calcification. JACC Cardiovasc Imaging. 20.

109870

Modality: E-Poster Young Researcher – Case Report

Category: CARDIOVASCULAR SURGERY

## Acute Pericarditis and Pericardial Effusion After COVID-19 Infection

RAQUEL SILVA BRITO DA LUZ^1^, Adnaldo da Silveira Maia^1^, Arturo Adrian Jara^1^, Janayna Rabelato^1^, Antonio Flavio Sanchez^1^

(1) Instituto Dante Pazzanese de Cardiologia

**Introduction:** COVID-19 infection has a wide spectrum of clinical presentations, including cardiovascular, including myocarditis and pericarditis. The prevalence and events associated with this condition still remain under analysis, as well as the repercussion of such complications after the viral infection.

**Case Description:** 74-year-old female patient, former smoker, bilateral carotid stenosis without hemodynamic repercussions, rheumatoid arthritis and diagnosis of viral pneumonia due to COVID-19 in January 2022. She was admitted, in March 2022, to a referral center in cardiovascular diseases, with a clinical picture of chest pain that worsened during inspiration with 2 months of evolution. Laboratory measurements of troponin and D-dimer within normal limits. Admission electrocardiogram suggestive of pericarditis. Transthoracic echocardiogram (TTECT) revealed preserved biventricular function and pericardial effusion with a greater depth of 11.0 mm. Despite the optimized clinical treatment for the pericarditis, the patient evolved with clinical worsening and rapid evolution of the pericardial effusion initially evident, with a 30.0 mm blade and signs of hemodynamic repercussion, requiring urgent drainage. On the 1st postoperative day in the intensive care unit, the patient evolved with severe acute biventricular dysfunction and need for vasoactive drugs, without response to the established clinical measures, progressing to death.

**Conclusion:** The prevalence of underlying pericarditis and pericardial effusion in patients with COVID-19, as well as its clinical significance remains the subject of clinical research. Thus, knowing risk factors and the pathophysiology of cardiovascular complications related to COVID-19 are sine qua non conditions for understanding the clinical presentation, prognosis and therapeutic management.

109879

Modality: E-Poster Young Researcher – Case Report

Category: HEMODYNAMICS AND INTERVENTIONAL CARDIOLOGY

## A Unique Case of Stent Kinking

SEPIDEH DARBANDI^1^, Samuel Congello^1^, Ali Mardan^1^

(1) MercyOne North Iowa Medical Center

Patient is a 62 year old male with past medical history of tobacco abuse who had presented with abrupt onset of pain and paralysis in his right arm. Of significance, he had suffered a motor vehicle accident two years prior but had refused medical treatment for the diagnosed right clavicle fracture at that time. On physical examination, patient had ischemic discoloration of his right arm and forearm and was noted to have absent pulses in that arm. CT angiogram of the right upper extremity was performed which showed an occlusion at the thoracic outlet with another obstruction down at the bifurcation of the distal brachial artery at about the level of the antecubital fossa. Embolectomy was initially performed. A EV3 Protege self-expanding stent was deployed within the distal subclavian artery and proximal axillary artery. The stent was postdilated with a EV3 EverCross balloon. Post stenting angiogram did not show any significant residual stenosis. There were no immediate post operative complications. To further investigate etiology of patient’s arterial thrombosis, transesophageal echocardiogram was performed that was negative for intracardiac thrombus, however atheromatous plaque lesion was present in aorta and CT angiogram of the chest was recommended for further characterization. CT angiogram of the chest showed new right subclavian artery stent placement with resolution of previous occlusion. Report of CT scan read as such: Stent is patent but buckled proximally with luminal narrowing in the area of buckling. He subsequently underwent examination of the right arm and shoulder under fluoroscopy and showed that the distal segment of the clavicle (due to untreated displaced fracture) impinged on the stent with motion. Interestingly enough, with patient’s arm hyperadducted, there was impingement on the subclavian stent, and patient would actually experience numbness in his right arm. As a result, patient underwent operation by orthopedics for right clavicle open reduction internal fixation, and his symptoms of right arm paresthesia was resolved as his stent remained patent without kinking.

**Conclusions:** The incidence of subclavian artery thrombosis is quite uncommon, although this risk is increased with risk factors such as peripheral vascular disease, obesity, and diabetes mellitus. Stent impingement should be on the differential in a patient that is admitting to motional symptoms that is related to the regional blood supply.

110595

Modality: E-Poster Young Researcher – Case Report

Category: COVID-19 AND CARDIOVASCULAR SYSTEM

## Acute Coronary Syndrome Correlation between Echocardiogram, Catheterization and COVID-19

THIAGO BURIL FONTES^1^, Rudyney Azevedo^1^, Maria Gorett W. Matioli^1^, Regina Adler^1^, Andre Luis Mendes Martins^1^

(1) Hospital Servidor Publico Municipal de São Paulo

**Background:** Acute coronary syndrome (ACS) is an event that happens because of rupture of an atherosclerotic plaque, leading to ischemia of the heart muscle. The Covid-19 pandemic raised questions related to the increase in the number of infarctions, perhaps due to the destabilization of plaques by the inflammatory substrate.

**Objectives:** To make a correlation between the clinical variables of the presentation, complementary exams such as ECG, echocardiogram and catheterization and the concomitant presentation with Covid-19.

**Methods:** We evaluated 62 patients consecutively admitted with the diagnosis of ACS to a public hospital in São Paulo in 2021, during the Covid-19 pandemic.

**Results:** The mean age of the patients was 66.9 years and standard deviation 10,44, 51.6% were men, 66.12% were hypertensive, 33.87% had diabetes, 14.51% had a documented history of coronary artery disease (CAD), 35% with a high risk GRACE score, 6.45% with Covid-19, of which 75% with STEMI. In the ECG, we obtained 11.29% of anterior ischemia, 12.9% of posterior ischemia and 20.96% without alteration. On the echocardiogram, the mean LVEF was 0.57 quantified by the Simpson method, 12.9% of regional change in the anterior wall and 24.19% of the posterior wall. In catheterization, the predominance was involvement of the anterior descending artery with 16.12% and only 3.22% without obstructive CAD. Only one patient with Covid-19 had a normal ECG.

**Conclusions:** In a population treated with the diagnosis of ACS during the Covid-19 pandemic, the prevalence of positive cases was low, however, the incidence of STEMI seems to be high in this population, which denotes greater attention to the care and clinical evolution of these patients.

109893

Modality: E-Poster Young Researcher – Case Report

Category: CARDIOVASCULAR SURGERY

## Takotsubo Syndrome After Mitral Valve Surgery

RAQUEL SILVA BRITO DA LUZ^1^, Adnaldo da Silveira Maia^1^, Jhonathan Gouveia da Mota^1^, Matheus Meirelles^1^, Mario Issa^1^

(1) Instituto Dante Pazzanese de Cardiologia

**Introduction:** Takotsubo Syndrome is a recurrent diagnosis, however, it’s rarely seen post cardiac surgery. The disease usually occurs after physical or emotional stress, most prevalent in post-menopausal women. The clinical features may present similar to acute coronary syndrome, with chest pain and electrocardiogram (ECG) alterations, but with this diagnosis being excluded after the absence of obstructive lesions in coronary angiography and encountering typical echocardiogram findings, such as compensatory apical akinesia and basal compensatory hypercontractility.

**Case Description:** A 56-year-old woman with a history of rheumatoid arthritis, active smoking and percutaneous mitral valvuloplasty in 2007, presents with functional class II dyspnea and transthoracic echocardiogram (TTECO) showing important mitral regurgitation. Patient was submitted to mitral valve replacement surgery with an mechanical prosthesis on March 2022, without complications. In the immediate postoperative care, the patient evolved with cardiogenic shock, requiring vasoactive drugs. The ECG presented with T-wave inversion of precordial leads and TTECO revealed hypercontractility of the basal segments and akinesia of medium-apical segments, with left ventricular ejection (LVEF) of 20%. During hospitalization the patient gradually improved from cardiogenic shock, with normalization of LVEF to 64%.

**Conclusion:** Takotsubo Cardiomyopathy is a reversible condition, characterised by transient ventricular dysfunction, with rare cases described in the context of postoperative care due to valve surgery. The surgical intervention can be considered as a trigger for the development of this syndrome.

109916

Modality: E-Poster Young Researcher – Case Report

Category: HEART FAILURE/CARDIOMYOPATHY/TRANSPLANT

## An Integrative Approach to Elucidate Exercise Intolerance Mechanisms in Heart Failure with Functional Mitral Regurgitation

PEDRO CASTILHOS DE FREITAS CRIVELARO^1^, Felipe Homem Valle^1^, Willian Roberto Menegazzo^1^, Anderson Donelli da Silveira^1^, Nadine Oliveira Clausell^1^

(1) Hospital de Clínicas de Porto Alegre HCPA

**Background:** Functional mitral regurgitation (fMR) is associated with worse prognosis in heart failure (HF). Furthermore, fMR severity worsening during exercise has been associated with even worse prognosis in this scenario.

**Report:** A 44-year-old female with post-chemotherapy HF and moderate fMR presented with progressive exercise intolerance. To further elucidate mechanisms of exercise intolerance, combined exercise invasive hemodynamic assessment and exercise echocardiography were carried. The procedure was performed by right brachial venous access with exercise at a cycle ergometer. Hemodynamics were evaluated at rest and during progressive incremental exercise, with combined transthoracic echocardiography at both stages. At exercise in a 20 Watts work-load, fMR worsening and increase in pulmonary artery pressures occurred. These findings suggest a potential role of fMR in exercise intolerance.

**Conclusion:** Exercise pulmonary hypertension is associated with worse prognosis in fMR. Whether transcatetheter mitral valve interventions may alter prognosis in this scenario is yet to be determined. We herein present a case of exercise intolerance that is potentially related to fMR worsening during exercise. Our group is prospectively evaluating the role of combined invasive exercise hemodynamic assessment and exercise echocardiography to elucidate mechanisms of exercise intolerance in fMR.


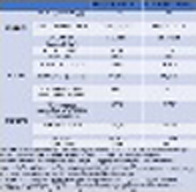


109927

Modality: E-Poster Young Researcher – Case Report

Category: CARDIOVASCULAR INTENSIVE CARE/CARDIOVASCULAR EMERGENCIES

## ST-Segment Elevation Myocardial Infarction (STEMI) and Pericarditis Epistenocardica Due to Coronary Thrombosis Associated with Cannabis use in a Patient with Low Cardiovascular Risk

PEDRO GUIMARÃES SILVA^1^, MELINA DE OLIVEIRA VALDO^1^, GUILHERME RAPOSO DE MEDEIROS^1^, MARILIA TAILY SOLIANI^1^, JOSE CARLOS NICOLAU^1^

(1) INSTITUTO DO CORAÇÃO DO HCFMUSP

**Introduction:** Certain habits and practices present in groups defined as low risk for cardiovascular disease may be associated with the occurrence of major clinical events, such as acute myocardial infarction (AMI) and stroke. The following report portrays the case of a low-risk patient who suffered from a STEMI followed by pericarditis and whose only risk factor would be recreational cannabis use.

**Case report:** A 35-year-old male with no previous comorbidities or positive family history of cardiovascular disease reported the onset of angina at 03pm on 02/26/2022, only seeking medical attention due to unrelenting thoracic discomfort by 5am on 02/27/2022. He admitted having an average alcohol consumption of 2–3 doses a day on weekends, and a daily cannabis use (up to 10 cigarettes a day), with no use of other drugs in the last 10 years. During assessment, he was in Killip class II and the ECG showed an anterior wall ST elevation, with high sensitivity troponin levels >25000. With the diagnosis of anterior STEMI he was referred to the quaternary reference hospital for a primary angioplasty, where he arrived at midday. The coronary angiography showed significant thrombosis in the proximal segment of the anterior descending artery *(ADA) and a stenosis of approximately 40%. There was also an occlusion in the distal segment, with features suggestive of thromboembolism. Other vascular beds had no lesions whatsoever. Thromboaspiration and angioplasty of the distal segment were performed, without any success in achieving reperfusion. Therapy with intravenous GpIIb/IIIa inhibitor infusion (Tirofiban) for 24 hours was undertaken due to the high thrombotic burden, The next day he evolved with recurrence of severe pleuritic chest pain and underwent a new angiography, with no new findings. Despite that, it was decided to treat the remaining stenosis in the ADA’s proximal segment. Subsequent ECGs presented diffuse ST-segment elevation associated with PR segment depression, suggestive of pericarditis epistenocardica. Echocardiographic data showed an ejection fraction of 40% and mild pericardial and pleural effusions. An association of Aspirin + clopidogrel and colchicine 0.5 mg of 12/12h was prescribed for the management of both STEMI and pericarditis. The patient evolved with an inactive electrical zone in the anterior wall and regression of the ST elevation, in addition to a recovery of the ejection fraction (48% on 03/09/22) and lessening of the symptoms.

109910

Modality: E-Poster Young Researcher – Case Report

Category: HEMODYNAMICS AND INTERVENTIONAL CARDIOLOGY

## Syncope Secondary to Carotid Sinus Syndrome in a Patient with Cervical Mass: A Case Report

HELENA ALVES DE ANDRADE RIBEIRO^1^, Bianca Sarria^1^, Vanessa Sanson Lani^1^, Karllayno Camatta Milleri^1^, Fernando Luiz Torres Gomes^1^

(1) Antônio Moraes University Hospital (HUCAM) – Federal University of Espírito Santo (UFES)

**Introduction:** Carotid sinus syndrome (CSS) is a complication of head and neck tumors, resulting from baroreceptor hyperexcitation. It was first recognized by Weiss and Baker in 1933, who established the role of this complex. Since then, there have been reports of the association of neck masses related to syncope. The case below describes a patient who presented with symptomatic bradycardia secondary to compression exerted by a neck mass.

**Case-report:** AJBS, 69 years old, female, was admitted to the emergency department with episodes of recurrent syncope, with intensification in the left lateral decubitus position, associated with profuse sweating, nausea, left peripheral facial palsy, and generalized weakness, starting one week before seeking for medical care. She had a large left neck mass and a history of evolution during 20 years, with progressive growth, especially 2 months before the symptomatic condition. The heart rate was 27 beats per minute, in sinus rhythm, with ventricular extrasystoles, with a good response to atropine and venous hydration. Neck computed tomography shows expansive formation with soft tissue density, necrosis, calcification and heterogeneous enhancement, affecting the left parotid, measuring 7.9 × 6.7 × 6.4 cm, with local bulging and parapharyngeal space involvement and multiple lymph node hypertrophy. As an immediate treatment, a transvenous pacemaker was implanted, with subsequent insertion of a definitive pacemaker. This intervention initially showed a positive impact, with heart rate stability, but there was a successive return of hypotension episodes, reaffirming the character of an independent local stimulus caused by the expansive formation. Fine needle aspiration puncture (FNA) of the lesion was performed, with material under analysis. The therapeutic under analysis is local treatment for mass reduction and interruption of the feedback caused by it.

**Conclusion:** SSC caused by the presence of neck masses is due to compression and invasion of the carotid sinus and nerve branches. This syndrome has two subtypes: cardioinhibitory and vasodepressor, and the differentiation is not always clear, since the abnormal stimulation of baroreceptors encompasses different cardiovascular phenotypes. Early diagnosis associated with proper management alters the course and outcomes of this syndrome, mitigating unfavorable outcomes resulting from the condition and having a positive impact on morbimortality.

109912

Modality: E-Poster Young Researcher – Case Report

Category: COVID-19 AND CARDIOVASCULAR SYSTEM

## Successful Coronary Artery Bypass Graft Surgery After Recent use of Tocilizumab During the Covid19 Pandemic

VERÔNICA HOMEM DE CARVALHO E SILVA^1^, João Pedro Soares Costa^1^, Yasmeen Salah Mustapha El Qatta^1^, Victor Marçal Carvalho Mendes^1^, Alexandre Anderson de Sousa Munhoz Soares^2^

(1) Universidade de Brasília (UnB); (2) Cardiologia DASA Hospital Brasília Unidade Águas Claras

COVID-19 infection triggers a cytokine storm that includes a high expression of interleukin-6 (IL-6). Therefore, anticytokine therapy in patients with acute heart failure associated with SARS-COV2 demonstrated to be a good option of treatment. The case report in question is a case in which the use of tocilizumab with myocardial revascularization was successful. A 48-years-old man with past medical history of obesity, systemic arterial hypertension, type 2 diabetes and non-dialysis chronic kidney disease. Presented with complaints of progressive dyspnea for 10 days, decubitus intolerance and low oxygen saturation. At admission, blood exams demonstrated NT-proBNP 6825 pg/ml, D-dimer 425 ng/mL and creatinine 3,04 mg/dl. Next day, troponin curve was on the rise (519–542–671), which led to the suspicion of myocarditis due to COVID-19. After discussing the risks with the family, he was started on off-label treatment of tocilizumab intravenous, and methylprednisolone 2 mg/kg. Over the next days, exams demonstrated negative RT-PCR, pulmonary congestion on CT (moderate right and small left pleural effusion with restrictive atelectasis and multifocal and bilateral ground-glass opacities), compatible to hypertensive pulmonary acute edema, possibly by myocarditis, therefore tocilizumab and corticoid was suspended and cardiac MRI requested. The MRI resulted in a pattern of inferior and lateral fibrosis suggestive of ischemia, thus coronary angiography was requested. The angiography concluded the presence of severe coronary atherosclerotic disease with a three-vessel pattern, indicating the need of myocardial revascularization surgery. Due to the immunosuppression that tocilizumab causes, the infectious disease team was consulted to discuss the risks/benefits of delaying the surgery, it was agreed that it would be after the half-life of the product, which is 11 to 16 days. The surgery took place without complications. After surgery, the patient needed to use norepinephrine, dobutamine and vasopressin, reacting well to the treatment. After the episode, he spent another 9 days in the ICU with hemodialysis as his chronic renal disease worsened but quickly recovered, without other noteworthy complications, being discharged after the period. We report the successful myocardial revascularization surgery after the use of tocilizumab without any infectious complication. The surgery schedule after the half –life period may be considered to the treatment of this complication.

109961

Modality: E-Poster Young Researcher – Case Report

Category: ACUTE AND CHRONIC CORONARY DISEASE/THROMBOLYSIS

## Recurrence of Spontaneous Coronary Artery Dissection in a Puerperal Woman with COVID-19

VICTOR ALEXANDRE DOS SANTOS VALSECCHI^1^, Paula Santiago Teixeira^1^, Thalita Ruolla Barros^1^, Marcos Damião Cândido Ferreira^1^, Adriano Caixeta^1^

(1) Universidade Federal de São Paulo

**Introduction:** Spontaneous coronary artery dissection (SCAD) is more common in women, aged approximately 50 years. It accounts for approximately 15–20% of myocardial infarctions (MI) during pregnancy or the peripartum period. It is still a disease of unknown cause, but it is related to triggering factors such as emotional stress and pregnancy.

**Objective:** To report a case that occurred in a university hospital and emphasize the diagnostic importance.

**Case Report:** E.L.A., 36 years old, female, hypertension during a previous pregnancy. Three months ago during gestational period and infection by Influenzae presented coronary syndrome without ST-segment elevation (NSTEMI). Catheterization showed spontaneous coronary dissection of the anterior descending artery and circumflex both classified as Saw type 2 with conservative treatment. The patient was admitted in the third postoperative day of a cesarean section due to atypical chest pain. Electrocardiogram showed T-wave inversion in DI, AVL, V5 and V6. The curve of markers of myocardial necrosis was negative and COVID-19 was positive. Echocardiogram (ECO) without segmental dysfunction. During hospitalization for NSTEMI patient evolved with a new episode of chest pain and dynamic alteration of the ST segment. Catheterization showed new focal spontaneous dissection in the distal third of anterior descending artery and first diagonal dissection in all its extension, both with Saw type 2 classification. Improvement of circumflex and right coronary artery free of stenosis. In view of hemodynamic instability, coronary angioplasty of the LAD was performed with 1 drug-eluting stent, with angiographic success. The patient evolved with improvement of symptoms. New ECO showed anterior apical and septal apical akinesia.

**Conclusion:** It is critical that in young women with chest pain, SCAD is suspected because it is an underdiagnosed disease. Accurate and rapid diagnosis is paramount as the management of MI caused by SCAD differs from that of an atherosclerotic cause.

109964

Modality: E-Poster Young Researcher – Case Report

Category: CARDIO-ONCOLOGY

## Combined Heart-Kidney Transplantation in Congestive Heart Failure in Onco-Cardiology

AMANDA DE ANDRADE CAMPELLO GIROTTO^1^, Luisa Wagner do Rego Barros^1^, Juliana dos Santos Macaciel^1^, Jacqueline Sampaio Miranda^1^, Antônio Fatorelli^1^

(1) Instituto Nacional de Cardiologia

**Introduction:** Heart transplantation is the definitive therapy for eligible patients with end-stage heart failure. This subgroup of patients often develops with concomitant kidney injury and, in this context, treatment options become limited. Combined heart and kidney transplantation is a growing practice in recent years in large centers, with increasingly promising results.

**Case Report:** L.R.G, female, 22 years old, born in Rio de Janeiro, with dilated cardiomyopathy secondary to anthracycline cardiotoxicity, being followed up for heart failure with reduced ejection fraction with good functional class. Refers progression of the condition 3 months after pregnancy, when she started to present dyspnea on minor exertion. In use of Carvedilol 50 mg, Sacubitril/Valsartan 98/103 mg 2x, Spironolactone 25 mg, Furosemide 80 mg. Undergoing CPET: VO2 10.7|VEVCO2 36.3|R: 1.17, being referred for evaluation of heart transplantation. Loss of outpatient follow-up in the following months, when developed osteomyelitis in the left lower limb prosthesis, and the need to use broad-spectrum antibiotics. She presented Acute Kidney Injury with evolution to daily hemodialysis. Clinical intractability and need for transfemoral limb amputation were defined. She maintains anuria and anasarca despite dialysis therapy, with the need for inotropes for clinical compensation. Dobutamine was started for type 2 cardiorenal component test, with partial response. SHe evolves with clinical stability on INTERMACS III, with improvement of anasarca. After discussion in the Heart Team, was opted for a combined heart and kidney transplant. Submitted to the procedure on 06/22/21. She was discharged from the hospital with satisfactory diuresis and preserved systolic function.

**Conclusion:** The clinical case reinforces the importance of clinical identification of patients with signs of advanced heart failure, to refer them as soon as possible to specialized centers, with early treatment being an important prognostic marker. Combined heart transplantation is a worldwide trend approach, and this strategy is increasingly used in relation to the sequential transplantation strategy (kidney after heart). The database is still limited and has some retrospective studies, highlighting the importance of new studies with high scientific rigor to guide complex cases.

109969

Modality: E-Poster Young Researcher – Case Report

Category: COVID-19 AND CARDIOVASCULAR SYSTEM

## Case Report: Post-Acute COVID-19 Myocarditis and Long-Term Effects on Reduced Functional Capacity

BRUNO LINHARES AZEREDO CORRÊA^1^, Fábio Lucas Bassini e Silva^1^, Jaime Lobo FIgueiredo^1^, Renata Rodrigues Teixeira de Castro^1^, Fábio Akio Nishijuka^1^

(1) Hospital Naval Marcílio Dias

**Introduction:** COVID-19 infection has become a multiorgan disease, which is already well documented in the literature. 2 years after the beginning of the pandemic, we are knowing the long-term effects. As if that wasn‘t enough, vaccines against COVID-19 still had important side effects on the cardiovascular system.

**Objective:** The objective of this report is to discuss the chronic cardiological form of COVID-19, its pathophysiological mechanisms and propose ideas for an approach.

**Case report:** A 30-year-old male, physically active and without comorbidities, with confirmed COVID-19 infection at the end of 2020 and in August 2021, both with a mild condition, was hospitalized with acute myocarditis in September 2021. 3 weeks after discharge, he received SARS vaccine -CoV-2 (Pfizer), when the chest pain intensified, becoming daily. Hospitalized in November 2021 for tachycardia associated with lipothymia. Investigated with 24-hour holter that did not demonstrate arrhythmias and exercise test without criteria for ischemia. Magnetic resonance imaging showed worsening of mesocardial enhancement from 2% (Sep21) to 4% (Jan22) and edema. At follow-up, he presented with progressive dyspnea on exertion with intolerance to physical exercise, in addition to atypical chest pain that led to several visits to the emergency, attributed to chronic pericarditis. In March 2022, he performed an exercise stress test reaching 10.2 METS, and an echocardiogram with strain, without changes in function or contractility. This is why this service referred the patient to cardiac rehabilitation in order to improve functional capacity.

**Conclusion:** Understand the pathophysiological mechanisms and develop strategies to mitigate the chronic and/or late cardiological effects of COVID-19. The symptoms of long-COVID cause multiple emergency visit, in addition to several exams that financially burden the patient and the health system. Similar cases could generate an unprecedented impact on the functionality of the economically active population.


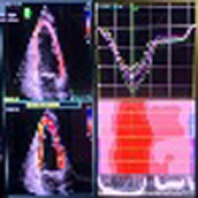


109979

Modality: E-Poster Young Researcher – Case Report

Category: CONGENITAL AND PEDIATRIC CARDIOLOGY

## Extrinsic Compression of the Left Main Coronary Artery Secondary to the Common Atrium

VÍTOR BONIATTI NEVES^1^, Deborah Valente Ramos Rocha^1^, Vítor Boniatti Neves^2^, Juan Tiago Nunes Pagnussat^1^, Tiago Vendruscolo^3^

(1) Universidade Federal da Fronteira Sul – RS; (2) Faculdade IMED – RS; (3) Instituto do Coração de Passo Fundo – RS

**Introduction:** Common atrium is characterized by complete absence of the interatrial septum, the presence of small strads of tissue presente at the superior atrial wall of the common chamber, absence of interventricular communication and accompanying atrioventricular cushion defect.

**Objective:** Present a report of a rare case of interatrial communication with absence of interatrial septum and complications.

**Case Report:** N.R.M.O, 54 years old, female. Hypertensive and chronic obstructive pulmonary disease. Arives at the emergency room with precordial pain for 1 year, worsening in the last 5 days, associated with dyspnea. Management for acute coronary syndrome was initiated and the patient was referred for invasive stratification. Physical examination: regular rhythm, 2 clicks, systolic murmur 3/6+ at pulmonary focus. Oxygen saturation: 88%. Digital clubbing, but labial cyanosis at rest was not observed. Laboratory data revealed an elevated ultrasensitive troponin – 1941. Electrocardiography: showed left atrial overload. Echocardiogram; complete absence of the interatrial septum. The right ventricle was markedly enlarged with mild pulmonary hypertension (estimated right ventricular pressure, 68 mmHg). Coronary cineangiography demonstrated a LMCA with moderate/severe proximal lesion. An angiography of the ostial left main coronary artery for probable extrinsic compression of the pulmonary artery was performed (IVUS) for anatomical evaluation of significant luminal reduction. The first measurement identified a minimum luminal area of 8.77 mm^2^, without evidencing atherosclerotic disease. The heart team still discussing the therapy plain.

**Conclusion:** The difficult diagnosis, which begins with investigation of other pathologies, leads to clinical and myocardial worsening of the patient. In the case of this patient, the common atrium led to pulmonary hypertension, with significant dilation of the pulmonary artery, causing extrinsic compression of the left coronary trunk. There are few case reports of older patients with such condition and the therapeutic approach still a challenge.


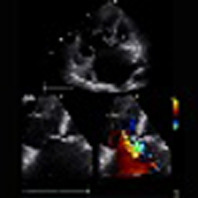


110038

Modality: E-Poster Young Researcher – Case Report

Category: HEART FAILURE/CARDIOMYOPATHY/TRANSPLANT

## Eosinophilic Cardiomyopathy Secondary to Chemotherapy: A Case Report

ADOZINA MARQUES DE SOUZA NETA^1^, Victor Agueda Salles^1^, Luciana da Rocha Ferreira Lobbe Cotta^1^, Bruno Miranda Marques^1^, Jacqueline Sampaio dos Santos Miranda^1^

(1) HOSPITAL COPA STAR

Hypereosinophilic syndrome (HES) is rare and defined as blood eosinophil count >1.5 × 109/L and eosinophilia-related organ damage. According to Mankad et al. in 2015, the heart is an affected organ with an incidence of up to 50% of cases. HES, it is classified into hereditary, undetermined significance, primary (clonal/neoplastic) and secondary (reactive) variants. Diagnosing and stratifying the disease is essential to start treatment at an early. Male patient, 65 years old, with dyspnea and with pulmonary congestion for three weeks. Previous history of lymphoproliferative disease, Waldenstrom’s Macroglobulinemia, treated with cyclophosphamide, doxorubicin, vincristine, prednisone and rituximab R-CHOP in 2013. In 2018, due to disease progression, he used R-bendamustine + lenalidomide 6 cycles ending 04/15/2019. Currently in remission. Previous lab history of leukogram with eosinophilia ranging from 19.1% on to 47.3%. He sought the emergency department with a picture of decompensated heart failure, associated with elevated troponin and BNP, being referred for coronary angiography, which showed no significant coronary artery disease, however, ventriculography with left ventricular tip obliteration. In the 48 hours after admission, the patient hemodynamic instability, requiring inotropes and mechanical ventilation. Leukogram with eosinophil of 1,160/mm^3^ (10%). Cardiac NMR imaging showed mild myocardial fibrosis and left ventricular apex occupied by solid content. With the possibility of HES, he underwent pulse therapy with 1g methylprednisolone for 3 days and underwent surgery to remove the mass from the left ventricle. The histopathology of the material showed a fibrin thrombus with numerous eosinophils, thus confirming the SHE. Faced with the possibility of recurrence of the lymphoproliferative disease, a bone marrow biopsy was performed to rule out the presence of B lymphocytes and a negative karyotype for the FIP1L1-PDGFRA mutation. Hereditary causes and reactivation of the neoplasm were ruled out, it confirms the hypothesis of SHE secondary to chemotherapy. The patient evolved with recovery of ventricular function and he was discharged from the hospital with corticosteroids and anticoagulation. In view of this report, the importance of performing routine echocardiography in patients with persistent eosinophilia is evident, given the high prevalence of cardiac involvement in HES. Early diagnosis and treatment improve quality of life reduce mortality.

110055

Modality: E-Poster Young Researcher – Case Report

Category: COVID-19 AND CARDIOVASCULAR SYSTEM

## COVID-19 and Primary Angioplasty: Importance of the Door-to-Balloon Time

ARGILA GONÇALVES DE CARVALHO SANTANA^1^, Árgila Gonçalves de Carvalho Santana^1^, Patrícia Veiga Nascimento^1^, Simone Letícia Souza Querino^3^, Thayná Oliveira Militão^1^

(1) Universidade Federal do Recôncavo da Bahia; (2) Hospital Ana Nery; (3) Universidade Federal da Bahia

**Background:** Covid-19 infection exposes patients to several complications, including a greater predisposition to Acute Myocardial Infarction (AMI). Thrombotic complications occur as a result of the inflammatory process, triggering platelet activation, endothelial dysfunction and stasis.

**Objective:** To report a case of a young patient, with no associated comorbidities, with ST-segment elevation AMI who underwent Primary Angioplasty.

**Methods:** Retrospective study research, based on chart analysis, case study type. Case report supported by the Ethics Committee with CAAE 44553421.0.0000.0056.

**Results:** Patient, 45 years old, male, black, general services assistant, smoker and without other previous comorbidities, complaining of fever, malaise associated with 5 days, taking the 2nd dose of the vaccine against SARs CoV2 – Oxford even 2 days ago showing typical symptoms of Covid-19 infection. On the morning of 07/18 at 5:30 am, he presented oppressive chest pain associated with upper limb paresthesia, without respiratory distress. Admitted to the hemodynamics of the HAN on an emergency basis at 09:40h, coming together with the SAMU, TR for SARS CoV2 negative, ECG performed with supra inferior wall, V3R, V4R, triggered AMI protocol, performed 300 mg of ASS, 600 mg of clopidogrel and morphine 2 mg. Undergoing coronary cineangiography and primary angioplasty: Right Coronary Artery with 100% lesion in the middle third by a thrombus. At the end of the procedure, there was good angiographic appearance of the treated arteries, TIMI 3 flow. Echocardiogram: Simpson EF: 42%, with segmental alteration: Akinesia of the basal and middle segments of the inferior and inferoseptal walls. The patient evolved without complications and was discharged after 2 days of hospitalization.

**Conclusion:** Despite a door-to-balloon time of 4h10min, due to the prolonged ischemia time and high thrombotic load, although asymptomatic, the patient evolved with left ventricular segmental dysfunction, which demonstrates that “time is myocardium”.

110067

Modality: E-Poster Young Researcher – Case Report

Category: ACUTE AND CHRONIC CORONARY DISEASE/THROMBOLYSIS

## Embolic Myocardial Infarction Due to Aortic Valve Infective Endocarditis Complication in a Previously Healthy Young Patient

ANANDA RIBEIRO FRETES^1^, Matheus Lima Lula Guimarães^1^, Paula Maria Pinheiro Miranda^1^, Leticia Aparecida Braga da Silva^1^

(1) Universidade Federal do Mato Grosso

**Introduction:** Coronary artery embolism is an uncommon cause of acute myocardial infarction and one of the most important causes of non-atherosclerotic ST-segment-elevation myocardial infarction. The most common causes are: Infectious Endocarditis (IE), Atrial Fibrillation and Prosthetic Heart Valve Thrombosis.

**Case Report:** Patient, 24 years old, male, smoker, alcoholic, with no previous diagnosed pathologies, with moderate intensity retrosternal pain in the last 30 days, dyspnea on moderate exertion, daily fever, sweating, chills and weight loss of 7 kg in the period. Admitted to an emergency care unit with significant worsening of severe retrosternal pain, worsening with exertion and associated with dyspnea on minor exertion. Physical examination showed an abscess in the left upper molar, cardiac auscultation with a more audible systolic murmur in a 4+/6+ aortic focus radiating to the carotids. The electrocardiogram showed ST-segment elevation in V2, V3, V4, V5 and V6 and troponin: 210 (reference value: <0.03). After 5 hours of evolution, catheterization was performed demonstrating left anterior descending artery (LAD) occluded in the middle third, right coronary artery and circumflex coronary artery free of obstructive lesions, followed by primary angioplasty with implantation of 01 drug-eluting stent in the LAD. An echocardiogram was performed with evidence of vegetation in the aortic valve and an image of paravalvular abscess. Infective endocarditis was confirmed with initiation of antibiotic therapy. Subsequently, during the same hospitalization, aortic valve replacement surgery was performed with implantation of a metallic prosthesis, correction of paravalvular abscess with a pericardium flap and enlargement of the aortic root. The patient evolved in the postoperative period without intercurrences, being discharged after the end of antibiotic therapy, with resolution of the previously presented complaints.

**Conclusion:** Embolic myocardial infarction is a rare but increasingly recognized complication of infective endocarditis and it has increased risk of heart failure and a high mortality rate. Septic embolization resulting in acute coronary syndrome has an incidence of 2.2%. It should be suspected in young patients with a diagnosis of endocarditis or suggestive clinical signs.

110223

Modality: E-Poster Young Researcher – Case Report

Category: HEART FAILURE/CARDIOMYOPATHY/TRANSPLANT

## Carcinoid Heart Disease: A Condition to Remember in Cases of Right-Sided Heart Failure

DOUGLAS BORGES DA COSTA FILHO^1^, Euton Freitas de Castro Junior^1^, Ana Carolina Brito de Alcântara^2^, Danielli Oliveira da Costa Lino^3^, Ane Karoline Medina Néri^4^

(1) Universidade Federal do Ceará, Hospital Universitário Walter Cantídio; (2) Hospital Geral Dr. Waldemar de Alcântara; (3) Hospital de Messejana Dr. Carlos Alberto Studart Gomes; (4) Universidade de Fortaleza, Postgraduate Program in Collective Health

**Introduction:** Although uncommon, carcinoid heart disease is one of the main causes of morbidity and mortality in the context of carcinoid syndrome and can occur as the initial presentation of this neuroendocrine tumour. Among the cardiac impairments are the thickening of valves and right chambers due to infiltration of the parenchyma by carcinoid plaques and the high circulating concentration of serotonin, which can lead to severe dysfunctions. The aim of this study is to share a case series of individuals with carcinoid heart disease and to discuss the main clinical features related to this condition.

**Case Report:** Three cases of carcinoid heart disease were treated in reference hospitals in Cardiology in the city of Fortaleza, state of Ceará, Brazil. The patients were female, were in adulthood, had intestinal carcinoid tumor with liver metastasis, presented with tricuspid valve involvement and a clinical presentation of right-sided heart failure (HF) at diagnosis. The first patient, a 59-year-old female, had insufficiency of the tricuspid and pulmonary valves with the latter also presenting stenotic lesion demonstrated by transthoracic echocardiography (TTE). The second patient, a 48-year-old woman, sought medical assistance complaining of gastrointestinal discomfort. A neuroendocrine tumour was diagnosed in the terminal ileum, with secondary lung implants. TTE showed a tricuspid valve with thickened leaflets, reduced mobility, moderate regurgitation and mild stenosis. The third patient, a 55-year-old female, sought an evaluation complaining of abdominal discomfort and cervical and facial flush. She was diagnosed with a well-differentiated neuroendocrine tumour in the colon and a TTE showed a tricuspid valve with thickened leaflets and mobility restriction with significant coaptation failure and torrential tricuspid insufficiency.

**Conclusions:** This study reported the cases of three patients with carcinoid syndrome with heart involvement, describing their main clinical presentations and aspects in common between them, such as female gender, adulthood, the presence of intestinal carcinoid tumor with liver metastasis, tricuspid valve involvement and a clinical presentation compatible with right-sided HF. Despite being infrequent, carcinoid heart disease deserves attention and should generally be screened for among patients with carcinoid tumors, especially in those individuals who present with a condition compatible with right-sided HF.

110330

Modality: E-Poster Young Researcher – Case Report

Category: ACUTE AND CHRONIC CORONARY DISEASE/THROMBOLYSIS

## Carfilzomib as a Cause of ST-Segment Elevation

DANIELA BARBOSA MATEUS^1^, António Carvalho^1^, Rita Gomes^1^, Rita Duarte^1^, Carlos Rabaçal^1^

(1) Hospital Vila Franca de Xira – Portugal

**Introduction:** Chemotherapeutic agents, especially proteasome inhibitors, used in the treatment of Multiple Myeloma, have a high risk of cardiovascular disease. The authors report a case of ST-segment elevation secondary to vasospastic angina resulting from the use of carfilzomib.

**Case Report:** 63-year-old man, melanodermic, with a personal history of IgG lambda multiple myeloma diagnosed in February 2012 (stage IIA), under chemotherapy with carfilzomib 120 mg weekly, since May 2021. Admitted in the emergency department due to retrosternal pain with left upper limb irradiation, which started at rest, ten hours prior to presentation. Hemodynamically stable, with an unremarkable physical exam. Initial investigation revealed: electrocardiogram with sinus tachycardia, ST elevation (>2 mm from point J) in V2-V4 with hyperacute T waves; echocardiogram with hypokinesia of the apex and apical segments of the septum, anterior wall and inferior wall; preserved systolic function; non-dilated cardiac chambers; valve structures without morphofunctional changes. The patient was transferred to a center of emergency cardiac catheterization. The coronary arteries presented no angiographically apparent lesions; ventriculography revealed good ventricular function with apical hypokinesia and functional study of the anterior descending artery demonstrated reactivity to acetylcholine, favoring the diagnostic hypothesis of epicardial coronary vasospastic angina. Subsequently, analytically, troponin I (high sensibility) was 5,4 pg/mL, reaching a maximum value of 58 pg/ml. Vasospastic angina secondary to carfilzomib was assumed. Vasodilator therapy was started with good clinical evolution, without recurrence of angor and and maintaining the previously described electrocardiographic changes.

**Discussion:** The authors intend to alert to the harmful cardiovascular effects with the use of chemotherapeutic agents, in this case carfilzomib.

110267

Modality: E-Poster Young Researcher – Case Report

Category: CONGENITAL AND PEDIATRIC CARDIOLOGY

## Don’t be Blinded by an Obvious Diagnosis – Pulmonary Hypertension with Multifactorial Mechanisms

BRUNO GONÇALVES GARCIA^1^, Thaíssa Santos Monteiro^1^, Fábio Akio Nishijuca^1^, Jamili Zanon Bonicenha^1^, Maria Carolina Terra Cola^1^

(1) National Institute of Cardiology (INC) – Rio de Janeiro, Brazil

**Introduction:** Atrial septal defect (ASD) is a congenital heart disease (CHD) in which blood flows between the atria, primarily with a left-right shunt leading to pulmonary hyperflow and right ventricle (RV) volume overload and consequently pulmonary hypertension (PH) type I and RV enlargement and dysfunction. Eisenmenger syndrome occurs when, because of the PH and RV dysfunction, the shunt flow turns right-left and cyanosis appears. However, there are many other causes for PH, which is classified by 5 different types.

**Case Report:** Female patient, 41 year-old, with low educational level, former smoker and history of asthma, was referred to an adult CHD center after being initially diagnosed with ASD with Eisenmenger syndrome. The patient had clubbing and central cyanosis, a palpable RV and systolic murmur in the tricuspid focus. Echocardiogram identified RV dysfunction, bidirectional shunt in the interatrial septum, tricuspide valve regurgitation, pulmonary artery systolic pressure of 128 mmHg and significant enlargement of the pulmonary artery (PA), with thrombus in situ. Although there was a shunt in the interatrial septum, the ASD observed was small. Angiotomography of the pulmonary arteries confirmed bilateral chronic thromboembolism. Rheumatological assessment was negative, as well the thrombophilic panel. Late syphilis was diagnosed by antitreponemic antibody, and treatment was instituted. PA catheterization was not performed due to risk of distal embolization of thrombus present in the PA. The heart team concluded it was a multifactorial PH, including type I and type IV. Syphilitic arteritis of the PA was also remembered, but could not be confirmed. Closure of the ASD was considered contraindicated because of the PH, and anticoagulation was initiated as well as sildenafil, and the patient followed with improvement of oxygen saturation and exercise capacity.

**Conclusion:** Facing a patient with PH and a late diagnosis of ASD, could have mislead to the conclusion of this being a case of PH type I with Eisenmenger syndrome purely. ASD is one of the most common cause of adult CHD, as there are other common causes for PH. One should not be blinded by the combination of both, and a thorough assessment should be done to insure there is indeed a cause-and-effect relationship.

110369

Modality: E-Poster Young Researcher – Case Report

Category: CARDIOVASCULAR INTENSIVE CARE/CARDIOVASCULAR EMERGENCIES

## Systemic Fibrinolysis for Treatment of Obstructive Shock in a Patient with Aortic Bioprosthetic Valve-in-Valve Thrombosis

JIAN CHU^1^, Nikitaa Nath^1^, Natasha Rana^2^

(1) Rush University Medical Center, Department of Internal Medicine; (2) Rush University Medical Center, Department of Internal Medicine, Section of Cardiology

An 81-year-old man with ischemic heart failure and aortic valve-in-valve bioprosthesis presented with acute dyspnea. On exam, he was afebrile, but hypotensive, tachycardic, and tachypneic. Labs were notable for a BNP of 3056 pg/mL and venous lactate of 2.1 mmol/L. Due to suspicion for cardiogenic shock, he underwent emergent cardiac catheterization, which revealed elevated left cardiac filling pressures. He was started on inotropes and vasopressors with placement of an intra-aortic balloon pump. A transthoracic echocardiogram showed a LV ejection fraction of 10–15% and prosthetic aortic valve dysfunction with a peak velocity of 3.7 m/s and mean gradient of 32 mm Hg (markedly higher than baseline). A 9 × 17 mm echodensity was seen on the valve leaflet consistent with bioprosthetic valve thrombosis (BPVT). After multidisciplinary discussion with cardiothoracic surgery and interventional cardiology, he was administered systemic tissue plasminogen activator with a 10 mg load followed by 40 mg over 4 hours. Within 24 hours, he was able to wean off all pressors with improved echocardiographic parameters. The data behind fibrinolysis for aortic BPVT is largely derived from the PROMETEE study, which supports ultraslow fibrinolysis and the present case shows that faster infusions may be safe and effective in decompensated patients. However, more data is needed to guide choice of surgical vs. non-surgical management of patients with decompensation from BPVT.


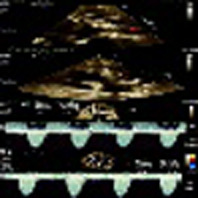


110485

Modality: E-Poster Young Researcher – Case Report

Category: PERICARDIUM/ENDOCARDIUM/VALVOPATHIES

## Infectious Tricuspid Valve Endocarditis and Septic Pulmonary Embolism in a Young Male with Suspected Antiphospholipid Syndrome

ADRIANO LOPES BATISTA^1^, Gracylma Guimarães Rocha^1^, Ingrid Loureiro de Queiroz Lima^1^, Marlúcia do Nascimento Nobre^1^, Augusto de Carvalho Bezerra^3^

(1) Hospital Universitário Getúlio Vargas – HUGV; (2) Universidade Federal do Amazonas – UFAM; (3) Hospital Universitário Francisca Mendes – HUFM

Antiphospholipid syndrome is an autoimmune disease characterized by recurrent episodes of vascular thrombosis, predominantly in females. Infective endocarditis associated with this disease is uncommon, with more prevalent aseptic endocarditis. However, there is potential for this collagenosis to lead to pulmonary hypertension, which in turn causes cardiac dysfunction in the right chambers, predisposing the endocardium to infection and, consequently, the lung to septic embolism. The case report is of a 23 years-old male with a clinical history since adolescence of recurrent deep venous and arterial thrombosis, lower limb edema, and progressive dyspnea. Eight years after the onset of symptoms, he developed a condition of digestive hemorrhage associated with thrombocytopenia where, during blood product transfusion, he showed signs of bacteremia. Since then, daily, with mild symptoms of chills, low-grade fever, sweating, arthralgia, and progressive worsening of dyspnea. After 30 days, the patient evolved with pulmonary focus septic shock and was then transferred to our hospital. Chest tomography showed nodules with necrosis and central cavitation compatible with septic emboli(1). Echocardiography showed dilatation of the right chambers, significant pulmonary hypertension, insufficient tricuspid valve, perforated, and a mobile vegetative lesion measuring 1.7 × 1.4 cm adhered to the anterior leaflet compatible with endocarditis. He received antibiotic therapy and valve replacement surgery. All collected blood cultures since admission were negative. We are not aware of cultures performed at the hospital where he came from. He was discharged from the Intensive Care Unit (ICU) and was awaiting the results of investigative tests for the underlying thrombogenic disease. Suddenly, he presented dyspnea and chest pain, evolving into cardiac arrest due to pulseless electrical activity resulting in death. After death, we received the tests that were positive for Antiphospholipid Syndrome. However, for the definitive diagnosis of this collagenosis, the antibodies must remain positive after 12 weeks of the first collection.


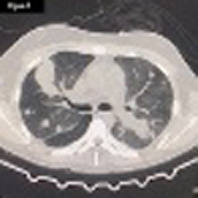


110493

Modality: E-Poster Young Researcher – Case Report

Category: HEART FAILURE/CARDIOMYOPATHY/TRANSPLANT

## Adverse Effect of Vaccination for COVID-19: Myocarditis Case Report

FELIPE MONGE TEIXEIRA^1^, Paulo Vitor da Silva Gonçalves^1^, Diego Bianchi Braga Campos^1^, Marcelo Imbroinise Bittencourt^1^, Ricardo Mourilhe Rocha^1^

(1) Cardiology Service – Pedro Ernesto University Hospital – UERJ

**Introduction:** In 2020, the WHO declared COVID-19 a pandemic. In the same year, the US Food and Drug Administration (FDA) granted emergency use authorization for the Pfizer/BioNTech and Moderna vaccines. Despite the benefits of vaccination, it can also pose potential risks such as myocarditis.

**Case Report:** Female, 37 years old, without comorbidities, denies alcohol consumption, smoking, and recent infections. She presented mild COVID in January 2021, and after 7 months, she was vaccinated with 2 doses of Pfizer/BioNTech. After vaccination, she developed oppressive type A chest pain, VAS 8/10, radiating to the left upper limb and nausea, seeking emergency care. An ECG was performed, without significant changes, but with high ultrasensitive troponin values (12,500|RV < 5), motivating his hospitalization. She underwent transthoracic echocardiography (TE), which demonstrated septo-apical hypokinesia with preserved function. Coronary angiography was performed on the first day after admission, which showed no obstructions. She had a laboratory with still high troponin, but falling, in addition to a negative PCR for COVID. A new TE was performed, which showed no segmental lesions or changes. Cardiac resonance imaging was requested, demonstrating moderate myocardial fibrosis (11%) suggestive of myopericarditis, as shown in Figures 1A and 1B. In view of this, he started treatment with colchicine and ASA, which led to a drop in troponin after 7 days of hospitalization, and he was discharged asymptomatic.

**Conclusion:** Vaccination for COVID-19 continues to be recommended for all people, clearly outweighing the risks in all populations. As observed in the case described, most individuals with myocarditis had a full recovery of ventricular function, with few reports of major complications.


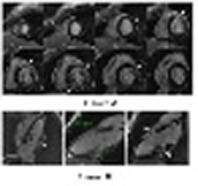


110515

Modality: E-Poster Young Researcher – Case Report

Category: CARDIOVASCULAR IMAGING

## Echodopplercardiography as a Marker of Malignancy in Intracardiac Tumors: Case Report

VINÍCIUS STÉFANO BARROS DA ROCHA^1^, Anita de Oliveira e Souza Fragoso^1^, Maria Luciana Zacharias Hannouche da Trindade^2^, Ana Clara Tude Rodrigues^1^

(1) Hospital das Clínicas da Faculdade de Medicina da Universidade de São Paulo (HCFMUSP); (2) Instituto de Radiologia do Hospital das Clínicas da Faculdade de Medicina da Universidade de São Paulo (InRad)

**Introduction:** Leiomyosarcoma is one of the most common malignant tumors among retroperitoneal tumors, corresponding to approximately 15% of these. Involvement of the inferior vena cava (IVC) and right heart chambers is rare and related to a worse prognosis. Ultrasound-enhancing agent echocardiography (ECHO with enhancement) has been used to identify intracardiac tumors with high accuracy. However, this exam may eventually present limitations in relation to the definition of malignancy.

**Case Report:** A 53-year-old female patient with diabetes and dyslipidemia sought medical care with left low back pain for about 5 months, associated with bulging in the right flank region and weight loss of 5 kg. Investigation with computed tomography (CT) of the abdomen from an external service showed a retroperitoneal mass in the IVC and right atrium (RA), and the biopsy with guided puncture was compatible with high-grade leiomyosarcoma. The enhanced echo showed partial perfusion of the mass in the RA, while the three-dimensional transesophageal echocardiogram showed a heterogeneous multilobulated mass extending from the most apical portion of the IVC to the RA, close to the tricuspid valve, with vacuolar areas in its interior, both tests suggesting a benign process. The patient was scheduled for vascular and cardiac surgery for definitive treatment.

**Conclusion:** Despite studies showing good accuracy of echocardiography with enhancement for differentiating between malignant and benign tumors, according to the characteristics of the perfusion, malignant tumors with necrotic process inside them, such as leiomyosarcomas, may present with partial perfusion of the tumor, limiting the accuracy of the method.


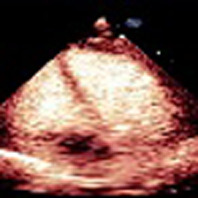


110539

Modality: E-Poster Young Researcher – Case Report

Category: CARDIOVASCULAR INTENSIVE CARE/CARDIOVASCULAR EMERGENCIES

## Uncommon Presentation of Arterial Thrombosis in a Young Male with Systemic Lupus Erythematosus

GABRIEL RIBEIRO ROSA^1^, Elisa Carolina de Almeida Negrello^1^, Alcirley de Almeida Luiz^1^, Pedro Henrique Bonifácio Shiino^1^

(1) Hospital Universitário do Oeste do Paraná (HUOP)

**Introduction:** Systemic lupus erythematosus (SLE) is associated with severe cardiovascular manifestations, including myocardial infarction (MI), which is an important cause of premature mortality and morbidity. Accelerated atherosclerosis is the main mechanism involved. We report a case of a young male with an acute MI with ST segment elevation, secondary to coronary thrombosis, which led to the diagnosis of SLE-associated antiphospholipid syndrome (APS).

**Case Report:** A 24-year-old male was admitted to the hospital with typical chest pain, lasting 6 hours. The electrocardiogram in the emergency room (ER) showed ST segment elevation in the anterior wall. The patient had a history of SLE, which was diagnosed when he was 15 years old, and was undergoing investigation for APS due to the positivity of IgG anti-β2 glycoprotein-1 antibody. He had traditional risk factors, such as hypertension and class I obesity. He received treatment in the ER and was referred to Interventional Cardiology. A coronary angiogram showed complete occlusion proximally in the left anterior descending artery. A percutaneous angioplasty with no stent was performed, due to angiographic signs of thrombosis without associated atherosclerotic disease in other vessels. The patient had a good recovery without other complications.

**Conclusions:** SLE is an autoimmune disease commonly associated with APS. This association increases mortality, and the positivity of antiphospholipid antibodies in SLE patients’ serum is an independent risk factor for premature death. Deep venous thrombosis and stroke, differently than the reported case, are the most common cardiovascular events in these patients.


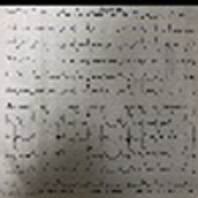


110546

Modality: E-Poster Young Researcher – Case Report

Category: ACUTE AND CHRONIC CORONARY DISEASE/THROMBOLYSIS

## Post-Infarction Left Ventricular Pseudoaneurysm Treated with Percutaneous Transcatheter Occlusion: A Case Report

ISABELA GALIZZI FAÉ^1^, Carolina Kuchenbecker Soares^1^, Luís Henrique Coelho Pinto^1^, Fernanda Araújo Sá^1^, Anderson Ferreira Leite^1^

(1) Hospital das Clínicas da Universidade Federal de Minas Gerais

**Introduction:** Left ventricular (LV) pseudoaneurysm is a rare complication after myocardial infarction (MI) characterized by rupture of the LV wall contained by pericardium, thrombus or scar tissue. If not treated properly, it can lead to cardiac tamponade and death. We aim to describe a case of LV pseudoaneurysm after MI treated with percutaneous closure.

**Case Report:** A 57-year-old man presented with chest pain and extensive anterior ST-segment elevation with right bundle-branch block. Acute MI protocol and thrombolysis was performed, without reperfusion criteria. Rescue angioplasty was not available that day. Cineangiocoronariography in the next day revealed severe occlusion (90%) in descending artery (DA) ostium, so angioplasty with pharmacological stent in the left main artery (LMA) towards DA was performed. Echocardiogram showed ejection fraction of 38%, segmentary defect of contractility and an image suggesting pseudoaneurysm in apex of LV, which was confirmed in cardiac resonance and angiotomography. The opted approach was percutaneous transcatheter closure due to high risk of rupture. Surgery was dismissed, as the patient was clinically stable and had elevated risk surgery, owing to MI and recent angioplasty to LMA towards DA. An interatrial communication prosthesis was implanted, without any flow inside the pseudoaneurysm orifice. The patient remains asymptomatic after six months of follow-up.

**Conclusion:** High suspicion is required for the diagnosis of post-infarction LV pseudoaneurysm. Non-invasive imaging is indispensable in stable patients. Surgical treatment is usually recommended, notwithstanding, percutaneous transcatheter closure is growing in importance as it has lower morbimortality compared to surgery, with multiple successful case reports being described.


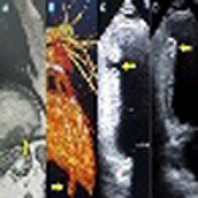


110790

Modality: E-Poster Young Researcher – Case Report

Category: CARDIOVASCULAR IMAGING

## Coronary Fistula, Right Ventricle Epicardial Pseudoaneurysm and Acute Myocardial Infarction – “Three in a Row”

JULIANA JANGELAVICIN BARBOSA^1^, Otavio Augusto Oliveira de Carvalho^1^, Thiago Abizaid Kleinsorge^1^, Ruldney Ray dos Santos Oliveira^1^, Antonio Tito Paladino Filho^1^

(1) Hospital SEPACO (SEPACO)

**Introduction:** Coronary artery fistula (CAF) is a rare abnormality characterized by the connection between one or more coronary arteries and a cardiac cavity or great vessel. Depending on several factors can present without clinical repercussions or cause significant hemodynamics complications.

**Case description:** A 33-year-old male patient presented with chest pain and ST-segment elevation in leads DII, DIII and aVF at the ECG. Troponin was elevated (Pocket). Referred for urgent cardiac catheterization, we not identify obstructive coronary lesions, but observed the presence of a fistula with extravasation of contrast into a neo-cavity. Cardiac magnetic resonance imaging was performed with findings suggestive of acute myocardial infarction (AMI) in the inferior wall and a “mass” adjacent to the right atrium with an image of a thrombus in its interior. The investigation was complemented with coronary CT angiography, bringing the hypotheses of a pseudoaneurysm and evidencing the feeding origin from the right coronary fistula. This “mass” exerted a compression effect on the right coronary artery (RCA), resulting in inferior wall infarction. The patient was referred for surgical intervention, confirming the hypotheses of a right ventricular (RV) pseudoaneurysm with a thrombus inside it.

**Conclusion:** The diagnosis of CAF is rare, and in the clinical case, it was responsible for the formation of a pseudoaneurysm that expanded, evolving with AMI due to compression of the RCA. The case brings together other findings – CAF and RV epicardial pseudoaneurysm – which, despite being rare, presented with a common condition in cardiac emergencies: AMI. Advances in cardiovascular imaging contribute in such types of clinical cases with early diagnosis and effective management, leading to favorable clinical outcomes.


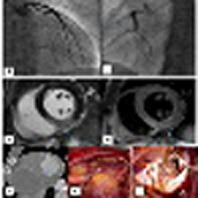


110810

Modality: E-Poster Young Researcher – Case Report

Category: CARDIO-ONCOLOGY

## Case Report: Ventricular Dysfunction Associated with Pazopanib

LAÍSSE BARRETO FERREIRA REIS^1^, Maíra de Melo Ibrahim Nogueira^2^, Valmir de Freitas Costa^1^, Rafael Alves da Silva^1^, Douglas Celestino Fróes^3^

(1) Instituto do Coração/FMUSP; (2) Hospital Felício Rocho; (3) Centro Hospitalar do Município de Santo André

**Introduction:** Pazopanib is a tyrosine kinase inhibitor whose main therapeutic targets are PDGF (platelet-derived growth factor) and, mainly, VEGF (endothelial growth factor). Its use in oncology is in the treatment of renal cell carcinoma, sarcomas and, less commonly, thyroid cancer. This medication has arterial hypertension as its main cardiovascular side effect, but it can also cause arrhythmias, QT interval prolongation and heart failure.

**Case description:** A 64-year-old patient with a personal history of arterial hypertension, dyslipidemia and non-insulin-requiring diabetes, had no history of heart failure and had an echocardiogram with a left ventricular ejection fraction of 60%. Diagnosed with non-metastatic renal cell carcinoma, she started using pazopanib at a dose of 800 mg daily. After one month of use, he started to experience dyspnea on minor exertion, orthopnea and lower limb edema. She was hospitalized for evaluation and management of the condition, her NT-Pro-BNP was 4,500, the electrocardiogram had left bundle branch block and the echocardiogram showed an ejection fraction of 35% with an area of septal and inferior akinesia and hypokinesia of the other walls. In view of this finding, pazopanib was discontinued and the use of enalapril, carvedilol, spironolactone and furosemide was started. An investigation of the etiology of ventricular dysfunction was carried out with the following findings: negative serology for Chagas disease, cardiac catheterization showed no obstructive lesions, cardiac magnetic resonance imaging with no edema, late enhancement or perfusion defects at rest and stress. Therefore, pazopanib was considered the cause of her heart failure. After discontinuation of chemotherapy and initiation of treatment for heart failure, the patient showed clinical improvement and a new echocardiogram showed an ejection fraction of 45%. In a decision shared between the oncology and cardiology teams and with the patient, it was decided to keep the medications for ventricular dysfunction and pazopanib at a lower dose, 400 mg once a day.

**Conclusion:** Pazopanib plays a very important role in the survival of patients with renal cell carcinoma, the incidence of ventricular dysfunction is low and it is a diagnosis of exclusion. Management is challenging and requires shared decision-making between oncology and cardiology, respect for patient values and preferences, and frequent clinical surveillance.

110861

Modality: E-Poster Young Researcher – Case Report

Category: CARDIOLOGY OF SPORTS, EXERCISE, ERGOMETRY AND CARDIOVASCULAR REHABILITATION

## The Importance of a Close Follow up for Athletes with Congenital Third Degree Block

GABRIELA MELCHUNA MADRUGA^1^, Rodrigo Otávio Bougleux Alô^1^, Lucas Yuiti Mori^1^, Camila Mota Guida^1^, Marco Antônio Smiderle Gelain^1^

(1) INSTITUTO DANTE PAZZANESE DE CARDIOLOGIA

Congenital third degree block (CHB) is a relative rare disorder. The manifestations of CHB can vary according to many aspects, including ventricular rate of the escape rhythm and ventricular function. The main therapeutic decision is the need for and the ideal timing of permanent pacemaker insertion. Patients with adequate ventricular heart rate increasing appropriately during exertion and no symptoms such as syncope or near syncope and a structurally normal heart and cardiac function can usually be followed without intervention, and therefore, they can selectively participate in competitive sports. Although, this is not a permanent recommendation, and these patients should be followed closely.

**Case Report:** 13-year-old asymptomatic male patient, with a previous diagnosis of CHB, who practices competitive soccer, in yearly follow up at the sports cardiology outpatient clinic, who presented a normal echocardiography, without structural alterations, normal Left Ventricle (LV) dimensions, a Left Ventricle End Diastolic Diameter (LVEDD) of 48 mm, and preserved LV function. His exercise test showed an excellent functional capacity (15 METS) despite a chronotropic insufficiency. However, on the 2021 clinical evaluation, he presented a significant worsening of the tests results, although remained asymptomatic. The new echocardiography presented LV dilatation with a LVEDD of 63 mm, but with preserved ejection fraction. The exercise test showed a worse functional capacity of 11 METS and chronotropic deficit. Therefore, it was indicated the insertion of a pacemaker. New tests were performed two months after the procedure. The echocardiography showed normal LV dimensions (LVEDD: 46 mm). At the cardiopulmonary exercise test, the patient reached 103% of the predicted VO2, showing a normal functional capacity. Although all these improvements, the practice of soccer was contraindicated due to the high risk of thoracic trauma. Nevertheless, the patient still may participate of other competitive and high intensity activities with low risk of lead dislocation and thoracic trauma. In conclusion, the CHB is not a prohibitive condition of high intensity sports, as long as the patient remains without structural cardiac alterations, normal LV function, asymptomatic and with a normal functional capacity. However, these conditions can change in a relative short period, so the follow up must be done closely.

110891

Modality: E-Poster Young Researcher – Case Report

Category: PERICARDIUM/ENDOCARDIUM/VALVOPATHIES

## Libman-Sacks Endocarditis Associated with Mitral Regurgitation in a SLE Patient: A Case Report

FLÁVIA CORRÊA DE OLIVEIRA LIMA^1^, Marília Spolador Marrafão^1^, Camila Clara da Costa Geraldo^1^, Maria Beatriz Marcussi Moretti^1^, Rafael Cecílio de Lima^1^

(1) Hospital Regional de Presidente Prudente – HRPP

**Introduction:** Libman-Sacks endocarditis (LSE) – thrombotic, non-bacterial, marantic or verrucous – is characterized by inflammation of the endocardium and sterile vegetations in the heart valves. It is present in 6 to 11% of patients with Systemic Lupus Erythematosus (SLE), with a significant correlation of duration and severity. The development possibly occurs by endothelial injury of circulating cytokines in a patient in a hypercoagulable state, which results in the deposition of platelet thrombi and inflammatory molecules in the valves.

**Case description:** Female, 18 years old, was hospitalized with dyspnea, weakness and bruising for 30 days. Personal history of active SLE with grade III lupus nephritis, hypertension, severe depressive disorder and drug addiction. Patient evolved with persistent high fever, neutropenia, thrombocytopenia, blood pressure peaks and significant worsening of renal function; referred to the intensive care unit. Physical examination: diffuse edema; cardiac auscultation without murmurs and pulmonary auscultation with crackling rales in bilateral bases. Acute intubation with heart failure due to advanced hemodialysis pulse patient edema. Four blood cultures were performed within 30 days, all negative. At the entrance performed Transesophageal Echocardiography (TEECHO), inlet ventricular hypertrophy, mitral function, va a to bicuspid with thickening of the inferior important and echogenic image adhered, filamentous, with random movements, suggestive of endocardial selection. Due to severe thrombocytopenia, it was decided not to perform anticoagulation; clinical support and multidisciplinary follow-up. After 30 exams that showed increased ventricular hypertrophy, increased repetition of myopia, insufficiency and absence of vegetations. In medical records, 9 years ago, ET ECHO with important mitral indication found and leaflet in anterior leaflet with later normalization. At the clinic, a cardiac surgery equipment opted for the conservative approach.

**Conclusions:** Cardiac complications are the ones that most attribute morbidity and mortality to SLE cases. The treatment of the underlying disease with an interdisciplinary approach that included prevention, health education (therapeutic adherence and health care), individualized treatments and diagnosis of complications, such as LSE, can lead to an early risk of negative cardiovascular problems and a better in patient survival.

110960

Modality: E-Poster Young Researcher – Case Report

Category: ACUTE AND CHRONIC CORONARY DISEASE/THROMBOLYSIS

## Spontaneous Coronary Artery Dissection with Systemic Lupus Erythematosus

NOURHAN CHAABAN^1^, Shilpa Kshatriya^2^

(1) University of Kansas; (2) Heartland Cardiology

Spontaneous coronary artery dissection (SCAD) has been reported to be a rare cause of acute coronary syndrome (ACS) and sudden cardiac death (SCD). We report a case here to emphasize on the benefit of early recognition of SCAD, and to highlight on its association with systemic lupus disease. A 37-year-old female patient known to have hypertension, systemic lupus erythematosus, a history of pre-eclampsia with 3 C-section deliveries, and an active smoking habit presented to the emergency department with chest pain. On arrival, the patient was hypertensive to 152/122 mmHg, with a normal heart rate and respiratory rate. An electrocardiogram (EKG) showed minimum ST elevation in lead V2. Initial troponin was 0.1 ng/mL (normal <0.4 ng/mL) with a peak of 54.5 ng/mL after 6 hours. Aspirin 325 mg was given, and the patient underwent an urgent cardiac catheterization. Intravascular ultrasound was performed that showed evidence of intimal flap (mid LAD) spontaneous dissection with subintimal hematoma. The angioplasty resulted in successful stent placement in the mid-left anterior descending artery. SCAD diagnosis is challenging and requires a high index of suspicion. We aim to elaborate on the association of SCAD with systemic lupus disease and add to the literature this unusual case.


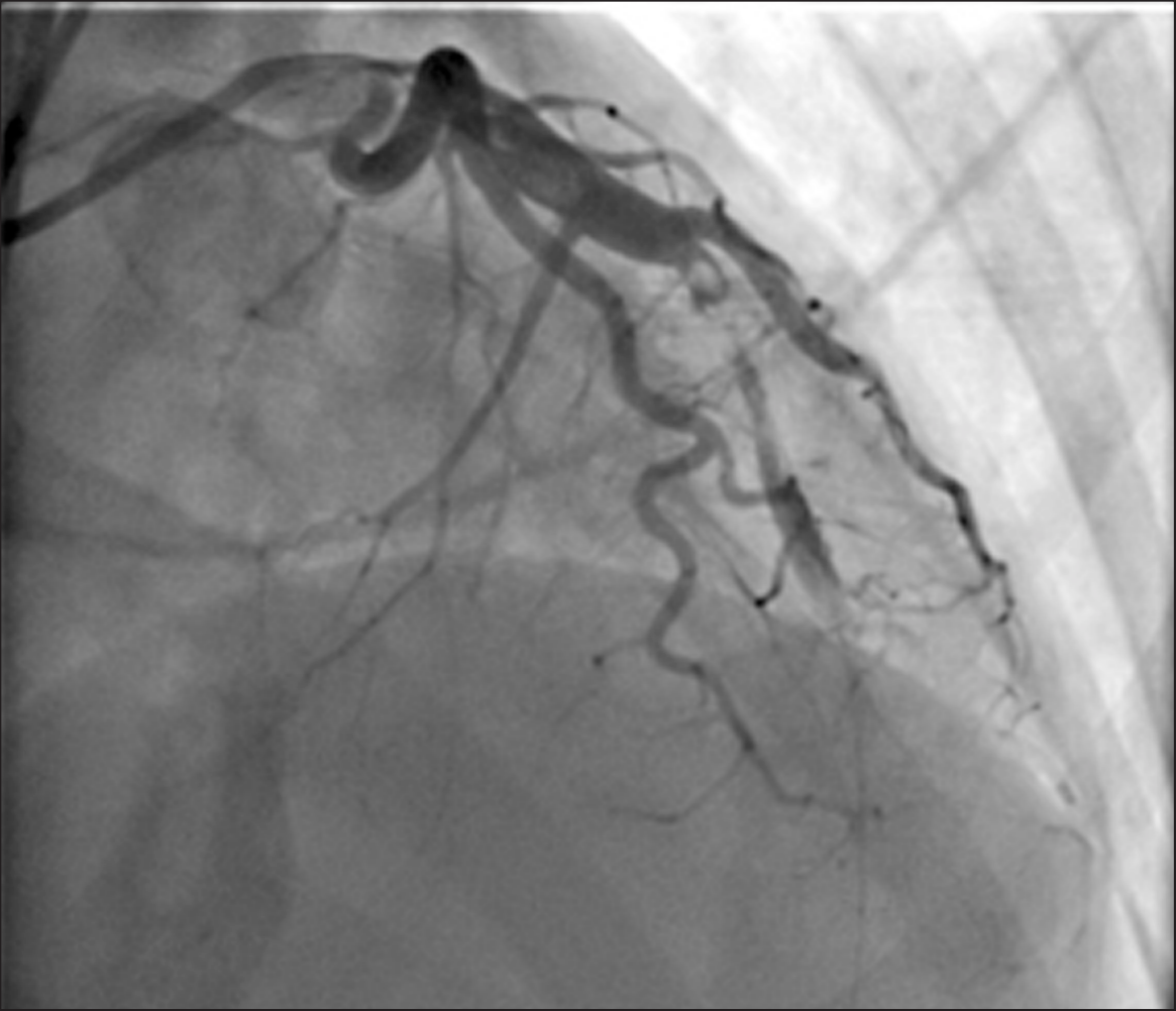


110999

Modality: E-Poster Young Researcher – Case Report

Category: PERICARDIUM/ENDOCARDIUM/VALVOPATHIES

## Purulent Pericarditis Associated with Coronary Thrombosis Due to Escherichia Coli: Hemorrhoidal Disease as the Infection’s Origin?

POLIANA FERREIRA STROLIGO DIAS^1^, Paula Bline Lazzari^1^, Bruno de Souza Paolino^1^

(1) Hospital Universitário Pedro Ernesto (HUPE)

**Introduction:** Purulent pericarditis is a pericardium located infection characterized by pus. The most common pathophysiology is through continuity or hematogenous dissemination of another infection. It’s usually associated with pneumonia and other infection sites are rare. Although the disease is manifested by chest pain, differential diagnosis with acute coronary syndrome (ACS) is made to exclude one of the conditions, since the concomitant presence of pericarditis and ACS is even rarer.

**Case:** 52-year-old smoker and alcoholic male with hypertension and hemorrhoids seeks Emergency Care (EC) with typical chest pain, is diagnosed with inferior STEMI and treated by thrombolysis. He reported anal pain with purulent discharge 15 days before and fever 2 days before but did not use antibiotics. Patient transferred to our institution, still febrile, for invasive stratification after 4 days of EC hospitalization. Coronary angiography showed thrombotic occlusion of posterior ventricular branch (PV), with no atherosclerosis in other vessels. PV angioplasty and thrombus aspiration performed without flow return. Evolution to hemodynamic instability, orotracheal intubation and cardiopulmonary arrest with pulseless electrical activity for 6 min, reversed after CPR. TTE showed pericardial effusion with cardiac tamponade signs, with purulent fluid’s output of 300 mL in pericardiocentesis. He was referred to the surgical center, where pericardial window evidenced smooth pericardium. There whas leukocytosis with deviation up to metamyelocyte and CRP elevation in admission exams. Pericardial fluid analysis showed 68600 leukocytes/μl, numerous polymorphonuclear and gram-negative bacilli. Ventilatory and hemodynamic supports were kept and empirical antibiotic therapy with Vancomycin and Meropenem was initiated. The condition worsened, with fever despite antibiotics and antipyretics, progressing to death after 3 days. Later, culture results showed multi-sensitive E. Coli growth in all blood and pericardial fluid samples.

**Discussion:** Purulent pericarditis is a rare and severe disease with mortality up to 30% even with pericardial drainage and adequate antibiotic treatment. Possibly, the infectious condition triggered concomitant coronary thrombosis, another rare but plausible association. Rapid recognition and appropriate treatment are essential for better outcomes and to prevent constrictive pericarditis.

111188

Modality: E-Poster Young Researcher – Case Report

Category: HEART FAILURE/CARDIOMYOPATHY/TRANSPLANT

## Case Report: Male Patient, Adult, 31 Years Old, with Single Ventricle, Membranous IVC and Severe Pulmonary Hypertension

LARISSA SEBOLD^1^, Abiran Dalri Merizio^1^, Bárbara Dalri Andreghetoni^1^, Tiago Gonçalves Pereira^1^, James Alberton^1^

(1) Cardiological department – Santa Isabel Hospital

**Introduction:** As a congenital heart disease, the univentricular heart may be related to other pathological conditions, such as: pulmonary artery stenosis and aortic obstruction. Within this complex group, the left ventricle hypoplasia (HLHS) is the most common disorder, occurring when there is an underdevelopment of the left ventricle, mitral valve, aortic valve and aortic arch. Typically, it presents in the first 24 to 48 hours of life, due to the constriction of the ductus arteriosus. Adult patients not submitted to repairs usually develop pulmonary stenosis or pulmonary vascular disease.

**Case Description:** AS, 31 years old, male, with congenital heart disease: Single ventricle of left morphology, membranous interventricular communication (IVC), ventriculoarterial connection type “Transposition of the Great Arteries” (TGA) – with pulmonary artery connected to SV, and aorta connected to the hypoplastic chamber – and presence of patent foramen ovale, maintaining signs and symptoms of Heart Failure, with Transthoracic Echocardiogram demonstrating complex cyanotic congenital heart disease (single ventricle + IVC + TGA + Patent Foramen Ovale). Mitral Insufficiency and mild to moderate Tricuspid. Mild Aortic and Pulmonary Insufficiency. Late postoperative of Pulmonary Artery Banding, apparently effective. Moderate enlargement of both Atria. SV with significant enlargement and dysfunction (Ejection fraction:27%). Medication optimization was done and A.S. was listed for performing Heart Transplantation.

**Conclusion:** According to the case above, the clinical manifestations of SV depend on associated cardiac anomalies: if obstructed pulmonary flow, Tetralogy of Fallot clinic, with important cyanosis and without HF; if unobstructed flow, clinical of transposition of great vessels with intraventricular communication, with minimal cyanosis and important HF. When non-operated, many patients succumb to HF from birth, however, the rapid evolution of successful palliation, with surgical approach in stages, has led to a population of adults with only one ventricle.


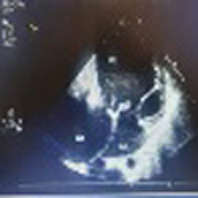


111026

Modality: E-Poster Young Researcher – Case Report

Category: ACUTE AND CHRONIC CORONARY DISEASE/THROMBOLYSIS

## Coronary Thrombosis as a Cause of Chest Pain in a Young Anabolic Users: Case Report

ANA LUÍZA SILVEIRA BORELA PELLIZZER^1^, Reno Leite Zamignan^1^, Kamilla Guimarães Dias^1^, Pedro Leonel de Paiva Neto^1^, Ana Paula Lindoso Lima Barreiro^1^

(1) Santa Casa de Misericórdia de Goiânia

**Background:** Coronary thrombosis is a serious cause of chest pain and needs to be promptly diagnosed and treated in time, as it leads the patient to the risk of death. One of the causes of coronary thrombosis is the indiscriminate use of anabolic steroids, in most documented reports is the combination of various anabolic steroids. Stanozolol is an anabolic considered the safest among the others, with little probability of complications, for oral use, and which is even approved by the FDA and has grown widely in its use. There are no reports in the literature with the isolated use of Stanozolol associated with serious cardiovascular complications.

**Case Report:** Patient, 25 years old, previously healthy, evolving with tight chest pain, irradiating to the left lower limb, during physical activity (bodybuilding), in which he sought medical attention, and an electrocardiographic diagnosis of acute myocardial infarction with ST-segment elevation on the anterior wall was made. He reports that he had been on regular use of Stanozolol 10 mg once a day for 1 month, denying the use of any other associated medication, such as testosterone, oxandrolone and/or GH. He underwent cardiac catheterization that showed an occluded anterior descending artery with proximal thrombus. Angioplasty and recanalization with intracoronary thrombus aspiration were performed. Due to the presence of residual thrombi and TIMI-II flow, Agrastrat was started. Control catheterization was performed after 5 days in which no intracoronary thrombi were observed, and was then discharged from hospital with outpatient return.

**Discussion and Conclusion:** Stanozolol, the only medication used by the aforementioned patient, is widely used by nutrologists and endocrinologists due to its good efficacy in gaining lean mass and the low probability of complications. The complications documented in publications and articles are neurological changes and hepatotoxicity. This case is of paramount importance, since there are no reports of serious coronary complications in the presence of the use of Stanozolol yet, which should serve as a warning to the general population and health professionals for its use.

112359

Modality: E-Poster Young Researcher – Case Report

Category: DYSLIPIDEMIA

## Impact of C681X Mutation on Treatment of Homozygous Familial Hypercholesterolemia: Review and Case Report

GIOVANNA BOLINI BRAZÃO^1^, Isabella Rocha Gonçalves^1^, Eduarda Gabriel Mafra^1^, Samuel Sabbá Fadul^1^, Ana Augusta Motta Oliveira Valente^1^

(1) Centro Universitário do Estado do Pará (CESUPA)

**Introduction:** Familial hypercholesterolemia (FH) is a genetic disease resulting from the mutation of one of the critical genes for the formation or catabolism of Low-Density Lipoprotein (LDL). Transmission is autosomal dominant and can be heterozygous or homozygous. In about 80% of patients with a definitive diagnosis, mutation can be observed in one of 3 genes: LDL receptor gene (LDLR), gain-of-function mutation in the Protein Convertase Subtilisin/Cexin Type 9 gene (PCSK9) and mutation in the apolipoprotein B (Apo B) gene. Homozygotes will invariably need multiple drugs to control cholesterol, and receptor negative patients will not respond to drugs that act directly or indirectly on LDL receptors.

**Case details:** A 15-year-old man with a diagnosis of homozygous familial hypercholesterolemia by C681X mutation in LDL receptor, as known as Lebanese mutation, one of the most prevalent in Brazil. He has a family history of hypercholesterolemia (father, mother and siblings) and grandmother and uncles who died after AMI under 50 years. On physical examination, corneal arches with cholesterol deposits and tuberous xanthomas on knees, elbows and heels were observed. The treatment was conducted with two distinct pharmacological combined therapy: Therapy 1: rosuvastatin 40 mg daily, ezetimibe 10 mg daily and mipomersen 200 mg every two weeks, for one year. Therapy 2: rosuvastatin 40 mg daily, ezetimibe 10 mg daily and evolocumab 140 mg every two weeks, for one year. The result suggests that the second therapy is better suited to reach the target of 50% reduction in LDL, even without plasmapheresis and in low doses. This corroborates with recent findings that defines C681X mutation as null-receptor mutation, where PCSK9 inhibitors have low efficacy.

**Conclusion:** Studies should be carried out to accumulate data on the distribution of the C681X mutation throughout the Brazilian territory, to pressure public health agencies to create treatment protocols aimed at the Lebanese mutation.


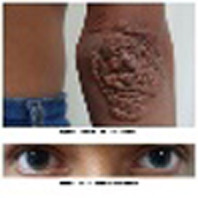


111041

Modality: E-Poster Young Researcher – Case Report

Category: COVID-19 AND CARDIOVASCULAR SYSTEM

## Myocarditis After Vaccine for COVID-19

MARIA CLARA SILVEIRA MARQUES PEREIRA^1^, Thaisa Paula Perini^1^, Mayara Aparecida da Silva Oliveira^1^, Juliana Tasso Cândido de Lima^1^, Lucas Simões de Mello^1^

(1) Hospital Universitário de Londrina – HU Londrina/UEL

**Introduction:** The coronavirus disease-2019 (COVID-19) pandemic has quickly become a global health problem with high rates of mortality and morbidity. The improvement of the world scenario was possible after the development and wide application of vaccines. A possible complication after vaccination is myocarditis. We report a case of myocarditis after inactivated SARS CoV-2 virus antigen vaccine.

**Case Report:** An 18-year-old male patient attended the emergency department complaining of self-limited episodes of retrosternal chest pain, unrelated to exertion or rest, without irradiation, without other associated symptoms, and lasting up to 1 hour. He denied fever, dyspnea, flu-like symptoms, syncope or other complaints. Personal history of obesity grade III. He received a vaccine for Covid-19 15 days before the onset of the condition. An electrocardiogram was performed on admission, showing sinus tachycardia, positive ultrasensitive troponin result (1.887 – reference value <50 ng/L), NT-pro-BNP 100 (reference value <450 pg/ml). Investigation with chest angiotomography without evidence of pulmonary embolism, chest tomography without alterations. During evolution, the patient showed improvement in chest pain episodes, but with a significant ultrasensitive troponin curve (reaching 3.459 ng/L), without dynamic changes on the electrocardiogram. Considering the hypothesis of post-vaccine myocarditis, cardiac resonance was performed, showing an ejection fraction of 62.9%; area of hypersignal in the inferoseptal wall of the apical segment of the left ventricle (edema); preserved biventricular function; normal-sized cardiac chambers; absence of signs of ischemia at rest or areas of fibrosis (Figure 1). Patient presented spontaneous improvement of pain, being discharged from hospital after investigation.

**Conclusion:** Myocarditis after vaccination is a possible reaction, but with a very low incidence, being more reported among young males, with a good prognosis and a mild and self-limiting course. Therefore the benefits of vaccination outweigh the morbidity and mortality rates associated with COVID-19 infection.


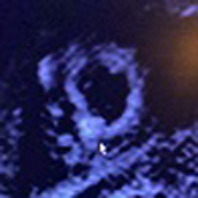


111079

Modality: E-Poster Young Researcher – Case Report

Category: HEART FAILURE/CARDIOMYOPATHY/TRANSPLANT

## Sitosterolemia, Advanced Heart Failure, and Mechanical Circulatory Support Implant: A Case Report

KAREN KATCHVARTANIAN^1^, Luis Henrique Silveira Moreira^1^, Armindo Jreige Junior^1^, Renan Cintra de Alvarenga Oliveira^1^, Camila Vilela Vieira^1^

(1) Instituto do coração – INCOR-SP

L.P., age 46, female, with a previous history of benign familial thrombocytopenia, entry into the InCor emergency room in 2017, receiving a primary diagnosis of acute myocardial infarction with ST segment elevation. Being submitted to an investigation of primary dyslipidemia, sitosterolemia was identified, a rare genetic condition in which lipid metabolism is altered. During hospitalization, the patient developed critical segmental dysfunction and left ventricular failure, with drug therapy being optimized for HF conditions and regular patient follow-up with a specialized team. During hospitalization, progressive worsening conditions evolved presenting multiple organic dysfunctions despite the inotropic drug administration. Chosen MCS (HeartMate 2) as destination therapy, since the patient presented heart transplantation contraindication, because of the elevated immunologic panel due to multiple transfusions by benign familial thrombocytopenia. Sitosterolemia is characterized by hyperabsorption and lowered biliary excretion of dietary sterols, including the phytosterol beta-sitosterol, by plant ingestion. Healthy individuals absorb only about 5% of dietary phytosterols, but sitosterolemia-affected individuals absorb around 15% to 60%. Thus, there is a higher atherosclerosis risk in these patients, and the diagnosis delay has limiting consequences, amongst them, acute myocardial infarction and the subsequent progression to advanced HF when early diagnosis is not achieved. Advanced HF is characterized by heart dysfunction associated with severe symptoms such as dyspnea and fatigue at rest or with minimal physical effort (NYHA III or IV), of life, even with optimized drug therapy, in which life expectancy might be less than two years. At this stage, advanced therapies are considered, including heart transplantation, continuous inotropic therapy, MCS, or palliative care. In 2020, around 3 thousand patients were treated with MCS, and, more than 3 thousand patients had heart transplantation done in the United States, with additional 3,5 thousand patients awaiting transplantation. The importance of support devices as a destination therapy for left ventricularfailure grows incessantly due to organ donor scarcity. However, despite its dissemination, MCS implants still represent a high-risk, burdensome procedure, related to serious adverse events such as right HF, stroke, infection, pump thrombosis, and hemolysis.

111083

Modality: E-Poster Young Researcher – Case Report

Category: HEART FAILURE/CARDIOMYOPATHY/TRANSPLANT

## Sitosterolemia, Advanced Heart Failure, and Left Ventricular Assist Device Implant: A Case Report

KAREN KATCHVARTANIAN^1^, Camila Vilela Vieira^1^, Luis Henrique Silveira Moreira^1^, Renan Cintra de Alvarenga Oliveira^1^, Armindo Jreige Junior^1^

(1) Instituto do coração – INCOR-SP

Heart failure (HF) showed an exponential increase in the last years, affecting about 1–2% of the world’sadult population, and having a significant impact on morbidity and mortality of patients. At the advanced stages of the disease after optimized drug therapy, both a heart transplant and left ventricular assist device (LVAD) implant must be considered for treatment. L.P., age 46, female, with a previous history of benign familial thrombocytopenia, was treated in the Instituto do Coração de São Paulo (InCor) emergency room in 2017, with a diagnosis of acute myocardial infarction, with left ventricular dysfunction development during hospitalization. Was discharged from hospitalization with optimized drug therapy with the outpatient return. At the followup was submitted to an investigation of primary dyslipidemia and sitosterolemia was identified, a rare genetic condition in which lipid metabolism is altered. In 2021 returned to the emergency room due to HF decompensation. During hospitalization, progressive worsening conditions evolved presenting multiple organic dysfunctions despite the inotropic drug administration. Chosen LVAD (HeartMate 2) implantation as destination therapy, since the patient presented heart transplantation contraindication, because of the elevated immunologic panel due to multiple platelet transfusions received. Sitosterolemia is a genetic, autosomal recessive condition, with identified loss-of-function variants in ABCG5 and ABCG8 genes, leading to absorption increase and reduction in the biliary excretion of phytosterol and cholesterol, resulting in prominently elevated serum concentrations. Thus, these patients present tendinous or tuberous xanthomas and early atherosclerosis, similar to familial hypercholesterolemia. The association between the clinical condition of serum sitosterol increase to levels above 1 mg/dl, and pathogenic variant confirmation in the associated genes listed above confirms the diagnosis. The treatment consists of sterol ingestion reduction, so these patients must avoid ingestion of sterol-rich plants such as corn, sesame seeds and oil, peanut, soy, margarine, avocado, and chocolate, associated with standard drug therapy such as ezetimibe and bile acid sequestrants. Sitosterolemia is a rare disease, being of paramount importance its identification to avoid early atherosclerosis. Diagnosis delay has limiting consequences, amongst them, acute myocardial infarction and subsequent progression to advanced HF.

111095

Modality: E-Poster Young Researcher – Case Report

Category: ACUTE AND CHRONIC CORONARY DISEASE/THROMBOLYSIS

## Acute Coronary Syndrome (ACS) in a Young Woman – Spontaneous Coronary Dissection: A Challenge for the Heart Team

SÉRGIO LAÉLIO PEREIRA DA SILVA JÚNIOR^1^, Bruno Gonçalves Garcia^1^, Mariana Mancilha Carvalho dos Santos^1^, Carlos Henrique Oliveira Frango da Silva^1^, Sérgio Laélio Pereira da Silva Júnior^1^

(1) National Institute of Cardiology - NIC

**Introduction:** Spontaneous coronary artery dissection (SCAD) has a low incidence – it represents 0.1 to 4% of ACS cases. However, the cases are underdiagnosed, especially in the early stages of presentation. The evolution of this entity is unpredictable. Keywords: Abstract: A 42-year-old woman with low cardiovascular risk, hospitalized after typical chest pain, with an initial diagnosis of acute myocardial infarction with ST-segment elevation, not thrombolysed, Killip I. Transferred to tertiary hospital for invasive anatomical stratification two weeks after initial event. She presented contractile alteration in the anterior and anteroseptal walls, with global systolic dysfunction of the left ventricle. Invasive anatomical evaluation showed SCAD, involving the left main coronary artery (LMCA) up to the middle third of the anterior descending artery, with TIMI 2 flow. Due to the technical difficulty of percutaneous resolution, clinical and hemodynamic stability of the patient, conservative treatment was initially chosen. After the reappearance of angina at rest, during hospitalization, the Heart Team indicated coronary artery bypass graft surgery, considering the feasibility of the surgical technique of vascular graft anastomosis. The postoperative course was satisfactory, without complications.

**Conclusions:** The diagnostic difficulty of SCAD is a big challenge for Heart Team. The clinical presentation of ACS and/or cardiac arrest in a young patient with low cardiovascular risk strengthens the hypothesis. The diagnosis is mostly angiographic. It includes differentiating iatrogenic and secondary entities to coronary atherosclerosis. The classic angiographic feature – the radiolucid intimal flap is present in less than 30% of non-atherosclerotic cases, which underdiagnoses the condition. The prognosis is a reflection of the degree of involvement of SCAD and according to the modality of proposed therapeutic intervention. Estimates are drawn from retrospective series evaluating outcomes of heart failure, 10-year death, infarction, or dissection recurrence. Cases treated conservatively or with revascularization surgery had better outcomes when compared to percutaneous treatment.

111126

Modality: E-Poster Young Researcher – Case Report

Category: CONGENITAL AND PEDIATRIC CARDIOLOGY

## Giant Coronary Aneurysm Associated with Kawasaki Disease

MARIANA FERNANDES GUIMARÃES^1^, Carolina Resende Mariz^1^, Joaquim Márcio Duarte e Silva^1^, Viviane Campos Barbosa de Sena^1^, Aline Reis Bereta^1^

(1) Instituto Nacional de Cardiologia (INC)

**Introduction:** Kawasaki disease (KD) is an acute vasculitis of unknown etiology that affects medium-caliber arteries. Coronary artery aneurysm is the most feared complication.

**Case Description:** F.N.C, female, 10 years old, diagnosed with KD at nine months. Received immunoglobulin on the 24th day after the onset of symptoms and did not use acetylsalicylic acid. Four years after diagnosis, she evolved with a giant coronary aneurysm. Intake echocardiogram with normal cavitary dimensions and contractility, dilated left coronary artery (LC), greater aneurysm diameter of 0.7 cm. Angiotomography of the coronary arteries with aneurysm (0.63 × 0.55 cm) of the anterior descending (AD). Ectasia in circumflex (Cx) ostium. Giant aneurysm in right coronary (RC) (2.3 × 2.0 cm) with thrombus and subocclusion. Initially, opted for clinical follow-up, since the patient was asymptomatic and scintigraphy did not show stress-induced myocardial ischemia. After five years, the patient developed atypical chest pain and a new scintigraphy suggestive of stress-induced ischemia was performed in the anterior segments of the left ventricle. Coronary angiography presented mild to moderate lesion in the trunk and aneurysm in the LC. Bifurcation of AD with Diagonal Artery presenting significant stenosis. Ectasia in Cx. Occluded RC, with recanalization of the posterior ventricular branch from the collateral branch of Cx. The team indicated myocardial revascularization surgery. Patient awaiting procedure.

**Conclusion:** KD with coronary artery aneurysm is a cause of acute myocardial infarction in early adulthood. It is currently expected that more than 10,000 patients with cardiovascular sequelae have reached adulthood. The understanding of pediatricians and clinicians about this pathology is fundamental.


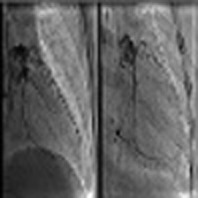


111155

Modality: E-Poster Young Researcher – Case Report

Category: HEART FAILURE/CARDIOMYOPATHY/TRANSPLANT

## Cardiac Lipomatosis is not so Benign Disease: A Case Report

KAROLYNE MOURA RIQUE DE OLIVEIRA^1^, Fábio Augusto de Luca^1^, Ernaque Viana Malta^1^, Ítalo Bruno dos Santos Sousa^1^, Júlia Galvani Nobre Ferraz^1^

(1) Hospital São Luiz – Rede D’or

**Introduction:** Cardiac tumors are benign or malignant neoplasms arising primarily in the inner lining, muscle layer, pericardium or the surrounding of the heart, and can be primary or metastatic. The incidence of cardiac tumors is observed to be 0.02%, based on 22 large autopsy data from a United Kingdom center. Almost 75% of all primary tumors of the heart are benign and include myxoma, papillary fibroelastoma, rhabdomyoma, fibroma, lipoma and hemangioma. The lipomas of the heart are encapsulated tumors that are composed primarily of mature fat cells and can originate either from subendocardium, subpericardium or myocardium. Clinical manifestations depend on tumor size and location. The main method for demonstrating lipomatous tumors consist in computed tomography (CT) and magnetic resonance imaging (MRI).

**Case Report:** 42-year-old obese woman with dyspnea on exertion for 8 months, in addition to lower limb edema, orthopnea, and paroxysmal nocturnal dyspnea. A transthoracic echocardiogram detected a mediastinal mass with extrinsic compression of the right atrium and interatrial septum of approximately 72 × 54 mm. Subsequently confirmed by cardiac CT and MRI, suggesting cardiac lipomatosis. On examinations, cardiac chambers had preserved dimensions and biventricular systolic function, without areas of infarction or myocardial fibrosis. The patient underwent thoracotomy for resection of the mass, however, in the intraoperative period, it was visualized multiple tumors with a lipomatous appearance, infiltration in the wall of the right atrium and ventricle, right coronary artery, pulmonary artery trunk extending to the left atrium wall, unresectable. Histopathological examination confirmed cardiac lipomatosis. Thus, the patient had a benign but unresectable primary cardiac tumor with an indication for heart transplantation as the only possibility of a cure.

**Conclusion:** Cardiac lipomas are observed rarely and mostly consist of benign tumors, frequently remaining asymptomatic during a patient’s lifetime, but our patient had heart failure-like symptoms. Surgical excision is the treat and has good long-term success and low morbidity. Unfortunately, our patient has an unresectable masses, requiring heart transplantation. It is imperative that cardiac tumors are treated early without making a benign-malignant distinction. In this case, although benign, the lipomatosis was not subject to resection due to the involvement of multiple vital structures.

111157

Modality: E-Poster Young Researcher – Case Report

Category: CARDIOVASCULAR IMAGING

## Coronary Anomaly by Coronary Angiotomography: A Study Case not Described in the Literature

ANNA XIMENES ALVIM^1^, Maicon Kirchmaier^2^, Weliton Mendes Apolinario^5^, Marcos Emanuel Rodrigues^2^, Thais Ortega Teixeira Guerra^6^

(1) Hospital São Jose do Avaí; (2) Universidade Iguaçu Campus V – Itaperuna (UNIG); (3) Faculdade Metropolitana São Carlos (FAMESC); (4) Cursos de Urgências Emergências (CUREM); (5) Universidade Federal Fluminense (UFF); (6) Faminas

**Introduction:** The estimated prevalence of coronary anomalies in the general population is 1.3%, however it is variable and has been described in the literature between 0.21% and 5.8%. In most cases, these are isolated findings, without clinical or hemodynamic repercussions. However, they may be associated with other heart defects, such as valvular heart disease and congenital heart diseases, which may be the cause of myocardial ischemia and sudden death, including sudden cardiac death in young athletes, so their diagnosis and early treatment are important.

**Case Report:** In normal coronary anatomy, the right coronary artery arises from the right coronary sinus (RCS), and the left main coronary arises from the left coronary sinus. It crosses the pulmonary main posteriorly, bifurcating into anterior descending and circumflex. There are reports in the literature of several coronary anomalies, among them, anomalous origin of the circumflex or anterior descending arteries from the RCS. However, there is no description in the literature of cases in which both coronary arteries (right coronary, circumflex and anterior descending arteries) leave the RCS in separate ostia. M.A.O, 61 years old, female, referred by the cardiologist for coronary angiotomography due to atypical chest pain. Physical examination without major changes. The exam performed did not show obstructive plaques, but a coronary anomaly not yet described in literature was found. All coronary arteries emerging from the RCS and with separate ostia. The course of the coronary arteries did not show any characteristics of malignancy, but the RCS was dilated in comparison to the non-coronary and left coronary sinuses. The patient is being followed up by a cardiologist who was alerted to the anomaly found.

**Conclusion:** Angiotomography of the coronary arteries has been essential in the propedeutic of patients with low to moderate cardiovascular risk to detection and exclusion of atherosclerotic plaque. In addition, it presents other highly relevant information, such as the evaluation of coronary anomalies, as described in the case above.


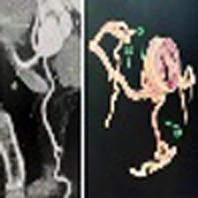


111169

Modality: E-Poster Young Researcher – Case Report

Category: PERICARDIUM/ENDOCARDIUM/VALVOPATHIES

## Fungal Endocarditis: What is the Best Time for Surgery? The Role of a Heart Team

CAMILA DALCOMUNI DOS SANTOS^1^, Igor Henrique Silva Leite^1^, Rafael Vieira Fernandes^1^, Airton Salviano de Sousa Júnior^1^, Barbara Porto Valente^1^

(1) Instituto Dante Pazzanese de Cardiologia – IDPC

Fungal endocarditis is an uncommon affliction with high mortality rate; it represents 2% of infectious endocarditis cases with a mortality rate of about 50% and many times diagnosed post-mortem.¹² It most frequently affects an adult’s aortic or mitral valves. Standardized treatment requires antifungal therapy associated with surgical treatment.^3^ If there is an uncontrolled fungal infection, an early valvular replacement is recommended (<7 days). Patients that have received only medical treatment have a 100% mortality rate.² 78-year-old male, diabetic, with a previous bioprosthetic aortic valve replacement due to severe aortic stenosis, attended the emergency department with fever for the past two months, initially once a week and recently twice daily, associated with nonspecific unwellness and chills. He brought transthoracic echocardiogram that suggested prosthetic valve endocarditis. The patient was hospitalized. Blood cultures were taken and a transesophageal echocardiogram done, which showed typical vegetation annexed to the prosthetic’s aortic ring (7 × 7 mm) with mobile component projecting out of the left ventricle and restricting the opening of the prosthetic valve in this region, causing an increase of its gradients. A multi-sensitive Candida parapsilosis was shown in the blood cultures and then Anidulafungin 200 mg/day was initiated for an estimated 42-day treatment. The case was discussed with Heart Team because of the high surgical risk and the early need of valve replacement. Initial course of action had us schedule surgery for the seventh day of the antifungal treatment. Patient maintained daily fever and blood cultures were collected every 72h showing increase in Candida parapsilosis in all samples and surgery was sped up. The patient was submitted to a bioprosthetic aortic valve replacement with amplification of the ring due to the presence of abscess evidenced in mid-surgery. The patient was admitted in critical condition to the ICU, hemodynamically unstable in use of ascending vasoactive drugs, in vasoplegic shock with refractory septic component. The patient remained refractory to instituted measures evolving to death 24h after surgery. Fungal endocarditis is a rare but extremely serious condition, and a multidisciplinary approach is extremely important. Discussing a case of reoperation with high surgical risk with all those responsible for the care is the role of Heart Team and was essential to decide the best time for the surgery.

111174

Modality: E-Poster Young Researcher – Case Report

Category: ACUTE AND CHRONIC CORONARY DISEASE/THROMBOLYSIS

## Case Report: An Unexpected Case of Minoca in a 56-Year-Old Male with Myocardial Bridge

CAMILA DALCOMUNI DOS SANTOS^1^, Rafael Vieira Fernandes^1^, Airton Salviano de Sousa Júnior^1^, Igor Henrique Silva Leite^1^, Barbara Porto Valente^1^

(1) Instituto Dante Pazzanese de Cardiologia – IDPC

**Introduction:** A myocardial bridge (MB) is typically asymptomatic, but sometimes can lead to a myocardial infarction with several mechanisms such as: coronary spasm, thrombosis, coronary dissection, or the development of focal atherosclerosis immediately proximal to the MB. Sometimes more than one mechanism can be present. The use of intracoronary images modalities, as Optical Coherence Tomography (OCT) in patient with MB can accurately define the mechanism of the myocardial infarction and provide further guidance to management strategy.

**Case Report:** 56-year-old male with past medical history of systemic arterial hypertension and active tobacco use, attended the emergency department with severe oppressive chest pain after a long car trip in the heat. The initial electrocardiogram showed ST-elevation in anterior leads. Coronary angiography revealed a myocardial bridge with a systolic constriction of more than 90% in the proximal third of the left anterior descending artery (LAD), OCT demonstrate a lesion <20% with no signs of rupture. The patient was diagnosed with MINOCA with multiple mechanisms such as supply and demand imbalance, cigarette-induced vasospasm and hypovolemia. The patient was treated with beta blockers and antiplatelet therapy and discharged 3 days later. There was no recurrence of chest pain at follow-up appointments.

**Conclusion:** Myocardial bridge (MB) is a congenital anatomic anomaly whereby a length of the artery tunnels beneath a section of myocardium. It can potentially be associated with phasic arterial spasm and ischemia. About 67–98% of cases have their anatomical location in the LAD. The gold standard for diagnosis is autopsy, but angiography and OCT provides more information about MBs, such as assessment of vulnerable plaque and coronary morphology. Unlike classic atherosclerotic plaque that produces a fixed stenosis, MB produces a dynamic effect that varies with cardiac cycle, heart rate, and sympathetic tone. According to the classification by Schwarz 2009, this case is classified as type C, with altered intracoronary hemodynamics. The clinical management was maintained with pharmacological therapy, which is the mainstay treatment. Angioplasty would be suggested only for cases with refractory symptoms. The patient was conducted with medications for management of heart failure and general orientation, with no recurrence of chest pain.

111198

Modality: E-Poster Young Researcher – Case Report

Category: ANTICOAGULATION

## Thrombolysis in Metallic Prosthetic Valves: A Series of Cases in a Tertiary University Hospital

NATANAEL MENDES DE ARAUJO^1^, Natanael Mendes de Araújo^1^, Ana Paula Otaviano^1^, André Schmidt^1^

(1) HOSPITAL DAS CLÍNICAS DA FACULDADE DE MEDICINA DE RIBEIRÃO PRETO DA UNIVERSIDADE DE SÃO PAULO

**Background:** Prosthetic valve thrombosis (PVT) is an important and severe complication of valvar replacement. Until today, treatment guidelines are scarce.

**Methods:** A consecutive series admitted to a tertiary university hospital is reported. All of them were presented in a Heart Team meeting and surgical intervention was denied by various reasons. Non-fractioned heparin was started and patients reevaluated five days later with Doppler echocardiography to identify signs of improvement. Patients with sustained leaflet impairment and/or significant transvalvar gradient, an ultra-slow thrombolytic infusion protocol (Alteplase 25 mg in 25hours) followed by non-fractioned heparin was started and after completion a new echocardiographic evaluation was performed. A descriptive analysis is presented.

**Results:** Six patients (4 females), 53 ± 9 years with a diagnosis of PVT admitted in two consecutive years were evaluated. Three (50%) of them had a metallic prosthesis in the mitral position, two (33%) in the aortic and one (17%) in the tricuspid position. All of them were using warfarin but the INR values were infra therapeutic (1.12 to 1.88). Two of the patients with mitral prosthesis had a significant reduction of transvalvar gradient (51% mean reduction) and good leaflet mobility and did not received thrombolytic. In the other 4 patients, thrombolysis was started and after completion of the protocol a mean gradient reduction of 69% was observed. Only the patient with a prosthesis in the tricuspid position had thrombolytic protocol repeated due to incomplete recover of leaflet mobility. No hemorrhagic or embolic complications occurred. All patients were discharged alive.

**Conclusion:** Thrombolytic treatment using an ultra-slow infusion protocol was safe and presented a high effectiveness, suggesting that large studies are needed to define its indications.


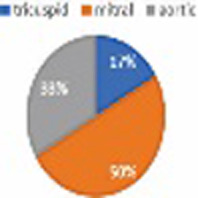


111200

Modality: E-Poster Young Researcher – Case Report

Category: ACUTE AND CHRONIC CORONARY DISEASE/THROMBOLYSIS

## Interventricular Septum Rupture After Acute Myocardial Infarction: Late Diagnosis and Favorable Outcome

LETICIA MACACCHERO MOREIRAO^1^, Leonardo Giglio Gonçalves de Souza^1^, Gisele Messias Mattioli^1^, Alex dos Santos Felix^1^, Matheus Burigo Oliveira^1^

(1) Instituto Nacional de Cardiologia (INC)

**Introduction:** Rupture of the interventricular septum (IVS) is a surgical medical emergency and it is part of mechanical complications of Acute Myocardial Infarction (AMI), which can occur between 2 and 14 days after the event. Like the other ones, it becomes increasingly rare in the “post reperfusion” era. An incidence of 0.2% is estimated in the US, mainly when associated with single-vessel transmural infarction or the first event. Even with surgical treatment, it is still followed by high rates of mortality, reaching 80% in the first 30 days if not corrected.

**Clinical Case:** R.S.M., 62 years old, male, white. Carrier of Diabetes Mellitus and dyslipidemia, he reports a sudden episode in October/2020 of chest pain. Diagnosed with AMI at the time, not reperfused. In the following two weeks, the pacient developed dyspnea on exertion, orthopnea associated with worsening of functional class (NYHA II). On physical examination, he presented a holosystolic murmur in the lower left parasternal border. A transthoracic echocardiogram was performed, with diagnosis of ventricular septal defect measuring 1.8 cm associated with multiple communicating orifices, LV > RV shunt showing a gradient of 85 mmHg. Coronary angiography showed severe subtotal lesion in the right coronary artery. The patient was hospitalized after a year and a half of the acute event for evaluation of surgical treatment, maintaining the symptoms described. After discussion with Heart Team, we opted to correct communication with bovine patch.

**Conclusion:** Rupture of the IVS is a rare mechanical complication of AMI, with high mortality rates if not corrected early. The illustrated case presents a patient with a late diagnosis of this complication, a large area of communication and a favorable outcome even without initial surgical correction, which is rare according to the literature. BIBLIOGRAPHY: ABDULA, A.D. et al. Mechanical Complications of Acute Myocardial Infarction: A Scientific Statement From the American Heart Association. American Heart Association. Jul/21. THIELE H., ABBOTT J.D. Acute myocardial infarction: Mechanical complications. UpToDate, 2022.


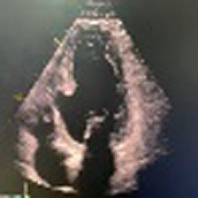


111205

Modality: E-Poster Young Researcher – Case Report

Category: CONGENITAL AND PEDIATRIC CARDIOLOGY

## Aortic Dissection in Childhood: A Case Report and Literature Review

LEONARDO PAIVA OHASHI^1^, Luiz Augusto de Andrade Costa^1^, José Cícero Stocco Guilhen^1^, José Honório de Almeida Palma da Fonseca^1^, Célia Maria Camelo Silva^1^

(1) Escola Paulista de Medicina/Universidade Federal de São Paulo

Aortic dissection is a serious disease with a high mortality, characterized by the delamination of the media and intima layers of the vascular wall, promoting a rupture in the endothelium of this vessel. Despite of being studied in the adult population, it is rare in the pediatric population and there are few findings in the literature, which makes the management of these cases quite challenging. In this case report, a patient 9 years and 9 months old, male, 28 kg, student, was admitted on 24 of april 2021 to the emergency department of a tertiary hospital with a complaint of abdominal pain within the last 3 hours. He was accompanied by his mother, who revealed that the patient woke up with severe abdominal pain in the upper abdomen region (9 on a scale of 10). The pain radiated to the back and substernal region, and was accompanied by nausea and three episodes of vomiting. He denied similar previous events and any change in bowel habits, as well as fever or urinary symptoms. At birth, he was diagnosed with polycystic kidney disease and evolved with the need for a kidney transplant performed at the of four. There was no family history of aortic disease or a family history of sudden death. On admission physical examination, the physical examination was innocent, except for a absent peripheal pulse on the left upper limb. The admission tests showed mild leukocytosis with no deviations, with other laboratory tests within the normal range. Acute abdome routine X-rays were performed that only showed the presence of mediastinal widening. Aortic CT was then performed (figure 01), with the presence of a dissection flap that extended from the aortic arch to the bifurcation of the common iliac arteries. After a hypertensive peak, the patient evolved for a cardiorespiratory arrest anda despite all efforts during the CPR the death was confirmed at 00:00. This pathology can occur at this age group in 22% of cases, but, classically is relates to congenital anomalies, mainly those of the connective tissue. This way, early diagnosis and treatment are essential for better clinical outcomes.


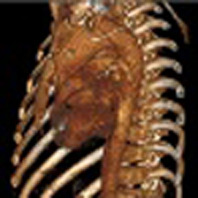


111213

Modality: E-Poster Young Researcher – Case Report

Category: CARDIOVASCULAR SURGERY

## Konno-Rastan Procedure for Correction of Aortic Prosthesis Mismatch and Left Ventricular Outflow Tract Stenosis

LUÍS HENRIQUE COELHO PINTO^1^, Gabriela Zamunaro Lopes Ruiz^1^, Claudio Leo Gelape^1^, Luiz Guilherme Passaglia^1^, Maria Letícia Leão Lana^1^

(1) Hospital das Clínicas da Universidade Federal de Minas Gerais – HC-UFMG

**Introduction:** Konno-Rastan procedure is an anterior aortoventriculoplasty originally described for the correction of congenital aortic stenosis, whether subvalvular, valvular or supravalvular. We report a case of an adult patient evolving with a major mismatch of a mechanical aortic prosthesis after 5 invasive surgical procedures due to congenital stenosis of the aortic valve and left ventricular outflow tract (LVOT).

**Case Description:** Female patient, 37 years old, with congenital aortic valve disease associated with LVOT stenosis, who underwent 5 other surgical procedures – aortic commissurotomy in 1996 and 1999, valve replacement for a biological prosthesis in 2009, valve replacement for a mechanical prosthesis in 2013 (No. 19 – Standard), exploration of the valve with 3 linear incisions in the fibrosis of the interventricular septum (subvalvular) – evolving with NYHA functional class III dyspnea, chest pain, palpitations and presyncope on exertion. Transthoracic echocardiogram (TTE) showed a 40 mm left atrium, 25 mm aorta, left ventricle with mild hypertrophy, normal cavity and preserved ejection fraction. Mechanical aortic prosthesis with thickened discs, preserved opening and mobility. Maximum gradient of 87 and mean of 52 mmHg, valve area of 0.9 cm^2^. The patient underwent cardiac surgery in January 2022, using the Konno-Rastan technique, in which a section of the valve annulus, enlargement of the annulus and LVOT with section of the interventricular septum and septal myectomy were performed, followed by reconstruction of the annulus and septum with bovine pericardium patch and St. Jude model Regent No. 21 mechanical aortic prosthesis implantation. There were 134 minutes of cardiopulmonary bypass and 116 minutes of aortic clamping. The patient evolved in the postoperative period with severe vasoplegia and complete atrioventricular block. Bichamber pacemaker implantation was performed 8 days after surgery. Postoperative TTE showed a reduction in the mean transprosthetic gradient to 10 mmHg. She was discharged from hospital 16 days after surgery. The patient remains under follow-up, asymptomatic, maintaining echocardiographic parameters within normal limits.

**Conclusion:** The Konno-Rastan procedure is a complex tecnique that proved to be an adequate option for the correction of the LVOT stenosis and mismatch presented by this patient, since it involved valvular, subvalvular and supravalvular stenosis not corrected in previous approaches.

111221

Modality: E-Poster Young Researcher – Case Report

Category: ACUTE AND CHRONIC CORONARY DISEASE/THROMBOLYSIS

## Spontaneous Coronary and Carotid Arteries Dissection Associated with Cerebral Aneurysm in a Young Woman

ISABELLE MENDES RODRIGUES SALOMÃO^1^, Alessandra Arnez Pacheco^1^, Daniel Xavier de Brito Setta^1^, Julia Paulo Mourilhe Rocha^1^, Ricardo Mourilhe-Rocha^1^

(1) Hospital Pró-Cardíaco

**Introduction:** Spontaneous coronary artery dissection (SCAD) is an uncommon cause of myocardial infarction (AMI), reaching approximately 1% of cases and may be associated and may be rarely associated with carotid dissection. In most cases, it is underdiagnosed and may evolve to worse prognosis and even sudden death.

**Case report:** Female, 36 years old, white, with a history of arterial hypertension, gestational diabetes and overweight. She reported stabbing chest pain, 10+/10, for 40 minutes, at rest, without irradiation, associated with sudden dyspnea, nausea and sweating. Normal electrocardiogram (ECG) and positive troponin I (TnI). Reported improvement in her symptoms after aspirin and clopidogrel and was transferred to another hospital asymptomatic. A new ECG was performed, which showed T wave inversion in the anterior and inferior walls and TnI us = 3,950 ng/mL. Echocardiogram (ECO) with severe left ventricular (LV) systolic dysfunction, hypokinesia of the basal segments and akinesia of the middle and apical portions of the LV. Coronary angiography (CAT) was performed, which showed spontaneous anterior descending dissection (ADA), 1st. diagonal, septal and 1st. marginal with TIMI III flow. One day after the CAT, she evolved into a new chest pain with hemodynamic stability and ECG with a new ST elevation in the inferolateral wall, being maintained with ASA, clopidogrel, statin and associated with bisoprolol. Cardiac resonance was performed, which showed recent transmural AMI affecting the anteroseptal wall of the LV (7% infarcted mass) and subendocardial AMI in the inferolateral wall (3% infarcted mass). Coronary CT angiography with absence of atherosclerosis with images of dissections in the middle third of the ADA and moderate dissections in the distal third of the right coronary artery. Angiotomography of the neck and skull showed saccular aneurysmal dilatation in the ophthalmic segment of the right internal carotid artery (ICA) measuring 6 mm and dissection in the distal cervical segment of the left ICA. After clinical control, she was discharged from the hospital in NYHA functional class 1 with improvement in LV function by ECO.

**Conclusion:** SCAD is more prevalent in young women with few risk factors for coronary artery disease and may be related to fibromuscular dysplasia, pregnancy and the puerperium. The association with vascular alterations in other sites must be resherched in those patients. Conservative treatment had been chosen in most cases.

111223

Modality: E-Poster Young Researcher – Case Report

Category: NURSING

## Valve-in-Valve Tavi: First Case of Balloon-Expandable Prosthesis in Self-Expanding Prosthesis Performed at a University Hospital in Southern Brazil

EMILY JUSTINIANO^1^, Rejane Reich^1^, Juliana Kruger^1^, Dulce Daise Guimaraes^1^, Paola Severo Romero^1^

(1) Hospital de Clínicas de Porto Alegre

**Introduction:** Aortic stenosis is the most prevalent acquired valvular heart disease in the Brazilian population and has high morbidity and mortality. Transcatheter aortic valve implantation (TAVI) is an established procedure for the treatment of symptomatic stenosis for patients considered inoperable, at high risk and intermediate surgical risk.

**Objective:** To report the first case of balloon-expandable prosthesis implantation in a degenerated self-expanding prosthesis performed in a public university hospital in southern Brazil.

**Case description:** An 86-year-old female patient with a history of TAVI CoreValve prosthesis in 2012 was hospitalized with an echocardiogram showing aortic prosthesis with severe stenosis and moderate insufficiency, and a new TAVI (valve in valve) was chosen. The catheterization laboratory team previously performed combinations with the operating room team due to the possibility of conversion to a surgical approach. In addition to alignments with regard to materials, a team of professionals developed strategies to deal with intercurrences. The procedure was performed in September 2021, under general anesthesia, with transesophageal echocardiography follow-up. A balloon-expanded prosthesis, Sapien 3 23 mm, was implanted inside a previous prosthesis, by puncture in the right femoral artery (7 and 14 French introducer) and left femoral artery (7 French introducer). Passed 6 French introducer in the right femoral vein for transvenous pacemaker and in the right radial artery for catheterization of the right coronary artery. Hemostasis was performed with a suture closure device (Perclose) in the right femoral artery and by compression in the other access sites. Patient transferred to intensive care unit extubated and without vasopressor. The procedure time was 215 minutes and the room time was 337 minutes. Four medical specialties participated in the procedure, a nurse, two nursing technicians, a radiology technician, a prosthetic representative, in addition to a perfusionist and an instrumentation specialist in cardiac surgery present at the unit. The patient evolved satisfactorily and was discharged from the hospital on the tenth day after the procedure.

**Conclusions:** The nurses of the catheterization laboratory, together with a team of professionals, actively participated in the organization of material resources and the logistics outlined for the care of a possible surgical approach. The procedure, although complex, was uneventful.

111234

Modality: E-Poster Young Researcher – Case Report

Category: PERICARDIUM/ENDOCARDIUM/VALVOPATHIES

## Quadricuspid Aortic Valve in a Patient with Mucopolysaccharidosis Type 1: A Case Report

DANIEL FIORAVANTI GIMENEZ^1^, Elaine dos Reis Coutinho^1^, Eduardo Hadad Cherulli^1^, Marina Romano Capellini^1^, Aloísio Marchi Rocha^1^

(1) Hospital da PUC- Campinas

**Introduction:** The Mucopolysaccharidosis is a group of rare autosomal recessive metabolic disorders which affects multiple systems, including cardiovascular. It’s characterized by deficiency of lysosomal enzymes (alpha-L-iduronidase) that leads to accumulation of glycosaminoglycans in tissues and in consequence, tissue dysfunction. Mucopolysaccharidosis type 1 (MPS1) has an estimated prevalence of 1:100.000 births. This case reports a patient with MPS1, Scheie phenotype, with severe aortic stenosis.

**Case Report:** A 47-year-old woman with MPS1 diagnosis since birth and with preserved ejection fraction heart failure (echocardiogram 03/21: left ventricular ejection fraction (LVEF): 51%) was admitted at emergency room with low cardiac output accompanied by pulmonary congestion due to COVID and Bacterial Pneumonia. The echocardiogram revealed: LVEF:25%, globally reduced, absence of segmental disorders, left chambers enlargement with Left Ventricular hypertrophy, malformed aortic valve (quadricuspid aspect) with mild thickening and calcification, critical stenosis and subtle insufficiency. Also, there was an severe mitral insufficiency with ring dilatation, apical systolic closure and posterior leaflet prolapse. The cardiac surgery team was opposed to an aortic valve replacement procedure due to high surgery risk. TAVI was not possible due to an unfavorable anatomy for the procedure and iodine allergy. Regarding her decreased mobility and NYHA functional class I, she was discharged and keeps her follow up in the ambulatory with optimized clinical therapy. In less severe MPS phenotypes, there may be valvular disorders independent of existing risk factors as congenital or rheumatic atherosclerosis. Therefore, the cardiological follow up is important for those patients, as pointed out in the case, in which an adult patient presents severe aortic stenosis with ventricular function loss secondary to MPS 1.

**Conclusion:** The cardiovascular disease emerges subtly in attenuated phenotypic presentations of MPS, contributing to the premature mortality of these patients. Besides being rare, MPS 1 may present late cardiovascular implications, even in broader cases, and severe consequences that compromise the patient’s lifestyle. The case is even more uncommon not only for its underlying disease, MPS 1, but for the aortic valve’s anatomic modification (quadricuspid), not often reported in literature in this syndrome.

111233

Modality: E-Poster Young Researcher – Case Report

Category: CARDIOVASCULAR INTENSIVE CARE/CARDIOVASCULAR EMERGENCIES

## The Role of Non-Invasive Intracranial Pressure Monitoring in Hemodynamic Management of Critical Care Patients

AMANDA AYAKO MINEMURA ORDINOLA^1^, Salomon Soriano Ordinola Rojas^1^, Caroline Spagnol^1^, Viviane Cordeiro Veiga^1^

(1) Beneficência Portuguesa de São Paulo

Intracranial pressure (ICP) assessment is a method that can guide the hemodynamic management of patients undergoing procedures and conditions associated with neurological disturbances. In this paper, two cases were reported. The first case is a 40-year-old male with Marfan’s syndrome, admitted with aortic endocarditis which underwent valved conduit exchange with extracorporeal circulation (ECC) 182 minutes, the patient presented one episode of tonic-clonic seizure. Non-invasive ICP (NIICP) showed altered brain compliance (figure 1). The management included increase in sedation and prevention of secondary cerebral ischemia. New NIICP showed well response to the neuroprotection management (figure 2). The second is a 64-year-old female diabetic patient admitted for cytoreductive surgery due to colon adenocarcinoma. Edema and facial plethora were presented on admission, associated with high central venous pressure and high systolic volume variation. The hypothesis of superior vena cava syndrome was raised and confirmed by evidence of thrombosis related to catheter. The NIICP showed altered brain compliance (figure 3), with improvement after cerebral protection measures. In all cases reported, NIICP identified morphological changes in the ICP waveform and throughout patient management in the intensive care unit. Noninvasive method assisted in clinical decision-making regarding the optimization of protocols adapted for neuroprotection.


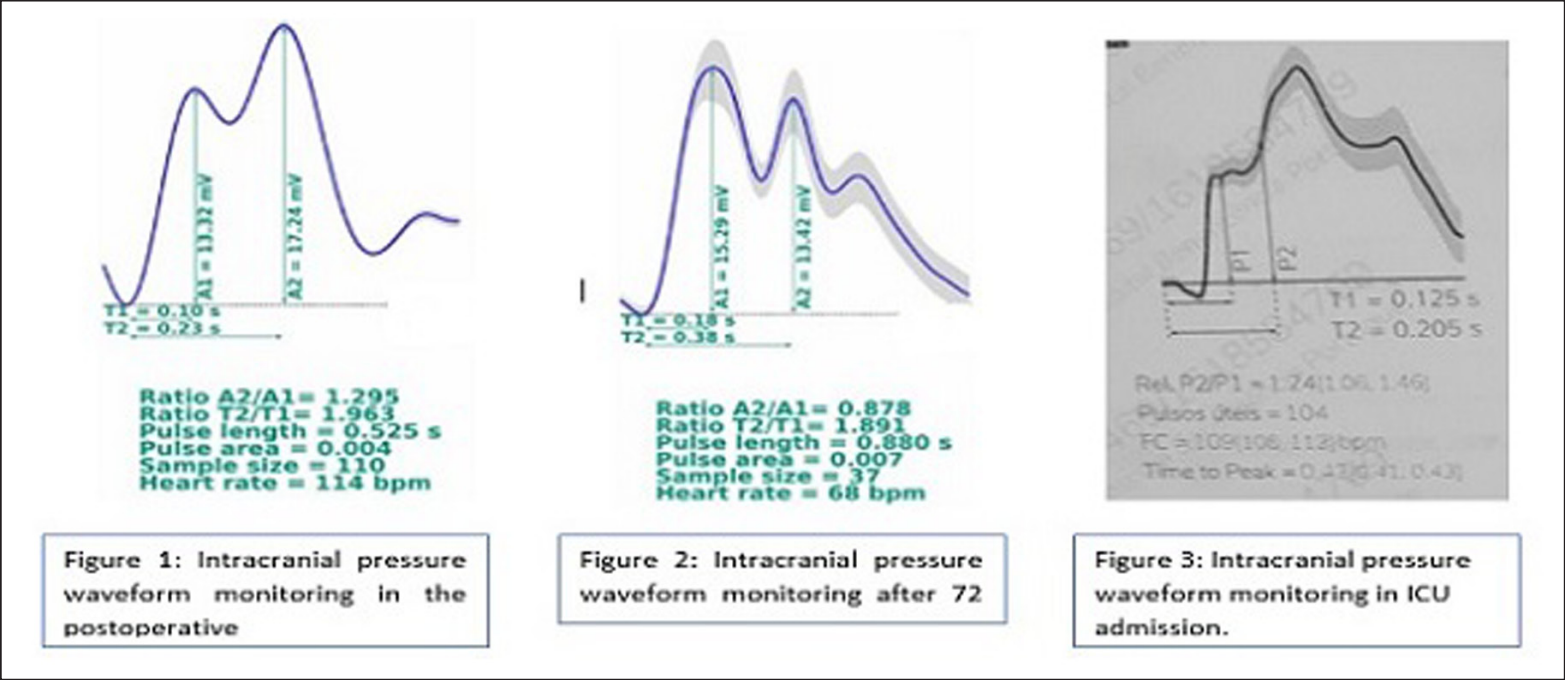


111236

Modality: E-Poster Young Researcher – Case Report

Category: COVID-19 AND CARDIOVASCULAR SYSTEM

## Aortic-Right Atrium Fistula and Pulmonary Tromboembolism After COVID-19 Infection: Case-Report

YURI CAVALCANTI ALBUQUERQUE TENORIO^1^, Priscila Alves da Silva^2^, Stephanny Isabelly Pessôa Neri de Araujo^2^, Igor Vieira Lima Alexandre^2^, Francisco de Assis Costa^2^

(1) Hospital Veredas; (2) Centro Universitário Tiradentes

**Introduction:** Several cardiac complications have been described after the beginning of pandemic Covid-19, such as myocardial ischemia, acute cardiac heart failure, arrhythmia and myocarditis.

**Case description:** M.J.F.S., female, 43 years old, denied any chronic disease or use of medication, admitted to the ward of a high complexity hospital presenting cardiac heart failure (NYHA IV) and oedema in the lower limbs. The patient had Covid-19 infection two months before and presented a normal routine transthoracic echocardiography (TT-ECHO). After the admission, a new TT-ECO presented global dilatation of cardiac chambers, specially left atrium (LA) and signs of coronary sinus of valsalva rupture and Aortic-Right Atrium (Ao-RA) fistula. These findings were confirmed in a transesophageal echocardiography, that presented left ventricle ejection fraction of 69%, rupture of sinus of valsalva aneurysm at aortic root promoting strong shunt from aorta to right atrium and cardiac chambers dilatation. Thoracic angiotomography described bigger dimensions of the right atrium and signs of pulmonary thromboembolism. Therapeutic decision included surgical correction of Ao-RA fistula, which occurred with complication of complete heart block requiring transitory pacemaker and cardiac arrest successfully reverted to systemic circulation. Definitive cardiac pacemaker was implanted and the patient was discharged in a stable clinical situation after 59 days of hospital stay.

**Conclusion:** The clinical report presents rupture of sinus of valsalva aneurysm leading to Ao-RA fistula and pulmonary thromboembolism two months after Covid-19 infection. As a consequence, the authors suggest linkage between Covid-19 and thrombogenic state and inflammatory process leading to endomyocardial inflammation.

111245

Modality: E-Poster Young Researcher – Case Report

Category: PERICARDIUM/ENDOCARDIUM/VALVOPATHIES

## Thrombolysis in Biological Mitral Valve on a Patient with High Surgical Risk

ARIANE ZONHO WOGEL^1^, Andrei Carvalho Sposito^1^, Marcelo Sousa^1^, Renata Muller Couto^1^, Silvério Fernandes^1^

(1) Universidade Estadual de Campinas (UNICAMP)

**Introduction:** Prosthetic valve thrombosis is a rare and highly lethal complication, more frequently found in mechanical valves and in the mitral position. The treatment involves systemic anticoagulation and valve replacement surgery. Thrombolysis appears as a second option, due to its complication rates and low efficacy.

**Case Report:** A 65-year-old man who underwent mitral bioprosthesis implantation for severe secondary mitral regurgitation returns after 3 months with dyspnea and, on transthoracic echocardiography (TTE), prosthesis dysfunction due to thrombosis (Image 1: A-C). There was also a global dilation and left ventricular systolic dysfunction (ejection fraction of 17%). Due to a prohibitive surgical risk (EUROSCORE 34%), alteplase 25 mg was administered for 6 hours followed by full anticoagulation with intravenous unfractionated heparin. TTE 24 hours post thrombolysis showed thrombus reduction, improvement in valve motion, and a drop in the transvalvular gradient (Image 1: D–E). The same dose of alteplase was repeated after 48 hours, resulting in improvement in dyspnea, and in valve opening and mobility on TTE (Image 1:F–G). There was no bleeding or adverse effect and he was discharged with Warfarin.

**Conclusion:** Fibrinolytic therapy for prosthetic biological valve thrombosis remains controversial due to the lack of evidence of its benefit and safety. Hence, its use has been sporadic and restricted to critically ill patients who are at high surgical risk. The case presented here stands out for the efficacy and safety of fibrinolysis in severe thrombosis in a mitral bioprosthesis. This report deserves consideration as a therapeutic possibility either in clinical research or in similarly unfavorable clinical conditions.


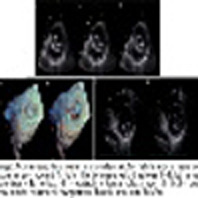


111265

Modality: E-Poster Young Researcher – Case Report

Category: CARDIOLOGY OF SPORTS, EXERCISE, ERGOMETRY AND CARDIOVASCULAR REHABILITATION

## Mechanical Ventricular Assistance Devices: A Case Report

PALOMA DE BORBA SCHNEIDERS^1^, Heloíse Benvenutti^1^, Ane Glauce Freitas Margarites^1^, Mauren Porto Haeffner^1^, Fernanda Cecília dos Santos de Vasconcellos^1^

(1) Hospital de Clínicas de Porto Alegre

**Introduction:** Heart Failure is the final pathway for heart diseases. Considering limiting situations such as lack of donors and contraindications to the Heart Transplant (HTx) new strategies are needed. Mechanical Ventricular Assistance Devices allow patients to be able to perform physical exercises in Cardiac Rehabilitation.

**Description of Cases:** Case 1: FPB, male, 54 years old, ischemic etiology. Ejection Fraction 32%, HTx contraindicated due to immunological hypersensitivity. After HeartMate II, had reduced respiratory muscle strength – RMS (PiMax: 18/PeMax: 37) and cardiorespiratory fitness – CRF (distance in six meters walking test: 261 m). Preserved peripheral muscle strength – PMS (28 KgF) and no self-care limitations (SCL). Case 2: JSP, female, 50 years old, chagasic etiology. Ejection Fraction 16%, HTx contraindicated due to immunological hypersensitivity. After HeartMate II, had reduced RMS (PiMax: 34/PeMax: 46) and CRF (383 m). Preserved PMS (18 KgF). After hospital discharge showed improvement in CRF (429 m). Case 3: MHPD, female, 61 years old, Dilated Cardiomyopathy etiology, worsened after chemotherapy. Ejection Fraction 13%, HTx contraindicated due to pulmonary hypertension. After HeartMate III, had reductions in RMS (PiMax: 50/PeMax: 56), PMS (17 KgF), CRF (273 m) and SCL (Katz: 2). Case 4: LBF, female, 63 years old, Dilated Cardiomyopathy etiology. Ejection Fraction 16%, HTx contraindicated due to pulmonary hypertension. After HeartMate II, had SCL (Katz: 2) and present reductions in RMS (PiMax:75/PeMax:109), PMS (28 KgF) and CRF (390 m). Case 5: AAAV, male, 61 years old. Dilated and non-ischemic etiology. Ejection Fraction 20%. After HeartMate II, had some complications (driveline infection, massive pleural effusion, multifactorial anemia) and prolonged stay (154 days). At hospital discharge, he could walk more than 30 m, but failed to perform the walking test; SCL (Katz: 3); adequate PMS (25 kgF).

**Conclusion:** Hospital Cardiac Rehabilitation was performed with aerobic, respiratory/peripheral strength and balance training. All patients had reductions in RMS and PMS. Two of them had functional SCL and two of them had low exercise tolerance (<300 m). These patients‘ CRF may be reduced, due to low adaptive capacity to increase cardiac output during physical exercise. Potentially reversible extracardiac factors such as muscle atrophy and deconditioning seem to improve patients‘ functionality.

111274

Modality: E-Poster Young Researcher – Case Report

Category: ACUTE AND CHRONIC CORONARY DISEASE/THROMBOLYSIS

## Coronary Ectasia as a Cause of Acute Myocardial Infarction

ISMAR JUNIOR PEINADO LIJERON^1^, Larissa Kaline Santana Diniz^1^, Marina Albanez Albuquerque de Medeiros^1^, Soraia Rachid Youssef de Campos^1^, Valeria Mozetic de Barros^1^

(1) Instituto Dante Pazzanese de Cardiologia IDPC

**Introduction:** Aneurysmal dilatation of the coronary arteries is found in up to 5% of the patients undergoing coronary angiography, with higher incidence in men and proximal segments of the coronary artery. The right coronary artery (RCA) is more affected (40%), followed by the left Anterior Descending (AD) (32%). Atherosclerosis, vasculitis, genetic susceptibility and post infection are reported as possible etiologies. The presence of coronary aneurysm or ectasia has been associated with a worse long-term prognosis.

**Case Description:** Female, 48 years old, with no previous diseases, presented with an unprecedented typical chest pain after performing moderate physical exertion. On the electrocardiogram it was observed sinus rhythm with electrically inactive area in the inferior wall and positive troponin. Performed coronary angiography that showed important ectasia of RCA, AD with mild ectasia in the proximal third and other arteries with discrete parietal irregularities. Echocardiogram shows akinesia of the inferolateral wall and of the middle and basal segments of the inferior wall and ejection fraction 58%. The angiotomography of coronary arteries confirmed ectasia of RCA, measuring in its largest diameters 10.8 × 10.4 mm and thrombosis in the distal third. Started anticoagulation with warfarin and clopidogrel together with antianginal drugs and after treatment she got angina improvement and had no new thromboembolic events.

**Conclusion:** Aneurysmal dilatation of the coronary vessels is an unusual. Antiplatelets and anticoagulation are the most addressed therapy, but there are possibilities of performing percutaneous coronary intervention or surgical resection. The treatment is still controversial, requiring more randomized studies to define the best and safest treatment strategy.


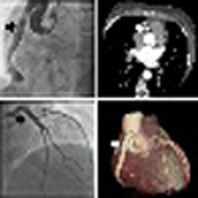


111515

Modality: E-Poster Young Researcher – Case Report

Category: HEART FAILURE/CARDIOMYOPATHY/TRANSPLANT

## The Importance of Genetic Testing and Early Diagnosis of Cardiac Amyloidosis: A Case Report

ANTONIO FELICIANO FATORELLI^1^, Vitor Agueda Salles^1^, Elisangela Cordeiro Reis^1^, Letícia Macacchero Moreirão^1^, Jacqueline Sampaio dos Santos Miranda^1^

(1) Instituto Nacional de Cardiologia

**Introduction:** Cardiac amyloidosis (CA) is a systemic disease caused by tissue deposition of fibrillar and insoluble protein aggregates in different organs, including the heart, leading to organic dysfunction. Different subtypes of amyloidosis can give rise to overlapping clinical manifestations. The importance of carrying out the genetic test is due to the need to correctly characterize the precursor protein for the institution of specific treatment. Thus, the present report aims to describe the case of two patients diagnosed with transthyretin-linked (TTR) AC and discuss the importance of genetic testing, as well as early diagnosis for a better prognosis.

**Case Report:** These are two elderly patients, 71 and 83 years old, with a clinical picture of advanced heart failure, who had indicators in the anamnesis, physical examination and complementary exams of CA and whose etiology was confirmed during the workup. Both had immunoglobulin heavy and light chain form (AL) ruled out with investigation for monoclonal light chains by means of serum and urinary immunofixation, in addition to measurement of the serum kappa/lambda ratio. On pyrophosphate scintigraphy, grade III uptake was identified, one of them with extensive involvement of the right ventricle, corroborating an advanced stage of the disease. Despite the high prevalence of wild type ATTR CA in the elderly population, both were tested for mutation of the ATTR V122l gene. This mutation is well established as a pathogenic variant, with heterozygosity being the most common. It presents after the age of 60 with predominantly cardiological involvement, with a higher frequency in Afro-descendants, which possibly makes it frequent in our country. Specific therapy was instituted with selective stabilizers of TTR tetramers with good tolerance and the patients are in follow up.

**Conclusion:** Thus, CA could be considered an underdiagnosed condition, rather than a rare disease. The patient’s journey to diagnosis is long; it is estimated that there is a delay of more than 2 years from symptom onset to diagnosis, with the involvement of an average of five different professionals. Thus, it is essential to disseminate knowledge about CA; clinicians and cardiologists must give greater consideration to this entity, aiming at earlier diagnosis and adequate therapeutic guidance, thus improving patient prognosis and survival.

111931

Modality: E-Poster Young Researcher – Case Report

Category: CARDIOVASCULAR SURGERY

## Video-Assisted Pericardial Window as an Alternative in Patient with Thoracic Deformity: A Case Report

LEONARDO PAIVA OHASHI ^1^, Diego Felipe Gaia dos Santos^1^, Paulo Matheus Sanches de Souza^1^, Aislan Henrique Bezerra Pinheiro^1^, Pedro Reges Pereira Meira^1^

(1) Escola Paulista de Medicina/Universidade Federal de São Paulo

Pericardial effusion is a common finding in clinical practice with a spectrum manifestations ranging from asymptomatic mild effusions to cardiac tamponade. As a minimally invasive form of treatment, video-assisted pericardial window is an alternative intervention. A patient, 73 years old, male, referred to a tertiary hospital for investigation of Asthenia, dyspnea, chronic low back pain, loss of 10 kg in 3 months and solid expansive lesion in the pre-sacral region. The patient had a history of hypertension, Kyphoscoliosis, Pectus Carinatum, Alcoholism for 15 years and previous treatment of gastric ulcer. Laboratory and imaging findings at admission and during hospitalization culminated in a diagnosis of Multiple Myeloma. During the institution of chemotherapy, an echocardiographic follow-up was performed, which showed growth of the pericardial effusion lamina, which progressed from asymptomatic to the presence of signs of ventricular restriction and symptoms of cardiac tamponade. As a result, it was decided to perform a pericardial window before hospital discharge. However, the presence of previously described thoracolumbar deformities, added to the massive abdominal hernia caused by diastasis of the rectus abdominis, precluded the classic subxiphoid surgical approach or through lateral thoracotomy. It was then decided that the only therapeutic possibility would be the use of videothoracoscopy (figure 1). In perioperative care, multiparametric monitoring, invasive blood pressure acquisition through the right radial artery puncture, central venous access in the right Femoral Vein, and Orotracheal Intubation were performed. Intraoperatively, 400 ml of serohematic fluid was released, with a drain in the chest on the right. After the procedure, the patient improved with dyspnea, maintaining chest drainage for 5 days. This is a patient diagnosed with Multiple Myeloma who evolved during hospitalization with significant pericardial effusion and signs and symptoms of cardiac tamponade. Due to the chest deformity that made conventional drainage unfeasible, a video-assisted intervention was chosen.


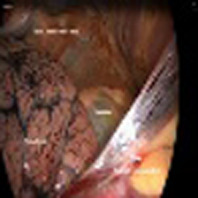


111330

Modality: E-Poster Young Researcher – Case Report

Category: CARDIOVASCULAR INTENSIVE CARE/CARDIOVASCULAR EMERGENCIES

## Extracorporeal Membrane Oxygenation as a Bridge to Transplantation: A Successful Case Report

RAIANE FONSECA SILVA HERDY^1^, Douglas Fernandes de Souza^1^, Nathalia Targa Silva^1^, Alina de Souza dos Santos^1^, Marcia Barbosa de Freitas^1^

(1) Instituto Nacional de Cardiologia

**Introduction:** Mechanical circulatory support has been increasingly used in refractory cardiogenic shock associated with conventional treatment in patients with severe heart failure (HF). The literature indicates use as a bridge to recovery for transplantation (Tx) for decision or target therapy. We report the case of using venous-arterial extracorporeal membrane oxygenation (ECMO) in a young patient with cardiac Tx indicated already in use intra-aortic balloon (IAB) and maintenance of low cardiac output that recovered his clinical condition and subjected to Tx.

**Case Description:** A 38-year-old male patient with dilated cardiomyopathy for 2 years developed severe heart failure in NYHA III–IV despite optimized medical therapy. He was indicated cardiac Tx due to disease progression. The patient was hospitalized several times due to HF decompensation in a cold and wet hemodynamic profile, requiring high-dose inotropic support. In the current hospitalization, even with inotropic, his case deteriorated, with several parameters of poor tissue perfusion (lactate increase, renal function worsening, increased transaminases and bilirubins). Already with association of 2 inotropic (Dobutamine and Milinone), as well as vasopressor (norepinephrine and vasopressin), was indicated a device of short permanence circulatory support, IAB. The next day there was maintenance of critical state, which led to an indication of ECMO. There was a significant improvement of oliguria, significant decrease in lactate and plasma creatinine. Two days later, a compatible organ emerged, the patient underwent Tx, extubated six days after the procedure and recovers well from necrotizing pneumonia acquired during hospitalization.

**Conclusions:** ECMO is indicated as a bridge to another device or to Tx in severe heart failure when the patient has refractory cardiogenic shock. Therefore, patients with non-recoverable cardiac function should not be selected. The success of the therapy involves a multidisciplinary team trained with experience in this technology. In the case presented, the use of ECMO associated with systematized care was fundamental to keep the patient viable until the emergence of a compatible organ. Thus, heart transplantation was possible and the patient recovered from the baseline clinical condition, illustrating a good example of the successful use of ECMO as a bridge to cardiac Tx.

111332

Modality: E-Poster Young Researcher – Case Report

Category: PERICARDIUM/ENDOCARDIUM/VALVOPATHIES

## Infective Endocarditis of the Right Heart: Second Case Report in the Literature of Tricuspid Involvement by Staphylococcus Caprae

FELIPE KESSLER PEREIRA^1^, Luanna Damasceno Amaral de Sousa^1^, Julia Paulo Mourilhe Rocha^1^, Daniel Xavier de Brito Setta^1^, Ricardo Mourilhe-Rocha^1^

(1) Hospital Pró Cardíaco

**Introduction:** Infective endocarditis of the right side (IELD) of the heart is an entity where there is microbial involvement of the tricuspid or pulmonary valves and is responsible for 10% of all cases of infective endocarditis (IE).

**Case Report:** M.B.B, 59 years old, male, with arterial hypertension, dyslipidemia, type I diabetes mellitus, hypothyroidism, coronary artery disease, was admitted to the hospital with atypical chest pain associated with chills, fever, and vomiting. Acute coronary syndrome was ruled out and the hypothesis of myopericarditis was raised, and pharmacological treatment with colchicine was initiated. Despite the established treatment, the patient continued to experience chills and vomiting. Blood cultures were collected and antimicrobial therapy with vancomycin was administered empirically. There was growth of vancomycin-sensitive Staphylococcus caprae, but in the investigation of IE with transesophageal echocardiography, there was no evidence of vegetations. A scintigraphy with labeled leukocytes was then performed to screen for an infectious focus and the tricuspid valve was the only point of evidence of an inflammatory process, confirming the diagnosis of IE, subsequently being treated with antibiotic therapy for 6 weeks.

**Discussion:** Staphylococcus caprae is part of the group of coagulase negative staphylococci (CoNS) and was isolated for the first time in goat milk. Later, it was described as a component of the microbiota of healthy human skin, nails, and nasal mucosa, living symbiotically with its host. S. caprae is a rare cause of infective endocarditis, even more so of native valve. In the literature review, we found a single case report, published in 1995 by Vandenesch et al, of infective endocarditis caused by S. caprae. The case was reported in situ mitral valve involvement, which was treated with intravenous antibiotics for 2 weeks and early surgical removal of the vegetation, preserving the valve, with a good clinical outcome. The main predisposing factors for S. caprae infection are recent use of antibiotics, malignancies, diabetes mellitus, chronic renal failure, open fractures, immunodeficiency, and immunosuppression associated with systemic or local corticosteroids, chemotherapy, radiotherapy. Seng et al. observed that 21 of the 25 patients with S. caprae infection were male, supporting the sex preference hypothesis previously made by Shuttleworth et al.

111348

Modality: E-Poster Young Researcher – Case Report

Category: ACUTE AND CHRONIC CORONARY DISEASE/THROMBOLYSIS

## Coronary Reperfusion Dilemas from Admission to Outpacient Follow-Up

THALES CARDOSO WHATELY^1^, João Gabriel Monteiro Junqueira^1^, Ana Salomé Eurico^1^, José Ary Boechat e Salles^1^, Esmeralci Ferreira^1^

(1) Hospital Universitário Pedro Ernesto; (2) Universidade do Estado do Rio de Janeiro

**Introduction:** The main etiology of acute ST-segment elevation myocardial infarction (STEMI) is the rupture of atherosclerotic plaque with thrombus formation. However, other less common etiologies, such as vasospasm, COVID 19 and coronary embolism, should be considered and investigated when there are risk factors, being important to define the best treatment for each patient.

**Case Report:** Female, 56 years old, former smoker, with systemic arterial hypertension, ischemic stroke (CVA) for one month with motor sequelae (right hemiparesis) and aphasia. Sought emergency for typical anginal chest pain. An electrocardiogram was performed, which showed atrial fibrillation rhythm and ST-segment elevation from V1 to V6, DI and AVL (extensive anterior wall STEMI). She was not submitted to fibrinolytic therapy due to absolute contraindication for stroke in less than 3 months. She was referred to the hemodynamics department, and occlusion of the anterior descending artery was evidenced with a negative image, suggestive of a thrombus, without severe obstructive lesion. As reperfusion therapy, mechanical thrombus aspiration was performed followed by implantation of a drug-eluting stent, reestablishing the coronary flow. Transesophageal echocardiogram showed severe left ventricular dysfunction with anterior hypokinesia and presence of thrombus in the auricle. Cranial resonance was performed with ischemic images suggestive of thromboembolism. In view of the results of the complementary exams, the most likely hypotheses for acute coronary syndrome are atherosclerosis associated with coronary embolism caused by thrombus in the auricle related to unkown atrial fibrillation. Dual antiplatelet therapy (clopidogrel and ASA) and full anticoagulation were initially started, with outpatient and echocardiographic follow-up to assess cardioversion and adjusts in antiplatelet therapy.

**Conclusion:** The authors describe a patient with STEMI due to high thrombotic load, recent stroke and presence of atrial thrombus, whose treatment dilemmas begin with the impossibility of using fibrinolytic therapy, the decision to perform thrombus aspiration and the definition of the best post-reperfusion pharmacolocgical therapy.

111756

Modality: E-Poster Young Researcher – Case Report

Category: CARDIOVASCULAR INTENSIVE CARE/CARDIOVASCULAR EMERGENCIES

## Coronary Vasospasm as a Cardiovascular Repercussion of Severe Obstructive Sleep Apnea: A Case Report

CATHARINA TUROLLA DE ANDRADE ^1^, Roberta Dutra Fortes^1^, Luiza Gondim Toledo^4^, David Nigri^3^, Erverton Gregório de Andrade^2^

(1) Hospital de Força Aérea do Galeão; (2) Clinica São Vicente da Gávea – Rede dOr São Luiz; (3) Hospital Copa Star – Rede dOr São Luiz; (4) Universidade Federal do Rio de Janeiro – UFRJ

**Introduction:** Obstructive sleep apnea (OSA) is a disorder characterized by obstructive apneas, hypopneas or respiratory effort-related caused by repetitive collapse of the upper airway during sleep. The estimated prevalence is 30 percent in males. It is essential to recognize OSA as a predisposing factor for cardiovascular disorders. This case report aims to show the occurrence of coronary vasospasm (CV) in a patient with OSA.

**Case Report:** A 67-year-old male, body mass index 26, ex-smoker, systemic arterial hypertension, chronic coronary artery disease (three previous stents), with a known high rate of admissions in the emergency room for acute pulmonary edema (APE), has been readmitted at the hospital with similar acute symptoms started during sleep. The vital signs showed desaturation, tachypnea and high blood pressure. Respiratory auscultation with bibasal crackles. The patient has been hospitalized for clinical surveillance and further investigation for APE. Troponin was elevated in one dosage, electrocardiogram showed no acute alterations, transthoracic echocardiogram was normal and a chest computerized tomography with a pattern of congestion. Despite the initial treatment, the patient continued to have episodes of hypoxemia and dyspnea, especially during sleep, and further complementary tests were requested. The patient underwent coronary angiography, which revealed vasospasm in the circumflex artery and marginal branch with a focal lesion of 70 percent, reversing during the exam with intracoronary mononitrate infusion [figure1]. Polysomnography showed severe OSA. The patient received specific treatment – continuous positive airway pressure and oral nitrate – with significant clinical improvement, remained asymptomatic after discharge.

**Conclusion:** This report presented demonstrates CV as a cardiovascular repercussion possibly caused by severe OSA. Recognizing OSA as predisposing factor for cardiovascular dysfunction is essential to provide adequate therapy, prevent complications, reduce symptom exacerbation and morbidity for these patients.


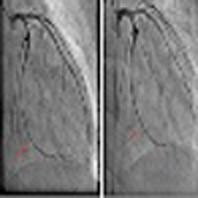


111377

Modality: E-Poster Young Researcher – Case Report

Category: PERICARDIUM/ENDOCARDIUM/VALVOPATHIES

## Whipple’s Endicarditis: A Case Report

CAROLINA KUCHENBECKER SOARES^1^, Claudio Leo Gelape^1^, Robson de Souza Almeida Junior^1^, Geraldo Brasileiro Filho^1^, Luiz Guilherme Passaglia^1^

(1) Hospital das Clinicas da UFMG

**Introduction:** Whipple’s disease (WD) is a rare chronic multisystem illness caused by the bacterium Tropheryma whipplei. Most patients have polyarthritis, malabsorption and anemia. Up to 50% can present with cardiac manifestations, being infective endocarditis (IE) with negative blood culture considered the most frequent and aortic valve the most affected.

**Case description:** A 63-year-old woman, previously healthy, is admitted in the Emergence Department with sudden left hemiplegia. Head tomography confirmed stroke showing acute hypodensity in right corona radiata. Carotid doppler was normal and transthoracic echocardiogram showed a filamentous structure in the ventricular face of the right coronary leaflet of the aortic valve, 9.3 mm long, with moderate regurgitation (confirmed by transesophageal exam). There were no clinical or laboratory signs of infection. Serial blood cultures, rheumatoid factor, abdominal ultrasound, and urinalysis were normal. In view of the possible differential diagnosis of IE, fibroelastoma and Lambl excrescences, as well as the concern for further embolization, cardiac surgery and excision of the material was chosen for anatomopathological diagnosis. She was then submitted to aortic valve replacement with a bioprosthetic valve, an uneventful procedure. The slide evaluation showed mononuclear cell infiltrate with foamy macrophages containing PAS-positive granulations, diagnosing WD.

**Conclusion:** Although rare, WD can be prone to recurrences and its recognition is important to initiate target therapy with parenteral streptomycin and benzylpenicillin for 14 days, followed by oral trimethoprim-sulfamethoxazole for 1 year. Unlike common IE, WD occurs mainly in native valves, without underlying disease and the anatomopathological pattern of inflammation is fundamental, as Trophyerma whipplei cannot be cultured using standard techniques. The presence of prodromes such as gastrointestinal symptoms or arthritis should also be considered in the suspicion.


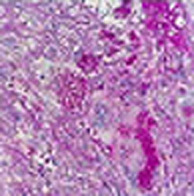


111911

Modality: E-Poster Young Researcher – Case Report

Category: CARDIOVASCULAR IMAGING

## Case Report: The Efficacy of PET-CT in Prosthetic Valve Endocarditis

ERICA SILVA^1^, Bernardo Santos Resende^1^, Ingred Hellen de Resende Andrade^1^, Henrique Patrus Mundim Pena^1^, Carlos Eduardo Ornelas^1^

(1) Rede Mater Dei de Saúde

**Introduction:** Infective endocarditis (IE) is a high mortality rate (30–50%) disease with many clinical features. Thus, some diagnoses may be challenging. Increase in the use of prosthetic materials and intracardiac devices (ICD) follows the importance of complementary tests, as 20% of EI patients have valve prosthesis or ICD. Duke criteria, traditional in IE diagnosis, are based on the presence of typical microorganisms in hemoculture and suggestive valve alteration in echocardiogram. However, in over 20% of cases, these may be inconclusive, requiring extended propaedeutics with more accurate exams. Thus, in other suspicious cases, more exams are essential for a precise diagnosis, such as Positron emission tomography (PET-CT).

**Case Report:** F.J.S.M., 59 years old, 15 years with an aortic biologic prosthesis, was admitted to ER reporting paresthesia of left upper limb, lips and tongue, of short duration and spontaneous recovery. Neurology assessed the case and nuclear MR of the skull showed cerebral ischemia area suggesting embolic etiology. Cardiological follow-up and complementary propaedeutics were started for etiologic investigation. Transesophageal echocardiogram image suggested aortic biologic valve periprosthetic vegetation. Patient had no symptoms of infection. Serial hemoculture results showed no bacterial growth. So, under Duke criteria, IE diagnosis was inconclusive (1 major, 2 minor criteria). PET-CT with radiopharmaceutical FDG-18F aimed at cardiac assessment confirmed hypothesis showing hypercaptation of the radiopharmaceutical, circumferential, in the aortic position biologic prosthesis, compatible with valve infective endocarditis. Directed treatment initiated with empiric antibiotic therapy and heart surgery for valve replacement.

**Conclusions:** As the use of prosthetic materials and intracardiac devices (ICD) advances, it is necessary to perform exams other than the traditional doppler echocardiography. Such information follows the European IE Treatment Guidelines (ESC 2015), which cites use of PET-CT in patients with clinical suspicion, when the evidence of valve periprosthetic abnormal activity is a major criterion in IE diagnosis. In the case described, PET-CT was essential to confirm IE diagnosis in patient with valve prosthesis, when Duke criteria were inconclusive. It is a high mortality disease, so, when it is suspected, even with atypical signs, investigation must be exhaustive to minimize risk of improper treatment.

111434

Modality: E-Poster Young Researcher – Case Report

Category: CONGENITAL AND PEDIATRIC CARDIOLOGY

## Atrial Septal Defects, Arrhythmias and a Novel Tbx5 Gene Variant

ANNE KATHRINE MØLLER NIELSEN^1^, Lars Allan Larsen^2^, Vibeke Hjortdal^1^

(1) Department of cardiothoracicsurgery, Rigshospitalet, Copenhagen, Denmark; (2) Institute of cellular and Molecular Medicinen, University of Copenhagen, Denmark

**Background and aims:** Patients with atrial septal defect (ASD) have increased mortality and morbidity. This can only be partly explained by hemodynamic changes caused by the ASD, suggesting additional underlying causes. Patients with an ASD have an increased burden of pathogenic gene variants in ASD related genes, indicating genetics as an important etiologic factor. This study aimed to investigate genetic associations in familial ASD and comorbidities.

**Methods:** In a cohort with familial ASD we identified a family with ASD present in 12 family members in three generations. We performed whole exome sequencing on five family members older than 18 years to detect the causative gene variant in this family and evaluated phenotype.

**Results:** We identified a novel pathogenic variant within the T-box domain of TBX5 (F232L) in all affected family members over 18 years of age. They presented with diverse cardiac phenotypes including heart failure and arrhythmias. Two carriers needed a pacemaker. Skeletal malformations where subtle with small hands as the only visible malformation. These findings propose Holt-Oram syndrome.

**Conclusions:** We report a novel variant in TBX5 in a family with Holt-Oram Syndrome, characterized by septal defects, severe cardiac arrhythmias, and mild skeletal malformations. Clinical awareness of family history, arrhythmias, and heart failure in patients with familial ASD is important and may lead to timely treatment and uncover patients with Holt-Oram Syndrome.

111478

Modality: E-Poster Young Researcher – Case Report

Category: PERICARDIUM/ENDOCARDIUM/VALVOPATHIES

## Octogenarian with Refractory Heart Failure, Dextrocardia and Multivalvular Disease – A Complex Case Report with a Successful Percutaneous Approach

HENRIQUE TROMBINI PINESI^1^, Vinicius Esteves^1^, Francisco Monteiro de Almeida Magalhães^1^, Rafael Alves Franco^1^, Carlos Vicente Serrano Junior^2^

(1) Rede D’OR São Luiz; (2) Instituto do Coracao, Faculdade de Medicina, Universidade de Sao Paulo

**Introduction:** The percutaneous approach of valvular diseases has gained increasing importance in recent years, especially in frail, elderly and high surgical risk patients. However, procedures in the tricuspid valve are still rare.

**Case Description:** Female, 87 years old, previously independent for instrumental activities of daily living, with a history of dextrocardia, dual-chamber pacemaker and difficult-to-manage atrial fibrillation requiring atrioventricular node ablation. She presented with progressively worsening dyspnea up to NYHA class III/IV, fatigue, and progressive swelling of the extremities in the last 4 months, with two hospitalizations due to acute heart failure in this period. Transthoracic echocardiogram identified preserved biventricular function, with significant biatrial and right ventricular dilatation. The Mitral valve showed significant regurgitation with prolapse and the tricuspid valve severe regurgitation with failed coaptation and torrential reflux secondary to annular dilation. A transesophageal echocardiogram identified dilatation of the valve annulus with a slight thickening of the cusps in addition to prolapse of segments A1/A2 of the anterior cusp and P1/P2 of the posterior cusp, with significant reflux. After optimization of medical treatment, she remained symptomatic with signs of congestion, associated with worsening of renal function. After discussion with the Heart Team, considering the patient’s elevated surgical risk (STS mortality of 17,29%), a less invasive treatment was chosen. A combined procedure was successfully performed, with a percutaneous mitral valve edge to edge repair (MitraClip™) and heterotopic tricuspid treatment with percutaneous bicaval valve implantation (TricValve™). The procedure was a success despite the difficulties caused by the simultaneous multivalvular approach in a patient with dextrocardia. The patient evolved with clinical improvement and normalization of ventricular function. She was discharged with a great enhancement in functional capacity.

**Conclusion:** We present a rare and up to the moment, the first description case of an elderly patient with dextrocardia, high surgical risk and refractory heart failure who underwent successful percutaneous treatment of the mitral and tricuspid valves simultaneously.

111481

Modality: E-Poster Young Researcher – Case Report

Category: PERICARDIUM/ENDOCARDIUM/VALVOPATHIES

## Subacute Lactobacillus Endocarditis in Blalock Prosthesis: A Case Report

RAPHAELA TEREZA BRIGOLIN GAROFO^1^, GUILHERME COELHO FORTES^1^, ISABELLE DE OLIVEIRA PARAHYBA^1^, CAMILA VIEIRA VILELA^1^, RAYZA KARLLA SALLES ARAUJO^1^

(1) HOSPITAL DO CORAÇÃO – ASSOCIAÇÃO DO SANATÓRIO SÍRIO

**Introduction:** The diagnosis of infective endocarditis is often difficult. We found diagnostic limitations especially in patients with prosthetic valves and implantable cardiac electronic devices.

**Case Report:** A 37-years-old man, diagnosed at birth with tricuspid hypoplasia, significant right ventricle hypoplasia and wide atrial septal defect with bidirectional shunt. He has been submitted to Blalock-Thomas-Taussig surgery on the right at 2 years old and on the left at 20 years old. Between 2019 and 2020 he was diagnosed with pneumonia on 4 occasions. After six months he continued to present with weight loss, low fever at night and asthenia, with elevated serum inflammatory markers. In July 2020, he sought the Emergency complaining of ventilatory-dependent chest pain and worsening of asthenia. Chest tomography showed a consolidation area in the right lung and antibiotic therapy was initiated. Even after the end of treatment, the patient remained subfebrile and with elevated C-Reactive Protein. Blood cultures were positive for Lactobacillus casei, paracasei and Rhamnosus. Due to the clinical suspicion of an undetermined infectious focus and with a negative investigation for Infective Endocarditis, an 18FDG PET/CT was performed, which showed an increase in glycolytic metabolism in a focal area next to the vascular prosthesis interposed on the left in the pulmonary systemic anastomosis. The diagnosis of prosthetic valve infective endocarditis was confirmed. After antibiotic therapy guided by blood cultures, the patient showed clinical and laboratory improvement. He was discharged asymptomatic, with a negative blood culture and normalized inflammatory markers. Lactobacillus are commensal microorganisms of humans and are recognized as a rare cause of endocarditis in people with previous comorbidities. When questioned, the patient reported daily ingestion of Lactobacillus-based probiotics.

**Conclusion:** The FDG-18F PET/CT should be used when there is a high clinical suspicion without diagnostic confirmation with traditional methods in a patient with a prosthetic valve or intracardiac device.


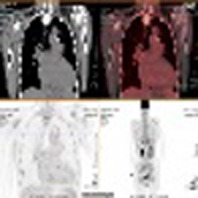


111485

Modality: E-Poster Young Researcher – Case Report

Category: COVID-19 AND CARDIOVASCULAR SYSTEM

## Myocarditis Associated with Coronary Thrombosis After Vaccine for COVID-19: Case Report

RAPHAELA TEREZA BRIGOLIN GAROFO^1^, GUILHERME COELHO FORTES^1^, THAÍS DOS SANTOS VIEIRA^1^, LAURO MARTINS NETO^1^, CAROLINA TOSI PIRES^1^

(1) HOSPITAL DO CORAÇÃO – ASSOCIAÇÃO DO SANATÓRIO SÍRIO

**Introduction:** Chest pain in young patients without comorbidities is a challenge. This report is relevant since the patient in the case presented with acute myocarditis associated with acute coronary thrombosis.

**Case Report:** A 20-year-old male patient without previous diseases and with a history of myocarditis 4 years ago. He went to the emergency room with type C chest pain for four hours. An electrocardiogram was performed with no changes. Laboratory tests with troponin curve (0.638 and 2.570 – Reference 0.034). Due to the hypothesis of myocarditis, a Cardiac Magnetic Resonance (CMR) was performed, which showed anterior and anterolateral hypokinesia associated with myocardial edema, and non-ischemic late mesoepicardial enhancement in these segments. It was decided to perform coronary CT angiography due to the segmental alteration on CMR, which showed proximal occlusion in the Anterior Descending Coronary Artery (ADCA) and its diagonal branches. Coronary Angiography was performed in association with Optical Coherence Tomography, which showed acute thrombosis in ADCA without signs of atherosclerosis. The thrombus was aspirated and a stent was implanted. After these findings, the patient reported a dose of mRNA vaccine for COVID-19 21 days before admission. The coronavirus causes a condition of hyperinflammation and hypercoagulability, leading to dysfunctions of various organs, including the heart. Among the cardiac dysfunctions we can mention myocarditis and thromboembolic events. Myocarditis should be considered in young people with chest pain after mRNA vaccine. He had an episode of acute myocarditis and ADCA occlusion with high thrombotic burden without evidence of atherosclerosis, other etiologies for myocarditis and rheumatologic disease. Due to its recent vaccination history for COVID-19, it is likely that the vaccine was the protagonist of the activation of the coagulation cascade and the inflammatory response.

**Conclusion:** This case reinforces the importance of differential diagnoses in young patients with atypical chest pain in order to promote adequate treatment.


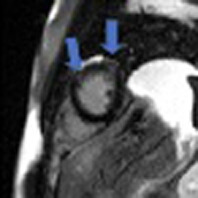


111500

Modality: E-Poster Young Researcher – Case Report

Category: CARDIOVASCULAR INTENSIVE CARE/CARDIOVASCULAR EMERGENCIES

## Case Report: Amniotic Fluid Embolism in a Public Maternity Hospital in the State of Amazonas

RODRIGO BINDA DE MAGALHÃES LOIOLA ^1^, Emerson Oliveira Lise^3^, Laura Barbosa David^4^, Gisele Tezolin Menezes^5^, Alexandre Lobato Rodrigues Guimarães^6^

(1) Faculdade Metropolitana de Manaus; (3) Faculdade Metropolitana de Manaus; (4) Faculdade Metropolitana de Manaus; (5) Faculdade Metropolitana de Manaus; (6) Maternidade Estadual Balbina Mestrinho

Amniotic fluid embolism (AFE) is a rare and exclusive complication of pregnancy, and is characterized by the sudden onset of maternal impairment. It usually involves the cardiorespiratory and hematological systems, and can progress to cardiac arrest and coagulopathy, leading to death. The estimated incidence of AFE is 1:15,200 and 1:53,800 births in North America and Europe, respectively. The mortality rate associated with AFE is 30%. The objective of this report is to emphasize the need for knowledge of this serious clinical entity. Patient 21 years, G2P2A0C2, GA 38 weeks and 1 day, admitted to the maternity hospital. Ultrasound with Doppler was requested, by which signs of fetal centralization were found, and cesarean section was indicated. On the 1st day post cesarean section (PCS), in the operating room, she suddenly developed acute respiratory distress and cardiorespiratory arrest (CRA), with return of spontaneous circulation after the first cycle. Sent to the ICU, where she presented 2 more CRAs, both with return in the first cycle. On the same day, the patient developed cardiogenic shock, requiring the use of vasoactive drugs for hemodynamic compensation. Wells criteria: with a high probability of PTE. Laboratory tests and echocardiogram (ECG) were requested, which revealed an increase in right chambers, pulmonary artery systolic pressure (PASP) 68 mmHg. On the 2nd day PCS, ECG showed dilatation of the right ventricle (RV) and right atrium (RA), left artery collapse, dilated inferior vena cava, dilated superior vena cava, presence of pulmonary hypertension with PASP: 60 mmHg. On the 3rd day PCS, she suffered 2 more CRAs, with return in the first cycle. On the 4th day PCS, weaning of vasoactive drugs and sedation was initiated, ECG was requested, dilated inferior vena cava not collapsing with inspiration, RV in “D” due to high pressure, PASP: 60 mmHg, RA increased in size. On the 5th day PCS, extubation was performed. On the 6th day, patient was hemodynamically stable without the use of vasoactive drugs, in good general condition. Presented edema of upper limbs+++/4+ and lower limbs ++/4+. Started on furosemide. On the 8th day, she started captopril due to BP of 149/85 mmHg. On the 9th day PCS, the patient was discharged from the ICU. Although rare, AFE should be maintained as a differential diagnosis for intensivists and obstetricians. Their knowledge is of paramount importance for the early recognition and proper management of the patient.

111528

Modality: E-Poster Young Researcher – Case Report

Category: COVID-19 AND CARDIOVASCULAR SYSTEM

## Heart Failure with Reduced Ejection Fraction After Biontech-Pfizer Vaccine

BÁRBARA MARIANA DOS SANTOS SILVA^1^, Carlos Eduardo Lucena Montenegro^1^, Fiamma Ferreira Nogueira^1^, Raquel Cristina Farias de Medeiros Queiroz^1^, Claudia Carolina Mendonça Campos^1^

(1) Pronto Socorro Cardiológico de Pernambuco (PROCAPE)

**Introduction:** Cardiac complications such as post-vaccination myocarditis from BioNTech-Pfizer have been reported. There is prevalent in young men, evolving with fever, chest pain and dyspnea in the first days after the vaccine. Active infection is ruled out by negative RT-PCR and in all cases MRI showed delayed myocardial enhancement after contrast. Most of the reported cases showed complete resolution of symptoms and absence of sequelae after supportive therapy. Cases of myocarditis have a difficult etiological diagnosis, but they should be considered as part of the clinical diagnosis.

**Case Report:** A 42-year-old woman, previously diagnosed with hypertension, dyslipidemia and overweight, with no other comorbidities, presented to the emergency department with progressive dyspnea started about 2 months ago, progressing to dyspnea on minimal exertion, orthopnea and paroxysmal nocturnal dyspnea. On physical examination, the patient presented with signs of pulmonary and systemic congestion and cardiac auscultation without alterations. She reported onset of symptoms after 1 week of taking the BioNTech-Pfizer COVID-19 vaccine, reporting only a few episodes of associated retrosternal chest pain. She had normal laboratory tests, ECG with sinus rhythm and left bundle branch block pattern, chest X-ray that showed signs of pulmonary congestion, transthoracic echocardiogram with left ventricle showing moderate enlargement, walls with diffuse hypokinesia and markedly reduced systolic function (SIMPSON ejection fraction 25%), cardiac catheterization without evidence of atherosclerotic disease and cardiac MRI with late mesocardial enhancement after gadolinium infusion, with signs of non-ischemic fibrosis in the septal-lower segments and inferior basal and middle, suggestive of sequel of previous inflammatory/infectious process. Optimized treatment was started for heart failure with reduced ejection fraction (sacubitril-valsartan, bisoprolol, spironolactone and furosemide), the patient evolved with significant symptomatic improvement. The patient has had no recurrent symptoms since hospital discharge and is currently under the care of the heart failure center.

**Conclusion:** Post-vaccination myocarditis is a rare event, but it should be considered a differential diagnosis after vaccine for COVID-19, especially when it has a temporal relationship with the BioNTech-Pfizer vaccine. Its evolution is still uncertain and clinical follow-up in specialized centers is necessary.

111532

Modality: E-Poster Young Researcher – Case Report

Category: ACUTE AND CHRONIC CORONARY DISEASE/THROMBOLYSIS

## Research of Thrombus in the Left Ventricle in a Patient After Acute Myocardial Infarction: A Case Report

ANDRÉ DIAS NASSAR NABACK^1^, Anderson Ferreira Leite^1^, Lucas Queiroz Fernandes Campos^1^, Larissa Barroso Mayrink^2^, Thales Moura De Battisti^1^

(1) Hospital das Clínicas da Universidade Federal de Minas Gerais, HC-UFMG; (2) Centro Universitário de Belo Horizonte, UniBH

**Introduction:** Left ventricular (LV) thrombus is one of the complications after acute ST-segment elevation myocardial infarction (STEMI), mostly in cases of anterior wall STEMI with LV ejection fraction <50%. For its diagnosis, transthoracic echocardiography (TTE) is the initial method of choice.

**Clinical case:** Male, 76 years old, diagnosed with STEMI of the anterior wall and submitted to primary angioplasty of the left anterior descending coronary artery (LAD) three and a half hours after the onset of symptoms. One day after the procedure, he presented with transient mental confusion and rotational vertigo. Cranial computed tomography showed a dubious image of an ischemic area in the posterior fossa region. ECOTT did not show intracavitary thrombus, despite an LV ejection fraction of 44%. Nine days after the STEMI, he again presented chest pain and V2–V6 ST-segment elevation on the electrocardiogram. Coronary angiography showed proximal thrombosis of drug-eluting stent in LAD requiring balloon angioplasty and tirofiban infusion. One day later, he recurred with chest pain and worsening of the ST-segment elevation, requiring a new balloon angioplasty due to a new stent thrombosis in the LAD. Changed P2Y12 receptor inhibitor. Two new TTE did not show intracavitary thrombus. It was decided to perform magnetic resonance imaging (MRI) of the heart, which confirmed an image compatible with a thrombus adhered to the apex of the LV, measuring 18 × 10 × 4 mm. Brain MRI confirmed cerebellar ischemic area. Then, anticoagulation with warfarin was started.

**Conclusion:** Intracavitary thrombi are commonly underdiagnosed. Although TTE is the most accessible test, its sensitivity is 35%. The use of ultrasound contrast is an alternative to improve the performance of the exam (sensitivity of 64%). However, despite being less accessible and more expensive, MRI is the gold standard for the diagnosis (sensitivity of 82%–88% and specificity close to 100%), being indicated for patients with high probability of LV thrombus and TTE without thrombus.


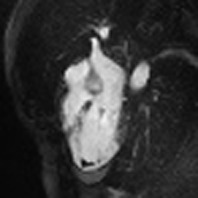


111938

Modality: E-Poster Young Researcher – Case Report

Category: CARDIOVASCULAR SURGERY

## Acute Type a Aortic Dissection Secondary to Closed Thoracic Trauma: Two Stage Approach, a Case Report

ELISA ITO MENDES DE ANDRADE^1^, José Carone Filho^1^, Márcio Luiz Roldi^1^, José Carone Júnior^1^, Carlos Alberto Sancio Júnior^1^

(1) Hospital Evangélico de Vila Velha

**Introduction:** Acute type A aortic dissection secondary to trauma is a potentially fatal cardiovascular emergency that needs to be diagnosed early and treated effectively. Redirection of blood flow to the true lumen with prosthetic grafts is the mainstay of treatment.

**Method:** A 58-year-old man was admitted to the hospital in his city after a power pole fell. A CT scan was requested due to a suspected fracture of the facial bones. In addition to confirmed bone lesion, he showed acute type A (Stanford) aortic dissection secondary to trauma. The patient was transferred to the referral service in cardiovascular surgery, where computed tomography angiography (CT Angio) of the thoracic aorta showed “Flap in the aortic arch and emergence of the brachiocephalic trunk, with flow maintained in them. The other segments of the thoracic aorta had normal caliber, walls and flows”. He underwent transsternal thoracotomy, and aortic derivation from the proximal aorta to the carotids with a bifurcated graft (Debranching) and implantation of an endoprotheses with antegrade approach without cardiopulmonary bypass (CPB). He is discharged from the Intensive Care Unit (ICU) on the fourth day after surgery, being transferred for surgical treatment of mandibular fracture. Returns to the emergency room 49 days after discharge, complaining of atypical chest pain, which started one week ago, with significant worsening that day. A new CT angiography of the aorta showed the presence of an endoprosthesis that extended from the emergence of the brachycephalic trunk to the descending portion of the thoracic aorta, with contrasted content outside the proximal portion of the endoprosthesis and in apparent continuity with the intimal flap, suggesting an II/IA ? endoleak. He underwent transfemoral implantation of a 36/36/150 endoprosthesis in the ascending aorta and arch, requiring the use of an accommodation balloon with excellent results. The patient progressed satisfactorily in the postoperative period, being discharged on the third day after the approach.

**Discussion:** It is estimated that about 80% of patients with acute type A aortic dissection secondary to trauma die even before hospital care. Endovascular treatment is not the first option for type A aortic dissection, but in this case the patient was at high risk of serious complications if submitted to heparinization and cardiopulmonary bypass. A study with the objective of long-term follow-up of patients undergoing endovascular treatme.

111565

Modality: E-Poster Young Researcher – Case Report

Category: PERICARDIUM/ENDOCARDIUM/VALVOPATHIES

## Atypical Manifestation of Infective Endocarditis

BÁRBARA MARIANA DOS SANTOS SILVA^1^, Diana Patricia Lamprea Sepulveda^1^, Raquel Cristina Farias de Medeiros Queiroz^1^, Suéllen Lídia da Silva Barbosa^1^, Déborah Rodovalho de Menezes^1^

(1) Pronto Socorro Cardiológico de Pernambuco (PROCAPE)

**Introduction:** Infective endocarditis has different forms of presentation. Signs and symptoms result from either local destructive effects of the cardiac endothelial surface, metastatic embolization of infected fragments, hematogenous seeding of other sites or antibody formation. Typical clinical features are fever, weight loss, malaise, pulmonary or peripheral congestion and new or changing heart murmur. In rare cases, it may be associated with cutaneous vasculitis or hepatitis.

**Case Report:** A 36-year-old man with a rheumatic heart disease, has undergone mitral and aortic valve replacement in 2008 and aortic valve replacement with tricuspid valve repair in 2018, referred progressive dyspnea for about 3 months associated with febrile episodes and jaundice. His initial laboratory panel showed a marked increase in leukocytes with neutrophilia, thrombocytopenia, elevated CRP, liver dysfunction (AST 528/ALP 357) and hyperbilirubinemia (TB 15,1/DB 12,5). Abdominal imaging exams without changes. On examination, the patient was emaciated, with moderate intensity jaundice, diffuse purpuric lesions, signs of peripheral embolization and jugular distension. On cardiac auscultation, important systolic and diastolic murmurs in the mitral focus and systolic murmurs in the aortic and tricuspid areas. Pulmonary auscultation with crackling rales in lung bases. Transesophageal echocardiography revealed moving filamentous images suggestive of vegetation on the atrial face of the mitral bioprosthesis, in addition to signs of mitral, aortic and tricuspid valve dysfunction. Antibiotic treatment with oxacillin, ceftriaxone, gentamicin and rifampicin was started, with gradual improvement in a few days of the purpuric lesions and the icteric syndrome. Initial blood cultures with negative results. The patient underwent a new surgery to replace the aortic and mitral valves (mechanical) and tricuspid repair. During hospitalization, positive control blood culture samples for Candida albicans and Candida haemulonii were evidenced. Antibiogram-guided treatment with micafungin was performed. Patient was discharged clinically stable and with improvement of all initial clinical and laboratory findings.

**Conclusion:** Infective endocarditis can appear in an atypical manner and can be a diagnostic challenge in such cases that initial signs are these rare complications, such as hepatic failure or vasculitis. Late diagnosis worsens prognosis and leads to increased mortality.

111579

Modality: E-Poster Young Researcher – Case Report

Category: CARDIOVASCULAR SURGERY

## Congenital Anomaly Type Single Ostum of Coronary Artery with Origin in the Right Coronary Sinus

NICOLAS BIONI STEFANO ^1^, Elisa Souza Lini^2^, Alexandre Quadros^1^, Renato Kalil^1^, João Carlos Vieira da Costa Guaragna^1^

(1) Hospital Divina Providência; (2) Pontifícia Universidade Católica do Rio Grande do Sul

**Introduction:** Coronary artery single ostium cardiac anomaly represents a rare congenital alteration of unknown etiology. This condition presents clinically from asymptomatic forms to cardiac ischemia and manifestations of sudden cardiac death, especially in young adults. The incidence of this pathology is variable, affecting 0.03% to 0.4% of patients undergoing cardiac catheterization.

**Case Report:** Patient LVAG, 69 years old, male, hypertensive, dyslipidemic, heart failure, hypothyroidism and COPD. Hospitalized for salmonella colitis. During hospitalization, he presented anginal chest pain. He already had similar episodes at home with small efforts, with gradually worsening in the last few weeks. ECG showed sinus rhythm and septal inactive zone, there was elevation of troponin levels, being taken to coronary angiography. During evaluation, an anomalous coronary path was visualized, with the anterior descending artery and circumflex artery originating from the right coronary sinus, together with the right coronary artery. There was also an ulcerated lesion in the middle segment of the circumflex artery, but with a complicated anatomy and path to perform angioplasty. Coronary computed tomography angiography showed a malignant path of the circumflex artery, with passage between the aorta and the pulmonary artery. Echocardiogram showed mild systolic dysfunction with an ejection fraction of 42%. Heart Team defined coronary artery bypass graft surgery for definitive treatment.

**Conclusion:** Congenital coronary artery anomalies are subdivided into seven categories, highlighting the single-ostium coronary artery anomaly, in which four paths can be followed: retroaortic, septal, anterior and interarterial. Its clinical presentation includes angina, ventricular arrhythmias, dyspnea, syncope, heart failure and sudden death. The occurrence of ischemia associated with this anomalous condition, in this case, consists of the path of the vessel between the pulmonary artery and the aorta, which would eventually suffer extrinsic compression. In the present case, the patient had an anomalous interarterial course (between the pulmonary artery and the aorta), considered a malignant type. In these cases, the patient may initially undergo coronary angioplasty and, later, surgical treatment, through reimplantation or ligation of the coronary arteries, followed by grafting of the saphenous vein or internal thoracic artery.

111591

Modality: E-Poster Young Researcher – Case Report

Category: ACUTE AND CHRONIC CORONARY DISEASE/THROMBOLYSIS

## Chronic Total Occlusion of the Left Main Coronary Artery with Normal Left Ventricular Function and Stable Angina: A Case Report

MATHEUS RAMOS DAL PIAZ^1^, Caio Menezes Machado de Mendonça^1^, Bruno Mahler Mioto^1^, Luis Henrique Wolff Gowdak^1^

(1) Instituto do Coração (InCor) do Hospital das Clínicas da Faculdade de Medicina da Universidade de São Paulo (HCFMUSP)

**Introduction:** Severe stenosis of the left main coronary artery (LMCA) in patients with stable symptoms is uncommon and usually associated with a dominant right coronary artery (RCA) and rich collateral circulation. The incidence of total occlusion has been reported to be between 0.04% to 0.43%. The impact on the left ventricle (LV) function is often significant with systolic dysfunction, especially with significant involvement of the RCA. Surgical myocardial revascularization remains the best treatment for chronic total occlusion (CTO) of the LMCA.

**Case Report:** A 69-year-old woman with hypertension, dyslipidemia, and obesity, without previous acute myocardial infarction, presented with CCS III angina, associated with dyspnea on exertion for a year. A myocardial perfusion scan revealed extensive stress-induced ischemia in the anterior, anterolateral, lateral, and inferolateral walls. A transthoracic echocardiogram showed a globally preserved ventricular function (LVEF = 65%). We found a CTO of the LMCA and a 70% stenosis of the middle RCA during an elective coronary angiography, with grade III collateral circulation from the RCA to the LMCA. The patient was admitted for surgical myocardial revascularization. A successful triple bypass was performed (left internal thoracic artery-left anterior descending artery, radial artery-left obtuse marginal branch, and saphenous vein graft-posterior descending artery).

**Conclusion:** CTO of LMCA is a rare manifestation in patients presenting with stable symptoms. Two conditions are related to a higher probability of patient survival and maintenance of LV function: a dominant RCA without significant stenosis and an extensive collateral circulation to the left system, conditions only partially found in our patient. Despite having significant stenosis of the RCA, LV function was preserved, and the patient had stable symptoms for a year.


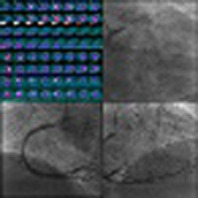


111598

Modality: E-Poster Young Researcher – Case Report

Category: HEMODYNAMICS AND INTERVENTIONAL CARDIOLOGY

## Report of Three Cases Submitted to Percutaneous Embolization of Pulmonary Arteriovenous Fistulas

LARISSA XAVIER ALVES DE OLIVEIRA^1^, Debora Rodrigues^1^, Mauricio Lopes Prudente^1^, Adriano Gonçalves de Araújo^1^, Giulliano Gardenghi^1^

(1) Hospital Encore

**Introduction:** Pulmonary arteriovenous malformations (PAVMs) consist in an abnormal communication between an artery and a vein, causing clinical manifestations as chronic hypoxia or embolic events. Most cases of PAVMs are represented by hemorrhagic telangiectasia, a rare congenital disease. In the past, pulmonary fistulas were treated only by open surgery, with an expressive rate of complications. In the 70s, the first percutaneous catheter embolization was performed with coils. Currently percutaneous embolization has become the treatment of choice. We report three different cases of percutaneous correction of PAVMs using coils and vascular plugs.

**Cases Description:** First patient, 47 years old, had an 8 mm diameter fistular sub pleural sac. Complete embolization was performed without residual shunt. The second patient, 48 years old presented a pulmonary fistula associated with chronic hypoxemia. The fistula came from several branches of the right pulmonary artery. After embolization was shown immediate improvement of central oxygen saturation. Third patient, 30 years old, the PAVM had a pseudoaneurysm raising next to the first branches of the left pulmonary artery to the upper lobe of the left lung, and two important fistulas in the right inferior lobar arteries forming a shunt right to left. Embolization of the PAVM and pseudoaneurysm was performed successfully.

**Conclusion:** The percutaneous treatment of PAVMs is effective and safe in these cases.


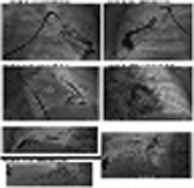


111629

Modality: E-Poster Young Researcher – Case Report

Category: ACUTE AND CHRONIC CORONARY DISEASE/THROMBOLYSIS

## Early Hemorrhagic Pericarditis After Thrombolisys in Acute Myocardial Infarction: A Case Report

CAIO MENEZES MACHADO DE MENDONÇA^1^, Matheus Ramos Dal Piaz^1^, Antônio Carlos Fonseca de Queiroz Filho^1^, Matheus Barbosa Gastaldo^1^, Luis Henrique Wolff Gowdak^1^

(1) Instituto do Coração (InCor) do Hospital das Clínicas da Faculdade de Medicina da Universidade de São Paulo (HCFMUSP)

**Introduction:** Post–myocardial infarction pericarditis occurs in approximately 5% to 6% of patients who receive thrombolytic agents. It develops in the first 2 to 4 days following infarction, mainly in the first 24 hours, and usually produces only mild symptoms. Nonetheless, rare complications include hemopericardium, cardiac tamponade, and constrictive pericarditis. Treatment is primarily supportive, with appropriate use of nonsteroidal anti-inflammatory agents. However, pericardiocentesis is indicated to treat a large pericardial effusion, particularly if accompanied by tamponade.

**Case Report:** A 63-year-old male patient with a history of hypertension and smoking was transferred to our hospital 24 hours after an extensive anterior ST-elevation myocardial infarction (STEMI). The patient was initially treated in another facility with ASA + clopidogrel and underwent thrombolysis with tenecteplase, with signs of reperfusion. The patient was brought to the cath lab, significant 3-vessel disease was found, and surgical myocardial revascularization was recommended. Clopidogrel was withdrawn, and full anticoagulation with enoxaparin was initiated. The patient remained asymptomatic, but a transthoracic echocardiogram on the second-day post-MI revealed a pericardial effusion of 25 to 30 mm with signs of ventricular filling restriction with no evidence of left ventricular (LV) dysfunction or LV free wall rupture. The patient developed severe hypotension on the same day, requiring vasoactive drugs. Urgent pericardiocentesis was performed, and 900 mL of hematic fluid was recovered. Right after, the patient evolved with significant hemodynamic improvement. Histopathological examination of a pericardial fragment revealed the diagnosis of acute fibrinohemorrhagic pericarditis.

**Conclusion:** Acute hemorrhagic pericardial tamponade after infarction rarely occurs, at a reported incidence of only 1% of STEMI patients treated by thrombolysis, often within the first 24h. Despite being a rare complication, the present case calls attention to the fact that hemopericardium and cardiac tamponade can occur in patients who have suffered an acute MI and should be included in the differential diagnosis in those presenting with hemodynamic instability. Prompt echocardiography assessment may lead to early diagnosis and better outcomes in these patients.

111883

Modality: E-Poster Young Researcher – Case Report

Category: CARDIOVASCULAR IMAGING

## Right Ventricle Intracardiac Mass of Unknown Origin Presenting as Right-Sided Heart Failure- A Case Report

MAYARA DE SOUZA VASCONCELOS^1^, PEDRO RAFAEL VIEIRA DE OLIVEIRA SALERNO^1^, LUCIA MARIA VIEIRA DE OLIVEIRA SALERNO^1^, EVELINE BARROS CALADO^1^

(1) HOSPITAL DAS CLÍNICA DA UNIVERSIDADE FEDERAL DE PERNAMBUCO

**Introduction:** Intracardiac masses are rare. Causes include neoplasic and non-neoplasic conditions. Diagnosis is challenging due to nonspecific clinical presentation and commonly occurs incidentally. Echocardiography (ECHO) is usually the initial method of assessing the size, location, fixation, mobility, and hemodynamic repercussion of the mass. Computed tomography (CT) and magnetic resonance imaging (MRI) further complement the mass evaluation.

**Case Description:** A 54-year-old female presented with facial edema and conjunctival hyperemia that progressed to diarrhea, lower limbs edema, ascites, abdominal pain, jaundice, and dyspnea at moderate efforts. After a syncopal event with no post-ictal period, she was admitted for investigation. Past medical history included a stroke in 2019 and schistosomiasis that was treated in 2000. After admission, discrete leukocytosis, with significant eosinophilia, elevated C-reactive protein, high aminotransferases, and high lactate dehydrogenase were identified. Blood cultures were negative. Urine culture was positive for Escherichia coli and ceftriaxone + metronidazole were initiated. Transthoracic and transesophageal ECHOs displayed a large right ventricle (RV) mass that occupied most of the chamber and was attached to the tricuspid valve (TV), enlargement of the right atrium, inferior vena cava, and hepatic veins were also seen. Cardiac MRI confirmed the mass that measured 21 × 28,5 × 33,4 mm, attached to the TV and projecting towards the RV outlet. Metastatic disease screening with imaging studies provided non-significant findings. Lower limbs USG with Doppler identified bilateral deep venous thrombosis. Tumor markers and investigation for thrombophilia and collagen disease were negative. Cardiac surgery for the removal of the mass was done, intraoperative findings included a mass involving the papillary muscles and the leaflets of the TV with a caseous appearance. After excision, a biologic prosthesis was inserted in TV position. Histopathological analysis showed a material containing fibrin and leukocytes, and endomyocardial necrosis without significant eosinophil infiltration—no signs of neoplasia, bacteria, or fungus. After discharge, previous blood tests were identified and showed chronic fluctuating eosinophilia (largest count 4.190/mm^3^, 27,3%).

**Conclusion:** Unique features of this case are the large thrombus-like RV mass causing obstructive symptoms and the presence of persistent eosinophilia perhaps suggesting Loeffler’s endocarditis.

111622

Modality: E-Poster Young Researcher – Case Report

Category: CARDIOVASCULAR IMAGING

## Evolution of a Case of Serratia Marcescens Endocarditis – The Role of 3D Transesophageal Echocardiography

THIAGO BURIL FONTES^1^, Pedro Henrique Pereira Inglez^1^, Rudyney Azevedo^1^, Cássia Cardoso Costa^1^, Carolina Frezzatti de Andrade Neves^1^

(1) Hospital do Servidor Público Municipal de São Paulo

**Introduction:** Infective endocarditis (IE) is a cardiovascular disease. The etiology is predominantly gram-positive cocci, but it has other infectious agents: HACEK group, fungi and other atypical germs. IE caused by Serratia marcescens is rare, documented in less than 1% of cases. Pathophysiology is multifactorial.

**Case Report:** E.G.V., 58 years old, history of aortic valve disease due to rheumatic fever and permanent pacemaker (PM). PM, on 03/05/22, evolving after ten days of the exchange with phlogistic signs, fever and fluctuation in the generator store region. Opted for cleaning the PM pocket with secretion collection for culture. Febrile peaks remained,37.7°C, after PM cleaning. White blood cell 13 (4.8–10.8) with absolute neutrophils of 11 (1.4–6.5). Cultures with the presence of Serratia marcescens in secretion of PM store and in blood culture. Treatment for bloodstream infection with Meropenem and Teicoplanin. The electrodes were then removed from the PM, with laser, and sent for culture. In the culture, 04/01/22, there was growth of Serratia marcescens on PM electrodes. Because of this, a 2D TEE was performed, showing a mass adhered to the tricuspid valve, which may correspond to vegetation. Changed Daptomycin and maintained Meropenem. The definitive PM generator implanted on 04/19/22, maintaining afebrile condition on the 6th PO and obtaining negative results from the 6 control blood cultures. 3D TEE was performed on 05/11/22 showing persistence of the image of the mass in the right ventricle, this time being interpreted as a residual image due to the removal of the PM.

**Conclusion:** It is a rare case of Serratia marcescens infection and the challenge of interpreting an image of an intracardiac mass, the 3D TEE can help in the follow-up of the case.


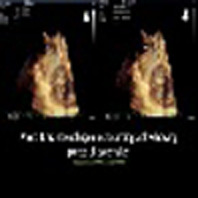


111626

Modality: E-Poster Young Researcher – Case Report

Category: CARDIOVASCULAR SURGERY

## Aortoesophageal Fistula in Post-Operative Dissection of Chronic Ascending and Descending Aorta: A Case Report

SABAH ABAZAR YOUSIF MOHAMED ALI^1^, Paulo Rafael Gonçalves da Silva Von Zuben^1^, Gabriel Queiroz de Abreu^1^, Ricardo Ribeiro Dias^2^, Georgina del Cisne Jadán Luzuriaga^2^

(1) Centro universitário São Camilo (CUSC); (2) Instituto do coração do Hospital das Clínicas (InCor-HC)

Aortic dissection is a disease, which presents with chest discomfort, acute hemodynamic compromise and high risk of mortality, requiring surgical management, with endovascular repair of the aorta. However, the prosthesis used in the procedure can be considered a possible etiology of aortoesophageal fistula. LF, female, 64 years old, with a history of systemic arterial hypertension (SAH) for 6 years, hysterectomy for 30 years, with diagnostic hypotheses of aortic aneurysm and chronic dissection of the ascending aorta (55 mm) and descending (50 mm). Replacement of the aortic branch and reimplantation of supra-aortic island branches was performed. Three months after the first surgery, the patient reported daily low-grade fever, malaise, dyspnea on minor exertion, and hyporexia for two months, in addition to hemoptysis, cough, and diffuse chest pain. According to tomography angiography, a large thrombus was identified in the aorta associated with a dissection blade in the abdominal aorta, from the celiac trunk to the bifurcation, extending along the entire length of the superior mesenteric artery. Upper gastrointestinal endoscopy (UGE) showed ulceration in the esophagus with a fistulous orifice in the upper thoracic esophagus. An aortic endoprosthesis was implanted. Two weeks after the second surgery, the aortoesophageal fistula was repaired. The surgeries were a success, however, the patient developed urinary tract infection, acute respiratory failure, pneumothorax, bacteremia and acute kidney injury. The condition evolved with septic shock due to mediastinitis caused by Pseudomonas aeruginosa due to the aortoesophageal fistula, resulting in death. In the literature, aortoesophageal fistula in the postoperative period of chronic aortic dissection is rarer than aortic dissection. Based on the findings of this study, it was possible to observe the severity of such a complication, such as mediastinitis secondary to bacterial infection, which rapidly progresses to death. The situation reported, similarly to previous studies, describes presentations with the same outcome of death, which had some factors similar to that of this patient, such as chronic dissection, uneventful postoperative period and need for reintervention. Patient with an uncommon complication of aortoesophageal fistula after correction of chronic aortic dissection, however, with extreme severity of infectious complications, such as mediastinitis and death.

112133

Modality: E-Poster Young Researcher – Case Report

Category: CARDIOVASCULAR SURGERY

## Anomalous Origin of Right Coronary Artery and Malignant Path with Late Repercussion: Case Report

ANAÍS CONCEPCION MARINHO ANDRADE DE MOURA^1^, Cítara Trindade de Queiroz^1^, Dr. Marcos Antonio Ferreira Lima^2^, Dr. Marcel Delafiori Hikiji^2^, Dra. Aline Hofmann Baiao^2^

(1) Universidade Potiguar; (2) Hospital do Coração de Natal

**Introduction:** The coronary arteries‘ anomalous origin is rare, with a frequency of reported cases of 0.64% in neonates and 0.17% in asymptomatic children and adolescents who were discovered through an echocardiogram. Its late discovery may occur in symptomatic patients with hemodynamic repercussions, therefore, early intervention is necessary and performed with surgical correction.

**Case Report:** A 69-year-old female, diabetic, hypertensive, and with chronic coronary artery disease was diagnosed with an anomalous origin of the right coronary artery (RCA) with a malignant path. Symptoms of typical angina and recurrent syncope started in 2012. In 2013, after an anginal condition, she underwent an elective angioplasty, with a complication that led to coronary dissection and brief cardiorespiratory arrest, requiring implantation of 2 stents in the proximal third and mean RCA, however, the artery anomaly was not described in the procedure report. Even after the intervention, the patient maintained the same clinical picture with optimized therapy for 9 years. After a coronary angiotomography in 2021, the anomalous origin of the RCA emerging in the upper portion of the left coronary sinus and its interatrial course, associated with ostial cleft compression, was described, and then coronary artery bypass graft surgery was indicated. The surgical approach was performed in 2022, through an aortic bypass graft to the middle third of the RCA and closure of its proximal third, where the previous stent was located. The patient developed with a total improvement of angina and syncope.

**Conclusion:** Patient with a rare ACD anomaly with a malignant path, which was only discovered when she was elderly, because of symptoms of ischemia that occurred through compression of the artery, which passed between the ascending aorta artery and the pulmonary trunk artery, during ventricular systole. It is worth emphasizing the importance of early diagnosis and therapeutic intervention for better patient survival because of the high risk of sudden death.


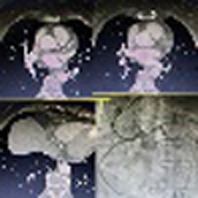


111646

Modality: E-Poster Young Researcher – Case Report

Category: HEMODYNAMICS AND INTERVENTIONAL CARDIOLOGY

## Evolution of Coronary Thromboaspiration: Penumbra CAT RX System

MURYELLE ROSA COELHO^1^, Rogerio Las Casas^1^, Vinicius Daher Vaz^1^, Fabricio Las Casas^1^, Tannas Jatene^1^

(1) Hospital do Coração Anis Rassi (HCAR)

**Introduction:** Acute Myocardial Infarction (MI) occurs due to atherosclerotic plaque rupture, and consequent coronary thrombosis. A large amount of intracoronary thrombus is a challenge for the interventional cardiologist as it difficult to restore TIMI III flow and predisposes embolization to distal microcirculation (no-reflow). Coronary thromboaspiration reduces the thrombotic load, improves coronary perfusion and reduces fibrosis area.

**Case Description:** Male, 72 years old, smoking history, hypertension, dyslipidemia, diabetes mellitus and chronic kidney disease. Admitted at emergency room with ST-segment elevation myocardial infarction on inferior wall and 7-hour pain. A loading dose of acetylsalicylic acid and ticagrelor was administered followed by cardiac catheterization that revealed acute total occlusion in the middle third of the right coronary artery. After balloon recanalization, high thrombotic load was observed. A glycoprotein IIbIIIa inhibitor was administered, followed by unsuccessful conventional mechanical thrombus aspiration and TIMI I distal flow. In the face of failure, a Penumbra CAT RX mechanical thrombus aspiration system was used. It contains a catheter with a larger internal lumen and coupled to a pressure aspirator, unlike the conventional modality that uses a manual suction syringe system. TIMI III flow was reestablished and drug-eluting stent was successfully implanted, followed by transfer to the coronary care unit. The evolution was uneventful, echocardiogram showed preserved ejection fraction and inferior wall hypokinesia. Hospital discharge after four days of hospitalization, using dual antiplatelet therapy, statin and beta-blocker.

**Conclusions:** Although TAPAS trial suggests a reduction in major cardiovascular events with routine mechanical thrombus aspiration in MI, larger randomized trials such as TASTE and TOTAL turned this therapy a class III recommendation, because it did not demonstrate clinical benefits and increased the risk of stroke. As the favorable results of mechanical thrombus aspiration on TOTAL trial subgroup with high thrombotic load, the Penumbra CAT RX system was developed. This system contains features as continuous vacuum, making aspiration more effective and with a lower incidence of stroke. The ongoing CHEETAH trial exclusively evaluates patients with high thrombotic burden using the Penumbra CAT RX, as in the case described, to answer the real benefit on mechanical thrombus aspiration.

111654

Modality: E-Poster Young Researcher – Case Report

Category: CARDIOVASCULAR SURGERY

## Type a Aortic Dissection (Stanford) in the 36th Week of Pregnancy: Case Report of a Multidisciplinary Emergency

RAMON OTT VARGAS^1^, Elisa Ito Mendes de Andrade^1^, José Carone Filho^1^, Assad Miguel Sassine^1^, José Carone Júnior^1^

(1) Hospital Evangélico de Vila Velha

**Introduction:** Acute aortic dissection is a rare, but lethal entity during pregnancy. Difficulties in early diagnosis and small number of studies available in this group of patients are complicating factors.

**Case Description:** Female, 30 years old, hipertensive, at 35 weeks 6 days of pregnancy, coming from high-risk pregnancy referral service, where she was hospitalized due to restricted intrauterine growth. One week after admission, she developed severe chest pain and syncope. Electrocardiogram and lab work showed no myocardial ischemia. Transthoracic Echocardiogram was performed, suggesting ascending aortic dissection affecting the aortic valve, with mild regurgitation. A computed tomography scan confirmed the diagnosis of Stanford type A aortic dissection. Patient was transferred for cardiovascular surgery, accompanied by all the necessary equipment and medical personnel to perform emergency delivery. Admitted to this service with chest pain, using sodium nitroprusside. In the operating room, patient was submitted to orotracheal intubation with care to avoid cardiorespiratory depression of the fetus. Emergency C-section was performed, neonate with good vitality, Apgar 8/9. Immediately after the C-section, aortic surgery began. Dissection of the common carotid artery was performed for arterial cannulation. Deep hypothermia was induced in the patient. After aortotomy, a dissection flap was detected in the non-coronary sinus. The diseased aortic segment was excised, and both ends were reinforced with teflon matresses, and ultimately anastomosed to one another, obtaining excellent results. Patient resumes spontaneous rhythm after opening of the aortic clamp. Cardiopulmonary bypass time was 100 minutes and aortic cross clamp was 80 minutes. Patient developed sinus tachycardia and hypertension, requiring use of beta-blockers and intravenous vasodilators. Extubated after 48 hours. Patient progresses well, discharge from the Intensive Care Unit on the 6th day after surgery and hospital discharge seven days later.

**Conclusion:** Despite the dismal diagnosis of acute type A aortic dissection in pregnancy and the challenges of keeping both mother and fetus alive in the context of a complex surgery such as the one described, both the patient and the neonate showed good outcome after the approach. Accurate diagnosis and timely multidisciplinary action were decisive for the result obtained in this case.

111663

Modality: E-Poster Young Researcher – Case Report

Category: CARDIOVASCULAR SURGERY

## Staged Hybrid Approach for Treatment of Very High Risk Octogenarian Patient

JOSÉ CARONE FILHO^1^, Roberto Ramos Barbosa^1^, Elisa Ito Mendes de Andrade^1^, Ramon Ott Vargas^1^, Roger Alain Pantoja Ribera^1^

(1) Hospital da Santa Casa de Misericórdia de Vitória – ES

**Introduction:** Acute cardiac failure is a high mortality syndrome with varied ethiology. Mitral regurgitation following acute coronary syndrome is one of its causes and necessary management requires exceptional attention to haemodynamic patient balance. This paper describes the in-hospital events of a patient on which a intra-aortic ballon pump was implemented, after acute mitral insufficiency and cardiogenic shock, until surgery for valve replacement.

**Case Description:** Woman, 84 years old, hypertensive, presenting with oppressive chest pain, radiating to the jaw, even at rest. Physical exam: diaphoretic, regular general state. Irregular cardiac rhythm, normal S1 and S2, holosystolic murmur heard on cardiac apex (2+/6+). Pulmonary auscultation with bilateral rales, respiratory rate of 32 and pulse oximetry saturation of 76%. Acute miocardial infarction (without elevated ST segment) was diagnosed (Killip 3, Timi Risk 4). Transthoracic echocardiography (TTE) was performed and showed chordae rupture resulting in severe regurgitation. Left ventricular ejection fraction was 62%. Patient evolved with cardiogenic shock, respiratory failure with endotracheal intubation, requiring vasopressor. Case was discussed between a multidisciplinary team and intra-aortic balloon pump (IABP) implantation was indicated for salvage and support for percutaneous coronary intervention. Patient was at hight operative risk at that moment (EuroSCOREII: 28%). On the eighth day after admission, percutaneous coronary intervention was performed, with implantation of 3 stents (anterior descending, circumflex and right coronary arteries). After 20 days in-hospital, mitral valve replacement surgery was performed, and a bioprosthesis (size 29) was implanted. IABP was removed after termination of cardiopulmonary by-pass. Patient was discharged 40 days after admission. Control TTE showed functioning bioprosthesis and no sign of intracavitary thrombus. Patient is clinically well, receiving optimized medical therapy and no signs of decompensation.

**Conclusion:** Presence of increasingly high risk patients with multiple comorbidities and complex conditions is rising by the day in our Cardiology practices. This case shows how staged hybrid approach can be performed in high operative risk patients obtaining satisfactory results.

111666

Modality: E-Poster Young Researcher – Case Report

Category: ACUTE AND CHRONIC CORONARY DISEASE/THROMBOLYSIS

## Minoca: Always use Dapt?

RAQUEL CRISTINA FARIAS DE MEDEIROS QUEIROZ^1^, Bárbara Mariana dos Santos Silva^1^, Isly Maria Lucena de Barros^1^, Rafael Dayves Medeiros de Queiroz^1^, Suéllen Lidia da Silva Barbosa^1^

(1) Pronto Socorro Cardiológico de Pernambuco – PROCAPE/UPE

**Introduction:** Some patients presenting with ST elevation myocardial infarction had a syndrome named myocardial infarction with non-obstructive coronary arteries (MINOCA). This diagnosis requires an AMI and absence of obstructive CAD on angiography and may present with or without ST segment elevation on the ECG. Thrombosis may be a mechanism and the prevalence is low. It may arise from thrombotic disorders, atherosclerotic plaque disruption or coronary dissection with associated thrombosis or coronary emboli. To aid in diagnosis, we can use angiography with intravascular ultrasound or OCT (40% of patients with MINOCA have some evidence of plaque disruption). In this scenario, the inpatient treatment usually involves anticoagulation and the outpatient treatment may vary depending on the underlying etiology, but we know that DAPT (dual antiplatelet therapy) may be useful in those cases where plaque disruption is possible.

**Case Report:** Male patient, 39-year-old, with no previous comorbidities and denying the use of illicit substances or medications. He sought emergency care complaining of intense retrosternal pain, which had started forty minutes before, associated with sweating and vomiting. Electrocardiogram showed ST-segment elevation in anterior leads. The patient was referred for invasive stratification and received DAPT. The coronary angiography showed an image suggestive of thrombus presence in the proximal segment of the anterior descending artery, with Thrombolysis in Myocardial Infarction – TIMI 2 – distal flow, and all the vessels were free of significant stenosis. It was decided not to perform a percutaneous intervention at that time; abciximab was administered by 24 hours and anticoagulation was maintained with unfractionated heparin, as well as the concomitant use of DAPT. The new catheterization ten days later did not reveal the same image compatible with thrombus presence and showed a small plaque in the same proximal segment of the anterior descending artery. He showed clinical improvement and was discharged with a prescription of DAPT.

**Conclusion:** This case illustrates the importance of identifying the causes of MINOCA to facilitate more definitive and specific treatment strategies, such as the use of DAPT. Multimodality imaging with OCT and CMR can help in our understanding of the underlying pathophysiological mechanisms within and beyond the context of coronary atherosclerotic disease, however, are not available in our practice.

111687

Modality: E-Poster Young Researcher – Case Report

Category: CARDIOVASCULAR IMAGING

## Coronary Computed Tomography Angiography in the Evaluation of Kawasaki Disease Aneurism

GABRIELA RIBEIRO PRATA LEITE BARROS^1^, Juliana Pato Serra Souza^1^, Kevin Rafael De Paula Morales^1^, Jürgen Beuther^1^, Diana Rodrigues de Araujo^1^

(1) Instituto do Coração do Hospital das Clínicas da Faculdade de Medicina da Universidade de Sao Paulo.

**Introduction:** Kawasaki disease (KD) is an acute vasculitis that preferentially affects medium-sized arteries, particularly the coronary arteries. Patients with coronary artery aneurysms (CAA) are at risk for cardiac events including coronary artery thrombosis or stenosis, myocardial infarction and death. Coronary computed tomography angiography (CCTA) has been shown to be superior to echocardiography for the detection of CAA in distal portions of the vessel. It also a reliable modality for follow-up of CCA, with close correlation with catheter angiography.

**Case report:** A 9-year-old patient with previous diagnosis of kawasaki disease treated with immunoglobulin and history of giant coronary aneurism seven years ago, presented to the pediatric cardiology consult complaining of chest pain on exertion. Previous CCTA showed coronary dilatation in of the right coronary artery (RCA) measuring and left anterior descending artery (LAD), both without luminal reduction. A new CCTA was performed. The LAD showed a large aneurysmal dilatation in the proximal segment, measuring 10 × 10 mm with proximal luminal evaluation limited by intense calcification (Figure A). The RCA showed a fusiform aneurysmal dilatation measuring 8 × 7 mm, with mural thrombi and diffuse parietal calcifications, highlighting an area of critical luminal reduction right at the origin of the aneurysm (Figure B). Cardiac catheterization was performed, proving significant luminal reduction in RCA, and balloon angioplasty was successfully performed.

**Conclusion:** Aneurysms in Kawasaki Disease can evolve with difficult-to-treat stenosis. Coronary computed tomography angiography is an attractive alternative to catheter angiography for the assessment of coronary manifestations of the disease.


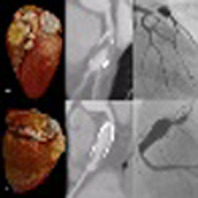


111690

Modality: E-Poster Young Researcher – Case Report

Category: ACUTE AND CHRONIC CORONARY DISEASE/THROMBOLYSIS

## Extrinsic Compression of the Left Main Coronary Artery by Pulmonary Artery as Cause of Chest Pain in a Young Patient with Pulmonary Arterial Hypertension

LARISSA MARIA VOSGERAU^1^, Roberto de Ávila Martins^1^, Pedro Felipe Gomes Nicz^1^, Paulo Roberto Cruz Marquetti^1^, Eduardo Leal Adam^1^

(1) Hospital de Clínicas da Universidade Federal do Paraná (HC-UFPR)

**Introduction:** Chest pain in patients with pulmonary arterial hypertension (PAH) is usually attributed to the imbalance oxygen supply demand to the right ventricle, which can lead to subendocardial hypoperfusion. It is known that the pulmonary artery (PA) can induce extrinsic compression of the left main coronary artery (LMCA) in these patients, and it is described that a PA trunk greater than 40 mm in diameter with typical angina is correlated with compression of LMCA in most patients. This luminal reduction can lead to acute myocardial infarction, left ventricular dysfunction, arrhythmias and even sudden death. The evaluation of coronary arteries is often not performed routinely in these patients, so the incidence of LMCA compression by the PA is not well established in literature. There is no consensus about the best therapeutic strategy, but percutaneous coronary intervention is a viable option to relief symptoms and improve outcomes with a high success rate.

**Case Report:** A 30-year-old female patient diagnosed with idiopathic PAH for 9 years and with optimized treatment seeks medical service for progressive chest pain with minimal efforts and quality-of-life impairment. During investigation, a coronary computed tomography was performed and showed a 56 mm PA trunk with a left main coronary artery ostial angulation (17.9°) and significant luminal reduction (>70%) due to extrinsic compression (figure 1). Coronary angiography shows a critical stenosis in the LMCA. A percutaneous angioplasty with implantation of two drug-eluting stents (stent-in-stent) was successfully performed and the patient evolved with complete improvement of chest pain after revascularization. Treatment of PAH was associated with dual antiplatelet therapy with subsequent outpatient reassessment.

**Conclusion:** Extrinsic compression of the LMCA should be considered as a differential diagnosis in patients with PAH and angina, especially in patients with a PA trunk greater than 40 mm. Percutaneous coronary intervention is a safe and effective option to improving symptoms and reduce cardiovascular outcomes with a low rate of complications.


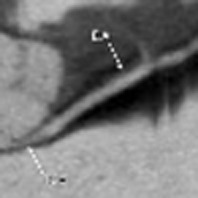


111725

Modality: E-Poster Young Researcher – Case Report

Category: NEGLECTED CARDIOVASCULAR DISEASES

## Coronary Embolism – Challenges in Diagnosis and Treatment

MATHEUS BURIGO OLIVEIRA^1^, Gisele Messias Mattioli^1^, Fernanda Nascimento Dourado^1^, Leticia Macacchero Moreirão^1^

(1) Instituto Nacional de Cardiologia

**Introduction:** Coronary embolism is a rare cause of myocardial infarction (MI). Unlike type I MI (plaque instability), its pathophysiology and etiology are not yet well defined. Some studies show it is the underlying cause of 3% of MI, but often neglected.

**Case Report:** A 31-year-old male diagnosed with dilated cardiomyopathy and hypertension was admitted with typical chest pain and Q waves in leads V1–V6 on the electrocardiogram and submitted to coronary angiography, which showed thrombi in the left anterior descending artery and second diagonal branch without other obstructive lesions. Investigation ruled out the presence of risk factors for atherosclerosis (coronary calcium score 0 and normal lipid profile), left ventricular thrombus, atrial fibrillation and paradoxical embolism. Research for thrombophilia showed antinuclear antibodies (ANA) 1:180 of pathological pattern (nucleolar homogeneous) which did not meet the criteria for diagnosis of lupus or antiphospholipid antibody syndrome (APS). We opted for conservative therapy with enoxaparin 1 mg/kg twice a day. A new coronary angiography 1 week after therapy showed a considerable reduction in thrombi.

**Conclusion:** MI caused by coronary embolism should be considered in young patients with few risk factors for atherosclerotic disease. This case illustrates the need to search for triggers of this event, notably arrhythmias, severe left ventricular dysfunction, thrombophilia and rheumatological diseases. The lack of randomized trials on this subject makes it difficult to decide on the type of anticoagulation and the duration of therapy. Finally, the underlying diagnosis is impaired by acute MI either by changes in the coagulation cascade or by the need for rapid use of anticoagulation, which may alter the results of laboratory tests.


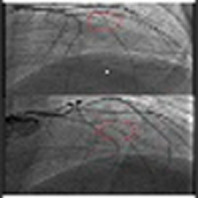


111737

Modality: E-Poster Young Researcher – Case Report

Category: PERICARDIUM/ENDOCARDIUM/VALVOPATHIES

## Dilated Cardiomyopathy with Reduced Ejection Fraction Secondary to Rheumatic Myocarditis

ARMINDO JREIGE JÚNIOR^1^, Karen Katchvartanian^1^, Luis Henrique Silveira Moreira^1^, Renan Cintra de Alvarenga Oliveira^1^, Bruno Biselli^1^

(1) Instituto do Coração do Hospital das Clínicas da FMUSP (InCor/HCFMUSP)

**Introduction:** Rheumatic fever is a possible late complication secondary to group A streptococcus infection. Repeated episodes of acute outbreaks can progress to rheumatic heart disease, which is associated with significant morbimortality in children and young adults.

**Case Description:** A 46-year-old male patient with history of rheumatic fever underwent valve replacement with a caged-ball metallic prosthesis in 1992 due to combined aortic stenosis and regurgitation. He remained asymptomatic until 2020, evolving after that with symptoms of heart failure (HF). The initial hypothesis was dysfunction of the metallic prosthesis. Transesophageal echocardiogram did not reveal prosthetic alterations, showing the presence of ventricular dysfunction, reduction of ejection fraction to 25% (previously normal), dilatation of left cavities and areas of segmental alteration. Coronary angiography showed no obstructive lesions and serology for Chagas disease was negative. Magnetic resonance imaging (MRI) of the heart showed areas of late transmural enhancement in the apical and subendocardial segments in the lateral, anterior and middle wall segments. In view of the MRI findings and the previous diagnosis of rheumatic fever, the main hypothesis was active rheumatic myocarditis. Gallium-67 scintigraphy identified the presence of increased uptake in a projection of the cardiac area with a diffuse pattern and mild degree. This result corroborated the existence of an active cardiac inflammatory process. Patient currently under outpatient follow-up, undergoing clinical evaluation for corticosteroid therapy or inclusion in the heart transplant waiting list.

**Conclusions:** The treatment of rheumatic carditis lacks consensus, with little evidence. Regarding the diagnosis and treatment of rheumatic myocarditis, the scenario is even worse, with only a few observational studies. The therapy currently proposed for severe and refractory cases, as described, is based on the use of corticosteroids. There is great variation between the data in the literature, related to the dosage of the drug to be used, treatment duration and associated pulse therapy. Heart transplantation is also a possibility for cases of terminal and refractory HF. Due to the high prevalence of rheumatic fever in our country and the clinical severity associated with rheumatic myocarditis, further studies must be done for adequate therapeutic management and reduction of morbimortality secondary to this clinical entity.

111742

Modality: E-Poster Young Researcher – Case Report

Category: CONGENITAL AND PEDIATRIC CARDIOLOGY

## Cardiac Complications After SARS-CoV-2 Infection

CAROLINA RESENDE MARIZ^1^, Catharina de Almeida Serra Faria^1^, Julia Colonese Serra^1^, Viviane Campos Barbosa de Sena^1^, Joaquim Marcio Duarte e Silva^1^

(1) INSTITUTO NACIONAL DE CARDIOLOGIA

**Introduction:** Since the beginning of SARS-CoV-2 pandemic in 2020, several consequences resulting from this infection have been reported, including myocarditis. The mechanism’s of myocardial injury are not fully understood yet, but seems to be related to direct damage to cardiomyocytes, systemic inflammation, interstitial fibrosis, mediated immune response, and exaggerated cytokine response.

**Case Description:** G.D.M, male, 4 months old, previously healthy, admitted to Intensive Cardiac Unit in acute congestive heart failure, requiring invasive mechanical ventilation, inotropic and diuretic support for 15 days. Echocardiogram revealed severe mitral insufficiency, left ventricular(LV) dilation and severe dysfunction, with ejection fraction (EF) of 18%. Angiotomography ruled out coronary artery anomalies and cardiac resonance magnetic revealed evidence of myocarditis. Laboratory tests showed elevated BNP, D-dimmer as well as troponin. SARS-CoV-2 PCR and IgM were negative, but SARS-CoV-2 IgG was positive. Also, respiratory syncytial virus and Influenza PCRs were also negative. After the diagnosis of Myocarditis related to SARS-CoV-2 infection was made, therapy with immunoglobulin, enoxaparin and acetylsalicylic acid was initiated. After this therapy, the patient improved clinically as well as laboratory and was discharged with medications. Follow up made with echocardiogram at 2 months showed significant LV dilation, with only mild improvement of EF (Teicholtz and Simpson, respectively, 27% and 24%).

**Conclusion:** Pediatric post viral myocarditis usually has a benign and self-limited course, but in some cases, it results in severe cardiac dysfunction. Given the ongoing pandemic, patients with cardiovascular dysfunction should raise suspicion for SARS-CoV-2 infection and its complications.


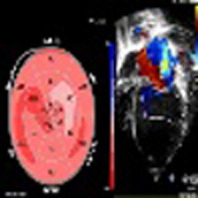


111744

Modality: E-Poster Young Researcher – Case Report

Category: CARDIOVASCULAR IMAGING

## Multilobulate Left Ventricular Pseudoaneurysm in Late Mitral Valve Replacement

GABRIELA RIBEIRO PRATA LEITE BARROS^1^, Juliana Pato Serra Souza^1^, Kevin Rafael De Paula Morales^1^, Max Walter Reyes Barrenechea^1^, Eduardo Kaiser Ururahy Nunes Fonseca^1^

(1) Instituto do Coração do Hospital das Clínicas da Faculdade de Medicina da Universidade de Sao Paulo.

**Introduction:** Left ventricular pseudoaneurysm (LVP) results from a cardiac-free wall rupture contained by adherent pericardium or scar tissue without any involvement of myocardium or endocardium. It frequently occurs in the setting of myocardial infarction, cardiac surgery and endocarditis. Patients with LVP can report symptoms of heart failure, dyspnea, or chest pain. Left ventriculography is the gold standard for diagnosis, but coronary computed tomography angiography has been increasingly used as noninvasive diagnostic technique.

**Case Report:** A 68-year-old female patient, with a previous history of mitral valve replacement, developed dyspnea and atypical chest pain. During investigation, a transthoracic echocardiogram was performed and showed an oval image with flow inside and aparent communication with the right atrium. Heart and coronary tomography showed a multiloculated pseudoaneurysm (*) with origin in the basal inferoseptal wall of the left ventricle, close to the mitral valve prosthesis. This formation maintained ample contact with the middle and distal segments of the right coronary artery, measuring about 64 × 32 × 27 mm. The image corresponded to a left ventricular pseudoaneurysm of apparent late postoperative etiology, determining compression of the of the venous sinus and in intimate communication with the right coronary artery.

**Conclusion:** Heart and coronary computed tomography is a non-invasive method for diagnosis and evaluation of LVP.


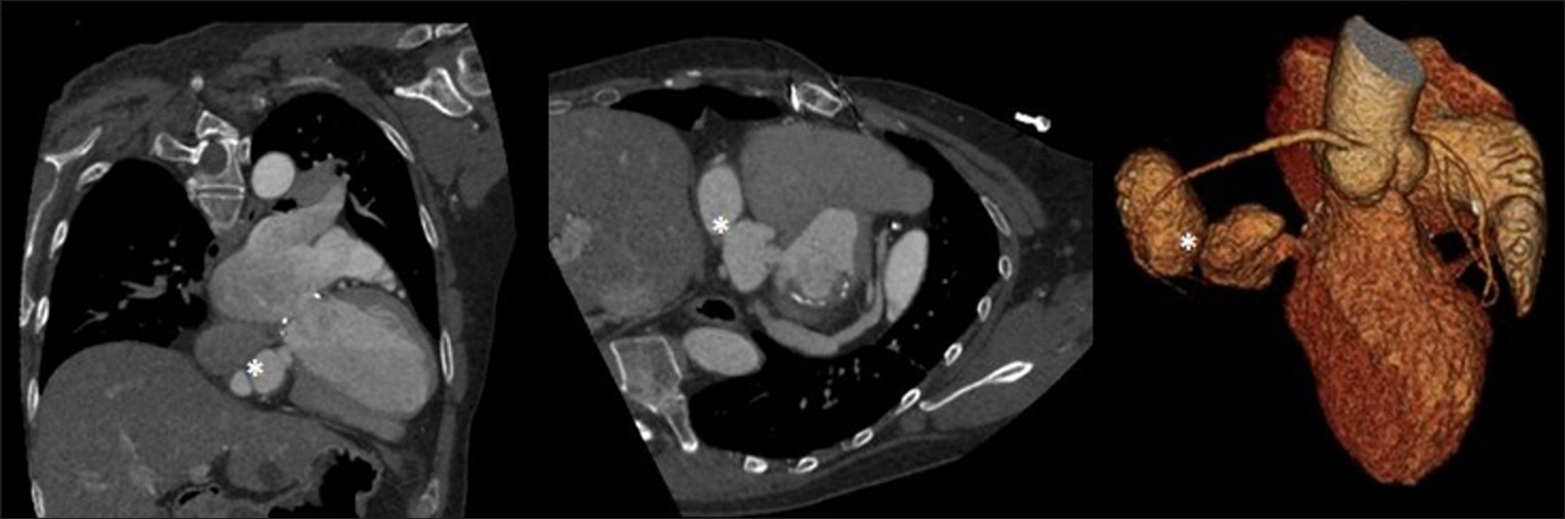


111768

Modality: E-Poster Young Researcher – Case Report

Category: PERICARDIUM/ENDOCARDIUM/VALVOPATHIES

## Complications of Lutembacher Syndrome: A Case Report

CAROLINA JANUARIO DA SILVA^1^, Diana Patricia Lamprea Sepulveda^1^, Flávio Hilton Feijó Cavalcanti Silva^1^, Raquel Cristina Farias de Medeiros Queiroz^1^

(1) PRONTO SOCORRO CARDIOLÓGICO DE PERNAMBUCO – PROCAPE

**Introduction:** Lutembacher Syndrome (LS) is a rare condition and is defined as a combination of ostium secundum atrial septal defect (ASD) and mitral valve stenosis and both can be congenital or acquired. The hemodynamic repercussions of this syndrome result from the severity of the valve stenosis and ASD sizes. This can lead to numerous symptoms and complications, such as intolerance to exertion, palpitation, pulmonary hypertension, left atrium and right chamber dilatation, atrial arrhythmias and tricuspid valve failure. The treatment can happen through open surgical correction (with ASD closure and mitral commissurotomy or valve replacement) or percutaneous therapy (with balloon mitral valvuloplasty and septal occlusion with a prosthetic device) in eligible patients. We report a case in a mild age male, with late diagnosis and complicated by atrial fibrillation and pulmonary hypertension.

**Case Report:** Male patient, 46-year-old, with previous diagnosis of systemic arterial hypertension. In 2011, he sought emergency care complaining of dyspnea on exertion and orthopnea. The echocardiogram showed an important mitral stenosis; ostium secundum atrial septal defect with a shunt from the left atrium to the right atrium; preserved left ventricle function and moderate left atrium dilatation. Based on the clinical and imaging findings, LS was diagnosed and was opted for the isolated mitral valve repair, with subsequent correction of interatrial communication – which occurred in 2013. In april 2022, the patient was hospitalized for the same symptoms he had 11 years before. The new echocardiogram showed mitral valve with reduced opening and thickened cusps, average gradient of 5 mmHg and valvular area calculated by the planimetry of 1 cm², representing an important mitral stenosis associated with a moderate mitral regurgitation; important left and right atrium dilatation; preserved left ventricle function and a moderate pulmonary arterial hypertension (with pulmonary artery systolic pressure estimated at 47 mmHg). The electrocardiogram showed a previously unknown atrial fibrillation. Clinical treatment was optimized and the patient was referred for mitral valve replacement.

**Conclusion:** Early diagnosis with correction of mitral stenosis and closure of ASD indicate a good prognosis. However, in the cases that the diagnosis occurs late, the presence of heart failure, atrial fibrillation and pulmonary hypertension is more common and the prognosis is reserved.

111776

Modality: E-Poster Young Researcher – Case Report

Category: ACUTE AND CHRONIC CORONARY DISEASE/THROMBOLYSIS

## Aneurysmal Dilation of the Coronaries: A Case Series

CAMILA DALCOMUNI DOS SANTOS^1^, Rafael Vieira Fernandes^1^, Airton Salviano de Sousa Júnior^1^, Igor Henrique Silva Leite^1^, Barbara Porto Valente^1^

(1) Instituto Dante Pazzanese de Cardiologia – IDPC

Aneurysmal dilation of the coronaries arteries has a prevalence rate between 1–3%(1). It can be classified in aneurism or ectasia. The first, defined as a focal dilation of about 1.5× the normal diameter and the second as a diffuse dilation The right coronary is the most targeted with 40% of cases, followed by 32% of the left anterior descending (2). Fifty percent are associated with atherosclerosis, the rest is caused by congenital anomalies and inflammatory diseases, such as Kawasaki’s (1). Most are clinically silent and are only detected incidentally during coronary angiography or computed tomography. Due to the lack of evidence, management is challenging. Usually, these patients are more susceptible to presenting complications like local thrombosis, distal embolization and myocardial infarction (5). The use of anticoagulants in the context of the acute coronary syndrome (ACS) is implicated in the reduction of major adverse cardiovascular events (MACE) in observational studies. Similar evidence sustains the use of dual antiplatelets (2,4). We present here a serie of 9 cases with aneurysm/coronary ectasia with median follow up of 3,5 years. All 9 patients had Myocardial infarction with non-obstructive coronary artery, 7 with ST-Elevation, due to aneurysm/coronary ectasia, of which 6 were men and 3 women, median age 54,5 years. Two patients had a family history of early coronary artery disease and previous infarction. Three patients had diabetes, history of smoking and obesity. Four patients were dyslipidemic and six had history of hypertension. One patient had atrial fibrillation/Flutter. The ejection fraction ranged from 40 to 60%, with an average of 52.7%. In the treatment, all patients were using statins and beta-blockers. Regarding antiplatelet and anticoagulation of these patients, 2 were using warfarin associated with P2Y12 inhibitors (iP2Y12), 1 was using warfarin alone, 1 was using direct-acting oral anticoagulant alone, 1 was using aspirin alone, 1 was using warfarin associated with aspirin and iP2Y12, and 2 used aspirin associated with iP2Y12. One of the patients evolved with dyspneia and 3 with angina. Three patients had another ACS. No deaths were recorded. Aneurysmal dilation of coronary vessels is a challenging and misdiagnosed entity, with lack of evidence, supporting an individualized management.

111812

Modality: E-Poster Young Researcher – Case Report

Category: ACUTE AND CHRONIC CORONARY DISEASE/THROMBOLYSIS

## Coronary Artery Vasospasm in a Patient with Chron‘s Disease

FELIPE ROSSI LORO^1^, CARLOS ALBERTO CAMPELLO JORGE^2^, GUILHERME DE MORAES FAVERO^1^, GABRIELLE MOLINA PINTO^1^, FERNANDA FURTADO REGATIERI^1^

(1) UNIVERSIDADE FEDERAL DE MATO GROSSO DO SUL – UFMS; (2) Hospital Militar de Área de Campo Grande – HMILACG

Vasospastic angina, also known as Prinzmetal angina, corresponds to a form of angina pectoris related to coronary artery spasm, which can lead to transient ST-segment elevation on the electrocardiogram. We documented a case of a 62-year-old male, a former smoker, with hypertension, with a history of atypical chest pain during exercise for 11 years, and a previous investigation with catheterization showing no findings. In the meantime, he was also diagnosed with Crohn’s Disease due to gastrointestinal symptoms. He presented to our emergency department due to typical chest pain, which had been lasting 45 minutes and started after a positive treadmill exercise stress test for ischemia. The electrocardiogram and echocardiogram performed in the emergency room showed no abnormalities. He performed catheterization that showed severe stenoses in the right coronary and the left anterior descending arteries, but while preparing for angioplasty, the stenoses resolved after intracoronary nitroglycerin injection, thereby confirming the vasospasm. The case represents an atypical presentation of variant angina. Our hypothesis is that vascular hyperreactivity caused by Crohn’s disease acted as a precipitating factor for the patient coronary spasms.


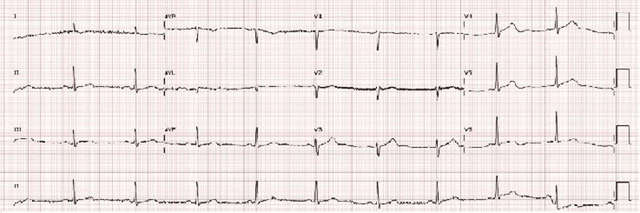


111822

Modality: E-Poster Young Researcher – Case Report

Category: COVID-19 AND CARDIOVASCULAR SYSTEM

## COVID-19, An Independent Cardiovascular Risk Factor? Case Report of a Patient with Rapid Progression of Coronary Artery Disease

JOÃO DE AZEVEDO^1^, Marcelo Kirschbaum^1^, Silvio Marques Póvoa Júnior^1^, Breno Oliveira Almeida^1^, Claudio Cirenza^1^

(1) Hospital Israelita Albert Einstein

**Introduction:** Complications of the post-acute phase of COVID-19 can manifest in several organs and systems, mainly in the lung, but also in the cardiovascular and other organs. This is an uncommon clinical case of a patient with previous history of coronary artery disease and recent angioplasty, who was infected by COVID-19. This patient evolved with rapid and significant progression of underlying atherosclerotic disease resulting in an acute coronary syndrome and sub occlusion of the main left coronary artery.

**Case Report:** A 58-year-old male patient was admitted to the emergency room with intermittent left arm pain, worsening on exertion and improving at rest, starting 24 hours before presentation. He had a history of coronary artery disease with elective angioplasty of the left anterior descending artery 2 months before in the context of stable angina, with no other coronary lesions. Later he had mild COVID 19 infection. He presented a dynamic change in the EKG, with ST-segment depression greater than 2.0 mm in DI, DII, AVF, V2–V6, in addition to ST-segment elevation in AVR, ultrasensitive troponin of 19 (VR ≤ 5). He was referred to the hemodynamics department for early invasive stratification and underwent angioplasty of the left main coronary artery due a sub occlusive lesion in that artery.

**Conclusion:** Cardiovascular risk may increase in post-acute phase of COVID-19. This is an important issue that should draw health system attention to improve assistance either for mild or aggressive post-infection patients.


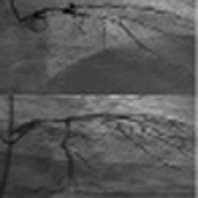


111828

Modality: E-Poster Young Researcher – Case Report

Category: CARDIOVASCULAR SURGERY

## Subaortic Ventricular Septal Defect as a Complication of Native Valve Endocarditis

EVELYN AMBROSANO ANTONIO^1^, JOSÉ EDUARDO DE LIMA BORRELLI FILHO^1^, LUCIANE FRANCISCHINI GOTTSCHALL ODONE^1^, FERNANDA MENDES^1^, VINICIUS MEARIM ODONE^1^

(1) HOSPITAL DOS FORNECEDORES DE CANA

Despite the technological and therapeutic advances, infective endocarditis (IE) remains a pathology with high morbidity and mortality. On the other hand, there is a change in its epidemiological profile, with an increase in cases related to the presence of invasive devices and valve degeneration, with staphylococcus being the main infectious agent. We report a successful case in the treatment of a severe aortic valve IE that resulted, in addition to multiple sites of embolization and cardiogenic shock resulting from acute and severe aortic insufficiency (AOI), in coronary leaflet perforation, extensive valve abscess and consequent ventricular septal defect (VSD) subaortic COP, 73, male, with indolent lymphocytic lymphoma and non-dialytic chronic renal failure. Admitted with poor general condition, fever, abdominal pain and dizziness. Submitted to infectious screening and initially diagnosed with urinary tract infection, without improvement despite empirical antibiotic therapy. After neurological worsening, associated with septic shock of abdominal focus, multiple sites of embolization and hematogenous dissemination were visualized on tomography, including pyelonephritis, splenic abscess, cerebellar ischemia and skin lesions characterized as Janeway lesions. Identified in paired blood cultures S. Aureus infection. He underwent transesophageal echocardiography, which confirmed filamentous vegetation in a 10 mm native valve associated with valve dysfunction – moderate aortic insufficiency. On the 45th day of treatment, he presented rapid clinical deterioration to cardiogenic shock and cardiorenal syndrome, secondary to acute and severe AOI, and urgent surgical treatment was chosen. He underwent aortic valve replacement with a biological prosthesis, with intraoperative visualization of right coronary sinus perforation, extensive valve abscess and contiguity to the septum, resulting in a VSD approximately 1 cm below the aortic valve, which was corrected at the same surgical time. Maintained in intensive support and prolonged antibiotic therapy, with resolution of the infection, cardiac compensation and recovery of renal function We report the case of successful clinical and surgical treatment in a severe IE of native aortic valve, with positivity in all diagnostic criteria, multiple embolizations and rapid evolution to valve insufficiency, associated with abscess and consequent subaortic VSD, an uncommon but serious and potentially fatal local complication.

111870

Modality: E-Poster Young Researcher – Case Report

Category: HEART FAILURE/CARDIOMYOPATHY/TRANSPLANT

## Focal Contraction Pattern in Takotsubo Syndrome: A Case Report

FELIPE HADDAD LOVATO^1^, Arthur Antunes Silva Castro^1^, Maria Julia Palitot de Melo^2^, Isabella Secol Corteze^1^, Isabella Souza Oliveira^1^

(1) Hospital Sírio-Libanês (HSL); (2) Pontifícia Universidade Católica de Campinas (PUCCAMP)

**Background:** Takotsubo cardiomyopathy (TCM) is a reversible left ventricular dysfunction triggered by emotional or physical stress, which closely mimics acute coronary syndrome (ACS) and can presents with chest pain and dyspnea, dynamic ST/T changes, elevated myocardial necrosis biomarkers and and regional left ventricular (LV) wall motion abnormalities, without significant coronary artery disease. It is particularly frequent in 65–70 year-old female patients and should be considered as a differential diagnosis in episodes of chest pain. We describe the case of a 69 year-old woman who presented with chest pain while under stress and was then diagnosed with a focal variant of TCM, which represents 1.5% of patients in the International Takotsubo Registry study. SUMMARY M.E., 69 year-old female, with previous diagnosis of hypertension, presented to the emergency department with a 3 hour long chest pain during emotionally stressful event. On admission, ECG showed ST-segment depression in the inferior wall and V4–V6, with asymmetric negative T-wave in V2–V3, and laboratory tests showed high troponin. With the suspection of ACS, cardiac catheterization was indicated and showed no obstructive lesions. Echocardiogram showed hypokinesia in the anterior basal region. During investigation, the myocardial MRI showed a focal akinesia of the anterior middle segment of the LV. After these findings, we diagnosed TCM with focal involvement.

**Conclusion:** TCM is an underdiagnosed cause of chest pain. It should be considered a differential diagnosis especially in postmenopausal women presenting with symptoms similar to ACS. This cardiomyopathy is often delayed, so it is important that physicians maintain a high index of suspicion for TCM, mainly in patients with few cardiovascular risk factors.


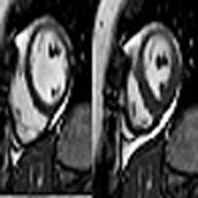


111877

Modality: E-Poster Young Researcher – Case Report

Category: CARDIOVASCULAR SURGERY

## Recurrent Left Ventricular Pseudoaneurysm Secondary to Myopericarditis – Case Report

MARIANA SILVEIRA DE ALCANTARA CHAUD^1^, Laís Olivo Rossi^1^, Felipe Lopes Malafaia^1^, Pedro Gabriel de Melo Barros e Silva^1^, Mucio Tavares de Oliveira Junior^2^

(1) Hospital Samaritano Paulista; (2) Instituto do Coração do Hospital das Clínicas da Faculdade de Medicina da Universidade de São Paulo

**Introduction:** Left ventricular (LV) pseudoaneurysm is a rare disorder. It results from the rupture of the LV free wall and leads to hemorrhagic process contained by pericardium or scar tissue. LV pseudoaneurysms are most commonly caused by myocardial infarction. It can also be identified after cardiac interventions, infections or trauma. We describe a rare case of LV pseudoaneurysm secondary to myopericarditis with recurrences after first approach.

**Case Report:** A 36-year-old man, with past medical history of hypertension, presented to our hospital with a 3-year complaint of chest pain, worsening in the last thirty days. Discomfort was described as sharp and radiated to left shoulder. It was associated to dyspnea on moderate exercion. Admission tests revealed negative troponin. Coronary computed tomography angiography (CTA) had no obstructive lesions. Echocardiogram confirmed LV pseudoaneurysm. At magnetic ressonance imaging, LV pericardial and mesoepicardial thickening was observed, with delayed enhancement suggesting myopericarditis, in addition to apical thrombus image. Coronary angiography confirmed the diagnosis, and aneurysmectomy was indicated. Surgery was uneventful and he was discharged in good condition twelve days after surgery. Five months after surgery, patient presented with chest pain and fever. Chest CTA showed a saccular formation at the apex of the LV. The patient was admitted for re-approach. Aneurysmal sac was sewn with anchored stitches and it was closed over the orifice without complications. He was discharged on the seventh postoperative day with good clinical evolution. Six months after the second intervention, patient had chest pain again. Echocardiogram showed LV rupture and communication to a neocavity with thrombus formation, which led to a new surgical approach with a vascular plug. On the fifth postoperative day, he was discharged with a well-positioned apical plug, obliterating the orifice. Six months later, a control echocardiogram was performed with an apical occluder device without shunt, apical hypokinesia and preserved LV ejection fraction. At one-year follow-up the patient remains asymptomatic and practicing physical activity.

**Conclusion:** The case demonstrates that the presence of LV pseudoaneurysm resulting from myopericarditis, although rare, must be identified soon with multimodality tests. Surgical correction must be performed before long in order to prevent its rupture and provide quality of life for the patient.

111879

Modality: E-Poster Young Researcher – Case Report

Category: CARDIOVASCULAR SURGERY

## A Case of Tricuspid Insufficiency with Late Evolution

ANA LUIZA DE OLIVEIRA QUEIROZ TEOFILO^1^, WILSON ANDRÉS MENA SILVA^1^, Amanda de Miranda Constantino^1^, Luiz MInuzzo^1^, Daniel Dantas^1^

(1) INSTITUTO DANTE PAZZANESE DE CARDIOLOGIA

**Introduction:** Tricuspid insufficiency (TI) is thought to be a rare complication of blunt, non-penetrating chest trauma. The right ventricle (RV) is immediately behind the sternum, predisposing it to an anteroposterior compression type of injury, especially during the end diastolic phase.

**Case Description:** A previously healthy 39-year-old male without cardiac history was admitted to the emergency room with symptoms of heart failure and atrial flutter. Transthoracic echocardiogram showed significant tricuspid regurgitation, with an image suggestive of chordae rupture related to the anterior leaflet, with significant enlargement of the right chambers (right atrium indexed volume 115 ml/m²), moderate RV systolic dysfunction. After clinical stabilization, a history of high-energy automobile trauma occurred 20 years ago was identified, which resulted in traumatic brain injury and blunt abdominal trauma, requering abdominal surgeries; did not have any cardiovascular symptoms. Since then, he has not performed any cardiovascular follow-up. However, about three years ago, after starting to practice intense sports, he noticed of fatigue and dyspnea. He noticed progression of symptoms, seeking care in the emergency room with dyspnea on minimal exertion, palpitations and chest pain. In the heart team it was indicated surgical correction for severe TI. In the intraoperative, rupture of the chordae was observed, with calcification of the related papillary muscle and significant dilation of the tricuspid ring (60 mm);he was underwent valve replacement for a biological prosthesis in the tricuspid position. Postoperative echocardiogram showed the prosthesis in tricuspid position without reflux and maximum gradient of 8 mmHg.

**Conclusion:** We report a case of traumatic TI, manifesting symptoms 20 years after the trauma. The interval between the traumatic event and the appearance of symptoms is variable, possibly due to the variability of possible associated valve lesions. While papillary muscle rupture is associated with more severe and acute symptoms (weeks to months), chordae rupture more commonly causes a slower course –10 to 25 years. The timing and appropriate treatment of traumatic TI depends on the natural course of the disease. Most cases do not require surgical intervention in the immediate period after the event. However, in the medium and long term, surgical correction of the tricuspid valve -by replacement or valve repair- is indicated to improve symptoms and avoid permanent impairment of the RV.

111887

Modality: E-Poster Young Researcher – Case Report

Category: ACUTE AND CHRONIC CORONARY DISEASE/THROMBOLYSIS

## Interventricular Communication After Acute Inferior Wall Myocardial Infarction with Good Clinical Outcome: A Case Report

MARIANA DE PAULA PERES^1^, Clarissa San Thiago^1^, Eduardo Rosa da Silva^1^, Camila Bussolo Schmitt^1^, Evandro de Campos Albino^1^

(1) Instituto de Cardiologia de Santa Catarina

Interventricular communication (IVC) after acute myocardial infarction (AMI) is more common after anterior wall infarction, in the apical septal region, but, less commonly, it can occur in inferior wall AMI, at the base of the septum. It is a serious and potentially fatal complication that can suddenly lead to cardiogenic shock. Female patient, 56 years old, cleaning lady, is admitted to the emergency department with burning chest pain, of strong intensity, beginning 2 hours ago, irradiated to the left upper limb. She had arterial hypertension (SAH), diabetes mellitus, dyslipidemia and obesity, in addition to being an active smoker, with early family history of acute coronary syndrome. On admission, she was hemodynamically stable, respiratory and cardiac auscultation without alterations, as well as the remainder of the physical examination. The electrocardiogram (ECG) presented a tracing with sinus rhythm and ST-segment elevations in leads referring to lower walls. Coronary angiography visualized an obstruction of the distal third of the right coronary artery (RCA). Primary angioplasty of the lesion in the RCA was successfully performed. Transthoracic echocardiogram (TTE) showed aneurysm in the inferior wall of left ventricle (LV), LV concentric remodeling, grade I diastolic dysfunction and ejection fraction preserved despite segmental myocardial involvement and mild mitral regurgitation. Five days after hospitalization, she evolved with the presence of a 4+/6+ systolic murmur in the lower left sternal border, and a new TTE was requested, which showed IVC in mid-inferobasal segment from the base of the aneurysm, neck diameter estimated in 1.5 cm and diameter of the outlet in the right ventricule estimated at 0.6 cm, with mild hemodynamic repercussion. After one day, the patient started with symptoms of pulmonary congestion and showed clinical improvement with furosemide. Correction of post-AMI IVC with bovine pericardium was successfully performed twice weeks after identification via TTE. A new TTE was performed, and showed an intact interventricular septum (IVS). Currently, the surgery is indicated even in hemodynamically stable pacientes and with preserved left ventricular function, due to the possibility of abrupt expansion of the local rupture, resulting in sudden hemodynamic collapse. The shutdown surgery remains the procedure of choice, but the timing is controversial, and it should be performed as early as possible in cases of cardiogenic shock.

111893

Modality: E-Poster Young Researcher – Case Report

Category: PERICARDIUM/ENDOCARDIUM/VALVOPATHIES

## Cardiac Tamponade as the First Manifestation of Systemic Histoplasmosis

FLÁVIA DE MORAES PEDRO MOISÉS^1^, Bernardo Cleto Teles e Silva^1^, Andrea Haddad^1^, Alessandra Godomiczer^1^, João Felipe Tamiozzo Reis^1^

(1) Hospital Unimed Rio

Histoplasmosis is an infection caused by inoculation of the fungus Histoplasma capsulatum into soil or by dust contaminated with bird or bat feces. Prolonged and intense exposure, age and immunosuppression are risk factors. It may be restricted to the lung or spread hematogenously to other organs. Most cases are asymptomatic or mild, progressing without diagnosis. Cardiac manifestations are endocarditis, mediastinal granuloma, fibrosing mediastinitis, pericarditis and cardiac tamponade. Patient 27 a, male, without comorbidities, denies drinking and smoking, delivery man at a food distributor. He started having an afternoon fever for 1 month, dry cough, hyporexia, sweating, weight loss and pain in the right hypochondrium. Tachycardic and feverish, pulmonary auscultation reduced in bases. Chest CT with consolidations and nodules with subpleural soft tissue density in the right lower lobe, right pleural effusion with a heterogeneous mass in the right paratracheal chain and voluminous pericardial effusion. CT abdomen with lymph node enlargement at the height of the hepatic hilum. Laboratory without leukocytosis, lymphopenia, hepatogram without alterations, normal ESR and C-reactive protein. Echocardiogram with significant pericardial effusion, signs of tamponade with diastolic restriction, inferior vena cava without variation. Drainage 750 ml of pericardial fluid and make a pleuropericardial window with biopsy. Pericardial fluid with hemorrhagic appearance, lactate dehydrogenase 689, glucose 74, proteins 5.4 g/dl, adenosine deaminase 69 g/dl, BAAR negative, cytology without cell atypia, PCR for M. tuberculosis and negative fungal culture. Serology for dengue, human immunodeficiency virus, hepatitis B and C and COVID negative. A lymph node biopsy, right paratracheal mass and parietal pleura showed a granulomatous chronic inflammatory process involving mediastinal and pleural fibroadipose connective tissue with areas of central necrosis, presence of rounded and minute structures in the necrotic central portion of the granulomas suggestive of fungal yeasts from Histoplasma sp with absence of AFB. Although the patient did not present risk factors, did not present pulmonary lesions and presented a non-specific condition, the diagnosis was suspected due to paratracheal lymph node enlargement with a granulomatous aspect, increased incidence in the state and after exclusion of common causes of tamponade. Stable discharge for outpatient follow-up.

111958

Modality: E-Poster Young Researcher – Case Report

Category: CONGENITAL AND PEDIATRIC CARDIOLOGY

## Transient Ischemic Attack + Acute Myocardial Infarction + Pulmonary Embolism – Three Comcomitant Embolic Events in a Patient with Patent Foramen Ovale

BARBARA VIDIGAL DOS SANTOS^1^, Laís Villela Costa Vazquez^1^, Rayane Fontoura Koch^1^, Mariane Higa Shinzato^1^, Maria Júlia Silveira Souto^1^

(1) Instituto Dante Pazzanese de Cardiologia

**Introduction:** Patent foramen ovale (PFO) is the persistence of the opening between the atrial septum primum and atrial septum secundum at the location of the fossa ovalis. It is present in about 25% of adults and is a casual finding without hemodynamic repercussions. However, studies prove an association between PFO and potentially severe clinical conditions, such as ischemic stroke, pulmonary embolism (PE), and, more rarely, acute myocardial infarction (MI). Despite well-documented consequences of paradoxical embolism through the PFO, the passage of venous clot from a patent foramen has been rarely described in the literature. We report a case of a patient with PFO and manifestation of three concomitant embolic events: venous thromboembolism, transient ischemic attack, and MI.

**Case Description:** A 49-year-old woman with a history of idiopathic pulmonary arterial hypertension, asthma, PFO, and two previous episodes of PE sought emergency care with dyspnea on mild exertion for 2 weeks and oxygen desaturation on room air. She was admitted for compensation and clinical investigation. Chest CT angiography revealed dilatation of the pulmonary trunk and filling defects in the segmental and subsegmental arteries bilaterally. Full anticoagulation was started with enoxaparin. On the fourth day of hospitalization, the patient presented sudden burning chest pain, frontal headache, decreased consciousness, and tachypnea. ECG showed ST-segment elevation inferior MI and complete heart block, which spontaneously reverted to sinus rhythm. Patient also developed sudden left-sided hemiparesis and ipsilateral hemineglect. Non-contrast brain CT ruled out hemorrhage and aortic angiotomography excluded dissection. Due to the reported PFO, an embolic etiology was hypothesized and it was decided not to perform a coronary cineangiography and also to suspend antiplatelet drugs. The patient had complete reversal of the deficits within hours and no changes were noted in a subsequent brain CT. Cardiac magnetic resonance imaging confirmed inferior acute MI, and echocardiogram revealed PFO with small right-to-left shunt. After 18 days, the patient was discharged under oral anticoagulation and outpatient follow-up revealed no further complications.

**Conclusion:** We report a case of paradoxical embolism manifested in distinct sites in a young patient with PFO, with good outcomes with anticoagulant therapy.

111964

Modality: E-Poster Young Researcher – Case Report

Category: ACUTE AND CHRONIC CORONARY DISEASE/THROMBOLYSIS

## Inverted Takotsubo Cardiomiopathy in Middle Aged Man: Case Report

JOÃO VICTOR LIMA DANTAS ^1^, Hellen Dutra Passos^1^, Josefa Camila Menezes Reis Carvalho^1^, Antonio Carlos Sobral Sousa^1^, Joselina Luzia Meneses Oliveira^2^

(1) Hospital São Lucas – HSL; (2) Universidade Federal de Sergipe – UFS

Takotsubo’s cardiomyopathy is defined as a transient abnormality of left ventricular wall movement, triggered by severe emotional or physical stress, in the absence of coronary obstruction. It is estimated that the syndrome is present in 2% of all patients who come to the emergency room for suspected ACS. Multiple variants have been described, classified according to the site of involvement of the left ventricular wall. The inverted form (Inverted Takotsubo), which corresponds to basal hypokinesia/akinesia, associated with apical hyperkinesia has a prevalence of 2.2%, occupying the 3rd place among the 4 described forms, therefore, a rare condition. We report the case of a previously healthy male patient, 56 years old, hospitalized due to left tibial fracture after falling from his own height, which evolved with hemodynamic instability followed by CRP during anesthetic induction to correct the trauma. The patient was submitted to the investigation and received the diagnosis of inverted Takotsubo. Although the inverted variant is more prevalent in women and young people, the greater knowledge of the pathology has shown increased incidence in men, with physical stress being the main triggering factor. The pathophysiological process of inverted Takotsubo is similar to its typical variant and not completely understood, but there is considerable evidence that sympathetic stimulation is the central mechanism of its pathogenesis.

111970

Modality: E-Poster Young Researcher – Case Report

Category: CONGENITAL AND PEDIATRIC CARDIOLOGY

## Scimitar Syndrome: Is the Adult Always Asymptomatic?

AMANDA DE MIRANDA CONSTANTINO^1^, Vivian De Biase^1^, Beatriz Moura Sucupira Tajra^1^, Alana Osterno Moreira Linhares^1^, Mardelson Nery de Souza^1^

(1) Instituto Dante Pazzanese de Cardiologia

**Introduction:** Scimitar syndrome (CS) is a rare congenital cardiac malformation that occurs in 2/100,000 live births with a 2:1 predominance in females, it belongs to the group of partial anomalous pulmonary venous connections, which has numerous described variations, being a subtype that constitutes only 3–5% of congenital cardiac anomalies. It is classified as a heart disease with increased pulmonary and cyanogenic flow, its diagnosis is usually early, but we will highlight the importance of attention to this pathology in adult patients without a previous diagnosis.

**Case Report:** Female patient, 39 years old, with dyspnea for 3 years, with progressive worsening of functional class. On physical examination, pulmonary auscultation was normal with 98% saturation. On cardiac auscultation, regular rhythm, with fixed split S2 and ejective systolic murmur 2+/4+ in the middle right sternal border. A chest X-ray was performed during the investigation, which showed a slightly arched tubular structure towards the right atrium with dextrocardia. In transthoracic echocardiogram evidenced anomalous pulmonary venous connections: the right pulmonary veins drain into the right atrium at the mouth of the inferior vena cava, with free flow, and the left into the left atrium. Ostium secundum interatrial communication of 13.5 mm with bidirectional flow, significant dilation of the right cavities, with right ventricular myocardium with mild hypertrophy, with preserved function. PSVD 51 mmHg and hypoplastic right pulmonary artery.

**Discussion:** SC is a rare disease characterized by anomalous venous drainage from the lung directly into the inferior vena cava. The disease may be associated with right pulmonary hypoplasia, dextrocardia, cardiac abnormalities, and systemic pulmonary collaterals. The present case report draws attention to the late discovery of a congenital heart disease, with the patient already symptomatic and with possible important hemodynamic repercussions, highlighting the importance of always evaluating the clinical picture of dyspnea and heart failure in adults during the routine investigation. the presence of a congenital heart disease.

112001

Modality: E-Poster Young Researcher – Case Report

Category: CARDIOVASCULAR IMAGING

## Role of Cardiac Magnetic Resonance in Cardiac Sarcoidosis Diagnosis

YURI RHIOS ARAÚJO SANTOS^1^, Luiz Henrique de Oliveira Bernardino^1^, Yuli Mendes de Souza^1^, Victor dos Reis Cunha^1^, Thaiz Ruberti Schmal^1^

(1) Universidade Federal de Juiz de Fora, UFJF

**Introduction:** Sarcoidosis is an idiopathic systemic inflammatory disease marked by the formation of non-caseating granulomas, most prevalent in middle aged women and afro descendants. Diagnosis is made based on clinical, imaging, and histological findings after excluding other causes of granulomatous diseases. Although rare, Cardiac Sarcoidosis (CS) is the second cause of death in affected patients. Poor prognosis is related to ventricular dysfunction and severe arrhythmias. Although endomyocardial biopsy allows final diagnosis, guidelines using clinical and imaging criteria have been proposed to avoid invasive procedures.

**Case Report:** A 41-year-old black woman complaining of headache develops amaurosis, hypoacusis, cutaneous lesions, dyspnea, hypotension, and tachycardia. Meningeal biopsy revealed non necrotizing granulomatous inflammation, leading to the diagnosis of neurosarcoidosis. In face of cardiac symptoms, cardiac involvement was also suspected. The electrocardiogram showed sinus tachycardia. Echocardiogram revealed moderate left ventricle (LV) systolic dysfunction, reduced Global Longitudinal Strain and hypokinesia of multiple wall segments. Magnetic Resonance Imaging exhibited mild LV global dysfunction and segmental wall dysfunction of multiple segments. Specific T2 weighted sequences showed small regions of myocardial edema and the specific sequences for late enhancement demonstrated subepicardial fibrosis of the septal apical and anterolateral basal segments and transmural fibrosis of the inferolateral medial wall as seen in the annexed image. The diagnosis of sarcoidosis with meningeal and cardiac involvement was made and imunossupressor systemic therapy was started with standard heart failure medication, leading to clinical stabilization. The patient was discharged and continued its follow-up as an outpatient.

**Conclusion:** The current report shows that it is possible to diagnose CS in patients with a pre-established diagnosis of sarcoidosis through clinical and imaging findings. As a result, adequate treatment may be started as soon as the diagnosis is made, avoiding disease progression.


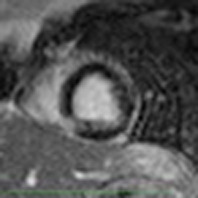


112012

Modality: E-Poster Young Researcher – Case Report

Category: CARDIOVASCULAR INTENSIVE CARE/CARDIOVASCULAR EMERGENCIES

## Aortic Dissection as a Manifestation of Lupic Disease Activity: Case Report

RAYANNE KALINNE NEVES DANTAS^1^, Dieison Pedro Tomaz da Silva^1^, Ramon Felix de Avila^1^, Eduardo Caetano Abujamra^1^, Luciano Vaccari Grassi^1^

(1) Hospital de Base São José do Rio Preto; (2) Faculdade de Medicina de São José do Rio Preto

**Introduction:** Systemic lupus erythematosus (SLE) is often associated with cardiovascular lesions, and may evolve mainly with pericarditis and myocarditis. Aortitis is a rare complication in SLE and can present itself in a variety of ways, from minimal aortic valve regurgitation to aneurysm with risk of dissection. This is an extremely rare complication, being more commonly found in patients with arterial hypertension and prolonged use of corticosteroids, and, in many cases, they are not diagnosed early and are documented during the perioperative period or by autopsy.

**Case Report:** Female patient, 49 years old, with a previous diagnosis of systemic lupus erythematosus, systemic arterial hypertension and membranous nephropathy, using prednisone 5 mg/day, hospitalized after an outpatient consultation due to pancytopenia, arthritis and worsening of renal dysfunction, hemodynamically stable. One day after hospital admission, he developed tight chest pain, started at rest, of strong intensity and with spontaneous improvement. An electrocardiogram showed diffuse changes in ventricular repolarization and, on laboratory examination, he showed an ascending troponin curve. The possibility of lupus myocarditis was questioned and the decision was made to start pulse therapy with methylprednisolone. The patient evolved with orthopnea and jugular distension, without improvement with intense diuretic therapy, muffled heart sounds and desaturation, requiring supplemental oxygen, and was transferred to the intensive care unit. An emergency transthoracic echocardiogram was performed and confirmed the diagnosis of cardiac tamponade, probably due to lupus pericarditis. During the surgical approach for pericardiocentesis, an important acute aortic dissection was visualized. Surgical approaches for both conditions were uneventful, and today, the patient is clinically and hemodynamically stable from a cardiovascular point of view.

**Conclusion:** Thoracic aneurysms and vascular dissections, mainly aortic, in SLE have a greater correlation with disease activity, in addition to a high risk of fatal outcome. Given this scenario, it is important to remember that aortitis is a cardiovascular manifestation in these patients, aiming for timely treatment to reduce death and disabling sequelae.

112028

Modality: E-Poster Young Researcher – Case Report

Category: CARDIOVASCULAR INTENSIVE CARE/CARDIOVASCULAR EMERGENCIES

## Initiating Sacubitril-Valsartan in Patient with Cardiogenic Shock in use of Inotropic Medication

ANDRESSA DE MEDEIROS PULCHERIO TOSETTO^1^, Joana Carolina Junqueira Brum^1^, Vanessa Grings^1^, Luiz Claudio Danzmann^1^

(1) Hospital São Lucas PUCRS – HSL PUCRS; (2) Santa Casa de Misericórdia de Porto Alegre

**Introduction:** The withdrawal of inotropic drugs in patients with heart failure with reduced ejection fraction (HFrEF) is frequently challenging. In this group of patients, peripheral vasodilatation contributes for clinic and hemodynamic improvement (1). For this reason, the vasodilatory properties of neprilysin inhibitors can be of great value in this scenario. However, there’s little data available in regard to the use of neprilysin inhibitors in patients receiving inotropic drugs.

**Case Report:** A 71-year-old male patient with previous diagnosis of HFrEF with ischemic etiology (Simpson EF 31%) was admitted in a hospital in south of Brazil after cardiac arrest in a public street. The cardiac arrest rhythm was identified by first responders as ventricular fibrillation. Patient was immediately referred to coronary angiography which showed total occlusion of the circumflex coronary artery, subocclusion of the right coronary artery and severe stenosis of the anterior descending coronary artery. Performed angioplasty of the anterior descending coronary artery and intra aortic balloon and Swan-Ganz catheter insertion. Simultaneously, he needs hemodynamic support with noradrenaline, vasopressin and dobutamine. After four days of intensive care he presents hemodynamic and laboratory improvement, withdrawal of noradrenaline and vasopressin and maintenance of dobutamine at moderate doses. At this moment, sacubitril-valsartan is added to the therapeutic regimen. Initially, there were some episodes of hypotension (systolic arterial pressure 80–85 mmHg), but after 48 hours of medication was observed improvement in lactatemia and central venous oxygen saturation without hiperkalemia or worsening of renal function. In this context, it was possible to withdrawal dobutamine and discharge patient from ICU. Later, patient was discharged from the hospital with an optimized dose of sacubitril-valsartan and after 2 months, he maintained clinical stability and functional class NYHA II.

**Conclusion:** The PIONEER-HF study (2) showed the safety of starting neprilysin inhibitors in a hospital setting, excluding patients in use of vasoactive drugs. In this case report, it was decided to start the medication in a patient while in use of an inotropic medication. This approach was taken considering data of a small retrospective analysis of 25 patients with HFrEF in use of vasodilators and/or inotropes (3). In this study, the use of neprilysin inhibitors was well tolerated and impro.

112057

Modality: E-Poster Young Researcher – Case Report

Category: ACUTE AND CHRONIC CORONARY DISEASE/THROMBOLYSIS

## ST-Elevation Myocardial Infarction with Two Culprit Arteries

MILENA JOLY KULICZ^1^, Alana Ohashi^1^, Isadora Daleffi Zocoler^1^, Daniel Hideki Tashima^1^, Talita Beithum Ribeiro Mialski^1^

(1) Complexo Hospital de Clínicas UFPR (CHC-UFPR)

**Introduction:** The ischemic heart disease is the main cause of death in Brazil and worldwide. The following case illustrates a diagnoses of ST-elevation myocardial infarction (STEMI) with two culprit arteries.

**Case Report:** A 53-year-old man, obese and active smoker (40 pack-years), admitted at a tertiary hospital with typical chest pain for 10 hours. The admission electrocardiogram showed right bundle branch block and ST-elevation on anterior wall, DIII and aVF leads. The patient received loading doses of aspirin and clopidogrel and was referred to invasive coronary angiography, which demonstrated proximal occlusion of the right coronary artery (RCA), 99% stenosis with thrombus inside of proximal anterior descending coronary artery (LAD), and 80% stenosis of distal left coronary artery (LCA). Percutaneous coronary intervention (PCI) was performed with stent placement in the RCA and LAD. The patient was referred to the cardiac ICU requiring doses of vasoactive drugs for 24 hours, presenting significant clinical and hemodynamic improvement. After Heart Team discussion, it was performed PCI with stent placement in the LCA. The patient was discharged asymptomatic from hospital, with left ventricular ejection fraction of 39%, in addition to septal, apical, mid anterior and inferior akinesia, receiving STEMI and heart failure treatment.

**Discussion:** Multiple Culprit Arteries are rare in STEMI and associated with worse outcomes, death and high incidence of complications, including cardiogenic shock and ventricular arrhythmias. The presented case highlights the STEMI with two culprit vessels (RCA and LAD), severe stenosis of distal LCA and good evolution after PCI.


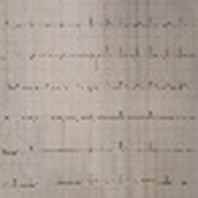


112060

Modality: E-Poster Young Researcher – Case Report

Category: CARDIOVASCULAR IMAGING

## Left Ventricular Pseudoaneurysm After Acute Myocardial Infarction: Case Report

HEBER HENRIQUE ALVES LESSI^1^, Santiago Andres Castro Vintimilla^1^

(1) Instituto do Coração

Left ventricular pseudoaneurysm is a very rare complication after acute myocardial infarction, which results from ventricular wall rupture. Although rare, it can be catastrophic due to imminent risk of rupture and death. The differential diagnosis with true aneurysm is very important because it implies the patient’s prognoses. We report an interesting case of presentation of left ventricular giant pseudoaneurysm after myocardial infarction in a 72-year-old man, with a history of acute coronary syndrome eighty days before he sought our service, admitted to the emergency unit with new chest pain that had started 24 hours ago. About 48 hours after admission, the patient underwent cardiac catheterization, showing multivessel disease and inferior aneurysm on cardiac ventriculography. A transthoracic echocardiogram was performed that showed an pseudoaneurysm in the basal segment of the inferior wall. The inferobasal pseudoaneurysm has a neck of 2.8 cm, a length of 4.7 cm and a distal width of 3.8 cm. Cardiac Magnetic Resonance confirmed the diagnostic in the left ventricle with increased dimensions at the expense of a giant pseudoaneurysm in the basal segment of the inferior wall and the presence of intracavitary thrombus measuring 16 × 5 mm, in the lower basal wall. The patient underwent surgical correction of the pseudoaneurysm with geometric reconstruction of the left ventricular due to free wall rupture and coronary arterial bypass grafting. A favorable hemodynamic evolution and discharge from the intensive care unit on the third postoperative day. About one week after discharge from the ICU he developed infectious complications and required readmission to the intensive care unit and died 30 days after cardiac surgery.


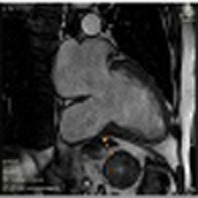


112066

Modality: E-Poster Young Researcher – Case Report

Category: CARDIOVASCULAR IMAGING

## Prosthetic Valve Uptake on FDG PET/CT in Absence of Endocarditis: What are the Possibilities?

LEONCIO BEM SIDRIM^1^, Simone Cristina Soares Brandão^2^, Diana Patrícia Lamprea^1^, Thais Araújo Nóbrega^1^

(1) Pronto Socorro Cardiológico de Pernambuco – PROCAPE/UPE; (2) Hospital das Clínicas – UFPE

**Introduction:** Positron emission tomography with 18F-fluorodeoxyglucose (FDG PET/CT) is increasingly used in cases of Prosthetic Valve Endocarditis (PVE). However, the specificity of the method still represents a limitation. It is recognized that an uptake in the perivalvular area can occur in the absence of infection. The persistence of uptake in the periprosthetic area can be explained by the persistence of a healing process around the seam ring, with endothelization. Such a process can even trigger marked fibroblast proliferation, leading to obstructive pannus in the most severe cases.

**Case Description:** Male, 51 years old, with a history of rheumatic heart disease, with two biological aortic and mitral valve prostheses implanted 2 years ago. Admitted with paresthesia and labial commissure deviation for 3 weeks. He denied fever. Cardiac auscultation without murmurs. Cranial tomography showed hypodensity in the caudate nucleus related to the sequel of an ischemic insult. A transesophageal echocardiogram was performed, which showed a dysfunctional mitral bioprosthesis with a filamentous image measuring 3.0 cm, adhered to one of the leaflets. Blood cultures did not show bacterial or fungal growth. FDG PET/CT showed homogeneous hyperuptake in mitral and aortic perivalvular topography (maximum SUV of 5.49 and 5.28, respectively). The patient underwent surgery for valve replacement due to prosthetic dysfunction. The intraoperative finding was of vegetating lesions adhered to the prosthesis. The culture of the prosthesis was negative.

**Conclusion:** Caution is needed when interpreting FDG PET/CT in suspected PVE, and special attention should be paid to the CVP uptake pattern and implantation time. Finally, the need to integrate FDG PET/CT results with the clinical context is highlighted.


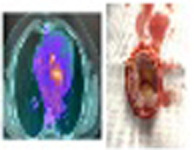


112081

Modality: E-Poster Young Researcher – Case Report

Category: COVID-19 AND CARDIOVASCULAR SYSTEM

## COVID-19 is the New Lupus? Chronic Pericarditis as Another Presentation of SARS-COV-2 Infection

BRUNO LINHARES AZEREDO CORRÊA^1^, Jaime Lobo Figueiredo^1^, Sabrina Pedrosa Lima^1^, Fabio Akio Nishijuka^1^, Renata Rodrigues Teixeira de Castro^1^

(1) Hospital Naval Marcílio Dias

**Introduction:** Lupus is an autoimmune disease with multiple presentations, several signs and symptoms and involvement of various organs and systems. Like Lupus, Coronavirus-19 is no longer an acute lung disease to become a multiorgan disease. The involvement of multiple systems is already well described in the literature. 2 years after the beginning of the pandemic, we are knowing its long-term effects. The cardiological spectrum has been gaining prominence after the chronification of the disease because it has a great impact on the quality of life of patients.

**Case Report:** Female, 33 years old, no previous comorbidities, with a history of COVID-19 in April 2020 without hospitalization. Started home treatment with symptomatic and oral corticosteroids with partial improvement of symptoms. Evolving with exercise intolerance, palpitation, chest pain and symptoms of postural orthostatic tachycardia syndrome (POTs). In March 2021, after remission, she takes 2 doses of Coronavac, and returned with cardiovascular symptoms, associated of low output. She performed cardiac magnetic resonance imaging (cMRI) in April 2021 with a pattern of inflammatory injury in the pericardium. The 24-hour holter resulted sinus rhythm, sinus arrhythmias, and mean heart-rate of 104. Treated with ibuprofen for 30 days and colchicine for 6 months. In October 2021, the control cMRI showed significant improvement in pericarditis. In January 2022, a new oligosymptomatic SARS-Cov-2 infection. In March 2022 she returned with symptoms. In a new study with cMRI, the pattern of pericarditis returned. Ibuprofen was restarted for 30 days and colchicine for 6 months, maintaining medical follow-up until the present moment.

**Conclusion:** Time has consolidated COVID-19 as another multiorgan disease. In this context, it’s important to understand the pathophysiological mechanisms in the heart, generated by the long term COVID-19, as well as to develop strategies to mitigate the impacts on the functionality and quality of life of the economically active population.


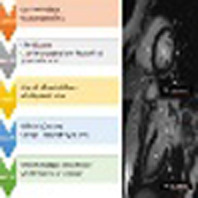


112087

Modality: E-Poster Young Researcher – Case Report

Category: CONGENITAL AND PEDIATRIC CARDIOLOGY

## Anomalous Pacemaker Lead Placement – a Report of Extrinsic Compression of the Left Pulmonary Artery

AMANDA DE MIRANDA CONSTANTINO^1^, Vivian De Biase^1^, Beatriz Moura Sucupira Tajra^1^, Thauan Paulúcio de Oliveira^1^, Mardelson Nery de Souza^1^

(1) Instituto Dante Pazzanese de Cardiologia

**Introduction:** The Atrioventricular Septal Defect (AVSD) corresponds to 3% of congenital heart diseases, being associated with genetic syndromes and other congenital malformations. One of the anatomical alterations present in AVSD is the posterior displacement of the atrioventricular node, located between the coronary sinus and the annulus of the atrioventricular valve, increasing the risk of injury to the same during mitral cleft repair, generating atrioventricular block (AVB), Such a complication may evolve with the need for definitive implantation of a pacemaker.

**Abstract:** A 27-year-old female patient with complex heart disease (left atrial isomerism, partial AVSD, muscular interventricular communication, single atrium, pulmonary hypertension), underwent atrial septation surgery, mitral cleft repair and ventriculoseptoplasty in the first year of life, evolving in the postoperative period with total AVB, requiring implantation of a permanent pacemaker 1 month after the surgical procedure. In follow-up, she demonstrated heart rate reversibility, being submitted to extraction of the pacemaker generator and plasty of the abdominal pocket at age 22, remaining with the endocardial electrode. The patient evolved with symptoms of right heart failure (dyspnea, with worsening of the functional class). During the current diagnostic investigation, a significant pulmonary gradient was evidenced by the transthoracic echocardiogram, but without valvular lesion. An angiotomography of the heart and basal vessels was performed, which showed extrinsic compression of the right pulmonary artery by the pacemaker wire, which generated the pulmonary stenosis evidenced by the echocardiogram.

**Conclusion:** Extrinsic compression by endocardial pacemaker lead is a rare phenomenon that can be difficult to recognize. In the case reported, due to the left atrial isomerism and the need to implant a permanent pacemaker during childhood in the abdominal cavity, the electrode was implanted through the azygos venous system. With the development and growth of the patient, the fixed electrode implanted in the right ventricle pulled the azygos vein under the right pulmonary artery, generating extrinsic compression and pulmonary stenosis evidenced both by imaging methods and by the patient’s clinical condition. It is clear the importance of late follow-up of patients who still have implanted electrodes, even without the use of a pacemaker, in complex anatomical situations.

112084

Modality: E-Poster Young Researcher – Case Report

Category: CARDIAC ARRHYTHMIAS/ELECTROPHYSIOLOGY/ELECTROCARDIOGRAPHY

## Pacemaker Lead Prolapse Through the Pulmonary Valve in Young Patient: Case Report

MONIQUE ALMEIDA VAZ^1^, Gustavo Lara Moscardi^1^

(1) Hospital Universitário de Brasília (HUB-UnB)

**Introduction:** The permanent pacemakers (PPM) are most commonly implanted via an epicardial approach in pediatric patients, due to transvenous route limitations. The advent of generators and thinner leads has increased the implantation of PPM with transvenous leads over the last three decades. Growth of children causes stress on leads, which do not tolerate ongoing linear strain. Aiming to reduce conductor fractures, some redundancy should be left as slack to avoid tension, or the use of an atrial loop. However, the extra loop of lead can result in mechanical and hemodynamic complications, as lead prolapse through the pulmonary valve (PV) and its insufficiency.

**Case Report:** A 16-year-old male with a history of congenital complete heart block, underwent dual chamber epichardical pacemaker implant on the first day of life. At age 10, transvenous atrial and ventricular leads were implanted along with pulse generator in thoracic site, left an atrial loop. During routine follow-up, he was asymptomatic and with normal average growth. Over the next year, transthoracic echocardiogram (TTE) showed prominent lead slack prolapsing across the PV, into the pulmonary artery (PA), mild tricuspid regurgitation, mild pulmonary regurgitation and normal left ventricular systolic function (56%). At age 13, TTE elucidated prolapsed lead into pulmonary valve, causing moderate regurgitation, and signs of interventricular dyssynchrony. During device interrogation, at age 16, evidence of lead fracture was found. A new TTE showed right ventricular (RV) hypertrophy and dysfunction, and redundant lead with a long loop, located inside the PA, causing double pulmonary valve lesion. Removal and replacement of the ventricular lead was indicated.

**Conclusion:** Prolapse of transvenous lead through the PV can occur when excess slack is left for growth. The tip of ventricular lead fixated in the apex may facilitate its prolapse into PA when it is closer to the outflow tract, because of alterations in contractility and vortex flow patterns. Therefore, it is recommended to place RV leads in septal locations to avoid that complication. The treatment consists in repositioning leads, through extraction and replacement, particularly if adherent to valve apparatus. However, the pulmonary valve disfunction may be irreversible. Periodic radiographic and echocardiographic monitoring is essential in pediatric patients, allowing a preclinical detection of valvular dysfunction secondary to lead prolapse.

112120

Modality: E-Poster Young Researcher – Case Report

Category: CARDIOVASCULAR IMAGING

## A Case of Acute Myocardial Infarction as a Complication of Atrial Myxoma

JÉSSICA MAYARA DE FIGUEIRÊDO OSÉAS^1^, Nestor Rodrigues de Oliveira Neto^1^, Jessione de Carvalho Lima Neto^1^, Matheus Fontes Leite^1^, Camila Albuquerque Coelho Lopes^2^

(1) Universidade Federal do Rio Grande do Norte, UFRN; (2) Universidade Potiguar, UnP

This study aims through a case report of an unusual pathology to recall the importance of considering the importance of differential diagnosis in clinical reasoning in the care of patients with acute myocardial infarction, especially considering the possible etiologies of a type 2 infarction. A 42-year-old male patient, with no history of comorbidities, was admitted to UPA 30/02/2022 due to severe precordial pain, in sudden onset grip when performing physical exercise. Requested propaedeutics of infarction. ECG with lower wall ST segment unevenness and elevated troponin, compatible with AMI. He was referred to the university hospital for cardiac catheterization on the same day, which did not show any athertortotic lesions in coronary arteries. However, due to the abnormal vascular path observed on the examination, hemodinamycrist suggested the presence of tumor lesion in the left atrium. On 4/6, a transesophageal echocardiogram showed a mass in both atria of 6.4 × 2.6 cm, suggestive of myxoma. Surgery was performed on 4/29, and complete resection of the myxoma was performed in need of resection of the entire interatrial septum. The patient evolved clinically stable and discharged from the hospital on 05/05/2022 for outpatient follow-up with clinical treatment after AMI. This report of a type 2 infarction by an atheroembolic event due to atrial myxoma in a young patient without comorbidities exemplifies why we should not limit the diagnosis of infarctions to type 1, even if it is the most common.


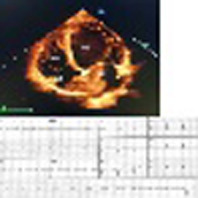


112111

Modality: E-Poster Young Researcher – Case Report

Category: CONGENITAL AND PEDIATRIC CARDIOLOGY

## Ischemic Cardiomyopathy Due to Late Diagnosis of Alcapa Syndrome

AMANDA DE MIRANDA CONSTANTINO^1^, Vivian De Biase^1^, Beatriz Moura Sucupira Tajra^1^, Thauan Paulucio de Oliveira^1^, Mardelson Nery de Souza^1^

(1) Instituto Dante Pazzanese de Cardiologia

**Introduction:** ALCAPA Syndrome (Anomalous Left Coronary from the Pulmonary Artery) is the anomalous origin of the left coronary artery from the pulmonary artery. Also known as Bland-White-Garland Syndrome, it is a rare congenital heart defect, present in 0.25–0.5% of patients with congenital heart defects. It can occur in isolation or be associated with other birth defects. Usually the diagnosis is early, made in the prenatal period and the need for its correction is immediate.

**Case Report:** A 63-year-old female patient sought emergency care with chest pain, dyspnea on moderate exertion, and palpitations. Myocardial scintigraphy was positive for ischemia, corresponding to the territory of the anterior descending artery. Cardiac catheterization was requested, in which an anomalous left coronary artery was observed in the pulmonary artery trunk. The transthoracic echocardiogram showed the following findings: akinesia of the apical and anteroapical septum, with hypokinesia of the anterolateral wall and of the mid-basal segments of the anterior wall, moderate left ventricular systolic dysfunction (ejection fraction of 37%), pulmonary artery systolic pressure of 62 mmHg. It has magnetic resonance imaging of the heart, which shows the areas of infarction mentioned in the echocardiogram, without myocardial viability.

**Discussion:** ALCAPA syndrome is difficult to diagnose, and should be suspected in children with dilated cardiomyopathy. About 85% of cases manifest up to 2 months of age, but symptoms can be misinterpreted and the disease may be underdiagnosed. The disease requires surgical treatment to restore the flow of oxygenated blood to the myocardium. The procedure of choice consists of reimplanting the coronary artery in the aorta and it is estimated that the surgical mortality is less than 5–10%, with a good prognosis after reimplantation. The peculiarity of the case reported is due to the fact that the diagnosis was extremely late and with important irreversible hemodynamic repercussions: the patient has an ischemic cardiomyopathy without myocardial viability, a condition usually related to chronic coronary artery disease or acute coronary syndromes, rarely related to ALCAPA, since due to the severity of the syndrome, its diagnosis is early. Currently, due to the lack of myocardial viability, surgical correction would not bring benefits or recovery of heart muscle, opting for clinical treatment for cardiac remodeling.

112145

Modality: E-Poster Young Researcher – Case Report

Category: HEMODYNAMICS AND INTERVENTIONAL CARDIOLOGY

## Mitral Valve in Valve Replacement Through a Bovine Pericardial Septal Patch, a Case Description of a Challenging Approach

ANDRES FELIPE VALENCIA RENDON^1^, Marcello Augustus de Sena^2^, Murilo Castro^3^, Bernardo Kremer^1^

(1) Federal University of Rio de Janeiro; (2) ITPAC Palmas; (3) Palmas Medical Center

43 years old female patient, with controlled Hypertension, without any other known comorbidities. Two years ago, she was submitted to an open biological Mitral valve replacement due to severe rheumatic mitral valve stenosis. During the procedure, a highly redundant atrial septum was noted, hence an atrioseptoplasty was performed using bovine pericardium. In the recent year, she evolved with worsening dyspnea, platipnea, with a Trans Thoracic Echocardiogram showing a severe Mitral stenosis with a mean gradient of 20 mmHg. The patient declined another open-heart surgery, therefore a transcatheter approach was selected. Under general anesthesia, under guidance of Trans Esophageal Echocardiography (TEE), a Brokenbrough catheter with needle was advanced though the right femoral vein reaching the right atrial septum. Not having a clear reference of the Fossa Ovalis due to the previous atrioseptoplasty, the anatomical navigation for the septal puncture was performed under the TEE landmarks. The penetration of the bovine pericardial patch presented a high resistance with the classical approach; hence the needle was connected to an electro surgical scalpel console using 30 mV, having success at the first attempt, with no anatomical alterations or hemodynamic instability. A sapiens 3 valve was advanced to the mitral valve position with no issues after deployment, presenting a minimal atrio ventricular pressure gradient. To our knowledge, this is one of the first reports of a mitral valve in valve intervention through a previous a bovine pericardial septal patch.

112151

Modality: E-Poster Young Researcher – Case Report

Category: COVID-19 AND CARDIOVASCULAR SYSTEM

## Impact of COVID-19 Infection on Post-Infectious Cardiomyocyte Compromise in a Healthy Middle-Aged Women: A Case Report

GIOVANNA BOLINI BRAZÃO^1^, Fábio Vasconcellos Brazão^3^, Maria Angélica Bolini Brazão^2^, Patrícia Brazão Cohen^4^

(1) Centro Universitário do Estado do Pará (CESUPA); (2) Universidade Federal do Pará (UFPA); (3) Laboratório Ruth Brazão, LTDA; (4) Universidade do Estado do Pará (UEPA)

**Introduction:** In the last two years, several post-infectious cardiac repercussions have been observed in SARS-CoV-2 infections. In severe cases, the infection can cause pneumonia, severe acute respiratory syndrome, cardiomyopathies, kidney failure and even death, with an impact on public health.

**Case Description:** Female, 51 years old, without comorbidities, remained in prolonged hospitalization due to COVID-19 in a private hospital in São Paulo (SP). Upon admission, developed a Severe Acute Respiratory Infection, requiring admission to the ICU-Adult and later orotracheal intubation and mechanical ventilation (MV). Remained on MV for a long time and presented complications such as septic shock from ventilator-associated pneumonia, bronchitis obliterans with organizing pneumonia, which required high-dose corticosteroids, in addition to a short-term catheter-related bloodstream infection [antimicrobials used: Ceftriaxone; Cefepime; Meropenem + Vancomycin; Ceftazidime + Vancomycin; Polymyxin + Amikacin; single dose ivermectin]. At that time, presented a chest computed tomography with a report showing more than 90% of pulmonary involvement. Due to the prolonged time on MV, she needed a Tracheostomy to wean off sedatives and MV. After adequate awakening, started a process of respiratory, cognitive, muscular and sensory rehabilitation due to the critically ill patient’s polyneuromyopathy. About a month after awakening, during the rehabilitation process, had an episode of chest discomfort investigated for suspected acute coronary syndrome. A transthoracic echocardiogram was performed, which showed mild hypokinesia of the anterior wall and segmental myocardial involvement of the left ventricle – non-existent changes in an exam performed one month before – in addition to the dosage of troponin (18 pg/mL) and CPK (21 U/L). The investigation and risk stratification showed no high cardiovascular risk, since they related changes to psychological stress due to the death of the patient’s mother in this interval. Therefore, was released for activities and for hospital discharge. She was discharged to continue outpatient follow-up and home rehabilitation, which lasted for about 6 months.

**Conclusion:** Studies should be carried out to accumulate more data on the cardiac repercussions of the new coronavirus and its variants in Brazil and in the world, in order to pressure public health agencies to create protocols aimed at the prevention and treatment of such cardiomyopathies.

112177

Modality: E-Poster Young Researcher – Case Report

Category: HEART FAILURE/CARDIOMYOPATHY/TRANSPLANT

## The Age of Genes: The Importance of Genetic Research in Defining the Etiology of Heart Failure

BRUNO LINHARES AZEREDO CORRÊA^1^, Sabrina Pedrosa Lima^1^, Fabio Lucas Bassini e Silva^1^, Fabio Akio Nishijuka^1^, Renata Rodrigues Teixeira de Castro^1^

(1) Hospital Naval Marcílio Dias

**Introduction:** Heart failure is not a pathology that usually affects young adults. However, once manifested, ischemic etiologies or those associated with infectious myocarditis should be ruled out. In parallel, hereditary etiologies must be considered, in which genetic tests are of enormous importance, and which are gaining more and more space in current medicine.

**Case Report:** A 22-year-old man presented with progressive dyspnea until minimal exertion, associated with paroxysmal nocturnal dyspnea and orthopnea. Admitted with heart failure with reduced ejection fraction (EF) (admission echocardiogram EF 09% by Teichozl) hemodynamic profile B. MAGGIC Score 29 points. After the condition was compensated, triple therapy with beta-blockers, spironolactone and enalapril (ACEI) was started. Subsequently, the ACEI is replaced by the angiotensin and neprilysin receptor inhibitor (INRA) for hospital discharge. 3 weeks after discharge, she was readmitted due to decompensation associated with poor adherence to treatment. There was no water restriction. A new drug adjustment was performed, with sequential nephron block and inclusion of dapagliflozin, with a good response. During the diagnostic investigation, there was a positive family history of dilated cardiomyopathy on the part of the mother at age 25 years. Cardiac magnetic resonance imaging (cMRI) showed late enhancement with a pattern of myocardial injury of non-ischemic origin with EF 10%. Six months after admission, a new cMRI showed the same pattern, but with EF 21%, and ergospirometry with VO2max of 35.36 ml/kg/min. The genetic test showed the presence, in heterozygosity, of the variant described as NM_001458.5(FLNC):c.6976C > T;p.(Arg2326Ter), classified as pathogenic, in the FLNC gene (Filamin C), associated with Familial Restrictive Cardiomyopathy of the type 5 (OMIM:617047), of autosomal dominant inheritance.

**Conclusion:** In young patients with heart failure, in addition to investigating the ischemic etiology or related to infectious myocarditis, one should also consider hereditary etiologies. At this point, genetic research provides important information about the definitive diagnosis and therapeutic choice. However, the high cost of genetic sequencing makes this test little available. Making it accessible will allow testing the source patient’s family members and offering patient-directed therapy, ensuring improvement in symptoms and, consequently, quality of life.

112226

Modality: E-Poster Young Researcher – Case Report

Category: ACUTE AND CHRONIC CORONARY DISEASE/THROMBOLYSIS

## Minoca in a Patient with APS After Prolonged Air Travel

MILENE FERNANDES FARIAS^1^, Adriel Alves de Paiva; Emídio Almeida Júnior Jr; João Marcos Bemfica Barbosa Ferreira; Katia do Nascimento Couceiro.^2^

(1) Fundação Hospital do Coração Francisca Mendes – Amazonas, Brasil.; (2) Universidade do Estado do Amazonas

**Introduction:** Myocardial infarction without obstructive coronary atherosclerosis (MINOCA) is a syndrome with many causes, representing 10 to 15% of all diagnoses of acute myocardial infarction (AMI). Antiphospholipid antibody syndrome (APS) is a systemic autoimmune disease characterized by the plasma detection of antiphospholipid antibodies, such as anticardiolipin and lupus anticoagulant, which clinically manifests itself mainly as recurrent arterial and/or venous thrombosis. It is the main acquired cause of hypercoagulability, occurring in 2% of the general population and has high morbidity and mortality. Myocardial involvement, however, is rarely described in this pathology.

**Case Report:** The present report describes the case of an elderly, non-sedentary, hypertensive patient, using Nebivolol 5 mg, who, after a flight to Switzerland, presented on the seventh day in that country with typical chest pain, without dyspnea, syncope or associated palpitations, electrocardiogram with millimeter elevation of V3, with flat T waves, even negative in aVL, elevated troponins, being diagnosed with acute coronary syndrome without ST-segment elevation. Echocardiogram showing normal ventricular ejection fraction (estimated at 60%), mild inferior hypokinesia. After coronary angiography, thrombosis of the posterior ventricular branches was evidenced with thrombus aspiration by microcatheter and thrombectomy with a stent retriever. Lower limb venous thrombosis was ruled out by angiographic evaluation, and treatment with low-molecular-weight heparin was initiated. Upon returning to Brazil, three months after the event, the patient was treated at a referral hospital, and a diagnostic investigation was carried out, including APS, deep vein thrombosis of the lower limbs and patent foramen ovale (PFO).

**Conclusion:** We report the favorable outcome of primary APS with cardiological complications, which after its diagnosis determined the institution of full anticoagulation with oral anticoagulant. Clinical management of hypertension was maintained with beta-blocker therapy, with no recurrence of chest pain. Therefore, he continues to be followed up with a hematologist and cardiologist, with the aim of reducing the morbidities that may eventually exist.

112232

Modality: E-Poster Young Researcher – Case Report

Category: CARDIOVASCULAR SURGERY

## Thrombus in the Ascending Aorta and Aortic Arch – in Association with COVID-19

ALBERT SALVIANO DOS SANTOS ^1^, Ana Luiza Scholl Giaretta^1^, Ana Maria Rocha e Pinto^1^, Valquíria Pelisser Campagnucci^1^, Camila de Queiros Mattoso Archela dos Santos^1^

(1) Irmandade da Santa Casa de Misericórdia de São Paulo

**Introduction:** Primary aortic thrombosis is a rare condition, with incidence of 0.45% in aortas with no prior lesions. It’s usually presented as embolization and has a high mortality rate. Its correlation with COVID-19 was described in six cases in the literature and in our service, in three patients.

**Cases:** The first case a 57-year-old man, a former smoker, with DM2 and thyroid cancer. Diagnosed a thrombus in the ascending aorta, received parenteral anticoagulation and after 13 days the thrombus has completely resolved. The second patient, 47 years old, smoker, hypertensive, dyslipidemic, with a history of stroke a year prior, sought medical attention for a subocclusion of the axillary artery and thrombus in the aortic arch. The patient was anticoagulated and evolved with the resolution of the thrombus. The last case, a 56-year-old female, smoker, arrived at the ER with acute myocardial infarction and acute arterial occlusion of the left upper extremity. A thrombus was found in the ascending aorta and aortic arch. The patient underwent emergency surgery. The patient progressed with biventricular dysfunction, need for an intra-aortic balloon, high doses of vasoactive drugs, and died from mixed shock and electrical instability 24 hours after surgery.

**Conclusion:** The formation of thrombus in COVID-19 is related to endothelial inflammation due to hypoxia, increased concentration of clotting factors, and its high specificity in binding to ACE-2 receptors present in the endothelium. Primary aortic diseases are the most frequent risk factors for thrombus formation. In COVID-19-related cases, smoking was a common risk factor. Our patients had additional risk factors that, associated with the thrombogenicity of SARS-CoV-19, increased the risk of aortic thrombosis even in the absence of lesions, such as plaques or ulcers. In the surgery of the 3rd case, we observed thrombi pedunculated in smooth endothelium, without lesions. There is no consensus on treatment. Most authors advocate anticoagulation, with surgery being reserved for cases of persistence, recurrence of the thrombus or embolization. Others argue that aortotomy should be the immediate treatment to prevent complications. There is a trend toward early surgical treatment in young patients, thrombi larger than 3 cm, location in the ascending aorta or arch, recurrence of embolic events, and high thrombus mobility.

107750

Modality: E-Poster Scientific Initiation – Non-case Report

Category: CARDIOVASCULAR SURGERY

## Assessment of Arterial Rigidity in Heart Transplanted Patients

HENRIQUE ULISSES DUARTE DE CASTRO^1^, Caio Caldas Couto^1^, Bruno Almeida Rezende^1^

(1) Faculdade Ciências Médicas de Minas Gerais (FCMMG); (2) Universidade Federal de Minas Gerais (UFMG)

**Introduction:** Heart failure is an extremely important clinical condition whose symptoms are the result of a structural or functional disorder of the heart. Heart transplantation is a complex and high-risk procedure, but when successfully performed demonstrates a considerable improvement in the clinical condition and in the patient’s quality of life. However, the vascular changes of such a procedure are not fully known.

**Objective:** Collect and analyze data related to arterial stiffness in patients undergoing heart transplants from March 2020 to January 2021 at the Hospital das Clínicas de Belo Horizonte.

**Methods:** The Mobil-O-Graph 24H device was used, which makes it possible to measure hemodynamic parameters of arterial rigidity that is estimated by evaluating the variables pulse wave velocity (PWV) in the brachial artery, augmentation index (AIx) adjusted for a heart rate of 75 beats per minute (Aix@75), central and peripheral arterial pressure. The measurements were made immediately before surgery and 60 days after.

**Results and Discussion:** There was an increase in blood pressure levels after transplantation surgery for both systolic (SBP), diastolic (DBP), and mean (MAP) blood pressure (p < 0.031). The values of central arterial pressure also increased significantly (p < 0.031). Cardiac output and cardiac index increased (p < 0.031 and p < 0.036). PWV was also increased after surgery (p < 0.031). The increase in PWV can be related to the medications used to control the rejection of the transplanted organ and hemodynamic changes. The data obtained in the study showed that there is a trend in the increase of Aix@75 after heart transplantation (which happened with all the patients studied so far). However, the inconstancy of this variable made it not possible to demonstrate that this increase was statistically significant due to the low sampling. Possibly, with the increase in the sample, it will be possible to show a significant increase in Aix@75 in the short term after heart transplantation.

**Conclusion:** The non-invasive assessment of hemodynamic parameters can contribute to better control of the evolution of patients, in order to prevent cardiovascular events related to vascular remodeling. As it is an easily applicable method, it could be used for the continuous monitoring of the patient in different phases of the post-transplant recovery process. A longer follow-up period and a larger sample are needed to reinforce the applicability of the proposed method.

107773

Modality: E-Poster Scientific Initiation – Non-case Report

Category: PERICARDIUM/ENDOCARDIUM/VALVOPATHIES

## Epidemiology Profile of Patients with Infective Endocarditis in Three Tertiary Centers in Brazil

LUCAS SOUSA SALGADO^1^, Renato Lott Bezerra^2^, Yago Machado da Silva^3^, Gustavo Guimarães Rocha Figueiredo^3^, Raimundo Matos Bezerra Filho^3^, Eduardo Luís Guimarães Machado^3^, Isabel Cristina Gomes^3^, Ângelo Geraldo José Cunha^1^

(1) União Educacional do Vale do Aço, UNIVAÇO, Ipatinga, MG.; (2) Hospital João XXIII, Belo Horizonte, MG; (3) Complexo de Saúde São João de Deus, 3 Divinópolis, MG – Brazil

**Introduction:** Infective endocarditis (IE) is a high morbimortality disease with increasing incidence. With medical improvement in diagnosis and treatment, several epidemiological changes have been reported over time, however, most of the studies were conducted in developed countries, and it is questionable whether developing countries are susceptible to this epidemiological transition.

**Objectives:** It was sought to describe the epidemiological profile, mortality predictors, and analysis of possible microbiological transition from patients admitted to three tertiary centers in Brazil.

**Methods:** In this cross-sectional and retrospective study, data from 211 patients with definite or probable IE were analyzed according to the modified Duke criteria, between 2003 and 2017. The association between categorical variables was assessed using Chi-square or Fisher’s exact test and binary logistic models were built to investigate the death outcome. We considered p < 0.05 as statistically significant.

**Results:** Median age of the sample was 48.00 (33–59) years old, 70.6% were men and the most prevalent pathogen was Staphylococcus spp. (19%). Mortality was 22.3% and it was observed that increasing age is the leading risk factor for death (p = 0.028). With regard to the location of the disease, native valves were the most affected, with aortic valve being more affected in men when compared to women (p = 0.017). The average number of cases of Staphylococcus spp. (τ = 0.293, p = 0.148) and Streptococcus spp. (τ = –0.078, p = 0.727) has remained stable over the years.

**Conclusion:** No trend towards a reduction or increase in mortality between 2003 and 2017 was evident. Although Staphylococcus spp. was the most prevalent pathogen, it was not possible to observe the expected epidemiological transition.


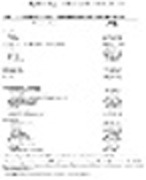


107794

Modality: E-Poster Scientific Initiation – Non-case Report

Category: CARDIOVASCULAR SURGERY

## Predictors of Late Extubation from Mechanical Ventilation After Cardiac Surgery

Maria Luísa Souza Santana^1^, Rodrigo Carvalho de Menezes^2^, Isabella Bonifácio Brige Ferreira^3^, Kaique Vinicius da Cruz Santos Aguiar^4^, Raissa Barreto Lima^3^, Ana Luísa de Aguiar Almeida Silva^5^, Elias Soares Roseira^5^, Luiz Carlos Santana Passos^6^, Rodrigo Morel Vieira de Melo^6^, Nivaldo Menezes Filgueiras Filho^1^

(1) Medicine, Salvador University, C ampus Teacher Barros, Salvador, Brazil; (2) Human pathology, Federal University of Bahia Ondina C ampus, Salvador, Brazil; (3) Medicina e saúde, Bahia School of Medicine and Public Health – C abula, Salvador, Brazil; (4) Gemini, Grupo de Estudos em Medicina Intensiva, Salvador, Brazil; (5) Medicine, Federal University of Bahia Ondina C ampus, Salvador, Brazil; (6) Medical board, Hospital Ana Nery, Salvador, Brazil

**Introduction:** Prolonged use of mechanical ventilation has been associated with poor clinical outcomes, and about 10% of patients who undergo heart surgery are part of this group. Identifying those who are at a higher risk for late weaning may assist in the management of these patients, decreasing the risk of death and complications.

**Objectives:** To determine factors associated with late extubation (>6 hours) after cardiac surgery.

**Methods:** Retrospective cohort study, with secondary data analysis from Ana Nery Hospital’s, Bahia, Brazil, patients who underwent cardiac surgery between 2018 and 2021. The D‘agostino test was used to assess normality. Continuous variables were assessed using the Mann-Whitney test, categorical variables using Fisher’s exact test. Variables that showed statistical relevance in the univariate analysis were included in a binary logistic regression model.

**Results:** A total of 1594 patients were admitted to the study, with a mean age of 56 years (45–66) and a slight predominance of male patients. The most performed surgery was coronary artery bypass grafting (635 [40.1%]). Median Society of Thoracic Surgeons (STS) score of 1.17 (0.70–2.08) and a EUROscore of 1.30 (0.86–2.15). Among admitted patients, 860 had late extubation (>6 hours) MV, with a median of 12 (9–18) hours on the machine while patients who had early extubation had a median of 4 (2.5–5) hours on MV. 74.2% had type 2 diabetes. They also had more noradrenaline use – 55.3% – less vasopressin use (6.86%), with a median peak troponin of 3.51 (1.67–8.24).

**Conclusions:** The presence of congenital valvulopathy, history of endocarditis, troponin and lactate values, presence of previous type 2 diabetes, and use of vasopressin and debutamine were independently associated with late extubation.


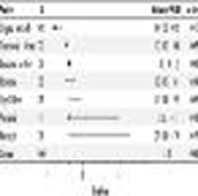


107813

Modality: E-Poster Scientific Initiation – Non-case Report

Category: CARDIOVASCULAR SURGERY

## Predictors of Intra-ICU Complications After Cardiac Surgery

ANA CAROLINA RIOS CARVALHO^1^, Maria Luisa Souza Santana^1^, Rodrigo Carvalho de Menezes^2^, Isabella Bonifácio Brige Ferreira^3^, Kaique Vinicius da Cruz Santos Aguiar^4^, Raissa Barreto Lima^3^, Ana Luísa de Aguiar Almeida Silva^5^, Elias Soares Roseira^5^, Luiz Carlos Santana Passos^6^, Rodrigo Morel Vieira de Melo^6^, Nivaldo Menezes Filgueiras Filho^1^

(1) Medicine, Salvador University, C ampus Teacher Barros, Salvador, Brazil; (2) Human pathology, Federal University of Bahia Ondina C ampus, Salvador, Brazil; (3) Medicina e saúde, Bahia School of Medicine and Public Health – C abula, Salvador, Brazil; (4) Gemini, Grupo de Estudos em Medicina Intensiva, Salvador, Brazil; (5) Medicine, Federal University of Bahia Ondina C ampus, Salvador, Brazil; (6) Medical board, Hospital Ana Nery, Salvador, Brazil

**Introduction:** Cardiac surgery aims to restore the functional capacity of the heart and reduce symptoms. Complications have a negative and variable effect on outcome after cardiac surgery. Therefore, the measurement of its effect is of fundamental importance.

**Objectives:** To determine patient characteristics associated with complications post-cardiac surgery.

**Methods:** Retrospective cohort study, with data analysis from Hospital Ana Nery’s, Bahia, Brazil patients who underwent cardiac surgery between 2018 and 2021. The D‘agostino test was used to assess normality. Continuous variables were evaluated using the Mann-Whitney test, and categorical variables evaluated using Fisher’s exact test. Variables that showed statistical relevance in the univariate analysis were included in a binary logistic regression model.

**Results:** A total of 1594 patients were admitted to the study, with a mean age of 56 years (45–66) and a lower predominance of male patients. The most performed surgery was coronary artery bypass grafting (635 [40.1%]). The study population has a median Society of Thoracic Surgeons (STS) score of 1.17 (0.70–2.08) and a EUROscore of 1.30 (0.86–2.15). 279 patients had intra-ICU complications. These were older (58 [48–67] vs. 56 [45–65]), median 85 (64–110) min on cardiopulmonary bypass, 62.2% used dobutamine, 64.4% used noradrenaline. Among complications, 183 (65.6%) had infection, 103 (37.3%) required reoperation, 56 (20.1%) had acute kidney injury, 11 (9.73%) had stroke, and 23 (8.30%) required dialysis.

**Conclusions:** Cardiopulmonary by-pass time, debutamine use, presence of type 2 diabetes, chronic kidney disease, and use of intra-aortic balloon pumps were identified as independent risk factors for the development of postsurgical complications.


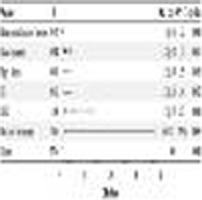


107814

Modality: E-Poster Scientific Initiation – Non-case Report

Category: CARDIOVASCULAR SURGERY

## Mortality Predictors After Cardiac Surgery

ANA CAROLINA RIOS CARVALHO^1^, Maria Luísa Souza Santana^1^, Rodrigo Carvalho de Menezes^2^, Isabella Bonifácio Brige Ferreira^3^, Kaique Vinicius da Cruz Santos Aguiar^4^, Raissa Barreto Lima^3^, Ana Luísa de Aguiar Almeida Silva^5^, Elias Soares Roseira^5^, Luiz Carlos Santana Passos^6^, Rodrigo Morel Vieira de Melo^6^, Nivaldo Menezes Filgueiras Filho^1^

(1) Medicine, Salvador University, C ampus Teacher Barros, Salvador, Brazil; (2) Human pathology, Federal University of Bahia Ondina C ampus, Salvador, Brazil; (3) Medicina e saúde, Bahia School of Medicine and Public Health – C abula, Salvador, Brazil; (4) Gemini, Grupo de Estudos em Medicina Intensiva, Salvador, Brazil; (5) Medicine, Federal University of Bahia Ondina C ampus, Salvador, Brazil; (6) Medical board, Hospital Ana Nery, Salvador, Brazil

**Introduction:** Risk prediction models have been developed to provide information about risk for both physicians and patients, as well as to guide decision making. Likewise, knowing a patient’s risk may allow the implementation of individualized strategies, aimed at preventing mortality.

**Objectives:** To determine patient characteristics associated with post-cardiac surgery Mortality.

**Methods:** Retrospective cohort study, with secondary data analysis from Ana Nery Hospital’s, Bahia, Brazil, patients who underwent cardiac surgery between 2018 and 2021. The D‘agostino test was used to assess normality. Continuous variables were evaluated using the Mann-Whitney test, and categorical variables evaluated using Fisher’s exact test. Variables that showed statistical relevance in the univariate analysis were included in a binary logistic regression model.

**Results:** A total of 1594 patients were admitted to the study, with a median age of 56 years (45–66) and a lower predominance of male patients [RCdM1] [MS2] The most performed surgery was coronary artery bypass grafting (635 [40.1%]). The study population has a mean Society of Thoracic Surgeons (STS) score of 1.17 (0.70–2.08) and a EUROscore of 1.30 (0.86–2.15). Among the patients who did not survive, it was observed that a median age of 66 (53.3–71), BMI with a median index of 24.4 (21.5–27.6), higher rate of CKD and 41.6% had previous AMI. They had a higher EUROscore – 2.58 (1.45–3.76) – and STS – 2.05 (1.31–3.75), needed more supportive medications, and had more postoperative complications, measuring up to 51.1%.

**Conclusions:** BMI, peak 24-hour lactate, CKD, presence of complications in the intensive care unit, use of norepinephrine and vasopressors were identified as risk factors associated with postoperative patient mortality.


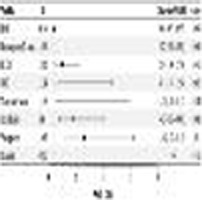


107822

Modality: E-Poster Scientific Initiation – Non-case Report

Category: CARDIO-ONCOLOGY

## Echocardiographic Evaluation of Secondary Cardiotoxicity in Women‘s Breast Cancer Treatment

IVANA BECKER^1^, Bruna Strube Lima^1^, João Pedro Dutra^2^, Samantha Cristiane Lopes^1^, Susane Fanton^3^, Franciani Rodrigues da Rocha^1^, Marcelo Vier Gambetta^1^, Luiz Eduardo Bacca^1^, Caroline de Oliveira Fischer Bacca^1^

(1) UNIDAVI – Centro Universitário para o Desenvolvimento do Alto Vale do Itajaí; (2) CEPON – Centro de Pesquisas Oncológicas; (3) FURB – Universidade Regional de Blumenau

**Background:** The introduction of new chemotherapy drugs has improved overall survival for patients fighting breast cancer. They might however be the cause of secondary structural or functional alterations to the heart, defined as cardiotoxicity.

**Objectives:** To identify the incidence of secondary cardiotoxicity in breast cancer treatment while emphasizing its demographical, clinical, and echocardiographic characteristics.

**Methods:** Observational, analytical, and cross-sectional study in the oncology sector of a reference hospital in Alto Vale do Itajaí, Santa Catarina, Brazil. We analyzed 238 patient’s records of women under medical echocardiographic supervision from August/2018 until June/2021. It was defined as cardiotoxicity the presence of left ventricular dysfunction, represented by the reduction of at least 10% in the ejection fraction (LVEF) to values of lower limits of normality (LVEF <50%). It was employed the SPSS software, Kolmogorov-Smirnov normality, and Fisher exact tests. The chosen level of significance was p < 0.05.

**Results:** The sample counted on 118 women who met eligibility criteria. Average age was 52.73 ± 12.35 years. The incidence of cardiotoxicity was 2.5% (4 patients), meaning significance to the association between treatment with trastuzumab and radiotherapy (p < 0.05). There wasn’t any statistical relevance in the analysis of sociodemographic parameters, as well as in the clinical data related to CT outcomes.

**Conclusion:** The incidence of cardiotoxicity in women under medical echocardiographic supervision during breast cancer treatment was 2.5%, with statistical relevance to the association between treatment with trastuzumab and radiotherapy.


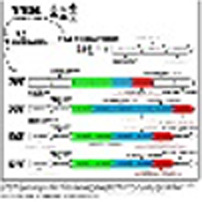


107900

Modality: E-Poster Scientific Initiation – Non-case Report

Category: CARDIO-ONCOLOGY

## An Evaluation of the Recommendations for the Blood Pressure Measurement in Patients who Underwent Mastectomy

JULIA DE CONTI PELANDA^1^, André Vinícius de Oliveira^1^, Annelise de Jesus Oliveira^1^, Mateus Rodrigues Alessi^2^, Alexandre Alessi^1^

(1) Universidade Federal do Paraná (UFPR); (2) Universidade Positivo

**Introduction:** Patients who underwent mastectomy as cancer treatment are often advised not to measure their blood pressure (BP) in the ipsilateral arm and to avoid many daily-life activities, in order to prevent lymphedema. These recommendations are based on a small number of studies with widely controversial results. Hence, a thorough review of the literature is imperative to better evaluate the adequacy of the current guidelines.

**Methods:** A cross-sectional online survey with cardiologists and mastologists, and a semi-systematic literature review were performed. Data were cross-linked to assess the degree of concordance between clinical practice and guidelines. Survey results were evaluated by Chi-Square test, with a significance level of 95%.

**Results:** From the 334 physicians surveyed, 206 (62%) were mastologists and 128 (28%) were cardiologist. Whereas cardiologists contraindicated measuring the BP after mastectomy (Chi² 20.1, p < 0.001), sentinel lymph node biopsy (Chi² 25.6, p < 0.001) and radiotherapy (Chi² 18.5, p < 0.001), mastologists did not. According to the literature, there is no data supporting the maleficence of such procedure in mastectomy patients.

**Conclusion:** These results highlight the discrepancy of recommendations among physicians of different specialties, which can increase patients’ illness anxiety. In this study, mastologists showed greater awareness of risks and benefits of measuring the BP in the ipsilateral arm of the procedure.


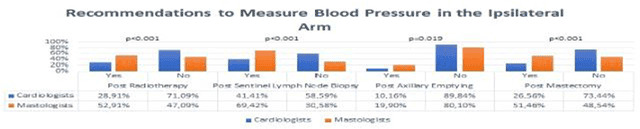


107945

Modality: E-Poster Scientific Initiation – Non-case Report

Category: ATHEROSCLEROSIS/CARDIOVASCULAR RISK FACTORS/CARDIOVASCULAR PREVENTION

## Risk Reclassification by Subclinical Atherosclerotic Disease in the Brazilian Diabetes Study

SOFIA HELENA VITTE^1^, Joaquim Barreto^1^, Andrei C. Sposito^1^

(1) Universidade Estadual de Campinas

**Background:** Cardiovascular risk estimates assist healthcare providers in targeted selection of type 2 diabetes (T2D) individuals who may benefit the most from stricter control of risk factors. Subclinical cardiovascular disease (SCD) either as carotid plaque or coronary calcifications is a surrogate marker of increased incidence of cardiovascular events and is thus employed as a classifying feature. Whether screening SCD significantly reclassifies the estimated risk of T2D individuals assessed by risk engine equations at the primary prevention level remains unexplored.

**Objectives:** We sought to determine whether SCD significantly reclassified the UKPDS-based risk estimates in T2D individuals at primary prevention.

**Methods:** This is a cross-sectional study with data collected by the national prospective cohort Brazilian Diabetes Study. Briefly, in this cohort, patients with DM2, aged ≥30 years, underwent carotid Doppler ultrasound for common carotid intima-media thickness (CIMT) measurement and computed tomography angiography to calculate coronary calcium score (CAC). SCD was considered in those who had at least one of the following: (i) carotid atherosclerotic plaque; (ii) CIMT > percentile 75; (iii) CAC > 0 Agatston. The UKPDS risk engine was used to estimate the 10-year risk of CV events classified as low (<15%), intermediate (15–30%) and high-risk (30%). Receiver operator curve (ROC) was used to evaluate the predictive value of UKPDS value. The proportion of individuals at each risk class were compared between the UKPDS and the UKPDS + SCD groups by chi-square test.

**Results:** Among 371 patients included in this analysis (Mean age: 58 + 7.5 years; T2DM duration: 9 + 6.7 years; 59.8% male), 302 (81.4%) had SCD defined by either CIMT (72.4%) or CAC score (71.6%). The UKPDS had an area under the curve of 0.66 (95%CI: 0.60, 0.74; p < 0.001). Adding SCD data to the UKDPS risk engine significantly change the proportion of individuals classified as low- (80.32% vs 17.25%), intermediate (15.36% vs 1.08%) and high-risk (4.31% vs 81.67%) (p < 0.001 for all). Only 5% of SCD individuals were classified as high-risk by UKPDS.


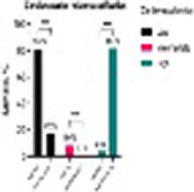


108555

Modality: E-Poster Scientific Initiation – Non-case Report

Category: EPIDEMIOLOGY AND HEALTH POLICIES/GLOBAL HEALTH

## Epidemiological Aspects and Number of People Affected by Malignant Neoplasm of the Heart, Mediastinum and Pleura in Brazil from 2019 to 2021

AUREA NATHALLIA GOMES DE SOUZA^1^, Bianca Paula Miranda Martins^1^, Camila Silva de Oliveira^1^, Cecília Rodrigues Viana^1^, Larissa Silva Ferreira^1^, Luiz Felipe Façanha Ramos^1^, Marcos Roberto Marques da Silva Júnior^1^, Vinícius Maciel Vilhena^1^, Reny Wane Vieira dos Santos^1^

(1) Universidade Federal do Amapá UNIFAP

**Introduction:** Malignant Neoplasm of the Heart, Mediastinum and Pleura (MNHMP) is considered a rare condition, which could only be better studied after improving diagnostic methods. Heart tumors originate mainly in internal tissues, such as the muscular layer and the pericardium, presenting with symptoms common to other cardiovascular diseases, requiring a differential diagnosis.

**Objective:** To analyze the epidemiological aspects and number of people affected by MNHMP, in the years 2019, 2020 and 2021, observing the variation of numbers over the years and main epidemiological findings.

**Methods:** We used the data available in the PAINEL-ONCOLOGIA, from the Department of Informatics of the Unified Health System (DATA-SUS), which comes from the Outpatient Information System (SIA).

**Results:** It was observed, a reduction in the number of cases of people diagnosed with MNHMP over the years. In 2019, 1420 cases were recorded, in 2020, there were 1273 and in 2021, the decrease was more pronounced, totaling 621 cases. Concerning gender, it was found that the frequency in females is slightly bigger, in 2019, 50.53% were females, in 2020, they corresponded to 53.18% of cases and in 2021, they represented 52.17%. Furthermore, another important epidemiological aspect is the age group of these patients, where it was possible to observe that in 2019, in the age group from 0–19 there were 103 cases, and a larger group, which comprises 45–79 years old, represents 58.87% of the cases that year, where those aged 65–69 reached 178 cases. In 2020, the age group from 50–74 years old stood out, with greater attention to 55–59 years old, with 180 cases. In 2021, with the decrease in the total number, no age group exceeded 100 cases, with the highest number, 77 cases, patients between 60 and 64 years old. It was also possible to analyze the distribution of these cases throughout Brazilian territory, some states maintained higher numbers of MNHMP diagnoses during the analyzed period: Bahia, Minas Gerais, Rio de Janeiro, São Paulo and Rio Grande do Sul.

**Conclusions:** It is possible to sketch epidemiologically, from the data, that MNHMP affects more females than male, from the fifth decade of life, who live mainly in the states of Bahia, Minas Gerais, Rio de Janeiro, São Paulo and Rio Grande do Sul. It is necessary to pay attention to the sudden decrease that occurred from 2020 to 2021 and not rule out the possibility of underreporting, mainly because of the current pandemic.

108595

Modality: E-Poster Scientific Initiation – Non-case Report

Category: HYPERTENSION/RENAL DENERVATION

## Mortality Due to Essential Hypertension in the Northeast Compared to Brazil and Schooling

LARISSA DE OLIVEIRA BELTRÃO^1^, Ricardo Henrique Freitas Tavares^2^, Maria Keyllane Vasconcelos de Miranda^2^, Isabella Carla Barbosa Lima Angelo^2^, Ieda Fernanda da Silva Santos^2^, Henrique Pessoa Tseng^2^, João Henrique Neves Ferreira^2^, Carlos Henrique Pereira da Silva^2^, Matheus Guilherme de Assunção França^2^, Maria Luisa Lopes Rodrigues^2^, Erinaldo Siqueira de Medeiros^2^, Pedro Rafael Salerno^2^

(1) Faculdade Pernambucana de Saúde (FPS); (2) Universidade Católica de Pernambuco (UNICAP)

**Introduction:** Systemic Arterial Hypertension (SAH) is a chronic and non-communicable disease of multifactorial origin, characterized by sustained elevation of systolic blood pressure ≥140 mmHg or diastolic blood pressure ≥90 mmHg, when it has no clearly identifiable cause, it is called essential hypertension. It was estimated that almost a quarter of the Brazilian population has hypertension.

**Objectives:** This study aims to compare the mortality rate from essential hypertension and relate it to years of study between the Northeast Region and Brazil.

**Results:** It was observed that there were 241,797 deaths resulting from essential hypertension in Brazil from 2010 to 2019, with 78,134 cases in the Northeast region, characterizing it as the second region with the most deaths due to this pathology, representing 32.31%. In addition, regarding education, in individuals who had no years of study, there were 33,641 deaths in the Northeast, representing a rate of 51.31%. Furthermore, among patients with 1 to 3 years of study in the Northeast, there were 16,062 deaths, with 26.42% of the total in Brazil. As for the population with 4 to 7 years of study, 8,049 deaths were obtained in the Northeast, representing 20.44%. In relation to 8 to 11 years of study, 3,704 deaths were recorded in the Northeast, constituting 19.61%. In addition, in individuals with 12 or more years of study, the Northeast represented a total of 906 deaths, with 16.17% of the total. Finally, in some notifications, the degree of education was not reported, which represented 15,772 deaths in the Northeast, representing 30.57%.

**Conclusion:** The schooling factor is a variable to be considered when assessing death from SAH, highlighting the Northeast region of Brazil. It was observed that mortality rate was inversely proportional to the level of education, with half of the national cases occurring in individuals who did not have training, demonstrating the relevance of this variable. Thus, health prevention and promotion measures are needed for patients with low schooling to control essential hypertension in order to reduce complications resulting from this disease.

107996

Modality: E-Poster Scientific Initiation – Non-case Report

Category: HEART FAILURE/CARDIOMYOPATHY/TRANSPLANT

## Outcomes of Palliative Care Interventions in Patients with Heart Failure: A Systematic Review of the Literature

HITESH BABANI ^1^, Hitesh Babani^1^, Giovana De Oliveira Sarubi^1^, Marjorie Bindá Leite^1^, Guilherme Henrique Souza^1^, Rania Gabriele Said^1^, Ornella Aquino Da Silva^1^, Lorrana de Oliveira Teixeira^1^, Adria Melissa Silva Campos^1^, Jéssica Alessandra Cruz Dos Santos^1^, Rodrigo de Souza Leitão^2^

(1) Centro Universitário Fametro; (2) Fundação Hospitalar de Hematologia e Hemoterapia do Amazonas (HEMOAM)

**Background:** Heart failure (HF) is a progressive life-limiting condition affecting 1–2% of the general adult population in high-income countries. By 2030, it is estimated to affect 8 million people, consuming more than $69 billion in medical costs. Patients with advanced HF have a risk of premature death and report physical symptoms, psychosocial burdens, and spiritual needs, making necessary the interventions of PC (palliative care). PC offers total care for a person with an incurable disease which may still respond to disease-modifying treatments, but is nonetheless progressive and life-shortening and is not limited to a specific diagnosis, nor to a particular prognosis.

**Aims:** This document aims to identify outcomes of PC intervention in patients with HF and ascertain the efficacy of management.

**Methods:** A systematic literature review was developed in three steps: Development of the research question, search for scientific articles in the Pubmed database and critical analysis of included articles. The search was conducted in March 2021, and articles between 2019 and 2022 were selected, for a total of 74 articles, of which 19 were used.

**Results:** PC interventions improved quality of life, symptom burden, physical symptoms, mental symptoms, and patients were able to end their life with dignity. The PC needs to be based on multidisciplinary team management for end-stage HF with the purpose of improving quality of life and reducing healthcare costs. In addition, the presence of a multidisciplinary team was associated with multiple positive results. The outcomes of improving access to palliative care for people with advanced HF might reduce their suffering and of their loved ones, as well as decrease hospital readmissions.

**Conclusions:** PC aims at integral care and approaches the patient as a whole (spiritually, socially, physically, and emotionally) these interventions should be provided alongside optimal cardiologic management. This study indicates that there are associations between PC and positive outcomes for patients with end-stage heart failure, resulting in a substantial improvement in quality of life as well as comfort and dignity whilst dying.

108575

Modality: E-Poster Scientific Initiation – Non-case Report

Category: CONGENITAL AND PEDIATRIC CARDIOLOGY

## Trisomy 21 and Congenital Heart Defects: Frequency and Types Verified in a Sample of 299 Patients

ISADORA BUELONI GHIORZI^1^, Maurício Rouvel Nunes^1^, Mateus dos Santos Taiarol^1^, Marina da Rocha Besson^1^, Eliaquim Beck Fernandes^1^, Adriano Louro Moreira^1^, Juliana Bergmann^1^, Giulia Righetti Tuppini Vargas^1^, Konopka^1^, Valberto Sanha^1^, Thais Vanessa Salvador^1^, Rafael Fabiano Machado Rosa^1^

(1) Universidade Federal de Ciências da Saúde de Porto Alegre

**Introduction:** Patients with trisomy 21, or Down syndrome (DS), have an increased risk of presenting congenital heart defects (CHDs). They have been described in around 40–50% of the patients.

**Objective:** To detect the frequency and types of CHDs in a sample of patients with DS.

**Methods:** The sample was composed by patients seen at a Genetics Clinic Department, in a period between 1994 and 2008. Data was collected retrospectively, emphasizing the cardiological assessment. All patients underwent cytogenetic evaluation through GTG-Banding karyotype.

**Results:** Among the 299 patients from the sample, 166 (55.5%) were male. The mean age at first evaluation was 23.4 days. The predominant karyotypic abnormality was trisomy 21 (96%). Structural abnormalities were found in 3% of patients of the sample, and mosaicism was found in 1% of them. Two hundred and thirty two patients (77.6%) underwent cardiological assessment, and 160 of them (69%) presented abnormal physical examination findings. Among them, 151 underwent echocardiography, and malformations were detected in all of the patients. Among the 72 patients with normal findings (31%), 41 underwent echocardiography, and malformations were detected in 3. Therefore, CHDs were detected in 154 patients among the 232 who underwent cardiological assessment (66%). The most common CHDs observed were: atrioventricular septal defect (n = 61; 39.6%), atrial septal defect (n = 60; 39%), ventricular septal defect (n = 38; 24.7%), and patent ductus arteriosus (n = 38; 24,7%). Other CHDs consisted of Fallot tetralogy (n = 12; 7.8%), pulmonary stenosis (n = 7; 4.5%), pulmonary atresia (n = 1; 0.65%), and Ebstein abnormality (n = 1; 0.65%).

**Conclusions:** Data of this study confirm the increased prevalence of CHD in patients with DS. Therefore, as opposed to what was observed in this sample, in which 22.4% were not assessed, this study reinforces the importance of cardiological assessment in every child with DS, since it may have a significant impact on their prognosis.

108730

Modality: E-Poster Scientific Initiation – Non-case Report

Category: CARDIOVASCULAR SURGERY

## Effects of Strengthening the Surae Triceps Muscle on Venous Pump Function in Chronic Venous Insufficiency

LUISA PEREIRA DE OLIVEIRA ZANETTI GOMES^1^, Ana Carla Schmidt^1^, Camila Martins Marinelli^1^, Ricardo Zanetti Gomes^1^

(1) Universidade estadual de ponta grossa; (2) Universidade da região de Joinville

Chronic venous insufficiency (CVI) is a common disease that causes calf muscle pump dysfunction and has repercussions for the hemodynamics of the structures involved. To analyze the effects on venous hemodynamics of exercises to strengthen the calf muscles in patients with CVI. This non-randomized clinical trial analyzed 25 lower limbs with CVI, classified from C1 to C5 according to CEAP, Were included if they had achieved at least 60% attendance in the calf strengthening group session. The variables analyzed were collected at at baseline, after 1 month and 2 months and at the end of the exercise protocol. The isometric dynamometry, goniometry, leg circumference and adipometry of the calf The intervention duration 24 session. Both open and closed kinetic chain exercises were performed, involving plantar and dorsal flexion of the ankle joint. Initially, a descriptive analysis of patient variables was conducted, estimating simple frequencies. The variables were analyzed with estimation of mean, median, standard deviation, and 25th and 75th percentiles for each data collection period. Differences between different data collection times were tested with the Friedman test followed Bonferroni correction. In this study were included 25 limbs, comprised of women, who had been diagnosed with CVI for a mean of 6.4. The results of the comparison between the first and the last section showed that there was an increase in the muscular strength of 0.5 kgf (p = 0,2) In the dorsiflexion and plantar flexion measurements increase by 5º (p < 0,0001). The leg circumference increases 0,6 cm (p = 0,215). The Adipometry reduce in 5 mm (p < 0.001). At the analysis of the dynamometry results, there were no statistically significant. The dorsiflexion and plantar flexion there was an increase of 5º at the C3 in the dorsiflexion, at the C2 group in the plantar flexion (p < 0,002). The leg circumference increase 1,2 cm in the group C1 (p = 0.045). The adipometry results showed a reduction at the C3 classification of 1 mm (p = 0,004), Administration of exercise protocols should be considered as a treatment option for CVI, since it has a positive impact on risk factors and on the functions that are impaired by this pathology. A positive impact was observed after the 3 month at the muscular strength, adipometry, range of movement in the patients.

108010

Modality: E-Poster Scientific Initiation – Non-case Report

Category: CARDIO-ONCOLOGY

## “Study of the Role of Focal Adhesion Kinase in the DNA Damage Response in Cardiomyocytes Exposed to Doxorubicin by Super-Resolution Structured Microscopy”

ANTONIO DOSUALDO NETO^1^, Aline Mara dos Santos^2^, Ana Paula Samogim^2^, Isabela Aparecida Moretto^2^, Gabriel Macherini Quaglia^2^, André Alexandre de Thomaz^2^

(1) Pontifícia Universidade Católica de Campinas (PUC-CAMPINAS); (2) Universidade Estadual de Campinas (UNICAMP)

**Introduction:** Cardiovascular complications of anticancer therapies are emerging as a major public health problem, considering that the cancer survival rate has increased considerably in recent years. Cardiotoxicity resulting from the action of chemotherapeutic agents, such as Doxorubicin (doxo), is a serious condition that can progress to chronic cardiomyopathy, congestive heart failure and patient death. Studies focusing on signaling activated by antineoplastic therapies have demonstrated the importance of focal adhesion kinase (FAK) for cell survival and resistance to this treatment, however, this signaling remains poorly understood. The present work aimed to characterize the effects of treatment with the chemotherapy drug doxo on the subcellular redistribution of FAK in H9C2 cardiac myocytes. Furthermore, we investigated the interaction between FAK and DNA damage response (DDR) related proteins after doxo treatment, such as DDX5, XRCC5 and DNA-PK which were previously identified in FAK co-immunoprecipitation experiments.

**Objective:** Investigate whether FAK is modulated by Doxo in cardiac myocytes and whether this protein co-localizes with DNA damage response (DDR) proteins by means of super-resolution microscopy.

**Methodology:** Culture of ventricular cardiomyoblasts (n = 80 cells), treatment with doxorubicin, immunostaining of proteins with subsequent analysis by Super Resolution Microscopy were performed. Finally, the T-Student distribution was used for the statistical data and the comparative effect between the control and chemotherapy groups was detailed by the ImageJ program.

**Results:** Among the main findings, it was found that there is an accumulation of activated FAK in the nucleus and the approximation between this kinase and the DDR proteins during genotoxic stress by doxo, indicative of mutual action to maintain cell viability. Furthermore, doxo stress did not cause a significant gain in fluorescence intensity of DDX5, but a subnuclear redistribution accompanied by FAK. XRCC5 and DNA-PK increased their intensities in cells treated with chemotherapy, with concomitant approximation of FAK.

**Conclusion:** The findings of this project contributed to the understanding of the mechanisms by which FAK promotes cell survival against treatment with doxo and, in addition, may contribute to the establishment of new therapeutic modalities for tumor treatment with reduction of the deleterious effects on cardiac function. of the patient.

108048

Modality: E-Poster Scientific Initiation – Non-case Report

Category: ATHEROSCLEROSIS/CARDIOVASCULAR RISK FACTORS/CARDIOVASCULAR PREVENTION

## Orthostatic Hypotension is Related to Subclinical Coronary Artery Disease in Type 2 Diabetes Individuals

ANA RAQUEL WHITAKER FILIPE^1^, Ana Raquel Whitaker Filipe^1^, Sofia Helena Vitte^1^, Daniel Campos de Jesus^1^, Joaquim Barreto^1^, Andrei C. Sposito^1^

(1) Universidade Estadual de Campinas (UNICAMP)

**Introduction:** Orthostatic hypotension (OH) is a surrogate marker of poor prognosis in type 2 diabetes (T2D) and results at least partially from arteriosclerosis, autonomic dysfunction, and glucotoxicity; all features involved also in the progression of atherosclerotic cardiovascular disease.

**Objective:** To determine whether OH is related to subclinical coronary artery disease (SCD) in T2D individuals.

**Methods:** This was a cross-sectional, predefined analysis of the Brazilian Diabetes Study, a single-center, prospective cohort of T2D. After 3-min resting sited with their arms at their heart level, participants had their blood pressure (BP) measured three times with a 1-min interval between each measurement and the mean value of the last 2 was considered. Orthostatic BP was then measured as the BP obtained after 1-min standing. OH was defined as an orthostatic systolic or diastolic BP drop greater than 20 mmHg and 10 mmH, respectively, when compared to sitting BP. SCD was a coronary calcium score above zero in individuals without prior acute coronary syndrome, coronary revascularization, or stable angina. Logistic regression was used to evaluate the influence of the independent variable, OH, on the dependent variable, SCD, and further adjustment by age, gender, and LDL-C was performed. To assess if OH was related to SCD severity, ordinal logistic regression was performed using OH as a predictor of higher CAC groups (0, 1–99 and > 100 Agatston).

**Results:** Among 366 individuals (mean age, 57 + 8 years; 58% male; T2D duration, 9 years), included in this analysis, 41 (11.2%) had OH and 260 (71%) had SCD. Compared with controls, HO group were older (57 + 7.4 vs 60 + 7.8 years; p = 0.034), had higher levels of LDL-C (106 + 38 vs 121 + 40.1, p = 0.033) and displayed a higher prevalence of SCD (69.2% vs 85.4%, p = 0.032). HO was related to SCD with an OR of 2.59 (95%CI: 1.06, 6.36; p = 0.037), which remained significant after adjustment (OR: 2.87; 95%CI: 1.03, 8.03, p = 0.044). OH was significantly related to the chance of being classified at a higher CAC group in logistic ordinal regression, with an OR of 2.34 (95%CI: 1.09, 5.01, p < 0.001).

**Conclusion:** T2D individuals with HO are at an increased risk of SCD. This should be borne in mind of healthcare providers when assessing CV risk in this population.

108065

Modality: E-Poster Scientific Initiation – Non-case Report

Category: ATHEROSCLEROSIS/CARDIOVASCULAR RISK FACTORS/CARDIOVASCULAR PREVENTION

## Cardiovascular Risks of Hormone Replacement Therapy in Transgender Women: A Systematic Review

PRISCILA PEREIRA ALBUQUERQUE^1^, Kassielly Melissa Ribeiro Rodrigues^1^, Nathalia Bianco Fabris^1^, Rodrigo Lage Carneiro^1^, João Gabriel Pacetti Capobianco^1^

(1) Pontifícia Universidade Católica de Minas Gerais campus Poços de Caldas

**Introduction:** There is a growing number of transgender women who use hormone replacement therapy (HRT), based mainly on the use of estrogen, so it’s necessary to understand their effects on the individual’s health. The main goal of this treatment is to adjust secondary sexual characteristics to be congruent with your gender. The impact of hormone therapy on cardiovascular health is not widely known.

**Objective:** The study aims to perform a systematic review on what are the cardiovascular risks caused by hormone therapy in transgender women.

**Methodology:** The search was performed using the following electronic databases: Pubmed, Scopus, BVS, Scielo, Web Of Science, Science Direct, Sage Journals, DOAJ, Oxford Journal, Lippincott Williams & Wilkins Journals from 2015 to 2020, according to the Preferred Reporting Items for Systematic Reviews and Meta-Analyses (PRISMA) methodology using the terms “Hormone treatment AND Transgender AND Cardiovascular diseases”. Only primary studies were included, which investigated the cardiovascular effects of HRT in transgender women. Reviews, meta-analyses, non-primary studies, animal studies, and adolescent studies were not included.

**Results:** Of the 228 articles found, after applying the inclusion criteria, 7 articles were selected for analysis. The results were heterogeneous and showed that cross-sex hormone therapy impacts the health of transgender women in several aspects, such as: changes in blood pressure (BP), lipid profile, body mass index (BMI) and vascular compromise. Regarding BP, 2 studies showed an increase in blood pressure levels and 1 observed reduction. As for changes in the lipid profile, 2 studies didn’t observe changes, however, 2 other studies reported a decrease in these parameters. Only 1 study showed an increase in BMI as a consequence of the use of HRT. Concerning, vascular compromise, 1 study reported the occurrence of deep vein thrombosis, on the other hand, another study with 676 transgender women showed only one case.

**Conclusion:** The heterogeneity of the results found in relation to the effects of HRT indicates the need for studies with larger samples that longitudinally follow up individuals involved in this process. The recent beginning of the use of this therapy and the neglect that this population group suffers from society are the factors identified by the authors as responsible for the lack of robust data regarding the safety of HRT for the health of transgender women.

108066

Modality: E-Poster Scientific Initiation – Non-case Report

Category: ATHEROSCLEROSIS/CARDIOVASCULAR RISK FACTORS/CARDIOVASCULAR PREVENTION

## Diabetic Polyneuropathy is Related to Carotid Artery Disease in Type 2 Diabetes Individuals

NATÁLIA CHICANI ZORZETO ^1^, Sofia Helena Vitte^1^, Daniel Campos de Jesus^1^, Ana Raquel Whitacker Filipe^1^, Joaquim Barreto^1^, Andrei Carvalho Sposito^1^

(1) Universidade Estadual de Campinas – UNICAMP

**Introduction:** Sensory-motor diabetic polyneuropathy (DP) is driven by axonal lesion, which is chiefly mediated by the activation of receptors for advanced glycation end-products (RAGE). RAGE are also expressed in the endothelial layer, where it intensifies the progression of atherosclerosis. Henceforth, plausibly DP may be a surrogate marker of atherosclerotic cardiovascular disease among T2D individuals.

**Objective:** We sought to investigate whether DP is related to the risk of atherosclerotic cardiovascular disease in T2D individuals.

**Methods:** This was a prespecified analysis of the Brazilian Diabetes Study dataset. T2D subjects were screened for DP using the Michigan Neuropathy Screening Instrument (MNSI), which was deemed altered in all participants scoring 4 or more points in a 15-questions questionnaire, or in those scoring 2 points in a directed foot examination. Carotid doppler ultrasound was performed for intima-media thickness (C-IMT) measurement, and carotid disease (SCD) was considered for all with C-IMT higher than the 75th percentile validated for our population by the ELSA-Brasil Study. The relationship between SCD and DP was estimated by binary logistic regression, adjusted by the covariates: age, gender, A1c, LDL-C, hypertension, and prior cardiovascular disease.

**Results:** Among 324 individuals (age: 58 + 7.1 years; 85% hypertensive; T2D duration: 9 + 6 years; 58.6% male; HbA1c: 7.8 + 1.6%), 134 (41.4%) had DP and 220 (67.9%) had SCD. Compared to non-DP, DP group had a higher prevalence of SCD (61.1% vs 77.5%; p = 0.002) and higher values of C-IMT (0.71 + 0.16 mm vs 0.78 + 0.19 mm; p < 0.001). DP was related to a SCD risk, with an OR of 2.21 (95%CI: 1.34, 3.64; p = 0.002), which remained significant after adjustment by age and gender (OR: 2.31; 95%CI: 1.28, 3.53; p = 0.003), and when further adjustment by A1c, LDL-C, hypertension and prior CVD was performed (OR: 3.38; 95%CI: 1.46, 7.81; p = 0.004).

**Conclusion:** In T2D individuals, DP is independently related to SCD. Healthcare providers should thus consider DP screening as a potential tool when assessing individuals at higher risk of cardiovascular events.

108069

Modality: E-Poster Scientific Initiation – Non-case Report

Category: CARDIAC ARRHYTHMIAS/ELECTROPHYSIOLOGY/ELECTROCARDIOGRAPHY

## Evaluation of the Clinical-Epidemiological Profile, Risk of Stroke and Bleeding in Patients with Atrial Fibrillation and End-Stage Renal Disease on Hemodialysis

LUCAS YUJI SONODA^1^, Maria da Graça Lepre Hawerroth^1^, Jean José Silva^2^, Walter Alvarenga de Oliveira^2^

(1) Minas Gerais State University; (2) Santa Casa de Misericórdia de Passos

**Introduction:** Atrial fibrillation (AF) is the most prevalent sustained cardiac arrhythmia and shares several common risk factors with end-stage renal disease (ESRD), as advanced age, hypertension, diabetes and vascular disease.

**Objective:** To assess the clinical-epidemiological profile, risk of stroke and bleeding in patients with AF on renal replacement therapy undergoing hemodialysis in a regional hospital in Minas Gerais.

**Methods:** A cross-sectional study, carried out in the nephrology sector of a hospital in the southwest of Minas Gerais, which included patients on renal replacement therapy on hemodialysis from January to March 2020. Patients with glomerular filtration rate (GFR) > 15 mL/min/1.73 m^2^, hemodialysis treatment for < 3 months, age < 18 years, and patients on peritoneal dialysis were excluded. All patients underwent a 12-lead electrocardiogram (ECG) during the hemodialysis session. The risk of stroke and major bleeding were calculated using the CHA2DS2VASC and HAS-BLED scores, respectively.

**Results:** 247 patients from the hemodialysis sector were screened, of which 17 (6.8%) had AF on the ECG. Among patients with AF, 76% were male, the mean age was 72.5 ± 7.6 years. The mean GFR was 6.8 ± 3.1 mL/min/1.73 m^2^. The most common comorbidities were hypertension 14 (82.4%), smoking history 13 (76.4%), heart failure 11 (64.7%), diabetes 5 (29.4%), coronary artery disease 5 (29.4%) and stroke 3 (17.6%). The CHA2DS2VASC was *≥* 2 in 94.1% (mean 3.8 ± 1.5) of these patients. HAS-BLED scored *≥* 3 in 76.4% (mean 3.2 ± 1.1) of the cases. 3 (17.6%) of the patients were taking oral anticoagulants.

**Discussion:** The prevalence of AF in patients with ESRD is approximately 11.6%, with a range from 4.5 to 27%. These patients have several conditions which increase the risk of stroke and also the risk of bleeding. However, to date, there is a lack of evidence on the safety and efficacy of oral anticoagulants in hemodialysis patients, remaining a dilemma in the management of these patients.

**Conclusion:** In the present study, there was a prevalence of 6.8% of patients with AF on hemodialysis, with multiple comorbidities, high risk for both stroke and major bleeding and most were not receiving oral anticoagulation.

108079

Modality: E-Poster Scientific Initiation – Non-case Report

Category: ATHEROSCLEROSIS/CARDIOVASCULAR RISK FACTORS/CARDIOVASCULAR PREVENTION

## Prevalence and Risk Factors for Orthostatic Hypotension in Individuals with Type 2 Diabetes

ANA RAQUEL WHITAKER FILIPE^1^, Ana Raquel Whitaker Filipe^1^, Sofia Helena Vitte^1^, Daniel Campos de Jesus^1^, Joaquim Barreto^1^, Andrei C. Sposito^1^

(1) Universidade Estadual de Campinas (UNICAMP)

**Introduction:** Orthostatic hypotension (OH) is a surrogate marker of cardiovascular morbimortality in type 2 diabetes (T2D). The prevalence of OH is influenced by characteristics that significantly vary across populations.

**Objective:** To determine the prevalence of OH and its risk factors in T2D Brazilian individuals.

**Methods:** This was a cross-sectional, predefined analysis of the Brazilian Diabetes Study, a prospective ongoing cohort of T2D. Blood pressure (BP) was measured after 3-min resting using appropriately sized cuffs and the mean value of the last two out of three measurements were considered. Enrolees stood for 1-min for orthostatic BP. OH was defined as systolic or diastolic BP drops greater than 20 mmHg and 10 mmHg, respectively, or any OH-related symptoms. Baseline demographic and anthropometric data were recorded, and blood samples collected for biochemical analysis. The median family income was considered to classify study sample as low- or high-income, whereas LDL-C > 100 mg/dL was deemed as high.

**Results:** Among 1030 patients (mean age: 57 years; T2D duration: 9 years; 59% male), 104 (10,09%) had HO. Compared with non-HO, HO group was older (58 + 8.1 vs 60.4 + 7.5 years; p = < 0.001) and had a higher prevalence of female individuals (38.7% vs 58.7%; p < 0.001), high LDL-C levels (53.0% vs 65.8%; p = 0.038), low-income (15.2% vs 35.6%; p < 0.001), and of individuals with less than primary school education (15.1% vs 27.2%; p < 0.001). In binary logistic regression, the characteristics related to HO were age (OR: 1.046, 95%CI: 1.02, 1.07; p < 0.001), female gender (OR: 2.25, 95%CI: 1.49, 3.39; p < 0.001), high LDL-C (OR: 1.70, 95%CI: 1.02, 2.84; p = 0.040), whilst high-income (OR: 0.33; 95%CI: 0.17, 0.62; p < 0.001) and secondary education (OR: 0.31; 95%CI: 0.17, 0.57; p < 0.001, compared with less than primary education) were protective. After adjustment by covariates, female (OR: 2.16; 95%CI: 1.08, 4.34; p = 0.030) and secondary education (OR: 0.29; 95%CI: 0.09, 0.85; p = 0.025) were independently related to HO risk.

**Conclusions:** OH affects 10% of Brazilian T2D subjects and its prevalence is higher among female and less educated individuals. This should be borne in mind when assessing OH status in this population.

108703

Modality: E-Poster Scientific Initiation – Non-case Report

Category: EPIDEMIOLOGY AND HEALTH POLICIES/GLOBAL HEALTH

## Fake News in Cardiology – Study on the Online Dissemination of Fake News About Vaccine and Sudden Death

ESTEPHANY DE JESUS SILVA^1^, Aline Goneli de Lacerda^3^, Claudio Tinoco Mesquita^1^

(1) Universidade Federal Fluminense – UFF; (2) Fundação Euclides Cunha – FEC; (3) Pós Graduação em Comunicação – UFF

**Introduction:** During the COVID-19 pandemic, the spread of fake news on social media took on such proportions that it was described by the World Health Organization as an “infodemic‘‘. Sudden deaths of artists and athletes or by stroke have become associated with Covid-19 vaccines in social networks and message groups. As YouTube is the largest online video platform, our goal is to evaluate the spread of Fake News linking vaccines and sudden death on YouTube in Portuguese language videos.

**Methodology:** We conducted a search for videos on Youtube Data Tools by the “Video Network”, from the junction of the keywords “vaccine” AND “sudden death”, from the crawl depth 0 of the tool to identify the most relevant actors (videos) on the topic on this platform. Then we used the software Gephi 0.9.2, for network analysis and visualization. Finally, we analyzed the content of the 99 most relevant videos to group them into 2 categories: “fake news” and “non-fake news”.

**Results:** Of the 99 videos, 17 were considered as fake news videos (18%), with a total of 2,289,355 views. The most watched fake news (673,175 views) related the death by stroke of a young man with the use of the COVID vaccine, although the hypothesis was ruled out by the health team in less than 6 months. In contrast we found 82 non-fake news videos, with the most viewed one accounting for 1,410,154 views. Videos with fake news often did not feature health professionals, and some were from mainstream media vehicles such as television newspapers.

**Conclusion:** One in five of the most watched videos on YouTube linking vaccines and sudden death contain fake news. Although most videos are scientifically based, the dissemination of misinformation can contribute negatively to the health of the population by increasing hesitancy to vaccinate. Strategies to increase scientifically based cardiology content should be encouraged.

108356

Modality: E-Poster Scientific Initiation – Non-case Report

Category: ATHEROSCLEROSIS/CARDIOVASCULAR RISK FACTORS/CARDIOVASCULAR PREVENTION

## D:A:Dr Seems to Agree Perfectly with Framingham Risk Score in a Brazilian Population Living with HIV

VITOR QUEIROZ DE CASTRO SOUZA^1^, Victória Valadares Andrade^1^, Thais Velasques Dias^1^, Carlos Roberto Brites Alves^2^, Eduardo Martins Netto^2^

(1) Faculdade de Medicina da Bahia (FMB/UFBA); (2) Laboratório de Pesquisa em Infectologia (LAPI), Hospital Universitário Professor Edgard Santos (HUPES), Salvador, Brazil

**Introduction:** People living with HIV (PLHIV) are twice as likely to develop cardiovascular disease. The identification of high cardiovascular risk (CVR) is essential to manage prevention strategies and can be performed through prediction equations. However, there is no validated CVR score for Brazilians living with HIV.

**Objective:** To assess the agreement between three CVR scores in PLHIV.

**Methods:** Cross-sectional study carried out with 265 Brazilians living with HIV, aged 40 to 74 years, using antiretroviral therapy. Ten-year CVR was calculated using two scores for the general population, the Framingham Risk Score (FRS) and the American College of Cardiology/American Heart Association (ASCVD), and one specific for PLHIV, Data Collection on Adverse Effects of Anti-HIV Drugs Cohort reduced (D:A:Dr). Multivariate logistic regression was performed to investigate the association of high CVR with statistically significant variables. Agreement between scores was assessed using the weighted Kappa coefficient and the Bland-Altman plot. The preventive recommendation with statins was analyzed, according to the Brazilian Clinical Protocol and Therapeutic Guidelines, through the use of scores.

**Results:** The median age was 52 years (47–58), 58.9% were men, 46% brown, 11.3% were smokers, 34% were hypertensive, 9.1% were diabetic and 50.2% were overweight/obese. The median time of antiretroviral use was 15 years (7–21), 8.3% had a detectable viral load. Multivariate analysis showed that age, current smoking and diabetes mellitus were independent predictors for high CVR in the three scores. The agreement between the D:A:Dr and the FRS was perfect (k = 0.82; 95% CI 0.77–0.87; p < 0.001), and substantial between the FRS and the ASCVD (k = 0, 74; 95% CI 0.69–0.79; p < 0.001) and between D:A:Dr and ASCVD (k = 0.70; 95% CI 0.64–0.76; p < 0.001). The Bland Altman plot revealed greater discordance between scores as the RCV increased. The use of statins would be recommended for 25 patients when using the FRS, 8 with the ASCVD and 23 with the D:A:Dr.

**Conclusion:** A high CVR was found in this study. The agreement between the FRS and the D:A:Dr suggests that both scores may be suitable for classifying the CVR in this population. There is still a need to develop calibrated equations for Brazilians living with HIV. Our results generate insights for the incorporation of D:A:Dr as an alternative to the FRS in the Brazilian protocol, as well as other international guidelines have been advocating.

108373

Modality: E-Poster Scientific Initiation – Non-case Report

Category: CARDIAC ARRHYTHMIAS/ELECTROPHYSIOLOGY/ELECTROCARDIOGRAPHY

## Prevalence of Esophageal Injury After Atrial Fibrillation Ablation in Hospital Israelita Albert Einstein

BEATRIZ HACHUL DE CAMPOS^1^, Denise Tessariol Hachul^2^, Cristiano Pisani^3^, Tan Chen Wu^3^, Alberto Ferraz^3^, Mauricio Ibrahim Scanavacca^2^

(1) FICSAE – São Paulo – São Paulo – Brasil; (2) Hospital Israelita Albert Einstein – São Paulo – São Paulo – Brasil; (3) INSTITUTO DO CORAÇÃO DO HCFMUSP – SP – BRASIL

**Introduction:** Catheter ablation of atrial fibrillation (AF) presents esophageal injury as a possible post-procedure complication. It is important to know its prevalence, to analyze the efficacy of protection techniques and establish early diagnosis and preventive treatment.

**Objective:** To identify the prevalence of esophageal lesions in patients who underwent AF catheter ablation at Hospital Israelita Albert Einstein in a determined period.

**Methods:** This is a retrospective study, with clinical follow-up based on the survey of medical records related to the perioperative period, of patients who underwent AF catheter ablation from 2016 to 2019. The esophageal mucosa evaluation was made by upper digestive endoscopy, performed between 24 and 72 hours after the procedure. Patients who showed severe injuries were selected to undergo a second endoscopy in the following week. The lesions observed were standardized by the Kansas City classification (KCC).

**Results:** Fifty-seven patients were included in the study. Most of them were male (75.4%) with a mean age of 60.1 years old. Of the patients included, 75.4% had paroxysmal AF. In most cases (86%), a linear thermometer was used to avoid critical increase in the temperature and the occurrence of injury. In the other cases, esophagus displacement with a transesophageal Echo probe was carried out. Considering the esophageal injuries, 36 (63.2%) patients had no lesions in the endoscopy. Among the lesions observed, 1 (1.8%) had erythema (KCC1), 7 (12.3%) had erosion (KCC2A), 3 (5.3%) had hematoma-ecchymosis and 8 (14%) had traumatic injury. The overall prevalence of esophageal lesions was 34.5%. In the control endoscopy, performed in the following week, most of the injuries healed spontaneously, and no patient had complications during the medium and long terms follow-up.

**Conclusion:** Despite protective measures, a considerable number of esophageal lesions was observed. Thus, this study suggests the need to improve the techniques of left atrium ablation and of esophageal protection.

108375

Modality: E-Poster Scientific Initiation – Non-case Report

Category: ATHEROSCLEROSIS/CARDIOVASCULAR RISK FACTORS/CARDIOVASCULAR PREVENTION

## Myocardial Insulin Resistance

GABRIELA CORREIA MATOS DE OLIVEIRA^1^, Luis Matos de Oliveira^2^, Luis Jesuino de Oliveira Andrade^3^

(1) Faculdade de Medicina – UniFTC – Salvador – Bahia – Brazil.; (2) Faculdade de Medicina – Salvador – Bahia – Brazil.; (3) Colegiado de Medicina – Universidade Estadual de Santa Cruz – Ilhéus – Bahia – Brazil.

**Background:** The low available of Glut-4 transporters in sarcolemma of the cardiac cells is what characterizes the myocardial insulin resistance (MIR), which is triggered separately of generalized insulin resistance. Insulin receptors are quite evident in the heart muscle and vessels, and mitochondrial activity performs a significant function in MIR preserving cellular homeostasis by cell reproduction, cells livelihoods, and energy generation.

**Objective:** It is to evaluate the MIR mechanism, and through the signaling pathway design.

**Methods:** PubMed database was employed to search for reviews publications with MIR. The referenced data of the signaling pathway was chosen aggregating references of the Kyoto Encyclopedia of Genes and Genomes (KEGG) database. A signaling pathway was designed based on MIR research manuscripts, where we show several mechanisms included in the MIR. The KEGG server was employed to exploit the interrelationship protein-protein, and elaborate signaling pathway diagram. The signaling pathway mapping was carried out with PathVisio software.

**Results:** We selected 42 articles from a total of 450 articles in the PubMed database that presented a significant association between the terms “insulin resistance myocardial” AND “signaling pathway”. Founded on database-validated research papers, we choose well-founded pathways and we succeeded representative description of these pathways. The reproduction contigs taken from the KEGG database designed the signaling pathway of the bio-molecules that lead to MIR. Thus, the acting among multiple mechanisms releases factors that participate of the development of MIR.

**Conclusion:** The interaction among various mechanisms and molecular interactions are important factors in development of MIR.


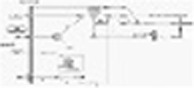


108661

Modality: E-Poster Scientific Initiation – Non-case Report

Category: HEMODYNAMICS AND INTERVENTIONAL CARDIOLOGY

## Mortality and Outcomes After Correction of Aortic Stenosis by Surgical Replacement and Transcateter in Sergipe – Smallest State of Brazil

GABRIELA DE OLIVEIRA SALAZAR^1^, José Icaro Nunes Cruz^1^, Cláudia Bispo Martins-Santos^1^, Mayara Evelyn Gomes Lopes^1^, Ullany Maria Lima Amorim Coelho de Albuquerque^1^, Marília Marques Aquino^1^, Juliana Maria Chianca Lira^1^, Nathalia Luiza Silva Sobral^1^, Lucas Villar Shan de Carvalho Cardoso^1^, Antônio Carlos Sobral Sousa^2^, Joselina Luzia Menezes Oliveira^2^, Eduardo José Pereira Ferreira^2^

(1) Federal University of Sergipe; (2) Rede D’Or São Lucas Hospital; (3) Primavera Hospital

**Introduction:** The symptomatic manifestation of aortic stenosis (AoS) represents a considerable increase in the risk of death and indicates valve replacement, either by transcatheter route (TAVR) or by conventional transthoracic surgery (SAVR). Recent studies seek to compare the results and complications between these procedures, with attractive results for TAVR, especially after the development of more modern prostheses.

**Objective:** To compare hemodynamic outcomes, mortality and in-hospital complications in patients with severe AoS treated in SAVR and TAVR in Sergipe (Brazil).

**Methods:** Retrospective, observational and analytical cohort study. It included data collected between 2013 to 2021 based on medical records. Fifty-five patients were included, divided into groups according to treatment for severe aortic stenosis, SAVR (17; 30.9%) or TAVR (38; 69.1%). Statistical analysis was performed using the Student’s T test, indicated by the Shapiro-Wilk test, in addition to the chi-square test or Fisher’s exact test. A significance level of 5% was adopted.

**Results:** The mean age of the sample was 76.6 ± 10.93 years. Of the total, 59.3% were male. Of the TAVRs (38), all were via transfemoral access and the most used prostheses were EVOLUT R (64.7%), followed by COREVALVE (17.6%) and SAPIEN 3 (8.8%). In SAVR (17), bovine pericardium bioprosthesis was the most used (80%). Patients in the TAVR group had a higher surgical risk profile according to the EuroSCORE I, II and STS scores (p < 0.05). The mean length of hospital stay was 11 days for both groups and hospital mortality was 8.5% for TAVR and 5.9% for SAVR (p > 0.05). There were no differences regarding the percentage of improvement in the echocardiographic parameters after the procedure, regarding the development of conduction disturbance or cardiac rhythm alteration (p > 0.05). However, there was more mild and moderate paravalvular regurgitation in the TAVR group (p = 0.03) and more major or minor bleeding complications (p = 0.046) and acute renal failure (p = 0.017) in the SAVR group.

**Conclusion:** Patients with severe AoS treated by SAVR and TAVR had similar results in terms of valvular hemodynamic performance, mortality rate and mean hospital stay. Patients in the TAVR group had a higher rate of paravalvular regurgitation and those in the SAVR group had more bleeding complications and acute kidney injury.

109089

Modality: E-Poster Scientific Initiation – Non-case Report

Category: ATHEROSCLEROSIS/CARDIOVASCULAR RISK FACTORS/CARDIOVASCULAR PREVENTION

## The Use of Electronic Cigarettes and Its Impacts on the Cardiovascular System

BRUNA GUIMARÃES AGUIAR^1^, Ana Christina Beltrão de Souza Guerra Curado^2^, José Felipe Valois Ribeiro Silva^1^, Paulo Borges Santana^3^

(1) Centro Universitário Maurício de Nassau (UNINASSAU); (2) Faculdade Pernambucana de Saúde (FPS); (3) Hospital Agamenon Magalhães (HAM)

**Introduction:** The use of traditional cigarettes (TC) is still a major public health problem, having negative effects on the cardiovascular system (CVS), despite incentives to reduce its use. The electronic cigarette (EC) appears aiming to replace the TC, releasing nicotine without containing the smoke of the latter. However, ECs are not pharmacologically controlled products, leading them to have an even higher nicotine content than CT.

**Objectives:** Analyze the cardiovascular effects of EC use, as well as the harm caused by the chemical products present in cigarettes.

**Methodology:** A systematic review was carried out using a search for scientific articles on Scielo and PubMed, in March 2022, without language and year restrictions, using the descriptors: “Cardiovascular System”, “Cardiovascular Disease”, “cigarette electronic” associated to the Boolean operator AND.

**Results and Discussion:** 98 articles were found, of which 83 were discarded by reading the titles, 10 by reading the abstracts, since they were not related to the research purpose, being selected 5 articles for complete reading, and all were used for the research. ECs work by heating and releasing vapors that produce toxic substances, including nicotine. Its use is related to high cytotoxicity and changes in cell morphology, which negatively affects endothelial function and the supply of nitric oxide, resulting in an increased risk on CVS. These users, when compared to non-smokers, have twice the risk of having a myocardial infarction, and may have worsened CVS ongoing pathologies, since nicotine, which is also related to dependence, has repercussions on hemodynamics, coronary blood flow, angiogenesis, hypertension and inflammation. Paradoxically, the concentration of nicotine in EC is higher than in CT, and can vary from 16 to 21 mg/mL. In addition, the use of EC increases the risk of atherosclerosis, due to changes in low-density lipoprotein (LDL).

**Conclusion:** Smoking is a relevant health problem, being an important risk factor for heart disease. EC may contain an even higher nicotine content than CT, due to the lack of pharmacological control. The risk of myocardial infarction is greater in smokers than in non-smokers. In addition, nicotine affects hemodynamics, angiogenesis and worsens atherosclerosis. Furthermore, further studies on this topic are needed to broaden the understanding of the EC’s repercussions on public health.

108460

Modality: E-Poster Scientific Initiation – Non-case Report

Category: COVID-19 AND CARDIOVASCULAR SYSTEM

## Remote Monitoring of Cardiac Devices During the COVID-19 Pandemic. Timely and Effective?

EDUARDO AUGUSTO QUIDUTE ARRAIS ROCHA^1^, Luis Gustavo Bastos Pinho^3^, Juvencio Santos Nobre^3^, Pedro Sales Pereira Gondim^1^, Arthur Holanda Dantas^1^, Pedro Barbosa Duarte Vidal^3^, Aston Alves de Freitas^1^, Vitor Olímpio Coimbra^1^, Davi Sales Pereira Gondim^2^, Bruna Sobreira Kubrusly^3^, Fernanda Pimentel Arraes Maia^3^, Francisca Tatiana Moreira Pereira^4^

(1) Centro Universitário Christus (Unichristus); (2) Universidade de Fortaleza (Unifor); (3) Universidade Federal do Ceará (UFC); (4) Centro de Arritmia do Ceará (CACE)

**Introduction:** The COVID-19 pandemic determined great difficulty or even the impossibility of ambulatory follow-up for several patients, including those with heart disease and those with implanted electronic cardiac devices (IECD). These patients need special monitoring for testing and programming their devices. The groups with remote monitoring (RM) could be followed during the pandemic at a distance. It is important to analyze the results of this population, including the possibility of expanding this form of monitoring in Brazil. The aim of this study was to analyze the findings of IECD followed by RM during the COVID-19 pandemic.

**Methods:** Cohort, observational, prospective study involving 119 patients. Events detected in the RM were: presence of atrial and ventricular arrhythmias, defibrillator therapies, changes in the battery, electrode or heart failure parameters. Comparisons were performed using the chi-square test and paired student t test, with p < 5% considered significant. The numbers of events during the pandemic (Group 1-G1) and before the pandemic (Group 2-G2) were compared, with the patient initially being used as his own control. Then, the event rates of G-1 were compared with the group followed just before the pandemic period (Group 3-G3).

**Results:** The patients were 30.2% female, with a mean age of 72 ± 14.2 years, ejection fraction of 55% (34.5/57%), and these types of devices: 30.2% pacemaker; 42, 8% with defibrillator, 3.3% biventricular pacemaker and 22.7% with defibrillator/biventricular pacemaker. 58.8% of the patients in the study had events in the pandemic. Group 1 hadn’t a higher number of events when compared to its own control (G2), RR 1.03 (CI 95% 0.83–1.28), or in relation to group 3, RR 1.02 (CI 95% 0.81–1.30). The groups followed during the pandemic and outside the pandemic showed no differences in characteristics such as age (p = 0.86), ejection fraction (p = 0.08) or functional class > II (p = 0.25).

**Conclusions:** During the COVID-19 pandemic, patients with IECD followed by RM had a high number of monitored events. The COVID-19 pandemic hasn’t determined increases in arrhythmic events in the population of this study. The RM should be considered as an additional form of follow-up for patients with IECD in Brazil.

108718

Modality: E-Poster Scientific Initiation – Non-case Report

Category: CARDIOLOGY OF SPORTS, EXERCISE, ERGOMETRY AND CARDIOVASCULAR REHABILITATION

## Independent Predictors of Low Cardiorespiratory Fitness in Patients with Myocardial Ischemia on Exercise Stress Echocardiography

JOSÉ ICARO NUNES CRUZ^1^, Gabriela de Oliveira Salazar^1^, Cláudia Bispo Martins-Santos^1^, Juliana Maria Chianca Lira^1^, Edvaldo Victor Gois Oliveira^1^, Lara Teles Alencar Duarte^1^, Ullany Maria Lima Amorim Coelho de Albuquerque^1^, Myllena Maria Santos Santana^1^, Lucas Villar Shan de Carvalho Cardoso^1^, Antônio Carlos Sobral Sousa^2^, Enaldo Vieira de Melo^1^, Joselina Luzia Menezes Oliveira^2^

(1) Federal University of Sergipe; (2) Rede D’Or São Luiz – São Lucas Hospital

**Introduction:** Cardiorespiratory fitness (CF), quantified in Metabolic Equivalent of Task (MET), has an inverse relationship with cardiovascular morbidity and mortality. It is estimated that an increase of 1 MET to the CF reduces mortality from all causes from 11.6% to 15.0% and from 16.1% to 19.0% mortality from cardiovascular causes.

**Objectives:** To determine the predictors of low CF in patients with myocardial ischemia.

**Methods:** Cross-sectional, analytical study, whose inclusion criterion was the diagnosis of myocardial ischemia on exercise stress echocardiography. The criterion for low CF was MET < 7.9. A multivariate analysis using binary logistic regression was performed to determine risk and protective factors for low CF in patients with myocardial ischemia. The Nagelkerke pseudo R-squared for the model was 39.3%. The analyses were performed using SPSS Statistics software, version 22.0.

**Results:** 1443 patients were included in the study, of which 56.1% were male (809) and 37.8% had low CF (545). Predictors of low CF were smoking or ex-smoking, chronotropic incompetence, female sex, sedentary lifestyle, typical chest pain, systemic arterial hypertension (SAH), diabetes mellitus (DM) and age. Individuals with asymptomatic myocardial ischemia were 57.3% less likely to have low CF. Left ventricular ejection fraction (LVEF) was also a protective factor – an increase of 1% in LVEF provided 4.2% greater protection against low CF (Table 1).

**Conclusions:** Smoking, female sex and sedentary lifestyle are strong predictors of low cardiorespiratory fitness. It has also been demonstrated that chronotropic incompetence, typical chest pain, SAH and DM are risk factors for low CF. In addition, the absence of symptoms and preserved LVEF confer a lower risk of low CF in ischemic patients.


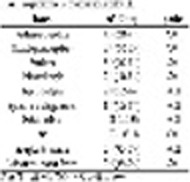


108927

Modality: E-Poster Scientific Initiation – Non-case Report

Category: ATHEROSCLEROSIS/CARDIOVASCULAR RISK FACTORS/CARDIOVASCULAR PREVENTION

## Cardiovascular Risk Profile of a Young Adult Population with High Risk for Obstructive Sleep Apnea Screened by Stop-Bang and Epworth Sleepiness Scale in a Primary Health Care Unit

TOMÁS DE SOUZA MELLO^1^, Karine da Silva Guimarães^1^, Gabriela Gama Zagni Jardim^1^, Ana Rachel Bucar Cervasio^1^, Clara Avelar Mendes de Vasconcellos^1^, Juliana Camara Garcia^1^, Rodrigo Eugenio Vinuto Borges^1^, Natalia Rossilho Moyses Ushijima^1^, Luiza Brandão Catharina^1^, Luísa Leite Vaz da Silva^1^, Ana Cristina Tenório da Costa Fernandes^1^, Elizabeth Silaid Muxfeldt^1^

(1) IDOMED – Universidade Estácio de Sá, Medicine School, Campus Vista Carioca, Rio de Janeiro, RJ, Brasil.

**Objective:** To establish the relationship between cardiovascular (CV) risk profile and detected risk of Obstructive Sleep Apnea (OSA) in two questionnaires – STOP-BANG (SB) and Epworth Sleepiness Scale (ESS), in a young population of adults registered in a Primary Health Care unit in Rio de Janeiro, Brazil.

**Design and Methods:** This cross-sectional population study enrolled adults between 20–50 years old, registered in a primary healthcare unit in Rio de Janeiro. A database is being developed including sociodemographic and anthropometric data, and CV risk factors. Office blood pressure and Home Blood Pressure Monitoring (7-day protocol) (Omron-705CP). Moreover, OSA was investigated by SB and ESS. Patients with a high risk for OSA in either of these two questionnaires were subsequently assigned for polysomnography.

**Results:** A total of 562 subjects were evaluated [40% males, 38.9 ± 8.8 years], where 151 (26.9%) were identified as high risk for OSA by the SB questionnaire and 210 (37.4%) by ESS. The most common CV risk factor was physical inactivity (43%), followed by dyslipidemia (38%) and obesity (28%). By Office blood pressure, the prevalence of hypertension was 13.4% while by Home Blood Pressure Monitoring was 18.6%, with a low concordance between them (kappa = 0.472). Subjects with a high risk at SB are older, with a higher prevalence of obesity, hypertension and higher Office blood pressure and Home Blood Pressure Monitoring. On the other hand, individuals with high-risk by ESS were more obese, with increased waist circumference, higher prevalence of dyslipidemia and metabolic syndrome. Nevertheless, there was no difference in Office blood pressure levels. Among the subjects submitted to polysomnography, 46% had a diagnosis of OSA (AHI ≥ 5/hour) and 23% of moderate/severe OSA (AHI > 15/hour). The best predictor of AOS was SB, positive in 100% of subjects with moderate/severe OSA, while ESS was positive in only 20% of them.

**Conclusion:** This young and apparently healthy population presented a high prevalence and risk for OSA. The SB had a higher association with higher Office blood pressure levels, while ESS was associated with a worse metabolic profile. SB questionnaire seems to be the best predictor for moderate/severe OSA in this young adult population.

108480

Modality: E-Poster Scientific Initiation – Non-case Report

Category: HEART FAILURE/CARDIOMYOPATHY/TRANSPLANT

## Gender Impact on Mortality Rates of Patients with Heart Failure Stratified by a New Classification Proposal: A 5-Year Follow-Up in a Hospital in Southern Brazil

ALINE PETRACCO PETZOLD^1^, Fernanda Lourega Chieza^3^, Luiz Cláudio Danzmann^2^, Luiz Carlos Bodanese^1^

(1) Pontifícia Universidade Católica do Rio Grande do Sul (PUCRS); (2) Universidade Luterana do Brasil (ULBRA); (3) Hospital São Lucas da PUCRS

**Introduction:** Gender differences in the epidemiology of heart failure (HF) have been suggested. Findings reinforce that women respond differently to left ventricular ejection fraction (LVEF) values and HF treatment.

**Objectives:** To compare survival between sexes in HF patients, stratified by LVEF values, in a new proposal (< 40%, between 40–59% and > 60%), in a 5-year cohort follow-up.

**Methods:** This is a retrospective cohort study in which the 5-year mortality rates of 385 patients, was analyzed. The sample was divided into three groups: LVEF ≤ 40% (n = 133), LVEF 40–59% (n = 145) and LVEF ≥ 60% (n = 107), differentiating the sexes in each group. Inclusion criteria were age > 18 years, previous diagnosis of HF and transthoracic echocardiogram. The logrank test and Kaplan-Meier curve were used.

**Results:** Patients with lower LVEF were younger, for both sexes. High mortality among all groups was observed. The main HF cause was, for women, hypertension, and, for men, ischemic heart disease. In a 5-year analysis, the mortality rates for each LVEF group and sex were compared (Table 1). The mortality rates found were similar between sexes, thus our results go against women responding differently to LVEF values and to HF treatment.

**Conclusions:** No statistically significant difference was found in survival between the sexes, comparing the different LVEF groups. There is a need for better assessment of the HF population, since all mortalities found were high.


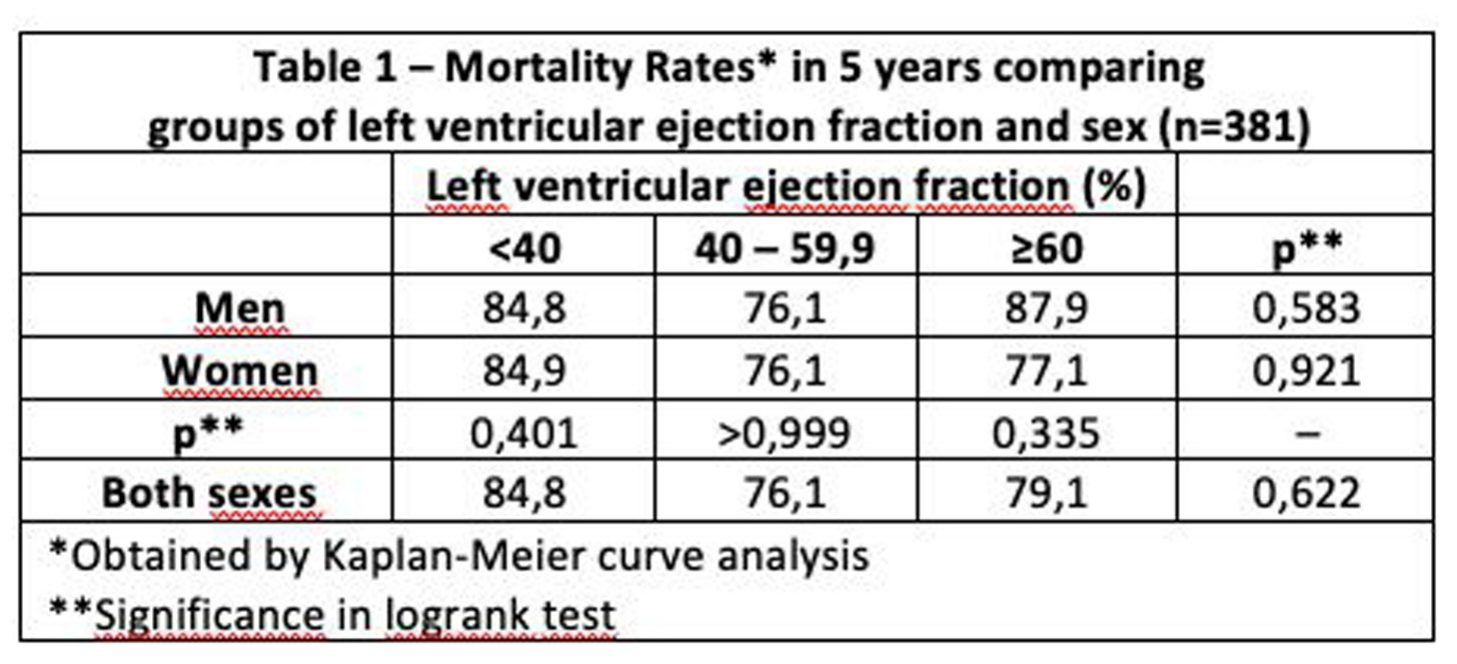


108512

Modality: E-Poster Scientific Initiation – Non-case Report

Category: NEGLECTED CARDIOVASCULAR DISEASES

## Reduction in Elective Cardiology Diagnostic Tests and Procedures During the COVID-19 Pandemic in Brazil

DANIEL MARQUES DA SILVA^1^, SEVERINO PEIXOTO NUNES NETTO^2^, LETÍCIA STEPHANIE DE SOUZA ARAÚJO^3^

(1) UNIVERSIDADE FEDERAL DO RIO GRANDE DO NORTE (UFRN); (2) UNIVERSIDADE FEDERAL DE UBERLÂNDIA (UFU); (3) UNIÃO SOCIAL CAMILIANA (USC)

**Introduction:** During the COVID-19 Pandemic’s first three months in Brazil, a decrease in Elective Cardiology Diagnostic Tests and Procedures (ECDTP) was reported, which can be associated with deficient planning of cardiovascular disease (CVD) management and may have contributed to the increase of in-hospital fatality due to CVDs.

**Objective:** To analyze the impact of the Pandemic on the amount of ECDTP performed at Brazilian Unified Public Health System (SUS) in 2020 and 2021.

**Methods:** We evaluated the number of ECDTP performed at SUS between March and December of each year from 2015 to 2021 from the Brazilian Outpatient Information System (SIA/SUS). The data until 2019 was used to project the number of ECDTP expected in 2020 and 2021 by a linear regression using a statistical significance level of 0.05 (P-value = 0.0026 for both angular and linear coefficients). The projection was compared to the real data from 2020 and 2021.

**Results:** The linear regression until 2019 presented a R-squared of 0.967, which showed a rising trend of ECDTP performed per year before the Pandemic. The data recorded in 2020 was 38.65% lower than the predicted. It showed that the cardiovascular care provision was notably affected by the pandemic due to SUS rearrangement to attend COVID-19 requirements. In 2021, after the mass immunization campaign in Brazil, the ECDTP returned to a rising trend, with 21.39% increase compared to 2020. Despite the improvement, in 2021 the real data was 29.14% lower than the amount predicted.

**Conclusions:** Rearrangements to address COVID-19 requirements affected the amount of ECDTP performed. This effect was mainly noted in 2020, but was still remarkable in 2021 even with the immunization campaign. This situation may have resulted in the neglection of cardiovascular diseases.


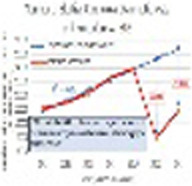


108536

Modality: E-Poster Scientific Initiation – Non-case Report

Category: ANTICOAGULATION

## Venous Thromboembolism in Patients Hospitalized for SARS- COV-2: A Systematic Review

ESTEVÃO OLIVEIRA CARVALHO^1^, João Ricardo Nogueira Perez^1^, Gabriela Billafan Ferreira^1^, Lucas Lagares Bragança Magami^1^, Luan de Castro França^1^, Leandro Guedes Santos^1^, Diana Aristotelis Rocha de Sá^1^

(1) ESCOLA SUPERIOR DE CIÊNCIAS DA SAÚDE (ESCS)

**Introduction:** The coronavirus disease 2019 (COVID 19), caused by Sars-CoV-2 virus, according to stipulations, claimed about 18,2 million lives until December 31, 2021 [1]. The COVID-19 generates a hypercoagulability state. Therefore, the main guidelines [2–3] recommend thromboprophylaxis in hospitalized COVID-19 patients. However, doubts remain about the use of low-molecular-weight heparin (LMWH).

**Aim:** To analyze the incidence of venous thromboembolism in hospitalized patients with COVID-19 in the regime of thromboprophylaxis with LMWH.

**Method:** This systematic review was conducted following the Preferred Reporting Items for Systematic Reviews and Meta-analyses (PRISMA) rules. EMBASE and PubMed were the selected databases. The following health descriptors were used: “venous thromboembolism” “COVID-19”, “coronavirus disease 2019”, “anticoagulant” and “anticoagulants agent”. Two authors independently performed a systematic literature search in databases. A search encompassed that was published between 01/01/2019 until 31/12/2021. The studies eligible for inclusion had a population over 18 years old, with confirmed diagnosis of COVID-19, hospitalized, and all population did use exclusively LMWH Articles were excluded if they did not provide data about prophylactic anticoagulation regimen (PAR) and if they are case reports. Two authors (E.O.C and J.R.N.P) realized the data collection process, in an independent form.

**Results:** Our search found 202 articles, of which 80 were duplicates. A total of 122 titles were excluded after reading the title and abstract. Therefore, 17 articles were selected for reading the full-text. In this step, 5 titles were excluded, because they don’t give information about the PAR and 3 because they don‘t use LMWH exclusively. So, we selected 9 titles [4–12]. A total of 1614 participants, from 8 countries, submitted to prophylaxis with LMWH. Being that 160 participants (9,91%) had a venous thromboembolic event. 6 articles [6–11] gave information about bleeding that occurred with 43 participants (3%).

**Conclusion:** The proportion of thrombotics events in hospitalized patients diagnosed with COVID-19 is 18% to 37%, without prophylactic anticoagulation [13]. Our research demonstrated that less than half of the population submitted to prophylaxis anticoagulation evolved to venous thromboembolic events.

109325

Modality: E-Poster Scientific Initiation – Non-case Report

Category: HEART FAILURE/CARDIOMYOPATHY/TRANSPLANT

## Prevalence of Transthyretin Cardiac Amyloidosis in a Population Previously Diagnosed with Hypertrophic Cardiomyopathy

LEONARDO SANTANA ANDRADE^1^, Emerson Santana Santos^1^, Braúlio Cruz Melo^1^, Antônio Guilherme Cunha de Almeida^1^, João Victor Andrade Pimentel^1^, Larissa Rebeca da Silva Tavares^2^, Vinícius Barbosa dos Santos Sales^1^, Júlia Souza Diniz^1^, João Paulo Dias Costa^1^, Irlaneide da Silva Tavares^1^, Enaldo Vieira de Melo^1^, Joselina Luzia Menezes Oliveira^1^

(1) Universidade Federal de Sergipe; (2) Universidade Tiradentes

**Introduction:** Transthyretin amyloidosis (ATTR) is one of the hypertrophic cardiomyopathy (HCM) phenocopies. It is caused by pathogenic variants in the transthyretin (TTR) gene and occurs in five per cent of patients with unexplained HCM.

**Aims:** Estabilish the prevalence of ATTR in a population previously diagnosed with HCM.

**Methods:** A prospective study was conducted and a 168 genes panel related to cardiomyopathy was performed. HCM was diagnosed according to the guidelines of the American Heart Association (AHA).

**Results:** The TTR gene was sequenced in 74 index patients referred to our cardiogenetics outpatient clinic between January 2021 and March 2022. Among the included patients, 4 had positive diagnosis for ATTR and other major clinical findings are summarized on Table 1.

**Conclusion:** The prevalence of ATTR was similar to literature. Our study highlights the importance of early diagnosis for ATTR since treatment has higher rates of effectiveness when administered in the early stages, it also reinforces that phenocopies, such as the ATTR, should always be included in differential diagnosis investigations for patients with unexplained HCM.

**Keywords:** Transthyretin amyloidosis, Hypertrophic Cardiomyopathy, Genetic Panel.


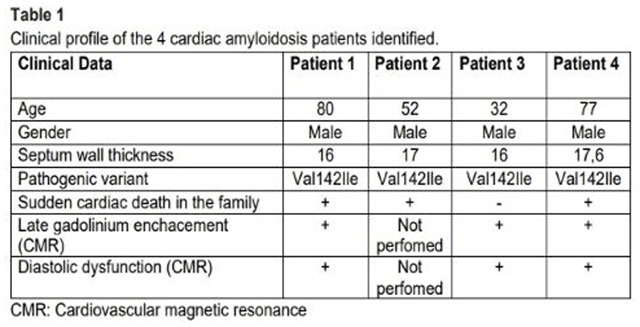


108685

Modality: E-Poster Scientific Initiation – Non-case Report

Category: CARDIOVASCULAR SURGERY

## Prevalence and Risk Factors of Delirium in Post-Operative Period of Heart Surgery

ARTHUR MAROT DE PAIVA^2^, Gabriel Baêta Branquinho Reis^2^, Luiz Fernando Sposito Ribeiro Baltazar^2^, Pedro Guimarães Moreira da Silva^2^, Walace Chaves dos Santos^1^, Maurício Lopes Prudente^1^, Abissay Francisco Dias^1^, Marcos Vinícius Pires Rodrigues^1^, Thainá Lopes de Souza^1^, João Alberto Pansani^1^, Artur Henrique de Souza^1^, Giulliano Gardenghi^1^

(1) Hospital ENCORE – Aparecida de Goiânia/GO; (2) Universidade Federal de Goiás- Goiânia/GO

**Introduction:** Delirium is an acute mental status change, with a fluctuating course and a high incidence in cardiac surgery post-operative (PO) period. Delirium can lead to both short and long-term consequences.

**Objective:** Describe the prevalence of delirium in patients summitted to cardiovascular surgery.

**Methods:** This is a descriptive cross-sectional study with patients summitted to cardiac surgery between January 2020 and February 2022. The assessment of delirium was performed on the 1st PO in the Intensive Care Unit using the CAM-ICU and RASS scales. The evaluation of epidemiological data, previous comorbidities, use of pre and post-operative medications, anthropometric measurements and information related to the surgery were collected from TASY® electronic medical record. Data collected and tabulated using specific spreadsheets with Excel 2010® software.

**Results:** 74 patients (39 men) were included. The prevalence of delirium was 16.2%. In patients with delirium, 50% were males and in those without delirium, 53.3% were males. The mean age in those with delirium was 72(±7.9) years old and the mean of BMI was 25.6(±2.89), while those without delirium was 57.2(±12.9) and 27.8(±4.1), respectively. In patients with delirium, 58.3% was summitted to general anesthesia associated with spinal anesthesia and in 41.6% general anesthesia was used alone. In patients without delirium, 61.3% underwent spinal anesthesia plus general anesthesia and in 38.7% only general anesthesia. In patients with delirium, the mean cardiopulmonary bypass (CPB) time was 100.7(±31.34) minutes, and the mean aortic clamping time was 70(±27.3) minutes. In those without delirium, the mean time of CPB was 97.5(±26.6) minutes and the aortic clamping time was 69.4(±22.6) minutes. Regarding comorbid diseases, the mean was 5.08(±2.27) in patients with delirium and 3.2(±2.2) in those without delirium. With respect of the number of medications used, in patients with delirium, the mean home medications used was 5(±2.1) and the mean of post-operative medications was 7.8(±1.9). While, in patients without delirium the mean number of medications used was 4.1(±2.1) and post-operative used was 7.5(±1.9).

**Conclusion:** The prevalence of delirium found in the sample was consistent with current literature data. Considering patients with delirium, multiple risks factors were observed, such as advanced age, greater number of comorbidities and pre and post-operative polypharmacy.

108757

Modality: E-Poster Scientific Initiation – Non-case Report

Category: HYPERTENSION/RENAL DENERVATION

## Are Elderly Patients with Hypertension Still using Potentially Inappropriate Medications? an Observational Study with Secondary Data

TAINÁ SILVEIRA ALANO^1^, Larissa Xavier Neves da Silva^3^, Guilherme Lucca Casali^3^, Jayne Santos Leite^3^, Andresa Conrado Ignacio^3^, Linda Ariene dos Santos Cardoso^1^, Daniel Umpierre^2^

(1) Universidade Federal de Ciências da Saúde de Porto Alegre (UFCSPA); (2) Hospital de Clínicas de Porto Alegre (HCPA); (3) Universidade Federal do Rio Grande do Sul (UFRGS); (4) Prefeitura Municipal de Porto Alegre (PMPA)

**Background:** The elderly are the most medicalized age group in society. Due to their metabolism changes, many commonly used drugs are no longer appropriate, with their risks outweighing the benefits. When the patient already has a chronic disease, the prescription should be even more responsible. The Beers Criteria for Potentially Inappropriate Medications (PIMs) is a systematic cataloging of the existing evidence in this field, aiming to improve the healthcare of the elderly.

**Objectives:** Assess the prevalence and characterize the use of PIMs in older adults with hypertension enrolled in research studies in the city of Porto Alegre.

**Methods:** Cross-sectional study of secondary data. The population was composed of individuals aged 60 years or older and diagnosed with systemic arterial hypertension (SAH). The data were retrieved from 2 studies carried out in the city of Porto Alegre between 2018 and 2020. Based on the answers to a General Health Questionnaire, the prevalence of PIMs was analyzed using the 2015 Beers Criteria. Drugs with minimum dosage requirements were excluded from the analysis. The data analysis was performed using Python 3.9. The pre-analysis plan, statistical codes, and dataset used for this study are available at osf.io/ksfg2.

**Results:** There were 318 hypertensive patients (71% female) included, with a mean age of 69 years old (standard deviation 6.4). A total of 32 different PIMs were found, with the most prevalent being omeprazole, used by 30 (9%) patients. A total of 88 (28%) patients were using at least one PIM. Considering sex, 23% of males and 30% of females were using PIMs. Patients aged from 60–69, 70–79, and 80–89 years old had a prevalence rate of using at least one PIM of 28%, 27%, and 30%, respectively.

**Conclusions:** There is still a notable usage of PIMs in the elderly with SAH, with the highest rates being in females and octogenarians. The existing literature on this subject is inconclusive, with prevalences ranging from 24% to 82%. The results reflect the need for better pharmacological and geriatrics training for healthcare professionals and the empowerment of the patient in the prescription process. This study presents some limitations, such as the absence of the full illness history and a possible selection bias, since the participants were already involved in health-related scenarios. Further studies could explore interventions of education on responsible prescribing and self-medication risks in hypertensive elderlies.

108639

Modality: E-Poster Scientific Initiation – Non-case Report

Category: CONGENITAL AND PEDIATRIC CARDIOLOGY

## Down Syndrome and Congenital Heart Diseases: Clinical and Cytogenetic Characteristics of Patients Admitted to a Cardiac Intensive Care Unit of a Pediatric Hospital

LETICIA VIEIRA SENGER^1^, Rafael Fabiano Machado Rosa^1^, Helena Guedes da Rocha^1^, Helena Marcon Bischoff^1^, Grasiele do Amaral Martins^1^, Georgia Marques Jardim^1^, Alexandre Perin Decol^1^, Matheus Ribeiro Fretes^1^, Bianca Brinques da Silva^2^, Estefany Karenine Rodriguez Casanova^1^, Carolina Feijó Bombana^1^, Carolina Andreatta Gottschall^2^

(1) Universidade Federal de Ciências da Saúde de Porto Alegre (UFCSPA); (2) Universidade Luterana do Brasil (ULBRA)

**Introduction:** In children with Down Syndrome, there is a high prevalence of congenital heart diseases, which characterize anomalies associated with hospitalization and surgical correction.

**Objectives:** To investigate incidence, clinical presentation and cytogenetic characteristics of patients with Down syndrome (DS) and congenital heart disease (CHD) who are admitted to a cardiac Intensive Care Unit (ICU) of a pediatric hospital.

**Methodology:** A prospective and consecutive cohort of patients hospitalized for the first time by CHD in the Cardiology ICU of the Santo Antônio Children’s Hospital was evaluated for 1 year. For each patient, a standard clinical protocol was applied, with abdominal ultrasound and karyotype examination.

**Results:** 207 patients composed the sample and karyotype could be performed in 204. Chromosomal alterations were observed in 29 individuals, 24 of them (12%) with DS (23 by trisomy free of chromosome 21 and 1 by isochromosome 21q). The main CHDs observed in these patients were septal defects (n = 18), especially the atrioventricular septal defect (AVSD), present in half of the cases. DS was the cause of AVSD in 55% of the cases of the sample, and the association of this defect with DS was statistically significant. There was no difference in the frequency of alterations detected on abdominal ultrasound, length of hospital stay and deaths among patients with DS and normal karyotype.

**Conclusions:** The frequency of DS found in our study, as well as the types of chromosomal abnormalities identified in these patients, were in accordant with the literature. In our series, patients with DS presented a good evolution, similar to patients with normal karyotype, as described in other studies.

108640

Modality: E-Poster Scientific Initiation – Non-case Report

Category: HEMODYNAMICS AND INTERVENTIONAL CARDIOLOGY

## Percutaneous Correction of Interatrial Communication (IAC) in Adults in a High Complexity Cardiology Service in Brazilian Western Amazon: A Case Series

LEO CHRISTYAN ALVES DE LIMA^1^, Leo Christyan Alves de Lima^1^, Elaine Largura Biazati^1^, Willian Kubo^1^, Hildeman Dias da Costa^2^, Alexandre Venturelli da Silva^3^, Fabiana Cristina Schabatoski Passos^3^, Luiz Henrique Gasparelo^3^

(1) Liga Rondoniense de Cardiologia Clínica e Cirúrgica (LICAR); (2) Universidade Federal de Rondônia; (3) Angiocenter

**Introduction:** Interatrial communication (IAC) is one of the most common congenital heart diseases, affecting one in 800 births, characterized by a defect in the closure of the interatrial septum. Due to its slow evolution, it is usually diagnosed late in adulthood, when symptoms result from right heart failure, pulmonary hypertension with cor pulmonale or atrial arrhythmias.

**Objectives:** This is a descriptive study of the registration of cases of IAC correction performed in a high-complexity service in the Western Amazon.

**Methods:** Data obtained through research via medical records and complementary exams were used. The database selection was through Pubmed and Scientific Electronic Library On-line (SCIELO).

**Results:** Five patients were followed, one male and four female, all of them with symptoms of dyspnea on exertion and fatigue. The transthoracic echocardiogram showed an ostium secundum (OS) IAC, with signs of right chamber overload and pulmonary hypertension (mild to moderate). The prostheses were implanted through the right femoral vein and with the aid of intracardiac echocardiography. Although vascular complications are known, in this small serious there were no complications during the procedures. The prostheses used were Coccum® in one case and Amplatz® in four cases. The patients had a hospital stay of less than two days and in the outpatient follow-up they showed a favorable evolution.

**Discussion:** The indication for percutaneous IAC closure are patients who have ostium secundum (OS) +96 with favorable anatomy associated with symptoms or with any degree of pulmonary hypertension, right chamber overload, as described in this work. In addition, percutaneous closure is a less invasive alternative and is associated with fewer thromboembolic complications compared to the traditional surgical method.

**Conclusion:** Despite a smaller number of cases than most large centers, positive results can be seen in this type of treatment. The percutaneous treatment of OS type IAC is a safe procedure and performed in the state without the need to transfer to other locations, improving the patient’s quality of life with a relatively low risk and increasing their life expectancy.

108643

Modality: E-Poster Scientific Initiation – Non-case Report

Category: HEMODYNAMICS AND INTERVENTIONAL CARDIOLOGY

## Influence of Clinical Characteristics on Therapeutic Indication for Aortic Stenosis in Sergipe – the Smallest Brazilian State

JOSÉ ICARO NUNES CRUZ^1^, Gabriela de Oliveria Salazar^1^, Cláudia Bispo Martins-Santos^1^, Mayara Evelyn Gomes Lopes^1^, Ullany Maria Lima Amorim Coelho de Albuquerque^1^, Marília Marques Aquino^1^, Lucas Villar Shan de Carvalho Cardoso^1^, Edvaldo Victor Gois Oliveira^1^, Joselina Luzia Menezes Oliveira^2^, Enaldo Vieira de Melo^1^, Eduardo José Pereira Ferreira^2^, Antônio Carlos Sobral Sousa^2^

(1) Federal University of Sergipe; (2) Rede D’Or São Luiz – São Lucas Hospital

**Introduction:** Aortic stenosis (AoS) is the restriction of blood outflow through the aortic valve leaflets during ventricular systole. When symptomatic, AoS can be treated with valve replacement, which can be performed percutaneously (TAVR) or by conventional transthoracic surgery (SAVR).

**Objectives:** To evaluate the influence of clinical variables and surgical risk on therapeutic indication for AoS in Sergipe – the smallest Brazilian state.

**Methods:** This is an observational, cross-sectional, analytical study, conducted in two private hospitals in Sergipe (Brazil). The sample included patients who underwent TAVR or SAVR after diagnosis of severe AoS defined by echocardiographic criteria and symptomatological assessment, between 2013 and 2021. The TAVR and SAVR groups were compared in terms of comorbidities and surgical risk scores. Categorical variables were analyzed using Fisher’s exact test, whereas quantitative variables were analyzed using Student’s t-test or Mann-Whitney U test. The statistical analysis was performed using SPSS Statistics software, version 22.0.

**Results:** The sample included 55 patients, of which 38 underwent TAVR and 17 underwent SAVR. The TAVR and SAVR treatment groups did not demonstrate differences in sex, height and weight distributions, however age was higher among those who underwent TAVR (88 vs. 62 years; p < 0.001). The TAVR group exhibited more patients in functional class III or IV (New York Heart Association) compared to the SAVR group (88.6% vs. 40.0%; p < 0.01), as well as higher prevalence for diabetes mellitus (46.2% vs. 5.9%), dyslipidemia (76.0% vs. 31.3%) and chronic kidney disease (78.6% vs. 33.3%) (p < 0.05). The four surgical risk scores assessed were also higher in the TAVR treatment group: logistic EuroSCORE I (15.6% vs. 6.7%; p < 0.05); EuroSCORE II (7.0% vs. 2.9%; p < 0.01); STS mortality score (5.3% vs. 1.7%; p < 0.05); and STS morbidity or mortality score (29.5% vs. 11.5%; p < 0.05).

**Conclusions:** Patients treated with TAVR presented older age, greater severity of heart failure, greater frequency of comorbidities, in addition to exhibiting a greater surgical risk profile according to the 30-day mortality prediction scores. Although the guidelines recommend TAVR for patients at higher surgical risk, this treatment was also expanded to intermediate-risk patients in Sergipe.

108657

Modality: E-Poster Scientific Initiation – Non-case Report

Category: CARDIOVASCULAR IMAGING

## Diagnostic Accuracy of Coronary Angiotomography for Coronary Artery Disease in Sergipe – the Smallest Brazilian State

JOSÉ ICARO NUNES CRUZ^1^, Cláudia Bispo Martins-Santos^1^, Gabriela de Oliveira Salazar^1^, Juliana Maria Chianca Lira^1^, Myllena Maria Santos Santana^1^, Ana Luísa Lisboa Prado^1^, Giulia Vieira Santos^1^, Letícia Luiza Gomes Marques^1^, Antônio Carlos Sobral Sousa^2^, Enaldo Vieira de Melo^1^, Joselina Luzia Menezes Oliveira^2^, Luiz Flávio Galvão Gonçalves^2^

(1) Federal University of Sergipe; (2) Rede D’Or São Luiz – São Lucas Hospital

**Introduction:** Coronary angiography is the gold standard test for the diagnosis of coronary artery disease (CAD). In recent years, with the advent of coronary computed tomography angiography (CCTA), the possibility of non-invasively diagnosing CAD has emerged. However, in order to validate CCTA locally, it is necessary to assess its diagnostic accuracy in a northeastern brazilian state, where the use of CCTA has recently been expanded.

**Objectives:** To determine the diagnostic accuracy of CCTA in a sample from Sergipe, considering coronary angiography as the gold standard test.

**Methods:** This is an observational, cross-sectional, analytical study. The sample included patients with cardiovascular complaints who underwent CCTA and coronary angiography at an interval of less than six months, in Sergipe (Brazil). The identification of plaques associated with luminal narrowing of any degree was considered positive for CAD. Sensitivity, specificity, positive predictive value (PPV), negative predictive value (NPV), accuracy and Receiver Operating Characteristic (ROC) curve were evaluated to determine the diagnostic potential of CCTA for CAD, considering coronary angiography as the gold standard method.

**Results:** 130 patients were included in the study. The median age was 62 years (IQR: 57.0–67.25). The prevalence of CAD determined by the gold standard test was 76.9% (100). The CCTA showed a sensitivity of 95.0%, specificity of 80.0%, PPV of 94.1%, NPV of 82.8% and accuracy of 91.5%. The area under the ROC curve demonstrated significant discriminatory capacity (0.875; CI95% 0.79–0.96; p < 0.001) (Figure 1).

**Conclusions:** The study results are consistent with those observed in meta-analytic studies and clinical trials. The high prevalence of CAD in the sample determined a higher PPV than NPV. It is concluded that, in Sergipe, CCTA has significant accuracy for the diagnosis of CAD, being recommended for the assessment of chest pain in patients at intermediate risk for CAD.


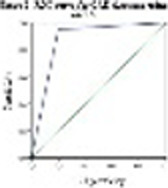


108672

Modality: E-Poster Scientific Initiation – Non-case Report

Category: COVID-19 AND CARDIOVASCULAR SYSTEM

## People with Previous Covid Positive Diagnose Have Higher Pulse Wave Values and Their Hemodynamic Parameters of Arterial Stiffness?

SARA CRISTINE MARQUES DOS SANTOS^1^, Luan Tardem Veloso Teixeira^1^, Thaís Lemos de Souza Macedo^1^, João Pedro de Resende Côrtes^1^, Ivan Lucas Picone Borges dos Anjos^1^, Paula Pitta de Resende Côrtes^1^, João Carlos de Souza Côrtes Júnior^1^, Eduardo Tavares de Lima Trajano^1^, Carlos Eduardo Cardoso^1^, Ivana Picone Borges de Aragão^1^

(1) Universidade de Vassouras

**Introduction:** In COVID infection, vascular stiffening may be induced due to indirect damage caused by the virus and its systemic inflammatory state and binding to angiotensin-converting enzyme 2, which cause cellular injury1. These mechanisms may contribute to the progression of atherosclerosis and increased cardiovascular risk, arterial stiffness, and augmentation index in previously infected individuals. The objective of the present study was to seek correlation between pulse wave analysis data in individuals up to 30 years of age who tested positive for COVID at least 15 days before the exam and individuals who were not infected.

**Methodology:** Observational and cross-sectional study, carried out from May to July 2021 in students aged over 20 and under 30 years, in accordance with the current hypertension guideline of the Brazilian Society of Cardiology. Of 59 participants, 4 were excluded for absence of answers about COVID infection and 10 for age >30 years. After selection, participants were divided into 2 groups: prior COVID (CoP) and non-infected (NI). We used an anonymous questionnaire and the values provided by the Arteris device by means of the oscillometric method: PWV, AIX@75, heart rate (HR), central systolic pressure (CSBP) and central diastolic pressure (CDBP). The mean, maximum and minimum values were calculated using Excel software. Evaluation of sample normality (Shapiro-Wilk) and unpaired Student’s T test (with Welch correction) were performed for parametric samples and Mann Whitney for non-parametric samples, with confidence level of 95% through GraphPad Prism Software version 9.2.

**Results:** In the CoP group, the means were: PWV 4.65 m/s (5.4 ± 4.2); AIX@75 23.22% (40.7 ± 9.3); HR 89.5 bpm (119 ± 71); CSBP 97.72 mmHg (118 ± 80); CDBP 76.68 mmHg (98 ± 61). While in the NI group, the means were: PWV 4.58 mmHg (5.2 ± 3.1); AIX@75 21.85% (41.7 ± 5.3); HR 86.3 bpm (128 ± 60); CSBP 97.6 mmHg (113 ± 80) and CDBP 73.8 mmHg (54 ± 91). There was no statistical difference between PWV (p = 0.95) and AIX@75 (p = 0.63) values between CoP and NI group.

**Conclusion:** Although higher values were observed for the CoP group in several hemodynamic and arterial stiffness parameters, no statistical difference was obtained between this group and NI group. However, it is valid to emphasize the importance of further studies in the area to be able to affirm or rule out the influence of the SARS-COV-2 virus on vascular integrity.

108678

Modality: E-Poster Scientific Initiation – Non-case Report

Category: CARDIOVASCULAR SURGERY

## Assessment of Pain in Patients Submitted to Conventional Valve Replacement Surgery Versus Minimally Invasive Cardiac Surgery

ARTHUR MAROT DE PAIVA^2^, Gabriel Baêta Branquinho Reis^2^, Luiz Fernando Sposito Ribeiro Baltazar^2^, Pedro Guimarães Moreira da Silva^2^, Thais Vieira de Araújo Rodrigues^1^, Lays de Souza Albuquerque Oliveira^1^, Abissay Francisco Dias^1^, Marcos Vinícius Pires Rodrigues^1^, João Alberto Pansani^1^, Maurício Lopes Prudente^1^, Artur Henrique de Souza^1^, Giulliano Gardenghi^1^

(1) Hospital ENCORE – Aparecida de Goiânia/GO; (2) Universidade Federal de Goiás- Goiânia/GO

**Introduction:** Minimally invasive cardiac surgery has becoming popular in the last two decades, associated with a better aesthetic effect and less surgical trauma, which may be related to a lower prevalence of pain or a different neuropathic component in pain.

**Objective:** The aim is to compare the prevalence of pain in the first postoperative day (PO1) of patients undergoing valve replacement between conventional and minimally invasive techniques.

**Methods:** Analytical cross-sectional study with patients undergoing heart valve replacement surgery between January 2020 and February 2022. Pain assessment was performed on the PO1 using the Visual Analog Pain Scale (VAS) and the Leeds Assessment of Neuropathic Symptoms and Signs (LANNS) Scale. For the analysis of independent samples, the t-student test was performed with 5% significance.

**Results:** 41 patients were included in the study. 33 patients underwent conventional heart valve replacement surgery (CONV group) and 8 patients underwent minimally invasive valve replacement surgery (MINI group). The mean age of the CONV group was 55.5 ± 12.5 years versus the MINI group: 62.9 ± 16.1 years (p = 0.19). Regarding pain intensity by VAS, the mean of the CONV group was 4.3 ± 3.4 points versus 4.1 ± 3.4 of the MINI group (p = 0.89). Regarding the number of painful areas, the CONV group had a mean of 1.3 ± 1.0 points versus 1.1 ± 0.8 of the MINI group (p = 0.59). In the CONV group, the main painful area was the sternal region and in the MINI group it was in the right thoracic region. According to the LANNS Scale in the CONV group, 72.7% of the patients had a score lower than 12, 15.2% did not report pain and could not be classified with the scale, and 12.1% had a score higher than 12. In the MINI group, 75% of patients scored less than 12 and 25% reported no pain, which means that no one in this group had a neuropathic pain. In the statistical analysis, there was no significant difference between the groups (p = 0.81).

**Conclusion:** There was no difference between conventional and minimally invasive surgeries in terms of pain assessed in the present sample.

108683

Modality: E-Poster Scientific Initiation – Non-case Report

Category: CARDIOVASCULAR SURGERY

## Prevalence of Delirium in Patients Undergoing Conventional Heart Valve Replacement Versus Minimally Invasive Cardiac Surgery

ARTHUR MAROT DE PAIVA^2^, Gabriel Baêta Branquinho Reis^2^, Luiz Fernando Sposito Ribeiro Baltazar^2^, Pedro Guimarães Moreira da Silva^2^, Thais Vieira de Araújo Rodrigues^1^, Abissay Francisco Dias^1^, Marcos Vinícius Pires Rodrigues^1^, Thainá Lopes de Souza^1^, Walace Chaves dos Santos^1^, João Alberto Pansani^1^, Artur Henrique de Souza^1^, Giulliano Gardenghi^1^

(1) Hospital ENCORE – Aparecida de Goiânia/GO; (2) Universidade Federal de Goiás- Goiânia/GO

**Introduction:** Minimally invasive cardiac surgery has been gaining popularity in the last two decades, associated with better aesthetic effect and decreased surgical trauma.

**Objective:** The present study aims to compare patients undergoing valve replacement between conventional method and minimally invasive cardiac surgeries.

**Methods:** Analytical cross-sectional study with patients undergoing heart valve replacement surgery between January 2020 and February 2022. Patients undergoing microsurgery were included from September 2021 onwards. The assessment of delirium was performed at first postoperative (PO1) day in an Intensive Care Unit (ICU) using the CAM-ICU and RASS scales. The evaluation of epidemiological data, previous comorbidities, use of pre and postoperative medications, anthropometric measurements and information related to the surgery were collected from TASY® electronic medical record system. The collected data were tabulated and calculated directly using specific spreadsheets with Excel 2010®. For the analysis of independent samples, the t-student test was performed.

**Results:** 41 patients were included in the study. 33 patients underwent heart valve replacement surgery by the conventional manner (CONV group) and 8 patients underwent minimally invasive cardiac surgery (MINI group). When comparing patients undergoing different surgical procedures, the mean age of patients in CONV group was 55.5 (±12.5) and in MINI group was 62.9 (±16.1) (p = 0.19). The mean BMI of CONV was 26.6 (±3.8) and of MINI 27.7 (±6) (p = 0.51). The mean of cardiopulmonary bypass (CPB) time in CONV was 99.2 (±19.8) minutes and the mean clamping time was 71 (±15.7) minutes and the mean CPB time of MINI was 115.4 (±32.8) minutes (p = 0.08) and the mean clamping time was 76.9 (±30.2) (p = 0.45). The mean number of comorbidities in CONV was 3.1 (±2) and in MINI was 3.9 (±2.5) (p = 0.33). In CONV, the mean of home medications was 4.1 (±2.3) and in the postoperative period was 7.7(±2.3), while in MINI the mean of home medications was 5.1(±2, 9) (p = 0.29) and of postoperative medications was 7.1 (±1) (p = 0.47). The overall incidence of delirium in the CONV was 15.2% and 12.5% in MINI (p = 0.85).

**Conclusions:** The observed delirium prevalence findings were consistent with the current literature in both groups. Risk factors and triggers classically related to delirium, such as age and polypharmacy, was not statistically significant between the two groups.

108694

Modality: E-Poster Scientific Initiation – Non-case Report

Category: ACUTE AND CHRONIC CORONARY DISEASE/THROMBOLYSIS

## Impact of On- vs. Off-Hours Admission of Patients with Stemi in Treatment and Hospital Outcomes in a Public University Hospital

JARBAS RYGOLL DE OLIVEIRA FILHO^1^, ALESSANDRO MENEGHETTI ANVERSA^2^, ISABELLA KLAFKE BRIXNER^3^, ANDRESSA DUARTE SEEHABER^3^, NATÁLIA DA SILVEIRA COLISSI^3^, ALESSANDRA REBELATTO BOESING^3^, MATHEUS WERLANG DONADEL^3^, BRUNA SANTI DOS SANTOS^3^, ALEXANDRA SEIDE CARDOSO^3^, ALEXANDRE SHAAN DE QUADROS^3^, MATEUS DINIZ MARQUES^3^, ANIBAL PEREIRA ABELIN^3^

(1) Universidade Federal da Fronteira Sul (UFFS), Campus Passo Fundo, BRASIL; (2) Instituto de Cardiologia do RS/FUC, Porto Alegre, RS, BRASIL; (3) UFSM, Santa Maria, RS, BRASIL

**Background:** ST-elevation myocardial infarction (STEMI) is a myocardial injury characterized by coronary artery occlusion. Treatment is rapid myocardial reperfusion performed through primary percutaneous coronary intervention (pPCI) or fibrinolytic therapy. Off-hours hospital presentation may impact the choice of reperfusion strategy, therefore increase cardiovascular outcomes.

**Objeticve:** The aim of this study was to compare the effect of different times of presentation (on- and off-hours) on reperfusion strategy and in-hospital outcomes.

**Methods:** Prospective cohort study, including patients diagnosed with STEMI between September/2016 and February/2020 in a public university hospital in southern Brazil. Patients were divided into on- and off-hours admission. Off-hours presentation was defined as hospital admission between 18:00 p.m. and 8:00 a.m. from Monday to Friday and weekends. In-hospital outcomes were evaluated.

**Results:** A total of 150 patients (56%) were admitted during off-hours, and 118 (44%) during on-hours. Baseline characteristics were balanced between the two groups, except that hypertension was more prevalent in the off-hour group. Patients admitted off-hours had a higher rate of non-reperfusion strategy (31.3% vs 22.9%), lower rate of fibrinolytic therapy (9.3% vs 22%) and similar pPCI (p = 0,011). Median door-to-balloon time was similar between groups (on-hours group: 119 min (IQR 56–175) vs. 109 min (IQR 59–161)) (p = 0,640). No difference was found in the rates of in-hospital mortality (p = 0,621) and MACCE (p = 0,16).

**Conclusions:** In our hospital we observed a difference in the reperfusion estrategy between patients admitted on and off-hours, with a higher rate of non-reperfusion. However, there was no difference in-hospital outcomes.

109405

Modality: E-Poster Scientific Initiation – Non-case Report

Category: COVID-19 AND CARDIOVASCULAR SYSTEM

## COVID-19 and Chronic Cardiovascular Diseases: Clinical and Epidemiological Indicators in Elderly People in Southern Brazil

JARBAS RYGOLL DE OLIVEIRA FILHO^1^, EVA BRENDA SANTOS SILVA^1^, THIAGO EMANUEL RODRIGUES NOVAES^1^, TASSO KFURI ARAÚJO MAFRA^1^, JOSSIMARA POLETTINI^1^, GUSTAVO OLSZANSKI ACRANI^1^, SHANA GINAR DA SILVA^1^, RENATA DOS SANTOS RABELLO^1^, IVANA LORAINE LINDEMANN^1^

(1) Universidade Federal da Fronteira Sul (UFFS), Campus Passo Fundo, BRASIL

**Introduction:** The new viral infection, caused by Severe Acute Respiratory Syndrome CoronaVirus 2 (SARS-CoV2), called Coronavirus Disease 2019 (COVID-19), presents different clinical conditions and levels of severity, like patients who develop Acute Respiratory Syndrome. Severe (SRAG). Such cases are mostly elderly, considered at risk because they are more vulnerable due to physiological changes inherent to the aging process.

**Objectives:** To identify the prevalence of hospitalization in the Intensive Care Unit (ICU), use of ventilatory support and case evolution in elderly patients diagnosed with COVID-19, carriers and non-carriers of chronic cardiovascular diseases (CVD).

**Methods:** Observational study based on data analysis from the Influenza Epidemiological Surveillance Information System (SIVEP-Gripe) in Passo Fundo, RS. The study population consisted of SARS cases due to COVID-19, confirmed and notified from January 1 to December 31, 2020. Sociodemographic and health data were collected from individuals aged 60 years or older. The use of ventilatory support, admission to the ICU and the evolution of the case (death/cure) were used as outcomes of interest, and the exposure analyzed was the diagnosis of CVD. The prevalence was verified with a 95% confidence interval (95%CI) of the outcomes and their distribution according to the predictor variable (chi-square test, admitting an α error of 5%).

**Results:** The sample consisted of 585 participants, most of them women (52.3%), aged up to 75 years (63.4%) and with CVD (59.0%). Of the total, 31.4% were admitted to the ICU (CI95 28–35), 74.2% used ventilatory support (CI95 71–78) and 41.2% (CI95 37–46) died. A higher prevalence of ICU admission was observed among patients with CVD (34.3%, p = 0.031), but no statistically significant difference was identified in the relationship between CVD and death (38.0%, p = 0.242) and use of ventilatory support (75.5%, p = 0.320).

**Conclusions:** Elderly people diagnosed with SARS by COVID-19, carriers of CVD, were more exposed to ICU admission, which reinforces that having CVD increases the complexity of the case. Although the mechanisms are not yet fully understood, it is possible that the fact is related to the action of the angiotensin-converting enzyme 2, the main gateway of SARS-COV2 in the body and also the target of treatments in patients with CVD. With regard to lethality and use of ventilatory support, it was not possible to establish a relationship.

108707

Modality: E-Poster Scientific Initiation – Non-case Report

Category: ATHEROSCLEROSIS/CARDIOVASCULAR RISK FACTORS/CARDIOVASCULAR PREVENTION

## Cardiovascular Diseases in Teachers of the Public Education: Prevalence of Risk Factors

JARBAS RYGOLL DE OLIVEIRA FILHO^1^, SILVIO LUIZ SIMOES ANCINES FILHO^1^, SABRINE AGUIAR DE SOUZA^1^, ALAN ROBSON FERREIRA DA PAZ JÚNIOR^1^, GUSTAVO OLSZANSKI ACRANI^1^

(1) Universidade Federal da Fronteira Sul (UFFS), Campus Passo Fundo, BRASIL.

**Introduction:** The relationship between risk factors (RF) for Cardiovascular Diseases (CVD) and patients workplace represents an important environmental factor. The Brazilian high school teacher is part of one of the classes that suffers most from environmental factors, occupying the second category of professionals with the highest presence of occupational diseases by the International Labour Organization (ILO).

**Objective:** To analyze the prevalence of risk factors for cardiovascular disease among teachers of basic education in public schools in the city of Passo Fundo-RS-Brazil as well as the variables related to the outcome.

**Methods:** This is a cross-sectional study, whose data were collected between August 2019 and February 2020 through a questionnaire sent to teachers by email and messages in mobile phone social network groups. Sociodemographic, life, health and employment data were collected. Cardiovascular risk stratification was calculated from the Brazilian Guidelines on Arterial Hypertension published by the Brazilian Society of Cardiology update 2020.

**Results:** The sample consisted of 225 teachers, 91.1% women, 64.9% over 40 years old, 89.3% white, 54.5% with monthly income below R$ 5,000, 52.8% with more than 16 years of career, 73.4% with more than 30 working hours per week. After the analysis, it was found that 32% of those surveyed have a high risk for cardiovascular comorbidities in 10 years. It was possible to verify a statistically significant relationship between the presence of high cardiovascular risk among male teachers (60%, p = 0.005), age over 60 years (71.4%, p = 0.013), obesity (44%, p = 0.001), anxiety (37.5%, p = 0.018), RSI/WRMD (45.6%, p = 0.001) and exhaustion (42.9%, p = 0.044).

**Conclusions:** The present study showed a high number of professors at high risk for cardiovascular disease in 10 years. It also found high rates of associated risk factors that denote negligence in the management of health care programs for teachers. The continuity in the production of studies on the relationship between teaching work and cardiovascular risk is fundamental for the development of measures that are, in fact, effective in combating the disease process, avoiding further complications in the future.

108709

Modality: E-Poster Scientific Initiation – Non-case Report

Category: HYPERTENSION/RENAL DENERVATION

## Influence of Blood Pressure Self Measurement on Pressure Control, Self-Medication and Emergency Visit

GABRIELA DE OLIVEIRA SALAZAR^1^, Glessiane de Oliveira Almeida^1^, Felipe J Aidar^1^, Marcos Antonio Almeida-Santos^1^, Ikaro Daniel de Carvalho Barreto^1^, Dihogo Gama de Matos^1^, Cláudia Bispo Martins-Santos^1^, José Icaro Nunes Cruz^1^, Joselina Luzia Menezes Oliveira^2^, Enaldo Vieira de Melo^1^, José Augusto Soares Barreto Filho^2^, Antônio Carlos Sobral Sousa^2^

(1) Federal University of Sergipe; (2) Rede D’Or São Luiz – São Lucas Hospital

**Introduction:** One of the strategies to control hypertension, recommended by some guidelines, is the self-measurement of blood pressure (BP). However, the benefits of this practice are not yet unanimous.

**Objective:** To assess whether there is an association between non-standardized BP self-measurement and the control of systemic arterial hypertension (SAH), self-medication and emergency department visit.

**Methodology:** Observational, analytical and cross-sectional study. Information was obtained through a standardized questionnaire and medical records of patients seen at cardiology outpatient clinics in Sergipe (Brazil). Adults with SAH were included. BP was considered controlled when systolic and diastolic pressure did not exceed 130 mmHg and 80 mmHg, respectively. The Shapiro Wilk, Chi-square with odds ratio and the Mann-Whitney U tests were performed. A two-tailed p ≤ 0.05 was set for statistical significance.

**Results:** The sample consisted of 1000 patients, with a mean age of 61 ± 12.5 years, 57.1% were women and 52.3% had comorbidities. Most (93.8%) used a digital device for self-measurement and 21.1% were aware of the need for annual calibration of the device, which was performed in 10.7% of the total. The highest frequency of self-measurement in the population was 1 to 3 times/day (35%). The symptom that most induced BP self-measurement was headache (44.4%; p < 0.0001). The most prevalent behaviors after verifying high BP were doing nothing (49.9%) and self-medication (26%), followed by looking for urgency and trying to contact the doctor (22.8% and 1.3%). 99.2% reported that there was no guidance on how to measure BP. There was no association between sex or age with BP self-measurement. Patients with comorbidities perform more self-assessment (p = 0.005), including diabetics, dyslipidemic patients, patients with obstructive arterial disease (p < 0.001) and stroke (p = 0.010). Self-measurement was associated with uncontrolled BP (p < 0.001; OR 1.953; 95% CI 1.707–2.233), emergency visits in the last 12 months for hypertensive causes (p < 0.001; OR 3.410; 95%CI 2.928–3.972) and to self-medication (p < 0.001; OR 1.958; 95% CI 1.703–2.251).

**Conclusion:** Non-guided BP self-measurement, practiced by 93.8% of the sample, was associated with non-control of SAH, self-medication and more visits to the emergency department in the last 12 months.

108711

Modality: E-Poster Scientific Initiation – Non-case Report

Category: EPIDEMIOLOGY AND HEALTH POLICIES/GLOBAL HEALTH

## Law Nº 12.732/12 and Analysis of Data Regarding the Time Interval between Diagnosis and Treatment of Malignant Neoplasm of the Heart, Mediastinum and Pleura in Brazil from 2019 to 2021

AUREA NATHALLIA GOMES DE SOUZA^1^, Bianca Paula Miranda Martins^1^, Camila Silva de Oliveira^1^, Cecília Rodrigues Viana^1^, Larissa Silva Ferreira^1^, Luiz Felipe Façanha Ramos^1^, Marcos Roberto Marques da Silva Júnior^1^, Reny Wane Vieira dos Santos^1^

(1) Universidade Federal do Amapá UNIFAP

**Introduction:** In 2012, law nº 12.732/12 was approved in Brazil, which provides for the first treatment of patients with proven malignant neoplasia and establishes a maximum period of 60 days from the diagnosis as the start date.

**Objective:** To analyze the data about the year of diagnosis and year of treatment, observing compliance with law nº 12.732/12 in force in Brazil and other related aspects.

**Method:** Were used the data available in PAINEL-ONCOLOGY, from the Department of Informatics of the Unified Health System (DATA-SUS), for more detail, in the detailed diagnosis category, C-38 (Malignant neoplasm of the heart, mediastinum and pleura), year of treatment and year of diagnosis and for a deeper analysis, the filters Month/Year of diagnosis and Month/Year of treatment were used, combining them and generating tables for analysis.

**Results:** It is important to highlight the absence of data registered in the Information System over the years analyzed. Of the patients who were diagnosed in 2019, there is no information about the year of treatment for about 52.36%. Those who received their diagnosis in 2020 and 2021, the numbers also exceeded 50% of patients, being, respectively, 51.76% and 53.84%, approximately. In addition, some data were filled in incorrectly, stating that the patient started treatment one month before being diagnosed. Using the filter Month/Year of diagnosis and Month/Year of treatment, it can be said that law nº 12,732/12 is complied with in most of the registered cases, but it was observed that in some cases there is a delay in starting treatment, overloading the subsequent months. The annual general data show that 26 people who were diagnosed in 2019 started their treatment in 2020, and when looking at the detailed data per month, only 8 were registered according to the month, of these, 5 in the period governed by the law. And 20 people diagnosed in 2020 started treatment in 2021, but in the detailed data per month, there is information about 4, and 3 started in the period governed by the law. There is no detailed information about the year 2021.

**Conclusion:** The analysis was hampered by the lack of data, which also has an impact on the absence of public policies aimed at the delay in starting the treatment of malignant neoplasms in general, which can have negative consequences for the prognosis of patients. The records show partial compliance with Law nº 12.732/12.

108721

Modality: E-Poster Scientific Initiation – Non-case Report

Category: HYPERTENSION/RENAL DENERVATION

## Factors Predicting Blood Pressure Control between Public and Private System Patients

GABRIELA DE OLIVEIRA SALAZAR^1^, Glessiane de Oliveira Almeida^1^, Felipe J Aidar^1^, Marcos Antonio Almeida-Santos^1^, Ikaro Daniel de Carvalho Barreto^1^, Dihogo Gama de Matos^1^, José Icaro Nunes Cruz^1^, Cláudia Bispo Martins-Santos^1^, Joselina Luzia Menezes Oliveira^2^, José Augusto Soares Barreto Filho^2^, Enaldo Vieira de Melo^1^, Antônio Carlos Sobral Sousa^2^

(1) Federal University of Sergipe; (2) Rede D’Or São Luiz – São Lucas Hospital

**Introduction:** The number of hypertensive people in the world has doubled to 1.28 billion since 1990. It is estimated that about 50% of hypertensive people are uncontrolled.

**Objectives:** To assess whether there are differences between the public and private systems in terms of blood pressure control and which factors predict non-control of systemic arterial hypertension (SAH) in these groups.

**Methods:** Observational, cross-sectional and analytical study including hypertensive patients treated at cardiology outpatient clinics of the Unified Health System (SUS) and the Supplementary Network (RS) in Sergipe (Brazil). Information was obtained using a standardized questionnaire and medical records. The Shapiro Wilk, Chi-square, Mann-Whitney U test and binary logistic regression (backward stepwise method) were performed, which considered the model entry a p ≤ 0.25 and permanence ≤ 0.05.

**Results:** Of the total, 50% of the patients were from the UHS and 50% from the SN. UHS users were more diabetic (24.6% vs. 18.6%; p = 0.021) and dyslipidemic (42.6% vs. 25.4% p < 0.001). UHS patients had more uncontrolled SAH, more visits to the emergency room in the last 12 months due to hypertension and fewer regular consultations with the cardiologist, while SN patients performed more self-medication. There was no difference regarding the self-measurement of blood pressure (BP). The predictors of uncontrolled SAH in both groups were emergency visits for hypertension and BP self-measurement. In the UHS, there were other factors, such as female sex and self-medication (Tables 1 and 2).

**Conclusion:** There was a higher prevalence of uncontrolled SAH in the UHS, with female sex and self-medication being predictors of uncontrolled BP in this group, and self-measurement and emergency room visits predictors in both groups.


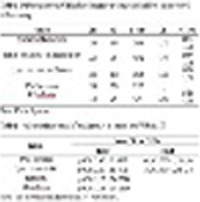


108727

Modality: E-Poster Scientific Initiation – Non-case Report

Category: CONGENITAL AND PEDIATRIC CARDIOLOGY

## Increase in Cases of Arterial Hypertension in Childhood: An Overview of the Last Decade

PRISCILA SANTOS MARIANO^1^, Amanda Cyntia Lima Fonseca Rodrigues^2^, Isabelly Salgado Marin^3^, Heloisa Belinati Pereira Perez^3^, Maria Carolina Casagrande^3^

(1) Universidade Federal de Ouro Preto; (2) Deutsche Hochdruckliga e.V. DHL® Deutsche Gesellschaft für Hypertonie und Prävention; (3) Centro Universitário Ingá

**Background and Aims:** Arterial hypertension (AH) has become a common childhood health condition. When compared to adulthood, screening for childhood AH is complicated and unstable. This stems from the misunderstanding of the effects of AH in childhood, due to the scarcity of information about the long-term consequences of this condition, proof of this is that many pediatric patients have altered blood pressure and are not diagnosed or treated. Based on this evidence, there is a need for in-depth studies about primary hypertension in childhood and how its diagnosis and treatment can act in the prevention of cardiovascular diseases in adult life.

**Methods:** This systematic review used the “PubMed” database to search for the descriptors “Arterial Hypertension” and “Childhood”, associated with articles from the last 10 years, finding 122 articles, which 38 were selected to perform the abstract.

**Results:** The prevalence of AH in the age group from 7 to 17 years remained stable, ranging from 4.4 to 6.4%. However, the risks of developing SAH increased considerably from 6.3% in 1995 to 19.2% in 2014, among the predisposing factors obesity stands out. In other analyses, other possible predisposing factors to childhood obesity, such as longitudinal effects of maternal depression, unhealthy lifestyle, high screen time and low levels of physical activity. In addition, it can also be mentioned that newborns of women with greater weight gain in the first trimester of pregnancy had an increased risk of overweight or obesity, as well as elevations in diastolic blood pressure. Children born to mothers who had gestational diabetes mellitus had significantly higher systolic and diastolic blood pressure.

**Conclusion:** Childhood hypertension is becoming progressively more common in the pediatric population, which demonstrates an enormous public health challenge worldwide. There is a correlation between BMI and AH level in overweight adolescents, and the obese BMI range in this age group is related to an increased risk of incidence of cardiovascular diseases, morbidity (ischemic heart disease and stroke) and mortality in adulthood. Early identification of abnormal AH at young ages is of utmost importance, as it is possible to increase its control and awareness, in addition to reducing progression to higher levels of AH and target organ damage.

108852

Modality: E-Poster Scientific Initiation – Non-case Report

Category: PERICARDIUM/ENDOCARDIUM/VALVOPATHIES

## Clinical Outcome of Patients with Rheumatic Tricuspid Valve Disease: Matched Cohort Study

IGOR MARQUES JORDÃO^1^, Alana Helen Dos Santos Matos^1^, Ana Beatriz Cordeiro Prates^1^, Beatriz Dias Pinheiro^1^, Andre Barbosa de Andrade^1^, Isadora Gonçalves Roque^1^, Lucas Lopes Toledo^1^, Isabel Pellegrinelli Bacelar^1^, Fernando Coletti Mazarão^1^, José Luiz Padilha da Silva^2^, William Antônio de Magalhães Esteves^1^, Maria do Carmo Pereira Nunes^1^

(1) Federal University of Minas Gerais (UFMG); (2) Federal University of Parana (UFPR)

**Background:** Rheumatic heart disease (RHD) is still a major health problem, especially in low- to mid-income countries, leading premature deaths owing to valvular disease (VD). Although left-sided valvular involvement is most commonly seen in RHD, the tricuspid valve can also be affected. However, there is a lack of information about the prognostic value of primary tricuspid valve disease (TVD) in RHD.

**Objective:** This study aimed to determine the impact of TVD on clinical outcome in RHD.

**Methods:** This prospective study enrolled patients with RHD referred to a tertiary center in Brazil for management of VD between 2011 and 2021. Primary rheumatic TVD was defined by echocardiographic features including thickening of leaflets associated with some degree of restricted mobility. Patients with rheumatic TVD were matched to patients with mitral valve disease (MVD) using 1:1 genetic matching algorithm that maximized balance of baseline covariates prior to exploring outcome differences. The main outcome was either need for MV replacement or death.

**Results:** Among 694 patients eligible for the study, age of 47 ± 13 years, 84% female, 39 patients (5.6%) had rheumatic TVD. After excluding patients with incomplete data, 33 patients with TVD were matched to 33 controls based on age, right-sided heart failure, atrial fibrillation, and MV area. Over a median follow-up of 28 months [interquartile range: 8–71], 28 underwent MV replacement, and 6 patients died. Kaplan–Meier survival estimates showed worse event-free survival in patients with TVD compared to matched controls (Figure 1). TVD was predictor of adverse events with a hazard ratio of 3.39 (95% CI: 1.56, 7.35, p = 0.002) in the genetic matched cohort with balance on baseline covariates of interest.

**Conclusions:** In the setting of RHD, rheumatic TVD significantly increased risk of adverse events compared with matched controls. The involvement of TV may express overall disease severity that adversely affects clinical outcome. Our study is the first to assess clinical outcome using genetic matching approach to account for baseline differences in patients characteristics.


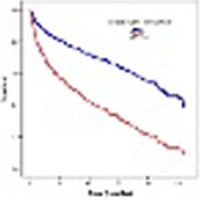


108743

Modality: E-Poster Scientific Initiation – Non-case Report

Category: COVID-19 AND CARDIOVASCULAR SYSTEM

## Takotsubo Cardiomyopathy and the COVID-19 Vaccine: A Systematic Review

JESSICA SILVA ROCHA^1^, Gabriel Silva Rocha^2^, André Moisés de Oliveira Nunes^4^, Geovanna Vitória da Cruz Xavier Silva^1^, Júlia Ranielly Oliveira Rios^1^, Lucas de Lacerda Ramos^3^

(1) Centro Universitário UniFTC (UniFTC); (2) Hospital Ana Nery (HAN); (3) Universidade Federal da Bahia (UFBA); (4) Escola Bahiana de Medicina e Saúde Pública (EBMSP)

**Introduction:** Takotsubo cardiomyopathy (TCM) is caused by catecholamine surge, which is also observed in COVID-19 disease due to cytokine storm. TCM refers to a transient left ventricular systolic dysfunction, usually with associated chest pain, whose main differential diagnosis is acute coronary syndromes. Although vaccination against SARS-CoV-2 is the primary factor in combating the pandemic, myocarditis has been demonstrated in reports as a side effect of the vaccine. And, despite being rare cases, a new category is being reported, with studies still limited, such as TCM.

**Objectives:** To evaluate the association between the incidence of TCM and vaccination against the COVID-19 virus, in addition to characterizing the rate of post-vaccination TCM hospitalizations.

**Methods:** This is a systematic review, in which the databases available on the platforms Pubmed, Lilacs, Medline were analyzed. For this, studies that address Takotsubo Syndrome related to COVID-19 vaccination were selected, following the PRISMA guidelines. As for the inclusion criteria, it consists of case reports presenting TCM as the only differential diagnosis, published in the last 2 years and available in the electronic version. The excluded studies were those that showed distance from the central objective of the research and duplication.

**Results:** Seven eligible studies were selected for data collection. Of the results obtained, the female sex predominated (85.75%), mean age 62.1 ± 17.8 years, the mean number of days from the vaccine to the event was 3 days and 71.4% of the cases had some comorbidity.

**Conclusion:** Typical epidemiological characteristics of Takotsubo Syndrome were evidenced, such as female gender, advanced age and the presence of comorbidities, in which the event was exclusively related to COVID-19 vaccination, subjectively excluding other causes. Therefore, the need for further studies characterizing TTK, as well as its relationship with mRNA vaccines is important, since there are also previous reports correlating TTK with the influenza vaccine. And from that, to evaluate the safety of mRNA vaccines and optimize patient outcomes.


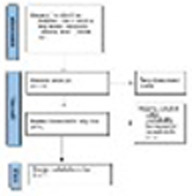


108748

Modality: E-Poster Scientific Initiation – Non-case Report

Category: CARDIOLOGY OF SPORTS, EXERCISE, ERGOMETRY AND CARDIOVASCULAR REHABILITATION

## Electrocardiographic Findings and Medical History of Female Soccer Professional Athletes

GIULIA VITORIA NASCIMENTO DA SILVA^1^, LARISSA DACIER LOBATO COMESANHA^2^, TEREZA MARIA MEIRELES FERNANDES DA SILVA^1^, MARIANA DOS SANTOS GUIMARÃES^2^, GABRIEL REZENDE NEVES^1^, MIGUEL CHAMOSO GILSANZ NETO^1^, PAULO CESAR LOBATO MAGALHÃES^2^, GABRIELA DE ARRUDA CUSTÓDIO^3^, HENRIQUE CUSTÓDIO DA SILVA^1^

(1) Universidade do Estado do Pará (UEPA); (2) Universidade Federal do Pará (UFPA); (3) Centro Universitário Metropolitano da Amazônia (UNIFAMAZ)

**Introduction:** Professional athletes constantly suffer physiological and anatomic alterations in the cardiovascular system in order to balance the intense cardiac activity during exercises. Although women develop less cardiac diseases in their life as a result of healthier habits of life than men, feminine athletes are not exempt from the constant cardiovascular adaptation inherent to high performance sports and its risks. In this regard, changes in the stimulant complex of the heart and in its structure may lead to some severe complications such as arrhythmias and cardiac insufficiency.

**Objective:** To identify electrocardiographic abnormalities in athletes from a professional women’s soccer team.

**Method:** Cross-sectional, analytical-descriptive and quantitative study. Data were obtained through medical records of 20 professional female soccer athletes, in the year 2020, in the city of Belém, Brazil. In addition to electrocardiographic records, information on age, family history, chest pain when practicing physical activity and consumption of alcohol drinks were also analyzed. Chi-square test and Fisher’s exact test were used for statistical analysis of variables.

**Results:** The average age of the athletes were 25.1 ± 3.9 years. In the study population, only 4 participants (20%) had ECG abnormalities, which were 2 right bundle branch conduction delay (50%), 1 left ventricular hypertrophy signs (25%) and 1 right bundle branch block (25%,). There was no statistical significance between alcohol consumption and the presence or absence of ECG changes (p > 0.05). Moreover, there were also no statistically significant differences associated with ECG changes when related to the presence of chest pain during physical activity or the existence of a family history of sudden death or heart disease (p > 0.05).

**Conclusion:** The findings of the present study showed electrocardiogram abnormalities in 20% of the athletes included, but not related to chest pain, family history of sudden death or heart disease, nor to the use of alcohol drinks, being in similarity with other studies available in the literature. Despite this, the importance of continuity of cardiological medical evaluation by athletes for the prevention of cardiovascular diseases and possible sudden death is highlighted.

108756

Modality: E-Poster Scientific Initiation – Non-case Report

Category: HYPERTENSION/RENAL DENERVATION

## Malaria Increases Blood Pressure and Risk of Hypertension in an Observational Study in the Brazilian Amazon

KARINE OLIVEIRA LIMA^1^, Laura Cordeiro Gomes^2^, Philip Brainin^3^, Marliton Vinicius Pedrosa Evangelista^1^, Isabelle Victória Martins Vieira^1^, Anna Engell Holm^3^, Luan Oliveira Matos^1^, Bianca Vasconcellos Rodrigues Lopes^4^, Iara Fernanda Vasconcelos de Oliveira e Silva^1^, Tor Bierning-Sorensen^5^, Cláudio Romero Farias Marinho^2^, Odilson Marcos Silvestre^5^

(1) Multidisciplinary Center, Federal University of Acre, Câmpus Floresta, Cruzeiro do Sul, Acre, Brazil; (2) Department of Parasitology, Institute of Biomedical Sciences, University of São Paulo, São Paulo, Brazil; (3) Department of Cardiology, Herlev-Gentofte University Hospital, Hellerup, Denmark; (4) Health and Sport Science Center, Federal University of Acre, Rio Branco, Acre, Brazil; (5) Faculty of Biomedical Sciences, Copenhagen University, Copenhagen, Denmark

**Background:** The prevalence of hypertension in malaria endemic areas seems to be higher than in malaria-free areas. In these regions, a higher rate of hypertension has been detected in younger people when compared to developed countries. We aimed to investigate the relationship between malaria episodes and hypertension in the Brazilian Amazon.

**Methods:** The study was conducted in the Brazilian Western Amazon from June 2020 to August 2021. Patients with a lifetime history of malaria, but not infected at inclusion had a cross-sectional assessment. However, those who were infected with Plasmodium spp. at enrollment (T0) had a follow-up evaluation (FUP) performed 34 ± 7.2 days later. All of them answered a questionnaire concerning clinical and sociodemographic information and underwent physical examination. Patients were classified as hypertensive through previous medical diagnosis and/or according to the International Society of Hypertension parameters: ≥140 systolic blood pressure (SBP) and/or ≥90 diastolic blood pressure (DBP). The mean between both arms were considered for SBP and DBP. Since variables were non-parametric, Wilcoxon signed-rank test was applied to compare blood pressure betweenT0 and FUP. Logistic regression was performed to assess the risk of hypertension as a function of the age at the first malaria episode.

**Results:** Seventy-six symptomatic malaria patients completed the follow-up evaluation. The median age was 37 years (IQR 26 to 48) and 59% were males. A significant increase in the SBP (median [IQR] T0 119.5 mmHg [111– 129.5] vs FUP 122.5 mmHg [113.75–134], P = 0.015) and DBP (median [IQR] T0 74.5 mmHg [68–82.75] vs FUP 77.5 mmHg [72.5–84], P < 0.001) was found in the follow-up when compared to enrollment. Additionally, a total of 430 patients were included in the cross-sectional study [53% males; median age 39 years (IQR 30 to 47)]. Median age of the first malaria episode was 17 (IQR 12 to 27) years. After adjusting for number of previous malaria episodes, age, abdominal circumference, income, sex, smoking and diabetes, age at first malaria infection was associated with higher risk of hypertension [Odds ratio 1.031 (95% CI 1.006 to 1.057), P = 0.014].

**Conclusion:** Age at the first malaria episode seems to influence blood pressure levels. Both SBP and DBP increased 34 days after infection. More studies to investigate this association are required to improve health policies on hypertension in communities living in malaria endemic areas.

109436

Modality: E-Poster Scientific Initiation – Non-case Report

Category: ATHEROSCLEROSIS/CARDIOVASCULAR RISK FACTORS/CARDIOVASCULAR PREVENTION

## Physical Exertion and Emotions as Triggers of Stroke: A Systematic Review

JOÃO PEDRO TORRES NEIVA RODRIGUES^1^, Raquel Vieira Torres^1^, Pedro Viana Diniz^1^

(1) Universidade Federal de Juiz de Fora

**Introduction:** Risk factors for stroke onset are well known. However, factors that may act as a trigger still need clarification.

**Objective:** We aimed to review data from the literature to assess the role of physical exertion or emotional distress as triggers of stroke.

**Methods:** Reviewers researched on Pubmed, Embase, and PsycINFO for cohort, case-control, or case-crossover studies with exposition to triggers within 3 months of stroke onset. PRISMA protocol was used.

**Results:** 323 articles were found, and after applying inclusion criteria, 17 studies remained. Combined, there were 1168152 cases of stroke. Physical exertion was defined in most articles as activities that require more than 5 METs to be performed, however, sexual activity, and lifting >23 kg, were also included. Eight studies analyzed this group, and 5 studies reported a correlation between physical exertion and the onset of stroke. 15 studies reported emotional triggers such as anger, psychological distress, startling, positive, and negative emotions were investigated. A correlation between emotions and the onset of stroke was found in 12 studies (Table 1). Different cutoffs were used to determine an emotional outburst, which could increase studies heterogeneity and could cause discordant results between them. Self-reported questionnaires can be prone to memory bias.

**Conclusion:** Physical exertion and emotional triggers were associated with an increased risk of stroke onset in most studies.


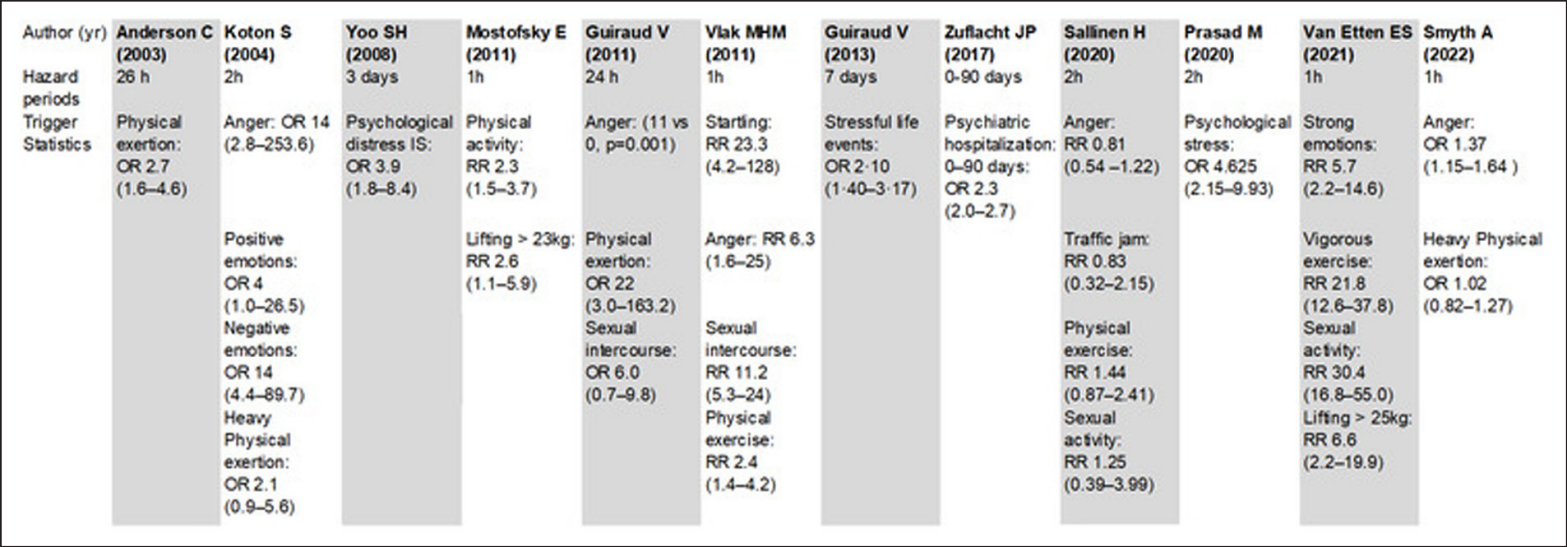


108793

Modality: E-Poster Scientific Initiation – Non-case Report

Category: DIGITAL HEALTH/INNOVATION

## Automatic Detection of Heart Diseases on Electrocardiograms using Machine Learning

GUSTAVO SANTOS PORFIRO^1^, Gustavo Santos Porfiro^1^, Leonardo Favaro Pereira^1^, Rosemberg Rodrigues Dal Gobbo^1^, Fernando Luiz Torres Gomes^1^

(1) Universidade Federal do Espírito Santo; (2) Universidade Federal do Espírito Santo; (3) Universidade Federal do Espírito Santo; (4) Universidade Federal do Espírito Santo; (5) Universidade Federal do Espírito Santo; (6) Universidade Federal do Espírito Santo

**Introduction:** Heart diseases are the leading cause of death in the world, affecting developed and underdeveloped countries, and have been presenting progressive and alarming growth. Early diagnosis of this type of condition is fundamental to reduce mortality. The main and most common way of diagnosing heart disease is the electrocardiogram (ECG), which is totally dependent on the supervision of a specialized professional. Consequently, it is pertinent to observe the need for preventive diagnosis in order to minimize the high mortality rates of these diseases.

**Objective:** To investigate the selection and prediction of Machine Learning (ML) algorithms to facilitate the process of identifying heart diseases from electrocardiogram signals.

**Methods:** We conducted a literature review, covering the period from January 2021 to January 2022, in the Pubmed, Cochrane and Physionet databases. The descriptors “machine learning”, “heart diseases”, “electrocardiogram”, separated by Boolean operators AND, were searched in the Medical Subject Heading. Among the inclusion criteria were full-text articles published from 2011 onwards.

**Results:** Performance evaluation of an ML algorithm was performed by means of statistical metrics relating overall prediction hit rate, accuracy and the confusion matrix to the treatment of tabular data. It was obtained from algorithms and solutions with open source code a hit rate higher than 80% both in predicting normal ECG exams and in predicting classes that presented heart disorders, such as myocardial infarction, being that in some cases the hit rates were higher than 75% for normal exams and almost 90% in relation to exams with myocardial infarction.

**Conclusion:** The use of intelligent software based on Machine Learning to facilitate the diagnosis of heart diseases is a promising advance, given the alarming growth of these diseases according to data from the World Health Organization. With increasingly advanced technology and improved resources, this software can partially reduce the dependence on analysis by a professional, and can indicate findings that may be overlooked by professionals. It is, then, an ally to the professionals who deal with these types of exams, and not their substitutes.

108798

Modality: E-Poster Scientific Initiation – Non-case Report

Category: CARDIOVASCULAR IMAGING

## Analysis of the Indication for Coronary Computed Tomography Angiography in Patients with Known or Suspected Coronary Artery Disease in a Particular Diagnostic Imaging Center in Curitiba

CAROLINA DOS ANJOS BASTOS^1^, Carolina dos Anjos Bastos^1^, Eduardo Pires dos Santos^1^, Anderson Rafael Pesamosca^1^, Tânia Zaleski^1^, Wilton Francisco Gomes^1^

(1) Faculdades Pequeno Príncipe (FPP)

**Background:** Cardiovascular diseases (CVD) remain the leading cause of premature death worldwide. There are several diagnostic methods for its detection, among them coronary computed tomography angiography (CTA), with increasing use in recent years. Defining the most appropriate complementary test, in terms of accuracy for clinical decision making and rationalization of resources, remains a challenge in medical practice.

**Objective:** To analyze the appropriateness of the indications for CTA in a sample from a private clinic in Curitiba, taking as reference the II Guidelines for Cardiovascular Magnetic Resonance and Computed Tomography of the Brazilian Society of Cardiology and Brazilian College of Radiology (IIDRT) and the Roll of the National Agency for Supplementary Health (ANS). As a secondary objective, to analyze the main variables that correlated with the presence of obstructive CAD in this sample.

**Method:** This is an observational and retrospective study, with the analysis of 2595 consecutive CTA exams for the evaluation of CAD, between January 2019 and December 2020. Indications were classified according to IIDRT recommendation classes and ANS Roll recommendations. Multivariate analysis was used for correlation between independent variables and outcome.

**Results:** Regarding the IIDRT indications for the performance of CTA, 1170 (45.1%) individuals fit into recommendation class I, 10 (0.4%) into IIa, 175 (6.7%) into IIb, 550 (21.2%) into III and 690 (26.6%) did not fit into any expected indication. As for the ANS Roll, 1620 (62.4%) individuals fit the indication criteria, while 975 (37.6%) did not. Risk factors such as dyslipidemia, diabetes and family medical history were associated with obstructive CAD in CTA (p < 0.001).

**Conclusion:** 47.8% of patients referred for CTA had a class III recommendation or did not have indications according to the IIDRT. By ANS criteria, over 37.6% of patients had non-predicted indications. This denotes that a critical analysis of current recommendations as well as clinical practice should probably be performed.

108803

Modality: E-Poster Scientific Initiation – Non-case Report

Category: PERIOPERATIVE EVALUATION

## Infections in the Postoperative Period of Cardiac Surgery: Incidence, Mortality and Hospitalization Time

EDUARDO PORTO SANTOS^1^, RAFAEL FORTES LOCATELI^1^, ANNA CAROLINA FLORES MARIATH^2^, VITÓRIA CAROLINA KOHLRAUSCH^1^, ISABELLA KLAFKE BRIXNER^1^, GABRIELLE LENZ DE ABREU^2^, ANÍBAL PEREIRA ABELIN^1^, DIEGO CHEMELLO^1^, MATEUS DINIZ MARQUES^1^

(1) UNIVERSIDADE FEDERAL DE SANTA MARIA; (2) HOSPITAL UNIVERSITÁRIO DE SANTA MARIA

**Background:** Infection is the most frequent non-cardiac complication after cardiac surgery. Its incidence is associated with increased mortality, hospitalization days and costs.

**Objective:** The aim of this study is to describe the incidence of infections in patients undergoing cardiac surgery at the University Hospital of Santa Maria and hospitalization days and mortality associated with.

**Methods:** This is a descriptive, longitudinal and retrospective study analyzing the medical records of patients undergoing cardiac surgery between September 2018 and April 2021. Patients undergoing pacemaker implantation or pericardial surgery or those who died intraoperatively were excluded. The analysis was performed using the software Stata version 15.1, and the results were expressed as percentage, mean and standard deviation.

**Results:** From a database of 200 patients, 194 filled the inclusion criteria. Of these, 60 patients (30.92%) were diagnosed with infection and used antibiotics postoperatively and 16 died (26.67%). The mortality among patients without nfection was 6.0% (8 patients). Infection was associated with death during hospitalization (x² = 16.37; p < 0.001), with 66.67% of deaths occurring among patients with infection. individuals with infection had higher mean hospitalization days than those without infection using the Mann Whitney U test (20 ± 15 days and 9 ± 5 days, respectively, U = 1465.500, Z = –7.099, p < 00.1).

**Conclusions:** The study portrayed the reality of a tertiary public hospital for heart surgery in the countryside, with a high incidence of postoperative infection. The diagnosis of infection was associated with longer hospital stay and higher mortality. The factors associated with higher incidence of infection and management factors need to be addressed in detail in future studies.

108808

Modality: E-Poster Scientific Initiation – Non-case Report

Category: ATHEROSCLEROSIS/CARDIOVASCULAR RISK FACTORS/CARDIOVASCULAR PREVENTION

## Female Sex is a Protective Factor Against Coronary Artery Disease

JOSÉ ICARO NUNES CRUZ^1^, Cláudia Bispo Martins-Santos^1^, Gabriela de Oliveira Salazar^1^, Juliana Maria Chianca Lira^1^, Mayara Evelyn Gomes Lopes^1^, Ana Luísa Lisboa Prado^1^, Marília Marques Aquino^1^, Nathalia Luiza Silva Sobral^1^, Arthur Leite Lessa^1^, Antônio Carlos Sobral Sousa^2^, Enaldo Vieira de Melo^1^, Joselina Luzia Menezes Oliveira^2^

(1) Federal University of Sergipe; (2) Rede D’Or – São Lucas Hospital

**Introduction:** Cardiovascular diseases are important causes of morbidity and mortality worldwide. Among these, coronary artery disease (CAD) stands out as a significant cause of death and health costs. Some studies demonstrated that female sex is a protective factor for CAD in the premenopausal period, suggesting a beneficial action of endogenous estrogens on the heart. In this context, it is essential to analyze the risk factors and prevalence of CAD between the sexes.

**Objectives:** To assess the frequency of CAD, as well as its characteristics, according to sex.

**Methods:** This is an observational, cross-sectional, analytical study. The sample included patients who underwent coronary computed tomography angiography (CCTA) in a private hospital. Coronary stenosis ≥ 50% was considered significant on CCTA. Statistical analysis was conducted using Chi-Square test, with significance level set at 5% for comparisons. Odds ratio (OR) and 95% confidence intervals (CI95%) were also described. All analyses were performed using SPSS Statistics software, version 22.0.

**Results:** The sample consisted of 1250 patients, of which 48.3% (604) were female. The mean age was 57.9 ± 12.9 years, with a non-significant difference between the sexes (p > 0.05). Women demonstrated higher frequencies of obesity (27.9% vs. 21.8%; p = 0.027) and sedentary lifestyle (75.3% vs. 65.3%; p = 0.001). Men demonstrated higher rates of smoking (4.6% vs. 3.8%; p = 0.041) and alcohol consumption (41.2% vs. 18.0%; p < 0.001). Male subjects exhibited higher prevalence of CAD (52.6% vs. 38.1%; p < 0.001), as well as higher chance of calcified plaques (OR: 1.95; CI95%: 1.40–2.72; p < 0.001) and non-calcified plaques (OR: 1.75; CI95%: 1.08–2.82; p = 0.021). In addition, men were more likely to have significant coronary stenosis (OR: 2.65; CI95%: 1.82–3.85; p < 0.001), as well as triple vessel CAD (OR: 3.88; CI95%: 2.02–7.46; p < 0.001) and double vessel CAD (OR: 2.76; CI95%: 1.72–4.43; p < 0.001).

**Conclusions:** The prevalence of CAD was lower among women. Men were more likely to have calcified and non-calcified plaques, significant coronary stenosis, double vessel CAD and triple vessel CAD. Thus, it is concluded that females are less affected by CAD.

108817

Modality: E-Poster Scientific Initiation – Non-case Report

Category: CARDIOLOGY OF SPORTS, EXERCISE, ERGOMETRY AND CARDIOVASCULAR REHABILITATION

## Alcohol Consumption and Cardiorespiratory Fitness in Echocardiogram Evaluated Women Under Physical Stress

GABRIELA DE OLIVEIRA SALAZAR^1^, José Icaro Nunes Cruz^1^, Cláudia Bispo Martins-Santos^1^, Juliana Maria Chianca Lira^1^, Lucas Villar Shan de Carvalho Cardoso^1^, Nathalia Luiza Silva Sobral^1^, Ana Luísa Lisboa Prado^1^, Giulia Vieira Santos^1^, Mayara Evelyn Gomes Lopes^1^, Enaldo Vieira de Melo^1^, Antônio Carlos Sobral Sousa^2^, Joselina Luzia Menezes Oliveira^2^

(1) Federal University of Sergipe; (2) Rede D’Or São Lucas Hospital

**Introduction:** Low cardiorespiratory fitness (CF) is a risk factor for cardiovascular morbidity and mortality. Women have significantly lower CFs than men. Furthermore, alcohol consumption is a known determinant of disease burden, although there is still no consensus on the influence of alcohol on CF. In recent years there has been an increase in alcohol consumption among women.

**Objective:** To identify predictors of low CF in women, with emphasis on the influence of social alcohol consumption on CF.

**Methods:** Observational, cross-sectional, analytical study, with data collection from record of echocardiogram exams under physical stress performed in Sergipe (Brazil). Female patients were included. The CF was classified according to the metabolic equivalent of task (MET) as low (MET < 7.9), intermediate (7.9 ≤ MET < 10.9) and high (MET ≥ 10.9). Statistical analysis included the chi-square test and multinomial logistic regression using SPSS Statistics software. A value of 5% was set for statistical significance.

**Results:** 2202 patients were included, with a mean age of 58.48 ± 10.9 years. Among the risk factors, 54.1% were hypertensive, 47.5% sedentary, 46.4% dyslipidemic, 21.9% obese, 17.1% alcoholic and 4.3% smokers. CF was considered low in 35%, intermediate in 37.5% and high in 27.5% of the population studied. Alcohol consumption was a predictor of low CF in women, compared to those with high and intermediate CF, as well as sedentary lifestyle. Systemic arterial hypertension (SAH) and obesity predicted low CF when compared to the high CF group (Table 1).

**Conclusion:** Alcohol consumption was an independent predictor associated with low CF in women. The same was verified with the presence of sedentary lifestyle, SAH and obesity.


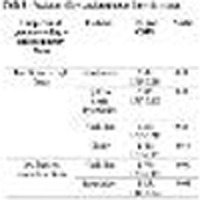


108835

Modality: E-Poster Scientific Initiation – Non-case Report

Category: CARDIOVASCULAR IMAGING

## Assessment of Left Ventricular Dysfunction using Native T1 Mapping Through Cardiac Magnetic Resonance Imaging

JOSÉ ICARO NUNES CRUZ^1^, Gabriela de Oliveira Salazar^1^, Cláudia Bispo Martins-Santos^1^, Lara Teles Alencar Duarte^1^, Nathalia Luiza Silva Sobral^1^, Juliana Maria Chianca Lira^1^, Raisan Almeida Santos^2^, Myllena Maria Santos Santana^1^, Antônio Carlos Sobral Sousa^2^, Enaldo Vieira de Melo^1^, Joselina Luzia Menezes Oliveira^2^, Luiz Flávio Galvão Gonçalves^2^

(1) Federal University of Sergipe; (2) Rede D’Or – São Lucas Hospital

**Introduction:** The pathophysiology of ventricular dysfunction comprises cardiac remodeling with formation of diffuse cardiac fibrosis, a marker of poor prognosis. Native T1 mapping, performed by cardiac magnetic resonance imaging (CMRI), is a non-invasive method of assessing compromised myocardial areas, such as fibrotic areas, being considered a sensitive marker of cardiovascular morbidity and mortality.

**Objectives:** To associate native T1 mapping with parameters related to left ventricular dysfunction on CMRI.

**Methods:** This is an observational, cross-sectional, analytical study. The sample included patients who underwent CMRI in a Phillips Ingenia 1.5T magnet, from a private hospital located in Sergipe (Brazil), in 2021. Native T1 mapping of the left ventricle was compared with other variables related to ventricular function, which were indexed to body surface when measurements were borderline. Statistical analysis was performed using the Mann-Whitney U test and one-way analysis of variance (ANOVA) adjusted by bootstrapping, followed by Games-Howell post-hoc. All analyses were performed using SPSS Statistics software, version 22.0.

**Results:** Among 415 patients who underwent CMRI in 2021, native T1 mapping was originally obtained for 225 patients (54.2%). The median native T1 mapping in the included sample was 1036 ms (IQR: 1008.0–1067.0 ms). Native T1 mapping values were significantly higher in patients with increased left ventricular (LV) end-diastolic volume (1055.5 vs. 1035.0 ms; p = 0.003), increased LV mass (1066.5 vs. 1034.0 ms; p < 0.001) and increased left atrium (LA) size (1053.0 vs. 1033.0 ms; p < 0.001). The native T1 mapping was also superior in patients with reduced left ventricular ejection fraction (LVEF) compared to patients with preserved LVEF (1143.53 vs. 1032.03 ms; p = 0.001), as well as among patients with LVEF borderline compared to those with preserved LVEF (1077.35 vs. 1032.03 ms; p = 0.007).

**Conclusions:** Elevated native T1 mapping was strongly associated with indicators of cardiac dysfunction, such as greater LV end-diastolic volume, greater LV mass, greater LA dimension and reduced LVEF. Thus, the study confirms the applicability of native T1 mapping on the assessment of heart failure.

108857

Modality: E-Poster Scientific Initiation – Non-case Report

Category: ATHEROSCLEROSIS/CARDIOVASCULAR RISK FACTORS/CARDIOVASCULAR PREVENTION

## Metabolically Healthy Obesity in a Young Population Assisted by the Family Health Strategy

ANA RACHEL BUCAR CERVASIO^1^, Clara Avelar Mendes Vasconcellos^1^, Carlos Augusto Parente Macedo Moura^1^, Daniel Barreto Kendler^1^, Deisianny Santos Ferreira^1^, Elizabeth Silaid Muxfeldt^1^, Karine da Silva Guimarães^1^, Gabriela Gama Zagni Jardim^1^, Lívia Lopes Monteiro de Barros Junqueira^1^, Rodney Barberá Boghossian^1^, Tomás de Souza Mello^1^, Vitória Santa Marinha Flumignan^1^

(1) IDOMED – Universidade Estácio de Sá, Medicine School, Campus Vista Carioca, Rio de Janeiro, RJ, Brasil – UNESA

**Background:** Obesity is increasing in younger populations, and is associated with a high cardiovascular (CV) risk, however, it is not clear whether metabolically healthy obesity (MHO) may have a lower CV risk or if it is just an earlier stage of the disease.

**Objective:** To evaluate the prevalence and cardiovascular (CV) risk factors associated with metabolically healthy obesity (MHO) in a young population assisted by a Family Health Care unit in a large urban center.

**Design and Methods:** A cross-sectional population study for CV risk assessment in adults aged 20–50 years old from a Family Health Care unit. Demographic, anthropometric data and CV risk factors were recorded. All underwent office blood pressure (BP) measurements, laboratory evaluation (lipid and glycidic profile). Obesity was defined as a body mass index (BMI) > 30 kg/m^2^ and MHO are those who have less than 3 of the following criteria for metabolic syndrome: office BP higher or equal to 130 × 85 mmHg, hypertension, fasting blood sugar higher or equal to 100 mg/dL, HDL < 40 mg/dL (men) and 50 mg/dL (women), triglycerides >150 mg/dL and waist circumference > 102 cm (men) and > 88 cm (women).

**Results:** A total of 632 individuals were evaluated (60% female; mean age 37 ± 9 years). The prevalence of obesity was 26%, of which 73% were classified as MHO. Obeses are older, with a higher prevalence of physical inactivity (51% vs 41%, p = 0.03), hypertension (44% vs 19%, p < 0.001), dyslipidemia (50% vs 36%, p = 0.002), and diabetes (7% vs 2%, p = 0.001) with higher systolic OBP. MHO compared to unhealthy ones are significantly younger and smoke less. Despite being obese, they have lower BMI (33.6 vs 35.2 kg/m^2^, p = 0.02) and abdominal circumference (102 vs 110 cm, p = 0.03), with lower diastolic BP.

**Conclusion:** MHO was more prevalent in this young population and seems to have a lower CV risk, however it is not clear whether these younger and less obese individuals are only at an earlier stage of the disease. Perhaps the CV diseases onset is postponed for a few years. Even so, these individuals should not be excluded from public health policies as a form of primary prevention.

108872

Modality: E-Poster Scientific Initiation – Non-case Report

Category: HYPERTENSION/RENAL DENERVATION

## Kidney Subclinical Lesions in Hypertension Phenotypes Defined by Home Blood Pressure Monitoring Among Young Adults Enrolled in a Family Health Strategy Unit

CLARA AVELAR MENDES DE VASCONCELLOS^1^, Tomás de Souza Mello^1^, Karine da Silva Guimarães^1^, Gabriela Gama Zagni Jardim^1^, Ana Rachel Bucar Cervasio^1^, Carlos Augusto Parente Macedo Moura^1^, Felipe Rey Costa Tostes^1^, Pedro Henrique dos Santos Lemos^1^, Clara Maria da Costa Muguet^1^, Joana Sauerbronn Corrêa da Costa^1^, Inah Maria Drummond Pecly^1^, Elizabeth Silaid Muxfeldt^1^

(1) IDOMED – Universidade Estácio de Sá, School of Medicine, Campus Vista Carioca, Rio de Janeiro, RJ, Brasil.

**Introduction:** The association between kidney lesion, hypertension (HTN) and cardiovascular risk (CV) is well established. Asymptomatic patients with decreased glomerular filtration rate (GFR) and/or increased albuminuria seem to have increased CV risk. The refinement of HTN diagnosis using Home Blood Pressure Monitoring (HBPM) allows to identify individuals with different phenotypes: normotension, sustained HTN, masked HTN and white-coat HTN, increasing diagnostic accuracy.

**Objective:** To assess kidney subclinical lesions among different HTN phenotypes identified by HBPM in an adult population enrolled in a Family Health Strategy unit.

**Design and Methods:** Cross-sectional, populational study with adults between 20–50 years old enrolled in a Family Health Strategy unit in a metropolis. Sociodemographic, anthropometric and CV risk factors were registered. Office blood pressure (BP) was the average of two consecutive measurements, and HBPM followed a 7-day protocol. HBPM < 135 × 85 mmHg and office BP < 140 × 90 mmHg were considered normal, identifying four phenotypes: normotension (controlled office BP and HBPM); white-coat HTN (uncontrolled office BP and controlled HBPM); masked HTN (controlled office BP and uncontrolled HBPM) and sustained HTN (uncontrolled office BP and HBPM). Albuminuria was dosed in an isolated urine sample whereas GFR was calculated by CKD Epi formula using serum creatinine.

**Results:** 483 individuals were evaluated (39% male; mean age: 37.6 ± 8.8 years). More often, individuals with white-coat HTN (9.3%) are male, with greater neck circumference and higher prevalence of metabolic syndrome. Individuals with masked HTN (10%) are more obese, with increased neck and waist circumferences whereas those who present sustained HTN are predominantly male, more obese, with increased neck and waist circumferences and higher prevalence of diabetes and metabolic syndrome. Analyzing four phenotypes, a progressive albuminuria increase was observed among normotensive patients: white-coat HTN 4.0 ± 2.2; masked HTN 5.3 ± 3.4 and sustained HTN 7.1 ± 1.4 mg/g creatinine. We also observed an increased serum creatinine and decreased GFR: white-coat HTN 0.74 ± 0.16/132 ± 34; masked HTN 0.79 ± 0.17/123 ± 28; sustained HTN 0.81 ± 0.32/120 ± 59, respectively, although without statistical significance.

**Conclusion:** The performance of HBPM refined HTN diagnosis and was able to early identify a progressive worsening of kidney function parameters in the different phenotypes.

110530

Modality: E-Poster Scientific Initiation – Non-case Report

Category: HYPERTENSION/RENAL DENERVATION

## Medication Adherence Rate of Apparent Resistant Hypertensive Patients in a Teaching-Care Outpatient Clinic

GABRIELA FREITAS VALVERDE ^1^, Gabriel Martins Nogueira^1^, Bruna Marmori Lima^1^, João Victor Araújo de Oliveira^1^, Adriana Santiago de Carvalho Borges^1^, Lorena de Souza Santos^1^, Gabriel Von Flach Sarmento^1^, Alexandra Brito Rocha da Silva^1^, Jéssica Reis de Jesus^1^, Ana Flávia de Souza Moura^1^, Constança Margarida Sampaio Cruz^2^

(1) Escola Bahiana de Medicina e Saúde Pública (EBMSP); (2) Obras Sociais Irmã Dulce (OSID)

**Introduction:** Resistant Arterial Hypertension (RHTN) is a clinical condition in which blood pressure (BP) remains above target even with the concomitant use of three specific antihypertensive drugs of different classes at maximum doses or within the target using 4 or more of those. The main factor interfering with BP control is drug non-adherence, which can be assessed by the 8-item Morisky Medication Adherence Scale (MMAS-8), an update of the four-item, still not widely found in national studies on RHTN. Thus, this study aims to estimate the rate of medication adherence in patients with apparent RHTN and correlated factors in a teaching-care outpatient clinic.

**Methods:** This is a cross-sectional study carried of patients treated at a cardiology teaching-care outpatient clinic, over 18 years old, diagnosed with hypertension and with at least six months of antihypertensive treatment. Patients with severe psychiatric and/or cognitive limitations, who did not meet RHTN criteria and with secondary hypertension were excluded from the sample. Medication adherence was collected through the application of the MMAS-8 questionnaire in person, in an outpatient clinic and by telepresence, through a virtual form. The score obtained for the therapeutic adherence rate ranges from zero to eight, with eight considered high adherence, ≥ 6 and < 8, medium adherence and < 6, low adherence.

**Results:** A total of 31 patients were included in the study, 23 (74.19%) women and a median age of 60 (IQR 53–65) years. Furthermore, 18 (58.06%) had a family history of hypertension, 20 (64.5%) had no BP control, 25 (80.64%) were sedentary, nine (29.03%) were alcoholics and the most prevalent comorbidity was Diabetes Mellitus (35.4%). The body mass index (BMI) was, on average, 32.37 (IQR 27.10–35.86), the median time of diagnosis of hypertension was 10 (IQR 4–15.5) years and the median number of antihypertensive drugs in use and the median total number of drugs were four (IQR 3–4.5) and seven (IQR 5–8.5), respectively. The median score obtained on the MMAS-8 was 5.75 (IQR 4.75–7.75), considered low adherence. It was found that the no BP control (p = 0.020), a higher BMI (p = 0.043) and higher serum urea values (p = 0.045) are correlated with a lower rate of adherence to antihypertensive drugs.

**Conclusion:** Low outpatient medication adherence to antihypertensive drugs was found. There was a correlation between adherence and the control variables of BP, BMI and serum urea values.

108904

Modality: E-Poster Scientific Initiation – Non-case Report

Category: ACUTE AND CHRONIC CORONARY DISEASE/THROMBOLYSIS

## Negative Correlation of Coronary Artery Disease in Patients with Chagas Disease: A Case-Control Study

FRANCISCO JEAN DE MOURA SANTOS FILHO^1^, Carlos Eduardo Batista de Lima^1^, André Gonçalves Honório Carvalho^1^, Maria Clara Lima Almeida^2^, Paulo Márcio de Sousa Nunes^1^, Antônio Maycon da Silva Sousa^1^, Patryck Araújo Dantas^1^, Mauro Guimarães Albuquerque^1^, Ginivaldo Victor Ribeiro do Nascimento^1^, Francisco César de Oliveira Gonçalves^1^, Arthur Oliveira Nogueira e Lago^1^, Jardel Silva Santos^3^

(1) Hospital Universitário da Universidade Federal do Piauí (HU-UFPI) – Empresa Brasileira de Serviços Hospitalares (EBSERH); (2) Instituto do Coração do Hospital das Clínicas da Universidade de São Paulo – InCor HCFMUSP; (3) Universidade Estadual do Piauí (UESPI) – Hospital Getúlio Vargas (HGV)

**Introduction:** Chagas cardiomyopathy is a myocarditis that can progress to dilated non-ischemic cardiomyopathy, heart failure, ventricular arrhythmias and thromboembolic complications. During the chronic phase, an important proportion of patients complain of atypical chest pain. Some studies showed a lower occurrence of chronic coronary disease in Chagas disease, but this fact is not clarified.

**Objective:** Comparative analysis of structural cardiac alterations and the presence of severe obstructive coronary artery disease in patients with and without Chagas disease.

**Methods:** A paired case-control study for atherosclerosis risk factors based on the Framingham risk score (FRS). From November 2014 to November 2019, all patients undergoing coronary angiography at the University Hospital of the Federal University of Piauí, in an elective basis for investigation of coronary disease, were evaluated and chagasic patients were selected, confirmed with two positive serological tests by different methods. The control group was selected consecutively in a 1:3 ratio. For statistical analysis, the chi-square test was used, considering the value of p < 0.05 for statistical significance.

**Results:** Among 1120 patients evaluated, 15 patients with Chagas disease were identified and 45 patients without a diagnosis of Chagas disease were selected. The FRS variables, including age, sex, arterial hypertension, diabetes, physical inactivity, smoking, dyslipidemia and ejection fraction showed no statistical difference between the groups. The occurrence rate of CAD was lower in chagasic patients: 13.3% versus 64.4%, and for severe CAD the percentages were 13.3% and 51.1% (p = 0.003). The presence of structural cardiac alterations was high in both groups, being 80% of the chagasic and 73.3% of the non-chagasic, with no statistical difference (p = 0.606).

**Conclusion:** In this series, the presence of segmental structural alterations of the left ventricle was high in both groups, while in the evaluation of coronary artery disease there was a lower occurrence in the group of patients with Chagas disease.

108910

Modality: E-Poster Scientific Initiation – Non-case Report

Category: CARDIOVASCULAR IMAGING

## Correlation between Remote Myocardial T1 Mapping with Morphological and Functional Parameters in Patients with Chronic Infarction

GABRIELA DE OLIVEIRA SALAZAR^1^, Cláudia Bispo Martins-Santos^1^, José Icaro Nunes Cruz^1^, Lara Teles Alencar Duarte^1^, Nathalia Luiza Silva Sobral^1^, Juliana Maria Chianca Lira^1^, Mayara Evelyn Gomes Lopes^1^, Raisan Almeida Santos^2^, Enaldo Vieira de Melo^1^, Antônio Carlos Sobral Sousa^2^, Joselina Luzia Menezes Oliveira^2^, Luiz Flávio Galvão Gonçalves^2^

(1) Federal University of Sergipe; (2) Rede D’Or São Lucas Hospital

**Introduction:** Risk stratification in patients with chronic infarction is based on left ventricular (LV) morphological and functional parameters, such as left ventricular ejection fraction (LVEF), left ventricular volumes, extent of delayed gadolinium enhancement, among others risk scores. However, native T1 and extracellular volume (ECV), tools for the assessment of interstitial fibrosis, when measured in the myocardium distant from the infarcted territory, present an association of poor prognosis in patients with chronic infarction.

**Objective:** To evaluate the correlation between native T1 and ECV with LV morphological and functional parameters in patients with chronic infarction.

**Methods:** Observational, cross-sectional, analytical study. Patients who underwent cardiac magnetic resonance imaging using a Philips Ingenia 1.5T magnet in 2021 in Sergipe (Brazil) were included. Patients with delayed transmural and/or subendocardial enhancement compatible with myocardial infarction were selected and those with acute infarction or other diagnoses were excluded. T1 measurements were performed in myocardium without delayed enhancement using specific software, and ECV was calculated using native and post-contrast T1 of the myocardium and cavity, in addition to hematocrit. The analysis included the execution of the Shapiro-Wilk tests, Spearman correlation and Mann Whitney U test. The significance level was set at 5%.

**Results:** Of the 75 patients with delayed subendocardial or transmural enhancement, 56 (74.7%) had chronic infarction. The mean age was 68.14 ± 10.24 years. The mean value of native T1 was 1044 ± 43 ms and ECV was 29.8% ± 4.7. There was a negative correlation between T1 and LVEF (ρ-0.374; p = 0.027). The group with increased T1, considering local reference value (>1030 ms), had a higher LV systolic diameter (Md 47.2 vs. 40.2; p = 0.049; TDE-LC 69.4%) and lower LVEF (Md 43 vs. 58; p = 0.038; TDE-LC 70.4%). The group with increased ECV (ECV > 28%) had higher LV mass (Md 96 vs. 120; p = 0.019; TDE-LC 78.4%).

**Conclusion:** The value of native T1 showed an inverse correlation with LVEF. The group with increased T1 had higher LV systolic diameters and lower LVEF. The group with increased ECV had higher LV mass. These findings suggest that the increase in T1 and ECV may be associated with LV remodeling, resulting in diffuse interstitial fibrosis, which may impact the long-term prognosis.

108914

Modality: E-Poster Scientific Initiation – Non-case Report

Category: HYPERTENSION/RENAL DENERVATION

## Inflammatory Biomarkers and Obesity in Resistant Hypertension

GABRIELA DA SILVA NASCIMENTO^1^, Fernanda Oliveira de Carvalho Carlos^1^, Carlos Filipe dos Santos Pimenta^1^, Victor Margallo^1^, João Gabriell Bezerra da Silva^1^, Bianca Zattar de Mello Barreto^1^, Carolina de Carvalho Fortes^1^, Marcus Vinicius Serejo Borges Vale da Silva^1^, Sofia Luz Coutinho Botelho Lobo^1^, Arthur Fernandes Cortez^1^, Bernardo Chedier^1^, Elizabeth Silaid Muxfeldt^1^

(1) UFRJ, Universidade Federal do Rio de Janeiro – Faculdade de Medicina, Hospital Universitário Clementino Fraga Filho – ProHart

**Inflammatory:** Biomarkers and Obesity in Resistant Hypertension Fundamental: In the last few years, obesity has acquired pandemic features, being an important public health’s problem and being strongly related to cardiovascular diseases, beyond of being responsible for the difficulty of blood pressure and metabolic control of patients with resistant hypertension (RHT) apparently due to an inflammatory process that underlines this unfavorable context.

**Objective:** To evaluate the relationship between inflammatory biomarkers and obesity in a large cohort of patients with RHT.

**Methods:** This cross-sectional study evaluated 423 subjects with RHT (30.5% male; 63.9 ± 10.8 years old), 215 (50,8%) of whom are obese. In all subjects the inflammatory biomarkers, TNF-α, MCP-1, E-Selectin and PAI-1, were dosed. Socio-demographic characteristics, anthropometric measurements and cardiovascular risk factors were recorded. Variance analysis compared the serum levels of 4 biomarkers inflammatory and the bivariate analysis compared resistant hypertensives with and without obesity.

**Results:** Obese subjects are younger, with a higher prevalence of peripheral obstructive arterial disease. No difference was found concerning to the blood pressure, neither to subclinical lesions. The values of PAI-1 (123 [107–164] vs 113 [89–138] and E-Selectin (53.2 [34.2–68.6] vs 44.6 [20.8–62.0] were significantly higher in patients with obesity. The other biomarkers evaluated did not evidence association with obesity.

**Conclusion:** Among the inflammatory biomarkers evaluated, which was most strongly correlated with obesity was PAI-1 and E-Selectin. According to literature PAI-1 is tightly linked to metabolic disorders, such as insulin resistance and central obesity, while E-Selectin has an important role on atherosclerosis’s development.

108916

Modality: E-Poster Scientific Initiation – Non-case Report

Category: HYPERTENSION/RENAL DENERVATION

## Effect of Long-Term use of Continuous Positive Airway Pressure Treatment on Arterial Stiffness in Patients with Obstructive Sleep Apnea and Resistant Hypertension

CAMILA BELLO NEMER^1^, Bianca Zattar de Mello Barreto^1^, Carolina de Carvalho Fortes^1^, Marcus Vinicius Serejo Borges Vale da Silva^1^, Sofia Luz Coutinho Botelho Lobo^1^, Aline de Hollanda Cavalcanti^1^, João Carlos Moreno de Azevedo^1^, Carlos Filipe dos Santos Pimenta^1^, Taissa Lorena dos Santos^1^, Bernardo Chedier^1^, Elizabeth Silaid Muxfeldt^1^, Christian Nejm Roderjan^1^

(1) Universidade Federal do Rio de Janeiro – Hospital Universitário Clementino Fraga Filho – ProHArt

**Objective:** To evaluate the impact of a long-term use of CPAP on arterial stiffness (AS) measured by pulse wave velocity (PWV) in patients with resistant hypertension (RHT) and moderate-severe obstructive sleep apnea (OSA).

**Design and Methods:** An observational prospective study was performed in 121 patients (38% male, mean age 60.7 ± 7.8 years) with RHT and moderate/severe OSA (AHI > 15/hour) divided in 2 groups using CPAP (CPAP group – n = 62) and not using CPAP (control group – n = 59) and followed for at least 12 months. Pulse wave velocityand ABPM were measured before and after follow-up. Primary outcomes were changes in aortic stiffness. Intergroup comparisons of differences in PWV were assessed by a general linear model with allocation group (CPAP or control) as a fixed factor and adjusted for their respective baseline PWV values in addition to gender, age, and 24-hour SBP values. Per-protocol analysis was performed excluding patients with poor CPAP adherence.

**Results:** Patients were followed-up for a median of 68 [49–81] months. They used a median of 5 [3–8] anti-hypertensive drugs and had mean PWV of 8.41 ± 1.52 m/s. CPAP and control groups were similar in their baseline demographic, anthropometric, office and ambulatory BP, PWV, and laboratory characteristics. Control group had a mean increase in PWV of 0.88 m/s (95% CI: 0.52–1.25 m/s, p < 0.001), whereas CPAP group had an average increase in PWV of 1.20 m/s (95% CI: 0.84–1.55 m/s; p < 0.001). After adjustment for initial PWV, gender, age and 24-systolic BP, the mean difference between CPAP and control groups was +0.32 (95%CI: 0.17–0.83; p = 0.20). In a subgroup analysis with patients with higher PWV (>10 m/s) the mean difference adjusted was –0.20 (95%CI: –2.17–1.75, p = 0.83).

**Conclusions:** Long-term treatment with CPAP did not reduce aortic stiffness in RHT with moderate/severe OSA, but it may prevent its worsening in relation to control group in those with baseline increased arterial stiffness.

108917

Modality: E-Poster Scientific Initiation – Non-case Report

Category: PERIOPERATIVE EVALUATION

## Incidence, Mortality and Hospitalization Time of Postoperative Atrial Fibrillation in Cardiac Surgery

RAFAEL FORTES LOCATELI^1^, Vitória Carolina Kohlrausch^1^, Eduardo Porto Santos^1^, Isabella Klafke Brixner^1^, Anna Carolina Flores Mariath^2^, Gabrielle Lenz de Abreu^2^, Aníbal Pereira Abelin^1^, Diego Chemello^1^, Mateus Diniz Marques^1^

(1) Universidade Federal de Santa Maria – UFSM; (2) Hospital Universitário de Santa Maria – HUSM

**Introduction:** Arrhythmias are one of the most frequent cardiac complications in the cardiac surgeries postoperative. Atrial fibrillation (AF) is the most common arrhythmia with 20 to 50% incidence. Most cases occur in the first five postoperative days, with a maximum incidence on the 2nd day. The consequences of AF are increased hospital stay, risk of recurrence and increased mortality.

**Objective:** This study aims to compare mortality and hospital stay among patients who presented AF and those who did not have AF in the postoperative period of cardiac surgery at the University Hospital of Santa Maria from January 2011 to April 2021.

**Methods:** This is a longitudinal, descriptive and retrospective study reviewing physical and electronic medical records of patients undergoing cardiac surgery at public tertiary hospital at Santa Maria, Brazil. Exclusion criteria are age younger than 18 years or a procedure involving only the pericardium or pacemaker implantation. The variables analyzed for this study were the occurrence of AF, period of occurrence and death during hospitalization.

**Results:** From a database of 856 patients, 30,96% patients were diagnosed with AF in the postoperative period (n = 265). The mean number of days for the occurrence was 3,35 and the day with the highest incidence was the 2nd. The total number of deaths in the study was 82 (9,57%). Among those who had AF, the mortality was 7,54% (n = 20) and the odds ratio was 0,6965 (CI 95% 0,41–1,17; p = 0,08). The mean hospitalization days for those who had AF was 14 ± 13 days, and 10 ± 9 days for those without AF. Those without AF had lower hospitalization days (–3.57 days, CI 95% – 5.13 – –2; p < 0.0001).

**Conclusions:** This analysis highlights the reality of a tertiary hospital in southern Brazil. AF occurrence was not associated with overall mortality during hospitalization despite a longer hospital stay. Data continues to be collected and future analyzes will show more accurate results.

108919

Modality: E-Poster Scientific Initiation – Non-case Report

Category: PERIOPERATIVE EVALUATION

## Incidence and Mortality of Stroke in the Postoperative Period of Cardiac Surgery in a Brazilian University Hospital

VITÓRIA CAROLINA KOHLRAUSCH^1^, Vitória Carolina Kohlrausch^1^, Rafael Fortes Locateli^1^, Eduardo Porto Santos^1^, Anna Carolina Flores Mariath^2^, Gabrielle Lenz de Abreu^2^, Anibal Pereira Abelin^1^, Diego Chemello^1^, Mateus Diniz Marques^1^

(1) Universidade Federal de Santa Maria – UFSM; (2) Hospital Universitário de Santa Maria – HUSM

**Introduction:** Stroke in the cardiac surgery postoperative is an important neurologic complication. Postoperative stroke is defined as occurring up to 30 days after the procedure, with 56% of them occurring within the first 24 hours. The incidence usually varies between 1.3 to 5% of the procedures and the mortality is from 13 to 41%.

**Objective:** The purpose of this study is to describe the incidence and mortality of stroke in the postoperative period of cardiac surgery at the University Hospital of Santa Maria from January 2011 to April 2021.

**Methods:** The physical and electronic medical records of patients undergoing cardiac procedures at the HUSM were reviewed, between January 2011 and April 2021. This is a retrospective and longitudinal study. and pericardium or only pacemaker implantation procedures were not included. A total of 857 medical records were reviewed and the variables of stroke presentation in the postoperative period, time in days of stroke after surgery and death were evaluated. Data were analyzed using EpiInfo software (version 7.2.5.0.) and comparisons were made based on articles indexed in the PubMed platform.

**Results:** Of the 857 patients included in the study, 3.73% had a stroke in the postoperative period (n = 32; SD = 2.66%–5.22%) – with 46.88% occurred in the first 24 hours after surgery (n = 15; SD = 29.09%–65.26%). The overall mortality was 9.57% (n = 82; SD = 7.78%–11.72%), and higher in the group with perioperative stroke (25%;n = 8; SD = 11.46%–43.40%) than in the group that did not develop this complication (8.97%; n = 74; SD = 7.21–11.11%), with OR = 3.3829, x2 = 9.13, p < 0,005.

**Conclusions:** Despite the low income and economical barriers faced by public hospitals in Brazil we showed that a tertiary public hospital in southern Brazil has similar results than general hospitals around the world regarding neurologic complications in the cardiac surgery postoperative. The database continues to be filled and new studies are being written, analyzing variables such as sex, age and previous diseases.

108926

Modality: E-Poster Scientific Initiation – Non-case Report

Category: HYPERTENSION/RENAL DENERVATION

## Cardiovascular Risk and Subclinical Target Organ Damage in a Young Adult Population Provided by a Primary Healthcare Unit

KARINE DA SILVA GUIMARAES^1^, Gabriela Gama Zagni Jardim^1^, Ana Rachel Bucar Cervasio^1^, Clara Avelar Mendes de Vasconcellos^1^, Tomás de Souza Mello^1^, Carlos Augusto Parente Macedo Moura^1^, Mariana Stutz Klen^1^, Heitor de Oliveira Moraes Rêgo Bandeira^1^, Diego Alves Calvão^1^, Ramon Narde Simão^1^, Ana Cristina Tenório da Costa Fernandes^1^, Elizabeth Silaid Muxfeldt^1^

(1) IDOMED – Universidade Estácio de Sá, Medicine School, Campus Vista Carioca, Rio de Janeiro – RJ

**Background:** In primary care it is necessary to identify early cardiovascular risk factors and target organ damage with low complexity procedures. The most available with low cost are Ankle-Brachial Index (ABI), left ventricular hypertrophy by voltage index in ECG and aortic stiffness by pulse pressure (PP) calculation.

**Objective:** To evaluate cardiovascular risk factors and subclinical target organ damage in a young and apparently healthy population assisted in a Primary Healthcare unit.

**Design and Methods:** A cross-sectional population study for cardiovascular risk assessment in adults aged 20–50 years old provided by a Primary Healthcare Unit. A total of 632 individuals were evaluated (40% male; mean age 36 ± 9 years). Sociodemographic, anthropometric data, and traditional cardiovascular risk factors were recorded. All underwent office Blood Pressure (BP) and pulse pressure (PP) (systolic BP minus diastolic BP) was calculated. Ankle-brachial index (ABI) was also calculated after BP measurements in the 4 limbs. The median of the ABI was 1.14 [1.08–1.22], which is the cutoff point used to define early changes. All participants were submitted to ECG to calculate Sokolow-Lyon Index (SLI) and Cornell Voltage Index (CVI) for left ventricular hypertrophy diagnosis. We considered the median of the SLI (20 mm) and the CVI (11 mm) as the cutoff point for early alterations.

**Results:** The prevalence of hypertension was 16%. The median [IQR] office PP was 46 [39–52] mmHg. Elevated Office PP (> 60 mmHg) were identified in 64 participants. High office PP was more frequent in men, obese, with increased neck circumference and lower ABI (1.07 vs 1.16, p < 0.001). It was also associated with higher voltage index: SLI 21.9 vs 19.8, p = 0.04 and CVI 13.4 vs 7.1, p = 0.03. Individuals with decreased ABI are more obese with smaller neck circumference (10 vs 5%, p = 0.03). They also had higher systolic BP (125 vs 119 mmHg, p < 0.001) and PP (49 vs 43 mmHg, p < 0.001). A total of 362 ECGs were performed. Increased SLI was more frequent in men, younger and overweight, in addition to a higher prevalence of hypertension and higher PP. Those with increased CVI are more often male, obese with higher BP levels.

**Conclusion:** In this young population, early changes in subclinical target organ damage already identify a higher cardiovascular risk profile, indicating the importance of implementing primary prevention measures to reduce this risk.

109374

Modality: E-Poster Scientific Initiation – Non-case Report

Category: CARDIOLOGY OF SPORTS, EXERCISE, ERGOMETRY AND CARDIOVASCULAR REHABILITATION

## Effects of High Intensity Interval Training and Moderate Intensity Continuous Training on the Right Ventricle in Obese Women

BRENDA BARETA SIRONI^1^, Wendell Arthur Lopes^1^, Caroline Ferraz Simões^1^, João Carlos Locatelli^1^, Higor Barbosa Reck^1^, Victor Hugo de Souza Mendes^1^, Gustavo Henrique de Oliveira^1^, Rogério Toshiro Passos Okawa^1^

(1) Universidade Estadual de Maringá

**Introduction:** Obesity appears to affect the right ventricle, considered the forgotten chamber in cardiac evaluation. The TAPSE (tricuspid annular plane systolic excursion) have been used for this evaluation, and more recently, new methods of evaluating the right ventricle function have been developed, in which the right free wall Longitudinal Strain, and the FAC (fractional area change), are highlighted. Studies have shown that aerobic training seems to have some effects on the right ventricular structure and function, however little is known about the impact of different types of training on it.

**Objective:** To investigate the effects of 8 weeks of HIIT (high intensity interval training) and MICT (moderate intensity continuous training) on right ventricle by three different methods (TAPSE, FAC and right strain) in obese women.

**Methods:** The sample consisted of 44 women with obesity grade I or II (age: 28.4 ± 4.8 years; BMI: 35.6 ± 3.1 kg/m^2^), who were randomly allocated to the HIIT group (n = 22) and MICT (n = 22). The training protocols were adjusted so that both promoted similar energy expenditure. Echocardiographic measurements of left and right ventricular function and morphology were evaluated using the Vivid T8 GE ultrasound system. The Vo2 max, was measured during a cardiopulmonary stress testing, with the Cortex device. Body composition was measured using a tetrapolar bioimpedance device. Data were analyzed using the Statistical Package for the Social Sciences (SPSS, IBM®). Differences between the HIIT and MICT groups and moments before and after 8 weeks of training were explored by the ANOVA test for repeated measures.

**Results:** Twenty-five obese women completed 8 weeks of HIIT (n = 11) or MICT (n = 14). Both HIIT and MICT training significantly increased the TAPSE (p = < 0.006; <0.026), FAC (p = 0.002; <0.001) and right free wall GLS (p = <0.003; <0.001). Only HIIT reduced body mass (p = 0.002), body mass index (p = 0.002), body fat percentage (BF% p = <0.001), and significantly increased VO2peak (p = 0.024.).

**Conclusion:** The results obtained with 8 weeks of training on the right ventricle function showed no difference between the two exercise training interventions, both with a positive impact in the right ventricle function parameters. Overall, aerobic training can be considered as a potential intervention for substantial improvements in the right ventricle function of young women with grade I and II obesity, however, HIIT may have additional benefits.

108978

Modality: E-Poster Scientific Initiation – Non-case Report

Category: HYPERTENSION/RENAL DENERVATION

## Impact of Long-Term use of CPAP Treatment on Blood Pressure Among Refractory Hypertensive Patients with Obstructive Sleep Apnea

CAROLINA DE CARVALHO FORTES^1^, Carolina de Carvalho Fortes^1^, Taissa Lorena Dos Santos^1^, Camila Bello Nemer^1^, Sofia Luz Coutinho Botelho Lobo^1^, Gabriela da Silva Nascimento^1^, Hugo Farah Affonso Alves^1^, Lucca Hiroshi de Sá Kimura^1^, Aline de Hollanda Cavalcanti^1^, João Carlos Moreno de Azevedo^1^, Bernardo Chedier^1^, Elizabeth Silaid Muxfeldt^1^

(1) Universidade Federal Do Rio de Janeiro – Hospital Universitário Clementino Fraga Filho – ProHArt

**Objective:** To assess blood pressure response to long-term continuous positive airway pressure (CPAP) treatment among patients with refractory hypertension (RfHTN) with moderate and severe obstructive sleep apnea (OSA).

**Design and Methods:** An observational prospective study was performed in 26 patients (35.7% male, average age 59.3 ± 7.9 years) with diagnostic of RfHTN and moderate and severe OSA who were divided in 2 groups to use CPAP (CPAP group, n = 13) or not (control group, n = 15). All of them were submitted to clinical follow-up and their anti-hypertensive medication was adjusted according to the assistant physician. 24-hour ABPM was performed both at the beginning and at the end of the study. Primary outcomes were changes in office and ambulatory blood pressure (BP) and BP control. The comparison between groups of BP changes were calculated by a general linear model with group allocation as a fixed factor and adjusted by its respective BP basal values in addition to gender and age.

**Results:** The median follow-up time was 68 months (IQR: 49–81 months). CPAP and control groups were similar in their baseline demographic, anthropometric, laboratory characteristics, and office and ambulatory BP. They had mean systolic and diastolic 24-hour BP of 142 (15) and 83 (14), respectively. At the end of follow-up, refractory patients using CPAP had a reduction of 8.5 and 5.0 mmHg of office systolic and diastolic BP with an intergroup difference (CPAP and control) of 13.5 (–15.2–42.3) mmHg and 10.1 (–7.5–5.4) mmHg, respectively. The most important difference between groups was noticed at nighttime with an intergroup difference of 10.0 (–8.6–28.7) mmHg of systolic BP and 6.7 (–3.9–17.3) mmHg of nighttime diastolic BP. Out of 28 initial refractory patients, 12 are not refractory anymore, being 3 (20%) in control group and 9 (69.2%) in CPAP group with a significant difference between the groups (p = 0.02)

**Conclusion:** Long-term use of CPAP among refractory hypertensive patients with moderate/severe OSA seems to be effective to reduce and control blood pressure measured both in the office BP and 24-hour ABPM, especially lowering nighttime blood pressure.

108960

Modality: E-Poster Scientific Initiation – Non-case Report

Category: ATHEROSCLEROSIS/CARDIOVASCULAR RISK FACTORS/CARDIOVASCULAR PREVENTION

## Cardiovascular Risk Factors, Lifestyle and Social Determinants in a Cross-Sectional Population Study- Laparc Study

GABRIELA GAMA ZAGNI JARDIM^1^, Ana Rachel Bucar Cervasio^1^, Clara Avelar Mendes de Vasconcellos^1^, Tomás de Souza Mello^1^, Karine da Silva Guimarães^1^, Giovanna Francesca Ferreira Maselli^1^, Michelle Felipe Falcão^1^, Ivan da Costa Velho Junior^1^, Raphaella Ferrão^1^, Inah Maria Drummond Pecly^1^, Rafael Bica^1^, Elizabeth Silaid Muxfeldt^1^

(1) IDOMED – Universidade Estácio de Sá, Medicine School, Campus Vista Carioca, Rio de Janeiro, RJ, Brasil.

**Objective:** To evaluate the relationship between the main CV risk factors and socioeconomic indicators in a population of adults registered in a Family Health Care (FHC) unit in the center of Rio de Janeiro.

**Design and Methods:** Cross-sectional population study that included adults aged between 20 and 50 years living in the area covered by the FHC in Rio de Janeiro. Demographic data (gender and age), socioeconomic data (education level, profession, employment), CV risk factors (smoking, sedentary lifestyle, obesity, hypertension, diabetes, dyslipidemia) were recorded. The metabolic profile is evaluated through laboratory tests. Those who studied up to high school were considered poorly educated.

**Results:** 604 individuals were enrolled [39% male, mean age: 38.8 ± 8,9 years] The median of schooling was 12 years. 288 individuals had high schooling, 44.5% were male. A total of 130 individuals did not study or work. Women with low education had a higher risk of smoking, obesity and hypertension with no difference regarding labor or study activities. Otherwise, men with low education had higher risk of sedentary lifestyle and hypertension. Among men, not working or studying increased the risk of smoking and hypertension.

**Conclusion:** We found an inverse association between socioeconomic conditions and the prevalence of CV risk factors. Women are more affected by low schooling, while men are more affected by their working occupation. The study suggests that socioeconomic factors influence the CV risk, affecting men and women differently, pointing to the need for public policies to reverse this situation.

109353

Modality: E-Poster Scientific Initiation – Non-case Report

Category: CARDIOLOGY OF SPORTS, EXERCISE, ERGOMETRY AND CARDIOVASCULAR REHABILITATION

## Dizziness Complaints and Cardiorespiratory Fitness Post Severe Forms of COVID-19 Among the Middle-Aged Population

GLÓRIA DE MORAES MARCHIORI^1^, Glória de Moraes Marchiori^1^, Bráulio Henrique Magnani Branco^1^, Mauricio Medeiros Lemos^1^, Daiane Soares de Almeida Ciquinato^1^, Elen Cristina Bressiano^1^, Luciana Lozza de Moraes Marchiori^1^

(1) Universidade UniCesumar UniCesumar

**Introduction:** Dizziness is a common nonspecific sensation of disorientation or impairment in spatial perception and stability. The pathophysiology of post COVID-19 rotatory dizziness is probably similar to that of other viral infections, with some of its manifestations such as hypercoagulability and microthrombus formation, causing significant circulatory disorders possibly affecting its pathogenesis. Cardiorespiratory fitness is one of the significant variables to monitor health, and the gold standard used has been maximal or peak consumption of oxygen (VO2max or VO2peak) through a direct analysis of gas exchange. Thus, VO2max demonstrates the maximum capacity of an individual to absorb, transport, and consume oxygen fitness may be used to identify those at the most significant risk for severe COVID-19 illness. Association between dizziness complaints and cardiorespiratory fitness has not been investigated in individuals with severe forms of COVID-19.

**Objective:** To verify whether there is a correlation between dizziness complaints and cardiorespiratory fitness among people with severe forms of COVID-19.

**Methods:** This study presents a cross-sectional design part of a broader research named the “Post COVID-19 project.” The human research ethics committee approved the project of the institution, with a sample of people post COVID-19 who responded to the Visual-Analog Scale (VAS) for dizziness. To evaluate cardiorespiratory fitness, it used the clinical assessment and Bruce test to measure oxygen consumption directly (via gas analyzer).

**Results:** Of the 133 participants, with a mean age of 49.8 ± 12.2 years old. The prevalence of self-reported dizziness was 51.1% (n = 68); of these, 9.8% (n = 13) were related to dizziness prior to the diagnosis of COVID-19 and 41.4% (n = 55) were related to dizziness during or after COVID-19. The subgroup analysis for age groups, a difference was found in the middle-age (45–64 years) group for the absolute VO2 peak (p = 0.027).

**Conclusion:** There was a statistically significant difference between dizziness complaints and the VO2 peak among the middle-aged population, with the dizziness group having a lower absolute VO2 peak than the non-dizziness group.

109001

Modality: E-Poster Scientific Initiation – Non-case Report

Category: ATHEROSCLEROSIS/CARDIOVASCULAR RISK FACTORS/CARDIOVASCULAR PREVENTION

## Comparative Study of Pulse Wave Analysis and Other Hemodynamic Parameters in Medical Students: Initial Data

SARA CRISTINE MARQUES DOS SANTOS^1^, Luan Tardem Veloso Teixeira^1^, Ivan Lucas Picone Borges dos Anjos^1^, Thaís Lemos de Souza Macedo^1^, João Pedro de Resende Côrtes^1^, Paula Pitta de Resende Côrtes^1^, João Carlos de Souza Côrtes Júnior^1^, Eduardo Tavares de Lima Trajano^1^, Carlos Eduardo Cardoso^1^, Ivana Picone Borges de Aragão^1^

(1) Universidade de Vassouras

**Introduction:** Pulse wave velocity is currently (PWV) considered a gold standard parameter in the assessment of arterial stiffness, and may be associated with cardiovascular risk in several groups. Stiffness is characterized by the decrease in arterial distensibility, being present in aging, diabetes, atherosclerosis and chronic renal disease1. The objective of the present study was to perform the analysis of central and peripheral hemodynamic values in men and women aged 30 years old or less.

**Methodology:** Observational and cross-sectional study, carried out from May to July 2021 in students aged up to 30 years (in accordance with the first group for pulse wave velocity of the current brazilian hypertension guideline). 49 participants were selected, 32 female (F) and 17 male (M). It was used an anonymous questionnaire and the values provided by the Arteris device through the oscillometric method: PWV; AIX@75; heart rate (HR); peripheral systolic blood pressure (SBP) and central (CSBP); and peripheral diastolic (DBP) and central (CDBP); centre pulse pressure (CPP), cardiac output (CO); vascular age (VA); peripheral vascular resistance (SVR) and cardiac index (CI). For the general group (G) mean, maximum and minimum values were calculated; while for M and F, only the mean was calculated.

**Results:** Mean age G was 23 years (29 ± 20), M 22 and F 23; PWV G of 4.65 m/s (5.4 ± 3.1), M 4.9 and F 4.5; AIX@75 G of 22.6% (41.7 ± 5.3), M 18.4% and F 25%; SBP G of 111 mmHg (137 ± 88), M 120 and F 107; DBP G 74.5 mmHg (96 ± 56), M 77 and F 73; CSPB G 98 mmHg (118 ± 80), M 103 and F 95; CDPB G 75.5 mmHg (98 ± 54), M 79 and F 73.5; HR G 88 bpm (128 ± 60), M 83 and F 90; CPP G 22 mmHg (36 ± 13), M 24.5 and F 20.5; VA G of 22.6 years (32 ± 18), M 25 and F 21; CO G 3.9 l/min (4.6 ± 2.9), M 4.1 and F 3.81; SVR G 1.30 mmHg/ml (1.58 ± 0.97), M 1.29 and F 1.30; CI G 3.9 l/min/m^2^ (4.6 ± 2.9), M 4.1 and F 3.8.

**Conclusion:** Men presented higher values of PWV, SBP, DBP, CSPB, CDPB, CPP, VA, CO and CI both in relation to the female group and to the general group. Meanwhile, women obtained higher values in SVR, HR and AIX@75. It is worth noting that the values of CSPB and PWV found in the study participants are within the normal range for the population without cardiovascular risk factor (established in the 2020 hypertension guideline of the Brazilian Society of Cardiology) while they are borderline in the European index of CSPB and adequate for PWV.

109005

Modality: E-Poster Scientific Initiation – Non-case Report

Category: HYPERTENSION/RENAL DENERVATION

## Pulse Wave Analysis in a Group of Individuals with and Without Family History of Arterial Hypertension: Preliminary Data

SARA CRISTINE MARQUES DOS SANTOS^1^, Luan Tardem Veloso Teixeira^1^, João Pedro de Resende Côrtes^1^, Thaís Lemos de Souza Macedo^1^, Ivan Lucas Picone Borges dos Anjos^1^, Paula Pitta de Resende Côrtes^1^, João Carlos de Souza Côrtes Júnior^1^, Eduardo Tavares de Lima Trajano^1^, Carlos Eduardo Cardoso^1^, Ivana Picone Borges de Aragão^1^

(1) Universidade de Vassouras

**Introduction:** Family history of hypertension (FHH) is a strong risk factor for the development of high blood pressure (HBP), where individuals of normotensive parents would have a lower occurrence of the stiffening process of the arteries1. It is believed that stiffness also has a genetic influence, as well as HBP and age2. The mechanism of pulse wave velocity (PWV) and HPB is not completely established; there is an increase in PWV with increased blood pressure, but it is still not possible to define which is the cause or consequence3. The objective of the present study was to analyze two groups: with positive family history of HBP (PFH) and the group with negative family history of HBP (NFH) through the answers of questionnaires about self-knowledge, life habits, the values provided by the Arteris device of central hemodynamics and arterial stiffness, such as PWV, AIX@75, central systolic pressure (CSP), central diastolic pressure (CDP), central pulse pressure (CPP) and vascular age (VA).

**Methodology:** Observational, cross-sectional study conducted from May to July 2021 in medical students. Done through an anonymous questionnaire and analysis of the pulse wave by the oscillometric method in the Arteris device. The mean was calculated using Excel. Evaluation of sample normality (Shapiro-Wilk), multiple logistic regression, with confidence level of 95% and calculated odds ratio by GraphPad Prism Software version 9.2.

**Results:** Total of 59 participants, mean age of 25.29 (20 ± 42), where 45 belonged to the PFH group and 14 to the NFH group. When analysing the values obtained by the device, the PFH group obtained averages of: PWV 4.82 m/s (6.6 ± 3.1); AIX@75 22.59% (41.67 ± 5.33); CSP 97.47 mmHg (124 ± 11); CDP 77.13 mmHg (106 ± 58); CPP 36.27 mmHg (61 ± 21) and VA 24.84 (45 ± 18). While in the NFH group, means of: PWV 4.76 m/s (5.4 ± 4.2); AIX@75 18.53% (37.67 ± 8.67); CSP 99.86 mmHg (118 ± 81); CDP 75.14 mmHg (98 ± 54); CPP 37.85 mmHg (52 ± 26) and VA 23.36 (32 ± 18). The lower the PWV, the lower the chance of having PFH (OR: 0.4) and the lower the CPP (OR = 0.7), CDP (OR = 0.9) and PWV (OR = 0.3); the lower the probability of being hypertensive.

**Conclusion:** In the PFH group, the values of PWV, AIX@75, CDP and VA were higher than those found in those NFH. Also, it was possible to show statistically that the PFH can influence the increase in the value of PWV, the reduction of the levels of PWV and other hemodynamic parameters are important for prevention of HBP.

109013

Modality: E-Poster Scientific Initiation – Non-case Report

Category: ATHEROSCLEROSIS/CARDIOVASCULAR RISK FACTORS/CARDIOVASCULAR PREVENTION

## Diagnostic Value of Atherogenic Indices in Atherosclerotic Cardiovascular Diseases

YURI BARBOSA ARAÚJO^1^, Glebson Santos Sobral^1^, Rafael Alexandre Meneguz Moreno^1^

(1) Universidade Federal de Sergipe

**Background:** For years, the search for clinically useful assessment methods with good accuracy, low cost, non-invasiveness and easy management has been stimulated, aiming to improve the prevention and treatment of atherosclerotic diseases.

**Objective:** To verify the diagnostic potential of the following indices in patients with clinical Atherosclerotic Cardiovascular Disease (ASCVD): Castelli I and II indices, Atherogenic Coefficient, Atherogenic Index of Plasma (AIP) and the variation of the perfusion index between 90 and 120 seconds after reactive hyperemia in the evaluated limb (ΔPI90–120).

**Methods:** A case-control study, of whom 161 patients were included so far, of which 44 (27.3%) were part of the ASCVD group, selected by means of a cinecoronariography, exercise stress test, coronary CTA, cranial CT, carotid or lower limb artery Doppler. To estimate the potential of these atherogenic indices, sensitivity, specificity, positive (PV+) and negative (PV–) predictive value, positive (LR+) and negative (LR-) likelihood ratios were estimated through receiver operating characteristic (ROC) curves. Additionally, multivariate logistic regression was performed to estimate the association of these indices with the outcome. Values of p < 0.05 were considered statistically significant.

**Results:** The number of patients with a history of smoking, hypertension, diabetes mellitus and dyslipidemia was higher in the ASCVD group, as well as mean age and some laboratory parameters: HDL-c, triglycerides, HbA1c and creatinine. The ROC analysis showed better performance with AIP and ΔPI90–120. For the former, a cut-off of (> 0.04) achieved an LR+ and LR- of 4.84 and 0.15, respectively (sensitivity 88.0%; specificity 81.8%; PV+ 64.7%; PV– 94.7%). For the latter, a cut-off of (≤ 56.6) achieved an LR+ and LR- of 5.76 and 0.30, respectively (sensitivity 73.6%; specificity 87.2%; PV+ 68.4%; PV– 89.8%). In multivariate analysis adjusted for age and comorbidities, both AIP and ΔPI90–120 were independently associated with ASCVD: respectively, OR 23.6 (95%CI 4.3–130.9; p < 0.001) and OR 17.8 (95%CI 3.3–97.0; p < 0.001).

**Conclusions:** AIP and ΔPI90–120 were independent predictors of ASCVD. In addition, both have better diagnostic performance for ASCVD compared to the other evaluated indices, making them attractive for future trials and possible contribution to the prevention and treatment of atherosclerotic diseases.

109020

Modality: E-Poster Scientific Initiation – Non-case Report

Category: EPIDEMIOLOGY AND HEALTH POLICIES/GLOBAL HEALTH

## Knowledge of Cardiopulmonary Resuscitation Manoeuvre and use of Automatic External Defibrillator by Medical Students

SARA CRISTINE MARQUES DOS SANTOS^1^, Thaís Lemos de Souza Macedo^1^, João Pedro de Resende Côrtes^1^, Ivan Lucas Picone Borges dos Anjos^1^, Eduardo Tavares de Lima Trajano^1^, Carlos Eduardo Cardoso^1^, João Carlos de Souza Côrtes Júnior^1^, Eucir Rabello^1^, Ivana Picone Borges de Aragão^1^

(1) Universidade de Vassouras

**Introduction:** With high mortality rates, the out-of-hospital cardiac arrest (OHCA) is a subject of discussion in the medical field and currently, it is emphasized the inclusion of CPR training for laymen and health professionals, in order to reduce the number of deaths and sequelae in victims. The objective of the present study was to analyze the medical students knowledge regarding the care of victims of CPR and their ability to perform this care.

**Methodology:** This is an observational and cross-sectional study, with quantitative data collection obtained through an anonymous questionnaire, containing questions related to the recognition of a CRA and the CPR maneuver procedures by medical students during the years 2018 to 2021.

**Results:** Of the total 468 students interviewed, 149(31.84%) answered correctly about maximum time limit from which a cardiac arrest can be considered irreversible. Regarding the use of the AED, 222(47.44%) said they knew how to use it, with 158(33.76%) reporting having learned to use it in college. Still on the AED, 265(56.62%) do not know the difference between shockable and non-shockable rhythms, but 307(65.60%) understand that this rhythm will influence in the conduct of care. Regarding the use of AED during CPR, 169(36.11%) answered correctly stating that it should be used as soon as possible. In pediatric CPR, 51(10.90%) could correctly answer regarding the depth of compressions. In the victim’s pulse reassessment, 203(43.38%) did not know the correct periodicity. At the end of the questionnaire, 206(44.02%) considered themselves able to perform a CPR.

**Conclusion:** It was possible to observe from the present study that more than half of the students do not know the ideal time to help the victim of CRA, the time of pulse reassessment during the assistance and about 90% of them, would not do the pediatric compression effectively. Regarding the AED, about 53% do not know, also not knowing the difference of the rhythm found and the need for its immediate use in a CRA. Less than half of the interviewees consider themselves capable of performing CPR. The need to implement courses in hands-on modality is emphasized, where students can realistically train the care and thus, fix it in their professional life, thus bringing benefits in post-CPR survival rates.

109023

Modality: E-Poster Scientific Initiation – Non-case Report

Category: EPIDEMIOLOGY AND HEALTH POLICIES/GLOBAL HEALTH

## Automatic External Defibrillator at Cardiac Arrest: Are Medicine Students Able to Use?

SARA CRISTINE MARQUES DOS SANTOS^1^, Thaís Lemos de Souza Macedo^1^, Ivan Lucas Picone Borges dos Anjos^1^, João Pedro de Resende Côrtes^1^, Eduardo Tavares de Lima Trajano^1^, Carlos Eduardo Cardoso^1^, Eucir Rabello^1^, Esmeralci Ferreira^2^, João Carlos de Souza Côrtes Júnior^1^, Paula Pitta de Resende Côrtes^1^, Ivana Picone Borges de Aragão^1^

(1) Universidade de Vassouras; (2) Universidade do Estado do Rio de Janeiro

**Introduction:** Cardiac arrest (CA) is defined as the sudden interruption of necessary mechanical ventricular activity and breathing. Clinically, pulse, breath, and consciousness are absent. Biologically, there is still a viable brain and other biological functions. (1) In Brazil, approximately 200 thousand individuals are victims per year of CA, 50% of them occur in extra-hospital environments such as homes, shopping centers, stadiums, airports, sports centers, among other spaces (2). In the United States of America, approximately 350,000 people die annually from CA, and, currently, the prevalence of training in cardiopulmonary resuscitation is low (3). Chest compressions associated with the use of an automatic external defibrillator (AED) are essential for survival and reducing post-CA sequelae. The present study aimed to analyze the knowledge of medical students about the use of DEA.

**Methods:** Observational and cross-sectional, quantitative, and qualitative study between 2018 and 2019, through an anonymous survey, distributed after approval by the Research Ethics Council, addressing questions related to the knowledge of medical students about handling an AED during a CA.

**Results:** Of 291 students interviewed, 162 (55.67%) knew how to handle an AED. Of these, 115 (39.52%) learned at college, 45 on courses (15.46%), 3 (1.03%) over the internet, 1 at work (0.34%), and 127 (43.65) did not inform or not applied. A total of 115 (39.52%) knew the difference between shockable and non-shockable rhythms. However, 190 (65.29%) reported knowing that the cardiac rhythm influences the conduct in the CA, 88 (30.24%) did not know and 13 (4.46%) did not inform. In assisting the victim of CA, 92 (31.62%) answered correctly that the AED should be used as soon as possible, 94 (32.3%) said that the use should be made later, 56 (19.24%). When asked about the locations that could identify the presence of the DEA, 92 (31.62%) mentioned shopping centers, 92 (31.62%) in universities and schools, 45 (15.46%) in gyms, 3 (1, 03%) in cinemas and 147 (50.52%) did not know.

**Conclusion:** It was observed that slightly more than half of the students consider themselves capable of using an AED in an emergency and less than half know the difference between heart rhythms. However, they know that it will influence the conduct of the CPR. Less than a third knew the correct moment to use the AED. It shows that is necessary to implement practical workshops on CA management through universities, targ.

109024

Modality: E-Poster Scientific Initiation – Non-case Report

Category: CARDIOVASCULAR SURGERY

## Efficacy of Thyroidal Hormones Supplementation in the Evaluation of Cardiac Surgeries in Pediatric Patients: A Systematic Review

ISRAEL FIGUEIRA LEMOS^1^, Israel Figueira Lemos^1^, Luiz Carlos Figueiredo Filho^1^, Maria Eduarda Dantas da Veiga^1^, Mariana Lassance Maya Palheta^1^, Juliana de Sousa Tavares^1^, Ivan Cuoco Sampaio^1^, Rafael Augusto Silva Cabeça^1^, João Lucas Silva Sales^1^, Luma Maria Favacho Bordalo^1^, Ingrid Jade Muniz Wanderley^1^, Paula Larissa Baía Lima^1^

(1) Universidade do Estado do Pará

**Introduction:** Extracorporeal circulation, especially associated with children undergoing cardiac surgeries, is related to situations of decline in serum levels of thyroid hormones. Studies show that the rates of improvement in prognosis in surgical outcome situation in these patients can be optimized by supplementation of these hormones, due to their importance in vital functions, becoming essential in the improvement in operative outcome.

**Objectives:** To evaluate the effectiveness of thyroid hormonal supplementation in pediatric cardiac surgeries for improvement of the operative prognosis.

**Methods:** Systematic review using the PICO strategy and guiding question: “Does the supplementation of thyroidal hormones influence the recovery from cardiac surgery in pediatric patients?”. Inclusion criteria: randomized clinical trials and articles that focus on the effect of thyroidal hormones supplementation in pediatric patients undergoing cardiac surgeries. Exclusion criteria: articles with methodological approach focused on surgical techniques rather than the supplementation of these hormones. The inclusion of articles followed the filtering protocol of the PRISMA diagram. The articles were selected from PubMed and VHL databases with the temporal delineation from 2000 to 2022. To reduce the risk of bias, Rob 2 software guidelines were used to analyze the articles. Furthermore, the descriptors used were: “thyroid hormones”, “heart surgeries” and “children”.

**Results:** At first, 8 articles were found in the databases by means of the descriptors, of these, 5 fit the selective criteria. In the postoperative period, with the administration of T4, there was no improvement in hemodynamic performance or less use of mechanical ventilation in patients. Moreover, 4 studies reported in common the role of improvement in myocardial function by T3 administration, highlighting: improvement in cardiac output, reduction of systemic vascular resistance and ischemia-reperfusion lesions. In pharmacokinetic terms, doses between 0.4–0.8 μg/kg are safe and effective.

**Conclusion:** Efficacy of thyroid hormone supplementation was evidenced in the prognostic evaluation of surgical pediatric conditions, which allowed for an improvement of the condition. A limitation of this study was the scarcity of studies, that still absent in the literature, for the comparison of the longer surgical evaluation time with the supplementation of thyroidal hormones.

109462

Modality: E-Poster Scientific Initiation – Non-case Report

Category: CARDIOVASCULAR SURGERY

## Difference between Clinical, Surgical and Echocardiographic Profiles of Patients Undergoing Aortic Valve Replacement Procedure for Rapid Implant Prosthesis and Conventional Bioprosthesis

PEDRO AUGUSTO MOREIRA ZAPPA^1^, Sérgio Lima de Almeida^1^, Fernando Graça Aranha^1^, Luis Enrique Portugal^1^, Marli Annes^1^, Giani Osni Alves^1^, Giovanna Grunewald Vietta^2^, Portiuncola Gorini^1^, Adriana Ferraz Mrtins^1^, José Fernando Arruda^1^, Roberta Luiza Salum^1^

(1) Hospital SOS Cárdio; (2) Universidade do Sul de Santa Catarina

**Background:** The high prevalence of patients with aortic stenosis has led to the development of new surgical treatment options, such as rapid implant valves. These can reduce transoperative times, which are crucial for reducing complications for patients benefiting from this type of prosthesis.

**Objective:** To evaluate the difference between the clinical, surgical and echocardiographic profile of patients undergoing aortic valve replacement procedure using rapid implant prosthesis and conventional bioprosthesis.

**Method:** Case-control study, carried out at Hospital SOS-Cárdio in Florianópolis; population of 60 cases and 119 controls, the controls were selected at random. The variables studied were demographic, clinical, surgical and echocardiographic factors, in order to identify differences between the times of aortic clamping and extracorporeal circulation.

**Results:** 73.3% of patients in the case group were at high risk, compared to 39.5% of controls (p < 0.001). Mortality was similar between groups (p > 0.999). The CPB time was on average shorter for the case group (72.35 ± 18.459 minutes), as well as the Clamp time (62.87 ± 16.802) (p < 0.001). The mean transvalvular gradient differed between groups: 7.95 ± 2.74 mmHg for cases, and 13.19 ± 13.142 mmHg for controls (p < 0.001).

**Conclusion:** The study demonstrated a reduction in cardiopulmonary bypass times and aortic clamping in the case group, as well as, this same group showed better hemodynamic performance in the prosthesis implanted in it. There was no difference in mortality between the groups, which demonstrated a better result when using the rapid implant prosthesis, as it presented significantly increased risks.

109032

Modality: E-Poster Scientific Initiation – Non-case Report

Category: SPIRITUALITY AND CARDIOVASCULAR MEDICINE

## Humanized Medicine. What is the Impact on the Quality of the Patient‘s Hospitalization?

SARA CRISTINE MARQUES DOS SANTOS^1^, Ivan Lucas Picone Borges dos Anjos^1^, Lívia Liberata Barbosa Bandeira^1^, Thaís Lemos de Souza Macedo^1^, Eucir Rabello^1^, Ivana Picone Borges de Aragão^1^

(1) Universidade de Vassouras

**Introduction:** Promoting the approach of medical students to patients aims to encourage the implementation of humanization in the doctor-patient relationship1. In a study carried out in 2008, a total of 82.5% of respondents who were under hospitalization, considered themselves stressed3. Because of the stress observed during hospitalization and the benefit that humanization of care can bring to patients, the objective was to bring first-time medical students closer to patients admitted to a teaching hospital with a humanized focus and to evaluate through questionnaires anonymously.

**Methodology:** Prospective, observational, and cross-sectional study, with the opinion of the Research Ethics Council (CEP) No. 1,963,944 and carried out from 2017–2019 under the guidance of teachers to first-time medical students in accompanying hospitalized patients with vision humanized as a person. An anonymous questionnaire of quick responses to patients was administered on the participation of students during hospitalization.

**Results:** A total of 1732 hospitalized patients were interviewed and 1156 (66.7%) considered the participation of students with a humanized focus as excellent; 492 (28.4%) as good; 69 (4%) as regular and only 09 (0.5%) as poor, with 06 (0.3%) not responding. They considered that the student’s participation during hospitalization was positive, bringing more comfort and tranquility to patients, according to the reports of the questionnaires, during hospitalization, seeking to supply problems reported by patients in various areas reported. Of the total group, 835 (48.2%) of them reported that their hospitalization was responsible for the occurrence of disorders in their routine, 351 (42%) being concerned about the disease, 235 (28.1%) reporting communication problems with family members, 232 (27.8%) with work, financial 151 (8.7%), 24 (1.4%) problems with personal hygiene and 48 (2.7%) did not report.

**Conclusion:** Humanization in hospitalized patients stands out as an important tool in the treatment because of the reduction of stress generated by the disease and hospitalization. The present study demonstrated that 95.15% of the patients perceived the student’s participation as positive from the humanized point of view. Humanized treatment should be encouraged by the doctor’s initial training, contributing to stress reduction, optimizing treatment.

109043

Modality: E-Poster Scientific Initiation – Non-case Report

Category: CARDIOLOGY OF SPORTS, EXERCISE, ERGOMETRY AND CARDIOVASCULAR REHABILITATION

## The Increment in Aerobic Capacity is Correlated with Lower Wall Motion Score Index After Exercise in Patients with Chronic Coronary Syndrome

CLÁUDIA BISPO MARTINS SANTOS^1^, Gabriela de Oliveira Salazar^1^, José Icaro Nunes Cruz^1^, Lucas Villar Shan de Carvalho Cardoso^1^, Ana Luísa Lisboa Prado^1^, Giulia Vieira Santos^1^, Letícia Luiza Gomes Marques^1^, Arthur Leite Lessa^1^, Edvaldo Victor Gois Oliveira^1^, Antônio Carlos Sobral Sousa^1^, Enaldo Vieira de Melo^1^, Joselina Luzia Menezes Oliveira^1^

(1) Federal University of Sergipe

**Introduction:** Physical exercise leads to cardiovascular and autonomic adaptations essential for most favorable prognosis of chronic coronary disease (CCD), increasing aerobic capacity expressed by maximal oxygen consumption (VO2MAX).

**Objective:** To analyze the behavior of cardiorespiratory capacity in patients with CCD undergoing Exercise Stress Echocardiography (ESE).

**Methods:** Cross-sectional study between January/2000 and January/2022 of patients with CCD who underwent ESE at a cardiology referral service in Sergipe (Brazil). A total of 2000 patients (61.04 ± 10.17 years) were categorized as sedentary or active according to the definitions of the World Health Organization. Student’s t test or Mann-Whitney test and chi-square test was used. The correlation of VO2MAX with clinical variables was evaluated using Pearson’s or Spearman’s tests. A significance level of 5% was adopted. Analyzes were performed using SPSS Statistics software.

**Results:** A total of 1033 (51.66%) sedentary and 967 (48.34%) active individuals were evaluated. The active patients reached higher VO2MAX (35.41 vs. 30.48 mg/kg.min; p < 0.001) and, the sedentary, higher diastolic pressures after exercise (85.05 vs. 83.99 mmHg; p = 0.003). Sedentary lifestyle was associated with the female sex (45.5% vs. 34.7%; p < 0.001). VO2MAX was negatively correlated with resting systolic blood pressure, wall motion score index after exercise and age – being those relationships stronger in the active group; resting diastolic blood pressure was positively correlated with VO2MAX only in sedentary individuals – Table 1.

**Conclusions:** Practicing physical exercise permitted the increase of aerobic capacity, reducing systolic pressure levels at rest and correlating with lower wall motion score index after exercise.


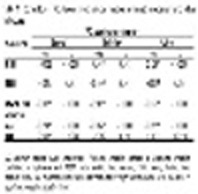


109048

Modality: E-Poster Scientific Initiation – Non-case Report

Category: ACUTE AND CHRONIC CORONARY DISEASE/THROMBOLYSIS

## Myocardial Ischemia and Smoking on Post-Acute Myocardial Infarction Patients Undergoing Exercise Stress Echocardiography

CLÁUDIA BISPO MARTINS-SANTOS^1^, José Icaro Nunes Cruz^1^, Gabriela de Oliveira Salazar^1^, Juliana Maria Chianca Lira^1^, Lucas Villar Shan de Carvalho Cardoso^1^, Mayara Evelyn Gomes Lopes^1^, Marília Marques Aquino^1^, Octavio Morais Veloso^1^, Giulia Vieira Santos^1^, Antônio Carlos Sobral Sousa^1^, Enaldo Vieira de Melo^1^, Joselina Luzia Menezes Oliveira^1^

(1) Federal University of Sergipe

**Introduction:** Despite the clear association between smoking and atherosclerotic diseases, the discussion about the smoker’s paradox persists, given the low reactivity of platelets induced by smoking.

**Objective:** To evaluate the occurrence of smoking-related myocardial ischemia (MI) in patients with previous Acute Myocardial Infarction (AMI) submitted to Exercise Stress Echocardiography (ESE).

**Methods:** Cross-sectional study between January/2000 and January/2022 with individuals presenting a positive history for AMI who underwent ESE at a cardiology referral service in Sergipe (Brazil). A total of 737 patients (61.01 ± 10.78 years) were categorized according to tobacco exposure: smokers, former smokers and non-smokers. Analysis of variance (ANOVA) was used for multiple comparisons with post-hoc Bonferroni and the chi-square test were applied. A significance level of 5% was adopted. Binary logistic regression was performed to identify whether smoking was independently associated with MI. To enter the model, the significance level was p < 0.10 and, to remain, p < 0.05. Analyzes were performed using SPSS Statistics software.

**Results:** Fifty-four (7.33%) smokers, 581 (78.83%) ex-smokers and 102 (13.84%) non-smokers were evaluated, which did not presented differences in the distribution of sex, systemic arterial hypertension, family history and systolic and diastolic blood pressures at rest (p > 0.05). The age was higher on the non-smoker group when compared to smokers (63 vs. 58 years; p = 0.022). Smokers were more likely to be diabetics (p = 0.0037), and ex-smokers, non-diabetics (p = 0.0010). Former smokers were generally non-dyslipidemic (p = 0.0051), while non-smokers were dyslipidemic (p < 0.0001). The chance of having MI was higher among smokers (OR = 2.44; 95%CI 1.14–5.22; p = 0.022) and former smokers (OR = 2.24, 95%CI 1.42–3.53, p = 0.001).

**Conclusions:** When evaluating post-AMI patients, being a former smoker was independently associated with MI. The predictive model also showed that the chance of having MI was even greater among those who continued to smoke.

109050

Modality: E-Poster Scientific Initiation – Non-case Report

Category: ACUTE AND CHRONIC CORONARY DISEASE/THROMBOLYSIS

## Associated Factors on Myocardial Ischemia Without Obstructed Coronary Arteries (INOCA)

CLÁUDIA BISPO MARTINS-SANTOS^1^, José Icaro Nunes Cruz^1^, Gabriela de Oliveira Salazar^1^, Ullany Maria Lima Amorim Coelho de Albuquerque^1^, Myllena Maria Santos Santana^1^, Lucas Villar Shan de Carvalho Cardoso^1^, Marília Marques Aquino^1^, Nathalia Luiza Silva Sobral^1^, Lara Teles Alencar Duarte^1^, Antônio Carlos Sobral Sousa^1^, Enaldo Vieira de Melo^1^, Joselina Luzia Menezes Oliveira^1^

(1) Federal University of Sergipe

**Introduction:** When myocardial ischemia (MI) is documented and there is no obstruction of coronary arteries, the diagnosis of ischemia with no obstructive coronary artery disease (INOCA) is established, which has an adverse prognosis.

**Objective:** To analyze the factors associated with the absence of obstructive coronary artery disease (CAD) in patients with MI.

**Methods:** Cross-sectional study between January/2000 and January/2022 with individuals presenting MI on Exercise Stress Echocardiography carried out at a cardiology referral service in Sergipe (Brazil). A total of 1631 patients (59.80 ± 10.56 years) were analyzed, divided into patients with INOCA – defined by coronary angiography – and with obstructive CAD. Mann-Whitney and chi-square tests were applied. A significance level of 5% was adopted. Analyzes were performed using SPSS Statistics software.

**Results:** 500 (30.66%) individuals had INOCA and 1131 (69.34%) had CAD. Patients with INOCA were more likely to be female (56.6% vs. 35.6%; p < 0.0001). The INOCA group showed a lower tendency to have dyslipidemia (57.0% vs. 70.4%; p < 0.0001) and a positive family history of cardiovascular diseases (54.6% vs. 64.2%; p < 0.0001). There was no association between absence of CAD on MI and the factors: hypertension, diabetes mellitus and obesity (p > 0.05). Patients with INOCA tended to be younger (58 vs. 60 years; p < 0.0001) and the analyses of wall motion score index at rest and after exercise dit not presented significant differences (p > 0.05) between the groups. Systolic blood pressure levels at rest and after exercise did not differ between INOCA and CAD (p > 0.05).

**Conclusions:** Female sex, absence of dyslipidemia and negative family history for cardiovascular events were factors associated with INOCA. There was no difference in the involvement of left ventricular motility, which suggests that ischemia in patients with CAD and INOCA has a similar degree.

109052

Modality: E-Poster Scientific Initiation – Non-case Report

Category: CARDIOVASCULAR IMAGING

## Elevated Levels of Native T1 Myocardial Mapping in Patients Without Late Enhancement

CLÁUDIA BISPO MARTINS-SANTOS^1^, Gabriela de Oliveira Salazar^1^, José Icaro Nunes Cruz^1^, Nathalia Luiza Silva Sobral^1^, Juliana Maria Chianca Lira^1^, Lara Teles Alencar Duarte^1^, Raisan Almeida Santos^2^, Giulia Vieira Santos^1^, Antônio Carlos Sobral Sousa^1^, Enaldo Vieira de Melo^1^, Joselina Luzia Menezes Oliveira^1^, Luiz Flávio Galvão Gonçalves^2^

(1) Federal University of Sergipe; (2) Hospital São Lucas

**Introduction:** Cardiovascular magnetic resonance (CMR), through the myocardial T1 time mapping technique, provides the tissue characterization of myocardial fibrosis, both localized and diffuse, in a quantitative manner, in order to overcome some limitations of the evaluation of late enhancement.

**Objective:** To analyze CMR parameters in relation to the increase in native T1 map levels in patients without delayed enhancement, in order to demonstrate its role as a marker of advanced heart disease.

**Methods:** Cross-sectional study between January and December 2021 with individuals undergoing CMR who did not present late enhancement at a cardiology referral service in Sergipe (Brazil). A total of 131 patients (54.54 ± 16.96 years) were analyzed, divided into two groups according to the increase (≥1030 ms) or not (<1030 ms) in the levels of native T1 acquired by the modified look locker method (MOLLI) in a magnetic field of 1.5 Tesla. Student’s t, Mann-Whitney and chi-square tests were applied. A significance level of 5% was adopted. Analyzes were performed using SPSS Statistics software.

**Results:** 47 (35.88%) individuals had increased levels of native T1 and 84 (64.12%) presented levels below 1030 ms. The group with increased T1 had more females than the group without increased T1 (72.3% vs. 32.1%; p < 0.0001). Patients with increased native T1 levels had lower left ventricular ejection fraction values when compared to those with normal T1 levels (56.32% vs. 62.35%; p = 0.014). The group with high native T1 had even lower values of left ventricular mass (95.34 g vs. 105.41 g; p = 0.027), right ventricular end-diastolic volume (108.89 ml vs. 129.52 ml; p = 0.007) and right ventricular measurements at short axis orientation (3.66 cm vs. 3.99 cm; p = 0.001).

**Conclusions:** Elevated levels of native T1 mapping were associated with female sex, as well with lower left ventricular mass and ejection fraction values in those patients without late enhancement. Therefore, the role of CMR in the assessment of ventricular function is reiterated and its application in patients without late enhancement is demonstrated.

109069

Modality: E-Poster Scientific Initiation – Non-case Report

Category: ACUTE AND CHRONIC CORONARY DISEASE/THROMBOLYSIS

## Epidemiological Study of Morbidity and Mortality from Acute Myocardial Infarction Reported in Brazil between 1999 and 2019

FILIPE ALVES RAMOS ^1^, Augusto Dê Marco Martins^2^

(1) Catholic University of Brasília; (2) Biocárdio Institute

**Introduction:** Acute myocardial infarction is one of the main causes of death in Brazil. According to the Ministry of Health, risk factors such as (1) smoking, (2) dyslipidemia, (3) hypertension, (4) obesity, (5) diabetes, and (6) stress have contributed to an increase in the number of deaths associated with infarction, and thus, generating social and economic impacts on the Brazilian society.

**Objective:** To investigate the number of deaths associated with Acute Myocardial Infarction in Brazil between 1999 and 2019.

**Methods:** This is a cross-sectional epidemiological study carried out between 1999 and 2019 on the number of deaths from acute myocardial infarction reported in Brazil. The analysis of information had been extracted from the Department of Informatics of the Brazilian Unified National Health System (DATASUS).

**Results:** In the analyzed period, 1,610,781 deaths from acute myocardial infarction were reported in Brazil. There was a 28.42% increase in the mortality rate in 2019 when compared with the rate reported in 1999. The number of deaths in Brazil in 2019 (95,557 deaths) was 65.00% higher than in 1999 (57,913 deaths). The male sex was the most afflicted and corresponded to 59.03% of the deaths reported in that period. Regarding the deaths by race, it was observed that white people (57.03%) and pardo people (29.48%) were the most affected. When grouped by age, it was observed a higher frequency of mortality rate between 70 to 79 years old: 411,161 (25.53%); followed by 80-year-olds: 380,371 (23.61%); 60 to 69 years: 378,933 (23.52%); 50 to 59 years: 263,790 (16.38%); 40 to 49 years: 126,090 (7.83%); 30 to 39 years: 37,176 (2.31%) and under 30 years old: 11,244 (0.70%). In 2019, the mortality rate per 100,000 people in Brazil was 45.36. Regarding the numbers by region, it was noted that the southeast region was responsible for 48.45% of deaths reported in Brazil in the analyzed period.

**Conclusions:** In the light of the results obtained, it was verified that the mortality rate and the number of deaths due to acute myocardial infarction increased over the years. In this study, it was observed a higher frequency of deaths from acute myocardial infarction among white men over the age of 50. Therefore, it is essential to promote health campaigns against stress, sedentary lifestyle, and poor diet, which are aimed at the prevention of coronary artery disease.

109074

Modality: E-Poster Scientific Initiation – Non-case Report

Category: EPIDEMIOLOGY AND HEALTH POLICIES/GLOBAL HEALTH

## What Factors Influence Brazilian Medical Students to Pursue a Career in Cardiology?

MARYANA HELENA DE SOUZA MENDONÇA RIBEIRO^1^, Jeanne du Fay de Lavalla^2^, Luciene Ferreira Oliveira Mota^3^, Annabelle Santos Volgman^4^

(1) Medicine College of Itajubá, Itajubá, MG, Brazil; (2) Department of Internal Medicine, Rush University Medical Center, Chicago, IL, USA.; (3) Department of Cardiology, Medicine College of Itajubá, Itajubá, MG, Brazil; (4) Division of Cardiology, Rush University Medical Center, Chicago, IL USA

**Introduction:** While a diverse workforce improves the care of patients, encourages different views, and reduces bias in the working environment, female cardiologists continue to be <30% of the workforce in the United States and Brazil. Therefore, including women’s recruitment as a priority for the cardiology community through understanding and discovering what inspires women to follow a cardiology career is essential. We conducted a survey of students from a Brazilian medical school to ascertain their motivations to consider cardiology and sought to find sex differences among the students.

**Objective:** Identify factors which affect medical students’ interest in cardiology and assess any sex differences.

**Methods:** Medical students answered a digital survey based on the science interest survey, validated by the Rasch scale and modified to address cardiology interest questions. The survey consisted of 20 questions, answered in the Likert scale and divided into five categories: class, family, friends, informal (learning outside the classroom), and professors. Altogether, 100 medical students (28 men/72 women) from the first to sixth year answered this survey. p < 0.05 was considered significant.

**Results:** In both sexes the influence of professors had higher median ratings compared to the classes, family, friends, and informal. Friends had the least influence on students’ interest in cardiology. There were no significant sex differences.

**Conclusion:** In this cohort of Brazilian medical students, professors had the most influence on encouraging medical students into pursuing cardiology. In the same way, practical classes in cardiology wards and visits to specialized centers appeared to be a good alternative to inspire men and women on becoming cardiologists.


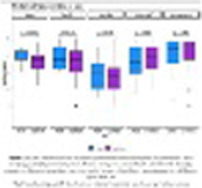


109075

Modality: E-Poster Scientific Initiation – Non-case Report

Category: COVID-19 AND CARDIOVASCULAR SYSTEM

## Safety of COVID-19 Vaccines Among Individuals with Cardiopathies Assisted in an Outpatient Facility in Brazil

ANA LUISA SOARES CHIARETTI^1^, Bianca de Almeida Nunes^2^, Livia Brito Oliveira^2^, Glicia Gleide Gonçalves Gama^2^, Fábio Figueiredo Costa^2^, Adriana Lopes Latado^2^

(1) Faculdade de Medicina da Bahia da Universidade Federal da Bahia – FMB-UFBA; (2) Hospital Universitário Professor Edgar Santos da Universidade Federal da Bahia/Empresa Brasileira de Serviços Hospitalares – HUPES-UFBA/EBSERH

**Introduction:** The rapid development of effective and safe vaccines was set as a priority due to the COVID-19 pandemic. This acceleration generated an infodemic, raising questions about the safety of the vaccines between the population, including cardiac patients.

**Objectives:** Evaluate the safety of COVID-19 vaccination in patients with cardiopathies.

**Methodology:** Unicentric prospective observational cohort that evaluated vaccination cover, frequency and severity of adverse events (AE) associated with the COVID-19 vaccines between patients with cardiopathies followed up in a cardiology referral service, in Salvador, Bahia, Brazil, between 2021 and 2022. AE were defined as any unwanted medical occurrence after the vaccination, which were categorized as severe (SE) or non-severe (NSE) events. Descriptive analysis of the data were performed and the inferential analyses were exploratory. Significance level adopted was 5%.

**Results:** 259 patients were included, 65,4% were women, 83,3% were black or brown. The average age was 62.4 (+15.8) years). Among morbidities, 81,4% had arterial hypertension, 44,6% had diabetes mellitus, 23% had chronic kidney disease, 78,7% had dyslipidemia, 16,3% had atrial fibrillation, 11,2% had previous myocardial infarction and 36,6% were diagnosed with heart failure. With regard to vaccination, 84,3% (204) had received at least two doses of vaccine, of which 38,0% received the CoronaVac, 30,2% (78) received the AstraZeneca, and 15,1% received Pfizer. 1,9% received a single dose of Janssen and 14,7% (38) did not know about vaccine type. By the end of the study, 35,6% had received a third dose of vaccine, 97,5% (81) were given Pfizer and 2,5% were given CoronaVac. Of total vaccinated, 30% reported NSE (local soreness, fever, myalgia or headache, during less than 3 days) and no SE (life-threatening events, requirement of hospitalization or causes of permanent sequelae) were reported. Patients who reported AE were younger (56,5 + 15,1 vs 65,5 + 14,9 years; p < 0,01), mostly women (36,8% vs 18,3% p = 0,01) and without hypertension (40,6% vs 34%, p = 0,04). The AE were more frequently reported after vaccination with adenovirus carrier or RNA vaccines (AstraZeneca, Janssen and Pfizer) than with Coronavac (p < 0,01).

**Conclusion:** The COVID-19 vaccines available in Brazil are safe for application in patients with cardiopathies. AE were frequent but without gravity, being more frequent in women and after vaccination with adenovirus carrier or RNA immunizers.

109122

Modality: E-Poster Scientific Initiation – Non-case Report

Category: EPIDEMIOLOGY AND HEALTH POLICIES/GLOBAL HEALTH

## Chronic Rheumatic Cardiopathy in the Northern Region of Brazil: A Ten-Year Analysis in the Single Health System

LEO CHRISTYAN ALVES DE LIMA^1^, Hildeman Dias da Costa^2^, Laura Jane França Lacerda^1^, Matheus Akira Suzuki de Oliveira^2^, Leticia Luana Alves Ferreira^1^, Emanuelle Toneto Souza Silva^1^, Andressa Flores da Costa^1^, Carla Cibelly Mesquita Almeida^1^, João Paulo Lucena^1^

(1) Centro Universitário São Lucas (UniSL); (2) Universidade Federal de Rondônia (UNIR)

**Introduction:** Chronic rheumatic heart disease (CRHD) is a non-suppurative complication of pharyngotonsillitis caused by group A beta-hemolytic streptococcus and results from a delayed immune response in genetically predisposed populations. It is a leading cause of cardiovascular disease in developing countries, accounting for approximately 15 percent of all heart failure patients in endemic countries.

**Objectives:** To describe the epidemiological profile of hospitalizations for Chronic Rheumatic Heart Disease in adults between the years 2009 and 2019.

**Methodology:** Epidemiological, descriptive, retrospective study. Data were obtained from the IT department of the Unified Health System – DATASUS, from January 2009 to December 2019, with individuals aged 20 to 79 years, evaluating hospitalization rates, mortality and pattern of carriers: range age, race and sex.

**Results:** In the analyzed period, 3830 hospitalizations were observed, 2052 were female and 1778 were male. Regarding race, there were 2089 occurrences in pardos, 272 in whites, 76 in blacks, 20 in yellows, 7 in indigenous people. The age group with the highest number of cases was between 40 and 49 years, with 777 individuals. The average total hospital stay was 14 days, representing a total expense of R$31,020,178.05, with 2017 being the year with the highest number of hospitalizations (534) and 2010 the lowest number (295). The total mortality rate was 9.63, with the state of Amapá having the highest rate (18.37), followed by Rondônia (12.27), with a peak in 2014 (17.49). The total number of deaths corresponded to 369 deaths, with the state of Pará holding the highest number (143), followed by Amazonas (87).

**Conclusion:** CRHD is still a major public health problem, especially in developing countries, resulting in huge health expenditures. The present study demonstrated a large number of cases of the disease in the northern region of Brazil. Between the years 2009 to 2019, the epidemiological profile was characterized by females, brown, aged between 40 and 49 years. The state with the highest number of cases was Pará and the highest mortality rate found was in Amapá. The description of these sociodemographic and epidemiological characteristics guide the planning of health actions in order to improve the population’s quality of life.

109124

Modality: E-Poster Scientific Initiation – Non-case Report

Category: EPIDEMIOLOGY AND HEALTH POLICIES/GLOBAL HEALTH

## Hypertension and Heart Rate: Insights from a National Study with Brazilian Adolescents

DOMINGOS ALVES DE SANTANA NETO^1^, Anna Mayse Feitosa da Silva^1^, Mariana Evaristo Leite^2^, Sarah Mariani Rocha Oliveira^1^, Ana Beatriz Vaz de Araújo^1^, Katia Vergetti Bloch^1^

(1) Universidade Federal do Rio de Janeiro (UFRJ); (2) Universidade Federal de São João del-Rei (UFSJ)

**Introduction:** Hypertension (HTN) is one of the main risk factors for cardiovascular diseases. Hypertensive adolescents are at higher risk of becoming hypertensive adults. Heart rate (HR) is a simple measure associated with sympathetic activity and can be influenced by numerous environmental factors. Elevated resting HR in adolescents can be a predictor of HTN in adults.

**Objective:** To explore the association of Hypertension with Heart rate in Brazilian adolescents. We also aimed to investigate the influence (confounding or effect modification) of sex, age, physical activity, and obesity in this association.

**Methods:** The Study of Cardiovascular Risks in Adolescents (ERICA) was a national, school-based, cross-sectional study. The sample has a complex design, being composed of adolescents aged between 12 and 17 years old from public and private schools located in Brazilian municipalities with more than 100,000 inhabitants. Data were collected using a self-administered questionnaire on an electronic device. Blood pressure and heart rate at rest were measured with an oscillometric device. Weight and height were measured to calculate the body mass index (weight/height2), classified according to WHO reference curves by sex and age. Physical activity was evaluated using a list of 24 modalities, including information on frequency (days) and time (hours and minutes) of practice during the last week. Adolescents who accumulated more than 300 min/week of physical activity were classified as active. Raw and adjusted linear regression models were performed in Stata 15.0.

**Results:** Age and obesity were confounding variables, while sex and physical activity were effect modifiers for the association between Hypertension and Heart rate (p < 0.001 for interaction). Girls and hypertensive adolescents had the highest Heart rate levels (88.5 bpm; 95%CI 87.0 to 90.0), while boys and non-hypertensive adolescents had the lowest (78.1 bpm; 95%CI 77.6 to 78.5) adjusted for age and obesity. The Heart rate of hypertensive and inactive adolescents was 87.6 bpm (95%CI 86.0 to 89.1), and non-hypertensive and active adolescents was 79.1 bpm (95%CI 78.6 to 79.5).

**Conclusion:** The observed association of Hypertension with Heart rate was independent of age and obesity. The effects of sex of physical activity on this association should be considered in longitudinal studies to estimate the prediction.

109217

Modality: E-Poster Scientific Initiation – Non-case Report

Category: HYPERTENSION/RENAL DENERVATION

## Acetylsalicylic Acid for Preventing Preeclampsia and Complications: A Systematic Review

RAYANNE ALESSANDRA DA SILVA BARRETO^1^, Anderson Brasileiro de Araújo^1^, Ana lívia Gadelha Xavier da Nóbrega^1^, Beatriz Gadelha e Xavier^1^, Bianca Vasconcelos Braga Cavalcante^1^, Letícia Lacerda Burity^1^, Vinicius Vieira Leandro da Silva^1^

(1) Faculdade de medicina Nova Esperança-FAMENE

**Introduction:** Preeclampsia is defined as hypertension (systolic blood pressure ≥140 mmHg and/or diastolic blood pressure ≥90 mmHg), arising after 20 weeks of gestational age with proteinuria (300 mg) or other signs of end-organ damage and is an important cause of maternal and perinatal morbimortality, particularly when of early onset associated with deficient intravascular production of prostacyclin, a vasodilator, and excessive production of thromboxane, a vasoconstrictor and stimulant of platelet aggregation. These observations led to the hypotheses that doses greater than 80 mg/day of acetylsalicylic acid, might delay or prevent development of preeclampsia. Prophylaxis is recommended in women at high risk of this disease that includes history of preeclampsia, chronic hypertension, family history of preeclampsia, renal disease, a body mass index greater than 30, multifetal gestation, and sociodemographic characteristics. Should be initiated between 12 weeks and 28 weeks of gestation, optimally before 16 weeks, and continued daily until delivery.

**Objective:** To assess the effectiveness and safety of doses greater than 80 mg/day of acetylsalicylic acid (ASA), in early pregnancy with increased risk of preeclampsia with the objective of delaying or preventing this disease.

**Methods:** Keywords were combined for electronic databases search. Relevant citations were extracted from PubMed, Elsevier and web science from 2007 to 2021. This systematic review includes only randomized, controlled trials. The population in the studies involved pregnant women at risk of preeclampsia treated with aspirin low-dose initiated at or before 16 weeks of gestation.

**Results:** 17 trials (n = 15.908) to assess maternal and perinatal health outcomes and 15 trials (n = 15.767; 10 good-quality) to assess prevention of preeclampsia. All trials were placebo-controlled. ASA treatment beginning in early gestation was associated with a greater reduction in the incidence of preeclampsia than treatment beginning in late gestation.

**Conclusions:** Daily low-dose acetylsalicylic acid use in pregnancy is considered safe. Treatment with this drug initiated early in pregnancy is an efficient method of reducing the incidence of preeclampsia and is associated with a low likelihood of serious maternal, or fetal complications, or both, related to use.

109129

Modality: E-Poster Scientific Initiation – Non-case Report

Category: ACUTE AND CHRONIC CORONARY DISEASE/THROMBOLYSIS

## Overview of Acute Myocardial Infarction (AMI) Cases in the Regions of Brazil: A Comparative Analysis between a Period Before and During the COVID-19 Pandemic

MARINA DE ANDRADE BATISTA^1^, Ana Verena Silva Santos^1^, Daiane Lima dos Santos^2^

(1) Cetro universitário UniFTC; (2) Universidade Estadual da Bahia

**Introduction:** According to the Brazilian Society of Cardiology (2015), there are about 350 thousand new cases of Acute Myocardial Infarction (AMI) per year and 40 to 65% of them evolve to death in the first hours. Among cardiovascular diseases, AMI has one of the highest rates of morbidity and mortality. In the period of the COVID-19 pandemic, given the restrictions to seek health services, the possibility of underreporting of AMI cases in the country is raised. In addition, from 2019 to 2020, there was a decline of 89.97% in patients who sought the cardiology outpatient clinic. Therefore, given this public health problem, there is an urgent need to analyze the registration of these cases in regional units in Brazil.

**Objective:** To analyze the number of confirmed cases of acute myocardial infarction, before and during the Covid-19 pandemic, among the Regional Units of the Brazilian territory, comparing the periods from 2017 to 2019 and 2019 to 2022.

**Method:** This is a retrospective epidemiological study of an analytical nature, carried out using data published on the Ministry of Health portal from 2017 to 2022. For this study, confirmed cases of AMI were analyzed by Regional Unit from 2017 to 2022. Appraisal by the Research Ethics Committee is unnecessary because this research used public data, without identification of the participants.

**Results:** In the analyzed period, 738,196 cases of AMI were confirmed in Brazil, with 362,649 in the pre-pandemic period (1) 2017 to 02/2019 and 370,547 in the pandemic period (2) from 03/2019 to January 2022. Studying the regional units in period 1, the following stand out: Southeast with 49.3% (n = 178,942), Northeast with 19.7% (n = 71,509) and South with 19.3% (n = 70,021) of cases. In period 2, regional highlights were in charge of the same regional units, respectively, with 48.8% (n = 180,945) of cases, 19.8% (n = 73,724) and 18.8% (n = 69,868). Therefore, during the pandemic, there was an increase of 2.1% (n = 7,898) compared to the pre-pandemic period.

**Conclusion:** While access to health services has declined during the pandemic, AMI records have increased. However, given the possible underreporting, it is inferred that the increase was even more significant. Since AMI has high morbidity and mortality, many individuals were left without proper assistance during this period, increasing their susceptibility to AMI and its complications.

109131

Modality: E-Poster Scientific Initiation – Non-case Report

Category: CARDIOVASCULAR SURGERY

## Impact of Atrial Fibrillation on Left Atrial Function Following Percutaneous Mitral Valvuloplasty

ANA CLARA DE PAULA CALDAS^1^, Ana Clara de Paula Caldas^1^, Juliana Rodrigues Soares^2^, Vinicius Tostes Carvalho^2^, Lucas Lodi-Junqueira^2^, Guilherme Rafael Sant’Anna Athayde^2^, Paulo Henrique Melo^1^, Fernando Coutinho^1^, Lucas Posoli^1^, Pedro Emanuel Carvalho^1^, William Antonio M. Esteves^1^, Maria Carmo Pereira Nunes^2^

(1) Universidade Federal de Minas Gerais (UFMG); (2) Hospital das Clínicas da Universidade Federal de Minas Gerais (HC – UFMG)

**Background:** Rheumatic mitral stenosis (MS) results in elevated left atrial (LA) pressures and subsequent atrial remodeling, atrial fibrillation (AF), and thrombus formation. Progressive LA enlargement can occur, despite the relief of valvular obstruction with valve intervention. This study evaluates LA function by three-dimensional echocardiography (3DE) in rheumatic MS patients undergoing percutaneous mitral valvuloplasty (PMV) to assess the impact of AF on LA function.

**Methods:** A total of 160 MS patients referred to PMV were prospectively enrolled. LA volumes and function were measured by 3DE pre, immediately after PMV, and at 1 year follow-up.

**Results:** Mean age was 44 ± 12 years, and 134 (84%) patients were women. Pre PMV, 44 (28%) were in AF, and 66 (41%) were in NYHA functional class III or IV. LA maximum volume decreased from 55.5 ± 23.1 ml/m² to 53.5 ± 29.7 ml/m² 48 hours after the procedure (p = 0.147), and to 50.5 ± 24.6 ml/m² at 1 year follow-up (p = 0.011). LA emptying fraction increased from 20.4 ± 10.1 ml/m², to 28.7 ± 11.4 ml/m² after 48 hours of procedure (p < 0.001), and to 32.6 ± 13.3 ml/m² at 1 year follow-up (p = 0.003). In AF patients, LA emptying fraction was 13.8 ± 7.5% at baseline, 21 ± 9.3% 48 hours after the procedure (p = 0.039) and 20.8 ± 8.7% at follow-up (p = 0.946) (Figure 1). Age, initial presence of AF, and postprocedural mean gradient were identified as the most significant predictors of late LA function.

**Conclusions:** LA presents reverse remodeling after PMV. This remodeling is higher immediately after the procedure, but continues to occur in the following months. In patients with MS and AF, volumes remained unchanged after PMV, either at 48 hours and at follow up, and only the emptying fraction improves at 48 hours, without further improvement at follow up.


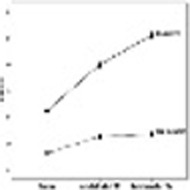


109143

Modality: E-Poster Scientific Initiation – Non-case Report

Category: COVID-19 AND CARDIOVASCULAR SYSTEM

## Comparative Analysis of the Epidemiological Panorama of Hospitalizations and Deaths in Patients with Pulmonary Embolism in the Periods Pre, During and Post-Covid 19 Pandemic

ERICK BRODER BICHARA^1^, Victor Arthur Soares Costa Araújo^1^, Diego Moreira Arruda^1^

(1) Escola Bahiana de Medicina e Saúde Pública

**Background:** Pulmonary embolism (PE) is one of the most frequent underdiagnosed acute cardiovascular diseases. In the COVID-19 first wave, especially, there were many disorders with similar symptoms. The pandemic has created an atmosphere of uncertainty among healthcare professionals and this fear leaded to a pulmonary embolism diagnosis even more underdiagnosed within the focus on COVID cases. Increased D-dimer works as a predictor for the presence of COVID. This indicator is still an essential factor for diagnosis PE, and it’s increase in patients with COVID can result in prothrombotic state.

**Methods:** Quantitative, descriptive study, carried out using secondary data from the Hospital Information System of DataSUS, about the monthly frequencies of hospitalizations and deaths related to pulmonary embolism.

**Objective:** Describe the epidemiological profile of hospitalizations and deaths resulting of pulmonary embolism in periods pre, during and post-COVID-19 pandemics in Brazil.

**Results:** In pre-pandemic period (Apr/19–Jan/20), there were 7915 hospitalizations in the country, 1948 in capitals. 2979 hospitalizations occurred in men and 4936 in women. Age group 60–69 years (1496) led. As for deaths, there were 1387 in country, 344 in capitals. 560 deaths in men and 827 in women. The age group 80 years and over (358) led. In 1st wave period (Feb/20–Nov/20), there were 7173 hospitalizations in country, 1777 in capitals. An increasing pattern was observed between Dec/19–Feb/20 (28, 141 and 222) – pandemic beginning – and a decreasing between Oct/20–Nov/20 (187 and 79) – end of the first wave. 2803 hospitalizations occurred in men and 4370 in women. Age group 60–69 years (1406) led. As for deaths, there were 1289 in country, 351 in capitals. 527 deaths in men and 762 in women. Age 80 and over (333) led. During vaccine period (Jan/21–Oct 2021), there were 8087 hospitalizations in country, 1936 in capitals. This period had the highest number among the three periods (1042 in Jul/21). There was an increasing pattern from Aug/20–Apr/21 (4, 6, 23, 47, 109, 178, 182, 185, 232) and a decreasing from Jun/2021–Oct/21 (256, 253, 196, 151, 97). 3440 hospitalizations occurred in men and 4647 in women. Age group from 60–69 years (1565) led the cases. As for deaths, there were 1585 in country and 387 in capitals. 692 deaths in men and 893 in women. Age 80 and over (402) led.

**Conclusion:** Both cases and deaths decreased during 1st wave period and increased during vaccine period.

109708

Modality: E-Poster Scientific Initiation – Non-case Report

Category: PSYCHOLOGY

## The Relationship and Benefits of Psychotherapy in Patients After Cardiac Surgery

VINICIUS VIEIRA LEANDRO DA SILVA^1^, Leticia Lacerda Burity^1^, Beatriz Gadelha e Xavier^1^, Bianca Vasconcelos Braga Cavalcante^1^, Ana Lívia Gadelha Xavier da Nóbrega Pereira^1^, Rayanne Alessandra da Silva Barreto^1^, Giovanna Alves de Souza^2^, Eduardo Franco Correia Cruz Filho^3^, Salmo Vasser Paranhos de Oliveira^4^, Beatriz Calsolari Ranha^5^, Idemar Luis Cover Filho^6^, Luís Fernando Brito Ferreira^7^

(1) Faculdade de Medicina Nova Esperança (FAMENE); (2) Universidade Cidade de São Paulo (UNICID); (3) Centro Universitário de João Pessoa (UNIPE); (4) Universidade: Instituto de Ensino Metropolitano de Ensino Superior (IMES – Univaço); (5) Universidade do Estado do Rio de Janeiro (UERJ); (6) Faculdade Ciências Médicas (FCM); (7) Centro Universitario Facisa (FACISA)

**Introduction:** Cardiovascular diseases (CVDs) are the leading cause of death in the world, bringing concern to the main global health agencies. Among the risk factors for CVDs, emotional aspects are increasingly being associated with increased cardiovascular risk. In the clinical practice of cardiac surgical patients, it is observed that psychological factors such as depressed mood, anxiety and fear of dying influence the patient’s recovery.

**Objective:** This abstract aims to show the benefits of psychotherapy in patients after cardiac surgery, and its relationship with cardiovascular disease.

**Methods:** This is an integrative review based on relevant publications on databases of the Latin American and Caribbean Literature on Health Sciences Information (LILACS), Scientific Electronic Library Online (SciELO), Virtual Health Library (BVS) and PubMed, in English and Portuguese from 2007 to 2020.

**Results:** It is known that the frequency of psychiatric disorders in hospitalized patients after cardiac surgery is between 20 and 60%, where anxious and depressive disorders due to fear of loss of autonomy or death stand out. Psychotherapy has brought several benefits to hospitalized patients, where there has been a reduction in the symptoms of psychological disorders, greater adherence to medication and diet, and an improvement in the acceptance of the procedures performed, leading to a reduction in the number of hospitalization days, helping in a better patient recovery.

**Conclusions:** Therefore, we can conclude that the support of psychotherapy in patients who have undergone cardiovascular surgery has brought relevant results, causing a faster and more comfortable recovery for hospitalized patients.

109182

Modality: E-Poster Scientific Initiation – Non-case Report

Category: CARDIOLOGY OF SPORTS, EXERCISE, ERGOMETRY AND CARDIOVASCULAR REHABILITATION

## Chronotropic Index as a Predictor of Mortality in Patients with Coexisting Heart Failure and Atrial Fibrillation

GUILHERME OLIVEIRA MAGALHÃES COSTA^1^, Bruno Oneto Y Viana Pintos^2^, Maurício Pimentel^2^, Anderson Donelli da Silveira^2^

(1) Universidade Federal do Rio Grande do Sul (UFRGS); (2) Hospital de Clínicas de Porto Alegre (HCPA)

**Introduction:** Atrial fibrillation (AF) is associated with a worse prognosis in patients with heart failure (HF). A blunted chronotropic response seems to be related to worse prognosis and functional capacity in those patients, independently of beta-blocker use.

**Objectives:** To evaluate heart rate response during cardiopulmonary exercise testing (CPET) in patients with both HF and AF and its association with cardiovascular events.

**Methods:** Retrospective cohort study including patients with HF and AF assessed by CPET, followed-up in an outpatient clinic at a public university hospital. The primary endpoint was death or need for heart transplant. T-Test for independent samples or Mann-Whitney U test were used when appropriate. The best cut-off point for the chronotropic index was obtained by a ROC curve analysis. Univariate and multivariate survival analysis was performed using Cox regression and Kaplan-Meier curve.

**Results:** A total of 1083 patients with HF comprised the entire cohort, 208 of those with coexisting AF. Mean age was 58 (±11) years and average follow-up time was 42 (±20) months. 31% were female, 19% ischemic, 15% had HFpEF, 85% had HFrEF and 93% were receiving beta-blockers. Primary outcome occurred in 21.4% of patients. It was more frequent in patients who had lower peak HR (123 vs 141 bpm, P < 0.001), ΔHR (47 vs 58 bpm, P = 0.004), chronotropic index (0,57 vs 0,78, P < 0.001), peak VO2 (15.4 vs 16.9 ml/kg/min, P = 0.03) and OUES (1.18 vs 1.39, P = 0.002). Univariate survival analysis showed that the same variables remained as outcome predictors. A chronotropic index <0.6 was associated with an HR 2.7 (IC 95% 1.5 –4.7) to the primary endpoint in the univariate analysis and an HR 3,1 (IC 95% 1,2–7,8) in the multivariate adjusted for sex, age, CKD, DM and use of beta-blockers, remaing as the strongest risk predictor in our sample.

**Conclusion:** A blunted HR response in patients with HF and AF, evidenced by lower chronotropic index, ΔHR and HR peak, was associated with an increased risk of death or need for heart transplant in our sample. These findings question the real benefit of beta-blocker use in this scenario. Further studies evaluating the reduction or withdrawal of these medications could help to answer our question.

109185

Modality: E-Poster Scientific Initiation – Non-case Report

Category: CONGENITAL AND PEDIATRIC CARDIOLOGY

## Correlation between Antenatal Diagnosis and Origin of Critical Congenital Heart Defects in a Neonatal Intensive Care Unit

NICOLE DE OLIVEIRA SANTOS^1^, Giovana Rezende Fernandes Costa^1^, Jennifer Klassen Boeing^1^, Luís Antonio Zorzi Santin^1^, Adriana Saito Jasper^2^

(1) Universidade Positivo (UP); (2) Associação Hospitalar de Proteção à Infância Dr. Raul Carneiro/Hospital Pequeno Príncipe (HPP)

**Introduction:** Critical congenital heart diseases (CCHD) require intervention as early as possible. The delay in its diagnosis increases the risk of morbidity and mortality in newborns (NB). Prenatal diagnosis of CCHDs is at first done using morphological ultrasound, and then more thoroughly by fetal echocardiography, allowing for a more deliberate management planning of birth conditions with the family and health-care providers. However, these exams are not offered to all pregnant women by the Healthcare System in Brazil.

**Objective:** To correlate the antenatal diagnosis with the origin of newborns admitted for critical congenital heart defects in a neonatal intensive care unit of a tertiary hospital in Curitiba, Paraná, Brazil.

**Methods:** This was a retrospective study reviewing medical records covering the period from January 1st 2015 to December 31st 2019 of patients diagnosed with critical congenital heart disease who were admitted to the Neonatal Intensive Care Unit. A descriptive analysis of the collected data was performed.

**Results:** The study included 127 patients. The CCHD type incidence found in our sample was: coarctation of the Aorta (20.5%), transposition of great arteries (15.7%), pulmonary atresia (13.4%), hypoplastic left heart syndrome (11.8%), tetralogy of Fallot (3.1%), anomalous pulmonary vein drainage (3.1%), Ebstein anomaly (3.1%), tricuspid atresia (2.4%), truncus arteriosus (2.4%), double outlet right ventricle (1.6%), aortic stenosis (0.8%) and two or more heart defects combined (22%). Prenatal diagnosis, performed on 41.7% of the patients, showed statistically significant relation with the origin of the NB (p < 0,0001). Curitiba, capital of the state of Paraná, had the highest proportion of prenatal diagnosis (69%) when compared to the Greater Curitiba region (25.9%), the countryside region of Paraná (25%) and other Brazilian states (50%).

**Conclusion:** Most patients with CCHD weren’t diagnosed prenatally and, amongst the NB with a prenatal diagnosis, most were from Curitiba – PR. Due to the available infrastructure in the capital, Curitiba has the necessary technology and professionals qualified to perform diagnosis, scanning protocols and practice related to prenatal screening, suggesting an important role in their implementation.

109188

Modality: E-Poster Scientific Initiation – Non-case Report

Category: PERICARDIUM/ENDOCARDIUM/VALVOPATHIES

## Value of Atrial Fibrillation in Progression of Mitral Regurgitation in Rheumatic Heart Disease

ALEXANDRE NEGRÃO PANTALEÃO^1^, Nayana FA Gomes^1^, Vicente Rezende Silva^1^, William AM Esteves^1^, Marildes Luiza de Castro^1^, Lucas Chaves Diamante^1^, Fernando Cunha Ruffo^1^, Matheus de Oliveira Ferreira^1^, Luís Felipe Rezende de Almeida^1^, Maria Carmo P Nunes^1^

(1) School of Medicine, Federal University of Minas Gerais (UFMG)

**Introduction:** Rheumatic heart disease (RHD) is the leading cause of cardiovascular death in children and young adults, and disproportionally affects low- and middle-income countries. Mitral regurgitation (MR) is the most common valve abnormality in RHD and it is often associated with atrial fibrillation (AF). AF is the most prevalent sustained arrhythmia in RHD and it is a marker of disease severity with poor prognosis.

**Objective:** This study aims to investigate the association between AF and MR progression in patients with RHD.

**Methods:** Consecutive RHD patients with non-severe MR associated with any degree of mitral stenosis were selected between 2011 and 2021. Primary endpoint was progression of MR, which was defined as an increase of one grade in MR severity from baseline to the last follow-up echocardiogram. AF diagnosis was based on a history of permanent AF supported by past 12-lead electrocardiogram (ECG). Diagnosis of new-onset AF in patients with sinus rhythm at baseline was confirmed by 12-lead ECG.

**Results:** 539 patients were included, aged 46.2 ± 12 years, and 83% were women. At a mean follow-up time of 4.2 years (IQR: 1.2 to 6.9 years), 54 patients (10%) displayed MR progression with an overall incidence of 2.4 per 100 patient-years. Patients who had new-onset AF during the follow-up were at risk for progression (adjusted hazard ratio [HR] 2.447, 95% CI 1.035–5.788) and patients with permanent AF were at the highest risk (HR 4.459, 95% CI 2.148–9.631) compared with patients in sinus rhythm.

**Conclusions:** In RHD patients with a full spectrum of MR severity, progression of MR occurs over time, associated with the presence of AF. AF burden either permanent or new-onset AF led to an increased risk of progression of MR.


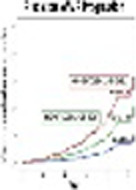


109194

Modality: E-Poster Scientific Initiation – Non-case Report

Category: HEART FAILURE/CARDIOMYOPATHY/TRANSPLANT

## Cross Sectional Study of Heart Failure Mortality Reported in Brazil between 1999 and 2018

FILIPE ALVES RAMOS ^1^, ROSANGELES KONRAD^2^

(1) Catholic University of Brasilia; (2) Hospital Anchieta – Cárddio Clinic

**Introduction:** Heart failure is a cardiovascular pathology that presents a high mortality rate. According to the World Health Organization (OMS), 23 million people live with this disease in the world. Hypertension, as well as diabetes and coronary artery disease are the main risk factors for the development of heart failure and are related to their worst prognosis.

**Objective:** Analyzing the evolution of the number of deaths from heart failure in Brazil between 1999 and 2018.

**Methods:** This is a descriptive and quantitative cross sectional study conducted between 1999 and 2018 on the number of deaths from heart failure documented in Brazil. The analyzed data were obtained through the Department of Informatics of the Brazilian Unified National Health System (DATASUS).

**Results:** From 1999 to 2018, 550,969 deaths from heart failure were recorded in Brazil. The mortality rate in 2018 decreased 29.71% compared to 1999. The number of deaths in Brazil in 2018 (26.482 deaths) was 10.32% lower than the recorded in 1999 (29.531 deaths). In relation to race/skin color deaths, it was found that white people 297.198 (53.94%) and pardo people 157.282 (28.55%) were the most affected. In the distribution by age groups there was a higher prevalence of mortality over 80 years: 233.283 (42.34%); followed by 70 to 79 years: 144.164 (26.16%); 60 to 69 years: 89.072 (16.17%); 50 to 59 years: 46.643 (8.46%); 40 to 49 years: 20.871 (3.79%); 30 to 39 years: 8.589 (1.56%) and those under 30: 7.747 (1.41%). There was a 29.71% reduction in the mortality rate for every 100,000 people, when compared to the one registered in 2018 (12.66%) with that documented in 1999 (18.01%).

**Conclusions:** Through the results presented, it is noted that the mortality rate, as well as the number of deaths from heart failure has decreased in Brazil over the years. This reduction is justified by the greater use of beta-blockers and angiotensin-converting enzyme, which reduce the morbidity and mortality associated with heart failure. Add to this, the reduced selection of heart failure as a basic cause of death in the face of other cardiovascular diseases, such as hypertension. In the present study, there was a higher frequency of heart failure deaths among white men over the age 60.

109196

Modality: E-Poster Scientific Initiation – Non-case Report

Category: EPIDEMIOLOGY AND HEALTH POLICIES/GLOBAL HEALTH

## Epidemiological Study of Morbimortality from Heart Arrhythmia and the Relationship with the use of Chloroquine and Hydroxychloroquine as Treatment of COVID-19 in Brazil

LARISSA SILVA FERREIRA^1^, Larissa Silva Ferreira^1^, Aurea Nathallia Gomes de Souza^1^, Bianca Paula Miranda Martins^1^, Camila Silva de Oliveira^1^, Cecília Rodrigues Viana^1^, Luiz Felipe Façanha Ramos^1^, Marcos Roberto Marques da Silva Júnior^1^, Vinícius Maciel Vilhena^1^, Reny Wane Vieira dos Santos^1^

(1) Universidade Federal do Amapá (UNIFAP)

**Introduction:** Torsades de pointes is a rare type of ventricular cardiac arrhythmia that is characterized by an increase in the QT interval on the electrocardiogram (ECG). After the beginning of the COVID-19 pandemic, the use of chloroquine and hydroxychloroquine (HCQ) became part of treatment protocols. However, later studies demonstrated the low efficacy of these drugs for the treatment and the increase of cardiovascular risks such as angina, arrhythmias and heart failure.

**Objective:** To analyze the mortality trend due to cardiac arrhythmias in the northern region between the periods of 2020 and 2022.

**Methods:** Epidemiological study with a cross-sectional design of mortality and arrhythmia in Brazil between 2020 and 2022. The investigated data were extracted from the Department of Informatics of the Unified Health System (DATASUS).

**Results:** In the period investigated, 18,741 deaths from arrhythmias were documented in Brazil. The growth in the mortality rate was from 11.64% in January 2020 to 15.62% in January 2022. It was observed that the highest mortality rate occurred in the Midwest region (35.04%). Furthermore, the number of hospitalizations for arrhythmias in this period was 27,887, being higher in the Southeast region, which represents 47.7% of hospitalizations. Regarding deaths by sex, it was found that males were the most affected (15.43%) as well as in the number of hospitalizations (56.03%). In the distribution by age groups, higher mortality was observed in individuals aged 80 years or older (16.83%) compared to those aged 55 to 59 years (14.80%). It was also noted an increase in cases of hospitalization in this period in the age group from 65 to 69 years, representing 53.3% of the total number.

**Conclusions:** Based on the results obtained, there was an increase in cases of hospitalizations and deaths due to arrhythmias in the period studied. This mortality was higher in males and in the age group from 65 years old. Thus, HCQ can prolong the ECG QT interval and can trigger an arrhythmia called torsades de pointes. Thus, the use of this drug during the COVID-19 pandemic may, indirectly, have been one of the aggravating factors in this arrhythmia mortality rate.

109241

Modality: E-Poster Scientific Initiation – Non-case Report

Category: CARDIOVASCULAR PHARMACOLOGY

## The Effect of Medicinal Plants in Prevention and Treatment of Cardiovascular Diseases: A Systematic Review

LEONARDO YOHAN SANTOS CHUCRE DE LIMA^1^, Elizama Raquel de Souza Silva^1^, Bruna Lisboa Nunes^1^, Isabela Ferreira de Freitas^1^, Luiz Felipe Leão Lima^1^, Micandria Yanka Fender Lobato^1^, Luma Maria Favacho Bordalo^1^, Fernando Gabriel dos Santos Santiago^1^, Caio Vinicius Botelho Brito^1^

(1) Laboratório de Cirurgia Experimental da Universidade do Estado do Pará

**Introduction:** Noncommunicable diseases (NCDs) are responsible for 70% of global deaths and about 45% of those are of cardiovascular origin. In Brazil, 72% of deaths result from NCDs and 30% are by cardiovascular diseases (CVDs). Thus, the importance of finding therapeutical strategies and new pharmacological models is evident. In this context, a great number of traditional herbs which have high anti-oxidative and anti-inflammatory capacities have been referred to as effective and might prove beneficial against CVDs.

**Objective:** To assess the existence of cardioprotective effects in medicinal plants.

**Methods:** This study is a systematic review with meta-analysis through the PRISMA method. Searches were conducted in the MedLine and LILACS databases, through the “Medicinal plants” and “Cardiovascular diseases” descriptors and their synonyms indexed in DeCS/MeSH. Randomised studies, case reports and observational studies published in the last 5 years in english and portuguese were included. Integrative reviews and articles without free full text available were excluded. Research resulted in 290 articles that were filtered for analysis by 4 reviewers.

**Results:** The primary outcomes showed important effects of different medicinal plants over congenital cardiopathies’ treatment, highlighting its antiatherogenic potencial and reduction of cardiovascular risk factors, especially through lowering body mass index (BMI), waist circumference and cholesterol levels. Furthermore, as secondary outcomes, it was possible to observe a hypotensive action of Acacia longifolia, Urtica Dioica and Viola Odorata extracts, and the regularization of biomarkers and inflammatory response as a result of citrus flavonoids and polyphenols combination, which improved both lipid panel and flow-mediated dilation in studied patients. Moreover, the use of oils, such as Brazil nuts and coconut combined with epigallocatechin gallate, is effective against lipid peroxidation and decreases risk of CVDs.

**Conclusions:** Medicinal plants are a great alternative for treating and preventing CVDs and might be used to develop future studies for promising medicines.


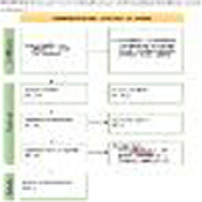


112393

Modality: E-Poster Scientific Initiation – Non-case Report

Category: PSYCHOLOGY

## Psychological Distress at Work: A Cardiovascular Risk Neglected in Public Safety Professionals

SUELLEN KEYZE ALMEIDA LIMA^1^, Dra. Antoinette Oliveira Blackman^1^, Beatriz Montenegro Oliveira^1^, Dra. Solange Lopes da Silva^1^, Adda Cecília Batista de Carvalho Vieira^1^, David Ricardo Bernal Lima Hernandez^1^, Pedro Henrique Alves Miranda^1^, Patrícia Brito de Almeida Borges^1^

(1) Centro Universitário do Planalto Central Apparecido dos Santos

**Introduction:** Remarkable changes in the work situation have led to the increasing importance of psychomental and socioemotionally demanding conditions at work. Psychic distress at work can encompass stressful situations and numerous consequences that compromise the worker’s lifestyle, including cardiovascular risk.

**Objective:** To understand the main triggers of uneasiness at work and relationships between psychic suffering in public security personnel and the risk of cardiovascular diseases among these professionals.

**Method:** An integrative literature review was carried out in the databases BVS-PSI, Scientific Eletronic Library Online (SCIELO), PubMed/MEDLINE, Google Scholar, UptoDate, PePSIC.

**Results:** This study analyzed the association between work-related behavioral characteristics and the main triggers related to illness, such as load, pace, schedule, work environment, among others.

**Final Considerations:** We present reflections about behaviors adopted by the servers, as tools to their process of getting sick; we also point out aspects to be highlighted in prevention and assistance actions. And it links an immediate alert system for anticipation of care to the health of these servers, because they are getting sick.

109224

Modality: E-Poster Scientific Initiation – Non-case Report

Category: CONGENITAL AND PEDIATRIC CARDIOLOGY

## Overview of Fallot Tetralogy Correction Procedures and Variants in Children and Adolescents in Brazilian Regions in 10 Years

JULIANA DE ALMEIDA SILVEIRA^1^, Sara Cristine Marques dos Santos^1^, Anna Júlia Tamiozzo Reis^1^, Girley Cordeiro de Sousa^1^, Juliana Alves Costa^1^

(1) Universidade de Vassouras

**Introduction:** Among the congenital abnormalities, cardiac malformations are the most responsible for death, being the main cause of infant death in first world countries. Tetralogy of Fallot (TF) is the most frequent cyanotic congenital heart disease, with an incidence of 10% of congenital heart defects1. TF consists of four concomitant anomalies, namely: right ventricular outflow tract obstruction (RVOT), sub-aortic interventricular communication (VSD), aortic dextroposition associated with overriding over the interventricular septum and right ventricular hypertrophy. The importance of TF lies not only in its incidence, but also in the severity of symptoms and the effectiveness of surgical correction, especially when performed in childhood.

**Objective:** To analyze the current panorama of Tetralogy of Fallot procedures and variants in children and adolescents performed in Brazil for 10 years and to correlate the current epidemiology with the results obtained.

**Methods:** A systematic review of the literature and an observational, descriptive and cross-sectional collection of data on correction of Tetralogy of Fallot and variants in children and adolescents, available in DATASUS – Hospital Information System of the SUS (SIH/SUS) were carried out for a ten-year period – December 2011 to December 2021 – evaluating the value of public spending, complexity, mortality rate, deaths, permanence and character of care and articles available in Scielo and PubMed.

**Results:** In the analyzed period, 2,777 hospitalizations were observed for the performance of correction procedures for Tetralogy of Fallot and variants in children and adolescents, representing a total expense of R$ 65,165,447.56. The total mortality rate in the 10 years studied was 10.44, corresponding to 290 deaths, with 2011 being the year with the highest mortality rate, 12.99, while 2018 had the lowest rate, 8.16. The Brazilian region with the highest number of hospitalizations was the Southeast with 1,265 hospitalizations, followed by the Northeast region with 627. The Midwest region had the highest mortality rate 20.29, while the Southeast region had the lowest rate, with a value of 7.67.

**Conclusions:** The present study identified that the rate dropped from 8.89 in 2009 to 8.76 in 2019. It is worth noting that the Southeast region, despite having the highest number of hospitalizations, has the lowest mortality rate, evidencing the effectiveness of the services performed.

109230

Modality: E-Poster Scientific Initiation – Non-case Report

Category: HYPERTENSION/RENAL DENERVATION

## Epidemiological Analysis of Mortality from Hypertensive Diseases in Brazil, by Region, from 2009 to 2019

CECÍLIA RODRIGUES VIANA ^1^, Aurea Nathallia Gomes de Souza^1^, Bianca Paula Miranda Martins^1^, Camila Silva de Oliveira^1^, Larissa Silva Ferreira^1^, Luiz Felipe Façanha Ramos^1^, Marcos Roberto Marques da Silva Júnior^1^, Vinícius Maciel Vilhena^1^, Reny Wane Vieira dos Santos^1^

(1) UNIVERSIDADE FEDERAL DO AMAPÁ (UNIFAP)

**Introduction:** Systemic arterial hypertension (SAH) consists of a chronic increase in blood pressure to values greater than or equal to 140 mmHg for systolic pressure and 90 mmHg for diastolic pressure. It is considered a multifactorial condition, which presents important risk factors, such as obesity and sedentary lifestyle. It represents one of the most relevant conditions for the incidence of cerebrovascular diseases.

**Objective:** To analyze the epidemiological aspects related to mortality from hypertensive diseases in Brazil, by region, from 2009 to 2019.

**Methodology:** Cross-sectional epidemiological study of mortality from hypertensive diseases by Brazilian region, during the years 2009 to 2019, from the data analysis from the SUS Hospital Information System (DATASUS).

**Results:** In the analyzed period, Brazil recorded 530,382 deaths from hypertensive diseases, with 29,149 in the North region (5.49%), 165,717 in the Northeast (31.24%), 228,553 in the Southeast (43.09%), 73,711 in the South (13.89%) and 33,252 in the Midwest (6.26%). In relation to deaths by race/color, white individuals were the most affected in Brazil (n = 250,330), however, when analyzing by region, changes in this situation are noted, since in the North region, the predominance was of pardos (66.45%), as well as in the Northeast region (62.2%) and in the Center-West region (48.07%). The Southeast and South regions followed the trend of Brazil with the predominance of the white population, corresponding to 57.39% and 84.85%, respectively. Regarding gender, in Brazil, women were the most affected (53.05%), however, when analyzing by region, there are differences in the North and Center-West regions, in which the male population had the highest rates., 52.83% and 51.56%, respectively. The Northeast, Southeast and South regions maintained the Brazilian trend and their female population was the most affected, representing 53.08%; 53.95% and 54.63% of the total, respectively. Regarding the age group, the population aged 80 years and over is the one that dies the most from hypertensive diseases (n = 222,829) in the country. This holds true in all regions, with the Southeast having the highest number of cases (39.17%), followed by the Northeast (35.44%), South (14.68%), Central-West (5.39%) and North (5.29%).

**Conclusion:** It’s concluded that SAH mortality in Brazil presents a heterogeneous aspect when analyzed by region, since, generally, Brazilian prevalence trends were not maintained for all.

109231

Modality: E-Poster Scientific Initiation – Non-case Report

Category: EPIDEMIOLOGY AND HEALTH POLICIES/GLOBAL HEALTH

## Sex and Obesity Modify the Association of Physical Activity with Heart Rate in Brazilian Adolescents

MARIANA EVARISTO LEITE^1^, Domingos Alves de Santana Neto^2^, Sarah Mariani Rocha Oliveira^2^, Anna Mayse Feitosa da Silva^2^, Ana Beatriz Vaz de Araújo^2^, Katia Vergetti Bloch^2^

(1) Universidade Federal de São João del-Rei (UFSJ); (2) Universidade Federal do Rio de Janeiro (UFRJ)

**Introduction:** Physical activity is defined as any bodily movement generated by the skeletal muscles that increase energy expenditure. During physical activity, the autonomic nervous system promotes changes in the cardiovascular system to compensate for the increased metabolic demand required during the effort, including increased heart rate and stroke volume. Resting heart rate usually decreases with physical conditioning, and the stroke volume increases. Therefore, heart rate can be a marker of physical conditioning that accurately assesses physical activity levels.

**Objective:** The study aimed to analyze the association of resting heart rate with physical activity. We also tested the effect of sex and obesity on this association in Brazilian adolescents.

**Methods:** The Study of Cardiovascular Risks in Adolescents was a cross-sectional, national, school-based study with adolescents aged 12 to 17 years old from public and private schools in municipalities with more than 100 inhabitants. An oscillometric blood pressure monitor assessed heart rate. Leisure-time physical activity was categorized according to the week volume (active ≥ 300 min and inactive = zero min). Obesity was classified by body mass index (BMI = weight/height2) according to age and sex. Data analysis was performed using Stata 15.0, estimating raw and adjusted linear regression models.

**Results:** A total of 73,399 adolescents were evaluated, of which 49.6% were considered inactive (60.2% were girls and 36.9% were boys). The mean heart rate of the inactive group was 83.4 beats per minute, and that of active adolescents was 79.7 beats per minute, p < 0.001. Adolescents with obesity had higher heart rate than those without obesity (83.7 vs. 81.2 beats per minute, respectively; p < 0.001). Adjusting for age, sex and obesity modified the association of physical activity and heart rate (p < 0.001 and p = 0.03 for interaction, respectively).

**Conclusion:** Active adolescents have lower resting heart rate than inactive ones, indicating a predominance of parasympathetic modulation instead of sympathetic modulation. These results suggest that the physical activity classification adopted in the study reflects the physical conditioning of adolescents and that sex and obesity modify the effect of the relationship between physical activity and heart rate.

109247

Modality: E-Poster Scientific Initiation – Non-case Report

Category: COVID-19 AND CARDIOVASCULAR SYSTEM

## The use of Chloroquine and Hydroxychloroquine in Hospitalized COVID-19 Patients: A Retrospective Analysis

DANIELLA NUNES PEREIRA^1^, Maíra Viana Rego Souza-Silva^1^, Magda Carvalho Pires^1^, Lucas Emanuel Pereira Ramos^1^, Maria Aparecida Camargos Bicalho^2^, Fernando Anschau^3^, Yara Cristina Neves Marques Barbosa Ribeiro^4^, Milton Henriques Guimarães Júnior^5^, Luanna Silva Monteiro Menezes^6^, Neimy Ramos de Oliveira^7^, José Miguel Chatkin^8^, Milena Soriano Marcolino^1^

(1) Universidade Federal de Minas Gerais; (2) Fundação Hospitalar do Estado de Minas Gerais; (3) Grupo Hospitalar Conceição; (4) Hospital Metropolitano Dr. Célio de Castro; (5) Hospital Márcio Cunha; (6) Hospital Metropolitano Odilon Behrens; (7) Hospital Eduardo de Menezes; (8) Hospital São Lucas da PUCRS

**Introduction:** Despite the evidence of lack of benefit, chloroquine has still been prescribed by some doctors to treat coronavirus disease 2019 (COVID-19) patients.

**Objectives:** To describe and analyze the side effects and outcomes associated with the use of chloroquine and hydroxychloroquine in COVID-19 therapy.

**Methods:** This retrospective cohort of a substudy of the multicentric cohort Brazilian COVID-19 Registry, which included consecutive COVID-19 patients, admitted to 37 hospitals in 17 Brazilian cities, from March to September 2020. A propensity score was used to select the sample, matched by age, sex, cardiovascular comorbidities and hospital. Chi-square or Fisher tests were used to compare categorical variables and the Wilcoxon test for numerical variables.

**Results:** From 7,732 COVID-19 patients, 280 (89.7%) were treated with hydroxychloroquine and 32 with chloroquine (10.3%) and 515 were randomly selected as controls. Demographic data and comorbidities are shown in Table 1. During hospitalization, 3.2% of the chloroquine patients presented side-effects to the medication and 40.0% of them required therapy interruption. Electrocardiography abnormalities were more prevalent in patients using chloroquine than controls (36.5% vs 26.9%). With regards to patient outcomes, patients who used chloroquine/hydroxychloroquine had a higher frequency of admission in intensive care units (45.2 vs. 34.0%, p = 0.002) and mechanical ventilation (34.6 vs. 25.5% p = 0.008). Mortality was not significantly different between groups.

**Conclusion:** Patients undergoing COVID-19 treatment with chloroquine/hydroxychloroquine presented higher frequency of intensive care unit and mechanical ventilation requirement than matched controls who did not use the medications. In-hospital death rates were similar.


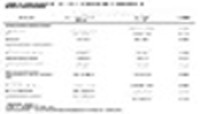


109250

Modality: E-Poster Scientific Initiation – Non-case Report

Category: CONGENITAL AND PEDIATRIC CARDIOLOGY

## Clinical and Epidemiological Analysis of Critical Congenital Heart Diseases in a Neonatal Intensive Care Unit

GIOVANA REZENDE FERNANDES COSTA ^1^, Jennifer Klassen Boeing^1^, Luís Antonio Zorzi Santin^1^, Nicole de Oliveira Santos^1^, Adriana Saito Jasper^2^

(1) Universidade Positivo (UP); (2) Associação Hospitalar de Proteção à Infância Dr. Raul Carneiro/Hospital Pequeno Príncipe (HPP)

**Introduction:** Congenital heart disease (CHD) is the most common congenital disease among newborns (NBs). CHDs defined as critical, generally dependent on the ductus arteriosus’ permeability, account for around 20% to 25% of heart diseases. The definition of the clinical-epidemiological profile of these patients can help in proposals and optimization of treatments.

**Objective:** Perform an epidemiological analysis of critical congenital heart diseases (CCHDs) in NBs admitted to the Neonatal Intensive Care Unit of a tertiary care hospital in Curitiba/PR.

**Methods:** This was a retrospective study reviewing medical records covering the period from January 1st 2015 to December 31st 2019 of patients diagnosed with CCHDs who were admitted to the Neonatal Intensive Care Unit.

**Results:** The sample consisted of 127 patients. The incidence of diagnoses found is represented in the graph. Sixty-five (51.2%) male and 62 (48.8%) female patients were identified. Regarding gestational age, 0.8% of NBs were born at less than 30 weeks, 7.1% between 30 and 33 weeks, 13.4% between 34 and 36 weeks, 47.2% 37 to 38 weeks and 31.5% over 39 weeks. Considering the clinical outcomes, 38.6% died, of which 73.5% of deaths were due to cardiogenic shock, 24.5% due to septic shock and 2% due to other causes. When relating the type of CCHD with the number of deaths, the mortality rate was higher in cases of hypoplastic left heart syndrome (p < 0.017) and tricuspid atresia (p < 0.027).

**Conclusion:** Within our sample, the main diagnoses were hypoplastic left heart syndrome, coarctation of the aorta, transposition of the great arteries, tetralogy of Fallot, and pulmonary atresia, following what is detailed in literature. No level of significant correlation was found between the type of CCHD and gender and between the type of CCHD and gestational age at birth. There was a statistical significance in the correlation between mortality and the two heart diseases: hypoplastic left heart syndrome and tricuspid atresia, in most cases resulting in death. These data should be evaluated as opportunities to improve care for heart disease.


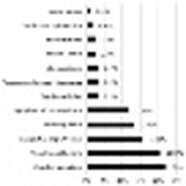


109256

Modality: E-Poster Scientific Initiation – Non-case Report

Category: CONGENITAL AND PEDIATRIC CARDIOLOGY

## Epidemiological Aspects of Congenital Heart Diseases as a Cause of Infant Death in the Northern Region of Brazil between 2016 and 2019

CECÍLIA RODRIGUES VIANA ^1^, Aurea Nathallia Gomes de Souza^1^, Bianca Paula Miranda Martins^1^, Camila Silva de Oliveira^1^, Larissa Silva Ferreira^1^, Luiz Felipe Façanha Ramos^1^, Marcos Roberto Marques da Silva Júnior^1^, Vinícius Maciel Vilhena^1^, Reny Wane Vieira dos Santos^1^

(1) UNIVERSIDADE FEDERAL DO AMAPÁ (UNIFAP)

**Introduction:** Congenital heart diseases (CHD) consist of macroscopic abnormalities of the heart or great vessels and constitute an important cause of infant death, death occurring in live-born children from the moment of birth to an incomplete year of age. These malformations can occur in the first eight weeks of pregnancy, in which the cardiac system begins its formation. The patient may have respiratory and circulatory failure as the main complications resulting from this pathology, which are decisive for the compromise of the child’s quality of life.

**Objective:** To analyze the epidemiological aspects regarding infant death due to congenital heart disease in the North region, from 2016 to 2019.

**Methods:** Cross-sectional epidemiological study of infant mortality due to congenital heart disease in the North region between 2016 and 2019, based on the analysis of data from the Hospital Information System (SIH) from the DATASUS database.

**Results:** During the study period, 1,342 infant deaths from congenital heart disease were reported in the North region, which corresponds to 11.9% of the total number of deaths from this cause in Brazil (11,277 deaths). In 2016, the number of deaths was 301 children, in 2019 this figure was 349, which represents an increase of 15.95% during the years analyzed. Regarding deaths by color/race, it was noted that brown individuals were the most affected: 830 (61.85%), followed by declared whites: 354 (26.37%). With regard to maternal schooling, it was observed that mothers with schooling between 8 and 11 years are the most affected (37.77%) by this fatality. In terms of gender, males had a higher number of cases: 705 (52.53%) versus 630 (46.94%) throughout the analyzed period.

**Conclusions:** Based on the data obtained, it can be concluded that the highest incidence of infant deaths from congenital heart disease in the North region occurs in mixed-race male individuals whose mother has between 8 and 11 years of schooling. There was also an increase in the number of cases between 2016 and 2019.


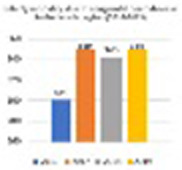


109257

Modality: E-Poster Scientific Initiation – Non-case Report

Category: COVID-19 AND CARDIOVASCULAR SYSTEM

## Association of Myocardial Injury with Mortality in COVID-19 Patients

EDUARDO BARROS SCHAUSTZ^1^, João Dario Martins de Mattos^1^, Andréa Silvestre de Sousa^3^, Emiliano Horacio Medei^2^, Denílson C. de Albuquerque^1^, Olga Ferreira de Souza^1^, Fernando A. Bozza^1^, Gabriel C Camargo^1^, Paulo Henrique Rosado de Castro^1^, Ronir Raggio Luiz^2^, Renata Moll-Bernardes^1^

(1) D’Or Institute for Research and Education (IDOR); (2) Federal University of Rio de Janeiro (UFRJ); (3) Evandro Chagas National Institute of Infectious Diseases (INI)

**Background:** Myocardial injury has been associated with adverse outcomes and mortality in patients with Coronavirus disease 2019 (COVID-19); however, there are still important knowledge gaps regarding its pathogenesis and clinical implications.

**Aim:** To assess the prevalence and mortality associated with myocardial injury in a large population of hospitalized patients with COVID-19.

**Methods:** Consecutive adult patients hospitalized with COVID-19 were prospectively included in a multicenter registry (n = 3246). Troponin levels were measured in the first week of hospitalization (n = 1476) and normalized according to the 99th percentile upper reference limit (URL). Demographic, clinical, and laboratory data were collected, and a logistic regression model was used to quantify the association with the primary outcome.

**Results:** Myocardial injury (troponin levels > URL) was detected in 353 cases (23.9%). Total in-hospital mortality was 10.9% (IC95%: 9,8% – 12,0%). The mortality rate was higher among patients with cardiac injury (22,7% vs 5.5%) and increased for each level of troponin (Figure 1). In-hospital mortality and myocardial injury were associated with older age, reduced oxygen saturation, diabetes, hypertension, heart failure, coronary artery disease, and chronic renal disease, but were not associated with sex. After adjusting for age and comorbidities, a multivariable logistic model showed a higher risk of death in patients with cardiac injury with an odds ratio of 2.99 (95% CI: 2,06–4,36). According to the model, age, oxygen saturation ≤93% and diabetes were also independent risk factors for mortality with COVID-19.

**Conclusions:** Myocardial injury was detected in 23.9% of the patients hospitalized with COVID-19 and was an independent predictor of in-hospital mortality. Mortality rate increased for each higher troponin category level. Myocardial injury and mortality were also associated with age, reduced oxygen saturation and cardiovascular comorbidities.


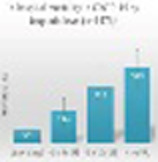


109266

Modality: E-Poster Scientific Initiation – Non-case Report

Category: ANTICOAGULATION

## Fondaparinux Versus Enoxaparin in the Treatment of Obese Patients with Acute Coronary Syndrome

BEATRIZ ROCHA DARZÉ^1^, Carolina Costa da Silva Souza^1^, João Victor Santos Pereira Ramos^1^, Queila Borges de Oliveira^2^, Mateus Santos Viana^2^, Eduardo Sahade Darzé^2^, Luiz Eduardo Fonteles Ritt^2^

(1) Escola Bahiana de Medicina e Saúde Pública; (2) Instituto Dor de Pesquisa e Ensino, Hospital Cárdio Pulmonar

**Introduction/Background:** Fondaparinux is an effective and safe anticoagulant in the treatment of non-ST segment elevation acute coronary syndromes (NSTE-ACS), when compared to enoxaparin. However, due to the underrepresentation of the obese population in clinical trials, the application of these results to obese patients is still uncertain.

**Purpose:** To compare fondaparinux to enoxaparin in the treatment of obese patients with NSTE-ACS.

**Methods:** This is a retrospective cohort study including obese patients (BMI ≥ 30 kg/m^2^) admitted with a diagnosis of non-ST segment elevation myocardial infarction (NSTEMI) or unstable angina (UA) and treated with fondaparinux or enoxaparin at a tertiary hospital, between 2010 and 2017. The study compared clinical and laboratory characteristics between the fondaparinux and enoxaparin groups using the Chi-square and Mann Whitney tests, when appropriate. The incidence of the primary outcome (death, reinfarction, stroke or major bleeding) was compared between groups and a p-value < 0.05 was used for all analyses. The study complied with the Declaration of Helsinki and was approved by the institutional review board.

**Results:** A total of 335 patients with obesity and NSTE-ACS were included, of which, 238 used fondaparinux and 97, enoxaparin. The mean age was 64 ± 12 and 52.5% were male. The prevalence of diabetes, hypertension, dyslipidemia, previous diagnosis of coronary artery disease, previous stroke and use of an invasive strategy were similar between fondaparinux and enoxaparin groups. The incidence of the primary outcome was 4.2% in the fondaparinux group and 5.2% in the enoxaparin group (p = 0.702). There were no significant differences between groups when the primary outcome components were analyzed separately. The incidence of death, stroke, myocardial infarction, and major bleeding in the fondaparinux and the enoxaparin groups were, respectively, 1.3% vs 3.1% (p = 0.251), 0% vs 1% (p = 0.117), 1.3% vs 3.1% (p = 0.251), 2.1% vs 0% (p = 0.355).

**Conclusion:** In a sample of patients with NSTE-ACS and obesity, there was no difference in the occurrence of the composite outcome (death, stroke, reinfarction and major bleeding) between patients using fondaparinux or enoxaparin. Randomized trials are needed to confirm these findings.

109267

Modality: E-Poster Scientific Initiation – Non-case Report

Category: CONGENITAL AND PEDIATRIC CARDIOLOGY

## Analysis of Correction Procedures for Interventricular Communication in Brazilian Regions in 10 Years

JULIANA DE ALMEIDA SILVEIRA^1^, Sara Cristine Marques dos Santos^1^, Girley Cordeiro de Sousa^1^, Anna Júlia Tamiozzo Reis^1^, Yasmin Monteiro Mandu^1^

(1) Universidade de Vassouras

**Introduction:** Interventricular communication (IVC) is defined as the absence of septal tissue, which allows communication between the ventricles. Among the congenital heart diseases, it is the most frequent, corresponding to about 35%. In Brazil, data indicate an incidence variation of 0.6/1000 live births and it is estimated that 28,000 new cases will appear per year, of which 23,000 will require surgical intervention. In view of the above, a more in-depth knowledge about this anomaly becomes, therefore, a subject of great interest not only for its high frequency, high number of hospitalizations and expenses for the health system, but also serves as an alert for the need of more comprehensive research in order to know the possible risk factors and make an early diagnosis.

**Objective:** To analyze the current panorama of CIV Correction procedures performed in Brazil for 10 years.

**Methods:** A systematic literature review and observational, descriptive and cross-sectional collection of IVC Correction data, available in DATASUS – SUS Hospital Information System (SIH/SUS) for a period of ten years – December 2011 were carried out. to December 2021 – evaluating the value of public spending, complexity, mortality rate, deaths, permanence and character of care and articles available in Scielo and PubMed.

**Results:** In the analyzed period, there were 2,951 hospitalizations for IVC Correction procedures, representing a total expense of R$55,801,572.62. The Brazilian region with the highest number of hospitalizations was the Southeast with 1,228 hospitalizations. The region with the highest number of deaths was the Southeast with 79 cases, while the North region had the lowest number, with 6 deaths recorded. The North region had the highest mortality rate (8.96). The Northeast region had the lowest rate, with a value of 5.58.

**Conclusions:** In view of the data, there is a higher prevalence of hospitalizations in the three most industrialized Brazilian regions (Southeast, Northeast and South). On the other hand, emergency procedures have the highest mortality rate. Furthermore, the highest mortality rate occurs in the regions with the lowest prevalence, the North and Midwest regions, and the deaths are higher in the Southeast and Northeast regions. Thus, there is a need to expand data collection in order to improve the current epidemiological analysis.

109272

Modality: E-Poster Scientific Initiation – Non-case Report

Category: EPIDEMIOLOGY AND HEALTH POLICIES/GLOBAL HEALTH

## Hypertension and Diabetes: An Analysis of its Prevalence in Women in a Brazilian State Over a Decade

DANIEL DE OLIVEIRA MEIRELES ^1^, Sara Cristine Marques dos Santos^1^, Thaís Lemos de Souza Macêdo^1^, Ivan Lucas Picone Borges dos Anjos^1^, Patrícia Rangel Sobral Dantas^1^, Ivana Picone Borges de Aragão^1^

(1) Universidade de Vassouras

**Introduction:** High blood pressure (HTN) and diabetes mellitus (DM) are two common diseases today. Complications of DM and HTN can include chronic kidney disease, cardiovascular, or cerebrovascular disease. The association of these diseases increases cardiovascular risk and morbidity and mortality1. The present study aimed to analyze the current panorama of cases of hypertensive and also diabetic patients in the state of Paraíba for 10 years and to correlate the current epidemiology with the results obtained.

**Methods:** A systematic review of the literature and an observational, descriptive and cross-sectional collection of data on HTN and DM, available at DATASUS – System for registration and monitoring of (HYPERDIA) for a period of ten years – December 2002 to December 2012 – and articles available in Scielo, Lilacs, and PubMed.

**Results:** In the analyzed period, 55,993 follow-up records of patients with HTN and DM were observed, of which 38,103 were female and 17,890 were male. Of the 55,993, 27,486 are considered sedentary, 19,008 of whom are women. Overweight in 25,635 cases. According to the age group, there is a greater number of cases from 55 to 69 years old, with an average of 7,461 cases (obtained through the arithmetic mean of the values shown by DATASUS). There are 14,569 smokers out of the total. Among the total cases, 5,222 patients with previous acute myocardial infarction and 6,513 with stroke were identified. 3,026 cases of diabetic foot were recorded. Counting 1,859 cases of amputation due to DM. Of 55,993 patients, 3,344 have chronic kidney disease. As for risk, 27,784 are considered high risk, 10,217 are very high risk and 17,992 have no calculated risk.

**Conclusions:** From the present study, the prevalence of SAH and DM in women is higher than in men. It is worth emphasizing the need to invest in primary care to control and treat these patients, also preventing new ones. Besides, there is a need for correct notification of procedures, to improve the current epidemiological analysis.

109273

Modality: E-Poster Scientific Initiation – Non-case Report

Category: CONGENITAL AND PEDIATRIC CARDIOLOGY

## Peripartum Cardiomyopathy and the Relationship of Oxidative Stress and Prolactin Levels

LARA ALÍPIO PEDROSA^1^, Ariana Lacerda Garcia^1^, Beatriz Ribeiro Coutinho de Mendonça Furtado^1^, Carolina Gonçalves da Cunha Lima^1^, Gabriella de Figueiredo Falcão^1^, Laís Nóbrega Diniz^1^

(1) Faculdade de Medicina Nova Esperança (FAMENE)

**Introduction:** Peripartum cardiomyopathy is a form of dilated cardiomyopathy of uncertain etiology, characterized by reduced left ventricular ejection fraction, which affects women without preexisting heart disease during the last month of gestation or until the fifth month of puerperium. The factors identified as the greatest risk for its development include advanced maternal age, black race, gestational hypertension and family history of cardiomyopathies. Clinical presentation includes signs and symptoms of congestive heart failure with tachycardia, tachydyspnea, edema, orthopnea, swollen jugular veins, and nocturnal cough.

**Objectives:** To discuss the clinical manifestations of peripartum cardiomyopathy, in addition to the link between prolactin levels in the genesis of this cardiomyopathic condition.

**Methods:** This bibliographic review had a qualitative character based on exploratory and selective readings of articles and scientific websites (Sciello, PubMed, UpToDate) referring to the topic in question.

**Results:** The hemodynamic stress of pregnancy on the heart can trigger the clinical manifestation of genetic forms of previously silent cardiomyopathy. Studies show identified evidence of MCP carriers with a family history of cardiomyopathy and a subgroup of patients with mutations in genes related to familial forms of cardiomyopathy. New pathophysiological concepts were recently presented in the literature, involving oxidative stress and prolactin levels in the genesis of MCP. Oxidative stress activates cathepsin D in cardiomyocytes, an enzyme that promotes proteolytic cleavage of prolactin resulting in the 16 KDa fragment, which is a potent factor with pro-inflammatory, antiangiogenic, pro-apoptotic, vasoconstrictor and cardiomyocyte depressant characteristics. In agreement with these findings, inhibition of prolactin secretion with bromocriptine, a dopamine receptor agonist, prevents the development of MCP.

**Conclusion:** Peripartum cardiomyopathy is a condition with several risk factors and without a defined cause. Treatment uses drugs for heart failure in general and the prognosis is still poor. With therapeutic advances, the mortality rate of peripartum cardiomyopathy has decreased, among these, those related to the study of the association of prolactin. Research with a view to clarifying the etiopathogenesis of this disease should be implemented, which may facilitate preventive measures.

109288

Modality: E-Poster Scientific Initiation – Non-case Report

Category: ANTICOAGULATION

## Influence of Obesity on the Safety and Efficacy of Antithrombotic Therapy: A Systematic Review and Meta-Analysis

BEATRIZ ROCHA DARZÉ^1^, Queila Borges de Oliveira^2^, Mateus Santos Viana^2^, Eduardo Sahade Darzé^2^, Luiz Eduardo Fonteles Ritt^2^

(1) Escola Bahiana de Medicina e Saúde Pública; (2) Instituto Dor de Pesquisa e Ensino, Hospital Cárdio Pulmonar

**Background/Introduction:** The prevalence of obesity has increased in recent decades and, considering its association with a higher risk of Venous Thromboembolism (VTE) and Acute Coronary Syndromes (ACS), it is necessary to establish an anticoagulation regimen suitable for this group of patients. The obese population is historically underrepresented in clinical trials, so the effect of obesity on the efficacy and safety of antithrombotic treatment is still uncertain.

**Purpose:** To evaluate the influence of obesity on the safety and efficacy of antithrombotic therapy in patients with ACS or VTE.

**Methods:** This is a systematic review and meta-analysis, conducted according to the PRISMA methodology, using the MEDLINE/PubMed, Scielo, Cochrane, EMBASE and Lilacs databases. Articles characterized as randomized, cohort or case-control studies that compared the occurrence of clinical outcomes (mortality or bleeding) between obese and non-obese patients using parenteral anticoagulants for the treatment of ACS or VTE were selected. To assess the risk of bias, the Cochrane Risk of Bias Tool and the Newcastle-Ottawa Scale were used. Statistical analysis was performed using Revman 5.4 software with relative risk and 95% CI as analytical parameters.

**Results:** Six articles, with a total of 40.939 patients, were eligible for this review, being 3 randomized clinical trials and 3 retrospective cohorts. Of the patients, 87.7% had ACS. The mortality rate was 2.6% (95% CI 2.2% –3.0%) in obese patients and 3.6% (95% CI 3.3% –4.0%) in non-obese patients, while the rate of major bleeding was 6.0% (95% CI 5.6% –6.4%) in the obese group and 6.2% (95% CI 5.9% –6.5%) in the non-obese group. The incidence of major bleeding was similar between groups with RR: 0.90, 95% CI: 0.77–1.04, p = 0.14. The outcome incidence remained comparable when studies were analyzed separately by anticoagulant: enoxaparin (RR: 0.87, 95% CI, 0.70–1.08, p = 0.21) or UFH (RR: 0.96, 95% CI, 0.79–1.17, p = 0.67). The mortality rate, measured by only 2 studies, was lower in obese patients, with RR: 0.71, 95% CI 0.59–0.87, p = 0.0007.

**Conclusion:** In patients treated for VTE or ACS, rates of thrombotic complications, death and bleeding were comparable between obese and non-obese patients, regardless of the antithrombotic used.

109317

Modality: E-Poster Scientific Initiation – Non-case Report

Category: CARDIOVASCULAR PHARMACOLOGY

## Mavacamtem: A New Perspective in the Treatment of Hypertrophic Cardiomyopathy

ANA LÍVIA GADELHA XAVIER DA NÓBREGA PEREIRA^1^, Beatriz Gadelha e Xavier^1^, Bianca Vasconcelos Braga Cavalvante^1^, Rayanne Alessandra da Silva Barreto^1^, Leticia Lacerda Burity^1^, Vinicius Vieira Leandro da Silva^1^

(1) Faculdade de Medicina Nova Esperança (FAMENE)

**Introduction:** Hypertrophic cardiomyopathy (HCM) is a heart disease that causes anatomical changes in the cardiac structure, the main one being left ventricular hypertrophy. Mavacamten is a molecule capable of inhibiting myosin ATPase and aims to assist in the treatment of hypertrophic heart disease, reducing contractility, improving diastolic function and promoting a better energetic response in the myocardium. The drug was tested in preclinical studies, demonstrating safety and reduction of obstructive symptoms, in addition to improvement in left ventricular filling pressures.

**Objectives:** This article aims to systematically review the existing literature with the results of the use of the drug mavacamtem for the treatment of hypertrophic cardiomyopathy.

**Methods:** This is a systematic review of the literature indexed between 2018 and 2021, published in the Scientific Electronic Library and PubMed databases. Unfinished and inaccessible articles for free were excluded.

**Results:** The clinical presentation of HCM can vary from asymptomatic to advanced heart failure. Its diagnosis is confirmed by echocardiography and/or vascular magnetic resonance imaging with the observation of left ventricular hypertrophy. Currently, HCM therapy is based on the use of beta-blockers as a first line in symptomatic patients and disopyramide as a second option. Mavacamtem was recently tested in phase three of a randomized double-blind placebo-controlled trial with 251 patients enrolled in the program, where 123 were selected for use of the drug. It was evidenced that 45 patients using mavacamten reached the primary goal against the 22 who reached the use of placebo. Patients using the medication had significant reductions in left ventricular outflow tract obstruction after exercise, a greater increase in pVO2, and improvement in symptoms and quality of life compared to those who did not use the drug. In addition, there was a NYHA class reduction in at least 80 patients. In addition, the drug proved to be tolerable and without important adverse manifestations, being similar to placebo.

**Conclusion:** Currently, the use of macavamten must be associated with previously established pharmacological therapy, in an effective, safe and tolerable way, in order to act directly in the pathophysiology of the disease and to avoid or postpone the need for invasive processes in patients. However, additional studies are needed to determine the best therapy for patients with HCM.

109302

Modality: E-Poster Scientific Initiation – Non-case Report

Category: ACUTE AND CHRONIC CORONARY DISEASE/THROMBOLYSIS

## Accuracy of Clinical Judgment in the Evaluation of Specific Characteristics of Acute Chest Pain Predicting Coronary Artery Disease: A Systematic Review

CAROLINA COSTA DA SILVA SOUZA^1^, Cláudio Marcelo Bittencourt das Virgens^1^, Mateus dos Santos Viana^1^

(1) Escola Bahiana de Medicina e Saúde Pública (EBMSP)

**Background:** Faced with a patient with acute chest pain, the need to rule out the etiology of coronary artery disease (CAD) is substantial due to the high associated mortality. In a scenario of abundant and often unnecessary expenses in the medical field, the need for an initial reasoning to determine which patients need confirmatory tests is highlighted. Because of this, health professionals seek support in the specific characteristics of acute chest pain through the gestalt to determine the pre-test probability of each patient. In view of the wide applicability of this methodology, its validation becomes essential for medical practice.

**Purpose:** To assess the accuracy of clinical judgment in interpreting specific characteristics of acute chest pain for the diagnostic prediction of CAD.

**Methods:** A systematic literature search was carried out through PUBMED, EMBASE, LILACS, SCIELO and CENTRAL databases and manual search. The selected articles were evaluated using the QUADAS-2 tool, and the collected data were used for a qualitative and quantitative analysis of the data, with meta-analysis built according to the fixed effects model, considering the measures of sensitivity, specificity, odds ratio, and diagnostic odds ratio (DOR). The present research was formulated according to PRISMA methodology.

**Results:** Two studies with low risk of bias and good applicability for this systematic review were selected, with a total of 487 patients evaluated in terms of coronary obstruction in an emergency setting. Studies’ results were comparable, with values of sensibility and specificity ranging from 0,49 to 0,47 and 0,52 to 0,66, respectively. The calculated DOR was 1.47 (95%CI, 1.02–2.11), p = 0.04. The McNemar test for heterogeneity was 6.91 (p = 0.009).

**Conclusion:** The gestalt of acute chest pain in an emergency context is not a good accuracy method for the prediction of coronary artery disease. New research is necessary to reinforce these findings.

109346

Modality: E-Poster Scientific Initiation – Non-case Report

Category: CARDIOGERIATRICS

## Screening for Sarcopenia in Elderly Patients with Chronic Kidney Disease and Heart Failure

ELLEN BEATRIZ SOBRAL^2^, Carolina Jeronimo Magalhaes^3^, Maria Eduarda Borges Matias^2^, Sabrina Barreto Braga Pires^2^, Carolina de Carvalho Moury Fernandes^2^, Matheus Dantas Soeiro^2^, Jessica Myrian de Amorim Garcia^1^, Francisco Bandeira^3^

(1) Hospital Agamenon Magalhães (HAM); (2) Faculdade Pernambucana de Saúde (FPS); (3) Universidade de Pernambuco (UPE)

**Introduction:** The reduction of muscle strength, mass and function, known as sarcopenia, has a significant association with physiologic aging process. As the world population has aged, multiple comorbidities such as heart failure (HF) and chronic kidney disease (CKD) are frequent in the elderly, which has correlation with inflammatory factors and sarcopenia. Sarcopenia influences quality of life, morbidity, and mortality.

**Objectives:** Evaluate the screening of high risk for sarcopenia in elderly in-patients with CKD and HF.

**Methods:** Cross sectional observational study from August 2020 to December 2021 in a tertiary cardiology center with in-patients ≥65 years. Diagnosis and classification (stage 1 to 5) of CKD was based on CKD epidemiology collaboration equation to estimate glomerular filtration rate (GFR). For this study we established a considered renal disfunction patients with GFR < 60 mL/min/1.73 m^2^. Sarcopenia screening was performed with Sarc-f and patients with score >5 were defined in a high risk for sarcopenia.

**Results:** A total of 190 patients (53,6% female) were studied. Mean age of 72.9 ± 5.9 years. A total of 49,73% elderly patients had renal disfunction. Stage 3 CKD elderly patients were 31,6% of the population study; 8,9% stage 4; 8,4% stage 5. In elderly patients with both renal disfunction and HF, 36,8% had positive screening for sarcopenia with Sarc-f > 5. The presence of CKD (stages 3 to 5) and HF had no association with the prevalence of high risk for sarcopenia.

**Conclusions:** The screening with Sarc-f in elderly patients with CKD and HF in this study has not shown a significant high risk for sarcopenia. However, screening Sarc-f has providing identification of in-patients at risk of developing sarcopenia, in addition to practicality and specificity in medical practice, which shows the importance of more studies and further analysis.

109543

Modality: E-Poster Scientific Initiation – Non-case Report

Category: CARDIOVASCULAR PHARMACOLOGY

## Reduction of Cardiac and Vascular Remodeling in Pulmonary Hypertension Induced by Agonist of A2A Adenosine Receptor

GABRIEL FONSECA GOMIDE^1^, Jaqueline S Da Silva^1^, Bruna Rocha^1^, Tadeu Montagnoli^1^, Bruno Eduardo Dematté^1^, Rodolfo Maia^1^, Eliezer J Barreiro^1^, Gisele Zapata-Sudo^1^

(1) Universidade Federal do Rio de Janeiro UFRJ

**Introduction:** Pulmonary hypertension (PH) is a chronic progressive disease characterized by extensive pulmonary vascular remodeling, leading to right ventricle (RV) hypertrophy and failure. Activation of adenosine receptor A2A (A2A-AR) can limit the progression of PH by attenuating vascular and cardiac inflammation and remodeling. This work aims to evaluate the effects of a new A2A-AR agonist, LASBio-1900, in cardiac and vascular dysfunctions on monocrotaline (MCT)-induced PH in male Wistar rats.

**Methods:** After a single injection of MCT (60 mg/kg i.p.), twelve rats were randomly divided in groups and treated orally with either vehicle or LASSBio-1900. Hemodynamic parameters were obtained by Doppler echocardiography and intraventricular pressure measurement. Pulmonary arteries (PA) muscularization was determined through the percentage of the wall portion positively stained with alpha smooth muscle actin (α-SMA) relative to the total transversal area. Perivascular collagen in PA was evaluated using picrosirius red staining and interstitial fibrosis was measured by obtaining the total collagen area per arteriole area. Evaluation of inflammatory process in perivascular PA was performed by immunohistochemistry to iNOS content in as the ratio of stained area to the arteriole area.

**Results:** PH promotes increased PA vascular resistance, since the ratio of pulmonary acceleration time to total ejection time (PAT/TET) changed from 34.35 ± 0.85 in control rats to 23.17 ± 1.51 in MCT and improved in MCT-LASSBio-1900 to 30.86 ± 2.74 (p < 0.05). LASSBio-1900 reduced RV hypertrophy, as it reduced from 55.4 ± 2.3 to 35.3 ± 5.9% (p < 0.05). PH increased RV systolic and diastolic pressures, 19.21 ± 2.04 and 4.73 ± 1.04 mmHg in control to 51.5 ± 5.2 and 11.9 ± 1.3 mmHg, respectively. LASSBio-1900 recovered RV function reducing systolic and diastolic pressures to 28.4 ± 4.0 and 6.51 ± 0.84 mmHg. The increase in wall thickness observed in MCT 61.70 ± 1.11 to 83.24 ± 1.82% was improved by LASSBio-1900 to 75.24 ± 3.46% (p < 0.05). Perivascular fibrosis in PH rats was 15.06 ± 1.34% and LASSBio-1900 reduced the PA collagen deposition to 8.7 ± 1.4% (p < 0.05). iNOS expression evaluated in control of 4.5 ± 0.3 and MCT of 19.0 ± 0.9% (p < 0.05) in iNOS protein content were observed in PH and LASSBio-1900 reduced to 8.7 ± 1.4% (p < 0.05).

**Conclusion:** A2A-AR activation by LASSBio-1900 improved RV hemodynamics and pulmonary vascular structure in PH, indicating a new alternative treatment.

109378

Modality: E-Poster Scientific Initiation – Non-case Report

Category: CARDIOVASCULAR PHARMACOLOGY

## Systematic Review of the Efficacy of Anacetrapib in Reducing Cardiovascular Events in Patients with Dyslipidemia or Atherosclerosis

BERNARDO BALSAN CAMILLO ^1^, Carolina Martinez Teixeira^1^, Rodolfo Balsan Camillo^2^, Bruna Rocha Mezzomo^1^, Giuseppe Morales Gentilini^1^, Joel Soares Dahne^1^

(1) Universidade Católica de Pelotas; (2) Santa casa de misericórdia de Porto Alegre

**Introduction:** Among the causes of global morbidity and mortality, cardiovascular diseases are among the most prevalent. For the purpose of reducing cardiovascular risk, the use of cholesterol-lowering medications, such as Anacetrapib, has gained prominence in relation to other therapeutic options.

**Objective:** Description of the importance of Anacetrapib in the reduction of cardiovascular events associated with dyslipidemia and atherosclerosis.

**Method:** Systematic review (PRISMA) based on scientific literature from 2011 to 2019, published in PubMed databases. The descriptor used was “Anacetrapib”. Articles that show information about the choice of their choice were removed, and those whose content was not related to the proposed topic were removed. 249 titles were found. Of these, 15 were selected, 7 abstracts were read and 7 papers were selected for this study.

**Result:** The results demonstrate that dyslipidemic patients undergoing cholesterol ester transfer protein (CETP) inhibitors, a key protein involved in reverse cholesterol transport, especially Anacetrapib, had a reduction in mortality associated with atherosclerosis and dyslipidemia. The reduction in mortality is due to the significant reduction in LDL, triglyceride and HDL levels in patients who used the drug as a single therapy. Furthermore, in patients on dual therapy, associated with statins, they have a greater ability to reduce cardiovascular events. Although other CEPTs have been analyzed, the only one that has so far not demonstrated adverse effects such as death from cardiovascular and non-cardiovascular causes was Anacetrapib. In addition to being able to be used in replacement therapy for statins, this medication can be used both in patients who do not reach LDL levels adequate for their risk, and in patients who do not tolerate statin therapy.

**Conclusion:** Despite the occurrence of drugs that there are more studies on the subject, and ignoring the high cost factor, Anacetrapib is a drug that seems to reduce death from cardiovascular causes in patients with dyslipidemia or atherosclerosis, without causing significant adverse effects.

109369

Modality: E-Poster Scientific Initiation – Non-case Report

Category: COVID-19 AND CARDIOVASCULAR SYSTEM

## Admissional D-Dimer Level as a Biomarker for Assessment of COVID-19 Prognosis in a University Hospital

JOÃO PEDRO COSTA DOS SANTOS^1^, João Pedro Costa dos Santos^1^, Mariana Ranucci da Cunha^1^, Lucas Narciso Balchiunas^1^, Pedro Henrique Yukio Miyaji^1^, Henrique Thadeu Periard Mussi^1^

(1) Universidade Federal Fluminense

**Introduction:** COVID-19 is a respitarory disease that has a physiopathology highly associated with thromboembolic disorders and complications. Some studies suggest that elevated d-dimer can predict poor prognosis in this patients, although cut-off levels are not consensual yet.

**Objetives:** This study aims to correlate admissional d-dimer levels with the need for endotracheal intubation and mortality in COVID-19 patients.

**Methods:** Retrospective cohort study with 142 patients with laboratorial diagnosis of COVID-19 admited at Hospital Universitário Antônio Pedro. We compared mean serum levels of d-dimer between patients submitted to mechanical ventilation or not and with mortality in this group, searching for the association of this variables, using the t-test for this purpose. Statistical analysis was performed in Microsoft Office Excel 2016 and Jamovi 1.6.23.

**Results:** We enrolled 142 patients with laboratorial diagnosis of COVID-19, 53,5% were male, the mean age was 60,3 years, 62,6% were diagnosed with arterial hypertension and 37,3% with diabetes. The mean admissional d-dimer level in the whole population was 2289 ng/ml. In the patients that needed endotracheal intubation the admissional mean levels were 3275 ng/ml and 1809 ng/ml in those who were not intubated. Besides that, the mean admissional levels of d-dimer in the patients that died were 3556 ng/ml and 1834 ng/ml in those that did not have the same outcome. According to the t-test results, high d-dimer admissional serum levels were associated with the need of endotracheal intubation (Statistic = –3,52; p < 0,01) and with mortality (Statistic = –3,96; p < 0,01).

**Conclusions:** This studie concludes that high admissional levels of the d-dimer showed association with poor prognosis in patiets diagnosed with COVID-19 in Hospital Universitário Antonio Pedro. More studies are needed to define a cut-off point and confirm the association between these variables.

109377

Modality: E-Poster Scientific Initiation – Non-case Report

Category: HYPERTENSION/RENAL DENERVATION

## Probable Association between Body Mass Index and Hypertension in Elementary and High School Teachers

GLÓRIA DE MORAES MARCHIORI^1^, Glória de Moraes Marchiori^1^, Daiane Soares de Almeida Ciquinato^1^, Braulio Henrique Magnani Branco^1^, Luciana Lozza de Moraes Marchiori^1^

(1) Universidade UniCesumar UniCesumar

**Introduction:** Obesity and overweight are an important public health problem in society, due to their growth in all age groups and their association with various chronic diseases, especially arterial hypertension. The school constitutes an important environment in the configuration of the teaching staff’s reality of life and aspects related to the conditions and organization of the teacher’s work, which have repercussions on the health-disease process. The working conditions of teachers can contribute to the development of lifestyle-related diseases, including obesity and high blood pressure. High sedentary behavior has been associated with abdominal obesity, and high sedentary leisure breaks have been associated with lower chances of hypertension among public school teachers.

**Objective:** The objective of the present research was to compare hypertension with the body mass index (BMI) in a sample of teachers.

**Methods:** This is a cross-sectional study, part of a thematic project, part of a broader research called PRO-MESTRE. The project was approved by the Humana Research Ethics Committee, in which all participants were previously informed about its purpose and procedures and signed the Free and Informed Consent Form. Inclusion criteria included being elementary and high school teachers, being actively teaching and being in charge of a subject in class, working in the classroom for more than 12 months, in addition to not having had a leave of absence for >30 days in the 12 months previous. The sample of elementary and high school teachers, who answered the questionnaire with objective questions to verify hypertension. BMI (kg/m^2^) was calculated based on self-reported body weight (in kilograms) divided by height (in square meters), with the following classification: <18.5: low weight; 18.5 to 24.99: normal or eutrophic; 25.0 to 29.99: overweight; and 30.0: obese. The BMI calculation and classification followed the National Center for the Prevention of Chronic Diseases. Appropriate statistical tests were applied adopting a significance level lower than 0.05%.

**Conclusion:** In the present study, there was a significant association between hypertension and BMI in the teacher population. These observations can help health professionals in the diagnosis of arterial hypertension in teachers and in the approach of anthropometric factors and other comorbidities in the clinical evaluation of these professionals, considering the lifestyle.

109393

Modality: E-Poster Scientific Initiation – Non-case Report

Category: CONGENITAL AND PEDIATRIC CARDIOLOGY

## Epidemiology of Deaths by Congenital Malformations of Cardiac Septa in the North Region in the Years 2014 to 2019

JOSÉ GUILHERME MELO SILVA^1^, Eduardo Fernandes de Oliveira^1^, Newton Pessoa de Oliveira Neto^1^, Ana Beatriz Estrela de Sá^1^, Giordana do Nascimento Nunes^1^, Victor Matheus Costa Cardoso^1^

(1) Centro Universitário Tocantinense Presidente Antônio Carlos- UNITPAC

**Objective:** Congenital heart disease is considered the most common malformation and the leading cause of death among birth defects, despite being underdiagnosed in Brazil. The aim of this study was to describe the epidemiological profile of deaths that occurred due to congenital heart septal malformation from 2014 to 2019.

**Methods:** This is a cross-sectional, descriptive and quantitative epidemiological study using data of deaths due to congenital heart septal malformation in the Northern region in the period 2014–2019, which were obtained through the computer department of the single health system (DATASUS).

**Discussion and Results:** According to the age group, most cases of death due to cardiac septal malformation occurred in the first year of life, especially in the first month. Comparing the genders, females were slightly more prevalent than males. Comparing the races/colors, the prevalence occurred mainly in those who considered themselves as brown, followed by white, and thus, gathering more than 90% of the cases of deaths due to congenital malformation of the heart septum.

**Conclusion:** It is evident, therefore, that the research findings point to the importance of an active search for congenital heart diseases in the neonatal period and a greater attention to socioeconomic groups that historically are more exposed. Considering that this is one of the main causes of death in the first year of life. In addition, more comprehensive and detailed research is needed to understand the risk factors and develop preventive plans for some of these disabilities.

109411

Modality: E-Poster Scientific Initiation – Non-case Report

Category: HEMODYNAMICS AND INTERVENTIONAL CARDIOLOGY

## Association between Delayed Door to Ballon Time and “Off-Hours” Primary Percutaneous Coronary Intervention

BRUNO CARDOSO SCHMOELLER^1^, Leonardo Sinnott Silva^1^, Italo Mattos Rinaldi^1^, Gabriel Fernando Elias dos Reis^1^

(1) Universidade do Sul de Santa Catarina (UNISUL)

**Background:** Primary percutaneous coronary intervention (PCI) is currently the therapy of choice for the treatment of acute ST-segment elevation myocardial infarction (STEMI). However, randomized clinical trials have shown that when the door-to-balloon time exceeds 100 minutes, the benefits of PCI over fibrinolytic therapy is drastically reduced or even eliminated.

**Objective:** To verify the association of door-to-balloon time with patients admitted in business hours and non-business hours.

**Material & Methods:** 57 STEMI patients who underwent primary PCI between April and August 2021 were included.

**Results:** The average door-to-balloon time (D2B) found was 132.16 ± 68.96 minutes. For better characterization, the D2B time was converted into a categorical variable and separated into two groups: below 90 minutes (26.30%) and above 90 minutes (73.70%). Patients undergoing primary PCI outside of business hours, at night and on weekends, had a higher prevalence of D2B time over 90 minutes (66.60%), with a statistical difference compared to patients admitted during business hours (33.40%) (p = 0,027). It is easily noticeable that the longer D2B time at night and on weekends are related to logistical issues.

**Conclusion:** The average D2B time found is above what is recommended. It was evidenced that the delayed PCIs are mostly concentrated in non-business hours, which negatively affects the prognosis of treated patients.


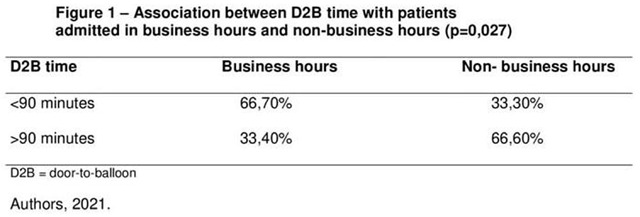


109478

Modality: E-Poster Scientific Initiation – Non-case Report

Category: EPIDEMIOLOGY AND HEALTH POLICIES/GLOBAL HEALTH

## Total Cost Estimate of Acute Coronary Syndrome (ACS) in the State of Piauí in the Period from 2011 to 2020

FRANCISCO ELTON COELHO DA SILVA FILHO^1^, Giuseppe Marques Alencar^1^, Lidia Lillian Santos Barbosa^2^, Antonio Maycon da Silva Sousa^1^, Carlos Eduardo Batista De Lima^1^

(1) Universidade Federal do Piauí – UFPI; (2) Centro Universitário UniFacid – UniFacid

**Objective:** To estimate the total cost of Acute Coronary Syndrome (ACS) in the State of Piauí, between January 2011 and December 2020.

**Methodology:** For the calculation of direct adirect cost we obtained the number and cost of hospitalizations for Acute Myocardial Infarction and Unstable Angina in Piauí, for the Unified Health System (SUS) through DATASUS/TABNET as well as the values related to angioplasties and revascularization surgeries. From this, data for the Supplementary Health System (SSS) were estimated based on specific annual coverage rates. To estimate indirect costs they were incorporated due to loss of productivity associated with early mortality and absenteeism in patients belonging to the occupied population.

**Results:** The sum of the amounts of hospitalizations by ACS and the procedures performed in the period (total direct cost) was R$ 178,943,799.06 (U$ 35,189,136.92). The sum (total indirect cost) of the estimated costs for absenteeism and early mortality was R$ 1,176,271,322.60 (U$ 231,312,696.18). Therefore, the total estimated cost was R$ 1,355,215,121.66 (U$ 266,501,833.10).

**Conclusion:** The estimated average annual cost of ACS in Piauí was R$ 135,521,512.16 (U$ 26,650,183.31) and showed a downward trend in the last five years of the period.


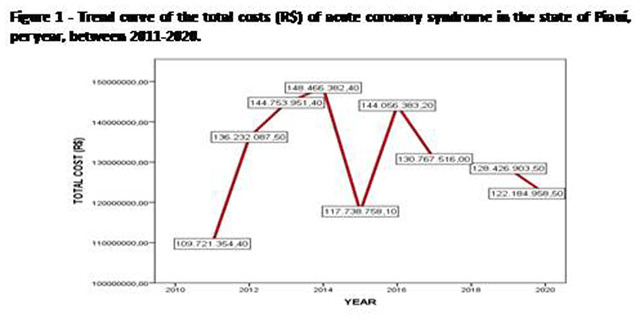


109414

Modality: E-Poster Scientific Initiation – Non-case Report

Category: COVID-19 AND CARDIOVASCULAR SYSTEM

## COVID-19 and Chronic Cardiovascular Diseases: Clinical and Epidemiological Indicators in Adults in Southern Brazil

JARBAS RYGOLL DE OLIVEIRA FILHO^1^, EVA BRENDA SANTOS SILVA^1^, THIAGO EMANUEL RODRIGUES NOVAES^1^, TASSO KFURI ARAÚJO MAFRA^1^, JOSSIMARA POLETTINI^1^, GUSTAVO OLSZANSKI ACRANI^1^, SHANA GINAR DA SILVA^1^, RENATA DOS SANTOS RABELLO^1^, IVANA LORAINE LINDEMANN^1^

(1) Universidade Federal da Fronteira Sul (UFFS), Campus Passo Fundo, BRASIL.

**Introduction:** The Coronavirus Disease 2019 (COVID-19) still represents a global health problem. It is known that such an infection, caused by Severe Acute Respiratory Syndrome CoronaVirus 2 (SARS-CoV2), can progress to complex clinical conditions, including Severe Acute Respiratory Syndrome (SARS), requiring hospitalization in the Intensive Care Unit (ICU) and use of ventilatory support. It is also clear that patients with previous cardiovascular diseases, as well as other comorbidities, are at greater risk of developing severe symptoms and progressing to death.

**Objectives:** To identify the prevalence of hospitalization in the Intensive Care Unit (ICU), use of ventilatory support and case evolution in adult patients diagnosed with COVID-19, carriers and non-carriers of chronic cardiovascular diseases (CVD).

**Methods:** Observational study based on data analysis from the Influenza Epidemiological Surveillance Information System (SIVEP-Gripe) in Passo Fundo, RS. The study population consisted of SARS cases due to COVID-19, confirmed and reported from January 1 to December 31, 2020. Sociodemographic and health data were collected from individuals aged between 20 and 59 years. The use of ventilatory support, admission to the ICU and the evolution of the case (death/cure) were used as outcomes of interest, and the exposure analyzed was the diagnosis of CVD. The prevalence was verified with a 95% confidence interval (95%CI) of the outcomes and their distribution according to the predictor variable (chi-square test, admitting an α error of 5%).

**Results:** The sample consisted of 574 participants, with a predominance of men (58.0%) and aged between 41 and 59 years (69.0%). The diagnosis of CVD was observed in 25.3%. The prevalence of ICU admission was 21.0% (CI95 18–24), use of ventilatory support was 65.1% (CI95 61–69) and 12.2% (CI95 9–12) died. A statistically significant relationship was found between CVD and ICU admission (29.0%, p = 0.002) and use of ventilatory support (77.4%, p < 0.001), but not between CVD and deaths (15.6%, p = 0.051).

**Conclusions:** There was a higher prevalence of ICU admission and use of ventilatory support in adults with CVD, a fact that attests to the greater complexity of managing the COVID-19 patient with CVD. The relationship of angiotensin-converting enzyme 2 – the main gateway of SARS-CoV2 – which is also related to CVD, is evaluated. As for lethality, no statistically significant relationship was identified.

109446

Modality: E-Poster Scientific Initiation – Non-case Report

Category: COVID-19 AND CARDIOVASCULAR SYSTEM

## The COVID-19 Pandemic and its Impact on Deaths from Cardiovascular Causes in Brazi

GUSTAVO FIALHO COELHO^1^, Mariana Moreira Vannier^2^, Tamara Tâmara de Souza^1^, Caroline Melo Jordão Reis^2^, Karla Santa Cruz Coelho^1^

(1) Universidade Federal do Rio de Janeiro campus Macaé; (2) Centro Universitário Serra dos Órgãos

**Introduction:** The first confirmed death by COVID-19 in Brazil was on 12th March 2020, in the city of São Paulo. From then on, the country’s health system was affected by the pandemic with the occurrence of direct and indirect deaths. Therefore, an analysis of the pandemic’s impacts on other health aspects such as deaths from cardiovascular causes is necessary.

**Objectives:** Analyze the pandemic’s impact on mortality from cardiovascular causes in Brazil, as a whole and at home, using death data from the Civil Registry in 2019, 2020 and 2021.

**Methods:** The Civil Registry Portal was accessed on March 28th 2022 and death data from unspecific (UCC) and specific cardiovascular causes – acute myocardial infarction and cerebrovascular accident (CVA), in the years 2019, 2020 and 2021, referring respectively to the pre-pandemic, 1st year of pandemic and 2nd year of pandemic were collected.

**Results:** When comparing the sequence of years there was a single reduction in deaths from infarction from 2019 to 2020 (–4.28%) with an increase in all other analyzed aspects. From 2019 to 2020 there was an increase in deaths from CVA (0.97%) and UCC (31.17%). While from 2020 to 2021 there was an increase in deaths from infarction (6.40%), CVA (3.55%) and UCC (15.94%). When analyzing deaths at home, there was an increase in all death causes; infarction (4.35%), CVA (26.67%) and UCC (69.89%) from 2019 to 2020 and infarction (4.16%), CVA (4.06%) and UCC (14.65%) from 2020 to 2021. There was also a more accentuated growth in deaths from unspecific than specific cardiovascular causes, especially in deaths at home.

**Conclusion:** Deaths from different cardiovascular causes increased when comparing the pre-pandemic period to the pandemic period and persisted from the first year of the pandemic to the second, almost in its entirety. Deaths from unspecific cardiovascular causes and at home had a significant increase, showing a probable influence of the COVID-19 pandemic on the Brazilian health system. Thus, the pandemic’s evolution in different phases and the adaptation of the health system to it, demonstrated an influence on the number of deaths from cardiovascular diseases. New studies should be carried out, analyzing the deaths registered in registry offices and the hospital admissions data.

109430

Modality: E-Poster Scientific Initiation – Non-case Report

Category: HYPERTENSION/RENAL DENERVATION

## Descriptive Analysis of Arterial Stiffness Data in a Cohort of Resistant Hypertensives: A Possible Predictor of Cardiovascular Risk

GABRIELA GAMA ZAGNI JARDIM^1^, Antonio Carlos Eberienos Assad Filho^2^, Julia Resende de Oliveira^2^, David Ferreira de Lima Duarte^2^, Maria Gabriela Pimenta dos Santos^2^, Paola Pugian Jardim^3^, Andréa Vaospasse Cocco Faria^3^, Lílian Soares da Costa^4^

(1) IDOMED – Universidade Estácio de Sá, Campus Vista Carioca, Rio de Janeiro, RJ, Brasil/Instituto Estadual de Cardiologia Aloysio de Castro/IECAC; (2) IDOMED – Universidade Estácio de Sá, Campus Cittá, Rio de Janeiro, RJ, Brasil/Instituto Estadual de Cardiologia Aloysio de Castro/IECAC; (3) Instituto Estadual de Cardiologia Aloysio de Castro/IECAC; (4) IDOMED – Universidade Estácio de Sá, Campus Vista Carioca, Rio de Janeiro, RJ, Brasil/IDOMED – Universidade Estácio de Sá, Campus Cittá, Rio de Janeiro, RJ, Brasil/Instituto Estadual de Cardiologia Aloysio de Castro/IECAC

**Background:** It is estimated that the prevalence of resistant arterial hypertension (RAH) is 200 million individuals worldwide. Compared to controlled hypertensives, a higher arterial stiffness is observed, and the measurement of pulse wave velocity (PWV) has been, in addition to being a predictor of vascular stiffness, a prognostic marker and increased cardiovascular (CV) risk, especially in low/medium risk populations.

**Objective:** Descriptively analyze the demographics and PWV findings of an RAH cohort.

**Materials and Methods:** Prospective observational study of a consecutive cohort of RAH, who are part of a cohort of individuals at high CV risk (n 163), followed up at a tertiary cardiology unit in Rio de Janeiro. A questionnaire was applied containing sociodemographic data, CV risk factors, comorbidities, medications in use and therapeutic optimization, in addition to reviewing medical records to collect data from complementary exams performed within 12 months. In this group, casual systolic (SBP) and diastolic blood pressure (DBP), central aortic pressure, amplification index and PWV were measured.

**Results:** Fifty-two individuals with RAH were evaluated. The mean age was 65.3 years (42–85 years) and 53.85% (n 28) were female. The most relevant data found were: 75% dyslipidemia, 75% confirmed coronary disease, 55.7% diabetes mellitus, 5.8% chronic kidney disease, 63.8% reported snoring with 25% untreated sleep apnea. In oscillometric analysis, the mean BP was 130.2 × 82.8 mmHg (SBP 91–220 and DBP 68 × 108 mmHg) and PWV 9.45 m/s (6.3–13.3 m/s). Clinical evaluation showed that blood pressure values were elevated by 28.8% for SBP and/or DBP (n 15) and 55.8% for PWV (n 29), using median values as a standard after adjustment for sex, age, and presence of additional risk factors.

**Conclusion:** The data indicate PWV values similar to those of studies with a RAH population of the same age group, emphasizing that a statistically significant percentage presented high PWV values, even in patients with RAH and controlled BP in oscillometric measurements. Additional studies are needed to demonstrate that PWV can be an independent prognostic marker also in high CV risk individuals, which may contribute to the management and pharmacological adjustment of this RAH population.

109454

Modality: E-Poster Scientific Initiation – Non-case Report

Category: CARDIOVASCULAR SURGERY

## Comparative Analysis between Myocardial Revascularization Procedures using Extracorporeal Circulation by Region between 2018 and 2021 in Brazil

MARCIO CÉSAR RIBEIRO MARVAO^1^, Márcio César Ribeiro Marvão^1^, Mariana Kondo Obara^2^, Alice Barroso Guimarães^2^, Lucas Campos Maia^3^, Teresa Giovanna do Carmo Alves^4^, Danillo Monteiro Porfírio^2^, Maria Clara Hollanda Cecim^4^

(1) Instituto Evandro Chagas (IEC); (2) Universidade Federal do Pará (UFPA); (3) Universidade do Estado do Pará (UEPA); (4) Centro Universitário do Pará (CESUPA)

**Introduction:** Among cardiovascular diseases, coronary artery disease (CAD) has been the leading cause of death in Brazil for half a century. Myocardial revascularization (RM) is the most performed cardiac surgery in the country, and aims to improve the blood supply of coronary arteries that have a narrowed or blocked portion, through the implantation of grafts, which can be performed with or without extracorporeal circulation (ECC).

**Objectives:** To quantitatively analyze data on MR using ECC performed in Brazil from 2018 to 2021 and analyze the percentage rate of surgeries by region and national and regional morbidity and mortality rates.

**Methodology:** Cross-sectional, observational and descriptive study, with data from the Hospital Information System of the SUS IT department (SIH/DATASUS) about MR with the use of ECC, including those in which 2 or more grafts were used, performed between 2018 and 2021.

**Results:** There were 67,122 hospitalizations for the procedure studied, with a peak in 2019 (28.7%) and concentrated in the Southeast (42.9%) and South (31.9%) regions. The North region had the lowest value (3.9%). There were 4,003 deaths, with the Southeast region presenting the most expressive amount (39.5%), followed by the South region (32%). The North region presented the lowest numbers (5.5%). The national mortality rate was 5.64 in 2018 and 5.53 in 2019 (a drop of 1.9%), however, during the period of the pandemic, there was a recorded value of 6.51 in 2020 (an increase of 18% compared to 2019). Taking into account the relative mortality rate in the period, the Central-West and North regions had the highest indicators (10.6 and 8.48, respectively).

**Conclusion:** There were 67,122 hospitalizations for coronary revascularization surgery, mainly in the Southeast and South regions, with the lowest rates in the North region. These data may be related to the availability of resources and the greater number of specialized centers in the richest regions. A total of 4,003 deaths were recorded, with the Southeast and South presenting the highest values, which may reflect the number of procedures being proportional to the number of deaths. However, when analyzing relative mortality, the Midwest and North regions led, which implies that there are other factors that increase mortality in these places. During the COVID-19 pandemic, there was an 18% increase in the death rate, suggesting that there may be a relationship between Sars-Cov-2 and CAD complications.

109492

Modality: E-Poster Scientific Initiation – Non-case Report

Category: HEART FAILURE/CARDIOMYOPATHY/TRANSPLANT

## Analysis of Hospital Admissions and Mortality Rate Due to Cardiac Insufficiency Amongst Elder Patients in Brazil’s Regions from 2009 to 2019

IVAN CUOCO SAMPAIO^1^, Israel Figueira Lemos^1^, Ingrid Jade Muniz Wanderley^1^, João Lucas Silva Sales^1^, Juliana de Sousa Tavares^1^, Luiz Carlos Figueiredo Filho^1^, Luma Maria Favacho Bordalo^1^, Maria Eduarda Dantas da Veiga^1^, Mariana Lassance Maya Palheta^1^, Paula Larissa Baía Lima^1^, Rafael Augusto Silva Cabeça^1^, Ivan Cuoco Sampaio^1^

(1) Universidade do Estado do Pará

**Introduction:** Cardiac insufficiency, also known as heart failure, occurs when the heart cannot pump strongly enough to make sufficient blood reach the entire body, causing several secondary effects on patients, including death. Recently, an increase in the number of cases and mortality related to this pathology has been observed, especially amongst the elderly, due to their vulnerability to this disease because of their age.

**Objective:** Carry out a comparative analysis of death and hospital admission numbers on elderly people, caused by Cardiac Insufficiency in Brazil’s regions.

**Method:** This is a quantitative-focused, retrospective cross-sectional study which uses the Brazilian public health system hospital database (“Sistema de Informações Hospitalares do SUS – SIH/SUS”), alongside ICD-10: Cardiac Insufficiency. The data searched refers to elder patients – 60 years or more – who were hospitalized or died between the years 2009 and 2019 in the five Brazilian regions.

**Results:** Regarding the data collected, there was a slightly higher frequency of female hospitalization (50.5%) compared to male (49.5%). The Southeastern Region concentrated the highest number of hospitalizations (41.5%), followed by the South (23.6%), Northeast (22.7%), Midwest (7%) and North (5%). The age group that accumulates the highest number of hospitalizations is 70 to 79 years (36.9%), followed by 60 to 69 years (32.8%), finally, 80 years and more (30.1%). In total, 259.416 patients died due to Cardiac Insufficiency. The Brazilian region with most death rates was Southeast (47.1%), followed by Northeast (24.1%), South (18.1%), Midwest (5.9%) and North (4.8%) regions. The age group that concentrated the most mortality was 80 years or more (52.8%), followed by 70 to 79 years (29.2%) and 60 to 69 years (18.0%).

**Conclusion:** According to the data collected, Southeast region has the highest number of admissions and deaths regarding Cardiac insufficiency, this happens due that region’s population density. Also, as patients grow older, more female patients suffer from this disease. It has been shown that even though the South has the 2nd highest number of hospital admissions, the Northeast possesses the 2nd highest mortality, possibly due to demographic density or poor infrastructure of the public health system, when compared to the South region.

109472

Modality: E-Poster Scientific Initiation – Non-case Report

Category: ATHEROSCLEROSIS/CARDIOVASCULAR RISK FACTORS/CARDIOVASCULAR PREVENTION

## Cardiovascular Risk Stratification of Patients Care at a Secondary Health Service

LAURA MINELLI CANTOIA^1^, Isabella Molina Silva^1^, Cláudia Helena Cury Domingos^1^

(1) Universidade de Ribeirão Preto- UNAERP

**Introduction:** Cardiovascular Diseases (CVD) are the leading cause of death in Brazil and worldwide. In Brazil, CVD continue to be the main cause of death and hospitalizations in the country, reaching the mark of 31% of deaths in the adult population. Risk factors for CVD have been in previous work from the Framingham Heart Study. In Brazil, the prevalence of these cardiovascular risk factors (CV) shows regional variation and an inverse relationship between socioeconomic status and cardiovascular mortality.

**Objectives:** Due to the impact of CVD on public health, nationally and internationally, this study aims to know the variables that make up the CV risk of patients, in order to stratify them into risk groups, identifying the most vulnerable patients and those with greater need for care attention.

**Methodology:** This is an observational, descriptive and transversal study. Data from medical records of patients being followed up at cardiology outpatient clinic of a secondary hospital in Ribeirão Preto, São Paulo, between 2019 and 2022 were included. Individuals aged between 49 and 95 years were included in the study. For CV risk stratification, the “Cardiovascular Risk Stratification Calculator” of the Brazilian Society of Cardiology was used, which allows inferring the patient’s prognosis over the next 10 years.

**Results:** A total of 182 medical records of patients undergoing follow-up at the outpatient clinic were studied. For CV risk stratification, the following variables were used: Presence of Clinical or Subclinical Atherosclerotic Disease, Type 1 or Type 2 Diabetes Mellitus, Gender, Age, Arterial Hypertension, Smoking and Dyslipidemia. Of the study patients, 40 of them (22%) were classified as very high CV risk, 124 (68.1%) as high CV risk, 13 (7.1%) as medium CV risk and 5 (2.7%) as low CV risk.

**Conclusion:** Cardiovascular risk stratification allows establishing a more assertive and individualized clinical approach. When analyzing the clinical profile of these patients, it is observed that most are at high risk for cardiovascular events and, therefore, will need a multiprofessional approach for adherence to lifestyle changes and prescription of adequate drugs, in order to reduce the chances of CV events in the next 10 years.

109490

Modality: E-Poster Scientific Initiation – Non-case Report

Category: EPIDEMIOLOGY AND HEALTH POLICIES/GLOBAL HEALTH

## Heart Failure Morbidity in the North of Brazil from 2017–2021: An Epidemiological Study

ÁDRIA RAYANE LIMA CASCAES^1^, Ana Josefina Gonçalves Salomâo^2^, Antônia Evelyn Albuquerque Costa^1^, Fernando Maia Coutinho^3^, Vinícius Queiroz Silva^1^

(1) Universidade do Estado do Pará UEPA; (2) Centro Universitário do Estado do Pará CESUPA; (3) Universidade Federal do Pará UFPA

**Background:** Cardiovascular diseases are one of the main causes for mortality and hospitalizations worldwide. In Brazil this tendency started in the decade of 1960 and persists until nowadays. The increasing incidence of admissions by heart failure from 2008 to 2018 shows the importance of analyzing the scenario of morbidity in the north of Brazil.

**Objective:** Review the epidemiological profile of heart failure morbidity between 2017–2021 in the north of brazil.

**Methods:** Epidemiological and Ecological descriptive study. Data extracted from the Department of Informatics of the Unified Health System (DataSUS) through health information (TABNET), considering age, sex, race and year the patients receive care.

**Results:** From 2017 to 2021, there were 50,264 hospitalizations for heart failure in the northern region of Brazil. The highest number occurred in 2018 (11,255), followed by 2019 (11,078) and 2017 (10,961), the lowest occurred in 2021 (8,176). Analysing distribution by sex, the predominance of admissions was among men with a range of 29,449 (58%), compared to women who total around 20,815 (41%), demostraiting that heart failure affects more in men’s health. The prevalence of the disease can be considered of great importance in the state of Pará, where the number of hospitalizations was the highest in the North, totaling 22,161 during the study period. Pará was not the one with the lowest mortality rate, obtaining about 10%, in comparison, the state of Acre had a much lower number of admissions (1,655) and the highest mortality rate among Brazilian states (17.95%). Ranging from younger than 1 to older than 80 years, elderly between 70 and 79 years old, followed by those between 60 and 69 years old, showed the higher incidence of heart failure hospitalizations. In opposition, the younger ones have the lowest values, and it was observed that hospitalizations begin to decline considerably from those under 30 years of age. In relation to color/race, the prevalence occurs in people self-declared to be brown (30,915) and to a lesser extent in indigenous people.

**Conclusions:** The epidemiological profile identified a higher alarm on the populations of men, self-declared to be brown, elderly between 70 and 79 years and residents of the state of Pará. Therefore, these groups need to be a distinct focus of the health system attention to the prevention of this morbidity, for a more effective management of resources and better care for patients with their specificities.

109494

Modality: E-Poster Scientific Initiation – Non-case Report

Category: ANTICOAGULATION

## Telemedicine-Based Management of Oral Anticoagulation: Impact on Thromboembolic and Bleeding Events – Systematic Review and Meta-Analysis

LAURA CAETANO DE SÁ ^1^, Letícia Santos Dias Norberto Ferreira^1^, Alair Junio Rocha Arantes^2^, Hebatullah Abdulazeem^3^, Ishanka Weerasekara^4^, Raissa Carolina Fonseca Cândido^2^, Rodrigo Lanna de Almeida^5^, Samuel Rosa Ferreira^2^, Tati Guerra Pezzini Assis^2^, Thais Marques^2^, Isabela Muzzi^1^, Milena Soriano Marcolino^1^

(1) Telehealth Center, University Hospital, Universidade Federal de Minas Gerais; (2) Medical School and University Hospital, Universidade Federal de Minas Gerais; (3) Department of sport and health sciences, Technical University of Minich, Germany; (4) School of Health Sciences, College of Health, Medicine and Wellbeing, The University of Newcastle, Australia; (5) Laboratory of Respiratory Physiology, University of Brasilia

Oral anticoagulation is the cornerstone treatment of several cardiovascular diseases, and due to its large applications, different e-health strategies have been proposed and implemented to support the management of such treatment. However, data on the effect of e-health-based anticoagulation management on clinical outcomes are very scarce. Therefore a summary of the best available evidence on the topic is warranted and very appropriate for the current moment, due to the explosion in adoptance of telehealth strategies during COVID-19 pandemic. Our goal was to systematically review the evidence on the impact of telemedicine-based management of oral anticoagulation therapy (OAT) compared to usual care (UC) on thromboembolic and bleeding events. Medline, EMBASE, Cochrane library, LILACS and Google scholar databases were searched from inception to September 2021 for randomized controlled trials (RCTs) comparing telemedicine to UC for OAT management in adult outpatients and reporting any of our outcomes of interest: total thromboembolic events (TTE), major bleeding (MB), mortality or time in therapeutic range (TTR). Risk of bias of included studies was assessed using the Cochrane risk-of-bias tool. Dichotomous outcomes were pooled as relative risks (RR) and continuous outcomes as mean differences (MD), using random-effects models. Regarding the results, twenty-seven RCTs were included, comprising 27 758 patients. Atrial fibrillation and venous thromboembolism were the most common indications for anticoagulation. Different telemedicine strategies were evaluated, such as electronic algorithms for anticoagulant dose adjustment, self-testing and self-management of anticoagulation with remote support, mobile applications for OAT management, among others. Telemedicine resulted in similar rates of TTE (9 studies, RR: 0.88 95% CI: 0.73, 1.06; I²: 0%), MB (9 studies, RR: 0.94, 95% CI 0.82, 1.07; I²: 0%) and mortality (10 studies, RR: 0.98, 95% CI 0.83, 1.17; I²: 0%), when compared to UC, and in slightly improved TTR (15 studies, MD: 2.76, 95% CI 0.37, 5.15; I²: 91%). Concluding, telemedicine-based management of OAT appears to be at least as good as UC when it concerns clinical outcomes, and may be slightly better in regard to quality of anticoagulation, although the confidence in those estimates was moderate or low.

109509

Modality: E-Poster Scientific Initiation – Non-case Report

Category: EPIDEMIOLOGY AND HEALTH POLICIES/GLOBAL HEALTH

## Epidemiological Profile of Hospitalizations by Myocardial Infarction in Northern Region of Brazil: A Ten Years Analysis

ADRIANO LEITÃO DE ALMEIDA^1^, Ana Carolina de Sousa^2^, Carlos Eduardo Alexandre Silva^2^, Vivian de Lima Brabo^1^, Larissa da Silva Cambraia^1^

(1) Universidade do Estado do Pará; (2) Universidade Federal do Pará

**Introduction:** Acute myocardial infarction (AMI) is defined as the presence of acute myocardial injury associated with the clinical manifestation of ischemia. In Brazil, from 1996 to 2016, the North region was the second region with the highest growth in the mortality rate from AMI.

**Objective:** To describe the epidemiological data regarding acute myocardial infarction hospitalizations in northern region of Brazil between 2012 and 2021.

**Method:** This research is a cross-sectional descriptive study, carried out under a quantitative approach. Data was collected on the website of Hospitalar information system (SIH), from Brazil’s healthcare information department, including the hospitalizations on patients living in north region from 2012 to 2021. This study evaluated the number of hospitalizations and its mean length of stay, which were compared by state, gender, age group, race/ethnicity and character of care. The data was analyzed using Microsoft Office Excel 2017 software.

**Results:** During the period studied, there were a total of 45890 cases of hospitalizations for AMI, with emphasis on the year 2021, which represented about 13.23% of all hospitalizations, with a mean length of 8.2 days. The state of Pará held the higher hospitalization rates, with 19075 cases (41.57%). The highest number of hospitalizations for AMI occurred between 60 and 64 years old, with 7170 cases (15.62%), however, the longest mean length of stay was in the 65–69 age group, lasting 8.8 days. Men had more hospitalizations due to AMI than women, with 69% of cases, as well as a longer permanence time on hospital, with 8.3 days. In people of brownish color, representing 65.79% of hospitalizations for this disease. Regarding the character of care, urgency was the predominant one, with 86.77% of the total hospitalizations.

**Conclusion:** Thus, this study found higher hospitalizations of AMI on brown men above 60 years old who lived in State of Pará, admitted on urgency care. The longer permanence time among advanced age men might suggest a higher morbidity on this group. During the pandemic, the number of hospitalizations was initially stable, however had a significant increase in 2021, which differ from other studies that reported a decrease on hospitalizations due to bigger demand on COVID-19 management, so that could be explained by the allocation of beds initially created for COVID when the incidence decreased, although this relation can be better evaluated on future studies.

109514

Modality: E-Poster Scientific Initiation – Non-case Report

Category: COVID-19 AND CARDIOVASCULAR SYSTEM

## Complication of Decompensated Heart Failure in COVID-19 Hospitalized Patients

GABRIELA SILVA SANTOS^1^, Gabriel Barbosa Figueira dos Santos^1^, Rafael Alexandre Meneguz-Moreno^1^, Gilberto Andrade Tavares^1^, Viviane Correia Campos^1^

(1) Universidade Federal de Sergipe (UFS) – Campus Lagarto

**Introduction:** Patients hospitalized with COVID-19 and heart failure (HF) have higher in-hospital mortality and complications. Besides, HF as a risk factor for severe SARS-CoV-2 infection could be a complication triggered by COVID-19. OBJECTIVE To analyze the complications developed during hospitalization of patients with COVID-19 and HF.

**Materials and Methods:** It was a retrospective study based on review of electronic medical records of adult patients admitted to the Respiratory Disease Unit (RDU) of a unique hospital from northeast Brazil with RT-PCR reagent for SARS-COV2 and HF diagnosis according to the Framingham criteria. Patients were divided into two groups, one with acute heart failure (AHF) and the other with acute chronic heart failure (ACHF), regarding if they presented with HF for the first time or if they already had this diagnosis, respectively. We have analyzed the comorbidities, complications and mortality between groups and complications according to New York Heart Association (NYHA) functional class and mortality as a hard outcome.

**Results:** The sample consisted of 77 participants, 59.7% (n = 46) male, age 67.1 ± 16.4 years, 33.8% (n = 26) with AHF and 66.2% (n = 51), ACHF. The AHF group had as previous comorbidities mostly diabetes (pdee = 0.019) and chronic lung disease (p = 0.017). There was no difference regarding complications and mortality among the groups. Those with NYHA classification IV and positive SARS-COV2 had an increased risk of developing shock (p = 0.001), cardiopulmonary arrest (CA) (p = 0.01), acute respiratory distress syndrome (ARDS) (p < 0.0001), bacteremia (p = 0.008), hemorrhage (p = 0.04), and liver damage (p = 0.04). In-hospital mortality rate was 39% (n = 30). Mortality was higher in patients who presented as complications: shock (p < 0.0001), CA (p < 0.0001), pneumonia (p = 0.009), ARDS (p < 0.0001), bacteremia (p = 0.0003), hemorrhage (p = 0.02), anemia (p = 0.02), cardiac arrhythmia (p = 0.03), and liver damage (p = 0.04).

**Conclusion:** NYHA functional class IV HF and COVID-19 have a higher risk of developing complications and death during hospitalization, especially when they develop pneumonia, ARDS, shock, CA, anemia, hemorrhage, arrhythmia, liver damage, and/or bacteremia.

109698

Modality: E-Poster Scientific Initiation – Non-case Report

Category: EPIDEMIOLOGY AND HEALTH POLICIES/GLOBAL HEALTH

## Impact of COVID-19 Pandemic on Admission Rates for Acute Myocardial Infarction and on Management with Coronary Artery Bypass Graft Surgery Through Brazil’s Public Health Care System

LARISSA DE OLIVEIRA BELTRÃO^1^, Beatriz Ximenes Bandeira de Morais^1^, Maria Letícia Garrote Bulhões^2^, Leticia Ferreira Leal^3^, Catiane Kelly Schaefer^4^, Isabela Torres Castro^1^, Vauma Garrote Bulhões Barros^5^, Rafael José Coelho Maia^6^, Maria de Fatima Nunes de Oliveira Mesquita^6^

(1) Faculdade Pernambucana de Saúde; (2) Universidade Estácio de Sá; (3) Universidade de Pernambuco; (4) Universidade de Santa Cruz do Sul; (5) Hospital do Coração de Alagoas; (6) Hospital Agamenon Magalhães

**Background:** Acute Myocardial Infarction (AMI), is the major cause of death worldwide, having Coronary Artery Bypass Graft (CABG) Surgery as a therapeutic option. In 2020, with the outbreak of the COVID-19 pandemic, there was a decrease in the number of patients admitted for AMI symptoms, as well as in the number of CABGs.

**Objective:** This study aims to demonstrate the impact of the COVID-19 pandemic changed the main indicators of hospitalizations of patients with AMI in hospitals of the Unified Health System (SUS) in Brazil, their length of stay, mortality rate, number of CABGs, correlation with sex, age and condition of the procedure and hospital stay, and if there is a tendency for return to normality.

**Methods:** This is a descriptive, cross-sectional study conducted using data from SUS Data Transfer Service, about hospital admissions for AMI and CABGs performed between 2019 and 2021. We analyzed the numbers of admissions and procedures undertaken, as well as, percentage reductions in each year across subgroups.

**Results:** 132,173 patients with AMI were admitted to SUS hospitals in 2019, decreasing by 23,95% in 2020, totaling 129,637. The following year it reduced by 13,25%, amounting to 125,916 patients (Tab1). This decrease was proportional between genders and was more prevalent between 50 and 70 years of age. Additionally, the amount of CABG decreased by 1.92% from 2019 to 2020 and 2.87% in 2021. It was accompanied by a decline in the length of hospital stay in 2020 (11,3 days), when compared to 2019 (11,85 days), followed by an increase in 2021 (11,6 days). Furthermore, there was a slight increase in the mortality rate of patients undergoing CABG, both elective and emergency. In 2019, this rate was 5.95, increasing to 7.11 in 2020 and decreasing to 6.84 in 2021.

**Conclusions:** Hospitalizations for AMI continue to decrease at lower intensity, showing a reduction of almost 50% in the trend of previous year, which leads to belief in a full recovery, if this trend continues, in 2022 or 2023. In CABG, we have a continued decrease, however with signs of an imminent recovery in mortality rate and days of hospitalization.


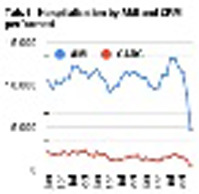


109516

Modality: E-Poster Scientific Initiation – Non-case Report

Category: HEMODYNAMICS AND INTERVENTIONAL CARDIOLOGY

## Primary Percutaneous Coronary Intervention During Off-Hours: One Decade of Experience from a Tertiary Cardiovascular Centre

PEDRO HENRIQUE TORRES TIETZ^1^, Filipe Cirne^1^, Márcia Moura Schmidt^1^, Cristiano de Oliveira Cardoso^1^, Alexandre Schaan de Quadros^1^

(1) Instituto de Cardiologia do Rio Grande do Sul (IC-FUC RS)

**Introduction:** The gold standard treatment for ST elevation myocardial infarction (STEMI) is primary percutaneous coronary intervention (pPCI), but the clinical impact of performing pPCI off-hours is controversial.

**Objectives:** To compare characteristics and major adverse cardiovascular events (MACE) of STEMI patients submitted to pPCI performed off-hours versus on-hours in a reference cardiology center.

**Methods:** Prospective cohort of patients who underwent pPCI for STEMI from 2009 to 2019 in our institution. Patients treated on- and off-hours were compared as to baseline characteristics and short and long-term events. Clinical events were assessed by Kaplan Meier survival curves and Cox regression analysis. A p value <0.05 indicated statistical significance.

**Results:** 2560 patients were treated off-hours and 1876 patients treated on-hours. Most of the baseline characteristics were well balanced between the groups, including similar door-to-balloon times (70min. × 69min; p = 0.15). Patients treated off-hours had a higher thrombus burden (49.6% × 45.5%; p < 0.01). Radial access was more frequently used off-hours (62.1% × 57.6%; p = 0.01). Procedural success (95.7% × 96.4%; p = 0.21) and peri-procedural complications were similar between the groups(p = 0,97). The off-hours groups had higher rates of MACE at 30 days (10.2% × 8.5%; p = 0.04) and 1 year(15.4% × 13.1%; p = 0.03), as well as higher rates of death at 30 days(7.8% × 6.1%; p = 0.03) and 1 year(11.1% × 9.0%; p = 0.02). No statistically significant differences in ischemic events were seen.

**Conclusion:** In our institution, clinical characteristics, door-to-balloon time, pPCI results and short and long-term ischemic events of STEMI patients treated on and off-hours were similar, but off-hours patients presented higher mortality.


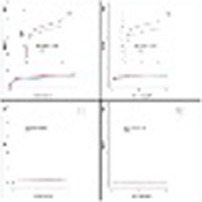


109521

Modality: E-Poster Scientific Initiation – Non-case Report

Category: HEART FAILURE/CARDIOMYOPATHY/TRANSPLANT

## Profile of Hypertrophic Cardiomyopathy Patients in a Cardiogenetic Unit

ANTONIO GUILHERME CUNHA DE ALMEIDA^1^, Bráulio Cruz Melo^1^, Emerson de Santana Santos^1^, João Victor Andrade Pimentel^1^, Beatriz Luduvice Soares^1^, Júlia Souza Diniz^1^, João Paulo Dias Costa^1^, Giovanna Medeiros Resende^2^, Irlaneide da Silva Tavares^1^, Enaldo Vieira de Melo^1^, Antônio Carlos Sobral Sousa^1^, Joselina Luzia Menezes de Oliveira^1^

(1) Universidade Federal de Sergipe; (2) Universidade Tiradentes

**Introduction:** Hypertrophic cardiomyopathy (HCM) is the most common cardiac genetic disease in the world. The diagnosis is made by echocardiography and cardiac magnetic resonance (CMR), and its genetic etiology is defined as pathogenic variations in sarcomeric genes causing left ventricular hypertrophy without any other plausible etiology.

**Methods:** A prospective study was conducted with patients referred to our cardiogenetic outpatient service between January 2021 and March 2022. Patients who met the American Heart Association hypertrophic cardiomyopathy diagnostic criteria had DNA sequenced using Next-Generation Sequencing technique. Statistical analysis was performed using R software version 4.0.1.

**Objective:** To describe the population of patients under investigation for HCM.

**Results:** A total of 74 patients were referred and 52 (70.2%) met HCM diagnostic criteria and had a mean age of 49.7 ± 18.9 years and 71.2% were men. A family history of sudden cardiac death was present in 59.6% patients. Beta-blocking agents were the most commonly used medications (44.2%); 7.7% of patients reported having an implantable cardioverter-defibrillator (ICD). Palpitations were the most reported symptom (69.2%). Rest echocardiogram showed a mean septum wall thickness of 16.6 ± 6.6 millimeters. CMR showed a maximal wall thickness of 17.4 ± 7.4 millimeters. 38.4% of patients had a pathogenic variant in gene panel, 70% of those presented pathogenic sarcomeric variants and the 30% presented a pathogenic variant of a phenocopy.

**Conclusion:** The present study corroborates previous findings. Genetic testing has enabled the distinction between HCM and its phenocopies and accurate genetic counseling for the patients’ families.


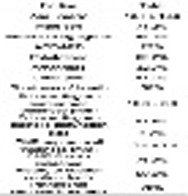


109522

Modality: E-Poster Scientific Initiation – Non-case Report

Category: HEMODYNAMICS AND INTERVENTIONAL CARDIOLOGY

## Overview of Coronary Angioplasty Procedures with Stenting in the City of Vassouras in 10 Years

JULIANA DE ALMEIDA SILVEIRA^1^, Sara Cristine Marques dos Santos^1^, Daniel de Oliveira Meireles^1^, Júlia Bardela de Oliveira^1^, Thaís Lemos de Souza Macêdo^1^, Ivan Lucas Picone Borges dos Anjos^1^, Isabela Costa Linhares^1^, João Paulo Norá Muñoz^1^, Anna Júlia Tamiozzo Reis^1^, Anderlúcia Côrrea Guedes^1^, Girley Cordeiro de Sousa^1^, Ivana Picone Borges de Aragão^1^

(1) Universidade de Vassouras

**Introduction:** Coronary angioplasty is a procedure performed by percutaneous coronary artery revascularization, decreasing myocardial ischemia. Technically, access is via a percutaneous artery, without the need for a thoracotomy. Stent device implantation was used most of the time. The present study aimed to analyze the current panorama of coronary angioplasty procedures with stent implantation performed in Vassouras – Rio de Janeiro, for 10 years and to correlate the current epidemiology with the results obtained.

**Methods:** A systematic literature review and an observational, descriptive and cross-sectional collection of data on coronary angioplasty with stent implantation, available at DATASUS – SUS Hospital Information System (SIH/SUS) for a period of ten years – December 2008 to December 2018 – evaluating the value of public spending, complexity, mortality rate, deaths, permanence, and character of care and articles available in Scielo, Lilacs, and PubMed.

**Results:** In the analyzed period, 1,675 hospitalizations were identified for the performance of coronary angioplasty procedures with Stent implantation, representing a total expenditure of R $ 8,086,346.37, with 2018 being the year with the highest number of hospitalizations (263) and the year of 2008 with the lowest number of hospitalizations (6). With 2018 as the year responsible for the largest amount spent during the period (R $ 1,173,571.59). Of these admissions, 1,025 were in private. Of the total procedures, 1,112 were performed on an elective basis and 563 on an urgent basis, with 1,675 being considered highly complex. The total mortality rate in the 10 years studied was 1.73, corresponding to 29 deaths, with 2011 being the year with the highest mortality rate, 4.17, with 8 deaths while 2015 had the lowest rate, 0,72, with 1 death. The mortality rate for elective procedures was 1.08 compared to 3.02 for urgent procedures. The average total hospital stay was 2.3 days.

**Conclusions:** It can be observed, from the present study, that there was an increase in the performance of the coronary angioplasty procedure with Stent implantation in the period of 10 years, starting in 2008, accompanied by a reduction in the number of deaths, showing the treatment success. Besides, there is a need for the correct notification of procedures, aiming to improve the current epidemiological analysis.

109529

Modality: E-Poster Scientific Initiation – Non-case Report

Category: CONGENITAL AND PEDIATRIC CARDIOLOGY

## 22Q11 Deletion Syndrome (Digeorge Syndrome/Velocardiofacial Syndrome) and its Correlation with Conotruncal Type Congenital Heart Diseases

WALDEMIR FERRARI JUNIOR^1^, Leonardo Nunes Sanson^1^, Mariana Castro Pires^1^, Capitulino Camargo Junior^1^, Vitor Reis de Souza^1^, Rafael Fabiano Machado Rosa^1^, Júlio Pasquali Andrade^1^, Mirian Francine Favero^1^, Laura Peroni Baldino^1^, Diego Seibel Júnior^1^, Pedro Enrico Ventura^1^, Maiquel André Teixera^1^

(1) Universidade Federal de Ciências da Saúde de Porto Alegre

**Introduction:** Congenital heart diseases (CHDs) represent an important public health problem. Although its etiology is not well understood, the 22q11 deletion syndrome (22q11DS) stands out among its known causes, also known as DiGeorge syndrome/Velocardiofacial syndrome.

**Objective:** The aim of this study was to verify the frequency of 22q11DS among patients with a conotruncal CHD.

**Methods:** The sample consisted of a prospective and consecutive cohort of patients in their first hospitalization in a cardiology intensive care unit (ICU) of a pediatric hospital, during a period of 1 year. An evaluation form was filled out for each patient with the collection of clinical data. ALL of them also underwent high resolution karyotype and 22q11 microdeletion investigation using the fluorescent in situ hybridization (FISH) technique. The classification into conotruncal defect was performed by a collaborating cardiologist based on the results of echocardiograms and catheterizations, and also the surgical descriptions

**Results:** Of all patients with CHD, 52 (25.1%) had a conotruncal defect. Thirty-two were male and their ages ranged from 1 day to 10 years (48% <1 month). The main reason for hospitalization was cardiac surgery (76.9%). The most observed CHD was tetralogy of Fallot (TOF) (40.4%). There were no cases of interruption of the aortic arch. Karyotypic abnormalities were observed in 5 patients (9.6%); however, none of them had 22q11DS. The analysis by the FISH technique could be performed successfully in 51 patients, and the 22q11 microdeletion was identified in 2 cases (3.9%) (both with TOF).

**Conclusions:** The frequency of 22q11DS verified in our study was similar to studies that found values ranging from 4 to 15% and different from others that detected rates between 17 and 48%. These differences seem to be related, especially, with the method of patient selection adopted in the studies. Patients with 22q11DS often have complex heart defects and extracardiac malformations, which can have direct implications in their treatment (especially the surgical one) and prognosis.

109536

Modality: E-Poster Scientific Initiation – Non-case Report

Category: COVID-19 AND CARDIOVASCULAR SYSTEM

## Impact of Coronavirus Infection in Patients with Cardiovascular Diseases: A Systematic Review

LUÍS FILIPE RIBAS SOUSA^1^, Brenno Laerth Neves Sousa^1^, Gabriel Lino Ribas Sousa^1^, Rafaela França da Silva^1^, Yana Mara de Oliveira Coelho Mendes^1^, Elyanne dos Santos Gomes^1^, Marcus Vinicius Moreira Barbosa^1^

(1) Instituto Tocantinense Presidente Antônio Carlos de Porto Nacional – ITPAC Porto

In December 2019, a city of China called Wuhan onset a new coronavirus infection, named Coronavirus Desease 19 (Covid-19), caused by Severe Acute Respiratory Syndrome-2 (SARS-CoV-2). It has affected more than 457 million people wordwide. The transmission occours person-to-person through close- range contact to respiratory particles. Although SARS-CoV-2 is willing to cause respiratory síndromes, It may cause cardiovascular complications, even in those who do not have past medical history. Thereby, it is intended to do a systematic review, using the PRISMA methodology to identify cardiovascular outcomes in pacientes infected with Covid-19. Scielo and Pubmed are the bases used in this review. Free access articles published in English and Portuguese were included, according to the purpose of the study. It was analyzed 1.817 pacientes infected by Covid-19, where 62.5% male with mean age of 58,8 years-old. According to the database, cardiac manifestations of Covid-19 happened in those who had previous comorbidy, like essential hypertension, diabetes mellitus, dyslipidemia or smoking and alchol using. Miocardical injury are the most common complications, ranging from 8.1% to 36.6%. Arrhythimias were found in 12.7% to 17,6% and myocardical infaction in 0,4% to 9,9%. From this, the studies were able to asses point of care susceptible to cardiovascular complications in people with Covid-19. Among them, men aged over 60 years-old, who chronicle cardiac deasease and white blood cells (WBC) contage ≤1,1 × 10^9 cells/L. It was pointed out that advanced age, especially in men, with risk factons, such as: smoking, diabetes mellitus, hypertension, dyslipdemia, chronic heart desease, chronic kidney desease with glomerular filtration rate <90l/day and WBC ≤1,1 × 10^9 cells/L. It all worsens patient’s prognosis.

109548

Modality: E-Poster Scientific Initiation – Non-case Report

Category: CARDIOVASCULAR INTENSIVE CARE/CARDIOVASCULAR EMERGENCIES

## Advanced Cardiac Life Support in Simulation for Undergraduate Students: There is Beneficial Data?

LUCAS LUCENA BEZERRA^1^, Guilherme Aragão Melo^1^, Bruna Ferreira Apolinário Costa^1^, Maria Júlia Pimentel de Albuquerque^1^, Cícero Samuel Tavares de Souza^1^, Lara Guedes Bezerra^1^, Mariana Diniz de Souza^1^, Natália Lisboa de Carvalho Wanderley Cavalcanti^1^, Michelle Alves de Farias^1^, Antonia Raiane Silva Claudino^1^, Isabella Cristina Oliveira Pacheco^1^, Diego Felipe Ferrão Pereira de Andrade Barros^1^

(1) FACULDADE DE MEDICINA DE OLINDA- FMO

**Introduction:** In-hospital cardiac arrest (IHCA) is an emergency health issue that can lead to morbidity and mortality in a varied number of patients. In Brazil, there is not much data on IHCA found in the literature, but it is known that cases have a bad prognosis and low survival rates. For a better outcome for patients who need a good resuscitation at the IHCA, undergraduate students must go through adequate training to improve their knowledge in Advanced Cardiac Life Support (ACLS) when dealing with cardiac arrest.

**Objective:** The main goal was to review and synthesize evidence on the following question: “What is the scientific evidence on the benefits of educational simulation interventions in advanced cardiology life support training in undergraduate students?”.

**Methods:** This systematic review follows the Preferred Reporting Items for Systematic Reviews and Meta-Analyses (PRISMA) guidelines. The literature research that was performed covered 3 databases (PUBMED, EBSCOHOST, AND ScienceDirect). The period of publication of the articles was from 2018 to 2022.

**Results:** Among 350 articles retrieved, 9 papers were included and totalizes 1152 undergraduate students. Most studies used multiple-choice questionnaires to evaluate knowledge retention and cardiac arrest simulation or other skills tests to evaluate skills retention. The interventions, which were delivered in a variety of ways, have seen improvements such as: better patient outcomes, improved skills, and increased teamwork performance. In one of the articles, the pre-test score and post-test for ACLS knowledge had an 88% improvement. The performance of ACLS maneuvers that most follows the guidelines of the main world institutions, were in the high-fidelity simulation training scenarios.

**Conclusions:** Simulation technology for resuscitation training as an innovative strategy for students, has brought great benefits to the ACLS learning process, helping bring better results in death prevention. However, more high-quality studies are needed, especially in Brazil.

109732

Modality: E-Poster Scientific Initiation – Non-case Report

Category: HEART FAILURE/CARDIOMYOPATHY/TRANSPLANT

## Risk Behavior in Sexual Activity in Patients Submitted to Heart Transplantation

GABRIEL SOUSA SANTOS^1^, Danielli Oliveira da Costa Lino^2^, Ane Karoline Medina Néri^3^, Carlos José Mota de Lima^2^, Glauber Gean de Vasconcelos^2^

(1) Universidade federal do Ceará, School of Medicine.; (2) Hospital de Messejana Dr. Carlos Alberto Studart Gomes.; (3) Universidade de Fortaleza, Postgraduate Program in Collective Health

Heart transplantation is a surgical alternative used to treat heart failure unresponsive to clinical treatment, being responsible for improving the expectation and quality of life of patients who present such a condition. After a cardiovascular event, patients and their families deal with numerous changes, including the consequences of the disease or its treatment on everyday functioning. Individuals assume behaviors for which they are not prepared, such as initiating sexual intercourse early, often due to anxiety about living quickly and intensely. The onset of early sexual activity increases vulnerability to sexually transmitted diseases, which interferes with post-transplant survival. Sexual activity is an aspect of quality of life that is important to many patients and partners who may be adversely affected by a cardiac event. Although healthy sexual activity integrates quality of life, few studies addressing this theme have been developed in heart transplant patients. To assess risky behaviors in sexual activity in individuals undergoing heart transplantation. Descriptive research with a qualitative approach, with the interview of 30 heart transplant patients treated at the outpatient clinic specializing in Cardiac Transplantation in 2018. It was observed that 25 patients (83.3%) did not receive guidance on sexual activity. In 66.7% of respondents, risky behaviors were present, such as not using condoms reported by 50% of respondents, having multiple partners reported by 17.9% and early return to sexual activity, contrary to the recommendation of the cardiac transplant team, by 7.1% of the evaluated individuals. We evaluated the presence of risky sexual behaviors among individuals undergoing heart transplantation and observed that most participants had not received guidance regarding sexual activity in the post-transplant period and that most individuals practiced risky sexual behaviors. The contribution of the transplant team in the guidelines related to sexual activity is shown in a very timid way in the post-transplant guidelines, which may be contributing to a considerable prevalence of risky behaviors in these individuals. Supporting post-transplant providing guidance regarding sexual activity proves a coherent decision as a humanized practice. The deleterious clinical consequences of sexual risky behaviors, such as exposure to infections, can lead to cardiac impairment and reduced life expectancy in these patients.

109590

Modality: E-Poster Scientific Initiation – Non-case Report

Category: CARDIOVASCULAR IMAGING

## Point-of-Care Ultrasound in Cardiopulmonary Arrest: A Systematic Review

LUCAS LUCENA BEZERRA^1^, Mariana Diniz de Souza^1^, Guilherme Aragão Melo^1^, Cícero Samuel Tavares de Souza^1^, Maria Júlia Pimentel de Albuquerque^1^, Bruna Ferreira Apolinário Costa^1^, Lara Guedes Bezerra^1^, Michelle Alves de Farias^1^, Natália Lisboa de Carvalho Wanderley Cavalcanti^1^, Isabella Cristina Oliveira Pacheco^1^, Antonia Raiane Silva Claudino^1^, Diego Felipe Ferrão Pereira de Andrade Barros^1^

(1) FACULDADE DE MEDICINA DE OLINDA- FMO

**Introduction:** Despite significant improvements in cardiopulmonary resuscitation (CPR), sudden cardiac arrest is one of the leading causes of mortality. Nowadays, Ultrasound is a widely available tool that can be used to evaluate the presence of cardiac wall motion during cardiac arrest, which can be used to determine prognostic of the patient during CPR. We performed a systematic literature review of the existing evidence of point-of-care ultrasound (POCUS) in Cardiopulmonary arrest (CPA).

**Objectives:** The main objective of this study was to systematically review and synthesize the published literature on the benefits of POCUS in CPA.

**Methods:** The reporting of this study follows PRISMA guidelines (Preferred Reporting Items for Systematic Reviews and Meta-analyses). The literature research that we performed covered 3 large databases (PUBMED, EBSCOHOST and ScienceDirect). The research was conducted gathering the articles from 2018–2022. We performed a systematic search in those databases up to 03/03/2022.

**Results:** Initially, the literature search identified 120 articles, EBSCOHOST: 36; SCIENCEDIRECT: 15 and PubMed: 69. Furthermore, the updated withdrawal of duplicate articles was 74, leaving 22 articles selected. Finally, after the last selection, 6 articles were included in our review Among 15 papers, 9 were included and totalizes 356 patients. As for the articles included in our review, POCUS was used in order to identify the cause of CPA and the return of spontaneous circulation. When identified early, it increases patient survival. Thus, achieving greater diagnostic accuracy associated with less neurological and cardiac damage. Despite this, 2 of them suggest that POCUS should not be integrated into the protocols, so as to prolong pauses in CPR. They all also point out that POCUS depends on operator training and experience. Likewise, in one of the reports the instrumentator was trained in a four-hour training session, which included a didactic lecture and hands-on POCUS instruction. After this training, paramedics are prescribed to operate and enter the exam.

**Conclusions:** POCUS has the potential to be an important tool in CPA and could potentially reduce health care costs. We conclude that POCUS is a powerful tool to assess reversible causes of cardiac arrest. However, it must be used in a protocolized and efficient way so as not to cause damage.

109592

Modality: E-Poster Scientific Initiation – Non-case Report

Category: COVID-19 AND CARDIOVASCULAR SYSTEM

## Assessing the Prevalence and Impact of Fake News About COVID-19 Vaccines and Heart Attack: A Youtube Study

LUANA DE OLIVEIRA RIBAS^1^, Luana de Oliveira Ribas^1^, Silvia Nazaré Braga Pereira^1^, Rebeca Bittencourt Jaqueira Rios^1^, Aline Goneli de Lacerda^1^, Claudio Tinoco Mesquita^1^

(1) Universidade Federal Fluminense- UFF

**Introduction:** In today’s globalized world, the dissemination of Fake News in the medical field represents a great risk for the population’s health. With the COVID-19 pandemic and the increasing of fake news circulation, including those of a general interest of medical nature, this phenomenon took on gigantic proportions, dominating social media. This scenario is propitious for the emergence of conspiratorial theories, pseudoscience and other misinformative speeches that represent a risk for public health. In this context, the purpose of this research is to survey and map the circulation of Fake News through videos in Portuguese that correlate COVID-19 vaccines and heart attack available on YouTube, popular and easily accessible data.

**Objective:** Evaluate the veracity of Portuguese-language videos on the YouTube platform after a search relating the COVID-19 vaccine to heart attack. The initial objective was to evaluate the first 200 videos found.

**Methods:** The data extraction was made through the YoutubeData Tools software through “Vídeo Network”, by the union of the key-words “vaccine” and “heart attack” utilizing the crawl depth 0 to identify the actors (videos) of most relevance about the subject. Then, the generated GDG file was imported to the software Gephi 0.9.2 responsible for the network analysis and visualization. Lastly, the 200 more relevant videos were analyzed for their content and labeled as truthful or containing Fake News.

**Results:** 200 videos in Portuguese on the YouTube platform have been analyzed, obtained through the GDF file generated by the YoutubeData Tools. Of these, only 171 were classified as “True” or “Containing Fake News”. Of the classified videos, 7 of them had fake news in their content, representing approximately 4.09% of the total videos analyzed. The authors of these videos almost always used persuasion strategies, such as the use of technical terms, doctoral degrees or the use of lab coats, as a way to convey a sense of trust. At the time of the survey, the total views of videos classified as “Containing Fake News” was 2,944,884.

**Conclusion:** In the midst of the advancement of technology and disinformation detection tools, Fake News are still a reality in our daily lives and a threat to Public Health. Although most of the videos analyzed have a scientific basis, the existence of Fake News in the health sector can represent a serious danger to the general population, being therefore a topic of scientific and social relevance.

109594

Modality: E-Poster Scientific Initiation – Non-case Report

Category: EPIDEMIOLOGY AND HEALTH POLICIES/GLOBAL HEALTH

## Epidemiological Profile of Hospitalizations for Phlebitis, Venous Thrombophlebitis and Venous Thrombosis in Brazil from 2017 to 2021

CAMILA SILVA DE OLIVEIRA^1^, Luiz Felipe Façanha Ramos^1^, Cecília Rodrigues Viana^1^, Aurea Nathalia Gomes de Souza^1^, Bianca Paula Miranda Martins^1^, Vinicius Maciel Vilhena^1^, Larissa Silva Ferreira,^1^, Marcos Roberto Marques da Silva Júnior^1^, Reny Wane Vieira dos Santos^1^

(1) Universidade Federal do Amapá (UNIFAP)

**Introduction:** Phlebitis presents as an inflammation of the intima of the veins, being caused by mechanical irritation, chemical or bacterial infection. Deep Vein Thrombosis (DVT), according to the Brazilian Society of Angiology and Vascular Surgery, is a disease caused by blood clotting inside the veins at an inappropriate place or time. And thrombophlebitis, according to the Brazilian Ministry of Health, consists of the inflammation of a clot formed when there is a thrombosis. In Brazil, data from the Ministry of Health put the three pathologies together and point out that women are the most affected due to risk factors such as pregnancy and use of contraceptives.

**Objective:** To analyze the epidemiological profile for phlebitis, in all age groups, in Brazil, from 2017 to 2021.

**Method:** Epidemiological study with a cross-sectional design of hospitalizations for phlebitis, venous thrombophlebitis and venous thrombosis in the regions of Brazil from 2017 and 2021. The investigated data were extracted from the SUS Hospital Morbidity System (SIM/SUS), from the database of the Department of Informatics of the Unified Health System (DATASUS).

**Results:** In the investigated period, a total of 205,029 were documented. hospitalizations for phlebitis, venous thrombophlebitis and venous thrombosis. In 2017, the total was 40,238. The sum of hospitalization rates was higher in the Southeast region, which has about 55% of total hospitalizations in the period from 2017 to 2021, followed by the South region with 21.21%, the Northeast region with 15.93%, the Midwest and North region with7.74%. In the distribution of age groups, below 1 year to 19 years old are the least affected age with 1.61%, from 20 to 59 years old with 57.20% and 50 years or older with 40.85% of the total number of hospitalizations. It is worth mentioning that women are the most affected with about 154.34% more than men. When crossing the data with color/race, about 21.99% of the total hospitalizations were not identified, indigenous and yellow people with 1.97%, white people with 41.13% and black and brown people with 34% of cases registered. In total, 5292 deaths were recorded.

**Conclusion:** Through the analyzed results, it was concluded that there was an increase in the rate of morbidity and mortality from phlebitis, venous thrombophlebitis and venous thrombosis in the period from 2017 to 2021. A higher prevalence was observed in white women aged 30 to 80 years.

109598

Modality: E-Poster Scientific Initiation – Non-case Report

Category: CARDIOVASCULAR PHARMACOLOGY

## The Flavonoid Kamatakenin is a New Calcium Channel Blocker: Docking Molecular Analyzes

CLARA DE ASSIS KAROLINE OLIVEIRA^1^, Clara de Assis Karoline Oliveira^1^, Adilson Lima dos Santos Júnior^1^, Marcus Vinicius Guerra Canto^1^, Thácia Kiara Beserra de Oliveira^2^, Joelmir Lucena Veiga da Silva^2^

(1) PRODIIC- Faculdade de Medicina de Olinda; (2) DOCENTE – Faculdade de Medicina de Olinda

**Introduction:** The cardiovascular diseases are evaluating due to risks and prevalence in world, as hypertension. The calcium-channel blockers (CCB) antihypertensives agents inhibit calcium-channel in the membrane of the vascular smooth muscle cells, it reduces calcium availability into cells impairing muscle contraction and decreasing vascular peripheral resistance. The flavonoid kumatakenin (5-hydroxy-2-(4-hydroxyphenyl)-3,7-dimethoxychromen-4-one), isolated from species vegetal Solanum paludosum, presented vasorelaxant effect in vitro by blocking the calcium channel (CaV). Thus, the researching by news drugs is a continuous process and natural products are a putative resource of medicines.

**Aim:** To analyze the conformers and binding sites of kumatakenin on voltage-gated calcium channel by molecular docking.

**Methods:** A quantitative and experimental research with an in silico approach that used the kumatakenin (CID 5318869) and nifedipine (CID 4485), standard drug, ligands from PubChem. To docking studies, atomics coordinates for the CaV, a structural basis of Ca2+ selectivity of a voltage-gated calcium channel, were retrieved from Protein Data Bank (ID 4MS2). The output conformers from Dockthor (GMMSB version 2.0), a receptor-ligand docking program, were ranked in order of increasing affinity with the protein. The affinity bonds (Ab) were obtained and compare using Test-t, where values p < 0.05 were significant.

**Results:** There were three conformers that showed greater binding affinity with CaV and kumatakenin or nifedipine. The kumatakenin presented Ab of –8.353, –8.304 and –8.283, as the nifedipine of –8.021, –8.024 and –8.049. The comparing Ab into kumatakenin and nifedipine revealed significant difference (p < 0.05), showing that the flavonoid had more affinity than standard drug. The flavonoid conformer with the highest binding affinity occupies the most central region of the constriction, occupying a larger volume and justifying the events of greater amplitude of CaV blocker and greater reduction in the calcium conductance. The types of interaction involved in the connection between the three best docking solutions (flavonoid or nifedipine) and the CaV could be polar interactions, as salt bridge and carbon-hydrogen bond.

**Conclusion:** The data confirm the blocking of voltage-gated calcium channel by kumatakenin in molecular insight, a putative CCB. It showed most affinity than nifedipine, a CCB antihypertensive.

109603

Modality: E-Poster Scientific Initiation – Non-case Report

Category: PHYSIOTHERAPY

## Relationship between Waist Circumference and the Occurrence of Signs and Symptoms in Cardiac Rehabilitation Programs: An Observational Longitudinal Study

ANNA JÚLIA LEAL RODRIGUES^1^, Heloisa Balotari Valente^1^, Lara Júlia Montezori Costa^1^, Felipe Ribeiro^1^, Carolina Takahashi^1^, Laís Manata Vanzella^1^, Luiz Carlos Marques Vanderlei^1^

(1) Universidade Estadual Paulista “Júlio de Mesquita Filho” (UNESP)

**Introduction:** During cardiac rehabilitation programs (CRP) sessions patients may present signs and symptoms, which may be manifest prior to major adverse events, such as acute myocardial infarction and sudden cardiac death. Considering that high waist circumference is associated with a higher risk of major adverse events, it is important to investigate the relationship between waist circumference and the occurrence of signs and symptoms in CRP, since it may contribute to better conditions of cardiac risk stratification and to increased patient safety.

**Objectives:** To investigate the relationship between waist circumference and the occurrence of signs and symptoms in CRP.

**Methods:** 68 participants (65.81 ± 11.67 years) from an exercise-based CRP, diagnosed with cardiovascular disease and/or cardiovascular risk factors were included. Waist circumference was measured using a tape measure at the smallest point between the lower ribs and the iliac crest. The occurrence of signs and symptoms was assessed during 24 CRP sessions. The following signs and symptoms were assessed at the end of each step of the CRP session (initial rest, warm-up, resistance, and relaxation), and counted by session: arrhythmias, increased systolic (SBP) (>200 mmHg) and/or diastolic (DBP) (>120 mmHg) blood pressure during the exercise, tachypnea, pallor, angina, cramp, muscle pain, fatigue, dizziness, and nausea. Pearson’s correlation or Spearman’s correlation were used to evaluate the relationship between waist circumference and the occurrence of signs and symptoms, depending on the normality of the data (Shapiro-Wilk). The statistical significance level was set at <0.05. (CAAE: 79213417.0.0000.5402).

**Results:** The average waist circumference found for the sample was 95.82 ± 9.72 centimeters (minimum: 70 centimeters; maximum: 119 centimeters). In total, 528 signs and symptoms were observed, which generated an average occurrence of 1 sign and symptom for each 3.09 hours of CRP session. No significant correlations were observed between waist circumference with total signs and symptoms (r = 0.111; p = 0.37), and with the main signs and symptoms observed in the 24 sessions: arrhythmias (r = 0.139; p = 0.26), muscle pain (r = 0.114; p = 0.35) and fatigue (r = –0.110; p = 0.37).

**Conclusion:** Waist circumference is not related to the occurrence of signs and symptoms in CRP.

109610

Modality: E-Poster Scientific Initiation – Non-case Report

Category: CARDIOVASCULAR SURGERY

## Analysis of Endovascular Repair of Abdominal Aortic Aneurysm in Brazil from 2016 to 2022

ISABELA ARAGÃO COLARES^1^, Mariana Salles Ballalai^1^, Helena Raquel Nogueira de Oliveira^1^, Gabriel Sousa Santos^1^, Renata Pinheiro Martins de Melo^1^, Gabriel Coelho Brito Dias^1^, José Levi Tavares Cavalcante^1^, Ane Karoline Medina Néri^2^, Weiber Silva Xavier^3^, João Luiz de Alencar Araripe Falcão^1^, Sandra Nívea dos Reis Saraiva Falcão^1^

(1) Universidade Federal do Ceará, Faculdade de Medicina, Curso de Medicina; (2) Universidade Federal do Ceará, Faculdade de Medicina, Hospital Universitário Walter Cantídio; (3) Programa de Educação em Reanimação Cardiorrespiratória

**Background:** Abdominal Aortic Aneurysm (AAA) is a dilatation of that artery when its normal limit diameter of 2 cm is exceeded by 50%. It is considered a lethal event because of its risk of rupture, therefore Endovascular Aneurysm Repair (EVAR) is considered to be an important treatment for this condition, as it is indicated in the treatment of ruptured aneurysms and in elective endovascular procedure, if anatomically viable, according to the August 2016 recommendations of Brazilian Guideline for the treatment of AAA.

**Objectives:** Observe the aspect of health care in the performance of EVAR, as well as in each Brazilian geographic region by verifying the data on hospitalizations, average hospital stay, mortality rate and death toll.

**Methods:** The data available at DATASUS platform, provided by the Hospital Information System (SIH), from August 2016 to January 2022, were quantitatively and descriptively analyzed.

**Results:** The EVAR with straight or conical endoprosthesis, of this period, performed electively, represents 135 hospitalizations, with a mean stay of 9.9 days, with a mortality rate of 5.10 and 7 deaths. As for the urgent care, there were 303 hospitalizations, with an average length of stay of 8.5 days, a mortality rate of 9.9, and 30 deaths. Furthermore, it was possible to verify the following information about each geographical region: the South had the highest number of hospitalizations, with 202 patients, and a mortality rate of 13.52, while the North had the lowest number of hospitalizations, 10 in total, and the second highest mortality rate, 14.82. Still, the Midwest Region showed the highest mortality rate, 15.12, among the 20 hospitalized patients. The Southeast presented 167 hospitalizations for the procedure in question, with a mortality rate of 13.02. The Northeast, with 42 hospitalizations, had a mortality rate of 13.03.

**Conclusion:** EVAR is recommended for reducing early morbidity and mortality and is a technique that can be increasingly improved, as well as the care after the procedure. Moreover, it has a significant mortality rate in emergency treatment compared to elective due to the patient’s clinical condition, which is directly related to the anatomical evaluation and success of EVAR. Additionally, the differences in mortality rates in each Brazilian region may be related to the character of the care, the clinical status and recovery of the patient, as well as the existence of local reference services.

109635

Modality: E-Poster Scientific Initiation – Non-case Report

Category: SPIRITUALITY AND CARDIOVASCULAR MEDICINE

## Association between Spirituality and Depression Among Hypertensive Outpatients

VITHORIA VIDOTTI NEVES^1^, Julio Cesar Tolentino Junior^1^, Victor Pacheco Zanela Monte^1^

(1) Hospital Universitário Gaffrée e Guinle

**Introduction:** Depression is associated with greater morbidity and mortality in hypertensive patients. Then, it is important to know potential protective factors for depression in these individuals. Studies have demonstrated that spirituality protects against depression in the general population. Therefore, we hypothesized that spirituality would be a predictor of depression. Additionally, higher spirituality could be related to less depression severity in this population.

**Objectives:** 1) Investigate spirituality as a predictor of depression among hypertensive patients; 2) Analyze the association between spirituality and depression severity.

**Method:** This cross-section study was carried out in hypertensive outpatients at a University Hospital. Depression was diagnosed by DSM-5 criteria. The Patient Health Questionnaire-9 evaluated depression severity. Spirituality was assessed through the Functional Assessment of Chronic Illness Therapy -Spiritual Well-Being (FACIT-Sp). In this widely validated questionnaire, total scores range from 0 to 48, with higher scores indicating higher spiritual well-being (SWB). With SPSS 25®, continuous variables were analyzed using the t-test and linear regression. The binary logistic regression model was performed to predict depression using sex, age, religious affiliation, and FACIT-Sp score as predictors. Data were presented with their respective 95% confidence intervals (95%CI) and odds ratios (OR), considering a significance level of 5%.

**Results:** We included 150 hypertensive outpatients. The age ranged from 28 to 80 years (53.5 ± 14.9), and 69.3% were female. The prevalence of depression was 29.3%. The mean FACIT-Sp score was significantly lower in depressive patients than those without depression (39.5 and 30.2, respectively; p < 0.001). The logistic regression model was statistically significant [χ2(2) = 34.0, p < 0.001] and the Wald test indicated that female sex (OR = 3.884; 95%CI: 1.231–12.253; p = 0.02) and FACIT-Sp score (OR = 0.882; 95%CI: 0.836–0.930; p < 0.001) added significantly to the model. Then, SWB was the strongest depression predictor. The FACIT-Sp has a significant negative association with the PHQ-9 (F(1,148) = 52.205; p < 0.001; R = 0.51; R2 = 0.261).

**Conclusion:** Spirituality was considered a protective factor for depression in hypertension outpatients. In addition, the higher SWB was associated with lower depression severity. These results highlight the importance of spirituality in the approach of hypertensive patients.

109638

Modality: E-Poster Scientific Initiation – Non-case Report

Category: EPIDEMIOLOGY AND HEALTH POLICIES/GLOBAL HEALTH

## Epidemiology of Hospitalization and Costs of Heart Failure Treatment in the Northern Region of Brazil from 2017 to 2021

LUIZ FELIPE FAÇANHA RAMOS^1^, Karen Tássia Façanha Ramos^1^, Hildeman Dias da Costa^2^, Aurea Nathallia Gomes de Souza^1^, Bianca Paula Miranda Martins^1^, Camila Silva de Oliveira^1^, Cecília Rodrigues Viana^1^, Larissa Silva Ferreira^1^, Marcos Roberto Marques da Silva Júnior^1^, Vinícius Maciel Vilhena^1^, Reny Wane Vieira dos Santos^1^

(1) Universidade Federal do Amapá; (2) Universidade Federal de Rondônia

**Introduction:** Heart failure (HF) is characterized by complex syndromes that compromise tissue metabolic demands. There are about two million new diagnosed cases of HF per year in the world, constituting the leading cause of hospital admission in the elderly in Brazil. In this sense, it impacts on the increase in the costs of treating patients, thus being an important public health problem.

**Objective:** To trace the epidemiology and treatment costs of HF from 2017 to 2021 in the northern region of Brazil.

**Methodology:** This is an analytical ecological study of time series and geographical distribution about hospitalization cases and HF treatment costs in the northern region of the country, using secondary data by place of residence (from 2017–2021) from the Department of Informatics of the Unified Health System (DATASUS), through the Hospital Information System and the Hospital Morbidity System, for the evaluation of frequencies, case rates and association factors.

**Results:** In the period 2017–2021, there were 52,963 hospitalizations for HF treatment in the North region of Brazil, which represented a cost of BRL 78,913,522.84 (with an average cost of BRL 1,489.97 per hospitalization) and with a growing trend in average spending on these services (1,658.32 in 2021). There was a drop in these hospitalizations in the period, and the State of Pará and Amapá, respectively, had the highest (42.14%) and lowest (2.2%) percentages of hospitalizations. In the region, there was an average length of stay of 8.2 days of hospitalization (ranged from 8.1 to 8.4 days) and a mortality rate of 11.97% (n = 6,319), with the year 2019 had the highest number of deaths (n = 1,415). The state of Roraima had the highest mortality rate from HF treatment (16.09%). It is noteworthy that associated factors that were more expressive are people of mixed race (n = 31,456), aged between 70 and 79 years (n = 12,608) and males (n = 29,921).

**Conclusions:** During the study period, HF had a high number of hospitalizations in the northern region of the country, with an increase in average treatment costs over the years, with the highest average cost in 2021, although the number of hospitalizations has decreased. It is worth noting the State of Pará with the highest number of hospitalizations and the State of Roraima with the highest mortality rate due to HF, and 2019 was the most lethal year for HF. Finally, it was noticed that HF affects more brown men aged 70 to 79 years.

109642

Modality: E-Poster Scientific Initiation – Non-case Report

Category: HEART FAILURE/CARDIOMYOPATHY/TRANSPLANT

## The Presence of the Third Heart Sound as a Predictor of Mortality in Patients with Heart Failure in a Referral Hospital in the North of Ceará

FELIPE SALIM HABIB BUHAMARA ALVES NASSER GURJÃO^1^, Felipe Salim Habib Buhamara Alves Nasser Gurjão^1^, Mateus de Sousa Cavalcante^1^, Bruna de Almeida Freixedelo^1^, Dara Medeiros Mendes^1^, Benedito Mesley Lima Portela^1^, João Marcos de Fontes Carneiro^1^, Daniel Salmito Chaves^1^, Paulo Roberto Matos Neto^1^, Luís Eduardo Rodrigues Reis^1^, Leandro Cordeiro Portela^1^

(1) Universidade Federal do Ceará – Campus Sobral

**Introduction:** The third heart sound (S3) corresponds to a protodiastolic gallop, which results from the ventricular resistance to the rapid filling phase of diastole. In heart failure, S3 is a marker that indicates systolic dysfunction. Thus, the presence of S3 may be an important feature in the diagnosis and prognosis of patients with heart failure.

**Means:** In this study, the objective is to analyze the relevance of the presence of S3 during hospital admission as a marker of mortality in patients at Hospital do Coração in Sobral with heart failure.

**Methodology:** This is a prospective cohort study obtained with primary data, using as a sample patients admitted to the Hospital do Coração de Sobral with heart failure between the years 2015 to 2019. Thus, at moment of hospital admission the patients who participated in the study had auscultation performed, in order to determine the presence or absence of S3. Then the data was correlated with the in-hospital mortality of these patients. The informations were submitted to statistical analysis using the Chi-Square test, using the OpenEpi program, with a significance value of p = 0.0001.

**Results and discussion:** This study obtained relevant data from 314 patients during this period. From those pacientes, only 21 (6.68%) had S3 at the time of hospital admission, from which 7 (2.23%) died. On the other hand, from the other 293 (93.31%) patients who were denied the presence of S3 at hospital admission, only 16 (5.09%) died. Thus, the risk of death in patients who were admitted with the presence of S3 in auscultation was 33.33%, while the risk of patients who were not admitted with S3 was 5.42%. Therefore, patients admitted with the presence of S3 on auscultation are 6.1 times more likely to die than patients without the presence of S3 during hospital admission.

**Conclusion:** Based on the data analyzed, it can be seen that the presence of S3 during hospital admission of patients with heart failure may be an important marker of in-hospital mortality. Thus, larger studies on the subject are necessary.

109647

Modality: E-Poster Scientific Initiation – Non-case Report

Category: EPIDEMIOLOGY AND HEALTH POLICIES/GLOBAL HEALTH

## Number of Deaths from Acute Rheumatic Fever between the Years 2010 to 2019

ORLANDO SEIXAS ROCHA NETO^2^, Orlando Rocha Seixas neto^2^, Ellen Magnavita Seixas Santos^2^, Luiz Augusto Ferreira Alvarez^2^, Katarina Pereira do Lago^2^, Cinayra Daisy Fraga de Souza^2^, Ingrid Porto Gomes^2^, Beatriz Souza Bastos^2^, Ian Felipe Mariano Gonçalves^2^, Letícia Barbosa Machado de Lima^2^, Mauricio Santa Fé Lins Júnior^2^, Bruno Cunha Freitas^2^

(1) Universidade Salvador; (2) Liga Baiana de Cirurgia Cardiovascular

**Introduction:** Rheumatic fever (RF) is an inflammatory, systemic disease triggered after an infection in pharyngotonsillitis and its etiology is group A beta-hemolytic streptococcus (ECGA). Early attention to pharyngitis coupled with antibiotic use can greatly reduce the risk of RF and its complications. In view of this scenario, one of the factors that may have implied a significant increase in this pathology in the national territory during the period 2010–2019 is the shortage of the main antimicrobial used in prophylaxis: benzathine penicillin.

**Objective:** To assess the number of deaths from acute rheumatic fever in Brazil from 2010 to 2019 and to correlate the shortage of the main antibiotic used for the disease, benzathine penicillin, and poor adherence to treatment.

**Methods:** A cross-sectional observational study of aggregated data, collected through the collection of information through the SUS Hospital Information System (SIH-SUS) by the SUS Department of Informatics (DATASUS), where the number of deaths in years from 2010 to 2019 related to acute rheumatic fever by macroregions of Brazil. The Kolmogorov-Smirnov test was performed for the subsequent adequacy of the tests of comparison of parametric or non-parametric groups. A linear regression in the 10 years surveyed was also performed to assess the increase in deaths in the Brazilian territory in this period.

**Results:** In the linear regression from 2010 to 2019, throughout Brazil, there was evidence for an increase in deaths related to acute rheumatic fever, with the following results: β = –0.848; R² = 0.719; p = 0.002.

**Conclusion:** Finally, it was possible to observe that, with regard to the mortality rate, the studies showed that in the period in which there was a shortage, the numbers of deaths increased, demonstrating a failure to adhere to treatment or a low continuity. of Streptococcus pyogenes prophylaxis in patients. However, even based on this assumption, the adoption of an adequate clinical approach for continuity in prophylaxis in order to avoid cardiac impairment and access to health resources for treatment is the most recommended, in order to modify these statistics.

110035

Modality: E-Poster Scientific Initiation – Non-case Report

Category: HYPERTENSION/RENAL DENERVATION

## Golden Ratio Deviation, SBP/DBP and DBP/PP Coefficients in Normotensive, Pre-Hypertensive, Hypertensive and Diabetic Patients – Clinical Applicability

LOUISE BUONALUMI TÁCITO YUGAR^1^, Tatiana Rubio Azevedo^2^, Larissa Costa Morete^2^, Beatriz Vaz Domingues Moreno^1^, Bruno Rodrigues^1^, José Fernando Vilela Martin^2^, Heitor Moreno Júnior^1^, Lúcia Helena Bonalume Tácito^2^, Juan Carlos Yugar Toledo^2^

(1) Faculdade de Ciências Médicas, UNICAMP; (2) Faculdade de Medicina de São José do Rio Preto, FAMERP

**Introduction:** The golden ratio (φ, phi) is defined as a line divided into two parts of different size (a and b, in which a > b) so that the ratio of the whole length to the larger one is equal to the ratio of the larger number to the smaller. It is approximately 1,61. This number is found throughout the body as in fingers, uterus and the cardiovascular system. The blood pressure curve can be segmented in three parts: pulse pressure (PP), diastolic blood pressure (DBP) and systolic blood pressure (SBP), which is the sum of DBP and PP. In this paper, we aimed to analyze geometrically these values in the form of proportions (DBP/PP and SBP/DBP) to assess if the deviations from phi can be used as additional markers of cardiovascular risk.

**Material and methods:** This retrospective study analyzed data of 171 normotensive patients (NT), 78 pre-hypertensive patients (PHTN), 46 hypertensive people (HTN) and 81 diabetic people (T2DM) from the study “Vascular hemodynamic alterations in HTN and T2DM type 2 patients”. The protocol was approved by the local ethical committee. We used age, sex, SBP, DBP, PP and pulse wave velocity (PWV) for the analysis. We made a descriptive statistical analysis along with t test and ANOVA in SPSS 24 (USA).

**Results:** We observed lower SBP/DBP in PHTN and HTN when compared to NT (p = 0,0002 e p < 0,0001, respectively). Between PHTN and HTN, PHTN and T2DM, there was a significant difference. The DBP/PP ratio showed greater values in PHTN and HTN when compared to HTN (p < 0,0001 in both) and lower and we compared T2DM and NT (p = 0,0495). The comparison between PHTN and HTN, PHTN and T2DM, as well as HTN and T2DM showed significant difference. Multivariate regression in the different groups demonstrated that age and PWV are correlated to SBP/DBP in HTN and there’s a correlation with sex, age and PWV in T2DM. Additionally, we found significant correlation between SBP/DBP and DBP/PP with PWV and PP in the four groups.

**Conclusion:** Deviations from phi were detected in PHTN, HTN and T2DM with different pattern among the groups and can be used as additional risk evaluators as well as markers of cardiovascular damage and are closely related to vascular accelerated ageing for its relation with PP and PWV.

109660

Modality: E-Poster Scientific Initiation – Non-case Report

Category: COVID-19 AND CARDIOVASCULAR SYSTEM

## Changes in the Epidemiological Profile of Cardiovascular Mortality in the Face of the COVID-19 Pandemic Scenario: A Focus on the Brazilian Reality

GABRIELA CUNHA FERNANDES^2^, Sylton Arruda de Melo^1^, Juliana Maria Gurgel Guimarães de Oliveira^1^, Ana Isabelly De Medeiros Tomaz^1^, Arthur Felipe Tertulino Cunha^1^, Pedro Nonato Silveira Costa^1^, Mateus Bessa Nogueira^2^, Sara Araújo de Oliveira Lima^2^, Maria Teresa da Fonseca Madruga^2^, Maria Laura Torres e Araújo^2^, Joice Raquel Urbano Nascimento^2^, Flávia Diógenes Forte Melo^2^

(1) Universidade Potiguar – UnP; (2) Liga Acadêmica de Cardiologia do Rio Grande do Norte (LiCordis-RN)

**Introduction:** In view of the pandemic caused by SARS-Cov 2, a broad approach to the disease and its repercussions is extremely important. Unquestionably, COVID-19 has potentially serious outcomes: cardiovascular, pulmonary, systemic repercussions and death, but we must also consider its impact on the health system in general.

**Objective:** To analyze the mortality rate from cardiovascular diseases (CVD) before and during the pandemic, noting the epidemiological situation in Brazil.

**Methods:** Cross-sectional, retrospective and portal study, through the analysis of transparency data – civil registry. It was a survey on the number of deaths from CVD in Brazil, considering the pre-pandemic period of the year 2019 (from January), and the pandemic scenario of 2020, 2021 until March 15, 2022.

**Results:** The period from January 2019 to March 15, 2022: in 2019 there were 275,032 deaths from cardiovascular causes, of which 37.2% (102,361) were due to stroke, while 36.4% (100,378) were due to acute myocardial infarction (AMI) and 26.2% (72,293) due to unspecific cardiovascular causes. In 2020, 293,963 deaths were observed, with 35.1% (103,238) from stroke, 32.6% (96,023) from AMI and 32.2% (94,702) from unspecific cardiovascular causes. In 2021, there were 318,602 deaths, 33.5% (106,817) from stroke, 32% (102,098) from infarction and 34.4% (109,687) from unspecific cardiovascular causes. In the year 2022, until March, 62,310 deaths were recorded, with 34.5% (21,522) deaths from stroke, 31.8% (19,831) from AMI and 33.6% (20,957) from unspecific cardiovascular causes.

**Conclusion:** An increase in the number of deaths from cardiovascular causes was observed between 2019 and 2022, with a significant increase in the number of deaths from nonspecific cardiovascular diseases (26.2% to 33.6%). However, in 2022, until the analyzed period, a predominance of mortality from stroke was evidenced and that nonspecific cardiovascular causes remained close to the percentage numbers between 2020 and 2021, still above the observed in 2019. It’s concluded that there was an increase in deaths from CVD in a pandemic scenario in Brazil, which can be attributed to the fear of patients to seek medical care in the face of the risk of contamination, reaching more advanced stages or even dying outside the hospital environment. In addition, the increase in the number of deaths from unspecific cardiovascular causes can be largely attributed to mortality from the cardiovascular repercussions of COVID-19.

109663

Modality: E-Poster Scientific Initiation – Non-case Report

Category: NURSING

## Nursing Diagnoses and Cardiovascular Risk Factors Related to Oral Health in Homeless Women in São Paulo City, Brazil

AMANDA CIRILO SILVA^1^, Andreia Correia de Menezes^1^, Ingrid Milani Nacaratto de Freitas^1^, Claudia Cristina Soares Muniz^1^, Everaldo Muniz Oliveira^1^, Fernanda de Mello Demai^1^

(1) Universidade Nove de Julho

**Introduction:** The homeless population in Brazil is minority composed of women, which represent only 14.6% in the city of São Paulo¹, where they are commonly affected by the scarcity in which they live, without health information and lack of access to personal and oral hygiene. We used the nursing diagnoses and possible interventions aiming to produce quality of life and reduction of damage to their health.

**Objective:** To evaluate cardiovascular risks to oral health in homeless women in São Paulo, associated with the Taxonomy of International Nursing Diagnoses Nanda I 2018/2019.

**Methods:** This is a field research with quantitative method, exploratory and cross-sectional nature, approved by the Institutional Ethics Committee under protocol 036417, CAAE:21519413.40000.5511. Conducted in downtown São Paulo, the research counted among 173 evaluated, it was found 18 women volunteers in street situation between the months of November 2019 to March 2020, having between 18 and 59 years, previously selected by convenience and submitted to a questionnaire, being evaluated sociodemographic data pointing the risk factors for cardiovascular diseases (CVD), measurement of blood pressure (BP) and heart rate (HR), subsequently associated with the International Nursing Taxonomy Nanda I.

**Results:** Of the women studied the mean blood pressure (BP) of 128 × 85 mmhg, and Heart Rate (HR) of 91bpm. Being that 18.1% reported having previous history for stroke, 9% for AMI and 76.6% could not inform previous history for AMI. Of these women, 18.1% reported having presented an abscess or edema in the last 6 months. And 27.2% reported toothache or pain in the mucosa region. It was observed that 72.70% of the women answered that yes, they use some illicit substance, 18.1% never used and 9% do not use. Thus, nursing diagnoses were listed during the analysis, using the NANDA I taxonomy, are: Related Ineffective Health Control, Impaired Dentition, and Risk of Impaired Oral Mucous Membrane Integrity.

**Conclusion:** However, it is evidenced that oral hygiene has a direct connection with cardiovascular alterations in this population associated with AMI, stroke, and atherosclerosis. Thus, the importance of public politics for the improvement and eradication of this problem was observed, reducing the aggravations of CVD and aiming to improve the quality of life for this often underprivileged part of society.

109752

Modality: E-Poster Scientific Initiation – Non-case Report

Category: EPIDEMIOLOGY AND HEALTH POLICIES/GLOBAL HEALTH

## Evolution of Mortality from Cardiomyopathies in Brazil

LUIZ FELIPE FAÇANHA RAMOS^1^, Karen Tássia Façanha Ramos^1^, Hildeman Dias da Costa^2^, Aurea Nathallia Gomes de Souza^1^, Bianca Paula Miranda Martins^1^, Camila Silva de Oliveira^1^, Cecília Rodrigues Viana^1^, Larissa Silva Ferreira^1^, Leo Christyan Alves de Lima^3^, Marcos Roberto Marques da Silva Júnior^1^, Vinícius Maciel Vilhena^1^, Reny Wane Vieira dos Santos^1^

(1) Universidade Federal do Amapá; (2) Universidade Federal de Rondônia; (3) Centro Universitário São Lucas

**Introduction:** Cardiovascular diseases are an important cause of mortality in the country, representing the main cause of mortality from all causes, especially cardiomyopathies.

**Objective:** To analyze the evolution and profile of mortality from cardiomyopathies in Brazil in 3 consecutive years.

**Methods:** This is an epidemiological, cross-sectional study on mortality from cardiomyopathies (ICD I42) from 2017 to 2019, in Brazil, using secondary data from the Department of Informatics of the Unified Health System (DATASUS).

**Results:** In the analyzed period, there was a 2% increase in mortality from cardiomyopathies, from 2017 to 2019, totaling 23,061 deaths, with the year 2018 having the highest mortality in the period (n = 7,834). During this period, the southern region of the country had the highest number of deaths (n = 14,658) and the northern region had the lowest number (n = 651). Analyzing the age group, marital status, sex and color, there were more deaths, respectively, among people aged 60 to 69 years (n = 7,412), single (n = 8,683), male (n = 15,904) and white people. (n = 10,387).

**Conclusion:** Thus, there was an increase in the number of deaths during the period studied, especially in 2018. In addition, there were more deaths in the south of the country, in the elderly, single and white. Therefore, there is an urgent need for public health policies for the southern region of the country to contain the advance of mortality from cardiomyopathies.


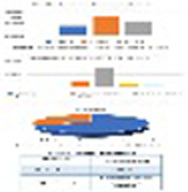


109687

Modality: E-Poster Scientific Initiation – Non-case Report

Category: EPIDEMIOLOGY AND HEALTH POLICIES/GLOBAL HEALTH

## Indirect Effect of the COVID-19 Pandemic on Mortality from Arterial Embolia and Thrombosis in Brazil

LUIZ FELIPE FAÇANHA RAMOS^1^, Karen Tássia Façanha Ramos^1^, Hildeman Dias da Costa^2^, Aurea Nathallia Gomes de Souza^1^, Bianca Paula Miranda Martins^1^, Camila Silva de Oliveira^1^, Cecília Rodrigues Viana^1^, Larissa Silva Ferreira^1^, Marcos Roberto Marques da Silva Júnior^1^, Vinícius Maciel Vilhena^1^, Reny Wane Vieira dos Santos^1^

(1) Universidade Federal do Amapá; (2) Universidade Federal de Rondônia

**Introduction:** The disease caused by the SARS-CoV-2 virus (COVID-19) has been responsible for high rates of hospitalization and high mortality. In addition to respiratory failure, coagulopathy has become a recurrent complication in infected patients, especially the most severe, thus increasing the number of arterial embolisms and thrombosis in the country.

**Objective:** To analyze the effect of the COVID-19 pandemic on cases of arterial embolism and thrombosis in Brazil.

**Methodology:** This is an ecological time series analytical study, using secondary data on arterial embolism and thrombosis (from 2017–2021) in the country based on the Department of Informatics of the Unified Health System (DATASUS), through the Information System of the Unified Health System (DATASUS). Hospital Morbidity. The data were tabulated in two spreadsheets – period 1 (P1): data from 2018–2019; period 2 (P2): 2020–2021 – of the Microsoft Excel software, which were analyzed by the GraphPad Prism software version 9, using the test of Wilcoxon and Pearson’s chi-square test to assess differences in rates, association factors, risk ratio (RiR) and their 95% CI, with a significance level of 5%.

**Results:** Analyzing the two groups of data, there was an increase in mortality from arterial embolism and thrombosis in Brazil during the pandemic period (from n = 3736 to n = 4215; p = 0.05; RiR 1.00; 95% CI 0.99–1.00), also expressed by the difference in standardized mortality rates, since P2 had a higher rate than P1 (8.76 and 8.37, respectively; p = 0.18). It should be noted that the number of deaths was higher in the elderly in both periods (P1, n = 3125; P2, n = 3474), with P2 being the most lethal for people aged 60 years or older (p = 0.14; RiR 0, 95; 95% CI 0.89–1.01). It is noteworthy that the number of deaths occurred more in females in both temporal groups (p = 0.07; RiR: 0.95; 95% CI 0.91–1.00) and in white people (p < 0.0001; RiR: 0.89; 95% CI 0.85–0.93).

**Conclusions:** During the pandemic, the indirect effect was the increase in the number of deaths and mortality rates from arterial embolism and thrombosis in the country, when compared to the same period 2 years before the beginning of the pandemic period. In addition, in the two analyzed periods, deaths occurred more among women, elderly people aged 60 years or older and white people. Thus, we can see the importance of investing in surveillance and effective treatment of this morbidity to possibly reduce the number of deaths.

110173

Modality: E-Poster Scientific Initiation – Non-case Report

Category: EPIDEMIOLOGY AND HEALTH POLICIES/GLOBAL HEALTH

## Analysis of the Number of Healthy Metrics in Women with Chronic Ischemic Heart Disease According to Current Therapeutic Recommendations

MARIA EDUARDA BERGAMO^1^, Maria Eduarda Bergamo^1^, Gustavo Henrique Ferreira Gonçalinho^1^, Nathalia Ferreira de Oliveira Faria^1^, Karen Lika Kuwabara^1^, José Rafael de Oliveira Nascimento^1^, Gean dos Santos de Sales^1^, Antonio de Padua Mansur^1^

(1) Insituto do Coração – HC FMUSP

**Introduction:** Ischemic heart disease (IHD) is the leading cause of death in women. Despite advances in the treatment of IHD, little is known about how this knowledge is applied in current outpatient clinical practice. This study aimed to analyze the number of healthy metrics (HM) the clinical response to the recommended treatment in women with IHD.

**Methods:** This cross-sectional study analyzed 462 women with IHD in outpatient care aged ≥30 years. IHD was diagnosed by cardiac catheterization for coronary lesions >70%. The response to clinical treatment was analyzed by quantifying the number of HM, namely: healthy eating (DASH-Dietary Approaches to Stop Hypertension diet), physical activity (PA), body mass index (BMI = 18.5–24.9 Kg/m^2^), blood pressure (SBP ≤140 and DBP ≤90 mmHg), non-smokers, LDL-cholesterol ≤50 mg/dL, glucose <126 mg/dl.

**Results:** Mean age was 66.5 years; 340 (74%) women with chronic IHD had 3 and 4 HM outside the targets established by current guidelines; 47 (10%) had none or one HM; only 0.4% (n = 3) with all HM. Only 17 (3.7%) of the patients practiced physical activity (PA) regularly, and the entire study population did not have a healthy eating pattern assessed by the DASH diet. The table shows the prevalence of HM analyzed and the mean values of clinical and metabolic variables.

**Conclusion:** The quality of outpatient care for women with chronic IHD did not meet the strategies recommended by current guidelines.


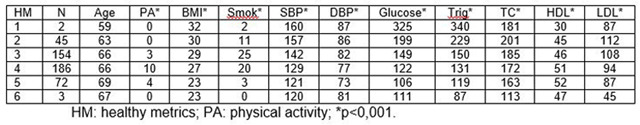


109693

Modality: E-Poster Scientific Initiation – Non-case Report

Category: HEART FAILURE/CARDIOMYOPATHY/TRANSPLANT

## Cardiovascular Continuum – is There Room for Analysis of Arterial Stiffness in the Assessment of Heart Failure with Reduced Ejection Fraction?

MARIA GABRIELA PIMENTA DOS SANTOS^1^, David Ferreira de Lima Duarte^1^, Gabriela Gama Zagni Jardim^2^, Julia Resende de Oliveira^1^, Antônio Carlos Eberienos Assad Filho^1^, Paola Pugian Jardim^3^, Andréa Vaospasse Cocco Faria^3^, Lilian Soares da Costa^4^

(1) Universidade Estácio de Sá/IDOMED, Campus Città; (2) Universidade Estácio de Sá/IDOMED, Campus Vista Carioca; (3) Instituto Estadual de Cardiologia Aloysio de Castro/IECAC; (4) Universidade Estácio de Sá/IDOMED, Campus Città e Vista Carioca; Instituto Estadual de Cardiologia Aloysio de Castro/IECAC

**Introduction:** Arterial stiffness measured by pulse wave velocity (PWV) is considered the gold standard in different national and international guidelines, being a parameter of significant prognostic value of cardiovascular (CV) risk. Although the assertion that elevation of PWV increases the occurrence of CV events, CV mortality and death from all causes, its prognostic value in very high-risk individuals, such as heart failure with reduced ejection fraction (HFrEF), is not yet well established.

**Objective:** Evaluate the oscillometric analysis of vascular pulsatile hemodynamics, using PWV, in patients with HFrEF.

**Materials and Methods:** A cohort study evaluating patients with HFrEF, followed up at a tertiary cardiology hospital in the State of Rio de Janeiro. Blood pressure measurements, PWV, anthropometric measurements and chart analysis were performed.

**Results:** PWV was analyzed in a series of sixteen consecutive cases of individuals with compensated HF and severe systolic dysfunction, comprising a cohort of 180 individuals at high CV risk, which 56% was male and mean age was 64.6 years (36–87 years). The subgroup is composed of sixteen patients, 75% male, mean age 63.4 years (44–78 years), HFrEF with mean EF 30.3% (18–40%). We emphasize that twelve individuals had three or more major risk factors or comorbidities, such as diabetes mellitus, arterial hypertension, dyslipidemia and coronary artery disease. A risk score for arterial stiffness (SAGE Score) was applied on the study population and demonstrated values within normal limits in 14/16 individuals. Blood pressure values within normal parameters were found in 5/16 participants and, in 2/16, arterial stiffness parameters were recorded above the median standards considered normal for the Brazilian population, adjusted for age, sex and presence of factors of risk.

**Conclusion:** As assumed in the literature, a greater number of individuals is needed to confirm the role of this tool as a CV prognostic marker in this particular group of very high-risk individuals with HFrEF. The future perspective of our cohort is that the possibility of pharmacological interventions in the short and medium term can modify these parameters, in order to assess outcomes, for possible assessments of inferences about the role of PWV in CV prognosis.

109700

Modality: E-Poster Scientific Initiation – Non-case Report

Category: CONGENITAL AND PEDIATRIC CARDIOLOGY

## Gastrointestinal Tract Malformations Among Patients with Congenital Heart Disease

GUILHERME RODRIGUES VIANA^1^, Grasiele do Amaral Martins^1^, Marco Antônio Vinciprova Dall Agnese^1^, Adriano Louro Moreira^1^, Pedro Henrique Torres Tietz^1^, Bianca Brinques da Silva^2^, Caroline Engster da Silva^1^, Eric Seiji Kanai^1^, Estefany Karenine Rodriguez Casanova^1^, Letícia Vieira Senger^1^, Matheus Ribeiro Fretes^1^, Rafael Fabiano Machado Rosa^1^

(1) Universidade Federal de Ciências da Saúde de Porto Alegre; (2) Universidade Luterana do Brasil

**Introduction:** Extracardiac malformations (ECMs) associated with congenital heart disease (CHD) may increase the risk of morbidity and mortality of the child, often making surgical intervention risky. This association between ECMs and CHD may involve defects of different systems or tracts, such as the gastrointestinal tract (GIT).

**Purpose:** To determine the frequency and types of GIT malformation in a sample of patients with CC.

**Methods:** A retrospective study using Clinical Data from patients hospitalized for the first time in a cardiac intensive care unit of a reference pediatric hospital in southern Brazil. In the present study, ECMs and CHD data were obtained by filling out a standard protocol.

**Results:** The sample was composed of 343 patients, 182 (53.1%) males, ages ranging from 1 day to 14 years and 6 months (60.1% <1 year). GIT changes were evident in 6 patients (1.7%) and consisted of esophageal atresia (n = 2), duodenal stenosis (n = 1), multiseptated gallbladder (n = 1), anteriorized anal canal (n = 1), and imperforate anus (n = 1). The most commonly observed CC among patients with GIT malformations were septal defects (n = 3), in particular ventricular septal defect (n = 2). Four patients were syndromic, and chromosomal alterations were observed in 5 patients.

**Conclusion:** From the information of the presence of an associated GIT alteration, health professionals can perform a more detailed and directed evaluation of patients with CC, aiming at the best management of these patients and preventing future complications, mainly related to their prognosis.

109714

Modality: E-Poster Scientific Initiation – Non-case Report

Category: EPIDEMIOLOGY AND HEALTH POLICIES/GLOBAL HEALTH

## Mortality from Circulation Diseases and the Evolution of Family Health in Brazil: An Ecological Study

LUIZ FELIPE FAÇANHA RAMOS^1^, Karen Tássia Façanha Ramos^1^, Hildeman Dias da Costa^2^, Aurea Nathallia Gomes de Souza^1^, Bianca Paula Miranda Martins^1^, Camila Silva de Oliveira^1^, Cecília Rodrigues Viana^1^, Larissa Silva Ferreira^1^, Marcos Roberto Marques da Silva Júnior^1^, Vinícius Maciel Vilhena^1^, Reny Wane Vieira dos Santos^1^

(1) Universidade Federal do Amapá; (2) Universidade Federal de Rondônia

**Introduction:** The Family Health Strategy (FHS) stands out, among many factors, for its comprehensive care for people living with non-infectious chronic diseases, especially circulatory diseases, which represent the main cause of death and physical disability in the world. However, there are few studies that associate the advancement of the FHS to the parameters of these diseases, making systematic evaluations of its expansion necessary.

**Objectives:** The objective of this study was to analyze mortality from circulatory diseases in parallel with the evolution of the FHS from 2017 to 2020 in Brazil.

**Methods:** This is an ecological, retrospective study, based on the temporal evolution of the FHS, inhabitant coverage x FHS, and on standardized rates (per 10,000 inhabitants) of mortality from circulatory diseases in Brazil. Secondary data were taken from the SUS Hospital Morbidity System, from the Information Technology Department of the Unified Health System (DATASUS), and from the Primary Care Information and Management System, using the Spearman Correlation test, through the GraphPad Prism software version 9.3.1, for statistical association.

**Results:** During the analyzed period, there was a 2% population increase in Brazil, a 4.7% increase in the number of FHS and a 20.5% increase in mortality from circulatory diseases. The population x FHS ratio increased from 4,965 (2017) to 4,834 (2020) people assisted (2.6% higher coverage). In 2020, the northeast region had the best population coverage by FHS, reaching 3,520 people per implemented unit. Even with the expansion of the FHS in the country, mortality from circulatory diseases increased in all regions, with the northern region having the highest increase (34%). When analyzing the data from the regions, it was noticed that in three there was a positive correlation between FHS and mortality from circulatory diseases (r > 0.7; p < 0.05) and the south and southeast did not present a significant correlation (r: 0, 2; p = 0.91). Overall, Brazil showed a positive correlation between FHS and mortality from circulatory diseases (r: 0.8; p = 0.33).

**Conclusion:** Thus, this study demonstrated that, even with the increase in population coverage by the FHS in Brazil, there was no reduction in mortality from circulatory diseases and that they are a challenge for public health policies. In addition, there was a large increase in mortality from 2017 to 2021 that did not follow the growth of FHS coverage in the same proportion.

109745

Modality: E-Poster Scientific Initiation – Non-case Report

Category: COVID-19 AND CARDIOVASCULAR SYSTEM

## Analysis of the Clinical Profile and Outcomes of Patients with Previous Cardiovascular Disease and COVID-19

CAMILA THAYNÁ DE MENEZES CLEMENTE^1^, Mariane Leandro Ferro de Sousa^1^, Matheus Dantas Soeiro^1^, Carolina Jeronimo Magalhaes^3^, Jessica Myrian de Amorim Garcia^1^

(1) Faculdade Pernambucana de Saúde; (2) Real Hospital Português de Beneficência em Pernambuco; (3) Universidade de Pernambuco

**Introduction:** Cardiovascular diseases (CV) is the most prevalent chronic conditions worldwide. The coronavirus disease 2019 (COVID-19) infection leads to heterogeneous clinical manifestations throughout the population. Therefore, it was necessary to establish the interplay between cardiovascular disease and COVID-19.

**Objective:** Analyze the clinical profile and outcome of patients with COVID- 19 and previous CV disease compared with patients with COVID-19 without these comorbidities.

**Methods:** A cross-sectional observational study of inpatients presenting with COVID-19. Conducted in a private Brazilian hospital from August 2020 and August 2021. The diagnostic criteria were a positive reverse transcriptase-polymerase chain reaction (RT-PCR) or ground-glass opacities on computerized chest tomography with compatible clinical manifestations of dyspnea, fever, and cough. The data were obtained from online medical records.

**Results:** We studied 212 patients with COVID-19. The sample had a mean age of 58.4 years, and 55.7% were males. There was a 50.5% prevalence of previous CV disease, 98.1% of these group of patients had systemic arterial hypertension, 11.2% acute myocardial infarction, and 13.0% congestive heart failure. The patients with both COVID-19 and CV disease were associated with greater mean inflammatory markers D-Dimer (p = 0.03) and troponin (p < 0.01) (TABLE 1). Patients with COVID-19 and CV disease had a greater incidence of shock (p = 0.01), anemia (p < 0.01), bacteremia (p = 0.03), and worse outcomes marked by an extended hospital stay (p = 0.04) as well as 80% of the deaths (TABLE 2).

**Conclusion:** Patients with previous cardiovascular disease infected by COVID-19 had a poorer prognosis and were subject to worse clinical course and complications during of hospitalization.


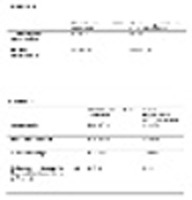


109755

Modality: E-Poster Scientific Initiation – Non-case Report

Category: EPIDEMIOLOGY AND HEALTH POLICIES/GLOBAL HEALTH

## Incidence of Acute Myocardial Infarction in Young Population

DANIEL DE OLIVEIRA MEIRELES ^1^, Sara Cristine Marques dos Santos^1^, Aline de Jesus Oliveira^1^, Thaís Lemos de Souza Macêdo^1^, Ivan Lucas Picone Borges dos Anjos^1^, Júlia Bardela de Oliveira^1^, Anderlúcia Côrrea Guedes^1^, Patrícia Rangel Sobral Dantas^1^, Ivana Picone Borges de Aragão^1^

(1) Universidade de Vassouras

**Introduction:** Acute myocardial infarction is when an obstruction of the coronary artery leads to oxygen deficiency in the myocardial supply, causing necrosis1. According to the Ministry of Health records, there was an increase in the occurrence of AMI in young people (aged 20 to 39 years) and a potential responsible for these cases, which despite being considered small when compared to the national context, are the unhealthy lifestyle habits associated with physical inactivity2. Infarctions in young people are more lethal due to the rapid evolution and their exuberant clinical condition. If there is a delay in starting treatment, complications such as heart failure and arrhythmias may occur2,3.

**Objectives:** To analyze the panorama of the occurrence of AMI in people aged 20 to 39 years.

**Methodology:** A literature review and an observational, descriptive, and cross-sectional collection of data on the occurrence of AMI, available at DATASUS – SUS Hospital Information System – December 2004 to December 2018, were carried out.

**Results:** In the analyzed period, if 45,883 hospitalizations for acute myocardial infarction in people aged between 20 and 39 years, where the year responsible for the largest number was 2018, with 3,823, followed by 2017 with 3,778; on the other hand, the lowest number was 2004 with 2,351, followed by 2005 with 2,518. The total number of deaths was 2,704 in 15 years studied. As for sex, 33,462 affected males while 12,421 were females. The Southeast with 23,901 cases, the Northeast with 8,139, the South with 8,088, the Midwest with 3,295, and the North with 2,460. Among the federation units, São Paulo led with 13,377 hospitalizations. As for the character of the hospitalizations, 33,289 were considered urgent, where they obtained 1,946 deaths (mortality rate of 5.85), the electives totaled 2,373 with 73 deaths (mortality rate of 3.08) and for other causes, 3 with 50% mortality rate. Of the 1946 deaths in emergency care, 1,569 were in patients aged 30 to 39 years, corresponding to 80.6% of cases.

**Conclusion:** It was possible to observe the percentage increase of 62% of the cases of AMI in individuals aged 20 to 39 years in 10 years, where 72.5% was attended to on an urgent basis, which obtained the highest mortality rate, mainly in the age range from 30 to 39 years. It is a disease prevalent in males, which accounts for 72.9% of cases. Where São Paulo has more than half of admissions across the Southeast.

109765

Modality: E-Poster Scientific Initiation – Non-case Report

Category: CARDIOVASCULAR SURGERY

## Impact of the COVID-19 Pandemic on the Performance of Myocardial Revascularizations using Extracorporeal Circulation in the State of Rio de Janeiro

GIULIA VITORIA NASCIMENTO DA SILVA^1^, GABRIEL REZENDE NEVES^1^, LUCAS DIAS SILVA^1^, SAMARA GUILHERMINA DE SOUSA^1^, PAULO CESAR LOBATO MAGALHÃES^2^, LETICIA RIBEIRO DOS SANTOS^1^, MARIANA DOS SANTOS GUIMARÃES^2^, LARISSA DACIER LOBATO COMESANHA^2^, TEREZA MARIA MEIRELES FERNANDES DA SILVA^1^, MARCONDES TAVARES NEVES JUNIOR^1^

(1) Universidade do Estado do Pará (UEPA); (2) Universidade Federal do Pará (UFPA)

**Introduction:** Due to the COVID-19 pandemic, logistical changes have occurred to meet the demand of patients affected by the SARS-CoV-2 virus. However, the expansion of beds for the hospitalization of infected people and the suspension of elective surgeries contributed to modifying the profile of cardiovascular interventions performed during the period.

**Objective:** To analyze the impact of the COVID-19 pandemic on myocardial revascularization using extracorporeal circulation in the state of Rio de Janeiro (RJ).

**Methods:** Observational, cross-sectional, analytical-descriptive and quantitative study. The numbers of myocardial revascularization surgeries using extracorporeal circulation, in RJ, from 2019 to 2021, were obtained via DATASUS – Hospital Information System of the Unified Health System (SIH/SUS). The amount of confirmed COVID-19 cases per month in RJ, from March 2020 to December 2021, were collected via Painel COVID-19 – the virtual platform of the Secretary of Health of the State of Rio de Janeiro.

**Results:** COVID-19 cases in RJ emerged in March 2020 and the number of notifications grew abruptly in May, a period in which there was an increase of 44,687 cases when compared to the previous month. During this period, 77 myocardial revascularizations were performed with the use of extracorporeal circulation, 37.4% less than in the same month of the previous year. In May 2021, the second month with the highest number of new cases of infections in RJ throughout the pandemic, 88 procedures were performed, representing an increase of 14.3% when compared to the same month in 2020 and a decrease of 28.4% compared to the same period in 2019. In addition, in September 2021, the period with the highest peak of new cases of COVID-19 in RJ, 97 myocardial revascularizations were performed, 6.7% more than the same month in 2020 and 33.1% less than in 2019. September 2021 values were succeeced by only 64 procedures in the following month, October 2021, which configured a 34% decrease in surgeries performed between these months. Comparing October 2021 with the same month in 2020, there was a reduction of 27.3%, and in relation to the same period in 2019, a decrease of 56.7%.

**Conclusion:** In view of the increase in cases of COVID-19, there is a significant decline in the volume of myocardial revascularization surgeries performed in the state of RJ, consequently hindering the medical therapy of atherosclerotic coronary disease.

109769

Modality: E-Poster Scientific Initiation – Non-case Report

Category: HEART FAILURE/CARDIOMYOPATHY/TRANSPLANT

## Jugular Vein Ultrasound: A Central Venous Pressure Predictor for Patients Hospitalized with Heart Failure – a Systematic Review

CAIO PLUVIER DUARTE COSTA^1^, Mayara Gabriele Toledo^1^, Eduardo Thadeu de Oliveira Correia^1^

(1) Universidade Federal Fluminense

**Introduction:** Monitoring volume status in patients hospitalized for heart failure (HF) is fundamental to provide therapeutic and prognostic guidance. Recently, the measurement of the internal jugular vein (IJV) by ultrasonography has been object of investigation by several studies. Therefore we aim to systematically review if IJV ultrasound is an useful parameter to predict central venous pressure (CVP) in patients hospitalized for HF.

**Methods:** We conducted a systematic review following the PRISMA guidelines. Original studies indexed by Embase, Pubmed, Cochrane and LiLACS with the keywords: “ultrasound” or “ultrasonography” and “heart failure” and “jugular vein” published until March, 2022 and which matched the inclusion criteria were included. Two authors (C.P and M.T.) performed the screening and data extraction, in cases of discordance, a third author (E.T.) made the decision.

**Results:** Thirteen studies were included, with a total of 898 patients analyzed. Due to the significant heterogeneity between studies, a meta-analysis was not conducted. In all included studies CVP was measured by right heart catheterization. For the ultrasound evaluation of CVP, 5 used the diameter of the IJV; another 4 used the cross-sectional area (CSA) of the IJV and 4 studies used the height of IJV collapse and added 5 centimeters to this measurement (uJVP). Studies that analyzed the diameter of the IJV showed an AUC of 0,74 to 0,84 to predict CVP. Regarding CSA, all 4 articles showed that the IJV ultrasound was useful to predict CVP. Zamboni et al. showed that the CSA was 50–60% better than clinical visualization of jugular vein, while Simon et al. demonstrated an AUC of 0,86 for a CSA variation higher than 66% during Valsalva, compared with rest. Moreover, for the uJVP method, all the included studies concluded that it was a significant predictor of CVP. Significantly, Wang et al. showed that uJVP was a significant predictor of CVP in obese patients, with an AUC of 0,81, although it had a better accuracy among non-obese patients.

**Conclusion:** This systematic review shows that IJV ultrasound is a significant predictor of CVP measured by right heart catheterization. Also, this demonstrates the need for a standard ultrasonographic measurement method of the IJV, to diminish the heterogeneity of future studies. In conclusion, this study paves the way for future randomized trials that aim to investigate if IJV ultrasound can be used to guide diuretic therapy and discharge.

109776

Modality: E-Poster Scientific Initiation – Non-case Report

Category: HYPERTENSION/RENAL DENERVATION

## Echocardiographic Profile of Patients with Resistant Arterial Hypertension: Preserved or Reduced Ejection Fraction?

OSVALDO CARLOS SILVA LEOPOLDINO^1^, Osvaldo Carlos Silva Leopoldino^1^, Camila Orge Rodrigues^2^, Jayne Milly Queiroz Santana^1^, Bernardo Oliveira Torres^3^, Juliana Almeida Frank^3^, Amanda Gabriela Rodrigues dos Santos de Souza^1^, Pedro Henrique Souza de Aragão^3^, Ana Luisa Soares Chiaretti^3^, Victória Valadares Andrade^3^, Cristiano Ricardo Bastos de Macedo^4^, Roque Aras Júnior^4^

(1) Universidade Salvador (UNIFACS); (2) Escola Bahiana de Medicina e Saúde Pública (EBMSP); (3) Universidade Federal da Bahia (UFBA); (4) Hospital Universitário Professor Edgard Santos (HUPES)

**Introduction:** The relationship between hypertension and diastolic dysfunction with preserved ejection fraction (EF) is well established, yet this data is still nebulous in patients with Resistant Arterial Hypertension (RAH). It is uncertain whether other particulates in this group, such as high afterload, may interfere with EF or diastolic dysfunction.

**Objectives:** Analyze the epidemiological and echocardiographic profile of patients with RAH. Methods. Open, Transversal, and Unicenter study from a University Hospital set in Brazil from June 2018 to March 2022 (last update). Eligible patients were those over 18 years and diagnosed with RAH (defined as medical office Blood Pressure (BP) ≥140/90 mmHg despite the use of ≥3 antihypertensive drugs, one of those being a diuretic, or controlled BP using ≥4 medication). Statistical tests (Chi-square test and Z-test) and dispersion measurements were performed using the graph pad and SPSS app. The BP was taken by standardized automated devices, and the echocardiographic exam was performed by the hospital’s staff.

**Results:** There were evaluated 118 hypertensive patients, mostly black (n = 64) and brown (n = 48), with a mean age of 65.28 (±11.14) years and a predominance of women (n = 91). The mean systolic/diastolic BP values were 143.6 (±22.09 [P < 0.01])/82.98 (±16.42 [P < 0.01]) mmHg. At the echocardiographic, the mean EF was 66.00% (±13.05 [P < 0.01]), with only 6 (5.08%) having EF < 40% and 6 (5.08%) with EF 40–50%. The interquartile range of EF (Q1–Q3) was 9,19. Of this sample, 63.5% (n = 75) manifested diastolic dysfunction, with 93.33% (n = 70) being type 1 dysfunction and 6.66% (n = 5) type 2. Regarding the clinical profile, 55 patients declared themselves sedentary, 73 presented with metabolic syndrome, 23 with Chronic Kidney Disease, and 48 with Type 2 Diabetes Mellitus.

**Conclusion:** This study presents some limitations, such as the low sample size and the echocardiogram limit (it is operator-dependent). Nevertheless, this work shows a high prevalence of preserved EF at the echocardiogram, revealing preservation of the left ventricle systolic function. A large proportion of the population had diastolic dysfunctions at an initial state, suggesting that, despite the severity of hypertension, the compromise to the cardiac functionality occurs at a rather late point in the natural history of the disease. At last, some individuals had low EF which suggests independent factors for systolic impairment.

109788

Modality: E-Poster Scientific Initiation – Non-case Report

Category: HEART FAILURE/CARDIOMYOPATHY/TRANSPLANT

## Deaths by Heart Failure in Northern Brazil in 2020

LEANDRO LOURENÇO SILVA MONTEIRO^1^, Luiz Fernando Leite da Silva Neto^1^, Luig Matias Barreiros Pires^2^, Ana Beatriz Rezende dos Santos Corrêa^2^, Talles Levi Pereira Nogueira^3^, Ana Paula Correa de Lima^2^, Igor Lucas Farias Lima^1^, Fernando Tavares Brasil Teixeira^1^

(1) Universidade do Estado do Pará (UEPA); (2) Centro Universitário do Estado do Pará (CESUPA); (3) Universidade Federal do Pará (UFPA)

**Introduction:** Cardiac Insufficiency, marked by heart failure in blood pumping, is still a common outcome for heart diseases of several etiologies and, despite optimized therapy, maintains high levels of hospitalization and in-hospital mortality of patients. In this sense, cardiac insufficiency has a high impact, since social determinants such as gender, age and socioeconomic conditions influence the clinical outcome of disease.

**Objective:** To trace the epidemiological characteristics of deaths by Heart Failure in the Northern region of Brazil in 2020.

**Method:** The study has ecological, descriptive and retrospective character. Data were collected from the Departamento de Informática do Sistema Único de Saúde (DATASUS) in the Sistema de Informação sobre Mortalidade (SIM) as to the number of deaths by Heart Failure in the Northern Region, and epidemiological characteristics of patients, in 2020. The following variables were analyzed: number of deaths, federation units, gender, age group, race/color, schooling and location.

**Results:** In 2020, 1.510 deaths were recorded. The Federation Units with highest records are from Pará (46,42%), Amazonas (17,68%) and Rôndonia (15,56%), and Amapá with the lowest value (2,98%). Relating to age groups, the most prevalent were over 80 years (39,07%), 70 to 79 years (25,58%) and 60 to 69 years (18,27%). In addition, men received quantitative prominence with 58,14%. Regarding race/color, the most notified were brown (65,29%) and white (22,58%). A significant number of deaths occurred in individuals without schooling (32,05%), followed by 1 to 3 years of schooling (21,45%) and 4 to 7 years (16,88%), with the lower value in the classification corresponding to school formation over 12 years (3,5%). Finally, most occurred in a hospital environment (73,04%), followed by home location (19,4%).

**Conclusion:** Cardiac Insufficiency is a disease with a five-year survival rate that is worrisome, because it generates functional loss and quality of life, gradually and eventually evolving to death. And yet, it is possible to notice that death from Heart Failure is prevalent in the Northern region, whose epidemiology is mainly related to age, gender, race/color and intra hospital environment according to data collected from DATASUS. Therefore, it is confirmed the importance of identifying the epidemiological characteristics of deaths for the implementation of effective public health policies in the prevention of such a pathology.

109795

Modality: E-Poster Scientific Initiation – Non-case Report

Category: HYPERTENSION/RENAL DENERVATION

## The Influence of Systemic Arterial Hypertension on the Pathophysiology of Acute Aortic Dissection

DAVI DE SOUZA CATABRIGA^1^, Lucas Dalvi Armond Rezende^2^, Janaína Rodrigues Barbosa^1^, Beatriz Contarini Peluzzo Moraes^1^, Caio Lucas Franco Inocêncio^1^, Lorena Pádua de Moura Duarte^3^, Karolini Zuqui Nunes^2^

(1) Higher School of Sciences of the Santa Casa de Misericórdia de Vitória – EMESCAM; (2) Nursing Department, Health Sciences Center, Federal University of Espírito Santo – UFES; (3) Vila Velha University – UVV

**Introduction:** Systemic arterial hypertension (SAH) is a risk factor for several diseases, including acute aortic dissection (AAD). AAD is defined by the entry of blood into the aortic wall, usually between the outer third and the inner two thirds of the tunica media, due to rupture of the intima.

**Objective:** To elucidate the pathophysiology of aortic dissection and its relationship with systemic arterial hypertension, through the guiding question “What is the pathophysiological relationship of acute aortic dissection with hypertension?”

**Methods:** A systematic review was performed, without meta-analysis, using the PRISMA protocol. Thus, the PubMed database and the Virtual Health Library (BVS) were used during the period of May 2021. The DeCS/MeSH descriptors “”Aneurysm, Dissecting”, “hypertension” and “Pathophysiology” were used. combined using the Boolean operator “AND”.

**Results:** A total of 270 articles were found; of which, after applying the criteria for inclusion, exclusion of duplicates and exclusion by analysis of title, abstract and full text, 13 articles remained for analysis, all in English. AAD comprises the rupture of the tunica intima, which causes an infiltration of blood into the tunica media, so that this blood continues to flow and creates a false lumen. SAH decreases the blood flow to the vasa vasorum, responsible for nourishing the middle layer of the aorta. This situation results in an increasing stiffness of this vessel, which can cause a degeneration of the middle layer of the aorta and increase shear stresses, situations that favor the AAD. This degeneration involves mechanisms of loss of smooth muscle cells and cellular matrix and production of pro-inflammatory cytokines and metalloproteins. On the other hand, the rupture is related to sites exposed to greater shear stresses, which promote an endothelial alteration that delays the proliferative, apoptotic and remodeling processes of the aorta.

**Conclusion:** It was found that the pathophysiological mechanism of AAD of hypertensive etiology is the degeneration of the aortic middle layer and the increased shear stress. These factors are caused by reduced flow of vasa vasorum, loss of smooth muscle cells, production of pro-inflammatory cytokinins and metalloproteins, and changes in proliferative, apoptotic and remodeling processes. We emphasize the need for further research on the pathophysiology of this process in order to define a better diagnostic and therapeutic approach to AAD.

109817

Modality: E-Poster Scientific Initiation – Non-case Report

Category: SPIRITUALITY AND CARDIOVASCULAR MEDICINE

## Spirituality and a New Look at Takotsubo Syndrome

BEATRIZ MONTENEGRO OLIVEIRA^1^, Dra. Antoinette Oliveira Blackman^1^, Adda Cecília Batista de Carvalho Vieira^1^, André Luiz Vergamini Dias^2^, David Ricardo Bernal Lima Hernandez^1^, Felipe Romério Marques Durães Barbosa^1^, Maria Gabriela Alves da Silva^1^, Patrícia Brito de Almeida Borges^1^, Pedro Henrique Alves Miranda^1^, Suellen Keyze Almeida Lima^1^, Warllson Jesus dos Santos^1^, Wenderval Borges Carvalho Júnior^3^

(1) Centro Universitário do Planalto Central Apparecido dos Santos; (2) Universidade Nove de Julho; (3) Hospital Universitário de Brasília

**Introduction:** Takotsubo Syndrome (TS), characterized by transient systolic dysfunction of apical contraction, which simulates Acute Myocardial Infarction (AMI) without obstruction in the corresponding territory, has in its management focus on pharmacological treatment. The pathogenesis is not completely known and the mechanisms suggested are: coronary spasm, microvascular dysfunction and excess of catecholamines, the latter being triggered by emotional stress. Thus, special attention must be given to the integral health of patients, corroborating the concept of health of the World Health Organization (WHO).

**Objective:** To highlight the importance of Spirituality in the complementary management of TS.

**Methods:** Narrative review with search in Pubmed and Scielo databases including case reports, cohort studies, randomized clinical trials and systematic reviews about TS in Portuguese and English languages.

**Results:** Data on non-pharmacological treatment of TS are limited and do not show a relationship between spirituality and TS. Initially more prevalent (90%) in women with a mean age of 68.5 years, with the pandemic of COVID-19 there was an increase in prevalence in men (about 30% of cases), due to stress caused directly by myocardial injury resulting from SARS-CoV-2 or indirectly by the socioeconomic influences of the pandemic. The INTERHEART study (conducted in 52 countries) assessed the impact of cardiovascular risk factors for AMI and found that psychosocial factors (stress, depression, and life events) have a 32.5% risk. The PEACE-III study with post-AMI patients and positive psychology interventions showed substantial improvement in psychological and behavioral outcomes. For the spiritual anamnesis, we have the FICA, HOPE, SPIRIT and FAITH tools, besides the DUREL index; the FACIT-Sp-12 scale; the WHO quality of life instrument (WHOQOL-SRPB); the Spiritual Well-Being scale; among others, as instruments to assess the dimensions of spirituality. Final considerations: The importance of integrating Spirituality in a systematic way in clinical practice and the expansion of the look at the patient (integrality) to improve cardiovascular outcomes is evident, and the application of the WHOQOL – SRPB to measure the dimensions of spirituality should be enforced. More robust studies in the population in question are recommended in order to explore opportunities for future research on TS integrated with Spirituality.

109818

Modality: E-Poster Scientific Initiation – Non-case Report

Category: HYPERTENSION/RENAL DENERVATION

## Analysis of Hospital Morbidity of Individuals with Primary Hypertension in Northern Brazil from 2017 to 2021

IGOR LUCAS FARIAS LIMA^1^, Luiz Fernando Leite da Silva Neto^1^, Wadilla Fiuza da Silva^2^, Larissa da Silva Cambraia^1^, Alexandre D‘Annibale Cartágenes^1^, Pietro Chaves Amaral Miralha^3^, Talles Levi Pereira Nogueira^2^, Vinícius Queiroz Silva^1^

(1) UNIVERSIDADE DO ESTADO DO PARÁ – UEPA; (2) UNIVERSIDADE FEDERAL DO PARÁ – UFPA; (3) CENTRO UNIVERSITÁRIO DO ESTADO DO PARÁ – CESUPA

**Introduction:** Primary Hypertension is the term used to characterize Systemic Arterial Hypertension with no known cause, accounting for about 95% of cases. In this clinical entity, the patient has blood pressure levels that confer a significant increase in the risk of clinical events in the short and long term. As it is a highly prevalent disease, it is necessary to understand the hospital morbidity of this disease in the North region of Brazil.

**Objective:** To characterize the epidemiological profile of patients with hospitalization outcome due to Primary Hypertension in the North region of Brazil between 2017 and 2021.

**Method:** The following study is ecological, descriptive and retrospective. Data were sourced from the Departamento de Informática do Sistema Único de Saúde (DATASUS) regarding the epidemiological characteristics of hospital morbidity due to primary hypertension in Northern Brazil between 2017 and 2021. The following variables were analyzed: number of hospitalizations, year of hospitalization, federation units, sex, age group, hospitalization cost, hospitalization time and mortality.

**Results:** A total of 28,331 hospitalizations were recorded in the verified period. The main years that received the most hospitalizations were: 2017 (22.24%), 2019 (22.07%) and 2018 (21.99%). As for the federation units, the most remarkable were: Pará (57.88%), Rondônia (17.38%) and Amazonas (14.57%). In concern to the profile of the hospitalized patient, the most prevalent were women (56.34%), with the most prevalent age groups from 60 to 69 years old (22.11%) and from 70 to 79 years old (19.66%). Furthermore, the medium cost of hospitalization was R$ 263.06, with an increase of 22.86% from 2017 to 2021. The average hospital internment was 3.3 days, with a higher value for males compared to females. Finally, the mortality rate is 2.02, with emphasis on the age groups over 80 years and 70 to 79 years.

**Conclusion:** Besides noting that the number of hospitalizations showed an uneven distribution among the states in the region, there was an increase in the average cost of hospitalization for the health system during the period. Concerning the profile of patients, most are female, but males had higher mortality, in addition to a clear correlation between age and the outcome of death. This study presents data that can guide not only clinical decisions focused on populations at risk, but also provide the basis for public health actions and resource allocation.

109834

Modality: E-Poster Scientific Initiation – Non-case Report

Category: HEART FAILURE/CARDIOMYOPATHY/TRANSPLANT

## Correlation between B-Lines After Submaximal Exercise Test and Nt-Probnp in Outpatients with Heart Failure with Reduced Ejection Fraction

ALICE ZANETTI DUSSIN^1^, Anna Paula Tscheika^2^, Luiz Claudio Danzmann^3^, Marcus Vinicius Simões^4^, Andrielle Dias Pinheiro^2^, Luiz Carlos Bodanese^1^

(1) Pontifícia Universidade Católica do Rio Grande do Sul (PUCRS); (2) Hospital São Lucas da PUCRS (HSL); (3) Universidade Luterana do Brasil (ULBRA); (4) Hospital das Clínicas da Faculdade de Medicina da Universidade de São Paulo (HC-FMUSP)

**Background:** Congestion detected by lung ultrasound (LUS) through B-lines is related to prognosis in patients with heart failure (HF); performing it after physical stress tests can detect congestion even earlier. However, stress tests require equipment and specialized professionals. The 6-minute walk test (6MWT) is a submaximal exercise test; it is a feasible, inexpensive, and reproducible test to evaluate patients with HF. The dosage of NT-proBNP is also useful in the diagnosis and prognosis of patients with HF. There are no studies evaluating the use of lung US after 6MWT.

**Aims:** To evaluate the correlation of the number of B-lines on LUS before and after the 6MWT with the measurement of the NT-pro-BNP in outpatients with heart failure with reduced ejection fraction (HFrEF).

**Methods:** It is a cross-sectional analytical study with prospective inclusion of patients from three HF outpatient clinics. Inclusion criteria were: age ≥18 years, to have the diagnosis of HF for at least 6 months and to have echocardiogram with ejection fraction <40%. Blood samples were collected for NT-proBNP analysis before the 6MWT. Patients were submitted to the LUS before and after 6MWT.

**Results:** From September 15, 2020 to November 30, 2021, 188 patients were included in the study. The mean age of the patients was 61.83 years (±12.13 years); most patients were male (63.8%); the median left ventricular ejection fraction was 31.73% (interquartile range 25–75% [IQR] 28–37%); the most prevalent etiology of HF was ischemic (52.7%). The NT-proBNP median value was 1,017.5 pg/mL (IQR 331.5–3,482.5); the median number of B-lines was 3 (IQR 1–9) at rest and 6 (IQR 2–13) after submaximal stress. The NT-proBNP value was correlated with the total number of B-lines at rest (r = 0.517; p < 0.001) and after submaximal stress (r = 0.585; p < 0.001), as shown in Figure 1.

**Conclusion:** B-lines in LUS after submaximal stress and at rest were correlated to NT-proBNP in outpatients with HFrEF. Since congestion is one of the main factors that precedes hospitalization, the use of LUS seems to be a useful and easy-to-learn tool to evaluate outpatients with HF.


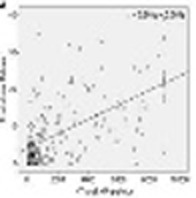


109839

Modality: E-Poster Scientific Initiation – Non-case Report

Category: ACUTE AND CHRONIC CORONARY DISEASE/THROMBOLYSIS

## Acute Myocardial Infarction and the Single Health System: An Analysis of Hospitalization and Death for this Pathology in Brazil

LUIZ FERNANDO LEITE DA SILVA NETO^1^, Leticia Lima Branco^1^, Kennedy Medeiros Cavalcante^1^, Adriano Leitão de Almeida^1^, Daniel Oliveira da Costa^1^, Maria Eduarda Silveira Bührnheim^1^

(1) Universidade do Estado do Pará

**Introduction:** Acute Myocardial Infarction (AMI) is responsible for 12.1% of all cardiovascular diseases (CVD) deaths. This disease is marked by the impairment of the cardiac heart by the minimal blood supply due to total or partial obstruction of a coronary artery. In this context, an expressive severity of the pathology generates hospitalizations and deaths, denoting the need for epidemiological studies in the Brazilian territory to understand its prevalence.

**Objective:** To evaluate the epidemiological profile of hospitalizations and deaths in Brazil due to myocardial infarction from January 2016 to December 2020.

**Methods:** This study has a descriptive, transversal, quantitative and ecological character. Data was collected on Unified Health System Information Department, including information from january 2016 to december 2020. Thus, there were analyzed variables such as hospitalization year, region, sex, age group, color, type of care and deaths.

**Results:** There were a total of 600.752 hospitalizations by AMI. The higher prevalence was found in the Southeast region (49,47%). Regarding the most affected group of people, white people (40,98%), from male sex (56,02%) and aged between 60 and 69 years old (30,75%) were in evidence. Furthermore, the hospitalization quantity increased from 2016 to 2019, although it decreased in 2020. Finally, most hospitalizations were categorized as emergencies (95.88%), and the number of deaths represented 10.31% of the total hospitalizations.

**Conclusion:** Hospitalizations caused by AMI increased from 2016 to 2019 due to demographic transition, characterized by a life expectancy increase in the population. However, there was a decrease in cases in 2020 due to the COVID-19 pandemic, marked by a reluctance to seek medical care and structural limitations in hospital centers, hampering notification. Therefore, with these epidemiological data, it is possible to determine populations at risk and the location with the highest prevalence, guiding future research in this area and the creation of public health actions as well as new financial investments.

109850

Modality: E-Poster Scientific Initiation – Non-case Report

Category: CARDIOGERIATRICS

## Resistance Exercises and Pressure Control in Hypertensive Elderly: A Systematic Review

BRENO VINICIUS DIAS DE SOUZA^1^, Lauanda Ênia de Medeiros Rocha^1^, Emilie Queiroga Queiroga^1^, Gerson Barbosa do Nascimento^1^

(1) Universidade Federal do Rio Grande do Norte (UFRN)

**Introduction:** Systemic Arterial Hypertension (SAH) is one of the main risk factors for cardiovascular diseases, which can lead to severe complications, target organ damage (TOD) and death, especially in the elderly. The disease control and consequent prevention of complications requires changes in lifestyle, with adherence to appropriate physical exercises for the age group.

**Objective:** To review, based on the scientific literature, the impact of resistance exercise on blood pressure reduction in elderly hypertensive individuals.

**Method:** Systematic review of articles published between 2011 and April 2021, in the BVS, LILACS, Cochrane and Pubmed databases with the descriptors “hypertension”; “resistance training” and “elderly”, with 579 articles found. There was the exclusion of duplicate articles, independent evaluation by two researchers of title and abstract, and analysis of the full text of original studies, according to the inclusion and exclusion criteria.

**Results:** 10 articles were included and analyzed. All were unanimous in defending the safety of strength training in hypertension and 9 attested to its direct hypotensive effect in the elderly. The evaluation of resistance exercise in elderly hypertensive women stands out, with a relevant reduction in systolic blood pressure (BP). A 2016 study attested chronic reduction in systolic, diastolic and mean BP in women who acutely already had the hypotensive effect, establishing a relationship between them. It was also evidenced that the greater the duration or volume of exercises, the greater the hypotensive effect. These findings are in line with Brazilian Directive on Arterial Hypertension’s recommendations.

**Conclusion:** Within the complexity of SAH treatment, the practice of resistance physical exercises was crucial for blood pressure control. Last decade’s studies with methodological rigor confirmed its role in the non-drug treatment of SAH, with safety, reduced BP and improvement in the hemodynamic profile in the elderly.

109852

Modality: E-Poster Scientific Initiation – Non-case Report

Category: EPIDEMIOLOGY AND HEALTH POLICIES/GLOBAL HEALTH

## Trend of Deaths from Valvular Heart Diseases in Brazil from 2000 to 2019

RENAN GERONIMO SOUZA DA SILVA^1^, Renan Geronimo Souza da Silva^1^, Alexandre Akio Majima^1^, Ana Paula Cassetta dos Santos Nucera^1^, Fábio de Souza^1^, Wilson Braz Corrêa Filho^1^, Eduardo Marinho Tassi^1^, Davi da Silveira Barroso Alves^1^, Paulo Henrique Godoy^1^

(1) Federal University of the Rio de Janeiro State (UNIRIO)

**Introduction:** Prevalence of heart valve diseases (HVD) is increasing and its etiological profile has changed over the last few decades.

**Objective:** To analyze the trend of deaths due to HVD, according to gender and age group in Brazil, from 2000 to 2019.

**Method:** Ecological study, in which information was collected from the Mortality Information System. The causes of death were categorized into: rheumatic valve disease (RVD); non-rheumatic valve disease (NRDV) and congenital valve disease (CVD), according to ICD-10. Age groups were: 0–29, 30–59, 60 or older. Crude and standardized rates were estimated according to sex and age.

**Results:** A total of 113,570 deaths due HVD were found, 54.93% were female. The distribution by causes was: RVD – 36,018, RVD – 71,477 and CVD – 3,583. The female gender was predominant in the RVD, while in the CVD the male gender was prevalent. In the DVNR, the standardized rate was higher in males, although a greater magnitude of deaths occurred in females. The highest mortality rates due DVR and DVNR were observed in the group aged 60 years and over. In CVD, the mortality rate was higher in the younger age group. The trend for deaths due DVNR and CVD was increasing, while from DVR was decreasing.

**Conclusions:** Trends in deaths from RVD and NRVD seem to reflect an epidemiological transition from these causes. The trend for DVC deserves attention. It seems to reflect higher mortality from congenital heart disease in the country.


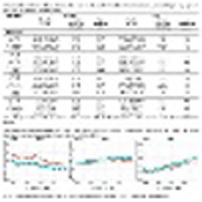


109859

Modality: E-Poster Scientific Initiation – Non-case Report

Category: EPIDEMIOLOGY AND HEALTH POLICIES/GLOBAL HEALTH

## Temporal Analysis of Mortality Rate and Hospital Profile of Hospitalized Patients with Systemic Arterial Hypertension in Brazil between 2012 and 2021

HILDEBRANDO ANTUNES DE CARVALHO NETO^1^, Bruno Oliveira Souza Prates^1^, Beatriz Souza Martins^1^, Beatriz Catarina dos Santos de Oliveira^1^, Gabriela Chateaubriand Campos^1^, Rafael Carlos Pereira^1^

(1) Universidade Federal da Bahia – UFBA

**Introduction:** Systemic Arterial Hypertension (SAH) is a chronic multifactorial condition that constitutes the main modifiable risk factor for cardiovascular complications. It is estimated that this disease has high morbidity and mortality and affects about 30% of the world’s adult population, with higher prevalence in low- and middle-income countries, such as Brazil. In this sense, the epidemiological study of the disease presents itself as an important instrument for this control and prevention.

**Objective:** To analyze the mortality rate and epidemiological profile of hospitalized patients with SAH in Brazil, between 2012 and 2021.

**Methods:** This is an ecological time series study from secondary data collected by the Hospital Information System (SIH-SUS), between the years 2012 to 2021. The total and percentage distribution of elective and urgent care, mortality rate and demographic data were included.

**Results:** In Brazil, in the years analyzed, 608,045 cases of patients hospitalized for SAH were reported, 93.9% of which were on an emergency basis. The year 2012 had the highest number of cases (85,646), and year 2021 had the lowest number (35,472). Females were the most prevalent (85.75%) and the age group most affected was the elderly between 60 and 69 years old (22.42%), followed by elderly people between 70 and 79 years old (20.34%). Most of the admissions were of brown patients (38.38%), followed by whites (24.24%). Within the years under review, the mortality rate ranged from 1.41 in 2012 and 2013 to 2.1 in 2021.

**Conclusions:** Based on the data analyzed in a temporal sequence, there is a tendency to decrease the number of hospitalized patients each year, with the year 2021 having a total number of hospitalizations 58.58% lower than the beginning of the historical series. This decrease in hospital admissions happened gradually, and had its most significant drop in 2020 (24.99%). Elderly, female and brown patients had a higher prevalence in all analyzed years. Regarding the mortality rate attributed to SAH, among hospitalized patients, there was a trend towards stability, with an increase in the year (27.27%).


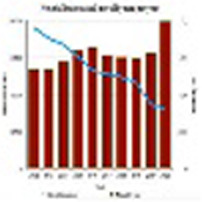


109863

Modality: E-Poster Scientific Initiation – Non-case Report

Category: CARDIOVASCULAR PHARMACOLOGY

## The Impact of Vericiguat as a Reducer of Mortality and Hospitalization in Patients with Heart Failure with Reduced Ejection Fraction: A Systematic Review

ENZZO BARROZO MARRAZZO^1^, Luiz Fernando Moraes Pereira^1^

(1) Pontifícia Universidade Católica de Minas Gerais

**Background:** Heart failure (HF) is occasioned by a functional or structural cardiac change. HF has been during years the major cause of mortality in the world. The treatment of chronic heart failure with reduced ejection fraction is mainly diuretics, neurohormonal antagonists and devices therapies. New pharmacological therapies have been developed as sodium-glucose cotransporter 2 inhibitors and vericiguat, which are promises a better prognosis.

**Aim:** To clarify the impact of Vericiguat use on mortality and on hospitalization rates of patients with heart failure with reduced ejection fraction.

**Methods:** A systematic review was carried out by using a sheared in the PubMed, Lilacs, and Embase databases to search for the descriptors “Vericiguat” e “Heart Failure, Reduced Ejection Fraction”. Only studies conducted on humans, clinical trials where participants were over 18 years old had been selected for inclusion. Studies, where was including patients with HF with preserved ejection fraction and studies focused on sacubitril/valsartan, were not included. To avoid bias was made a double-check in the entire filtering process by different authors.

**Results:** A total of 116 articles were identified and analyzed, of which only 7 were eligible for this review. The use of vericiguat after 3 months of follow-up demonstrated a decrease in the incidence of cardiovascular deaths or hospitalization for HF, reducing these primary outcomes by 4.2 events per 100 patient-years. Despite the few studies, it is suggested that these results can be generalized to patients with worsening HF events in clinical practice. Furthermore, the use of vericiguat was not related to the occurrence of atrial fibrillation events after randomization, although these events are related to the number of deaths and hospitalizations for HF. The effect of vericiguat on the assessed outcomes appears to be better in younger patients, however there is no heterogeneity in the treatment effect of vericiguat compared to placebo on the composite primary endpoint of patients according to baseline systolic blood pressure.

**Conclusion:** The use of vericiguat appears to be promising in reducing mortality and hospitalization rates in patients that developed HF with reduced ejection fraction. Despite recent and scarce studies, the drug demonstrates good safety in clinical management, not inducing the occurrence of events such as syncope, clinical manifestations resulting from hypotension and atrial fibrillation.

109868

Modality: E-Poster Scientific Initiation – Non-case Report

Category: COVID-19 AND CARDIOVASCULAR SYSTEM

## Impact of the COVID-19 Pandemic on Mortality from Heart Failure in Southern Region of Brazil

LARISSA ALMEIDA BUSNELLO^1^, Paola Gonçalves Moreira de Oliveira^1^, Bruna Karas^1^, Ana Carla Dlugosz^1^, Alice Magro Koscianski^1^, Francielle Nocera Viechineski^1^, Camilla Mattia Calixto^1^, Júlia Henneberg Hessman^1^, Mário Claudio Soares Sturzeneker^1^

(1) Universidade Estadual de Ponta Grossa (UEPG)

**Introduction:** Heart failure (HF) has an estimated worldwide prevalence of 1–2%, reaching ≥10% in elderly people ≥70 years. In Brazil, hospitalizations for HF correspond to 4% of general hospitalizations and have high morbidity and mortality. During the COVID-19 pandemic, there was congestion of the health system, but during the 2nd half of 2021, about 70% of the population was vaccinated with the 1st dose and 40% with the 2nd dose.

**Objective:** Evaluate the impact of the pandemic on HF mortality in the southern region of Brazil through the analysis of DATASUS data.

**Methods:** Data regarding HF in the South region were collected in the DATASUS system: hospitalizations, deaths and average hospital stay. Lethality was calculated by dividing the number (N) of deaths by the N of hospitalizations. The periods of pre-pandemic (02/17–02/20), pandemic (03/20–06/21), peak of the pandemic (12/20–05/21), 1st and 2nd quarter of the peak (12/20–02/21 and 03–05/21) and mass vaccination (06–12/21) were evaluated. The means of each variable were calculated by sex and age, and each vaccination period was compared with the others, with the results manifested as a percentage.

**Results:** There was a reduction in the number of hospitalizations in the period of mass vaccination in relation to the pre-pandemic period (25.88%) and an increase of 44,76% in comparison with 2nd trimester of the peak (2TP), and about hospitalizations and deaths, the largest percentage difference evaluated was seen in the 2TP. The evaluation by gender and age groups showed similar behaviour, including the largest increase in relation to the peak of the pandemic, reaching 55.06% in the population >80 years. Regarding the average hospital stay, there was an increase in the vaccination period compared to all other periods, except in the 20–29 age group. Regarding deaths, during the mass vaccination period, there was little variation when compared to the pre-pandemic period, but with significant increases compared to pandemic periods, especially when compared to the peak. There was a significant increase in lethality during the vaccination period when compared to the pre-pandemic period (39.42%), mainly in the age group of 40–49 years (62.43%) and 60–69 years (59.99%).

**Conclusion:** Hospitalizations and inpatient deaths reduced during the COVID-19 pandemics, especially at the peak of the pandemy. However, currently, with mass vaccination, there is a tendency to return the parameters to pre-pandemic values.

109869

Modality: E-Poster Scientific Initiation – Non-case Report

Category: COVID-19 AND CARDIOVASCULAR SYSTEM

## Impact of the COVID-19 Pandemic on Morbidity and Mortality from Heart Failure in the Southeast Region of Brazil

ANA CARLA DLUGOSZ^1^, Larissa Almeida Busnello^1^, Camilla Mattia Calixto^1^, Bruna Karas^1^, Júlia Henneberg Hessman^1^, Alice Magro Koscianski^1^, Mário Claudio Soares Sturzeneker^1^, Paola Gonçalves Moreira de Oliveira^1^, Francielle Nocera Viechineski^1^

(1) Universidade Estadual de Ponta Grossa (UEPG)

**Introduction:** The SARS-CoV-2 pandemic may have influenced heart failure (HF) morbidity and mortality due to a lack of vacancies or fear of contamination in the hospital.

**Objective:** Assess the influence of the SARS-CoV-2 pandemic on HF morbidity and mortality in the Southeast region.

**Method:** Data from 2017 to 2021 were collected on the DATASUS platform. Lethality was calculated by dividing the number (N) of deaths by the N of hospitalizations and expressed as a percentage. The periods denominated pre-pandemic (02/2017 to 02/2020), pandemic (03/2020 to 06/2021), peak of the pandemic (12/2020 to 05/2021), the peak’s 1st and 2nd quarter (12/2020 to 02/2021 and 03/2021 to 05/2021) and mass vaccination (06/2021 to 12/2021) were evaluated. The means of each variable were calculated in general by sex and age group. The pre-pandemic and mass vaccination periods were compared to the others, and the results were expressed as a percentage.

**Results:** Compared to the pre-pandemic period, there was a reduction in the number of hospitalizations in the pandemic period (20.56%), mainly at the peak (34.31%). There was a reduction in the average hospitalization length in all periods of the pandemic, except for the 20–29 age group in the peak’s 1st quarter. There was also a reduction in deaths numbers, mostly in the peak’s 2nd quarter. There were increases in lethality in all periods of the pandemic. During the mass vaccination period, there were reductions in hospitalizations (15.77%), hospitalization length (3.05%) and mortality (3.06%) compared to the pre-pandemic period. However, compared to the pandemic period, there was an increase in hospitalizations (6.03%), hospitalization length (3.96%) and mortality (11.12%). As for lethality, there was an increase of around 15% in both genders and in all age groups compared to the pre-pandemic period and 6.32% compared to the pandemic period, except for women.

**Conclusions:** Data suggests a significant impact of the pandemic on HF lethality in the Southeast region. The expressive reduction in the number of hospitalizations, associated with the reduction in the average hospitalization length, could justify a lower number of deaths and the divergent behaviour of lethality during the pandemic. Mass vaccination allowed these patients to have greater access to hospitals and, probably due to the increase in demand caused by the pandemic, there was an increase in hospitalizations, hospitalization length and mortality.

109874

Modality: E-Poster Scientific Initiation – Non-case Report

Category: EPIDEMIOLOGY AND HEALTH POLICIES/GLOBAL HEALTH

## Hospitalization and Mortality Index on Acute Myocardial Infarction in Brazil in the Last Decade

MARIANA SALLES BALLALAI^1^, Helena Raquel Nogueira de Oliveira^1^, Isabela Aragão Colares^1^, Gabriel Sousa Santos^1^, Renata Pinheiro Martins de Melo^1^, Gabriel Coelho Brito Dias^1^, José Levi Tavares Cavalcante^1^, Ane Karoline Medina Néri^2^, Weiber Silva Xavier^3^, João Luiz de Alencar Araripe Falcão^1^, Sandra Nívea dos Reis Saraiva Falcão^1^

(1) Faculdade de Medicina, Federal University of Ceará (UFC); (2) Hospital Universitário Walter Cantídio (HUWC), Federal University of Ceará (UFC); (3) Programa de Educação em Reanimação Cardiorrespiratória (PERC)

**Introduction:** Cardiovascular diseases are the leading cause of morbidity and mortality worldwide. Acute chest pain is responsible for more than 5% of all emergency department visits. Although most of these patients are discharged with a diagnosis of non-cardiac cause, about 25% are affected with acute myocardial infarction (AMI).

**Objectives:** To analyze the behavior of hospitalizations and deaths numbers due to acute myocardial infarction in the last decade in Brazil.

**Methods:** Retrospective descriptive study with data from the Brazilian Unified Health System database(Tabnet-DATASUS) on hospital admissions and deaths from AMI, by region, in Brazil, from January 2012 to January 2022.

**Results:** Annually, an average of 107,522.8 (±17,350.5) hospitalizations due to AMI occur in Brazil, and the southeast region has the highest average number of hospitalizations (55,005 ± 8003.8). There was an increase of 64% in the number of hospitalizations in 10 years, except in the year 2020 when there was a discrete reduction of 0.6%, which could be explained by the pandemic context. The most considerable increase occurred in the mid-western region, with 160.7% increase in the number of hospitalizations, followed by the northern region with 88.1%. On the other hand, the southern region had the shortest variation, with an increase of 51.7% in 10 years. About mortality, an average of 11,981.4 deaths (±936.26) occurred annually due to AMI. In the last decade, there was an increase of 28% in the number of deaths, except for the intervals 2016/2017 and 2019/2020, in which there was a subtle reduction respectively of 2.0% and 3.8% in the numbers of deaths. The period of greatest increase was 2020/2021 (8.6%). The region with the highest increase in mortality was the mid-west (37.8%). Although the Southeast region had the highest percentage of deaths, this region had the lowest variation in the number of deaths over the years (22.8%).

**Conclusion:** We analyzed the numbers of hospitalizations and deaths due to AMI in the last decade and we found that AMI is still very prevalent in Brazil and that it is an important cause of death in this country. We also noted that there was a considerable increase in hospitalizations during the evaluated period, especially in the southeastern region. Thus, a greater strict prevention in populations at risk is necessary, and the importance of early recognition and adequate treatment of these patients is reaffirmed, to avoid worse prognosis.

109876

Modality: E-Poster Scientific Initiation – Non-case Report

Category: CONGENITAL AND PEDIATRIC CARDIOLOGY

## Congenital Coronary Fistula: The Diagnosis Method and Its Treatment, An Experience After Correction with Minimally Invasive Videothoracoscopy

BRENDA GABRIELE SMANIOTTO RAULIK^1^, Jeronimo Antonio Fortunato Junior^2^, Juliana Fortunato^2^, Shema El-laden hammoud^1^, Letícia Gusso Scremin^1^, Nikolai Cernescu Neto^1^, Gabriel Fontana de Melo^1^, Thaísa Rodrigues Ferreira Basaglia^1^

(1) Universidade Positivo; (2) Hospital da Cruz Vermelha Brasileira – Filial Paraná

**Introduction:** Coronary artery fistula (CAF) is defined as an abnormal communication of coronary arterial blood with other capillary structure, without passing through myocardial capillaries. The congenital origin is the most common, despite accounting for only 0.2 to 0.4% of congenital heart defects. Although rare, they can be fatal. Cardiac surgery allows the correction of most coronary anomalies, but thoracotomy and cardiopulmonary bypass support are associated with a higher number of per- and postoperative complications compared to less invasive videothoracoscopy techniques of surgical treatment.

**Objectives:** Report 5 cases of patients diagnosed with CAF and describe the methodology used for diagnosis, the 5-year evolution of the patients, and the description of the first cases reported in the literature who underwent minimally invasive video-assisted thoracoscopic surgery (VATS).

**Methodology:** We selected the medical records of five medical with CAF diagnosed by two-dimensional echocardiography, coronary angiotomography and cineangiocoronariography.

**Results:** Patients who underwent VATS procedure had to sign an Informed Consent Form. In this study the male gender was more frequent, with 82% of cases, and the mean age was 56.8 years. All patients presented clinics of obstructive coronary insufficiency and only one patient evolved to heart failure NYHA III functional class, this same patient in angiographic studies presented, besides the CAF, an intramyocardial tract of the anterior descending artery. There were no intercurrences during the procedures and the primary success was confirmed with coronary angiographic studies to evaluate remaining fistulous branches or residual shunts. There was no mortality after 5 years of outpatient follow-up.

**Conclusion:** The association of 3 diagnostic methods has fundamental importance to guide the treatment to be followed. With the minimally invasive technique and ligation of the CAF via thoracoscopy, there is a satisfactory surgical result, associated with better long-term clinical outcome and low morbidity.

109900

Modality: E-Poster Scientific Initiation – Non-case Report

Category: CARDIOVASCULAR INTENSIVE CARE/CARDIOVASCULAR EMERGENCIES

## Preliminary Analysis of the Satisfaction Level and Perception of Change in the use of an Adapted Diving Mask for Non-Invasive Ventilation Evaluated by the Global Perception of Change (PGIC) in the Postoperative Period of Cardiac Surgery

EDUARDA CHAVES SILVEIRA^1^, Mariana Goulart Almiron^3^, Jessica Luiza Pedroso da Silva^1^, Bruna Diehl^1^, Tiago da Rosa Rambo^1^, Helena Rocha Machado^3^, Alexander Romão Vieira Morinélli^1^, Patrícia Érika de Melo Marinho^2^, Dulciane Nunes Paiva^1^

(1) Universidade de Santa Cruz do Sul (UNISC); (2) Universidade Federal de Pernambuco (UFPE); (3) Hospital Santa Cruz (HSC)

**Introduction:** Non-invasive ventilation (NIV) is widely used in the postoperative period (PO) of cardiac surgery (CS) to promote lung reexpansion. The orofacial mask, interface usually used in NIV, can cause air leakage and, in the Covid-19 pandemic, avoid aerosolization to the environment has become a priority focus. The adapted diving mask (Owner Mask) emerged as an alternative NIV interface and its effectiveness has been evaluated.

**Objective:** To assess the satisfaction level and the perception of change in patients undergoing NIV with an Owner mask in the PO of CS.

**Methods:** A cross-sectional study that evaluated patients undergoing coronary artery bypass graft or valve replacement surgery with clinical indication for NIV. The level of satisfaction and the perception of change was assessed using the Patients‘ Global Impression of Change questionnaire at discharge from the ICU. This instrument classifies the perception of improvement in 7 items: 1 = No changes, 2 = Almost the same, without any visible change, 3 = Slightly better, but without considerable changes, 4 = With some improvement, but the change did not represent any real difference, 5 = Moderately better, with slight but significant change, 6 = Better, and with improvements that made a real and useful difference, 7 = Much better, and with a considerable improvement that made all the difference. Data described in frequency of occurrence.

**Results:** Sample (n = 29; 52% male) had comorbidities such as SAH (66.7%), diabetes mellitus (24.8%), ischemic heart disease (19.4%), CHF (16.7%) COPD (11.1%), dyslipidemia (11.1%) and obesity (11.1%). Of the sample evaluated, 17.2% reported “no changes”, 3.4% “Almost the same, no visible changes”, 20.7% “Slightly better, but without considerable changes”, 10.3% “With some improvements, but the change didn‘t make any real difference”, 10.3% Moderately better, with slight but significant change, 20.7% “Better, and with improvements that made a real and useful difference” and 17.2% reported as “Much better, and with a considerable improvement that made all the difference”.

**Conclusion:** The level of satisfaction and the perception of change with the use of the Owner mask went from “Slightly better, but without considerable changes” to “Better, and with improvements that made a real and useful difference” in 41.4% of the sample evaluated. The comparison of these outcomes in relation to the conventional orofacial mask will be carried out with the expansion of the research.

109905

Modality: E-Poster Scientific Initiation – Non-case Report

Category: NUTRITION

## The COVID-19 Pandemic and Adolescents and Children Brazilian Lifestyles: A Cross-Sectional Study, 2020

RAQUEL SANTIAGO VITORINO^1^, Luana Azevedo de Aquino^1^, Letícia Martins Raposo^1^, Simone Augusta Ribas^1^, Michelle Teixeira Teixeira^1^

(1) Universidade Federal do Estado do Rio de Janeiro UNIRIO

In an overwhelming way, the Sars-Cov2 virus changed the planetary routine starting in 2020. In order to contain the advance and mortality of the pandemic, the WHO and several entities and governments recommended restriction in social contact, resulting from the closing of schools, commerce and leisure areas. The domestic environment becomes the only space for social coexistence, limiting the activities of children and adolescents. The vulnerability of development in this age group maximizes the risks by interrupting the children’s routine.

**Objective:** To describe the lifestyle of Brazilian children and adolescents, regarding food, physical activity, sleep quality, screen time, in the period of social restriction resulting from the COVID-19 pandemic.

**Methods:** Cross-sectional study carried out between May and June 2020, with data collected through online forms, intended for parents and/or guardians of children aged 2 to 9 years and adolescents aged 10 to 18 years, in a random and non-probabilistic manner, in all Brazilian regions. Prevalence calculations were performed, with a 95% confidence interval, with the R software.

**Results:** 1309 participated in the survey, 589 children and 720 adolescents. Of the total, half of the families were in social isolation (52%) and belong to the middle social class (56%). As for the practice of physical activity, 72% did not perform or performed less than one hour a day. In addition, the sedentary behavior of excessive use of electronic devices occurred among 75% of those evaluated, with adolescents being the users with the highest reported screen time. There was a change in sleep among 55%, with inadequacy for more (17%) or less (24%). As for eating habits, 30% used to replace meals with snacks, and there was a significant increase in the consumption of sugary drinks (25%), sweets (45%) and quick snacks (36%), especially among adolescents (p < 0.001). On the other hand, there was a reduction in the consumption of beans (10%), fruits (13%), vegetables (20%) and dairy products (10%).

**Conclusions:** The social restriction imposed by the COVID-19 pandemic has imposed negative changes in diet and physical activity level that could possibly have a long-term impact on the health of children and adolescents.

109909

Modality: E-Poster Scientific Initiation – Non-case Report

Category: COVID-19 AND CARDIOVASCULAR SYSTEM

## Ami Hospitalizations in the Current Context of the COVID-19 Pandemic: A Comparative Study between Demographic Regions in Brazil

BIANCA MENDONÇA REIS^1^, Gabriela El Bazi^1^, Ravy Soares Álvares^1^, Millena Batistela Pereira^1^, Caroline Borges de Assis^1^, Bruno Carraro^1^, Humberto Graner Moreira^1^

(1) Universidade Evangélica de Goiás

**Introduction:** The access to hospital care in Brazil has always been conditioned to each geographic region’s socioeconomic discrepancies. Following the isolation resulting of the pandemic of the new coronavirus, the access to health care has been compromised even further, especially regarding treatment for acute myocardial infarction (AMI). Until 2019, AMI represented the leading cause of deaths in Brazil, however, in 2020, with the unfolding of the pandemic, some countries observed a drop in the AMI notifications.

**Objectives:** To describe the reduction in reported cases of AMI among the geographic regions of Brazil.

**Methods:** Observational and descriptive study with a quantitative approach. Data collected using the databases of the Department of Informatics of the Unified Health System (DATASUS) from the Hospital Information System of the SUS (SIH/SUS) and the Mortality Information System (SIM) from January 2017 to December 2020, using the variables: number of hospital admissions; average length of hospitalization; hospital mortality rate and as the underlying cause myocardial infarction, codes I21 to I24 of the 10th International Statistical Classification of Diseases and Related Health Problems (ICD-10). Patients younger than 20 years old were excluded from the study.

**Results:** Comparing data from 2020 to 2019, there was a decrease in cases of AMI in the North, Northeast and Southeast regions, while the South and Midwest presented an increase. In the North region, there was a progressive increase in cases between 2017 and 2018. In 2020, the reduction was of 1.61% cases compared to 2019. In the Northeast region, the reduction of 7.13% cases from 2019 to 2020, was more significant. However, in the Midwest region, there was an increase of 948 cases from 2019 to 2020 while in the Southeast region, there was a decrease of 427 cases. In the South, there was an increase of 3.01% from 2019 to 2020.

**Conclusion:** Despite the expectations of a decrease in AMI cases in all geographic regions the Midwest and South regions presented increasing data. The reduction noticed in other regions is directly related to a sudden drop in the search for AMI treatment due to the imposed social isolation and the fear of contracting a respiratory infection in the hospital environment. Socioeconomic disparities between regions directly interfere with the result.

109915

Modality: E-Poster Scientific Initiation – Non-case Report

Category: CARDIAC ARRHYTHMIAS/ELECTROPHYSIOLOGY/ELECTROCARDIOGRAPHY

## Relation of Alcoholic Beverage Ingestion as a Risk Factor for Atrial Fibrillation in Patients with Heart Failure

JOÃO MARCOS DE FONTES CARNEIRO^1^, João Marcos de Fontes Carneiro^1^, Daniel Salmito Chaves^1^, Paulo Roberto Matos Neto^1^, Felipe Salim Habib Buhamara Alves Nasser Gurjão^1^, Mateus de Sousa Cavalcante^1^, Bruna de Almeida Freixedelo^1^, Benedito Mesley Lima Portela^1^, Leandro Cordeiro Portela^1^, David Carneiro Neto^1^

(1) Universidade Federal do Ceará – campus Sobral

**Introduction:** Sustained alcohol consumption is closely linked to atrial fibrillation (AF), which is the most common heart rhythm disorder. This is due to electrical remodeling in the atrium, which facilitates the formation of arrhythmogenic portions. This condition is of great social relevance, as it is associated with the deterioration of the hemodynamic profile and cardiac output in affected patients, which is, therefore, a worsening factor in heart failure (HF).

**Objectives:** To evaluate the relationship between alcohol intake and the incidence of AF in patients with decompensated HF.

**Methods:** A cross-sectional study was carried out with 322 patients admitted to a cardiology hospital for acute HF decompensation. Information was obtained from a database collected by properly trained health professionals present at the time of hospital admission, with a signed consent form. Participants were divided into two groups: those who had the habit of drinking alcohol and those who did not. Electrocardiographic data were collected in both groups, and the appearance of Atrial Fibrillation was recorded. To verify the significance of the association between alcohol intake and the onset of AF, Fisher’s Exact Test was used. The calculation of statistical significance was performed on the OpenEpi platform, using a significance level of p < 0.05.

**Results:** Of the 67 patients with atrial fibrillation at hospital admission, a total of 25 (37.31%) had the habit of drinking alcohol. On the other hand, of the 255 patients who were admitted without atrial fibrillation, only 65 (25.5%) reported drinking alcohol. Thus, there was a significant influence in both groups regarding the consumption of alcoholic beverages as a risk factor for the onset of atrial fibrillation at hospital admission, with a considerable significance level, with p = 0.04062.

**Conclusion:** The habit of drinking alcoholic beverages is an important propensity factor for the development of AF in patients with acute decompensated HF, evidencing the importance of correct control of certain lifestyle habits for the prevention and control of the prevalence of factors that influence the aggravation of the symptoms and greater chance of complications that culminate in a worse prognosis of HF.

111117

Modality: E-Poster Scientific Initiation – Non-case Report

Category: ATHEROSCLEROSIS/CARDIOVASCULAR RISK FACTORS/CARDIOVASCULAR PREVENTION

## High Risk for Obstructive Sleep Apnea Syndrome and Diastolic Function on Echocardiography

AHYSLA GONÇALVES DURÃES^1^, Adson Renato Leite^2^, Antônio José Lagoeiro Jorge^2^, César Augusto da Silva Nascimento^2^, Maria Luiza Garcia Rosa^2^, Wolney de Andrade Martins^2^, Ana Paula Arriaga Carvalho^2^, Clara Mônica Figueredo de Lima^1^, Luan Andrade Carvalho^3^, Emilly Vendramini Mello^1^, Ana Leticia dos Anjos Sampaio^1^, Nathália Felícia Silva Frias^1^

(1) Universidade Federal do Sul da Bahia (UFSB); (2) Universidade Federal Fluminense (UFF); (3) Universidade Federal do Vale do São Francisco (UNIVASF)

Obstructive sleep apnea (OSA) is a chronic, progressive disorder with high mortality and morbidity associated with cardiovascular diseases (CVD), including heart failure (HF). OSA-related pathophysiological alterations can directly impact the diastolic function of the left ventricle.

**Objective:** To evaluate the relation between the risk of OSA, considered by the Berlin Questionnaire (BQ), and echocardiogram parameters, related to diastolic function, in individuals without HF assisted by the Family Medical Program of Niterói (PMF).

**Methods:** Cross-sectional study that included 354 individuals (51% women) aged 45 years or older. The exclusion criteria were the presence of HF, non-completion of the BQ and obese patients with arterial hypertension and not classified as being at risk for OSA by another criterion. All the individuals selected for the study underwent an evaluation, performed in a single day, that consisted of the following procedures: consultation, filling out the BQ and clinical examination, performing laboratory tests and transthoracic doppler echocardiogram (TDE).

**Results:** Of the 354 individuals analyzed, 63% were classified as being at risk for OSA. Patients at risk for OSA present significant alterations in the parameters that assess the diastolic function, which may indicate a worsening of this function in these patients. An adjusted multivariate gamma regression was performed. After adjustment (gender, age, BMI, fasting glucose, triglycerides, serum uric acid, urea albumin/creatinine ratio, systolic and diastolic blood pressure in their continuous forms), risk for OSA confirmed its positive and statistically significant association with indicators of diastolic dysfunction, LAV-i (p = 0.02); E‘/A‘(p < 0.01), A (p = 0.02), E/A (p < 0.01).

**Conclusion:** Our data show that patients at risk for OSA present a worsening in the diastolic function parameters measured by the TDE.

109931

Modality: E-Poster Scientific Initiation – Non-case Report

Category: EPIDEMIOLOGY AND HEALTH POLICIES/GLOBAL HEALTH

## Heart Failure Morbidity and Mortality Profile in Brazil in the Last 5 Years

GABRIEL COELHO BRITO DIAS^1^, Mariana Salles Ballalai^1^, José Levi Tavares Cavalcante^1^, Helena Raquel Nogueira de Oliveira^1^, Isabela Aragão Colares^1^, Gabriel Sousa Santos^1^, Renata Pinheiro Martins de Melo^1^, Ane Karoline Medina Néri^2^, Weiber Silva Xavier^3^, João Luiz de Alencar Araripe Falcão^1^, Sandra Nívea dos Reis Saraiva Falcão^1^

(1) Universidade Federal do Ceará (UFC); (2) Hospital Universitário Walter Cantídio (HUWC); (3) Programa de Educação em Reanimação Cardiorrespiratória (PERC)

**Introduction:** Heart Failure (HF) is a syndrome in which the heart is unable to adequately pump blood to meet organic needs. Despite clinical advances, HF is the leading cause of hospitalization among circulatory system diseases.

**Objectives:** To describe the morbidity of HF in Brazil and its impact on public health in the country.

**Methods:** Observational descriptive study conducted using data obtained from DATASUS. The variables gender, age group, death and hospitalization costs of patients with HF between 2017 and 2021 were analyzed.

**Results:** In the period, the total number of hospitalizations was 928,541, with the highest number occurring in 2017 recording 209,162 (22.53%) and decreasing annually until the lowest value in 2021 (16.27%). There was a predominance of male patients representing 480,691 (51.77%). The most affected age group was the elderly aged 70 to 79 years with 26.40% of hospitalizations. In addition, 108,692 deaths were registered in the period, totaling 11.71% mortality rate for HF within this time frame. Finally, the average amount spent per hospitalization was R$ 1,649.47.

**Conclusion:** We analyzed the numbers of hospitalization and deaths due to HF in the last five years and we found that HF is still very prevalent in Brazil and, in despite of the decrease in the mean number of hospitalizations in 2021, it is an important cause of death in the country and a significant source of state expenses. Furthermore, we noticed that the hospitalized patients tended to be male and aged 70 to 79 years. Thus, a greater strict prevention in populations at risk is necessary and the importance of early recognition associated with an adequate treatment strategy is reaffirmed.

109933

Modality: E-Poster Scientific Initiation – Non-case Report

Category: COVID-19 AND CARDIOVASCULAR SYSTEM

## Ami Mortality During the First Year of the COVID-19 Pandemic: Lifestyle Improvements or Distraction?

RAVY SOARES ÁLVARES^1^, Gabriela El Bazi^1^, Bianca Mendonça Reis^1^, Millena Batistela Pereira^1^, Caroline Borges de Assis^1^, Bruno Carraro^1^, Humberto Graner Moreira^1^

(1) Universidade Evangélica de Goiás

**Introduction:** The search for medical assistance during the coronavirus pandemic in Brazil has declined significantly. Furthermore, the fear of contracting covid in hospitals and the prioritization of urgency and emergency care over elective consultations resulted in a decrease in the search for medical assistance during the pandemic. Faced with this new reality, some changes were noticed in the country’s morbidity and mortality rates, since the Acute Myocardial Infarction (AMI), the leading cause of death in the country until 2019, presented significant decrease during the pandemic.

**Objectives:** To describe the incidence of AMI by sex and age group during the pandemic.

**Methods:** Observational and descriptive study with a quantitative approach. Data was collected using the databases of the Department of Informatics of the Unified Health System (DATASUS) from the Hospital Information System of the SUS (SIH/SUS) and the Mortality Information System (SIM) from January 2017 to December 2020, using the variables: number of hospital admissions; average length of hospitalization; hospital mortality rate and as the underlying cause myocardial infarction and then stratified by age group, and sex, encompassing only individuals over 20 years old.

**Results:** In 2020, the age group with the highest hospitalization rate for AMI was the one of 60 to 69 years old, while the lowest rate belongs to the age group of 80 years and older. In the age group of 80 years and older, females had the most significant reduction (3.3%) in hospitalizations while males decreased in 1.5% Previously to the pandemic, there was an annual progressive increase in mortality by AMI, however in 2020 there was an overall reduction of 6118 deaths when compared to 2019, being that the age group of 80 years and older presented a decrease of 56.15% of total deaths. Females presented a reduction of 2886 deaths in 2020 when compared to 2019 while males, in the age group of 80 years and older, presented a reduction of 30.9% of cases

**Conclusion:** A reduction in hospitalizations and mortality by AMI was expected and confirmed in this study. However, it could be noticed that the main reason behind said reduction was not the improvement in people’s lifestyles and subsequent reduction in cardiovascular diseases, but the association between the lower search for hospital assistance and the fact that the AMI symptoms can be disguised by the coronavirus infection due to the latest’s greater expressiveness.

111850

Modality: E-Poster Scientific Initiation – Non-case Report

Category: CARDIOGERIATRICS

## Low Aerobic Capacity as a Predictive Factor for Ventricular Dysfunction in Elderly Undergoing Stress Echocardiography

ALLEXA GABRIELE TEIXEIRA FEITOSA^1^, Gabriela de Oliveira Salazar^1^, José Icaro Nunes Cruz^1^, Cláudia Bispo Martins-Santos^1^, Lara Teles Alencar Duarte^1^, Cleovaldo Ribeiro Ferreira-Júnior^1^, Edvaldo Victor Gois Oliveira^1^, André Pinheiro Zylberman^1^, Eduardo José Pereira Ferreira^1^, Antônio Carlos Sobral Sousa^1^, Enaldo Vieira de Melo^1^, Joselina Luzia Menezes Oliveira^1^

(1) Universidade Federal de Sergipe – UFS

**Introduction:** The advance of age is an important risk factor for cardiovascular diseases, including ventricular dysfunction, which makes the elderly an important research and control group.

**Objective:** To identify predictors of ventricular dysfunction in a population of elderly people and to evaluate the level of aerobic capacity of these patients.

**Methods:** Cross-sectional study between January 2000 and January 2022 with individuals over the age of 60 years undergoing Physical Stress Echocardiography (SE) in a respected cardiological service. 1644 (68 ± 6 years) patients were categorized according to cardiorespiratory capacity (CRC) into low (MET < 7,9), intermediate (7,9 ≤ MET < 10,9) and high (MET ≥ 10,9) fitness. The chi-square test and logistic regression were used using SPSS Statistics software version 22.0. A significance level of 5% was admitted.

**Results:** Among the patients analyzed, 40,8% had low CRC, 34,8% intermediate and 24,4% had high aerobic capacity. Of the total, 8,2% (135) had ventricular dysfunction. Low CRC was associated with the presence of: systemic arterial hypertension, sedentary lifestyle, obesity, alcohol consumption, female sex (p < 0,001) and family history (p = 0,01). Male sex (OR = 1,950; IC95% = 1,328–2,863; p = 0,001), diabetes mellitus (OR = 1,908; IC95% = 1,267–2,874; p = 0,002), smoking (OR = 2,540; IC95% = 1,409–4,577; p = 0,002), dyslipidemia (OR = 1,588; IC95% = 1,092–2,307; p = 0,015), low CCR (OR = 3,218; IC95% = 1,846–5,611; p < 0,001) and intermediate CCR (OR = 1,989; IC95%1,129–3,503; p = 0,017) were predictors of ventricular dysfunction in the elderly.

**Conclusions:** Low aerobic capacity was associated with systemic arterial hypertension, sedentary lifestyle, obesity, alcohol consumption and the female sex, as well as being a predictor of ventricular dysfunction in the elderly.

110112

Modality: E-Poster Scientific Initiation – Non-case Report

Category: ATHEROSCLEROSIS/CARDIOVASCULAR RISK FACTORS/CARDIOVASCULAR PREVENTION

## The Association between Arterial Stiffness Through Aortic Pulse Wave Velocity and Increased Heart Disease in Copd Patients

BIANCA VASCONCELOS BRAGA CAVALCANTE^1^, Ana Lívia Gadelha Xavier da Nóbrega Pereira^1^, Beatriz Gadelha e Xavier^1^, Leticia Lacerda Burity^1^, Rayanne Alessandra da Silva Barreto^1^, Vinicius Vieira Leandro da Silva^1^

(1) FACULDADE DE MEDICINA NOVA ESPERANÇA – FAMENE

**Introduction:** Chronic obstructive pulmonary disease is one of the main causes of mortality in the world and is closely related to cardiovascular diseases, with a risk of 4.5 times greater compared to the healthy population. There is a correlation between heart failure (HF) and COPD, which has been extensively investigated in recent years.

**Objective:** To correlate arterial stiffness in patients diagnosed with COPD. Method and materials: This is a systematic review of the literature indexed between 2018 and 2022, published in the Scientific Electronic Library and PubMed databases. The following terms were applied to the Health Science Descriptors (DeCS): “Chronic Obstructive Pulmonary Disease” and “Aortic diseases”. Articles unrelated to the topic and inaccessible for free were excluded.

**Results:** It is known that about 30% of patients with HF are also diagnosed with COPD, which corroborates the prognosis of increased hospitalizations and mortality in these people. There are few studies that report which biomarkers are related to the two diseases, but it has been seen that aortic pulse wave velocity (aPWV) is the gold standard measure to determine arterial stiffness and, therefore, is a predictor for elevated cardiovascular events. in COPD patients (defined as +0.7 m/s). In a meta-analysis, a +1 m/s increase in aPWV was reported to determine a 15% increase in the risk of heart disease. A plausible explanation for the relationship between aPWV and COPD is the pathological mechanism of degradation of elastin fibers in the lungs and large arteries, such as the aorta, since patients with COPD lose a large part of the cutaneous elastin, which generates severity in emphysema. and arterial stiffness.

**Conclusion:** It is known that these data can influence the determination of the negative impacts of the two pathologies. Thus, there is a clear need for further studies on biomarkers related to COPD and heart disease.

111395

Modality: E-Poster Scientific Initiation – Non-case Report

Category: EPIDEMIOLOGY AND HEALTH POLICIES/GLOBAL HEALTH

## Epidemiological Profile of Deaths Caused by Acute Myocardial Infarction in a State of the Brazilian Amazon from 2016 to 2020

AMANDA GABRIELA FREITAS RODRIGUES^1^, Matheus Ricardo Malveira Camacho^2^, Lucas dos Santos Fontes^1^

(1) Universidade Federal do Pará; (2) Universidade Estadual do Pará

**Introduction and/or Fundamentals:** Acute myocardial infarction (AMI) is responsible for significant mortality rates in Brazil and worldwide. Despite the implementation of national policies to prevent precursor diseases of cardiovascular importance such as hypertension and diabetes, mortality rates from AMI remain high compared to the rates of developed countries. Therefore, the analysis of the epidemiological profile of deaths caused by AMI in Brazil is crucial, so that the strategy towards reducing mortality from this disease is more accurate. 2.

**Objectives:** Trace an epidemiological profile of deaths caused by acute myocardial infarction in the state of Pará, Brazil, from 2016 to 2020. 3.

**Methods:** Analysis of data provided by the digital platform of the Department of Informatics of the Unified Health System (DATASUS), in the category “deaths from preventable causes from 5 to 74 years old”, ICD-10 category “I21 Acute myocardial infarction”, selecting the sections according to the search parameters, such as “sex” and “education”. 4.

**Results:** In the period evaluated, there was a trend of growth in the number of cases of deaths caused by AMI; 1577 were registered in 2016; 1709, in 2017; 1721, in 2018; 1840, in 2019; and 1863, in 2020; totaling 8710 deaths. There was a greater involvement of men (n = 5953/68.4%) and less of women (n = 2757/31.6%). Regarding self-declaration of color, most deaths were registered in brown people (n = 6702/76.9%), followed by white (n = 1181/13.5%) and black (n = 605/6.9%). People aged between 60 and 69 years were more affected (n = 3343/38.4%), followed by people aged between 50 and 59 years (n = 2159/24.8%) and people aged between 70 and 74 years old (n = 1697/19.5%). In addition, in relation to schooling, there was a higher occurrence among people with 4 to 7 years of schooling (n = 2193/25.2%), followed by people with 1 to 3 years of schooling (n = 2138/24.5%) and people with 8 to 11 years of schooling (n = 1614/18.5%). 5.

**Conclusion:** This study has found an important social stratum in the data in question; people with up to 7 years of schooling symbolize 49.7% of the total number of cases registered and analyzed, indicating an influence of social status in this scenario, as well a stronger the prevalence of AIM between males. Therefore, public policy should be more aware of epidemiological data to define strategies to prevent AIM.

109957

Modality: E-Poster Scientific Initiation – Non-case Report

Category: ATHEROSCLEROSIS/CARDIOVASCULAR RISK FACTORS/CARDIOVASCULAR PREVENTION

## Disorders Caused by Hellp Syndrome in Women During Gestational and Post Childbirth Period: A Systematic Review

RAFAEL AUGUSTO SILVA CABEÇA^1^, Ingrid Jade Muniz Wanderley^1^, João Lucas Silva Sales^1^, Israel Figueira Lemos^1^, Ivan Cuoco Sampaio^1^, Maria Eduarda Dantas da Veiga^1^, Mariana Lassance Maya Palheta^1^, Juliana de Sousa Tavares^1^, Luiz Carlos Figueiredo Filho^1^, Luma Maria Favacho Bordalo^1^, Paula Larissa Baía Lima^1^

(1) Universidade do Estado do Pará

**Background:** HELLP Syndrome (hemolysis, elevated liver enzymes and low platelets) is a multisystemic disorder that usually arises from a serious condition of pre-eclampsia, which is present in 7,1% of pregnant women with hypertensive disorder. The presence of this condition is responsible for the development of endovascular endothelial damage by the intravascular platelet activation, which leads to many cases of maternal, perinatal and neonatal morbidity and morbimortality.

**Objective:** Describe, through a systematic review, the available evidence about the maternal disorders arising from HELLP syndrome.

**Methods:** A systematic review with articles collected at MEDLINE, Scielo and LILACS databases published from 2017 to 2022, using the descriptors “HELLP” and “HELLP Syndrome”. Were included cohort retrospective studies, full text available, in english, spanish and portuguese languages, while studies with tematic deviation, duplicated and articles that haven‘t published results. This review was based on preferred reporting items for systematic reviews and meta-analysis (PRISMA) standards and procedures.

**Results:** 310 articles were collected, and after the selection, analysis and the application of exclusion and inclusion criteria, 11 articles were selected. In the period of study, the analysis of publications demonstrated the prevalence of three poor outcomes on pregnant women’s clinical conditions: liver damage, kidney damage and hypertension, which were classified as primary outcomes. Furthermore, the studies described the following secondary outcomes: premature birth, placental abruption and pulmonary infection, uterine infection and gastrointestinal infection. Were observed the presence of gestational diabetes and congenital abnormalities caused by placental disorders. In addition, cerebrovascular morbidities were detected in patients with HELLP syndrome, and epigastric pain was the main isolated symptom on pregnant women, despite some patients related to having both symptoms in considerable part of the cases. The outcomes associated with cardiac disorders were concentric hypertrophy of the left ventricle and diastolic dysfunction.

**Conclusion:** In this aspect, although HELLP syndrome is a rare obstetric condition, it was possible to observe that the outcomes resulted by, particularly related to hepatic, kidney and vascular damages, may leave after-effects at short and long term in the life of the pregnant.

109965

Modality: E-Poster Scientific Initiation – Non-case Report

Category: EPIDEMIOLOGY AND HEALTH POLICIES/GLOBAL HEALTH

## Temporal Analysis of the Mortality Rate and Epidemiological Profile of Patients who Died from Atherosclerotic Disease in Brazil between 2012 and 2021

BRUNO OLIVEIRA SOUZA PRATES^1^, Hildebrando Antunes de Carvalho Neto^1^, Beatriz Souza Martins^1^, Beatriz Catarina dos Santos de Oliveira^1^, Gabriela Chateaubriand Campos^1^

(1) Universidade Federal da Bahia – UFBA

**Introduction:** Atherosclerosis is a chronic inflammatory disease of multifactorial origin and is one of the main causes of morbidity and mortality worldwide. Its prevalence has been increasing rapidly in developing countries, such as Brazil, and this growth is closely linked to the lifestyle adopted by the population. In this way, the epidemiological study of deaths associated with atherosclerosis is presented as a tool to assess the efficiency of the treatment instituted.

**Objective:** To analyze the epidemiological profile of patients who died from atherosclerotic disease in Brazil, between 2012 and 2021.

**Methods:** This is an ecological time series study from secondary data collected by the Hospital Information System (SIH-SUS), between the years 2012 to 2021. The total and percentage distribution of deaths, the mortality rate and sociodemographic data were included.

**Results:** A total of 7.927 deaths were recorded during the analyzed period, with 2012 presenting 572 cases and 2021 with 1.006. The mortality rate presented an oscillatory character, varying in a range between 3.63 (in 2017) and 4.11 (in 2021). There was no significant difference between genders, but the age group most affected was the elderly over 60 years (89.29%). Furthermore, the Southeast region has the highest number of cases with 3,967 (50.04%) followed by the Northeast region with 2,169 (27.36%).

**Conclusions:** As can be seen from the data obtained, despite the fact that the main risk factors for the development of atherosclerosis, as well as their treatment, are currently very well established, the numbers of deaths continue to increase in Brazil. And this increase is not just due to population growth, since the highest mortality rate in the historical series was recorded in 2021. As for sociodemographic data, the need for greater attention to elderly patients was once again reinforced, since they account for almost 90% of deaths. Finally, the data obtained prove that atherosclerosis remains an important and preventable cause of death in Brazil.


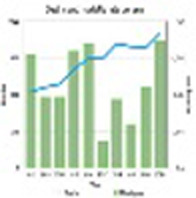


109968

Modality: E-Poster Scientific Initiation – Non-case Report

Category: COVID-19 AND CARDIOVASCULAR SYSTEM

## SARS-COV-2 Pandemic and Mass Vaccination: Impact on Morbidity and Mortality from Acute Myocardial Infarction in the Brazilian Southeast

CAMILLA MATTIA CALIXTO^1^, Alice Magro Koscianski^1^, Ana Carla Dlugosz^1^, Bruna Karas^1^, Francielle Nocera Viechineski^1^, Julia Henneberg Hessman^1^, Larissa Almeida Busnello^1^, Paola Gonçalves Moreira de Oliveira^1^, Mário Claudio Soares Sturzeneker^1^

(1) Universidade Estadual de Ponta Grossa (UEPG)

**Introduction:** Acute myocardial infarction (MI) is the primary global cause of death, and accounted for 7.06% of all deaths in Brazil in 2017. Chest pain is the cause of up to 40% of hospital admissions; and 10% of non-traumatic visits to the emergency department. The pandemic could reduce access to healthcare due to hospital overcrowding and the reduced seeking of the healthcare system, although the evidence is limited.

**Objective:** Estimating the possible influence of the SARS-CoV-2 pandemic on morbimortality related to acute MI in the Southeast region through analysis of DATASUS data from 2017 to 2021.

**Methods:** Data on the number of admissions, mortality, lethality, and duration of inpatient stay was collected. The lethality was calculated as the ratio of deaths over admissions and expressed as a percentage. We studied the periods pre-pandemic (02/2017 to 02/2020), pandemic (03/2020 to 06/2021), pandemic’s peak (12/2020 to 05/2021), peak’s 1st trimester (12/2020 a 01/2021), peak’s 2nd trimester (03/2021 a 06/2021), and mass vaccination (06/2021 a 12/2021). We calculated the mean of each variable for sex and age group. We compared the mass vaccination period to the others and recorded the difference between means as a percentage.

**Results:** During mass vaccination, there was an increase of 6.69% in admissions compared to the pre-pandemic period, 10.35% compared to the peak, and 20.4% for the 2nd trimester, particularly among women (5.87%, 12.03%, and 23.06% respectively). The duration of stay dropped by 15.34% compared to the pre-pandemic period in a similar way between the sexes. Regarding the pandemic’s peak, the 2nd trimester showed a 4.66% increase. The mortality increased, especially comparing the peak’s 2nd trimester with the vaccination period (18.66%). The lethality showed mixed results with a slight upward tendency. There was a 9.44% increase compared to the 1st trimester and a 1.46% drop for the 2nd trimester. The sex and age analysis had similar results, except for an increase in all comparisons in the age group 40–49 and a reduction in the 30–39 age group.

**Conclusion:** After the mass vaccination period, there was an increase in mortality and hospital admissions. The duration of inpatient stay decreased compared to the rates before the pandemic and increased compared to the pandemic’s peak. The lethality had a slight upward tendency.

110011

Modality: E-Poster Scientific Initiation – Non-case Report

Category: CARDIOVASCULAR SURGERY

## Results of the Surgical Treatment of the Mitral Valve by a Minimally Invasive Procedure in Rheumatic and Degenerative Disease, Comparison of Results

SHEMA EL-LADEN HAMMOUD^1^, Juliana Fortunato^2^, Jeronimo Antonio Fortunato Junior^2^, Leticia Gusso Scremin^1^, Nikolai Cernesco Neto^1^, Gabriel Fontana de Melo^1^, Brenda Gabriele Smaniotto Raulik^1^

(1) Universidade Positivo; (2) Hospital Cruz Vermelha Brasileira – Filial do Paraná

**Introduction:** In Brazil, valvulopathies represents a significant portion of hospitalizations by cardiovascular disease. Rheumatic Fever is the main etiology, representing 70% of all cases, unlike developed nations, where degenerative diseases (DD) are the main cause. For patients that have DD, mitral valvuloplasty with a ballon catheter (MVBC) is still the most recommended treatment. However, in the last decades the minimally invasive cardiologic surgery (MICS) assisted by video, recieved a major boost and has become a possible choice of treatment to the diseases of the mitral valve. In pacients with a rheumatic disease (RD), the inicial treatment is farmacologic, reserving the surgical intervention on refratary cases.

**Objective:** To compare the results of mitral valvuloplasty performed using the MICS techinique in patients with DD and RD; and also assess the initial surgical results with a five year evolution from the same patients.

**Methods:** Between 2005 and 2019, patientes with Degenerative Desease (43) and Rheumatic Desease (36) undergoing MICS were selected totalizing 79 patients.

**Results:** Among the patients selected, 56% were female and the average age was 49 years. Of all valve alterations, the rheumatic etiology prevailed in mitral valve stenosis while the degenerative disease prevailed in mitral valve insufficiency. There were 97,7% of plasties in DD, against 72,2% in RD. Sequential echocardiography showed minimal mitral regurgitation in 88,4% in patients with DD, versus 61,5% in RD. Left ventricular diastolic dimension was 57,8 mm in DD pacients, versus 51 mm in RD. Valve replacement after plasty failure occurred in the initial postoperative period from one pacient with RD and another pacient with DD, one year later the procedure. Five years later, one pacient with DD was submitted to a new surgical procedure due to mitral endocarditis. Late survival after 10 years of follow-up was 91,67% in RD versus 88,4% in DD.

**Conclusion:** As observed in the world literature, the surgical results in pacients with DD was satisfactory. In pacients with RD the surgical results were also promising, with increased survival.

110013

Modality: E-Poster Scientific Initiation – Non-case Report

Category: CARDIAC ARRHYTHMIAS/ELECTROPHYSIOLOGY/ELECTROCARDIOGRAPHY

## Assessment of Discordances and Causes of Pathological Q Wave Discordance, using the Minnesota Code, in Patients from the Longitudinal Study of Adult Health (Elsa-Brasil)

GABRIEL FELIPE GONZAGA SILVEIRA^1^, Antonio Luiz Pinho Ribeiro^1^, Marcelo Martins Pinto-Filho^1^

(1) Universidade Federal de Minas Gerais UFMG; (2) Universidade Federal de Minas Gerais UFMG; (3) Universidade Federal de Minas Gerais UFMG

The electrocardiogram (ECG) is a simple and useful clinical tool, used in population studies as a tool for disease prognosis estimation. The Minnesota code (CM) is used for better standardization of the ecg reading. The degree of ECG variability in the same patient (as well as the variation of codes) over time is not well established, with some reporting significant variations including pathological Q waves. The aim of the present study is to evaluate the discordance and causes of these discordances in major Q-wave CM in patients from The Longitudinal Study of Adult Health (ELSA-Brasil) over a 4-year follow-up period. The ELSA-Brasil is a multicenter cohort that included more than 15,000 participants aged between 35 and 74 years at baseline. The ECG is one of the tests performed in the study. For the present study, the presence of pathological Q wave was evaluated in a total of 26,516 ECGs performed in the years 2013 (13,877) and 2018 (12,639), using the major criteria of group 1 of the Minnesota Code (CM 1.1 a 1.2). The ECGs were performed in a sequence called waves 1, 2 and 3. The ECGs of wave 3 were fully lauded and coded with manual review and therefore were considered the gold standard for comparison. Waves 2 and 3 ECGs were compared in order to observe the discordance and causes of these discordances in the larger Q wave CM. We called Major Disagreement when there were changes in the pathological Q wave category, that is, the major Q wave pathological changes changed to minor or non-existent pathological changes between the groups in the CM. Minor disagreement: when there are changes in the CM, but no change in the Q wave category. Total agreement when the CM is identical and New pathological Q wave: when new pathological Q wave events have occurred. Patients without ECG were excluded. From the 26,516 electrocardiograms performed, we found 1,562 major changes in the Q wave (CM 1.1 and 2.1). When comparing the electrocardiograms performed in 2013 (13,877) and 2018 (12,639) we found 121 (1.0%) total agreement. Minor disagreement: 94 (0,7%). Major disagreement: 5 (0.04%) and New pathological Q wave:713 (5.6%). In conclusion, there was little Total agreement between the 2013 and 2018 ECGs. Most of the disagreements are due to Minor disagreement, which is possibly due to millimeter variations in measurement and the New pathological Q wave, which possibly occurred due to the aging of the population of the study.

110032

Modality: E-Poster Scientific Initiation – Non-case Report

Category: HEART FAILURE/CARDIOMYOPATHY/TRANSPLANT

## Impacts of the Pandemic on the Increase in Mortality from Heart Failure in a Developing Country

SOFIA HELENA VITTE^1^, Idrys Henrique Leite Guedes^2^, Joanna Sousa da Fonsêca Santana^3^, Marclébia Quesado Borges^4^, Marcelle Rodrigues Carneiro de Souza Reis^5^

(1) Pontifícia Universidade Católica de Campinas; (2) Universidade Federal de Campina Grande; (3) Escola Bahiana de Medicina e Saúde Pública; (4) Universidade Federal do Recôncavo da Bahia; (5) Centro Universitário do Planalto Central Apparecido dos Santos

**Introduction:** Congestive heart failure is a complex syndrome that is a result of either functional or structural disorder, which can be divided into caused by heart failure such as ischemic heart disease and systemic hypertension. During the pandemic, it was possible to see a scenario that got worse in a country that faced serious consequences due to the coronavirus onset, with higher mortality data. Thus, our main goal was to show this impact on the statics of heart failure in an emergent country.

**Methodology:** A cross-sectional quantitative study was carried out using the open database of the Brazilian Ministry of Health (DATASUS). Information on hospitalizations and mortality rates for heart failure, as well as hospitalizations for ischemic diseases and primary hypertension from January 2018 to December 2021, were analyzed.

**Results:** There were 201,040 and 199,858 hospitalizations for heart failure in Brazil in 2018 and 2019, respectively, while in 2020 and 2021 these values were 167,375 and 151,106, which represents a reduction of 20.5% between the averages of the two biennia. In these same periods, heart failure mortality rates ranged from 11.12 and 11.41 in 2018–2019 to 12.11 and 13.54 in 2020–2021, resulting in a 13.8% increase between the two periods. Finally, average hospitalizations for other ischemic diseases and primary hypertension in Brazil decreased by 23.7% and 30.7% in 2020–2021 compared to 2018–2019.

**Conclusion:** From the available data, a significant reduction in the number of hospitalizations for heart failure after the beginning of the pandemic, as well as for hypertension and ischemia, can be observed. Conversely, the mortality rate for the pathology grew, which demonstrates a possible relationship between the limitations imposed in the period on the adequate treatment of the two main etiologies of heart failure in the country.

110945

Modality: E-Poster Scientific Initiation – Non-case Report

Category: ATHEROSCLEROSIS/CARDIOVASCULAR RISK FACTORS/CARDIOVASCULAR PREVENTION

## Prevalence of Orthostatic Hypotension in the Municipality of Sertã

CATARINA RAQUEL GASPAR DOS SANTOS^1^, Patrícia Coelho^1^, Francisco Rodrigues^1^

(1) Instituto Politécnico de Castelo Branco

**Introduction:** It is characterized as Orthostatic Hypotension when there is a drop in systolic blood pressure greater than 20 mmHg and/or 10 mmHg in diastolic blood pressure. Its symptoms are related to cerebral hypoperfusion, such as blurred vision, nausea, palpitations, headache, neurological changes, and loss of balance. Its prevalence is associated with increasing age.

**Objective:** To determine the prevalence of orthostatic hypotension in the population of Sertã.

**Methodology:** Prospective study with sample collection based on the cluster method, through the random selection of streets in the municipality of Sertã. Initially, anthropometric data were collected and, as a base, an assessment of blood pressure was carried out with the individual sitting and at rest, then, with an interval of 3 minutes, after this medication, blood pressure was reassessed in the sitting position. orthostatic. Statistical treatment and data analysis were performed using the SPSS Statistics program.

**Results:** The sample is composed of 1000 individuals, of legal age and residing in the municipality, 515 (52%) male and 485 (48%) female. The mean age was 56.04 years with a standard deviation of ±16.04 years. It was found that 7.4% of the sample has orthostatic hypotension, with 54.1% being male and 45.9% female. Regarding the age groups, it was between 61 and 70 years old that stood out the most with this diagnosis, with a represented value of 41.9%.

**Discussion:** Like the results found in the present study, there is an article on the study of Orthostatic Hypotension in the municipality of Proença-a-Nova, in which a prevalence of 5.3% of the population with this diagnosis was found, having been more prevalent in females, contrary to what was observed in the municipality of Sertã.

**Conclusions:** Despite the low prevalence, it is still important to carry out its diagnosis in health units since this risk factor is also associated with cardiovascular disease.

110072

Modality: E-Poster Scientific Initiation – Non-case Report

Category: HEART FAILURE/CARDIOMYOPATHY/TRANSPLANT

## Jugular Venous Ultrasound for the Diagnosis of Heart Failure in the Emergency Department – a Systematic Review

MAYARA GABRIELE TOLEDO^1^, Caio Pluvier Duarte Costa^1^, Eduardo Thadeu de Oliveira Correia^1^

(1) Universidade Federal Fluminense

**Introduction:** Tools that aid the differentiation of dyspnea from cardiovascular and noncardiovascular etiology are fundamental in the emergency department (ED). In this study we will systematically review if internal jugular vein (IJV) ultrasound can serve as a screening method for the diagnosis of heart failure in the ED.

**Methods:** We conducted a systematic review following the PRISMA guidelines. Original studies indexed by Embase, Pubmed, Cochrane and LiLACS with the keywords: “ultrasound” or “ultrasonography” and “heart failure” and “jugular vein” published until March, 2022 and which matched the inclusion criteria were included. Two authors (C.P and M.T.) performed the screening and data extraction, in cases of discordance, a third author (E.T.) made the decision.

**Results:** Eight studies were included, with a total of 931 patients analyzed. The ultrasound examination of the IJV was used to evaluate patients with dyspnea in ED, using different methods. Due to significant heterogeneity between studies, a meta-analysis was not performed. All of the included studies showed that IJV ultrasound had a high sensitivity for differentiation of dyspnea on the ED. Jang et al defined 10 cmH2O of central venous pressure (CVP) as a good cutoff to define HF by IJV ultrasound for patients without jugular vein distension on physical examination, showing a sensitivity of 98.2% and a specificity of 42.9% for IJV ultrasound in dyspneic patients comparing with pulmonary edema on initial Chest x-ray, a sensitivity of 99%, specificity of 59% compared with echocardiography, and finally a sensitivity of 100%, specificity of 43% compared with a BNP >= 500 pg/ml. Tzadok’s study evaluated the use of the change of the IJV area with respiration in patients with dyspnea to detect acute HF, showing that the respiratory area change of the IJV had a specificity and sensitivity of nearly 70% rate to identify HF.

**Conclusion:** Our systematic review demonstrates that IJV ultrasound is a complementary tool that provides important information for the diagnosis of HF in patients with dyspnea in the ED. Further studies are needed to define a standard measurement method to apply IJV ultrasound in clinical practice.

110064

Modality: E-Poster Scientific Initiation – Non-case Report

Category: CARDIO-ONCOLOGY

## Cardio Oncology Applied to a Ecological Study in a Emergent Country

SOFIA HELENA VITTE^1^, Idrys Henrique Leite Guedes^2^, Joanna Sousa da Fonsêca Santana^3^, Marclébia Quesado Borges^4^, Marcelle Rodrigues Carneiro de Souza Reis^5^, Allan Christian Cardozo Cembrane^6^

(1) Pontifícia Universidade Católica de Campinas; (2) Universidade Federal de Campina Grande; (3) Escola Bahiana de Medicina e Saúde Pública; (4) Universidade Federal do Recôncavo da Bahia; (5) Centro Universitário do Planalto Central Apparecido dos Santos; (6) Hospital Alvorada Brasília

Cardio-oncology is a field focused on detection, monitoring and treatment of cardiovascular disease occuring due to chemotherapy cardiotoxicity. Not only that, but cardiovascular diseases and cancer share multiple risk factors, such as increased age, smoking and obesity, and the presence of one condition worsens the other. Even though cardio-oncology is a new field, the most researched topics are focused on chemotherapy’ toxicity and cardiac assessment during treatment, leaving very little room to discuss heart malignancies. 25% of malignant cardiac tumors are cardiac sarcoma, and secondary tumors are 40 times more likely to appear. Therefore, the aim of this study is to analyze the profile of heart, mediastinum and pleural cancer in Brazil. This is a cross-sectional descriptive population based study with values obtained from the database of the Ministry of Health of Brazil (DATASUS) during the period from 2018 to 2021. The analyzed variables were: treatment time, sex, year and age group. The tables were obtained and exported to the IBM SPSS 26 statistical software for the purpose of comparing the variables. A total of 4438 diagnoses were accounted, with 2143 corresponding to males and 2295 to females. In the annual distribution, we can observe an increase of approximately 93.6% from 2018 to 2019, and a decrease of 12% from 2019 to 2020 and of 16.5% from 2020 to 2021. The most affected age group was from 60 to 69 years old. Finally, regarding the average treatment time, it can be observed that in 2,020 cases, treatments lasted up to 30 days, while 71 cases were between 31 and 60 days and 123 cases were for more than 60 days. Data shows a decrease in the number of diagnosed neoplasms in Brazil in the post-pandemic period, with a small increase between 2020 and 2021. Among those affected, elderly people, under 70 years old, are the age group with greater expression of the disease. However, it is not possible to establish a relationship between sexes, due to the small difference in cases. Regarding the treatment, most of it was done within a period of up to 30 days, showing a small time rate of tratament.

110065

Modality: E-Poster Scientific Initiation – Non-case Report

Category: EPIDEMIOLOGY AND HEALTH POLICIES/GLOBAL HEALTH

## Comparative Cross-Sectional Study of the Incidence of Chagas Disease with Deforestation in the Amazon Region of the State of Pará

MARCIO CÉSAR RIBEIRO MARVAO^1^, Giovanna Coutinho Jardim^2^

(1) Instituto Evandro Chagas (IEC); (2) Universidade Federal do Pará (UFPA)

**Introduction:** Chagas disease is an anthropozoonosis caused by the parasite Trypanosoma cruzi that is considered a public health issue in several countries of The Americas, being endemic in 21 countries of the continent, reaching about 7 million people. There are several pathophysiological mechanisms that surround Chagas disease, so that there is permanent tissue repercussion, autosomal abnormality and impaired coronary microcirculation, the latter can generate decreased myocardial performance and impair the functional capacity of the affected person. The relationship between Chagas and deforestation can be addressed by the theme of destruction of the vector habitat, causing its migration to areas with human presence. The state of Pará, northern Brazil, leads deforestation rates in the Amazon region, in addition to suffering from chagas disease endemics.

**Objective:** To perform a comparative analysis between deforestation data and incidence of acute chagas disease in the state of Pará from 2015 to 2020.

**Methodology:** Cross-sectional, observational and descriptive study, with data from the Notifiable Diseases Information System (SINAN/DATASUS) on the frequency of acute chagas disease in the state of Pará. The linear regression test was used using the PASR4.03 software to verify the statistical relationship. The variables of annual forest loss, forest cover rate and forest deforestation rate were analyzed.

**Results:** During the period described, the highest incidence occurred in 2016, marking 3.3 new cases per 100,000 inhabitants. In relation to the forest situation, the greatest loss of forest cover (hectare) marked the year 2020, representing a loss of 675591.3, with a forest deforestation rate of 24.93%, followed by 2016, with 597199 of area loss and with a rate of 24.04%. The statistical correlation showed R2 = 37% of events, demonstrating a variability of 63% of the relationship occurring by other factors. Non-significant P-value (0.19997).

**Conclusion:** Although deforestation is a major problem for the onset of diseases and a decrease in the quality of life of the population. The data collected did not bring a significant relationship between deforestation and the incidence of chagas in the described region, not ruling out this relationship in other locations.

111668

Modality: E-Poster Scientific Initiation – Non-case Report

Category: EPIDEMIOLOGY AND HEALTH POLICIES/GLOBAL HEALTH

## Profile of Patients who Died from Aortic Valve Stenosis (AVS) in Brazil in the Last 10 Years

VALENTINA BRATTI DE NADAL^1^, Pedro Van Der Sand Germani^2^, Juliana Nichterwitz Scherer^1^

(1) Universidade do Vale do Rio dos Sinos (UNISINOS); (2) Pontifícia Universidade Católica do Rio Grande do Sul

**Introduction:** AVS is, in children and adults, the most common cause of left ventricular outflow obstruction. When the severity of Aortic Valve Stenosis is low to moderate, the disease is well tolerated and is usually not accompanied by symptoms. When it progresses to severe, AVS is associated with significant mortality. The classic manifestations when the disease is in terminal stage are dyspnea on exertion, syncope and angina. It is important to identify the profile of patients who died from such condition in order to provide more adequate screening and to identify risky groups.

**Objective:** To describe the profile of patients who died from AVS in Brazil in the last 10 years.

**Methodology:** An ecological study was conducted through the collection and analysis of data from the Departamento de Análise de Saúde e Vigilância de Doenças Não Transmissíveis (DASNT) system, covering the period from January 2011 to December 2021. The variables evaluated were: number of deaths for AVS, geographic region and place of occurrence, age group, race/color and sex. All data were extracted into an excel spreadsheet, and the descriptions of the variables were performed by analyzing absolute and relative frequencies.

**Results:** A total of 32,094 deaths due to AVS were recorded in Brazil between 2011 and 2021. Of these, 53.6% were females and 46.3% were males. Considering age group, 96.1% of deaths occurred in individuals over 50 years old, with 47.3% of total deaths being aged 80 years old or over. The lowest rate occurred in patients aged 05 to 09 years, totalizing 0.06%. About race/color, 73.8% of deaths occurred in white patients, 17.8% in brown and 3.5% in black, while the lowest rate occurred in indigenous people, 0.09%. Regarding region, the Southeast had the highest rate of deaths (50.9%), followed by the South (26.3%) and the Northeast (14.7%). The North and Midwest accounted for 8.1% of deaths.

**Conclusions:** As 96.1% of deaths caused by AVS occurred in patients over 50 years old, it should be noted that there is a need for medical management to identify these patients before they become symptomatic, which improves survival. Public policies that propose regular monitoring of these patients are extremely important to monitor hemodynamic progression and the development, mainly in the Southeast, where there were 50.9%% of deaths.

110074

Modality: E-Poster Scientific Initiation – Non-case Report

Category: HYPERTENSION/RENAL DENERVATION

## Golden Ratio Deviation: SBP/DBP and DBP/PP Coefficients During Ambulatory Blood Pressure Monitoring in Diabetics and Resistant Hypertensive Patients

LOUISE BUONALUMI TÁCITO YUGAR^1^, Larissa Costa Morete^2^, Tatiana Rubio Azevedo^2^, Elizabeth do Espírito Santo Cestario^2^, Bruno Rodrigues^1^, Sílvia Elaine Ferreira Souza Melo^1^, Carolina Manzano^2^, Lúcia Helena Bonalume Tácito^2^, José Fernando Vilela Martin^2^, Heitor Moreno Júnior^1^, Juan Carlos Yugar Toledo^2^

(1) Faculdade de Ciências Médicas, UNICAMP; (2) Faculdade de Medicina de São José do Rio Preto, FAMERP

**Introduction:** The wave pressure profile can be separated into three segments: pulse pressure (PP), diastolic blood pressure (DBP) and systolic blood pressure (SBP), which corresponds to the sum of the previous ones. Although the measurement of these values has advanced, there are still few studies on the relationship between them. The golden ratio (φ, phi, or Fibonacci number) is present in several organisms and is defined by dividing a straight line into two segments a and b (a being greater than b) so that the ratio between a and b is equal to the ratio of the sum of the quantities by the larger segment. The value of this equality is approximately 1.61. In this sense, we try to relate SBP, DBP and PP to phi. In this study, we evaluated and compared the deviations from the golden ratio during the periods of ABPM: 24h, daytime and nighttime in resistant hypertensives (RHTN) and type 2 diabetics (T2DM) using the SBP/DBP and DBP/PP ratios for comparison.

**Material and Methods:** We retrospectively analyzed data from RHTN participants (n = 54) from the ResHypOT ClinicalTrials.gov study, ID: NCT 02832973 and T2DM patients (n = 81) from the study “Changes in vascular hemodynamics in patients with RHTN and T2DM. The data used were: age, sex, SBP, DBP and PP. Descriptive statistical analysis, t-test and ANOVA were performed with SPSS 24 softwares (IBM-USA).

**Results:** We observed elevated blood pressure values in RHTN and normal values in T2DM in the 24-hour periods, daytime and nighttime with attenuation of the systolic and diastolic nocturnal dipping, being more pronounced in the RHTN group. The SBP/DPB ratio in the 24-hour period and nighttime was significantly higher in the T2DM group when compared to the RHTN group (p = 0.0063 and p = 0.044, respectively), above phi in both. The SBP/DBP ratio in daytime was higher in the RHTN group (p < 0.0001) (figure 1). The DBP/PP ratio in RHTN was lower in the 24-hour period and during nighttime in T2DM (p = 0.0012; p = 0.0189, respectively) (figure 2). There was no significant difference between T2DM and RHTN groups for DBP/PP ratio in daytime.

**Conclusion:** The phi deviations found in T2DM are greater than those observed in RHTN. These differences may be attributed to the difference in blood pressure values between the groups and reduced nocturnal SBP dipping in patients with RHTN. Future studies using the deviations of the golden ratio are needed to validate the information obtained from this mathematical proposal.

110081

Modality: E-Poster Scientific Initiation – Non-case Report

Category: EPIDEMIOLOGY AND HEALTH POLICIES/GLOBAL HEALTH

## Epidemiology of Risk Factors for Heart Failure in Children and Adolescents in Brazil between 2021 and 2022

LARISSA SILVA FERREIRA^1^, Aurea Nathallia Gomes de Souza (Souza, ANG)^1^, Bianca Paula Miranda Martins^1^, Camila Silva de Oliveira (Oliveira, CS)^1^, Cecília Rodrigues Viana^1^, Luiz Felipe Façanha Ramos^1^, Marcos Roberto Marques da Silva Júnior^1^, Vinícius Maciel Vilhena^1^, Reny Wane Vieira dos Santos^1^

(1) Universidade Federal do Amapá (UNIFAP)

**Introduction:** Cardiovascular diseases are the major causes of death in Brazil. Among the most likely risk factors for the development of these diseases are: arterial hypertension (SAH), dyslipidemia, diabetes mellitus (DM), obesity and physical inactivity. With the changes in life habits in Brazil and in the world, high-calorie dietary changes and the decrease in physical activity among children and young people stand out, which raises an alert for the increased probability of developing diseases in the cardiovascular system in the first two decades of life.

**Objective:** To analyze the mortality rate of children and adolescents due to heart failure in children and adolescents in Brazil, with data from 2021 and 2022.

**Method:** Cross-sectional epidemiological study on the mortality rate of individuals aged 0 to 19 years, due to heart failure cardiac arrest, based on data extracted from the Information Technology Department of the Unified Health System (DATASUS).

**Results:** Between 2021 and 2022, the mortality rate from heart failure before the age of 19 was 7.51% and increased numbers were observed in children under 1 year (9.18%) and between 15 and 19 years (9.83%). With regard to Brazilian regions, the Midwest region has the highest mortality rate (10%), while the Northeast region has the lowest percentage (6.97%). In addition, the rate is higher in children under 1 year old (10.67%) and decreases with age, increasing again from 10 to 14 years old (6.29%) and until reaching 15 to 19 years old (8.75%). This rate is higher in males (8.57%). Finally, the indigenous are the most affected (18.75%) and the yellow ones the least (5.26%).

**Conclusions:** In the child and adolescent population, there was a predominance of males and individuals aged less than 1 year and 15 to 19 years in all data evaluated. Thus, it is understood that changes in habits are increasingly affecting a younger population. This shows the need to invest in health promotion policies for the prevention of cardiovascular diseases, a need reinforced by the high cost of assisting modifiable factors such as diet and physical activities.

110360

Modality: E-Poster Scientific Initiation – Non-case Report

Category: ATHEROSCLEROSIS/CARDIOVASCULAR RISK FACTORS/CARDIOVASCULAR PREVENTION

## Tongue Inflammation is Associated with Higher Intensity of Immunostaining for IL-1 in Aortic Arteries: Autopsy Study

BEATRIZ JUNQUEIRA MATTAR^1^, Bruno Henrique dos Reis Souza Oliveira^1^, Káthia Maria Duarte Ono^1^, Thiago Lima Pereira^2^, Renata Margarida Etchebehere^2^, Rodrigo César Rosa^2^, Sanívia Aparecida de Lima Pereira^1^

(1) Universidade de Uberaba UNIUBE; (2) Universidade Federal Do Triângulo Mineiro UFTM

**Introduction:** Atherosclerosis is an inflammatory disease of the arteries responsible for a high rate of morbidity and mortality worldwide.

**Objective:** To associate the histopathological and immunohistochemical aspects of tongue inflammation with aortic and coronary atherosclerosis in autopsied humans.

**Methodology:** A total of 4,378 autopsy reports were analyzed and all cases where there were stored fragments of the tongue, aorta and coronary artery of the same individual were selected (n = 16). Morphological and immunohistochemical evaluation was performed for IL-1 beta, IL-6, TNF alpha and IFN gamma.

**Results:** IL-1 in the aorta was associated with the following parameters evaluated in the tongue: IL-6 (p = 0.031); inflammation (p = 0.047); spongiosis (p = 0.018); lymphocytic exocytosis (0.003). IL-6 in the tongue was associated with IL-1 (p = 0.031), IL-6 (p = 0.016) and TNF-alpha (p = 0.016) in the aorta. Tongue exocytosis was associated with IFN gamma in the aorta (p = 0.003).

**Conclusion:** Inflammation and higher immunostaining by IL-6, TNF-alpha and IFN gamma on the tongue are associated with higher immunostaining by IL-1 in atherosclerotic plaques of the aorta arteries. Therefore, knowing that IL-1 is a pro-inflammatory mediator associated with the development of atherosclerosis and that infectious agents are the major causes of inflammation in the tongue, we suggest that prevention and treatment of lingual infections could reduce the risk of atherosclerosis. Thus, these simple and low-cost measures could help prevent the atherosclerotic process, reducing the morbidity and mortality rates associated with this disease. However, further studies are needed in order to corroborate our findings.

110356

Modality: E-Poster Scientific Initiation – Non-case Report

Category: DIGITAL HEALTH/INNOVATION

## 3D Printing in Teaching Cardiac Embryological Development

CAMILA PIENTZNAUER SOARES MONTE VIANNA^1^, Claudio Tinoco Mesquita^1^, Juliana Cadilho da Silva Abrantes^1^

(1) Universidade Federal Fluminense (UFF)

**Introduction and/or Fundamentals:** 3D printing has proven to be a resource in healthcare through the creation of models of organs and structures with various applications. In cardiology it is possible to use this technology both in the educational area and in surgical planning. In education, an important application of this tool is the printing of three-dimensional embryological models of the formation of the heart, in order to provide a better understanding of human embryology and congenital heart defects. Thus, 3D printed embryological models can provide active learning by visualizing the stages of heart formation in a three-dimensional way, being an innovative didactic resource in embryology and cardiology classes.

**Objective:** We aimed to describe and analyze the improvements of the application of 3D printing in teaching cardiac embryology to health care students.

**Materials and Methods:** At the university we have a digital fabrication laboratory (Fab Lab) that has a GT MAX 3D FDM printer, which builds models by heating and extruding PLA and ABS filaments. These models (Figure 1) were digitally modeled by the lab’s multidisciplinary team using the Sculptris software and saved in STL format by analyzing images from books and articles in the area. Three embryological cardiac structures have already been printed: cardiac tubes and cardiac tube folding. The printed models are being used for teaching embryology to undergraduate students through face-to-face seminars in the lab.

**Results:** The classes resulted in in-depth teaching of the structures of the heart at different stages of its formation, focusing on the visualization of structures, as well as stimulating the sciences and technologies applied to medicine. Through the three-dimensional visualization of the structures, the students obtained a better understanding of the embryological development of the heart, as well as of its malformations due to failures in this process. In addition, the understanding of the initial formation of the heart helped to better understand the anatomy of the adult heart.

**Conclusions:** The Fab Lab has been of great value and interest to the academic-scientific community. The experience with the 3D models of the heart in teaching embryology and anatomy shows the great potential of this tool allowing a multidisciplinary integration that serves as a stimulus to attract young students.

110371

Modality: E-Poster Scientific Initiation – Non-case Report

Category: COVID-19 AND CARDIOVASCULAR SYSTEM

## Elevated D-Dimer as a Marker for Thromboembolic Events in Pediatric Patients with COVID-19: A Systematic Review

JADE ZARICHTA COSTA^1^, Pietro Preis Casagrande^2^, Vitor Henrique Macarini^2^, Josué Barbosa^2^, Franciely Vanessa Costa^3^, Maíra Cola^4^, Roberta de Paula Martins^1^

(1) Laboratório de Bioquímica Patológica, LABIP, Departamento de Ciências da Saúde, Centro de Ciências, Tecnologias e Saúde, Universidade Federal de Santa Catarina, UFSC, Campus Araranguá.; (2) Acadêmico do Curso de Graduação em Medicina, Departamento de Ciências da Saúde, Centro de Ciências, Tecnologias e Saúde, Universidade Federal de Santa Catarina, UFSC, Campus Araranguá.; (3) Departamento de Ciências da Saúde, Centro de Ciências, Tecnologias e Saúde, Universidade Federal de Santa Catarina, UFSC, Campus Araranguá.; (4) Grupo de Gestão em Riscos e Desastres, Departamento de Ciências da Saúde, Centro de Ciências, Tecnologias e Saúde, Universidade Federal de Santa Catarina, UFSC, Campus Araranguá.

**Introduction:** Pediatric patients may develop Multisystem Inflammatory Syndrome (MIS-C) in severe cases of COVID-19, a clinical condition that is poorly described yet and may cause cardiovascular changes, persistent fever, skin manifestations, and coagulopathies.

**Objective:** To determine whether elevated D-dimer levels are a risk marker for the development of thromboembolic events in the pediatric population with COVID-19. If so, D-dimer levels could be used to determine prophylactic anticoagulation measures if needed.

**Methods:** This systematic review was conducted in accordance with the recommendations of the Preferred Reporting Items for Systematic Reviews and Meta-Analyzes (PRISMA) and registered in the PROSPERO Database of Systematic Reviews under CRD42022298330. The last update of the database search was on December 14, 2021, resulting in 79 documents for analysis. Inclusion criteria were: academic articles (case series, cohort studies, and case controls) on patients aged up to 21 years, in English, Portuguese, and Spanish; the databases used were PubMed, Scopus, MEDLINE/Bireme (Virtual Library of Health – VLH), Web of Science, and EMBASE, queried by topic, keywords, or abstract. Bibliographic reviews, books, nonacademic research, case reports, and content in languages other than those indicated were excluded. All articles were independently reviewed by all authors using the Joanna Briggs Institute (JBI) checklist and critical appraisal for risk of bias. Disagreements were resolved through discussion within the group. Six of the articles used were rated as low risk of bias and one was rated as moderate risk.

**Results:** From the 79 articles found, only 7 were selected for analysis. Among these seven articles, only one had patients with thromboembolic events. In the other articles, D-dimer levels were elevated but were considered controversial in predicting events, with no clear association between the magnitude of D-dimer change and the magnitude of thrombosis risk, as the level tended to increase in all inflammatory diseases.

**Conclusions:** Although D-dimer is used for adults, it was not a good parameter for assessing the risk of thromboembolic events in individuals younger than 21 years. The main shortcomings are that D-dimer is elevated in many patients even without the occurrence of thromboembolic events and increases with any type of inflammation, therefore is not a specific marker.

110836

Modality: E-Poster Scientific Initiation – Non-case Report

Category: ATHEROSCLEROSIS/CARDIOVASCULAR RISK FACTORS/CARDIOVASCULAR PREVENTION

## The Prevalence of Diabetes in Adults of Santiago Island -Cabo Verde

KELLY ANNY FERNANDES MASCARENHAS ^1^

(1) Instituto Politécnico de Castelo Branco

**Introduction:** Diabetes are defined as a metabolic disorder characterized by insufficient insulin production or by the incapacity of the body in using them. Consequently, it results in an increase in glucose levels in the blood, being type II diabetes the most common one. It’s considered an important risk factor for cerebrovascular pathologies development such as acute myocardial infarction and cerebral vascular accident. In Cabo Verde, according to the National Survey on the Risk Factors of the Non-Transmitted Diseases (SNTD II) 2020, 3.9% of the adult population of Cabo Verde was diagnosed with diabetes, being the females the most predominant one, and 16.6% of them with the range between 60–69 years of age.

**Objectives:** To compare the results of measured diabetes through questionnaire application as well as evaluated diabetes through capillary glycemia of the adults from the island of Santiago – Cabo Verde.

**Methodology:** The transversal study of population background of 599 participants over 18 years old, from both genders. The data collection took place in October and November 2021, in the nine municipalities of Santiago Island- Cabo Verde. It was excluded from the study the individuals of Cape Verdean nationality who have been living for over a year abroad and that have just arrived on Santiago Island in less than 30 days, as well as the individuals with any kind of disabilities that may affect their participation in the study. The data were collected through questionnaires; As for diabetes, it was evaluated through a glucometer.

**Results:** The sample was made of 599 individuals with the age range between 18 and 99 years old with an age average of 42 years of age, 54.8% were female and 45.2% were male. Through a questionnaire, it was possible to verify that 7.5% of the participant population answered they have a diabetes diagnosis 71.1% of females. In the diabetes evaluation carried out through capillary glycemia, we found a prevalence of 4.5% of diabetes, of which 63% are females. In both cases, it was verified that there’s a meaningful statistical relationship between diabetes and the age group. Furthermore, only measured diabetes by questionnaire showed there’s a meaningful statistical relationship with gender. Thus, we could infer that 3% of diabetics have controlled glycemia.

**Conclusion:** There is a greater rate of prevalence in evaluated diabetes by questionnaire, with the women presenting greater predominance in the adminis.

110459

Modality: E-Poster Scientific Initiation – Non-case Report

Category: CARDIOVASCULAR SURGERY

## Association of Morbimortality and Lymphocytes and Neutrophils in Patients Undergoing Cardiovascular Surgery with Cardiopulmonary Bypass in a Public Hospital in Belo Horizonte

ISABELLA VICTÓRIA DA CUNHA PEIXOTO RIBEIRO^1^, Eduardo Augusto Victor Rocha^1^, Maria Paula Parreira^1^, Arthur de Vasconcellos Rocha^2^, Vitor Lanza Avelar Almeida^2^

(1) Faculdade Ciências Médicas de Minas Gerais – CMMG; (2) Faculdade da Saúde e Ecologia Humana – FASEH

**Introduction:** Cardiovascular surgeries with cardiopulmonary bypass (CPB) have great associated risk, therefore prognostic markers are very important to help in medical decision. In this study we tried to search for markers that help in the decision making process.

**Objective:** To evaluate variables and relate them to morbidity/mortality in order to find prognostic markers for cardiovascular surgery with CPB.

**Method:** Observational retrospective cohort study through consecutive analysis of 113 medical records of patients over 18 years old undergoing cardiovascular surgery with cardiopulmonary bypass from January 1 to December 31, 2015, at the Hospital Universitário Ciências Médicas de Minas Gerais, in the city of Belo Horizonte, MG – Brazil. We evaluated 32 variables relating them to intensive care stay, hospital stay and mortality.

**Results:** EuroScore II, preoperative creatinine, on intensive care unit (ICU) admission and at hospital discharge, reintubation, surgical infection and return to CPB were statistically significant variables. The number of lymphocytes at ICU admission, above 1263.9 was a statistically significant variable in relation to mortality. These results, except the lymphocyte values, were expected to be related to death. However, the values of lymphocytes collected at the ICU entrance, is an interesting finding and is not in the algorithms for prognostic evaluation in cardiovascular surgery. The study has some limitations due to the small number of participants in the study, because it was done in a single center, and because it has only one year of data collection. However, this study brings a new data, which may be an important prognostic marker in cardiac surgery.

**Conclusion:** The value of lymphocytes above 1263.9/mm^3^, in addition to Euroscore II, Creatinine in the pre, per and postoperative, infection, return to CPB, showed a significant relation with mortality in cardiovascular surgery with CPB.

110468

Modality: E-Poster Scientific Initiation – Non-case Report

Category: CARDIAC ARRHYTHMIAS/ELECTROPHYSIOLOGY/ELECTROCARDIOGRAPHY

## Determinants and Prognostic Value of In-Hospital Infection in Patients Waiting for Permanent Pacemaker Implantation

MATHEUS CANDIDO BARBOSA^1^, Willian Cirillo^1^, Fernando Piza^1^, Marcio J.O. Figueiredo^1^, Odilson M. Silvestre^2^, Miguel M. Fernandes-Silva^3^, Roberto Schreiber^1^, Matheus F.R.A. Oliveira^1^, Otavio R. Coelho-Filho^1^, José R. Matos-Souza^1^, Andrei C. Sposito^1^, Wilson Nadruz Jr.^1^

(1) Department of Internal Medicine, School of Medical Sciences, State University of Campinas, São Paulo, Brazil.; (2) Federal University of Acre, Rio Branco, Brazil.; (3) Federal University of Parana, Curitiba, Brazil.

**Background:** In-hospital delays in permanent cardiac pacemaker (PPM) implantation are common and may result in in-hospital infection among patients waiting for PPM implantation (pre-PPM-HI). This study investigated the predictors and prognostic impact of these events.

**Methods:** We retrospectively evaluated 905 consecutive patients (68.2 ± 16.0 years; 54% males) who underwent PPM implantation. Clinical characteristics, pre-PPM-HI and 30-day mortality were recorded and a risk score for pre-PPM-HI was generated using multivariable logistic regression coefficients.

**Results:** Eigthy-nine patients (10% of the sample) developed pre-PPM-HI. Multivariable logistic regression analysis identified implantation of temporary pacemaker, diabetes, hospitalization >3 days, heart failure and complete atrioventricular block as independent predictors of pre-PPM-HI. The generated score (range 0–9.9) played a better role in predicting pre-PPM-HI than individual factors, yielding an area under the curve [95%CI] of 0.702 [0.653–0.751]. Patients with score ≥7.5 had 12-fold greater risk of developing pre-PPM-HI than those with score <2.5. Furthermore, multivariable Cox-regression analysis showed that patients who developed pre-PPM-HI had greater 30-day mortality after PPM implantation (hazard ratio [95%CI] = 2.86 [1.16–7.06], p = 0.022) as compared with their counterparts.

**Conclusions:** This study reveals that pre-PPM-HI is an independent predictor of early mortality after PPM implantation. In addition, a clinical score developed from simple clinical variables accurately identified patients at high risk of pre-PPM-HI. In scenarios where delays in PPM implantation are unavoidable, such as reference hospitals with high demand, the use of this tool can potentially help in the hierarchy of patients and in the reduction of this adverse event.


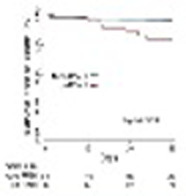


110476

Modality: E-Poster Scientific Initiation – Non-case Report

Category: PHYSIOTHERAPY

## Photobiomodulation at 808nm in the Induced Fatigue of the Triceps Sural Muscle of Physically Active People. Double-Blind Randomized Study

LUCAS CONSOLAIO DE ASSIS^1^, Lucas Consolaio de Assis^1^, Liz Maria Xavier de Oliveira^2^, Cristiane Rodrigues Pedroni^3^

(1) Universidade Estadual Paulista “Júlio de Mesquita Filho” – UNESP, Marília, Faculdade de Filosofia e Ciências (FFC – Marília, SP); (2) Centro de Estudos e Reabilitação (CER – UNESP, Marília – SP); (3) Laboratório de Pesquisa em Ortopedia e Recursos Terapêuticos (LAPORT – Unesp, Marília)

Dysfunction or fatigue of the triceps surae muscles can lead to a defect posture, muscle contracture, fiber ruptures, contusion and injuries related to excessive and repetitive dorsiflexion. Photobiomodulation is a well-studied resource for reduce fatigue and the risk of muscle injuries, being a treatment option and a effective method also for improving muscle performance.

**Purpose:** To investigate the effect of photobiomodulation on muscle performance and recovery after the installation of triceps surae fatigue in physically active people.

**Methods:** Randomized double-blind study, with 14 healthy and physically active subjects, of both sexes, aged between 18 and 30 years, randomly divided into two groups: irradiation and placebo. were carried out the Y Balance Test, manual dynamometry and the electromyographic exam to verify the lower limb performance, strength and electrical activity respectively. In Then, the adapted Bruce protocol and fatigue protocol were applied to the triceps surae, and then active photobiomodulation or placebo according to the group. used the equipment Cluster LASER infrared with 4 diodes, wavelength 808 nm, 120 mW output power, continuous mode emission and total energy (cluster – 4 points) equal to 60J. The effect of therapy between the groups was verified through the values of performance on YBT, strength values by dynamometry and by RMS and index values of fatigue calculated by the median frequency of the electromyographic signal.

**Results:** No significant differences were observed in the intergroup comparison and when compared pre and post in the evaluations of YBT (p = 0.46), muscular strength (p = 0.73) and in the electromyographic behavior of mean RMS values (p > 0.05) and fatigue index (p > 0.05).

**Conclusion:** The photobiomodulation protocol applied after muscle fatigue proposed for the gastrocnemius muscle was not enough to change the parameters of lower limb performance, muscle strength and electromyography.

112438

Modality: E-Poster Scientific Initiation – Non-case Report

Category: CONGENITAL AND PEDIATRIC CARDIOLOGY

## Effect of Beta-Blockers and Losartan for Preventing Aortic Dissection in Marfan Syndrome: A Systematic Review

MARIA HELOÍSA BEZERRA VILHENA^1^, Bárbara Vilhena Montenegro^1^, Lorena Souza dos Santos Lima^1^, Hillary Ferreira Parnaiba^1^, Natália Fernandes Ribeiro^1^, Michelle Sales Barros de Aguiar^1^

(1) Instituto Michelle Sales

**Introduction:** Use of antihypertensive medication in Marfan Syndrome (MFS) aims to prevent one of the worst outcomes, aortic dissection. Beta-blockers are well-established drugs in this context, however, a possible response to the use of losartan has attracted the attention of researchers.

**Objective:** To describe the effect of beta-blockers and losartan for preventing aortic dissection in MFS.

**Methods:** This systematic review is based on the PRISMA (Preferred Reporting Items for Systematic Reviews and Meta-Analyses) protocol. PUBMED and Cochrane databases were searched, using the descriptors “Aortic dissection”, “Beta-blocker” and “Marfan Syndrome” combined with the Boolean operator “AND”. Inclusion criteria were: published in the last 5 years, full text available, clinical trials, randomized trials, books and documents. Exclusion criteria were articles on Loeys-Dietz Syndrome. 116 articles were identified, of which 7 met the inclusion criteria. After checking the studies, a careful reading was carried out, verifying the PRISMA protocol checklist, in order to obtain the qualitative results of the systematic review.

**Results:** According to a randomized study carried out in 2018, the use of atenolol for 3 years by patients with MFS caused a decrease in aortic root stiffness by lowering heart rate (P = 0.01). In the losartan group, no significant differences were observed (p = 0.31). However, a clinical trial was carried out in 2018, in which no significant differences were observed in the increase in the diameter of the aortic root between losartan group (95% CI: 0.2 to 0.5 mm/year) and atenolol group (95% CI: 0.3 to 0.6 mm/year). A study carried out in 2020 in the Netherlands reports that the group of patients using beta-blockers exclusively (n = 124) had 11 cases of aortic dissection, while the group using combined therapy with beta-blockers and losartan had 3 evolutions for this event during the eight years of study. In another 2020 finding, patients with MFS who had the CC genotype and were in use of atenolol had a better rate of improvement than patients who were using losartan (interaction P = 0.002; –0.20 ± 0.02 vs –0.07 ± 0.02; P < .001).

**Conclusion:** Combined therapy of atenolol and losartan has beneficial effects when compared to losartan alone, although there are no significant clinical differences on the exclusive use of losartan. There are insufficient randomized studies to prove the effectiveness of this therapy in acute aortic dissection in MFS.

110549

Modality: E-Poster Scientific Initiation – Non-case Report

Category: ATHEROSCLEROSIS/CARDIOVASCULAR RISK FACTORS/CARDIOVASCULAR PREVENTION

## Factors Associated with Carotid Intima-Media Thickness in Adults and the Elderly in a German City in Southern Brazil

NATÁLIA MERHEB HADDAD^1^, Suhen Aquino de Liz^2^, Marcello Ricardo Paulista Markus^3^, Maria Eduarda Venera^4^, Ernani Tiaraju de Santa Helena^5^, Clóvis Arlindo de Sousa^6^, Alan de Jesus Pires de Moraes^1^

(1) Universidade do Vale do Itajaí; (2) Programa de Pós-Graduação Stricto Sensu em Saúde Coletiva. Universidade Regional de Blumenau; (3) Department of Internal Medicine B, University Medicine Greifswald, Greifswald, Germany. German Centre for Cardiovascular Research (DZHK), partner site Greifswald, Greifswald, Germany. German Center for Diabetes Research (DZD), partner site Greifswald, Gre; (4) Departamento de Educação Física. Universidade Regional de Blumenau, Blumenau, Santa Catarina, Brasil; (5) Departamento de Medicina. Programa de Pós-Graduação Stricto Sensu em Saúde Coletiva, Universidade Regional de Blumenau; (6) Departamento de Educação Física, Programa de Pós-Graduação Stricto Sensu em Saúde Coletiva, Universidade Regional de Blumenau

**Background:** Carotid intima-media thickness (CIMT) is a marker of atherosclerosis and a predictor of cardiovascular disease, however, there are few studies on factors associated with CIMT in specific populations.

**Objective:** The goal was to analyze the prevalence and factors associated with CIMT among adults and the elderly people in a German city in southern Brazil.

**Method:** Cross-sectional population-based study, with 2488 people between 20 and 79 years old, from the Study of Health in Pomerode – SHIP-Brazil. The CIMT was evaluated by ultrasonography and the altered thickening was values ≥0.90 mm. The independent variables involved sociodemographic aspects, lifestyle, health conditions and chronic diseases. Crude and adjusted Poisson regression with estimates of prevalence ratios (PR) and confidence intervals were used.

**Results:** The prevalence of high CIMT was 10.9%, higher in the elderly (42.5%) when compared to adults (4.7%). Among adults, male (PR = 1.75), ex-smoker (PR = 2.06), smoker (PR = 2.15), high waist-to-hip ratio (PR = 2.09), hypertension (PR = 2.19), and carotid plaque (PR = 2.34) were associated with elevated EMIC. In the elderly, male (PR = 1.33), insufficiently active (PR = 1.39), high waist-to-hip ratio (PR = 1.34) and plaque (PR = 1.54).

**Conclusion:** Actions to prevent and control high CIMT involve increase the level of physical activity, smoking cessation, preventing of hypertension and excess abdominal fat.

111061

Modality: E-Poster Scientific Initiation – Non-case Report

Category: SPIRITUALITY AND CARDIOVASCULAR MEDICINE

## Resilience in Patients with Chronic Coronary Syndrome Assisted in Public and Private Healthcare Networks: Are there Differences?

JOSÉ ICARO NUNES CRUZ^1^, Adelle Cristine Lima Cardozo^1^, Giulia Vieira Santos^1^, Jade Soares Dória^1^, Mariano César de Souza Reis^1^, Gabriela de Oliveira Salazar^1^, Bruna Souza Matos de Oliveira^1^, Juliana Maria Chianca Lira^1^, Diego Maldini Borba de Lima^1^, Antônio Carlos Sobral Sousa^2^, Enaldo Vieira de Melo^1^, Joselina Luzia Menezes Oliveira^2^

(1) Federal University of Sergipe; (2) Rede D’Or São Luiz – São Lucas Hospital; (3) Primavera Hospital

**Introduction:** Resilience refers to the ability to develop adequately, even when facing difficulties, and can be an important factor in health promotion, especially in chronic diseases.

**Objectives:** To evaluate how resilience behaves among patients with Chronic Coronary Syndrome (CCS) seen in the public and private healthcare networks.

**Methods:** This is a cross-sectional, analytical study, whose sample included patients with CCS assisted at cardiology outpatient clinics of two public hospitals and one private hospital. The patients were divided into two groups, according to the healthcare system: I) Public; II) Private. Participants completed the Connor-Davidson Resilience Scale (CD-RISC-10), composed of ten items, each one presented as an ordinal scale from 0 to 4. The total resilience level comprises the sum of the responses from each item, and thus ranges from 0 to 40. The results were described in terms of means and standard deviations and the statistical analysis was performed using Fisher’s Exact test and Student’s T-test, with significance level set at 0.05.

**Results:** 53 patients with CCS were included, 69.8% from the Public group and 30.2% from the Private group. The gender distributions were not different between groups (p > 0.05), however the Public group had a lower mean age than the Private group (60.2 vs. 66.9 years; p < 0.05). Patients with CCS assisted at the public system showed a lower level of total resilience than patients assisted at the private healthcare system (26.4 vs. 31.9; p < 0.05). The CD-RISC-10 showed that patients in the Public group believed less that they could achieve their goals compared to the Private group (3.0 vs. 3.8; p < 0.05). When presented with the statement “I am not easily discouraged by failures”, Public group patients had lower mean scores on the scale (2.9 vs. 3.7; p < 0.05), which denotes that this statement is less true for this group compared to the Private group.

**Conclusions:** The results show that patients with CCS assisted in the public healthcare system have a lower level of resilience than patients assisted in private settings. Differences in resilience levels between the groups are concentrated in the belief in the possibility of achieving personal goals (lower for the Public group), and in discouragement in the face of failures (higher for patients in the Public group).

110589

Modality: E-Poster Scientific Initiation – Non-case Report

Category: CONGENITAL AND PEDIATRIC CARDIOLOGY

## Risk Factors Related to Congenital Heart Diseases: An Analysis of the Unified Health System Database in the State of Paraná, Brazil (Datasus)

MARIA JÚLIA DA SILVEIRA MARQUES^1^, Maria Júlia da Silveira Marques^1^, Audrei Pavanello^1^

(1) Unicesumar

Congenital heart diseases (CHDs) describe a set of abnormalities of the heart and great vessels which are present at birth and are associated with great physiological impact on the neonate. The etiology of those malformations is multifactorial, including genetic and environmental factors, maternal obesity, pre-gestational diabetes mellitus and the use of potentially teratogenic drugs like retinoic acid. These causes seem to influence the development of the fetal cardiovascular system, causing cardiac and great vessel anomalies and consequently, a functional disorder of this system. Considering these factors, the objective of this study is to correlate the risk factors associated with poor fetal cardiovascular development, through an analysis of the Unified Health System database (DATASUS). The data used were collected from the DATASUS Information System for Live Births (SINASC) platform with the help of Software R (version 4.1.3) and RStudio (version 2021.09.0 + 351). For data collection, the R microdatasus package was used, with the referring period of 2007 to 2020 in the state of Paraná. Data were filtered using the R tidyverse package, with 1135 births with congenital heart anomalies registered during this period (ICD Q200 to Q250). For comparison, 5676 observations of non-anomaly neonates chosen from random data were used. The analysis was performed using the rpart package, where a classification tree was constructed crossing the variables: weeks of pregnancy, maternal age, birth weight, sex and race against the presence of congenital anomalies. Data were plotted using the rpart.plot package. With the analysis of the data, through the softwares R and RStudio, the following results were obtained: in order of greater to lesser relevance, the factors that influence the development of CHD are the birth weight (184.497256), the type of delivery (40.688717), gestational age (29.954032), mother’s age (21.551755), race (2.277806) and sex of the neonate (1.405119). Greater attention is necessary to these factors, aiming possible prevention and early management of this condition.

110627

Modality: E-Poster Scientific Initiation – Non-case Report

Category: ATHEROSCLEROSIS/CARDIOVASCULAR RISK FACTORS/CARDIOVASCULAR PREVENTION

## Cardiovascular Risk Profile in Patients with Metabolic Dysfunction-Associated Liver Disease

MARIA EDUARDA DE ALMEIDA OLIVEIRA^1^, Daniele Araujo de Azevedo Coutinho^2^, Sergio Augusto Antonio^3^, Davi Cassiano Costa^4^, Vanessa da Costa Rodrigues^5^, Raphael Carreiro Moura^6^, Maria Auxiliadora Saad^7^, Carlos Roberto Moraes de Andrade Junior^8^, Priscila Pollo Flores^9^, Débora Vieira Soares^10^

(1) Universidade Federal Fluminense; (2) Universidade Federal Fluminense; (3) Universidade Federal Fluminense; (4) Universidade Federal Fluminense; (5) Universidade Federal Fluminense; (6) Universidade Federal Fluminense; (7) Universidade Federal Fluminense; (8) Universidade Federal Fluminense; (9) Universidade Federal Fluminense; (10) Universidade Federal Fluminense

**Background and Aims:** Non- alcoholic fatty liver desease (NAFLD) is the most frequent cause of hepatic disease, with a world prevalence of 25%. There seems to be a connection between the gravity of NAFLD, endothelial dysfunction, atherosclerosis and the increase in cardiovascular mortality events. This study aimed to asses cardiovascular risk profile of individuals with NAFLD.

**Methods:** Prospective observational analytical study. Adults with established risk for the development of NAFLD were selected, such as: type 2 Diabetes Mellitus (DM), metabolic syndrome and obesity are selected. Non-invasive assessment of liver steatosis and fibrosis was performed by hepatic ultrasound (FLI-score) and transient elastography. We assessed the frequency of the cardiovascular disease, according to the clinical history, common carotid artery intima-media thickness (IMT) using an ultrasound examination of the carotids and a stratification of the cardiovascular risk by a specific algorithm.

**Results:** All data are presented in median (IQR) and n(%): 31 individuals, 25(80,65) female, performed the ultrasound examination of the carotids. Chronological age 67,5(53,75–66,5) years, vascular age 85(46–85)years. Right IMT 0,805(0,54–0,79)cm and left IMT 0,85(0,53–0,75)cm, 8(25,81) had atherosclerotic plaques. We observed: Hepatic Steatosis in 28(90,32), being 6 (19,35) mild, 12(38,71) moderate and 10(32,25) severe. Liver Fibrosis in 8(25,81), being 2(25,00) moderate and 6(75,00) advanced or cirrhosis. Systemic Arterial Hypertension 24(77,42), DM 18(58,06) and Dyslipidemia 22(70,97).

**Conclusion:** Our data shows a high frequency of hepatic fibrosis and atherosclerotic disease in the collected sample.

110660

Modality: E-Poster Scientific Initiation – Non-case Report

Category: CARDIOVASCULAR SURGERY

## Mechanical or Biological Valve Prostheses for Aortic Valve and Mitral Valve Replacement: Analysis of the Patients Operated in a Tertiary Health Center between 2010–2021

AMANDA BERGAMO MAZETTO^1^, Eduarda Druck Magadan^2^, Rafael Aprecido Garcia^2^, Hellen Cristina Cinesio^1^, Abdalla Hoelz^1^, Julien Ramos Stein^1^, Berta Paula Napchan Boer^1^, Paula Lavitola^1^, Ana Flávia Diez de Andrade^1^, Flávio Tarasoutchi^1^

(1) Instituto do Coração da Faculdade de Medicina da USP; (2) Escola de Medicina da PUCRS

**Introduction:** Valvular heart disease is a public health problem in Brazil, mainly due to the high prevalence of rheumatic fever, resulting in the need for surgical procedures to repair the valvular disease. Choosing the type of prosthesis in some situations remains a challenge, since it is necessary to consider the patient’s age, preferences and socioeconomic conditions, besides thromboembolic, hemorrhagic and new valve replacement risks. With the development of the Valve-in-Valve procedure, the risk of reoperation has decreased considerably and, therefore, the ideal type of prosthesis for the patient remains a controversial topic.

**Objectives:** The objective of the present study is to analyze the type of prosthesis that is most chosen in the surgical treatment of valve replacement in aortic or mitral position in patients operated in a tertiary health center.

**Methods:** Medical records were reviewed of patients undergoing valve replacement surgery in aortic and mitral position between 2010 and 2021.

**Results:** There were 5333 patients with a mean age at the time of surgery of 57.8 years. Among the analyzed patients, 49.9% of the individuals were female. Regarding the indication of the procedure, elective surgery was the indication in 82.2% of the cases, and urgent and emergency cases, in 17.8% of the cases. In total, 91.4% of biological prostheses and 8.6% of mechanical prostheses were placed. A total of 3042 (57%) aortic valve replacements were performed alone, of which 2876 (94.5%) were biological and 166 were mechanical (5.4%). In the mitral position, there were 1889 (35.42%) valve replacements alone, of which 1654 (87.5%) were biological and 235 (12.4%) were mechanical. In the study, 402 (7.53%) patients with double lesions (aortic and mitral) underwent surgery, 354 (88.1%) being biological and 48 (11.9%) mechanical.

**Conclusion:** Biological prosthesis is the model of choice in our service. The main factors that justify this choice are the age of our patients, the socioeconomic and educational conditions that often limit the possibility of anticoagulation, the better quality of life without the need for anticoagulation, and the lower risk of current reoperation with the Valve-in-Valve procedure in biological prostheses.

110676

Modality: E-Poster Scientific Initiation – Non-case Report

Category: CARDIOVASCULAR INTENSIVE CARE/CARDIOVASCULAR EMERGENCIES

## Prevalence in the Number of Hospitalizations for the Treatment of Aortic Aneurysms in Brazil between January of 2011 and December of 2021

PABLO RIBEIRO MIRANDA BARBOSA^1^, Brunna Machado Medeiros^1^, Gabriel Dias de Oliveira^1^, Gustavo Fornachari^1^, Matheus Giacomelli da Trindade^1^, Marcelle Telasca Patzlaff^1^, Lucas Álvares de Souza^1^, Ricardo Augusto Oliveira Mendes^1^, Mikaelle Tainá Bertoli^1^, Bruno Noschang Blaas^2^

(1) Federal University of Pelotas (UFPEL); (2) Federal University of São Paulo (UNIFESP)

**Introduction:** An aneurysm is the dilation of a blood vessel that is greater than 50% of its normal diameter. It induces a fragility in the arterial wall and can trigger severe outcomes, such as the compression of adjacent structures or rupture of the blood vessel itself[1]. In Brazil, the most prevalent cases of aneurysms come in the form of an abdominal aortic aneurysm (AAA). This is more frequently found in elderly patients, with other risk factors in addition to age being: smoking, systemic arterial hypertension (SAH), dyslipidemia and diabetes mellitus. It is 4 to 6 times more frequent in males. Furthermore, ultrasonography (USG) AAA screening is recommended for men between the ages of 65 and 75, who are smokers or who have first-degree relatives with a history of AAA. As for women, screenings are recommended for those with a family history of AAA where a 1st degree relative has had a diagnosis.[2]

**Objective:** Describe the annual prevalence in the number of hospitalizations for the treatment of aortic aneurysms (AA) in Brazil between January of 2011 and December of 2021.

**Methodology:** Descriptive cross-sectional study based on a quantitative approach to the frequency of hospital admissions for treatment of aortic aneurysm in Brazil between January of 2011 and December of 2021. Data regarding hospitalizations was collected from the Hospital Information System of the Unified Health System in Brazil, colloquially known as SIH/SUS.

**Results:** In 2011 there were 2,892 registered hospitalizations for the treatment of AA, followed by 2,906 in 2012, 3,184 in 2013, 3,440 in 2014, 3,815 in 2015, 4,061 in 2016, 3,907 in 2017, 4,184 in 2018, 4,456 in 2019, 3,733 in 2020 and 4,092 cases in 2021. Thus, we conclude an increase of 41.4% in the number of cases between 2011 and 2021.

**Conclusion:** We observed an increase of more than 40% in hospitalizations for the treatment of AA during the study period. However, during the same period, it is important to note that there was a population increase of 10.9%.[5] As a result, we can speculate that there was an increase in diagnostic screening, in addition to a higher incidence of comorbidity such as obesity, diabetes mellitus, dyslipidemia and hypertension. In conclusion, this article is not able to infer specific causality for the number of hospitalizations, thus suggesting that further studies in this topic are needed.

110768

Modality: E-Poster Scientific Initiation – Non-case Report

Category: COVID-19 AND CARDIOVASCULAR SYSTEM

## SARS-COV-2 Pandemic and the Morbimortality of Acute Myocardial Infarction in the Northeast Region of Brazil

ANA FLÁVIA BOTELHO^1^, Caroline Link^1^, Larissa Almeida Busnello^1^, Paola Gonçalves Moreira de Oliveira^1^, Francielle Nocera Viechineski^1^, Bruna Kara^1^, Ana Carla Dlugosz^1^, Alice Magro Koscianski^1^, Camilla Mattia Calixto^1^, Julia Henneberg Hessman^1^, Mário Claudio Soares Sturzeneker^1^

(1) Universidade Estadual de Ponta Grossa

**Introduction:** Worldwide, ischemic heart disease is the most common cause of death, and, in this context, acute myocardial infarction (AMI) is a significant public health problem. The SARS-CoV-2 pandemic caused changes in the dynamics of care in emergency units and consequently influenced morbidity and mortality from AMI.

**Purpose:** To estimate the impact of the SARS-CoV-2 pandemic on morbidity and mortality from AMI in the Northeast region through the analysis of DATASUS data.

**Method:** In the period from 2018 to 2021, subdivided into pre-pandemic (11/2018 to 02/2020), pandemic (03/2020 to 06/2021), the peak of the pandemic (12/2020 to 05/2021), first and second trimester of the peak (12/2020 to 02/2021 and 03/2021 to 05/2021) and mass vaccination period (06/2021 to 12/2021), were evaluated in general and by gender, hospitalizations, deaths, mortality rate (percentage obtained by dividing the number of deaths by the number of hospitalizations), calculating the average of each variable and comparing the respective periods, with differences expressed as percentages.

**Results:** In the pandemic period, the number of hospitalizations reduced by approximately 4% compared to the pre-pandemic period. There is an increase in the number of hospitalizations in the second trimester of the peak and the period of mass vaccination, with an increase of 2% and 15%, respectively. Compared to the pre-pandemic period, the average hospital stay was lower in the pandemic and first trimesters of the peak periods (12.3%). There was a reduction in the number of deaths in the pandemic (1%) and the first trimester of the peak (5%) compared to the pre-pandemic period. There is an increase in the number of deaths in the second trimesters of the peak, especially in the mass vaccination period (13%) compared to the pre-pandemic period. Regarding gender, the number of hospitalizations was higher in men, and the mortality rate was higher in women throughout the period evaluated.

**Conclusions:** Social isolation and functional changes in the health system caused by the SARS-CoV-2 pandemic influenced morbidity and mortality from AMI in the Northeast region of Brazil, and mass vaccination culminated in the return to characteristics similar to the pre-pandemic period.

110789

Modality: E-Poster Scientific Initiation – Non-case Report

Category: ATHEROSCLEROSIS/CARDIOVASCULAR RISK FACTORS/CARDIOVASCULAR PREVENTION

## Prevalence of Subclinical Atherosclerosis with Altered Coronary Calcium Score in a Santa Catarina Microregion

JULIA FARENZENA ZAPELINI^1^, Manuela Matheus^1^, Samantha Cristiane Lopes^1^, Franciani Rodrigues da Rocha^1^, Ottávia de Vasconcelos Zainho Helbok^1^, Caroline de Oliveira Fischer Bacca^1^

(1) UNIDAVI – Centro Universitário para o Desenvolvimento do Alto Vale do Itajaí

**Introduction:** Considering that some cardiac death occurs in asymptomatic patients, it is very important to identify subgroups of people with a high risk of cardiovascular arterial diseases (CAD). The coronary artery calcium (CAC) is a marker that identifies the atherosclerotic burden, which is related to the incidence of CAD.

**Objectives:** Evaluate the prevalence of subclinical atherosclerosis defined as altered CAC, recognizing the most prevalent risk factors.

**Methods:** Cross-sectional study with 547 patients who underwent CAC between Jan/16 and Dec/21. Statistical analyses were performed using SPSS employing Fisher’s exact test or Pearson’s chi-square test. The level of statistical significance was set at p < 0.05.

**Results:** The mean age was 52.3 ± 8.9 years. Including CAC, the mean age was 70.5 ± 13.4 years and the CAD risk was 9.5 ± 8.8%. There was correlation between hypertension (SAH) and CAC > 400 (p = 0,04; p < 0,05); and diabetes (DM) and CAC > 400 (p = 0,01; p < 0,01). When the CAC was adjusted in percentiles, the presence of SAH was relevant with the percentile >75 (ra = 4,8; p < 0,01), and dyslipidemia with the percentile 50–75 (ra = 2,7; p < 0,01) and >75 (ra = 2,5; p < 0,01). The absence of SAH (ra = 4,3; p < 0,01) and dyslipidemia (ra = 4,1; p < 0,01) were protective factors, since they showed significance with the 0 percentile.

**Conclusions:** Elevated CAC showed relevance with DM, SAH and dyslipidemia, while the absence of SAH and dyslipidemia were cardiovascular protection factors.


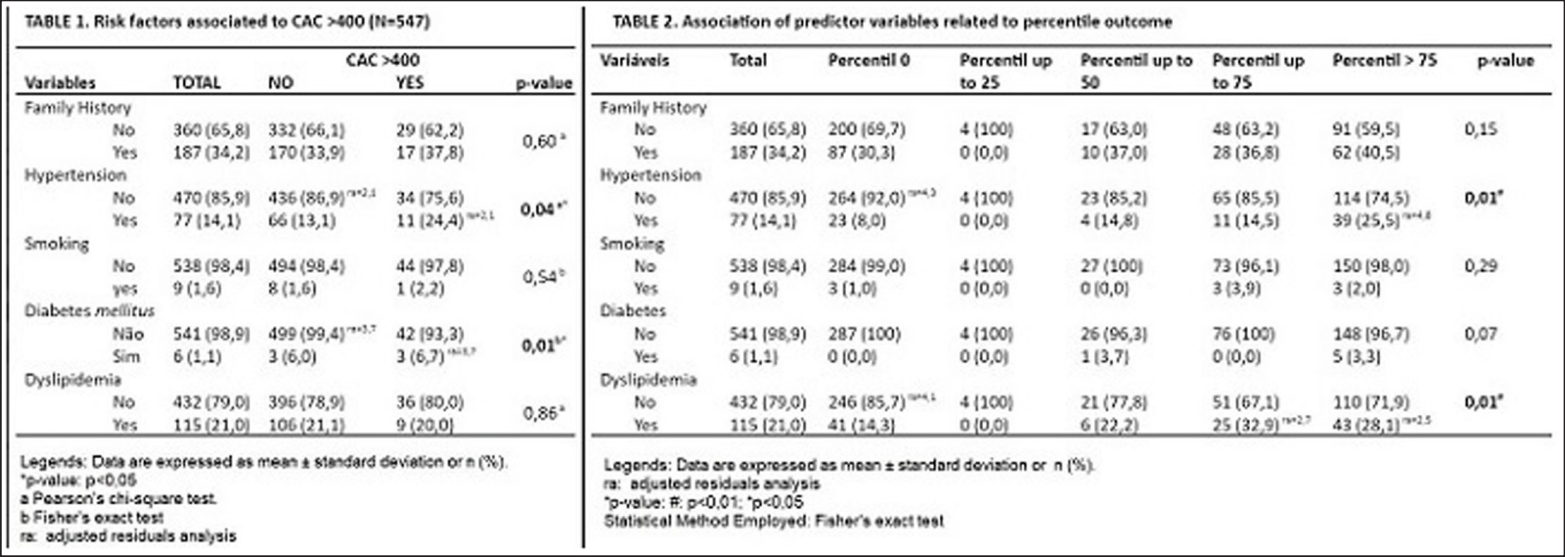


110797

Modality: E-Poster Scientific Initiation – Non-case Report

Category: CARDIOGERIATRICS

## What are the Results and Characteristics of the Head-Up Tilt Test in the Elderly?

ANA GABRIELA PONTE FARIAS^1^, Arnóbio Dias da Ponte Filho^4^, Francisca Tatiana Moreira Pereira^4^, Vera Marques^4^, Luís Gustavo Bastos Pinho^1^, Maria Jacqueline Batista^1^, Eduardo Augusto Quidute Arrais Rocha^3^, Ana Rosa Pinto Quidute^1^, Marcela Albuquerque de Holanda^3^, Aston Alves de Freitas^3^, Arthur Holanda Dantas^3^, Eduardo Arrais Rocha^4^

(1) Universidade Federal do Ceará; (2) Universidade de Fortaleza; (3) Centro Universitário Christus; (4) Centro de Arritmia do Ceará

**Introduction:** In the elderly, there are several causes of syncope or presyncope, making etiological diagnosis challenging. The relevance of the Head-Up Tilt Test (HUT) in this population has been questioned because of a high incidence of cardiac causes with risk of sudden death. However, dysautonomic causes are also common in this age group, affecting morbidity and mortality.

**Objective:** The aim of this study was to compare the results of the HUT between the elderly (≥60 years old) and non-elderly age groups.

**Methods:** This was a retrospective cohort study, carried out from 2016 to 2021. For comparisons, non-parametric Mann-Whitney/Wilcoxon tests were used, with a significant p-value <5%. The protocols were Westminster or Italian, with the sensibilization phase (1.25 mg sublingual isosorbide) used according to the medical decision during the examination.

**Results:** A total of 2347 tilt tests were analyzed, 61.7% were female, with a median age of 51.1 (31–71). The overall positivity rate was 33.3%, with 43.3% with pharmacological sensibilization (p < 0.01). There were 972 (41.82%) in the elderly group (EG) and 1352 (58.18%) in the non-elderly group (NEG). The positivity rate of EG was lower (p < 0.01); 266 (11.45%) in the EG had positive HUT × 496 (21.24%) in the NEG. The positivity rate with sensibilization in the EG was 27.37% × 20.52% in the NEG (p = 0.008). In the EG, 48 patients (5.04%) had a dysautonomic response × 10 (0.73%) in the NEG (p < 0.001). In the EG, the rates of vasovagal response were 209 (21.97%), distributed as follows: vasodepressor (15.23%); cardioinhibitory (0.92%); mixed (5.86%), while in the NEG, the rates were 466 (33.94%), being vasodepressor (16.94%), mixed (15.90%), and cardioinhibitory (1.26%) (p < 0.001). The rate of complications in the EG was 1.54% × 1.63% in the NEG (p = 0.02), but without serious events. Prodromes were more common 39.86% (539) in the NEG × 31.10% (302) in the EG (p < 0.001).

**Conclusion:** The HUT in the elderly population showed a lower incidence of positivity in the passive phase, however with a higher rate in the sensibilization phase, compared to the non-elderly. The elderly had a higher incidence of dysautonomic responses and a lower rate of prodromes and complications. This method should remain, therefore, as a complementary test in the syncope or presyncope investigation also in the elderly.

110799

Modality: E-Poster Scientific Initiation – Non-case Report

Category: HEMODYNAMICS AND INTERVENTIONAL CARDIOLOGY

## Angiography Quantitative Flow Ratio: A Systematic Review and Analysis of Diagnostic Accuracy

LUCAS BONACOSSA SANT’ANNA^1^, Eduardo Amar Ferreira^1^, Sérgio Lívio Menezes Couceiro^3^, Daniel Chamié^4^, Fernando Mendes Sant‘Anna^2^

(1) Fundação Técnico-Educacional Souza Marques (RJ); (2) Universidade Federal do Rio de Janeiro (Macaé); (3) Hospital Santa Izabel (Cabo Frio); (4) Instituto Dante Pazzanese de Cardiologia (SP)

**Introduction:** Fractional flow reserve (FFR) is the gold standard to evaluate severity of coronary stenosis. Quantitative flow ratio (QFR) is a new angiography-based method used to infer FFR. Studies have shown >90% agreement between QFR and FFR.

**Objective:** To conduct a systematic review and diagnostic accuracy analysis of QFR using individual vessel data.

**Methods:** This review follows PRISMA guidelines. MEDLINE, EMBASE and Cochrane Library of Clinical Trials were searched for QFR accuracy studies published until Oct 2020. Inclusion criteria: (a) QFR vs FFR; (b) QFR diagnostic capacity; (c) agreement data between QFR/FFR expressed as dot plots or individual data tables. Graphic data were digitized using a semiautomatic software. QFR/FFR values were dichotomized using cutoff values of ≤0.80 for ischemia. QFR diagnostic accuracy was assessed by two logistic regressions superimposed on the same graph to ensure the probability of agreement between QFR and FFR for any QFR value. FFR was the reference standard.

**Results:** 20 studies comprising 5,318 vessels from 4,429 patients were included. Most patients were male (64%) at an age of 66.8 ± 5.2 years. Figure 1A shows FFR distribution and QFR diagnostic accuracy for different QFR ranges. QFR overall accuracy, sensitivity, specificity, PPV and NPV are displayed in Figure 1B. A diagnostic accuracy of 87% was reached for QFR cutoff values <0.71 or >0.86, and 95% or 98% with cutoffs <0.66 or >0.91 and <0.62 or >0.94, respectively (Figure 1B).

**Conclusions:** A very good diagnostic accuracy of QFR measures was observed using individual vessel data. QFR can be used to evaluate the severity of coronary stenosis. At less accurate values addition of FFR can improve precision.


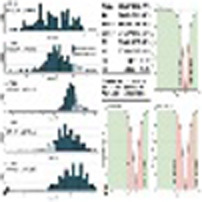


110835

Modality: E-Poster Scientific Initiation – Non-case Report

Category: ATHEROSCLEROSIS/CARDIOVASCULAR RISK FACTORS/CARDIOVASCULAR PREVENTION

## The Prevalence of Hypertension in Santiago Island- Cabo Verde

KELLY ANNY FERNANDES MASCARENHAS MASCARENHAS^1^

(1) Instituto Politécnico de Castelo Branco

**Introduction:** It is estimated that about 1.13 thousand million people around the world are affected by Hypertension (HTN), which causes about 45% of heart disease deaths and 51% of the CVA deaths, more than 8 million deaths per year, and 92 million years of life disability. In Cabo Verde, according to the National Survey about the Risk Factors of Non-Transmitted Diseases (NTDS II) 2020, 20,1% of the population suffer from hypertension.

**Objectives:** To study the prevalence of hypertension in adults residing in Santiago Island – Cabo Verde.

**Methodology:** The transversal Study of the population background in which the data collection took place in October and November 2021, in the municipality of Santiago IslandCabo Verde. It was excluded from the study the individuals of Cape Verdean nationality who have been living for over a year abroad and that have just arrived on Santiago Island in less than 30 days, as well as the individuals with any kind of disabilities that may affect their participation in the study. The data were collected through questionnaires; As for hypertension, it was evaluated using an automated sphygmomanometer.

**Results:** It was studied 599 individuals with the age range between 18 and 99 years old with the age average of 42 years of age, while 54.8% female and 45.2% male. The findings showed that 32.6% of the participants in the study had high blood pressure; 53.8% are female and 46.2% are male. Out of 195 hypertensive participants, it’s realized that 34.6% had stage I HTN, 26.2% had stage II HT, 14.1% had stage III HTN and 25.1% had systolic Isolated hypertension. Thus, it was also found that there is a meaningful statistical relationship between HTN, diabetes, and the age group.

**Conclusion:** There is a high prevalence of HTN in the population of Santiago Island – Cabo Verde.

110867

Modality: E-Poster Scientific Initiation – Non-case Report

Category: NEGLECTED CARDIOVASCULAR DISEASES

## Nets Release from Peripheral Blood Neutrophils from Patients with Chagas Disease: Correlation with Ecocardiogram Parameters

LUANN GABRIEL PORPINO SALES^1^, Marcelo Augusto Araújo Castro^1^, Pedro Felipe Alves de Souza^1^, Valéria Duarte de Almeida^1^, Eduardo Bulhões Leopoldo da Câmara^1^, Suyane Maria Paiva Pimenta^1^, Micássio Fernandes de Andrade^1^, Cléber Mesquita Andrade^1^, Thales Allyrio Araujo de Medeiros Fernandes^1^, Amanda Estevam Carvalho^1^, Felipe Alves de Lima^1^

(1) Universidade do Estado do Rio Grande do Norte (UERN)

**Introduction:** The neutrophils are capable of interacting with Trypanosoma cruzi, the etiological agent of Chagas Disease (CD), and release NETs (extracellular Neutrophils traps) causing damage to the myocardium.

**Objective:** To evaluate the correlations between the amounts of NETs released by patients with CD, their clinical forms, with the echocardiographic parameters of these patients.

**Methods:** Retrospective cross-sectional study with conventional sampling. A total of 35 volunteers participated in the research, being 11 healthy individuals and 24 patients with Chagas disease (15 of the indeterminate clinical form and 9 of the cardiac form). The quantification of the formation and release of NETs was performed by means of fluorescence. The identification of clinical forms and the analysis of the echocardiogram were performed by analyzing the filed medical records. The data obtained were processed and statistically analyzed using the GraPad Prism 7.00.

**Results:** Neutrophils from healthy individuals, when stimulated with soluble T. cruzi antigen, release NETs greater than the cells of chagasic patients, both with the clinical cardiac form (* p = 0.0014), as for the indeterminate clinical form (* p = 0.0142). When correlating the concentration of NETs released with the parameters of the Echocardiogram, a negative correlation was observed with the systolic diameter of the left ventricle (r = –0.07998; * p = 0.0097) and with the left ventricular mass index (r = –0.7743; * p = 0.0086), in addition to a positive correlation with the left ventricular ejection fraction (r = 0.6539; * p = 0.0403).

**Conclusions:** Neutrophils from patients with CD release less NETs than neutrophils from healthy individuals and may be associated and contribute to the clinical course of Chagas cardiomyopathy.


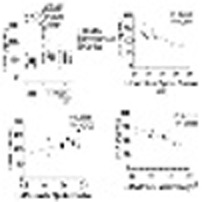


110877

Modality: E-Poster Scientific Initiation – Non-case Report

Category: CARDIOGERIATRICS

## Prevalence of Cognitive Dysfunction in Elderly Patients with Ischemic Heart Failure

CAROLINA DE CARVALHO MOURY FERNANDES^1^, Maria Eduarda Borges Matias^1^, Carolina Jeronimo Magalhães^3^, Matheus Dantas Soeiro^1^, Sabrina Barreto Braga Pires^1^, Ellen Beatriz Sobral^1^, Jessica Myrian de Amorim Garcia^2^, Francisco Bandeira^3^

(1) Faculdade Pernambucana de Saúde; (2) Hospital Agamenon Magalhães; (3) Universidade de Pernambuco

**Introduction:** The incidence of heart failure (HF) has steadily increased especially due to the aging of the population. One of its main etiologies is the coronary artery disease (CAD). In the elderly there is a high prevalence of cognitive dysfunction, which can be analyzed by mini cog, a short cognitive impairment screening exam. This cognitive dysfunction may be associated with both coronary and cerebrovascular disfunction.

**Objectives:** Analyze the prevalence of cognitive dysfunction in hospitalized patients with ischemic heart failure.

**Methods:** Cross-sectional observational study of inpatients aged ≥65 years, hospitalized with HF. The study was conducted in a public Brazilian cardiology teaching center from August 2020 to December 2021. The diagnostic criteria for ischemic heart failure was based on coronary angiography findings, while cognitive dysfunction was evaluated using the Mini-Cog.

**Results:** Our total sample consisted of 198 elderly patients diagnosed with HF, we studied 98 of them that also had CAD, showing the prevalence of ischemic heart failure. The mean age was 72.7 years ranging from 65 to 90, and 56.1% were male. Among the comorbidities, 42.9% had diabetes and 89.8% hypertension. The prevalence of cognitive dysfunction was 59.2% based on the Mini-Cog.

**Conclusion:** There was a high prevalence of cognitive dysfunction among the elderly patients with CAD and HF, that was evident when analyzing using the Mini-Cog.

111798

Modality: E-Poster Scientific Initiation – Non-case Report

Category: EPIDEMIOLOGY AND HEALTH POLICIES/GLOBAL HEALTH

## Profile of Hospitalization in the Health Unic System in the Municipality of São Paulo for Acute Myocardial Infarction in the Year 2021

MAYURI AKEMI RODRIGUES HIGASHI^1^, Gabriel Ribeiro de Souza^1^, Rafaela Andrade Penalva Freitas^2^

(1) UNIVERSIDADE DE SANTO AMARO; (2) INSTITUTO DANTE PAZZANESE DE CARDIOLOGIA

**Introduction:** Despite significant scientific advances and public prevention policies, acute myocardial infarction corresponds to a high rate of hospitalizations and morbidity and mortality.

**Objective:** To analyze and describe the profile of patients who are hospitalized in the city of São Paulo for Acute Myocardial Infarction in the year 2021.

**Methodology:** It consists of a descriptive, retrospective, longitudinal epidemiological study, based on the analysis of a secondary source of data, with the aim of the studies being hospital admissions in the Unified Health System of the city of São Paulo, in the year 2021, for causes related to diseases. hypertensive As inclusion criteria, all hospital admissions were selected through the Hospital Information System (SIH), through DATASUS. The period studied was the year 2021. The morbidities were separated and selected according to the main hospitalization diagnosis and classified according to the International Disease Code 10 (ICD10), the following being selected: I10 -Essential hypertension, I11 – Hypertensive heart disease, I12 – Hypertensive kidney disease, I13 – Hypertensive heart and kidney disease, I15 – Secondary hypertension. Gender, race/color and age group were also taken into account.

**Results:** During the study period, 2219 hospitalizations were performed for hypertensive diseases, of which 49.2% were male and 50.8% female. Among the target audience of the study, when considering race/color, 35.8% of patients consider themselves white, 8% black, 29.5% brown and 0.2% yellow. 26.5% of the registered admissions had no description of race/color. Regarding the age groups studied, there is a gradual increase in the number of hospitalizations as the observed age group also increases, with the group of patients over 80 years of age having the highest number of hospitalizations, corresponding to 14% of all the hospitalizations.

**Conclusion:** Hospitalizations in the city of São Paulo due to hypertensive syndromes were slightly higher in males, most of whom were white and aged over 80 years.

110903

Modality: E-Poster Scientific Initiation – Non-case Report

Category: PERICARDIUM/ENDOCARDIUM/VALVOPATHIES

## Features of Enterococcal Endocarditis in a Cardiology Surgical Center in Brazil: A Comparative Analysis

NÍCOLAS DE ALBUQUERQUE PEREIRA FEIJÓO^1^, Mariana Giorgi Barroso de Carvalho^1^, Thatyane Veloso de Paula Amaral de Almeida^1^, Ingrid Paiva Duarte^1^, Léo Rodrigo Abrahão dos Santos^1^, Rafael Quaresma Garrido^2^, Giovanna Ferraiuoli Barbosa^2^, Clara Weskler^2^, Wilma Golebiovski^2^, Bruno Zappa^2^, Marcelo Goulart Correia^2^, Cristiane Lamas^2^

(1) Unigranrio/Afya, Rio de Janeiro, Brasil; (2) Instituto Nacional de Cardiologia, Rio de Janeiro, Brasil; (3) Instituto Nacional de Infectologia Evandro Chagas, Fiocruz, Rio de Janeiro, Brasil

**Introduction/Objectives:** Enterococci have increasing frequency as agents in infective endocarditis (IE) due to the population’s increasing life expectancy. They are also strongly related to infections acquired in healthcare scenarios. The present study aims to describe episodes of Enterococcal Endocarditis (EE) in a cardiac quaternary referral center, while also comparing them to other cases IE within the cohort.

**Materials and Methods:** Adult patients with definite IE according to the modified Duke criteria were included from 2006–2021 using the International Collaboration in Endocarditis case report form. Patients were identified prospectively. Categorical variables of the EE group were compared to the other patients IE in the cohort by test of proportions. Statistical significance was set in p < 0,05 threshold.

**Results:** Enterococcal Endocarditis was observed in 48 out of 435 (11%) cases, in which 2 out of 48 (4.2%) cases were identified as E.faecium, while E.faecalis accounted for the vast majority (46/48, 95.8%). Patients with EE were significantly older (median age 59 years, IQR 46–65.3) than the remaining of the cohort (median age 46.0, IQR 32.5 –61.0). A higher incidence of EE was observed in patients with coronary artery disease (13/48, 27.1% vs 47/380, 12.4%, p = 0.006), previous cardiovascular surgery (26/48, 54.2% vs 144/385, 37.4%, p = 0.025), coronary artery bypass graft surgery (6/47, 12.8% vs 19/383, 5%, p = 0.031), diabetes mellitus (12/48, 25% vs 43/387, 11.1%, p = 0.006), chronic renal failure, CRF (16/48, 33,3% vs 75/385, 19.5%, p = 0.026), and cerebrovascular disease (6/48, 12.5% vs 23/386, 6%, p = 0.087). EE patients often acquired the infection in hospital (23/48, 47.9% vs 89/386, 23.1%, p < 0.001). Furthermore, data demonstrated that early Prosthetic Valve Endocarditis (PVE) was proportionally more common in patients with EE (9/48, 18.8% vs 38/387, 9.8%, p = 0.06). New conduction abnormalities (11/43, 25.6% vs 43/349, 8.3%, p = 0.017) were more frequent in patients with EE.

**Discussion:** The frequency of EE in our cohort was similar to the literature (5 to 20%). The results define a profile of older patients with multiple comorbidities, especially CRF and diabetes, and a higher proportion of early-PVE. The observed complication of new conduction disturbances may result from PVE. We hypothesize vascular accesses were the portal of entry for enterococci, and special care must be taken with with them, especially in the perioperative period.

110905

Modality: E-Poster Scientific Initiation – Non-case Report

Category: PERICARDIUM/ENDOCARDIUM/VALVOPATHIES

## Features of Fungal Endocarditis in a Cardiac Referral Center in Rio de Janeiro, Brazil

NÍCOLAS DE ALBUQUERQUE PEREIRA FEIJÓO^1^, Thatyane Veloso de Paula Amaral de Almeida^1^, Mariana Giorgi Barroso de Carvalho^1^, Ingrid Paiva Duarte^1^, Léo Rodrigo Abrahão dos Santos^1^, Rafael Quaresma Garrido^1^, Giovanna Ferraiuoli Barbosa^2^, Clara Weksler^2^, Wilma Golebiovski^2^, Bruno Zappa^2^, Marcelo Goulart Correia^2^, Cristiane Lamas^2^

(1) Unigranrio/Afya, Rio de Janeiro, Brasil; (2) Instituto Nacional de Cardiologia, Rio de Janeiro, Brasil; (3) Instituto Nacional de Infectologia Evandro Chagas, Fiocruz

**Introduction/Objectives:** Fungi, especially candida spp., have become a frequent cause of bloodstream infections, and Fungal Endocarditis (FE) has a bad prognosis with high in-hospital mortality rates. This study aims to describe cases of FE in a cardiac quaternary referral center and compare them to other infective endocarditis (IE) cases within the cohort.

**Materials and methods:** Adult patients with definite IE according to the modified Duke criteria were included from 2006–2021 using the International Collaboration in Endocarditis case report form. Patients were identified prospectively. Categorical variables of the EF group were compared to the other patients with IE within cohort by test of proportions. Statistical significance was set in p < 0,05 threshold.

**Results:** FE occurred in 14/434 (3.2%) of IE cases; Candida spp accounted for 12/434 (2.7%), of which 8/12 (66.6%) were C. parapsilosis, 2/12 (16.6%) C. albicans, 1 (8.3%) C. tropicalis and 1 (8.3%) C.famata, and Trichosporum spp for 2/434 (0.4%). A higher frequency of FE was observed in patients who had previously been submitted to cardiovascular surgery (8/12, 66.7% vs 162/421, 38.5%, p = 0.07). Patients with FE often acquired the infection in hospital (7/12, 58.3% vs 105/422, 24.9%, p = 0.016). Vascular embolic events were more frequent in FE (9/12, 75% vs 196/412, 47.6%, p = 0.079). Specifically, peripheral embolization (3/12, 25% vs 35/421, 8.3%, p = 0.079) and mycotic aneurisms (4/12, 33,3% vs 43/420, 10.2%, p = 0.032). A higher rate of mortality was observed in patients with Fungal Endocarditis (6/12, 50% vs 103/413, 24.9%, p = 0.05). There was no difference between the rates of surgical indication (91.7% vs 86%) and performed surgery (63.6% vs 80.3%).

**Discussion:** The incidence of FE within the cohort of IE in adults was similar to studies published by other centres (2 to 4%). Non-albicans candida predominated (91.7%), especially C.parapsilosis, which differs from international studies but is similar to other centres in South America. This is probably related to hospital acquisition and the affinity of parapsilosis to catheters and prosthetic material. In fact, previous heart surgery and nosocomial IE were more frequent in FE. Emboli, particularly manifested as mycotic aneurisms, and peripheral emboli were two common complications of FE, which highlights the strong emboligenic dissemination of these agents. Mortality was higher in FE, at 50%, which is similar to the published literature.

110908

Modality: E-Poster Scientific Initiation – Non-case Report

Category: PERICARDIUM/ENDOCARDIUM/VALVOPATHIES

## Comparison of Features of S.Aureus Endocarditis to Non-S.Aureus Endocarditis in a Cardiac Referral Center in Brazil

NÍCOLAS DE ALBUQUERQUE PEREIRA FEIJÓO^1^, Thatyane Veloso de Paula Amaral de Almeida^1^, Mariana Giorgi Barroso de Carvalho^1^, Ingrid Paiva Duarte^1^, Léo Rodrigo Abrahão dos Santos^1^, Rafael Quaresma Garrido^2^, Giovanna Ferraiuoli Barbosa^2^, Clara Weksler^2^, Wilma Félix Golebiovski^2^, Bruno Zappa^2^, Marcelo Goulart Correia^2^, Cristiane C. Lamas^2^

(1) Unigranrio/Afya, Rio de Janeiro, Brasil; (2) Instituto Nacional de Cardiologia, Rio de Janeiro, Brasil; (3) Instituto Nacional de Infectologia Evandro Chagas, Fiocruz, Rio de Janeiro, Brasil

**Introduction/Objectives:** S.aureus has become the mostly encountered pathogen in series of patients with infective endocarditis (IE) from developed countries in recent years, but its role and behaviour is not so well defined in developing countries. S.aureus IE (SAIE) is described as more severe and lethal. Our aim was to describe cases of SAIE in a developing country and compare it to other cases of IE in a cohort presenting to a cardiac surgical referral center.

**Materials and methods:** Adult patients with definite IE according to the modified Duke criteria were included from 2006–2021 using the International Collaboration in Endocarditis case report form. Patients were identified prospectively. Categorical variables were presented as frequencies and percentages, and the SAIE group was compared to the other patients with IE in the cohort by test of proportions. A p-value less than 0.05 was considered statistically significant.

**Results:** S.aureus accounted for 49/434(11.3%) episodes of IE. Resistance to methicillin was seen in 8/49(16.3%) strains (5 MRSA, 3 community-acquired MRSA or ca-MRSA). Patients with SAIE more often had chronic renal failure (15/49, 30.6% vs 76/384, 19.8%, p = 0.08). Rheumatic valve disease (RVD) was less often seen in SAIE (16.3% vs 33.5%, p = 0.015). Patients with SAIE more often had hospital-acquired IE (46.9% vs 23.1%, p < .001), and devices were more often affected (24.5% vs 5.5%, p < 0.001). No differences were seen regarding the frequency of paravalvular abscess (18.4% vs 15.1%), valve perforation (14.3% vs 18.8%) or fistulae (4.1% vs 4.2%). Complications more often found in SAIE were persistent bacteremia (23.9% vs 5.6%, p < .001), peripheral emboli (18.8% vs 7.5%, p = 0.01) and recurrent emboli (19.1% vs 5.1%, p < .001). Acute renal failure 44.7% vs 32.5%, p = 0.097) and need for hemodialysis (52.2% vs 25.0%, p = 0.008) were more often seen in SAIE. Rates of surgical indication (83.3% vs 86.5%), surgery (72.7% vs 80.7%) and mortality (28.6% vs 25.3%) were similar between groups.

**Discussion:** S.aureus was the third mostly encountered causative agent in IE in our center, and was mostly associated with healthcare acquisition and intracardiac devices. Surgical rates and mortality were similar to other IE, possibly because of referral bias, but recurrent emboli, persistent bacteremia and acute renal failure were more frequent in SAIE, highlighting its acute presentation with sepsis.

110914

Modality: E-Poster Scientific Initiation – Non-case Report

Category: ATHEROSCLEROSIS/CARDIOVASCULAR RISK FACTORS/CARDIOVASCULAR PREVENTION

## Cardiovascular Risk in the Municipality of Sertã

CATARINA RAQUEL GASPAR DOS SANTOS^1^, Catarina Santos^1^, Patrícia Coelho^1^, Francisco Rodrigues^1^

(1) Instituto Politécnico de Castelo Branco

Arterial hypertension (HTA) is one of the main risk factors for cerebrovascular diseases, representing a high incidence rate in the world population. In this way, it becomes increasingly important to make people aware of the importance of hypertension and the risk factors that it entails. As such, the objective of this investigation was to assess the prevalence of hypertension in the municipality of Sertã. The present research study is of the prospective type. The sample collection was based on the cluster method, through the random selection of streets in the municipality of Sertã. Anthropometric data were collected, 3 blood pressure assessments were performed, with an interval of 3 minutes between each, and a survey on risk factors was applied. Statistical treatment and data analysis were performed using the SPSS Statistics program. The sample is composed of 1000 individuals, of legal age and residing in the municipality under study, with 515 (52%) of the participants being male and 485 (48%) females. The mean age was between 56.04 years. Regarding the prevalence of arterial hypertension, a percentage of 43.6% was found, and this result includes all individuals who presented mean SBP and/or DBP values above normal values (140/90 mmHg, respectively) and all individuals who take antihypertensive medication. About individuals who reported performing associated therapy, 165 individuals (60.2%) had controlled hypertension, however, 109 (39.8%) had uncontrolled hypertension. Regarding risk factors, the most affluent were obesity (61.7%), physical inactivity (48.2%) and family history of hypertension (42.3%). It is concluded that the present study found that there is a significant prevalence of hypertensive patients in this region, which may be related to the lack of control of risk factors.

110920

Modality: E-Poster Scientific Initiation – Non-case Report

Category: CARDIOVASCULAR PHARMACOLOGY

## Analysis of the Effects of Antidepressive Agents on Blood Pressure: A Systematic Review

GABRIELA DANTAS MACHADO LEAL^1^, Fernanda Dantas Machado Leal^1^, Marília Menezes Gusmão^1^

(1) Escola Bahiana de Medicina e Saúde Pública

**Introduction:** The prevalence of depression and the use of antidepressive agents have been increasing worldwide over the last few years. Hypertension is another clincal condition of high prevelence in which it is common to be associated with drepression.

**Objective:** Avaluate by a systematic review, the likelihood of antidepressive agents effecting blood pressure, the study will also evaluate what are the classes of antidepressive agents that are related to the deacrease or increase of blood pressure value. Methods and materials: searches were conducted in databases such as PubMed and Scielo, and selected papers in humans, which included the use of antidepressive agents for depression treatment and blood pressure mesurement. Researches that have used other medications that were not antihipertensive agents, but could have potentially altered blood pressure measurement, were excluded. The review protocol was registeres in PROSPERO. The protocol registration number is CRD42020194118.

**Results:** The final sample consisted of seven articles. In three of them, the use of antidepressive agents did not cause significant changes on the value of blood pressure. One study detected blood pressure drop after the use of antidepressive agents. While three other studies detected blood pressure spike after the use of antidepressive agents.

**Conclusion:** Selective serotonin reuptake inhibitors (SSRIs) e serotonina and norepinephrine reuptake inhibitors (SNRIs) are not capable of influencing and altering blood pressure value, however, GSK372475 is capable of increase blood pressure value.

111006

Modality: E-Poster Scientific Initiation – Non-case Report

Category: CARDIOVASCULAR IMAGING

## Comprehensive Echocardiographic Assessment with Global Longitudinal Strain in a Cohort of On-Treatment Acromegalic Patients

CAROLINA MENDES PEREIRA^1^, Camila Pereira de Carvalho^2^, Clara Alice Lima Leal^1^, Luis Augusto Oliveira Santos^1^, Breno Carvalho Cirne de Simas^1^, Ferdinand Gilbert Saraiva da Silva Maia^1^, Josivan Gomes de Lima^1^, Lucia Helena Coelho Nóbrega^2^, Ricardo Luiz de Medeiros Lima^2^

(1) Federal University of Rio Grande do Norte; (2) University Hospital Onofre Lopes

**Introduction:** Acromegaly is a rare disease characterized by high levels of growth hormone (GH) and insulin-like growth factor (IGF-1). Cardiovascular disease, especially heart failure, is a common complication in acromegaly.

**Objectives:** Perform a comprehensive echocardiographic assessment of acromegalic patients and relate systolic function indexes with disease activity.

**Methods:** Bidimensional echocardiographic assessment of left ventricular geometry, Ejection Fraction (EF, determined by Simpson’s method), Global Longitudinal Strain (GLS, using speckle-tracking technique) and diastolic function were performed in on-treatment acromegalic patients without established cardiovascular disease. Patients with poor acoustic window, atrial fibrillation and oncologic treatment (systemic chemotherapy or thoracic radiotherapy) were excluded. Ejection fraction and GLS were compared in patients with active and remitting disease.

**Results:** 28 acromegalic patients were included (17 women and 11 men; mean age 42,8 ± 11,5 years; mean time from diagnosis 7 ± 4.9 years). Thirteen patients were in biochemical remission, 11 had active disease and 4 had elevated GH level with normal IGF-1 (indeterminate pattern). Structural and/or functional cardiac alterations were present in 16 patients (57.8%). Eleven patients (39.28%) exhibited abnormal geometry (7 with eccentric hypertrophy, 3 with concentric hypertrophy and 1 with concentric remodeling). Mean EF was 58.8% (±6.3). Only one patient had mildly reduced EF. Mean GLS was 20.5 ± 2.8. Seven patients (25%) had below normal GLS (6 patients had borderline and 1 had reduced GLS). Four patients (14.28%) presented alteration in diastolic function indexes (2 with type 1 and 2 with indeterminate diastolic dysfunction). EF (57.4 ± 7.39 vs 59.5 ± 6.2, p = 0.44) and GLS (19.8 ± 3.1 vs 20.4 ± 2.7, p –0.60) were not different in patients with active or remitting disease.

**Conclusions:** In this cohort of on-treatment acromegalic patients without established cardiovascular disease, structural and/or functional echocardiographic alterations were frequent. Left ventricular hypertrophy is the most frequent alteration. Ejection fraction and global longitudinal strain were not different in patients with active or remitting disease.

110923

Modality: E-Poster Scientific Initiation – Non-case Report

Category: NEGLECTED CARDIOVASCULAR DISEASES

## Ring Shaped Neutrophils from Peripheral Blood of Patients with Chagas Disease:Correlations with Echocardiogram Parameters

LUANN GABRIEL PORPINO SALES^1^, Pedro Felipe Alves de Souza^1^, Felipe Alves de Lima^1^, Marcelo Augusto Araújo De Castro^1^, Henrique Rangelly Gabriel de Melo^1^, Leonardo Lacerda do Amaral^1^, Rafaela Germana Cavalcanti da Nóbrega^2^, Micássio Fernandes de Andrade^1^, Amanda Estevam Carvalho^1^, Valéria Duarte de Almeida^1^, Cléber Mesquita Andrade^1^

(1) Universidade do Estado do Rio Grande do Norte (UERN); (2) Centro Universitário de João Pessoa (UNIPÊ)

**Introduction:** Neutrophils are cells with a typical polylobulated nucleus, capable of modulating the immune response in Chagas disease (CD). In some occasions, these cells may have a ring shaped nucleus, typical of immature leukocytes with different performing mechanisms, and your greater incidence be associated with solid tumor cancers and CD.

**Objective:** Evaluate the morphology of the peripheral blood neutrophils nucleus of patients with Chagas disease, in the different clinical forms of the disease.

**Methods:** Transversal study with non-probabilistic sampling. A total of 32 volunteers participated in the research, being 11 individuals with indeterminate clinical form of the disease and 21 individuals with cardiac clinical form. The analysis of the morphology of the nucleus of the polymorphonuclear cells were performed by light microscopy. The identification of the clinical forms and the analysis of the echocardiogram were performed by analyzing the filed medical records. Spearman and Pearson tests were used to determine the correlations between the absolute number of ring neutrophils and echocardiographic parameters.

**Results:** A negative correlation was observed between the absolute number of ring neutrophils in the patients‘ peripheral blood and the left ventricular (LV) ejection fraction (*p = 0.0031 and r = –0.7087). However, regarding the other echocardiographic parameters of LV Systolic Diameter (*p = 0.0009 and r = 0.8040), LV Diastolic Diameter (*p = 0.0250 and r = 0.5942) and Mass Index of the LV (*p = 0.0351 and r = 0.5655), a positive correlation was noticed concerning the absolute number of annular neutrophils.

**Conclusion:** Ring shaped neutrophils from patients with CD may contribute to the physiopathology of Chronic Chagas Heart Disease.


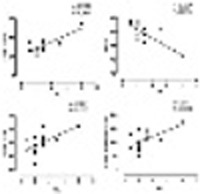


110948

Modality: E-Poster Scientific Initiation – Non-case Report

Category: ATHEROSCLEROSIS/CARDIOVASCULAR RISK FACTORS/CARDIOVASCULAR PREVENTION

## Study Prev.Cardio.Cv – Ilha De Santiago

KELLY ANNY FERNANDES MASCARENHAS^1^, Patrícia Coelho^1^, Francisco Rodrigues^1^, Júlio Rodrigues^2^

(1) Instituto Politécnico de Castelo Branco; (2) Ministério de Saúde Cabo Verde

**Introduction:** Cerebrocardiovascular diseases represent a meaningful death rate worldwide, making a big challenge to public health and the economy. Many African countries like Cabo Verde are facing an epidemiologic transition from transmitted diseases to non-transmitted diseases, demographic changes as well as socio-economic changes, and are facing rapid growth in terms of cardiovascular diseases and are linked to rising in cardiovascular risk.

**Objectives:** This present study had as its objective to identify the prevalence of risk factors of cardiovascular diseases in adults residing on the island of Santiago-Cabo Verde.

**Methodology:** The transversal study of a population background of individuals over 18 years of age, from both genders. The data collection took place in October and November 2021, in the nine municipalities of Santiago Island- Cabo Verde. It was excluded from the study the individuals of Cape Verdean nationality who have been living for over a year abroad and that have just arrived on Santiago Island in less than 30 days, as well as the individuals with any kind of disabilities that may affect their participation in the study. The sample size was calculated based on the population projection of the year of Santiago Island, for those over 18 years of age according to the appropriate epidemiologic sample. The field data (domiciliary) were obtained through a questionnaire application where ages, sex, race, weight, height, risk factors, (diabetes presence, dyslipidaemias, hereditariness, physical exercise practices, smoking habits, cerebrovascular antecedent events, weight control habits, and blood pressure) are considered.

**Results:** The sample of the study is mostly made of females representing 54.8%. Furthermore, the age group with the highest sample number was between 18–27 years old, corresponding to (21%). 42.2% with BMI ≥ 25 kg/m^2^; 65.1% of physical inactivity; 7.3% with Tabaco, 14.4% with alcohol, 6.5% with cardiac diagnosed diseases, 19.9% with close family with cardiac pathology, 2.7% have had cerebral vascular, 0.1 has already suffered an ischemic transitory accident, 0.5% myocardial infarction, and 23.5% had a periodic habit of checking the blood pressure, 32.6% hypertensives and 4.5% of diabetics.

**Conclusion:** The result of the study allowed us to affirm that there’s a high prevalence of risk factors for cerebrovascular accidents in the island of Santiago-Cabo Verde, especially among women.

110988

Modality: E-Poster Scientific Initiation – Non-case Report

Category: PERICARDIUM/ENDOCARDIUM/VALVOPATHIES

## Late Prosthetic Valve Endocarditis in a Cohort of Adults in a Quaternary Surgical Referral Center in Brazil

THATYANE VELOSO DE PAULA AMARAL DE ALMEIDA^1^, Mariana Giorgi Barroso de Carvalho^1^, Nícolas de Albuquerque Pereira Feijóo^1^, Ingrid Paiva Duarte^1^, Léo Rodrigo Abrahão dos Santos^1^, Rafael Quaresma Garrido^2^, Giovanna Ferraiuoli Barbosa^2^, Clara Weksler^2^, Wilma Golebiovski^2^, BRUNO ZAPPA^2^, Marcelo Goulart Correia^2^, Cristiane C. Lamas^3^

(1) Unigranrio/Afya, Rio de Janeiro, Brasil; (2) Instituto Nacional de Cardiologia, Rio de Janeiro, Brasil; (3) Instituto Nacional de Infectologia Evandro Chagas, Fiocruz, Rio de Janeiro, Brasil

**Introduction:** Prosthetic valve infective endocarditis (IE) has high morbidity and mortality. Late prosthetic valve IE (LPVE), defined as that acquired 12 or more months after prosthesis implantation, often has as causative agents’ community pathogens. Our objective was to describe cases of LPVE and compare them to other cases of IE.

**Methods:** Adult patients with definite IE according to the modified Duke criteria were included from 2006–2021 using the International Collaboration in Endocarditis case report form. Statistical analysis was performed using the Jamovi and R software.

**Results:** Of the 435 patients with IE, 77 (17.7%) had LPVE. They were older, with a median age of 52 years (IQR 38–68), compared to 47 years (IQR 32–61). Enterococci were more frequent in LPVE (29.2% vs 16.3%, p = 0.027), but negative blood cultures were less frequent (11.9% vs 20.3%, p = 0.036). In LPVE, infection was community-acquired in 75.3%, and hospital-acquired (HA) in 16.9%. Patients with LPVE more often had a history of coronary artery disease (21.1% vs 12.5%, p = 0.051), coronary artery bypass surgery (12.2% vs 4.5%, p = 0.01), congestive heart failure (59.2% vs 35.9%, p < 0.001), and cerebrovascular disease (17.1% vs 4.5%, p < 0.001); chronic renal failure was less frequent in LPVE (11.7% vs 23.0%, p = 0.027). Rheumatic valvular heart disease (RVD) was observed more frequently in LPVE (57.9% vs 25.7%, p < 0.001). Aortic and mitral involvement was frequent in both groups. The frequency of abscess (23.7% vs 13.7%, p = 0.029) and conduction disturbances (25.7% vs 11.2% p = 0.001) was higher in LPVE. Heart failure secondary to acute valve regurgitation was equally frequent (56.6% vs. 61.6%), as were neurological events (40.5% vs. 50.0%), including mycotic aneurysms (6.5% vs 11.8%). Splenic emboli were frequent in both groups (32.5% vs. 35.8%). Surgical indication occurred less frequently (78.9% vs 87.7%, p = 0.046), as well as surgery (67.2% vs 82.4%, p = 0.005). In-hospital death was higher in LPVE (39.5% vs 22.6%, p = 0.002).

**Conclusion:** LPVE had a frequency of 17.7%, like other series in the literature (10–30%). However, it showed a high prevalence of VHD, as in other developing countries where it is the main reason for valve replacement. Clinical features were like other IE, except for the presence of abscesses, and mortality was very high, possibly due to patients being older, having more underlying illnesses and less often having valve replacement surgery for IE.

110991

Modality: E-Poster Scientific Initiation – Non-case Report

Category: NEGLECTED CARDIOVASCULAR DISEASES

## The Prognostic Value of the Electrocardiogram in Chagas Disease Serology Evolution

GUILHERME DANTAS CAMPOS PINTO^1^, Silvia Marinho Martins^2^, Cristina de Fátima Velloso Carrazzone^2^, Maria das Neves Dantas da Silveira Barros^1^, Carolina de Araújo Medeiros^2^, Gabriela Arcoverde Wanderley^1^, Gustavo Sales Santa Cruz^1^, Gabrielly Nascimento de Lima^1^, Virginia Maria Barros de Lorena^3^, Luciane de Freitas Firmino^3^, Maria da Gloria Aureliano de Melo Cavalcanti^2^, Wilson Alves de Oliveira Júnior^2^

(1) Universidade de Pernambuco (UPE); (2) Ambulatório de Doença de Chagas e Insuficiência Cardíaca – PROCAPE/UPE; (3) Fundação Oswaldo Cruz (Fiocruz)

**Introduction:** Changes in the electrocardiogram (ECG) are markers of severity and prognosis in chronic Chagas disease (CD). However, in the acute form, the prognostic value of the ECG before and after treatment with benznidazole, as a tool to analyze serological evolution, has not been well established yet.

**Objective:** To verify the association between electrocardiographic changes and serology evolution in patients with acute CD.

**Methodology:** This is a cohort study with 25 patients infected by T. cruzi in an outbreak in a city in the Brazilian Northeast. The population was followed up at a referral outpatient clinic in Pernambuco State, from acute phase treatment until 24 months (+/- 6) after benznidazole administration. The diagnosis was confirmed by serological methods and/or molecular tests. ECGs and serological tests were performed before and after treatment. Then, the remission, maintenance, and appearance of electrocardiographic changes were determined, as well as the serological evolution of each patient. The ECGs were evaluated by two professionals – one of whom is an expert in the area. P-value < 0.05 indicates a statistically significant association.

**Results:** The population was mostly female (56%), with a mean age of 30 years (SD = 11.5) and without comorbidities (92%). The prevalence of ECG changes after 30 and 60 days of the infection was 56%. They were: altered cardiac repolarization (ACR) (20%), electrical axis deviation (20%), low QRS voltage (20%), incomplete right bundle branch block (IRBBB) (8%), left anterior fascicular block (LAFB) (8%) and first-degree atrioventricular block (8%). On reassessment of the ECGs after 2 years, 48% had some alteration: low QRS voltage (32%), IRBBB (12%), LAFB (8%), left anterior fascicular block (8%), and chaotic atrial rhythm (8%). When follow-up ECG was compared to pretreatment exam, 7 remained normal; 5 remained altered; 6 which were previously altered were normalized, and 7 presented some new alteration. Fifteen patients (60%) showed positive IgG for T. cruzi. In the acute phase, 100% of patients who had ACR before treatment had positive serology tests after 2 years (p = 0,061). There was no statistical association between serology results and the follow-up ECG.

**Conclusions:** ECG changes in the acute phase showed no power of association with post-treatment CD serology. It is considered relevant to elucidate the predictive value of ACR in the acute phase for seroconversion, through novel studies.

111000

Modality: E-Poster Scientific Initiation – Non-case Report

Category: NEGLECTED CARDIOVASCULAR DISEASES

## Prognostic Value of Brain Natriuretic Peptide in Patients with Chagas Cardiomyopathy from Endemic Areas: Sami-Trop Cohort Study

ADMILSON LEMOS DA COSTA FILHO^1^, Marcelo Alves Maia^2^, Ester Cerdeira Sabino^3^, Ana Isabel Nobre Maia^4^, Artur Lima Sendin^1^, Kássia Burini^1^, Fellipe Colares P G Versiani^4^, Antonio Luiz P Ribeiro^2^, Maria Carmo P Nunes^2^

(1) School of Medicine, Universidade Federal de Minas Gerais, Belo Horizonte, MG, Brazil.; (2) Post Graduation Program in Infectious Diseases and Tropical Medicine, School of Medicine, Universidade Federal de Minas Gerais, Belo Horizonte, MG, Brazil.; (3) Instituto de Medicina Tropical e Departamento de Moléstias Infecciosas e Parasitarias da Faculdade de Medicina da Universidade de São Paulo, São Paulo, Brazil.; (4) Health Science Program, Universidade Estadual de Montes Claros, Montes Claros, Brazil.

**Background:** Chagas cardiomyopathy is an important cause of heart failure in endemic areas. Natriuretic peptides are well established in the diagnostic of heart failure, but their prognostic value is not well defined, especially in community-based Chagas patients.

**Objective:** This study aims to assess the prognostic value of N-terminal pro-brain natriuretic peptide (NT-proBNP) in predicting mortality in patients with Chagas cardiomyopathy from remote areas.

**Methods:** Patients with Chagas disease from endemic areas who had heart failure defined as left ventricular ejection fraction (LVEF) <50% and/or NT-ProBNP >300 pg/ml were eligible for the study. Clinical data were obtained using a standardized questionnaire. A resting 12-lead electrocardiogram was recorded at baseline. A range of readily obtained echocardiographic measures were collected using portable equipment at public health primary centers. The end point was all-cause mortality.

**Results:** The study cohort consisted of 370 patients with a mean age of 66.2 ± 12.7 years, and 210 patients (57%) were women. The majority of the patients had left ventricular (LV) systolic dysfunction with mean ejection fraction of 41 ± 12%. During a mean follow-up of 31 months, 133 patients died (36%) with overall mortality incidence rate of 39.6 deaths per 100 patient-years. Four key echocardiographic parameters were predictors of mortality, including LVEF (HR 0.97, 95% CI 0.95 to 0.98), right ventricular (RV) end-diastolic area (HR 1.06, 95% CI 1.03 to 1.09), E/e’ ratio (HR 1.03, 95% CI: 1.01 to 1.05), and left atrial (LA) volume (HR 1.01, 95% CI 1.00 to 1.01). The inclusion of NT-ProBNP on top of echocardiographic parameters resulted in significant improvement in model performance.

**Conclusions:** In a contemporary cohort of patients with Chagas cardiomyopathy, NT-proBNP was a strong predictor of death, independently of LV dysfunction severity and RV involvement. NT-proBNP assessment may be used in a clinical setting to improve the risk prediction model for mortality in patients with Chagas cardiomyopathy.

111010

Modality: E-Poster Scientific Initiation – Non-case Report

Category: CARDIOVASCULAR INTENSIVE CARE/CARDIOVASCULAR EMERGENCIES

## Consensus and Disparities Regarding Clinical Protocols for Cardiogenic Shock: A Systematic Review

THIAGO FONSECA DE AZEVEDO^1^, Ádria Rayane Lima Cascaes^1^, Leonardo Rodrigues Ferreira Diogo^1^, Pablo Rodrigues Nunes de Souza^1^

(1) Universidade do Estado do Pará (UEPA)

**Introduction:** In the research about cardiac pathologies, cardiogenic shock (CS) stands out, given its complexity and high incidence (between 5 and 15% of patients with acute myocardial infarction). In general, CS is the inability of the heart to perform a tolerable cardiac output to oxygenate and nourish tissues. In this sense, understanding the processes of this cardiological condition is important, as there is still a high mortality associated with it (about 50%). Despite this, it is observed that the treatment of cardiogenic shock is not standardized and doubts persist regarding its therapeutic protocol. Therefore, it is important to understand the therapeutic methods associated with cardiogenic shock, in order to manifest a logical and temporal framework in the progression of the disease and in the improvement or worsening of the patient.

**Objective:** To assess the most used clinical treatment protocols for cardiogenic shock.

**Methods:** Systematic review without meta-analysis, using the descriptors “Clinical protocol” and “Cardiogenic shock”. The search was carried out in the PubMed, LILACS and Cochrane databases. As inclusion criteria, articles in English limited to the last five years (2018–2022) were accepted. Studies such as randomized clinical trials, cohort, case-control and observational studies were selected. Articles without abstract or full text available and duplicates were excluded.

**Results:** Critical analysis of the collected data revealed the absence of a standardized protocol for CS across centers, together with a high post-discharge mortality despite advances in the field; however, some factors, such as early invasive hemodynamics and percutaneous coronary intervention, as well as shock team-based protocols, were correlated with higher rates of survival and better prognosis, while the use of intra-aortic balloon pump didn’t show advantages in several studies, and is accordingly not recommended anymore for management of CS.

**Conclusion:** The present study concludes that ECMO, Inotropic Agents, Impella 5.0 and percutaneous hemodynamic support were prevalent in the studies analyzed. In Addition, intra-aortic balloon pump and the creation of a Shock Team were used as a way to improve prognosis in patients with cardiogenic shock. The disparity of results showed the necessity of clinical trials and a clearer Cardiogenic Shock clinical protocol in order to establish a better CS clinical management.

111002

Modality: E-Poster Scientific Initiation – Non-case Report

Category: CARDIAC ARRHYTHMIAS/ELECTROPHYSIOLOGY/ELECTROCARDIOGRAPHY

## Postural Orthostatic Tachycardia Syndrome: A Challenging Diagnosis

EDUARDO AUGUSTO QUIDUTE ARRAIS ROCHA^1^, Marcela Sobreira Kubrusly^1^, Luís Gustavo Bastos Pinho^3^, Vera Marques^4^, Francisca Tatiana Moreira Pereira^4^, Bruna Sobreira Kubrusly^3^, Davi Sales Pereira Gondim^2^, Fernanda Pimentel Arraes Maia^3^, Rodrigo Carvalho Paiva^1^, Maria Eduarda Quidute Arrais Rocha^2^, Arnóbio Dias da Ponte Filho^4^, Roberto Lima Farias^3^

(1) Centro Universitário Christus (Unichristus); (2) Universidade de Fortaleza (Unifor); (3) Universidade Federal do Ceará (UFC); (4) Centro de Arritmia do Ceará (CACE)

**Introduction:** Postural Orthostatic Tachycardia Syndrome (POTS) represents one form of clinical presentation among neurally mediated syndromes, with several peculiar characteristics. Many aspects of this syndrome still remain unknown. This study aimed to analyze the characteristics of patients with clinical suspicion of POTS.

**Methods:** Retrospective cohort study, conducted in the period 2016–22. Statistical analysis was performed in the stats models module. To obtain odds ratios, we used logistic regression models (for positivity as a response) and multinomial logistic regression models (for the type of response). Other comparisons were made using the Mann-Whitney test and Fisher’s exact test, with p-value of 5% considered significant.

**Results:** The mean age for the 36 patients with a POTS response was 35.3 ± 0.45 years; 72.2% (26) were male. Among the symptoms that motivated the TT, in the POTS group, we had 69.4% (25) with syncope, 8.3% (3) presyncope, 8.3% (3) with a previous suspicion of POTS, 8.3% (3) with dizziness and 5.5% (2) unknown. None of the patients had associated pathology or reported previous medication use. The complication rate was 2.7% (1). The total number of tilt tests performed were 2462, with 115 exclusions, 37.5% of which were male, with a median age of 51.1 (31–71) years, with 66.31% TT with normal response and 33.69% positive TT. The types of response were 16.22% (377) hypotensive, 11.7% (272) mixed, 2.49% (58) dysautonomic, 1.55% (36) POTS, 1.11% (26) cardioinhibitory, 0.47% (11) psychogenic. A total of 14 patients had a previous suspicion of POTS, with 42.8% (6) presenting a positive TT, while 57.1% (8) a negative one. In the group that did not have a suspicion of POTS, the values were, respectively, 32.7% (756) and 67.2% (1554) (p = 0.47). The symptom reproduction rate in patients with a POTS response was 52.7% (19) versus 47.2% (17).

**Conclusion:** The Postural Orthostatic Tachycardia Syndrome represented a small percentage of the TT exams performed, predominating in young patients, with a positive response on the TT, even in the absence of clinical suspicion, with reproduction of symptoms in most cases and a low rate of complications in the exam. POTS is usually a challenging diagnosis.

111057

Modality: E-Poster Scientific Initiation – Non-case Report

Category: HYPERTENSION/RENAL DENERVATION

## Inflammatory Dynamics in Brazilian Indigenous People with and Without Arterial Hypertension: A Multivariate Network Approach in the Project of Atherosclerosis Among Indigenous Populations (PAI)

MANOEL PEREIRA GUIMARÃES^1^, Carlos Dornels Freire de Souza^1^, Rodrigo Feliciano do Carmo^1^, Ricardo Khouri^2^, Sávio Luiz Pereira Nunes^3^, Jandir Mendonça Nicacio^1^, Ana Marice Teixeira Ladeia^4^, João Augusto Costa Lima^5^, Anderson da Costa Armstrong^1^, Manoel Barral-Netto^2^

(1) Universidade Federal do Vale do São Francisco (UNIVASF); (2) Instituto Gonçalo Moniz/FIOCRUZ; (3) Universidade de Pernambuco (UPE); (4) Escola Bahiana de Medicina e Saúde Pública; (5) Johns Hopkins University

**Introduction:** Atherosclerotic disease and its risk factors show relationships with inflammatory changes. The inflammatory mediation of cardiovascular risk in indigenous groups undergoing a rapid process of urbanization is still unknown.

**Objective:** To describe the inflammatory dynamics of two indigenous groups in Northeast Brazil with and without arterial hypertension.

**Methods:** Cross-sectional ancillary study (2016–2017) involving 302 indigenous people from the Fulniô (n = 210) and Truká (n = 92) ethnic groups (Pernambuco, Brazil). Sociodemographic data and samples of blood to measure the inflammatory markers IL-8, MPO, Tenascin-C2, Trail, FGF-2 and Lox-1. For the analysis, multivariate network analysis was used by the Leats absolute shrinkage and selector operator (LASSO)- with non-parametric transformation. This project is part of the PAI-Study (Integral Indigenous Care Project).

**Results:** The prevalence of arterial hypertension was 23.5%. (n = 71) [20.0% in Fulniô and 31.5% in Truká] in hypertensive patients, a positive correlation was observed between Tenascin-C2 and FGF-2, which was not present in non-hypertensive indigenous people. There is a stronger positive correlation between TRAIL and IL-8 than the obs herbed in non-hypertensive patients. On the other hand, the correlation between FGF-2 and PAI-1 was stronger in non-hypertensive individuals.

**Conclusions:** Differences were found in the inflammatory dynamics of hypertensive and non-hypertensive indigenous people, with emphasis on the positive correlation between Tenascin-C2 and FGF-2.


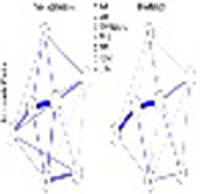


111062

Modality: E-Poster Scientific Initiation – Non-case Report

Category: HYPERTENSION/RENAL DENERVATION

## Differences in Hypertension Diagnosis and Control in Public and Private Health Sectors in Northeast Brazil using Home Blood Pressure Monitoring

MANOEL PEREIRA GUIMARÃES^1^, Antônio Marconi Leandro da Silva^1^, Elder Gil Alves da Cruz^2^, Matheus Pereira Barreira^1^, Everardo Joaquim Gonçalves dos Santos^1^, Frank Land Carvalho^1^, Fernando Marcos França^3^, Audes Diógenes de Magalhães Feitosa^4^, Rodrigo Feliciano do Carmo^1^, Jeová Cordeiro de Morais Júnior^1^, Carlos Dornels Freire de Souza^1^, Anderson da Costa Armstrong^1^

(1) Universidade Federal do Vale do São Francisco (UNIVASF); (2) Clínica do Coração Dr. Elder Gil; (3) CARDIOVASF – Instituto do Coração do Vale do São Francisco; (4) Universidade Federal de Pernambuco (UPE)

**Introduction:** Validated home blood pressure monitoring (HBPM) is not widely available in Brazil. In the places HBPM is available, it is unknown how Brazilian inequalities in access to health may impact on hypertension diagnosis and control.

**Objectives:** To compare hypertension diagnosis and control in public and private health sectors in an unfavored outland area in Northeast Brazil (Sertão; Pernambuco).

**Methods:** Longitudinal, multicenter study. Information on sex, age, body mass index (BMI), antihypertensive medications, office BP and HBPM was collected using the TeleMRPA platform, between September 2017 and February 2022. All equipment was validated and calibrated. All participants were classified according to the Brazilian Guidelines on Hypertension and grouped following the health sector (public vs. private). Data was also collected in recurrent exams up to six months. For data analysis, the Shapiro-Wilk, Mann-Whitney and chi-square tests were used.

**Results:** 2,261 individuals were included. People from the public sector were younger (58.3 ± 15.7 vs 55.5 ± 15.2 years; p = 0.001), had higher BMI (29.6 ± 5.8 vs 28.1 ± 5.2 g/m^2^; p < 0.001), and used higher number of BP pills (1.6 ± 1.3 vs 0.9 ± 1.0 pill; p < 0.001). Male participants were more prevalent in the private sector (37.2%vs31.4%; p = 0.019). In the first HBPM performed, 54.9% of the public sector group had BP out of control, compared to 44.0% (p < 0.001) in the private sector. During follow-up, 50 participants enrolled in the new HBPM within six months of the first exam. For the second HMBP, 47.1% of the public sector group had BP out of control compared to 54.5% of the private sector (p = 0.616).

**Conclusion:** Hypertensive participants enrolled from the public health sector had a more severe disease profile in the initial assessment but were similar to those from private sector in the 6-month follow-up period, when HBPM was used. The wide use of HBPM may aid reducing inequalities in hypertension treatment in Brazil.

111103

Modality: E-Poster Scientific Initiation – Non-case Report

Category: HEART FAILURE/CARDIOMYOPATHY/TRANSPLANT

## The Prognostic Value of QRS Axis in Chronic Heart Failure with Reduced Ejection Fraction

GABRIELA ARCOVERDE WANDERLEY^1^, Guilherme Dantas Campos Pinto^1^, Gustavo Sales Santa Cruz^1^, Felipe José Gomes Pereira de Lucena^1^, Larissa Cassiano de Araújo^1^, Maria da Glória Aureliano Melo^2^, Maria Elisa Lucena Alves^2^, Maria das Neves Dantas Silveira Barros^2^, Wilson Alves de Oliveira Júnior^2^, Silvia Marinho Martins Alves^2^

(1) Universidade de Pernambuco; (2) Casa do Paciente Portador de Doença de Chagas e Insuficiência Cardíaca (PROCAPE)

**Introduction:** Heart failure is a high mortality clinical syndrome. The electrocardiogram (EKG) is an assessable exam with low cost, very useful in the evaluation of heart failure patients. The QRS axis, determined by EKG, is a possible prognostic tool.

**Objective:** To verify the prognostic value of the QRS axis in patients with heart failure with reduced ejection fraction (HFrEF).

**Methodology:** Cohort study with 110 patients with HFrEF (EF ≤40% in a last year echocardiogram -ECHO). QRS axis was determined by the Hexaxial Reference System and was classified in: normal (–30° to +90°), left axis deviation (–90° to –30°), right axis deviation (+90° to +180°) and northwest axis (–90° to –180°). To prognostic analysis was considered: funcional class (New York Heart Association), left ventricular end-diastolic diameter (LVEDD) on ECHO and right ventricle systolic function (RVSF), evaluated by tricuspid annular plane systolic excursion on ECHO and classified in reduced RVSF and normal RVSF. P-value <0,05 indicates a statistically significant association and a 95% confidence interval was considered.

**Results:** The population was predominantly male (58%) and brown (“pardos”, 68%) with a mean age of 57 years (29–82, SD = 11,5). Low education levels were present (70% of the patients dropped out in Primary or Secondary School). The most prevalent comorbidities were overweight/obesity (67%), arterial hypertension (61%), dyslipidemia (46%) and diabetes (31%). HFrEF most frequent etiologies were Chagas Cardiomyopathy (31%), Hypertensive Heart Disease (19%) and Isquemic Heart Disease (17%). Mean ejection fraction was 29% (12–40%, SD = 6,8). Mean LVEDD was 65 mm (30–90, SD = 8,5) and 38% of the patients had reduced RVSF. About QRS axis: 48% had normal axis, 36% had left axis deviation, 10% had right axis deviation and 5,5% had the northwest axis. Neither the functional class (p = 0,174) nor LVEDD (p = 0,623) were statistically associated with QRS deviation. QRS axis deviation was associated with RVSF (p = 0,013) and patients with right axis deviation (OR = 9,5) or left axis deviation (OR = 1,3) had more chances of presenting reduced RVSF when compared with patients with normal axis. However, the northwest axis was less likely to have reduced RVSF when compared with normal axis (OR = 0,03).

**Conclusion:** QRS axis deviation was only associated with RVSF.

111278

Modality: E-Poster Scientific Initiation – Non-case Report

Category: CARDIOVASCULAR PHARMACOLOGY

## Evaluation of the use of Antihypertensive Drugs by Mothers During Pregnancy of Children with Congenital Heart Disease

GEORGIA MARQUES JARDIM^1^, Bianca Brinques da Silva^2^, Guilherme Rodrigues Viana^1^, Carolina Guimarães Herzog^1^, Eric Seiji Kanai^1^, Grasiele do Amaral Martins^1^, Carolina Andreatta Gottschall^2^, Egídio Junior Lorenzetti Ruggini^1^, Helena Guedes da Rocha^1^, Letícia Vieira Senger^1^, Rafael Fabiano Machado Rosa^1^

(1) Universidade Federal de Ciências da Saúde de Porto Alegre- UFCSPA; (2) Universidade Luterana do Brasil – ULBRA

**Introduction:** Congenital heart diseases (CHDs) are abnormalities in the structures or functions of the heart that can compromise the individual in all age groups. In this sense, they are a public health problem, since early diagnosis is crucial for patient survival.

**Objective:** To evaluate the use of antihypertensive drugs according to their risk classification in pregnancies of fetal CHD patients.

**Method:** The study sample consisted of 198 patients, who were consecutively evaluated during their first hospitalization in a cardiac intensive care unit. The participants were submitted to a protocol that evaluated the use of antihypertensives during their pregnancy. These were divided according to their fetal risk, following the classification proposed by the Food and Drug Administration (FDA). The data analysis considers high risk the exposure of children to drugs that have pharmacological classes D and X.

**Results:** Of the total sample, 103 patients (52%) were male, with ages ranging from 1 to 4934 days. The interview for data collection was carried out with the patient’s mother in 48.5% of cases and with both parents in 42.9%. Most of the patients are users of the brazilian national health service. The most frequently observed CHDs were ventricular septal defect (16.1%) and atrial septal defect (16.1%). Furthermore, 124 patients (62.6%) were exposed to at least one drug during pregnancy. 18 mothers (9%) reported using antihypertensive drugs during pregnancy, which included methyldopa (n = 7–3.5%), enalapril (n = 4–2%), hydrochlorothiazide (n = 3–1.5%), verapamil (n = 2–1%) and propranolol (n = 2–1%). Regarding the use of enalapril (n = 4), two pregnant women used it in the first trimester of pregnancy and two in the second. As for propranolol, one did it in the first trimester and the other in the third. According to the FDA classification, 3 cases (16.7%) were class B medications, 12 (66.7%) class C and 3 (16.6%) class D (2 cases of enalapril, in which the use was performed in the second trimester and one of propranolol, in the third trimester).

**Conclusion:** The use of antihypertensives belonging to class D among pregnant women in our study, suggest that these exposures may have relevance in the origin of CHD detected in patients later. The incidence and severity of clinical cases of CHD highlight the importance of prevention and education of health professionals as well as patients regarding the use of these medications in pregnancy.

111131

Modality: E-Poster Scientific Initiation – Non-case Report

Category: COVID-19 AND CARDIOVASCULAR SYSTEM

## The Impact of the SARS-COV-2 Pandemic on Morbimortality from Heart Failure: The Northeastern Brazil Scenario

CAROLINE LINK^1^, Ana Flavia Botelho^1^, Francielle Nocera Viechineski^1^, Bruna Kara^1^, Ana Carla Dlugosz^1^, Alice Magro Koscianski^1^, Camilla Mattia Calixto^1^, Larissa Almeida Busnello^1^, Paola Gonçalves Moreira de Oliveira^1^, Julia Henneberg Hessman^1^, Mário Claudio Soares Sturzeneker^1^

(1) Universidade Estadual de Ponta Grossa – UEPG

**Introduction:** Heart failure (HF) remains poorly prognostic and has a high morbidity and mortality rate. The SARS-CoV-2 pandemic may have influenced these parameters mainly due to the overload of health services, although regional characteristics may have been influential.

**Objective:** To assess the impact of the SARS-CoV-2 pandemic on HF morbidity and mortality in Northeast Brazil through the analysis of DATASUS data.

**Method:** The average hospital stay, the number (No) of hospitalizations and deaths, and the mortality rate (calculated by dividing the number of deaths by the number of hospitalizations) were evaluated from 02/2017 to 12/2021. The respective period was subdivided into pre-pandemic (02/2017 to 02/2020), pandemic (03/2020 to 06/2021), peak (12/2020 to 05/2021), first and second trimester of the peak (12/2020 to 02/2021 and 03/2021 to 05/2021) and mass vaccination (06/2021 to 12/2021). The subdivisions of the total period evaluated, named pre-pandemic and mass vaccination, were compared with the others, and the results were expressed as a percentage.

**Results:** Compared to the pre-pandemic period, there was a reduction of approximately 33% in the pandemic period and 15% in the mass vaccination period. Compared to the peak period, there was a 50% increase in hospitalizations during the mass vaccination period. There was a reduction in the monthly average of deaths (21–28%) in the pandemic period when compared to the pre-pandemic period, with this average being similar in the mass vaccination and pre-pandemic periods. However, there was a significant increase in the mortality rate in the pandemic period (12.45%) when compared to the pre-pandemic period (10.9%), with the highest percentage observed in the mass vaccination period (12.79%). Mean hospital stay in days was highest in the first trimester (8.5) and lowest in the second trimester of the peak (7.7) and 8.1 in the pre-pandemic period. The mortality rate was higher, and the average length of stay was lower in all periods for the female gender.

**Conclusions:** There were relevant consequences of the SARS-CoV-2 pandemic on HF morbidity and mortality in the Northeast region of Brazil, with a lower number of hospitalizations and an increase in mortality compared to the pre-pandemic period. During the vaccination period, the increase in hospitalizations and the higher mortality rate may reflect both the difficulty in monitoring the disease in previous periods and the greater access to health services.

111146

Modality: E-Poster Scientific Initiation – Non-case Report

Category: HEART FAILURE/CARDIOMYOPATHY/TRANSPLANT

## Comparison between Clinical and Echocardiographic Profiles of Patients with Chagasic and Non-Chagasic Heart Failure in an University Hospital

ARTHUR MAROT DE PAIVA^1^, Ana Luísa Guedes de França-e-Silva^1^, Beatriz Caldas Gonçalves^1^, Felipe Thomé Arradi^1^, Gustavo Elias Gomes de Oliveira^1^, Daniela do Carmo Rassi^1^, Salvador Rassi^1^, Aguinaldo F. Freitas Jr^1^

(1) Universidade Federal de Goiás – UFG

**Introduction:** Chagas disease (CD) affects about 6 to 7 million people in Latin America with a dramatic increase in areas such as United States and Europe. Cardiomyopathy is the most frequent clinical form of chronic CD, occurring in 20% to 30% of chronically infected individuals, with heart failure (HF) being one of its main manifestations.

**Objective:** Compare echocardiographic and clinical profile of patients diagnosed with chagasic and non-chagasic HF.

**Methods:** Analytical cross-sectional study with outpatients treated at an University Hospital between June 2020 and February 2022. Were included patients with a previous diagnosis of HF whom underwent echocardiography in the last 5 years. For the analysis of independent samples, the student’s t test was performed (5% significance).

**Results:** A total of 91 patients were included, of which 51 have HF because of CD (CHAGAS group) and 40 have non-chagasic HF (NCE group). Of the NCE patients, 10 were ischemic etiology, 9 hypertensive, 1 post-chemotherapy, 13 idiopathic, 1 trachycardiomyopathy, 2 peripartum, 1 myocarditis, 10 valvular and 6 alcoholic. Comparing the age of the two groups, the mean of CHAGAS was 64.90 (±10.67) and NCE (±15.05) (p = 0.04). When analyzing the heart rate, the CHAGAS group had a mean of 64.72 (±10.81) and the NCE 72.37 (±12.92) (p = 0.0028). Regarding systolic blood pressure (SBP), the mean of CHAGAS group was 104.78 mmHg (±19.99) and of NCE group was 117.60 mmHg (±30.82) (p = 0.01). About the diastolic blood pressure (DBP), the mean of CHAGAS group was 67.74 mmHg (±11.37) and of NCE group was 75.27 mmHg (±21.77) (p = 0.03). Regarding the echocardiographic measurements, the mean of ejection fraction in CHAGAS group was 31.07% (±74.16) and in NCE group was 33.64% (±101.80) (p = 0.22), the left atrium in the CHAGAS group had an average of 43.77 mm (±7.79) and the NCE group 42.00 mm (±7.28) (p = 0.27). The right ventricle had an average of 29.06 mm (±8.04) in the CHAGAS group and 25.75 mm (±6.58) in the NCE group (p = 0.04). The mean of left ventricular mass index in CHAGAS group was 134.97(±39.40) and in the NCE group was 142.91 (±39.69) (p = 0.36).

**Conclusion:** About clinical measures, there was a significant difference in the values of heart rate, SBP and DBP between the two groups, with CHAGAS showing lower values than NCE group. Among the echocardiographic measurements, the measurement of the right ventricle was the only one that showed a significant difference, with CHAGAS group showing higher values.

111139

Modality: E-Poster Scientific Initiation – Non-case Report

Category: CARDIAC ARRHYTHMIAS/ELECTROPHYSIOLOGY/ELECTROCARDIOGRAPHY

## Tilt Table Test in the Evaluation of Syncope, Presyncope or Postural Dizziness. Analysis of a Cohort of 2462 Patients in Brazil

MARIA EDUARDA QUIDUTE ARRAIS ROCHA^1^, Luís Gustavo Bastos Pinho^2^, Bruna Sobreira Kubrusly^2^, Roberto Lima Farias^2^, Vera Marques^4^, Arnóbio Dias da Ponte Filho^4^, Francisca Tatiana Moreira Pereira^2^, Maria Jaqueline Batista^2^, Juvêncio Santos Nobre^2^, Maria Camila Timbó Rocha^3^, Camila Pinto Cavalcante^2^, Eduardo Arrais Rocha^2^

(1) Universidade de Fortaleza (Unifor); (2) Universidade Federal do Ceará (UFC); (3) Centro Universitário Christus (UNICHRISTUS); (4) Centro de Arritmia do Ceará (CACE)

**Introduction:** The tilt table test (TT) provides relevant information on individual susceptibility to neuro-mediated hypotension and bradycardia. Its importance has been questioned with the advent of implantable cardiac monitors. In this work, we analyzed the results and safety of the Tilt Test in the investigation of syncope, presyncope or postural dizziness in the general population.

**Methods:** Retrospective cohort study, with analyzes of TT exams performed by 5 specialists in cardiac arrhythmias, from 2016 to 2021, in a syncope unit. Comparative analyses were performed using the Mann-Whitney tests, multiple logistic regression and ROC curves, with a p-value of 5% considered significant. The protocols used were the Westminster or Italian protocol, with a sensitization phase with 1.25 mg of sublingual isosorbide, used as assessed by the physician during the examination.

**Results:** A total of 2462 tilt tests performed were analyzed, with 115 exclusions, 61.7% of which were female, with a median age of 51.1(31–71) years. The overall positivity rate was 33.3%, with 43.3% with pharmacological sensitization (p < 0.01). In patients with tests requested to investigate syncope, positivity was 34.2% (477) × 30.65% (285) for other symptoms, with a significant difference in relation to the overall rate (p < 0.001), while evaluating syncope and pre-syncope together, the difference was 37.55%(623) × 20.9%(139) for other symptoms (p < 0.001). Positivity rates were higher in: males (p < 0.01; OR = 1.40 (1.16–1.69)), older patients (p < 0.01; 1.01(1.009–1.02)), in the sensitized tests (p < 0.01; 2.01(1.64–2.38)), in patients with early orthostatic hypotension with symptoms (p < 0.01; 9.68 (4.13–27.44)) or without symptoms (p < 0.01; 2.93(2.07–4.18). Previous use of hypotensive drugs or the pathologies presented were not significant as predictors of response. The complication rate was 4.85%, without hospitalization or death.

**Conclusions:** The Tilt Table Test continues to be an important and safe methodology in the investigation of patients with suspected neurally mediated syndromes in clinical practice in Brazil.

111142

Modality: E-Poster Scientific Initiation – Non-case Report

Category: HEART FAILURE/CARDIOMYOPATHY/TRANSPLANT

## Tolerability of Beta-Blockers: Comparison between Patients with Chagasic and Non-Chagasic Heart Failure

ARTHUR MAROT DE PAIVA^1^, Ana Luísa Guedes de França-e-Silva^1^, Beatriz Caldas Gonçalves^1^, Felipe Thomé Arradi^1^, Gustavo Elias Gomes de Oliveira^1^, Daniela do Carmo Rassi^1^, Salvador Rassi^1^, Aguinaldo F. Freitas Jr^1^

(1) Universidade Federal de Goiás – UFG

**Introduction:** Chagas disease (CD) is a zoonosis whose main manifestation is heart failure (HF). One of the drugs most related to a better prognosis in HF are beta-blockers. However, the use of this class in patients with heart failure because of CD may be limited due to the patient’s heart rate.

**Objective:** Compare the tolerability of beta-blockers in patients with chagasic and non-chagasic HF

**Methods:** Analytical cross-sectional study with patients with HF who used beta-blockers in outpatient clinic of an University Hospital between June 2020 and February 2022. For the analysis of independent samples, the student’s t test was performed (5% significance).

**Results:** 78 patients were included in the study, being 27 male and 51 female. The non-chagasic etiology group (NCE) had 35 patients and the chagasic etiology group (CHAGAS) 43. Of the NCE group, 8 of them were ischemic etiology, 1 valvular, 6 alcoholic, 8 hypertensive, 1 post-chemotherapy, 6 idiopathic, 2 peripartum and 1 myocarditis. In CHAGAS group, 33 were in use of metoprolol with a mean dose of 67 mg and 10 carvedilol with a mean dose of 6 mg. In the NCE group, 27 were in use of metoprolol with an average dose of 90 mg and 8 carvedilol with an average dose of 6 mg. In the CHAGAS group, 48% were in use of amiodarone, 4% calcium channel blockers, 25% angiotensin receptor blockers (ARB), 20% digoxin, 83% loop diuretic, 86% spironolactone, 32% ACE inhibitor, 11% SGLT-2 inhibitor and 23% sarcobitril-valsartan. In the NCE group, 17% were in use of amiodarone, 14% calcium channel blockers, 34% ARB, 11% digoxin, 71% loop diuretic, 82% spironolactone, 45% ACE inhibitors, 20% ISGLT-2 and 17% used sarcobitril -valsartan. When comparing heart rate, the NCE group had a mean of 72.94 (±13.46) and the CHAGAS group 64.67 (±10.59) p = 0.0033. The NCE group had a mean systolic blood pressure (SBP) of 117.8 (±32.13) and the CHAGAS group had a mean SBP of 107.18 (±19.82) p = 0.07. The mean NCE ejection fraction was 33.64% (±10.09) and the CHAGAS mean was 31.07% (±8.61) p = 0.22. Of the patients in the CHAGAS group, 6 were on a maximum dose of beta-blocker and 37 were not. In the NCE group, 9 were on the maximum dose and 26 were not, with a prevalence ratio of 0.86 (0.6–1.0).

**Conclusion:** The CHAGAS group had a lower heart rate than NCE group. SBP of CHAGAS group showed a tendence to have lower values than NCE group. The number of patients using maximum dose of beta-blockers was lower in CHAGAS group than NCE group.

111144

Modality: E-Poster Scientific Initiation – Non-case Report

Category: EPIDEMIOLOGY AND HEALTH POLICIES/GLOBAL HEALTH

## The Impact of the COVID-19 Pandemic on the Care and Mortality of Patients with Rheumatic Heart Disease in Brazil: A Comparative Study and Time Series Analysis

BEATRIZ XIMENES BANDEIRA DE MORAIS^1^, Larissa de Oliveira Beltrão^1^, Luiza Dias Aguiar^1^, Bianca Guirra Matos de Oliveira^1^, Arycia Laís Nascimento Cunha^1^, Paloma Gomes Tavares Sette^1^, Michelle Lima de Carvalho Silva^1^, Lucas Sandes de Lima^1^, Renato Dias Aguiar^1^, Augusto França Cruz Ximenes^2^, Andreza Leite Marques de Sá Souza^1^, Alexandre Duram de Lima Júnior^3^

(1) Faculdade Pernambucana de Saúde (FPS); (2) Universidade Católica de Pernambuco (UNICAP); (3) Instituto de Medicina Integral Professor Fernando Figueira (IMIP)

**Background:** Rheumatic Heart Disease (RHD) is the major cause of morbidity and mortality from severe or multiple episodes of acute rheumatic fever (ARF), a serious public health problem, especially in low and middle-income countries. These patients need continuous cardiac care and, in severe cases, heart valve surgery. During the COVID-19 pandemic, access to these services was limited, leading to adverse clinical outcomes to many patients living with RHD.

**Objectives:** This study aims to assess the impact of the COVID-19 pandemic on Hospital Admissions (HA), In-Hospital Morbidity and Mortality Rate from RHD in Brazil.

**Methods:** A time-series, observational study, using the public database from the Brazilian Unified Public Health System (DATASUS), to conduct a comparative analysis of HA, Morbidity and Mortality Rate from RHD, in Brazil, in 2020 and 2021, using as reference values from 2016 to 2019, and linear regression projection for 2020 and 2021.

**Results:** Compared to 2019, HA for RHD decreased in 31.5% in 2020 and 30.65% in 2021, In-Hospital Morbidity decreased in 19.15% in 2020 and 28.24% in 2021, In-Hospital Mortality Rate was 9.21 in 2020, a 16.43% increase, and 8.05 in 2021, a 1.77% increment. The number of valvuloplasties and valve replacement surgery fell, there was a reduction of 31.66% in 2020 and 34.64% in 2021, compared to 2019, whilst their mortality rate grew from 12.02 in 2019 to 13.59 in 2020, 13.05% increase, and was at 12.26 in 2021. All the 2020–2021 rates differed from their projected trend, specially 2020 values.

**Conclusion:** Resource prioritization and COVID-19 contention strategies, paired with patient’s reluctance to seek medical help, corroborated with precautionary postponement of elective procedures and reduction in HA and In-hospital morbidity for RHD, leading to delays in care and assistance, and a consequent increase of in-hospital mortality rates. Meanwhile, postponement of procedures implicated in the delay and decrease of valvuloplasties and heart valve replacement surgeries, leading to higher procedural mortality rates and severe consequences to the clinical outcome of RHD patients.


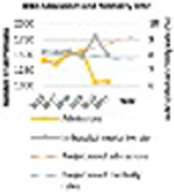


111161

Modality: E-Poster Scientific Initiation – Non-case Report

Category: COVID-19 AND CARDIOVASCULAR SYSTEM

## Cardiovascular Manifestations in Previously Healthy Individuals with COVID-19: A Sub-Analysis of Coronaheart Study

GEORGIA FIGUEIREDO RAMOS^1^, Francis Ribeiro de Souza^2^, Patricia O. Guimaraes^3^, Renato D. Lopes^4^, Cicero P. Albuquerque^2^, Alexandre Soeiro^2^, Renata M. do Val^2^, Ludhmila A. Hajjar^2^, Maria Janieire de Nazaré Nunes Alves^2^, Roberto Kalil Filho^2^

(1) University Santo Amaro (UNISA), São Paulo, SP, Brazil; (2) Heart Institute (InCor), Hospital das Clínicas da Faculdade de Medicina da Universidade de São Paulo, São Paulo, SP, Brazil; (3) Hospital Israelita Albert Einstein, São Paulo, SP, Brazil; (4) Duke University Medical Center – Duke Clinical Research Institute, Duke Health, Durham, United States of America; (5) Hospital Beneficência Portuguesa Mirante, São Paulo, SP, Brazil; (6) Hospital Sírio Libanês, São Paulo, SP, Brazil

**Introduction:** Previous studies showed that patients with prior history of cardiovascular disease present worst clinical outcomes when infected by Coronavirus Disease 2019 (COVID-19). Moreover, elevated levels of troponin and N-terminal pro B-type natriuretic peptide (NT-proBNP), are important prognostic markers associated with poor outcomes. However, whether patients with COVID-19 without prior structural heart disease may present cardiovascular alterations are poorly understood.

**Objectives:** We aimed to evaluate if individuals with COVID-19 without prior history of structural heart disease may present cardiovascular manifestations and/or cardiac dysfunction. In addition, we also evaluated the factors that could be associated with a worst clinical outcome.

**Methods:** This is an unicentric, retrospective, secondary analysis of the previous study (CoronaHeart study). For this analysis, 1198 patients were assessed. However, only those patients without prior history of structural heart disease who presented alterations in echocardiogram (ECHO) at admission were included. Additionally, logistic regression models to evaluate the association between clinical variables and in-hospital mortality were performed.

**Results:** A total of 40 participants had cardiovascular manifestations and were included (mean age 60 ± 14 years; 62% male). Overall, at hospital admission, 60% were admitted to intensive care units (ICU), and needed mechanical ventilation, 75% had elevated troponin levels, and 60% had elevated D-dimer levels. In-hospital mortality was 48%. The strongest factors associated with in-hospital mortality were elevated NT-proBNP levels (hazard ratio [HR] 1.0, 95% confidence interval [CI] 1.0–1.0 p = 0.002) and length of ICU stay (HR 0.87, 95% CI 0.77–0.98 p = 0.024). Additionally, in the hospital admission ECHO, 30% had acute pulmonary hypertension in the absence of thromboembolic events. Further analyses showed a higher rate of mortality in those individuals who presented an increased Systolic Pulmonary Artery Pressure (PAPs) compared to those individuals who presented normal values of PAPs [median, 95% CI, 44 (34–51) vs 30 (24–40) mmHg, respectively, p = 0.027].

**Conclusion:** Patients with COVID-19 even in the absence of prior history of structural heart disease may present cardiovascular manifestations and cardiac dysfunction associated with higher rate of mortality. Additionally, the increasing PAPs was more evident in the subjects with worst outcome.

111173

Modality: E-Poster Scientific Initiation – Non-case Report

Category: CARDIOLOGY OF SPORTS, EXERCISE, ERGOMETRY AND CARDIOVASCULAR REHABILITATION

## Cardiac Autonomic Response to Isometric Exercise in Elderly Subjects with Frailty Syndrome

GIULIA MICALI^1^, Tuany Mageste Limongi Zamperlim^2^, Luciana Gonzales Auad Viscardi^1^, Camila Martins^1^, Giovanna Dolder^1^, Pricila Helena de Souza^1^, Rodrigo Moreno de Oliveira^1^, Thamyres Oliva Bueno^1^, Higor Quintas Gonçalves^1^, Leila Dal Poggetto Moreira^1^, Adriana Sarmento de Oliveira^1^

(1) UNIVERSIDADE ANHEMBI MORUMBI -UAM; (2) UNIVERSIDADE FEDERAL DE JUIZ DE FORA-UFJF

**Introduction:** Frailty syndrome is the progressive decline of physiological systems. One of its dysfunctions is reduction of heart rate variability (HRV), which is related to adverse cardiovascular effects, a leading cause of death in the elderly.

**Objective:** To evaluate the impact of advanced fragility syndrome on cardiac autonomic control in the elderly.

**Methods:** In this cross-sectional prospective study, 18 Pre-Fragile (PF) and 9 Fragile (F) patients, of both genders aged over 65 years, were evaluated at Centro Integrado de Saúde. Using Fried’s criteria, participants were grouped as F, PF or NF if presenting with 3, ≤2, or nil criteria features, respectively. Staging focused on unintentional weight loss, low level of physical activity, exhaustion, reduced walking speed, and decreased grip strength. Isometric exercise protocol was performed at 30% maximum handgrip strength with participants in supine position during a 3 minutes isometric exercise. Analyses in the time domain used heart rate indices (MNN, SDNN, RMSSD, and pNN50). Low frequency (LF: 0.04–0.15 Hz) and high frequency (HF: 0.15–0.49 Hz) indices in absolute (m2) and normalized (un) units and the LF/HF ratio representing the sympathovagal balance were used in frequency domain analyses. Student t-test and Mann Whitney U test were used to compare differences between two groups. Statistical significance was set at p < 0.05.

**Results:** For time domain, there were no significant difference between groups for interval between heart beats (Mean RR; PF: 829.2 ± 61.4 vs F: 828.5 ± 85.7;p = 0.98), heart rate (Mean HR; PF: 72.8 ± 5.3 vs F: 73.4 ± 8.4;p = 0.83) and in parasympathetic modulation indices like SDNN [PF: 17.4 (13.1–19.5) vs F: 17.0 (10.0–20.1);p = 0.41], RMSSD [(PF: 17.9 (12.6–19.0) vs F: 15.6 (12.9–17.9);p = 0.41] and pNN50 [(PF: 1.6 (0.0–5.1) vs F: 0.4 (0.0–1.2);p = 0.28]. Conversely, for frequency domain parasympathetic modulation was lower in the F group compared to PF group [HFms; PF: 106.1 (56.8–165.7) vs F: 67.6 (19.8–85.6); p = 0.03]. There were no significant differences between groups for sympathetic modulation [LFms; PF: 144.8 (84.7–163.0) vs F: 86.9 (36.9–129.7); p = 0.12], LFun (PF: 56.2 ± 11.4 vs F: 63.5 ± 10.9; p = 0.11), HFun related to parasympathetic modulation (PF: 43.7 ± 11.5 vs. F: 36.4 ± 10.9; p = 0.11) and the sympathetic-vagal balance LF/HF ratio (PF: 1.5 ± 0.8 vs. F: 2.0 ± 0.9; p = 0.12).

**Conclusion:** During exercise, fragile individuals show poorer parasympathetic modulation compared to pre-fragile elderly.

111182

Modality: E-Poster Scientific Initiation – Non-case Report

Category: HEART FAILURE/CARDIOMYOPATHY/TRANSPLANT

## Epidemiological Profile of Hospital Morbimortality Due to Chronic Rheumatic Heart Disease in Northern Brazil from 2011 to 2021

VANDO DELGADO DE SOUZA SANTOS^1^, Elisama Quintino Sales^1^, Saul Rassy Carneiro^1^

(1) Universidade Federal do Pará – UFPA

**Introduction:** Chronic Rheumatic Heart Disease (RHD), associated with reduced socioeconomic development in a given country, is a sequela of rheumatic fever, which is a complication of pharyngotonsillitis caused by Streptococcus pyogenes and results from a late immune response to this infection in populations genetically predisposed.

**Objectives:** To analyze the epidemiological profile of hospital admissions and the RHD mortality rate in the northern region of Brazil from 2011 to 2021.

**Methods:** This is a descriptive longitudinal epidemiological study based on secondary data from the Department of Informatics of the Unified Health System regarding the number of hospitalizations, gender, age group and RHD mortality rate in northern Brazil, between January from 2011 to December 2021.

**Results:** In the analyzed period, 3,841 hospitalizations for RHD were recorded in the northern region, with the highest number of hospitalizations in 2017 (n = 468) and the lowest in 2021 (n = 246). The state of Pará had the highest rate of hospitalization for CRC (39.47%), followed by the state of Amazonas (30.82%), the state of Roraima had only 14 cases (0.36%). Females were the most affected (52.17%), the state of Amazonas was the only one with the highest rate of hospitalizations of the male population compared to females (59.97%) and the state of Tocantins was the one with the highest rate of hospitalizations females compared to males (59%), the age group most affected was 50 to 59 years (17.65%). Regarding the number of deaths, a total of 361 deaths from RHD were recorded, the year 2014 had the highest number (n = 55) and 2013 the lowest (n = 21), the state of Roraima had the highest mortality rate (21, 43%) and the state of Tocantins the lowest (9.06%). The female population had the highest mortality rates (54.02%) compared to the male population (45.98%), the most affected age group was 50 to 59 years (21.33%).

**Conclusion:** The study showed that the states Pará and Amazonas had the highest numbers of hospitalizations and deaths in the analyzed period. The low rate of hospitalizations and mortality in the most geographically distant states was noticeable, indicating an inefficiency in access to health. The data showed a higher number of hospitalizations and deaths in women and in the population over 30 years of age. With this, the impact of RHD on the health of population and the need for prevention, diagnosis and early treatment policies can be seen.

111183

Modality: E-Poster Scientific Initiation – Non-case Report

Category: ACUTE AND CHRONIC CORONARY DISEASE/THROMBOLYSIS

## Smoking Load as a Predictor of Cardiovascular Events in 30 Days After the First Myocardial Infarction

MARIA LUIZA DE CASTRO AMARAL^1^, Maria Luiza de Castro Amaral^1^, Manuella Bernardo Ferreira^1^, Maria Eduarda Valente da Luz Fontes^1^, Cecília Meirelles Gaspar Coelho Tomazzoni^1^, Larissa Rovaris de Quevedo^1^, Roberto Léo da Silva^2^, Tammuz Fattah^2^, Daniel Medeiros Moreira^2^

(1) Universidade do Sul de Santa Catarina (UNISUL); (2) Instituto de Cardiologia de Santa Catarina (ICSC)

**Objective:** This study evaluated whether there is an association between previous smoking load and the incidence of cardiovascular events 30 days after the first myocardial infarction (AMI).

**Methods:** This is a preliminary analysis of data from the Catarina Heart Study, a prospective cohort that assesses patients diagnosed with first-time AMI.

**Results:** There was a significant association between smokers and non-smokers age (56 ± 9.2 vs 62.0 ± 11.5) and Body Mass Index (BMI) [26.6 (23.6–29.3) vs. 27.9 (25.0–31.1)]. There was an increase in thrombosis and reinfarction (AMI) at 30 days in smokers vs. non-smokers, with an incidence (2.5% vs. 0.2%) and (3.7% vs. 0.7%), respectively. There was an association between previous smoking load to the first AMI with reinfarction [OR 1.028 (CI 1.013–1.043)] and in-stent thrombosis [OR 1.023 (CI 1.004–1.043] at 30 days among smokers. In the smoking population, there was a significant association between previous smoking load and post-infarction ventricular function evaluated with left ventricular ejection fraction (LVEF) (r = 0.093, P = 0.047).

**Conclusion:** Patients with previous smoking load had more re-infarction and in-stent thrombosis. Smokers had better post-infarction LVEF, fitting into the smoking paradox.

111191

Modality: E-Poster Scientific Initiation – Non-case Report

Category: CONGENITAL AND PEDIATRIC CARDIOLOGY

## Infective Endocarditis Associated with Congenital Heart Disease in a Cardiac Surgery Referral Center in Brazil

MARIANA GIORGI BARROSO DE CARVALHO^1^, Thaissa dos Santos Monteiro^2^, Thatyane Veloso de Paula Amaral de Almeida^1^, Nícolas de Albuquerque Pereira Feijóo^1^, Leo Rodrigo Abrahão dos Santos^1^, Wilma Golebiovki^2^, Clara Weksler^2^, Giovanna Ferraiuoli Barbosa^2^, Bruno Zappa^2^, Rafael Quaresma Garrido^2^, Marcelo Goulart Correia^2^, Cristiane C. Lamas^3^

(1) Universidade do Grande Rio – Unigranrio; (2) Instituto Nacional de Cardiologia – INC; (3) Instituto Nacional de Doenças Infecciosas Evandro Chagas – Fiocruz

**Introduction:** Congenital heart disease is among the several risk factors for infective endocarditis (IE) and it has distinct clinical features.

**Objectives:** Our objective was to describe cases of IE in patients with congenital heart disease (CHDIE) in a developing country and compare it with other cases of IE in a cohort presented to a referral center for cardiac surgery.

**Methods:** Adult patients with definite IE according to the modified Duke criteria were included from 2006–2021 using the International Collaboration in Endocarditis case report form. Statistical analysis was performed using the Jamovi and R software.

**Results:** There were 435 episodes of IE, of which 61 (14%) were CHDIE. Of these, 21/61(34.4%) were bicuspid aortic valves. There was no difference in the proportion of gender between CHDIE and other IE. Coronary artery disease, diabetes mellitus, systemic arterial hypertension and chronic renal failure were observed less frequently in CHDIE (3.3% vs 15.8%, p. = 0.008; 4.9% vs 13.9%, p = 0.06; 27.6% vs 51.6%, p < 0.001; 6.6% vs 23.4%, p = 0.002, respectively), but previous heart failure was frequent for both groups (39.3% vs 40.1%). Patients with CHDIE less frequently had hospital-acquired IE (14.8% vs 27.6%, p = 0.033). Aortic regurgitation was more frequent in CHDIE (51.7% vs 36.7%, p = 0.027) as were aortic vegetations (62.3% vs 38.2%, p < 0.001), while mitral regurgitation was less frequent (14.8% vs 50.7% p < 0.001). No differences were observed regarding the frequency of valve perforation (18% vs 18.3%) or fistulae (4.9% vs 4%), however paravalvular abscess was more frequent (32.8% vs 20.9% p = 0.004). The frequency of fever, embolic and immunological phenomena, splenomegaly, CRP and ESR levels were not different between groups. There was no difference in the proportion of microorganisms (S.aureus, viridans group streptococci, enterococci or fungi) identified in blood cultures. The rates of surgical indication (91.7% vs 85.3%) and surgery performed (81% vs 79.6%) were similar. Mortality was lower for CHDIE (19.3% vs 26.6%) but this was not statistically significant (p = 0.238).

**Conclusion:** In our cohort, bicuspid aortic valves were the most frequent CHD found. There were fewer comorbidities in patients with CHDIE, and a lower mortality, possibly because it presents usually in young adults. The aortic valve was more affected, corroborating the most common malformation in congenital heart diseases, which is the bicuspid aortic valve.

111203

Modality: E-Poster Scientific Initiation – Non-case Report

Category: EPIDEMIOLOGY AND HEALTH POLICIES/GLOBAL HEALTH

## Effects of Hiperdia Program on Mortality from Hypertension, Diabetes Mellitus and Cardiovascular Diseases in Bahia, Brazil

DAVI FIALHO SILVA^1^, Davi Fialho Silva^1^, Fabio Leandro dos Santos Correia^1^, Anselmo Araújo Oliveira^1^, Lucas Perales Borbon^1^, João Gabriel Neves Lopes^1^

(1) Universidade do Estado da Bahia

**Introduction:** Cardiovascular diseases (CVD) and Diabetes Mellitus (DM) are currently among the main challenges for Public Health in Brazil. Systemic Arterial Hypertension (SAH) is a multifactorial disease that affects the blood circulation, causing serious damage to target organs. DM is an endocrine-metabolic disease that mainly affects carbohydrate metabolism, causing systemic and target organ impairment. The proper diagnosis and treatment of these diseases, as well as an effective therapeutic adherence is essential for the patient’s quality of life. Failures resulting from incorrect management demand high public expenditures. To reduce the impact of these comorbidities, the HiperDia program was created through Ordinance No. 371/2002 of the Ministry of Health.

**Objective:** To verify the effect of the HiperDia Program on mortality from SAH, DM and CVD in Bahia.

**Methods:** Data were obtained from the Mortality Information System database (SIM-DATASUS), and deaths related to codes I10, E10, E11, E13, E14 and chapter IX of the ICD-10 were collected. Mortality coefficients and annual percentage change rates (APC) of SAH, DM and CVD were calculated in Bahia in the period, before and after the HiperDia Program.

**Result:** There was an increase in the median mortality rate in all the variables studied after the implementation of the program (Table 1). There was a statistically significant reduction in the growth of deaths from DM and CVD (Table 2).

**Conclusion:** The increase in the median mortality coefficients indicates how challenging it is for the Health System to properly manage these diseases. However, there was a deceleration in the growth of mortality from CVD and DM in Bahia after Hiperdia, suggesting a possible effect of the program on mortality from these diseases.


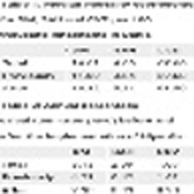


111211

Modality: E-Poster Scientific Initiation – Non-case Report

Category: ATHEROSCLEROSIS/CARDIOVASCULAR RISK FACTORS/CARDIOVASCULAR PREVENTION

## Menopause: A Pillar for Assessing Myocardial Ischemia in Women

CLEOVALDO RIBEIRO FERREIRA-JUNIOR^1^, Cláudia Bispo Martins-Santos^1^, Lara Teles Alencar Duarte^1^, Allexa Gabriele Teixeira Feitosa^1^, Edvaldo Victor Gois Oliveira^1^, Enaldo Vieira de Melo^1^, Antônio Carlos Sobral Sousa^1^, Joselina Luzia Menezes Oliveira^1^

(1) Universidade Federal de Sergipe (UFS)

**Introduction:** Cardiovascular disease (CVD) in women differs from CVD in men because it has sex-specific risk factors related to reproductive health and hormonal changes. In the menopausal transition phase, estrogen levels decrease, and this is a moment of acceleration of the risk of CVD, with CVD being the main cause of morbidity and mortality in postmenopausal women. According to a meta-analysis involving studies from 24 countries on 6 continents, the global mean age of menopause is 48,78 years.

**Objective:** To evaluate menopause as an additional factor to the classic risk factors for myocardial ischemia (MI) in women undergoing Physical Stress Echocardiography (ESE).

**Methods:** Cross-sectional study performed between January 2000 and January 2022 with women who underwent ESE at a cardiology referral service in Sergipe. A total of 7276 women (age: 58.6 ± 10.9 years) were included, divided into two categories according to the ESE result: positive or negative for MI. Patients over 48 years of age were considered menopausal women. For analysis, binary logistic regression was used to identify whether menopause and other risk factors were independently associated with MI. To enter the model, the significance level was p < 0.10 and, to remain, p < 0.05. Finally, the chi-square test was performed to assess factors associated with menopause.

**Results:** The frequency of MI was 19.5% with a 95% confidence interval of 18.6–20.3. The factors independently associated with MI were: menopause (OR = 1.72; 95%CI 1.42–2.08), dyslipidemia (OR: 1.90; 95%CI 1.66–2.16), diabetes (OR = 1.54; 95%CI 1.30–1.82), hypertension (OR = 1.22; 95%CI 1.07–1.39) and family history (OR = 1.58; 95%CI 1.39–1.79). The group of postmenopausal women had a higher presence of comorbidities, such as hypertension (p < 0.0001), diabetes (p < 0.0001) and dyslipidemia (p < 0.0001); however, it presented better habits regarding the practice of physical activity (p = 0.002) and a lower frequency of smoking (p = 0.007). There was no difference between women before and after menopause in the distribution of obesity and family history (p > 0.05).

**Conclusions:** Menopause is an additional factor to consider when evaluating for MI in women. The classic risk factors dyslipidemia, diabetes, hypertension and family history were also associated with MI.

111218

Modality: E-Poster Scientific Initiation – Non-case Report

Category: PERICARDIUM/ENDOCARDIUM/VALVOPATHIES

## Early Prosthetic Valve Infective Endocarditis in a Cardiology Referral Center Cohort of Adult Patients

MARIANA GIORGI BARROSO DE CARVALHO^1^, Thatyane Veloso de Paula Amaral de Almeida^1^, Nícolas de Albuquerque Pereira Feijóo^1^, Ingrid Paiva Duarte^1^, Leo Rodrigo Abrahão dos Santos^1^, Wilma Golebiovki^2^, Clara Weksler^2^, Giovanna Ferraiuoli Barbosa^2^, Bruno Zappa^2^, Rafael Quaresma Garrido^2^, Marcelo Goulart Correia^2^, Cristiane C. Lamas^3^

(1) Universidade do Grande Rio – Unigranrio; (2) Instituto Nacional de Cardiologia – INC; (3) Instituto Nacional de Doenças Infecciosas Evandro Chagas – Fiocruz

**Introduction:** Early Prosthetic Valve Infective Endocarditis (EPVIE), defined as infective endocarditis (IE) occurring up to 1 year after valve replacement surgery, is a feared complication and usually involves hospital pathogens. Our aim was to describe cases of EPVIE in a developing country and compare it with other cases of IE in our cohort.

**Methods:** Adult patients with definite IE according to the modified Duke criteria were included from 2006–2021 using the International Collaboration in Endocarditis case report form. Statistical analysis was performed using the Jamovi and R software.

**Results:** EPVIE was responsible for 10.8% of episodes of IE (47/435). Median age of patients with EPVIE was 54 years [34.5–64.5], significantly different to other IE, where it was 47 years [33–61.3]. No differences were found regarding gender, diabetes mellitus, chronic renal failure, congenital heart disease and rheumatic valve disease (RVD). However, patients with EPVIE more frequently had coronary artery disease (25.5% vs 12.6%, p = 0.016), coronary artery bypass graft surgery (15.2% vs 4.7% p = 0.004), heart failure (58.7% vs 37.7% p = 0.006), cerebrovascular disease (12.8% vs 5.9% p = 0.077) and arterial hypertension (69.8% vs 45.5% p = 0.003). Fever, embolic and immunological phenomena, splenomegaly, CRP and ESR levels were not different between EPVIE and other cases of IE, although the presence of new murmurs was less frequent (28.3% vs 57.8%, p < 0. 01). Most found complications in EPVIE were myocardial or paravalvular abscess (32.6% vs 21.4%, p < 0.01), acute renal failure (ARF) (48.9% vs 32%, p = 0.024) and need for hemodialysis (HD) (45.8% vs 82.4%, p < 0.01). The microorganisms most frequently associated with EPVIE were enterococci (18.8% vs 9.8%, p = 0.06) and coagulase-negative staphylococci, but not S.aureus (4.1 vs 11.7%). Surgical indication rate was 58.7% (vs 89.4%, p < .001) and surgery (56.4% vs 80.7%, p < .001) in patients with EPVIE were lower when compared to other cases of IE, while the mortality rate (31.1% vs 25%) was higher, but not statistically different between groups.

**Conclusions:** Myocardial abscess, which is an absolute surgical indication, occurred in almost 1/3 of EPVIE. Possibly because of poor clinical status and frequent comorbidities, patients with EPVIE had less surgical indication and less surgery than the others in the cohort, evolving with high mortality.

111227

Modality: E-Poster Scientific Initiation – Non-case Report

Category: CARDIOLOGY OF SPORTS, EXERCISE, ERGOMETRY AND CARDIOVASCULAR REHABILITATION

## Clinical Factors Associated with Chronotropic Incompetence in Women

CLEOVALDO RIBEIRO FERREIRA-JUNIOR^1^, Lara Teles Alencar Duarte^1^, Allexa Gabriele Teixeira Feitosa^1^, Edvaldo Victor Gois Oliveira^1^, Cláudia Bispo Martins-Santos^1^, Enaldo Vieira de Melo^1^, Antônio Carlos Sobral Sousa^1^, Joselina Luzia Menezes Oliveira^1^

(1) Universidade Federal de Sergipe (UFS)

**Introduction:** Cardiovascular disease (CVD) has become increasingly prevalent in women, although it is underestimated when compared to other diseases specific to women. It differs from CVD in men in terms of pathophysiology, with traditional risk factors predicting higher or lower risk of CVD in women than in men.

**Objective:** To evaluate the clinical factors associated with Chronotropic Incompetence (CI) in women undergoing Exercise Stress Echocardiography (ESE).

**Methods:** Cross-sectional study between January 2000 and January 2022 with women who underwent ESE at a cardiology referral service in Sergipe. A total of 7049 women (age: 58.6 ± 10.9 years) were included, divided into two categories according to the chronotropic index: chronotropic competent and incompetent. Patients with a chronotropic index less than 0.8 were considered chronotropic incompetent. For analysis, binary logistic regression was used to identify factors that were independently associated with CI.

**Results:** We compared 6430 (91.2%) competent women and 619 (8.8%) incompetent women. Dyslipidemia, diabetes, family history of CVD, obesity and beta-blocker use (withdrawn 3 days before the exam) were not independently associated with CI (p > 0.05). Factors independently associated with CI were: sedentary lifestyle (OR = 2.49; 95%CI 1.93–3.21), smoking (OR: 2.84; 95%CI 1.60–5.05), former smoking (OR = 1.90; 95%CI 1.29–2.79), age 60 years or older (OR = 1.55; 95%CI 1.24–1.95), hypertension (OR = 1,39; 95%CI 1.09–1.78) and previous presence of symptoms such as chest pain – typical or atypical – or dyspnea in the clinical history (OR = 1.45; 95%CI 1.14–1.85).

**Conclusions:** Bad habits such as smoking and physical inactivity had the highest odds ratios for CI. Patients who did not quit smoking were more likely to have CI than former smokers. Beta-blocker discontinued 3 days before the exam was not independently associated with CI.

111658

Modality: E-Poster Scientific Initiation – Non-case Report

Category: EPIDEMIOLOGY AND HEALTH POLICIES/GLOBAL HEALTH

## Number of Deaths from Ischemic Cardiovascular Diseases in Brazil: An Epidemiological Analysis

BEATRIZ CUNHA LISBOA DE MEDEIROS NUNES^1^, Luísa Silva de Azevedo^2^, Júlia Freitas Carneiro^2^, Kimberlly Aparecida de Sousa Ferreira^2^

(1) Universidade Potiguar – UNP; (2) Universidade Do Estado Do Rio Grande Do Norte – UERN

**Introduction:** Chronic non-communicable diseases (CNCD) have gained space in the epidemiological and social context, constituting a global problem responsible for 74% of annual deaths. In Brazil, which follows this pattern, it is still possible to single out ischemic heart diseases (IHD) as the main cause of death in all states. To endure this reality, the Strategic Action Plan for Combating CNCD was established which was not able to achieve its goals.

**Objective:** To analyze the number of deaths due to IHD in Brazil, from 2010 to 2020, and correlate it with the goals of the Strategic Action Plan to Combat CNCDs in force.

**Methods:** Based on a time-series study on the number of deaths from IHD that occurred in Brazilian macro-regions, from 2010 to 2020. Data were taken from the Mortality Information System of the Informatics Department of the Unified Health System. To obtain the approach, the International Classification of Diseases was used, specifically the codes I20 to I25.

**Results:** From 2010 to 2020, 1,208,022 total deaths from IHD were registered in Brazil, with a percentage increase of approximately 10% of the cases recorded in the studied period. Furthermore, there is an increase in the number of deaths recorded by four out of the five regions in the country, with emphasis on the North, which shows an increase of 56.13% in its percentage of deaths. In 2011, the Health Ministry of Brazil launched the Strategic Action Plan to Combat CNCD as a way to contain the rising trend predicted in 2010 for the following decades. In the opposite direction of the efforts, it can be observed, by the exposed data, that the purpose is not close to being achieved. On the contrary, the numbers in most Brazilian regions are in full advance, as can be seen in the figures in Table 1.

**Conclusions:** IHDs still represent an important obstacle to public health in Brazil. The disparities in rates among the states reveal the need for actions consistent with the reality and local singularities. Not reaching the goals established in the plan has undeniable impacts on the health of the Brazilian population.


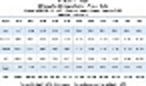


111253

Modality: E-Poster Scientific Initiation – Non-case Report

Category: CARDIO-ONCOLOGY

## Diagnosis of Heart Neoplasia, Mediastinum and Pleura in Brazil between the Years of 2013 and 2020

PAOLA BITAR DE MESQUITA ABINADER^1^, Ana Flávia Oliveira de Souza^2^, Ana Josefina Gonçalves Salomão^3^, Camila Rodrigues Maciel^4^, Cecília Gomes da Silva^5^, Daniel Oliveira da Costa^6^, Davi Gabriel Barbosa^7^, Gabriel de Siqueira Mendes Lauria^8^, Juliana Ayumi Azevedo Kurosawa^9^, Lucas Guimarães Junqueira^10^, Rafaela Oliveira Cardoso^11^, Luis Eduardo Werneck de Carvalho^12^

(1) Centro Universitário do Estado do Pará (CESUPA); (2) Centro Universitário do Estado do Pará (CESUPA); (3) Centro Universitário do Estado do Pará (CESUPA); (4) Centro Universitário do Estado do Pará (CESUPA); (5) Universidade Federal do Pará (UFPA); (6) Universidade Estadual do Pará (UEPA); (7) Universidade Estadual do Pará (UEPA); (8) Centro Universitário Metropolitano da Amazônia (UNIFAMAZ); (9) Centro Universitário do Estado do Pará (CESUPA); (10) Universidade Estadual do Pará (UEPA); (11) Centro Universitário do Estado do Pará (CESUPA); (12) Clínica Oncológica do Brasil

**Introduction:** The cardiac tumors can begging in the cardiac tissues of the internal coating, muscular layer and pericardium. Such neoplasms can show secondary symptoms and pathologies, such as: obstructions to the blood flow, dyspnea, chest pains or syncope, embolizations and arrhythmias. These clinical pictures usually show up late, and are non- specific to characterize these tumors, therefore hampering the clinical diagnosis. However, the image examinations crucial in this diagnosis are: transesophageal echocardiography, thorax tomography, magnetic resonance of the heart and even the fetal echocardiography, which allows for a diagnosis in the intrauterine phase. Therefore, the diagnosis methods in conjunction with the clinical interfere directly on the early choice of treatment and prognosis of the patients, showing the importance of the epidemiology on this pathology.

**Objectives:** Characterize the epidemiology of the diagnosis of heart neoplasia, mediastinum and pleura in Brazil between the years of 2013 and 2020.

**Method:** It’s and epidemiological study about malignant neoplasias of the heart, mediastinum and pleura in Brazil between the years of 2013 and 2020. The data was collected on the database of the Departamento de Informática do Sistema Único de Saúde (DATASUS). The variables analyzed here were the year and number of notifications, gender and age group.

**Results:** In total, 3954 cases of malignant neoplasias of the heart, mediastinum and pleura were registered between the years of 2013 and 2020, with a greater incidence on males (50,9%) and a lesser in females (49,01%). Regarding the age groups, in males there was a greater prevalence of cases between 60–64 years (48,3%), while in females, the cases concentrated on the age group of 55–59 years (56%). Besides that, it was observed that 2019 was the year with the biggest number of cases, counting 1420 cases (35,9%), distributed between males and females.

**Conclusion:** Based on the results obtained, malignant neoplasias of the heart, mediastinum and pleura affect, most of all, elderly men, with the biggest number of cases being observed in 2019. Taking on account the noted epidemiology and the difficult of identifying the risk factors and symptomatology of this type of cancer, this work puts emphasis on the need of more studies on the topic aiming to diagnose and treat the afflicted individuals early. Besides that, is primordial that the population achieves a greater access to the means of diagnosis.

111564

Modality: E-Poster Scientific Initiation – Non-case Report

Category: EPIDEMIOLOGY AND HEALTH POLICIES/GLOBAL HEALTH

## Delayed Percutaneous Coronary Intervention in ST Elevation Myocardial Infarction Patients in the Public Health System in a City of the South of Brazil

LUCAS MÜLLER PRADO^1^, Jessica Tamires Reichert^1^, Gustavo Sarot Pereira^1^, Jorge Tadashi Daikubara^1^, Michelle Bozko Collini^1^, Rafael Moretti^1^, Matheus Bissa Duarte Ferreira^1^, Eduardo Leal Adam^2^, Miguel Morita Fernandes da Silva^2^

(1) Universidade Federal do Paraná; (2) Complexo Hospital de Clínicas, Universidade Federal do Paraná.

**Background:** In patients with acute ST elevation myocardial infarction (STEMI), primary percutaneous coronary intervention (PCI) is the first-choice reperfusion therapy, but fibrinolysis is recommended in non-PCI centers, when the expected time-to-PCI is expected to be more than 120 minutes (min).

**Objectives:** To assess the time from chest pain (CP) to PCI (CP-to-PCI time) and the reasons for delay in patients with STEMI undergoing PCI in a tertiary hospital from the public health system in Brazil.

**Methods:** We prospectively included patients admitted for STEMI in a Tertiary Hospital in Curitiba, Brazil, from Oct/2019 to Dec/2021. CP onset was the beginning of the last symptom that motivated the patient seeking care. For patients first admitted in non-PCI centers, we collected the moment the patient arrived in the emergency care unit (ECU) before being referred to the tertiary hospital.

**Results:** We included 71 patients (61 ± 13 years old, 31% women), with 46 (65%) referred from non-PCI centers. Overall, the CP-to-PCI time was 378 [272.0, 628.0] min, and there was no significant difference between patients referred from non-PCI centers and those directly admitted in the tertiary hospital (p = 0.54). CP-to-ECU time was shorter in patients in non-PCI centers than those directly admitted in the tertiary hospital (104.5 [47.0, 206.5] vs 170.0 [123.0, 339.0] min, p = 0.012), but this was offset by a delay between the non-PCI center ECU and the tertiary hospital (172 [116.0, 335.0] min), with 74% of patients taking longer than 120 min (Figure 1).

**Conclusion:** We found important delays in primary PCI in patients with STEMI treated in the public health system in a city of Brazil, with time of referral from non-PCI centers to the tertiary hospital being largely responsible for these delays.


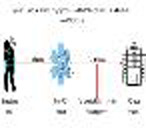


111957

Modality: E-Poster Scientific Initiation – Non-case Report

Category: DYSLIPIDEMIA

## Screening for Dyslipidemia and its Risk Factors in Children and Adolescents Residents in Riverside Communities in the Western Amazon

GABRIEL IRISMAR RODRIGUES SCHWAMBACK^1^, RAIMUNDO BENÍCIO DE VASCONCELOS NETO^1^, REBECCA SHAIANE SOARES NUNES RIVOREDO^1^, BRENDA DOS SANTOS RODRIGUES^1^, SOFIA DOS SANTOS SOUZA^1^, JADE GOMES DA COSTA MEDEIROS^1^, LIANA MIRANDA PEREIRA^1^, ANA JÚLIA OMODEI RODRIGUES MARTIM^1^, ANTONIETA RELVAS PEREIRA^2^, JULIANA DE SOUZA ALMEIDA ARANHA CAMARGO^1^, SERGIO DE ALMEIDA BASANO^1^, LUIS MARCELO ARANHA CAMARGO^1^

(1) Centro Universitário São Lucas – UNISL; (2) Centro Universitário Aparício Carvalho – FIMCA

**Introduction:** Measuring the prevalence of dyslipidemia and its risk factors is necessary to enable the design of interventions and management of population health. However, it is observed that these data are scarce in the Western Amazon, especially regarding the monitoring of children living in riverside communities.

**Objectives:** To estimate the prevalence of dyslipidemia and associated risk factors in children and adolescents residing in riverside communities in the Western Amazon, Brazil.

**Methods:** The present cross-sectional study was carried out with individuals aged between 6 and 16 years, living in the riverside region of the Madeira River, in Humaitá (S 6o 58’62” and W 62o 50” 08W), Amazonas State, Brazil. The project has been approved by the research committee of the Brazilian Research Center for Tropical Medicine in Rondônia (CEPEM). For the research, a clinical-epidemiological questionnaire was applied, as well as 10 mL of blood samples were collected and analyzed, as established in the parameters of the V Brazilian Directive on Dyslipidemia and Prevention of Atherosclerosis. The analysis was performed by applying the prevalence ratio (PR), Mantel-Haenszel chi-square test, with statistical significance p < 5%.

**Results:** It was possible to analyze the data of 131 (82%), among 160 children and adolescents, being 63 females and 68 males. Eleven blood samples were found with serum values consistent with dyslipidemia, representing an absolute prevalence of 8.4%. The mean age of the sample was 11 years, ranging from 6 to 16 years. The average family income reported was calculated at R$ 809.16. For risk factors, the PR between dyslipidemia and female sex was equal to 2.0 (p = 14%); for a family history of dyslipidemia, the PR was 1.66 (p = 21%); for obesity the PR was 1.9 (p = 28%); and for sedentary lifestyle and systemic arterial hypertension, PR values were lower than 1, with p > 5%.

**Conclusion:** Dyslipidemia is poorly studied disease in remote communities, especially when the target audience is children and adolescents. The data point to a prevalence of dyslipidemia of almost 10%, considered high for children and requiring intervention by the health system to mitigate the occurrence of chronic non-communicable diseases in adulthood. In this study, no proven risk factors associated with dyslipidemia were found.

111633

Modality: E-Poster Scientific Initiation – Non-case Report

Category: CARDIAC ARRHYTHMIAS/ELECTROPHYSIOLOGY/ELECTROCARDIOGRAPHY

## Impact of COVID-19 on the Treatment of Conduction Disorders in Brazilian Public Health System

IDRYS HENRIQUE LEITE GUEDES^1^, Kleber Oliveira de Souza^1^

(1) Universidade Federal de Campina Grande

**Introduction:** In the global pandemic scenario and crisis in the Brazilian health system, the restriction of medical care for diseases other than COVID-19 infection triggered vast effects in the treatment of various pathologies. Therefore, it is important to analyze the consequences of this situation specifically on the treatment and management of patients with conduction disorders and cardiac arrhythmias, given the complexity of these conditions.

**Objectives:** Collect and analyze hospitalization, hospital stay, and death data for conduction disorders and cardiac arrhythmias in Brazil.

**Methodology:** Cross-sectional quantitative study on the treatment of conduction disorders and cardiac arrhythmias in Brazil from January 2018 to December 2021, based on data obtained from the SUS Hospital Information System (SIH/SUS), through the Ministry of Health database (DATASUS), and analyzed using IBM SPSS statistical software. The following variables were included: year of care, hospitalizations, the average length of stay, and the number of deaths.

**Results:** There were 64790 and 69673 hospitalizations due to conduction disorders and cardiac arrhythmias in Brazil in 2018 and 2019, respectively, while in 2020 and 2021 there were 60362 and 59834 hospitalizations, resulting in a 14% difference between the highest and lowest values and a P-value <0,001. The average hospital stay in the pre-pandemic biennium increased from 4.75 days to 4.3 days (P-value of 0.978), while the number of deaths increased from 7153 and 8500 to 8576 and 8674, with a respective P-value < 0,001.

**Conclusion:** According to the analysis, there was an indication of a significant reduction in the number of hospitalizations, a likely result of the low demand for medical care in the pandemic period. It is still possible to observe a lower number of days of hospital stay in the post-pandemic period, probably contributing to the growth in the number of deaths observed, which is also favored by the greater severity in the evolution of patients who delayed medical follow-up for too long.


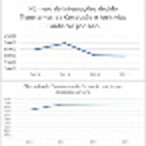


111513

Modality: E-Poster Scientific Initiation – Non-case Report

Category: HYPERTENSION/RENAL DENERVATION

## The Impact of Renal Sympathetic Denervation to the Treatment of Resistant Hypertension

MARIA RAFAELA ALVES NASCIMENTO^1^, Yure Batista Sousa^1^, Letícia Rego Borborema^1^, Victoria Liery Ribeiro Alves^1^, Lara Carneiro Magalhães^1^, Livia Caroline Bemquerer Veloso^1^, Karolina Campos Sampaio Lopes^1^, Fernando Guimarães Fonseca^1^, Lanuza Borges Oliveira^1^, Maria das Mercês Borem Correa Machado^1^

(1) Centro Universitário FIPMOC – Afya

**Introduction:** Systemic arterial hypertension (SAH) is a considerable risk factor for cardiovascular diseases. Resistant hypertension (RH) diagnosis can be reached through the management of elevated levels of blood pressure (BP), despite the use of three or more antihypertensive medications of different classes, including diuretic ones. The sympathetic nervous system (SNS) plays an essential role in elevating BP through peripheral and central mechanisms which have an impact on the kidneys, the heart and blood vessels. The use of invasive procedures for RH treatment has recently been proven to be effective. Renal denervation modifies the sympathetic interactions between the kidneys and the central nervous system through a percutaneous procedure by connecting a catheter to a radiofrequency device.

**Objective:** To observe the impact of renal sympathetic denervation to the treatment of resistant hypertension.

**Method:** Systematic review has been used, with articles found in the SciELO database as well as in the Biblioteca virtual em saúde, LILACS, and Medline databases. The keywords “renal denervation”, “resistant hypertension”, and “sympathetic nervous system” have been used in research, with the use of PRISMA methodology. Nineteen papers published in the last ten years have been selected; ten of which published in Brazil and nine others published internationally.

**Results:** Based on the positive results of the pilot study, a new study has been encouraged: the Symplicity HTN-2. In this trial, 106 RH patients were randomly selected for renal sympathetic denervation (RSD) (n = 52, initial average BP of 178/96 mmHg) or underwent previous treatment (n = 54, initial average BP of 178/97 mmHg). After six months of observation, it was possible to determine a decrease of systolic blood pressure higher than 10 in 84% of patients subjected to RSD. In the control group, only 35% of patients showed positive results. Finally, 20% of patients from the intervention group showed decrease in the amount of medication used, as compared to the control group, which showed only 6% of patients.

**Conclusion:** With the data collected from the study, it is possible to conclude that RSD is an efficient procedure in light of RH diagnosis, which showed no relevant complications or adverse effects and has proven to provide significant increase in the quality of life of patients who have been subjected to such treatment.

111479

Modality: E-Poster Scientific Initiation – Non-case Report

Category: SPIRITUALITY AND CARDIOVASCULAR MEDICINE

## Association between Spirituality/Religiosity and Risk Factors for Cardiovascular Diseases

BRUNA SOUZA MATOS DE OLIVEIRA^1^, Adelle Cristine Lima Cardozo^1^, José Icaro Nunes Cruz^1^, Camille Marques Aquino^1^, Jade Soares Dória^1^, Giulia Vieira Santos^1^, Juliana Maria Chianca Lira^1^, Gabriela de Oliveira Salazar^1^, Philipi Santos Soares^1^, Antônio Carlos Sobral Sousa^2^, Enaldo Vieira de Melo^1^, Joselina Luzia Menezes Oliveira^2^

(1) Federal University of Sergipe; (2) Rede D’Or São Luiz – São Lucas Hospital; (3) Primavera Hospital

**Introduction:** Several studies analyze the influence of spirituality and religiosity (S/R) on the lifestyle of individuals. It is known that sedentarism, smoking and high alcohol consumption are risk factors for cardiovascular disease, the leading cause of global morbidity and mortality.

**Objectives:** To analyze the relationship between S/R levels with the variables sedentarism, smoking and alcohol consumption.

**Methods:** Observational, cross-sectional, analytical study. The sample included patients from cardiology outpatient clinics of three hospitals in Sergipe. Three natures of religiosity were assessed according to Duke Religiosity Index (DUREL): organizational (OR), non-organizational (NOR) and intrinsic (IR). The cut-off point for defining low or high level of OR and NOR was 4 (<4: low; ≥4: high), while the cut-off point for low or high level of IR was 13 (<13: low; ≥13: high). The variables alcohol consumption, smoking, and sedentary lifestyle were assessed by applying a self-questionnaire and their prevalence was compared between the OR, NOR and IR groups using Fisher’s exact test, with the significance level set at 0.05.

**Results:** 130 patients were included in the study, of which 62.3% were female. The mean age was 60.6 ± 11.2 years. In this sample, 73.8% were hypertensive, 59.2% were dyslipidemic, 40.8% had chronic coronary syndrome, and 35.4% had diabetes mellitus. Patients with low level of NOR had higher prevalence of alcohol consumption when compared to high level patients (71.4% vs. 28.9%; p < 0.05). Individuals with low level of IR showed higher prevalence of alcohol consumption compared to individuals with high level (61.9% vs. 25.0%; p < 0.05). There was no difference between patients with low or high level of OR for the prevalence of alcohol consumption, smoking, and sedentary lifestyle (p > 0.05). There was no difference between patients with low or high level of religiosity for the prevalence of smoking and sedentary lifestyle (p > 0.05).

**Conclusions:** Patients with high levels of NOR and IR had lower prevalence of alcohol consumption compared to patients with low levels. The prevalences of the risk factors evaluated were not different between patients with high and low OR. There was no difference between patients with low or high levels of religiousness for the prevalence of smoking and sedentary lifestyle. Future studies with larger numbers of participants are needed to better understand this association.

111580

Modality: E-Poster Scientific Initiation – Non-case Report

Category: EPIDEMIOLOGY AND HEALTH POLICIES/GLOBAL HEALTH

## Trend in Mortality Due to Hypertensive Diseases According to Underlying Cause and Mentioned Cause in Brazil from 2000 to 2019

RENATTA KAROLINE BEKMAN VOGAS^1^, Jonatas Benarroz da Silva^1^, Davi da Silveira Barroso Alves^1^, Paulo Henrique Godoy^1^

(1) Federal University of the State of Rio de Janeiro – UNIRIO

**Introduction:** Hypertensive diseases (HD) have a high prevalence and are a risk factor for cerebrovascular and coronary diseases. They are one of the main causes of death in Brazil, however, its participation as the underlying cause of death (UCD) may be underestimated due to the selection rules of the International Classification of Diseases (ICD-10).

**Objective:** To analyze the trend in mortality by underlying cause and by mentioned cause due to hypertensive disease, according to age and sex, in Brazil, from 2000 to 2019.

**Methods:** Ecological study with a description of a historical series on deaths due to HD, reported as UCD or mentioned cause (MC) in lines A to D, recorded in Mortality Information System databases, in Brazil, from 2000 to 2019, based on ICD-10 codes for HD (I10–I15). Crude and standardized mortality rates were estimated for UCD and MC, according to sex and age groups.

**Results:** There were 814,722 deaths whose UCD was HD and 3,714,982 deaths recorded as MC. The number of deaths was 4.5 times higher in the case of MC than when considered the UCD. Mortality rates as MC were significantly higher than when considered as UCD and there was a proportional increase according to age groups, especially in males, aged 30–59 years. Trends observed in the 0–29 age group were the ones that most differed, for the sexes, according to UCD and MC.

**Conclusions:** It seems reasonable to use mentioned cause of death as a method to analyze mortality due to HD and minimize possible underestimations due to UCD selection rules, especially when considering younger groups.


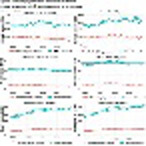


111358

Modality: E-Poster Scientific Initiation – Non-case Report

Category: HEART FAILURE/CARDIOMYOPATHY/TRANSPLANT

## The use of Ambulatory Internal Jugular Vein Ultrasound to Evaluate Congestion in Patients with Heart Failure – A Systematic Review

MAYARA GABRIELE TOLEDO^1^, Mayara Gabriele Toledo^1^, Caio Pluvier Duarte Costa^1^, Eduardo Thadeu de Oliveira Correia^1^

(1) Universidade Federal Fluminense

**Introduction:** One of the key therapeutic aims of the outpatient management of heart failure (HF) is to treat peripheral and pulmonary edema. However, although clinical signs can identify patients with congestion, it may still be present subclinically, which is an indicator of a worse prognosis. Therefore, this study aims to systematically evaluate the use of internal jugular vein (IJV) ultrasound to detect congested HF patients seen in outpatient clinics.

**Methods:** We conducted a systematic review following the PRISMA guidelines. Original studies indexed by Embase, Pubmed, Cochrane and LiLACS with the keywords: “ultrasound” or “ultrasonography” and “heart failure” and “jugular vein” published until March, 2022 that matched the inclusion criteria were included. Two authors (C.P and M.T.) performed the screening and data extraction, in cases of discordance, a third author (E.T.) made the decision.

**Results:** Six studies were included with a total of 1236 patients analyzed, excluding control patients. Due to the significant heterogeneity between studies, a meta-analysis was not conducted. For the ultrasonic assessment of volume status, 4 studies used the variation of the IJV diameter at rest and during the Valsalva maneuver, 1 used the variation of cross-sectional area (CSA) at rest and after the Valsalva maneuver before and after the use of diuretics, and 1 study compared the measurement of right atrial pressure (RAP) estimated by IJV ultrasound with the value obtained during right heart catheterization. One of the studies that analyzed the variation of IJV diameter at rest and during the Valsalva maneuver showed that jugular vein distention (JVD) is a valid option to assess fluid status of patients and provides diagnostic and prognostic information similar to natriuretic peptides. Pellicori et al, in their 2 studies showed that resting JVD was lower in controls (0.16 and 0.17 cm) than in HF patients (both 0.23 cm) and the JVD ratio was higher in controls (both 6.3) than in HF patients (4.5 and 4.4). In one of his studies, he showed that JVD was similar during Valsalva (1.03 cm vs 1.08 cm) in controls and HF.

**Conclusion:** This systematic review shows that IJV ultrasound is useful for examining intravascular volume status and identifying congestion. Future clinical trials evaluating whether IJV ultrasound could be used to guide diuretic therapy are needed. Finally, further studies are needed to define a standard IJV ultrasound technique to examine volume statu.

111754

Modality: E-Poster Scientific Initiation – Non-case Report

Category: HEMODYNAMICS AND INTERVENTIONAL CARDIOLOGY

## Transcateter Aortic Valve Implantation (TAVI) in a High Complexity Center in the Western Brazilian Amazon: A Case Series

REBECCA SHAIANE SOARES NUNES RIVOREDO ^1^, Rebecca Shaiane Soares Nunes Rivoredo^1^, Raimundo Benício de Vasconcelos Neto^1^, Gabriel Irismar Rodrigues Schwamback^1^, Paulo Roberto de Oliveira Molina^4^, Isabel Bussinguer Gomes^1^, Ingrid da Silva Morais Freitas^4^, Francisco Siosney Almeida Pinto^2^

(1) Centro Universitário São Lucas/Afya UNISL; (2) Instituto Cardiovascular- INCARDIO; (3) Centro Universitário Aparício Carvalho

**Introduction:** Transcatheter aortic valve implantation (TAVI) is a minimally invasive procedure that, since its debut in 2002, evolved to become the standard care for patients with aortic stenosis (AS) at intermediate and severe risk to surgery once conventional surgery as the femoral approach can be safely implemented. Over the years, there has been a significant improvement in the development of transcatheter heart valves (THV), in addition to the refinement of the skills involved in the implant process, which made it possible to increase the spectrum of patients who benefit from TAVI. Currently, studies are investigating the role of TAVI in patients at low surgical risk, new indications and new alternatives for perioperative management.

**Objectives:** This is a descriptive study of TAVI cases performed in a high complexity hospital located in the Western Amazon.

**Methods:** The data presented were obtained through research via medical records, complementary exams and databases such as PubMed and Google Academy.

**Results:** In the period between September 2019 and November 2021, 11 TAVIs were performed by the cardiology team of a high complexity hospital in the Western Amazon. Patients undergoing TAVI are 5 women and 6 men, with an average age of 65.8 years, of which only one underwent the procedure on an emergency basis. The main symptom reported by patients before implantation was dyspnea on minor exertion and at rest, this symptomatology is justified by the diagnosis of severe aortic stenosis associated with heart failure NYHA > 3. The implants were performed using the Inovare® expandable balloon prostheses, introduced by catheter via the femoral artery with a unanimous duration of approximately 2 hours per procedure. A total of 7 patients evolved with a good prognosis without aortic regurgitation and after 24 hours of hospitalization in the intensive care unit, they were discharged from the hospital six days after the procedure and consequent progressive decrease in associated symptoms. From the sample space, 4 patients died, due to non-surgical causes.

**Conclusion:** The data presented show the effectiveness of the procedure in patients of high and medium complexity. Therefore, the preparation and efficiency of the cardiology team responsible for managing cases and modifying the prognosis of patients who benefit from TAVI in this region of the country is of remarkable importance.

111405

Modality: E-Poster Scientific Initiation – Non-case Report

Category: HEMODYNAMICS AND INTERVENTIONAL CARDIOLOGY

## Recanalization of Coronary Chronic Total Occlusions in the Daily Practice: Insights from a Multicenter Registry in Latin America

FRANCIELE ROSA DA SILVA^1^, Lucio Padilla^2^, Antonio Carlos Botelho^3^, João Eduardo Tinoco de Paula^4^, Carlos Augusto Homem de Magalhães Campos,^5^, Marco Alcantara^6^, Ricardo Santiago^7^, Franklin Leonardo Hanna Quesada^8^, Félix Damas de Los Santos^9^, Marcia Moura^1^, Pedro Piccaro de Oliveira^1^, Alexandre Schaan de Quadros^1^

(1) Instituto de Cardiologia do Rio Grande do Sul (ICFUC); (2) Instituto Cardiovascular de Buenos Aires; (3) Hospital São Jose do Avai; (4) Instituto Cardiovascular de Linhares (UNICOR); (5) InCor; (6) Centro Médico 20 de Noviembre ISSSTE; (7) Hospital Pavia Santurce; (8) Clinica Comfamiliar; (9) Instituto Nacional de Cardiologia Ignacio Chavez

**Background:** Chronic total occlusion (CTO) percutaneous coronary interventions (PCI) has been increasingly performed worldwide, but there is few information in Latin America.

**Objectives:** To assess contemporary CTO PCI in the daily practice of Latin America.

**Methods:** Centers volunteered to participate, and no minimal number of procedures was required. The databank was managed in a REDCap platform, and clinical, procedural and outcome data were evaluated. Predictors of unsuccessful procedures were assessed by multivariable analysis.

**Results:** Data from 2.968 CTO PCIs performed in 63 hospitals from Argentina, Brazil, Bolivia, Chile, Colombia, Costa Rica, Ecuador, Mexico and Puerto Rico was analyzed. Most patients were male (78%) with a mean age of 64.0 ± 10.7 years. CTO PCI was performed mainly for angina control (83%) and/or to treat significant ischemia (30%). Radial access, dual injections and a microcatheter were used in most cases. The overall success rate of CTO PCI was 85%. The successful strategy was antegrade wire escalation in 80%, retrograde approach in 10% and antegrade dissection reentry in 10%. Proximal cap ambiguity, blunt stump, calcification, and previous attempt were predictors of unsuccessful procedures. In-hospital major adverse cardiovascular events occurred in 1.6% of the cases, and at the one-year follow-up 82% of the patients remained free of angina.

**Conclusions:** CTO PCI in Latin America has achieved a high rate of angiographic and clinical success with low complication rates in centers dedicated to these procedures.

111406

Modality: E-Poster Scientific Initiation – Non-case Report

Category: EPIDEMIOLOGY AND HEALTH POLICIES/GLOBAL HEALTH

## Relationship between Smoking and Occurrence of Amputation in Diabetes Associated with Systemic Arterial Hypertension: An Epidemiological Analysis in the State of Pará

MATHEUS RICARDO MALVEIRA CAMACHO^1^, Amanda Gabriela Freitas Rodrigues^2^, Wellen Sampaio Ferreira^1^, Izaura Maria Vieira Cayres Vallinoto^2^

(1) Universidade do Estado do Pará; (2) Universidade Federal do Pará

**Introduction:** The association between diabetes and hypertension is a public health problem with great socioeconomic impact, being responsible for great morbidity and mortality – generating high rates of hospitalization and burden on the Brazilian Unified Health System. Among the most common late complications of hypertensive and diabetic patients is amputation, a condition that requires a considerable amount of resources in its treatment and is associated with a significant reduction in the quality of life of patients. Added to this, recent studies demonstrate that the association between smoking, diabetes and hypertension may be responsible for early death due to macrovascular complications.

**Objectives:** To analyze the influence of smoking on the occurrence of amputations among the population that has both hypertension and diabetes.

**Methods:** A quantitative research was carried out in a secondary database using the digital platform of the Department of Informatics of the Unified Health System (DATASUS). Data referring to hypertension associated with diabetes between the years 2003 and 2013 in the geographic scope of the state of Pará were selected, analyzing the following variables: smoking and amputation. The data were worked with the help of tabulators for the Internet and for Windows (TABNET and TABWIN). The data found were analyzed by Pearson’s chi-square statistical test using the Biostat software.

**Results:** During the study period, 56,974 cases of hypertension concurrent with diabetes were reported in the state of Pará. Among the registered cases, 1632 (2.86%) were smokers, while 55,342 (97.14%) were non-smokers. Among the total of smokers, 578 progressed to amputation, making 35.42% of the participants in this group. On the other hand, among non-smokers, amputation occurred in 12,049 patients, a figure that represents 27.83% of this group. When calculating the p-value of the data, a value <0.0001 was obtained; therefore, there is a statistically significant difference in the occurrence of amputations between smokers and non-smokers.

**Conclusions:** There is an important relationship between the occurrence of amputations in patients with hypertension concomitant to diabetes and smoking in Pará. This data is in agreement with the current literature and signals the need for public policies related to the prevention of hypertension and diabetes, as well as encouraging smoking cessation in the state.

111407

Modality: E-Poster Scientific Initiation – Non-case Report

Category: CARDIOVASCULAR SURGERY

## Impact on Short-Term Outcomes of using a Coated Cardiopulmonary Bypass Circuit in Cardiac Surgery at a Referral Hospital

KARLA SANTOS PINTO^1^, Luiz Carlos Passos^1^, Rodrigo Morel^1^, Diogo Azevedo^1^, Jackson Brandão^1^, Clara Salles^1^, Mariana Baraúna da Silva^1^, João Pedro Martins Moreira Granja^1^, Beatriz Barbosa Viana^1^, Elias Soares Roseira^1^, Livia Maria Goes Lemos^1^, Raissa Barreto Lima^1^

(1) Hospital Ana Nery

**Introduction:** The surgical technique with Extracorporeal Circulation (ECC) is used as the technique of choice in most cardiac surgeries, whether myocardial revascularization or valvular revascularization. However, CEC acts as an aggressor agent due to the diversion of blood to an artificial circuit. This tool puts the patient at a greater risk of developing coagulopathies, infection and vasoplegia, due to the inflammatory response. Therefore, new technologies are being developed to minimize these risks, such as ECC with endothelial biological material coating.

**Goal:** To assess the impact on mortality and in-hospital outcomes of the use of a covered circuit for cardiopulmonary bypass

**Methods:** This is a prospective cohort with all patients undergoing cardiac surgery using ECC from March 2021 to February 2022, in a referral hospital in Salvador, Bahia. For analysis, they were divided into group 1 (use of the coated circuit) and group 2 (use of the standard circuit); there was no prerequisite for the use of the coated circuit, it was defined in a semi-random way in the distribution of operating rooms. Surgical mortality, length of stay in the intensive care unit (ICU), length of mechanical ventilation (MV) and time of vasoactive drug use (VAD) were evaluated. Statistical significance was considered for p < 0.05.

**Results:** A total of 292 patients were included, 104 (40.5%) using an ECC coated circuit. Of the patients, 128 (43.8%) were female and 152 (52.0%) of the surgeries involved valve approach and 140 (48.0%) underwent myocardial revascularization. The Euroscore II mean was 1.77% (±1.5). There was no difference in mortality, in the duration of mechanical ventilation: 7 (4–12) versus 5 (3.8–9); p = 0.058, DVA use: 23 (0–46.5) versus 24 (2.8–48); p = 0.950 or length of ICU stay: 3 (2–4) versus 3 (2–3); p = 0.057, respectively, for groups 1 and 2.

**Conclusion:** The use of a coated cardiopulmonary bypass circuit did not reduce mortality, duration of vasoactive drug use, length of stay in the ICU or time of use of mechanical ventilation.

111821

Modality: E-Poster Scientific Initiation – Non-case Report

Category: EPIDEMIOLOGY AND HEALTH POLICIES/GLOBAL HEALTH

## Epidemiological Profile of Correction by Cor Triatriatum in Brazilian Regions in 10 Years

SARA CRISTINE MARQUES DOS SANTOS^1^, Maria Luiza Silva Barbosa^1^, Alice Machado de Sales Silva^1^, Ivana Picone Borges de Aragão^1^

(1) Universidade de Vassouras UV

**Introduction:** Cor triatriatum, or triatriated heart, is a rare congenital anomaly, representing 0.1% to 0.4% of congenital heart diseases¹. Embryologically, it occurs when the pulmonary vein leaves a remnant in the left atrium, dividing it into three chambers. Physiologically, there is a similarity to mitral stenosis and other obstructive pathologies of the right ventricle2. This study aims to analyze the current panorama of Cor Triatriatum correction procedures performed in Brazil for 10 years and to correlate the current epidemiology with the results obtained.

**Methods:** A systematic literature review and an observational, descriptive, and cross-sectional collection of triatriatum color correction data, available at DATASUS – SUS Hospital Information System (SIH/SUS) for a period of ten years – December 2008 to December 2018.

**Results:** In the analyzed period, 61 hospitalizations were observed for the performance of color correction triatriatum procedures. The total expense was R $ 937,420.58, with the year 2017 being responsible for the highest cost: R $ 179,379.02. The 61 procedures were considered highly complex, of which 31 were performed on an elective basis and 30 were urgent. The total mortality rate in the 10 years studied was 12.90, corresponding to 8 deaths, a mortality rate of 50 was identified in the years 2010 and 2018, representing the highest, while the years 2009 and 2017 had the lowest rate, 11,11. The average total hospital stay was 14.6 days. The Brazilian region with the highest number of hospitalizations was the Southeast with 17 hospitalizations, followed by the Northeast region with 15, South, and Midwest with 12 and, finally, the North region with 5 hospitalizations. Among the units of the federation, the states of São Paulo and Minas Gerais concentrated the majority of hospitalizations, accounting for 8 each. The North region had the highest mortality rate (20.0), followed by the South region (16.67). The Midwest region had the lowest rate, with a value of 7.69.

**Conclusions:** From the present study, it can be observed that the North region, despite having the lowest number of hospitalizations, has the highest mortality rate when compared to other regions. It is worth noting that it is a rare congenital malformation and therefore, little discussed. Also, highlight the need for the correct notification of procedures, due to the absence of certain information, to improve the current epidemiological analysis.

111418

Modality: E-Poster Scientific Initiation – Non-case Report

Category: CARDIOVASCULAR SURGERY

## Association of Severe Pulmonary Hypertension and Mortality in Heart Valve Surgery in a Referral Center

KARLA SANTOS PINTO^1^, Elias Soares Roseira^1^, João Pedro Martins Moreira Granja^1^, Mariana Baraúna da Silva^1^, Beatriz Barbosa Viana^1^, Taina Viana^1^, Rodrigo Morel^1^, Ana Karenina^1^, Luiz Carlos Passos^1^, Clara Salles^1^, Livia Maria Goes Lemos^1^, Raissa Barreto Lima^1^

(1) Hospital Ana Nery

**Introduction:** Pulmonary hypertension (PH) is one of the factors associated with high morbidity and mortality, especially in valve surgeries. It is generally associated with more severe valve disease or with an evolved natural history.

**Objective:** To describe the association between PH and mortality in heart valve surgery, with main emphasis on patients with suprasystemic pulmonary artery pressure (PASP) with values above 100 mmHg.

**Methods:** This is a prospective cohort including all individuals over 18 years of age who underwent heart valve surgery using ECC from January 2020 to December 2021 in a referral hospital in the city of Salvador-Ba. For univariate analysis, chi-square test was used for categorical variables and t test for continuous variables. Multivariate analysis with logistic regression was performed, in which predictors with p < 0.05 in univariate analysis or those with biological plausibility were included. A value of p < 0.05 was considered statistically significant.

**Results:** A total of 474 individuals were included, with a mean age of 48.1 ± 14.4 years, 180 (38%) were male and the main etiology of valvular heart disease was rheumatic heart disease: 244 (51.5%), mortality estimated by Euroscore II was on average 2.34 ± 2.53. Mean PASP was 49.8 ± 19.6 and mean ejection fraction was 60.3 ± 12.1. PASP as a continuous variable was associated with higher mortality: RR 1.06 (CI 1.05–1.07); p < 0.001 and severe PH (considered as PASP > 70 mmHg) was associated with higher mortality: RR 3.7 (1.6–8.5); p = 0.005. Seven patients presented PASP above 100 mmHg, none of which evolved to surgical death.

**Conclusion:** Severe pulmonary hypertension was independently associated with higher mortality in heart valve surgery, which is an important factor to be considered in decision making regarding the surgical risk of patients who are candidates for surgery, with perspectives of specific conducts directed to the care of pulmonary circulation and function. of right chambers in the perioperative period.

111427

Modality: E-Poster Scientific Initiation – Non-case Report

Category: CARDIOVASCULAR SURGERY

## Predictors of Blood Transfusion in the Postoperative Period of Cardiovascular Surgery with Cardiopulmonary Bypass

KARLA SANTOS PINTO^1^, Aurea Maria Lago Novais^1^, Elias Soares Roseira^1^, Larissa Xavier Gomes da Silva^1^, Raissa Barreto Lima^1^, Ana Luísa de Aguiar Almeida Silva^1^, Tainá Viana^1^, Rodrigo Morel^1^, Luiz Carlos Passos^1^, Clara Salles^1^, Diogo Azevedo^1^, Ana Karenina^1^

(1) Hospital Ana Nery

**Introduction:** The need for blood transfusion in the postoperative period of cardiac surgery is associated with increased surgical morbidity and mortality. For this reason, it is important to identify predictors so that we can try to optimize them or try to solve them before the procedure.

**Objective:** To identify predictors of need for blood transfusion in the postoperative period of cardiovascular surgery using cardiopulmonary bypass (CPB).

**Methods:** This is a prospective cohort including all individuals over 18 years of age who underwent cardiac surgery using ECC from January 2020 to December 2021 in a referral hospital in the city of Salvador-BA. For univariate analysis, the chi-square test was used for categorical variables and the t test for continuous variables. Multivariate analysis with logistic regression was performed, in which predictors with p < 0.05 in univariate analysis or those with biological plausibility were included. A value of p < 0.05 was considered statistically significant.

**Results:** A total of 1668 individuals were included, with a mean age of 54.5 ± 14.4 years, 889 (53%) were male, 412 (25%). The majority underwent isolated valve surgery 738 (44.2%), followed by coronary artery bypass graft surgery in 672 (40.3%). The mean EuroSCORE II was 1.96 ± 2.26 and the mean baseline blood count (Hb) was 12.9 ± 2.0. There was a need for blood transfusion in 506 (30.3%). Blood transfusion was associated with higher mortality: RR 2.7 (CI 1.8–4.0); p < 0.001. The predictors of blood transfusion in the multivariate analysis were male gender: RR 1.66 (1.31–2.11) and preoperative Hb: RR 1.19 (1.10–1.28).

**Conclusion:** Blood transfusion in the postoperative period of cardiovascular surgery with the use of ECC was associated with higher mortality. Transfusion predictors were sex and preoperative hemoglobin. Therefore, it is important to identify patients with lower levels of basal hemoglobin to attempt optimization, with possible impact on surgical outcomes

111443

Modality: E-Poster Scientific Initiation – Non-case Report

Category: CARDIAC ARRHYTHMIAS/ELECTROPHYSIOLOGY/ELECTROCARDIOGRAPHY

## Electrocardiographic Findings Recognize Left Ventricular Aneurysm in Patients with Chagas Disease

LETÍCIA RODRIGUES GATTI PEREZ^1^, Carlos Alberto Pastore^2^, Nelson Samesima^2^, Leonardo Paschoal Camacho Varoni^2^, Mirella E. Facin^2^

(1) Universidade Municipal de São Caetano do Sul – USCS; (2) Instituto do Coração (InCor) – Hospital das Clínicas HCFMUSP, Faculdade de Medicina, Universidade de São Paulo

**Background:** The clinical presentations during the chronic phase of Chagas disease may be chest pain, thromboembolism, cardiac arrhythmias, sudden death and chronic heart failure. Although the electrocardiogram (ECG) has a crucial role in the diagnosis/prognosis of chronic Chagas cardiomyopathy (CCC) complications, there is no report of any ECG aspect associated with left ventricular (LV) apical aneurysms (present in 21%–46% of cases).

**Objective:** To characterize the ECG pattern of LV apical aneurysms in the chronic chagasic cardiomyopathy.

**Methods:** Cross-sectional study of patients with CCC, with and without LV aneurysm. From 2012–2019, 3377 patients were consecutively enrolled. Those with a time interval of ≤6 months between cardiac magnetic resonance imaging and ECG were included. Exclusion criteria were pacemaker, left bundle-branch blocks (LBBB), tachyarrhythmias, premature ventricular contractions, aneurysm in inferior wall, previous cardiac surgery and manifest coronary artery disease. Seventy-six patients were studied, 34% with aneurysm. The ECG analysis evaluated the presence of T-wave with plus-minus pattern in V2 and/or V3 and/or V4, in addition to DI and aVL leads, to identify those with LV aneurysm. In patients with LBBB+LSFB, the aneurysm was identified by the finding of ST-segment elevation in V2 ≥ 1 mm and a positive T-wave.

**Results:** A T-wave plus-minus pattern in V2 and/or V3 and/or V4 and/or DI and aVL identified LV apical aneurysm with sensitivity, specificity, positive (PPV), and negative predictive value (NPV) of 58%, 100%, 100% and 82%, respectively. ST-segment elevation in V2 ≥ 1 mm (in the presence of LBBB+LSFB) showed respectively 45%, 98%, 83% and 89%. The concomitant utilization of both criteria resulted in 77%, 98%, 95%, 89%, 91%, and 38 for sensitivity, specificity, PPV, NPV, accuracy and likelihood ratio, respectively.

**Conclusions:** The presence of a T-wave with plus-minus pattern in V2 and/or V3 and/or V4, and/or DI and aVL leads, in addition to ST-segment elevation in V2 ≥ 1 mm with positive T-wave (in the presence of LBBB+LSFB) showed high accuracy to identify LV apical aneurysm in patients with chronic Chagas cardiomyopathy. Further prospective studies should evaluate the relationship between these new ECG findings and morbi-mortality clinical outcomes.

111446

Modality: E-Poster Scientific Initiation – Non-case Report

Category: NEGLECTED CARDIOVASCULAR DISEASES

## Obstructive Sleep Apnea in a Smoking Cessation Group

RAFAEL MATOSO DE OLIVEIRA FIGUEIREDO^1^, Paula Gouvêa Abrantes^1^, Samuel Barud Massensine^1^, Rodrigo Máximo Silveira^1^, Moisés de Toledo Vilela^1^, Gabriela Godinho Rezende^1^, Amanda Gonçalves Vieira Martins^1^, Eliane Ferreira Carvalho Banhato^1^, Arise Garcia de Siqueira Galil^1^

(1) Cardiology Department, Medical School, Federal University of Juiz de Fora, Minas Gerais – UFJF

**Introduction:** Obstructive sleep apnea (OSA) and smoking have been established as prevalent and relevant risk factors for cardiovascular outcomes, such as stroke, acute myocardial infarction and death. Although their reported synergic effect, OSA can be an underdiagnosed and undervalued as a co-risk factor.

**Objective:** Assess the prevalence of OSA in a smoking cessation group and the correlations with comorbidities and other known risk factors.

**Methods:** A transversal study was conducted from 2021 to 2022, with six consecutive smoking cessation groups. The participants received comprehensive evidence-based support, including a multidisciplinary team care with pharmacological treatment. A STOP-Bang score of at least 5 was defined as high-risk screening for OSA, due to its high sensibility (>90%) and accuracy. Other clinical data were collected and analyzed for knowledge between the OSA positive and negative screening groups.

**Results:** A sample of 36 smokers, 66.7% female, 56.63 ± 10.8 years old and 42.9% with low schooling (<8 years). Comparing users with measured positive OSA with the negative OSA, it was possible to verify that the prevelance of arterial hypertension (p < 0.0001), cardiac arrhythmias (p < 0.012), abdominal obesity (p < 0.049), cognitive decline (p < 0.001), anxiety (p < 0.035) and a high tendency for depression (p < 0.077) were significantly higher for the OSA group. Regarding smoking history, nicotine dependence (p < 0.002), prevalence of the smoking triggers: stress, habits and coffee use (p < 0.001, p < 0.021 and p < 0.013, respectively) and use of marijuana and crack (p < 0.002) were significantly higher in the positive screening for OSA group.

**Conclusion:** We observed that tobacco use might be a potential factor for aggravating or developing obstructive sleep apnea. Therefore, the synergic effect goes beyond both being relevant for cardiovascular outcomes and death. These findings may suggest how both can impact the global patient health, as isolated or, more importantly, combined risk factors.

111448

Modality: E-Poster Scientific Initiation – Non-case Report

Category: ATHEROSCLEROSIS/CARDIOVASCULAR RISK FACTORS/CARDIOVASCULAR PREVENTION

## E-Cigarette use as a Cardiovascular Risk Factor: A Systematic Review

MARIA GABRIELA MEDEIROS CUNHA DE ARAUJO^1^, Anna Luiza Fragoso Guimarães Costa^2^, Beatriz Cunha Lisboa de Medeiros Nunes^3^, Eduarda Caroline Lopes de Freitas^4^, Felipe Andrade de Lima Trindade^2^, Felipe José Cavalcanti de Albuquerque Holanda^2^, João Victor Bezerra Ramos^1^, Lívia Farias de Holanda Furtado^1^, Mateus Ribeiro Fernandes Teixeira^5^, Quezia Valerio Brito^6^

(1) Universidade Federal da Paraíba; (2) Faculdade de Ciências Médicas da Paraíba; (3) Universidade Potiguar – UnP; (4) Centro Universitário Fametro; (5) Centro Universitário de João Pessoa – UNIPÊ; (6) Universidade Nilton Lins

**Introduction:** Electronic cigarettes or e-cigarettes (EC), used as an alternative to smoking cessation, have become popular despite the uncertain cardiovascular risk. Although toxic-carcinogenic substances are present at lower levels in ECs compared to tobacco cigarette (TC) smoking, the products in e-liquid can be harmful to health and worryingly are often consumed by a nonsmoking public, especially young people.

**Objective:** To provide current insight into the relation of e-cigarettes and their effects and or risks of developing cardiovascular disease.

**Methods:** This is a systematic review, using the PubMed and Scopus databases. The Mesh descriptors used were: Electronic Nicotine Delivery Systems, Heart Disease Risk Factors, Cardiovascular Diseases and their synonyms. The initial search generated a total of 1301 articles, which after using filters resulted in 113 and after inclusion and exclusion criteria, 35 articles, used for the present study, for which the PRISMA methodology was followed.

**Results:** It is notorious the divergence on the effects and cardiovascular risks of ECs. Some studies show damage in cellular repolarization, increase in heart rate and rhythm, and mean arterial pressure, while others do not. Furthermore, most users make dual use of electronic cigarettes and combustible cigarettes, making them more vulnerable to cardiovascular risk factors than smokers of tobacco alone or never smokers, with a higher prevalence of Metabolic Syndrome, for example. Switching from conventional smoking to the use of electronic devices is associated with a potential lower cardiovascular risk compared to continuing conventional tobacco, but some studies show no useful change in biomarkers of oxidative stress. On the other hand, users who started electronic devices after conventional ones, have a substantial higher cardiovascular risk than those who have ceased smoking. However, according to imaging methods, both conventional cigarette smokers and EC users have more carotid plaque disposition and similar alteration of vascular stiffness, with no difference in inflammatory degree between them, when compared to nonsmokers.

**Conclusion:** The use of tobacco and the use of ECs cause damage to the cardiovascular system and alter inflammatory biomarkers. However, there is still a lack of evidence-based studies on this subject, since many of the studies are based only on acute and not chronic alterations, and the irreversibility of the disease is unknown.

111449

Modality: E-Poster Scientific Initiation – Non-case Report

Category: ATHEROSCLEROSIS/CARDIOVASCULAR RISK FACTORS/CARDIOVASCULAR PREVENTION

## Relation between Pulse Wave Speed and Cardiovascular Risks Variables

FERNANDA ABRAHAM LEÃO^1^, Maria Eduarda S. Barbosa^1^, Raquel P. Cabús^1^, Marco Antonio Mota Gomes^1^, Waléria Dantas Pereira^1^, Márcia Sâmia Fidelix^1^, Annelise Machado Gomes de Paiva^1^

(1) Centro Universitário Cesmac

**Background:** The onset of CVD is most often associated with genetic predisposition, sex and age, in addition to a lifestyle that includes alcoholism, smoking, physical inactivity and diet; factors that can also influence the development of Type 2 Diabetes, Dyslipidemia and Arterial Hypertension. Given this situation, it is pertinent to analyze the relation between blood pressure parameters and cardiovascular risk variables.

**Objectives:** To analyze the relation of different risk factors with PWV as an outcome.

**Methodology:** Identification and behavioral data were formed, risk factors were verified, and the study variables were: age, sex, smoking, sedentary lifestyle, alcoholic beverages, presence of hypertension, diabetes, dyslipidemia, in addition to peripheral parameters and blood pressure (blood pressure and pulse wave velocity) which was created by the Mobil-O-Graph® equipment. An anthropometric assessment (weight, height) was performed and then the calculation of the body mass index (BMI) was verified.

**Results:** When all risk factors were observed, 58.3% of the sample had hypertension, one of the most relevant pathologies for increasing PWV and corrected for age, it was noted that SBP with a p-value of <0.01, alcoholism with a p-value of 0.04 and smoking with a p-value of 0.02 were significant in relation to PWV.

**Conclusion:** It appears that age and sex are the main causes of increased PWV, but they are not unique components, analyzed as risk factors, and it was noted that it is not an innate factor, but a set of mortifiable conditions.


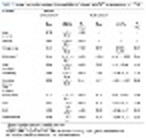


112103

Modality: E-Poster Scientific Initiation – Non-case Report

Category: CARDIOLOGY OF SPORTS, EXERCISE, ERGOMETRY AND CARDIOVASCULAR REHABILITATION

## Relevance of Myocardial Changes in Functional Capacity in Patients with Chronic Chagas Cardiomyopathy

ENRICO DE FRANCISCO MAGNANI^1^, Jhessica Macieira Pereira^1^, Danielle Aparecida Gomes Pereira^1^, Rafael Dias de Brito Oliveira^1^, Thayrine Rosa Damasceno^1^, Suelen Cristina Silva de Oliveira^1^, Denise Mayumi Tanaka^2^, Eduardo Elias Vieira de Carvalho^2^, Júlio César Crescêncio^2^, Eduardo Rubio Azevedo^2^, Marcus Vinícius Simões^2^, Luciano Fonseca Lemos de Oliveira^1^

(1) Universidade Federal de Minas Gerais – UFMG; (2) Faculdade de Medicina de Ribeirão Preto – FMRP/USP

**Introduction:** Heart failure (HF) is one of the main disorders that characterize chronic Chagas cardiomyopathy (CCC), and it is directly related to diffuse myocarditis, ventricular dilatation and reparative fibrosis. With its development, there is a consequent reduction in functional capacity and the appearance of symptoms such as fatigue and dyspnea on exertion. However, little is known about the factors that determine such reduction in functional capacity in CCC.

**Objective:** To evaluate the functional capacity and identify the determining factors of the reduction of the maximum oxygen consumption (VO2peak) in patients with CCC.

**Methods:** Observational study using 101 patients with CCC, who underwent clinical examination, cardiopulmonary exercise test (CPET) and echocardiography as part of the standard clinical evaluation. Data normality was analyzed using the Kolmogorov-Smirnov test. The association between VO2peak and clinical or echocardiographic variables were analyzed by Pearson or Spearman correlation coefficients. Multivariable linear regression analysis was used in order to identify independent clinical and echocardiographic predictors associated with peak VO2max.

**Results:** Mean age of study patients was 56.2 ± 13.4 years. The mean left ventricular ejection fraction (LVEF) was 42.6 ± 18%, and mean VO2peak was 16.8 ± 6.1 ml/kg/min–1. VO2peak was associated with age (r = 0.44; p < 0.001), male gender (p < 0.001), functional class (FC-NYHA, r = 0.53; p < 0.001), LVEF (r = 0.51; p < 0.001), LV diastolic diameter (r = –0.38; p < 0.001), left atrium diameter (r = –0.34; p < 0.05), E wave (r = –0.32; p = 0.01) and LV mass index (r = –0.28; p = 0.006). In the multivariate analysis, only age, male gender, LVEF and E wave remained independently associated with VO2peak with adjusted R2 = 0.66. In patients who were able to assess pulmonary artery systolic pressure (PASP, n = 45) and tricuspid annular plane systolic excursion (TAPSE, n = 55), the VO2peak correlated significantly with PASP (r = –0.60, p < 0.001) and with TAPSE (r = 0.28, p = 0.04).

**Conclusion:** In patients with CCC, disease severity (FC-NYHA), male gender, LV systolic and diastolic function, as well as pulmonary hypertension influence functional capacity.

111520

Modality: E-Poster Scientific Initiation – Non-case Report

Category: EPIDEMIOLOGY AND HEALTH POLICIES/GLOBAL HEALTH

## Epidemiological Study of Congenital Heart Diseases in Brazil

VANESSA FARIA DE ALMEIDA SCHNEIDER^1^, Larissa Lima Nunes^1^, Inaiê Maiala De Almeida Miranda^1^, Ana Gabriela da Silva Farias^1^, Leandro Rozin^1^

(1) Faculdades Pequeno Príncipe

**Introduction:** Congenital Heart Diseases (CHD) are abnormalities in cardiocirculatory structure or function that occur in the intrauterine period, being diagnosed during pregnancy or after birth. They range from communications between cavities that regress spontaneously, to large malformations that require several invasive procedures, which can result in intrauterine death, in childhood or even in adulthood.

**Objective:** To estimate the prevalence of CHDs and their most frequent subtypes in Brazil, from the years 2017 to 2021.

**Methods:** An observational epidemiological survey was carried out using data from the Information System on Live Births (SINASC) and DataSUS, available by the Ministry of Health. We analyzed, through descriptive statistics, the incidences at birth and the prevalence in that period, bringing information according to the country’s regions for every 10,000 live births.

**Results:** In the total of 5 years researched, 14,748 cases of Congenital Malformations Of The Circulatory System (Q20–Q28) occurred in Brazil. Its highest incidence (18.59) was in 2017 in the Southeast region. Among the subtypes of the disease, the Southeast region continues in highlight due to Congenital Malformations Of The Cardiac Septum (Q21), with a prevalence of 7.79, reaching its highest incidence (8.87) in 2017. The greatest rates of this subtype correspond to Atrial Septal Defect (Q21.1), with a prevalence of 5.09 and its highest incidence of 6.04 occurred in 2020. Another highlighted subtype concerns Other Congenital Heart Malformations (Q24), which reached a prevalence of 4.74 in the South of the country and its highest incidence of 5.17 in 2020 – its highest rates account for Unspecified Heart Malformations (Q24.9), with a prevalence of 3.77 and its highest incidence of 4.02 occurred in 2018. A fact that draws attention is that, in the sum of the 5 years, more than 65% of cases of CHD occurred in the Southeast region, information that opens valuable precedents for new research.

**Conclusions:** It is a fact that the difficulty in diagnosing Congenital Heart Diseases represents a significant Global Health Problem, mobilizing public policies for early diagnosis, which in Brazil has the “Teste do coraçãozinho” (Little Heart Test), Morphological Ultrasound Exams, and Fetal Echocardiography. Despite this, it is worth mentioning that there is a disparity in access to these resources, which represents another challenge to the issue and may indicate underreporting of cases.

111550

Modality: E-Poster Scientific Initiation – Non-case Report

Category: HEMODYNAMICS AND INTERVENTIONAL CARDIOLOGY

## Case Series of Percutaneous Balloon Mitral Valvuloplasty for Severe Mitral Stenosis Correction in a High Cardiological Complexity Service in the Western Amazon

CAMILA ALENCAR DE ANDRADE^1^, Gabriel Irismar Rodrigues Schwamback^1^, Bruna Katharine Cavalcante Nascimento^1^, João Pedro de Oliveira Vicente^1^, Leo Christyan Alves de Lima^1^, Fabiana Cristina Schabatoski Passos^2^, Alexandre Venturelli da Silva^2^, Luiz Henrique Gasparelo^2^

(1) Centro Universitário São Lucas – UNISL; (2) Angiocenter

**Introduction:** First introduced more than 40 years ago by Japanese surgeon Kanji Inoue, percutaneous balloon mitral valvuloplasty (PBMV) is performed using a balloon inflated with high pressure in a stenotic heart valve. Mitral stenosis (MS) is a serious disease that can progress to elevated left atrial pressure and pulmonary hypertension, formation of emboli, among other complications.

**Objectives:** This is a descriptive study of registration of cases of percutaneous mitral valvuloplasty with Inoue balloon, performed in a highly complex service in the state of Rondônia, in the Western Amazon.

**Methods:** Data obtained through research via medical records and complementary exams were used.

**Results:** In this case series, four patients were followed, three female aged 24, 42 and 54 years and one male aged 28 years. All patients were symptomatic (complaints of fatigue and/or dyspnea). On echocardiography, they showed significant MS with a mean gradient of 13 to 20 mmHg. The procedures were performed through the right femoral vein with an Inoue balloon, through transseptal puncture performed with the aid of transesophageal echocardiography. Patients had a hospital stay of less than two days. In the one-year follow-up with echocardiographic control, they had a favorable evolution of less than moderate stenosis and insufficiency.

**Discussion:** Correction of MS is indicated for patients with severe symptomatic mitral stenosis (dyspnea with New York Heart Association II to IV) or asymptomatic patients with complicating factors, including pulmonary hypertension or new-onset atrial fibrillation. The patient should preferably have an echocardiographic score less than or equal to 8 (with subvalvular apparatus and calcification less than or equal to 2). Patients who present pre-procedure echocardiographic score greater than 8, post-procedure mitral valve area with a value of less than 1.5 or post-procedure mitral regurgitation with a value greater than or equal to 2, will have the need for of mitral valve replacement surgery at 2 years after of the procedure.

**Conclusion:** Although it is a recent procedure for the region when compared to large centers, performing PBMV for the treatment of severe MS in the state of Rondônia promotes a safe procedure, capable of improving the patient’s quality of life and increasing life expectancy, with a relatively low risk.

111936

Modality: E-Poster Scientific Initiation – Non-case Report

Category: CONGENITAL AND PEDIATRIC CARDIOLOGY

## Incidence of Dextrocardia in Brazil in the Last Decade: An Epidemiological Analysis

BRUNA VIEIRA SILVA OLIVEIRA^2^, Ana Paula Fernandes Pereira^2^, Marla Thais Fernandes Teodoro^3^, Davi Marques de Souza^1^, João Victor Silva Souza^1^, Lara Oliveira Santana Rocha^1^

(1) Universidade estadual do sudoeste da Bahia; (2) Universidade federal da Bahia; (3) Faculdade Santo Agostinho

**Introduction:** Dextrocardia is a congenital malformation that consists of a shift of the heart axis to the right side of the chest, contrary to physiological and derives from a change in the embryological process. In the literature, the overall incidence is about 10:100,000, and may be underdiagnosed in most cases.

**Objectives:** To determine the annual incidence of the malformation in each Brazilian region during the last 10 years.

**Methods:** Data collection in the platform of the SUS Department of Informatics – Information System on Live Births (DATASUS – SINASC), separated by region – north, northeast, south, southeast, and center-west – and year – 2010 to 2019 – with statistical calculations of the Brazilian incidence rate per 100,000 inhabitants, in order to make the data more tangible.

**Results:** The Brazilian region with the highest incidence was the southeast, with 92 cases, and the lowest was the midwest, with 8. The year 2016 had the highest number of cases, totaling 16. Table 1 shows the relative data by region in each year. The incidence rate was about 0.5:100,000 population.

**Conclusions:** Dextrocardia is still an underdiagnosed condition in Brazil, since the incidence rate was lower than the global rate. A result that corroborates this statement is that the southeastern region had the highest incidence in all years, perhaps due to greater access to diagnostic methods or because of a lower prevalence of complications related to the condition.


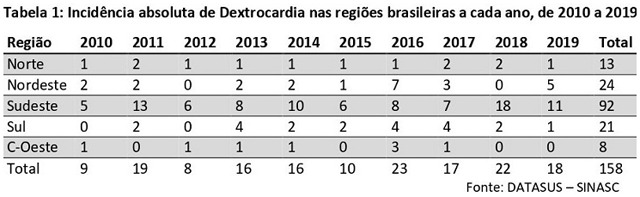


111573

Modality: E-Poster Scientific Initiation – Non-case Report

Category: EPIDEMIOLOGY AND HEALTH POLICIES/GLOBAL HEALTH

## Initial Experience of the Telehealth Nucleus of the University Hospital of the Federal University of Piauí: Results of the Tele-Electrocardiogram

ANTONIO MAYCON DA SILVA SOUSA^1^, Carlos Eduardo Batista de Lima^2^, Victor Eulálio Campelo^2^, Ginivaldo Victor Ribeiro do Nascimento^2^, Newton Nunes de Lima Filho^2^, Thiago Nunes Pereira Leite^2^, Jônatas Melo Neto^2^, Maurício Giraldi^2^, Lucas Teixeira Dias^2^, Paulo Márcio Sousa Nunes^2^

(1) Universidade Federal do Piauí (UFPI); (2) Hospital Universitário da Universidade Federal do Piauí (HU-UFPI)

**Introduction:** Telemedicine experienced strong growth after the Covid-19 pandemic.

**Objective:** Evaluate the clinical demographic profile, rate and probability of occurrence of electrocardiographic abnormalities in the population studied in relation to sex and age.

**Methods:** The telehealth center of the University Hospital of the Federal University of Piauí (THC UH-FUPI) uses an analysis system that allows receiving exams from peripheral units. From November 2020 to March 2022, data from patients who underwent electrocardiogram (ECG) in basic health units sent to THC UH-FUPI were evaluated. The variables analyzed were: age, gender and electrocardiographic diagnosis: unspecific changes in ventricular repolarization (UCVR), old infarction, bundle branch block, ischemia, intraventricular conduction disorder (IVCD), arrhythmias and chamber overloads. For statistical analysis, Anderson-Darling, Mann-Whitney and logistic regression tests were used with a significant p-value <0.05.

**Results:** ECG of 27955 patients were evaluated, aged between 51.9 ± 17.3 years and predominantly female (63.0%). Of the ECGs analyzed, 6075 (21.7%) had an abnormality, with a higher occurrence with advancing age (p < 0.0001). UCVRs were more prevalent (2337; 7.96%), followed by conduction disorders (1949; 6.63%), arrhythmias (1401; 4.77%), bundle branch block (594; 2.02%), chamber overload (481; 1.64%), old infarction (359; 1.22%) and ischemia (30; 0.1%). Atrial fibrillation (AF) was observed in 138 cases (0.5%); 13% in <60 years; 87% in >60 years. Male patients were more likely to have an ECG change (OR 2.25, 95%CI 1.77–2.78), however, adjusted for advancing age, this probability cancels out. As a factor regardless of age, males had a lower chance of UCVR (OR 0.85; 95%CI 0.78–0.93), and a higher chance of arrhythmias (OR 1.29; 95%CI 1.15–1, 44), old infarction (OR 1.69; 95%CI 1.37–2.09), and ischemia (OR 1.93; 95%CI 0.93–3.99).

**Conclusion:** In the population studied, the rate of abnormal ECGs was high (21.7%), increasing with age, with females being more prevalent. The rates of occurrence of AF and ischemia were low (0.5% and 0.1%, respectively). The main alteration was UCVR with a higher chance of occurrence in females and males were more likely to have arrhythmias, old infarction and ischemia. In this initial experience, it was observed that the tele-ECG is feasible and can be implemented, with a wide reach, even in regions where there is greater difficulty in accessing health care.

111571

Modality: E-Poster Scientific Initiation – Non-case Report

Category: EPIDEMIOLOGY AND HEALTH POLICIES/GLOBAL HEALTH

## Mortality Due to Secondary Hypertension as Underlying Cause and Mentioned Cause in Brazil and Regions from 2000 to 2019

RENATTA KAROLINE BEKMAN VOGAS^1^, Jonatas Benarroz da Silva^1^, Davi da Silveira Barroso Alves^1^, Paulo Henrique Godoy^1^

(1) Federal University of the State of Rio de Janeiro – UNIRIO

**Introduction:** Secondary hypertension (SH) is a condition in the group of hypertensive diseases, which has a lower prevalence. Knowing mortality from SH is important, however, the lower prevalence can influence its analysis in death records.

**Objective:** To analyze mortality due to SH, when registered as the underlying cause of death (UCD) and mentioned cause (MC), according to age group and sex, in Brazil and regions, from 2000 to 2019.

**Methods:** Ecological study on deaths due to SH, registered as UCD and MC in lines A to D, of death certificates, from the Brazilian Mortality Information System. Code I15 of the International Code of Diseases 10 (ICD-10) was used. Crude and standardized mortality rates were estimated for UCD and MC, for Brazil and regions, from 2000 to 2019, according to sex and age groups.

**Results:** For UCD 14 deaths were recorded, the standardized rates were similar for both sexes. The highest frequency of deaths occurred in the age group of 60 years or older. In the Midwest region, no deaths were recorded, while the Northeast accounted for 57% of deaths. As for MC, 16,892 were registered due to HS. This result is 1,200 times greater than that found in the records by UCD. There was greater disparity between the sexes than in UCD analysis, with 43% of the standardized rate for males higher than females. Standardized rates were lower in women and crude rates increased with age. The North region presented the highest standardized rate and the Southeast the lowest, in both sexes.

**Conclusions:** It is possible that the selection rules, defined by the ICD-10, for UCD underestimate deaths from HS. Thus, it may be useful to consider MC as a method for analyzing mortality from less prevalent diseases.


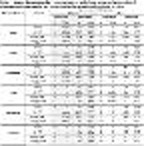


111701

Modality: E-Poster Scientific Initiation – Non-case Report

Category: CARDIO-ONCOLOGY

## Mortality Due to Malignant Neoplasms of the Heart, Mediastinum and Pleura in Brazil

MARCELLA OLIVEIRA MONTE SANTO^1^, Afonso Moraes Melo Junior^3^, Ana Flávia Oliveira de Souza^3^, Camila Rodrigues Maciel^3^, Cecília Gomes da Silva^1^, Lucas Guimarães Junqueira^2^, Mainã Cristina Santos dos Santos^2^, Rafael Silva Lemos^2^, Rafaela Oliveira Cardoso^3^, Paola Bitar de Mesquita Abinader^3^, Luis Eduardo Werneck de Carvalho^4^, Davi Gabriel Barbosa^2^

(1) UNIVERSIDADE FEDERAL DO PARÁ (UFPA); (2) UNIVERSIDADE ESTADUAL DO PARÁ (UEPA); (3) CENTRO UNIVERSITÁRIO DO PARÁ (CESUPA); (4) ONCOLÓGICA DO BRASIL

**Introduction:** Within the thoracic region there are 3 main sites of development and metastasis of malignant neoplasms: the heart, the mediastinum and the pleura. Although neoplasms in the cardiac muscle tissue are rare, there are cases of primary and secondary tumours, representing 0.05% and 1–2% of neoplasms. In contrast, pleural tumours are more common, the main cancer of which is mesothelioma in its diffuse form. In general, most tumours present in these organs are metastases from other neoplasms in advanced stages.

**Objectives:** To establish an overview of mortality from malignant neoplasms of the heart, mediastinum and pleura in Brazil, through a time-series study, between 2011 and 2020.

**Methodology:** This is a cross-sectional study, evaluating the information on mortality from malignant neoplasms of the heart, mediastinum and pleura in the years 2011 to 2020. A search was performed in the tabnet DATASUS system. The ICD C38 (heart, pleura and mediastinum) was selected to perform the search and comparison between the regions of Brazil. It established the stratification by age range affected, the division by gender and the total number of cases per year in the selected period.

**Results:** In the period 2011–2020 about 7585 deaths from cancer of the heart, mediastinum and pleura, and 2019 was the year with the highest number of deaths; 867 were recorded. In 2019, the southeastern region had the highest number of deaths (45.7%). Of the total absolute deaths in the regions, 5859 (77.3%) were in ages 50 years and above, with the most prominent age group being 60–69 years with 1809. Comparing the sexes, there were more male deaths, with 4,249 (56.01%) registered, compared to the female, with 3,332 deaths (43.92%). Both genders presented a higher number of deaths in 2019, with men representing 54.09% and women 45.91%. Moreover, the highest number of deaths of both sexes was registered in the Southeastern region and the most affected age group was 60–69 years. Women presented a greater constant of deaths along the years than men.

**Conclusion:** It appears that neoplasms of the heart, mediastinum and pleura in Brazil have a higher incidence at ages between 60–69 years. There was a predominance of male deaths, besides the southeastern region accounting for most of the deaths. Thus, it is necessary to invest in prevention actions directed to this public, in order to reduce the number of deaths from these neoplasms.

111604

Modality: E-Poster Scientific Initiation – Non-case Report

Category: CARDIOVASCULAR SURGERY

## Assessment of Postoperative Pain on Cardiac Surgery Patients Randomized between Preemptive Pregabalin and Placebo: Preliminary Analysis of Pregaba-Heart Study

FABIO ANTONIO SERRA DE LIMA JUNIOR^1^, Fábio Antônio Serra de Lima Júnior^1^, Ana Beatriz Venâncio de Paula Bezerra^1^, Renan Furtado de Almeida Mendes^1^, David Cesarino de Sousa^1^, Raphael Patrik Borges da Costa^1^, Isaac Newton Guimarães de Andrade^1^, André Telis de Vilela Araújo^1^

(1) Universidade Federal da Paraíba

**Introduction:** Cardiac surgery, through sternotomy, can cause intense postoperative pain, most commonly in the immediate postoperative period, affecting 50% of patients and may lead to significant short- and long-term consequences, as well as chronification. Despite this, between 50% and 75% of patients do not receive adequate analgesic management. Few randomized controlled trials have addressed the efficacy of pregabalin administration in the perioperative period for pain control, delirium, and postoperative recovery, obtaining variable results.

**Objectives:** This study aimed to evaluate the preemptive use of pregabalin to decrease pain perception in patients undergoing cardiac surgery in the first three postoperative days.

**Methodology:** Patients, undergoing cardiac surgery, were randomized into two groups: one group will preemptively use pregabalin 75 mg on the day of surgery until the second postoperative day, and the other group will use placebo for the same period. After postoperative extubation, they were evaluated at 24, 48, and 72 hours for visual analog scale (VAS) and analgesic use. The study was registered in ClinicalTrials.gov through NCT04173390.

**Results:** Twenty-three patients were included, with 11 receiving pregabalin and 12 placebo, presenting, respectively, visual pain scale means of 1.75 ± 2.188 vs. 2.27 ± 2.412 (p = 0.634) at 1st PO day; 0.88 ± 1.246 vs. 2 ± 2.049 (p = 0.188) at 2nd PO day; and 1.75 ± 2.915 vs. 1.64 ± 1.804 (p = 0.918) at 3rd PO day. There was no statistically significant difference in the use of simple analgesics, weak or strong opioids on any of the postoperative days.

**Conclusion:** In this partial evaluation of the study, pregabalin did not affect patients‘ postoperative pain scores, nor did it affect their analgesic consumption. The study will continue to randomize and collect patients until it reaches the anticipated sample size for more adequate statistical power.

111609

Modality: E-Poster Scientific Initiation – Non-case Report

Category: CARDIO-ONCOLOGY

## Myocardial FDG Uptake Variation and its Relationship with Survival During Immunotherapy

MATHEUS COELHO TORRES^1^, Juliana Góes Martins Fagundes^2^, Luis Fabio Barbosa Botelho^1^, Thiago Lins Fagundes de Sousa^5^, Alinne Fernanda Amaral Verçosa^3^, Rodrigo de Carvalho Flamini^3^, Eudanusia Guilherme de Figueiredo^2^, Emilio Carlos De Arruda Lacerda^2^, Igor Lemos Duarte^2^, Jean Fabrício De Lima Pereira^2^, Marcelo Dantas Tavares De Melo^1^, Silvia Moreira Ayub Ferreira^4^

(1) Hospital Universitário Lauro Wanderley – UFPB, João Pessoa, Brazil; (2) Oncoclinicas Paraiba (Centro Paraibano de Oncologia), Joao Pessoa, Brazil; (3) Nova Diagnostico por Imagem, Joao Pessoa, Brazil; (4) Instituto do Coração, Sao Paulo, Brazil; (5) Hospital Universitário Alcides Carneiro, UFCG, Campina Grande, Brazil

**Background:** The rise of Immune Checkpoint Inhibitors (ICI) as a cancer treatment has been followed by the increase of cardiotoxicity events reports. Retrospective studies with anthracycline revealed a relationship between increased myocardial 18F-FDG uptake (MGU) and a slight drop in Left Ventricular Ejection Fraction values, suggesting that this may be a useful tool for early detection of cardiotoxicity. Our study is intended to evaluate the prognostic impact of the variation in MGU in advanced lung cancer undergoing treatment with ICI.

**Methods:** This is a unicentric, retrospective study, that evaluated lung cancer patients treated with ICI from 2016 to 2021 and submitted to at least two positron emission tomography (PET-CT). The primary objective was to evaluate the MGU variation during ICI treatment (ΔSUV) and its impact on survival outcomes.

**Results:** 59 patients fulfilled all inclusion criteria. 51% received Immunotherapy in combination with chemotherapy and 49% received only immunotherapy. Global median ΔSUV was +0.05; Among patients in the disease control group, the median ΔSUV was +0.48, while the median ΔSUV of the disease progression group was –0.66. Considering patients with positive myocardial ΔSUV versus negative ΔSUV, there was a median increase of progression-free survival (PFS) of 161 days in favour of the positive myocardial ΔSUV group (p = 0.066) and increase of 281 days on Overal Survival (p = 0.256). There was an increase of 759 days in the OS of diabetic patients (p = 0.023), and better results in metformin users (p = 0.015), in addition to an increase of 285 days in favor of beta-blocker (BB) users (p = 0,886). The trend data found indicate that BB and/or metformin could perhaps have a supporting role in the treatment of ICI. These findings correlate with recent evidence showing an increase in MGU after BB use and that BB may function as immunomodulatory agents improving immunotherapy results.

**Conclusions:** The increase in MGU was associated with better outcomes, suggesting that perhaps it could be studied as a predictor of treatment response, in addition to the trend of benefit also shown with the use of metformin and BB, which strengthens their possible synergistic effect in treatment. However, more robust studies are needed to better understand the MGU role on ICI treatment.

111642

Modality: E-Poster Scientific Initiation – Non-case Report

Category: ATHEROSCLEROSIS/CARDIOVASCULAR RISK FACTORS/CARDIOVASCULAR PREVENTION

## Detection and Monitoring of Arterial Hypertension Through Residential Monitoring of Blood Pressure (MRPA) in Adolescents: A Literature Review

PAOLA DE OLIVEIRA DAS CHAGAS^1^, Camila Alvarez Alonso^1^, Luz Alcira Avila Rincon Alves^1^, Fabiane Rosa Rezende Honda Marui^1^

(1) Universidade Municipal de São Caetano do Sul – USCS

**Introduction:** Hypertension in adolescents has become more and more frequent due to the increase in the childhood obesity rate. In addition to obesity and overweight, stress and excessive salt intake are also factors related to increased blood pressure in this age group. The increase in cases of hypertension in adolescence has been a recent concern, since until the mid-1970s, the measurement of Blood pressure in asymptomatic children was not frequently performed, after updates to the guidelines, it is recommended that in the absence of comorbidities, the Blood pressure is measured annually. ABPM, which is the gold standard for monitoring, has been the most recommended for confirming the diagnosis of AH, but despite the recommendation, ABPM has high costs and risks of failure. Home blood pressure monitoring has shown great efficacy and sensitivity when compared to ABPM, although there are not enough studies.

**Method:** The bibliographic survey was carried out from articles collected on the LILACS and MEDLINE platforms. Articles published between 2012 and 2021, in Portuguese, English and Spanish were eligible. The articles were initially selected by reading the titles and abstracts for categorization according to the study objectives. Subsequently, the selected ones were read in full.

**Results:** Considering the analysis of the studies, it was identified that the ABPM obtained values similar to the ABPM when performed in adolescents and presented a better cost-benefit ratio, in addition to being more effective in identifying White apron effect and masked hypertension. Studies have shown that this accuracy prevents teenagers from being misdiagnosed and that they consequently involve unnecessary treatment with antihypertensive drugs.

**Final considerations:** Although evidence on HBPM is still limited, the literature review points out that the values obtained through home monitoring proved to be accurate when compared to other methods and also provided information on the daily variability of the assessed public, showing be a precise alternative in confirming the diagnosis of hypertension and young people.

111648

Modality: E-Poster Scientific Initiation – Non-case Report

Category: EPIDEMIOLOGY AND HEALTH POLICIES/GLOBAL HEALTH

## Epidemiological Profile of Hospitalizations and Deaths Due to Systemic Arterial Hypertension in a Brazilian Amazon’s State

GABRIEL NERY LIMA^1^, Arthur Afonso Ferreira Rebelo^1^, Thassio Marcyal Bento Ferreira^1^, Gisely Seguchi Spinassé^1^, Matheus Ricardo Malveira Camacho^1^, Amanda Gabriela Freitas Rodrigues^2^, Israel Figueira Lemos^1^

(1) Faculdade de Medicina da Universidade do Estado do Pará; (2) Faculdade de Medicina e Cirurgia do Pará

**Introduction:** Systemic Arterial Hypertension (SAH) is a clinical condition that consists in high and sustained levels of blood pressure, generating effects in the heart, brain, kidney and blood vessels. The SAH is related with several metabolic disorders, which leads to elevated risk of cardiovascular fatal and non-fatal events. Nowadays, in Brazil, the SAH affects approximately 30% of the population and corresponds to 40% of deaths from stroke and 25% of deaths from Chronic Arterial Disease. Therefore, there is great relevance in studying the epidemiology of this disease in the country, seeking to understand which social groups are most affected by it and, thus, need greater attention from Public Health.

**Objective:** To describe the epidemiological profile of hospitalizations and deaths due to Systemic Arterial Hypertension in the state of Pará.

**Methods:** A cross-sectional, descriptive and quantitative study was carried out using the database of the Department of Informatics of the Unified Health System (DATASUS). Data referring to patients hospitalized or who died from SAH in the period from 2008 to 2020 in the state of Pará were selected, considering the following variables: age, sex and race. Data were processed using Excel and Biostat software.

**Results:** During the study period, 55,503 hospitalizations and 508 deaths from SAH were reported in the state of Pará. Hospitalizations were more frequent among women (58.5%), black and brown people (57.6%), and in the 50–69 age group (42.6%). On the other hand, deaths occurred mainly among men (51.9%), black and brown people (43%), and aged over 70 years (54.6%). It is worth mentioning that the analysis of the “race” criterion was hampered by a large number of cases without information in the system (23,097 records).

**Conclusion:** There is greater morbidity and mortality among the non-white population, which is in agreement with the current literature, which states that there is a greater prevalence and severity of SAH among black and brown people, mainly due to genetic factors. However, it is known that in Brazil there is a historical marginalization of this population; this factor may be related to the difficulty in accessing the right to health, aggravating the risk factors for the development of SAH in this social group. In this sense, greater efforts must be made in order to reduce social inequality and promote universal access to heahealth thus minimizing unfavorable outcomes due to lack of assistance.

111649

Modality: E-Poster Scientific Initiation – Non-case Report

Category: EPIDEMIOLOGY AND HEALTH POLICIES/GLOBAL HEALTH

## Mortality Due to Acute Myocardial Infarction Among Indigenous and Non-Indigenous: The Brazilian Scenario Over the Past Decade

MARCOS OLIVEIRA^1^, Solange Lima Gomes^1^, Franciane de Paula Fernandes^1^

(1) Universidade do Estado do Pará (UEPA)

**Introduction:** Acute Myocardial Infarction (AMI) is an important cause of mortality in society. In indigenous peoples, this illness gains prominence due to barriers that hinder access to health. Despite advances, AMI remains among the main causes of death in these peoples. In view of this, it is necessary to investigate how this situation affects the occurrence of this disorder in traditional populations.

**Objective:** To analyze the mortality due to AMI in indigenous and non-indigenous peoples from Brazil over the past decade.

**Methods:** This is a descriptive study, with a quantitative, cross-sectional, and retrospective approach. Data from public domains of the Mortality Information System of the Informatic Department of the Unified Health System (SIM/Datasus) were analyzed.

**Results:** 974,862 deaths were reported in non-indigenous peoples and 1,775 in indigenous peoples. Among non-indigenous peoples, the highest number occurred in 2019. In the same group, 59% were male and 40.98% female. Furthermore, the age group of 80 years or older was predominant (26.04%) and the majority of deaths occurred in hospitals (51.24%). Among non-indigenous peoples, the highest number of deaths occurred in 2020. There was a predominance of males with 59.15%, while 40.78% were female. The most affected age group was 80 years or older (29.35%). It was also noted, a higher frequency of notifications at home (47.49%).

**Conclusion:** This context may be a reflection of greater difficulty in accessing health facilities for the traditional population. It is evident the need for health access strategies that consider the cultural particularities of each group, in addition to awareness campaigns with a preventive approach, and research that explores prevention strategies and control of risk factors in health units.


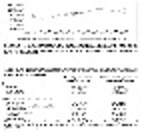


111680

Modality: E-Poster Scientific Initiation – Non-case Report

Category: HEART FAILURE/CARDIOMYOPATHY/TRANSPLANT

## The Impact of Comorbidities on the Prognosis of Patients with Acute Heart Failure

JOSÉ RENAN DE MATOS PAIN^1^, José Renan de Matos Pain^1^, Jorge Tadashi Daikubara Neto^1^, Matheus Bissa Duarte Ferreira^1^, Rafael Moretti^1^, Jessica Tamires Reichert^1^, Lucas Muller Prado^1^, Gustavo Sarot Pereira da Cunha^1^, Leornardo Henrique dos S. Melo^1^, Michelle Bozko Collini^1^, Raphael Henrique Déa Cirino^1^, Miguel Morita Fernandes da Silva^1^

(1) Universidade Federal do Paraná (UFPR)

**Background:** Comorbidities are associated with worse quality of life and prognosis in chronic heart failure. However, there is lack of data on the impact of comorbidities in acute heart failure (AHF).

**Objective:** To assess the association between comorbidities and the and prognosis of patients with AHF.

**Methods:** Patients diagnosed with AHF, admitted to a tertiary hospital in southern Brazil, between 2019 and 2021 participated in the study. We selected the following comorbidities: hypertension, diabetes mellitus, dyslipidemia, obesity, smoking, alcohol consumption, anemia, stroke, atrial fibrillation, coronary artery disease (CAD), peripheral vascular disease (PVD), chronic obstructive pulmonary disease (COPD) and chronic kidney disease (CKD). The outcome was a composed of all-cause death or re-hospitalization within 6 months. We performed a stepwise forward selection with Cox regression analysis to identify the comorbidities independente associated with the outcome.

**Results:** We analyzed 175 patients with AHF were included, of which 66 died or were re-hospitalized during the study period. The mean age of the patients was 67 years, 88 (50%) patients were female, the mean ejection fraction was 43.8, and the ejection fraction extremes were 61.6–26.1. The five most prevalent comorbidities were: hypertension (79%), diabetes (38%), atrial fibrillation (36%), dyslipidemia (31%), CAD (29%) and COPD (18%). In the stepwise multivariate regression, the only comorbidity that was independently associated with the composite outcome was COPD (Hazard ratio 1.83, 95% confidence interval, 1.04–3.23, p = 0.035). Ejection fraction (EF) was not significantly associated with the outcome (p = 0.48).

**Conclusion:** In patients with AHF, COPD was the only comorbidity independently associated with the risk of events in a six month follow up.

111691

Modality: E-Poster Scientific Initiation – Non-case Report

Category: COVID-19 AND CARDIOVASCULAR SYSTEM

## Is There a Relationship between the COVID-19 Pandemic and the Epidemiological Behavior of Acute Coronary Syndromes in Salvador-Bahia?

JÚLIA SCHOUCAIR NEVES^1^, Marília Menezes Gusmão^1^, Laís Araújo Carneiro de Campos^1^

(1) Escola Bahiana de Medicina e Saúde Pública – EBMSP

**Introduction:** The COVID-19 pandemic brought impacts that may have had a great influence on the incidence and lethality of Acute Coronary Syndromes (ACS). Since the actions to contain the pandemic have regional differences, it is necessary to know if the increase in the number of cases of COVID-19 was linked to an increase in the number of cases and mortality from ACS in Salvador.

**Objective:** To investigate whether there was an association between cases of ACS and cases of SARS-CoV-2 infection, during the first wave of the COVID-19 pandemic, in Salvador.

**Methodology:** Observational study of comparative incidence and temporal trend carried out using secondary data from a local public ambulance service (SAMU) registries and the COVID-19 database provided by Health Secretary of Bahia State. The monthly incidence, lethality and assessment of the association between its incidences and Pearson’s correlation coefficient were performed in Excel software.

**Results:** From April to December 2020, there were 490 new cases of ACS, attended by SAMU, in Salvador. Compared with the number of cases in the same period of pre-pandemic years (2017, 2018 and 2019), respectively 341; 484 and 496 cases, it was observed that the incidence remained constant. There was no significant correlation between the monthly incidence of COVID-19 and ACS (r = –0.1) or lethality among these two variables (r = –0.13), from April 2020 to April 2021.

**Conclusion:** There was no increase in ACS cases registered by SAMU between April 2020 and April 2021, during the COVID-19 pandemic, in Salvador, when compared to the same period of the last 3 years and there was no significant correlation between the incidence of ACS cases and COVID-19 cases.


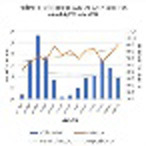


111705

Modality: E-Poster Scientific Initiation – Non-case Report

Category: HYPERTENSION/RENAL DENERVATION

## Screening for Systemic Arterial Hypertension and Its Risk Factors in Students Resident in a Riverside Community in the Western Amazon

RAIMUNDO BENÍCIO DE VASCONCELOS NETO^1^, Raimundo Benício De Vasconcelos Neto^1^, Rebecca Shaiane Soares Nunes Rivoredo^1^, Gabriel Irismar Rodrigues Schwamback^1^, Brenda dos Santos Rodrigues^1^, Sofia dos Santos Souza^1^, Maria Eduarda Brotto de Souza^1^, Bárbara Barbosa Pires^1^, Antonieta Relvas Pereira^2^, Sergio de Almeida Basano^3^, Juliana de Souza Almeida Aranha Camargo^4^, LUÍS MARCELO ARANHA CAMARGO^4^

(1) Centro Universitário São Lucas- UNISL; (2) Centro Universitário Aparício Carvalho- UniFIMCA; (3) CEMETRON; (4) ICB-5 USP

**Introduction:** Systemic arterial hypertension (SAH) is a chronic non-communicable disease (CNCD) with high worldwide prevalence. It is a disease that has its onset, often in childhood and adolescence, and can lead to serious future consequences. However, it appears that there is a data scarcity about the occurrence of SAH in Western Amazon communities, especially riverside communities.

**Objectives:** To estimate the prevalence of SAH and associated risk factors in public school students residing in riverside communities in the Western Amazon, Brazil.

**Methods:** This work is a cross-sectional study, carried out with individuals aged between 4 and 21 years old, without gender distinction, living in a riverside region in Humaitá (S 6o 58’62’’ re W 62o 50’’ 08W), Amazonas State, Brazil. The project was approved by the research committee of the Brazillian Center for Research in Tropical Medicine (CEPEM). For the blood pressure measurement, the patients were prepared according to the protocol of the Brazilian Ministry of Health, 3 measurements were performed, excluding the first and applying the average of the last two in each arm. The analysis was performed using the prevalence ratio (PR), Mantel-Haenszel chi-square test, with statistical significance p < 5%.

**Results:** 160 public school students were randomly selected for the study, of which 152 (95%) had their data analyzed, 82 male and 70 female. Twenty-five individuals were found with blood pressure levels consistent with SAH, representing an absolute prevalence of 16.5%. The mean age of hypertensive students was 12 years, ranging from 5 to 21 years. The average income reported was calculated at R$ 862.00. Regarding risk factors, the PR between SAH and males was 1.5 (p = 13%); for family history of SAH, the PR was 1.2 (p = 35%); for SAH and sedentary lifestyle, the PR was 1.3 (p = 30%); for the association between SAH and obesity in this population and SAH and dyslipidemia, the PR was less than 1, but with p > 5%.

**Conclusions:** A few decades ago, CNCD, such as SAH had significance only among the adult population, now it also affect children and adolescents in a similar way. The data from this analysis point to a prevalence of more than 16% of SAH among students aged 5 to 21 years, which is considered high for this age group, requiring an intervention by the health system in order to attenuate the occurrence of NCDs in adulthood. There was no association between risk factors and SAH in this study.

111733

Modality: E-Poster Scientific Initiation – Non-case Report

Category: ATHEROSCLEROSIS/CARDIOVASCULAR RISK FACTORS/CARDIOVASCULAR PREVENTION

## Obesity Screening and Risk Factors in Riverside Schools in the Western Amazon

REBECCA SHAIANE SOARES NUNES RIVOREDO^1^, Rebecca Shaiane Soares Nunes Rivoredo^1^, Raimundo Benício de Vasconcelos Neto^1^, Gabriel Irismar Rodrigues Schwamback^1^, Jade Gomes da Costa Medeiros^1^, Liana Miranda Pereira^1^, Bárbara Barbosa Pires^1^, Ana Júlia Omodei Rodrigues Martim^1^, Antonieta Relvas Pereira^2^, Sergio de Almeida Basano^3^, Juliana de Souza Almeida Aranha Camargo^4^, LUÍS MARCELO ARANHA CAMARGO^4^

(1) Centro Universitário São Lucas/Afya UNISL; (2) Centro Universitário Aparício Carvalho FIMCA; (3) CEMETRON; (4) ICB-5

**Schools in the Western Amazon Introduction:** The current Brazilian epidemiological profile is undergoing a process of transition in the most gentrified regions of the country, where infectious diseases predominated, has been showing an increase in non-communicable chronic diseases. Obesity is a chronic disease considered a serious public health problem, defined by excess body fat. Its prevalence may contribute to the emergence of cardiovascular diseases. This study aimed to identify the prevalence of obesity and its risk factors in riverine schoolchildren in the Western Amazon, Brazil.

**Methodology:** This is a cross-sectional study, carried out with individuals aged between 6 and 16 years old who attend public schools. The sample space consisted of 152 individuals belonging to 22 riverside communities along the Madeira River in Humaitá, Amazonas State, Brazil. For the research, a clinical-epidemiological questionnaire was applied, in addition to obtaining anthropometric data (weight, height, waist circumference), body mass indices (BMI) were calculated, considering the guidelines of the Brazilian Ministry of Health as a standard of normality. The statistical analyzes performed were the prevalence ratio (PR), Mantel-Haenszel chi-square test, with statistical significance p < 5%.

**Result:** The 152 individuals evaluated corresponded to 95% of the sample population aged between 6 and 16 years. The study showed 7 individuals with BMI values consistent with obesity, demonstrating an absolute prevalence of 4.6%. The age of obese individuals ranges from 6 to 16 years old, and the average family income reported was R$ 809.16, most of which came from financial Federal Government Programs. Among the risk factors, the association between obesity and family history for obesity, the PR is 0.4 (p = 40%), the PR for males is 4.84 (p = 5.7%) and finally, sedentary lifestyle and obesity have a PR that is below 1 (p = 13%).

**Conclusion:** Little is known about the culture and epidemiological profiles of riverside communities, but with the data collected, it is observed that even in remote areas, cases of obesity are already identified. It is important to develop public policies aimed at these populations, with the objective of improving knowledge about nutrition and health, physical activity and diet, ensuring good nutrition throughout life, promoting health in addition to reducing public spending on health problems. triggered by excess weight.

111718

Modality: E-Poster Scientific Initiation – Non-case Report

Category: EPIDEMIOLOGY AND HEALTH POLICIES/GLOBAL HEALTH

## Study of the Overview of Hospitalizations for Acute Myocardial Infarction between the Regions of Brazil from 2011 to 2021

NESLAYNE LOUISE CAMPIOL^1^, Andreisa Prieb^1^, Thiago Santos Souza^1^, Monique Veloso Lima^1^, Nathalia Freire Gilo^1^, Maurício Antônio Batista Cavalcante^1^

(1) Universidade de Gurupi – UNIRG

**Introduction:** Non-transmissible cardiovascular diseases represent a major public health problem worldwide. In Brazil, Acute Myocardial Infarction (AMI) is the leading cause of death in the country, according to DATASUS, and constitutes a process of ischemic necrosis caused by acute obstruction of a coronary artery. Therefore, a greater awareness of the measures for prevention and control of AMI is necessary, in addition to the stratification of cardiovascular risk in the population.

**Objective:** Analyze the epidemiological profile of hospitalizations for AMI among Brazilian regions from 2011 to 2021.

**Methods:** A retrospective, observational, descriptive, cross-sectional study was conducted on data from hospitalizations for AMI, available at DATASUS – SUS Hospital Information System (SIH/SUS), in the period from January 2011 to December 2021, evaluating several variables such as the number of hospitalizations, mean value, mean length of stay, number of deaths and mortality rate.

**Results:** In the period studied, 1,187,982 hospitalizations for procedures to treat AMI were observed, representing a total expenditure of R$ 4,378,770,679.45. The Southeast region recorded 593,441 hospitalizations, corresponding to half of the entire country, followed by 233,365 in the Northeast (19.6%), 232,330 in the South (19.6%), 81,086 in the Midwest (6.8%), and 47,760 in the North (4.02%). The total number of deaths in Brazil was 130,209, being higher in the Southeast with 63,517 (48.8%) and in the Northeast with 28,506 (21.9%). The mortality rate in Brazil was 10.96, with the Northern and Northeastern regions above the national average, 12.22 and 12.04, respectively; and the other regions below the national average, Southeast (10.70), South (10.36), and Center-West (10.32). The national average stay was 7.3 days, while in the North it was 8.2, followed by 7.7 in the Northeast and Southeast, 7.3 in the Midwest, and 5.6 in the South. Concerning the average value of hospitalization, the highest value was in the South, R$4,417.68, followed by the Southeast R$3,695.58, Center-West R$3,503.92, Northeast R$3,194.34, and North R$2,716.46.

**Conclusions:** It is important to emphasize the importance of promotion and prevention in primary health care and early diagnosis to avoid sequelae and reduce the risk of death in patients. In addition, it reinforces the need for correct notification so that the information can help in the development of public health policies.

111726

Modality: E-Poster Scientific Initiation – Non-case Report

Category: EPIDEMIOLOGY AND HEALTH POLICIES/GLOBAL HEALTH

## Epidemiological Study of Pacemaker Implants in the City of Rio de Janeiro

LÉO RODRIGO ABRAHÃO DOS SANTOS^1^, INGRID PAIVA DUARTE^1^, ANDRÉ MELO DE FARIA^1^, MATHEUS SOUZA DE MOURA RIBEIRO^1^, NATALIA MELO DUARTE DE ALMEIDA^1^, LUANA TEIXEIRA DA SILVA^1^, LUIZA FURTADO AREAS^1^, THATIANA DA SILVA PIRES^1^, DANIEL ALBUQUERQUE MARTINS ZANIBONE^1^, THIAGO CAVALHEIRO TAVARES^1^, LUIS ORLANDO POZES PEREIRA FILHO^1^, OGI JANDERSON ANTUNES DE CASTRO BRITO^1^

(1) Unigranrio-Afya, Rio de Janeiro, Brazil

**Introduction/Objectives:** Artificial Cardiac Pacemakers are devices capable of replacing electrical impulses through multiprogrammable stimulation to achieve physiological cardiac electrical activity. They are classified by clinical need as Temporary or definitive, by transcutaneous route, transvenous or thoracotomy, by the types of endocavitary and epicardial cable, single chamber stimulated chambers, double, triple or the four chambers. The study aims to know epidemiologically the population that underwent pacemaker implantation in thecity of Rio de Janeiro.

**Materials/methods:** The collection of data was made in the hospital information system of the municipality of Rio de Janeiro from January 2010 to December 2020, researching race, age, type of pacemaker performed, hospitalizations, mortality and expenses. The present study is observational, descriptive and cross-sectional.

**Results:** The study found records of 5031 patients who underwent the procedure, 4063 (81%) were Transvenous Double Chamber Pacemakers, 564 (11%) of Transvenous Single Chamber, 219 (4%) of Transvenous Multi-site, 73 (2%), of Epimyocardial Double Chamber, 56 (1%) Temporary Transvenous, 30 (1%) of Single Epimyocardial Chamber, 21 (<1%) of Multi-site Epimyocardial by Thoracotomy for Thoracotomy for electrode implant and 5 (<1%) multi-site Endocavitary with reversal to epimyocardial. The majority were attended at the Instituto de Cardiologia Aloysio de Castro (IECAC) which totaled 1524 (30.3%) procedures. 3238 (64%) performed the implant electively. 73% were over 65 years old. 44% were White. 2547 (51%) were Men. The average length of hospital stay was 5.44 days and an average expenditure of US$ 2,552. In addition, 2499 (49.7%) had a daily ICU with an average expenditure of US$ 3,125. The most expensive procedure was the Transvenous Multi-site pacemaker with an average cost of US$ 5875.6. There were 84 deaths in general, 61% in Transvenous Double Chamber Pacemakers, the highest mortality rate was found in patients who underwent temporary transvenous pacemaker implantation with 25% of deaths.

**Discussion/Conclusion:** Patients undergoing pacemaker implantation have a profile mainly of elderly males, whites who perform Transvenous Double Chamber Pacemakers mostly in high complexity cardiac hospitals. These data, in addition to characterizing users, show how costly, complex and common the use of these devices is, and the need for investment in cardiology services in the city.

111746

Modality: E-Poster Scientific Initiation – Non-case Report

Category: EPIDEMIOLOGY AND HEALTH POLICIES/GLOBAL HEALTH

## Epidemiological Evaluation of Chronic Rheumatic Heart Disease Admissions in the Northern Region of Brazil between 2017 and 2021

IGOR LUCAS FARIAS LIMA^1^, Luciano Moura de Assunção^3^, Giulia Vitoria Nascimento da Silva^1^, Ariane Lobato Moraes^2^, Marcos Aurélio Vieira da Costa Filho^2^, Wanda Maria de França Pires^2^, Larissa Dacier Lobato Comesanha^2^, Júlia de Moura Carvalho Faria^1^

(1) UNIVERSIDADE DO ESTADO DO PARÁ – UEPA; (2) UNIVERSIDADE FEDERAL DO PARÁ – UFPA; (3) FUNDAÇÃO SANTA CASA DE MISERICÓRDIA DO PARÁ

**Introduction:** Chronic rheumatic heart disease (RHD), a sequel of rheumatic fever (RF), is caused by lesions that mainly affect mitral and aortic valves, and late diagnosis results in major complications such as heart failure, pulmonary hypertension and atrial fibrillation¹. The primary prevention of RHD consists of the diagnosis and treatment of streptococcal pharyngitis and secondary prevention in long-term antimicrobial prophylaxis in individuals with a previous diagnosis of RF².

**Objective:** To assess the prevalence and analyze the epidemiological profile of RHD hospitalizations in the Northern Region of Brazil from 2017 to 2021.

**Methods:** Observational, cross-sectional, descriptive and quantitative study based on data from the SUS Hospital Information System (SIH/SUS) – DATASUS. The variables analysed were: number of hospitalizations, age, sex, average hospital stay, average cost per hospitalization and mortality rate, referring to hospitalizations due to RHD in the Northern region of Brazil between 2017 and 2021.

**Results:** In the analyzed period, 1744 hospitalizations for RHD were reported in the Northern Region of Brazil, with the highest number of hospitalizations in the state of Pará (697). In the North Region, the largest amount occurred in 2017 with 468 cases, with a gradual decrease until 2021 with 246 hospitalizations. Regarding gender, 51.15% of the hospitalized patients were female and 48.85% were male. The most affected age groups were 50 to 59 years old, with 308 (17.6%) notifications, and 40 to 49 years old, with 297 (17%) cases. The average length of stay in 2017 to 2021 was 13.1 days, being highest in Rondônia (15.1 days) and lowest in Roraima (7.3 days). In the North region, the lowest average time (11.1 days) occurred in 2020 and the highest (14.7 days) in 2017. The mortality rate was 7.91%, being the lowest in 2018 (6.14%) with gradual growth until 2021 (10.16%). The state with the highest mortality rate was Roraima (28.57%) and the lowest was Amazonas (6.53%). A total of R$16,878,115.65 was spent in the considered period, with an average cost of R$9,677.82 per hospitalization.

**Conclusion:** The study reports that the highest number of hospitalizations occurred in 2017, mostly in the state of Pará. Mortality rate due to RHD is rising and the highest rate comes from Roraima. In addition, the most affected age group was 50 to 59 years, and there was no significant difference in prevalence between genders.

111751

Modality: E-Poster Scientific Initiation – Non-case Report

Category: COVID-19 AND CARDIOVASCULAR SYSTEM

## Long-Term Impact of COVID-19 on the Practice of Physical Exercise in Patients with Heart Disease: A Cohort Study in the Brazilian Amazon

LUANA LIVELLI BECKER^1^, Kletey Mendes Silva^1^, Odilson Marcos Silvestre^1^, Miguel Fernandes da Silva Morita^2^, Dhayn Cassi Freitas^1^, Roberta Gabriela da Silva^1^, Laís Vitória de Andrade Miranda^1^, Sabrina da Silva Medeiros^1^, Emanuel Silva de Abreu^1^, Jéssica Borsoi Maia do Carmo^1^, Leonardo Buranello^1^, Wilson Nadruz^3^

(1) Federal University of Acre; (2) Federal University of Parana; (3) University of Campinas

**Introduction:** The impact of COVID-19 infection in patients with heart disease remains little explored.

**Object:** To investigate the incidence of physical inactivity in individuals with heart disease after 12 months of SARS-CoV-2 infection.

**Methods:** We prospectively evaluated 837 individuals who practiced regular physical exercise infected with SARS-CoV-2 in Rio Branco, Acre. Sociodemographic information, diseases of interest (cardiopathies, hypertension and diabetes mellitus) and details about the acute phase of the disease were collected after COVID-19 infection. In a second 12-month follow-up questionnaire, participants were asked about their physical exercise. To identify independent predictors of physical inactivity, multivariate logistic regression with stepwise strategy was used.

**Results:** The mean age of the sample was 39 ± 11 years, 54% were male. A total of 14 participants were identified with heart disease (cardiac arrhythmia, n = 7; heart failure, n = 5; congenital heart disease, n = 1; third-degree atrioventricular block and pacemaker, (n = 1) and 42 participants with diabetes mellitus. A total of 243 (29%) participants were inactive after 12 months of SARS-CoV-2 infection. Compared to individuals without heart disease, those with heart disease had a higher occurrence of physical inactivity after 12 months of COVID-19 (28% vs 71%, respectively). After adjusting for multiple confounders, heart disease, diabetes mellitus, and female gender were predictors of long-term physical inactivity (figure 01).

**Conclusion:** After 12 months of infection by SARS-CoV-2, 71% of patients with heart disease did not practice physical exercises. Female sex, diabetes mellitus and heart disease were independent predictors of long term physical inactivity.


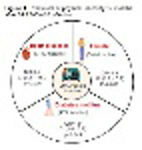


111752

Modality: E-Poster Scientific Initiation – Non-case Report

Category: HYPERTENSION/RENAL DENERVATION

## Screening a Diagnosed Population with Systemic Arterial Hypertension and Diabetes Mellitus in the Amazon Riverside Community

RAIMUNDO BENÍCIO DE VASCONCELOS NETO^1^, Gabriel Irismar Rodrigues Schwamback^1^, Rebecca Shaiane Soares Nunes Rivoredo^1^, Maria Eduarda Brotto de Souza^1^, Archimedes Fernandes Alves de Santana^1^, Julia Maria de Lourdes Balsan^1^, Sofia dos Santos Souza^1^, Renata Gonçalves Silva Santos^1^, Izabella Gurgel do Amaral Pini^1^, Gesanaje da Paz Carvalho^1^, Fernanda Gabry Scazuza Gomes de Souza^1^, Byron Maia Feitosa^1^

(1) Centro Universitário São Lucas- UNISL

**Introduction:** Characterized as Chronic Non-Communicable Diseases (CNCD), Systemic Arterial Hypertension (SAH) and Diabetes Mellitus (DM) affect a large part of the Brazilian population, even if undiagnosed. Despite having different mechanisms of action, when associated, these CNCD considerably increase the risk of developing cardiovascular and renal pathologies. Therefore, assessing the prevalence of these comorbidities is necessary to establish intervention and management strategies for the population, especially in remote communities.

**Objectives:** To estimate the prevalence of the association of Systemic Arterial Hypertension and Diabetes Mellitus in individuals living in a riverside community in the Western Amazon, Brazil.

**Methods:** This is a cross-sectional, quantitative, retrospective study carried out with individuals aged between 1 and 87 years, regardless of gender, living in a riverside region in Porto Velho, Rondônia, Brazil. Home visits were carried out in which the residents answered an individual epidemiological questionnaire, each form had the information tabulated.

**Results:** 268 individuals were randomly selected for the study, of which 55 (20.52%) had informed SAH, 26 (9.7%) had informed DM and 23 (8.58%) claimed to have both associated chronic non-communicable diseases. Females had the highest prevalence of the association between Systemic Arterial Hypertension and Diabetes Mellitus, with 14 cases (60.86%). The average age of individuals with both chronic non-communicable diseases is 58 years old.

**Conclusion:** Systemic Arterial Hypertension and Diabetes Mellitus are the most frequent non-communicable chronic comorbidities in the world population. Nevertheless, the significant prevalence of these health conditions in the Amazon riverside population observed in this study reinforce the importance of monitoring cardiovascular health and associated risk factors.

111763

Modality: E-Poster Scientific Initiation – Non-case Report

Category: ATHEROSCLEROSIS/CARDIOVASCULAR RISK FACTORS/CARDIOVASCULAR PREVENTION

## Association between Subjective Sleep Quality and Peripheral and Central Blood Pressure Values of Patients in a Cardiology Clinic

CLARISSA MARIA TITO BELTRÃO^1^, Waléria Dantas Pereira Gusmão^2^, Alana Costa Machado Gomes^1^, Fernanda Abraham Leão^1^, Silvia Dandara Coutinho de Souza Lins Machado^1^, Giulia Abraham Leão^1^, Isabele Rejane de Oliveira Maranhão Pureza^1^, Annelise Machado Gomes de Paiva^1^, Marco Antonio Mota Gomes^1^, Claudia Roberta de Castro Moreno^2^

(1) Centro Universitário CESMAC; (2) Universidade de São Paulo

**Introduction:** Blood pressure (BP) oscillations are regulated by neural, endocrine, endothelial and hemodynamic pathways in a circadian pattern. Although environmental and behavioral phenomena can mask the circadian rhythm of BP, circadian variability has higher levels in the morning, a plateau throughout the day and lower values at night during sleep. Poor quality or sleep deprivation can potentiate greater insulin resistance, chronic inflammatory processes, increased cortisol and catecholamines and exacerbation of stress, can disorganize the structure of the vascular wall, which can increase pressure.

**Objective:** To determine if there is an association between subjective sleep quality and BP values.

**Methods:** This is a cross-sectional study, carried out in a cardiology outpatient clinic, from Sep. to Dec. 2021, in which sociodemographic and clinical data were collected and a visual analogue sleep quality scale was applied through a structured questionnaire. Pressure values were estimated using an Arteris-AOP® oscillometric device.

**Results:** 133 adults (19–89 years, mean 53.49 ± 14.01 years) were included in the study, 77.44% were female and 22.56% were male, the mean body mass index (BMI) was 29.30 ± 4.60 Kg/m^2^ (18.7–42.02 Kg/m²), the mean peripheral BP values were 128.51 ± 15.91 (99–181) mmHg of systolic BP, 83.05 ± 9.74 (62–115) mmHg of diastolic BP and 45.46 ± 13.55 (25–94) mmHg of pulse pressure. The BP central mean values were 118.63 ± 14.25 (116.19–121.08) mmHg of systolic BP, 84.62 ± 9.79 (64–116) mmHg of diastolic BP and 31.04 ± 11.25 (32.08–35.94) mmHg pulse pressure. To analyze the association between subjective sleep quality and peripheral and BP central values, linear regressions were performed with adjustment for age, sex and BMI. Subjective sleep quality was inversely proportional to systolic BP (β: –0.16; 95%CI: –2.76; –0.05; R2 = 0.19) and diastolic BP (β: –0. 28; 95%CI: –2.36; –0.59; R2 = 0.08), as well as systolic centrals BP (β: –081; 95%CI: –2.58; –0.20; R2 = 0.22) and diastolic centrals BP (β: –0.278; 95%CI: –2.35; –0.57; R2 = 0.08) with no effect on peripheral or central pulse pressure. The findings indicated an association between subjective sleep quality and peripheral BP levels in the studied population, that is, the worse the sleep quality, the higher the BP, constituting a promising area for study.

**Conclusion:** Hypertensive patients should investigate sleep quality, since there is an association between poor sleep quality and high BP.

111932

Modality: E-Poster Scientific Initiation – Non-case Report

Category: EPIDEMIOLOGY AND HEALTH POLICIES/GLOBAL HEALTH

## Financial Resources and Hospital Morbimortality from Chronic Rheumatic Heart Disease in Northern Brazil

VANDO DELGADO DE SOUZA SANTOS^1^, Saul Rassy Carneiro^1^

(1) Universidade Federal do Pará – UFPA

**Introduction:** Chronic Rheumatic Heart Disease (RHD) is a sequela of rheumatic fever, which is a complication of pharyngotonsillitis caused by Streptococcus pyogenes and results from a late immune response to this infection in genetically predisposed populations, having a direct relationship with the development of services. of health in the country. All the financial resources made available to the Unified Health System are made directly by the National Health Fund (NHF) for each Federation Unit.

**Objectives:** To relate the total financial transfers from the NHF and the amount of the Family Health Strategy (FHS) with the number of hospital admissions for RHD in northern Brazil from 2011 to 2020.

**Methods:** This is a descriptive longitudinal epidemiological study based on secondary data from the Department of Informatics of the Unified Health System, the Portal of the National Health Fund of the Federal Government and the Portal of the Secretariat of Primary Health Care. RHD hospital morbidity and mortality data were correlated with the financial resources allocated to each FU and with the total number of FHS in each FU.

**Results:** In the analyzed period, there were a total of 3.595 hospitalizations for RHD and a total of 336 deaths in the northern region, that is, a mortality rate of 9.35%. The state of Pará had the highest rates of hospitalizations (38.58%), followed by the state of Amazonas (32.54%), and the state of Roraima had the lowest rates (0.39%). In the same period, Pará and Amazonas were the ones that received the most financial transfers and had the largest number of FHS, and Roraima was the state that received the least financial resources and had the lowest number of FHS.

**Conclusion:** The study showed that the greater the availability of NHF resources for the FU and the greater the number of FHS, the greater the hospitalization rates and, consequently, the mortality rate, this is due to the greater screening of heart diseases and greater access to health.

111777

Modality: E-Poster Scientific Initiation – Non-case Report

Category: CARDIOVASCULAR SURGERY

## Profile of Myocardial Revascularization Procedures in the City of Vassouras for 10 Years

SARA CRISTINE MARQUES DOS SANTOS^1^, Maria Luiza Silva Barbosa^1^, Alice Machado de Sales Silva^1^, Ivana Picone Borges de Aragão^1^

(1) Universidade de Vassouras

**Introduction:** Myocardial revascularization surgery (CABG) is one of the treatments for obstructive atherosclerotic coronary artery disease accompanied by myocardial ischemia. The procedure can be performed with or without cardiopulmonary bypass (CPB). The present study aimed to analyze the current panorama of CRM procedures, with and without CPB and use of 1 or more grafts performed in the city of Vassouras for 10 years and correlate the current epidemiology with the results obtained.

**Methods:** A systematic literature review and observational, descriptive and cross-sectional data collection of myocardial revascularization procedures, available at DATASUS – SUS Hospital Information System (SIH/SUS) for a period of ten years – December 2008 to December 2018 – evaluating the value of public spending, complexity, mortality rate, deaths, permanence, and character of care and articles available in Scielo, Lilacs, and PubMed.

**Results:** In the analyzed period, 306 hospitalizations were observed for the performance of myocardial revascularization procedures, representing a total expenditure of R $ 3,228,341.33, with 2010 being the year with the highest number of hospitalizations (80) and 2011 being the year responsible for the largest amount spent during the period (R $ 687,969.79). Of the total procedures, 162 were performed on an elective basis and 144 on an urgent basis, with 306 being considered highly complex. The total mortality rate in the 10 years studied was 10.46, corresponding to 32 deaths, 2016 being the year with the highest mortality rate, 18.18, while 2012 had the lowest rate, 4.00. The mortality rate for elective procedures was 9.26 compared to 11.81 for urgent procedures. The average total hospital stay was 14.2 days.

**Conclusions:** It can be seen, from the present study, that CABG is a procedure considered to be highly complex, regardless of whether it is performed urgently or not. A progressively considerably high mortality rate has been identified. Also, the need for correct notification of procedures is highlighted, aiming to improve the current epidemiological analysis.

111780

Modality: E-Poster Scientific Initiation – Non-case Report

Category: ATHEROSCLEROSIS/CARDIOVASCULAR RISK FACTORS/CARDIOVASCULAR PREVENTION

## “Livres Do Tabaco”, Helping Smokers to Quit Smoking and Getting to Know Their Profile in the Smoking Cessation Process

BIANCA DE FÁTIMA PEREIRA^1^, Ramon José Moreira Silva^1^, Beatriz Stephan Farhat Jorge^1^, Pedro Drumond Maia^1^, Fernanda Silva Mota^1^, Gabriela Godinho Rezende^1^, Moisés de Toledo Vilela^1^, Paula Gouvea Abrantes^1^, Marcela Rabelo^1^, Hélio Brito de Lima Júnior^1^, Eliane Ferreira Carvalho Banhato^1^, Arise Garcia de Siqueira Galil^1^

(1) Cardiology Department, Medical School, Federal University of Juiz de Fora, Juiz de Fora – Minas Gerais.

**Introduction:** Smoking is a preventable and controllable chronic disease, in addition to high morbidity and mortality. Encouraging smoking cessation is a priority in Public Health and represents a cost-effective intervention.

**Objective:** To describe the profile of smokers assisted by a treatment group for smoking cessation.

**Methodology:** Longitudinal study, “Livres do Tabaco” group. Users with multimorbidities were assisted between September/2021 and March/2022, in consecutive treatment groups, mixed intervention (face-to-face and telemedicine), multidisciplinary team, behavioral cognitive approach sessions, drug treatment and follow-up. Definitions: Low level of schooling, <8 years. Normal systolic blood pressure (SBP) <130 mmHg. Depression, score Patient Health Questionnaire (PHQ-9) ≥9 points. High risk for Obstructive Sleep Apnea Syndrome (OSAS), Stop Bang questionnaire ≥5 points. Abusive use of alcohol, Audit-C ≥5 points. Cognitive deficit, Montreal Cognitive Assessment <26 points. High nicotine dependence, Fagerstrom test ≥5 points.

**Results:** Sample with 36 patients, 6 consecutive treatment groups. Age, 56.6 ± 10.8 years; elderly, 44%; female, 66.70%; low level of schooling, 42.9%. Dyslipidemia, 52.90%; sedentary lifestyle, 61.5%; family history of coronary artery disease, 88.20%. Arterial hypertension, 68.6%; coronary insufficiency, 25.7%; diabetes mellitus, 17.1%; chronic obstructive pulmonary disease; 31.4%; previous history of cancer, 8.6%. High risk for OSAS, 31.4%, depression, 55.90%, cognitive impairment, 79.4%, alcohol abuse, 20%. Daily consumption of 17.9 ± 12.2 cigarettes; pack-years, 40.7 ± 29.3; high nicotine dependence, 68.6% and 55.6%, heavy smokers. Smoking triggers, stress, 85.7%; coffee, 60% and alcohol intake, 48.6%, were the most frequent. Smoking cessation until the 12th week of accumulated treatment, 33.3%.

**Conclusion:** The initial data showed a high prevalence of both risk factors and comorbidities that most increase morbidity and mortality worldwide. It was also found a high prevalence of women, depression, cognitive impairment, in addition to high nicotine dependence and heavy smokers, data considered as weaknesses within the smoking cessation process. Even so, smoking cessation was observed to be within the average for smokers without associated comorbidities.

111783

Modality: E-Poster Scientific Initiation – Non-case Report

Category: DIGITAL HEALTH/INNOVATION

## Prototype of Electronic Stethoscope for Continuous Monitoring

GUSTAVO PEDROZO^1^, Sérgio Eduardo Soares Fernandes^1^, Pedro Henrique Santos de Medeiros^1^

(1) Escola Superior de Ciências da Saúde ESCS

**Introduction:** Cardiac auscultation as a semiological tool has been widespread for more than four centuries, but only recently, with the popularization of computer processors, some of them with GPIO (General Purpose Input/Output) interfaces, it was possible to bring technological evolution to low-cost research centers.

**Objectives:** To develop a low-cost cardiac and pulmonary audio signal capture prototype for future tests with continuous monitoring.

**Method:** A long literature review was carried out about concepts of acoustics, stethoscope chest-piece modelling, sound signal capture and articles with similar prototypes of digital stethoscopes. Then, a chest-piece was built with Autodesk Inventor® software and printed with antimicrobial PLA (Polylactic Acid) filament. The prototype was assembled with a digital microphone INMP441® connected to a raspberry® pi 3b board (figure 1). The signal capture was performed using Python 3.10.4 code with pyaudio and mathplot libraries. The tests were carried out exclusively among the research team and validation tests about the method have not yet been carried out until this submission date.

**Results:** The prototype was able to capture satisfactorily cardiac and pulmonary signals which were converted into phonograms (figure 1). The cost of materials used with the initial prototype was $67.32 including taxes and shipping. The cost proved that the reproduction of this prototype is viable for future larger tests. Likewise, it was possible to create new conceptual prototypes with potential for significant cost reduction, which will be produced in later phases of the study.

**Conclusion:** The prototype allowed the analysis of production feasibility, however, to proceed the project with validation of methods for continuous monitoring, safety studies will still be necessary and, as well, the development of a continuous analysis code with intelligence to alert critical findings. This study allowed not only the production of a viable prototype, but also perspectives of cost reduction and the means for the continuity of the project with the validation of a monitoring system by auscultation.


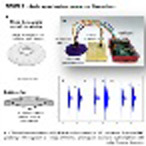


111869

Modality: E-Poster Scientific Initiation – Non-case Report

Category: HEART FAILURE/CARDIOMYOPATHY/TRANSPLANT

## Profile of Patients with Severe Heart Failure in a Tertiary Hospital in Salvador

KARLA SANTOS PINTO^1^, Tainá Viana^1^, Raissa Barreto Lima^1^, Ana Luísa de Aguiar Almeida Silva^1^, Eduarda Luciana Mendes Borges^1^, Larissa Xavier Gomes da Silva^1^, Aurea Maria Lago Novais^1^, Luiz Carlos Passos^1^, Mariana Baraúna da Silva^1^, Elias Soares Roseira^1^, Karla Santos Pinto^1^, João Pedro Martins Moreira Granja^1^

(1) Hospital Ana Nery

**Introduction:** Heart failure (HF) is a complex and highly prevalent clinical syndrome that limits the quality of life of many of its patients. Patients with heart failure reduced ejection fraction (HFrEF) are subject to higher mortality, given the potential reduction in cardiac function. The objective of this study is to describe the clinical and epidemiological profile of patients with HFrEF in a reference hospital in Salvador-BA.

**Methods:** This is a prospective cohort that included patients with HFrEF treated at a heart failure outpatient clinic of a cardiology referral hospital in Salvador from September 2020 to July 2021. The outcome variables evaluated were mortality and hospitalization for any cause in 1 year. Variables with nonparametric distribution were described by median and interquartile range and categorical variables were described as frequency and percentage.

**Results:** We included 196 patients with HFrEF with a median age of 60.5 (49–68) years, most of them men (54.1%), and a median LVEF of 29% (23–35). Ischemic and chagasic etiologies were the most prevalent, with 46 (23.5%) and 40 (20.4%) patients, respectively. Among the associated comorbidities, arterial hypertension (63.8%) and diabetes mellitus (30.6%) were the most observed. As for the medications in use, 93.9% of the patients used beta-blockers, 74% used ACEI/ARB, 13.3% used sacubitril/valsartan and 75% used spironolactone. After a 1-year follow-up, it was found that 25 (12.8%) patients died and 50 (25.5%) were hospitalized during the period. In the HF grading by the New York Heart Association (NYHA), 57 (34.5%) patients belonged to functional classes III-IV.

**Conclusion:** The present study showed that the prevalence of HFrEF in the studied population reflects a considerable degree of hospitalization and death. It is expected that the results found can improve health policies that help in the care of patients with HFrEF, as well as in the prevention of unfavorable outcomes.

111788

Modality: E-Poster Scientific Initiation – Non-case Report

Category: EPIDEMIOLOGY AND HEALTH POLICIES/GLOBAL HEALTH

## Prevalence of Diseases and Factors Associated with Cardiovascular Diseases in a Cardiology Ambulatory Service in the Brazilian Amazon: Evidence of a Potential Tool of Management in Primary Assistance

ANNA LUIZA ALVES DE OLIVEIRA MIRANDA^1^, Matheus Vinícius Mourão Parente^1^, Daniel Chagas Barreto^1^, José Pedro da Silva Sousa^1^, Beatriz Siems Tholius^1^, José Wilker Gomes de Castro Júnior^1^, Cláudio Eduardo Corrêa Teixeira^1^, Fabíola de Carvalho Chaves de Siqueira Mendes^1^

(1) Centro Universitário do Estado do Pará (CESUPA)

**Introduction:** CardioVascular Diseases (CVD) are a public health problem, representing more than 30% of all deaths worldwide. In this context, evidencing the prevalence of the main diseases associated with CVDs and the profile of these patients is necessary, in order to guide the management of public health services that receive this demand.

**Objectives:** In the present study, we aimed to highlight the prevalence of diseases and factors associated with CVDs in the population fraction admitted, via the Brazilian Unified Health System (Serviço ùnico de Saúde, SUS), at the Medical Specialties Center of the Pará State University Center, in the city of Belém (Brazilian Amazon).

**Methods:** A cross-sectional study was carried out based on the analysis of the medical records of patients seen at a Cardiology outpatient clinic for a period of 1 year.

**Results:** Of 202 patients seen at the outpatient clinic, it was observed that 68.3% were female. Regarding the age group, the group between 55 and 64 years old predominated (29.2%), followed by those over 65 years old (28.7%). The main complaint reported by patients was chest pain (11.5%), followed by dyspnea (10.9%) and Systemic Arterial Hypertension (SAH, 10.45%). In addition, physical inactivity (23.8%), smoking (18.1%) and alcohol consumption (16%) were observed, which are important risk factors for CVD. Regarding family history, SAH (35%) stood out, followed by cancer (17.3%), Diabetes Mellitus (15.22%), Stroke (12.75%) and Acute Myocardial Infarction. (10.3%). Consistently and as expected from the results cited, the most common diagnoses among these patients were: SAH (31.8%), DM2 (7.7%) and Dyslipidemia (7.3%). The most prescribed drugs were Losartan (18.6%), Hydrochlorothiazide (13.3%) and Simvastatin (9.9%). The main tests requested were the echocardiogram (9.5%) and electrocardiogram (5.4%).

**Conclusion:** We conclude that the observed frequency of diseases and factors associated with the development of CVDs is of significant prevalence, and such data should be valued and used as support tools in the management of public health services, especially in primary care.

111792

Modality: E-Poster Scientific Initiation – Non-case Report

Category: EPIDEMIOLOGY AND HEALTH POLICIES/GLOBAL HEALTH

## Hospitalization in the Public Health System in the Municipality of São Paulo Resulting from Hypertensive Diseases in the Year of 2021

GABRIEL RIBEIRO DE SOUZA^1^, Mayuri Akemi Rodrigues Higashi^1^, Rafaela Andrade Penalva Freitas^2^

(1) Universidade de Santo Amaro; (2) Instituto Dante Pazzanese de Cardiologia

**Introduction:** In Brazil, arterial hypertension has a high prevalence and low control rate, being a risk factor for cardiovascular diseases, which implies large public health expenses related to hospitalizations resulting from complications.

**Objective:** To quantitatively analyze the hospitalizations resulting from hypertensive syndromes in the city of São Paulo in the year 2021.

**Methodology:** It consists of a descriptive, retrospective, longitudinal epidemiological study, based on the analysis of a econdary source of data, with the aim of the studies being hospital admissions in the Unified Health System of the city of São Paulo, in the year 2021, for causes related to diseases. Hypertensive as inclusion criteria, all hospital admissions were selected through the Hospital Information System (SIH), through DATASUS. The period studied was the year 2021. The morbidities were separated and selected according to the main hospitalization diagnosis and classified according to the International Disease Code 10 (ICD10), the following being selected: I10 – Essential hypertension, I11 – Hypertensive heart disease, I12 – Hypertensive kidney disease, I13 – Hypertensive heart and kidney disease, I15 – Secondary hypertension. Gender, race/color and age group were also taken into account.

**Results:** During the study period, 2219 hospitalizations were performed for hypertensive diseases, of which 49.2% were male and 50.8% female. Among the target audience of the study, when considering race/color, 35.8% of patients consider themselves white, 8% black, 29.5% brown and 0.2% yellow. 26.5% of the registered admissions had no description of race/color. Regarding the age groups studied, there is a gradual increase in the number of hospitalizations as the observed age group also increases, with the group of patients over 80 years of age having the highest number of hospitalizations, corresponding to 14% of all the hospitalizations.

**Conclusions:** Hospitalizations in the city of São Paulo due to hypertensive syndromes were slightly higher in males, most of whom were white and aged over 80 years.

111842

Modality: E-Poster Scientific Initiation – Non-case Report

Category: CARDIOVASCULAR IMAGING

## The Influence of Previous Acute Myocardial Infarction on Exercise Stress Echocardiography Results and Risk Factors, Among Chronic Coronary Syndrome Patients

LARA TELES ALENCAR DUARTE^1^, Cláudia Bispo Martins-Santos^1^, José Icaro Nunes Cruz^1^, Allexa Gabriele Teixeira Feitosa^1^, Gabriela de Oliveira Salazar^1^, Cleovaldo Ribeiro Ferreira Júnior^1^, Danilo Brito Silva de Oliveira^1^, Victor Ravel Santos Macedo^1^, Eduardo Jose Pereira Ferreira^1^, Antônio Carlos Sobral Sousa^1^, Enaldo Vieira de Melo^1^, Joselina Luzia Menezes Oliveira^1^

(1) Universidade Federal de Sergipe (UFS)

**Introduction:** Considering the epidemiological relevance of Chronic Coronary Syndrome (CCS) and Acute Myocardial Infarction (AMI), studies that statistically associate the two groups with their main outcome, Myocardial Ischemia (MI), are still rare.

**Objective:** To assess the occurrence of MI related to the presence or absence of previous AMI, among patients with CCS undergoing Exercise Stress Echocardiography (ESE).

**Methods:** A cross-sectional study between January 2000 and January 2022 with individuals aged 40 years or older and with a positive history of CCS who underwent ESE at a cardiology referral service in Sergipe. A total of 1956 patients (X̅ = 62 ± 9.6 years) were categorized according to the presence or absence of a history of previous AMI. Student’s t test and chi-square test were used. A significance level of 5% was assumed. Analyzes were performed using SPSS Statistics software.

**Results:** 735 patients (37.6%) with previous AMI and 1221 patients (62.4%) without the ischemic event were evaluated. There was no difference between the mean age of the groups. Male sex was associated with previous AMI (75.9% vs. 65.7%; p < 0.001), as well as diabetes (23.6% vs. 19.9%; p = 0.05) and dyslipidemia (76.7% vs. 71.2%; p = 0.008). On the other hand, statin use was positively associated with the group of patients without previous AMI (80.6% vs. 59.9%; p < 0.001). When the ESE results were analyzed, there was an association between MI (ischemia, fixed ischemia or fixed and induced ischemia) with the presence of previous AMI (71.8% vs. 41.4%; p < 0.001). However, an association was found between the result of new ischemia (ie, induced) and the group with no history of previous AMI (14.7% vs. 7.2%; p < 0.001). There was no significant difference between the distributions of: obesity (p = 0.207), arterial hypertension (p = 0.12), family history (p = 0.159), use of oral antidiabetics (p = 0.283) and the presence of symptoms (p = 0.261).

**Conclusions:** MI in CCS patients was associated with patients with previous AMI in a fixed and fixed and induced manner, while new ischemia was associated with patients with no history of AMI. The relevance of statins as a protective factor for MI is also confirmed.

111802

Modality: E-Poster Scientific Initiation – Non-case Report

Category: CARDIOVASCULAR INTENSIVE CARE/CARDIOVASCULAR EMERGENCIES

## Analysis of Hypertensive Crisis Treatment in the Municipality of Vassouras in 10 Years

SARA CRISTINE MARQUES DOS SANTOS^1^, Maria Luiza Silva Barbosa^1^, Alice Machado de Sales Silva^1^, Ivana Picone Borges de Aragão^1^

(1) Universidade de Vassouras

**Introduction:** The hypertensive crisis is the rapid increase in systemic arterial pressure, which can occur in people with systemic arterial hypertension or those with normotension, potentially complicated with target organ damage. Divided into two categories, such as hypertensive urgency and hypertensive emergency. The present study aimed to analyze the current panorama of hypertensive crisis treatment procedures performed in the city of Vassouras for 10 years and to correlate the current epidemiology with the results obtained.

**Methods:** A systematic review of the literature and an observational, descriptive and cross-sectional collection of hypertensive crisis treatment data, available at DATASUS – SUS Hospital Information System (SIH/SUS) for a period of ten years – December 2008 to December 2018 – evaluating the value of public spending, complexity, mortality rate, deaths, permanence, and character of care and articles available in Scielo, Lilacs, and PubMed.

**Results:** In the analyzed period, there were 213 hospitalizations for the performance of hypertensive crisis treatment procedures, representing a total expenditure of R $ 82,593.96, 2009 being the year with the highest number of hospitalizations (66) and 2009 the year responsible for the highest amount spent during the period (R $ 28,385.62). Of the total procedures, 6 were performed on an elective basis and 207 on an urgent basis, with all 213 considered to be of medium complexity. The total mortality rate in the 10 years studied was 1.41, corresponding to 3 deaths, with the years 2008 and 2014 having the highest mortality rate, 9.09, while the year 2009 had the lowest rate, 1,52. The mortality rate for elective procedures was 0 compared to 1.45 for urgent procedures. Death cases covered only the years 2008, 2009, and 2014, with 1 death each. The average total hospital stay was 5.7 days.

**Conclusions:** Low mortality was demonstrated, with seven cases in 10 years analyzed. It is worth noting the greater occurrence of hospitalizations on an urgent basis, highlighting the need for primary and secondary prevention, in addition to investing in early recognition by the patient. It is important to have correct notification of the procedures, aiming to improve the current epidemiological analysis.

111803

Modality: E-Poster Scientific Initiation – Non-case Report

Category: ATHEROSCLEROSIS/CARDIOVASCULAR RISK FACTORS/CARDIOVASCULAR PREVENTION

## Low Aerobic Capacity Predictors Under Physiological Stress Echocardiography

LARISSA REBECA DA SILVA TAVARES^1^, Larissa Rebeca da Silva Tavares^1^, Claudia Bispo Martis-Santos^2^, Irlaneide da Silva Tavares^2^, José Icaro Nunes Cruz^2^, Gabriela de Oliveira Salazar^2^, Silvia Regina Freire Oliveira Melo^3^, Lais Oliveira Melo^1^, Stephanie Macedo Andrade^4^, Antônio Carlos Sobral Sousa^2^, Joselina Luzia Menezes Oliveira^2^, Diego Henrique da Silva Tavares^1^

(1) Universidade Tiradentes – UNIT; (2) Universidade Federal de Sergipe – UFS; (3) Hospital Primavera; (4) Hospital São Lucas

**Introduction:** The metabolic equivalent intensity levels (MET) are a way of expressing cardiorespiratory fitness (CRF). High risk cardiovascular profiles are related to low aerobic capacity.

**Objective:** To evaluate low cardiorespiratory fitness under stress during Physiological Stress Echocardiography (PSE).

**Methods:** Transversal study between January 20000 and January 2022 with individuals who have been through PSE at a cardiology reference service. 3894 Patients were categorised (57 ± 11 years) under low (MET < 7,9), intermediary (7,9 ≤ MET < 10,9) or high (MET ≥ 10,9) CRF. The chi-square test and multinomial logistic regression were used with the SPSS Statistics software version 22.0. Admitting a significance level of 5%.

**Results:** In the sample, 56.5% (2202) were female; 25.7% (1002) had low CRF, 33.5% (1306) intermediate and 40.7% (1586) high CRF. The comparison between high and low CRF groups show that older age (OR = 1.126; 95%CI = 1.113–1.139; p < 0.001), arterial hypertension (OR = 1.886; 95%CI = 1.501–2.369; p < 0.001), diabetes mellitus (OR = 1.447; 95%CI = 1.043–2.007; p = 0.027), obesity (OR = 1.936; 95%CI = 1.496–2.506; p < 0.001), sedentary lifestyle (OR = 3.18; 95%CI = 2. 55–3.966; p < 0.001), alcoholism (OR = 1.781; 95%CI = 1.387–2.286; p < 0.001) and segmental alteration during exertion (OR = 2.127; 95%CI = 1.562–2.896; p < 0.001) were predictors of low CRF. When compared to the intermediate CRF group, older age were predictors of low CRF (OR = 1.069; 95%CI = 1.058–1.08; p < 0.001); sedentary lifestyle (OR = 1.614; 95%CI = 1.32–1.975; p < 0.001), alcoholism (OR = 1.499; 95%CI = 1.177–1.908; p = 0.001), obesity (OR = 1.35; 95%CI = 1.076–1.694; p = 0.009) and segmental alteration during exertion (OR = 1.526; 95%CI = 1.168–1.996; p = 0.002). Male sex protected against low CRF in comparisons to the intermediate (OR = 0.491; 95%CI = 0.390–0.618; p < 0.001) and high cardiorespiratory fitness (OR = 0.164; 95%CI = 0.128–0.209; p < 0.001) groups.

**Conclusion:** When evaluating patients with low CRF, older age, sedentary lifestyle, obesity, alcohol consumption and segmental alteration on exertion were predictors. The predictive model also showed that physical inactivity increased the chance of low CRF by three times, being the main predictor.

111809

Modality: E-Poster Scientific Initiation – Non-case Report

Category: CARDIOVASCULAR SURGERY

## Profile of Transvenous Defibrillator Cardioverter Implant Procedures in Brazilian Regions for 10 Years

SARA CRISTINE MARQUES DOS SANTOS^1^, Maria Luiza Silva Barbosa^1^, Alice Machado de Sales Silva^1^, Ivana Picone Borges de Aragão^1^

(1) Universidade de Vassouras

**Introduction:** The defibrillator cardioverter implantation is intended to diagnose and treat rhythmic heart changes, preventing death. It is a minimally invasive and fast recovery procedure. The objective was to analyze the current panorama of single-chamber and transvenous double-chamber defibrillator implant procedures performed in Brazil and correlate the current epidemiology with the results obtained.

**Methods:** A literature review and observational, descriptive, and cross-sectional collection of single-chamber and transvenous double-chamber cardioverter implant data, available at DATASUS – SUS Hospital Information System (SIH/SUS) – December 2008 to December 2018.

**Results:** In the period analyzed, there were 10,736 hospitalizations for performing single-chamber and transvenous double-chamber cardioverter implantation procedures, representing a total expense of R$ 428,781,939.15, 2018 being the year with the highest number of hospitalizations (1,238) and the highest amount spent (R$ 54,823,826.53). The total mortality rate in the 10 years studied was 0.46, corresponding to 49 deaths, with 2008 being the year with the highest mortality rate, 3.17, while 2018 had the lowest rate, 0.24. The region with the largest number of hospitalizations was the Southeast with 4,837 hospitalizations, followed by the South with 2,570, the Northeast with 1,728, the Midwest with 1,434, and, finally, the North with 167 hospitalizations. The region with the highest number of deaths was the Southeast with 22 cases, while the North region had the lowest number, with 3 registered deaths. The northern region had the highest mortality rate (1.80) and the southern region had the lowest rate, with a value of 0.35.

**Conclusions:** It can be observed from the present study that this is a procedure with a considerably low mortality rate with a progressive reduction in the period of 10 years from 2008. It is worth noting that the northern region despite having the lowest incidence of performing the procedure has the highest mortality rate.

111813

Modality: E-Poster Scientific Initiation – Non-case Report

Category: HEMODYNAMICS AND INTERVENTIONAL CARDIOLOGY

## Comparison of Mortality Rates between Treatments for Acute Myocardial Infarction: Angioplasty and the use of Fibrinolytic

LUIZ FELIPE SILVA MARCIÃO^1^, Mateus Itiro Tamazawskas Otake^2^, Micaella Yanne Fender Lobato^2^, Leonardo Barbosa Duarte^2^, Nyara Rodrigues Conde de Almeida^3^, Victor Matheus Mendonça de Araújo^2^, Rafael Silva Lemos^2^, Lívia Guerreiro de Barros Bentes^2^, Danusa Neves Somensi^2^, Herick Pampolha Huet de Bacelar^2^, Edmundo Frota de Almeida Sobrinho^2^, Deivid Ramos dos Santos^2^

(1) Centro Universitário do Estado do Pará; (2) Universidade do Estado do Pará; (3) Universidade Federal do Pará

**Introduction:** The most serious representative of acute coronary syndromes is acute myocardial infarction, which is an extremely serious complication caused by the interruption of blood flow to the myocardium. Therefore, the therapeutic objective is arterial clearance and cardiac reperfusion; for this, there is the use of fibrinolytic and/or angioplasty, the therapeutic methods can be used exclusively or associated, depending on the patient’s clinic and the time of Evolution.

**Objective:** To analyze the mortality rate between Coronary Angioplasty and Catheter-Directed Thrombolysis with fibrinolytic procedures in Brazil in the period 2011–2021.

**Methods:** It is characterized as descriptive, quantitative, and retrospective performed by collecting data from the Hospital Information System of the Unified Health System (SIH/SUS-DATASUS) during May 2022. Data were organized according to the procedure performed in the Microsoft Excel program.

**Results:** In the period from 2011 to 2021, 33.867 Coronary Angioplasty (CA) surgeries were performed, with the Southeast region having the largest number of procedures performed (41.6%), in addition to an average annual growth rate (AAGR) of 3.4% between 2011–2016 and an annual decrease of 2.2% between 2016–2021. Furthermore, 2.179 deaths were associated with the procedure, and the mortality rate (MR) was 6.42 ± 1.32. For the Catheter-Directed Thrombolysis with fibrinolytic (CDT), 1168 were performed between 2011 and 2021, with an AAGR of 26.8% between 2011–2016 and 1.7% between 2016–2021, and the majority in the Southeast (51.7%). There were 49 deaths recorded in this period and an MR of 4.17 ± 2.11. Therefore, for CA, the number of deaths in 2011 was 126, in 2016 it was 205, and in 2021 it was 243, with an AAGR of 10.2% between 2011–2016 and 3.5% between 2016–2021. Regarding CDT, for the same variable, 3 records in 2011, 6 in 2016, and 2021 were 7 deaths, with an AAGR of 14.9% between 2011–2016 and 3.1% between 2016–2021. Both procedures showed an increase in deaths in the number of deaths vs a number of procedures, with emphasis on the correlation made in the CA, where the increase did not follow the decrease of the last five years, in addition to the fact that the MR was higher in the CA compared to CDT.

**Conclusion:** The Southeast region had the highest number of CA and CDT procedures performed in the period evaluated. In addition, a higher MR was observed for CA when compared to CDT and a decreasing AAGR between 2016–2021.

111815

Modality: E-Poster Scientific Initiation – Non-case Report

Category: CARDIOVASCULAR IMAGING

## Percutaneous Treatment with Coronary Stents Guarantees Patients with Previous Acute Myocardial Infarction Better Conservation of Echocardiographic Parameters at Rest and During Exercise

LARA TELES ALENCAR DUARTE^1^, José Icaro Nunes Cruz^1^, Cláudia Bispo Martins-Santos^1^, Allexa Gabriele Teixeira Feitosa^1^, Gabriela de Oliveira Salazar^1^, Cleovaldo Ribeiro Ferreira Júnior^1^, André Pinheiro Zylberman^1^, Edvaldo Victor Gois Oliveira^1^, Eduardo Jose Pereira Ferreira^1^, Antônio Carlos Sobral Sousa^1^, Enaldo Vieira de Melo^1^, Joselina Luzia Menezes Oliveira^1^

(1) Universidade Federal de Sergipe (UFS)

**Introduction:** Chronic Coronary Syndrome (CCS) appears frequently to clinical cardiologists, but the literature still lacks data on the evolution of its complex pathology.

**Objective:** To analyze, by Exercise Stress Echocardiography, the behavior of myocardial ischemia in CCS patients with previous Acute Myocardial Infarction (AMI), according to the given treatment.

**Methods:** A cross-sectional study from January 2000 to January 2022 with individuals aged ≥ 40 years (X = 62 ± 9.6 years) who underwent the exam at a cardiology referral service in Sergipe. They were grouped by the intervention performed after the AMI: surgical revascularization, Stent, or clinical treatment. Patients with CCS with no history of AMI were also considered. The behavior of the left ventricle was evaluated considering the Left Ventricular Mass Index (LVMI) and the Ejection Fraction (EF) while the behavior of the myocardial ischemia, considering the Wall Motion Score Index (WMSI). Chi-square test and one-way ANOVA analysis for multiple comparisons with Tukey’s post-hoc were used. The significance level was set to 5%. Analyzes were performed using SPSS Statistics.

**Results:** 727 patients (37.3%) with previous AMI were studied: 142 (7.3%) underwent surgical revascularization, 284 (14.6%), Stent, and 301 (15.5%), clinical treatment. 1221 patients (62.7%) without previous AMI were evaluated. On average, the increase in LVMI was greater among those who received only clinical treatment, compared to those who had no previous AMI (108g vs. 91.3g; p = 0.010); the other groups showed no significant difference. Regarding EF, individuals treated with surgical revascularization (14.4% vs. 3.5%; p < 0.001), with Stent (10.8% vs. 3.5%; p < 0.001) and clinically (9.5% vs. 3.5%; p < 0.001) had a higher percentage of reduced EF compared to patients with CCS without previous AMI. As for resting and exercise WMSI, the surgical revascularization group (averages 1.21 and 1.22 respectively) and the clinically treated group (averages 1.17 and 1.20) were statistically different from the Stent group (averages 1.11 and 1.10) (p < 0.001) and the SCC group without previous AMI (averages 1.04 and 1.05) – those last also different among themselves.

**Conclusions:** In the stratification of patients with CCS with previous AMI, treatment with the Stent showed better evolution in the behavior of the LVMI, EF and WMSI on the Exercise Stress Echocardiogram at rest and exercise, when compared to patients with CCS without previous infarction.

111819

Modality: E-Poster Scientific Initiation – Non-case Report

Category: COVID-19 AND CARDIOVASCULAR SYSTEM

## Myocarditis Related to Vaccine Against COVID-19 in Adults

VITÓRIA CALDAS GONÇALVES^1^, Beatriz Caldas Gonçalves^2^, Ana Elisa Caldas Gonçalves^3^, Gabriela Magalhães Bandeira Gomes^1^, Isadora Vilela Rodovalho^1^, Rodrigo Rodrigues Pache^4^, Ana Luiza Espíndula Rocha^1^, Paulo Henrique Rodrigues^1^

(1) Universidade Evangélica de Goiás – UniEvangélica; (2) Universidade Federal de Goiás – UFG; (3) Universidade Federal de Uberlândia – UFU; (4) Universidade Federal do Tocantins – UFT

**Introduction:** The mRNA vaccines developed against the SARS-CoV-2 virus, despite being well tolerated in most cases, are related to serious and potentially fatal reactions, such as myocarditis. This is characterized as an inflammation of the heart muscle resulting from the action of toxins, through hypersensitivity reactions or by an autoimmune mechanism. In order to reduce clinical complications and improve functional prognosis, diagnosis and treatment must be performed quickly and accurately.

**Objective:** To understand the development of myocarditis as an adverse effect of the vaccine against the SARS-CoV-2 virus, as well as its main prognostic aspects.

**Methods:** This is an integrative review with a search in the databases PubMed and The Virtual Health Library (VHL) using the Health Sciences Descriptors “myocarditis”, “covid-19” and “vaccine”, combined with the boolean operator AND. We selected 10 original articles, whose inclusion criteria consisted of recent publication date (last 5 years) and languages in Portuguese and English.

**Results:** With regard to immunization against COVID-19, myocarditis should be suspected when symptoms of heart failure such as progressive chest pain, dizziness, fever, nausea and vomiting begin about five days after vaccination. It is common the elevation of cardiac biomarkers in the complementary exams, such as troponin I, in addition to evidence of changes in ventricular repolarization on the electrocardiogram. Typically, an increase in the plasma concentration of N-terminal pro-B-type natriuretic peptide (NTproBNP) and C-reactive protein (CRP) is found. A possible pathophysiological justification for the relationship studied is the fact that cardiac myocytes express ACE2 receptors, which can bind to the spike glycoprotein, translated from the mRNA vaccine, and trigger cardiac complications. However, there is still not enough evidence to establish this causal association.

**Conclusion:** Myocarditis after vaccination against COVID-19 usually follows a benign course; however, medical monitoring is necessary due to the high rate of morbidity and mortality, even though it is an infrequent and poorly understood adverse effect. It is important to note that the benefits of vaccination outweigh the risks of developing myocarditis since COVID-19 infection carries a much higher rate of acute and chronic cardiovascular sequelae, including arrhythmias and thromboembolic events.

111827

Modality: E-Poster Scientific Initiation – Non-case Report

Category: ATHEROSCLEROSIS/CARDIOVASCULAR RISK FACTORS/CARDIOVASCULAR PREVENTION

## Impact of Diabetes Mellitus in Young Individuals with Acute Coronary Syndromes on Cardiovascular Outcomes and the Direct and Indirect Health-Related Costs

MARIANA GUIMARÃES SOUZA DE OLIVEIRA^1^, Ana Carolina Augusto Rocha^1^, Eduardo Souza Amancio da Costa^1^, Marina Miguel de Lucena^1^, Alexandre Anderson de Souza Munhoz Soares^2^, Luiz Sergio Fernandes de Carvalho^3^

(1) Universidade Católica de Brasília UCB; (2) Instituto Aramari Apo; (3) Clarity Healthcare Intelligence

**Objectives:** We seek to compare the prevalence of diabetes (DM) and cardiovascular outcomes (CO) associated with groups of young individuals who presented their first acute coronary syndromes (ACS) with ages <55 y/o (Premature ACS, pACS). In parallel, we sought to comprehend the economic impact of the pACS and DM association. 2.

**Methods:** We analyzed the registers retrospectively with the first ASC aged <55 y/o from 2013 to 2015 within the B-CaRE:QCO study, a registry that includes all the admissions to public hospitals of Federal District, Brazil. DM has been defined as hemoglobin A1c >6.5%, documented diagnostics, or the reception of diabetes treatment. Cause of death was adjudicated and all cause-specific hospitalizations were retrieved from digital records. The primary outcome was overall health care spending (sum of the cost of lost productivity [CLP], ambulatory costs, and the cost of recurrent hospitalizations [CRH] in international dollars [Int$] per year), and the secondary outcomes were the mortality due to all causes of the recurrent ACS. 3.

**Results:** Among 6,341 individuals with ACS, 2.242 were pACS (mean age 47.9 ± 5.5 years), diabetes was present in 514 individuals (29.7%), of whom 137 (27%) used insulin. During median follow-up of 6.5 (IQR 1.9) years, DM was associated with higher all-cause mortality (HR 1.58[95%CI 1.3–1.9]; p < 0.001) and recurrent ACS (1.48[95%CI 1.3–1.7]; p < 0.001) relative to young and non-diabetic individuals. These associations persisted after the adjustment for baseline covariates (all-cause mortality: 1.69; p = 0.003; recurrent ACS: 1.72; p < 0.001). Compared to older individuals, the pASC group had lower all-cause death rates (p < 0.001), but higher rates of recurrent ACS (p = 0.012) during the monitoring, particularly in diabetics. Driven primarily by CRH and CLP, young people with diabetes spent the highest amounts (Int$16,350 [12,545–23,740]/year in pACS+DM+ vs Int$14,555[12,593–20,354]/year in prACS+DM- vs Int$12,183 [10,082–17,416]/year in older individuals, p < 0.001). 4.

**Conclusions:** Diabetes was present in 29.7% of patients presenting ACS at age <55 y/o and was associated with higher all-cause mortality at long-term as well as a more recurrent ACS in comparison to young non-diabetic individuals, but otherwise lower when compared to older individuals. However, young diabetics have shown the highest overall healthcare expenditures. These findings highlight the need for implementation of heftier therapies aimed at preventing.

111834

Modality: E-Poster Scientific Initiation – Non-case Report

Category: HEART FAILURE/CARDIOMYOPATHY/TRANSPLANT

## Characteristics and Prognosis of Patients with Acute Heart Failure According to the Universal Ejection Fraction Classification

MICHELLE BOZKO COLLINI^1^, Gustavo Sarot Pereira da Cunha^1^, Leonardo Henrique dos Santos de Melo^1^, Matheus Bissa Duarte^1^, Jorge Tadashi Daikubara Neto^1^, Rafael Moretti^1^, Carolina Ruschel Senger^1^, Karoline Cordeiro Vercka^1^, Jessica Tamires Reichert^1^, Lucas Muller Prado^1^, Jamilly Giuriatti Anziliero^1^, Miguel Morita Fernandes-Silva^1^

(1) Universidade Federal do Paraná

**Introduction:** The universal classification of heart failure defined four classes according to the ejection fraction (EF). These classes have different characteristics and prognosis in chronic patients, but the information in the literature on acute heart failure is scarce.

**Objectives:** To compare the prognosis of the EF classes in patients admitted for acute heart failure.

**Methods:** We studied patients admitted for acute heart failure in a tertiary hospital in Curitiba, Brazil, from October 2019 to July 2021. EF classes were defined as: reduced EF (HFrEF): EF ≤40%, mildly reduced EF (HFmrEF): EF 41–49%, preserved EF (HFpEF): EF ≥50% and improved EF (HFimpEF): baseline EF ≤40%, with ≥10 point increase from baseline, and a second measurement of EF >40%. The outcome was all-cause mortality in a six-month follow-up.

**Results:** From 153 patients (67.21 ± 14.86 years, 50.3% female, EF = 43.79 ± 17.62%), 52% had HFrEF, 33% HfpEF and 12% HFmrEF. The proportion of patients with HFimpEF was very small (3%). As expected, HFrEF patients were mostly male (66.2%) and more likely to have an ischemic etiology (42.5%); and HFpEF patients were more likely female (67.3%), and had hypertension (90.9%) and atrial fibrillation (49.1%), as compared with the other categories. The 6-month survival rate did not significantly differed among the EF classes (p = 0.55) (figure 1).

**Conclusions:** In patients with acute heart failure, the proportion of HFimpEF was small and the 6-month all-cause mortality was similar among the EF classes.


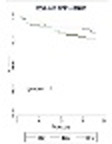


111837

Modality: E-Poster Scientific Initiation – Non-case Report

Category: HYPERTENSION/RENAL DENERVATION

## Prevalence of Arterial Hypertension in a Community Riverside of the Rio Jamari in the Amazon

ANA CAROLINE LEITE GUEDES^1^, Maria Eduarda Brotto de Souza^1^, Fernanda Dias de Oliveira^1^, Roberta Cristiane Oliveira da Silva^1^, Gesanaje da Paz Carvalho^1^, Jade Gomes da Costa Medeiros^1^, Caroline de Souza Alovisi^1^, Ana Júlia Omodei Rodrigues Martim^1^, Renata Gonçalves Silva Santos^1^, Lucas Vieira Amorim^1^, Sofia dos Santos Souza^1^, Fernanda Gabry Scazuza Gomes de Souza^1^

(1) Centro Universitário São Lucas- UNISL

**Introduction:** Systemic arterial hypertension (SAH) is a multifactorial condition, which depends on genetic/epigenetic, environmental and social factors, characterized by persistent elevation of blood pressure (BP). The diversity of causes for the development of SAH is extensive and, in most cases, it is associated with alcoholism, smoking and physical inactivity, in the entire population, with a special focus on remote communities. The health of the Amazon riverside population has been the focus of special attention, mainly related to factors such as changes in the level of physical activity in their daily lives (fewer active individuals) due to the so-called “facilities” of the modern world, and changes in their patterns of physical activity. In this way, the present work aims to analyze the prevalence of this comorbidity in riverine people and highlight the relevance of the problem.

**Objectives:** To describe the prevalence of SAH in a riverside community on the Jamari River in the Amazon, Brazil.

**Methods:** The present work is a cross-sectional and quantitative cohort study, through the application of a questionnaire to the population, between the ages of 1 to 87 years, residents of the region corresponding to the district of São Carlos do Jamari, in Rondônia. The collected data were described and tabulated for further analysis.

**Results:** 269 individuals from the region in question were randomly and unintentionally selected. Of these, 78 people (29%) reported having Systemic arterial hypertension, among them the highest prevalence for women. Social habits include drinking (15.3%) and smoking (10%), as well as unspecified heart problems (10%).

**Conclusion:** The aforementioned study brings the discussion about the prevalence of SAH in riverside populations in the Amazon, which presents an expressive percentage, with an association of multiple factors, proving the necessary detailed analysis of the situation aiming at reducing risks and improving the population’s quality of life.

111841

Modality: E-Poster Scientific Initiation – Non-case Report

Category: DIGITAL HEALTH/INNOVATION

## Accuracy of Cardiac Arrhythmias and Conduction Disorders Diagnosis using a Smartwatch

GUILHERME NUNES MIRANDA^1^, Daniel Italiano de Araujo Filho^1^, Francisco José de Almeida Cruz Júnior^2^, Hermano Rodrigues Pinheiro^3^, Maria Clara Almeida^4^, Carlos Eduardo Batista de Lima^2^

(1) Universidade Federal do Piauí; (2) Hospital Universitário do Piauí; (3) Centro de Pesquisa Cardiolima; (4) Instituto do Coração – FMUSP

**Introduction:** Implantable devices increase the detection of silent atrial fibrillation in high-risk populations, being a useful tool in the early diagnosis of these patients, allowing monitoring for a longer time. The Smartwatch could be a useful, easy tool for the daily cardiological propaedeutics. However, there is just a little information on the limitations of analysis on the watch’s Electrocardiogram (ECG) in comparison to the conventional one.

**Objective:** To analyze, in patients with cardiovascular disease, the electrocardiographic aspects detected from the device in comparison to the conventional ECG, identifying aspects of similarity between the tracings.

**Method:** A cross-sectional observational study of diagnostic accuracy is currently being carried out in a Hospital since July 2021. Each patient did three ECGs, ECG 1 = 12-lead conventional; ECG 2 = rhythm strip DI single lead conventional; ECG 3 = DI single lead on the smartwatch. The ECGs were randomly delivered for 3 cardiologists, the 3 examiners were blinded. The data was analyzed comparatively between the 3 measures in each patient and between each other. Data were analyzed using the Statistical Package for the Social Science program (SPSS®, version 20.0). For statistical analysis, the Kappa statistics, ANOVA test, Turkey post-test were used, considering a significant p-value <0.05.

**Results:** 19 patients were analyzed between July 2021 and April 2022. Fifty five ECGs were collected and the 3 examiners registered a total of 165 reports. The average age was 61,68 and male gender was more prevalent (78,9%), the most common diseases were angina (57,9%) and Atherosclerosis (57,9%). The results showed the sinusal rhythm detection Kappa between examiners 1, 2, 3 in the Smartwatch’s ECG were (0,799; 0,895; 0,712) and the sinusal rhythm detection Kappa in the conventional 12-lead ECG were (1,000; 1,000; 1,000). Some data showed that the Smartwatches ECG’s could detect abnormal rhythms, even when we could not specify, nonspecific changes in ventricular repolarization (AIRV) were detected.

**Conclusions:** In this 19 patient analysis, the Smartwatch has shown a good accuracy to detect if the patient had a normal rhythm. Even though the wereables presented some data capture limitations but, as we educate patients on how to use the 1-lead ECG, add more data and new technologies in each patient analysis, it could be a useful tool for monitoring and possible early diagnosis of abnormalities, such as arrhythmia.

111848

Modality: E-Poster Scientific Initiation – Non-case Report

Category: DIGITAL HEALTH/INNOVATION

## Association of Wearable Devices and Cardiovascular Risk Reduction

ANA ELISA BARRETO CALIXTO^1^, Ana Elisa Barreto Calixto^1^, Guilherme Rufino Marques Pellegrin^1^, Nathalia Noyma Sampaio Magalhães^1^, Luiza Dahbar Rodrigues^1^, Matheus Pericles Belcavello^1^, Patrick Ribeiro Reis^1^, Lucas Nicolato Almada^2^

(1) Faculdade de Ciências Médicas e da Saúde de Juiz de Fora – Suprema; (2) Hospital e Maternidade Therezinha de Jesus – HMTJ

**Introduction:** Cardiovascular diseases are the leading cause of death globally. They are preventable by changing risk behaviors, such as a sedentary lifestyle and poor sleep quality. The implementation of low-cost technology-based interventions can contribute not only to improve health outcomes, but also to reduce public health costs related to cardiovascular disease. Wearable devices (WD) are gadgets that can be worn anywhere on the body, allowing the generation of data on physical activity, heart rate and rhythm and, more recently, impedance and thoracic fluid data. The assessment of these data is an important challenge in the clinical setting.

**Objectives:** To evaluate the impact of WD, associated with physical exercises, in reducing cardiovascular risk.

**Methods:** Literature review was carried out in the MedLine database using the descriptors: “Wearable Devices”, “Cardiovascular Risk” and their variations according to MeSH. Controlled and randomized clinical trials carried out in humans and published in the English language in the last 10 years were included. The PRISMA scale was used to improve the reporting of this review.

**Results:** Two studies analyzed the outcomes of WD in patients with elevated cardiovascular risk. In the first one, 144 patients with a mean age of 61 years were recruited. 70 patients participated in the experimental group (EG) and, as an intervention, they used WD, resulting in a significant increase in the number of steps and activity level over time (p < 0.001 and p = 0.01, respectively). No significant changes were observed in blood pressure, weight, and sleep quality. In the second study, 40 individuals with a mean age of 72 years were randomized into two groups. Both of them received instructions concerning physical activity practice, but the EG also received WD. 12 weeks after randomization, the EG presented a significant increase in the number of steps (p < 0,05) while in the control group (CG) this number actually decreased. In EG, there was a reduction of 4 mmHg in blood pressure levels and the CG suffered an increase in this parameter. Sedentary behavior did not suffer any alteration in either group.

**Conclusion:** The results of the studies showed that the reduction of cardiovascular risk was optimized by the association of WD with regular practice of physical activities, which suggests that the use of these devices has the potential to help in the prevention and treatment of cardiovascular diseases.

111865

Modality: E-Poster Scientific Initiation – Non-case Report

Category: NUTRITION

## Effects of Energy Drinks on the Cardiovascular System and Their Long Therm Repercussions

GABRIEL JUNQUEIRA JÚLIO^1^, Giuliano Fernando da Silva Júlio^2^, Sofia Sousa Alexandre^1^, Katarina Masciano Pereira^1^, Sevana Valadão Naves^1^, Danilo Cezar Aguiar de Souza Filho^1^, Douglas Araujo Menezes Filho^1^, Renato Tassi de Lima^1^, Isadora Silva de Sousa^1^, Lorena Costa de Holanda^1^, Giovanna Cristina Silveira Corrêa^1^, Mariana Mello Menezes^1^

(1) Centro universitário euro americano – UNIEURO; (2) Prevencor

**Introduction:** The increased consumption of energy drinks (ED) in recent years has encouraged studies with the purpose of identifying possible damage caused by indiscriminate and prolonged use. The increase in consumption is associated with the intention to improve performance in daily activities. The compounds present in ED show that the levels of caffeine and sugar ingested can generate cardiovascular, behavioral and metabolic repercussions when used for a long time.

**Objectives:** Acknowledge the risks of indiscriminate consumption of abusive doses of ED and the effects caused by the chronicity of use.

**Methodology:** A systematic literature review was performed. In data collection, a bibliographic and documentary search was carried out on the subject, using articles that discuss the cardiovascular repercussions associated with the use of energy drinks. The search platform was PubMed, using the terms “energy drink” and “cardiovascular” as descriptors. 16 articles were selected, and the selection criteria were free systematic review studies or clinical trials, with 5 years of publication, in English or Portuguese, the articles that didn’t match the theme were eliminated. For analysis, the different results of the studies were systematized and compared.

**Results:** When comparing the studies, it was found that the abusive use of ED increases the probability of cardiovascular repercussions such as tachycardia, arrhythmia, ischemia, increased systemic blood pressure, changes in repolarization, conduction disturbances and prolongation of QT intervals on the electrocardiogram. In addition to behavioral changes, such as stress, increased anxiety attacks and insomnia. The metabolism is altered by elevated cortisol levels and increased sugar consumption, providing greater risk for developing type 2 diabetes mellitus. Caffeine is the main compound that influences the perceived changes and its association with alcoholic beverages generates more serious and faster-evolving repercussions.

**Conclusion:** The effects of the use of ED are already present in the studies carried out and show consequences in the lives of users who often don’t have the necessary knowledge about the drink and its long-term damage remains uncertain. Thus, the cardiovascular and metabolic systems are affected, but also the psychological system undergoes negative changes, demonstrating the need for studies with longer follow-up.

111907

Modality: E-Poster Scientific Initiation – Non-case Report

Category: HEART FAILURE/CARDIOMYOPATHY/TRANSPLANT

## Mortality Predictors in Patients with Heart Failure in a Referral Hospital

KARLA SANTOS PINTO^1^, Raissa Barreto Lima^1^, Ana Luísa de Aguiar Almeida Silva^1^, Eduarda Luciana Mendes Borges^1^, Elias Soares Roseira^1^, Livia Maria Goes Lemos^1^, Aurea Maria Lago Novais^1^, Beatriz Barbosa Viana^1^, João Pedro Martins Moreira Granja^1^, Karla Santos Pinto^1^, Larissa Xavier Gomes da Silva^1^, Clara Salles^1^

(1) Hospital Ana Nery

**Introduction:** Chronic heart failure is a condition associated with high rates of morbidity and mortality. Despite recent advances in optimized drug therapy for patients with reduced ejection fraction (HFrEF), they still have a significant risk of death. Therefore, the objective of this study is to identify predictors of death among individuals with HFrEF in a referral hospital in the city of Salvador, Bahia.

**Methods:** This is a prospective cohort of patients with HFrEF, treated at a heart failure outpatient clinic from September 2020 to July 2021. The outcome variables evaluated were mortality and hospitalization for any cause in one year. For data analysis, the Odds Ratio (OR) was used as a measure of association between categorical variables and 95% Confidence Intervals (CI), using the chi-square test in the bivariate analysis. Continuous variables with normal distribution were compared using Student’s t test. Multivariate analysis with logistic regression was used to assess independent predictors of mortality. A value of p < 0.05 was considered for statistical significance.

**Results:** We included 196 patients with heart failure with a median age of 60.5 (49–68) years, most of them men (54.1%) and a median LVEF of 29% (23–35). Ischemic and chagasic etiologies were the most prevalent, with 46 (23.5%) and 40 (20.4%) patients, respectively. After 1 year of follow-up, it was found that 25 (12.8%) died, 50 (25.5%) were hospitalized and NYHA III-IV totaled 57 (34.5%) patients. Most patients who died were male (64%) and 49% had a history of hospitalization <1 year. In the multivariate logistic regression model, Chagas‘ etiology remained the only independent predictor of mortality (OR 1.36; 95% CI 1.52–10.11; p = 0.005). In the univariate analysis, the mortality predictors were: DM2, CAD, hospitalization <1 year and Chagas‘ etiology (Table 1).

**Conclusion:** Chagas’ etiology was the only independent predictor of mortality in patients with HFrEF.

111875

Modality: E-Poster Scientific Initiation – Non-case Report

Category: HEART FAILURE/CARDIOMYOPATHY/TRANSPLANT

## The use of Colchicine in Acute Myocarditis – A Systematic Review

EDUARDA CORRÊA MAIA^1^, Evandro Tinoco Mesquita^1^

(1) Universidade Federal Fluminense

**Fundamentals:** Myocarditis is an inflammation of the myocardium that can affect cardiac function, reducing ejection fraction and causing arrhythmias, leading to heart failure syndrome, cardiogenic shock and sudden death. This condition can be caused by a viral infection, an immune-mediated response due a late viral response, inflammatory autoimmune diseases or an auto-inflammatory response, making myocarditis even more relevant within the COVID-19 pandemic. Colchicine is a low-cost anti-inflammatory, already known and used in rheumatologic diseases, and, now, used in cardiology for pericarditis and coronary disease. In cases of colchicine use for myocarditis there have been publications with positive results, however its effects in patients with acute myocarditis are not clearly demonstrated. Goal Systematically review the literature to assess the effects of colchicine use in patients with acute myocarditis.

**Methods:** We performed a systematic review of the effects of colchicine in patients with myocarditis. Case series, randomized studies and case controls published on PubMed, SciELO and Google Scholar platforms were selected, from January 2010 to January 2022, in Portuguese, English, Spanish and French.

**Results:** Initially, a total of 62 studies were found. After exclusions, 30 articles remained, with a total of 128 patients with acute myocarditis who used colchicine. Of this group, 10.93% (14 patients) had myocarditis in association with another disease, 3.9% (5 patients) in association with autoimmune diseases, and 16.4% (21 patients) with myopericarditis. In the only study using a control group consisting of 60 patients (didn’t use colchicine), 31.6% (19 patients) had complete resolution of symptoms. In the end, a positive association was demonstrated in the use of colchicine in the treatment of myocarditis, with 75.78% (97 of 128 patients) of patients presenting resolution of symptoms within 14 months after starting treatment.

**Conclusion:** The results found in this systematic review, so far, suggest that the use of colchicine in the treatment of acute myocarditis has been effective. The study also showed the absence of randomized studies that analyze the effectiveness of colchicine in cases of acute myocarditis, with only one study using a control group and showing significant improvement in the clinical picture. In addition, the little evidence related to cases of post-covid myocarditis is even more relevant in the current pandemic context.

111897

Modality: E-Poster Scientific Initiation – Non-case Report

Category: COVID-19 AND CARDIOVASCULAR SYSTEM

## COVID-19 Cardiovascular Outcomes in People Living with HIV in Brazil

MANUELA FURTADO SACIOTO^1^, Thaís Lorenna Souza Sales^2^, João Victor Baroni Neves^1^, Vivian Costa Morais de Assis^1^, Maíra Viana Rego Souza-Silva^3^, Polianna Delfino-Pereira^3^, Amanda Rabello Conceição^3^, Anna Luiza Homan Araújo Carvalho^1^, Isabela Moraes Gomes^3^, Lucas Emanuel Ferreira Ramos^3^, Magda Carvalho Pires^3^, Milena Soriano Marcolino^3^

(1) Faculdade Ciências Médicas de Minas Gerais (FCMMG); (2) Universidade Federal de São João Del-Rei (UFSJ); (3) Universidade Federal de Minas Gerais (UFMG)

**Introduction:** There is a lack of studies investigating the influence of HIV infection, on the cardiovascular outcomes, in COVID-19 patients in Latin America.

**Objective:** To compare cardiovascular outcomes, between patients with COVID-19 with and without HIV infection, in two consecutive years.

**Methods:** This is a substudy of a multicentric cohort, which included 27 Brazilian hospitals, comprising March to September 2020 and March to December 2021. Cardiovascular outcomes included acute myocardial infarction (AMI), pulmonary thromboembolism (PTE), deep vein thrombosis (DVT) and stroke. Patients with COVID-19 and HIV coinfection were matched for age, sex, number of comorbidities and hospital. Groups were compared using the Chi-Square Test for categorical variables and the Wilcoxon test for continuous variables. Results were considered statistically significant at a significance level of p < 0.05.

**Results:** From 17,101 COVID-19 patients, 130 were people living with HIV (PLHIV) (0.76%). Of these, 86 were hospitalized in 2020 and 44 in 2021. The median age in 2020 and 2021 periods was 53 and 54 years, respectively, with no significant differences between groups. The most frequent COVID-19 symptoms in both groups were dyspnea (2020: 52 [60.5%] vs 227 [67.0%]; p = 0.314/2021: 30 [68.2%] vs 122 [69.7%]; p = 0.989) and fever (2020: 50 [58.1%] vs 207 [61.1%]; p = 0.710/2021: 20 [45.5%] vs 76 [43.4%]; p = 0.942). The main cardiovascular outcomes between PLHIV in 2020 were PTE (4.7%), DVT (2.3%), AIM (2.3%), and stroke (1.2%). Non-HIV infected patients showed similar prevalence for the outcomes under study, with no significant difference. PTE (13.6%) and DVT (2.3%) were also the most common cardiovascular outcomes in PLHIV in 2021, however, no cases of AIM and stroke were observed in this group. Comparison of outcomes with the controls also showed no significant difference in this period. When observing the years of 2020 and 2021, there was a total reduction of cardiovascular outcomes, except for PTE.

**Conclusion:** There were no significant differences in the cardiovascular outcomes between PLHIV and their controls. However, the reduction of the cardiovascular outcomes between 2020 and 2021 indicates the importance of vaccination in the prognosis of COVID-19 in HIV coinfected patients.

111913

Modality: E-Poster Scientific Initiation – Non-case Report

Category: DIGITAL HEALTH/INNOVATION

## Diagnostic Accuracy of Smartwaches for Detection of Atrial Fibrilation: A Systematic Review

LAÍS ARAÚJO CARNEIRO DE CAMPOS^1^, Laís Araújo Carneiro de Campos^1^, Marília Menezes Gusmão^1^, Marília Menezes Gusmão^2^, Júlia Schoucair Neves^1^

(1) Escola Bahiana de Medicina e Saúde Pública (EBMSP); (2) Escola Bahiana de Medicina e Saúde Pública (EBMSP)

**Introduction:** Atrial fibrillation is a common cardiac arrhythmia that affects about 59 million people worldwide. In many cases its diagnosis occurs late, due investigation of an ischemic stroke. In other situations, it presents a paroxysmal behavior, which makes it difficult to detect this arrhythmia in conventional electrocardiography exams. Among recent technologies being used to monitor and detect cardiac arrhythmias, the smartwatches are becoming very popular. For that reason, it is important to verify if those devices are accurate to detect atrial fibrillation.

**Objective:** To describe the diagnostic accuracy of smartwatch-like devices in detecting atrial fibrillation.

**Methods:** A systematic review was conducted using PRISMA methodology, between June and September 2021, in the Databases of Pubmed/Medline and Virtual Health Library. The descriptors used included the terms “watch”, “wearable electronic devices”, “photopletysmography” and “atrial fibrillation”. After removal of duplicates, the results were screened for inclusion and exclusion criteria.

**Results:** 409 articles were returned by the search, of which, 120 were duplicates. After screening the remaining 289 titles and abstracts for inclusion and exclusion criteria, 96 were selected for the second screening (full article read), of which 10 studies matched the criteria and were included in the review. The accuracy was measured on 10 different devices models produced by 5 companies. The sensitivity of the smartwatches included in the review varied between 67–100%, the specificity 67–99%, positive predictive value 80–90% and negative predictive value 85–100%. The accuracy was available in only two studies (70% and 97,5%).

**Conclusion:** The smartwatches models reviewed by this study are accurate to detect atrial fibrillation.

111915

Modality: E-Poster Scientific Initiation – Non-case Report

Category: EPIDEMIOLOGY AND HEALTH POLICIES/GLOBAL HEALTH

## Hospitalizations for Cardiovascular Conditions Sensitive to Primary Health Care in Betim, Minas Gerais 2018–2020

FERNANDA AMPARO RIBEIRO^1^, SUELLEN MOURÃO SILVA^1^, GILBERTO ANTONIO REIS^1^, GILBERTO ANTONIO REIS^1^, FERNANDA AMPARO RIBEIRO^1^, FERNANDA AMPARO RIBEIRO^1^

(1) PONTIFICIA UNIVERSIDADE CATOLICA DE MINAS GERAIS – PUC-MG; (2) Fundação de Amparo à Pesquisa do Estado de Minas Gerais – FAPEMIG

Hospitalizations for Primary care-sensitive conditions are preventable health complications through disease prevention, diagnosis and care of acute conditions, monitoring of chronic conditions. The use of this indicator is based on the principle that effective and effective primary health care can prevent hospitalizations. Thus, a high number of Hospitalizations for primary care-sensitive conditions in a population points to difficulties in accessing and operating health services. Different activities of primary health care and specialized outpatient and hospital care were interrupted or paralyzed due to the priority given to COVID-19. For different reasons, both professionals and primary care users have dangerously delayed non-COVID-19 procedures, which could interfere with the effectiveness and effectiveness of primary health care and which, in turn, would reflect on Hospitalizations for primary care-sensitive conditions. Thus, the hypothesis presented is that the incidence of Hospitalizations for Primary care-sensitive conditions care in the population over 34 years of age in the city of Betim-MG may vary between the periods before and during the COVID-19 pandemic.

**Objective:** To measure the occurrence of hospitalizations for cardiovascular conditions sensitive to primary care in adults over 34 years of age living in the city of Betim – Minas Gerais and to estimate the variability between the previous period and during the COVID-19 pandemic.

**Methods:** This is an ecological, descriptive, longitudinal study with a quantitative approach. Data were extracted from the Hospital Information System available on the DATASUS, portal of the Ministry of Health. Descriptive statistical measures of data were used to analyze the variables included in the study.

**Results:** Among hospitalizations for cardiovascular conditions sensitive to primary care in the period between 2018–2021 in the city of Betim-MG, it was observed that the total number of hospitalizations behaved in a stationary way year after year. At the same time, there was a drop in the number of emergency admissions in 2021 compared to 2018, 2019 and 2020. The population most affected by cardiovascular and cerebrovascular causes were black and brown people and men. Stroke was the main cause of elective and emergency admissions.

111919

Modality: E-Poster Scientific Initiation – Non-case Report

Category: CONGENITAL AND PEDIATRIC CARDIOLOGY

## Epidemiological Profile of Live Births with Tetralogy of Fallot During the Period from 2016 to 2020 in Brazil

LAURA COUTINHO VIANA^1^, Laura Coutinho Viana^1^, José Wilker Gomes de Castro Júnior^1^, Marcello Vieira dos Santos^1^, Pedro Arthur Rodrigues de Oliveira^1^, Daniely Maués Beliqui^1^

(1) Centro Universitário do Estado do Pará – Cesupa

**Introduction:** Tetralogy of Fallot (TF) is a complex cyanotic congenital heart disease that involves changes in 4 cardiac segments in the newborn. They are: Right ventricular hypertrophy, aortic overriding of the interventricular septum, pulmonary artery stenosis, and sub-aortic interventricular communication (IVC). Its cause is multifactorial and affected pediatric patients may experience intense hypoxemic crises.

**Objective:** This work aims to perform an evaluation of the epidemiological profile of live births with tetralogy of Fallot in Brazil in the period from 2016 to 2020.

**Methodology:** A descriptive, retrospective and quantitative study was conducted based on secondary data provided by the Information System on Live Births (SINASC), SUS Department of Informatics (DATASUS). The collected information was stored and tabulated in Microsoft Office Excel™ program.

**Results:** Among the 527 cases found after analyzing the evaluated period, higher incidence is found in the years 2018, with 136 cases (25.8%) and 2019, with 126 cases (23.9%). The Brazilian region with the highest number of those born with TF was the Southeast region with 358 cases (67.9%) with the highest rate in the state of São Paulo with 286 cases (54.2%). Regarding racial profiles, whites were the highest number, with 310 cases (58.8%), followed by mixed race, with 158 cases (29.9%). Regarding gestational age, there were 369 cases (70.1%) of term pregnancy (37–41 weeks) and 134 cases (25.4) of premature pregnancy (before 37 weeks).

**Conclusion:** Therefore, we observed a higher incidence profile of TF in children born in the state of São Paulo, white and born at term. The possible cause would be success in screening and identification of the disease, which is indifferent to early birth.

111942

Modality: E-Poster Scientific Initiation – Non-case Report

Category: EPIDEMIOLOGY AND HEALTH POLICIES/GLOBAL HEALTH

## Chagas Disease: A Brazilian Epidemiological Overview

BRUNO ENÉAS ROLIM PAIVA^1^, Monique Benemérita Vilela Gomes^1^, Isabella Aparecida Abreu Garcia^1^, Isabela Rezende Costa^1^, Paulo Henrique Tenório Queiroz^1^, Renata Adélia Alves de Oliveira^1^, Giovanna Maria Lopes Magalhães^1^, Cléio Pereira dos Santos^1^, Paulo José da Silva^1^, Nisandra Pereira da Silva^1^, Ana Caroline Borges Lustosa^1^, Isabella de Almeida Nascimento^1^

(1) Universidade Federal do Piauí (Campus Senador Helvídio Nunes de Barros)

**Introduction:** Chagas disease is a clinical condition caused by the protozoan Trypanosoma cruzi. This pathology is an important public health problem in Brazil, especially due to its cardiovascular complications. In average, between 10 and 30 years after the acute infection, 30% of the infected have chronic Chagas disease cardiomyopathy, which is the main cause of morbidity in this pathology, and sudden death is the main cause of mortality in those cases.

**Goals:** To assemble an epidemiological overview of Chagas disease in Brazil.

**Methods:** For this analysis, an epidemiological study was made using the data about acute Chagas disease in the Notifiable Diseases Information System (SINAN/DataSUS). The search considered the data from 2016 to 2020, grouping the number of confirmed cases by region of notification and sex. Hereafter, a data search of the number of deaths due to acute Chagas disease was performed using the same filters. Moreover, this study determined the Brazillian population by using the demographic census of 2010 from the Brazilian Institute of Geography and Statistic (IBGE).

**Results:** According to DataSUS, between 2016 and 2020, there were 1662 notified cases of Chagas disease, affecting mostly and being more deadly in males. Between 2017 and 2019, an increase of 12.94% occurred, and 2019 was the year with most notifications, representing 23.1% of the cases between 2016 and 2020. The North region showed relatively more cases, representing 91.66% of deaths and 94.64% of the notified cases, with approximately 0.012% of the population being infected.

**Conclusion:** The majority of the cases of Chagas disease occurs in the North region, therefore, the most cases of Chagas disease cardiomyopathy. Furthermore, it was observed that many regions do not notify new cases of Chagas disease, making it harder to paint a real epidemiological estimative and intervention necessity throughout the country. Besides that, in 2020 there was a decrease in notifications, explained by the Covid-19 pandemic, that caused a reduction in the notification of diseases non-related to respiratory syndromes.


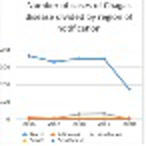


111949

Modality: E-Poster Scientific Initiation – Non-case Report

Category: CARDIOVASCULAR SURGERY

## The Decrease of Heart Transplantation During the COVID-19 Pandemic in Brazilian Reality: A Comparative Study between Socio-Demographic Regions

LARISSA DE OLIVEIRA BELTRÃO^1^, Beatriz Ximenes Bandeira de Morais^1^, Maria Letícia Garrote Bulhões^2^, Andreza Leite Marques de Sá Souza^1^, Luiza Dias Aguiar^1^, Leticia Ferreira Leal^3^, Isabela Torres Castro^1^, Juliana Mirelly Moura de Oliveira^1^, Catiane Kelly Schaefer^4^

(1) Faculdade Pernambucana de Saúde (FPS); (2) Universidade Estácio de Sá (UNESA); (3) Universidade de Pernambuco (UPE); (4) Universidade de Santa Cruz do Sul (UNISC)

**Background:** Heart transplantation is the therapeutic option for refractory heart failure cases and is a procedure with limited access in many underdeveloped countries. With the COVID-19 pandemic, health resources were directed to contention plans and patient management, leading to a global decrease in transplantations.

**Objectives:** This study aims to demonstrate the disparity in the impact of the COVID-19 pandemic on heart transplantation among the sociodemographic regions of Brazil.

**Methods:** This is a retrospective descriptive study with data from the Department of Informatics of the Unified Health System from 2018 to 2021. A comparative analysis was performed between the average of the first two years of study and the two subsequent years, before and during the pandemic, respectively.

**Results:** Between 2018 and 2021, 1,146 Brazilians underwent heart transplantation. Before the COVID-19 pandemic, 159.5 transplants were achieved in the Southeast region, the most developed in Brazil, 53.5 in the South, 31.5 in Midwest, and 80 transplants in the Northeast. With the COVID-19 pandemic, there was a drop to 41 procedures executed in the Northeast, 24.5 in the Midwest, 25.5 in the South, and 157.5 in the Southeast.

**Conclusion:** There was a decrease of approximately 50% of heart transplants achieved in the Northeast and South, while there was maintenance in the numbers of other regions, which are two of the three most developed regions of Brazil.


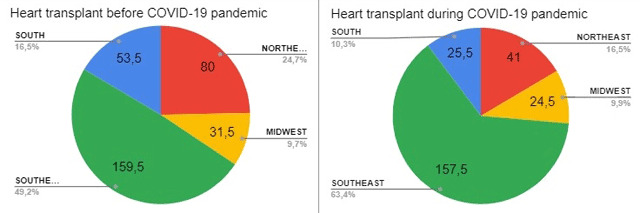


111953

Modality: E-Poster Scientific Initiation – Non-case Report

Category: COVID-19 AND CARDIOVASCULAR SYSTEM

## Central Pressure, Arterial Stiffness and Sympathovagal Balance After SARS-COV-2 Infection: Difference between Sexes

ANA LUIZA DE ARAUJO GARCIA^1^, Ana Luiza de Araujo Garcia^1^, Maria Luiza Rangel de Vasconcellos Guerra^1^, Michelle Rabello da Cunha^1^, Samanta de Souza Mattos^1^, Mario Fritsch Toros Neves^1^

(1) Universidade do Estado do Rio de Janeiro (UERJ)

**Introduction:** Acute infection caused by the SARS-CoV-2 can generate functional changes in the vascular endothelium but structural vascular alterations and long-term effects on the sympathovagal balance are still unknown.

**Objective:** To compare central hemodynamic parameters, arterial stiffness, and autonomic activity between men and women with a history of symptomatic COVID-19. Materials and

**Methods:** Cross-sectional study with patients aged between 30 and 70 years, both sexes, submitted to clinical evaluation, central hemodynamic parameters and pulse wave velocity (PWV) by Mobil-O-Graph, and sympathetic tone by heart rate variability (HRV). Patients were divided into two groups: Control (n = 25) with patients with no history of COVID-19, and post symptomatic COVID (n = 29), which included patients with positive RT-PCR in the last 12 months before enrollment in the study. An intragroup analysis was performed to verify the differences between men and women.

**Results:** Patients were predominantly female in both groups (64 vs 62%, p = 0.883) and with similar ages (55 ± 6 vs 51 ± 10 years, p = 0.088). In the analysis by sex, the frequency of hypertension was similar between men and women in the control group (100 vs 81%, p = 0.243) and in the post-COVID group (60 vs 56%, p = 0.570). The post-COVID group presented, in females, higher peripheral systolic and diastolic pressures (114 ± 15 vs 131 ± 23 mmHg, p = 0.040; 72 ± 9 vs 85 ± 16 mmHg, p = 0.013), central systolic pressure (106 ± 15 vs 122 ± 23 mmHg, p = 0.037), augmentation pressure (5 ± 3 vs 11 ± 7 mmHg, p = 0.018), augmentation index (16 ± 7 vs 30 ± 12%, p = 0.006) and pulse wave velocity (6.7 ± 0.9 vs 7.8 ± 1.6 m/s, p = 0.033). The control group showed no significant difference between sexes in these parameters. HRV indices were similar for both sexes in both groups. The sympathovagal balance, represented by the low frequency/high frequency (LF/HF) ratio, was higher in men in the post-COVID group (4.7 ± 8.6 vs 1.2 ± 1.5, p = 0.185), although not reaching statistical significance.

**Conclusion:** Early vascular aging, characterized by higher arterial stiffness and increased central pressure, was more evident in women who had symptomatic COVID-19 compared with men in the same group, even without differences in sympathovagal balance.

111963

Modality: E-Poster Scientific Initiation – Non-case Report

Category: CARDIOVASCULAR PHARMACOLOGY

## The Effectiveness of Beta-Blockers in the Treatment of Hypertensive Patients

ISRAEL FIGUEIRA LEMOS^1^, Alice Jacomini Barcos^1^, Gabriel do Nascimento Oliveira^1^, Ana Lucy Peixoto Rabelo^1^, Camila Namie Seki Garzon^1^, Edson Luis de Barros Siqueira^1^, Ana Luiza Nepomuceno Sampaio^1^, Caroline Hipólito Pires^1^, Luma Maria Favacho Bordalo^1^, Maria Eduarda Dantas da Veiga^1^, Juliana de Sousa Tavares^1^, Erick Clayton Gonçalves Feio^1^

(1) Universidade do Estado do Pará

**Introduction:** Beta-blocker drugs are widely used in the treatment of systemic arterial hypertension, being able to be used in monotherapy treatments or combined with other medications. However, due to their complex pharmacological actions and adverse effects, the effectiveness of beta-blockers raises uncertainties.

**Objectives:** Evaluate the effectiveness of beta-blockers in the control of hypertension.

**Methods:** A systematic review was conducted following the Preferred Reporting Items for Systematic Reviews and Meta-Analyses (PRISMA) recommendations, using the PICO strategy to elaborate the guiding question “Have beta-blockers been effective in hypertensive people?”. The data collection was based on the use of the following keywords: “beta blockers‘‘ AND “effectiveness” AND “systemic arterial hypertension”, which were obtained in English and Portuguese on the Medical Subject Headings (DeCS/MeSH). The articles were searched on the PubMed and Biblioteca Virtual em Saúde (BVS) databases, including those that approach the treatment of beta-blockers in systemic arterial hypertension, indexed in the selected databases and published between 2012 and 2022, in Portuguese and English. All studies that are not randomized clinical trials were excluded.

**Results:** A total of 20 articles were selected to integrate this study. Traditional beta-blockers (BB) are efficient agents to reduce BP, but they‘re not recommended as standard therapy to uncomplicated essential hypertension, for being less efficient in reducing cardiac events. However, new generation BB‘s, nebivolol for example, has auxiliary vasodilating properties. Compared to ACE inhibitor therapy, BB therapy was superior in hypertensive patients and required fewer antihypertensives to control BP. Furthermore, BB effects had different outcomes in regard to age, sex, ethnicity and daily habits.

**Conclusion:** In general lines, a positive response was seen on the hypertensives’ treatment with beta-blockers, considering the singular effects of the medicine’s actions of vasodilation and complacency on hypertensive patients, according to the drug manipulation, different treatments and varied physiologies.

111971

Modality: E-Poster Scientific Initiation – Non-case Report

Category: HEART FAILURE/CARDIOMYOPATHY/TRANSPLANT

## Decompensated HF in the Extreme Elderly: Characteristics and Outcomes. Data from a Hospital Record

LARISSA MELO TARGINO^1^, Claudio Lucas Silva Cunha^1^, Larissa Melo Targino^1^, Helen de Araújo Alves^2^, Luiz Eduardo Fonteles Ritt^2^

(1) Escola Bahiana de Medicina e Saúde Pública (EBMSP); (2) Hospital Cárdio Pulmonar da Bahia (HCP)

**Introduction:** The trend towards population aging and its direct association with the prevalence of heart disease has resulted in a higher incidence of Heart Failure (HF). However, extreme old patients are still underrepresented in clinical studies and few data regarding their profile and prognosis is present in the literature.

**Objective:** To describe and compare clinical characteristics and clinical outcomes between extreme elderly (≥80 years-of-age) and their counterparts patients hospitalized for decompensated HF.

**Methods:** Prospective cohort study in a single-center registry model. Patients hospitalized for HF at a private institution in Salvador, Bahia, Brazil, were included. Data were obtained from hospital records from 2019 to 2022. In addition, telephone follow-up was carried out 30 days after discharge, when the outcomes of death or readmission were measured. A p < 0.05 was set as a level of significance for all analyses.

**Results:** We analyzed 356 patients hospitalized for HF, who were divided into groups of extreme elderly (n = 159) and non-extremely elderly (n = 197). Compared to the non-extremely elderly group, the extreme elderly group were more likely to be female, had hypertension and was less likely to have obesity, smoking, use of a pacemaker, had paroxysmal nocturnal dyspnea and third beat extra heart sounds. The group ≥80 years had higher values of Left Ventricular Ejection Fraction (mean = 48.56, 95% CI [46.16–50.96] versus mean = 41.43, 95% CI [38.9–43.9]; p < 0.001), as well as higher levels of urea and brain natriuretic peptide (BNP) at discharge. There was no difference in the rate of betablocker or ACE inhibitor/angiotensin receptor blocker usage between groups. During hospitalization and the 30-day follow-up, the mortality in the extreme elderly group was significantly higher (28.9% versus 14.4%; p = 0.047). Within 30 days after discharge, 7.9% of patients <80 years were readmitted, while 13.4% of patients ≥80 years-of-age, although without statistical significance.

**Conclusion:** Extremely elderly patients with HF are susceptible to higher mortality rates and have a particular clinical profile. Future studies focusing on the treatment of these subgroup are warranted.

111974

Modality: E-Poster Scientific Initiation – Non-case Report

Category: HEMODYNAMICS AND INTERVENTIONAL CARDIOLOGY

## Clinical and Epidemiological Profile of Patients with Stemi Treated at a Referral Hospital

KARLA SANTOS PINTO^1^, Beatriz Barbosa Viana^1^, Larissa Xavier Gomes da Silva^1^, João Pedro Martins Moreira Granja^1^, Elias Soares Roseira^1^, Tainá Viana^1^, Luiz Carlos Passos^1^, Pollianna Roriz^1^, Adriano Tamazato^1^, Raissa Barreto Lima^1^, Ana Luísa de Aguiar Almeida Silva^1^, Eduarda Luciana Mendes Borges^1^

(1) Hospital Ana Nery

**Introduction:** Coronary Artery Diseases (CAD) are responsible for the highest mortality rate in the world. Acute ST-segment elevation myocardial infarction (STEMI) corresponds to one third of CAD and has a higher mortality among the disease subtypes. Early diagnosis and prompt reperfusion are effective ways to limit ischemia, reducing the risk of complications. Angioplasty is the main reperfusion strategy and, if it is not performed within 120 minutes, fibrinolysis therapy must be managed.

**Objective:** To describe the epidemiological and therapeutic clinical profile and clinical consequences in a tertiary referral hospital in the city of Salvador – BA, which receives an average of 50 patients per month in this condition.

**Methodology:** Descriptive study with 329 patients treated at health units in Salvador and the metropolitan region with suspected AMI, transferred through the AMI Protocol – SAMU for angioplasty proposal in a referral hospital, between January 2021 and April 2022. demographic variables, risk factors, first care, angiographic data, and outcomes. Categorical variables were presented as frequencies and continuous variables as mean or median.

**Results and Discussion:** There were 329 patients with STEMI, 60% of whom were men. The mean age was 61 years. Previous diseases were SAH (64%), DM (32%) and obesity (15%). 19% were smokers and 16% were ex-smokers. Most with Killip I in the first evaluation (84.8%). The most affected wall was the anterior one (46%). The most performed reperfusion was primary angioplasty (71.4%). The average time for the balloon holder was 354 minutes. The most frequent culprit artery was the anterior descending artery (47.1%), followed by the right coronary artery (28.2%). There was occlusion in 43.4% and none in 12% of the cases. Most access was via the Radial artery (76%). 40% had an initial TIMI of 0 and 20% had a TIMI of 3. 65% of the patients had a final TIMI of 3 and only 4.3% had a final TIMI of 0. Angioplasty was successful in about 70% of cases. As an outcome, there was death of 6, 6% during hospitalization.

**Conclusion:** Patients undergoing primary angioplasty were men with anterior wall AMI. Despite the long door-to-balloon time, a high success rate was obtained in restoring normal flow after the procedure. These results corroborate the generation of strategies to reduce injuries in patients undergoing angioplasty.

111985

Modality: E-Poster Scientific Initiation – Non-case Report

Category: EPIDEMIOLOGY AND HEALTH POLICIES/GLOBAL HEALTH

## Hospitalizations for Cardiovascular Conditions Sensitive to Primary Health Care in Betim, Minas Gerais 2018–2021

FERNANDA AMPARO RIBEIRO^1^, FERNANDA AMPARO RIBEIRO^1^, SUELLEN MOURÃO SILVA^1^, GILBERTO ANTONIO REIS^1^

(1) PONTIFICIA UNIVERSIDADE CATOLICA DE MINAS GERAIS – PUC-MG; (2) Fundação de Amparo à Pesquisa do Estado de Minas Gerais – FAPEMIG

Hospitalizations for Primary care-sensitive conditions are preventable health complications through disease prevention, diagnosis and care of acute conditions, monitoring of chronic conditions. The use of this indicator is based on the principle that effective and effective primary health care can prevent hospitalizations. Different activities of primary health care and specialized outpatient and hospital care were interrupted or paralyzed due to the priority given to COVID-19. For different reasons, both professionals and primary care users have dangerously delayed non-COVID-19 procedures, which could interfere with the effectiveness and effectiveness of primary health care and which, in turn, would reflect on Hospitalizations for cardiovascular conditions sensitive to primary care. Patients with cardiovascular diseases are more vulnerable to hospitalizations due to adverse events related to poor symptom control. Thus, the hypothesis presented is that the incidence of Hospitalizations for cardiovascular conditions sensitive to primary care in the population over 34 years of age in the city of Betim-MG may vary between the periods before and during the COVID-19 pandemic.

**Objective:** To measure the occurrence of hospitalizations for cardiovascular conditions sensitive to primary care in adults over 34 years of age living in the city of Betim – Minas Gerais and to estimate the variability between the previous period and during the COVID-19 pandemic.

**Methods:** This is an ecological, descriptive, study with a quantitative approach. Data were extracted from the Hospital Information System available on the DATASUS, portal of the Ministry of Health. Descriptive statistical measures of data were used to analyze the variables included in the study.

**Results:** Among hospitalizations for cardiovascular conditions sensitive to primary care in the period between 2018–2021 in the city of Betim-MG, it was observed that the total number of hospitalizations behaved in a stationary way year after year. At the same time, there was a drop in the number of emergency admissions in 2021 compared to 2018, 2019 and 2020. The population most affected by cardiovascular and cerebrovascular causes were black and brown people and men. Stroke was the main cause of elective and emergency admissions. The COVID-19 pandemic had an important impact on the care routine of all health service users. It is necessary to carefully investigate the long-term impacts of this health crisis.

111986

Modality: E-Poster Scientific Initiation – Non-case Report

Category: HEART FAILURE/CARDIOMYOPATHY/TRANSPLANT

## Predictors of Heart Failure with Improved Ejection Fraction on Patients with Chagas Disease

GABRIELLY NASCIMENTO^1^, Celina Maria de Carvalho Guimarães^1^, Silvia Marinho Martins^1^, Luis Eduardo Martins Alves^1^, Taciane Queiroz Medeiros Gomes^1^, Maria Elisa Lucena Sales de Melo^1^, Maria da Glória Aureliano de Melo^1^, Carolina de Araújo Medeiros^1^, Maria das Neves Dantas da Silveira Barros^1^, Maria da Piedade Costa Reis de Albuquerque^1^, Cristina de Fátima Velloso Carrazzone^1^, Wilson Alves de Oliveira Júnior^1^

(1) Pronto Socorro Cardiológico de Pernambuco – PROCAPE

**Introduction:** Heart failure with improved ejection fraction (HFimpEF) is associated with improved clinical outcomes[1,2], however little is known about HFimpEF of chagasic etiology[3].

**Objective:** Identify predictors of progression to HFimpEF in Chagas‘ etiology.

**Methodology:** Cohort study in a referral outpatient clinic for Chagas Disease admitted between march-2016 and august-2019. From the 862 included in database, 86 were selected, under two criteria from two different echocardiograms (echos), the first was the admission one, with left ventricular ejection fraction (LVEF) <40%, and the second one, the follow-up. The mean time interval between the echos was 3.13 years (1 ± 5 years). HFimpEF was defined as LVEF ≥40% on the follow-up echo. The variables analyzed were sex, age, presence of comorbities (diabetes mellitus, dyslipidemia and arterial hypertension), medications: spironolactone, beta-blockers, loop and thiazide diuretics, angiotensin II receptor blocker/angiotensin converting enzyme inhibitor and sacubitril valsartan, left ventricle end-diastolic diameter (LVEDD) and right ventricule function to the Echo, QRS duration, presence of right bundle branch block and left bundle branch block on the electrocardiogram. For data analysis the technique utilized was a logistic regression with variables that presented p-value < 0,15 in the univariate analyses.

**Results:** On admission, mean age was 60.9 years (standard deviation 11.5, minimum 30 and maximum 80), 52% women, 77.7% had arterial hypertension, 19.7%: diabetes, 33.7%: dyslipidemia. On the electrocardiogram: 43% right bundle branch block, 37.2% left bundle branch block. On the admission echo, LVEF with median 33% (minimum 16%, maximum 39%), LVEDD average of 65(standard deviation: 1,25, minimum 45, maximum 81) and 25.5% with right ventricle dysfunction. On therapeutics: 95.3% beta blockers, 77.9% used loop and thiazide diuretics, 74.4% angiotensin II receptor blocker/angiotensin converting enzyme inhibitor, 67.4% spironolactone, 8.1% sacubitril valsartan. In the univariate analysis, use of spironolactone (p = 0.027) and LVEDD (p = 0.001) were shown to be factors for improving LVEF, but in the multivariate analysis, only LVEDD (Odds Ratio 0.87, 95% CI 0.80–0.95, P = 0.002) was an independent predictor associated with improvement in LVEF.

**Conclusion:** In agreement with the literature when analyzing different etiologies, in Chagas‘ etiology, only LVEDD was able to predict progression to HFimpEF.

111990

Modality: E-Poster Scientific Initiation – Non-case Report

Category: EPIDEMIOLOGY AND HEALTH POLICIES/GLOBAL HEALTH

## Acute Rheumatic Fever: An Epidemiological Analysis of Hospitalizations in the Northern Region of Brazil between 2017 and 2021

GIULIA VITORIA NASCIMENTO DA SILVA^1^, IGOR LUCAS FARIAS LIMA^1^, MARCOS AURÉLIO VIEIRA DA COSTA FILHO^2^, JÚLIA DE MOURA CARVALHO FARIA^1^, WANDA MARIA DE FRANÇA PIRES^2^, ARIANE LOBATO MORAES^2^, LARISSA DACIER LOBATO COMESANHA^2^, LUCIANO MOURA DE ASSUNÇÃO^3^

(1) Universidade do Estado do Pará (UEPA); (2) Universidade Federal do Pará (UFPA); (3) Fundação Santa Casa de Misericórdia do Pará

**Introduction:** Rheumatic fever (RF) is a pathology that follows as an immune response to an infection from group A β-hemolytic Streptococcus. Its prevalence is higher in sub-evolved countries, being that around 15 million cases in the world are linked to valve damage, a characteristic of chronic rheumatic heart disease – most serious manifestation of the disease.

**Objective:** To analyze the epidemiological profile of hospital admissions, mortality and cost per hospitalization resulting from acute RF with cardiac in the northern region of Brazil, from 2017 to 2021.

**Methods:** Observational, cross-sectional, analytical-descriptive and quantitative study based on data from Sistema de Informações Hospitalares do SUS (SIH/SUS) – DATASUS. The number of hospitalizations, age, sex, average hospital stay, average cost per hospitalization and mortality rate were analyzed, referring to hospitalizations due to acute RF in the North region of Brazil, from 2017 to 2021.

**Results:** In the period described, 868 hospitalizations for acute RF were reported in the Northern region of Brazil. The lowest number occurred in 2021, with 99 admissions and the highest in 2018 and 2019, with 219 and 218 cases, respectively. The average number of hospitalizations per year was 173.6. Females represented 53.6% (465) of the hospitalizations and males 46.4% (405). The most affected age groups were 5 to 14 years old, with 263 (30.2%), and 40–49 years old, with 101 cases (11.6%) of the total. Children under 5 years had 44 admissions and adults over 20 years old had 515 cases. The average length of hospitalizations during 2017 and 2021 was 6.1 days and the standard deviation was 0.67 between the annual values. The shortest admission time was in 2018, with an average of 5.2 days, and the longest was in 2020 with 7.2 days. The mortality rate was 1.14% with the lowest in 2019 (0.46%) and the highest in 2021 (3.03%). R$328,658.56 was spent on hospitalizations with an average cost of R$373.04.

**Conclusion:** The number of hospitalizations gradually decreased during the study period. Among these hospitalizations, most were women and the main age group affected was children between 5 to 14 years old. The mortality rate followed a pattern contrary to the hospitalization rate, increasing gradually until 2021. It is notorious for the necessity of affirmative actions in public health to prevent the disease and reduce its impact on the brazilian health and economic system.

111996

Modality: E-Poster Scientific Initiation – Non-case Report

Category: ACUTE AND CHRONIC CORONARY DISEASE/THROMBOLYSIS

## The Influence of Educational Level on the Suspicion of Acute Myocardial Infarction (AMI) in Patients of the Public Health System of Salvador – BA

JÚLIA SCHOUCAIR NEVES^1^, Pollianna de Souza Roriz^1^, Fabiana Benevides Pontes^1^, Mariana Mendonça de Almeida^1^, Frederico Gesteira de Viveiros Júnior^1^, João Gabriel Batista Simon Viana^1^, Franciele Mascarenhas Alves Luz^1^, Marianne Soglia Calixto Costa^1^, Luiz Ricardo Cerqueira Freitas Junior^1^, Victoria Kelly Lima de Castilho^1^

(1) Serviço de Atendimento Móvel de Urgência – SAMU

**Introduction:** Social determinants of health –such as the level of one’s education – as risk factors for cardiovascular diseases have been highlightened by several studies. These determinants are directly related to the prognosis, and may impact the patient’s identification of the disease itself. In this context, the present study aims to evaluate the influence of the level of education on the self-perception of disease in patients with ST-segment elevation Acute Myocardial Infarction (STEMI) treated in the public health system of the city of Salvador – BA.

**Methods:** A cross-sectional study, carried out with data collected from the STEMI survey from Salvador (PERSISST), which analyzed the association between suspected AMI in patients who had a heart attack between January/2019 and December/2021 and their corresponding level of education. The sample of 859 people was divided into Group I – patients who did not suspect AMI – and Group II – patients who suspected AMI –. Each group was subdivided into 5 different levels of education. Such division of groups aimed to possibly identify an association between the lowest level of education with a lower suspicion of the condition in question.

**Results:** 859 patients were included in the survey (590 in Group I and 269 in Group II). In both groups there was a predominance of male patients, with no difference between the two groups in terms of gender and age. In Group I, 26.8% (158) had gone to school until the 4th year of elementary school; 27.1% (160) had finished elementary school (up to 5th grade); 18.6% (110) had finished middle school (up to 9th grade); 21.2% (125) finished high school and 6.3% (37) had graduated from college. While in Group II, the following distribution was observed in terms of education: 23.8% (64) up to 4th grade; 20.4% (51) finished elementary school, 18.2% (49) finished middle school; 27,9% (75) finished high school and 9.7% (26) finished college. After comparing the groups, it was found that patients with a lower level of education had a lower degree of suspicion, pointing to a statistically significant difference of p = 0.034.

**Conclusion:** In our sample, composed by STEMI patients treated by the public health system of Salvador, the low level of education was related to the patient’s lower self-suspicion of heart attack. This analysis reinforces the need for educational measures, which could positively influence the early recognition of time-dependent diseases.

112013

Modality: E-Poster Scientific Initiation – Non-case Report

Category: EPIDEMIOLOGY AND HEALTH POLICIES/GLOBAL HEALTH

## Epidemiological Profile of Patients Affected by Chagas Disease in the State of Pará between 2010 a 2020

ARIANE LOBATO MORAES^1^, Ariane Lobato Moraes^1^, Icaro Jose Araújo de Souza^1^, Cassio Kenzo Câmara Yamada^2^, Jayssa Leite Freitas^3^, Bruna Araujo Smith^3^, Giovana Fonseca Pontes^3^, Rayane Calandrini de Azevedo^3^, Luciano Moura de Assunção^4^

(1) Universidade Federal do Pará (UFPA); (2) Universidade do Estado do Pará (UEPA); (3) Centro Universitário do Pará (CESUPA); (4) Fundação Santa Casa de Misericórdia do Pará (FSCMPA)

**Introduction:** Chagas disease or American trypanosomiasis is a parasitic infection caused by the protozoan Trypanosoma cruzi. Transmission occurs from the contact of the injured skin or mucous membranes with contaminated feces, mainly through the ingestion of contaminated food, such as açaí. Affected individuals may develop Chagas myocarditis, with possible electrocardiographic changes.

**Objectives:** To analyze the epidemiology of Chagas disease cases in the state of Pará, from 2010 to 2020, analyzing the following variables: sex, race, age group, area of residence, probable mode of infection and disease evolution.

**Methods:** This is an epidemiological, descriptive and analytical study, whose data were collected in the Notifiable Diseases Information System (SINAN) made available by the Department of Informatics of the Unified Health System (DATASUS), referring to reported cases of Acute Chagas, in the state of Pará, from 2010 to 2020, totaling 2,221 cases. For the statistical analysis, the Chi-Square test of equal expected proportions was used, through the Bioestat 5.3 program, in order to calculate the p-value of the data found. Data with p value < 0.05 were considered significant. There was no need for submission to the research ethics committee.

**Results:** This study showed that, of the 2.221 reported cases, there was a higher frequency of Acute Chagas Disease in brown patients (80.10%), male (55.16%) and in adults between 20 and 59 years old (58, 89%). The most frequent route of transmission was oral (79.78%). The significant number of unreported data prevents a more detailed analysis. Finally, the predominant evolution was the survival of 86% of the total cases in the analyzed period.

**Conclusion:** The study reports that the epidemiological profile of the patient with Chagas cardiomyopathy between the years 2010 to 2020 is a male patient, brown and aged between 20 and 59 years. In addition, underreporting and missing data are relevant features. Thus, we identified the demand for new studies in order to generate more data and promote public policies aimed at reducing the incidence of Chagas Disease in the region.


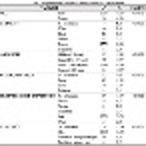


112017

Modality: E-Poster Scientific Initiation – Non-case Report

Category: CARDIAC ARRHYTHMIAS/ELECTROPHYSIOLOGY/ELECTROCARDIOGRAPHY

## Alcohol Consumption and Atrial Fibrillation Incidence in Brazilian Capitals: A Population-Level Study

ROBERTA GABRIELA DA SILVA^1^, Laís Vitória de Andrade Miranda^1^, Roberto Vieira Botelho^4^, Rodrigo Alves da Silva^4^, Marcelo Vieira Damião^5^, Marcio Sommer Bittencourt^3^, Miguel Morita Fernandes da Silva^2^, Odilson Marcos Silvestre^1^

(1) Universidade Federal do Acre; (2) Universidade Federal do Paraná; (3) University of Pittsburgh; (4) Instituto do Coração do Triângulo; (5) ITMS Telemedicina Brasil

**Introduction:** Alcohol consumption can lead to tachyarrhythmias, particularly atrial fibrillation.

**Aim:** We evaluated the association between alcohol consumption at the population level and the incidence of atrial fibrillation/flutter (AF) in Brazilian capitals.

**Methods:** We performed an ecological time-series study using electrocardiogram (EKG) reports in all Brazilian capitals from 2008 to 2013. EKG data were provided by ITMS Telemedicine Network, and the diagnosis of AF was defined according to the EKG medical report. Abusive alcohol consumption at each capital, defined as the estimated proportion of individuals who binge drinking (≥4 doses for women and ≥5 doses for men, on an occasion), was estimated from the Surveillance of Risk and Protective Factors for Chronic Diseases by Telephone Survey (VIGITEL) in the respective year and divided in terciles: 1st tercile (10.6–14.6%); 2nd tercile (14.7–16.9%); 3rd tercile (17.1–26.6%). A stratified two-stage weighted survey analysis was performed to estimate the incidence of AF at each tercile of alcohol consumption.

**Results:** We evaluated 474,357 EKGs (48.8 ± 18.5 years old, 50% women) performed in all 27 Brazilian capitals. The incidence of AF was significantly higher in the 3rd tercile (5.3%, 95% Confidence interval [CI] 1.6, 9.0%) as compared with the 2nd (1.5%, 95%CI 1.2, 1.7%) and 1st (2.1%, 95%CI 1.7, 2.6%, p < 0.001, figure) terciles of alcohol consumption, respectively.

**Conclusion:** In this population-level study of Brazilian capitals, higher alcohol consumption was associated with increased incidence of AF.


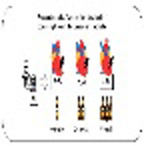


112027

Modality: E-Poster Scientific Initiation – Non-case Report

Category: EPIDEMIOLOGY AND HEALTH POLICIES/GLOBAL HEALTH

## Prevalence of Electrocardiographic Abnormalities Detected by a Telediagnosis Service in Primary Care in a Municipality in Southern Brazil

HENRIQUE GUIMARÃES AIRES E SILVA^1^, Pedro Ermel Martins^1^, Márcia Barbosa de Freitas^2^, Chaiana Esmeraldino Mendes Marcon^1^

(1) Universidade do Sul de Santa Catarina; (2) Instituto Nacional de Cardiologia

**Background:** The 12-lead electrocardiogram (ECG) is one of the most used tests in the evaluation of cardiovascular diseases (CVD) in primary health care (PHC) because it is cheap and available in the Sistema Único de Saúde (Brazil’s health care model). However, particularities in the frequency of electrocardiographic findings are observed when population characteristics are considered.

**Objective:** To identify the main electrocardiographic abnormalities and the clinical and epidemiological factors associated with their occurrence in primary health care.

**Methods:** This study analyzed all ECG and application forms of patients submitted to primary care examination over 20 years of age in a municipality of Santa Catarina – Brazil – in 2020. Descriptive analysis of the data and statistical inference was performed. The level of significance adopted was 5%.

**Results:** ECGs of 1907 patients were analyzed, 57.37% of them abnormal. The mean age of the patients was 54,8 ± 15,0 years-old. The abnormalities found varied according to age group, however the most common were nonspecific changes in ventricular repolarization (52.93%), right branch conduction block (28.52%), left ventricular overload (12.07%), electrically inactive areas (10.42%) and divisional blocks of the left branch (8.96%). The main clinical and epidemiological factors related to the presence of an abnormal ECG were age over 60 years (OR 2.17 [CI 95% 1,79–2,62]), systemic arterial hypertension (OR 1.76 [CI 95% 1,46–2,11]), diabetes mellitus (OR 1.78 [CI 95% 1,38–2,30]), dyslipidemia (OR 1.87 [CI 95% 1,49–2,34]), previous acute myocardial infarction (OR 5.63 [CI 95% 1,28–24,72]), previous myocardial revascularization surgery (OR 8.24 [CI 95% 1,06–64,01]), or the use of any medication (OR 1.55 [CI 95% 1,27–1,88]). The report of the electrocardiogram by the cardiologist was made available with less than two days in more than 70% of the tests.

**Conclusion:** The evaluation of the prevalence of electrocardiographic findings and associated factors allows the construction of pre-test probability, which is essential in the context of public health for the indication of the examination rationally and without burdening Brazil’s health care system. The use of telediagnostic resources in this setting allowed to minimize the effects of the large geographic dimentions and social disparitys that exist in Brazil.

112030

Modality: E-Poster Scientific Initiation – Non-case Report

Category: CARDIO-ONCOLOGY

## Cardiotoxicity by Anthracyclines in Cancer Treatment: Systematic Review

DAVI GABRIEL BARBOSA^1^, Paola Bitar de Mesquita Abinader^2^, Ana Paula Correa de Lima^2^, Ana Cláudia Reis Guilhon^2^, Camila Rodrigues Maciel^2^, Fernando Gabriel Rodrigues Pereira^1^, Isabella Soares Souza^3^, Luiz Fernando Leite da Silva Neto^1^, Fernando Tavares Brasil Teixeira^1^, Luis Eduardo Werneck de Carvalho^4^

(1) Universidade do Estado do Pará – UEPA; (2) Centro Universitário do Pará – CESUPA; (3) Universidade Federal do Pará – UFPA; (4) Sociedade Brasileira de Cancerologia – SBC

**Introduction:** Immunotherapies have demonstrated a relevant effect on the prognosis of neoplasms over the years. However, antineoplastic agents increase the risk of cardiovascular disease by inducing cardiotoxicity.

**Objective:** To analyze the induction of cardiovascular diseases by cardiotoxicity caused by antineoplastic drugs in cancer patients.

**Methods:** Systematic review about cardiotoxicity due to antineoplastic treatment, whose data were obtained through the Biblioteca Virtual da Saúde (BVS) from the last 5 years, using the PICO with PRISMA strategy, excluding case reports. The descriptors used were antineoplastic, cardiotoxicity, and anthracycline.

**Results:** On the clinical aspects, no deaths were reported due to cardiotoxicity, i.e., anthracyclines did not cause any effect on mortality, and cardiotoxicity with the occurrence of heart failure was not statistically significant in treatment with anthracyclines. But 4 studies have analyzed the occurrence of arrhythmias, presenting statistically significant numbers, occurring in 317 patients. 3 studies have addressed the side effects of cardioprotective drugs: hypotension, acute pulmonary edema (enalapril), and bradycardia (metropol). About cardiac markers the changes in CK-MB, Troponin I and NT-proBNP were statistically significant along with markers of inflammation and oxidative stress having increased IL-6 and ROS1 with anthracycline treatment. On LVEF The cardioprotective drugs showed a statistically significant benefit in preventing LVEF reduction compared to the control group (MD 3.57, 95% CI 93%). Also, analyzing the cardioprotective drugs, a positive trend was found with the studies involving beta-blockers. The SRAA blockers as a cardioprotective drug also had positive results regarding LVEF. The cardioprotective effects were positive and similar in the various types of chemotherapeutic drugs: doxorubicin and epirubicin, especially in a study addressing breast cancer patients where LVEF obtained a considerabldifference, The cardioprotective drugs showed a possible benefit in preventing the reduction of Mitral Peak E/A Wave Velocity.

**Conclusion:** In summary, there was an increase in inflammatory and cardiac markers and the presence of cardiotoxicity with the occurrence of arrhythmias by the use of anthracyclines. Moreover, the use of drugs with cardioprotective potential has efficacy in preventing the reduction of left ventricular ejection fraction, such as angiotensin-converting enzyme inhibitors.

112031

Modality: E-Poster Scientific Initiation – Non-case Report

Category: HEART FAILURE/CARDIOMYOPATHY/TRANSPLANT

## Medical Therapy Optimization During Hospitalization for Acute Heart Failure Relates to Better Prognosis

RAFAEL MORETTI^1^, Michelle Bozko Collini^1^, Karoline Cordeiro Vercka^1^, Carolina Ruschel Senger^1^, Jorge Tadashi Daikubara Neto^1^, Matheus Bissa Duarte Ferreira^1^, Jessica Tamires Reichert^1^, Lucas Müller Prado^1^, Gustavo Sarot Pereira da Cunha^1^, Leonardo Henrique dos Santos Melo^1^, José Renan de Matos Pain^1^, Miguel Morita Fernandes da Silva^1^

(1) Complexo Hospital de Clínicas da Universidade Federal do Paraná (CHC-UFPR)

**Background:** Initiating or uptitrating doses of modifying-disease drugs for heart failure (HF) is recommended during hospitalization for decompensated HF. However, data on the impact of HF medical therapy optimization (MTO) during hospitalization on long-term prognosis is scarce.

**Objectives:** This study aims to evaluate the association of MTO during hospitalization and the composite outcome of re-hospitalization and death in patients with decompensated HF.

**Methods:** Prospective cohort study that included patients >18 years old admitted for decompensated HF in a tertiary hospital from October 2019 to February 2022. Patients with ejection fraction >50% or who died during hospitalization were excluded. MTO was defined as any of the following during hospitalization: 1) addition of one of the following classes: ACE-I (Angiotensin-Converting Enzyme Inhibitors)/ARB (Angiotensin II Receptor Blockers)/ARNI (Angiotensin Receptor-Neprilysin Inhibitor), beta-blocker, spironolactone and SGLT2 inhibitor; 2) exchange of ACE-I/ARB for ARNI; or 3) reaching the target dose of one of these medications already in use. The outcome was the composite of re-hospitalization or death from any cause during a follow-up of up to 180 days. Survival analysis was performed by COX regression adjusted for confounding factors.

**Results:** 109 patients were analyzed (66 ± 14 years old, 40% women, ejection fraction: 33 ± 9%), with 36 (33%) presenting the composite outcome. Compared to patients without MTO (n = 44), those with MTO (n = 65) had lower serum creatinine values [1.1 (0.8–1.5) vs 1.4 (0.9–2.0) mg/Dl; p = 0.014) and higher systolic blood pressure [129.7 ± 23.9 vs 119.8 ± 22.8 mmHg; p = 0.035] at hospital admission, and were less likely to have decompensated due to infectious causes (7.7% vs 20.5%; p = 0.05) or non-adherence to sodium and water restriction (1.5% vs. 18.2%, p = 0.002), respectively. After adjusting for these confounders, therapeutic optimization was associated with a lower incidence of the composite outcome (Hazard Ratio: 0.42; 95% Confidence Interval: 0.18–0.96, p = 0.039).

**Conclusion:** In patients with decompensated HF, therapeutic optimization during hospitalization was associated with a lower incidence of the composite outcome of death or re-hospitalization in the 6-month follow-up.

112040

Modality: E-Poster Scientific Initiation – Non-case Report

Category: HEART FAILURE/CARDIOMYOPATHY/TRANSPLANT

## Correlation between Distance Covered in the Stress Test and B-Lines Detected on Lung Ultrasound in Outpatients with Heart Failure with Reduced Ejection Fraction

ALICE ZANETTI DUSSIN^1^, Anna Paula Tscheika^2^, Luiz Claudio Danzmann^3^, Marcus Vinicius Simões^4^, Andrielle Dias Pinheiro^2^, Luiz Carlos Bodanese^1^

(1) Pontifícia Universidade Católica do Rio Grande do Sul (PUCRS); (2) Hospital São Lucas da PUCRS (HSL); (3) Universidade Luterana do Brasil (ULBRA); (4) Hospital das Clínicas da Faculdade de Medicina da Universidade de São Paulo (HC-FMUSP)

**Background:** Congestion detected by lung ultrasound (LUS) through B-lines is related to prognosis in HF (heart failure) patients; performing it after a physical stress test can detect congestion even earlier. The 6-minute walk test (6MWT) is a submaximal stress test used to assess response after treatment, functional capacity, and prognosis in HF patients. There are no studies evaluating the use of lung US after the 6MWT.

**Aim:** To evaluate the correlation of the number of B-lines on LUS before and after the 6MWT with the distance covered in the stress test in outpatients with heart failure with reduced ejection fraction (HFrEF).

**Methods:** It is a cross-sectional analytical study with prospective inclusion of patients from three HF outpatient clinics. Inclusion criteria were: age ≥18 years, to have the diagnosis of HF for at least 6 months and to have echocardiogram with ejection fraction <40%. Patients were submitted to the LUS before and after 6MWT.

**Results:** From September 15, 2020 to November 30, 2021, 188 patients were included in the study. The mean age of the patients was 61.83 years (±12.13 years); most patients were male (63.8%); the median left ventricular ejection fraction was 31.73% (interquartile range 25–75% [IQR] 28–37%); the most prevalent etiology of HF was ischemic (52.7%); the median number of B-lines was 3 (IQR 1–9) at rest and 6 (IQR 2–13) after submaximal stress; the median distance covered on 6MWT was 363.5 m (IQR 270–436). The distance covered on the 6MWT was inversely correlated with the total number of B-lines after submaximal stress (r = –0.235; p = 0.001) and at rest (r = –0.253; p = 0.001).

**Conclusion:** The distance covered on the 6MWT was inversely correlated with the total number of B-lines after submaximal stress and at rest in outpatients with HFrEF. There was no significant difference between stress and rest B-lines. More studies, with prognostic follow-up, are needed to assess whether LUS associated with the 6MWT adds more benefit in the evaluation of patients with HF.


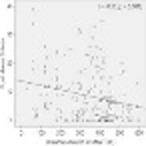


112049

Modality: E-Poster Scientific Initiation – Non-case Report

Category: EPIDEMIOLOGY AND HEALTH POLICIES/GLOBAL HEALTH

## Evaluation of the Behavior of the Number of Cases of Chagas Disease in Brazilian Regions between 2016 and 2020

ANNA LUIZA ALVES DE OLIVEIRA MIRANDA^1^, Maria Eduarda dos Santos Lopes Vasconcelos^1^, José Pedro da Silva Sousa^1^, José Wilker Gomes de Castro Júnior^1^, Beatriz Siems Tholius^1^, Evaldo da Costa Sá Borges de Rezende^2^, Matheus Rocha Maia^1^

(1) Centro Universitário do Estado do Pará (CESUPA); (2) Universidade Federal do Pará (UFPA)

**Introduction:** Chagas disease (CD) is a zoonosis caused by the protozoan Trypanosoma cruzi, whose vector is a hematophagous triatomine. The transmission mechanisms are vectorial, oral, vertical, accidental, by blood transfusion or organ transplantation.

**Objective:** To analyze the behavior of the annual number of cases of CD in Brazilian regions, between 2016 and 2020.

**Methods:** Descriptive, retrospective, and quantitative study, from 2016 to 2020. Data were extracted from the Notifiable Diseases Information System (SINAN) from the Department of Informatics of the SUS (DATASUS), with the variables: sex, probable mode of infection and confirmation criteria.

**Results:** From 2016 to 2020, were 1,662 new cases of CD in Brazil, 1,573 (94%) in the North, 75 (9%) in the Northeast, 7 (0.5%) in the Southeast and 7 (0.5%) Midwest. Regarding the year of detection, 359 (22%) occurred in 2016, 338 (21%) 2017, 384 (23.5%) 2018, 385 (23.5%) 2019 and 157 (10%) 2020. Gender, 897 (54%) were men and 765 (46%) were women. According to the probable mode of infection, 97 (6%) were: vectorial, 5 (0.3%) vertical, 5 (0.3%) accidental, 1,405 (84.4%) oral and 150 (9%) ignored. According to the confirmation criteria, 1,579 (95%) were performed by laboratory, 45 (2.5%) clinical-epidemiological, 7 (0.5%) remained under investigation, and 31 (2%) were ignored.

**Conclusion:** There was an increase in the annual number of CD cases in Brazil in the years studied, with a predominance in the North region, men, browns and oral mode of infection, the diagnosis being confirmed through laboratory tests, they are the research of the parasite and the identification of antibodies in the serum. In recent years, the average worldwide incidence of CD per year has remained around 30,000 cases/year, with Brazil being an endemic area, especially in the state of Pará, corresponding to 74% of cases in the five years evaluated, a state that has culture of consumption of açaí and sugarcane juice, which are the main sources of food contamination by deposition of feces of infected triatomines or crushing of the same during processing. In this way, it is evident that acting in the awareness of the population about the consumption and treatment of food, as well as greater participation of inspection in the places where potentially contaminated food is sold, contributes to the prevention of Chagas disease, a serious pathology that can lead to cardiomyopathy. and progress to heart failure.

112052

Modality: E-Poster Scientific Initiation – Non-case Report

Category: HYPERTENSION/RENAL DENERVATION

## Screening for Systemic Arterial Hypertension in Pre-Elderly and Elderly in an Amazon Riverside Community

GABRIEL IRISMAR RODRIGUES SCHWAMBACK^1^, RAIMUNDO BENÍCIO DE VASCONCELOS NETO^1^, Rebecca Shaiane Soares Nunes Rivoredo^1^, Ana Caroline Leite Guedes^1^, Jade Gomes da Costa Medeiros^1^, Lucas Vieira Amorim^1^, Archimedes Fernandes Alves de Santana^1^, Byron Maia Feitosa^1^, Maria Eduarda Brotto de Souza^1^, Renata Gonçalves Silva Santos^1^, Orisman Martins de Souza Rocha Filho^1^, Fernanda Gabry Scazuza Gomes^1^

(1) Centro Universitário São Lucas – UNISL

**Introduction:** Systemic Arterial Hypertension (SAH) is considered a chronic non-communicable disease (NCD) of insidious onset and that, if not diagnosed and treated, evolves as a precursor to other cardiovascular pathologies. With the aging process, it is common for this CNCD to become more frequent in the population, given the physiological processes of the heart that are affected by time and harmful lifestyle habits. Thus, tracking the number of pre-elderly people (between 55 and 64 years old) and elderly people (over 65 years old) with SAH contributes to highlighting the situation in which a given population finds itself, in addition to providing data for later health interventions.

**Objectives:** To estimate the prevalence of SAH in pre-elderly and elderly people residing in a riverside community in the Western Amazon, Brazil.

**Methods:** This is a cross-sectional, quantitative study carried out with individuals aged between 55 and 87 years, regardless of gender, residing in a riverside community in Porto Velho, Rondônia, Brazil. Data collection was made possible by the application of an individual epidemiological questionnaire during home visits. The analysis was performed by applying the prevalence ratio and the Mantel-Haenszel chi-square test, with statistical significance p < 5%.

**Results:** Data from 58 individuals were analyzed, of which 30 (52%) were male and 28 (48%) were female. We found 35 (60.3%) individuals with an informed prior diagnosis of SAH, with a mean age of 69 years, ranging from 55 to 87 years. As a risk factor for SAH, females have a prevalence ratio equal to 1.4 (variation from 0.93 to 2.19) compared to males (p = 4.9%).

**Conclusion:** SAH is a highly relevant disease to be studied in elderly populations, especially with regard to tracking this CNCD in remote communities, given the strong association between cardiovascular diseases and advanced age. The data point to a prevalence of more than 60% of respondents, an expected value for pre-elderly and elderly people, reinforcing the importance of monitoring the cardiovascular health of this population. No associations were found between sex and SAH in this study.

112054

Modality: E-Poster Scientific Initiation – Non-case Report

Category: PSYCHOLOGY

## Investigation Into the Influences of Depression on the Management of People with Heart Disease: A Systematic Review

LUMA MARIA FAVACHO BORDALO^1^, Yasmin Cavalleiro de Macedo Maranhão^1^, Paula Cordeiro Aguiar de Almeida^1^, Letícia Mariana Gomes Santiago Freire^1^, Glendse Giovanna Costa Pinheiro^1^, Luma Fleury de Figueiredo^1^, Ana Júlia Farache Cabral^1^, Heullem Uyhara da Silva Amorim^1^, Israel Figueira Lemos^1^, Maria Eduarda Dantas da Veiga^1^, Juliana Tavares de Sousa^1^, Erick Clayton Gonçalves Feio^1^

(1) Universidade do Estado do Pará

**Introduction:** In analyzing heart diseases, multiple factors are considered, such as the presence of Major Depressive Disorder (MDD) since pathologies can establish, among themselves, a syndemic relationship capable of aggravating the patients‘ condition. Therefore, the investigation of the impacts of this scenario on the quality of life of those affected is essential to establish improved management measures.

**Objective:** To verify the influence of depression in the treatment of people with heart disease.

**Methods:** The methodology was based on the PRISMA protocol and searches were conducted in the Virtual Health Library (VHL) database using the descriptors “treatment”, “heart diseases” and “depression”; observational studies, published between 2017 and 2022 in Portuguese and English were included. Those that do not relate to the central question, do not have full text available on the platform, or were duplicated were excluded.

**Results:** 6042 articles were obtained with the descriptors. After applying the filters 134 were selected and after deep reading, 4. The first literature indicated that among patients with cardiovascular disease (CVD), there is a prevalence of major depressive disorder (MDD) two or three times greater than the general population and that the possible mechanisms that justify this syndemic relationship between MDD and CVD include dysfunctional neurohormonal and autonomic nervous system effects, brain-derived neurotrophic factor and behavioral factors, as well as inflammation and increased platelet aggregation. Confirming the existence of this relationship, another article points out that individuals with MDD are more likely to develop CVD and associated cumulative diseases, in addition to being more vulnerable to subsequent mortality. Two articles show that patients with CVD and MDD represent a significantly higher financial cost for follow-up than patients without MDD. The fourth study indicates that motivational interviews and interventions based on the transtheoretical model are shown to be effective in improving depressive symptoms associated with coronary atherosclerosis.

**Conclusion:** Patients with CVD are more likely to have MDD and both health conditions have an imbricated relationship, in which the worsened manifestation of one tends to cause the same scenario in the other. In addition, TDM and CVD, when together, result in more financially expensive treatment.

112068

Modality: E-Poster Scientific Initiation – Non-case Report

Category: HEART FAILURE/CARDIOMYOPATHY/TRANSPLANT

## Cross-Sectional Study of Deaths Caused by Acute Myocardial Infarction in Brazil, Divided by Regions (2020)

VINÍCIUS MACIEL VILHENA^1^, Aurea Nathallia Gomes de Souza^1^, Bianca Paula Miranda Martins^1^, Camila Silva de Oliveira^1^, Cecília Rodrigues Viana^1^, Larissa Silva Ferreira^1^, Luiz Felipe Façanha Ramos^1^, Marcos Roberto Marques da Silva Júnior^1^, Reny Wane Vieira dos Santos^1^

(1) Universidade Federal do Amapá

**Introduction:** Acute myocardial infarction is an ischemic lesion of the heart muscle due to a severe alteration in the functioning of the coronary arteries, leading to decreased blood flow to the myocardium and consequent tissue necrosis. This event can lead to death if not diagnosed urgently. In addition, this disease represents one of the main causes of death in Brazil and in the world, with serious consequences for mortality, morbidity and cost to society.

**Objective:** To analyze the epidemiological profile of deaths caused by acute myocardial infarction in Brazil during 2020.

**Methods:** Epidemiological study with a cross-sectional design of mortality from acute myocardial infarction in Brazil during 2020, divided by regions. The data presented were extracted from the DATASUS database, operated by the Health Department.

**Results:** According to the data collected, in Brazil in 2020 there were 90.465 deaths caused by acute myocardial infarction. With a mortality rate of approximately 42.72 per 100.000 inhabitants. Among the Brazilian regions, the South region had the highest mortality rate, approximately 46.85 per 100,000 inhabitants, followed by the Northeast region with a rate of 43.67. The northern region had the lowest mortality rate, approximately 29.78. Regarding the states, those with the highest rate were: Mato Grosso do Sul (65.67), Rio de Janeiro (61.68) and Piauí (56.83). Those with the lowest rate were: Amazonas (19.03), Amapá (22.28) and Distrito Federal (23.63). According to sex, 53.925 men and 36.534 women died from acute myocardial infarction in 2020.

**Conclusions:** It was observed that Brazil presents different situations in relation to mortality from acute myocardial infarction in each region and state. It is possible to reflect on the quality of life in each region and the possible risk factors related to this disease and how this can change the data, which should be further studied. In addition, there is the possibility of underreporting in regions with lower infrastructure conditions for the collection of these data, which can make the management of this disease difficult.


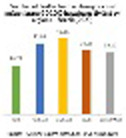


112088

Modality: E-Poster Scientific Initiation – Non-case Report

Category: HEART FAILURE/CARDIOMYOPATHY/TRANSPLANT

## COVID-19 Vaccine-Associated Takotsubo Cardiomyopathy: A Systematic Review

VINICYUS EDUARDO MELO AMORIM^1^, Ana Cecília Araújo Cabral^2^, Letícia Fagundes do Nascimento Silva^3^

(1) Faculdade Pernambucana de Saúde; (2) Faculdade Pernambucana de Saúde; (3) Faculdade Pernambucana de Saúde

**Background:** The coronavirus (COVID-19) pandemic caused by severe acute respiratory syndrome coronavirus-2 has negatively affected millions worldwide. There is an urgent need to advance vaccine campaigns, however, the adverse effects of these vaccines remain unclear. Some patients have reported chest pains and atypical symptoms characteristic of Takotsubo cardiomyopathy (TCM) shortly after receiving the vaccine. TCM is often triggered by intense emotional stress. It characteristically presents with features that mimic acute myocardial infarction.

**Objectives:** To assess the relationship between Takotsubo Cardiomyopathy and the Covid-19 vaccine through a systematic review.

**Methods:** The bibliographic search was performed, using the PRISMA protocol, from articles published in PubMed from January 2021 to April 2022 associating the key terms: Takotsubo Cardiomyopathy, Vaccination and COVID-19. From this, 154 articles were found. The articles included fulfilled the criteria for discussing the arising of TCM after vaccination for covid-19. Articles that only approach the increase in the incidence of TCM during the pandemic were excluded.

**Results:** After selection, nine case reports and one case series were identified through this search process totalling eleven patients. The median age of the 11 TCM patients was 62,27 years, with most being female [81,8%]. Vaccines received by patients were Pfizer-BioNTech Covid-19, mRNA-1273 and ChadOX1 nCOV-19. Ten patients developed atypical symptoms within the first 10 days post-vaccination. The most common symptoms were: body pain, chest pain, chest pressure, dyspnea, palpitation, malaise, nausea, fatigue, sweating, vomiting and hypotension. Eight patients had comorbidities, but only 1 had a previous history of Covid-19 infection. All patients had positive troponin and electrocardiogram changes, with a negative T wave being the most common finding. Coronary angiography and X-ray were used to rule out other possible diagnoses. Cardiac magnetic resonance, transthoracic echocardiography and ventriculography were used to diagnose TCM and the most common findings were left ventricular akinesia and hypokinesia, apical ballooning and ejection fraction reduction of less than 50%.

**Conclusion:** Despite the similarity between the cases, the causality of TCM triggered by vaccination cannot be proven, due to the small sample size; however, it appears to be the most likely cause, as the onset of symptoms occurred very soon after vaccination.

112100

Modality: E-Poster Scientific Initiation – Non-case Report

Category: HEMODYNAMICS AND INTERVENTIONAL CARDIOLOGY

## Left Atrial Appendage Occlusion During Cardiac Surgery as a Preventive Strategy for Stroke in Patients with Atrial Fibrillation: A Systematic Review and Meta-Analysis

LETÍCIA RIBEIRO DO VALE^1^, Elias Pereira de Lisboa^2^, Pedro Guido Rocha de Almeida^3^, Vitor Hugo Soares Rosa^4^, Maria Isabel Fortunato Cavalcante^5^, Felipe Eduardo campos da Silva^6^, Lucas Amaral da Silveira^7^, Elany Maria Ferreira Portela^8^, Helvecio Teixeira Mazon Junior^9^, Giovanna Vinhal Reis^10^, Brenna Pinheiro Zuttion^11^, Letícia Ribeiro do Vale^1^

(1) Letícia Ribeiro do Vale; (2) Elias Pereira de Lisboa; (3) Pedro Guido Rocha de Almeida; (4) Vitor Hugo Soares Rosa; (5) Maria Isabel Fortunato Cavalcante; (6) Felipe Eduardo campos da Silva; (7) Lucas Amaral da Silveira; (8) Elany Maria Ferreira Portela; (9) Helvecio Teixeira Mazon Junior; (10) Giovanna Vinhal Reis; (11) Brenna Pinheiro Zuttion

**Introduction:** Atrial fibrillation (AF) results from abnormalities in electrical conduction. The left atrial appendage (LAA) is the most recurrent site for thrombus formation and its occlusion is an intervention that reduces the risk of stroke.

**Objective:** To compare the results of LAA occlusion during cardiac surgery in patients with AF and the development of Ischemic Stroke.

**Method:** Systematic review using the PRISMA methodology and the PICO criteria in the MEDLINE, COCHRANE and LILACS databases, using the keywords “Atrial fibrillation”, “Left Atrial Appendix” and “Thromboembolism” to identify relevant articles. Five publications were considered for the meta-analysis, involving 15,595 patients with AF who underwent surgical cardiac interventions. The odds ratio (OR) values and their respective 95% confidence intervals (95% CI) were calculated using RStudio.

**Results:** Of the five studies, one presented an OR value greater than 1, rejecting the hypothesis. The overall OR was 0.68 [95% CI 0.57; 0.82]. The I² heterogeneity measure was 0%, a value considered as low heterogeneity. Publication bias was assessed using the funnel plot.

**Conclusion:** Patients with AF who underwent LAA occlusion reduced the chances of developing ischemic stroke when compared with patients with AF who did not undergo the procedure. This study presents evidence in favor of performing the LAA occlusion procedure as an effective alternative to reduce the risk of stroke.


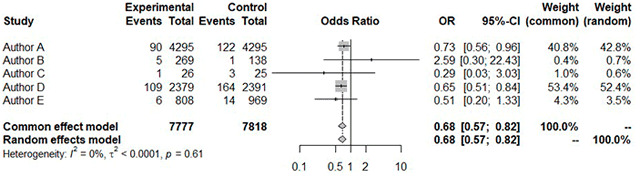


112094

Modality: E-Poster Scientific Initiation – Non-case Report

Category: CARDIO-ONCOLOGY

## Heart Failure Induced by Antineoplastic Treatment: A Systematic Review

DAVI GABRIEL BARBOSA^1^, Marcella Oliveira Monte Santo^2^, Paola Bitar de Mesquita Abinader^3^, Daniel Oliveira da Costa^1^, Afonso Moraes Melo Junior^3^, Camila Rodrigues Maciel^3^, João Lucas Watrin Braga^3^, Bruna Freitas Vinagre^3^, Igor Lucas Farias Lima^1^, Luig Matias Barreiros Pires^3^, Wadilla Fiuza da Silva^2^, Luis Eduardo Werneck de Carvalho^4^

(1) Universidade do Estado do Pará – UEPA; (2) Universidade Federal do Pará – UFPA; (3) Centro Universitário do Pará – CESUPA; (4) Sociedade Brasileira de Cancerologia – SBC

**Introduction:** Antineoplastic chemotherapy consists in the administration of drugs that act at the cellular level, destroying tumor cells. Because they act without specificity, they can generate undesirable effects, such as cardiotoxicity; presenting as main damages the higher frequency of moderate or severe heart failure associated with systolic or diastolic ventricular dysfunction.

**Objectives:** To analyze the induction of heart failure in patients on antineoplastic treatment by searching for scientific evidence in the database literature.

**Methods:** A systematic review was conducted through a bibliographic search of articles, using the Virtual Health Library database, whose filters were: articles published in the last 5 years, published freely and in full, and randomized clinical trials. The descriptors used comprised (Heart Failure) AND (Antineoplastics) AND (Cardiotoxicity) and the exclusion criteria were case reports and other systematic reviews. PICO and PRISMA methodology was employed.

**Results:** The use of trastuzumab can lead to decreased left ventricular function and moderate or severe HF. There is a decline in LVEF with improved survival with the use of ACEI and beta-blockers, reducing mortality and patient hospitalization. Crizotinib showed few events associated with HF. Anthracycline, used in first-line lymphoma treatment increases the risk of CHF in patients with no prior history of heart disease, particularly when treatment courses with more than 6 cycles of R-CHOP/CHOEP. The expression of TLR2 and TLR3 biomarkers is up- and down-regulated, respectively, in patients treated with doxorubin, causing cardiac dysfunction. ACE inhibitors in anthracycline-induced cardiotoxicity, did not develop arrhythmia or heart failure, and enalapril acted in reducing cardiac toxicity after anthracycline administration.

**Conclusion:** It is inferred that the antineoplastic drug with the highest relation to HF is anthracycline. However, the group of ACE inhibitors and enalapril decrease the cardiotoxicity of anthracycline. Moreover, trastuzumab is related to the onset of moderate or severe HF, and the ACE inhibitor drug groups IECA and beta-blockers decrease mortality in these patients. Doxorubin has predictive markers: TLR2, up-regulated, and TLR3, down-regulated.

112099

Modality: E-Poster Scientific Initiation – Non-case Report

Category: EPIDEMIOLOGY AND HEALTH POLICIES/GLOBAL HEALTH

## Mortality from Cerebrovascular Disorders in Women in the Northern Region of Brazil from 2010 to 2020

GABRIELLY CARVALHO LEÃO^1^, Gabrielly Carvalho Leão^1^, Saul Rassy Carneiro^1^, Vando Delgado de Souza Santos^1^, Bruno Patricio dos Santos Oliveira^1^

(1) Universidade Federal do Pará

**Introduction:** Cerebrovascular Disorders encompass several diseases with clinical manifestations resulting from the interruption of brain blood flow, which can manifest in an ischemic way, usually linked to circulation obstruction, or in a hemorrhagic way, when there is vascular rupture. In view of the wide occurrence of deaths and permanent sequelae that such diseases generate, investigation and understanding of them become indispensable.

**Objectives:** To identify and analyze the epidemiological profile linked to the mortality of female individuals due to Cerebrovascular Disorders in the northern region of Brazil, from 2010 to 2020, as well as to associate it with regional factors of a social and economic nature.

**Methods:** This is an observational and cross-sectional study, which used mortality records in cases of cerebrovascular diseases in northern women, between January 2010 and December 2020, obtained through the Departamento de Informática do Sistema Único de Saúde (DATASUS), using the variables age group, color, schooling and place of occurrence.

**Results:** The data gathered showed the occurrence of 30,490 deaths of women from cerebrovascular diseases in the region in question during the period considered, a value that corresponds to 45.94% of the total deaths of this category in the north of Brazil. Regarding the profile of these northern women, it should be noted that 79.96% of them were aged over 60 years. In addition, 33.47% of these women did not have any complete levels of education and 66.35% declared themselves to be brown. Finally, regarding the place of death, 74.55% took place in hospital settings.

**Conclusion:** Thus, it is evident that cerebrovascular disorders predominate in the female population in disadvantaged socioeconomic situations, especially elderly brown women, without a degree of education, with low income and possibly without access to health surveillance and primary prevention mechanisms (such as encouraging and subsidizing healthy eating and physical activity). In addition, the high death rates in the hospital context indicate the contribution of the diseases analyzed to the burden of the health system, reinforcing their severity and the need for preventive actions, in order to guarantee the well-being and longevity of women northerners.

112104

Modality: E-Poster Scientific Initiation – Non-case Report

Category: HEART FAILURE/CARDIOMYOPATHY/TRANSPLANT

## Population at Risk for Heart Failure in the Amazon Basin

BIANCA VASCONCELLOS RODRIGUES LOPES^1^, Luisa de Oliveira Pereira Vila Nova Ramalho^1^, Laura Cordeiro Gomes^3^, Philip Brainin^2^, Anna Engell Holm^2^, Karine Oliveira Lima^1^, Isabelle Victória Martins Vieira^1^, Marliton Vinicius Pedrosa Evangelhista^1^, Luan Oliveira Matos^1^, Iara Fernanda Vasconcelos de Oliveira e Silva^1^, Tor Biering-Sørensen^2^, Odilson Marcos Silvestre^1^

(1) Federal University of Acre; (2) Herley-Gentofte University Hospital; (3) University of São Paulo

**Introduction:** Neglected diseases, less access to health services and socioeconomic factors makes the Amazon Basin a vulnerable area for heart disease. There is no data concerning the risk for developing heart failure in this setting.

**Aim:** To assess the population at risk for developing heart failure in the Amazon Basin.

**Methods:** We performed a cross-sectional study in the Brazilian Amazon Basin. Patients were classified as at risk for HF according to the American College/American Heart Association staging system. For stage A, we considered the presence of at least one of the following factors: hypertension, diabetes, BMI > 30 kg/m^2^, hypercholesterolemia, moderate or intense alcohol use, smoking and history of rheumatic fever. We compared those at stage A with those with no risk factors (table).

**Results:** The mean age was 39 years and 47% were male. 70% of all patients were in stage A for HF. Among those, 29% had hypertension, 4% had diabetes, 24% were obese, 38% had hypercholesterolemia, 11% consumed alcohol at moderate or intense level, 11% smoked and 6% had a history of rheumatic fever. HF risk participants had lower educational levels, 214 (27.7%) participants did not complete elementary school vs. 43 (13.4%) in out of risk population (p < 0.001). A total of 385 (49.9%) stage A patients have lived in rural areas for the most part of their lives against 121 (37.7%) at non-risk group (p < 0.001). Median income was lower in stage A [median (IQR) R$ 360.0 (175 to 700.0)] when compared to the other group [median (IQR) R$ 400.0 (240.0 to 800)] (p < 0.004).

**Conclusion:** Most of Amazon Basin population is at risk for developing heart failure. Patients at stage A are older, have lower income and educational levels and have lived in rural areas for the most part of their lives.


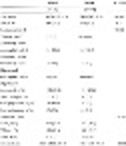


112112

Modality: E-Poster Scientific Initiation – Non-case Report

Category: CONGENITAL AND PEDIATRIC CARDIOLOGY

## Stent Implantation for the Treatment of Carotid Aortic Coarctation in Infants

LÍVIA FARIAS DE HOLANDA FURTADO^1^, Patrícia Oliveira Lima de Macedo^1^, Lais Vieira Araujo^1^, João Victor Bezerra Nunes^1^, Abel Belarmino de Amorim Neto^2^, Lara Andrade Dantas^2^, Ranise Nunes Pereira Moura^2^, Daniel Marcelo Silva Magalhaes^2^, Carlos Marximiliano Alves de Oliveira^2^, Juliana Sousa Soares de Araujo^1^, Cláudio Teixeira Regis^3^, Fabrício Leite Pereira^2^

(1) UFPB – Universidade Federal da Paraíba; (2) Hospital Metropolitano Dom José Maria Pires; (3) Complexo Pediátrico Arlinda Marques

**Introduction:** Coarctation of the aorta is a congenital heart disease in which there is narrowing of the aortic isthmus. Stenting for treatment is well established in adults and in children over 25 kg. However, in younger children the usual treatment is surgery or balloon recoarctation. Currently, this technique has been used in selected cases.

**Objective:** To demonstrate the early results of stent implantation for the treatment of carotid coarctation in infants.

**Methods:** This is a cross-sectional, observational, retrospective study of a case series of stenting in carotid coarctation in a public hospital in Paraíba.

**Results:** 4 infants underwent carotid stenting in infants with a mean age of 110 days (22 days to 12 months), mean weight 5.3 kg (2.9 to 10.6 kg) all male, and with another cardiac alteration. In the first case, the coarcted area increased from 2.6 to 7 mm; the second increased from 1.3 to 6 mm; the third increased from 3 to 8 mm, and the fourth increased from 3.4 to 8.5 mm. Thus, the pre-treatment mean was 2.58 mm and became 7.38 mm, with the size of the balloon chosen by measuring the abdominal aorta and the aortic arch. Only 1 patient presented as a complication a small aortic dissection, of expectant management. All patients presented normalization of the abdominal aortic flow in the control echo.

**Conclusion:** Stent implantation proved to be effective and safe in the short term, and may be a treatment option also at this age. However, further long-term studies are needed to monitor the effectiveness of the technique in terms of reinterventions required due to somatic growth of patients.

112116

Modality: E-Poster Scientific Initiation – Non-case Report

Category: CARDIO-ONCOLOGY

## Antineoplastic Protocols in Cardio-Oncology: Updates from the Past 5 Years

PAOLA BITAR DE MESQUITA ABINADER^3^, Marcella Oliveira Monte Santo^1^, Ana Josefina Gonçalves Salomão^3^, Davi Gabriel Barbosa^2^, Joao Lucas Watrin Braga^3^, Leandro Lourenço Silva Monteiro^2^, Manuela pedreira da Cruz Rocha^3^, Vinicius Queiroz Silva^2^, Alexandre D‘Annibale Cartágenes^2^, Luis Eduardo Werneck de Carvalho^4^, Luiz Fernando Leite da Silva Neto^2^

(1) UNIVERSIDADE FEDERAL DO PARÁ (UFPA); (2) UNIVERSIDADE ESTADUAL DO PARÁ (UEPA0; (3) CENTRO UNIVERSITÁRIO DO PARÁ (CESUPA); (4) ONCOLÓGICA DO BRASIL

**Introduction:** Antineoplastic protocols guarantee standards and criteria to treat different neoplasms, based on effectiveness and scientific evidence. Henceforth, it is possible to define the degree of validity in therapeutic category. In the cardio-oncology field, adopting these protocols is essential, since some pharmacological interventions can be cardiotoxic, such as the use of anthracyclines, becoming necessary the regulation of therapies for these pathologies.

**Objective:** Analyzing antineoplastic protocols in cardio-oncology searching for scientific evidence and database updates.

**Methods:** This is a systematic literature review with descriptive collection and a qualitative approach to data on antineoplastic protocols in cardio-oncology, available in the BVS database. The search formula used was: Antineoplastic Protocols AND Cardiac Neoplasms. Studies that presented a 5 years maximum gap since their publication, and published in completion and in English, were selected. Exclusion criteria were: other types of studies. The data extraction and synthesis strategy followed the PICO and PRISMA guidelines.

**Results:** 795 studies were found on the search platform with the descriptors, 113 remained after initial filtering, 40 of these were applicable in the inclusion criteria, 4 studies were selected for analysis. In the case of cardiac rhabdomyomas related to tuberous sclerosis, surgery is indicated, but it has a high mortality rate. Regarding pharmacological treatment for them, there are studies indicating that prolonged treatment with sirolimus has shown positive results and has been shown to be more beneficial in male patients. In the case of ventricular fibromas and cardiac myxomas, surgical resection with cardiopulmonary bypass showed benefits and a high survival rate with few complications.

**Conclusion:** In this viewpoint, it is noted a beneficial relationship in the use of antineoplastic drugs in patients affected by cardiac tumors, especially in male subjects, with the treatment being faster; aged over 4 years and fewer cardiovascular surgeries. Thus, even though the number of studies on this condition is scarce, there is an important possibility of pharmacological intervention in cardio-oncology services that needs further studies.

112123

Modality: E-Poster Scientific Initiation – Non-case Report

Category: CARDIOVASCULAR SURGERY

## Comparison of Electrocardiographic Changes Consistent with Pericarditis in Patients Undergoing Posterior Pericardiotomy During Coronary Artery Bypass Graft Surgery – A Randomized Double-Blinded Study

GUSTAVO GUIMARÃES OLIVEIRA^3^, Gustavo Guimarães Oliveira^3^, Carolina Maria Vianna Guimarães^2^, Jackson Brandão Lopes^2^, Tainá Teixeira Viana^1^, Diêgo Moreira Arruda^1^

(1) Hospital Ana Nery – HAN; (2) Universidade Federal da Bahia – UFBA; (3) Escola Bahiana de Medicina e Saúde Pública – EBMSP

**Background:** Posterior pericardiotomy (PP) is a surgical technique in which a 4 cm incision is made in the pericardium in order to avoid complications in the postoperative period (PO) of cardiac surgeries, such as pericardial effusion (PE) and atrial fibrillation (AF). There are, however, few studies addressing the presence of electrocardiographic findings of pericarditis in patients undergoing this procedure.

**Objective:** Compare the presence of electrocardiographic alterations compatible with pericarditis, AF and PE in the PO period of myocardial revascularization between the groups.

**Methods:** A double-blinded, single center, randomized study, conducted by Hospital Ana Nery – Bahia, with a sample size of 30 patients. Patients were randomized into two groups: intervention group (submitted to PP) and control group (CG), which did not undergo PP. Data collected for the study included pre-, intra- and postoperative data.

**Results:** In the sample, 62.5% are men, the PP group was composed by 66% male and the CG 80%, while the mean age of the study was 62.4 ± 8.9 years. The patient’s EUROSCORE II had a median of 0.76 with a maximum of 2.5 and a minimum of 0.5. All the patients analyzed used the internal mammary artery in the surgery, while 26.7% of the CG used double mammary against 6.7% in the PP group. The radial artery was used in 33.3% of patients in the PP group and 53.3% of the CG. In the PP group, a frequency of 6.7% of AF was observed in the PO, while in the CG 0.0% (p = 0.30). Regarding PE, there was a 26.7% incidence in the PP group and 40.0% in the CG (p = 0.43). Electrocardiographic findings of pericarditis were identified in 6.7% of patients in the CG and 20.0% of the PP group (p = 0.28). There was one death in the study that occurred in the CG on the 20th postoperative day (p = 0.30).

**Conclusion:** Although a lower incidence of PE was found in the PP group, it was not statistically significant. It was also not possible to identify a statistically significant difference in the occurrence of AF between the groups or of electrocardiographic changes compatible with pericarditis. Due to the low incidence of events in this study, the N was not powerful enough to identify the statistical differences already reported in the literature, which makes us believe that a larger sample is needed to corroborate or disprove previously published data.

112124

Modality: E-Poster Scientific Initiation – Non-case Report

Category: DYSLIPIDEMIA

## Prevalence of Left Ventricular Diastolic Dysfunction Among Patients with Familial Hypercholesterolemia Under Primary Prevention

AMANDA PEREIRA MATOS^1^, Rogério Krakauer^2^, Renato Jorge Alves^2^

(1) Faculdade de Ciências Médicas da Santa Casa de São Paulo; (2) Santa Casa de Misericórdia de São Paulo

**Background:** Familial Hypercholesterolemia (FH) is a disorder characterised by an increased serum LDL-cholesterol level (LDL-c > 190 mg/dL). It represents a high cardiovascular risk and leads to complications that can compromise the ventricular myocardium function. There are no studies on left ventricular (LV) diastolic function in patients with FH. The early detection of subclinical morphological changes could contribute to a more effective prevention strategy.

**Purpose:** This study aimed to obtain the prevalence of diastolic dysfunction among patients with FH under primary prevention (PP) and to epidemiologically compare PP and secondary prevention (SP) in patients with FH.

**Methods:** We performed a cross-sectional study with 42 patients with FH over 18 years old of a dyslipidemia outpatient clinic of a tertiary hospital. The patients were divided into two groups: PP and PS. We excluded those who, at the time of the echocardiographic assessment, presented: ejection fraction less than 50%, LV regional wall motion abnormality, significant valve dysfunction, cardiac arrhythmia, pericardial effusion or unavailable information to determine LV diastolic function. Each exam was performed on the same machine and analysed by the same physician.

**Results:** Out of 42 patients, 9 were excluded. Of a total remaining 33, 12 were included in the PP group and 21 in the SP. Therefore, 36,4% (12/33) underwent the transthoracic echocardiogram exam for diastolic function assessment. After exams‘ analysis: 75% (9/12) of the patients had normal diastolic function, 8.3% (1/12) indeterminate function and 16.7% (2/12) presented grade I diastolic dysfunction. Of those with normal function, one had diabetes and one, grade I obesity. Of those with the dysfunction, one had diabetes and one, diabetes and grade III obesity, both elderly. By comparing the two groups: PP had an average age of 60.5 years old and SP 67 years old; PP 91.7% women and SP 71.4%; PP 25% and SP 38.1% diabetes; PP 50% and SP 90.5% arterial hypertension (p = 0,015).

**Conclusions:** Patients with FH under PP did not show significant changes in LV diastolic function (low prevalence of grade I dysfunction only). Furthermore, patients under SP showed a higher frequency of arterial hypertension. However, new studies must be carried out, with a higher number of patients, for better clarification.

112125

Modality: E-Poster Scientific Initiation – Non-case Report

Category: CARDIOVASCULAR SURGERY

## Preoperative Electrocardiographic Parameters as Predictors of Atrial Fibrillation in the Postoperative Period of Cardiac Surgery

DAVID CESARINO DE SOUSA^1^, Fábio Antônio Serra de Lima Junior^1^, Renan Furtado de Almeida Mendes^1^, Andre Loureiro Fernandes^1^, Ana Beatriz Venancio de Paula Bezerra^1^, Raphael Patrik Borges da Costa^1^, Tiago Lucena de Brito Pereira^2^, João Bosco Ferreira Gadelha^2^, Isaac Newton Guimarães de Andrade^1^, André Telis de Vilela Araújo^1^

(1) Universidade Federal da Paraíba; (2) Clínica Dom Rodrigo

**Introduction:** Postoperative atrial fibrillation is responsible for important postoperative morbidity and mortality, and the ability to predict it could allow prophylactic measures and intensive monitoring in patients at higher risk.

**Objectives:** To relate preoperative ECG findings of patients undergoing cardiac surgery with the occurrence of postoperative arrhythmias, and compare the results with those found in national and international studies.

**Methods:** A longitudinal observational study was conducted with patients undergoing cardiac surgery between 2019 and 2021 in a cardiology referral hospital in João Pessoa. Pre- and postoperative 12-lead electrocardiogram data were collected and processed, in addition to patients‘ constitutive and surgical data.

**Results:** A total of 105 patients were observed, with the presence of supraventricular extrasystole (2 vs. 0 cases, p = 0.017), P-wave amplitude (0.993 ± 0.8426 mV vs. 0.318 ± 1.1467 mV, p = 0.015), the Morris index (–94.286 ± 78.2849 mV.ms vs. –36.951 ± 94.7297 mV.ms, p = 0.023) and the presence of left ventricular overload (6 vs. 12 cases, p = 0.015).

**Conclusion:** The presence of supraventricular extrasystole, P wave amplitude, Morris index, and the presence of electrocardiographic criteria of left ventricular overload were associated with a higher risk of developing postoperative atrial fibrillation. Larger samples may reveal yet other significant predictor variables.

112127

Modality: E-Poster Scientific Initiation – Non-case Report

Category: HYPERTENSION/RENAL DENERVATION

## Assessment of Adherence to Hypertension Treatment in People with Depressive Symptoms: A Systematic Review

ISRAEL FIGUEIRA LEMOS^1^, Alice Marcely dos Santos Tuñas^1^, Rafael Malcher Meira Rocha^1^, Giovanna Lopes Evangelista^2^, Ronaldo Monteiro Veras^3^, Guísela Gabriela Silveira de Souza^1^, Maria Giovanna Trindade Rocha^1^, Lucas Matheus Marinho de Miranda^1^, Maria Eduarda Dantas da Veiga^1^, Juliana de Sousa Tavares^1^, Erick Clayton Gonçalves Feio^1^, Luma Maria Favacho Bordalo^1^

(1) Universidade do Estado do Pará; (2) Centro Universitário do Pará; (3) Universidade Federal do Pará

**Introduction:** According to the new Brazilian Hypertension Guidelines (2020), the main reason for inadequate control of hypertension is noncompliance with long-term treatment. Some factors such as the absence of symptomatology and the requirement for continuous treatment, as well as changes in habits, are important in this statistic. Depression, however, can make the patient reduce his health care for a psychic issue, by the symptoms of discouragement, easy fatigue, and need for greater effort to do things.

**Objectives:** To evaluate the relationship between adherence to treatment for hypertension and depressive symptoms.

**Methodology:** This is a systematic literature review, it was used as a database the Virtual Health Library (VHL), with the descriptors “Hypertension”, “Depression” and “Treatment Adherence” between the years 2017 and 2022, in English and Portuguese, published free and fully, selected through the PRISMA method. Where articles that did not address the relationship to be evaluated and literature reviews were excluded.

**Results:** 283 articles were obtained with the use of the descriptors, 87 with the application of search filters, after detailed reading, 8 met the objectives of the study. From the literature, it is observed that depression negatively influences adherence to hypertension treatment. The chance of a patient being at the target pressure increases significantly with increased adherence to treatment for depression. In one study, with women only, depression was more reported by patients with worse adherence to AH treatment (p = 0.026). However, no differences in depression symptoms were observed between adherent and non-adherent patients seen in an emergency room. Hypertensives with depressive symptoms have a significantly higher relative risk of treatment noncompliance. In this sense, the negative impact of depression on the capacity for self-care and continuation of the prescribed drug therapy is noted, as well as the vulnerability and need for support.

**Conclusion:** In this article, we sought to evaluate adherence and continuity of pharmacological and non-pharmacological treatment for AH in patients with depressive symptoms. Therefore, expanding studies that propose and evaluate strategies for adherence to drug treatment is essential to ensure that individuals remain normotensive, with the potential to reduce the pressure on the health system and represent simultaneous gains in the quality of life of hypertensive individuals.

112134

Modality: E-Poster Scientific Initiation – Non-case Report

Category: COVID-19 AND CARDIOVASCULAR SYSTEM

## The Impact of COVID-19 Pandemic on Cases of Cardiac Arrest in Brazil

RAFAEL BRACCIO ZAWISLAK^1^, Mariana Saadi de Azevedo^1^, Alice Zanetti Dussin^1^, Thomás Ranquetat Andrade^1^, Mario Wiehe^1^

(1) Pontifícia Universidade Católica do Rio Grande do Sul (PUCRS)

**Background:** Coronavirus disease (COVID-19) is caused by SARS-CoV-2, in which the patient affected has different clinical manifestations. The infection may cause different cardiovascular complications, such as sudden death and cardiac arrest. Besides the pathophysiologic relation of the infection with cardiovascular diseases, these different bad outcomes may also have a social and political component, such as saturation of the health system and loss of patient follow-up.

**Aims:** To evaluate the impact of the Covid-19 pandemic on cases of cardiac arrest with death outcome in Brazil and verify the frequency according to in or out-hospital events.

**Methods:** The data of the last 4 years (2018 to 2021) of deaths due to cardiac arrest were collected using the Mortality Monitoring Panel of the Health Surveillance Department of Brazil. The number of deaths were separated into tables comparing the year of the deaths to location of event. This was conceived as a descriptive study.

**Results:** Pre-pandemic numbers (2018 and 2019) were very similar, averaging them for comparison. The number of deaths of the 2 years before the Covid-19 pandemic were quite similar in age groups and sex. The average number of deaths in the 2018–19 period was 1384, with an increase of 56.21% in 2020 and 130.41% in 2021. The cause of the cardiac arrest was described as sudden death in all periods analyzed. The average number of cardiac arrests in-hospital in the 2018–19 period was 383.5, with an increase of 32.72% in 2020 and 87.48% in 2021. In the 2018–19 period, out-hospital cardiac arrests were at an average number of 1000.5, with an increase of 65.21% in 2020 and 146.87% in 2021.

**Conclusion:** The number of cardiac arrests during the COVID-19 pandemic increased overall, in-hospital as well as out-hospital. This may be related to pathophysiological and social-political components. Events that occurred at home had a significant growth, which may be associated with lockdown protocols and difficulty in obtaining basic access to healthcare, possibly related to a hesitation of a possible acquisition of SARS-CoV-2 infection and the overload of hospitals.


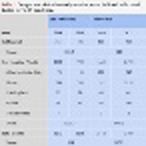


112144

Modality: E-Poster Scientific Initiation – Non-case Report

Category: COVID-19 AND CARDIOVASCULAR SYSTEM

## The Epidemiology of Deaths by Hypertensive Disease During the COVID-19 Pandemic in Brazil

MARIANA SAADI DE AZEVEDO^1^, Rafael Braccio Zawislak^1^, Alice Zanetti Dussin^1^, Rafael Vianna Behr^1^, Mário Wiehe^1^

(1) Pontifícia Universidade Católica do Rio Grande do Sul

**Background:** Covid-19, disease caused by Sars-CoV-2, is responsible for the pandemic that generated instability in health systems across the globe and over 66,0000 deaths in Brazil since 2020. There is evidence that arterial hypertension is associated with a more severe course and higher death rates. In addition, control of chronic diseases has been a challenge due to loss of patient follow up and failure to seek medical help in an acute condition.

**Aims:** To convey epidemiological data of the last 5 years of death by hypertensive disease in Brazil and to elucidate its possible relation to Sars-CoV-2 infection and social aspects of the Covid-19 pandemic.

**Methods:** This was conceived as a descriptive study. The Mortality Monitoring Panel of the Health Surveillance Department of Brazil was used to collect the data of the last 5 years (2017 to 2021) of deaths due to hypertensive disease. The data was organized in a table comparing the years pre and post Covid-19 advent.

**Results:** There has been a 19.98% increase in the average of death rates by hypertensive disease in Brazil post covid-19 (years 2020 and 2021) in relation to the years previous to the pandemic that were analyzed (2017–2019). In the years 2017–2019, the average of yearly deaths at home was 21440, while in the years 2020 and 2021 the average escalated to 30146 cases. This shows an increase of 40.6% in the average of deaths at home by hypertensive disease in Brazil post Covid-19. Concerning deaths in hospital, there was an increase of 0.72% in the yearly average post Covid-19.

**Conclusion:** There has been an increase of death by hypertensive disease in Brazil in the years of 2020 and 2021, which coincides with the Covid-19 pandemic. This can be attributed to a significant rise in domicile death rates. Future studies can evaluate factors involved in this increase and, consequently, the forms of preventing it in similar situations. In addition, it must be observed if, in the years following the pandemic, the number of out hospital deaths will return to that of previous years, or if they will still be affected by the pandemic period.


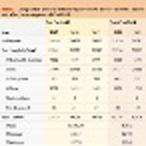


112152

Modality: E-Poster Scientific Initiation – Non-case Report

Category: EPIDEMIOLOGY AND HEALTH POLICIES/GLOBAL HEALTH

## Association between the Transfer of Resources via the National Health Fund and the Number of Hospitalizations for Diseases of the Circulatory System

VANDO DELGADO DE SOUZA SANTOS^1^, Saul Rassy Carneiro^1^

(1) Universidade Federal do Pará – UFPA

**Introduction:** Diseases of the circulatory system (DCS) are diseases that affect the heart and blood vessels. Since 1980, they have been the leading cause of death in the world. The analysis of the association between DCS and financial transfers becomes, therefore, essential for the implementation of health policies.

**Objectives:** To analyze the relationship between hospital admissions for DCS and transfer of financial resources via the National Health Fund (NHF) to the northern region of Brazil from 2011 to 2021.

**Methods:** This is a longitudinal epidemiological study carried out with secondary data from the Department of Informatics of the Unified Health System on the number of hospitalizations for diseases of the cardiocirculatory system and the transfer of resources via NHF to the states of the northern region between January 2011 to December 2021.

**Results:** In the analyzed period, a linear regression model was performed, where the transfer of funds via NHF was the independent predictor to analyze hospitalizations for DCS, under the formula y = 69–2.57e–9x. The allocation of resources to the states of the northern region resulted in a significant reduction in the number of hospitalizations (R = 0.748; p < 0.001). A model with the number of family health strategies was used, but it proved to be less adjusted than the transfer of resources, perhaps because the increase in liquidity in the states had a direct impact on the increase in family health teams.

**Conclusion:** There was an inversely proportional relationship between hospitalizations for DCS and financial transfers via NHF. It is important to emphasize the aspect of primary care performance in controlling these indicators, because, with efficient primary care integrated with reference services, the number of hospitalizations for CAD can be reduced.


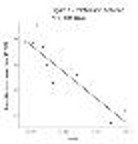


112170

Modality: E-Poster Scientific Initiation – Non-case Report

Category: ANTICOAGULATION

## Anticoagulant Therapy in COVID-19 Hospitalised Patients

REGINA MARIA ALEXANDRE FERNANDES DE OLIVEIRA^1^, Amanda Pereira Matos^1^, Ronaldo Fernandes Rosa^2^, Milton Luiz Gorzoni^2^, Renato Jorge Alves^2^

(1) Faculdade de Ciências Médicas da Santa Casa de São Paulo; (2) Hospital da Santa Casa de Misericórdia de São Paulo

**Background:** Coronavirus disease 2019 has been associated with a prothrombotic state, which puts into question whether a prophylactic anticoagulant therapy could be used to prevent bad outcomes in hospitalised patients since they represent the most severe cases of the disease. However, substantial studies have reported divergent results regarding its benefits and applicability in prevention strategies.

**Purpose:** To explore the incidence of bad outcomes in COVID-19 hospitalised patients that have received Anticoagulants or not.

**Methods:** This retrospective observational study analysed electronic medical records from hospitalised patients with laboratory-confirmed COVID-19 in a large tertiary hospital, between March 2020 and July 2020. The study sample was divided between those who received any dose of anticoagulation (low molecular weight or unfractionated heparin) and those who did not (control group). Our primary outcome was data on mortality, hospital length of stay, need for Intensive Care Unit admission and use of vasoactive medications between the two groups.

**Results:** Of a total 317 patients, 259 (37.8% females, 62.2% males) received Anticoagulants and 58 (37.9% females, 62.1% males) did not. The mortality rate was 49.4% in the Anticoagulant recipient group, and 58.6% in the control group. However, no association could be made between Anticoagulant use and hospital survival (odds ratio [OR] 1.4; 95% confidence interval [CI], 0.8–2.6). The length of hospital stay showed to be similar between the two groups, with an average of 17.8 days (95% CI, 15.3–20.2) for the Anticoagulant group and 16.2 days (95% CI, 9.5–22.8) for the control one. Additionally, lesser need of intensive care could not be associated with blood thinners use (OR 1.6; 95% CI, 0.8–3.1), whereas utilisation of vasoactive medications could (OR 6.5; 95% CI, 3.2–13.3).

**Conclusion:** The findings of our study suggest that the use of Anticoagulants in COVID-19 hospitalised patients is associated with reduced need for vasoactive drugs, with no impact, however, in mortality, hospital length of stay or need for Intensive Care Unit admission. We intend to reexamine the matter with a larger sample of patients.

112160

Modality: E-Poster Scientific Initiation – Non-case Report

Category: HYPERTENSION/RENAL DENERVATION

## Percutaneous Renal Sympathetic Denervation in the Treatment of Hypertension in Patients with Chronic Kidney Disease

LETICIA FAGUNDES DO NASCIMENTO SILVA^1^, Ana Cecília Araújo Cabral^2^, Vinicyus Eduardo Melo Amorim^3^

(1) Faculdade Pernambucana de Saúde – FPS; (2) Faculdade Pernambucana de Saúde – FPS; (3) Faculdade Pernambucana de Saúde – FPS

**Background:** Systemic arterial hypertension (SAH) is a highly prevalent disease that can be both the cause and the consequence of chronic kidney disease (CKD). Chronic kidney disease is defined when the glomerular filtration rate (GFR) is less than 60 mL/min/1.73 m2 for three or more months. In this way, the treatment objective to reduce the progression of kidney disease and cardiovascular risk. Given this, percutaneous sympathetic denervation emerges as a promising new therapy for patients with resistant hypertension when drug therapy fails. In this procedure, ablation of sympathetic fibers is performed through the application of radiofrequency transluminally in the renal arteries.

**Methods:** The study is designed as a systematic review and the articles used were collected through the “PUBMED” and “BVS” platforms and selected according to the PRISMA 2020 protocol. Using the descriptors “Renal denervation”, “Hypertension” and “Chronic kidney disease”, 105 articles were found. Those with full texts were selected while duplicate articles and that did not directly address the research topic were excluded, leaving 18 articles for review.

**Results:** 18 articles included in the review, including randomized clinical trials, clinical trials, meta-analyses, prognostic study, diagnostic study and etiology study. It was observed an improvement in central pressures and pulse wave velocity, a reduction in systolic pressure was observed in most patients after 3 years of treatment, in addition to renal protection in most of the studies performed. Changes in renal sensory function participate in the maintenance of high vasomotor activity and cardiorenal changes; as such, renal sensory fibers may be a potential therapeutic target for the treatment of cardiorenal diseases. However, results from some studies do not show a reduction in the degree of kidney injury. Furthermore, the change in aldosterone level evidenced reaffirms the effect of denervation on the renin-angiotensin-aldosterone system. It is necessary to point out that more clinical trials are needed to assess the safety and reproducibility of the method.

**Conclusion:** Renal denervation is safe for patients with resistant SAH and CKD, with a significant reduction in systolic pressure. However, further studies are needed to assess safety, application advantages and clinical impact. In addition, the mechanisms involved in cardiorenal protection by denervation have not yet been fully clarified.

112171

Modality: E-Poster Scientific Initiation – Non-case Report

Category: PSYCHOLOGY

## Psychological Impacts on Patients who have Suffered from Acute Myocardial Infarction: A Systematic Review

MARIA EDUARDA DANTAS DA VEIGA^1^, Juliana de Sousa Tavares^1^, Erick Clayton Gonçalves Feio^1^, Luma Maria Favacho Bordalo^1^, Israel Figueira Lemos^1^, Amanda de Queiroz Andrade^1^, Bruno Kauê Rodrigues Vilhena^1^, Chan David Ribeiro^1^, Greta Évelin da Silva Mota^1^, Luis Arthur Moreira Ferreira^1^, Victor Leno Silva Paes^1^, Alice Marcely dos Santos Tuñas^1^

(1) Universidade do Estado do Pará – UEPA

**Introduction:** Acute Myocardial Infarction (AMI) is a fatal condition, and survivors can present multiple complications related to mental health and quality of life. Thus, the psychological consequences in the individual who suffered AMI interfere with the prognosis of the disease, as there is an association between psychological and biological factors, such as depression and anxiety. Furthermore, these pathologies affect the patient’s recovery and life expectancy, increasing the risk of AMI recurrence.

**Objectives:** Identify the psychological impacts on patients after AMI.

**Methods:** Systematic literature review with searches in the Virtual Health Library (BVS) using the descriptors “Psychological distress”, “Psychological stress”, “Myocardial Infarction” and “Post-traumatic stress disorder”. The filters used were original articles, complete and open to public consultation from the last 10 years in Portuguese, English and Spanish. Case reports, notices, editorials, comments and literature reviews were excluded. The PRISMA protocol and the question formulated by the PICO methodology were used.

**Results:** 158 articles were obtained with the descriptors. After applying the filters and detailed reading of the data, 17 articles were included in the present study. The literature pointed to the occurrence of several psychological disorders in patients who suffered from AMI, which made recovery even more difficult. It was pointed out that individuals with Post Traumatic Stress Disorder (PTSD) after infarction had a greater stress response, suggesting an association between PTSD with or without cardiovascular outcomes. Based on this logic, it was found that patients with emotional distress are more likely to die after the AMI. Other results, in addition, indicated heterogeneity of emotional suffering, showing a higher incidence of stress related to youth, poverty and the female gender. Furthermore, high levels of anxiety and depression were noted as predictive factors for mortality and readmission to patients with symptoms of ischemic heart disease. Moreover, professional psychological support is also related to a reduction in the mortality rate and decrease in the recurrence of the cardiac condition.

**Conclusion:** It was identified that the psychological impacts, especially depression and anxiety, are evident in people who have suffered from AMI. In this sense, a more in-depth study of the topic and its relationship with the quality of life of these patients is necessary.

112176

Modality: E-Poster Scientific Initiation – Non-case Report

Category: CARDIAC ARRHYTHMIAS/ELECTROPHYSIOLOGY/ELECTROCARDIOGRAPHY

## Chagas Cardiomyopathy and its Prevalence as to the Type of Heart Device and its Outcomes

KARLA SANTOS PINTO^1^, Elias Soares Roseira^1^, Rafaela Ferreira dos Santos^1^, José Victor de Sá Santos^1^, GABRIEL SAPUCAIA DA SILVA DE QUEIROZ^1^, Pollianna Roriz^1^, Luiz Carlos Passos^1^, William de Carvalho^1^

(1) Hospital Ana Nery

**Introduction:** Chagas disease (CD) is the fourth disease with the greatest social impact in Latin America. It is associated with ventricular tachyarrhythmias – an important cause of sudden death (SD). Thus, the implantable cardioverter-defibrillator (ICD) appears as a treatment option for being able to reverse these arrhythmias. Despite this, there are gaps in the indication of the device with a focus on this population, especially in the context of primary prevention.

**Objective:** To describe the profile of CD patients with ICD implantation, prevalence of triggered therapies and post-implantation outcomes.

**Methods:** Descriptive study including patients treated at a tertiary hospital in Salvador-BA diagnosed with Chagas Disease, between January/2021 to April/2022. The variables described were age, sex, NYHA functional class, previous comorbidities, medications, Rassi score (in primary prevention), ICD indication for primary or secondary prophylaxis, number of ICD electrodes, Maggic ICD therapy score and deaths.

**Results:** Sample (n) included 234 CD patients, with a mean age of 60 years, 63.7% of whom were men. The most prevalent previous comorbidities were systemic arterial hypertension (61.1%) and type II diabetes mellitus (14.5%). 27.8% had atrial fibrillation, 15.4% and 9.4% had a history of AMI and CVA, respectively. 62% were classified as NYHA II. 90.2% implanted an ICD for secondary prophylaxis. 76.5% implanted a bicameral ICD. 13.7% had a history of arrhythmic syncope. 40.6% had episodes of unstable ventricular tachycardia (VT). 20.1% suffered cardiorespiratory arrest due to ventricular fibrillation or VT. 85, 8% did not present therapies applied by the ICD, in 13.8% appropriate therapies were applied; 0.4% took inappropriate therapies. 62.7% had high mortality by the Rassi Score. The average Maggic score was 13 points for 1 year and 29 points for 3 years. There was death in 19.7%.

**Conclusion:** Most patients who underwent ICD implantation were for secondary prevention, but there is room for primary prevention. This pathology continues to add high morbidity and cost to its treatment (like ICD), and it is important to act at all levels of care to minimize deaths from CD.

112179

Modality: E-Poster Scientific Initiation – Non-case Report

Category: COVID-19 AND CARDIOVASCULAR SYSTEM

## Mortality in Patients with Cardiometabolic Disease During the COVID-19 Pandemic: A Systematic Review and Meta-Analysis

GABRIELLA DE SOUSA CARBALLOSA GONZÁLEZ^1^, Elisabeth Uchoa de Melo^1^, Érika Thienne Lopes da Silva^1^, Luciana Andrade Tavares^1^, José Kaellyson Barbosa dos Santos Oliveira^1^, Carolline Araujo^1^

(1) Faculdade de Medicina de Olinda

The COVID-19 pandemic, responsible for the biggest health crisis today, has caused more than 6 million deaths worldwide, constituting a serious public health problem. Cardiovascular diseases, such as hypertension and dyslipidemia, associated with diabetes mellitus and obesity, characterizing the metabolic syndrome, have been associated with severe forms of the disease and death. We investigated mortality in patients with SARS-COV-2 infection and its association with cardiometabolic diseases. This is a systematic review and meta-analysis following the PRISMA recommendation. Cohort, case-control and cross-sectional studies on the investigated topic were considered. The MEDLINE/EBSCO, Cochrane Library, Pubmed and SciELO databases were consulted using strategies limited to English, Spanish and Portuguese, using the descriptors available in MeSH (Medical Subject Headings) and DeCS (Health Sciencies Descriptors) as keywords. The meta-analysis involved all studies that presented a measure of association (Odds ratio, relative risk and others) in their results. Heterogeneity was assessed using Pearson’s chi-square test. A p-value was adopted, at a significance level of 5%. Data analysis was performed using Stata software. From this search, 24 articles were included. The median number of people among the analyzed works was 382 (P25 = 154/P75 = 894) and involved older individuals with a mean age of 56 years (dP = 11.4), predominantly male (63.6%). Covid-19 infection was confirmed using the RT-PCR molecular test. The mortality rate in this population was 18.5%. Among the cardiometabolic comorbidities that were significantly associated with mortality (p < 0.005), hypertension was more prevalent (25%), followed by diabetes (19.9%), obesity (18.7%) and dyslipidemia (9.35%). The presence of cardiometabolic comorbidities constituted factors associated with mortality in patients with SARS-COV2 infection. Future studies are needed to accurately determine the pathogenic mechanism involving these patients, especially hypertensive, diabetic and obese men, and the development of severe forms of COVID-19 infection.

112194

Modality: E-Poster Scientific Initiation – Non-case Report

Category: CARDIOVASCULAR SURGERY

## Post Anesthesia Recovery After Preemptive Analgesia with Pregabalin on Cardiac Surgery: Randomized Controlled Trial – First Partial Analysis of the Pregaba-Heart Study

ANA BEATRIZ VENANCIO DE PAULA BEZERRA^1^, Fabio Antonio Serra de Lima Junior^1^, David Cesarino de Sousa^1^, Renan Furtado de Almeida Mendes^1^, Raphael Patrik Borges da Costa^1^, Ana Gabriela Venancio de Paula Bezerra^2^, Joao Bosco Ferreira Gadelha^3^, Tiago Lucena de Brito Pereira^3^, Isaac Newton Guimarães Andrade^1^, Andre Telis de Vilela Araujo^1^

(1) Universidade Federal da Paraíba; (2) Faculdade de Medicina Nova Esperança; (3) Clínica Dom Rodrigo

**Introduction:** Pain is one of the main morbidity factors associated with heart surgery, affecting about 50% of patients. Sternotomy can cause intense pain in the postoperative period, most commonly in the first 7 days, and can lead to significant short and long term consequences. Among the complications we can mention postoperative delirium, poor prognosis, increased length of stay in hospital and in the intensive care unit or in the post-anesthesia recovery unit, increased treatment costs and increased mortality. Few controlled and randomized clinical trials have addressed the efficacy of pregabalin administration in the perioperative period for pain control or recovery after surgery, obtaining variable results.

**Objectives:** This study aims to evaluate the partial results if the preemptive use of pregabalin reduces the perception of pain in the patient undergoing heart surgery in the first 72 hours.

**Results:** 23 patients submitted to cardiac surgery were randomized into two groups: 11 of them used preemptively 150 mg pregabalin starting 1 hour before surgery until the 3° postoperative day, the other 12 used a placebo for the same period. After extubation they were evaluated in 4 moments for the visual analog scale (VAS) and clinical parameters, at the 2° postoperative day the Qor-40 was applied. After analisis 70% were men, 78,2% had elevated blood-pressure, 39,1% had coronary arterial disease and 30,4% were diabetic. In relation to the clinical parameters studied, we verified that none showed statistically relevant differences between the use of pregabalin or placebo, but the pCO2 was the only one that was able to show a certain tendency that the pregabalin group accumulated less CO2 in the postoperative period (p = 0,074), which could mean a better thoracic expansion and therefore, less pain. The intervention group presented a mean QoR-40 of 180,17 + 17,88, while the control, 183,80 + 9,17 (p = 0,692). Revealing a non statistically significant better post-anesthesia recovery in the control group, which may be explained by an attrition bias while questioning, aimed to be ratified after team training in the remainder of this research.

**Conclusion:** This partial analysis suggests that there may be a difference in the preemptive use of pregabalin, but it may be better verified in a bigger sample of patients.

112195

Modality: E-Poster Scientific Initiation – Non-case Report

Category: EPIDEMIOLOGY AND HEALTH POLICIES/GLOBAL HEALTH

## The Prevalence of Death Resulting from the Specific Hypertensive Disease of Pregnancy (DHGE) in the Last Decade in Sus

YURI MAGALHÃES FERNANDES^1^, Bárbara Bernardes Magalhães^1^, Maria Inês Alves Brasil^1^, Rafaela Frota Malheiro^2^

(1) Universidade Estadual do Sudoeste da Bahia – Campus de Vitória da Conquista (Bahia); (2) Faculdade Santo Agostinho – Campus Vitória da Conquista (Bahia)

**Introduction:** The gestational hypertensive syndrome (defined as systolic blood pressure ≥140 mmHg and/or diastolic blood pressure ≥90 mmHg between 20 weeks of gestation and eight weeks postpartum) as a quite prevalent condition in the Brazilian population, affecting about 10% of pregnant women in the country. Furthermore, data from the Ministry of Health shows that maternal mortality in the country related to Pregnancy-Specific Hypertensive Disease (DHGE) amounts to about 35% of all maternal deaths while perinatal mortality reaches about 150 for every 1000 deliveries performed. For this, it is necessary to know the prevalence of deaths and hospitalizations related to the pregnancy-specific hypertensive disease (DHGE) in the last decade (2011–2020) in the unified health system (SUS), in order to know a little more about these comorbidities that affects many women during their pregnancy.

**Objectives:** To analyze the prevalence of deaths and hospitalizations due to pregnancy-specific hypertensive disease in Brazil in the last decade (2011–2020) in the single health system (SUS).

**Methods:** This is a retrospective cross-sectional study of secondary data made available by the Department of Informatics of the SUS (DATASUS) about mortality and hospitalization for pregnancy-specific hypertensive disease (DHGE) In Brazil in the period of years 2011 to 2020.

**Results:** During this period (2011 to 2020) there were 1198 deaths across the country from pregnancy-specific hypertensive disease. The most recent data show the northeast and southeast regions with the largest number of deaths (420 and 421, respectively). It is worth mentioning the increase in the number of cases in 2011 (116) compared to 2020 (146) and the upward trend in the 2 previous years analyzed (2019, 2018), with, respectively, 118 and 112 deaths.

**Conclusions:** The analysis of the data collected and the epidemiological knowledge about DHGE that the rate of women who still die as a result of this pathology is still high in our country. However, the most recent rates (2020 and 2019) show a great increase in relation to the previous years analyzed, leading to speculations about interrupted care or little assistance in this period, which coincides with the COVID-19 pandemic. In this sense, the analysis of these data is an important tool for understanding the national reality about care and deaths from the DHGE, as well as stimulates intervention formulation to reduce mortality in these patients.

112208

Modality: E-Poster Scientific Initiation – Non-case Report

Category: CONGENITAL AND PEDIATRIC CARDIOLOGY

## Pulse Oximetry, Echocardiography, and Active Search for Patients: Screening for Congenital Heart Disease in the State of Paraíba

LÍVIA FARIAS DE HOLANDA FURTADO^1^, João Victor Bezerra Ramos^1^, Patrícia Oliveira Lima de Macedo^1^, Ingrid Gabriele de Souza^1^, Maria Gabriela Medeiros Cunha de Araújo^1^, Dionarte Dantas Araújo^3^, Lara Andrade Dantas^3^, Fabrício Leite Pereira^2^, Cláudio Teixeira Regis^4^, Juliana Sousa Soares de Araújo^1^

(1) UFPB – Universidade Federal da Paraíba; (2) Hospital Metropolitano Dom José Maria Pires; (3) Rede Cuidar; (4) Complexo Pediátrico Arlinda Marques

**Introduction:** The Rede Cuidar is a health care strategy developed in Paraíba since 2018 that has as one of its lines to ensure the therapeutic care and follow-up of children with congenital heart disease in the state. The network is a primary strategy for the early identification, correct care, and follow-up of this population in the state. For this it uses three strategies: pulse oximetry test, screening echocardiogram, and active search through the caravan in 13 cities in the state, for early identification of congenital heart disease and thus reducing mortality.

**Objective:** To describe the incidence of cardiac alterations through pulse oximetry, echocardiogram and active search of patients in Paraíba.

**Method:** This is a retrospective epidemiological study, of observational nature, with patients screened by the Rede Cuidar between the years 2020 and 2021 through pulse oximetry, echocardiogram and active search of patients in Paraíba.

**Results:** Over one year (November/2020 to October/2021), in 18 hospitals and maternity hospitals in Paraíba, 34978 pulse oximetries were performed, identifying 150 patients with indication for echocardiogram, which represents an incidence of 4.2 for every 1000 live births. Of those, 67 were female (44.7%), 82 (54.7%) male, and 1 (0.6%) intersex. Still, 110 (73.3%) were adequate, 19 (12.7%) large and 12 (8%) small for gestational age, 9 missing information. Throughout 2021, 379 echocardiograms were performed, of which 253 (66.7%) were abnormal, 4 (1.1%) had an inconclusive report, and 122 (32.2%) were normal. In addition, by May 2020, 596 heart patients were followed in the 13 Heart Rooms, of these 12.6% had complex type heart disease and 10.4% had obstructive type heart disease.

**Conclusions:** One realizes, then, the importance of the strategy created in the state of Paraíba for early detection of children with heart disease, not only by traditional methods, but by actively searching in areas with difficulty in accessing a specialized health service.

112217

Modality: E-Poster Scientific Initiation – Non-case Report

Category: HYPERTENSION/RENAL DENERVATION

## Pressure Control and Safety Profile of Antihypertensive Agents in the Puerperium: A Systematic Review

FABIO ANTONIO SERRA DE LIMA JUNIOR^1^, Ana Beatriz Venancio de Paula Bezerra^1^, Ana Beatriz Torres Figueiredo de Lacerda^1^, Isaac Newton Guimarães Andrade^1^, André Telis de Vilela Araújo^1^

(1) Universidade Federal da Paraiba

**Introduction:** In puerperal women who had gestational hypertension, there is uncertainty regarding the choice of maintaining methyldopa or replacing it with other drugs.

**Objectives:** This review seeks to evaluate the efficacy of different antihypertensive agents tested during the puerperium on pressure control and their safety.

**Methodology:** MEDLINE, Embase, LILACS, Web of Science, CENTRAL and ClinicalTrials were searched for articles with terms associated with postpartum period and hypertension, eclampsia or pre-eclampsia. Randomized clinical trials that evaluated puerperal women who used antihypertensive agents during the puerperium and had their blood pressure monitored during the period were included. The primary endpoint was blood pressure control, and the secondary endpoints were incidence of hypertensive spikes and medication-associated adverse events.

**Results:** Initially, 2470 studies were found, which were filtered by title and abstract and full text. After screening by the PRISMA method, 11 studies were included. In patients with severe preeclampsia, the most studied antihypertensive was nifedipine, which showed benefits in mean arterial pressure control and renal perfusion compared to placebo, was similar to methyldopa in duration of treatment and in need for other agents, but inferior to diltiazem and labetalol in both effectiveness and hypertensive crisis profile. Furosemide monotherapy may reduce the risk of postpartum hypertension and its faster resolution in puerperae who had non-severe gestational hypertension, while in those who had severe preeclampsia, its association with nifedipine may reduce the need for other doses of antihypertensive agents. Protocols with different durations of magnesium sulfate in the postpartum period have been evaluated, and maintaining it for 6 hours may be as effective as 24 hours, but reducing the time for bladder catheterization, the beginning of ambulation, and contact with the newborn. Clonidine and captopril had similar efficacy and safety in puerperae who had severe preeclampsia. Labetalol and nifedipine are equally effective, although the former is associated with fewer hypertensive peaks. Studies comparing methyldopa to captopril and magnesium sulfate are ongoing.

**Conclusion:** Antihypertensives in the puerperium of gestational hypertension should be chosen according to the patient’s clinical pattern, service availability, and desired profile of pressure control and side effects.

112219

Modality: E-Poster Scientific Initiation – Non-case Report

Category: CONGENITAL AND PEDIATRIC CARDIOLOGY

## Analysis of the Active Search for Children with Congenital Heart Disease in the State of Paraíba in the Year 2021

MARIA GABRIELA MEDEIROS CUNHA DE ARAUJO^1^, João Victor Bezerra Ramos^1^, Ana Quezia Bezerra de Holanda Sousa^1^, Júlia de Melo Nunes^1^, Lucas Emmanuel Freitas Mendes^1^, Pedro Nascimento Araujo Brito^1^, Patrícia Oliveira Lima de Macedo^1^, Dionarte Dantas Araujo^2^, Lara Andrade Dantas^2^, Fabricio Leite Pereira^3^, Juliana Sousa Soares de Araújo^2^

(1) Universidade Federal da Paraíba; (2) REDE CUIDAR; (3) Hospital Metropolitano Dom José Rodrigues

**Introduction:** In Brazil, 28.9 thousand children are born with congenital heart disease per year (1% of the total births). Of these, about 80% (23.8 thousand) need heart surgery, and half need surgery in the first year of life. In the context of the state of Paraíba, until 2010, there was no qualified team in the area of cardiopediatrics. Knowing this, pediatric cardiology network of services was created to work in heart disease screening programs, in order to reduce late diagnosis. To this end, the Caravan of the Caring Network is held annually, a screening program held in conjunction with the Secretary of Health of the State, which actively searches for children with heart diseases in 13 cities in the state, and refers these patients for specialized monitoring.

**Objective:** To analyze the active search for children with congenital heart disease in the state of Paraíba in the year 2021.

**Methods:** This is a retrospective descriptive epidemiological study referring to the records obtained by the Caravan in the year 2021. Between November and December 2021, the action traveled through the cities of Paraíba: Monteiro; Princesa Isabel; Itaporanga; Cajazeiras; Sousa; Catolé do Rocha; Pombal; Patos; Queimadas; Picuí; Guarabira; Mamanguape; Itabaiana. In these records, information about the echocardiogram performed on patients and the diagnosis defined by professionals were observed, in order to allow the categorizations: shunt heart disease; complex heart disease; obstructive heart disease; acquired heart disease, and arrhythmias.

**Results:** In 2021, the total number of visits was 6291. About 92 children (1.4%) were diagnosed with some heart disease. Analyzing the diagnosis of those children, 56 (60.86%) have shunt heart disease, 17 (18.47%) have obstructive heart disease, 8 (8.69%) have acquired heart disease, 6 (6.52%) have complex heart disease and 5 (5.43%) arrhythmia.

**Conclusion:** Therefore, the relevance of the Caravan of the Caring Network as a mechanism for the detection and diagnosis of congenital heart diseases in Paraíba was evidenced, being important for the epidemiological monitoring of the State and for ensuring early care for children with heart disease. From this perspective, a higher incidence of shunt-type heart diseases was observed in the 2021 cut-off, included in the study, representing about 60% of the diagnoses performed.

107873

Modality: E-Poster Scientific Initiation – Case Report

Category: PERICARDIUM/ENDOCARDIUM/VALVOPATHIES

## Pericarditis and Pericardial Effusion Secondary to Methotrexate Treatment: The First Case Report in Brazil of this Rare Side Effect

MONIREH ZIMMERMANN RAMEZANALI^1^, Amanda Cristina dos Santos^1^, Gabrielle Purnhagen^1^, Natasha Silva Constancio^2^, Caroline de Oliveira Fischer Bacca^1^

(1) UNIDAVI – Centro Universitário para o Desenvolvimento do Alto Vale do Itajaí; (2) Hospital Regional Alto Vale

**Introduction:** Methotrexate (MTX) is a folic acid analogue that inhibits DNA synthesis by causing an acute intracellular deficiency of folate coenzymes. It is a pillar in the treatment of various immune-mediated diseases. Among the side effects of the MTX therapy, mucositis and hematological symptoms are common. Besides, serositis is also a major adverse effect, presenting as pneumonia and pleural effusion. Pericarditis and pericardial effusion (PE) are uncommon ones. We present the first clinical case in Brazil of MTX-induced pericarditis with PE.

**Case Report:** 67-year-old man, with systemic atherosclerosis, started a dermatologic treatment for cutaneous psoriasis with MTX 15 mg/week. After 30 days, he complained of chest pain radiating to the right shoulder, dyspnea and hypotension. Echocardiography (ECHO) showed a moderate PE (14 millimeters), without cardiac tamponade signals. Computerized tomography revealed PE around the cardiac area, with no evidence of lynphonodes, neoplastic lesions nor infections, including tuberculosis. During hospitalization, PE was aspirated and a pericardial biopsy was made. The fluid did not reveal any abnormalities and biopsy showed a chronic inflammatory infiltrate. All the laboratory tests for rheumatoid diseases were within normal limits. The suspension of MTX therapy and use of Prednisone 40 mg daily produced improvement in symptoms within 14 days and follow-up ECHO showed complete resolution of the PE.

**Conclusion:** Although extremely rare, pericarditis and PE might be consequences of MTX treatment. For the right diagnosis, a careful analysis should be undertaken to discontinue the drug therapy. The use of ECHO provides important information on the severity of the clinical condition. The patient was treated with MTX withdrawal and steroid therapy.


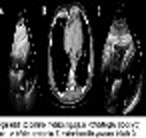


108354

Modality: E-Poster Scientific Initiation – Case Report

Category: CARDIOVASCULAR IMAGING

## Renovascular Hypertension Associated with an Anomalous Intrathoracic Originated Renal Artery

LARISSA ARAÚJO DE LUCENA^1^, Felipe Leite Guedes^2^, Fabiano César de Medeiros Júnior^1^, Márcio Vilar de Freitas^2^, Rodrigo Azevedo de Oliveira^1^

(1) Universidade Federal do Rio Grande do Norte; (2) Hospital Universitário Onofre Lopes

Renovascular disease (RVD) is responsible for 5.8% of secondary hypertension cases in young adults, caused mainly by obstructive lesions due to either atherosclerotic renal artery stenosis or fibromuscular dysplasia. Although the renal arteries’ supradiaphragmatic origin is exceedingly rare, up to date, three cases of secondary hypertension due to single ectopic renal arteries originating from the thoracic aorta have been reported in patients with customarily positioned kidneys. Herein we describe a case of a 21-year-old man with resistant hypertension whose investigation showed an ectopic right renal artery originated from the internal thoracic artery. Although both renal arteries were free of obstructive lesions, the right one was very long and tortuous, causing kidney hypoperfusion. A bypass surgery between the right renal artery and the aorta was performed uneventfully, leading to better blood press control. To the best of our knowledge, there are no previous reports of secondary hypertension due to the renal artery arising from the internal thoracic artery.


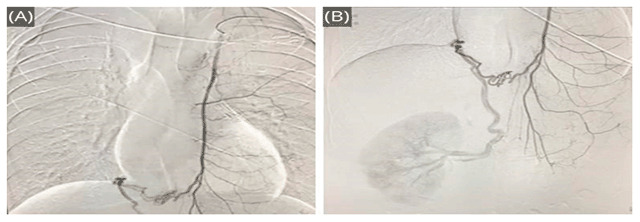


108454

Modality: E-Poster Scientific Initiation – Case Report

Category: COVID-19 AND CARDIOVASCULAR SYSTEM

## Unusual Cases of Remote Monitoring of Implantable Defibrillators During the COVID-19 Pandemic

DAVI SALES PEREIRA GONDIM^3^, Maria Eduarda Quidute Arrais Rocha^3^, Rodrigo Carvalho Paiva^2^, Marcela Albuquerque de Holanda^2^, Eduardo Arrais Rocha^4^

(1) Universidade Federal do Ceará-UFC; (2) Universidade Unichristus; (3) Universidade de Fortaleza-UNIFOR; (4) Centro de Arritmia do Ceará-CACE

**Introduction:** Implantable cardiac defibrillators (ICDs) represent the most effective therapy in the treatment of severe ventricular arrhythmias. Routine follow-up with periodic scheduled office visits may not be sufficient due to the complexity of the pathologies. The COVID-19 pandemic determined more limitations in this follow-up. In this work, we report a series of cases in which remote monitoring (RM) of ICDs were of great importance in treatment.

**Case 1:** Male patient, 65 years old, with ischemic cardiomyopathy, sustained ventricular tachycardia (SVT), with ICD implantation after reversed cardiac arrest. After 7 years, changes in the impedance of the shock electrode were detected, information transmitted remotely, requiring reprogramming in the shock vector. After 6 months, informations of changes in impedance of stimulation was received by e-mail as a red alert. Implantation of a new electrode was necessary. During the follow-up, he presented several SVTs and atrial fibrillation (AF), needing pharmacological adjustment and just one office visit for reprogramming.

**Case 2:** Female patient, 91 years old, with ICD with multisite pacemaker due to dilated cardiomyopathy. During acupuncture, there was an ICD shock. The professional had inadvertently used electro-acupuncture. Due to portable RM, the information was transmitted to the physician, who detected an inappropriate therapy and advised the professional not to use electrical stimulation during acupuncture.

**Case 3:** Female patient, 72 years old, with ischemic and valvar cardiomyopathy, hospitalized with SVT, when was implanted an ICD. After starting antiarrhythmics drugs, no new arrhythmias occurred in the short term. After months, she presented with several SVTs, some with high rate, reversed with anti-tachycardia pacing (ATP) and others SVTs with low rates, outside the therapy zones. AF was also detected, inappropriately recognized by the ICD as SVT and treated with inappropriate ATP therapies. Therapeutic adjustments were made and on 2 occasions there was a need for immediate reprogramming of the ICD.

**Discussion and Conclusion:** RM of patients with ICD during the COVID-19 pandemic allowed therapeutic optimization, reduction of scheduled office visits, hospitalizations and inappropriate shocks, with an improvement in the treatment and in the quality of life.

108936

Modality: E-Poster Scientific Initiation – Case Report

Category: CARDIOVASCULAR SURGERY

## Surgical Excision of a Large Left Ventricular Aneurysm Secondary to Chagas Cardiomyopathy Leading to Significant Clinical and Morphofunctional Improvement

MIKE VINICIUS CANTO DE ANDRADE^1^, Natália Barreira Silva^1^, Rodrigo Penha de Almeida^1^, Paulo Cesar Santos^1^, João Lucas O‘Connell^1^

(1) Universidade Federal de Uberlândia

**Introduction:** Left ventricular aneurysm (LVA) can be defined as a thin and dilated portion of the left ventricle (LV) due to the predominance of fibrosis and scar area. This phenomenon generates contractile disorders and, consequently, akinetic or dyskinetic ventricular segments observed during systole. They may develop after myocardial infarctions and secondary to some cardiomyopathies, such as chronic Chagas cardiomyopathy.

**Case Description:** Female patient, 61 years old, with Chagas cardiomyopathy and heart failure (HF) functional class III (NYHA) with low tolerance to drug therapy optimization due to arterial hypotension. A transthoracic echocardiogram and cardiac catheterization were performed. The exams identified significant left ventricular dysfunction with an image of a large aneurysm associated with a thrombus at the LV apex. Pointing out the refractoriness to clinical treatment, along with complex ventricular arrhythmias on Holter and the presence of a well-defined base, it was decided to perform an LV aneurysmectomy. A significant improvement was observed in the patient’s clinical state in the subsequent months. Currently, 2 years after the surgical treatment, he is in functional class I (NYHA), using Warfarin 5 mg/day, Enalapril 10 mg/day, Carvedilol 25 mg/day and Spironolactone 25 mg/day. There was a significant reduction in the size of the left ventricular cavity and a significant improvement in ventricular function (currently with normal LVEF). Not only pre- and post-surgical morphofunctional data from echocardiograms and holter will be presented, but also preoperative ventriculography and postoperative magnetic resonance imaging.

**Conclusion:** The treatment of patients with LV aneurysms is eminently clinical, through the optimization of drug therapy for HF. However, the surgical indication of aneurysmectomy should be considered for cases that develop refractory HF, angina, complex ventricular arrhythmia and systemic embolization of intraventricular thrombi. The reported case shows that the performance of aneurysmectomy, with preservation of viable myocardium in the basal and middle portions of the LV, is capable of significantly reducing the ventricular cavity and promoting a significant improvement in the global contractile function of the LV in selected cases. Surgical treatment can also provide an important improvement in the patient’s clinical condition.

108550

Modality: E-Poster Scientific Initiation – Case Report

Category: CONGENITAL AND PEDIATRIC CARDIOLOGY

## Pulmonary Artery Sling – Rare Diagnosis in a Wheezing Baby

BERNARDO MUSSI SOARES^1^, Andressa Mussi Soares^2^, Renata de Backer Pacífico^2^, Resi Apolinário^2^, Paulo José Ferreira Soares^2^

(1) Fundação Técnico Educacional Souza Marques – FTESM; (2) Hospital Evangélico de Cachoeiro de Itapemirim – HECI

Pulmonary artery sling – rare diagnosis in a wheezing baby.

**Introduction:** Pulmonary artery (PA) sling is a rare congenital vascular disease (59 cases per million children) in which the left pulmonary artery (LPA) derives from the right pulmonary artery (RPA), forming a ring that passes anteriorly to the esophagus and posteriorly to the trachea, which may compress mediastinal structures and cause severe upper airway symptoms in children.

**Case Description:** 2 years old male child, with respiratory distress at birth, evolved with frequent wheezing and tachydyspnea, without improvement. He presented several hospitalizations for respiratory symptoms, being diagnosed with allergy to cow’s milk protein. After evaluation by the immunoallergy, he was referred for echocardiographic evaluation. The echocardiogram (Echo) detected the APE emerging from the posterior wall of the RPA that originates from the pulmonary trunk and has a usual course, and the diagnosis of AP sling was made. Therefore, CT angiography of the chest was performed to better evaluate possible compressions in the trachea and esophagus. APE emerging from the dorsal surface of the RPA was detected, passing inferiorly to the origin of the upper lobar bronchus that exits directly from the trachea and over the right main bronchus, forming the vascular ring that causes a moderate degree of stenosis of the distal trachea. In addition, the APD presents an anatomical variation with anomalous segmentation at the hilum, where it trifurcates forming three main arterial trunks. This data is especially important at the time of the surgical approach to avoid incidental complications. CT angiography was performed with reconstruction focused on the air phase, allowing an excellent assessment of tracheal stenosis and thoracic esophageal distention. The child has just been diagnosed and will undergo surgery.

**Conclusions:** Due to the non-specific symptoms, the clinical diagnosis can be difficult and delayed, compromising the general condition and in some cases leading to death in the first months of life. Echo performed in detail is a robust tool for diagnosing PA sling. The other exams offer a better assessment of the relationships with neighboring structures. With an early diagnosis and surgical correction, the prognosis is generally good, but the anatomy of the structures must be very well evaluated because the risk of death during surgery increases when there is significant bronchial or tracheal stenosis.

108571

Modality: E-Poster Scientific Initiation – Case Report

Category: CARDIOVASCULAR SURGERY

## Importance of Cerebral Oximetry Monitoring in Cardiac Surgery with Cardiopulmonary Bypass: Case Report

RODRIGO BATISTA WARPECHOWSKI^1^, Emmanuela Flavia Alves Pinto^2^, Fabiana Rodrigues Philippsen^2^, Paulo Warpechowski^2^, Tiago Luz Leiria^1^

(1) Instituto de Cardiologia do Rio Grande do Sul; (2) Sociedade de Anestesiologia (SANE)

In cardiopulmonary bypass (CPB) surgeries, adverse events such as desaturation, hypoflow, hypoxia and brain injury may occur. Such situations may lead to complications such as cognitive decline (CO), observed in up to 50% of the patients. Herein, we present a case of hypoxia during cardiac surgery with CPB identified using the INVOS™ 5100 C Cerebral/Somatic Oximeter. Male patient, 77 years old, 80 kg, hypertensive, with root and ascending aortic aneurysm and aortic valve insufficiency. Scheduled aneurysm repair and valve replacement. No previous neurological deficits. Regional saturation (INVOS™) and anesthetic depth (Bispectral Index-BIS) monitors were installed. Regional brain O2 saturation (rSO2) was between 65–80 and BIS between 30–60 throughout the process. At thirty minutes of CPB, there was an abrupt drop in rSO2 (Image 1), reaching critical values <40, a drop in mean arterial pressure and a BIS reduction, with high suppression rates. Arterial blood gases revealed severe hypoxemia (PO2 of 40 mmHg and SO2 of 62%). A failure in the CPB gas mixer was identified by the perfusionist and it was replaced. Subsequently, rSO2 returns to values >55. The CPB time was 138 min, and aortic aneurysm repair was performed using the Bentall de Bono procedure. The patient showed no neurological damage and progressed satisfactorily, being discharged from the ICU after 3 days and discharged from the hospital 12 days after surgery. Studies such as those by Murkin et al and Slater et al, which evaluated the effectiveness of rSO2 monitoring by INVOS™, demonstrate that intraoperative cerebral O2 desaturation is significantly associated with an increased risk of CD and prolonged hospital stay after CPB, and its treatment was associated with a shorter length of stay in the ICU and a significantly reduced incidence of morbidity and mortality. Despite performing preventive maintenance procedures, equipment failure may occur. Thus, it is imperative that the anesthesiologist is aware and uses all available monitoring means to recognize possible failures and so that management can be carried out in the best way as possible.


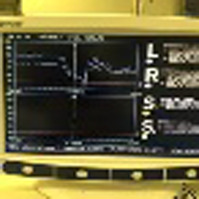


108581

Modality: E-Poster Scientific Initiation – Case Report

Category: CONGENITAL AND PEDIATRIC CARDIOLOGY

## Congenital Heart Disease and Its Association with the Fetal Valproate Syndrome

ISADORA BUELONI GHIORZI^1^, Adriano Louro Moreira^1^, Juliana Bergmann^1^, Mateus dos Santos Taiarol^1^, Marina da Rocha Besson^1^

(1) Universidade Federal de Ciências da Saúde de Porto Alegre

**Introduction:** Valproic acid is a medication frequently used in the treatment of epilepsy, and, among anticonvulsants, it has the highest incidence of major malformations. Our aim was to report a patient with fetal valproate syndrome (FVS) presenting congenital heart disease.

**Case Description:** The patient was the third child of a mother diagnosed with epilepsy. She used valproic acid (up to the third month) and phenobarbital (from the third month onwards) during pregnancy. She reported episodes of frequent seizures until the end of the third month of pregnancy. The female child was born by vaginal delivery, premature at 35 weeks, weighing 2450 grams and with Apgar scores of 8 at first minute and 9 at fifth minute. She had heart failure after birth. Echocardiography identified perimembranous ventricular septal defect (VSD) with a wide outflow tract, mild peripheral pulmonary stenosis, and left superior vena cava persistence draining into the coronary sinus. The evaluation also showed growth retardation, keeled skull, epicanthal folds, bilateral tear duct obstruction, well-marked infraorbital crease, anteverted nostrils, long philtrum, thin upper lip, retroverted and low-set ears, and accessory nipples to the right. Head computed tomography confirmed the diagnosis of trigonocephaly. The karyotype was normal. The child underwent surgery to correct the VSD at 8 months of age.

**Conclusions:** The findings presented by the patient were compatible with the diagnosis of FVS. Congenital heart defects may be part of the picture and they have been described in about 25% of patients. The VSDs were less common malformations.

108591

Modality: E-Poster Scientific Initiation – Case Report

Category: CARDIAC ARRHYTHMIAS/ELECTROPHYSIOLOGY/ELECTROCARDIOGRAPHY

## The Tilt Test in Munchhausen Syndrome

MARIA EDUARDA QUIDUTE ARRAIS ROCHA^1^, Pedro Sales Pereira Gondim^3^, Arthur Holanda Dantas^3^, Marcela Albuquerque de Holanda^3^, Camila Pinto Cavalcante Miná^2^

(1) Universidade de Fortaleza (Unifor); (2) Universidade Federal do Ceará (UFC); (3) Centro Universitário Christus (UNICHRISTUS)

The Tilt Test in Munchhausen Syndrome Introduction. The Tilt Test (TT) is a complementary method widely used to investigate the susceptibility to vasovagal phenomena or late orthostatic hypotension. Reproduction of clinical symptoms is essential to the true positivity of the test and its good accuracy. Patients may present with several symptoms during the test, without concomitant hemodynamic changes, called psychogenic response. We described a remarkable case of a patient with a suspected diagnosis of Munchhausen Syndrome, a more severe form of factitious disorder self-imposed, confirmed during the TT.

**Case:** Female patient, 22 years old, with a history of more than ten years of multiple symptoms of loss of consciousness, intensification in recent years, descriptions and videos showing tonic movements, with deviation of looking up, pallor, falls, palpitations, causing a worsening in her quality of life. She had a history of childhood sexual abuse, dependence on the use of morphine and psychotropic drugs for the treatment of fibromyalgia, herniated disk and depression. There were several hospitalizations, with suspected diagnoses of pheochromocytoma, hyperthyroidism, adrenal insufficiency, autoimmune diseases and dysautonomia, when she was referred to syncope unit for investigation. There was a hypothesis of her psychiatrist of the syndrome of post traumatic disorder. Laboratory tests showed nonspecific findings. The cardiologic tests: sinus tachycardia 120–151 bpm, even when lying down; normal 24-hour Holter with RR variability showing preserved autonomic balance. Neurologic tests were normal. In 2020, she underwent TT with a report of pseudo syncope. In 2021, during a recorded TT, there was full reproduction of the symptoms, without any correlation with hemodynamic changes. The diagnosis was made according to the criteria of the manual diagnostic for mental disorders (DSM-5), and she was forwarded to the psychiatrist. In the evolution, the patient no longer returned for revaluation, a situation described in this syndrome after confirmation of the diagnosis, remaining meanwhile with evaluations by other professionals.

**Discussion and Conclusion:** This work highlights the importance of knowledge of Munchhausen Syndrome due to the wealth and multiple symptoms presented and the role of the Tilt Test in excluding other etiologies, since the diagnosis is rarely done by the psychiatrist, but mainly by the specialist.

109134

Modality: E-Poster Scientific Initiation – Case Report

Category: COVID-19 AND CARDIOVASCULAR SYSTEM

## Acute Decompensation of Heart Failure by COVID-19 with Need for Heart Transplant: A Case Report

CAROLINA TISOTT BURTET^1^, Amanda Hillesheim Schuck^1^, Maria Constanza Cé Erig^1^, Rafaella Magni Berthier^1^, Olga Sergueevna Tairova^1^

(1) Universidade de Caxias do Sul (UCS)

**Introduction:** Coronavirus disease (COVID-19) is an infectious disease which is caused by severe acute respiratory syndrome coronavirus 2 (SARS-CoV-2). Patients with previously established comorbidities, such as heart failure, are at particularly high risk of morbidity and mortality from this infection – they generally have a worse prognosis and a mortality rate of more than 10%. Studies suggest that viral infections can exacerbate a preexisting cardiac insufficiency, with multiple mechanisms responsible for triggering and aggravating this process.

**Case Description:** Female patient, 34 years old, without comorbidities. History of mild blood pressure increase and left ventricular enlargement since 2015. Progression of loss of ejection fraction (EF) up to 20% after COVID-19 infection in December 2020. Evolved with worsening of the general condition, acute heart failure and need for sequential dialysis. After the condition, the patient became a candidate to receive a heart transplant, which was performed in February 2021, with good postoperative clinical evolution. Complementary tests after transplant: Electrocardiogram with regular rhythm, HR 95, without alterations; Echocardiogram with improvement in EF (69%), anomalous movement of the interventricular septum and left atrial enlargement.

**Stress Test:** Distance covered 0.86 km; Duration 11 minutes; maximum HR 129; Max Vo2 15.44; Maximum power: 29.9 W; NYHA III functional group; Also showing a very low cardiorespiratory fitness (AHA). The patient has been followed up on a weekly basis for physical and cardiopulmonary rehabilitation at the Sports Medicine outpatient clinic, with good evolution.

**Conclusion:** Acute infections result in an inflammatory cascade, leading to a severe inflammatory storm that can exaggerate the initial injury. Furthermore, it is already documented that increased metabolic demand can lead to cardiac depression and new-onset heart failure or acute decompensation of chronic cardiac insufficiency. The case above describes a severe acute decompensation in a young patient, without associated comorbidities with the need for immediate heart transplantation. Thus, we can conclude that it is essential to understand the interactions between heart disease and the virus to prevent unfavorable outcomes like this one and improve the management of these patients. In addition, there are yet few reports in the literature that demonstrate the need for heart transplantation after COVID-19.

108771

Modality: E-Poster Scientific Initiation – Case Report

Category: HYPERTENSION/RENAL DENERVATION

## Renal Artery Angioplasty and Stent Implantation in Patient with Refractory Arterial Hypertension Associated to Low Back Pain and Progressive Renal Dysfunction

DANIELLE CAMPOS DE ALMEIDA^1^, Benara Otoni de Siqueira^1^, Ebes Kléofas da Silva Magalhães^1^, Gabriel Alves Meneses^1^, João Lucas O’Connell^1^

(1) Faculdade de Medicina, Universidade Federal de Uberlândia

**Introduction:** Renal Artery Stenosis (RAS) in elderly is, in most cases, a consequence of a progressive atherosclerosis, that may be associated with other blood vessels atherosclerosis. The most common symptoms are related to tough control of blood pressure (an important cause of secondary hypertension) and renal insufficiency. It’s described a patient’s case with multiple comorbidities and systemic atherosclerosis, evolving with refractory arterial hypertension, low back pain and progressive renal disease, even as relevant right renal artery stenosis.

**Case Description:** 75-years-old woman, smoker, hipertensive and dyslipidemic, with previous transitory ischemic attack, bilateral carotid stenosis, carotid endarterectomies, acute myocardial infarction, coronary angioplasties and stents implantation, mesenteric artery angioplasty with two stent implantation. Presenting for six months with worsening of blood pressure control (maintaining levels higher than 170 mmHg), right low back colic-like pain episodes, and recent worsening of creatinine levels (from 1,6 to 2,5 mg/dL). It was requested an abdominal aorta angiotomography (and renal arteriography later) that identified important right renal artery stenosis. The Patient was subjected to a angioplasty and successful stent implantation in the right renal artery without any complications. There was improvement of low back pain, blood pressure control and creatinine levels (returning to normal values). Performed new abdominal aorta angiotomography that detected blood vessels permeability and absence of stenosis within previously implanted prosthesis.

**Conclusion:** Renal Artery Stenosis diagnosis can be done by renal artery doppler, renal scintigraphy and abdominal aorta angiotomography. Concerning treatment, literatura still is controversial about drug treatment and percutaneous modalities effectiveness. For some cases, as described here, interventionist approach, renal artery angioplasty and stent implantation, may be mandatory for blood pressure control, pain control, due to a renal ischemia and to avoid renal insufficiency.

108778

Modality: E-Poster Scientific Initiation – Case Report

Category: PERICARDIUM/ENDOCARDIUM/VALVOPATHIES

## Progressive Chest Pain Evolving to Pericardial Effusion and Cardiac Tamponade Due to a Cardiac Suture Needle Perforation Used in Maxillofacial Surgery Decades Before

DANIELLE CAMPOS DE ALMEIDA^1^, Karina Baltor Cabral^1^, Mike Vinicius Canto de Andrade^1^, Paulo Cesar Santos^1^, João Lucas O’Connell^1^

(1) Faculdade de Medicina, Universidade Federal de Uberlândia

**Introduction:** Cardiac perforations usually are due to stab wounds or bladed weapons, firearms or eventually due to chest trauma followed by penetration of foreign bodies inside the chest wall. Although most of cardiac perforations are life threatening, minor lacerations may evolve to tamponade and cardiac surgery can avoid death. Cardiac perforations because of sharp perforation material are rare. Even more uncommon when surgical material was used on procedures outside the chest.

**Case Description:** A female patient of 60-years-old woman had orthognathic surgery 36 year ago and post-surgery identification of a suture needle “forgotten” on cervical region. Comes into the Emergency Room complaining about a progressive chest pain ventilator-dependent, associated with dyspnea. Physical exams evidence pericardial friction rub. Electrocardiograms and myocardial necrosis markers had no major changes. Coronary angiography showed absence of coronary obstruction. However, a suture needle was identified in the topography of the right ventricle (RV). A chest tomography was performed and revealed pericardial effusion and thickening, as well as micro-perforations of RV free wall associated with needle suture image. After two days, the patient evolved with an increase in pericardial effusion and tamponade cardiac signs, requiring emergency surgery aiming to remove the foreign body, followed by ventricule suture and pericardiocentesis. Patient is asymptomatic six months after the event.

**Conclusion:** Cardiac perforations caused by “forgotten” foreign bodies in previous surgeries are rare. Furthermore, if associated with surgical material used in surgeries outside the chest. The needle displacement from cervical region to chest cavity was very likely provoked by shoulders and knees magnetic resonance imaging (MRI) performed four months before. The magnetic field produced by MRI equipaments is capable of shifting ferromagnetic objects first implanted, that may result in tissue injury. The fast and efficient cardiac surgery was essential to manage cardiac tamponade, foreign body removal, RV suture and to save the patient’s live.

108783

Modality: E-Poster Scientific Initiation – Case Report

Category: NEGLECTED CARDIOVASCULAR DISEASES

## Acute Coronary Syndrome Associated with Anomalous Origin of the Right Coronary from the Left Coronary Sinus and Interarterial Course: A Challenging Clinical Scenario

DANIELLE CAMPOS DE ALMEIDA^1^, Alice Mirane Malta Carrijo^1^, Marcela Gomes de Souza^1^, Gabriel Alves Meneses^1^, João Lucas O’Connell^1^

(1) Faculdade de Medicina, Universidade Federal de Uberlândia

**Introduction:** Coronary Artery Anomalies (ACA) are characterized by changes on these vessels origin, course or structure. Compose a rare condition: less than 1% of general population. Within these anomalies, there are anomalous origin of coronary arteries from aorta which can have retroaortic, subpulmonary, pre-pulmonary and interarterial courses. At large, these abnormalities remains asymptomatic until adult age and are incidental findings on complementary exams or necropsy. Yet, it can cause angina, syncope, ischemia, arrhythmia or sudden death, especially in young athletes.

**Case Description:** 56-years-old men, hypertensive, family history of coronary disease, dyslipidemic, pre-diabetic, sedentary and obese. Was admitted in the hospital for evaluation of mild unstable angina. Electrocardiogram (ECG): Sinus rhythm and left ventricular overload signs. There wasn’t any increase on cardiac enzymes. Coronary angiography highlighted the origin of the right coronary from the left coronary sinus, slit ostium and moderate ostial stenosis (for probable extrinsic compression). Left coronary without stenosis. Left ventricules‘ global and segmental systolic function were preserved. Coronary artery angiotomography confirmed inappropriate RCA origin from left valsalva sinus, interarterial course (between aorta and pulmonary artery) and proximal moderate luminal reduction (“slit-like orifice” course). The possibility of surgical treatment has been considered, however it was opted for myocardial scintigraphy which showed normal myocardial perfusion. The conduct taken was drug therapy maintenance. The Patient remains asymptomatic even after two years passed from initial diagnosis.

**Conclusions:** Although anomaly’s interarterial course is associated to higher incidence of sudden death, especially after vigorous exercises, low physical efforts diaries, associated with absence of detectable ischemia on appropriate exam, reduce risk for fatal events, as well corroborates the therapeutic option chosen on this case. Moreover, the absence of angina symptoms after drug treatment also reinforces the initial non-interventionist option for the case in discussion.

108789

Modality: E-Poster Scientific Initiation – Case Report

Category: ACUTE AND CHRONIC CORONARY DISEASE/THROMBOLYSIS

## Acute Myocardial Infarction in a Young Man Caused by Spiral Spontaneous Coronary-Artery Dissection of the Left Coronary Trunk, Anterior Descending and Circumflex: A Surprising Clinical and Angiographic Scenario

ISABELA MARTINS RODRIGUES^1^, Joaquim Adélio de Oliveira Neto^1^, Ana Júlia Carvalho Paulinelli^1^, Gustavo Nahuel Leyes Ontivero^1^, João Lucas O‘Connell^1^

(1) Faculdade de Medicina da Universidade Federal de Uberlândia

**Introduction:** Spontaneous Coronary-Artery Dissection (SCAD) is defined as the rupture of the intima and media layer of an epicardial coronary-artery wall by an intramural hemorrhage and is not secondary to atherosclerotic disease, aortic dissection, or intravascular trauma. SCAD occurs primarily in women, and the predisposing factors include intense emotional stress, intense physical activities (especially isometric exercises), hormone therapy, use of corticosteroids, use of cocaine, and smoking.

**Case Description:** A 39-year-old man, smoker, and cocaine user was referred to our hospital with intense chest pain at rest, associated with dyspnea, for more than 24 hours. He maintained mild chest discomfort at the time of admission and was hemodynamically stable. The electrocardiogram showed sinus rhythm, mild and diffuse ST-segment elevation. Coronary angiography revealed dissection in the left coronary trunk, involving the entire left anterior descending artery and the entire first left marginal branch with distal occlusion of these two vessels. Left ventriculography showed a significantly decreased global left ventricular systolic function, dyskinesia of the anterolateral wall, and akinesia of the apical and inferoapical walls. An attempt was made for coronary angioplasty in marginal branch, without success, because access to the true lumen of the vessel was not possible. Clinical treatment was performed and reassessment was scheduled with a new coronary angiography in 30 days or an emergency cardiac surgery in case of instability of the condition. The patient remained stable, and drug treatment was maintained for Coronary Artery Disease and Heart Failure. He is asymptomatic with partial recovery of ventricular function one year after the initial condition.

**Conclusion:** SCAD is a dramatic clinical and angiographic situation that can lead to death in young individuals without risk factors for coronary artery disease. There is a current trend towards adopting a conservative strategy, especially in those patients without recurrent ischemia. When attempting angioplasty, verification of the correct positioning of the guidewire in the true vessel lumen is mandatory. Stent angioplasty has failure rates that can reach 35.0%. The case reported is a SCAD, probably due to cocaine use, with spiral dissection involving multiple vessels. Despite the severity of the lesions, the conservative approach was successful, and the patient presented a satisfactory clinical response.

108790

Modality: E-Poster Scientific Initiation – Case Report

Category: NEGLECTED CARDIOVASCULAR DISEASES

## Left Ventricular Pseudoaneurysm Rupture: A Tragedy that Must be Avoided

MIKE VINICIUS CANTO DE ANDRADE^1^, Letícia Guimarães Mendonça^1^, Natália Barreira Silva^1^, Eduardo Henrique Costa Vitor^1^, João Lucas O‘Connell^1^

(1) Universidade Federal de Uberlândia

**Introduction:** Often, even patients with a favorable evolution in the first days after an Acute Myocardial Infarction (AMI) can develop important mechanical complications. These complications can be sudden and lethal, such as, for example, left ventricular (LV) free wall rupture. Another serious mechanical complication of patients with AMI is LV pseudoaneurysm, which develops due to rupture of the left ventricular (LV) wall contained by pericardial adhesions. When not diagnosed and treated early, the pseudoaneurysm can also have a catastrophic course.

**Case Description:** Male, 65 years old, presented with non-thrombolyzed inferolateral wall AMI. Coronary angiography, still in the hospital phase: three-vessel obstructive pattern with important and proximal stenosis in the anterior descending and circumflex arteries, in addition to total occlusion of the right coronary artery. Ventriculography showed a large extension of akinetic area in the inferior wall and “image suggestive of aneurysm in the inferior wall of the LV”. He was discharged from hospital on the sixth day; he was advised to look for a cardiac surgeon to schedule coronary artery bypass graft surgery (and possible LV aneurysmectomy). Outpatient echocardiogram (performed on the tenth day after AMI) identified the presence of a pseudoaneurysm of the inferior wall of the LV. Still in the echocardiography room, the patient had a cardiorespiratory arrest in a pulseless electrical activity (PEA) rhythm, which was not reversed after cardiopulmonary resuscitation (CPR) or puncture pericardiocentesis maneuvers. A new echocardiogram during cardiac arrest confirmed an image suggestive of significant pericardial effusion with signs of cardiac tamponade, not identified in the exam performed a few minutes earlier.

**Conclusion:** Left ventricular aneurysms and pseudoaneurysms are types of mechanical complications associated with AMI and the differential diagnosis between one situation and another is still a challenge in clinical practice. Unlike true aneurysms, false aneurysms have a high tendency to rupture and should be operated on soon after diagnosis. This report reinforces the importance of careful analysis of risk factors for the occurrence of mechanical complications after AMI and also of careful analysis of echocardiography and ventriculography images that allow early treatment of this potentially fatal clinical situation when the diagnosis is not made.

108799

Modality: E-Poster Scientific Initiation – Case Report

Category: ACUTE AND CHRONIC CORONARY DISEASE/THROMBOLYSIS

## Sudden and Unprecedented Mental Confusion as the Only Clinical Manifestation of Spontaneous Coronary-Artery Dissection in an Athlete

ISABELA MARTINS RODRIGUES^1^, Rodrigo Penha de Almeida^1^, Dennis Miguel Lemos da Silva^1^, Fabiane Mian de Souza^1^, João Lucas O‘Connell^1^

(1) Faculdade de Medicina da Universidade Federal de Uberlândia

**Introduction:** The occurrence of concomitant association between acute coronary syndrome and stroke is rare. Although several cases have been described in the literature, we did not find any case description of a patient with concomitant cerebrovascular accident (CVA) associated with Spontaneous Coronary-Artery Dissection.

**Case Description:** A 39-year-old man, tennis coach, and player without morbid history or classic risk factors for coronary artery disease presented to our hospital with sudden mental confusion, disorientation, and mild dysarthria, with partial reversal during the 7-day hospital stay. Magnetic Nuclear Brain Resonance showed alterations compatible with stroke. Electrocardiographic and echocardiographic alterations were seen suggestive of acute inferior wall myocardial infarction and led to the performance of coronary angiography, which revealed proximal occlusion of the right coronary artery, receiving grade II coronary collaterals left, without stenosis in other coronary arteries. Left ventriculography showed significant hypokinesia of the inferior wall with preserved global systolic function. We decided not to approach the right coronary artery at that time and the possibility of coronary dissection was considered a pathophysiological mechanism to explain the acute coronary syndrome and mobilization of the coronary thrombus to explain the consequent cerebral embolism. He returned 30 days after the initial condition and identified a recanalized right coronary artery, with subocclusive stenosis in the proximal segment and an image compatible with a long line of coronary dissection. Angioplasty of the right coronary artery was performed with successful implantation of two stents. The patient remains well and without further events two years after the initial event.

**Conclusion:** Several pathophysiological mechanisms can explain the occurrence of simultaneous ischemia in cardiac and cerebral territories. Firstly, the same traditional risk factors act for the genesis and rupture of obstructive atherosclerotic plaques in both territories. Secondly, one can also lead the other which justifies the simultaneous manifestation of the conditions. The initial percutaneous approach to a coronary vessel suspected of having dissection can be postponed to a second moment, in case of clinical stability of the patient. In the case reported, this initial clinical management proved to be very important.

108802

Modality: E-Poster Scientific Initiation – Case Report

Category: ACUTE AND CHRONIC CORONARY DISEASE/THROMBOLYSIS

## Myocardial Infarction Associated with Massive Right Coronary Aneurysm: A Challenging Case

LETÍCIA GUIMARÃES MENDONÇA^1^, Gustavo Nahuel Leyes Ontivero^1^, Victor Custódio Ribeiro^1^, Sthephany Yamaguchi de Melo^1^, João Lucas O‘Connell^1^

(1) Universidade Federal Uberlândia

**Introduction:** Coronary aneurysm (CA) is defined by the presence of a vessel segmental dilatation 1,5 times larger than the adjacent segment or as the largest vessel’s segment with the larger diameter of the vessel. Aneurysms incidence varies between 1,5% a 5% in adults and is more frequent in males. Also, this condition is related to Takayasu, Kawasaki arteritis, Polyarteritis Nodosa, and syphilitic, or previous exposure to trauma, dissection, angioplasty, atherectomy, and herbicides. The role of the classical risk factors in the genesis of aneurysms is controversial.

**Case Description:** Male, 49 years, class 3 obesity, hypertensive. Appeared with Non-ST segment elevation myocardial infarction (NSTEMI) of the inferior wall. Heart catheterization showed ectasias along the entire length of the right coronary, an aneurysm (vessel maximum diameter 12 mm), and an occlusive thrombus in the middle third. The exam also showed coronary ectasia, without significant stenosis in the anterior interventricular and circumflex branches. An attempt of primary balloon transluminal coronary angioplasty was made, it was associated with intracoronary thrombolysis and venous Alteplase maintenance, obtaining TIMI II coronary flow. A coronary stent wasn’t possible due to de excessive dilatation of the vessel. The patient evolved well until his hospital discharge and did not present new cardiological problems in the last 4 years. The patient took Aspirin for the first 28 days. Currently, he takes Atorvastatin (80 mg/day), Metoprolol (50 mg/day), Clopidogrel (75 mg/day), and Rivaroxaban (15 mg/day).

**Conclusions:** The pathophysiology of CA is related to multifactorial endothelial damage, which activates inflammatory mediators and vasodilator substances that lead to degeneration of the vessel layer and progressive coronary dilatation. Percutaneous revascularization can be associated with distal thrombus embolization, no-reflow phenomenon, malposition of the stent, dissection, and flow compromise. There are no specific guidelines for the management of CA in acute coronary syndrome and there is no data in the literature about the efficacy of thrombolytic therapy in this case. Finally, this case suggests that balloon angioplasty and the use of thrombolytic, followed by the clinical treatment optimized with high doses of statin, and antiplatelet aggregation associated with anticoagulation in the long term, can be good therapeutic choices for some selected cases of NSTEMI with CA.

108805

Modality: E-Poster Scientific Initiation – Case Report

Category: CONGENITAL AND PEDIATRIC CARDIOLOGY

## Silent Aortic Coarctation in Adulthood Associated with Bicuspid Aortic Valve, Aneurysm and Stanford B Aortic Dissection: A Case Report

CAROLINE DA SILVA TEIXEIRA^1^, Lucca Ziravello Elias Coelho^1^, Gabriela Rodrigues de Oliveira^2^, Ricardo Santiago Ferreira Coelho^2^, Juliano Novaes Cardoso^2^

(1) Faculdade Santa Marcelina; (2) Hospital Santa Marcelina

Coarctation of the aorta (CoA) is not only considered a narrowing of the aorta but part of a generalized arteriopathy. The incidence corresponds to 3 cases per 10.000 births. The associated lesions include a bicuspid aortic valve (BAV) in up to 85% of the cases. Patients with mild CoA may not manifest until adulthood, which can be accidentally diagnosed through clinical examinations as arterial hypertension. This case discusses a 36-year-old male who presented to the emergency department with a 1-hour history of sudden, tearing and severe (10/10 intensity) retrosternal pain radiating to his back. Initial electrocardiogram revealed first-degree AV block and left anterior superior divisional block. Blood pressure was different between the upper and lower limbs. His past medical history includes a non-investigated and non-treated asymptomatic arterial hypertension since he was seven years old. Aortic dissection was suspected and further investigation with angiotomography was realized. The image revealed an aortic coarctation after the emergence of the subclavian artery, associated with an aortic fusiform aneurysm after the stenotic region, parietal thrombus, aortic ulcer, and dissection flap. A transesophageal echocardiogram was performed, which detected a bicuspid valve with significant insufficiency. The patient was hospitalized, medicated and awaiting a surgical procedure. A medical history of re-coarctation, descending aortic aneurysms, hypertension, and recurrent procedures associated with congenital heart disease are related to higher cardiovascular morbidity. The diagnosis of CoA is the most commonly missed, being detected at the prenatal screening with less than one-third of the cases. In conclusion, early diagnosis is very important to reduce disease morbidity.


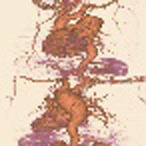


108816

Modality: E-Poster Scientific Initiation – Case Report

Category: CARDIOVASCULAR PHARMACOLOGY

## Blue-Gray Syndrome: A Rare Amiodarone-Induced Dermopathy

HELENA MARCON BISCHOFF^1^, Helena Marcon Bischoff^1^, Sérgio Ferreira de Ferreira Filho^2^, Raphael Boesche Guimaraes^2^, Mathias Silvestre de Brida^2^

(1) Universidade Federal de Ciências da Saúde de Porto Alegre (UFCSPA); (2) Instituto de Cardiologia do Rio Grande do Sul/Fundação Universitária de Cardiologia (IC-FUC)

**Introduction:** Amiodarone is a widely used drug in the treatment of arrhythmias, however, its metabolites are responsible for numerous side effects. We report a rare dermopathy secondary to the chronic use of amiodarone.

**Case Description:** Female patient, 57 years old, hypertensive and with a history of paroxysmal atrial fibrillation and mechanical mitral valve prosthesis, on chronic use of warfarin and amiodarone, sought consultation due to the appearance of bluish spots on her face and upper limbs in the last three months. She reported daily use of 400 mg of amiodarone for the last four years. Patient had lost medical follow-up, with no consultation in the last 4 years. On physical examination, areas of blue-gray hyperpigmentation were found in photoexposed areas, predominantly on the face and upper limbs. Chest X-ray and laboratory tests to evaluate other adverse effects were unremarkable. Considering the characteristic clinical findings, the hypothesis of amiodarone-induced skin disease, known as Blue-Gray Syndrome was raised. The patient was then instructed on the discontinuation of the drug, protection against sun exposure and the need for ophthalmological evaluation. After four months, she showed significant improvement in the lesions.

**Conclusions:** Blue-Gray Syndrome is an adverse effect that occurs in 1 to 7% of patients on chronic use of amiodarone. Differential diagnosis is made with other situations that cause facial discoloration, especially with the use of chlorpromazine and tricyclic antidepressants. Treatment is based on photoprotection and drug suspension, with usual resolution of the discoloration in 6 to 12 months. In conclusion, side effects of amiodarone should be widely known to the prescriber and follow-up is necessary in order to avoid unwanted events.


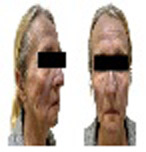


108822

Modality: E-Poster Scientific Initiation – Case Report

Category: ACUTE AND CHRONIC CORONARY DISEASE/THROMBOLYSIS

## Rupture of the Left Ventricle After the Acute Myocardial Infarction: A Dramatic Clinical Situation

VICTOR CUSTÓDIO RIBEIRO^1^, Huggo Santana Machado^1^, Rodrigo Penha de Almeida^1^, Dennis Miguel Lemos da Silva^1^, João Lucas O‘Connell^1^

(1) Universidade Federal Uberlândia

**Introduction:** Complications after myocardial infarction (MI) includes mechanical, arrhythmogenic, and hemorrhagic causes. In general, it happens in the first 5 days after the MI, which justifies the orientation of keeping the patients hospitalized during this period. However, the risk of complications remains high until the 14th day. The mechanical causes are more frequently associated with MI with ST-segment elevation, being responsible for 15% of the deaths by MI. There is a higher risk associated with a larger extension of the necrosis area, the longer period of time until the myocardial reperfusion, and the treatment with fibrinolytic (when compared to primary angioplasty).

**Case Description:** Female, 74 years, obese, smoker, family history of coronary heart disease. Presented with typical chest pain, being admitted 36 hours after the beginning of the symptoms, already asymptomatic. The electrocardiogram showed an ST-segment positive deflection in the inferolateral-dorsal wall and right ventricle (RV). The patient evolved with decompensated heart failure and improved after clinical support. The echocardiogram showed biventricular dysfunction with a left ventricular ejection fraction of around 50%, inferior akinesia, and inferolateral hypokinesia. The cineangiocoronariography indicated a multivessel obstructive pattern, and surgical treatment was chosen. On the fifth day of hospitalization, the patient presented with abdominal pain followed by a sudden loss of consciousness without pulse. Cardiopulmonary resuscitation and Marfan puncture were done, with a moderate volume of bloody secretion expelled. Unresponsive, the patient evolved to death. At necropsy, a rupture of the posterior wall of the left ventricle with hemopericardium was identified.

**Conclusion:** The most severe mechanical complication after MI is the rupture of the left ventricle. This condition is 7 times more common than RV rupture, with an incidence of up to 10%, especially when it affects territories supplied by the anterior descending artery. The acute form, with the presence of hemopericardium, progresses fast to death. The chronic form can be asymptomatic with the formation of pseudoaneurysms. Our case reinforces the importance of effective and early revascularization treatment. The persistence of ST-segment elevation after 72 hours attests to the risk of serious complications. Pericardiocentesis is an interim measure until clinical stabilization and referral for cardiac surgery.

108828

Modality: E-Poster Scientific Initiation – Case Report

Category: ATHEROSCLEROSIS/CARDIOVASCULAR RISK FACTORS/CARDIOVASCULAR PREVENTION

## Double Anterior Descending Artery Associated with Severe Atherosclerotic Disease Both in the Native Vessel and at the Origin of the Anomalous Anterior Descending Artery (In Bifurcation with the Right Coronary Artery)

RIZIA CARLA DA SILVA LEOPOLDINO^1^, Karina Baltor Cabral^1^, Fernanda Fernandes Gonçalves Ribeiro^1^, Caio Araújo da Cunha^1^, João Lucas O‘Connell^1^

(1) Universidade Federal Uberlândia

**Introduction:** Coronary artery anomalies have an incidence ranging from 0.1% to 1.4% in studies of necropsy and coronary angiography series. These anomalies are mostly benign and asymptomatic. However, they can cause symptoms when there are repercussions on coronary flow. The diagnosis of double Anterior Descending Artery (AD) occurs when identifying a short anterior descending artery that runs in the interventricular groove but does not reach the cardiac apex, and another long anterior descending artery, which has a variable proximal course and returns to the distal interventricular groove. The incidence of this type of anomaly is low: 0.017%. There is little data in the literature on the incidence of severe obstructive disease related to double AD.

**Case Description:** A 68-year-old female patient presenting myocardial ischemia in angina investigation. Coronary angiography was submitted that identified double AD and the presence of obstructive coronary artery disease associated with both the proximal segment of the AD (originated from the left coronary trunk) and the anomalous AD ostium (bifurcation stenosis with the right coronary artery (DC), which also presented important stenosis). The patient underwent Coronary Angioplasty for both branches of AD and DC, with good clinical and angiographic evolution. Images of coronary angiography will be presented before and after angioplasty treatment. The patient progressed well clinically and is asymptomatic, 2 years after angioplasties.

**Conclusion:** The diagnosis of double AD is given by coronary angiography or, more recently, by coronary tomography. Double AD should be suspected when there is a discrepancy between the apparent coronary distribution pattern and the motility of the ventricular walls. Dual Type IV AD is characterized by the origin of long AD in the right Valsalva sinus or right coronary artery. This anomaly is rare: 0.004% – 0.006%. Double AD can also be confused with occlusion of the middle third of AD, especially if only left coronary angiography is performed and long anomalous AD does not undergo selective catheterization. In addition, this anomaly may be associated with coronary obstructive atherosclerotic disease and the clinical aspects of the patient, angiographic complexity, and discussion among the members of the heart team should be considered in order to offer the best therapeutic proposal to patients with this pathology.

109111

Modality: E-Poster Scientific Initiation – Case Report

Category: HEMODYNAMICS AND INTERVENTIONAL CARDIOLOGY

## From Acute Myocardial Infarction to Intraventricular Communication: Case Study

INGRID SAGRILLO^1^, Júlia Carvalho Brasil Malaquias^1^, Cézar Ladeira Macedo Junior^2^, Juan Dias de Lima^2^

(1) Faculdade de Minas – Muriaé (FAMINAS – Muriaé); (2) Casa de Caridade Hospital São Paulo (HSP – Muriaé)

**Introduction:** The intraventricular communication (IVC) is among the main mechanical complications of acute myocardial infarction (AMI), with an incidence of 0.2%, next to free-wall papillary muscle rupture, all generally associated with hemodynamic instability and high mortality.

**Case Report:** J.H.V., a 59-year-old male, without previous comorbidities, came to the emergency room with typical chest pain for 20 hours, and electrocardiogram showed supra-ST-segment elevation in the inferior wall. He was immediately transferred to the hemodynamics department where primary angioplasty of the occlusive lesion of the right coronary artery was performed. On the third day after the onset of symptoms, the patient developed anginal pain, sweating, hypotension, and transthoracic echocardiogram showed IVC and Left Ventricular pseudoaneurysm (LVP). Thus, the patient was referred to a new hemodynamic procedure for percutaneous closure of the two IVC holes together with the LVP.

**Conclusion:** The main symptom of AMI is sudden and constant chest pain due to reduced blood flow. Diagnosis is through the electrocardiogram, which shows a ST-segment elevation. Left ventricular pseudoaneurysm is a rare complication after AMI that results in ventricular wall rupture without cardiac tamponade. It is diagnosed by echocardiography or nuclear magnetic resonance, but most are done in the chronic phase, which often evolves asymptomatically. However, once diagnosed, it must be treated surgically. Echocardiography is also the gold standard for the diagnosis of IVC, in which it causes significant occlusions, which with advancing age may arise adjuvant diseases. The treatment of choice is surgical, usually -pericardial patch-, with low operative mortality and good long-term results.

108846

Modality: E-Poster Scientific Initiation – Case Report

Category: NEGLECTED CARDIOVASCULAR DISEASES

## Severe Cor Pulmonale in a Patient with Pickwick Syndrome

VALENTINA BRATTI DE NADAL^1^, Pedro Boustany Escobar^1^, Isabelle Gambin Antonini^1^, Leonardo Mussoi^1^, Rafael Coimbra Ferreira Beltrame^2^

(1) Universidade do Vale do Rio dos Sinos (UNISINOS); (2) Hospital Dom João Becker

**Introduction:** Pickwick syndrome (PS) is defined by obesity, chronic hypoventilation and hypercapnia in absence of other secondary causes. Pulmonary hypertension with cor pulmonale is one of the consequences of severe untreated forms. Although well described, PS is often underdiagnosed. The following case illustrates the history of the untreated disease and the current challenges in its diagnosis.

**Case Description:** A 43-year-old male with grade 3 obesity, hypertension and obstructive sleep apnea syndrome with CPAP indication, was admitted due to generalized peripheral edema with spontaneous transudation through lower limbs skin, and dyspnea at rest initiated within the last 3 months. He was on continuous and multiprofissional care regarding his obesity for 8 years, being in pre-op evaluation for bariatric surgery. At admission he weighed 230 kg, oxygen saturation was 85% on room air, and cyanosis, jugular turgor, bilateral pulmonary crackles were present. Echocardiogram showed biventricular dysfunction (Figure 1). Abdominal tomography showed congestive hepatomegaly and splenomegaly. Managed with arterial and pulmonary vasodilators during hospitalization, CPAP and high dose diuretics, with complete improvement of congestion and 67 kg of fluid loss – discharge with 163 kg. Arterial blood gas analysis on room air after compensation showed a pCO2 of 63.3 mmHg and a pO2 of 52.6 mmHg, confirming the diagnosis of PS. Discharged with nutritional plan designed for obesity care, treatment for pulmonary hypertension and biventricular dysfunction, CPAP and home oxygen therapy.

**Conclusions:** Obesity is a public health epidemic. The patient above received long standing medical follow-up for obesity, without ever receiving specific evaluation for PS and its consequences, resulting in extremely advanced cardiopulmonary disease. Although a well-described entity, PS is rarely remembered and treated early by health care teams. The reasons are multifactorial, ranging from negative health care team biases related to the treatment of obese patients to lack of focus on the particularities of care for this group of patients.


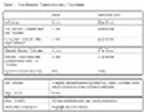


108892

Modality: E-Poster Scientific Initiation – Case Report

Category: HEMODYNAMICS AND INTERVENTIONAL CARDIOLOGY

## Acute Myocardial Infarct: Management of Coronary Events Due to use of Cocaine in Emergency Scenery

ELISA ROCHA NONEMACHER^2^, Elisa Rocha Nonemacher^2^, Gabriel Almeida Krul^2^, Rafaela de Oliveira Leite^2^, Marcelo Sabedotti^1^

(1) Hospital Geral; (2) Fundação Universidade de Caxias do Sul

**Introduction:** Cocaine is one of the most used illicit drugs worldwide. Its use can lead to acute or chronic cardiovascular intoxication – among them, the Acute Coronary Syndromes (ACS), aortic dissection and rupture, arrhythmias and cardiomyopathies stand out. Its effect is related to blocking adrenergic reuptake in the synaptic cleft in addition to blocking sodium channels. The acute myocardial infarction is present in 6% of the population with recent cocaine use.

**Clinic History:** 44-year-old woman, with a history of systemic arterial hypertension, smoker and cocaine user for 8 years. She sought emergency for medical care complaining of typical chest pain, associated with nausea, in 90 minutes of evolution. She was referred to the Coronary Intervention Unit of reference in the region, 4 hours after the onset of pain. On examination, she was with signs of hypertension and tachycardia. Electrocardiogram (ECG) with sinus tachycardia, ST elevated myocardial infarction (STEMI) in anteroseptal-lateral wall, being referred for early intervention. Coronary Coronary angiography demonstrated occlusion of the anterior descending artery. The thrombus burdens were aspirated resulting in restoration of coronary flow. Right after there was insertion of a drug-eluting stent in the affected artery, with clinical and hemodynamic improvement. Cocaine is a substance that, from a cardiological point of view, generates a cascade of events that is conducive to acute coronary syndromes, as it is a potent platelet activator, in addition to increasing heart rate and promoting vasoconstriction (mediated by alpha receptors). The blockade of sodium channels also contributes to a tendency to ventricular arrhythmias. Regarding drug therapy, it is prudent not to prescribe beta-blockers, especially in the acute phase, due to the risk of diverting all adrenergics from the synaptic cleft to alpha receptors, worsening the deleterious effects of cocaine.

**Conclusion:** Considering that ACS are an important cardiovascular consequence of cocaine use, proper investigation and early treatment are necessary. Even though protocol treatment is the most appropriate alternative, there are some important differences in relation to conventional drug treatment that should be considered.

108893

Modality: E-Poster Scientific Initiation – Case Report

Category: HEMODYNAMICS AND INTERVENTIONAL CARDIOLOGY

## Intranodal Lymphangiography in the Assessment and Treatment of Chylous Pleural Effusion in an Univentricular Patient- a Case Report

PEDRO RAFAEL VIEIRA DE OLIVEIRA SALERNO^1^, Santiago Raul Arrieta^2^, Mario Henrique Hattori^2^, Willian Yoshinori Kawakami^3^

(1) Faculdade Pernambucana de Saúde; (2) INCOR-SP; (3) Hospital das clínicas da faculdade de medicina da universidade de são paulo

**Introduction:** In univentricular patients, lymphatic complications, such as CPE, represent an important cause of morbidity and mortality.1 Identification of the source of the leak and management remains a challenge. ILG provides visualization of the lymphatic system and may represent a therapeutic alternative to surgical ligation of the TD2.

**Case Report:** 10-month-old patient, 6,6 kg, born full-term, with an intrauterine diagnosis of double-inlet single ventricle with aortic atresia and hypoplasia of the aortic arch presented with irritability. Patients‘ medical history included stage 1 hybrid palliative surgery with 7 days of life and Norwood-Glenn, atrioseptostomy, and stent placement in the left pulmonary artery at 8 months of life. CPE on the fifth postoperative day was reported. Physical examination: cyanotic child, dyspnea, and no fever. Gasometry with a Sat02 83,8%, lactate 18 mg/dL. Chest X-ray showed bilateral pleural effusion. Tube thoracostomy (TT) was performed, pleural fluid analysis: triglycerides 515 mg/dl and cholesterol <50 mg/dl. With a high bilateral TT drainage (46,66 ml/kg) and failure of clinical management, ILG was done. Guided by ultrasound, an inguinal lymph node was punctured bilaterally with 25G needles and ethiodized oil was injected. After visualization of the cisterna chyli, a 22G Chiba Needle was used to access it. Due to its reduced size, TD cannulation was not feasible. Therefore, further injection of ethiodized oil was performed and the lymphatic leak was observed. After 48 hours, TT drainage reduced (5 ml/kg) and computerized tomography showed a reduction of CPE. 1 week after the procedure, clinical and radiological improvement was observed.

**Conclusion:** CPE in univentricular patients results from iatrogenic lesions during palliative procedures or as an outcome of the increased lymph flow due to increased central venous pressure in patients with lymphatic anatomic variants.1 ILG is a low-risk procedure capable of identifying and treating the source of leakage.2 In this case, due to incapacity of TD cannulation, further use of ethiodized oil was implemented and successfully controlled the effusion.


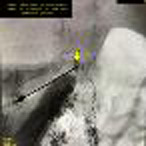


108944

Modality: E-Poster Scientific Initiation – Case Report

Category: HEART FAILURE/CARDIOMYOPATHY/TRANSPLANT

## Rare Association between Heart Failure and Late Onset Pompe Disease: A Case Report

CAROLINA TISOTT BURTET^1^, Amanda Hillesheim Schuck^1^, Luísa Gailhard Brito^1^, Rafaella Magni Berthier^1^, Olga Sergueevna Tairova^1^

(1) Universidade de Caxias do Sul (UCS)

**Introduction:** Pompe disease or glycogen storage disease type II (GSD II) is a rare hereditary autosomal disorder, whose incidence reaches 1:50.000. It’s caused by gene mutations that encodes the acid alpha-glucosidase enzyme that is responsible for the glycogen degradation – mainly in the muscles. Therefore, this hereditary disease may cause symptoms such as muscle weakness, unsteady gait and cardiorespiratory failure. It can be categorized as early onset Pompe disease (less than 12 months age) or late onset Pompe disease (more than 12 months age), and it is usually known based on the onset symptoms and on the presence or absence of cardiomyopathy.

**Case Description:** 53 year-old female patient with a heart failure history. The patient has been diagnosed with Pompe disease at 34 years of age, and has the family history of two brothers with the same disease. In the last 20 years, it progressed with dyspnea during physical exercise, global strength reduction with predominance in the right hemibody, associated with degenerative anatomical changes. In addition, it presented significant worsening in the lower limbs in the last 5 years. Physical examination: altered gait phases, decreased balance, altered cognitive and verbal level and decreased coordination of selectivity.

**Complementary Tests:** Echocardiogram with ejection fraction (EF) of 37%, eccentric hypertrophy, diastolic dysfunction and systolic dysfunction with a pattern of relaxation deficit; normal catheterization. Patient has no conditions of motor physiotherapy due to the lack of physical fitness.

**Conclusion:** Late-onset Pompe disease usually progresses with a less aggressive presentation and without major cardiac involvement. Despite that, the patient evolved into heart failure with reduced EF. Then, it is possible to propose an association between this condition and late Pompe disease. Although there are few reports in the literature that demonstrate this association, we can conclude that, even if it is rare, it may be possible to find this condition not only in early onset disease.

108971

Modality: E-Poster Scientific Initiation – Case Report

Category: HEMODYNAMICS AND INTERVENTIONAL CARDIOLOGY

## No-Reflow Phenomenon: Alternative Treatment Technique for Patients in the Public Health System

RAFAELA OLIVEIRA LEITE^1^, Bibiana Guimarães Maggi^2^, Marcelo Sabedotti^2^, Rafael Massuti^2^, Leandro Gazziero Rech^2^

(1) Fundação Universidade de Caxias do Sul; (2) Hospital Geral de Caxias do Sul

**Introduction:** The no-reflow phenomenon is defined by the lack of myocardial perfusion despite the opening of the coronary vessel in the context of primary percutaneous coronary intervention (PPCI) after stenting, with no presence of dissection, mechanical obstruction, significant residual stenosis, or spasm. Angiographic evaluation is done by assessing the contrast flow beyond the PPCI location to the distal vessel and is related to an increased risk of major cardiovascular events. To solve this phenomenon, intracoronary infusion of adenosine distal to the stent, by microcatheter, can improve the angiographic pattern. However, in the Brazilian public health care system, also known as Sistema ùnico de Saúde (SUS), PPCI doesn‘t contemplate microcatheter due to its high cost, making alternative techniques necessary.

**Case Report:** A 70-years-old male, with no previous comorbidity, was hospitalized due to typical chest pain without signs of hemodynamic instability. Complementary tests demonstrated an electrocardiogram with ST-segment elevation on the anterior-septal-lateral and high lateral walls, glomerular filtration rate of 43 mL/min/1,73 m2 and elevated troponin levels (3,9, for a reference of 0,014). Cardiac catheterization, performed through a puncture of the right radial artery, demonstrated ostial occlusion of the anterior descending artery. The left main coronary artery was cannulated with an EBU 4 (6F) catheter, followed by access of a 0,014 guidewire, with the thrombus burden aspiration with an Export® Medtronic aspiration catheter and circulation returned. When implanting the drug-eluting stent, the no-reflow phenomenon occurred. For its management, we positioned an aspiration catheter distal to the stent, and administered 1,2 mg of adenosine intracoronary, reestablishing the vessel flow (TIMI 3, BLUSH 3). The patient was transferred to the ward, where he remained asymptomatic until discharged after 6 days.

**Conclusion:** The technique of distal infusion of adenosine through a thrombus aspiration catheter is effective and can be used on the SUS as an alternative to the microcatheter.

108972

Modality: E-Poster Scientific Initiation – Case Report

Category: HEMODYNAMICS AND INTERVENTIONAL CARDIOLOGY

## Intraaortic Balloon: Therapeutic Alternative for Treatment of Patients with Acute Coronary Syndrome

RAFAELA OLIVEIRA LEITE ^1^, Elisa Rocha Nonemacher^1^, Gabriel Almeida Krul^1^, Marcelo Sabedotti^2^

(1) Fundação Universidade de Caxias do Sul; (2) Hospital Geral de Caxias do Sul

**Introduction:** Temporary short – and intermediate-term mechanical circulatory support devices (MCSD) may be used as an escalation strategy to manage situations like cardiogenic shock (CS). The selection of an MCSD depends on the specific indication for hemodynamic support, the urgency of need, costs, and patient-related or device-related risks. The main etiology of CS is acute myocardial infarction (AMI), with seven percent incidence and evolving with loss of up to forty percent of left ventricular function. This study aims to report and discuss a case of acute myocardial infarction with ST-segment elevation associated with occlusion of the left main coronary artery evolving to CC and the use of an Intraaortic balloon pump (IABP).

**Case Report:** A 40-years-old male, previously diagnosed with systemic arterial hypertension arrive at the hospital because of an acute myocardial infarction with ST-segment elevation. His physical examination demonstrated signs of hemodynamic instability, a Killip score of four (suggesting CS), and preserved neurologic status. High-sensitive troponin of the admission was 0,084 (reference of 0,014) and an electrocardiogram demonstrated sinus tachycardia, ST-segment elevation of 7 mm in aVR, and ST-segment depression in anterior-septal-lateral and inferior walls. The patient was promptly referred for early intervention. We performed coronary angiography, which demonstrated left main coronary artery occlusion. The thrombus burdens were aspirated with restauration the coronary flow, and then there was insertion a drug-eluting stent on the left main coronary artery towards the anterior descending artery. We opted for the implantation of an IABP after the procedure, for 24-hour duration support. The patient underwent intensive care for 72-hours. He was successfully discharged after ten days, asymptomatic, with orientation for optimized therapy, request for outpatient care, and cardiac rehabilitation.

**Conclusion:** Knowing that CS increases the morbidity and mortality of patients, it is necessary to evaluate the use of MCDS by the Heart Team. However, the available evidence does not support the routine use of IABP in most patients with AMI. There is evidence that IABP can be an extremely important adjunct in the treatment of these patients, reducing hospital mortality and myocardial injury. Therefore, the technique must be proposed in cases of AMI with rapid clinical deterioration.

108974

Modality: E-Poster Scientific Initiation – Case Report

Category: CARDIOVASCULAR SURGERY

## Multicavitary Mixoma: Case Report

LEO CHRISTYAN ALVES DE LIMA^1^, Leo Christyan Alves de Lima^1^, Hildeman Dias da Costa^2^, Laura Jane França Lacerda^1^, Milena Stephanie Alves Matos^3^

(1) Centro Universitário São Lucas (UniSL); (2) Universidade Federal de Rondônia (UNIR); (3) Secretaria de Estado da Saúde de Rondônia

**Introduction:** Primary cardiac tumors are extremely rare, with an incidence of less than 0.1%. In adults, most benign lesions of the heart are myxomas, accounting for more than 75% of cases.

**Case Description:** Male, 48 years old. A magnetic resonance imaging and transthoracic echocardiogram were performed for diagnostic investigation due to chest pain, which showed an enlarged right atrium (RA) with a mass pedunculated to the interatrial septum, irregular, mobile, invading the right ventricle (RV) in atrial systole, measuring 44 × 35 mm. In the left atrium (LA), oval mass, adhered to the interatrial septum, regular contours, measuring 15 × 11 mm. In RV, oval, subvalvular mass, measuring 19 × 14 mm. The images suggested myxoma in the right atrium and thrombus in the RV and LA, and surgical treatment was proposed. Intraoperatively, excision of the tumor that invaded the entire right atrium was performed, in addition to resection of the mass in the LA and RV, with resection of the atrial septum where the pedicle was located. The septal defect was closed with a continuous suture pericardium patch. He was taken to the ICU and discharged after 4 days. The anatomopathological result was positive for myxoma.

**Discussion:** Differently from the preoperative imaging exams, where two thrombi and one myxoma were suspected, they were confirmed and the three masses were confirmed for myxoma. The vast majority of myxomas are located in the left atrium (LA), the biatrial ones being infrequent and even rarer when associated with the LV location.

**Conclusion:** Due to the location of multicavitary myxoma and less prevalent prevalence, this report shows a rare and atypical morphological presentation of myxomas, with a classic diagnostic method, satisfactory recent postoperative surgical result and good prognosis.


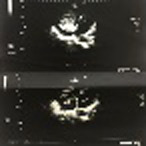


109045

Modality: E-Poster Scientific Initiation – Case Report

Category: CARDIOVASCULAR INTENSIVE CARE/CARDIOVASCULAR EMERGENCIES

## Use of Fibrinolytics 14 Days After an Episode of Pulmonary Thromboembolism Evolving with Hemodynamic Stability, But with Significant Pulmonary Arterial Hypertension and Limiting Symptoms

MIKE VINICIUS CANTO DE ANDRADE^1^, Karina Baltor Cabral^1^, Rizia Carla da Silva Leopoldino^1^, Sthephany Yamaguchi de Melo^1^, João Lucas O‘Connell^1^

(1) Universidade Federal de Uberlândia

**Introduction:** Pulmonary thromboembolism (PTE) has variable clinical conditions. They may be completely asymptomatic or there may even be situations in which massive emboli lead the patient to death, and the diagnosis only through necropsy is not uncommon. Fibrinolytic treatment is indicated for patients who develop clinical instability. However, the establishment of this therapy for hemodynamically stable patients with pulmonary hypertension and/or right ventricular dysfunction is controversial.

**Case Report:** Female, 34 years old, obese, no previous history, taking oral contraception. Reports an episode, 12 days before, of intense chest pain which radiated to her back along with pre-syncope and malaise. Since then, she has maintained dyspnea and fatigue on minor exertion. Physical examination: tachycardia and tachypnea, although hemodynamically stable; normal respiratory and cardiac auscultation. Electrocardiogram: sinus tachycardia (120 bpm) and S1Q3T3 pattern. Chest radiography: no changes. Arterial blood gases without hypoxemia. Elevated D-dimer, Troponin T and BNP. Transthoracic echocardiogram: right chamber dilation, significant right ventricular (RV) dysfunction, good left ventricular (LV) function. Pulmonary artery systolic pressure: 68 mmHg. Normal lower limb venous Doppler. Chest CT angiography: Signs of bilateral submassive thromboembolism. Full dose Enoxaparin was started on the 12th day and chemical thrombolysis was performed by an infusion of 100 mg of thrombolytic recombinant human tissue plasminogen activator (rTPA) in 2 hours (on the 14th day). She improved the symptoms, being discharged. Oral Rivaroxaban was prescribed. A transthoracic echocardiogram performed 3 days later revealed normalization of the right chambers, RV systolic function, and pulmonary artery pressure.

**Conclusion:** Patients with hemodynamically unstable PTE should receive thrombolytic treatment as early as possible; preferably within the first 48 hours. However, if the clinical picture persists, it can be performed up to 14 days after the acute event. In hemodynamically stable patients with signs of RV dysfunction on echocardiography, the use of fibrinolytics is controversial. The use of thrombolytic in this case allowed a significant improvement in the patient’s clinical condition, even though it was performed 14 days after the initial diagnosis. The use of the new oral anticoagulants also allows effective, safe anticoagulation and early hospital discharge.

109079

Modality: E-Poster Scientific Initiation – Case Report

Category: CARDIO-ONCOLOGY

## Right Atrial Metastasis of Renal Cell Carcinoma

NÍCOLAS HENRIQUE BORGES^1^, Ana Paula Guimarães Feuerschuette^1^, Jamylle Araújo Dias dos Santos^2^, Paulo André Bispo Machado-Júnior^1^, Gustavo Gavazzoni Blume^1^

(1) Pontifícia Universidade Católica do Paraná (PUC-PR); (2) Hospital Santa Casa de Misericórdia de Curitiba

**Introduction:** Renal cell carcinoma (RCC) is a solid lesion responsible for 90% of neoplasms of renal origin. It is classified as an aggressive, lethal tumor with high metastatic power in rare and uncommon locations. Tumor growth can extend to the intravascular environment through the renal veins (RVs) to the inferior vena cava (IVC) with an incidence of 15% in patients diagnosed with CRC, in addition, there is an additional extension of the tumor, which can reach the right atrium (RA), being considered rare and present in 1% of cases.

**Clinical Description:** Female patient, 68 years old, with a previous history of hypertension using hydrochlorothiazide 25 mg/day and hyperthyroidism using tapazole 10 mg/day, sought medical attention with complaints of palpitations and asthenia on minor exertion. In the investigation, exercise stress test was limited by fatigue, indicating functional class III (NYHA), and transthoracic echocardiogram showed a diastolic dysfunction with concentric remodeling of the left ventricle, in addition to the presence of a mobile mass inside the right cavities. On admission, she was dyspneic (RR: 20 mrpm), hypertensive (BP: 140 × 80) and presence of a murmur in the tricuspid focus. MR Angiography report an expansive cortico-medullary lesion in the lower 2/3 of the right kidney, with a central area of necrosis. There was involvement of the ipsilateral renal vein, which showed a filling, suggesting a tumor thrombus that extends to the inferior vena cava and into the right chambers. Cardiac MRI revealed dumbbell-shaped intracavitary mobile mass with protrusion through the tricuspid ring. The patient underwent a total nephrectomy, venous system thrombectomy and retroperitoneal lymphadenectomy. She remained under ICU for 3 days and stay under observation before, being discharged. Biopsy confirming a unifocal, histological grade G3, clear cell renal cell carcinoma of Fuhrmann. The patient presented an excellent evolution only taking medications.

**Conclusion:** Cardiac metastasis to the right atrium is rare in patients with renal cell carcinoma. In our case report, surgery was effective for clinical improvement.


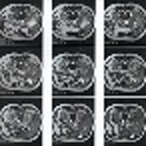


109175

Modality: E-Poster Scientific Initiation – Case Report

Category: ACUTE AND CHRONIC CORONARY DISEASE/THROMBOLYSIS

## Recurrent Takotsubo Syndrome in Postmenopausal Woman

PEDRO HENRIQUE ROSA E SILVA^2^, Pedro Henrique Rosa e Silva^2^, Jairo Rosa e Silva Junior^3^, Thiago Quinaglia^4^, Maria A Delbin^1^

(1) Department of Structural and Functional Biology, Institute of Biology, University of Campinas (UNICAMP), Campinas-SP; (2) School of Medical Sciences, University of Campinas (UNICAMP), Campinas-SP; (3) Climecárdio, Centro Integrado de Clínica Médica e Cardiologia, Ribeirão Preto-SP; (4) Discipline of Cardiology, School of Medical Sciences, University of Campinas (UNICAMP), Campinas-SP

**Introduction:** Takotsubo Syndrome (TTS) is an acute cardiac syndrome characterized by typical regional wall motion abnormalities that reflect impairment of myocardial contractility. TTS predominantly affects postmenopausal women and is often preceded by emotional stress. The case report was approved by the Ethics Committee (CAAE 45476521.9.0000.5404).

**Results:** A 69-year-old postmenopausal woman with hypothyroidism, type 2 diabetes mellitus, systemic hypertension, dyslipidemia and history of chronic anxiety had in 2/6/2013 an intermittent chest pain accompanied by dyspnea and triggered by emotional stress. The ECG showed an inverted and peaked T wave in the anterolateral and inferior wall, ECHO showed apical segments dyskinesia with aneurysmatic dilatation and coronary angiogram revealed no evidence of coronary artery disease, the patient was diagnosed with TTS. On 10/14/2015, the 71-year-old patient presented sudden symptoms of typical chest pain, accompanied by malaise and diaphoresis, also triggered by emotional stress. The ECG showed an inverted symmetrical and spiked T wave in anterior wall, the left ventricle (LV) ECHO showed the wall entire apex and basal portion hypokinesis, with global contractile function moderately depressed, and coronary angiogram revealed normal coronary arteries and alteration of LV segmental contractility with mild global systolic dysfunction, being diagnosed with TTS. At 74-year-old, on 11/25/2018 the patient presented typical chest pain, dyspnea and diaphoresis, triggered by emotional stress. The ECG showed an inverted and spiked T wave in the septal wall and ECHO showed alteration of segmental mobility in the septal wall associated with mitral regurgitation. Due to the previous medical history of TTS and to the similarity of the clinical conditions with previous events the coronary angiogram wat not performed, being diagnosed with TTS recurrence. In all events, the patient was admitted to the intensive therapy unit for five days with treatment for acute chest pain protocol and hemodynamic support. The ECG and ECHO changes were completely reversed. Years later a myocardial scintigraphy was performed showing no sign of sequels from the events.

**Conclusion:** The report presented a case of recurrent TTS triggered by emotionally stressful events in postmenopausal woman, two events with a differential diagnosis with coronary angiogram.

109176

Modality: E-Poster Scientific Initiation – Case Report

Category: HEMODYNAMICS AND INTERVENTIONAL CARDIOLOGY

## Transcathetter Implantation of Aortic Valve in a Patient with a Single Ostium Anomaly of the Coronary Artery with Left Sinus of Valsalva Origin and High Anatomical Risk for Coronary Occlusion

FRANCIELLE GODOI SANTOS^1^, ENZO OKU MARTINAZZO^1^, RÔMULO FRANCISCO DE ALMEIDA TORRES^2^

(1) Pontifícia Universidade Católica do Paraná; (2) Hospital Marcelino Champagnat

**Introduction:** Coronary obstruction (CO) during the transcatheter aortic valve implantation (TAVI) procedure remains an important complication due to a high mortality rate. Its main risk predictors are low origin of coronary ostia, narrow sinus of valsalva and valve-in-valve procedures. Coronary protection (CP) with guidewire and eventual stent implantation is used in patients at high risk for CO in order to increase the safety of the procedure. This study reports the case of a patient with symptomatic severe aortic stenosis who underwent TAVI with the CP technique. In the pre-intervention evaluation, she presented high risk predictors for CO and a rare anomaly of coronary origin.

**Case Description:** An 82-year-old patient was admitted to a tertiary hospital with heart failure. The transthoracic echocardiogram showed the presence of low-gradient normoflow aortic stenosis. Aortic valve calcium score was performed, which reinforced the presence of severe aortic disease. The evaluation with CT angiography showed the presence of a single ostium-like anomaly of the coronary artery originating in the left sinus of valsalva with a low origin in relation to the plane of the annulus. After evaluation by the institutional Heart Team, she underwent TAVI with the CP technique.

**Conclusions:** This report demonstrates the feasibility and safety of the coronary CP technique during the TAVI procedure in a patient with a rare coronary anomaly and at high risk for CO.


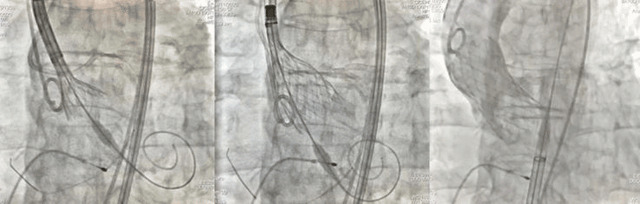


109237

Modality: E-Poster Scientific Initiation – Case Report

Category: PERICARDIUM/ENDOCARDIUM/VALVOPATHIES

## Infective Endocarditis by Proteus Mirabilis: A Rare and Defiant Association

NICHOLAS VINCENT LEE^1^, Nicolas Henrique Borges^1^, Rosane Carolina Paes de Lira^2^, Paulo André Bispo Machado Junior^1^, Gustavo Gavazzoni Blume^1^

(1) PONTIFICIA UNIVERSIDADE CATÓLICA DO PARANÁ – PUC/PR; (2) HOSPITAL SANTA CASA DE CURITIBA – HSC

**Introduction:** Infective Endocarditis (IE) is an uncommon disease with high morbidity and mortality, being commonly associated with gram-positive microorganisms. The IE by Proteus mirabilis, gram-negative, it’s rare, difficult to diagnose and the treatment is controversial, being necessary a fast intervention to avoid severe complications.

**Case Report:** Healthy young patient, come to the emergency room with symptoms of asthenia and progressive dyspnea for the last 2 months. On physical examination, the patient had a systolic murmur in aortic focus with irradiation to the carotid arteries. The echocardiogram shows us a severe double aortic valvar lesion and an importante systolic disfunction (Simpson’s Ejection Fraction of 29%), being indicated aortic valve replacement surgery. While waiting for the surgery, the patient developed bacteremia secondary to phlebitis in peripheric venous access, hemoculture with Staphylococcus epidermidis was positive, initiated a guided antibiotic regimen (oxacillin 500 mg 4/4 h IV). However, the patient remained feverish with high inflammatory markers, evolving to sepsis, and a new antibiotic regimen was started (vancomycin 1000 mg 12/12 h IV). Eight days after the diagnosis of the infeccion, news hemocultures revealed the presence of Proteus mirabilis in blood, being administrated a new antibiotic treatment (cefepime 2g 8/8 h IV) but without a good response. After multiples antibiotics and roled out other infectious foci, a new transesophageal ecocardiogram identify a laminar image in the aortic root suggestive of abscess without vegetations. Opted of emergency surgery, the diagnosis was confirmed of abscess valvar, but despite the intervention, the patient died.

**Conclusion:** The present report case demonstrated a rare and defiant association of IE by Proteus mirabilis with an atypical presentation in echocardiogram (abscess without vegetation) and even with the combined antimicrobial management and surgery, it did not present a favorable outcome.


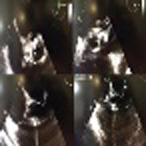


109261

Modality: E-Poster Scientific Initiation – Case Report

Category: ACUTE AND CHRONIC CORONARY DISEASE/THROMBOLYSIS

## Coronary Anomalies, a Rare Diagnosis and an Unfavorable Outcome: Case Report

SARA AYRES SOARES DE SOUZA^1^, Danielle Caiado de Castro Dragalzew^2^, Felipe Augusto Lima Rodrigues^2^

(1) Universidade Católica de Brasília – UCB; (2) Instituto Hospital de Base do Distrito Federal – IHBDF

**Introduction:** Coronary anomalies (CA) are a group of cardiac malformations that encompass variations as anomalous origin of the left coronary (AOLC) and coronaries fistulas. Those abnormalities are rare (0.2–1.2%) and difficult to diagnose, usually needing an imaging test. Patients with this condition are asymptomatic at onset, however they have a high risk of sudden death. Latest findings include valvular complications, myocardial ischemia, angina and congestive heart failure. As spontaneous resolutions are rare, it is essential to diagnose and treat these cases.

**Case Report:** JRSB 44-years-old woman, hypertensive, under treatment for squamous cell carcinoma of the cervix IIIB. Myocardial Scintigraphy (2018) shows preserved ejection fraction (EF), evidencing high degree of persistent hypoperfusion of the anterior wall and apex of the left ventricle, indicative of fibrosis/hibernating myocardium and apex myocardial ischemia. No follow-up was performed. She presented with acute coronary syndrome (ACS) in 2021. In the same year a coronary angiography showed right coronary artery (RCA) without obstruction, AOLC without possibility of selective catheterization and one fistula communication from the RCA to the pulmonary artery. Transthoracic echocardiogram with an EF of 65%, mild mitral, tricuspid and pulmonary valves insufficiency and septal dyskinesia. Electrocardiogram: diffuse repolarization abnormality and extrasystole. In 2022 patient presented with chest pain and tachycardia in moderate to mild intensity exercises, no findings on physical examination. The patient uses daily metoprolol 100 mg, enalapril 10 mg, simvastatin 20 mg, trimetazidine 80 mg. At the found diagnostic stage, the patient presented with symptomatic cardiac morphological change, and waits for surgical approach.

**Conclusion:** The diagnosis of CA is rare, but important as differential in cases of chest pain. In the present case, this is showed by the imaging tests demonstrating the presence of AOLC and coronary fistula, abnormalities that can evolve with ACS, myocardial ischemia and valve insufficiency as happened. This evolution was favored by the absence of follow-up and proper treatment. The patient is still waiting for surgical approach which will help to avoid unfavorable outcomes.

109297

Modality: E-Poster Scientific Initiation – Case Report

Category: CONGENITAL AND PEDIATRIC CARDIOLOGY

## Early Diagnosis and Intervention of Eisemenger Syndrome in a Six Months Old Child: A Case Report

ERYCLAUDIA CHRYSTIAN BRASILEIRO AGRIPINO^1^, Matheus Henrique Soares Neves da Silva^1^, Hanna da Costa Avelar Domingues^1^, Raquel Travassos Oliveira^1^, Estefane Ribeiro Melo^1^

(1) Universidade de Pernambuco – Campus Garanhuns (UPE)

**Introduction:** Eisenmenger syndrome (ES) represents the triad of pulmonary arterial disease, reversal of the central shunt’s flow and cyanosis resulting from systemic-to-pulmonary congenital cardiovascular communication. The key to decide management is the detection and treatment of the cardiac defect before development of pulmonary hypertension, preferably during early childhood. In this case-report, it’s described the diagnosis and following management of an ES secondary to intracardiac communication and a right-to-left shunt in a 6 months old child.

**Case Report:** A 6 months old girl presented with respiratory discomfort since birth, developmental delay and low weight gain was admitted to a tertiary hospital to undergo clinical evaluation. On examination, the patient presented a pansystolic murmur (5+/6+) and signs of respiratory failure. The remainder of the examination was normal. On the third day of hospitalization, it was ordered a transthoracic echocardiogram (TTE), that demonstrated interatrial communication with bidirectional shunt (right-to-left predominance), interventricular communication with bidirectional shunt (left-to-right predominance), moderate pulmonary hypertension (PAH), patent ductus arteriosus (PDA) with left-to-right shunt and relative mitral valve stenosis. On the fourth day, it was initiated therapy with spironolactone 1 mg/kg/day, digoxin 0,01 mg/kg/day and captopril 1 mg/kg/day. She was referred for a cardiological surgical service on the sixteenth hospital day. Atrial septoplasty and ventricular septoplasty with ligation of the PDA were performed. In the last week of hospitalization, a new TTE was ordered, which demonstrated intact atrioventricular septums, preserved biventricular systolic function and pulmonary pressure gradient unsuggestive of PAH. She was discharged after 43 days of hospitalization with a long-term therapy plan with furosemide 1 mg/kg/day, spironolactone 1 mg/kg/day and referral to ambulatory care.

**Conclusion:** ES is a truly devastating condition, mainly without the early diagnosis and adequate treatment. Most of the patients tend to suffer an early death due to heart failure, arrhythmias and thromboembolic cerebrovascular disease. In this case-report, the early diagnosis associated with clinical and surgical optimized intervention were decisives for the patient’s outcome to be modified following the literature recommendations regarding management in such conditions.

109327

Modality: E-Poster Scientific Initiation – Case Report

Category: HEART FAILURE/CARDIOMYOPATHY/TRANSPLANT

## Takotsubo Cardiomyopathy in a Paciente with Panhypopituitarism Resulting in QT Prolongation

FILIPE EVERTON SILVA RODRIGUES ^1^, João Gabriel Oliveira de Souza^1^, Ana Carolina Sampaio Freire^1^, Victor Oliveira da Cruz^1^, Alexandre Anderson de Sousa Munhoz Soares^1^

(1) Universidade de Brasília – UnB

**Introduction:** Takotsubo cardiomyopathy (TTC) is a reversible left ventricular dysfunction. The initial symptoms mimic acute coronary disease, as patients present with dyspnea, hypotension, syncope, elevated troponin levels and suggestive electrocardiographic changes. Predisposing factors include emotional stress, endocrine disease and adrenal insufficiency.

**Case Report:** A diabetic, hypertensive 50 year old woman with a history of chronic pericarditis and TTC seeked the emergency department presenting chest pain, sweating, hypotension and dyspnea. Transthoracic Echocardiogram showed regional changes in the left ventricle contractile function, suggesting TTC with a left ventricular ejection fraction of 47,6% and cardiac magnetic resonance imaging revealed pericarditis. After hospitalization, she had improvement in the symptoms, being discharged with further ambulatorial investigation scheduled. A week later, she presented with worsening symptoms, with high troponin and D-dimer levels. She developed an episode of unstable polymorphic ventricular tachycardia with QT prolongation, corrected QT (QTc) of 518 ms, needing electrical cardioversion. Upon investigation and questioning the patient revealed a visual impairment that began 6 months prior. With that information and imaging it was diagnosed a brain tumor (very likely a glioma) compressing the pituitary gland. After starting hormonal replacement therapy (HRT), there were no new episodes of arrhythmia. The patient was discharged waiting to begin tumor treatment.

**Conclusion:** Although the evolution of TTC in the majority of cases is favorable, prolonged QTc should be evaluated due to the potential of death. QT prolongation serves as a substrate for ventricular tachycardia and sudden cardiac death, but the occurrence of ventricular fibrillation and its predictors in the disease are still unknown. The exact pathophysiology of TTC is not fully understood. In this case, a panhypopituitarism due to tumor compression might of caused a secondary adrenal insufficiency, resulting in glucocorticoid deficiency, hyponatremia and related hypoglycemia, which may have induced a catecholamine release resulting arrhythmia and the recurrence of abnormal wall motion. To guide clinical decision-making, patients with cardiac abnormalities should be checked for hormonal alterations, knowing the possible connection between endocrine disorder in TTC and torsades de pointes, resulting in a potential therapeutic opportunity with HRT.

109329

Modality: E-Poster Scientific Initiation – Case Report

Category: CONGENITAL AND PEDIATRIC CARDIOLOGY

## 22Q11 Deletion Syndrome (Velocardiofacial/Digeorge Syndrome) and Congenital Heart Diseases: Report of Two Cases Together with a Literature Review

CAROLINA GUIMARÃES HERZOG^1^, Marco Antônio Vinciprova Dall’Agnese^1^, Pedro Dutra Batista^1^, Gabriel de Paula Alves^1^, Rafael Fabiano Machado Rosa^1^

(1) Universidade Federal de Ciências da Saúde de Porto Alegre – UFCSPA

**Introduction:** The 22q11 deletion syndrome (22q11DS) is considered the second most known genetic cause of congenital heart disease (CHD). Here we reported two patients with different types of CHD presenting 22q11DS.

**Cases Descriptions:** The first patient was a three-year-old girl, who was referred to the service due to a diagnosis of type A aortic arch interruption. She underwent cardiac surgery shortly afterwards, and developed hypoparathyroidism. Abdominal ultrasound revealed a left renal agenesis. The patient also presented intermittent strabismus, high palate, micrognathia, posteriorly rotated right ear, left ear in faun and umbilical hernia. Karyotypic analysis was normal. However, a posterior evaluation through the use of fluorescence in situ hybridization (FISH) technique confirmed the diagnosis of 22q11DS. The second patient was a two-day-old girl who presented an echocardiography with evidence of a type I truncus arteriosus. She had already been submitted to karyotypic evaluation during the prenatal period – due to an increased nuchal translucency measurement -, which was normal. The patient presented in her clinical evaluation blepharophimosis, posteriorly rotated and low-set ears, and clubfoot on the right. The use of the FISH technique verified the diagnosis of 22q11DS.

**Conclusion:** The 22q11DS presents an important association with CHDs, especially with the conotruncal ones. About half of the patients presenting type A interrupted aortic arch and one third of those with truncus arteriosus disclosed 22q11DS. As a consequence, patients with those CHDs should always be carefully evaluated regarding the existence of this syndrome.

109373

Modality: E-Poster Scientific Initiation – Case Report

Category: NEGLECTED CARDIOVASCULAR DISEASES

## Syphilitic Thoracic Aortic Aneurysm in a Young Patient – Case Report

MARIA CLARA SERAPIÃO FERREIRA^1^, Maria Clara Serapião Ferreira^1^, Alessandro da Costa Machado^2^, Fernanda Snovarski Mota^3^, Alberto Acosta Hermida^3^

(1) Universidade Federal da Integração Latino-Americana (UNILA); (2) Hospital Municipal Padre Germano Lauck (HMPGL); (3) Hospital Ministro Costa Cavalcanti (HMCC)

**Introduction:** Syphilis is a sexually transmitted infection (STI) caused by the bacterium Treponema pallidum; when not properly treated, it can progress to tertiary syphilis and produce cardiovascular lesions, mostly affecting the aorta. Syphilitic aortic aneurysm, although rare, is still a possibility. We report the case of a young patient with an aortic arch aneurysm susceptible to rupture of syphilitic origin.

**Case Description:** Male, 37 years old, with progressive dyspnea for 5 days, dry cough, mainly during inspiration, evolving with hoarseness for about 1 day. Hypertensive without treatment and smoker, associated with history of multiple sexual partners, not using protection. There is no history of STI or recent trauma. On physical examination, a hypertensive patient 140 × 80 mmHg (left upper limb) and 160 × 90 mmHg (right upper limb), normocardic, eupneic on room air. On cardiac auscultation, he had intense diastolic murmur (+4/+6) associated with thrill in aortic focus and hyperphonetic heart sounds. Chest angiotomography showed an aortic arch aneurysm, with an extension of 11,5 cm, with a mural thrombus in the right lateral portion, associated with tracheal deviation due to mass effect. Coronary computed tomography angiography showed no significant coronary lesions. Therefore, it was indicated a surgical approach for aneurysm repair, with aortic reconstruction and implantation of a 28 mm Dacron tube, without complications. Afterwards, considering the patient’s history and the aneurysm characteristics, especially its location, nontreponemal test was requested, being positive, with a VDRL of 1:128. For that reason, the treatment for tertiary syphilis with benzathine penicillin was initiated. The patient evolved uneventfully, was discharged six days after the operation, with outpatient follow-up.

**Conclusions:** Although the treatment of syphilis with benzathine penicillin is effective and widely available, and the manifestations of tertiary syphilis are uncommon, the diagnosis of syphilitic aortitis should be considered. We must consider that there is a direct relationship between the sociocultural level of the patient and the development of the disease. Thus, the case demonstrates that the late diagnosis of syphilis causes important cardiovascular repercussions, which require immediate recognition and surgical approach to obtain a favorable prognosis.

109420

Modality: E-Poster Scientific Initiation – Case Report

Category: HEMODYNAMICS AND INTERVENTIONAL CARDIOLOGY

## Aortic Perforation: A Rare but Serious Complication in Pacemaker Implantation

KESLLY KRAUSPENHAR CUCHINSKI^1^, Eduardo Bartholomay^1^, Liara Eickhoff Coppetti^1^, Melissa Nadal Duarte^1^, Tilae Steinmetz Soares^1^

(1) Universidade Luterana do Brasil – ULBRA

**Introduction:** Pacemaker implantation is a safe procedure and widely used in clinical practice. Intercurrences are infrequent, but a serious complication can occur, such as vessel perforation. The present study aims to report a case of cardiac pacemaker insertion with unfavorable outcome due to ascending thoracic aorta perforation and to discuss ways to avoid this rare complication.

**Case Description:** Male patient, 71 years old, submitted to pacemaker implantation due to sinus dysfunction and Wenckebach type 2 atrioventricular block with a narrow QRS. The device chosen was a DDD pacemaker with ventricular stimulation through the conduction system by the bundle of His. The implantation was performed by placing the atrial lead in the anteroseptal region through posteroanterior radiographic visualization and the ventricular lead by radiological orientation and by the His bundle electrogram in the septal region, close to the membranous septum. The procedure was performed with a duration of 1.5 hours with 7 min of radioscopy. After arriving in the intensive care unit (ICU), the patient showed cardiac tamponade detected by echocardiography. Pericardial drainage of 100 ml of blood was performed with resolution of the hemodynamic condition. The patient was referred for atrial lead replacement, since the His bundle lead was in the septum. During anesthetic preparation, the patient presented cough and sudden hemodynamic collapse, evolving to cardiorespiratory arrest. With the opening of the pericardium, a large amount of arterial blood was detected. It was opted for emergency sternotomy and direct cardiopulmonary resuscitation (CPR). A small screw tip of the atrial electrode was visualized, which had perforated the atrium and “clamped” the aorta. The bleeding was manually stopped and the spontaneous circulation returned after 60 minutes of CPR. The atrial electrode was repositioned in the anterior region of the right atrial appendage and the sternum was sutured. The patient was referred to the ICU, but evolved with hemodynamic shock due to systemic inflammatory response syndrome, passing away 4 days after the procedure.

**Conclusion:** Aortic perforation by atrial lead is a rare event described in few cases in literature; however, it is of extreme clinical relevance due to its high mortality. Therefore, we suwe suggest that the right oblique view should always be performed to certify the location of the anterior lead, aiming to avoid this rare, yet tragic complication.

109425

Modality: E-Poster Scientific Initiation – Case Report

Category: CONGENITAL AND PEDIATRIC CARDIOLOGY

## Congenital Complete Heart Block in a Newborn Due to Maternal Sjogren‘s Syndrome: A Case Report

PEDRO GUSTAVO STEVANATO DE OLIVEIRA^1^, Georgia Marques Jardim^1^, Nadine Edda Corrêa^2^, Gustavo Gianesini^1^, Neivis Cubillos^1^

(1) Universidade Federal de Ciências da Saúde de Porto Alegre; (2) Universidade Federal de Santa Catarina, Campus Araranguá

**Introduction:** Congenital complete heart block (CHB) occurs in approximately 1/20,000 live births and is associated with low ventricular rate and reduced cardiac output, leading to heart failure and fetal hydrops. Congenital CAVB is associated with a significant increase in mortality and morbidity and may or may not be secondary to immunological processes. The transplacental passage of maternal anti-RO and anti-La antibodies, which causes damage to the fetal conduction system, is responsible for congenital CHB of immunological etiology.

**Case Description:** Female patient, 31 years old, Sjogren’s syndrome with idiopathic thrombocytopenic purpura (remission for four years) and depression. The fetus was diagnosed with CHB during the prenatal follow-up of her fifth pregnancy. She followed up with fetal cardiology through echocardiograms that showed bradycardia around 42 bpm, ventricular rate around 47 and no signs of hydrops. The newborn (NB) was born full-term with high levels of anti-RO and anti-La, with a heart rate of 45 bpm without clinical hemodynamic repercussions. A postnatal echocardiogram showed ventricular chambers‘ enlargement and mild mitral and tricuspid regurgitation. A 24-hour holter monitoring confirmed CHB and QTC interval increase. After nine days, the patient underwent surgery to implant a pacemaker (Median Sternotomy: Implantation of an epicardial bipolar electrode in the left ventricular tip). He was presented with mild metabolic acidosis in the immediate postoperative period and progressed well, with a heart rate of 100 bpm, being extubated the next day, with hospital discharge one week after the procedure.

**Conclusion:** Congenital CABG is of significant risk to the newborn and may be secondary to immunological mechanisms, such as Sjogren’s syndrome, as seen in this case by the high anti-RO and anti-La levels in the NB. It can be treated by implanting a pacemaker with good evolution.

109438

Modality: E-Poster Scientific Initiation – Case Report

Category: CONGENITAL AND PEDIATRIC CARDIOLOGY

## Electrical Cardioversion in the Resolution of Atrial Flutter in a Newborn: A Case Report

PEDRO GUSTAVO STEVANATO DE OLIVEIRA^1^, Nadine Edda Corrêa^2^, Georgia Marques Jardim^1^, Gustavo Gianesini^1^, Bárbara Zottis^1^

(1) Universidade Federal de Ciências da Saúde de Porto Alegre; (2) Universidade Federal de Santa Catarina, Campus Araranguá

**Introduction:** Atrial flutter (AF) is a supraventricular tachyarrhythmia, rare in neonates, marked on the Electrocardiogram (ECG) by irregular, rapid atrial activity with 280–500 beats per minute (bpm), generally associated with 2:1 atrioventricular (AV) conduction. It usually affects children with cardiac malformations, but it can also develop in newborns with normal cardiac anatomy. Sinus rhythm can be restored with antiarrhythmic drugs, electrical cardioversion, or transesophageal stimulation.

**Case Description:** 8-day-old male newborn, cesarean delivery due to maternal desire, at term, without complications, with 24-hour Holter showing AF with ventricular response ranging between 105 and 235 bpm, with an average of 168 bpm, in addition to AV conduction in a 2:1 ratio. After five days of finding AF in the exam, propranolol was started, with no adequate response. The patient was transferred to the Pediatric Intensive Care Unit of the reference hospital in the city, where he arrived hemodynamically stable, without vasoactive drugs, on room air, and without the need for oxygen therapy. ECG and echocardiogram were requested, continued monitoring and anticoagulation with heparin 29IU/kg/h was initiated in case of intra-atrial thrombus. Echocardiogram performed the following day showed mild enlargement of the cardiac chambers, biventricular systolic function in the lower limits of normality, mild mitral regurgitation, and absence of intracardiac thrombi. Electrical cardioversion was chosen. Pre-procedure orotracheal intubation was performed with a rapid intubation sequence, an uneventful procedure. Two cardioversion attempts were performed with 2 Joules (0.5 joules/kg) each, successfully after the second. It was documented an electrocardiogram after the procedure with sinus rhythm. The patient was extubated throughout the day without complications. Anticoagulation was suspended 96 hours after cardioversion. He remained hospitalized, stable, without new arrhythmias for seven days after the procedure. He was discharged with a referral for cardiological follow-up within 30 days.

**Conclusion:** Despite being potentially fatal, atrial flutter in neonates has a good prognosis when diagnosed early, with electrical cardioversion representing an excellent therapeutic option, as evidenced in the above case.

109444

Modality: E-Poster Scientific Initiation – Case Report

Category: CONGENITAL AND PEDIATRIC CARDIOLOGY

## Congenital Atrioventricular Block: Diagnosis, Treatment and Follow-Up in a Child 01 Year and 8 Months Old

ISABELLA CAROLINE SOUZA DANTAS^1^, Marco Arthur Queiroz Passos^1^, Mary Anny Silva Fonseca^1^, Tarcisio Esdras Araujo Moura^2^, Jose Carlos Pachon Mateos^2^

(1) Faculdade Ages de Medicina; (2) Associação Beneficente Síria HCOR

**Introduction:** Bradyarrhythmias are rare in children, the main, of significance is the complete atrioventricular block (CAVB), whose reported incidence is one case for every 25,000 live births, being more predominent in females. Children with a normally structured heart, in general, have a congenital etiology and are often linked to the presence of maternal systemic lupus erythematosus (SLE).

**Case Report:** M.E.C.A.A.P., 01 year and 8 months old, presented dyspnea between feedings, low weight gain and syncope for +/–02 months. An electrocardiogram was performed, which showed a CAVB and a transthoracic echocardiogram (ECOTT) with a dilation of the left chambers. In the family history, mother with SLE. On 07/04/2018, an implantation of a definitive unicameral pacemaker by puncture of the left subclavian vein was performed, the electrode was positioned in the middle septum of the right ventricle and atrial loop was performed for growth curve. After implantation of the device, the patient evolved with significant improvement in dyspnea, with no recurrence of any syncope episodes and with the last ECOTT, performed four years after implantation, within normal limits.

**Conclusion:** Congenital CAVB is a rare disorder and occurs due to the pathological changes in the AV conduction system that can be associated or not with congenital heart disease. In 50% of cases, it occurs due to malformation of the conduction system and is associated with structural diseases, the most frequent being left atrial isomerism and corrected transposition of the great arteries. Autoimmune CAVB, associated with maternal anti-Ro and Anti-La antibodies, represents approximately 40% of cases. This pathology can evolve with ventricular dysfunction, severe heart failure and even fetal or neonatal death, justifying the importance of awakening the academic community for early diagnosis and adequate therapy. In the case described above, the patient had signs and symptoms of low cardiac output, syncope and growth issues. In addition, maternal autoantibodies (Anti-Ro and Anti-La) and heart rate lower than 70 bpm were still present with structural heart disease or ventricular dysfunction. For these reasons, a pacemaker was indicated, whose endovascular implant was performed with the atrial loop aiming at the child’s growth curve. After implantation of the device, the patient’s s symptoms and heart disease improved, in a four-year follow-up.

109474

Modality: E-Poster Scientific Initiation – Case Report

Category: CONGENITAL AND PEDIATRIC CARDIOLOGY

## Case Report of a Patient Diagnosed with Cat Eye Syndrome Presenting a Total Anomalous Pulmonary Venous Return (TAPVR)

WALDEMIR FERRARI JUNIOR^1^, Mariana Castro Pires^1^, Júlio Pasquali Andrade^1^, Leonardo Nunes Sanson^1^, Rafael Fabiano Machado Rosa^1^

(1) Universidade Federal de Ciências da Saúde de Porto Alegre

**Introduction:** Cat eye syndrome (CES) is a rare disease caused by a partial tetrasomy of chromosome 22. We aim to report a patient with CES presenting a total anomalous pulmonary venous return (TAPVR).

**Case Description:** The patient was the fourth child of a couple with no previous history of similar cases in the family. The child was a non-identical twin and was born by cesarean section, premature, weighing 2,240 g, with a length of 46 cm and a 6/8 Apgar score. The child needed oxygen therapy and mechanical ventilation immediately after birth, when a cardiac murmur was discovered. Due to an anal atresia with a vaginal fistula, the child required a colostomy surgery. Echocardiography revealed a non-obstructive TAPVR. The child underwent a cardiac surgery with 15 days of life, and kept the sternum open in the post-operative period. In the newborn evaluation, a right iris coloboma was also observed, in addition to both bilateral preauricular appendages and pits, micrognathia and a cutaneous appendage at the anus’ topology. The karyotype test revealed a partial tetrasomy of chromosome 22, resulting from a dicentric supernumerary marker chromosome: inv dup (22) (pter->q11.2::q11.2->pter). The child evolved with a persistent chylothorax, requiring thoracic drainage and pleurodesis, and died with 2 months old.

**Conclusion:** Congenital heart defects are observed in more than half of patients with CES, especially TAPVR. When patients present extracardiac manifestations, as coloboma, preauricular pits/tags and anal atresia, TAPVR should be investigated.

109582

Modality: E-Poster Scientific Initiation – Case Report

Category: HEART FAILURE/CARDIOMYOPATHY/TRANSPLANT

## Pediatric Hypertrophic Cardiomyopathy Due to Biallelic Pathogenic Variant in ALPK3: A Case Report

JOÃO VICTOR ANDRADE PIMENTEL^1^, Júlia Souza Diniz^1^, Beatriz Luduvice Soares^1^, João Paulo Dias Costa^1^, Emerson Santana Santos^1^

(1) Universidade Federal de Sergipe

**Introduction:** Alpha-protein kinase 3 (ALPK3) is one of the genes related to the development of cardiomyopathy. Patients with pathogenic variants in ALPK3 may present dilated cardiomyopathy that can progress to hypertrophic cardiomyopathy with poor systolic function. Biallelic pathogenic variants can cause several clinical features, with early-onset cardiomyopathy and extracardiac manifestations.

**Case Report:** A 16-year old male was referred to our cardiogenetics outpatient clinic due to the diagnosis of non-obstructive concentric hypertrophic cardiomyopathy (HCM) at the age of 10. Born from healthy and non-consanguineous parents. He has four siblings without any cardiac conditions. His maternal and paternal grandparents had sudden cardiac deaths. Extracardiac features were also observed (facial dysmorphism, downslanting ears, short neck). Patient has also depression and has been assisted by a psychiatrist. He has been using beta-blocker and fluoxetine. A HCM gene panel found two different pathogenic variants on ALPK3 gene. Genetic studies confirmed that both parents were carriers. Cardiac magnetic resonance showed a maximal wall thickness of 33 millimeters.

**Conclusion:** This case report highlights the importance of searching for the etiology of HCM and the genetic counseling for proper management of patients and their families.


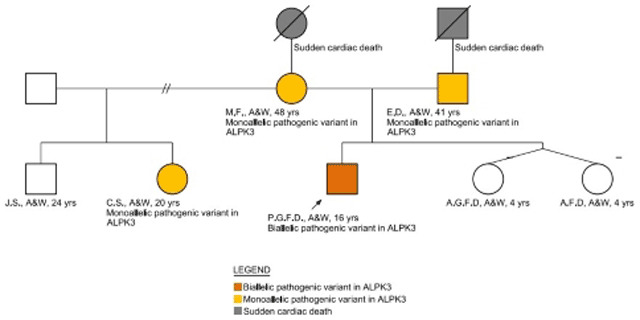


109604

Modality: E-Poster Scientific Initiation – Case Report

Category: PERICARDIUM/ENDOCARDIUM/VALVOPATHIES

## Subendocardial Calcification and Ventricular Tachycardia of Undetermined Etiology: A Case Report

DIEGO ARAÚJO SILVA^1^, Thaísa Moreira Cenção^1^, Gabriela Rodrigues de Oliveira^2^, Ricardo Santiago Ferreira Coelho^2^, Juliano Novaes Cardoso^2^

(1) Faculdade Santa Marcelina; (2) Hospital Santa Marcelina

Myocardial calcification, for the most part, has an etiology related to chronic pericardial diseases of multiple causes (viral, metabolic, rheumatologic). 67% of the patients with cardiac calcification have symptoms of heart failure; 8% have chest pain; 6%, abdominal pain; 4%, atrial arrhythmias; and 5%, symptoms suggestive of cardiac tamponade¹. The present study aims at reporting a case considered unprecedented in the literature because although most cases of cardiac calcification are related to constrictive pericarditis, the case in question is a calcification secondary to a subendocardial infarction, not correlated with any identified etiology. A 69-year-old female patient, seen due to complaint of acute vertigo, which evolved with unstable ventricular tachyarrhythmia and in need of electrical cardioversion. After cardiological evaluation, coronary cineangiography was submitted without alterations; However, ventriculography calcifications were identified leading to myocardial resonance imaging for differential diagnosis, which showed: subendocardial calcifications in inferolateral, basal, middle, and apical segments, compatible with subendocardial infarction. Due to the impossibility of surgical removal of the calcifications and the occurrence of ventricular tachycardia, implantation of an implantable cardioverter defibrillator was indicated. The patient presented stabilization of the condition and remains under outpatient follow-up. Unlike what is presented in the literature as the most common, the patient evolved with subendocardial calcifications of unspecified etiology, without a positive clinical history, nor infectious, metabolic, or rheumatological markers identified during the etymological evaluation performed. Subendocardial calcification is very rare, with few studies on this subject being found. Adequate planning and outpatient follow-up should be performed, considering subendocardial calcification, ICD, and the team’s experience.


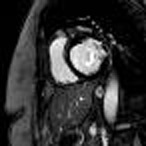


109652

Modality: E-Poster Scientific Initiation – Case Report

Category: CONGENITAL AND PEDIATRIC CARDIOLOGY

## Aortic Coarctation and Bicuspid Aortic Valve with Severe Regurgitation Being Diagnosed in Adulthood and Treated with Extra-Anatomical Tube Implantation and Valve Plasty

MARIA GABRIELA PIMENTA DOS SANTOS^1^, David Ferreira de Lima Duarte^1^, Maria Carolina Terra Cola^2^, Fabio Akio Nishijuka^2^, Thaíssa Santos Monteiro^2^

(1) Universidade Estacio de Sá/IDOMED; (2) Instituto Nacional de Cardiologia (INC)

**Introduction:** Aortic coarctation (AC) and bicuspid aortic valve (BAV) are commonly associated. Diagnosed of AC must be early.

**Case Report:** A 23 year-old patient diagnosed with AC and BAV after he had been hospitalized due to heart failure. Had many suggestive signs of AC in the physical exam, such as reduced pulse in the lower limbs, significant gradient of blood pressure between upper and lower limbs, and even a disproportion of upper and lower limbs development and signs of a significant aortic regurgitation as well. Echocardiogram (ECHO) identified BAV with severe regurgitation, left ventricle (LV) enlargement with moderate systolic dysfunction, monophasic blood flow in the distal descending aorta and abdominal aorta. Chest x-ray showed enlargement of the heart and erosions of the inferior border of the costal arches. Angiotomography (AT) showed a focal stenosis after the left subclavian artery, multiple collateral vessels and brachiocephalic trunk aneurysm. The initial strategy was to stent the AC and then treat the BAV surgically. The patient was submitted to an attempt of percutaneous treatment, but there was no success in passing the wire guide through the stenosis. It was then performed surgery with extra-anatomical tube implantation and aortic valve plasty with successful results and recovery.

**Conclusion:** AC associated with BAV with significant regurgitation can evolve with significant impairment of the LV, impacting the morbidity and mortality of a young patient. The physical exam can be very helpful in the suspicion of these diagnoses, highlighting the importance of a thorough physical exam. Confirmation with non invasive tests are required, such as ECHO and AT, but catheterization will evaluate the gradient of the AC and often treat the lesion in the same procedure.


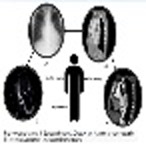


110116

Modality: E-Poster Scientific Initiation – Case Report

Category: CARDIOVASCULAR SURGERY

## Heyde‘s Syndrome: Presentation, Pathophysiology and Management

RODRIGO BATISTA WARPECHOWSKI^1^, Thomás Ranquetat Andrade^3^, Nicholas Travi Dornelles^3^, Iuri Schwaab^1^, Paulo Warpechowski^2^

(1) Instituto de Cardiologia do Rio Grande do Sul; (2) Sociedade de Anestesiologia (SANE); (3) Escola de Medicina da PUCRS

**Introduction:** Heyde’s syndrome (HS) is described as a multisystem disorder, characterized by a triad composed of aortic stenosis (AS), angiodysplasia (AD) of the gastrointestinal tract and acquired von Willebrand syndrome (AVWS) type IIA. In this case report, we present the evolution of a patient with HS presenting symptoms of congestive heart failure (CHF) and anemia.

**Case Report:** Female patient, 73 years old, hypertensive, diabetic, dyslipidemic, smoker, with a history of iron-deficiency anemia and atrial fibrillation, admitted for aortic valve replacement. Complains of dyspnea, chest pain and symptoms of CHF. Echocardiogram showed severe aortic valvular stenosis (0,6 cm²), calcified valve leaflets, biatrial overload, left ventricular concentric hipertrofy, and pulmonary hypertension. The patient presented anemia, (Hb 6.9 g/dL), and received packed red blood cells treatment. After 3 days, there was an improvement in her blood count, with acceptable haemoglobin levels. No coronary lesions. Blood transfusion and CHF therapy were performed, with correction of congestion and dyspnea. A valve replacement procedure with biological prosthesis was performed without intercurrences. A new episode of anemia (Hb 6,1 g/dL) due to gastrointestinal bleeding occurred, presenting melena and requiring a new blood transfusion. The patient was discharged with general improvement, with a request for colonoscopy in search of AD.

**Conclusion:** The most accepted AVWS mechanism proposes that in a situation in which shear forces are increased, as in AS, conformational changes occur in the multimers of von Willebrand Factor as it passes through the turbulent flow of a stenotic valve, causing interference in the control of angiogenesis and in the distribution of clotting factor VIII throughout the body. This association provides an important pathophysiological basis that supports valve replacement as a key strategy for long-term resolution of recurrent bleeding. Currently, most patients with HS after valve replacement have a reduction or disappearance of bleeding episodes. Furthermore, transcatheter aortic valve replacement (TAVR) has shown excellent results as well, with fewer perioperatory complications. Despite TAVR reduced risk, there are no differences in outcomes in terms of all-cause mortality in patients with HS undergoing these procedures. In addition, in patients who received bioprosthesis, the risk of recurrence of bleeding was 45% lower than in the mechanical ones group.

109729

Modality: E-Poster Scientific Initiation – Case Report

Category: CONGENITAL AND PEDIATRIC CARDIOLOGY

## Russell-Silver Syndrome and Congenital Heart Disease: Report of a Rare Association

WALDEMIR FERRARI JUNIOR^1^, Capitulino Camargo Junior^1^, Júlio Pasquali Andrade^1^, Vitor Reis de Souza^1^, Rafael Fabiano Machado Rosa^1^

(1) Universidade Federal de Ciências da Saúde de Porto Alegre

**Introduction:** Russell-Silver Syndrome (RSS) is a genetically heterogeneous condition characterized by the fault of the primordial growth. Our goal was to report a case of RSS presenting transposition of great vessels.

**Case Description:** The patient is the fifth child of a non-consanguineous couple, without history of similar cases in the family. The child was born of vaginal delivery, with 37 weeks of gestation, weighing 1,785 grams and with Apgar scores of 7/8. He was hospitalized in the neonatal intensive care unit requiring oxygen therapy. In this period, there was suspicion of a congenital heart disease. Echocardiography showed transposition of great vessels, persistence of the ductus arteriosus, ventricular septal defect and atrial septal defect of patent oval foramen type. The child underwent cardiac surgery and resection of the ductus arteriosus, as well as recovery and atrial septal defect repair on the thirteenth day of life. In the physical examination, with 11 months old, growth retardation was observed, upslanting palpebral fissures, epicanthic folds, wide and low nasal root, high arched palate, micrognathia, bilateral single palmar crease, fifth fingers clinodactyly, bilateral camptodactyly from the second to the fifth hand fingers, lower lombo asymmetry (the left member was smaller than the right), overlapping toes and hypotonia. He evolved with neuropsychomotor delay and feeding difficulties. His karyotype test was normal.

**Conclusion:** The sum of the presenting findings our patient was compatible with the diagnosis of RSS. The pre and postnatal growth retardation and limb asymmetry are considered major criteria for diagnosis. Congenital heart diseases are rarely described among these individuals.

109770

Modality: E-Poster Scientific Initiation – Case Report

Category: CARDIOVASCULAR SURGERY

## Necrotizing Aortitis with Aortic Dissection and Libman-S Sacks Endocarditis in a Lupus Patient

DAVID FERREIRA DE LIMA DUARTE^1^, Maria Gabriela Pimenta dos Santos^1^, Maria Carolina Terra Cola^2^, Fábio Akio Nishijuka^2^, Thaíssa Santos Monteiro^2^

(1) Universidade Estacio de Sá/IDOMED, Campus Città e Instituto Estadual de Cardiologia Aloysio de Castro/IECAC; (2) Instituto Nacional de Cardiologia

**Introduction:** Systemic lupus erythematosus (SLE) is an autoimmune disease. It is associated with multisystem organ damage mediated by autoantibodies and immune complexes. Lupus Aortitis is an uncommon and potentially fatal complication of SLE.

**Case Description:** Female 37-year-old, known to have SLE 15 years ago and Sjögren’s syndrome. Has had, lupus nephritis type IV, sensory motor polyneuropathy, idiopathic thrombocytopenic purpura and depression. Reports irregular use of azathioprine and hydroxychloroquine when presented a chest pain radiating to the neck and jaw after emotional stress, which lasted 15 minutes. Transesophageal echocardiographym identified an aneurysm of the ascending aorta measuring 4.9 cm with dissection 1.6 cm above the aortic valve, and severe tricuspid regurgitation with overload of the right ventricle. Angiotomography confirmed the diagnosis of aortic dissection Stanford A, Debakey I. was performed with replacement of the ascending aorta and arch, resection of vegetations in the tricuspid valve (TV) that was identified through histopatological analysis as thrombus, and plastic repair of TV. Controlled angiotomography after surgery identified several saccular aneurysms in the aortic root. FDG pet scan was performed to assess the SLE activity in the aorta, which identified inflammatory activity. Adjustments of the medical treatment were made in order to control lupus activity before the patient can be submitted to another surgery.

**Conclusions:** Lupus aortitis is a rare and severe complication of SLE, and surgical treatment as well as controlled of the rheumatological disease are required. The FDG pet scan can help in assessing the inflammatory activity of the disease, determining adjustments of the medical treatment and the best timing for surgery.


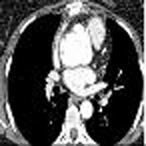


109783

Modality: E-Poster Scientific Initiation – Case Report

Category: HEART FAILURE/CARDIOMYOPATHY/TRANSPLANT

## Association between COVID-19 and Rapid Progression of Graft Vascular Disease in Late Heart Transplantation: Unexplored Link or Spurious Relationship?

BRUNA DE ALMEIDA FREIXEDELO^1^, Mateus de Sousa Cavalcante^1^, Dara Medeiros Mendes^1^, Jefferson Luís Vieira^2^, Leandro Cordeiro Portela^1^

(1) Universidade Federal do Ceará (UFC), campus Sobral, Sobral/CE-Brazil; (2) Hospital de Messejana Dr. Carlos Alberto Studart Gomes, Fortaleza/CE-Brazil

**Introduction:** Heart transplant (HT) recipients are at an increased risk of complications from COVID-19 due to chronic immunosuppression and comorbidities. Yet, no immunological link has been proven to explain an association between COVID-19 infection and late post-HT complications, including graft vascular disease (GVD) or late rejection.

**Case Description:** Male patient, 63 years old, with a history of HT for 18 years, admitted for HF and LVEF 35% one month after COVID-19 and change of immunosuppressive regimen due to unavailability of sirolimus in the health system. The pharmacological stress echocardiogram with dobutamine had been normal in the previous year. Diagnostic screening with endomyocardial biopsy (EMB) and panel of antibodies (including CD3, CD68, CD20 and C4D) ruled out cellular and/or humoral rejection, showing extensive interstitial and endocardial fibrosis, with myocytic hypertrophy and ischemic pattern; coronary angiography revealed significant multivessel coronary disease, with ectasias and rosary bead-like occlusions, confirming the clinical suspicion of GVD. Subsequent intracoronary ultrasound identified a minimum luminal area of 5 mm^2^ and the Heart Team opted for conservative management of the LVD.

**Conclusion:** GVD consists of the narrowing of the arterial lumen, due to the thickening and pathological remodeling of the tunica intima, due to the infiltration of mononuclear inflammatory cells. This condition has multiple possible etiologies, with immunological and non-immunological factors involved in the pathophysiology. The disease may result from a local and systemic inflammatory response initiated by alloantigen-dependent factors and non-immunological stress agents. The patient reported presented a clinical picture compatible with HFrEF due to decompensated GVD one month after infection by COVID-19 and change of the immunosuppressive regimen, without anatomopathological evidence of cellular or humoral rejection. In rare cases, there is evidence of accelerated progression of GVD from grade 0 (angiographically undetectable) to grade 3 (graft failure) in less than 4 months. Consequently, we believe that the systemic inflammatory response to SARS-Cov-2, with widespread cytokine production, direct myocytic damage, and vascular inflammation, may have played a key role in GVD progression. More studies with adequate methodology are needed to elucidate this potential association between GVD, myocardial damage and COVID-19 in HT recipients.

109816

Modality: E-Poster Scientific Initiation – Case Report

Category: HEMODYNAMICS AND INTERVENTIONAL CARDIOLOGY

## Spontaneous Coronary Dissection Angioplasty After Thrombolysis: Case Report

MATHEUS RODRIGUES TEIXEIRA NETTO^1^, Eduardo Comazzeto dos Reis^2^, Selma Rodrigues Chaves^2^, Alessandra Teixeira de Oliveira^2^, Valter Correia de Lima^2^

(1) Universidade Federal de Ciências da Saúde de Porto Alegre; (2) Santa Casa de Misericórdia de Porto Alegre

**Introduction:** Spontaneous coronary artery dissection (SCAD) is an important cause of myocardial infarction. The increase in its occurrence is related to the widespread use of coronary angiography in recent years. The etiology of SCAD is poorly understood. Its pathophysiology consists of separation of the arterial wall layers by intimal rupture or intraparietal hemorrhage, so that luminal narrowing and consequent coronary syndrome with elevation of biomarkers occur. Angiography shows the appearance of extraluminal contrast, multiple radiolucent lumens, and intraluminal filling defects. Conservative treatment is recommended, given the spontaneous healing of most lesions, while thrombolysis is contraindicated. Revascularization should be considered in case of ongoing ischemia, hemodynamic instability, arrhythmias or involvement of the left main coronary artery.

**Case Report:** Female patient, 54 years old, hypertensive and diabetic, presented an episode of oppressive retrosternal pain. In the first evaluation, electrocardiogram showed a current from a subepicardial lesion in the lower-dorsal wall. At the origin hospital, loading doses of ASA and Clopidogrel were administered, followed by thrombolysis with Alteplase and Enoxaparin. Due to the absence of reperfusion criteria, she was referred for rescue catheterization, which showed a left main coronary artery (LMCA) with 98% stenosis and occlusion of the circumflex (LCX) and anterior descending (LAD) branches. After LMCA predilation, LMCA and LAD dissection were diagnosed. Implantation of 3 drug-eluting stents was performed in LMCA, LAD and LCX, with good angiographic results. Follow-up was given with Aspirin, Clopidogrel, Simvastatin and Metoprolol Succinate.

**Conclusion:** The increase in the occurrence of SCAD brings with it the need for new studies to facilitate the understanding of how to manage it. Patients‘ inadvertent thrombolysis can worsen the condition and add to the need for new interventions. Therefore, whenever possible, all approaches should be based on the findings of coronary angiography.


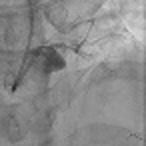


109825

Modality: E-Poster Scientific Initiation – Case Report

Category: HEART FAILURE/CARDIOMYOPATHY/TRANSPLANT

## Recurrent Gastrointestinal Bleeding in a Patient with Obstructive Asymmetric Septal Hypertrophic Cardiomyopathy, a Rare Form of Heyde Syndrome?

ANA OLÍVIA DANTAS^1^, KELSON KEMUEL CONFESSOR DE SOUSA^2^, THALINY BATISTA SARMENTO DE OLIVEIRA^1^, KELTON DANTAS PEREIRA^2^, FABIO MASTROCOLA^2^

(1) UNIVERSIDADE FEDERAL DO RIO GRANDE DO NORTE; (2) HOSPITAL UNIVERSITÁRIO ONOFRE LOPES

**Introdução:** Heyde’s Syndrome (HS) is classically described as an association between aortic stenosis and gastrointestinal bleeding from angiodysplastic lesions. Acquired von Willebrand factor (fVW) deficiency is related to its pathogenesis and results from hemodynamic stress on the stenotic valve. It is believed that valve replacement reduces the risk of bleeding, with success in 80% of cases.

**Relato De Caso:** Female patient, 60 years old, with chronic intestinal bleeding due to vascular ectasias in the gastrointestinal tract. She had clinical decompensation with major bleeding. Upper digestive endoscopy showed ectasia in the bulb and in the second and third duodenal portions without bleeding. Colonoscopy showed two angiectasia of the cecum and colon. On physical examination, aortic and mitral ejection systolic murmur was observed. Patient was unaware of previous heart disease. The ECG showed signs of left ventricular overload and the echocardiogram revealed asymmetric septal hypertrophy with left ventricular outflow tract (LVOT) obstruction – septum 26 mm and maximum systolic gradient 230 mmHg – and significant mitral regurgitation. Thus, the hypothesis of hypertrophic cardiomyopathy (HCM) with possible associated Heyde syndrome was raised, because, similarly to aortic stenosis, HCM in its obstructive asymmetric septal form could lead to the same mechanism of degradation of von Willebrand multimers of high molecular weight by the ADAMST13 enzyme due to the high shear stress generated by the interventricular septum in the LVOT. However, the dosage of Von Willebrand factor antigen (fVW) and ristocetin cofactor were normal.

**Conclusão:** We describe a case of probable SH in a patient with bleeding from angiodysplasias in the gastrointestinal tract associated with hypertrophic cardiomyopathy. Despite the normality of the VWf antigen, this data does not invalidate a possible association between MCH and SH, since the isolated dosage of the von Willebrand factor antigen may be normal in patients with acquired von Willebrand disease. The patient was discharged after stabilization of intestinal bleeding and correction of anemia, using a beta-blocker with reduced LVOT gradient. If bleeding recurs, specific therapies can be tried to reduce the diameter of the interventricular septum, such as alcohol ablation, myectomy and radiofrequency ablation, aiming to correct the pathophysiological substrate responsible for gastrointestinal bleeding.

109833

Modality: E-Poster Scientific Initiation – Case Report

Category: HEART FAILURE/CARDIOMYOPATHY/TRANSPLANT

## Neprilysin Inhibitor and Chagas Cardiomyopathy Improvement

JOÃO CLEITON MARTINS RODRIGUES^2^, João Cleiton Martins Rodrigues^2^, Talles Levi Pereira Nogueira^2^, Márcio César Ribeiro Marvão^3^, João Maria Silva Rodrigues^1^

(1) Instituto Clinicárdio; (2) Universidade Federal do Pará (UFPA); (3) Instituto Evandro Chagas (IEC)

**Introduction:** Chagas heart disease (ChHD) is caused by the protozoan Trypanossoma cruzi, was discovered in 1909 by Carlos Chagas and to this day is among the main endemic diseases of the Amazonic region. In the chronic phase the symptoms begin, resulting from the replication of protozoan amastigotes in myocytes, which causes mechanical cell’s rupture and release of pro-inflammatory cytokines, resulting in loss of tissue function and fibrosis. From this there is the process of cardiac remodeling and heart failure due to chagas disease. In 2014, the PARADIGM-HF study reported that the use of sacubitril/valsartan (neprilysin inhibitor and angiotensin II receptor blocker) significantly reduced mortality and hospitalization for heart failure with reduced ejection fraction (EF) compared to the use of enalapril. Another research was conducted with ChHD with reduced EF doing sacubitril/valsartan treatment for 6 months, which showed an improvement in the functional classification of NYHA of patients, without significant improvement of the EF on doppler echocardiography (DEC).

**Description:** A male patient M.J.P.S., 46 years old, 2 years ago sought clinical cardiology complaining of asthenia, epigastralgia, weight loss and insomnia. She had systemic arterial hypertension; heart failure (NYHA III); IgG indirect hemagglutination test and bioelisa reagents for chagas, cardiomegaly (+++) on chest x-ray PA; DEC showing diffuse involvement of an important degree in the left ventricle (LV), a image suggestive of thrombi at LV apex (approximately 20 × 21 mm²), EF of 29%, LV diastolic dimension of 69 mm (05/25/2020). After addicting sacubitril/valsartan in the treatment with carvedilol 6,25 mg/day, furosemide 20 mg/day, spironolactone 12,5 mg/day and sodic levothyroxine 50 mcg/day for approximately 6 months, a significant clinical improvement was noticed and DEC reported an enhancement in EF: preserved cardiac chambers, EF of 67%, diastolic dimension of LV of 53 mm (02/26/2022).

**Conclusion:** Therefore, the sacubitril/valsartan, associated with carvedilol, spironolactone and furosemide improved the clinical outcome of a ChHD patient, reverting a condition of reduced EF. Then, that medication eschedule is effective as a treatment for chagas cardiomyopathy.

109841

Modality: E-Poster Scientific Initiation – Case Report

Category: NEGLECTED CARDIOVASCULAR DISEASES

## Holocardiac Involvement in a Patient with Adult-Onset Still Disease: A Case Report

HENRIQUE KRICHENKO LEDRA^1^, Marcelo Vier Gambetta^1^, Franciani Rodrigues da Rocha^1^, Caroline de Oliveira Fischer Bacca^1^

(1) UNIDAVI – Centro Universitário para o Desenvolvimento do Alto Vale do Itajaí

**Introducion:** Adult-Onset Still’s Disease is a rare condition, with a prevalence of 1–34 cases per million people, classified as a multigenic auto-inflammatory disorder, negatively affecting the patient’s cardiovascular system through pericardial and myocardial damage mainly, considered an important risk factor for the development of cardiovascular diseases.

**Case Report:** We reported ten years of evolution of a male patient, 30 years old, hospitalized due to presence of low blood pressure, jugular veins distention, and muffled heart sounds on cardiac auscultation, diagnosed with cardiac tamponade. A successful emergency pericardial drainage was performed. A month later he was hospitalized again because of chest pain, fever and dyspnea. Diagnosed as aortic valve endocarditis with important aortic regurgitation after echocardiological evaluation. Aortic valve replacement surgery with metallic valve implant was performed and the vegetation biopsy showed chronic inflammatory lesion with no infeccion associated. He was referred to outpatient evaluation with a rheumatologist doctor, who diagnosed him with Adult-Onset Still’s Disease. Ten years after his first cardiovascular disability, the patient presented an important hemodynamic disturbance being hospitalized due to acute coronary syndrome because of the presence of thrombus in the descending and diagonal coronary arteries. He underwent successful balloon catheter angioplasty.

**Conclusion:** Our hypothesis for the patient’s cardiac involvement is based on the complex pathophysiology of the disease and it’s possible complications. Pericarditis is well documented as a quite common Still’s Disease complication. However the same does not apply to aortic valve vegetation since just a few reports bring the same association between those conditions. As far as we know, based on the literature, the occurrence of an intracoronary thrombus in a patient with Still’s Disease is unprecedented and we consider the coagulation disorder the best pathophysiological hypothesis.


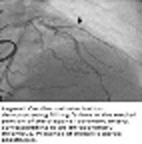


109856

Modality: E-Poster Scientific Initiation – Case Report

Category: ACUTE AND CHRONIC CORONARY DISEASE/THROMBOLYSIS

## Role of Pulse Wave Velocity in the Individual Relevance of Individuals at High Cardiovascular Risk – Case Report

DAVID FERREIRA DE LIMA DUARTE^1^, Maria Gabriela Pimenta dos Santos^1^, Julia Resende de Oliveira^1^, Gabriela Gama Zagni Jardim^3^, Lílian Soares da Costa^4^

(1) Universidade Estacio de Sá/IDOMED, Campus Città e Instituto Estadual de Cardiologia Aloysio de Castro/IECAC; (2) Universidade Estacio de Sá/IDOMED, Campus Città e Instituto Estadual de Cardiologia Aloysio de Castro/IECAC; (3) Universidade Estacio de Sá/IDOMED, Campus Città e Presidente Vargas e Instituto Estadual de Cardiologia Aloysio de Castro/IECAC; (4) Universidade Estacio de Sá/IDOMED, Campus Vista Carioca e Instituto Estadual de Cardiologia Aloysio de Castro/IECAC

**Introduction:** The analysis of arterial stiffness through the measurement of pulse wave velocity (PWV) has been listed as one of the oscillometric parameters among the best predictors of cardiovascular (CV) risk, when compared to the use of traditional scores and brachial artery pressure analysis, which, although consecrated in the literature, have numerous limitations and complexity in their evaluation. Different studies have shown that increasing PWV doubles the risk of CV events, CV mortality and death from all causes. In individuals at high CV risk, regardless their vascular age, this correlation is not entirely clear and defined.

**Case Report:** We report a cohort study of 163 individuals at high CV risk, object of a scientific initiation project at our institution. Male, 58 years old, white, hypertensive, obese, diabetic, submitted to percutaneous revascularization after acute coronary syndrome. Although with optimized prescription, it maintains irregular drug treatment and low adherence to lifestyle modifications. On examination, baseline blood pressure was 135 × 87 mmHg; FC 63 bpm; pulse pressure 46 mmHg. Vascular parameters showed central aortic pressure 120 mmHg and PWV 8.4 m/s, highlighting that the standards adopted for the medians adjusted for sex, age and presence of risk factor are 124 mmHg and 7.9 m/s, respectively. Remaining symptomatic in his outpatient risk stratification, he was submitted to a hemodynamic study, demonstrating severe coronary lesions (>80%) proximal in 4 vessels, and referred for surgical revascularization.

**Conclusion:** The analysis of PWV has been important as an additive value in the functional analysis of individuals with coronary artery disease (CAD), in different suggestions of new risk models. In this context, we believe that arterial stiffness, in addition to the possible early manifestation of atherosclerosis, as established in national and international guidelines, can be a CV prognostic marker of individual relevance in high CV risk, as demonstrated in this case, where the presence of high PWV could even be an additional factor in the severity and extent of CAD. Whether this finding could predict a higher risk of CAD evolution, its therapeutic response or even infer prognosis, so far there are no answers, but we hope to be able to contribute to future analyzes with our cohort study.

109919

Modality: E-Poster Scientific Initiation – Case Report

Category: CONGENITAL AND PEDIATRIC CARDIOLOGY

## Oral Facial Digital Syndrome: Report of a Patient Presenting an Atrioventricular Septal Defect Associated with Truncus Arteriosus

GUILHERME RODRIGUES VIANA^1^, Guilherme Taioqui Fioruci^1^, Helena Guedes da Rocha^1^, Rafael Fabiano Machado Rosa^1^

(1) UNIVERSIDADE FEDERAL DE CIÊNCIAS DA SAÚDE DE PORTO ALEGRE – UFCSPA

**Introduction:** Oral facial digital syndrome (OFDS) represents a heterogeneous group of disorders of genetic cause, characterized by oral, facial and limb anomalies with or without associated visceral malformations. Our aim was to describe a patient diagnosed with OFDS presenting an atrioventricular septal defect (AVSD) associated with truncus arteriosus.

**Case Description:** The pregnant, a 25-year-old woman, was in her third pregnancy and was referred at 20 weeks of pregnancy for presenting fetal ultrasound with description of congenital heart disease and polydactyly. The patient had a description of a previous pregnancy with a fetus with a history of hard palate and tongue abnormalities, polydactyly, syndactyly of fingers and toes, and suspected congenital heart disease. The child died on the first day of life. In the second trimester ultrasound performed in the current pregnancy at 20 weeks, the interatrial septum and the right ventricular outlet were not visualized. In addition, polydactyly of hands and feet was observed. Fetal echocardiography revealed a complete AVSD associated with pulmonary atresia. The fetal karyotype was normal (46, XY). The child was born by cesarean section at 38 weeks, weighing 3,200 g, with Apgar scores of 8 and 9. Physical examination revealed facial dysmorphia and tongue alteration indicative of the presence of hamartoma. There was also polydactyly of both hands, with the presence of 7 fingers, and syndactyly between the fourth and fifth fingers. There was also polydactyly of both feet, with partial syndactyly between the first two toes. Echocardiography performed shortly after birth revealed a complete type C AVSD and a ventriculo-atrial communication with a single right ventricular outflow valve. Computed angiotomography performed on the same day also showed the presence of a truncus arteriosus.

**Conclusion:** The clinical findings herein observed, including the cardiac ones, are compatible with the OFDS type II diagnosis, also known as Mohr syndrome. Cardiac malformations, overall ASD and VSD, have been reported in patients with this subtype of OFDS. Nevertheless, the truncus arteriosus, as observed in our patient, is considered to be a quite rare finding.

109982

Modality: E-Poster Scientific Initiation – Case Report

Category: CARDIOVASCULAR SURGERY

## Truncus Bovis, Abnormal Subclavian Artery and Aortic Aneurysm Without Genetic Inheritance: A Case Report

CAIO RESENDE DA COSTA PAIVA^1^, Brenna Pinheiro Zuttion^2^, Pedro Pimentel Rocha Faria^2^, Verônica Homem de Carvalho e Silva^1^, Ana Carolina Bezerra Goes^1^

(1) Universidade de Brasília; (2) Hospital Universitário de Brasília

**Introduction:** Among the most common congenital anomalies of the aortic arch, the retroesophageal right subclavian artery (RSA) (prevalence 0.5–1%) and Truncus bovis (prevalence 20–32%) stands out. These changes, mostly asymptomatic, are the result of defects in embryogenesis. Aortic aneurysm (AA) is an acquired anomaly and consists of a pathological dilation of this vessel. The case in question presents a patient in which these 3 alterations are present, without evidence of the presence of genetic syndrome (GS).

**Case Report:** Patient, female, 59 years old, complaining of paresthesia in the right upper limb and neck pain for 3 days. He has a history of arterial hypertension, surgical repair of ascending AA and aortic trunk in 2012, with a Dacron tube installed, without valve replacement. In use of atenolol, ASA, metoprolol, olmersartan + amlodipine. Physical examination without changes. He was admitted to the Hospital de Base de Brasília for investigation. An angiotomography of the chest and abdomen was performed, which showed type 1 thoracoabdominal AA, with a greater diameter of 75 mm in the descending segment, and intramural hematoma in this portion. Tortuous aorta except for the ascending portion, truncus bovis and retroesophageal ASD. Echocardiogram showed mild aortic insufficiency and coronary cineangiography showed stenosis of 60% in the anterior descending artery. SG were discarded. In view of this, we opted for a right carotid-subclavian bypass and, later, open surgery for thoracoabdominal AA, with cardiopulmonary bypass. Endovascular procedure was not performed due to impossibility due to aortic tortuosity. During the second operation, the patient evolved with myocardial infarction, refractory cardiogenic shock and death.

**Conclusion:** Anatomical changes of the aorta and its branches in combination are uncommon and poorly reported. When present, they occur mainly in patients with some genetic syndrome. Although usually asymptomatic, the congenital changes seen can lead to symptoms resulting from local compression, aneurysmal degeneration or atherosclerosis, causing dysphagia and paresthesias. In these cases, surgical intervention is the option of choice as well as for AA depending on its diameter and complications. Due to the high aortic tortuosity, percutaneous treatment was discarded, opting for open surgical repair of the vessel. The large scale of the surgery and the patient’s hemodynamic instability may have contributed to the outcome of the case.

110028

Modality: E-Poster Scientific Initiation – Case Report

Category: NEGLECTED CARDIOVASCULAR DISEASES

## Insidious Course of Infective Endocarditis in the Elderly: Considerations of the Diagnostic Challenge

BRENO VINICIUS DIAS DE SOUZA^1^, Humberto Cabral de Oliveira Filho^1^, Heron Alves Vale^1^, Gerson Barbosa do Nascimento^1^

(1) Universidade Federal do Rio Grande do Norte (UFRN)

**Introduction:** Infective endocarditis (IE) is characterized by inflammation of the endocardium, heart valves, or even an intracardiac device, such as pacemaker leads and prostheses. It is mainly by bacteria, and more, by fungi. Acute IE is usually caused by Staphylococcus aureus, and there is the subacute form, with slow evolution from weeks to months, with mild toxic, chronic fever and weight loss. A heart murmur may be present, as well as immunological and vascular phenomena.

**Case Report:** Male patient, 80-years-old, retired, is supported in a referral hospital with a weight loss of 12 kilograms in 5 months. During that time, he had sporadic low-grade fever, which gave way to the use of simple analgesia, followed by sweating. Still, had severe arthritis, with limited range of motion due to pain. He had anemia in this temporal cut. The patient is hypertensive and has a history of exchange cancer treated with radiotherapy and valve surgery by aortic operation 10 years ago. It evolved with persistence of the joint, with characteristics of the lower limbs. Still, general condition, suffered with feverish brand, without compromising the general condition. On examination, calculated oral without teeth, with unsatisfactory hygiene. There was a murmur in the aortic focus. It presented three changes persistently, with an echocardiogram showing vegetation in the aortic valve. He received antibiotic therapy with ceftriaxone and oxacillin, evolving with improvement.

**Conclusions:** The insidious course of the disease brought definitive treatment to establish a definitive diagnosis. The important echocardiogram image and the patient’s predisposition to valvular heart disease were used to guide the management, but it is noticed that there is a limitation for some times, since fever is poorly measured and that immunological manifestations are often not confirmed. Therefore, attention should be paid to non-positive germs such as blood cultures, such as Coxiella burnetii and fungi, which have a prolonged course.

111770

Modality: E-Poster Scientific Initiation – Case Report

Category: PERICARDIUM/ENDOCARDIUM/VALVOPATHIES

## 3D Printing in Mitral Stenosis’ Surgical Planning – Case Report

MATEUS DOS SANTOS BANDEIRA^1^, Ana Luísa Guedes de França-e-Silva^2^, Maria Luiza Novaes de Souza^1^, Eduarda Corrêa Maia^1^, Claudio Tinoco Mesquita^1^

(1) Universidade Federal Fluminense – UFF; (2) Universidade Federal de Goiás – UFG

**Introduction:** 3D printing is revolutionizing medical imaging. In cardiology, 3D printing is increasingly used by surgeons and health professionals, such as cardiovascular surgeons, in procedures with cardiac involvement, including mitral stenosis.

**Description:** A 57-year-old female patient reported dyspnea on moderate exertion, which began gradually and progressed to progressive worsening, being associated with orthopnea. She complained of chest discomfort. Patient had a history of interatrial communication corrected in 1987 associated with cleft repair of the anterior mitral leaflet, severe mitral regurgitation, tricuspid regurgitation, permanent atrial fibrillation, in addition to having systemic lupus erythematosus. A transthoracic echocardiogram demonstrated a cleft of the anterior leaflet of the mitral valve associated with severe regurgitation. 3D impression of the heart was performed with atrial and ventricular sections through preoperative CT images. The objective was to better understand the extent of the disease and assist in surgical planning, seeking to understand the potential benefit of the surgical approach. After 3D printing the selected approach was a median sternotomy with resection of the leaflets and implantation of a metallic mitral valvar prosthesis (numb er 3), associated with closure of the interatrial septum with a bovine pericardium patch and tricuspid annuloplasty with a number 28 ring. Unfortunately, patient died in 22nd postoperative day due to central venous catheter infection.

**Conclusion:** We describe the use of 3D printing to anticipate a realistic cardiac surgery, to guarantee a safe surgery, as well as to promote a reduction in the duration of the surgery. Due to the size of the IAC dimension, it is possible to allow a joint visualization of the defect size in the middle of a wide 3D impression of the target organ. This case reinforces the future promise of modeling for surgical planning purposes, in addition to the future of such modeling for education.


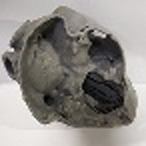


110385

Modality: E-Poster Scientific Initiation – Case Report

Category: NEGLECTED CARDIOVASCULAR DISEASES

## Clinical-Conservative Treatment Strategy After Coronary Dissection at the End of Pregnancy

GABRIEL ALVES MENESES^1^, Tauã Richel Belchior da Costa^1^, Kaio Saramago Mendonça^1^, Marcela Souza Carneiro^1^, João Lucas O‘Connell^1^

(1) Universidade Federal de Uberlândia

**Introduction:** Usually, the main causes of maternal mortality related or aggravated by pregnancy are hypertension, hemorrhage, puerperal infections, and miscarriage. However, other cardiovascular causes have contributed to increased pregnancy-related morbidity and mortality. In this scenario, arterial dissections (mainly of the aorta and coronary arteries) have an important role and also different therapeutic possibilities.

**Case Report:** Second-time pregnant, 34 years old, 35 weeks and 5 days, obese, without other comorbidities or gestational complications, presented with intense precordialgia with irradiation to the dorsum and left upper limb, lasting more than one hour after a light walk. Referred to the tertiary service with positive myocardial injury markers, dynamic T-wave in anterior wall on electrocardiogram (ECG), but without ST segment depression. Started propedeutics of Non-ST Segment Elevation Myocardial Infarction (NSTEMI). The hypothesis of coronary dissection was considered and initial drug treatment was chosen. Patient remained hospitalized and underwent cinecoronariography at 37 weeks of gestation, which showed dissection of the left main coronary artery involving the anterior descending and circumflex arteries with TIMI III coronary flow. Clinical treatment was maintained and caesarean section was performed at 38 weeks under general anesthesia. She presented, in the puerperium, a new presentation of chest pain, with dynamic ECG, without enzymatic elevation. Coronary angiography was repeated and improvement of the angiographic aspect of the coronary dissection was identified, and the expectant management was maintained. The binomial was discharged from hospital in good general condition and remained well for the last 6 months.

**Conclusion:** Due to hormonal changes and the hyperdynamic state, pregnancy alone increases the risk of acute myocardial infarction (AMI). Commonly, because we deal with young women, atherosclerotic diseases are not the main cause of AMI during pregnancy, but arterial dissections, which normally receive a differentiated therapeutic strategy. In addition, these dissections are associated with the occurrence of death or a reduction in maternal ejection fraction, which is directly related to maternal-fetal complications.

110398

Modality: E-Poster Scientific Initiation – Case Report

Category: HEART FAILURE/CARDIOMYOPATHY/TRANSPLANT

## A Novel Genetic BAG3 Gene Variant Associated with Peripartum Cardiomyopathy and Family History of Sudden Death

ANTONIO PEDRO LIMA COSTA PEREIRA^1^, Giovanna Lucieri Alonso Costa^1^, Ana Paula Cassetta dos Santos Nucera^1^, Maria Angelica de Faria Domingues de Lima^2^, Fábio de Souza^1^

(1) Universidade Federal do Estado do Rio de Janeiro (UNIRIO); (2) Hospital Universitário Gaffrée e Guinle (HUGG)

**Introduction:** Peripartum cardiomyopathy (PPCM) can be an initial manifestation of familial dilated cardiomyopathy (DCM). The BAG3 gene encodes a protein that is highly expressed in cardiac muscle and is involved in the anti-apoptosis effect. BAG3 pathogenic variant is associated with autosomal dominant DCM and has been reported in PPCM and in families undergoing sudden death.

**Case Description:** A 36-year-old black woman presented to the emergency department with palpitations while breastfeeding her 2-week-old daughter. She denied any other symptoms. Medical history included diabetes mellitus type 1 and preeclampsia. Electrocardiogram (ECG) exhibited ST segment deviation and inverted T-waves in the precordial leads. Echocardiogram revealed left ventricular enlargement with diffuse hypokinesis. There were no records of raised troponin levels. Weeks later, magnetic resonance (MRI) showed reduced left ventricular ejection fraction (29%) and late gadolinium enhancement detecting presence of fibrosis. In the patient’s family history, her father, brother and paternal half-brother died suddenly at 42, 28, and 26 years old, respectively. During a further investigation, a cardiomyopathy gene panel identified a heterozygous BAG3 pathogenic variant with a cytosine deletion at position 824 (c.824del). The result was consistent with the diagnosis of, BAG3-related conditions, including autosomal dominant DCM, and this variant has not been reported in the literature. Regarding her medical history, coronary artery disease was ruled out, and her coronary calcium score was 0. It was considered to place an implantable cardioverter-defibrillator, but myocardial findings on MRI were completely reverted as were ECG and echocardiogram repeated after three years. At present, the patient has no complaints. She is under clinical cardiology care, and genetic counseling will be provided for her relatives.

**Conclusion:** This case report highlights a rare occurrence of peripartum cardiomyopathy in a patient in which a novel pathogenic variant in the BAG3 gene was identified and related to a strong family history of sudden death.


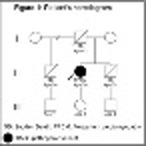


110547

Modality: E-Poster Scientific Initiation – Case Report

Category: CARDIAC ARRHYTHMIAS/ELECTROPHYSIOLOGY/ELECTROCARDIOGRAPHY

## Electrocardiographic Clues in a Rare Case of Hereditary Transthyretin Cardiac Amyloidosis

ANA GABRIELLA MEDEIROS^1^, Caroline Bittar Braune^2^, Marcio Aloysio Freitas Siqueira Junior^3^, Ana Paula Cassetta dos Santos Nucera^1^, Fábio de Souza^1^

(1) Universidade Federal do Estado do Rio de Janeiro; (2) Hospital Universitário Gaffrée e Guinle; (3) Hospital São Lucas

**Introduction:** Transthyretin cardiac amyloidosis (TTR-CA) is currently being discussed as a possible underlying cause of heart failure with preserved ejection fraction (HFpEF). Despite advances in cardiac imaging, monitoring electrocardiograms (ECGs) remains imperative for reducing TTR-CA misdiagnoses.

**Case Presentation:** A 78-year-old black man presented with breathlessness and progressive exercise intolerance. He had a medical history of mild hypertension controlled with losartan 50 mg once daily. Physical examination showed blood pressure of 120/80 mmHg, heart rate 66 bpm, and no edema or pulmonary congestion. Initial ECG was described as nonspecific and indicated a possible previous infarction. Two-dimensional echocardiogram (2D-Echo) revealed ejection fraction 55%, left ventricular (LV) hypertrophy (septum 13 mm), diastolic dysfunction grade 3, and no segmental changes. HFpEF was considered and a loop diuretic was prescribed. In fact, ECG demonstrated sinus rhythm, PR interval 220 ms, narrow QRS complex with left axis deviation, disproportional amplitude of QRS complex related to assumed LV hypertrophy by 2D-Echo, and absence of R-wave progression in the anterior leads, which were interpreted as a pseudo-infarction. A reduced global longitudinal strain (–13%) measurement was obtained with an “apical sparing” pattern. Scintigraphy with technetium pyrophosphate confirmed TTR-CA (Perugini grading scale = 3). Genetic testing detected a pathogenic variant in TTR gene (genotype V142I) in which cardiac involvement is predominant.

**Conclusion:** This report highlights ECG findings in a case of TTR-CA. Pseudo-infarction pattern and/or discordant amplitude of QRS compared to LV hypertrophy can be subtle clues indicating TTR-CA. Attention to the ECG is crucial to enable this challenging diagnosis.


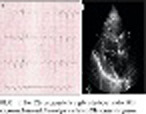


110896

Modality: E-Poster Scientific Initiation – Case Report

Category: CARDIOVASCULAR PHARMACOLOGY

## Marfan Syndrome: Importance of Drugs that Reduce Hemodynamic Stress in the Prevention of Aortic Aneurysm

GEORGIA MARQUES JARDIM ^1^, Rafael Fabiano Machado Rosa^1^, Marco Antônio Vinciprova Dall Agnese^1^, Rafaella Aléssio Naibo^2^, Alexandre Perin Decol^1^

(1) Universidade Federal de Ciências da Saúde de Porto Alegre- UFCSPA; (2) Universidade Federal do Rio Grande do Sul – UFRGS

**Introduction:** Marfan syndrome (MS), is a systemic connective tissue disorder with a high degree of clinical variability. The main manifestations consist of alterations involving the ocular, skeletal and cardiovascular systems. The short-term segment of a patient with MS, emphasize the importance of using propranolol in preventing the development of aortic aneurysm.

**Case Report:** A 13 years old female patient who had a paternal family history of cardiopathy in a cousin and grandparents. Both grandparents passed away due to heart disease. Patient full-term newborn, with 2,9 kg weight and 51 cm of height. The patient was forwarded for investigation of fainting and right facial paralysis within one week of evolution. The examination identified oblique palpebral slits, low ear implantation, important scoliosis, arachnodactyly in both hands and feet, joint hypermobility and Marfan’s habitus. In cardiac auscultation, was identified a systolic murmur 3+/6+. The electrocardiogram had a precordial repolarization alteration. The echocardiography showed normal systolic biventricular function, mild left ventricular enlargement, systolic prolapse of the mitral valve in both leaflets, moderate valve regurgitation, ectasia in the region of the sinuses of Valsalva, aortics and ascending aorta, and aortic regurgitation. Therefore, she started using propranolol 10 mg, 3 times a day. During his echocardiographic follow-up, no dilatation or appearance of aortic aneurysm was observed, in the period of three years. The spine radiography showed marked dextroconvex dorsal and left convex lumbar scoliosis. The ophthalmologic evaluation revealed bilateral iridodonesis, Bell’s palsy, alternating exotropia, and superior lens subluxation. All these findings led to the diagnosis of Marfan syndrome.

**Conclusions:** Drugs that decrease hemodynamic stress on the aortic wall, such as the use of β-blockers or angiotensin receptor blockers, are usually initiated at diagnosis or upon evidence of significant and/or progressive aortic dilatation in patients with MS. This therapy aims to prevent the development of aneurysms and the possible occurrence of aortic dissection, an important cause of death among these patients.

110607

Modality: E-Poster Scientific Initiation – Case Report

Category: CONGENITAL AND PEDIATRIC CARDIOLOGY

## Diabetic Embryopathy and its Relation with Congenital Heart Diseases

GRASIELE DO AMARAL MARTINS^1^, Carolina Guimarães Herzog^1^, Emanuella Lara Tarzo de Medina Coeli^1^, Rafaella Aléssio Naibo^2^, Rafael Fabiano Machado Rosa^1^

(1) Universidade Federal de Ciências da Saúde de Porto Alegre (UFCSPA); (2) Universidade Federal do Rio Grande do Sul

**Introduction:** Insulin-dependent diabetes mellitus (DM1) can lead to the presence of a spectrum of malformations known as diabetic embryopathy. The aim of this report was to discuss the findings of a fetus whose gestation course was characterized by diabetic embryopathy, highlighting its relationship with congenital heart disease (CHD).

**Case Description:** A 24-year-old, primiparous pregnant woman with a past medical history of DM1. Fetal ultrasound was performed outside the hospital at 22 weeks of pregnancy, the report described holoprosencephaly, congenital heart disease and single umbilical artery. Fetal echocardiography showed dextrocardia; double inflow tract atrioventricular connection to the left ventricle; single outflow tract ventriculoarterial connection and truncus arteriosus. Morphological ultrasound also showed increased amniotic fluid, single umbilical artery, and dilation of the third and lateral ventricles. The complementary examination also showed no visualization of the right kidney, the left kidney was small, and an abnormality was observed in the fetal spine. Fetal magnetic resonance imaging confirmed the finding of supratentorial hydrocephalus, in addition to the other malformations described on the ultrasound. The fetal karyotype was normal. The child was born by c-section at 33 weeks gestation, with a head circumference of 45 cm and Apgar scores of 3/7. Abdominal ultrasound showed that the right kidney was pelvic and the left kidney was multicystic dysplastic. Spinal x-ray showed hemivertebrae and butterfly vertebrae.

**Conclusion:** In the literature, there are reports that there is a relationship between diabetic embryopathy and fetal findings of VACTERL (vertebral defects, anal atresia, cardiac defects, tracheo-esophageal fistula, renal anomalies, and limb abnormalities) association, as observed in the present case report. CHD are part of the clinical spectrum of both conditions, with emphasis on those of the conotruncal type, i.e., involving the outflow tracts of the heart, as in the case of truncus arteriosus.

110855

Modality: E-Poster Scientific Initiation – Case Report

Category: NEGLECTED CARDIOVASCULAR DISEASES

## Takotsubo Syndrome Secondary to Catsaridaphobia

MIKE VINICIUS CANTO DE ANDRADE^1^, Danielle Campos de Almeida^1^, Gabriel Alves Meneses^1^, Fábio Vieira Fernandes^1^, João Lucas O’Connell^1^

(1) Universidade Federal de Uberlândia

**Introduction:** The cardiologic syndrome called “Takotsubo”, also called broken heart syndrome, is a type of stress-induced cardiomyopathy characterized by transient apical bulging and left ventricular dysfunction. The clinical picture usually mimics the same one of an acute myocardial infarction. In the absence of significant coronary obstruction, it courses with transient left ventricular dysfunction (usually apical). Characteristically, the clinical picture is preceded by intense emotional and/or physical stress. There is little data in the literature on fear or phobia reactions to insects or other potentially repulsive animals that lead to this condition.

**Case Report:** Female patient, 58 years old, previously healthy, asymptomatic. Started with typical intense and tight chest pain for 2 hours. She describes similar, less intense episodes in the 2 days prior to arrival at the hospital. Given a history of catsaridaphobia, she reported significant acute stress after contact with a cockroach 12 hours before the initial episode. Physical examination without changes. Electrocardiogram: sinus rhythm, anteroseptal inactive electrical zone and slight ST-segment elevation in the anterior wall. There were typical enzymatic alterations. Cardiac catheterization did not identify coronary stenoses, but a pattern of left ventricular apical akinesia characteristic of Takotsubo Syndrome. Echocardiogram confirmed apical akinesia. Cardiac Magnetic Nuclear Resonance, 5 days later, showed basal anterior myocardial edema and medial septum, without fibrosis and with improvement in ventricular function. The patient had a good clinical evolution and was discharged from the hospital on the 7th day, with normal global contractile function. She is asymptomatic one year after the initial event.

**Conclusion:** The reported clinical picture simulated an acute myocardial infarction, once absence of coronary obstruction was established, along with apical systolic dysfunction of the left ventricle and hyperkinesia of the basal portions. Transient acute ventricular dysfunction was clearly related to intense emotional stress, fulfilling current criteria for the diagnosis of stress-induced cardiomyopathy syndrome. The inducing stress of the condition was physical contact with an insect to which he has a phobia, whose occurrence may be the stressor mechanism for a part of the cases that develop Takotsubo syndrome.

110928

Modality: E-Poster Scientific Initiation – Case Report

Category: COVID-19 AND CARDIOVASCULAR SYSTEM

## The Need of Prompt Diagnosis of Mis-C to Prevent Cardiac Sequelae: A Case Report of a 2-Year-Old Patient

ALINE PETRACCO PETZOLD^1^, Bibiana Liberman Thomé^1^, Fernanda Serratte Warlet^2^, Larissa Arruda Ferreira^2^, Andrea Mabilde Petracco^2^

(1) Pontifícia Universidade Católica do Rio Grande do Sul; (2) Hospital Materno Infantil Presidente Vargas

**Introduction:** Kawasaki disease (KD) is one of the most common primary systemic vasculitis in childhood, being the main cause of acquired heart disease in children. Throughout the COVID-19 pandemic, cases with manifestations similar to KD were reported, being classified as Multisystem Inflammatory Syndrome (MIS-C) following SARS-CoV-2 infection. Most findings for the disease are nonspecific, such as high, persistent fever, and inflammatory findings without an infectious explanation. Despite usually being a self-limited disease, prompt diagnosis and management are fundamental to prevent cardiac sequelae, since coronary artery aneurysms occur in 25% of untreated or poorly treated cases.

**Case Report:** Male, 2-year-old patient, had contact with SARS-CoV-2 in January 2022. Admitted to the emergency for persistent fever for 20 days, with peaks every 2 days, associated with greenish coryza, rhinorrhea and eye discharge. A week before, was treated for otitis media with 3 doses of ceftriaxone, having momentary improvement, but further worsening. In the hospital, he presented fever, upper airway infection symptoms, conjunctival hyperemia with bilateral discharge, exsudate in oroscopy, opacity in the right tympanic membrane, hemoglobin of 10.2, and negative hemocultures and PCR for COVID-19. Echocardiography revealed patent foramen ovale and dilation of the anterior descending coronary artery, raising the hypothesis of post-COVID-19 MIS-C. The patient was started on acetilsalicylic acid (AAS) and methylprednisolone, persisting with mild fever. Since it was not available previously, he received intravenous immunoglobulin (IVIg) only 12 days after the diagnosis, showing clinical improvement. He was discharged with AAS and a scheduled echocardiogram in 15 days.

**Conclusion:** Since the findings for MIS-C are nonspecific and, in the absence of specific tests for the disease, its diagnosis relies on clinical manifestations, it is important to maintain a high degree of suspicion for the syndrome. Children with unremitting fever, an epidemiologic link to SARS-CoV-2 and suggestive clinical symptoms should be thoroughly evaluated. Prompt recognition and management are crucial to control the hyperinflammatory state and prevent severe organ dysfunction and potentially fatal complications, such as coronary aneurysms. Supportive care, IVIg, corticosteroids, biological immunomodulators, antiplatelet therapy, and prophylatic anticoagulation are key in the management of these childre.

110997

Modality: E-Poster Scientific Initiation – Case Report

Category: CARDIOVASCULAR INTENSIVE CARE/CARDIOVASCULAR EMERGENCIES

## Acute Aortic Root Dissection in Two Patients with Marfan Syndrome

LUANA DIAS XAVIER^1^, João Paulo Dias Costa^1^, Julia Sousa Diniz^1^, Beatriz Luduvice Soares^1^, Emerson De Santana Santos^1^

(1) Universidade Federal de Sergipe

**Introduction:** Aortic root dilatation/dissection is one of the cardinal features of Marfan syndrome (MS), a heritable disorder of the fibrillin 1 gene (FBN1), which leads to a systemic disorder of connective tissue, with a high degree of clinical variability manifestations and involves the ocular, skeletal, and cardiovascular systems. Abnormalities of the aortic wall are quite frequent in this population, causing progressive aortic dilatation, thus increasing the risk of acute aortic dissection.

**Case reports:** Two adult patients were referred from cardiothoracic surgery service to cardiogenetics evaluation after recovering from the successful intervention for acute aortic root dissection. Both patients were middle-aged males, not related, who fulfilled the Ghent criteria for MS. Patient 1 had no other affected member in the family and an FBN1 pathogenic variant was found. Patient 2 had an affected son and no molecular exam was performed. Other clinical data are summarized in the table.

**Conclusion:** Although other MS typical features were previously present, the two reported cases had the diagnosis confirmed only after cardiogenetics evaluation. The delay in diagnosis is quite harmful once, with proper management, the life expectancy of someone with Marfan syndrome approximates that of the general population. Genetic counseling was provided for both families and the patients have been followed by a multidisciplinary team.


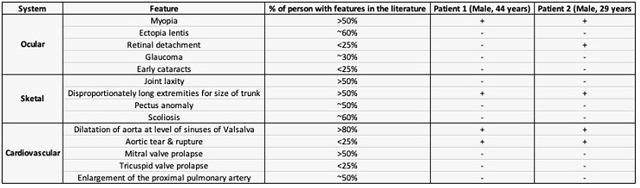


111085

Modality: E-Poster Scientific Initiation – Case Report

Category: CONGENITAL AND PEDIATRIC CARDIOLOGY

## 8 Years Old Patient with Cyanotic Congenital Heart Disease and Upper Extremity Deep Vein Thrombosis: A Case Report

LUÍSA RIGO LISE^1^, Luiza Fernandes Xavier^1^, Sabrina Comin Bizotto^1^, Júlia Helena Wegner^1^, Adriana Chassot Bresolin^2^

(1) Pontifícia Universidade Católica do Rio Grande do Sul (PUCRS); (2) Universidade Estadual do Oeste do Paraná

**Introduction:** The Complex Cyanotic Congenital Heart Disease is a deformation in the embryonic development of cardiac structures, causing an altered blood flow which may result in failures in the development of the circulatory system and systemic arterial oxygen desaturation. Its prevalence is aproximadely 1.45 per 1000 children.

**Description:** Female, 8 years old, diagnosed in intrauterine life by fetal echocardiography with situs inversus and cyanotic congenital heart disease. Father, 35 years old, and mother, 24 years old, both healthy with family history of second cousin with aortic coarctation. Full-term cesarean delivery with premature placental detachment. The echocardiography postnatal showed: situs inversus with levocardia; single left ventricle; hypoplastic right ventricle with double-outlet right ventricle; transposition of great arteries; total anomalous pulmonary venous connection (TAPVC); ostium primum; interventricular communication. At 1 year and 5 months, Glenn procedure and correction of TAPVC were performed without complications. At 6 years old, she developed upper extremity deep vein thrombosis, involving brachiocephalic, internal jugular and right subclavian. A computed tomography showed agenesis of inferior vena cava and overload of azygos-hemiazygos system that drains to right brachiocephalic vein. The patient progressed with more cyanosis, hepatomegaly and increased intensity of second sound and initiated Furosemide and Enoxaparin. Carvedilol, Enalapril, Dildenafil and Spironolactone were associated because of worsening signs of heart failure. 6 months after this episode, Enoxaparin was changed by Warfarin. Currently, at 8 years and 3 months, thrombosis of brachiocephalic and right subclavian veins are already resolved and thrombosis of internal jugular vein is in regression. In addition, she has diastolic dysfunction of ventricles, moderate insufficiency of atrioventricular valve and, due to the difficulty of controlling anticoagulation, warfarin was suspended and dabigatran was prescribed.

**Conclusion:** In this case, the association between anomalous venous return, blood hyperviscosity and hyperglobulinemia due to chronic hypoxia is observed as the cause of thromboembolism. Besides that, the Fontan Surgery was contraindicated due to agenesis of inferior vena. Therefore, early diagnosis, through fetal echocardiography and a careful medical follow-up, guarantees a better prognosis and lower morbidity and mortality in cases like this.

111168

Modality: E-Poster Scientific Initiation – Case Report

Category: PERICARDIUM/ENDOCARDIUM/VALVOPATHIES

## Atypical Presentation of Carcinoid Sindrom with Severe Tricuspid Regurgitation

LEONARDO PESSANHA CORDEIRO^1^, Juliana Fraga Soares^1^, Mellyssa Dias de Oliveira^2^, Pedro Hissa Monteiro^1^, Daniel J.M. Medeiros Lima^1^

(1) Faculdade de Medicina de Campos – FMC; (2) Centro Universitário Redentor – UniRedentor

Carcinoid tumors are tumors that originate most commonly in the enterochromaffin cells of the gastrointestinal tract, especially in the small intestine. In the presence of liver metastases or a production that exceeds the metabolism capacity, substances such as serotonin fall directly into the systemic circulation, causing the carcinoid syndrome. The prognosis is worse in patients with carcinoid syndrome and carcinoid heart. Here we report an atypical presentation of carcinoid syndrome with early involvement of the right heart chambers, without flushing or diarrhea: a 49-year-old woman with complaints of nausea and vomiting with one-month evolution, lower limb edema associated with increased abdominal volume, and an involuntary weight loss of 10% in 6 months. Physical examination showed important ascites with mobile dullness and positive Piparote sign, bilateral lower limb edema 2+/4, pathological jugular swelling, and systolic murmur in tricuspid focus 2+/6 with reinforcement after the Riveiro Carvalho maneuver. A magnetic resonance imaging revealed numerous hypervascular nodular lesions, suggestive of metastasis. A liver biopsy, immunohistochemistry, scintigraphy with a somatostatin analogue (Octreoscan), Chromogranin A and 5-hydroxy indole acetic acid levels were compatible with carcinoid syndrome resulting from a carcinoid tumor of the gastrointestinal tract. The echocardiogram showed an increase in the volume of the right atrium and ventricle with severe tricuspid regurgitation, the valve was thickened, with failure to coaptation of its leaflets. The main therapy for the carcinoid heart currently used is the somatostatin analogues. Surgical intervention as valvar replacement is also a possibility for a sectioned patient who is more likely to benefit from an invasive approach.


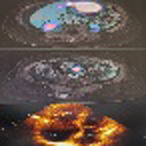


111275

Modality: E-Poster Scientific Initiation – Case Report

Category: CARDIOLOGY OF SPORTS, EXERCISE, ERGOMETRY AND CARDIOVASCULAR REHABILITATION

## Post – COVID-19 Cardiovascular Rehabilitation – Case Report

LUÍSA DE LAMARE DOS SANTOS PAULA ^1^, Gustavo Henrique de Oliveira^1^, Thiago Honório Dutra da Silva^2^, Jaqueline Lyrio Bermudes Okawa^2^, Rogério Toshiro Passos Okawa^1^

(1) Universidade Estadual de Maringá; (2) Avancor Cardiologia

R.A.F., male, 35 years old, businessman, born in Japurá-Paraná, from Santa Monica-Parana. Patient had a Covid-19 infection in March 2021, being admitted to an intensive care unit, for supplemental oxygen therapy in a high-flow mask. Thorax tomography showed 75% of pulmonary involvement. He had elevation of troponin and BNP during hospitalization. He was discharged with dyspnea on exertion, NYHA functional class II, being evaluated and requested complementary exams 30 days after hospital discharge, with echocardiogram with left ventricular ejection fraction with slight reduction: 42%, and reduced global longitudinal strain: – 12%. Cardiopulmonary stress test demonstrated Vo2: 24 mL/kg·min (72% of predicted), aerobic threshold 38% of predicted, with drop in oxygen pulse. He was medicated with Sacubitril/valsartan 49/51 mg every 12 hours, Bisoprolol 2.5 mg daily and Dapagliflozin 10 mg daily. Referred to cardiovascular rehabilitation, with 3 sessions per week, lasting 1 hour in each session. After 3 months of cardiovascular rehabilitation and pharmacological treatment, the symptoms improved, with a functional class I NYHA. The echocardiogram was repeated with improvement in left ventricular ejection fraction: 52%, improvement in global longitudinal strain: –16%, and improvement in ergospirometry parameters: Vo2: 37 mL/kg·min (90% of predicted), aerobic threshold 55% of predicted, oxygen pulse normalization. The present case report demonstrates a post-covid myocarditis, with left ventricular systolic dysfunction and the importance of pharmacological treatment with cardiovascular rehabilitation in this case.

111281

Modality: E-Poster Scientific Initiation – Case Report

Category: HEMODYNAMICS AND INTERVENTIONAL CARDIOLOGY

## POF as a Cause of Cryptogenic Ischemic Stroke in Elderly

MARIA GABRIELA PERERA^1^, Éveny Moraes Prola^1^, Helena Fussiger^1^

(1) Universidade Feevale

**Introduction:** Ischemic stroke (IS) is the leading cause of morbidity in the world. In 30–40% of cases, TOAST classification is from a cryptogenic etiology. In these cases, the patent oval foramen (POF) may be the etiologic agent through paradoxical embolism. In young patients (18–60 years), 10% of cases are associated with POF, in elderly, however, attributing the cause of the vascular event to FOP is still a major challenge.

**Case:** A previously healthy 70-year-old male presented with sudden onset of vertigo, nausea and vomiting, ataxia, hypoesthesia, and paresthesia of the left hemiface and left upper limb, lasting for 2 hours. His MRI scan showed no acute changes; only malacia in the left cerebellar hemisphere, but previous clinical history of focal neurological deficits. Then he was diagnosed with a transient ischemic attack (TIA). In the etiological investigation, transcranial Doppler identified passage of >20 HITs at rest, and after Valsalva maneuver >60 HITs, within the first 10 seconds of the infusion, considered a large shunt. Transesophageal echocardiography revealed interatrial septal aneurysm and POF with passage of a large amount of bubbles during the Valsalva maneuver. The rest of the investigation (with cervical Doppler, ECG, and laboratory tests) showed no changes that would justify the cerebral vascular event. RoPE score was 3 and PASCAL classified as a vascular event possibly related to POF. Treatment with Apixaban was initiated and a percutaneous endovascular closure procedure was performed for correction of POF.

**Discussion:** The association of POF with IS in young is already established. However, in case of elderly with cryptogenic event, especially in cases of TIA, where we don‘t have the imaging to indicate event suggestive of embolism, the connection becomes even more difficult. According to a systematic review of 2021, in patients with possible PASCAL classification, the absolute risk of ischemia recurrence in 2 years was 3.6 and 1.5 for those treated conservatively versus invasively, respectively. But the data are valid mainly for patients between 8–60 years. In elderly, more studies are needed to inform the therapeutic decision, which is still a challenge today.

**References:** Saver JL, et al. POF closure versus medical therapy for cryptogenic IS: topical review. 2018. Kent DM, et al. Heterogeneity of treatment effects in an analysis of pooled individual patient data from randomized trials of device closure of POF after stroke. JAMA. 2021.

111282

Modality: E-Poster Scientific Initiation – Case Report

Category: HEMODYNAMICS AND INTERVENTIONAL CARDIOLOGY

## Acute Reversible Aortic Insufficiency During Percutaneous Coronary Angioplasty: A Rare Mechanical Complication

ARNÓBIO ÂNGELO DE MARIZ NETO^1^, Carolline Araújo^2^, Luan Martins de Sousa^3^, Mateus Macena Correia de Lima^3^, Arnóbio A. Mariz Jr^3^

(1) UNINASSAU; (2) Faculdade de Medicina de Olinda – FMO; (3) Pronto Socorro Cardiológico Universitário de Pernambuco Prof. Luiz Tavares, PROCAPE/UPE

Percutaneous coronary angioplasty in patients with non-ST elevation acute myocardial infarction (NSTEMI) is a relatively common procedure and severe acute aortic regurgitation is a rare complication. We report a case of acute reversible aortic regurgitation during percutaneous coronary angioplasty in a 67-year-old woman, diabetic and hypertensive, with chronic coronary atheromatous disease who was admitted to hospital with NSTEMI. Subocclusive lesion in the circumflex artery and severe lesion in the anterior descending artery were evidenced in cardiac catheterization. During anterior descending angioplasty, a slowing of the flow through the vessel was observed (Figure 1), proceeded with the direct implantation of a drug-eluting stent with Sirolimus. At this moment, the patient developed oppressive chest pain, radiating to the upper limbs, profuse sweating, vomiting, sinus bradycardia and cardiogenic shock (BP = 64 × 42 mmHg). The images revealed significant acute aortic insufficiency (Figure 2) caused by the guiding catheter (Amplatz AL1) and reversed with the removal of the catheter. A new angiographic control revealed stents implanted with good results and a final TIMI III arterial flow (Figure 3). No dissection of the left main coronary artery was observed. Acute aortic regurgitation caused by coronary catheterization can be avoided through careful handling of the catheter, especially in challenging scenarios or in patients with an anomalous coronary origin.


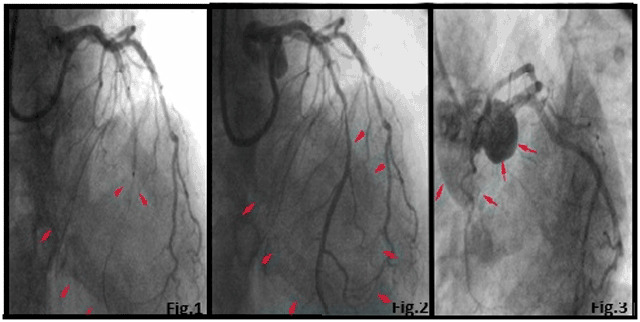


111785

Modality: E-Poster Scientific Initiation – Case Report

Category: HYPERTENSION/RENAL DENERVATION

## Preeclampsia and its Association with Patau Syndrome

PEDRO HENRIQUE TORRES TIETZ^1^, Laís Bettoni^1^, Carolina Feijó Bombana^1^, Guilherme Rodrigues Viana^1^, Rafael Fabiano Machado Rosa^1^

(1) Universidade Federal de Ciências da Saúde de Porto Alegre (UFCSPA)

**Introduction:** Trisomy 13 or Patau Syndrome (PS) is considered a chromosomal disease characterized by multiple malformations and a limited prognosis. The following reports indicate that pregnancies with trisomy 13 have higher risk of developing preeclampsia. This association is related to the placental abnormalities which are frequently found in pregnancies of PS fetuses, including reduced placental volume and vascularization and placental mesenchymal dysplasia.

**Case Description:** The first case reported a 30-year-old pregnant woman in her fifth pregnancy. She had a past medical history of one pregnancy loss. She was monitored by prenatal care and at the final stage of pregnancy she developed preeclampsia. She wasn’t submitted to fetal ultrasound at anytime of the pregnancy. The child was born prematurely at 36 weeks, weighing 2550 grams, by cesarean section. The Apgar score was 3/9. On examination, multiple alterations were observed, which included microcephaly, aplasia cutis on the scalp, cleft lip, micrognathia and hypertonia. Her karyotype indicated mosaic PS (47, XX, +13/46, XX). The child evolved with clinical worsening and died at 26 days of life. The pregnant woman in case 2 was 27 years old and in her second pregnancy. The pregnancy was accompanied by prenatal care, and it evolved with an episode of vaginal bleeding at 13 weeks. In addition, there was a description of an episode of urinary tract infection and preeclampsia. Due to the latter, the child was born by induced vaginal delivery. The other ultrasound exams performed during pregnancy were described as normal. The child was born at 37 weeks‘ gestation, weighing 2445 grams and with Apgar scores of 6 and 8. In her examination, there were alterations such as microcephaly, trigonocephaly, aplasia cutis on the scalp, microphthalmia, micrognathia, micropenis, empty scrotum, and polydactyly of hands and feet. The karyotype was compatible with PS (47, XY, +13). The child died at 12 days old.

**Conclusion:** Pregnant women with PS fetuses have a higher risk of developing preeclampsia, considered one of the main causes of maternal death during pregnancy. This may have important implications for the management and prognosis of these patients.

111333

Modality: E-Poster Scientific Initiation – Case Report

Category: HYPERTENSION/RENAL DENERVATION

## Secondary Arterial Hypertension by Aldosterone Producing Adenoma: A Case Report

IUGO ALVES DE SOUSA^1^, Filipe Batista de Brito^3^, Gabriela Menezes Gonçalves de Brito^2^, Matheus Araújo de Medeiros^1^, Renner Cassio Nunes de Lucena^1^

(1) Universidade Federal do Rio Grande do Norte; (2) Universidade Tiradentes-SE; (3) Hospital de Cirurgia-SE

**Introduction:** Adenomas are among the causes of increased aldosterone production by the adrenal gland. The prevalence in hypertensive patients varies from 3% to 22%, being higher in hypertensive patients who are difficult to control. In general, patients have stage 2 or 3 hypertension, which may be refractory to treatment. Currently, it is known that the prevalence of hypokalemia in primary hyperaldosteronism varies from 9% to 37% of cases. In the diagnosis of this condition, the differentiation between hyperplasia and adenoma is essential for proper treatment. From a clinical and laboratory point of view, patients with adenoma are, in general, younger, have more severe hypokalemia and higher aldosterone concentrations (> 25 ng/dl).

**Case Report:** Female, 32 years old, asymptomatic, with grade III arterial hypertension despite the use of three antihypertensive drugs of different classes. When performing abdominal ultrasound for ovarian evaluation, a random adenoma was observed in the left adrenal measuring approximately 3.5 × 2.9 × 2.5 cm with a volume of 19.2 cm², which was later confirmed by on total abdominal tomography. Aldosterone and renin levels were then requested and the results showed an aldosterone overproduction (37.2 ng/dL) not accompanied by a renin suppression (4.7 uIU/mL). A potassium dosage was also requested, which allowed the identification of hypokalemia (2.2 mEq/L). After confirmation, the patient followed for evaluation of cardiovascular alterations. Echocardiography showed eccentric left ventricular hypertrophy with mild mitral regurgitation, which suggests a functional impairment of the heart. Then, the patient was referred for unilateral adrenalectomy.

**Conclusion:** The case in question presents the diagnosis of a unilateral aldosterone-producing adenoma, in a young woman with a history of resistant arterial hypertension, accidentally identified in an imaging exam. The report aims to alert professionals about the need to screen this cause of secondary hypertension in all hypertensive patients with spontaneous or diuretic-induced hypokalemia and in hypertensive patients resistant to usual treatments, so that the disease is diagnosed early and treated according to guidelines.

111441

Modality: E-Poster Scientific Initiation – Case Report

Category: CARDIAC ARRHYTHMIAS/ELECTROPHYSIOLOGY/ELECTROCARDIOGRAPHY

## Bayés Syndrome in an Infant: Case Report with Atrial Fibrillation Associated with Bachmann Bundle Block and Tachycardiomyopathy

ANTONIO MAYCON DA SILVA SOUSA^1^, Carlos Eduardo Batista de Lima^2^, Patryck Araujo Dantas da Silva^1^, Marcelo Madeira Pinheiro Silva^2^

(1) Universidade Federal do Piauí (UFPI); (2) Hospital Universitário da Universidade Federal do Piauí – (HU-UFPI).

**Introduction:** Atrial fibrillation (AF) is the most frequent arrhythmia in clinical practice with a higher occurrence in the elderly and rarely occurs in pediatric patients with a structurally normal heart. Bayes syndrome has been described as the occurrence of AF in the elderly associated with electrocardiogram (ECG) findings of interatrial block by the Bachmann bundle, and there are no reports of cases in pediatrics so far. The reduced atrial myocardium mass in children makes the occurrence of AF difficult, reinforcing the mechanism triggered by the interatrial delay.

**Case Report:** S.R.C., male, 5 months old, in December 2018, admitted to a public hospital in Teresina-PI due to respiratory distress reported by the mother, which had been frequent since birth. Born at term, with no pathological antecedents. On physical examination, he had a 2+/4+ systolic murmur in the mitral area, irregular heart rhythm, high heart rate (HR), normal vesicular murmur, without adventitious sounds or signs of pulmonary edema. On admission, an echocardiogram (ECHO) was performed, which showed dilated left chambers, left ventricular ejection fraction of 55% and mild mitral and tricuspid regurgitation. The admission ECG showed AF with HR around 180 bpm and episodes characteristic of Ashman’s phenomenon. After reversal, in sinus rhythm, morphological alterations of the p wave characteristic of interatrial delay by Bachmann’s bundle were evidenced, a fixed alteration associated with a greater predisposition to the occurrence of AF. The patient used antiarrhythmic medications, with complete normalization of the ECHO and maintaining long-term sinus rhythm on an outpatient basis.

**Final Considerations:** We report a rare case of Bayes syndrome in an infant with AF and mild tachycardiomyopathy associated with an electrocardiographic alteration characteristic of a fixed conduction disturbance in the Bachmann fascicle due to interatrial delay. The presence of Bachmann’s bundle block on the electrocardiogram justifies the pathophysiological mechanism of the arrhythmia as a probable etiology, as it is a known risk factor for AF and no other clinical alteration was evidenced that would justify the occurrence of arrhythmia in this patient.

111450

Modality: E-Poster Scientific Initiation – Case Report

Category: CONGENITAL AND PEDIATRIC CARDIOLOGY

## Characteristics and Progress of a Twin Pregnancy with an Acardiac Fetus

PEDRO HENRIQUE TORRES TIETZ^1^, Vitor Agne Magnus^1^, Marco Antônio Vinciprova Dall‘Agnese^1^, Gabriel de Paula Alves^1^, Jorge Alberto Bianchi Telles^2^

(1) Universidade Federal de Ciências da Saúde de Porto Alegre (UFCSPA); (2) Hospital Materno Infantil Presidente Vargas (HMIPV)

**Introduction:** A twin pregnancy with an acardiac fetus is a rare event, with a low number of cases in the literature describing conservative treatments and parameters to be assessed.

**Case Description:** 29 years old female with a 14 week pregnancy presented to fetal medicine service due to an ultrasound evidencing a monochorionic and diamniotic twin pregnancy. One of the fetuses had a malformation in the superior part of the body, suggestive of an acardiac fetus. The patient was submitted to fetal ultrasound which confirmed this diagnosis of twin reversed arterial perfusion (TRAP). The patient was assessed for possible laser ablation of the umbilical cord. There weren‘t any signs of hemodynamic decompensation on the pump twin and a blood flow almost restricted to the umbilical cord on the acardiac fetus, without surgical indication at that moment. The 22 week ultrasound showed no morphologic alterations on the pump twin, which weighed 560 g, and a dysmorphic mass of 160 g representing the acardiac fetus. There was evidence of vascularization in its interior shown by a doppler ultrasound. The doppler echocardiogram and a magnetic resonance imaging were also normal for the pump fetus. The acardiac fetus still presented to be a misshapen mass with significantly peripheral edema. Ultrasounds were regularly made as of the 26 weeks of gestation, aiming at the early diagnosis of any sign of cardiac decompensation of the pump fetus. The evaluated parameters consisted on the relation between the weight of the acardiac and pump fetuses, cardiothoracic ratio, visualization of tricuspid regurgitation, presence of polyhydramnios and doppler ultrasound of the venous duct in the pump fetus. These parameters stayed normal and it was decided to hospitalize the patient with 30 weeks of gestation for an ultrasound follow-up and for daily cardiotocography (CTG). The CTGs were normal. The pump fetus, a girl, was born with 33 weeks of pregnancy and weighing 2.125 g, presenting an Apgar score of 8/9. The acardiac fetus weighed 1.090 g, being a misshapen mass, with more developed inferior limbs. The pump twin presented a respiratory failure. However, she progressed without major complications, being discharged 14 days after birth.

**Conclusion:** Although there is not a well defined pattern established for the management of twin pregnancy with an acardiac fetus, some factors like early diagnosis and hemodynamic assessment can help future cases to end successfully.

111476

Modality: E-Poster Scientific Initiation – Case Report

Category: CONGENITAL AND PEDIATRIC CARDIOLOGY

## Brugada Phenocopies in a Pediatric Patient with Ventricular Pre-Excitation: A Case Report

GABRIEL SILVA DE CARVALHO PONCIANO^1^, Amanda Metsa da Silva Cardoso^1^, Henrique Lobo Saraiva Barros^2^, Rafaela Marrocos Bezerra^2^, Sônia Maria Cavalcante da Rocha^2^

(1) Universidade do Estado do Rio Grande do Norte – UERN; (2) Policlínica dr. Luiz Carlos Fontenele – SPDM

**Introduction:** Brugada Syndrome (BrS) is a genetic disease of low worldwide prevalence and male preponderance, which can lead to severe arrhythmias. The diagnosis includes an electrocardiogram (EKG) with the presence of spontaneous or drug-induced ST elevation, followed by a symmetrical negative T wave in the right precordial leads and clinical features. Brugada phenocopies present an EKG pattern equivalent to BrS, but may arise from a previus ventricular repolarization alteration or associated with factors like hydroelectrolytic disorders. Such changes might generate a transmural myocardial gradient or delay the conduction around the right ventricular outflow tract, inducing the EKG pattern. This diagnosis includes EKG pattern, low pretest probability for the syndrome, drug and genetic testing.

**Case Description:** A 6 years old male child with psychomotor agitation was sent to cardiological evaluation in order to use methylfnidate. There were no heart complaints and a negative family history of sudden death or arrhythmic syncope. Short PR interval and delta wave were seen in all EKG leads. A 24H Holter monitoring was requested, which showed a short PR and delta wave throughout the tracing and no documented arrhythmias. Ventricular pre-excitation became the main diagnostic hypothesis. It was indicated a careful follow-up for the child. In early 2022, during a routine appointment, the child showed no heart complaints, but he was in a recovery from gastroenteritis. The EKG showed a pattern of type 1 BrS. The EKG was repeated after 2 days with no more BrS pattern. The possibility of BrS was ruled out due to the absence of clinical features, normalization of the EKG and negative family for BrS. For that ocasion the diagnosis of phenocopies seemed more likely to be suitable.

**Conclusion:** Considering phenocopies as a differential diagnosis of Brugada syndrome is important. A complete investigation of the patient is necessary for the diagnostic suspicion, considering the possible related factors for the condition. A careful follow-up along with a complete explanation of this entity is the best way of deal with this case.


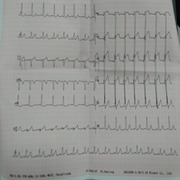


111594

Modality: E-Poster Scientific Initiation – Case Report

Category: HEART FAILURE/CARDIOMYOPATHY/TRANSPLANT

## Midventricular Form of Takotsubo Cardiomyopathy: A Clinical Entity that Needs to be Recognized

STHEPHANY YAMAGUCHI DE MELO^1^, Sthephany Yamaguchi de Melo^1^, Isabela Souza Cruvinel Borges^1^, Danielle Campos de Almeida^1^, João Lucas O‘Connell^1^

(1) Universidade Federal de Uberlândia – UFU

**Introduction:** Takotsubo cardiomyopathy (or broken heart syndrome) is characterized by transient left ventricular systolic and diastolic deficits, mimicking acute coronary syndrome (ACS). It is more common in women (9:1), elderly, with a history of psychiatric disorder and physical or emotional stress. Classically, the ventricular abnormality is analogous to the Japanese pot used to fish for octopus, which resembles the apical ballooning of the typical form of the disease. The description of this case highlights the possibility of other regional abnormalities (less common) that have also been described in the literature: the one that evolves with midventricular akinesia, with severe hypokinesia restricted to the middle ventricle.

**Case Description:** Female patient, 54 years old, hypertensive, smoker and anxious. She presented with syncope at home after emotional stress and being taken to the emergency room. Electrocardiogram: discrete infra-ST on the anterior wall. Elevation of myocardial necrosis markers. Coronary angiography: absence of obstructive coronary lesions; Significant hypokinesia of the middle segments with moderate global systolic dysfunction suggestive of Takotsubo cardiomyopathy of the midventricular form. He evolved well and was discharged with optimized clinical treatment for ventricular dysfunction. She is asymptomatic and with full recovery of contractility and ventricular function one year after the initial condition.

**Conclusion:** Although most cases are benign, Takotsubo heart disease is a potentially fatal disease, with a severe and irreversible course. Diagnostic criteria aim to identify acute left ventricular dysfunction and treatment is directed towards heart failure that sets in. Despite not being a diagnostic criterion, cardiac catheterization is commonly performed to rule out ACS. When performed, the absence of coronary artery disease is expected. Echocardiography and myocardial resonance assist in the diagnosis and identification of ventricular abnormalities that are sudden and without a defined time for reversibility. The mid-ventricular form represents a minority of cases (15%). Its benign character has been questioned in recent reviews due to the number of underestimated cases that may evolve unfavorably without a well-established prognosis.

111597

Modality: E-Poster Scientific Initiation – Case Report

Category: HEMODYNAMICS AND INTERVENTIONAL CARDIOLOGY

## “Valve-in-David”, a New Face of Structural Interventional Cardiology

GUSTAVO LUIZ MONTENEGRO DA COSTA^1^, Dany David Kruczan^2^, Lilian Soares da Costa^2^, Edgard Freitas Quintella^2^, Márcio José Montenegro da Costa^2^

(1) Faculdade Técnico-Educacional Souza Marques; (2) Instituto Estadual de Cardiologia Aloysio de Castro

**Introduction:** Valvular heart disease is an important contributor to morbimortality worldwide. This case report proposes to switch a redo operation on a valvular aortic regurgitation, in a 60-year-old man post David procedure for a transcatheter aortic valve replacement (TAVR-in-David).

**Case Description:** A 60-year-old man, after almost a 10-year outpatient follow-up due to a prior David procedure, presents onset of dyspnea with minimal exertion and chest pain in July 2021. Until March 2022, this patient was managed even optimal medical treatment has kept non-responsiveness and then was admitted in our hospital. The first choice of treatment during this admission was the conventional surgery for aortic valve replacement, but the surgical team denied the procedure due to the high risk of complications. Then we have implanted a balloon expandable valve with no leak and good final result. TAVR should be contemplate in the role of procedures as a final solution for severe aortic regurgitation in case of high surgical risk or inoperable patients, especially in centers of excellence.

**Conclusion:** The decision to treat a patient with a TAVR-in-David procedure is off label but we believe that with permission of the patient and, a multidisciplinary approach to get the best decision must be a rule inside Cardiology. In conclusion, we need a series of cases and a follow-up to validate this new proposal to switch a redo David procedure for a V-i-David transcatheter.


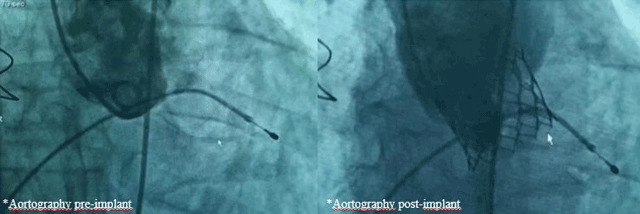


111600

Modality: E-Poster Scientific Initiation – Case Report

Category: ACUTE AND CHRONIC CORONARY DISEASE/THROMBOLYSIS

## Spontaneous Coronary Artery Dissection by a Woman in Early Postpartum

RODRIGO LACERDA GERVOU^1^, Daniel Luiz Messias Pereira^1^, Miguel Ângelo Ribeiro^2^, Natália Dominguez Paes Leme^2^, Nathalia Duarte Camisão^2^

(1) Universidade Federal do Estado do Rio de Janeiro UNIRIO; (2) Hospital Norte D’or

**Introduction:** Spontaneous coronary artery dissection (SCAD) is a rare cause of acute coronary syndrome (ACS), myocardial infarction and sudden death, but becomes more relevant in younger women, with multiple pregnancies, especially in the puerperium phase, which consists of 2–18% of all cases of SCAD. It has few association with atherosclerotical risk factors but emotional stress preceded in 50% of the cases, SCAD consists in a non-traumatic, non-iatrogenic separation of the coronary arterial wall resulting in an ischemic event and myocardium injury, that may represent up to 1–4% of total ACS events.

**Case Report:** A 39 years-old female patient arrives in the Emergency Room, reporting typical chest pain, started after the news that her newborn child had been transferred to the neonatal UCI, she referred of a similar pain one week ago, but with spontaneous cure. She has a history of arterial systemic hypertension but no other risk factors for atherosclerotic disease. An Electrocardiogram showed repolarization alteration in the Inferior wall and a small elevation of the ST segment in V2–V4. Echocardiogram stated a moderate dysfunction of Left Ventricular function (LVEF) and akinesis of apical segments. The patient was forwarded to coronary angiography that revealed SCAD of the left anterior descending artery, after diagonals branch. Due to the etiology of the coronary disease, opted for optical medical treatment, and no angioplasty was performed. Magnetic resonance Imaging showed poor LVEF of 40%, 11% infarcted area with disproportion between the ventricular dysfunction and the size of the ischemic area, suggesting a large stunned area that could become functional again. As the literature states, because of the risks of coronary angioplasty, especially without arterial oclusion, patient was discharged from hospital with optical medical treatment for heart failure.

**Conclusions:** SCAD is not a common cause of ACS, but is associated with great mortality and morbidity. It is an important etiology and deserves our attention so we can have it in mind, identify and to have the best approach to this pathology.

111608

Modality: E-Poster Scientific Initiation – Case Report

Category: CARDIOVASCULAR SURGERY

## Treatment of Superior Vena Cava Syndrome Caused by Tuberculous Lymphadenitis with Spiral Saphenous Vein Graft: A Case Report

FABIO ANTONIO SERRA DE LIMA JUNIOR^1^, André Loureiro Fernandes^1^, Isaac Newton Guimarães de Andrade^1^, Petrúcio Abrantes Sarmento^1^, André Telis de Vilela Araújo^1^

(1) Universidade Federal da Paraíba

**Introduction:** Tuberculous lymphadenitis is a rare cause of superior vena cava syndrome (SVCS). All of literature cases report a conservative or an endovascular approach to treat these cases.

**Case Description:** We aim to report a 42 years old female case who was allergic to two components of both standard and alternative tuberculosis treatment, and required a surgical approach. However, the same patient had a large and excessively calcified tuberculous node exponentially decreasing the preview success rate of a possible endovascular approach, due to the risk of drift. On the case, the surgical team chose to execute a spiral saphenous vein graft, a method previously reported to treat SVCS, but never on association to tuberculous lymphadenitis.

**Conclusion:** When treating superior vena cava syndrome, etiologies that induce intense vein fibrosis, such as mediastinal tuberculosis, may be unsuitable for endovascular treatment. In these cases, open surgical bypass using a spiral venous graft is an achievable and effective option for symptoms relief, with satisfactory long-term patency.

111758

Modality: E-Poster Scientific Initiation – Case Report

Category: HEMODYNAMICS AND INTERVENTIONAL CARDIOLOGY

## Chronic Total Occlusion Percutaneous Coronary Intervention for Correction of Ischemic Mitral Regurgitation: A Case Report

MONIQUE DE SOUSA LOPES^1^, Débora dos Santos Silva^1^, Bruno Gonçalves Machado^1^, Aline Viana Alves^1^, Ewerton de Souza Abreu^2^

(1) Centro Universitário Faminas Muriaé (FAMINAS); (2) Hospital Prontocor de Muriaé (PRONTOCOR)

The main indication for chronic total occlusion (CTO) percutaneous coronary intervention (PCI) is to improve anginal symptoms, being considerable when there is resistant angina or ischemia compatible with the territory of the occluded artery, however, there is no routine evidence of this approach for correction of ischemic mitral regurgitation (IMR). This study analyzed a 51-year-old patient, without previous comorbidities, with stage B Congestive Heart Failure. Transesophageal Echocardiogram showed severe Mitral Insufficiency, regurgitant jet and incomplete coaptation due to wide prolapse of the posterior leaflet, raising the hypothesis of IMR due to dysfunction of the posteromedial papillary muscle. Invasive coronary stratification revealed CTO in the middle third of the right coronary artery (RCA). Due to the persistence of symptoms despite optimized clinical treatment, a percutaneous approach to the RCA CTO was decided. With failure of an antegrade angioplasty, a retrograde approach was performed using the dissection and reentry technique, which was successful with positive results, including improvement of heart failure symptoms and immediate papillary dysfunction, confirming the diagnostic hypothesis. At ten-month follow-up after the procedure, the patient remains asymptomatic, with fully preserved physical and functional capacities and mild mitral regurgitation. Therefore, despite the technical limitations associated with the procedure and the need for further quality studies to better define the indications, specifically regarding the IMR, it is suggested that the approach for such purpose, when well indicated, may be a promising possibility within the interventional cardiology, impacting on the increase of survival and expanding the prospects of treatment.


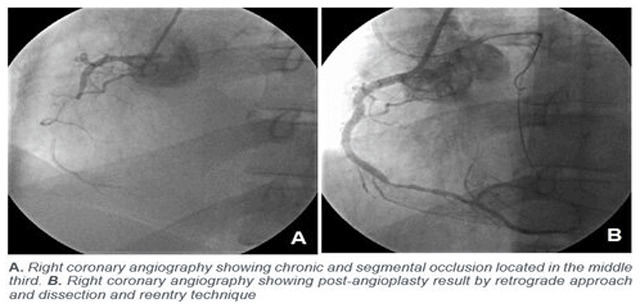


111779

Modality: E-Poster Scientific Initiation – Case Report

Category: HEMODYNAMICS AND INTERVENTIONAL CARDIOLOGY

## Effect of Lipid Therapy in a Patient with Cardiogenic Shock Due to Alpha and Beta-Blockers Intoxication: A Case Report

YURY PIFANO VARELA^1^, Marina Coelho Feitosa^1^, Caio Pessoa Cruz^2^, Isabelly Crysthynne Moreira da Luz^3^, Polianna Lemos Moura Moreira Albuquerque^4^

(1) Acadêmico do Curso de Medicina da Universidade de Fortaleza – UNIFOR; (2) Acadêmico do Curso de Medicina da Universidade Estadual do Ceará; (3) Acadêmico do Curso de Farmácia da Universidade Federal do Ceará; (4) Médica Nefrologista do Instituto Dr. José Frota e Coordenadora do Centro de Informação e Assistência Toxicológica (CIATox/IJF). Doutora em Ciências Médicas pela Universidade Federal do Ceará – UFC

**Introduction:** Intoxication by selective α–1 and β–1 adrenorreceptor blockers may cause serious repercussions in the cardiovascular system, that can progress to Cardiogenic Shock, which can lead to death in 80% to 90% of patients. There are successful reports using 20% lipid emulsion (20% ILE) as treatment after cardiovascular collapse caused by lipophilic agents such as tricyclic antidepressants, propranolol, verapamil, bupropion and barbiturates, although the literature still does not assign a standard protocol. This report case demonstrates the emergency use of 20% ILE in acute toxicity from attempted suicide through ingestion of doxazosin and metoprolol.

**Case Description:** A.H.M.L, 61 years old, male, admitted by a tertiary care hospital, reference in toxicological assistance, in Fortaleza/CE, in critical condition, 8 hours after intentional and unquantified ingestion of metoprolol and doxazosin. At admission, patient on mechanical ventilation, without sedoanalgesia, Glasgow Coma Scale 3, using high doses of vasoactive drugs (noradrenaline 40 ml/h + vasopressin 4 ml/h) (Table 1). With no response to the standard protocols for hemodynamic shock, it was suggested a therapeutic regimen with 20% ILE in 3 doses of 1 ml/kg (60 ml), implemented at intervals of 10 min, resulting in immediate hemodynamic upturn – distressed awakening and improvement in ventilatory parameters (Table 1). After 5 hours, it was necessary to repeat the 20% ILE regimen due to clinical instability. Over the 28 days of hospital stay, the initial high doses of vasoactive drugs were reduced without clinical complications. In addition, renal replacement therapy was performed (two sessions).

**Conclusion:** This is the first successful experience in the state of Ceará for the treatment of Cardiogenic Shock resulting from intoxication by selective α–1 and β–1 adrenorreceptor blockers agents. The study provides evidence on the use of 20% ILE as a relevant therapy in patients after cardiogenic shock due to high ingestion of alpha and beta blockers.


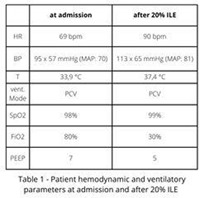


111794

Modality: E-Poster Scientific Initiation – Case Report

Category: PERICARDIUM/ENDOCARDIUM/VALVOPATHIES

## Viral Pericarditis Associated with Denge Infection: A Case Report

RAIMUNDO BENÍCIO DE VASCONCELOS NETO^1^, Ana Caroline Leite Guedes^1^, Rebecca Shaiane Soares Nunes Rivoredo^1^, Fernanda Gabry Scazuza Gomes de Souza^1^

(1) Centro Universitário São Lucas-UNISL

**Introdução:** Dengue is a viral infectious process transmitted by Aedes aegypti and most of it presents with a limited high condition, however, the endemic outbreaks of the disease open the way for the emergence of serious cases with complications. Cardiac involvement associated with dengue is a rare and broad-spectrum process, in which pericarditis and pericardial effusion have a lower occurrence. The pathophysiology of such involvement is still uncertain, but it is believed that viral infection in the pericardium can generate the release of inflammatory cytokines that cause dysfunction in the cardiac endothelium, and that promote increased vascular permeability, culminating in fluid leakage to the third space. and generating the process of pericardial effusion, which in most cases does not constitute an accentuated process. This article aims to report a case of a patient with pericarditis and mild pericardial effusion as a complication of dengue.

**Relato:** Female patient, 81 years old, hypertensive, with advanced stage of Alzheimer’s disease, was taken to the emergency room by family members who reported a decline in general condition, unmeasured fever and lowered level of consciousness. On admission, the patient presented Glasgow Coma Scale 14, was febrile, and diffuse rashes associated with significant swelling in the upper and lower limbs were observed. She was mildly dyspneic and hypoxemic, requiring the use of complementary oxygen therapy. In view of the symptoms presented, complementary exams and transthoracic echocardiography (TTE) were requested due to the suspicion of cardiac insufficiency. The hemogram results showed significant thrombocytopenia and leukometry within the normal range, serology was requested for dengue, which showed positive IgG and IgM for the viral infection. The TTE showed hyperrefringence of the pericardium and small effusion, concluding the diagnosis of viral pericarditis. The patient remained in the intensive care department under infectious, neurological and hemodynamic surveillance, with subsequent clinical improvement.

**Conclusão:** The present report points to the unusual association between pericarditis and dengue, which demonstrates the importance of a thorough clinical management and an accurate look at the individual, since the signs can vary from mild to severe, including pericardial effusion. If treated satisfactorily, they potentially increase the chances of restoring the patient to health.

111800

Modality: E-Poster Scientific Initiation – Case Report

Category: COVID-19 AND CARDIOVASCULAR SYSTEM

## Acute Myocardial Infarction Secondary to Coronary Thrombosis in a Patient After Mild COVID-19: Case Report

MARIANA FURTADO DE SÁ^1^, Vanessa Ortega^1^, Vinicius Eiji Kameoka^2^, Bianca Altrão Ratti Paglia^1^

(1) Universidade Cesumar – Unicesumar; (2) Hospital Paraná

**Introduction:** SARS-COV2 has been shown to be a virus that affects multiple organs, including the heart. This case report shows a severe cardiologic complication in a patient post mild COVID-19.

**Case Report:** L.M.B, 37 years old, female, former smoker (10 pack-years) and with no medical conditions, reports mild COVID-19 two months before an episode of intense retrosternal burning associated with diaphoresis. Initial electrocardiogram (ECG) without ischemic alterations, however, due to the recurrence of the pain, she returned to the emergency room after two days, in which diffuse ST elevation was evidenced in new ECG, associated with pleuritic chest pain. The patient was referred for coronary angiography (CA) and later cardiac magnetic resonance imaging (CMR). CA: Absence of atherosclerotic process. Distal occlusion of the left anterior descending. Residual thrombus in marginal branch. CMR: Apical transmural infarct. Intracavitary thrombus in apex. Acute Pericarditis.

**Conclusion:** Inflammation and hypercoagulability generated by COVID-19, increase the chances of cardiovascular events, such as the myocardial infarction evidenced in the case, even in the absence of significant risk factors. Therefore, the importance of studies is emphasized, to identify groups that will benefit from prophylactic therapies, avoiding similar outcomes.


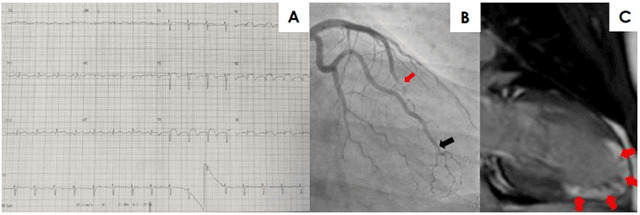


111825

Modality: E-Poster Scientific Initiation – Case Report

Category: NEGLECTED CARDIOVASCULAR DISEASES

## Atrial Mixoma with Cardioembolic Manifestations – When Should we Anticoagulate: A Case Report

JOYCE FERNANDES COSTA^1^, Thaíssa Santos Monteiro^2^, Fabio Akio Nishijuka^2^, Maria Carolina Terra Cola^2^

(1) Universidade Estácio de Sá – UNESA; (2) Instituto Nacional de Cardiologia – INC

**Introduction:** Cardiac tumors are rare, the majority being benign and atrial myxoma (AM) is the most common type. Most are symptomatic and may present Goodwin’s triad, with intracardiac obstruction, embolic manifestations and constitutional symptoms. The diagnostic method is echocardiography (ECO), assessing size and location of the tumor. Surgical resection is recommended. Anticoagulants may be indicated if embolic signs are present, although evidence points not to be an alternative to surgery, as half develop cerebral ischemia despite the anticoagulation. Local recurrence is uncommon and annual ECO follow-up is recommended for 4 years after tumor resection.

**Case Report:** Female, 35 year-old, hypertensive, smoker, obese grade I and family history of coronary artery disease. Admitted with severe chest pain, blood pressure of 220 × 100 mmHg, right bundle branch conduction disorder and positive myocardial necrosis markers, diagnosed with acute myocardial infarction type II. Recent history of transient focal deficit in the left upper limb. Presented cutaneous lesions resembling Janeway lesions in hands and feet, but tender. ECO observed an heterogeneous and irregular mass in the left atrium adhered to the atrial septum, measuring 5.2 × 3.6 cm, projecting to the mitral valve orifice, without significant obstruction, suggestive of AM. The patient was then transferred to our institution, and the screening for COVID-19 was positive, but with normal chest tomography (CT) and no related symptoms. Coronary angiotomography performed later showed no coronary obstructions, and head CT showed no signs of ischemia. Full anticoagulation was started due to the possible cardioembolic nature although there were no CT evidence for ischemia, while waiting for surgery. She underwent resection of the mass with atrial septoplasty. Postoperative ECO showed no shunt through the atrial septal and absence of intracavitary mass. There were no surgical complications on follow-up.

**Conclusion:** AM manifests itself in different ways and with imminent risks. Immediate surgical intervention is important, due to the risk of embolism, and in cases of previous embolic events, anticoagulation may also be considered, due to the risk of associated thrombi with the tumor.

111867

Modality: E-Poster Scientific Initiation – Case Report

Category: CONGENITAL AND PEDIATRIC CARDIOLOGY

## Type B Interrupted Aortic Arch with Ventricular Septal Defect in an Adult with Eisenmenger Syndrome and Features of Vacterl Association

HALAN VITOR CORREIA EVANGELISTA VIEIRA^1^, Catarina Vasconcelos Cavalcanti^1^, Paulo Ernando Ferraz Cavalcanti^1^, Ricardo Felipe de Albuquerque Lins^1^, Monica Cristina Rezende Fiore^1^

(1) Pronto Socorro Cardiológico de Pernambuco (PROCAPE); (2) Universidade de Pernambuco (UPE); (3) Faculdade de Ciências Médicas da Universidade de Pernambuco (FCM-UPE)

**Introduction:** Correlation between clinical findings and study with transthoracic echocardiography and angiotomography allow diagnosis of rare congenital heart disease in adult patient.

**Case Description:** A 42-year-old female patient with a long-standing history of cyanosis and abdominal discomfort, presenting worsening of symptoms in the last months was admitted at our center. The physical examination revealed cyanosis and digital clubbing with outstanding difference between upper and lower limbs, since the right hand was quite normal. Placing oximeter probe on her fingers and toes when breathing room air, differential peripheral oxygen saturation (SpO2) preserving the right hand was noted (Right fingers 99%, left fingers 94% and toes 88%). A loud second cardiac sound with fixed splitting was heard, as well as a systolic murmur in the left sternal border. Two-dimensional transthoracic echocardiography revealed a ventricular septal defect (12.1 mm) and the color Doppler showed bidirectional flow across the ventricular septal defect (VSD). The estimated systolic pulmonary artery pressure was 105 mmHg. An angiotomography was performed due to difficulties in the analysis of the aortic arch, revealing a type B aortic arch interruption associated with VSD and a patent ductus arteriosus as well as signs of severe pulmonary artery hypertension. Severe kyphoscoliosis for her age as well as incidental finding of ectopic kidney in the pelvis were also revealed. Deoxygenated blood from right ventricle flows into the descending aorta and left subclavian artery through the patent ductus arteriosus preserving the right hand from the long-term effects of deoxygenated blood. A hypothesis of VACTERL association was also formulated in view of the demonstrated features: V, vertebral anomaly by kyphoscoliosis; C, cardiac anomaly due to type B aortic arch interruption with interventricular communication; and R, renal anomaly with the left kidney in pelvic location.

**Conclusion:** The clinical and anatomical findings described and the rarity of the pathology in this adult patient (Not surgically treated) make this case report unique.


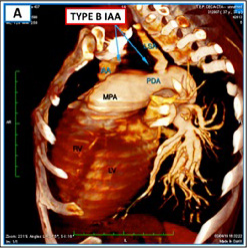


111876

Modality: E-Poster Scientific Initiation – Case Report

Category: CARDIOVASCULAR SURGERY

## Coronary Arteriovenous Fistula as a Cause of Angina: A Case Report

REBECCA SHAIANE SOARES NUNES RIVOREDO ^1^, Raimundo Benício de Vasconcelos Neto^1^, Ana Caroline Leite Guedes^1^, Fernanda Gabry Scazuza Gomes de Souza^1^

(1) Centro Universitário São Lucas/Afya UNISL

Coronary fistulas are rare congenital anomalies that constitute connections between the coronary artery and the heart chambers (coronary-cameral fistula) or with the venous system of the myocardium (coronary arteriovenous fistula). This fact is due to the malformation of the individual’s cardiac vascular system, which presents continuity of the embryonic sinusoids of the myocardium, which are responsible for the formation of the perforating arteries and the right and left coronary trunks, and should close during the gestational period. Coronary arteriovenous fistulas are one of the rarest cases of this pathology, usually causing symptoms only after the 4th decade of life, which course with heart failure, angina, arrhythmias or endocarditis. The intervention of the pathological condition is established according to the patient’s condition, consisting of factors such as cardiovascular symptoms, fistula drainage volume and myocardial ischemia. This is a case report of a patient with an arteriovenous fistula in the right and circumflex coronary artery leading to the coronary sinus.

**Report:** Female patient, 47 years old, hypertensive, diabetic and dyslipidemic. She sought medical care due to exertion angina, which started 3 months ago, of a progressive nature, having intensified in the week preceding hospitalization, which motivated her to seek care. She was received with type A chest pain, at rest, with an electrocardiogram showing anteroseptal subendocardial ischemia. At no time did she present positive myocardial necrosis markers, and she was treated as high-risk unstable angina and underwent coronary angiography that showed coronary arteriovenous fistulas (CAF) in the paths from the right coronary artery to the coronary vein and from the circumflex artery to the coronary vein. After diagnosis, in-hospital clinical treatment was optimized and the patient was referred to the cardiovascular surgery service for fistulization correction.

**Conclusion:** The aforementioned case brings to light a discussion about coronary arteriovenous fistula associated with anginal cause, which, although rare, when detected early and managed clinically and surgically in an adequate and satisfactory manner, evidences the relief of symptoms and improvement in the patient’s quality of life.

111929

Modality: E-Poster Scientific Initiation – Case Report

Category: NEGLECTED CARDIOVASCULAR DISEASES

## Takayasu’s Arteritis – When to Intervene?

PEDRO LUCAS CARDOZO BARROS^1^, Ana Flávia Silveira de Souza^1^, Joana Raquel Cardoso dos Santos^1^, Hellen Dutra Passos^2^, José Augusto Soares Barreto Filho^1^

(1) Hospital Universitário da Universidade Federal de Sergipe (HU-UFS); (2) Hospital São Lucas, Rede D’or São Luiz (HSL)

Takayasu’s arteritis (TA) is a chronic inflammatory disease of unknown etiology. It is a rare vasculitis involving the aorta and its main branches in which transmural granulomatous inflammation causes multiple arterial lesions. The present report is about a 32-year-old female which physical exam exposed asymmetric radial pulses and significant pressure difference between upper limbs. She was subjected to a tomography of thoracic and abdominal aorta for the investigation of an abdominal murmur and possible secondary arterial hypertension. Angiography then revealed diffuse arterial injuries, including dissection of right renal artery, stenosis of left renal artery and at infrarenal portion of abdominal aorta and fusiform aneurysm upstream. Outpatient follow-up almost 6 years after the diagnosis of TA reveals a completely asymptomatic individual without prior hospitalization or any other intervention besides conservative treatment. The woman has a history of two pregnancies among one abortion, practices regular physical activity and maintains all work performance. Her blood pressure is controlled; thoracic radiography, electrocardiogram and laboratory exams are all normal; and both transthoracic echocardiogram and Holter show discrete and unspecific cardiac alterations. Studies have demonstrated that few patients with symptomatic TA require surgery. The procedures indicated for occlusive disease are endovascular stent and arterial bypass; the aneurysmal form of TA may require arterial reconstruction by open surgery or endovascular approach. However, current literature lacks significant evidence to indicate most invasive treatments for asymptomatic individuals. Our patient’s clinical presentation is stable, thus not requiring invasive procedures despite of diffuse arterial injuries.

111939

Modality: E-Poster Scientific Initiation – Case Report

Category: HEART FAILURE/CARDIOMYOPATHY/TRANSPLANT

## Hyperthrophic Cardiomyopaty and Marfan Syndrome: Case Report of Complex Cardiopathy Association

GABRIELLA GOMES FERNANDES MAIA^1^, Rafael Souza Conceição da Silva^2^, Claudia Regina de Oliveira Cantanheda^1^, Dany David Kruczan^1^, Marcio Montenegro^1^

(1) Instituto Estadual de Cardiologia Aloysio de Castro – IECAC; (2) Unigranrio Barra da Tijuca

Marfan syndrome is an autosomal dominant multisystem connective tissue disorder caused by mutations in fibrillin. HCM is a inherited disease caused by mutations in genes encoding sarcomeric proteins in the heart cells. The estimated incidence rate for Marfan syndrome is 1:5000, while for HCM is 1:500. Both association has no incidence, which explains the motivation of this case report. A 39-year-old female patient, reporting dyspnea on exertion for 2 years and palpitations for 18 months, presents with HTN and a murmur, which progressed to dyspnea on moderate exertion, diaphoresis and lipothymia. After 6 months, she presented a dry cough and chest discomfort such as tingling, radiating to the left arm and jaw. She uses antihypertensive drugs. History of descending aortic endoprosthesis in 2016. On physical examination, normal HR and BP. Abnormal kyphoscoliosis. Ogival palate and Steinberg’s sign. Regular and wide pulses. Palpable Ictus without deviation. On auscultation, mitral focus with hypophonetic sounds, holosystolic murmur and S3. In the supine position, the systolic murmur increases with expiration. In left lateral decubitus, it increases after inspiration. Mesocardium with hypophonetic sounds, systolic murmur and gallop. Accessory aortic focus with systolic murmur radiating to clavicles, carotids, and suprasternal notch, and an diastolic murmur. Pulmonary focus with a rough protosystolic murmur, well audible diastolic murmur and split S2. The X-ray shows slight cardiac enlargement and aortic calcification. ECG shows sinus rhythm, cardiac axis at –30° and alteration in ventricular repolarization. ECHO shows increased of LA and RA, asymmetric LV hypertrophy with reduced contractility but preserved global systolic function; MR with intense anterior systolic motion, AR with failure of central coaptation; big dilation of the ascending aorta plus descending aortic dissection. Diagnosed with Marfan syndrome, severe AR and MR, dilated ascending aorta and HCM. Surgical correction with Myectomy, placement of Aortic Valve Tube and mitral valve replacement was indicated. However, due to technical problems, the responsible institution chose to perform only the aortic valve replacement with a biological prosthesis. Currently, the patient remains symptomatic and Percutaneous Septal Alcoholization is indicated, due to a safer and more effective conclusion for the case. It was preferred a less invasive technique, since the main outcome is to provide quality life.

111982

Modality: E-Poster Scientific Initiation – Case Report

Category: COVID-19 AND CARDIOVASCULAR SYSTEM

## Acute Pericarditis in Scholar 3 Weeks After Infection by COVID-19: A Case Report

GABRIELA ZIMMERMANN^1^, Melissa Dorneles de Carvalho^1^, Thamara Andressa Fagundes^1^, Fernando Caritas de Souza^1^, Giolana Mascarenhas da Cunha^1^

(1) Universidade Estadual do Oeste do Paraná (UNIOESTE)

Pediatric presentation of coronavirus disease 2019 (Covid-19) rarely includes severe complications in acute phase; however, characteristics of the condition among children are not yet fully established. We report a case of a scholar who presented developed acute pericarditis, tested positive for Covid-19, progressed to myopericarditis and had completely recovery after the therapeutic management. Male, 11 years old, previously healthy, referred chest pain, for over one hour, no radiating, that started suddenly in prone position and presented spontaneous remission. Fourteen hours later he felt the same symptoms, so he was taken to the emergency service. An electrocardiogram (EKG) showed alterations that could suggest infarction, nonetheless, considering the patient’s symptoms and age, the heart attack was rejected, and pericarditis was the most likely hypothesis. The patient denied having fever or dyspnea, but a flu episode about twenty-five days earlier. Physical exam had no irregular findings. Laboratory evaluation included leukocytes 12.820/mm^3^, erythrocyte sedimentation rate 20 mm/h, troponin I 19.572 ng/mL, pro B-type natriuretic peptide 790 pg/mL, C-reactive protein 5,9 mg/L, lactate dehydrogenase 335 mg/dL, D-dimer 222 ng/mL and COVID-19 serology results: IgG positive and IgM negative. With the orientation to make EKG’s series, another exam was performed (Figure 1), which presented ST-segment elevation in D2, D3, avF and precordial leads. Echocardiogram was conclusive with pericarditis. The treatment included nonsteroidal anti-inflammatory drugs (ibuprofen and high-dose aspirin) for two weeks. The hospital length of stay was 4 days, the scholar turned asymptomatic, and EKG was normal. The patient was not vaccinated against SARS-Cov-2. This is a case of pericarditis after SARS-Cov-2 infection that might be a useful reference to other clinicians caring for pediatric patients affected by COVID-19 and contribute to the differential diagnosis of chest pain. Despite the rapidly growing research on management of COVID-19 and its complications, there are many unanswered questions and areas to explore.


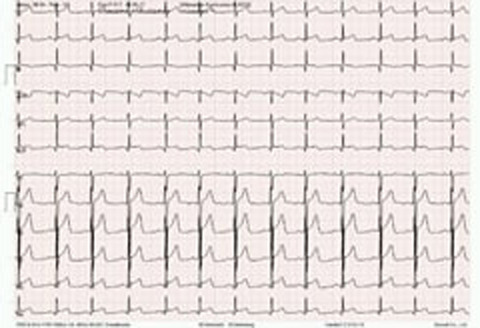


111984

Modality: E-Poster Scientific Initiation – Case Report

Category: CARDIOVASCULAR IMAGING

## Non-Invasive Diagnosis of Cardiac Amyloidosis by Technetium-Pyrophosphate Scintigraphy: A Case Report

ALICE ZANETTI DUSSIN^1^, Alice Einsfeld Britz^1^, Juliana Menezes Zacher^1^, Bárbara Dewes Silva^1^, João Carlos Vieira da Costa Guaragna^1^

(1) Pontifícia Universidade Católica do Rio Grande do Sul (PUCRS)

**Background:** Transthyretin amyloidosis (ATTR) is the main cause of cardiac amyloidosis and it is presented by clinical manifestations such as heart failure, diastolic dysfunction, left ventricular hypertrophy, atrial fibrillation (AF) and aortic stenosis. Technetium-pyrophosphate (Tc-99m-PYP) scintigraphy has a fundamental diagnostic role in differentiating types of amyloidosis, being able to detect the presence of transthyretin deposits.

**Case Report:** A 85-year-old diabetic female, with a history of multiple hospitalizations due to decompensated heart failure and chronic obstructive pulmonary disease since 2009. Patient brought a 2009 transthoracic echocardiogram (TTE) – ejection fraction (EF) of 74%, all cavities had normal dimensions – and two myocardial scintigraphy from the same period with low probability of ischemia. Five years ago, was presented with acute chest pain and underwent coronary angiography that showed coronary arteries without obstructions. Soon after, she started having syncopal episodes. Three years ago, started experiencing dyspnea on moderate efforts, and was diagnosed with AF; cardioversion was successfully performed. Thirty days later, she once again presented AF with worsening dyspnea, lower limb edema, jugular swelling and hepatomegaly. At the time, a chest tomography (cardiomegaly, right pleural effusion and enlargement of the pulmonary trunk) and a TTE (EF of 68%, left ventricle with normal diameters and biatrial overload) were executed. A cardiac magnetic resonance also showed biatrial enlargement. A Tc-99m-PYP scintigraphy was then requested, which diagnosed grade 2–3 ATTR.

**Conclusion:** Cardiac amyloidosis is a rare, progressive and lethal pathology; and its incidence has increased due to advances in cardiovascular imaging. A Tc-99m-PYP scintigraphy, for example, has a sensitivity of 90% and specificity of 97% for the diagnosis of ATTR. Therefore, it is extremely important for the cardiologist to be acquainted with this method, since this can allow an early diagnosis of ATTR without the need for invasive tests, such as biopsy, which is still considered the gold standard for diagnosis.


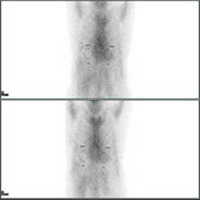


112065

Modality: E-Poster Scientific Initiation – Case Report

Category: CONGENITAL AND PEDIATRIC CARDIOLOGY

## Prenatal Findings of a Case of Imperfect Twinning

JULIANA ROSSI CATAO^1^, Guilherme Girardi May^1^, Eric Schwellberger Barbosa^1^, Henrique Perez Filik^1^, Rafael Fabiano Machado Rosa^1^

(1) Universidade Federal de Ciências da Saúde de Porto Alegre (UFCSPA)

**Introduction:** Imperfect twinning occurs in 1 in 100 monozygotic twins. Our aim was to report the prenatal findings of a case of conjoined thoracopagus twins.

**Case Presentation:** The patient was a 31-year-old pregnant woman in her fourth pregnancy. She was referred to the fetal medicine service due to an ultrasound at 12 weeks gestation, with a description of twins united in the abdomen. The examination at 14 weeks revealed that the twins were united at the chest and upper abdomen. There was apparent sharing of heart and liver. A single umbilical cord emerged caudally to the union of the fetuses. The emergence of the umbilical arteries was observed laterally to the bladders. The pregnancy was monochorionic and monoamniotic. Echocardiography revealed a normal fetus on the left and a fetus on the right with dextrocardia. Second trimester ultrasound, performed at 22 weeks of pregnancy, showed no morphological changes in the fetuses. Two venous ducts were visualized. The echocardiography performed soon after revealed an apparent communication of the hearts through the right atrium of the fetus located on the right with the left atrium of the fetus located on the left. The fetus on the left had a tetralogy of Fallot with pulmonary atresia. There was a large infundibular perimembranous ventricular septal defect with a large caliber aorta riding the trabecular septum in 50% of its annulus. Magnetic resonance imaging revealed fetuses fused through the thorax and upper abdomen, above the umbilical cord insertion. The right lobe of the liver of fetus one was related to the left lobe of the liver of fetus two. There were two stomachs and two gallbladders, as well as four kidneys. Close to the lower margin of the livers, small bowel loops were observed, which were closely related, making it difficult to characterize whether there was a crossing between them from one fetus to another.

**Discussion:** Although imperfect twinning is a rare condition, estimated at 1 in 75,000 births, its prenatal diagnosis is very important. It makes it possible to assess the fetal fusion site and its complexity to help define management and prognosis.

**Final Comments:** Thus, the evaluation of fetuses with imperfect twinning is usually multidisciplinary, involving mainly radiologists, obstetricians, pediatricians, and pediatric surgeons, intending to start birth planning during pregnancy, including a place that has adequate conditions for receiving and properly handling these babies.

112083

Modality: E-Poster Scientific Initiation – Case Report

Category: CONGENITAL AND PEDIATRIC CARDIOLOGY

## Hematological Alterations in a Patient with Congenital Heart Disease: Jacobsen Syndrome

HELENA GUEDES DA ROCHA^1^, Estefany Karenine Rodriguez Casanova^1^, Caroline Engster da Silva^1^, Laís Bettoni^1^, Rafael Fabiano Machado Rosa^1^

(1) Universidade Federal de Ciências da Saúde de Porto Alegre – UFCSPA

**Background:** Jacobsen syndrome is a chromosomal abnormality characterized by a deletion involving the long arm of chromosome 11 and is associated with different clinical findings.

**Objective:** To report the case of a patient with Jacobsen syndrome secondary to a translocation of paternal origin, highlighting its relationship with congenital heart diseases.

**Results/Case Report:** The patient, who is the second child of young parents, was born by cesarean delivery weighing 3500 g and with an Apgar score of 10 at the fifth minute. He had a history of surgery for correction of pyloric stenosis at 54 days of life. On physical examination, palpebral ptosis, right inguinal hernia, bilateral hallux valgus, clinodactyly of the 5th finger and syndactyly between the 2nd and 3rd pododactiles were observed. Echocardiography revealed an interventricular communication. The patient also had a history of epistaxis, ecchymosis and coagulation problems. He had a neuropsychomotor developmental delay that affected his school performance. In addition, the neurological evaluation found an attention deficit hyperactivity disorder. Computed tomography showed an epidermoid cyst in the skull. Audiometric evaluation revealed moderate sensorineural hearing loss in both ears. Karyotype showed a deletion of part of chromosome 11 (46, XY, del (11) (q23.1q25) [28]), compatible with the diagnosis of Jacobsen syndrome. The complementary examination of the father revealed the presence of a translocation between chromosomes 6 and 11.

**Conclusions:** Patients with Jacobsen syndrome may present a multisystemic involvement, which demands a multidisciplinary approach. Special attention should be dedicated to congenital heart diseases, since they are one of the main causes of death in these patients. Heart defects are observed in 56% of the cases and usually require drug treatment and/or surgical correction. The interventricular communications, such as the one observed in our patient, are the most common cardiac malformations. All this is of great importance, since hematological alterations are also common, which may corroborate to an increase in morbidity and mortality of the patients.

112102

Modality: E-Poster Scientific Initiation – Case Report

Category: HEART FAILURE/CARDIOMYOPATHY/TRANSPLANT

## Transthyretin Cardiac Amyloidosis Due to Homozygous Val142Ile Variant

LUANA DIAS XAVIER^1^, João Paulo Dias Costa^1^, Julia Souza Diniz^1^, Emerson De Santana Santos^1^, Joselina Luzia Menezes Oliveira^1^

(1) Universidade Federal de Sergipe

**Introduction:** Val142Ile, predominantly found among African descents, is the most common transthyretin (TTR) amyloidosis variant and predominantly develops a cardiomyopathy phenotype. Individuals with two variants in the TTR gene are extremely rare. They tend to manifest symptoms a mean of 10 years earlier than heterozygotes and it can be more severe. We report a Northeast Brazilian family with recurrence of homozygotes for Val142Ile due to consanguineous marriages.

**Case Report:** A 77-year-old male was referred to cardiogenetics evaluation due to progressive heart failure whose symptoms started at the age of 70 and cardiac amyloidosis was suspected. He had asymmetrical septal hypertrophy and arrhythmia. Holter showed sporadic ventricular ectopia and supraventricular ectopia. An echocardiogram revealed restrictive cardiomyopathy and left ventricle hypertrophy. 99mTc-Pyrophosphate scintigraphy was also suggestive of TTR cardiac amyloidosis. His cardiac magnetic resonance imaging (MRI) showed ventricular septal hypertrophy, perfusion deficit in stress, and delayed myocardial enhancement. A molecular exam detected Val142Ile in heterozygosity. His ex-wife (who is also his cousin), a 74-year-old lady who was born from a consanguineous marriage (her parents were first-grade cousins too), is also affected. Cardiac MRI showed asymmetric apical hypertrophy, stress echocardiogram was negative for ischemia, and the genetic test showed she was homozygous for the Val142Ile variant. Their older son had sudden cardiac death at the age of 52. Their younger daughter, a 42-year-old female, has no cardiac involvement yet and she is also homozygous for the Val142Ile variant. All reported patients had bilateral carpal tunnel syndrome and no other neurological manifestation.

**Conclusion:** The frequency of consanguineous marriages in Northeast Brazil is higher than in other regions, with rates as high as 6 to 12% in rural areas. Inbreeding has certainly contributed to the homozygosity in Val142Ile detected in two members of the family. Genetic counseling was provided to the family since early diagnosis may benefit affected members with the available treatments.

112204

Modality: E-Poster Scientific Initiation – Case Report

Category: CONGENITAL AND PEDIATRIC CARDIOLOGY

## Ebstein Syndrome in Elderly Patient

FELIPE SALIM HABIB BUHAMARA ALVES NASSER GURJÃO^1^, Felipe Salim Habib Buhamara Alves Nasser Gurjão^1^, Bruna de Almeida Freixedelo^1^, Mateus Sousa Cavalcante^1^, Leandro Cordeiro Portela^1^, Dara Medeiros Mendes^1^, Vicente Lopes Montes Neto^1^

(1) Universidade Federal do Ceará – Campus Sobral

**Introduction:** Ebstein’s anomaly corresponds to a set of malformations centered around the tricuspid valve. This syndrome corresponds to the most common congenital heart disease of the tricuspid valve. The severity of symptoms depends primarily on age and the degree of insufficiency presented. The clinical spectrum is variable and can range from death in 50% of cases in newborn patients to a silent disease with symptoms appearing only after decades.

**Case Description:** Male patient, 68 years, diagnosed with systemic arterial hypertension, type 2 diabetes and heart failure, since 2017, sought medical care at the Senador Carlos Jereissati Municipal Hospital on 04/18/2022 due to respiratory distress with progressive worsening for two days. He had BP: 70 × 40 mmHg, Capillary blood glucose: 180, RR: 30, HR: 111, T: 35.1°C and oxygen saturation: 85% in oxygenation of 15 L/min. Thus, due to the patient’s serious condition and the lack of stabilization in the hospital of origin, a transfer to the North Regional Hospital was requested, where the patient was admitted in the early hours of the following day. At the time of admission, the patient was tachypneic, diffusely diminished universal vesicular murmurs with diffuse crackles. An electrocardiogram was performed which reported low voltage and atrial fibrillation. The patient was then hospitalized under the action of vasoactive drugs and oxygen therapy for better hemodynamic stabilization and search for the cause of heart failure decompensation. Laboratory tests and a transthoracic echocardiogram were requested. Among these tests, the echocardiogram, performed on 04/21/2022, showed a significant increase in the right chambers, with atrialization of the right ventricle and 31 mm inferior detachment of the tricuspid valve septal cusp of the tricuspid valve, adhered to the contractile right ventricular wall e decreased right ventricle contractile function, which indicates Ebstein Syndrome.

**Conclusion:** Ebstein’s anomaly corresponds to a complex and rare disease that mainly affects newborns with greater severity. Knowing the anatomical changes and cardiac physiology is essential for the correct approach to this condition. Health professionals should be alert to detect symptoms suggestive of heart failure and request tests for confirmation. As it is characterized as an insidious disease, the early diagnosis of this condition corresponds to a point of great importance for the proper management of the patient.

112241

Modality: E-Poster Scientific Initiation – Case Report

Category: HEART FAILURE/CARDIOMYOPATHY/TRANSPLANT

## Peripartum Cardiomyopathy and the Hardships to Diagnose It: Case Report

ANA ROBERTA DE SOUSA ARAUJO^1^, Beatriz Nunes Deseyvan Rodrigues^2^, Rayanne Carvalho Vasconcelos de Azevedo^2^, Ana Roberta de Sousa Araujo^1^

(1) FIMCA; (2) FIMCA

**Opening:** The peripartum cardiomyopathy (PPCM) is a cardiac insufficiency secondary to systolic dysfunction of the left ventricle that affects women at the end of their pregnancy or even 6 months after giving birth. It has left ventricle ejection fraction (LVEF) < 45%, with a condition of dyspnea, fatigue and lower extremity edema (LEE). The risk factors include being an afro-descendant, multiparity, maternal old age and preeclampsia. Furthermore, it presents high and variable mortality rate, because it can be avoided with an early diagnosis and specific treatment. So, documenting PPCM is important, given that it often gets a late diagnosis. Thus, this study objectifies reporting the case of a woman diagnosed with PPCM 6 months after giving birth.

**Case Report:** 33-year-old woman, brown, married, home secretary, no prior diseases, multiparous, no cardiomyopathies in previous gestations, developed preeclampsia in the fifth gestation, which was treated. Her delivery occurred after a hypertensive peak via cesarean, ceasing the hypertensive disturbances. 6 months of gestation passed, reported tiredness, dyspnea and an edema on the lower limbs, that extended itself to the abdomen. There was a diagnosis of cardiomegaly, and with the condition getting worse, she was hospitalized in an intensive Therapy unit. It was identified a 120/94 mmHg blood pressure, pulmonary noises with sparse hisses, high troponin I in the laboratory tests and hepatomegaly in the total abdomen ultrasound. In the Echocardiogram, it was verified a LVEF of 30, left ventricle dilated, diffuse hypokinesia, sharp systolic dysfunction, right ventricle with moderate increase, significant aortic regurgitation and moderate mitral. In the cardiogram, it was observed sinus tachycardia (FC > 99 bpm), probable left ventricular hypertrophy and long QT interval. Hence, after clinical analyses the patient was diagnosed with PPCM, it was prescribed atorvastatin, carvedilol and enalapril. With a bad prognosis, the patient finds itself admitted to the transplant center.

**Conclusion:** Therefore, PPCM being a hard to diagnose disease, probable to adversely evolve, might be necessary to perform a transplant, as in the discussed case. However, controlling the appearance of signs and specific symptoms, besides doing early diagnostic tests, can effectively avoid complications. So, it’s indispensable to increase the scientific knowledge to improve the prognosis.

